# Gene conversion is a key driver of diversity hotspots in *M. tuberculosis* antigens and virulence-associated loci

**DOI:** 10.64898/2026.02.26.708061

**Published:** 2026-03-11

**Authors:** Maximillian G. Marin, Natalia Quinones-Olvera, Hu Jin, Michael A. Harris, Brendan M. Jeffrey, Alex Rosenthal, Kenan C. Murphy, Christopher Sassetti, Heng Li, Maha R. Farhat

**Affiliations:** 1 Department of Biomedical Informatics, Harvard Medical School, Boston, USA; 2 Department of Data Science, Dana-Farber Cancer Institute, Boston, USA; 3 Office of Cyber Infrastructure and Computational Biology, National Institute of Allergy and Infectious Diseases, National Institutes of Health, Bethesda, USA; 4 Department of Microbiology and Physiological Systems, University of Massachusetts Medical School, Worcester, USA; 5 Broad Institute of MIT and Harvard, Cambridge, United States; 6 Pulmonary and Critical Care Medicine, Massachusetts General Hospital, Boston, USA

## Abstract

Despite the long-held view of *Mycobacterium tuberculosis* (*Mtb*) as a genetically conserved pathogen, many genomic regions remain poorly resolved due to high sequence homology and repetitive content. Using complete genome assemblies generated from long-read sequencing of 151 globally representative clinical isolates, we comprehensively analyzed genome-wide patterns of genetic diversity and evolution across the *Mtb* genome. Our analysis uncovers pronounced diversity hotspots within paralogous regions generated by recurrent gene conversion between homologous genes. In many cases, these hotspots exhibit more than an order of magnitude greater genetic diversity than the rest of the *Mtb* genome, which is otherwise characterized by remarkably low variation. Mutations within these regions display clustered substitution patterns, excess paralog-matching variants, and distinct mutational spectra consistent with ongoing gene conversion. Our analysis identifies over 300 individual gene conversion events distributed throughout the *Mtb* phylogeny. These gene conversion events occur predominantly within gene families associated with virulence and host-pathogen interactions, including the PE, PPE, and ESX families. Several of the most pronounced diversity hotspots occur in antigens encoded within paralogous regions. Among these, the vaccine candidate PPE18 harbors mutations in validated epitope sequences and predicted alterations in HLA-II binding. Together, these findings demonstrate that gene conversion actively shapes antigenic and virulence-associated diversity in *Mtb*.

## Introduction

The bacteria of the *Mycobacterium tuberculosis* (*Mtb*) complex (Mtbc) are the causative agent of tuberculosis (TB), a disease responsible for over 1.2 million deaths per year (1). Understanding the forces driving *Mtb* genome evolution has advanced the development of DNA-based diagnostics for antibiotic resistance and allowed for improved detection of disease transmission in TB endemic areas (2). As an obligate human pathogen with no known environmental reservoir, *Mycobacterium tuberculosis* exhibits a strictly clonal population structure with low levels of genetic diversity and a limited accessory genome (3–5). Genome-wide population genomic analyses have found no measurable evidence of ongoing horizontal gene transfer among circulating *Mtb* strains(6). Experimental mating studies have likewise failed to demonstrate interstrain genetic exchange, further supporting the species’ minimal accessory genome (7, 8). Consequently, genetic variation within the species is thought to arise through endogenous mutational processes, dominated by single-nucleotide substitutions and small insertions or deletions, alongside occasional structural variants(5).

Most genomic analyses of *Mtb* have relied on short-read sequencing, which can limit the accuracy of read alignment and variant detection in repetitive or highly homologous genomic regions(9–11). As a result, these repetitive and paralogous regions, comprising nearly 10% of the *Mtb* genome, are frequently excluded from genomic analyses (9–11). Notably, these excluded regions are enriched for the PE, PPE, and Esx gene families, which encode substrates of the ESX secretion systems (3, 5, 12). These families are of particular interest because they expanded in copy number and diversified during the emergence of *Mtb* as an obligate pathogen from environmental mycobacterial ancestors(3, 13–18). Members of these protein families are frequently secreted or localized to the cell surface and have been implicated in diverse aspects of the host–pathogen interface, including modulation of cell wall composition, nutrient acquisition during infection, tissue tropism, and host immune responses (17–22). Despite their biological importance, our understanding of these genes derives largely from studies of a single *Mtb* reference strain (H37Rv), with limited assessment of their diversity across the species(18).

Recent advances in long-read sequencing now enable routine assembly of complete bacterial genomes (23–25), permitting confident resolution of repetitive and paralogous regions of the *Mtb* genome. Interrogating the mutational processes and evolutionary forces acting within these regions is therefore critical for understanding their function at the host-pathogen interface(5). The high sequence homology among paralogous loci creates the potential for gene conversion, a form of non-reciprocal intrachromosomal homologous recombination in which short tracts of sequence are copied from one paralogous locus to another (26–28). Gene conversion is a well-recognized evolutionary process among paralogous genes that can function both as a homogenizing force and as a diversity-generating mechanism through interlocus sequence transfer (26, 27, 29). Evidence for gene conversion among specific ESX, PE, and PPE genes has been reported using Sanger and short-read sequencing approaches, although these studies were largely restricted to individual loci or limited by the resolution of short-read data (30–32). More recently, long-read sequencing has enabled more confident detection of individual gene-conversion events in clinical *Mtb* isolates, providing direct evidence that gene conversion contributes to genetic diversification and suggesting a potential role in antigenic variation (33, 34).

However, the genome-wide prevalence, evolutionary timing, and functional impact of gene conversion across globally diverse *Mtb* lineages have not been systematically evaluated. Here, we analyze the genome-wide diversity across all major lineages of human-adapted *M. tuberculosis* using complete genome assemblies generated from matched long- and short-read sequencing data. Our analysis reveals gene conversion as a pervasive mutational mechanism across paralogous loci, with recurrent events generating localized diversity hotspots in genes implicated in host-pathogen interaction, including several experimentally validated T cell antigens. Together, these findings reveal gene conversion as a pervasive force shaping species-level diversity at the host-pathogen interface.

## Results

### Hotspots of extreme genetic diversity are found within paralogous genomic regions

We curated a dataset of complete genome assemblies from 151 clinical isolates spanning the global diversity of human adapted *Mtb* (lineages 1–6 and 8, Figure S1). All assemblies were generated using a hybrid *de novo* genome assembly approach that integrated both long- and short-read sequencing data to ensure high assembly accuracy and completeness (Methods). The final polished assemblies demonstrated the expected high similarity between *M. tuberculosis* genomes: average nucleotide identity (99.8–100%), genome size (4.38–4.44 Mb), and number of predicted coding sequences (4020 – 4135 CDSs) (Table S1).

**Figure 1.**
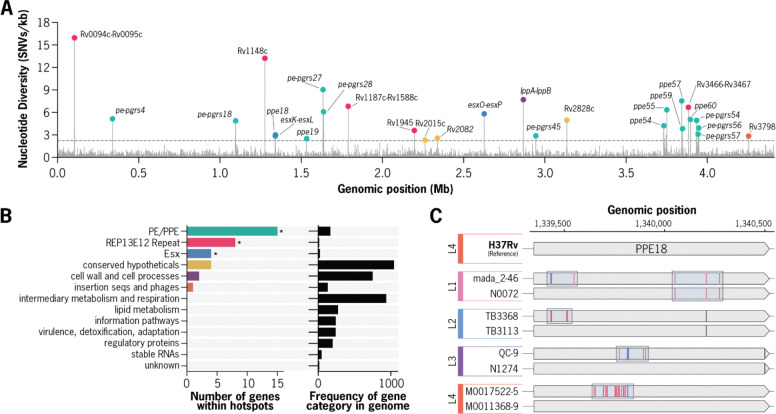
Characterization of nucleotide diversity hotspots within the *Mtb* genome. **a,** Genome-wide distribution of nucleotide diversity (π) across all non-overlapping 1-kb windows measured for complete Mycobacterium tuberculosis (Mtb) genome assemblies. A total of 37 1-kb windows were identified as diversity hotspots, with π values ranging from 2.3 to 15.5 substitutions per kb (up to 83-fold higher than the genome-wide median). Each diversity hotspot is colored based on the functional category of the associated gene(s). b, Functional classification of genes within diversity hotspots. **c,** Example of mutation patterns in the *ppe18* gene across representative *Mtb* isolates from distinct lineages. Red bars indicate substitution mutation positions relative to the H37Rv reference genome. Multiple dense tracts of substitutions are observed in different distinct of the *ppe18* gene across multiple *Mtb* genomes.

Across these high confidence assemblies, the median nucleotide diversity of the *Mtb* genome was low (0.2 substitutions/kb), however, a small subset of regions showed extreme diversity (Figure 1A) (Modified Z score > 10, 12–83x the genome median, Table S2, Methods). These diversity hotspots were located in 37 distinct 1-kb windows (2.3 to 15.5 substitutions/kb) and were significantly enriched for three gene categories: PE/PPE genes (15 genes, p < 8.4e-13), ESX genes (4 genes, p < 3.2e-05), and REP13E12 repeat regions (8 genes, p < 2.8e-14) (Figure 1B). Members of these gene families are often characterized by both multi-copy paralogs distributed throughout the genome and locally repetitive sequences (15, 18), leading to them being commonly excluded from genomic analyses using short-read sequencing (10).

We hypothesized that elevated diversification in these hotspots may be related to their non-unique sequence content, potentially through homology-mediated mutational processes. To evaluate this hypothesis, we systematically annotated multiple classes of non-unique sequence content across the *Mtb* genome, including multi-copy paralogous regions (PRs) as well as repetitive or low-complexity sequences (Supplementary Results, Figure S2, Table S3). Of the 37 diversity hotspots identified, 36 were specifically located within PRs (Table S3); moreover, the majority (31/37) of hotspots were themselves paralogous to at least one other hotspot (Figure S3).

Because non-unique sequences can be more prone to assembly errors, we manually examined read alignments to confirm assembly and variant call accuracy (Methods). During careful inspection of the data, we observed that mutations in these regions frequently occurred as clustered tracts of substitutions, as exemplified by the *ppe18* locus across a diverse panel of isolates (Figure 1C, Supplemental Data).

### Paralogous regions of the *Mtb* genome harbor distinct mutational patterns suggestive of ongoing gene conversion

Given the strong overlap between diversity hotspots and paralogous genomic regions, we hypothesized that elevated diversity in these regions is driven by gene conversion between paralogs rather than by rapid accumulation of independent point mutations. Frequent gene conversion would be expected to produce distinct mutational characteristics, including closely clustered substitutions that simultaneously match sequence variants found in other paralogs(35). To explore this hypothesis, we compared mutational characteristics of paralogous regions (PRs) to those of the rest of the *Mtb* genome. To enable phylogenetically informed analysis of substitution events across the evolutionary history of all 151 genomes, we reconstructed a genome-wide phylogeny and inferred ancestral states for all detected mutations (Methods).

**Figure 2.**
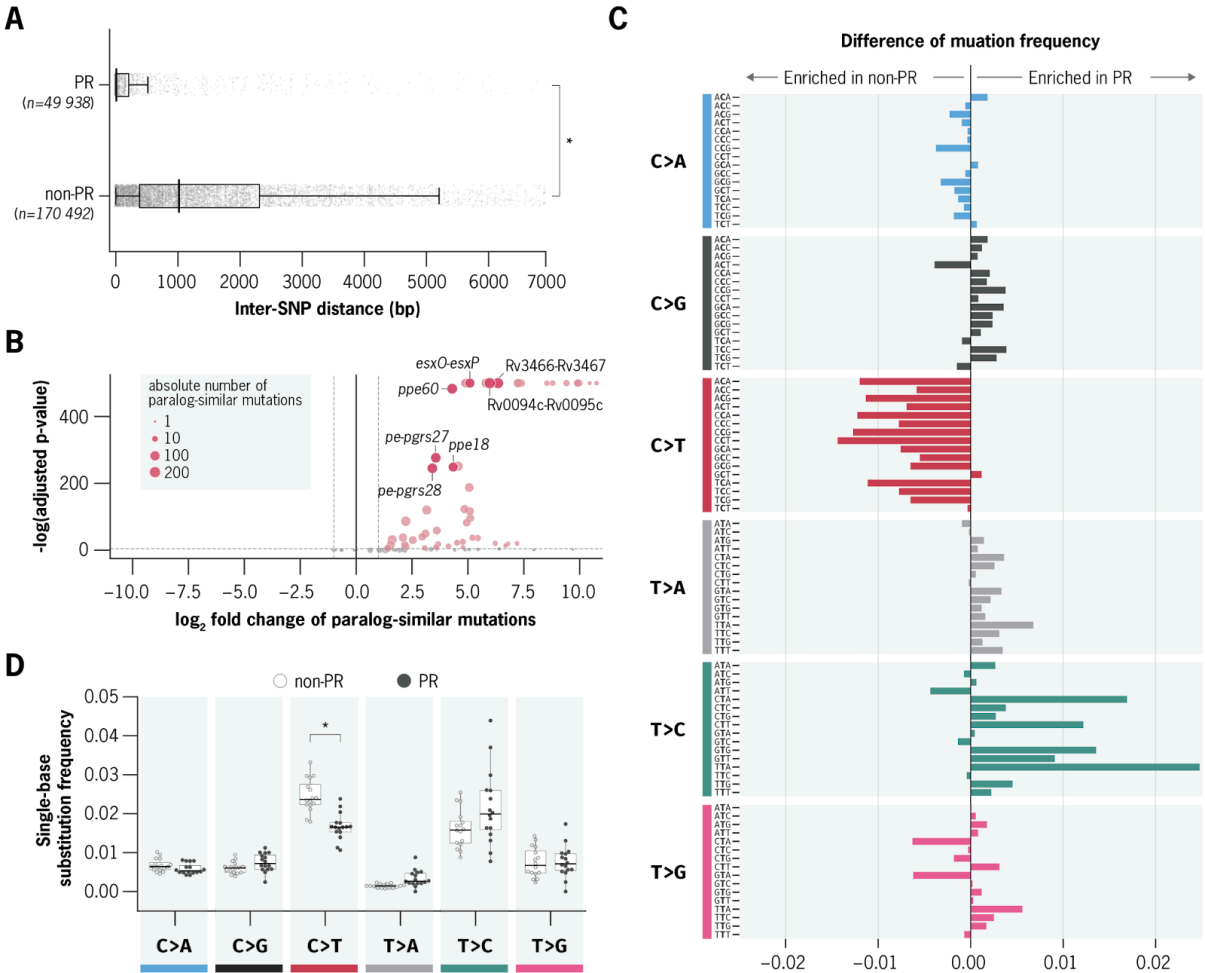
Paralogous regions of the *Mtb* genome harbor distinct mutational characteristics suggestive of gene conversion. **a,** Distribution of inter-SNP distances in paralogous regions (PRs, top) and non-PR regions (bottom) across all substitutions detected relative to the H37Rv reference genome. For each substitution, inter-SNP distance was defined as the distance to the nearest co-occurring substitution within the same genome. Mutations in PRs were significantly more clustered, with a median inter-SNP distance of 7 bp compared to 768 bp in non-PR regions (Wilcoxon rank-sum test, *p* < 4e-8). **b,** Quantification of paralog-similar mutations across PRs. Paralog-similar mutations were defined as substitutions that exactly match known sequence differences between paralogs in the reference genome. Each point represents one PR, with circle size indicating the absolute number of paralog-similar mutations. PRs highlighted in red are significantly enriched for paralog-similar mutations after multiple hypothesis correction (FDR < 0.05, Poisson test). **c,** Relative frequencies of substitution type across all trinucleotide contexts in PRs and non-PR regions. Boxplots summarize the distribution of normalized frequencies of each trinucleotide context within each substitution type. PRs exhibit a distinct mutational spectrum, including a depletion of C>T and an enrichment of T>C substitutions compared to non-PR regions. **d,** Visualization of the difference in trinucleotide substitution frequencies between PRs and non-PRs. Bars represent the Δ mutation rate (PR minus non-PR) for each substitution type.

We found that PRs harbor multiple distinct mutational characteristics consistent with gene conversion (Figure 2). First, substitutions in PRs exhibit significantly higher mutational density than those in non-PR regions (median inter-substitution distance of 7 bp versus 768 bp, respectively; Wilcoxon rank-sum test, p < 4 × 10^−8^; Figure 2A). Further, a subset (61/200) of PRs exhibited a significant mutational bias toward substitutions that match exactly variants found in other paralogs (q < 0.05; Figure 2B). Notably, these PRs encompassed nearly all (33/37) previously identified diversity hotspots, including *ppe18*, *ppe60*, *pe-pgrs27*, *pe-pgrs28*, *esxO-esxP*, *Rv0094c-Rv0095c*, and *Rv3466-Rv3467*. In parallel, mutational spectrum analysis revealed PRs to be significantly depleted in C>T/G>A and enriched in T>C/A>G substitutions compared to the rest of the genome (Figure 2C–D, Mann-Whitney U Test, p < 1.59e-5), suggesting the contribution of mutational processes beyond canonical point mutation.

Gene conversion relies on homologous recombination (HR) processes, which in mycobacteria predominantly proceeds through synthesis-dependent strand annealing (SDSA) (28, 36). To ensure that the repair pathways likely to mediate gene conversion are functional across the global *Mtb* population, we first confirmed the gene sequence integrity of all annotated DNA repair enzymes. We predicted loss-of-function (LoF) mutations in the 151 isolate dataset across 59 annotated genes associated with 13 DNA repair processes (Table S4). None of the 13 known SDSA/HR genes had any predicted LoF mutations. Only two DNA repair genes, *ligB* (Rv3062) and *radA* (Rv3585), were found to each contain a single LoF mutation in two independent isolates. Both of these genes are not related to SDSA/HR, and are non-essential for *in vitro* growth (36–38)(39, 40). Overall, these findings support that SDSA/HR pathways are functional across the global diversity of *M. tuberculosis* lineages.

### Frequent gene conversion between a small subset of paralogs drives their exceptional diversification

**Figure 3.**
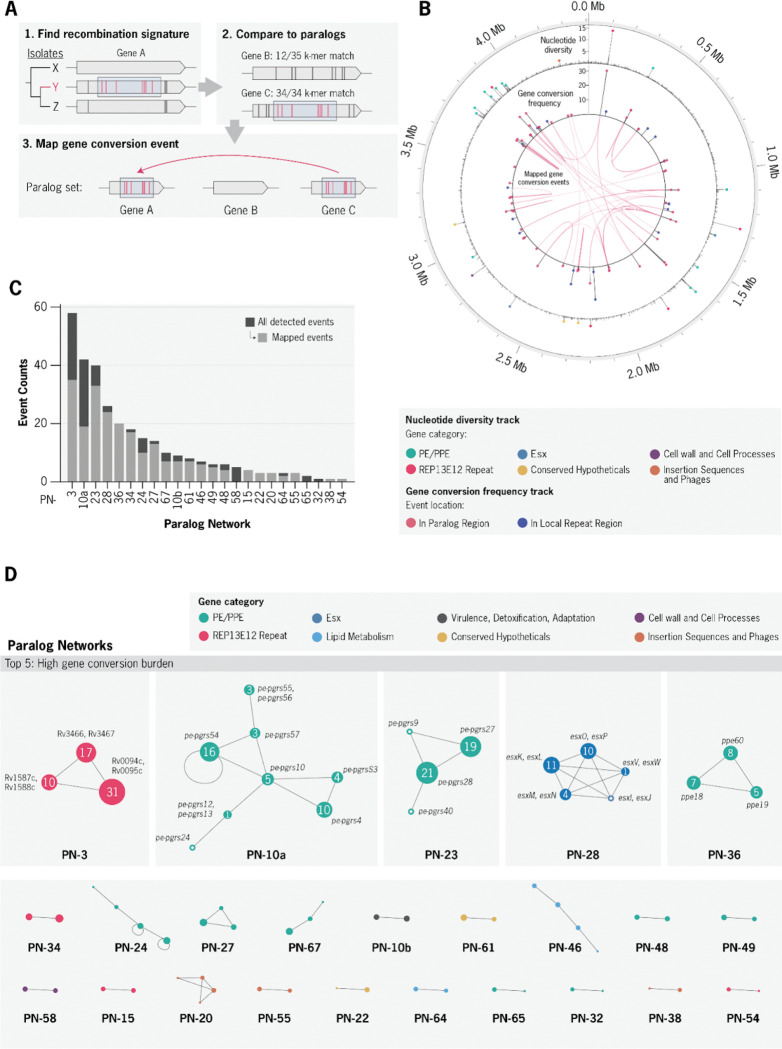
Systematic detection of gene conversion events through *Mtb* evolution. **a,** Overview of the computational pipeline to detect gene conversion (GC) events. **b,** Circular representation of the Mtb genome showing the genomic distribution of detected GCEs. Outer track: nucleotide diversity (π) per 1-kb window. Inner track: number of GCEs per genomic region. Red arcs represent donor–recipient pairs with at least one mapped GC event. **c,** Barplot showing the number of GCEs detected per paralog network. **d,** Network diagram of all recombining paralog networks. Each node represents a paralogous region (PR) with node size that reflects the total number of GCEs detected in that region. Edges represent homology between regions. Nodes are colored by the annotated gene category.

We next developed a multi-step phylogenetic workflow to study the dynamics of gene conversion through the evolutionary history of *M. tuberculosis*. To detect individual events, we first used Gubbins (41) to identify sets of clustered mutations that co-occurred contemporaneously along the phylogeny. Each of these detected gene conversion events (GCEs) is associated with both a set of mutations (>= 4 SNPs) and a specific branch of the phylogeny where the event is predicted to have occurred. For further validation, the associated recombinant sequence of each detected event was then compared to the sequence of all known paralogs using a k-mer based similarity metric (Methods, Figure 3A). All cases where a GCE’s mutations closely matched the reference sequence of known paralog(s) were further classified as mapped GCEs (mGCEs).

Using this framework, we detected 324 gene conversion events (GCEs) across the evolutionary history of our dataset (Figure 3). All GCEs were associated with PRs and/or locally repetitive regions of the genome. GCEs predominantly overlapped with known PRs (91%, 295 of 324), while the remainder were associated with locally repetitive regions (Figure S4A). Overall, detected GCEs were characterized by short, SNP-dense recombination tracts with a median length of 101 bp (IQR: 33–244 bp) and a median of 6 SNPs (IQR: 5–10) (Figure S5). A majority (74%, 217) of GCEs in PRs were mapped confidently to a reference paralog sequence (Figure S4B). Among the mapped GCEs, the genomic distances between inferred donor and acceptor paralogs spanned 1 kb to 2.14 Mb, supporting recombination between both adjacent sequences as well as distant regions of the chromosome (Figure S5). Across all the PRs there was a strong positive relationship between the GCE frequency and nucleotide diversity (Spearman ρ = 0.52, p = 1.4 × 10^−30^; Figure S6). Consistent with this pattern, a majority of detected GCEs (64%, 208) also directly mutated regions identified as diversity hotspots.

GCEs were detected across 76 distinct genes, with several gene categories showing clear enrichment in both the number of affected genes and the event frequency (Table 1). Significant enrichment was found for the following gene categories: PE/PPE (36 genes, 169 GCEs), ESX (7 genes, 36 GCEs), and REP13E12 repeat regions (5 genes, 87 GCEs) (Fisher’s exact test).

To characterize the distribution of GCEs across the *Mtb* genome, we constructed a genome-wide homology graph in which each PR is represented as a node and edges connect regions with detectable sequence homology (Methods). This approach grouped the 200 PRs into 71 discrete paralog networks. GCEs were highly concentrated in a subset of paralog networks (Gini = 0.85; Figure 3C–D), with only 24 networks containing any detected GCE activity. We further classified the 24 GCE-containing networks into 9 low-, 10 intermediate-, and 5 high-burden networks (Methods, Table 2).

**Figure 4.**
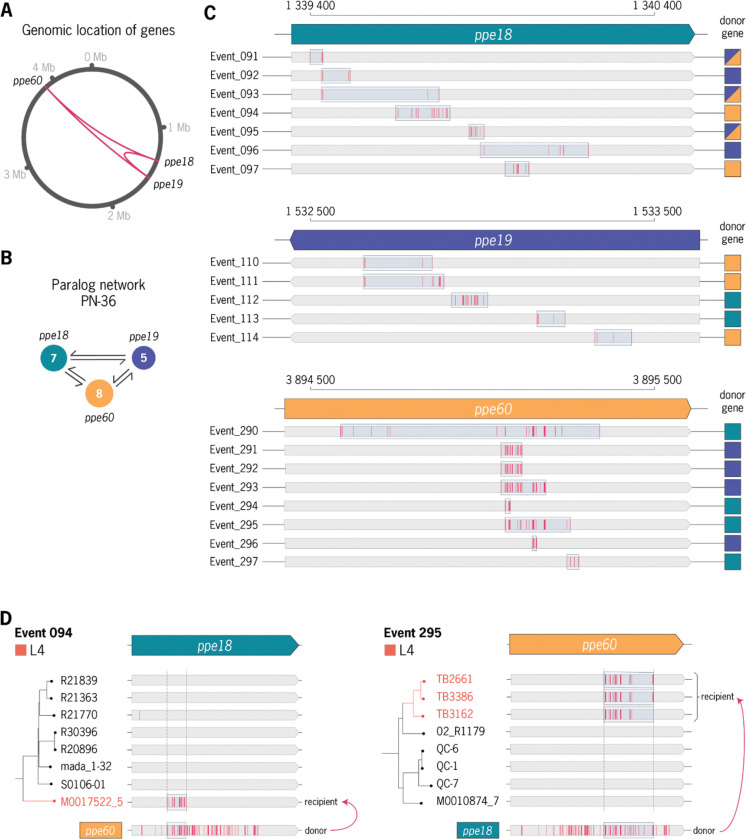
Detailed analysis of gene conversion signatures detected between ppe18, ppe19, ppe60 paralogs. **a,** Relative genomic location of PN-36 paralogs **b,** Paralog network graph labeled by number of detected GCEs per region. **c,** Overview of all gene conversion events detected per gene. Each event is labeled by the closest matching paralog(s) to the associated recombination tract. **d,** Phylogenetic context of mapped gene conversion events (Event-094 and Event-295) between *ppe18* and *ppe60*.

The five high-burden paralog networks, designated PN-3, PN-10-A, PN-23, PN-28, and PN-36, collectively accounted for 55% of all detected GCEs (Table 2, Methods). Collectively, these networks exemplified distinct gene categories that are strongly enriched for gene conversion. PN-3 involved multiple REP13E12 repeat loci, while PN-10-A and PN-23 each comprised networks of PE-PGRS paralogs. PN-28 contained five distinct pairs of *esx* paralogs. Finally, PN-36 involved three PPE paralogs, *ppe18*, *ppe19*, and *ppe60* (Figure 4). Beyond these high-burden networks, gene conversion was also detected in genes implicated in additional pathways relevant to virulence and immunogenicity. These notably include toxin-antitoxin modules (*vapB25-vapC25* and *vapB31-vapC31*) and lipid metabolism genes (*ppsA*, *ppsB*, and *pks12*) (42–44) (Figure S7).

### General sequence characteristics do not explain detected gene conversion frequency

To determine what proportion of variance in gene conversion frequency between PRs can be explained by broad sequence features, we examined whether sequence divergence, genomic distance, paralog copy number, or GC content were predictive of the number of mapped gene conversion events (mGCEs) detected between paralog pairs. We modeled mGCE counts using a generalized linear model with a negative binomial distribution to account for overdispersion (Methods). We found no statistical association between genomic distance, paralog copy number, and GC content with gene conversion frequency (p > 0.25; Figure S8, Table S5). However, higher sequence divergence between paralogs was associated with a reduction in the expected number of GCEs (incidence rate ratio = 0.983 per additional SNP per kb; p < 1 × 10^−4^). This observation is consistent with the homology dependence of gene conversion, whereby increasing sequence divergence between paralogs reduces their ability to pair during homologous recombination (45, 46). Overall, gene conversion frequency within a region was not strongly predicted by the general sequence features evaluated (McFadden’s pseudo-R^2^ = 0.14), suggesting that instead gene conversion activity varies in a locus-specific manner.

### Gene conversion is widespread throughout both recent and ancestral timescales of *Mtb* evolution

**Figure 5.**
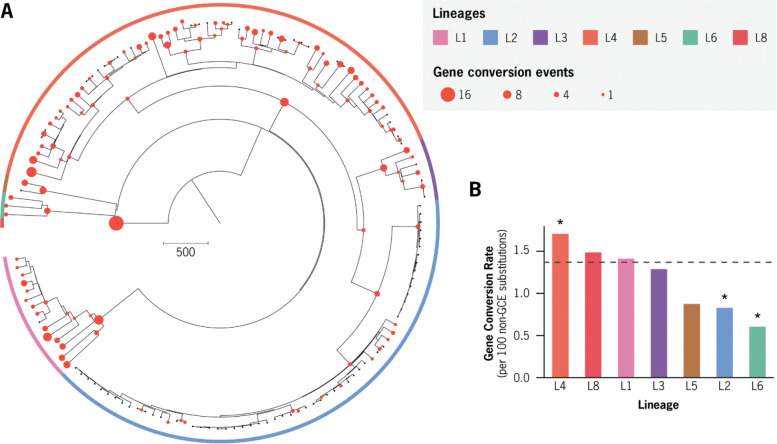
Signatures of gene conversion are widespread across the *Mtb* phylogeny. **a,** Maximum likelihood phylogeny of 151 complete *Mycobacterium tuberculosis* (Mtb) genomes, with branches colored by major phylogenetic lineage. Red circles indicate branches where mapped gene conversion (GC) events were confidently inferred. Circle size is proportional to the number of GC events detected per branch.**b,** Comparison of average GCE rates across all branches belonging to major Mtb lineages. The dashed horizontal line marks the phylogeny-wide average GCE rate.

Of the 324 detected GCEs across the *Mtb* phylogeny, 185 (57%) were inferred on terminal branches, consistent with more recent evolution, while 139 events (43%) occurred on internal branches, indicating that the associated mutations are shared across multiple descendant genomes (Figure 5A). In total, 32 GCEs affecting 20 unique genes were detected on the ancestral branches of lineages represented by two or more isolates (L1–L6). Several loci (i.e. *pe-pgrs27*, *ppe54*, and *ppe55*) underwent gene conversion convergently on the ancestral branches of multiple lineages (Table 4).

To contextualize gene conversion within the broader evolutionary dynamics of *Mtb*, we quantified GCE frequency relative to recombination-independent (non-GCE) SNPs widely used for molecular clock estimates of *Mtb* evolutionary history(47, 48). Phylogeny-wide, GCEs occurred at an average rate of 1.37 events per 100 non-GCE SNPs. At the substitution level, this corresponds to 0.124 GCE-associated SNPs per non-GCE fixed SNP.

We next evaluated lineage-specific variation in gene conversion rates by modeling expected GCE frequencies using a Poisson distribution parameterized by the phylogeny-wide average rate (Methods). Lineage 4 exhibited a significantly elevated rate of gene conversion, whereas Lineages 2 and 6 were characterized by significantly reduced rates (adjusted p < 0.05; Figure 5B, Table 3). Overall, these results indicate that gene conversion has acted as a persistent source of genetic variation throughout the evolutionary history of M. tuberculosis, while also exhibiting significant rate variation between lineages.

### Validation of individual gene conversion events in an independent dataset

To confirm that the detected GCEs were not driven by sequencing artifacts or dataset-specific biases, we first assessed whether gene conversion signatures could be detected in an independent short-read WGS dataset of *M. tuberculosis* isolates and then validated candidate events by long-read resequencing. We analyzed a dataset of 937 clinical *Mtb* isolates (Figure S9), previously sequenced with short-read WGS and for which genomic DNA was archived, for putative gene conversion signatures. Recognizing the limitations of short-read variant calling in paralogous regions, we used a conservative alignment-based variant detection strategy to focus on gene conversion signatures confidently resolved with short-read WGS data (Methods).

Across these 937 clinical isolates, we identified 27 putative gene conversion events using available short-read WGS (Table S6, Figures S10–11). Notably, 24 (89%) of these events occurred in genes that also harbored gene conversion events in our primary analysis . Of the 27 putative events, 12 occurred in isolates with sufficient DNA quality to enable PacBio HiFi long-read resequencing. For each putative event, we resequenced two clinical isolates: one predicted to harbor the recombinant sequence and a phylogenetically matched control lacking the predicted variants. In total, 22 *Mtb* isolates were resequenced using PacBio HiFi WGS to evaluate the accuracy of these 12 putative events. In all 12 cases, the mutational patterns initially detected using short-read data were validated by the long-read resequencing (100% precision; Table S7). However, among the resequenced genomes, substantially more gene conversion events could be resolved using long-read than short-read WGS. We identified an additional 72 gene conversion events using long-read data that were not detected in the short-read data, confirming the low recall (~14.2%) of short-read sequencing for gene conversion detection.

### Gene conversion drives elevated diversity in a select group of *Mtb* antigens

The prevailing view is that *M.tuberculosis* antigens contain little to no sequence variation (10, 49), unlike many other viral and bacterial antigens that frequently exhibit diversifying selection (50). Because many known M. tuberculosis antigens are encoded within paralogous regions (PRs), they have historically been excluded or poorly resolved in short-read-based analyses. We therefore used our dataset of complete genomes to examine whether antigens encoded in paralogous regions (PRs) exhibit distinct patterns of sequence diversity and mutational burden relative to antigens located in unique genomic regions.

To evaluate this comparison, we processed *M. tuberculosis* CD4+ T cell epitope-mapping data from two large-scale peptide screening studies (51, 52); 18,371 unique peptides were assayed across 3,840 annotated *Mtb* proteins. From these, we classified 53 proteins as high confidence T cell antigens containing a total of 226 experimentally validated epitope sequences (Figure 5, Figure S11, Methods). This list contains several well-characterized *Mtb* T cell antigens, including the IGRA diagnostic antigens (EsxA, EsxB) and 9 additional antigens targeted by various vaccine candidates (Table S8). Of these 53 validated T cell antigens, 20 (38%) are encoded in PRs (Figure S11).

We next quantified length-normalized mutational burden (missense mutation events per 100 amino acids) across all annotated coding sequences in the *Mtb* genome (Figure 5B, Figure S12), comparing four gene classes: PR-associated antigens, unique antigens, PR-associated non-antigens, and unique non-antigens (Table 5). We found that PR-antigens had the highest mutational burden (median = 4.1 missense/100AA) and were significantly more diverse than unique-antigens (median = 1.0 missense/100AA, Wilcoxon rank sum, p = 1e-05). In contrast, unique-antigens did not differ in mutational burden compared to unique non-antigens in diversity. Restricting the analysis to mutations within validated epitope sequences, PR-associated antigens showing higher mutational burden in epitopes (median = 1.5 missense/100AA) than those in unique antigens (median = 0.8 missense/100AA).(Wilcoxon Rank sum test, p = 1.e-05. Table 6).

Of the top ten antigens ranked by mutational burden, nine are encoded by paralogous genes belonging exclusively to two paralog networks (PN-28, PN-36) associated with frequent gene conversion (Figure 5, Table 5). Given that the most frequently mutated antigens are also those undergoing frequent gene conversion, we next examined whether GCEs preferentially affected epitope sequences encoded within PRs. Gene conversion events were significantly enriched within validated epitopes encoded in PRs: 25 (7%) GCEs directly mutated known epitope residues, despite epitopes accounting for only 3% of PR sequence by total length (OR = 5.0; Fisher’s exact test, p = 1 × 10^−5^).

Among the antigens characterized by both high mutational burden and frequent gene conversion, PPE18 stood out due to its clinical relevance as potent antigen and an important vaccine target. Within PPE18, 44 of 61 missense mutations occurred within experimentally validated T cell epitope sequences. Modeling mutation rates across PPE18, epitope regions exhibited a 1.73-fold higher nonsynonymous mutation rate than non-epitope regions (95% CI: 0.99–3.03; p = 0.055, Poisson regression), indicating a trend toward elevated epitope diversity that did not reach statistical significance. Using T cell response frequency data available for a subset of PPE18 peptides (n = 50), we evaluated whether mutational burden was associated with the proportion of responding donors (n= 63 individuals with latent TB). Overall, T cell responsiveness within the evaluated cohort was not associated with increased missense mutation burden (rate ratio = 1.14, 95% CI: 0.92–1.42; p = 0.223, negative binomial regression).

To further explore the impact of gene conversion mutations on PPE18 antigenicity, we performed *in silico* predictions of HLA-II binding using netMHCpanII (53). Binding predictions were generated for all possible 15-mer peptides within PPE18, both before and after mutation by each detected GCE, across a panel of 27 common HLA-II alleles (Figure S14, Methods). Of the seven GCEs detected in *ppe18*, five are predicted to change peptide binding for at least one HLA-II allele (Table S9). Across evaluated GCEs, both gains and losses in HLA-II binding were predicted, with gains occurring more often than losses overall (binomial test, p < 0.01). Notable among these, Event-094 introduced 17 missense mutations in PPE18 and yielded 41 predicted peptide-HLA binding changes (26 gains and 15 losses). To assess population-specific patterns, we re-evaluated Lineage 4 GCEs against the seven most frequent HLA-II alleles in European populations, yielding results consistent with predictions based on the 27 global alleles (Methods, Supplementary Results).

## Discussion

In this work, we analyzed genome-wide sequence diversity across a diverse collection of complete M. tuberculosis genomes and found gene conversion to be a pervasive and active mutational process shaping species evolution. Long-read genome sequencing enabled resolution of variation within repetitive and paralogous regions that were largely inaccessible to short-read–based analyses. Across the evolutionary history of our analyzed isolates, we identified hundreds of gene conversion events within paralogous loci throughout the M. tuberculosis genome. Our analysis revealed that recurrent gene conversion at a subset of paralogous loci drives the formation of pronounced diversity hotspots throughout the *Mtb* genome. These findings demonstrate that paralogous regions of the *Mtb* genome follow evolutionary dynamics distinct from the rest of the chromosome, with important implications for studies of selection, adaptation, host–pathogen interactions, and vaccine design.

Closer examination of the genomic and phylogenetic distribution of gene conversion revealed marked concentration within a limited subset of paralog networks (Figure 3). Gene conversion events were significantly enriched within the PE, PPE, and ESX gene families, with notable events also detected in other virulence-associated systems, including toxin–antitoxin modules and PDIM-associated lipid biosynthesis genes. The most prominent hotspots involved PE-PGRS and PPE paralog sets (*pe-pgrs17*, *pe-pgrs18*, *ppe18*, *ppe19*, and *ppe60*), as well as ESX paralog pairs (*esxK-L*, *esxO-P*, *esxM-N*). REP13E12 repeat regions, which contain a putative prophage attachment site but lack defined biological function, also ranked among the most frequently recombined loci. Together, these findings indicate that recurrent gene conversion promotes diversification within specific paralogous loci, with many of the most affected genes linked to host–pathogen interaction and virulence.

These findings also have important implications for the interpretation of selection in loci affected by gene conversion. Gene conversion can generate clustered tracts of substitutions that mimic convergent evolution, particularly when the same donor paralog serves as a template in independent recombination events. Although such mutation patterns are technically homoplastic, they arise through biased templated copying from donor paralogs rather than de novo point mutation, complicating their interpretation. More broadly, any form of recombination violates assumptions underlying selection metrics such as dN/dS because linked synonymous and nonsynonymous substitutions can be introduced simultaneously within conversion tracts, breaking the assumption that mutations accumulate independently(54). Consequently, dN/dS values in paralogous regions may not reliably reflect the strength or direction of selection. Thus, strong conclusions about adaptive evolution in paralogous loci should not be drawn without explicitly accounting for gene conversion.

Previous population genomic analyses have suggested that M. tuberculosis antigens are generally conserved and under purifying selection (10, 49). However, these conclusions were largely drawn from short-read–based analyses that excluded antigens encoded within paralogous regions. Consistent with prior work, our analysis found that antigens located in unique genomic regions exhibit low mutational burden. In contrast, our analysis identified several PR-encoded antigens with among the highest mutational burdens genome-wide that undergo recurrent gene conversion. Because gene conversion introduces clustered, linked substitutions, we did not apply conventional metrics such as dN/dS to these loci, as their underlying assumptions are violated. Although mutational burden does not directly measure selection, our analysis identifies specific PR-encoded antigens that harbor substantially more nonsynonymous substitutions than other antigens, in some cases differing by an order of magnitude.Together, these findings support a role for gene conversion in localized antigenic diversification within an otherwise low-diversity pathogen genome.

Among PR-encoded antigens characterized by a high mutational burden, *ppe18* and its paralogs *ppe19* and *ppe60* stood out as clinically relevant examples. The PPE18 protein has long been recognized as a potent T cell antigen and is a component of multiple vaccine candidates currently in clinical development (M72/AS01E and BNT164)(51, 52, 55–57). In our analysis, we detected multiple gene conversion events within PPE18 that directly altered experimentally validated T cell epitope sequences. Although enrichment of nonsynonymous substitutions within epitope regions did not reach conventional statistical significance (p = 0.055), the data was consistent with a trend for more diversification of epitope-containing regions. In addition, *in silico* modeling predicted that several gene conversion events may alter HLA-II binding across common human alleles. Together, these findings demonstrate that gene conversion can reshape epitope sequences within a vaccine-targeted antigen. However, sequence-based analyses alone cannot establish the immunological consequences of these variants. Determining how gene conversion–mediated diversity influences T cell recognition and vaccine efficacy will require integrated genomic, immunological, and clinical studies that account for both pathogen and host diversity.

Our identification of gene conversion as an active mutational process is further supported by mutational characteristics that align closely with established mechanisms of homologous recombination. For example, the tract lengths of detected events (median 101 bp) are consistent with RecA-mediated strand invasion and synthesis-dependent strand annealing, the homologous recombination pathway described in mycobacteria(36, 58). In addition, substitution patterns within paralogous regions reveal a distinct enrichment of T>C transitions relative to the genomic background. This bias is consistent with the observation in many species that mismatch repair dynamics during recombination-associated DNA synthesis differ from those operating during DNA replication(35, 59). Notably, the enrichment of T>C substitutions parallels mutation spectra observed in nucS-deficient mycobacterial strains, further supporting the possibility that NucS-mediated mismatch repair is reduced during homologous recombination in mycobacteria(60–62). Over evolutionary time, such recombination-associated repair asymmetries could contribute to reshaping nucleotide composition within paralogous genomic regions.

This study has limitations. Our detection framework identifies gene conversion events based on clustered substitutions and signatures of rapid sequence change; consequently, events occurring between nearly identical paralogs are likely underdetected. In addition, older gene conversion events may be progressively eroded by subsequent mutation, reducing the clustering signal required for detection and complicating inference of the original donor paralog. Reliance on a single reference genome may also introduce subtle biases in event localization and paralog assignment. Although the dataset spans major Mtb lineages, certain geographic regions and sub-lineages remain underrepresented. Larger collections of complete genomes and reference-independent approaches will be needed to refine estimates of gene conversion prevalence and evolutionary timing.

In summary, we identify gene conversion as a central endogenous mechanism shaping genetic diversity in M. tuberculosis. This work reframes assumptions about *Mtb* genomic stability and highlights the need for recombination-aware evolutionary analyses and lineage-informed vaccine design. Future studies integrating broader sampling, longitudinal data, and functional immunological assays will further clarify the role of gene conversion in pathogen evolution and host–pathogen interactions.

## Methods

### Dataset of clinical *Mtb* isolates with long- and short-read WGS

Our primary dataset comprised 151 complete *Mycobacterium tuberculosis* genomes previously generated using a hybrid assembly approach that integrated matched long-read (Oxford Nanopore, PacBio) and short-read (Illumina) whole-genome sequencing data. Long-read and short-read data had median genome-wide sequencing depth of 149x (IQR: 100–230x) and 56x (IQR: 47–100x) respectively. **File S2** details all relevant ENA/SRA run accessions, assembly accessions, and isolate metadata.

### Hybrid genome assembly using long and short-read whole genome sequencing data

The *de novo* genome assembly and polishing process was tailored to the specific requirements of various long-read platforms and chemistry versions used for analysis (PacBio subreads [RSII & Sequel II], ONT v9.4.1, PacBio CCS/HiFi [Sequel II] reads), as well as to the software versions available at the time of processing. In general, long-read data were first assembled and polished using the Flye assembler (v2.6 or v2.9) and then polished using matched short-read data with Pilon (v1.23). For Oxford Nanopore assemblies, additional long-read polishing was performed using Medaka, and PolyPolish was applied as an additional short-read polishing step. Refer to the supplemental methods for the exact combination of softwares used for each Assembly pipeline. All genome assemblies were annotated with the Bakta (v4.8) gene annotation pipeline.

### H37Rv reference genome and annotations

The H37Rv (NCBI Accession: NC_000962.3) genome sequence and annotations was used as the reference genome for all analyses. Functional category annotations for all genes of H37Rv were downloaded from Release 3 (2018–06-05) of MycoBrowser (https://mycobrowser.epfl.ch/releases).

### Variant detection and CDS effect annotation

Genetic variants relative to the H37Rv reference genome were inferred for each complete genome assembly using minimap2 & paftools.js (63). Variants were called from the resulting paf file using the paftool.js call command. Variant calls from all assemblies were processed uniformly and annotated using BioPython to infer coding sequence consequences relative to standard H37Rv gene annotations.

### Nucleotide diversity analysis

Variant calls from each sample were merged using bcftools to produce a single VCF file representing all observed small variants across the population. VCFtools was then used to calculate the nucleotide diversity across all 1-kb non-overlapping windows of the H37Rv genome.The nucleotide diversity (π) metric was calculated as the average number of substitutions that exist between all pairwise comparisons of genomes within the specific window of the genome being evaluated (64).

To identify outlier regions of elevated diversity, we used a robust statistical method based on the Modified Z Score(65). For each 1-kb window, the Modified Z Score $\left(Z_{i}\right)$ was calculated as:

$$Z_{i}=\left(x_{i}-\tilde{x}\right)/k*MAD$$

Where ${\text{x}}_{i}$ is the nucleotide diversity of the window, $\tilde{x}$ is the median nucleotide diversity across all windows, *MAD* is the median absolute deviation of the distribution, k=1.4826 is a scaling constant that makes the statistic comparable to a standard z-score under normality assumptions (65). This approach is robust to outliers and does not assume a normal distribution of values, making it well-suited for the highly skewed distribution of nucleotide diversity scores.

### Manual inspection of genome assemblies, variant calls, and read alignments in nucleotide diversity hotspots

To assess the accuracy of variant calls within regions of elevated nucleotide diversity, we performed manual inspection of all 37 identified diversity hotspots using the Integrative Genomics Viewer (IGV). For each hotspot, the consensus variant calls across all 151 genome assemblies were examined. In cases where multiple closely spaced variants were observed within a single isolate, we further inspected the whole-genome assembly alignment (produced by Minimap2) to assess the local structure and continuity of the assembled sequence. To verify that the identified variants were supported by the underlying sequencing data, we also examined long-read alignment pileups relative to each genome’s assembly as well as the reference genome.

### Phylogeny inference

Genetic variants relative to the H37Rv reference genome were inferred for each complete genome assembly using minimap2 & paftools.js (63). A concatenated SNP pseudoalignment was then generated by identifying and extracting single nucleotide polymorphisms (SNPs) from each genome assembly using bcftools(66). From the SNP alignment a maximum likelihood phylogeny was inferred using IQ-Tree with the general time reversible model and a SNP ascertainment bias correction(67).

### Defining all non-unique (repeats & paralogs) sequence content of the H37Rv genome

We sought to comprehensively identify all forms of repetitive and homologous sequence content within the *Mycobacterium tuberculosis* H37Rv reference genome (NC_000962.3). For this purpose, we defined four categories of sequence features: (i) paralogous regions (PRs), (ii) local repeat regions (LRRs), (iii) low-complexity regions (LCRs), and (iv) low-mappability regions (LowMapRegions). PRs were defined as non-overlapping homologous segments within the genome. Local repeat regions were defined as overlapping self-alignments, reflecting tandem or proximal repeats in which consecutive overlapping elements share homology. Low-complexity regions were defined as sequences enriched for short tandem motifs, including imperfect or approximate repeats. Low-mappability regions were defined as intervals with non-unique k-mer content, such that short reads cannot be aligned unambiguously.

### Detection of paralogous and local repeat regions via intra-genomic homology map

To generate a genome-wide homology map, we performed a self–self alignment of the H37Rv genome using minimap2 v2.26 with the parameters --MD -DP -k19 -w19 --cs, outputting results in both PAF and SAM formats. Alignments between non-overlapping regions of the genome were classified as paralogous regions (PRs), while alignments in which the query and target intervals overlapped were classified as local repeat regions (LRRs). Variants within each alignment were inferred using paftools.js with the command call -q 0 -l 100 -L 100. In total, 200 distinct PRs and 78 distinct LRRs were identified across the H37Rv reference genome.

### Detection of low-complexity regions

Low-complexity regions (LCRs) within H37Rv were identified using Longdust v1.2, run with the parameters -k6 -w1000 -t .5. Longdust scans genomic nucleotide sequences for intervals enriched in short tandem repeat motifs, and all reported intervals were reported as low-complexity regions(68).

### Detection of low-mappability regions

Low-mappability regions within H37Rv were detected using Pupmapper (https://github.com/maxgmarin/pupmapper), a pipeline that integrates Genmap-based k-mer uniqueness profiles with per-base pileup statistics. Genmap was run with a k-mer size of 50 bp, allowing up to 4 mismatches. Genomic positions with a non-perfect pileup mappability score (< 1) were designated as low k-mer mappability regions.

### Detection of gene conversion event signatures and donor-paralog mapping

Putative gene conversion events (GCEs) were identified using Gubbins (v3.3). For each inferred recombination tract, we sought to identify candidate donor paralog sequences within the *M. tuberculosis* genome using a k-mer–based similarity approach. We extracted all 31-mer k-mers overlapping each recombination tract and compared them to k-mer profiles of all reference paralogous sequences of the affected locus. Similarity between a recombination tract and each candidate paralog was quantified using the jaccard containment metric, defined as the proportion of recombination tract k-mers found in the target paralogous sequence. Containment scores range from 0 to 1, with higher values indicating greater sequence similarity. GCEs were classified as mapped to a paralog sequence if the k-mer jaccard containment was >= 0.5. For each mapped GCE, the paralogous region(s) with the highest k-mer containment score was assigned potential donor sequences.

### Evaluation of gene conversion frequency and lineage-specific rate variation across phylogeny

All gene conversion events (GCEs), GCE-associated SNPs, and recombination-independent (non-GCE) SNPs were inferred for every branch of the dataset phylogeny as part of the standard output of the Gubbins pipeline. Phylogeny-wide GCE frequency was calculated as the number of inferred GCEs per 100 non-GCE SNPs across all branches. To assess the relative contribution of gene conversion at the level of substitutions, we additionally quantified the ratio of GCE-associated SNPs to non-GCE SNPs. To evaluate lineage-specific variation in gene conversion rates, GCE counts and non-GCE SNP counts were summed across all phylogenetic branches assigned to each lineage Expected GCE counts were modeled using a Poisson distribution parameterized by the phylogeny-wide average GCE rate and scaled by the total number of estimated non-GCE SNPs per lineage to account for differences in evolutionary depth. Observed lineage-specific GCE counts were compared to expectations using Poisson tests with correction for multiple comparisons.

### Curation of validated T cell (CD4+/MHC-II) epitopes and antigens

We compiled CD4^+^ T cell epitope-mapping data from two large-scale peptide screening studies, each of which deposited their complete assay datasets in the Immune Epitope Database (IEDB). The first dataset, from Lindestam et al. 2016 (IEDB accession 1031648), assayed 693 unique 15-mer peptides derived from 88 *Mtb* proteins, identifying 207 unique positive epitope sequences. This study screened peptides with IFN-γ ELISPOT assays using PBMCs from 63 latently infected individuals in South Africa, targeting known immunogenic proteins and their homologs. The second dataset, from Panda et al. 2024 (IEDB accession 1000914), assayed 18,325 unique 15-mer peptides spanning the entire H37Rv proteome (with bias toward known antigens). This study screened peptides with IFN-γ ELISPOT assays of PBMCs from 21 patients with active tuberculosis in Peru, identifying 153 unique positive epitope sequences.

We merged the results of these two studies into a combined dataset (referred to as LPM), representing the union of all assayed peptides and their reactivity results (Positive epitope or non-reactive peptide). LPM contains 18,371 unique assayed peptides spanning 3,840 annotated protein-coding genes of H37Rv genome, with a total of 307 unique positive CD4+ epitope sequences (Table S1). All assayed peptide sequences were harmonized to H37Rv genomic coordinates using reference protein sequences, excluding any without a match to the reference proteome. Epitopes were considered experimentally validated if they were positive in at least one human CD4^+^ T cell assay (IFN-γ ELISPOT) and matched a reference protein sequence. Proteins were classified as high-confidence antigens if they contained ≥2 experimentally validated epitopes in the LPM dataset. This resulted in 53 high-confidence antigens containing a total of 226 validated epitope sequences. Antigens were also classified based on their overlap with annotated paralogous regions (PR).

### Regression analysis of paralogous region sequence features and GCE frequency

To evaluate whether general sequence features predict gene conversion frequency between PRs, we modeled the number of mapped gene conversion events (mGCEs) detected for each paralog pair using a generalized linear modeling framework. Analyses were restricted to paralog pairs that were not perfect repeats of each other (n = 284 pairs).

Because gene conversion counts are discrete and overdispersed, we fit a generalized linear model assuming a negative binomial distribution with a log link function. The response variable was the number of mapped gene conversion events detected between each paralog pair. Predictor variables included: 1) Sequence divergence, quantified as the number of SNPs per kilobase of aligned paralogous sequence. 2) Genomic distance between paralogs (kilobases). 3) Paralog copy number, measured as the number of overlapping paralogous alignments. 4) Local GC content

All predictors were included simultaneously in the model, and an intercept term was added. Robust (HC0) standard errors were used to account for potential heteroskedasticity. Model coefficients were exponentiated to obtain incidence rate ratios (IRRs), representing the multiplicative change in expected gene conversion counts per unit increase in each predictor. Model fitting and statistical inference were performed using the statsmodels Python package. Statistical significance was assessed using Wald tests, and results are reported in Table S5.

## Supplementary Material

Supplement 1

Supplement 2

Supplement 3

Supplement 4

Supplement 5

Supplement 6

Supplement 7

Supplement 8

Supplement 9

Supplement 10

Supplement 11

Supplement 12

Supplement 13

Supplement 14

Supplement 15

## Figures and Tables

**Figure 6.**
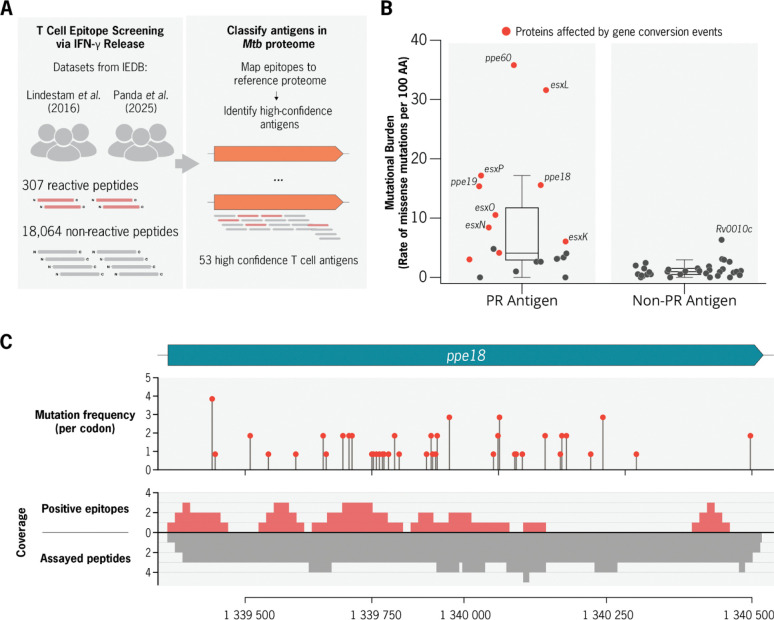
Evaluation of the mutational burden among validated T-cell antigens and epitopes. a, Overview of T-cell epitope data curation. Peptide reactivity data were obtained from two published CD4^+^ T-cell screening studies across TB patient cohorts, assaying 18,325 peptides spanning 3,840 Mtb proteins. **b,** Comparison of the mutational burden between antigens encoded within and outside paralogous regions. **c,** Visualization of missense mutation frequency and mapped epitope density within the PPE18 antigen. Top: frequency of missense mutations per codon across the PPE18 coding sequence. Bottom: mapped peptide screening data. The coverage of positive CD4^+^ T-cell epitopes (reactive in ≥1 patient) is shown above, while the overall coverage of assayed peptides per position is shown below in grey.

**Table 1.** Gene conversion event frequency across gene functional categories

Gene Category	# of detected GCEs	## unique genes wit detected GCE	Total annotated genes H37Rv genome
PE & PPE proteins	169	36	168
REP13E12 Repeat Regions	87	10	14
Esx protein family	36	7	23
conserved hypotheticals	19	7	1042
lipid metabolism	10	3	272
insertion sequences	7	5	133
cell wall and cell processes	6	3	749
virulence, detoxificatio adaptation	5	3	239
unknown	3	1	15
intermediary metabolism an respiration	1	1	936
information pathways	0	0	242
regulatory proteins	0	0	198
stable RNAs	0	0	48

**Table 2.** Gene conversion event frequency of among high-burden paralog networks

Paralog Network ID	Region ID	Annotated Gene(s)	Detected Gene Conversion Events		Gene Category
			# Total	# Mapped	
PN-3	PR-002	Rv0094c, Rv0095c	31	19	REP13E12 repeat
	PR-074	Rv1587c, Rv1588c	10	8	
	PR-180	Rv3466, Rv3467	15	8	
PN-10A	PR-183	*pe-pgrs54*	16	4	PE protein family
	PR-010	*pe-pgrs4*	10	6	
	PR-032	*pe-pgrs10*	5	3	
	PR-009	*pe-pgrs3*	4	4	
	PR-184	*pe-pgrs55, pe-pgrs56*	3	0	
	PR-185	*pe-pgrs57*	3	2	
	PR-037	*pe-pgrs12, pe-pgrs13*	1	0	
PN-23	PR-064	*pe-pgrs27*	19	14	PE protein family
	PR-065	*pe-pgrs28*	21	19	
PN-28	PR-054	*esxK, esxI*	11	9	Esx protein family
	PR-113	*esxO,esxP*	10	10	
	PR-087	*esxM,esxN*	4	4	
	PR-189	*esxv,esxW*	1	1	
	PR-042	*esxI, esxJ*	0	0	
PN-36	PR-182	*ppe60*	8	8	PPE protein family
	PR-053	*ppe18*	7	7	
	PR-060	*ppe19*	5	5	

**Table 3.** Comparison of detected gene conversion rates across phylogenetic branches of major *Mtb* lineages.

Lineage	Total Branches	# non-GC SNPs	# GC SNPs	Detected GCE	Detected GCE per 10 non-GCE SNPs	P-value	q-value (FDR Corrected)
1	29	4458	578	63	1.41	0.839	0.899
2	121	3018	221	25	0.828	**0.00864**	**0.0202**
3	13	1551	133	20	1.29	0.899	0.899
4	123	9775	1534	167	1.71	**0.00642**	**0.0202**
5	3	1257	93	11	0.875	0.154	0.267
6	5	1984	111	12	0.605	**0.00183**	**0.012794**
8	1	1076	172	16	1.49	0.811	0.899

**Table 4.** All gene conversion events detected on ancestral branches of each lineage within the phylogeny. For each event, the affected genes and the number of SNPs associated with the recombination tract are listed.

MTBC Lineage	# GC Events o ancestral branch	GC Event ID	Affected loci	# SNPs associated wit recombination tract
Lineage 1	9	Event-013	*Rv0094c*	*6*
		Event-076	*pe-pgrs18*	*7*
		Event-124	*pe-pgrs27*	*7*
		Event-229	*Rv2828c*	*14*
		Event-246	*PPE54*	*6*
		Event-254	*PPE55*	*11*
		Event-272	*PPE59*	*5*
		Event-297	*PPE60*	*5*
		Event-239	Intergenic	4
Lineage 2	4	Event-023	*Rv0095c*	*25*
		Event-132	*pe-pgrs27*	*5*
		Event-220	*lppA, lppB*	*10*
		Event-270	*ppe59*	*5*
Lineage 3	6	Event-105	*esxL*	*5*
		Event-152	*pe-pgrs28*	*5*
		Event-230	*Rv2828c*	*8*
		Event-236	*ppsA*	*5*
		Event-260	*PPE55*	*5*
		Event-225	Intergenic	4
Lineage 4	2	Event-125	*pe-pgrs27*	*4*
		Event-256	*ppe55*	*12*
Lineage 5	4	Event-010	*Rv0094c-Rv0095c*	*31*
		Event-053	*pe-pgrs6*	*5*
		Event-192	*Rv1945*	*14*
		Event-204	*Rv2082*	*6*
		Event-243	*ppe54*	*6*
		Event-266	*ppe56*	*4*
Lineage 6	5	Event-005	*Rv0094c*	*7*
		Event-050	*Rv0397*	*10*
		Event-057	*pe-pgrs10*	*7*
		Event-122	*pe-pgrs27*	*9*
		Event-252	*ppe54*	*8*

**Table 5.** Measured mutational burden compared across Antigens and non-antigens encoded in PR and non-PR regions

ID	Antigen Classification	Genomic context	# of CDSs	Normalized Overall Mutational Burden (Detected missense mutation events per 100 coding residues)	
				Median	Interquartile Range
Ag-PR	Antigen	PR	20	4.08	2.93 – 11.71
Ag-Unq	Antigen	Non-PR	33	0.96	0.49 – 1.51
NonAg-PR	Non-Antigen	PR	204	1.33	0.60 – 2.32
NonAg-Unq	Non-Antigen	Non-PR	3584	0.76	0.39 – 1.23

**Table 6.** Mutational burden within epitopes compared between antigens encoded in paralogous and non-paralogous regions of the genome

ID	Antigen Classification	Genomic context	# of CDSs	Epitope Mutational Burden (Detected missense mutation events in a validated T cell epitope per 100 coding residues)	
				Median	Interquartile Range
**Ag-PR**	**Antigen**	**PR Encoded**	20	**1.49**	**0.42 – 3.15**
Ag-Unq	Antigen	Non-PR Encoded	33	0.08	0.00 – 0.33

**Table 7.** Pairwise comparisons of normalized missense mutation rates across antigen and non-antigen groups stratified by genomic context.

Comparison	Test	p-value
NonPR-NonAg vs NonPR-Ag	Wilcoxon rank-sum	0.1922
NonPR-NonAg vs PR-NonAg	Wilcoxon rank-sum	**1.54E-15**
NonPR-NonAg vs PR-Ag	Wilcoxon rank-sum	**2.84E-09**
NonPR-Ag vs PR-NonAg	Wilcoxon rank-sum	0.0568
NonPR-Ag vs PR-Ag	Wilcoxon rank-sum	**1.26E-05**
PR-NonAg vs PR-Ag	Wilcoxon rank-sum	**3.67E-05**

## Data Availability

All SRA/ENA run accessions and associated metadata for all *M. tuberculosis* isolates used in this study can be found in Supplemental Data 1. Code for data processing and analysis is available from the following GitHub repository, https://github.com/maxgmarin/mtb-geneconv-manuscript/. The Snakemake workflow engine (v7.32.4) was used for sequencing data processing(69).
