# Supplementary material for: Gene conversion is a key driver of diversity hotspots in M. tuberculosis antigens and virulence-associated loci
Source: bioRxiv. 2026 Mar 11:2026.02.26.708061. Preprint. [Version 2] doi: 10.64898/2026.02.26.708061 (PMC13061034; doi:10.64898/2026.02.26.708061)
Supplement: Supplement 1 [file media-1.pdf]

Supporting Information for

Gene conversion is a key driver of diversity hotspots in *M. tuberculosis* antigens and virulence-associated loci

Maximillian G. Marin[1,2], Natalia Quinones-Olvera[1], Hu Jin[1], Michael A. Harris[3], Brendan M. Jeffrey[3], Alex Rosenthal[3], Kenan C. Murphy[4], Christopher Sassetti[4], Heng Li[1,2,5], Maha R. Farhat[1,6*]

1 Department of Biomedical Informatics, Harvard Medical School, Boston, USA
2 Department of Data Science, Dana-Farber Cancer Institute, Boston, USA
3 Office of Cyber Infrastructure and Computational Biology, National Institute of Allergy and Infectious Diseases, National Institutes of Health, Bethesda, USA
4 Department of Microbiology and Physiological Systems, University of Massachusetts Medical School, Worcester, USA
5 Broad Institute of MIT and Harvard, Cambridge, United States
6 Pulmonary and Critical Care Medicine, Massachusetts General Hospital, Boston, USA

*To whom correspondence may be addressed: Maha_Farhat@hms.harvard.edu

*To whom correspondence may be addressed.
Email: Maha_Farhat@hms.harvard.edu

This PDF file includes:

Overview of all supplementary materials
Supplemental Text
Figures S1 to S15
Tables S1 to S10

Supplementary Materials Overview

Supplementary Information: Supplemental Text, Figures (S1-15), and Tables (S1-10)

Supplementary Data 1: Isolate metadata, genome assemblies, and sequencing accessions for all data used.

Supplementary Data 2: Catalog of non-unique (high-homology or repetitive) sequence content annotated across the H37Rv reference genome

Supplementary Data 3: Genome-wide nucleotide diversity estimates and complete variant catalogs per genome assembly.

Supplementary Data 4: Mutation characteristics of paralogous regions compared to the rest of the genome

Supplementary Data 5: Catalog of detected gene conversion events (N=324) along with associated phylogenetic assignments, event-to-paralog mapping results, and quality-control metrics

Supplementary Data 6: Distribution of gene conversion events across paralogous regions and paralog networks in the H37Rv genome.

Supplementary Data 7: Supporting data for gene conversion analyses of TGEN-937-SR short-read WGS dataset and TBP-22 long-read (PacBio HiFi) resequencing dataset.

Supplementary Data 8: Curated T cell epitope mapping data from Lindestam et al. (2016) and Panda et al. (2024), and supporting analysis results.

Supplementary Data 9: *In silico* HLA class II binding predictions (netMHCpanII) and mutational effect analyses of PPE18 gene conversion events.

Supplementary Data 10: Phylogenies used in this study for the Mtb151CI, TBP-22CI, and TGEN-937CI datasets (Newick format).

Supplementary Data 11: Visualization of each detected nucleotide diversity hotspot with all detected variation shown in phylogenetic context.

Supplementary Data 12: Visualization of paralogous regions with all detected gene conversion events shown in both genomic and phylogenetic context.

Supplementary Data 13: Event-level visualizations of inferred gene conversion tracts, including variant patterns, phylogenetic context, and paralog sequence comparisons.

Supplementary Data 14: Visualization of per-codon mutation frequency and T cell epitope mapping coverage across selected antigens.

Supplemental Results

Systematic identification of high-homology, repetitive and low complexity sequences within the H37Rv Mtb genome

We systematically identified repetitive and homologous sequence features in the *Mycobacterium tuberculosis* H37Rv genome (NC_000962.3) based on four non-exclusive categories: **paralogous regions (PRs), local repeat regions (LRRs), low-complexity regions (LCRs), and low pileup-mappability regions (LowPmap)**. PRs were defined as distinct genomic segments with detectable homology elsewhere in the genome. Local repeat regions reflect cases where consecutive sequence elements share homology. Low-complexity regions are sequences enriched for short tandem motifs, including imperfect repeats. Low-mappability regions correspond to sequences with non-unique k-mer content (using a k-mer size of 50 bp and allowing up to 4 mismatches).

Across the 4.4 Mb H37Rv genome, PRs encompass 256.6 kb (~5%). Local repeat regions (LRRs) covered 149.8 kb (~3%). Low-complexity regions (LCRs) covered 127.2 kb (~2%). Regions of low mappability covered 189.1 kb (~4%). A summary of region categories and their definitions is provided in **Supplementary Table S3**, and their genomic distribution is shown in **Supplementary Figure S2**. The union of all four categories spanned a total of 447.1 kb (~10.1% of the genome) representing all regions associated with repetitive and/or high-homology sequence. Notably, these categories frequently overlapped with many PRs also containing local repeats, low-complexity motifs, or non-unique sequence content.

In silico analysis of effect of HLA binding of Gene Conversion events in PPE18 on HLA binding of european populations

As described in the main text (**Figure S10–11**, **Table S7–8**), we evaluated the predicted effects of gene conversion events in PPE18 on HLA-II binding using NetMHCpanII (50). We extended this analysis to specifically assess the impact of *PPE18* gene-conversion mutations on binding of the subset of HLA-II alleles most common in the context of European host populations (**Figure S15, Table S10**) Of the seven GCEs mutating PPE18, three (Events_091–093) introduced no amino acid changes in regions predicted to bind any of the tested HLA alleles. In contrast, the largest event (Event-94), which introduced 17 missense mutations, resulted in both predicted gains and losses of strong-binding interactions across multiple HLA alleles (10 gains, 4 losses). The remaining three events (Events 95–97) were each associated exclusively with gains in predicted HLA binding. These results indicate that some gene conversion events in PPE18 have the potential to alter the antigenic landscape by modifying HLA binding affinity.

Supplemental Figures & Tables

Figure S1

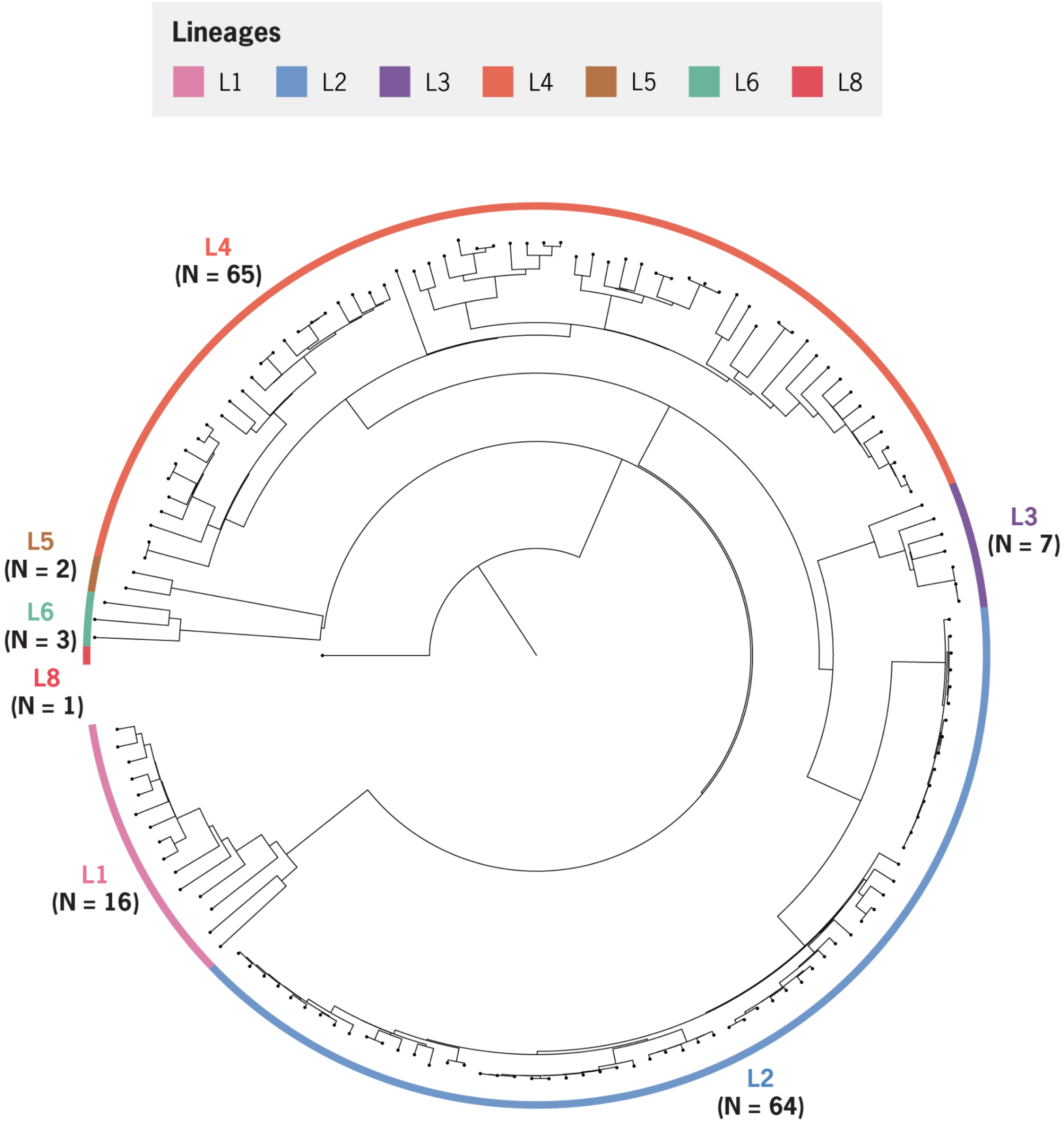

Figure S1. Maximum likelihood phylogeny of 151 *Mtb* isolates with complete genome assemblies. All assemblies were generated through *de novo* assembly and polishing with long-read whole genome sequencing data (Oxford Nanopore & PacBio), followed by polishing with short-read whole genome sequencing data.

Table S1

Stat	Median	Interquartile range	Min to Max range
BUSCO Completeness Score	99.4	99.3 - 99.5	98.6 - 99.6
Chromosome size	4,413 kb	4,407 - 4,421 kb	4,380 - 4,439
# of predicted CDSs<br>(*De novo* annotation via Bakta)	4074 CDSs	4065 - 4088	4020 - 4135
GC Content	65.6%	65.6 - 65.6%	65.6 - 65.6%

Table S1. Distribution of characteristics for dataset of 151 complete Mtb genome assemblies

Table S2

#	Coordinates (kb)	Affected gene(s)	π (SNPs/kb)	Gene Categories
1	103 - 104	Rv0094c, Rv0095c	4.05	REP13E12 repeat region
2	104 - 105		15.96	
3	105 -106		3.37	
4	338 - 339	PE_PGRS4	5.14	PE/PPE protein families
5	1,095 - 1,096	PE_PGRS18	3.10	PE/PPE protein families
6	1,096 - 1,097		4.88	
7	1,276 - 1,277	Rv1148c	13.22	REP13E12 repeat region
8	1,340 - 1,341	PPE18, esxK, esxL	2.84	PE/PPE protein families & esx
9	1,341 - 1,342		3.00	
10	1,533 - 1,534	PPE19	2.50	PE/PPE protein families
11	1,634 - 1,635	PE_PGRS27	9.04	PE/PPE protein families
12	1,637 - 1,638	PE_PGRS28	6.07	PE/PPE protein families
13	1,638 - 1,639		3.26	
14	1,788 - 1,789	Rv1587c,Rv1588c	2.28	REP13E12 repeat region
15	1,789 - 1,790		6.81	
16	2,196 - 2,197	Rv1945	3.59	REP13E12 repeat region
17	2,262 - 2,263	Rv2015c	2.30	conserved hypotheticals
18	2,338 - 2,339	Rv2082	2.56	conserved hypotheticals
19	2,339 - 2,340	Rv2082	2.41	
20	2,626 - 2,627	esxO,esxP	5.81	esx
21	2,867 - 2,868	lppA,lppB	7.70	cell wall and cell processes
22	2,944 - 2,945	PE_PGRS45	2.88	PE/PPE protein families
23	3,135 - 3,136	Rv2827c,Rv2828c	4.96	conserved hypotheticals
24	3,730 - 3,731	PPE54, PPE55	3.36	PE/PPE protein families
25	3,732 - 3,733		4.22	
26	3,735 - 3,736		2.56	
27	3,746 - 3,747		3.01	
28	3,750 - 3,751		6.34	
29	3,842 - 3,843	Rv3424c, PPE57	7.54	conserved hypotheticals & PE/PPE
30	3,847 - 3,848	PPE59	3.83	PE/PPE protein families
31	3,883 - 3,884	Rv3466,Rv3467	6.68	REP13E12 repeat region
32	3,895 - 3,896	PPE60	5.06	PE/PPE protein families
33	3,932 - 3,933	PE_PGRS54	3.94	PE/PPE protein families
34	3,934 - 3,935		4.93	
35	3,943 - 3,944	PE_PGRS56	3.10	PE/PPE protein families
36	3,947 - 3,948	PE_PGRS57	3.92	PE/ PPE protein families
37	4,254 - 4,255	Rv3798	2.84	Insertion sequences

Table S2. Summary of identified hotspots of nucleotide diversity (1-kb windows)

Table S3

**Category**	**Total size (bp)**	**% genome**	**Definition**
Paralogous Regions (PR)	256,568	5.8%	Non-overlapping self-alignments reflecting homologous segments between distinct genomic loci (paralogs)
Local Repeat Regions (LRR)	149,809	3.4%	Overlapping self-alignments reflecting tandem or proximal repeats
Low-Complexity Regions (LCR)	127,160	2.9%	Sequences enriched for short tandem motifs, including imperfect repeats
Low Mappability Regions (LowMap)	189,147	4.3%	Intervals overlapping with non-unique 50 bp k-mers (≤4 mismatches) within the genome
**Non-Unique (Union of all categories)**	447,067	10.1%	Union of all previous categories representing all genomic regions with any identified repetitive or high-homology sequence content

Table S3. Summary of types of repetitive and high-homology regions detected in the H37Rv genome

Figure S2

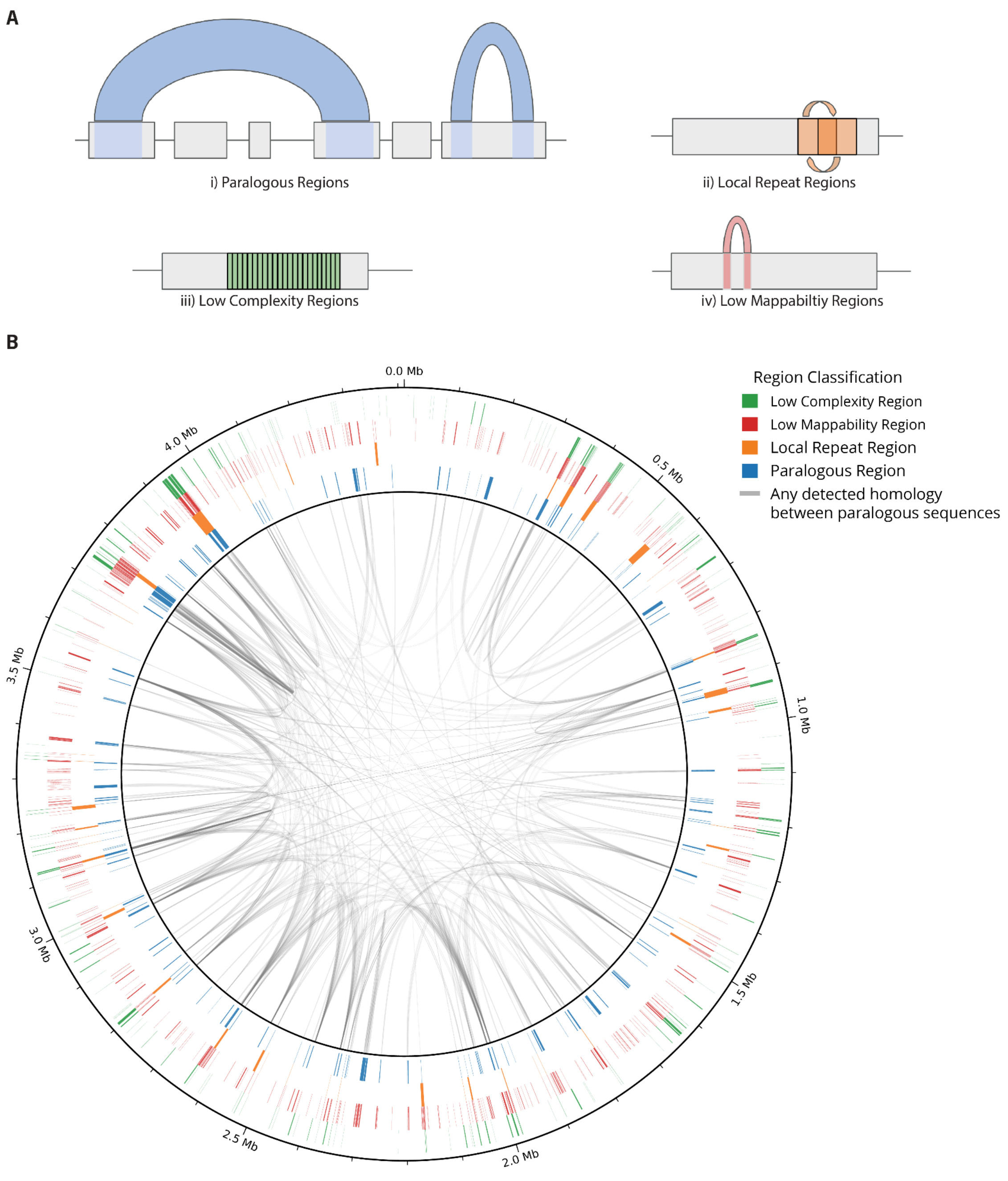

Figure S2. Distribution of repetitive and high-homology regions in the *M. tuberculosis* H37Rv genome. (**A)** Cartoon diagrams illustrate the four categories detected: paralogous regions (PRs), defined as distinct genomic segments with detectable homology; local repeat regions (LRRs), tandem overlapping repeats with detectable homology; low-complexity regions (LCRs), enriched for short tandem motifs; and low pileup-mappability regions (LowPmap), sequences with non-unique k-mer content. **(B)** The genomic distribution of these categories (PRs, blue; LRRs, orange; LCRs, green;

LowPmap, red), with inner links marking pairwise homology between paralogous loci. Many genomic regions fall into multiple categories, highlighting that non-unique sequence content in H37Rv is often simultaneously paralogous, repetitive, and low complexity.

Figure S3

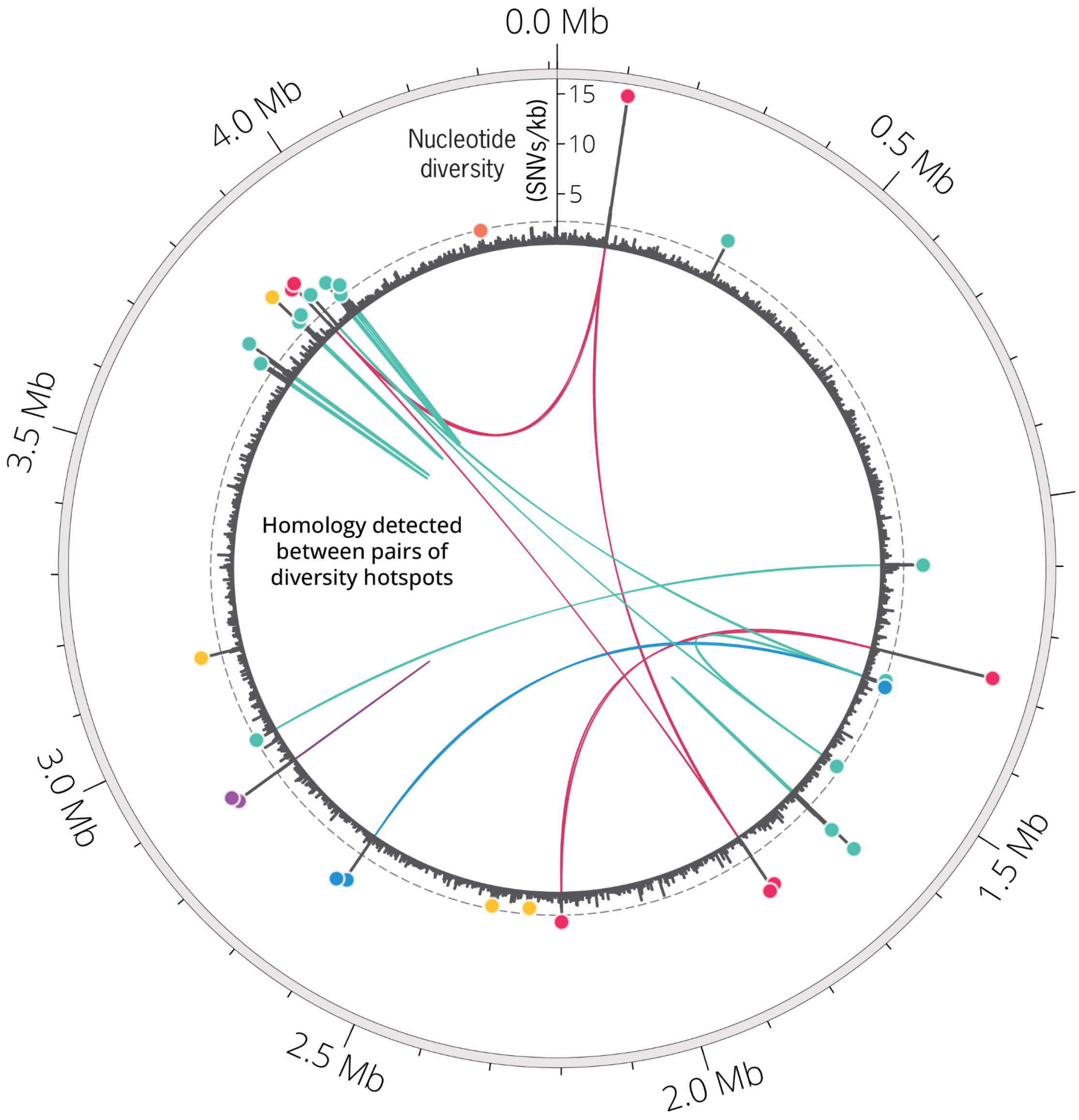

Figure S3. Highlighting pattern of homology between identified nucleotide diversity hotspots Visualization of genome-wide nucleotide diversity (π) across non-overlapping 1-kb windows in the H37Rv reference genome. Nucleotide diversity hotspots are indicated as colored points according to the functional category of the associated gene(s). Links within the center of the plot denote sequence homology between diversity hotspots. In total, 31 of the 37 identified hotspots are paralogous to at least one other hotspot, forming an interconnected network of homologous loci distributed across the genome.

Table S4

DNA Repair Pathway	# Genes	Genes (RvID; protein)	Loss-of-function variant summary
Homologous recombination: end resection and RecA loading	5	Rv3202c (AdnA); Rv3201c (AdnB); Rv0003 (RecF); Rv2362c (RecO); Rv3715c (RecR)	None detected across all 151 genomes
Homologous recombination: resolution	5	Rv2593c (RuvA); Rv2592c (RuvB); Rv2973c (RecG); Rv2594c (RuvC); Rv2554c (RuvX)	None detected across all 151 genomes
Homologous recombination: strand exchange	3	Rv2737c (RecA); Rv0054 (SSBa); Rv2478c (SSBb)	None detected across all 151 genomes
AP endonucleases	2	Rv0670 (End); Rv0427c (XthA)	None detected across all 151 genomes
Base excision repair – DNA glycosylases	10	Rv2924c (Fpg); Rv0944 (Fpg2); Rv3589 (MutY); Rv2976c (Ung); Rv1259 (UdgB); Rv1210 (TagA); Rv1317c (AlkA); Rv2464c (Nei1); Rv3297 (Nei2); Rv3674c (Nth)	None detected across all 151 genomes
DNA ligases	3	Rv3104c (LigA); Rv3062 (LigB); Rv3731 (LigC)	A single isolate (N1202, Lineage 6) was found to have a nonsense mutation (p.Trp435*) in ligB (Rv3062).
DNA polymerases	7	Rv1629 (PolA); Rv1547 (DnaE1); Rv3370c (DnaE2); Rv1537 (DinB1); Rv3056 (DinB2); Rv3730c (PolD1); Rv0269c (PolD2)	None detected across all 151 genomes
Mismatch repair	1	Rv1321 (NucS)	None detected across all 151 genomes
Non-homologous end joining	2	Rv0937c (Ku); Rv0938 (LigD)	None detected across all 151 genomes
Nucleotide excision repair	7	Rv1638 (UvrA); Rv1633 (UvrB); Rv1420 (UvrC); Rv1020 (Mfd); Rv2191 (Cho); Rv0949 (UvrD1); Rv3198c (UvrD2)	None detected across all 151 genomes
Nucleotide pool sanitization enzymes	6	Rv2985 (MutT1); Rv1160 (MutT2); Rv0413 (MutT3); Rv3908 (MutT4); Rv2697c (Dut); Rv1021 (MazG)	None detected across all 151 genomes
Other proteins	4	Rv1696 (RecN); Rv2736c (RecX); Rv3585 (RadA); Rv2694c (RecG)	A single isolate (R21770, Lineage 4.1.1.3) was found to have a 128 bp deletion (4,026,829–4,026,956) causing a frameshift (p.Gln129fs) in *radA* (Rv3585)
Ribonucleotide excision	2	Rv2228c (RNaseH1); Rv2902 (RNaseH2)	None detected across all 151 genomes
Single-strand annealing pathway	3	Rv0630c (RecB); Rv0631c (RecC); Rv0629c (RecD)	None detected across all 151 genomes

Table S4. Predicted loss-of-function variants across M. tuberculosis DNA repair genes. Genes are grouped by repair pathway (14 categories). Across all 60 genes, all 151 genomes were screened for predicted loss-of-function (nonsense/frameshift) variants. Only two LoF events were found: ligB (Rv3062; p.Trp435*) in one isolate (N1202, Lineage 6) and radA (Rv3585) with a 128 bp deletion (4,026,829–4,026,956) causing p.Gln129fs in one isolate (R21770, Lineage 4.1.1.3). Protein changes follow HGVS notation.

Figure S4

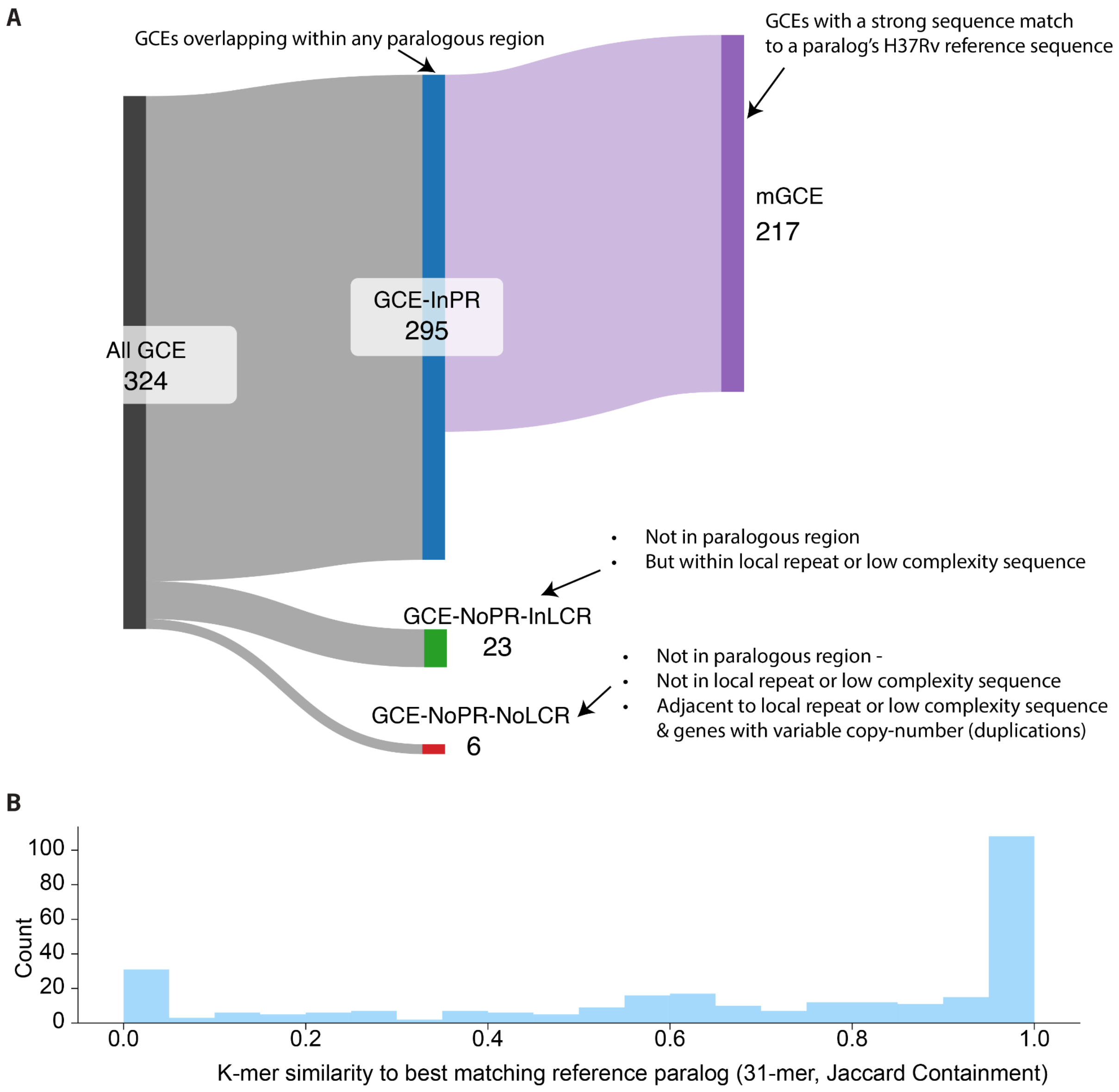

Figure S4. Breakdown of detected GCEs by overlap with non-unique features. a, Sankey diagram summarizing the classification of detected GCEs by overlap with paralogous regions, mapping status, and local repeat sequences. **b**, Histogram of k-mer similarity values between GCEs and their best-matching reference paralog.

Figure S5

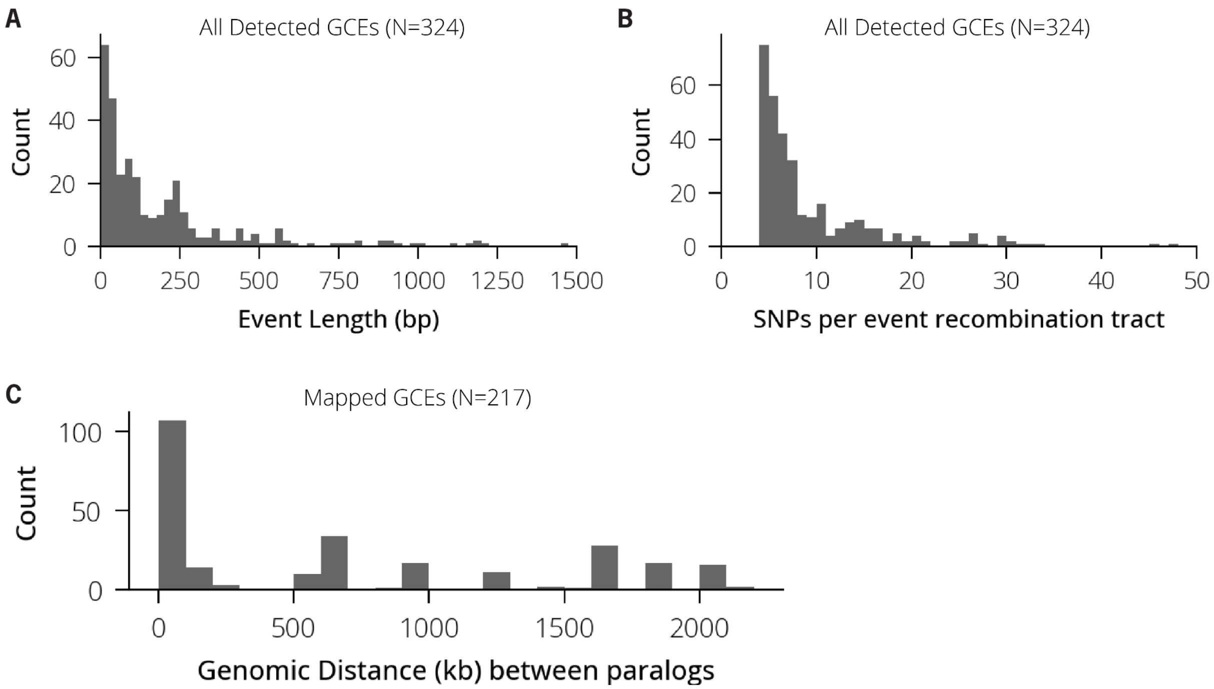

Figure S5. Overview of general characteristics of detected gene conversion events. a) Distribution of recombination tract lengths associated with detected GCEs. **b)** Distribution of number of substitutions associated with detected GCE. **c)** The distribution of genomic distances between the inferred donor paralog sequences and mapped GCEs.

Figure S6

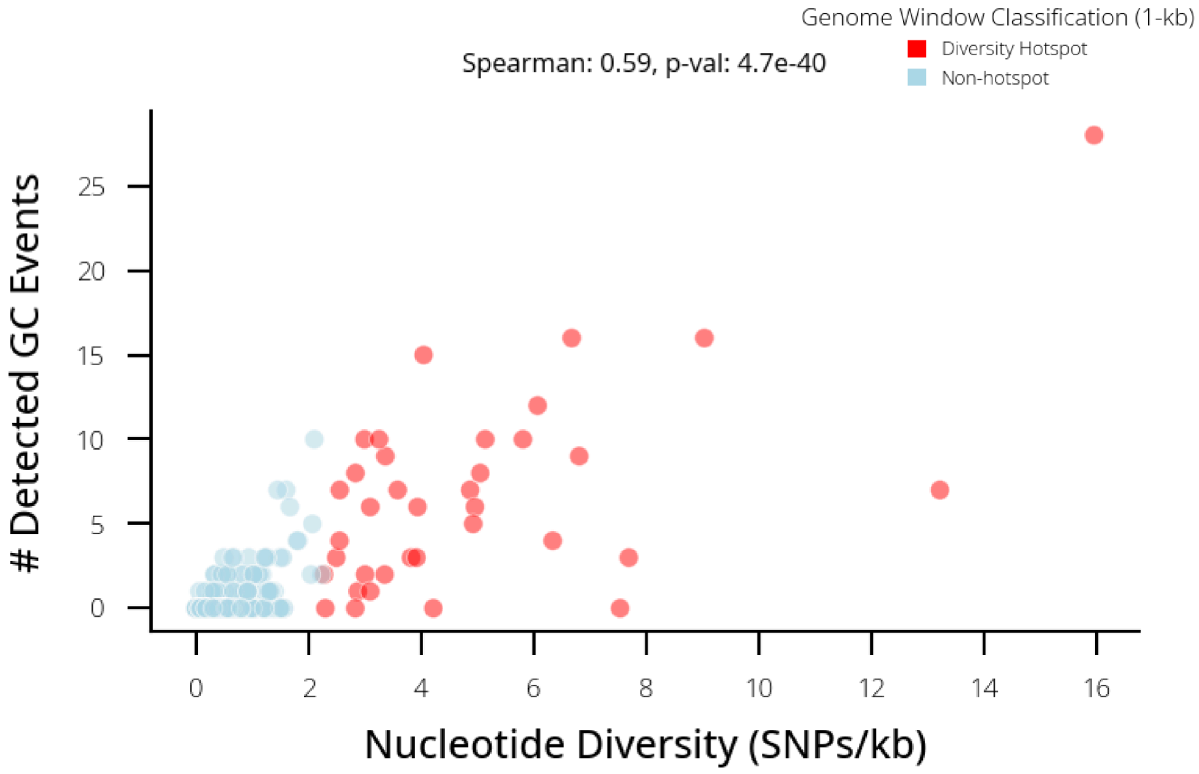

Figure S6. Scatterplot showing nucleotide diversity versus detected GCEs per 1-kb window overlapping paralogous regions. All windows classified as diversity hotspots are highlighted. Spearman's rank correlation: ρ = 0.59, p = 4.7×10^{-40}.

Figure S7

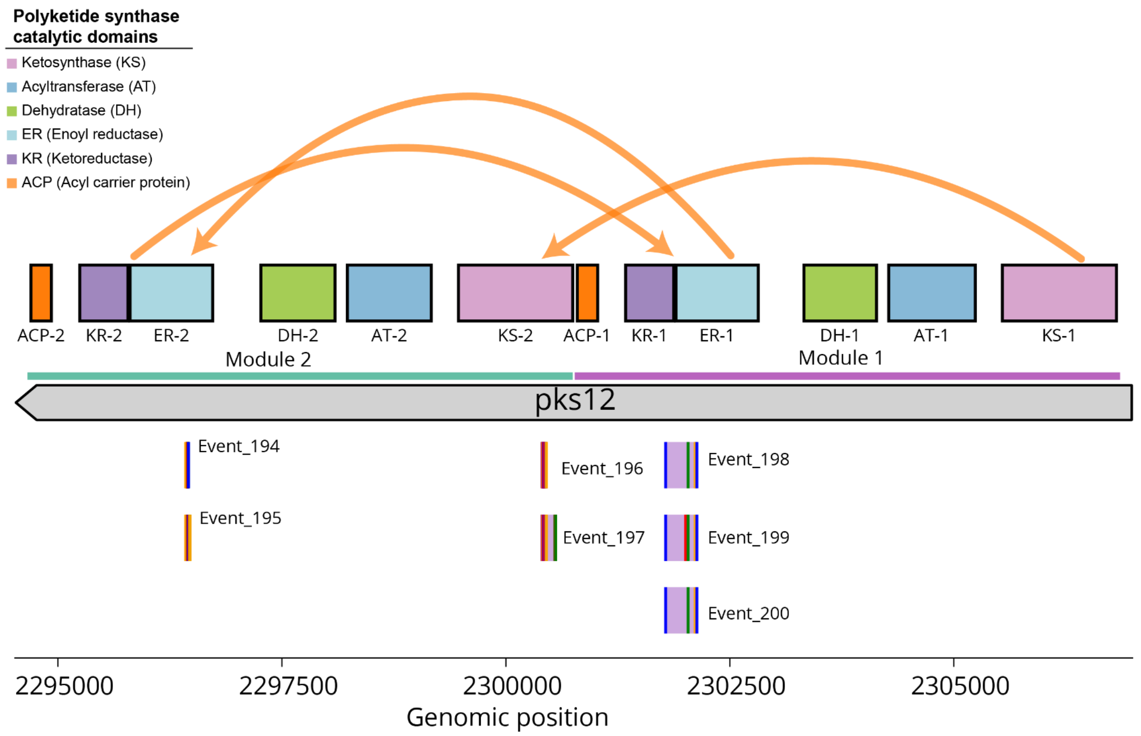

Figure S7. Overview of intragenic gene conversion events detected in pks12. Top: Schematic representation of the pks12 locus showing gene structure and annotated protein domains. pks12 comprises two modular units containing six homologous polyketide synthase catalytic domains. Bottom: Detected intragenic gene conversion events (n = 7) shown with associated substitution variants.

Table S5

Negative binomial regression of gene conversion frequency between paralogous regions				
**Predictor**	**Coefficient (log IRR)**	**IRR**	**95% CI (IRR)**	**p-value**
Sequence divergence (SNPs per kb)	−0.0171	**0.983**	0.978 – 0.988	**$<1\times10^{-4}$**
Genomic distance (kb)	−0.0002	0.9998	0.9994 – 1.0002	0.264
Paralog copy number	0.0153	1.015	0.880 – 1.171	0.834
GC content	0.0193	1.020	0.973 – 1.068	0.417
**GC Content (%)**	0.0218	0.017	1.302	0.194
**Model Summary Statistics**				
**McFadden pseudo-R^2: 0.1377**<br>**Sample Size (N) = 284 pairs of paralogous regions**				

Table S5. Negative binomial regression of gene conversion frequency between paralogous regions. A generalized linear model with a negative binomial distribution and log link was used to model the number of mapped gene conversion events (mGCEs) detected between non-perfect repeat paralog pairs (n = 284). Predictor variables included sequence divergence (SNPs per kb of aligned paralogous sequence), genomic distance between paralogs (kb), paralog copy number (number of overlapping paralogous alignments), and GC content. Reported values are incidence rate ratios (IRRs) with 95% confidence intervals, calculated using robust (HC0) standard errors. IRRs represent the multiplicative change in the expected number of mGCEs per unit increase in each predictor.

Figure S8

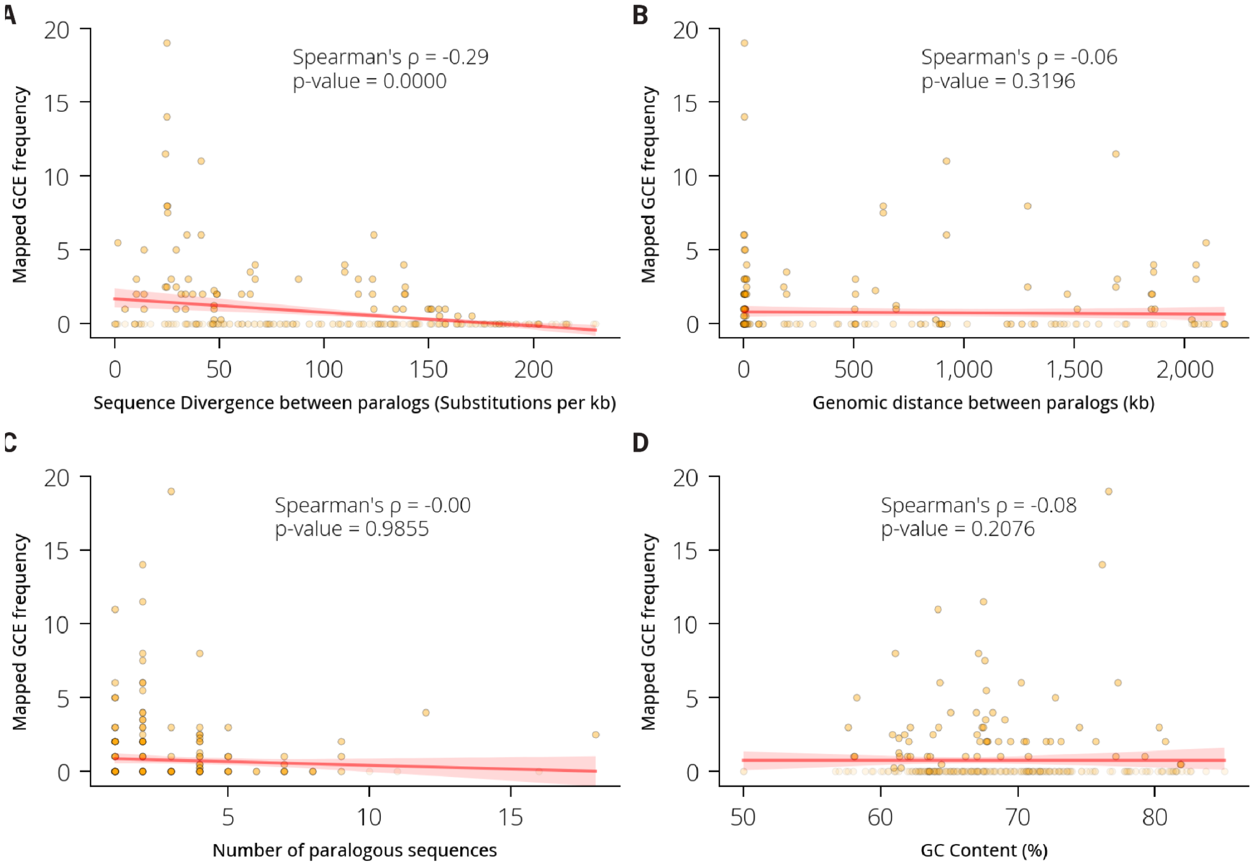

Figure S8. Relationship between gene conversion frequency and general sequence characteristics. (A–D) Scatterplots showing associations between mapped gene conversion event frequency (mGCEs) and general sequence or genomic characteristics across all evaluated paralog pairs (n = 284). Spearman's rank correlation coefficient (ρ) is shown for each comparison.

Figure S9

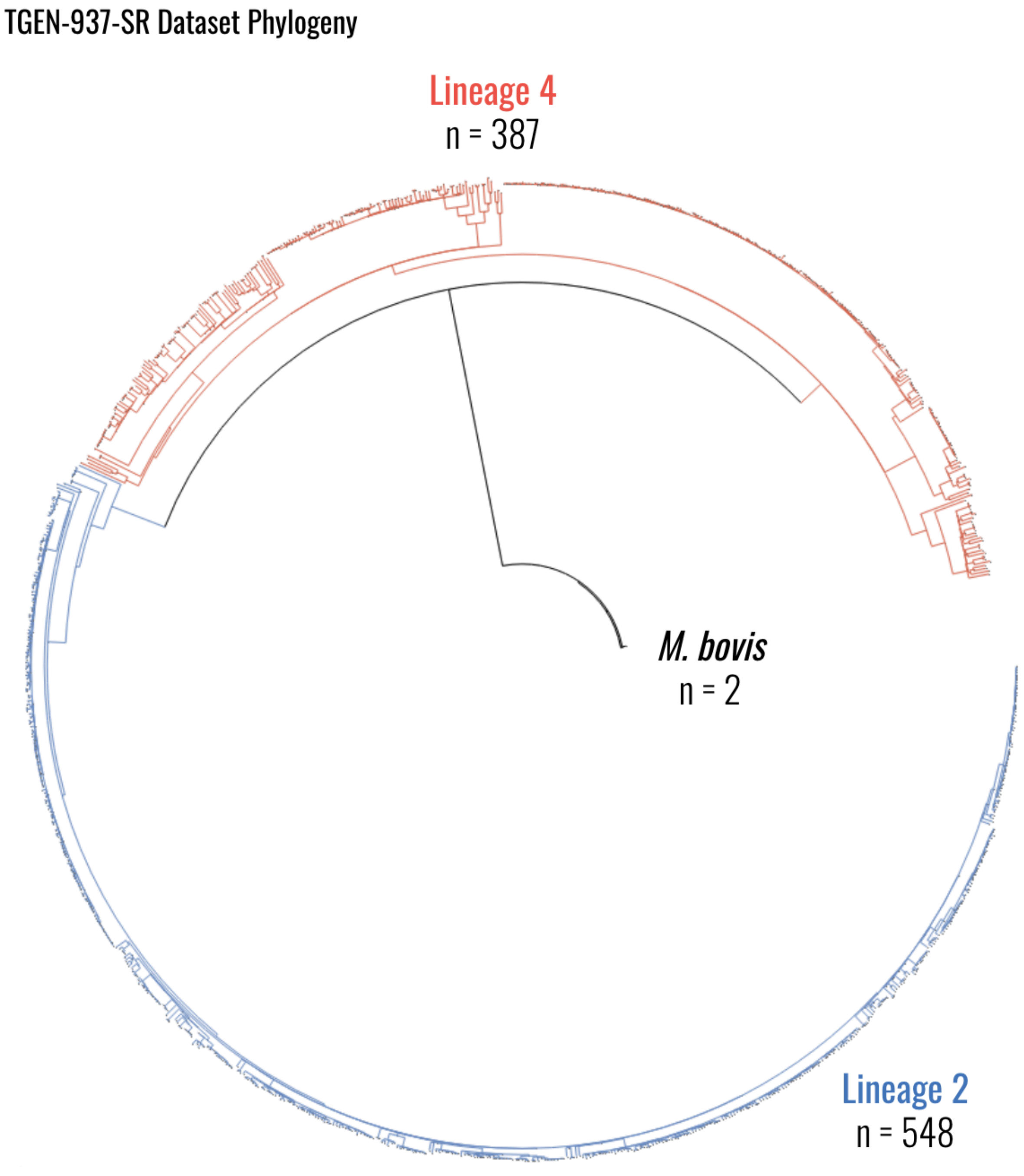

Figure S9. Maximum-likelihood phylogeny of 937 clinical isolates sequenced with short-read whole-genome sequencing (TGENSR dataset). The dataset comprises *M. tuberculosis* isolates from lineage 2 (n = 548) and lineage 4 (n = 387), with two additional isolates identified as Mycobacterium bovis.

Figure S10

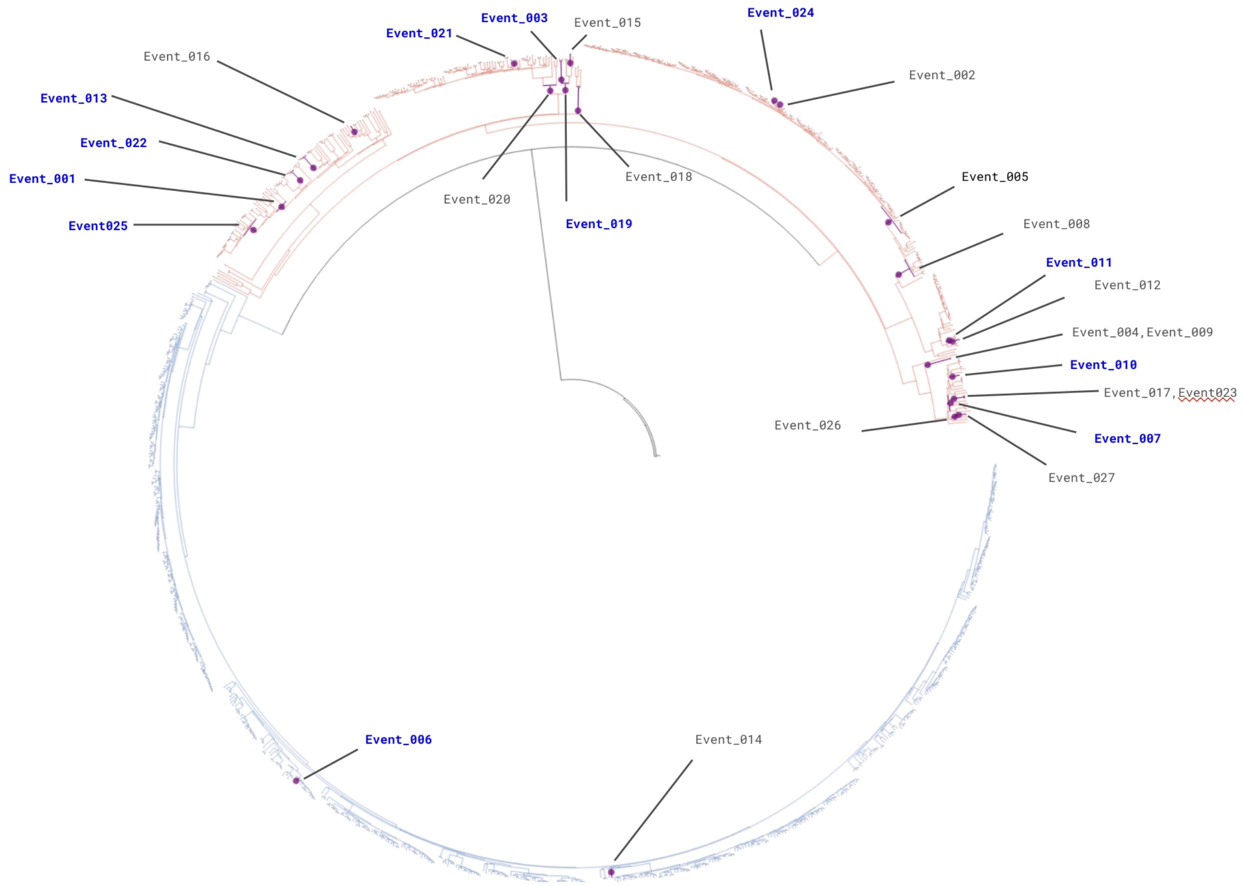

Figure S10. Phylogenetic distribution of putative gene conversion events detected in the TGEN-937-SR dataset. Phylogenetic branches inferred to harbor putative gene conversion events are highlighted in purple. Event labels shown in blue denote loci selected for long-read resequencing and validation.

Figure S11

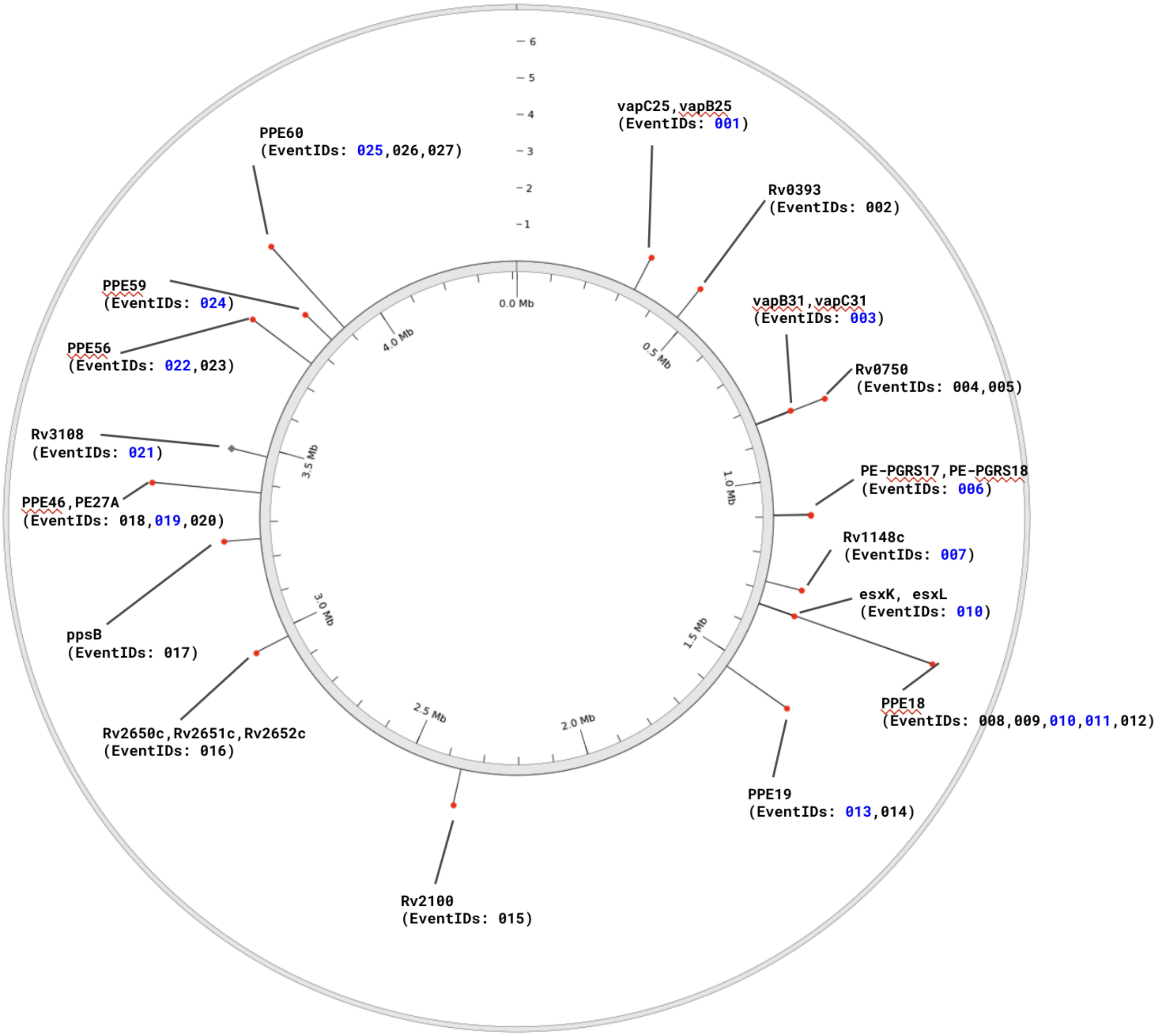

Figure S11. Genomic distribution of putative gene conversion events detected in the TGEN-937-SR dataset. Visualization of the genomic locations of all detected putative gene conversion events in the TGEN-937-SR dataset. Events selected for long-read resequencing are highlighted in blue.

Table S6

TGENSR EventID	Overlapping Genes	# of SNPs within recombination event signature	Lineage	Within a paralogous region?	In a region with detected GC events in primary analysis?
Event_001	vapC25,vapB25	8	L4	Yes	Yes
Event_002	Rv0393	6	L4	Yes	Yes
Event_003	vapB31,vapC31	14	L4	Yes	Yes
Event_004	Rv0750	9	L4	Yes	Yes
Event_005	Rv0750	7	L4	Yes	Yes
Event_006	PE_PGRS18	7	L2	Yes	Yes
Event_007	Rv1148c	5	L4	Yes	Yes
Event_008	PPE18	5	L4	Yes	Yes
Event_009	PPE18	8	L4	Yes	Yes
Event_010	PPE18, esxK, esxL	5	L4	Yes	Yes
Event_011	PPE18	7	L4	Yes	Yes
Event_012	PPE18	7	L4	Yes	Yes
Event_013	PPE19	6	L4	Yes	Yes
Event_014	PPE19	4	L2	Yes	Yes
Event_015	Rv2100	4	L4	Yes	No
Event_016	Rv2651c	5	L4	Yes	No
Event_017	ppsB	6	L4	Yes	Yes
Event_018	esxQ, PPE46	8	L4	Yes	Yes
Event_019	PPE46	8	L4	Yes	Yes
Event_020	PPE46	5	L4	Yes	Yes
Event_021	Rv3108	5	L4	No	No
Event_022	PPE56	7	L4	Yes	Yes
Event_023	PPE56, Rv3351c	7	L4	Yes	Yes
Event_024	PPE59, Rv3430c	8	L4	Yes	Yes
Event_025	PPE60	6	L4	Yes	Yes
Event_026	PPE60	5	L4	Yes	Yes
Event_027	PPE60	5	L4	Yes	Yes

Table S6. Putative gene conversion events detected in TGEN-SR dataset.

Table S7

TGEN-SR EventID	Overlapping Genes	In known PR?	Supported by PacBio WGS?	SNPs resolved by SR-WGS	SNPs resolved by PacBio (LR-WGS)	SR-SNPs agree with PacBio-SNPs
Event-001	*vapC25, vapB25*	Yes	Yes	8	19	8/8 (100%)
Event-003	*vapB31, vapC31*	Yes	Yes	14	21	14/14 (100%)
Event-006	*PE_PGRS18*	Yes	Yes	7	7	5/7 (71%)
Event-007	*Rv1148c*	Yes	Yes	5	42	5/5 (100%)
Event-010	*PPE18, esxK, esxL*	Yes	Yes	5	13	3/5 (60%)
Event-011	*PPE18*	Yes	Yes	7	8	7/7 (100%)
Event-013	*PPE19*	Yes	Yes	6	9	6/6 (100%)
Event-019	*PPE46*	Yes	Yes	8	13	8/8 (100%)
Event-021	*Rv3108*	No	Yes	5	5	5/5 (100%)
Event-022	*PPE56*	Yes	Yes	7	16	7/7 (100%)
Event-024	*PPE59*	Yes	Yes	8	29	8/8 (100%)
Event-025	*PPE60*	Yes	Yes	6	17	6/6 (100%)

Table S7. Validation results for all putative GC events with isolates resequenced with PacBio HiFi WGS.

Figure S12

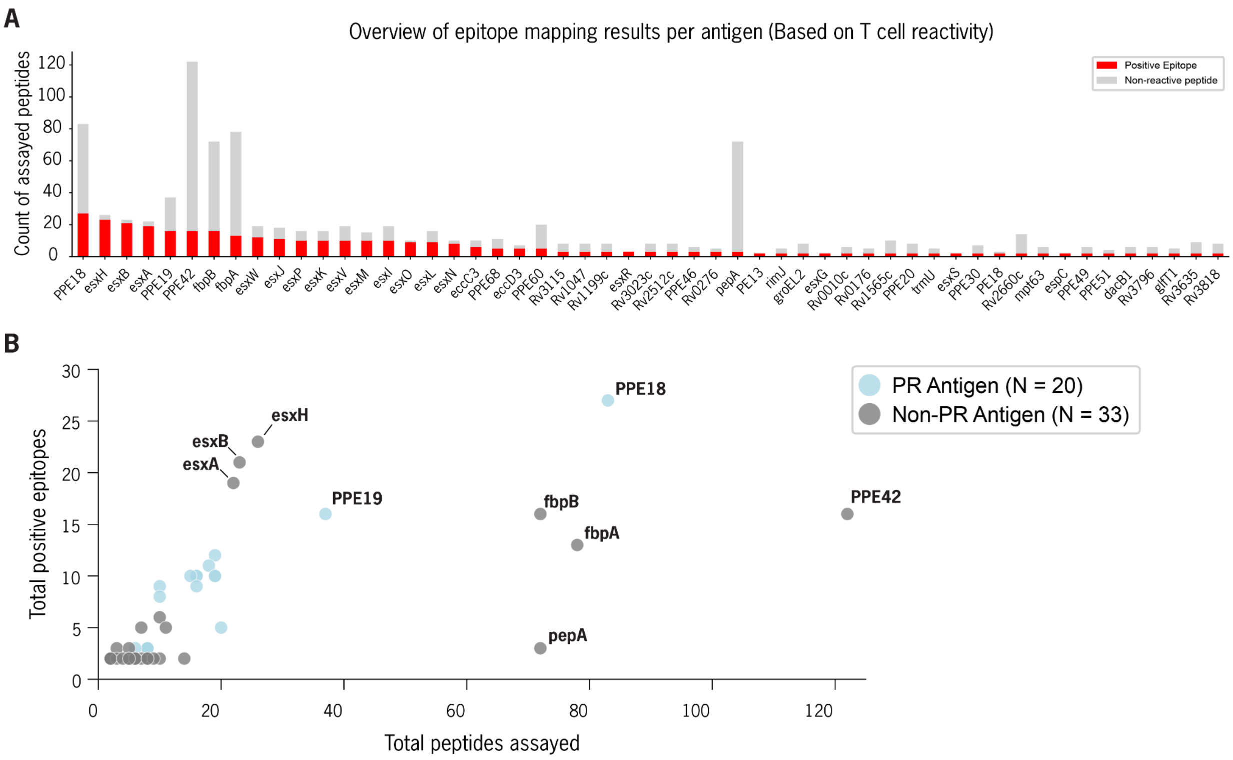

Figure S12. Overview of identified epitopes and assayed peptides per T cell antigen. a, Stacked bar plot showing the number of assayed peptides for each antigen (≥2 positive epitopes) in the curated epitope mapping dataset. Total bar height represents the total number of peptides assayed per antigen. Grey segments indicate non-reactive peptides, while red segments indicate reactive peptides (positive epitopes). b, Scatter plot of the same 53 T cell antigens, showing the relationship between the total number of peptides assayed (x-axis) and the total number of positive epitopes identified (y-axis).

Table S8

Antigen	Protein Length (aa)	# Positive Epitopes	# Negative Peptides	# Peptides Assayed	Region Type	Comments
pepA	356	3	69	72	Non-PR	M72/AS01E vaccine candidate
esxH	97	23	3	26	Non-PR	Aeras402, HyVac4 vaccine candidate
PPE18	392	27	56	83	PR	M72/AS01E vaccine candidate
fbpB (Ag85B)	326	16	56	72	Non-PR	Aeras402, H1, HyVac4 vaccine candidate
PPE42	581	16	106	122	Non-PR	ID93 vaccine candidate
Rv2660c	76	2	12	14	Non-PR	H56 vaccine candidate
esxV	95	10	9	19	PR	ID93 vaccine candidate
esxW	99	12	7	19	PR	ID93 vaccine candidate
fbpA (Ag85A)	339	13	65	78	Non-PR	MVA85A, Aeras402, Ad85A vaccine candidate
esxB (CFP10)	101	21	2	23	Non-PR	IGRA diagnostic antigen
esxA (ESAT-6)	96	19	3	22	Non-PR	IGRA diagnostic antigen; H1, H56 vaccine candidate

Table S8. T cell Epitope mapping statistics for IGRA diagnostic antigens and vaccine targets (N=11) within the set of T cell antigens. These 11 proteins are included in our set of 53 high-confidence T cell antigens that contain multiple experimentally validated $CD4^{+}$ T cell epitopes. Many are among the most extensively studied antigens in TB research, including IGRA diagnostic targets (EsxA, EsxB) and antigens incorporated into the following candidate vaccines: M72/AS01E, Aeras402, HyVac4, H1, H56, ID93, MVA85A, and Ad85A.

Figure S13

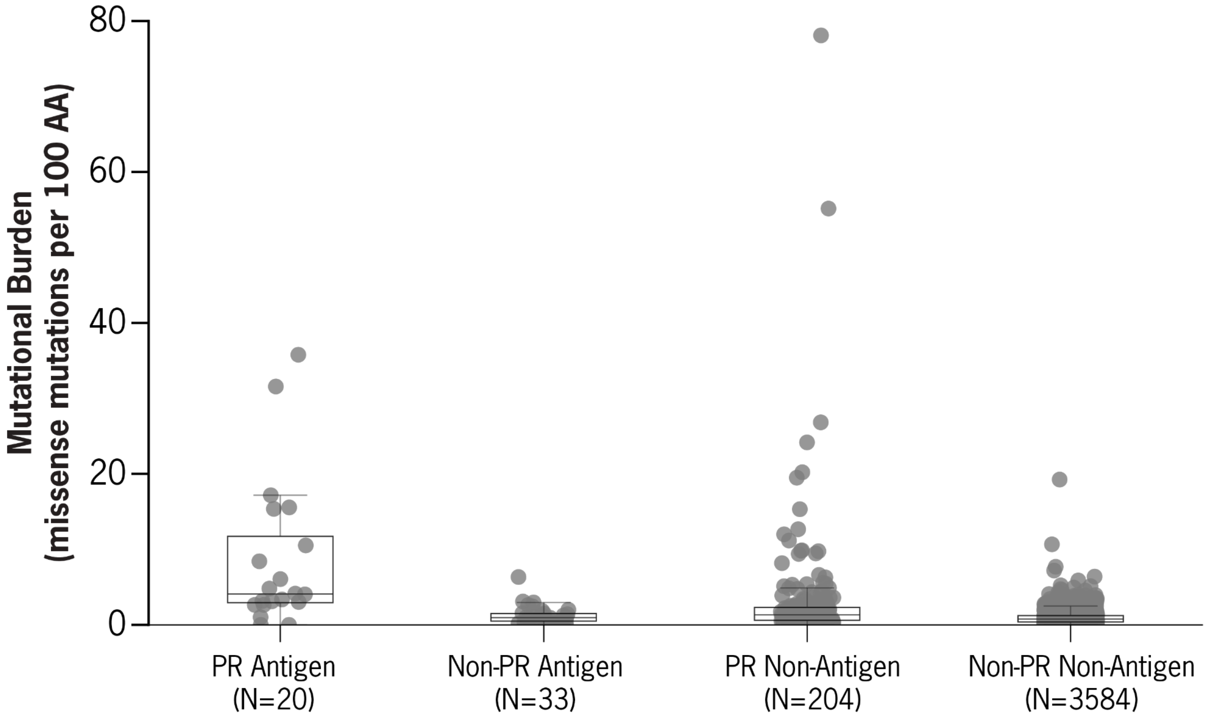

Figure S13. Mutational burden across antigens and non-antigens stratified by genomic context. Boxplot with overlaid scatter points showing normalized missense mutational burden (missense mutations per 100 amino acids) for four categories: PR-Antigen, Unique-Antigen, NonAntigen-PR, and NonAntigen-Unique. The number of coding sequences (CDSs) in each category is indicated on the x-axis. Box boundaries represent the IQR, horizontal lines denote the median, and whiskers extend to 1.5× IQR. Statistical comparisons between all category pairs are provided in Table S7.

Figure S14

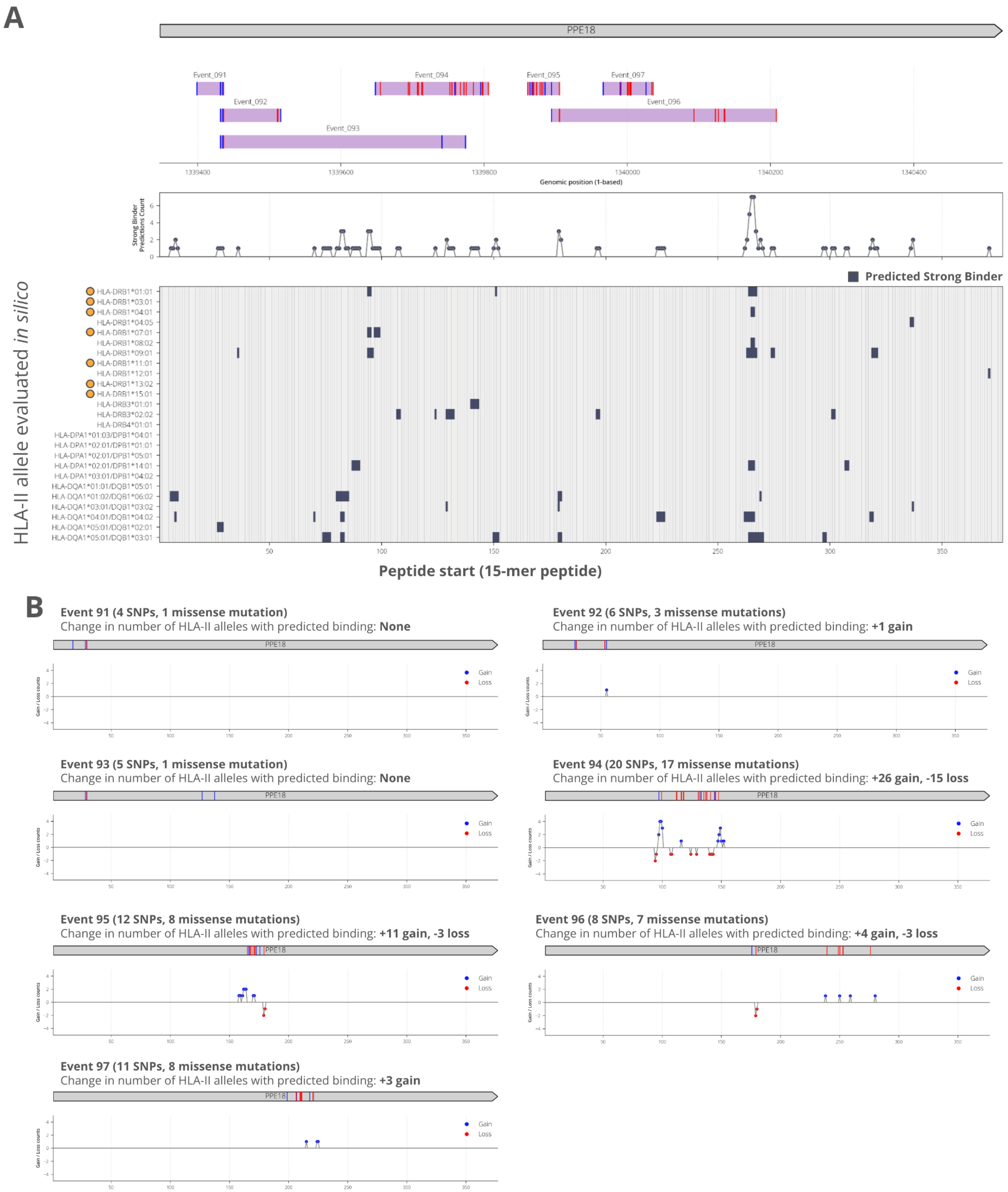

A
PPE18
Event_091
Event_092
Event_093
Event_094
Event_095
Event_096
Event_097
1339400
1339600
1339800
1340000
1340200
1340400
Genomic position (1-based)
Strong Binder Predictions Count
Predicted Strong Binder
HLA-II allele evaluated in silico
HLA-DRB1*01:01
HLA-DRB1*03:01
HLA-DRB1*04:01
HLA-DRB1*04:05
HLA-DRB1*07:01
HLA-DRB1*08:02
HLA-DRB1*09:01
HLA-DRB1*11:01
HLA-DRB1*12:01
HLA-DRB1*13:02
HLA-DRB1*15:01
HLA-DRB3*01:01
HLA-DRB3*02:02
HLA-DRB4*01:01
HLA-DPA1*01:03/DPB1*04:01
HLA-DPA1*02:01/DPB1*01:01
HLA-DPA1*02:01/DPB1*05:01
HLA-DPA1*02:01/DPB1*14:01
HLA-DPA1*03:01/DPB1*04:02
HLA-DQA1*01:01/DQB1*05:01
HLA-DQA1*01:02/DQB1*06:02
HLA-DQA1*03:01/DQB1*03:02
HLA-DQA1*04:01/DQB1*04:02
HLA-DQA1*05:01/DQB1*02:01
HLA-DQA1*05:01/DQB1*03:01
Peptide start (15-mer peptide)
B
Event 91 (4 SNPs, 1 missense mutation)
Change in number of HLA-II alleles with predicted binding: None
Event 92 (6 SNPs, 3 missense mutations)
Change in number of HLA-II alleles with predicted binding: +1 gain
Event 93 (5 SNPs, 1 missense mutation)
Change in number of HLA-II alleles with predicted binding: None
Event 94 (20 SNPs, 17 missense mutations)
Change in number of HLA-II alleles with predicted binding: +26 gain, -15 loss
Event 95 (12 SNPs, 8 missense mutations)
Change in number of HLA-II alleles with predicted binding: +11 gain, -3 loss
Event 96 (8 SNPs, 7 missense mutations)
Change in number of HLA-II alleles with predicted binding: +4 gain, -3 loss
Event 97 (11 SNPs, 8 missense mutations)
Change in number of HLA-II alleles with predicted binding: +3 gain
Gain
Loss
Gain / Loss counts

Figure S14. in silico analysis of the effect of PPE18 gene conversion events on HLA-II binding for 27 common HLA-II alleles. **A.** Heatmap of whether netMHCpanII predicted a strong binding interaction between each HLA-II allele from the IEDB reference HLA-II set (N= 27) for each 15-mer peptide of the PPE18 reference (H37Rv) protein sequence. Orange dots next highlight the most common HLA-II alleles across European populations (N = 7). **B.** For each gene conversion event in PPE18, the number of alleles with a predicted strong binding interaction with a 15-mer peptide sequence was plotted along the protein length of PPE18 before and after mutation.

Table S9

GC EventID	Mapped to Paralog?	# of SNPs	# of missense mutations	Change in predicted HLA-binding interactions (27 alleles)	
				# gained HLA-binding interactions	# loss HLA-binding interactions
**Event-091**	**Yes**	**4**	**1**	**0**	**0**
**Event-092**	**Yes**	**6**	**3**	**+1**	**0**
**Event-093**	**Yes**	**5**	**1**	**0**	**0**
**Event-094**	**Yes**	**20**	**17**	**+26**	**-15**
**Event-095**	**Yes**	**12**	**8**	**+11**	**-3**
**Event-096**	**Yes**	**8**	**7**	**+4**	**-3**
**Event-097**	**Yes**	**11**	**8**	**+3**	**0**

Table S9. Net effect of PPE18 gene conversion events on predicted HLA-II binding interactions for 27 common HLA-II alleles.

Table S10

GC EventID	# of SNPs	# of missense mutations	Change predicted HLA-binding interactions (7 common alleles in european populations)	
			# gained HLA-binding interactions	# loss HLA-binding interactions
Event-091	4	1	0	0
Event-092	6	3	0	0
Event-093	5	1	0	0
Event-094	20	17	+10	-4
Event-095	12	8	+3	0
Event-096	8	7	+2	0
Event-097	11	8	+1	0

Table S10. Net effect of PPE18 GC events on predicted HLA-II binding interactions for 7 common European HLA-II alleles.

Figure S15

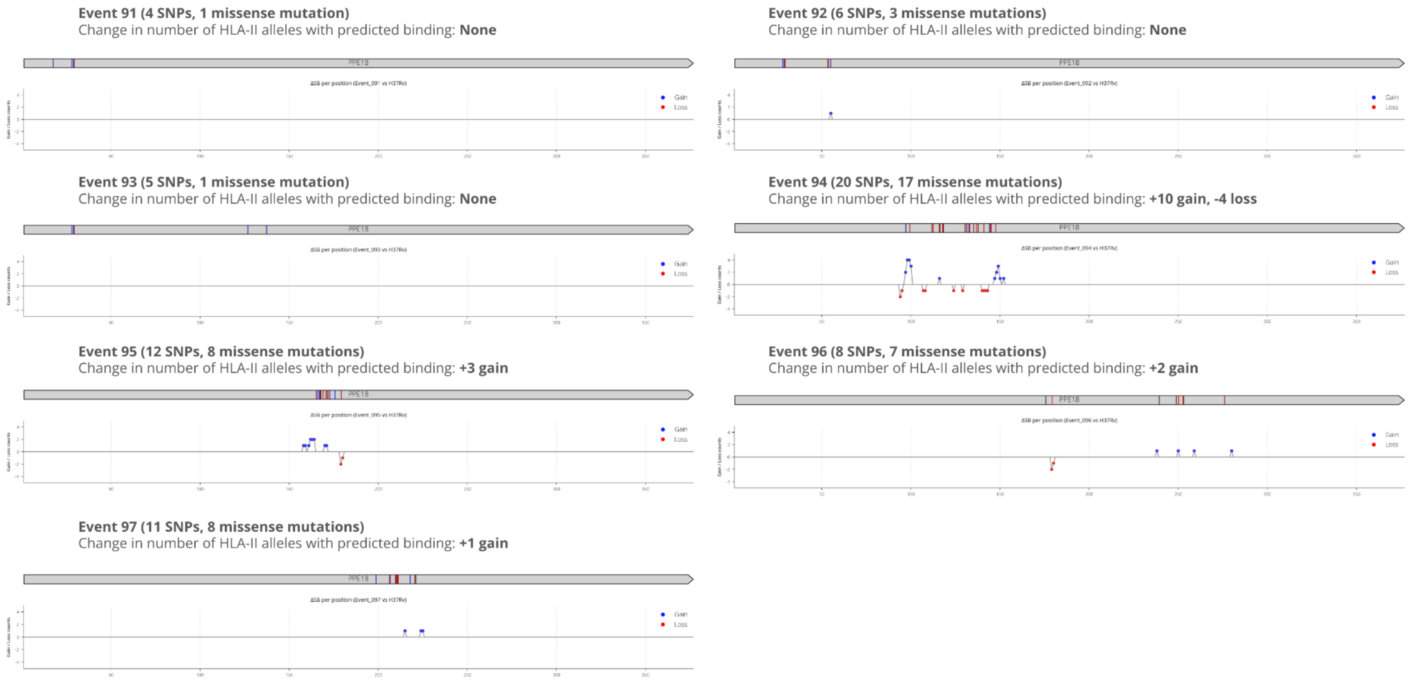

Figure S15. *in silico* analysis of the effect of PPE18 gene conversion events on HLA-II binding for 7 top HLA-II alleles in European populations. For each gene conversion event in PPE18, the number of alleles with a predicted strong binding interaction with a 15-mer peptide sequence was plotted along the protein length of PPE18 before and after mutation.
