# Supplementary material for: Gene conversion is a key driver of diversity hotspots in M. tuberculosis antigens and virulence-associated loci
Source: bioRxiv. 2026 Mar 11:2026.02.26.708061. Preprint. [Version 2] doi: 10.64898/2026.02.26.708061 (PMC13061034; doi:10.64898/2026.02.26.708061)

Diversity Hotspot View - 01:
Genomic range shown: NC_000962.3:102000-107000
Gene(s) of interest: Rv0094c,Rv0095c

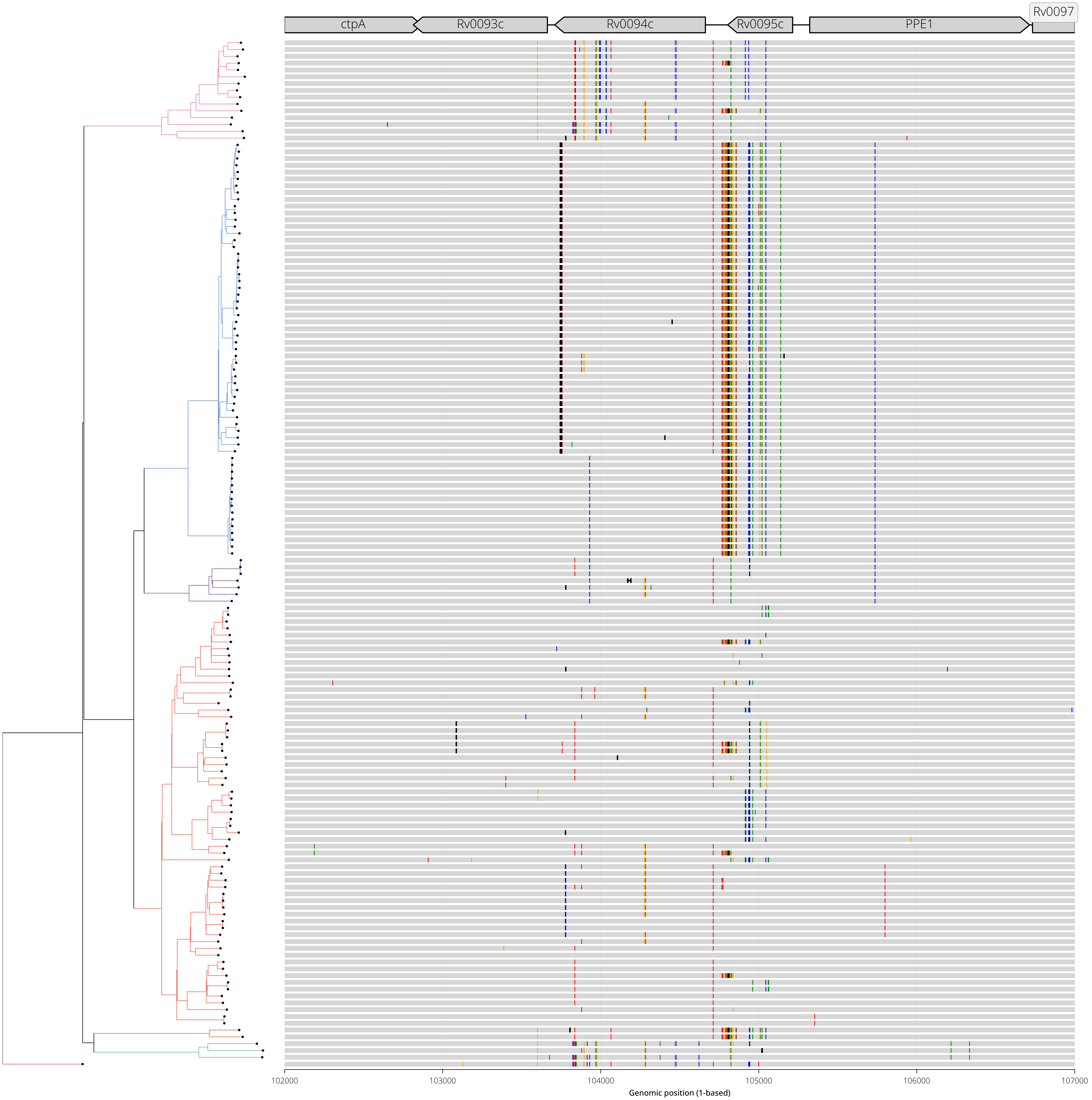

Diversity Hotspot View - 02:
Genomic range shown: NC_000962.3:336000-341000
Gene(s) of interest: PE_PGRS4

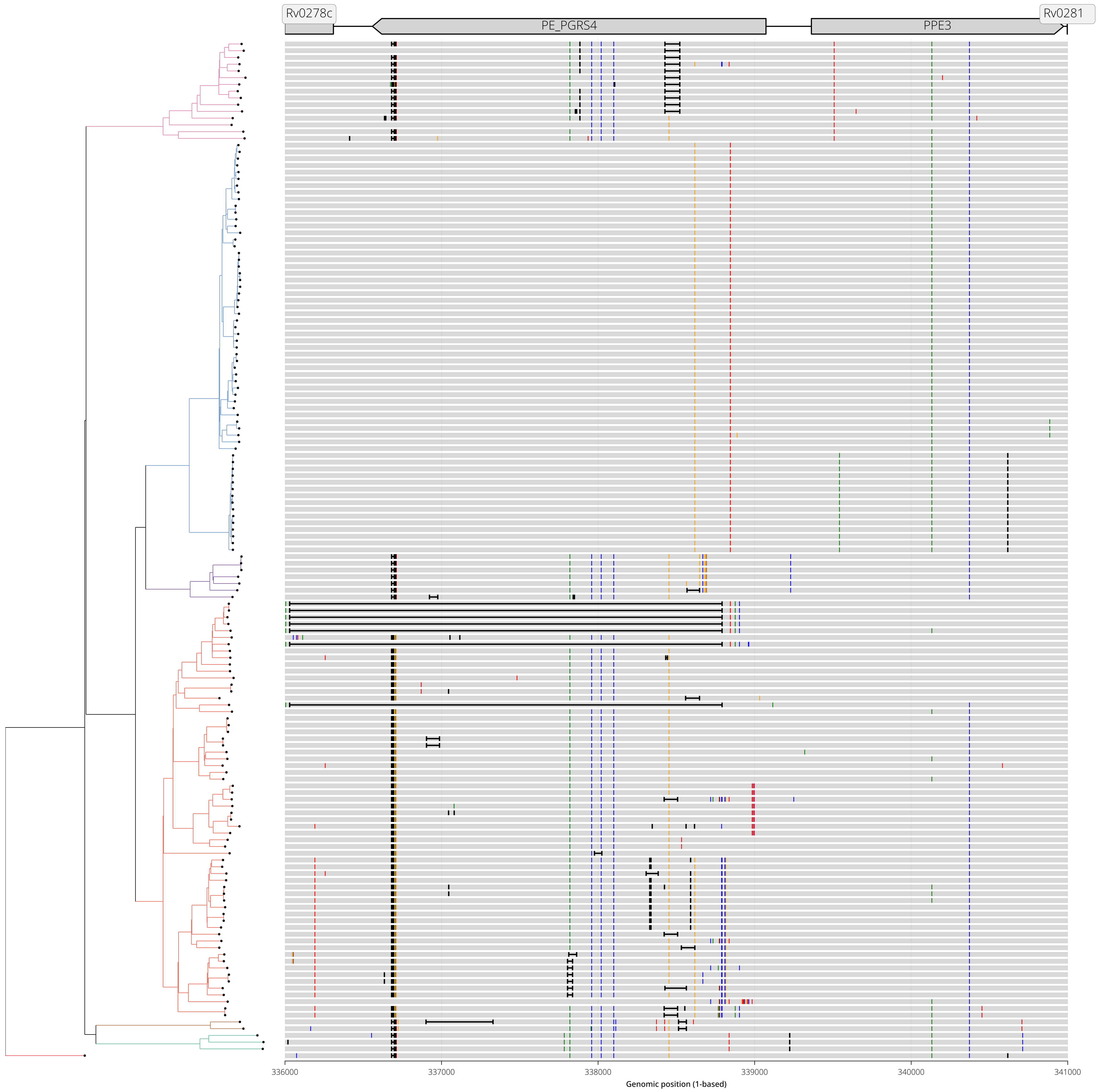

Diversity Hotspot View - 03:
Genomic range shown: NC_000962.3:1095000-1098000
Gene(s) of interest: PE_PGRS18,mprA

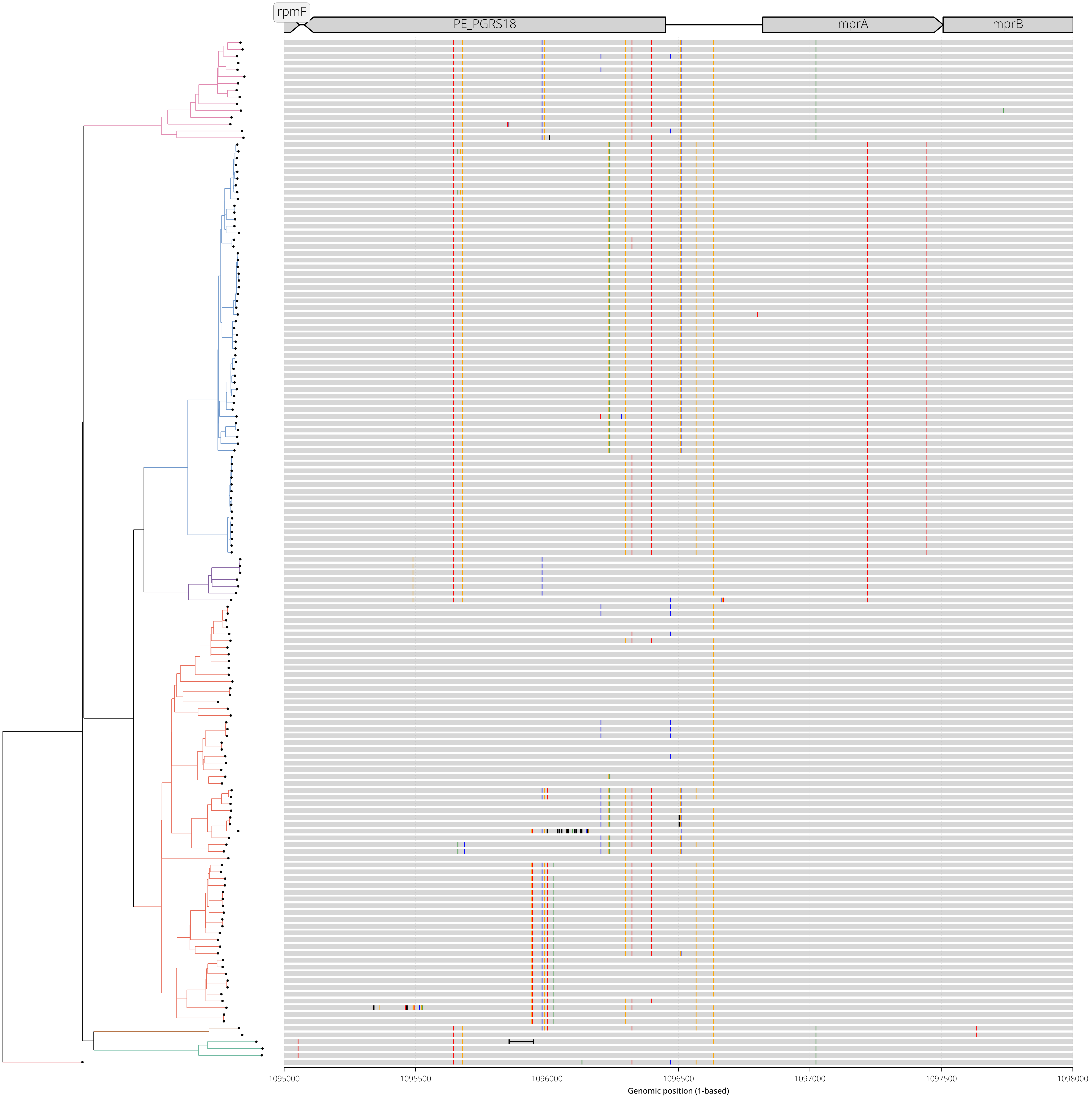

Diversity Hotspot View - 04:
Genomic range shown: NC_000962.3:1274000-1279000
Gene(s) of interest: Rv1148c

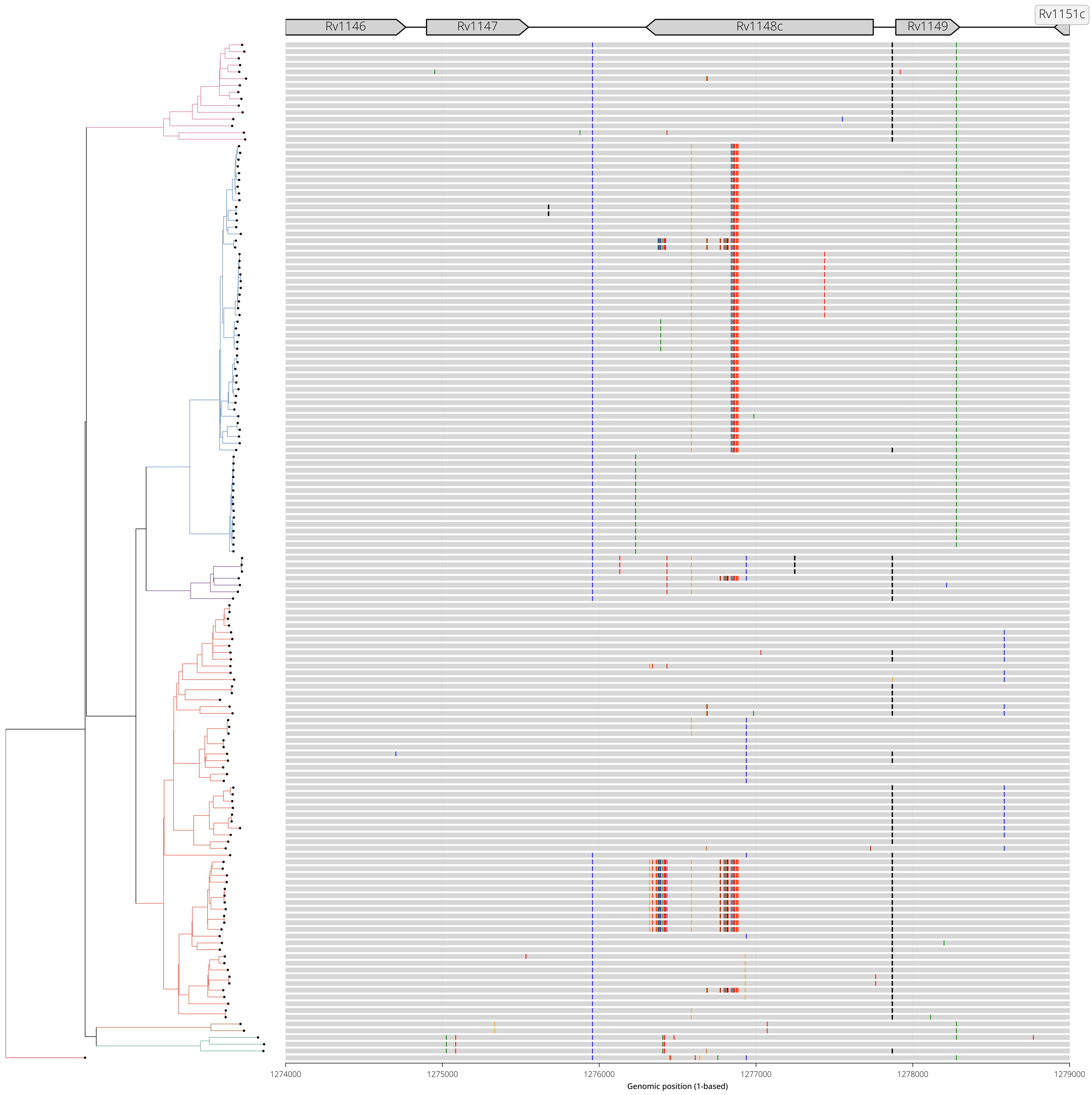

Diversity Hotspot View - 05:
Genomic range shown: NC_000962.3:1338000-1343000
Gene(s) of interest: PPE18,esxK

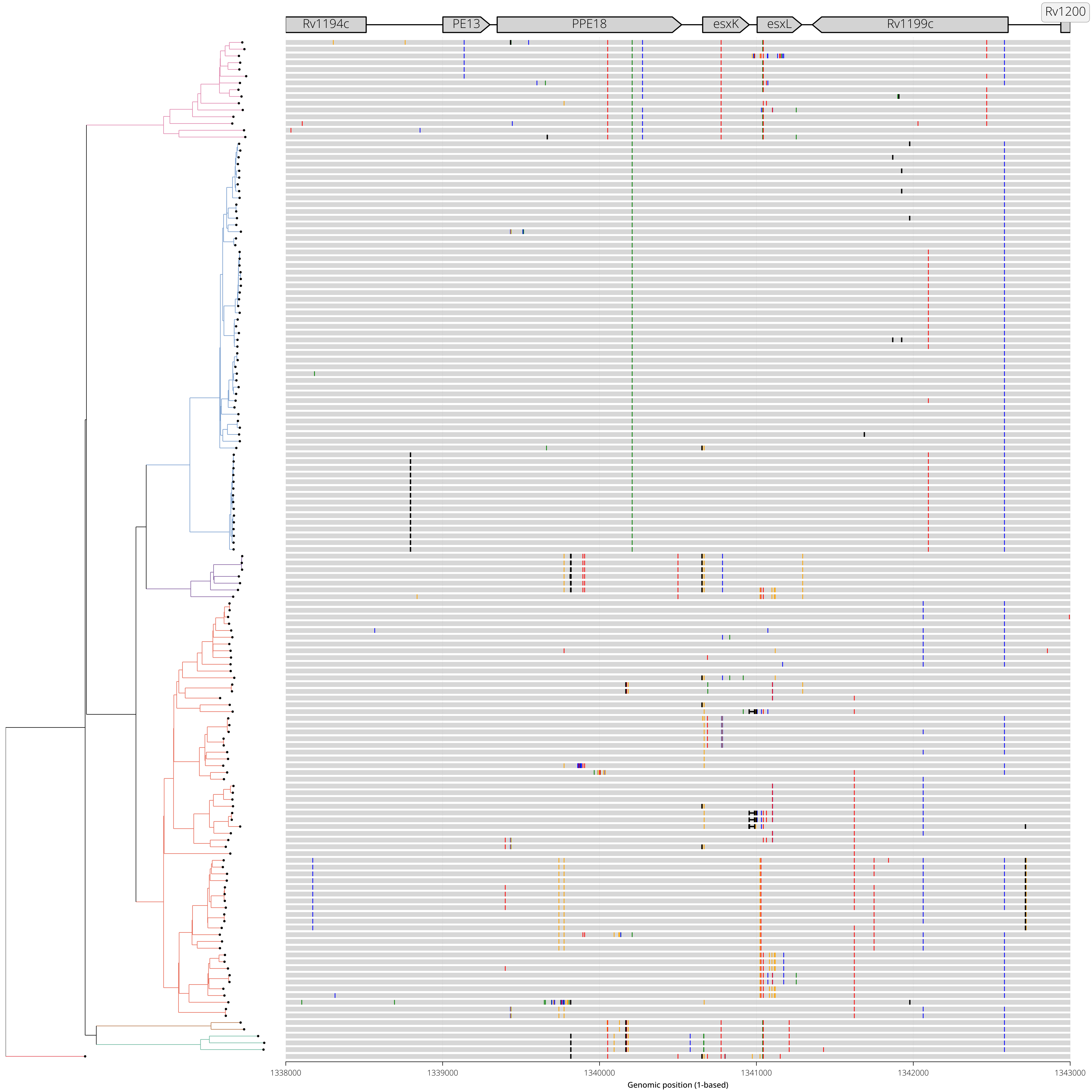

Diversity Hotspot View - 06:
Genomic range shown: NC_000962.3:1339000-1344000
Gene(s) of interest: esxL,Rv1199c

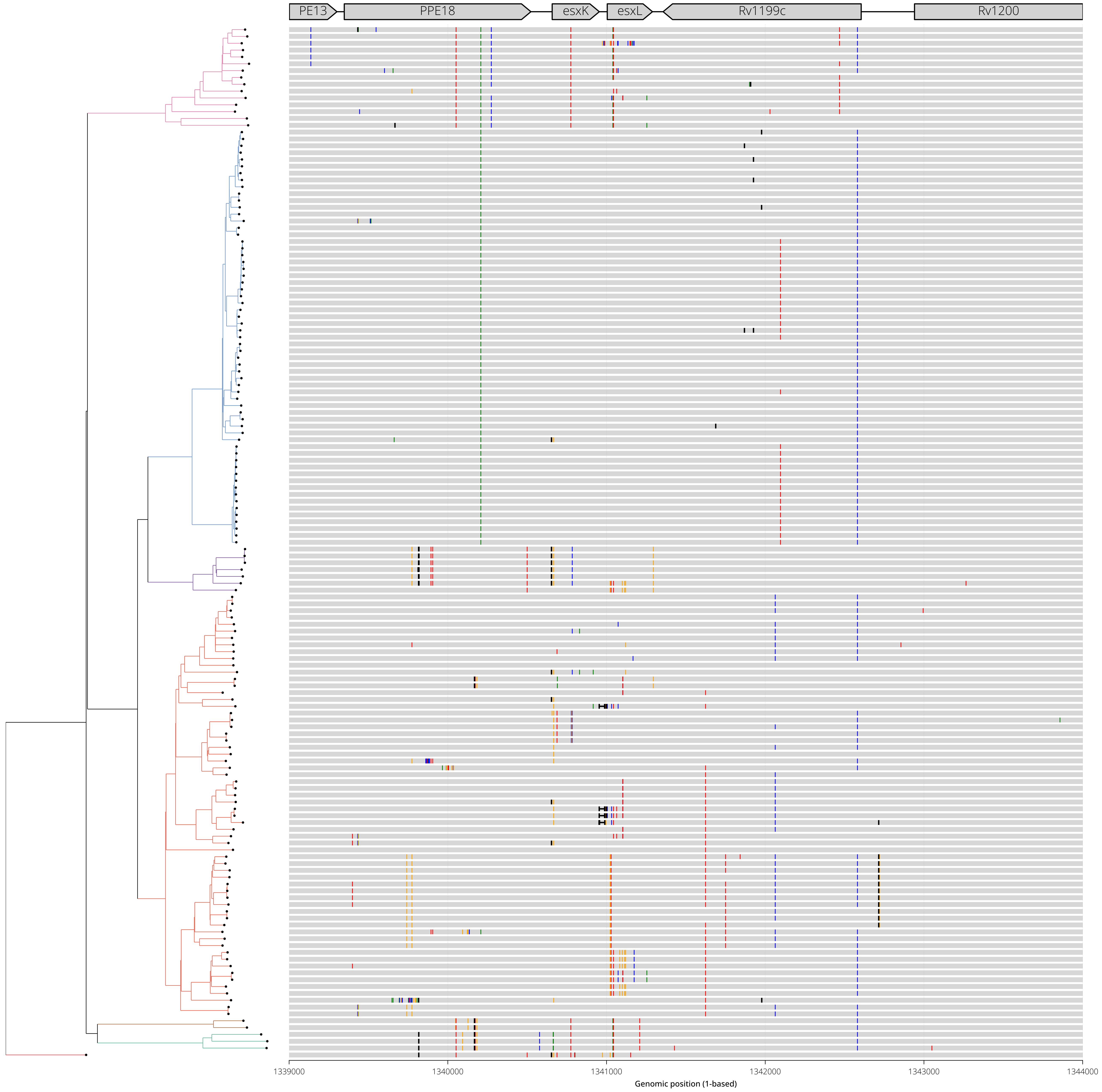

Diversity Hotspot View - 07:
Genomic range shown: NC_000962.3:1531000-1536000
Gene(s) of interest: PPE19,Rv1362c

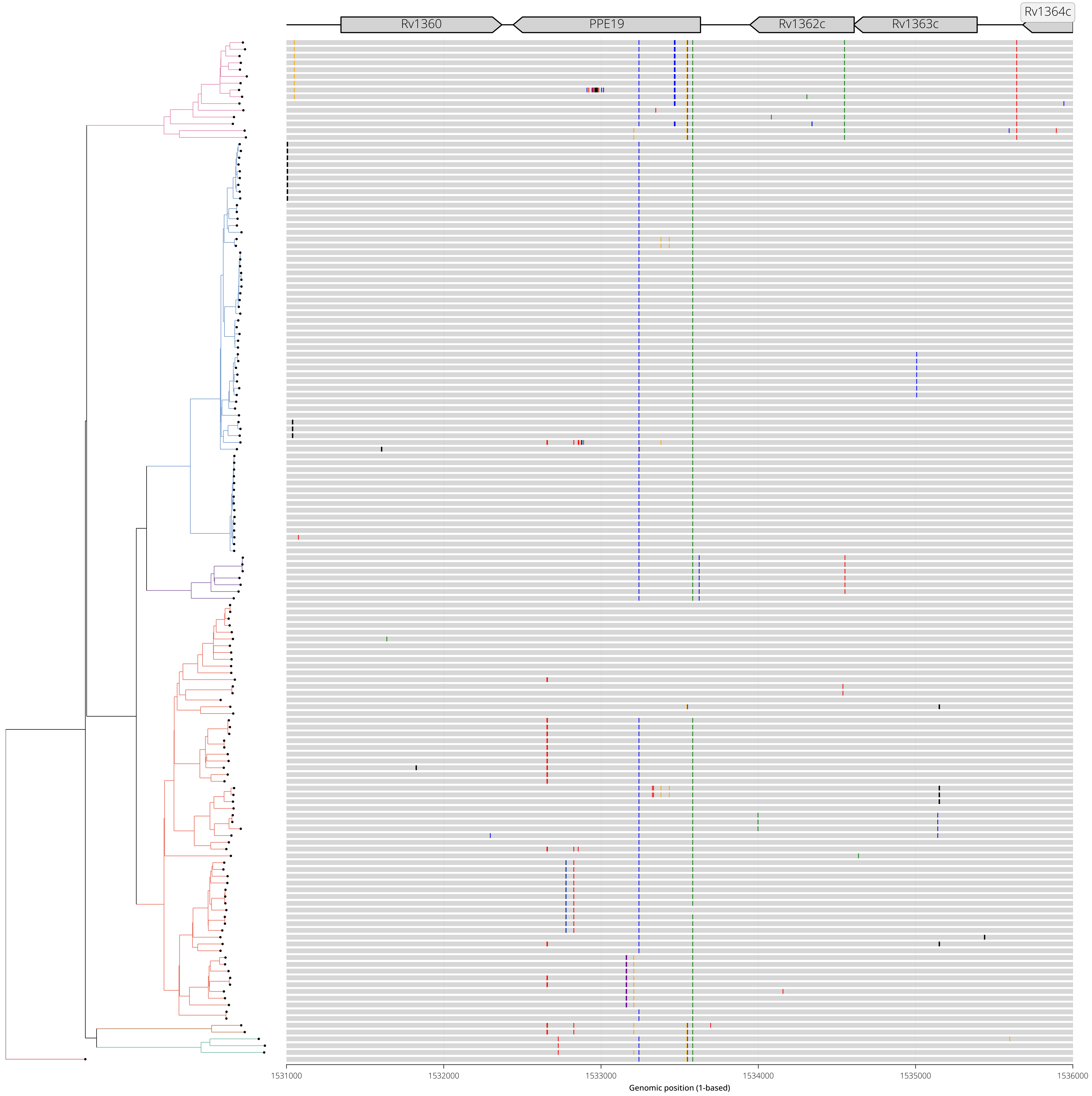

Diversity Hotspot View - 08:
Genomic range shown: NC_000962.3:1631000-1638000
Gene(s) of interest: PE_PGRS27

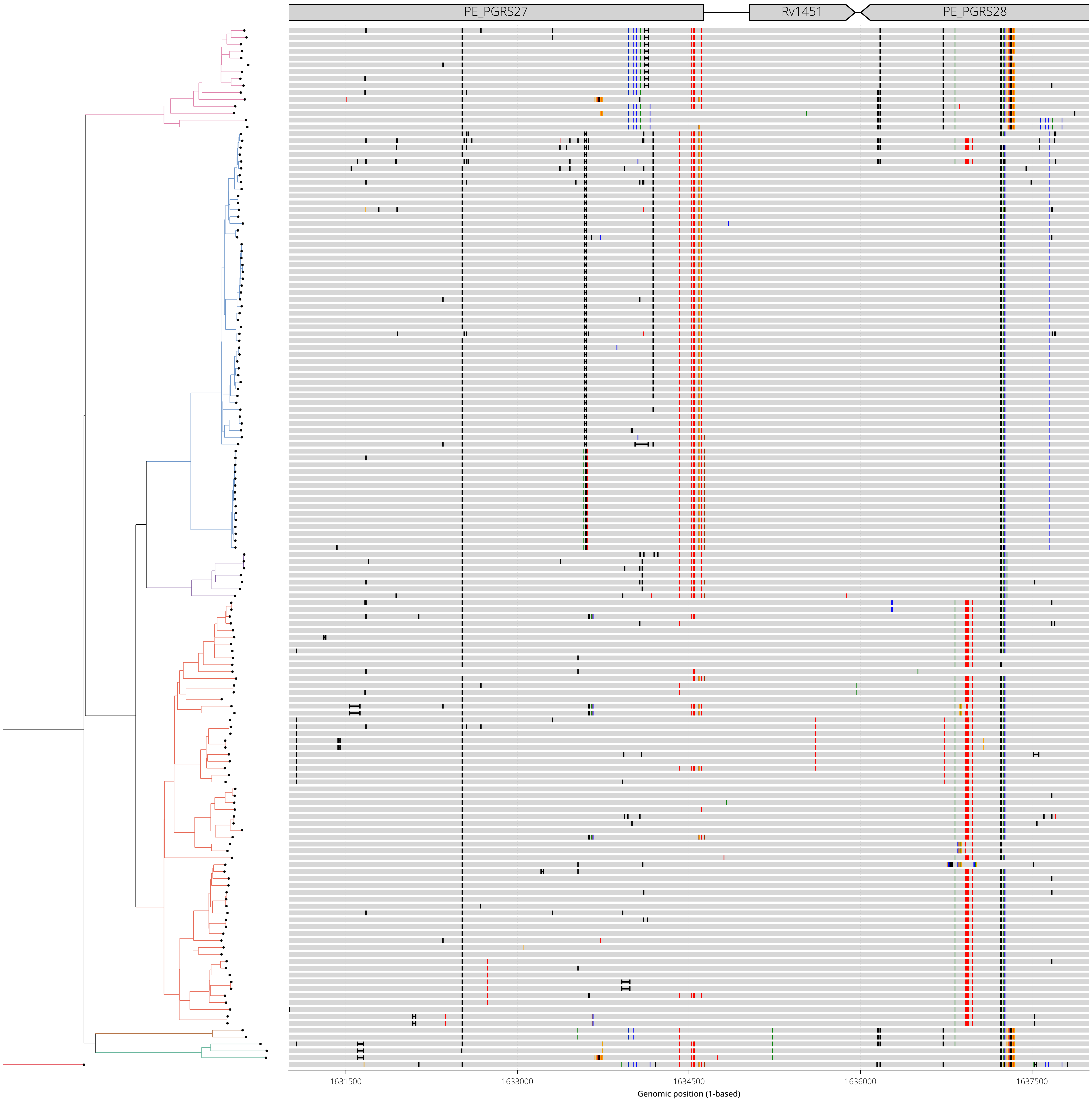

Diversity Hotspot View - 09:
Genomic range shown: NC_000962.3:1634000-1641000
Gene(s) of interest: PE_PGRS28

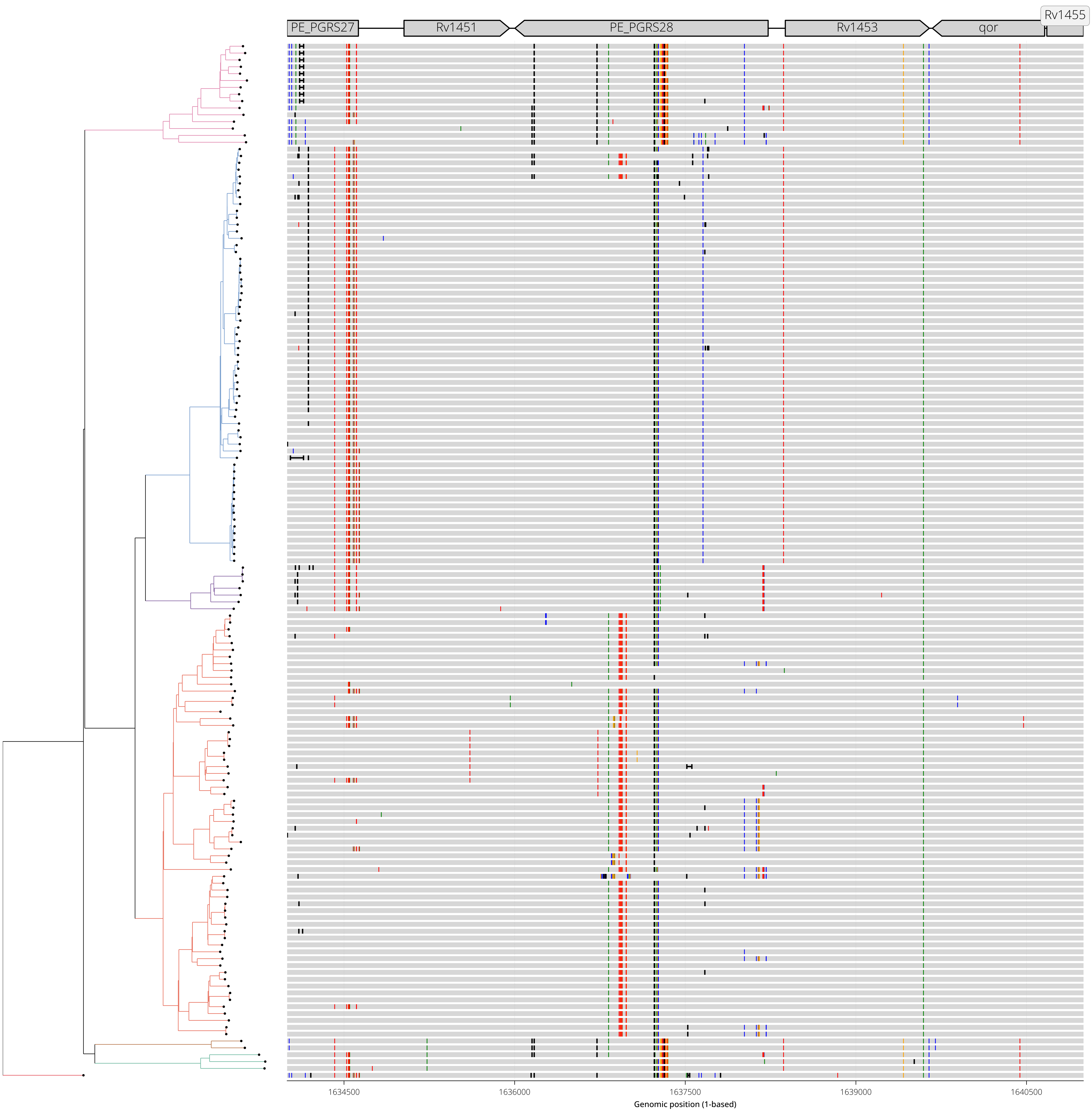

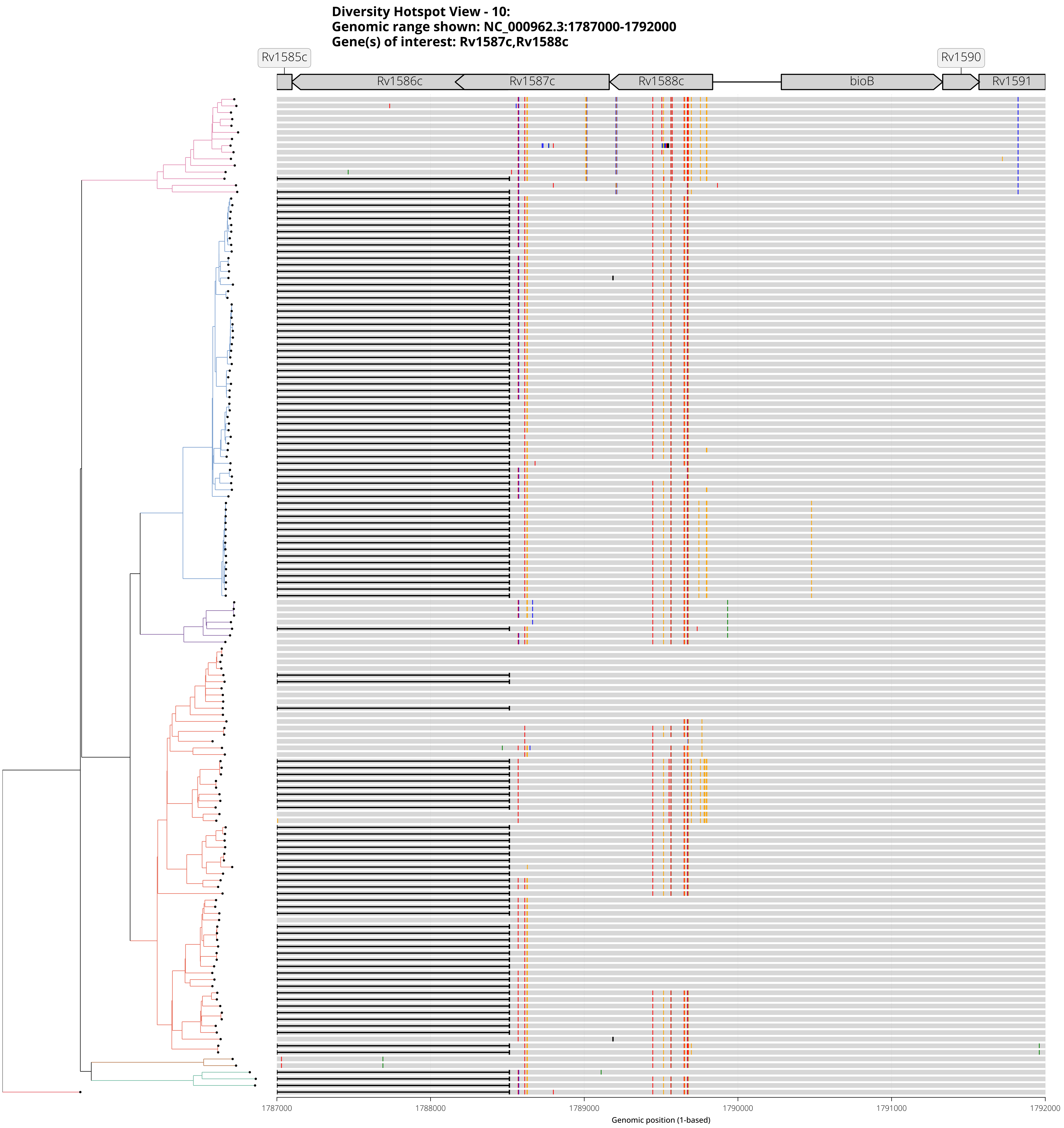

Diversity Hotspot View - 10:
Genomic range shown: NC_000962.3:1787000-1792000
Gene(s) of interest: Rv1587c,Rv1588c
Rv1585c
Rv1586c
Rv1587c
Rv1588c
bioB
Rv1590
Rv1591
1787000
1788000
1789000
1790000
1791000
1792000
Genomic position (1-based)

Diversity Hotspot View - 11:
Genomic range shown: NC_000962.3:2194000-2199000
Gene(s) of interest: Rv1945

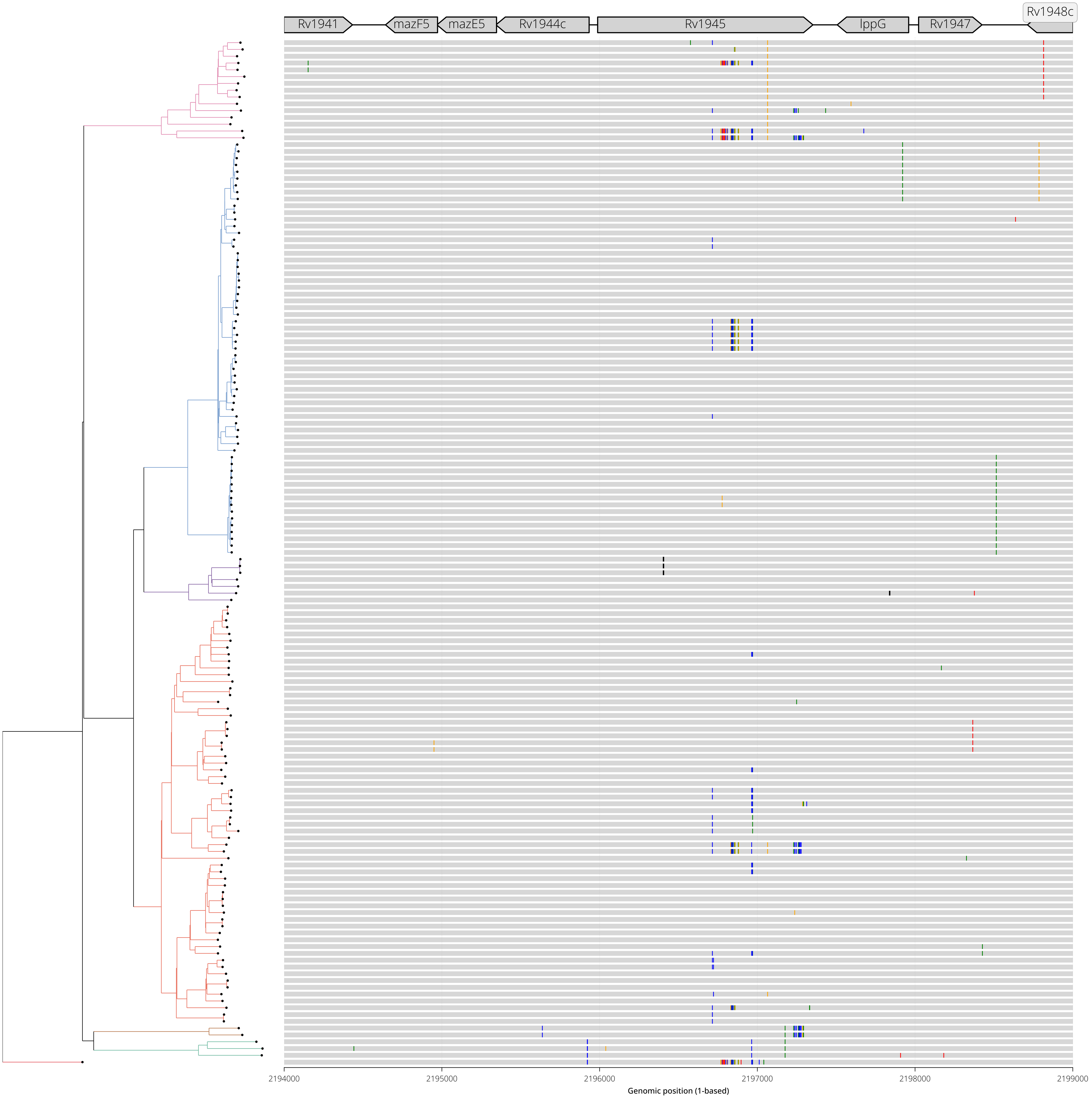

Diversity Hotspot View - 12:
Genomic range shown: NC_000962.3:2260000-2265000
Gene(s) of interest: Rv2015c

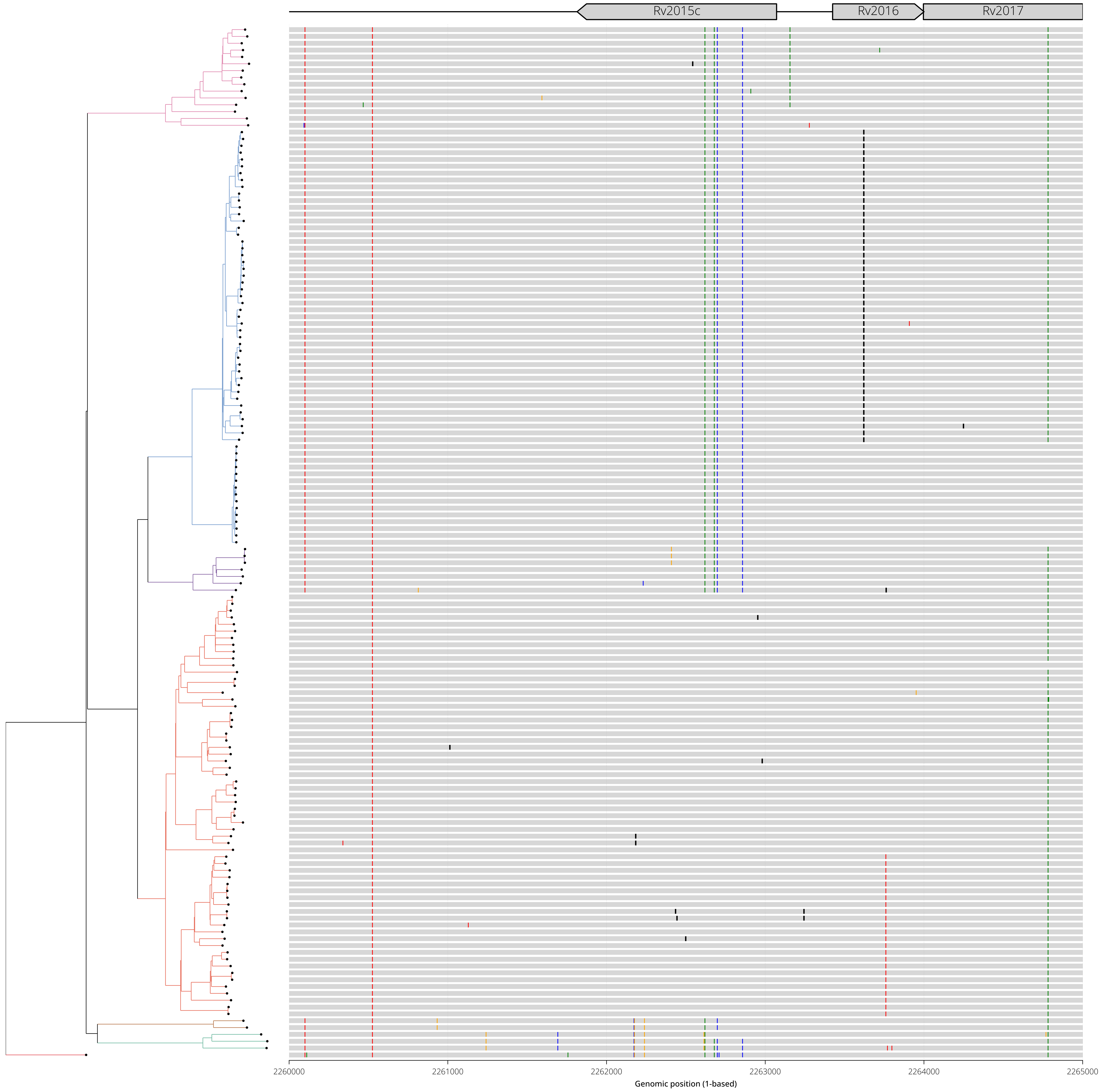

Diversity Hotspot View - 13:
Genomic range shown: NC_000962.3:2336000-2341000
Gene(s) of interest: Rv2081c,Rv2082

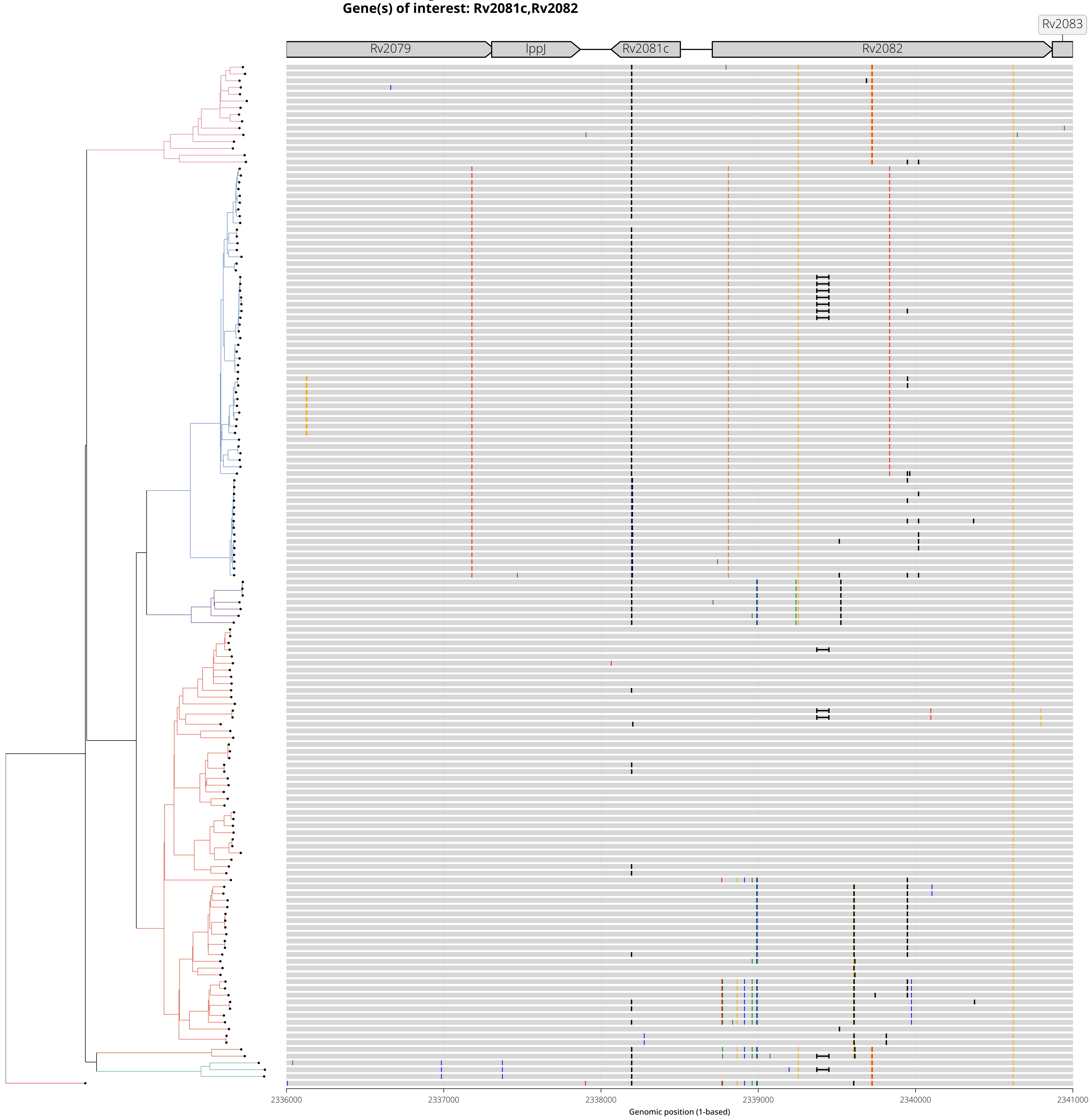

Diversity Hotspot View - 14:
Genomic range shown: NC_000962.3:2624000-2629000
Gene(s) of interest: esxO,esxP,Rv2348c

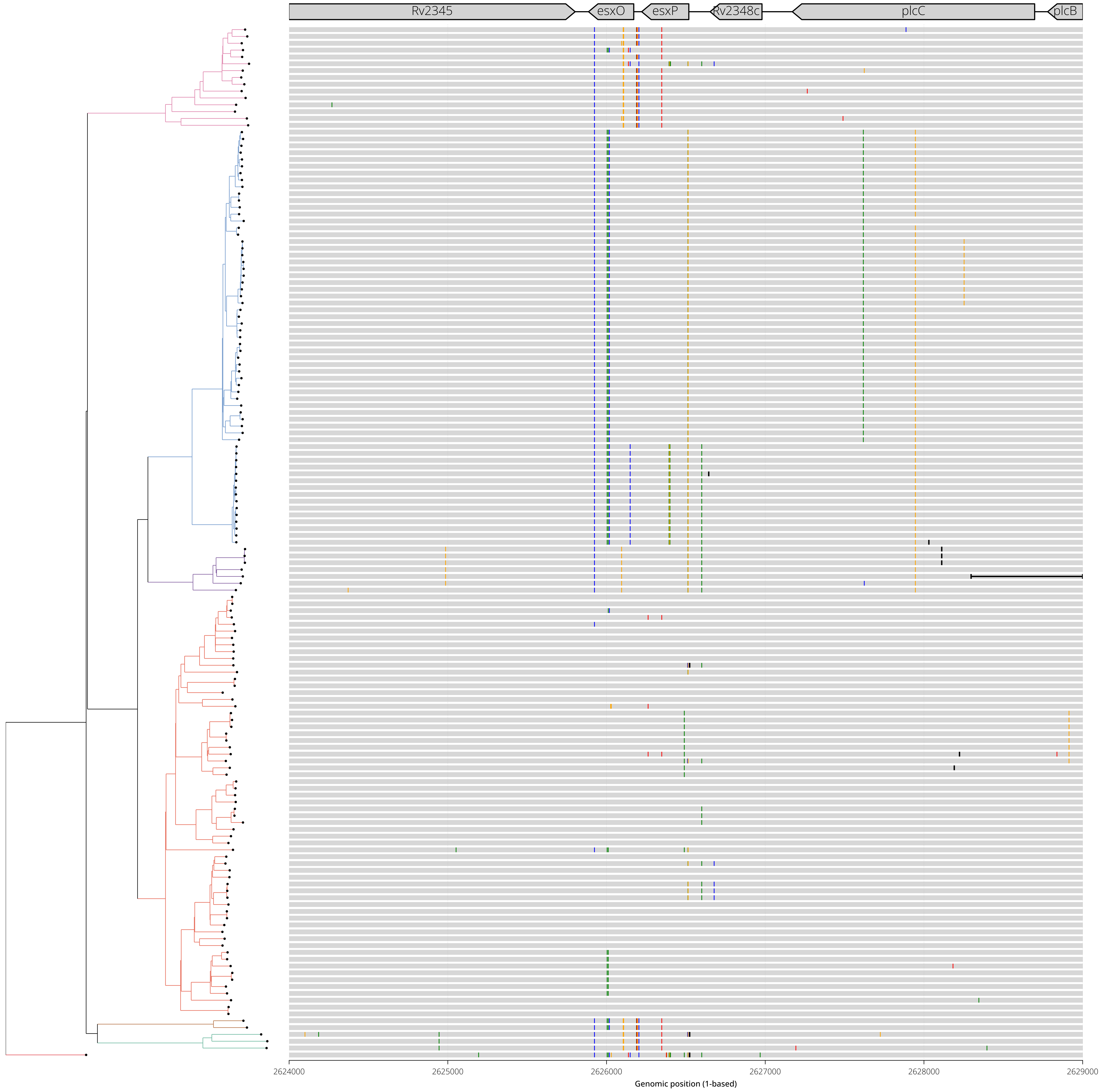

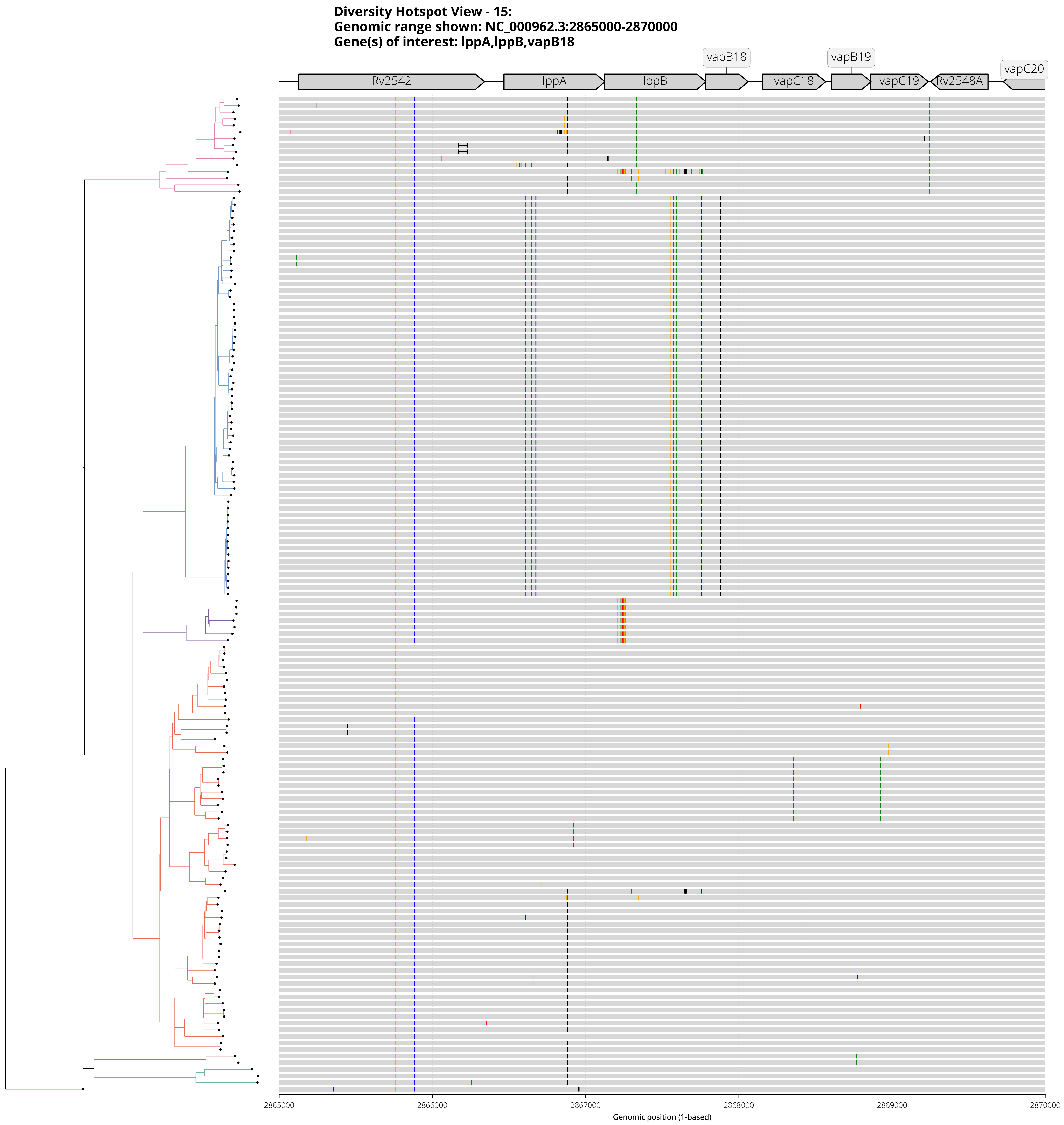

Diversity Hotspot View - 15:
Genomic range shown: NC_000962.3:2865000-2870000
Gene(s) of interest: lppA,lppB,vapB18
vapB18
vapB19
vapC20
Rv2542
lppA
lppB
vapC18
vapC19
Rv2548A
2865000
2866000
2867000
2868000
2869000
2870000
Genomic position (1-based)

Diversity Hotspot View - 16:
Genomic range shown: NC_000962.3:2942000-2947000
Gene(s) of interest: PE_PGRS45

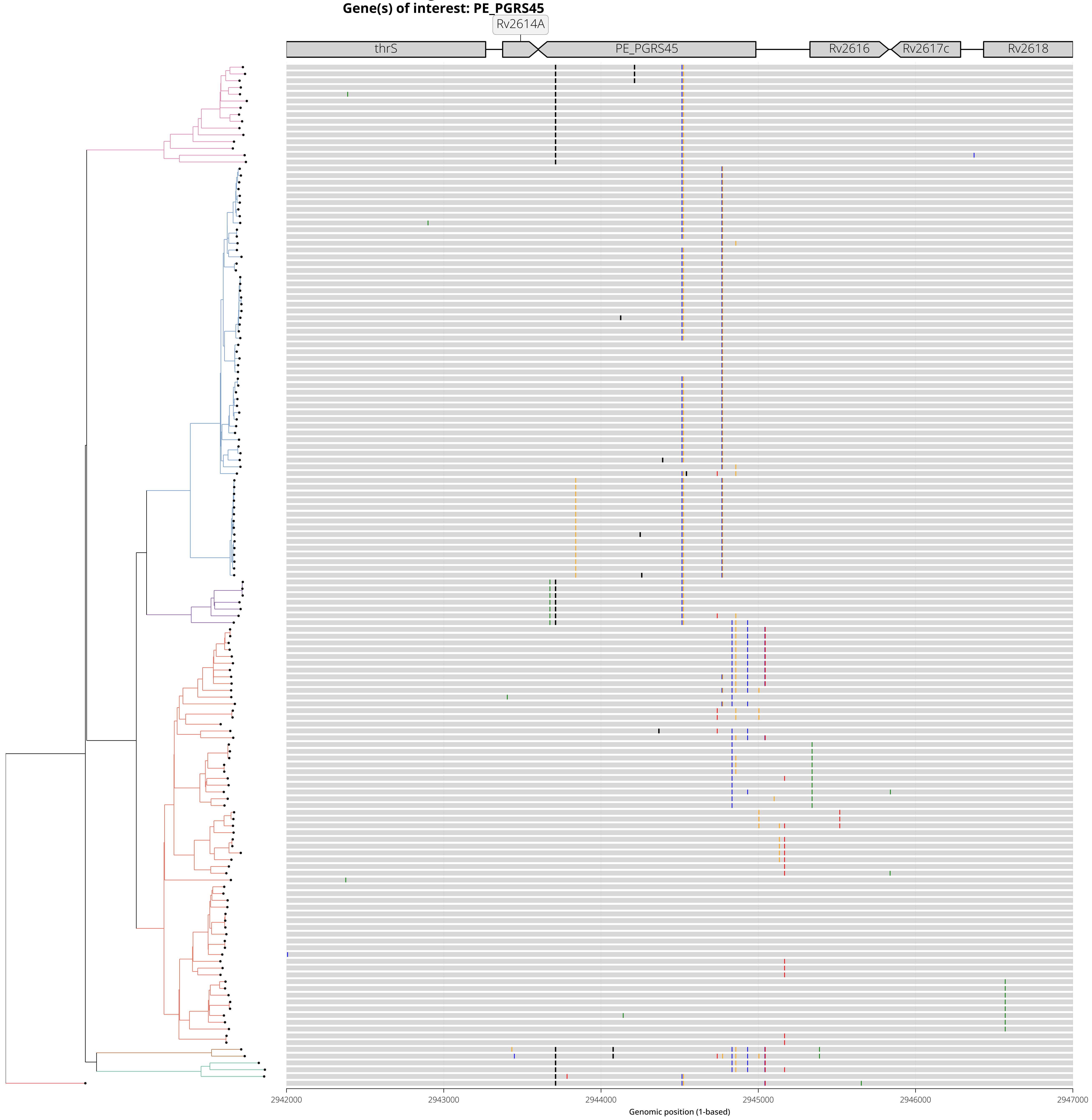

Diversity Hotspot View - 17:
Genomic range shown: NC_000962.3:3133000-3138000
Gene(s) of interest: Rv2827c,Rv2828c

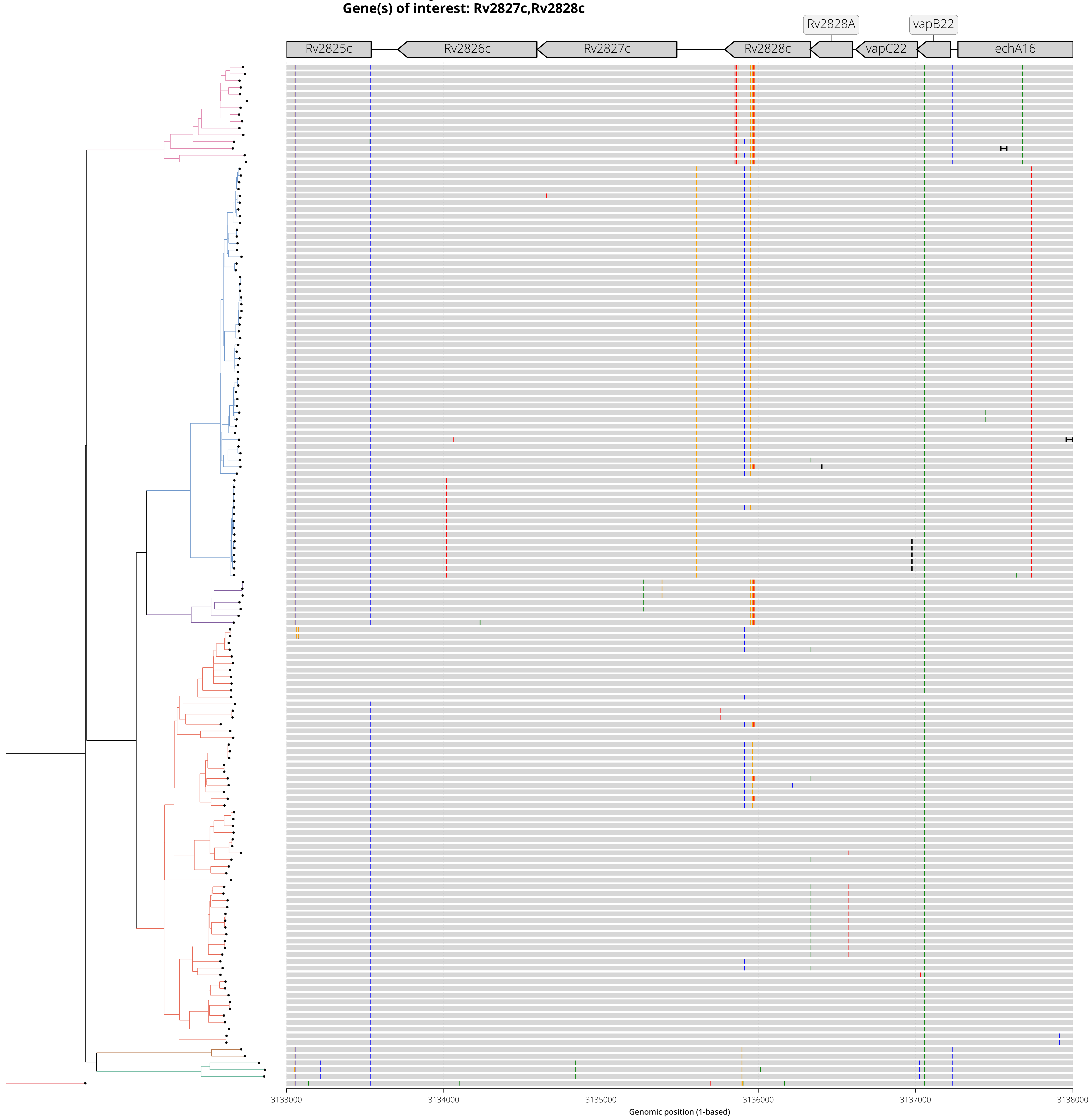

Diversity Hotspot View - 18:
Genomic range shown: NC_000962.3:3728000-3737000
Gene(s) of interest: PPE54

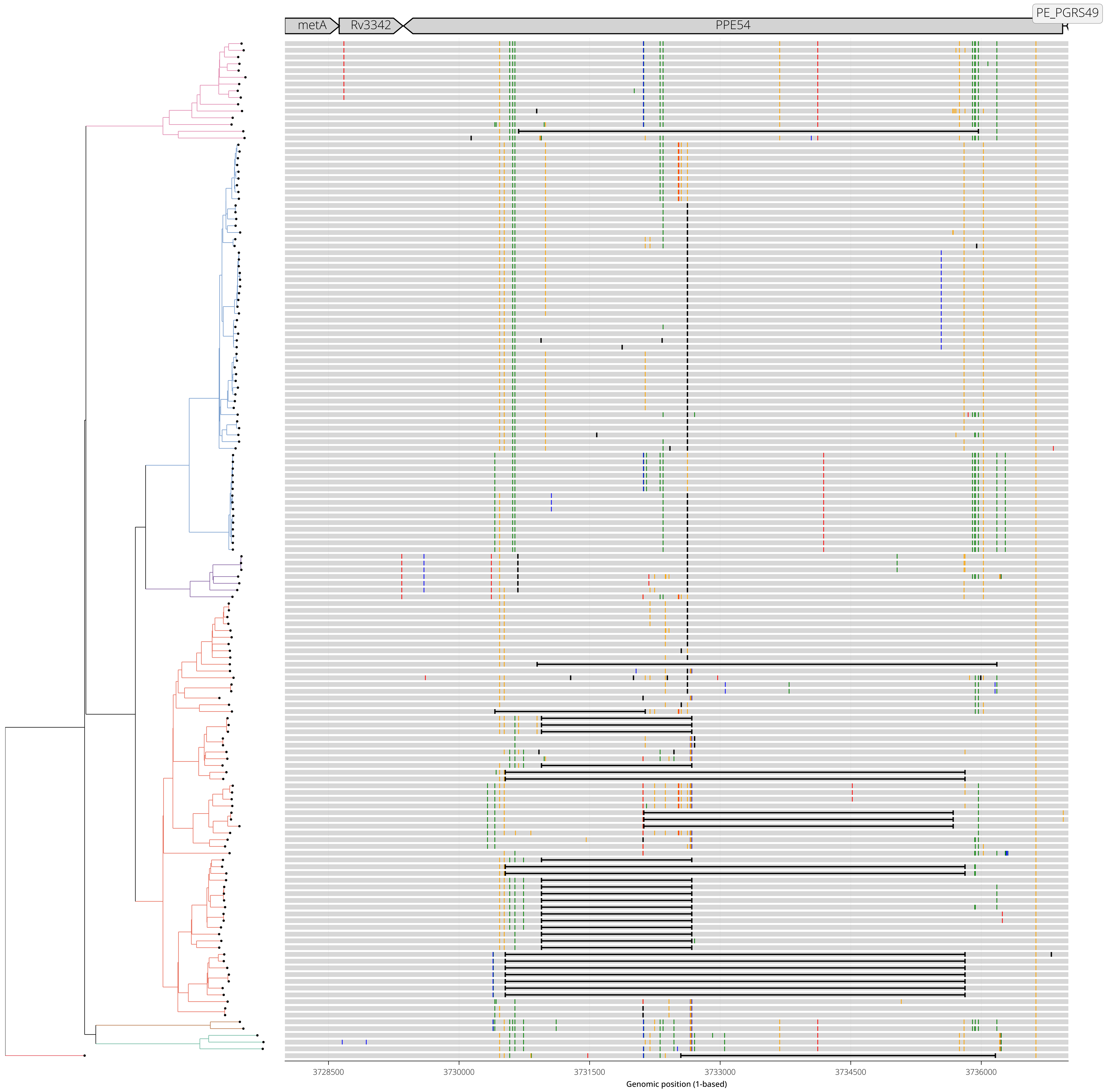

Diversity Hotspot View - 19:
Genomic range shown: NC_000962.3:3746000-3755000
Gene(s) of interest: PPE55

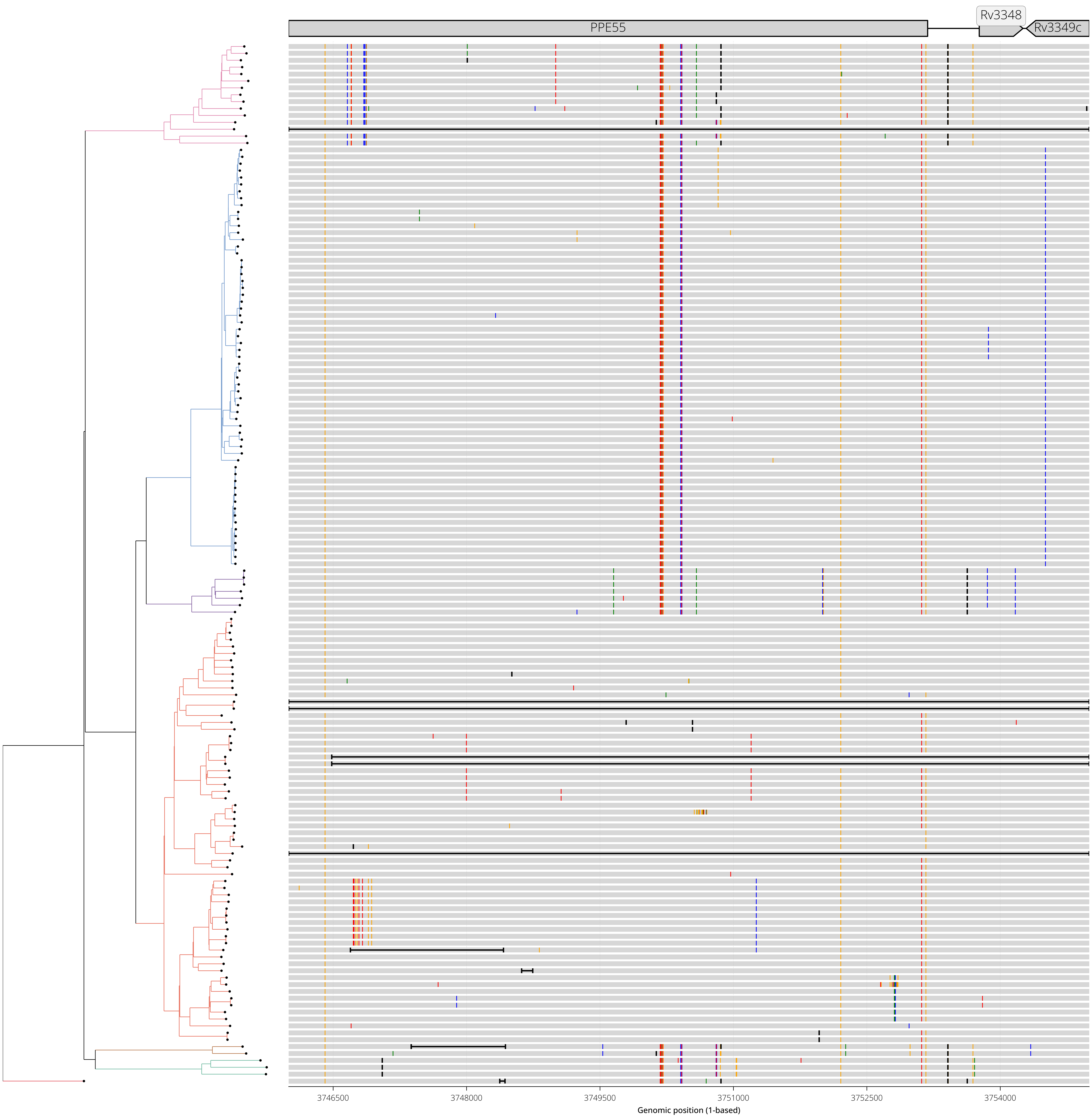

Diversity Hotspot View - 20:
Genomic range shown: NC_000962.3:3841000-3844000
Gene(s) of interest: Rv3424c,PPE57

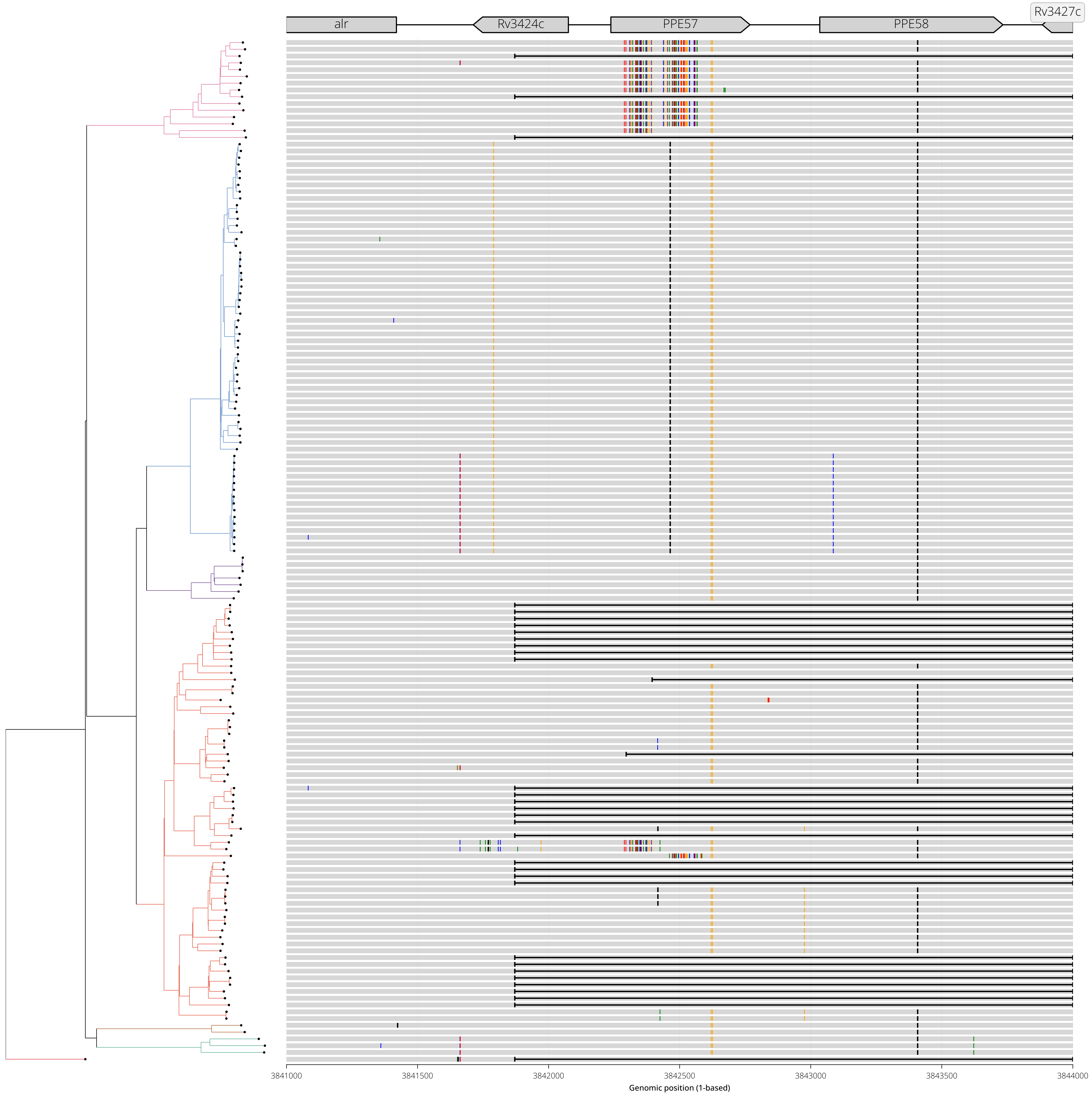

Diversity Hotspot View - 21:
Genomic range shown: NC_000962.3:3845000-3850000
Gene(s) of interest: PPE59,Rv3430c

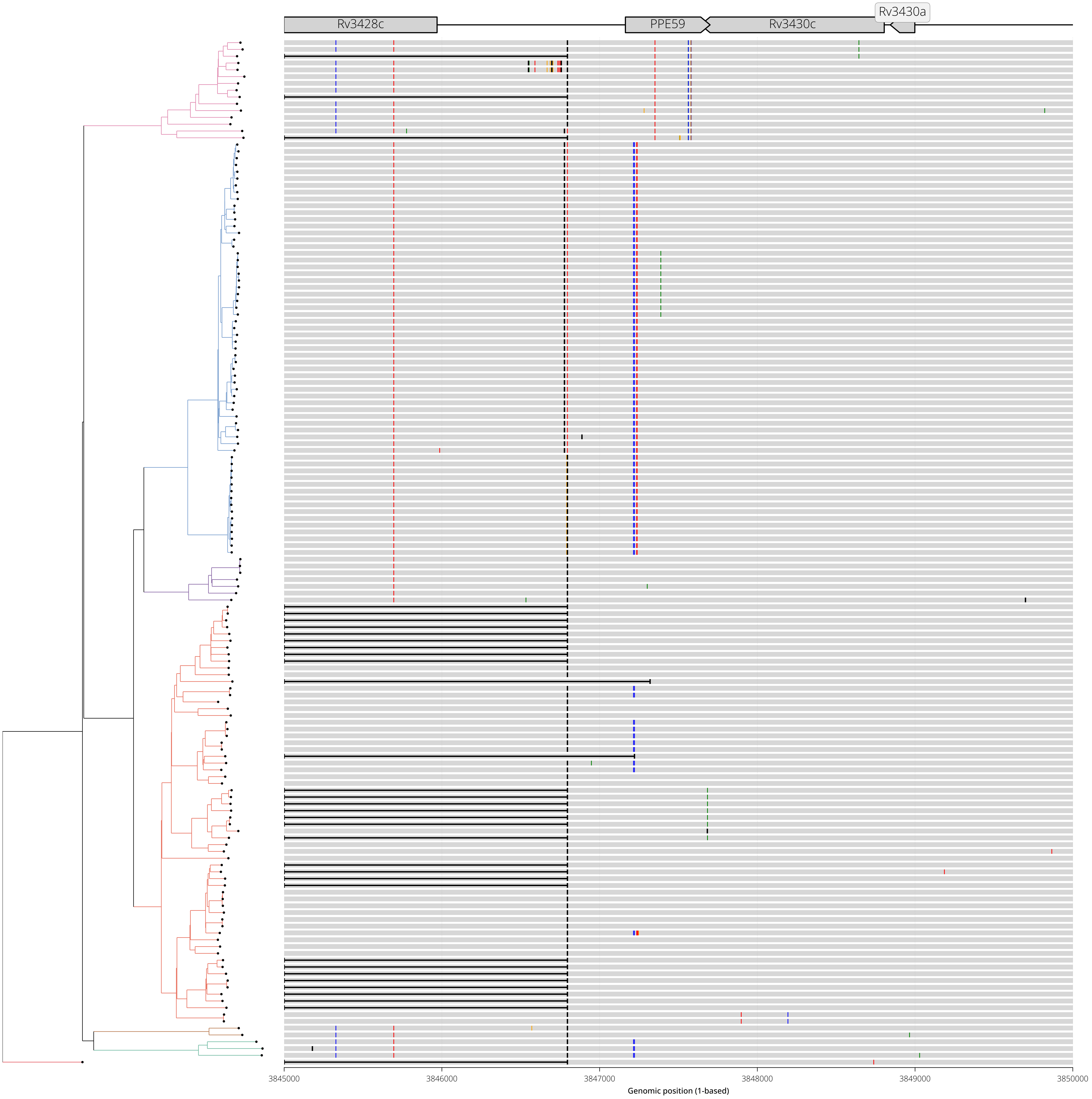

Diversity Hotspot View - 22:
Genomic range shown: NC_000962.3:3881000-3886000
Gene(s) of interest: rmlC,Rv3466,Rv3467

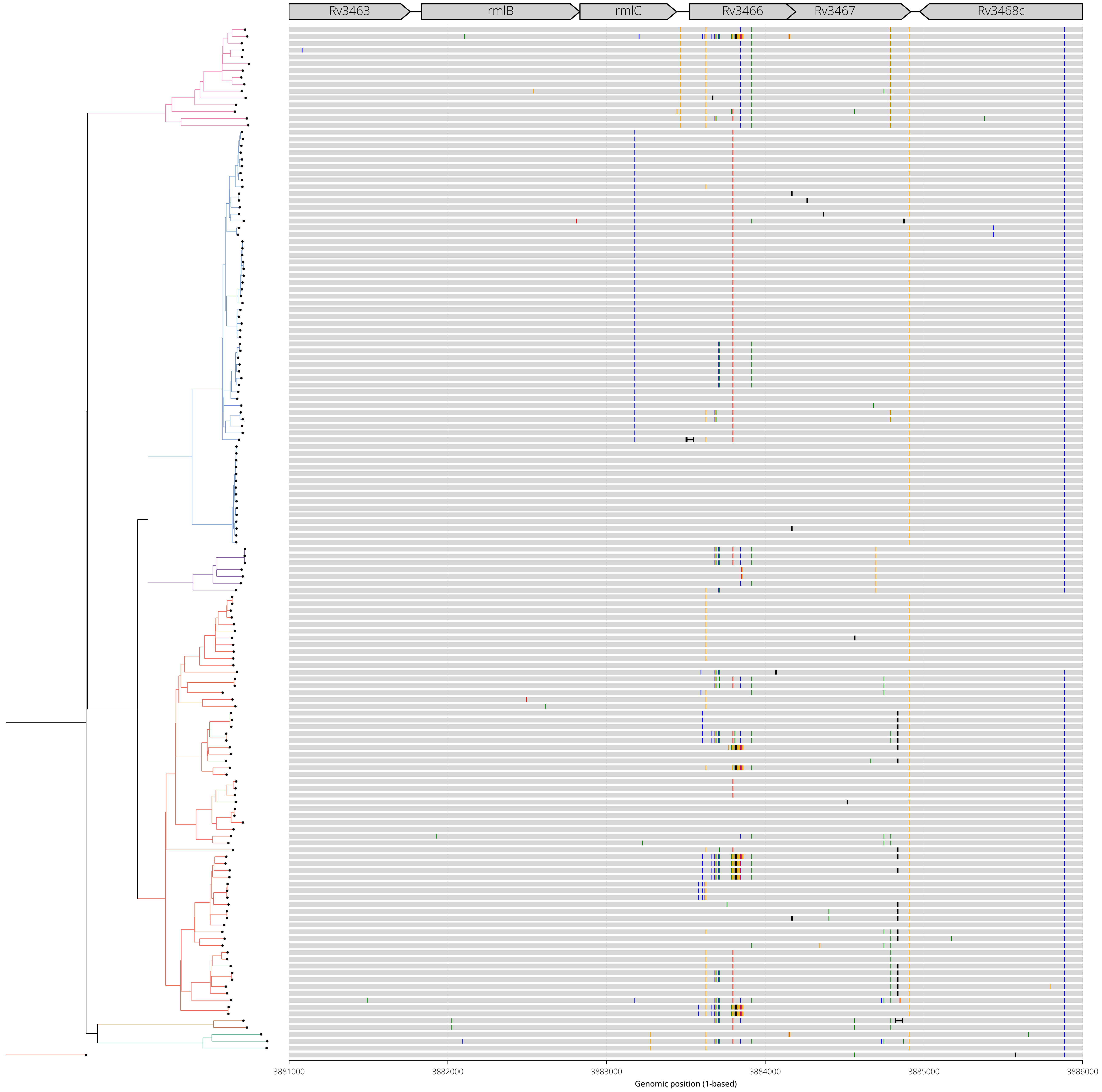

Diversity Hotspot View - 23:
Genomic range shown: NC_000962.3:3893000-3898000
Gene(s) of interest: PPE60,Rv3479

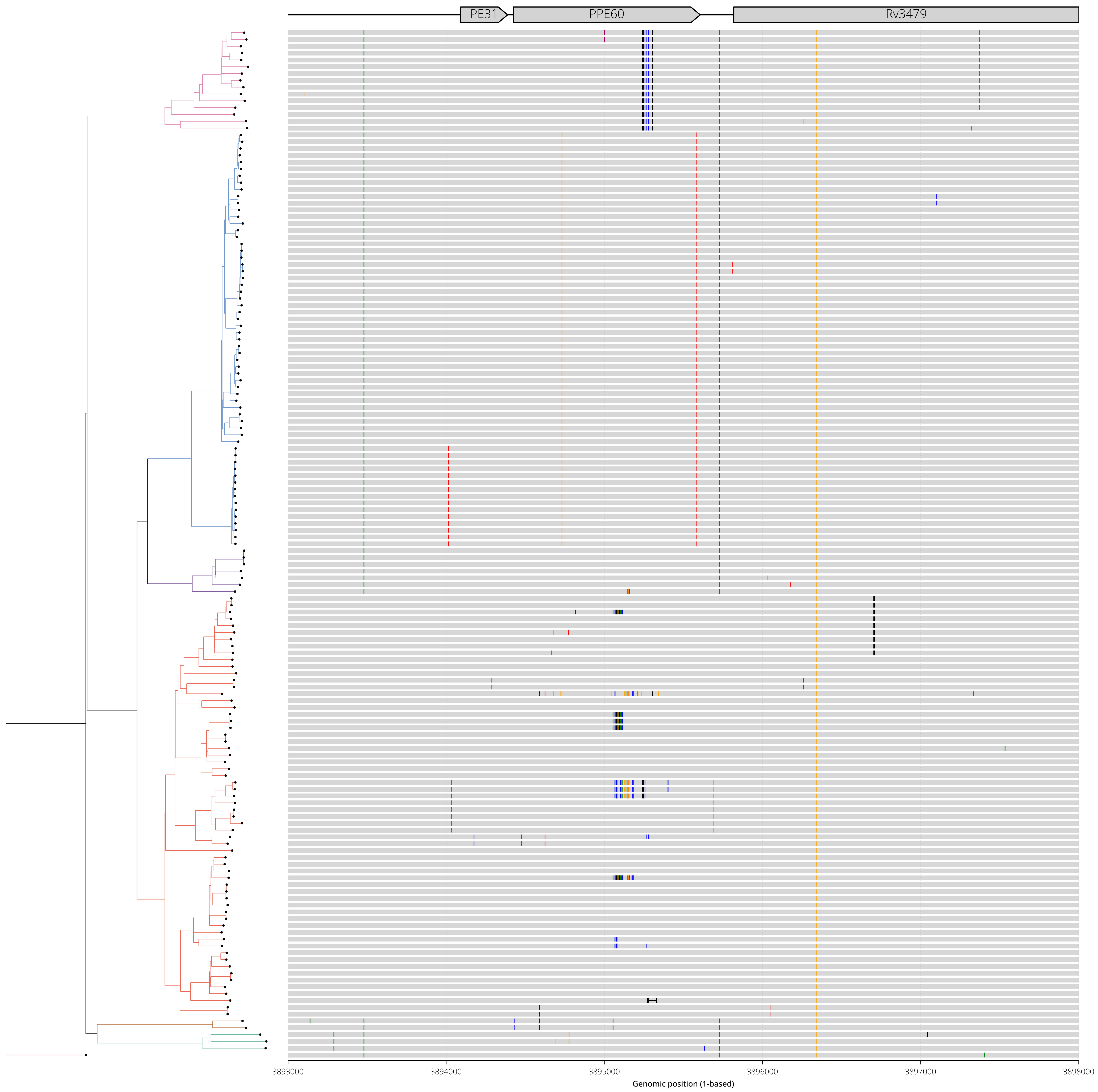

Diversity Hotspot View - 24:
Genomic range shown: NC_000962.3:3931000-3938000
Gene(s) of interest: PE_PGRS54

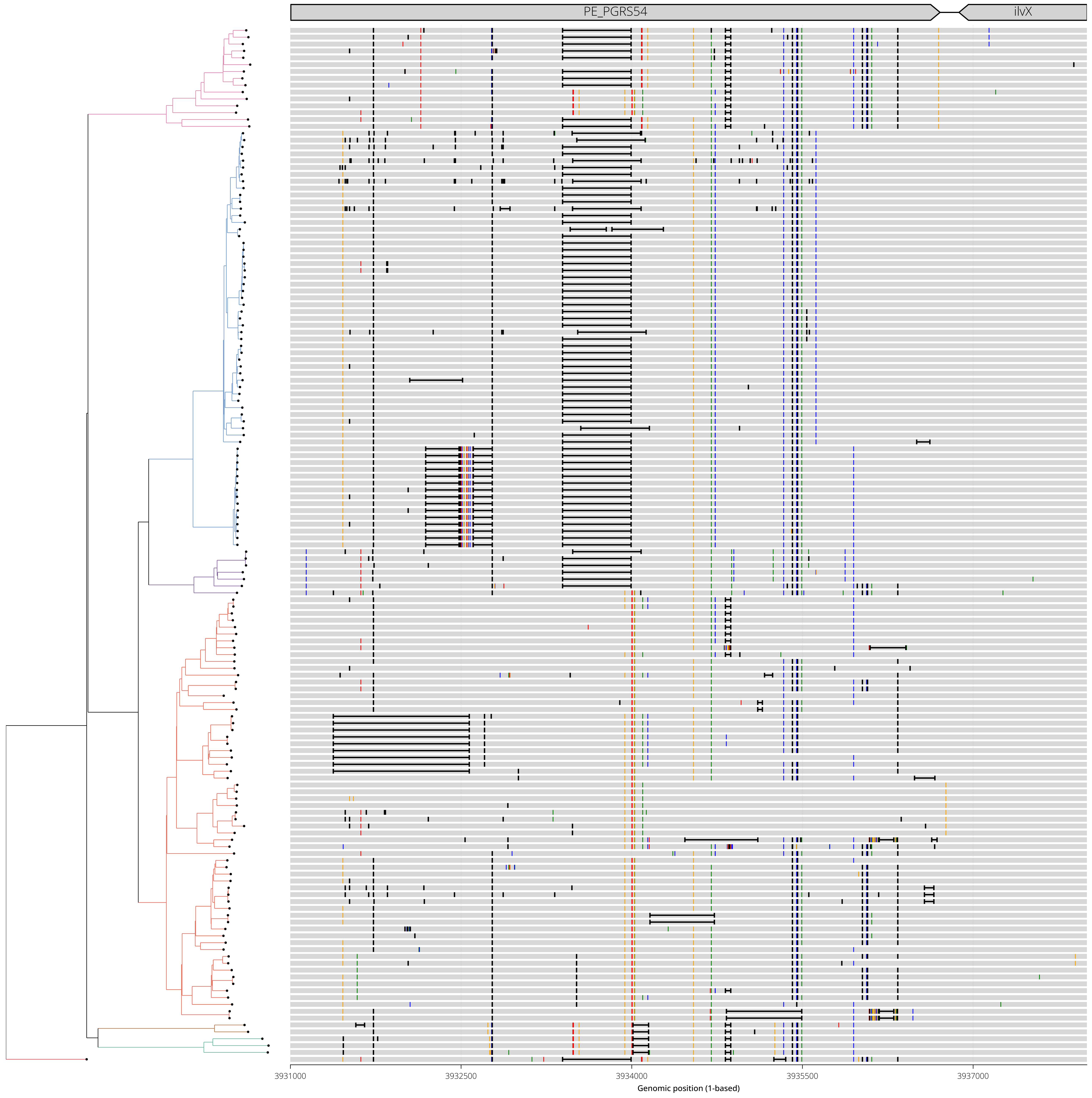

Diversity Hotspot View - 25:
Genomic range shown: NC_000962.3:3940000-3947000
Gene(s) of interest: PE_PGRS56

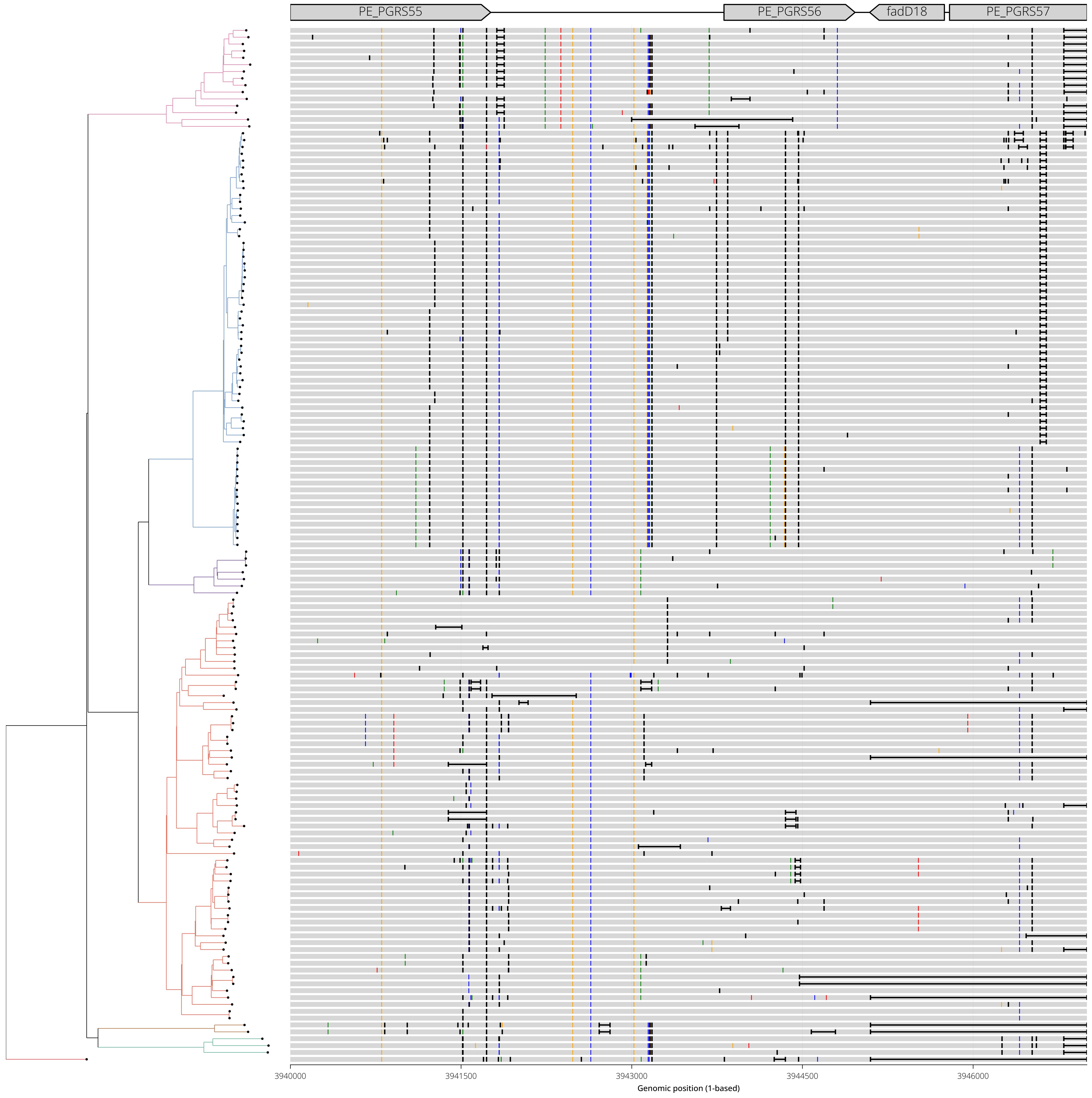

Diversity Hotspot View - 26:
Genomic range shown: NC_000962.3:3944000-3951000
Gene(s) of interest: PE_PGRS57

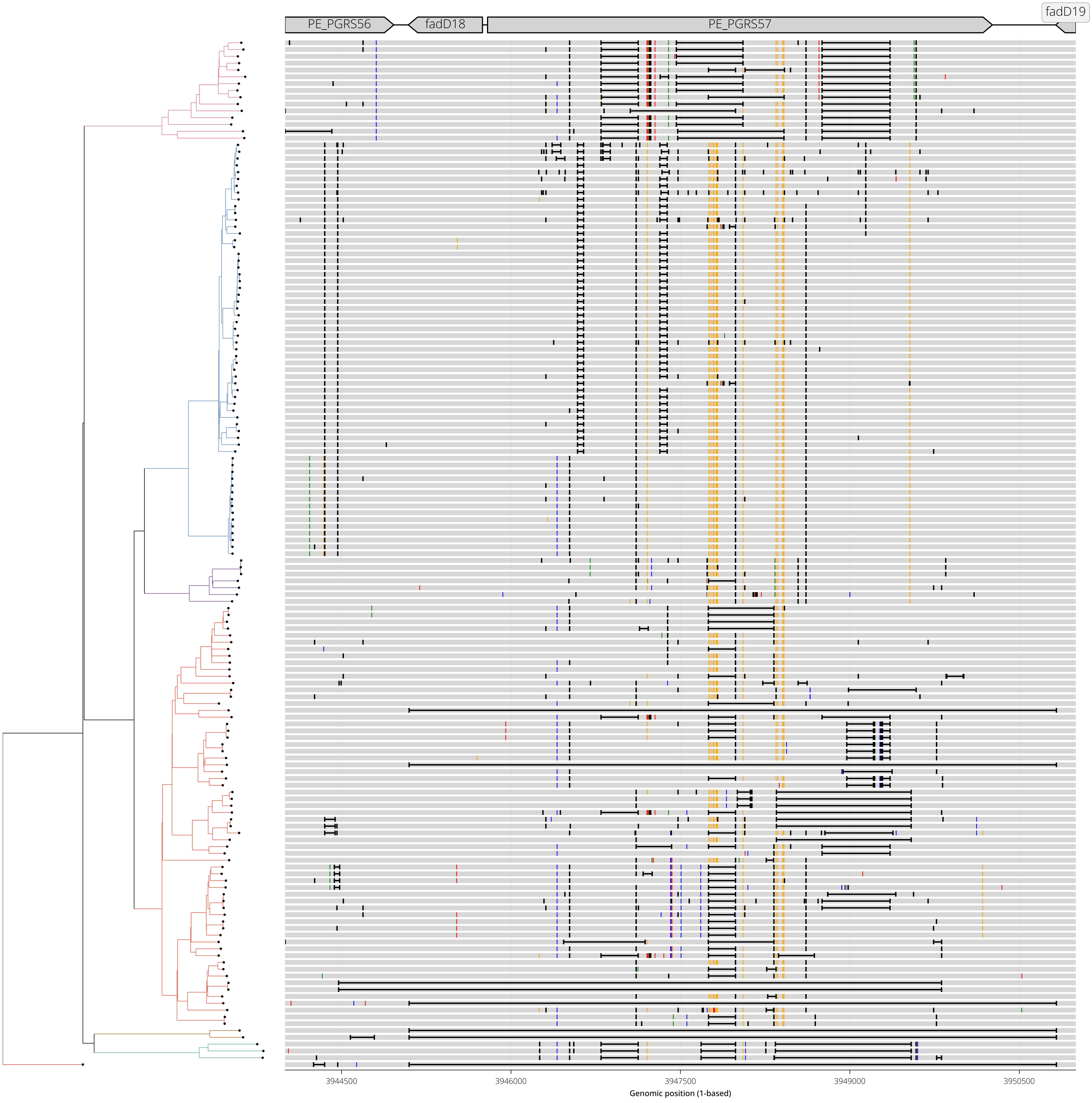

Diversity Hotspot View - 27:
Genomic range shown: NC_000962.3:4252000-4257000
Gene(s) of interest: Rv3798,accD4

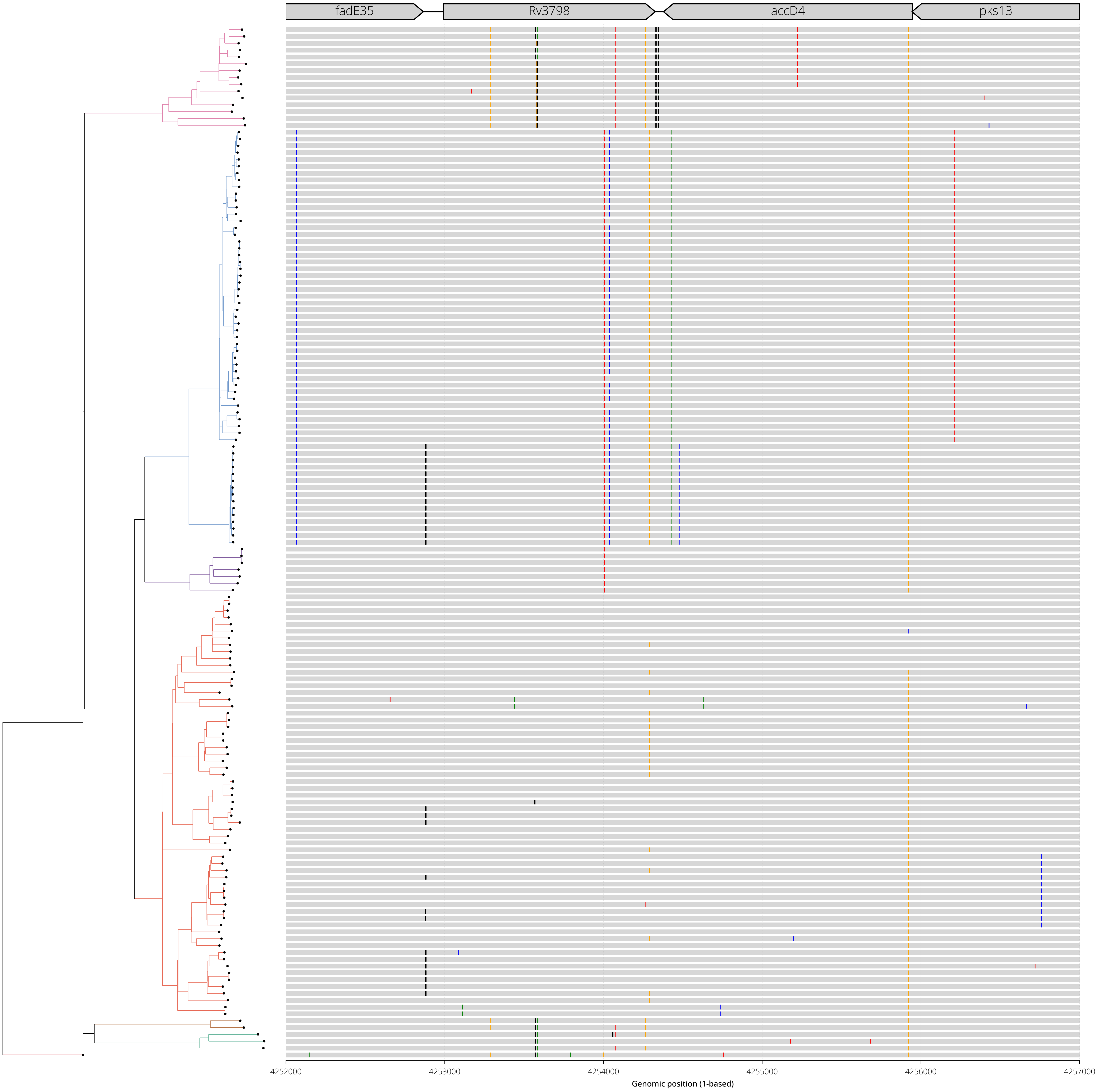

Supplement: Supplement 12 [file media-12.pdf]
