# Supplementary material for: Gene conversion is a key driver of diversity hotspots in M. tuberculosis antigens and virulence-associated loci
Source: bioRxiv. 2026 Mar 11:2026.02.26.708061. Preprint. [Version 2] doi: 10.64898/2026.02.26.708061 (PMC13061034; doi:10.64898/2026.02.26.708061)

RegionID: PR_HmRegion_002 | Paralog Network ID: PR_Set_3
Genes: Rv0094c,Rv0095c | NC_000962.3:102905-105930
Mapped GCEs: 19 | Putative GCEs: 31

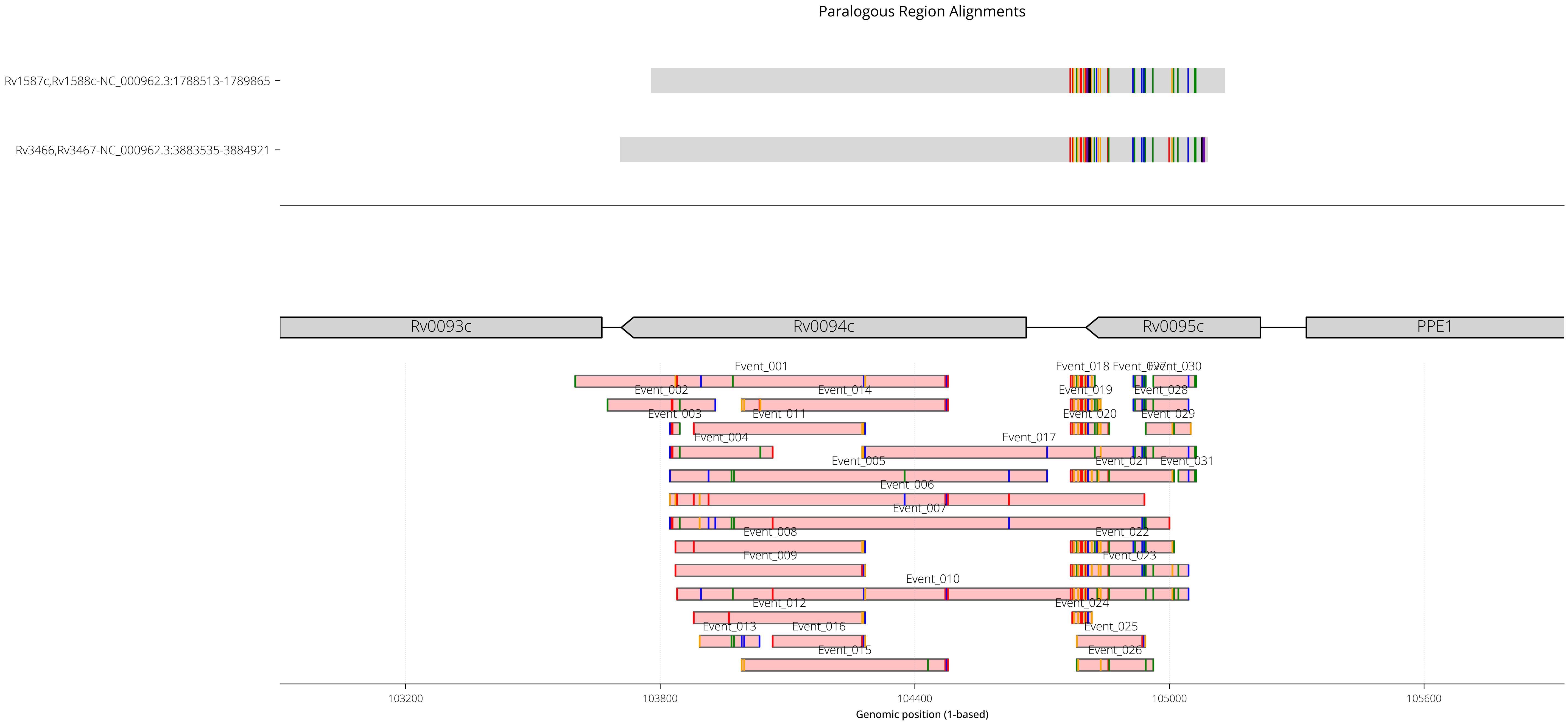

RegionID: PR_HmRegion_002 | Paralog Network ID: PR_Set_3
Genes: Rv0094c,Rv0095c | NC_000962.3:102905-105930
Mapped GCEs: 19 | Putative GCEs: 31

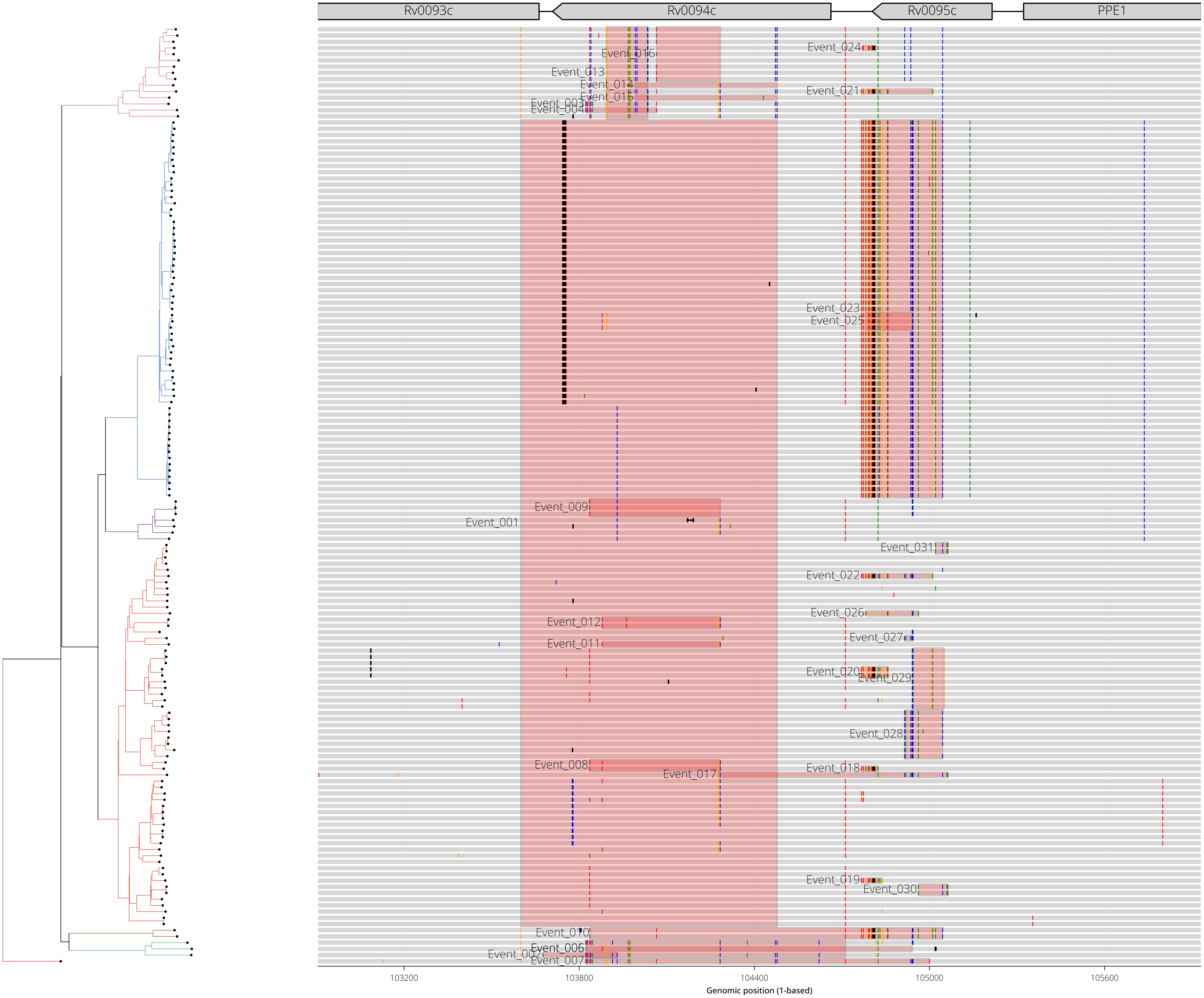

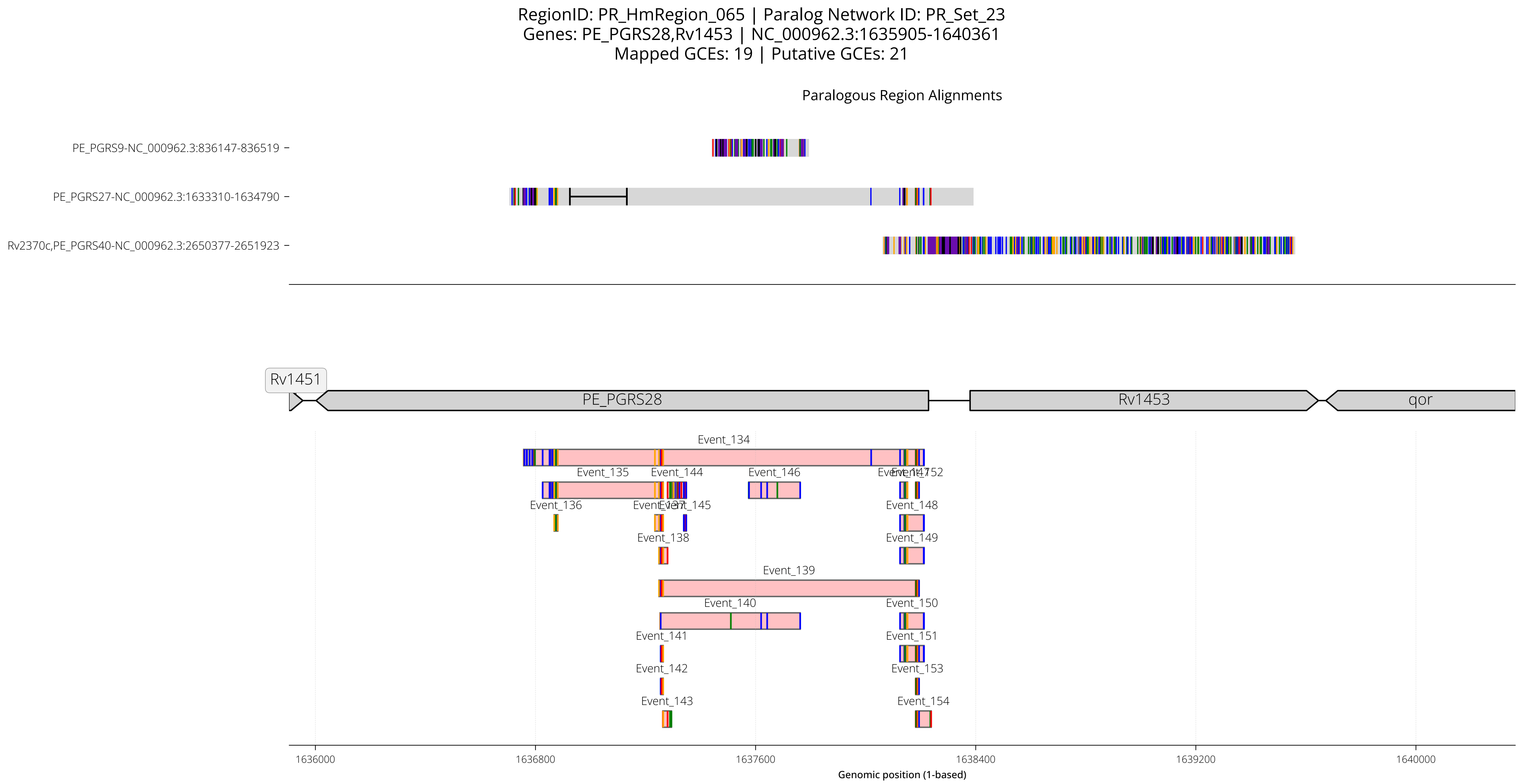
RegionID: PR_HmRegion_065 | Paralog Network ID: PR_Set_23
Genes: PE_PGRS28,Rv1453 | NC_000962.3:1635905-1640361
Mapped GCEs: 19 | Putative GCEs: 21
Paralogous Region Alignments
PE_PGRS9-NC_000962.3:836147-836519
PE_PGRS27-NC_000962.3:1633310-1634790
Rv2370c,PE_PGRS40-NC_000962.3:2650377-2651923
Rv1451
PE_PGRS28
Rv1453
qor
Event_134
Event_135
Event_144
Event_146
Event_136
Event_145
Event_148
Event_138
Event_149
Event_139
Event_140
Event_150
Event_141
Event_151
Event_142
Event_153
Event_143
Event_154
1636000
1636800
1637600
1638400
1639200
1640000
Genomic position (1-based)

RegionID: PR_HmRegion_065 | Paralog Network ID: PR_Set_23
Genes: PE_PGRS28,Rv1453 | NC_000962.3:1635905-1640361
Mapped GCEs: 19 | Putative GCEs: 21

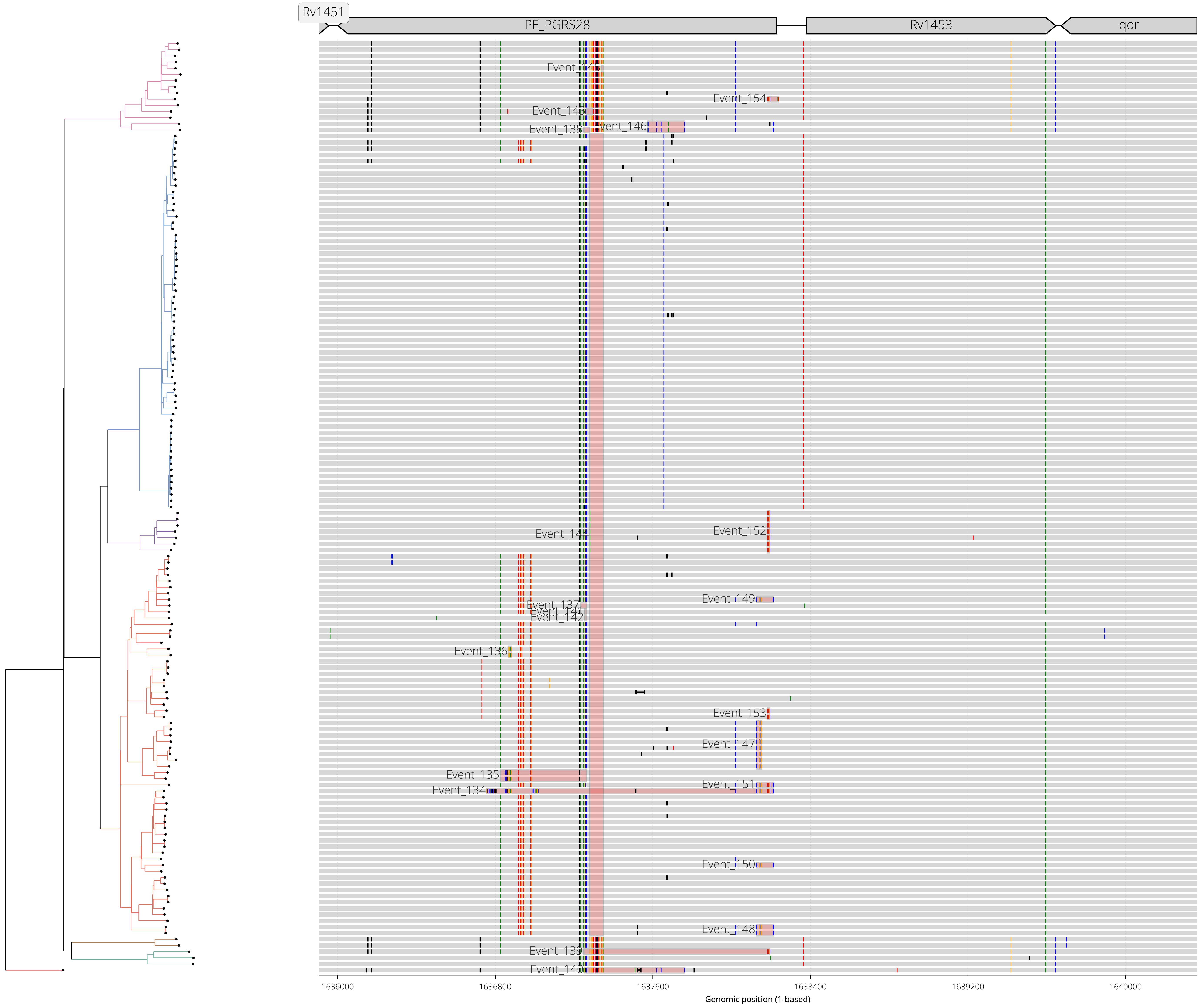

RegionID: PR_HmRegion_064 | Paralog Network ID: PR_Set_23
Genes: PE_PGRS27 | NC_000962.3:1632510-1635590
Mapped GCEs: 14 | Putative GCEs: 19

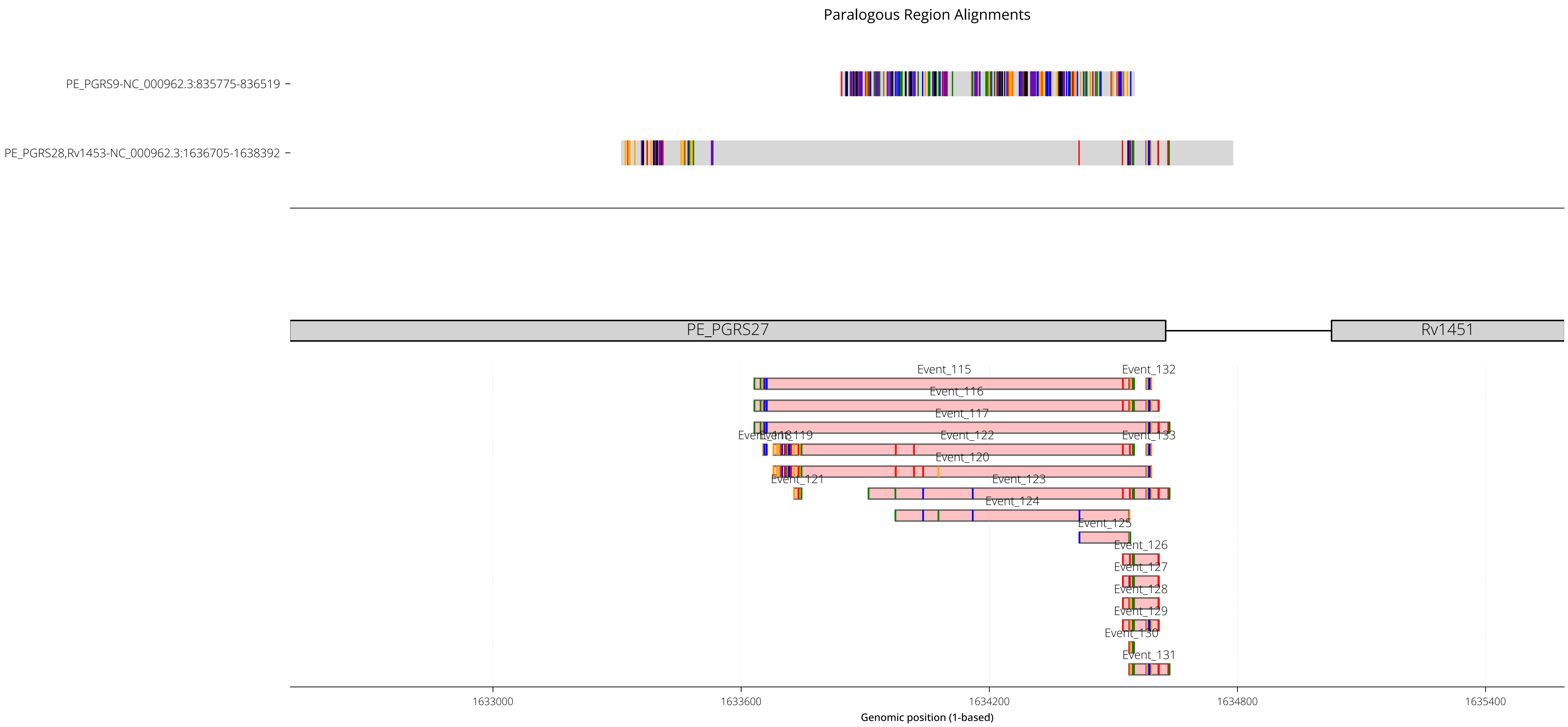

RegionID: PR_HmRegion_064 | Paralog Network ID: PR_Set_23
Genes: PE_PGRS27 | NC_000962.3:1632510-1635590
Mapped GCEs: 14 | Putative GCEs: 19

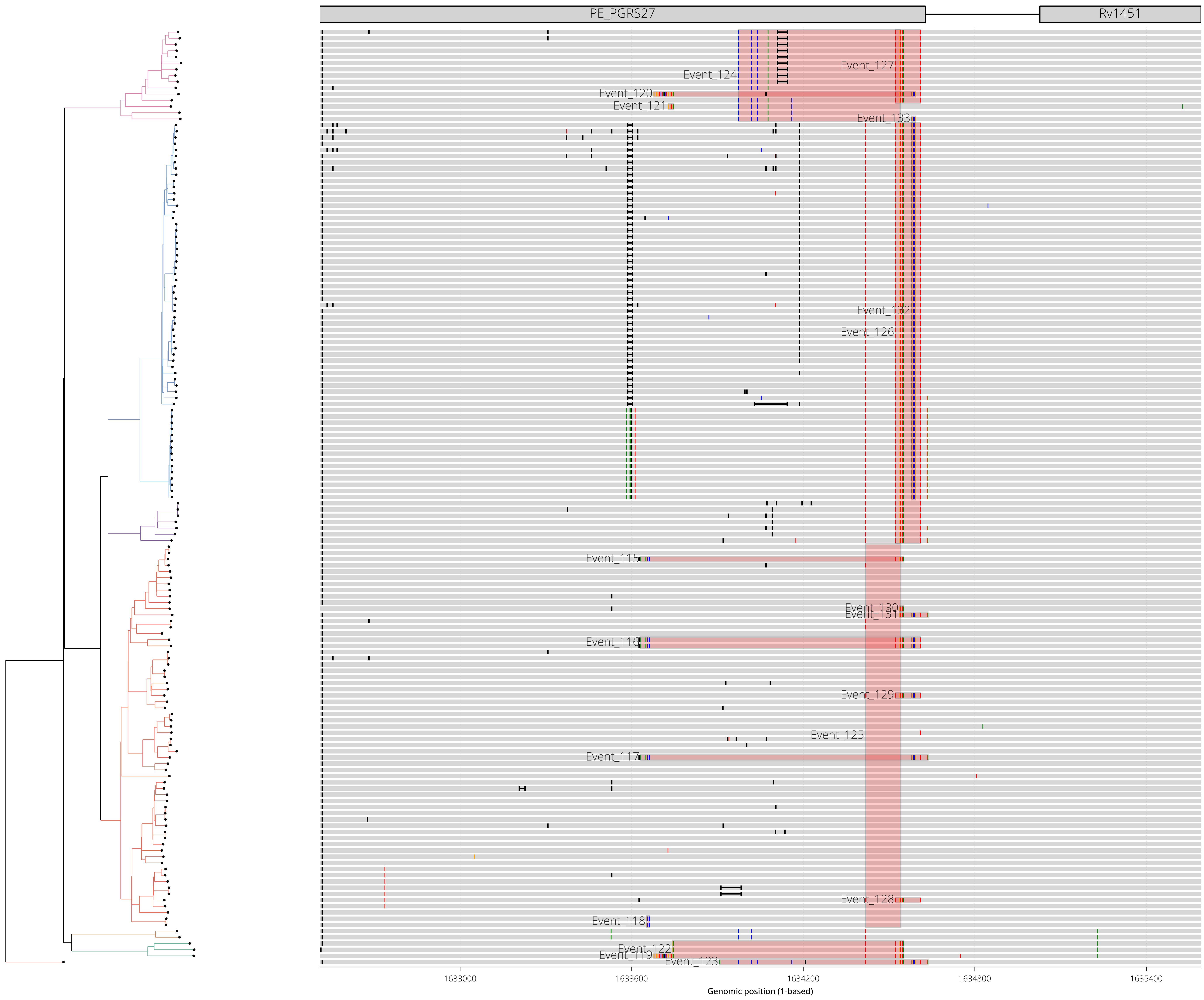

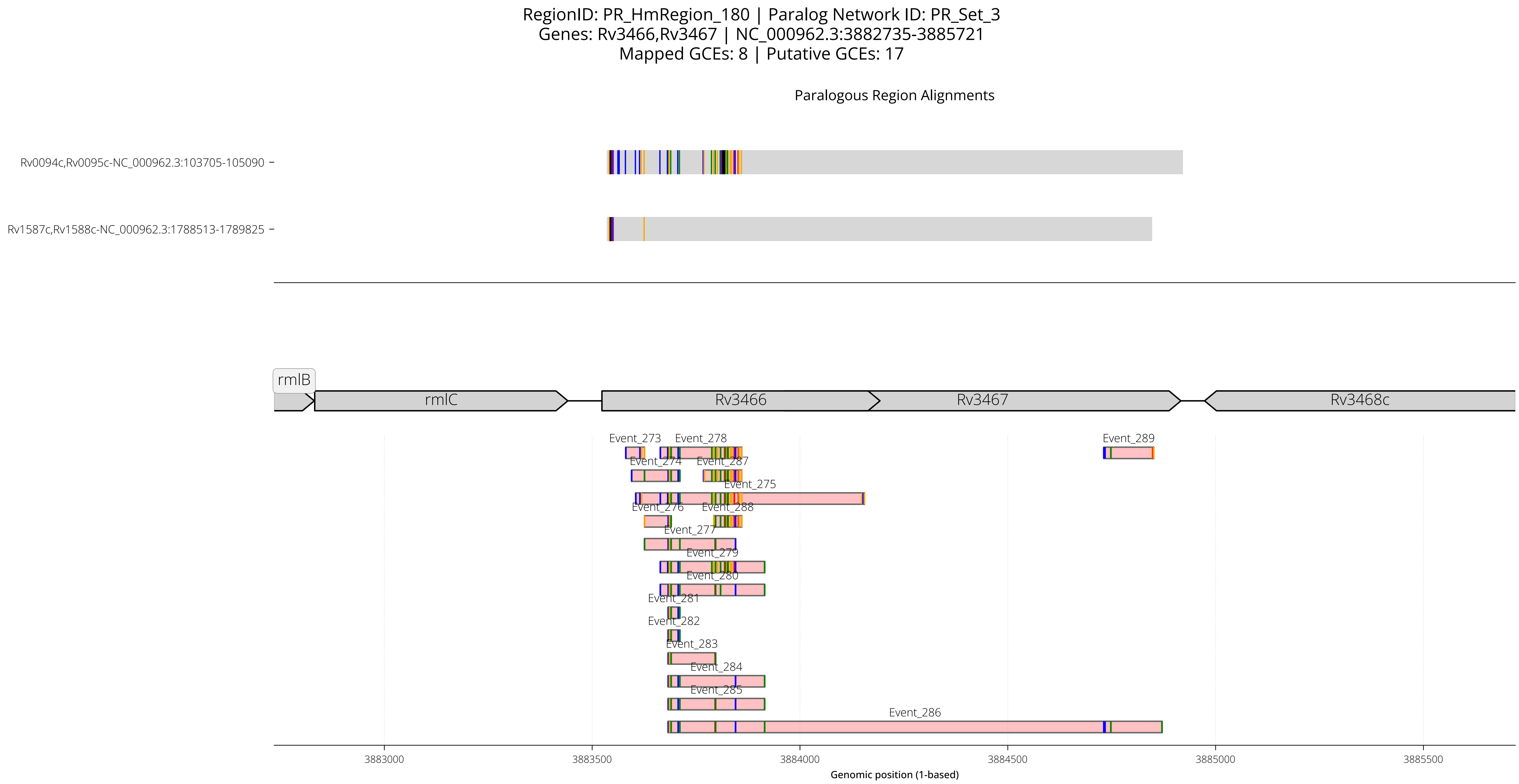

RegionID: PR_HmRegion_180 | Paralog Network ID: PR_Set_3
Genes: Rv3466,Rv3467 | NC_000962.3:3882735-3885721
Mapped GCEs: 8 | Putative GCEs: 17
Paralogous Region Alignments
Rv0094c,Rv0095c-NC_000962.3:103705-105090
Rv1587c,Rv1588c-NC_000962.3:1788513-1789825
rmlB
rmlC
Rv3466
Rv3467
Rv3468c
Event_273
Event_278
Event_289
Event_274
Event_287
Event_275
Event_276
Event_288
Event_277
Event_279
Event_280
Event_281
Event_282
Event_283
Event_284
Event_285
Event_286
3883000
3883500
3884000
3884500
3885000
3885500
Genomic position (1-based)

RegionID: PR_HmRegion_180 | Paralog Network ID: PR_Set_3
Genes: Rv3466,Rv3467 | NC_000962.3:3882735-3885721
Mapped GCEs: 8 | Putative GCEs: 17

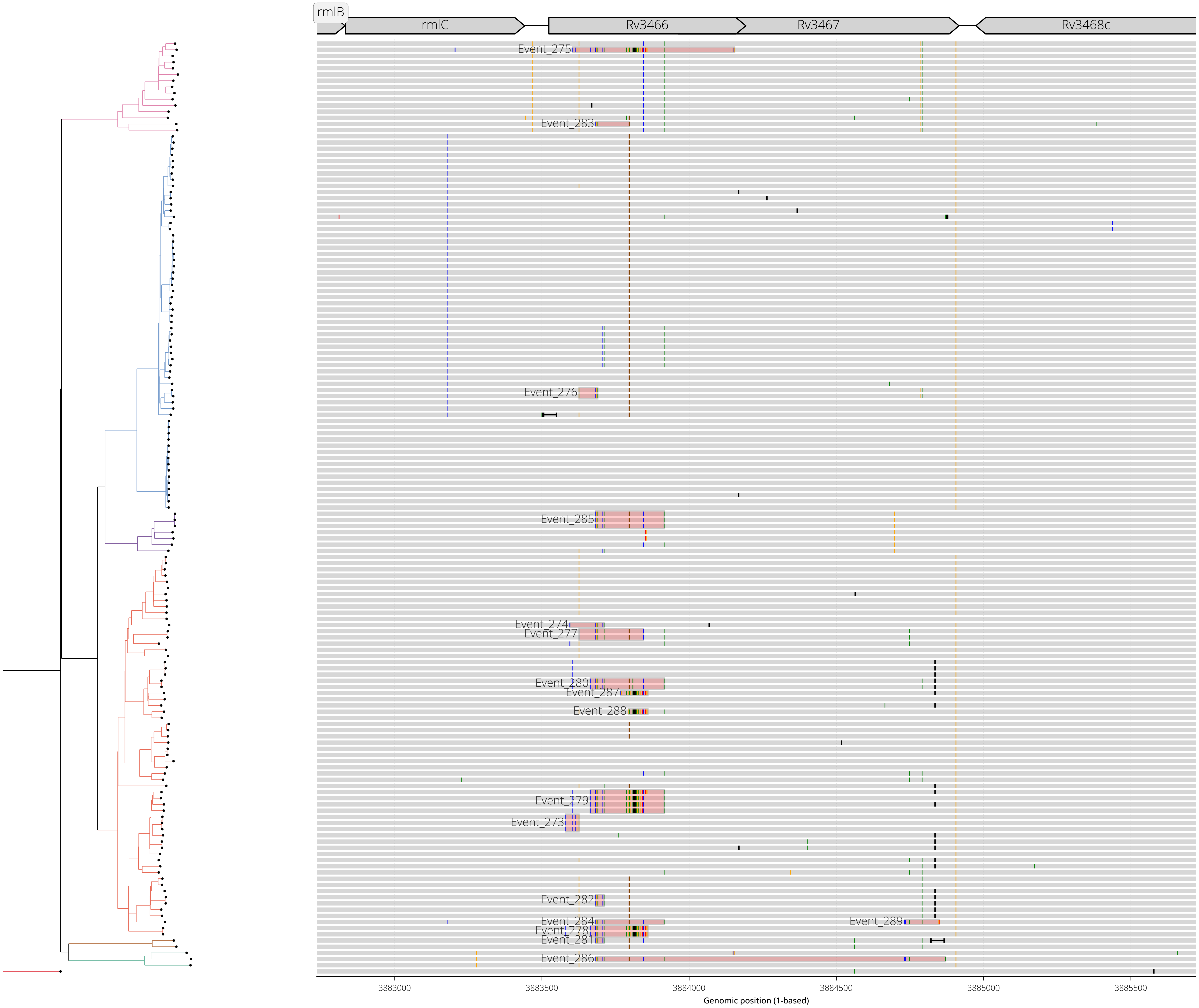

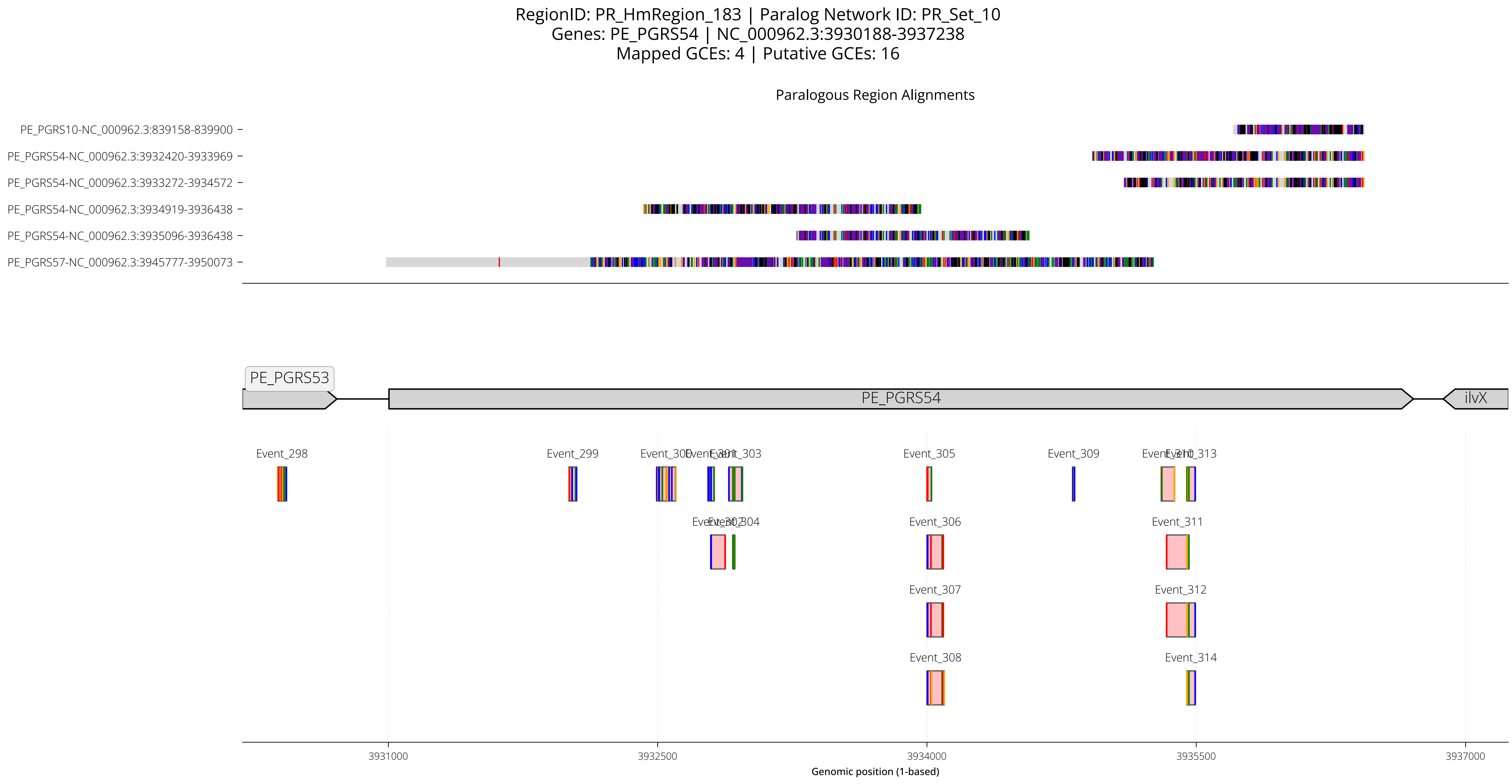
RegionID: PR_HmRegion_183 | Paralog Network ID: PR_Set_10
Genes: PE_PGRS54 | NC_000962.3:3930188-3937238
Mapped GCEs: 4 | Putative GCEs: 16
Paralogous Region Alignments
PE_PGRS10-NC_000962.3:839158-839900
PE_PGRS54-NC_000962.3:3932420-3933969
PE_PGRS54-NC_000962.3:3933272-3934572
PE_PGRS54-NC_000962.3:3934919-3936438
PE_PGRS54-NC_000962.3:3935096-3936438
PE_PGRS57-NC_000962.3:3945777-3950073
PE_PGRS53
PE_PGRS54
ilvX
Event_298
Event_299
Event_300
Event_301
Event_303
Event_302
Event_304
Event_305
Event_306
Event_307
Event_308
Event_309
Event_310
Event_313
Event_311
Event_312
Event_314
3931000
3932500
3934000
3935500
3937000
Genomic position (1-based)

RegionID: PR_HmRegion_183 | Paralog Network ID: PR_Set_10
Genes: PE_PGRS54 | NC_000962.3:3930188-3937238
Mapped GCEs: 4 | Putative GCEs: 16

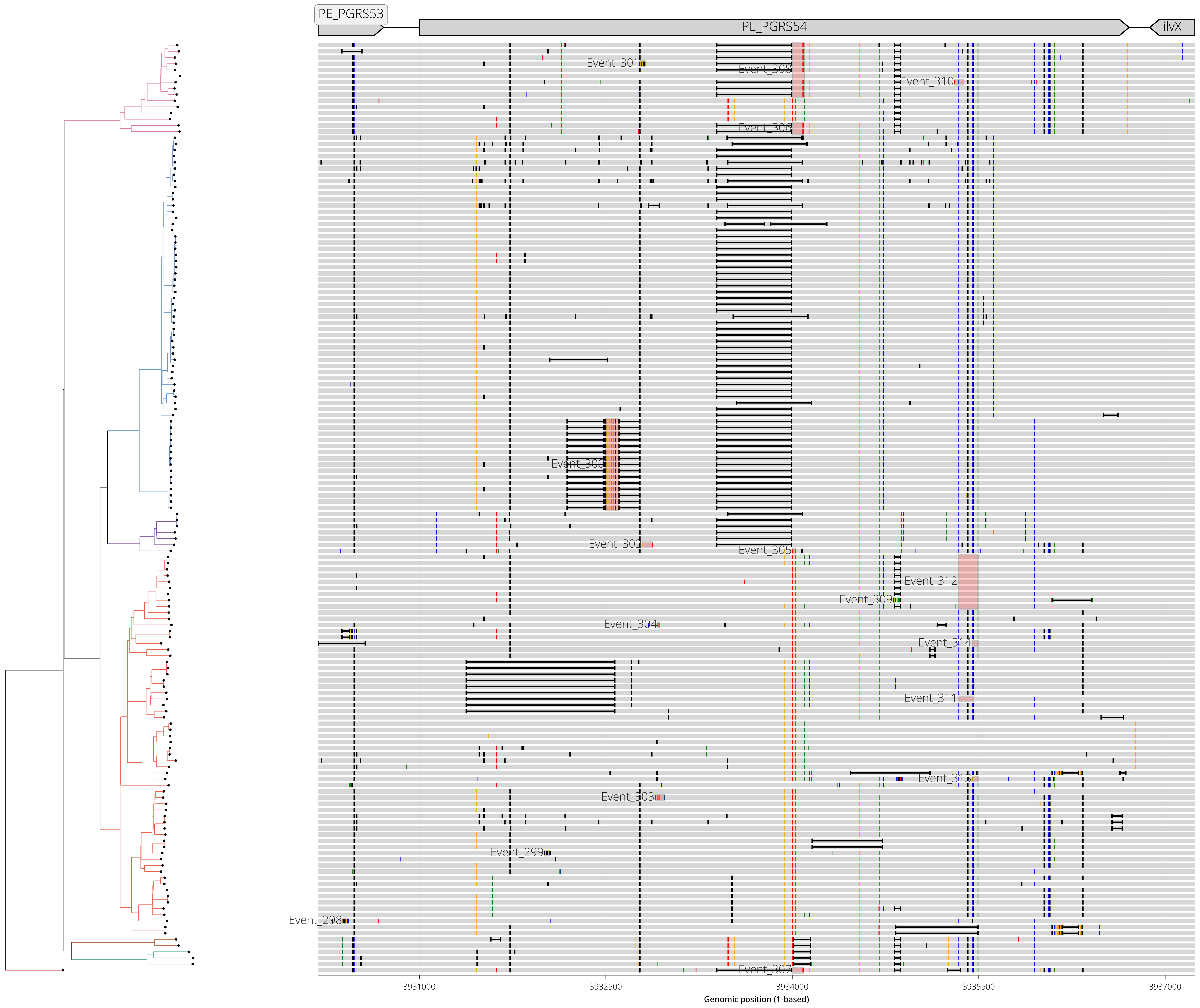

RegionID: PR_HmRegion_094 | Paralog Network ID: PR_Set_34
Genes: Rv1944c,Rv1945 | NC_000962.3:2195056-2198160
Mapped GCEs: 11 | Putative GCEs: 11

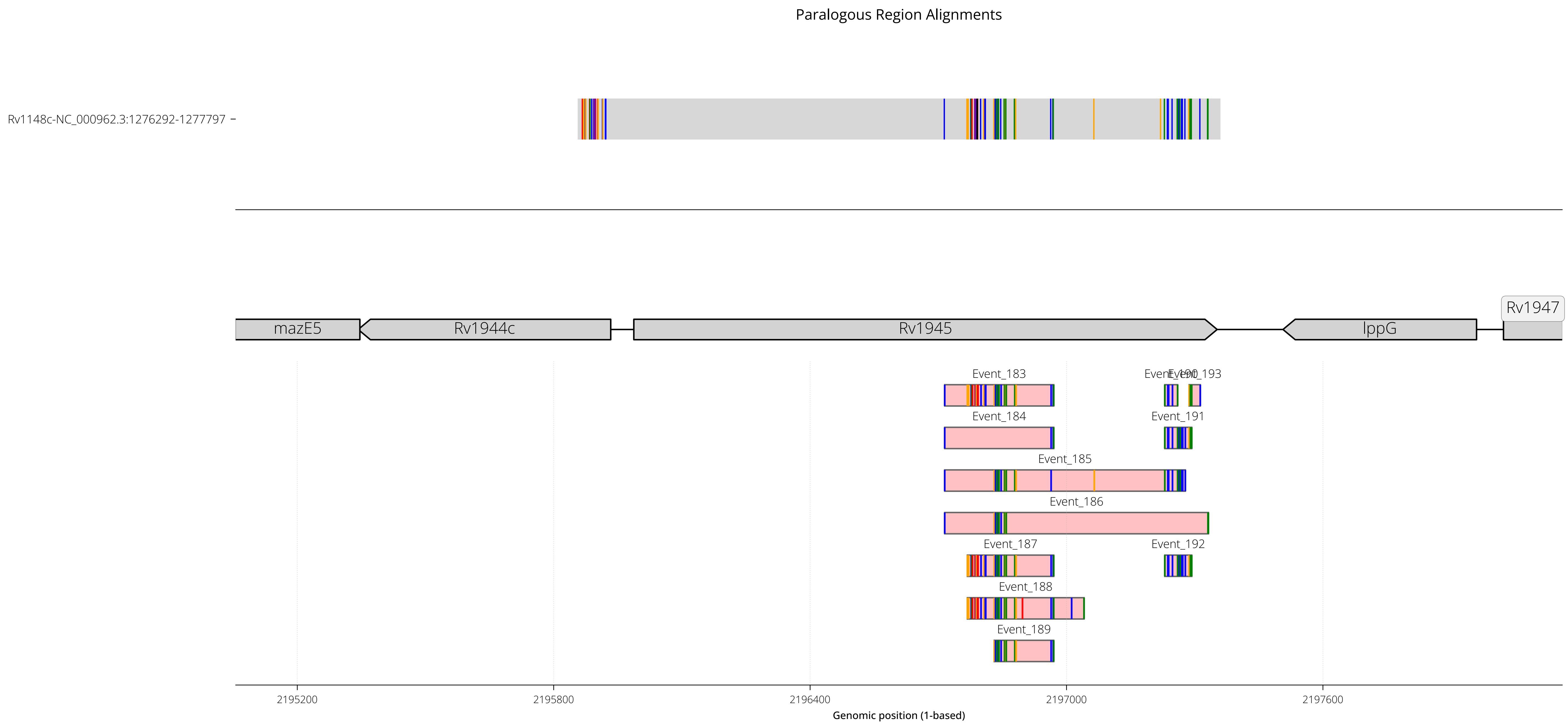

RegionID: PR_HmRegion_094 | Paralog Network ID: PR_Set_34
Genes: Rv1944c,Rv1945 | NC_000962.3:2195056-2198160
Mapped GCEs: 11 | Putative GCEs: 11

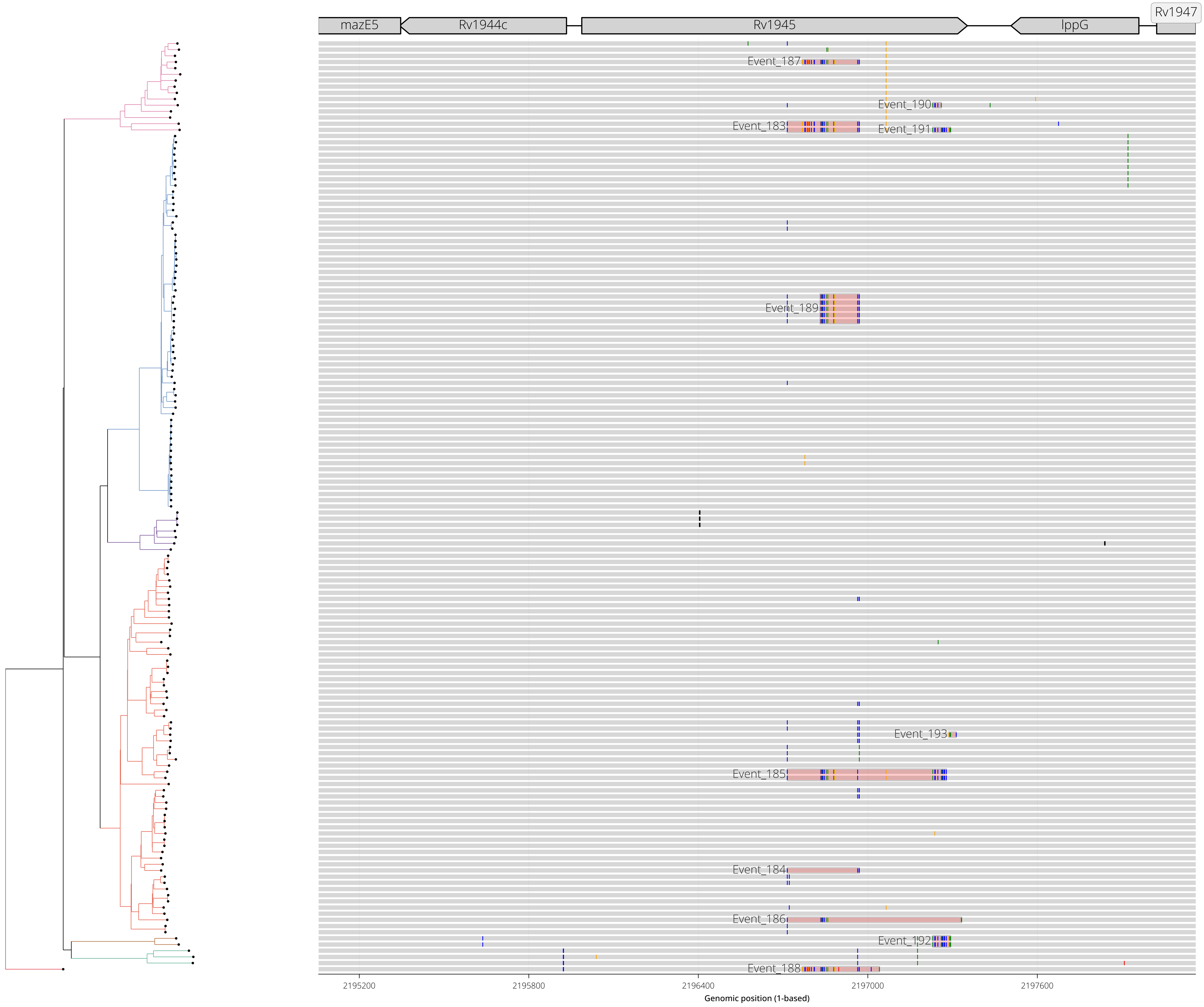

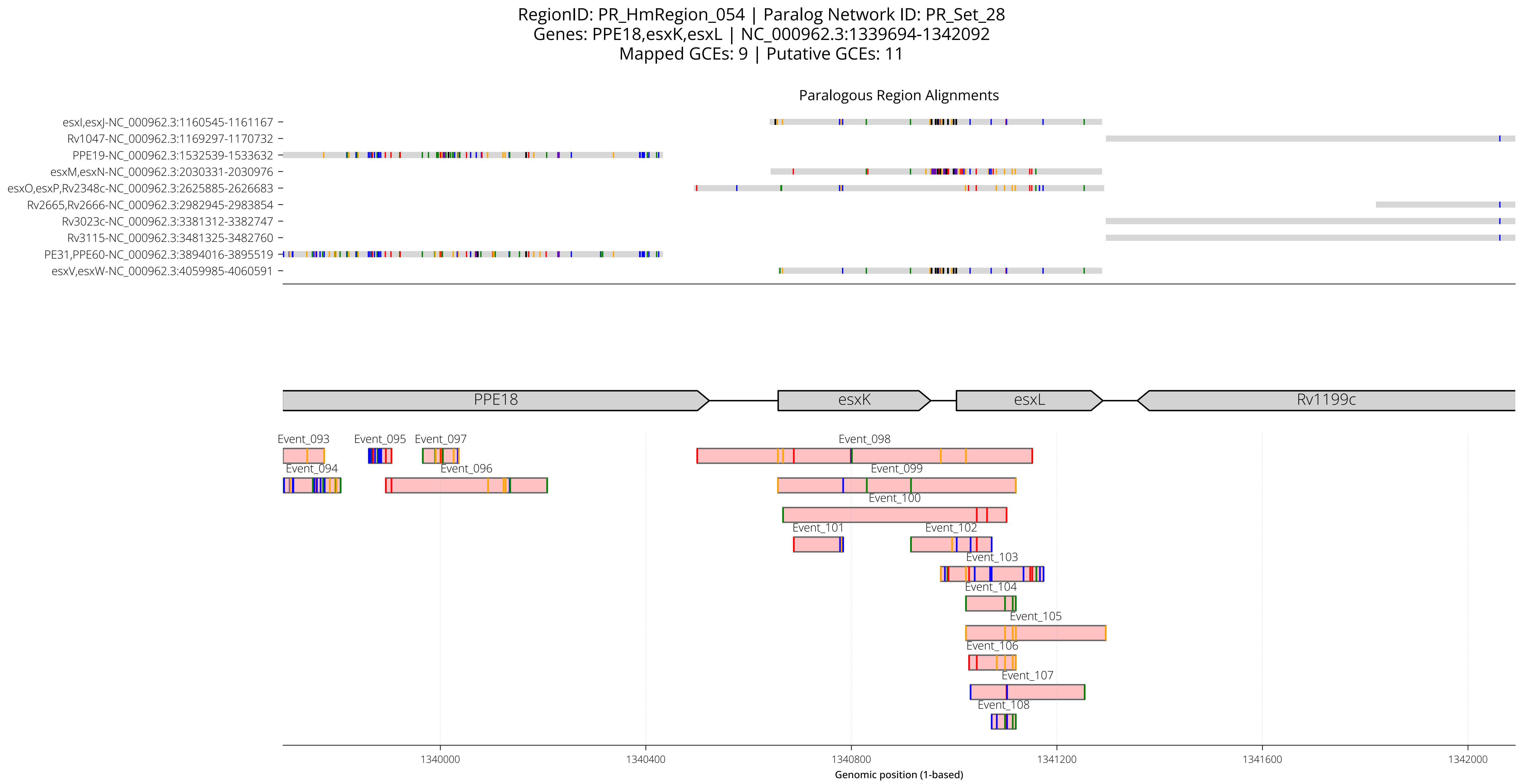
RegionID: PR_HmRegion_054 | Paralog Network ID: PR_Set_28
Genes: PPE18,esxK,esxL | NC_000962.3:1339694-1342092
Mapped GCEs: 9 | Putative GCEs: 11
Paralogous Region Alignments
esxI,esxJ-NC_000962.3:1160545-1161167
Rv1047-NC_000962.3:1169297-1170732
PPE19-NC_000962.3:1532539-1533632
esxM,esxN-NC_000962.3:2030331-2030976
esxO,esxP,Rv2348c-NC_000962.3:2625885-2626683
Rv2665,Rv2666-NC_000962.3:2982945-2983854
Rv3023c-NC_000962.3:3381312-3382747
Rv3115-NC_000962.3:3481325-3482760
PE31,PPE60-NC_000962.3:3894016-3895519
esxV,esxW-NC_000962.3:4059985-4060591
PPE18
esxK
esxL
Rv1199c
Event_093
Event_094
Event_095
Event_096
Event_097
Event_098
Event_099
Event_100
Event_101
Event_102
Event_103
Event_104
Event_105
Event_106
Event_107
Event_108
1340000
1340400
1340800
1341200
1341600
1342000
Genomic position (1-based)

RegionID: PR_HmRegion_054 | Paralog Network ID: PR_Set_28
Genes: PPE18,esxK,esxL | NC_000962.3:1339694-1342092
Mapped GCEs: 9 | Putative GCEs: 11

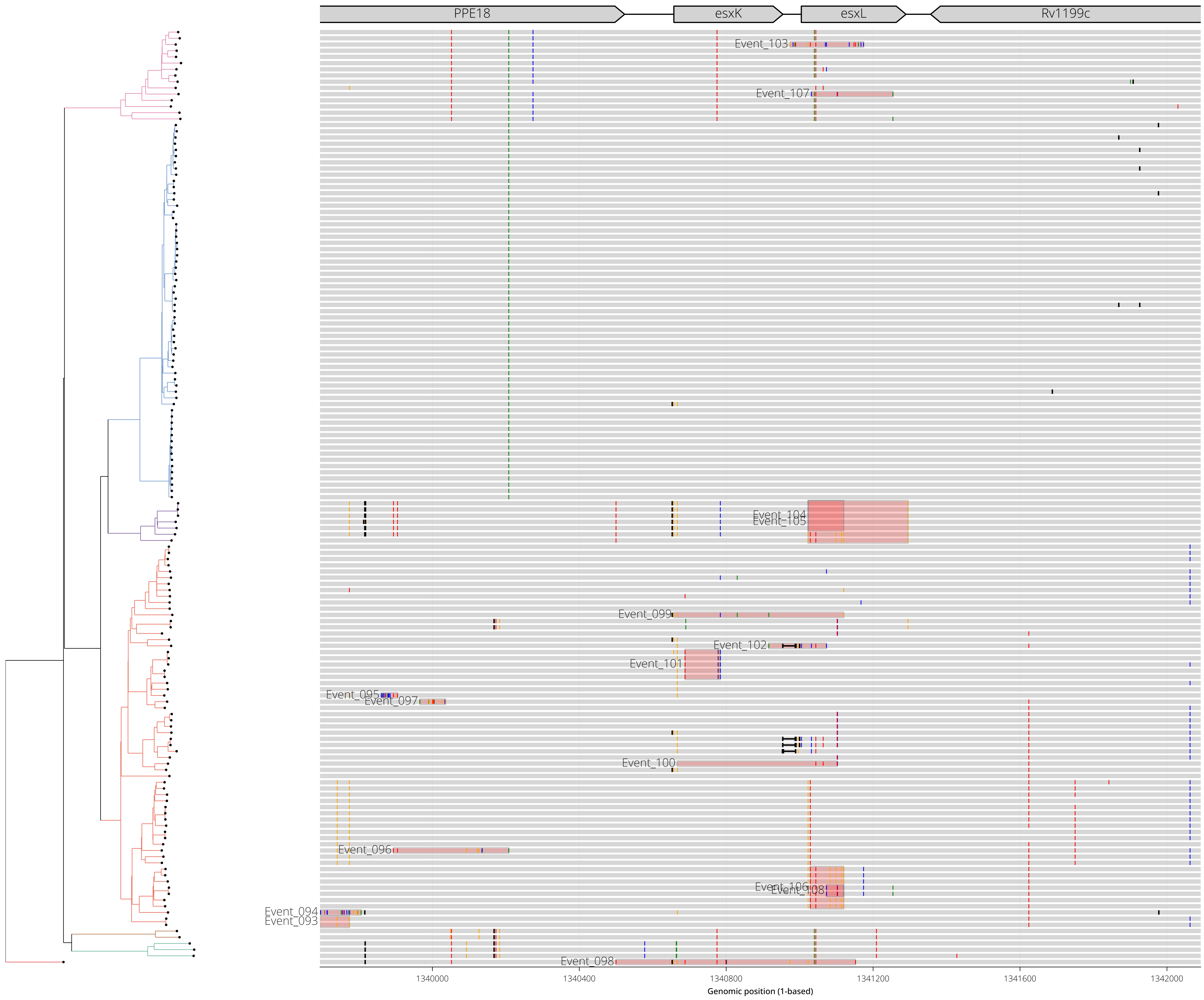

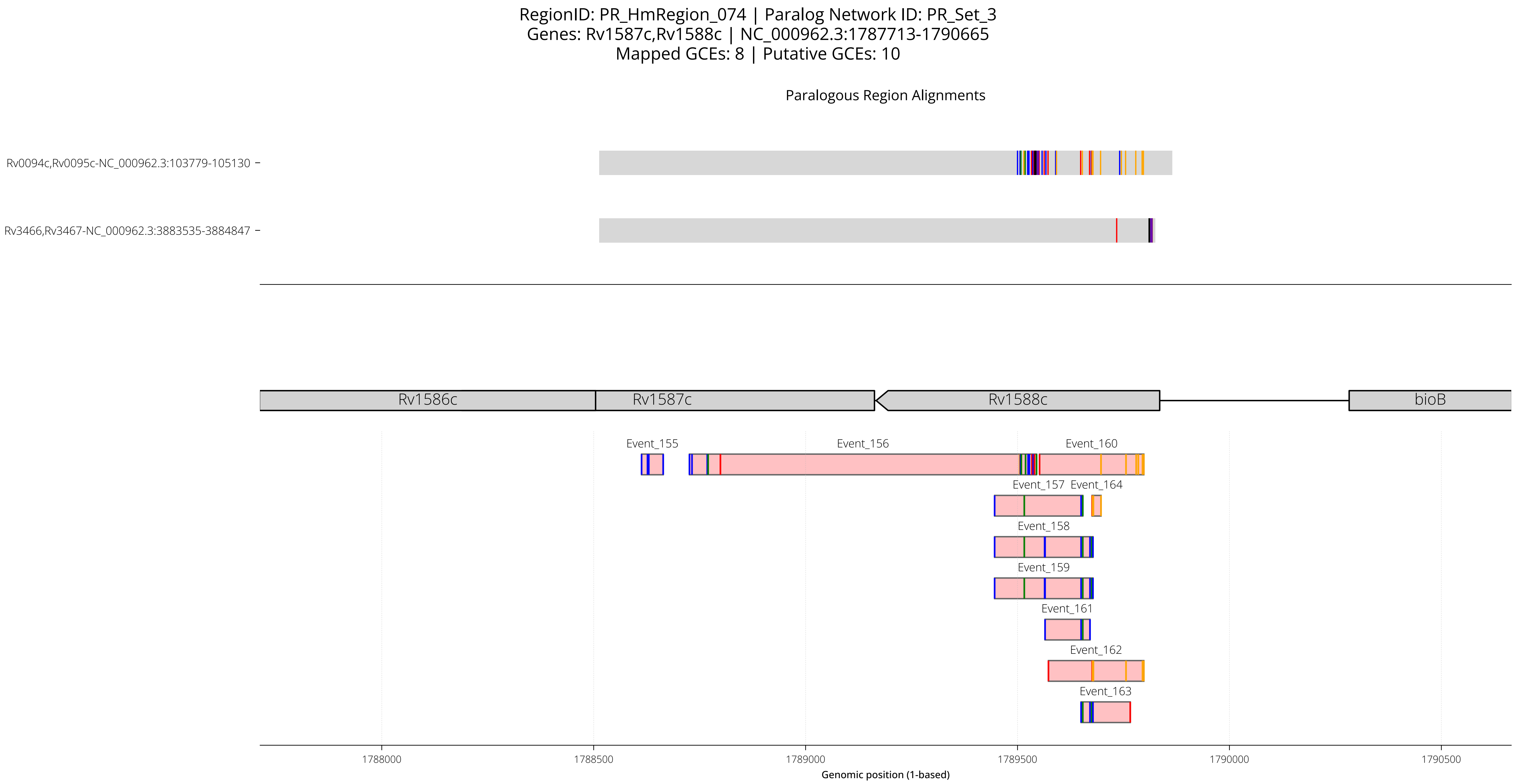

RegionID: PR_HmRegion_074 | Paralog Network ID: PR_Set_3
Genes: Rv1587c,Rv1588c | NC_000962.3:1787713-1790665
Mapped GCEs: 8 | Putative GCEs: 10
Paralogous Region Alignments
Rv0094c,Rv0095c-NC_000962.3:103779-105130
Rv3466,Rv3467-NC_000962.3:3883535-3884847
Rv1586c
Rv1587c
Rv1588c
bioB
Event_155
Event_156
Event_160
Event_157
Event_164
Event_158
Event_159
Event_161
Event_162
Event_163
1788000
1788500
1789000
1789500
1790000
1790500
Genomic position (1-based)

RegionID: PR_HmRegion_074 | Paralog Network ID: PR_Set_3
Genes: Rv1587c,Rv1588c | NC_000962.3:1787713-1790665
Mapped GCEs: 8 | Putative GCEs: 10

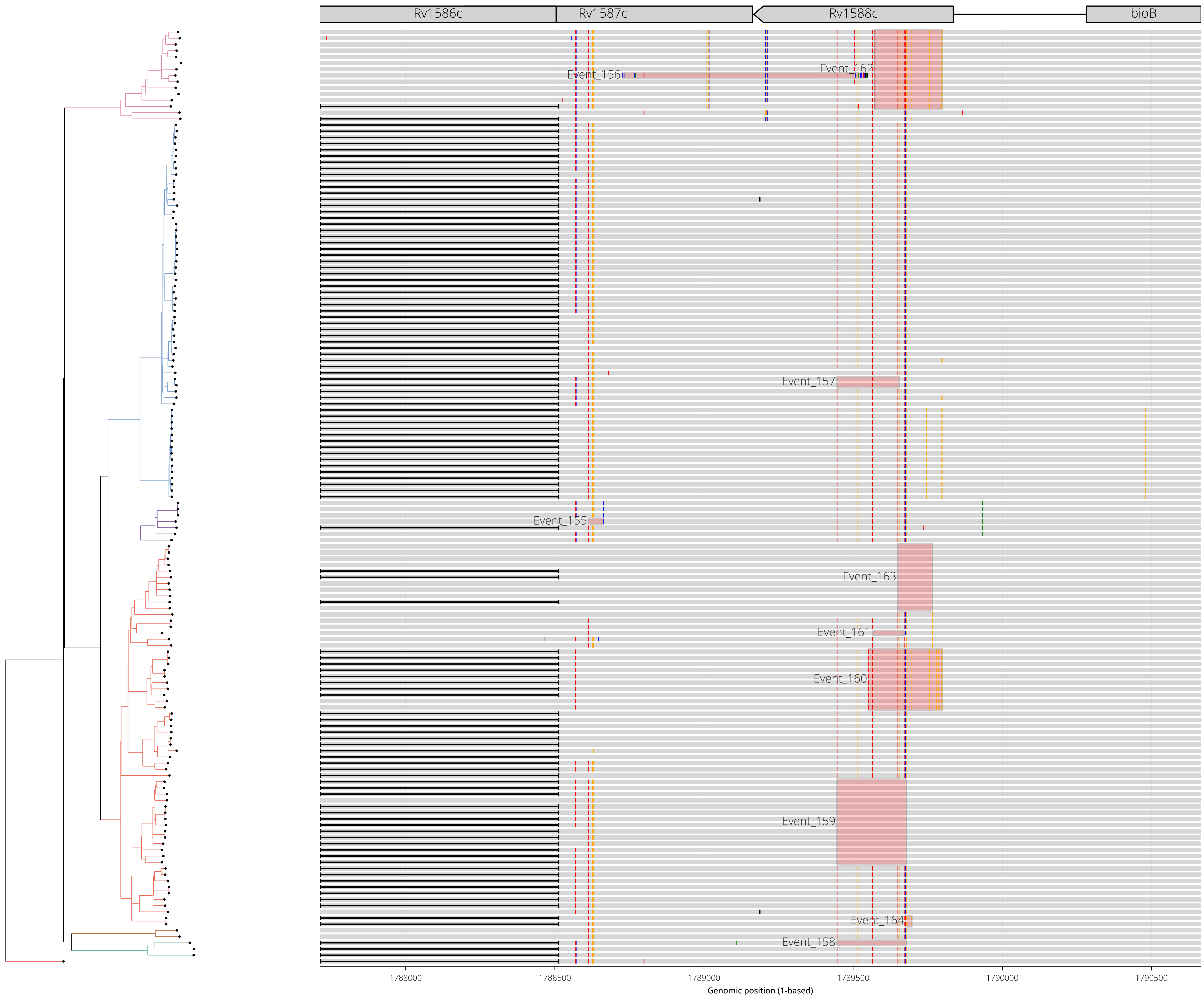

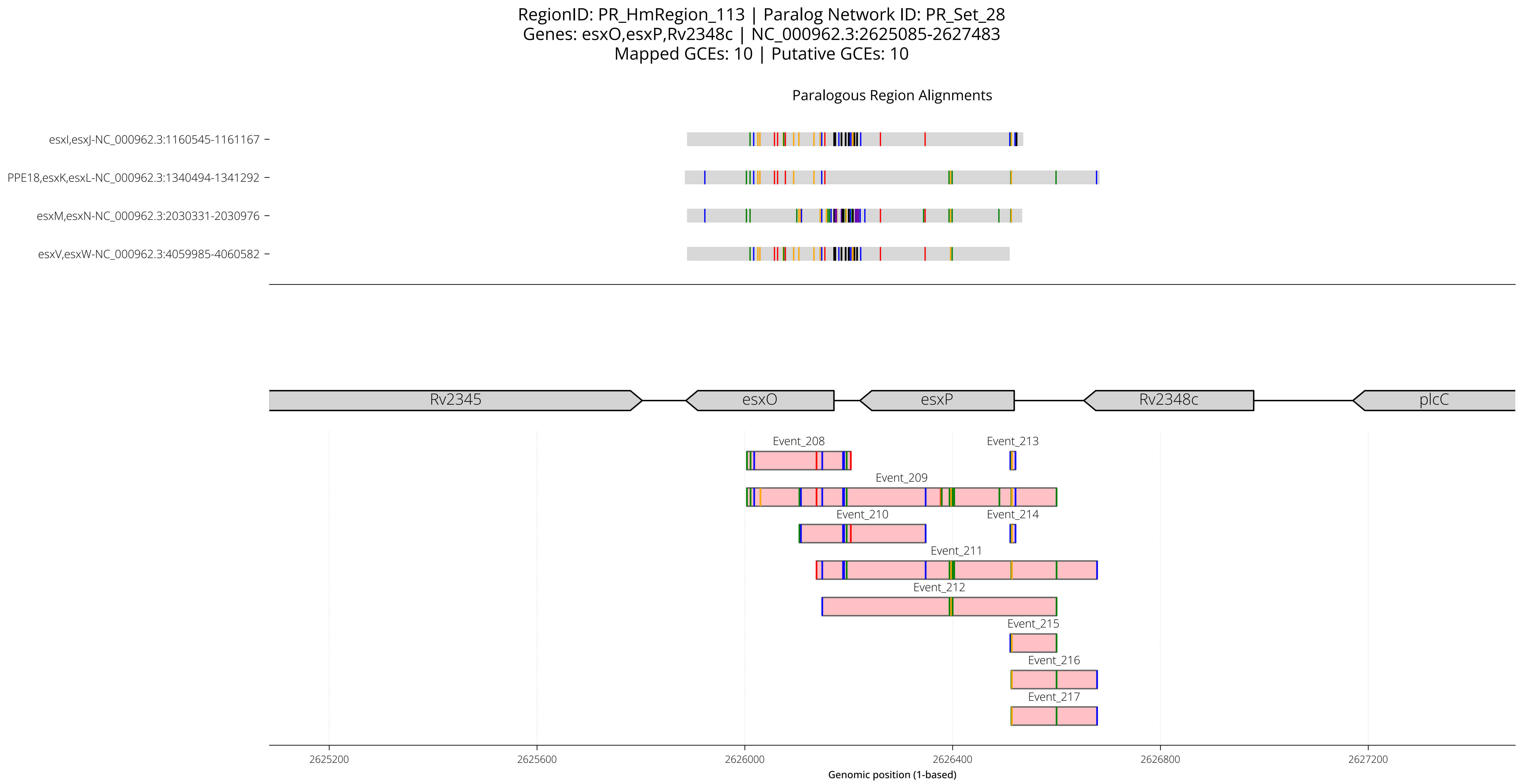
RegionID: PR_HmRegion_113 | Paralog Network ID: PR_Set_28
Genes: esxO,esxP,Rv2348c | NC_000962.3:2625085-2627483
Mapped GCEs: 10 | Putative GCEs: 10
Paralogous Region Alignments
esxI,esxJ-NC_000962.3:1160545-1161167
PPE18,esxK,esxL-NC_000962.3:1340494-1341292
esxM,esxN-NC_000962.3:2030331-2030976
esxV,esxW-NC_000962.3:4059985-4060582
Rv2345
esxO
esxP
Rv2348c
plcC
Event_208
Event_213
Event_209
Event_210
Event_214
Event_211
Event_212
Event_215
Event_216
Event_217
2625200
2625600
2626000
2626400
2626800
2627200
Genomic position (1-based)

RegionID: PR_HmRegion_113 | Paralog Network ID: PR_Set_28
Genes: esxO,esxP,Rv2348c | NC_000962.3:2625085-2627483
Mapped GCEs: 10 | Putative GCEs: 10

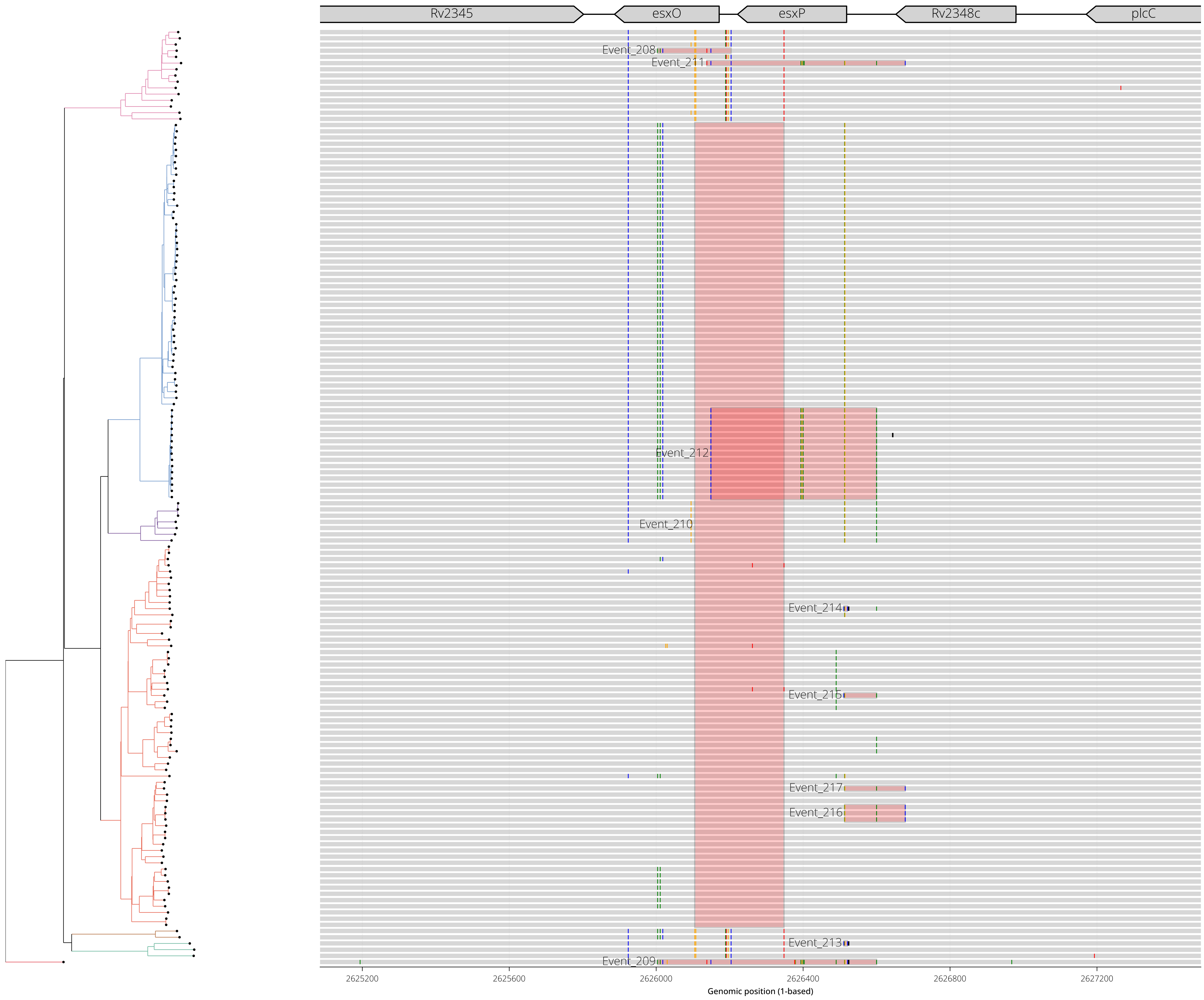

RegionID: PR_HmRegion_010 | Paralog Network ID: PR_Set_10
Genes: PE_PGRS4 | NC_000962.3:335765-339942
Mapped GCEs: 6 | Putative GCEs: 10

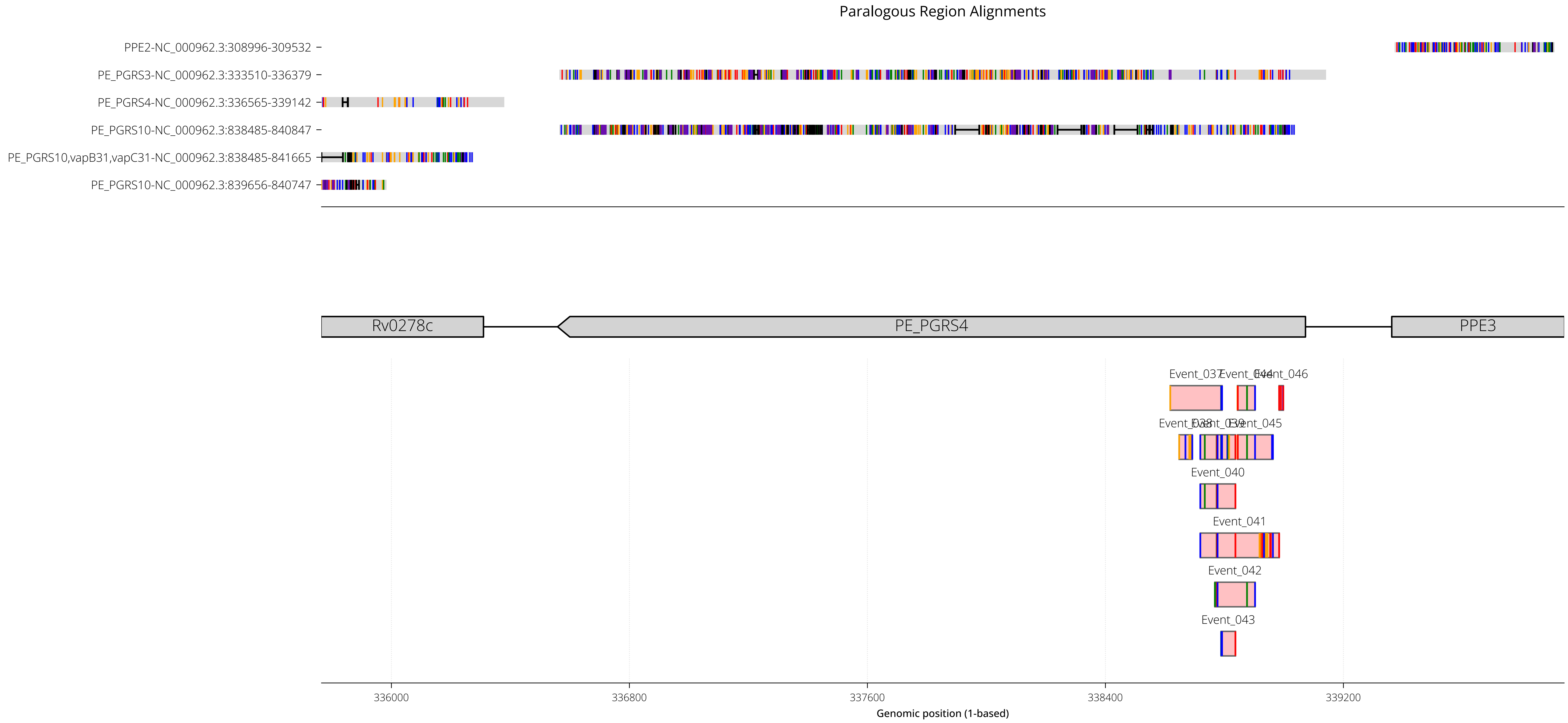

RegionID: PR_HmRegion_010 | Paralog Network ID: PR_Set_10
Genes: PE_PGRS4 | NC_000962.3:335765-339942
Mapped GCEs: 6 | Putative GCEs: 10

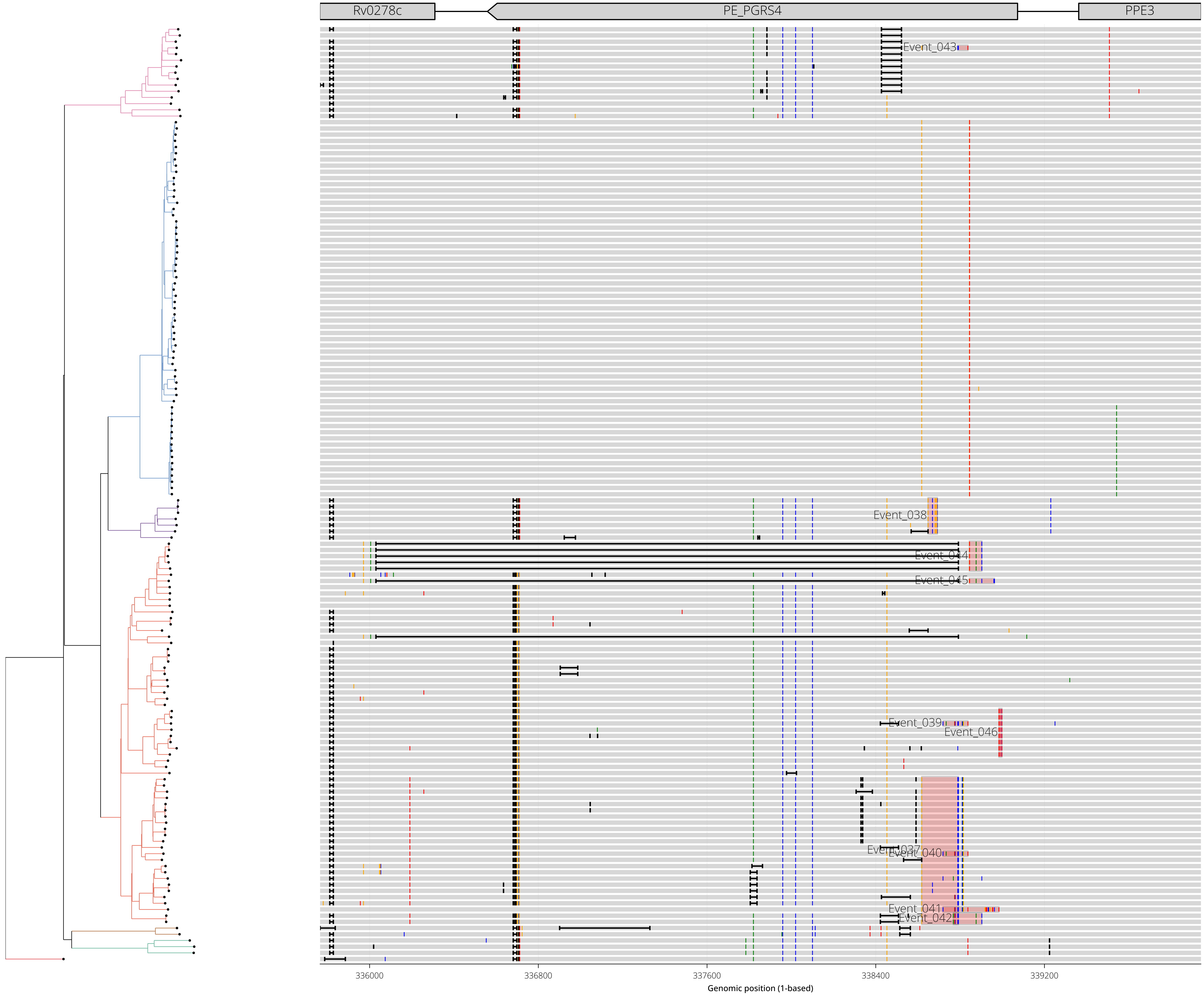

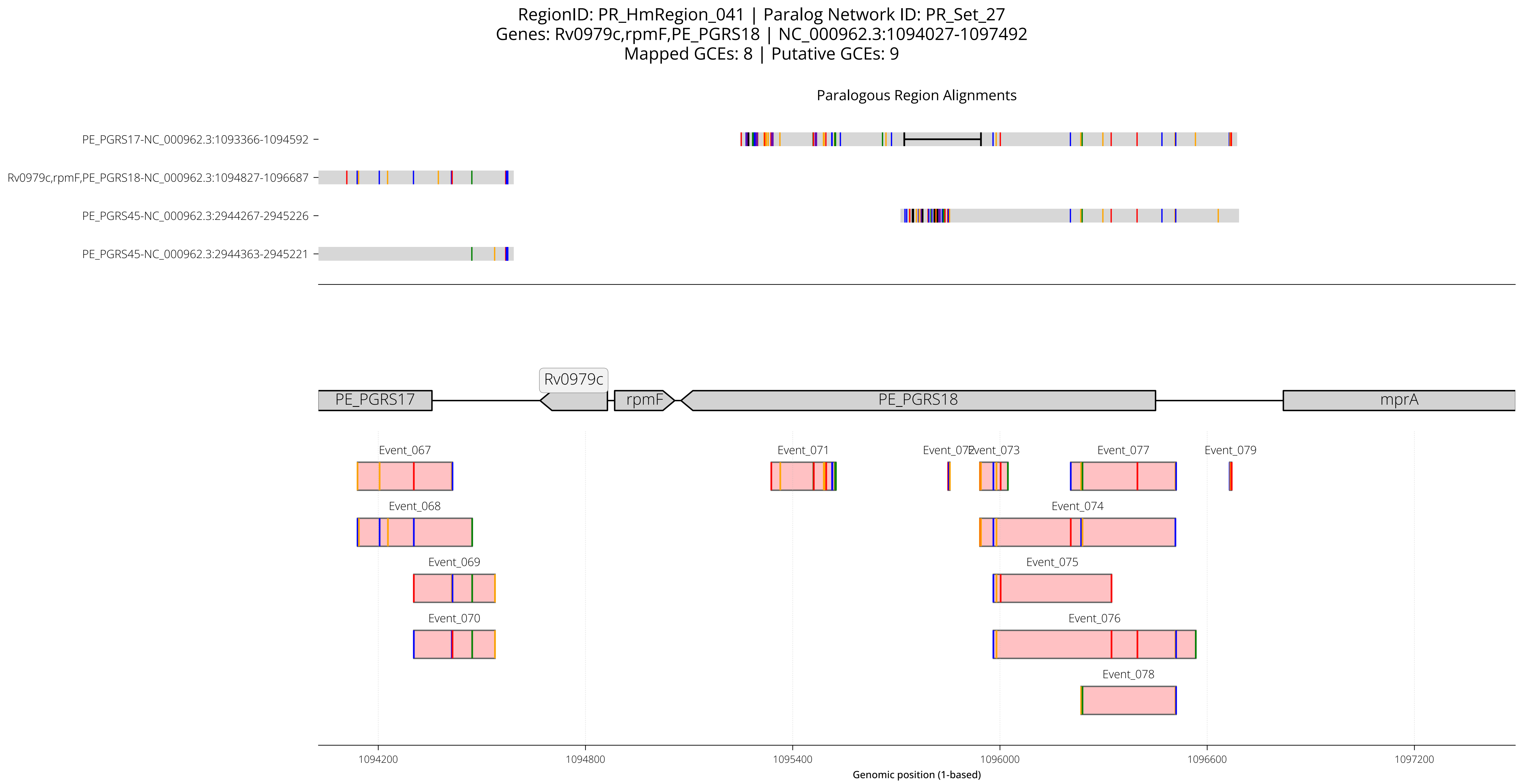
RegionID: PR_HmRegion_041 | Paralog Network ID: PR_Set_27
Genes: Rv0979c,rpmF,PE_PGRS18 | NC_000962.3:1094027-1097492
Mapped GCEs: 8 | Putative GCEs: 9
Paralogous Region Alignments
PE_PGRS17-NC_000962.3:1093366-1094592
Rv0979c,rpmF,PE_PGRS18-NC_000962.3:1094827-1096687
PE_PGRS45-NC_000962.3:2944267-2945226
PE_PGRS45-NC_000962.3:2944363-2945221
Rv0979c
PE_PGRS17
rpmF
PE_PGRS18
mprA
Event_067
Event_068
Event_069
Event_070
Event_071
Event_072
Event_073
Event_074
Event_075
Event_076
Event_077
Event_078
Event_079
1094200
1094800
1095400
1096000
1096600
1097200
Genomic position (1-based)

RegionID: PR_HmRegion_041 | Paralog Network ID: PR_Set_27
Genes: Rv0979c,rpmF,PE_PGRS18 | NC_000962.3:1094027-1097492
Mapped GCEs: 8 | Putative GCEs: 9

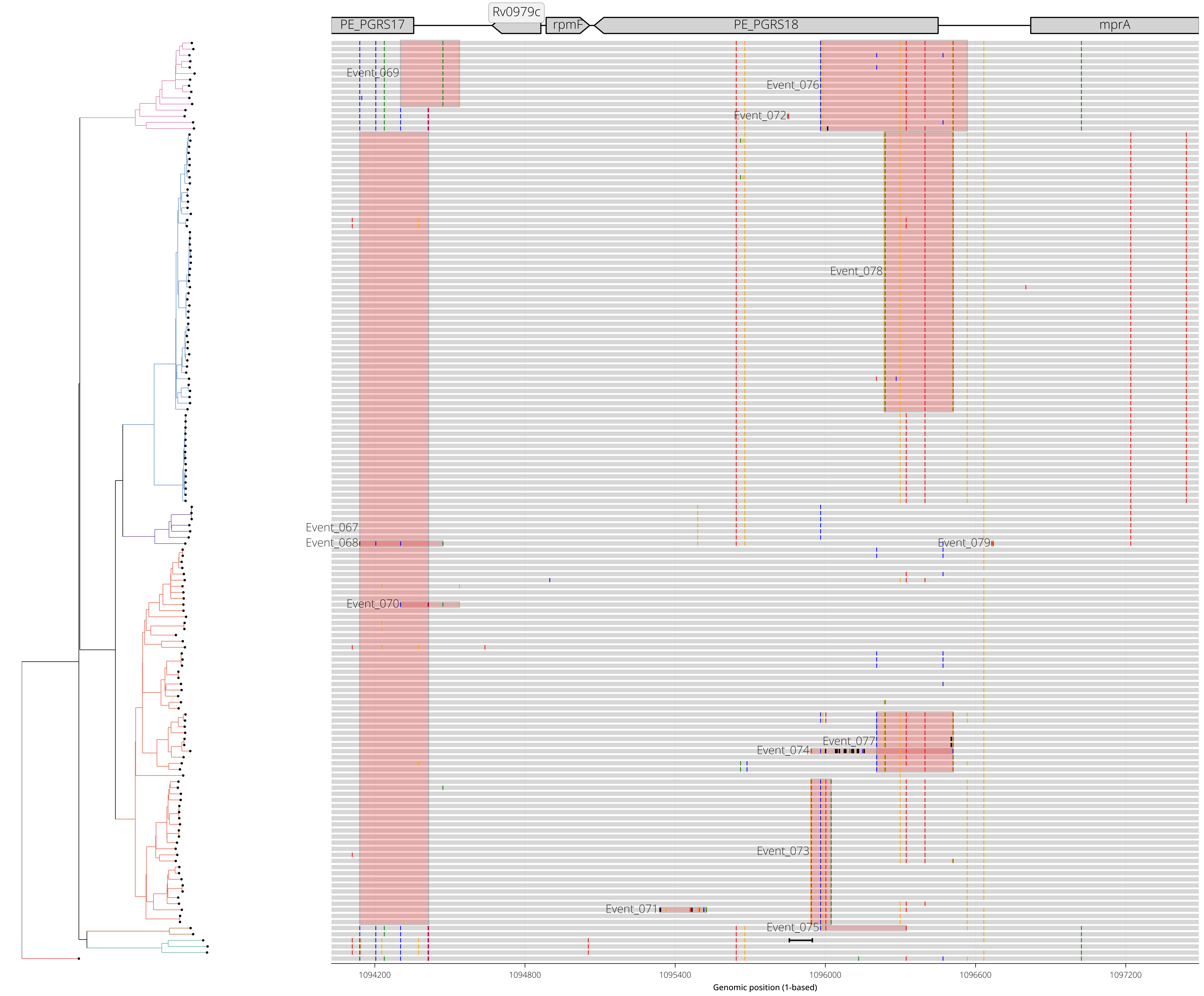

RegionID: PR_HmRegion_171 | Paralog Network ID: PR_Set_24
Genes: PPE55 | NC_000962.3:3742909-3753978
Mapped GCEs: 6 | Putative GCEs: 8

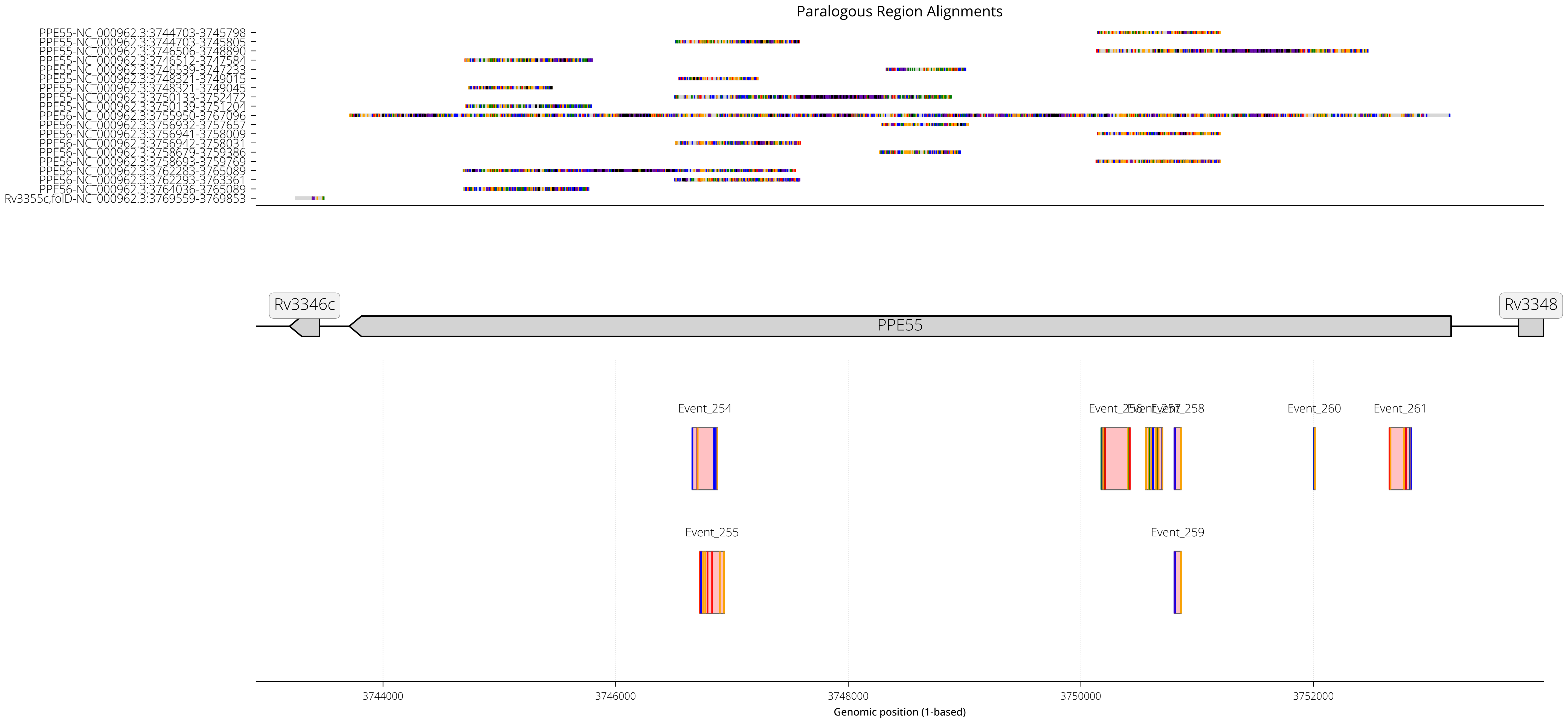

RegionID: PR_HmRegion_171 | Paralog Network ID: PR_Set_24
Genes: PPE55 | NC_000962.3:3742909-3753978
Mapped GCEs: 6 | Putative GCEs: 8

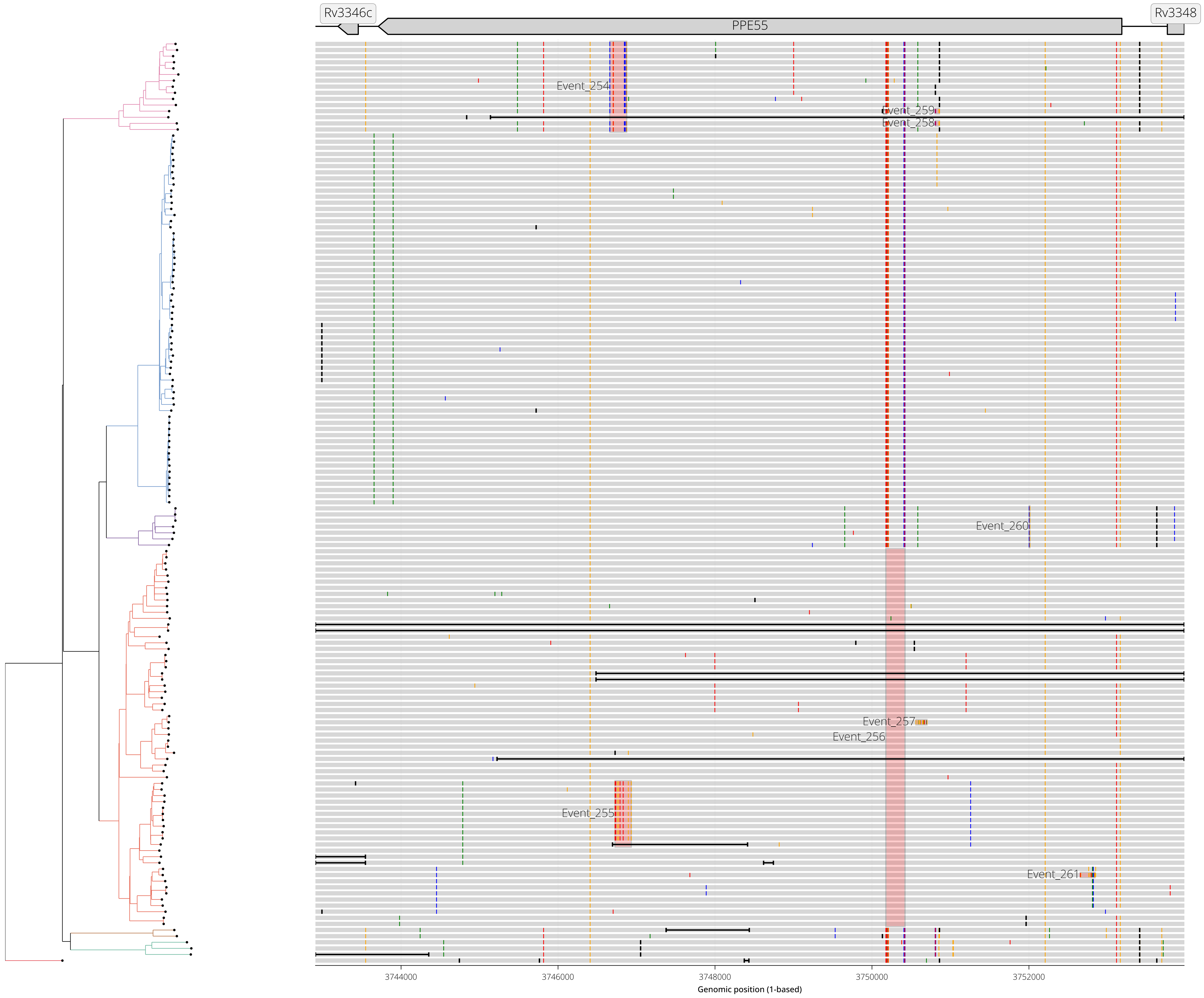

RegionID: PR_HmRegion_168 | Paralog Network ID: PR_Set_67
Genes: PPE54 | NC_000962.3:3734835-3737114
Mapped GCEs: 5 | Putative GCEs: 8

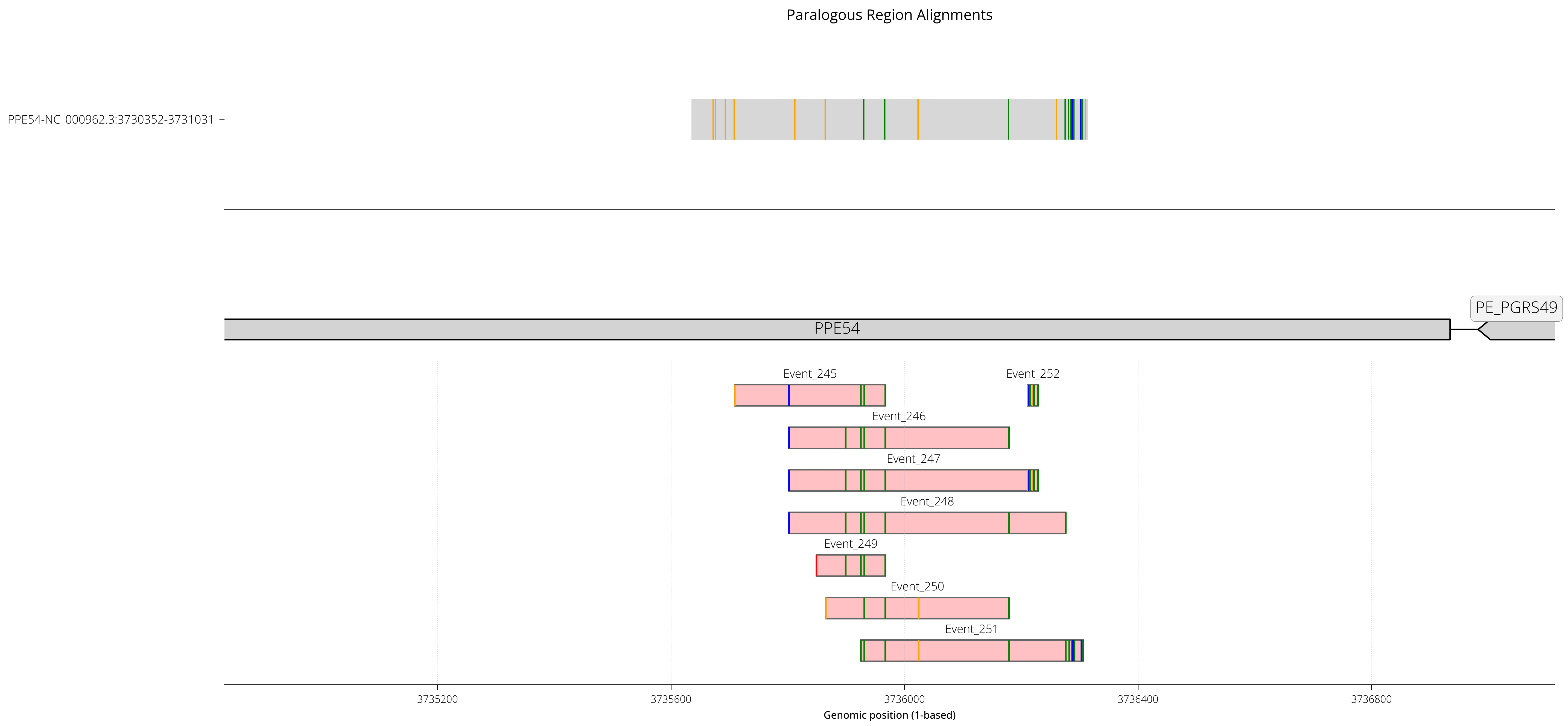

RegionID: PR_HmRegion_168 | Paralog Network ID: PR_Set_67
Genes: PPE54 | NC_000962.3:3734835-3737114
Mapped GCEs: 5 | Putative GCEs: 8

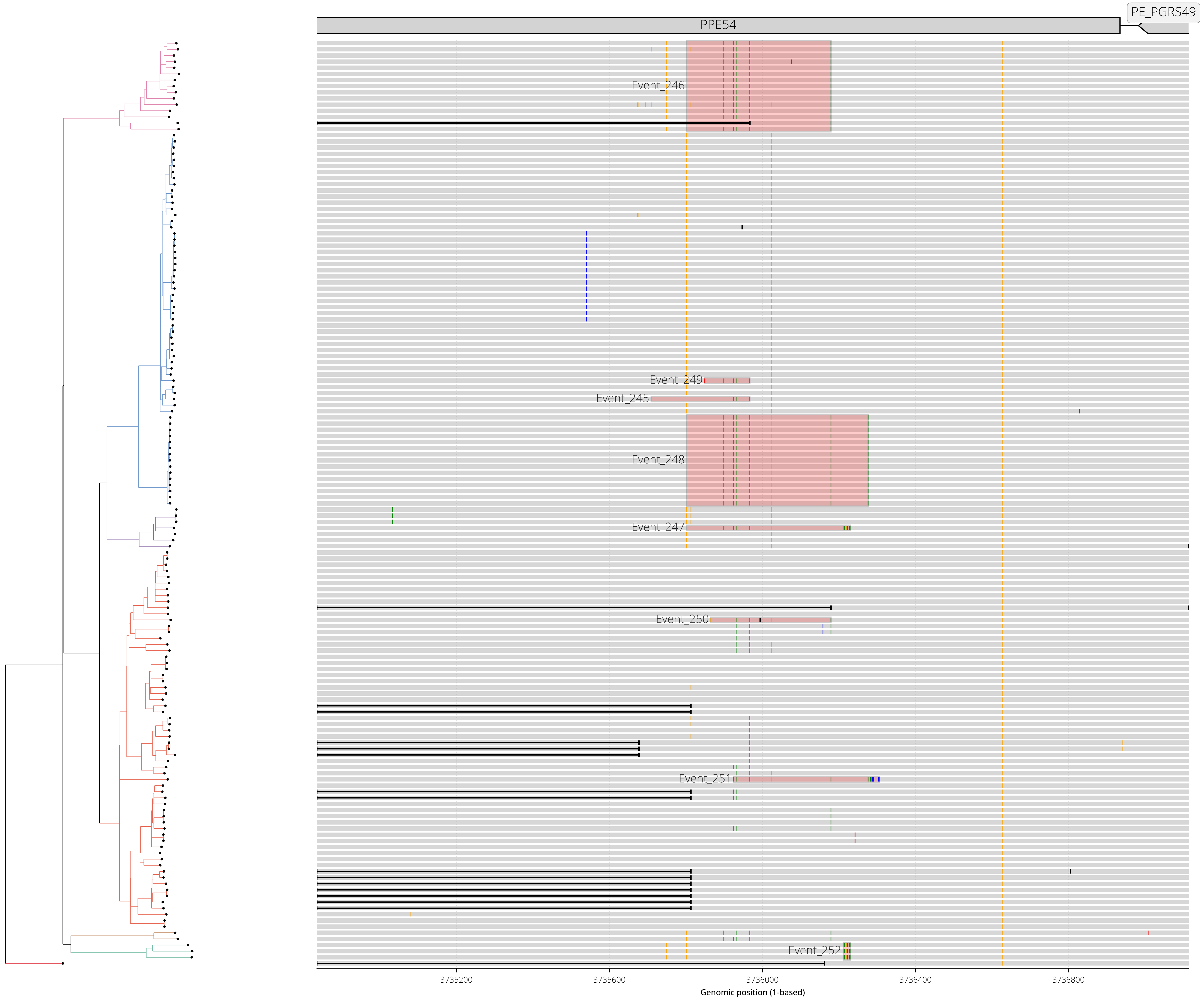

RegionID: PR_HmRegion_182 | Paralog Network ID: PR_Set_36
Genes: PE31,PPE60 | NC_000962.3:3893216-3896388
Mapped GCEs: 8 | Putative GCEs: 8

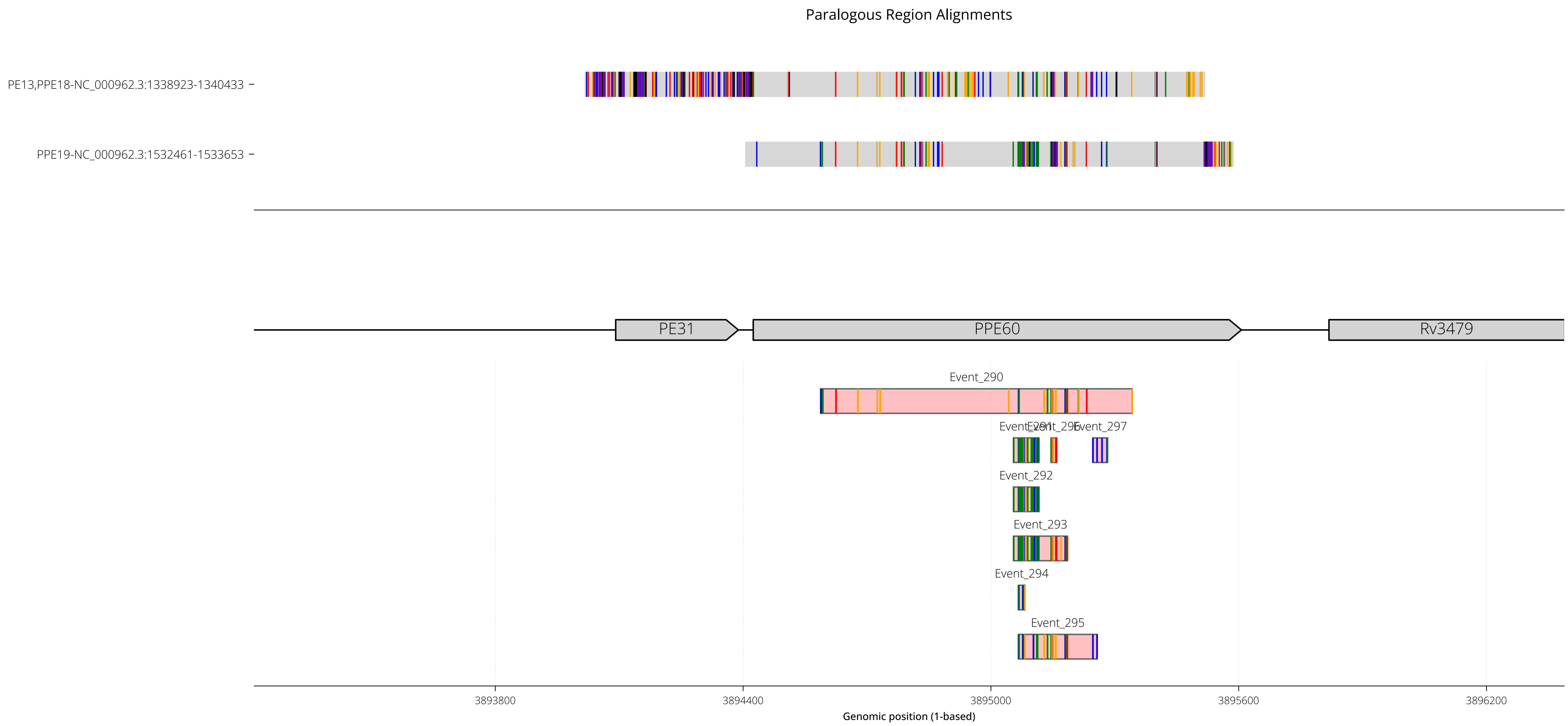

RegionID: PR_HmRegion_182 | Paralog Network ID: PR_Set_36
Genes: PE31,PPE60 | NC_000962.3:3893216-3896388
Mapped GCEs: 8 | Putative GCEs: 8

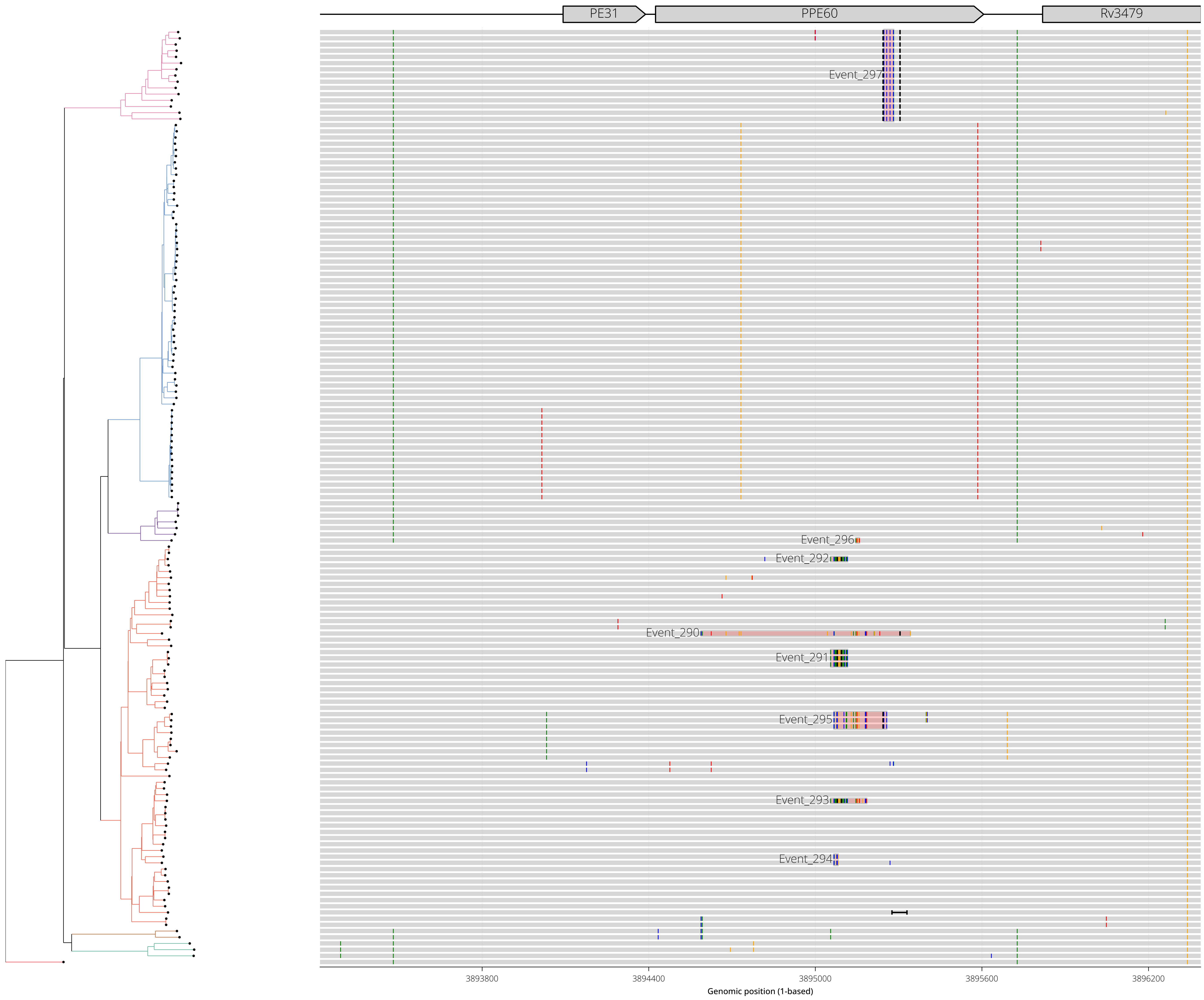

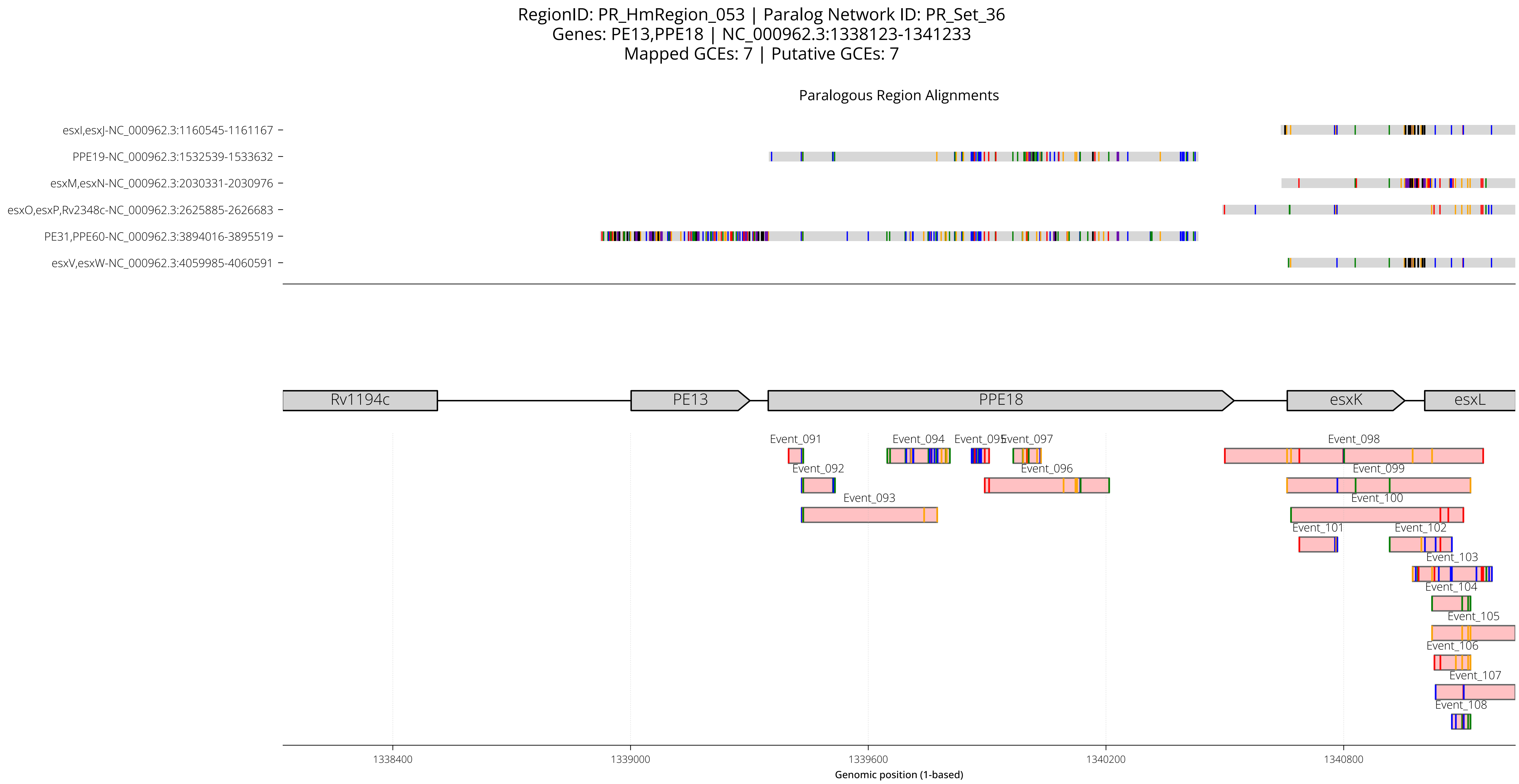
RegionID: PR_HmRegion_053 | Paralog Network ID: PR_Set_36
Genes: PE13,PPE18 | NC_000962.3:1338123-1341233
Mapped GCEs: 7 | Putative GCEs: 7
Paralogous Region Alignments
esxI,esxJ-NC_000962.3:1160545-1161167
PPE19-NC_000962.3:1532539-1533632
esxM,esxN-NC_000962.3:2030331-2030976
esxO,esxP,Rv2348c-NC_000962.3:2625885-2626683
PE31,PPE60-NC_000962.3:3894016-3895519
esxV,esxW-NC_000962.3:4059985-4060591
Rv1194c
PE13
PPE18
esxK
esxL
Event_091
Event_092
Event_093
Event_094
Event_095
Event_096
Event_097
Event_098
Event_099
Event_100
Event_101
Event_102
Event_103
Event_104
Event_105
Event_106
Event_107
Event_108
1338400
1339000
1339600
1340200
1340800
Genomic position (1-based)

RegionID: PR_HmRegion_053 | Paralog Network ID: PR_Set_36
Genes: PE13,PPE18 | NC_000962.3:1338123-1341233
Mapped GCEs: 7 | Putative GCEs: 7

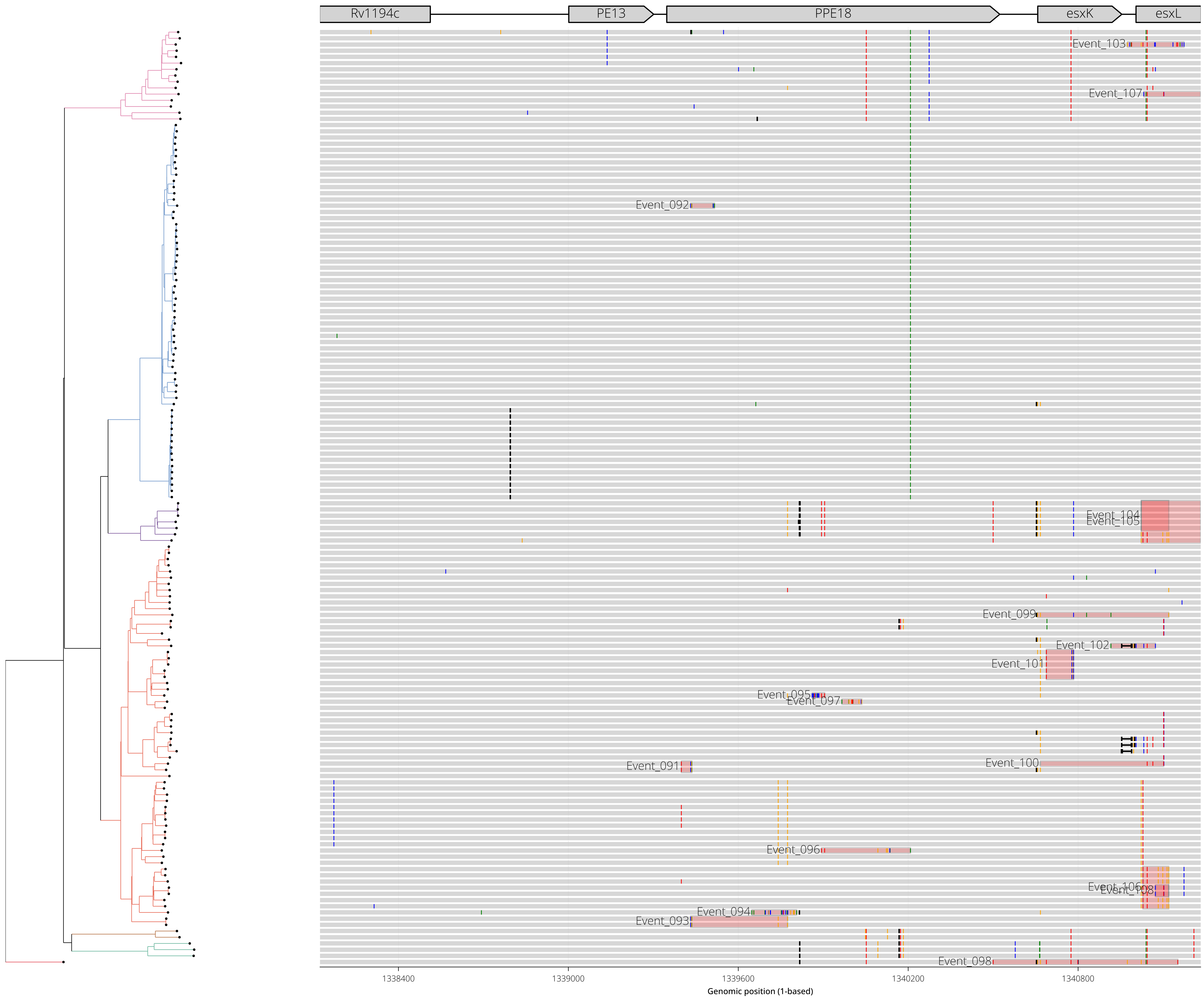

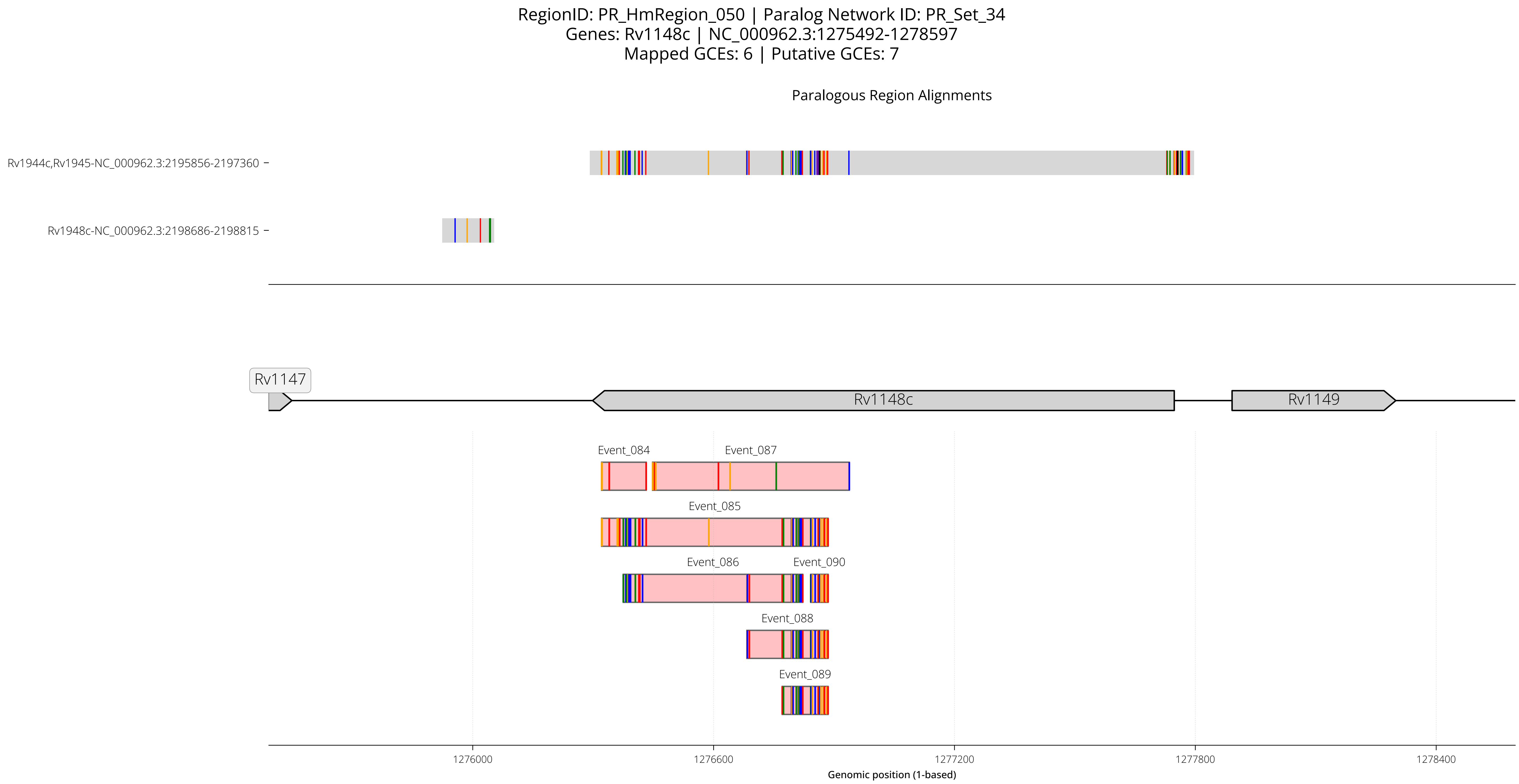
RegionID: PR_HmRegion_050 | Paralog Network ID: PR_Set_34
Genes: Rv1148c | NC_000962.3:1275492-1278597
Mapped GCEs: 6 | Putative GCEs: 7
Paralogous Region Alignments
Rv1944c,Rv1945-NC_000962.3:2195856-2197360
Rv1948c-NC_000962.3:2198686-2198815
Rv1147
Rv1148c
Rv1149
Event_084
Event_087
Event_085
Event_086
Event_090
Event_088
Event_089
1276000
1276600
1277200
1277800
1278400
Genomic position (1-based)

RegionID: PR_HmRegion_050 | Paralog Network ID: PR_Set_34
Genes: Rv1148c | NC_000962.3:1275492-1278597
Mapped GCEs: 6 | Putative GCEs: 7

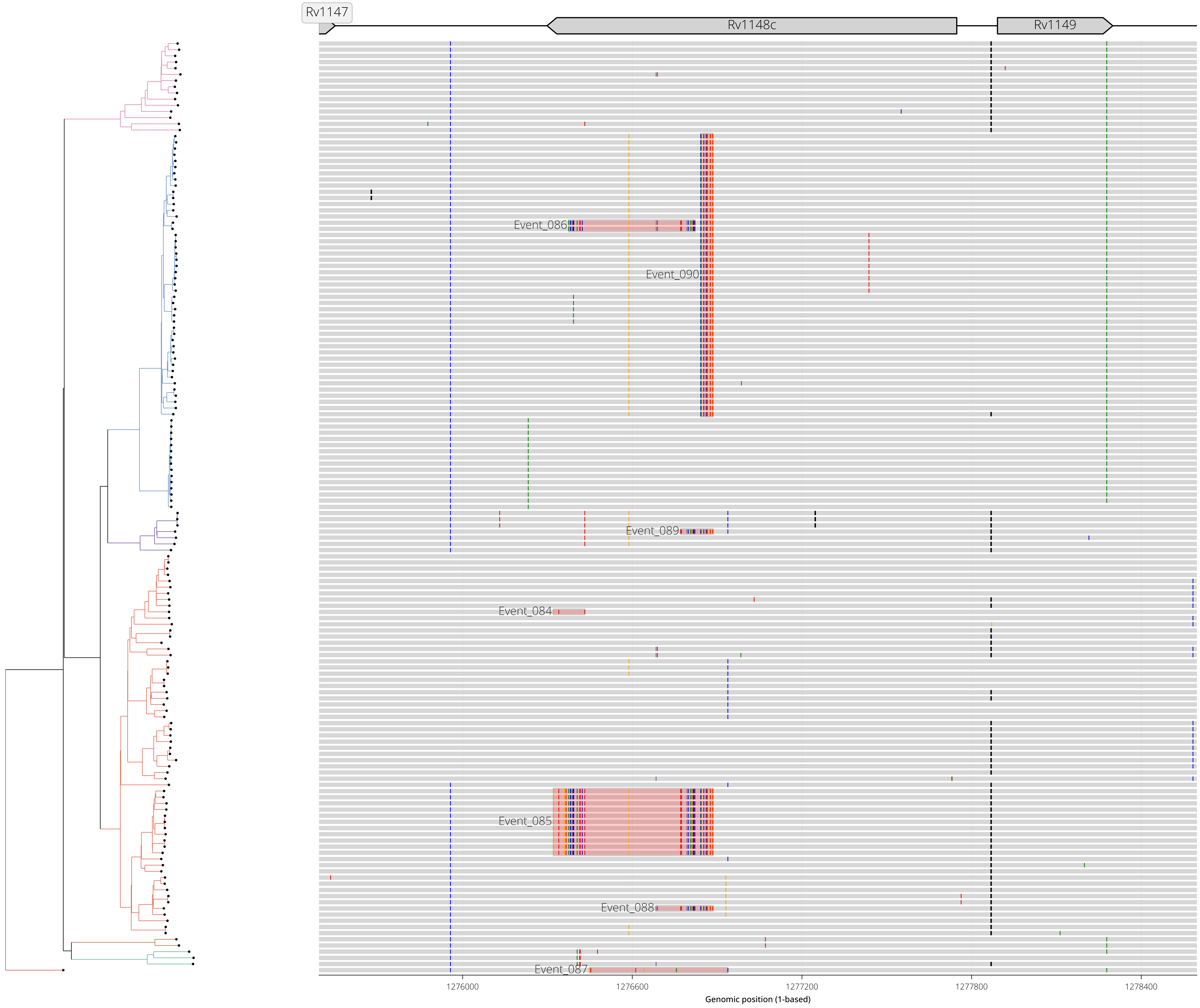

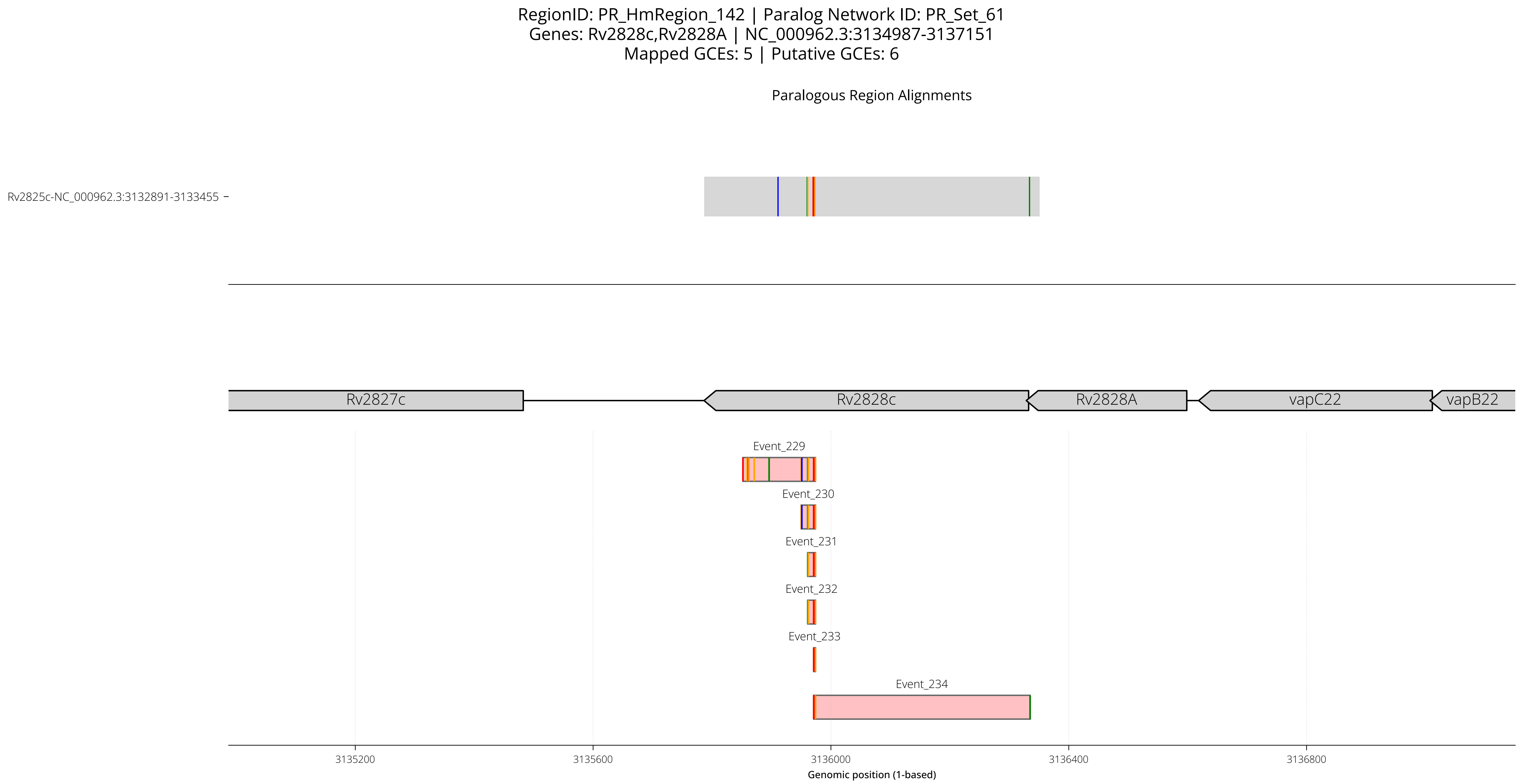
RegionID: PR_HmRegion_142 | Paralog Network ID: PR_Set_61
Genes: Rv2828c,Rv2828A | NC_000962.3:3134987-3137151
Mapped GCEs: 5 | Putative GCEs: 6
Paralogous Region Alignments
Rv2825c-NC_000962.3:3132891-3133455
Rv2827c
Rv2828c
Rv2828A
vapC22
vapB22
Event_229
Event_230
Event_231
Event_232
Event_233
Event_234
3135200
3135600
3136000
3136400
3136800
Genomic position (1-based)

RegionID: PR_HmRegion_142 | Paralog Network ID: PR_Set_61
Genes: Rv2828c,Rv2828A | NC_000962.3:3134987-3137151
Mapped GCEs: 5 | Putative GCEs: 6

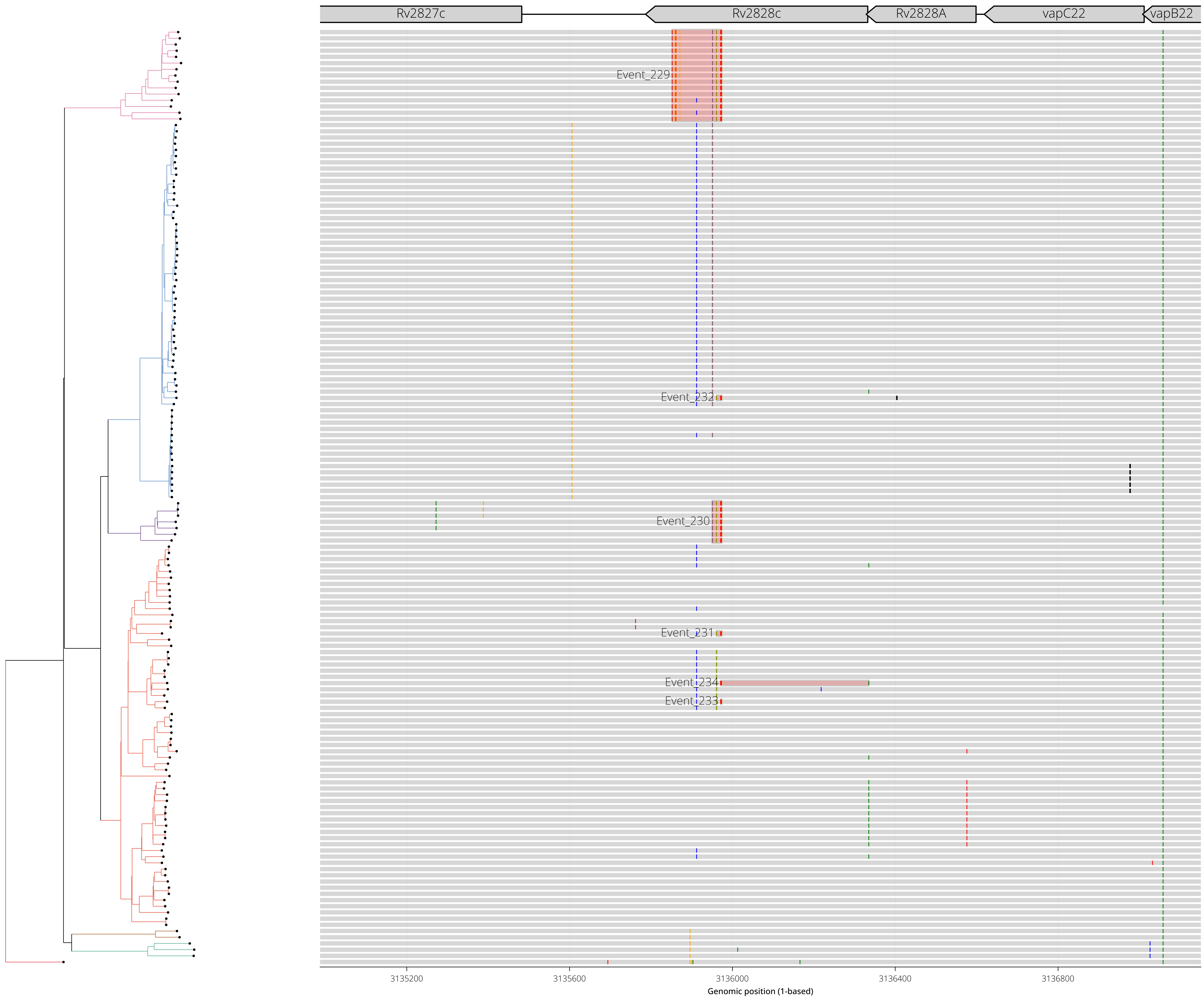

RegionID: PR_HmRegion_032 | Paralog Network ID: PR_Set_10
Genes: PE_PGRS10 | NC_000962.3:837685-841745
Mapped GCEs: 3 | Putative GCEs: 5

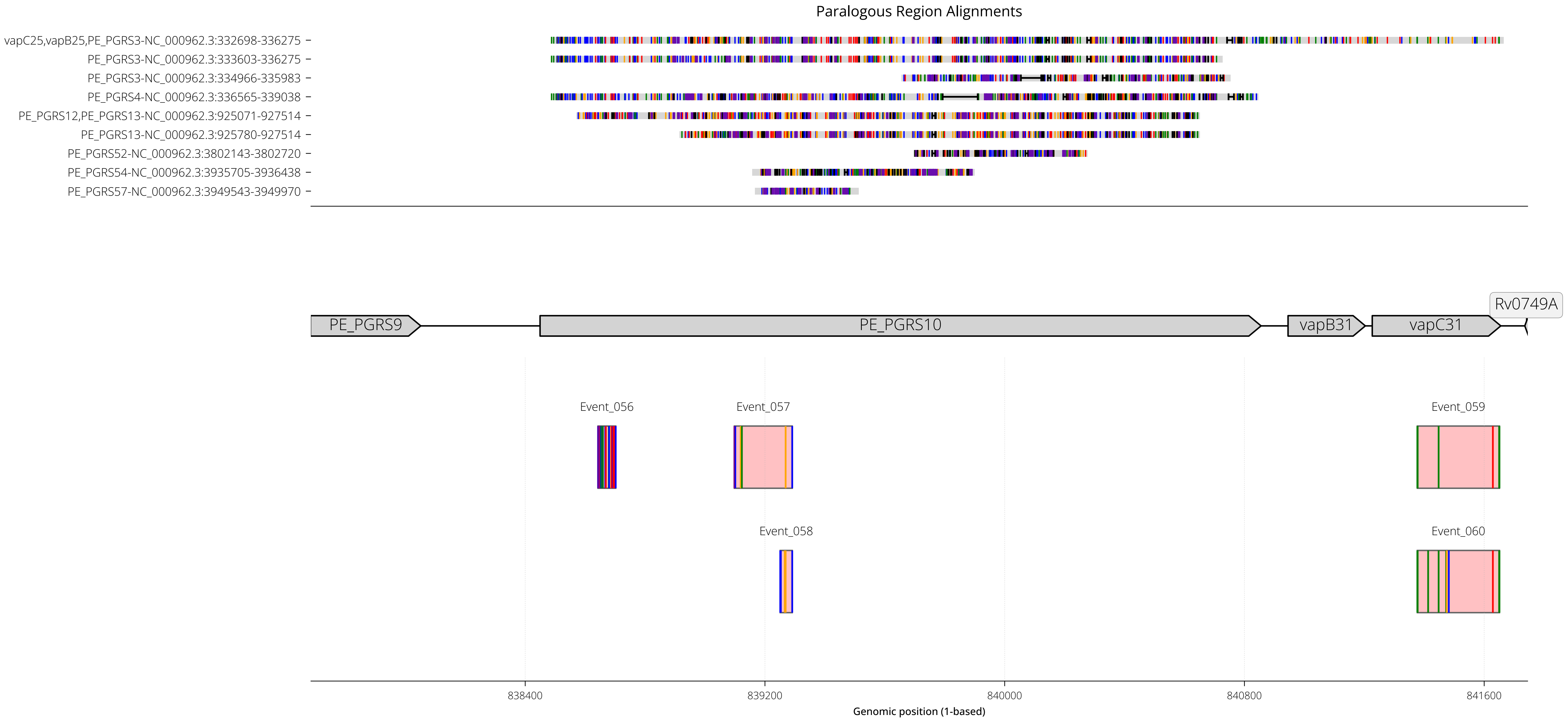

RegionID: PR_HmRegion_032 | Paralog Network ID: PR_Set_10
Genes: PE_PGRS10 | NC_000962.3:837685-841745
Mapped GCEs: 3 | Putative GCEs: 5

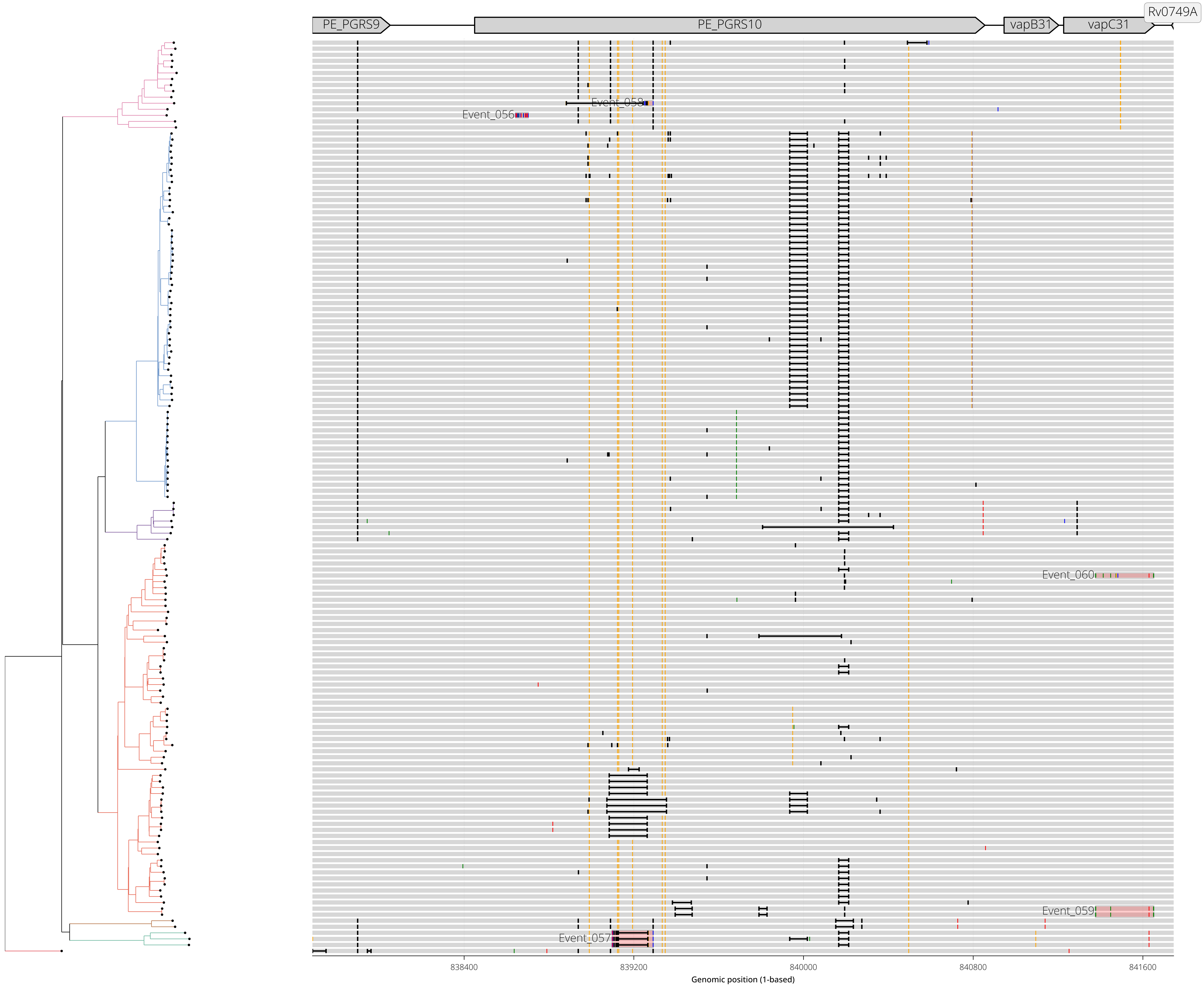

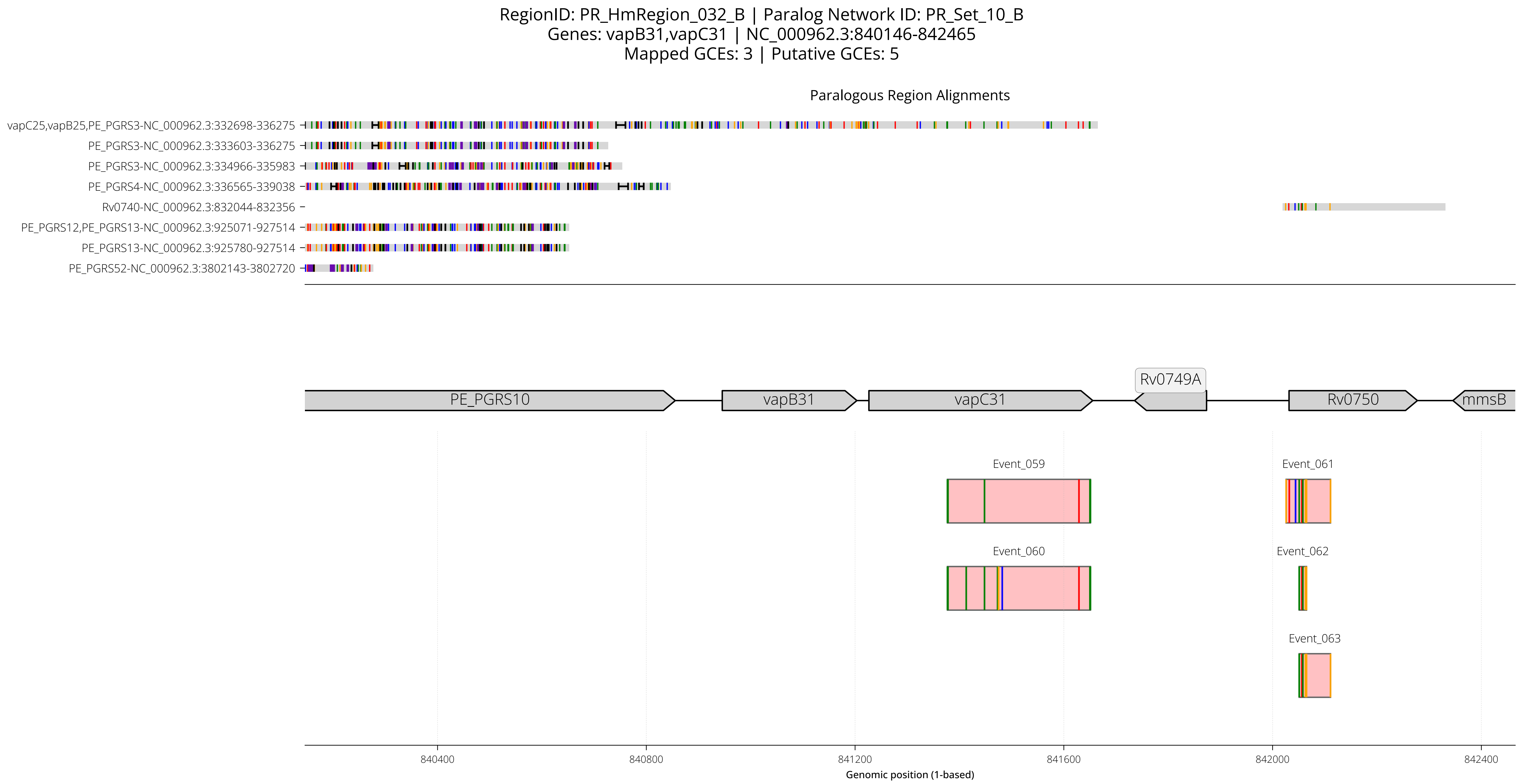
RegionID: PR_HmRegion_032_B | Paralog Network ID: PR_Set_10_B
Genes: vapB31,vapC31 | NC_000962.3:840146-842465
Mapped GCEs: 3 | Putative GCEs: 5
Paralogous Region Alignments
vapC25,vapB25,PE_PGRS3-NC_000962.3:332698-336275
PE_PGRS3-NC_000962.3:333603-336275
PE_PGRS3-NC_000962.3:334966-335983
PE_PGRS4-NC_000962.3:336565-339038
Rv0740-NC_000962.3:832044-832356
PE_PGRS12,PE_PGRS13-NC_000962.3:925071-927514
PE_PGRS13-NC_000962.3:925780-927514
PE_PGRS52-NC_000962.3:3802143-3802720
PE_PGRS10
vapB31
vapC31
Rv0749A
Rv0750
mmsB
Event_059
Event_061
Event_060
Event_062
Event_063
840400
840800
841200
841600
842000
842400
Genomic position (1-based)

RegionID: PR_HmRegion_032_B | Paralog Network ID: PR_Set_10_B
Genes: vapB31,vapC31 | NC_000962.3:840146-842465
Mapped GCEs: 3 | Putative GCEs: 5

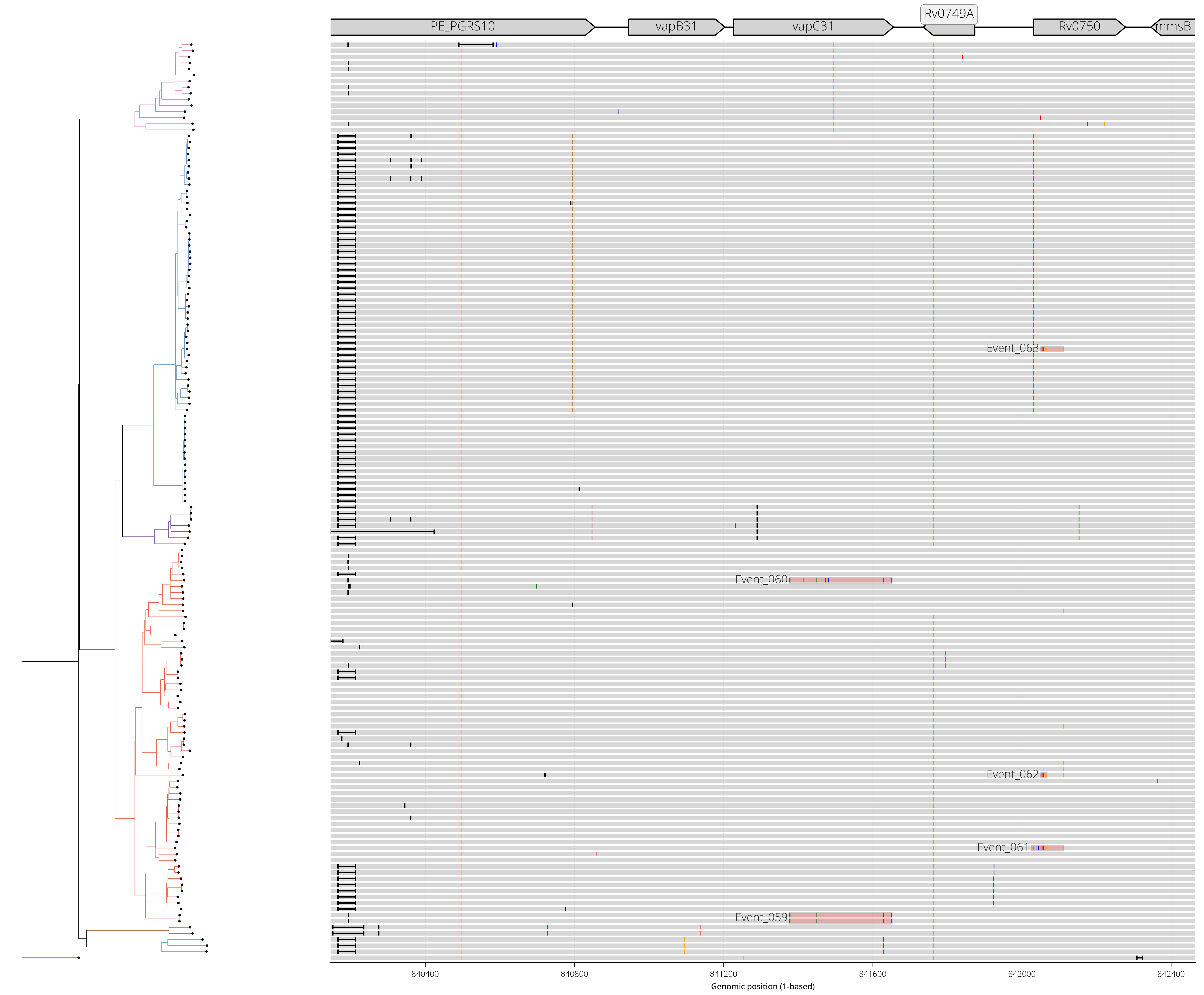

RegionID: PR_HmRegion_060 | Paralog Network ID: PR_Set_36
Genes: PPE19 | NC_000962.3:1531661-1534453
Mapped GCEs: 5 | Putative GCEs: 5

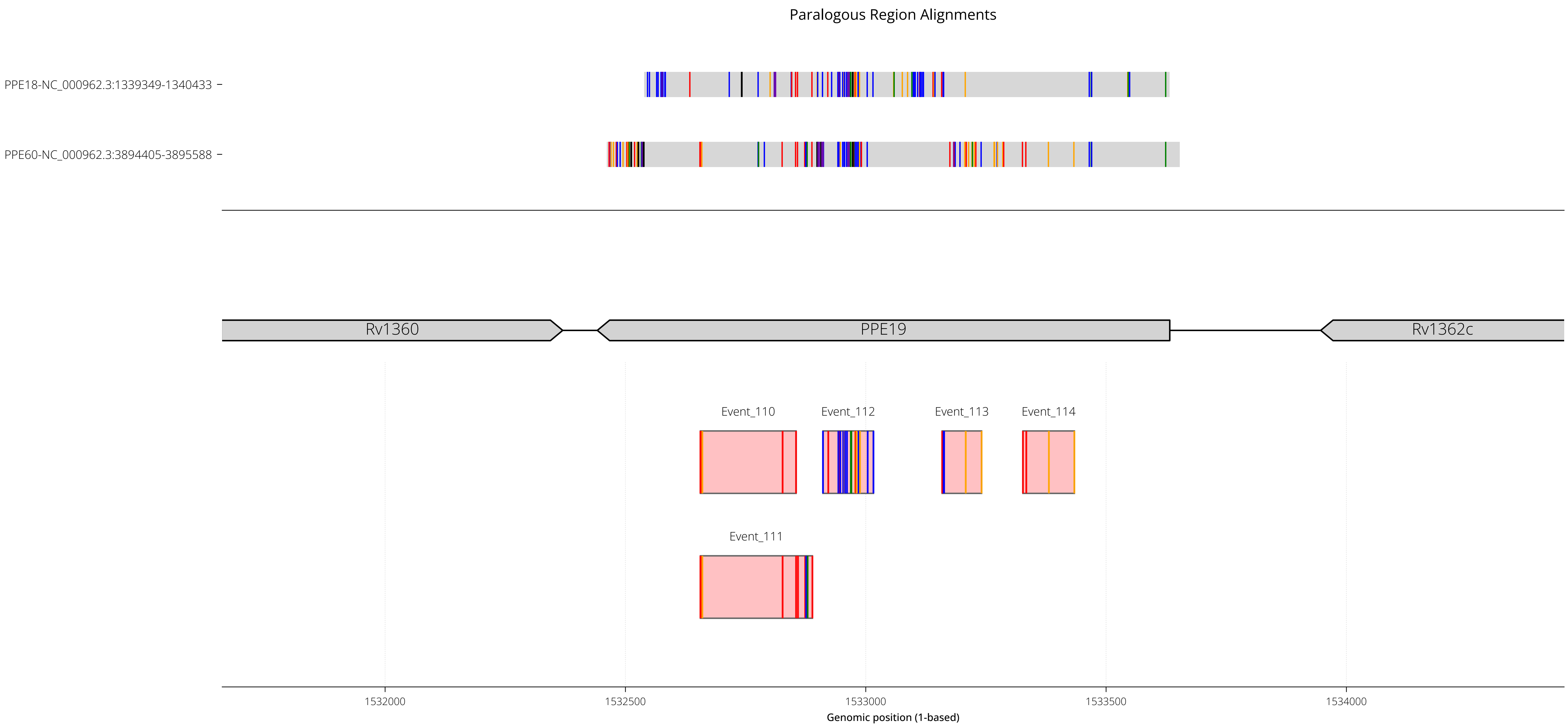

RegionID: PR_HmRegion_060 | Paralog Network ID: PR_Set_36
Genes: PPE19 | NC_000962.3:1531661-1534453
Mapped GCEs: 5 | Putative GCEs: 5

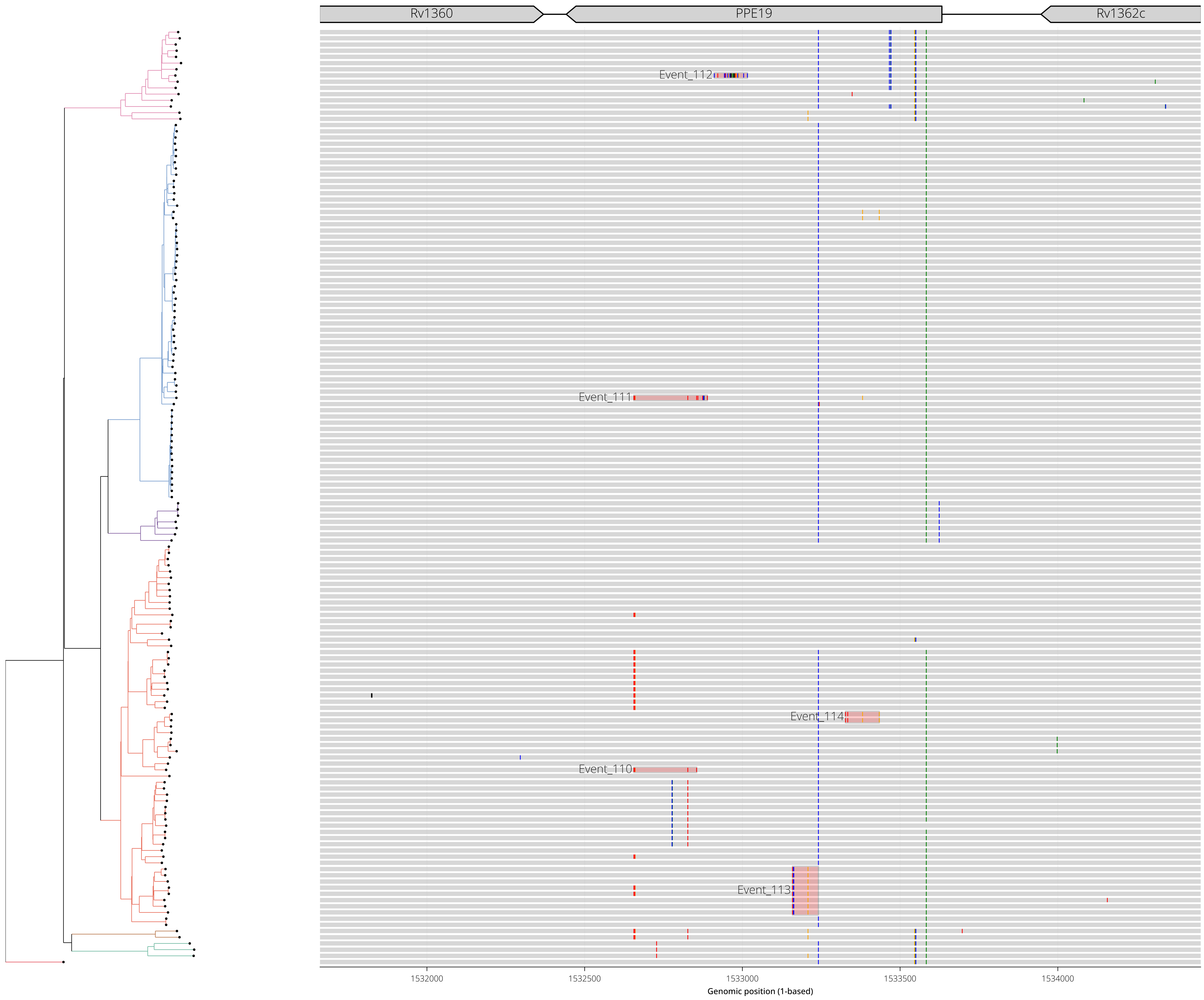

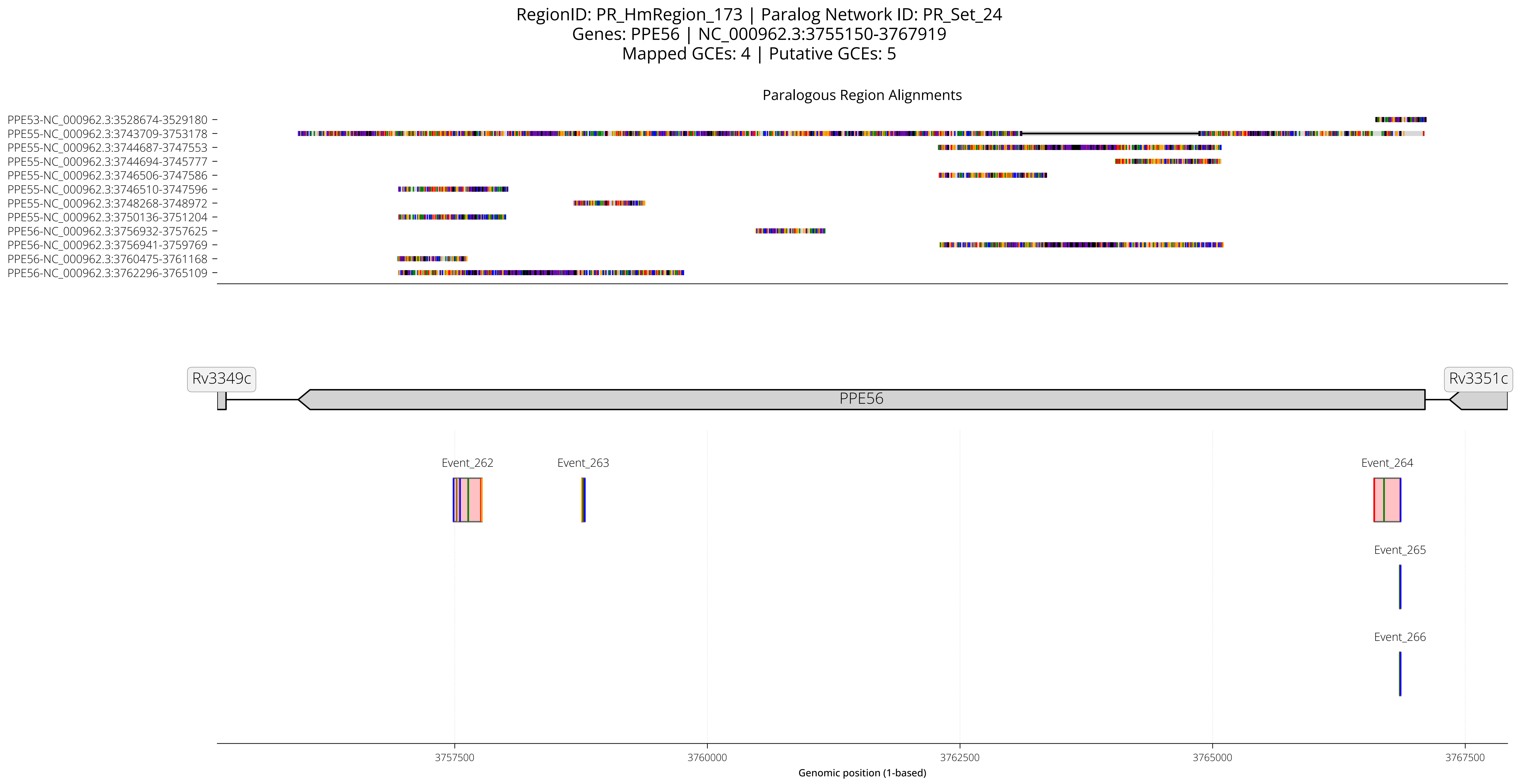
RegionID: PR_HmRegion_173 | Paralog Network ID: PR_Set_24
Genes: PPE56 | NC_000962.3:3755150-3767919
Mapped GCEs: 4 | Putative GCEs: 5
Paralogous Region Alignments
PPE53-NC_000962.3:3528674-3529180
PPE55-NC_000962.3:3743709-3753178
PPE55-NC_000962.3:3744687-3747553
PPE55-NC_000962.3:3744694-3745777
PPE55-NC_000962.3:3746506-3747586
PPE55-NC_000962.3:3746510-3747596
PPE55-NC_000962.3:3748268-3748972
PPE55-NC_000962.3:3750136-3751204
PPE56-NC_000962.3:3756932-3757625
PPE56-NC_000962.3:3756941-3759769
PPE56-NC_000962.3:3760475-3761168
PPE56-NC_000962.3:3762296-3765109
Rv3349c
PPE56
Rv3351c
Event_262
Event_263
Event_264
Event_265
Event_266
3757500
3760000
3762500
3765000
3767500
Genomic position (1-based)

RegionID: PR_HmRegion_173 | Paralog Network ID: PR_Set_24
Genes: PPE56 | NC_000962.3:3755150-3767919
Mapped GCEs: 4 | Putative GCEs: 5

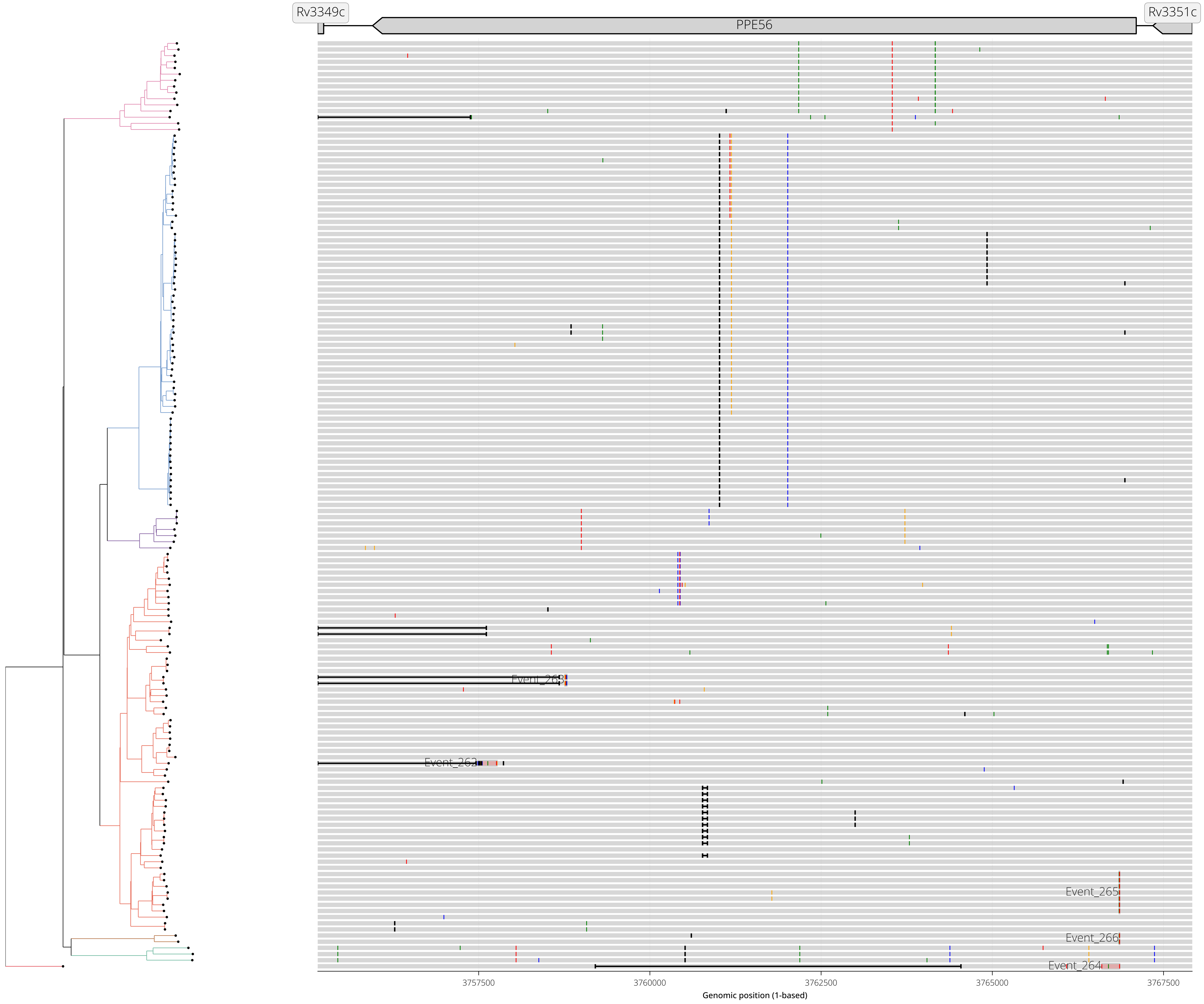

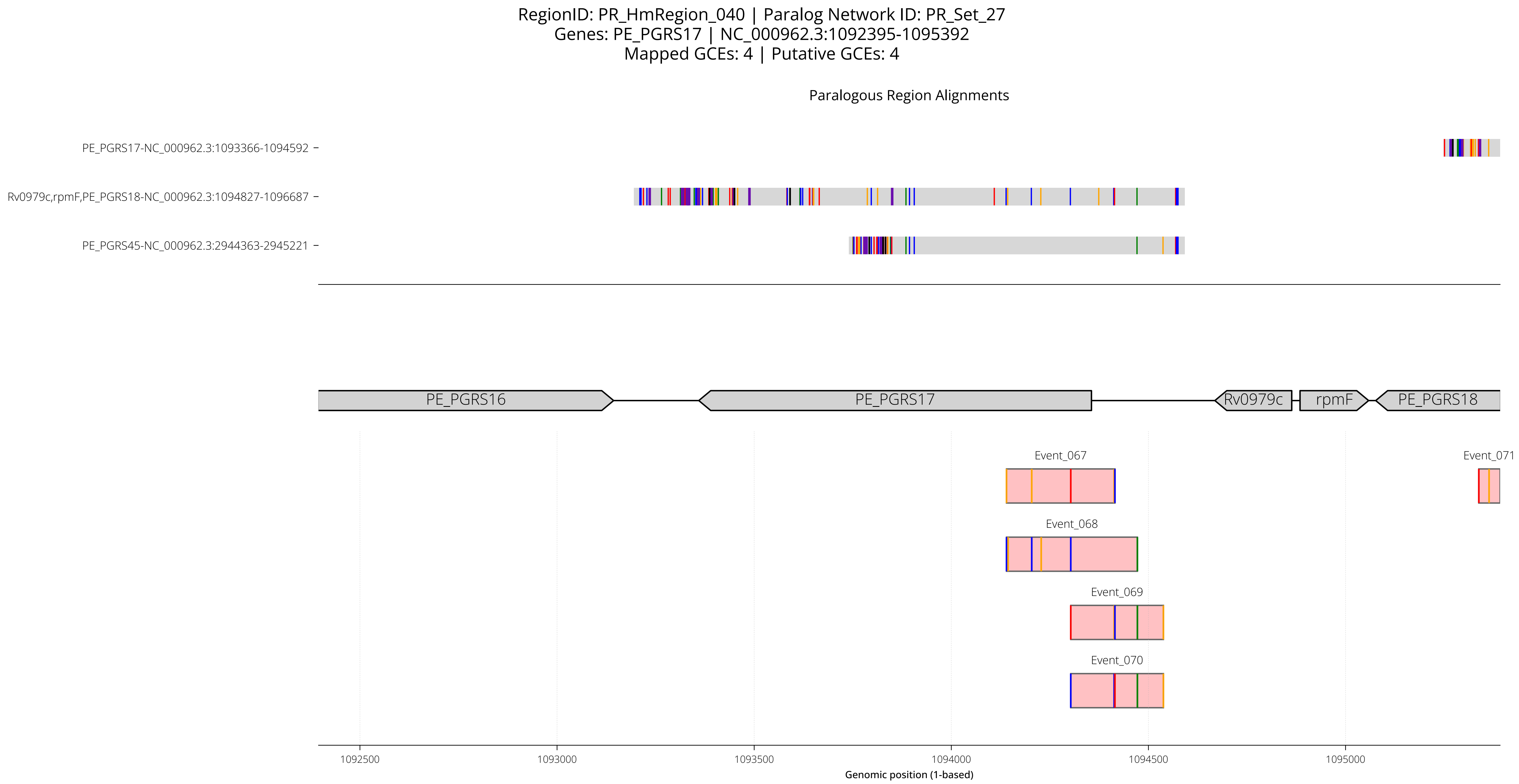

RegionID: PR_HmRegion_040 | Paralog Network ID: PR_Set_27
Genes: PE_PGRS17 | NC_000962.3:1092395-1095392
Mapped GCEs: 4 | Putative GCEs: 4
Paralogous Region Alignments
PE_PGRS17-NC_000962.3:1093366-1094592
Rv0979c,rpmF,PE_PGRS18-NC_000962.3:1094827-1096687
PE_PGRS45-NC_000962.3:2944363-2945221
PE_PGRS16
PE_PGRS17
Rv0979c
rpmF
PE_PGRS18
Event_067
Event_068
Event_069
Event_070
Event_071
1092500
1093000
1093500
1094000
1094500
1095000
Genomic position (1-based)

RegionID: PR_HmRegion_040 | Paralog Network ID: PR_Set_27
Genes: PE_PGRS17 | NC_000962.3:1092395-1095392
Mapped GCEs: 4 | Putative GCEs: 4

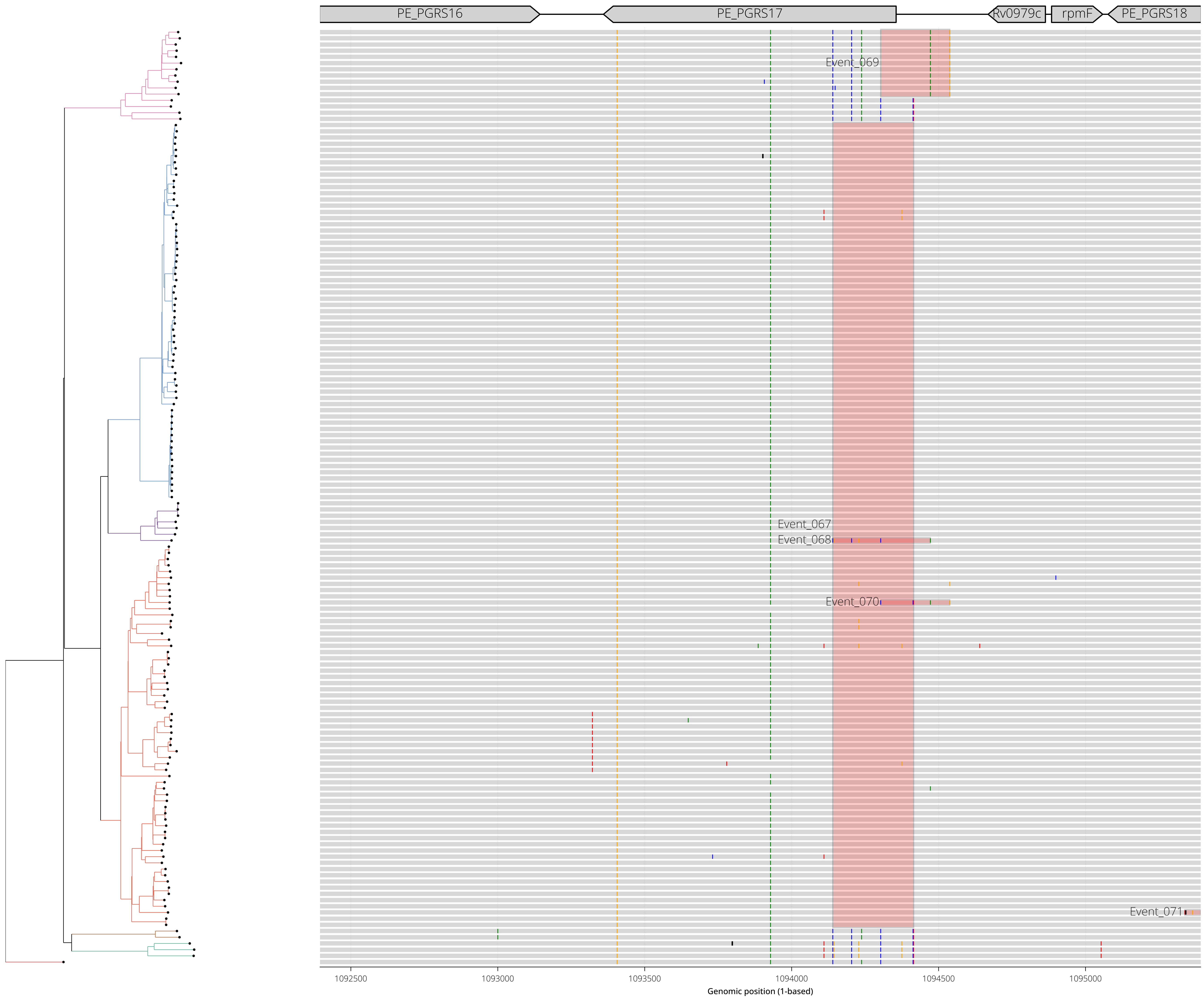

RegionID: PR_HmRegion_009_B | Paralog Network ID: PR_Set_10_B
Genes: vapC25,vapB25 | NC_000962.3:331898-334217
Mapped GCEs: 4 | Putative GCEs: 4

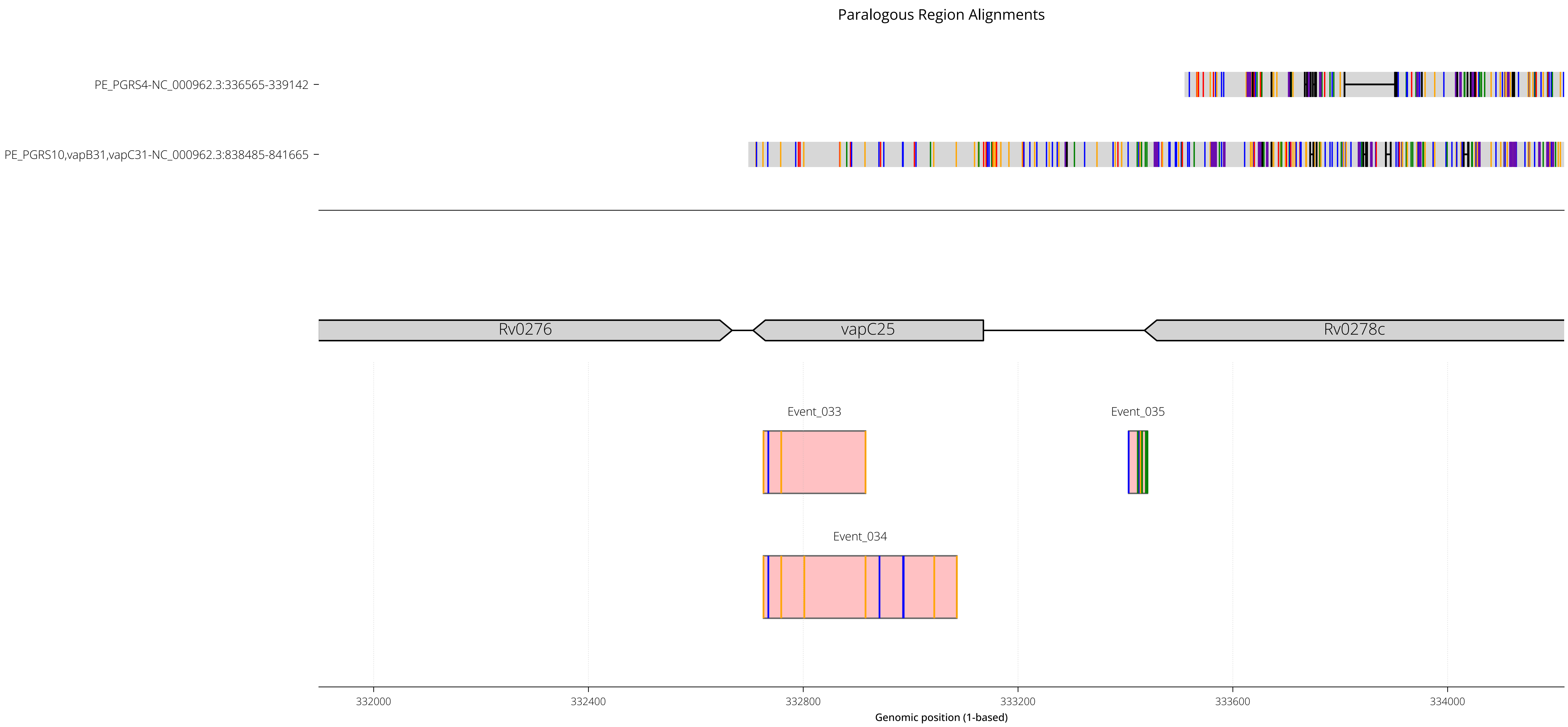

RegionID: PR_HmRegion_009_B | Paralog Network ID: PR_Set_10_B
Genes: vapC25,vapB25 | NC_000962.3:331898-334217
Mapped GCEs: 4 | Putative GCEs: 4

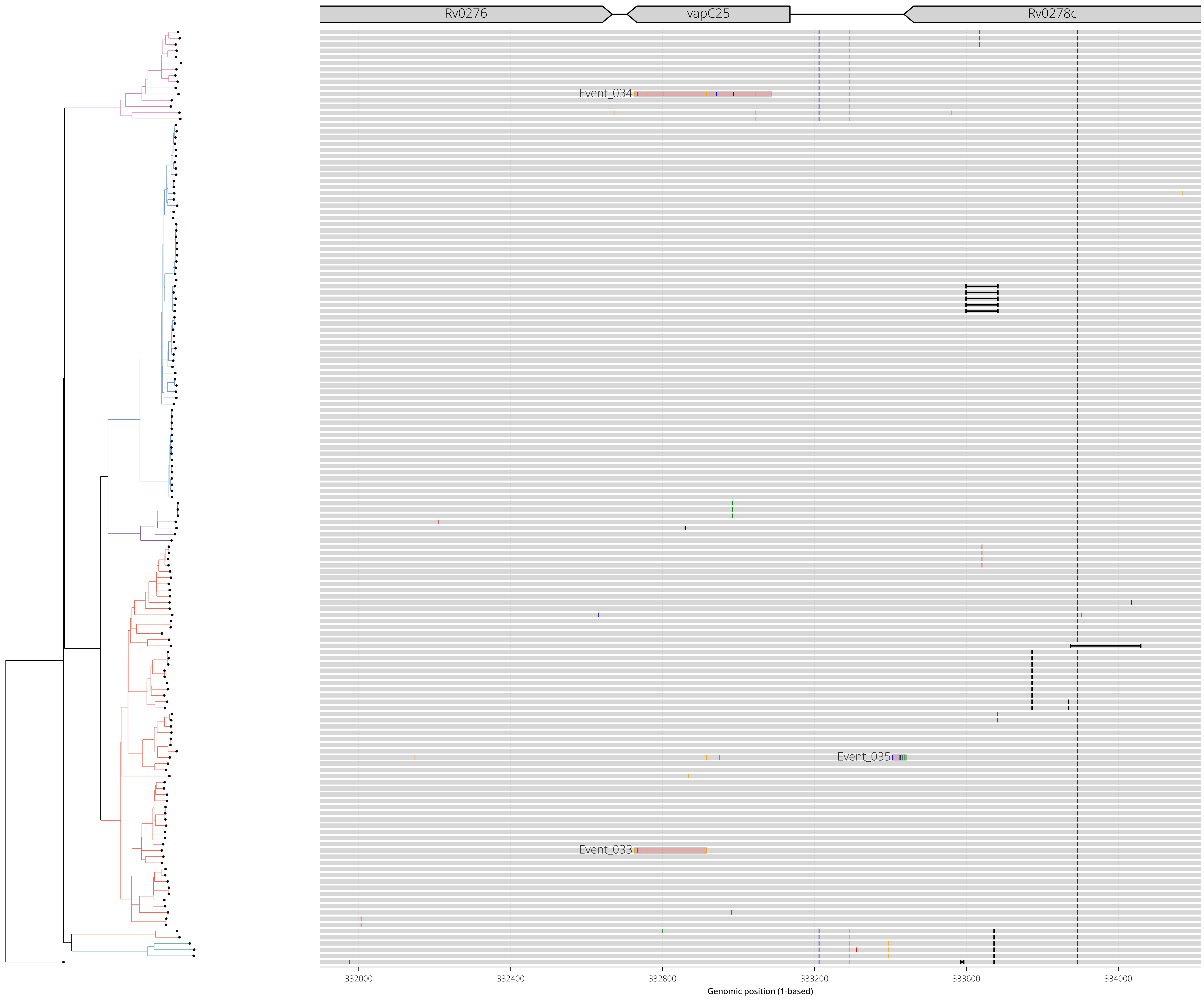

RegionID: PR_HmRegion_086 | Paralog Network ID: PR_Set_49
Genes: PPE27 | NC_000962.3:2027625-2030042
Mapped GCEs: 3 | Putative GCEs: 4

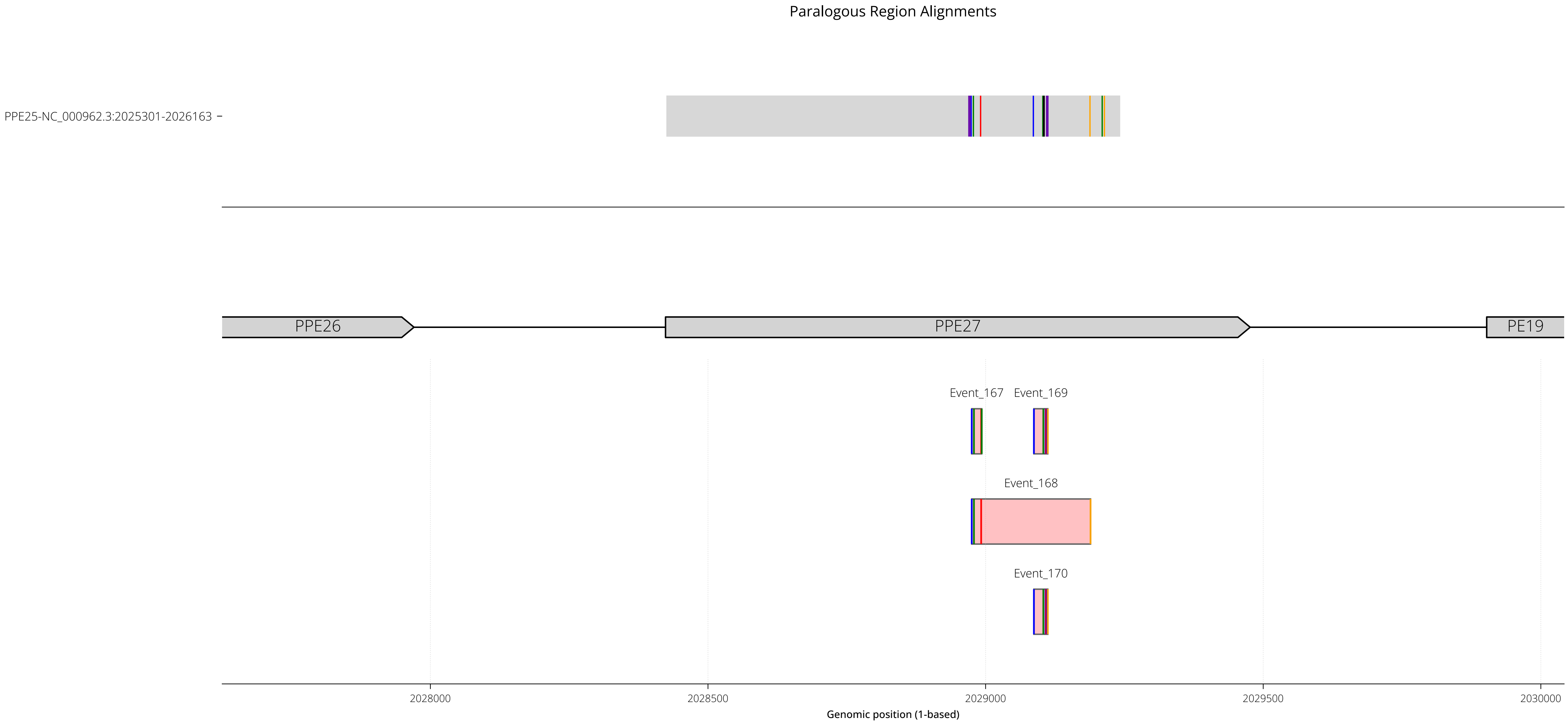

RegionID: PR_HmRegion_086 | Paralog Network ID: PR_Set_49
Genes: PPE27 | NC_000962.3:2027625-2030042
Mapped GCEs: 3 | Putative GCEs: 4

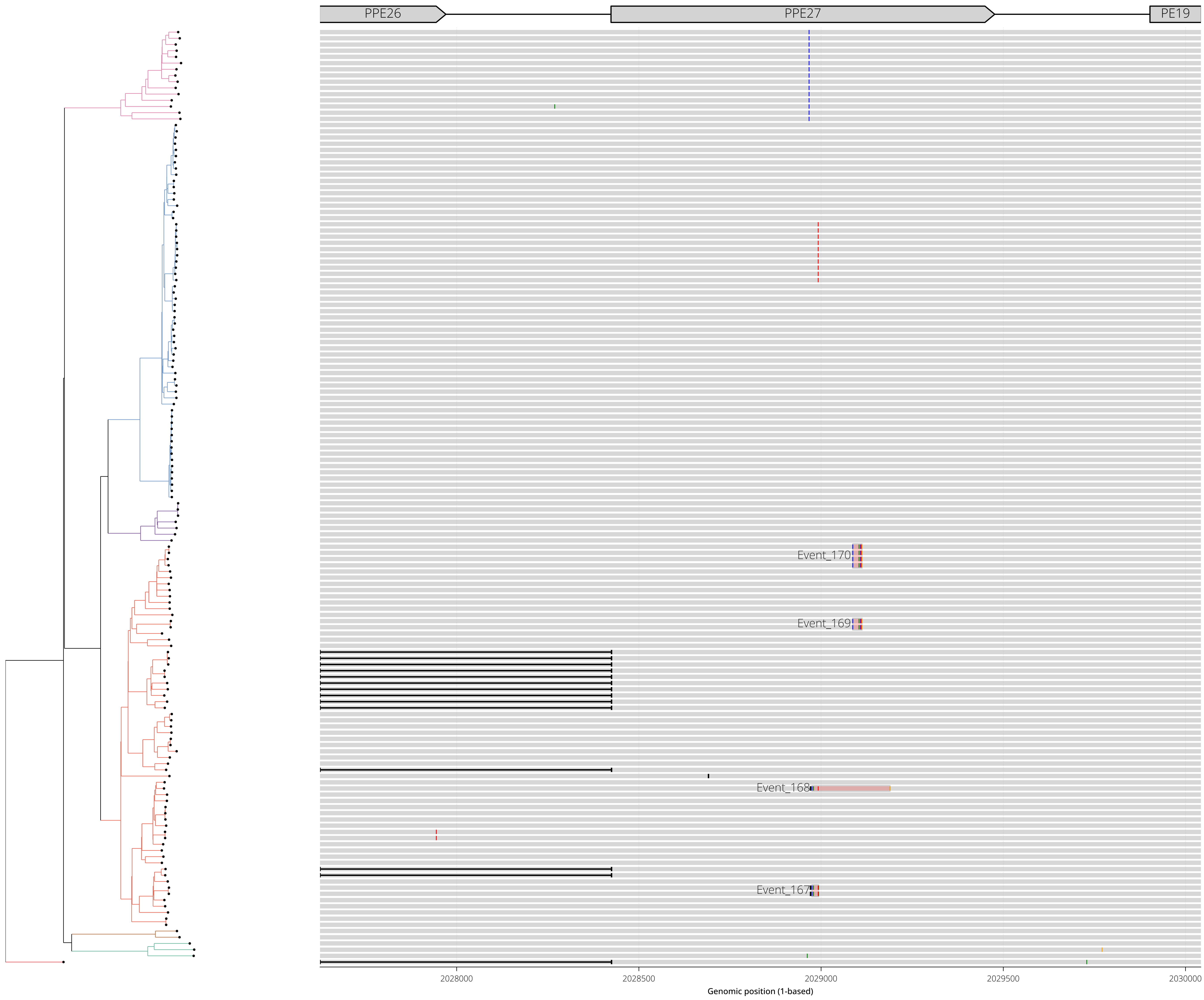

RegionID: PR_HmRegion_009 | Paralog Network ID: PR_Set_10
Genes: PE_PGRS3 | NC_000962.3:332618-337179
Mapped GCEs: 4 | Putative GCEs: 4

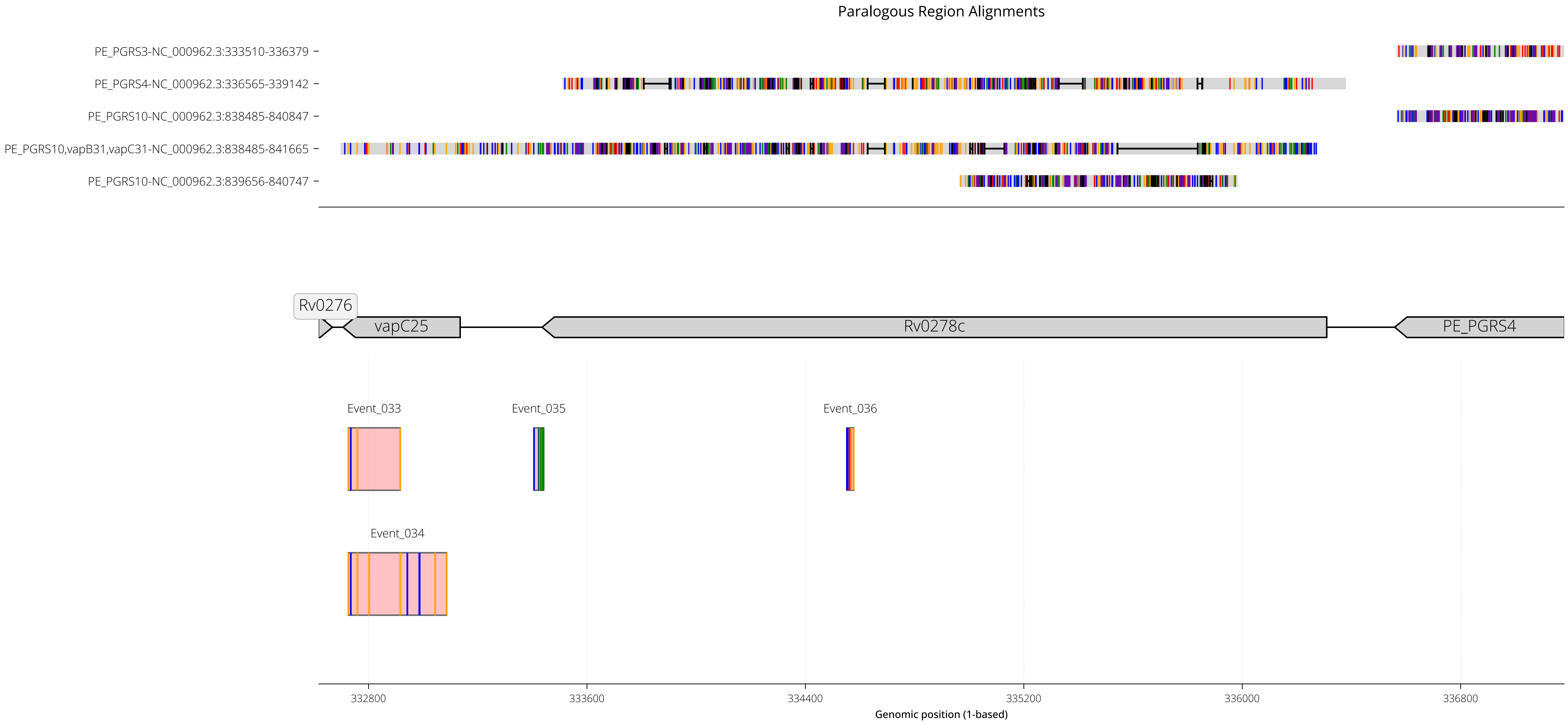

RegionID: PR_HmRegion_009 | Paralog Network ID: PR_Set_10
Genes: PE_PGRS3 | NC_000962.3:332618-337179
Mapped GCEs: 4 | Putative GCEs: 4

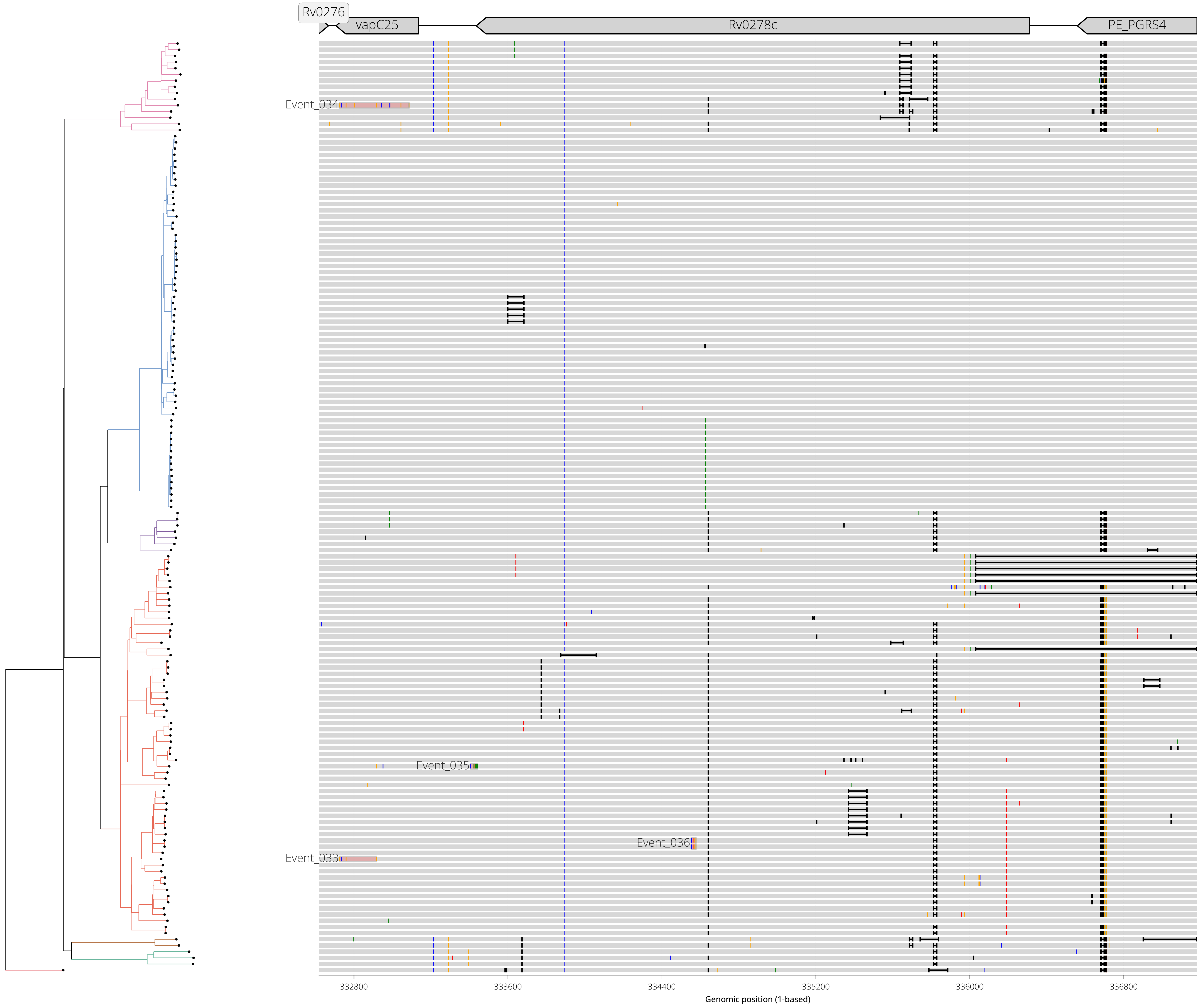

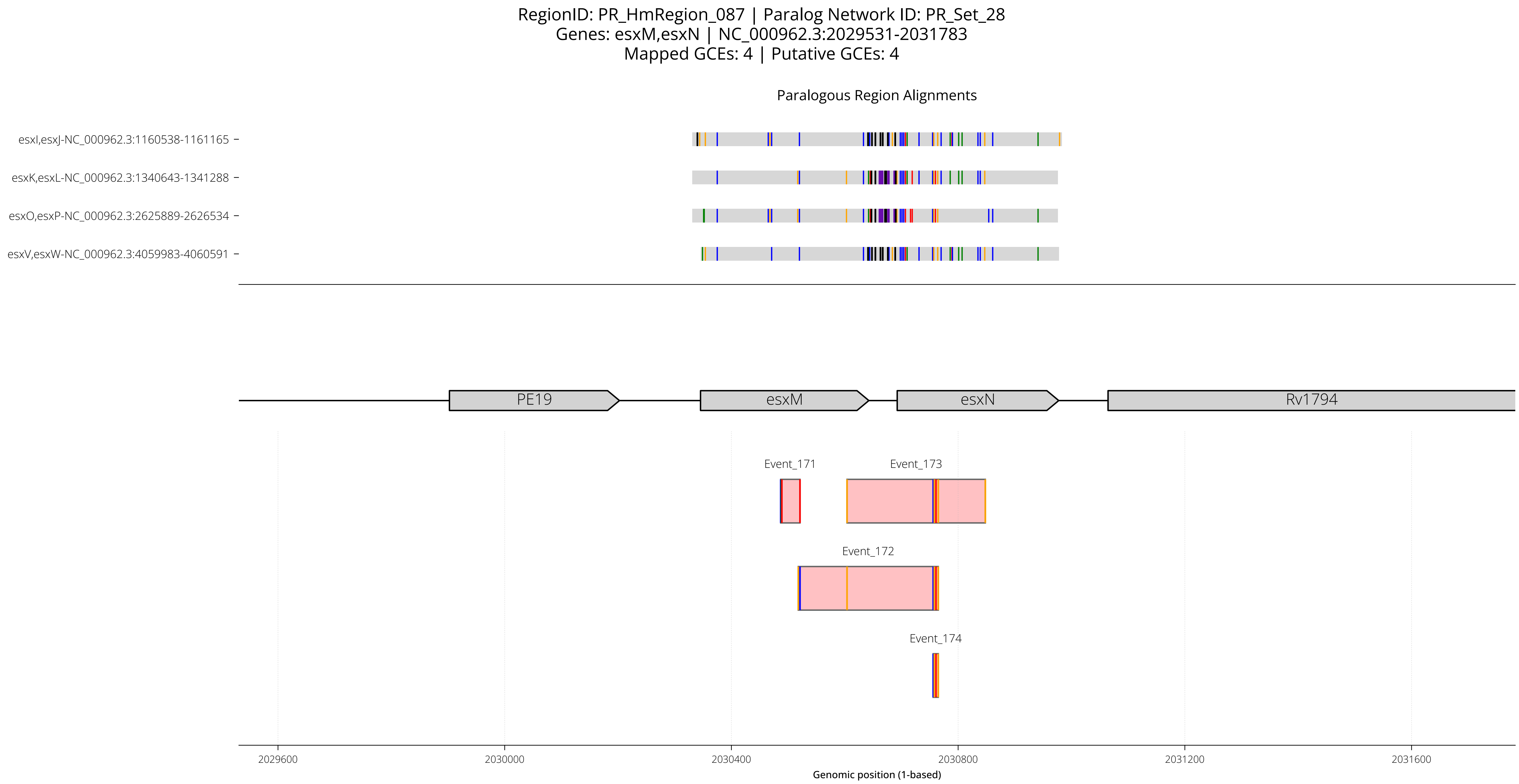
RegionID: PR_HmRegion_087 | Paralog Network ID: PR_Set_28
Genes: esxM,esxN | NC_000962.3:2029531-2031783
Mapped GCEs: 4 | Putative GCEs: 4
Paralogous Region Alignments
esxI,esxJ-NC_000962.3:1160538-1161165
esxK,esxL-NC_000962.3:1340643-1341288
esxO,esxP-NC_000962.3:2625889-2626534
esxV,esxW-NC_000962.3:4059983-4060591
PE19
esxM
esxN
Rv1794
Event_171
Event_173
Event_172
Event_174
2029600
2030000
2030400
2030800
2031200
2031600
Genomic position (1-based)

RegionID: PR_HmRegion_087 | Paralog Network ID: PR_Set_28
Genes: esxM,esxN | NC_000962.3:2029531-2031783
Mapped GCEs: 4 | Putative GCEs: 4

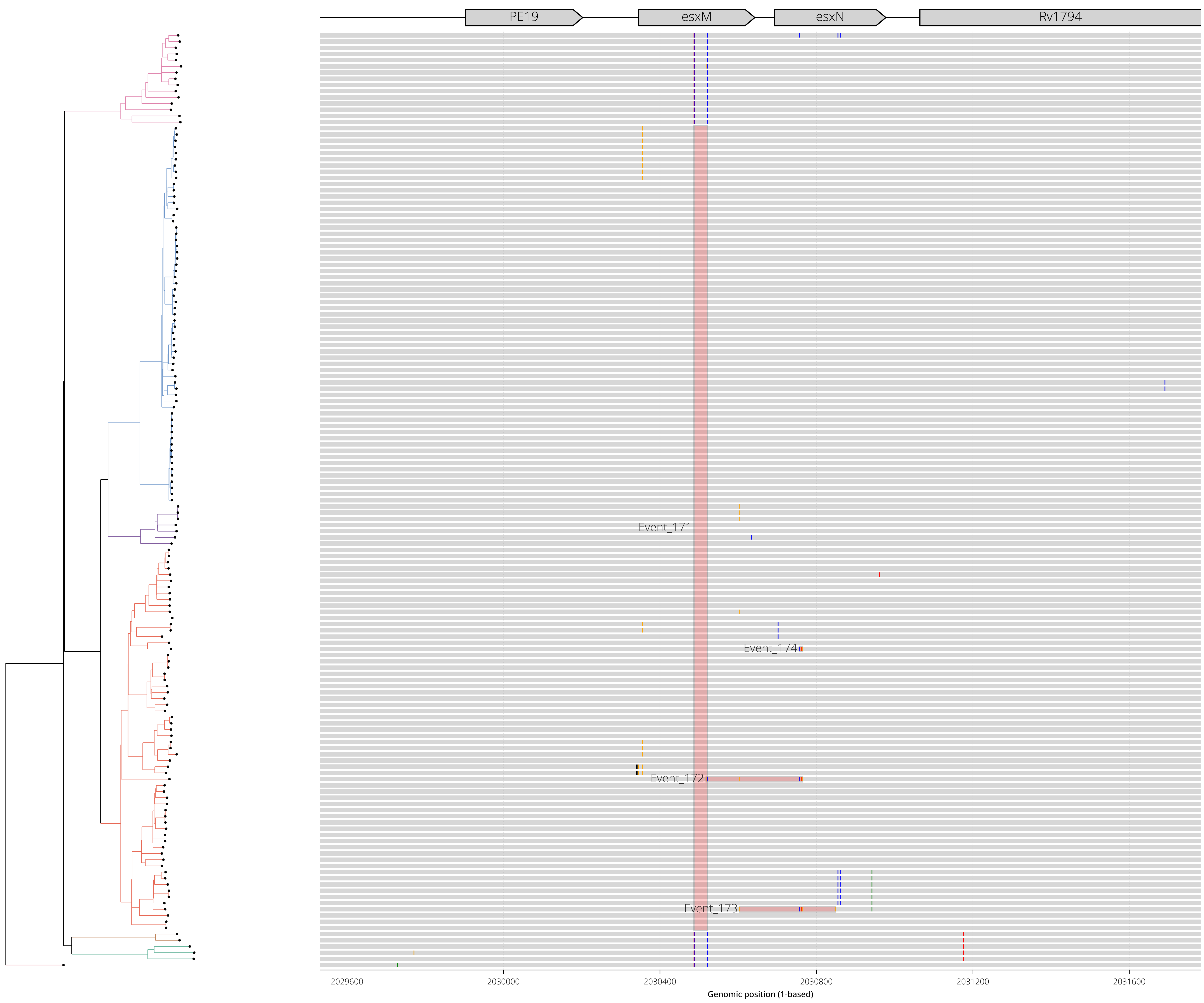

RegionID: PR_HmRegion_177 | Paralog Network ID: PR_Set_48
Genes: Rv3424c,PPE57 | NC_000962.3:3840732-3843704
Mapped GCEs: 1 | Putative GCEs: 3

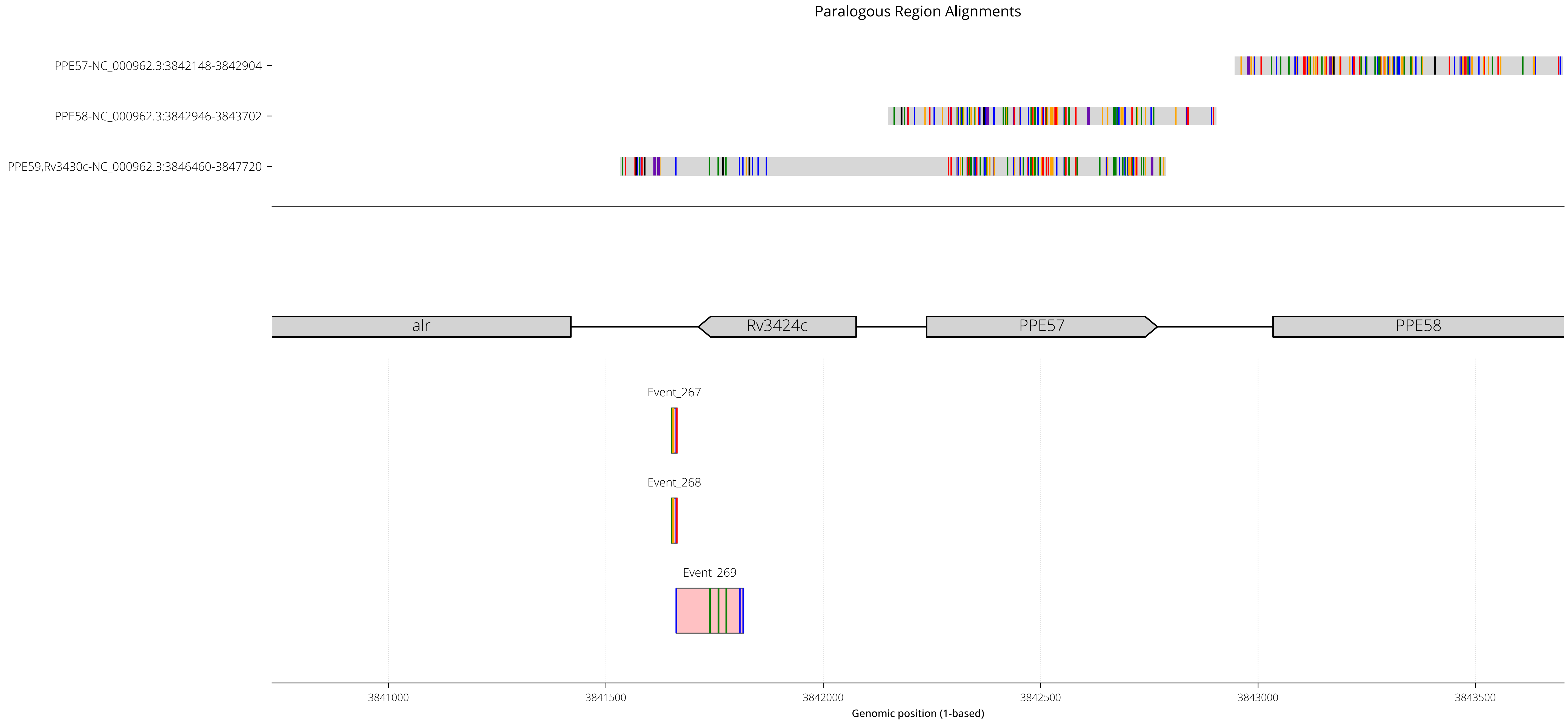

RegionID: PR_HmRegion_177 | Paralog Network ID: PR_Set_48
Genes: Rv3424c,PPE57 | NC_000962.3:3840732-3843704
Mapped GCEs: 1 | Putative GCEs: 3

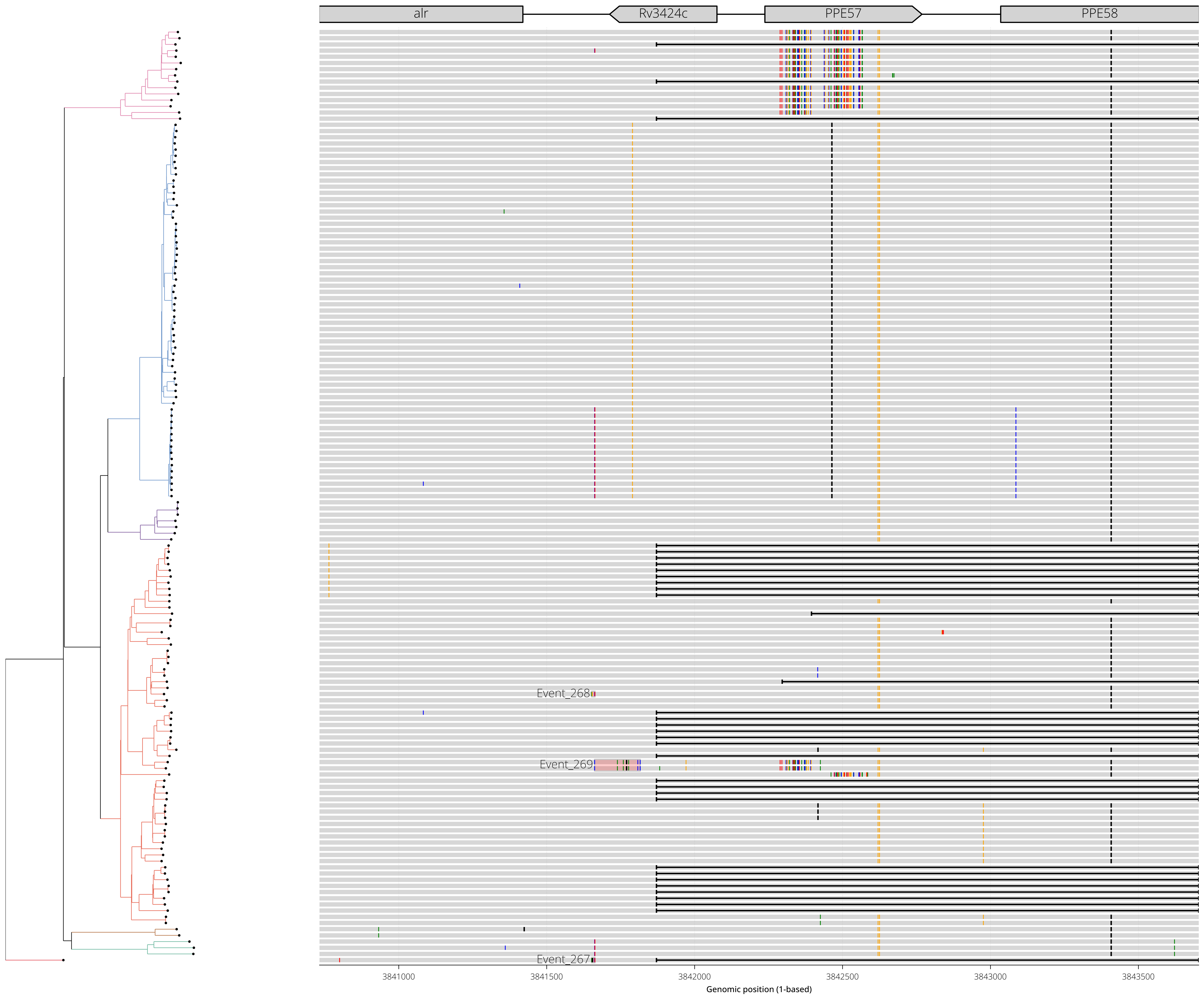

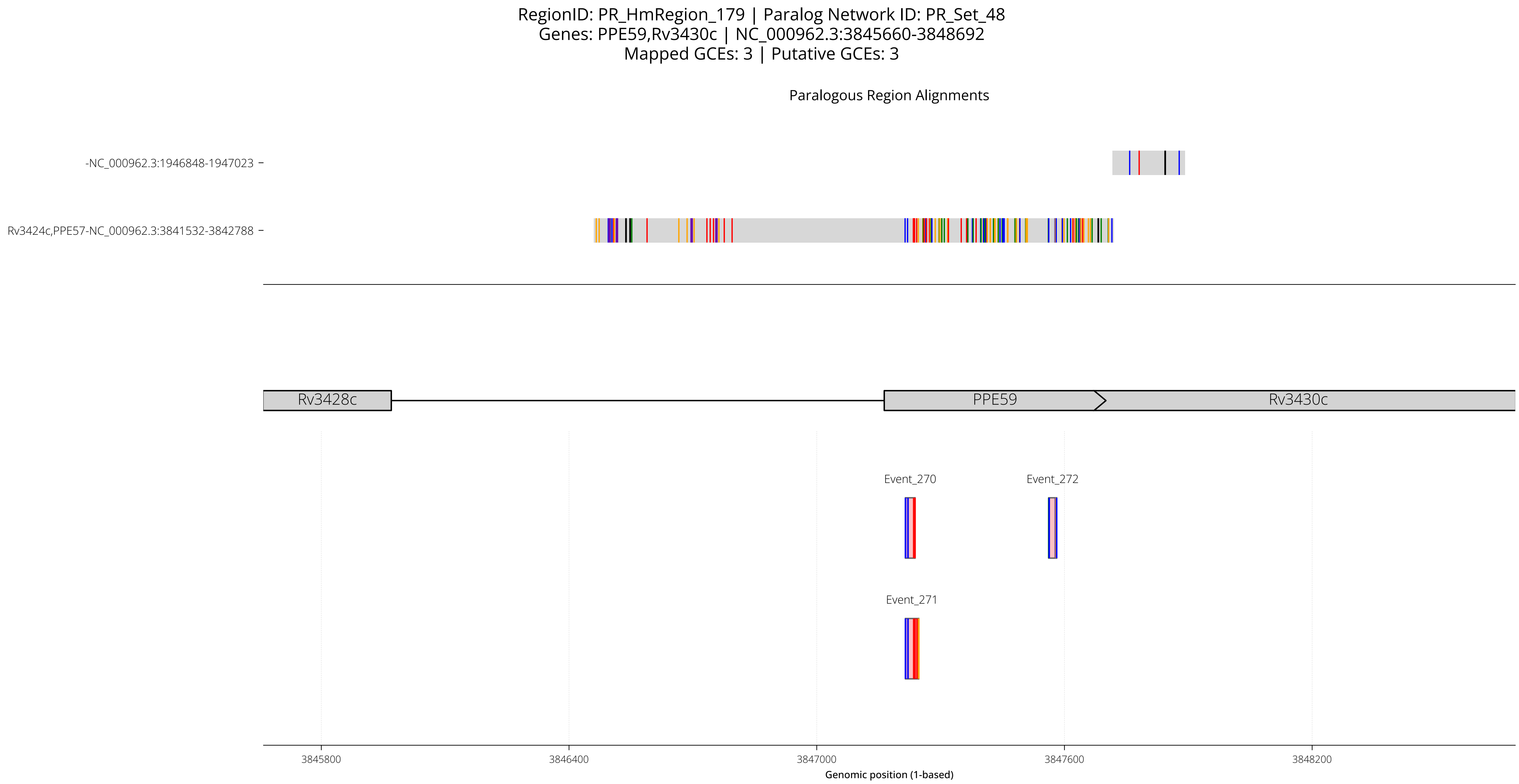
RegionID: PR_HmRegion_179 | Paralog Network ID: PR_Set_48
Genes: PPE59,Rv3430c | NC_000962.3:3845660-3848692
Mapped GCEs: 3 | Putative GCEs: 3
Paralogous Region Alignments
-NC_000962.3:1946848-1947023
Rv3424c,PPE57-NC_000962.3:3841532-3842788
Rv3428c
PPE59
Rv3430c
Event_270
Event_272
Event_271
3845800
3846400
3847000
3847600
3848200
Genomic position (1-based)

RegionID: PR_HmRegion_179 | Paralog Network ID: PR_Set_48
Genes: PPE59,Rv3430c | NC_000962.3:3845660-3848692
Mapped GCEs: 3 | Putative GCEs: 3

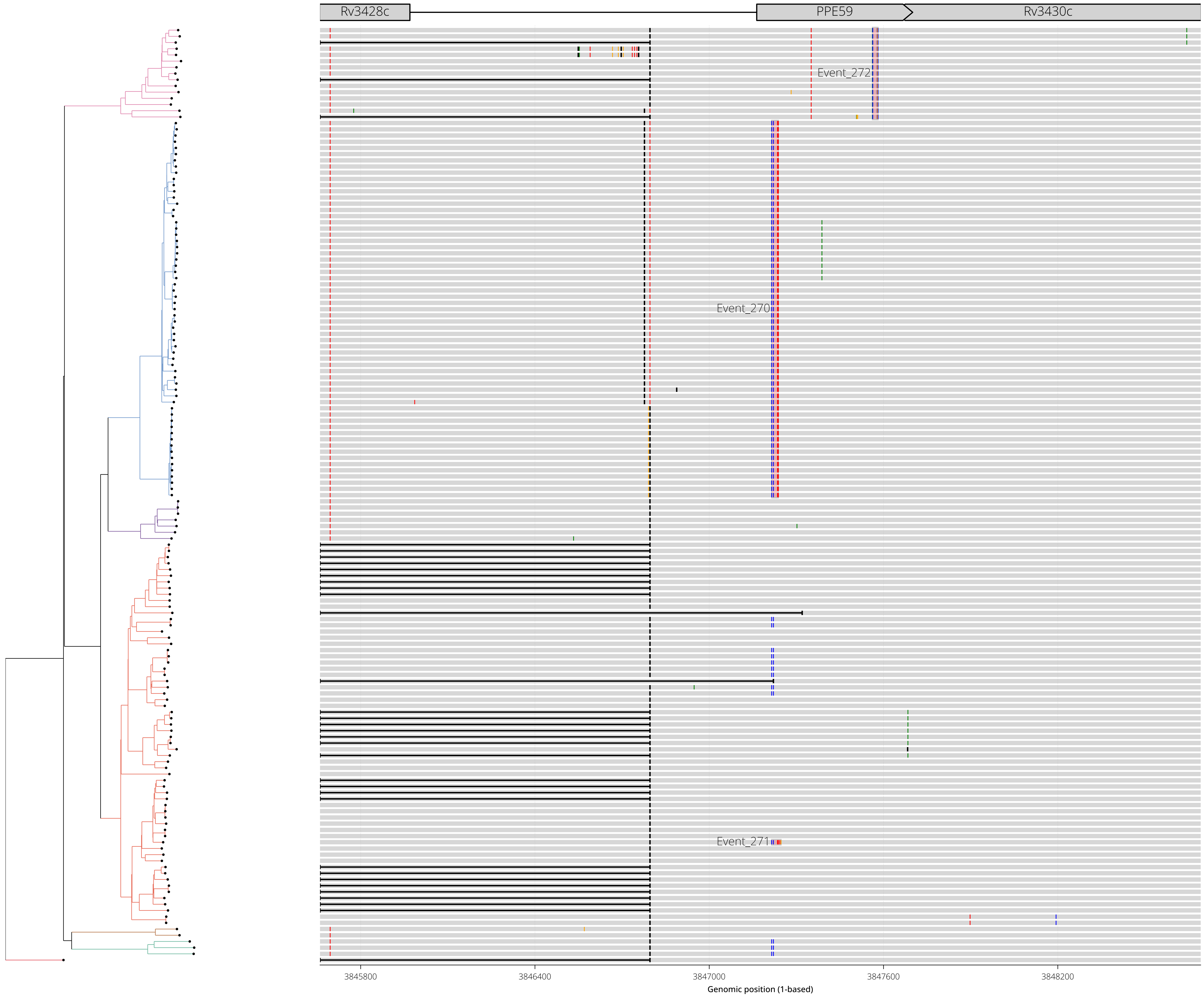

RegionID: PR_HmRegion_125 | Paralog Network ID: PR_Set_58
Genes: lppA,lppB | NC_000962.3:2866324-2868577
Mapped GCEs: 0 | Putative GCEs: 3

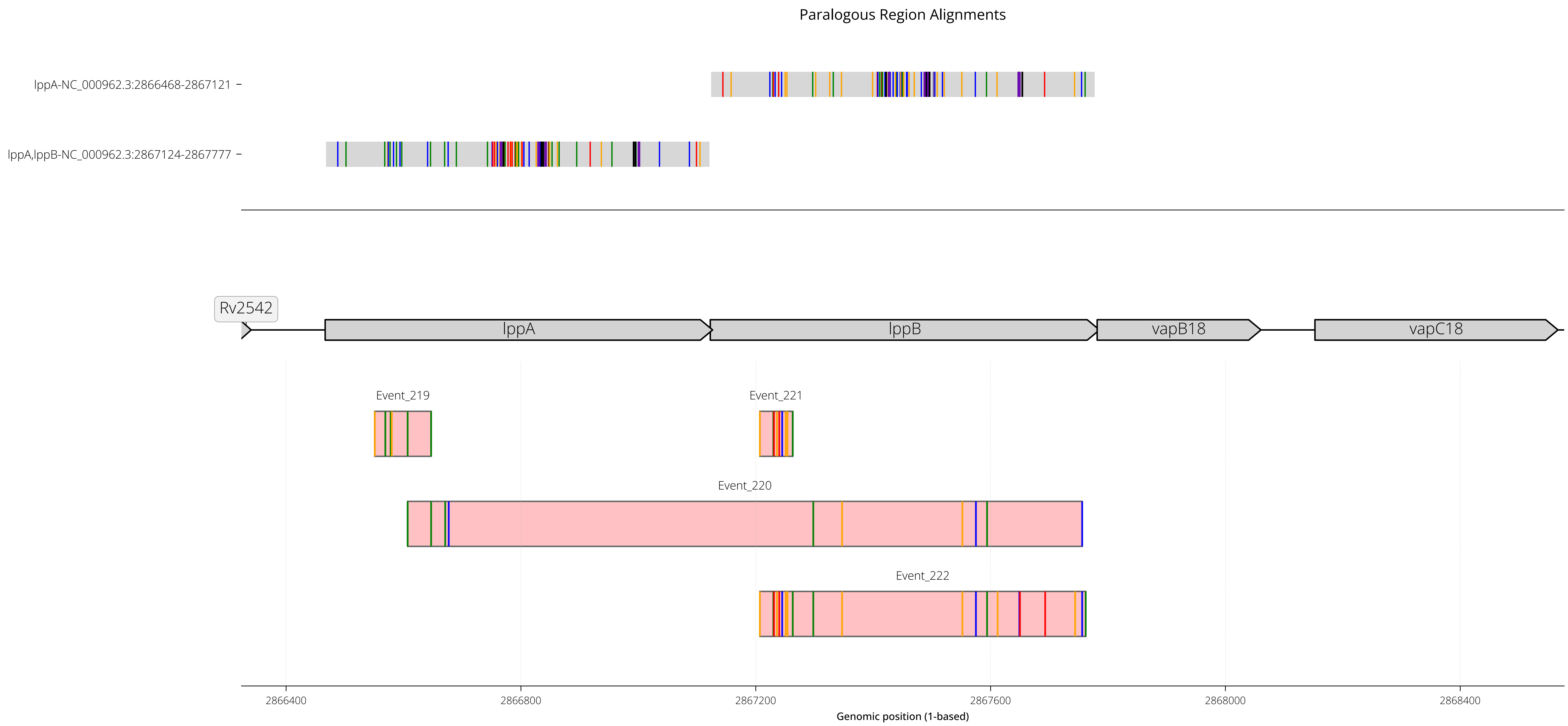

RegionID: PR_HmRegion_125 | Paralog Network ID: PR_Set_58
Genes: lppA,lppB | NC_000962.3:2866324-2868577
Mapped GCEs: 0 | Putative GCEs: 3

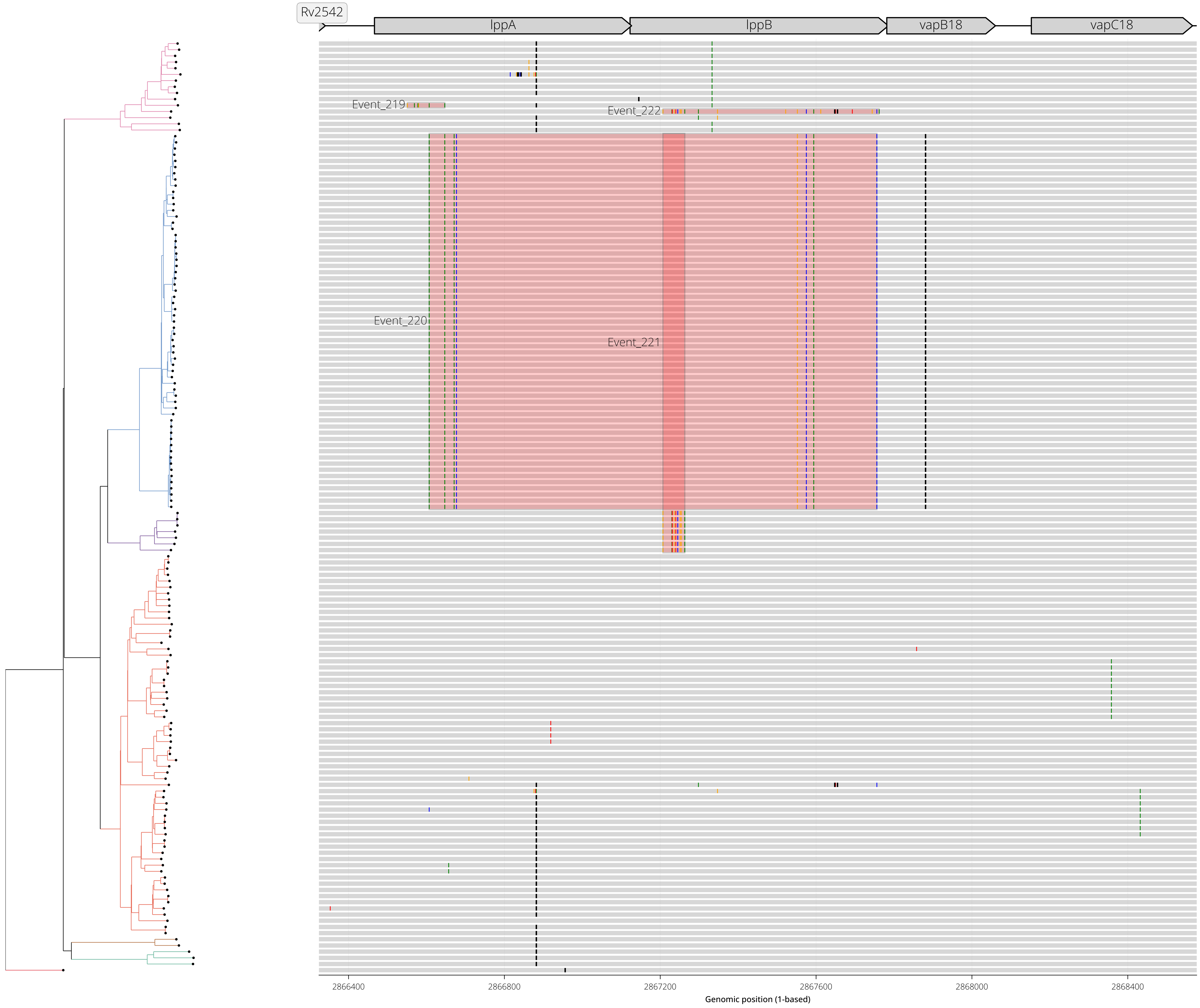

RegionID: PR_HmRegion_185 | Paralog Network ID: PR_Set_10
Genes: PE_PGRS57,fadD19 | NC_000962.3:3944977-3952129
Mapped GCEs: 2 | Putative GCEs: 3

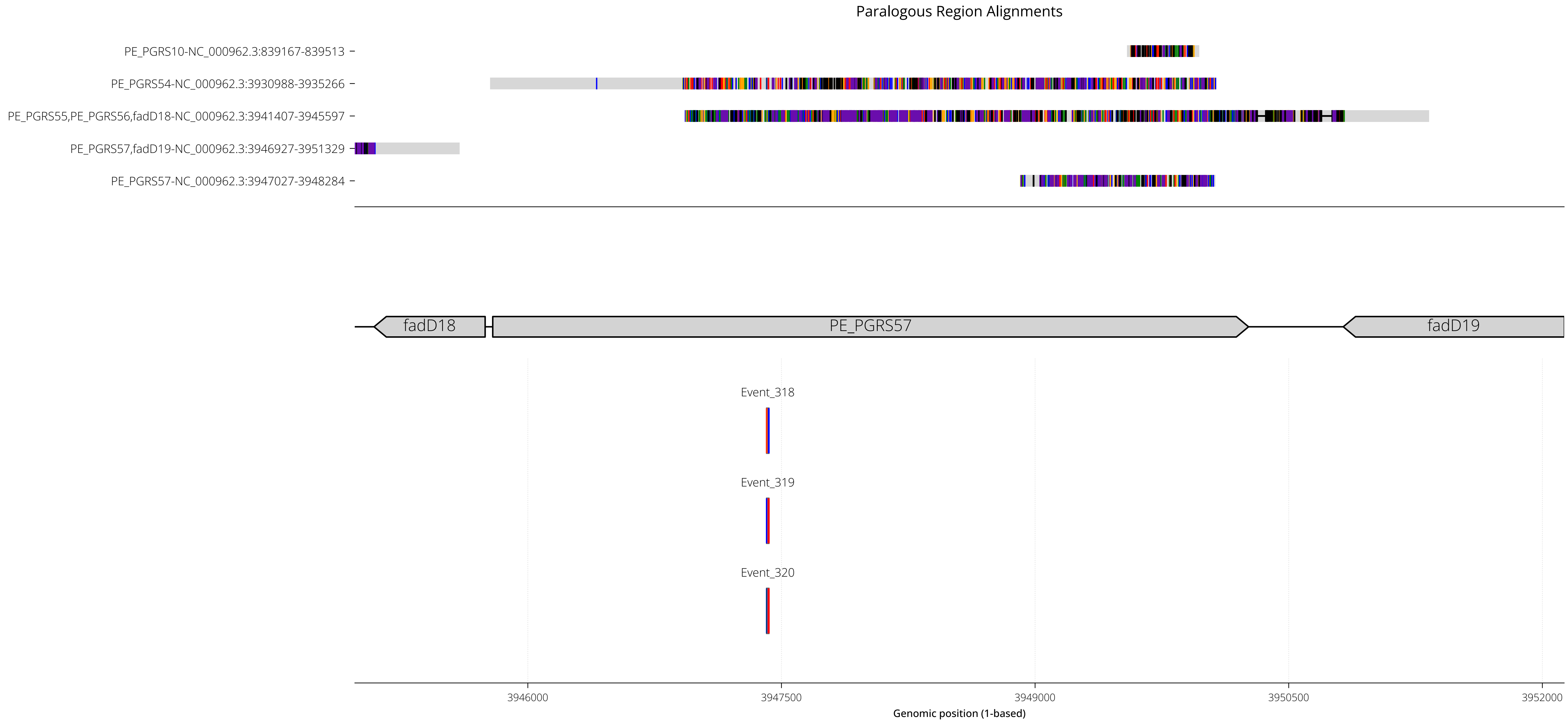

RegionID: PR_HmRegion_185 | Paralog Network ID: PR_Set_10
Genes: PE_PGRS57,fadD19 | NC_000962.3:3944977-3952129
Mapped GCEs: 2 | Putative GCEs: 3

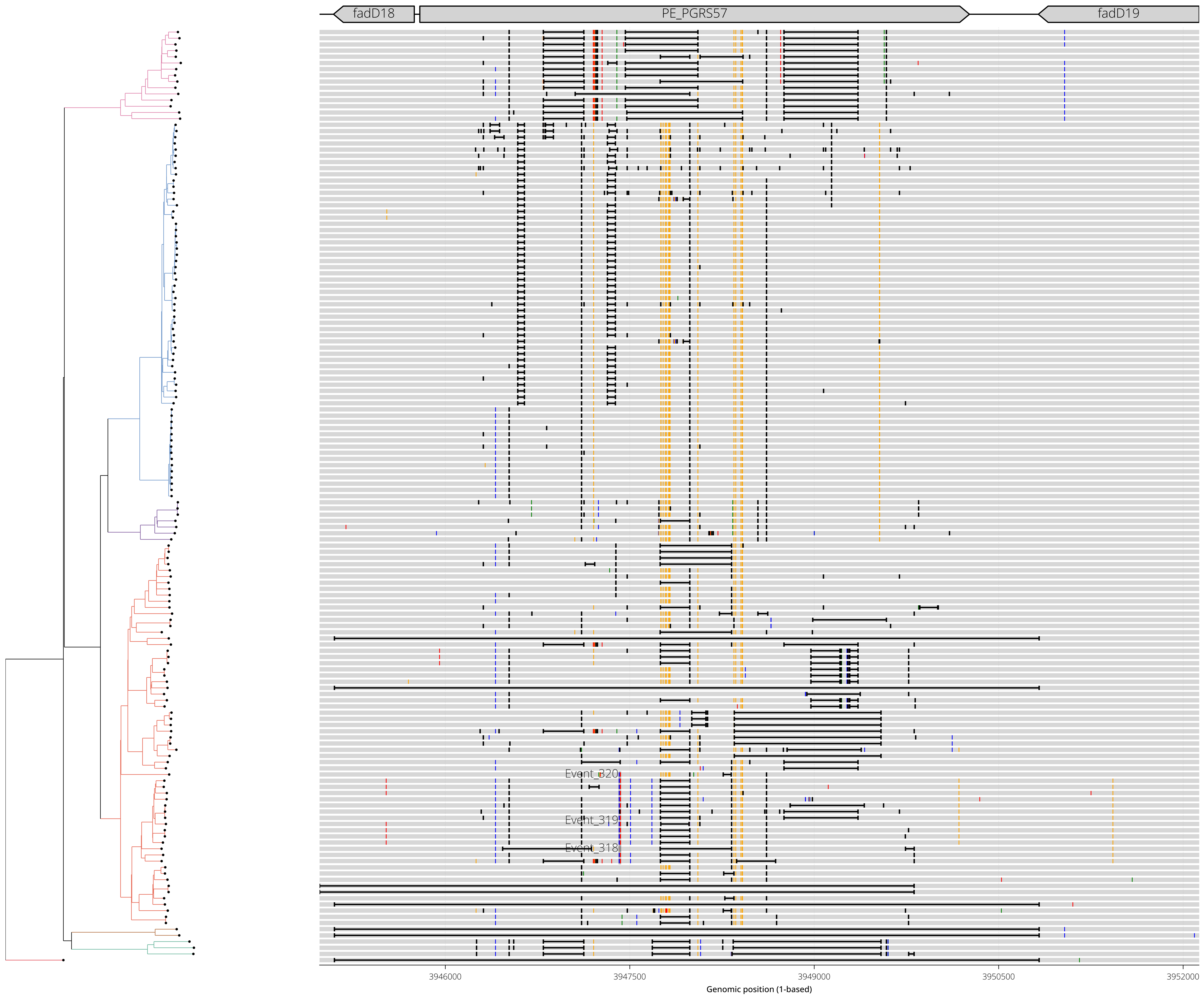

RegionID: PR_HmRegion_033 | Paralog Network ID: PR_Set_22
Genes: Rv0750 | NC_000962.3:841219-843131
Mapped GCEs: 3 | Putative GCEs: 3

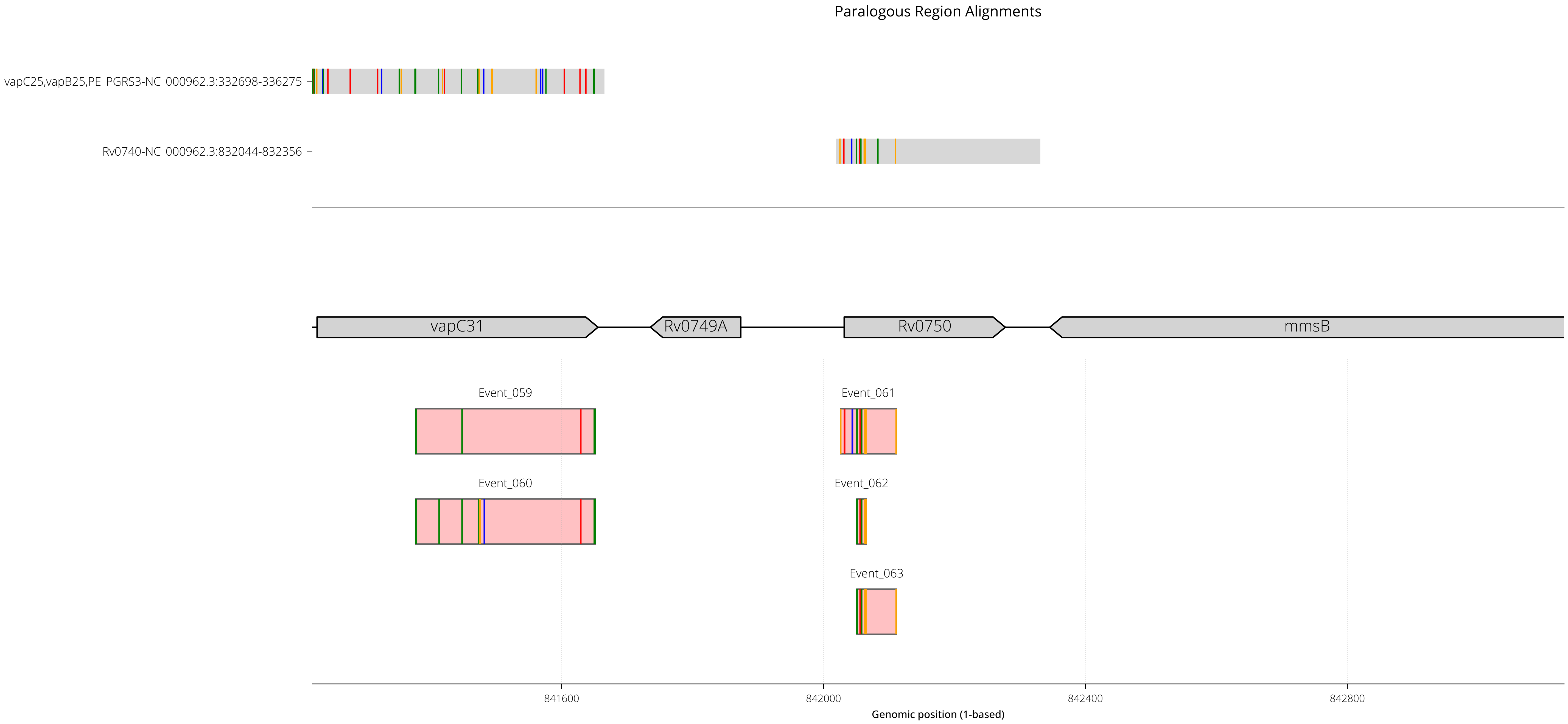

RegionID: PR_HmRegion_033 | Paralog Network ID: PR_Set_22
Genes: Rv0750 | NC_000962.3:841219-843131
Mapped GCEs: 3 | Putative GCEs: 3

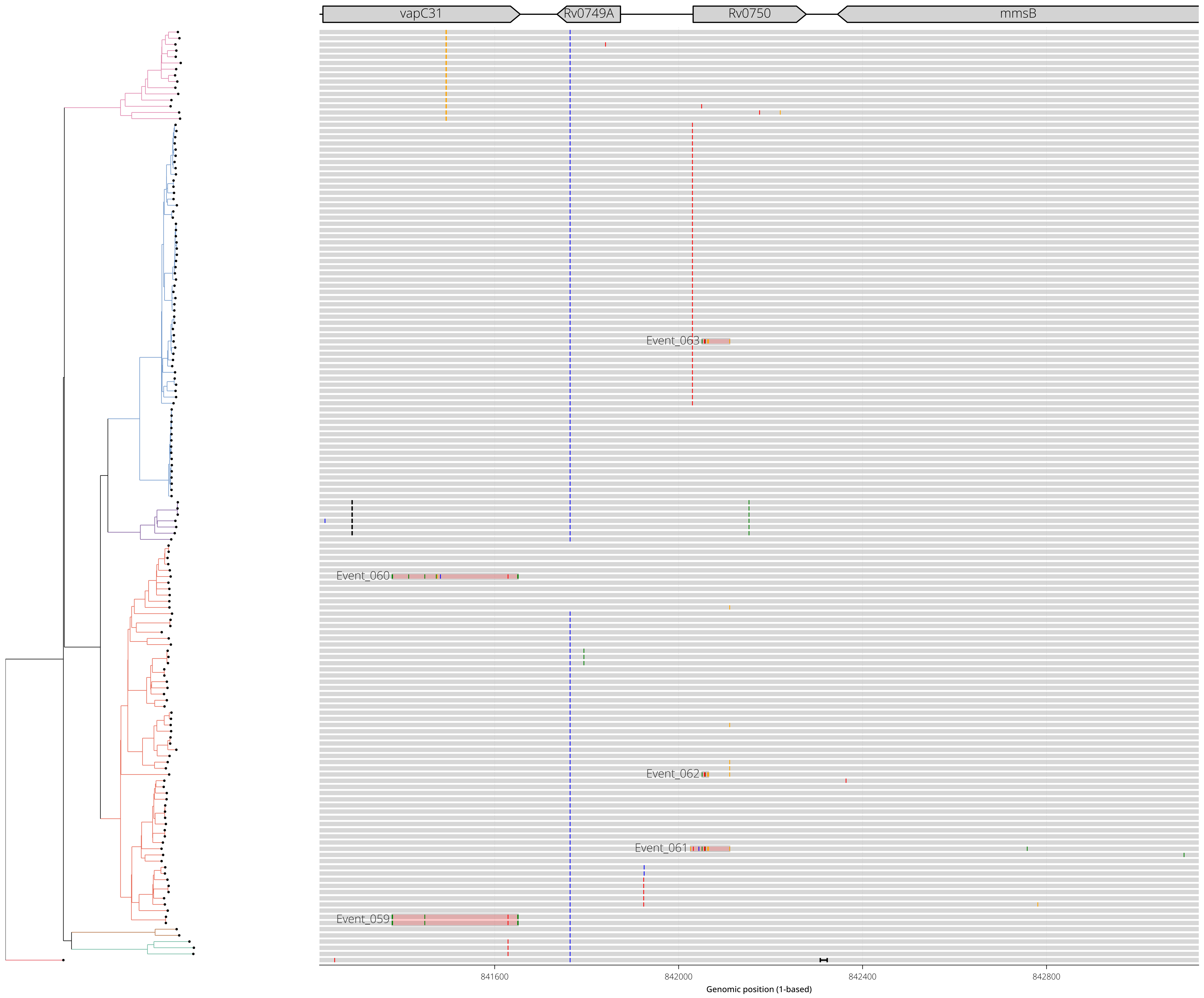

RegionID: PR_HmRegion_099 | Paralog Network ID: PR_Set_46
Genes: pks12 | NC_000962.3:2300112-2307689
Mapped GCEs: 2 | Putative GCEs: 3

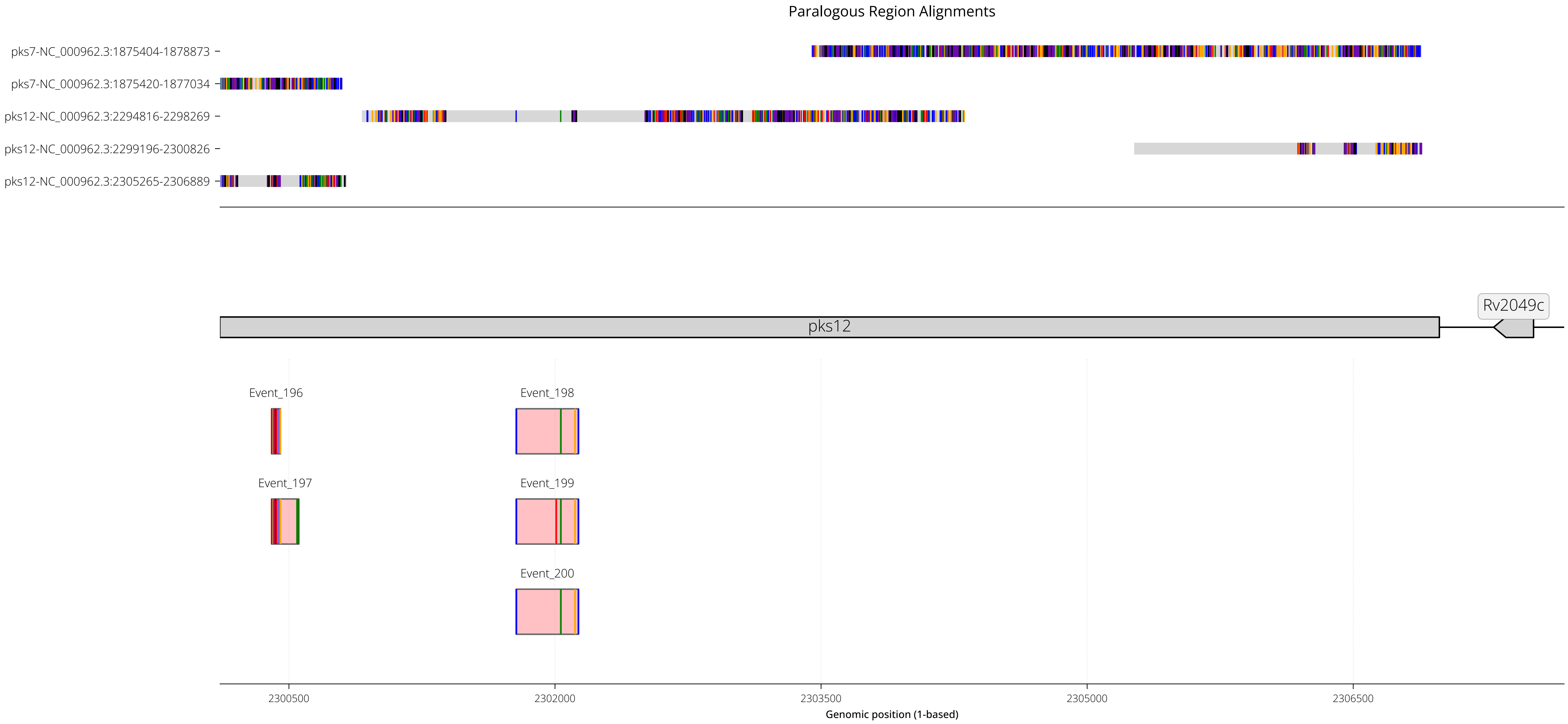

RegionID: PR_HmRegion_099 | Paralog Network ID: PR_Set_46
Genes: pks12 | NC_000962.3:2300112-2307689
Mapped GCEs: 2 | Putative GCEs: 3

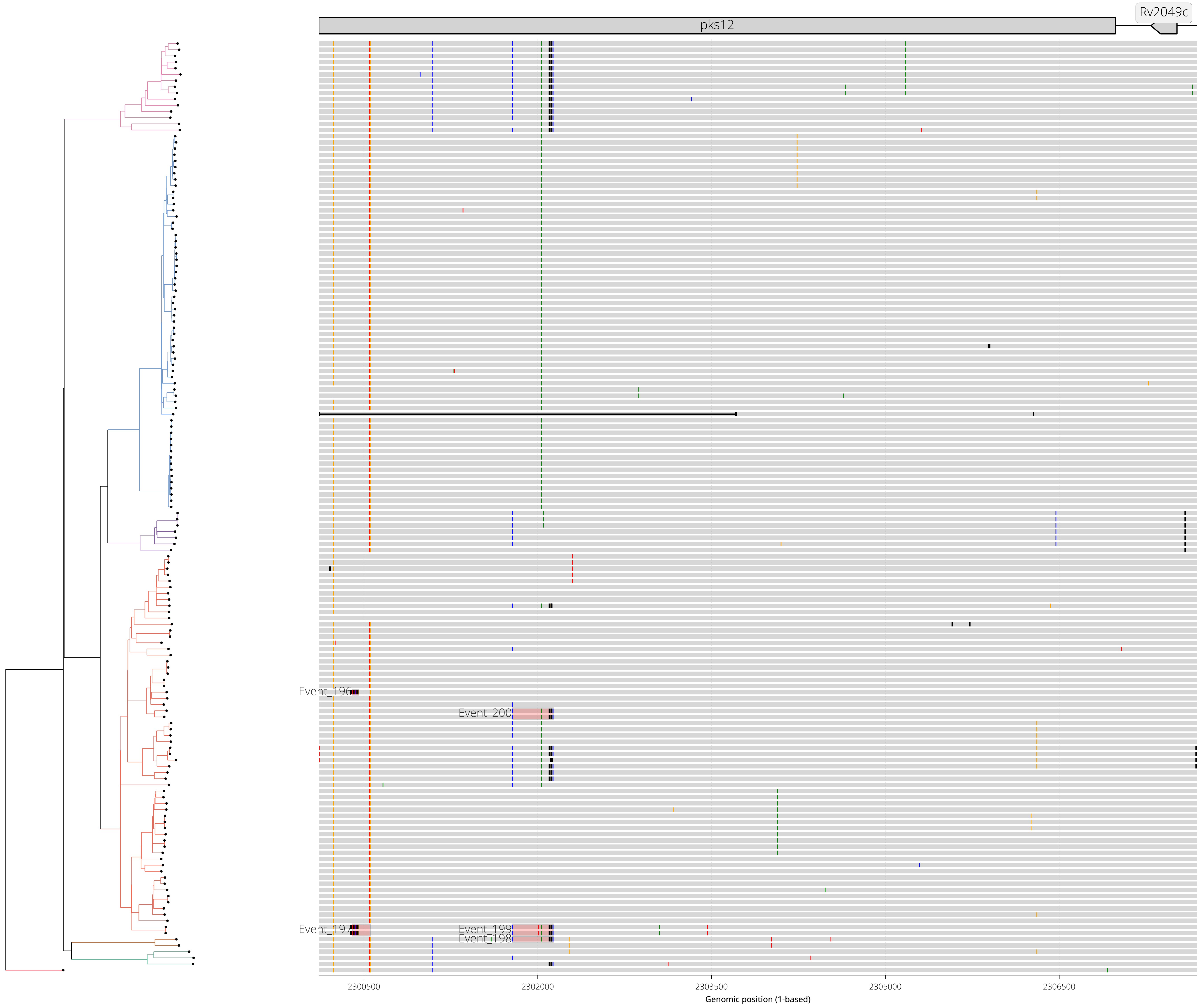

RegionID: PR_HmRegion_184 | Paralog Network ID: PR_Set_10
Genes: PE_PGRS55,PE_PGRS56,fadD18 | NC_000962.3:3940607-3946397
Mapped GCEs: 0 | Putative GCEs: 3

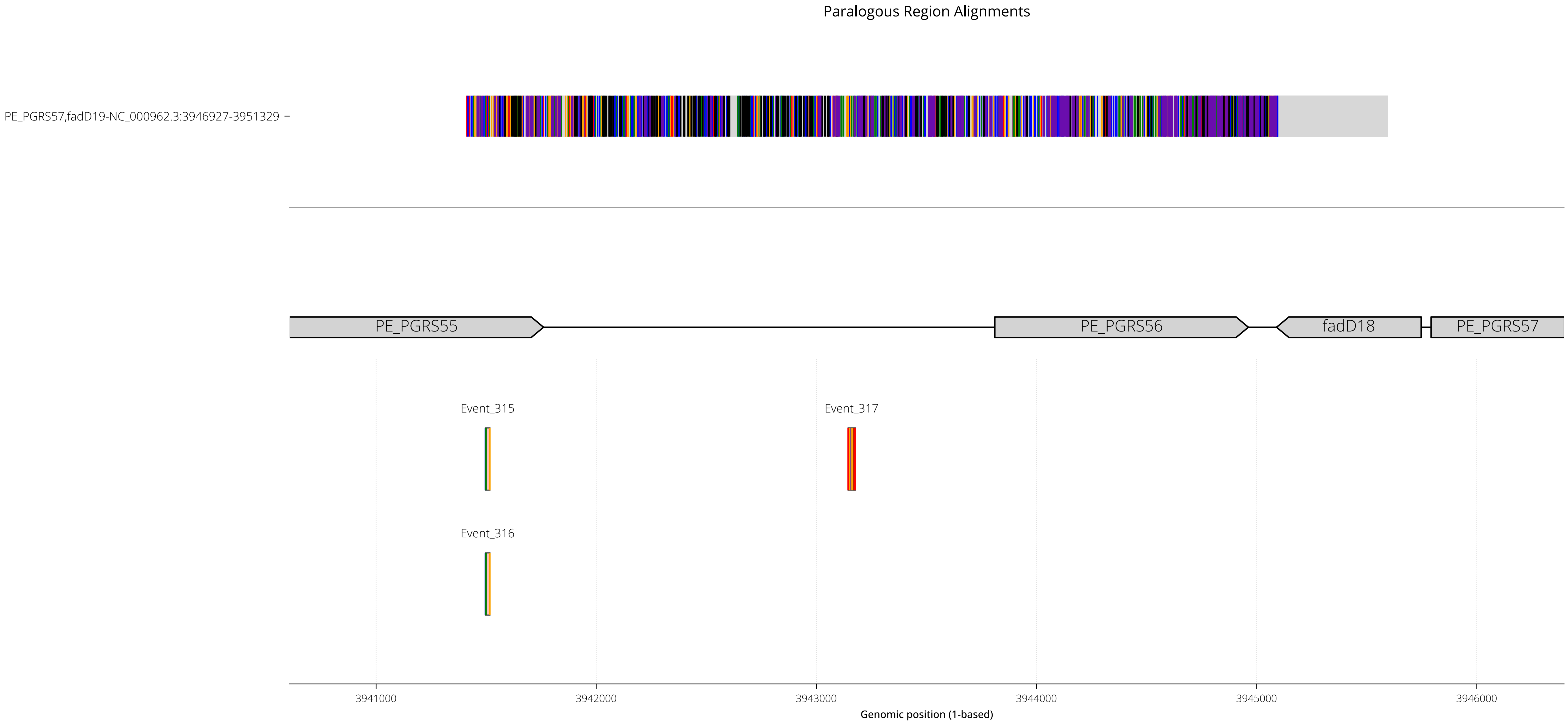

RegionID: PR_HmRegion_184 | Paralog Network ID: PR_Set_10
Genes: PE_PGRS55,PE_PGRS56,fadD18 | NC_000962.3:3940607-3946397
Mapped GCEs: 0 | Putative GCEs: 3

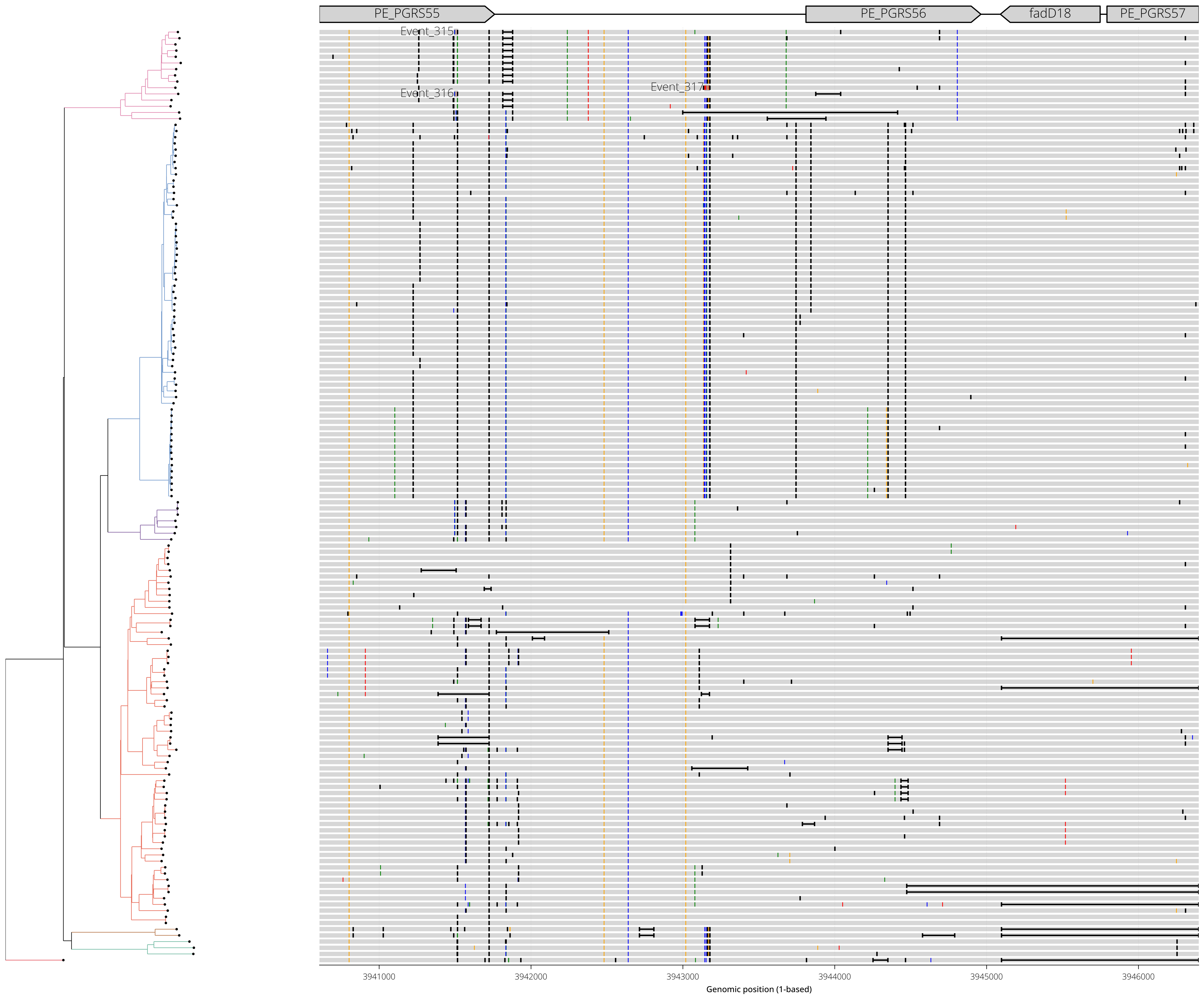

RegionID: PR_HmRegion_098 | Paralog Network ID: PR_Set_46
Genes: pks12 | NC_000962.3:2298396-2301626
Mapped GCEs: 2 | Putative GCEs: 2

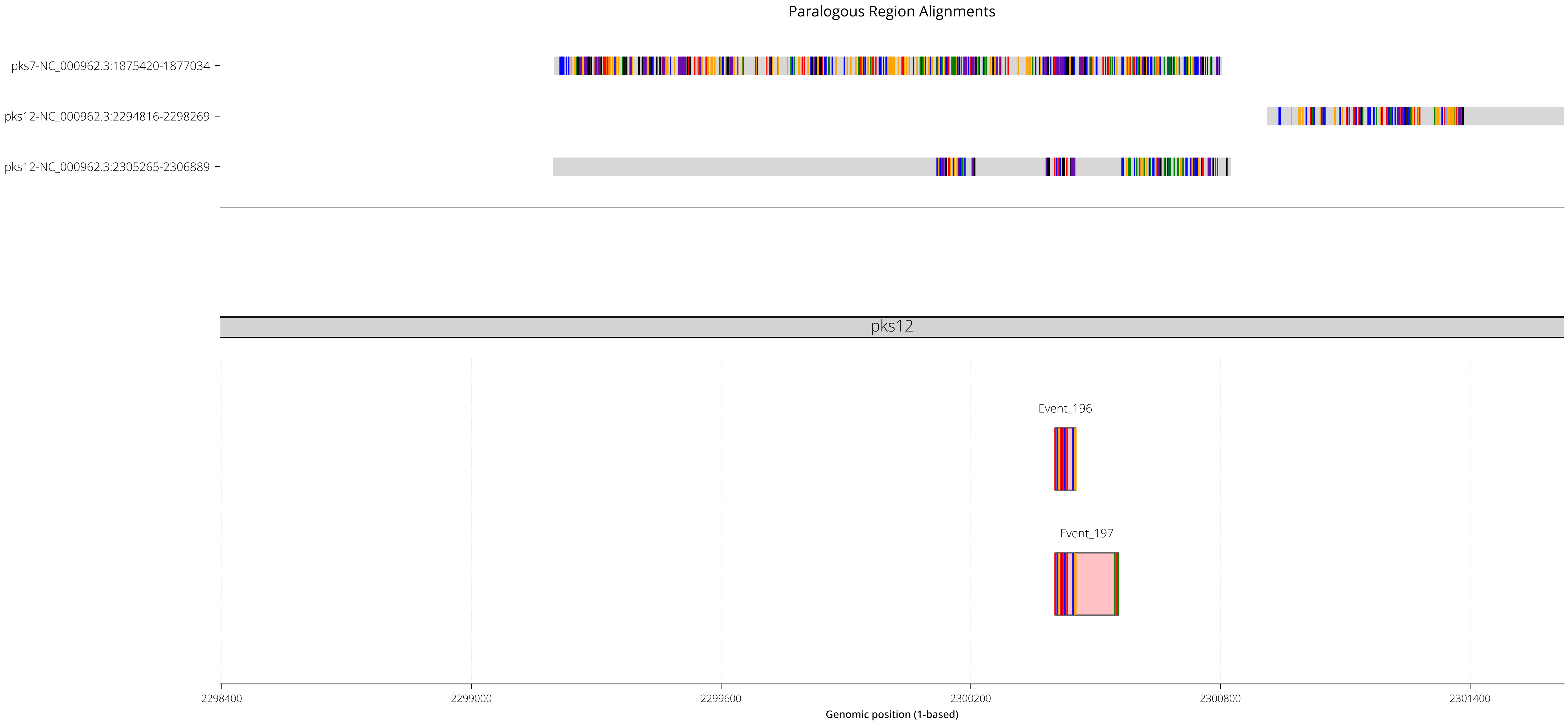

RegionID: PR_HmRegion_098 | Paralog Network ID: PR_Set_46
Genes: pks12 | NC_000962.3:2298396-2301626
Mapped GCEs: 2 | Putative GCEs: 2

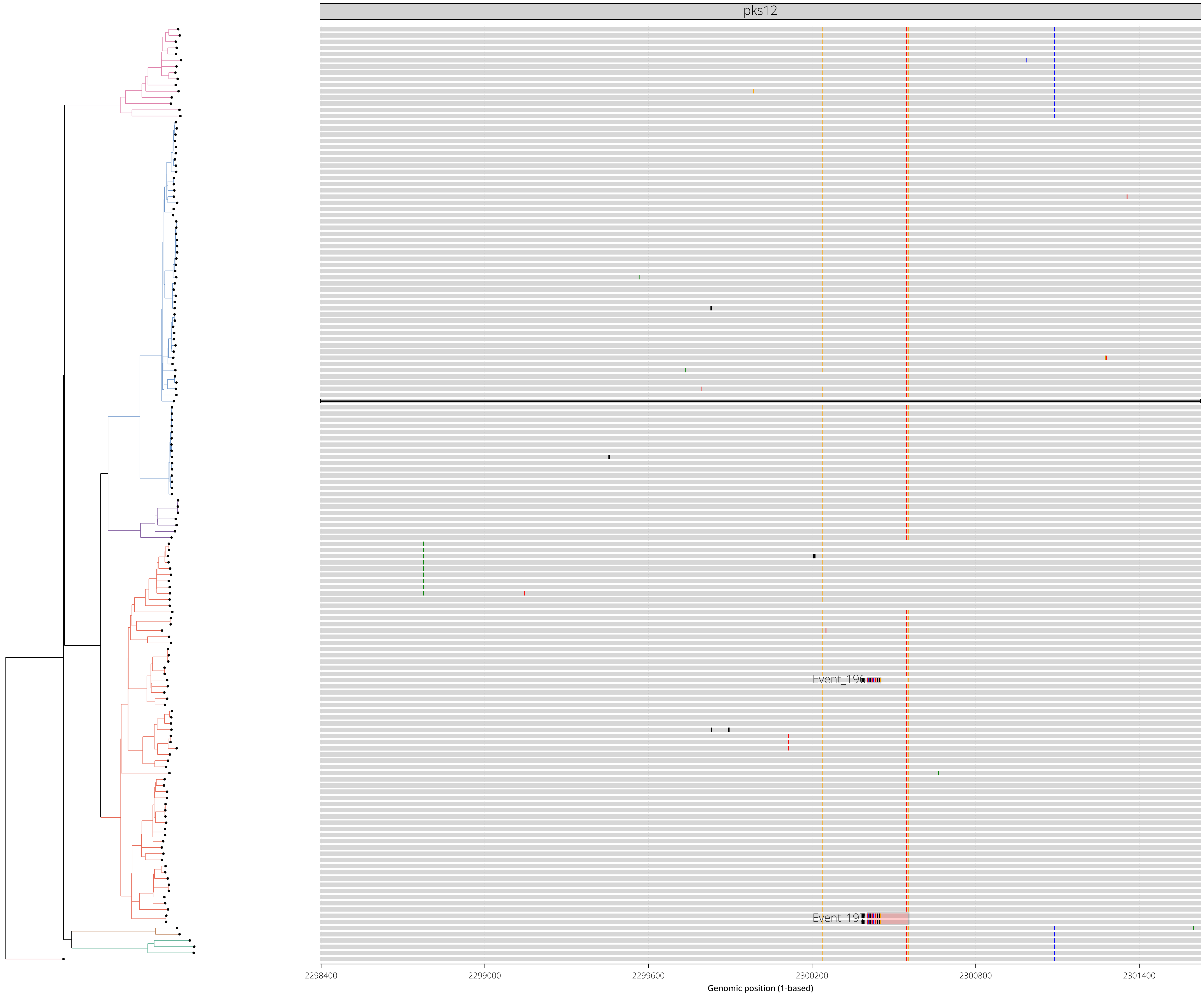

RegionID: PR_HmRegion_097 | Paralog Network ID: PR_Set_46
Genes: pks12 | NC_000962.3:2294016-2299069
Mapped GCEs: 2 | Putative GCEs: 2

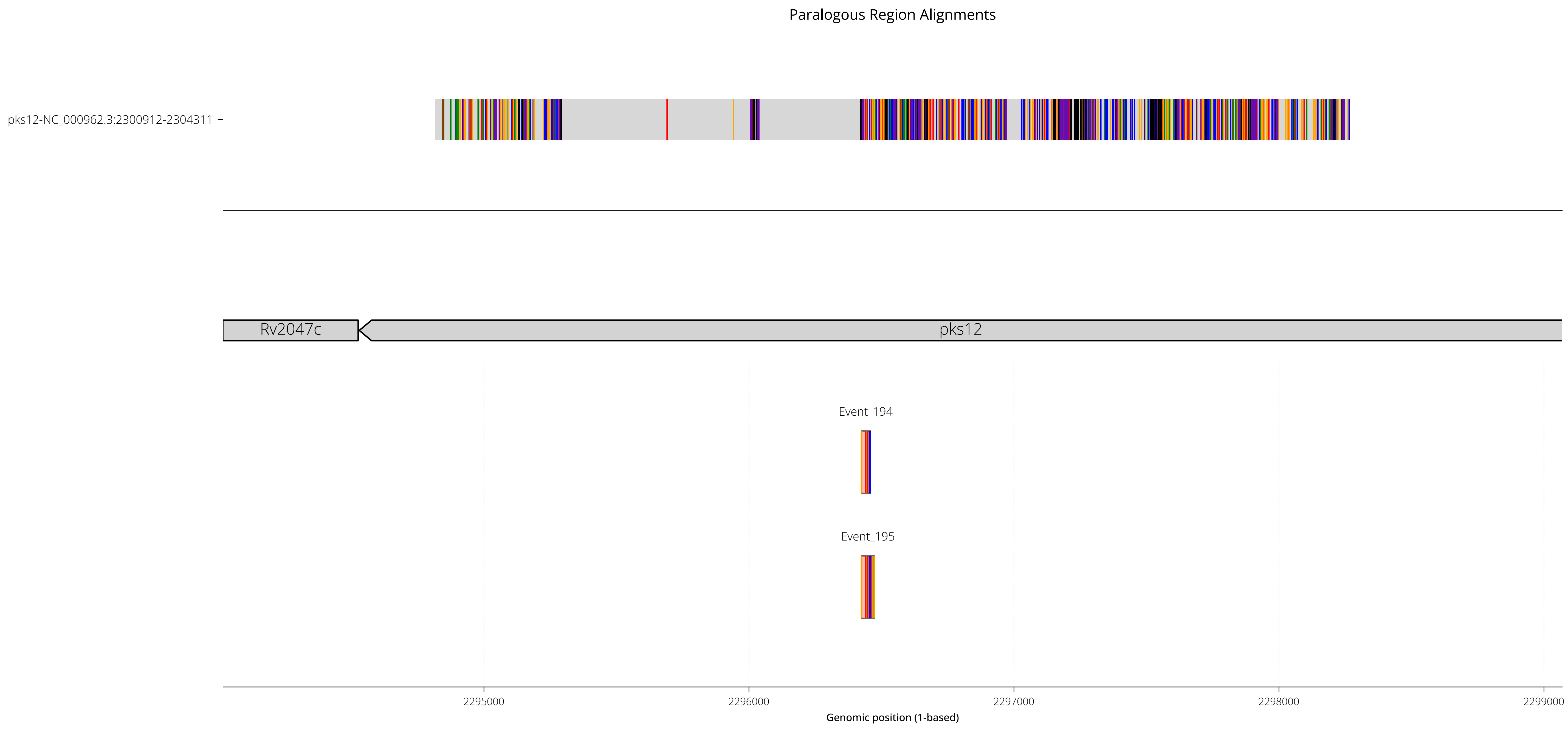

RegionID: PR_HmRegion_097 | Paralog Network ID: PR_Set_46
Genes: pks12 | NC_000962.3:2294016-2299069
Mapped GCEs: 2 | Putative GCEs: 2

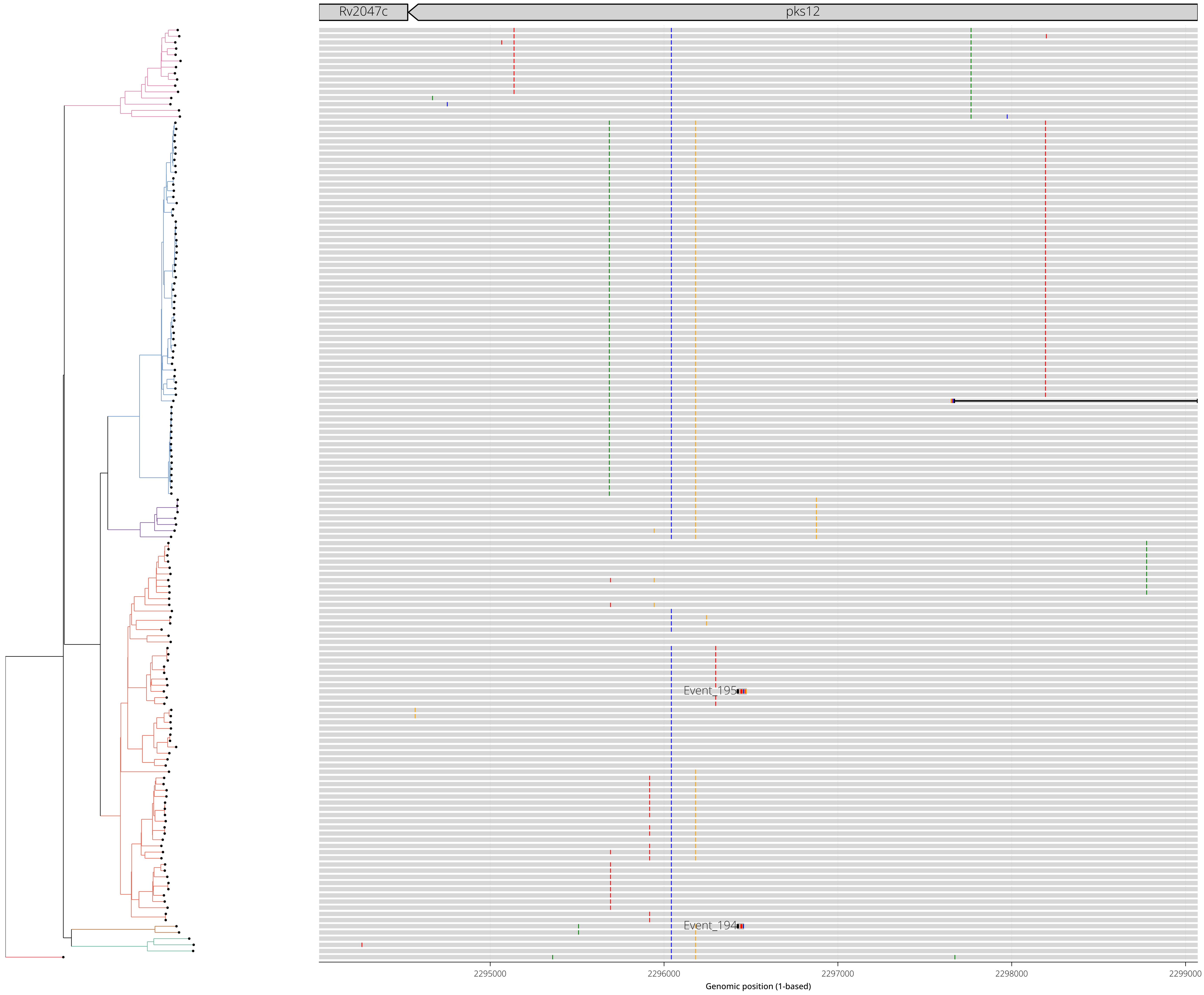

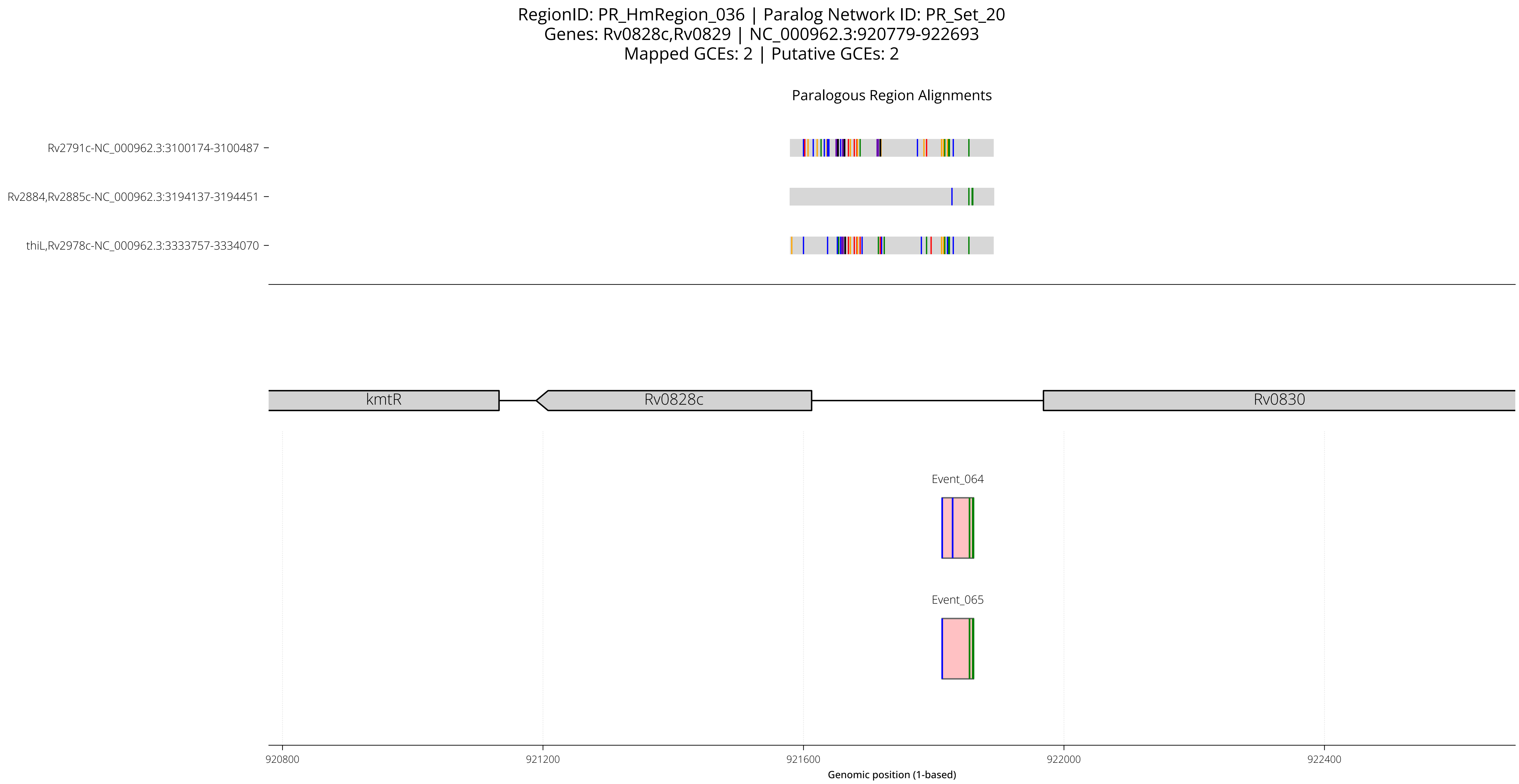
RegionID: PR_HmRegion_036 | Paralog Network ID: PR_Set_20
Genes: Rv0828c,Rv0829 | NC_000962.3:920779-922693
Mapped GCEs: 2 | Putative GCEs: 2
Paralogous Region Alignments
Rv2791c-NC_000962.3:3100174-3100487
Rv2884,Rv2885c-NC_000962.3:3194137-3194451
thiL,Rv2978c-NC_000962.3:3333757-3334070
kmtR
Rv0828c
Rv0830
Event_064
Event_065
920800
921200
921600
922000
922400
Genomic position (1-based)

RegionID: PR_HmRegion_036 | Paralog Network ID: PR_Set_20
Genes: Rv0828c,Rv0829 | NC_000962.3:920779-922693
Mapped GCEs: 2 | Putative GCEs: 2

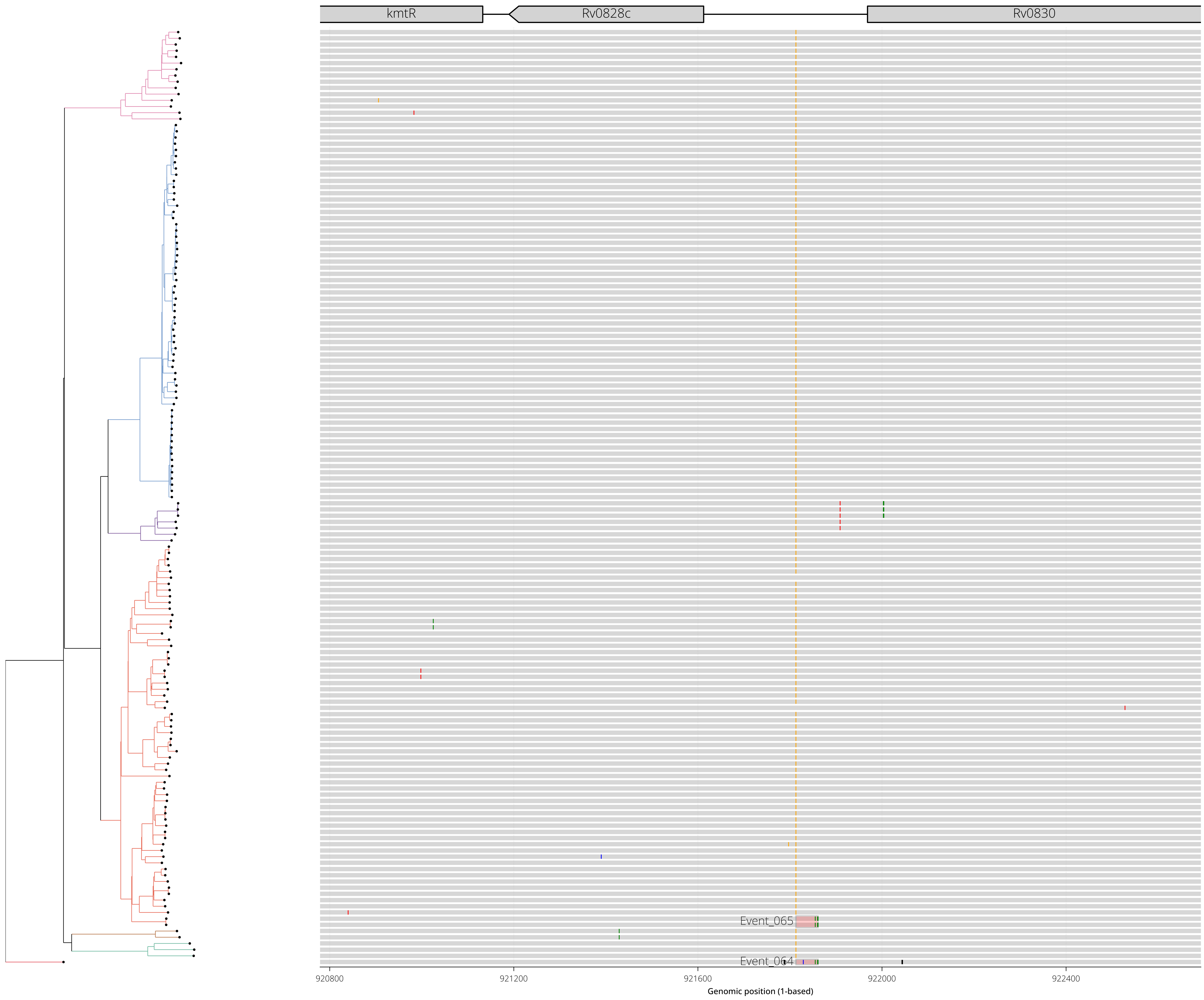

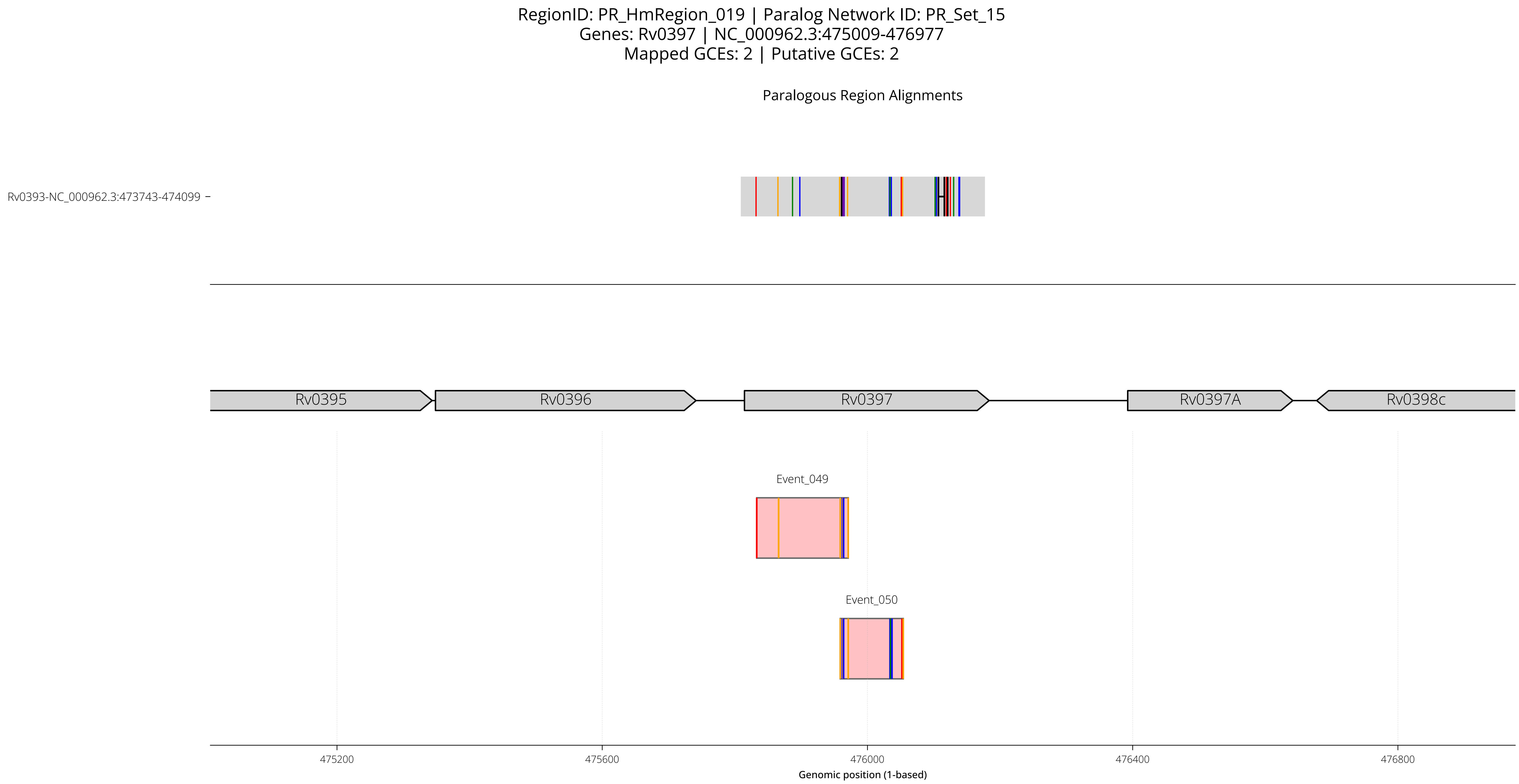
RegionID: PR_HmRegion_019 | Paralog Network ID: PR_Set_15
Genes: Rv0397 | NC_000962.3:475009-476977
Mapped GCEs: 2 | Putative GCEs: 2
Paralogous Region Alignments
Rv0393-NC_000962.3:473743-474099
Rv0395
Rv0396
Rv0397
Rv0397A
Rv0398c
Event_049
Event_050
475200
475600
476000
476400
476800
Genomic position (1-based)

RegionID: PR_HmRegion_019 | Paralog Network ID: PR_Set_15
Genes: Rv0397 | NC_000962.3:475009-476977
Mapped GCEs: 2 | Putative GCEs: 2

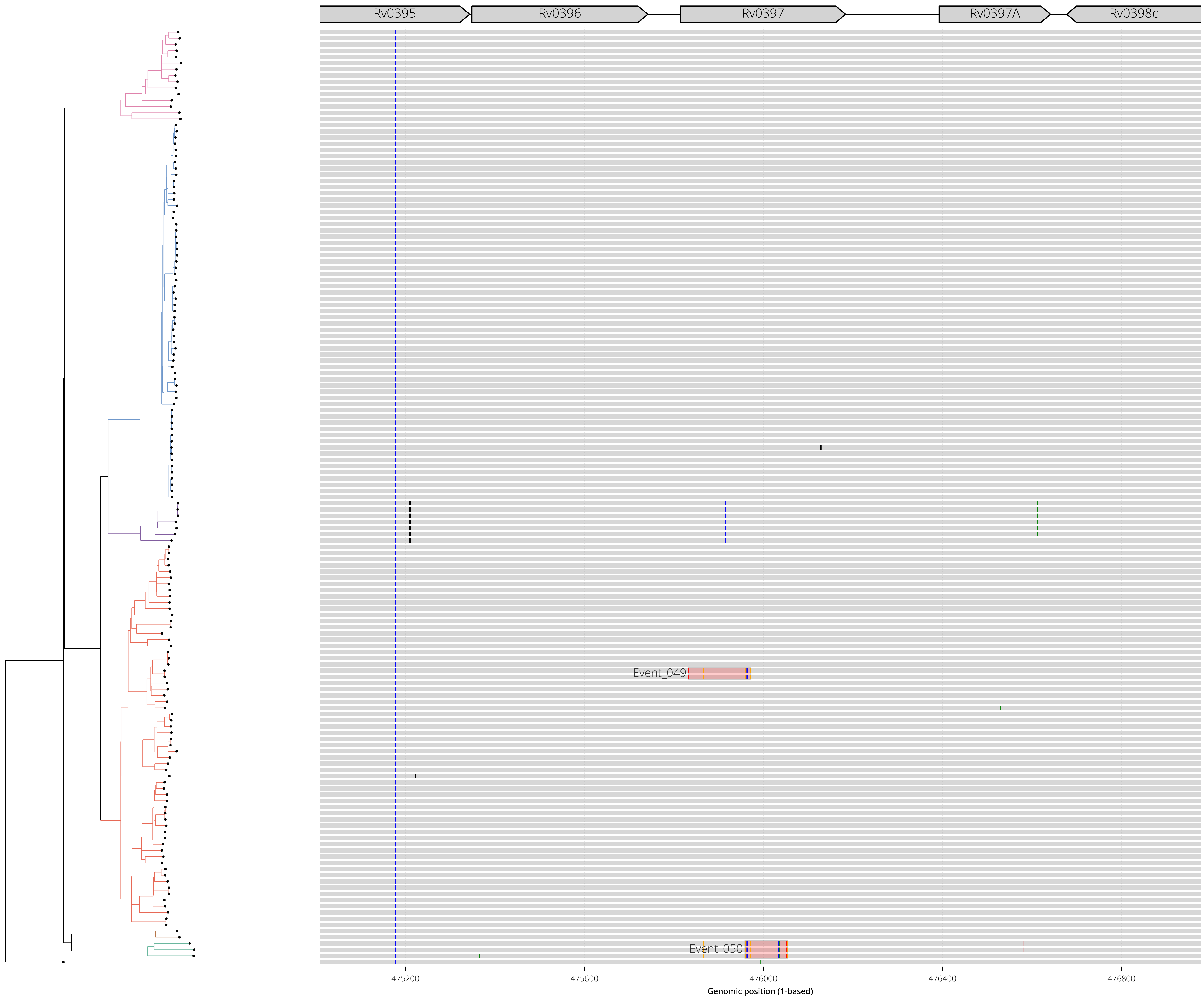

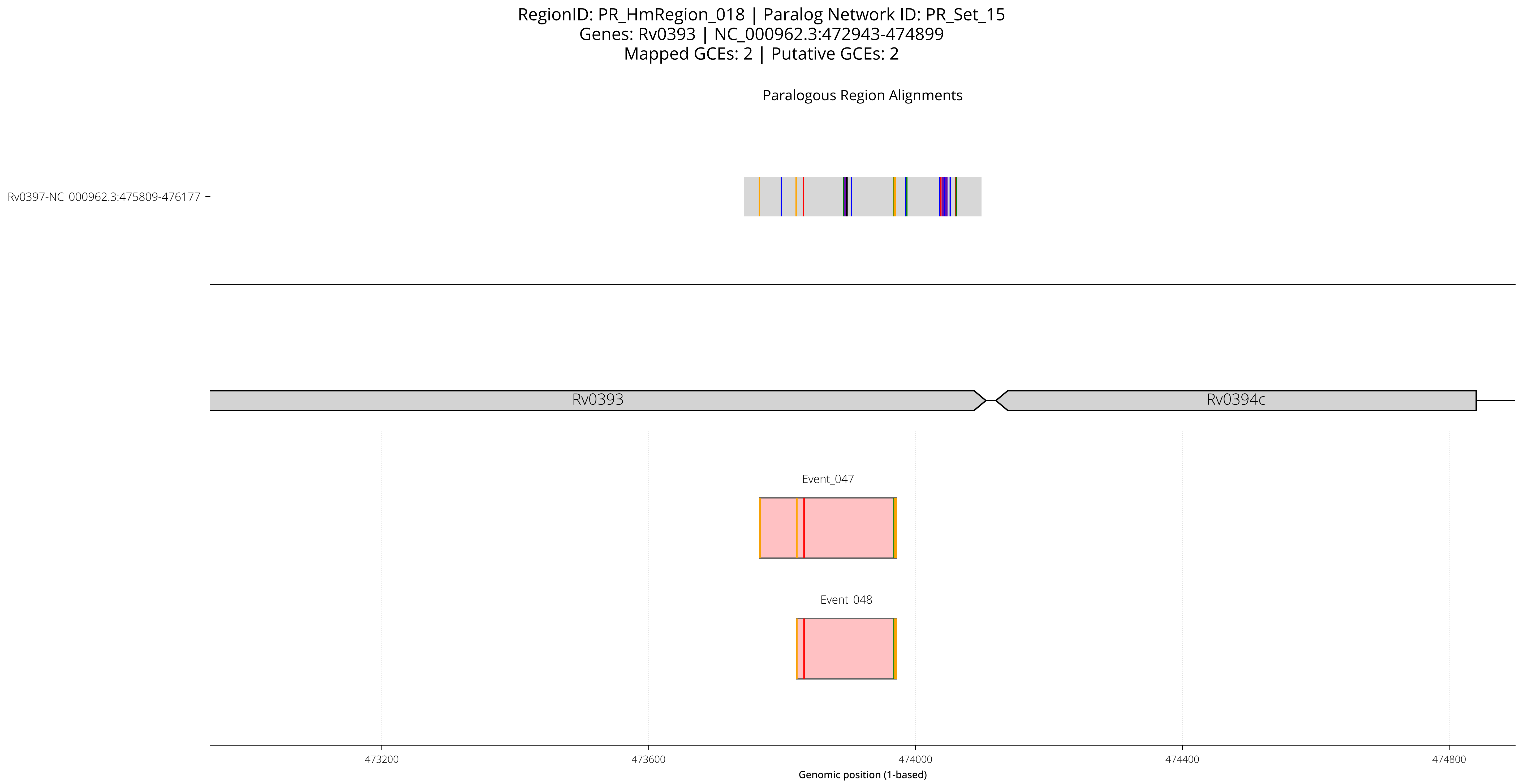
RegionID: PR_HmRegion_018 | Paralog Network ID: PR_Set_15
Genes: Rv0393 | NC_000962.3:472943-474899
Mapped GCEs: 2 | Putative GCEs: 2
Paralogous Region Alignments
Rv0397-NC_000962.3:475809-476177
Rv0393
Rv0394c
Event_047
Event_048
473200
473600
474000
474400
474800
Genomic position (1-based)

RegionID: PR_HmRegion_018 | Paralog Network ID: PR_Set_15
Genes: Rv0393 | NC_000962.3:472943-474899
Mapped GCEs: 2 | Putative GCEs: 2

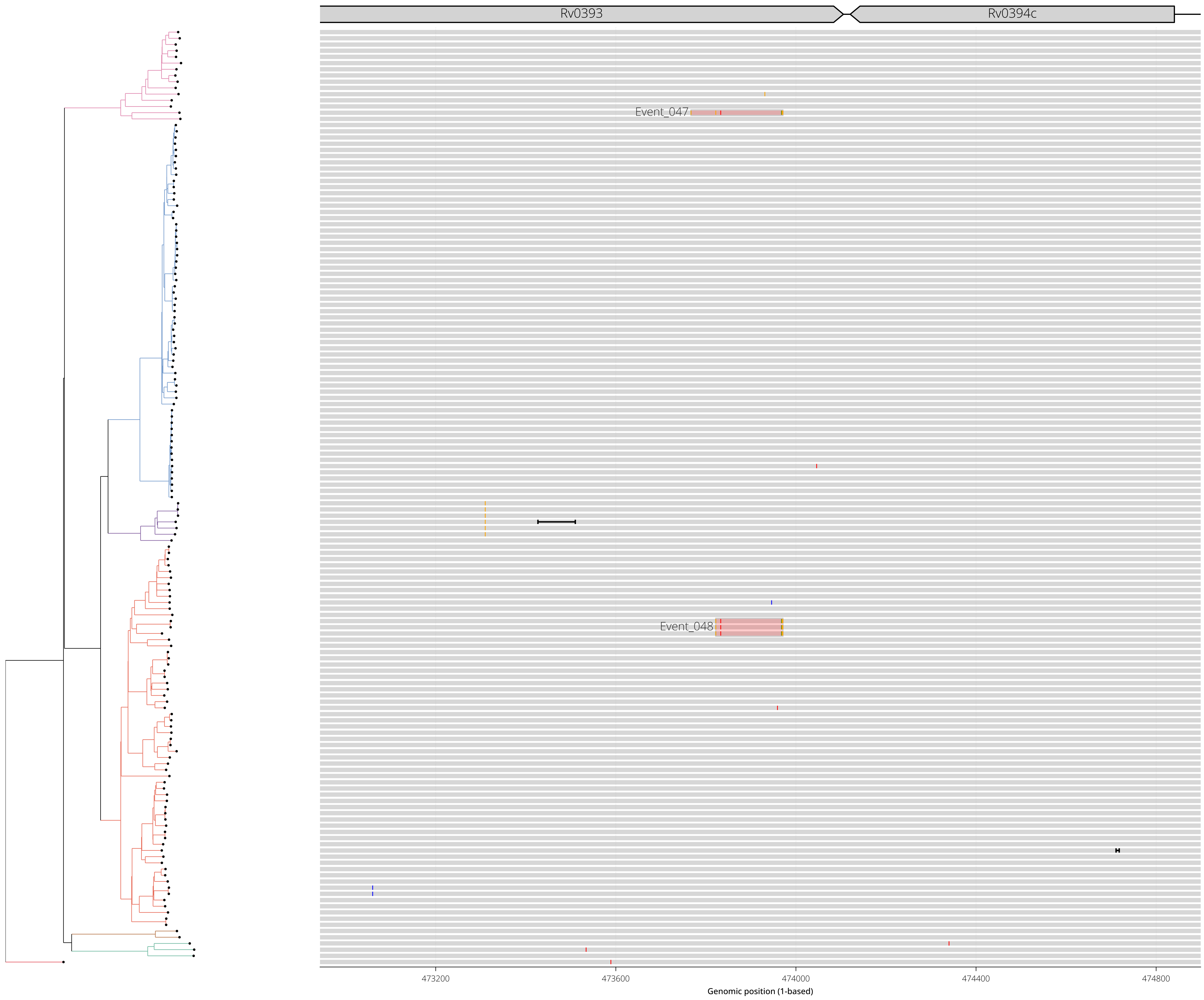

RegionID: PR_HmRegion_085 | Paralog Network ID: PR_Set_49
Genes: PPE25 | NC_000962.3:2024501-2026963
Mapped GCEs: 2 | Putative GCEs: 2

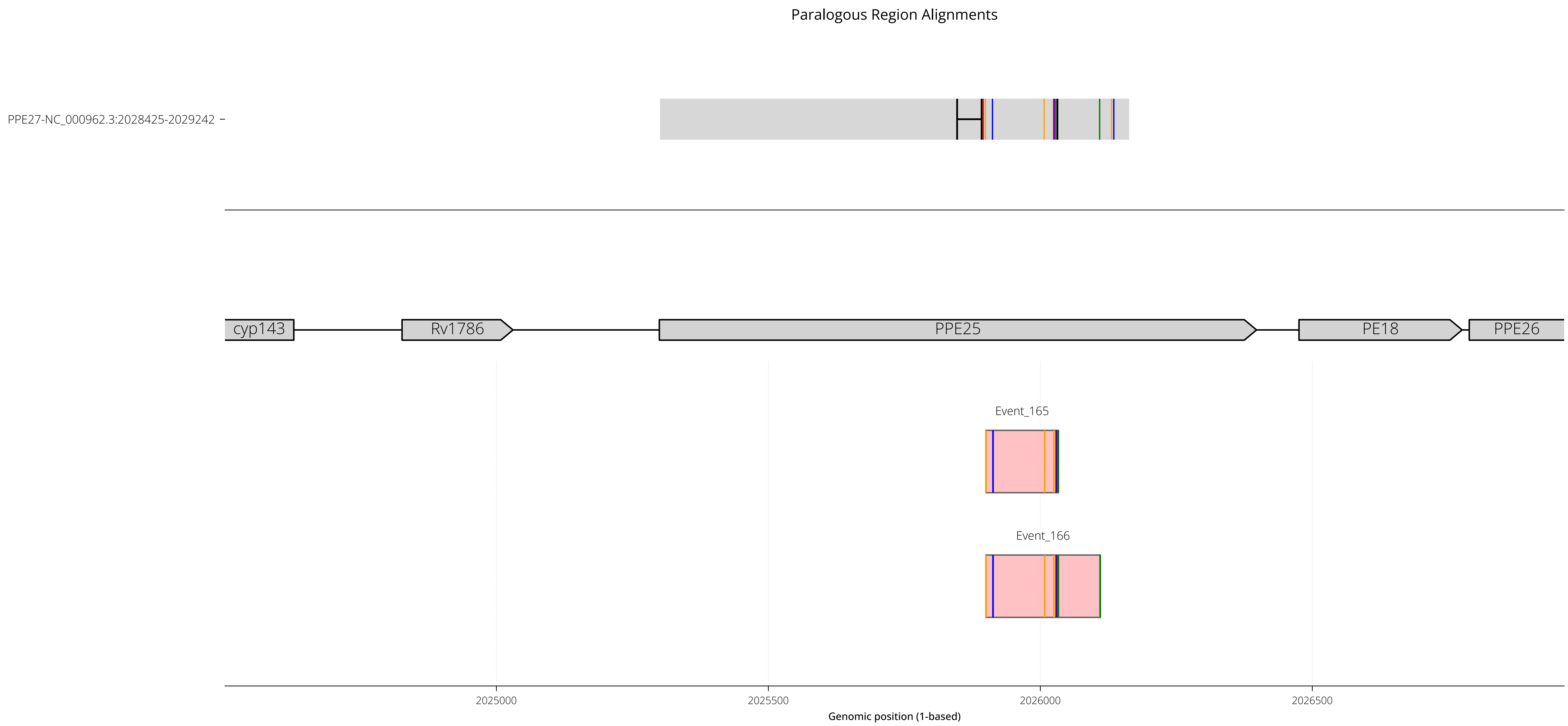

RegionID: PR_HmRegion_085 | Paralog Network ID: PR_Set_49
Genes: PPE25 | NC_000962.3:2024501-2026963
Mapped GCEs: 2 | Putative GCEs: 2

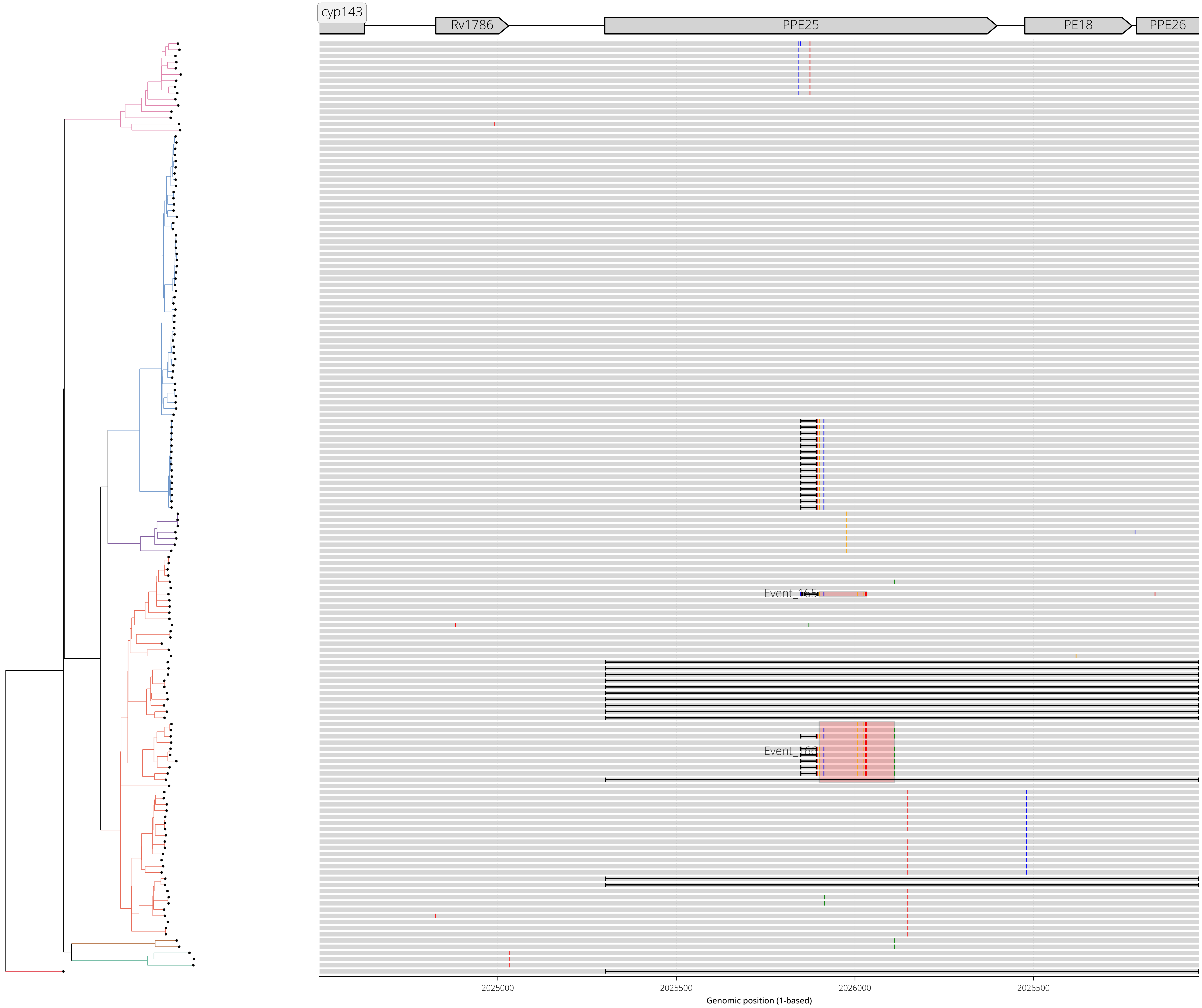

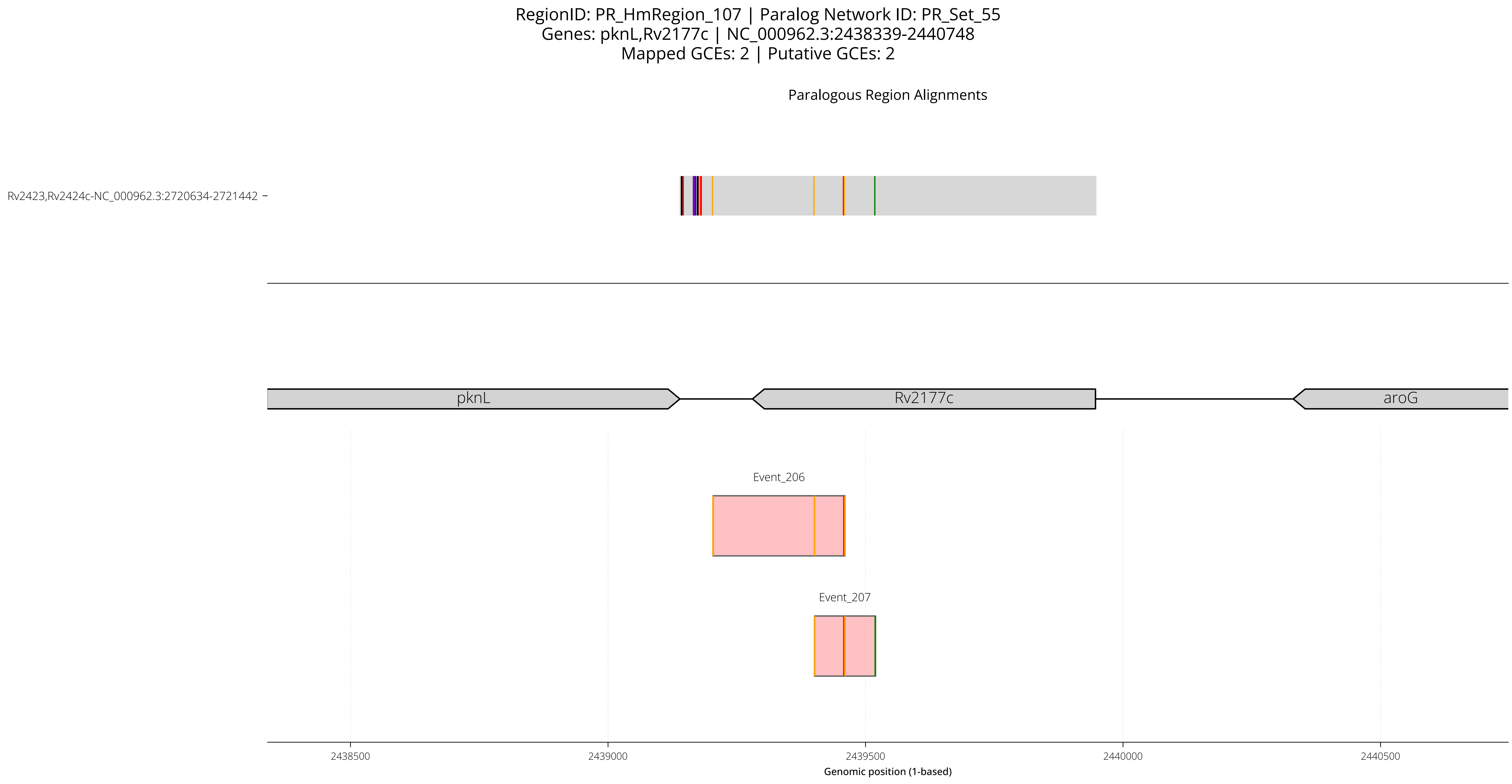
RegionID: PR_HmRegion_107 | Paralog Network ID: PR_Set_55
Genes: pknL,Rv2177c | NC_000962.3:2438339-2440748
Mapped GCEs: 2 | Putative GCEs: 2
Paralogous Region Alignments
Rv2423,Rv2424c-NC_000962.3:2720634-2721442
pknL
Rv2177c
aroG
Event_206
Event_207
2438500
2439000
2439500
2440000
2440500
Genomic position (1-based)

RegionID: PR_HmRegion_107 | Paralog Network ID: PR_Set_55
Genes: pknL,Rv2177c | NC_000962.3:2438339-2440748
Mapped GCEs: 2 | Putative GCEs: 2

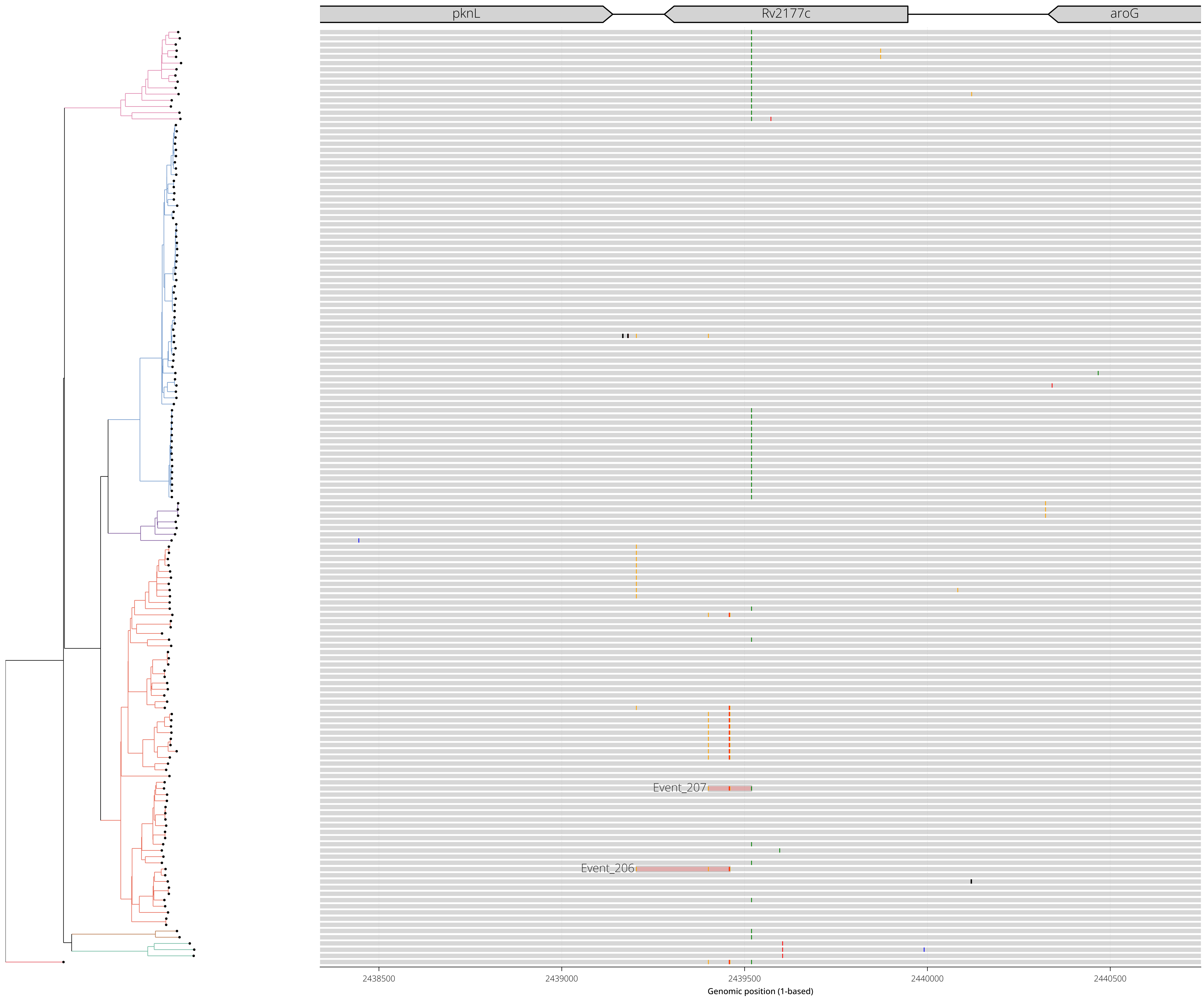

RegionID: PR_HmRegion_124 | Paralog Network ID: PR_Set_58
Genes: lppA | NC_000962.3:2865668-2867921
Mapped GCEs: 0 | Putative GCEs: 2

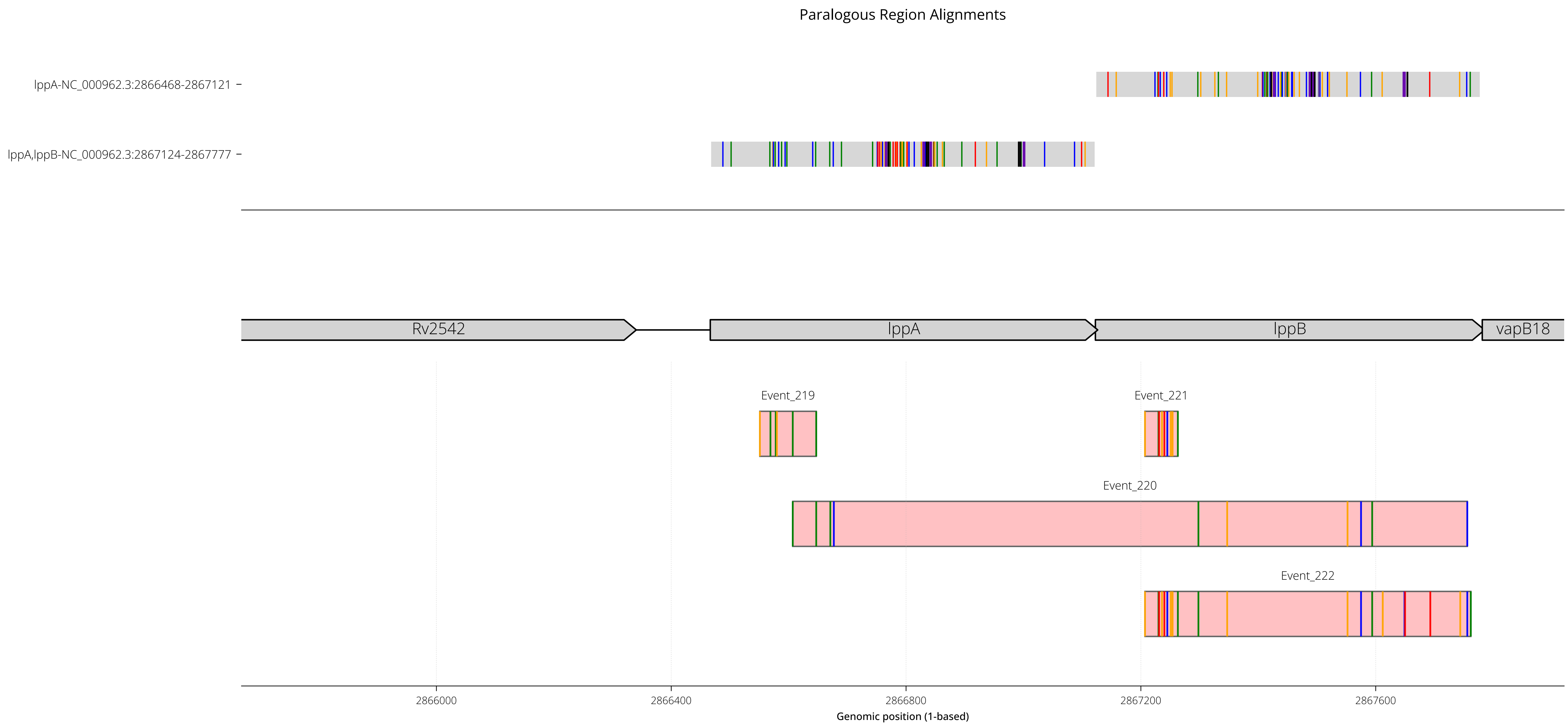

RegionID: PR_HmRegion_124 | Paralog Network ID: PR_Set_58
Genes: lppA | NC_000962.3:2865668-2867921
Mapped GCEs: 0 | Putative GCEs: 2

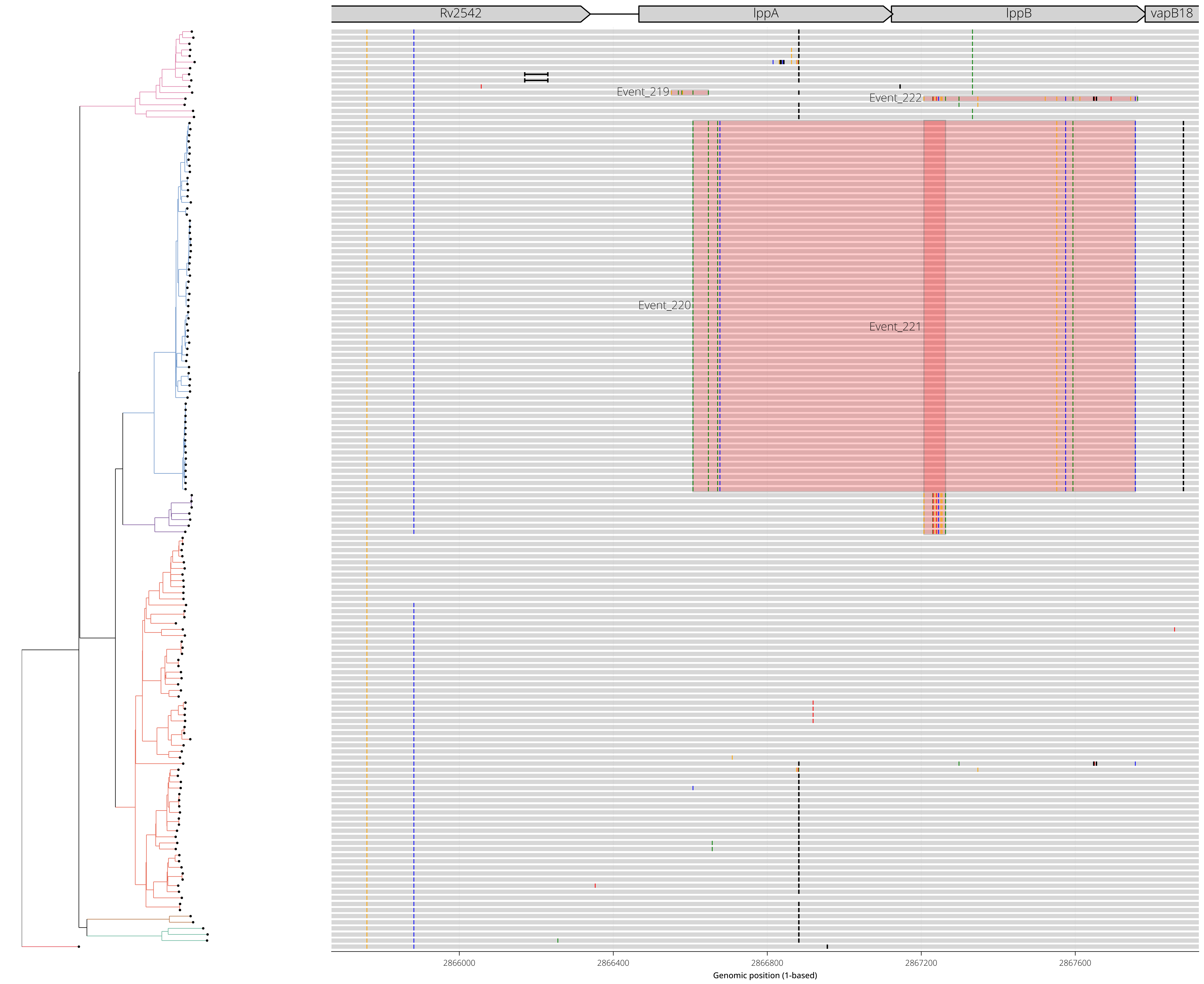

RegionID: PR_HmRegion_141 | Paralog Network ID: PR_Set_61
Genes: Rv2825c | NC_000962.3:3132091-3134255
Mapped GCEs: 2 | Putative GCEs: 2

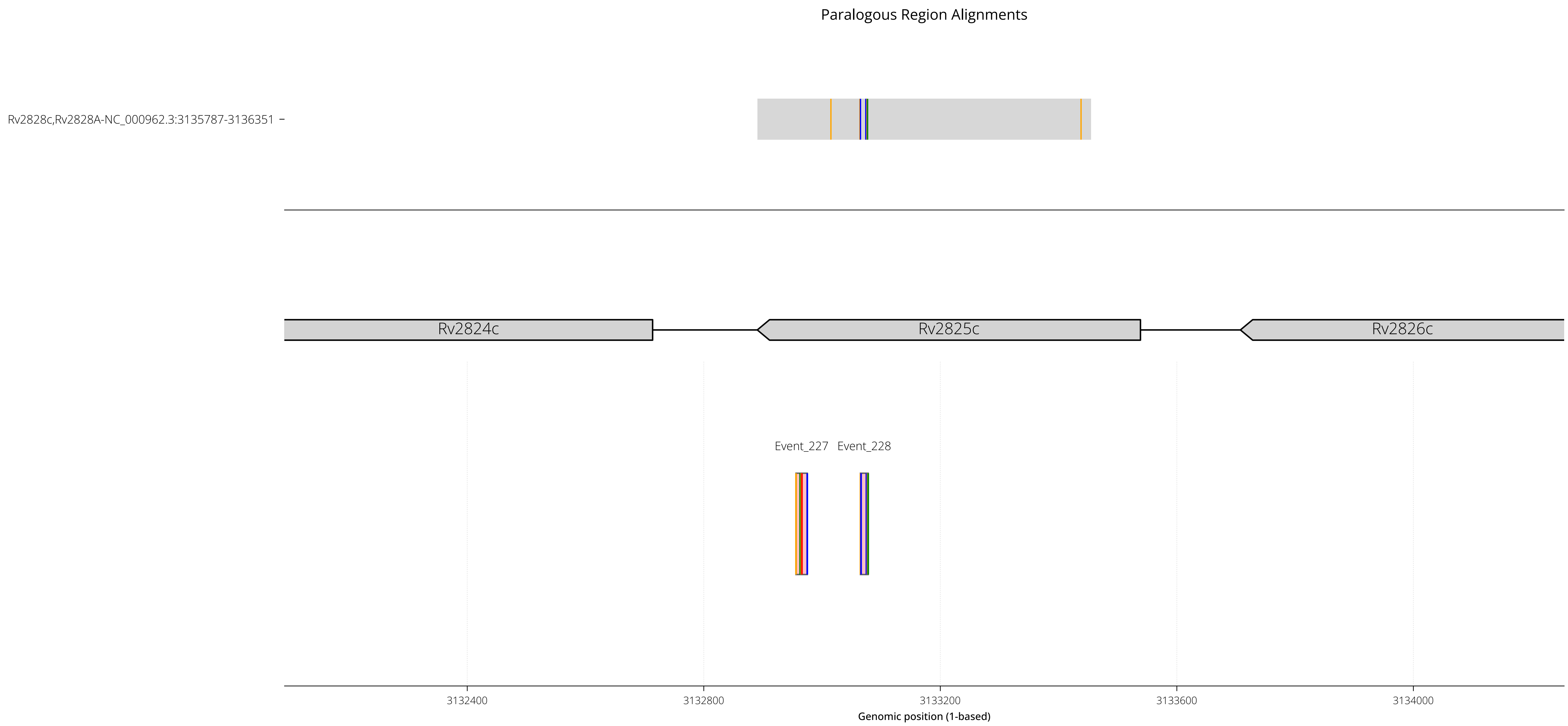

RegionID: PR_HmRegion_141 | Paralog Network ID: PR_Set_61
Genes: Rv2825c | NC_000962.3:3132091-3134255
Mapped GCEs: 2 | Putative GCEs: 2

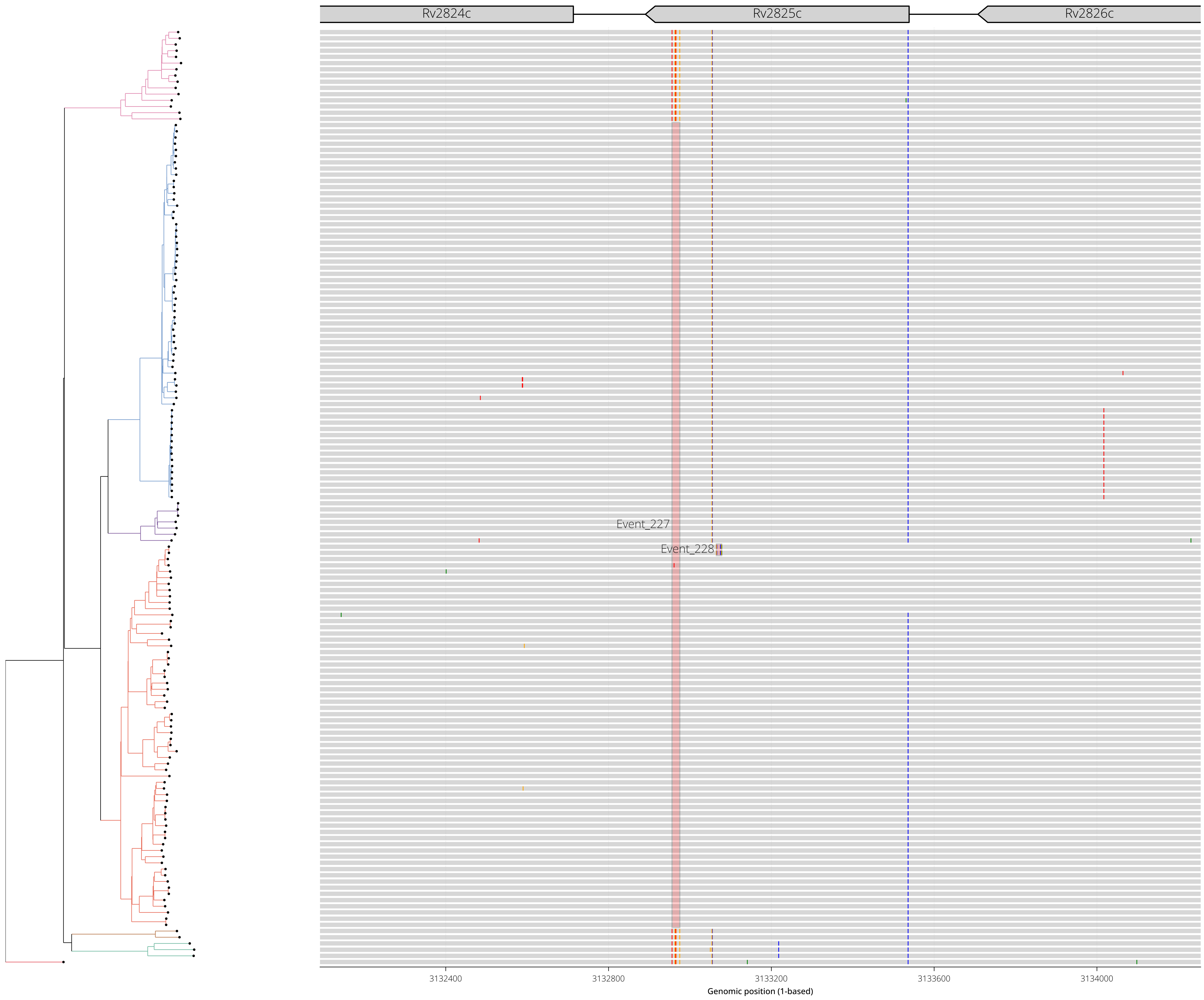

RegionID: PR_HmRegion_155 | Paralog Network ID: PR_Set_65
Genes: PPE46,PE27A | NC_000962.3:3376126-3379250
Mapped GCEs: 0 | Putative GCEs: 2

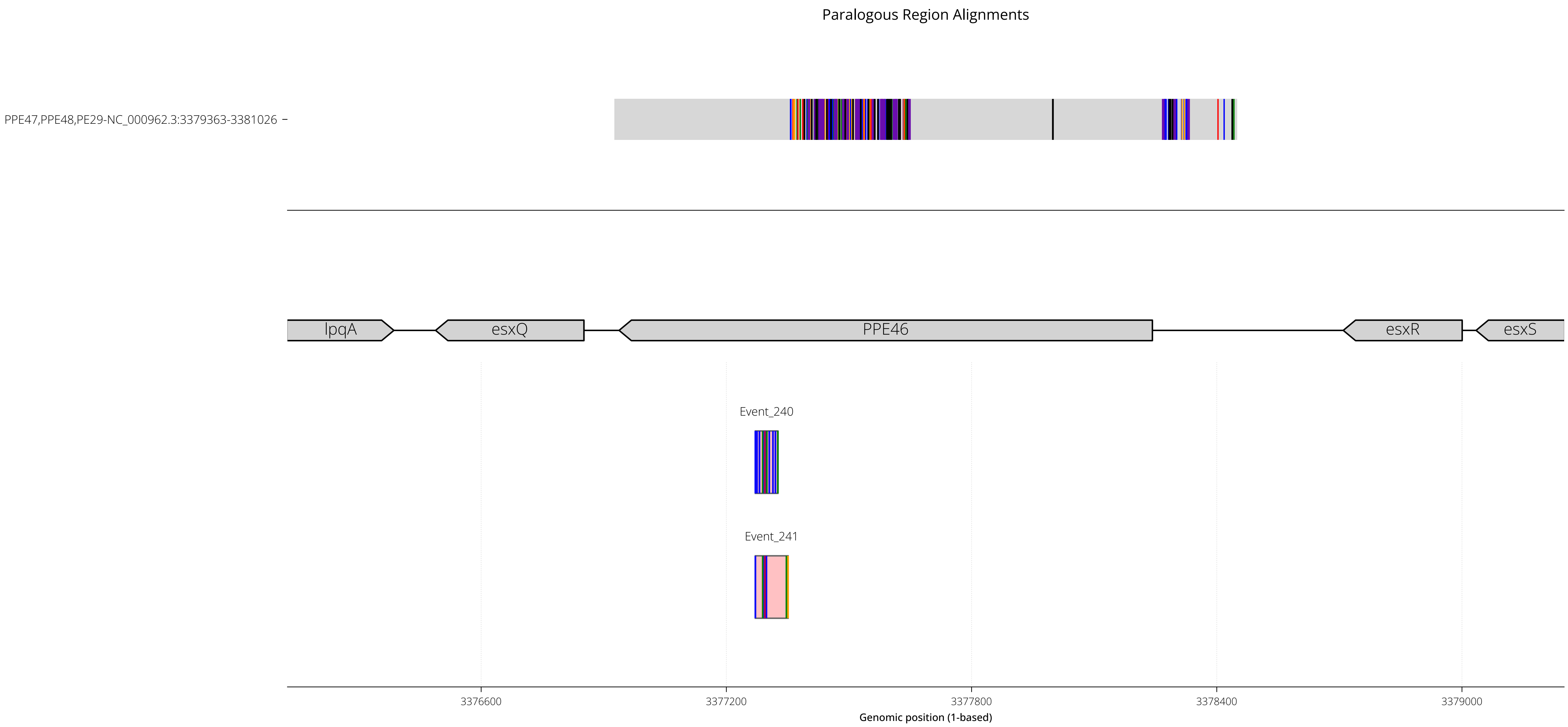

RegionID: PR_HmRegion_155 | Paralog Network ID: PR_Set_65
Genes: PPE46,PE27A | NC_000962.3:3376126-3379250
Mapped GCEs: 0 | Putative GCEs: 2

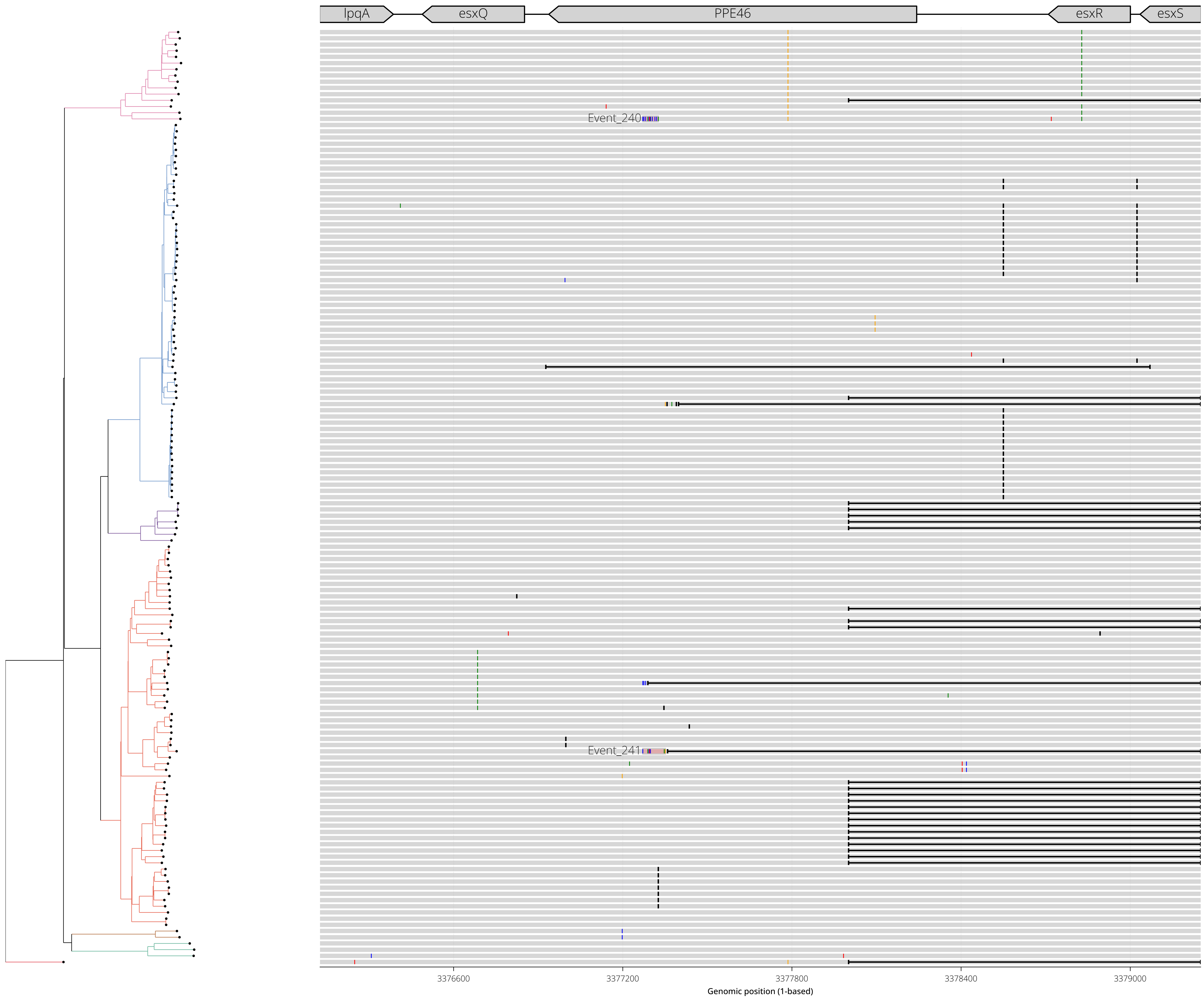

RegionID: PR_HmRegion_166 | Paralog Network ID: PR_Set_67
Genes: PPE54 | NC_000962.3:3729550-3731864
Mapped GCEs: 2 | Putative GCEs: 2

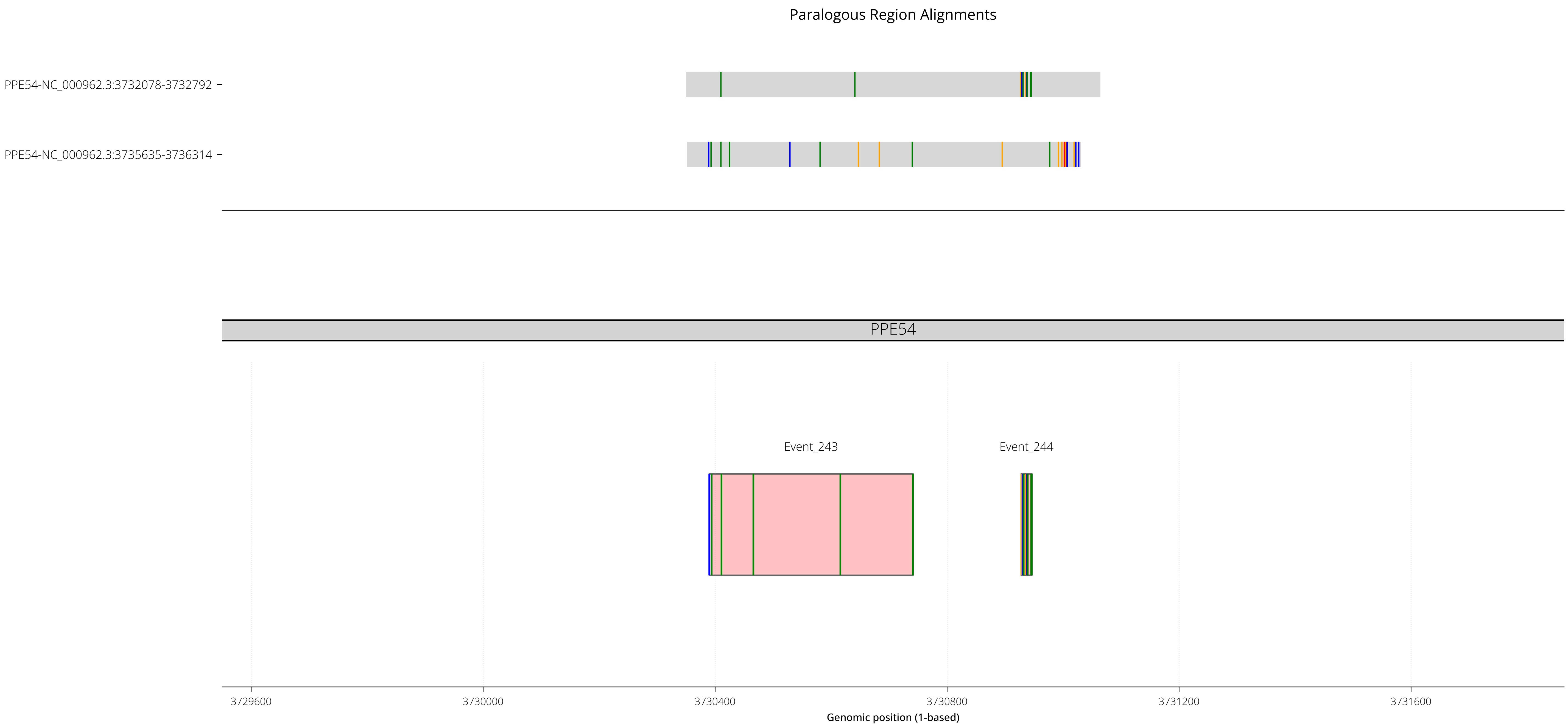

RegionID: PR_HmRegion_166 | Paralog Network ID: PR_Set_67
Genes: PPE54 | NC_000962.3:3729550-3731864
Mapped GCEs: 2 | Putative GCEs: 2

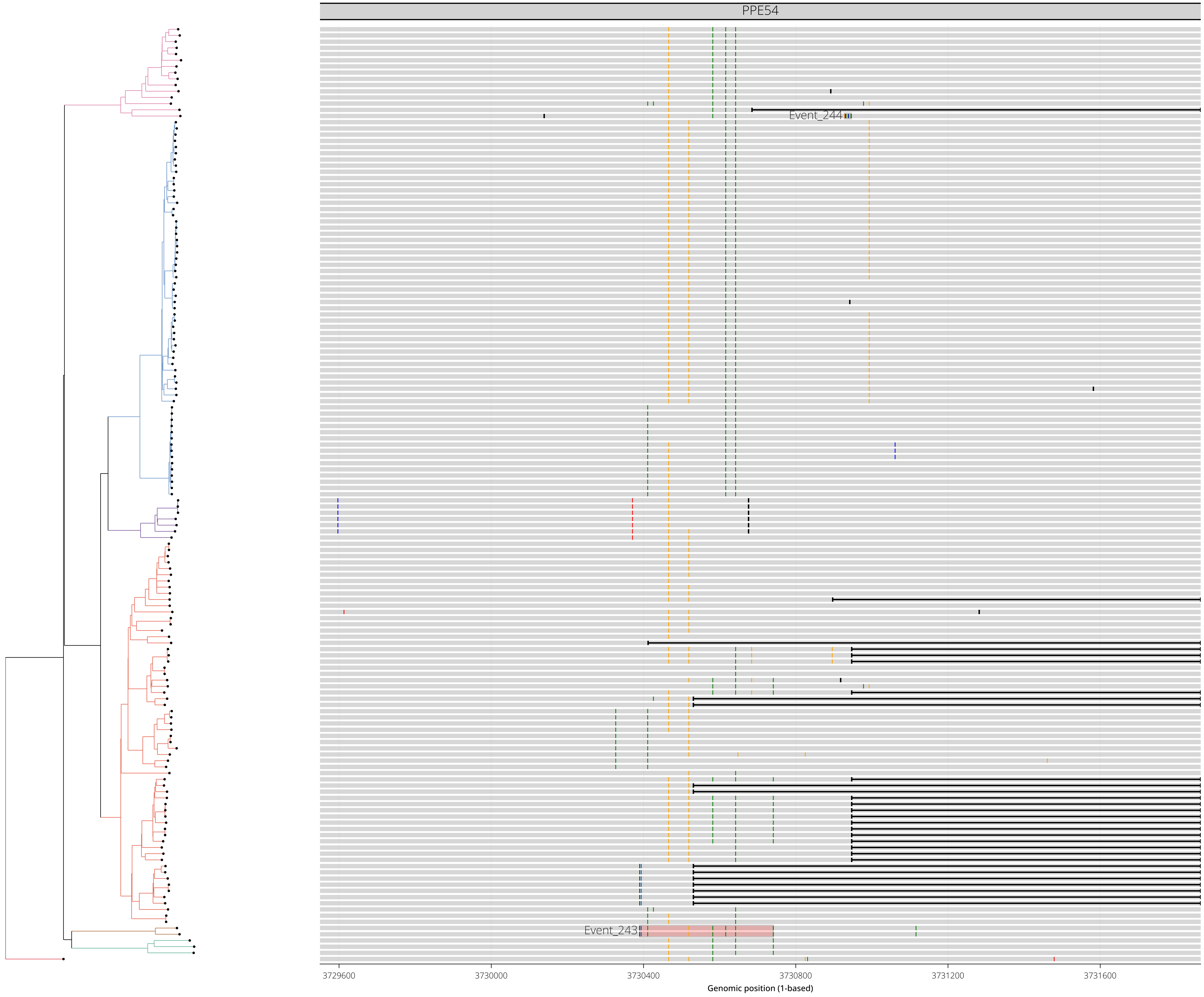

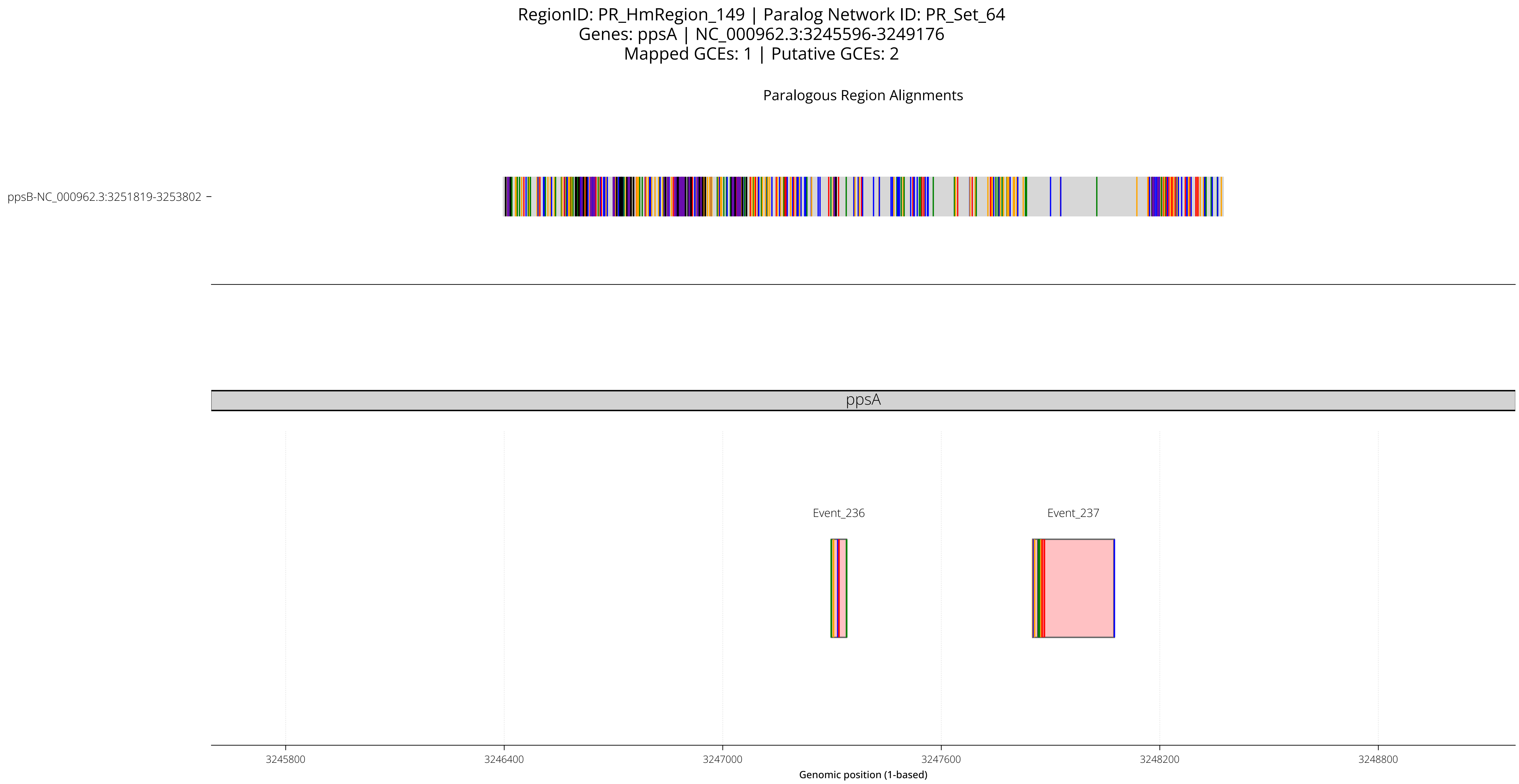
RegionID: PR_HmRegion_149 | Paralog Network ID: PR_Set_64
Genes: ppsA | NC_000962.3:3245596-3249176
Mapped GCEs: 1 | Putative GCEs: 2
Paralogous Region Alignments
ppsB-NC_000962.3:3251819-3253802
ppsA
Event_236
Event_237
3245800
3246400
3247000
3247600
3248200
3248800
Genomic position (1-based)

RegionID: PR_HmRegion_149 | Paralog Network ID: PR_Set_64
Genes: ppsA | NC_000962.3:3245596-3249176
Mapped GCEs: 1 | Putative GCEs: 2

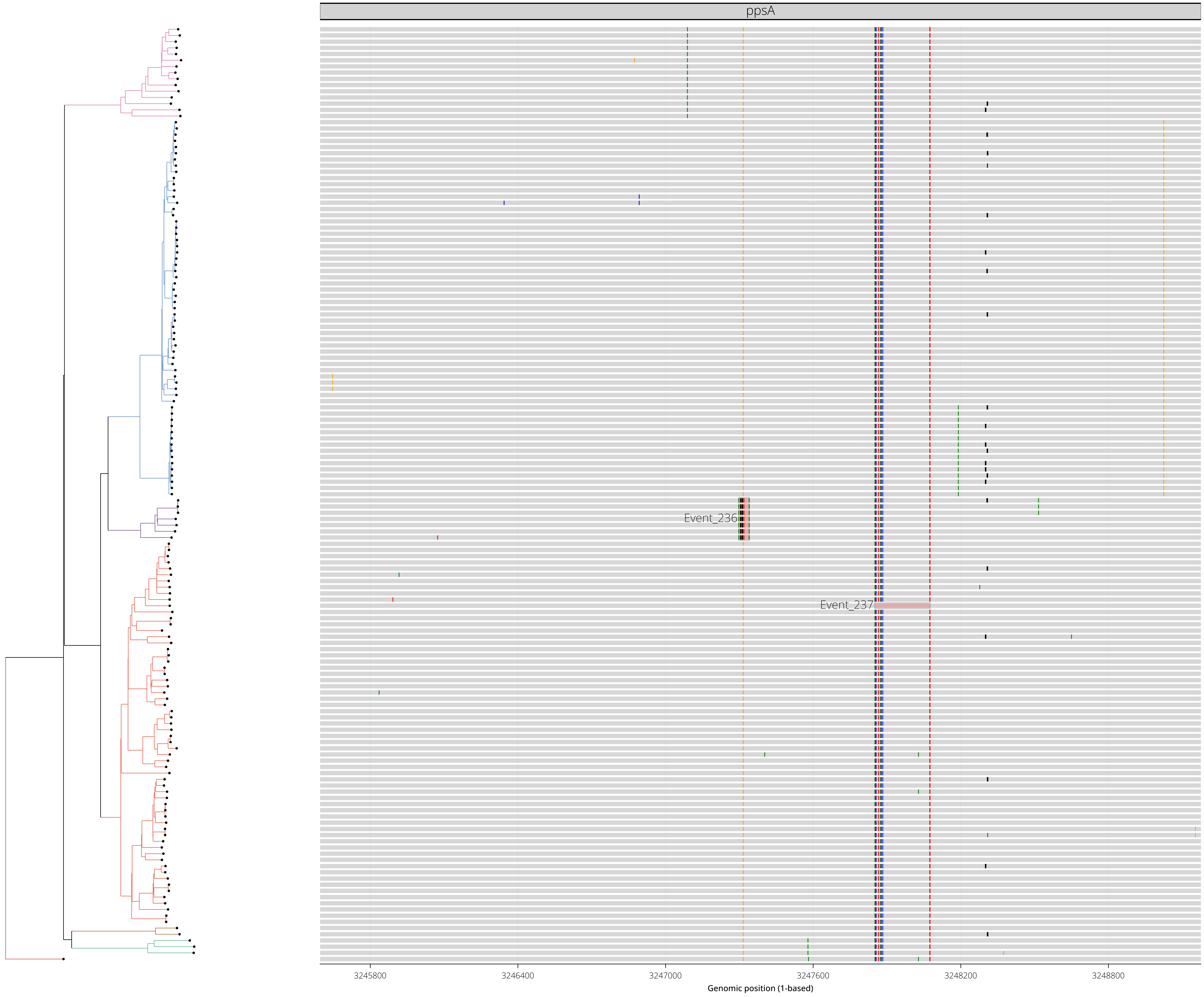

RegionID: PR_HmRegion_047 | Paralog Network ID: PR_Set_32
Genes: PE_PGRS21 | NC_000962.3:1210726-1213009
Mapped GCEs: 0 | Putative GCEs: 1

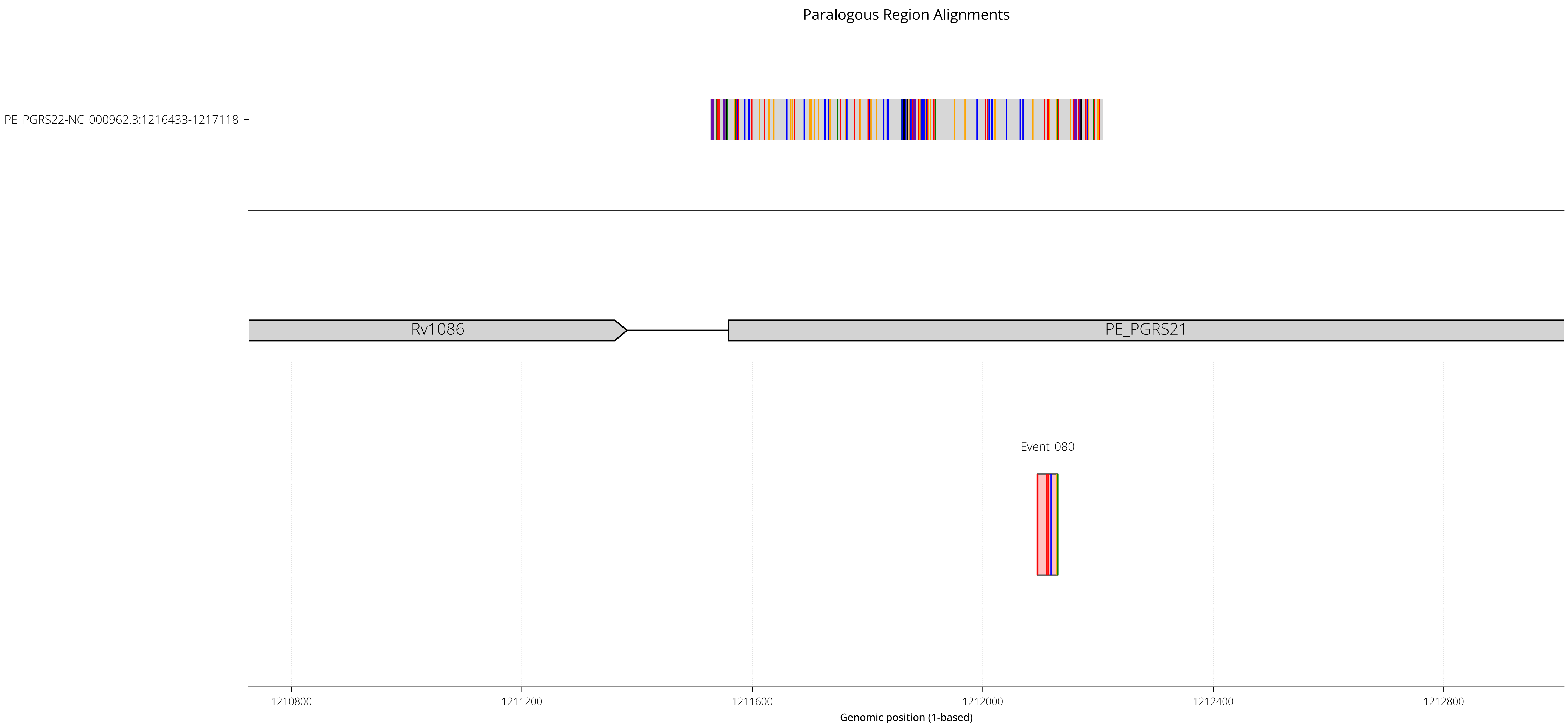

RegionID: PR_HmRegion_047 | Paralog Network ID: PR_Set_32
Genes: PE_PGRS21 | NC_000962.3:1210726-1213009
Mapped GCEs: 0 | Putative GCEs: 1

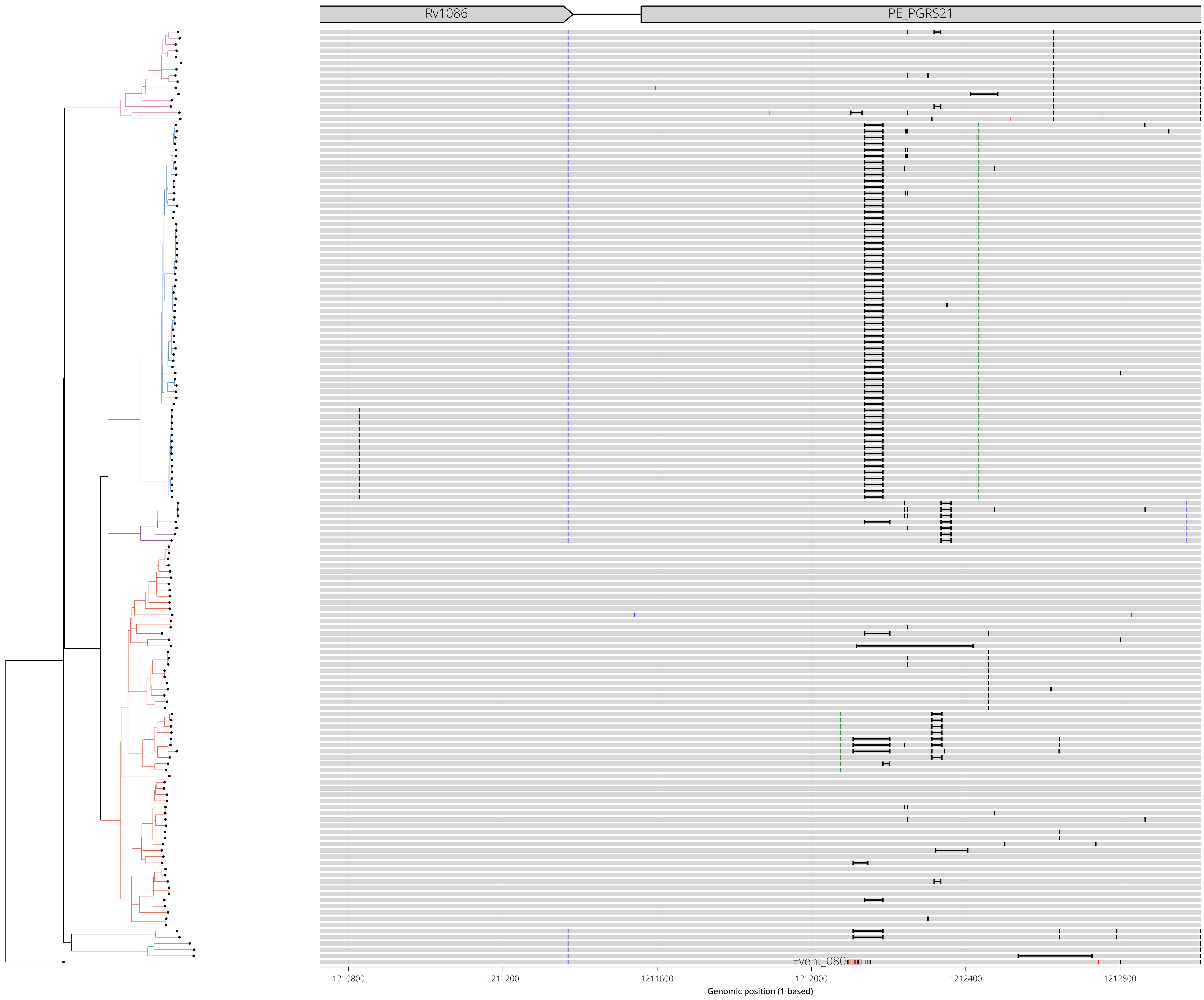

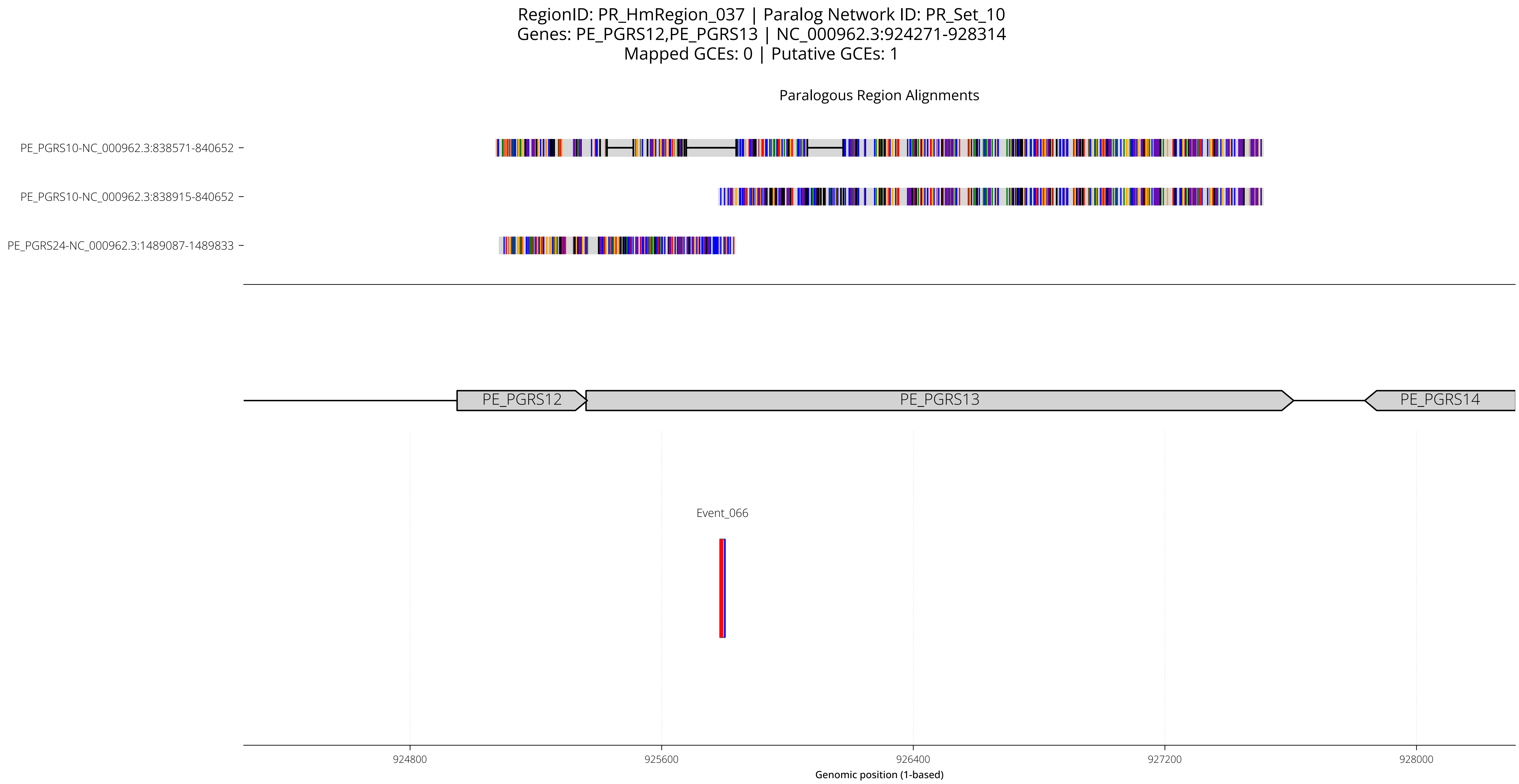
RegionID: PR_HmRegion_037 | Paralog Network ID: PR_Set_10
Genes: PE_PGRS12,PE_PGRS13 | NC_000962.3:924271-928314
Mapped GCEs: 0 | Putative GCEs: 1
Paralogous Region Alignments
PE_PGRS10-NC_000962.3:838571-840652
PE_PGRS10-NC_000962.3:838915-840652
PE_PGRS24-NC_000962.3:1489087-1489833
PE_PGRS12
PE_PGRS13
PE_PGRS14
Event_066
924800
925600
926400
927200
928000
Genomic position (1-based)

RegionID: PR_HmRegion_037 | Paralog Network ID: PR_Set_10
Genes: PE_PGRS12,PE_PGRS13 | NC_000962.3:924271-928314
Mapped GCEs: 0 | Putative GCEs: 1

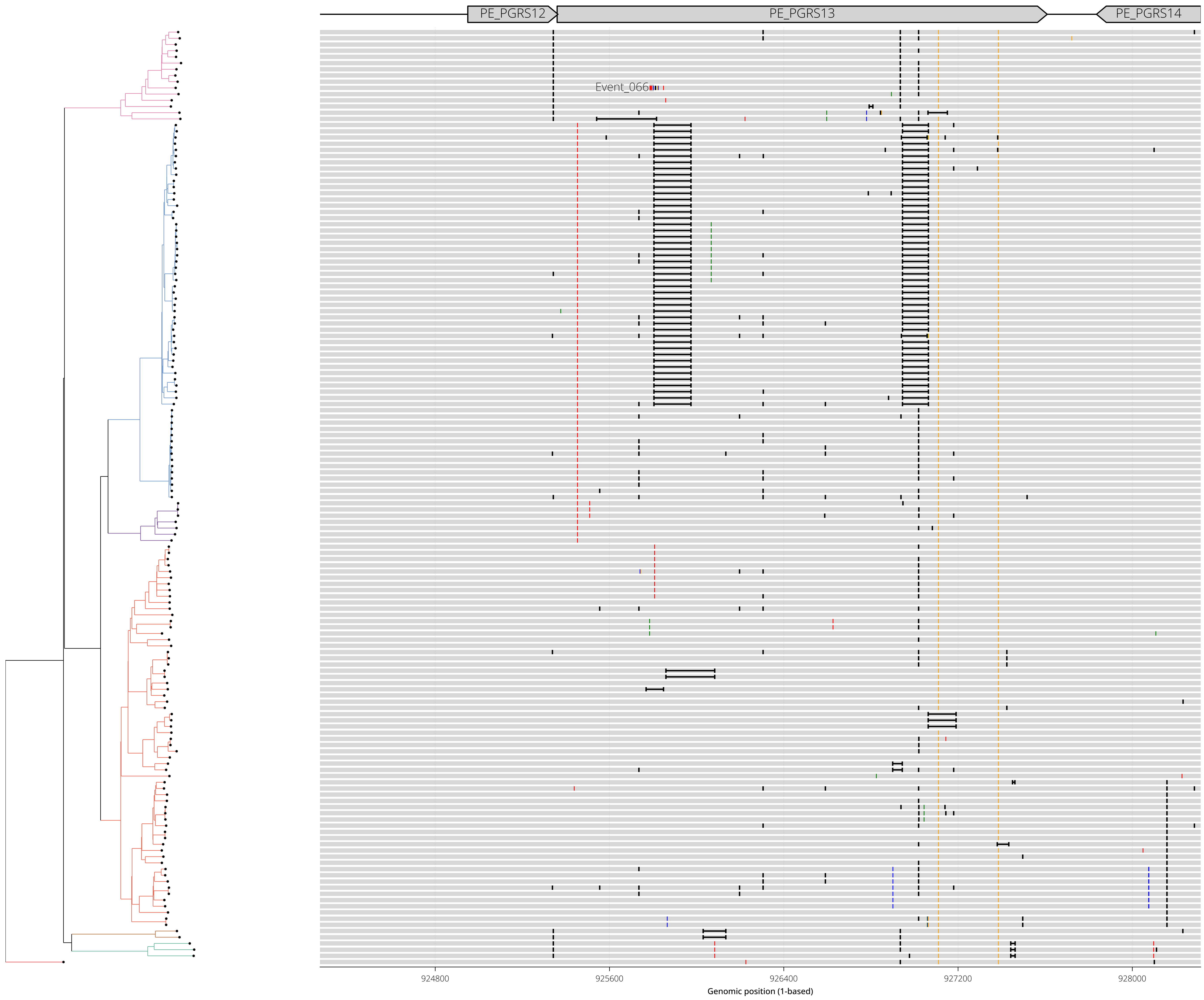

RegionID: PR_HmRegion_119 | Paralog Network ID: PR_Set_55
Genes: Rv2423,Rv2424c | NC_000962.3:2719834-2722242
Mapped GCEs: 1 | Putative GCEs: 1

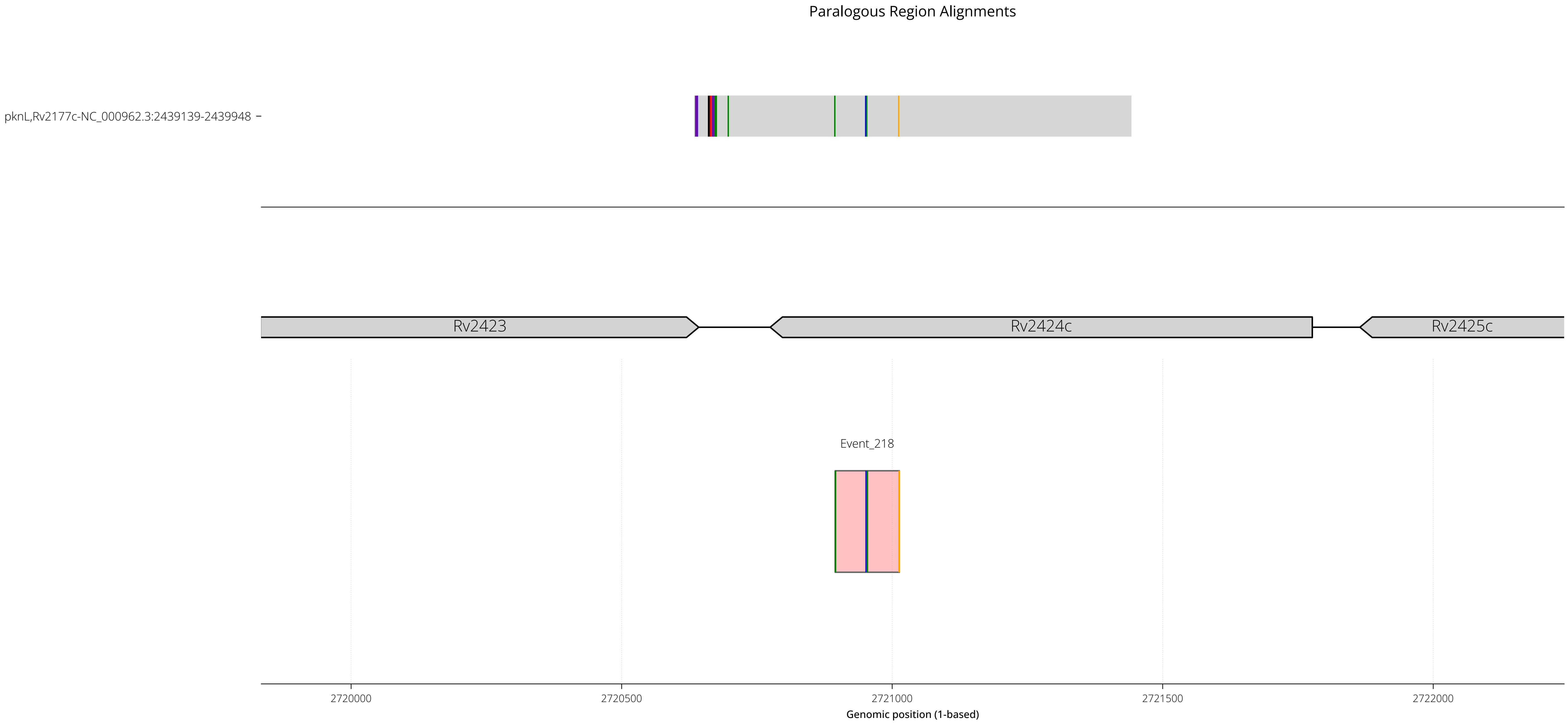

RegionID: PR_HmRegion_119 | Paralog Network ID: PR_Set_55
Genes: Rv2423,Rv2424c | NC_000962.3:2719834-2722242
Mapped GCEs: 1 | Putative GCEs: 1

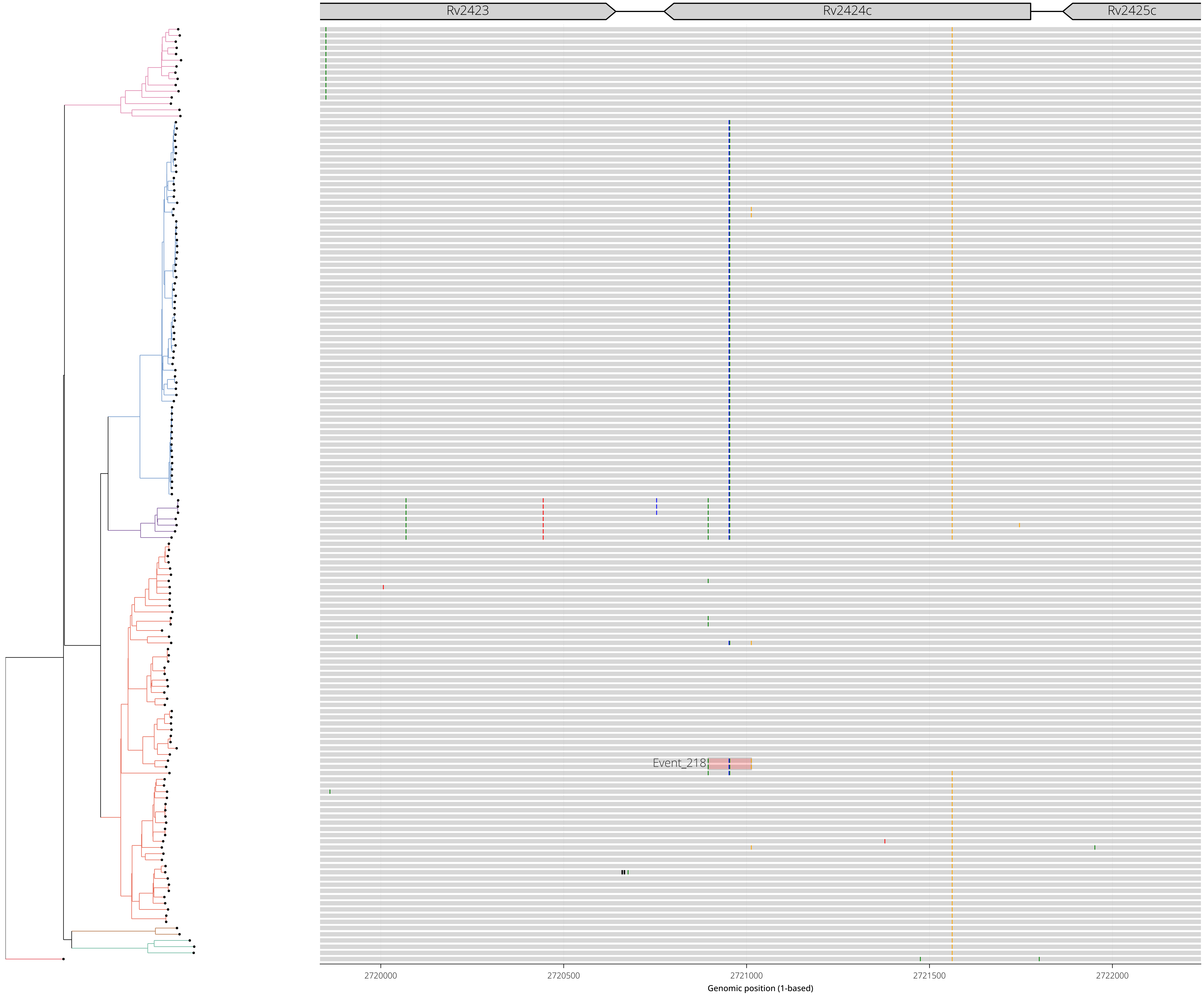

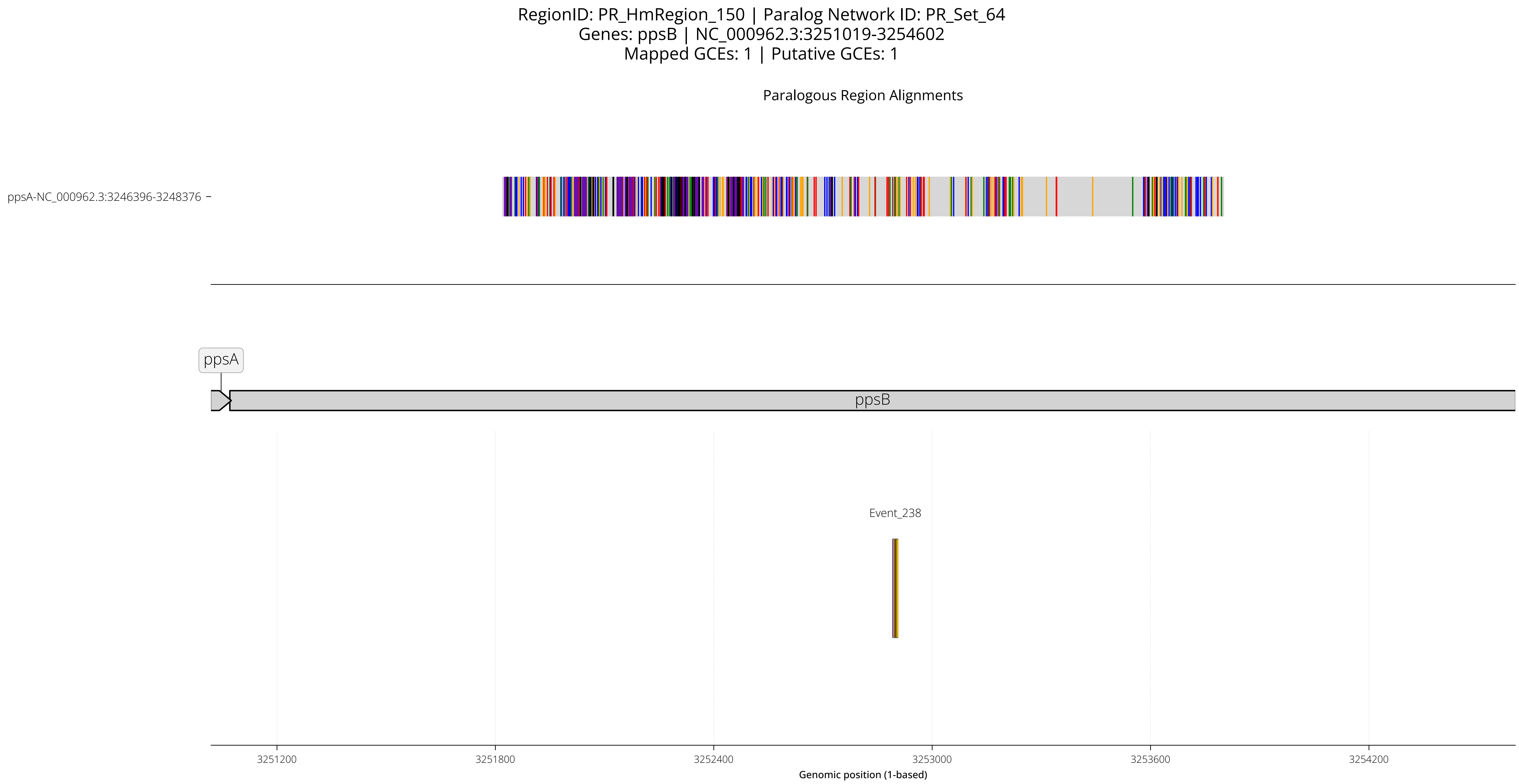

RegionID: PR_HmRegion_150 | Paralog Network ID: PR_Set_64
Genes: ppsB | NC_000962.3:3251019-3254602
Mapped GCEs: 1 | Putative GCEs: 1
Paralogous Region Alignments
ppsA-NC_000962.3:3246396-3248376
ppsA
ppsB
Event_238
3251200
3251800
3252400
3253000
3253600
3254200
Genomic position (1-based)

RegionID: PR_HmRegion_150 | Paralog Network ID: PR_Set_64
Genes: ppsB | NC_000962.3:3251019-3254602
Mapped GCEs: 1 | Putative GCEs: 1

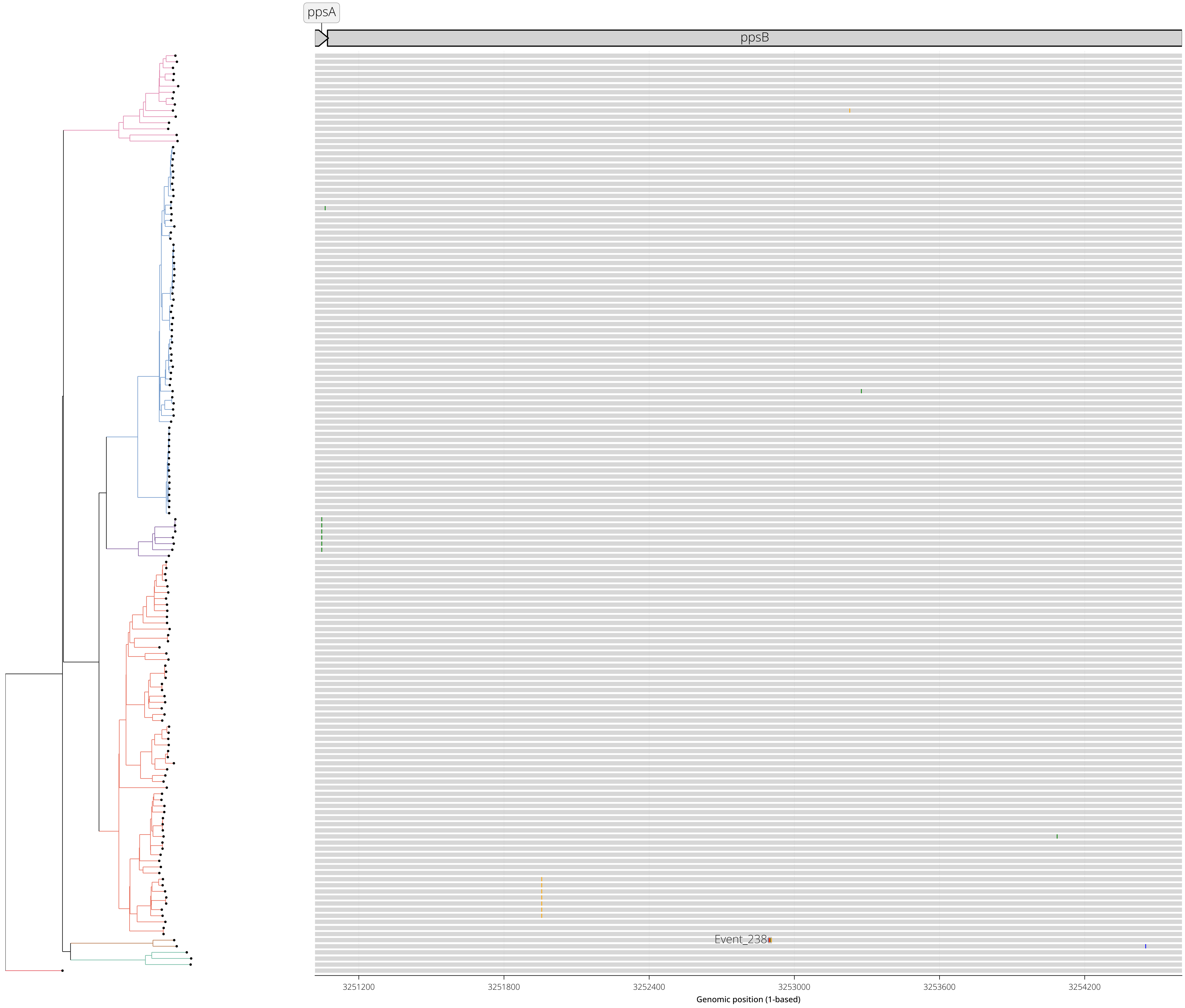

RegionID: PR_HmRegion_147 | Paralog Network ID: PR_Set_20
Genes: Rv2884,Rv2885c,Rv2886c | NC_000962.3:3193337-3197002
Mapped GCEs: 1 | Putative GCEs: 1

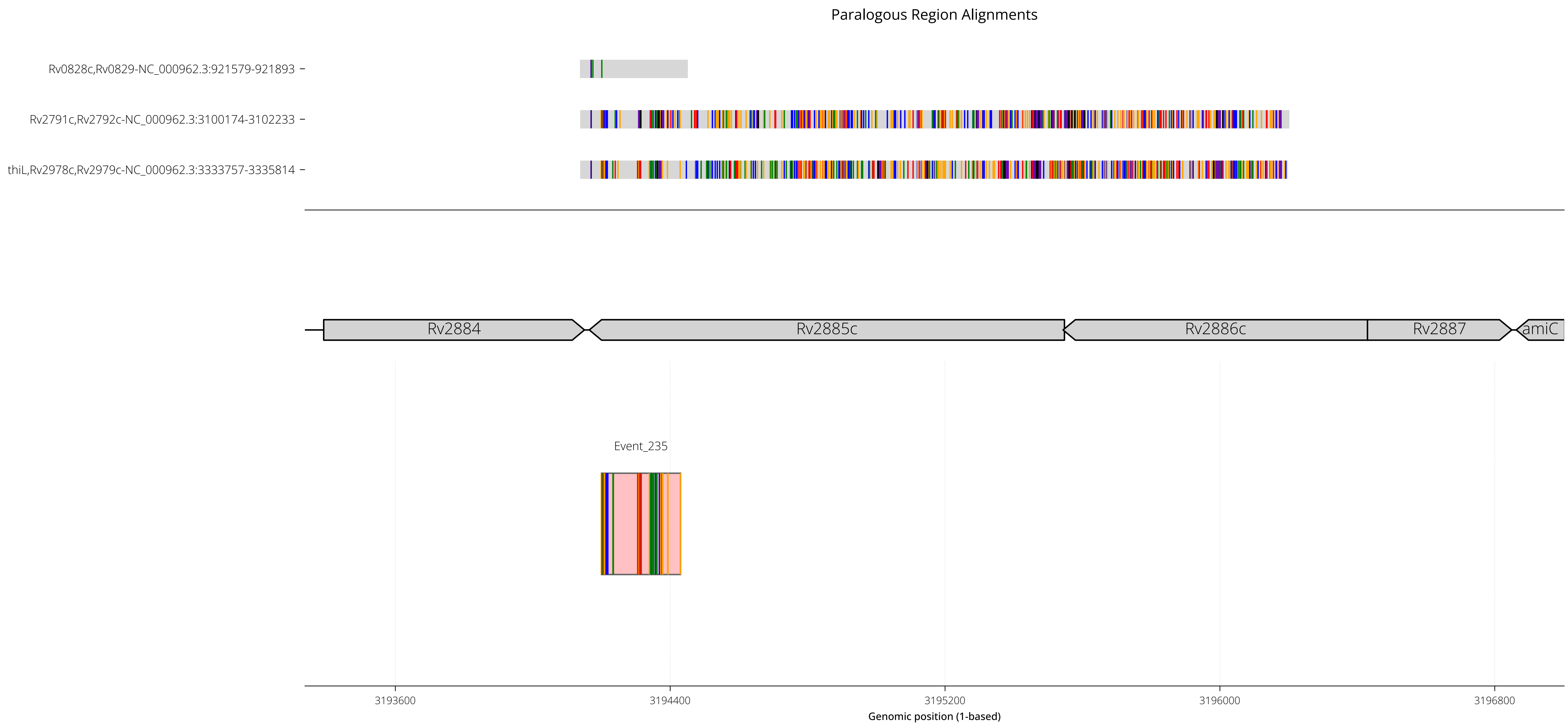

RegionID: PR_HmRegion_147 | Paralog Network ID: PR_Set_20
Genes: Rv2884,Rv2885c,Rv2886c | NC_000962.3:3193337-3197002
Mapped GCEs: 1 | Putative GCEs: 1

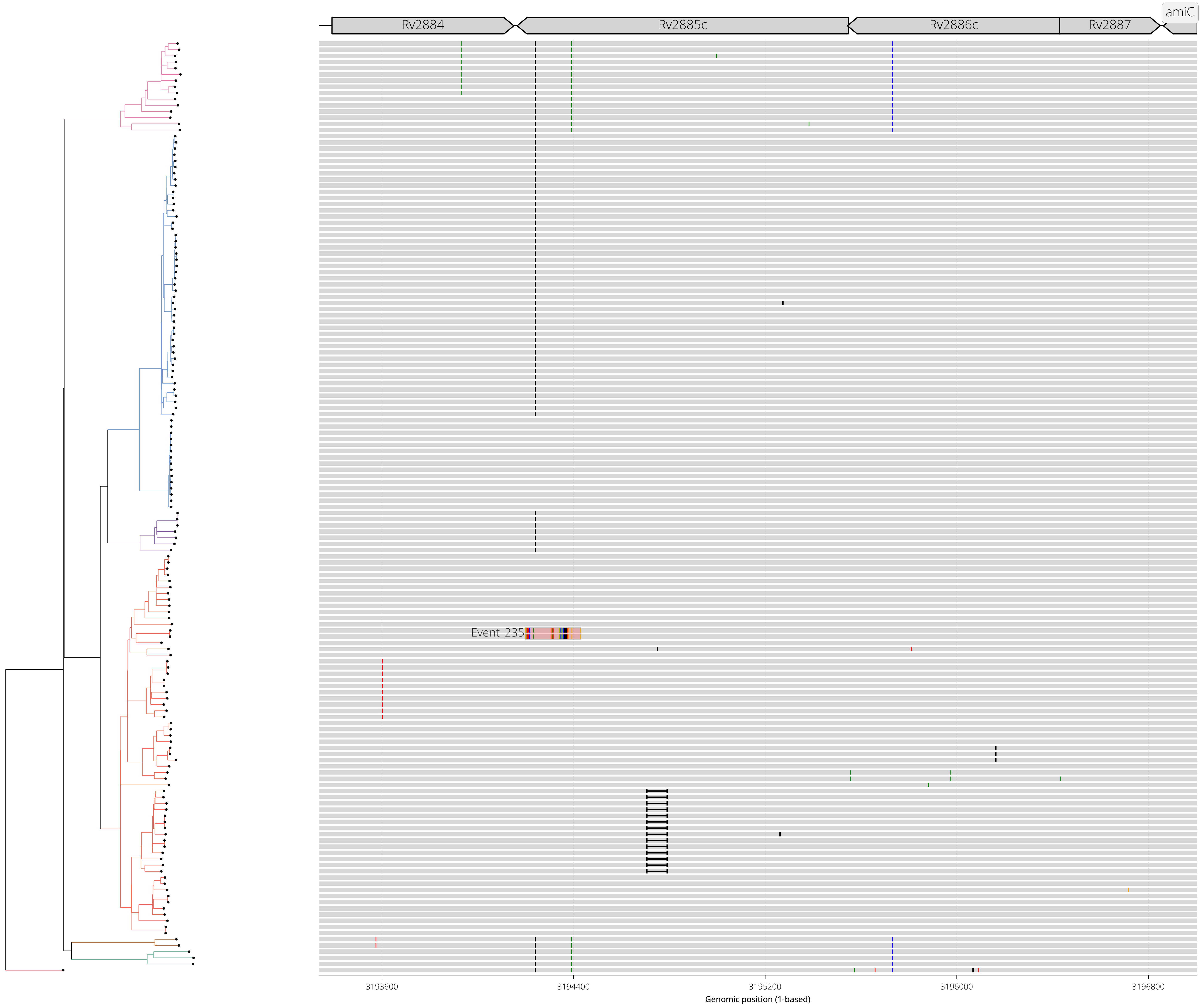

RegionID: PR_HmRegion_127 | Paralog Network ID: PR_Set_27
Genes: PE_PGRS45 | NC_000962.3:2943467-2946026
Mapped GCEs: 1 | Putative GCEs: 1

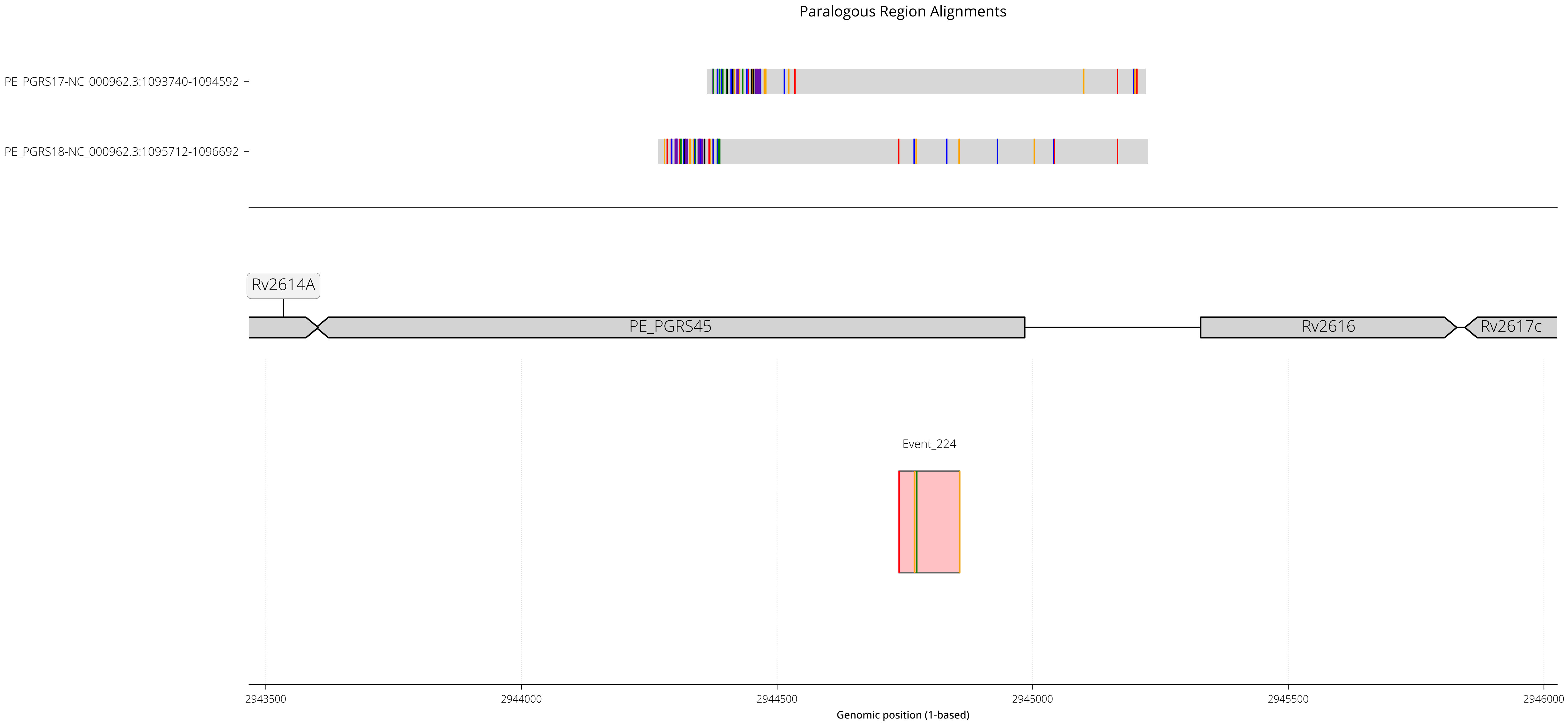

RegionID: PR_HmRegion_127 | Paralog Network ID: PR_Set_27
Genes: PE_PGRS45 | NC_000962.3:2943467-2946026
Mapped GCEs: 1 | Putative GCEs: 1

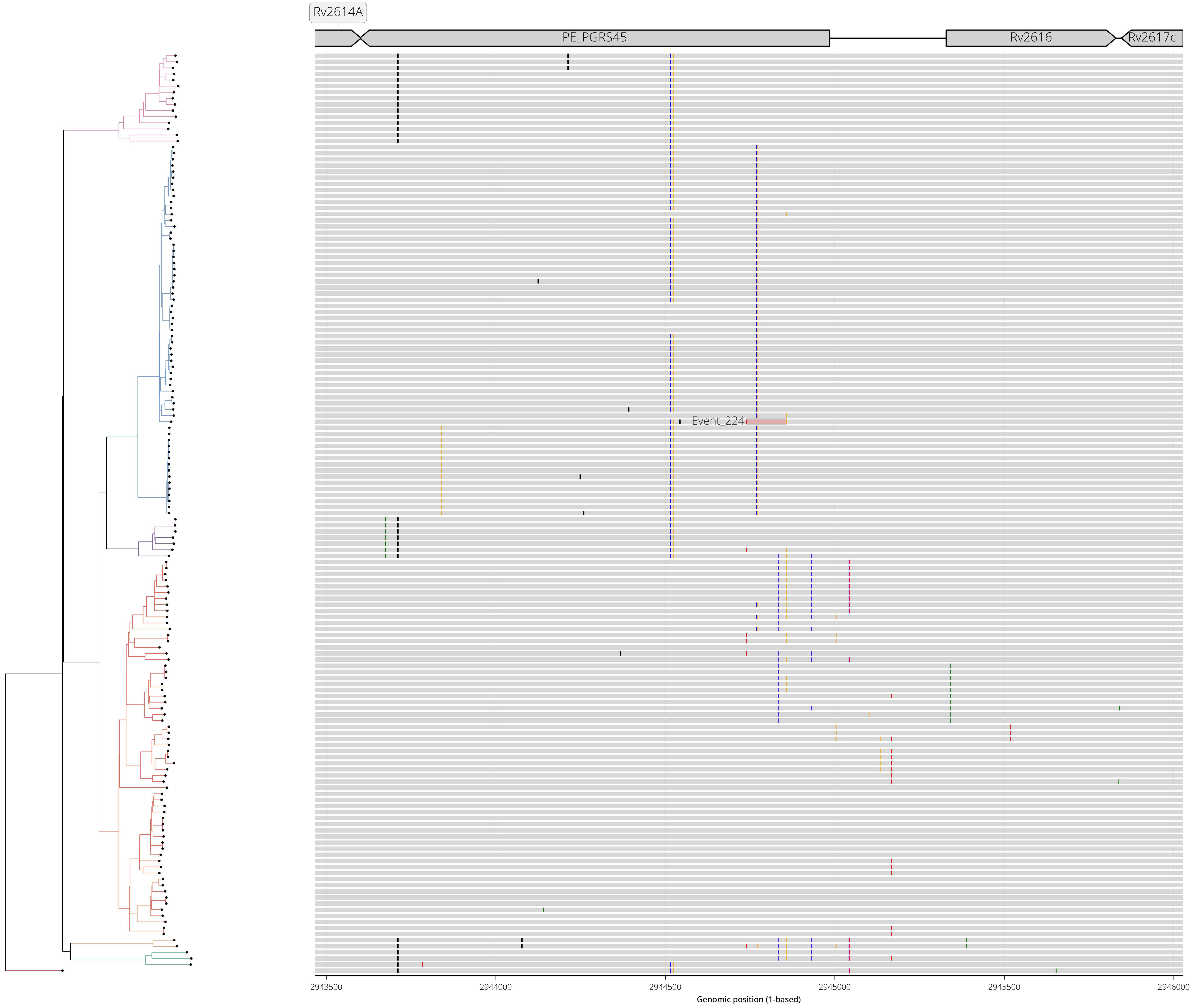

RegionID: PR_HmRegion_140 | Paralog Network ID: PR_Set_24
Genes: Rv2814c,Rv2815c | NC_000962.3:3118383-3124377
Mapped GCEs: 0 | Putative GCEs: 1

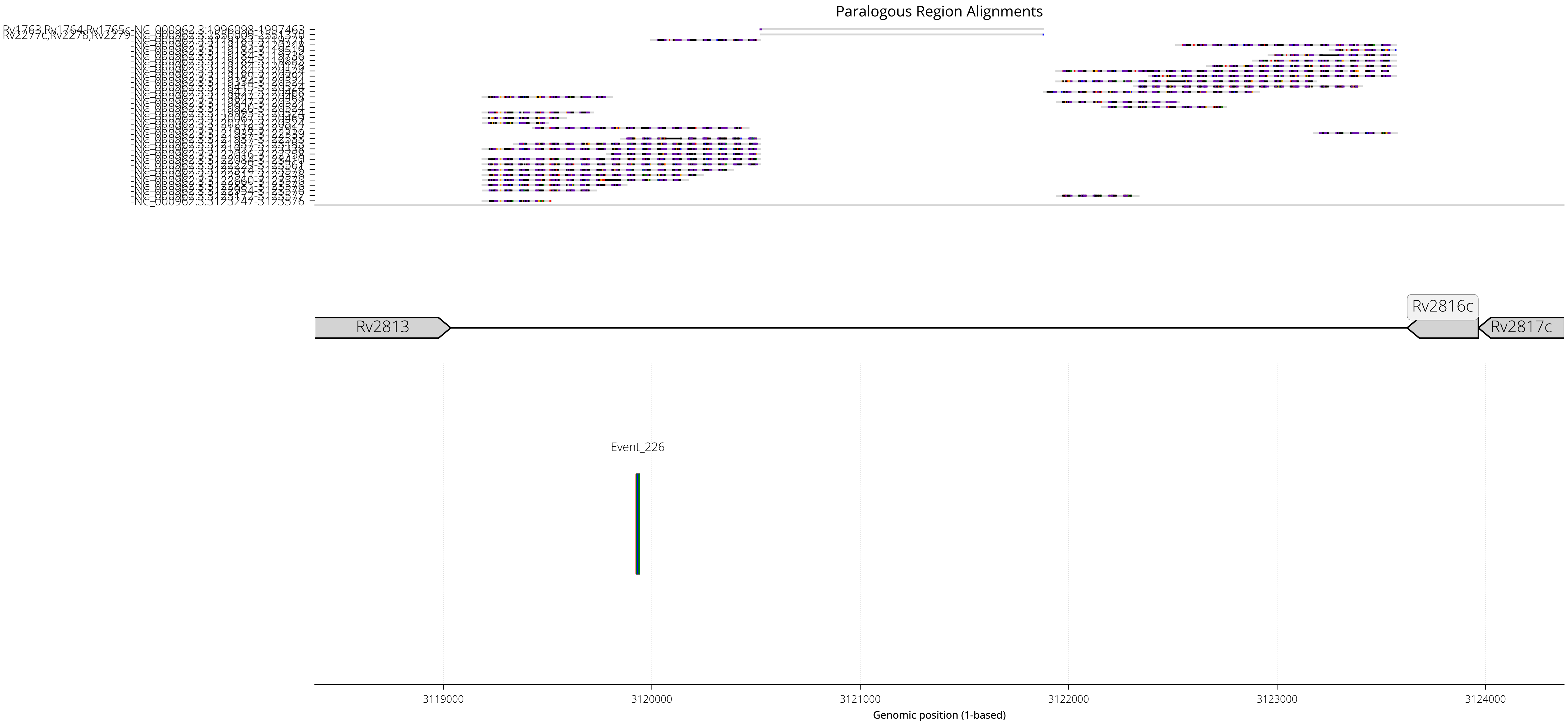

RegionID: PR_HmRegion_140 | Paralog Network ID: PR_Set_24
Genes: Rv2814c,Rv2815c | NC_000962.3:3118383-3124377
Mapped GCEs: 0 | Putative GCEs: 1

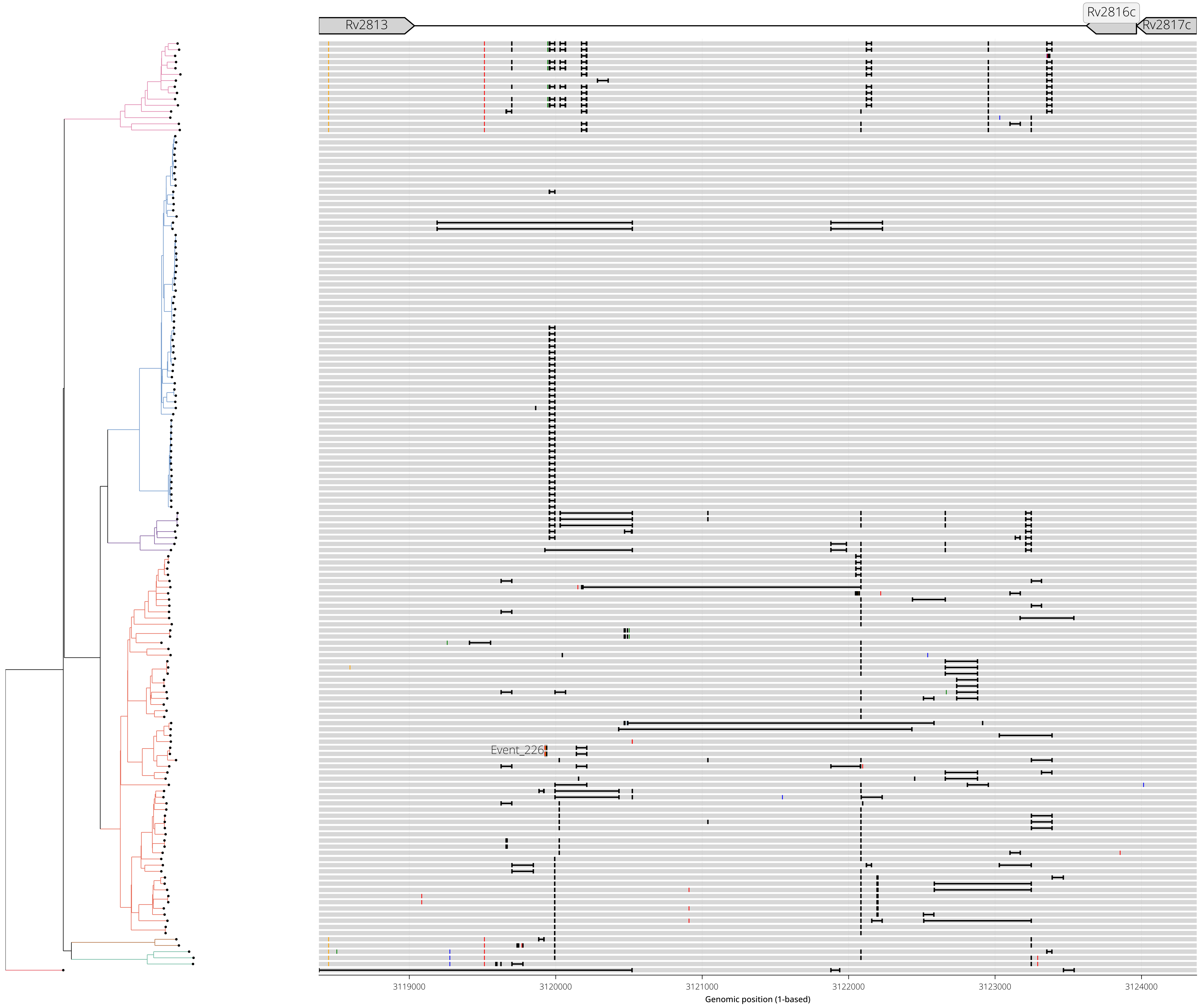

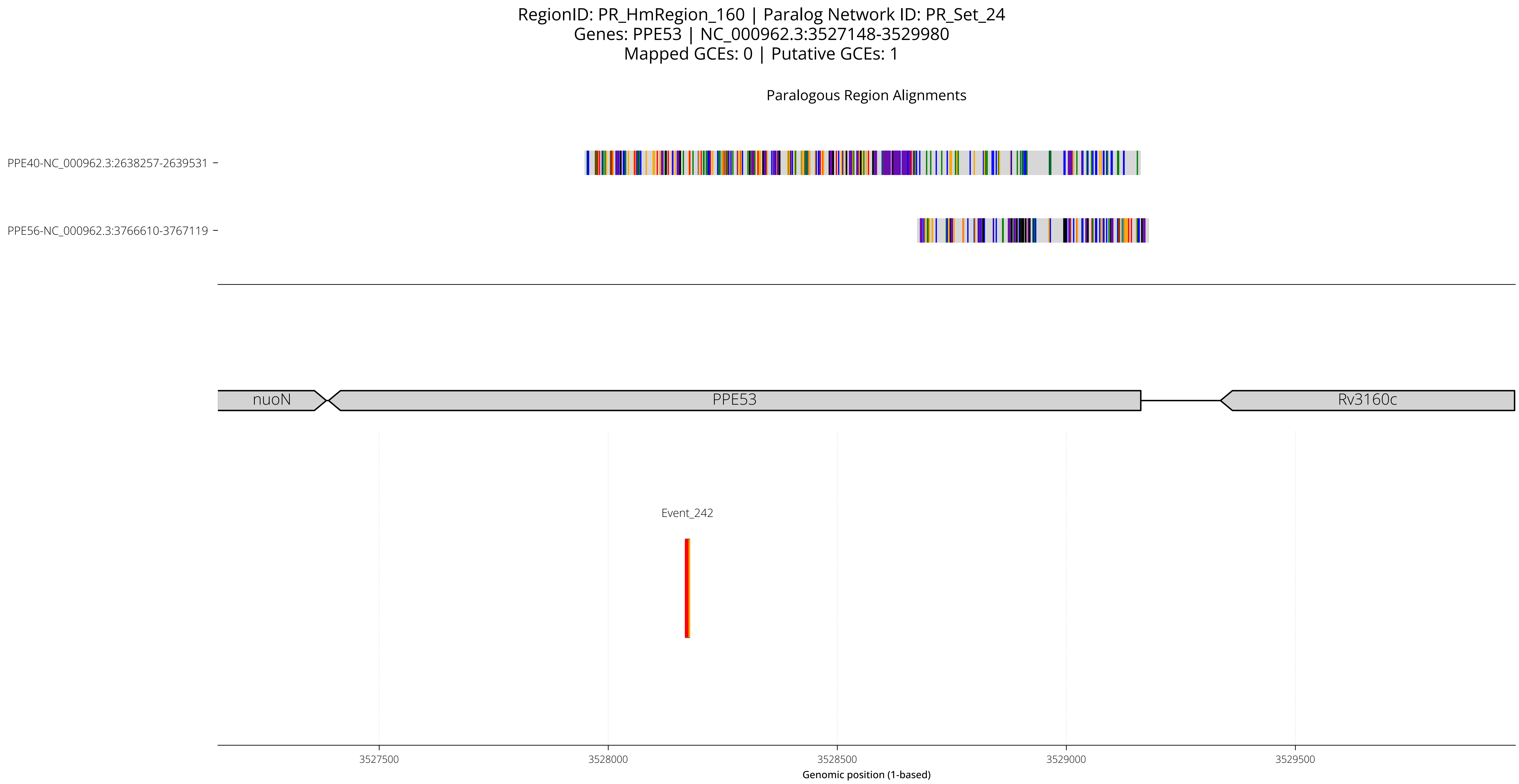
RegionID: PR_HmRegion_160 | Paralog Network ID: PR_Set_24
Genes: PPE53 | NC_000962.3:3527148-3529980
Mapped GCEs: 0 | Putative GCEs: 1
Paralogous Region Alignments
PPE40-NC_000962.3:2638257-2639531
PPE56-NC_000962.3:3766610-3767119
nuoN
PPE53
Rv3160c
Event_242
3527500
3528000
3528500
3529000
3529500
Genomic position (1-based)

RegionID: PR_HmRegion_160 | Paralog Network ID: PR_Set_24
Genes: PPE53 | NC_000962.3:3527148-3529980
Mapped GCEs: 0 | Putative GCEs: 1

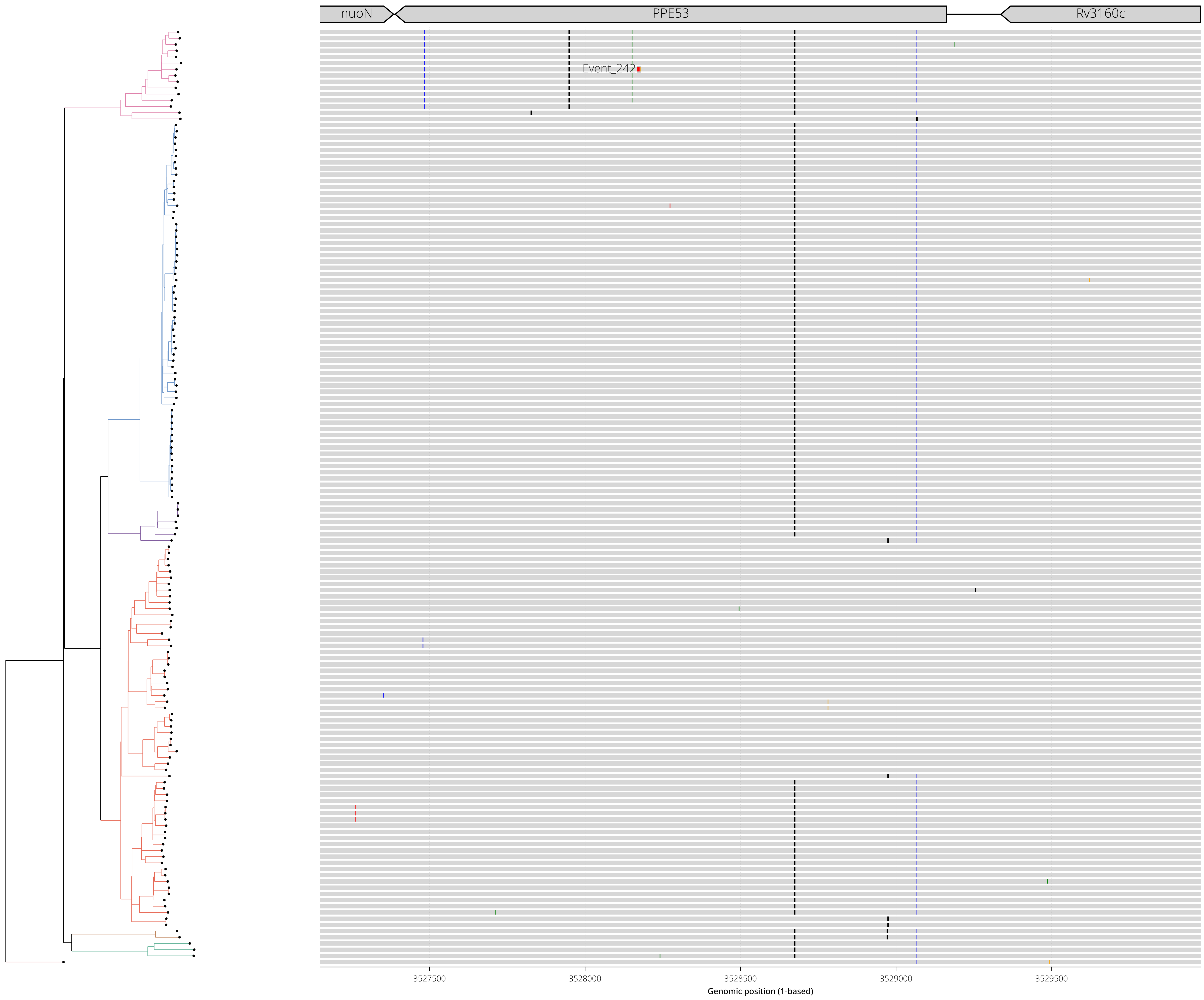

RegionID: PR_HmRegion_189 | Paralog Network ID: PR_Set_28
Genes: esxV,esxW | NC_000962.3:4059183-4061391
Mapped GCEs: 1 | Putative GCEs: 1

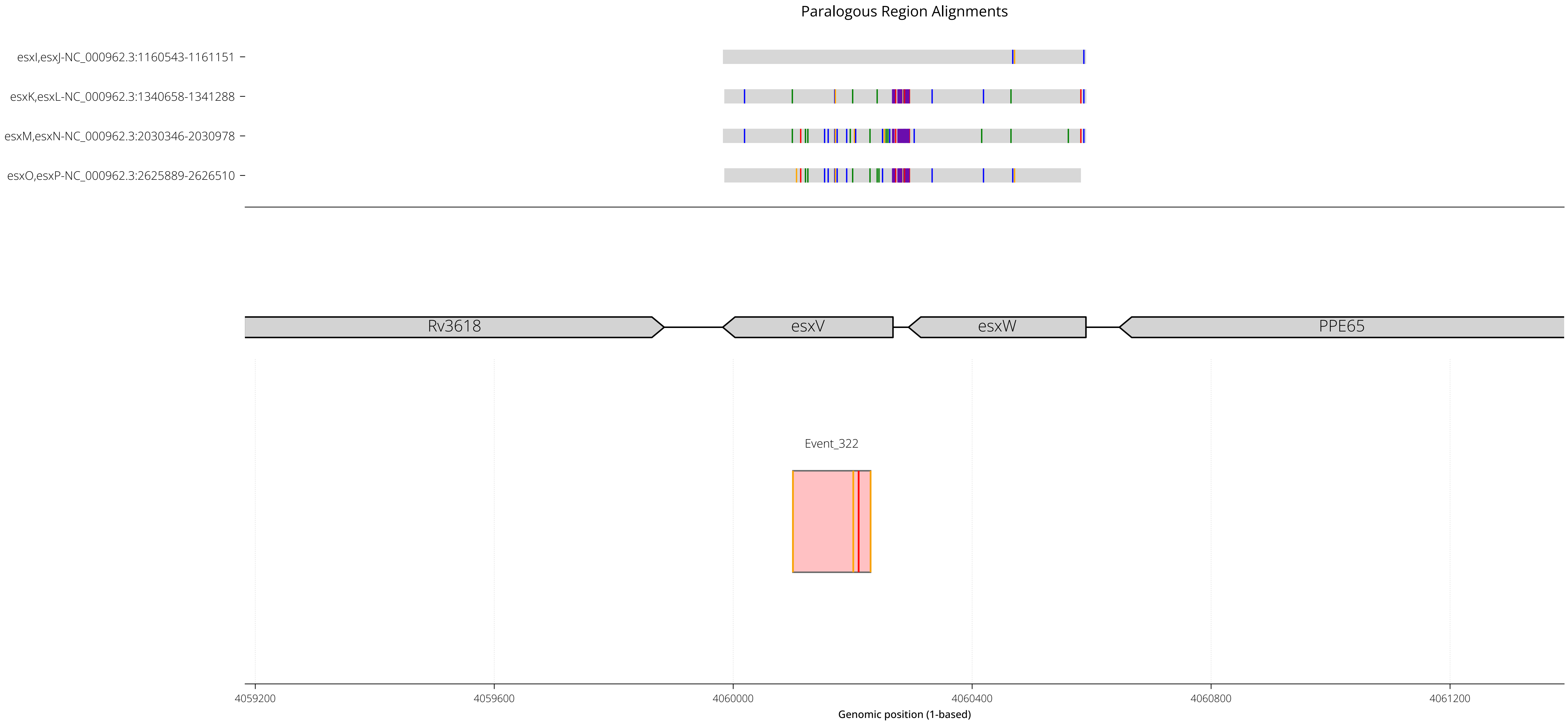

RegionID: PR_HmRegion_189 | Paralog Network ID: PR_Set_28
Genes: esxV,esxW | NC_000962.3:4059183-4061391
Mapped GCEs: 1 | Putative GCEs: 1

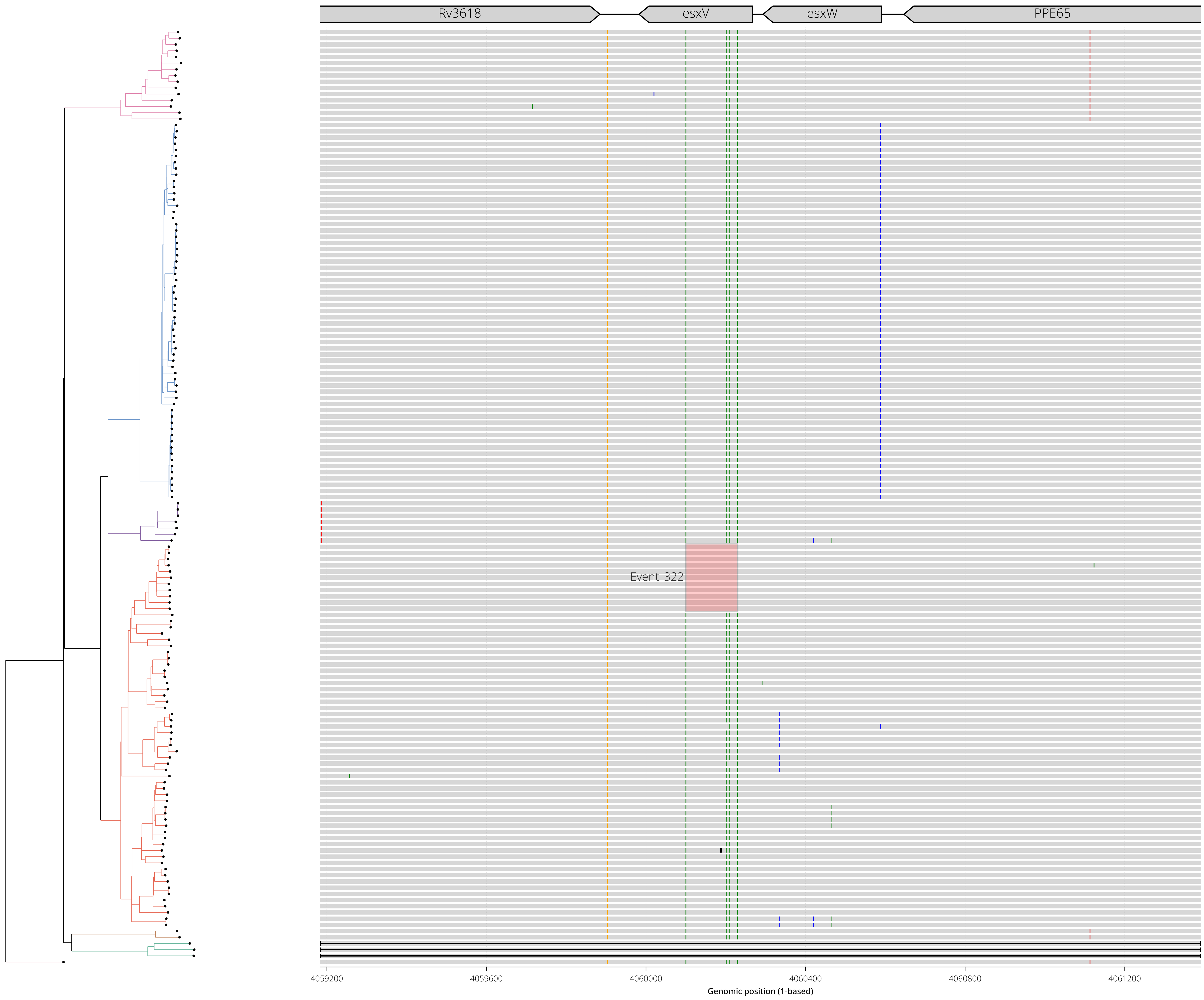

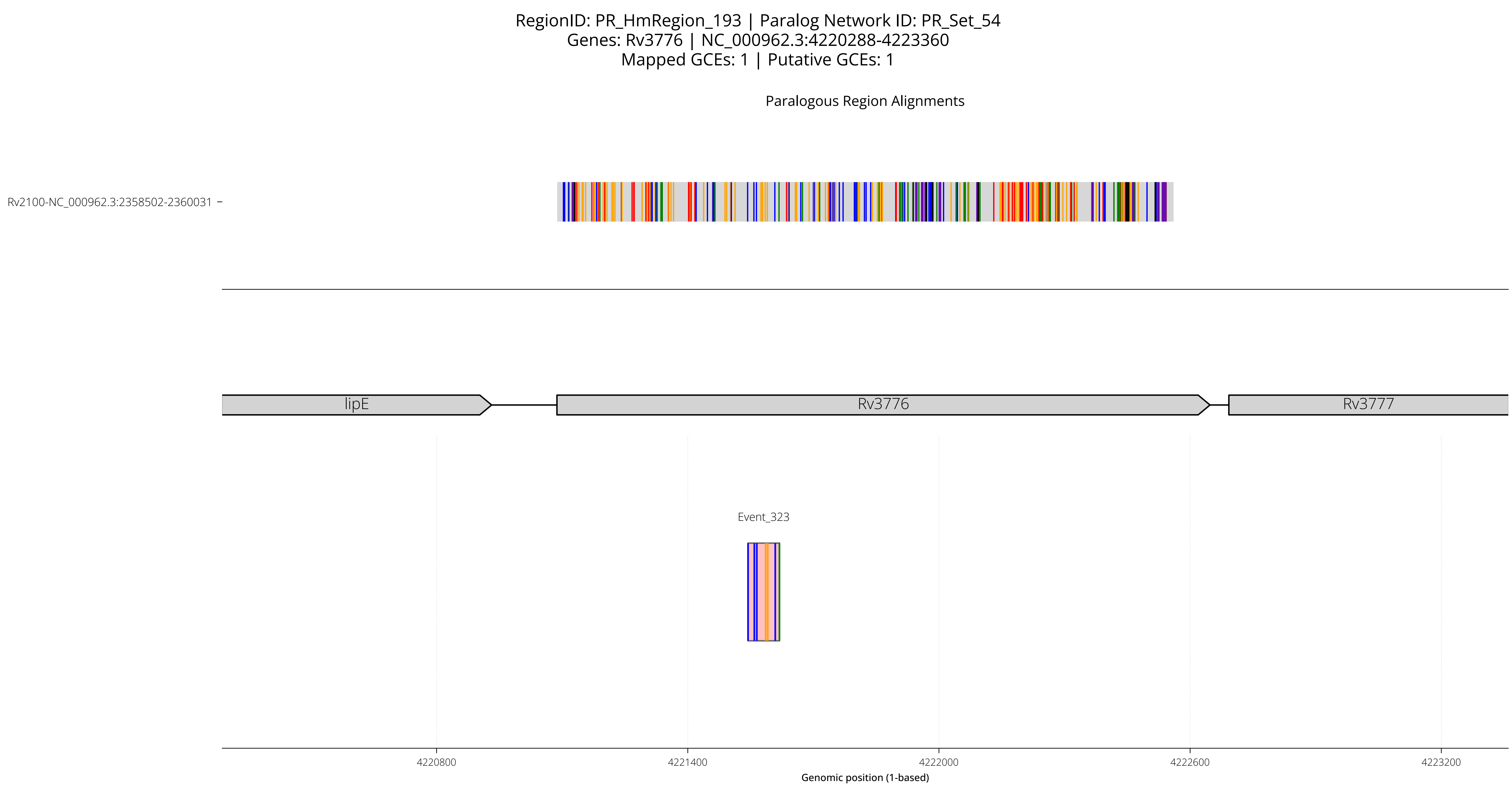

RegionID: PR_HmRegion_193 | Paralog Network ID: PR_Set_54
Genes: Rv3776 | NC_000962.3:4220288-4223360
Mapped GCEs: 1 | Putative GCEs: 1
Paralogous Region Alignments
Rv2100-NC_000962.3:2358502-2360031
lipE
Rv3776
Rv3777
Event_323
4220800
4221400
4222000
4222600
4223200
Genomic position (1-based)

RegionID: PR_HmRegion_193 | Paralog Network ID: PR_Set_54
Genes: Rv3776 | NC_000962.3:4220288-4223360
Mapped GCEs: 1 | Putative GCEs: 1

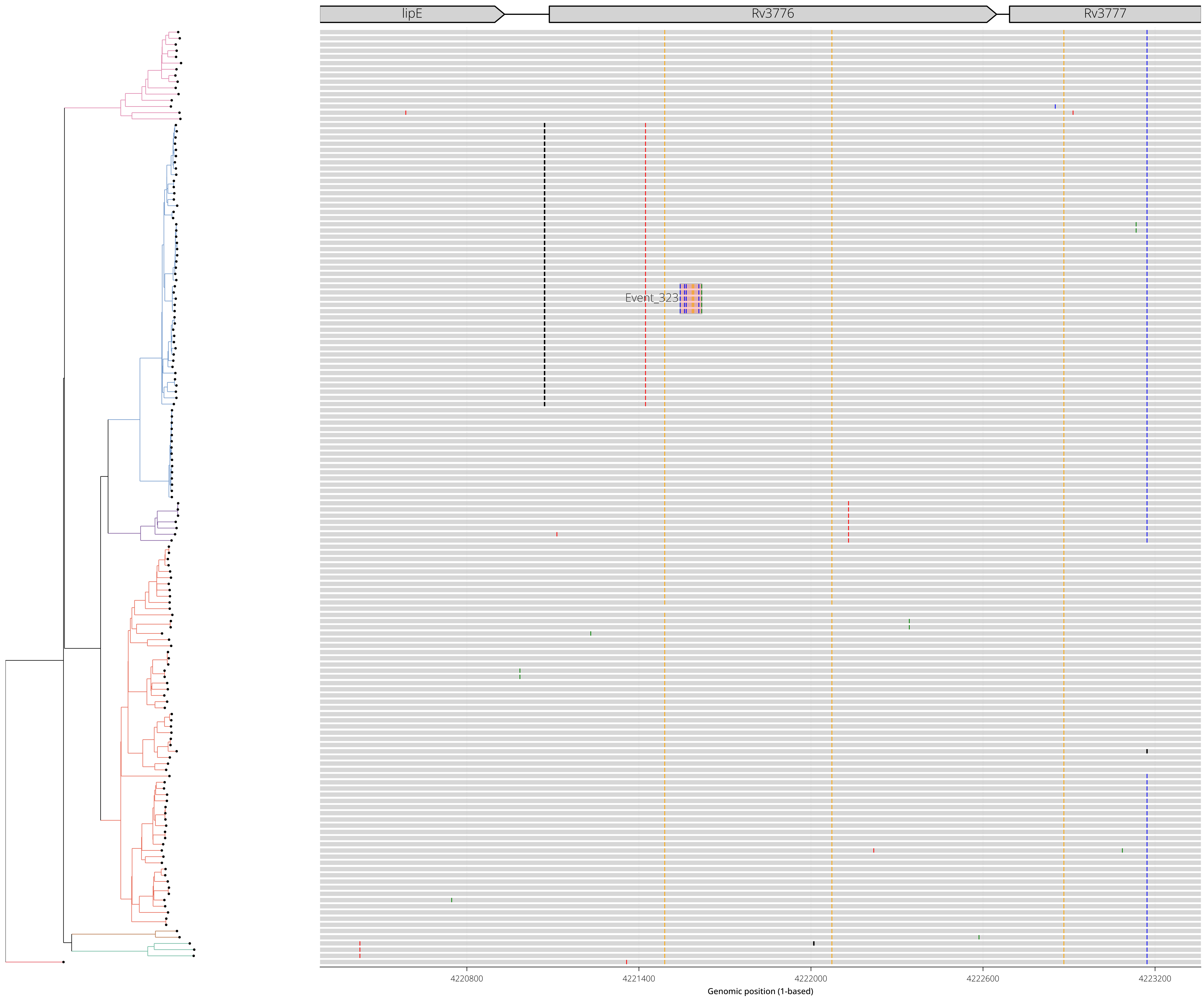

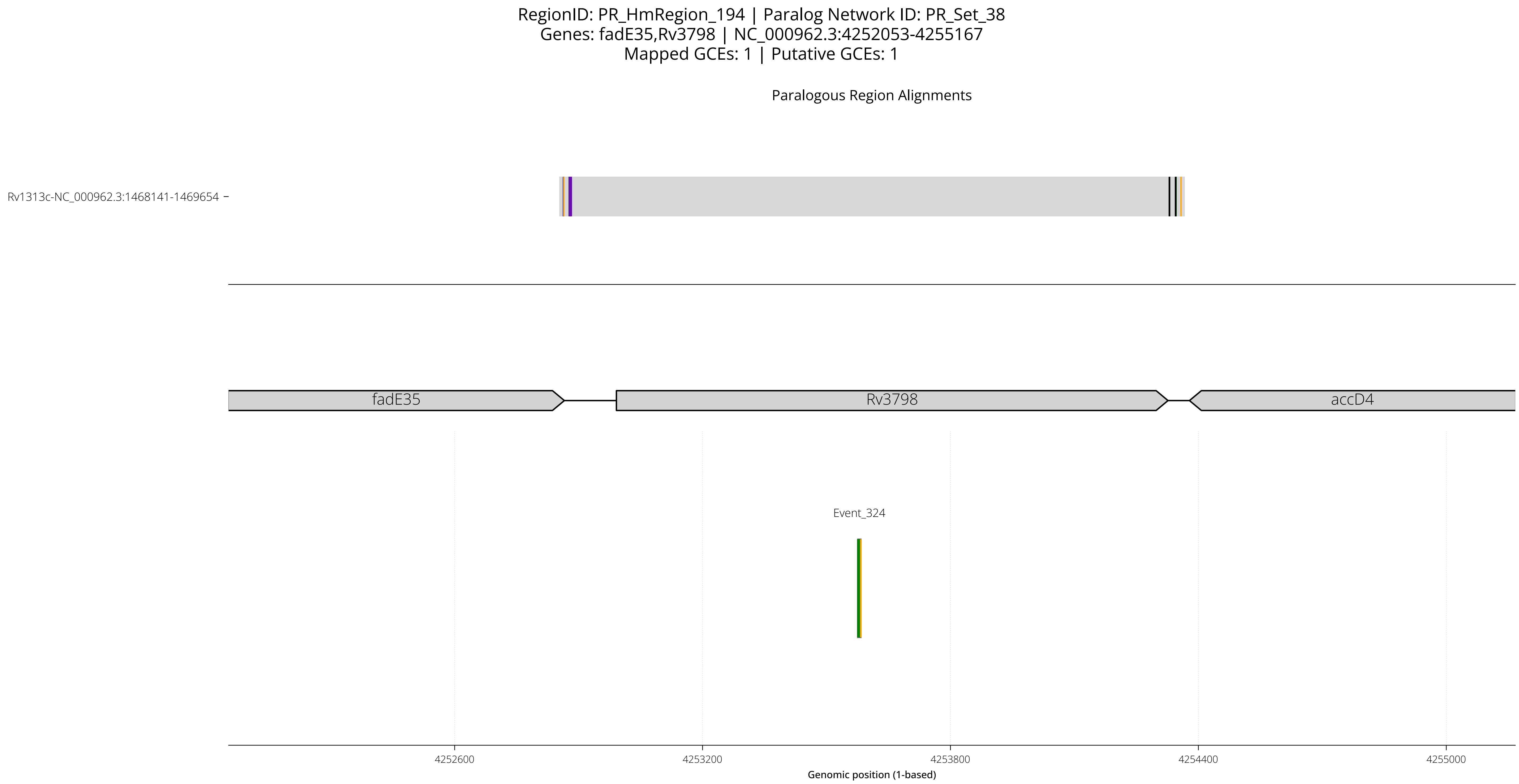
RegionID: PR_HmRegion_194 | Paralog Network ID: PR_Set_38
Genes: fadE35,Rv3798 | NC_000962.3:4252053-4255167
Mapped GCEs: 1 | Putative GCEs: 1
Paralogous Region Alignments
Rv1313c-NC_000962.3:1468141-1469654
fadE35
Rv3798
accD4
Event_324
4252600
4253200
4253800
4254400
4255000
Genomic position (1-based)

RegionID: PR_HmRegion_194 | Paralog Network ID: PR_Set_38
Genes: fadE35,Rv3798 | NC_000962.3:4252053-4255167
Mapped GCEs: 1 | Putative GCEs: 1

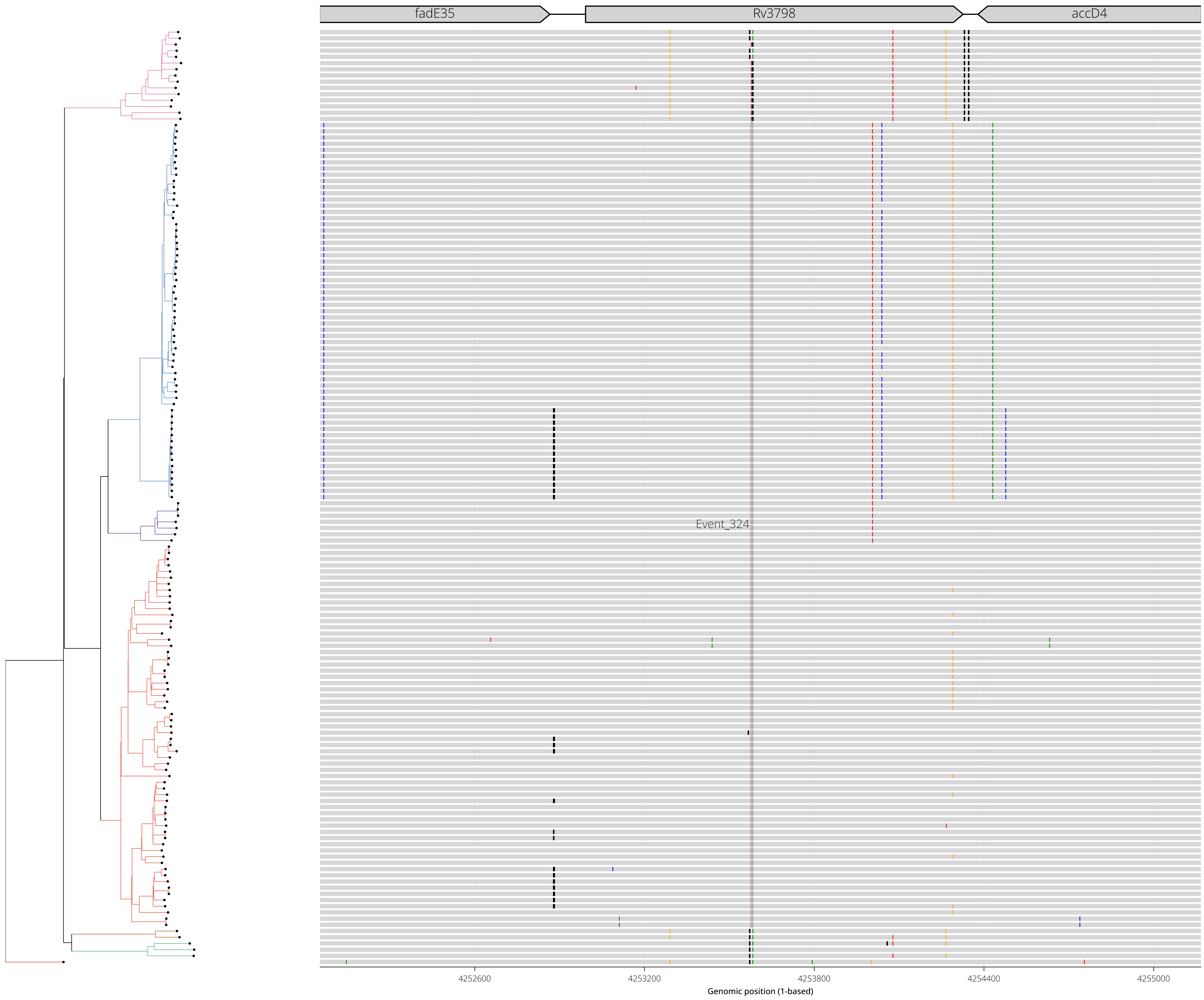

Supplement: Supplement 13 [file media-13.pdf]
