# Supplementary material for: Gene conversion is a key driver of diversity hotspots in M. tuberculosis antigens and virulence-associated loci
Source: bioRxiv. 2026 Mar 11:2026.02.26.708061. Preprint. [Version 2] doi: 10.64898/2026.02.26.708061 (PMC13061034; doi:10.64898/2026.02.26.708061)

Gene(s): PPE18
In Paralogous Region (PR)? : [True]
# of Gene Conversion Events (GCE) detected: 8
Missense Mutation Events Detected (Total): 61
Missense Mutation Events Detected (In Epitopes): 44
Total # of assayed peptides: 83
Positive T-cell epitopes: 27

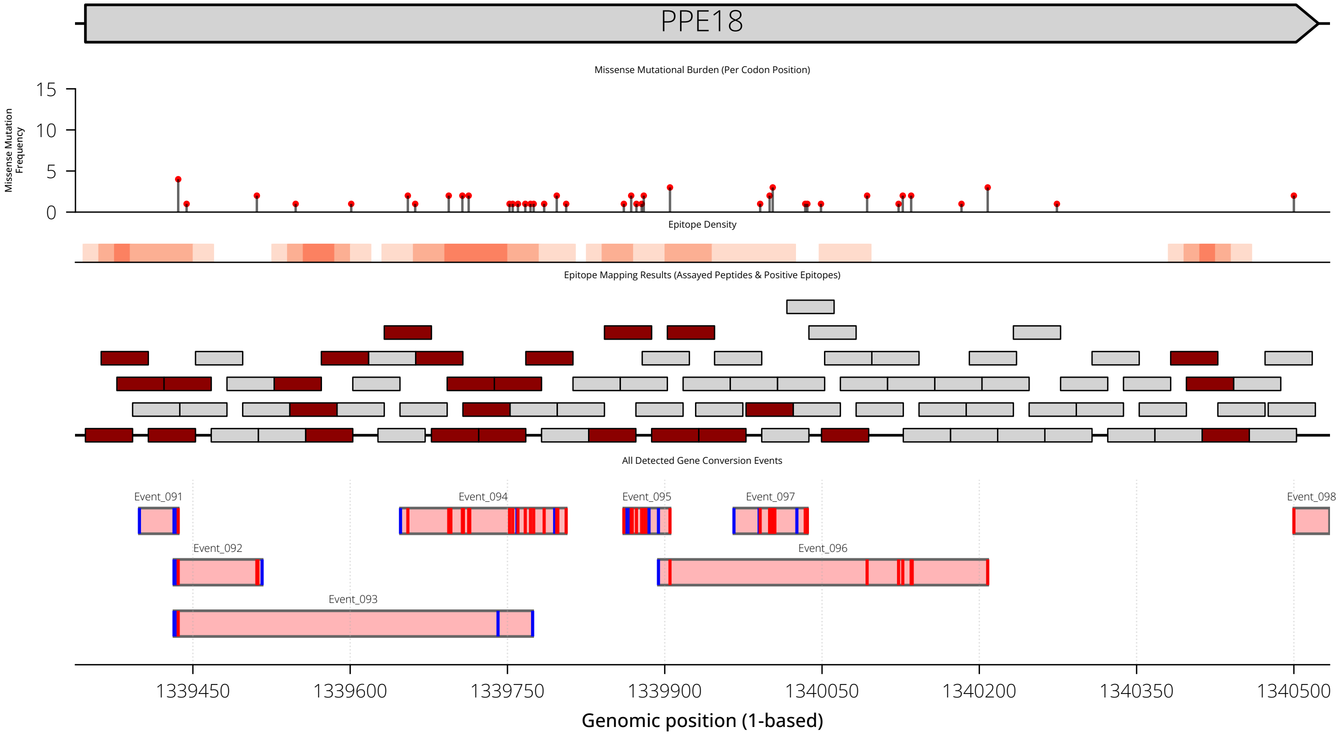

Gene(s): PPE19
In Paralogous Region (PR)? : [True]
# of Gene Conversion Events (GCE) detected: 5
Missense Mutation Events Detected (Total): 61
Missense Mutation Events Detected (In Epitopes): 9
Total # of assayed peptides: 37
Positive T-cell epitopes: 16

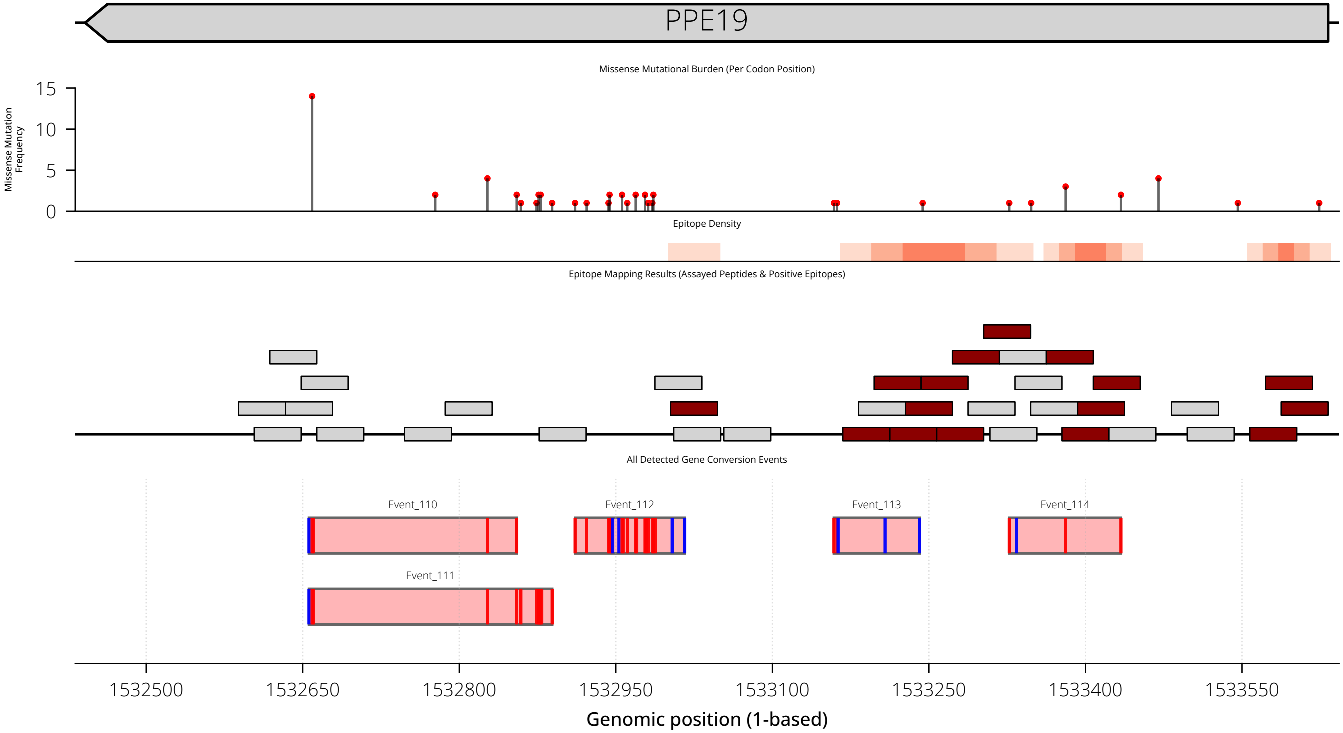

Gene(s): PPE60
In Paralogous Region (PR)? : [True]
# of Gene Conversion Events (GCE) detected: 8
Missense Mutation Events Detected (Total): 141
Missense Mutation Events Detected (In Epitopes): 24
Total # of assayed peptides: 20
Positive T-cell epitopes: 5

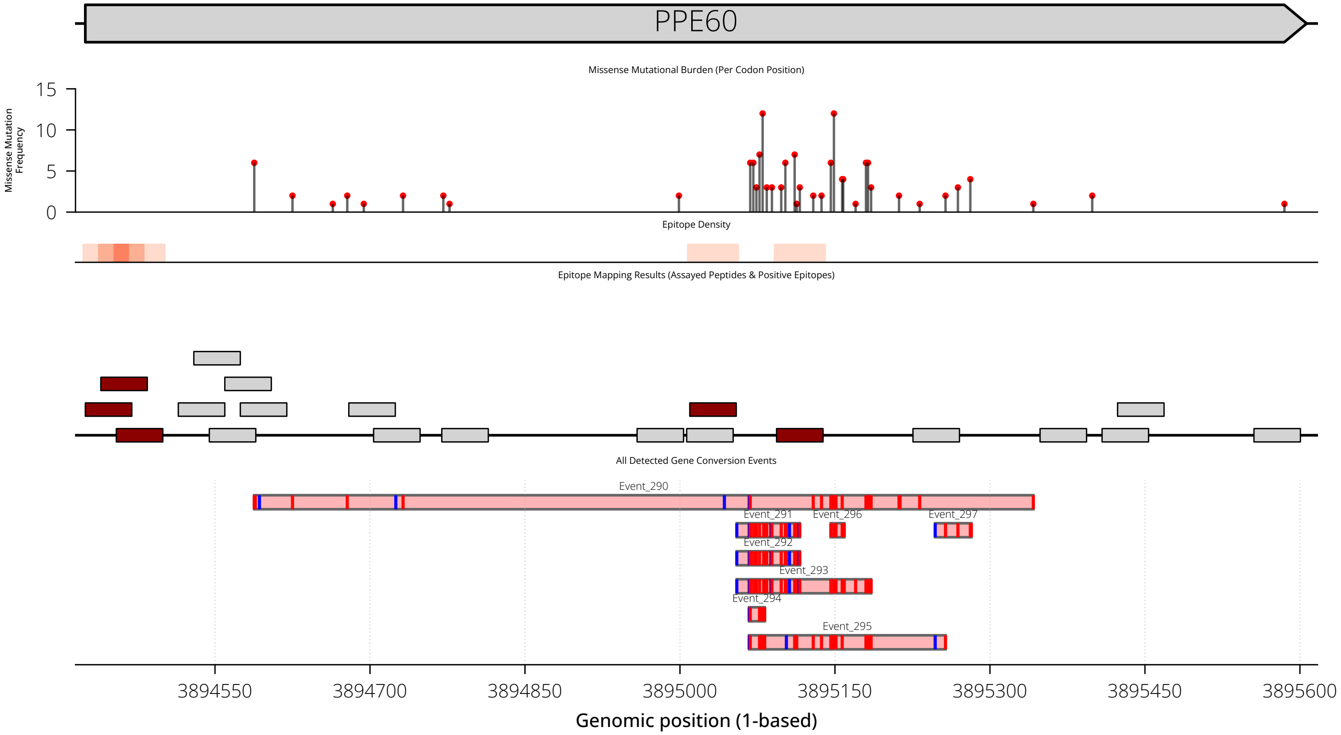

Gene(s): esxL,esxK
In Paralogous Region (PR)? : [True True]
# of Gene Conversion Events (GCE) detected: 15
Missense Mutation Events Detected (Total): 36
Missense Mutation Events Detected (In Epitopes): 30
Total # of assayed peptides: 32
Positive T-cell epitopes: 19

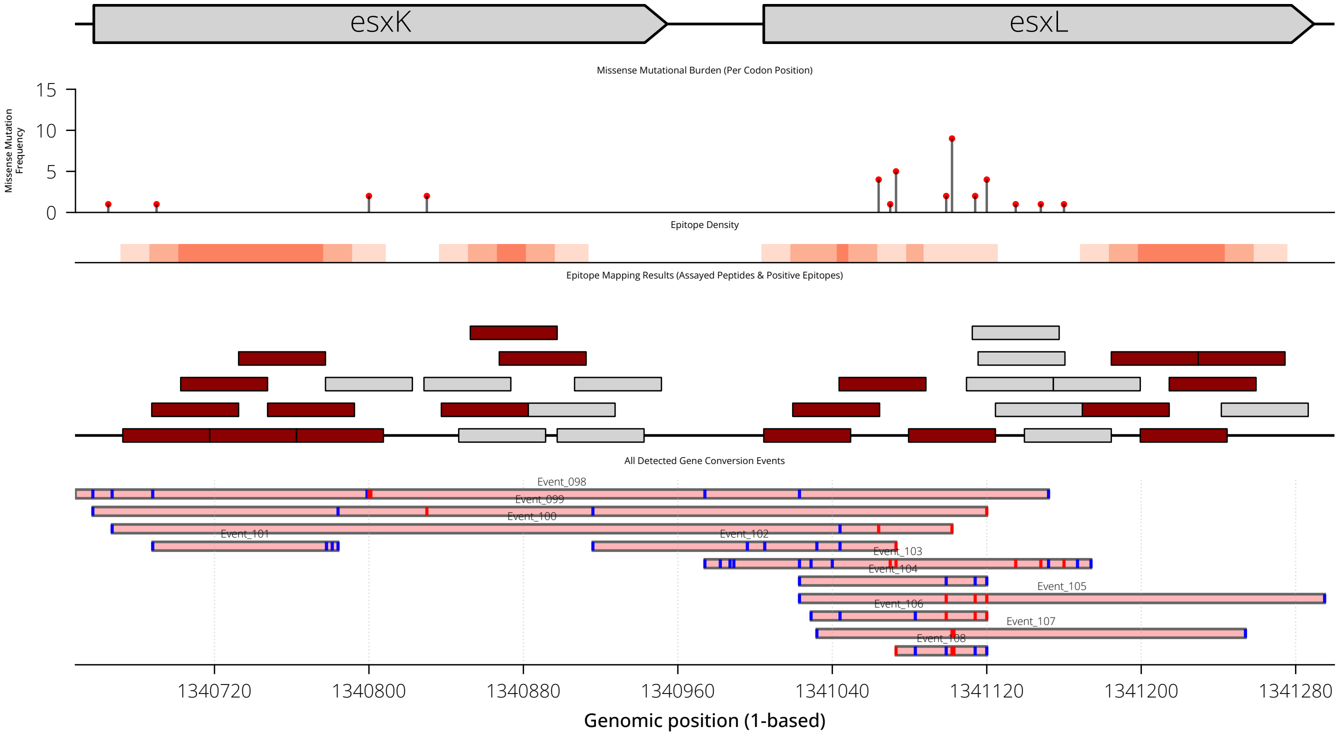

Gene(s): esxO,esxP
In Paralogous Region (PR)? : [True True]
# of Gene Conversion Events (GCE) detected: 14
Missense Mutation Events Detected (Total): 27
Missense Mutation Events Detected (In Epitopes): 5
Total # of assayed peptides: 26
Positive T-cell epitopes: 19

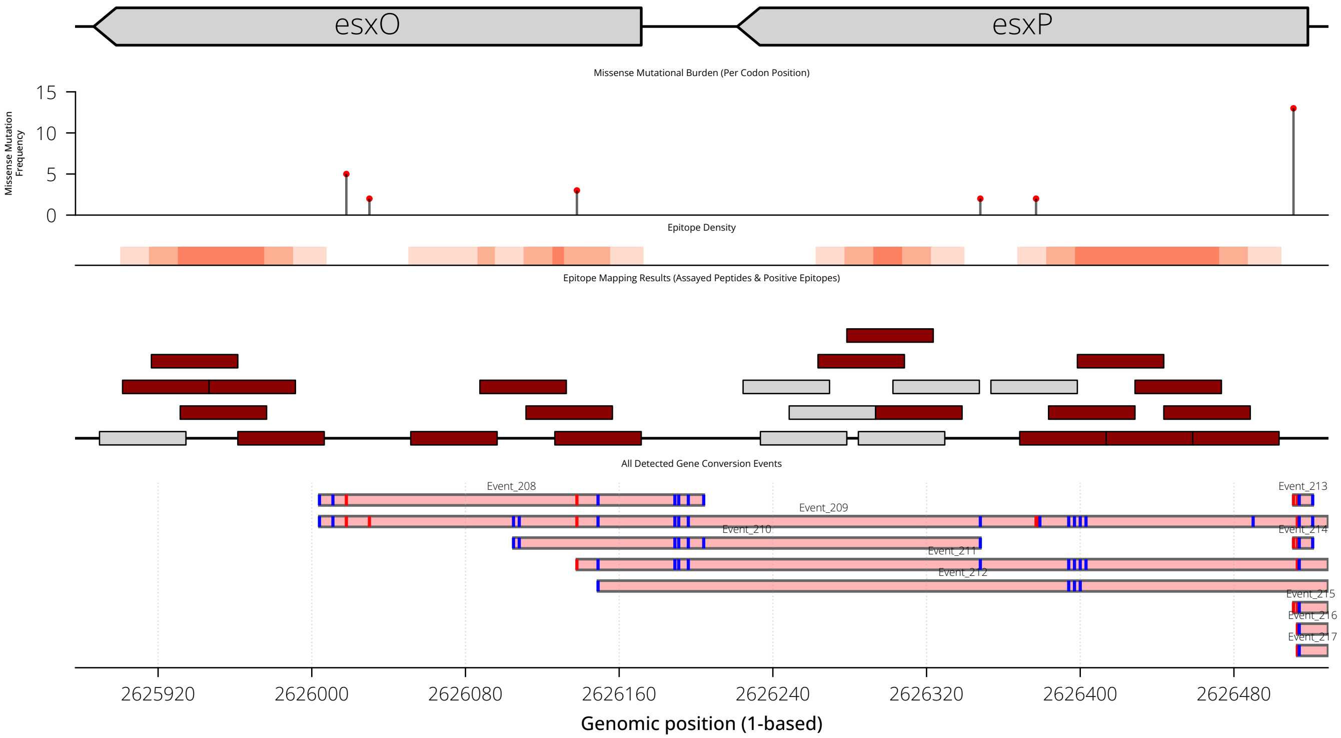

Gene(s): esxM,esxN
In Paralogous Region (PR)? : [True True]
# of Gene Conversion Events (GCE) detected: 6
Missense Mutation Events Detected (Total): 11
Missense Mutation Events Detected (In Epitopes): 1
Total # of assayed peptides: 25
Positive T-cell epitopes: 18

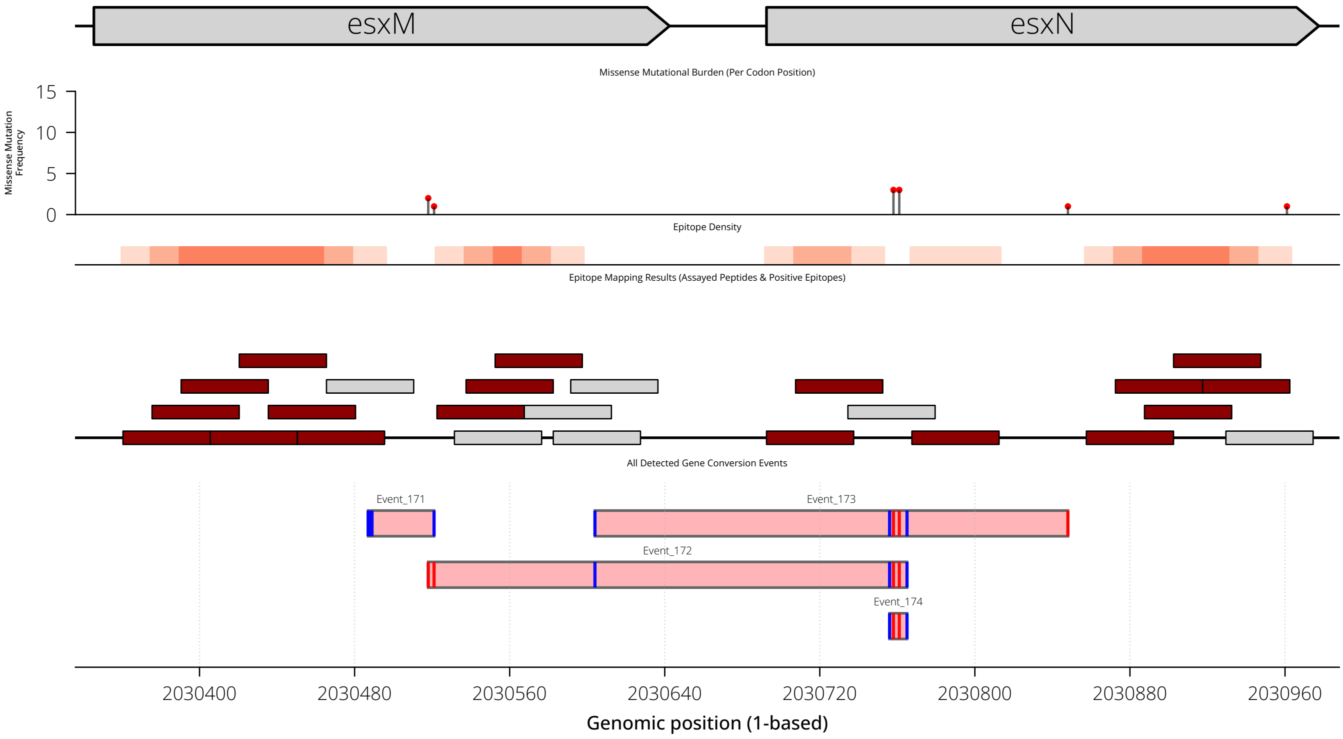

Gene(s): pepA
In Paralogous Region (PR)? : [False]
# of Gene Conversion Events (GCE) detected: 0
Missense Mutation Events Detected (Total): 5
Missense Mutation Events Detected (In Epitopes): 0
Total # of assayed peptides: 72
Positive T-cell epitopes: 3

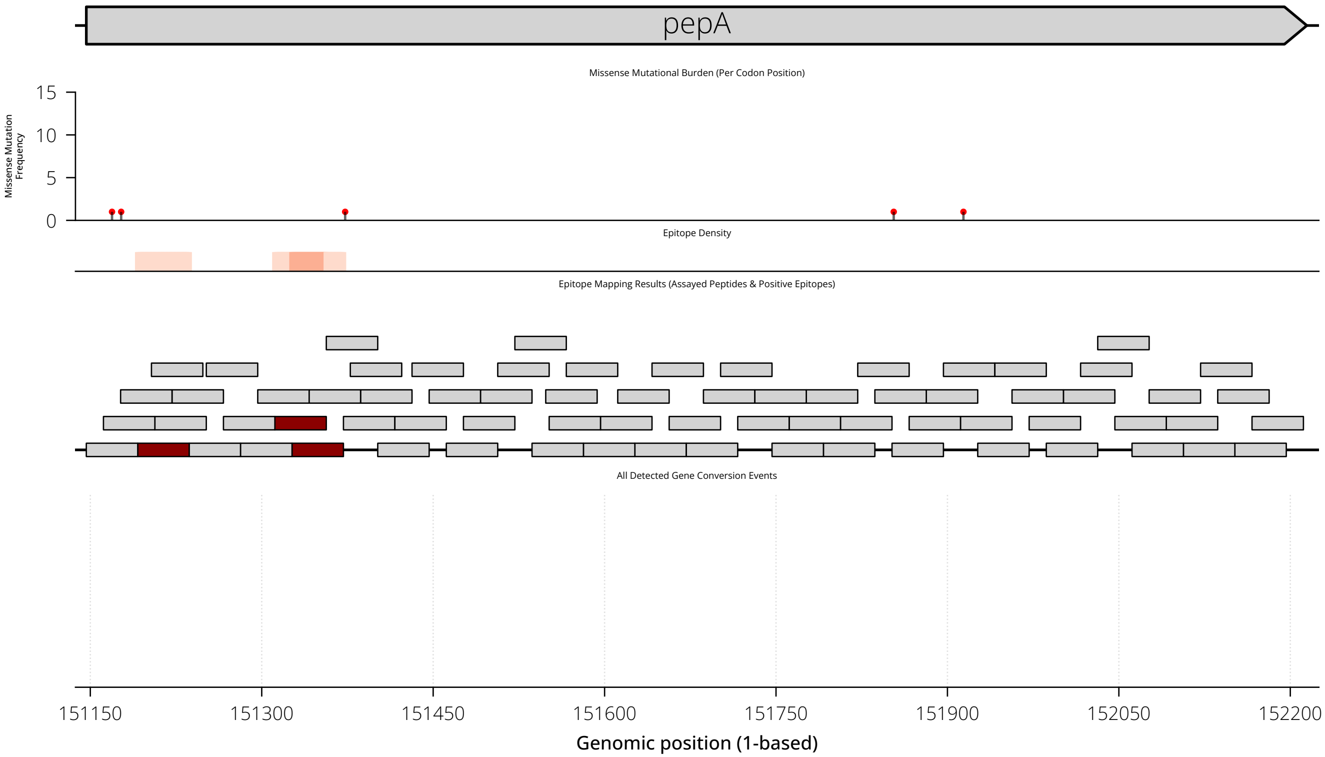

Gene(s): esxA,esxB
In Paralogous Region (PR)? : [False False]
# of Gene Conversion Events (GCE) detected: 0
Missense Mutation Events Detected (Total): 3
Missense Mutation Events Detected (In Epitopes): 3
Total # of assayed peptides: 45
Positive T-cell epitopes: 40

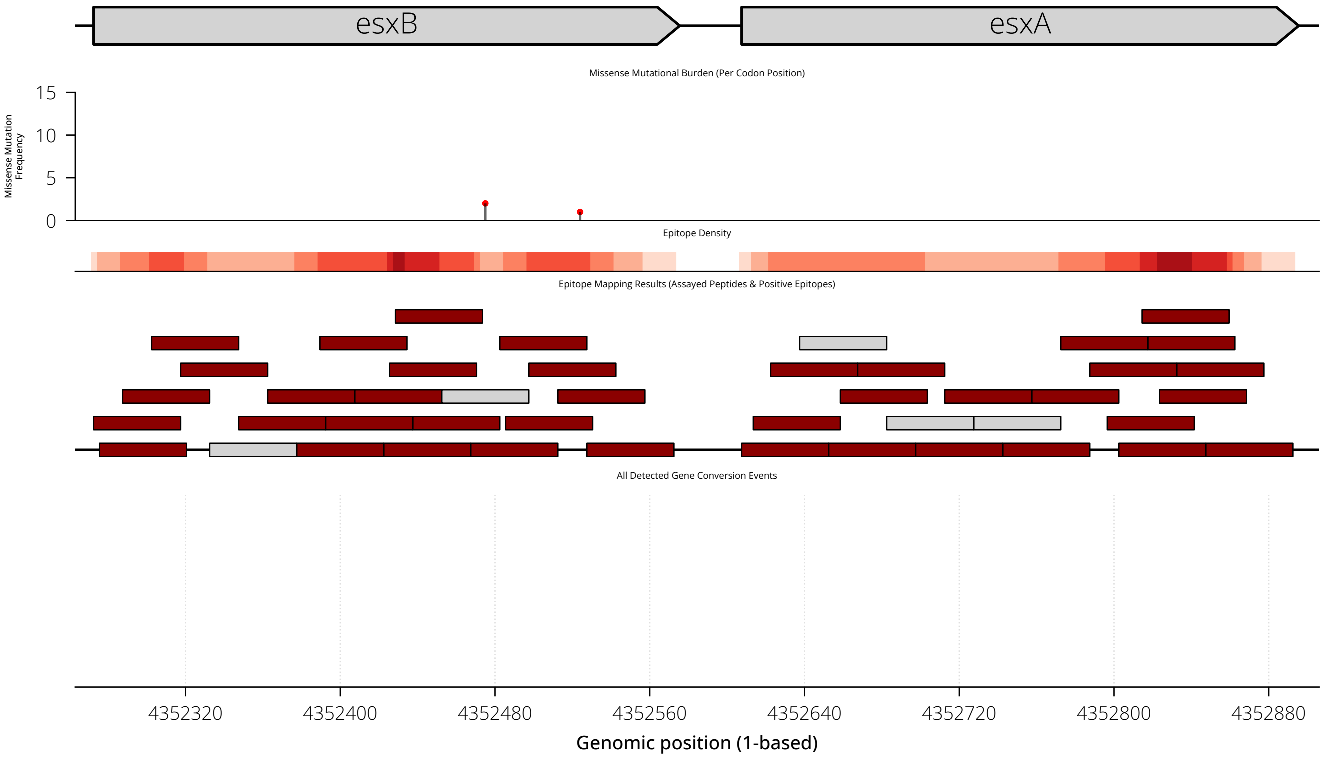

Gene(s): Rv0010c
In Paralogous Region (PR)? : [False]
# of Gene Conversion Events (GCE) detected: 0
Missense Mutation Events Detected (Total): 9
Missense Mutation Events Detected (In Epitopes): 2
Total # of assayed peptides: 6
Positive T-cell epitopes: 2

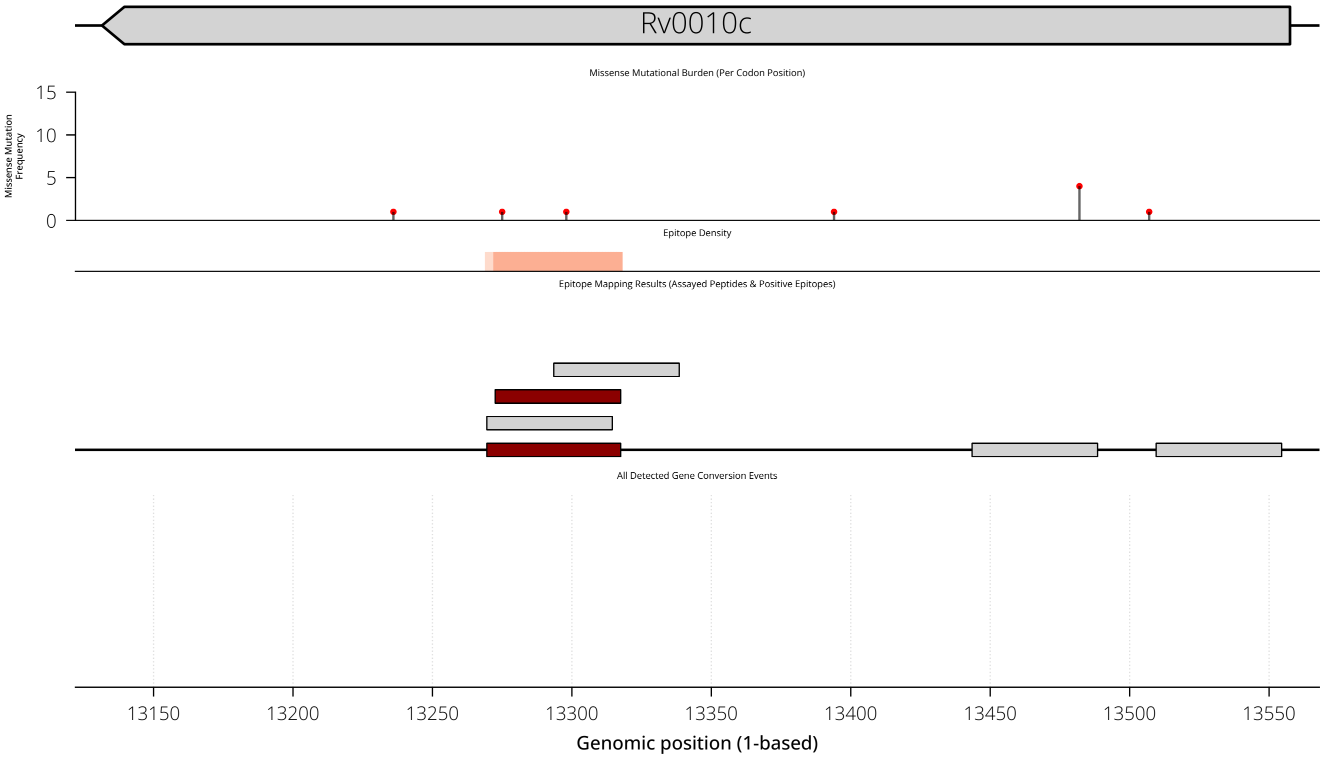

Gene(s): fbpA
In Paralogous Region (PR)? : [False]
# of Gene Conversion Events (GCE) detected: 0
Missense Mutation Events Detected (Total): 3
Missense Mutation Events Detected (In Epitopes): 1
Total # of assayed peptides: 78
Positive T-cell epitopes: 13

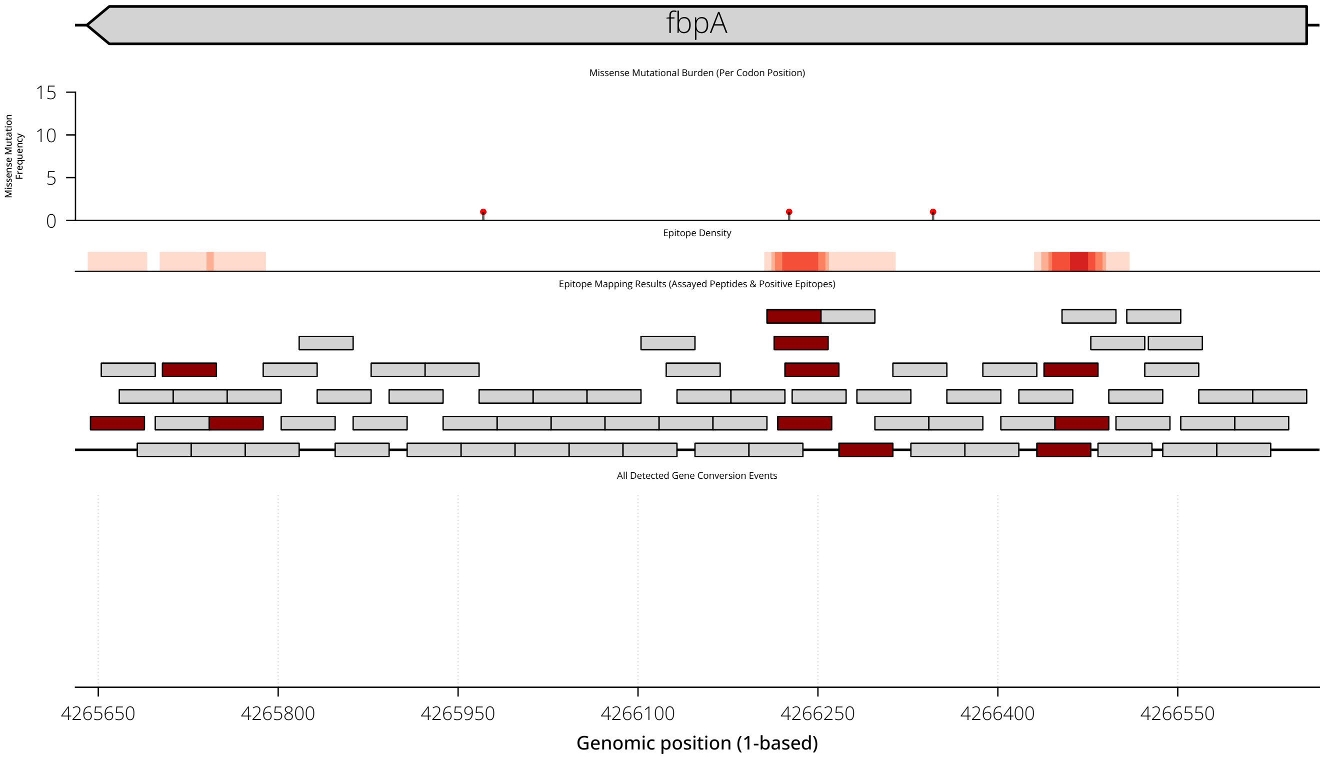

Gene(s): fbpB
In Paralogous Region (PR)? : [False]
# of Gene Conversion Events (GCE) detected: 0
Missense Mutation Events Detected (Total): 3
Missense Mutation Events Detected (In Epitopes): 2
Total # of assayed peptides: 72
Positive T-cell epitopes: 16

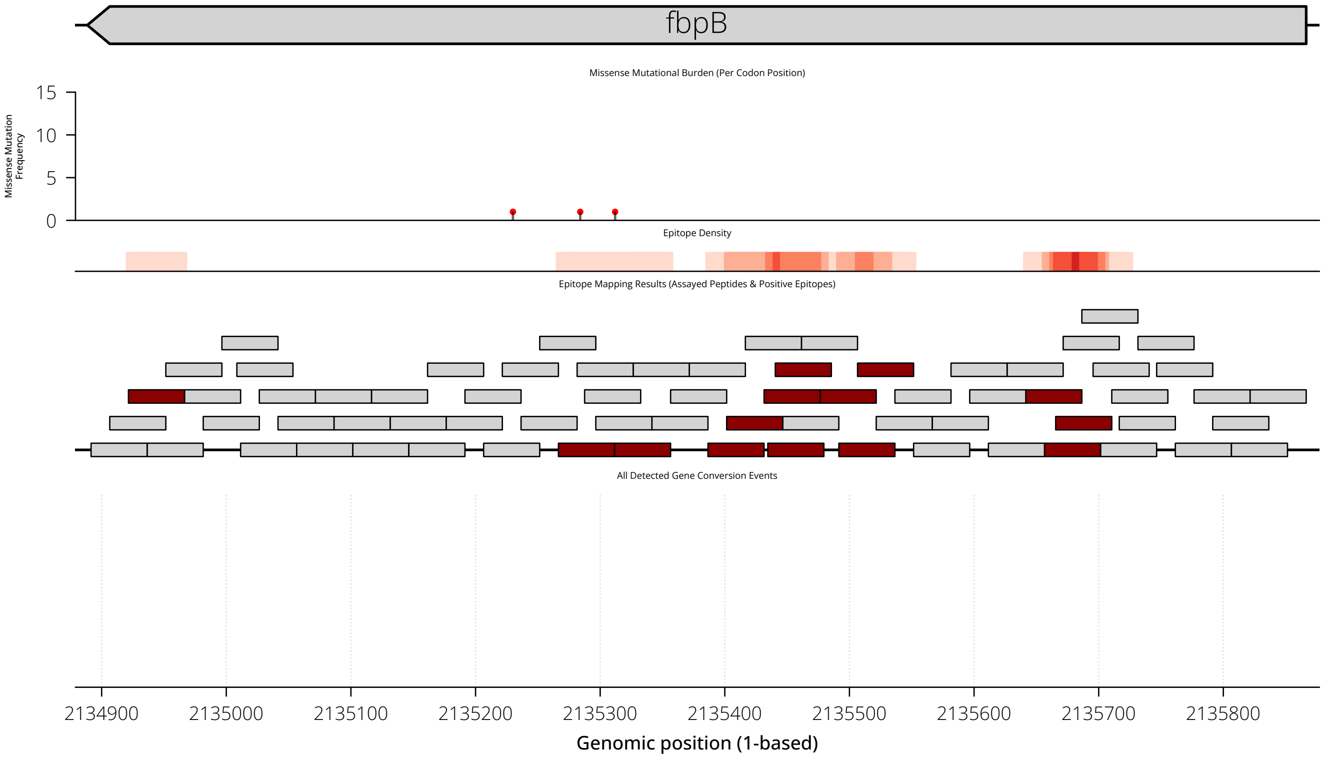

Supplement: Supplement 15 [file media-15.pdf]
