# Supplementary material for: Gene conversion is a key driver of diversity hotspots in M. tuberculosis antigens and virulence-associated loci
Source: bioRxiv. 2026 Mar 11:2026.02.26.708061. Preprint. [Version 2] doi: 10.64898/2026.02.26.708061 (PMC13061034; doi:10.64898/2026.02.26.708061)

# Individual GCE + Known Paralogs
GCE ID: Event_001

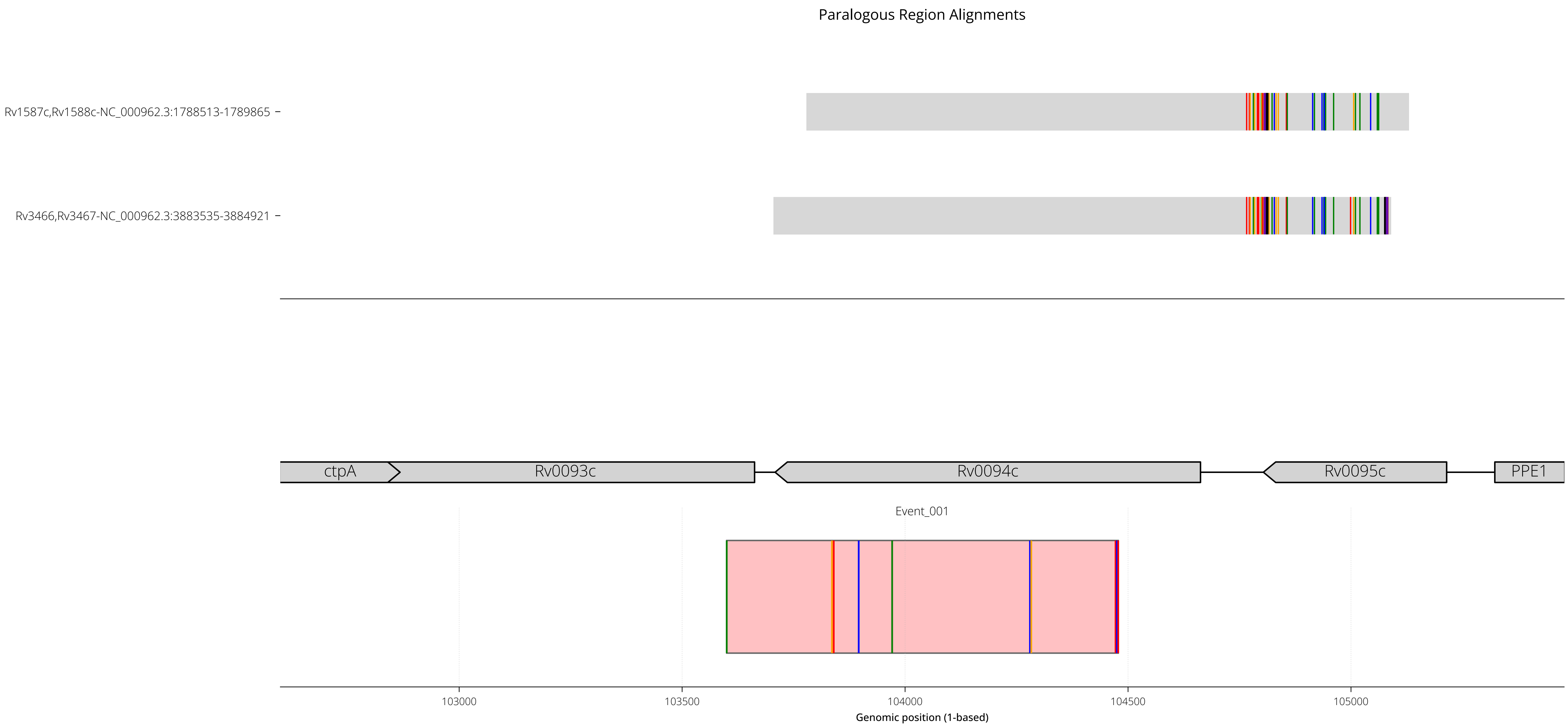

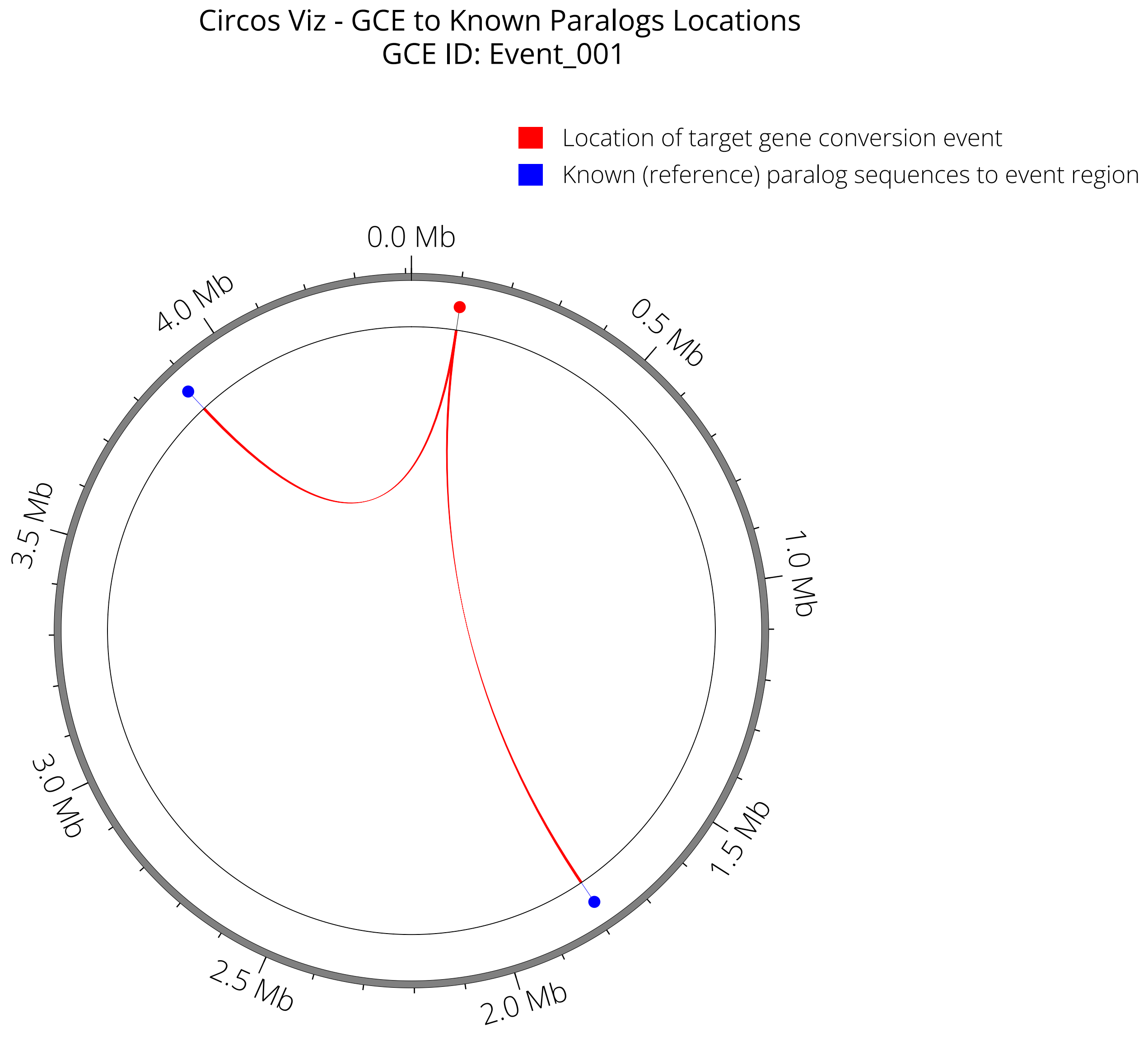
Circos Viz - GCE to Known Paralogs Locations
GCE ID: Event_001
Location of target gene conversion event
Known (reference) paralog sequences to event region
0.0 Mb
0.5 Mb
1.0 Mb
1.5 Mb
2.0 Mb
2.5 Mb
3.0 Mb
3.5 Mb
4.0 Mb

# Event_001 - 10 SNPs - k-mer analysis relative to paralogs

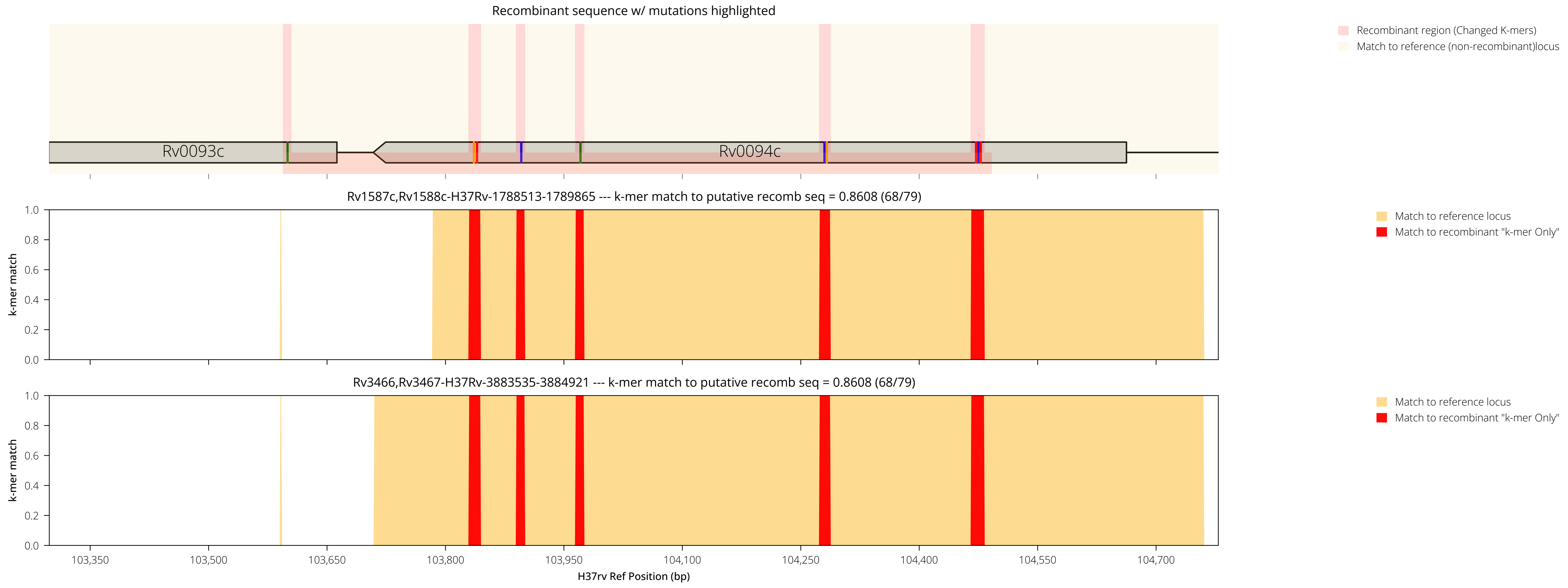

Sub-phylogeny Viz + Paralog Alignments
GCE ID: Event_001

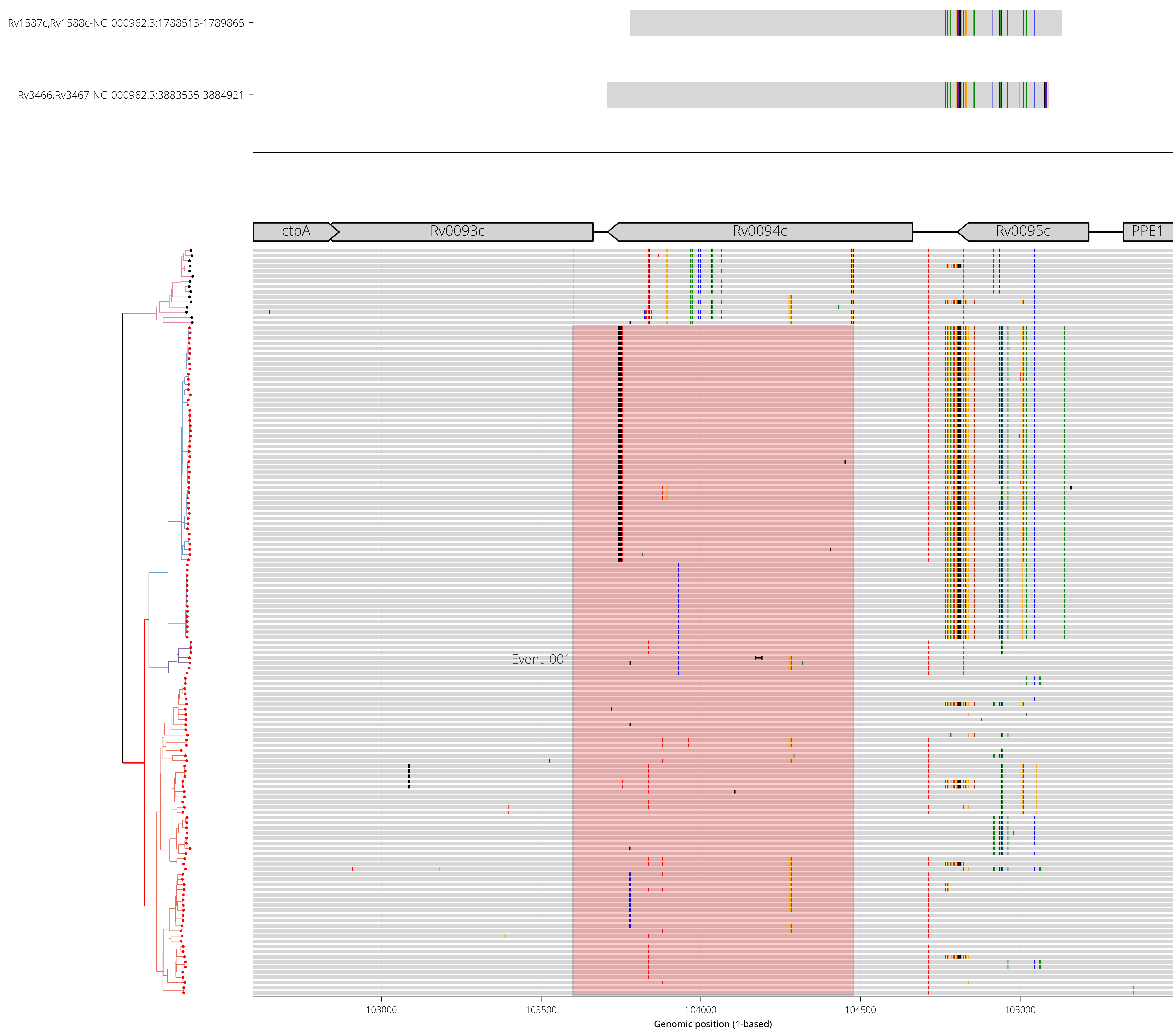

Full Phylogeny Viz + Paralog Alignments
GCE ID: Event_001

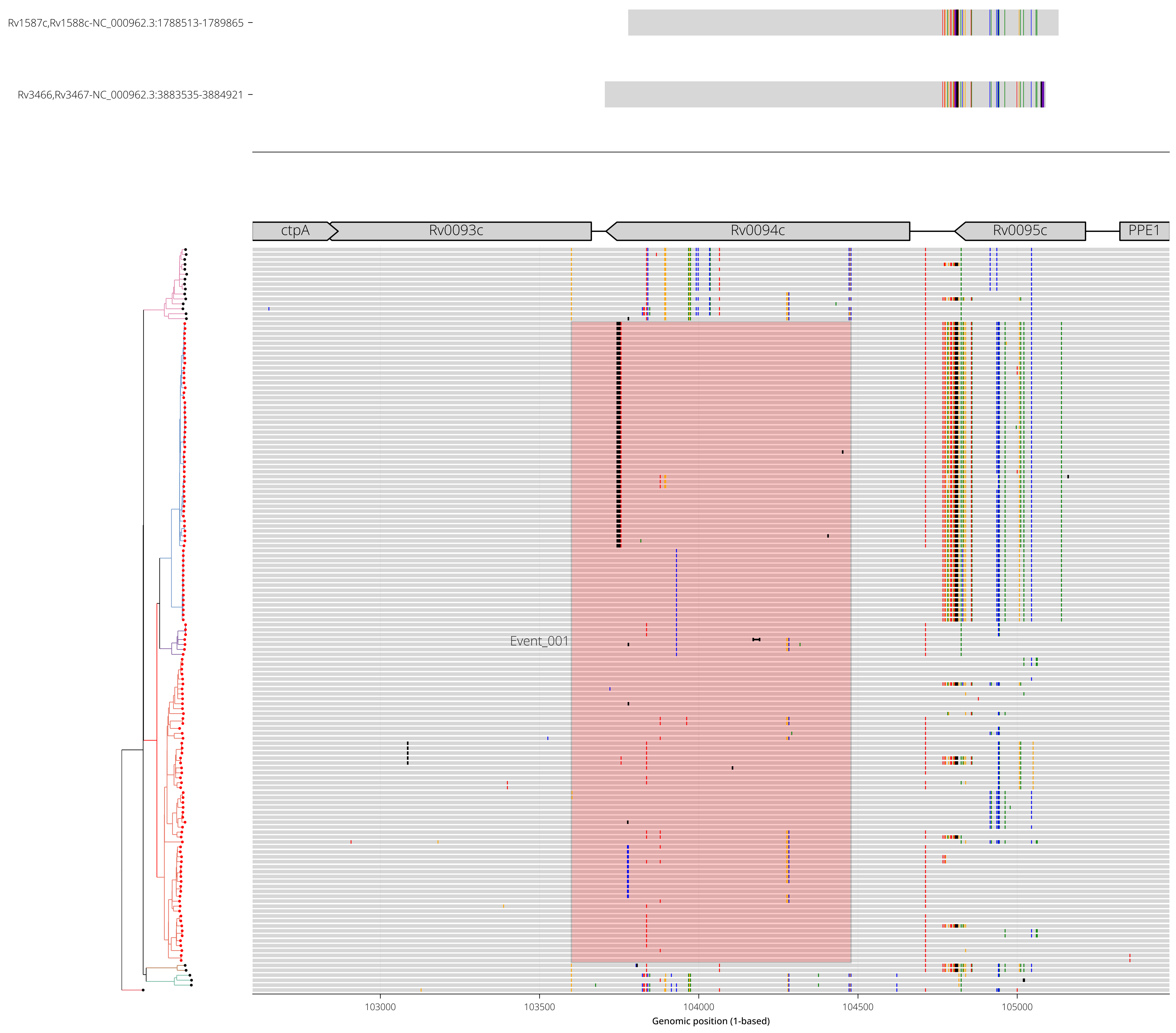

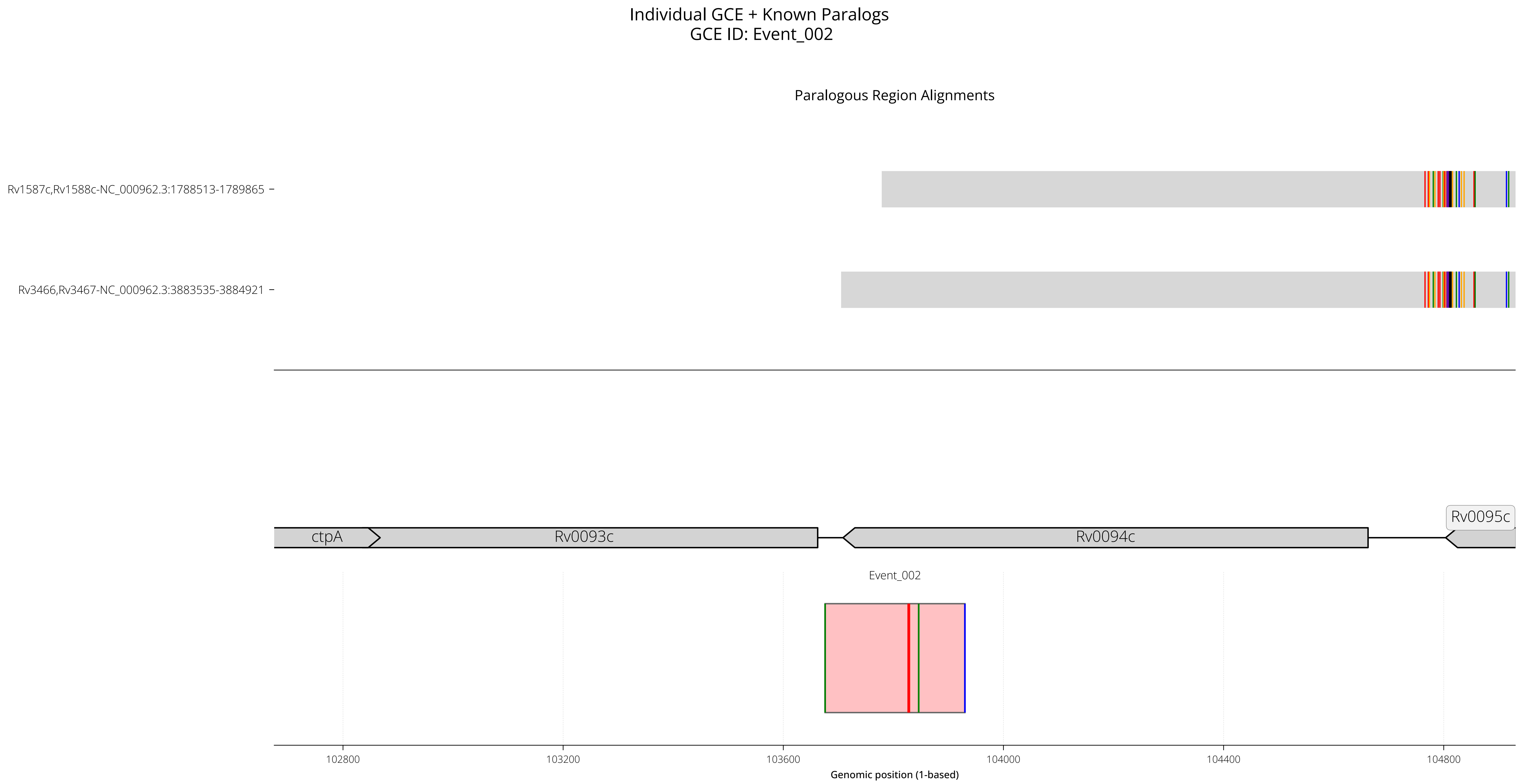

Individual GCE + Known Paralogs
GCE ID: Event_002
Paralogous Region Alignments
Rv1587c,Rv1588c-NC_000962.3:1788513-1789865
Rv3466,Rv3467-NC_000962.3:3883535-3884921
ctpA
Rv0093c
Rv0094c
Rv0095c
Event_002
102800
103200
103600
104000
104400
104800
Genomic position (1-based)

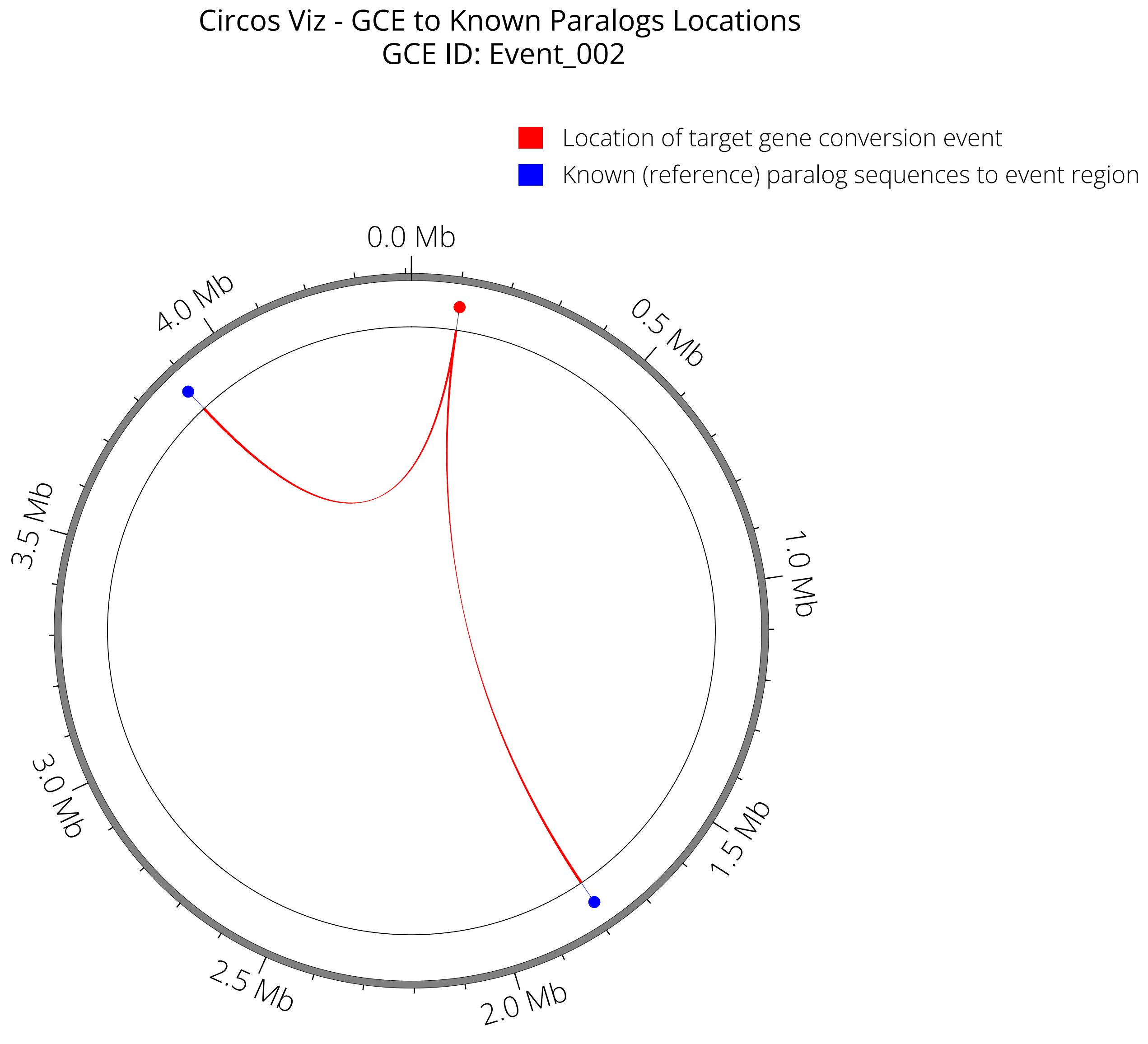

Circos Viz - GCE to Known Paralogs Locations
GCE ID: Event_002
Location of target gene conversion event
Known (reference) paralog sequences to event region
0.0 Mb
0.5 Mb
1.0 Mb
1.5 Mb
2.0 Mb
2.5 Mb
3.0 Mb
3.5 Mb
4.0 Mb

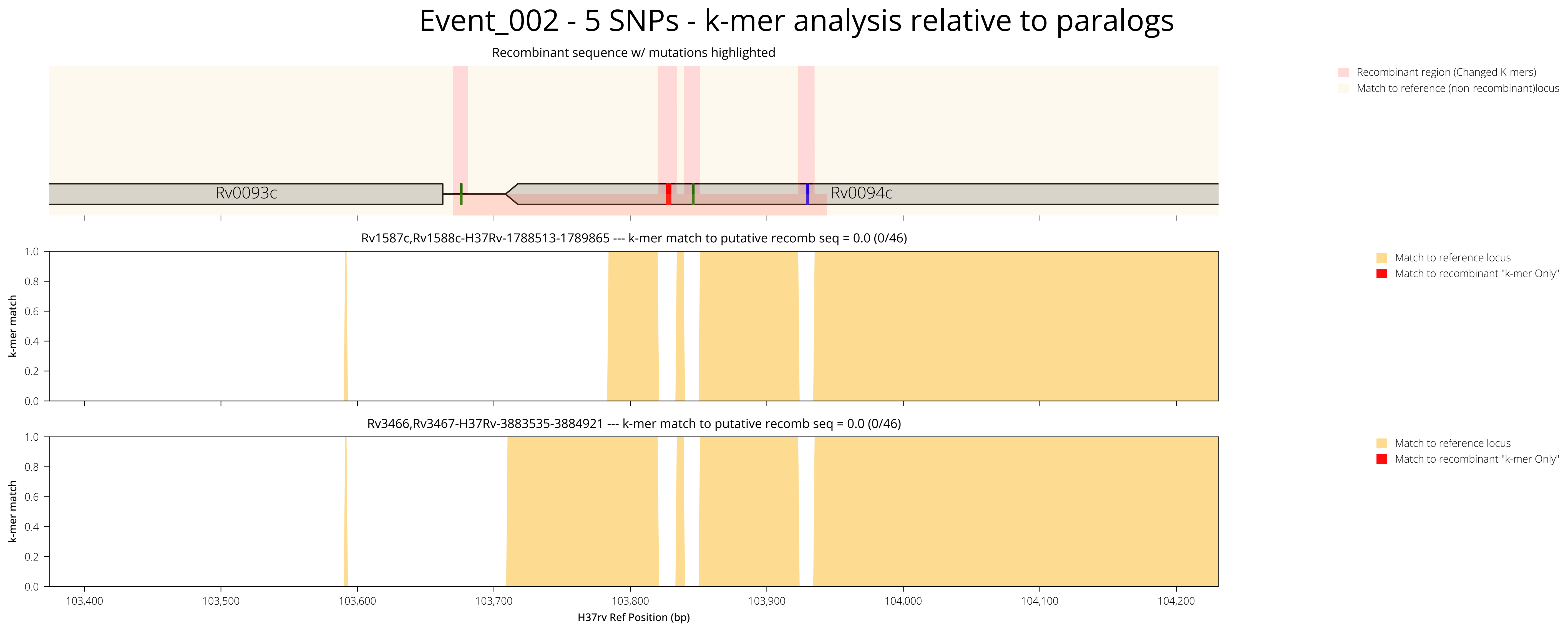

Event_002 - 5 SNPs - k-mer analysis relative to paralogs
Recombinant sequence w/ mutations highlighted
Recombinant region (Changed K-mers)
Match to reference (non-recombinant)locus
Rv0093c
Rv0094c
Rv1587c,Rv1588c-H37Rv-1788513-1789865 --- k-mer match to putative recomb seq = 0.0 (0/46)
Match to reference locus
Match to recombinant "k-mer Only"
k-mer match
1.0
0.8
0.6
0.4
0.2
0.0
Rv3466,Rv3467-H37Rv-3883535-3884921 --- k-mer match to putative recomb seq = 0.0 (0/46)
Match to reference locus
Match to recombinant "k-mer Only"
k-mer match
1.0
0.8
0.6
0.4
0.2
0.0
103,400
103,500
103,600
103,700
103,800
103,900
104,000
104,100
104,200
H37rv Ref Position (bp)

Sub-phylogeny Viz + Paralog Alignments
GCE ID: Event_002

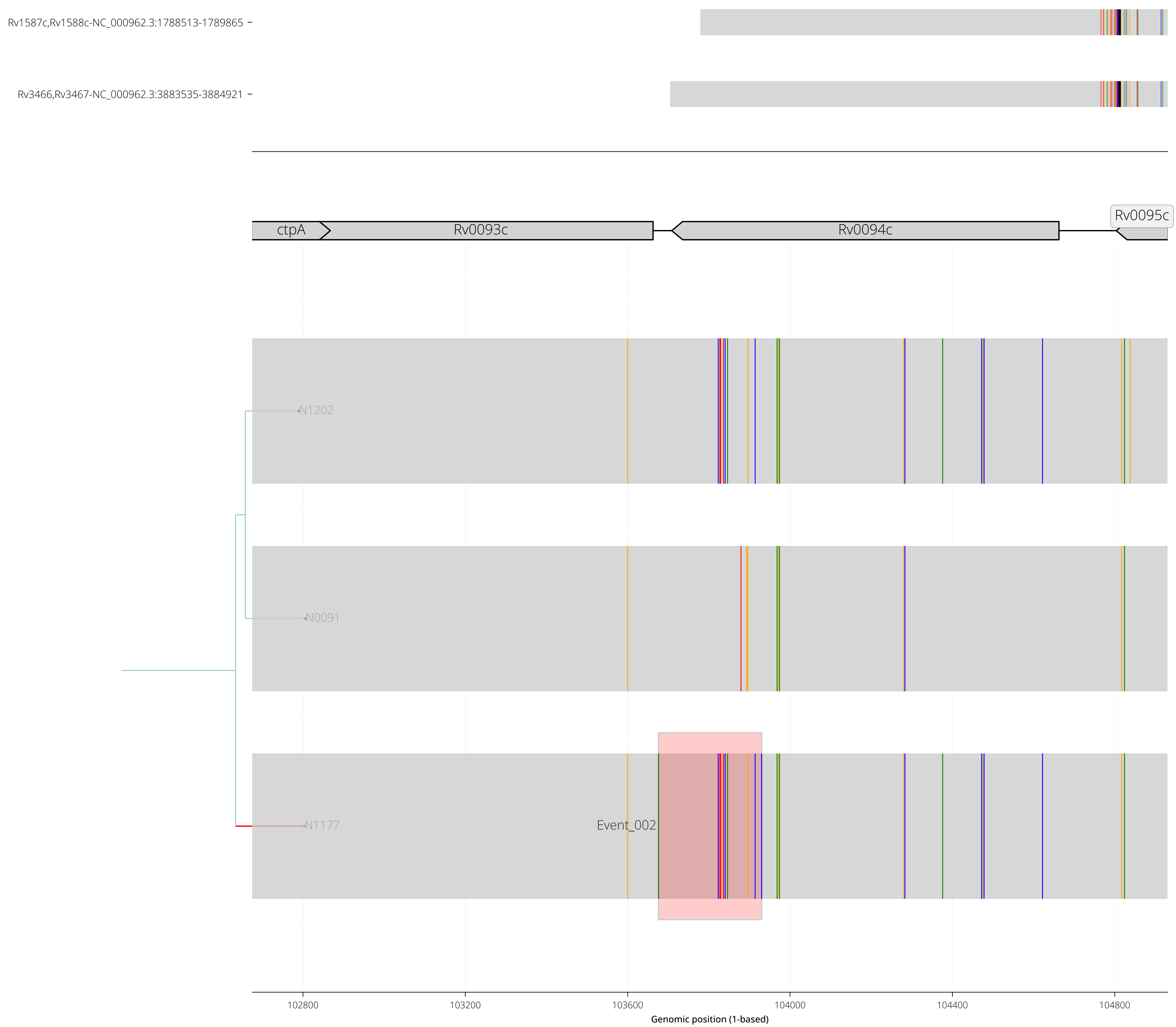

# Full Phylogeny Viz + Paralog Alignments
GCE ID: Event_002

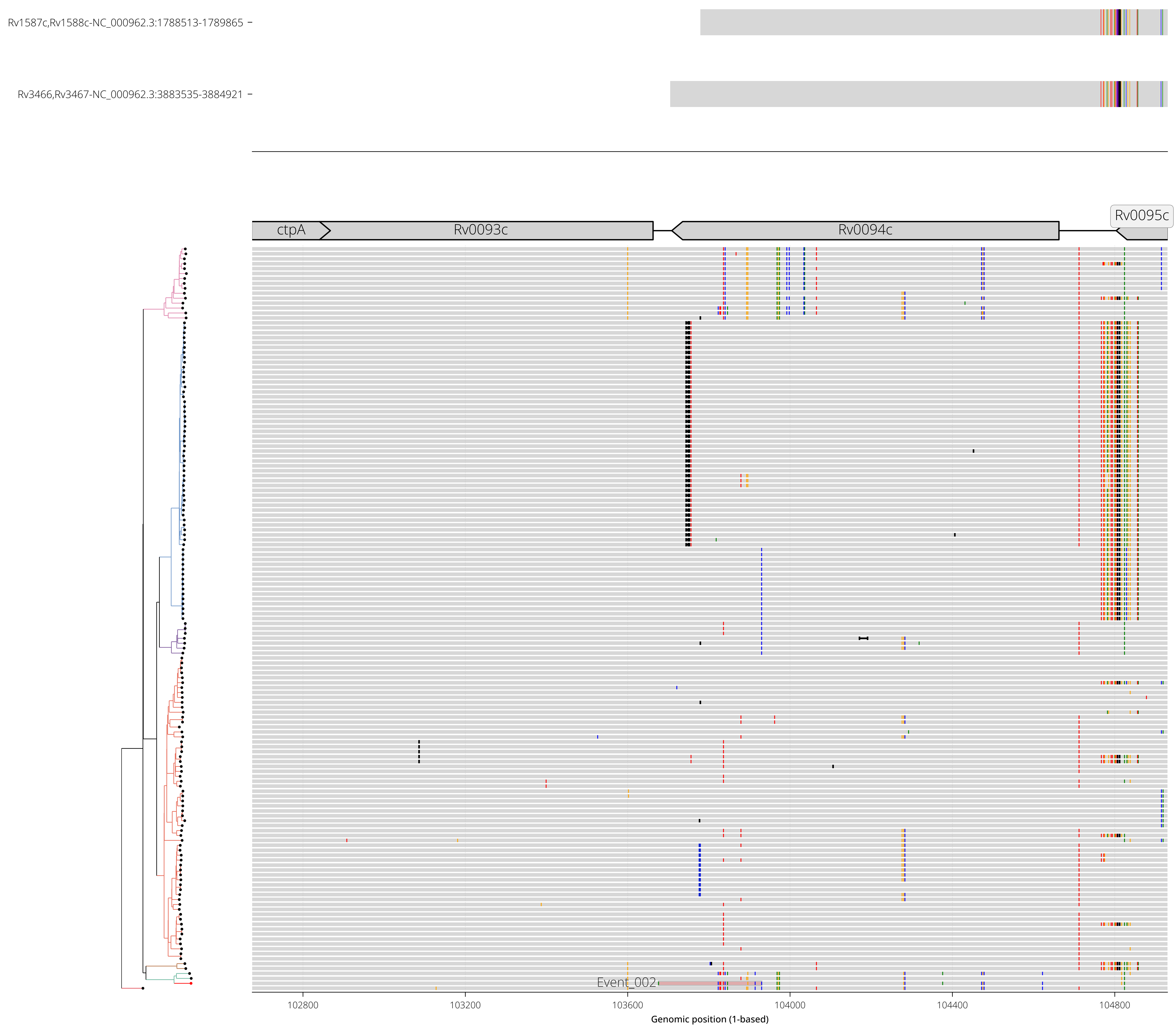

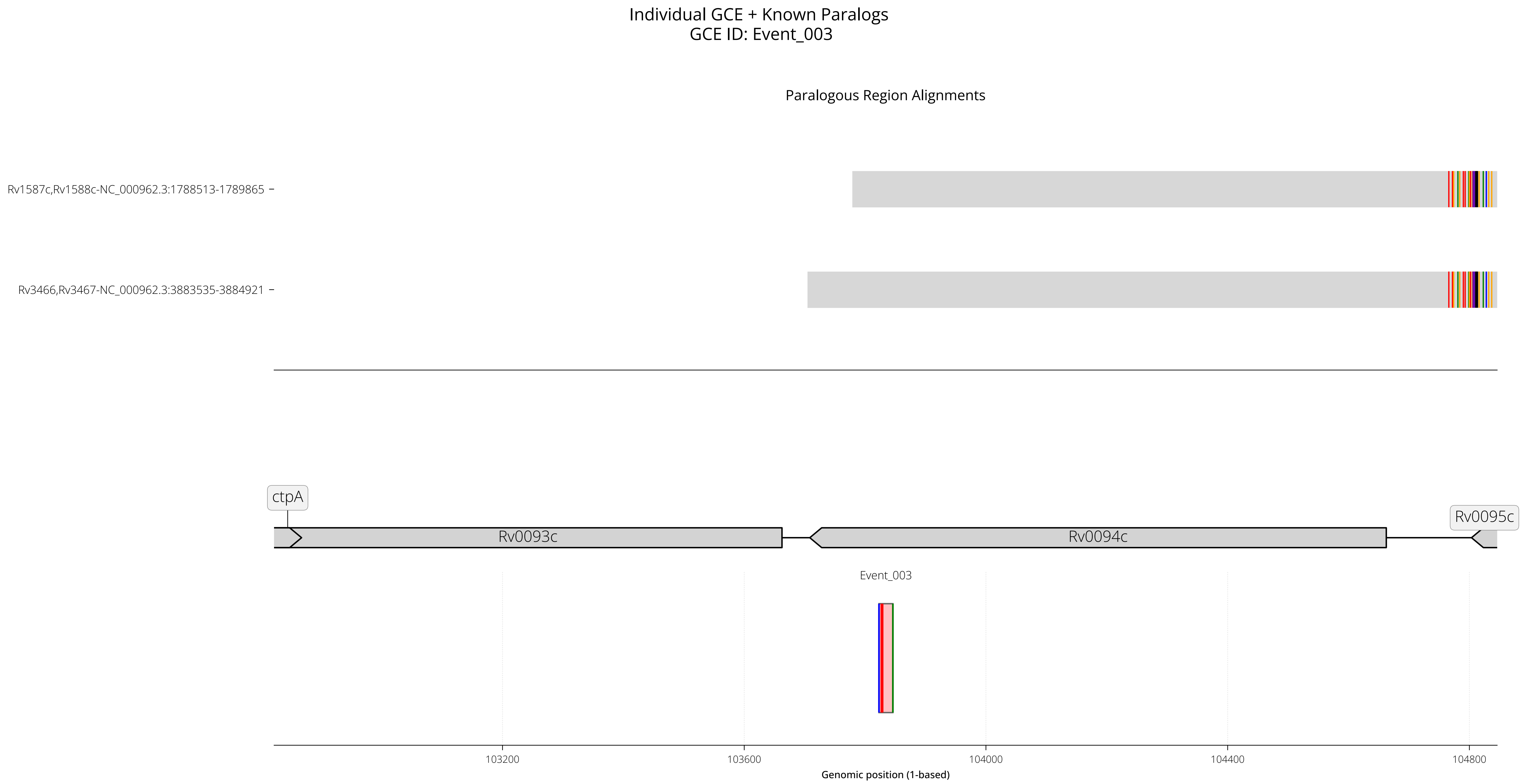
Individual GCE + Known Paralogs
GCE ID: Event_003
Paralogous Region Alignments
Rv1587c,Rv1588c-NC_000962.3:1788513-1789865
Rv3466,Rv3467-NC_000962.3:3883535-3884921
ctpA
Rv0093c
Rv0094c
Rv0095c
Event_003
103200
103600
104000
104400
104800
Genomic position (1-based)

Circos Viz - GCE to Known Paralogs Locations
GCE ID: Event_003

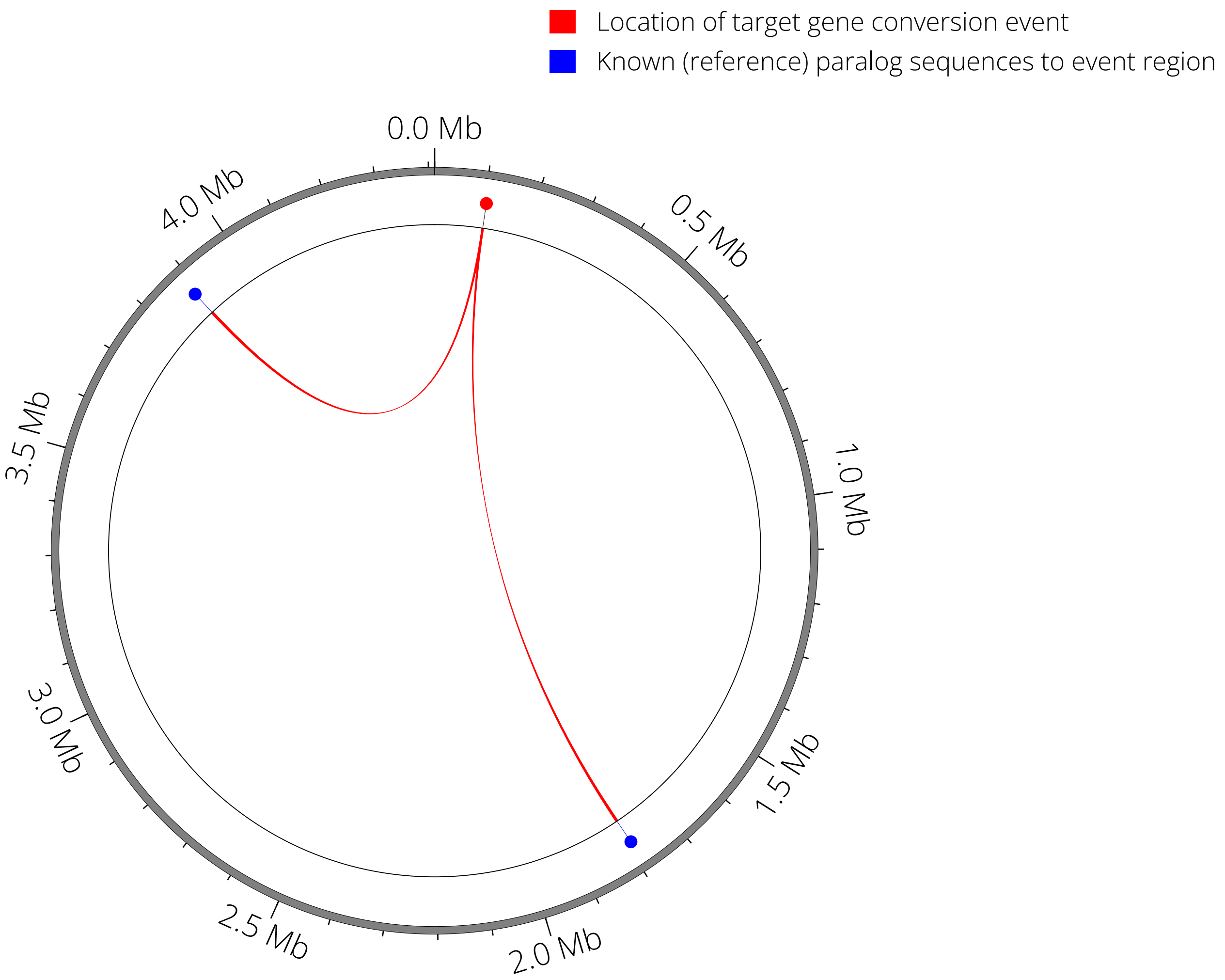

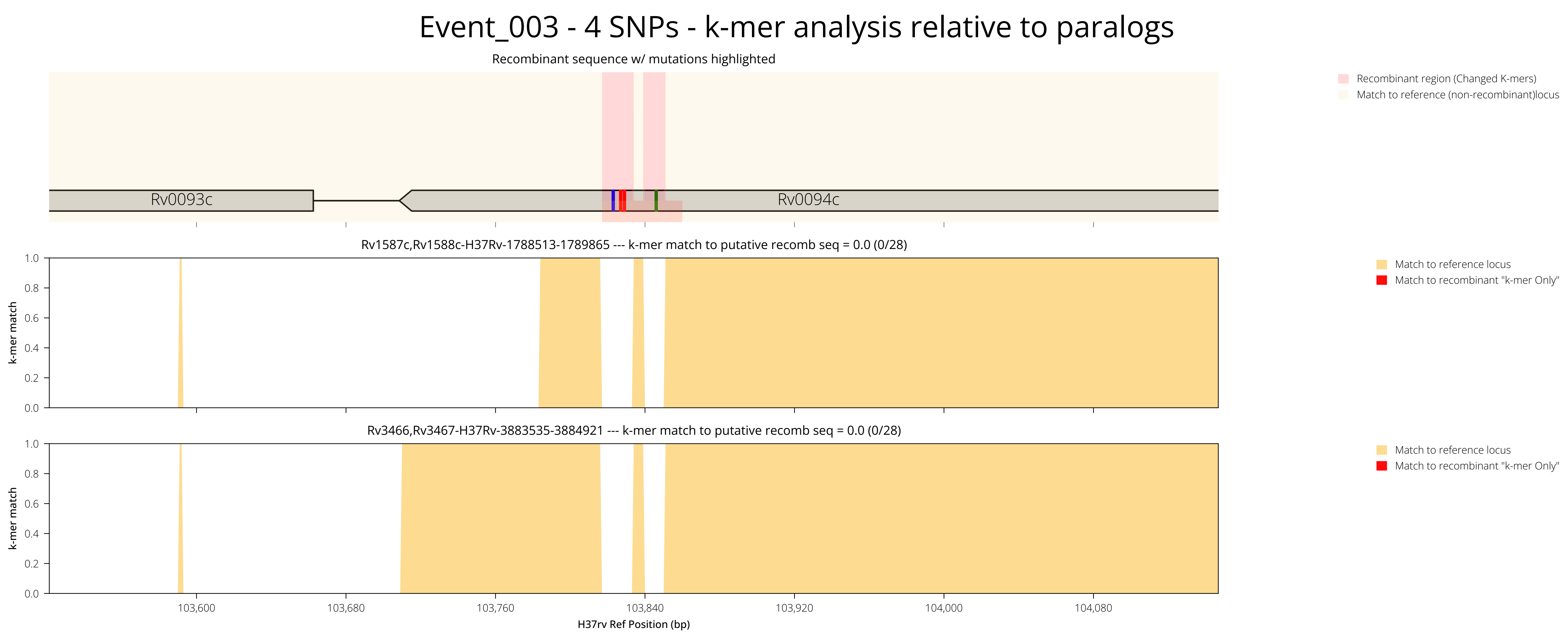
Event_003 - 4 SNPs - k-mer analysis relative to paralogs
Recombinant sequence w/ mutations highlighted
Recombinant region (Changed K-mers)
Match to reference (non-recombinant)locus
Rv0093c
Rv0094c
Rv1587c,Rv1588c-H37Rv-1788513-1789865 --- k-mer match to putative recomb seq = 0.0 (0/28)
Match to reference locus
Match to recombinant "k-mer Only"
k-mer match
1.0
0.8
0.6
0.4
0.2
0.0
Rv3466,Rv3467-H37Rv-3883535-3884921 --- k-mer match to putative recomb seq = 0.0 (0/28)
Match to reference locus
Match to recombinant "k-mer Only"
k-mer match
1.0
0.8
0.6
0.4
0.2
0.0
103,600
103,680
103,760
103,840
103,920
104,000
104,080
H37rv Ref Position (bp)

# Sub-phylogeny Viz + Paralog Alignments
GCE ID: Event_003

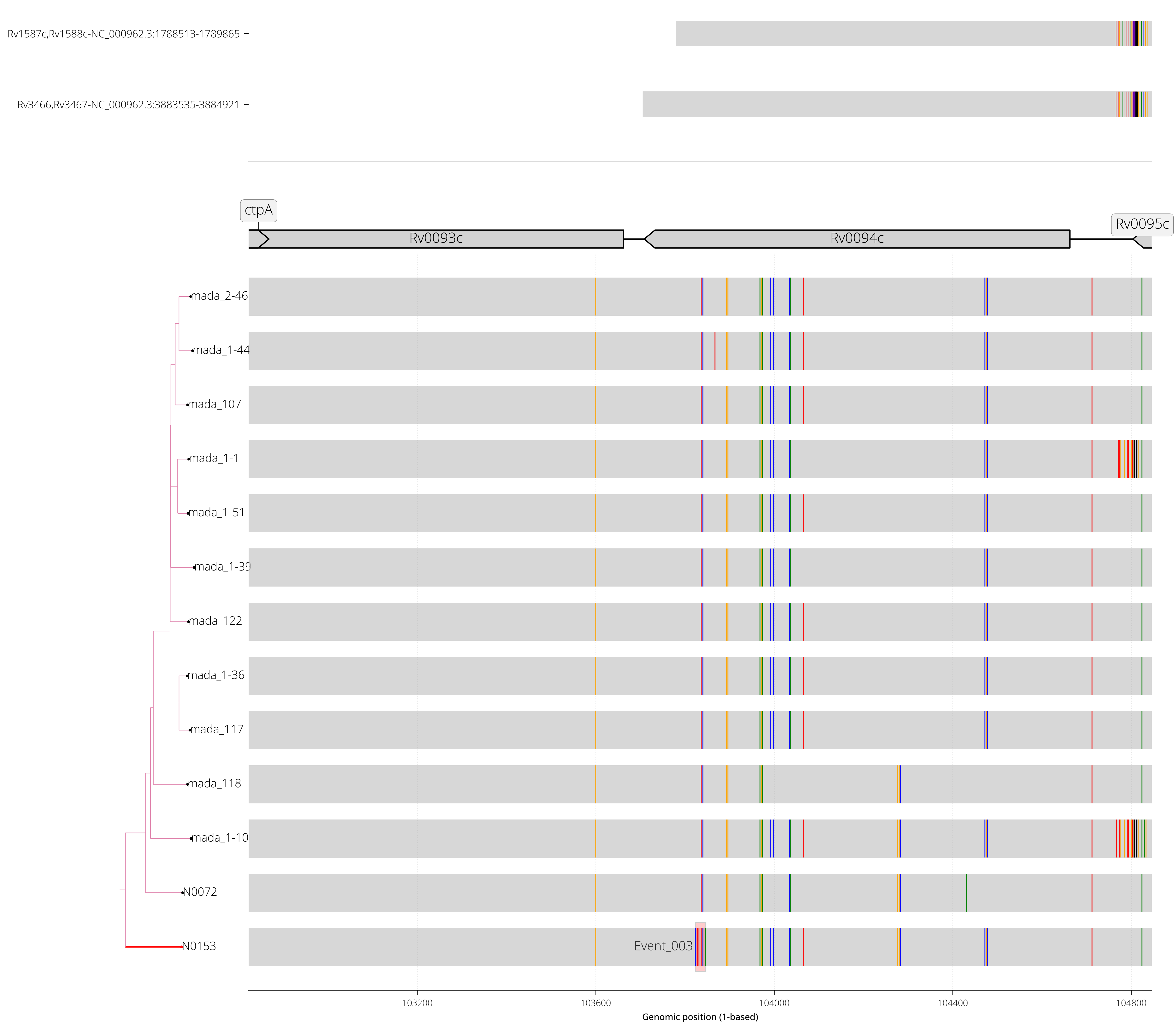

Full Phylogeny Viz + Paralog Alignments
GCE ID: Event_003

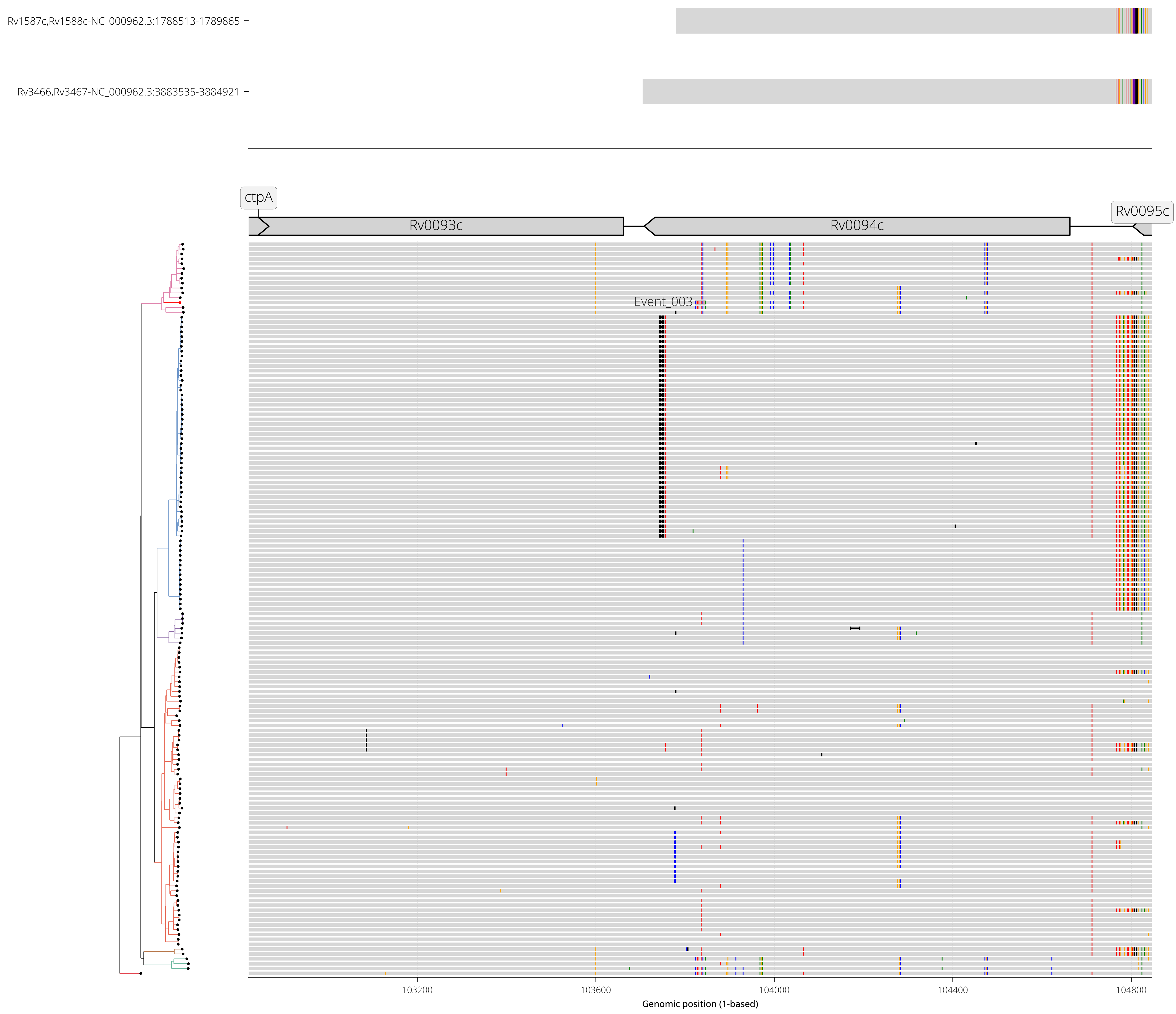

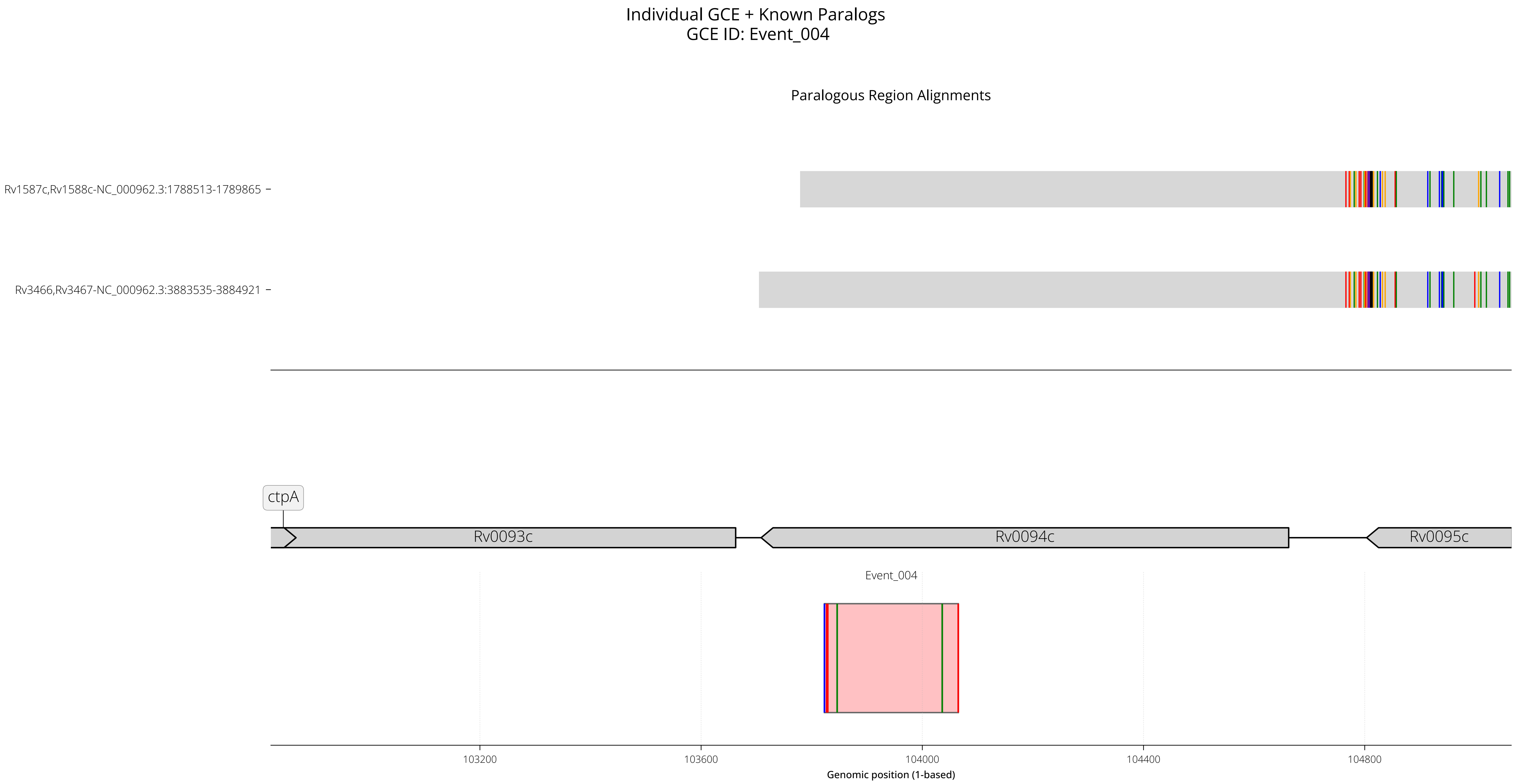
Individual GCE + Known Paralogs
GCE ID: Event_004
Paralogous Region Alignments
Rv1587c,Rv1588c-NC_000962.3:1788513-1789865
Rv3466,Rv3467-NC_000962.3:3883535-3884921
ctpA
Rv0093c
Rv0094c
Rv0095c
Event_004
103200
103600
104000
104400
104800
Genomic position (1-based)

Circos Viz - GCE to Known Paralogs Locations
GCE ID: Event_004

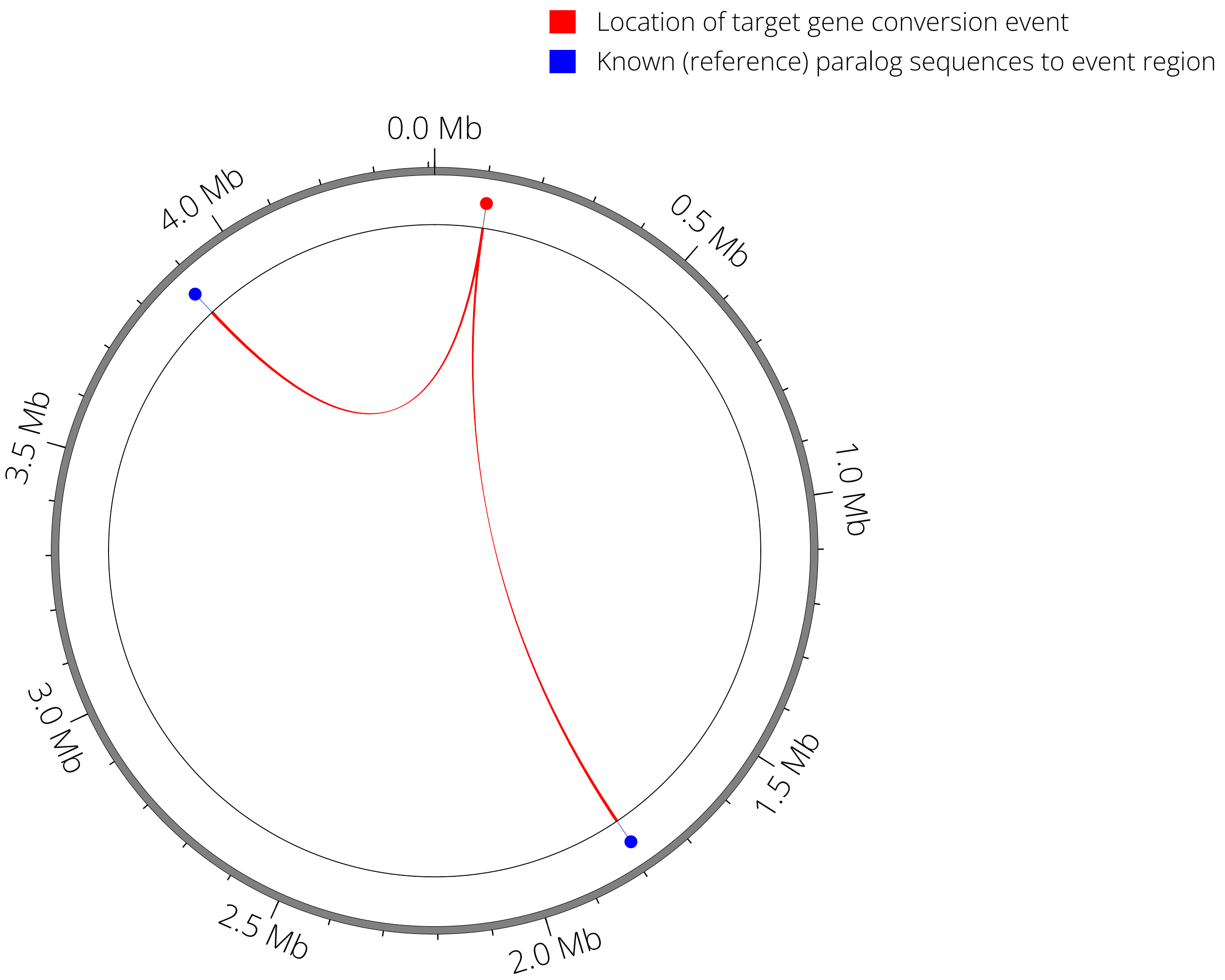

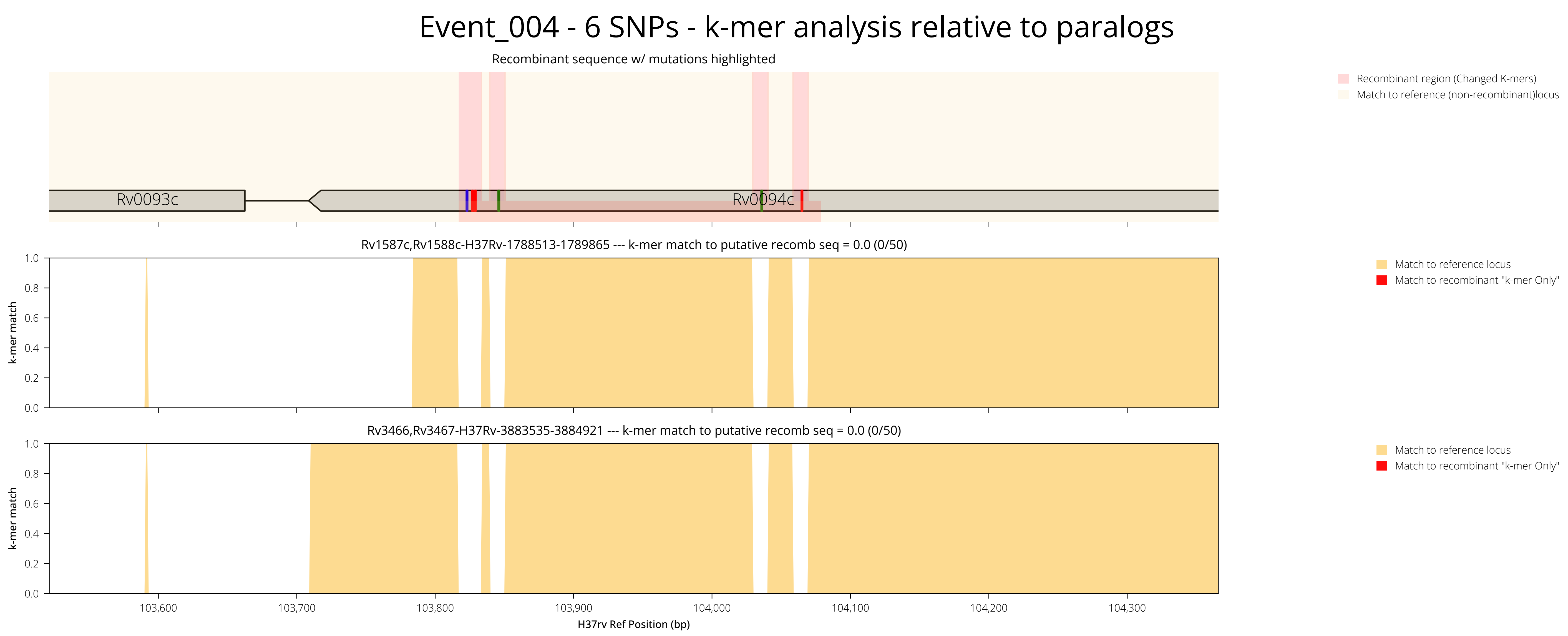
Event_004 - 6 SNPs - k-mer analysis relative to paralogs
Recombinant sequence w/ mutations highlighted
Recombinant region (Changed K-mers)
Match to reference (non-recombinant)locus
Rv0093c
Rv0094c
Rv1587c,Rv1588c-H37Rv-1788513-1789865 --- k-mer match to putative recomb seq = 0.0 (0/50)
k-mer match
1.0
0.8
0.6
0.4
0.2
0.0
Match to reference locus
Match to recombinant "k-mer Only"
Rv3466,Rv3467-H37Rv-3883535-3884921 --- k-mer match to putative recomb seq = 0.0 (0/50)
k-mer match
1.0
0.8
0.6
0.4
0.2
0.0
Match to reference locus
Match to recombinant "k-mer Only"
103,600
103,700
103,800
103,900
104,000
104,100
104,200
104,300
H37rv Ref Position (bp)

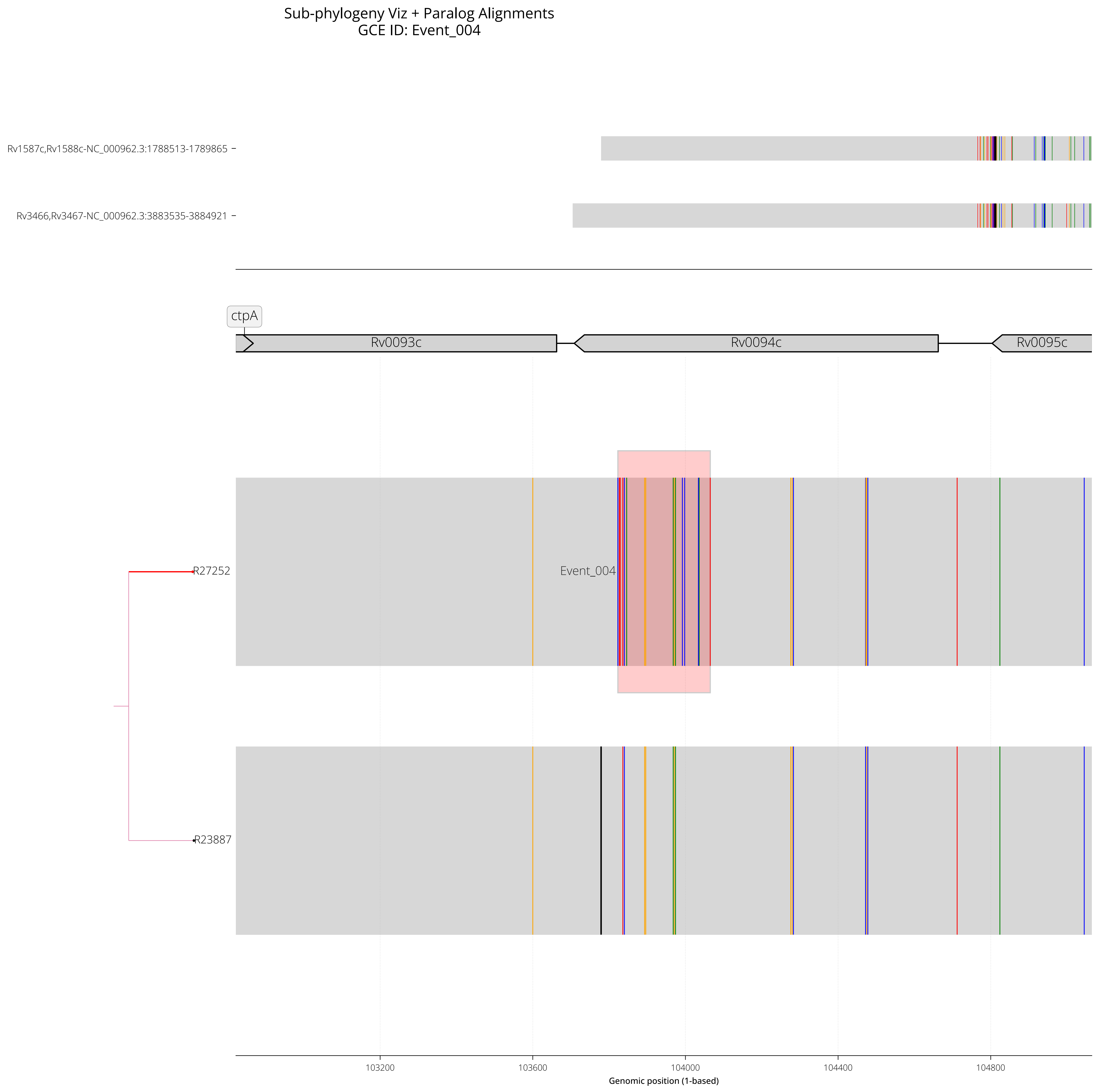

Sub-phylogeny Viz + Paralog Alignments
GCE ID: Event_004
Rv1587c,Rv1588c-NC_000962.3:1788513-1789865
Rv3466,Rv3467-NC_000962.3:3883535-3884921
ctpA
Rv0093c
Rv0094c
Rv0095c
Event_004
R27252
R23887
103200
103600
104000
104400
104800
Genomic position (1-based)

Full Phylogeny Viz + Paralog Alignments
GCE ID: Event_004

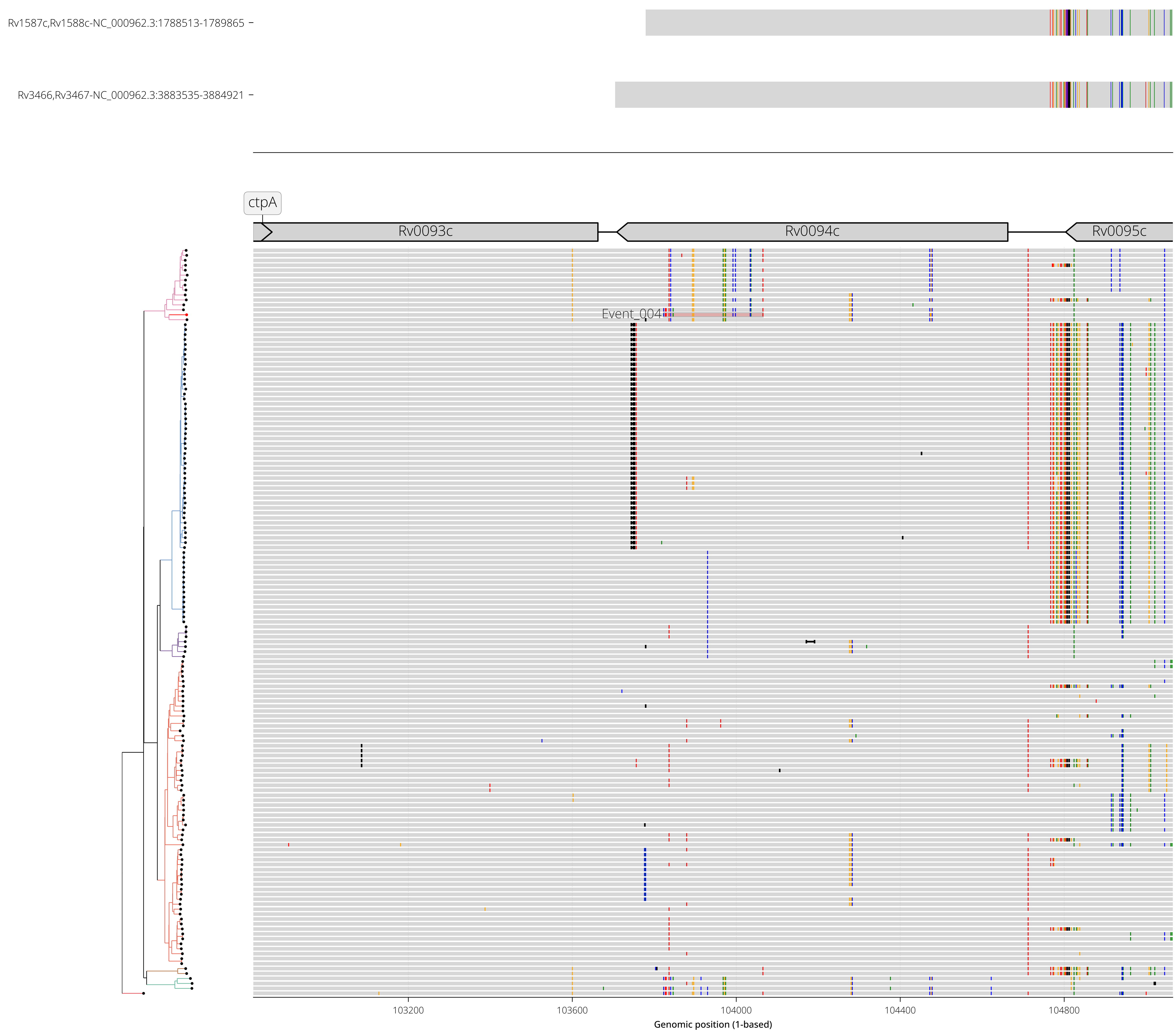

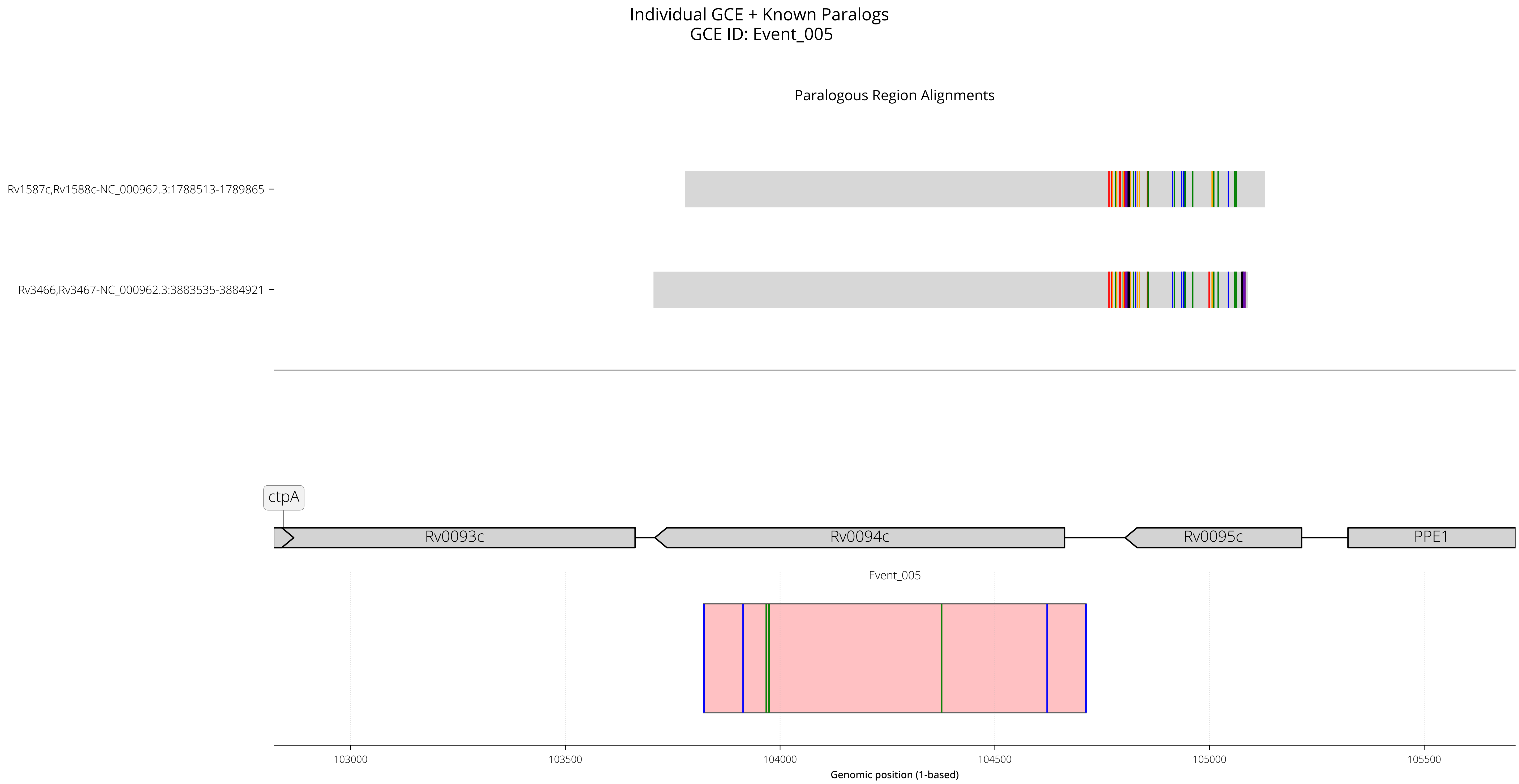
Individual GCE + Known Paralogs
GCE ID: Event_005
Paralogous Region Alignments
Rv1587c,Rv1588c-NC_000962.3:1788513-1789865
Rv3466,Rv3467-NC_000962.3:3883535-3884921
ctpA
Rv0093c
Rv0094c
Rv0095c
PPE1
Event_005
103000
103500
104000
104500
105000
105500
Genomic position (1-based)

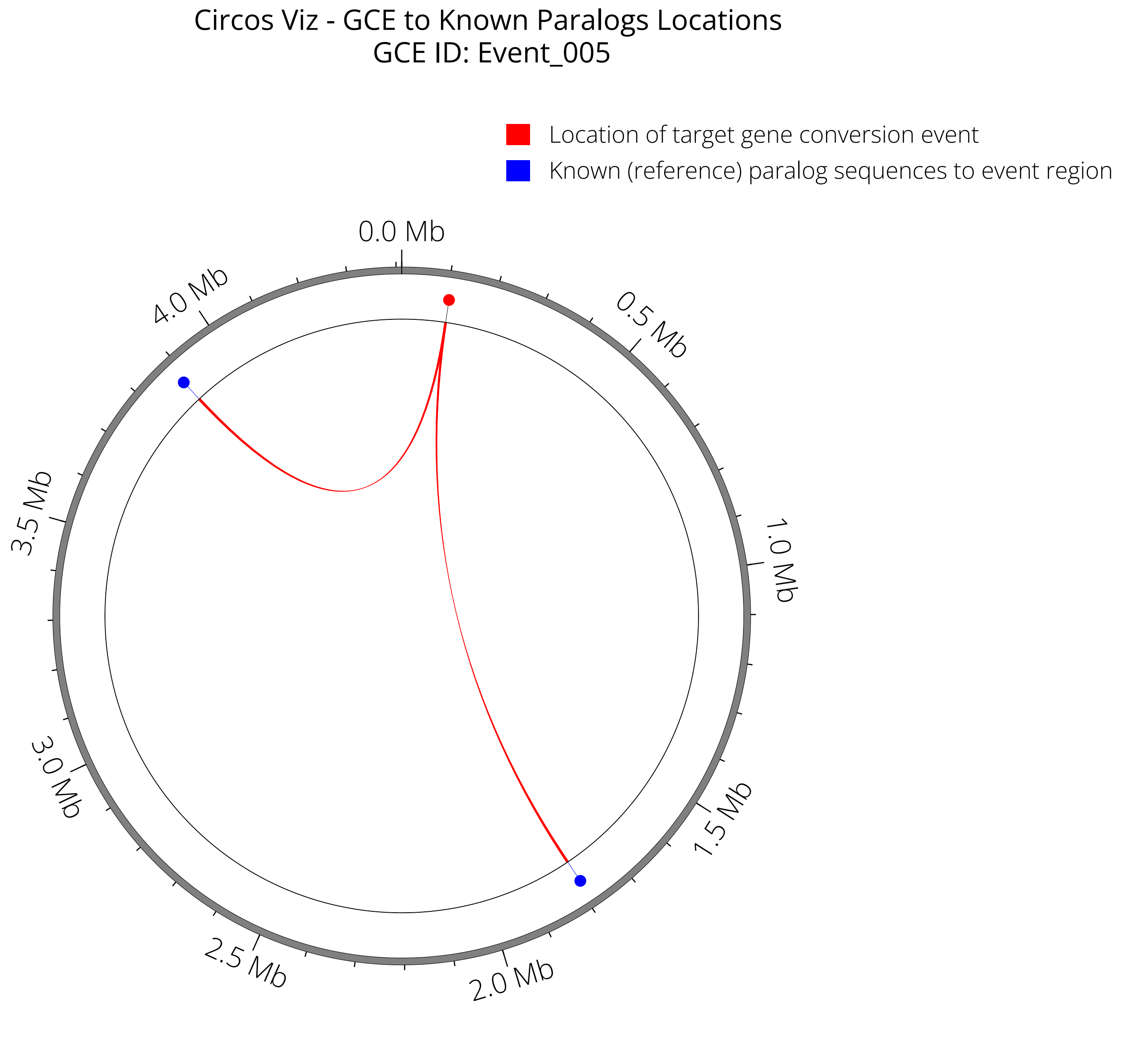
Circos Viz - GCE to Known Paralogs Locations
GCE ID: Event_005
Location of target gene conversion event
Known (reference) paralog sequences to event region
0.0 Mb
0.5 Mb
1.0 Mb
1.5 Mb
2.0 Mb
2.5 Mb
3.0 Mb
3.5 Mb
4.0 Mb

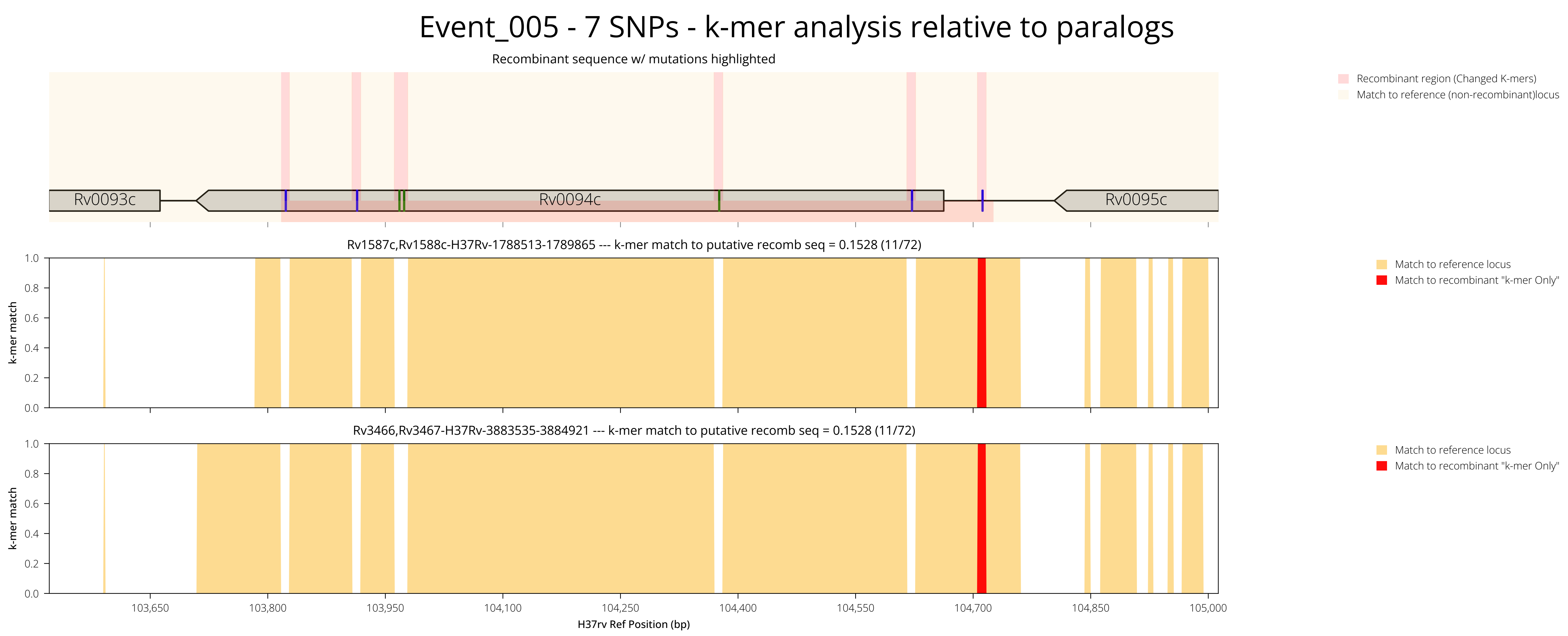

Event_005 - 7 SNPs - k-mer analysis relative to paralogs
Recombinant sequence w/ mutations highlighted
Recombinant region (Changed K-mers)
Match to reference (non-recombinant)locus
Rv0093c
Rv0094c
Rv0095c
Rv1587c,Rv1588c-H37Rv-1788513-1789865 --- k-mer match to putative recomb seq = 0.1528 (11/72)
Match to reference locus
Match to recombinant "k-mer Only"
k-mer match
1.0
0.8
0.6
0.4
0.2
0.0
Rv3466,Rv3467-H37Rv-3883535-3884921 --- k-mer match to putative recomb seq = 0.1528 (11/72)
Match to reference locus
Match to recombinant "k-mer Only"
k-mer match
1.0
0.8
0.6
0.4
0.2
0.0
103,650
103,800
103,950
104,100
104,250
104,400
104,550
104,700
104,850
105,000
H37rv Ref Position (bp)

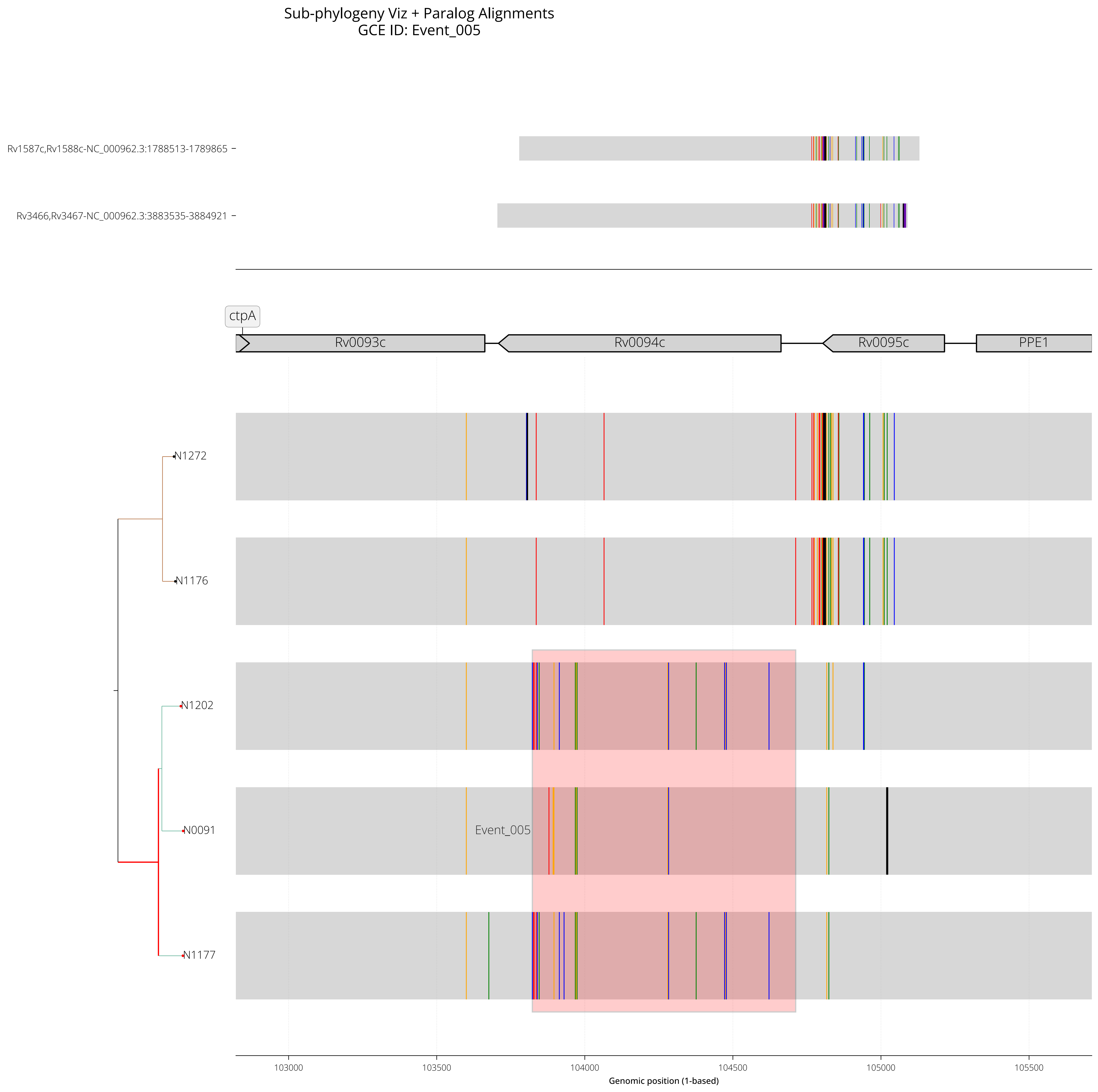

Sub-phylogeny Viz + Paralog Alignments
GCE ID: Event_005
Rv1587c,Rv1588c-NC_000962.3:1788513-1789865
Rv3466,Rv3467-NC_000962.3:3883535-3884921
ctpA
Rv0093c
Rv0094c
Rv0095c
PPE1
N1272
N1176
N1202
N0091
N1177
Event_005
103000
103500
104000
104500
105000
105500
Genomic position (1-based)

Full Phylogeny Viz + Paralog Alignments
GCE ID: Event_005

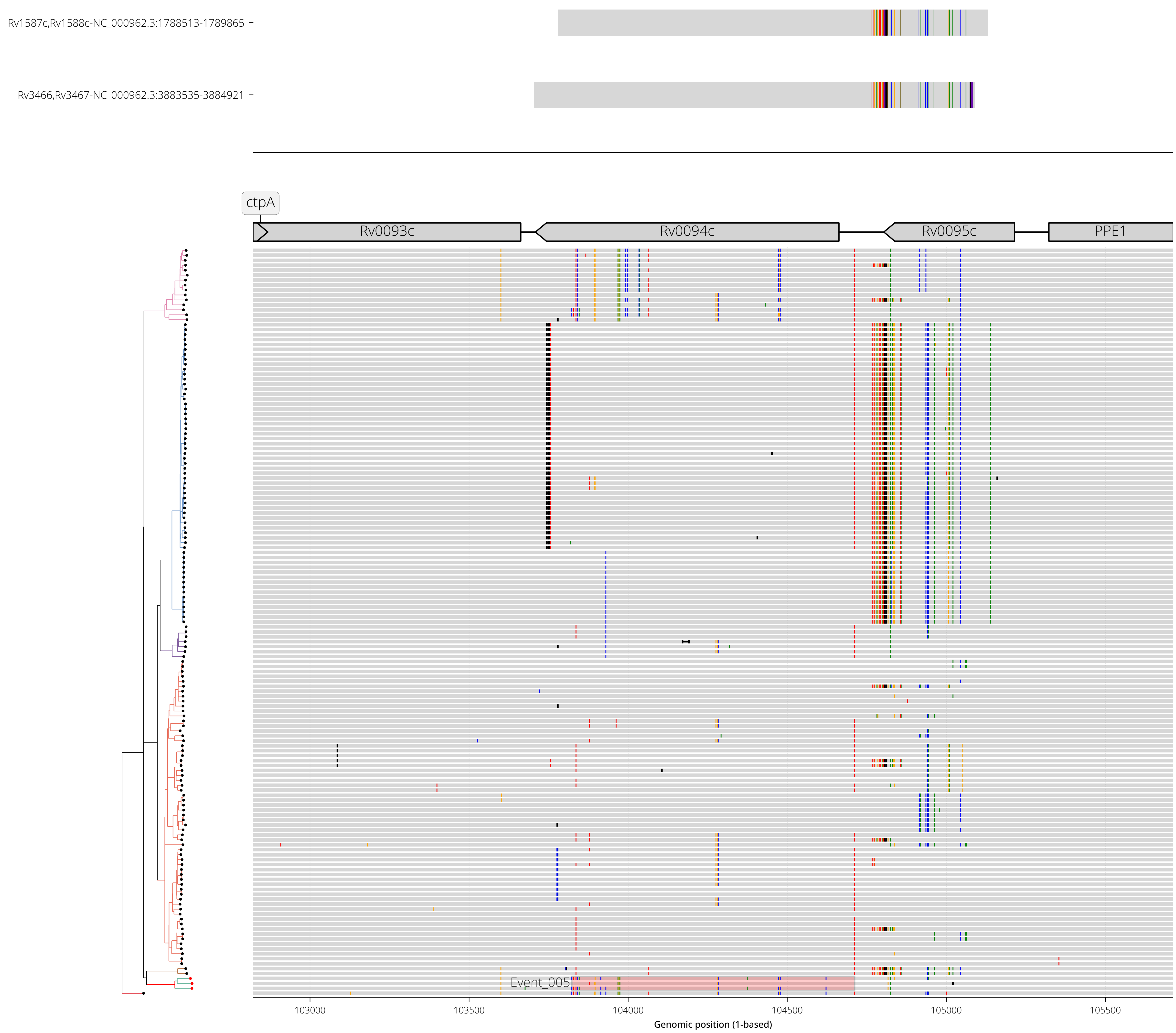

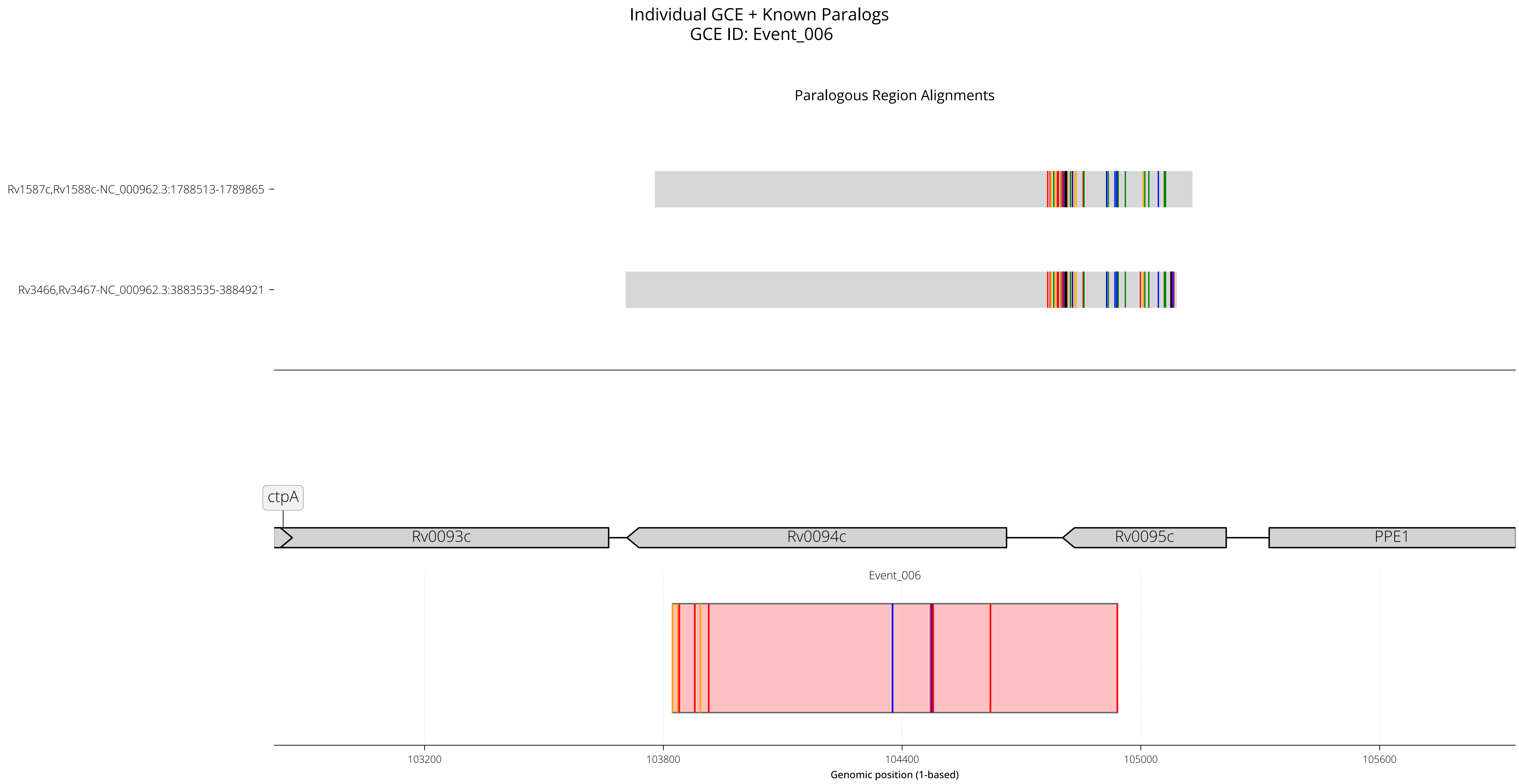
Individual GCE + Known Paralogs
GCE ID: Event_006
Paralogous Region Alignments
Rv1587c,Rv1588c-NC_000962.3:1788513-1789865
Rv3466,Rv3467-NC_000962.3:3883535-3884921
ctpA
Rv0093c
Rv0094c
Rv0095c
PPE1
Event_006
103200
103800
104400
105000
105600
Genomic position (1-based)

Circos Viz - GCE to Known Paralogs Locations
GCE ID: Event_006

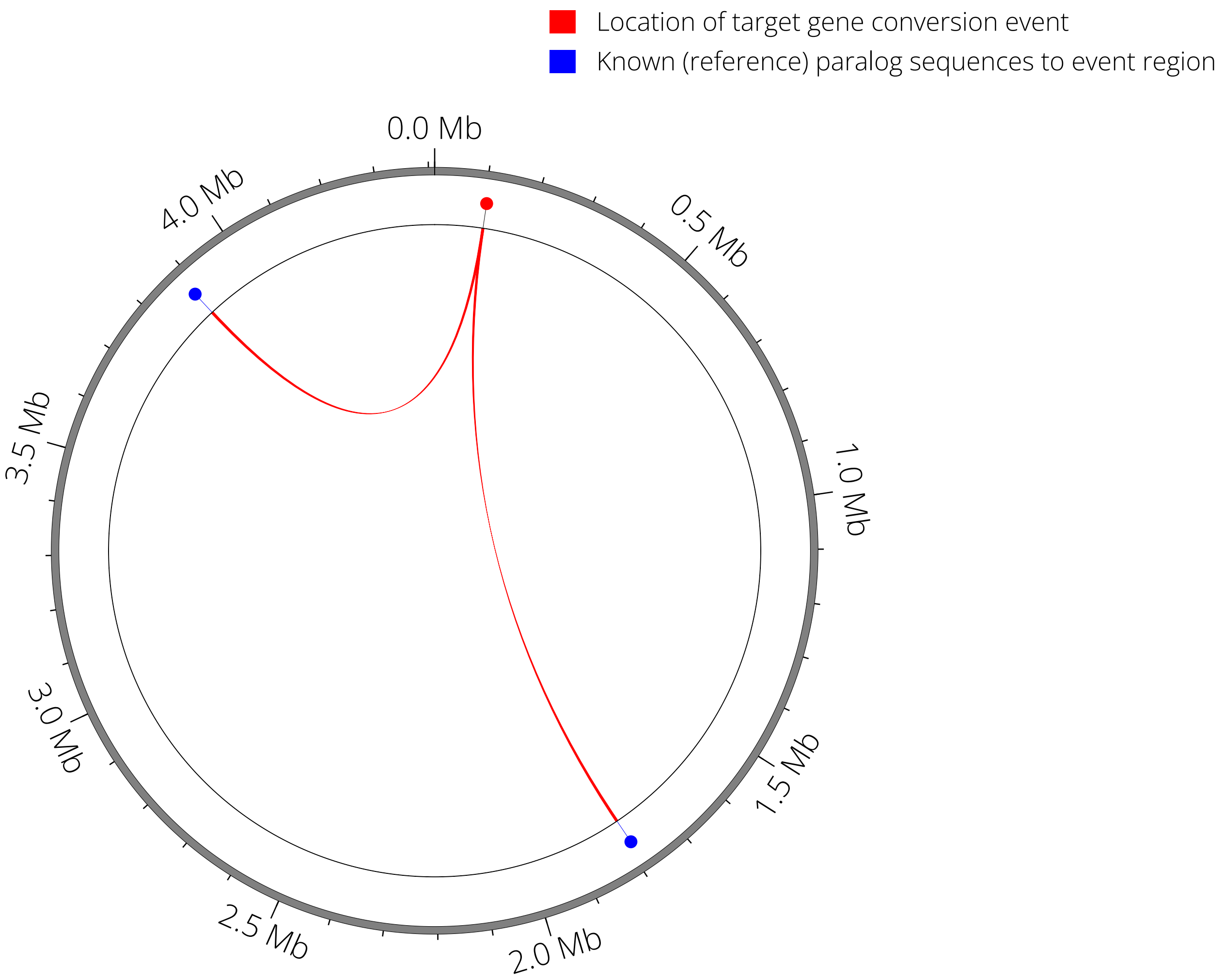

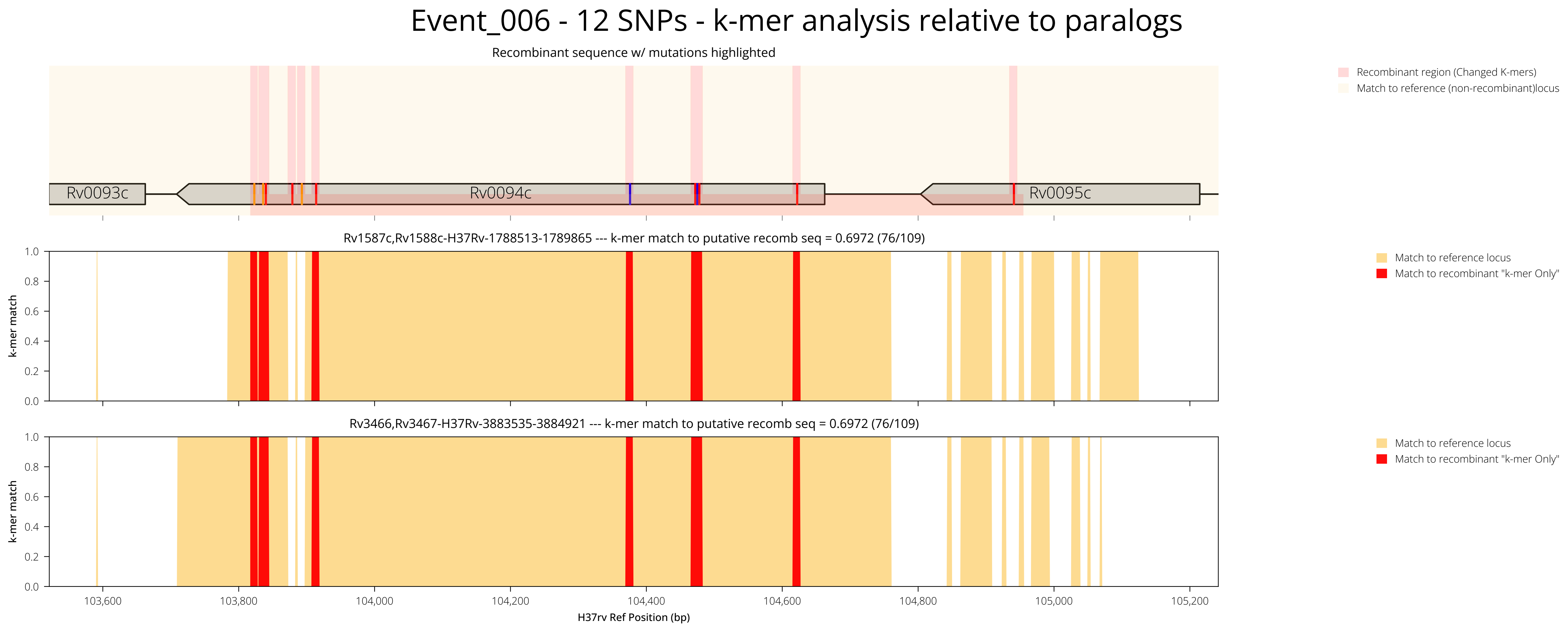
Event_006 - 12 SNPs - k-mer analysis relative to paralogs
Recombinant sequence w/ mutations highlighted
Rv0093c
Rv0094c
Rv0095c
Recombinant region (Changed K-mers)
Match to reference (non-recombinant)locus
Rv1587c,Rv1588c-H37Rv-1788513-1789865 --- k-mer match to putative recomb seq = 0.6972 (76/109)
Match to reference locus
Match to recombinant "k-mer Only"
Rv3466,Rv3467-H37Rv-3883535-3884921 --- k-mer match to putative recomb seq = 0.6972 (76/109)
Match to reference locus
Match to recombinant "k-mer Only"
k-mer match
1.0
0.8
0.6
0.4
0.2
0.0
103,600
103,800
104,000
104,200
104,400
104,600
104,800
105,000
105,200
H37rv Ref Position (bp)

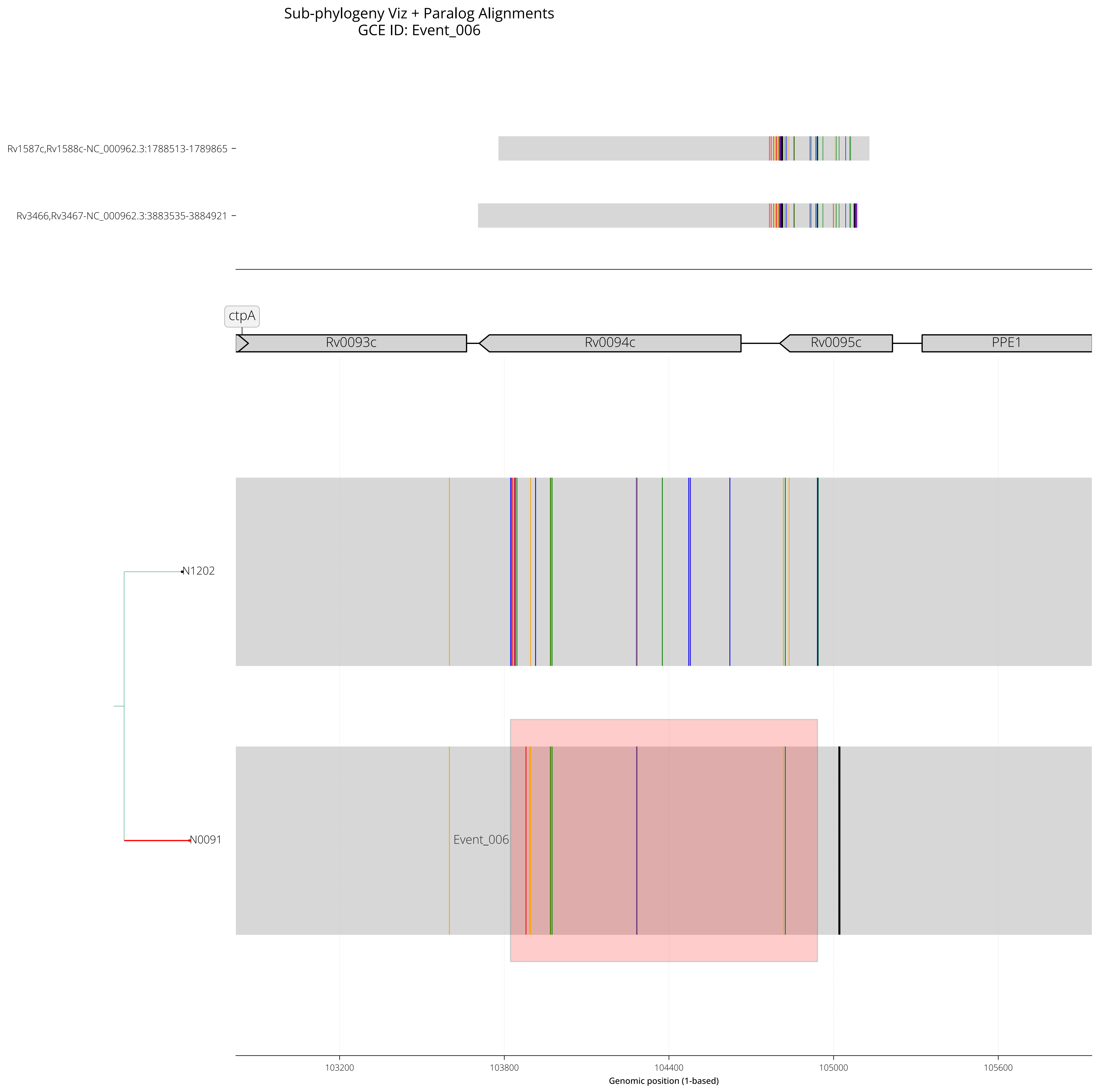

Sub-phylogeny Viz + Paralog Alignments
GCE ID: Event_006
Rv1587c,Rv1588c-NC_000962.3:1788513-1789865
Rv3466,Rv3467-NC_000962.3:3883535-3884921
ctpA
Rv0093c
Rv0094c
Rv0095c
PPE1
N1202
N0091
Event_006
103200
103800
104400
105000
105600
Genomic position (1-based)

Full Phylogeny Viz + Paralog Alignments
GCE ID: Event_006

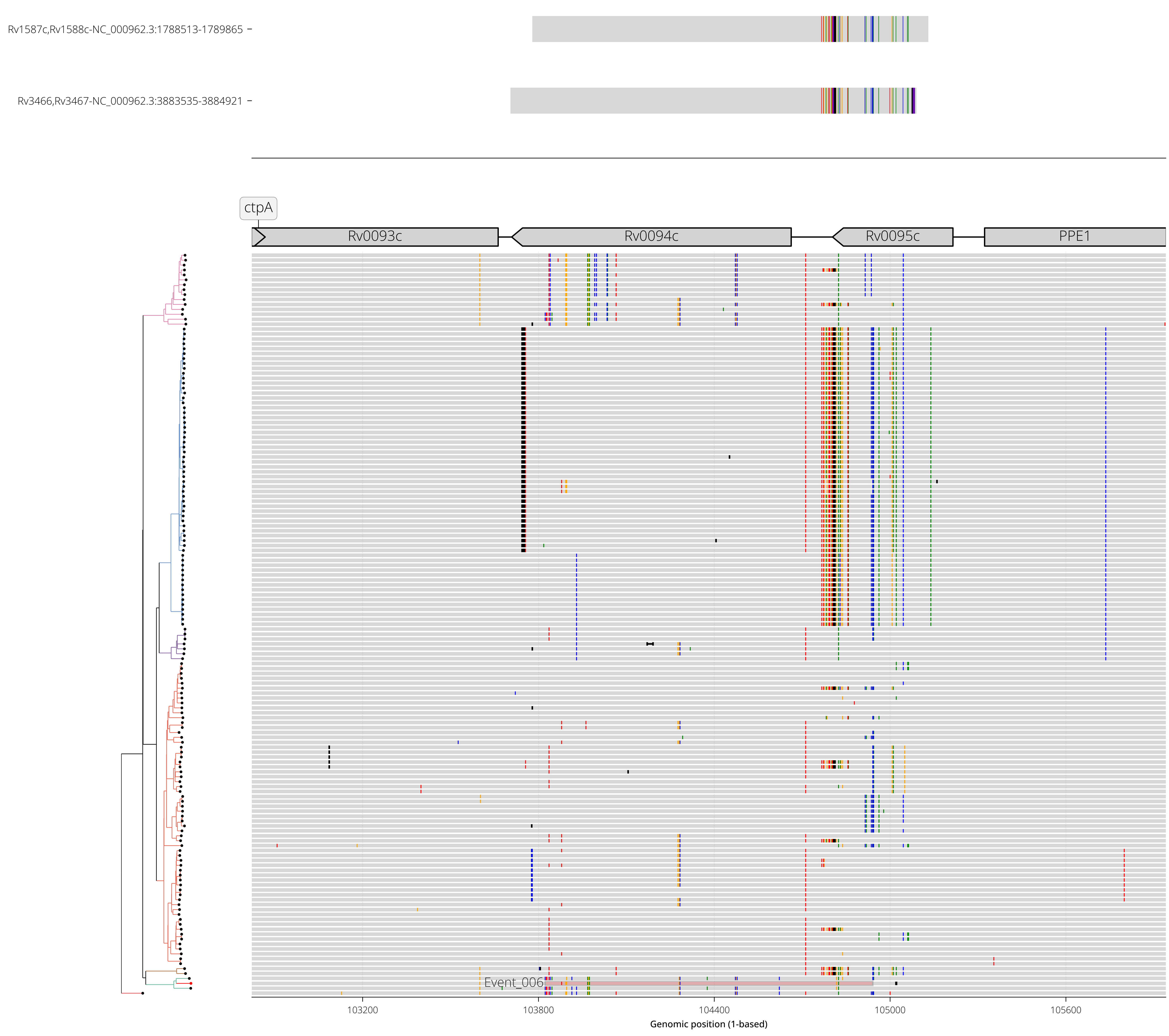

## Individual GCE + Known Paralogs
## GCE ID: Event_007

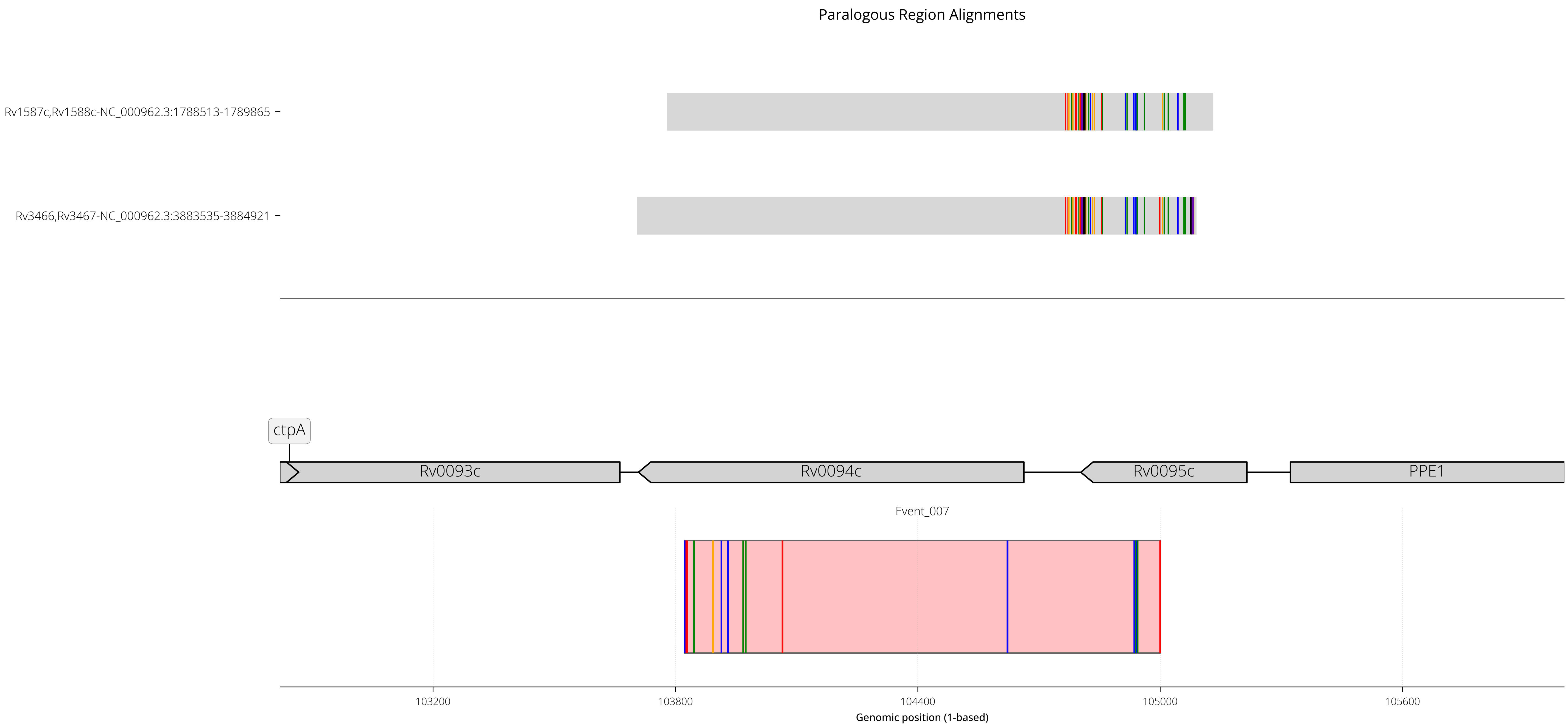

Circos Viz - GCE to Known Paralogs Locations
GCE ID: Event_007

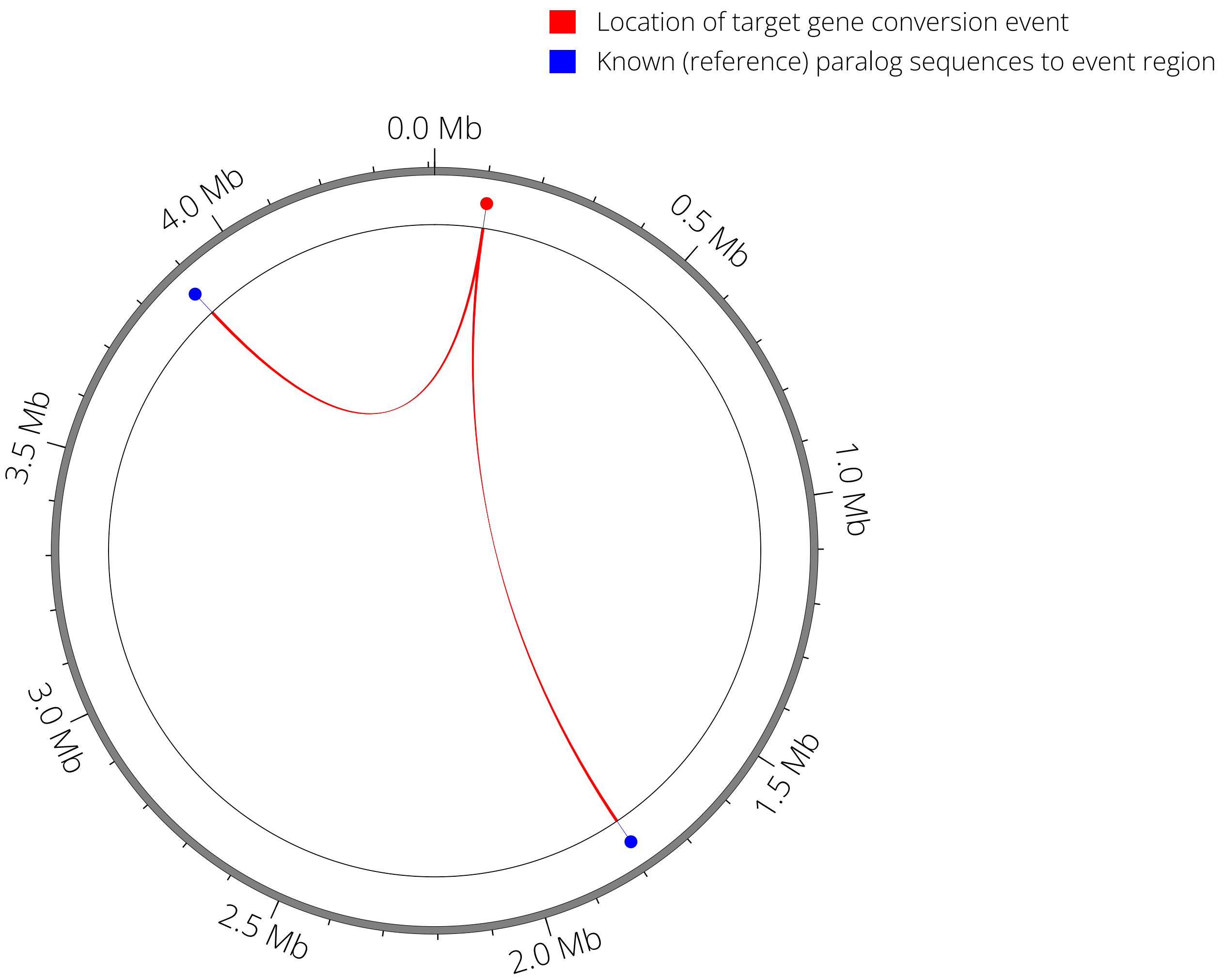

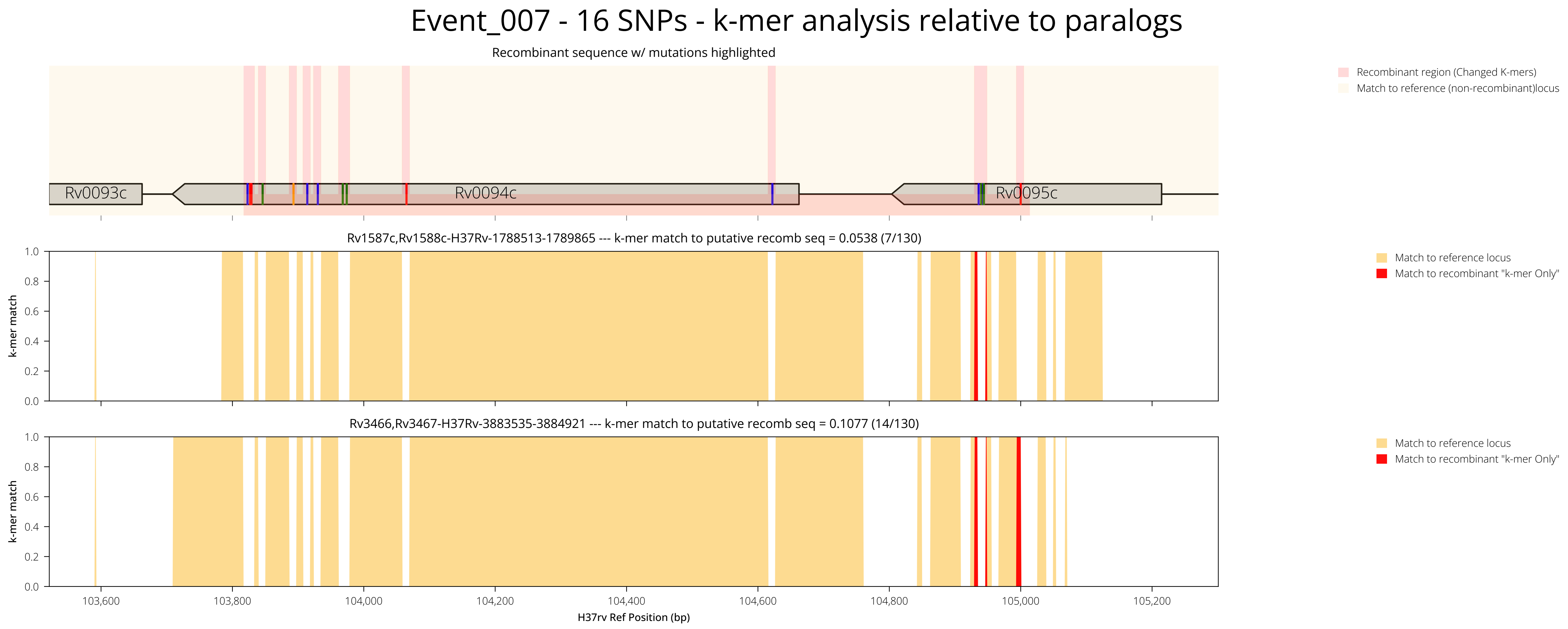
Event_007 - 16 SNPs - k-mer analysis relative to paralogs
Recombinant sequence w/ mutations highlighted
Rv0093c
Rv0094c
Rv0095c
Recombinant region (Changed K-mers)
Match to reference (non-recombinant)locus
Rv1587c,Rv1588c-H37Rv-1788513-1789865 --- k-mer match to putative recomb seq = 0.0538 (7/130)
Match to reference locus
Match to recombinant "k-mer Only"
Rv3466,Rv3467-H37Rv-3883535-3884921 --- k-mer match to putative recomb seq = 0.1077 (14/130)
Match to reference locus
Match to recombinant "k-mer Only"
k-mer match
1.0
0.8
0.6
0.4
0.2
0.0
103,600
103,800
104,000
104,200
104,400
104,600
104,800
105,000
105,200
H37rv Ref Position (bp)

Sub-phylogeny Viz + Paralog Alignments
GCE ID: Event_007

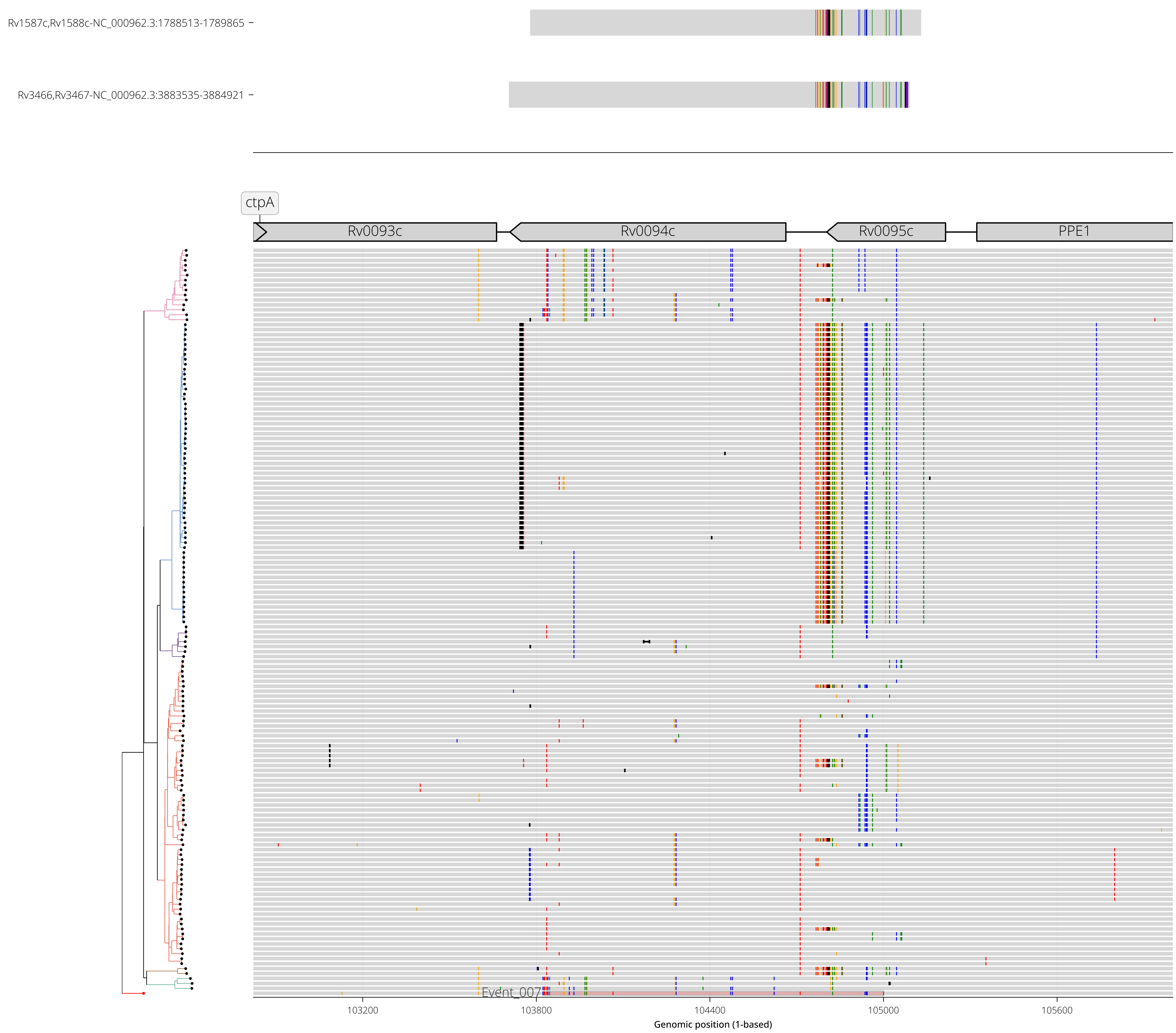

Full Phylogeny Viz + Paralog Alignments
GCE ID: Event_007

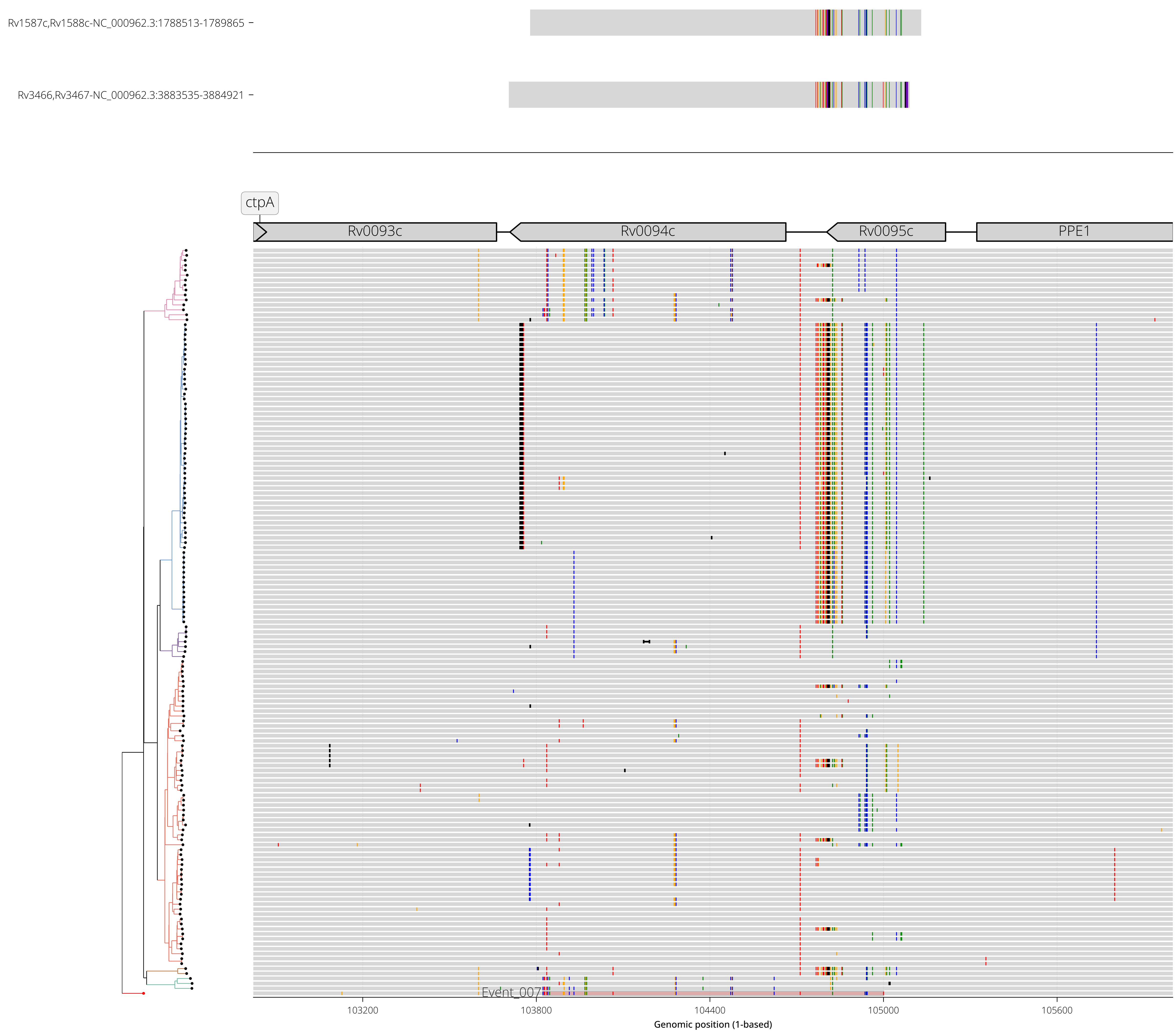

Individual GCE + Known Paralogs
GCE ID: Event_008

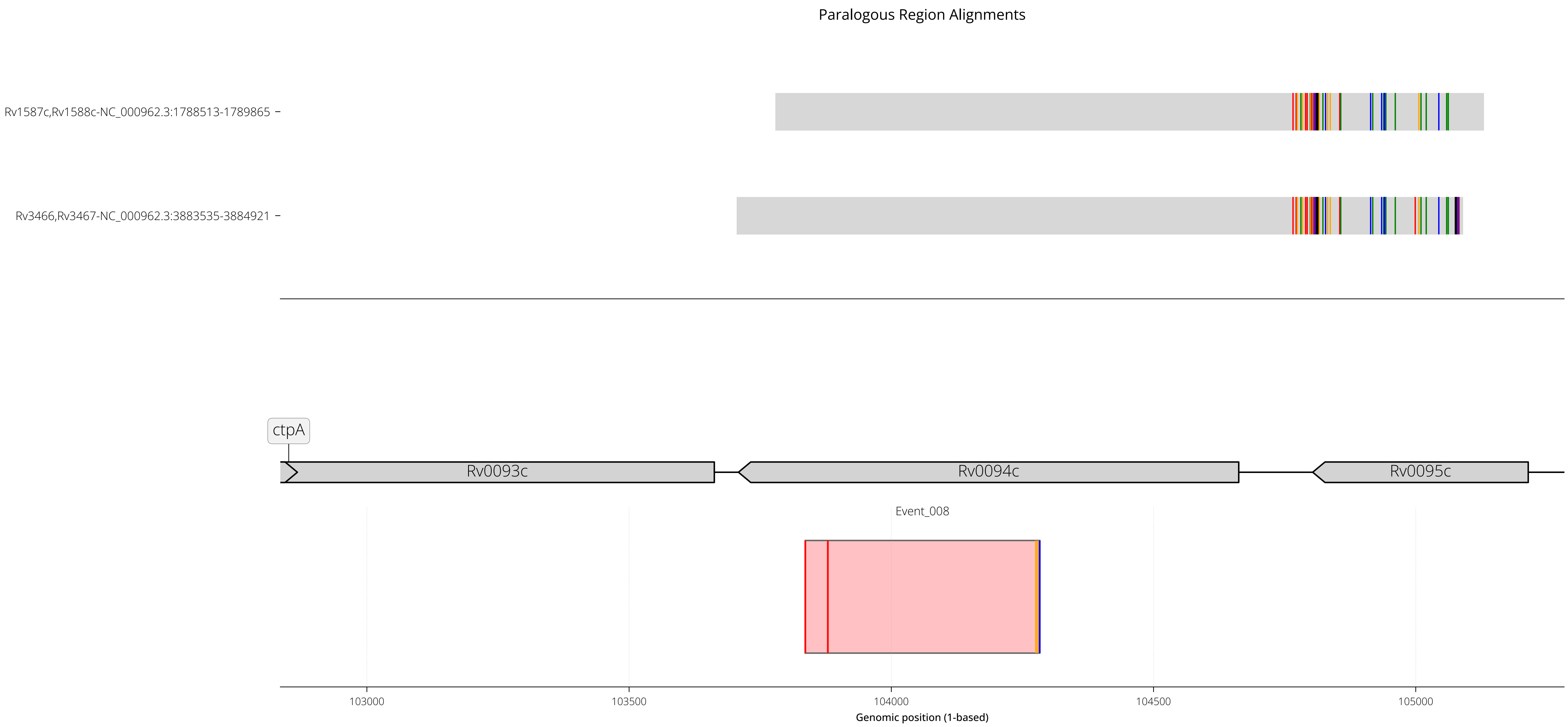

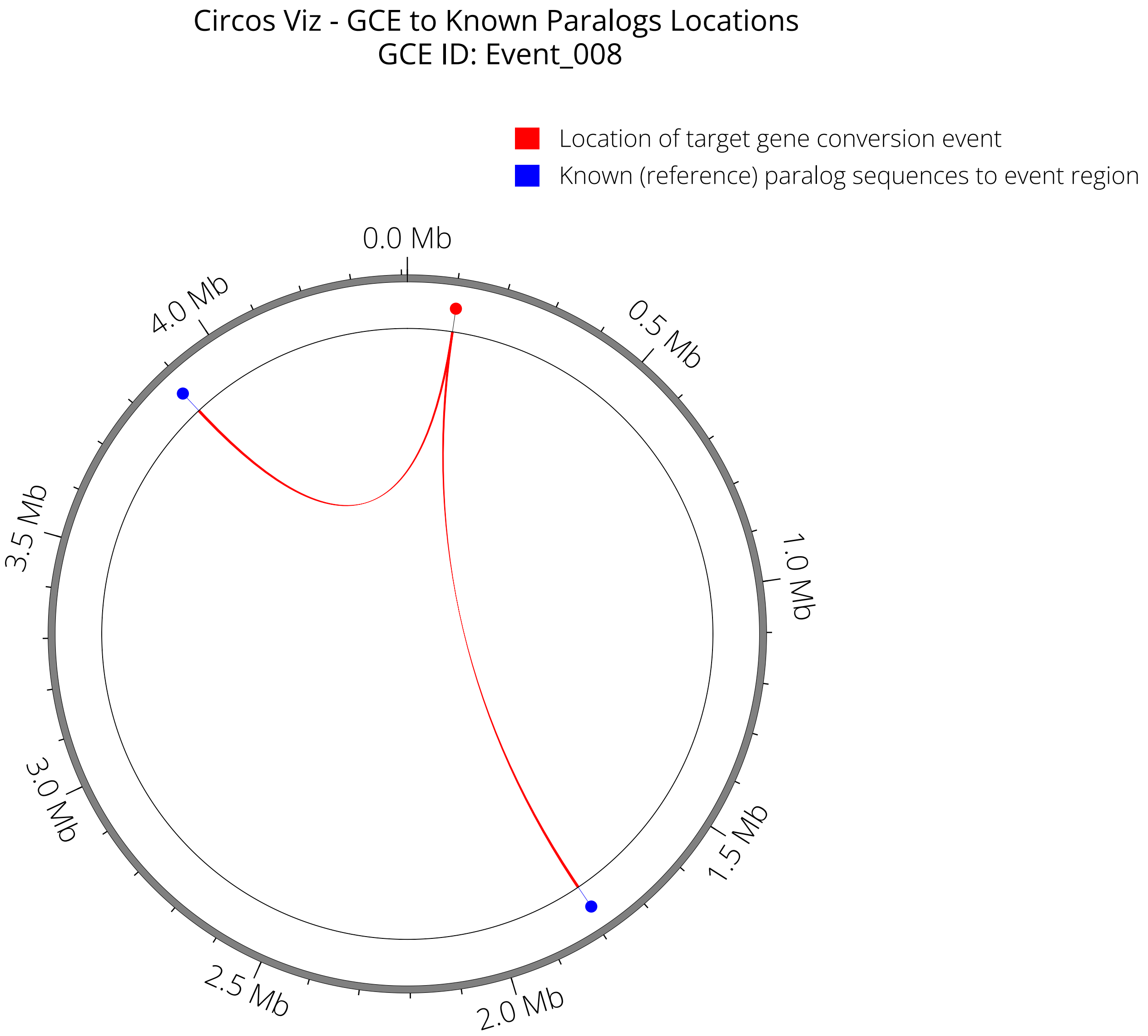
Circos Viz - GCE to Known Paralogs Locations
GCE ID: Event_008
Location of target gene conversion event
Known (reference) paralog sequences to event region
0.0 Mb
0.5 Mb
1.0 Mb
1.5 Mb
2.0 Mb
2.5 Mb
3.0 Mb
3.5 Mb
4.0 Mb

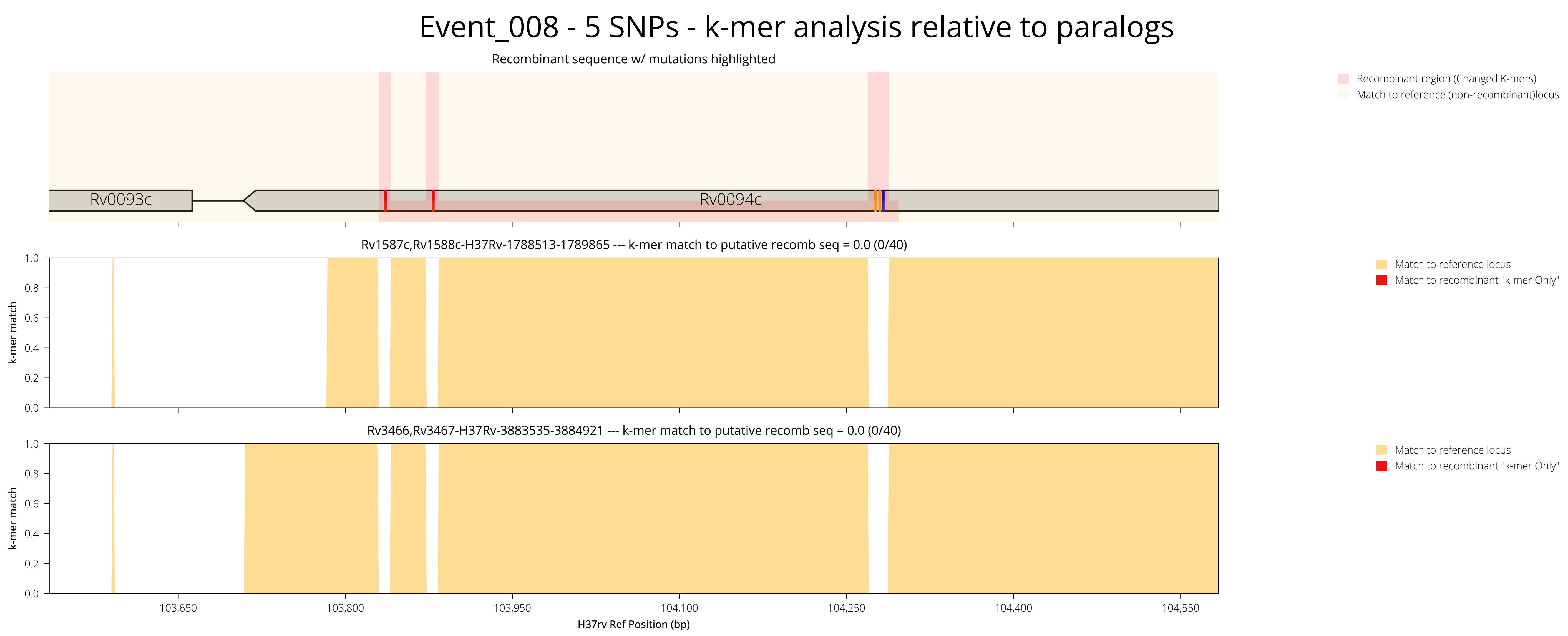
Event_008 - 5 SNPs - k-mer analysis relative to paralogs
Recombinant sequence w/ mutations highlighted
Recombinant region (Changed K-mers)
Match to reference (non-recombinant)locus
Rv0093c
Rv0094c
Rv1587c,Rv1588c-H37Rv-1788513-1789865 --- k-mer match to putative recomb seq = 0.0 (0/40)
Match to reference locus
Match to recombinant "k-mer Only"
k-mer match
1.0
0.8
0.6
0.4
0.2
0.0
Rv3466,Rv3467-H37Rv-3883535-3884921 --- k-mer match to putative recomb seq = 0.0 (0/40)
Match to reference locus
Match to recombinant "k-mer Only"
k-mer match
103,650
103,800
103,950
104,100
104,250
104,400
104,550
H37rv Ref Position (bp)

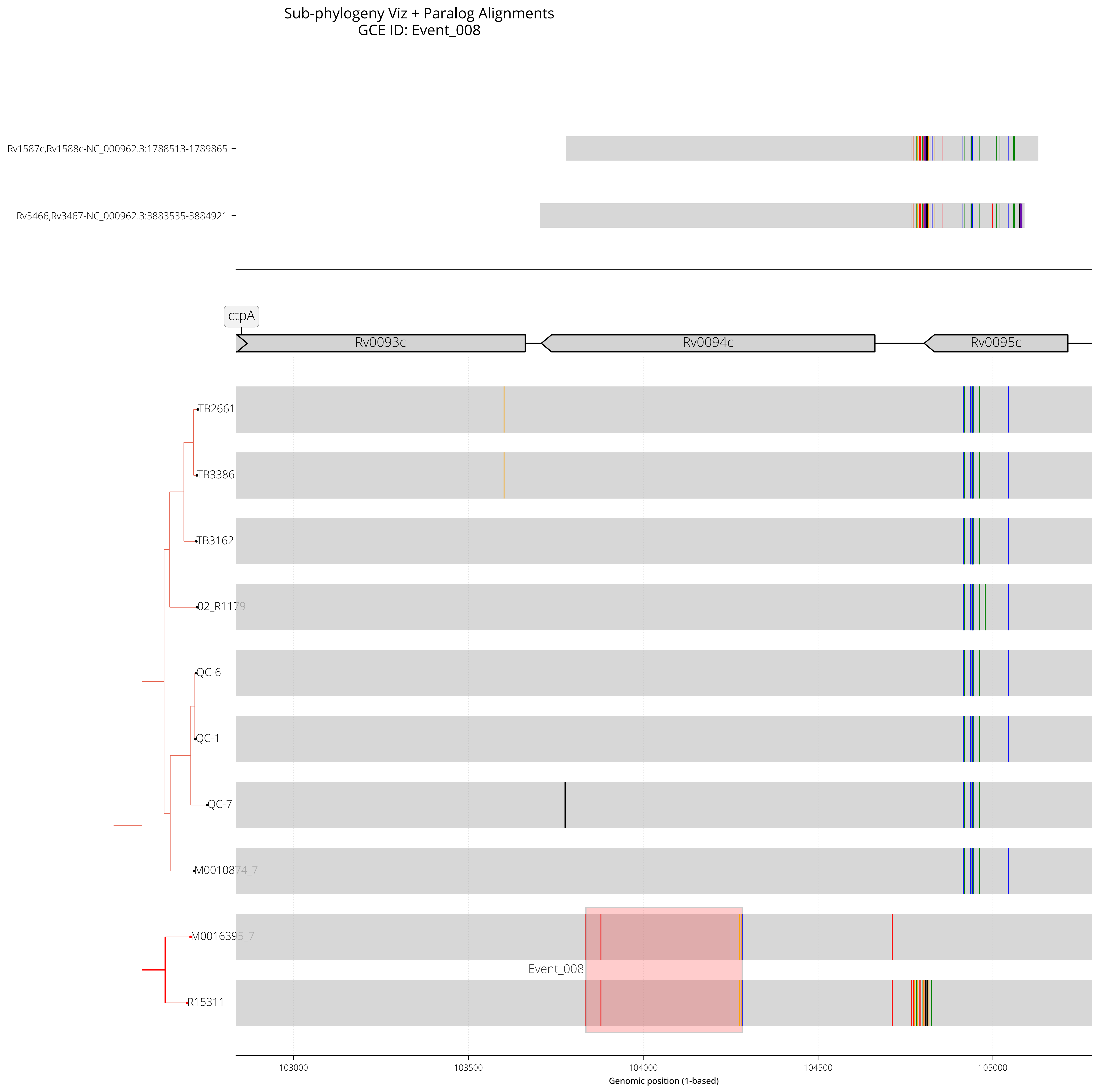

Sub-phylogeny Viz + Paralog Alignments
GCE ID: Event_008
Rv1587c,Rv1588c-NC_000962.3:1788513-1789865
Rv3466,Rv3467-NC_000962.3:3883535-3884921
ctpA
Rv0093c
Rv0094c
Rv0095c
TB2661
TB3386
TB3162
02_R1179
QC-6
QC-1
QC-7
M0010874_7
M0016395_7
R15311
Event_008
103000
103500
104000
104500
105000
Genomic position (1-based)

Full Phylogeny Viz + Paralog Alignments
GCE ID: Event_008

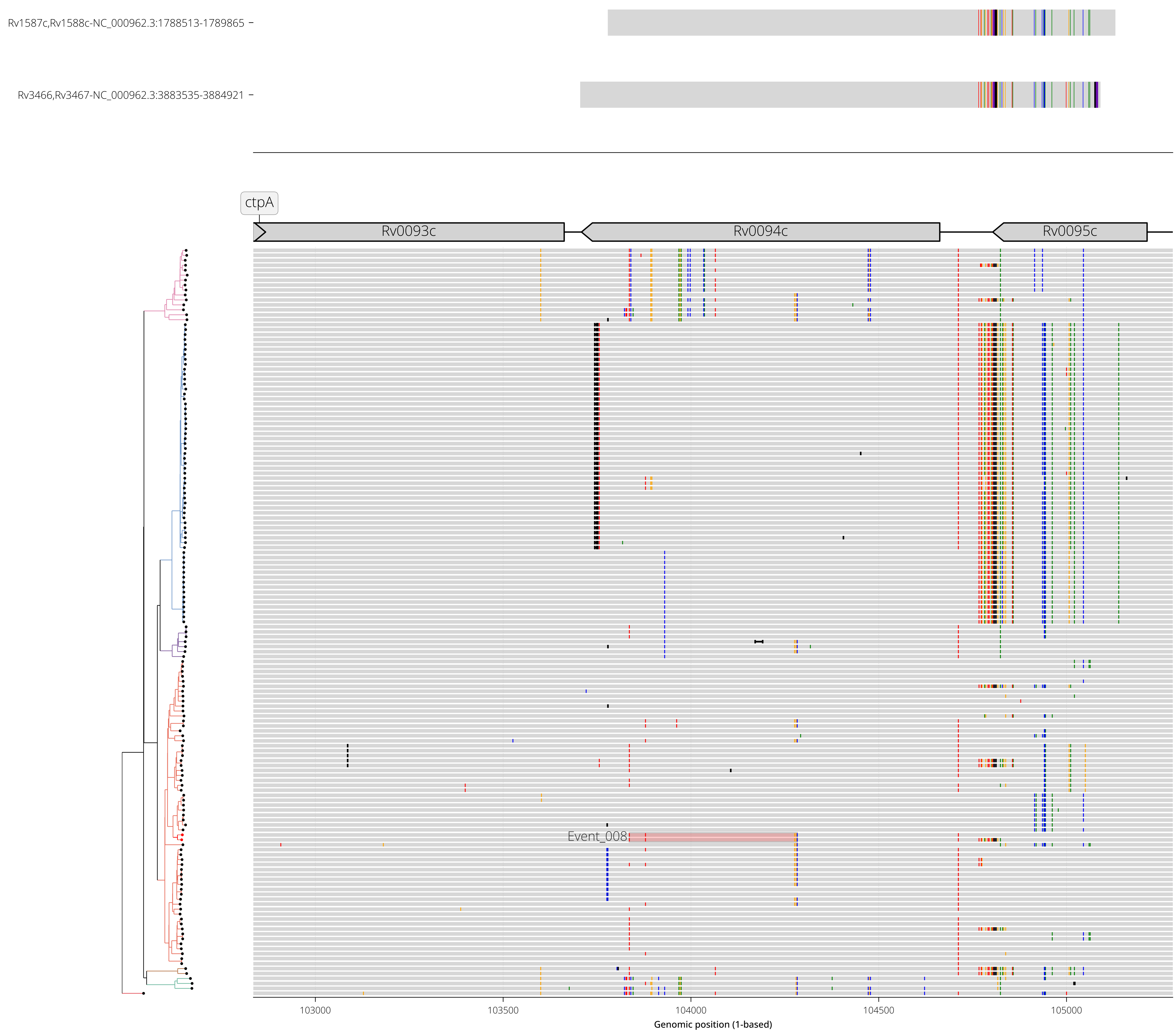

Individual GCE + Known Paralogs
GCE ID: Event_009

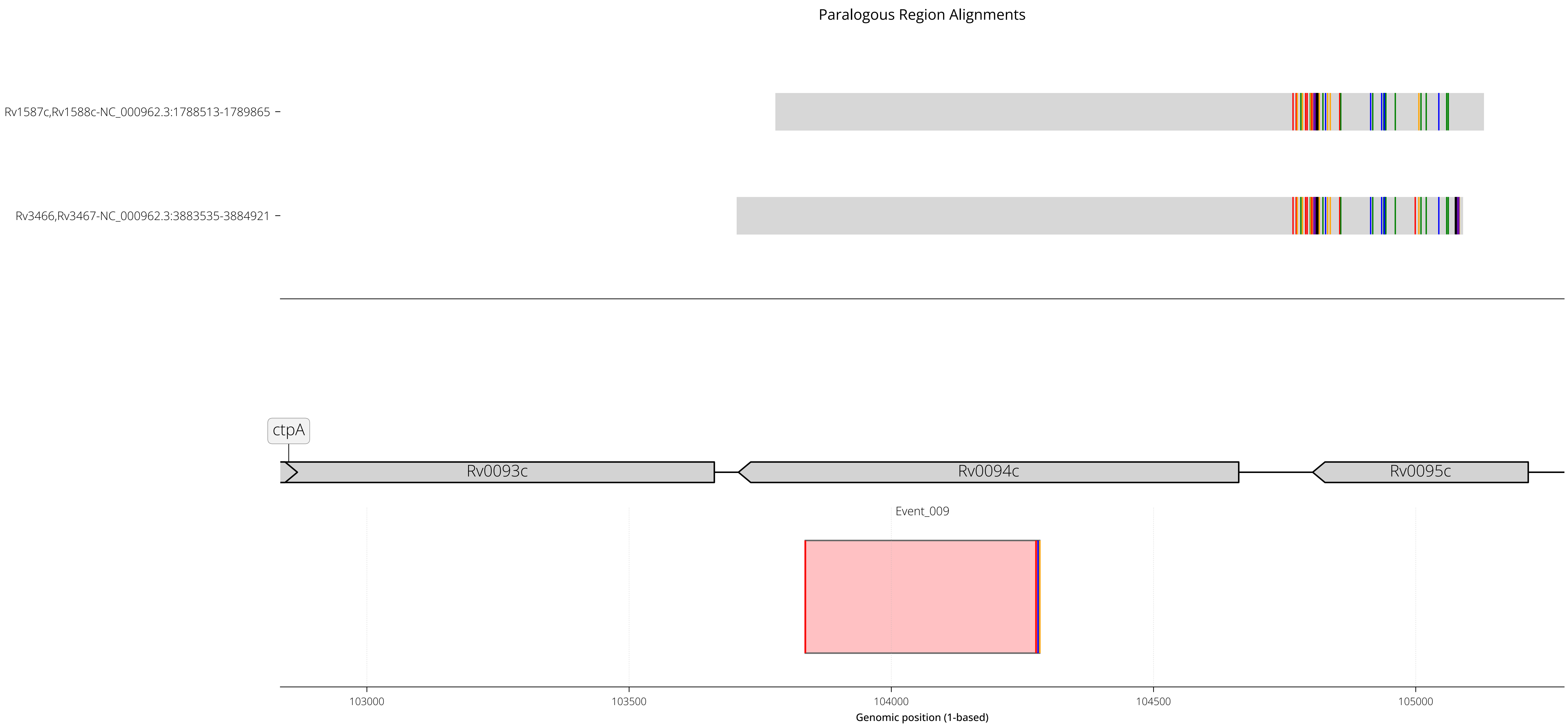

Circos Viz - GCE to Known Paralogs Locations
GCE ID: Event_009

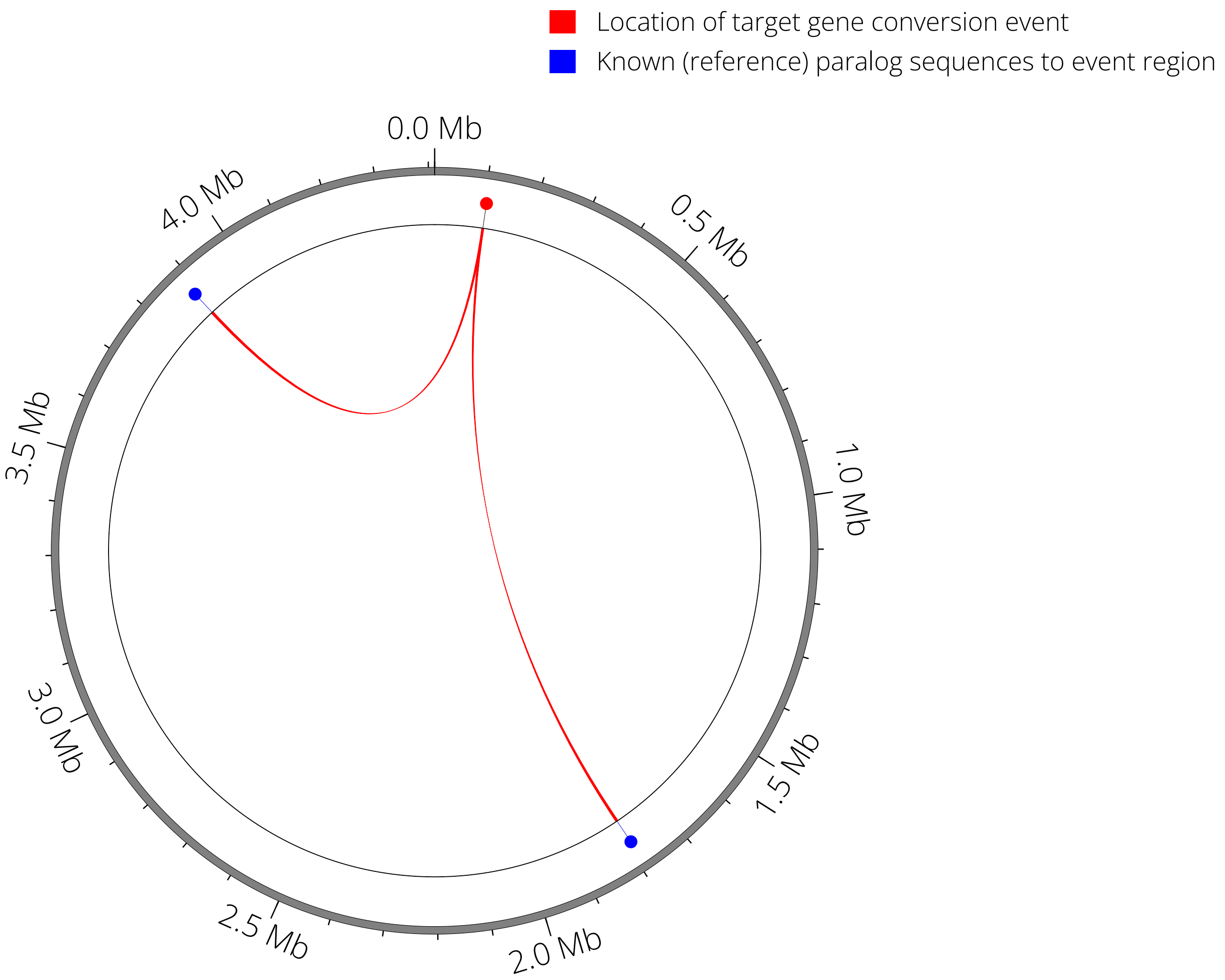

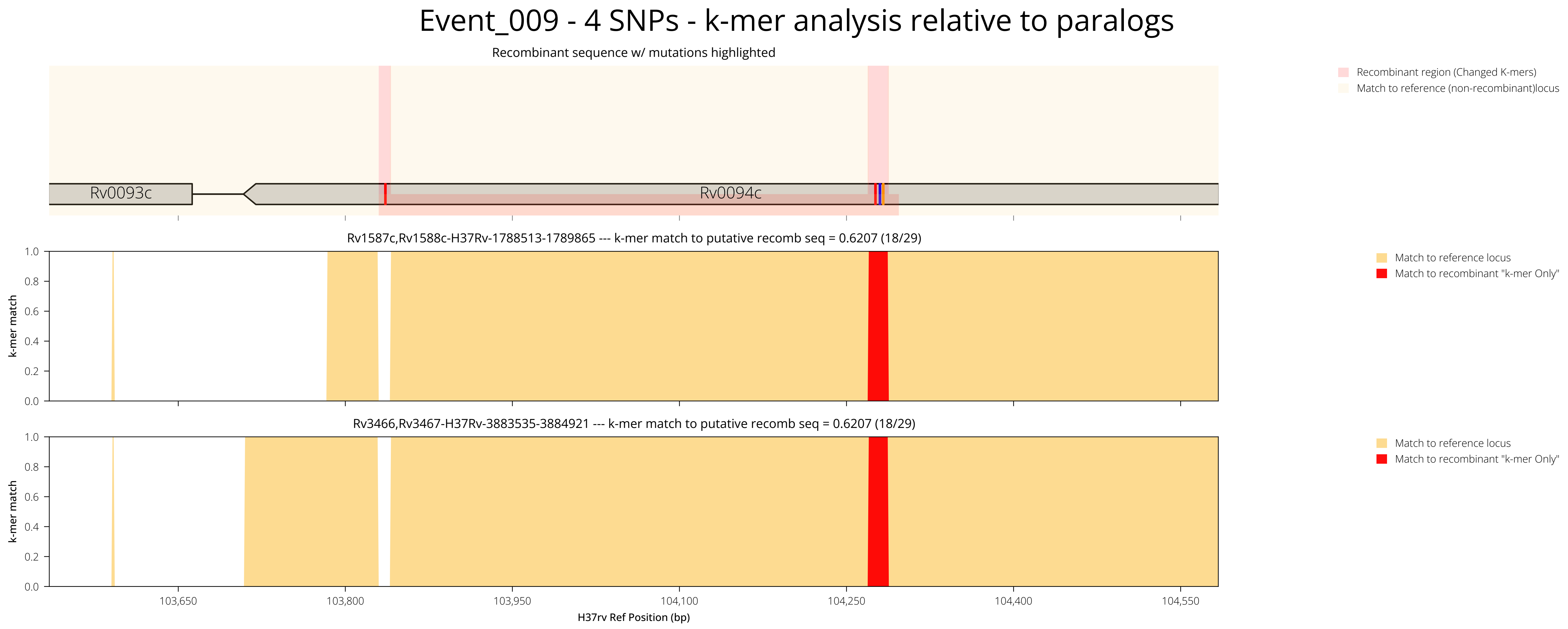
Event_009 - 4 SNPs - k-mer analysis relative to paralogs
Recombinant sequence w/ mutations highlighted
Recombinant region (Changed K-mers)
Match to reference (non-recombinant)locus
Rv0093c
Rv0094c
Rv1587c,Rv1588c-H37Rv-1788513-1789865 --- k-mer match to putative recomb seq = 0.6207 (18/29)
Match to reference locus
Match to recombinant "k-mer Only"
k-mer match
1.0
0.8
0.6
0.4
0.2
0.0
Rv3466,Rv3467-H37Rv-3883535-3884921 --- k-mer match to putative recomb seq = 0.6207 (18/29)
Match to reference locus
Match to recombinant "k-mer Only"
k-mer match
1.0
0.8
0.6
0.4
0.2
0.0
103,650
103,800
103,950
104,100
104,250
104,400
104,550
H37rv Ref Position (bp)

# Sub-phylogeny Viz + Paralog Alignments
GCE ID: Event_009

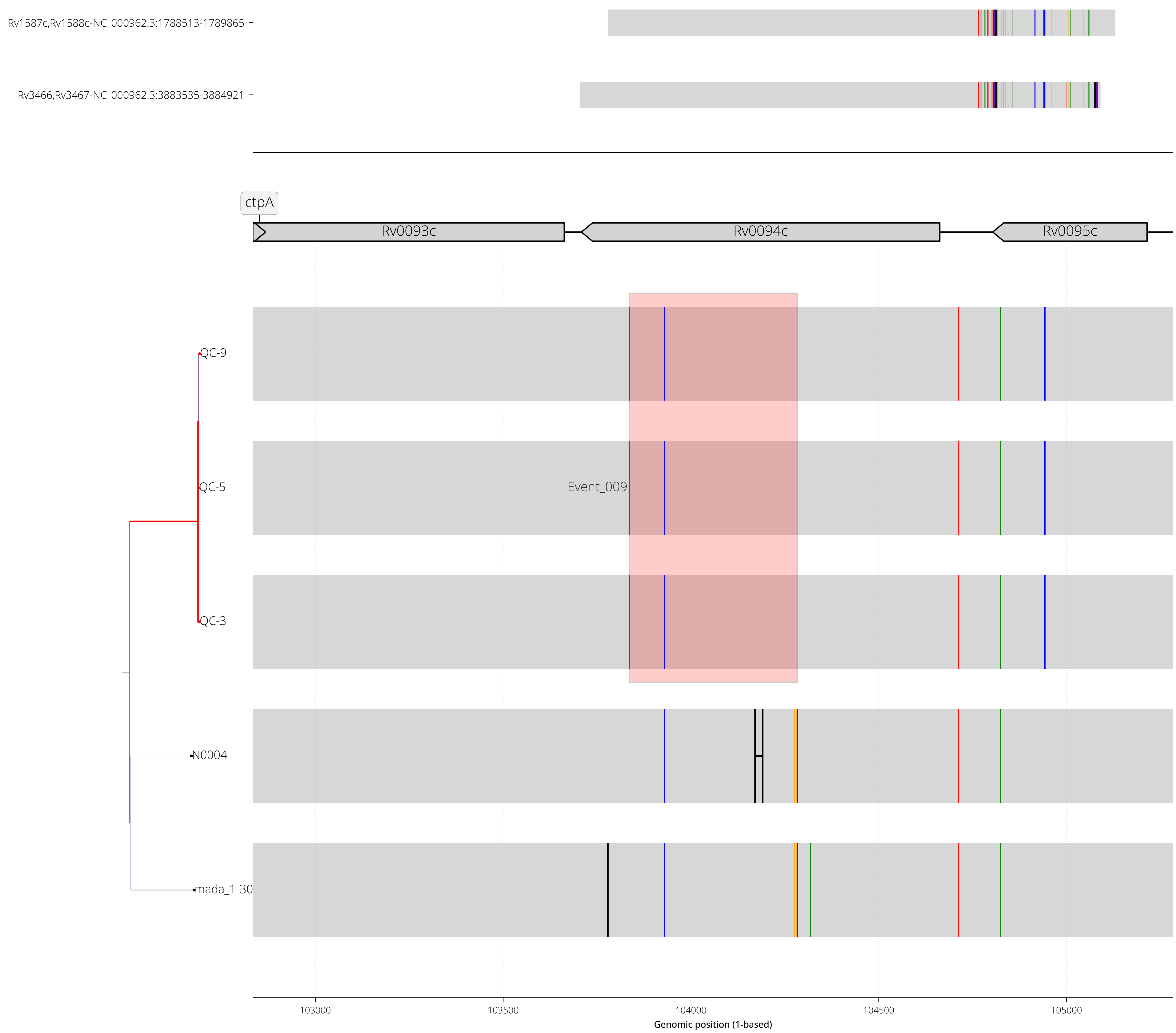

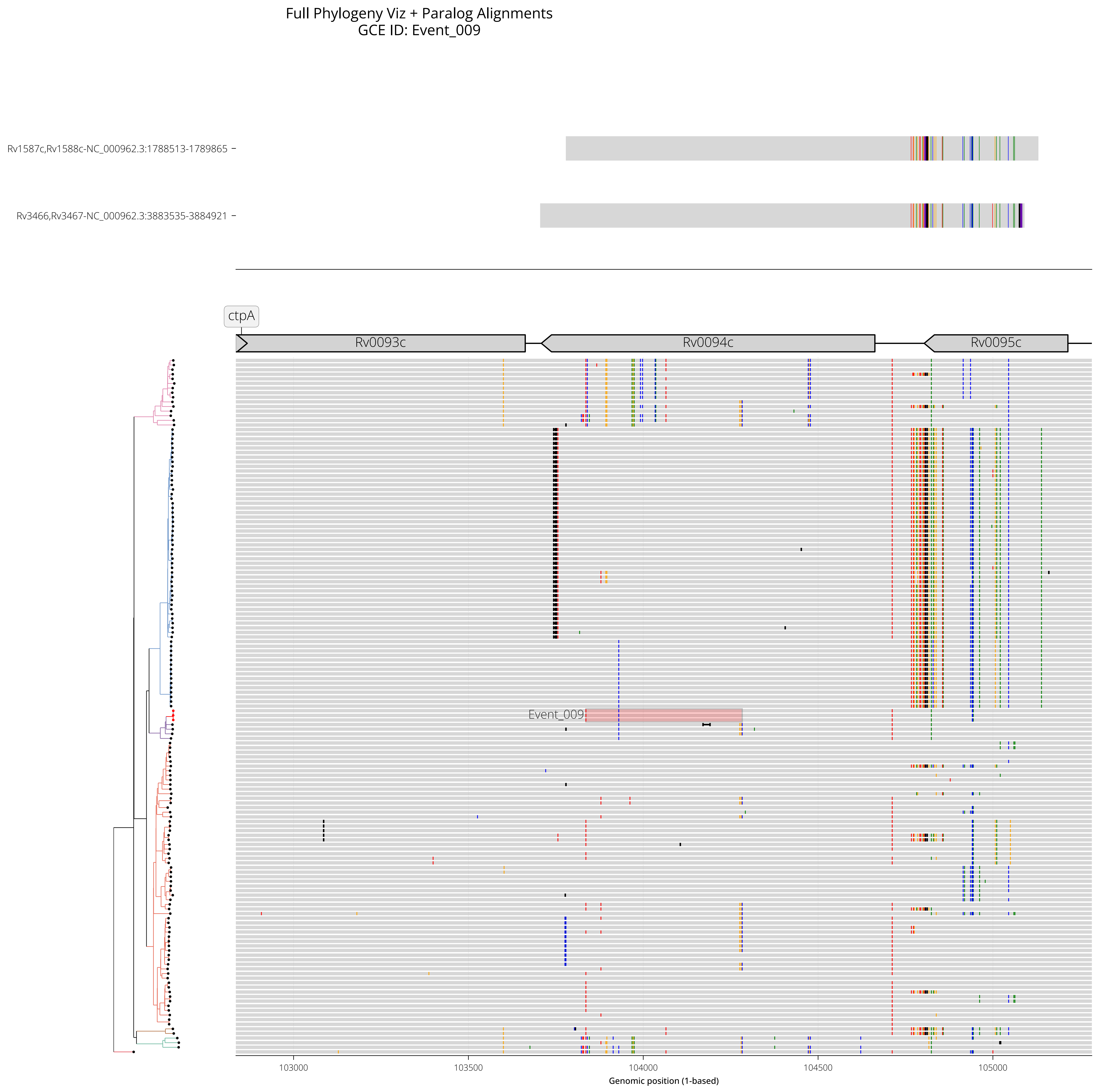

Full Phylogeny Viz + Paralog Alignments
GCE ID: Event_009
Rv1587c,Rv1588c-NC_000962.3:1788513-1789865
Rv3466,Rv3467-NC_000962.3:3883535-3884921
ctpA
Rv0093c
Rv0094c
Rv0095c
Event_009
103000
103500
104000
104500
105000
Genomic position (1-based)

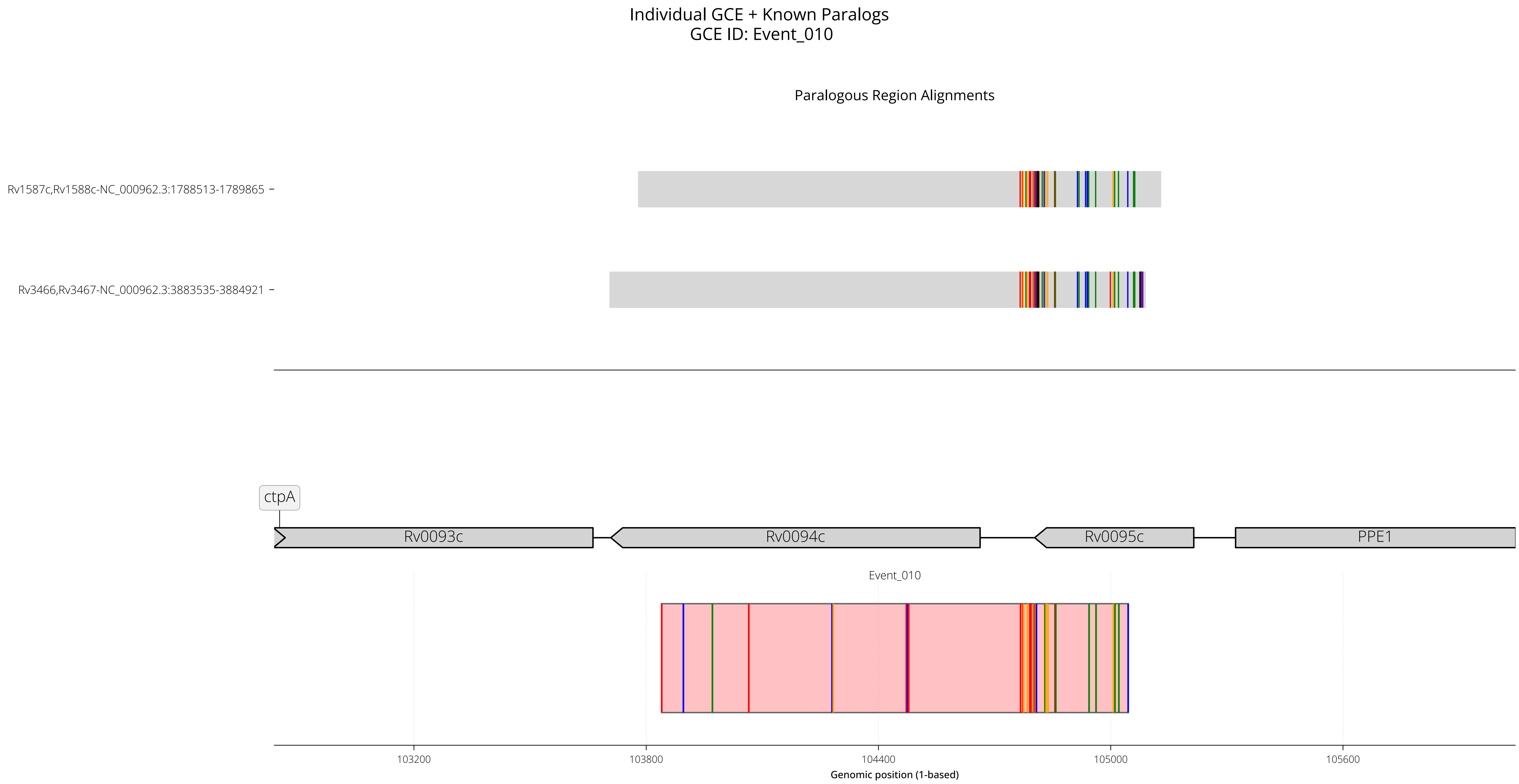
Individual GCE + Known Paralogs
GCE ID: Event_010
Paralogous Region Alignments
Rv1587c,Rv1588c-NC_000962.3:1788513-1789865
Rv3466,Rv3467-NC_000962.3:3883535-3884921
ctpA
Rv0093c
Rv0094c
Rv0095c
PPE1
Event_010
103200
103800
104400
105000
105600
Genomic position (1-based)

Circos Viz - GCE to Known Paralogs Locations
GCE ID: Event_010

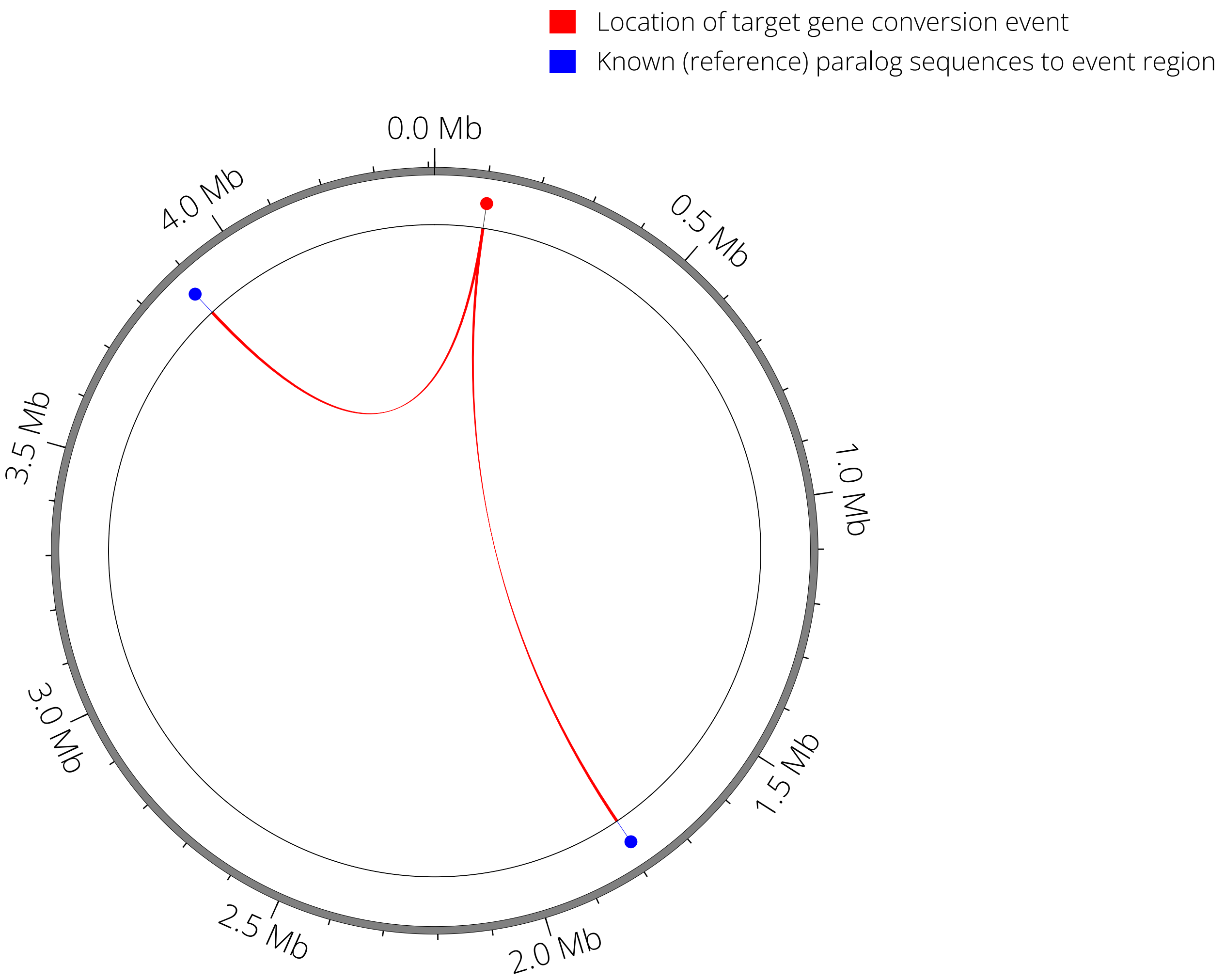

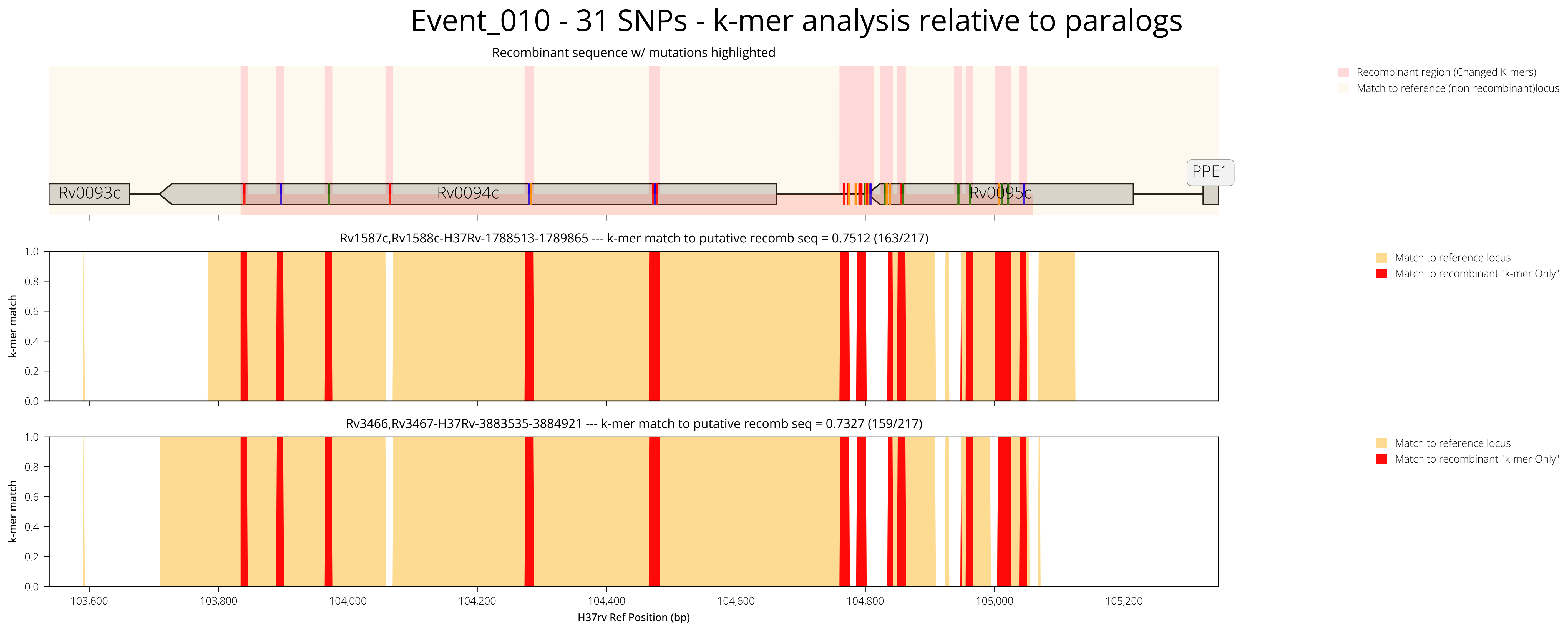

Event_010 - 31 SNPs - k-mer analysis relative to paralogs
Recombinant sequence w/ mutations highlighted
Recombinant region (Changed K-mers)
Match to reference (non-recombinant)locus
Rv0093c
Rv0094c
Rv0095c
PPE1
Rv1587c,Rv1588c-H37Rv-1788513-1789865 --- k-mer match to putative recomb seq = 0.7512 (163/217)
k-mer match
1.0
0.8
0.6
0.4
0.2
0.0
Match to reference locus
Match to recombinant "k-mer Only"
Rv3466,Rv3467-H37Rv-3883535-3884921 --- k-mer match to putative recomb seq = 0.7327 (159/217)
k-mer match
1.0
0.8
0.6
0.4
0.2
0.0
Match to reference locus
Match to recombinant "k-mer Only"
103,600
103,800
104,000
104,200
104,400
104,600
104,800
105,000
105,200
H37rv Ref Position (bp)

Sub-phylogeny Viz + Paralog Alignments
GCE ID: Event_010

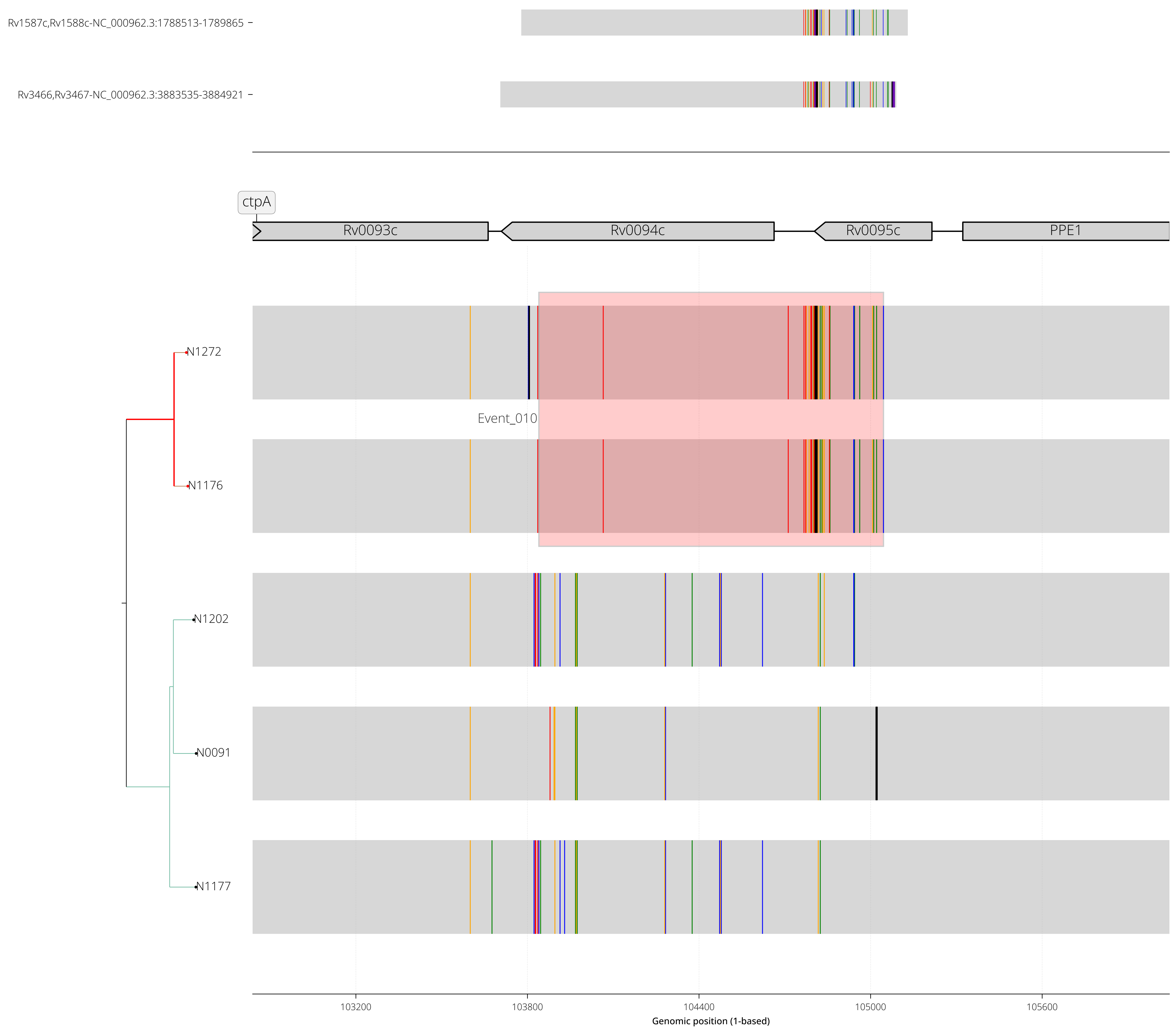

Full Phylogeny Viz + Paralog Alignments
GCE ID: Event_010

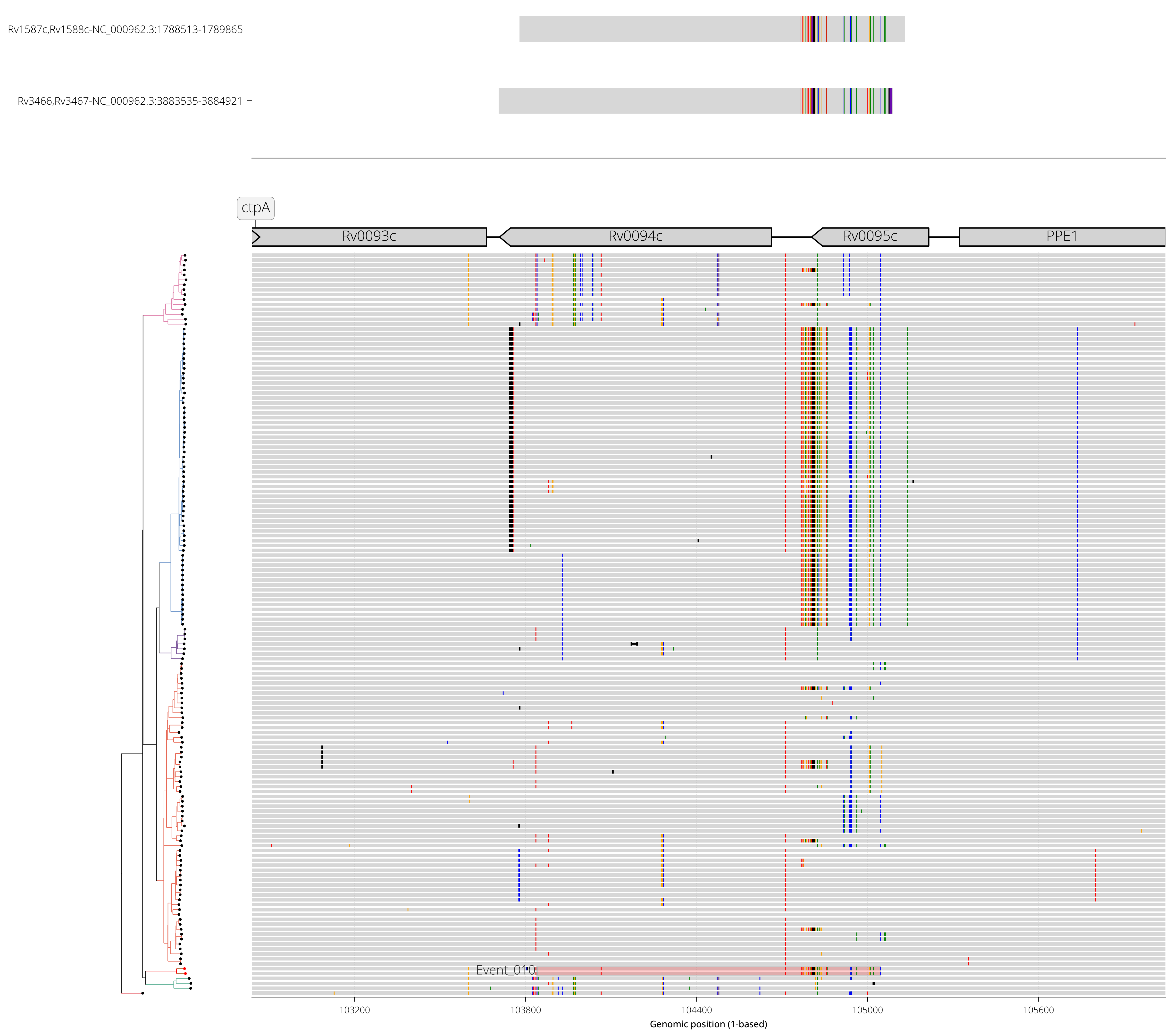

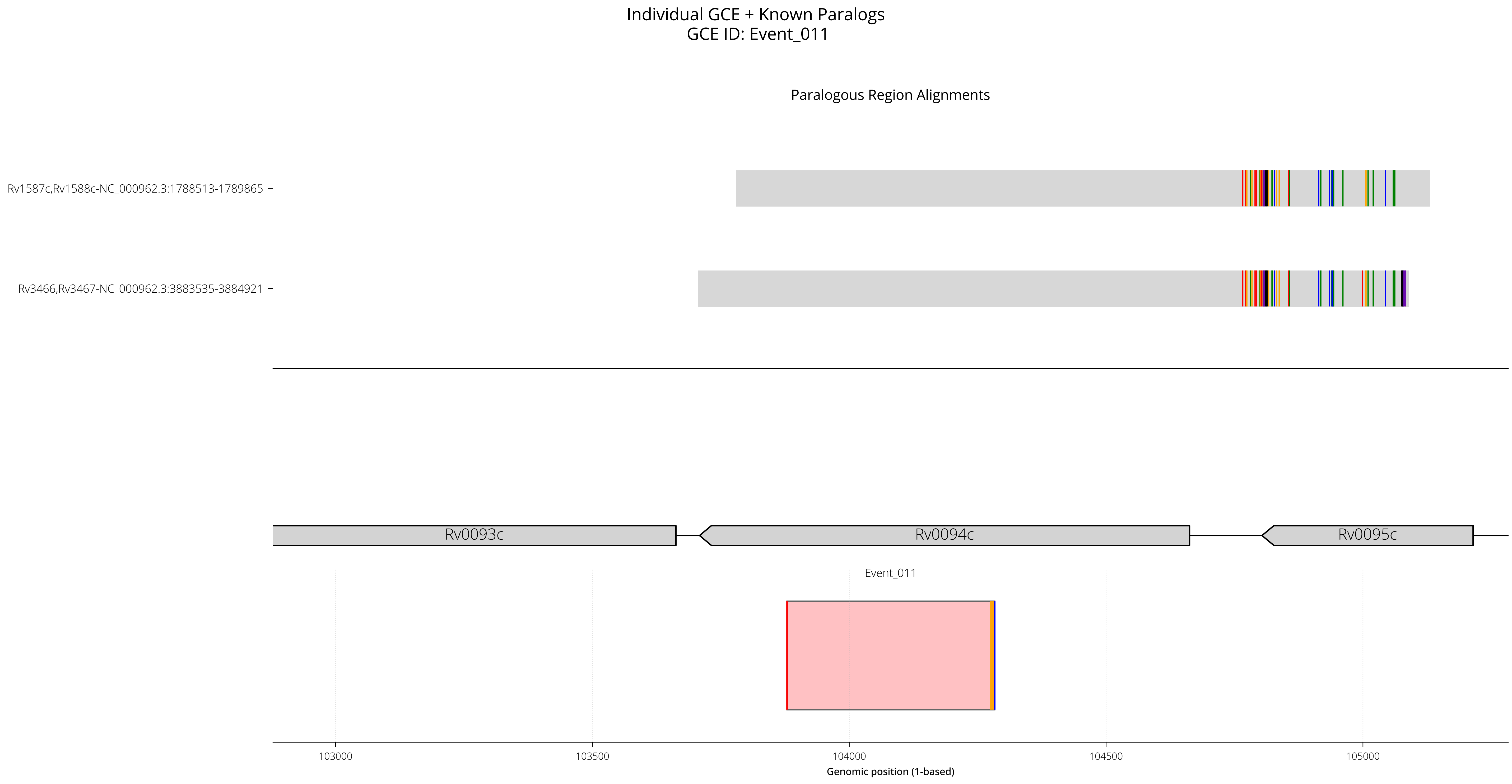
Individual GCE + Known Paralogs
GCE ID: Event_011
Paralogous Region Alignments
Rv1587c,Rv1588c-NC_000962.3:1788513-1789865
Rv3466,Rv3467-NC_000962.3:3883535-3884921
Rv0093c
Rv0094c
Rv0095c
Event_011
103000
103500
104000
104500
105000
Genomic position (1-based)

Circos Viz - GCE to Known Paralogs Locations
GCE ID: Event_011

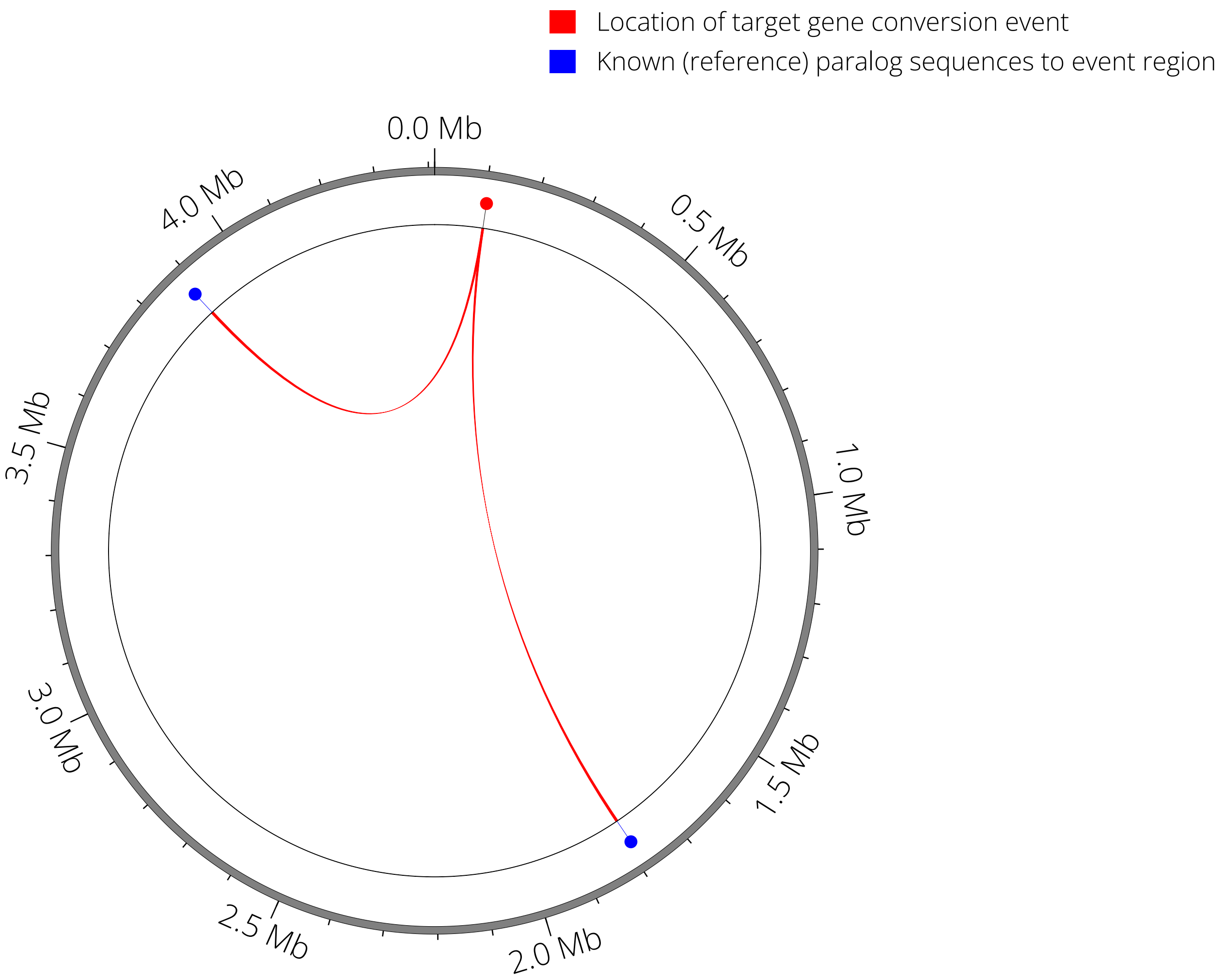

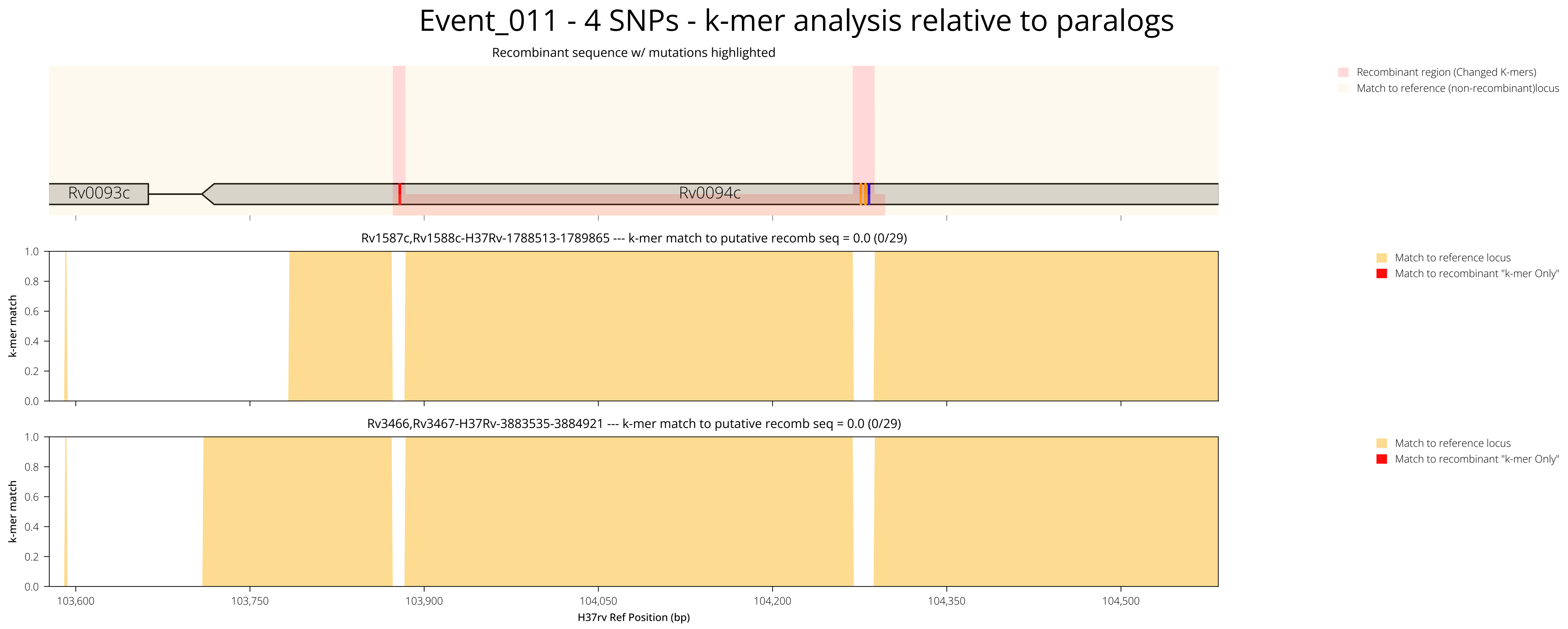

Event_011 - 4 SNPs - k-mer analysis relative to paralogs
Recombinant sequence w/ mutations highlighted
Recombinant region (Changed K-mers)
Match to reference (non-recombinant)locus
Rv0093c
Rv0094c
Rv1587c,Rv1588c-H37Rv-1788513-1789865 --- k-mer match to putative recomb seq = 0.0 (0/29)
Match to reference locus
Match to recombinant "k-mer Only"
k-mer match
Rv3466,Rv3467-H37Rv-3883535-3884921 --- k-mer match to putative recomb seq = 0.0 (0/29)
Match to reference locus
Match to recombinant "k-mer Only"
k-mer match
103,600
103,750
103,900
104,050
104,200
104,350
104,500
H37rv Ref Position (bp)

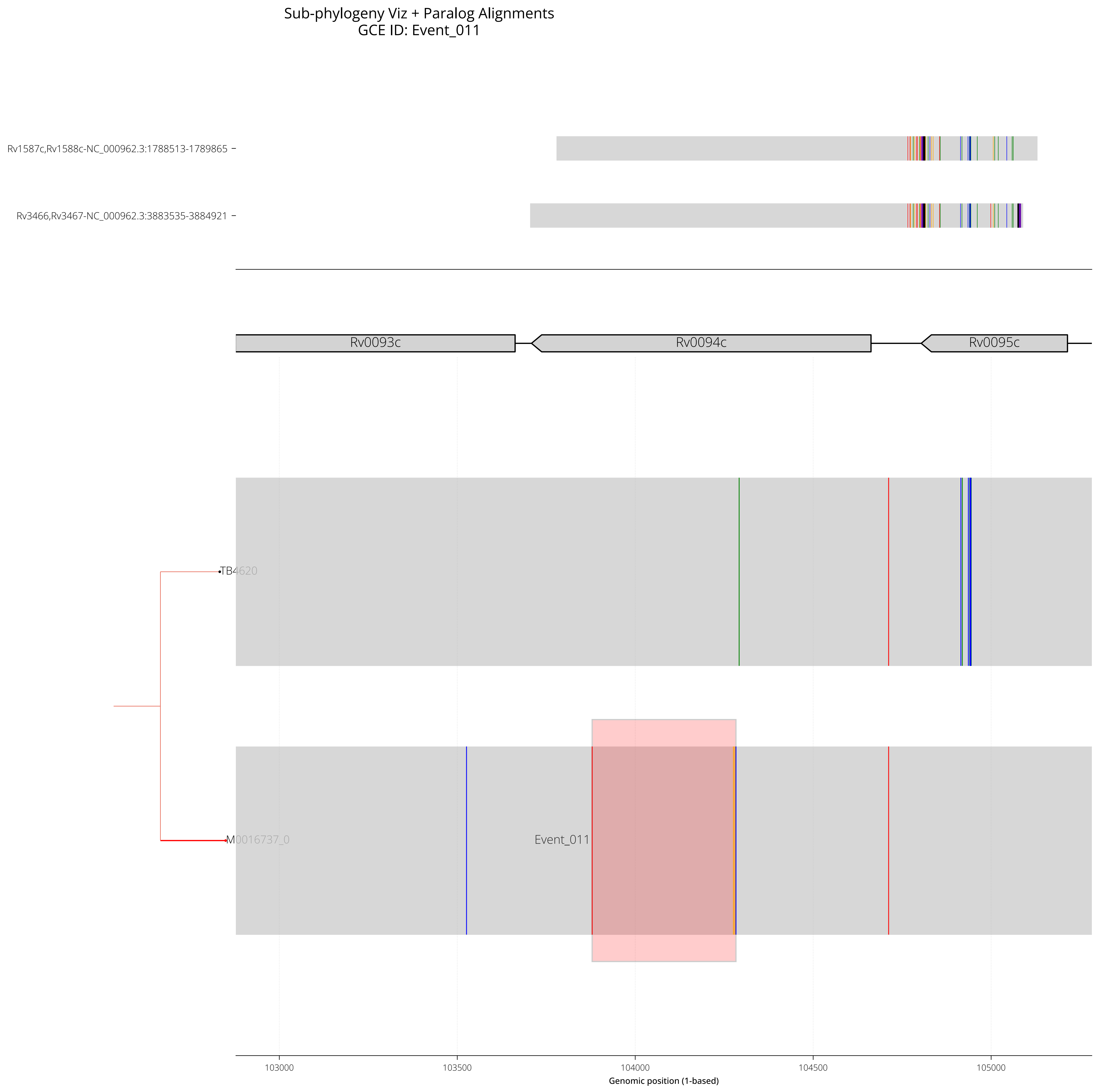

Sub-phylogeny Viz + Paralog Alignments
GCE ID: Event_011
Rv1587c,Rv1588c-NC_000962.3:1788513-1789865
Rv3466,Rv3467-NC_000962.3:3883535-3884921
Rv0093c
Rv0094c
Rv0095c
TB4620
M0016737_0
Event_011
103000
103500
104000
104500
105000
Genomic position (1-based)

Full Phylogeny Viz + Paralog Alignments
GCE ID: Event_011

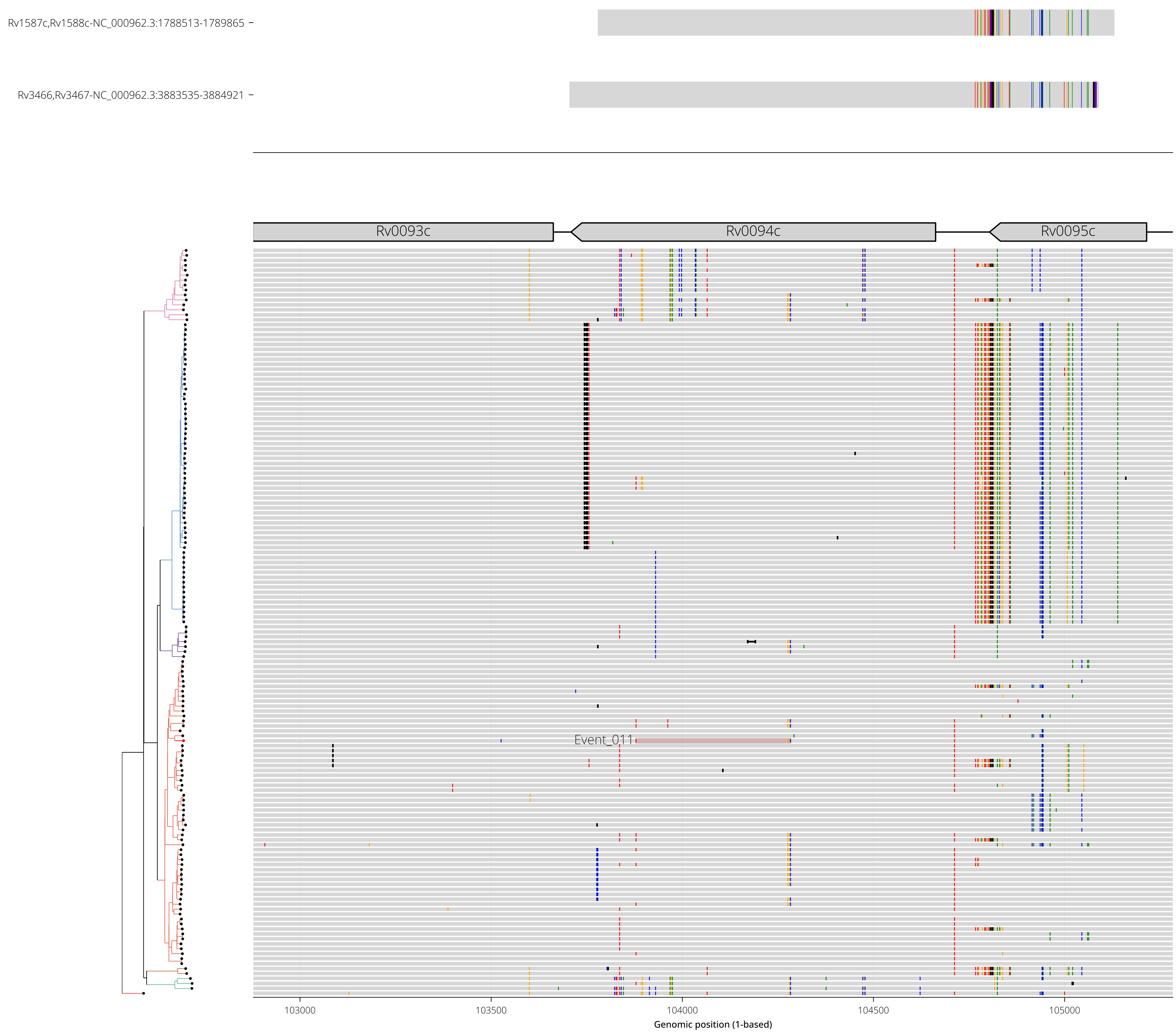

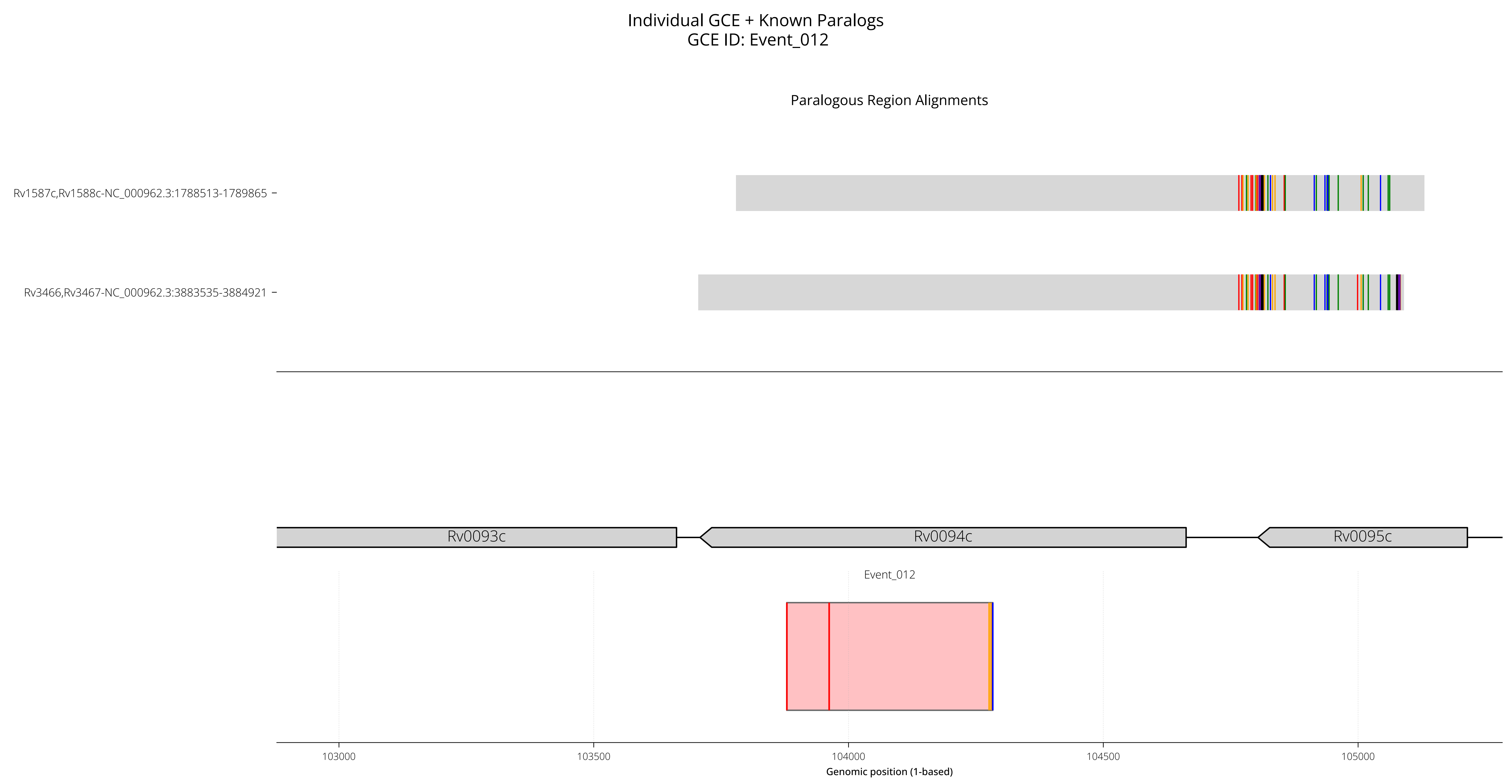
Individual GCE + Known Paralogs
GCE ID: Event_012
Paralogous Region Alignments
Rv1587c,Rv1588c-NC_000962.3:1788513-1789865
Rv3466,Rv3467-NC_000962.3:3883535-3884921
Rv0093c
Rv0094c
Rv0095c
Event_012
103000
103500
104000
104500
105000
Genomic position (1-based)

Circos Viz - GCE to Known Paralogs Locations
GCE ID: Event_012

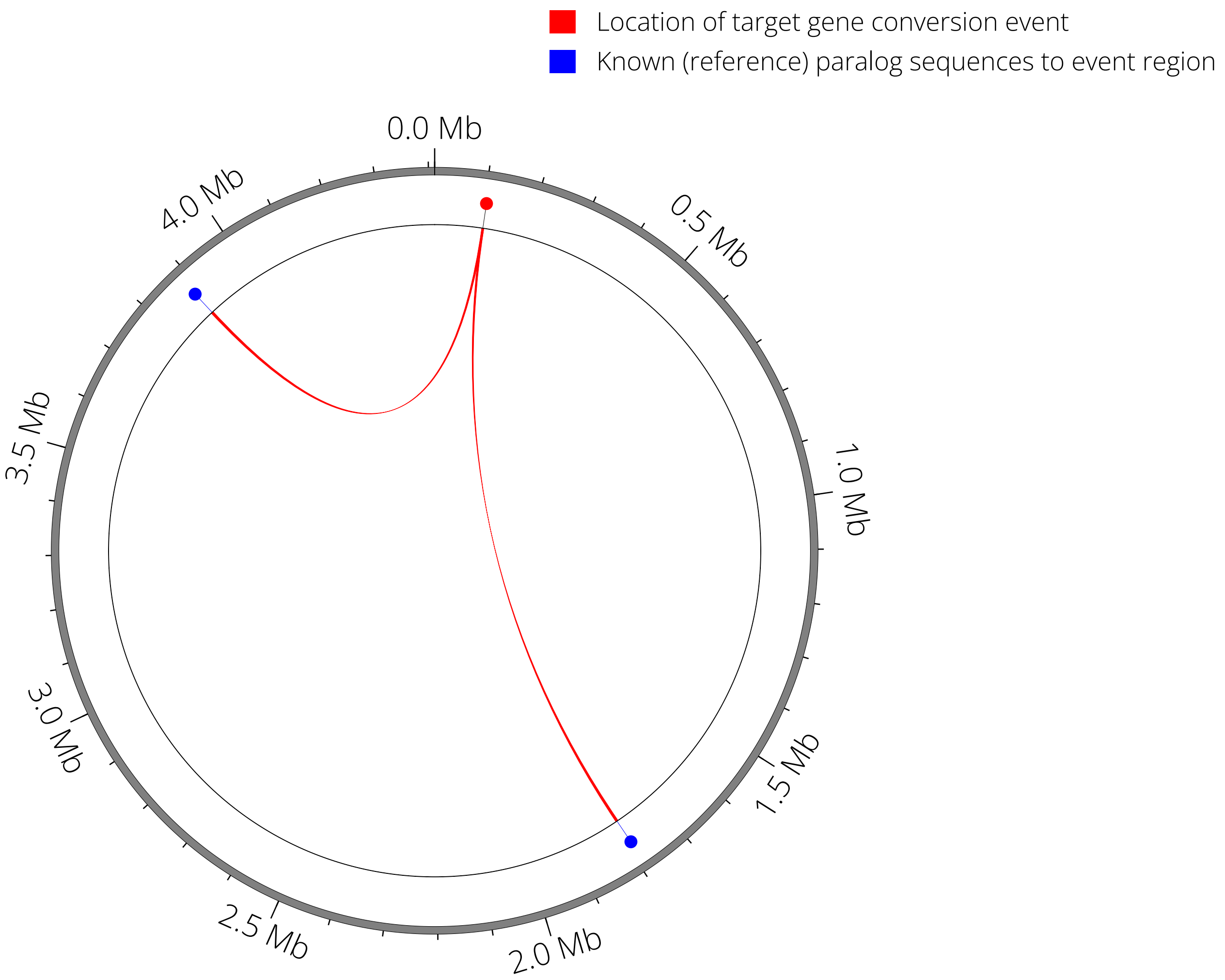

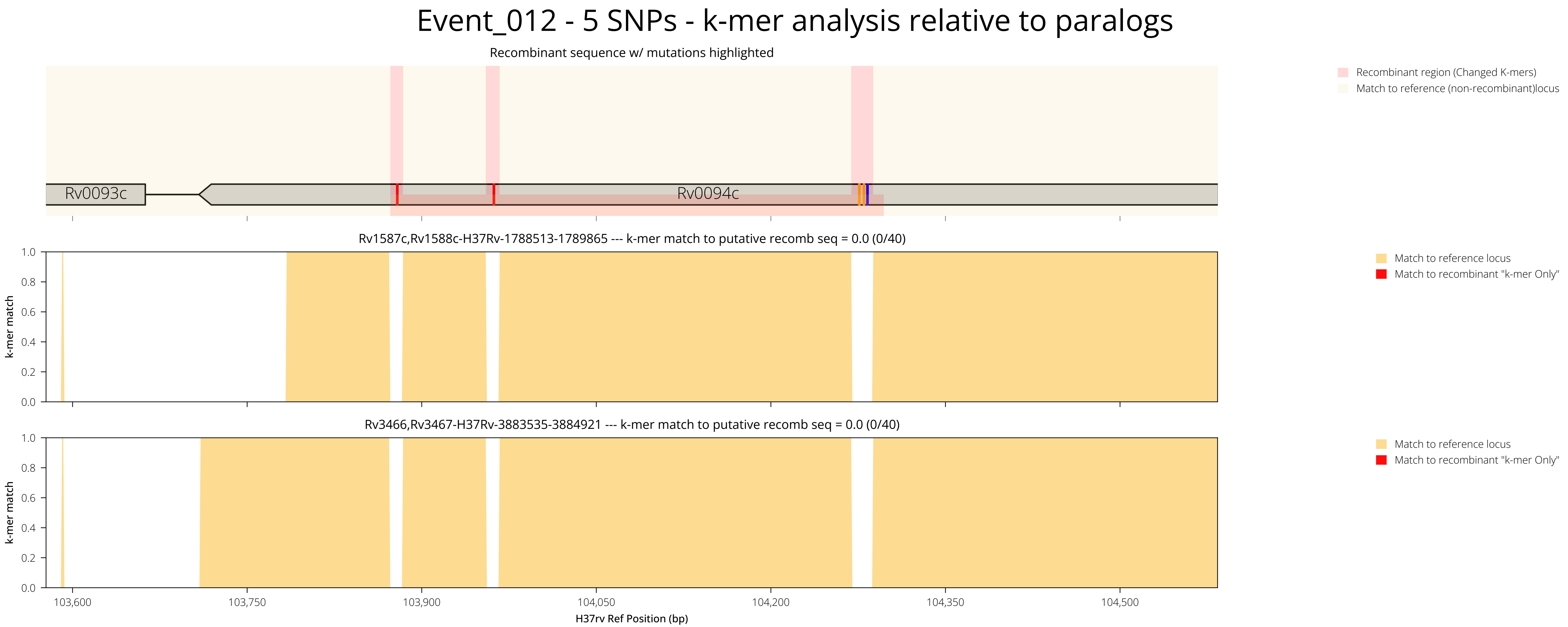

Event_012 - 5 SNPs - k-mer analysis relative to paralogs
Recombinant sequence w/ mutations highlighted
Recombinant region (Changed K-mers)
Match to reference (non-recombinant)locus
Rv0093c
Rv0094c
Rv1587c,Rv1588c-H37Rv-1788513-1789865 --- k-mer match to putative recomb seq = 0.0 (0/40)
Match to reference locus
Match to recombinant "k-mer Only"
k-mer match
1.0
0.8
0.6
0.4
0.2
0.0
Rv3466,Rv3467-H37Rv-3883535-3884921 --- k-mer match to putative recomb seq = 0.0 (0/40)
Match to reference locus
Match to recombinant "k-mer Only"
k-mer match
1.0
0.8
0.6
0.4
0.2
0.0
103,600
103,750
103,900
104,050
104,200
104,350
104,500
H37rv Ref Position (bp)

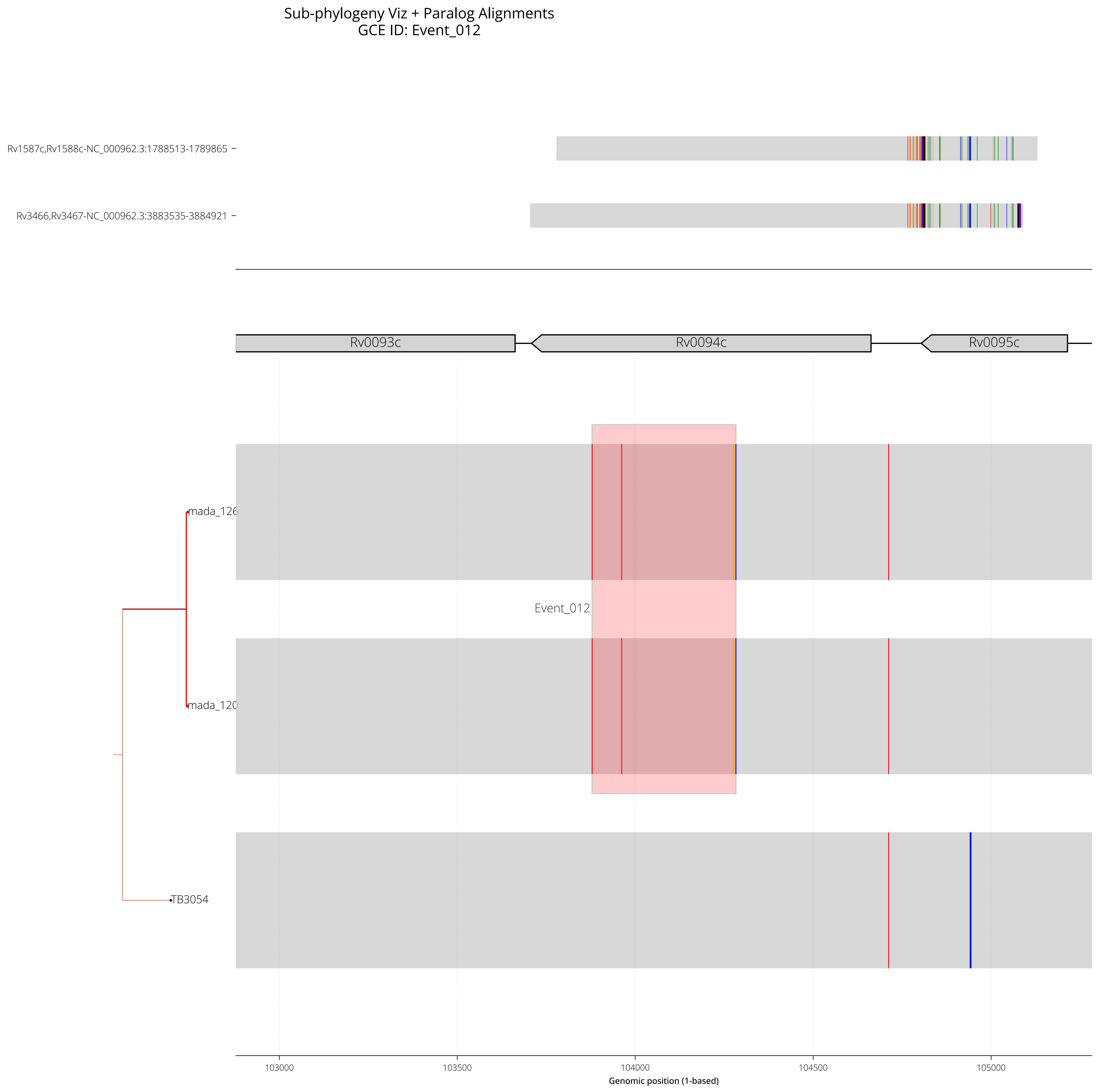

Sub-phylogeny Viz + Paralog Alignments
GCE ID: Event_012
Rv1587c,Rv1588c-NC_000962.3:1788513-1789865
Rv3466,Rv3467-NC_000962.3:3883535-3884921
Rv0093c
Rv0094c
Rv0095c
mada_126
mada_120
Event_012
TB3054
103000
103500
104000
104500
105000
Genomic position (1-based)

Full Phylogeny Viz + Paralog Alignments
GCE ID: Event_012

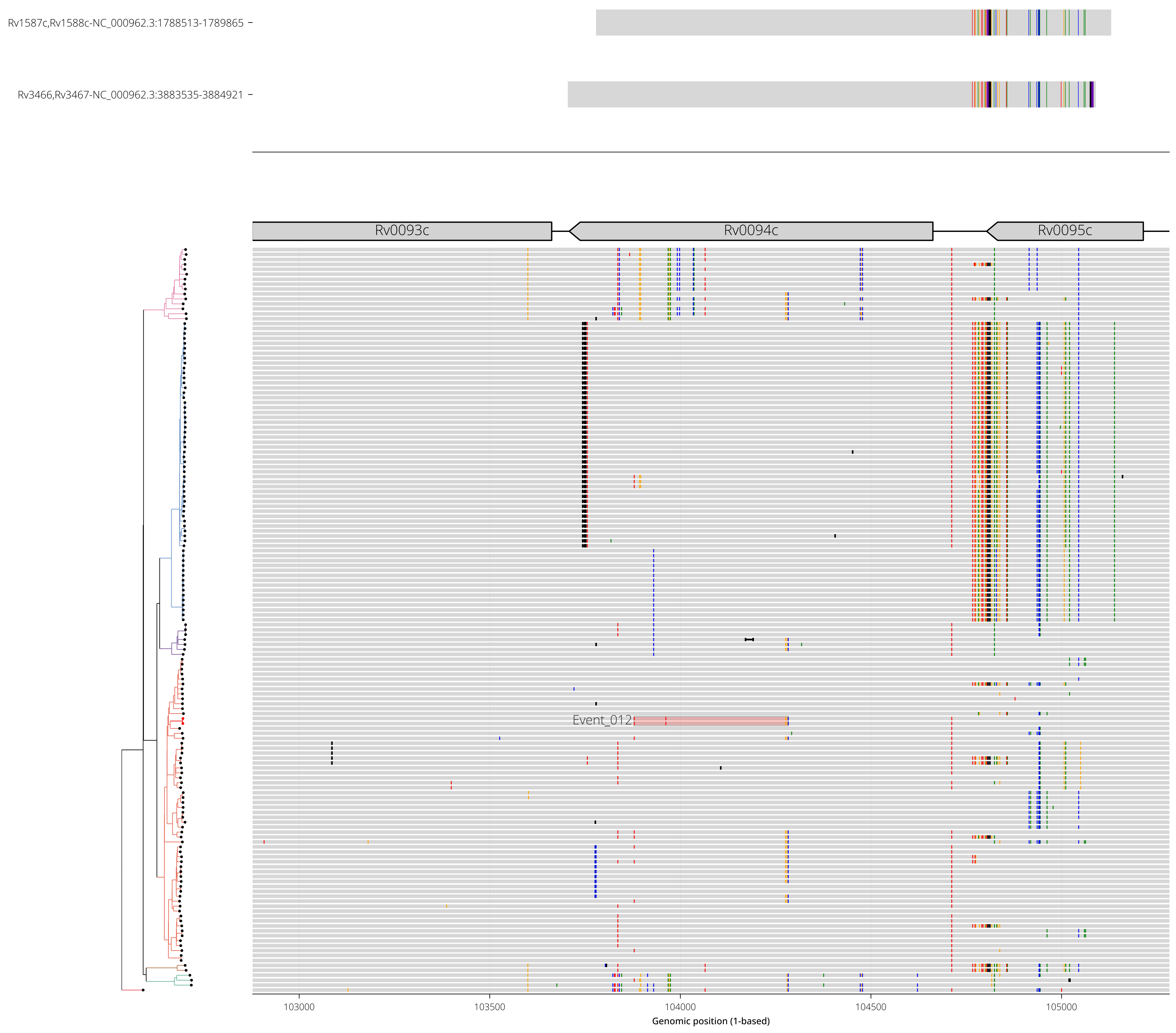

Individual GCE + Known Paralogs
GCE ID: Event_013

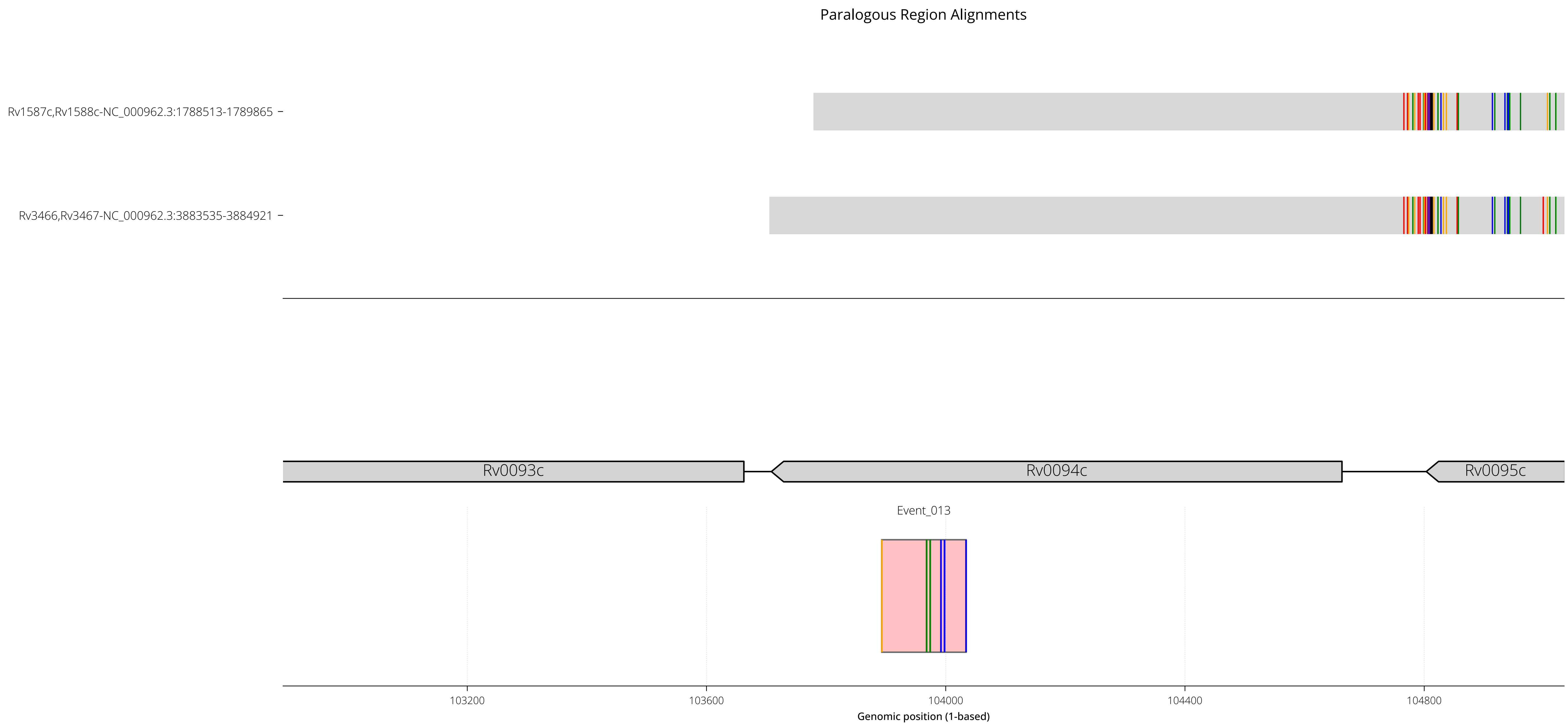

Circos Viz - GCE to Known Paralogs Locations
GCE ID: Event_013

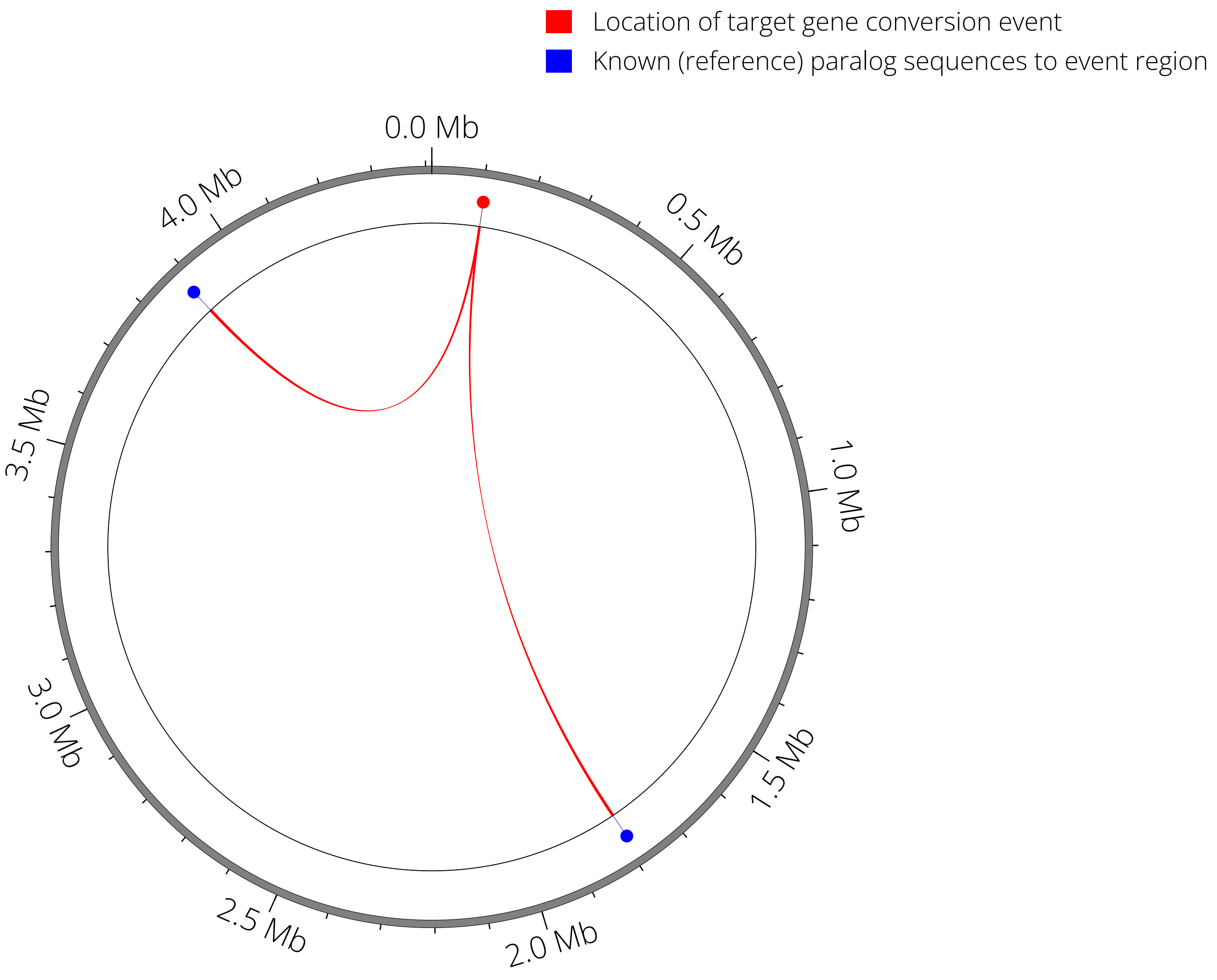

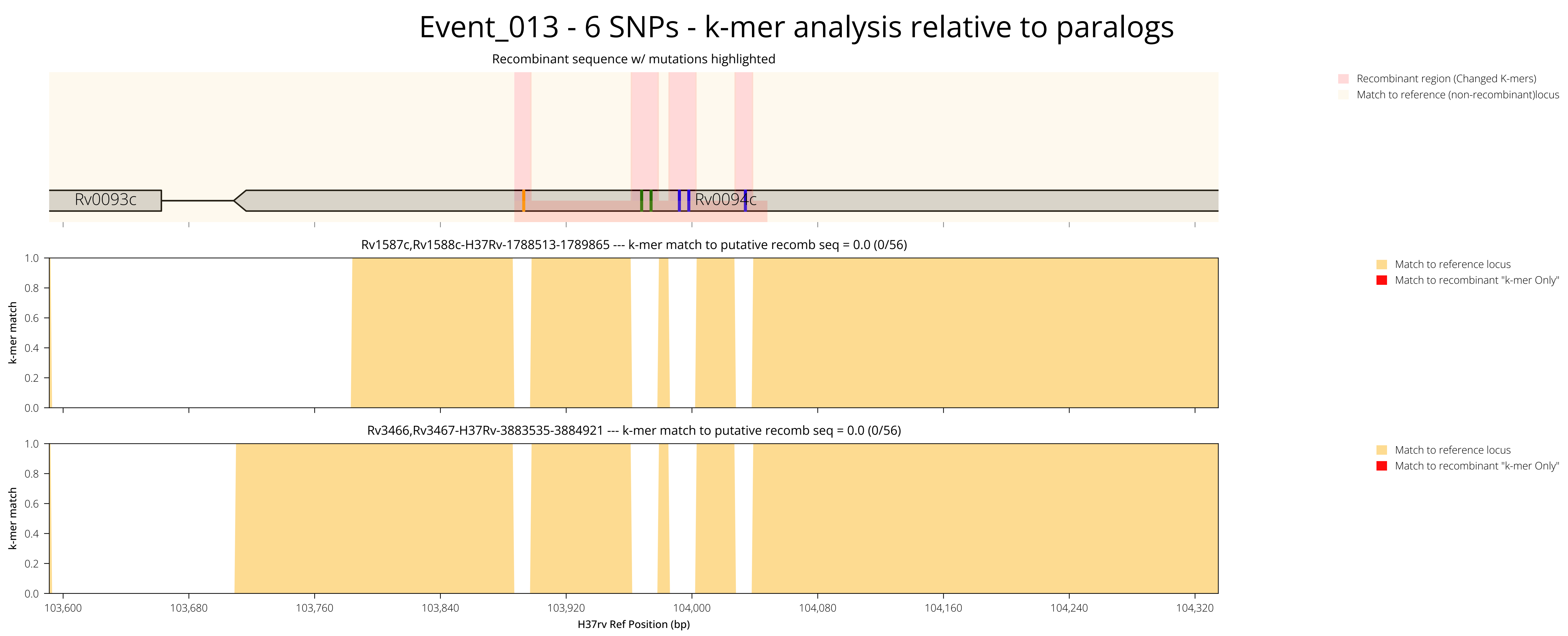
Event_013 - 6 SNPs - k-mer analysis relative to paralogs
Recombinant sequence w/ mutations highlighted
Rv0093c
Rv0094c
Recombinant region (Changed K-mers)
Match to reference (non-recombinant)locus
Rv1587c,Rv1588c-H37Rv-1788513-1789865 --- k-mer match to putative recomb seq = 0.0 (0/56)
Match to reference locus
Match to recombinant "k-mer Only"
Rv3466,Rv3467-H37Rv-3883535-3884921 --- k-mer match to putative recomb seq = 0.0 (0/56)
Match to reference locus
Match to recombinant "k-mer Only"
k-mer match
1.0
0.8
0.6
0.4
0.2
0.0
103,600
103,680
103,760
103,840
103,920
104,000
104,080
104,160
104,240
104,320
H37rv Ref Position (bp)

Sub-phylogeny Viz + Paralog Alignments
GCE ID: Event_013

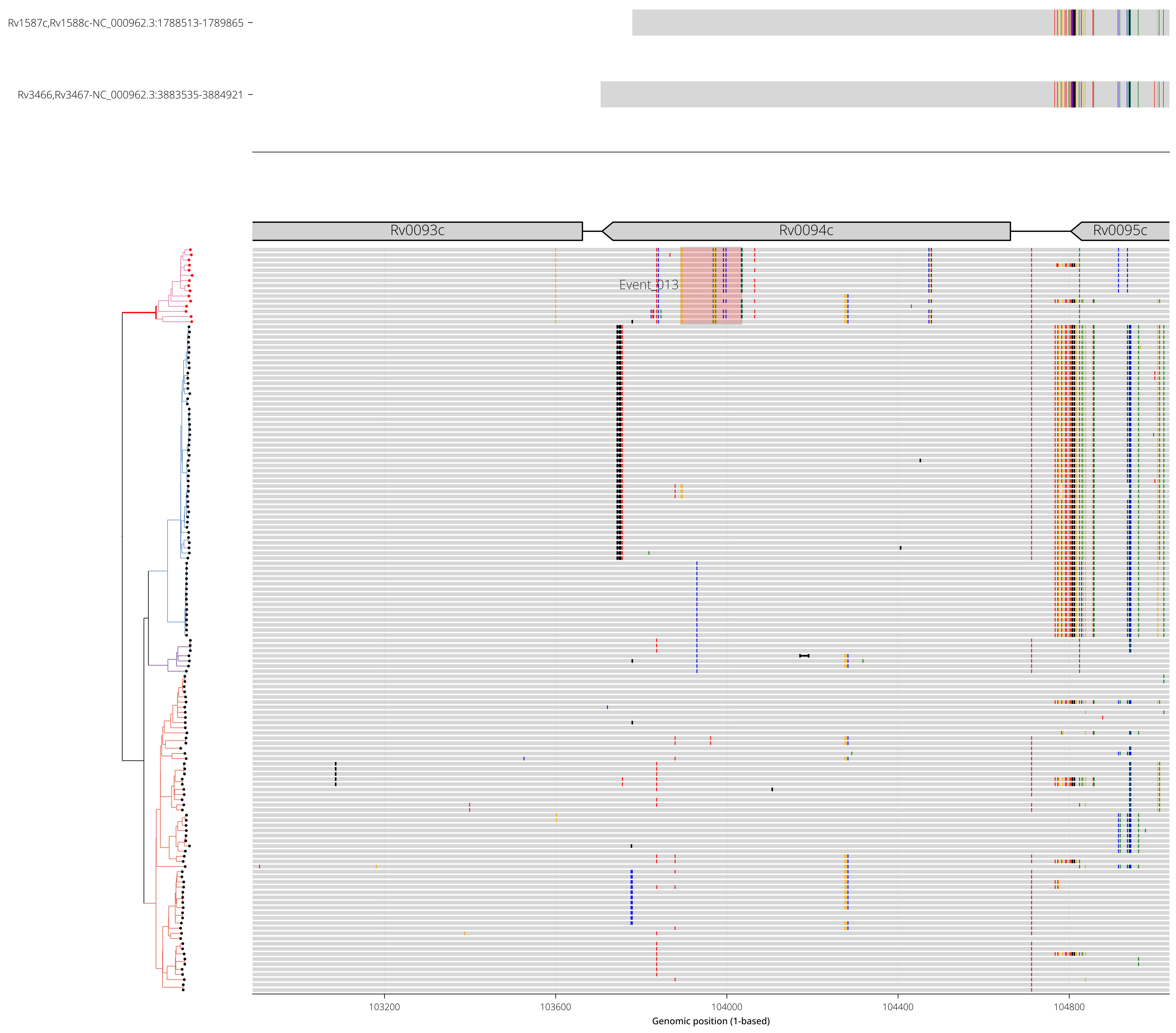

Full Phylogeny Viz + Paralog Alignments
GCE ID: Event_013

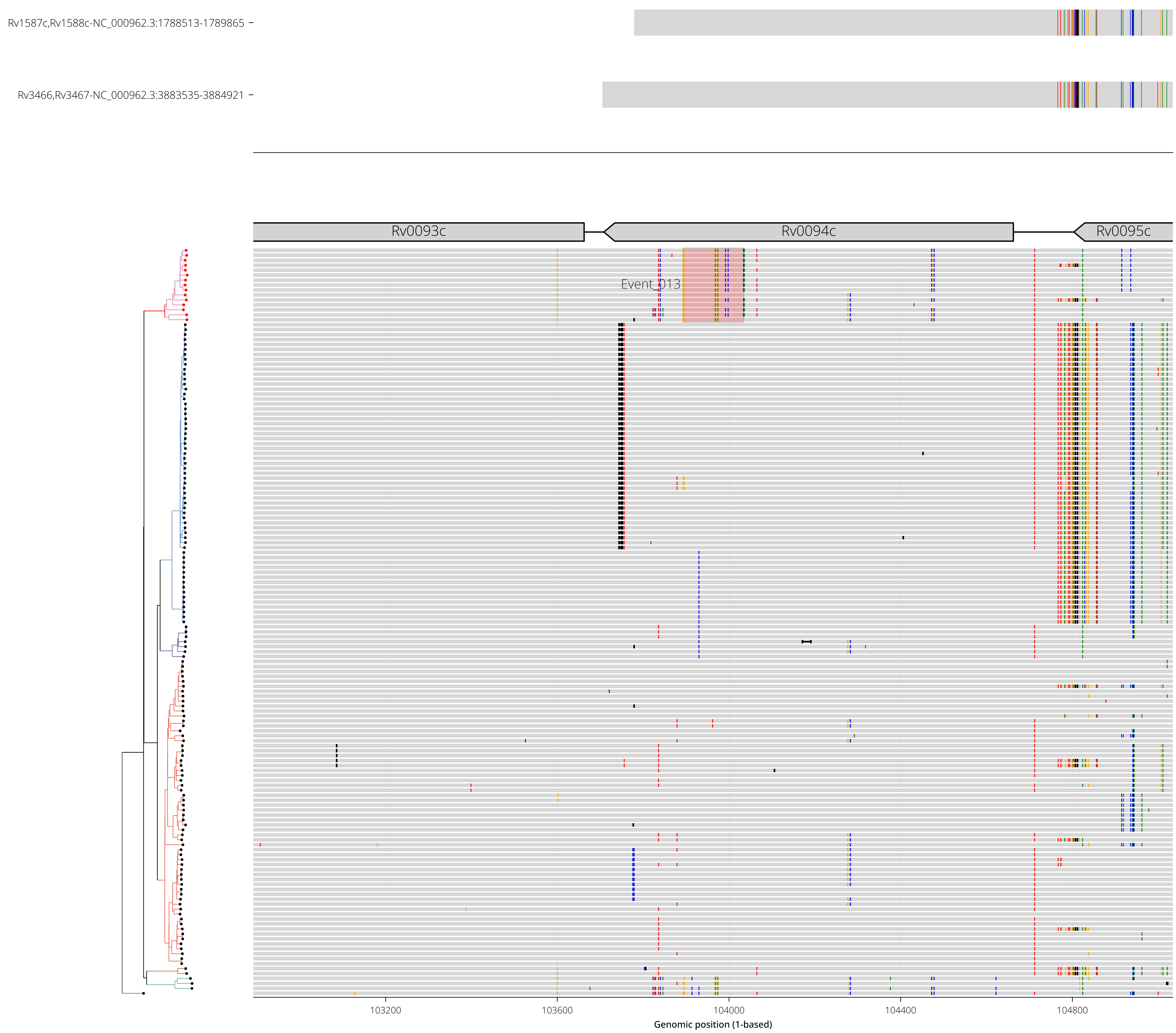

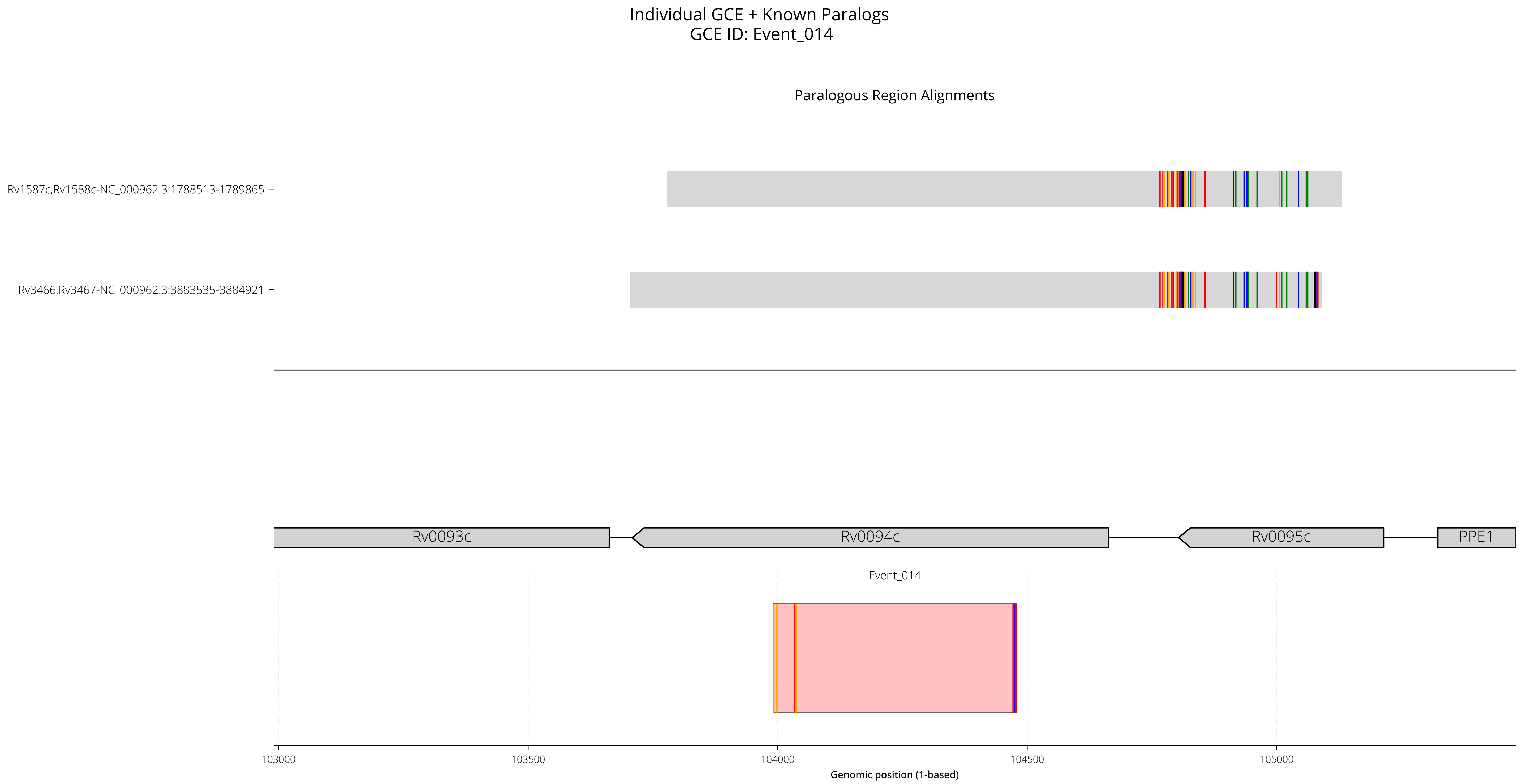

Individual GCE + Known Paralogs
GCE ID: Event_014
Paralogous Region Alignments
Rv1587c,Rv1588c-NC_000962.3:1788513-1789865
Rv3466,Rv3467-NC_000962.3:3883535-3884921
Rv0093c
Rv0094c
Rv0095c
PPE1
Event_014
103000
103500
104000
104500
105000
Genomic position (1-based)

Circos Viz - GCE to Known Paralogs Locations
GCE ID: Event_014

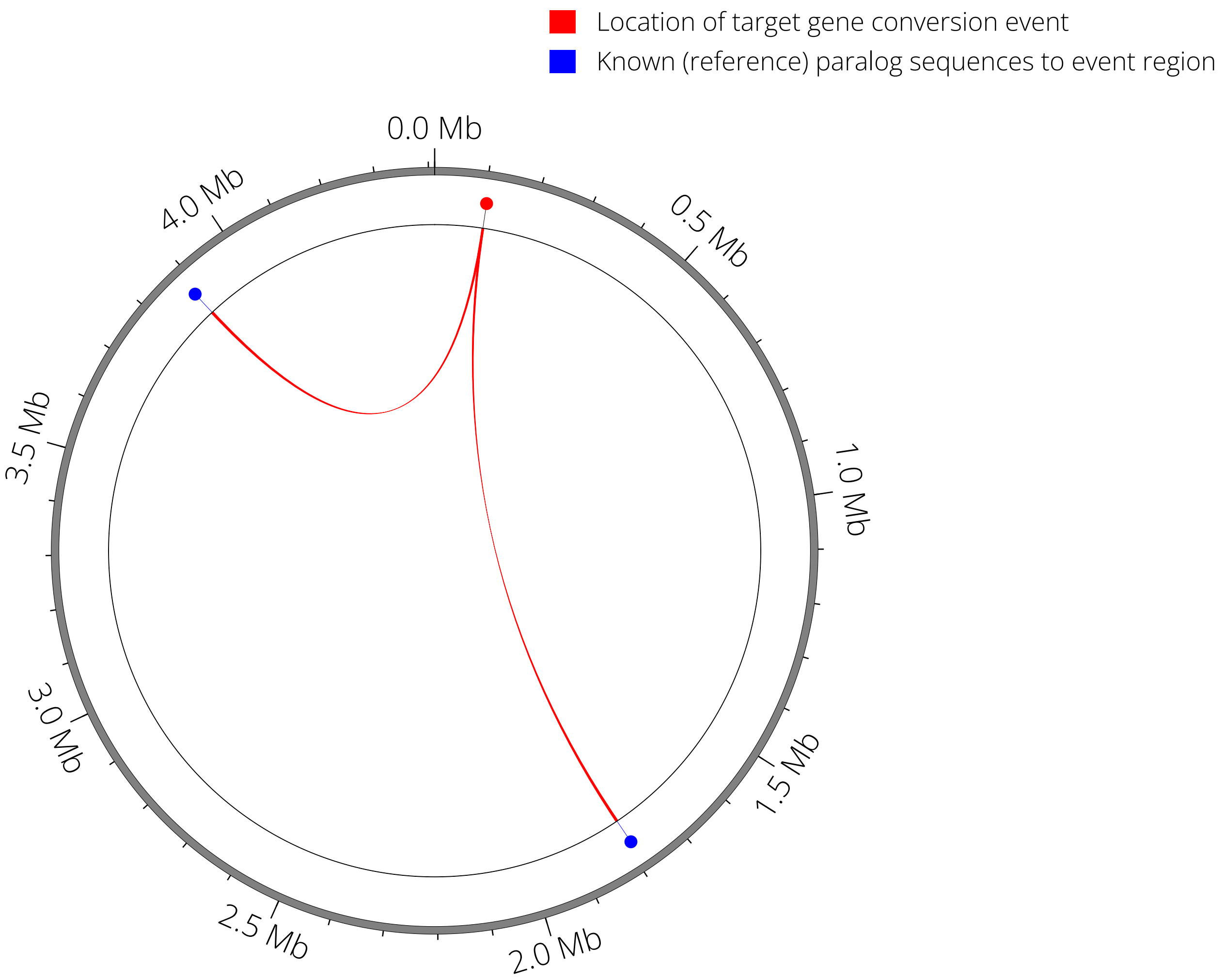

# Event_014 - 7 SNPs - k-mer analysis relative to paralogs

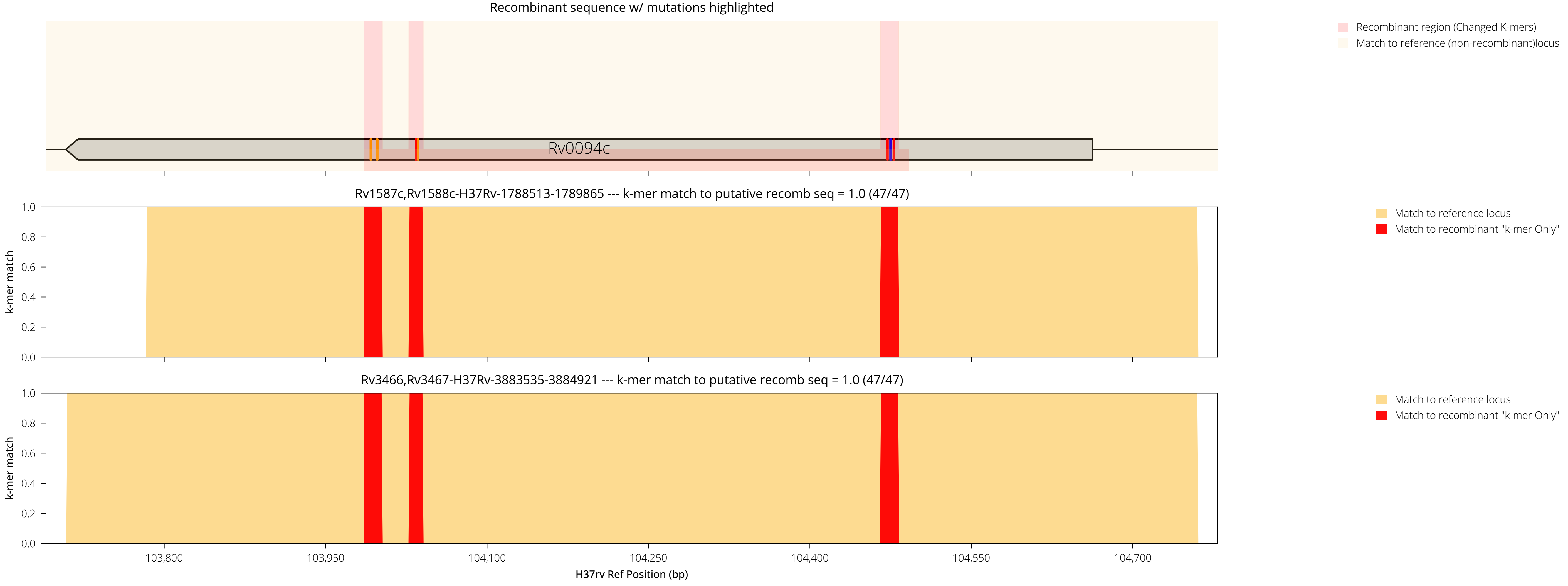

Sub-phylogeny Viz + Paralog Alignments
GCE ID: Event_014

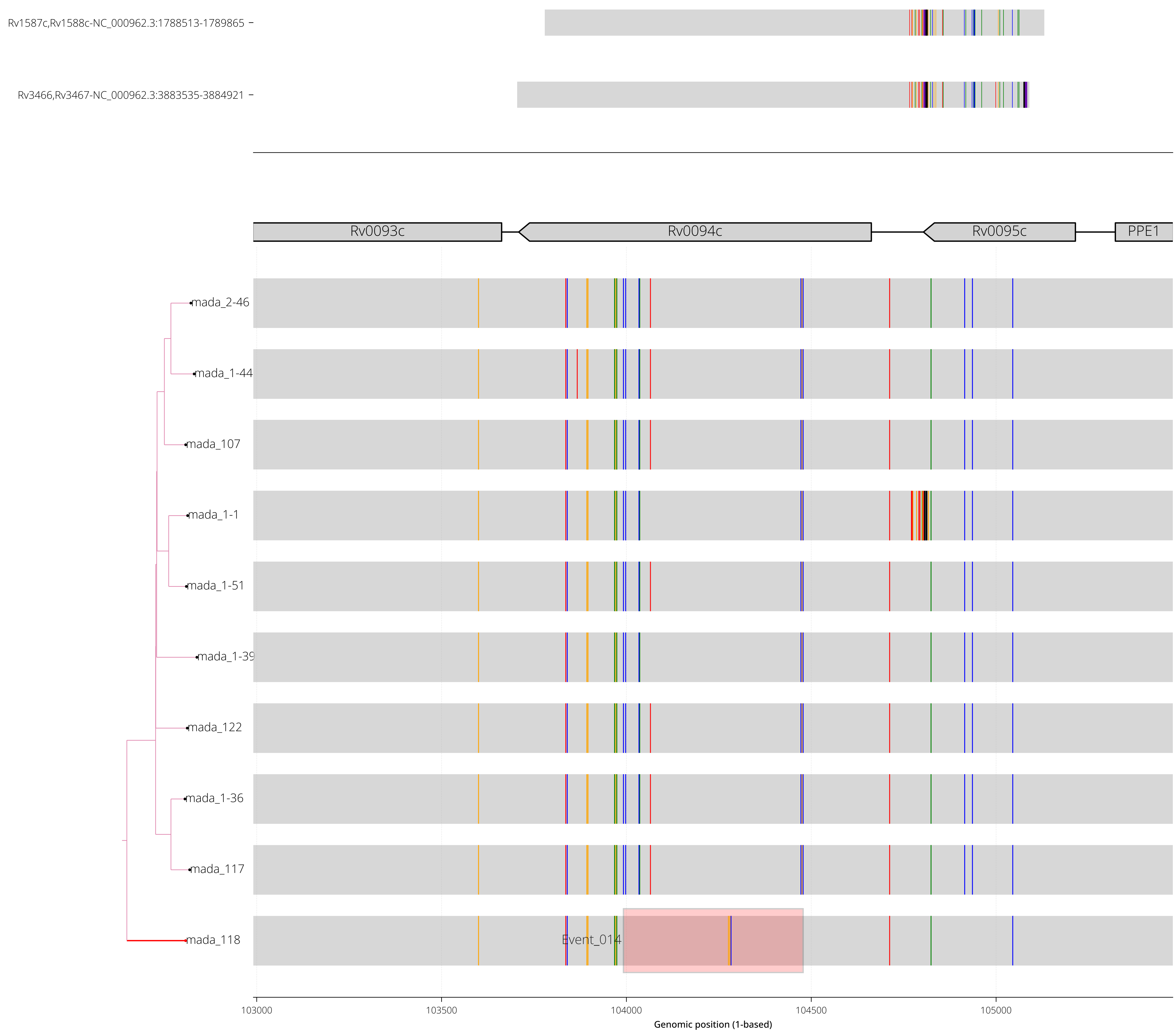

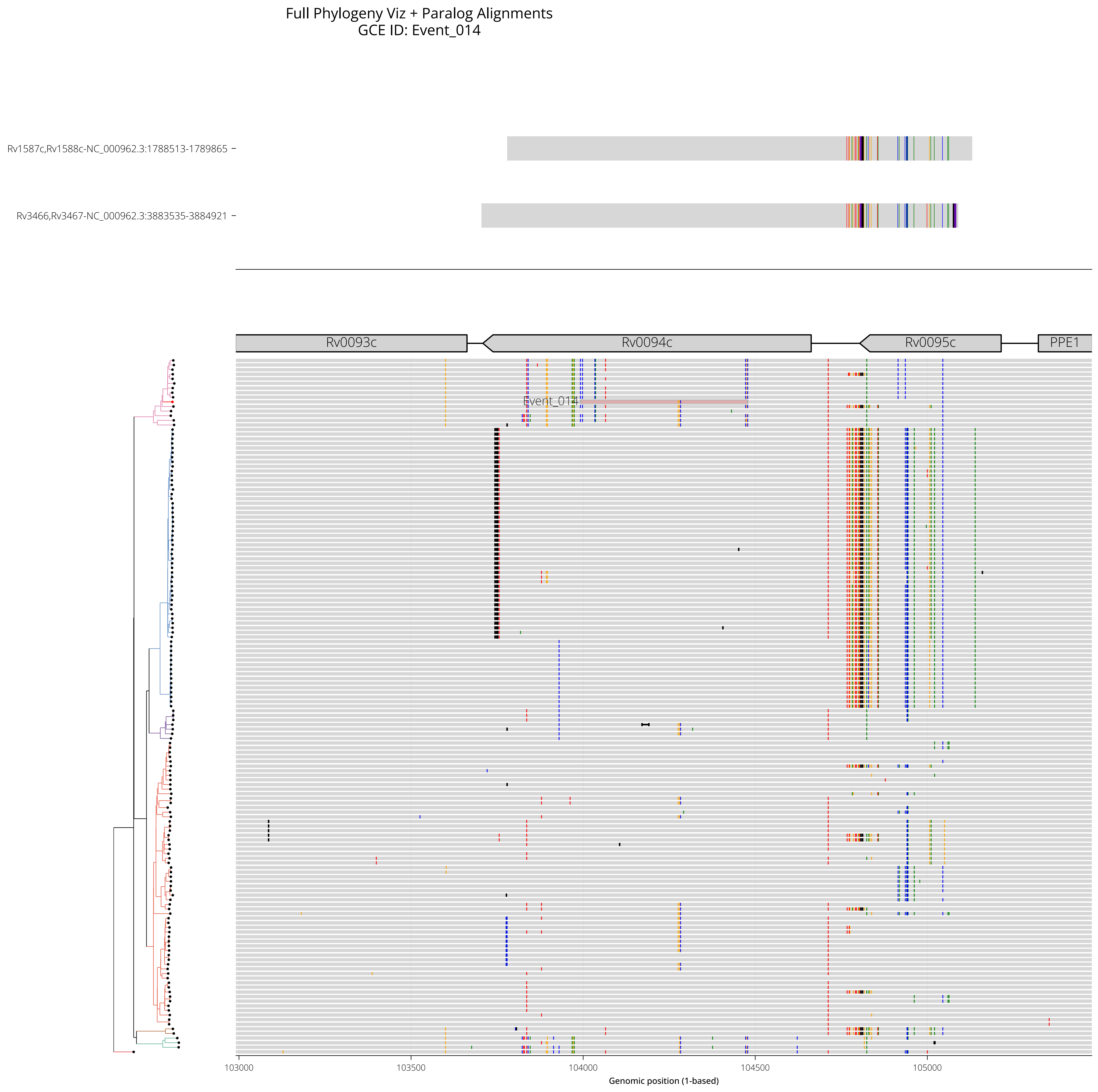

Full Phylogeny Viz + Paralog Alignments
GCE ID: Event_014
Rv1587c,Rv1588c-NC_000962.3:1788513-1789865
Rv3466,Rv3467-NC_000962.3:3883535-3884921
Rv0093c
Rv0094c
Rv0095c
PPE1
Event_014
103000
103500
104000
104500
105000
Genomic position (1-based)

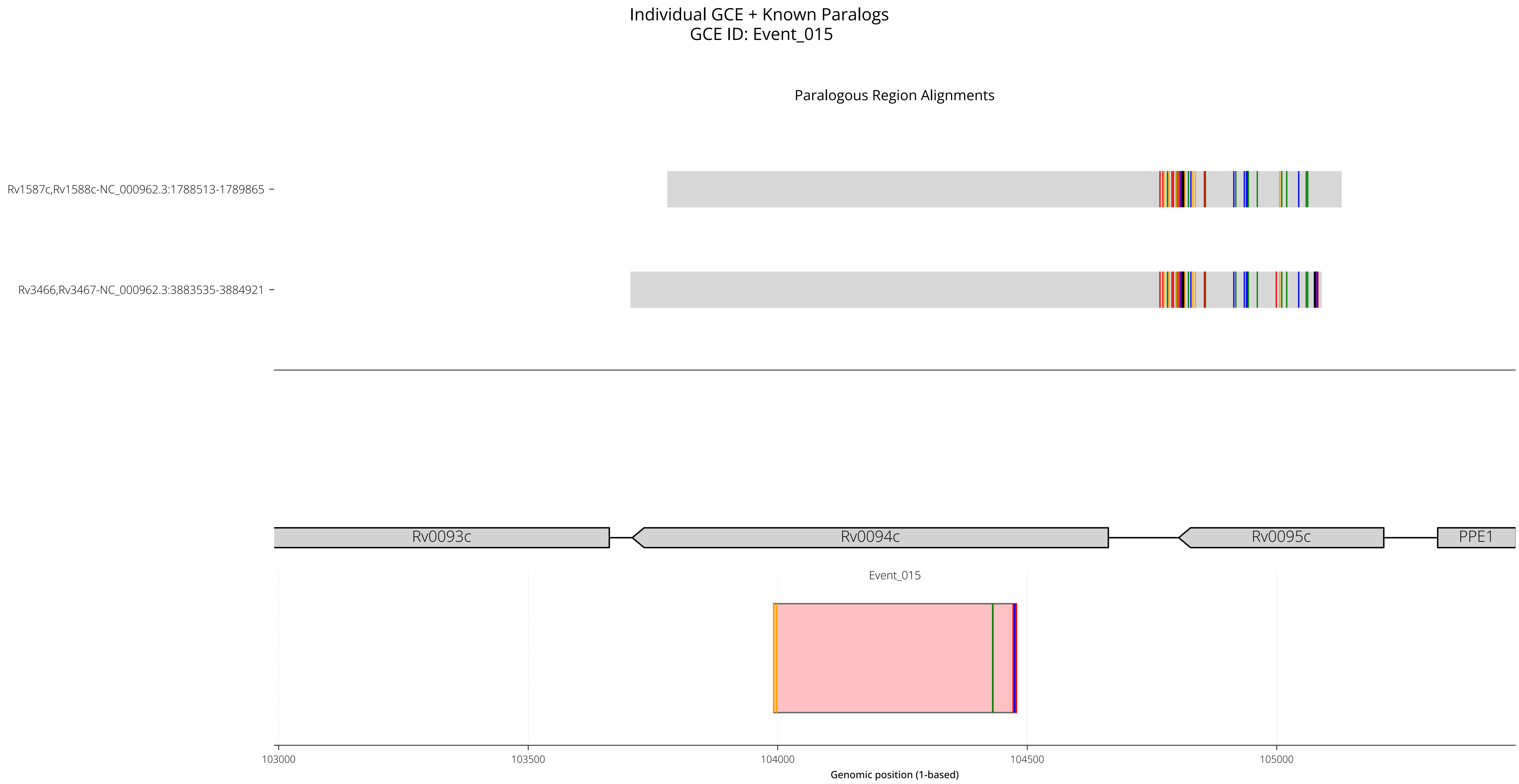
Individual GCE + Known Paralogs
GCE ID: Event_015
Paralogous Region Alignments
Rv1587c,Rv1588c-NC_000962.3:1788513-1789865
Rv3466,Rv3467-NC_000962.3:3883535-3884921
Rv0093c
Rv0094c
Rv0095c
PPE1
Event_015
103000
103500
104000
104500
105000
Genomic position (1-based)

# Circos Viz - GCE to Known Paralogs Locations
GCE ID: Event_015

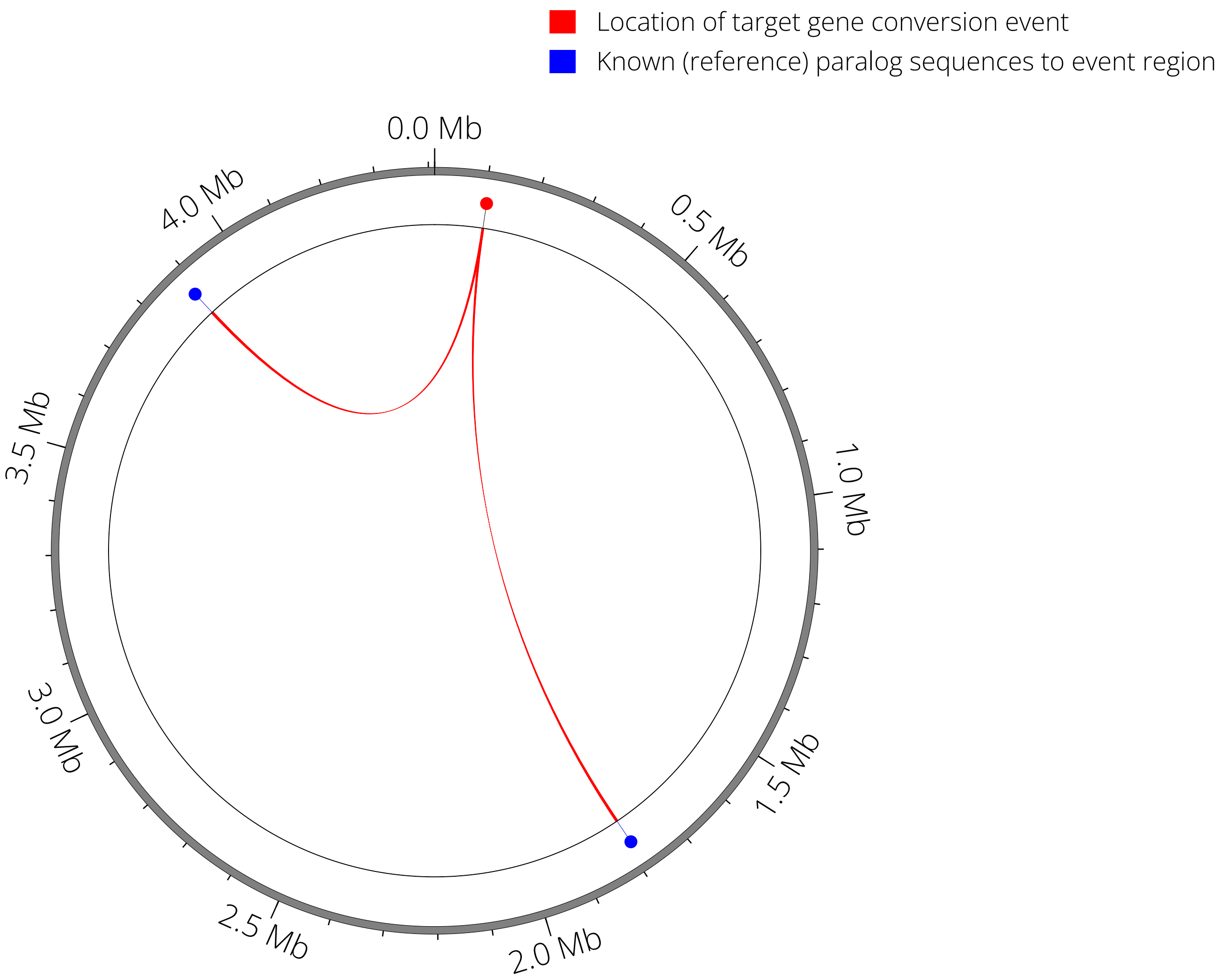

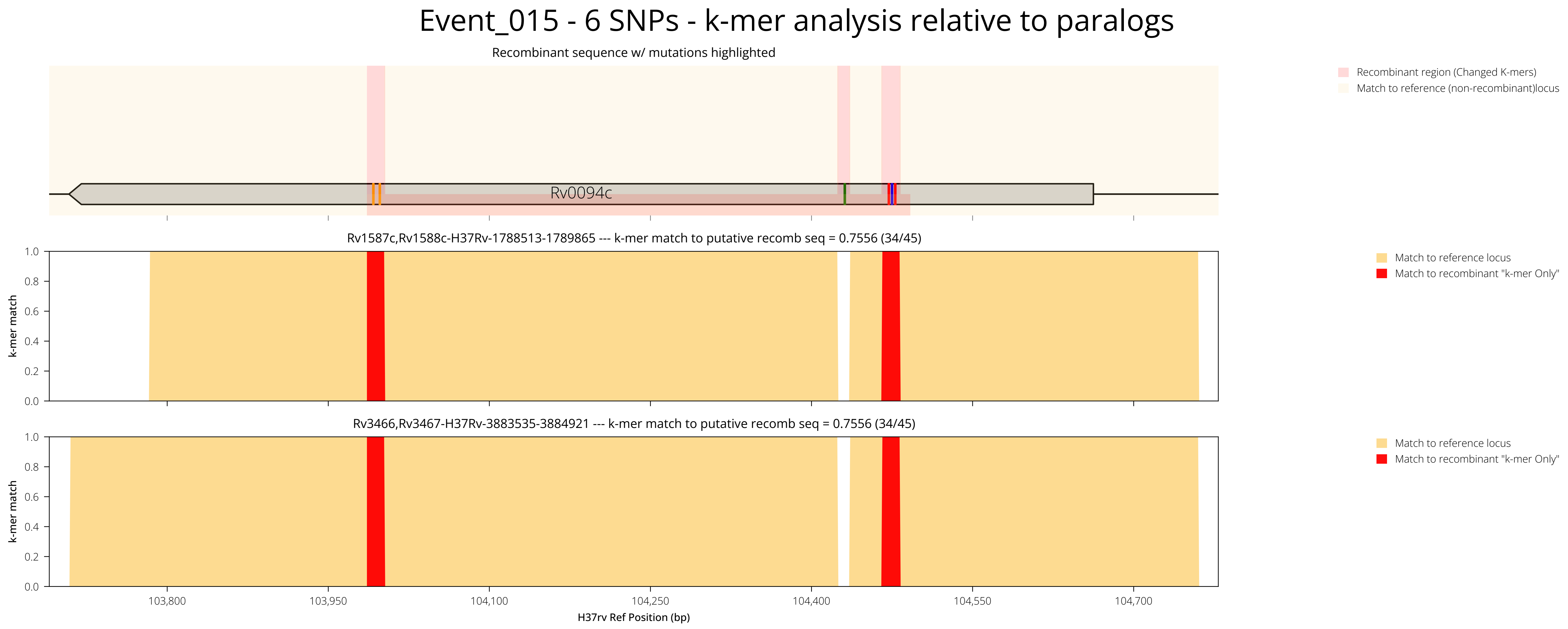
Event_015 - 6 SNPs - k-mer analysis relative to paralogs
Recombinant sequence w/ mutations highlighted
Recombinant region (Changed K-mers)
Match to reference (non-recombinant)locus
Rv0094c
Rv1587c,Rv1588c-H37Rv-1788513-1789865 --- k-mer match to putative recomb seq = 0.7556 (34/45)
Match to reference locus
Match to recombinant "k-mer Only"
k-mer match
1.0
0.8
0.6
0.4
0.2
0.0
Rv3466,Rv3467-H37Rv-3883535-3884921 --- k-mer match to putative recomb seq = 0.7556 (34/45)
Match to reference locus
Match to recombinant "k-mer Only"
k-mer match
1.0
0.8
0.6
0.4
0.2
0.0
103,800
103,950
104,100
104,250
104,400
104,550
104,700
H37rv Ref Position (bp)

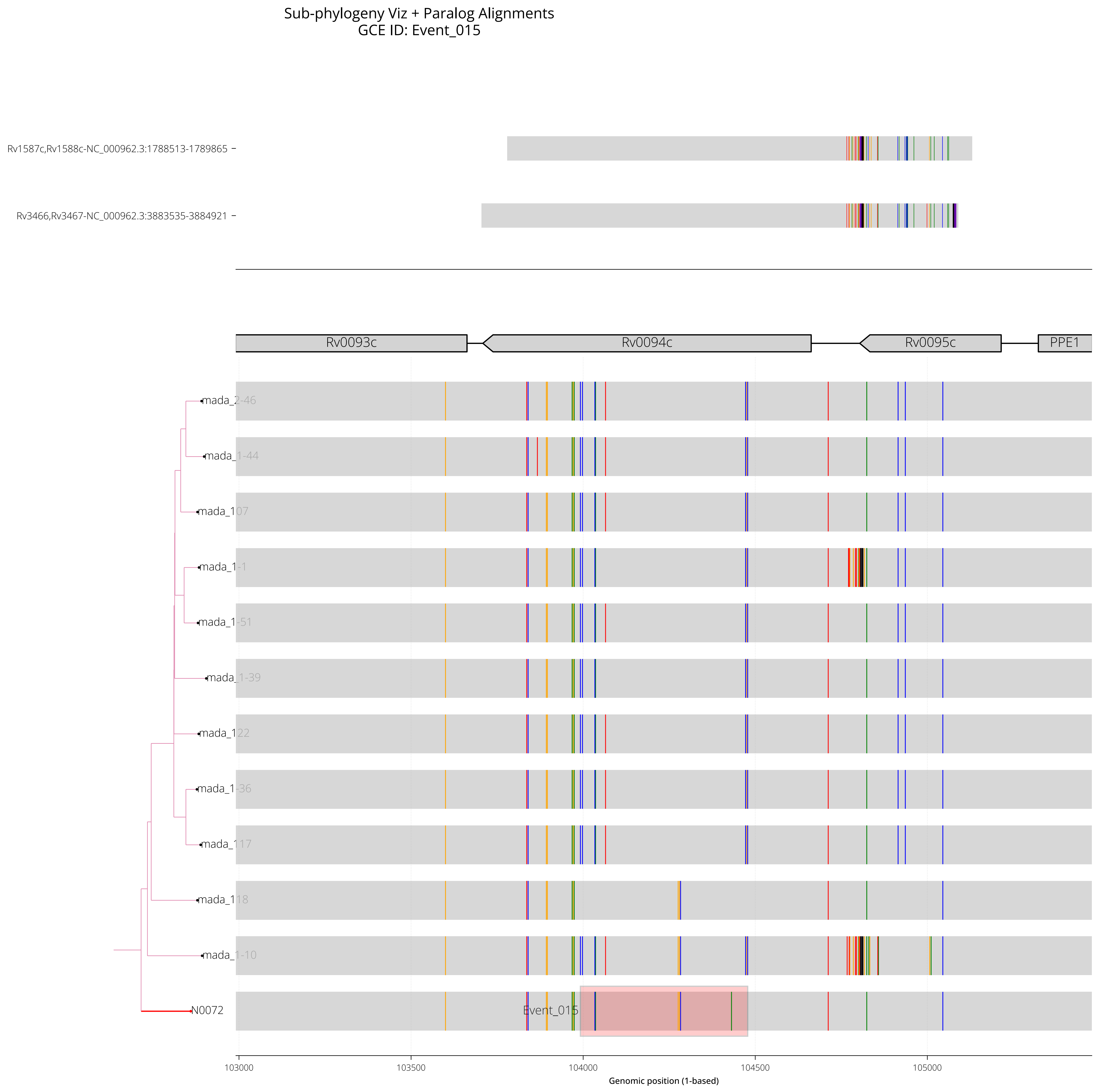

Sub-phylogeny Viz + Paralog Alignments
GCE ID: Event_015
Rv1587c,Rv1588c-NC_000962.3:1788513-1789865
Rv3466,Rv3467-NC_000962.3:3883535-3884921
Rv0093c
Rv0094c
Rv0095c
PPE1
mada_2-46
mada_1-44
mada_107
mada_1-1
mada_1-51
mada_1-39
mada_122
mada_1-36
mada_117
mada_118
mada_1-10
N0072
Event_015
103000
103500
104000
104500
105000
Genomic position (1-based)

Full Phylogeny Viz + Paralog Alignments
GCE ID: Event_015

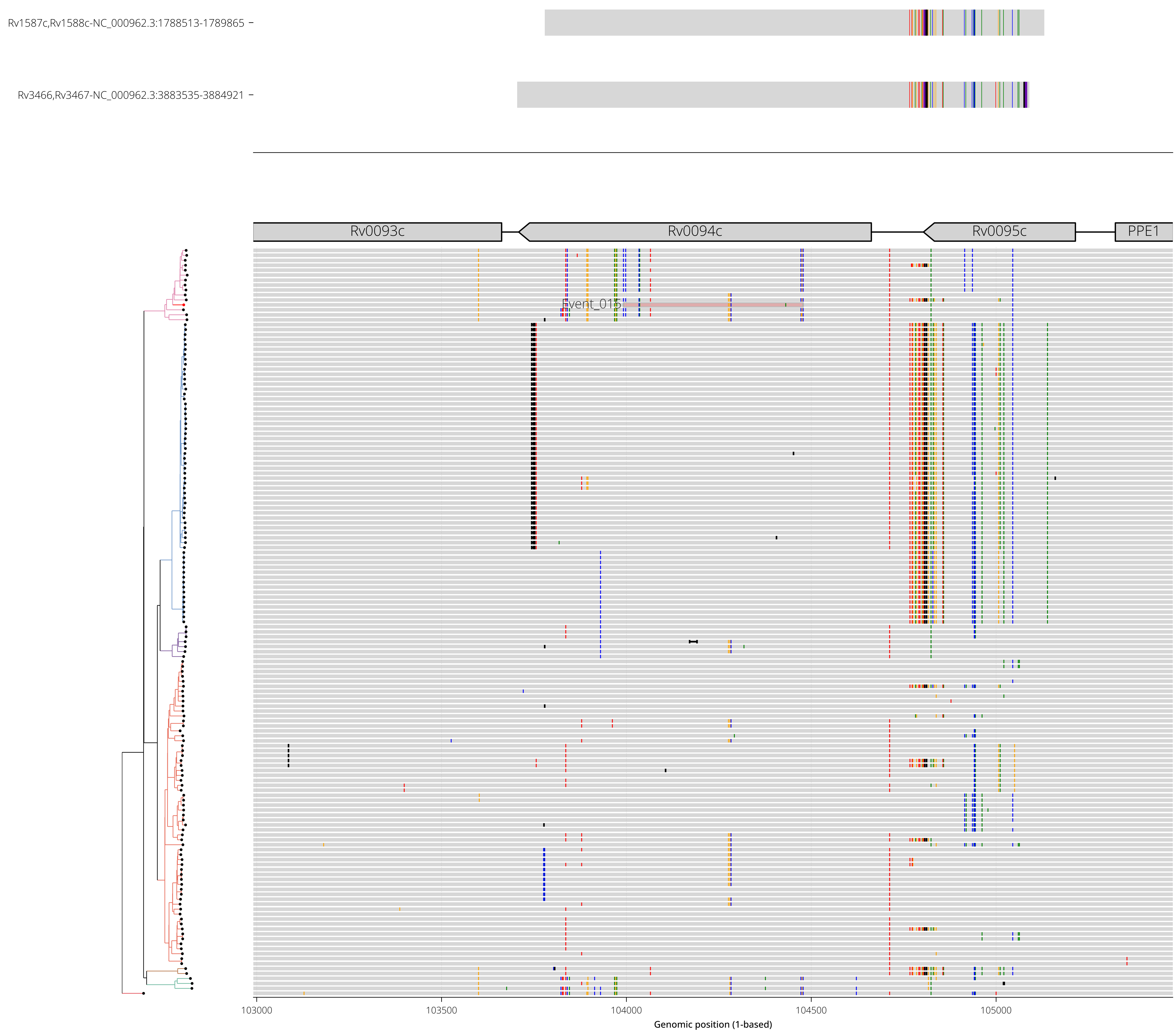

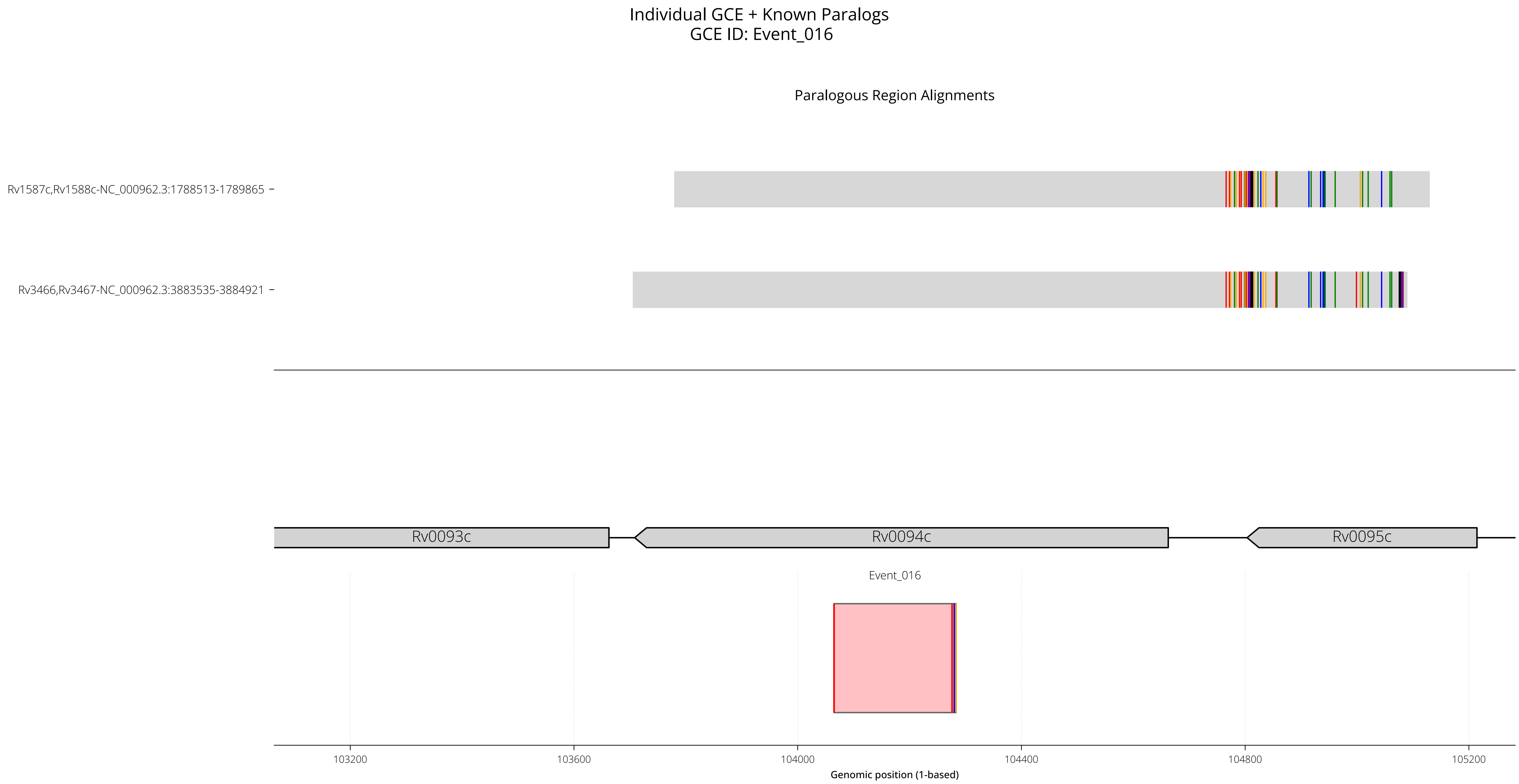

Individual GCE + Known Paralogs
GCE ID: Event_016
Paralogous Region Alignments
Rv1587c,Rv1588c-NC_000962.3:1788513-1789865
Rv3466,Rv3467-NC_000962.3:3883535-3884921
Rv0093c
Rv0094c
Rv0095c
Event_016
103200
103600
104000
104400
104800
105200
Genomic position (1-based)

Circos Viz - GCE to Known Paralogs Locations
GCE ID: Event_016

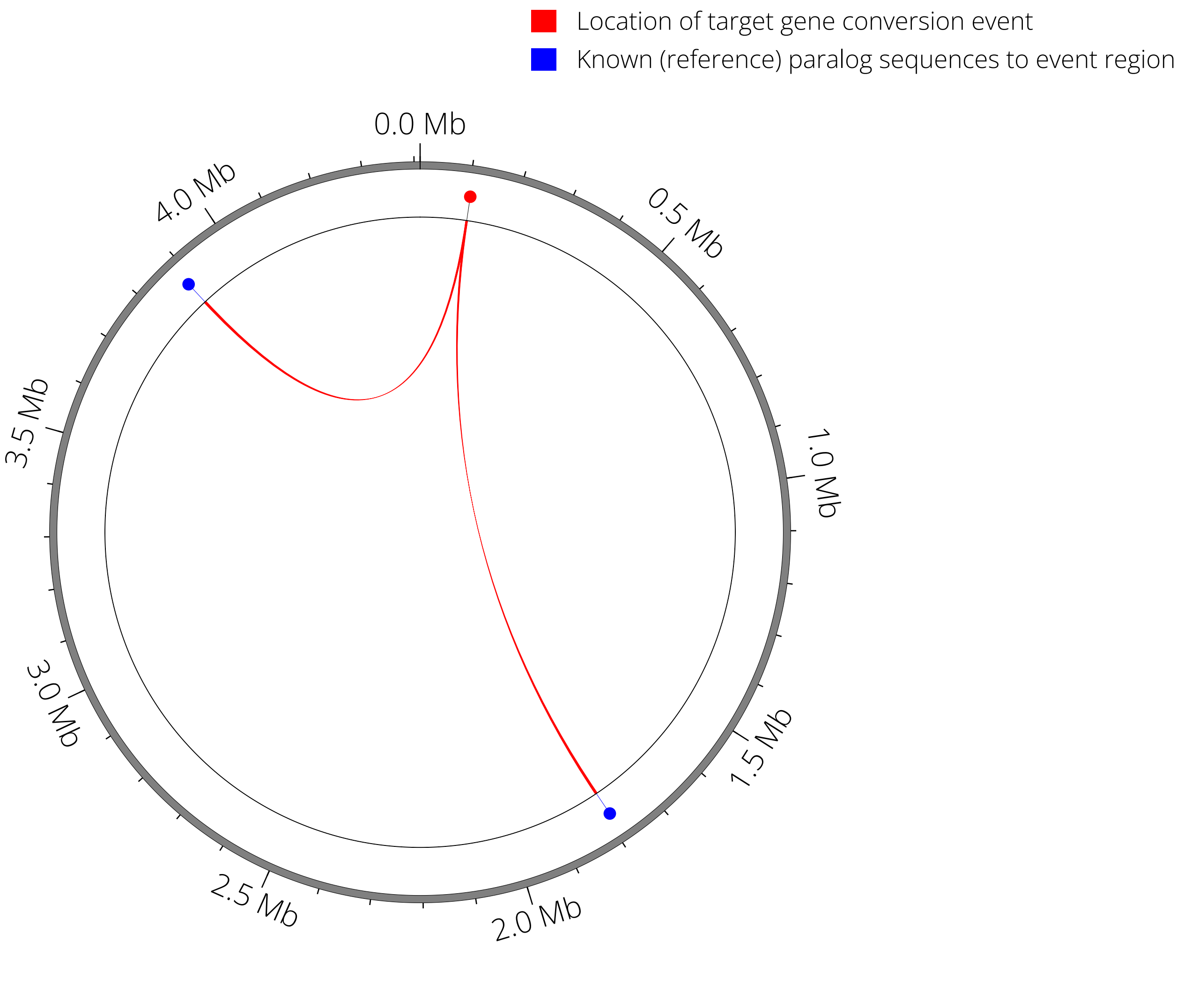

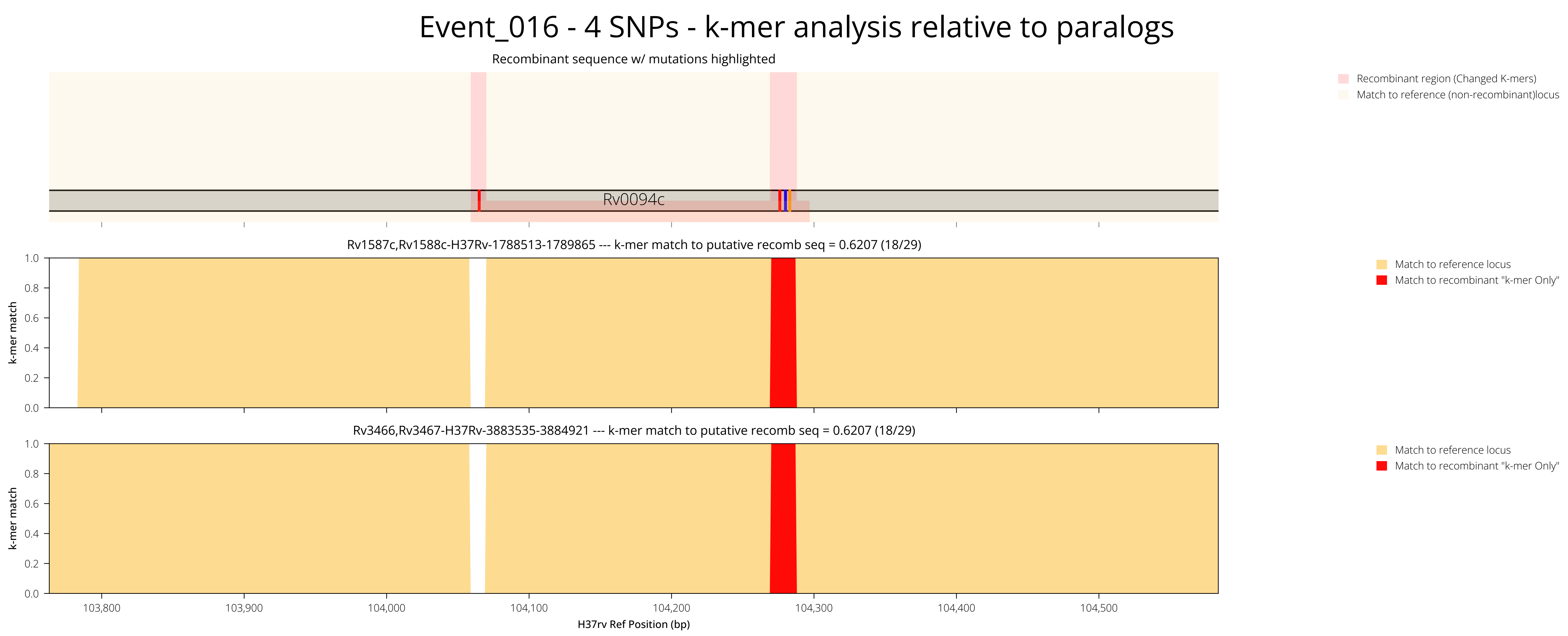

Event_016 - 4 SNPs - k-mer analysis relative to paralogs
Recombinant sequence w/ mutations highlighted
Rv0094c
Recombinant region (Changed K-mers)
Match to reference (non-recombinant)locus
Rv1587c,Rv1588c-H37Rv-1788513-1789865 --- k-mer match to putative recomb seq = 0.6207 (18/29)
Match to reference locus
Match to recombinant "k-mer Only"
Rv3466,Rv3467-H37Rv-3883535-3884921 --- k-mer match to putative recomb seq = 0.6207 (18/29)
Match to reference locus
Match to recombinant "k-mer Only"
k-mer match
1.0
0.8
0.6
0.4
0.2
0.0
103,800
103,900
104,000
104,100
104,200
104,300
104,400
104,500
H37rv Ref Position (bp)

Sub-phylogeny Viz + Paralog Alignments
GCE ID: Event_016

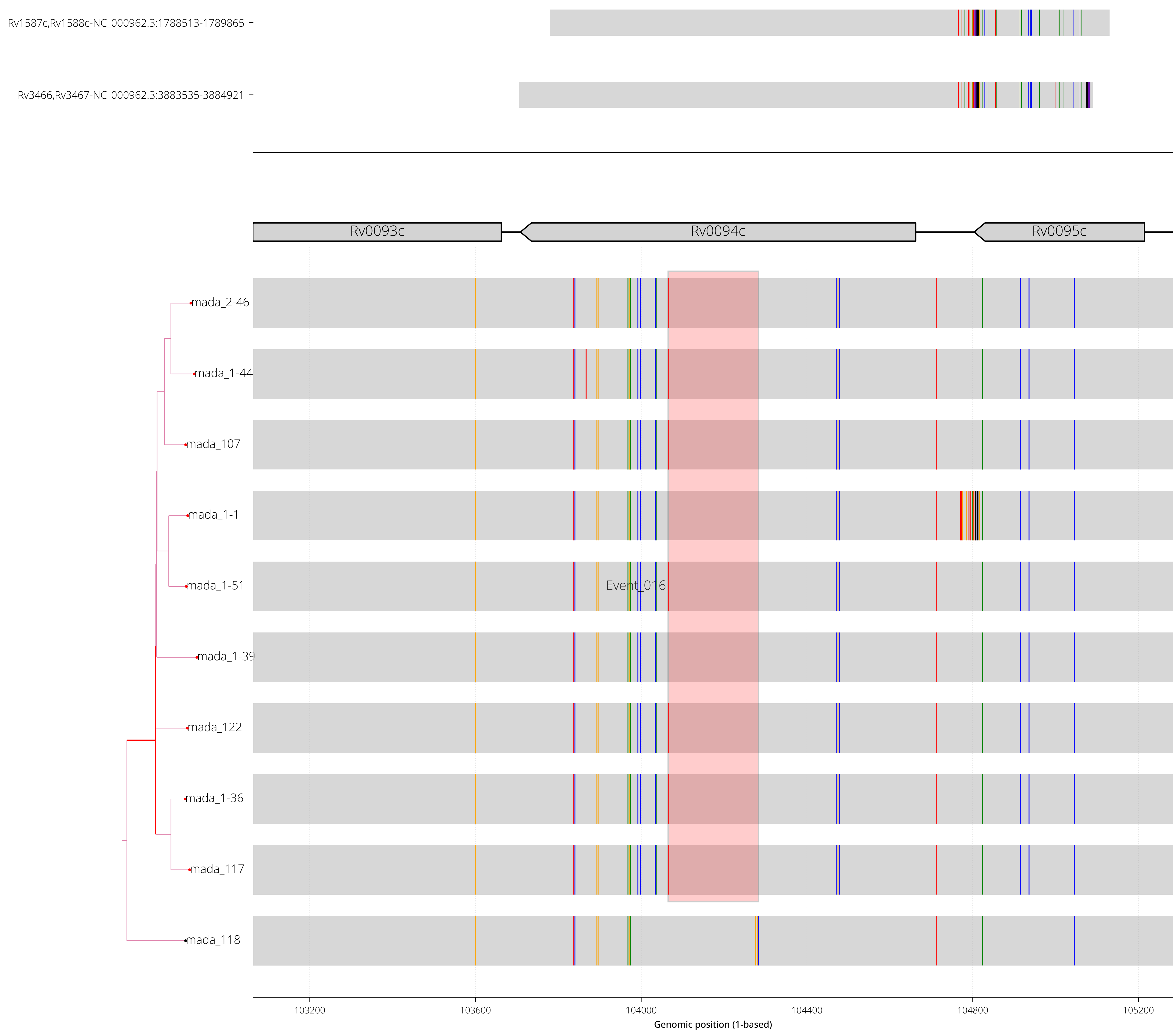

Full Phylogeny Viz + Paralog Alignments
GCE ID: Event_016

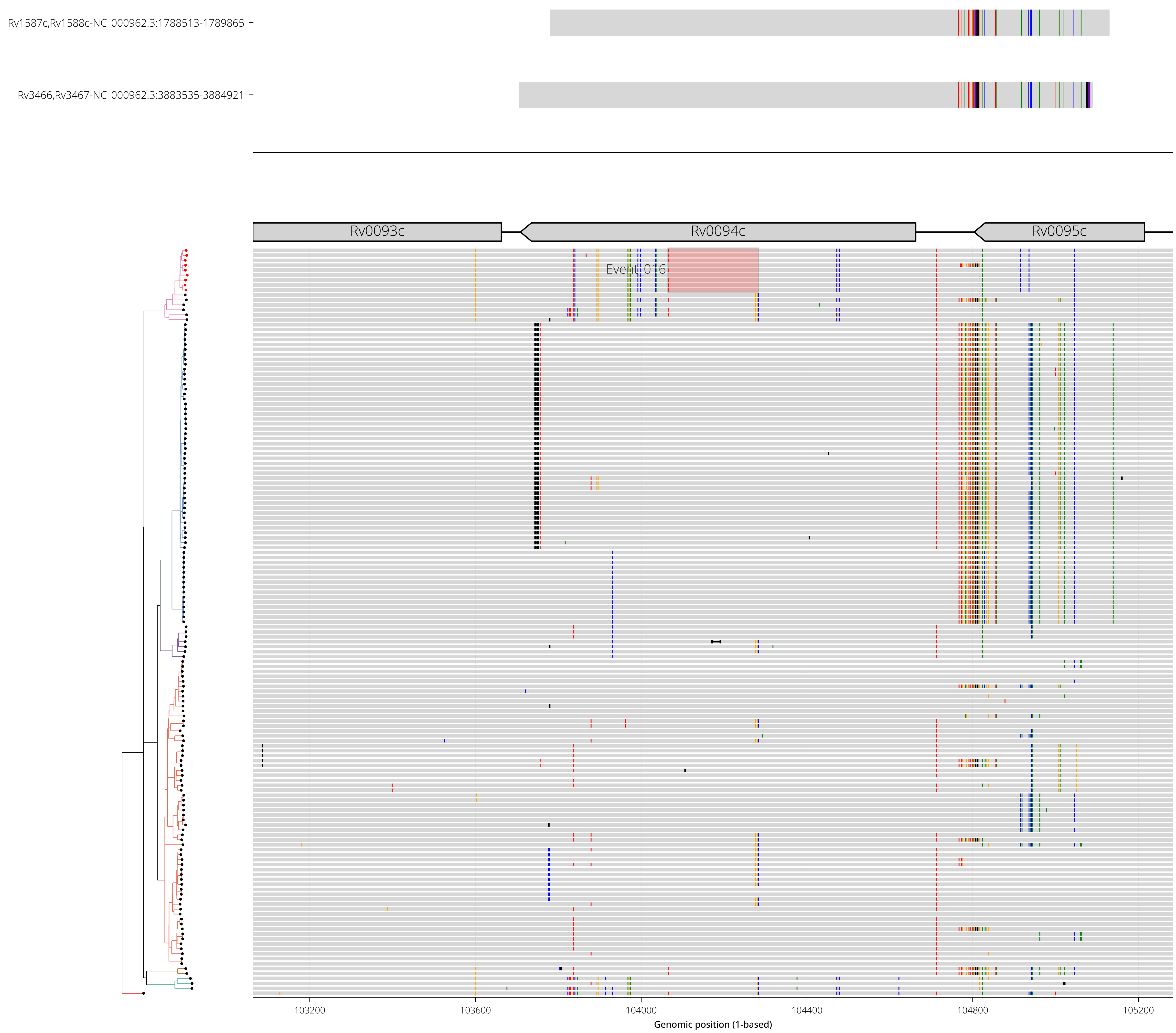

Individual GCE + Known Paralogs
GCE ID: Event_017

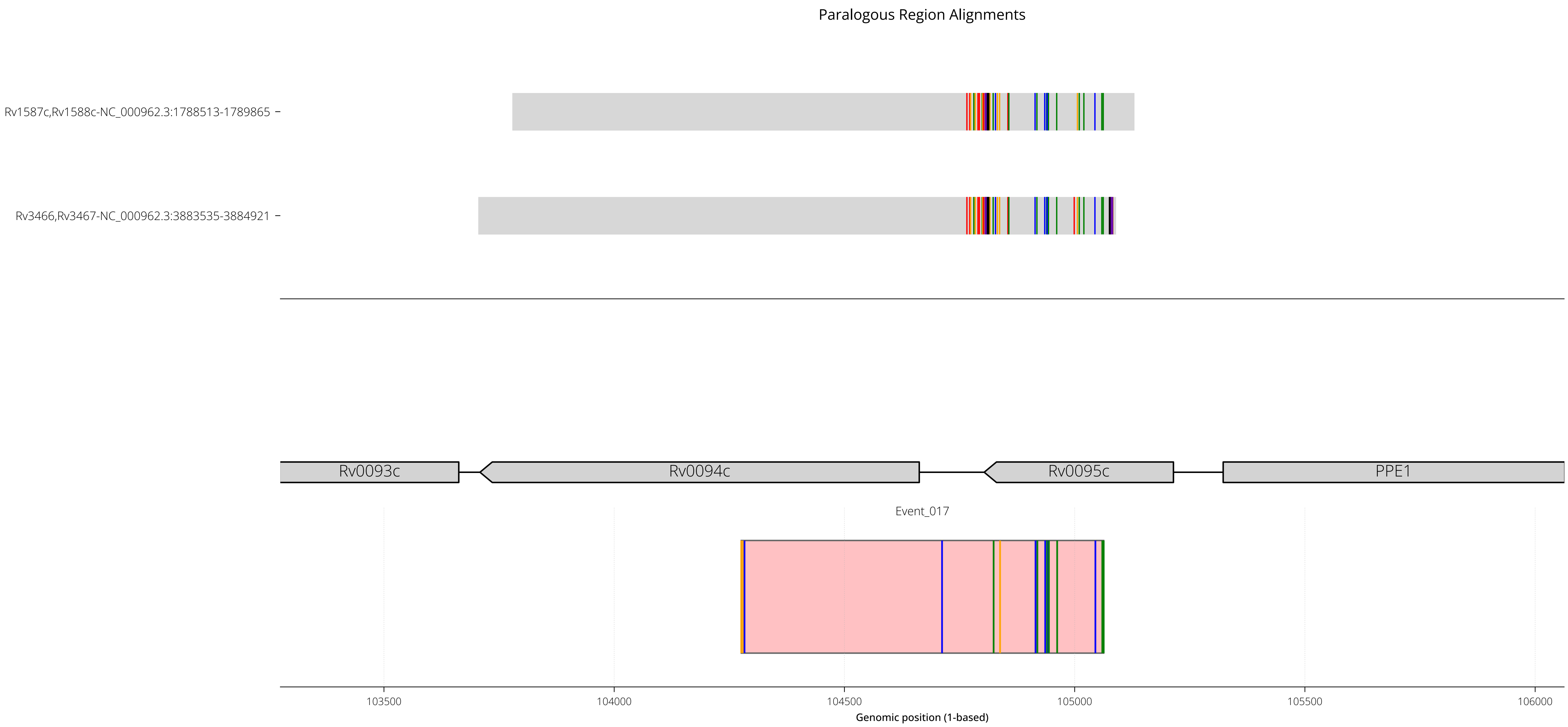

Circos Viz - GCE to Known Paralogs Locations
GCE ID: Event_017

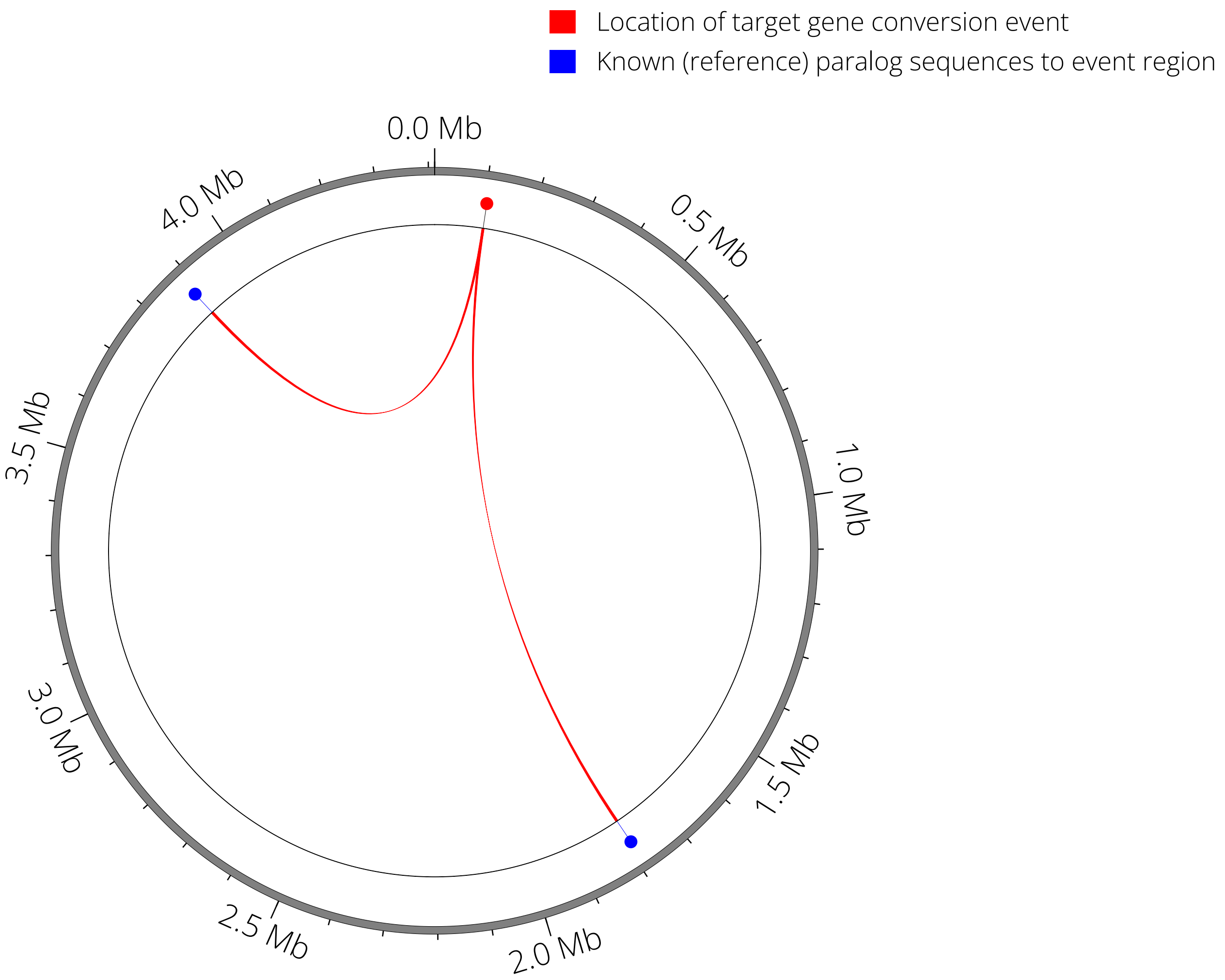

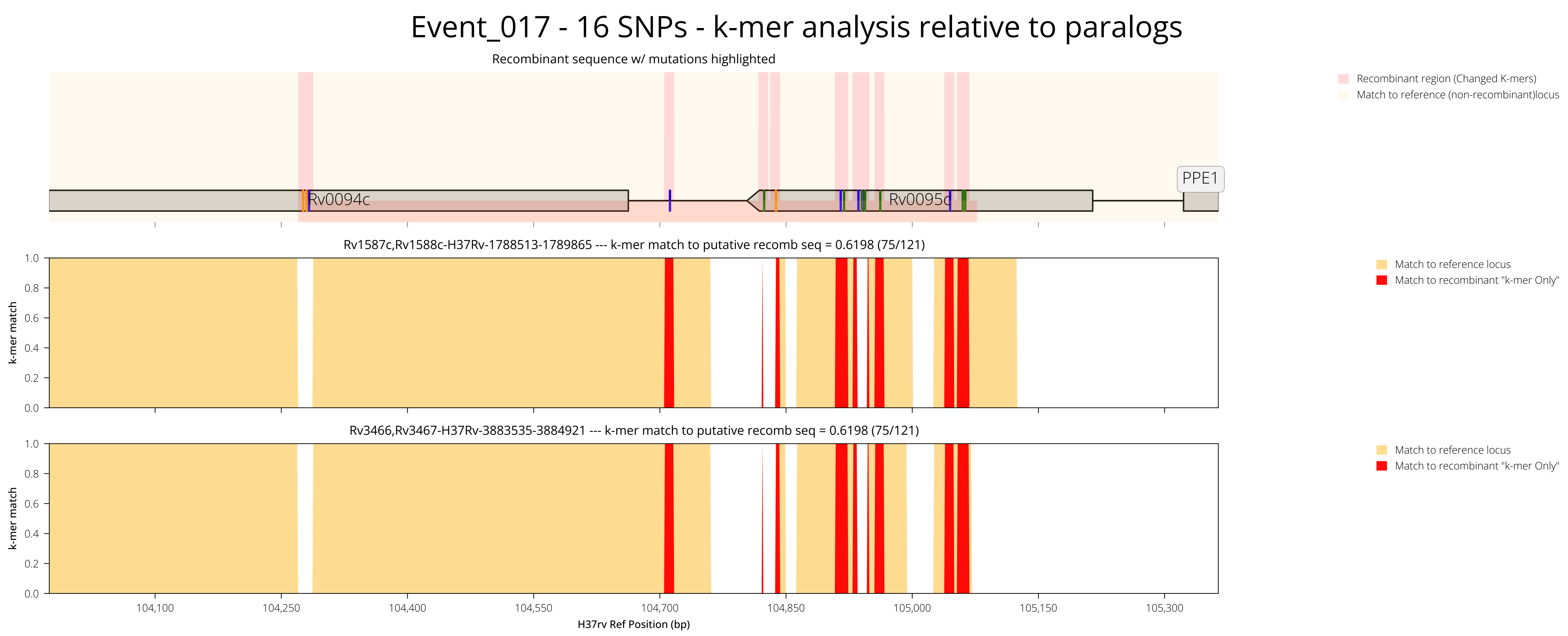
Event_017 - 16 SNPs - k-mer analysis relative to paralogs
Recombinant sequence w/ mutations highlighted
Rv0094c
Rv0095c
PPE1
Recombinant region (Changed K-mers)
Match to reference (non-recombinant)locus
Rv1587c,Rv1588c-H37Rv-1788513-1789865 --- k-mer match to putative recomb seq = 0.6198 (75/121)
Match to reference locus
Match to recombinant "k-mer Only"
k-mer match
Rv3466,Rv3467-H37Rv-3883535-3884921 --- k-mer match to putative recomb seq = 0.6198 (75/121)
Match to reference locus
Match to recombinant "k-mer Only"
k-mer match
0.0
0.2
0.4
0.6
0.8
1.0
104,100
104,250
104,400
104,550
104,700
104,850
105,000
105,150
105,300
H37rv Ref Position (bp)

Sub-phylogeny Viz + Paralog Alignments
GCE ID: Event_017

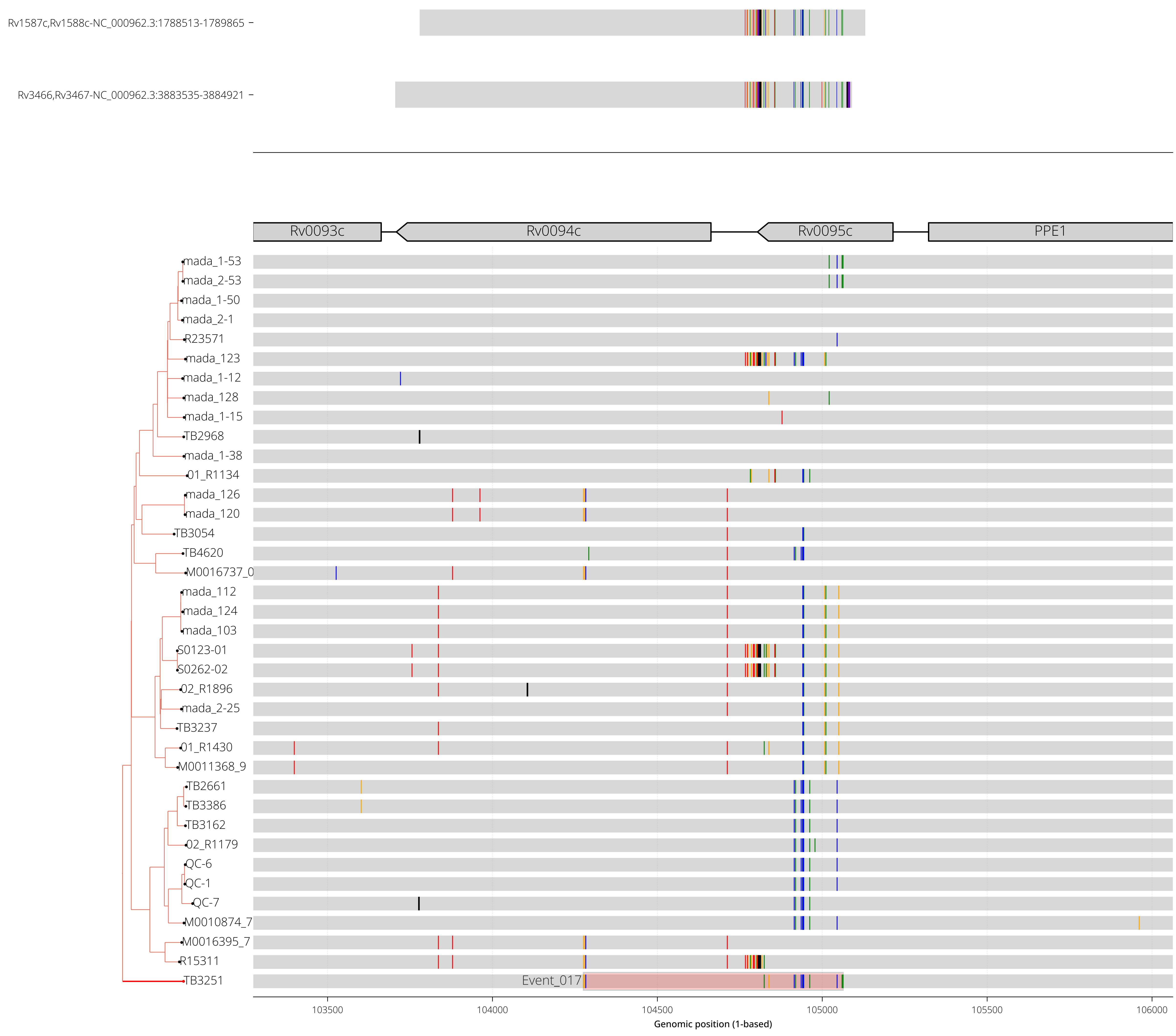

Full Phylogeny Viz + Paralog Alignments
GCE ID: Event_017

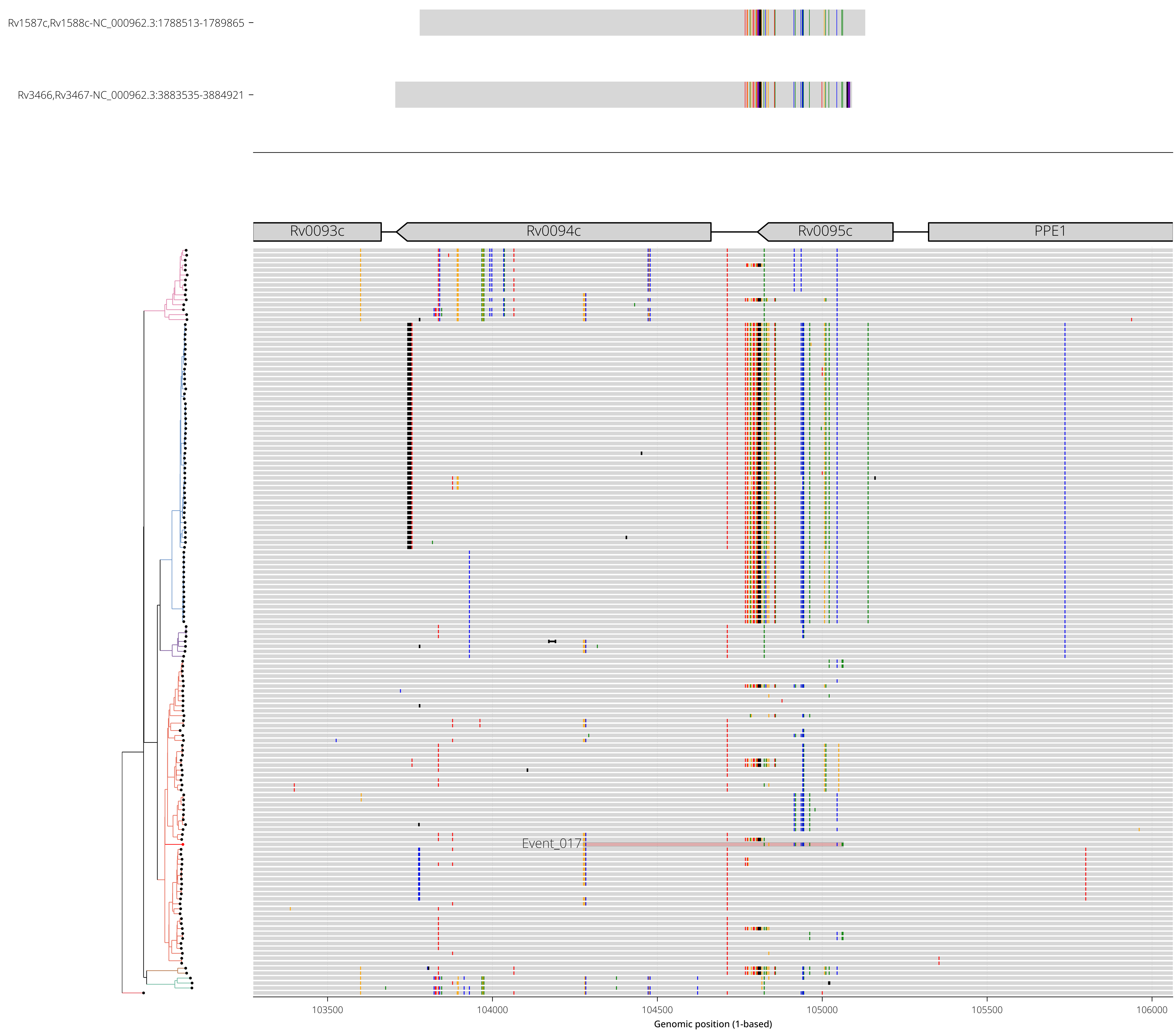

Individual GCE + Known Paralogs
GCE ID: Event_018

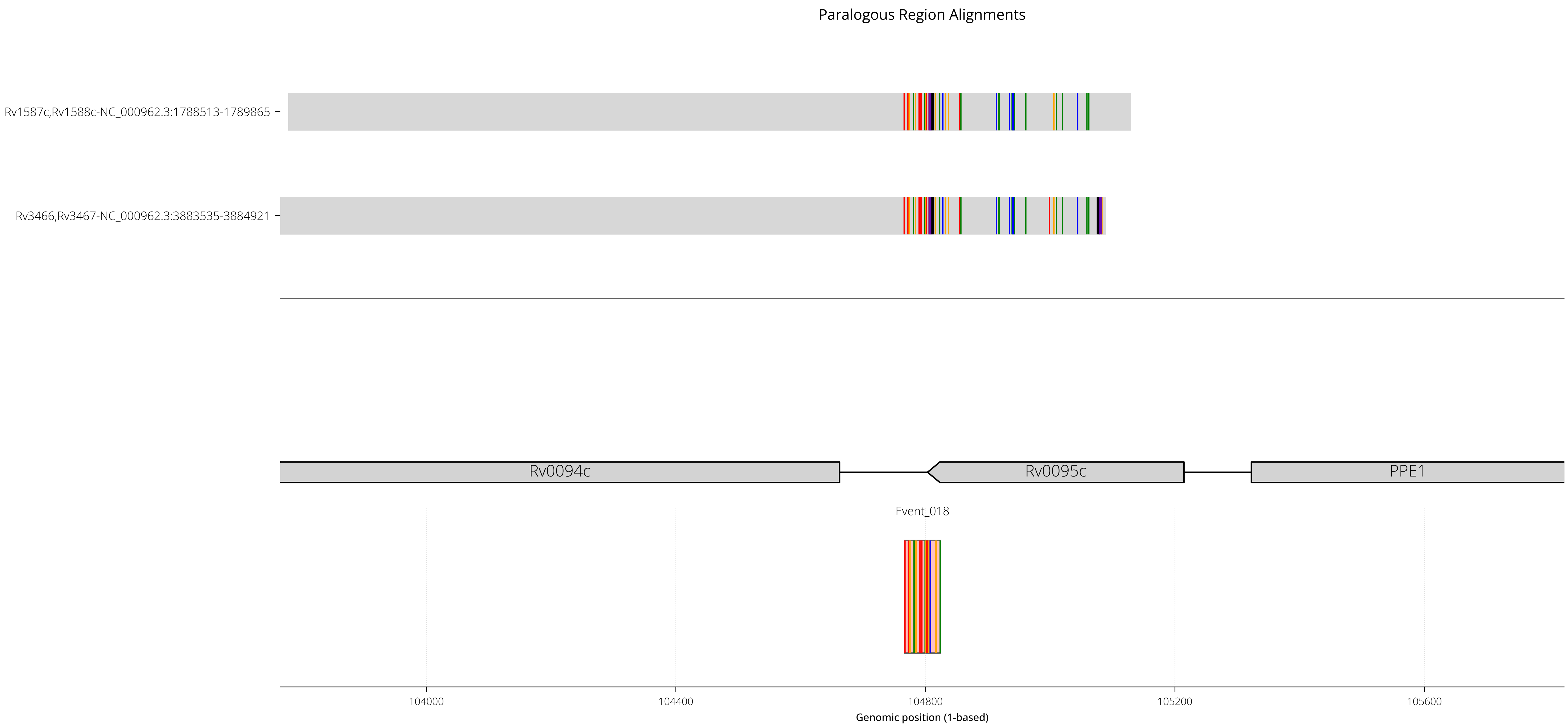

Circos Viz - GCE to Known Paralogs Locations
GCE ID: Event_018

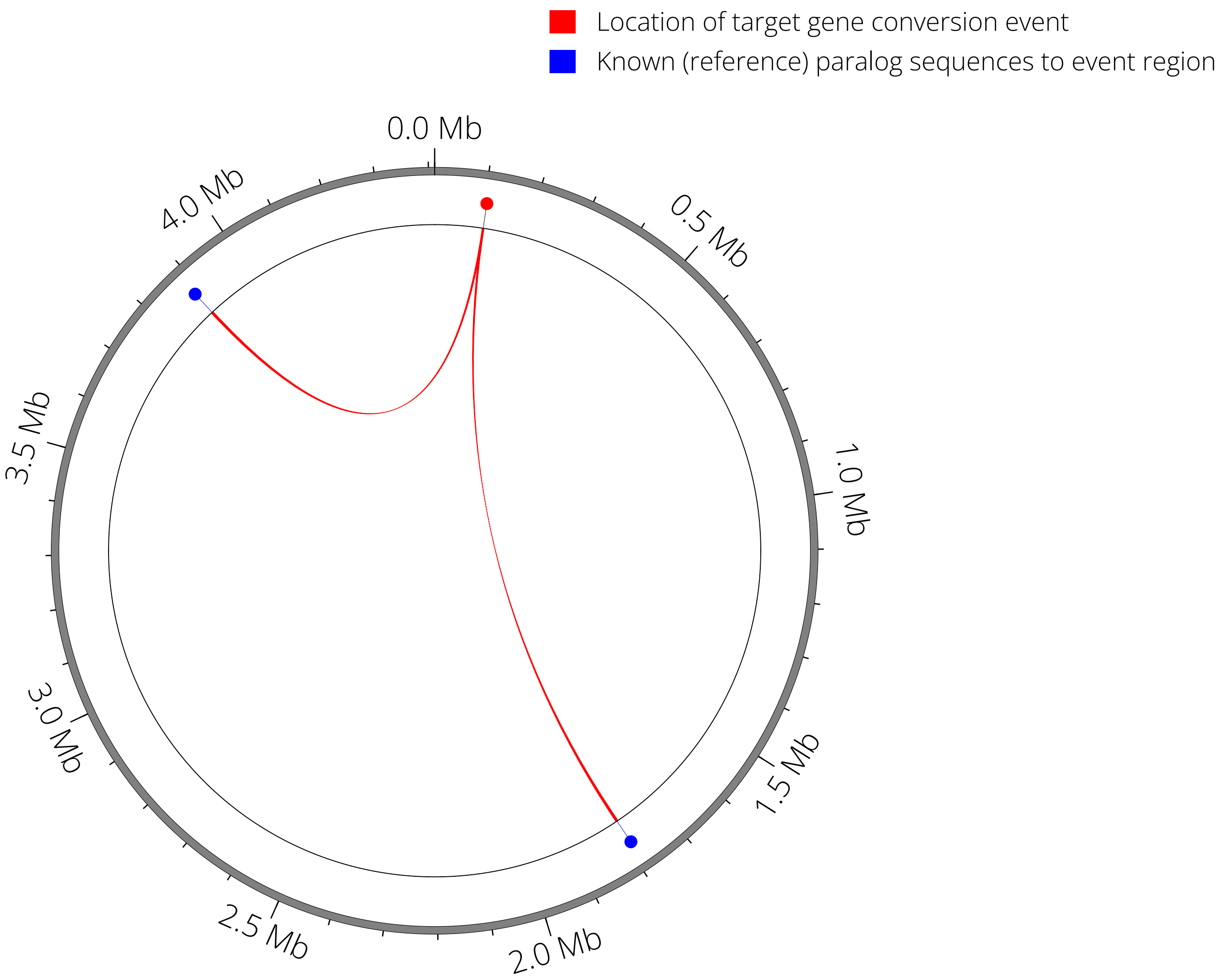

# Event_018 - 14 SNPs - k-mer analysis relative to paralogs

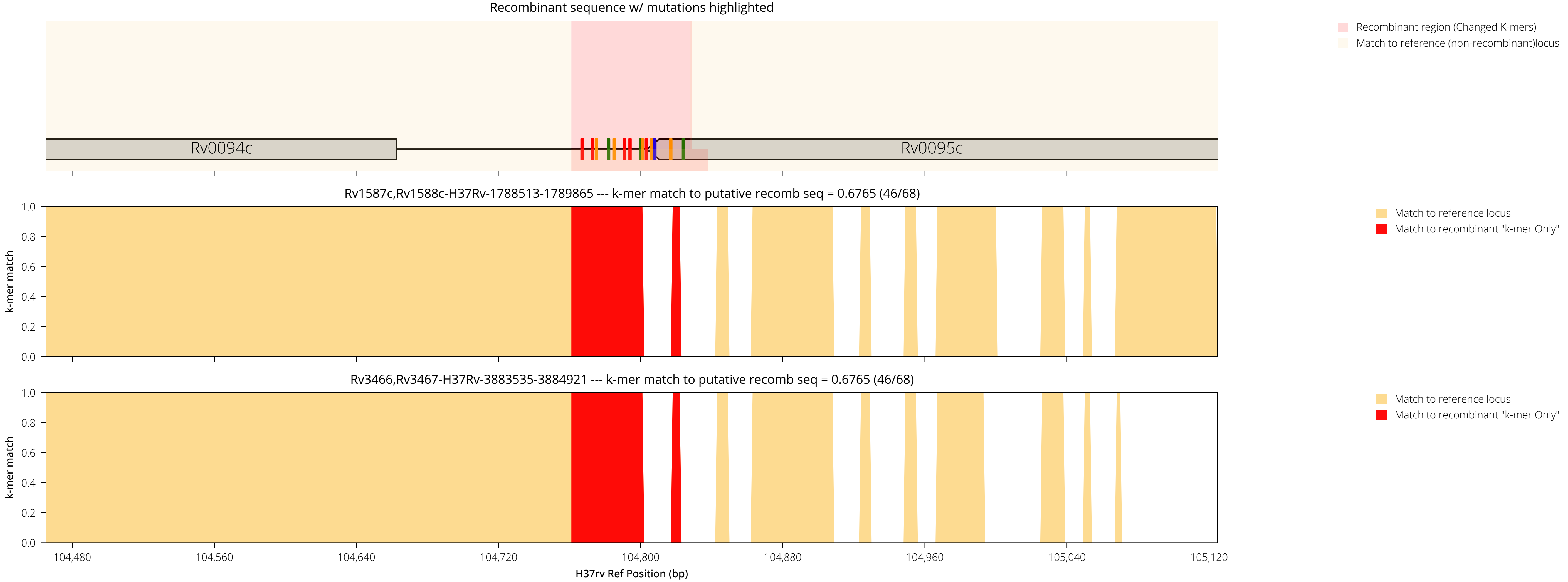

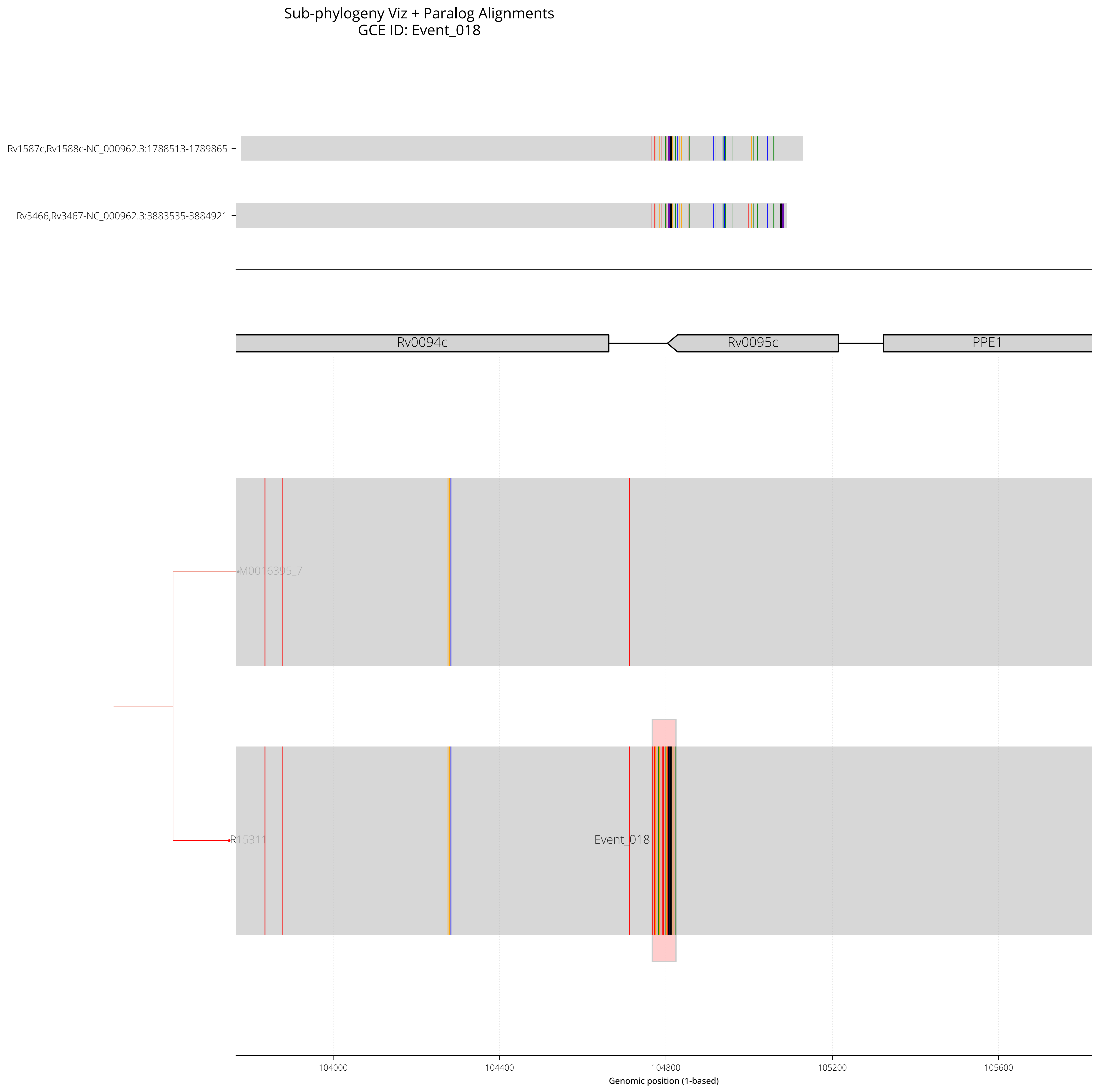
Sub-phylogeny Viz + Paralog Alignments
GCE ID: Event_018
Rv1587c,Rv1588c-NC_000962.3:1788513-1789865
Rv3466,Rv3467-NC_000962.3:3883535-3884921
Rv0094c
Rv0095c
PPE1
M0016395_7
R15311
Event_018
104000
104400
104800
105200
105600
Genomic position (1-based)

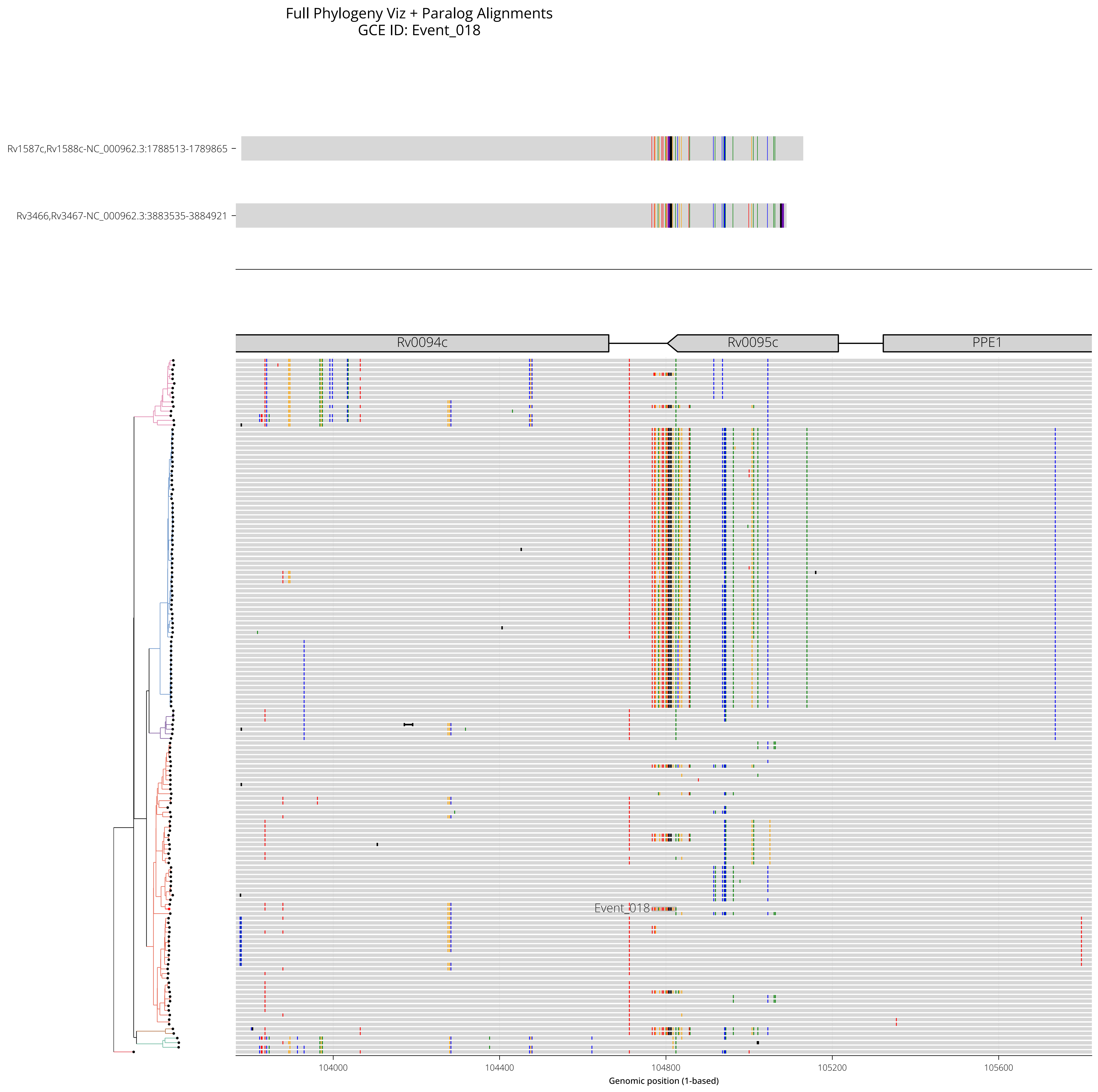

Full Phylogeny Viz + Paralog Alignments
GCE ID: Event_018
Rv1587c,Rv1588c-NC_000962.3:1788513-1789865
Rv3466,Rv3467-NC_000962.3:3883535-3884921
Rv0094c
Rv0095c
PPE1
Event_018
104000
104400
104800
105200
105600
Genomic position (1-based)

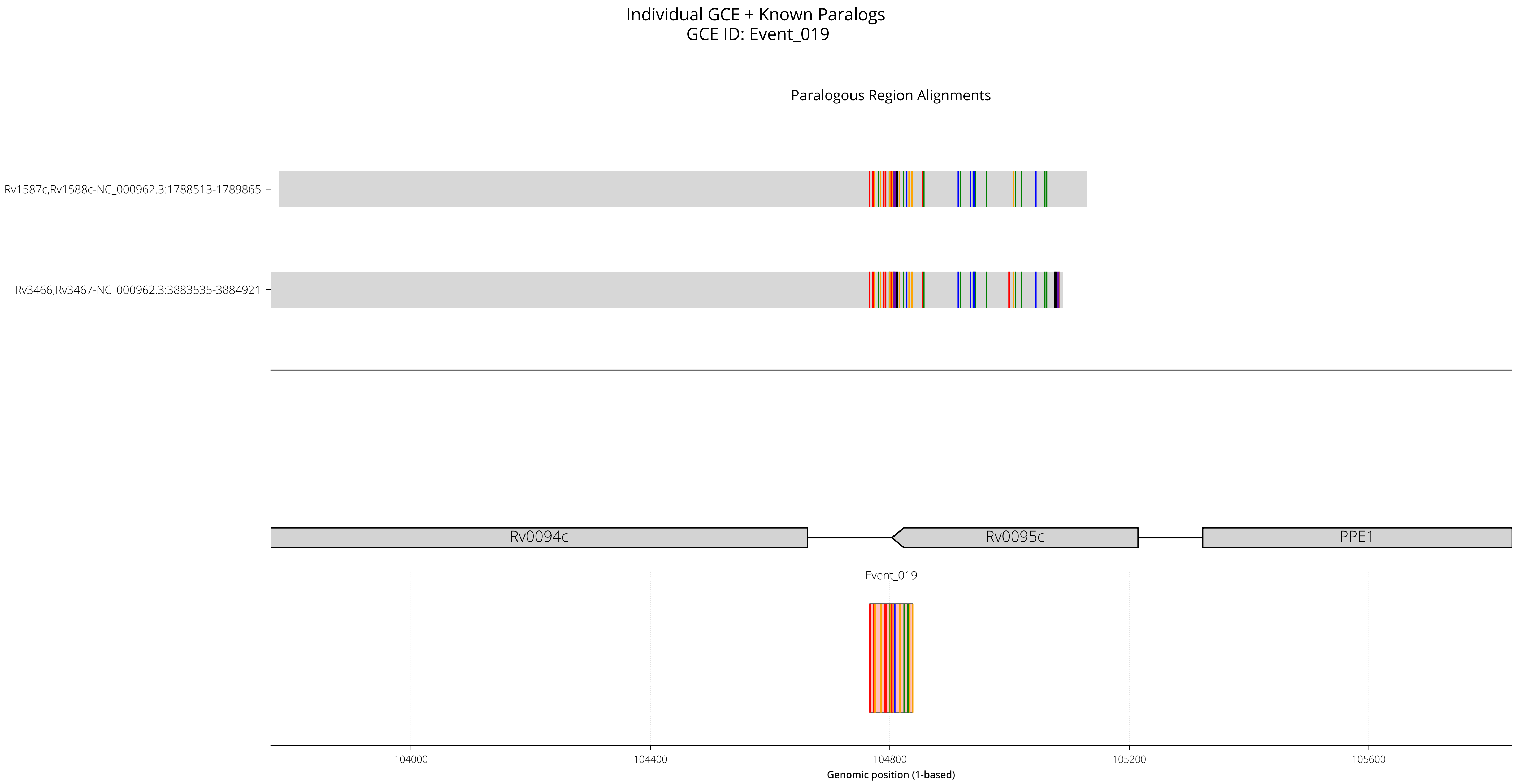
Individual GCE + Known Paralogs
GCE ID: Event_019
Paralogous Region Alignments
Rv1587c,Rv1588c-NC_000962.3:1788513-1789865
Rv3466,Rv3467-NC_000962.3:3883535-3884921
Rv0094c
Rv0095c
PPE1
Event_019
104000
104400
104800
105200
105600
Genomic position (1-based)

Circos Viz - GCE to Known Paralogs Locations
GCE ID: Event_019

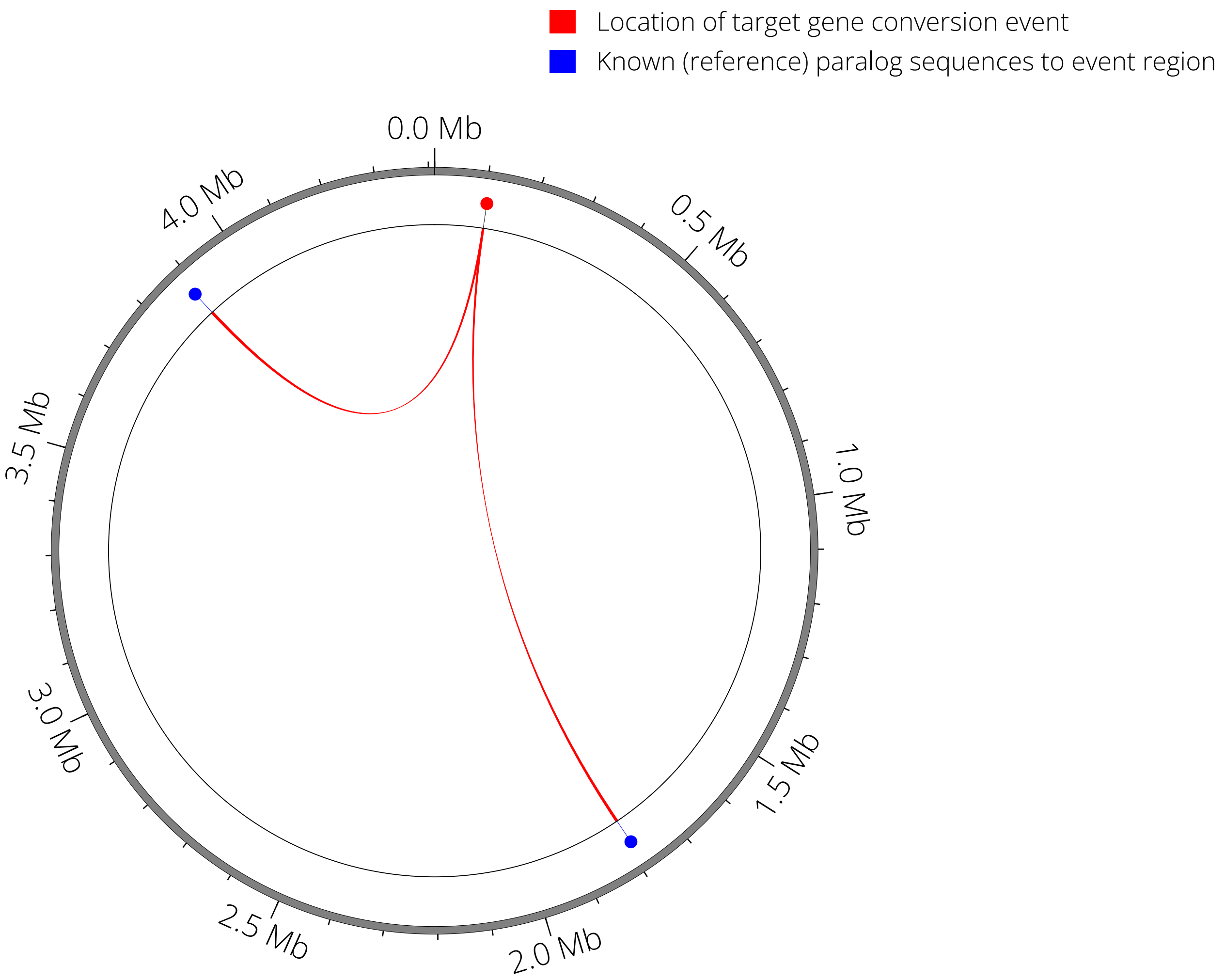

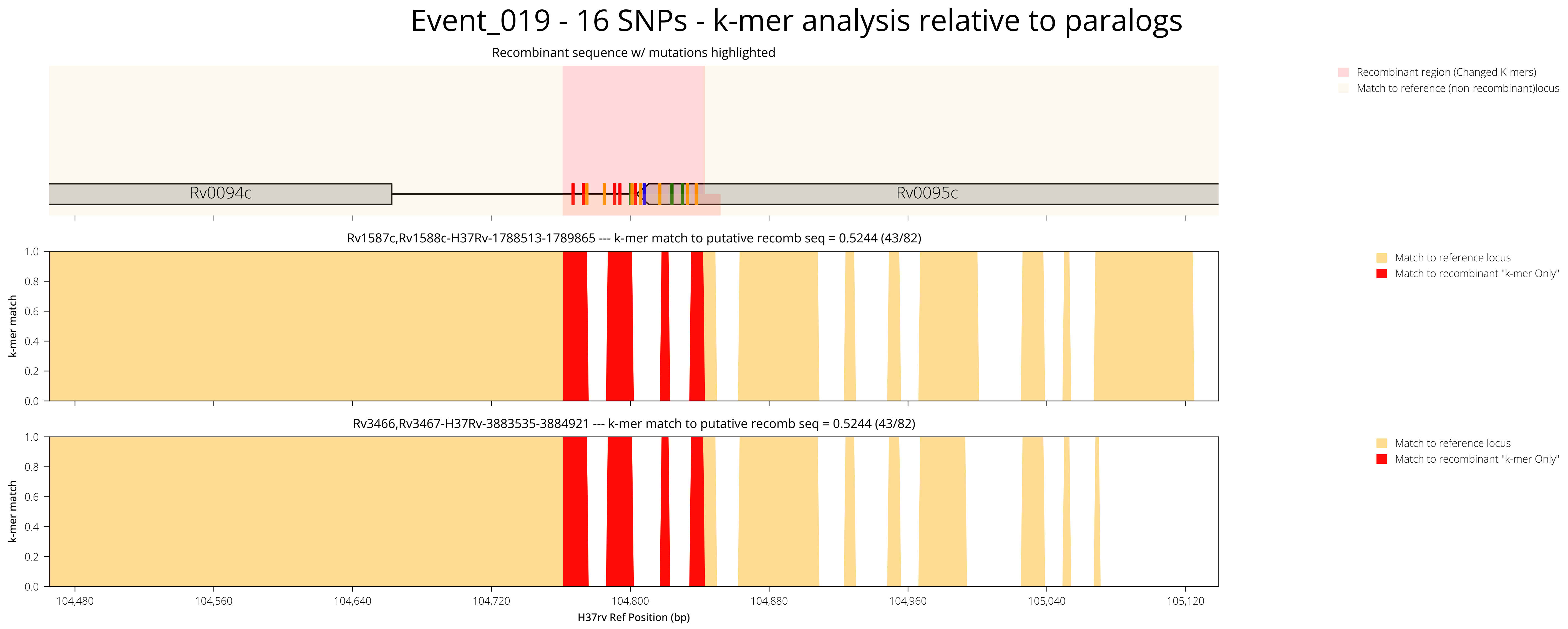

Event_019 - 16 SNPs - k-mer analysis relative to paralogs
Recombinant sequence w/ mutations highlighted
Rv0094c
Rv0095c
Recombinant region (Changed K-mers)
Match to reference (non-recombinant)locus
Rv1587c,Rv1588c-H37Rv-1788513-1789865 --- k-mer match to putative recomb seq = 0.5244 (43/82)
Match to reference locus
Match to recombinant "k-mer Only"
Rv3466,Rv3467-H37Rv-3883535-3884921 --- k-mer match to putative recomb seq = 0.5244 (43/82)
Match to reference locus
Match to recombinant "k-mer Only"
k-mer match
1.0
0.8
0.6
0.4
0.2
0.0
104,480
104,560
104,640
104,720
104,800
104,880
104,960
105,040
105,120
H37rv Ref Position (bp)

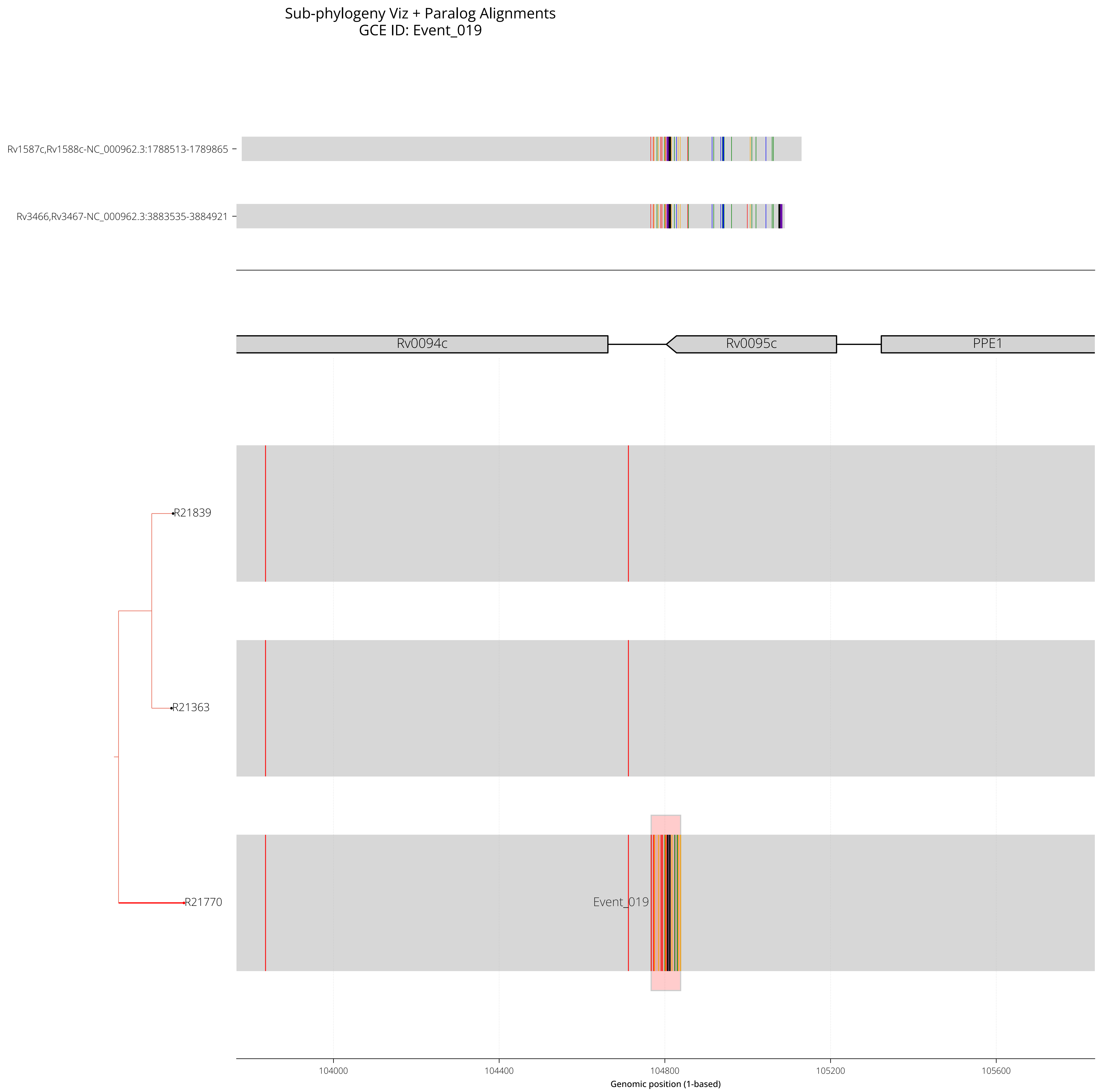
Sub-phylogeny Viz + Paralog Alignments
GCE ID: Event_019
Rv1587c,Rv1588c-NC_000962.3:1788513-1789865
Rv3466,Rv3467-NC_000962.3:3883535-3884921
Rv0094c
Rv0095c
PPE1
R21839
R21363
R21770
Event_019
104000
104400
104800
105200
105600
Genomic position (1-based)

Full Phylogeny Viz + Paralog Alignments
GCE ID: Event_019

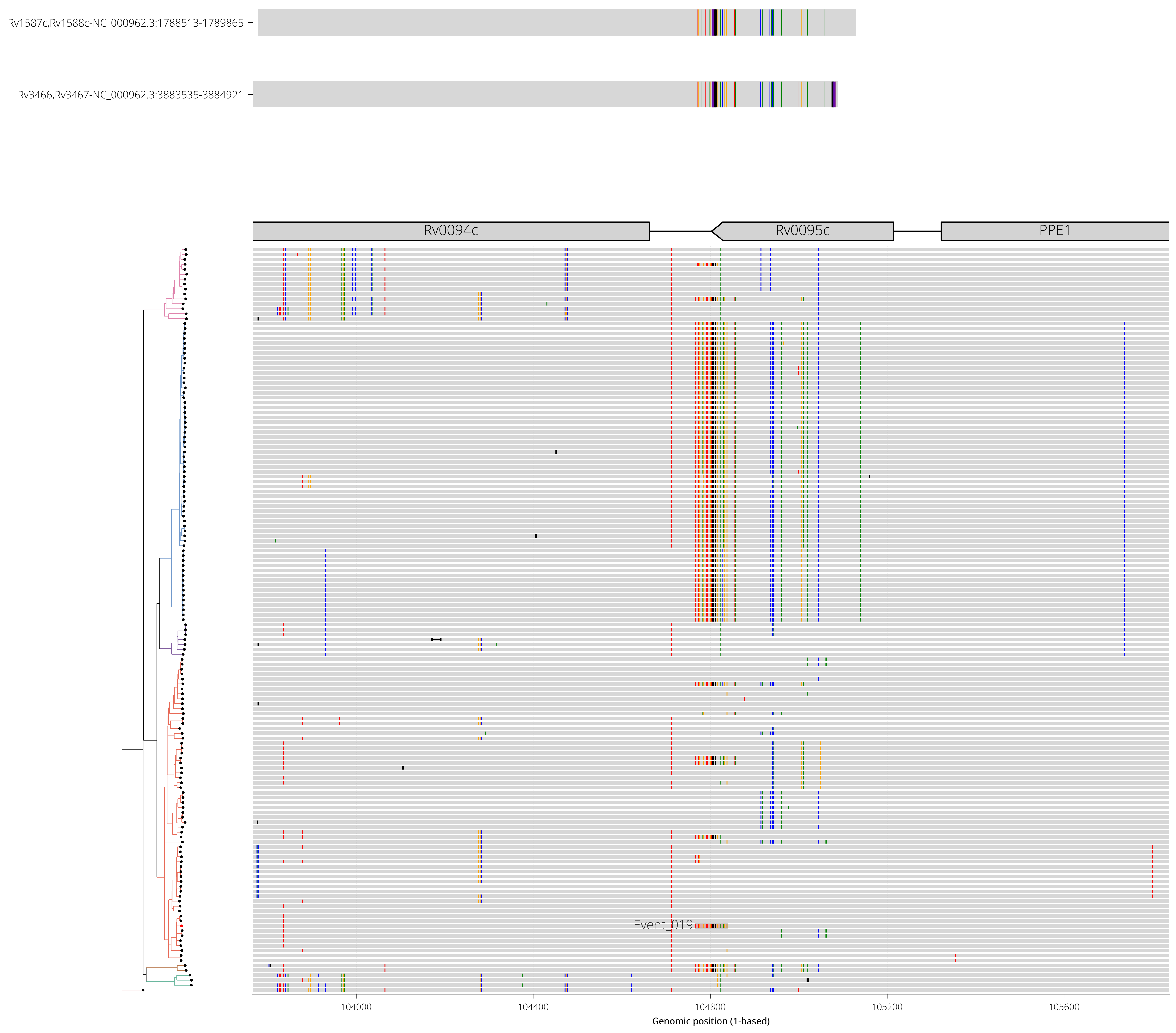

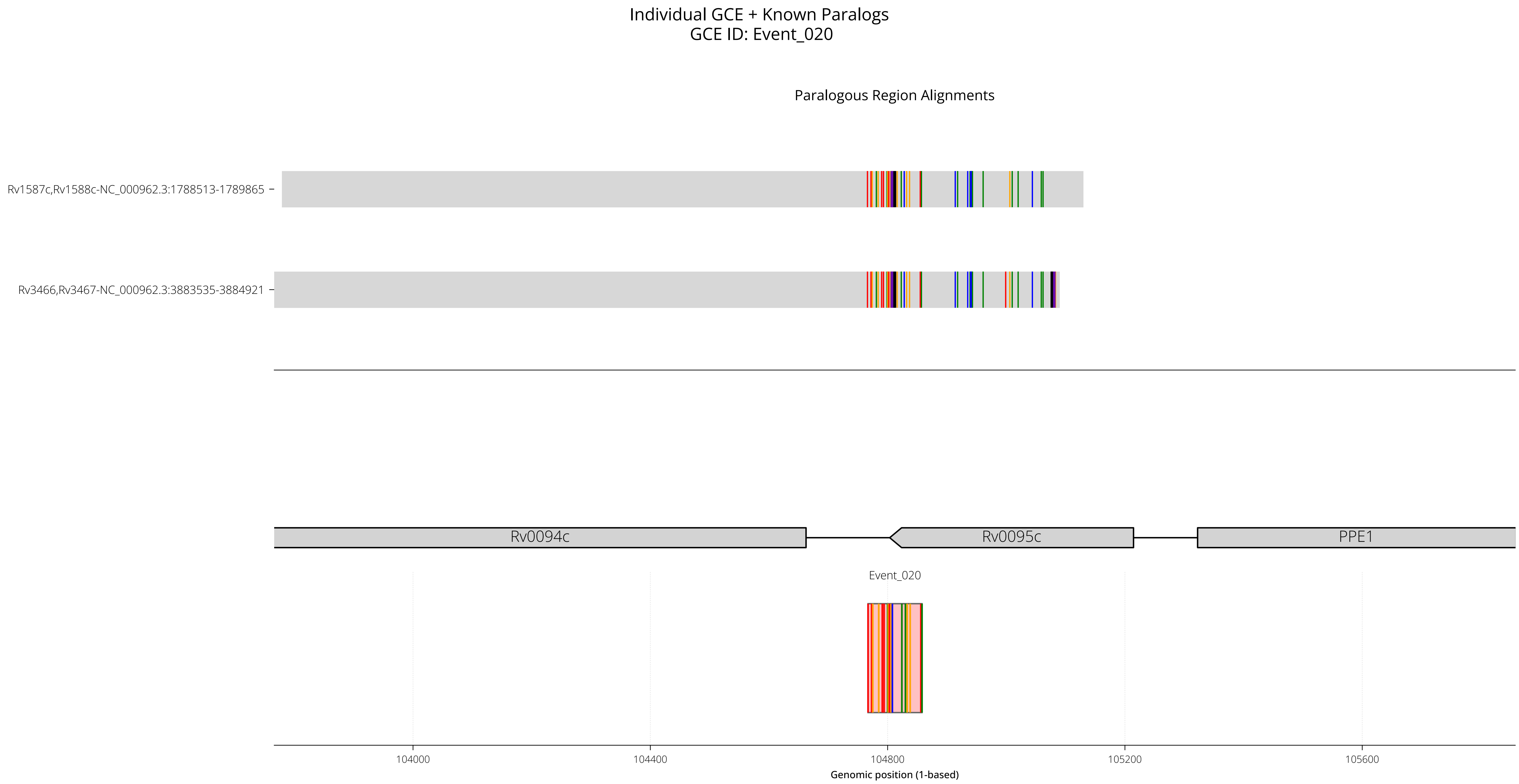

Individual GCE + Known Paralogs
GCE ID: Event_020
Paralogous Region Alignments
Rv1587c,Rv1588c-NC_000962.3:1788513-1789865
Rv3466,Rv3467-NC_000962.3:3883535-3884921
Rv0094c
Rv0095c
PPE1
Event_020
104000
104400
104800
105200
105600
Genomic position (1-based)

Circos Viz - GCE to Known Paralogs Locations
GCE ID: Event_020

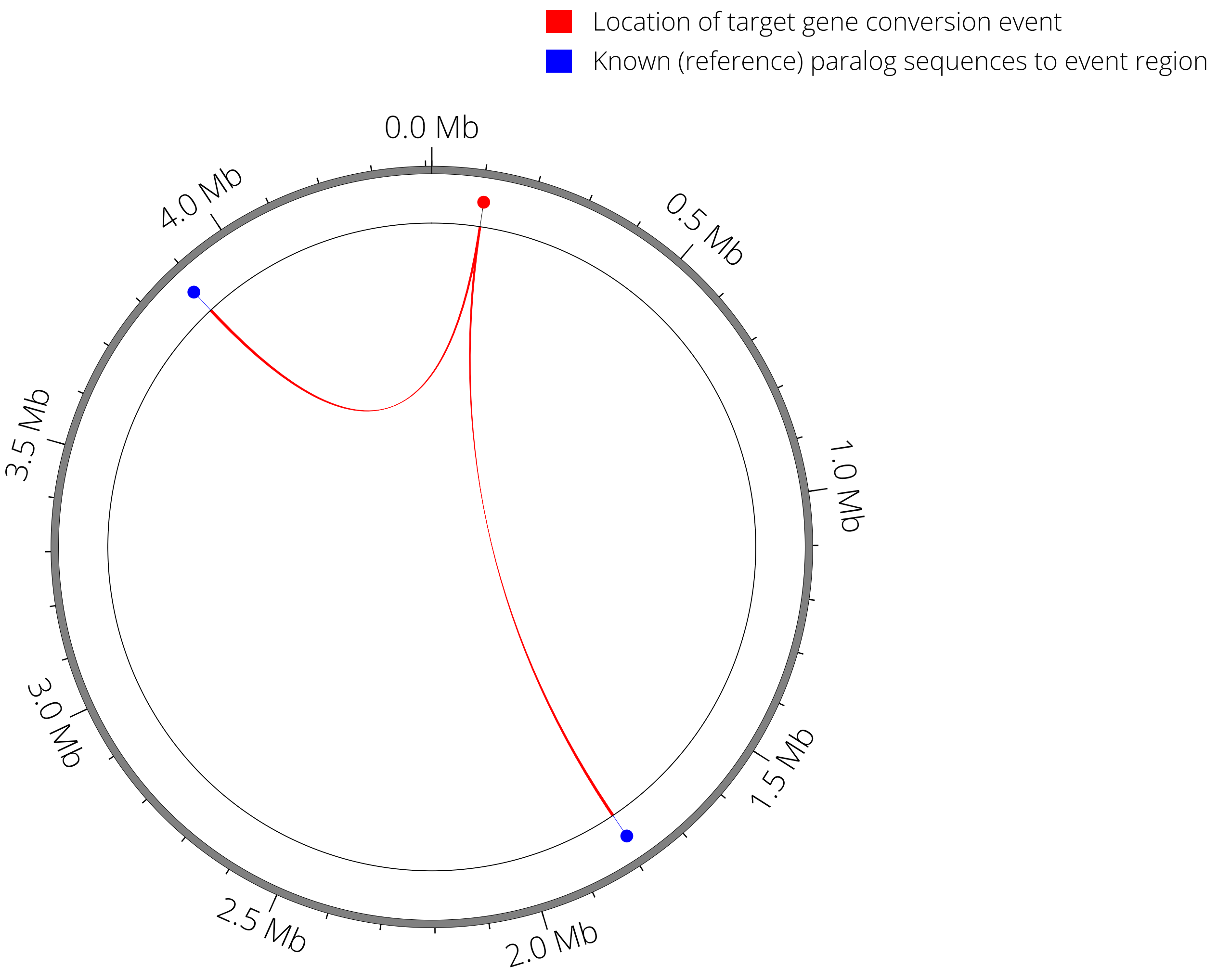

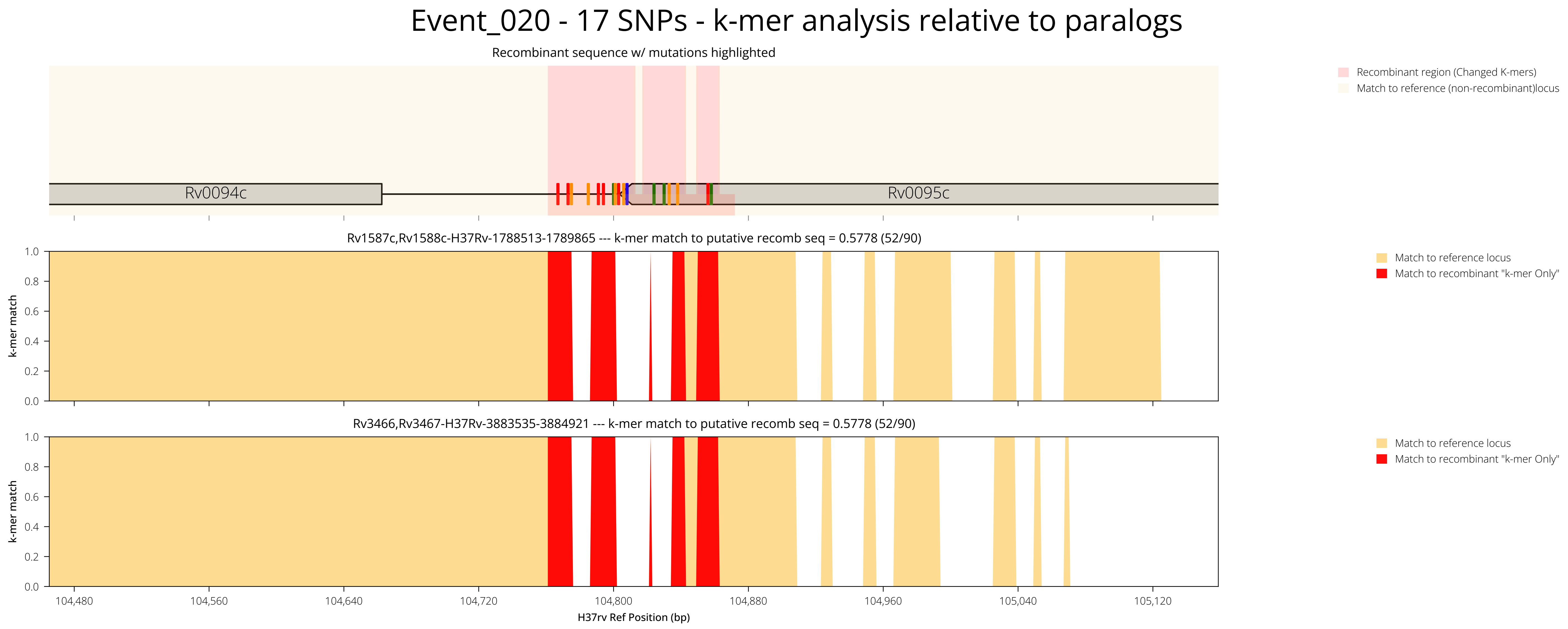

Event_020 - 17 SNPs - k-mer analysis relative to paralogs
Recombinant sequence w/ mutations highlighted
Recombinant region (Changed K-mers)
Match to reference (non-recombinant)locus
Rv0094c
Rv0095c
Rv1587c,Rv1588c-H37Rv-1788513-1789865 --- k-mer match to putative recomb seq = 0.5778 (52/90)
Match to reference locus
Match to recombinant "k-mer Only"
k-mer match
1.0
0.8
0.6
0.4
0.2
0.0
Rv3466,Rv3467-H37Rv-3883535-3884921 --- k-mer match to putative recomb seq = 0.5778 (52/90)
Match to reference locus
Match to recombinant "k-mer Only"
k-mer match
1.0
0.8
0.6
0.4
0.2
0.0
104,480
104,560
104,640
104,720
104,800
104,880
104,960
105,040
105,120
H37rv Ref Position (bp)

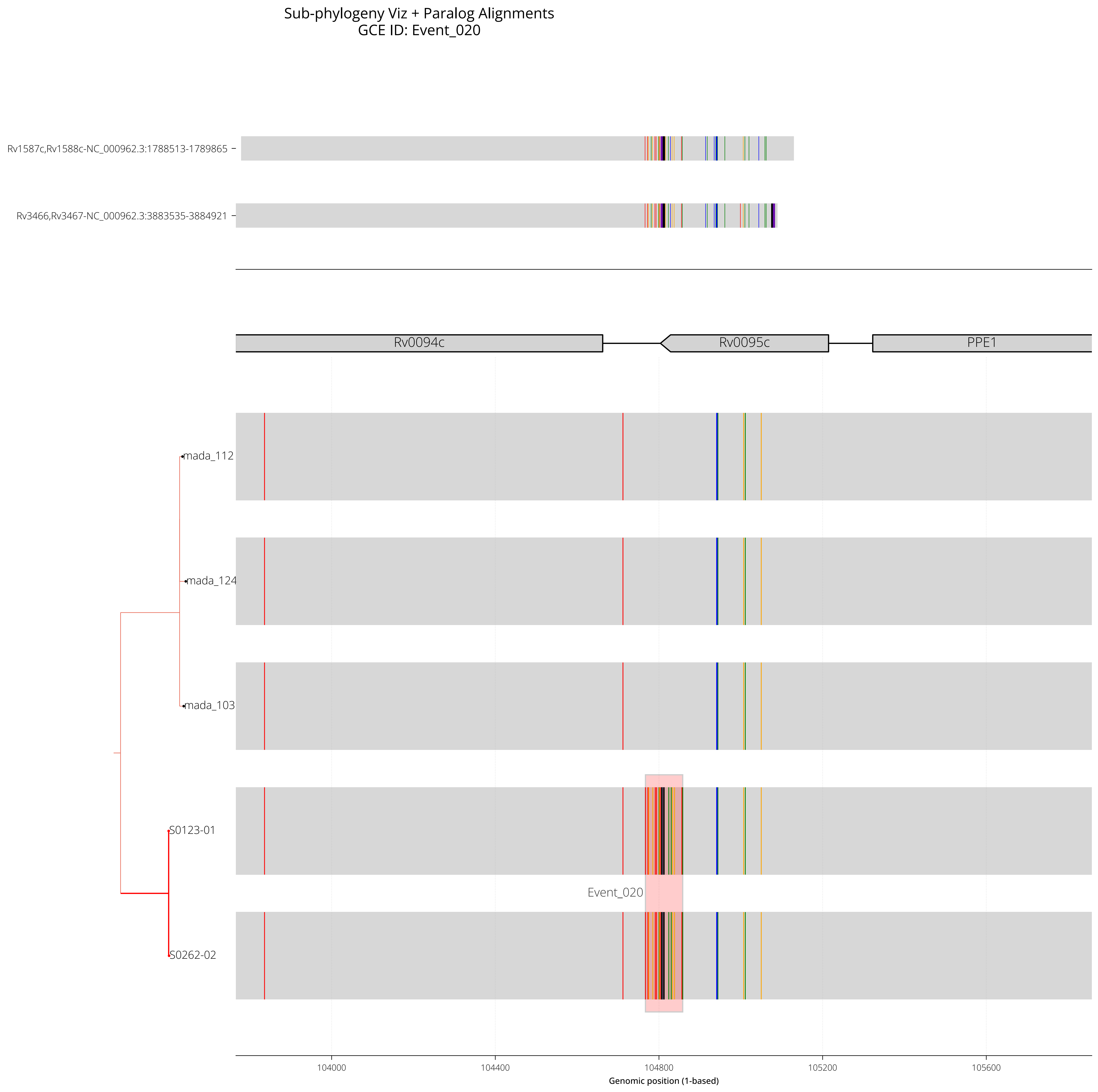

Sub-phylogeny Viz + Paralog Alignments
GCE ID: Event_020
Rv1587c,Rv1588c-NC_000962.3:1788513-1789865
Rv3466,Rv3467-NC_000962.3:3883535-3884921
Rv0094c
Rv0095c
PPE1
mada_112
mada_124
mada_103
S0123-01
Event_020
S0262-02
104000
104400
104800
105200
105600
Genomic position (1-based)

Full Phylogeny Viz + Paralog Alignments
GCE ID: Event_020

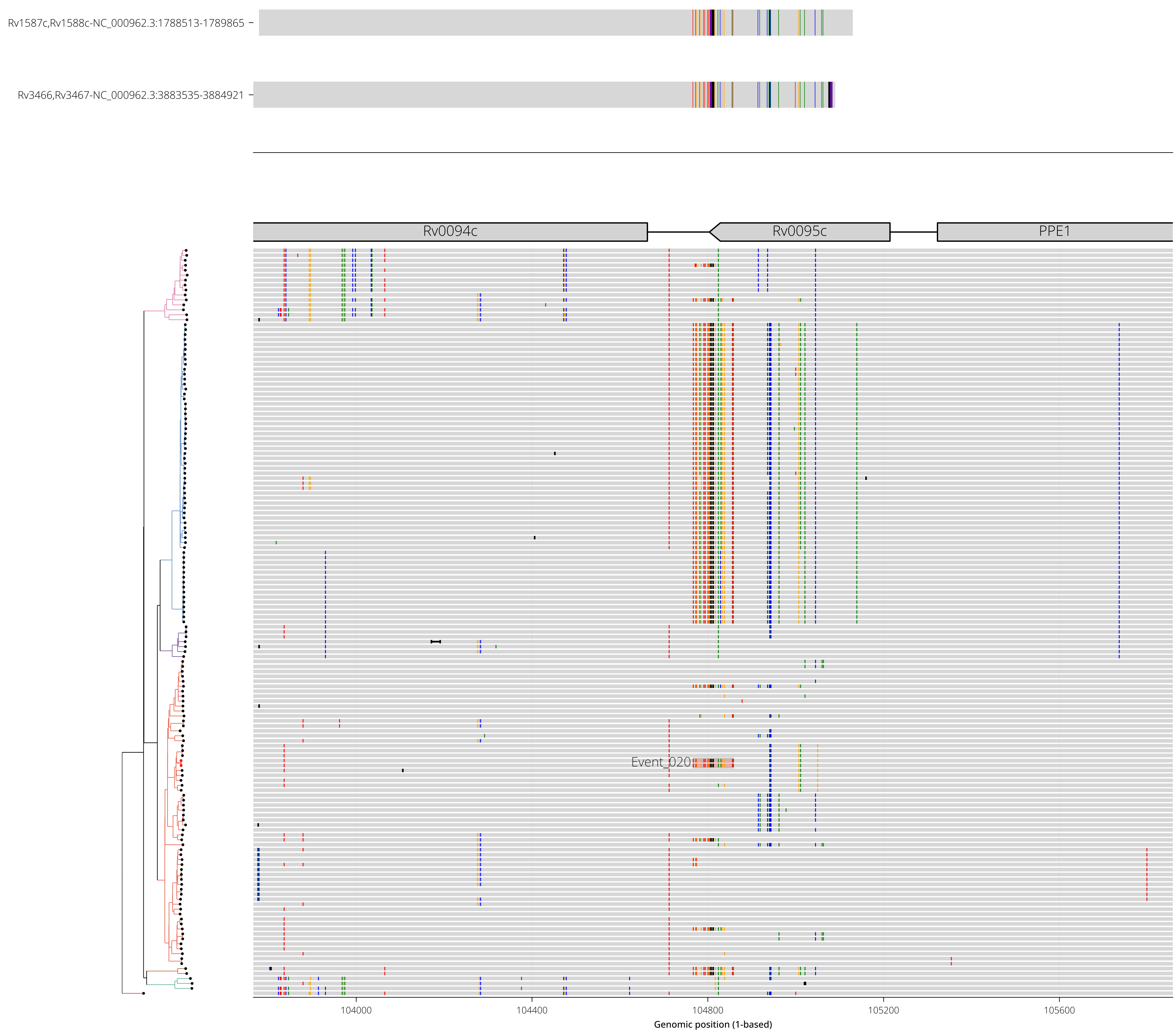

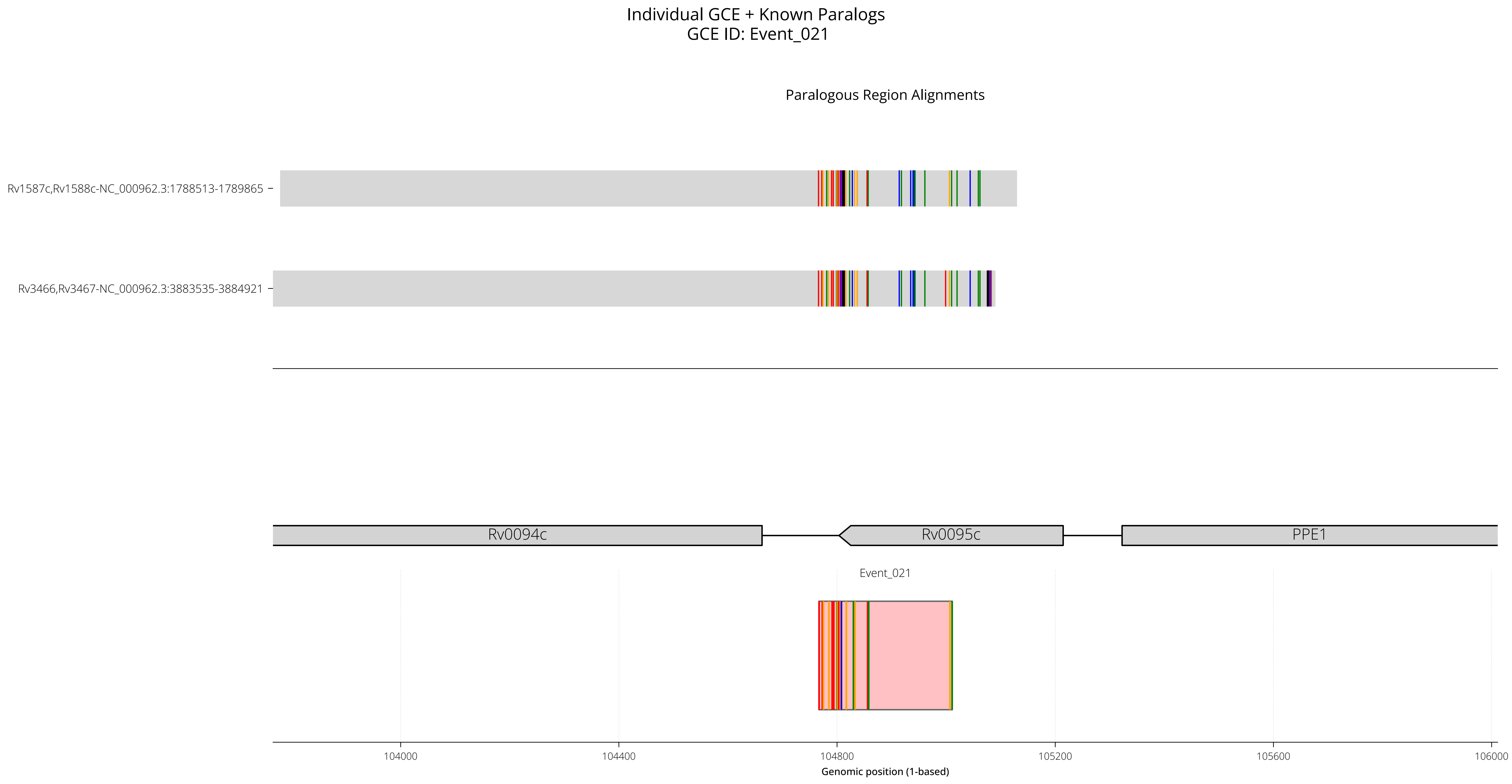
Individual GCE + Known Paralogs
GCE ID: Event_021
Paralogous Region Alignments
Rv1587c,Rv1588c-NC_000962.3:1788513-1789865
Rv3466,Rv3467-NC_000962.3:3883535-3884921
Rv0094c
Rv0095c
PPE1
Event_021
104000
104400
104800
105200
105600
106000
Genomic position (1-based)

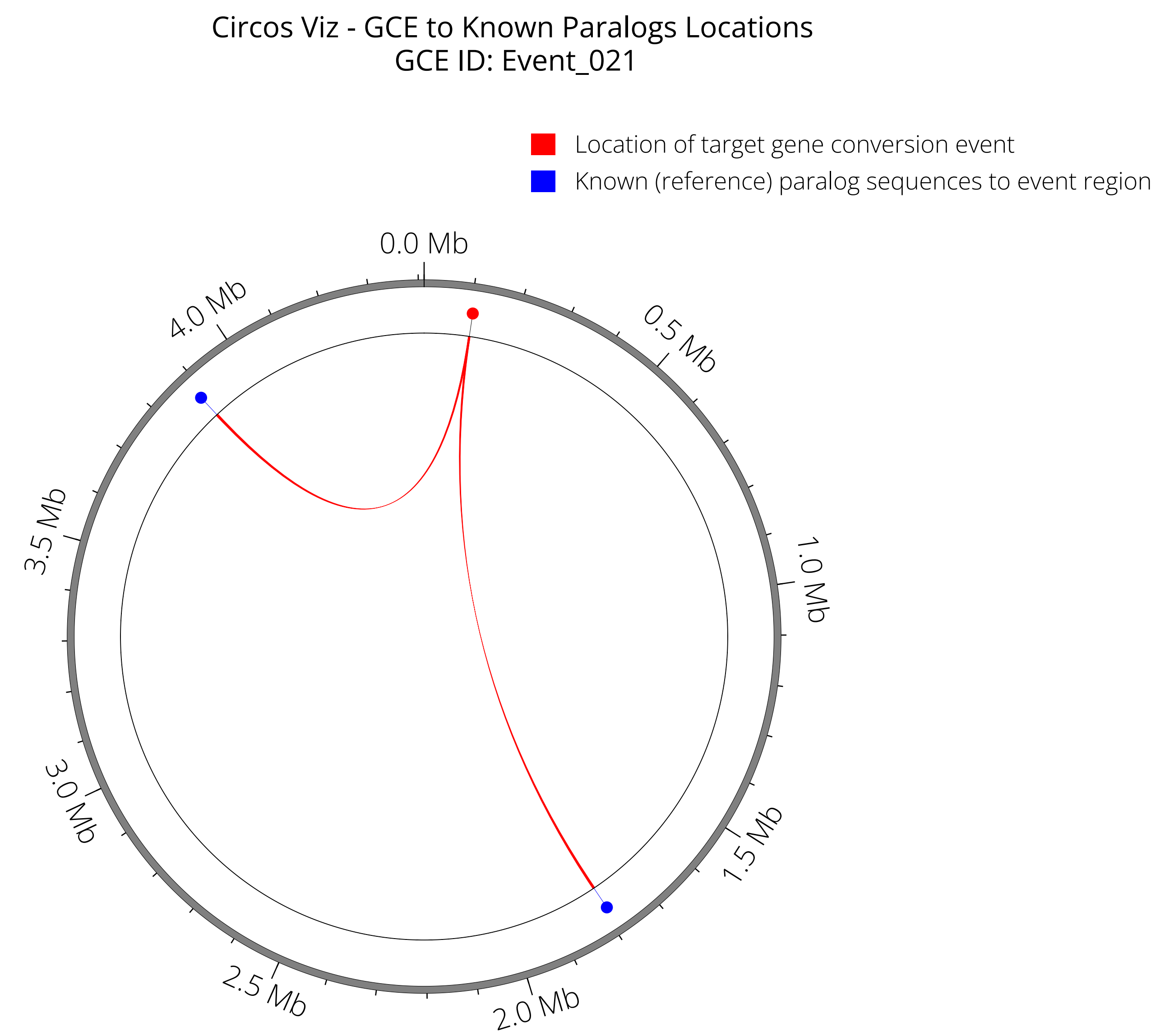
Circos Viz - GCE to Known Paralogs Locations
GCE ID: Event_021
Location of target gene conversion event
Known (reference) paralog sequences to event region
0.0 Mb
0.5 Mb
1.0 Mb
1.5 Mb
2.0 Mb
2.5 Mb
3.0 Mb
3.5 Mb
4.0 Mb

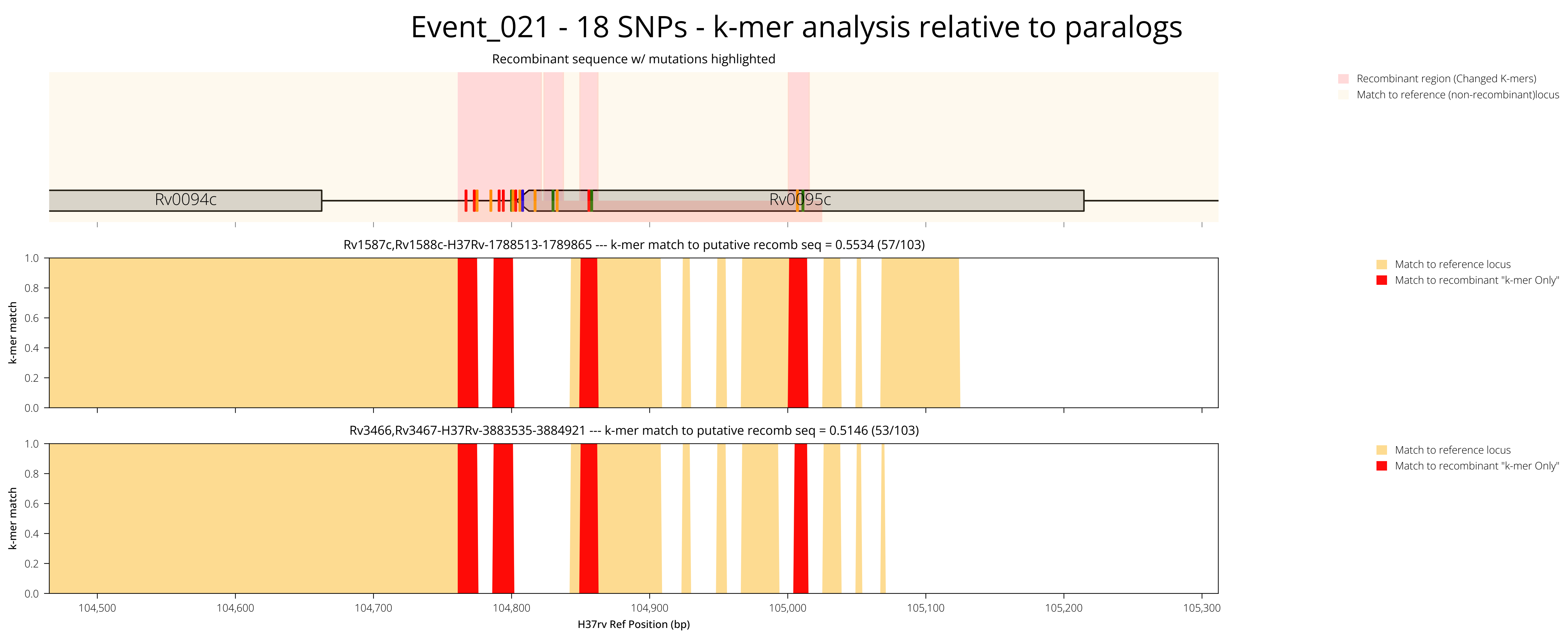

Event_021 - 18 SNPs - k-mer analysis relative to paralogs
Recombinant sequence w/ mutations highlighted
Recombinant region (Changed K-mers)
Match to reference (non-recombinant)locus
Rv0094c
Rv0095c
Rv1587c,Rv1588c-H37Rv-1788513-1789865 --- k-mer match to putative recomb seq = 0.5534 (57/103)
Match to reference locus
Match to recombinant "k-mer Only"
k-mer match
1.0
0.8
0.6
0.4
0.2
0.0
Rv3466,Rv3467-H37Rv-3883535-3884921 --- k-mer match to putative recomb seq = 0.5146 (53/103)
Match to reference locus
Match to recombinant "k-mer Only"
k-mer match
104,500
104,600
104,700
104,800
104,900
105,000
105,100
105,200
105,300
H37rv Ref Position (bp)

Sub-phylogeny Viz + Paralog Alignments
GCE ID: Event_021

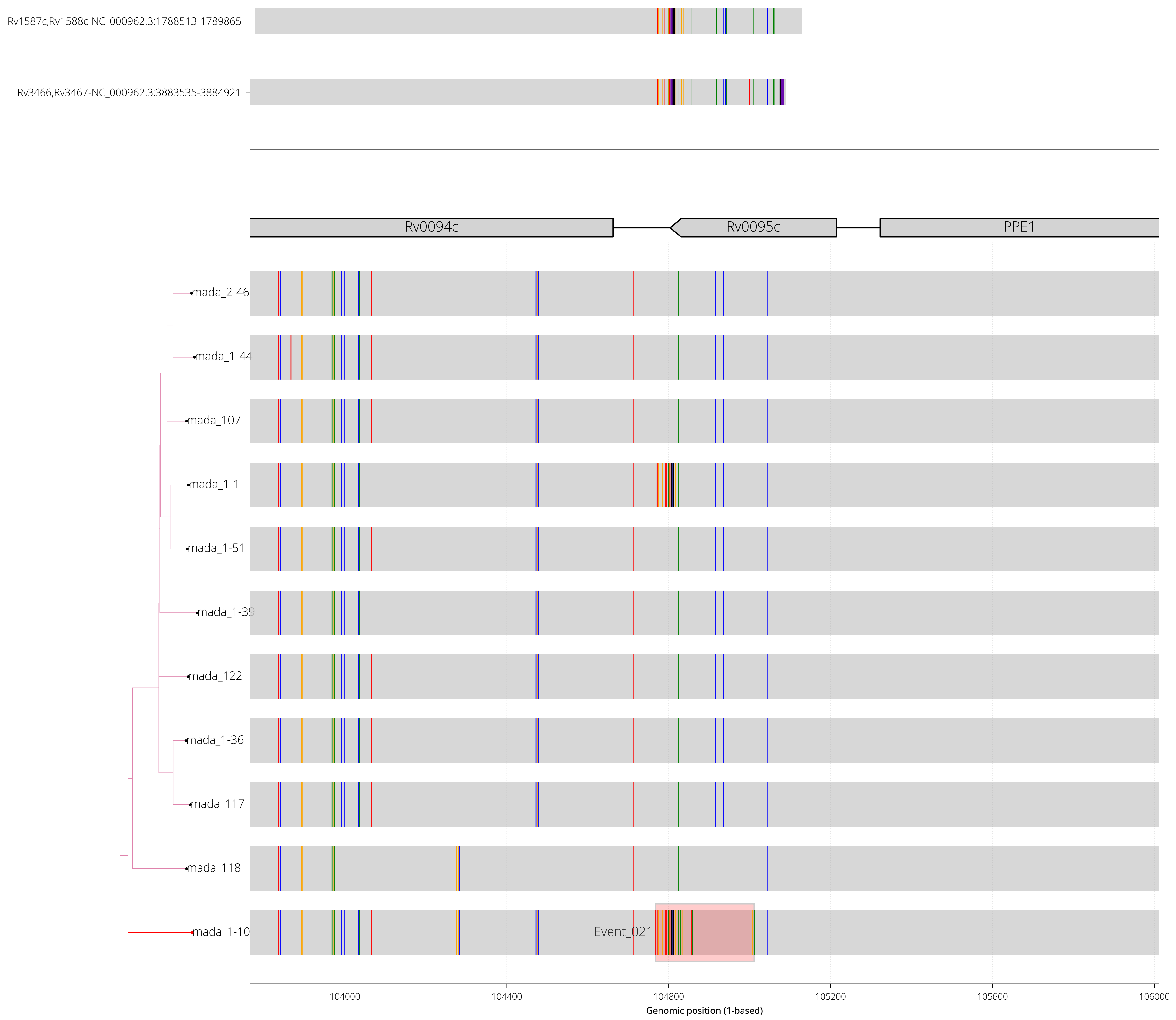

Full Phylogeny Viz + Paralog Alignments
GCE ID: Event_021

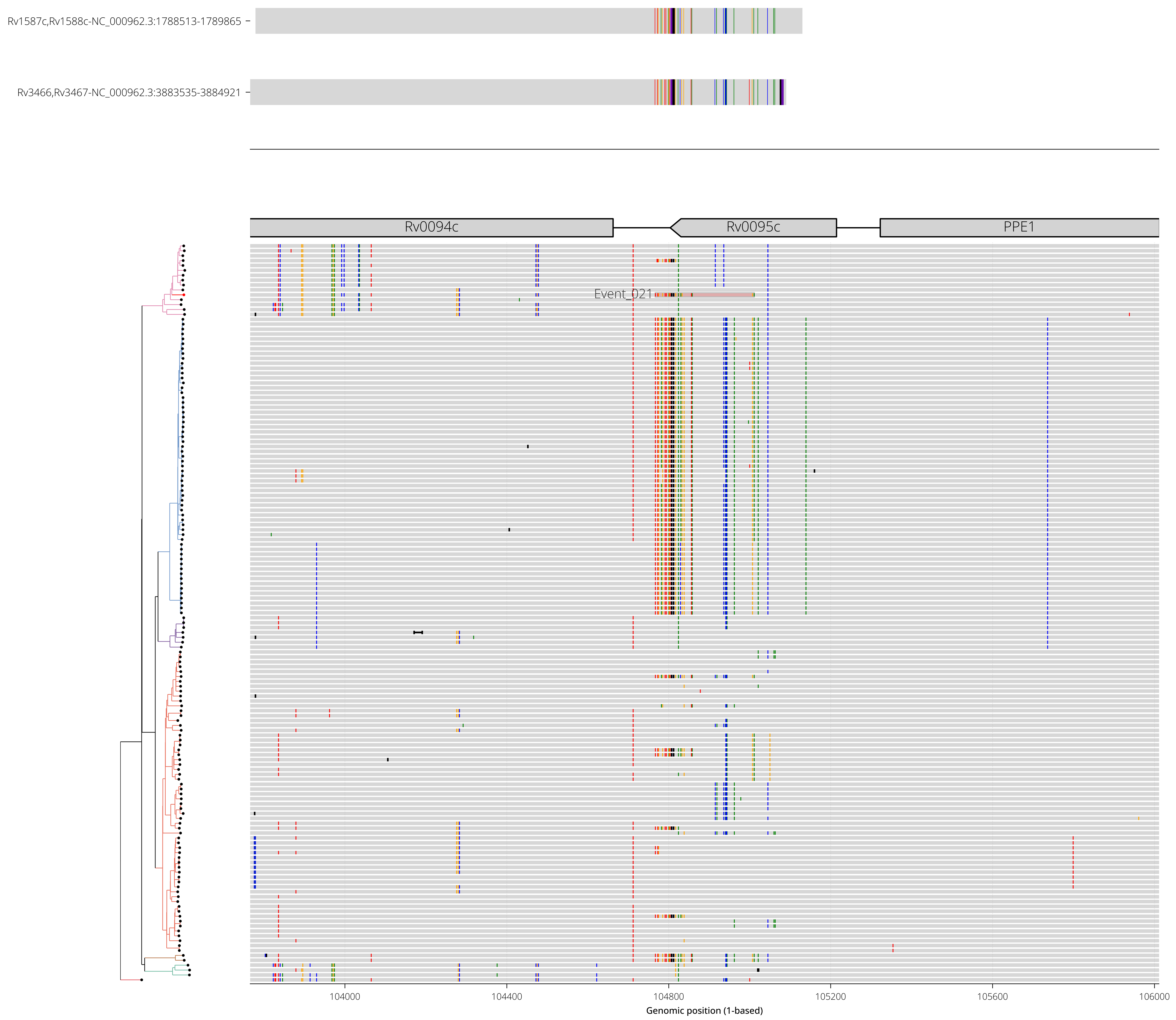

Individual GCE + Known Paralogs
GCE ID: Event_022

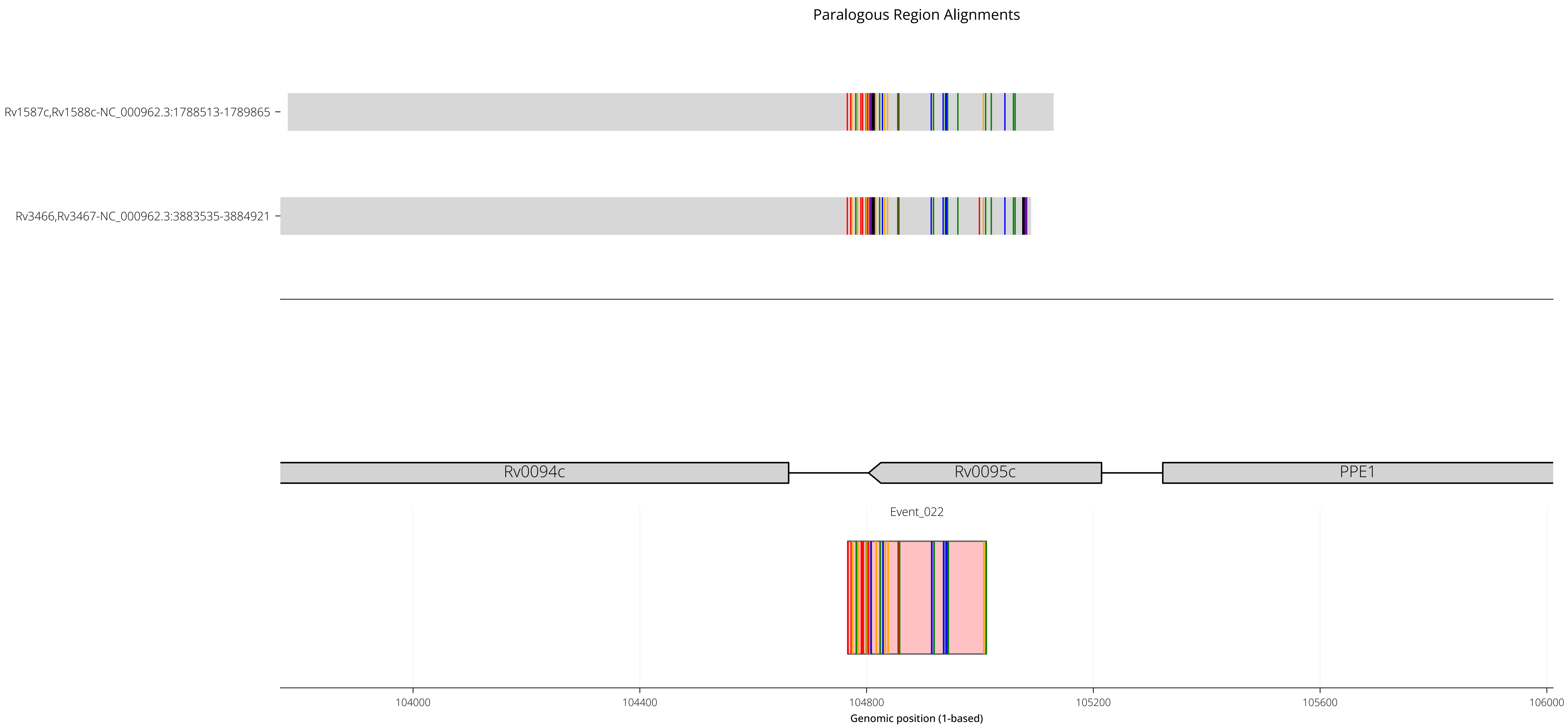

# Circos Viz - GCE to Known Paralogs Locations
GCE ID: Event_022

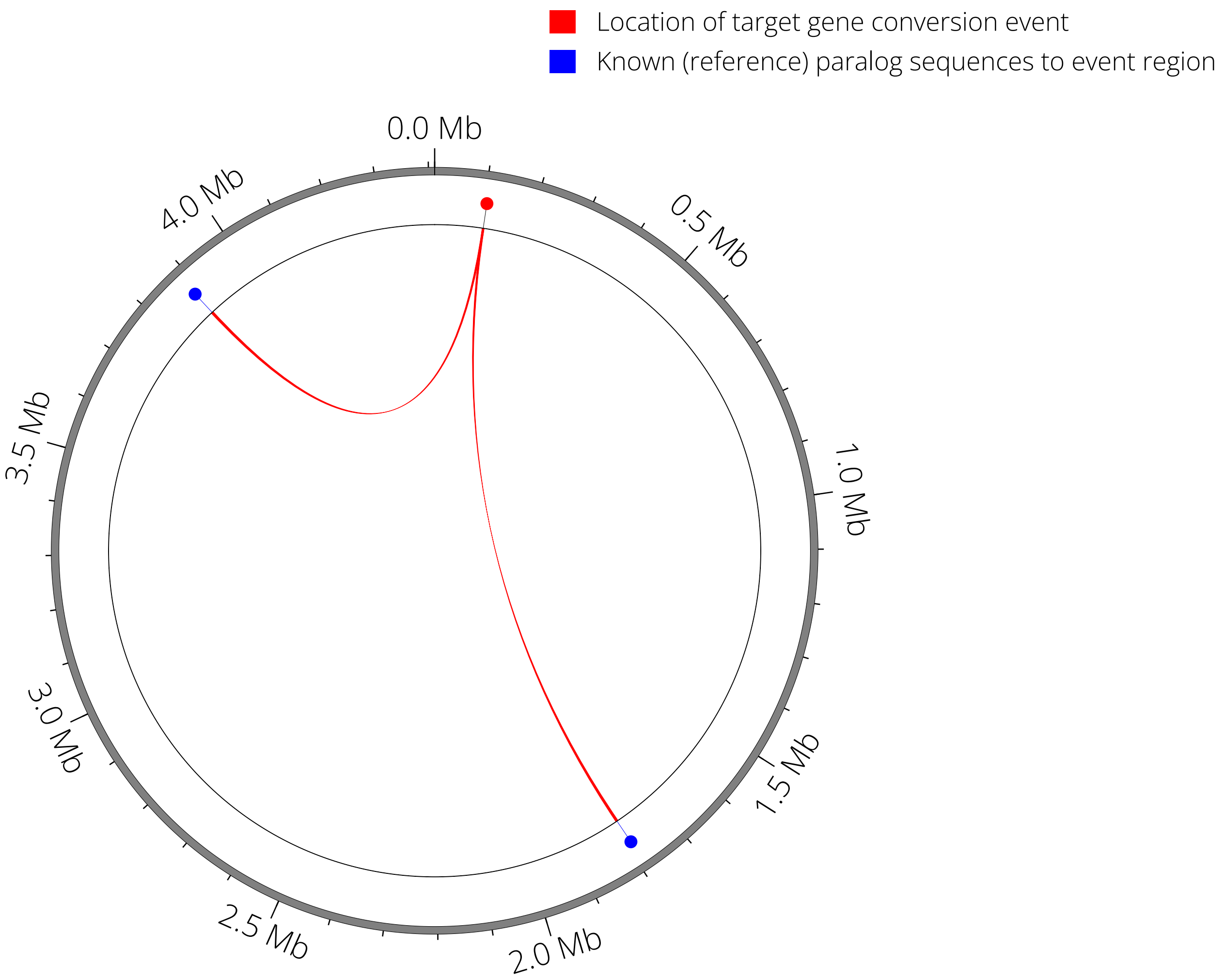

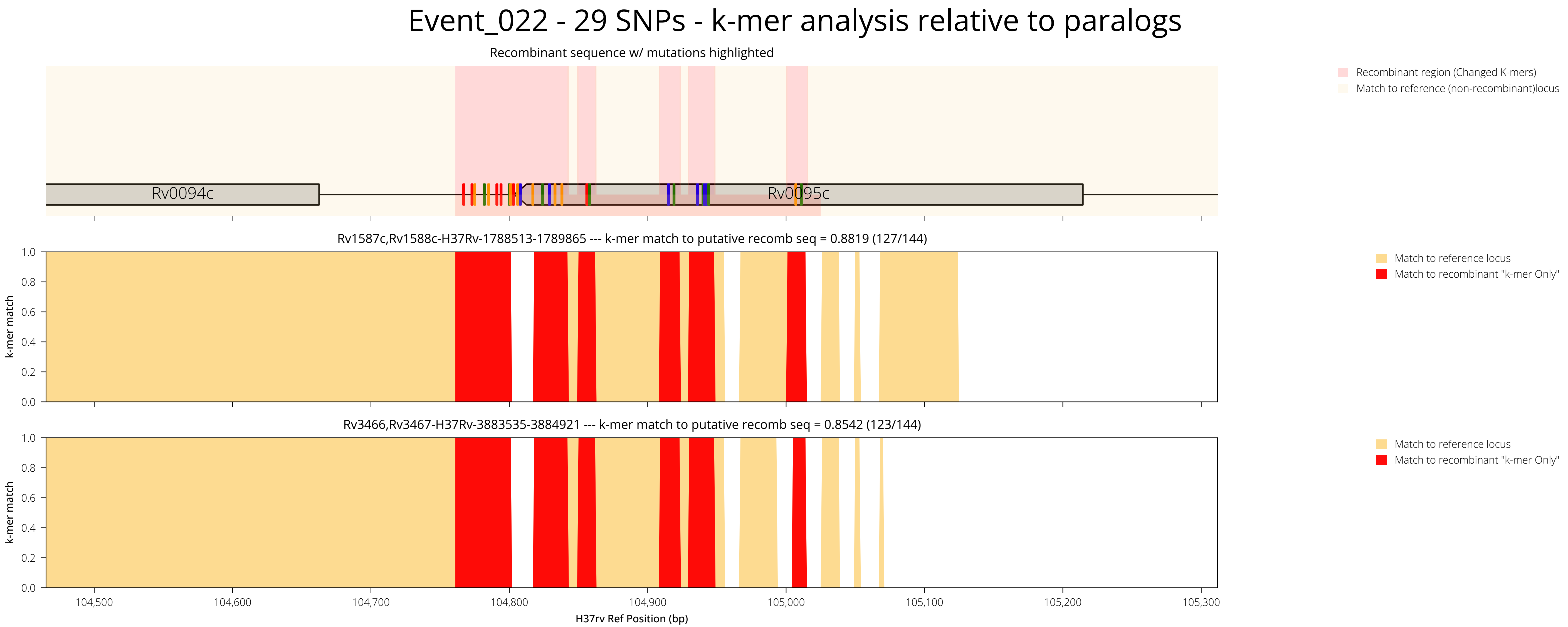
Event_022 - 29 SNPs - k-mer analysis relative to paralogs
Recombinant sequence w/ mutations highlighted
Recombinant region (Changed K-mers)
Match to reference (non-recombinant)locus
Rv0094c
Rv0095c
Rv1587c,Rv1588c-H37Rv-1788513-1789865 --- k-mer match to putative recomb seq = 0.8819 (127/144)
Match to reference locus
Match to recombinant "k-mer Only"
k-mer match
Rv3466,Rv3467-H37Rv-3883535-3884921 --- k-mer match to putative recomb seq = 0.8542 (123/144)
Match to reference locus
Match to recombinant "k-mer Only"
k-mer match
104,500
104,600
104,700
104,800
104,900
105,000
105,100
105,200
105,300
H37rv Ref Position (bp)

Sub-phylogeny Viz + Paralog Alignments
GCE ID: Event_022

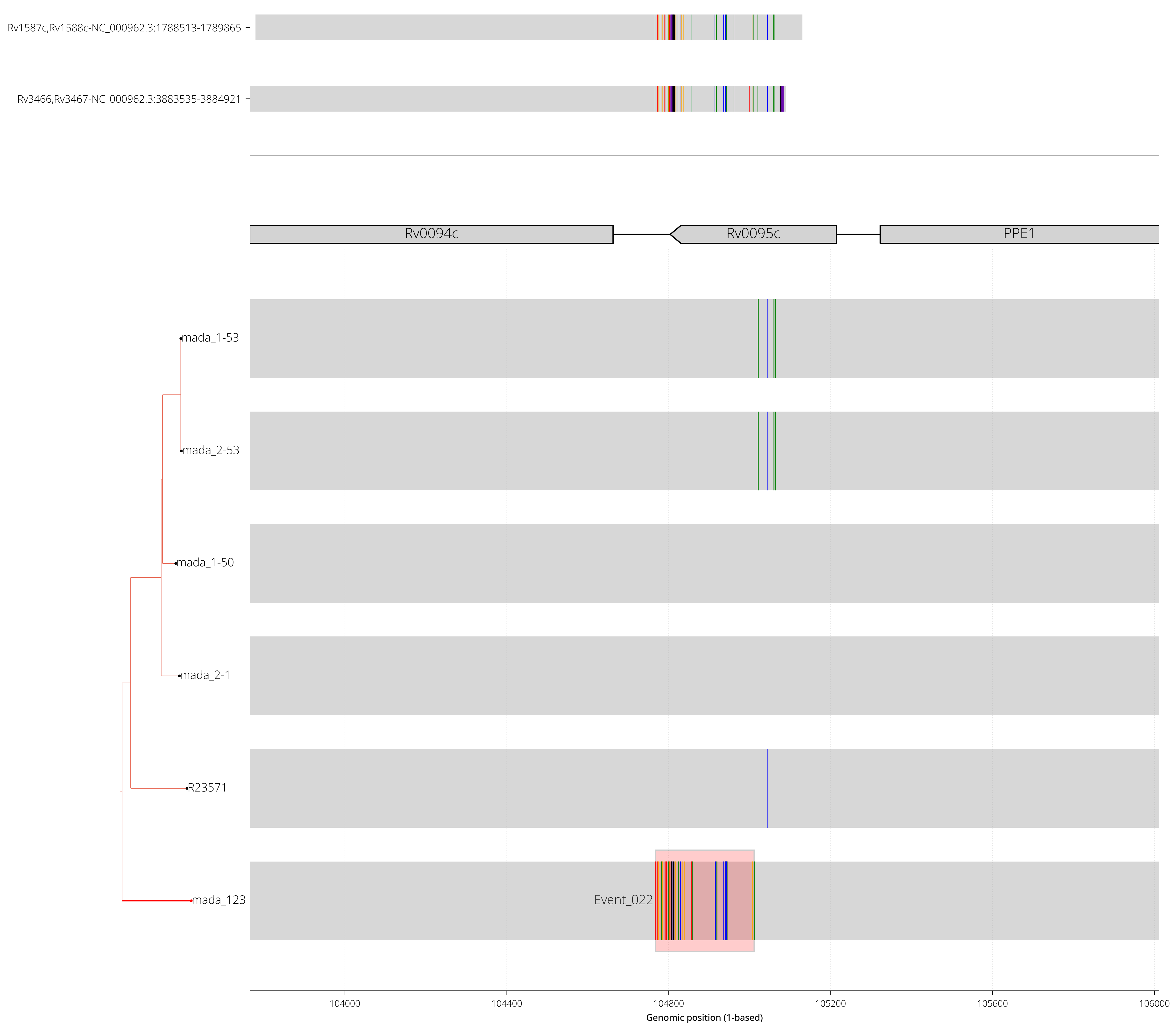

Full Phylogeny Viz + Paralog Alignments
GCE ID: Event_022

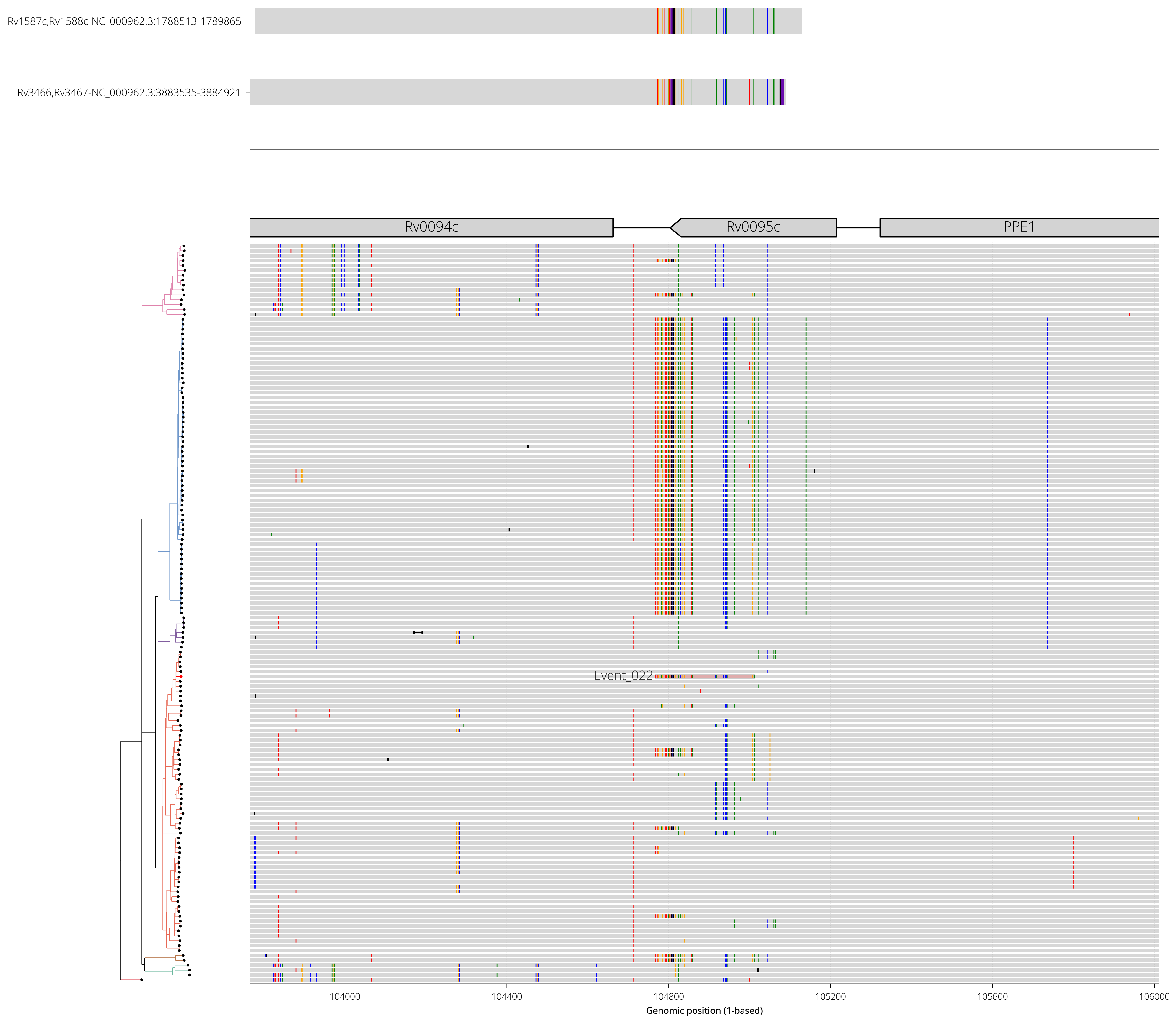

Individual GCE + Known Paralogs
GCE ID: Event_023

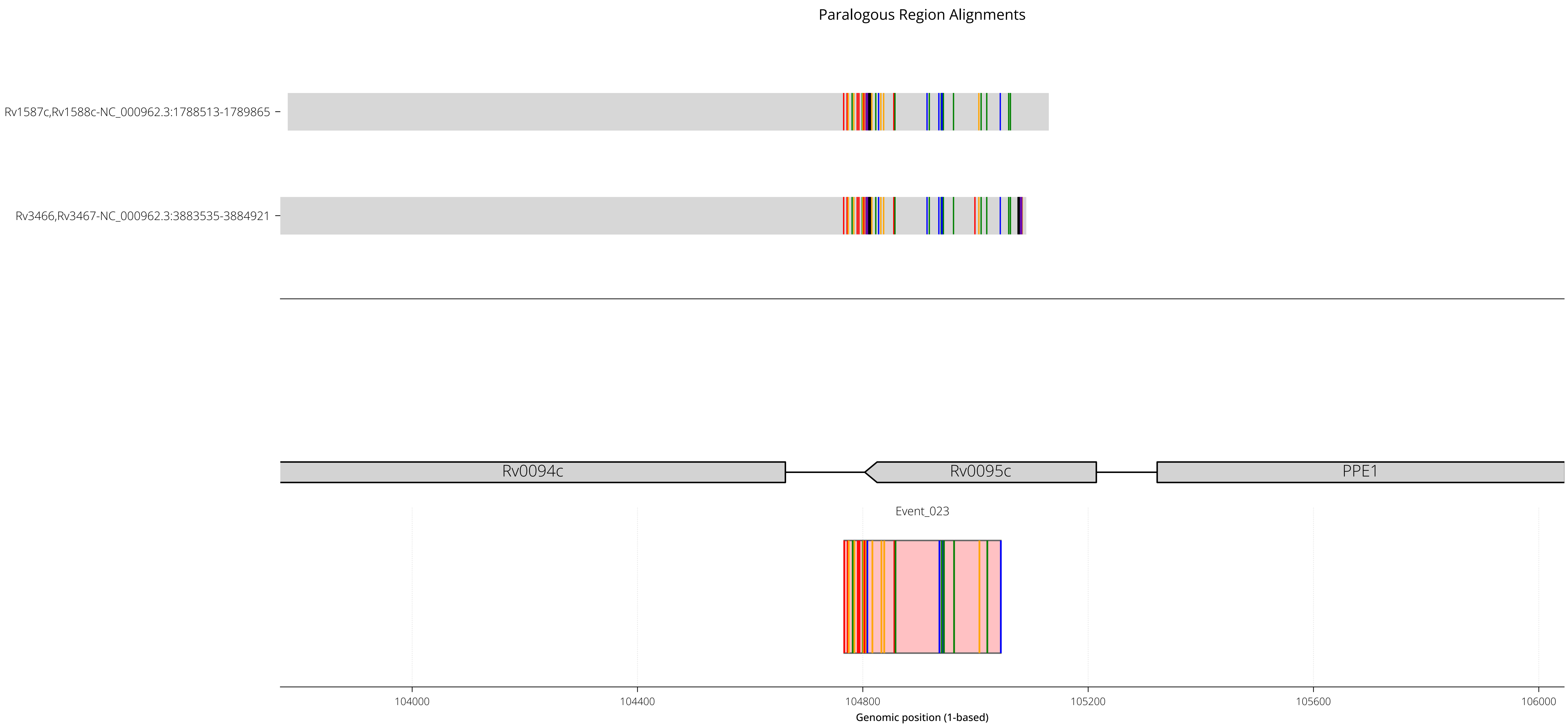

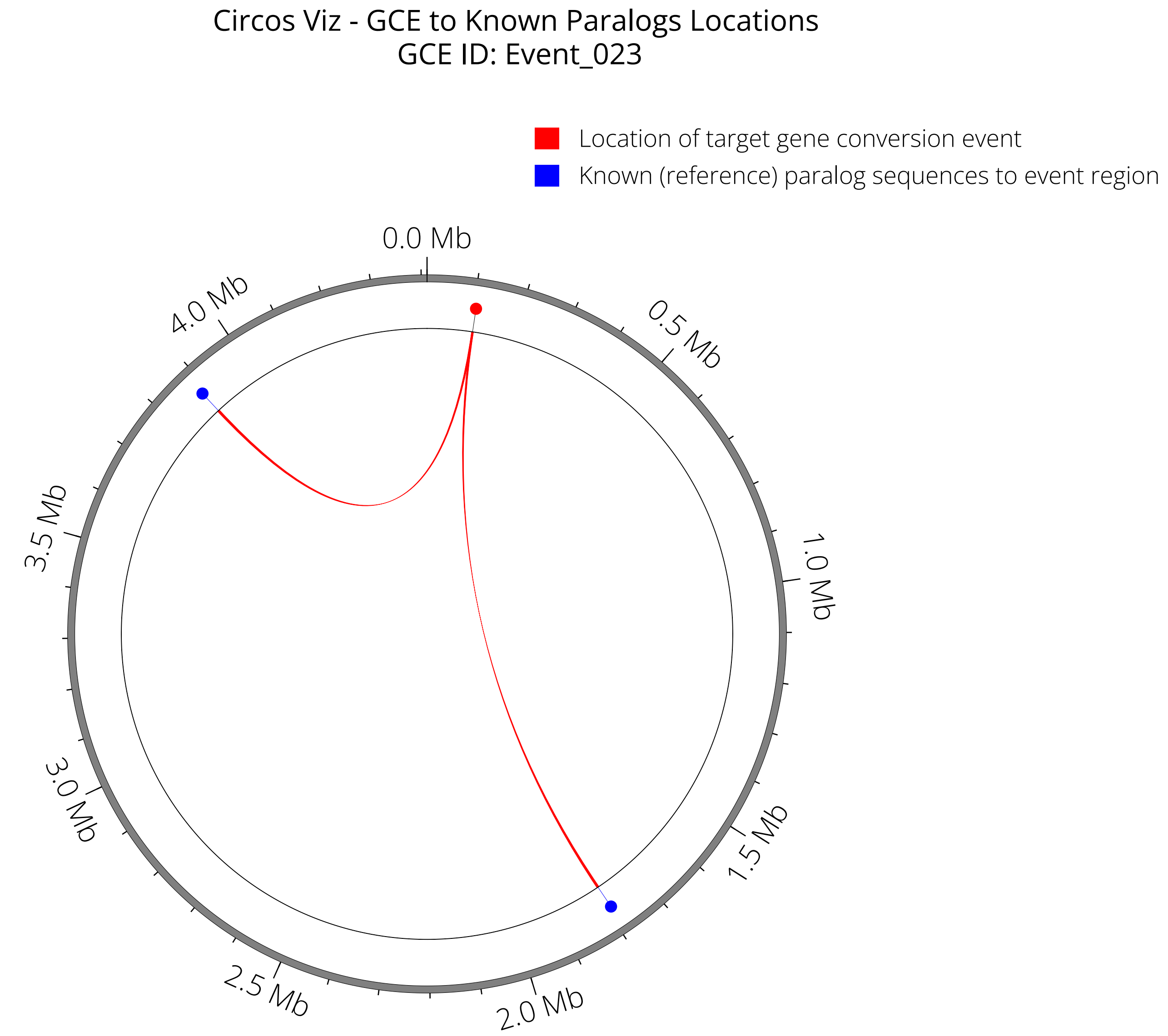
Circos Viz - GCE to Known Paralogs Locations
GCE ID: Event_023
Location of target gene conversion event
Known (reference) paralog sequences to event region
0.0 Mb
0.5 Mb
1.0 Mb
1.5 Mb
2.0 Mb
2.5 Mb
3.0 Mb
3.5 Mb
4.0 Mb

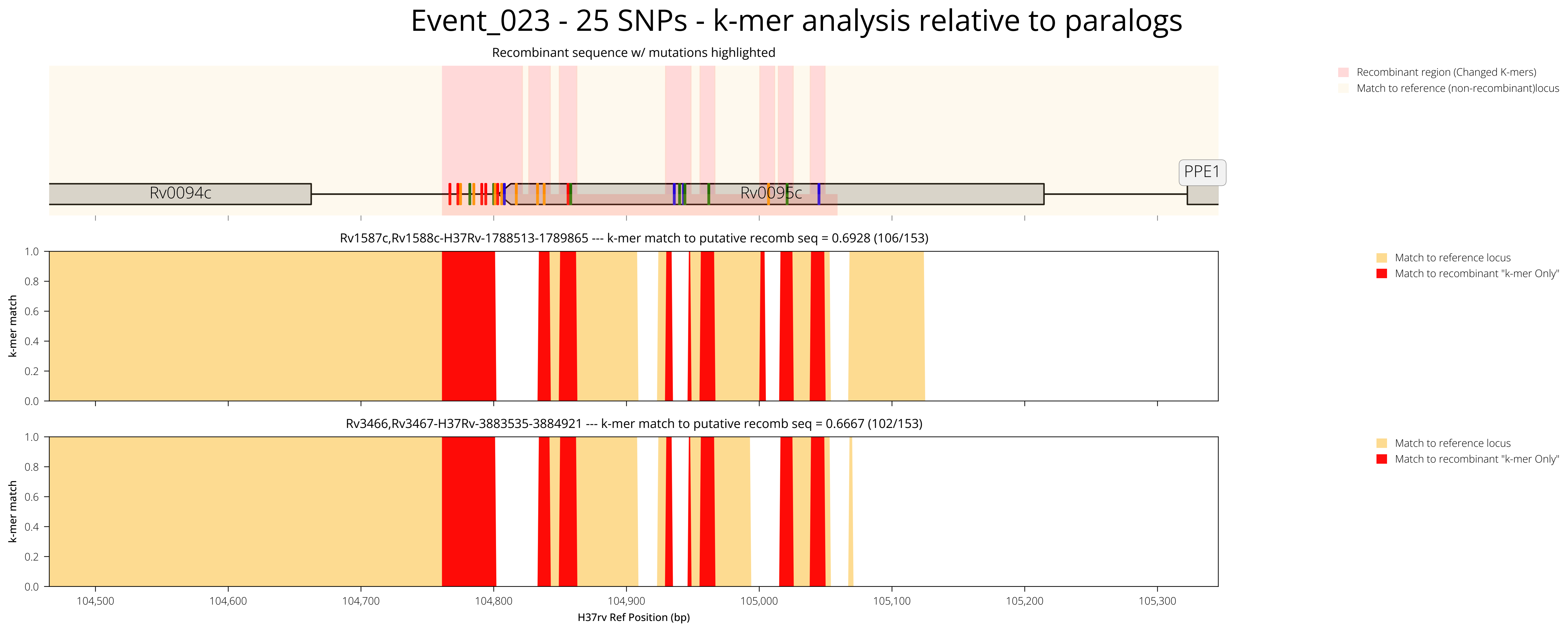

Event_023 - 25 SNPs - k-mer analysis relative to paralogs
Recombinant sequence w/ mutations highlighted
Recombinant region (Changed K-mers)
Match to reference (non-recombinant)locus
Rv0094c
Rv0095c
PPE1
Rv1587c,Rv1588c-H37Rv-1788513-1789865 --- k-mer match to putative recomb seq = 0.6928 (106/153)
Match to reference locus
Match to recombinant "k-mer Only"
k-mer match
1.0
0.8
0.6
0.4
0.2
0.0
Rv3466,Rv3467-H37Rv-3883535-3884921 --- k-mer match to putative recomb seq = 0.6667 (102/153)
Match to reference locus
Match to recombinant "k-mer Only"
104,500
104,600
104,700
104,800
104,900
105,000
105,100
105,200
105,300
H37rv Ref Position (bp)

Sub-phylogeny Viz + Paralog Alignments
GCE ID: Event_023

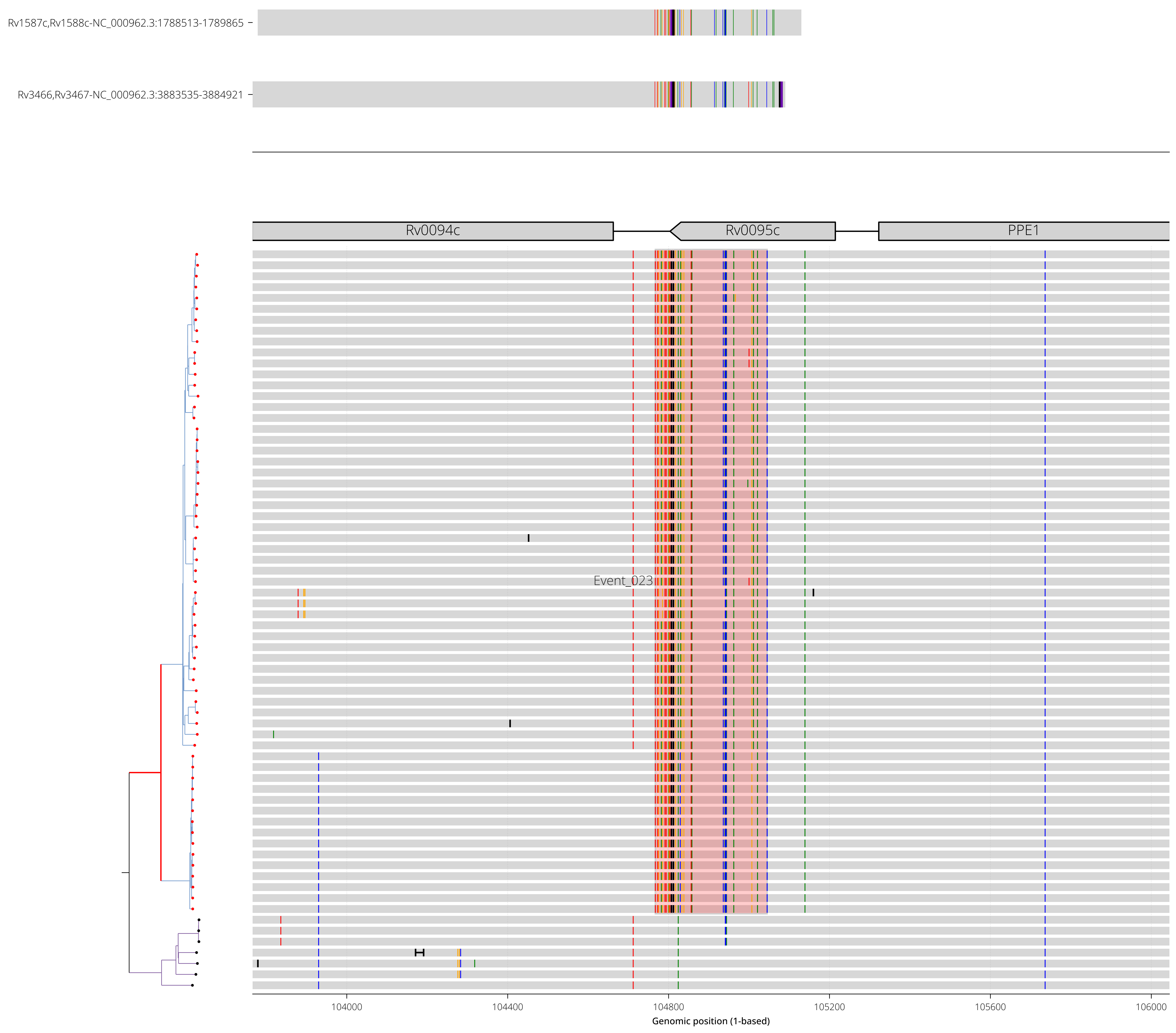

Full Phylogeny Viz + Paralog Alignments
GCE ID: Event_023

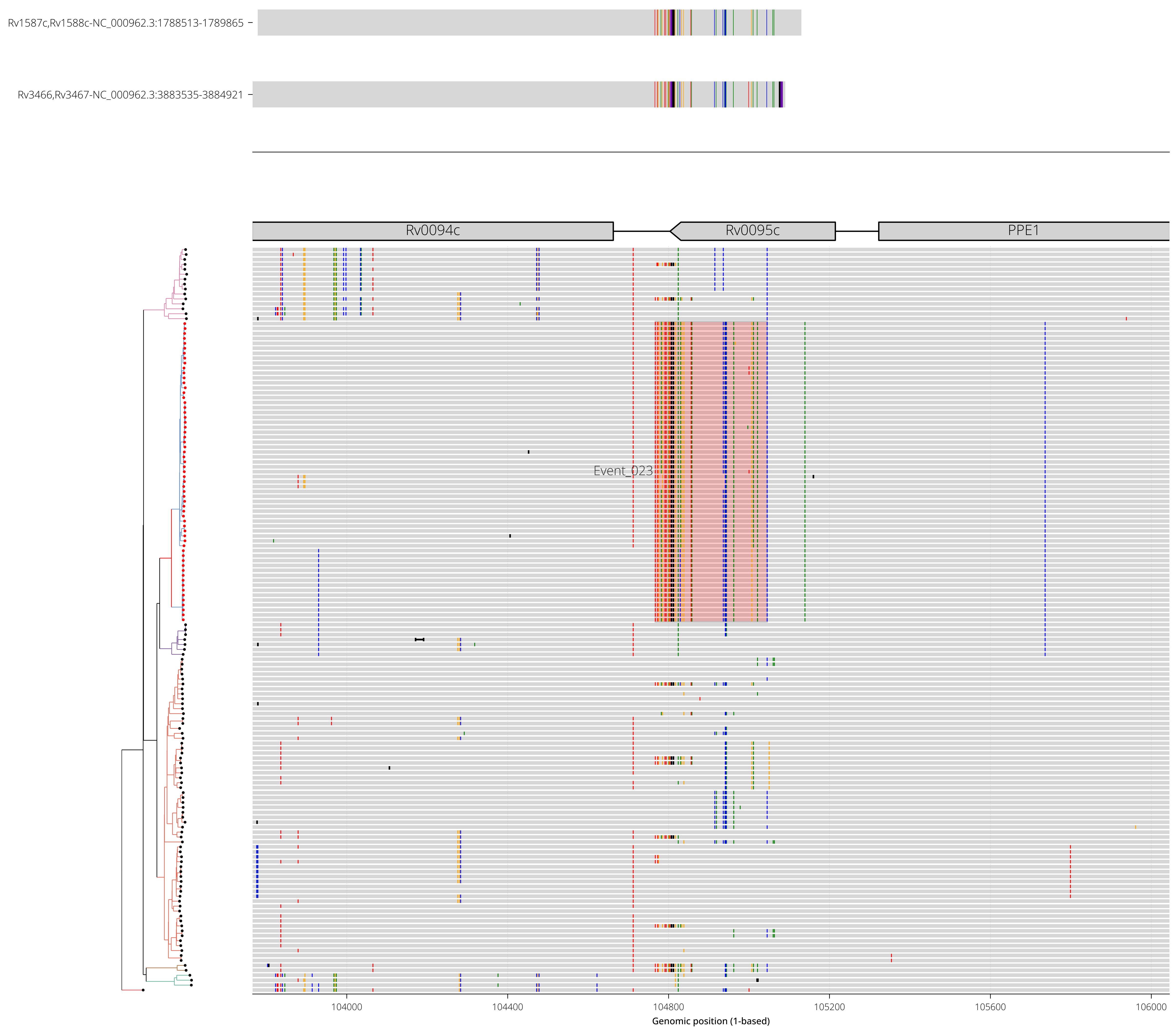

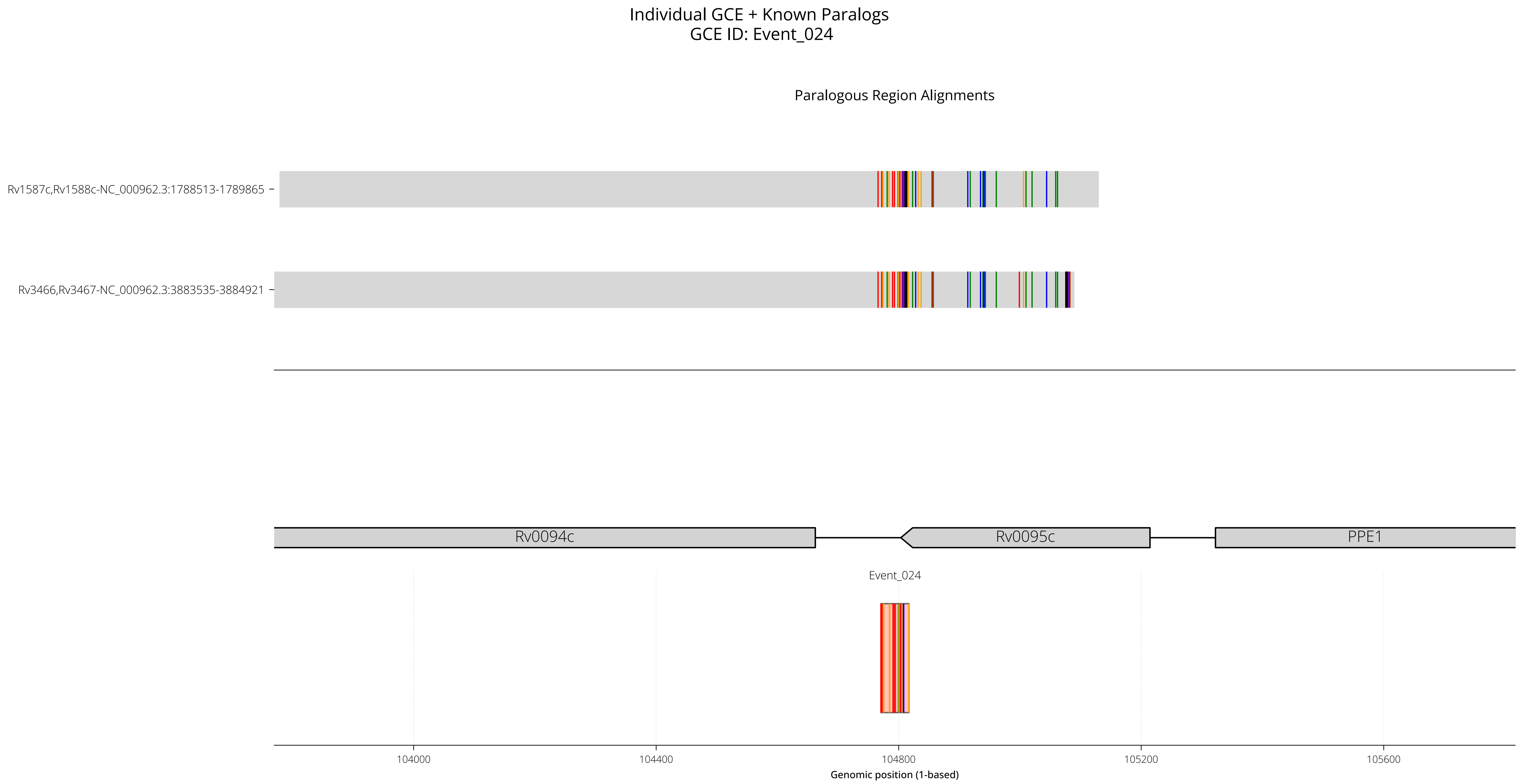
Individual GCE + Known Paralogs
GCE ID: Event_024
Paralogous Region Alignments
Rv1587c,Rv1588c-NC_000962.3:1788513-1789865
Rv3466,Rv3467-NC_000962.3:3883535-3884921
Rv0094c
Rv0095c
PPE1
Event_024
104000
104400
104800
105200
105600
Genomic position (1-based)

Circos Viz - GCE to Known Paralogs Locations
GCE ID: Event_024

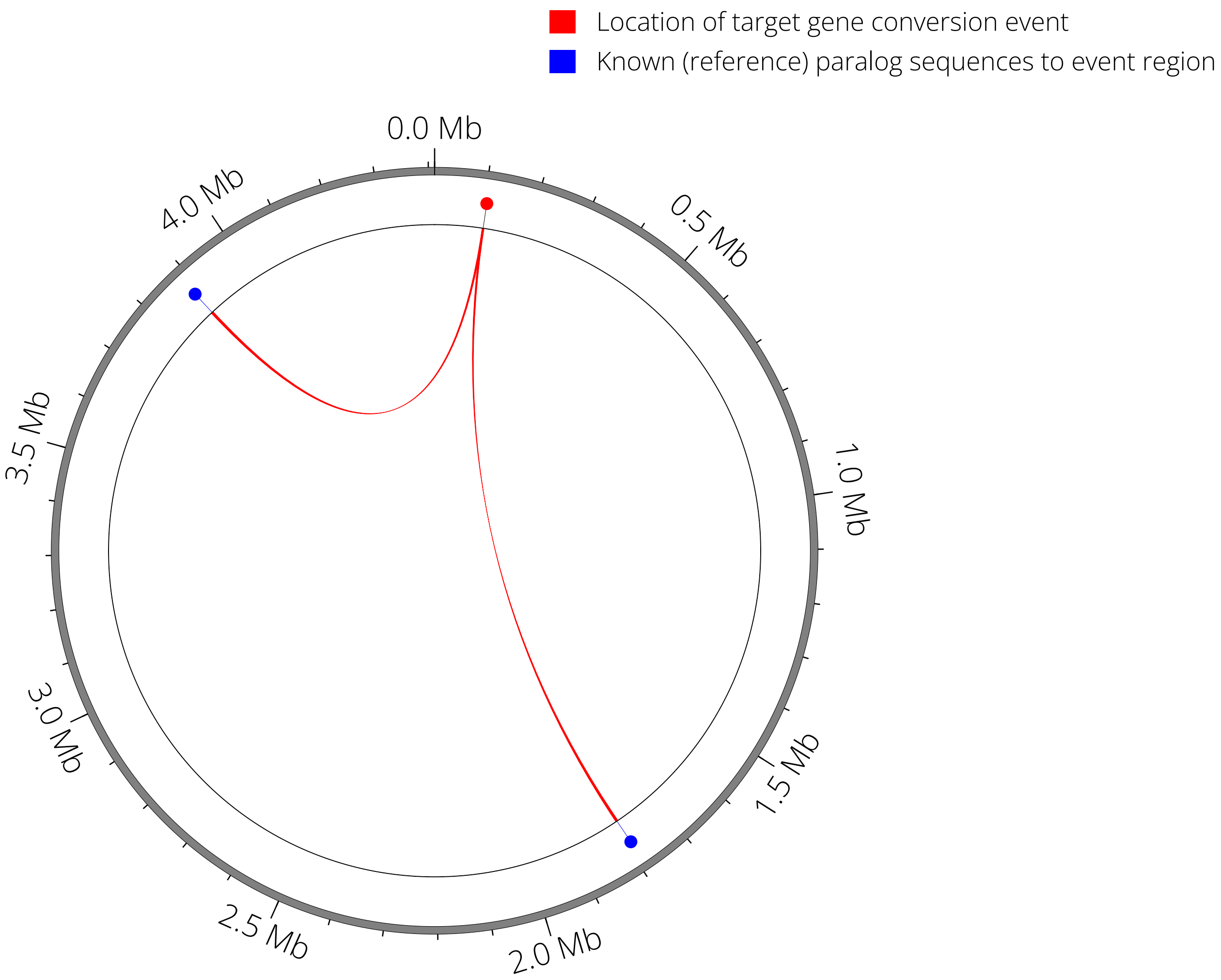

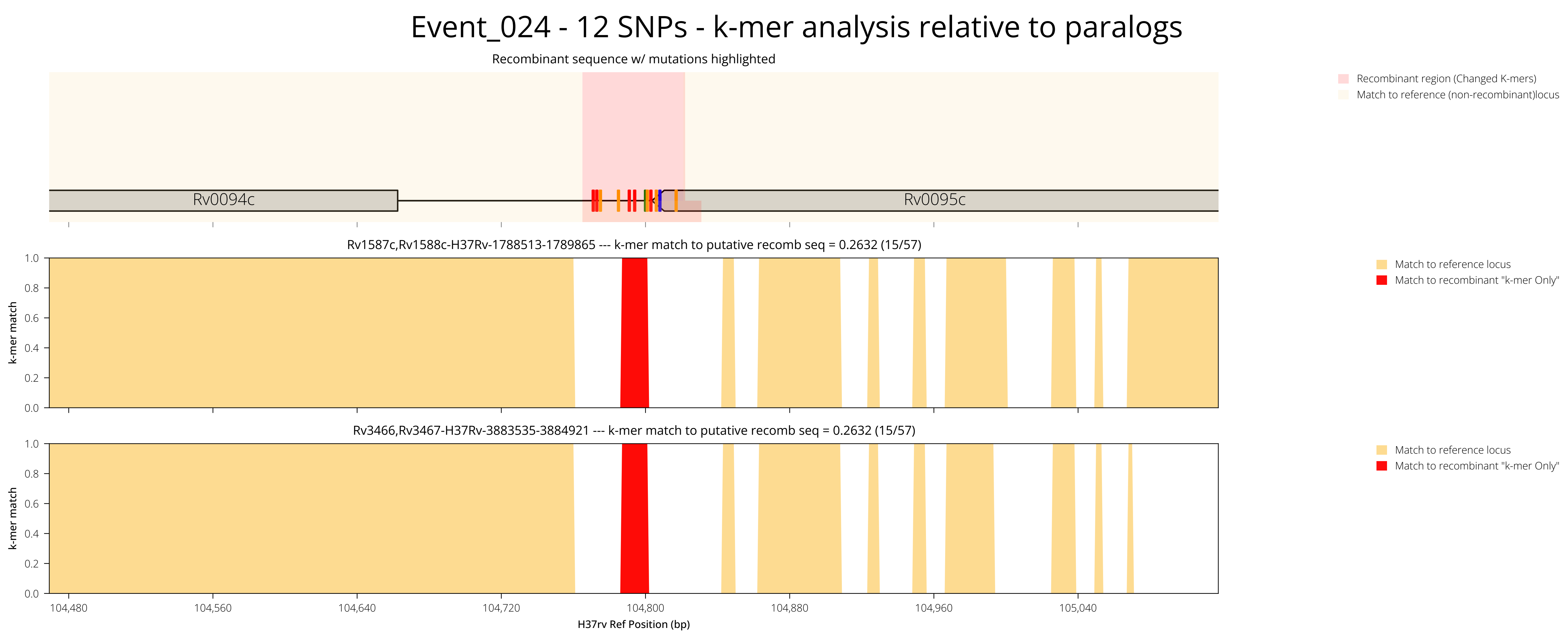
Event_024 - 12 SNPs - k-mer analysis relative to paralogs
Recombinant sequence w/ mutations highlighted
Rv0094c
Rv0095c
Recombinant region (Changed K-mers)
Match to reference (non-recombinant)locus
Rv1587c,Rv1588c-H37Rv-1788513-1789865 --- k-mer match to putative recomb seq = 0.2632 (15/57)
Match to reference locus
Match to recombinant "k-mer Only"
k-mer match
1.0
0.8
0.6
0.4
0.2
0.0
Rv3466,Rv3467-H37Rv-3883535-3884921 --- k-mer match to putative recomb seq = 0.2632 (15/57)
Match to reference locus
Match to recombinant "k-mer Only"
k-mer match
1.0
0.8
0.6
0.4
0.2
0.0
104,480
104,560
104,640
104,720
104,800
104,880
104,960
105,040
H37rv Ref Position (bp)

Sub-phylogeny Viz + Paralog Alignments
GCE ID: Event_024

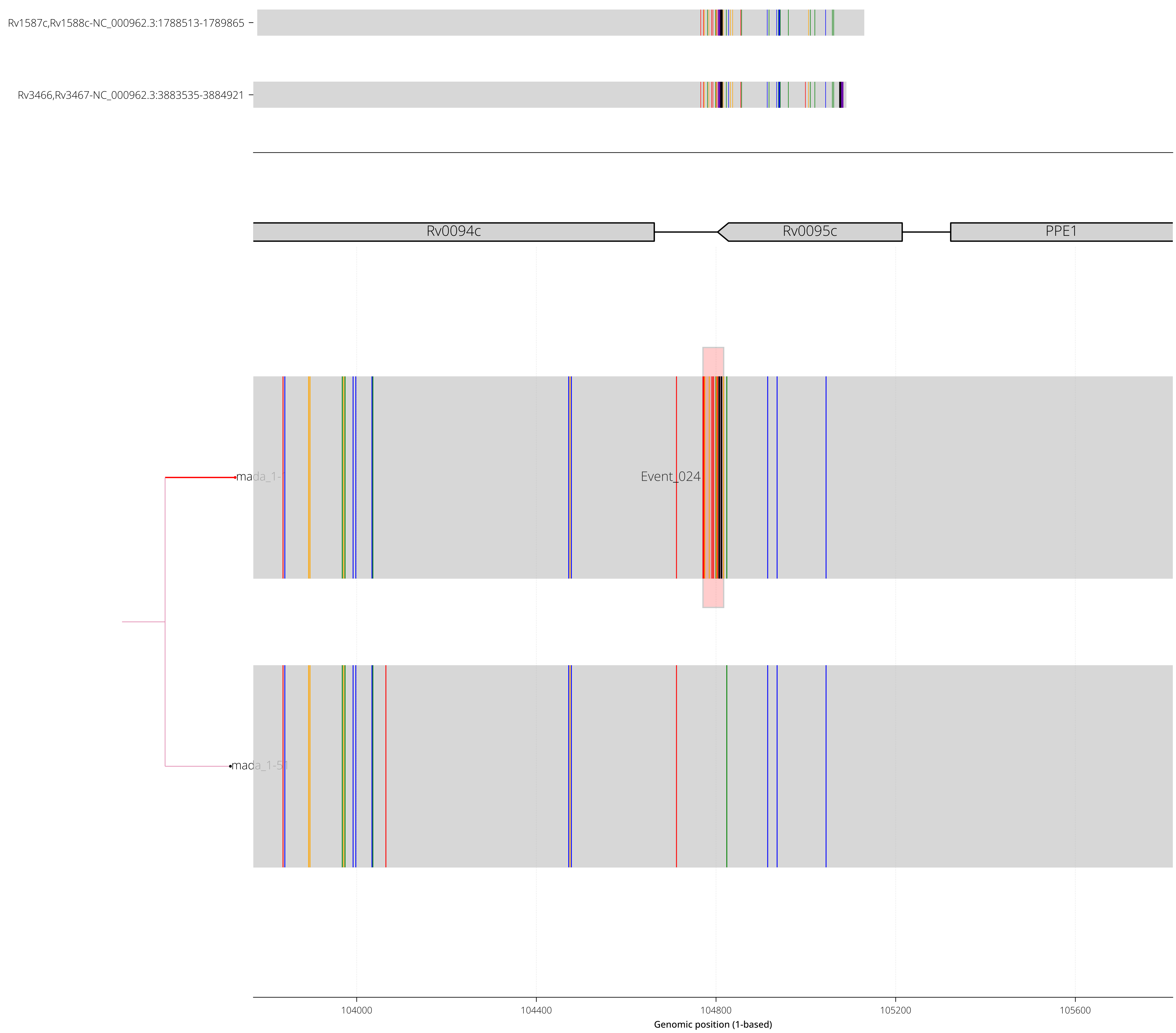

Full Phylogeny Viz + Paralog Alignments
GCE ID: Event_024

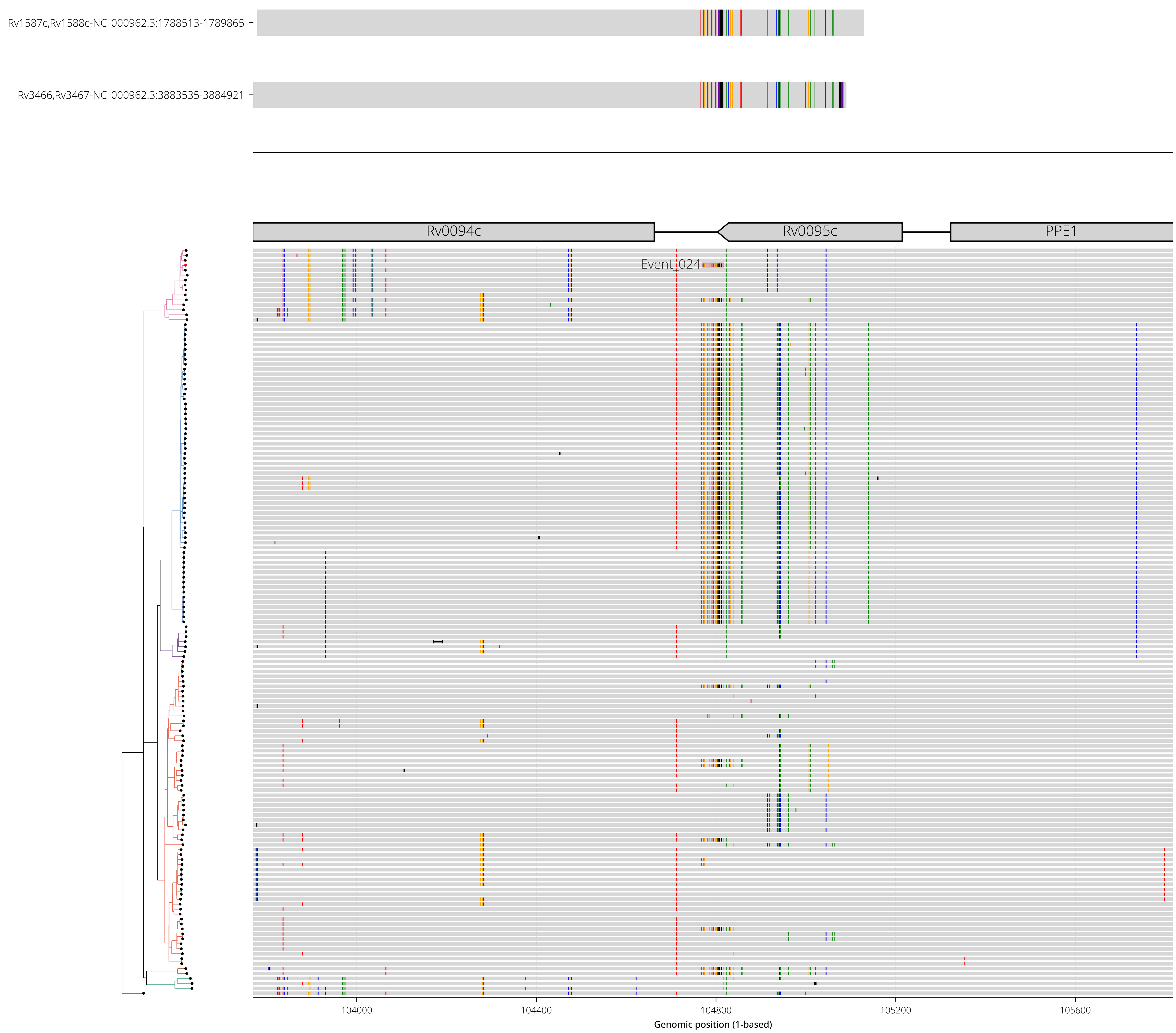

# Individual GCE + Known Paralogs
GCE ID: Event_025

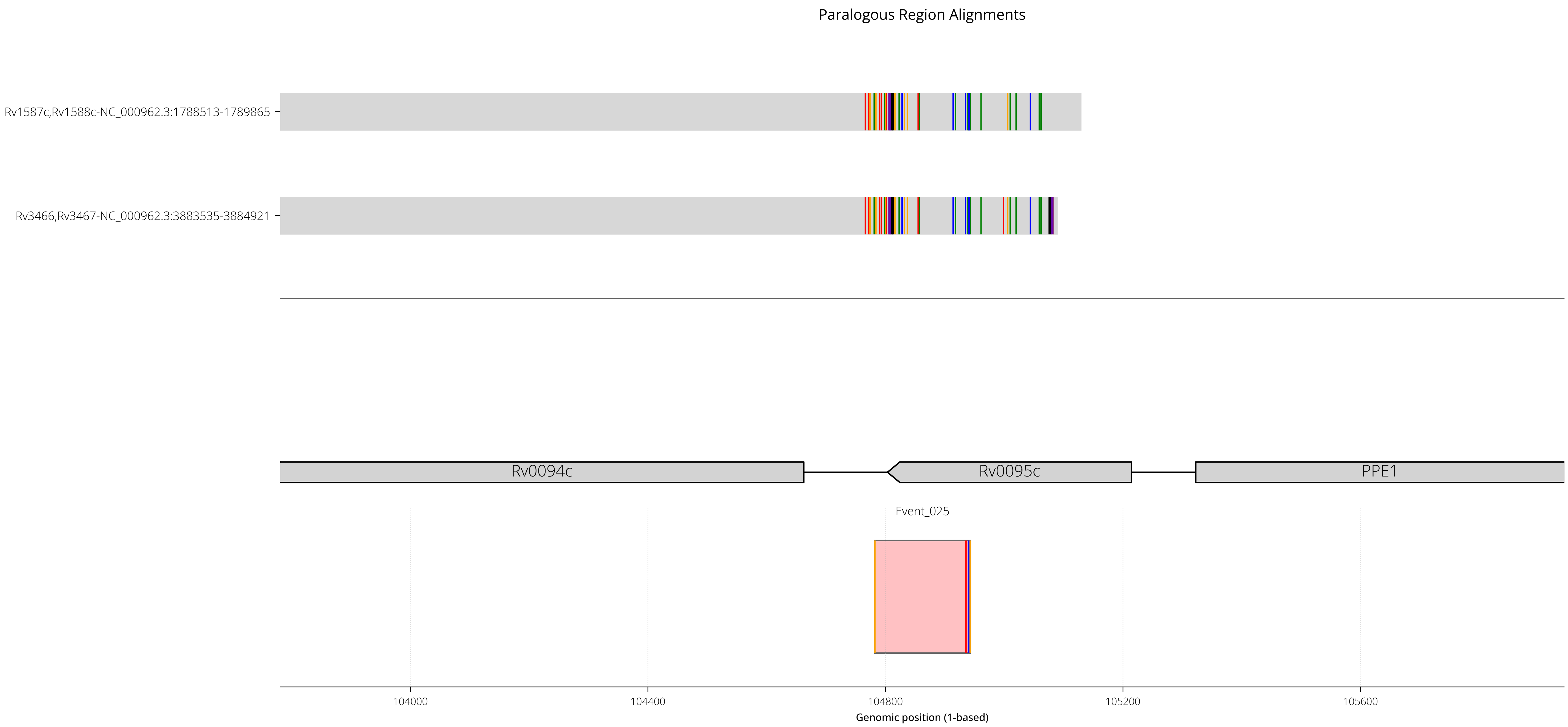

Circos Viz - GCE to Known Paralogs Locations
GCE ID: Event_025

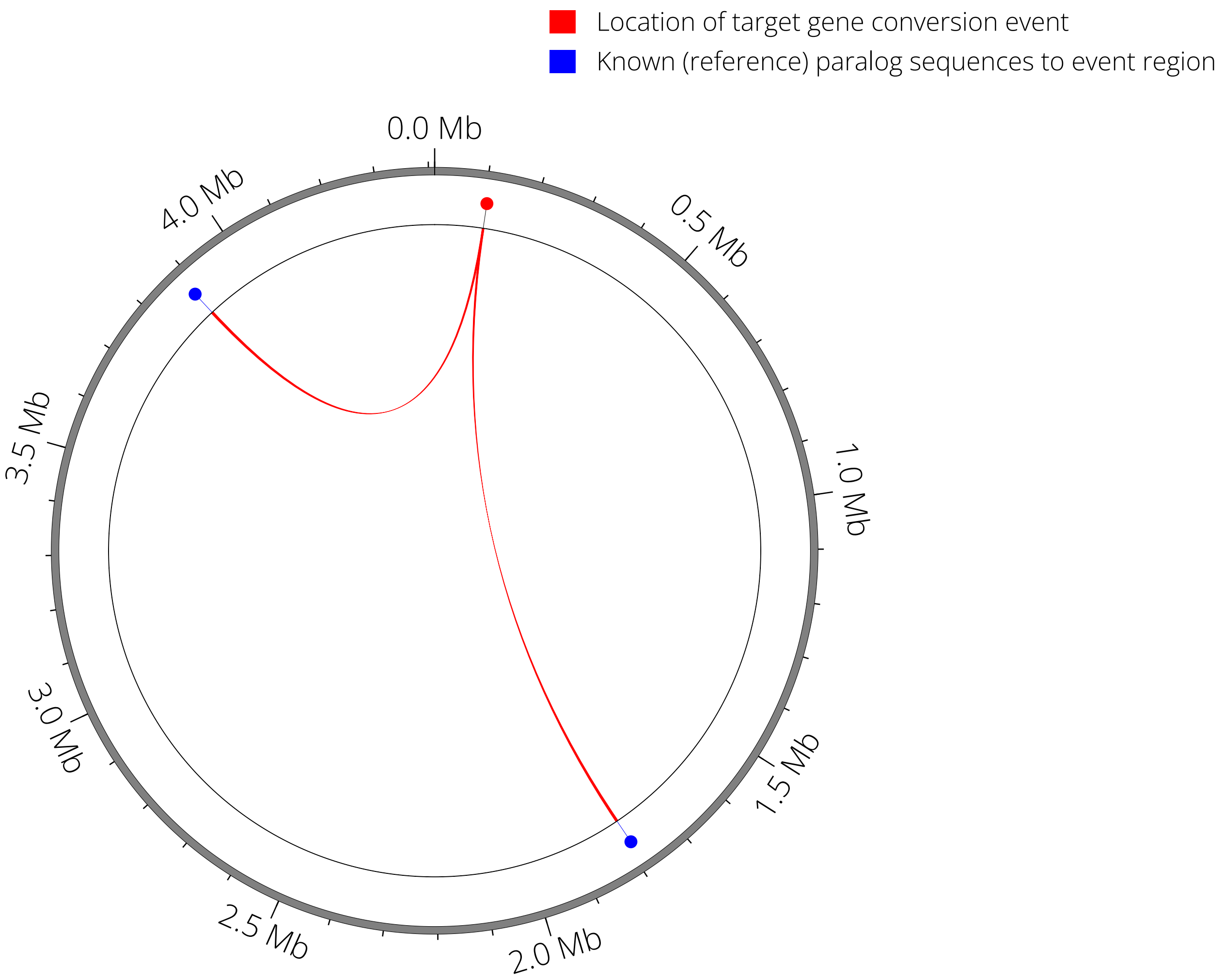

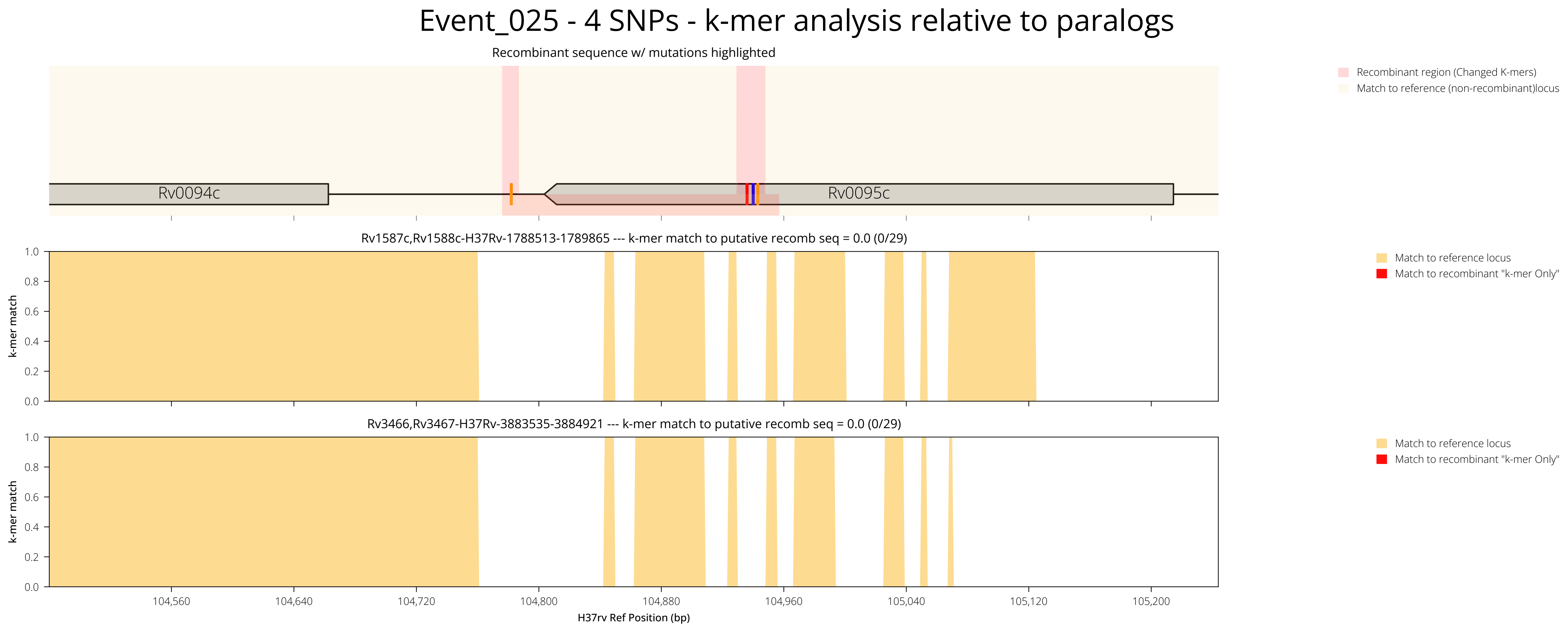
Event_025 - 4 SNPs - k-mer analysis relative to paralogs
Recombinant sequence w/ mutations highlighted
Rv0094c
Rv0095c
Recombinant region (Changed K-mers)
Match to reference (non-recombinant)locus
Rv1587c,Rv1588c-H37Rv-1788513-1789865 --- k-mer match to putative recomb seq = 0.0 (0/29)
k-mer match
1.0
0.8
0.6
0.4
0.2
0.0
Match to reference locus
Match to recombinant "k-mer Only"
Rv3466,Rv3467-H37Rv-3883535-3884921 --- k-mer match to putative recomb seq = 0.0 (0/29)
k-mer match
1.0
0.8
0.6
0.4
0.2
0.0
Match to reference locus
Match to recombinant "k-mer Only"
104,560
104,640
104,720
104,800
104,880
104,960
105,040
105,120
105,200
H37rv Ref Position (bp)

Sub-phylogeny Viz + Paralog Alignments
GCE ID: Event_025

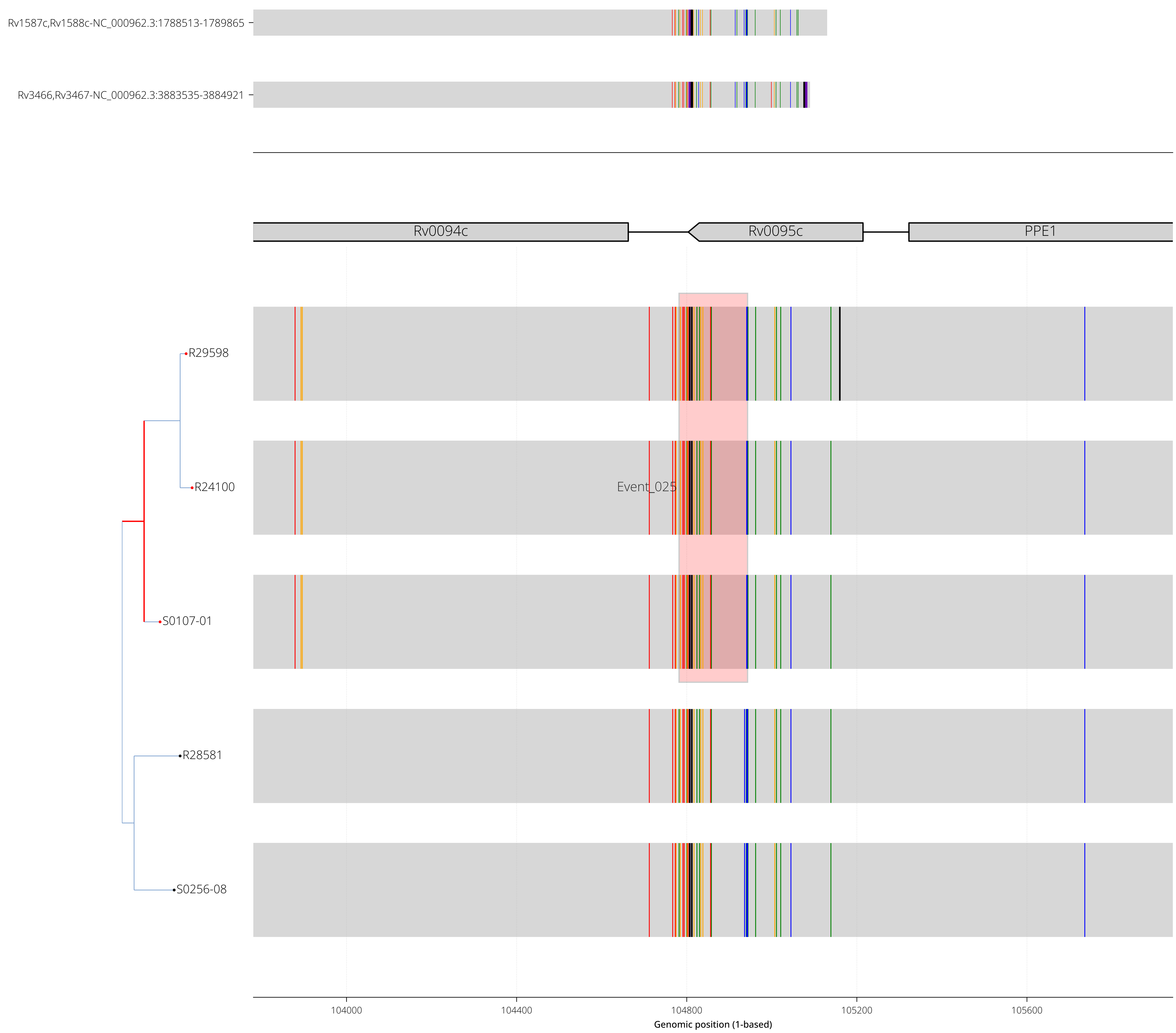

Full Phylogeny Viz + Paralog Alignments
GCE ID: Event_025

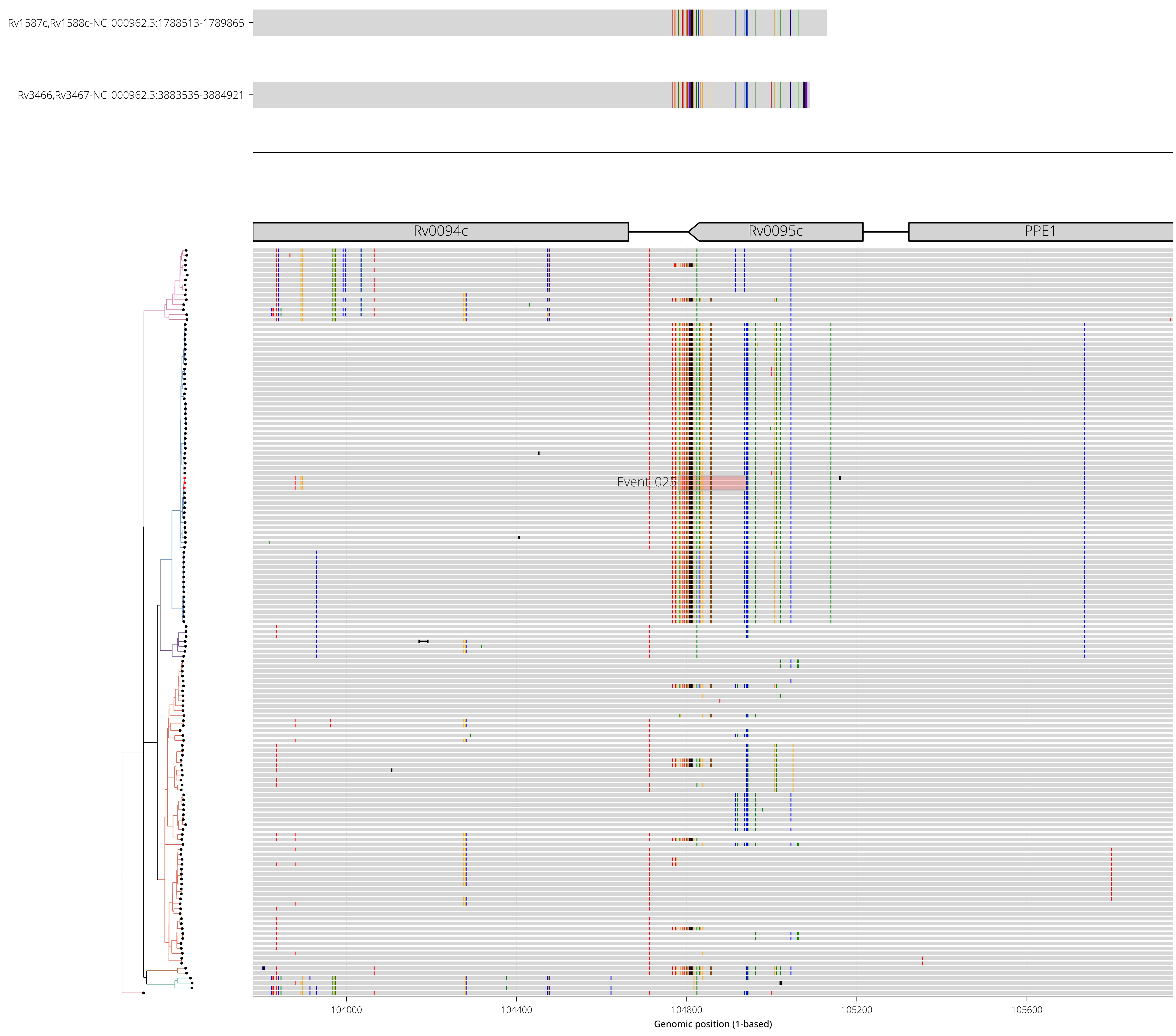

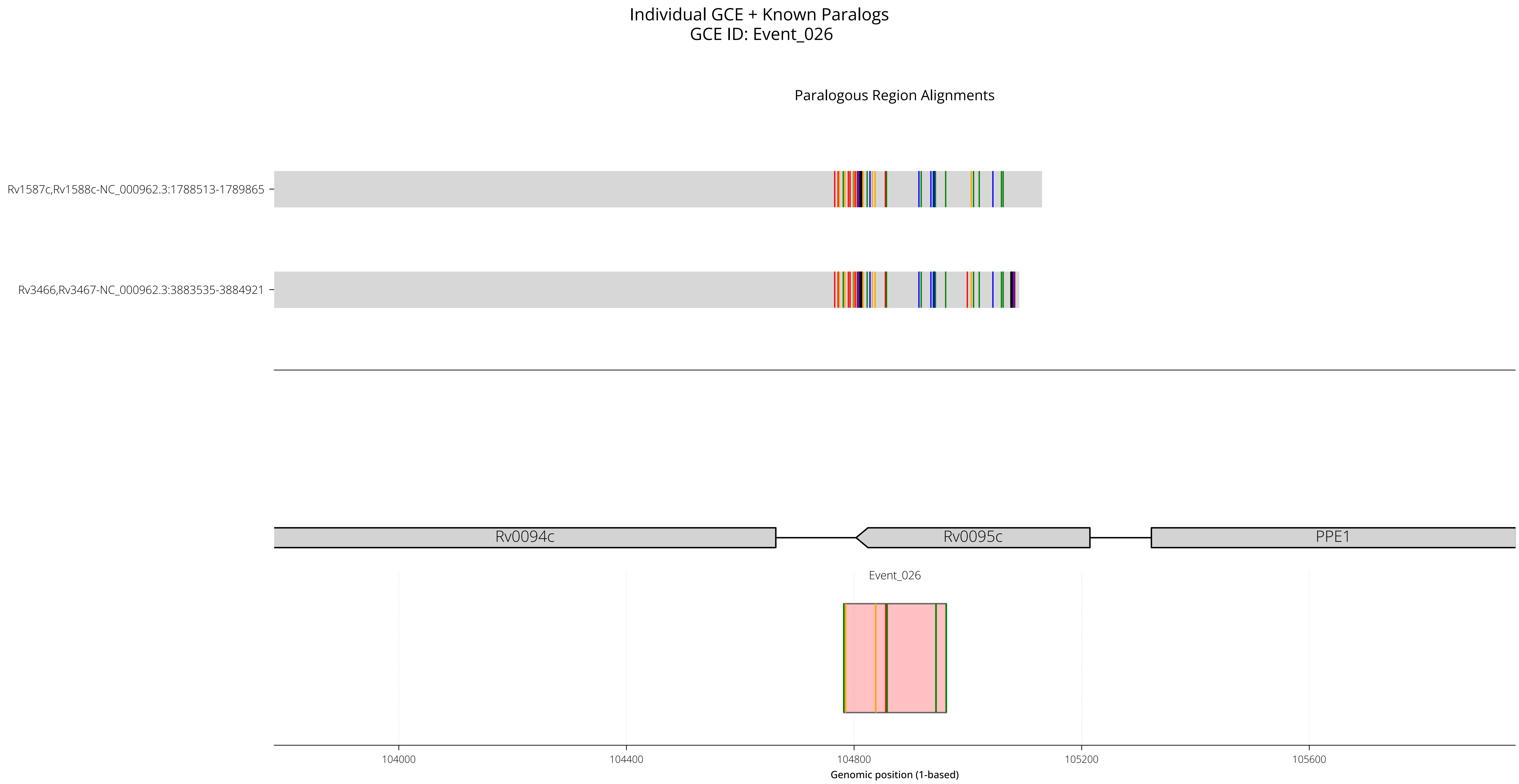
Individual GCE + Known Paralogs
GCE ID: Event_026
Paralogous Region Alignments
Rv1587c,Rv1588c-NC_000962.3:1788513-1789865
Rv3466,Rv3467-NC_000962.3:3883535-3884921
Rv0094c
Rv0095c
PPE1
Event_026
104000
104400
104800
105200
105600
Genomic position (1-based)

Circos Viz - GCE to Known Paralogs Locations
GCE ID: Event_026

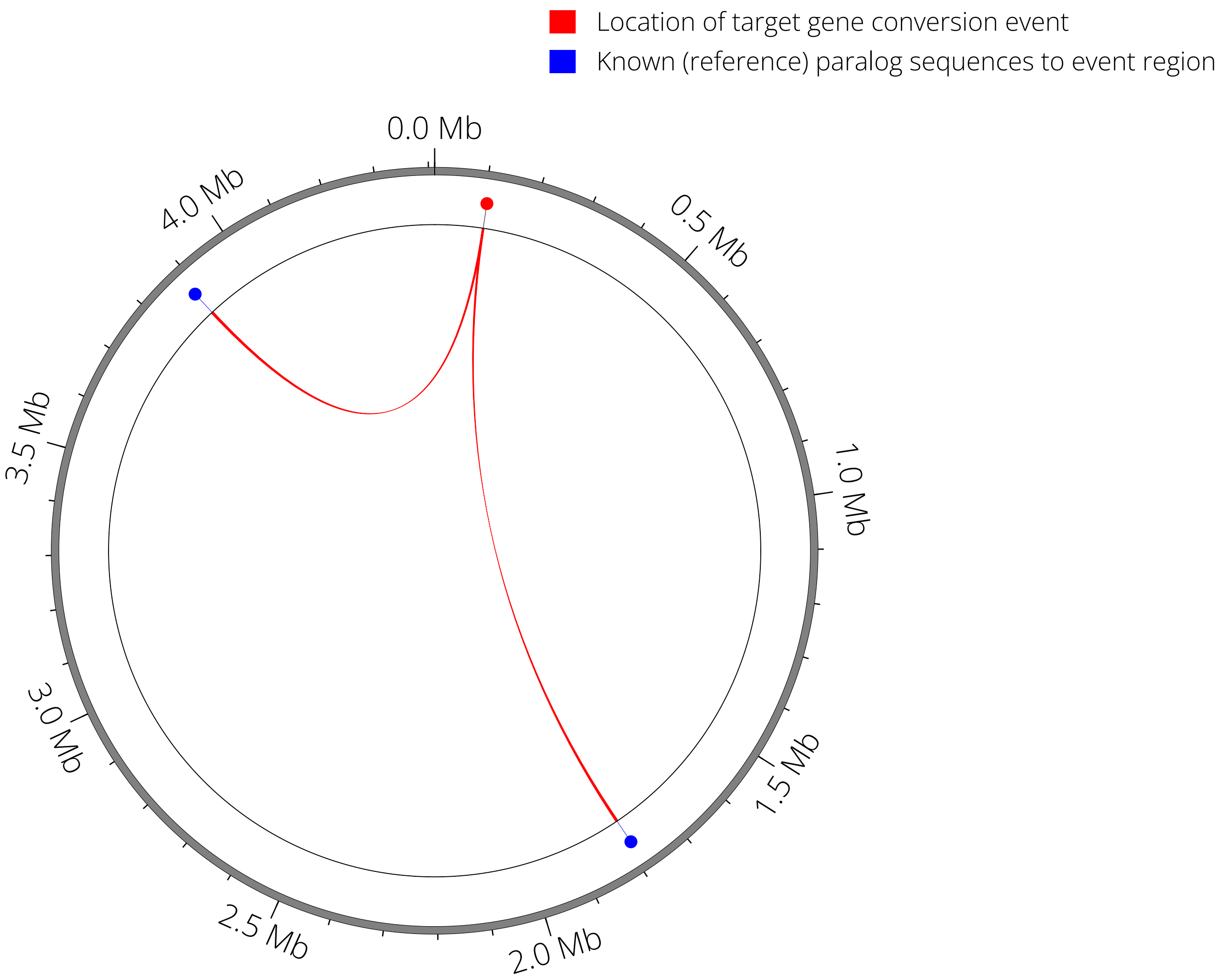

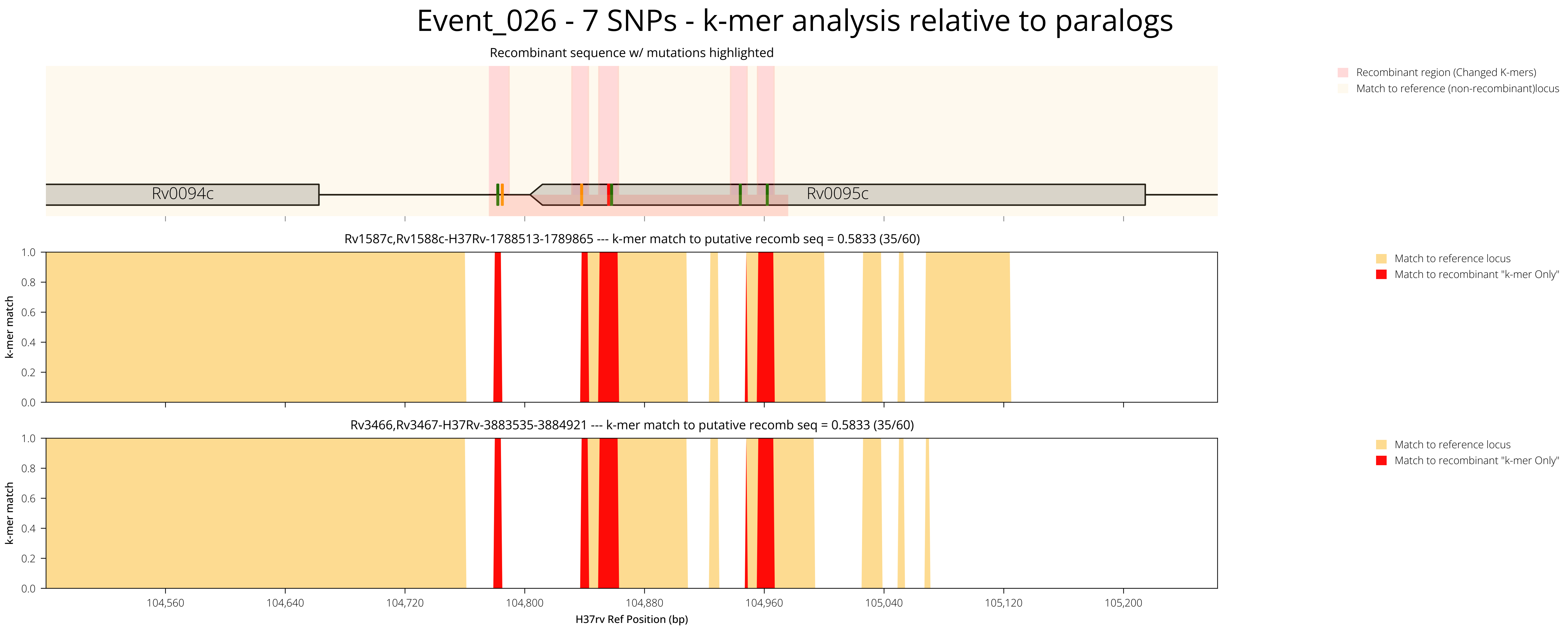

Event_026 - 7 SNPs - k-mer analysis relative to paralogs
Recombinant sequence w/ mutations highlighted
Rv0094c
Rv0095c
Recombinant region (Changed K-mers)
Match to reference (non-recombinant)locus
Rv1587c,Rv1588c-H37Rv-1788513-1789865 --- k-mer match to putative recomb seq = 0.5833 (35/60)
Match to reference locus
Match to recombinant "k-mer Only"
Rv3466,Rv3467-H37Rv-3883535-3884921 --- k-mer match to putative recomb seq = 0.5833 (35/60)
Match to reference locus
Match to recombinant "k-mer Only"
k-mer match
1.0
0.8
0.6
0.4
0.2
0.0
104,560
104,640
104,720
104,800
104,880
104,960
105,040
105,120
105,200
H37rv Ref Position (bp)

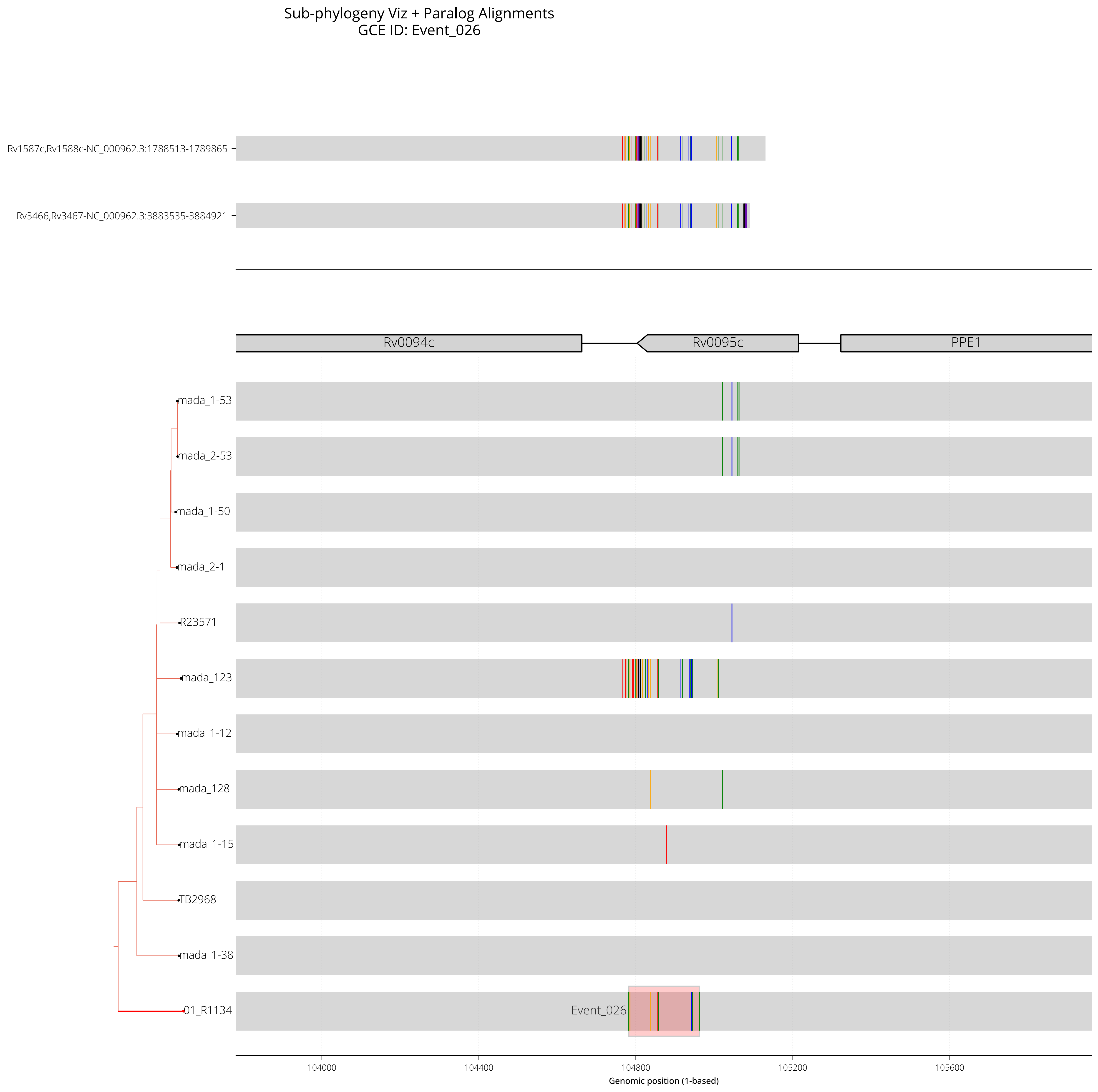

Sub-phylogeny Viz + Paralog Alignments
GCE ID: Event_026
Rv1587c,Rv1588c-NC_000962.3:1788513-1789865
Rv3466,Rv3467-NC_000962.3:3883535-3884921
Rv0094c
Rv0095c
PPE1
mada_1-53
mada_2-53
mada_1-50
mada_2-1
R23571
mada_123
mada_1-12
mada_128
mada_1-15
TB2968
mada_1-38
01_R1134
Event_026
104000
104400
104800
105200
105600
Genomic position (1-based)

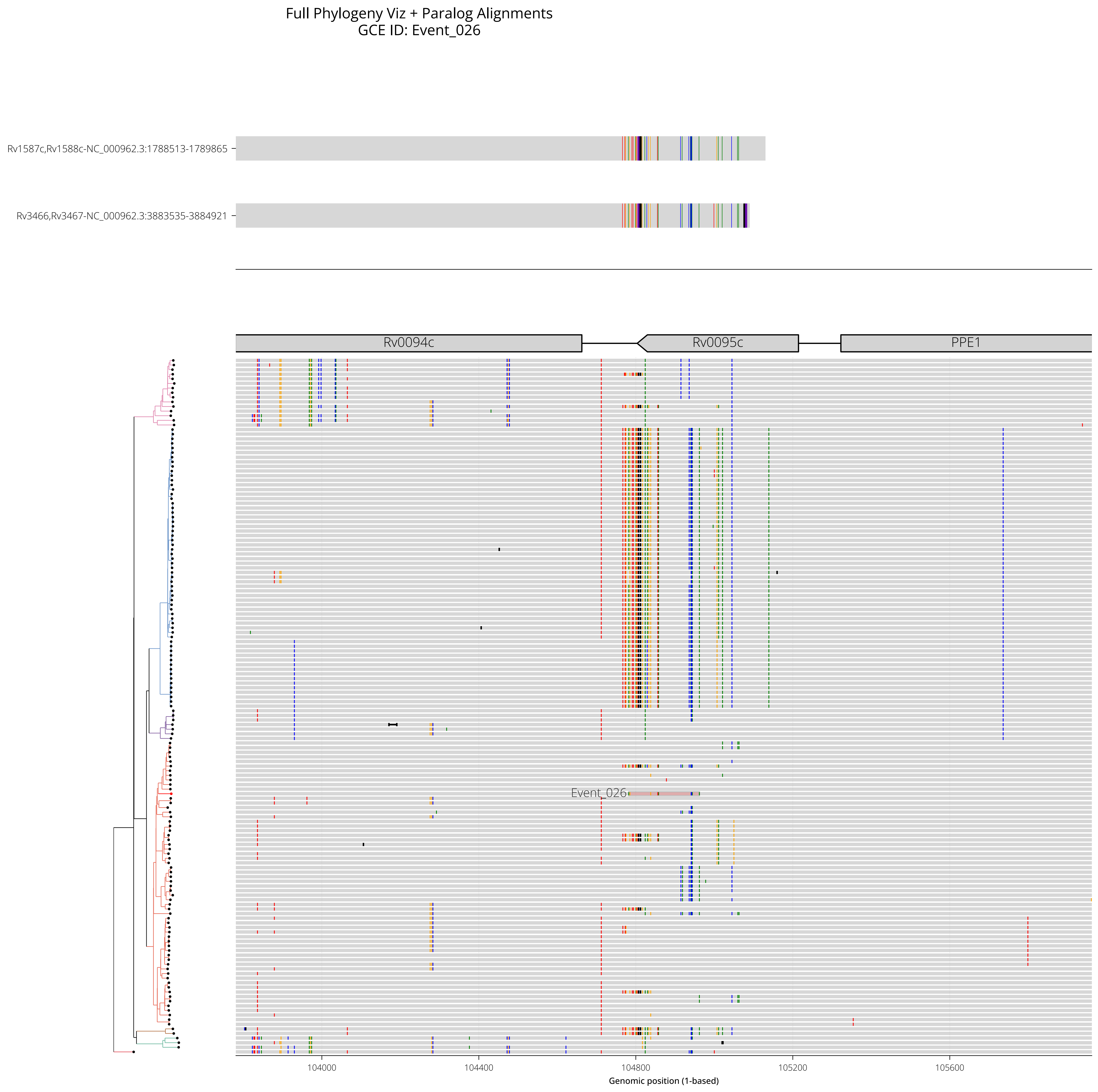

Full Phylogeny Viz + Paralog Alignments
GCE ID: Event_026
Rv1587c,Rv1588c-NC_000962.3:1788513-1789865
Rv3466,Rv3467-NC_000962.3:3883535-3884921
Rv0094c
Rv0095c
PPE1
Event_026
104000
104400
104800
105200
105600
Genomic position (1-based)

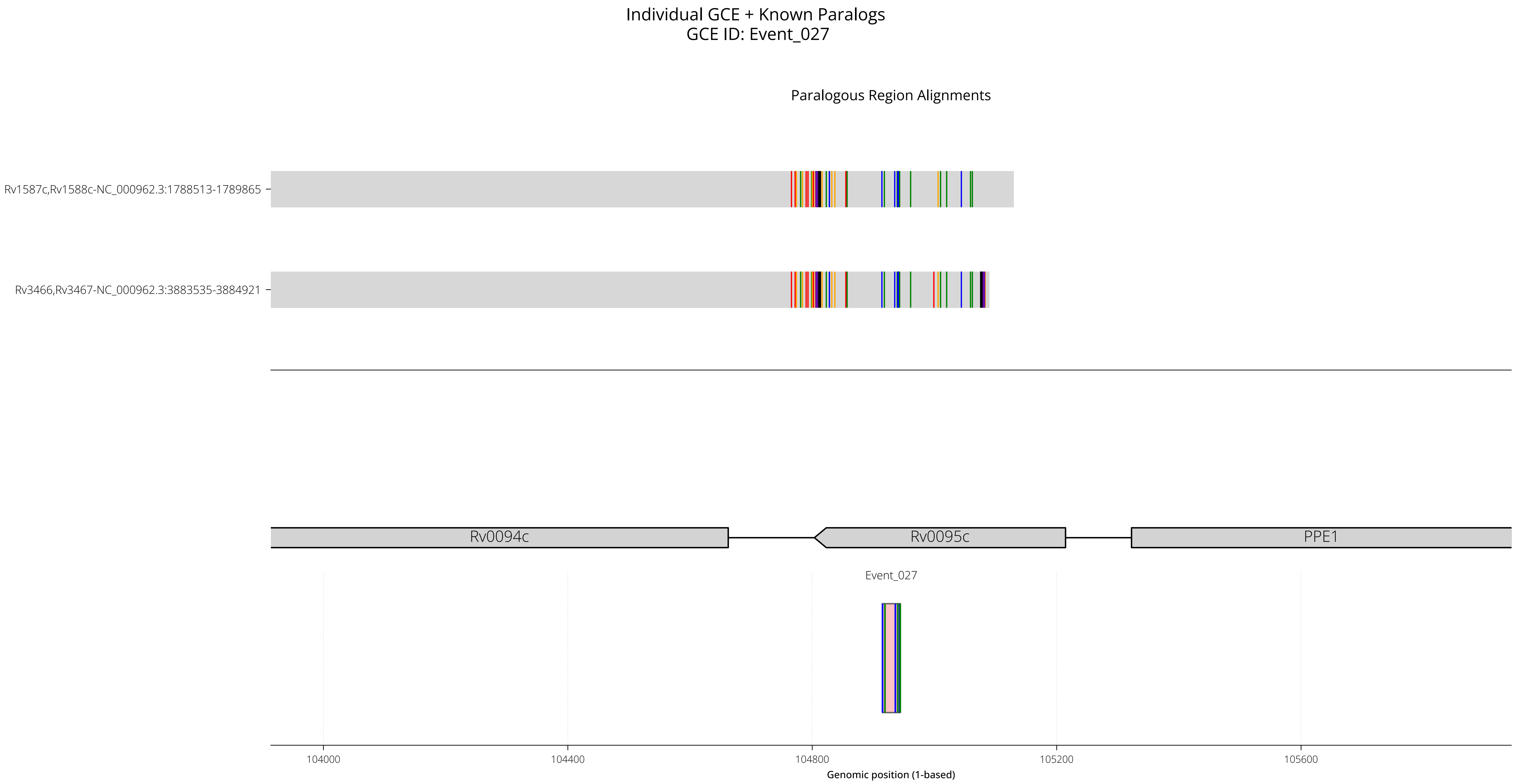
Individual GCE + Known Paralogs
GCE ID: Event_027
Paralogous Region Alignments
Rv1587c,Rv1588c-NC_000962.3:1788513-1789865
Rv3466,Rv3467-NC_000962.3:3883535-3884921
Rv0094c
Rv0095c
PPE1
Event_027
104000
104400
104800
105200
105600
Genomic position (1-based)

Circos Viz - GCE to Known Paralogs Locations
GCE ID: Event_027

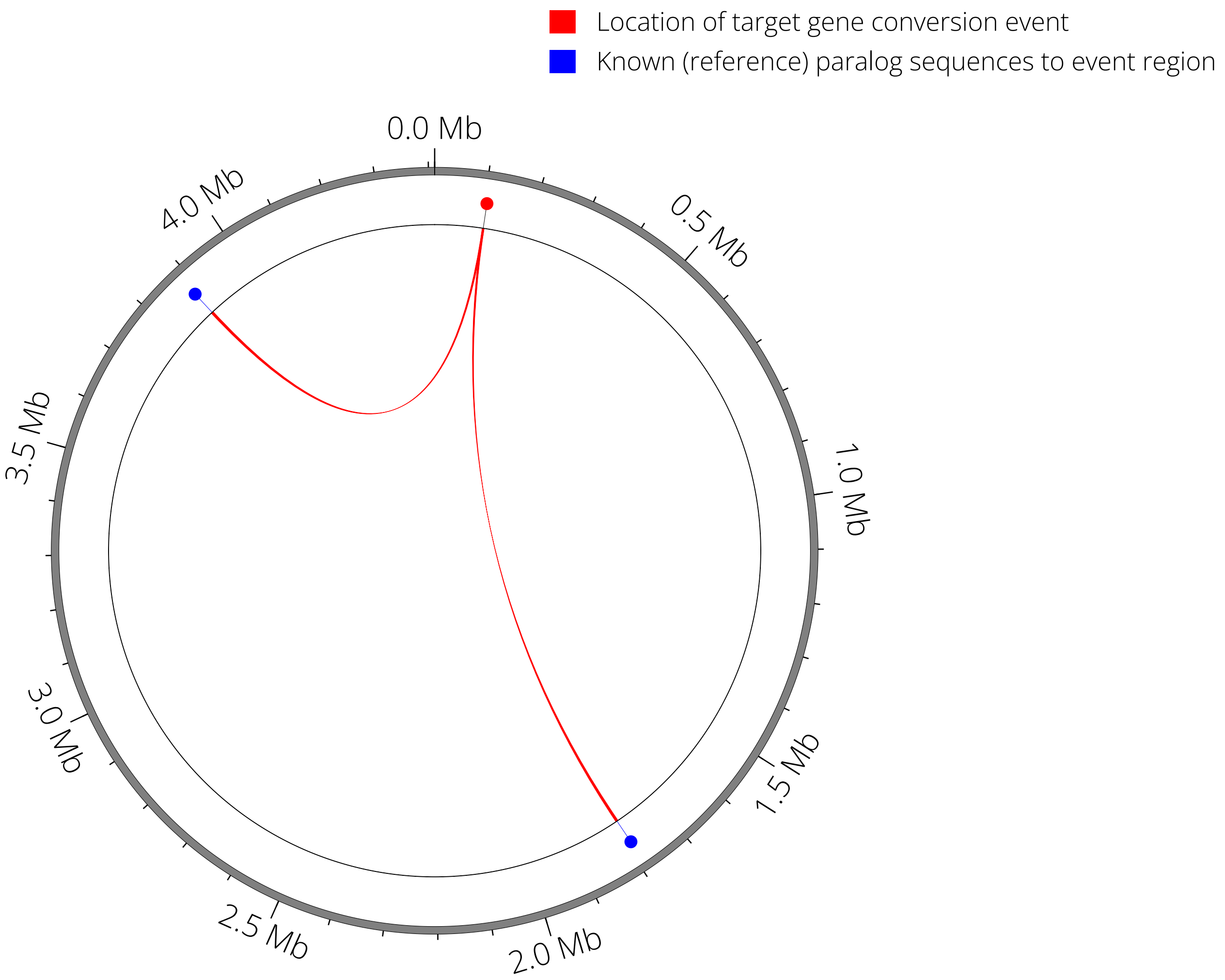

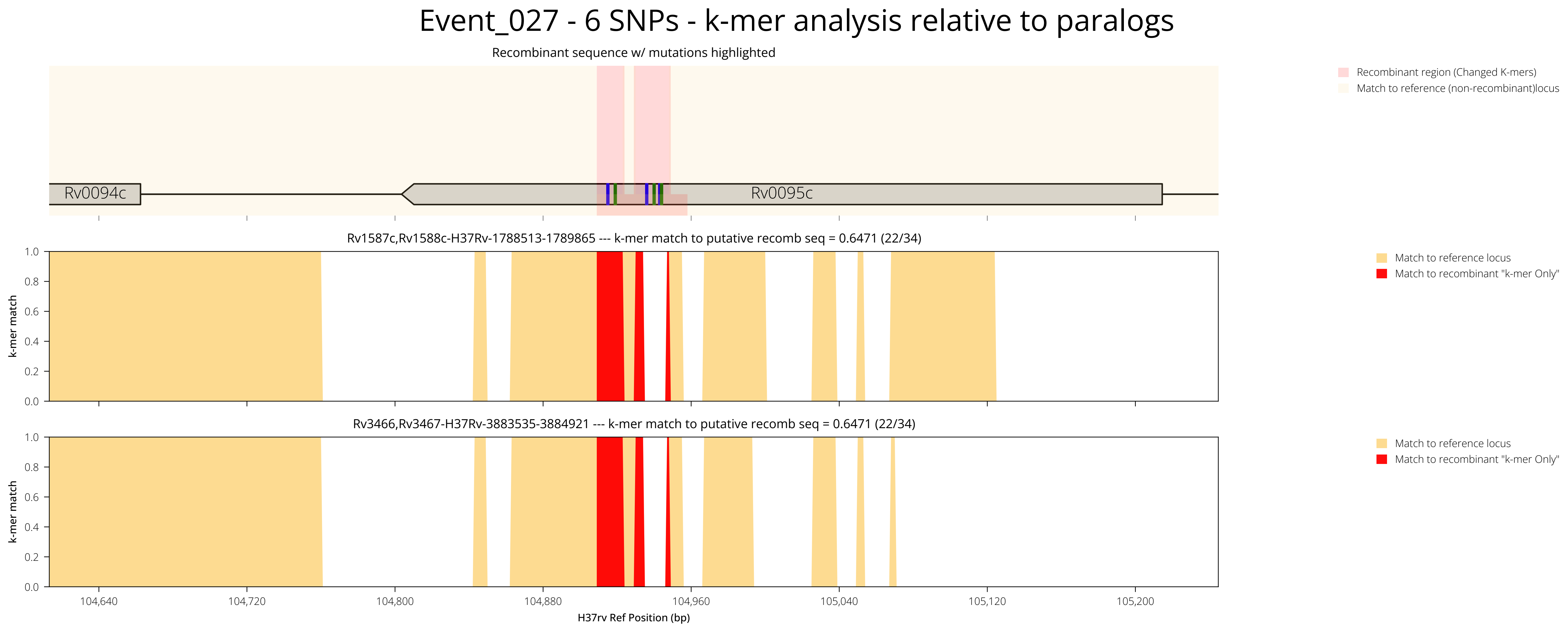

Event_027 - 6 SNPs - k-mer analysis relative to paralogs
Recombinant sequence w/ mutations highlighted
Recombinant region (Changed K-mers)
Match to reference (non-recombinant)locus
Rv0094c
Rv0095c
Rv1587c,Rv1588c-H37Rv-1788513-1789865 --- k-mer match to putative recomb seq = 0.6471 (22/34)
Match to reference locus
Match to recombinant "k-mer Only"
k-mer match
1.0
0.8
0.6
0.4
0.2
0.0
Rv3466,Rv3467-H37Rv-3883535-3884921 --- k-mer match to putative recomb seq = 0.6471 (22/34)
Match to reference locus
Match to recombinant "k-mer Only"
k-mer match
1.0
0.8
0.6
0.4
0.2
0.0
104,640
104,720
104,800
104,880
104,960
105,040
105,120
105,200
H37rv Ref Position (bp)

Sub-phylogeny Viz + Paralog Alignments
GCE ID: Event_027

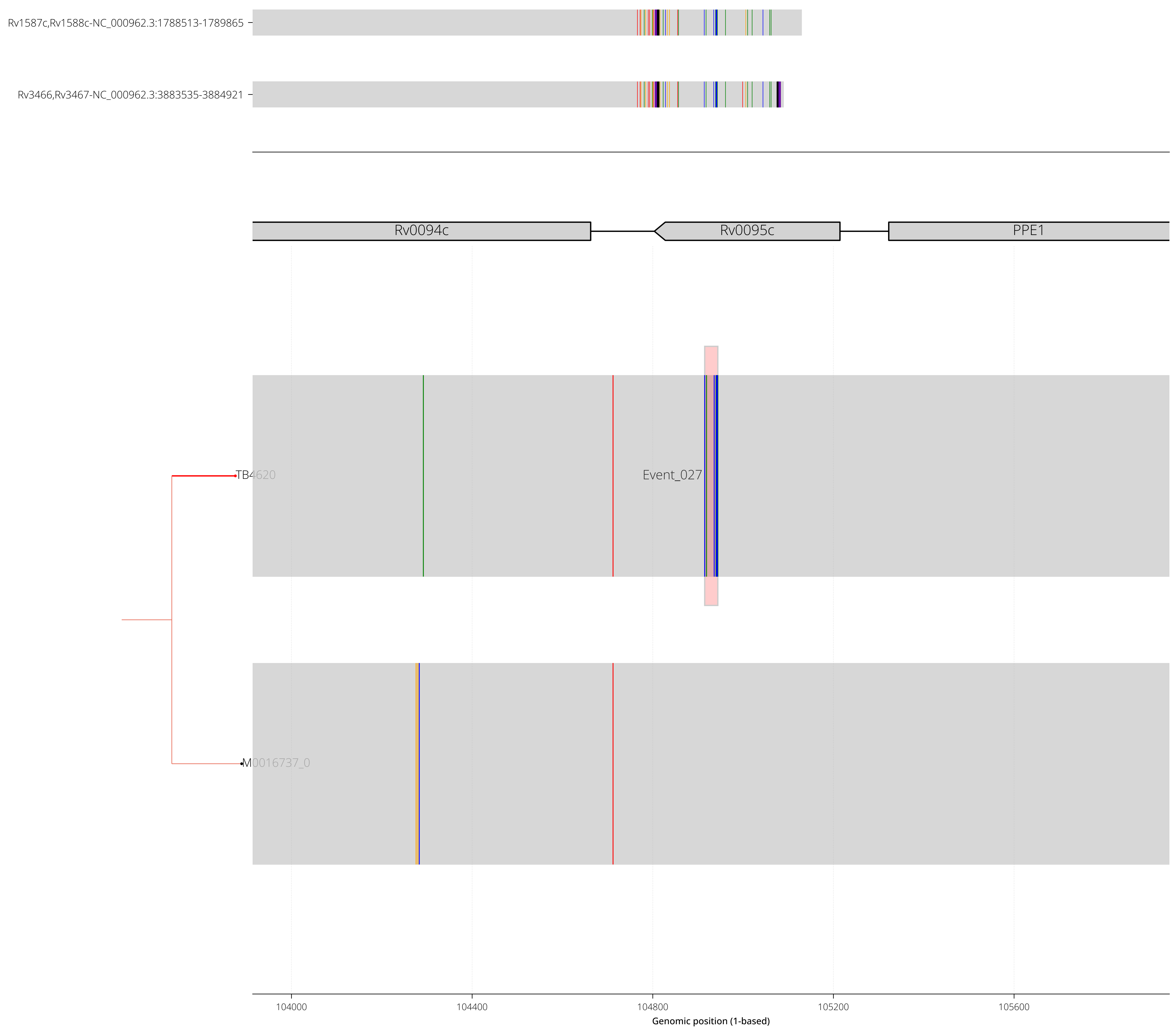

Full Phylogeny Viz + Paralog Alignments
GCE ID: Event_027

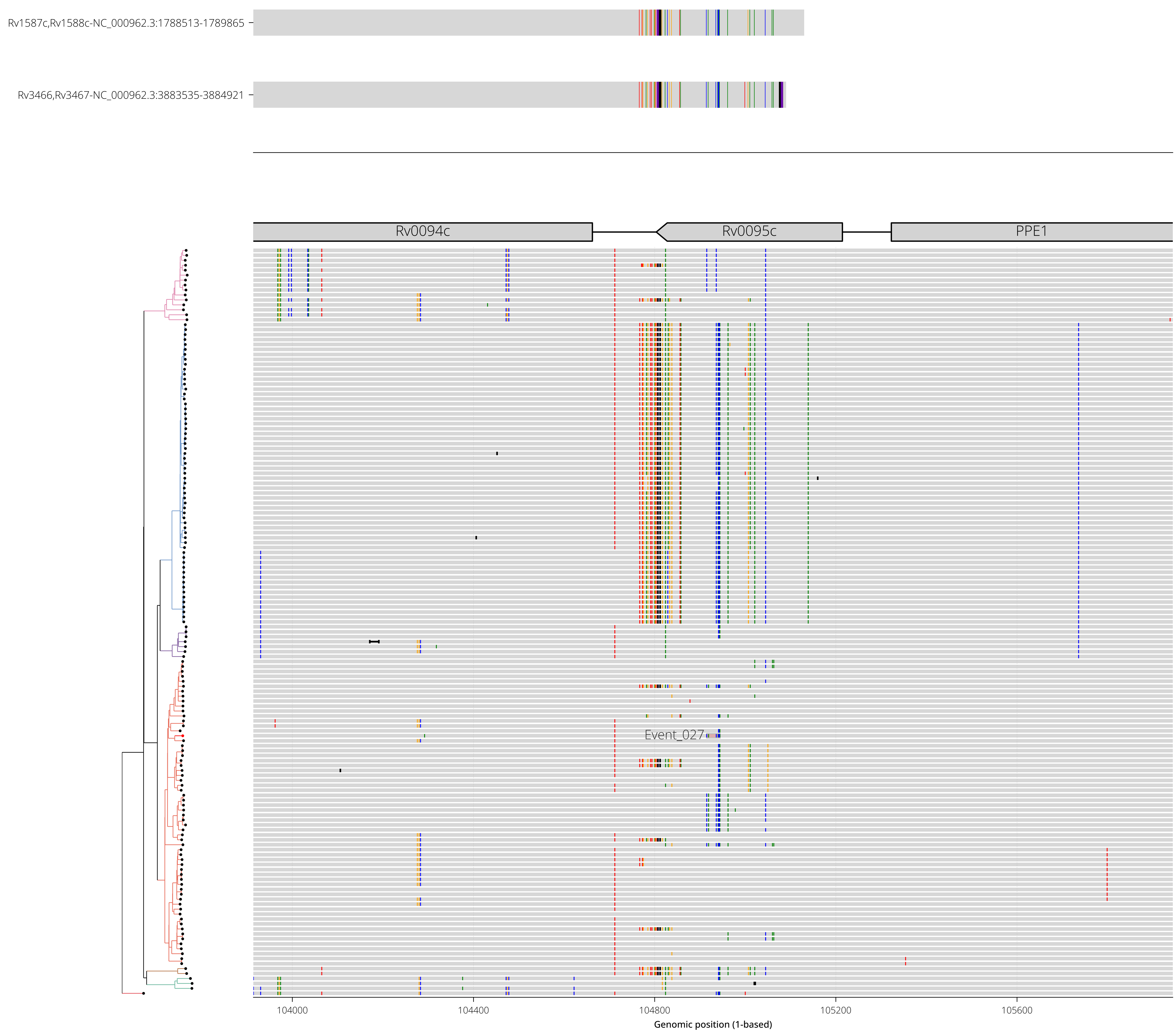

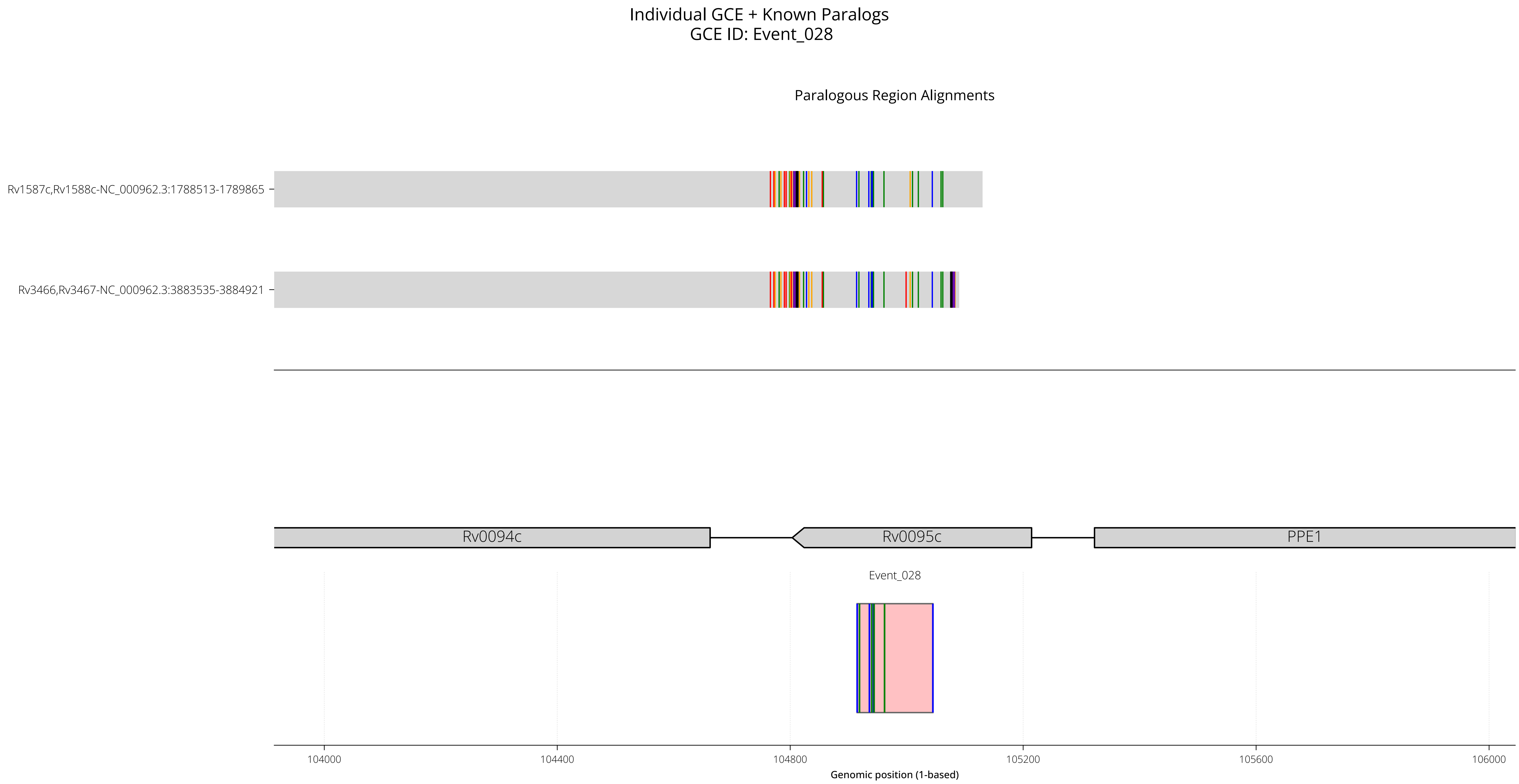
Individual GCE + Known Paralogs
GCE ID: Event_028
Paralogous Region Alignments
Rv1587c,Rv1588c-NC_000962.3:1788513-1789865
Rv3466,Rv3467-NC_000962.3:3883535-3884921
Rv0094c
Rv0095c
PPE1
Event_028
104000
104400
104800
105200
105600
106000
Genomic position (1-based)

Circos Viz - GCE to Known Paralogs Locations
GCE ID: Event_028

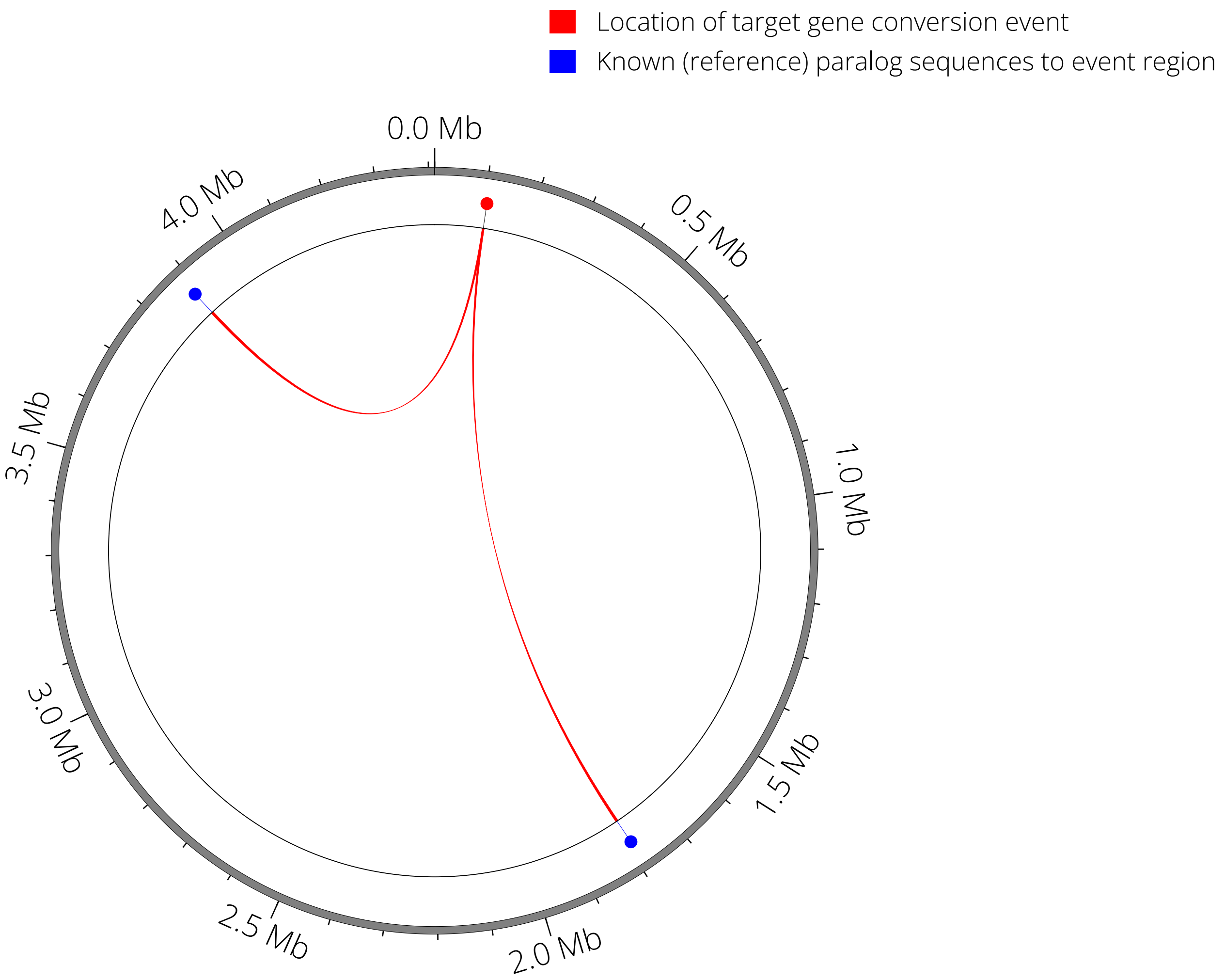

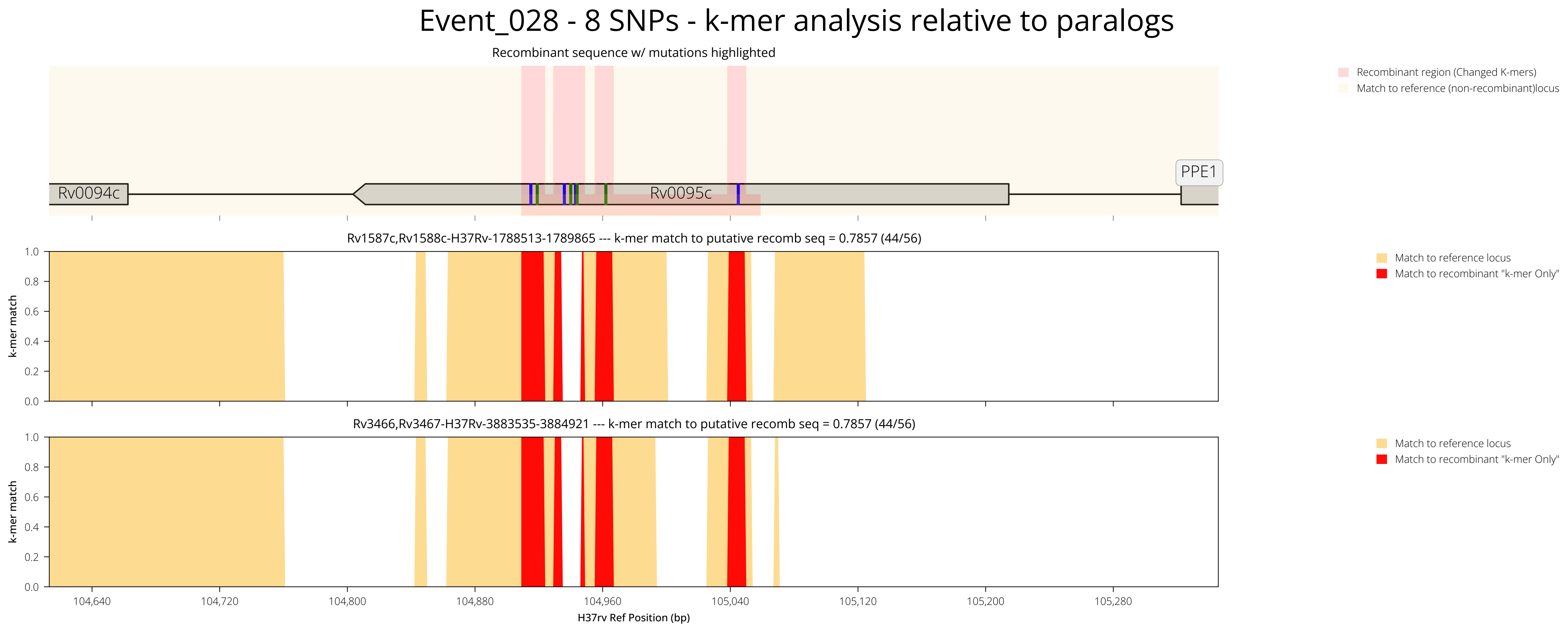
Event_028 - 8 SNPs - k-mer analysis relative to paralogs
Recombinant sequence w/ mutations highlighted
Recombinant region (Changed K-mers)
Match to reference (non-recombinant)locus
Rv0094c
Rv0095c
PPE1
Rv1587c,Rv1588c-H37Rv-1788513-1789865 --- k-mer match to putative recomb seq = 0.7857 (44/56)
Match to reference locus
Match to recombinant "k-mer Only"
k-mer match
1.0
0.8
0.6
0.4
0.2
0.0
Rv3466,Rv3467-H37Rv-3883535-3884921 --- k-mer match to putative recomb seq = 0.7857 (44/56)
104,640
104,720
104,800
104,880
104,960
105,040
105,120
105,200
105,280
H37rv Ref Position (bp)

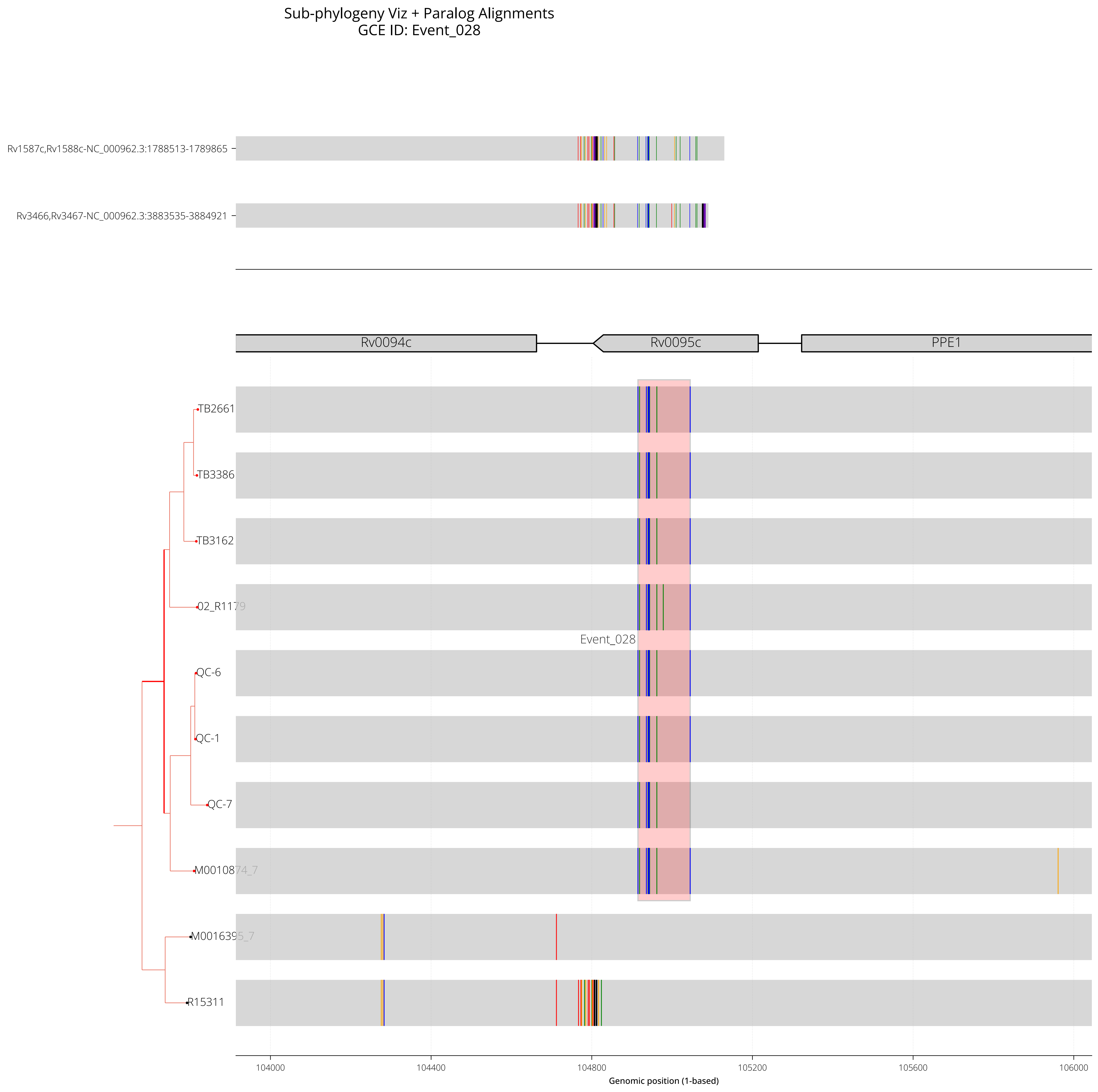

Sub-phylogeny Viz + Paralog Alignments
GCE ID: Event_028
Rv1587c,Rv1588c-NC_000962.3:1788513-1789865
Rv3466,Rv3467-NC_000962.3:3883535-3884921
Rv0094c
Rv0095c
PPE1
TB2661
TB3386
TB3162
02_R1179
Event_028
QC-6
QC-1
QC-7
M0010874_7
M0016395_7
R15311
104000
104400
104800
105200
105600
106000
Genomic position (1-based)

Full Phylogeny Viz + Paralog Alignments
GCE ID: Event_028

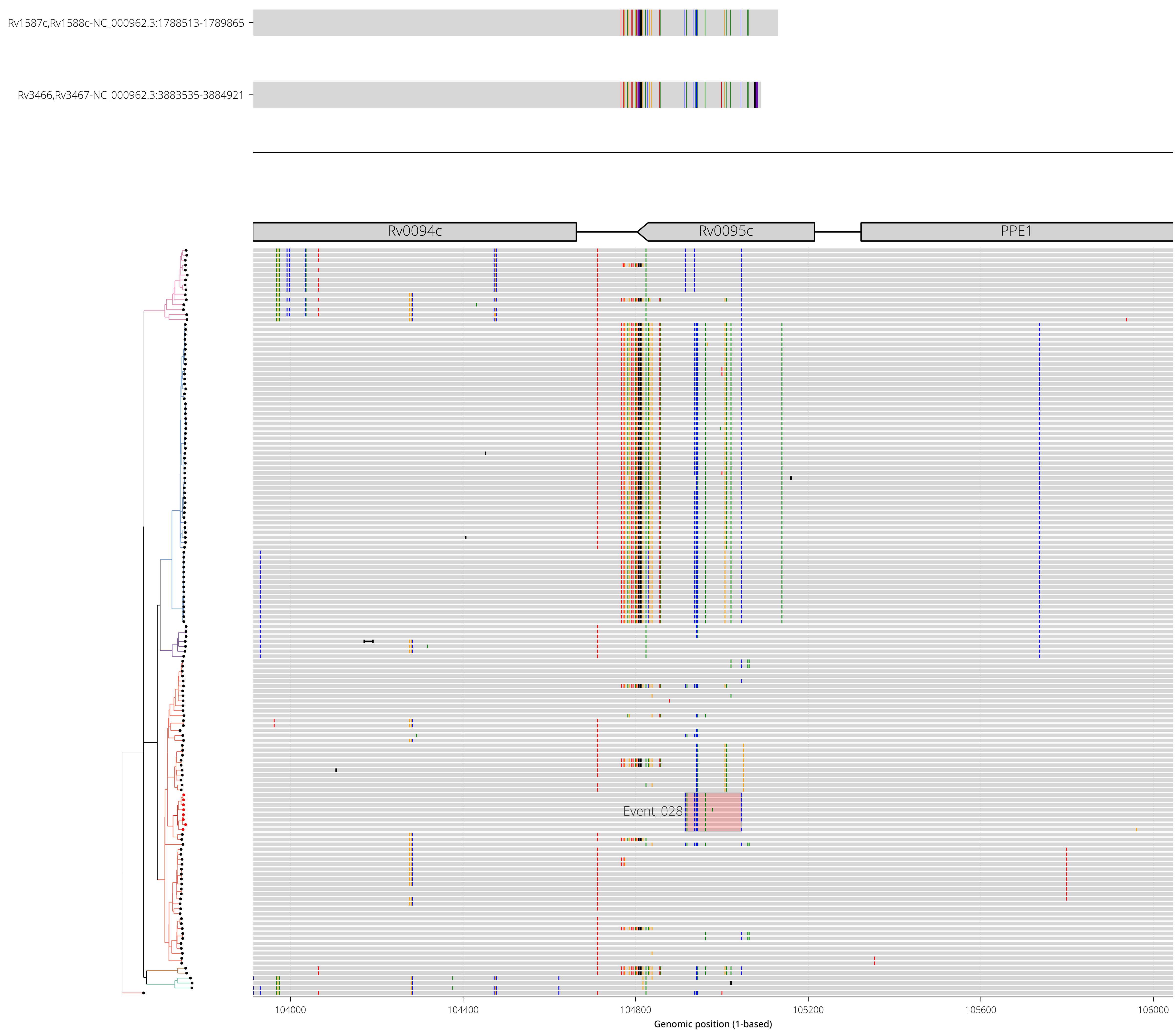

Individual GCE + Known Paralogs
GCE ID: Event_029

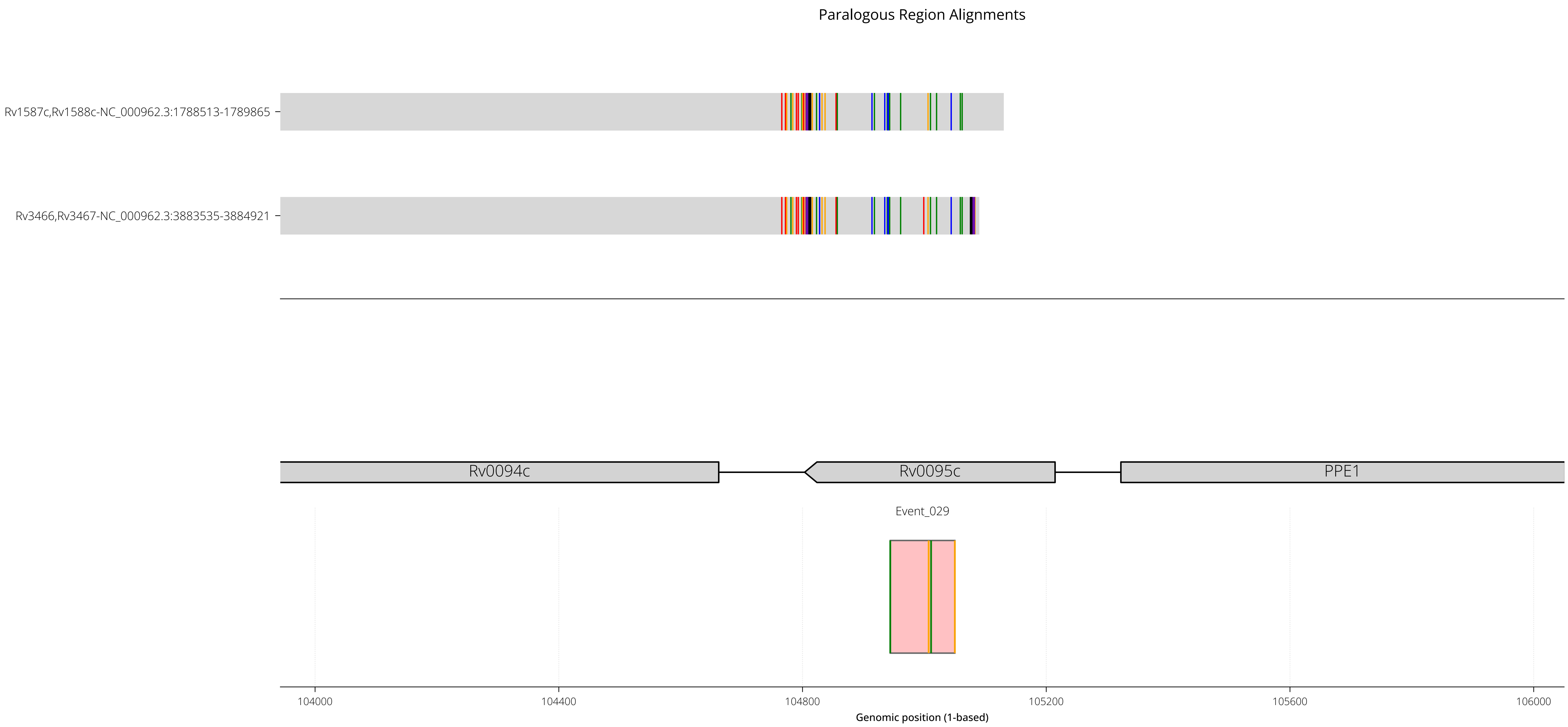

Circos Viz - GCE to Known Paralogs Locations
GCE ID: Event_029

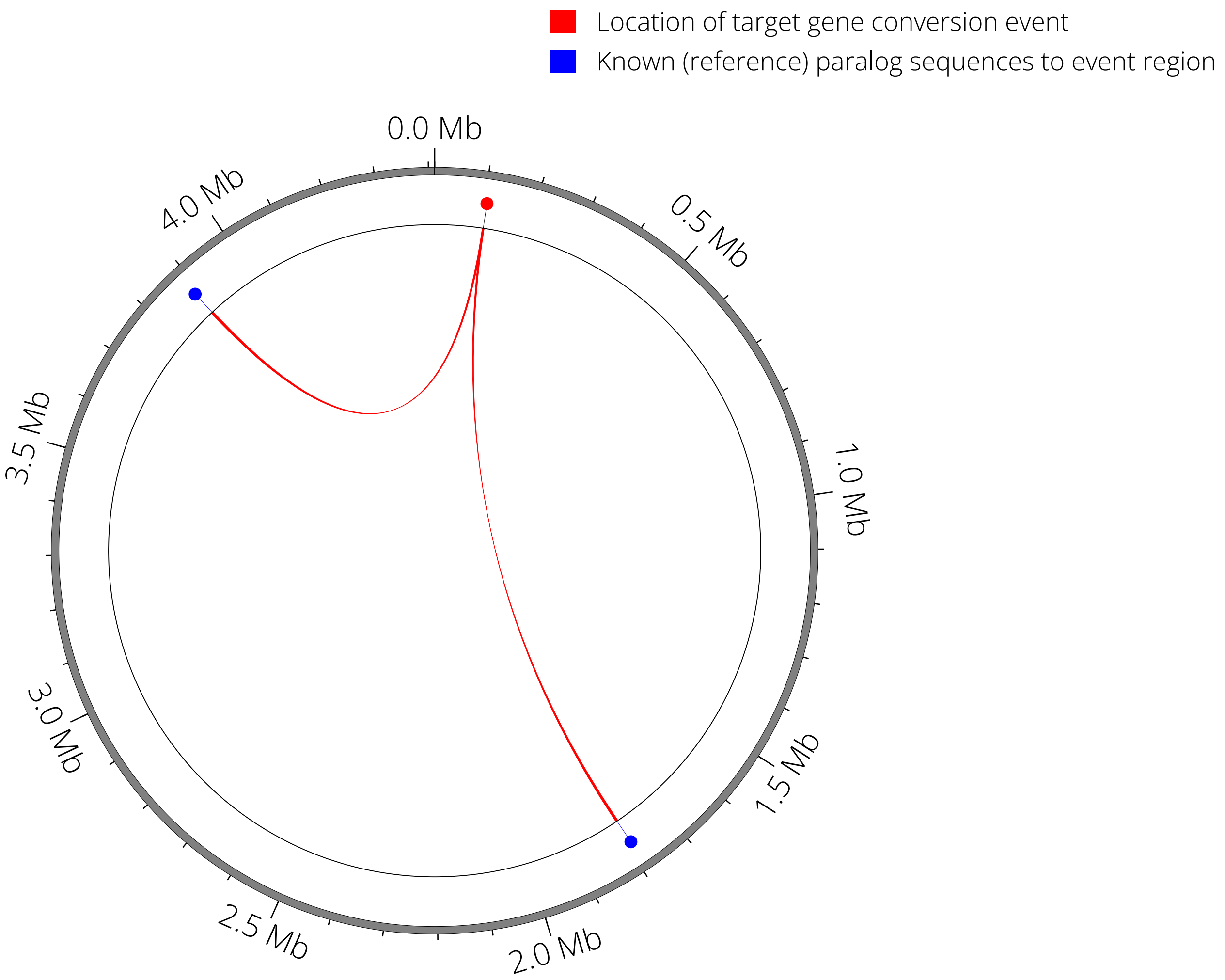

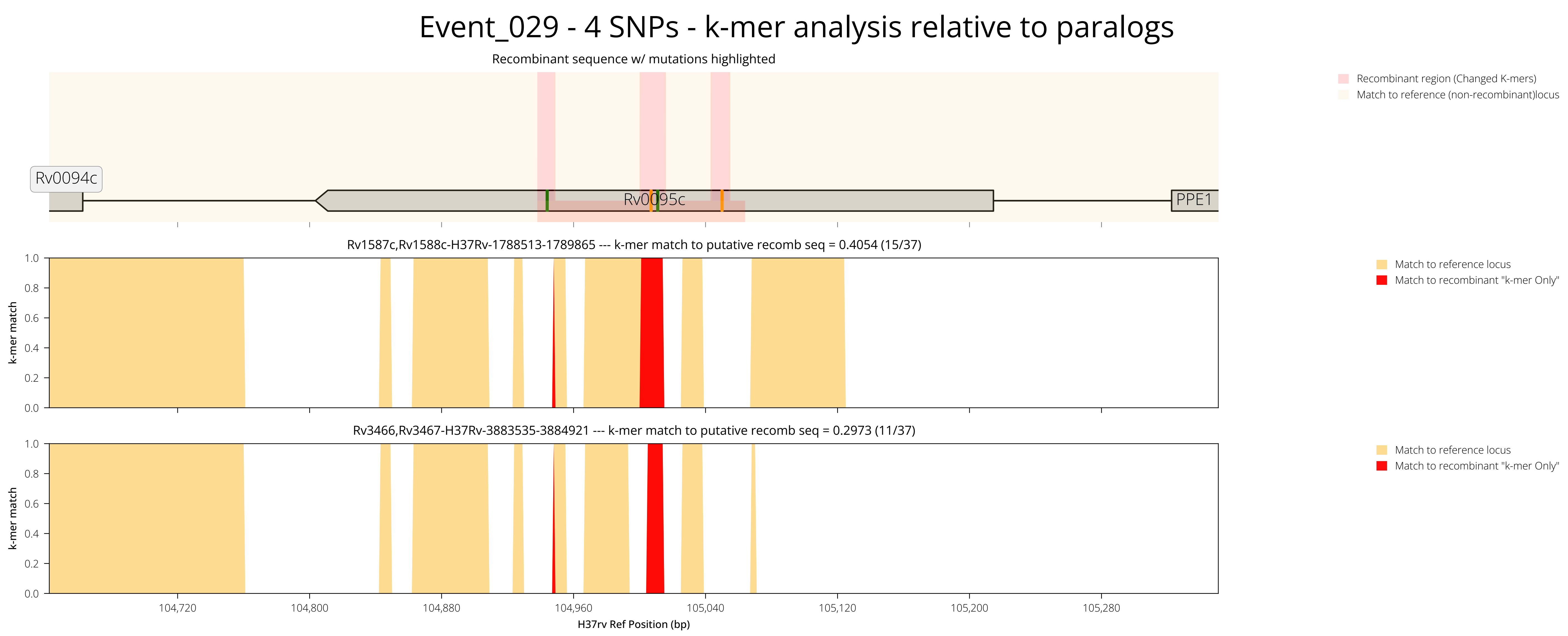

Event_029 - 4 SNPs - k-mer analysis relative to paralogs
Recombinant sequence w/ mutations highlighted
Recombinant region (Changed K-mers)
Match to reference (non-recombinant)locus
Rv0094c
Rv0095c
PPE1
Rv1587c,Rv1588c-H37Rv-1788513-1789865 --- k-mer match to putative recomb seq = 0.4054 (15/37)
k-mer match
1.0
0.8
0.6
0.4
0.2
0.0
Match to reference locus
Match to recombinant "k-mer Only"
Rv3466,Rv3467-H37Rv-3883535-3884921 --- k-mer match to putative recomb seq = 0.2973 (11/37)
k-mer match
1.0
0.8
0.6
0.4
0.2
0.0
Match to reference locus
Match to recombinant "k-mer Only"
104,720
104,800
104,880
104,960
105,040
105,120
105,200
105,280
H37rv Ref Position (bp)

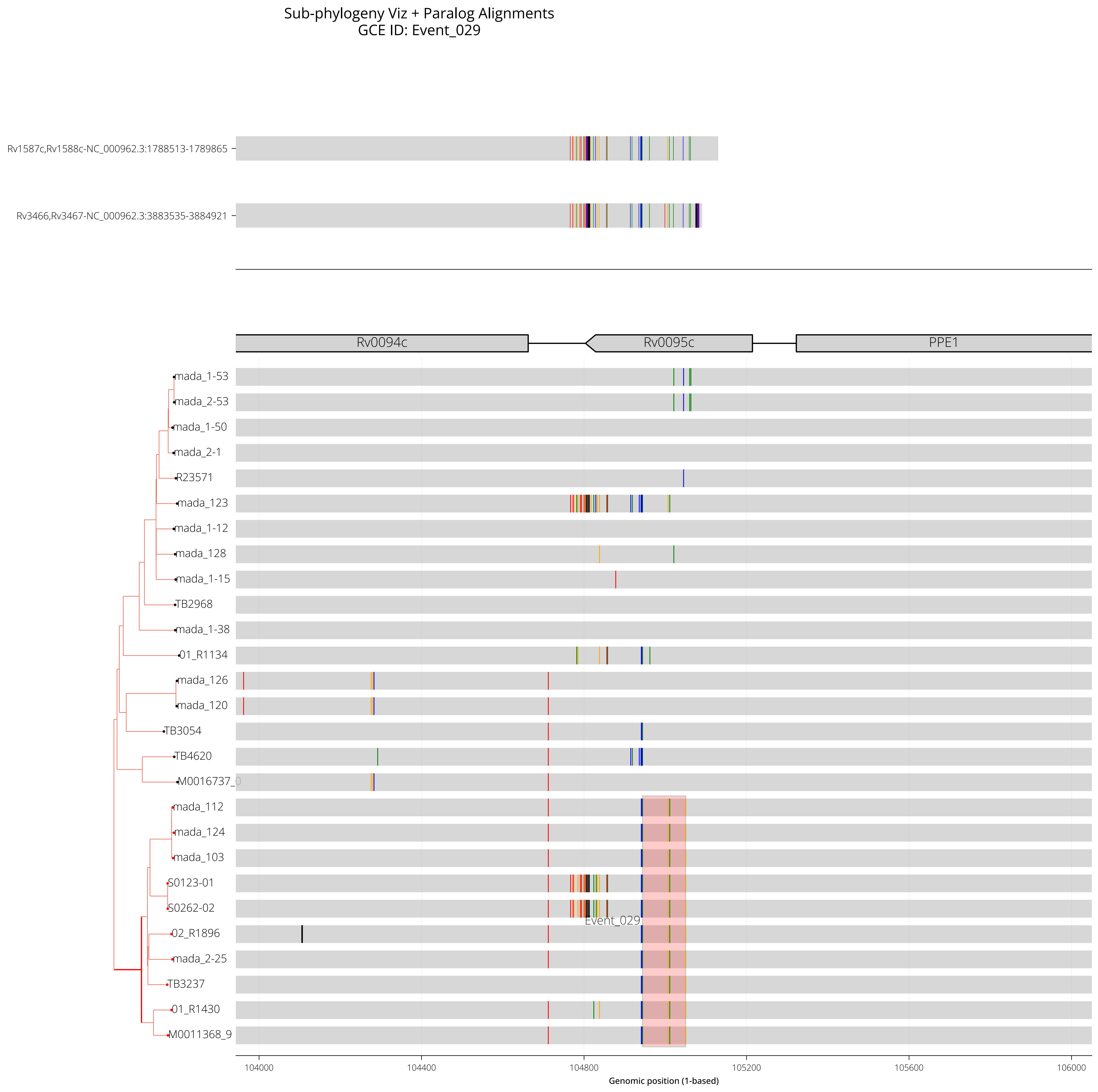

Sub-phylogeny Viz + Paralog Alignments
GCE ID: Event_029
Rv1587c,Rv1588c-NC_000962.3:1788513-1789865
Rv3466,Rv3467-NC_000962.3:3883535-3884921
Rv0094c
Rv0095c
PPE1
mada_1-53
mada_2-53
mada_1-50
mada_2-1
R23571
mada_123
mada_1-12
mada_128
mada_1-15
TB2968
mada_1-38
01_R1134
mada_126
mada_120
TB3054
TB4620
M0016737_0
mada_112
mada_124
mada_103
S0123-01
S0262-02
Event_029
02_R1896
mada_2-25
TB3237
01_R1430
M0011368_9
104000
104400
104800
105200
105600
106000
Genomic position (1-based)

Full Phylogeny Viz + Paralog Alignments
GCE ID: Event_029

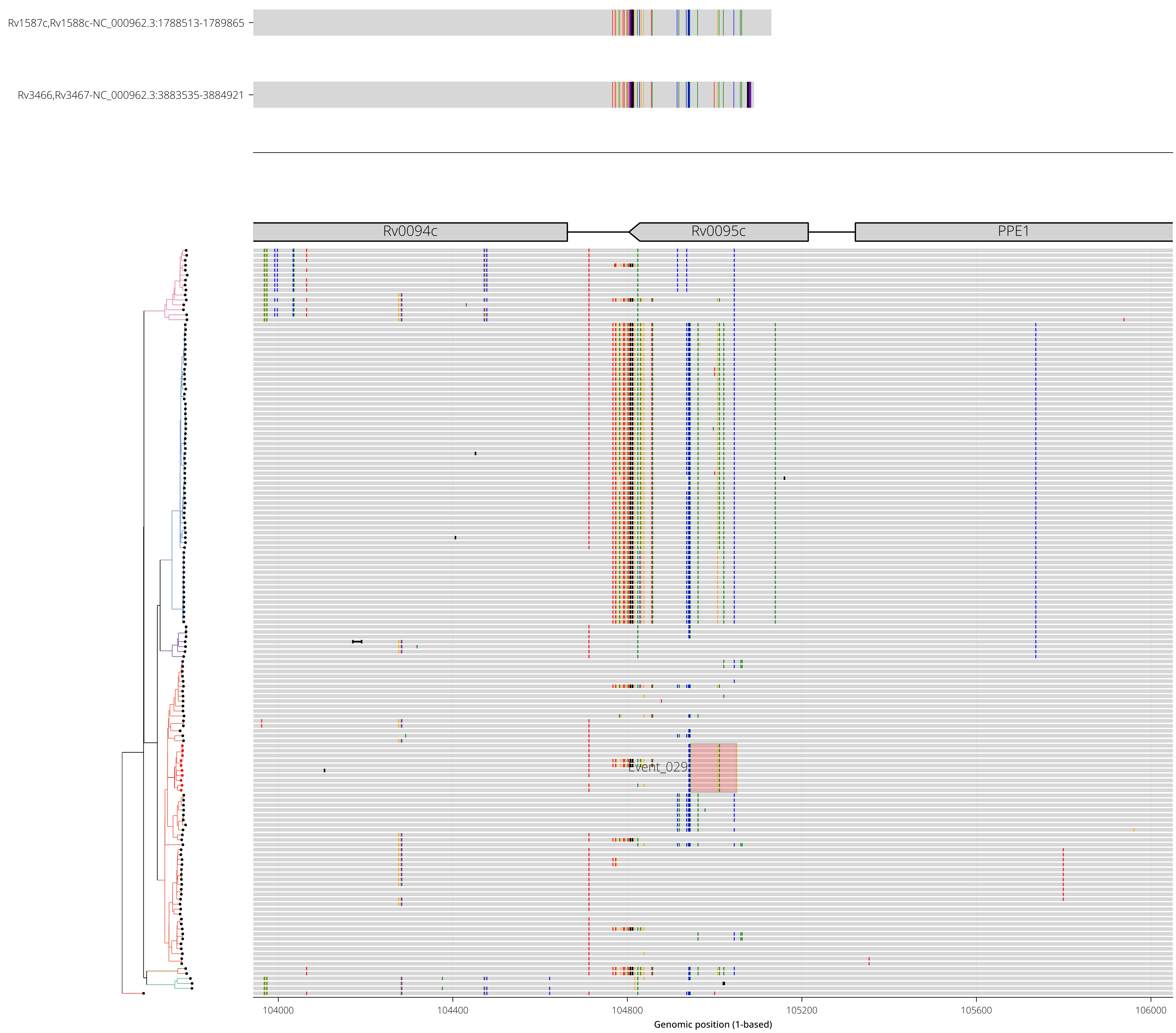

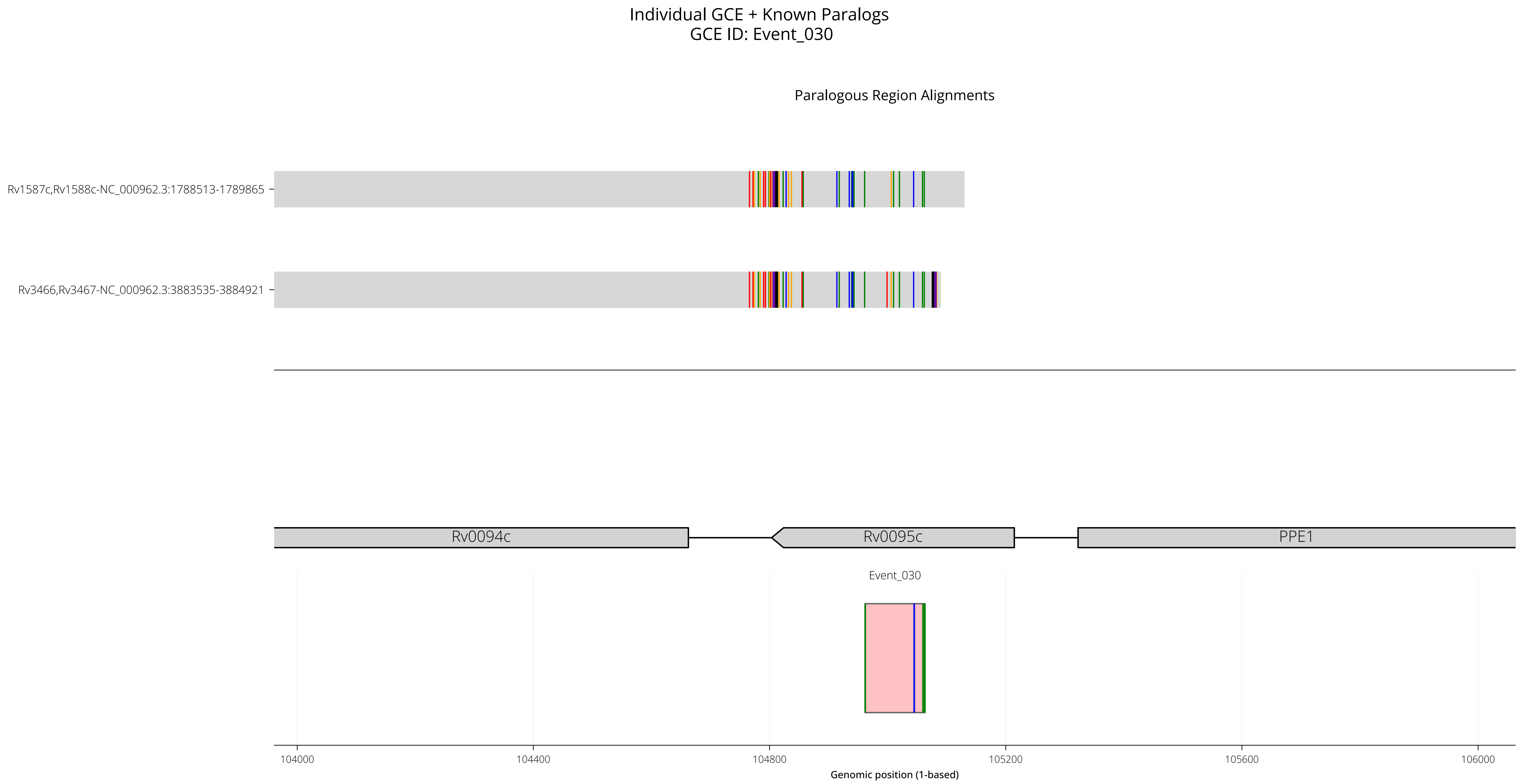
Individual GCE + Known Paralogs
GCE ID: Event_030
Paralogous Region Alignments
Rv1587c,Rv1588c-NC_000962.3:1788513-1789865
Rv3466,Rv3467-NC_000962.3:3883535-3884921
Rv0094c
Rv0095c
PPE1
Event_030
104000
104400
104800
105200
105600
106000
Genomic position (1-based)

Circos Viz - GCE to Known Paralogs Locations
GCE ID: Event_030

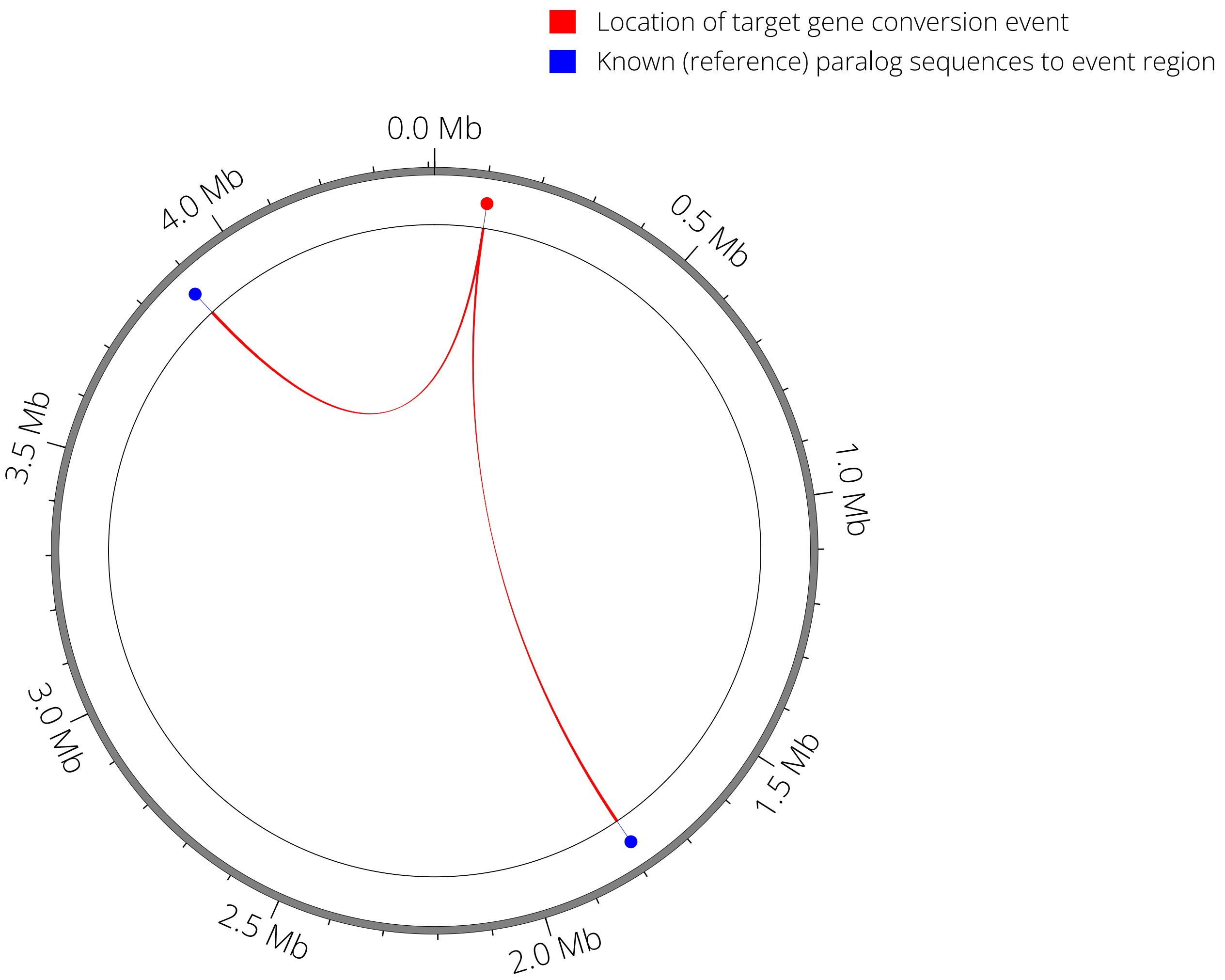

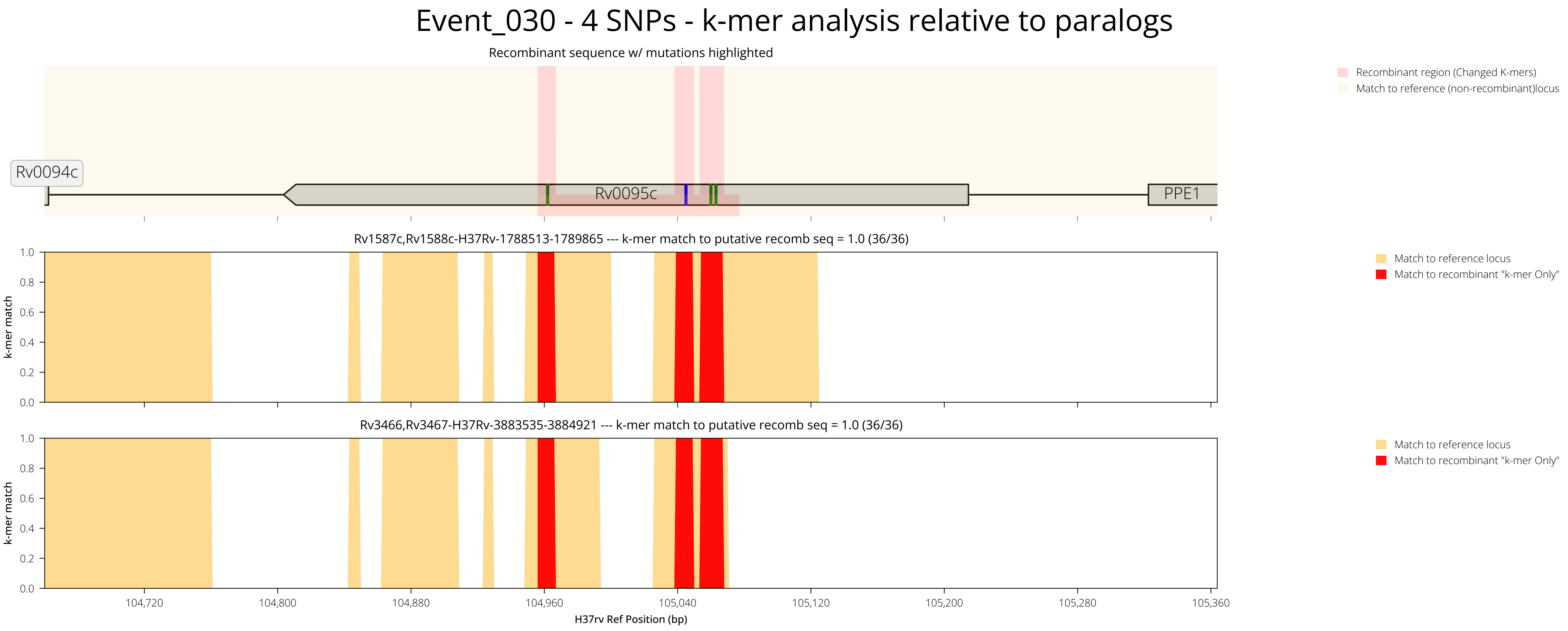
Event_030 - 4 SNPs - k-mer analysis relative to paralogs
Recombinant sequence w/ mutations highlighted
Recombinant region (Changed K-mers)
Match to reference (non-recombinant)locus
Rv0094c
Rv0095c
PPE1
Rv1587c,Rv1588c-H37Rv-1788513-1789865 --- k-mer match to putative recomb seq = 1.0 (36/36)
Match to reference locus
Match to recombinant "k-mer Only"
k-mer match
1.0
0.8
0.6
0.4
0.2
0.0
Rv3466,Rv3467-H37Rv-3883535-3884921 --- k-mer match to putative recomb seq = 1.0 (36/36)
Match to reference locus
Match to recombinant "k-mer Only"
k-mer match
1.0
0.8
0.6
0.4
0.2
0.0
104,720
104,800
104,880
104,960
105,040
105,120
105,200
105,280
105,360
H37rv Ref Position (bp)

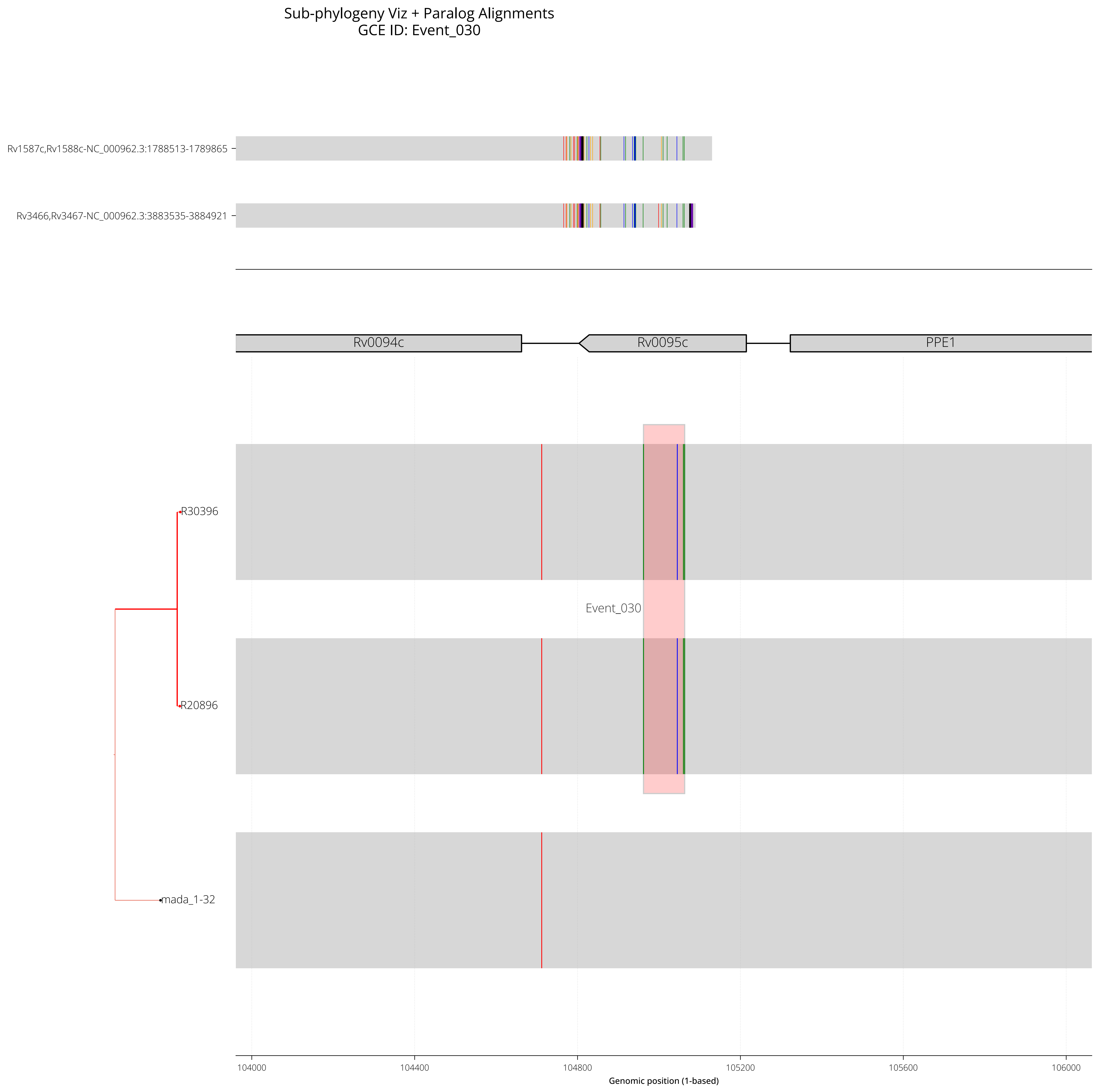

Sub-phylogeny Viz + Paralog Alignments
GCE ID: Event_030
Rv1587c,Rv1588c-NC_000962.3:1788513-1789865
Rv3466,Rv3467-NC_000962.3:3883535-3884921
Rv0094c
Rv0095c
PPE1
R30396
Event_030
R20896
mada_1-32
104000
104400
104800
105200
105600
106000
Genomic position (1-based)

Full Phylogeny Viz + Paralog Alignments
GCE ID: Event_030

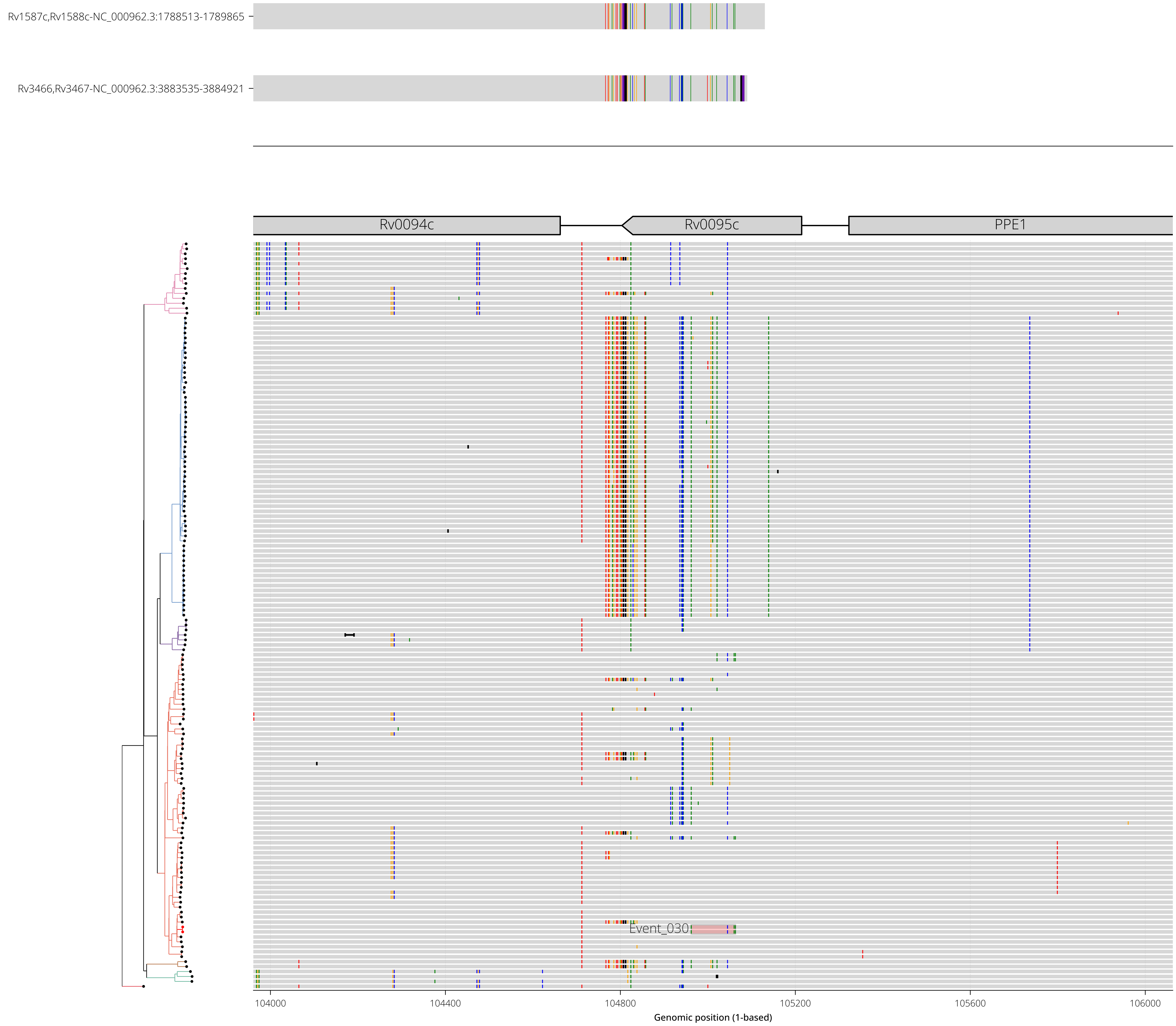

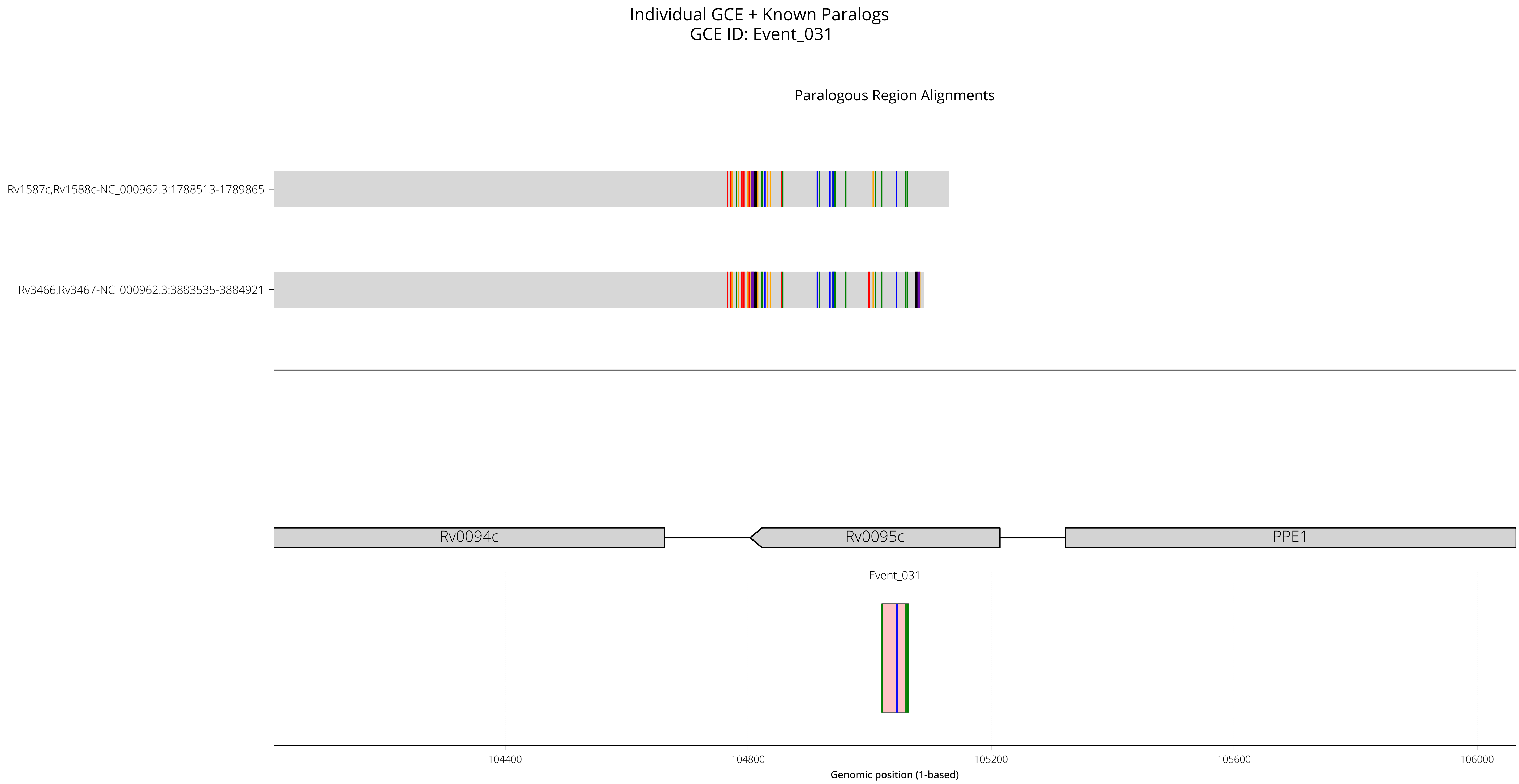

Individual GCE + Known Paralogs
GCE ID: Event_031
Paralogous Region Alignments
Rv1587c,Rv1588c-NC_000962.3:1788513-1789865
Rv3466,Rv3467-NC_000962.3:3883535-3884921
Rv0094c
Rv0095c
PPE1
Event_031
104400
104800
105200
105600
106000
Genomic position (1-based)

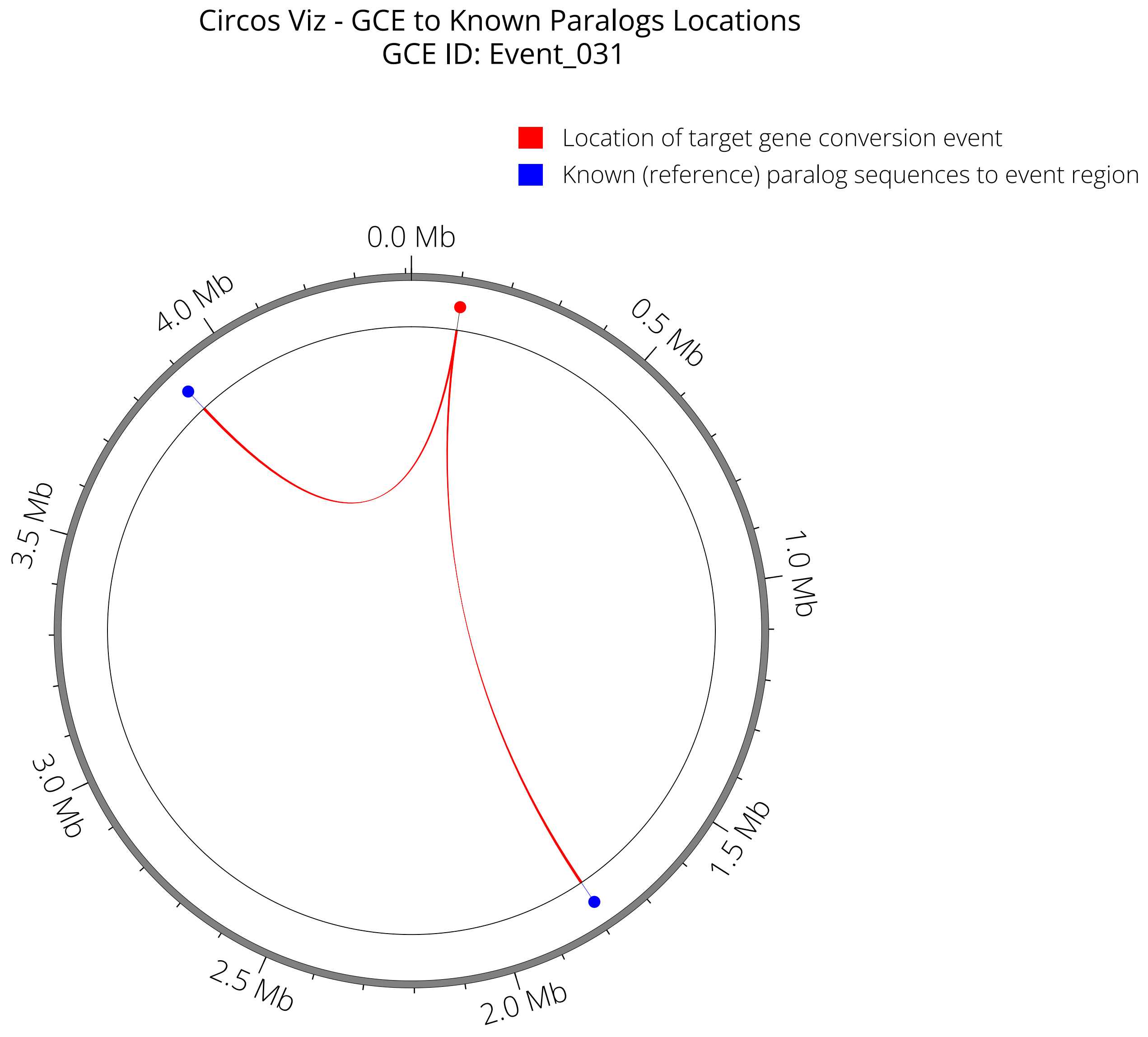

Circos Viz - GCE to Known Paralogs Locations
GCE ID: Event_031
Location of target gene conversion event
Known (reference) paralog sequences to event region
0.0 Mb
0.5 Mb
1.0 Mb
1.5 Mb
2.0 Mb
2.5 Mb
3.0 Mb
3.5 Mb
4.0 Mb

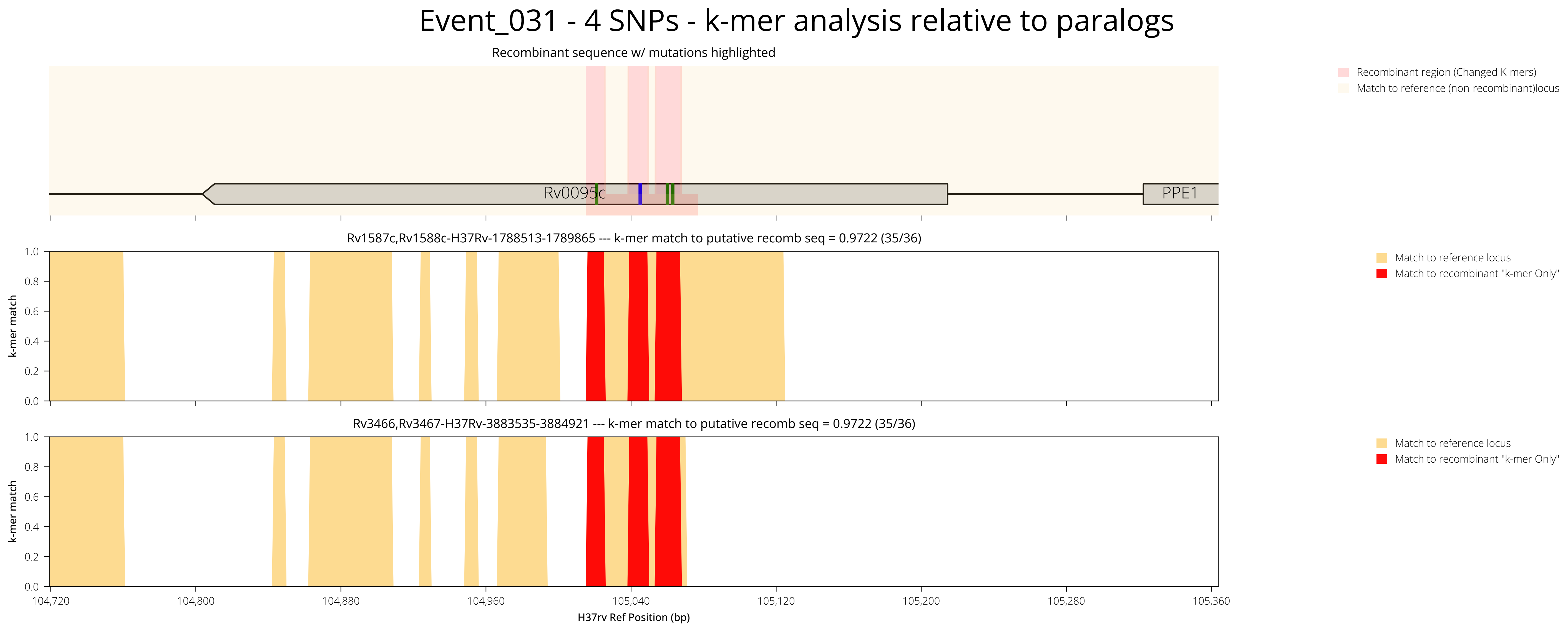
Event_031 - 4 SNPs - k-mer analysis relative to paralogs
Recombinant sequence w/ mutations highlighted
Rv0095c
PPE1
Recombinant region (Changed K-mers)
Match to reference (non-recombinant)locus
Rv1587c,Rv1588c-H37Rv-1788513-1789865 --- k-mer match to putative recomb seq = 0.9722 (35/36)
Match to reference locus
Match to recombinant "k-mer Only"
Rv3466,Rv3467-H37Rv-3883535-3884921 --- k-mer match to putative recomb seq = 0.9722 (35/36)
Match to reference locus
Match to recombinant "k-mer Only"
k-mer match
1.0
0.8
0.6
0.4
0.2
0.0
104,720
104,800
104,880
104,960
105,040
105,120
105,200
105,280
105,360
H37rv Ref Position (bp)

Sub-phylogeny Viz + Paralog Alignments
GCE ID: Event_031

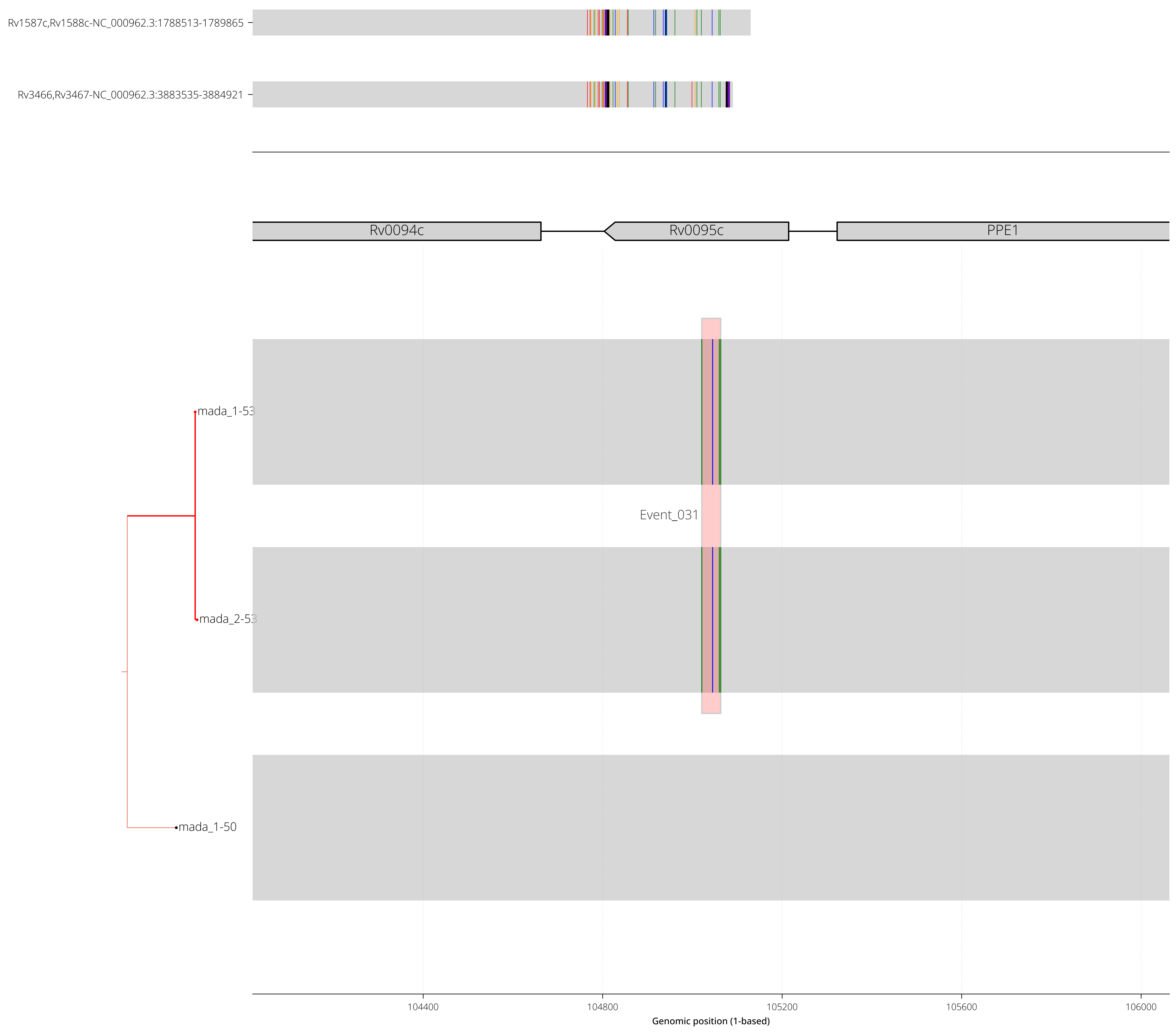

Full Phylogeny Viz + Paralog Alignments
GCE ID: Event_031

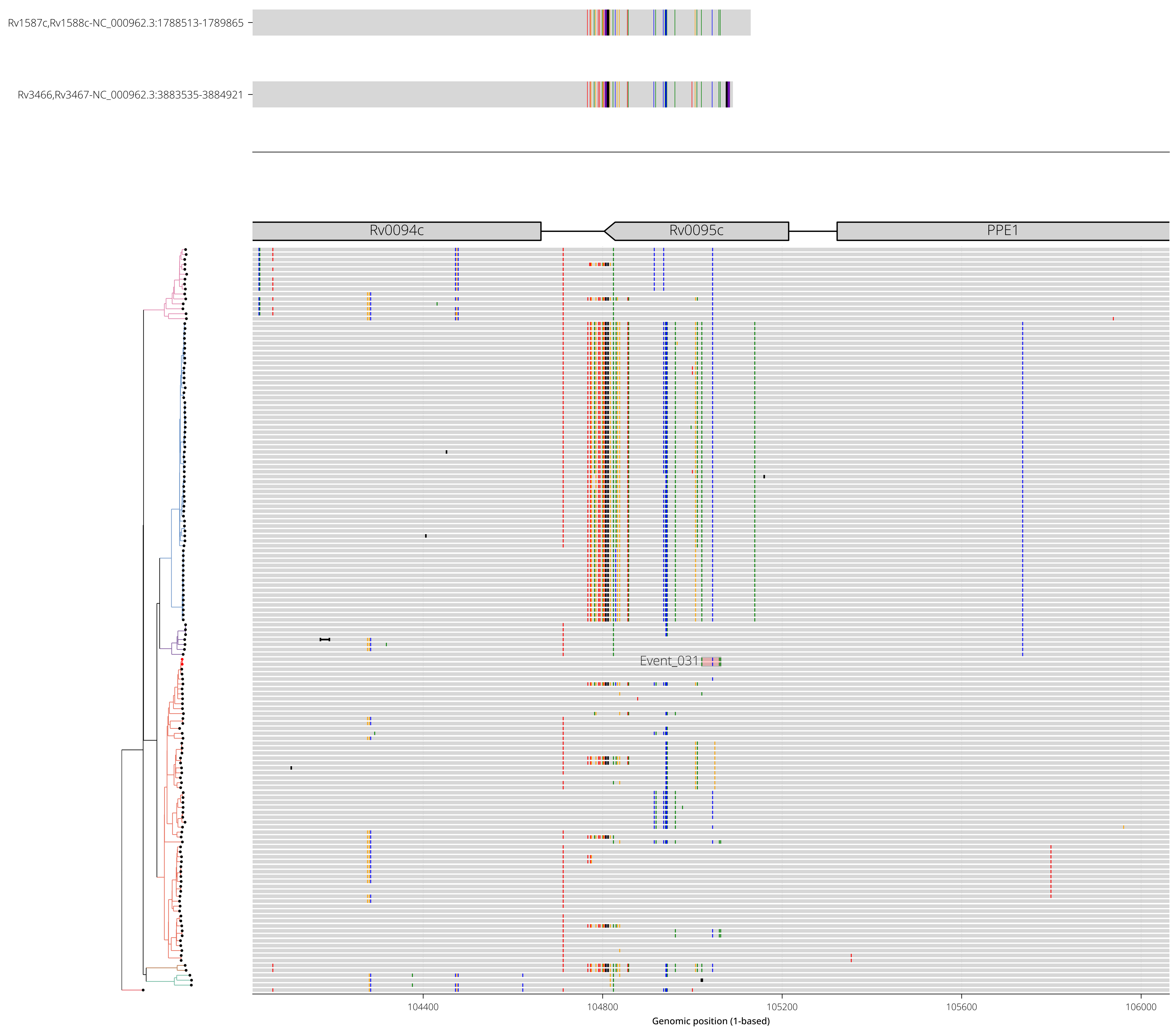

Individual GCE + Known Paralogs
GCE ID: Event_032

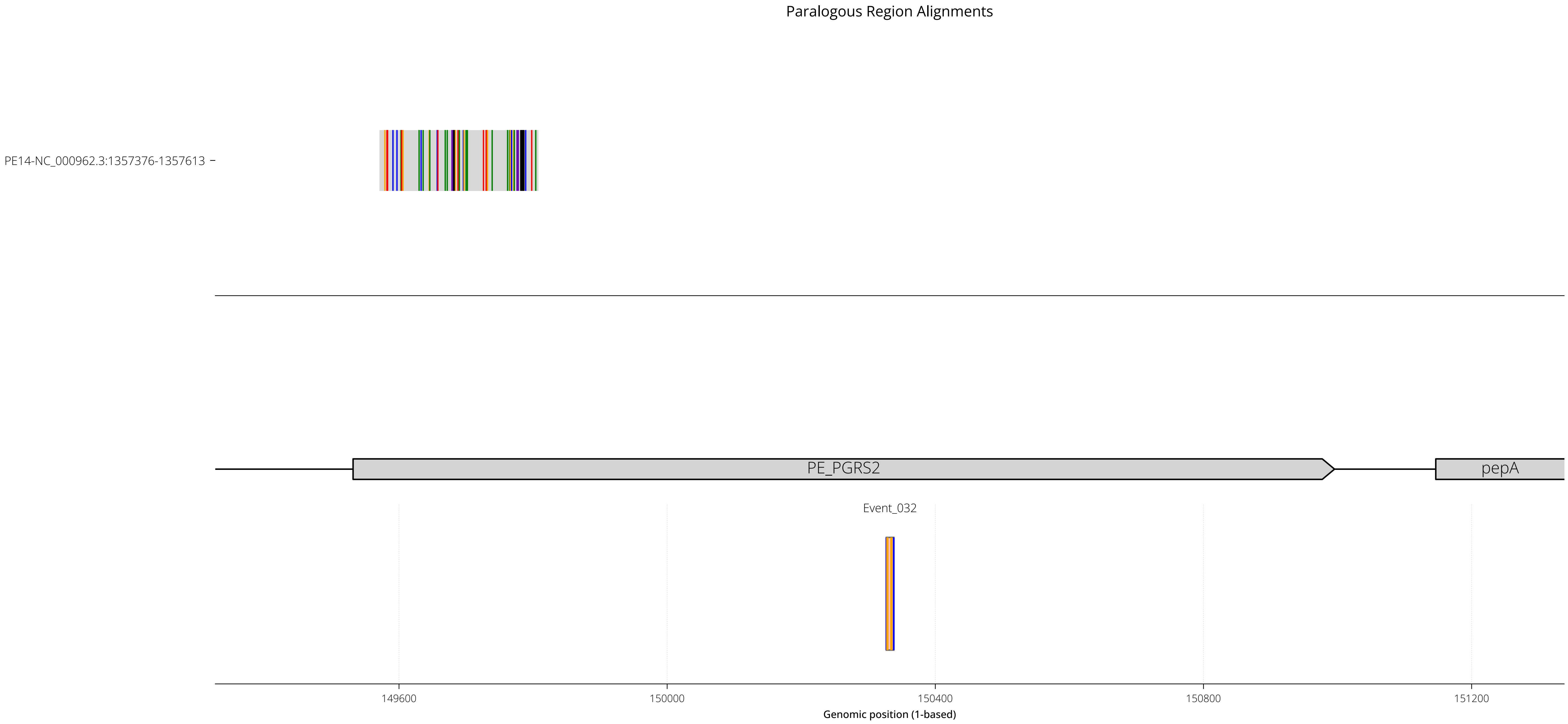

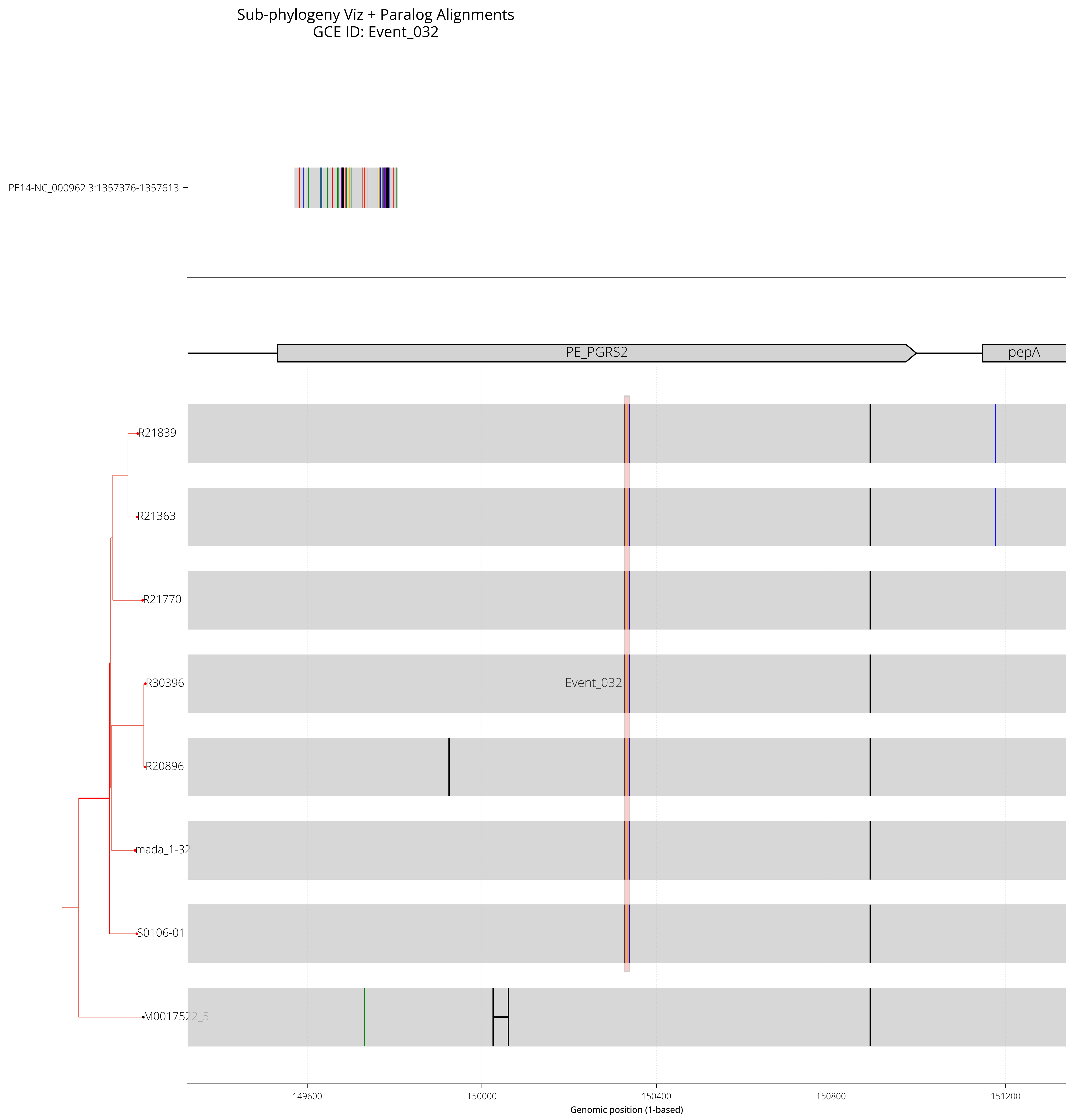

Sub-phylogeny Viz + Paralog Alignments
GCE ID: Event_032
PE14-NC_000962.3:1357376-1357613
PE_PGRS2
pepA
R21839
R21363
R21770
R30396
Event_032
R20896
mada_1-32
S0106-01
M0017522_5
149600
150000
150400
150800
151200
Genomic position (1-based)

Full Phylogeny Viz + Paralog Alignments
GCE ID: Event_032

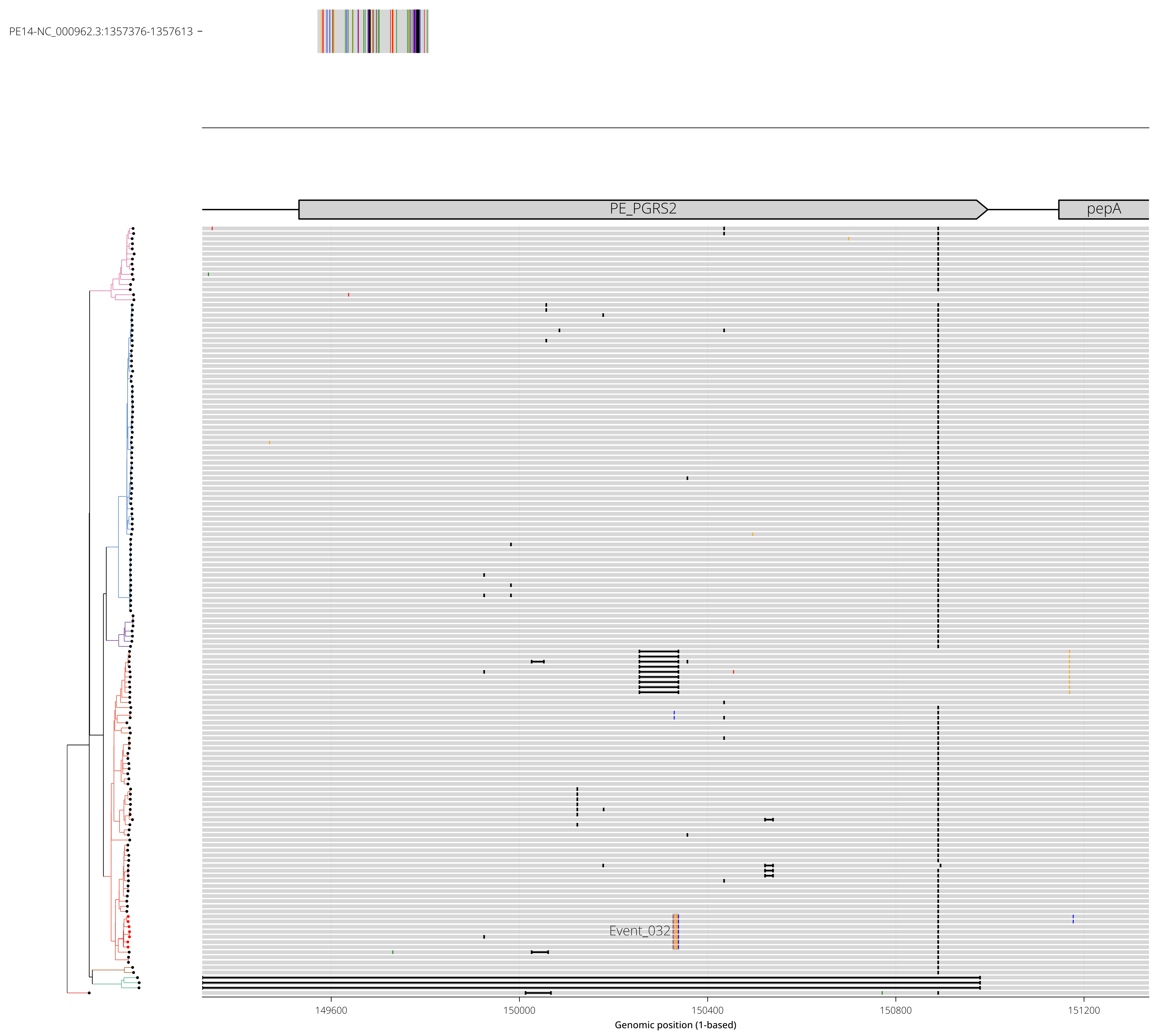

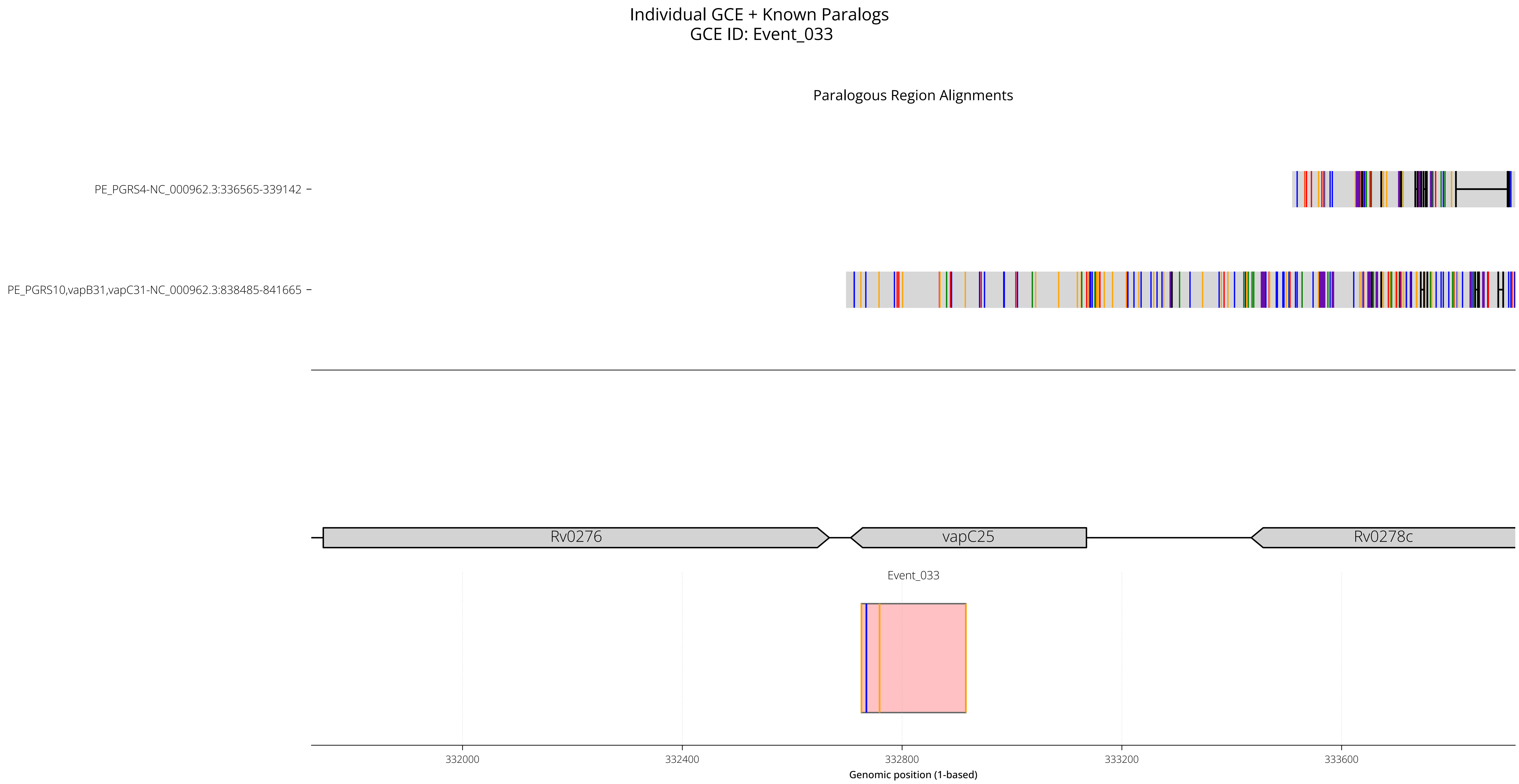
Individual GCE + Known Paralogs
GCE ID: Event_033
Paralogous Region Alignments
PE_PGRS4-NC_000962.3:336565-339142
PE_PGRS10,vapB31,vapC31-NC_000962.3:838485-841665
Rv0276
vapC25
Rv0278c
Event_033
332000
332400
332800
333200
333600
Genomic position (1-based)

Circos Viz - GCE to Known Paralogs Locations
GCE ID: Event_033

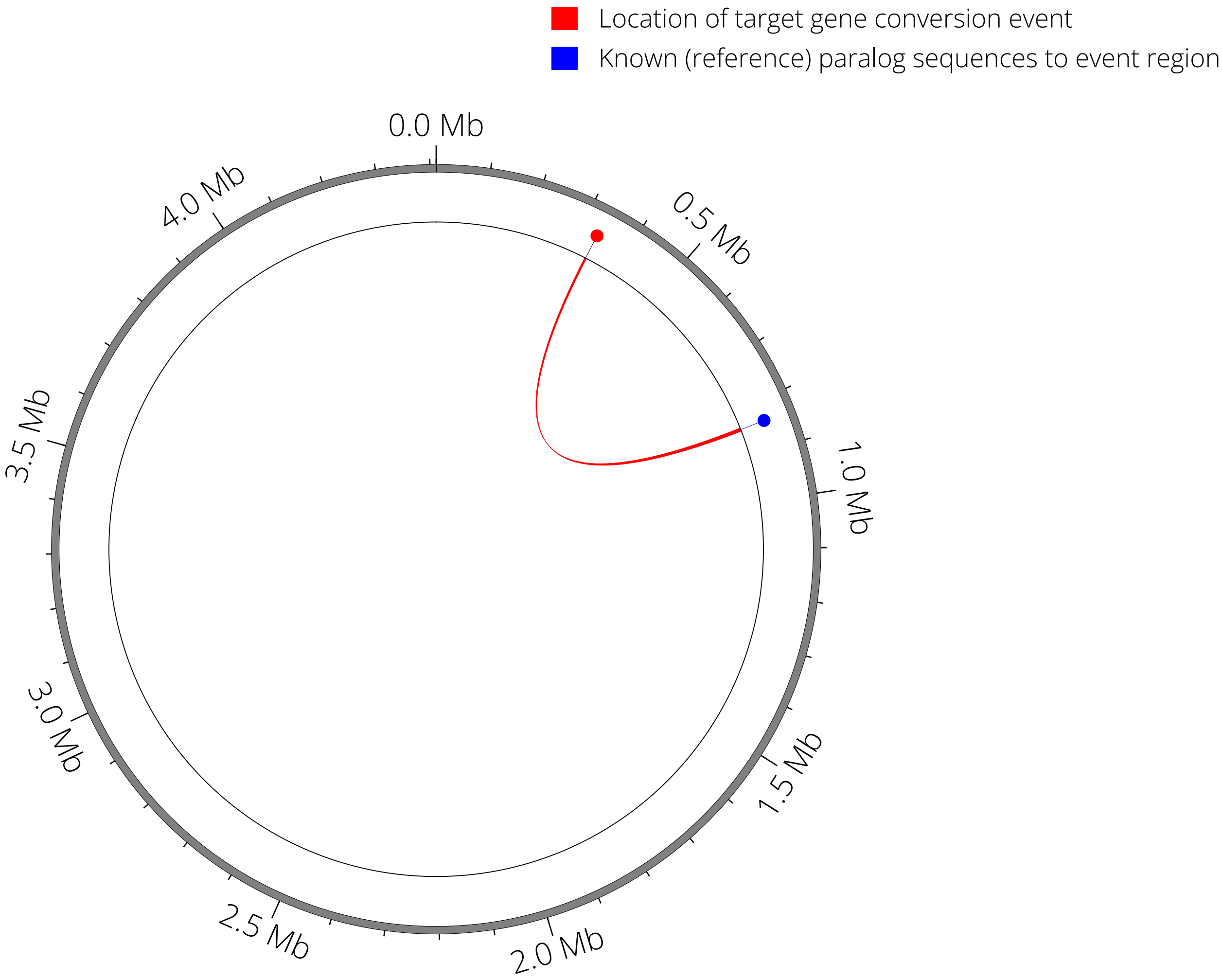

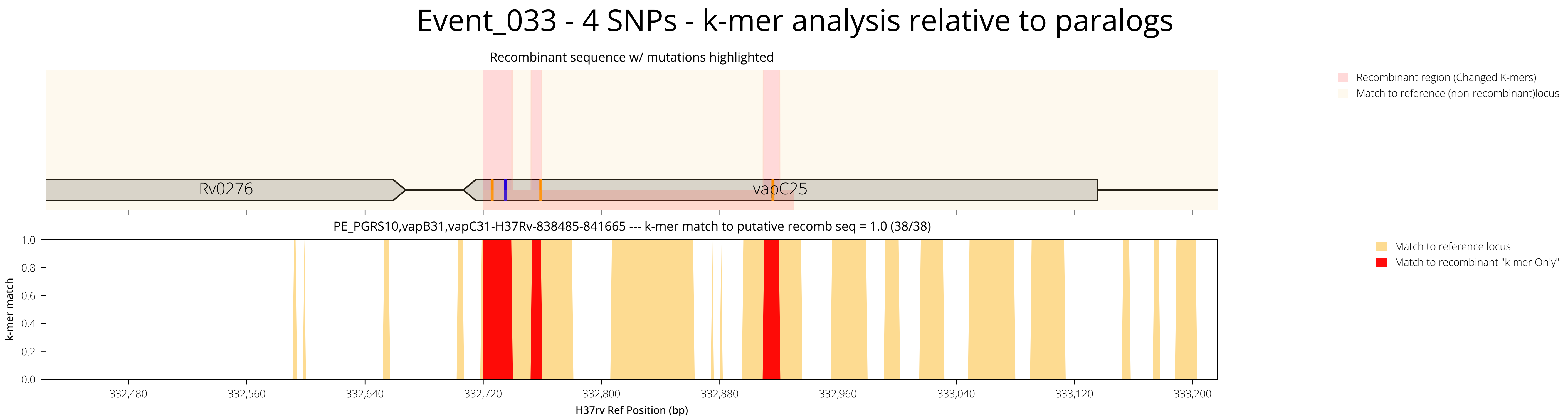

Event_033 - 4 SNPs - k-mer analysis relative to paralogs
Recombinant sequence w/ mutations highlighted
Recombinant region (Changed K-mers)
Match to reference (non-recombinant)locus
Rv0276
vapC25
PE_PGRS10,vapB31,vapC31-H37Rv-838485-841665 --- k-mer match to putative recomb seq = 1.0 (38/38)
Match to reference locus
Match to recombinant "k-mer Only"
k-mer match
1.0
0.8
0.6
0.4
0.2
0.0
332,480
332,560
332,640
332,720
332,800
332,880
332,960
333,040
333,120
333,200
H37rv Ref Position (bp)

Sub-phylogeny Viz + Paralog Alignments
GCE ID: Event_033

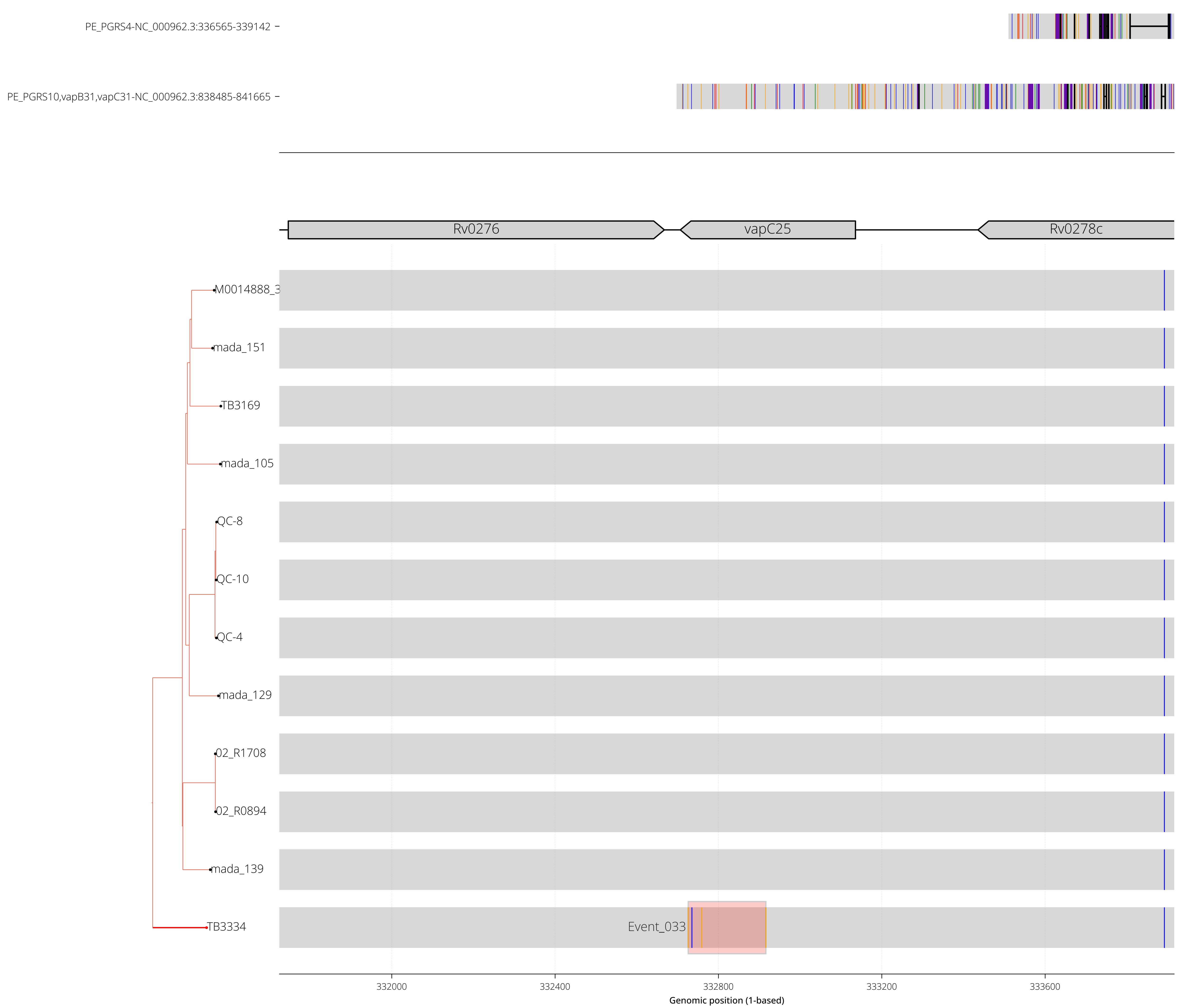

Full Phylogeny Viz + Paralog Alignments
GCE ID: Event_033

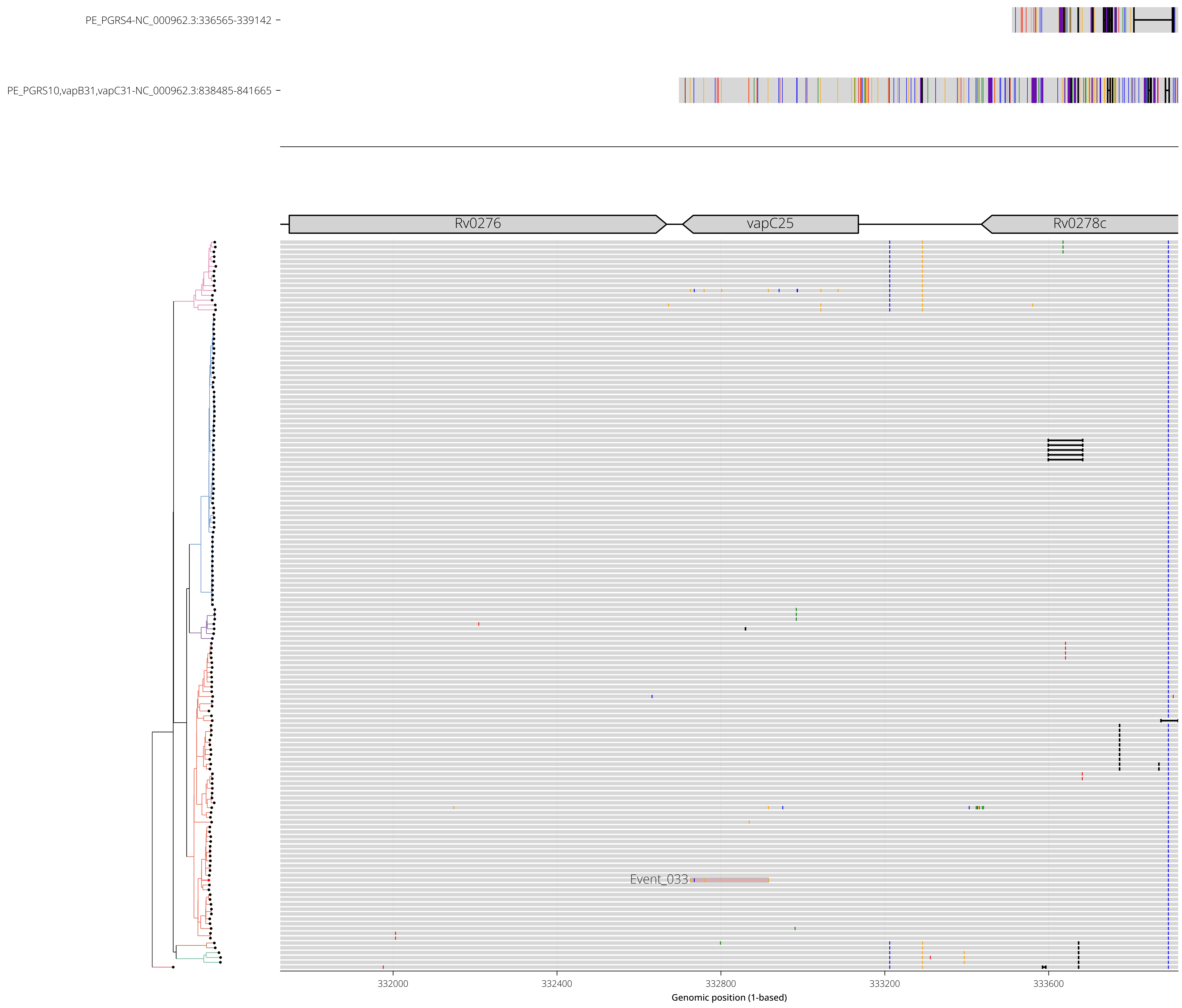

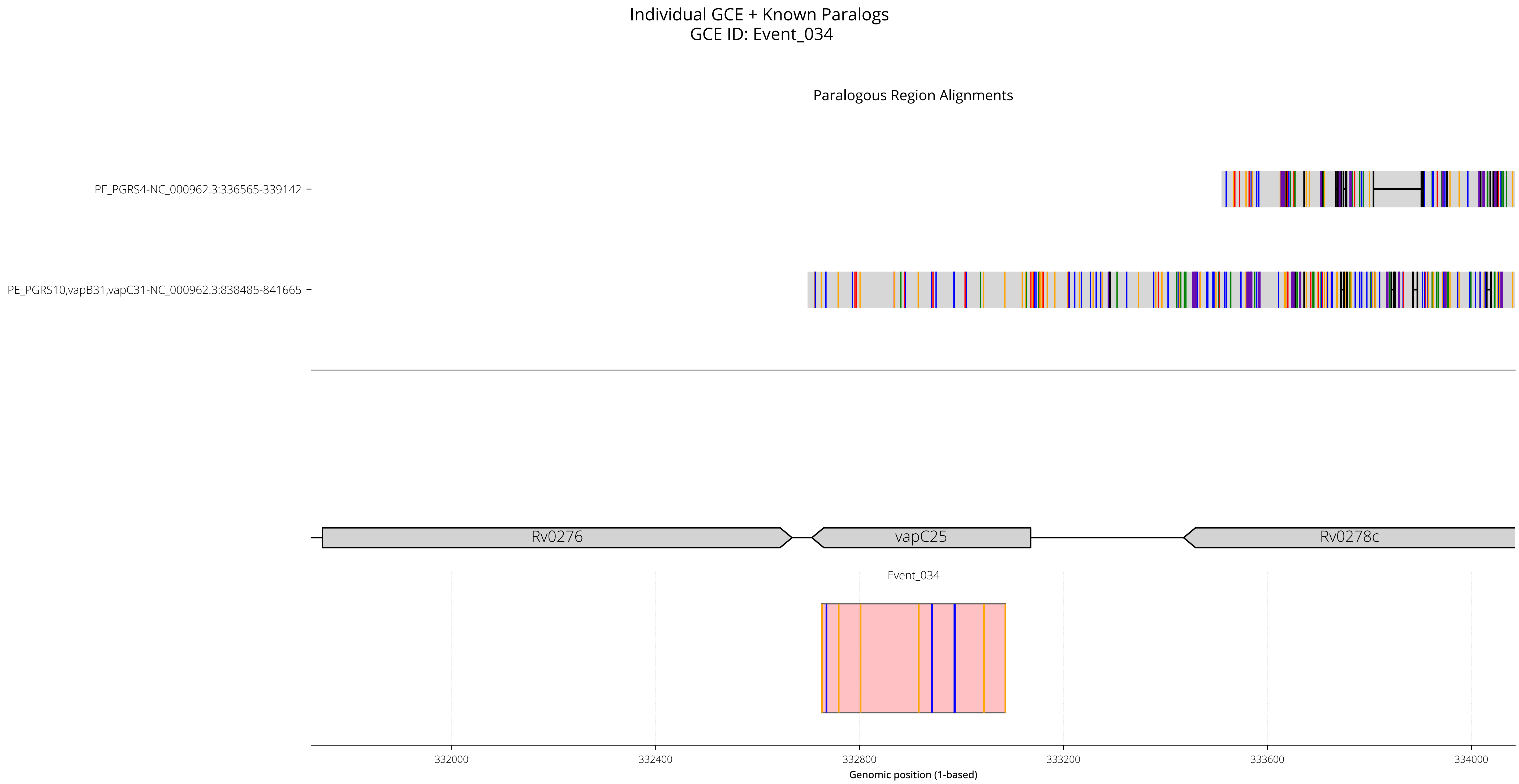

Individual GCE + Known Paralogs
GCE ID: Event_034
Paralogous Region Alignments
PE_PGRS4-NC_000962.3:336565-339142
PE_PGRS10,vapB31,vapC31-NC_000962.3:838485-841665
Rv0276
vapC25
Rv0278c
Event_034
332000
332400
332800
333200
333600
334000
Genomic position (1-based)

Circos Viz - GCE to Known Paralogs Locations
GCE ID: Event_034

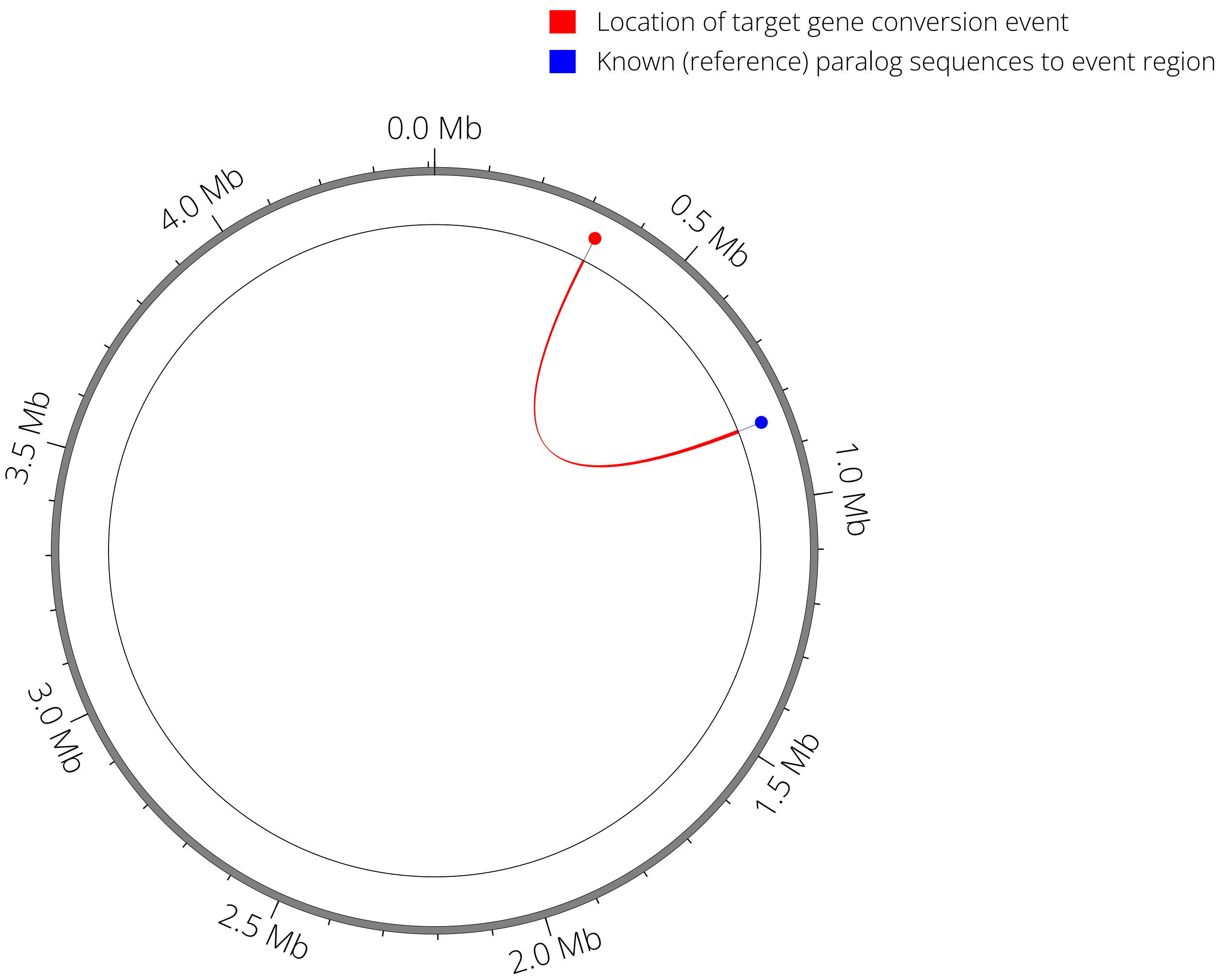

# Event_034 - 10 SNPs - k-mer analysis relative to paralogs

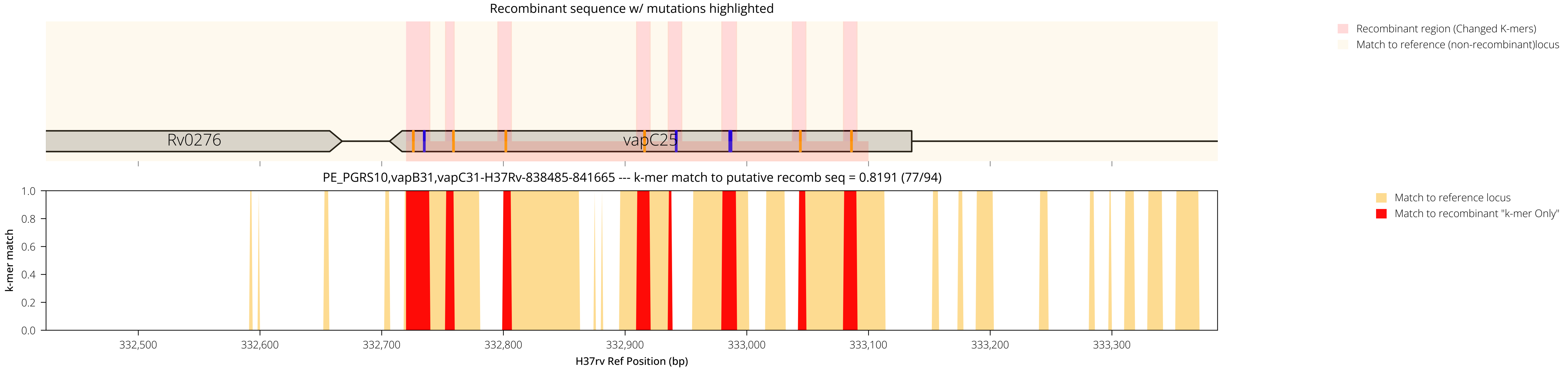

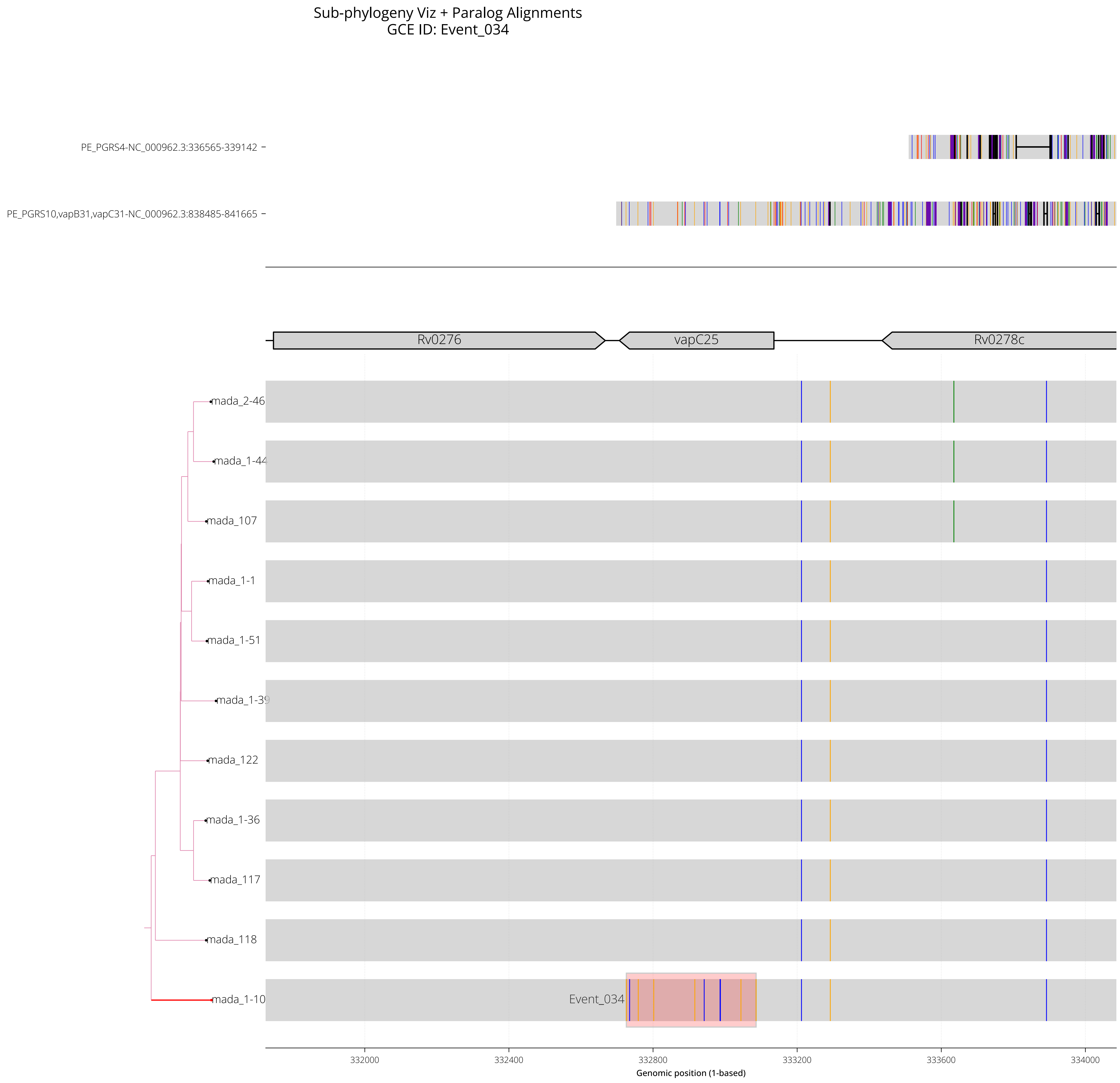

Sub-phylogeny Viz + Paralog Alignments
GCE ID: Event_034
PE_PGRS4-NC_000962.3:336565-339142
PE_PGRS10,vapB31,vapC31-NC_000962.3:838485-841665
Rv0276
vapC25
Rv0278c
mada_2-46
mada_1-44
mada_107
mada_1-1
mada_1-51
mada_1-39
mada_122
mada_1-36
mada_117
mada_118
mada_1-10
Event_034
332000
332400
332800
333200
333600
334000
Genomic position (1-based)

Full Phylogeny Viz + Paralog Alignments
GCE ID: Event_034

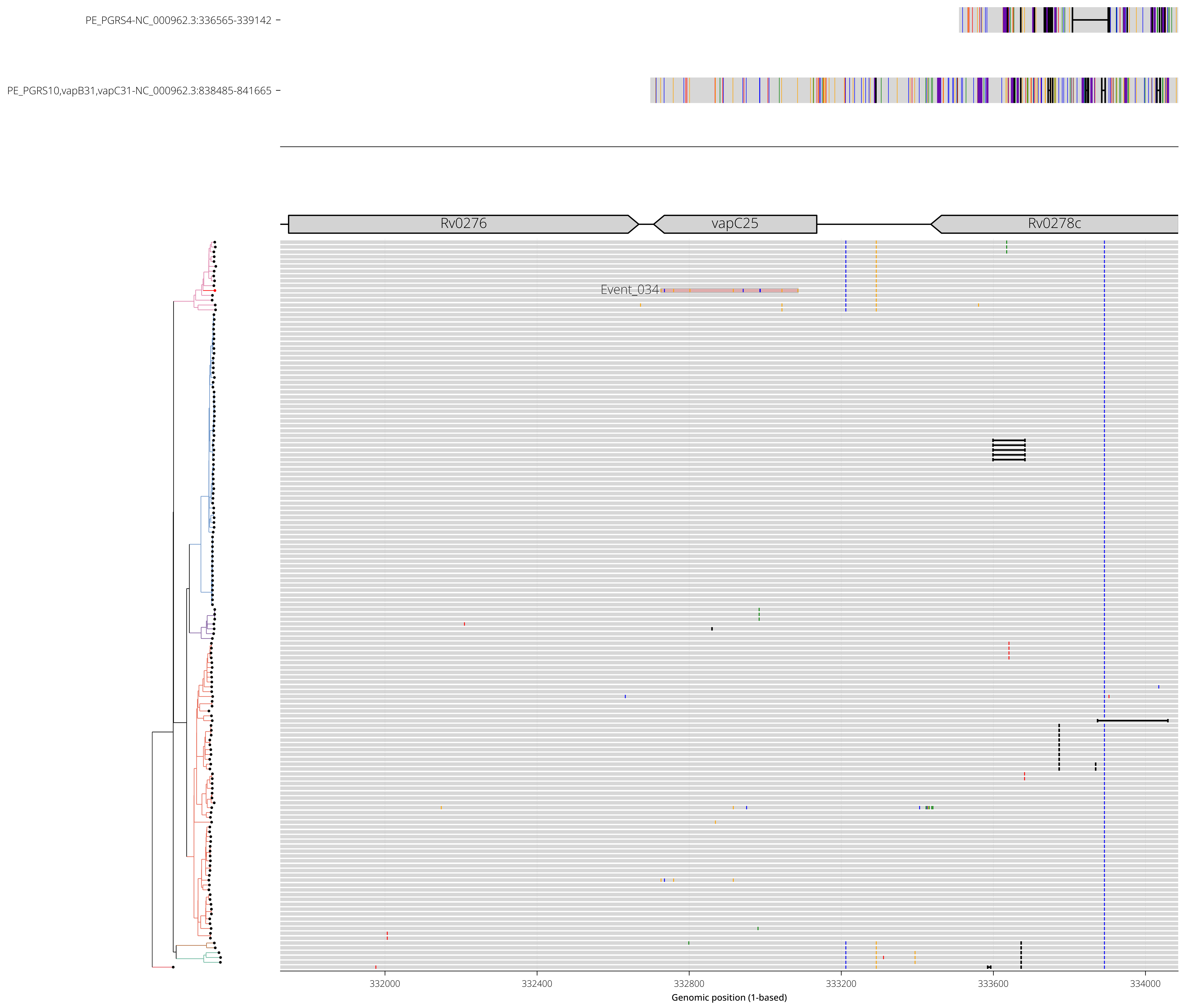

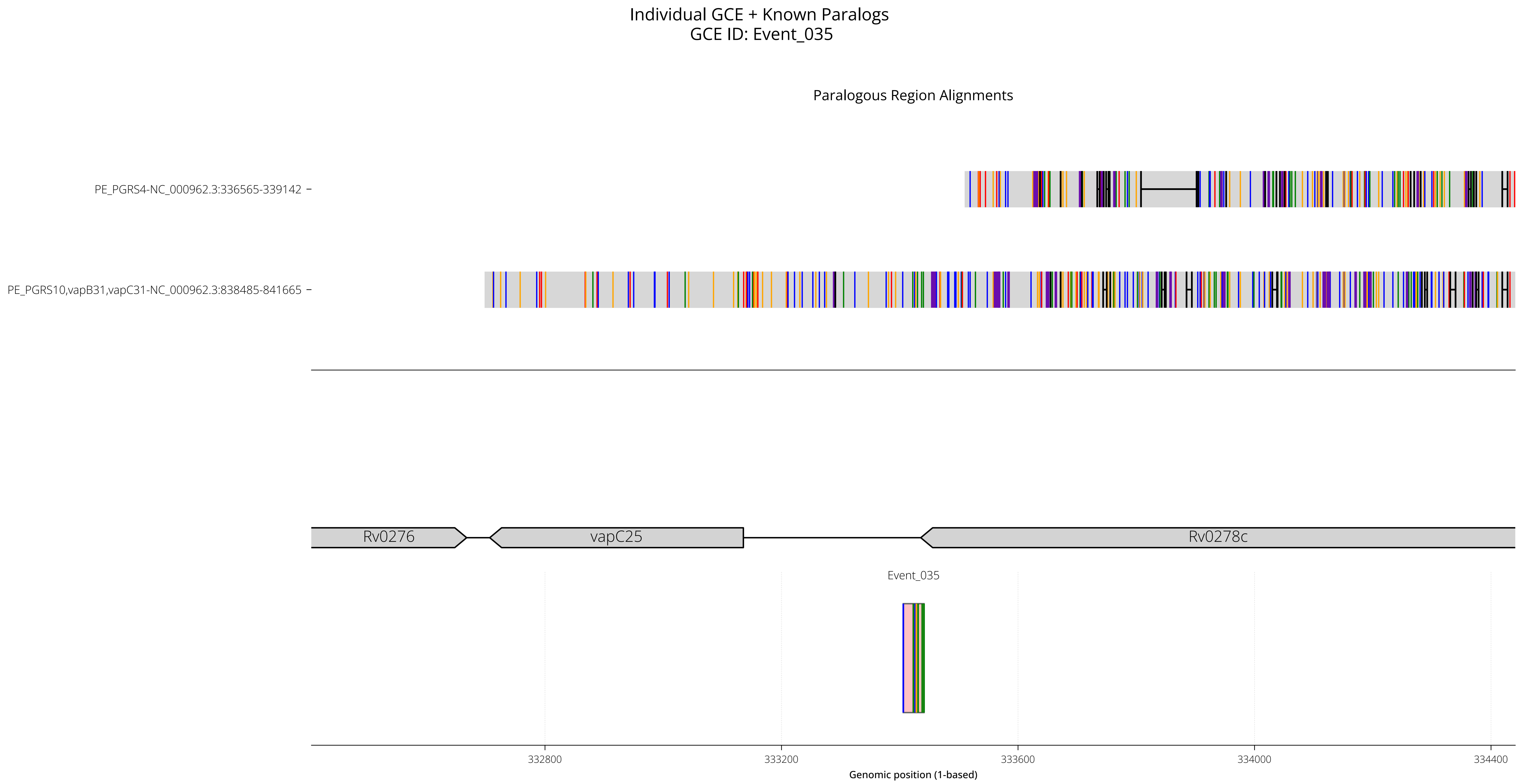

Individual GCE + Known Paralogs
GCE ID: Event_035
Paralogous Region Alignments
PE_PGRS4-NC_000962.3:336565-339142
PE_PGRS10,vapB31,vapC31-NC_000962.3:838485-841665
Rv0276
vapC25
Rv0278c
Event_035
332800
333200
333600
334000
334400
Genomic position (1-based)

Circos Viz - GCE to Known Paralogs Locations
GCE ID: Event_035

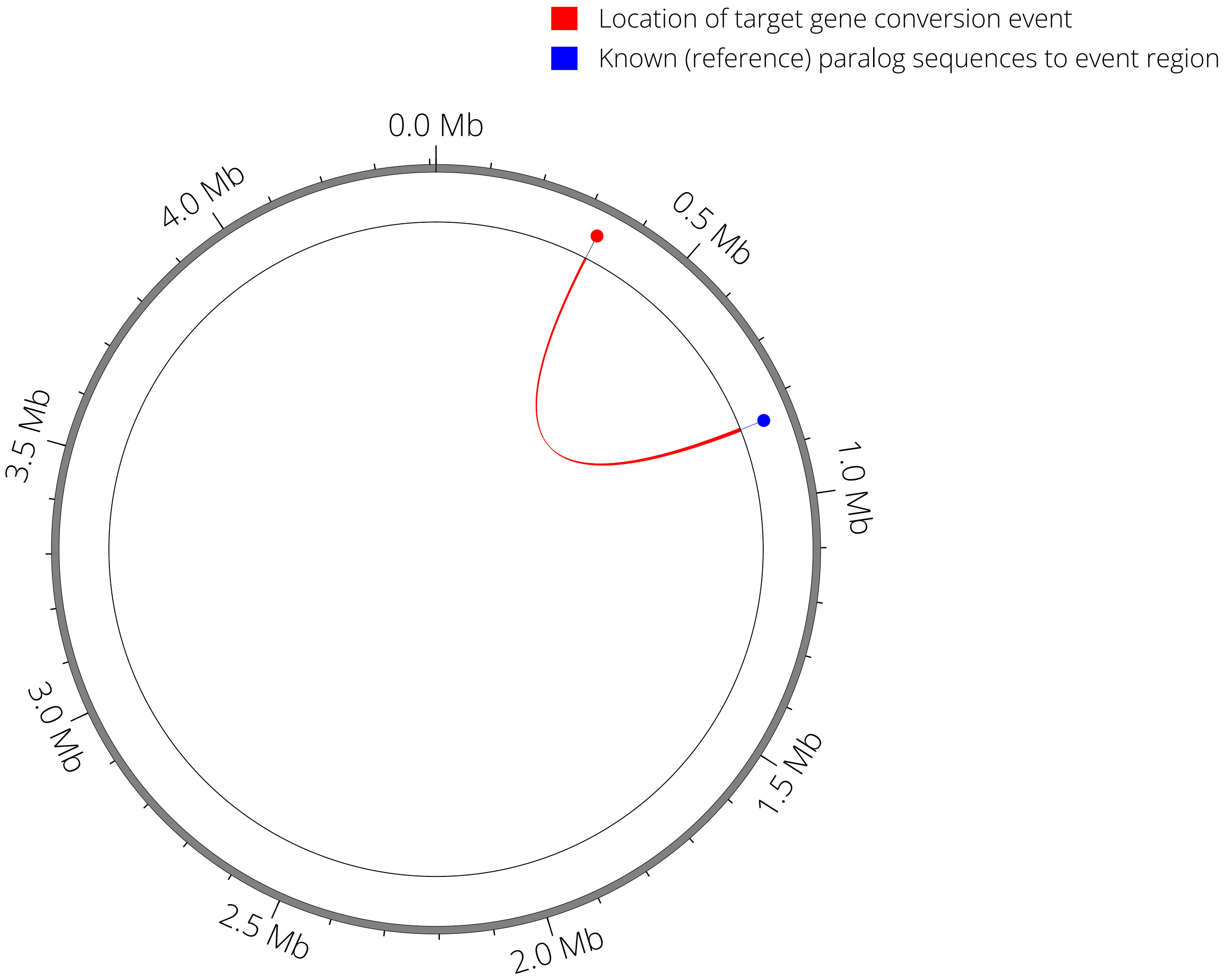

# Event_035 - 7 SNPs - k-mer analysis relative to paralogs

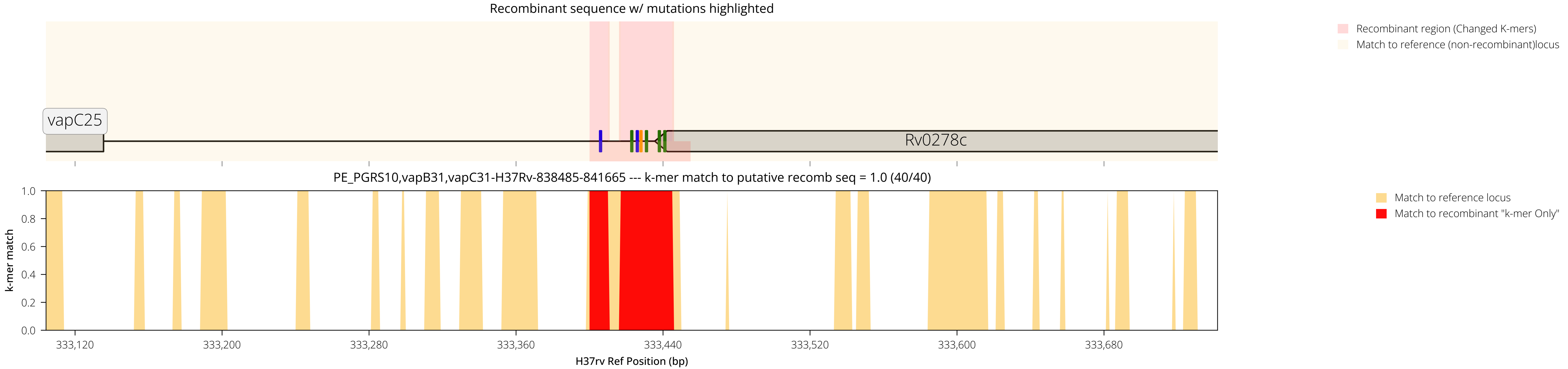

Sub-phylogeny Viz + Paralog Alignments
GCE ID: Event_035

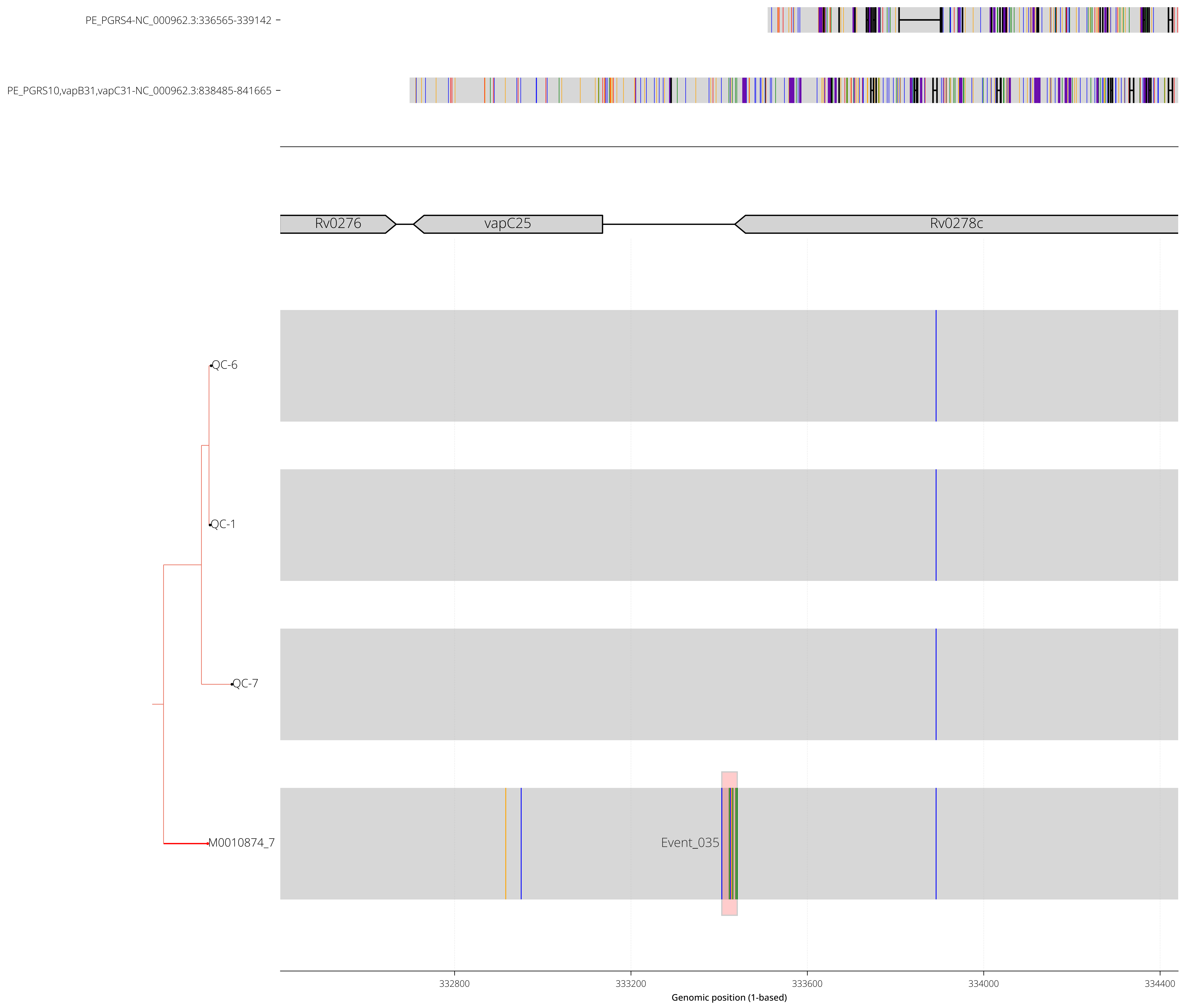

Full Phylogeny Viz + Paralog Alignments
GCE ID: Event_035

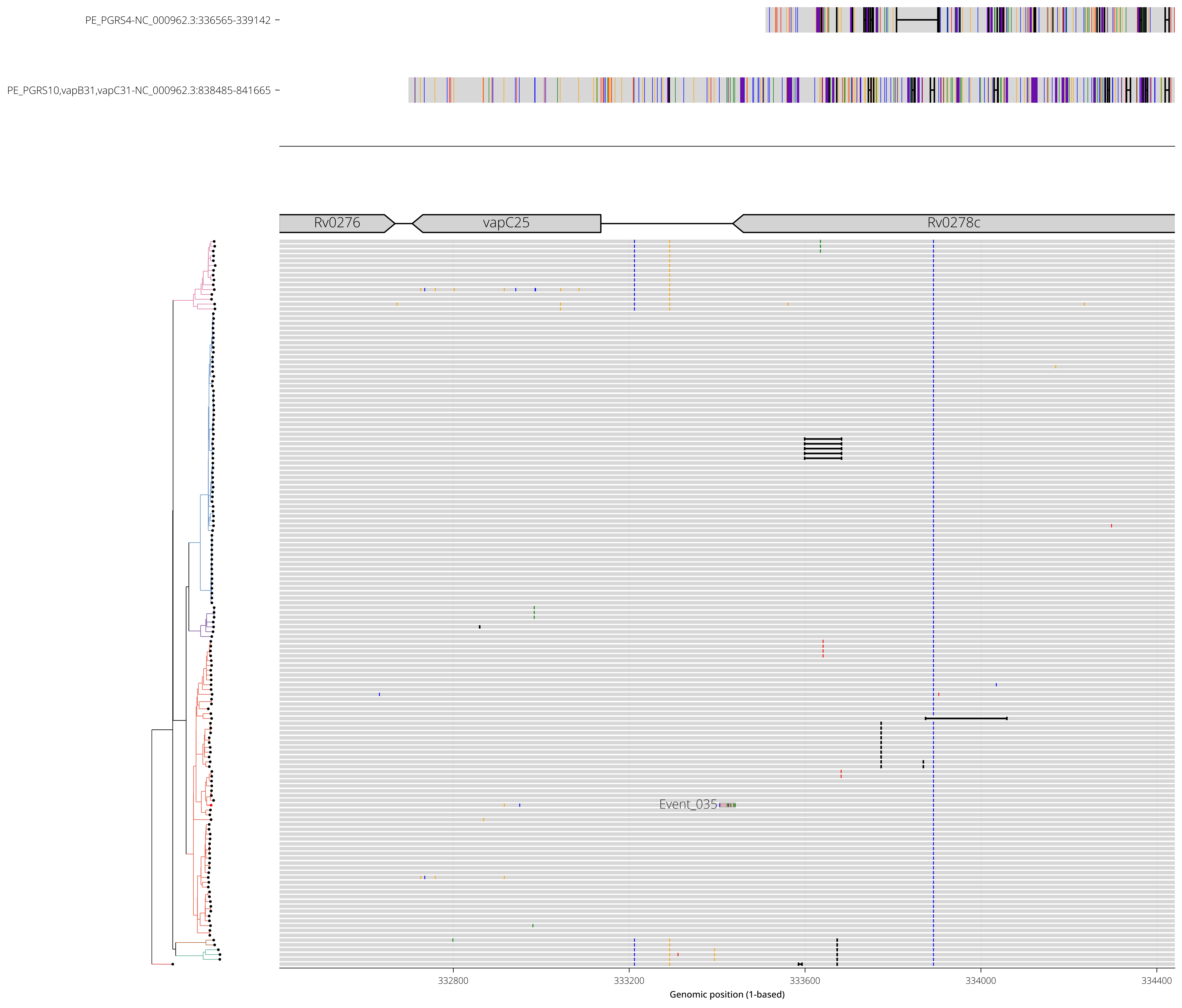

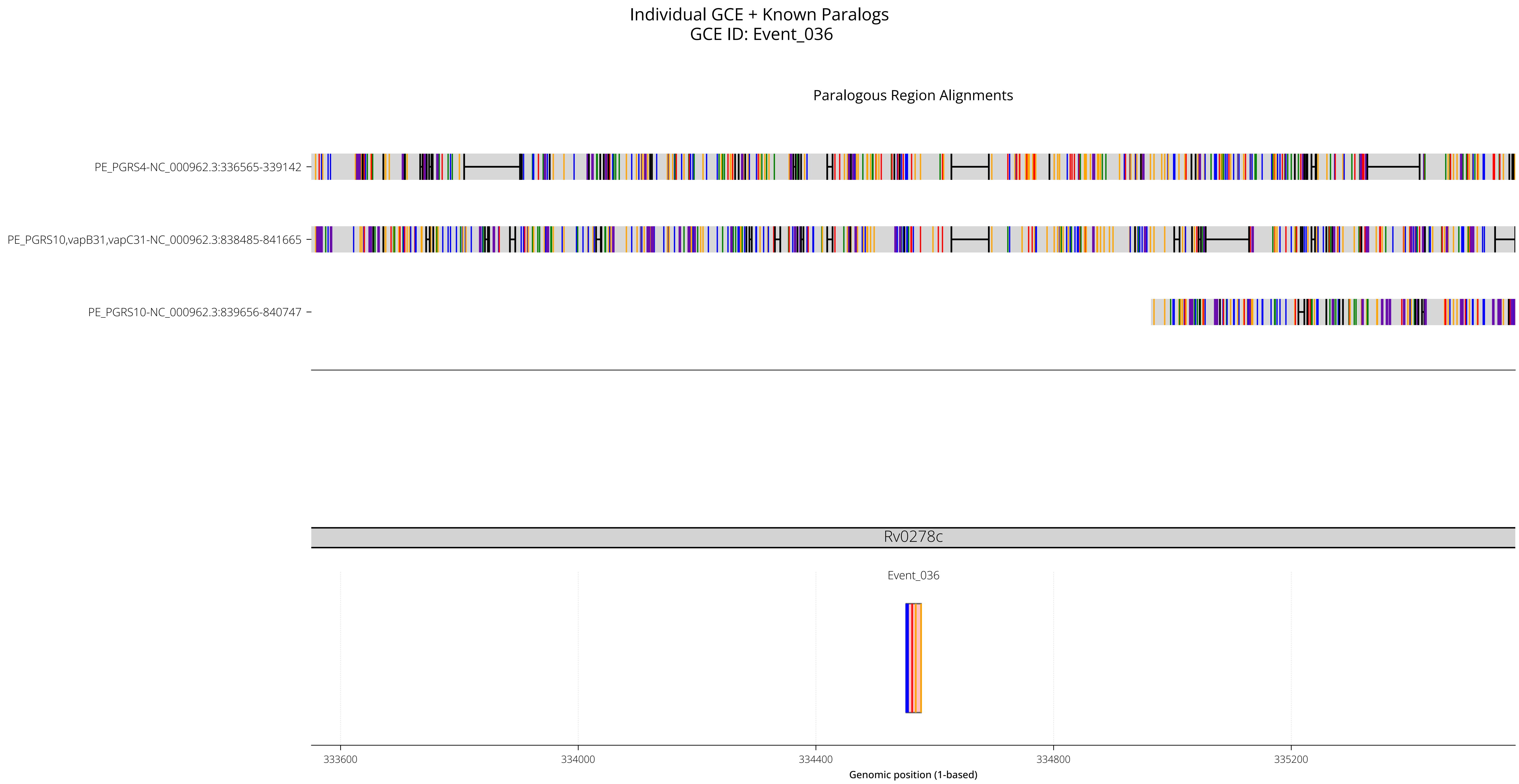
Individual GCE + Known Paralogs
GCE ID: Event_036
Paralogous Region Alignments
PE_PGRS4-NC_000962.3:336565-339142
PE_PGRS10,vapB31,vapC31-NC_000962.3:838485-841665
PE_PGRS10-NC_000962.3:839656-840747
Rv0278c
Event_036
333600
334000
334400
334800
335200
Genomic position (1-based)

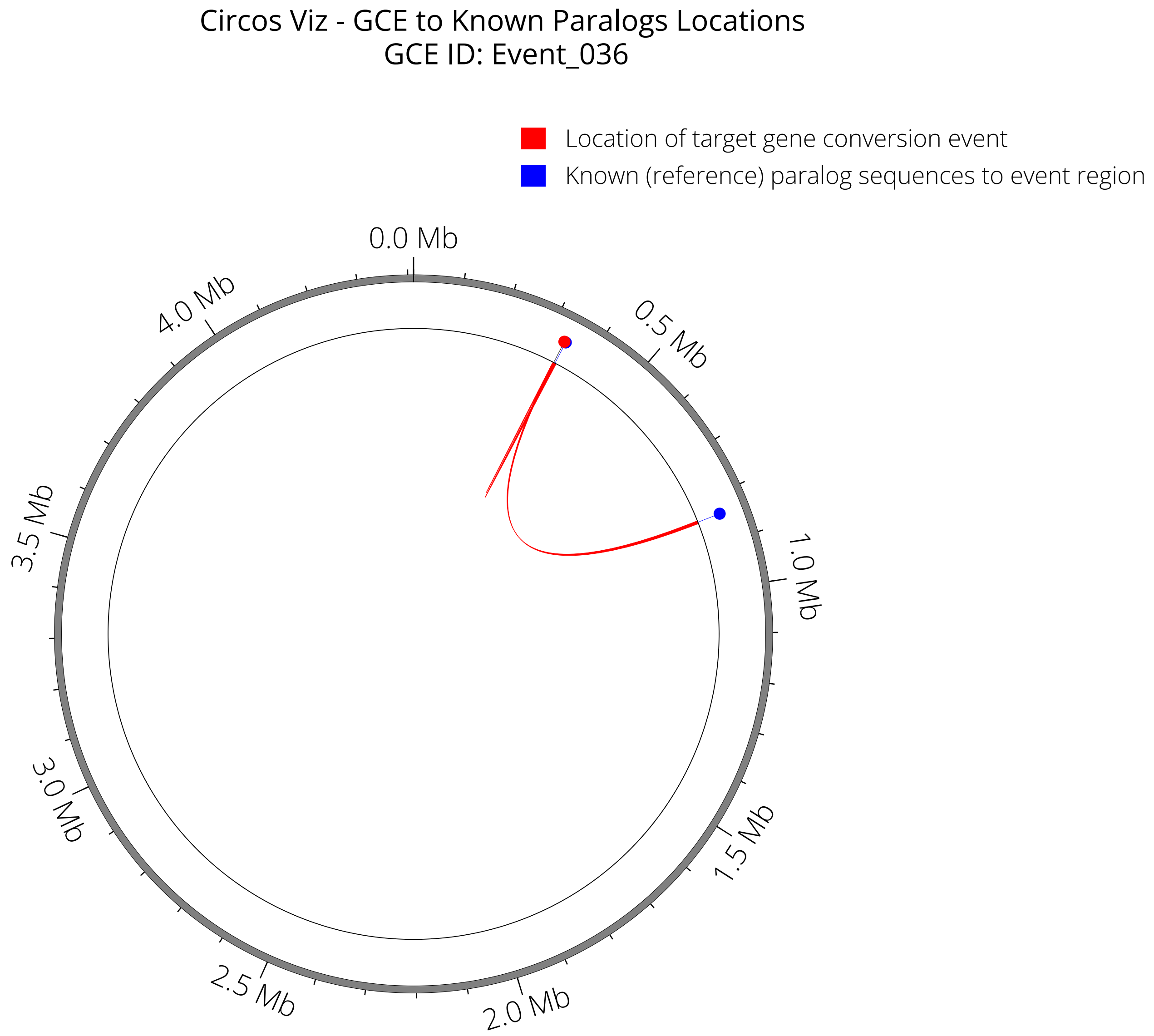

Circos Viz - GCE to Known Paralogs Locations
GCE ID: Event_036
Location of target gene conversion event
Known (reference) paralog sequences to event region
0.0 Mb
0.5 Mb
1.0 Mb
1.5 Mb
2.0 Mb
2.5 Mb
3.0 Mb
3.5 Mb
4.0 Mb

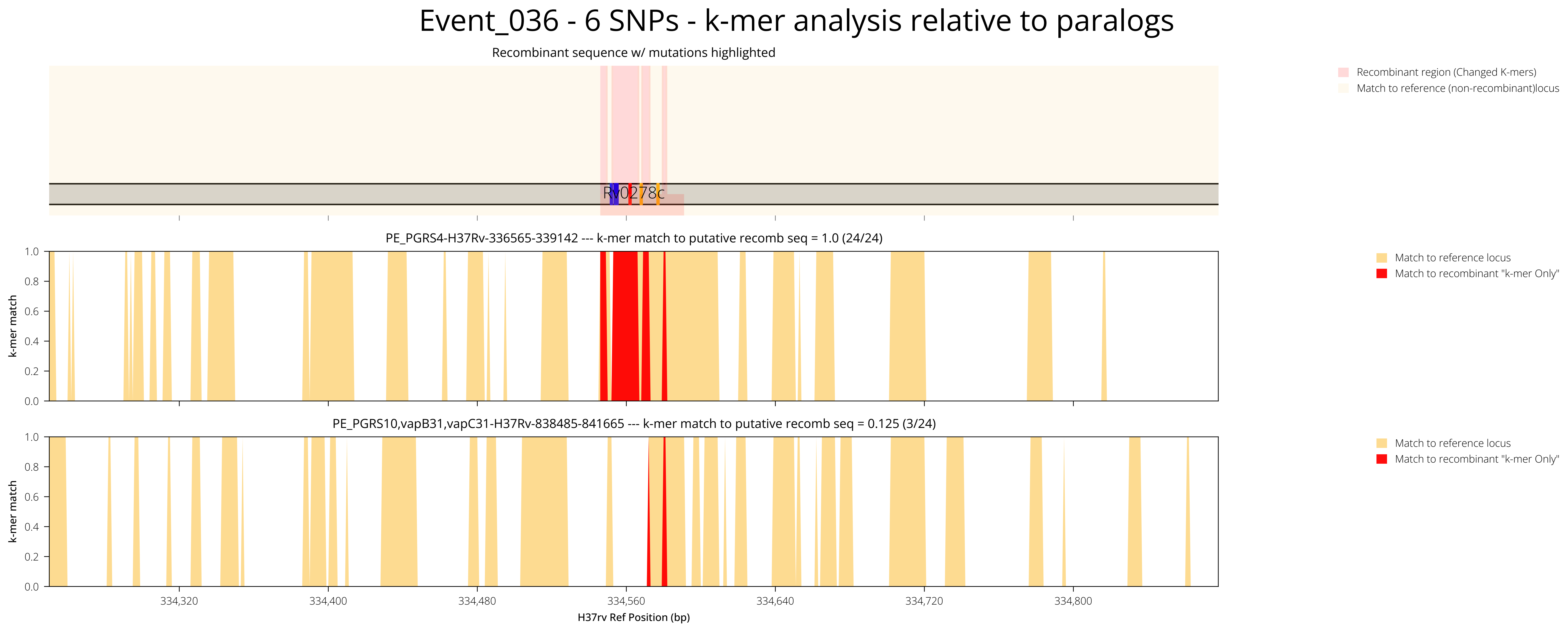
Event_036 - 6 SNPs - k-mer analysis relative to paralogs
Recombinant sequence w/ mutations highlighted
Rv0278c
Recombinant region (Changed K-mers)
Match to reference (non-recombinant)locus
PE_PGRS4-H37Rv-336565-339142 --- k-mer match to putative recomb seq = 1.0 (24/24)
k-mer match
1.0
0.8
0.6
0.4
0.2
0.0
Match to reference locus
Match to recombinant "k-mer Only"
PE_PGRS10,vapB31,vapC31-H37Rv-838485-841665 --- k-mer match to putative recomb seq = 0.125 (3/24)
k-mer match
1.0
0.8
0.6
0.4
0.2
0.0
Match to reference locus
Match to recombinant "k-mer Only"
334,320
334,400
334,480
334,560
334,640
334,720
334,800
H37rv Ref Position (bp)

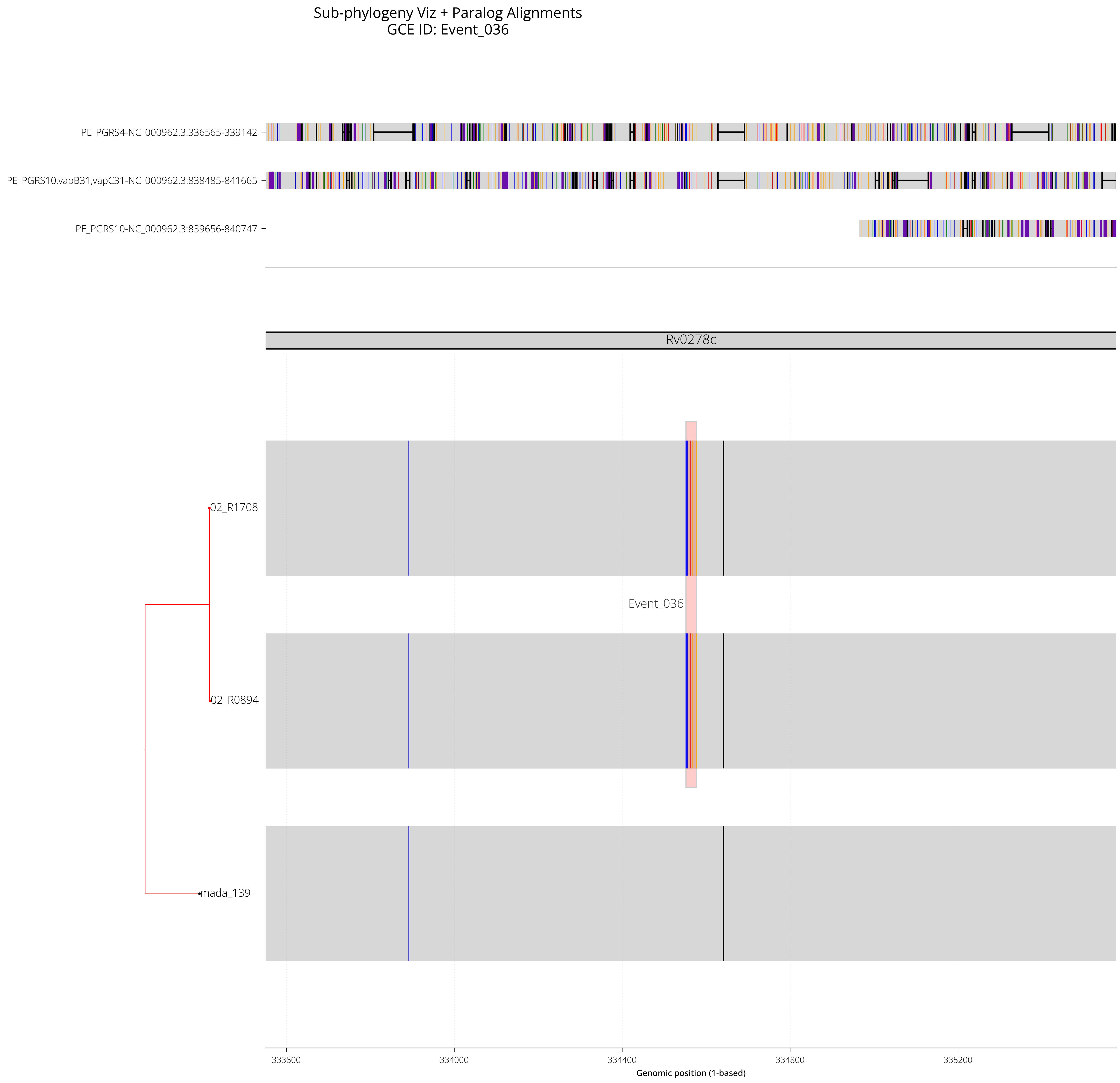

Sub-phylogeny Viz + Paralog Alignments
GCE ID: Event_036
PE_PGRS4-NC_000962.3:336565-339142
PE_PGRS10,vapB31,vapC31-NC_000962.3:838485-841665
PE_PGRS10-NC_000962.3:839656-840747
Rv0278c
02_R1708
Event_036
02_R0894
mada_139
333600
334000
334400
334800
335200
Genomic position (1-based)

Full Phylogeny Viz + Paralog Alignments
GCE ID: Event_036

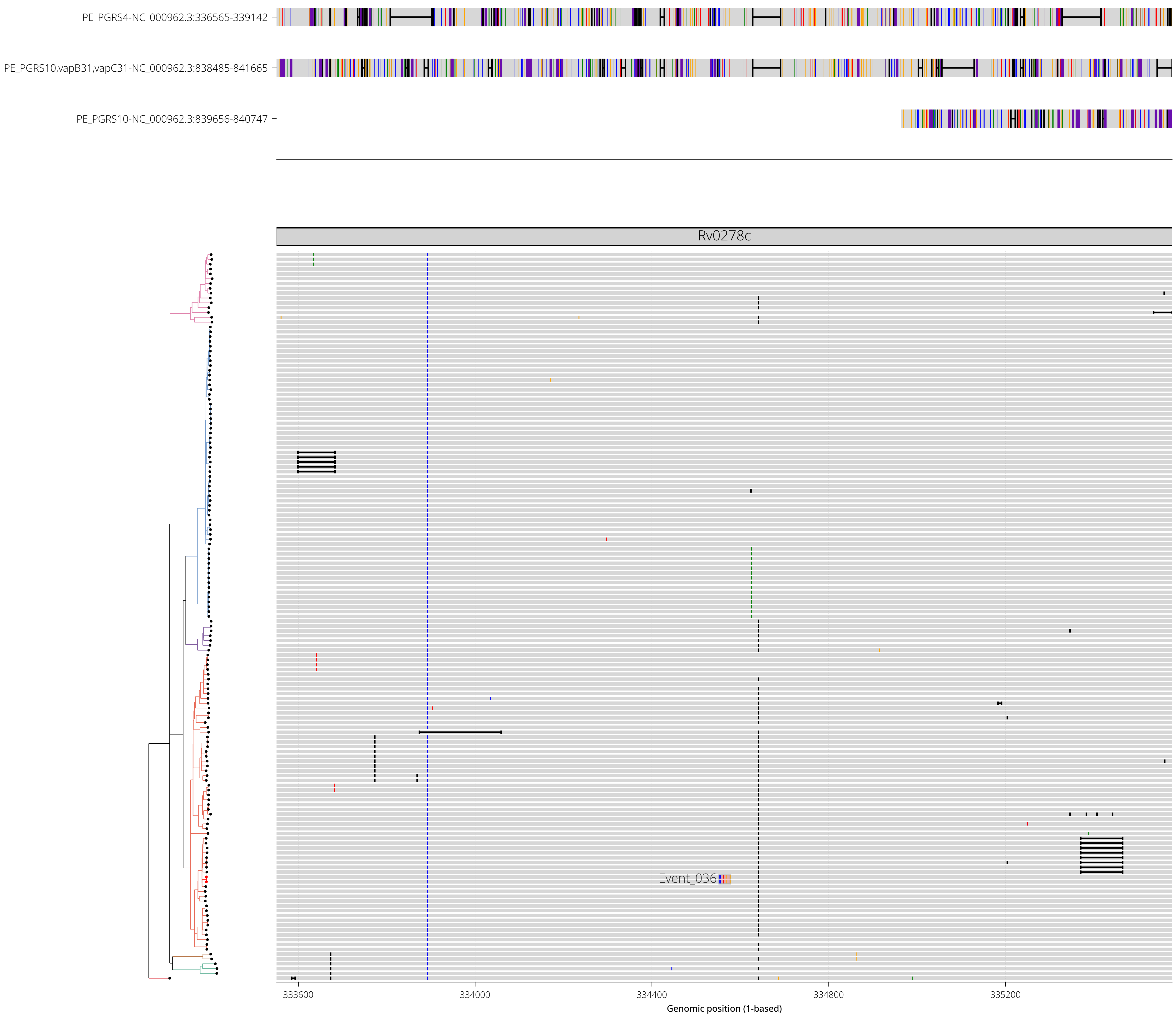

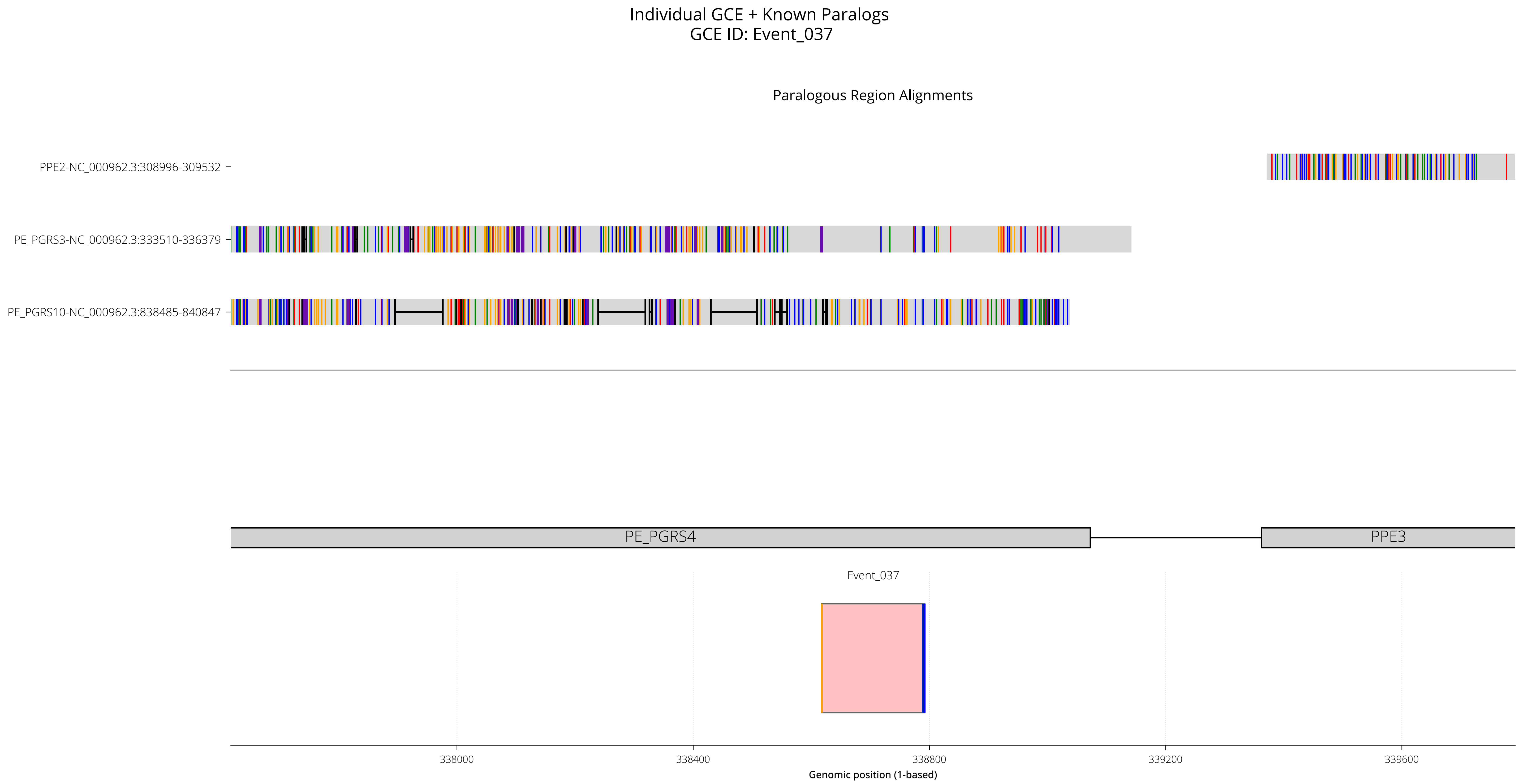
Individual GCE + Known Paralogs
GCE ID: Event_037
Paralogous Region Alignments
PPE2-NC_000962.3:308996-309532
PE_PGRS3-NC_000962.3:333510-336379
PE_PGRS10-NC_000962.3:838485-840847
PE_PGRS4
PPE3
Event_037
338000
338400
338800
339200
339600
Genomic position (1-based)

Circos Viz - GCE to Known Paralogs Locations
GCE ID: Event_037

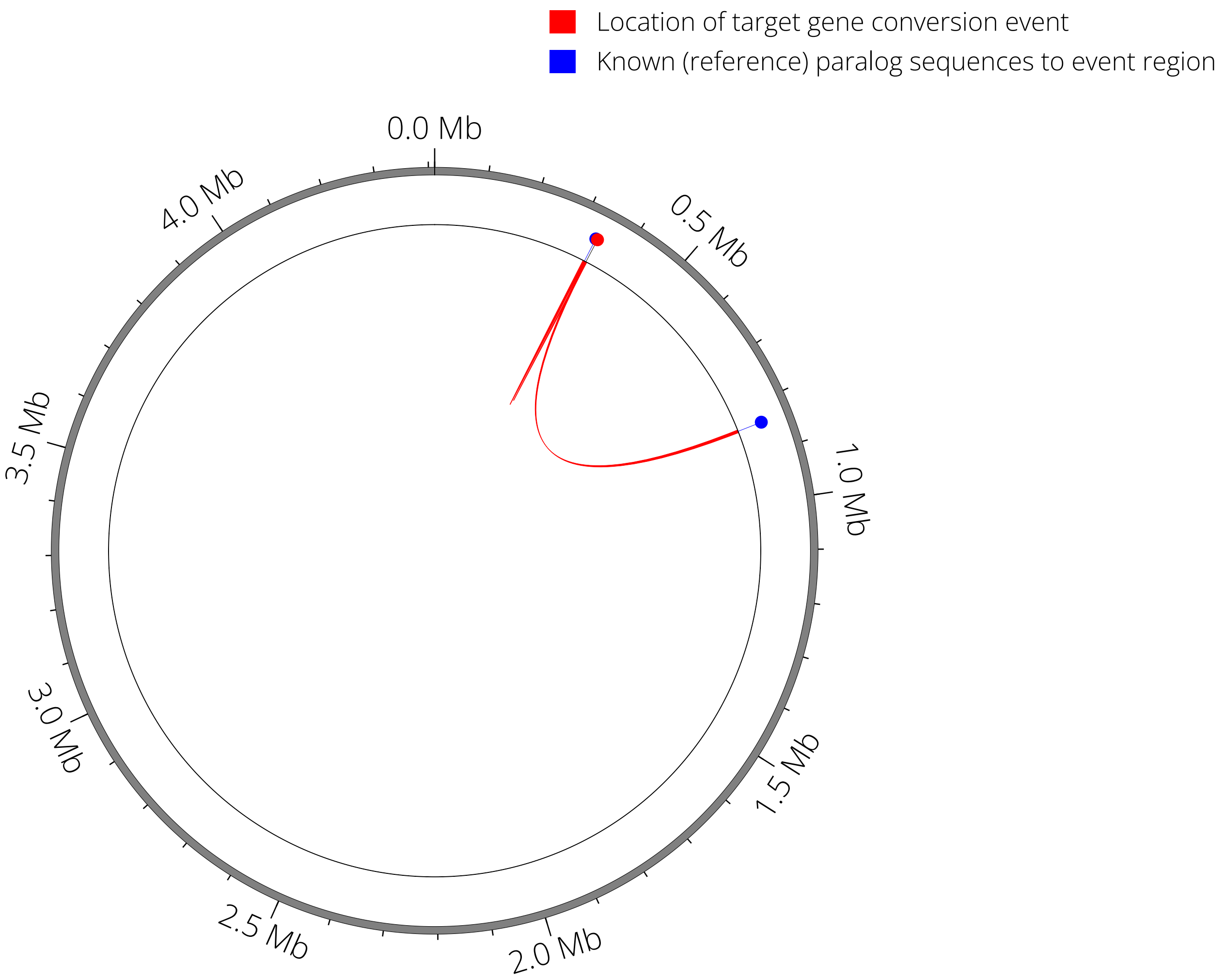

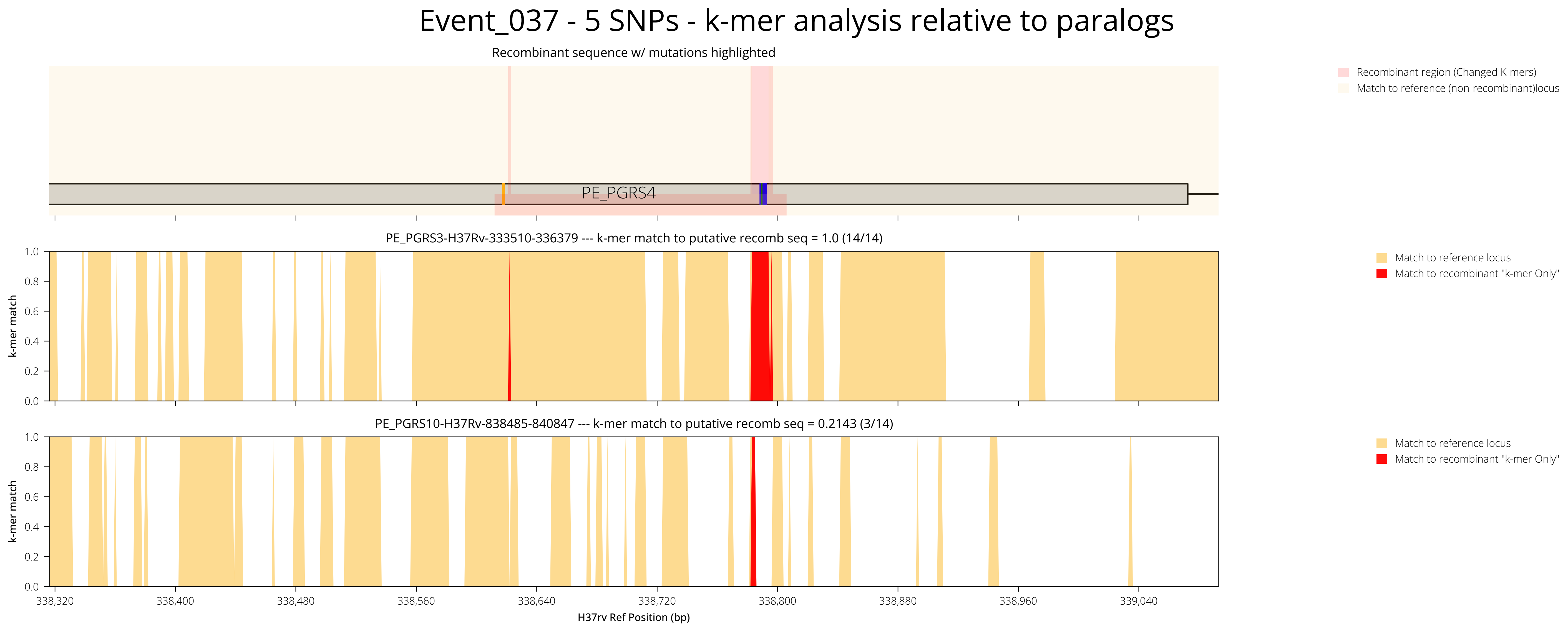

Event_037 - 5 SNPs - k-mer analysis relative to paralogs
Recombinant sequence w/ mutations highlighted
PE_PGRS4
Recombinant region (Changed K-mers)
Match to reference (non-recombinant)locus
PE_PGRS3-H37Rv-333510-336379 --- k-mer match to putative recomb seq = 1.0 (14/14)
Match to reference locus
Match to recombinant "k-mer Only"
PE_PGRS10-H37Rv-838485-840847 --- k-mer match to putative recomb seq = 0.2143 (3/14)
Match to reference locus
Match to recombinant "k-mer Only"
k-mer match
1.0
0.8
0.6
0.4
0.2
0.0
338,320
338,400
338,480
338,560
338,640
338,720
338,800
338,880
338,960
339,040
H37rv Ref Position (bp)

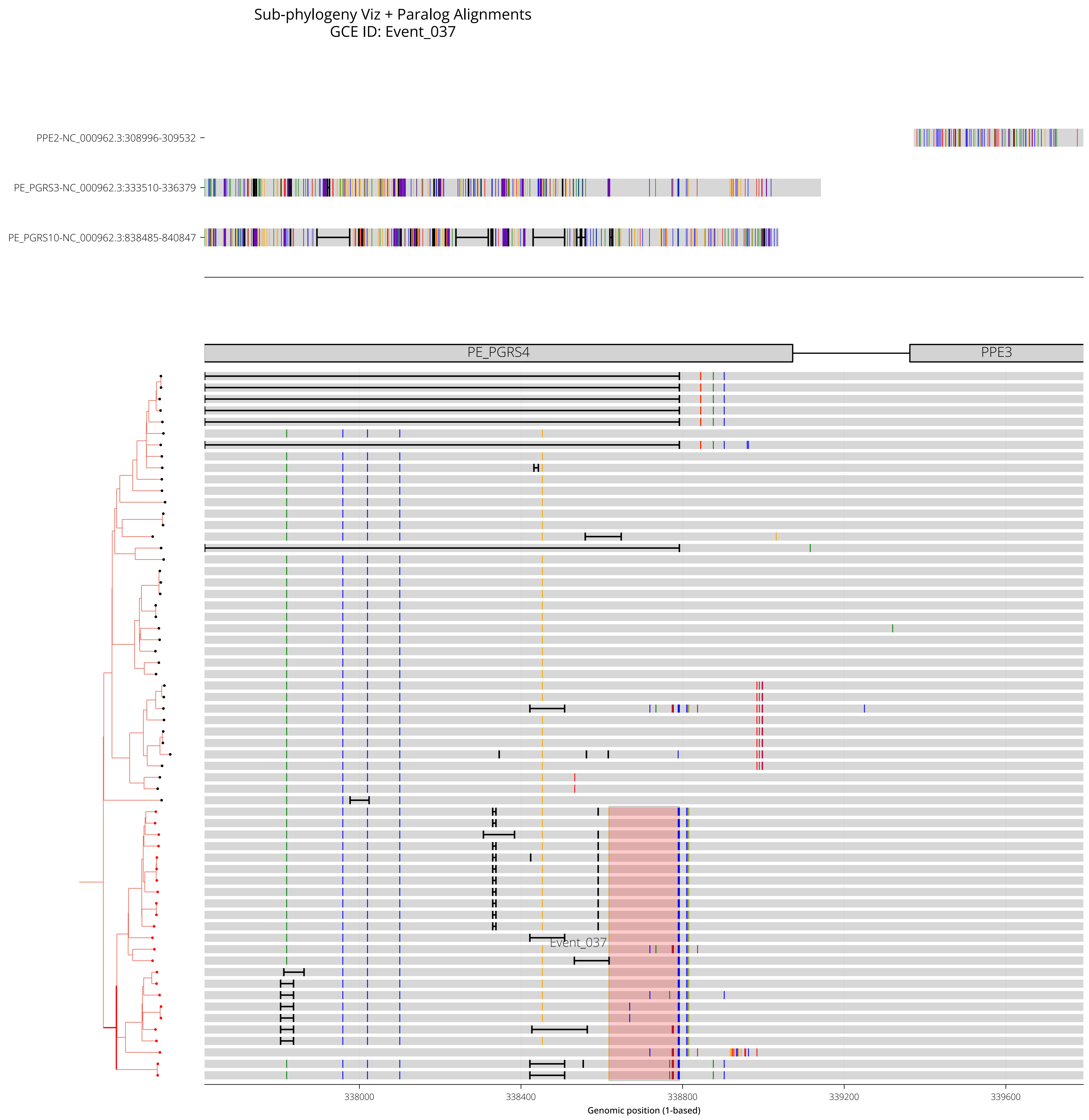

Sub-phylogeny Viz + Paralog Alignments
GCE ID: Event_037
PPE2-NC_000962.3:308996-309532
PE_PGRS3-NC_000962.3:333510-336379
PE_PGRS10-NC_000962.3:838485-840847
PE_PGRS4
PPE3
Event_037
338000
338400
338800
339200
339600
Genomic position (1-based)

Full Phylogeny Viz + Paralog Alignments
GCE ID: Event_037

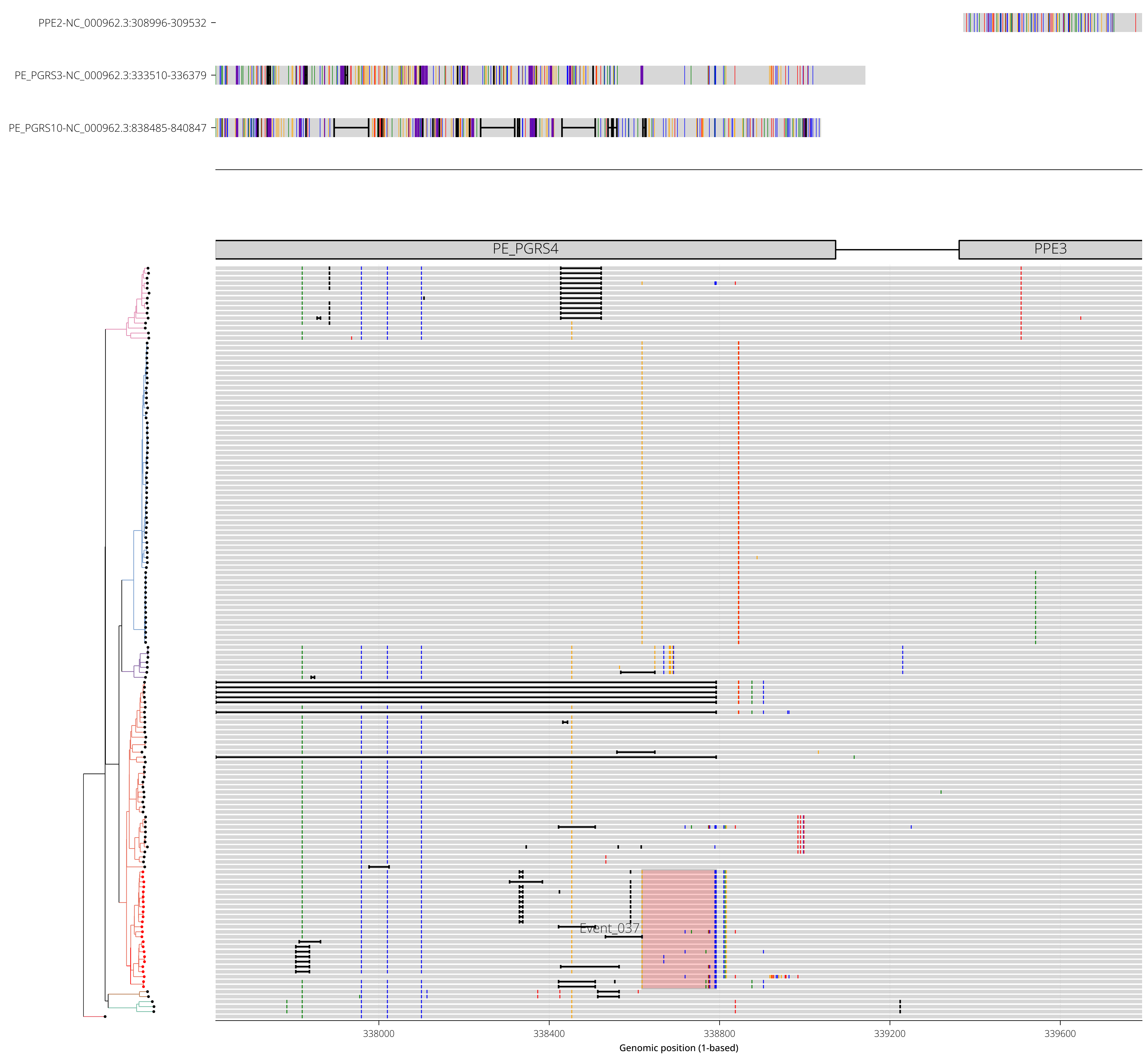

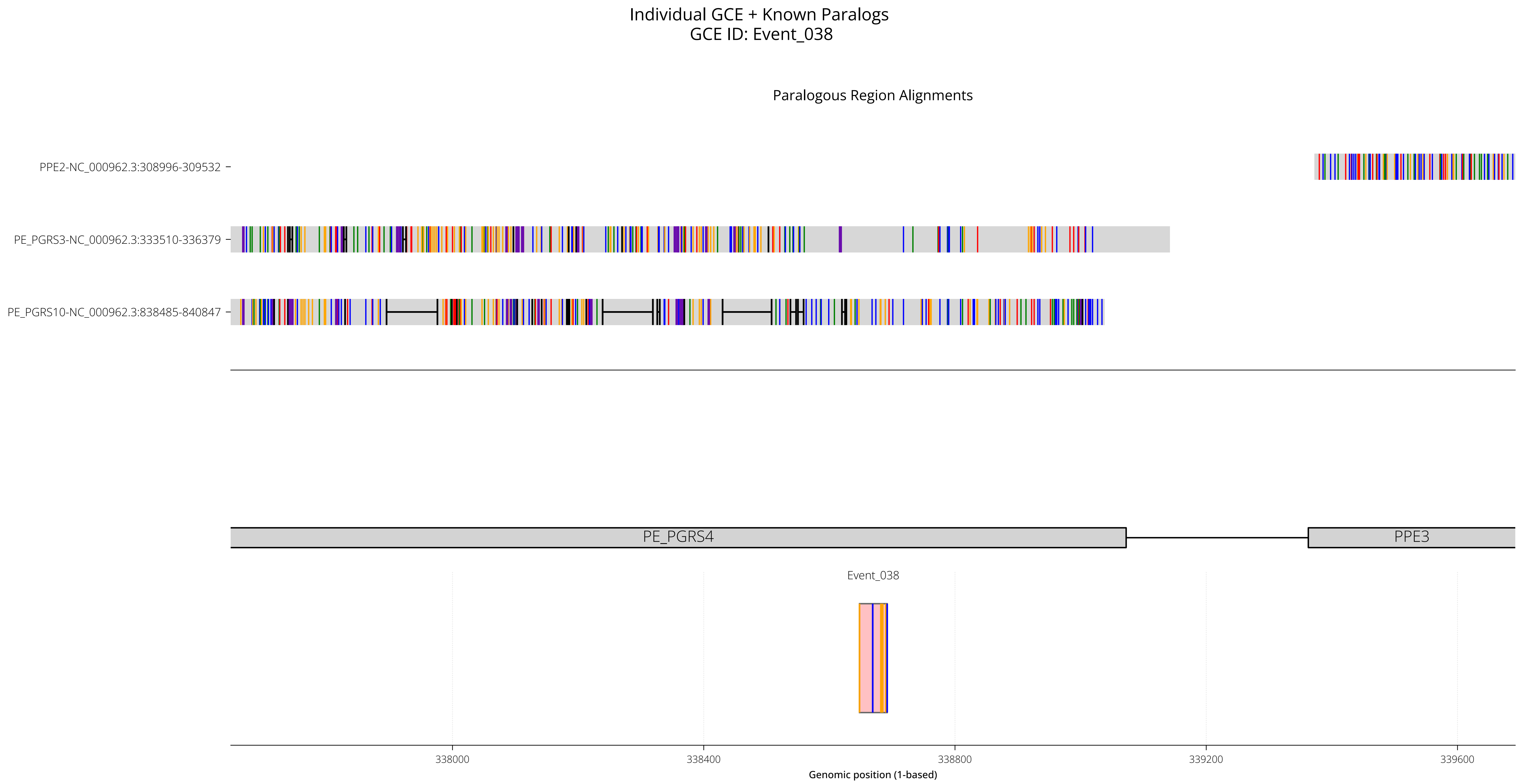

Individual GCE + Known Paralogs
GCE ID: Event_038
Paralogous Region Alignments
PPE2-NC_000962.3:308996-309532
PE_PGRS3-NC_000962.3:333510-336379
PE_PGRS10-NC_000962.3:838485-840847
PE_PGRS4
PPE3
Event_038
338000
338400
338800
339200
339600
Genomic position (1-based)

Circos Viz - GCE to Known Paralogs Locations
GCE ID: Event_038

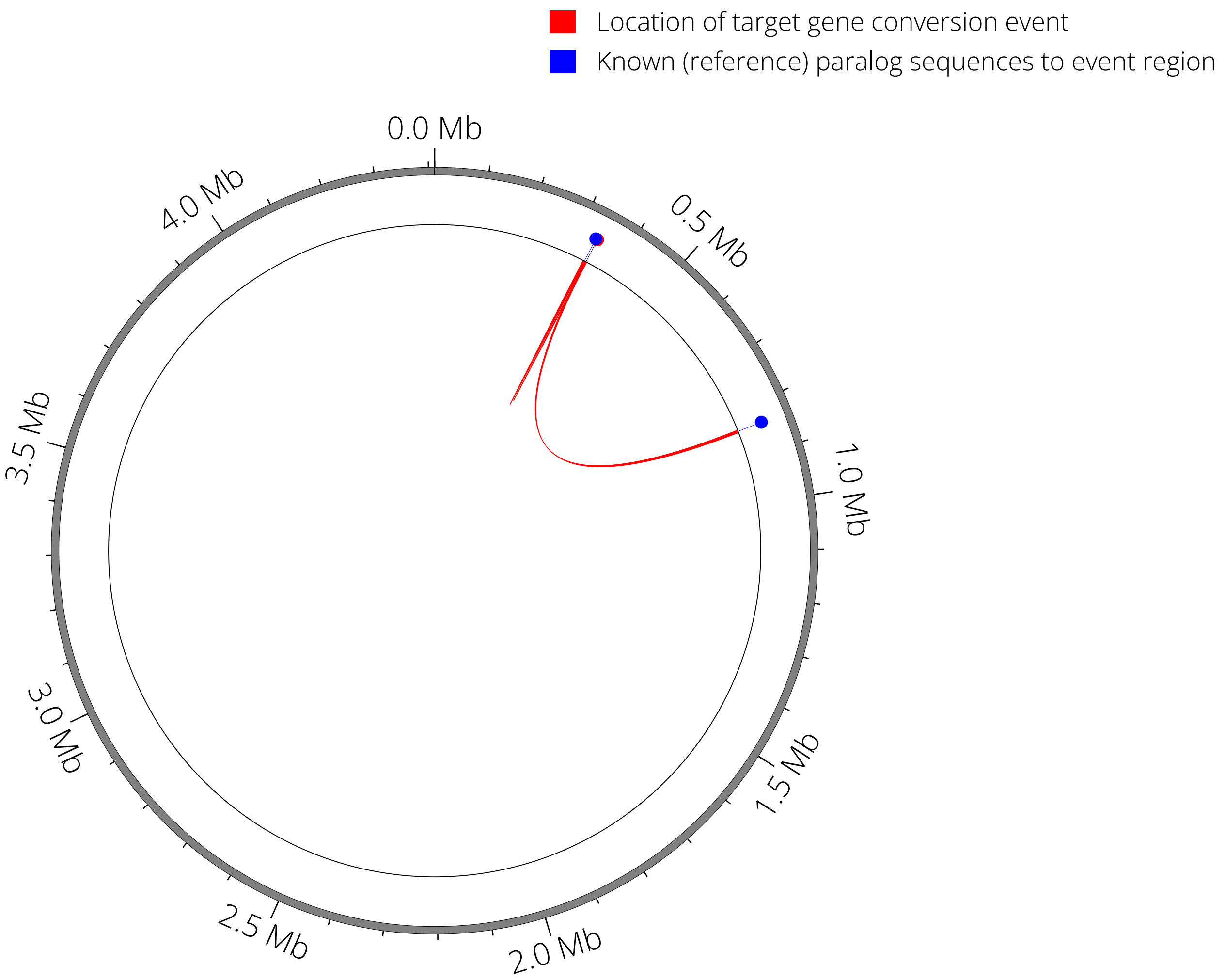

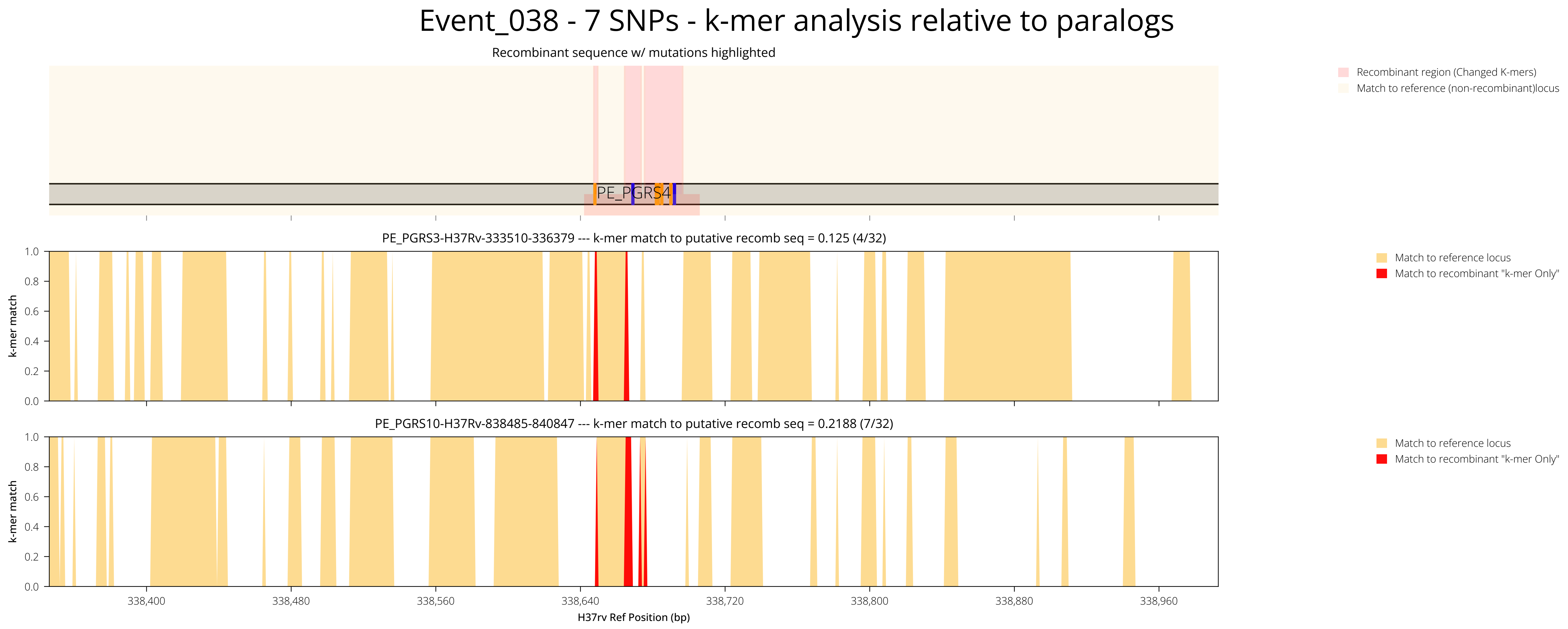
Event_038 - 7 SNPs - k-mer analysis relative to paralogs
Recombinant sequence w/ mutations highlighted
PE_PGRS4
Recombinant region (Changed K-mers)
Match to reference (non-recombinant)locus
PE_PGRS3-H37Rv-333510-336379 --- k-mer match to putative recomb seq = 0.125 (4/32)
Match to reference locus
Match to recombinant "k-mer Only"
k-mer match
PE_PGRS10-H37Rv-838485-840847 --- k-mer match to putative recomb seq = 0.2188 (7/32)
Match to reference locus
Match to recombinant "k-mer Only"
k-mer match
338,400
338,480
338,560
338,640
338,720
338,800
338,880
338,960
H37rv Ref Position (bp)

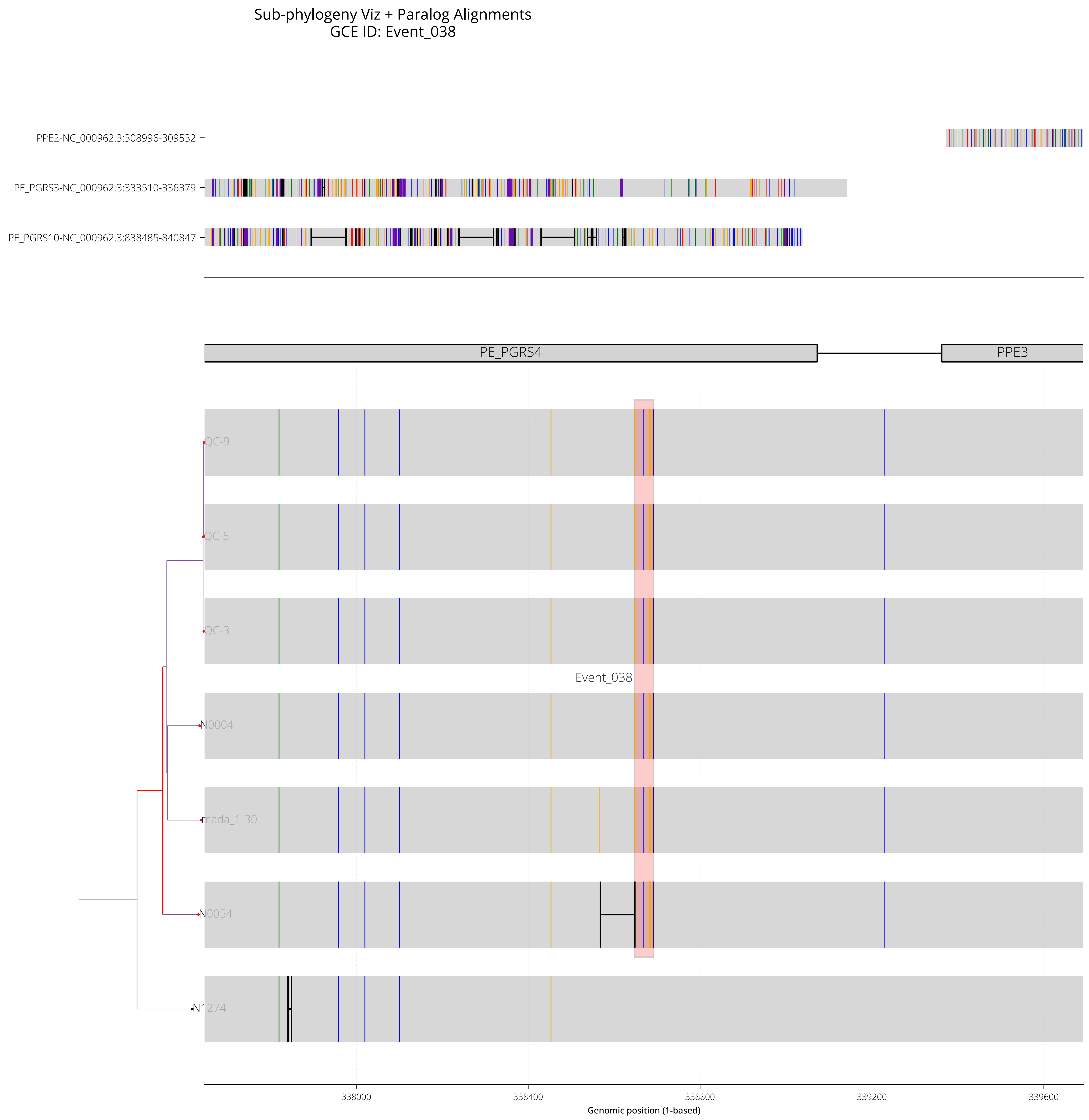

Sub-phylogeny Viz + Paralog Alignments
GCE ID: Event_038
PPE2-NC_000962.3:308996-309532
PE_PGRS3-NC_000962.3:333510-336379
PE_PGRS10-NC_000962.3:838485-840847
PE_PGRS4
PPE3
QC-9
QC-5
QC-3
Event_038
N0004
mada_1-30
N0054
N1274
338000
338400
338800
339200
339600
Genomic position (1-based)

Full Phylogeny Viz + Paralog Alignments
GCE ID: Event_038

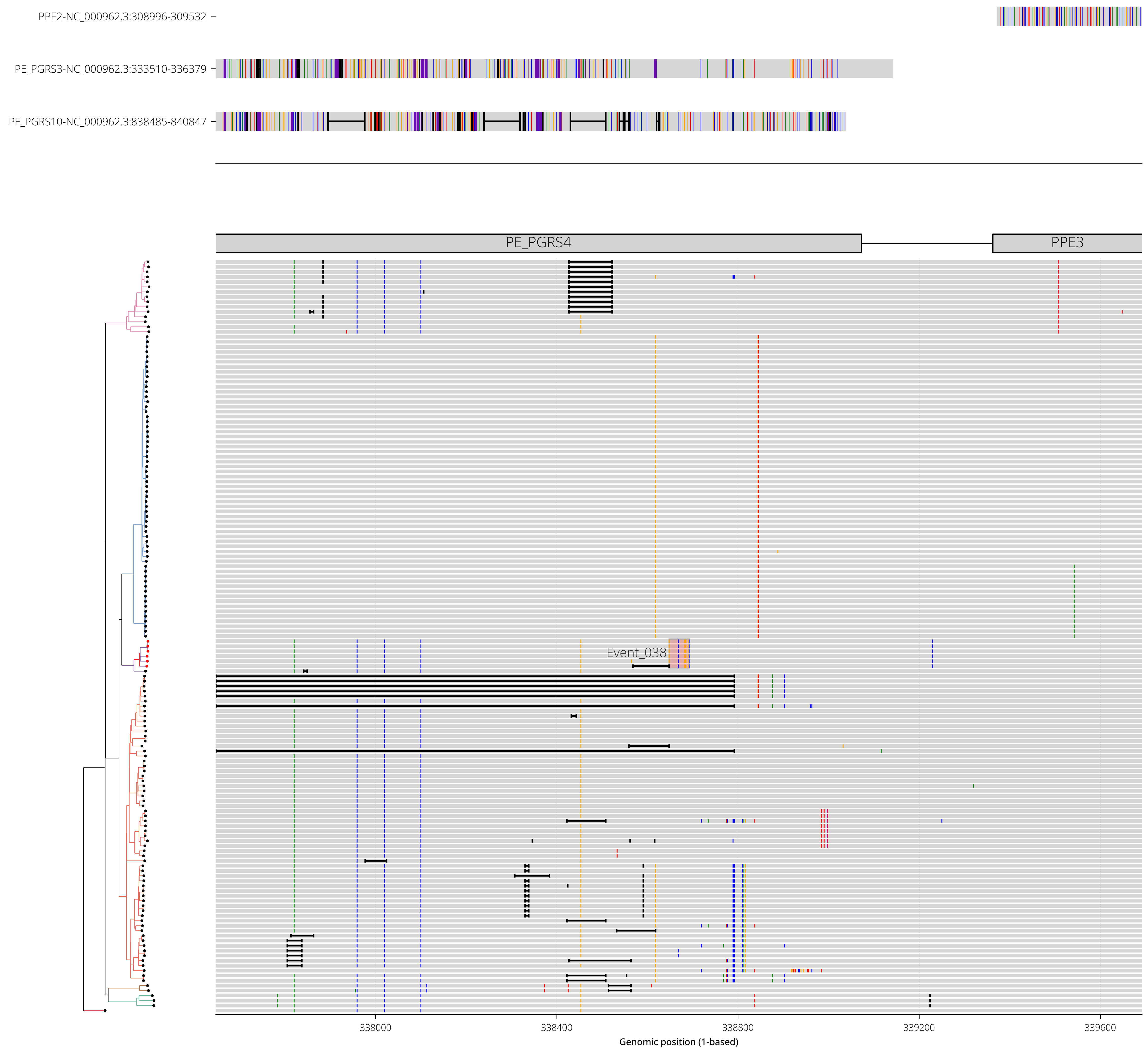

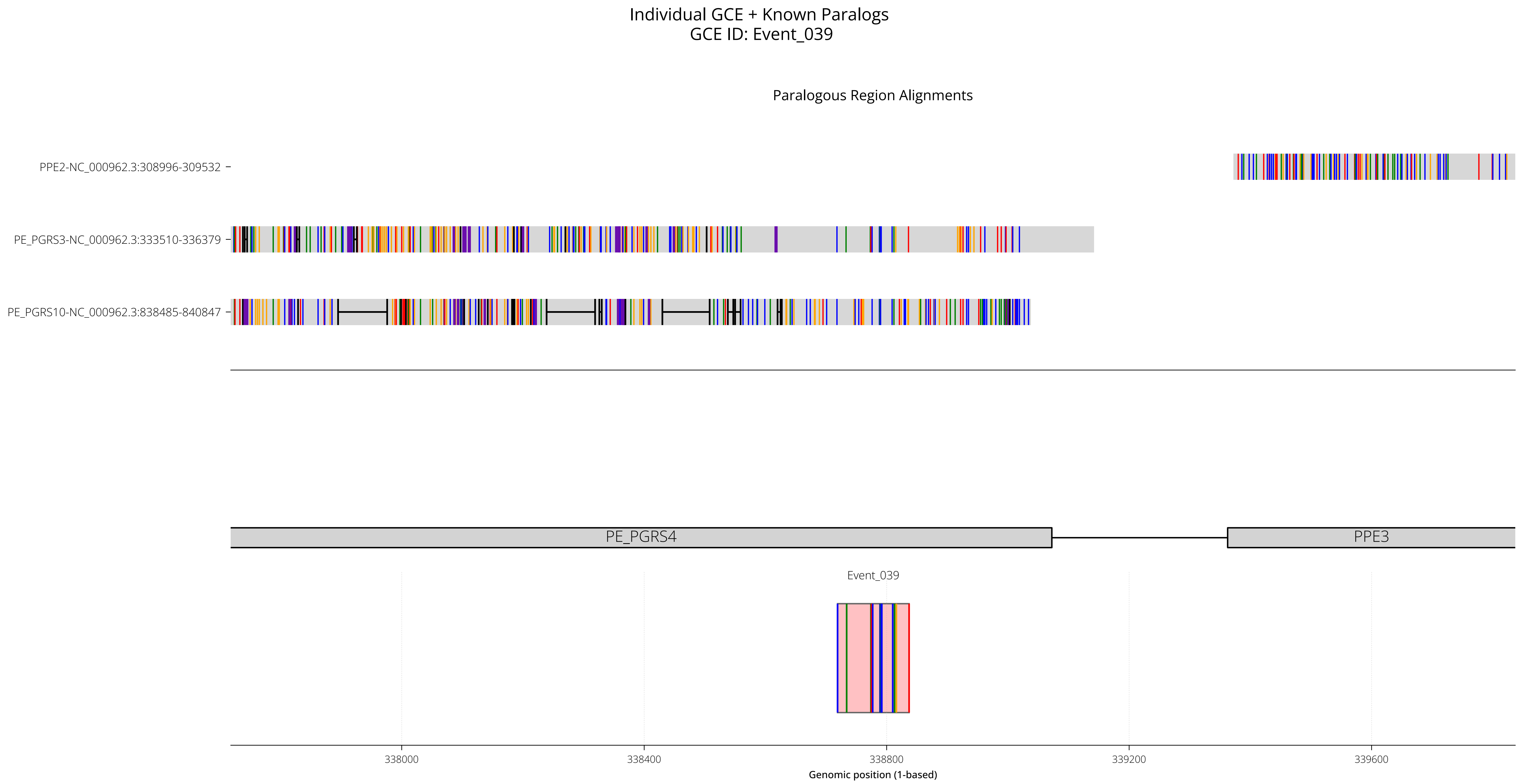
Individual GCE + Known Paralogs
GCE ID: Event_039
Paralogous Region Alignments
PPE2-NC_000962.3:308996-309532
PE_PGRS3-NC_000962.3:333510-336379
PE_PGRS10-NC_000962.3:838485-840847
PE_PGRS4
PPE3
Event_039
338000
338400
338800
339200
339600
Genomic position (1-based)

# Circos Viz - GCE to Known Paralogs Locations
GCE ID: Event_039

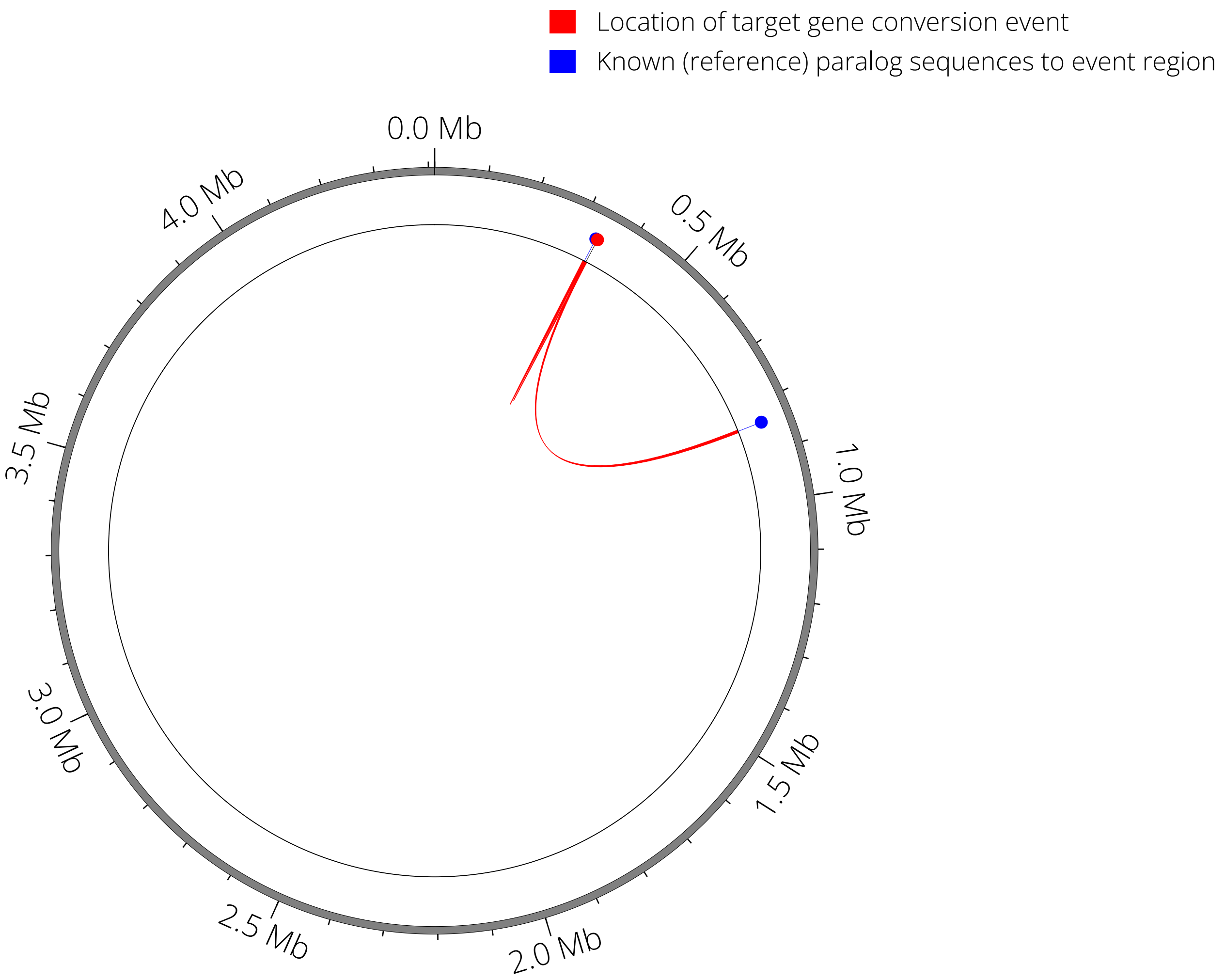

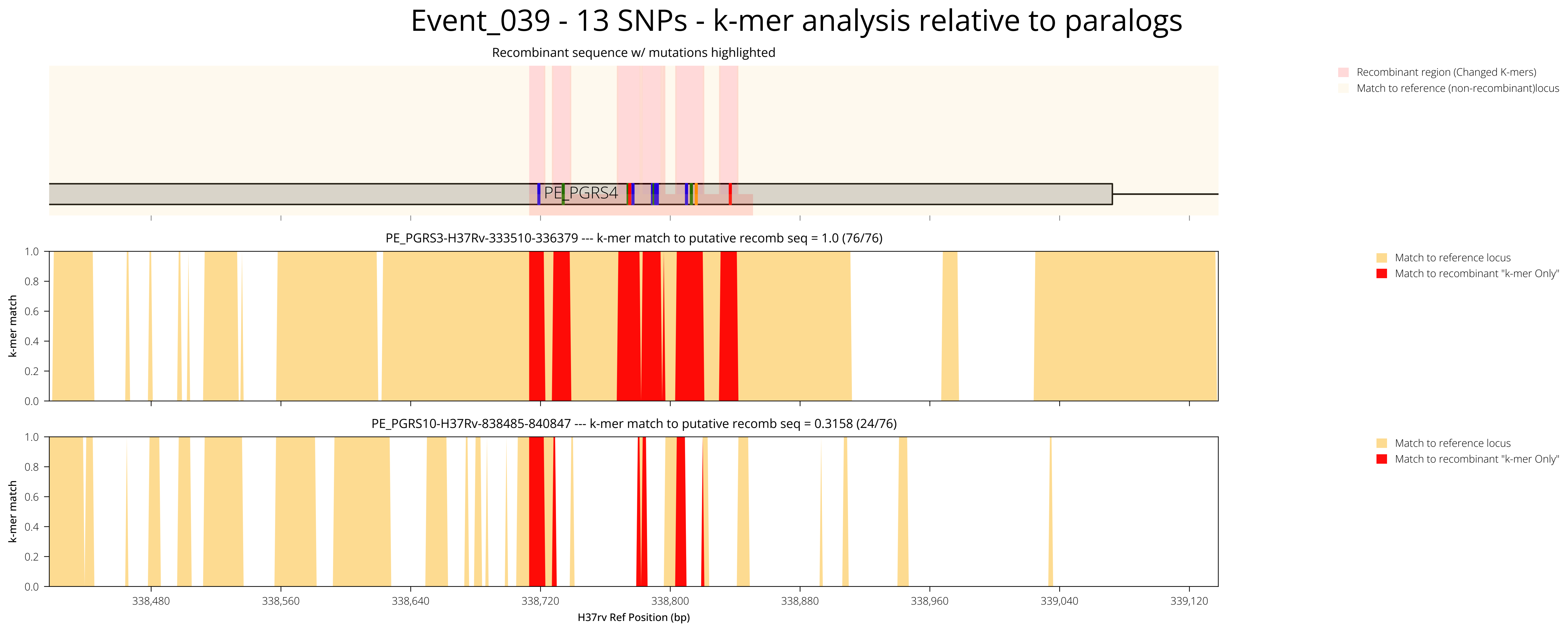
Event_039 - 13 SNPs - k-mer analysis relative to paralogs
Recombinant sequence w/ mutations highlighted
Recombinant region (Changed K-mers)
Match to reference (non-recombinant)locus
PE_PGRS4
PE_PGRS3-H37Rv-333510-336379 --- k-mer match to putative recomb seq = 1.0 (76/76)
Match to reference locus
Match to recombinant "k-mer Only"
PE_PGRS10-H37Rv-838485-840847 --- k-mer match to putative recomb seq = 0.3158 (24/76)
Match to reference locus
Match to recombinant "k-mer Only"
k-mer match
1.0
0.8
0.6
0.4
0.2
0.0
338,480
338,560
338,640
338,720
338,800
338,880
338,960
339,040
339,120
H37rv Ref Position (bp)

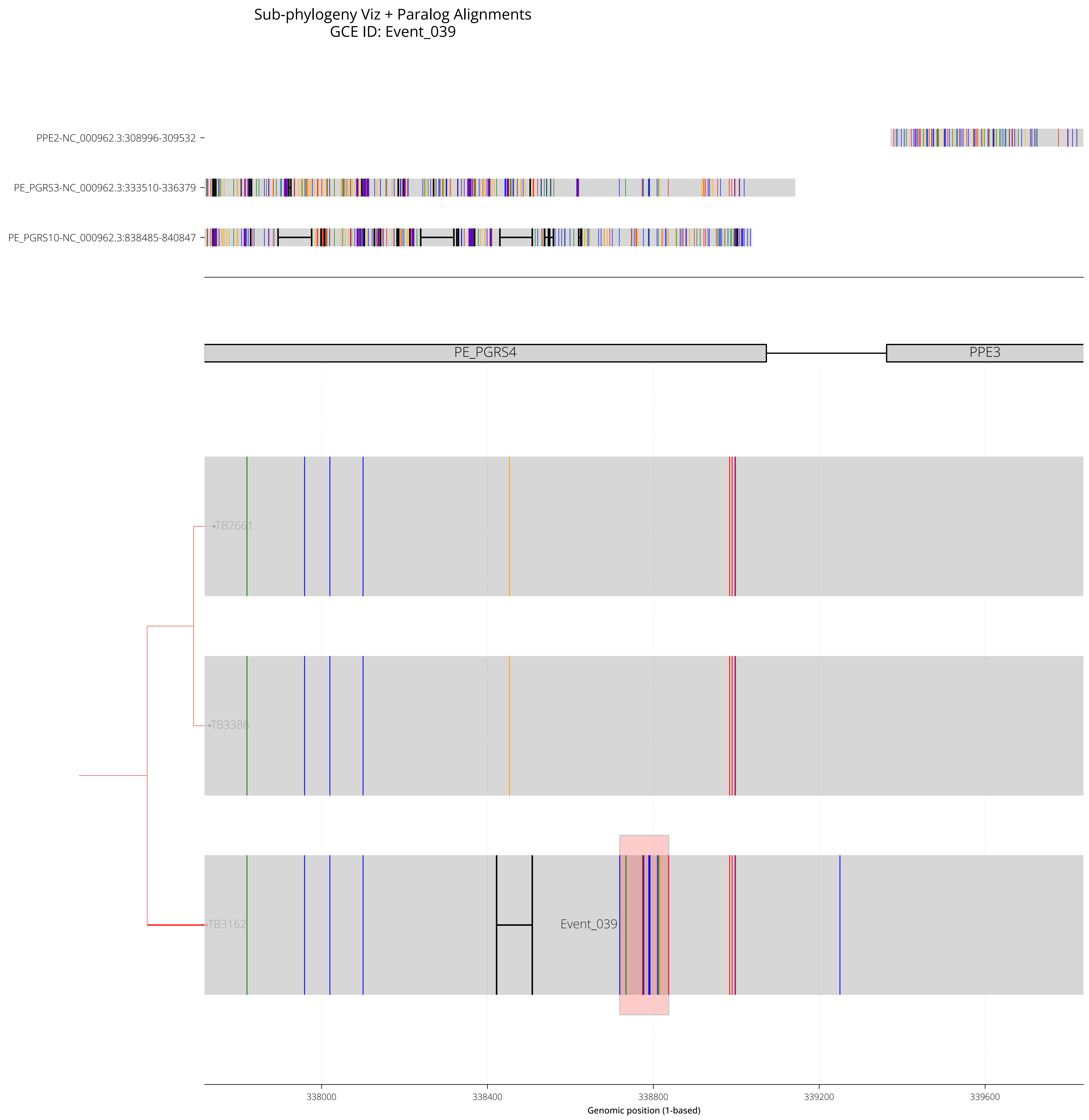

Sub-phylogeny Viz + Paralog Alignments
GCE ID: Event_039
PPE2-NC_000962.3:308996-309532
PE_PGRS3-NC_000962.3:333510-336379
PE_PGRS10-NC_000962.3:838485-840847
PE_PGRS4
PPE3
TB2661
TB3386
TB3162
Event_039
338000
338400
338800
339200
339600
Genomic position (1-based)

Full Phylogeny Viz + Paralog Alignments
GCE ID: Event_039

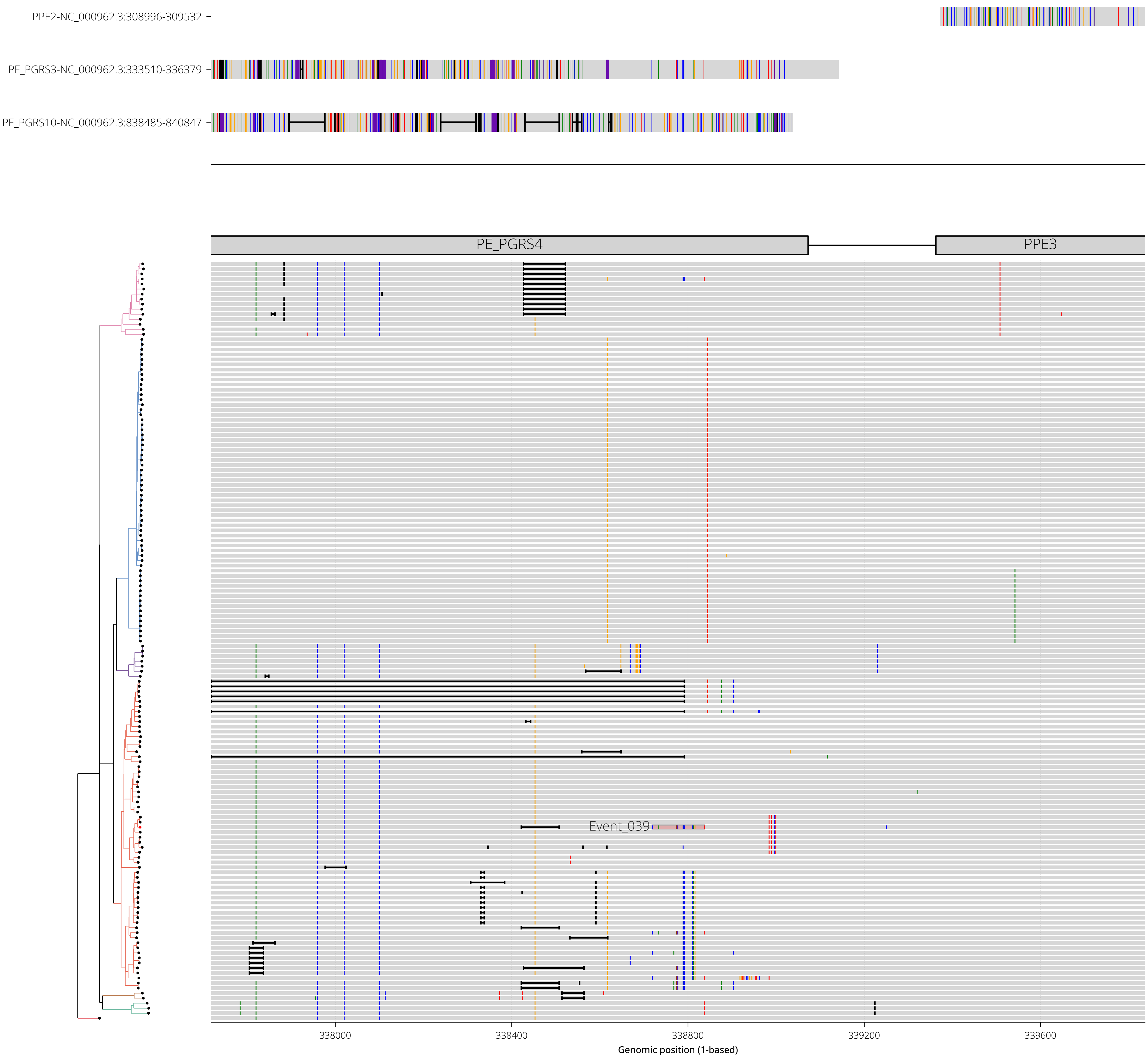

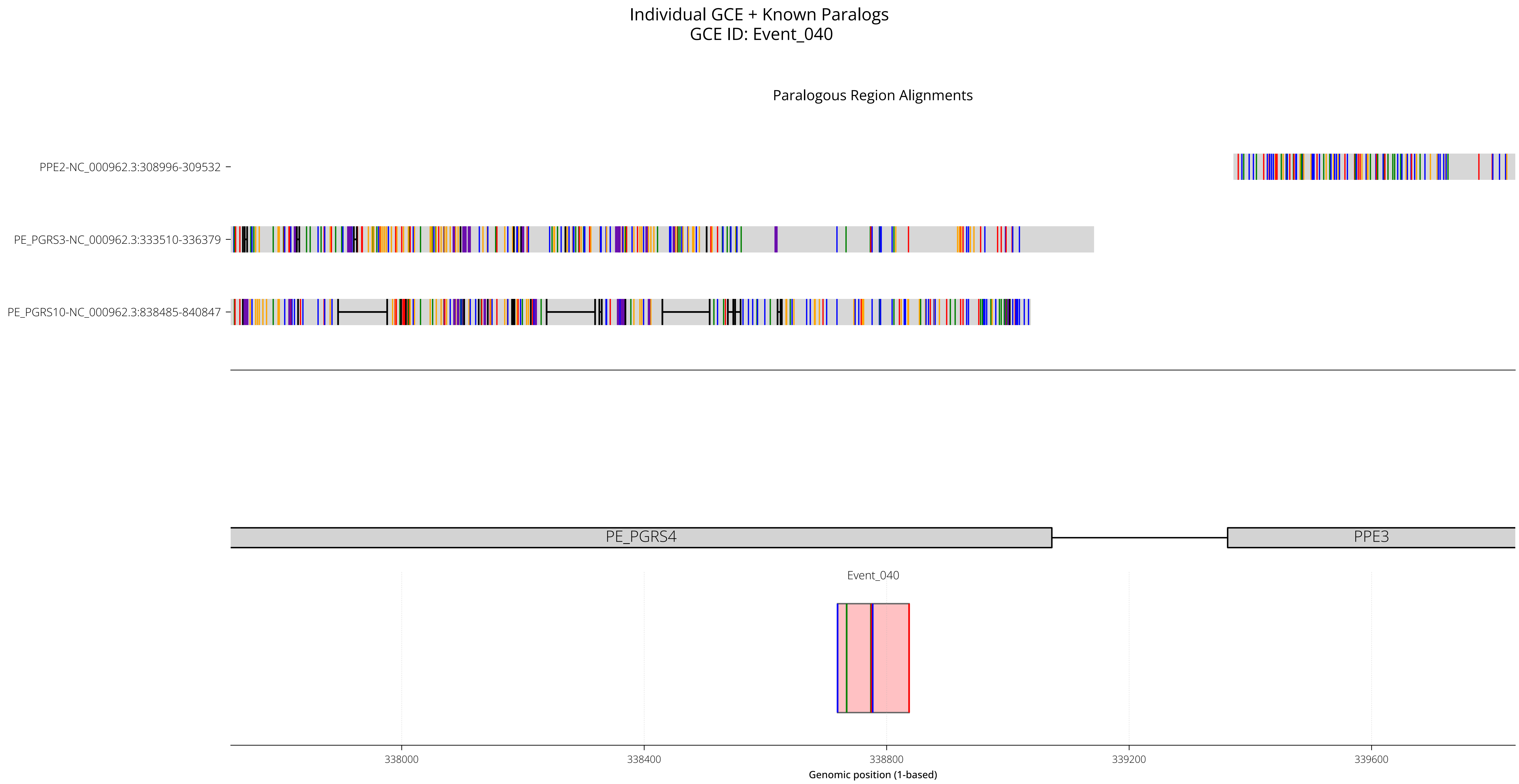
Individual GCE + Known Paralogs
GCE ID: Event_040
Paralogous Region Alignments
PPE2-NC_000962.3:308996-309532
PE_PGRS3-NC_000962.3:333510-336379
PE_PGRS10-NC_000962.3:838485-840847
PE_PGRS4
PPE3
Event_040
338000
338400
338800
339200
339600
Genomic position (1-based)

Circos Viz - GCE to Known Paralogs Locations
GCE ID: Event_040

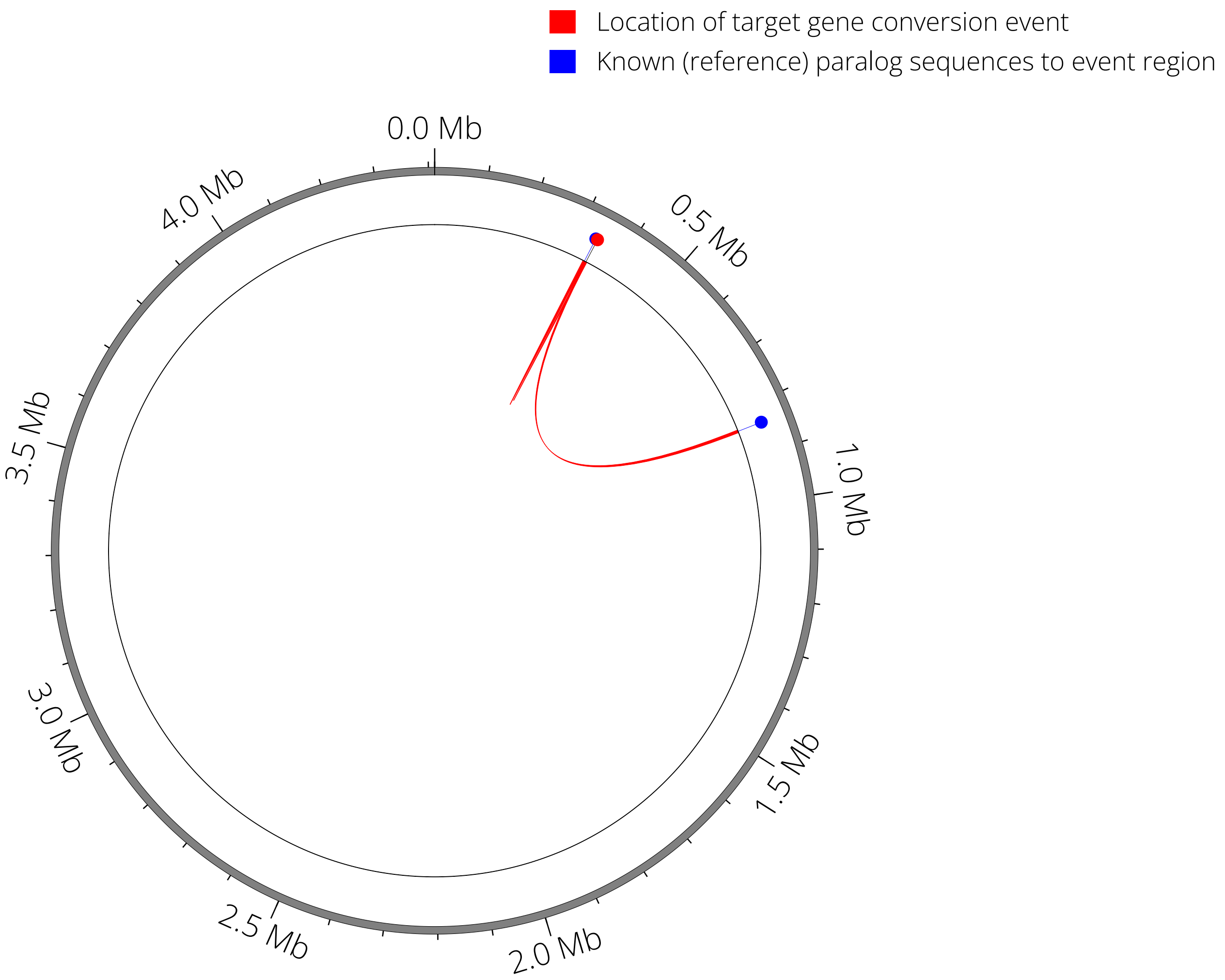

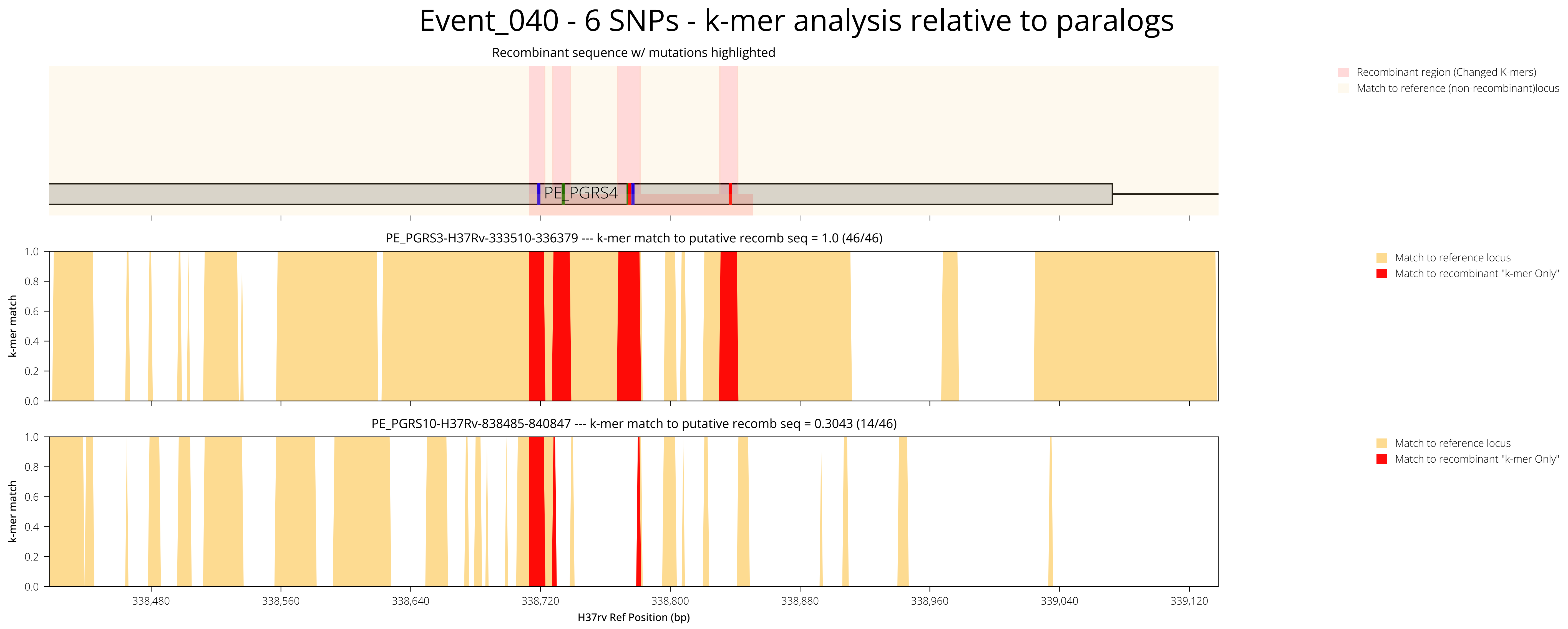

Event_040 - 6 SNPs - k-mer analysis relative to paralogs
Recombinant sequence w/ mutations highlighted
Recombinant region (Changed K-mers)
Match to reference (non-recombinant)locus
PE_PGRS4
PE_PGRS3-H37Rv-333510-336379 --- k-mer match to putative recomb seq = 1.0 (46/46)
Match to reference locus
Match to recombinant "k-mer Only"
k-mer match
PE_PGRS10-H37Rv-838485-840847 --- k-mer match to putative recomb seq = 0.3043 (14/46)
Match to reference locus
Match to recombinant "k-mer Only"
k-mer match
1.0
0.8
0.6
0.4
0.2
0.0
338,480
338,560
338,640
338,720
338,800
338,880
338,960
339,040
339,120
H37rv Ref Position (bp)

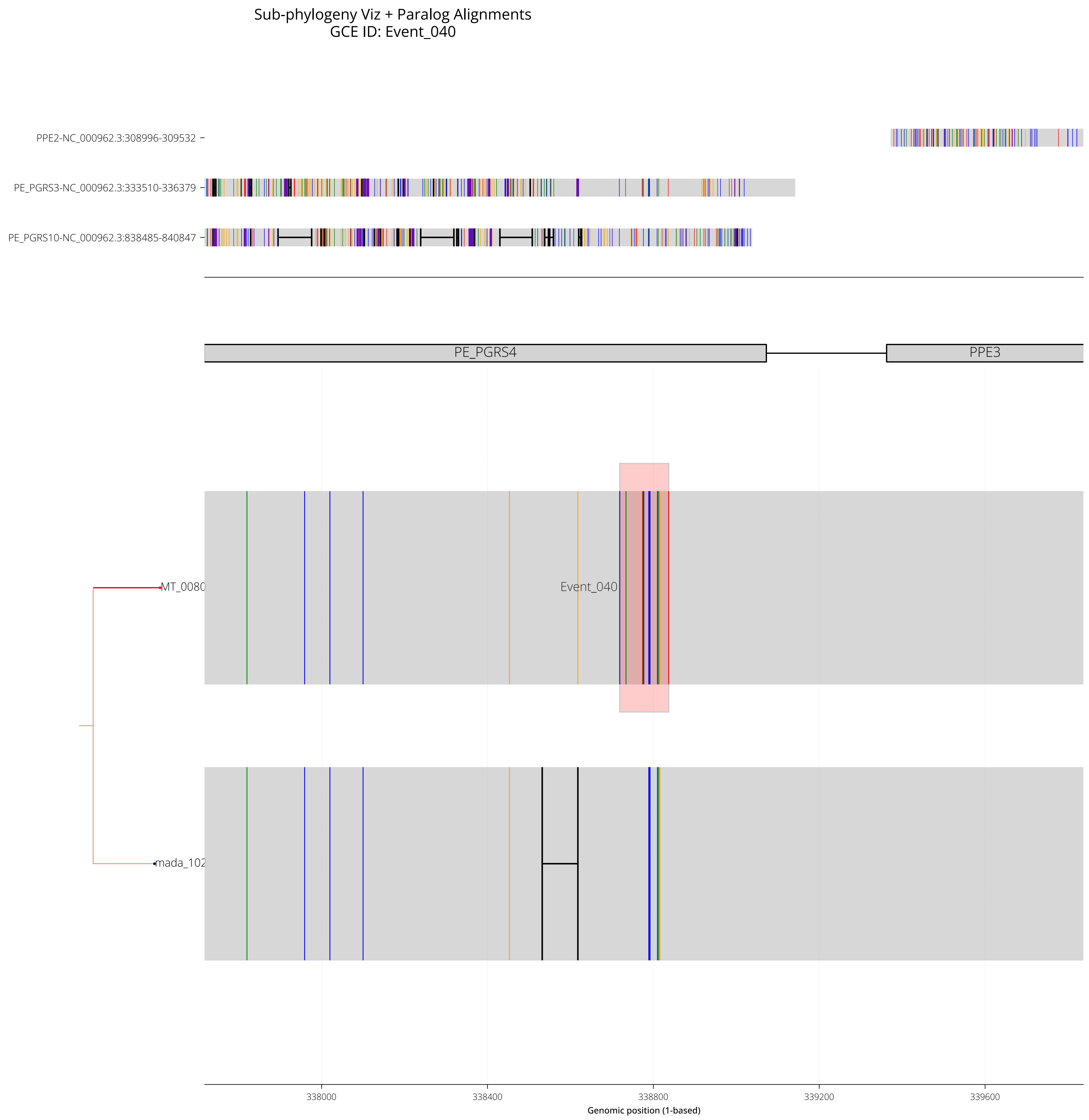

Sub-phylogeny Viz + Paralog Alignments
GCE ID: Event_040
PPE2-NC_000962.3:308996-309532
PE_PGRS3-NC_000962.3:333510-336379
PE_PGRS10-NC_000962.3:838485-840847
PE_PGRS4
PPE3
MT_0080
Event_040
mada_102
338000
338400
338800
339200
339600
Genomic position (1-based)

Full Phylogeny Viz + Paralog Alignments
GCE ID: Event_040

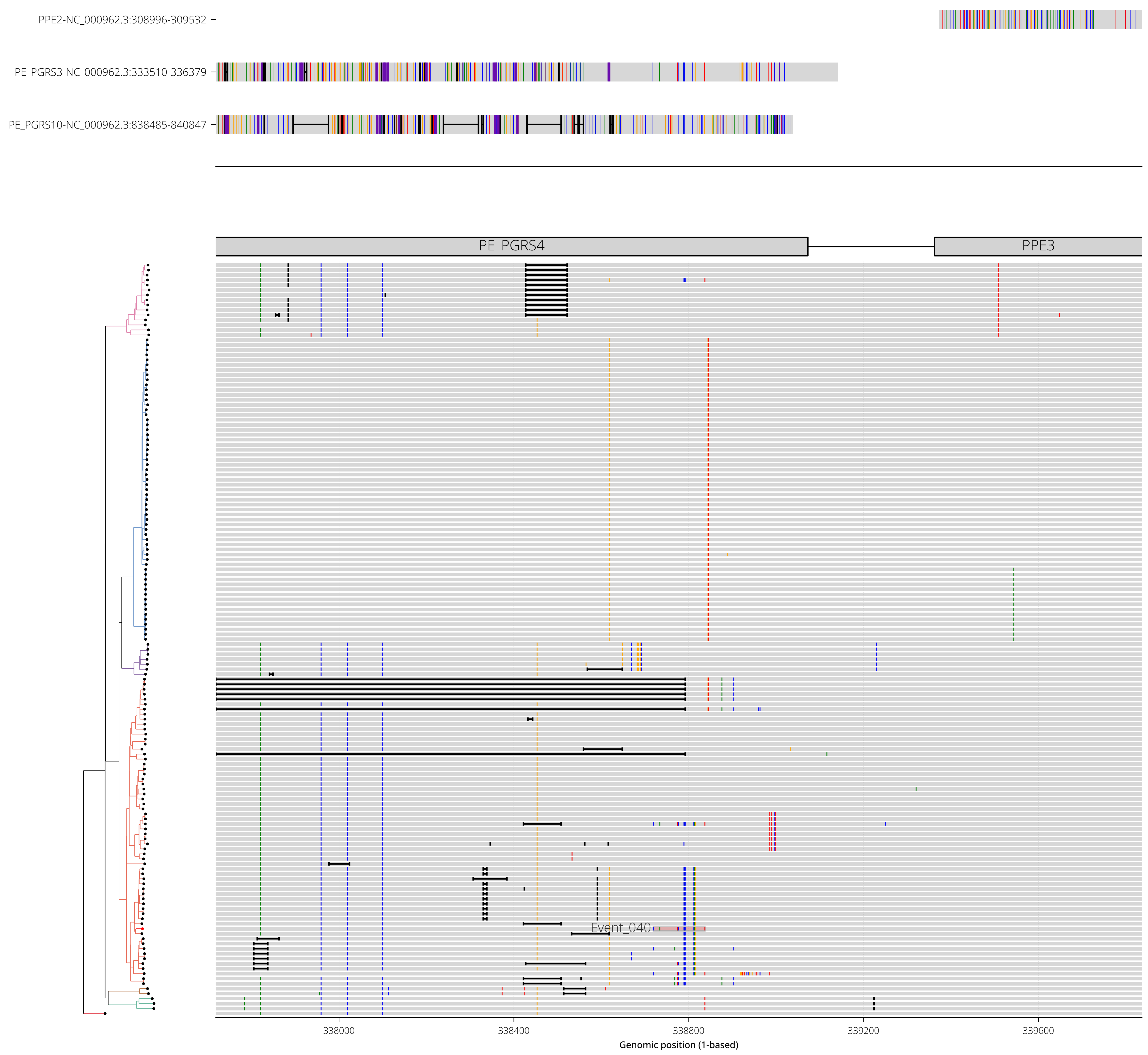

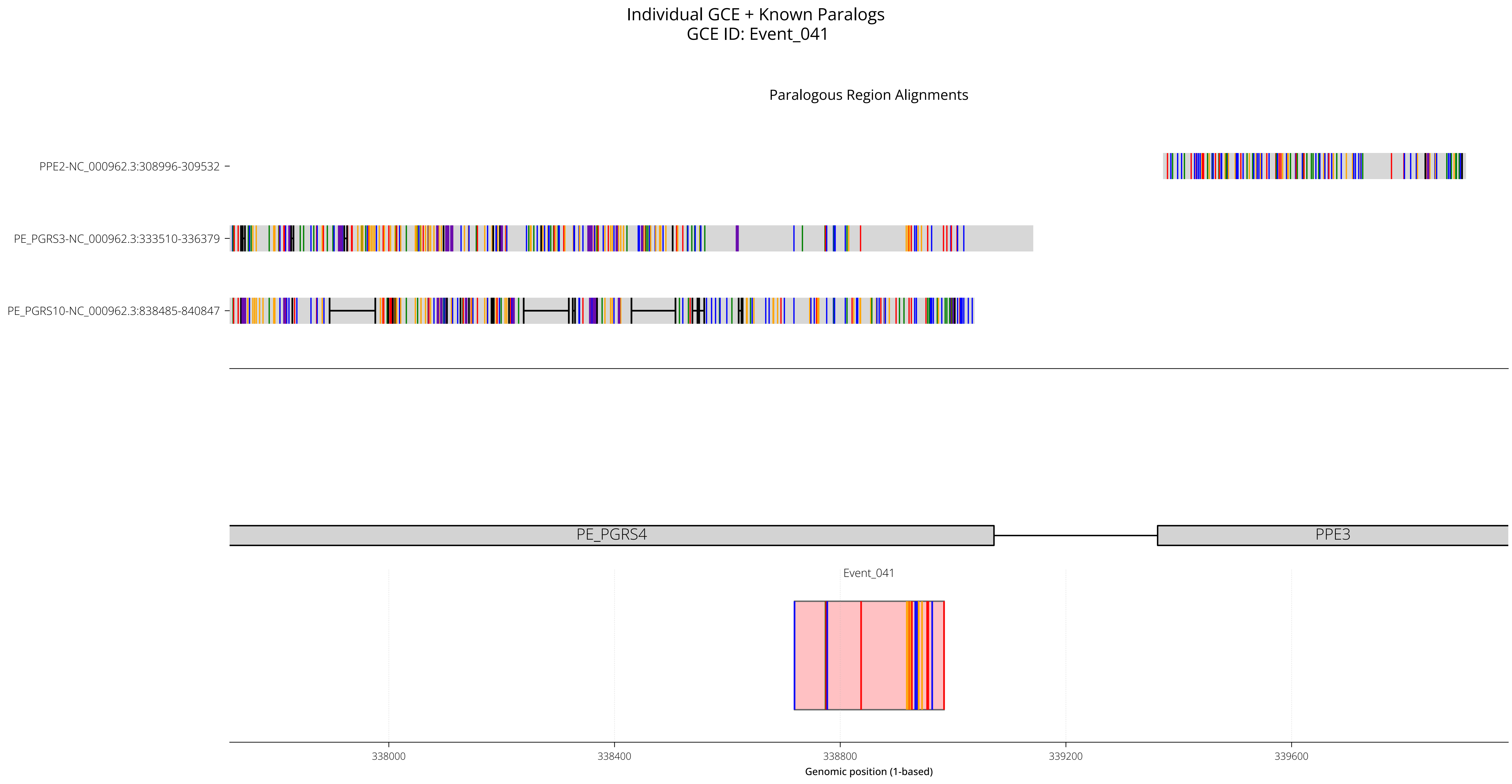

Individual GCE + Known Paralogs
GCE ID: Event_041
Paralogous Region Alignments
PPE2-NC_000962.3:308996-309532
PE_PGRS3-NC_000962.3:333510-336379
PE_PGRS10-NC_000962.3:838485-840847
PE_PGRS4
PPE3
Event_041
338000
338400
338800
339200
339600
Genomic position (1-based)

Circos Viz - GCE to Known Paralogs Locations
GCE ID: Event_041

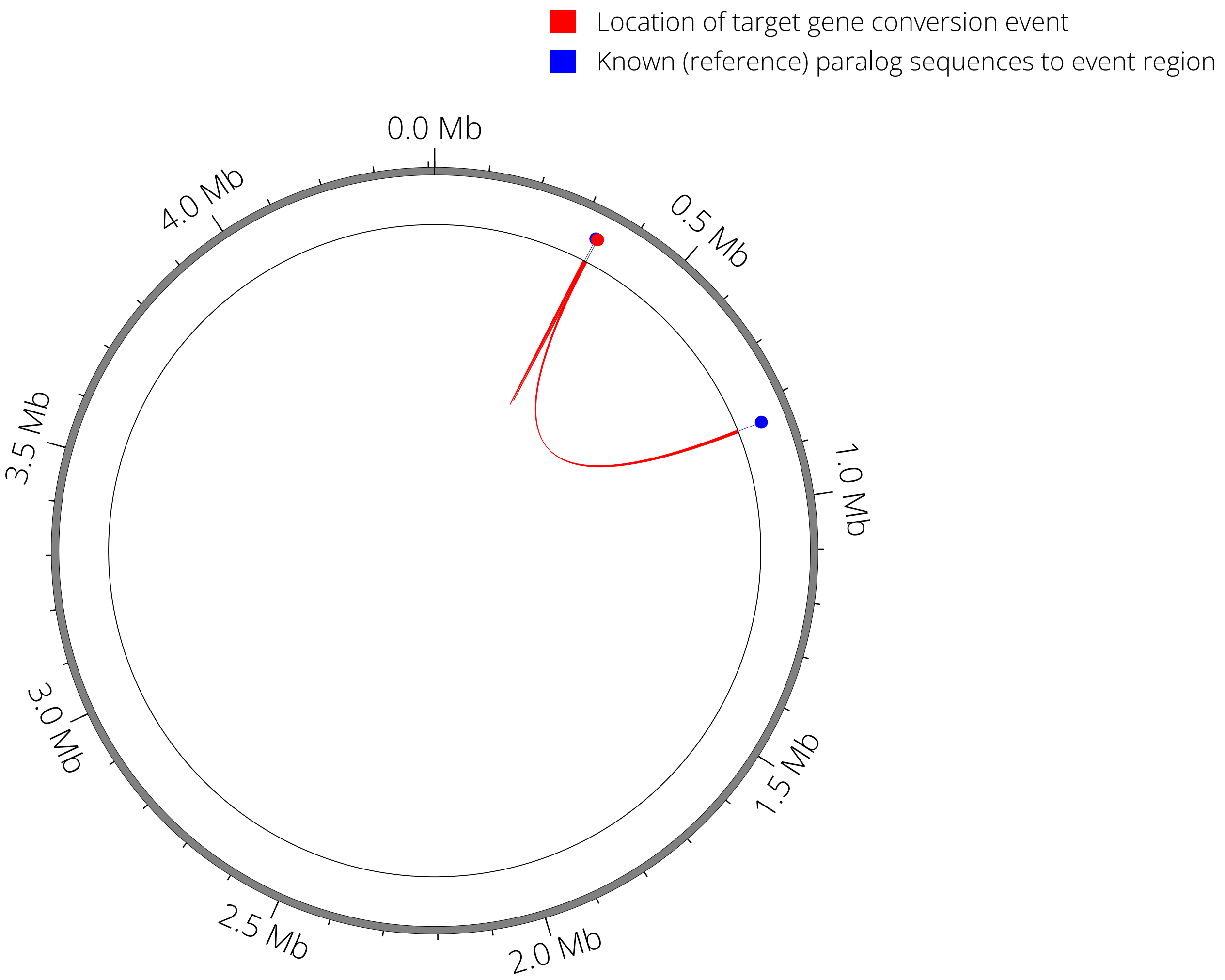

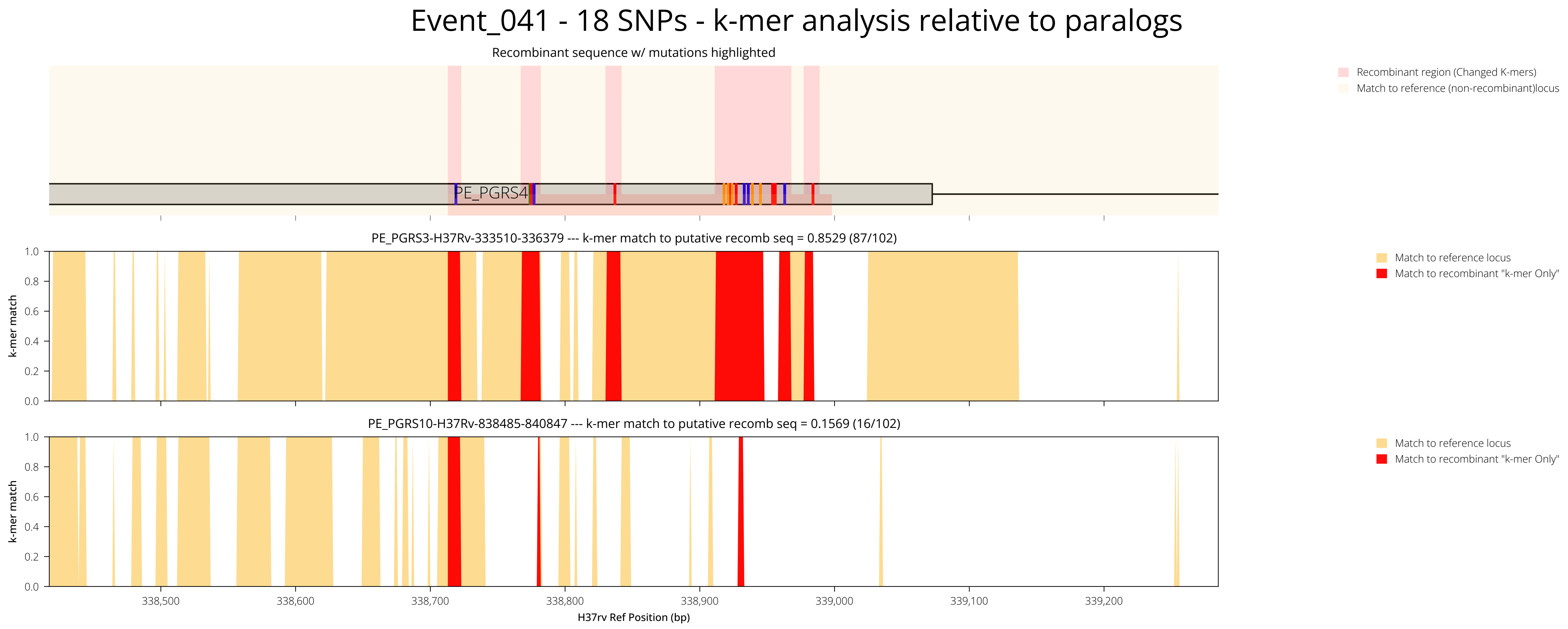

Event_041 - 18 SNPs - k-mer analysis relative to paralogs
Recombinant sequence w/ mutations highlighted
PE_PGRS4
Recombinant region (Changed K-mers)
Match to reference (non-recombinant)locus
PE_PGRS3-H37Rv-333510-336379 --- k-mer match to putative recomb seq = 0.8529 (87/102)
Match to reference locus
Match to recombinant "k-mer Only"
PE_PGRS10-H37Rv-838485-840847 --- k-mer match to putative recomb seq = 0.1569 (16/102)
Match to reference locus
Match to recombinant "k-mer Only"
k-mer match
1.0
0.8
0.6
0.4
0.2
0.0
338,500
338,600
338,700
338,800
338,900
339,000
339,100
339,200
H37rv Ref Position (bp)

Sub-phylogeny Viz + Paralog Alignments
GCE ID: Event_041

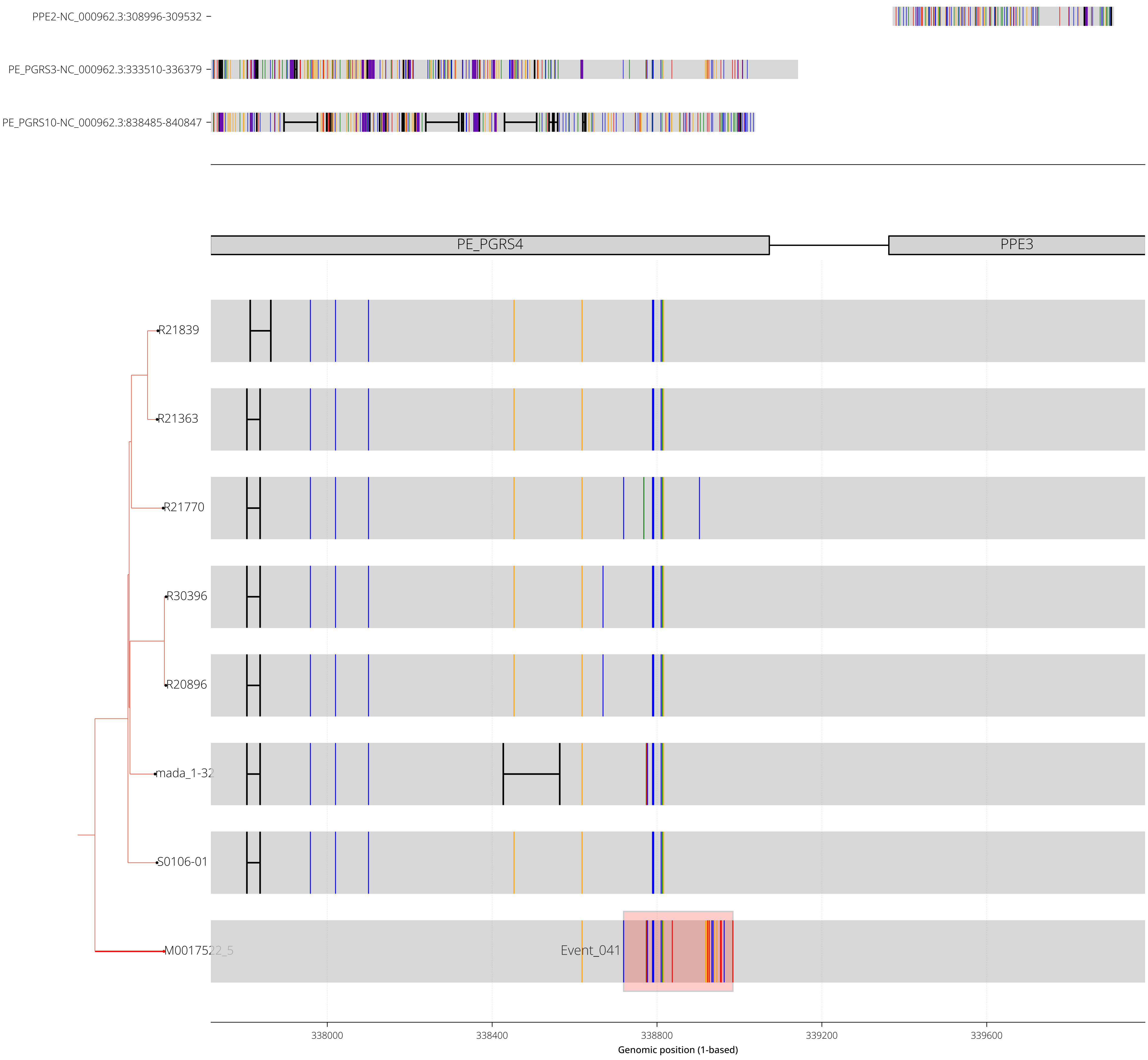

Full Phylogeny Viz + Paralog Alignments
GCE ID: Event_041

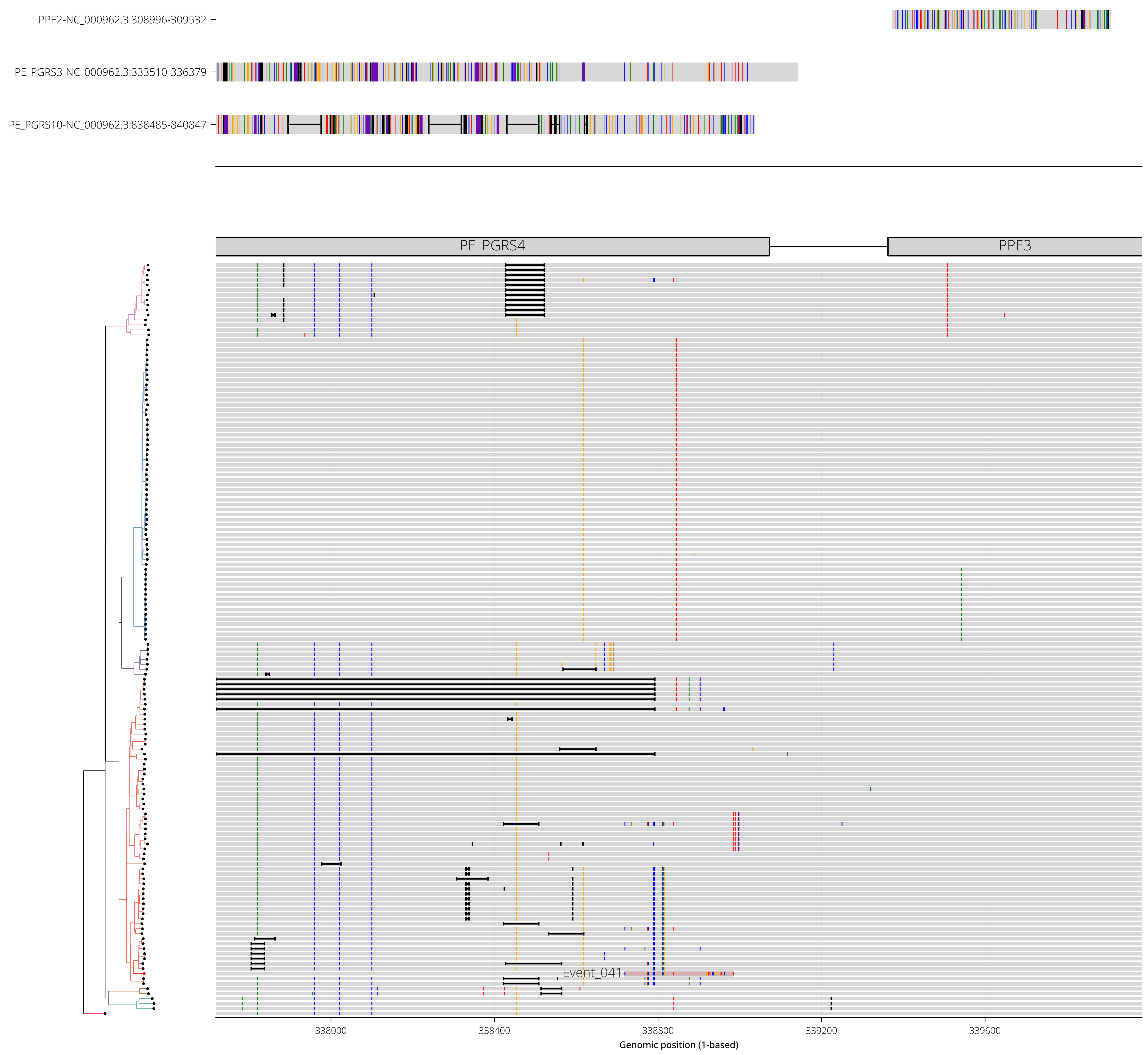

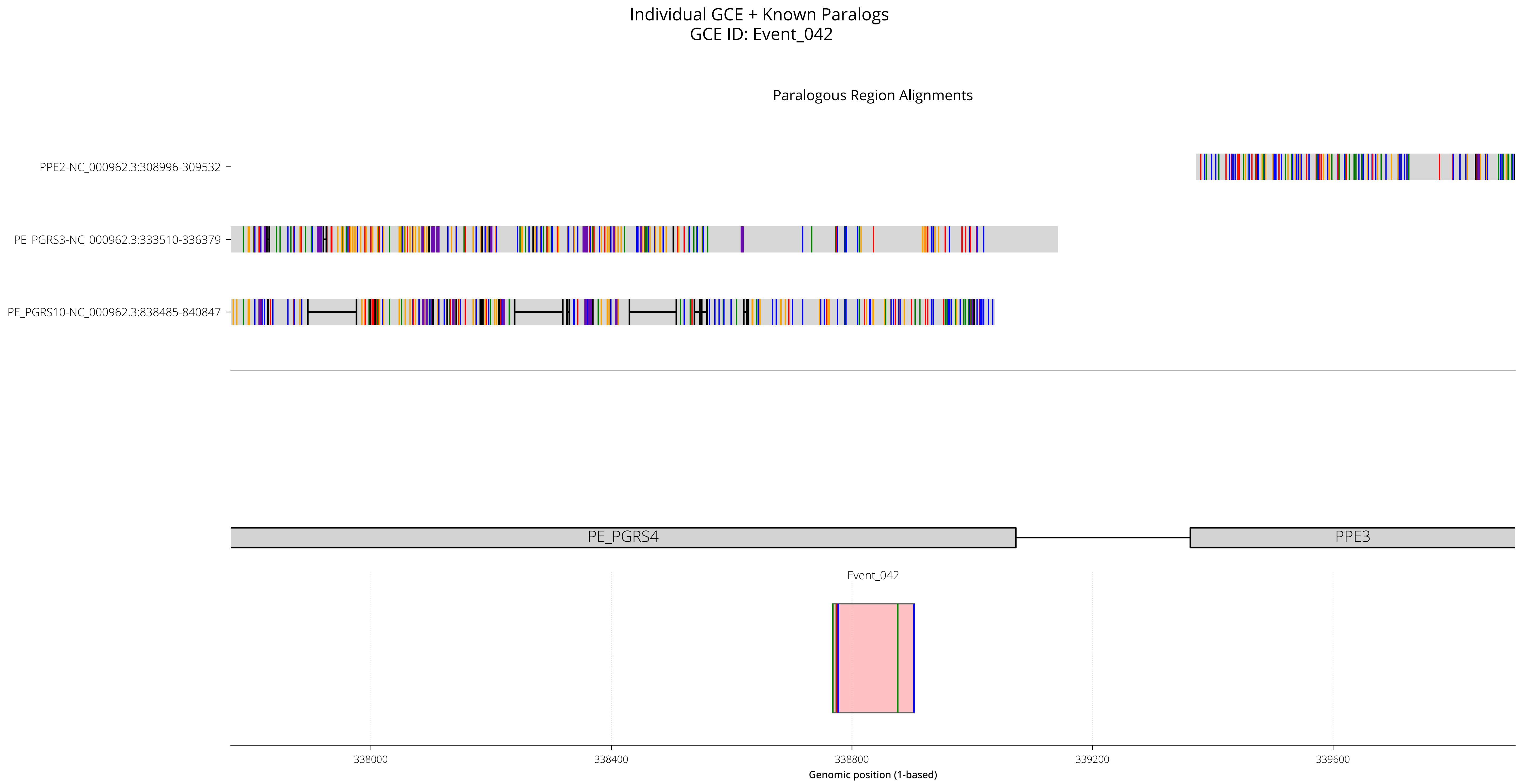

Individual GCE + Known Paralogs
GCE ID: Event_042
Paralogous Region Alignments
PPE2-NC_000962.3:308996-309532
PE_PGRS3-NC_000962.3:333510-336379
PE_PGRS10-NC_000962.3:838485-840847
PE_PGRS4
PPE3
Event_042
338000
338400
338800
339200
339600
Genomic position (1-based)

Circos Viz - GCE to Known Paralogs Locations
GCE ID: Event_042

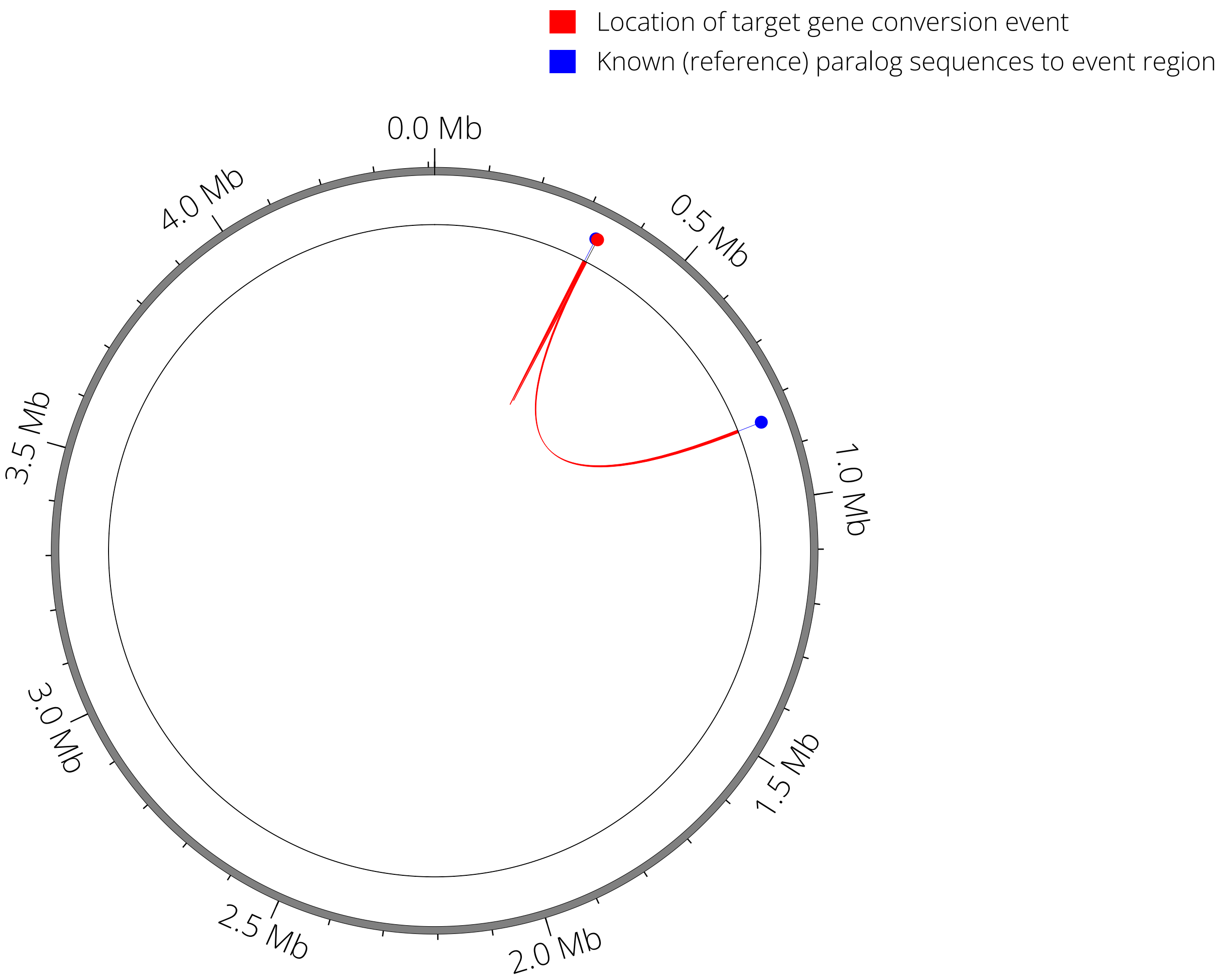

# Event_042 - 6 SNPs - k-mer analysis relative to paralogs

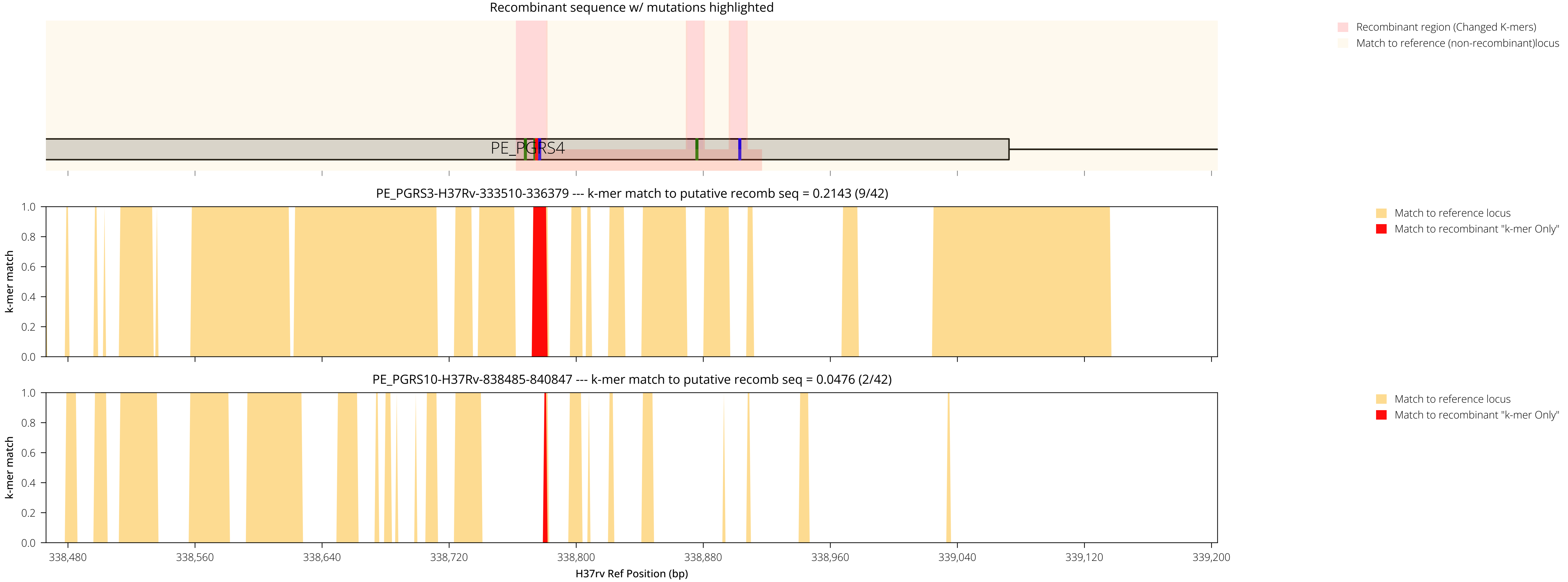

Sub-phylogeny Viz + Paralog Alignments
GCE ID: Event_042

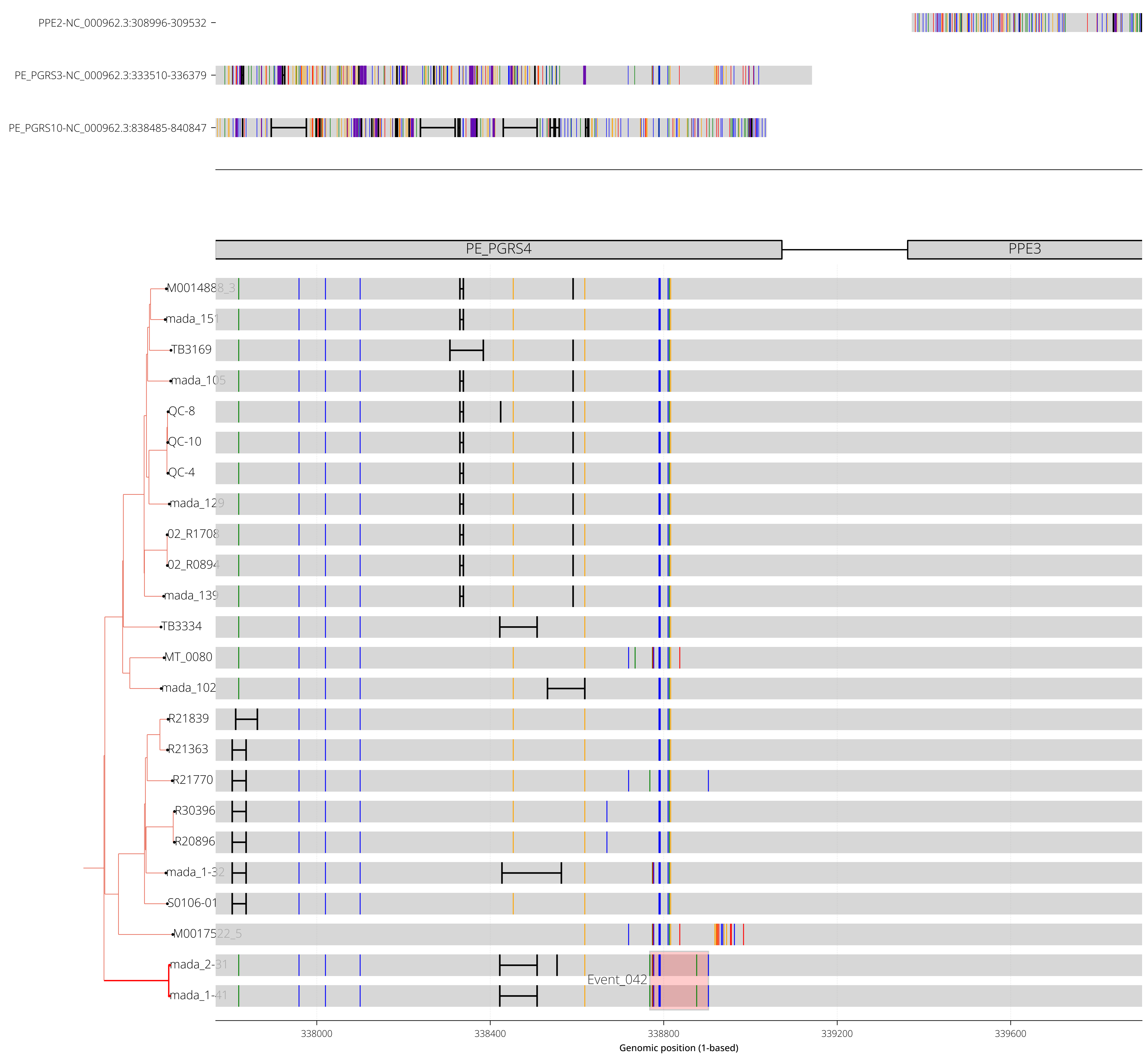

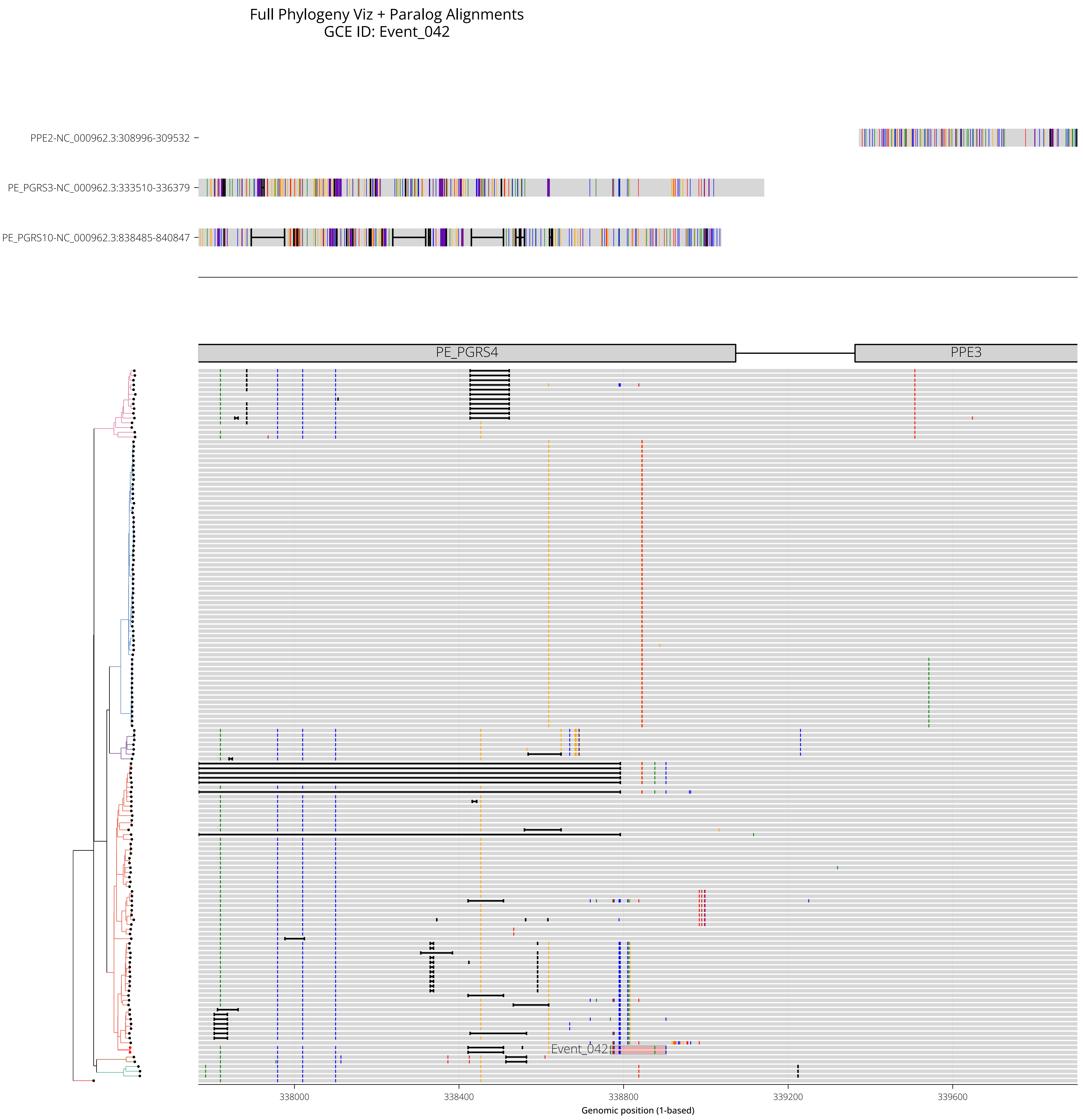

Full Phylogeny Viz + Paralog Alignments
GCE ID: Event_042
PPE2-NC_000962.3:308996-309532
PE_PGRS3-NC_000962.3:333510-336379
PE_PGRS10-NC_000962.3:838485-840847
PE_PGRS4
PPE3
Event_042
338000
338400
338800
339200
339600
Genomic position (1-based)

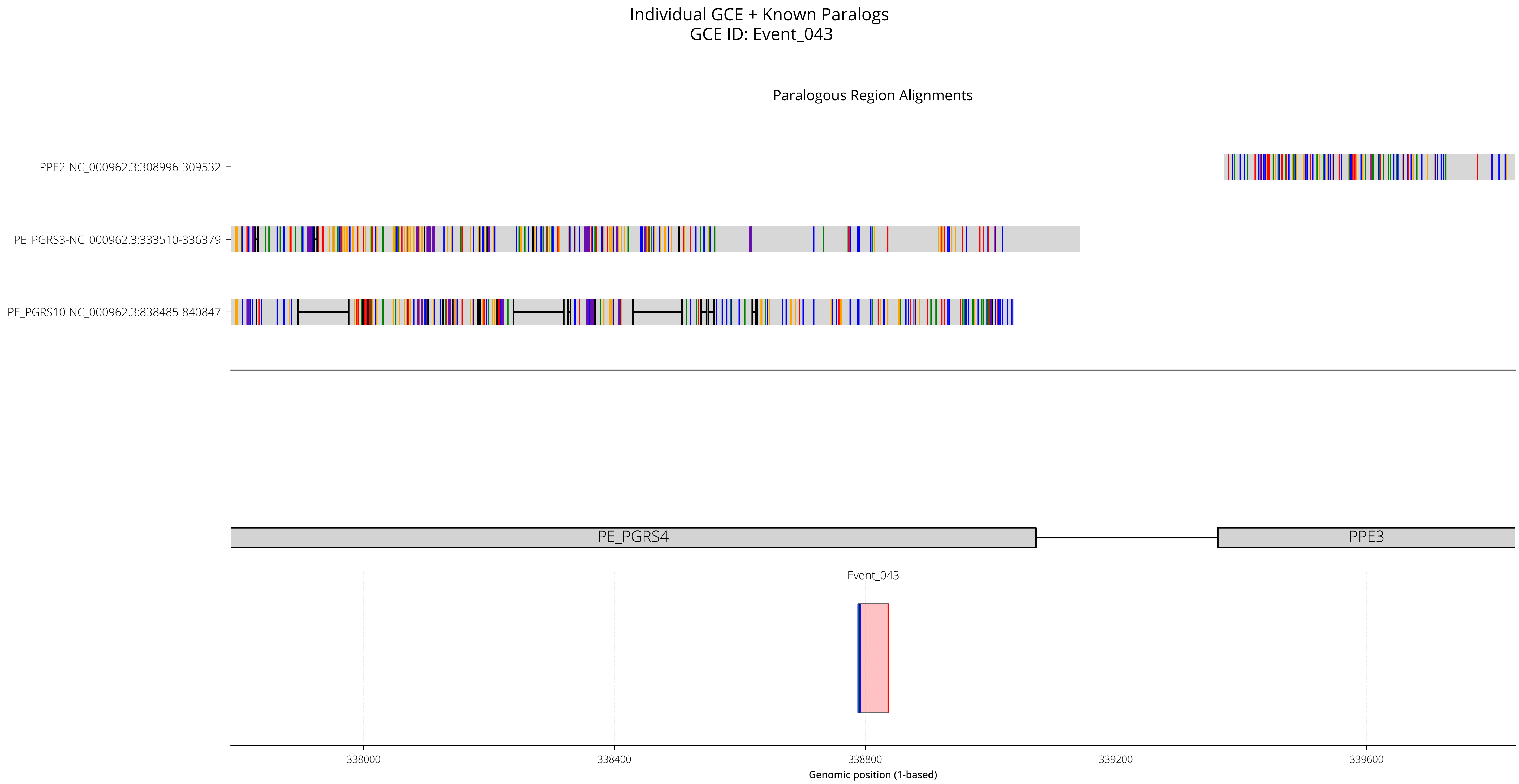

Individual GCE + Known Paralogs
GCE ID: Event_043
Paralogous Region Alignments
PPE2-NC_000962.3:308996-309532
PE_PGRS3-NC_000962.3:333510-336379
PE_PGRS10-NC_000962.3:838485-840847
PE_PGRS4
PPE3
Event_043
338000
338400
338800
339200
339600
Genomic position (1-based)

Circos Viz - GCE to Known Paralogs Locations
GCE ID: Event_043

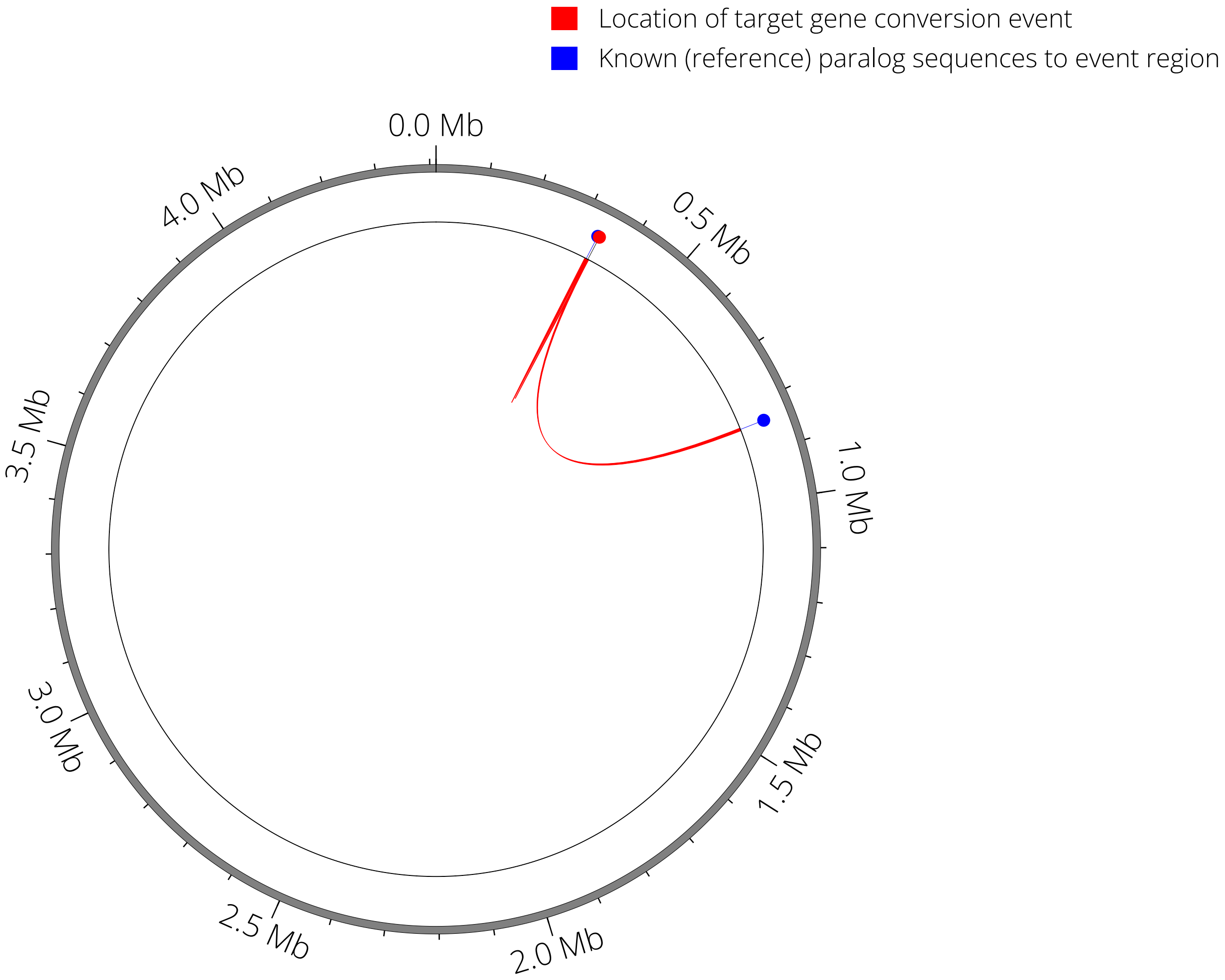

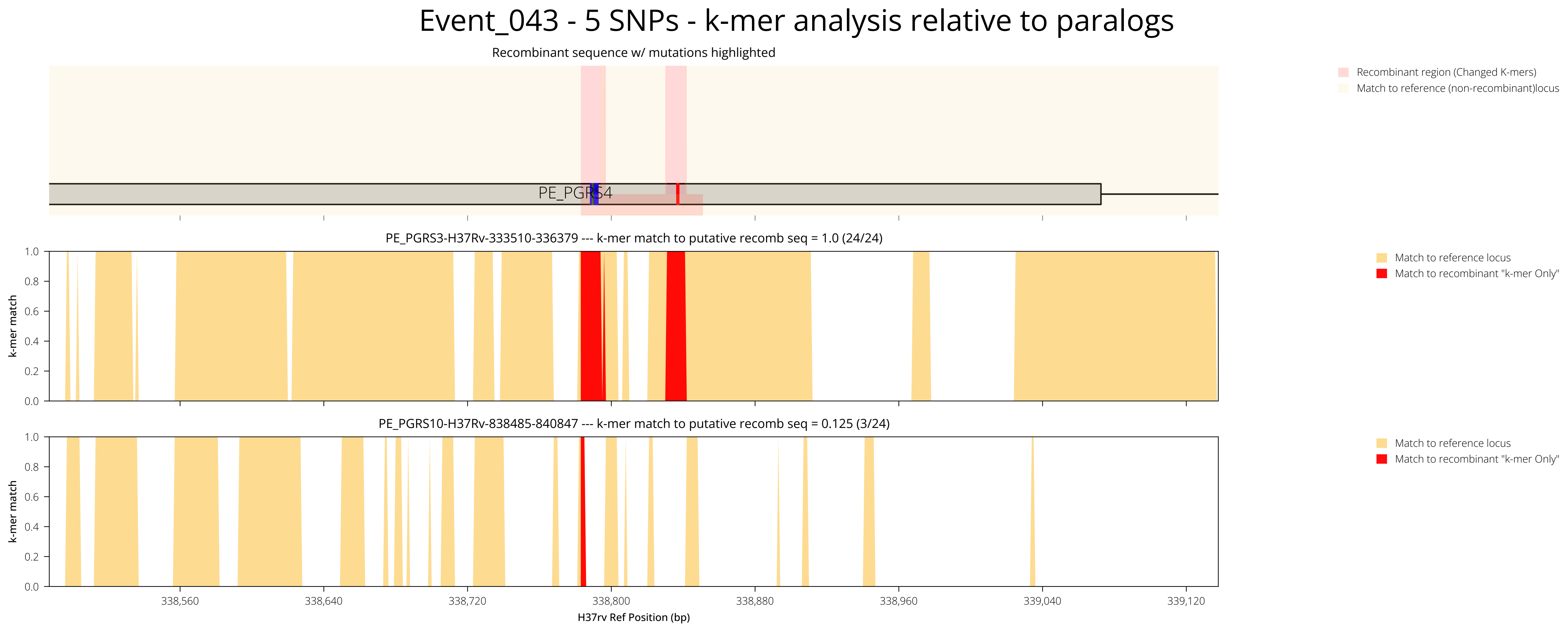

Event_043 - 5 SNPs - k-mer analysis relative to paralogs
Recombinant sequence w/ mutations highlighted
PE_PGRS4
Recombinant region (Changed K-mers)
Match to reference (non-recombinant)locus
PE_PGRS3-H37Rv-333510-336379 --- k-mer match to putative recomb seq = 1.0 (24/24)
Match to reference locus
Match to recombinant "k-mer Only"
PE_PGRS10-H37Rv-838485-840847 --- k-mer match to putative recomb seq = 0.125 (3/24)
Match to reference locus
Match to recombinant "k-mer Only"
k-mer match
1.0
0.8
0.6
0.4
0.2
0.0
338,560
338,640
338,720
338,800
338,880
338,960
339,040
339,120
H37rv Ref Position (bp)

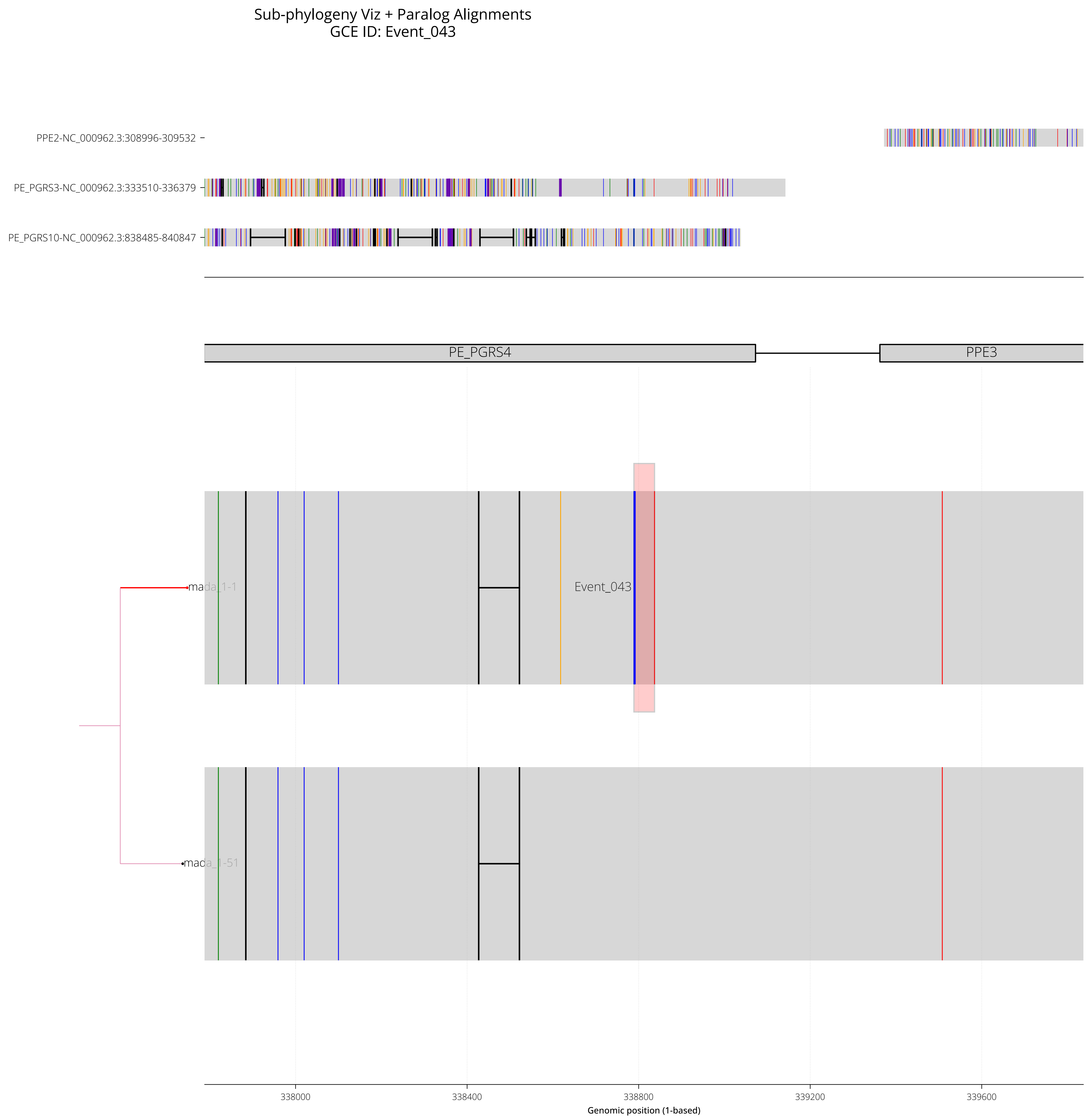

Sub-phylogeny Viz + Paralog Alignments
GCE ID: Event_043
PPE2-NC_000962.3:308996-309532
PE_PGRS3-NC_000962.3:333510-336379
PE_PGRS10-NC_000962.3:838485-840847
PE_PGRS4
PPE3
Event_043
mada_1-1
mada_1-51
338000
338400
338800
339200
339600
Genomic position (1-based)

Full Phylogeny Viz + Paralog Alignments
GCE ID: Event_043

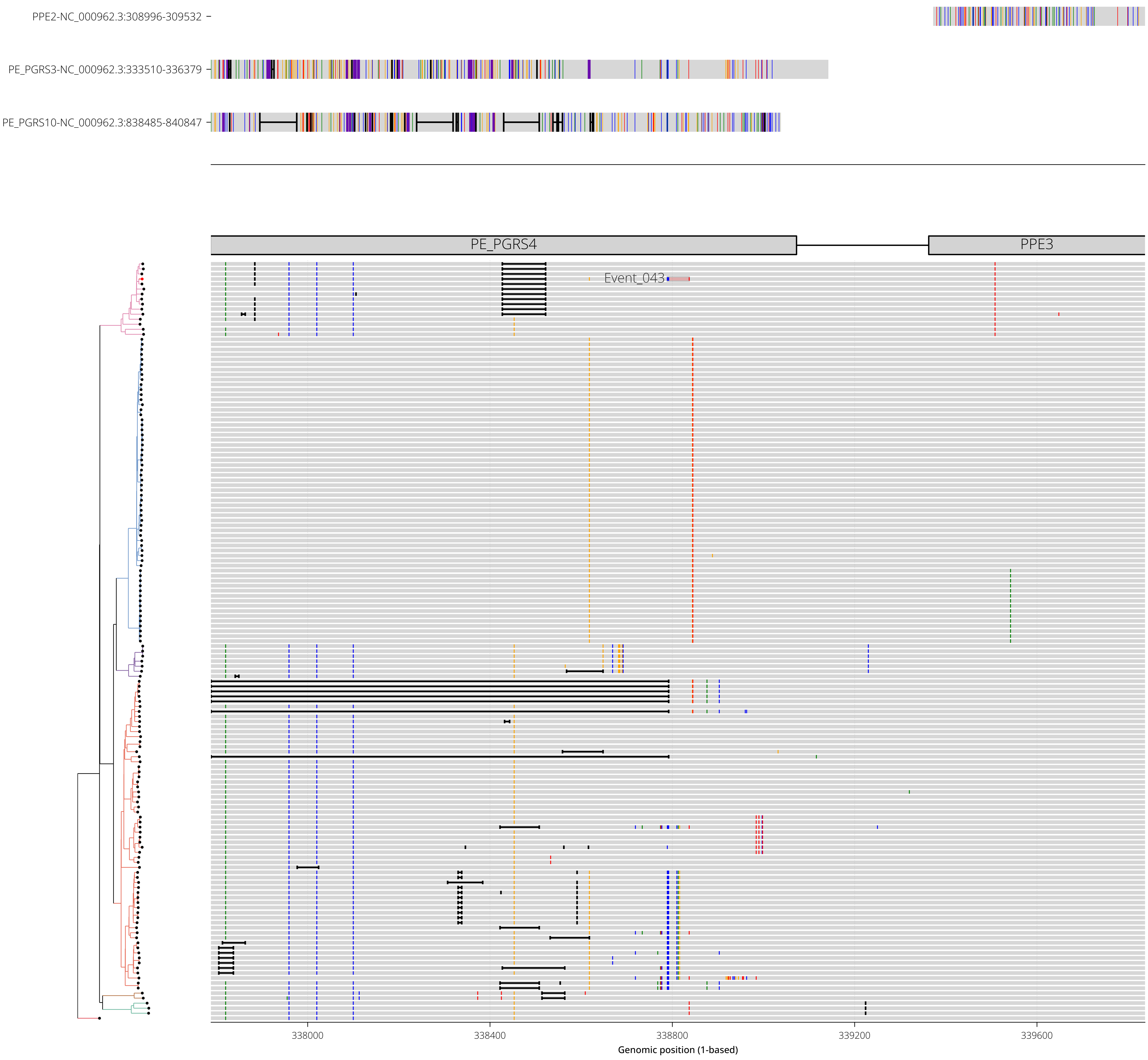

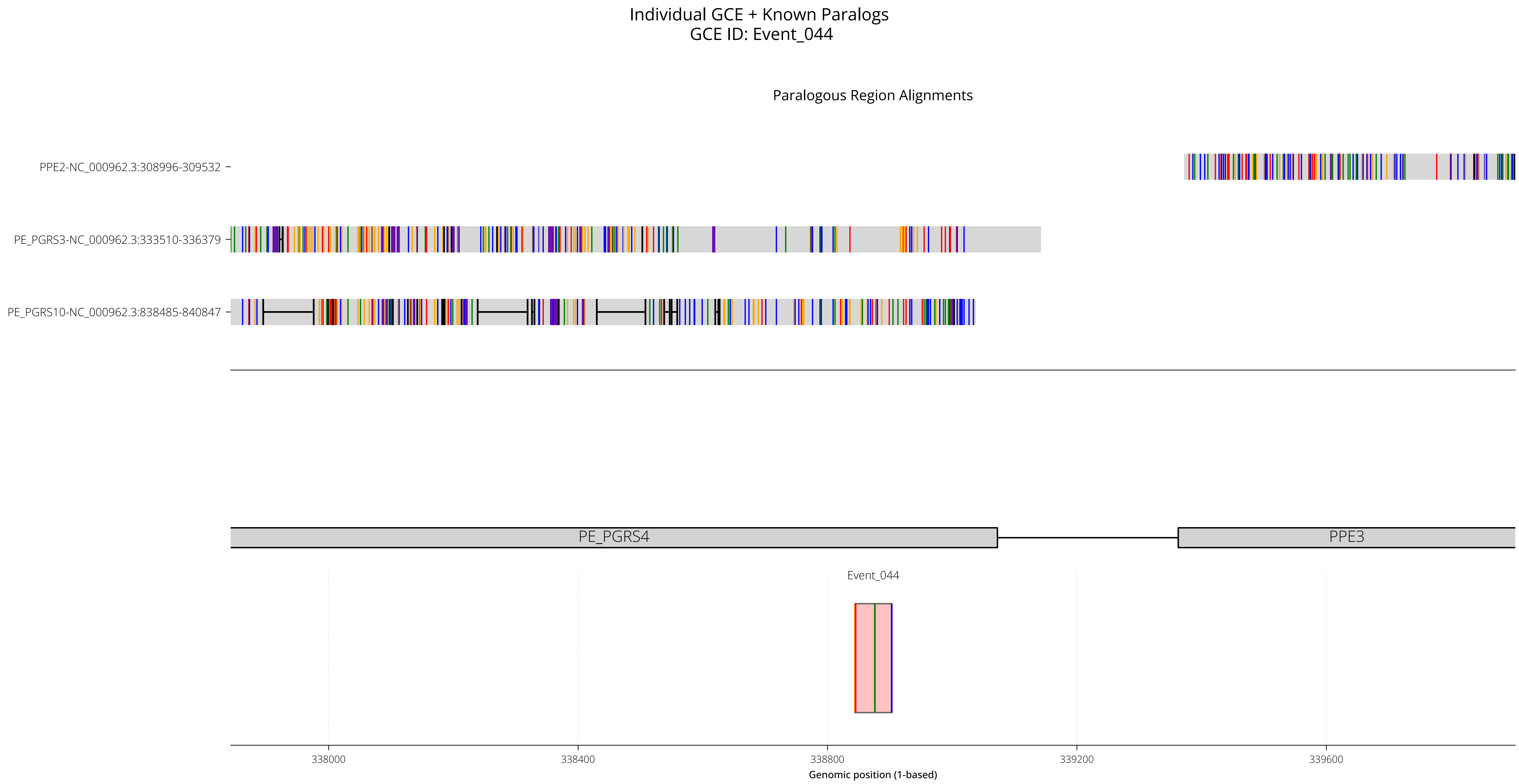
Individual GCE + Known Paralogs
GCE ID: Event_044
Paralogous Region Alignments
PPE2-NC_000962.3:308996-309532
PE_PGRS3-NC_000962.3:333510-336379
PE_PGRS10-NC_000962.3:838485-840847
PE_PGRS4
PPE3
Event_044
338000
338400
338800
339200
339600
Genomic position (1-based)

Circos Viz - GCE to Known Paralogs Locations
GCE ID: Event_044

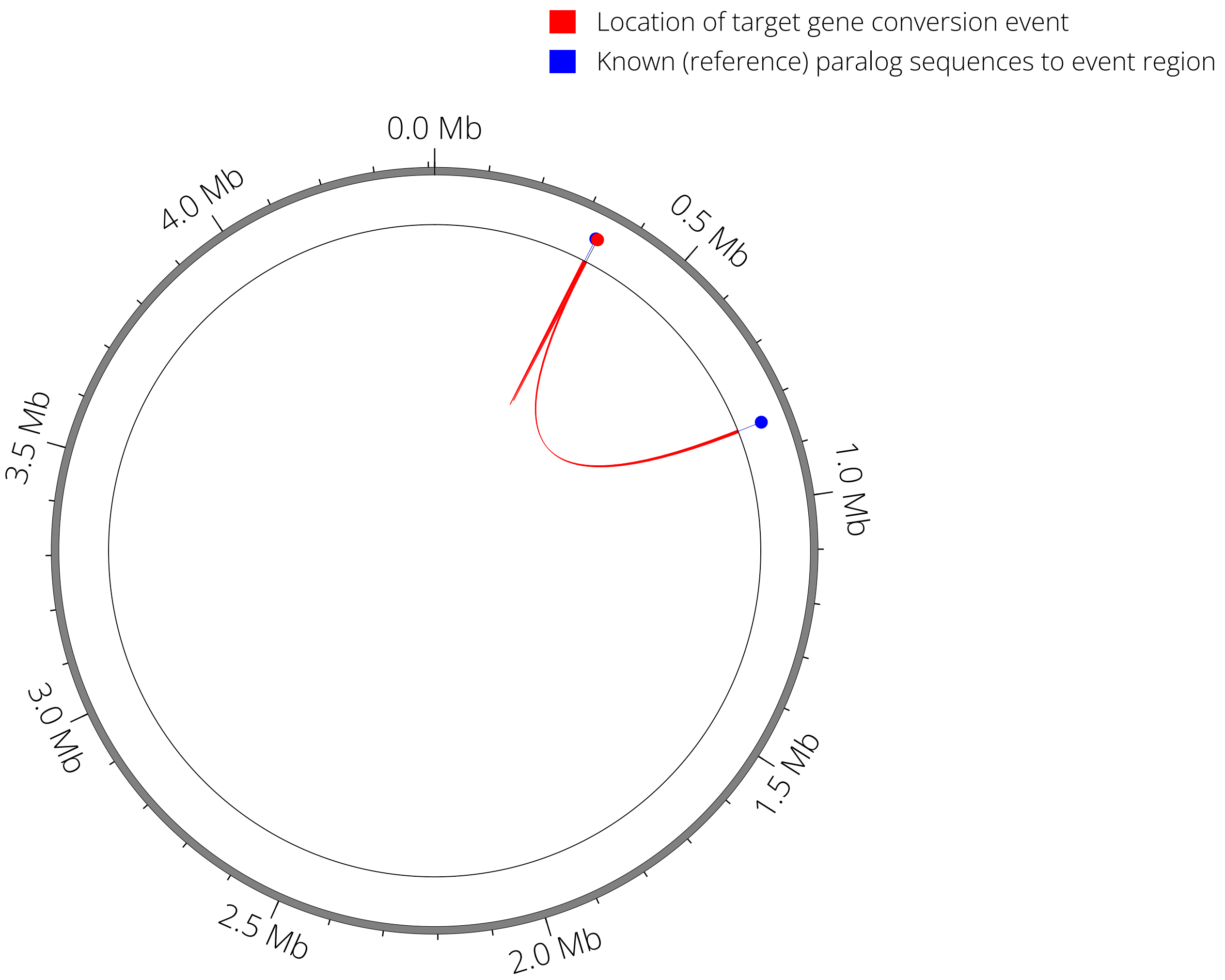

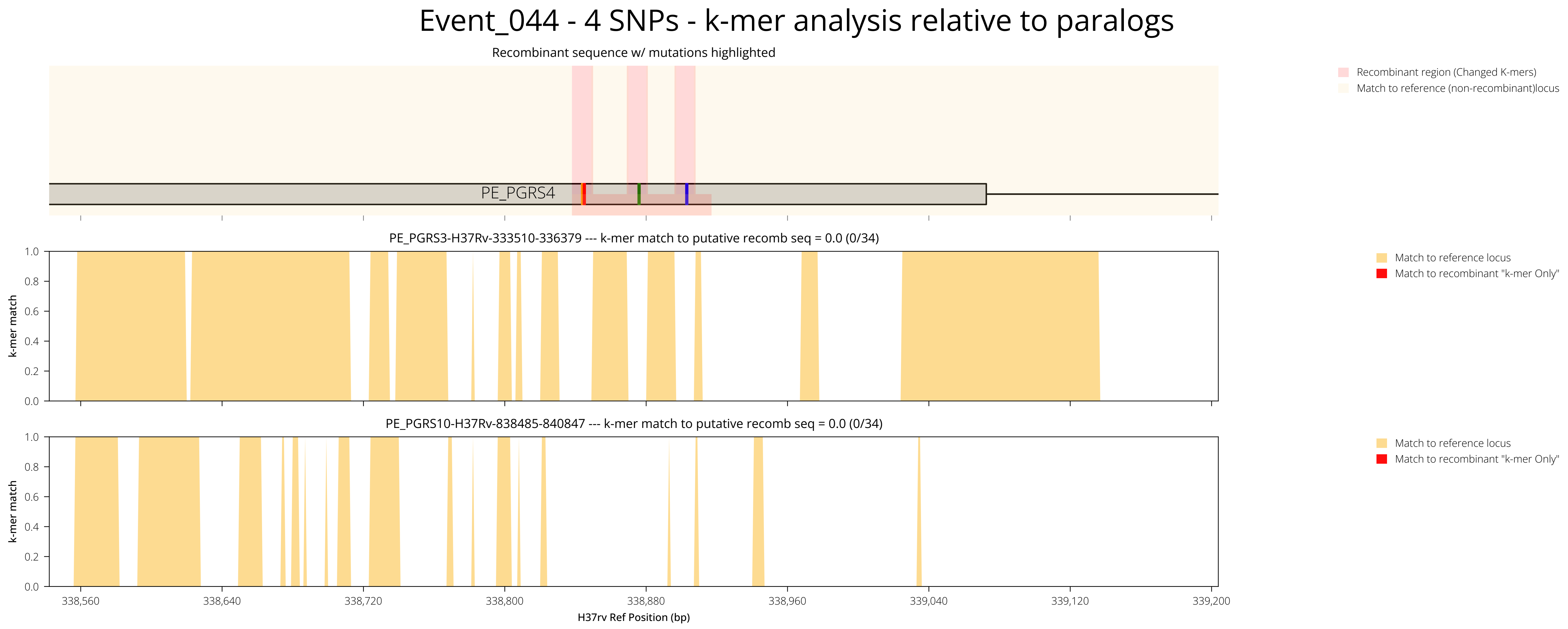

Event_044 - 4 SNPs - k-mer analysis relative to paralogs
Recombinant sequence w/ mutations highlighted
PE_PGRS4
Recombinant region (Changed K-mers)
Match to reference (non-recombinant)locus
PE_PGRS3-H37Rv-333510-336379 --- k-mer match to putative recomb seq = 0.0 (0/34)
Match to reference locus
Match to recombinant "k-mer Only"
PE_PGRS10-H37Rv-838485-840847 --- k-mer match to putative recomb seq = 0.0 (0/34)
Match to reference locus
Match to recombinant "k-mer Only"
k-mer match
1.0
0.8
0.6
0.4
0.2
0.0
338,560
338,640
338,720
338,800
338,880
338,960
339,040
339,120
339,200
H37rv Ref Position (bp)

Sub-phylogeny Viz + Paralog Alignments
GCE ID: Event_044

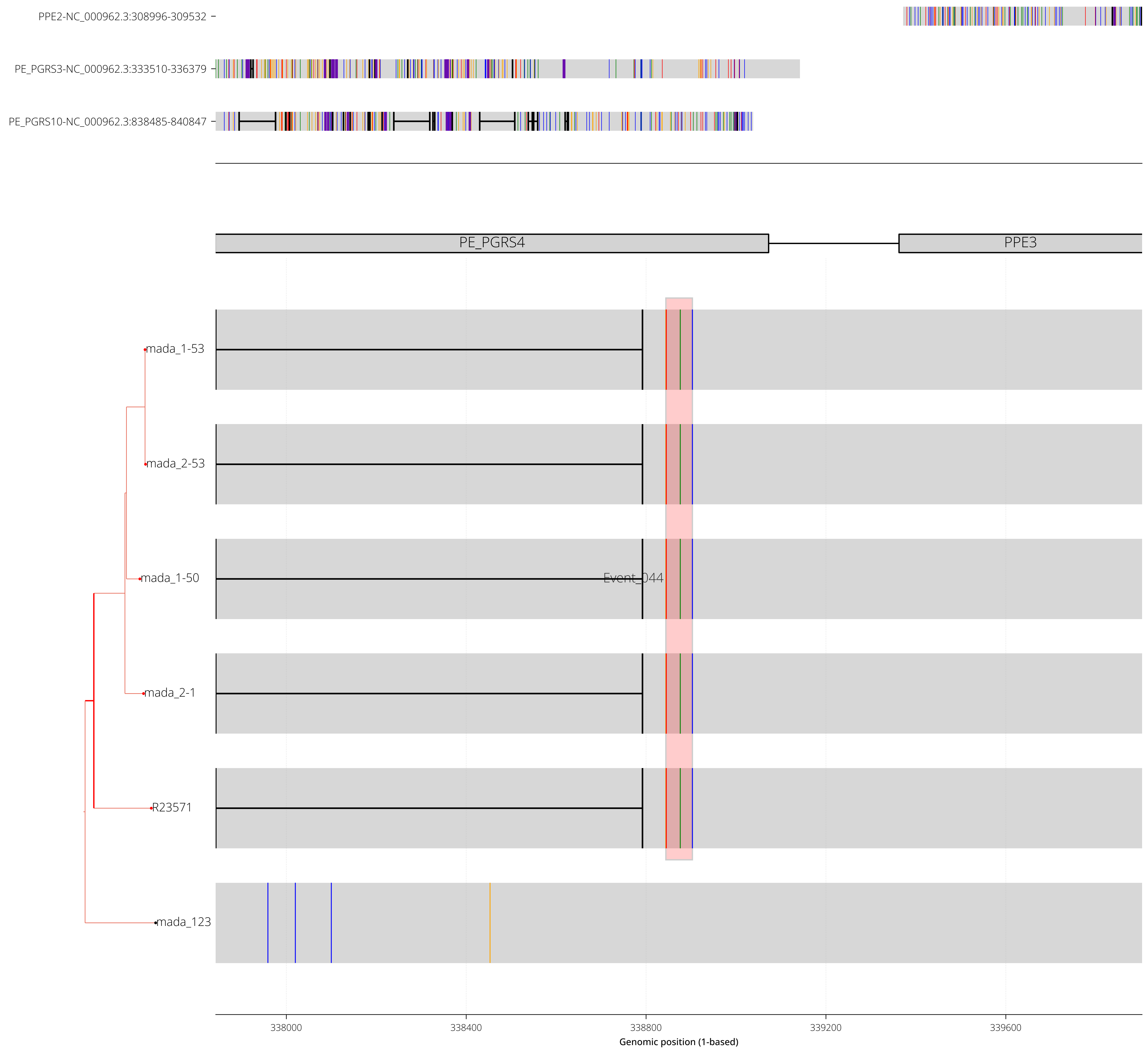

Full Phylogeny Viz + Paralog Alignments
GCE ID: Event_044

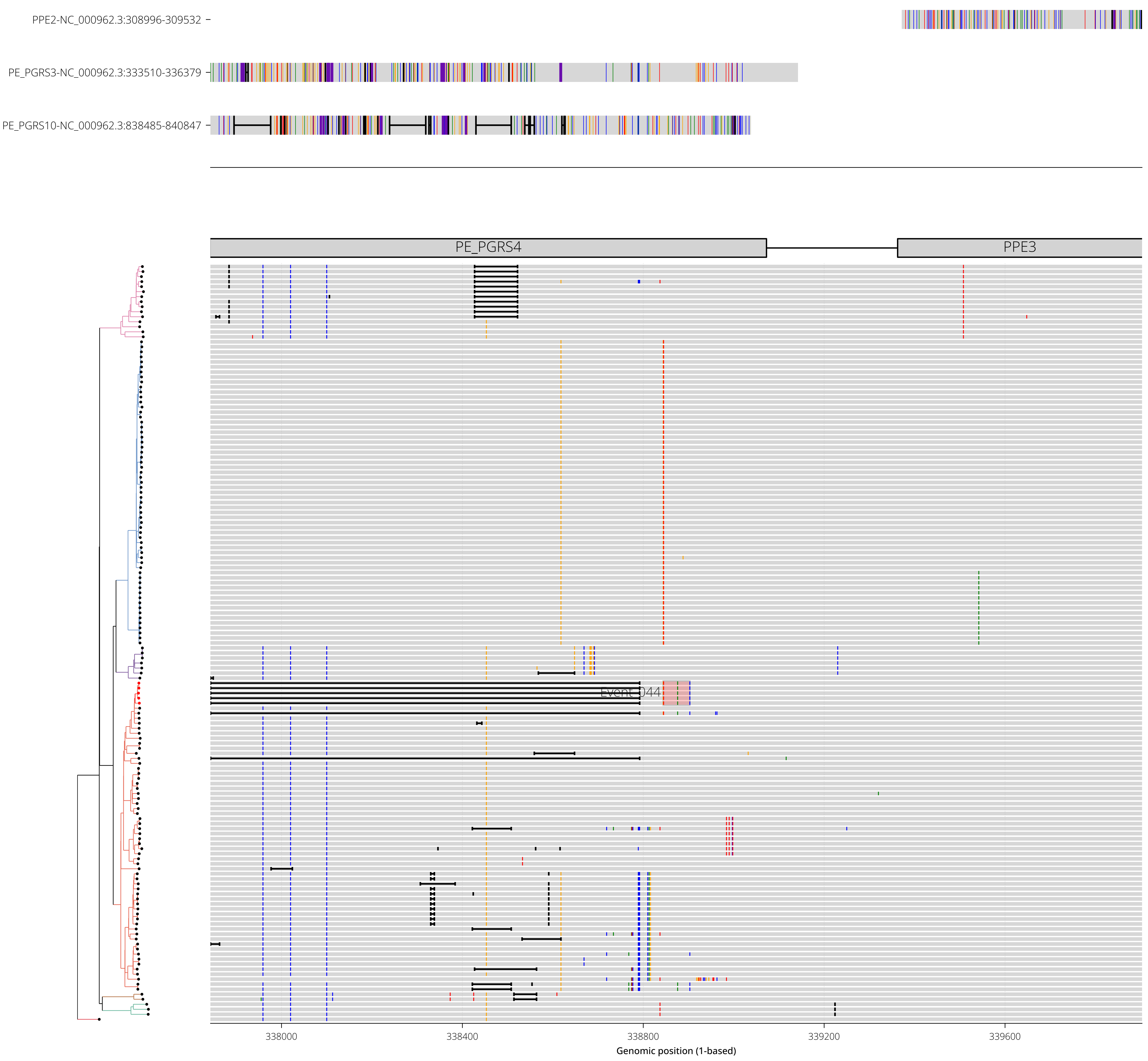

Individual GCE + Known Paralogs
GCE ID: Event_045

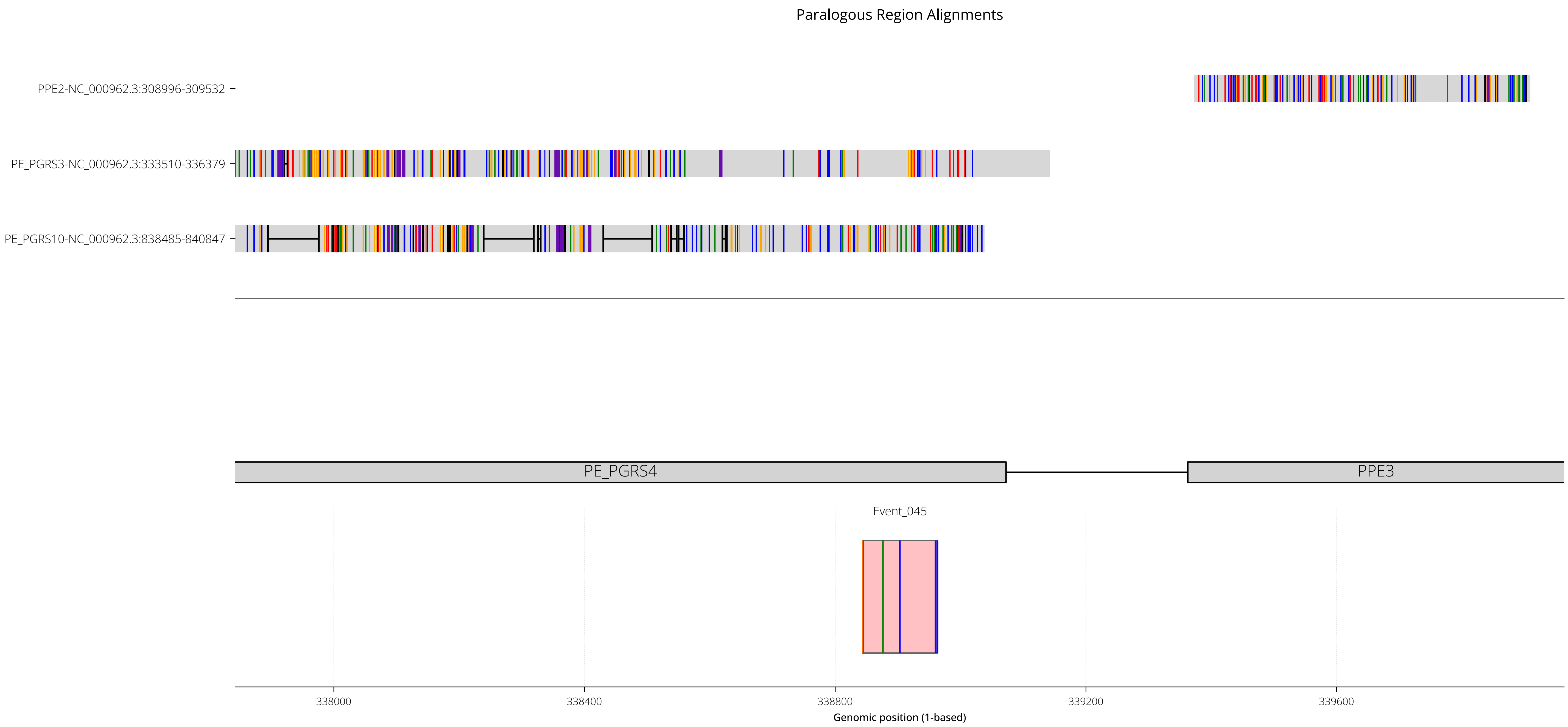

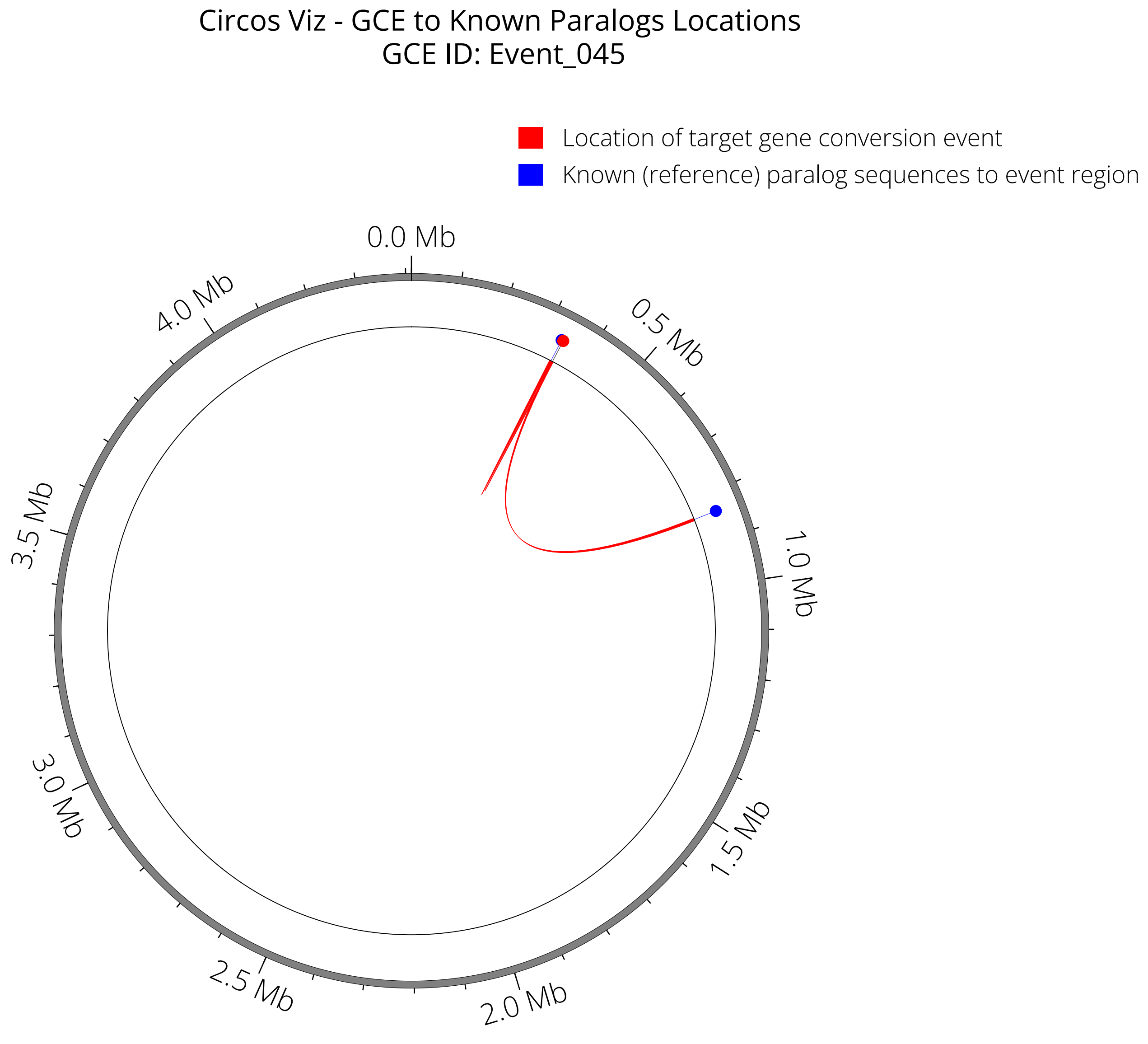
Circos Viz - GCE to Known Paralogs Locations
GCE ID: Event_045
Location of target gene conversion event
Known (reference) paralog sequences to event region
0.0 Mb
0.5 Mb
1.0 Mb
1.5 Mb
2.0 Mb
2.5 Mb
3.0 Mb
3.5 Mb
4.0 Mb

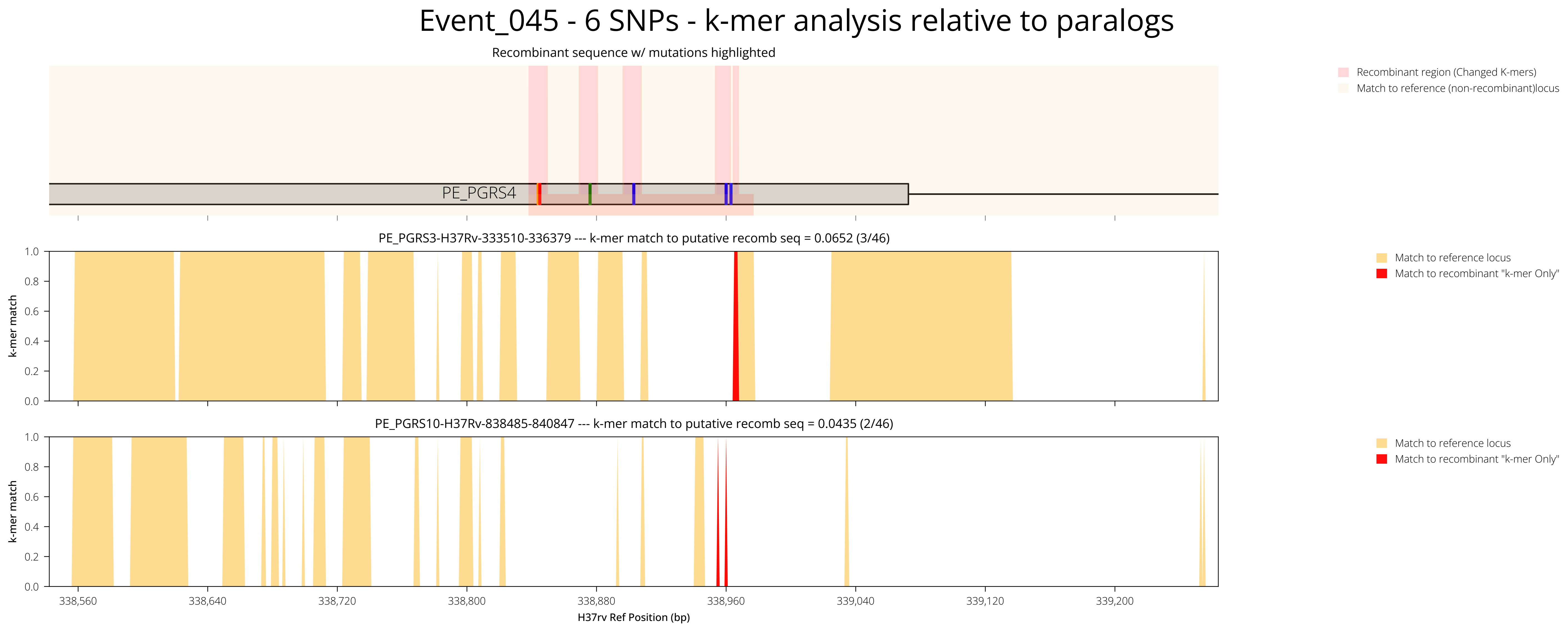

Event_045 - 6 SNPs - k-mer analysis relative to paralogs
Recombinant sequence w/ mutations highlighted
PE_PGRS4
Recombinant region (Changed K-mers)
Match to reference (non-recombinant)locus
PE_PGRS3-H37Rv-333510-336379 --- k-mer match to putative recomb seq = 0.0652 (3/46)
Match to reference locus
Match to recombinant "k-mer Only"
PE_PGRS10-H37Rv-838485-840847 --- k-mer match to putative recomb seq = 0.0435 (2/46)
Match to reference locus
Match to recombinant "k-mer Only"
k-mer match
1.0
0.8
0.6
0.4
0.2
0.0
338,560
338,640
338,720
338,800
338,880
338,960
339,040
339,120
339,200
H37rv Ref Position (bp)

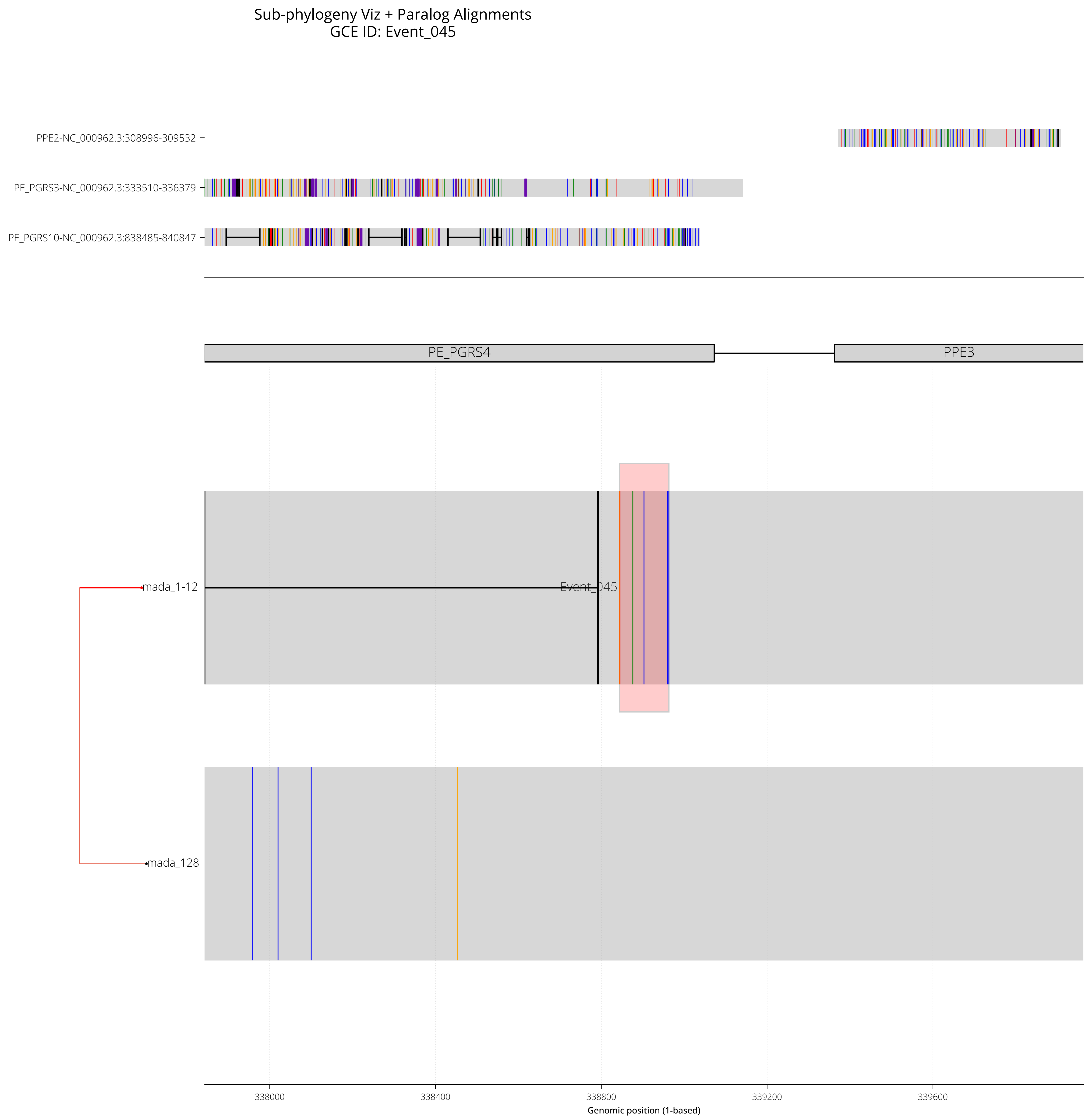

Sub-phylogeny Viz + Paralog Alignments
GCE ID: Event_045
PPE2-NC_000962.3:308996-309532
PE_PGRS3-NC_000962.3:333510-336379
PE_PGRS10-NC_000962.3:838485-840847
PE_PGRS4
PPE3
mada_1-12
Event_045
mada_128
338000
338400
338800
339200
339600
Genomic position (1-based)

Full Phylogeny Viz + Paralog Alignments
GCE ID: Event_045

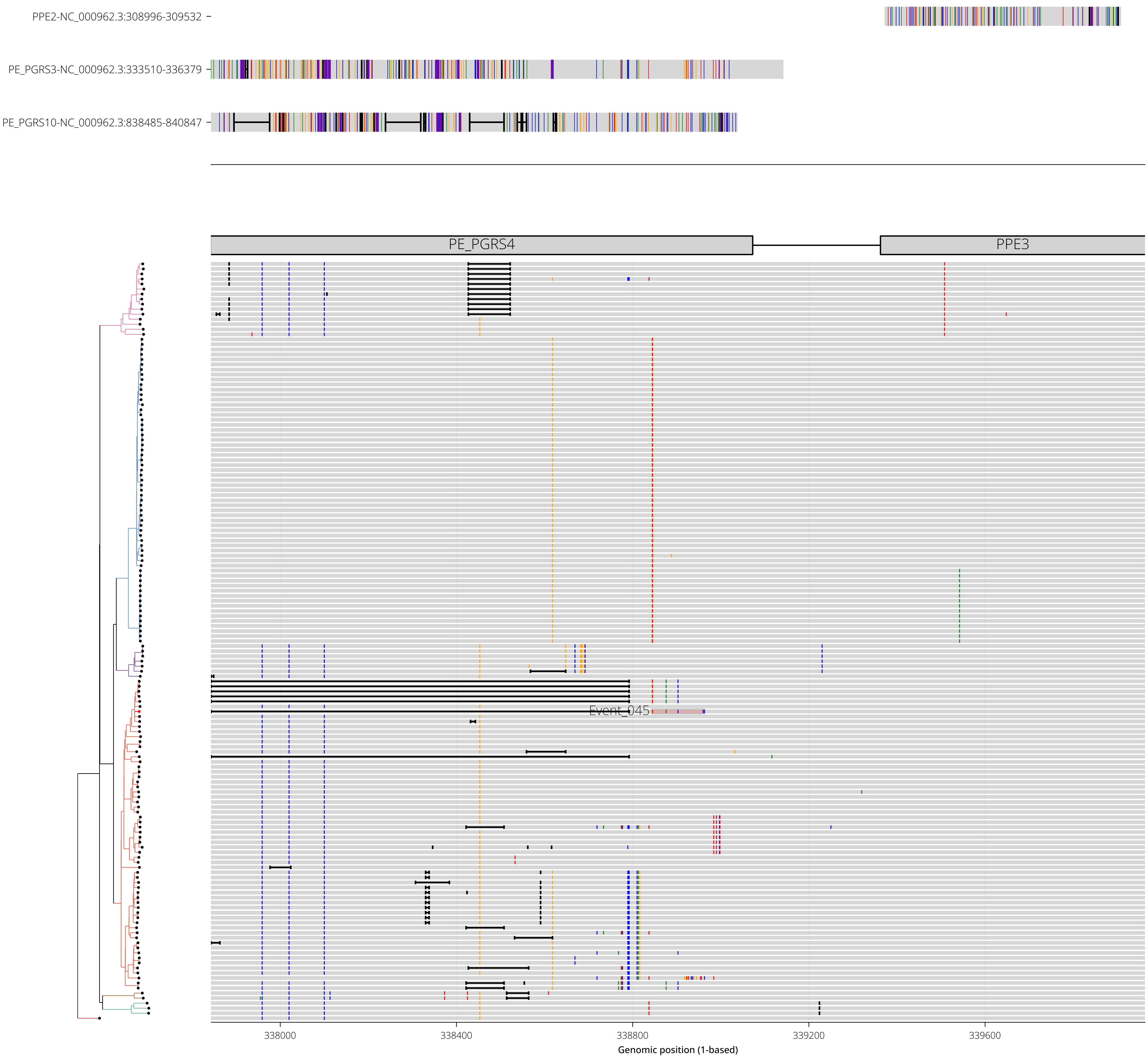

Individual GCE + Known Paralogs
GCE ID: Event_046

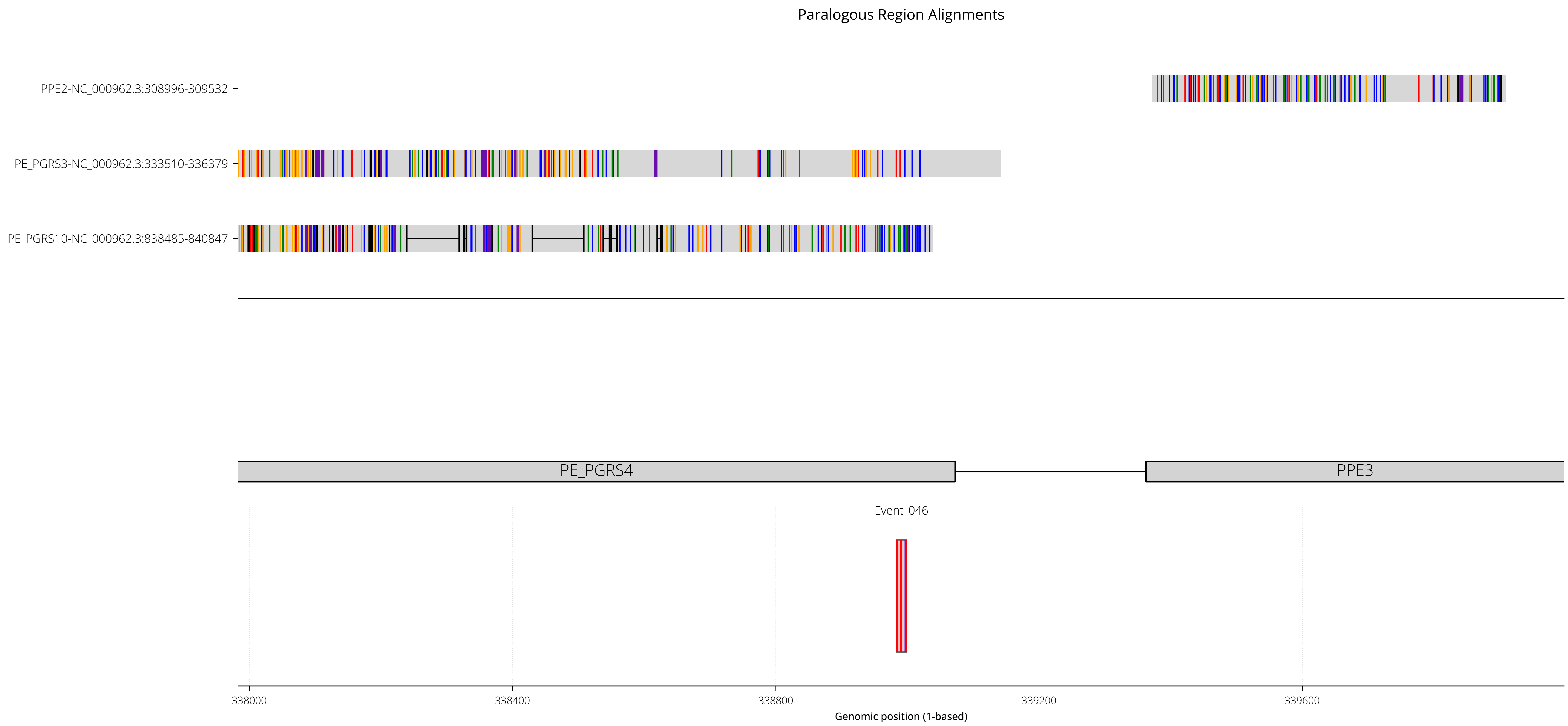

Circos Viz - GCE to Known Paralogs Locations
GCE ID: Event_046

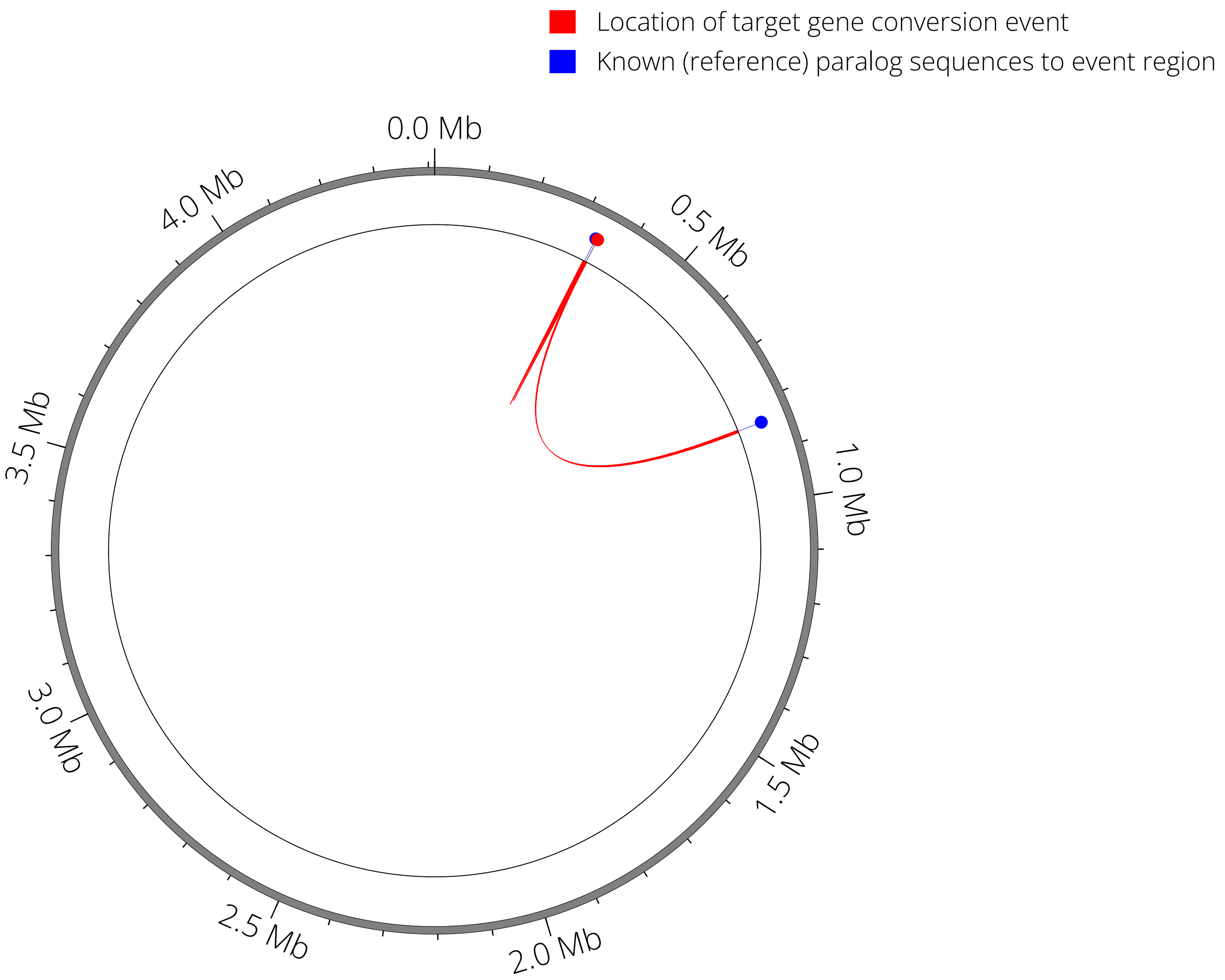

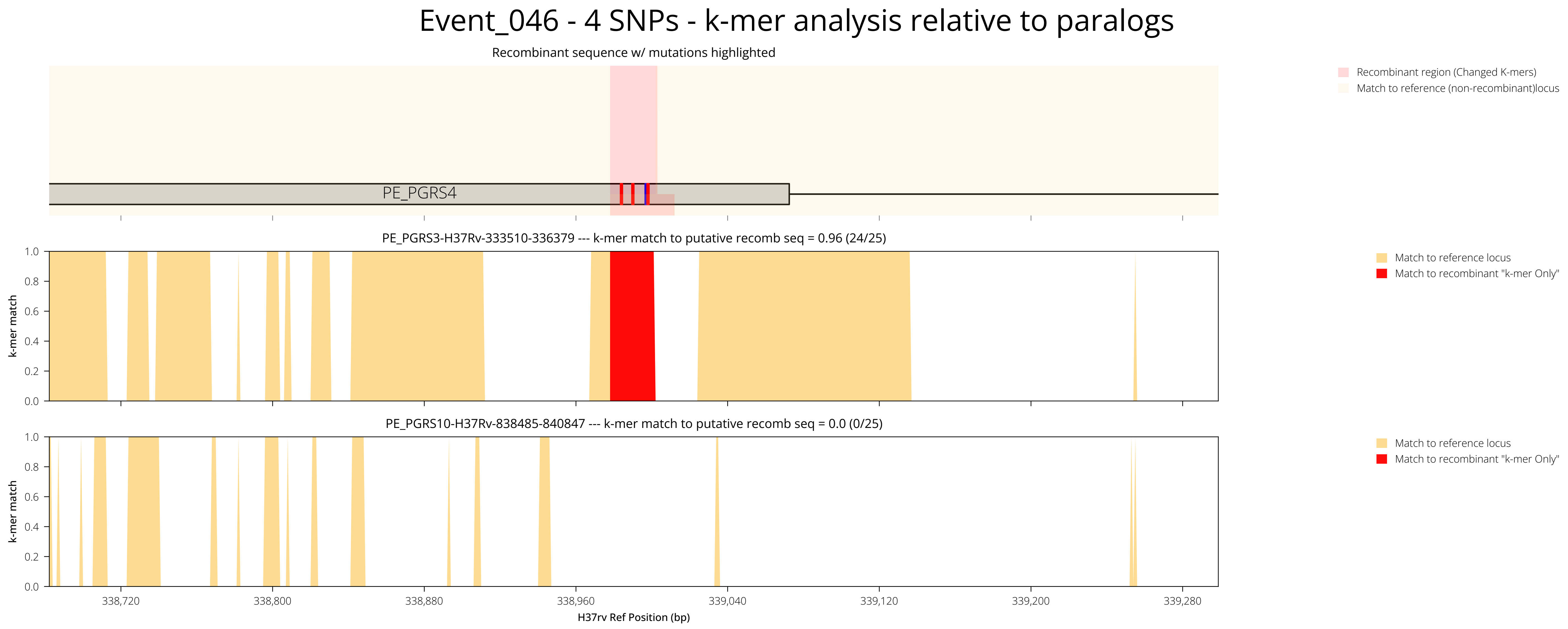

Event_046 - 4 SNPs - k-mer analysis relative to paralogs
Recombinant sequence w/ mutations highlighted
PE_PGRS4
Recombinant region (Changed K-mers)
Match to reference (non-recombinant)locus
PE_PGRS3-H37Rv-333510-336379 --- k-mer match to putative recomb seq = 0.96 (24/25)
k-mer match
1.0
0.8
0.6
0.4
0.2
0.0
Match to reference locus
Match to recombinant "k-mer Only"
PE_PGRS10-H37Rv-838485-840847 --- k-mer match to putative recomb seq = 0.0 (0/25)
k-mer match
1.0
0.8
0.6
0.4
0.2
0.0
Match to reference locus
Match to recombinant "k-mer Only"
338,720
338,800
338,880
338,960
339,040
339,120
339,200
339,280
H37rv Ref Position (bp)

Sub-phylogeny Viz + Paralog Alignments
GCE ID: Event_046

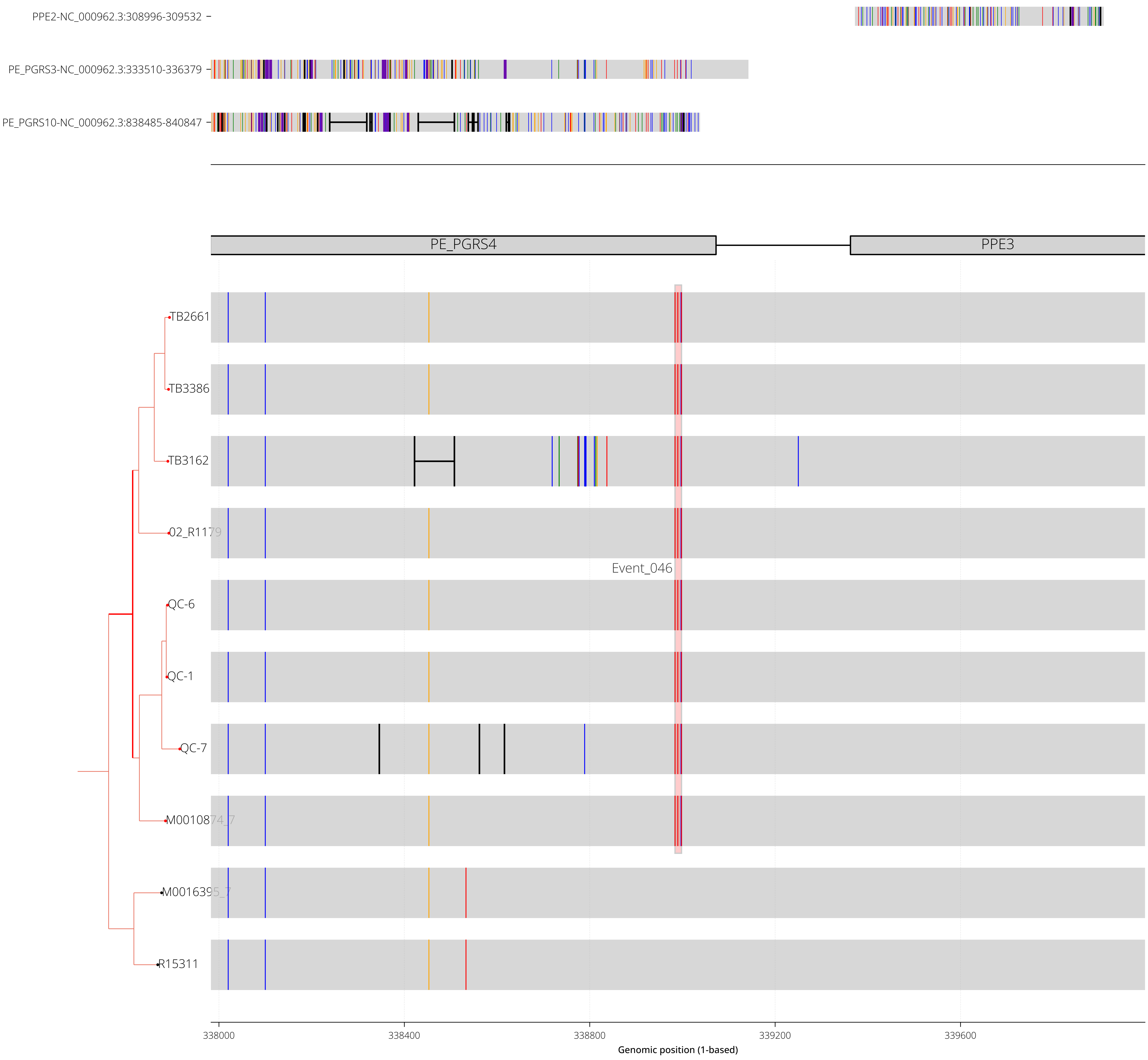

Full Phylogeny Viz + Paralog Alignments
GCE ID: Event_046

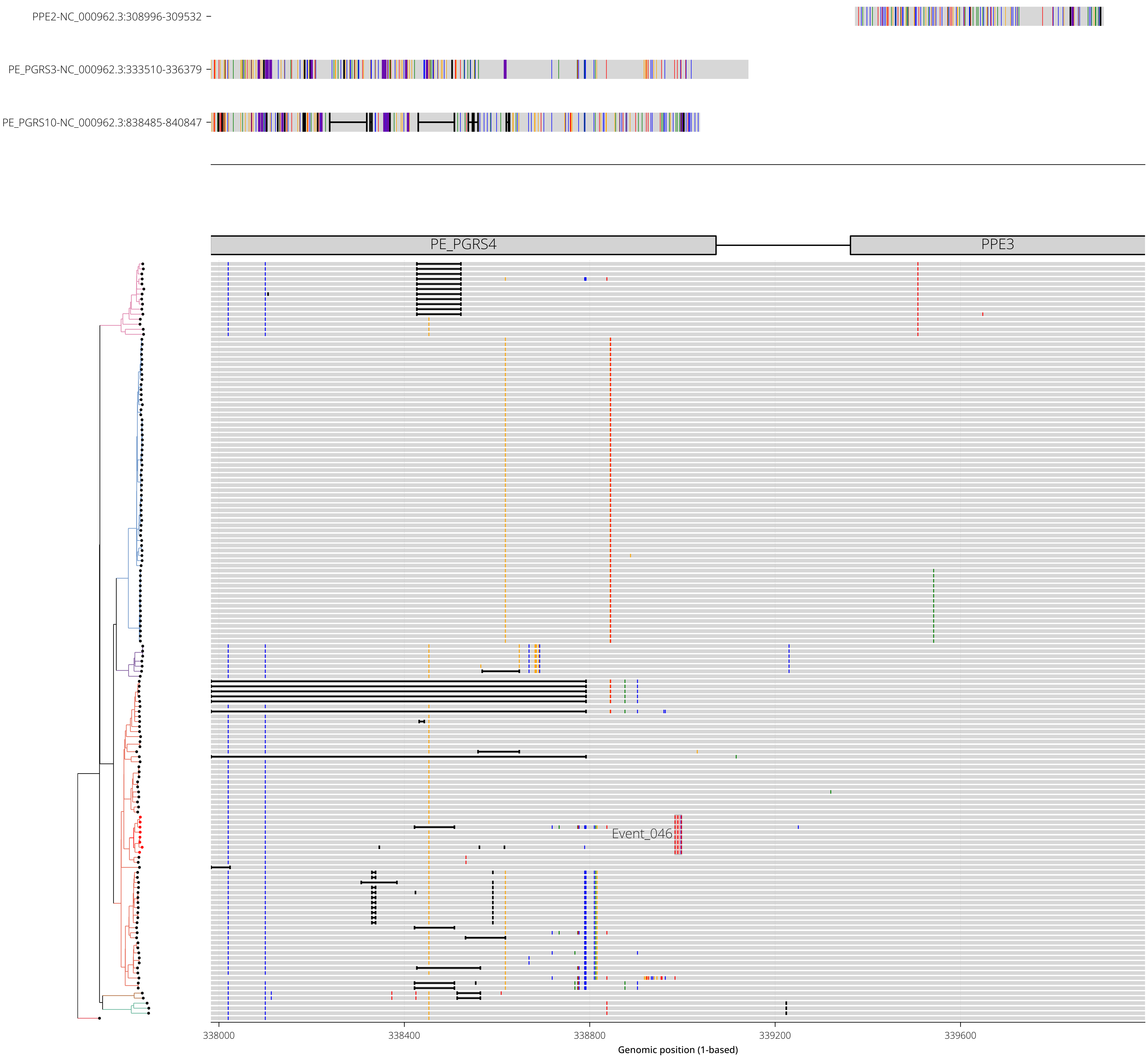

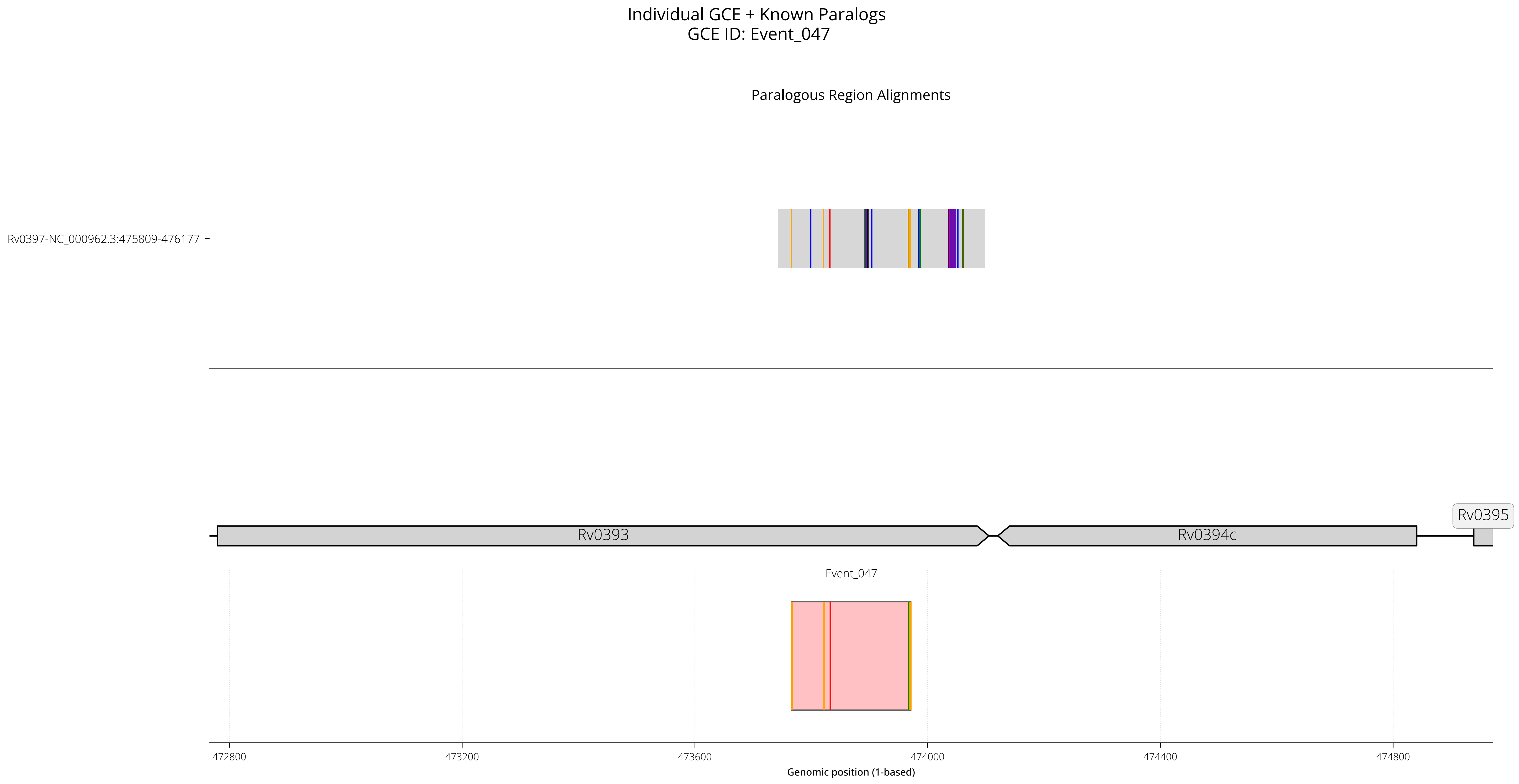

Individual GCE + Known Paralogs
GCE ID: Event_047
Paralogous Region Alignments
Rv0397-NC_000962.3:475809-476177
Rv0393
Rv0394c
Rv0395
Event_047
472800
473200
473600
474000
474400
474800
Genomic position (1-based)

Circos Viz - GCE to Known Paralogs Locations
GCE ID: Event_047

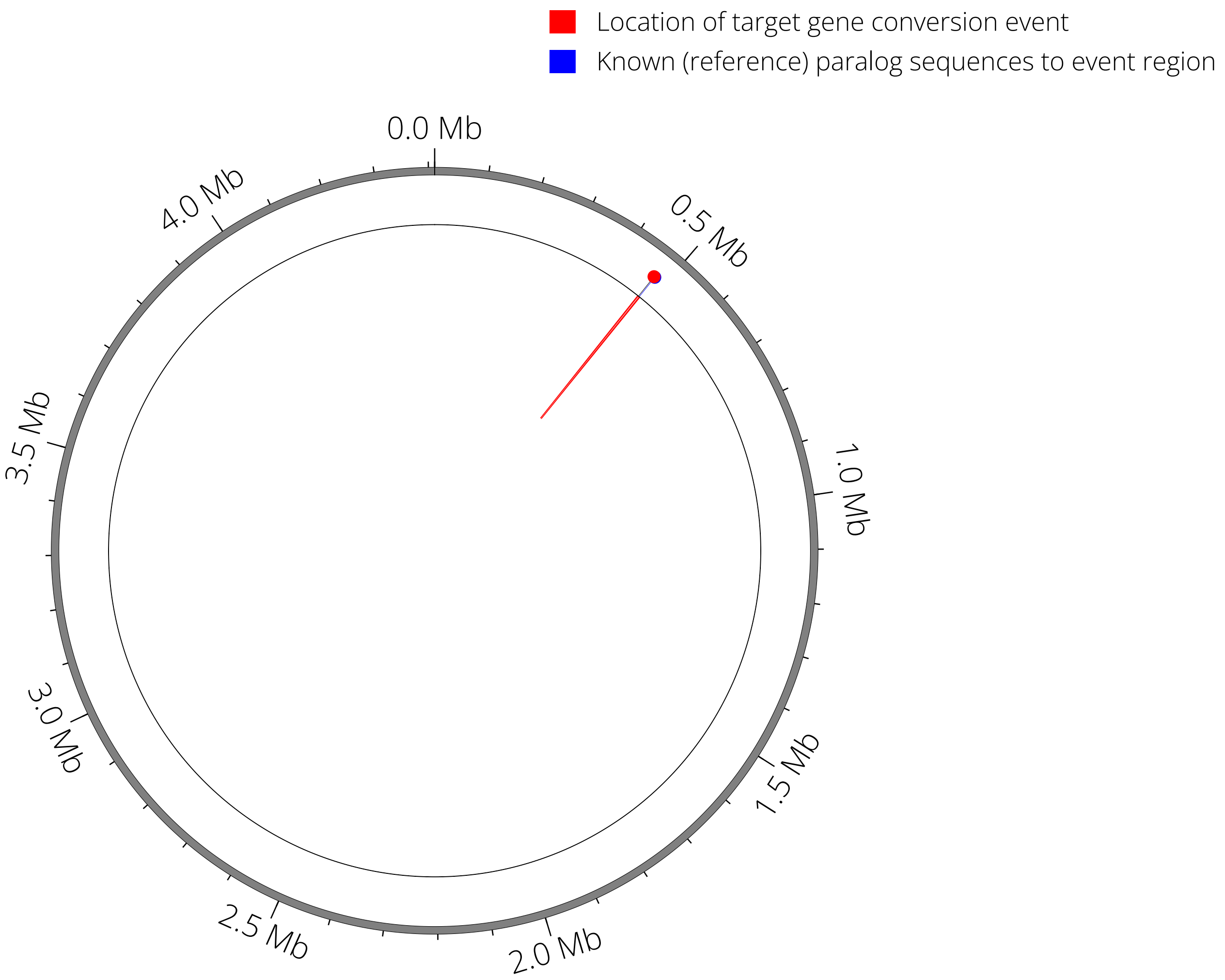

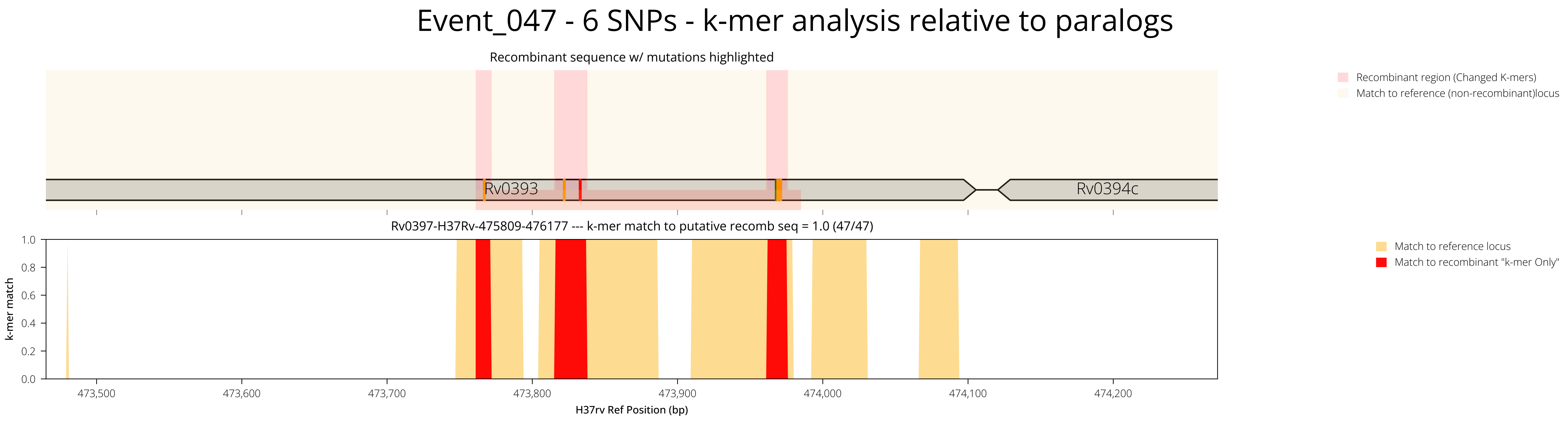

Event_047 - 6 SNPs - k-mer analysis relative to paralogs
Recombinant sequence w/ mutations highlighted
Rv0393
Rv0394c
Recombinant region (Changed K-mers)
Match to reference (non-recombinant)locus
Rv0397-H37Rv-475809-476177 --- k-mer match to putative recomb seq = 1.0 (47/47)
k-mer match
1.0
0.8
0.6
0.4
0.2
0.0
473,500
473,600
473,700
473,800
473,900
474,000
474,100
474,200
H37rv Ref Position (bp)
Match to reference locus
Match to recombinant "k-mer Only"

Sub-phylogeny Viz + Paralog Alignments
GCE ID: Event_047

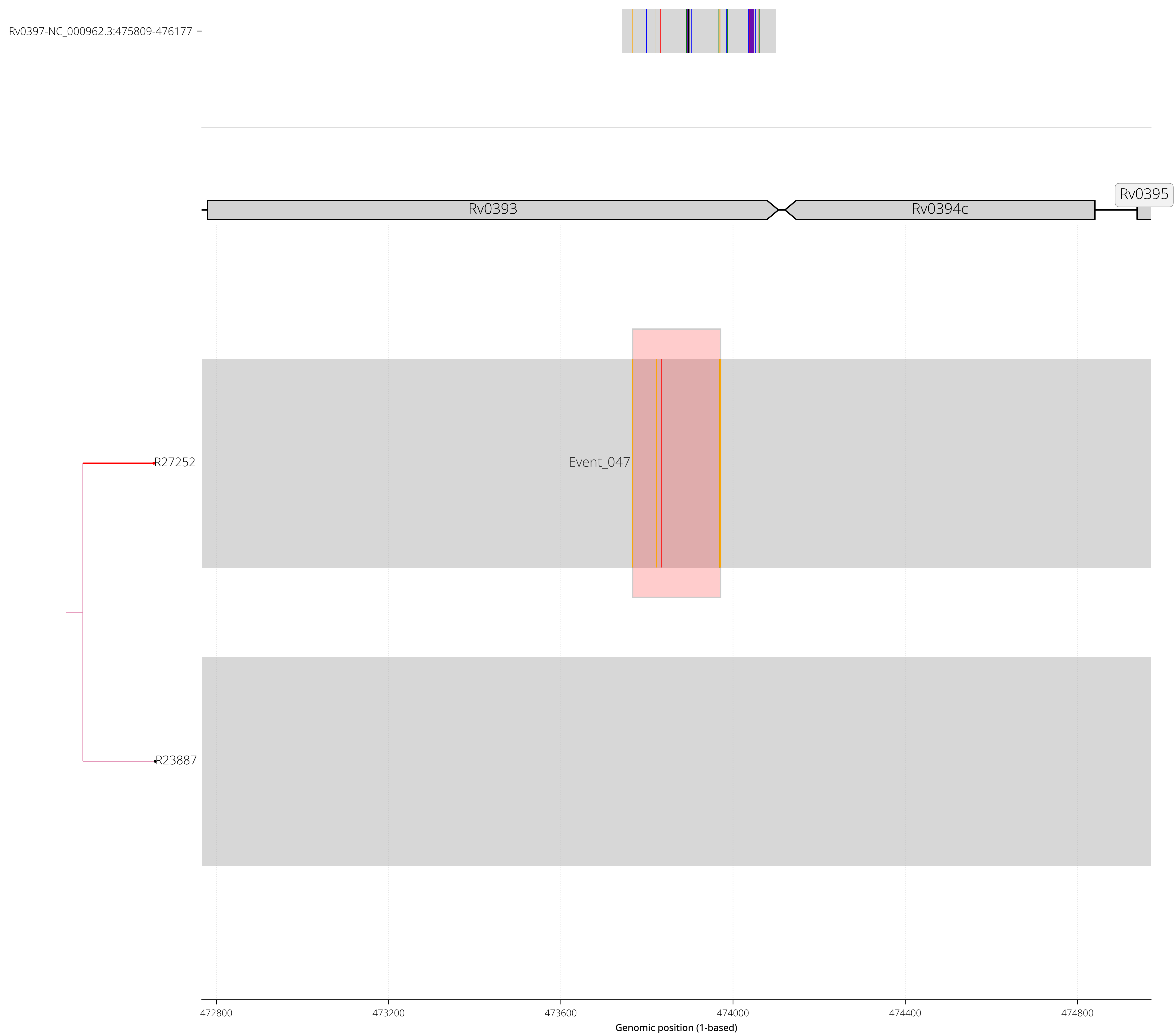

Full Phylogeny Viz + Paralog Alignments
GCE ID: Event_047

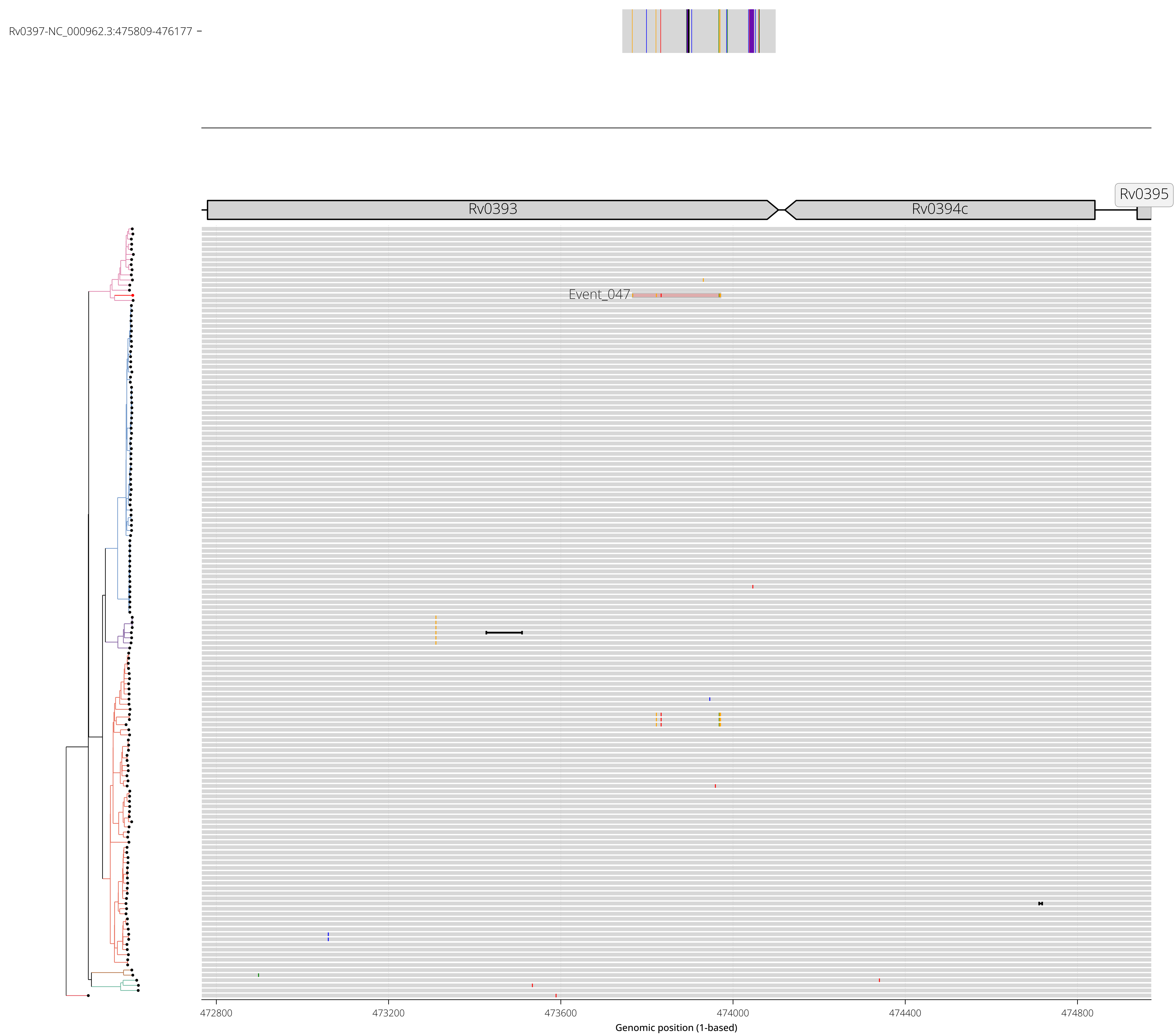

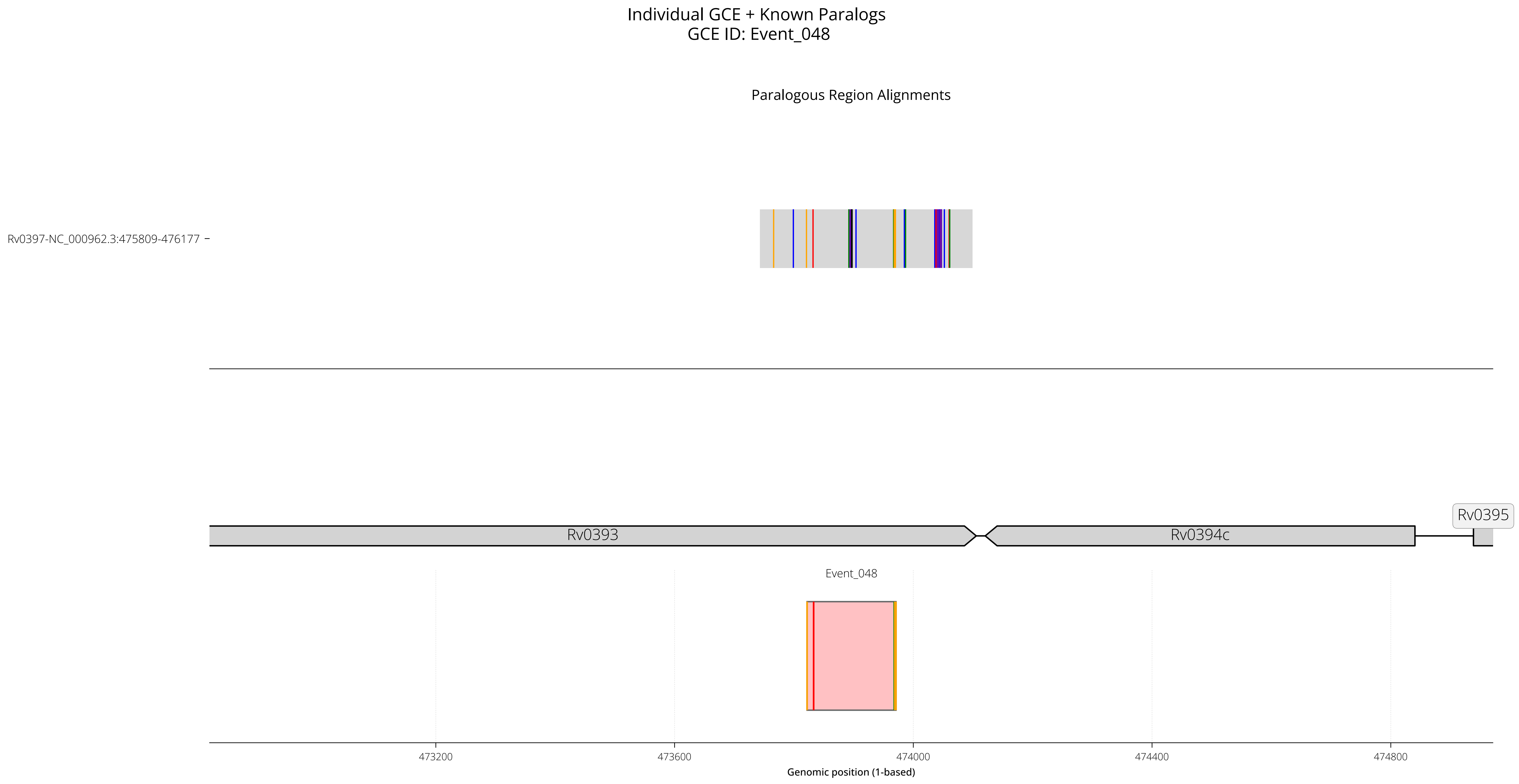
Individual GCE + Known Paralogs
GCE ID: Event_048
Paralogous Region Alignments
Rv0397-NC_000962.3:475809-476177
Rv0393
Rv0394c
Rv0395
Event_048
473200
473600
474000
474400
474800
Genomic position (1-based)

Circos Viz - GCE to Known Paralogs Locations
GCE ID: Event_048

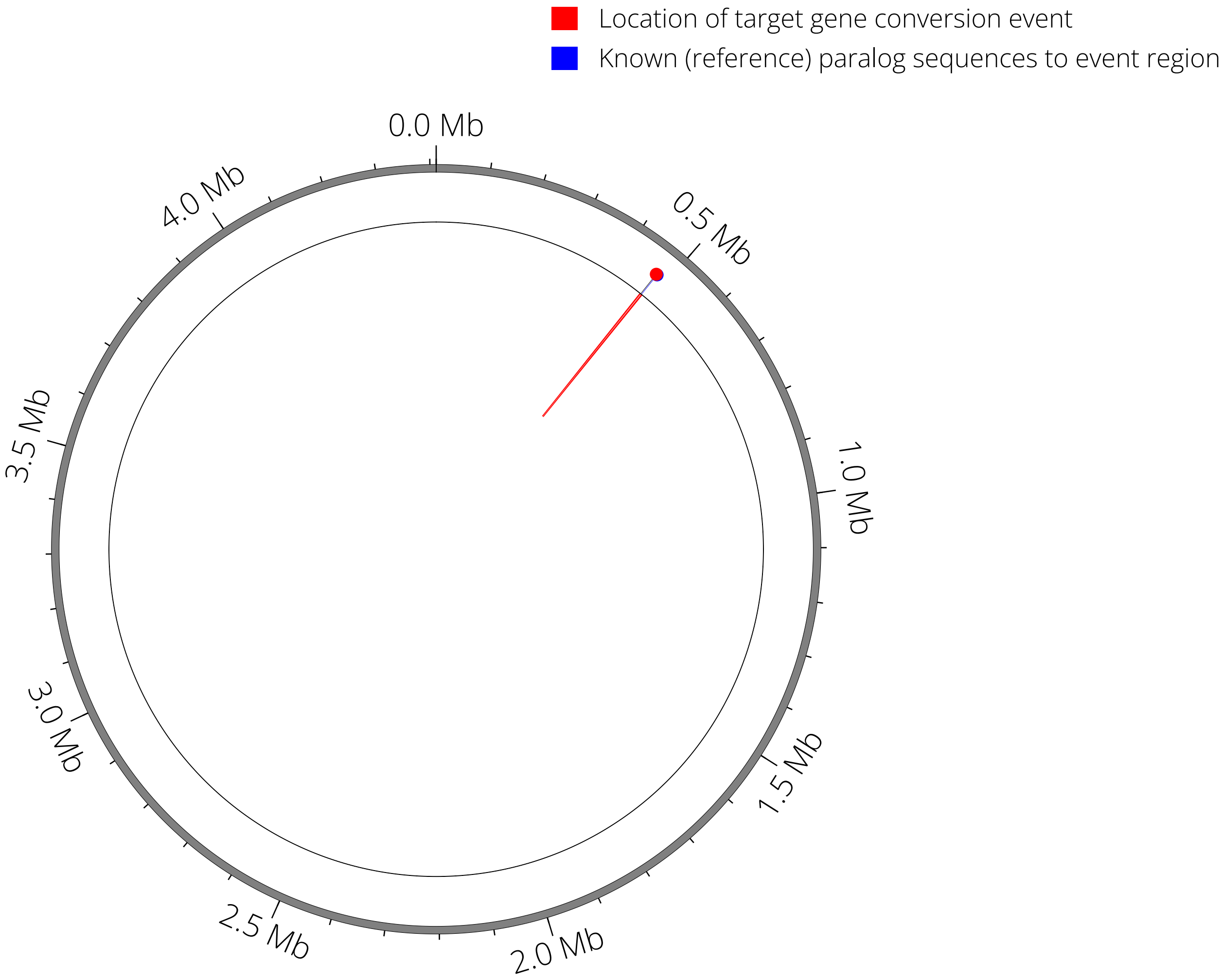

# Event_048 - 5 SNPs - k-mer analysis relative to paralogs

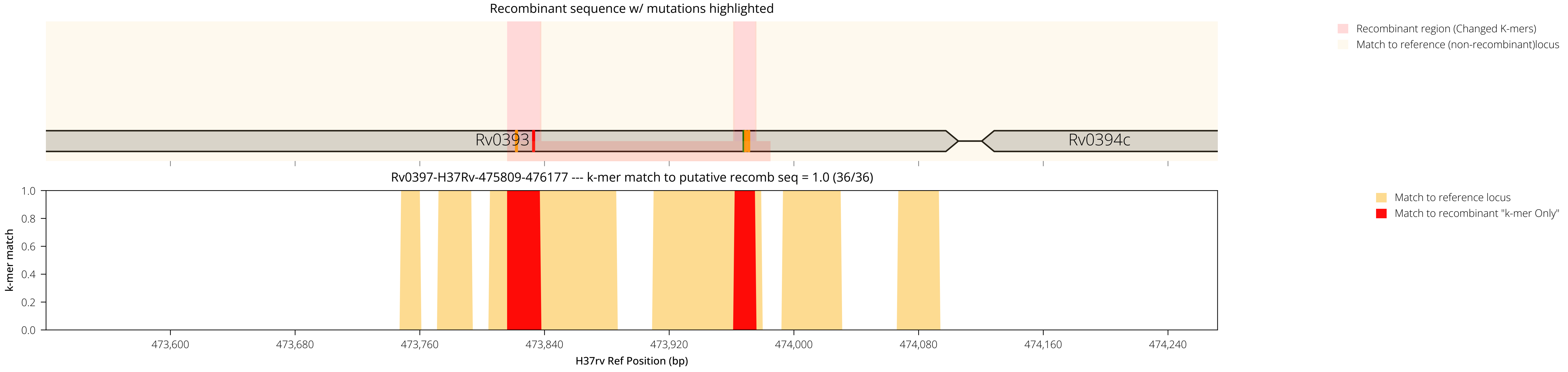

Sub-phylogeny Viz + Paralog Alignments
GCE ID: Event_048

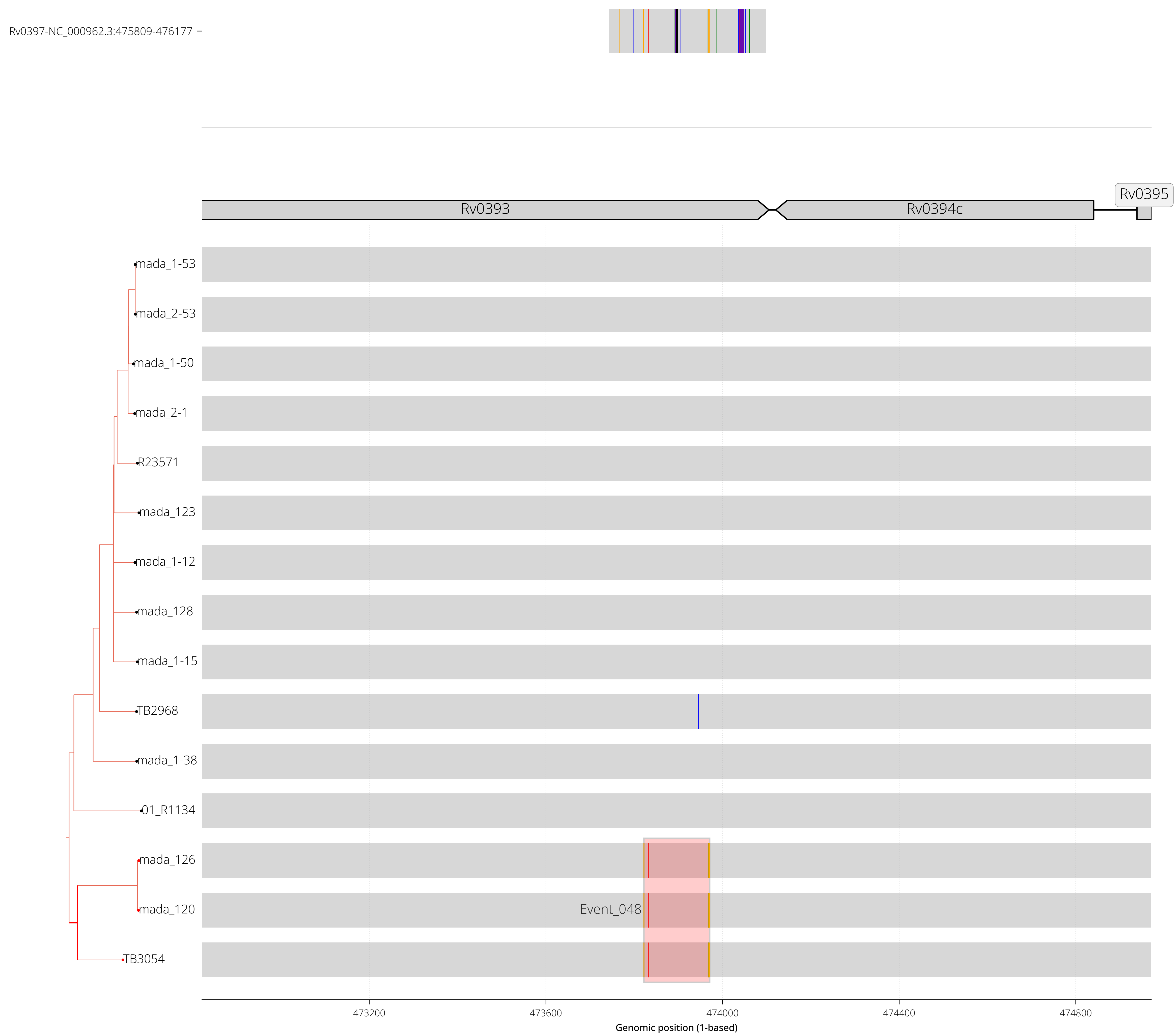

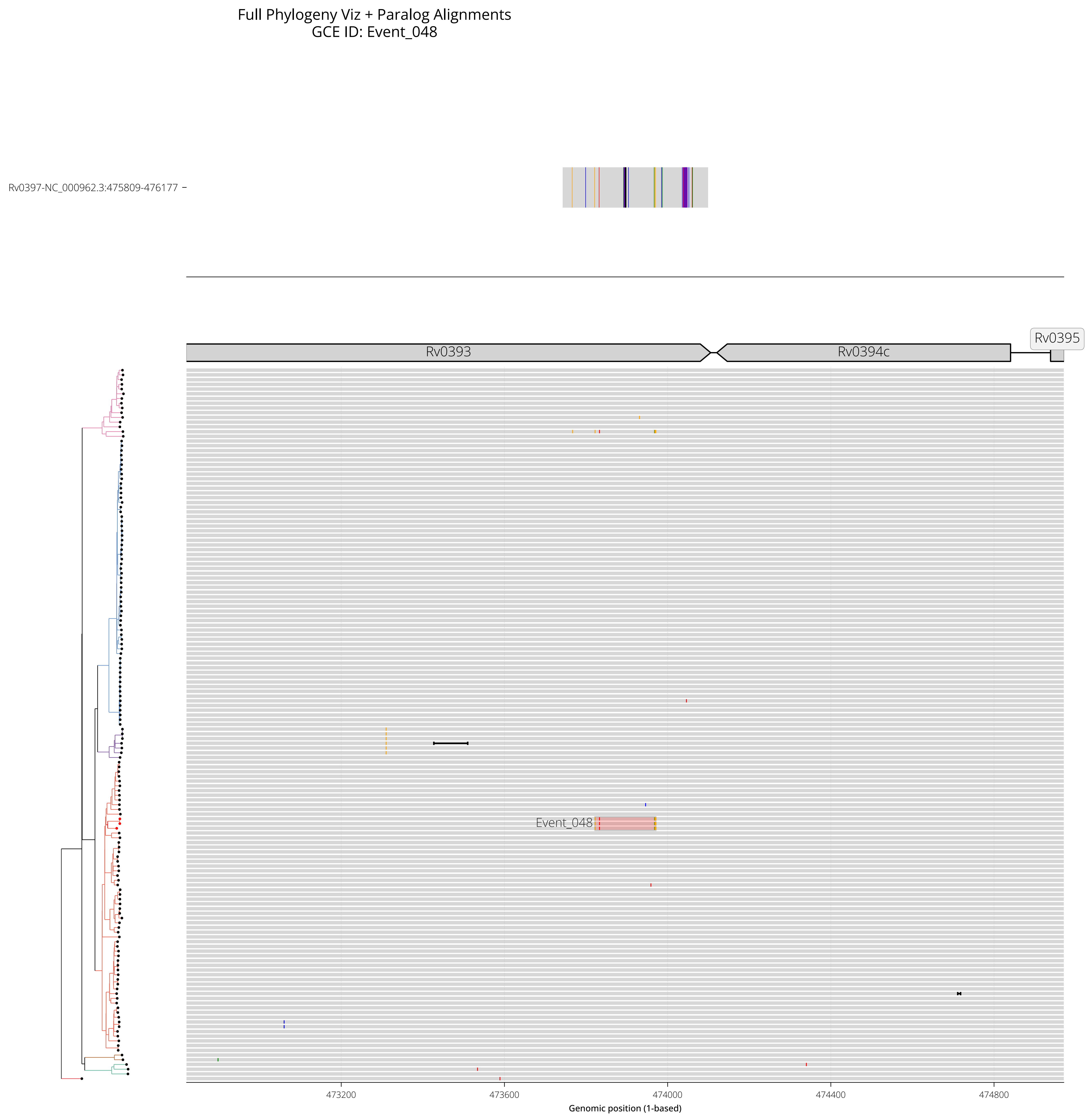

Full Phylogeny Viz + Paralog Alignments
GCE ID: Event_048
Rv0397-NC_000962.3:475809-476177
Rv0393
Rv0394c
Rv0395
Event_048
473200
473600
474000
474400
474800
Genomic position (1-based)

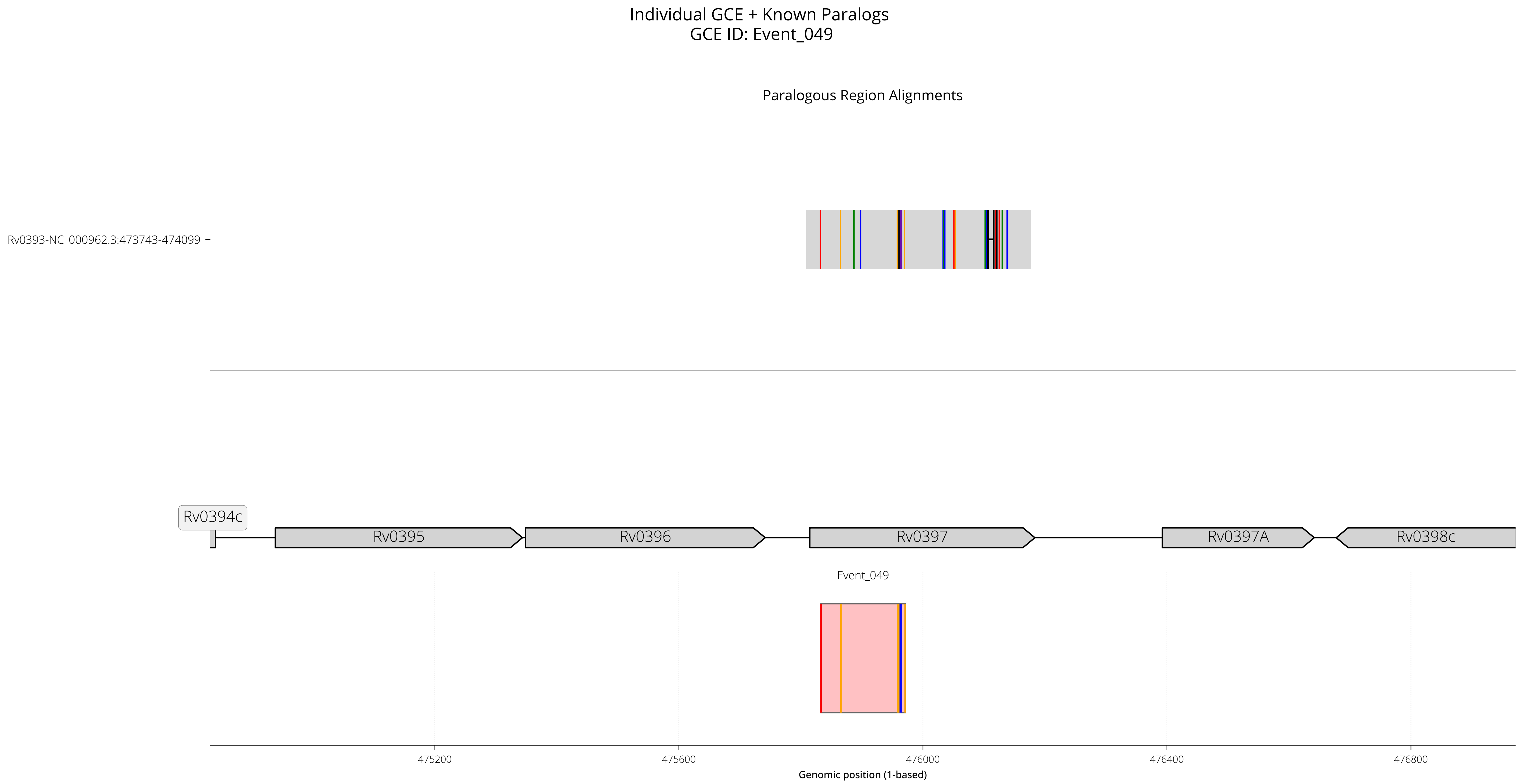

Individual GCE + Known Paralogs
GCE ID: Event_049
Paralogous Region Alignments
Rv0393-NC_000962.3:473743-474099
Rv0394c
Rv0395
Rv0396
Rv0397
Rv0397A
Rv0398c
Event_049
475200
475600
476000
476400
476800
Genomic position (1-based)

Circos Viz - GCE to Known Paralogs Locations
GCE ID: Event_049

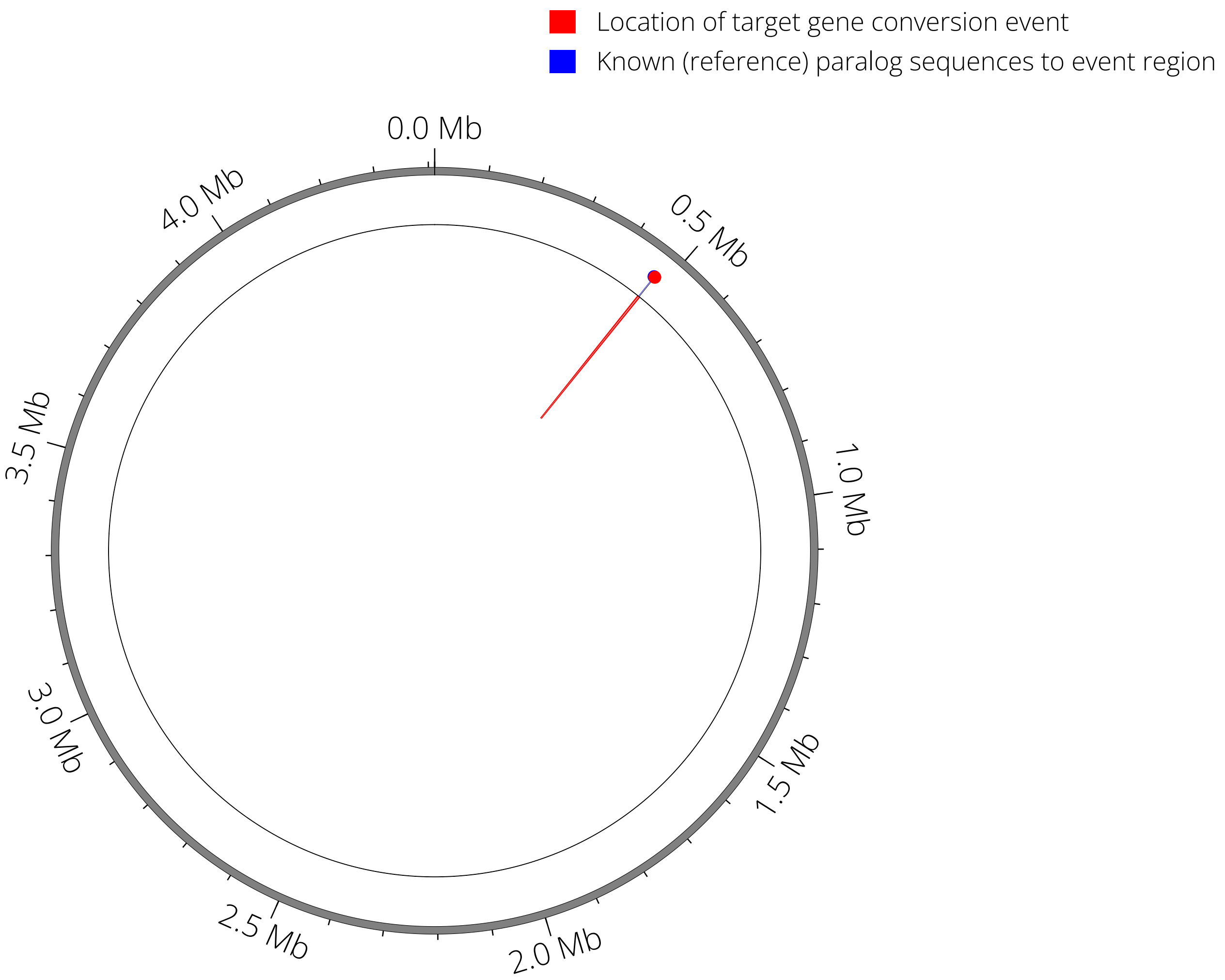

# Event_049 - 7 SNPs - k-mer analysis relative to paralogs

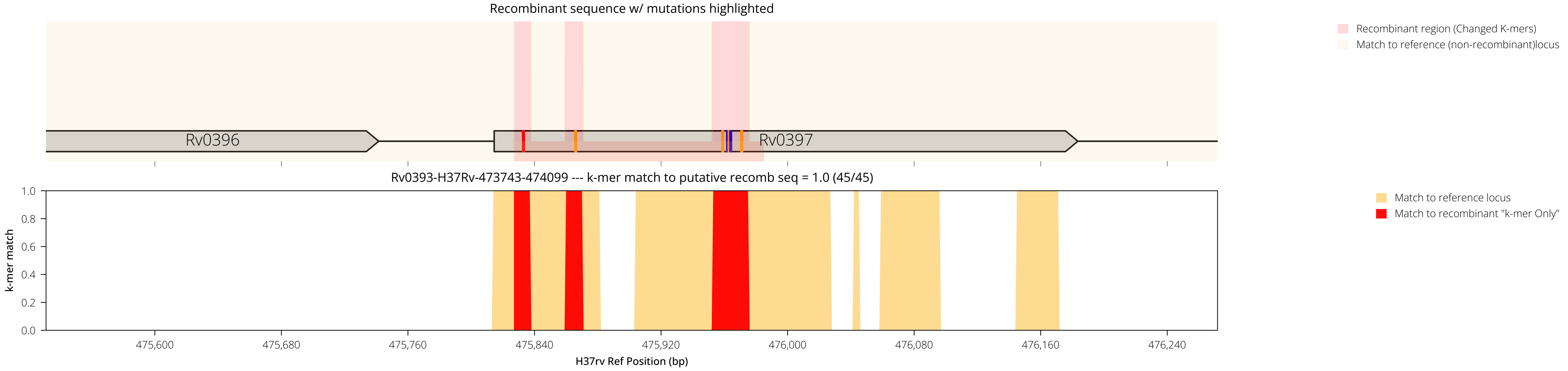

Sub-phylogeny Viz + Paralog Alignments
GCE ID: Event_049

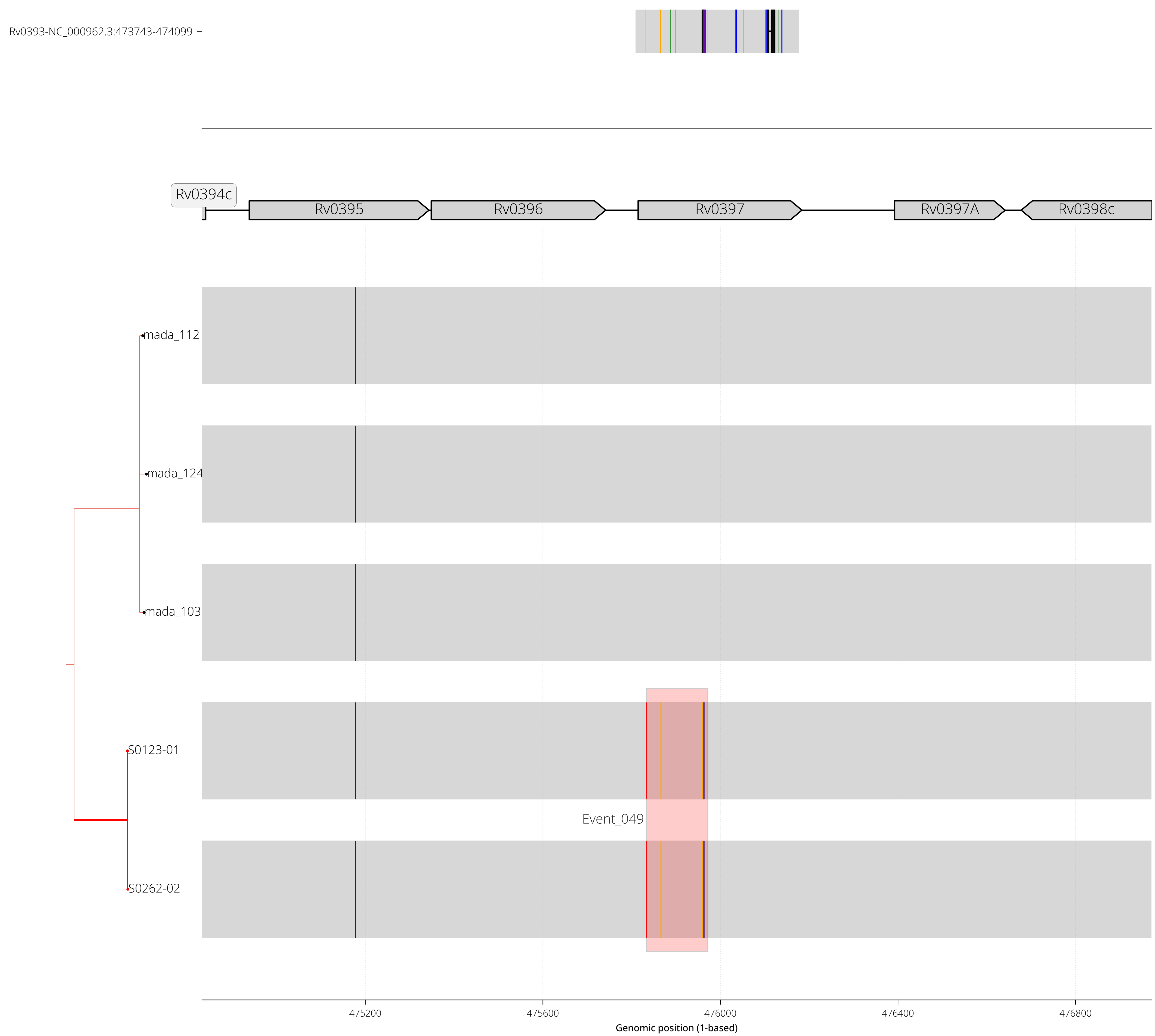

Full Phylogeny Viz + Paralog Alignments
GCE ID: Event_049

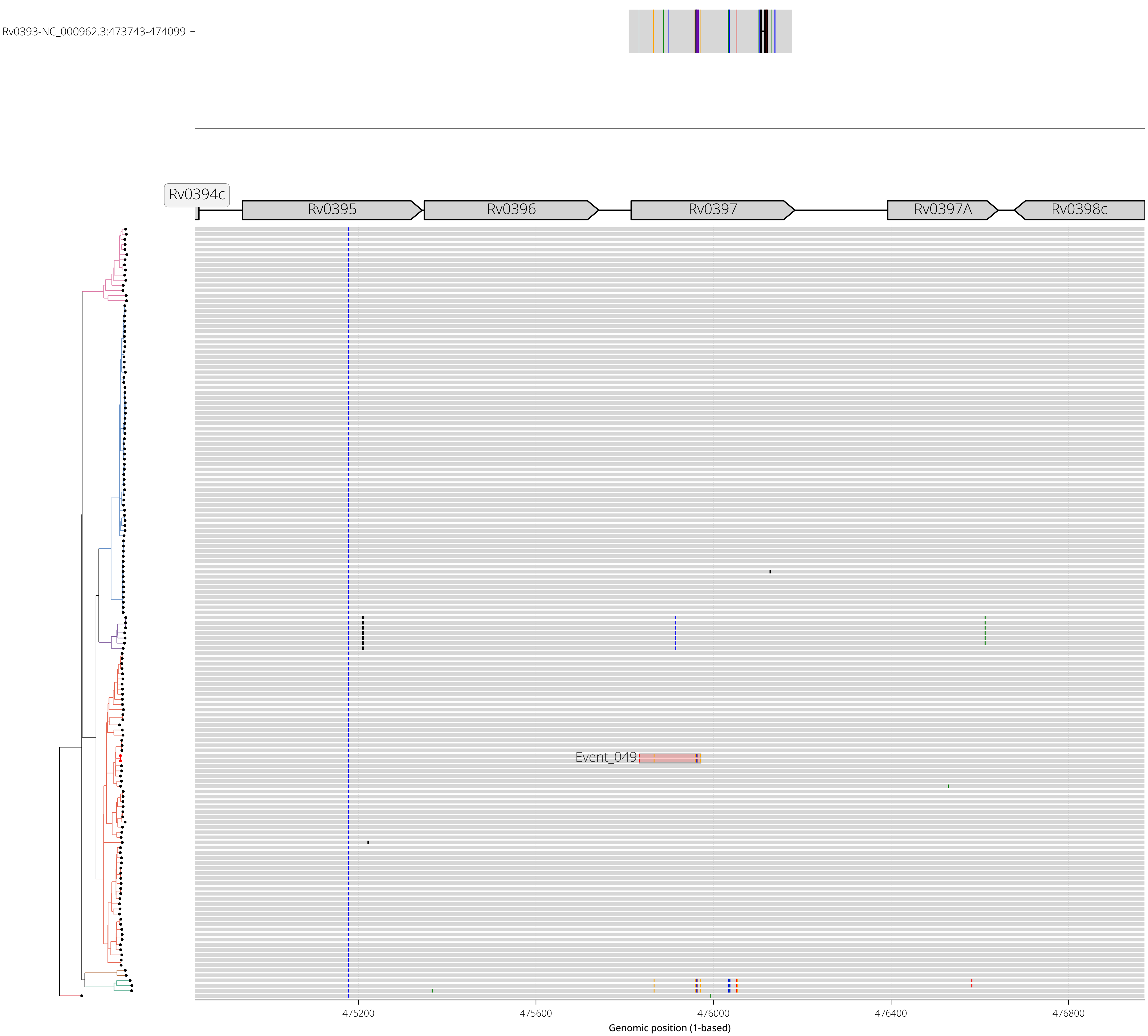

Individual GCE + Known Paralogs
GCE ID: Event_050

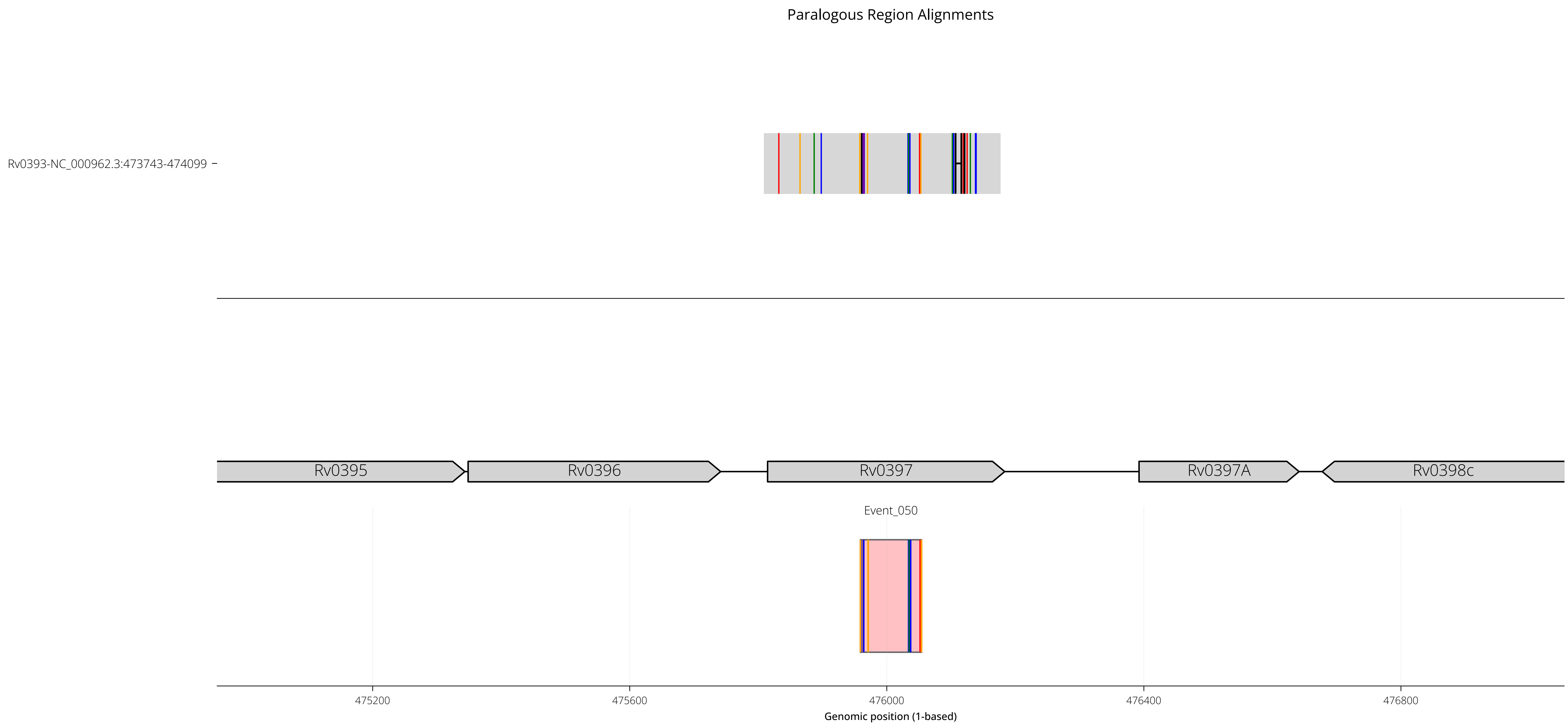

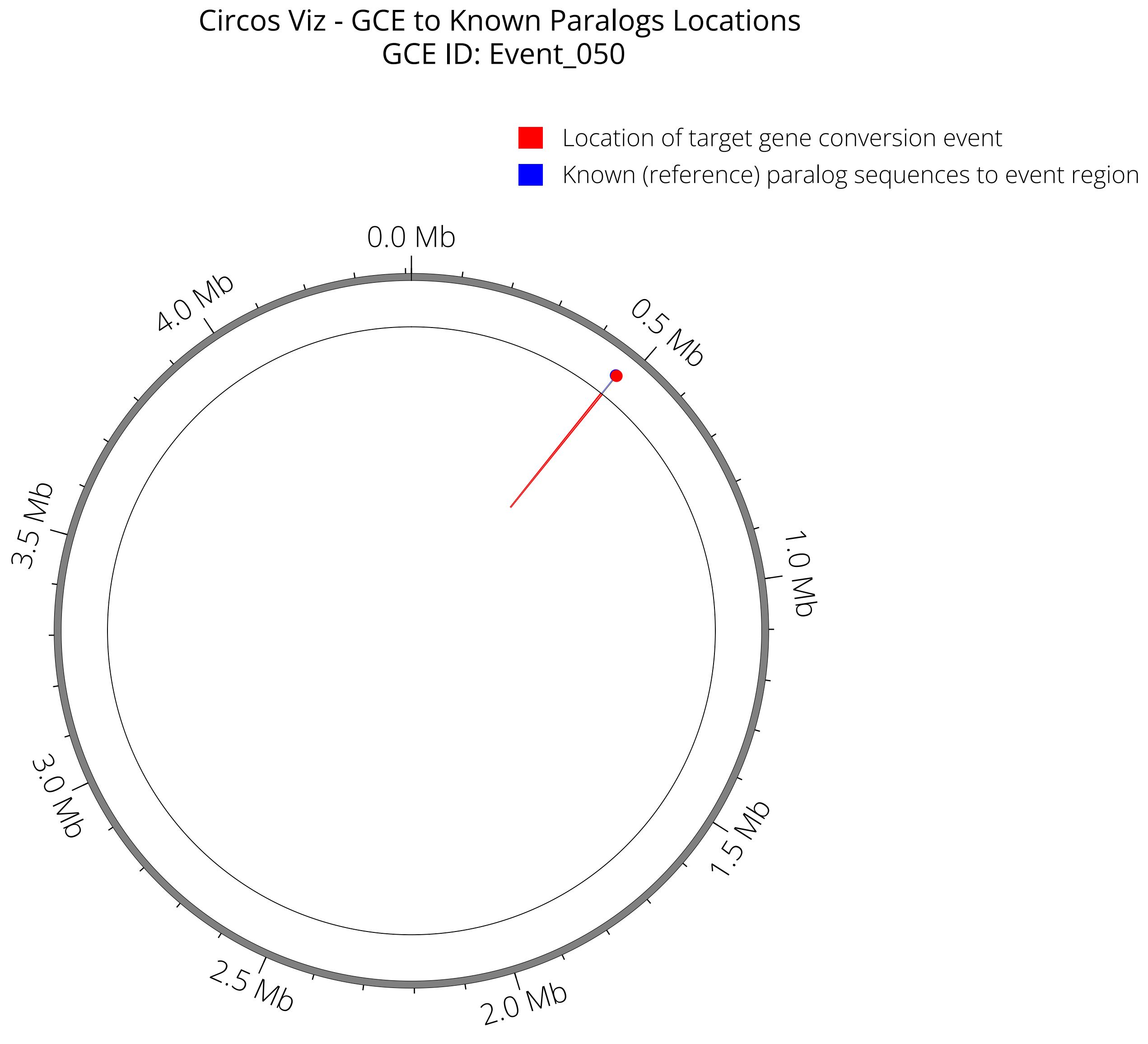
Circos Viz - GCE to Known Paralogs Locations
GCE ID: Event_050
Location of target gene conversion event
Known (reference) paralog sequences to event region
0.0 Mb
0.5 Mb
1.0 Mb
1.5 Mb
2.0 Mb
2.5 Mb
3.0 Mb
3.5 Mb
4.0 Mb

# Event_050 - 10 SNPs - k-mer analysis relative to paralogs

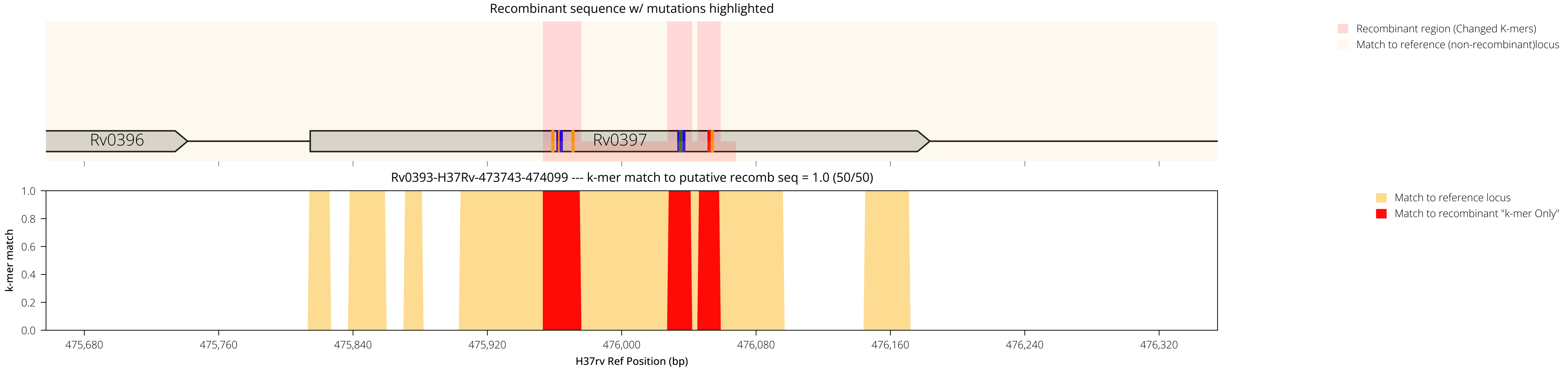

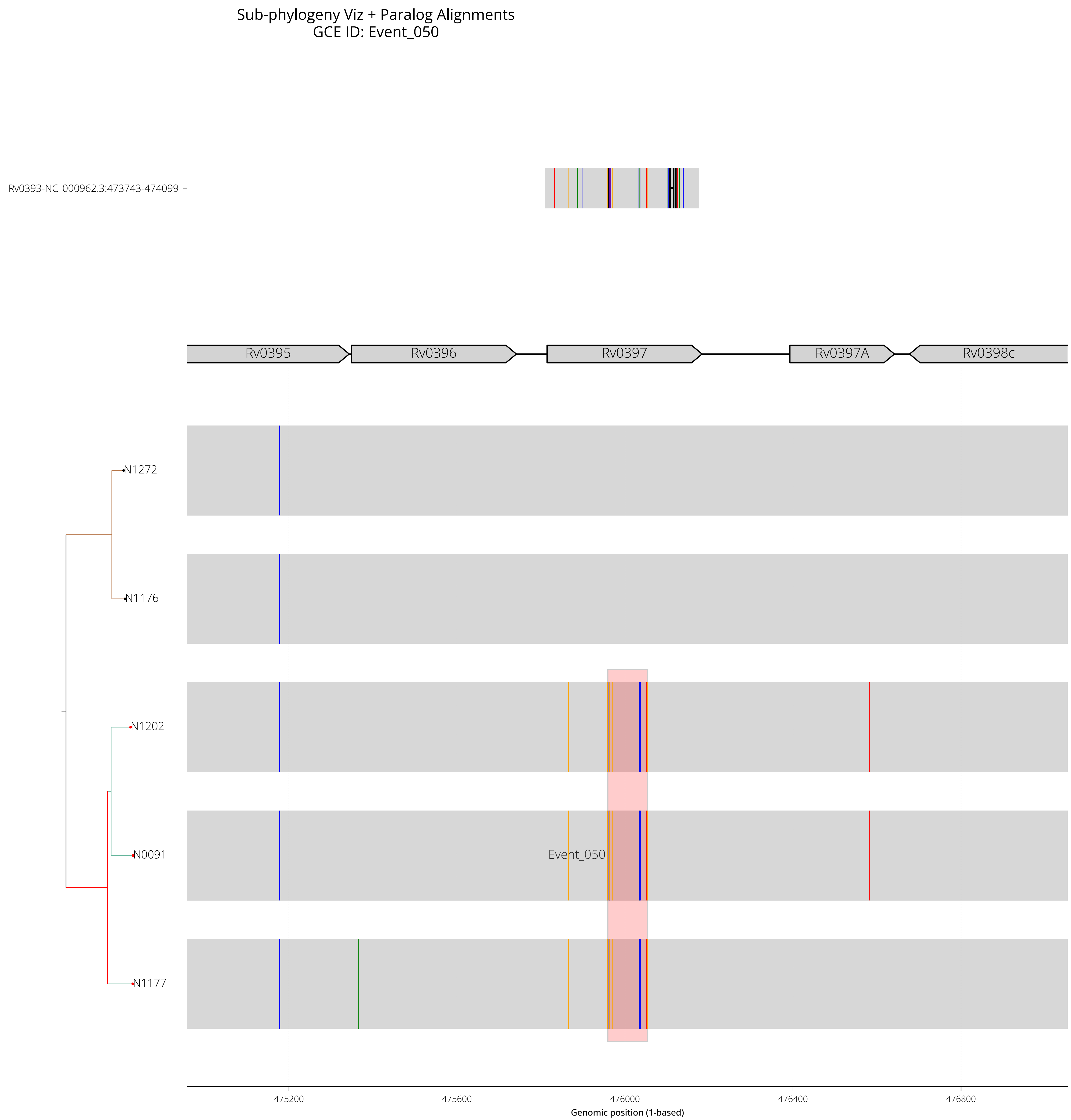

Sub-phylogeny Viz + Paralog Alignments
GCE ID: Event_050
Rv0393-NC_000962.3:473743-474099
Rv0395
Rv0396
Rv0397
Rv0397A
Rv0398c
N1272
N1176
N1202
N0091
N1177
Event_050
475200
475600
476000
476400
476800
Genomic position (1-based)

Full Phylogeny Viz + Paralog Alignments
GCE ID: Event_050

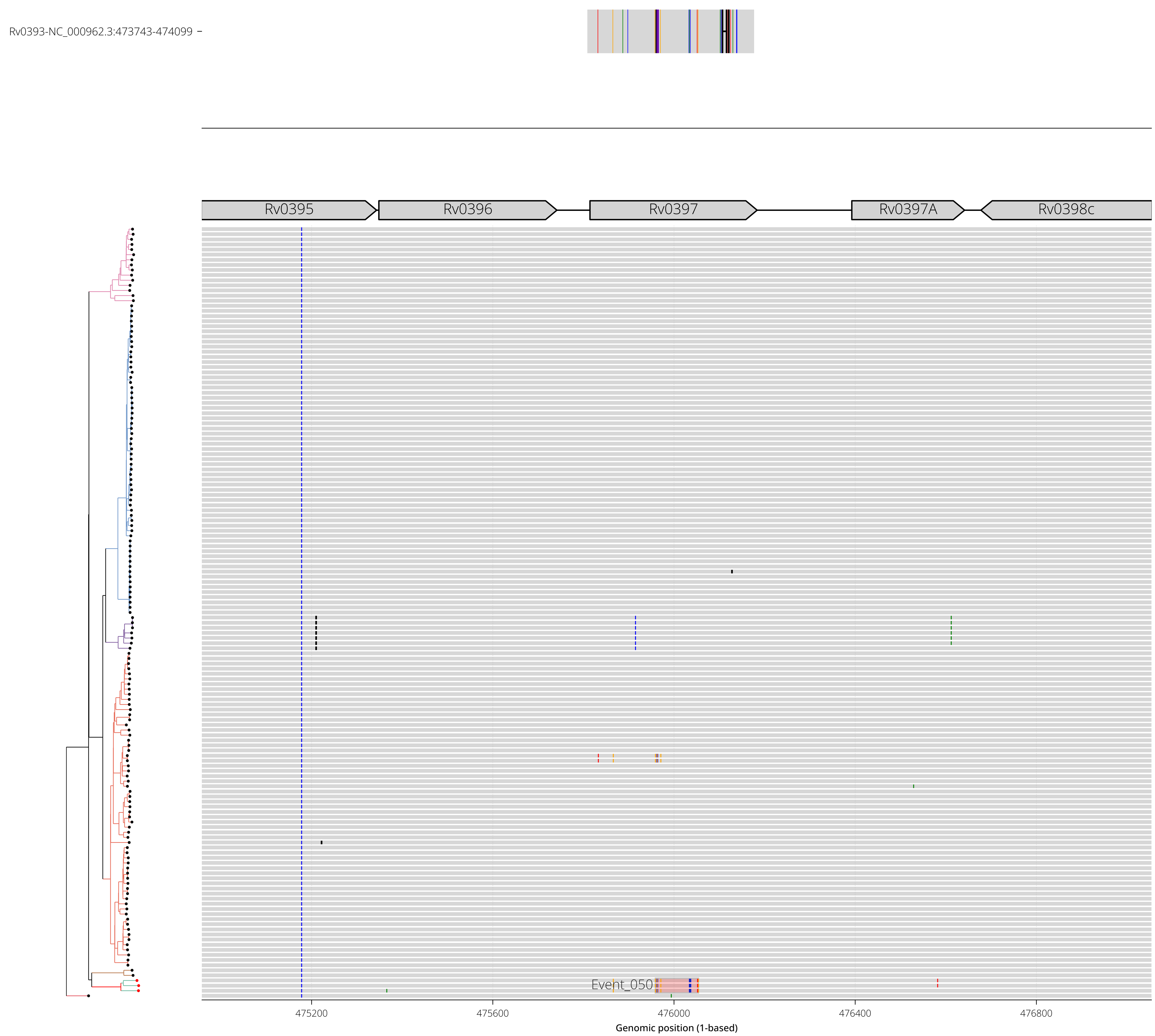

Individual GCE + Known Paralogs
GCE ID: Event_051

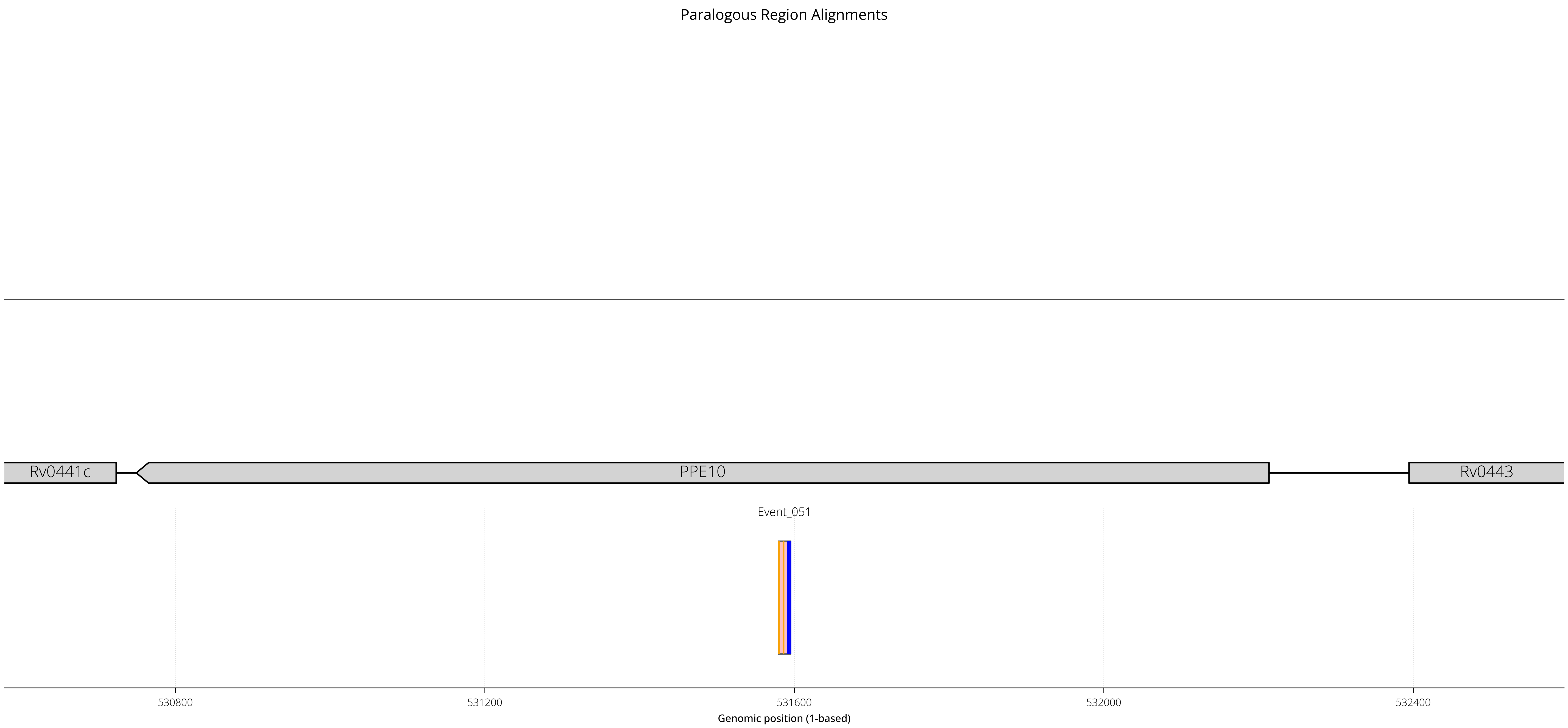

Sub-phylogeny Viz + Paralog Alignments
GCE ID: Event_051

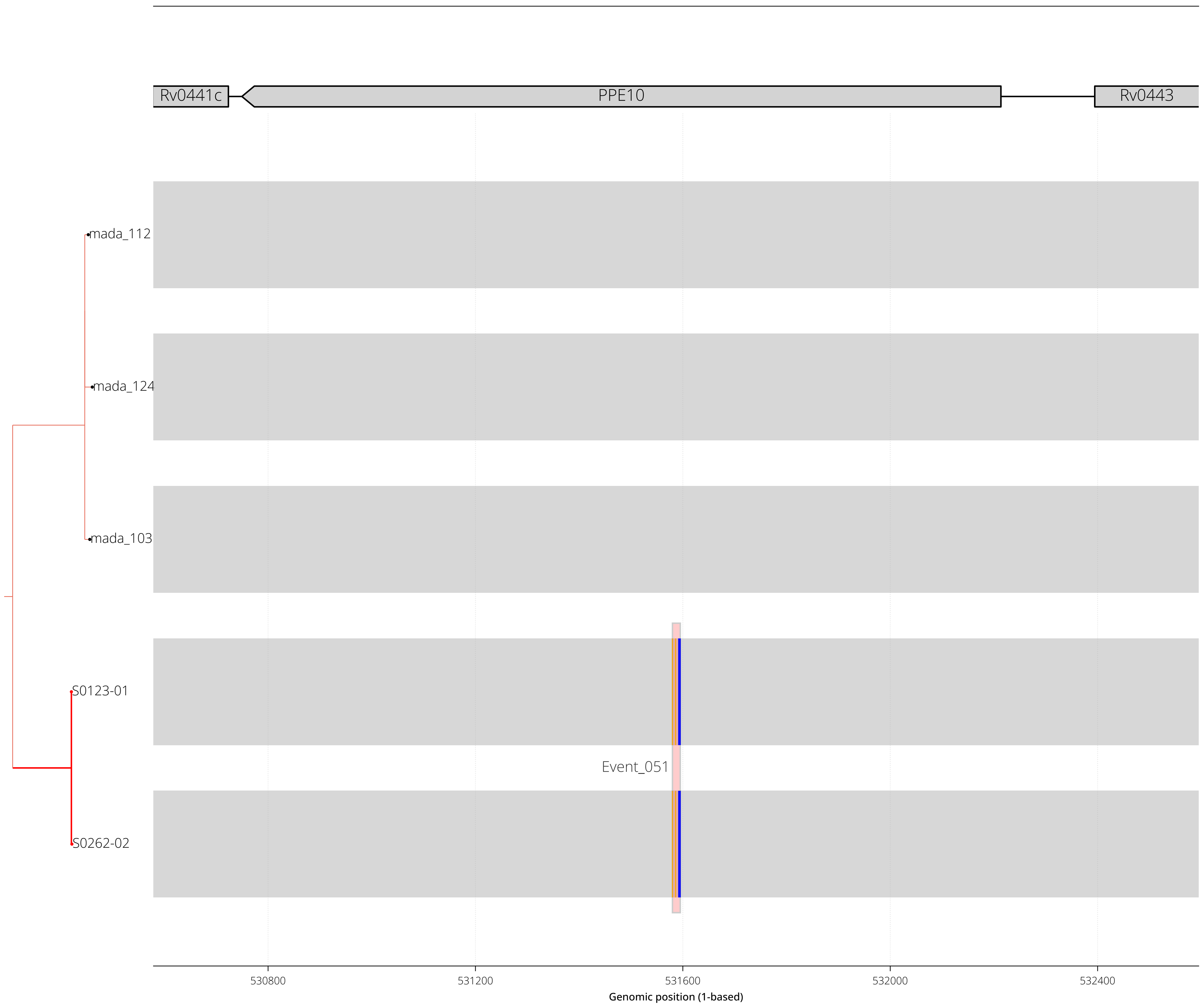

Full Phylogeny Viz + Paralog Alignments
GCE ID: Event_051

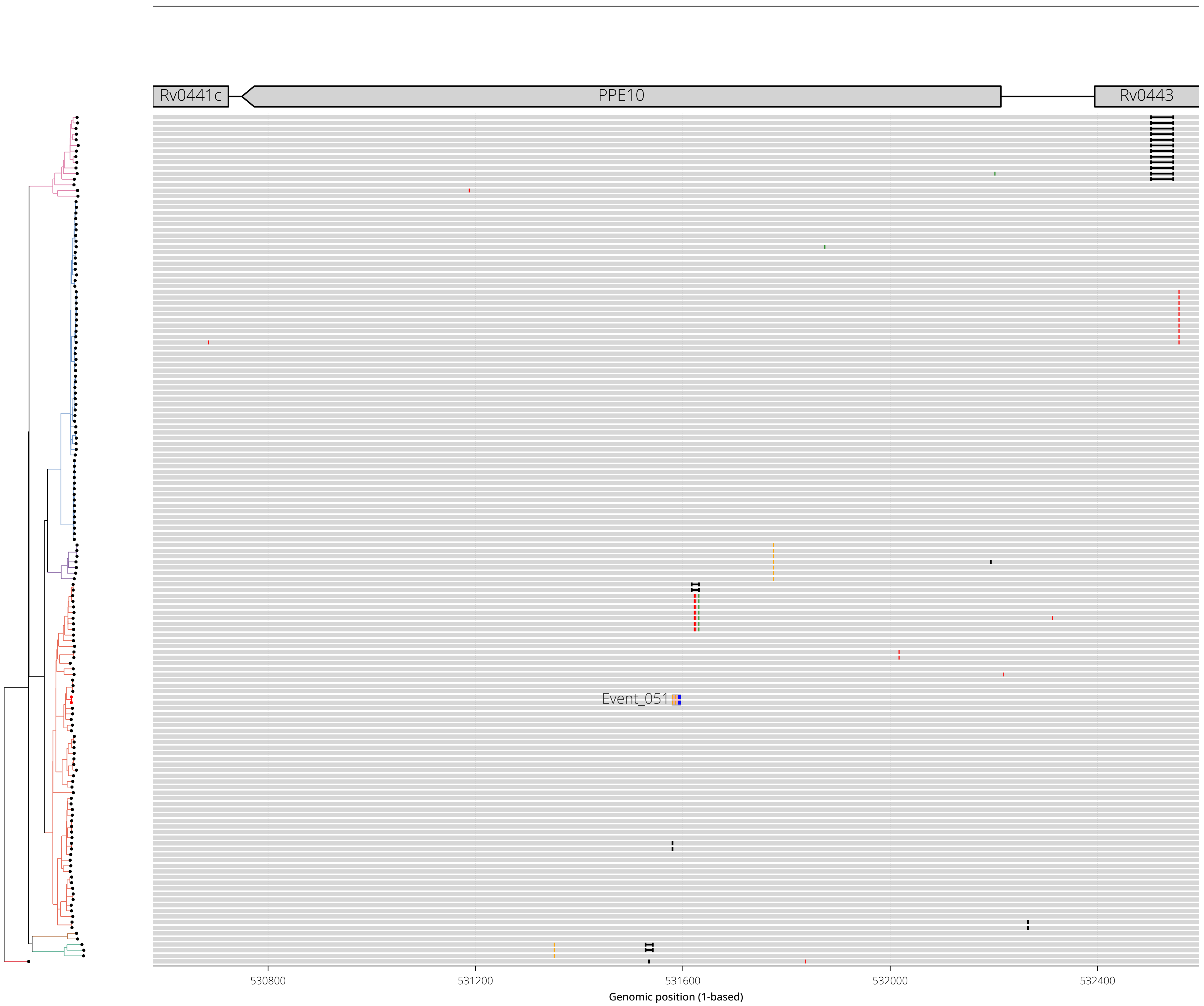

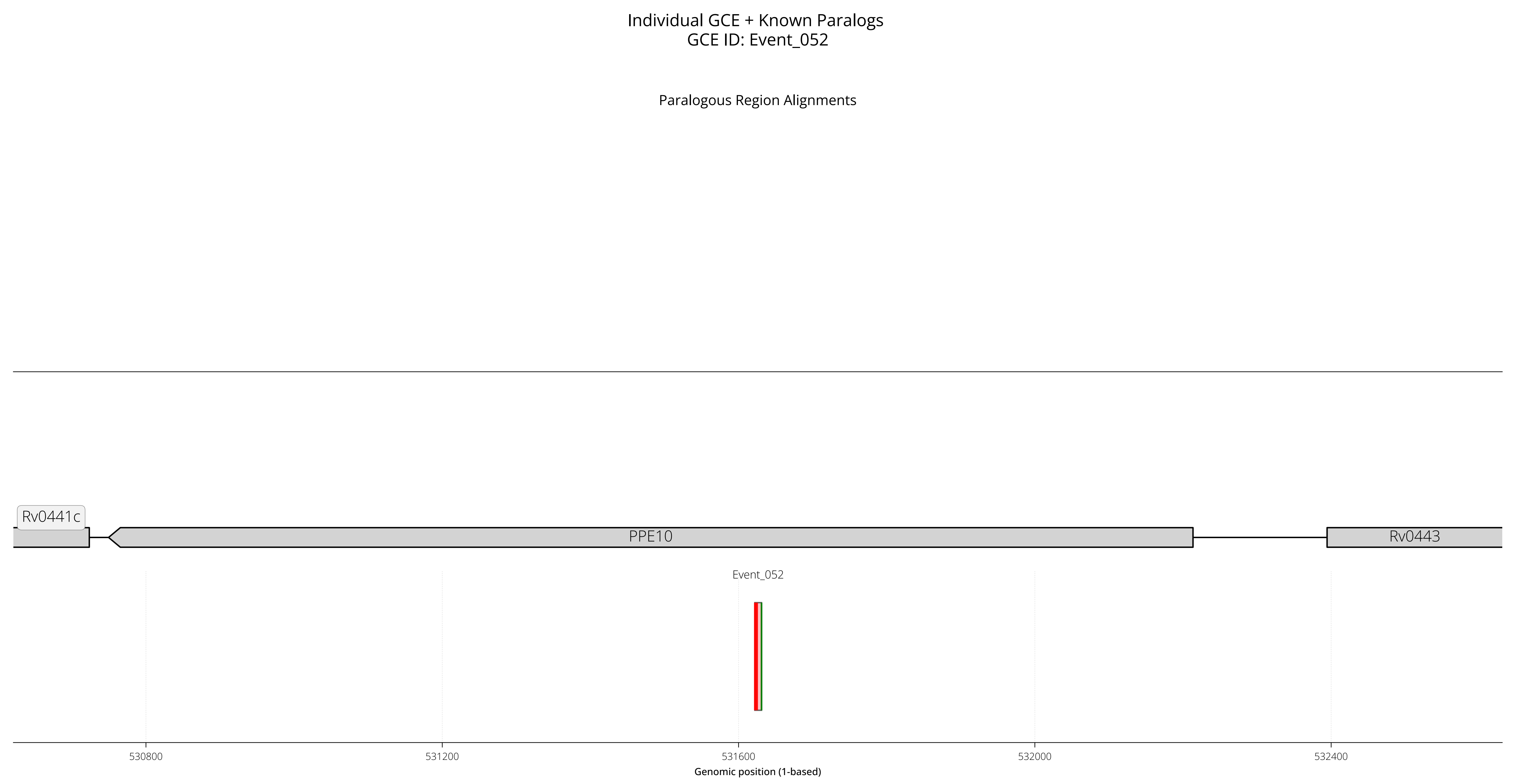

Individual GCE + Known Paralogs
GCE ID: Event_052
Paralogous Region Alignments
Rv0441c
PPE10
Rv0443
Event_052
530800
531200
531600
532000
532400
Genomic position (1-based)

Sub-phylogeny Viz + Paralog Alignments
GCE ID: Event_052

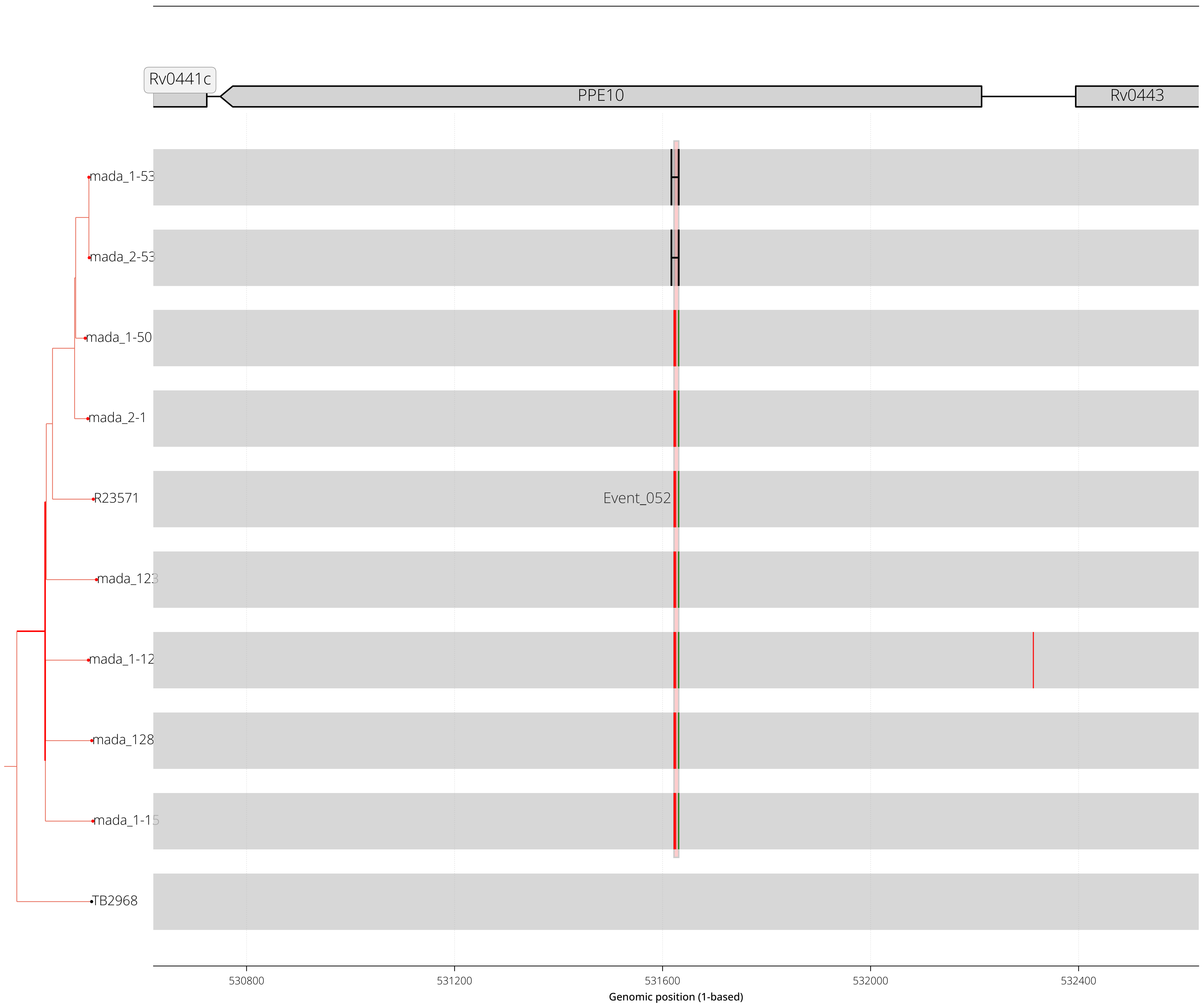

Full Phylogeny Viz + Paralog Alignments
GCE ID: Event_052

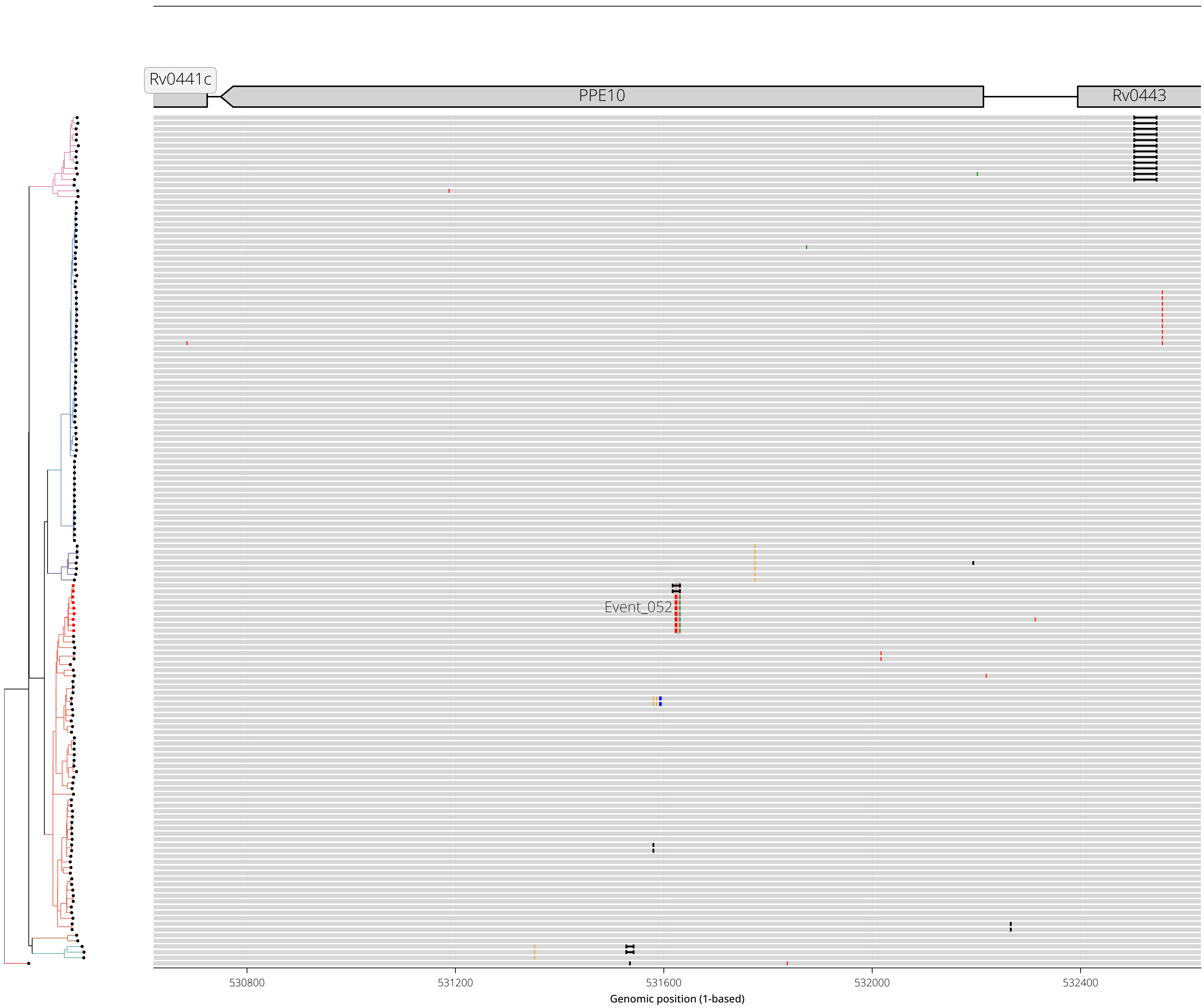

Individual GCE + Known Paralogs
GCE ID: Event_053

Paralogous Region Alignments

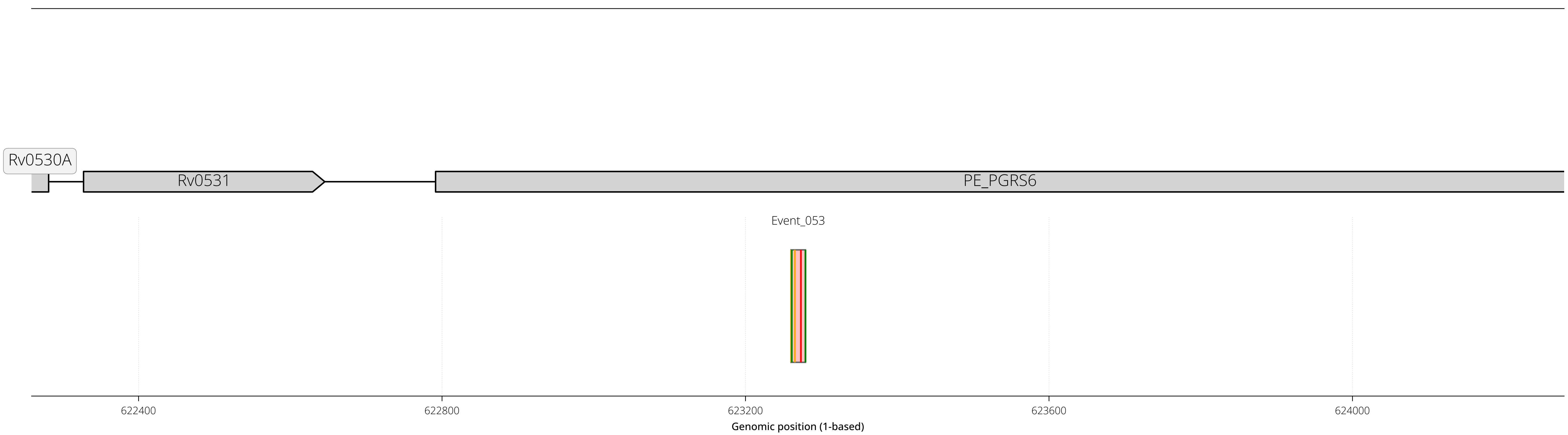

Sub-phylogeny Viz + Paralog Alignments
GCE ID: Event_053

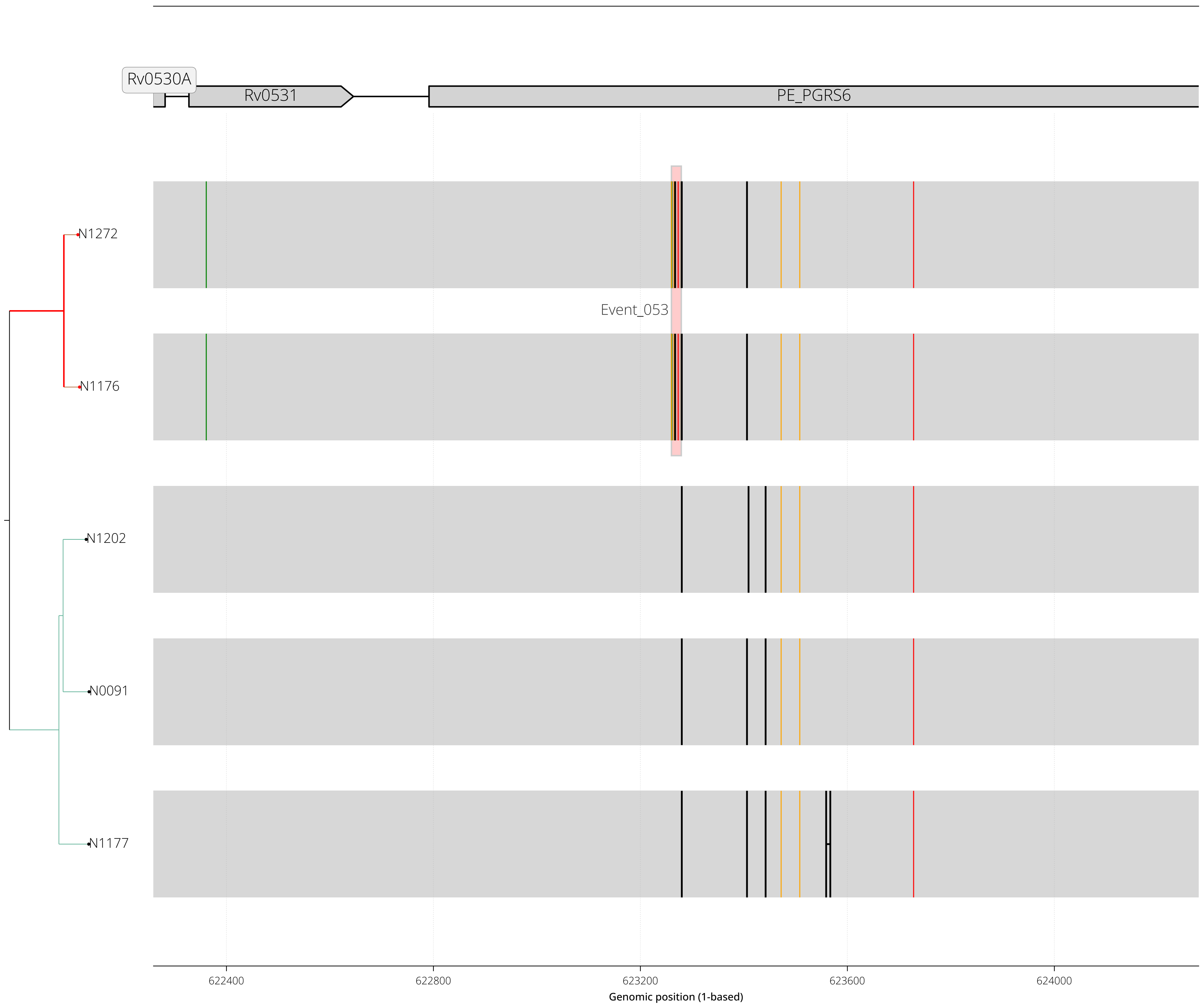

Full Phylogeny Viz + Paralog Alignments
GCE ID: Event_053

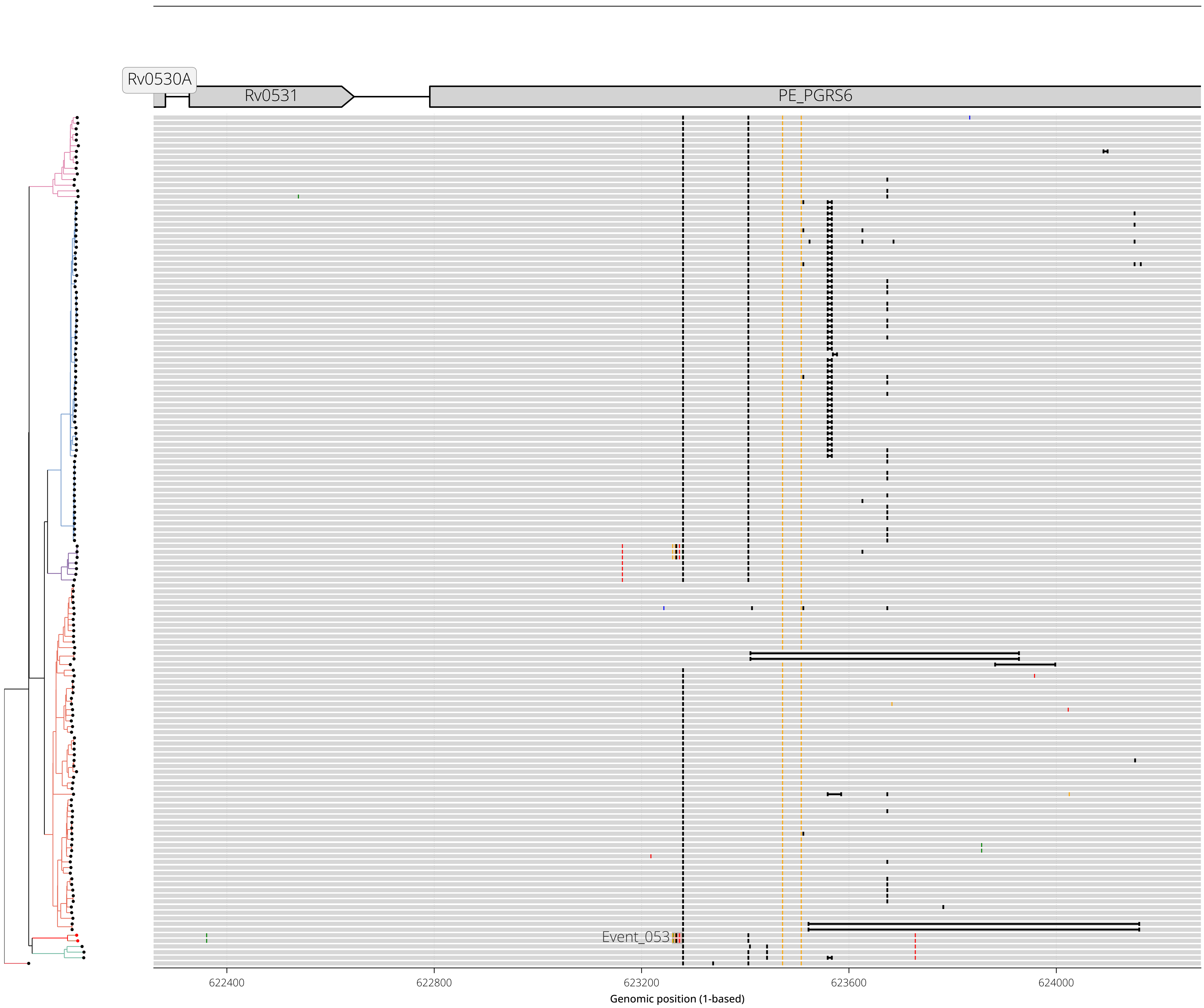

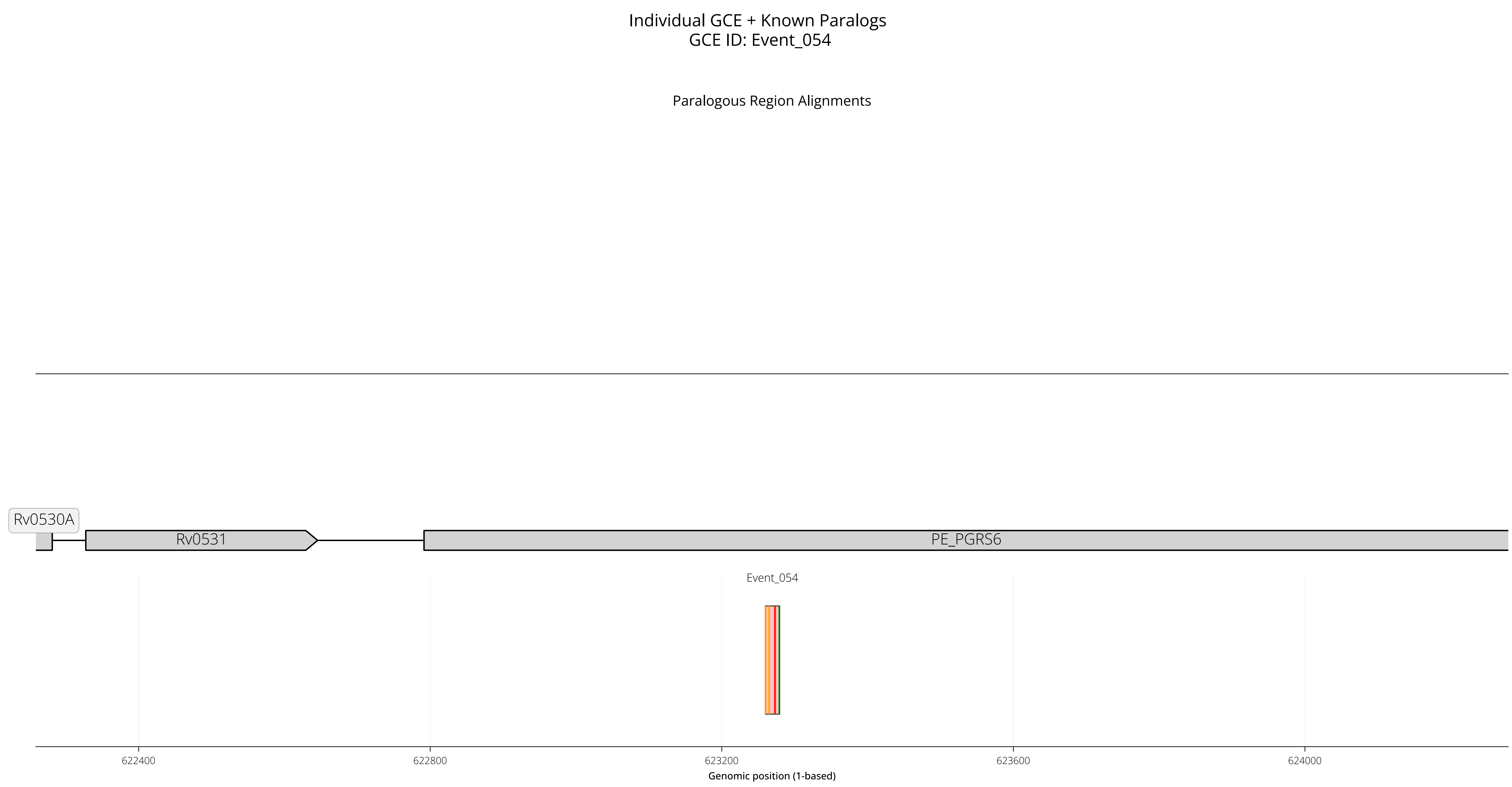
Individual GCE + Known Paralogs
GCE ID: Event_054
Paralogous Region Alignments
Rv0530A
Rv0531
PE_PGRS6
Event_054
622400
622800
623200
623600
624000
Genomic position (1-based)

Sub-phylogeny Viz + Paralog Alignments
GCE ID: Event_054

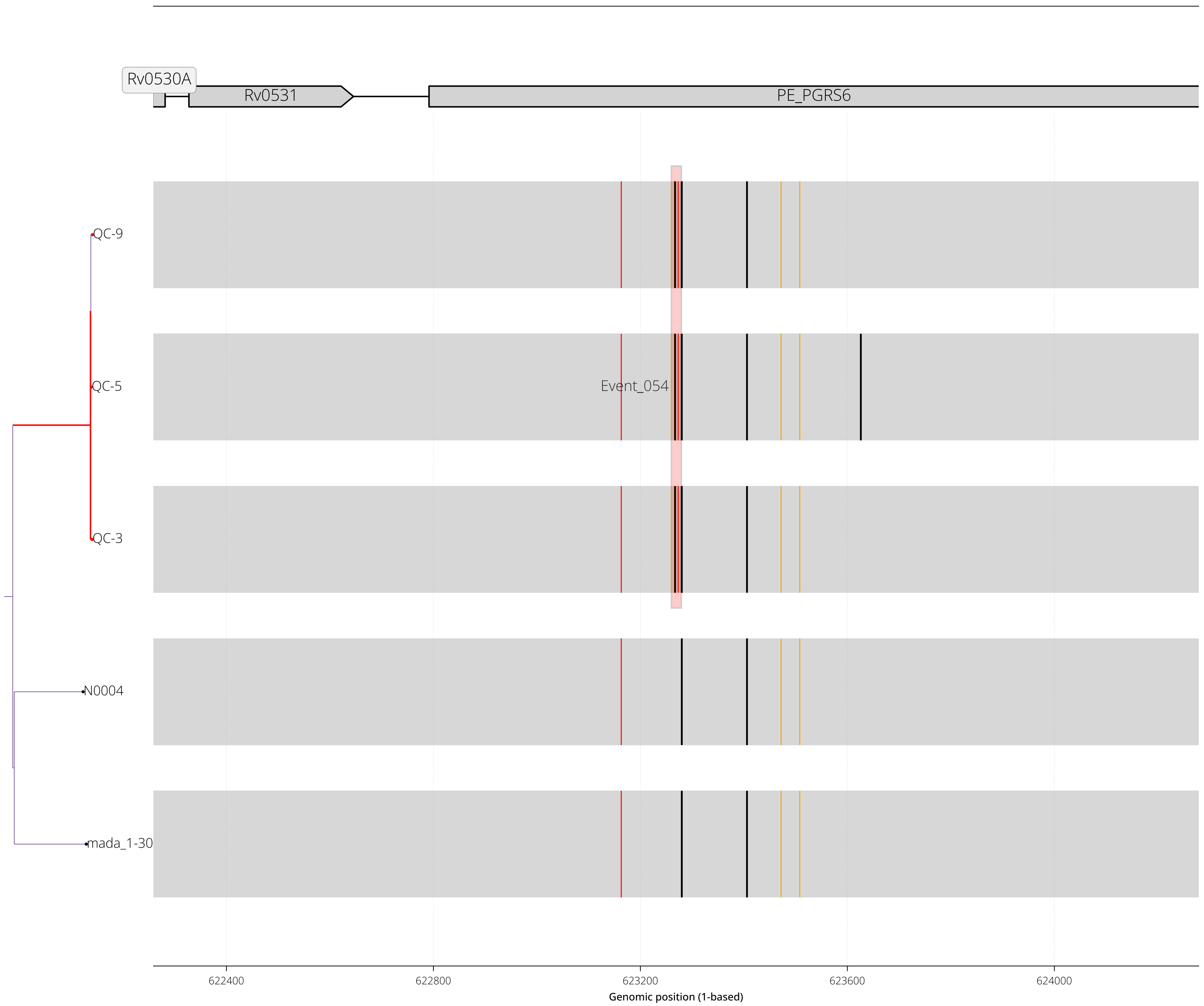

Full Phylogeny Viz + Paralog Alignments
GCE ID: Event_054

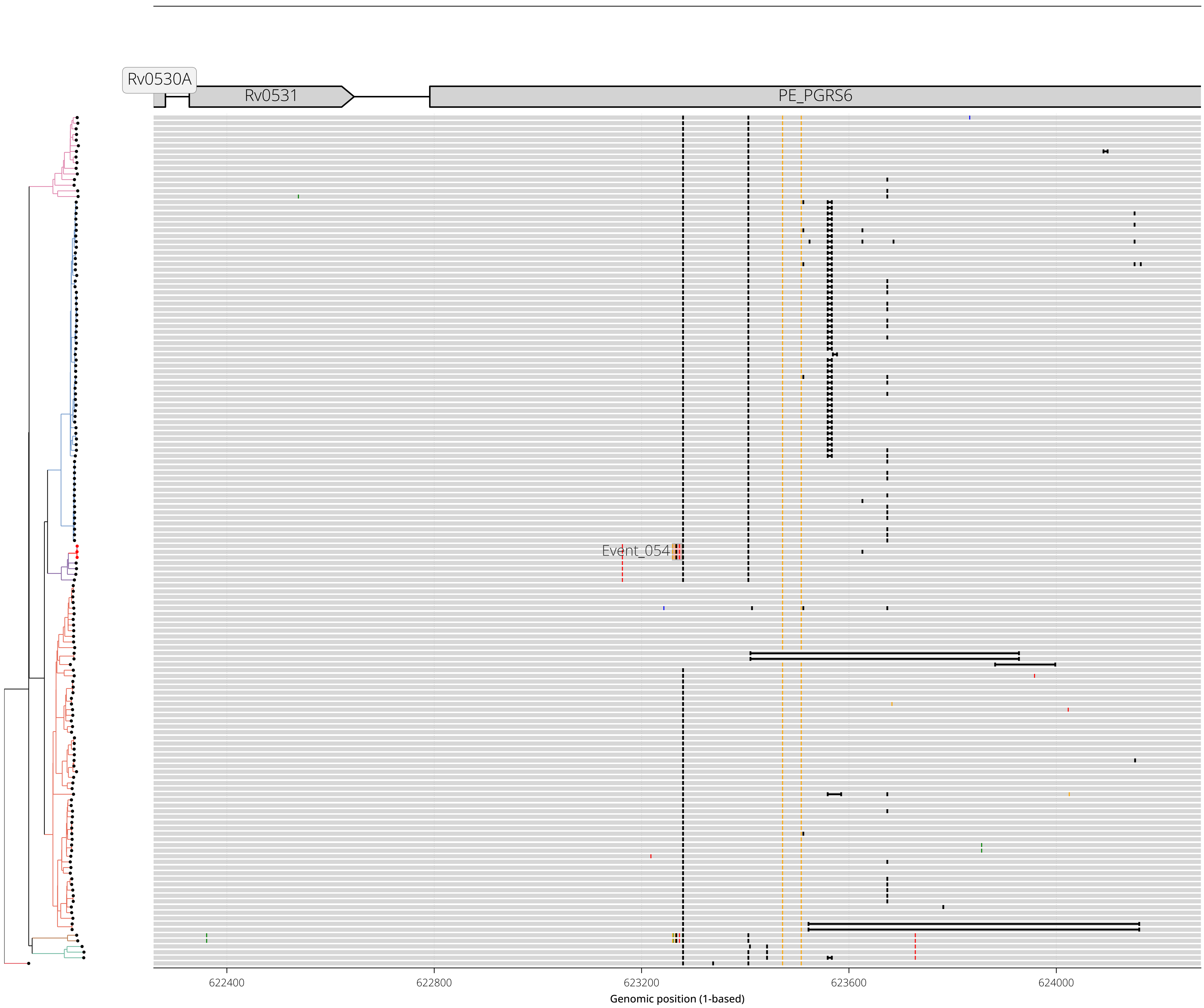

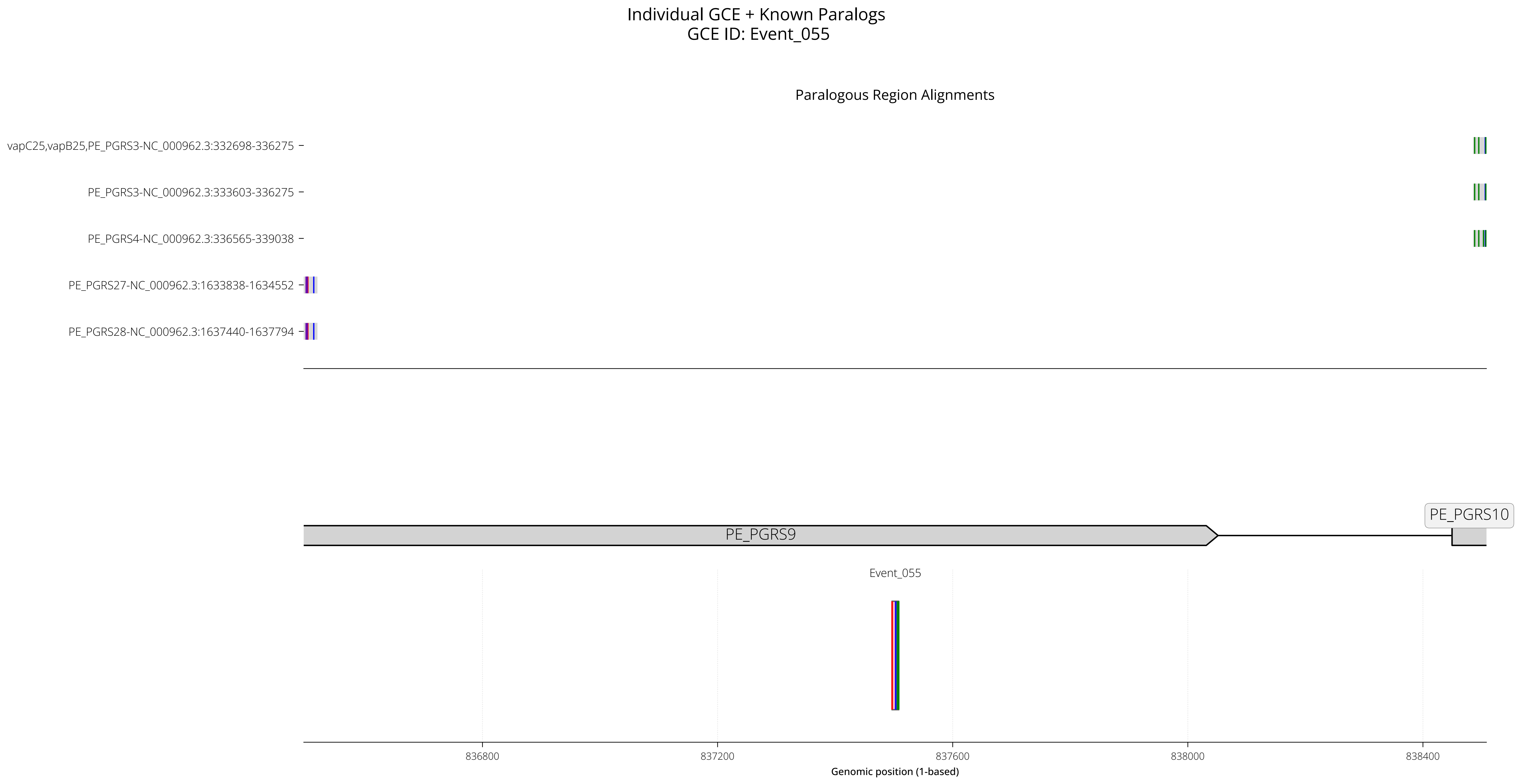

Individual GCE + Known Paralogs
GCE ID: Event_055
Paralogous Region Alignments
vapC25,vapB25,PE_PGRS3-NC_000962.3:332698-336275
PE_PGRS3-NC_000962.3:333603-336275
PE_PGRS4-NC_000962.3:336565-339038
PE_PGRS27-NC_000962.3:1633838-1634552
PE_PGRS28-NC_000962.3:1637440-1637794
PE_PGRS9
PE_PGRS10
Event_055
836800
837200
837600
838000
838400
Genomic position (1-based)

Sub-phylogeny Viz + Paralog Alignments
GCE ID: Event_055

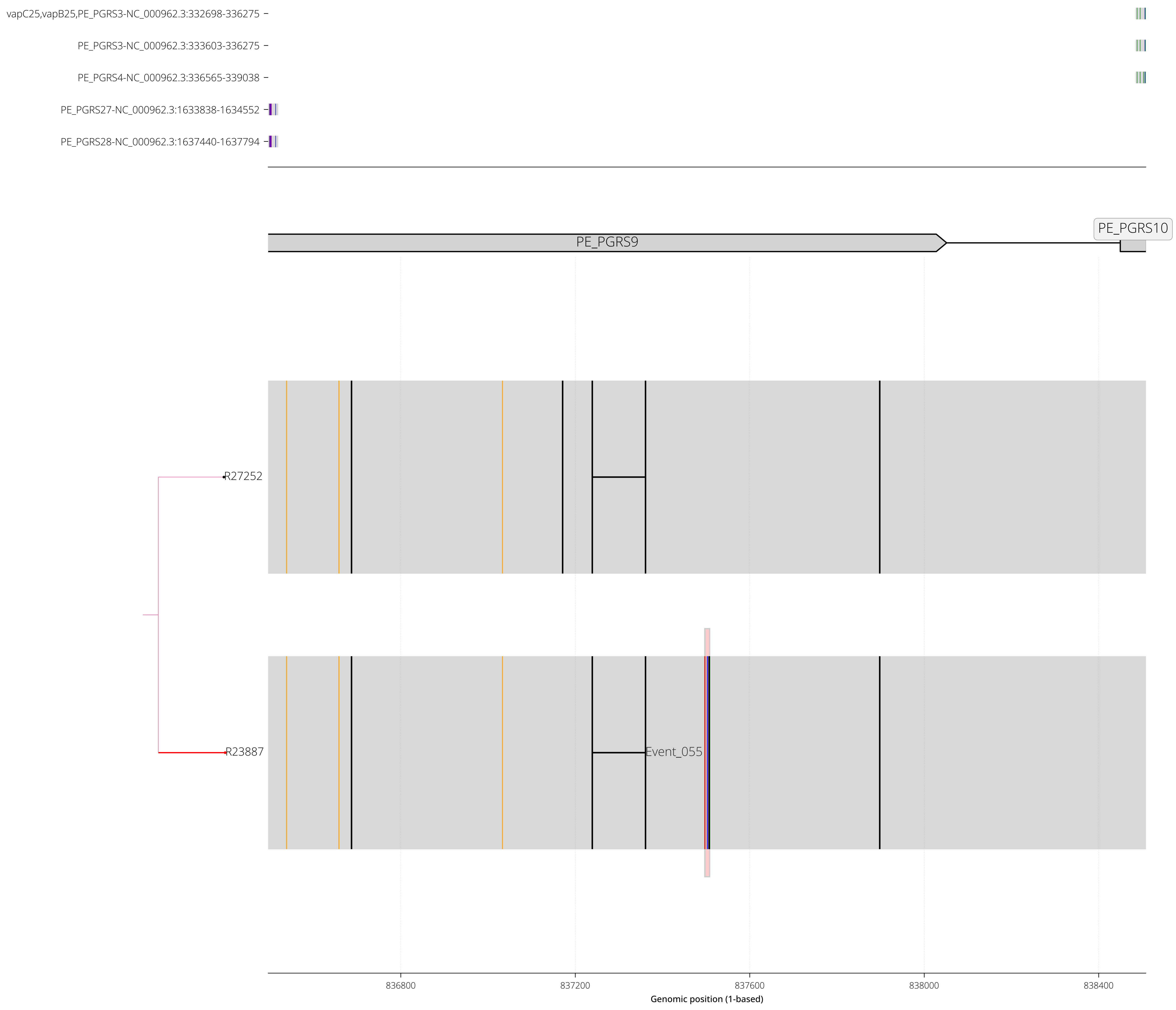

Full Phylogeny Viz + Paralog Alignments
GCE ID: Event_055

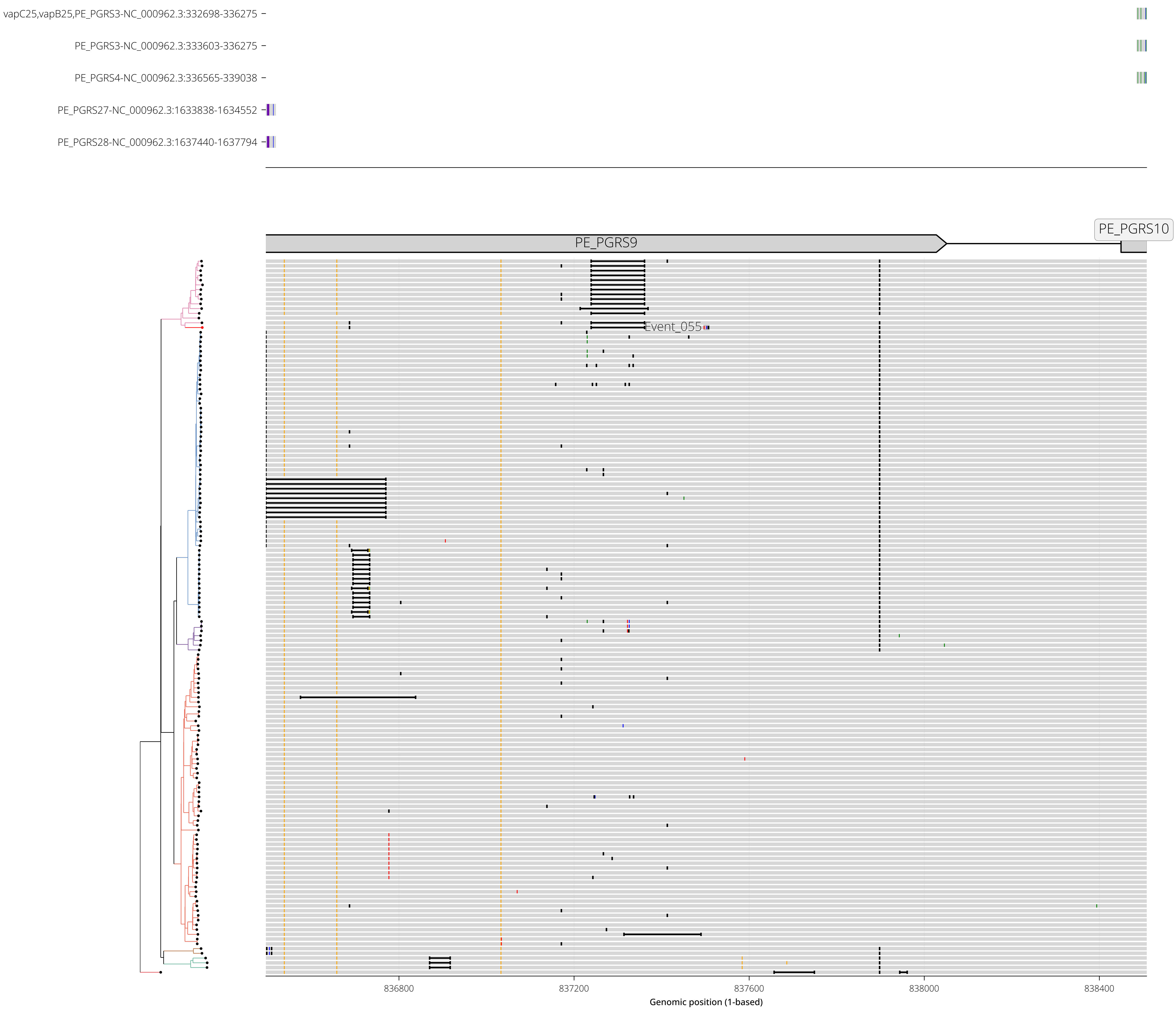

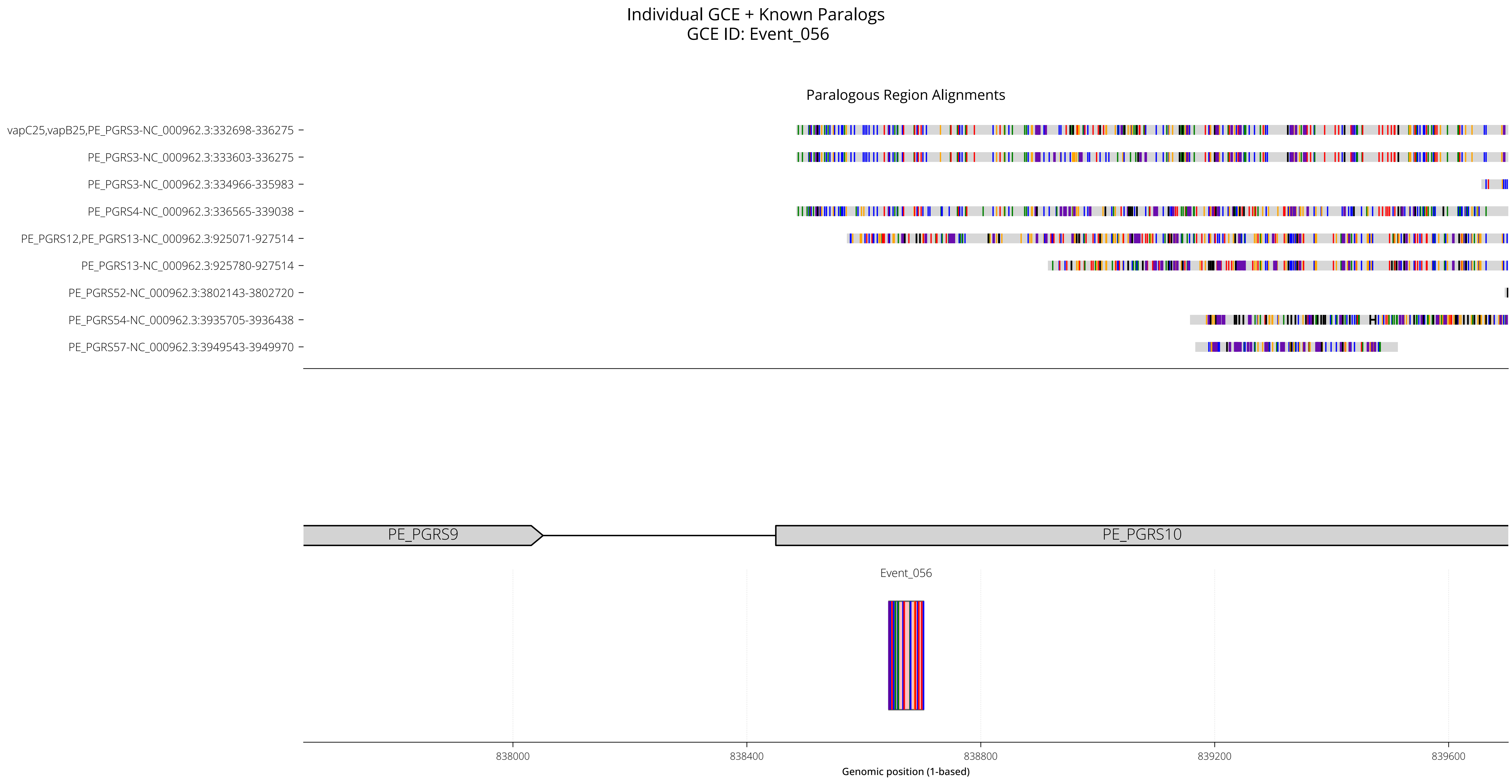

Individual GCE + Known Paralogs
GCE ID: Event_056
Paralogous Region Alignments
vapC25,vapB25,PE_PGRS3-NC_000962.3:332698-336275
PE_PGRS3-NC_000962.3:333603-336275
PE_PGRS3-NC_000962.3:334966-335983
PE_PGRS4-NC_000962.3:336565-339038
PE_PGRS12,PE_PGRS13-NC_000962.3:925071-927514
PE_PGRS13-NC_000962.3:925780-927514
PE_PGRS52-NC_000962.3:3802143-3802720
PE_PGRS54-NC_000962.3:3935705-3936438
PE_PGRS57-NC_000962.3:3949543-3949970
PE_PGRS9
PE_PGRS10
Event_056
838000
838400
838800
839200
839600
Genomic position (1-based)

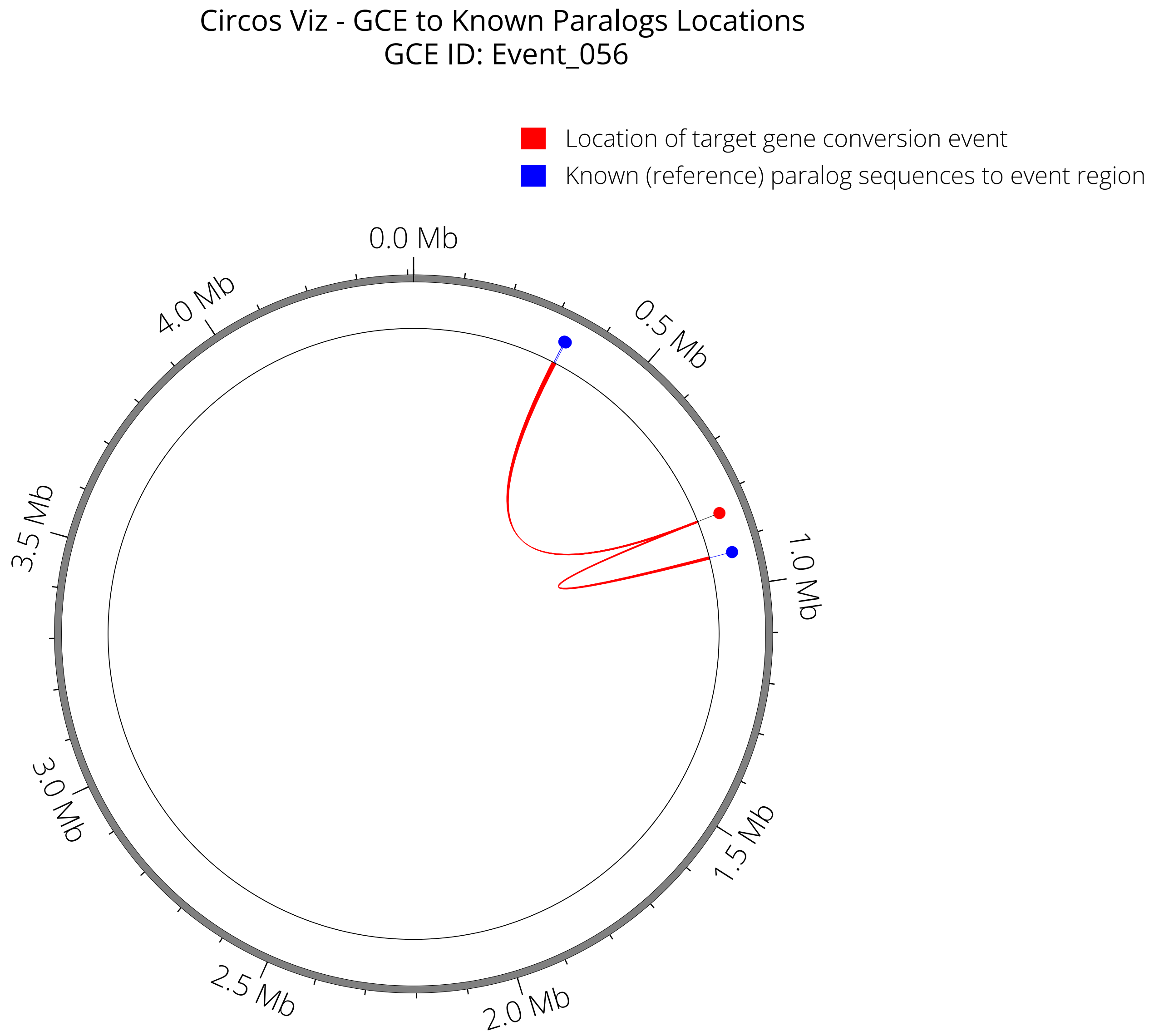

Circos Viz - GCE to Known Paralogs Locations
GCE ID: Event_056
Location of target gene conversion event
Known (reference) paralog sequences to event region
0.0 Mb
0.5 Mb
1.0 Mb
1.5 Mb
2.0 Mb
2.5 Mb
3.0 Mb
3.5 Mb
4.0 Mb

# Event_056 - 18 SNPs - k-mer analysis relative to paralogs

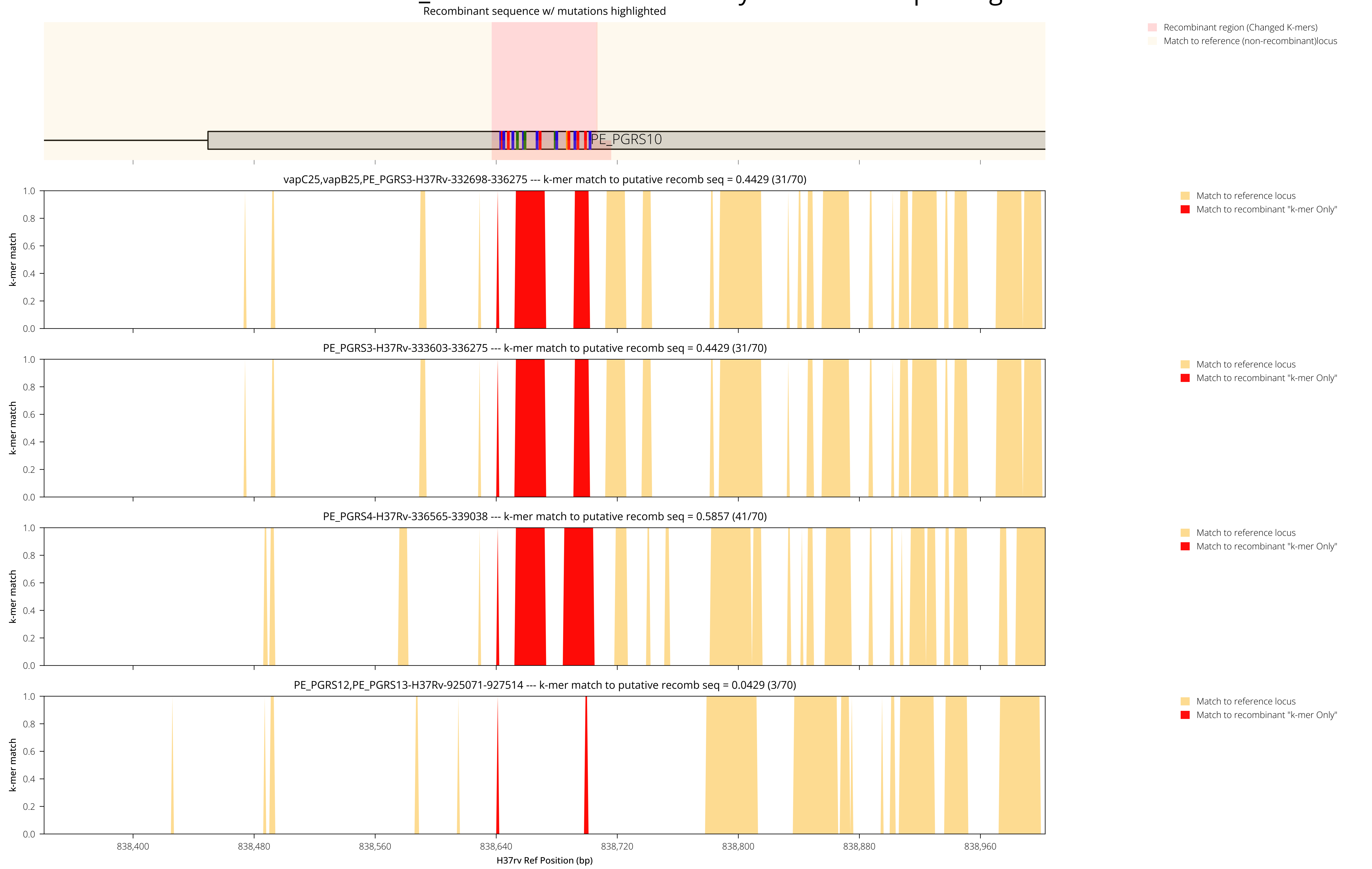

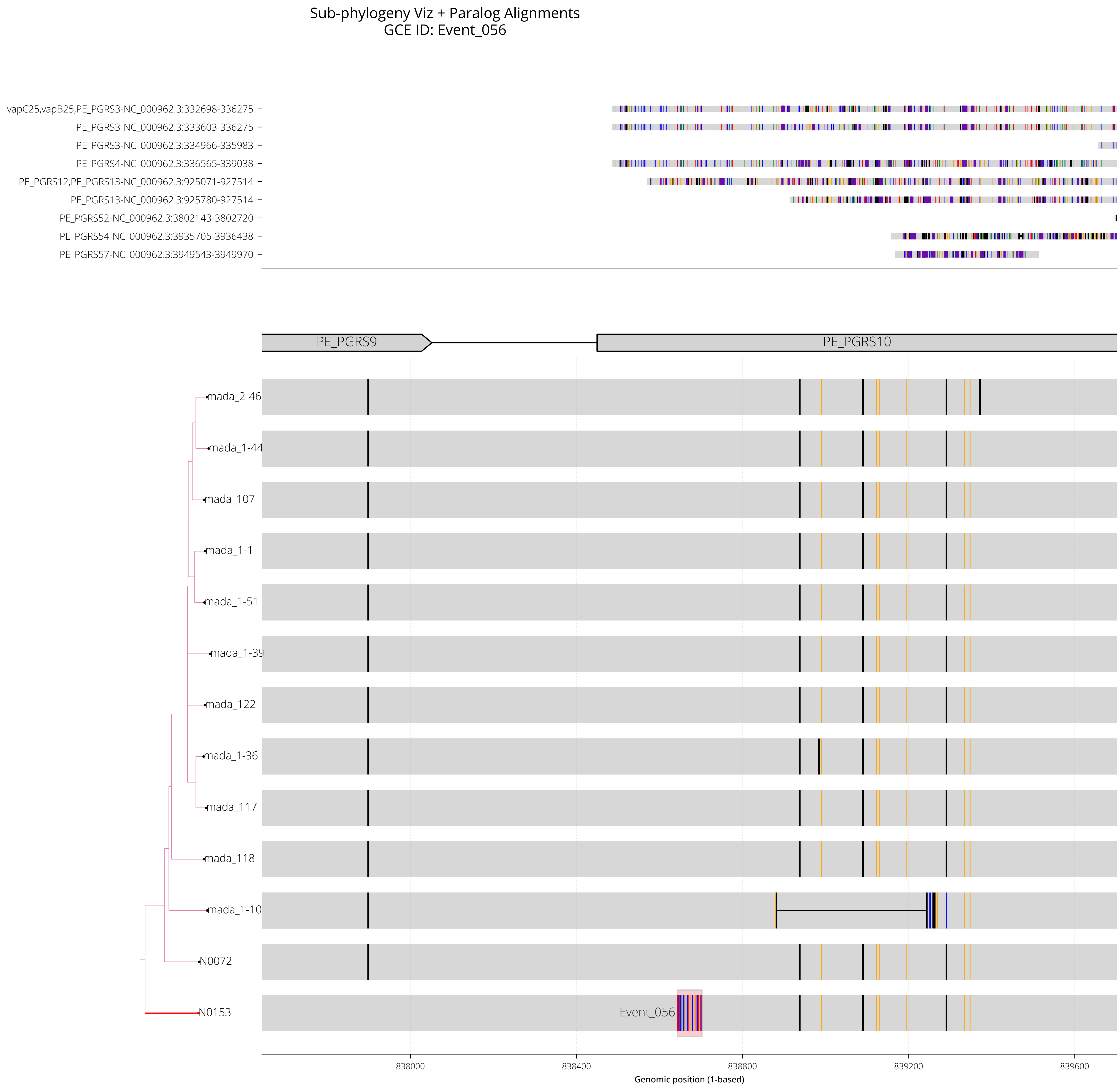

Sub-phylogeny Viz + Paralog Alignments
GCE ID: Event_056
vapC25,vapB25,PE_PGRS3-NC_000962.3:332698-336275
PE_PGRS3-NC_000962.3:333603-336275
PE_PGRS3-NC_000962.3:334966-335983
PE_PGRS4-NC_000962.3:336565-339038
PE_PGRS12,PE_PGRS13-NC_000962.3:925071-927514
PE_PGRS13-NC_000962.3:925780-927514
PE_PGRS52-NC_000962.3:3802143-3802720
PE_PGRS54-NC_000962.3:3935705-3936438
PE_PGRS57-NC_000962.3:3949543-3949970
PE_PGRS9
PE_PGRS10
mada_2-46
mada_1-44
mada_107
mada_1-1
mada_1-51
mada_1-39
mada_122
mada_1-36
mada_117
mada_118
mada_1-10
N0072
N0153
Event_056
838000
838400
838800
839200
839600
Genomic position (1-based)

Full Phylogeny Viz + Paralog Alignments
GCE ID: Event_056

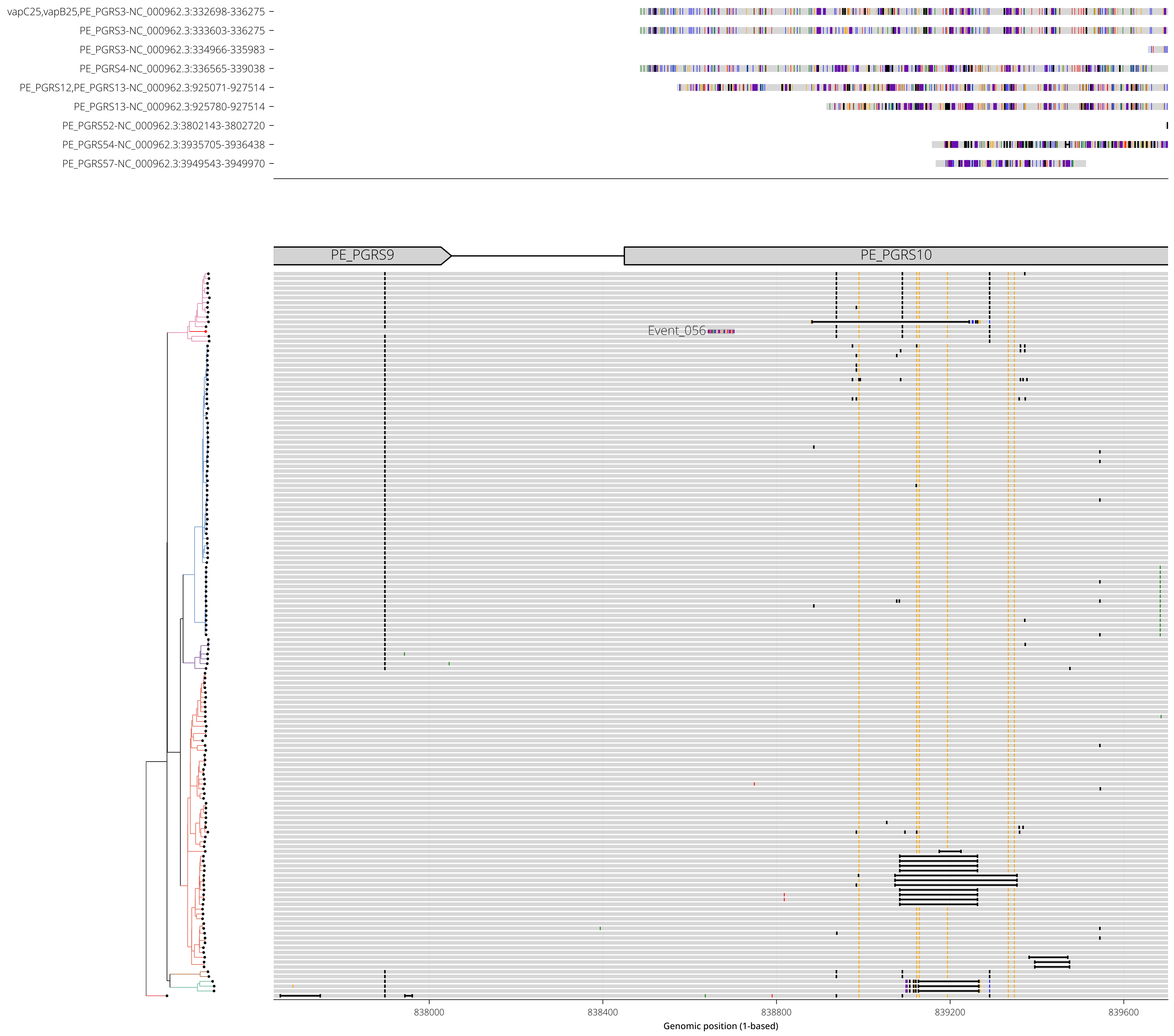

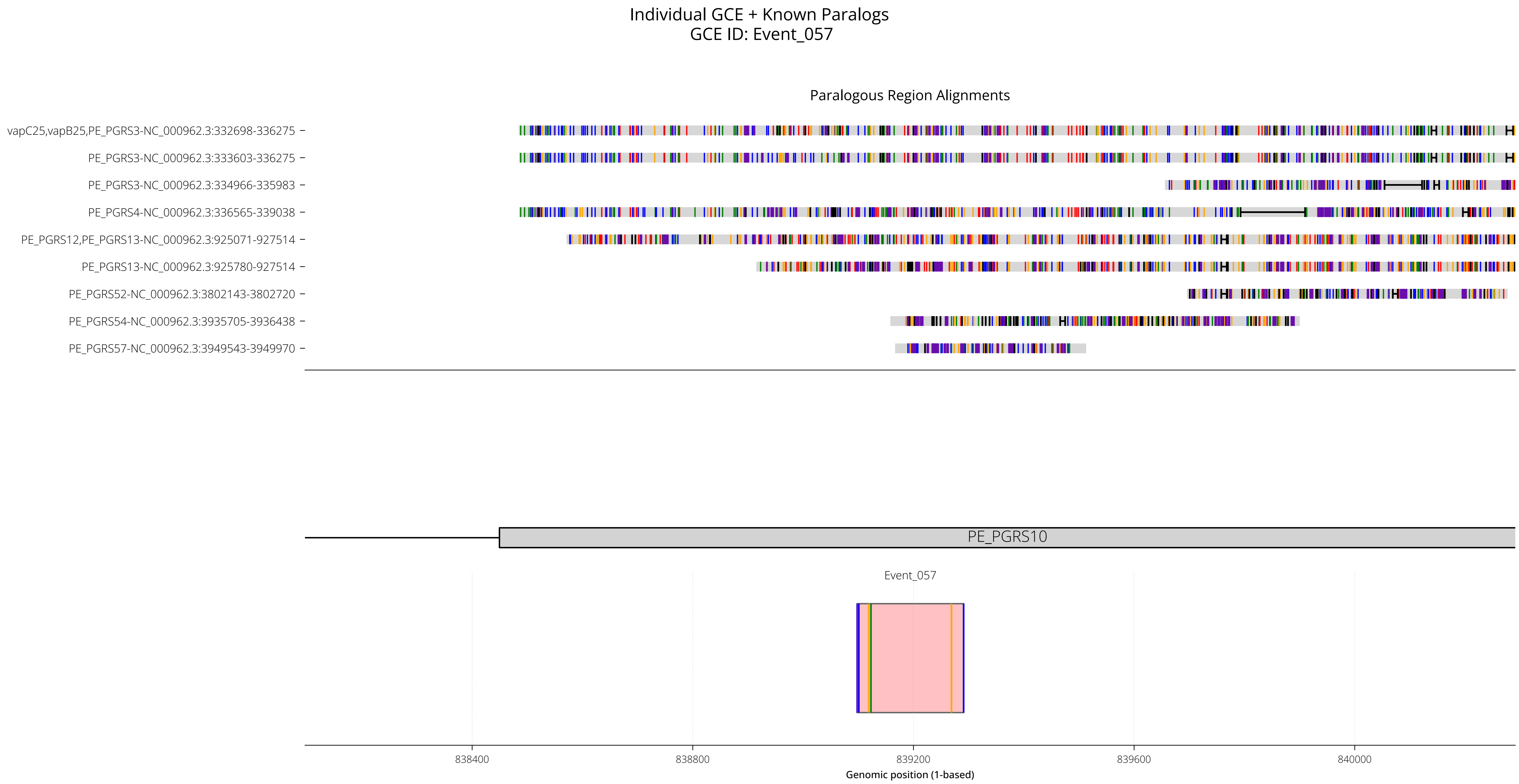
Individual GCE + Known Paralogs
GCE ID: Event_057
Paralogous Region Alignments
vapC25,vapB25,PE_PGRS3-NC_000962.3:332698-336275
PE_PGRS3-NC_000962.3:333603-336275
PE_PGRS3-NC_000962.3:334966-335983
PE_PGRS4-NC_000962.3:336565-339038
PE_PGRS12,PE_PGRS13-NC_000962.3:925071-927514
PE_PGRS13-NC_000962.3:925780-927514
PE_PGRS52-NC_000962.3:3802143-3802720
PE_PGRS54-NC_000962.3:3935705-3936438
PE_PGRS57-NC_000962.3:3949543-3949970
PE_PGRS10
Event_057
838400
838800
839200
839600
840000
Genomic position (1-based)

Circos Viz - GCE to Known Paralogs Locations
GCE ID: Event_057

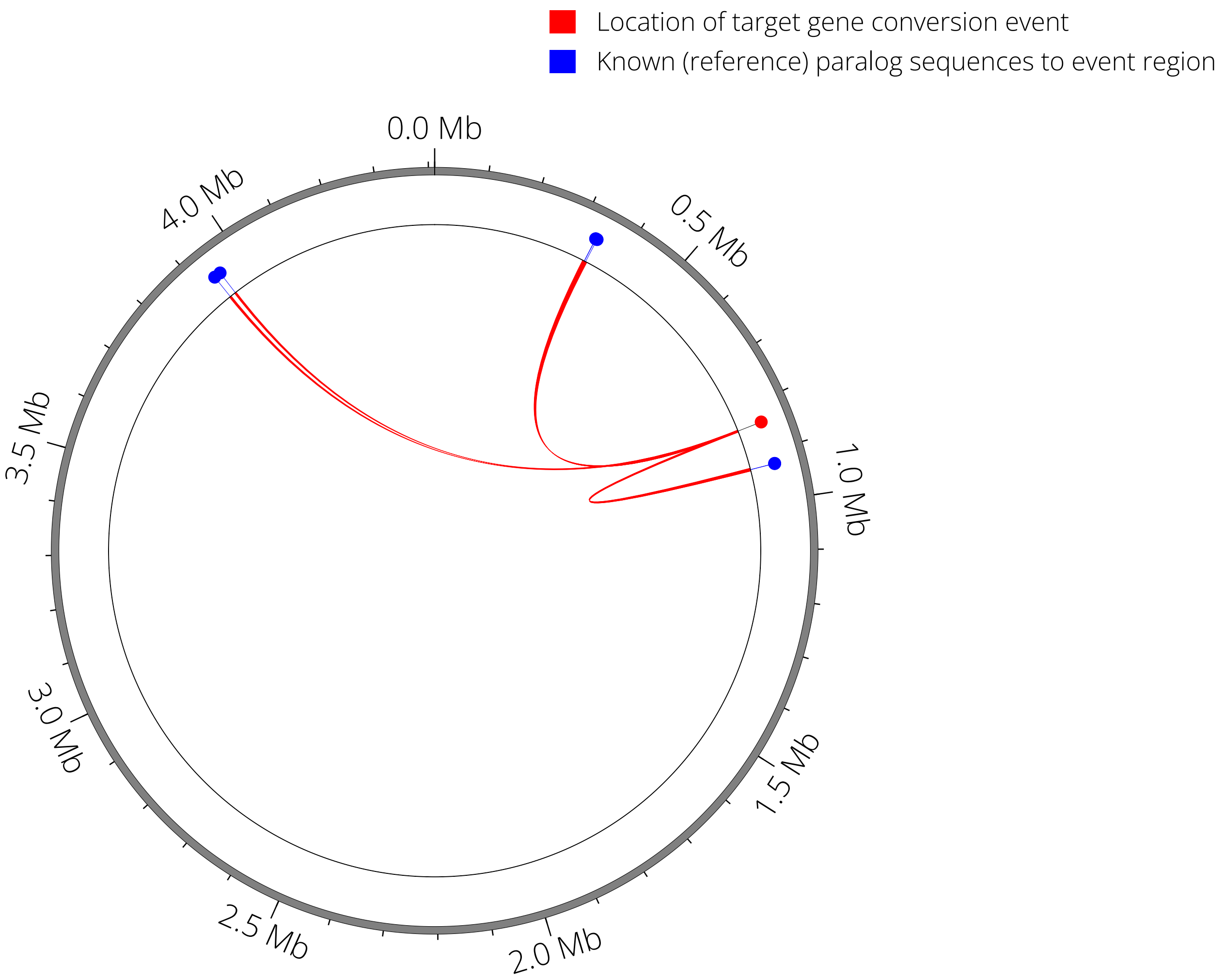

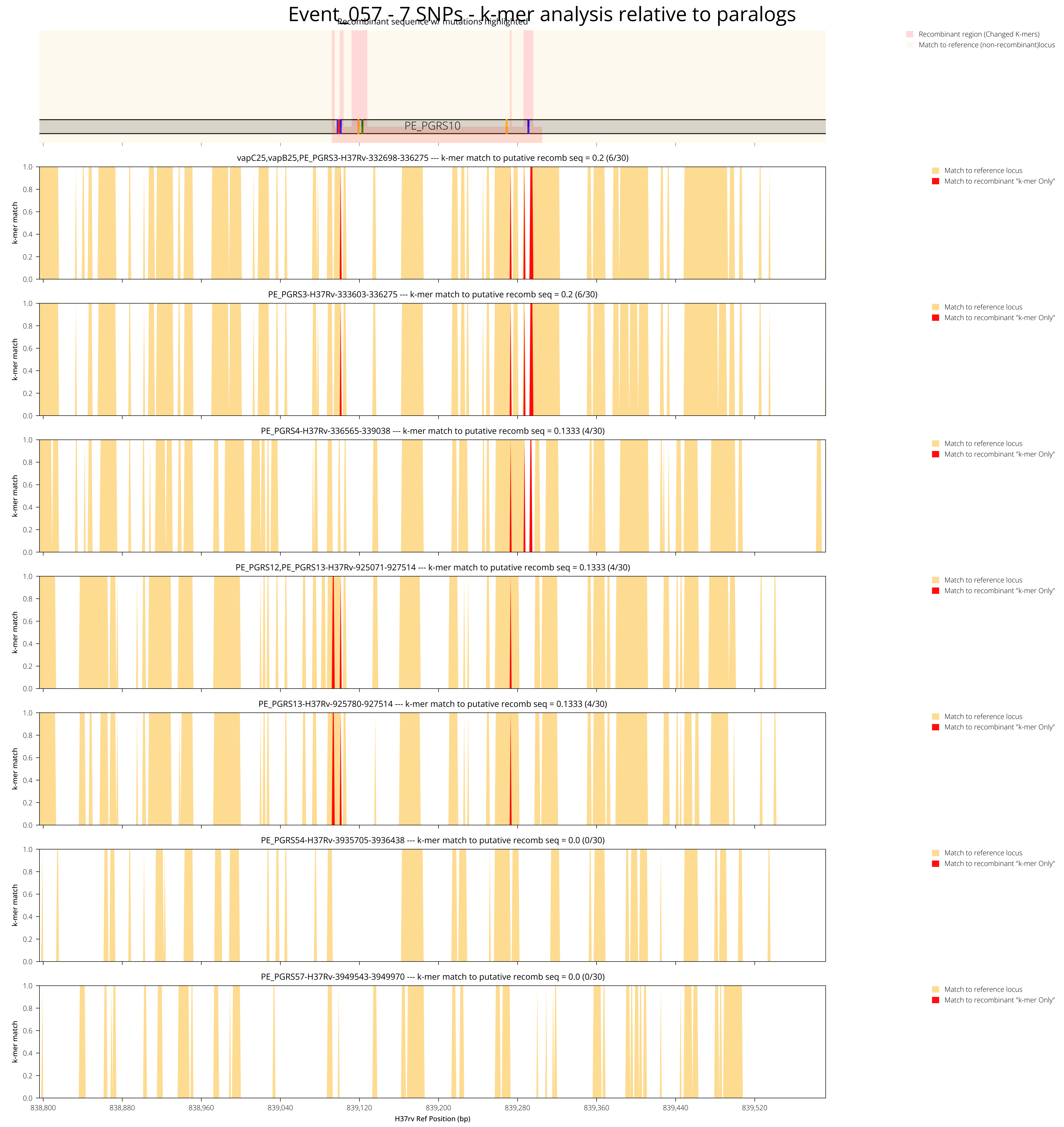

Event_057 - 7 SNPs - k-mer analysis relative to paralogs
Recombinant sequence w/ mutations highlighted
PE_PGRS10
Recombinant region (Changed K-mers)
Match to reference (non-recombinant)locus
vapC25,vapB25,PE_PGRS3-H37Rv-332698-336275 --- k-mer match to putative recomb seq = 0.2 (6/30)
PE_PGRS3-H37Rv-333603-336275 --- k-mer match to putative recomb seq = 0.2 (6/30)
PE_PGRS4-H37Rv-336565-339038 --- k-mer match to putative recomb seq = 0.1333 (4/30)
PE_PGRS12,PE_PGRS13-H37Rv-925071-927514 --- k-mer match to putative recomb seq = 0.1333 (4/30)
PE_PGRS13-H37Rv-925780-927514 --- k-mer match to putative recomb seq = 0.1333 (4/30)
PE_PGRS54-H37Rv-3935705-3936438 --- k-mer match to putative recomb seq = 0.0 (0/30)
PE_PGRS57-H37Rv-3949543-3949970 --- k-mer match to putative recomb seq = 0.0 (0/30)
Match to reference locus
Match to recombinant "k-mer Only"
k-mer match
0.0
0.2
0.4
0.6
0.8
1.0
838,800
838,880
838,960
839,040
839,120
839,200
839,280
839,360
839,440
839,520
H37rv Ref Position (bp)

Sub-phylogeny Viz + Paralog Alignments
GCE ID: Event_057

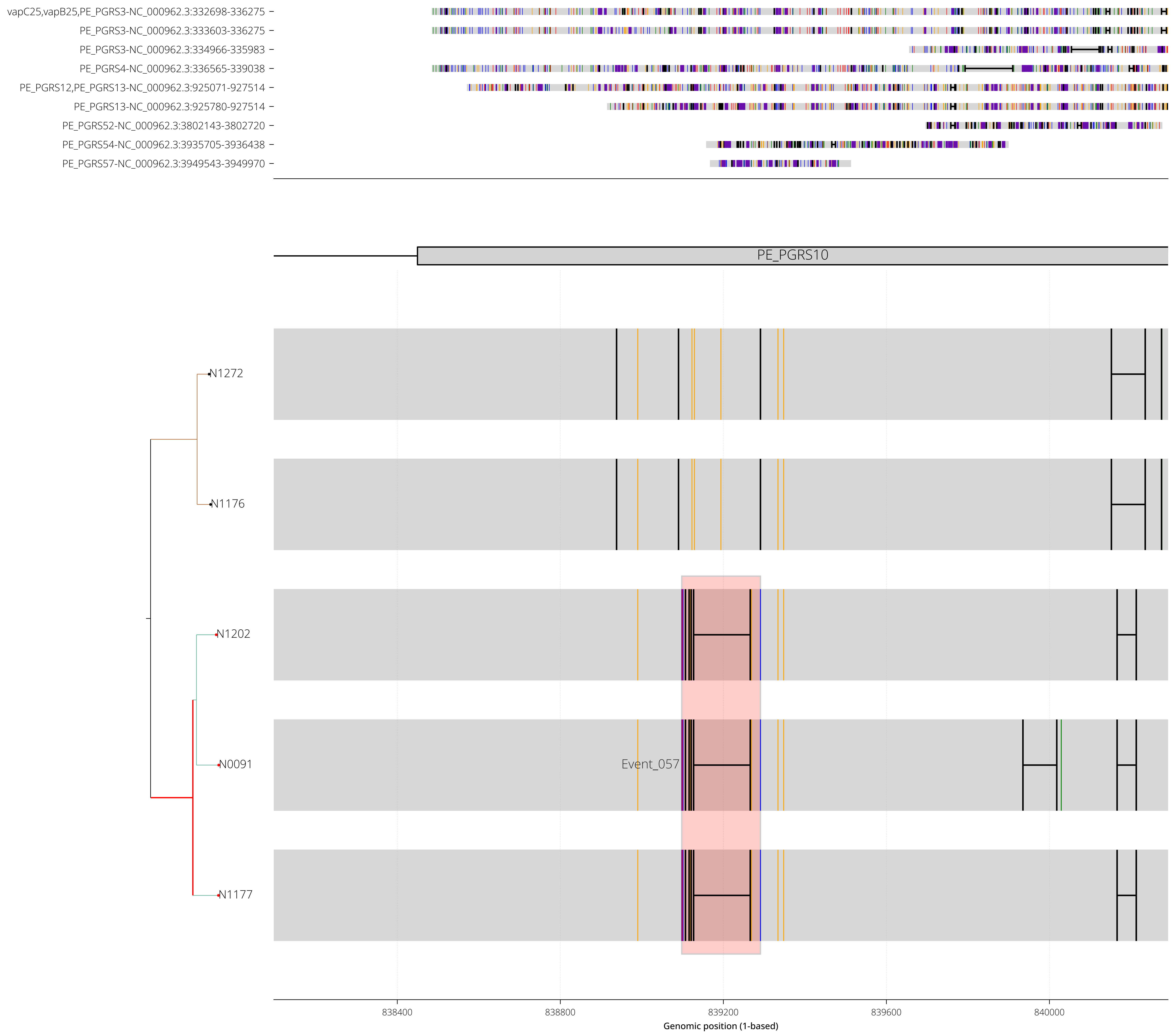

Full Phylogeny Viz + Paralog Alignments
GCE ID: Event_057

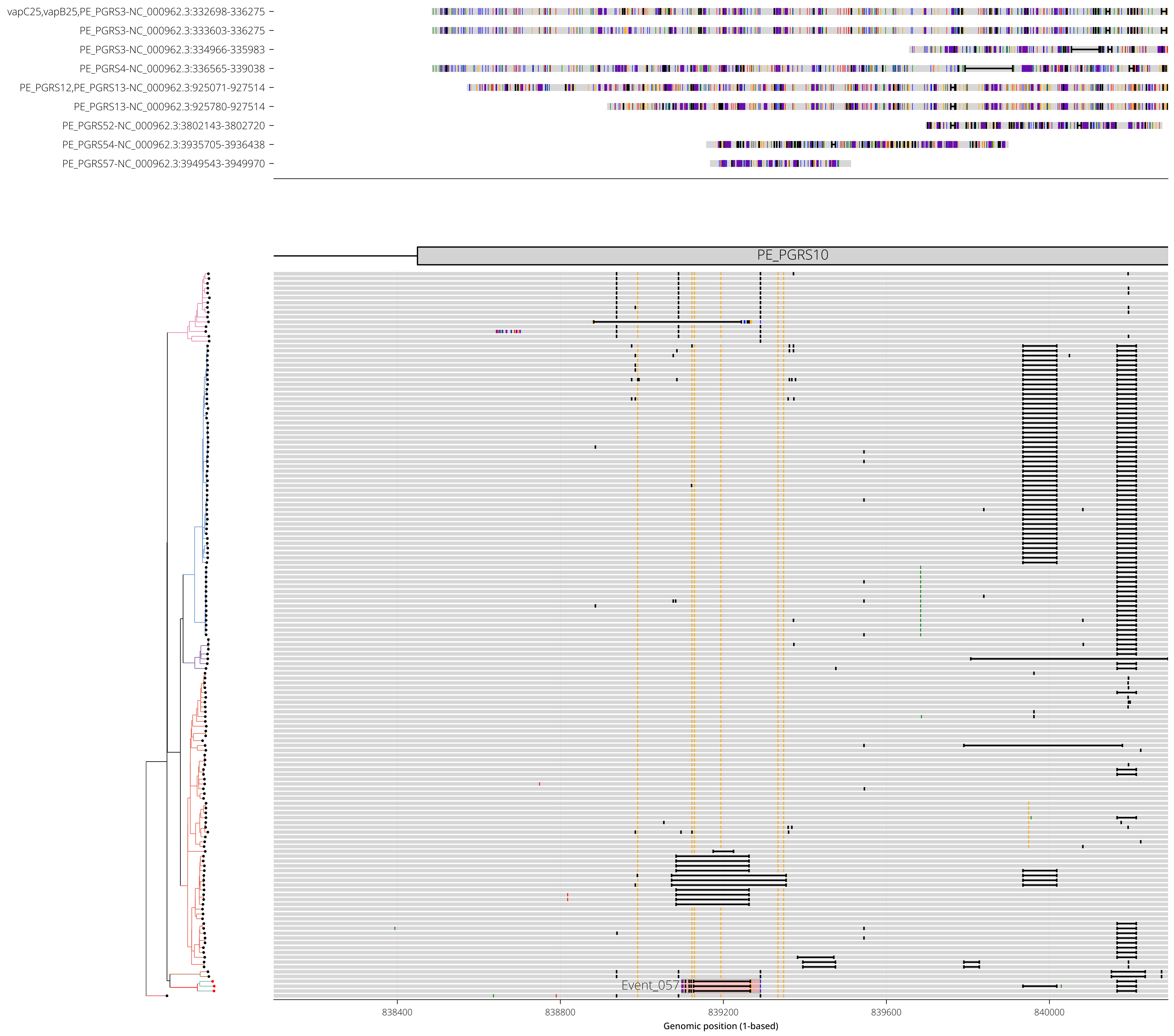

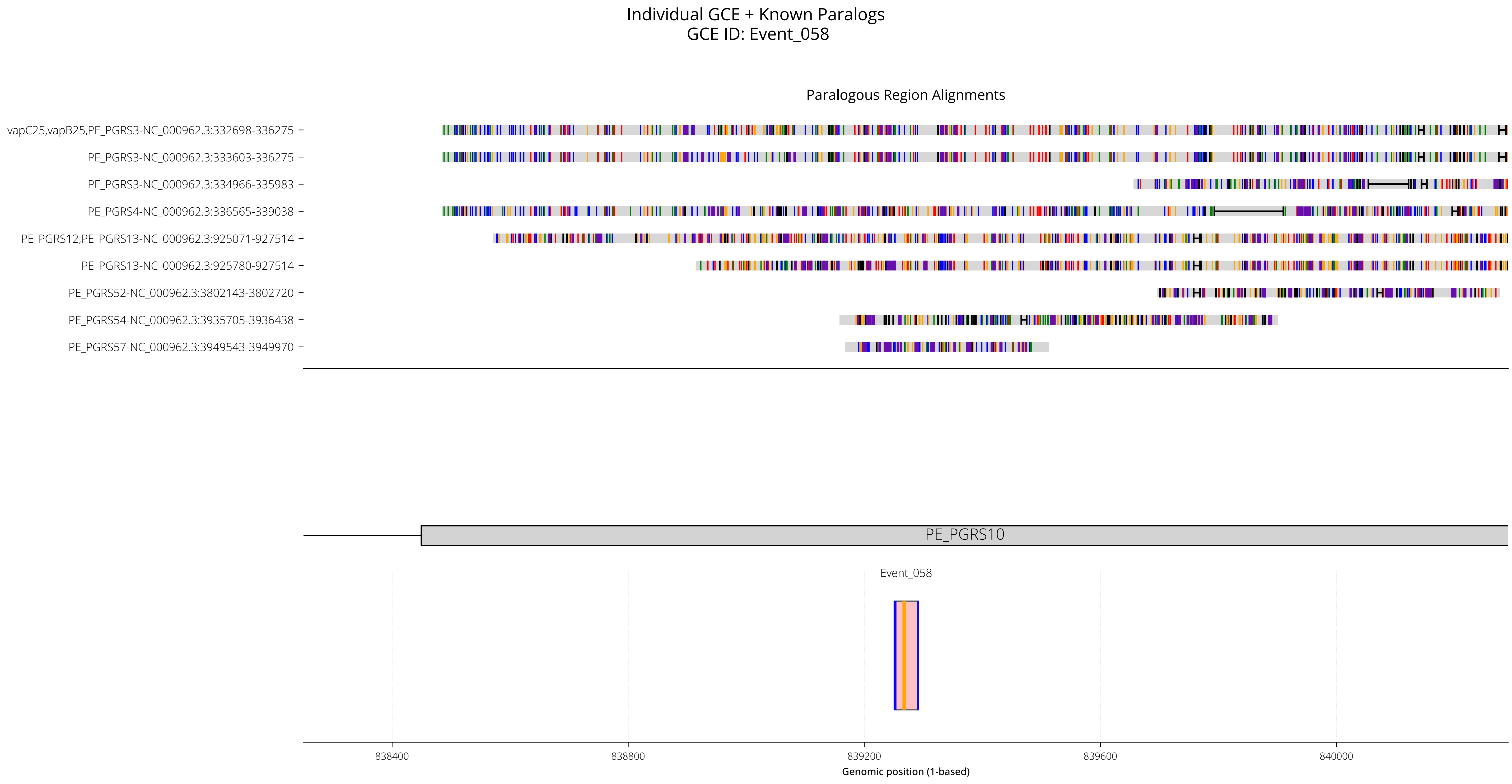

Individual GCE + Known Paralogs
GCE ID: Event_058
Paralogous Region Alignments
vapC25,vapB25,PE_PGRS3-NC_000962.3:332698-336275
PE_PGRS3-NC_000962.3:333603-336275
PE_PGRS3-NC_000962.3:334966-335983
PE_PGRS4-NC_000962.3:336565-339038
PE_PGRS12,PE_PGRS13-NC_000962.3:925071-927514
PE_PGRS13-NC_000962.3:925780-927514
PE_PGRS52-NC_000962.3:3802143-3802720
PE_PGRS54-NC_000962.3:3935705-3936438
PE_PGRS57-NC_000962.3:3949543-3949970
PE_PGRS10
Event_058
838400
838800
839200
839600
840000
Genomic position (1-based)

Circos Viz - GCE to Known Paralogs Locations
GCE ID: Event_058

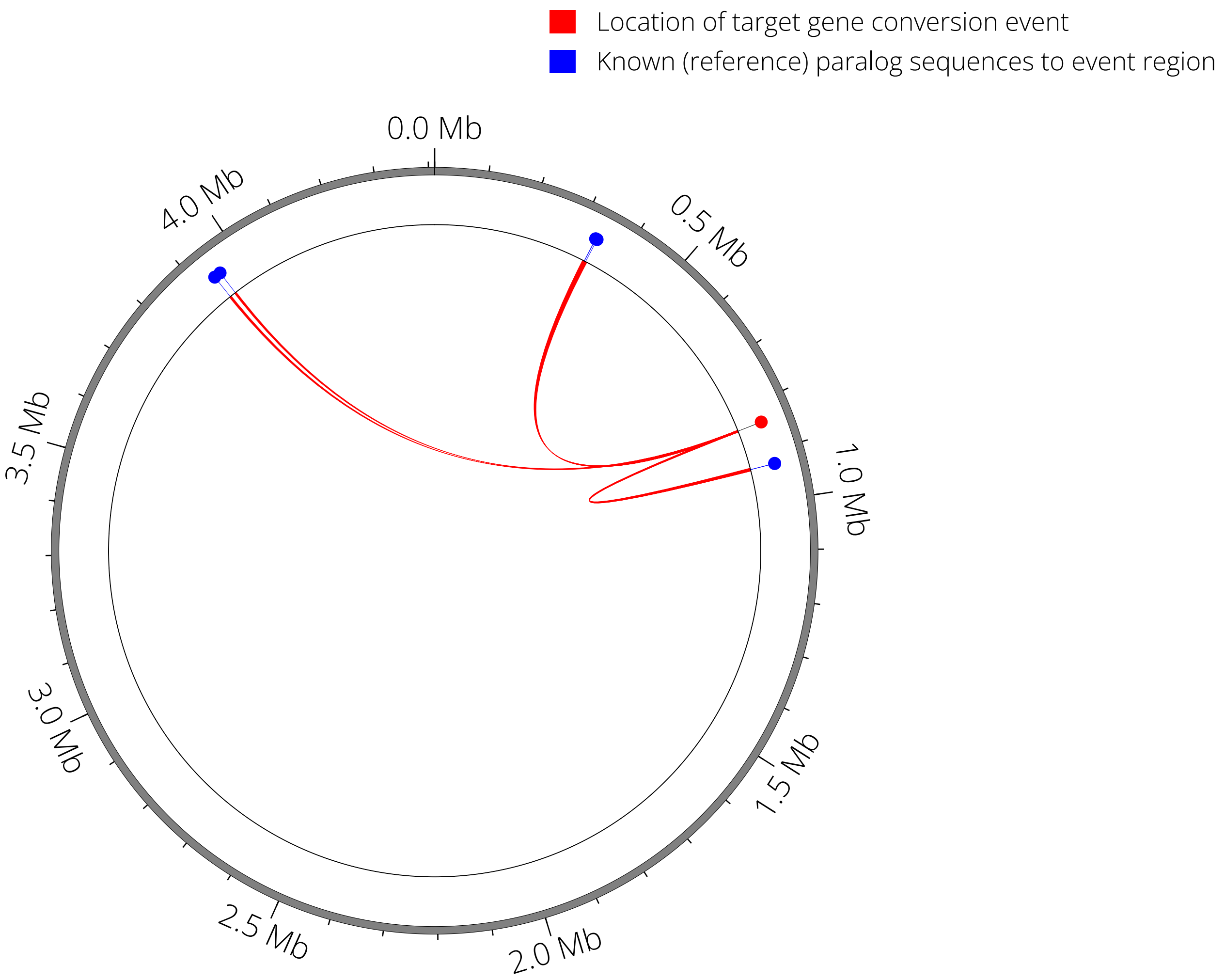

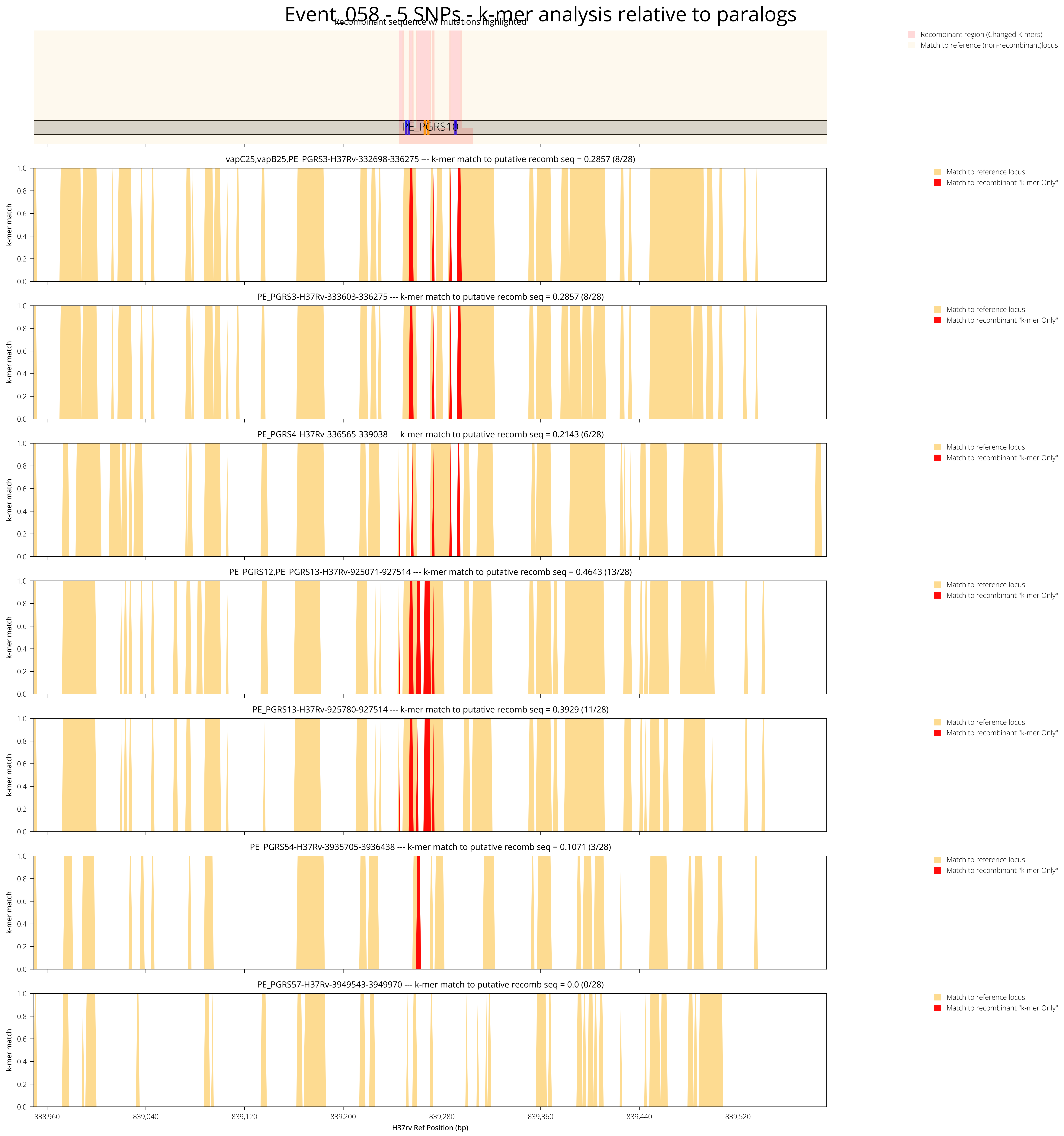

Event_058 - 5 SNPs - k-mer analysis relative to paralogs
Recombinant sequence w/ mutations highlighted
Recombinant region (Changed K-mers)
Match to reference (non-recombinant)locus
PE_PGRS10
vapC25,vapB25,PE_PGRS3-H37Rv-332698-336275 --- k-mer match to putative recomb seq = 0.2857 (8/28)
PE_PGRS3-H37Rv-333603-336275 --- k-mer match to putative recomb seq = 0.2857 (8/28)
PE_PGRS4-H37Rv-336565-339038 --- k-mer match to putative recomb seq = 0.2143 (6/28)
PE_PGRS12,PE_PGRS13-H37Rv-925071-927514 --- k-mer match to putative recomb seq = 0.4643 (13/28)
PE_PGRS13-H37Rv-925780-927514 --- k-mer match to putative recomb seq = 0.3929 (11/28)
PE_PGRS54-H37Rv-3935705-3936438 --- k-mer match to putative recomb seq = 0.1071 (3/28)
PE_PGRS57-H37Rv-3949543-3949970 --- k-mer match to putative recomb seq = 0.0 (0/28)
Match to reference locus
Match to recombinant "k-mer Only"
k-mer match
0.0
0.2
0.4
0.6
0.8
1.0
838,960
839,040
839,120
839,200
839,280
839,360
839,440
839,520
H37rv Ref Position (bp)

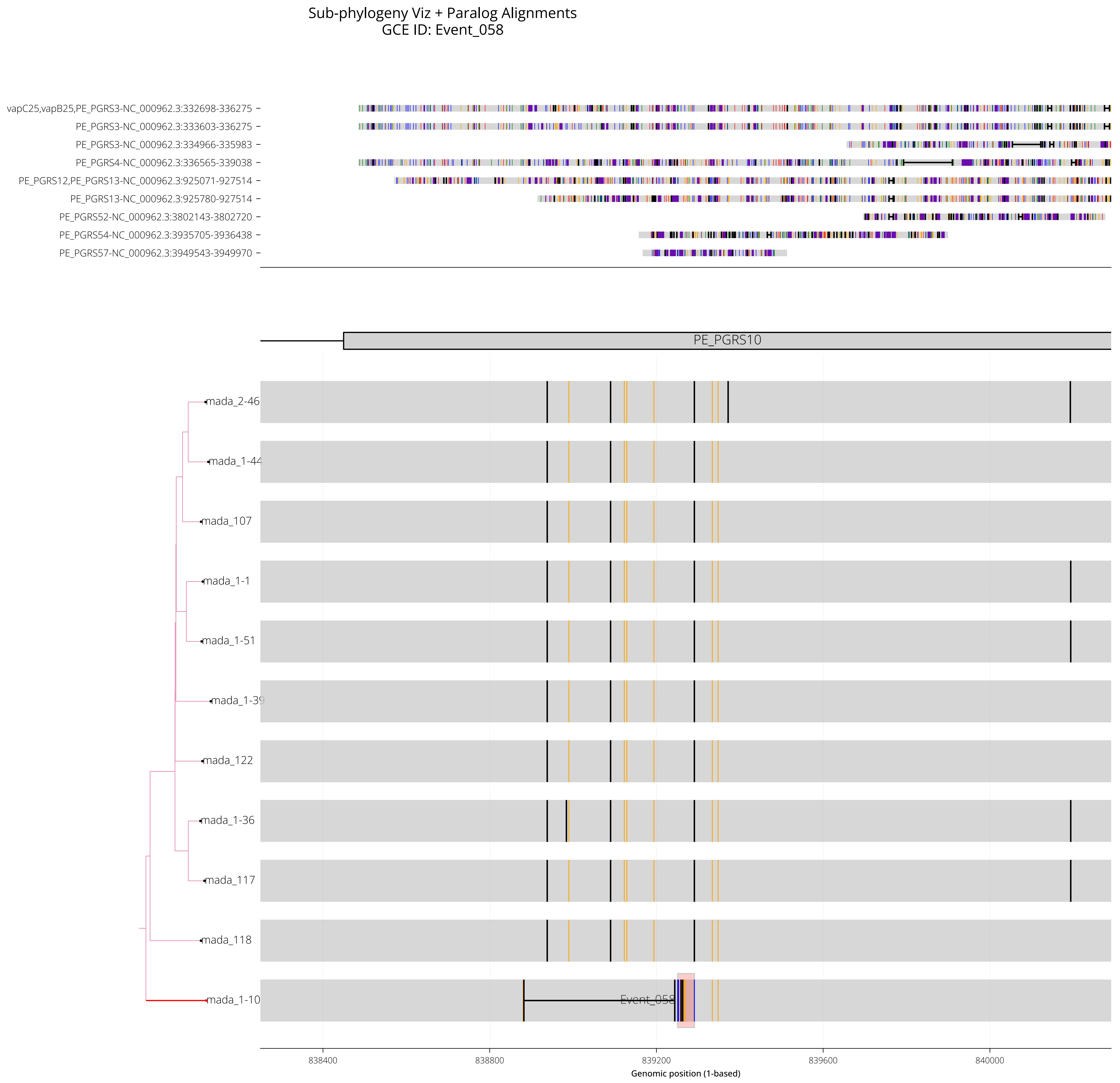

Sub-phylogeny Viz + Paralog Alignments
GCE ID: Event_058
vapC25,vapB25,PE_PGRS3-NC_000962.3:332698-336275
PE_PGRS3-NC_000962.3:333603-336275
PE_PGRS3-NC_000962.3:334966-335983
PE_PGRS4-NC_000962.3:336565-339038
PE_PGRS12,PE_PGRS13-NC_000962.3:925071-927514
PE_PGRS13-NC_000962.3:925780-927514
PE_PGRS52-NC_000962.3:3802143-3802720
PE_PGRS54-NC_000962.3:3935705-3936438
PE_PGRS57-NC_000962.3:3949543-3949970
PE_PGRS10
mada_2-46
mada_1-44
mada_107
mada_1-1
mada_1-51
mada_1-39
mada_122
mada_1-36
mada_117
mada_118
mada_1-10
Event_058
838400
838800
839200
839600
840000
Genomic position (1-based)

Full Phylogeny Viz + Paralog Alignments
GCE ID: Event_058

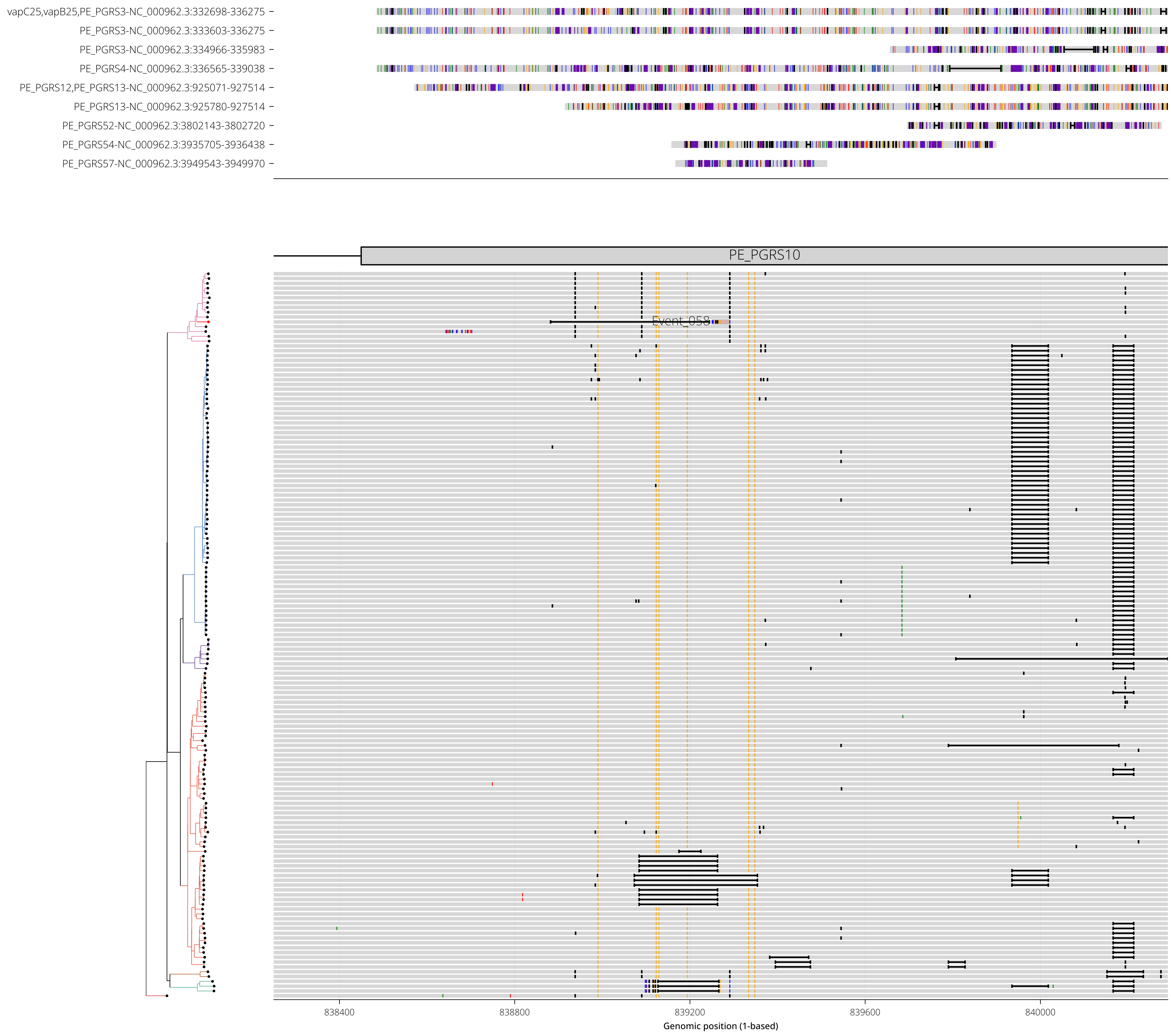

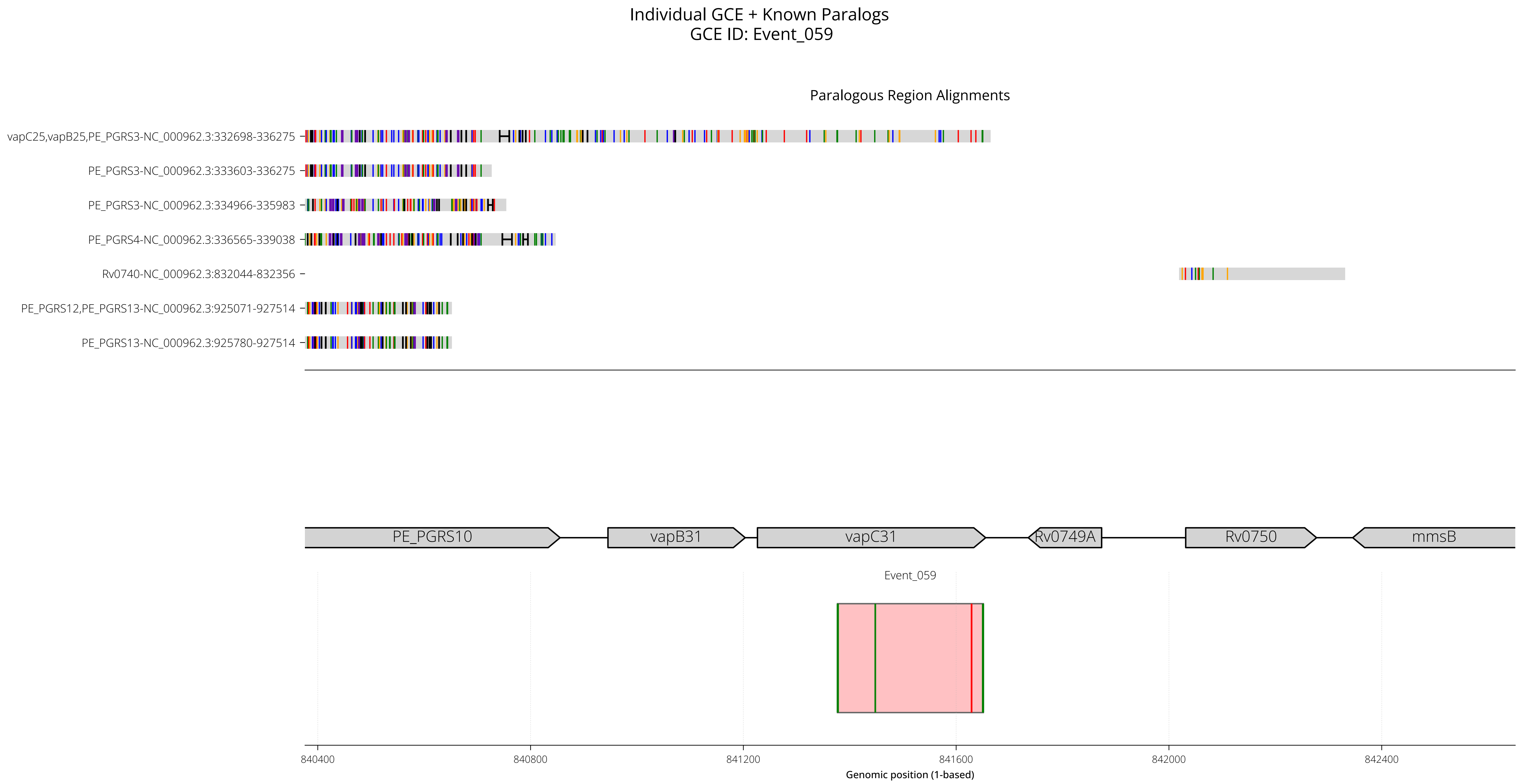
Individual GCE + Known Paralogs
GCE ID: Event_059
Paralogous Region Alignments
vapC25,vapB25,PE_PGRS3-NC_000962.3:332698-336275
PE_PGRS3-NC_000962.3:333603-336275
PE_PGRS3-NC_000962.3:334966-335983
PE_PGRS4-NC_000962.3:336565-339038
Rv0740-NC_000962.3:832044-832356
PE_PGRS12,PE_PGRS13-NC_000962.3:925071-927514
PE_PGRS13-NC_000962.3:925780-927514
PE_PGRS10
vapB31
vapC31
Rv0749A
Rv0750
mmsB
Event_059
840400
840800
841200
841600
842000
842400
Genomic position (1-based)

Circos Viz - GCE to Known Paralogs Locations
GCE ID: Event_059

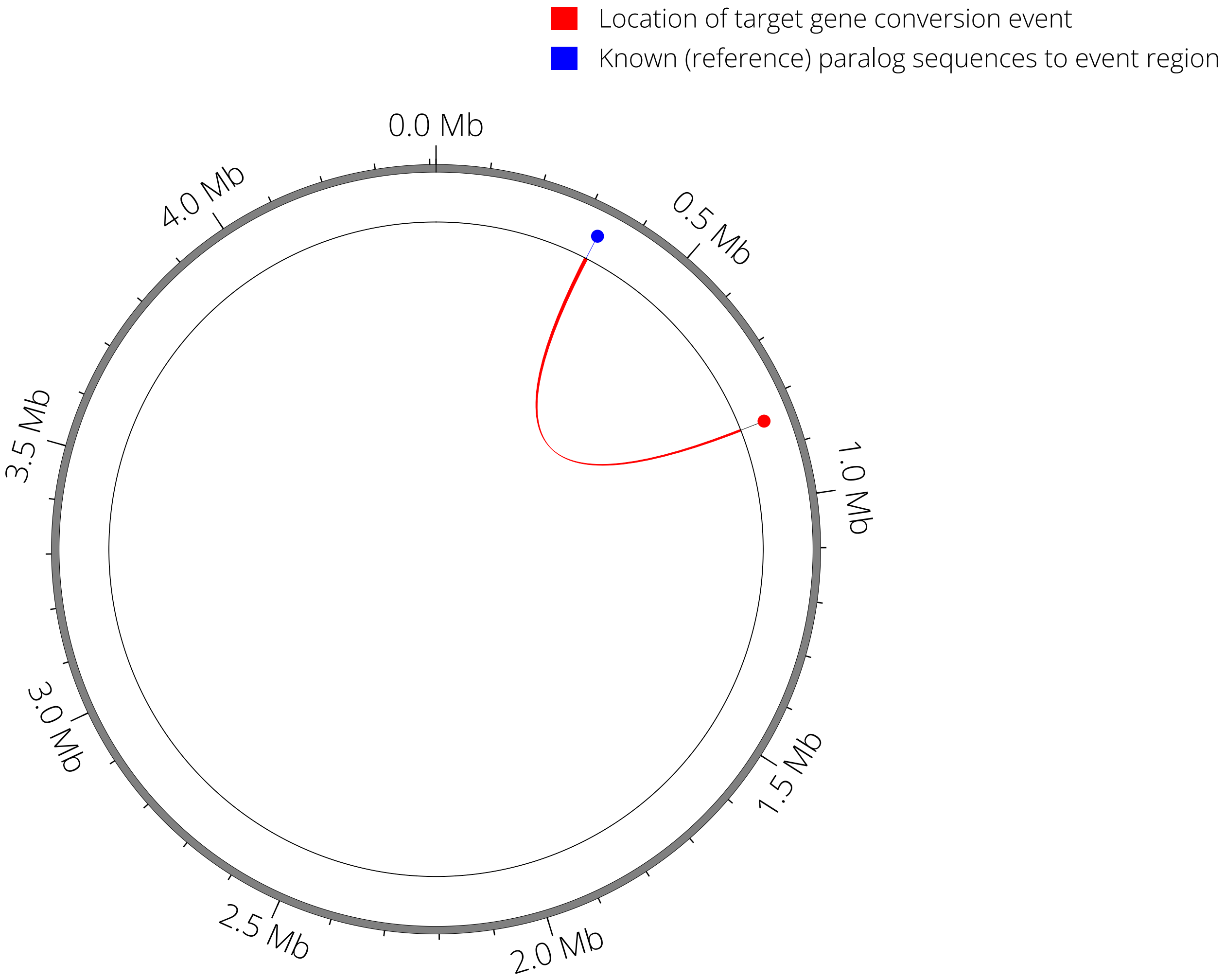

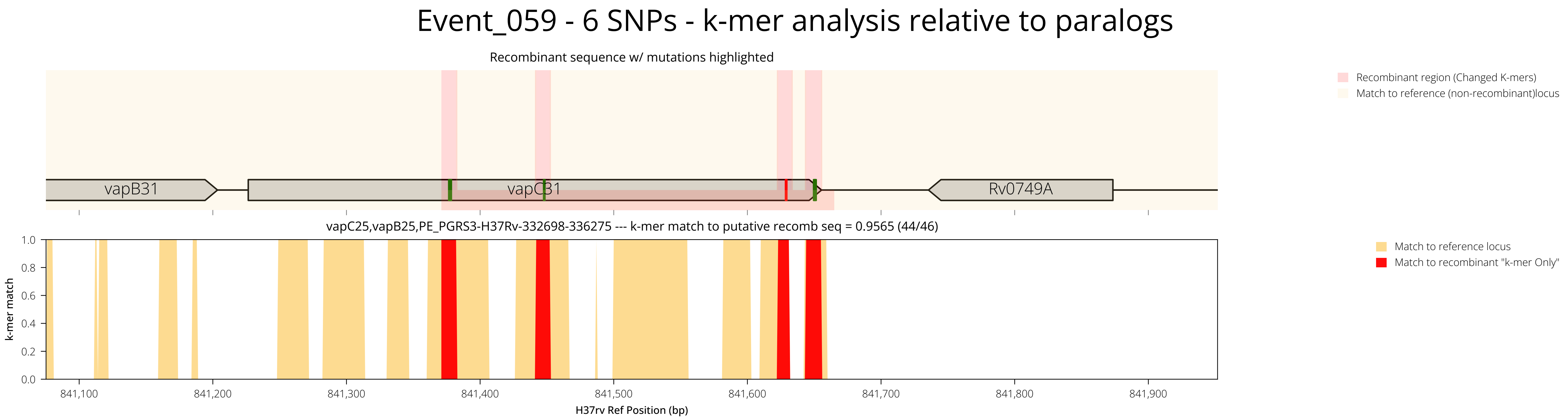
Event_059 - 6 SNPs - k-mer analysis relative to paralogs
Recombinant sequence w/ mutations highlighted
vapB31
vapC31
Rv0749A
Recombinant region (Changed K-mers)
Match to reference (non-recombinant)locus
vapC25,vapB25,PE_PGRS3-H37Rv-332698-336275 --- k-mer match to putative recomb seq = 0.9565 (44/46)
k-mer match
1.0
0.8
0.6
0.4
0.2
0.0
841,100
841,200
841,300
841,400
841,500
841,600
841,700
841,800
841,900
H37rv Ref Position (bp)
Match to reference locus
Match to recombinant "k-mer Only"

Sub-phylogeny Viz + Paralog Alignments
GCE ID: Event_059

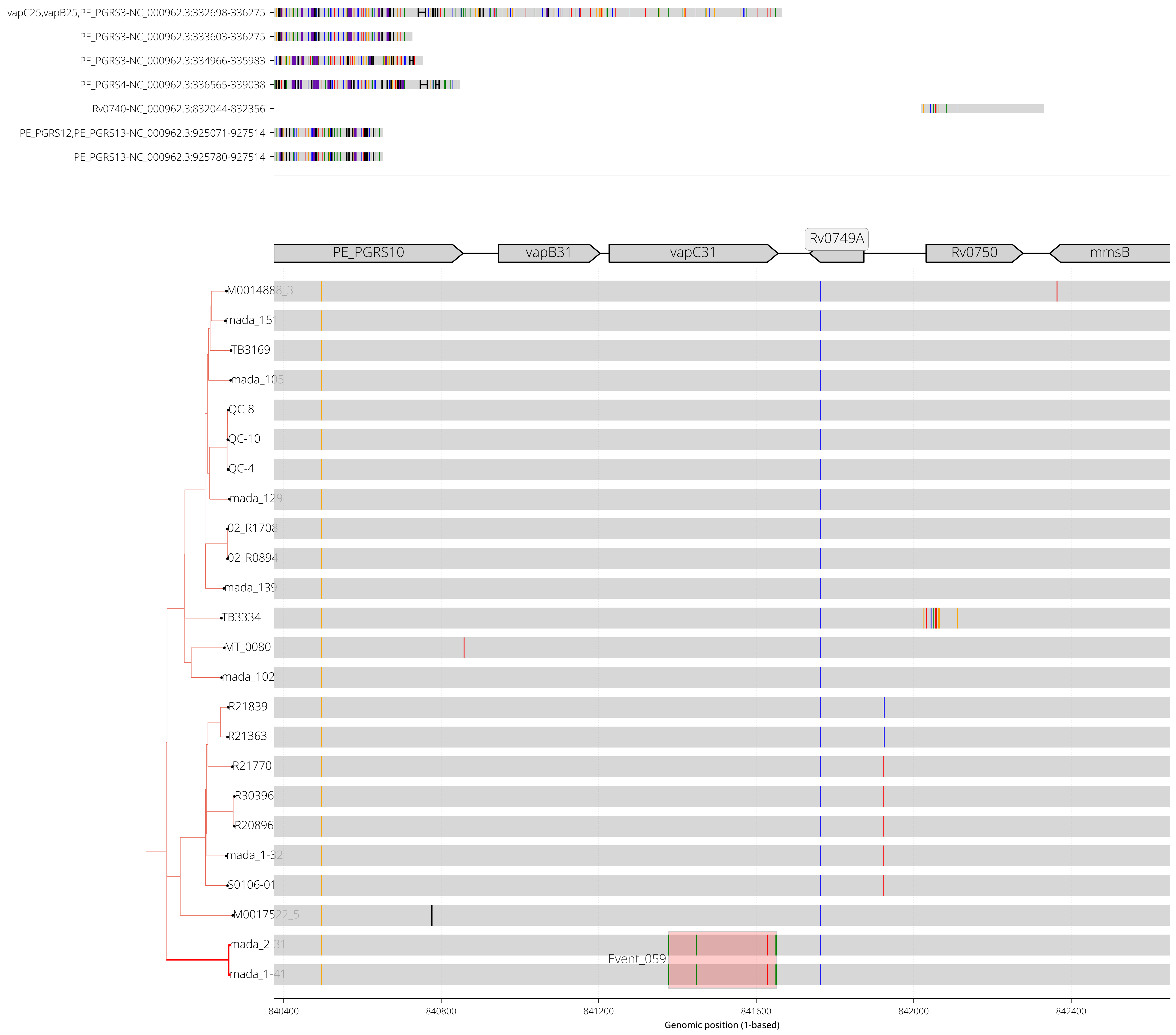

Full Phylogeny Viz + Paralog Alignments
GCE ID: Event_059

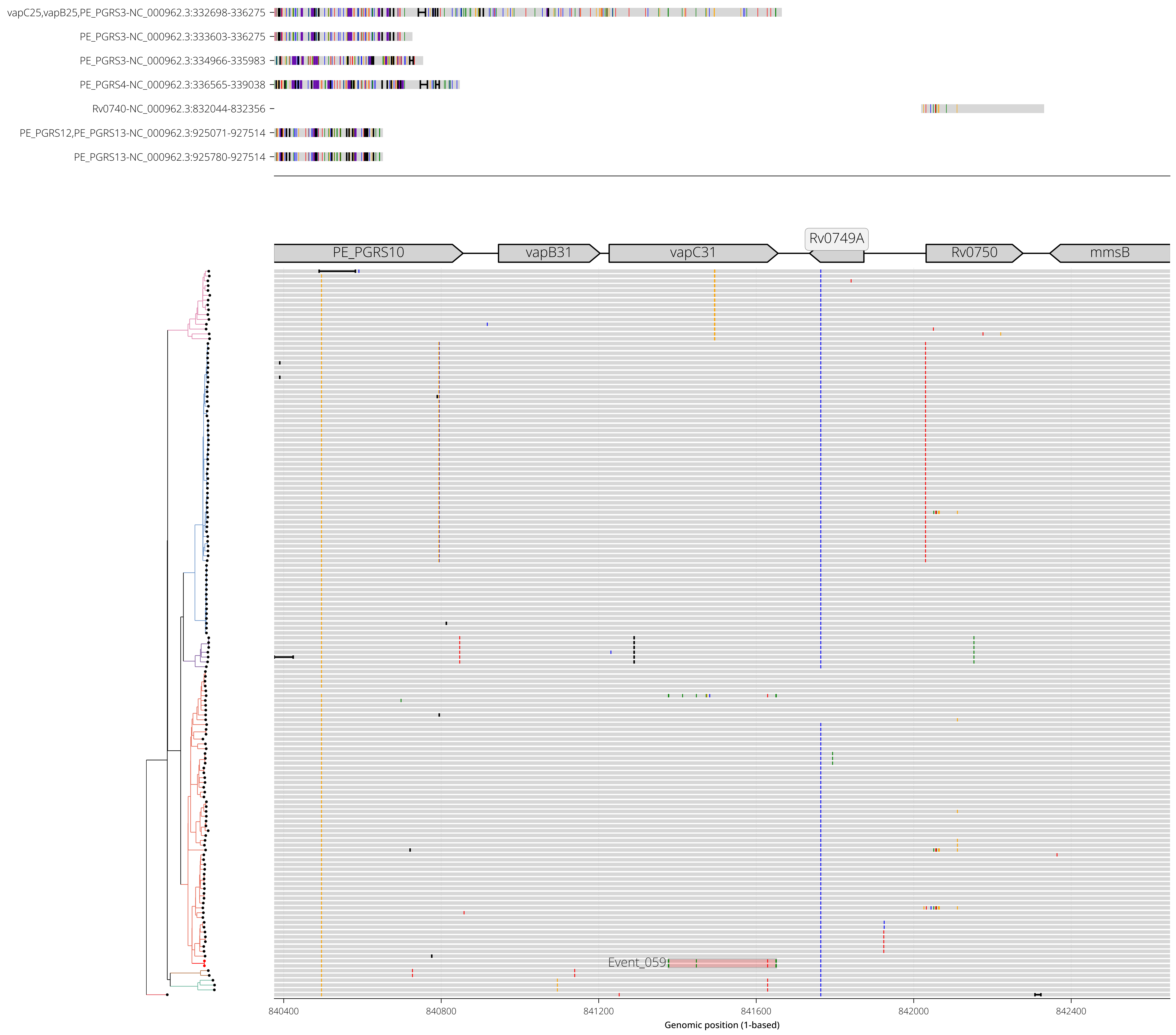

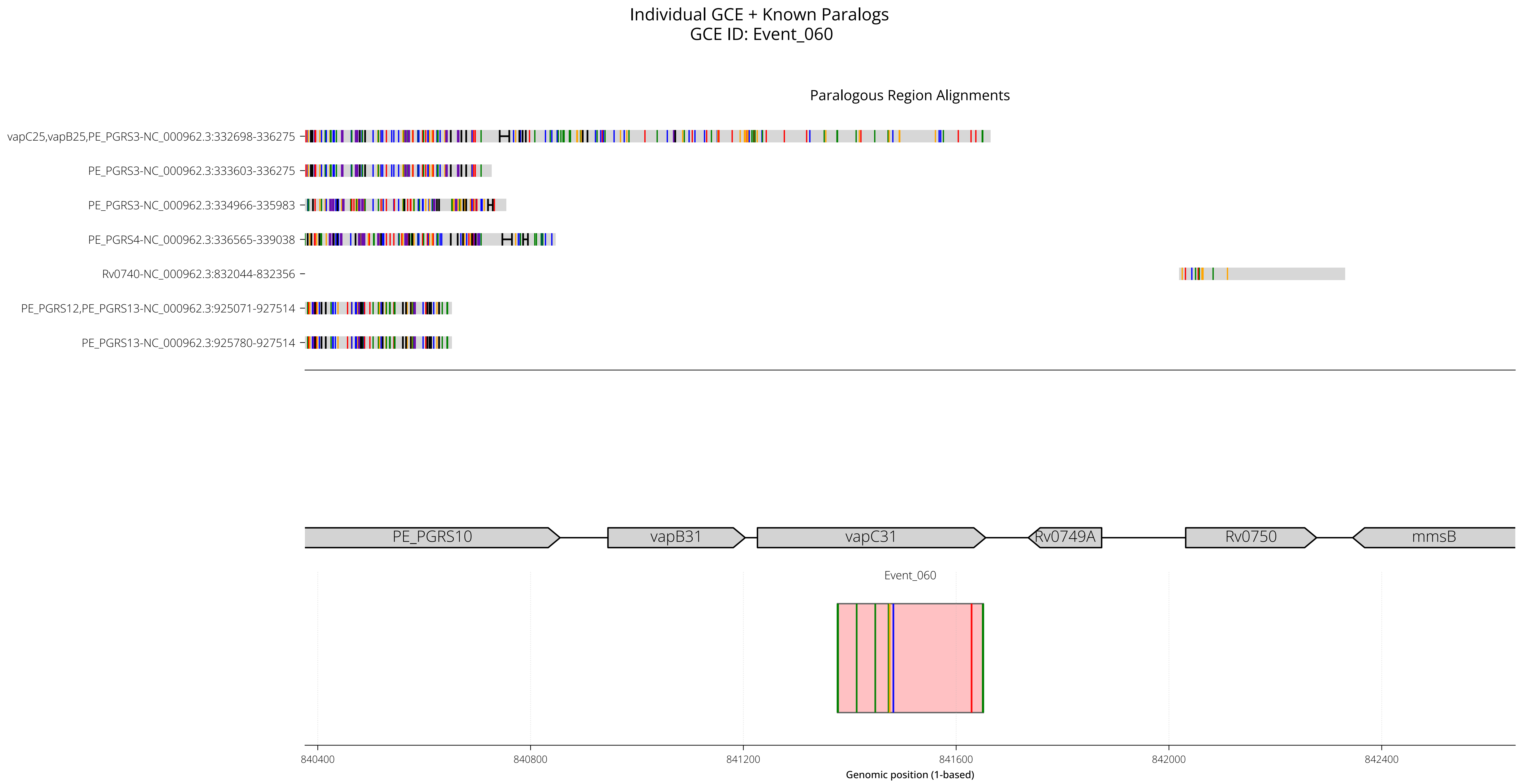

Individual GCE + Known Paralogs
GCE ID: Event_060
Paralogous Region Alignments
vapC25,vapB25,PE_PGRS3-NC_000962.3:332698-336275
PE_PGRS3-NC_000962.3:333603-336275
PE_PGRS3-NC_000962.3:334966-335983
PE_PGRS4-NC_000962.3:336565-339038
Rv0740-NC_000962.3:832044-832356
PE_PGRS12,PE_PGRS13-NC_000962.3:925071-927514
PE_PGRS13-NC_000962.3:925780-927514
PE_PGRS10
vapB31
vapC31
Rv0749A
Rv0750
mmsB
Event_060
840400
840800
841200
841600
842000
842400
Genomic position (1-based)

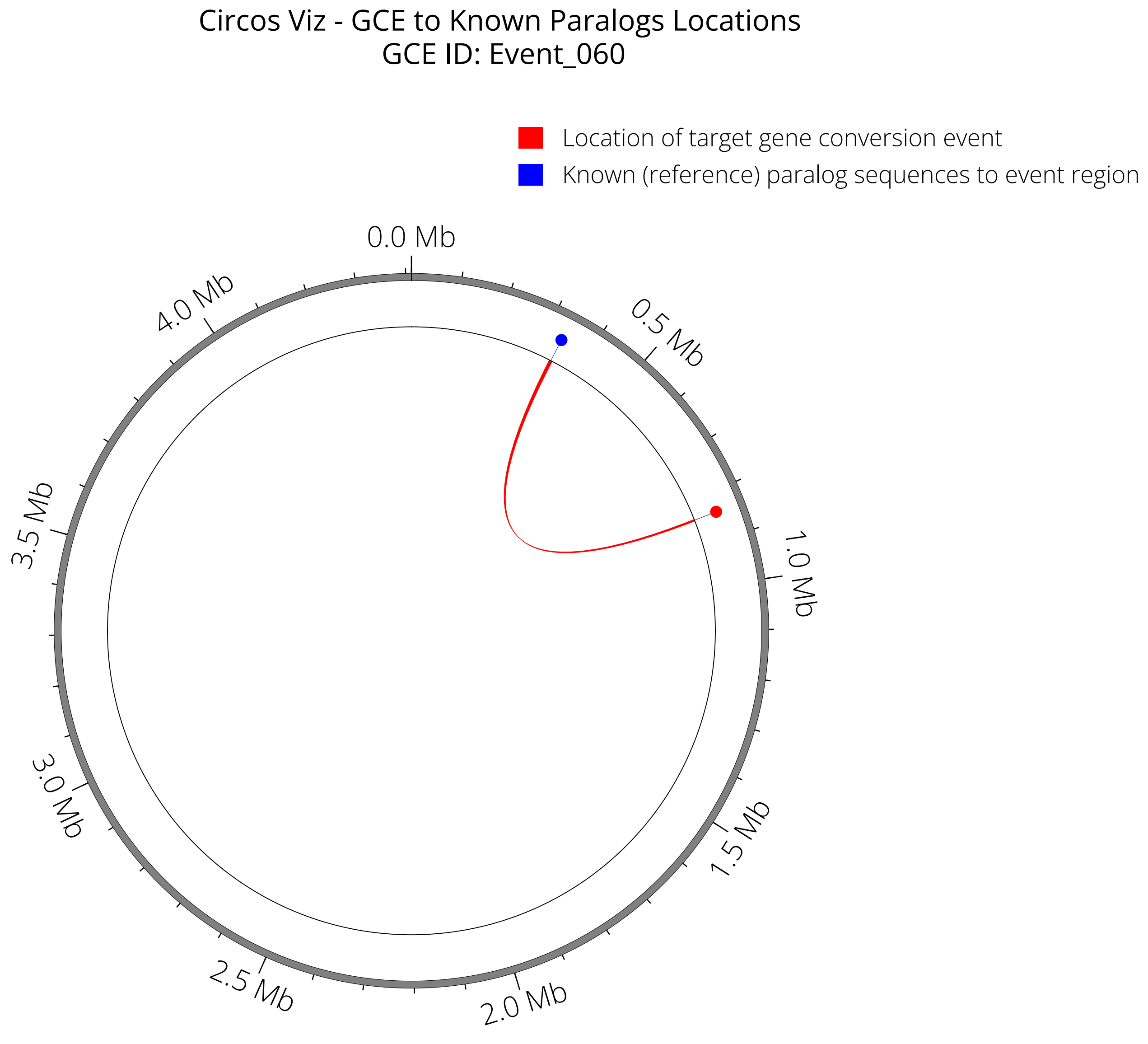

Circos Viz - GCE to Known Paralogs Locations
GCE ID: Event_060
Location of target gene conversion event
Known (reference) paralog sequences to event region
0.0 Mb
0.5 Mb
1.0 Mb
1.5 Mb
2.0 Mb
2.5 Mb
3.0 Mb
3.5 Mb
4.0 Mb

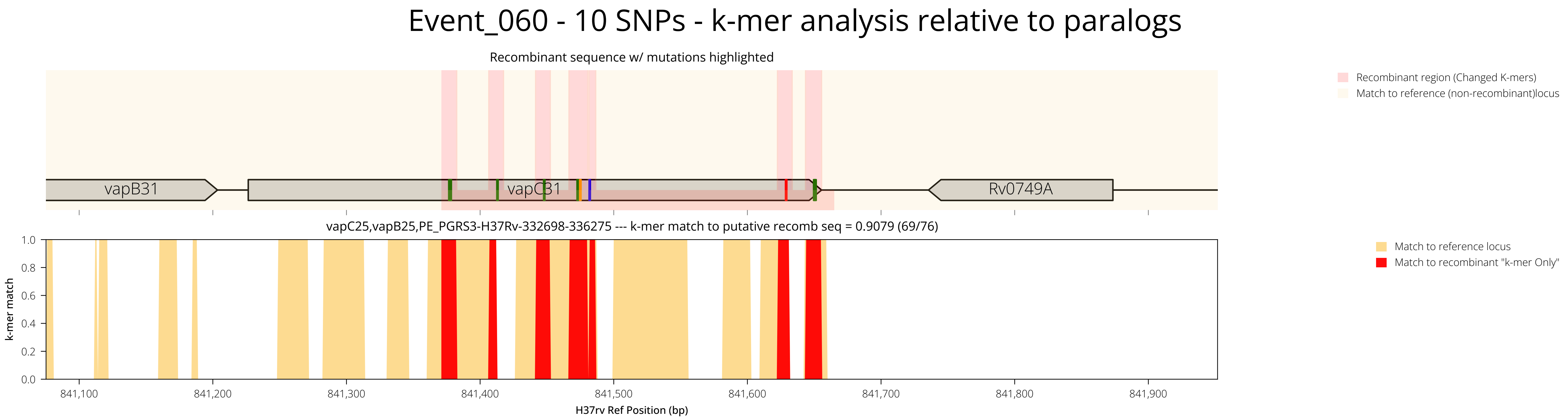

Event_060 - 10 SNPs - k-mer analysis relative to paralogs
Recombinant sequence w/ mutations highlighted
Recombinant region (Changed K-mers)
Match to reference (non-recombinant)locus
vapB31
vapC31
Rv0749A
vapC25,vapB25,PE_PGRS3-H37Rv-332698-336275 --- k-mer match to putative recomb seq = 0.9079 (69/76)
Match to reference locus
Match to recombinant "k-mer Only"
k-mer match
1.0
0.8
0.6
0.4
0.2
0.0
841,100
841,200
841,300
841,400
841,500
841,600
841,700
841,800
841,900
H37rv Ref Position (bp)

Sub-phylogeny Viz + Paralog Alignments
GCE ID: Event_060

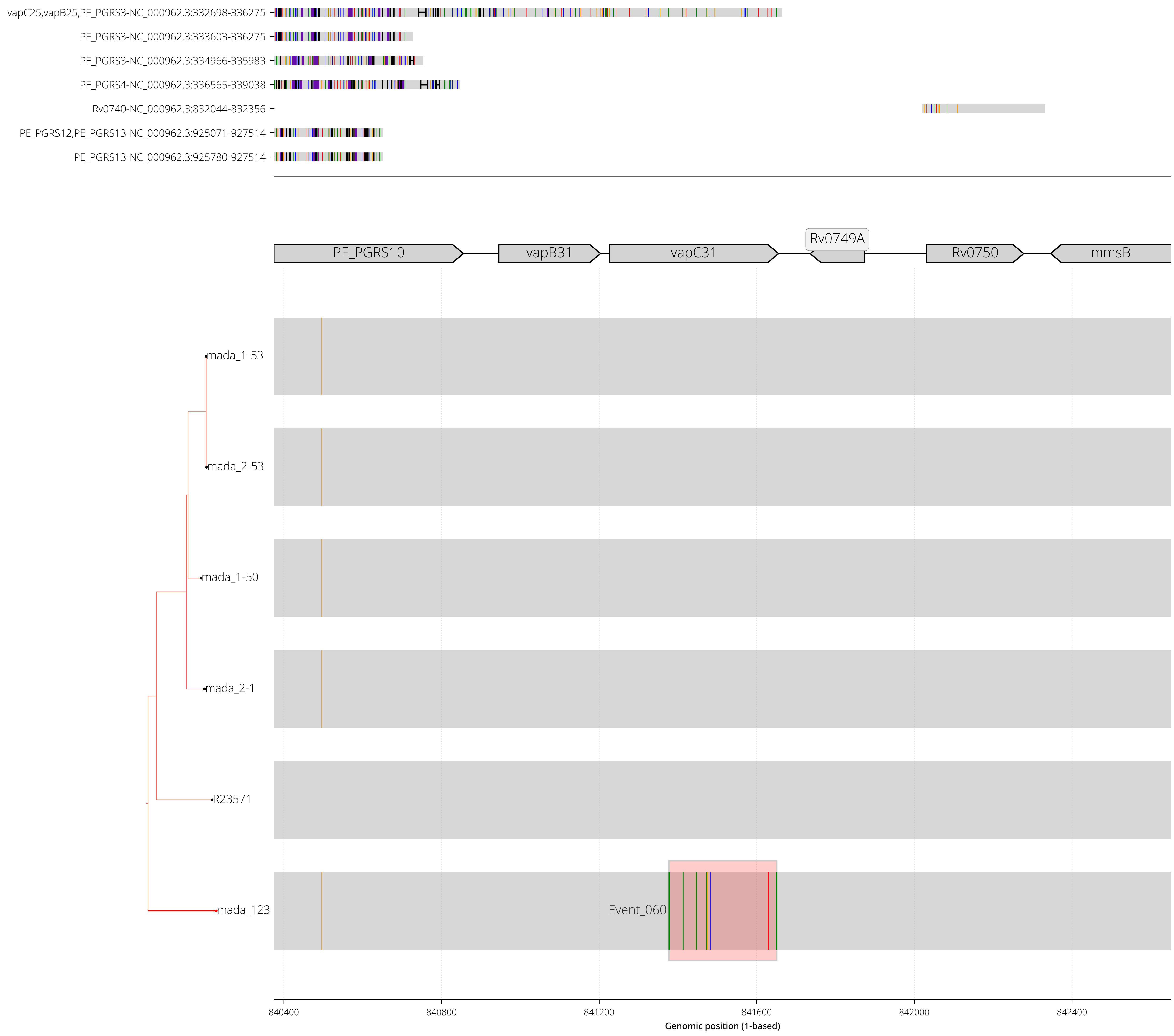

Full Phylogeny Viz + Paralog Alignments
GCE ID: Event_060

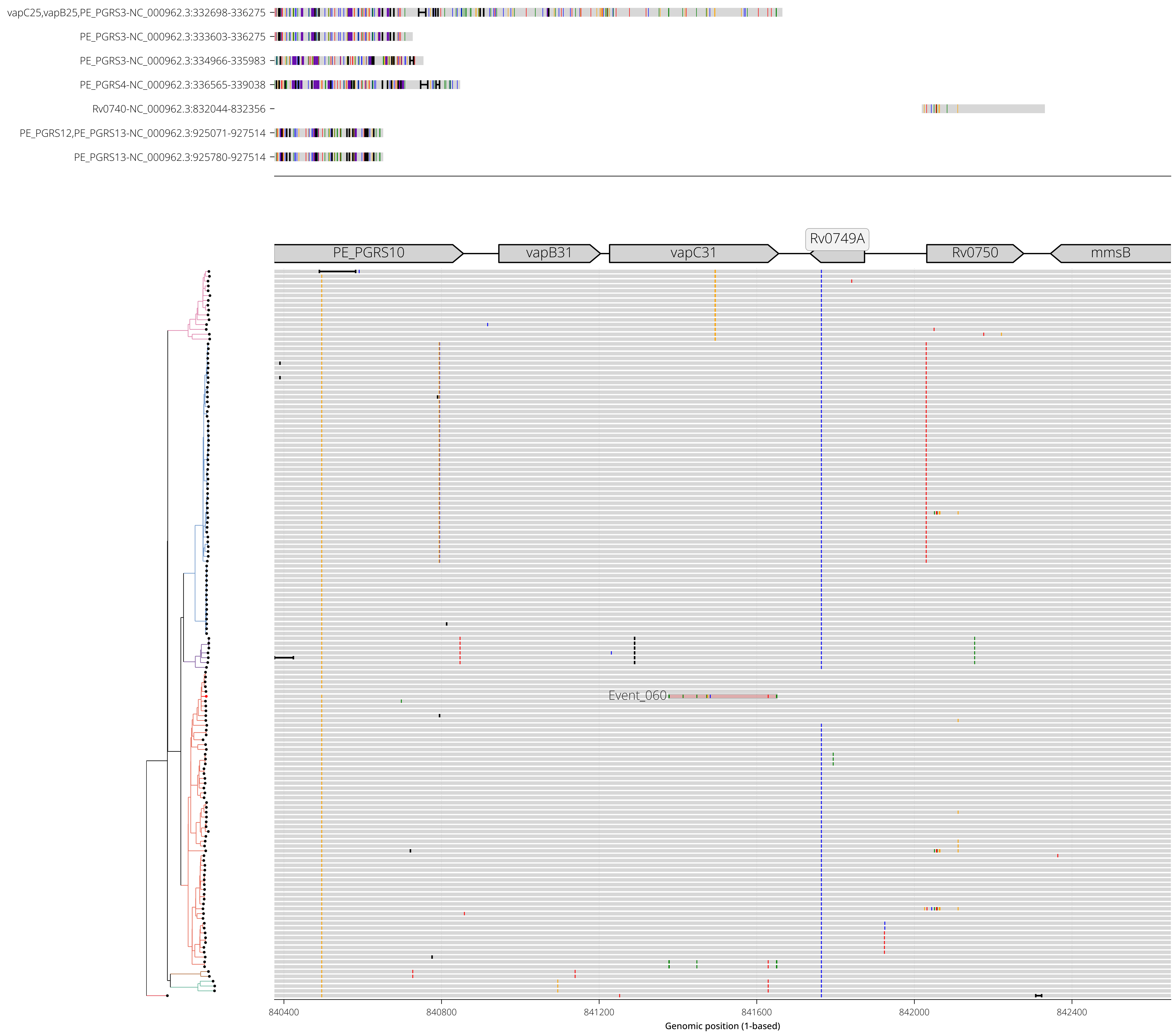

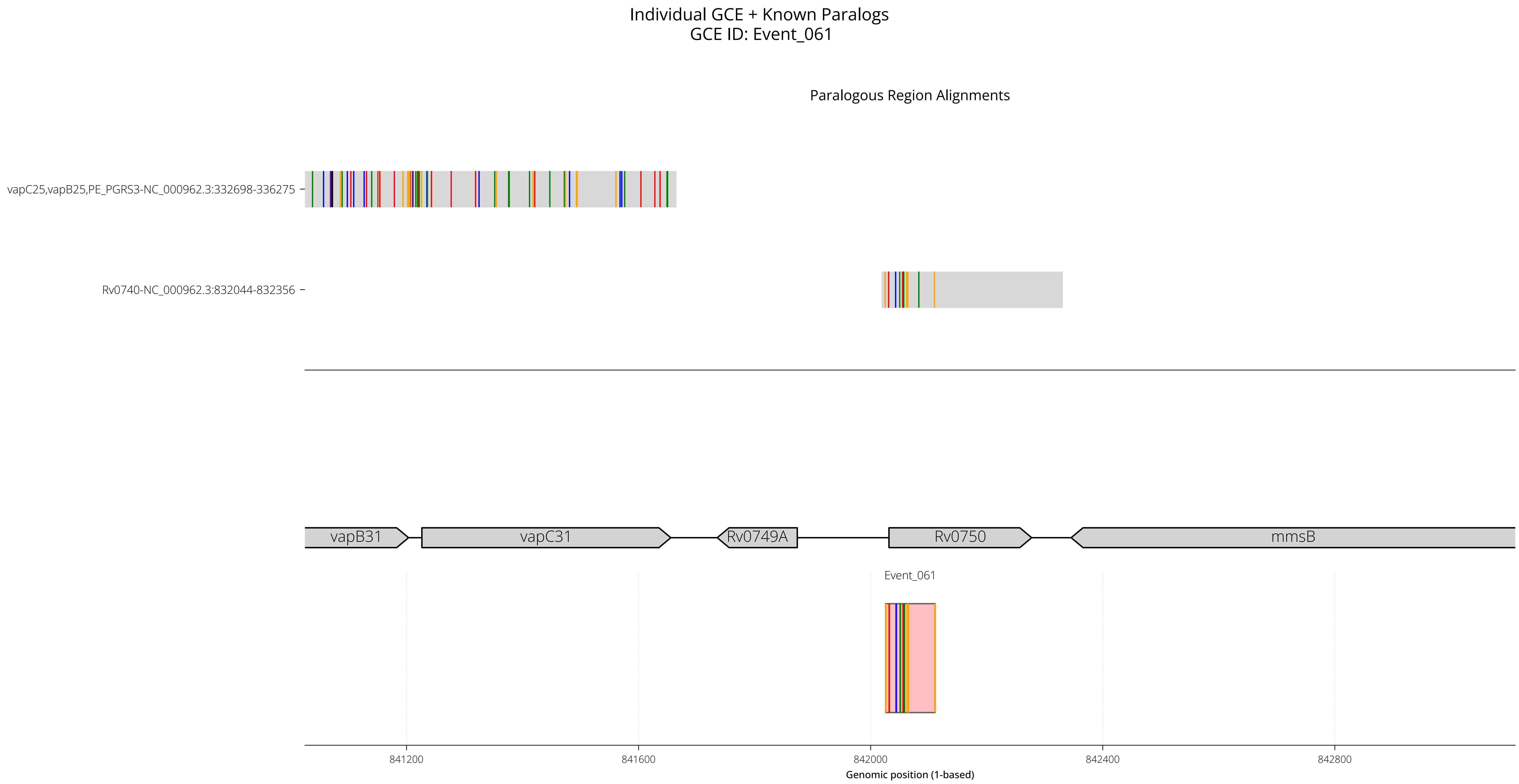
Individual GCE + Known Paralogs
GCE ID: Event_061
Paralogous Region Alignments
vapC25,vapB25,PE_PGRS3-NC_000962.3:332698-336275
Rv0740-NC_000962.3:832044-832356
vapB31
vapC31
Rv0749A
Rv0750
mmsB
Event_061
841200
841600
842000
842400
842800
Genomic position (1-based)

Circos Viz - GCE to Known Paralogs Locations
GCE ID: Event_061

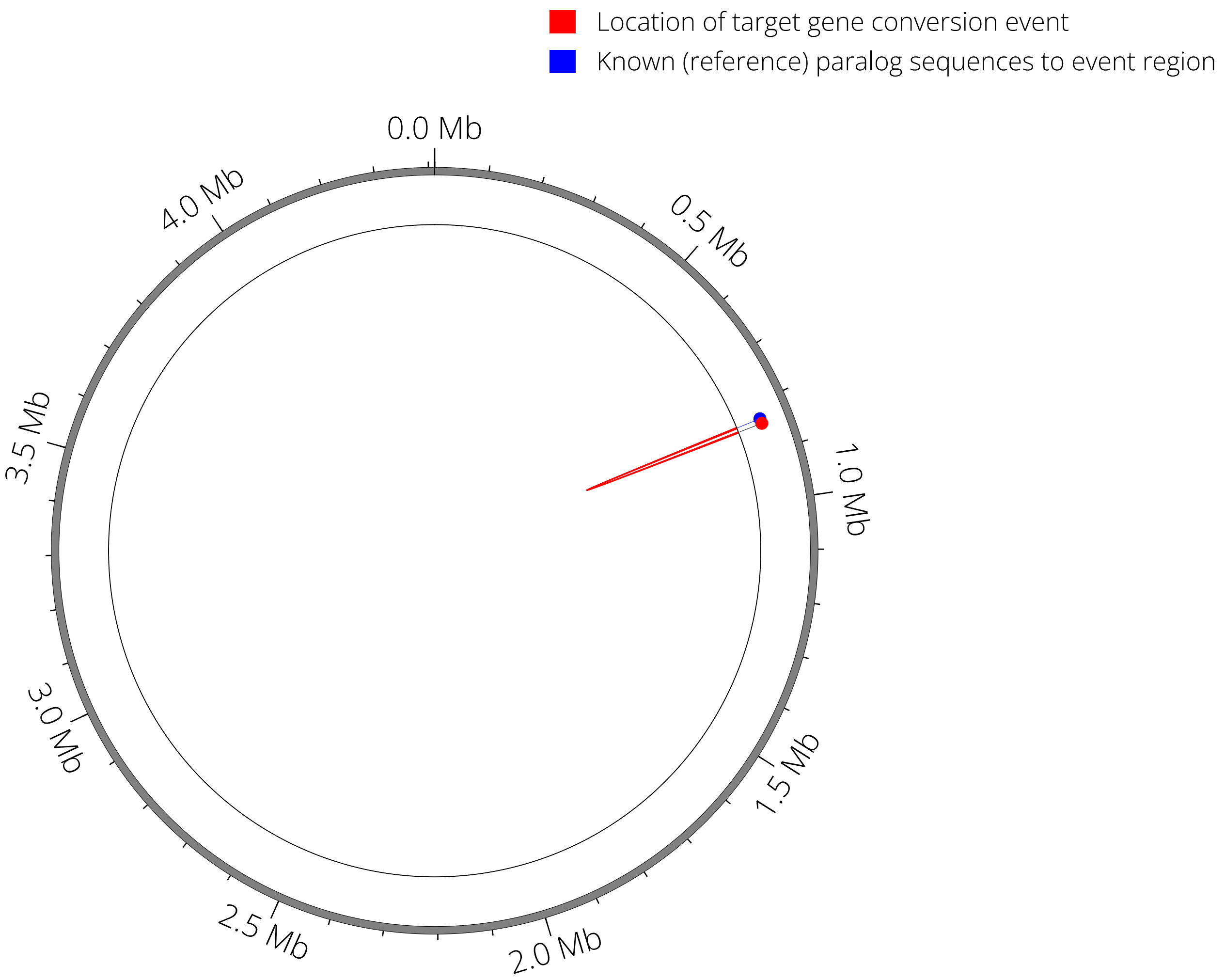

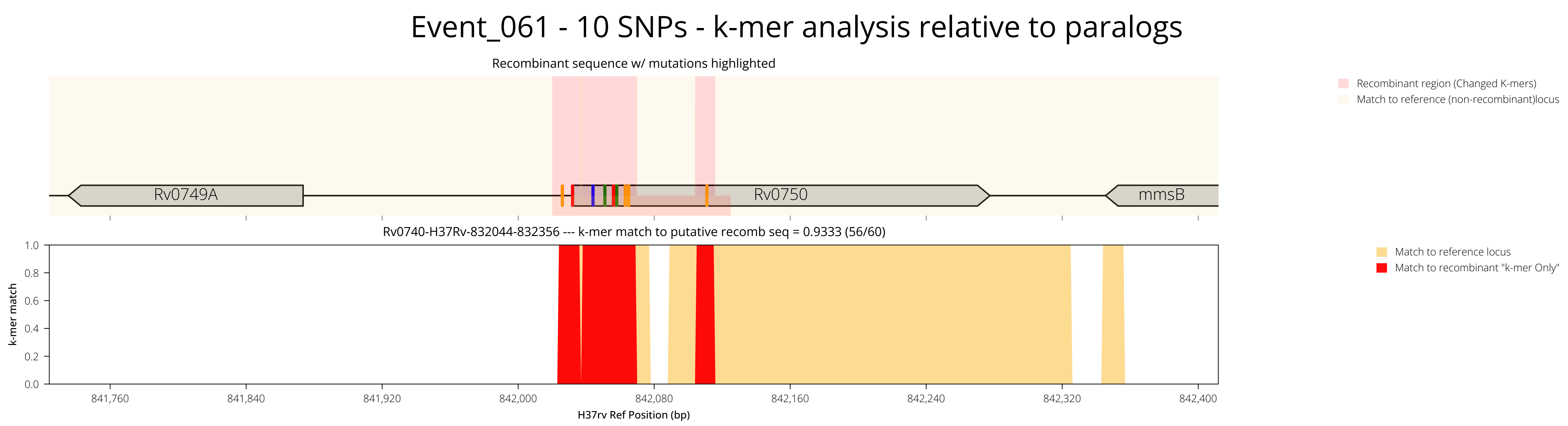
Event_061 - 10 SNPs - k-mer analysis relative to paralogs
Recombinant sequence w/ mutations highlighted
Rv0749A
Rv0750
mmsB
Recombinant region (Changed K-mers)
Match to reference (non-recombinant)locus
Rv0740-H37Rv-832044-832356 --- k-mer match to putative recomb seq = 0.9333 (56/60)
k-mer match
1.0
0.8
0.6
0.4
0.2
0.0
841,760
841,840
841,920
842,000
842,080
842,160
842,240
842,320
842,400
H37rv Ref Position (bp)
Match to reference locus
Match to recombinant "k-mer Only"

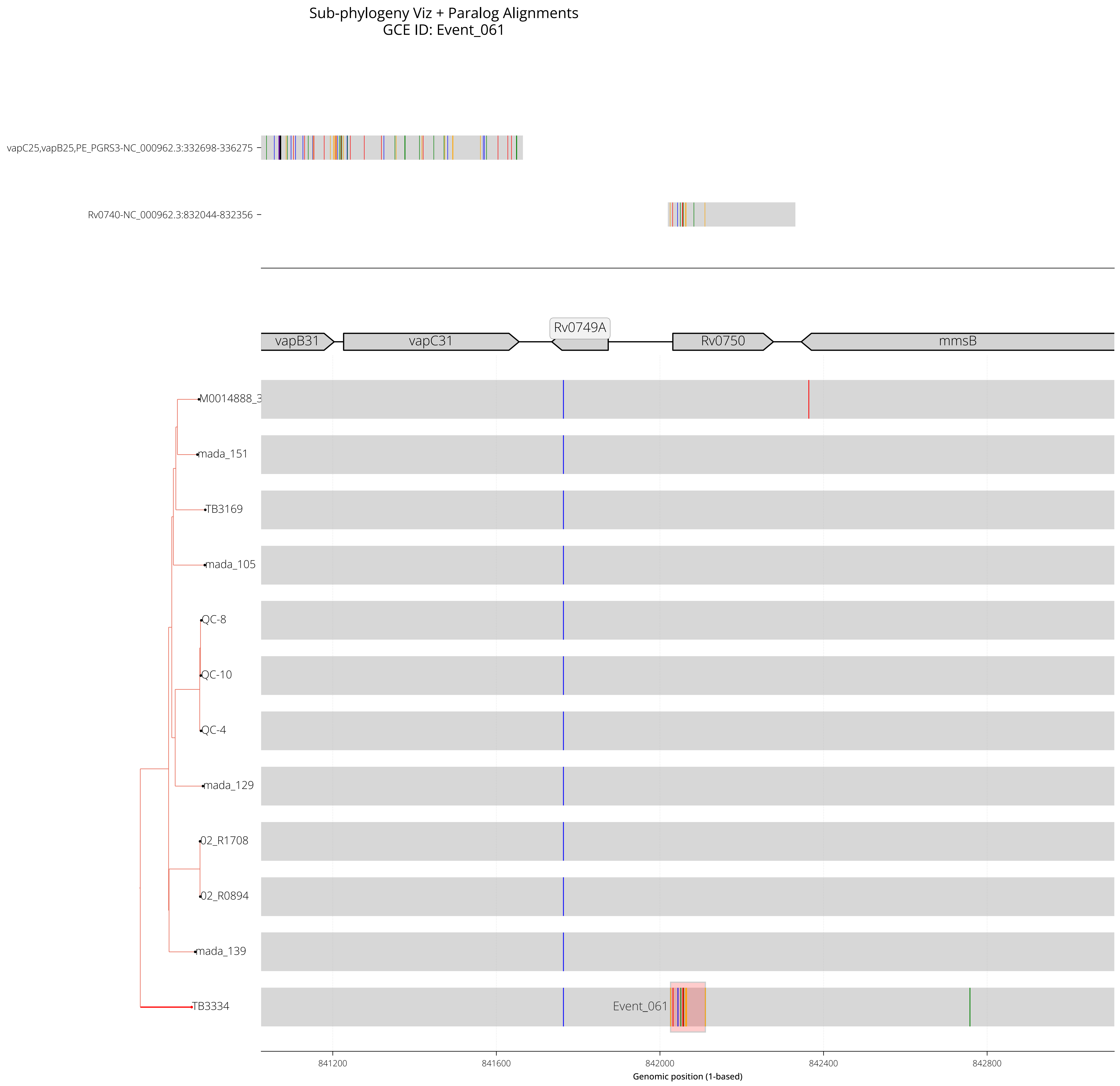

Sub-phylogeny Viz + Paralog Alignments
GCE ID: Event_061
vapC25,vapB25,PE_PGRS3-NC_000962.3:332698-336275
Rv0740-NC_000962.3:832044-832356
vapB31
vapC31
Rv0749A
Rv0750
mmsB
M0014888_3
mada_151
TB3169
mada_105
QC-8
QC-10
QC-4
mada_129
02_R1708
02_R0894
mada_139
TB3334
Event_061
841200
841600
842000
842400
842800
Genomic position (1-based)

Full Phylogeny Viz + Paralog Alignments
GCE ID: Event_061

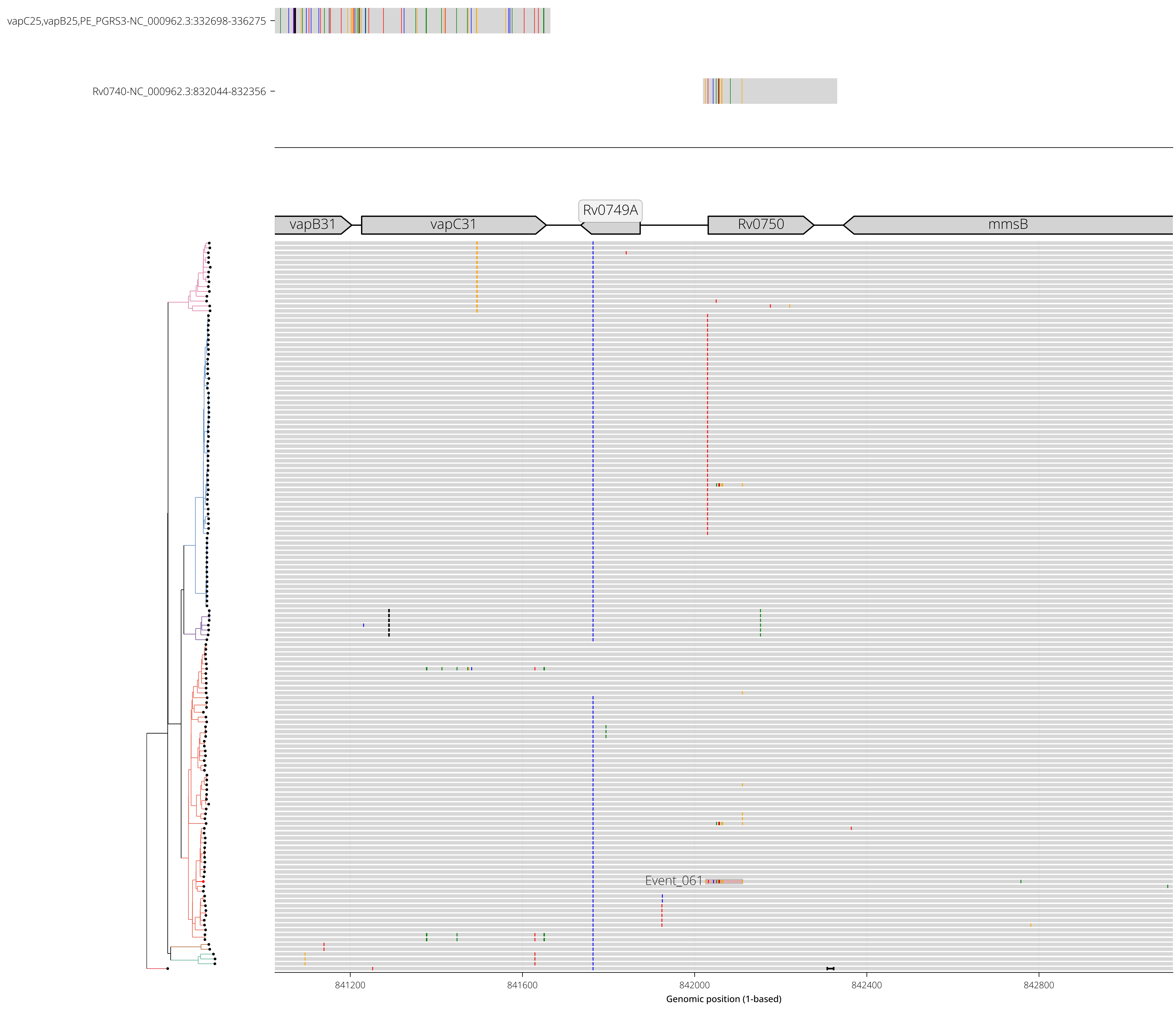

Individual GCE + Known Paralogs
GCE ID: Event_062

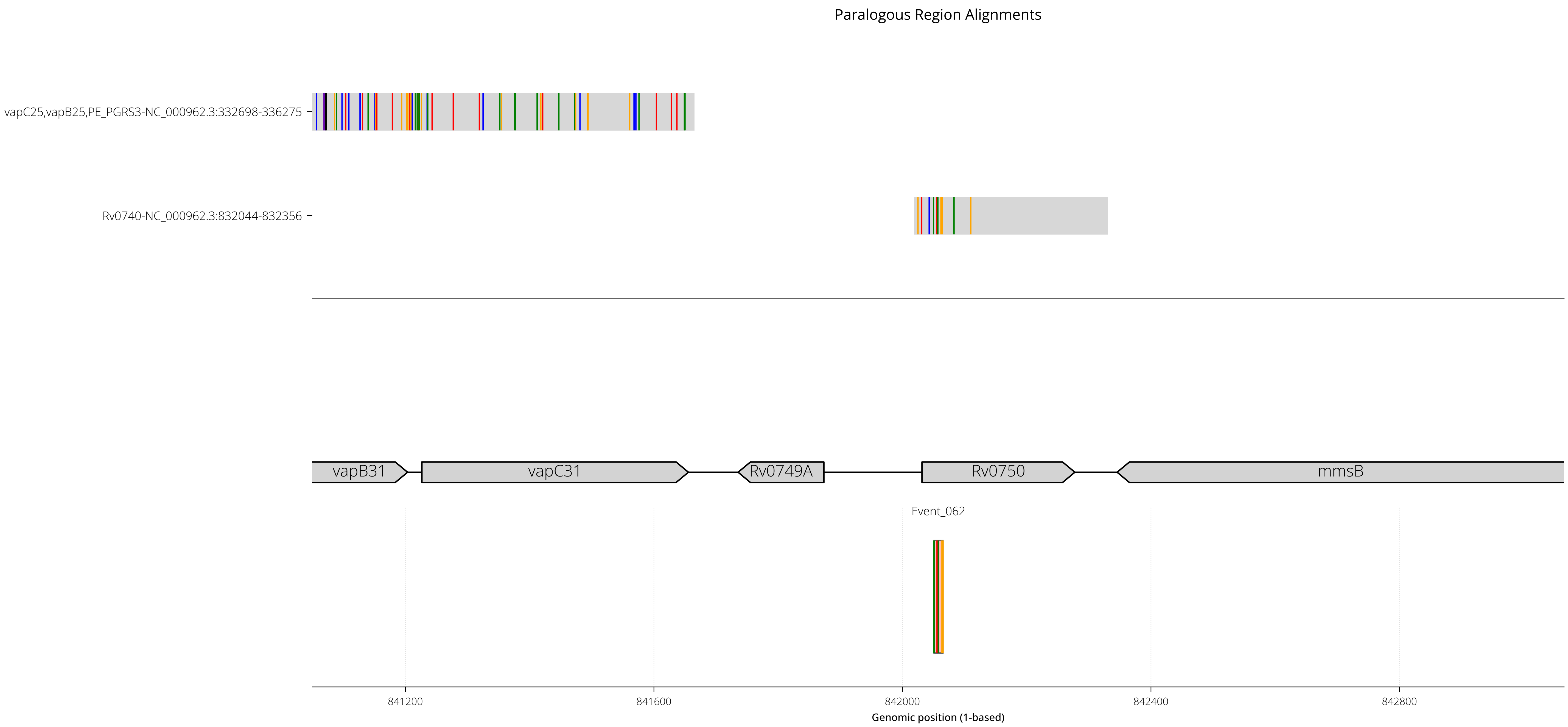

# Circos Viz - GCE to Known Paralogs Locations
GCE ID: Event_062

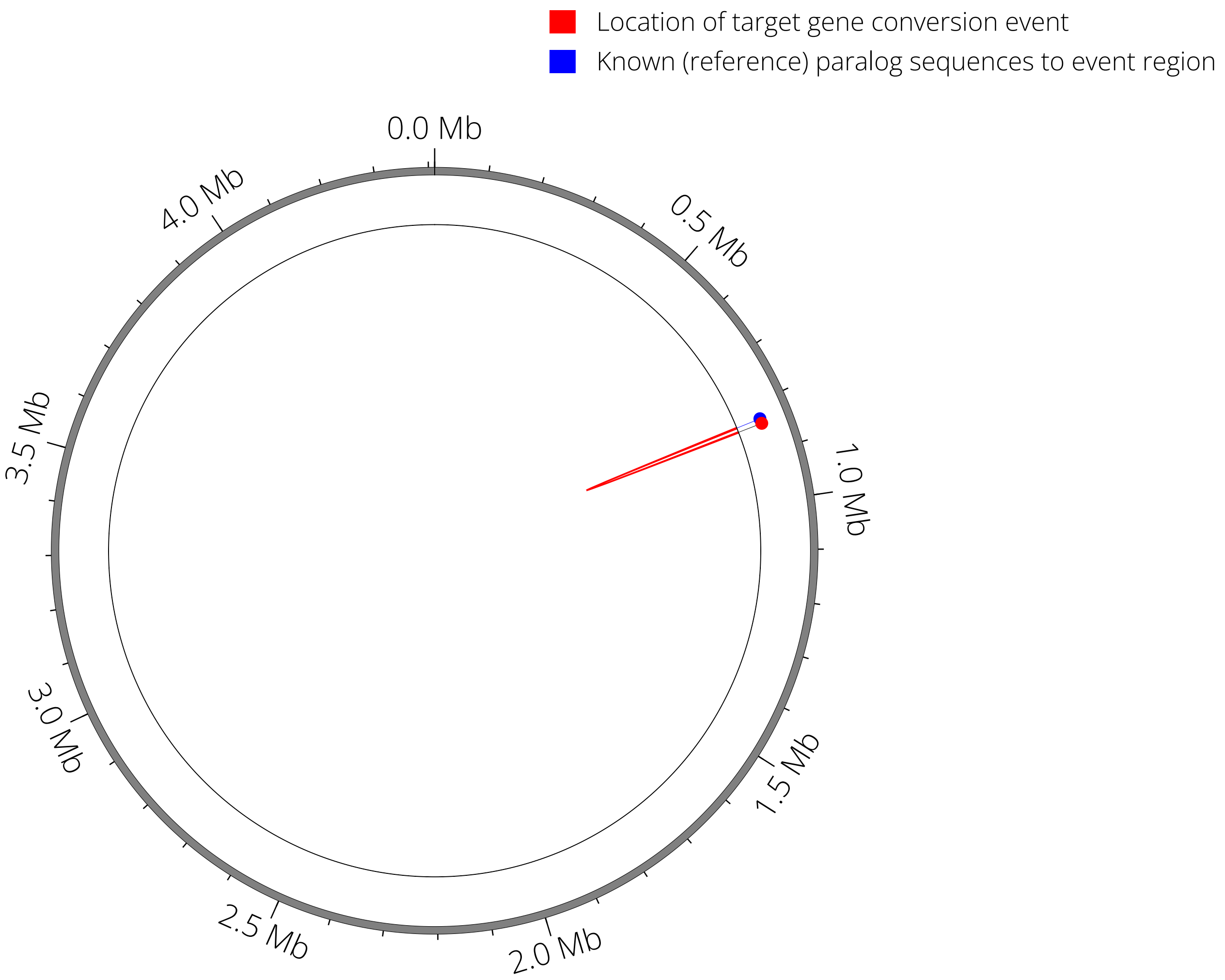

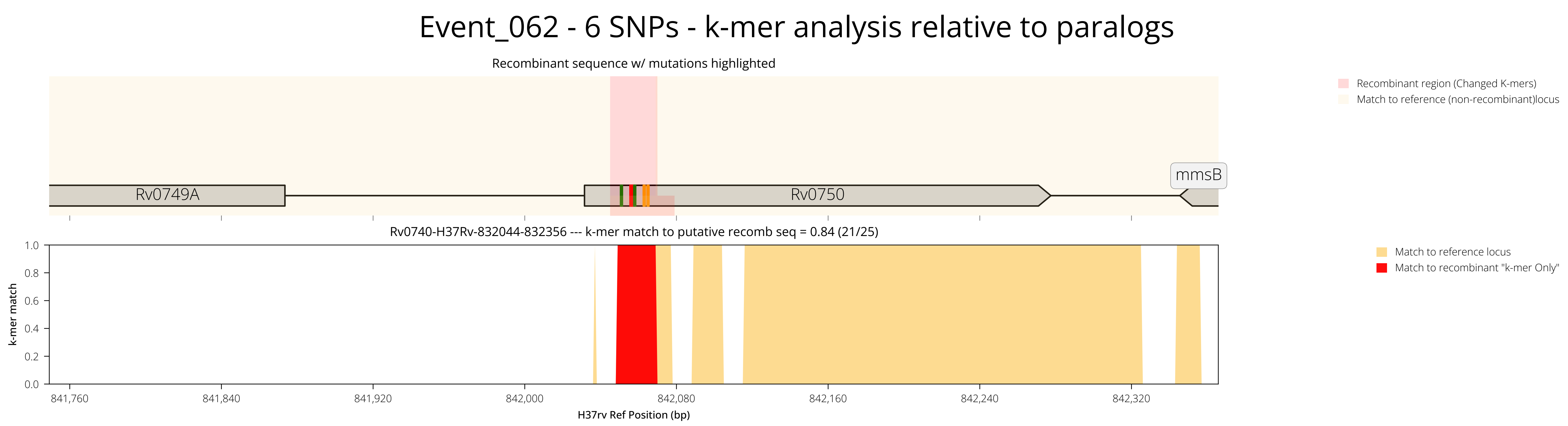
Event_062 - 6 SNPs - k-mer analysis relative to paralogs
Recombinant sequence w/ mutations highlighted
Recombinant region (Changed K-mers)
Match to reference (non-recombinant)locus
Rv0749A
Rv0750
mmsB
Rv0740-H37Rv-832044-832356 --- k-mer match to putative recomb seq = 0.84 (21/25)
Match to reference locus
Match to recombinant "k-mer Only"
k-mer match
1.0
0.8
0.6
0.4
0.2
0.0
841,760
841,840
841,920
842,000
842,080
842,160
842,240
842,320
H37rv Ref Position (bp)

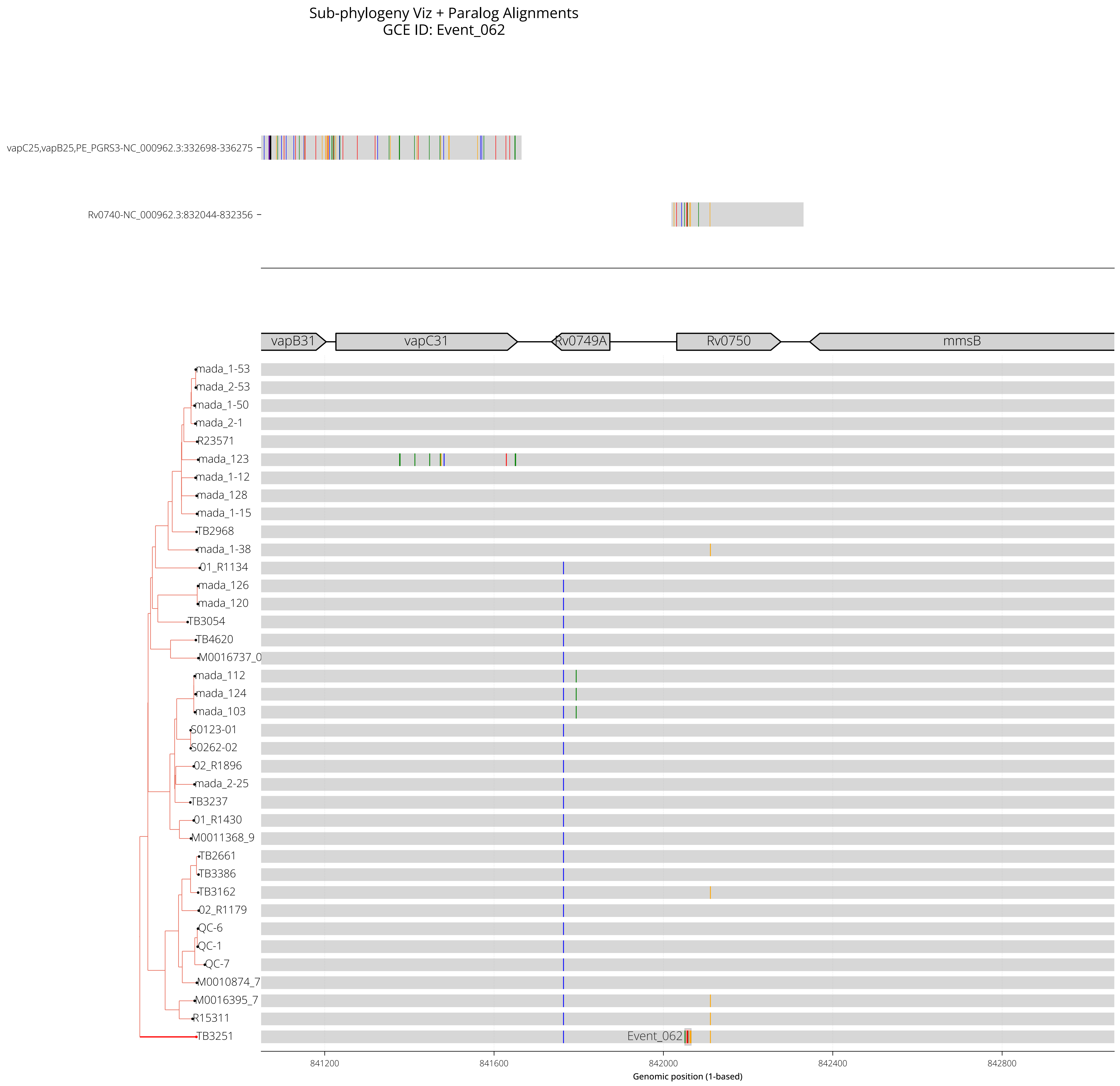
Sub-phylogeny Viz + Paralog Alignments
GCE ID: Event_062
vapC25,vapB25,PE_PGRS3-NC_000962.3:332698-336275
Rv0740-NC_000962.3:832044-832356
vapB31
vapC31
Rv0749A
Rv0750
mmsB
mada_1-53
mada_2-53
mada_1-50
mada_2-1
R23571
mada_123
mada_1-12
mada_128
mada_1-15
TB2968
mada_1-38
01_R1134
mada_126
mada_120
TB3054
TB4620
M0016737_0
mada_112
mada_124
mada_103
S0123-01
S0262-02
02_R1896
mada_2-25
TB3237
01_R1430
M0011368_9
TB2661
TB3386
TB3162
02_R1179
QC-6
QC-1
QC-7
M0010874_7
M0016395_7
R15311
TB3251
Event_062
841200
841600
842000
842400
842800
Genomic position (1-based)

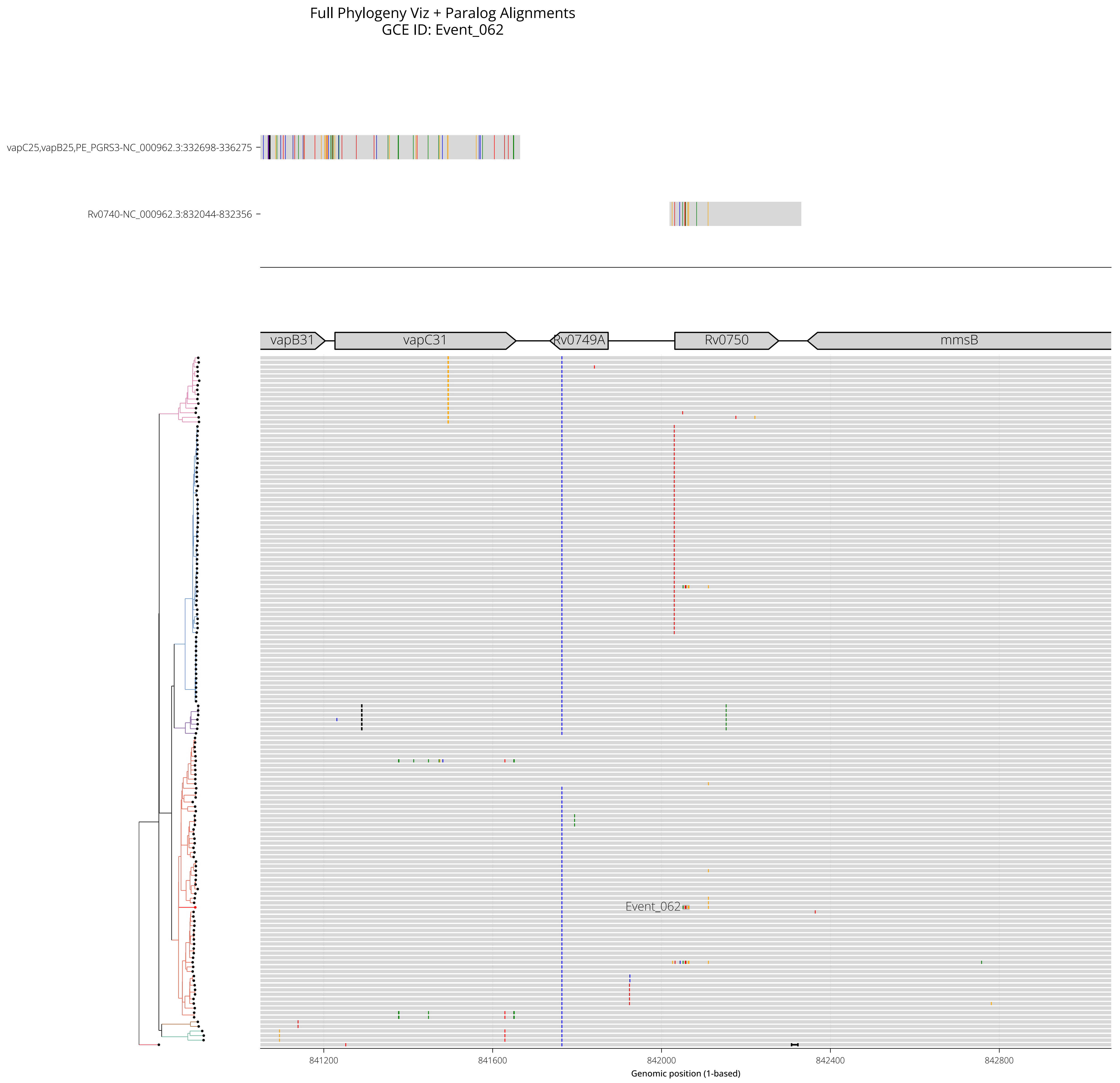

Full Phylogeny Viz + Paralog Alignments
GCE ID: Event_062
vapC25,vapB25,PE_PGRS3-NC_000962.3:332698-336275
Rv0740-NC_000962.3:832044-832356
vapB31
vapC31
Rv0749A
Rv0750
mmsB
Event_062
841200
841600
842000
842400
842800
Genomic position (1-based)

Individual GCE + Known Paralogs
GCE ID: Event_063

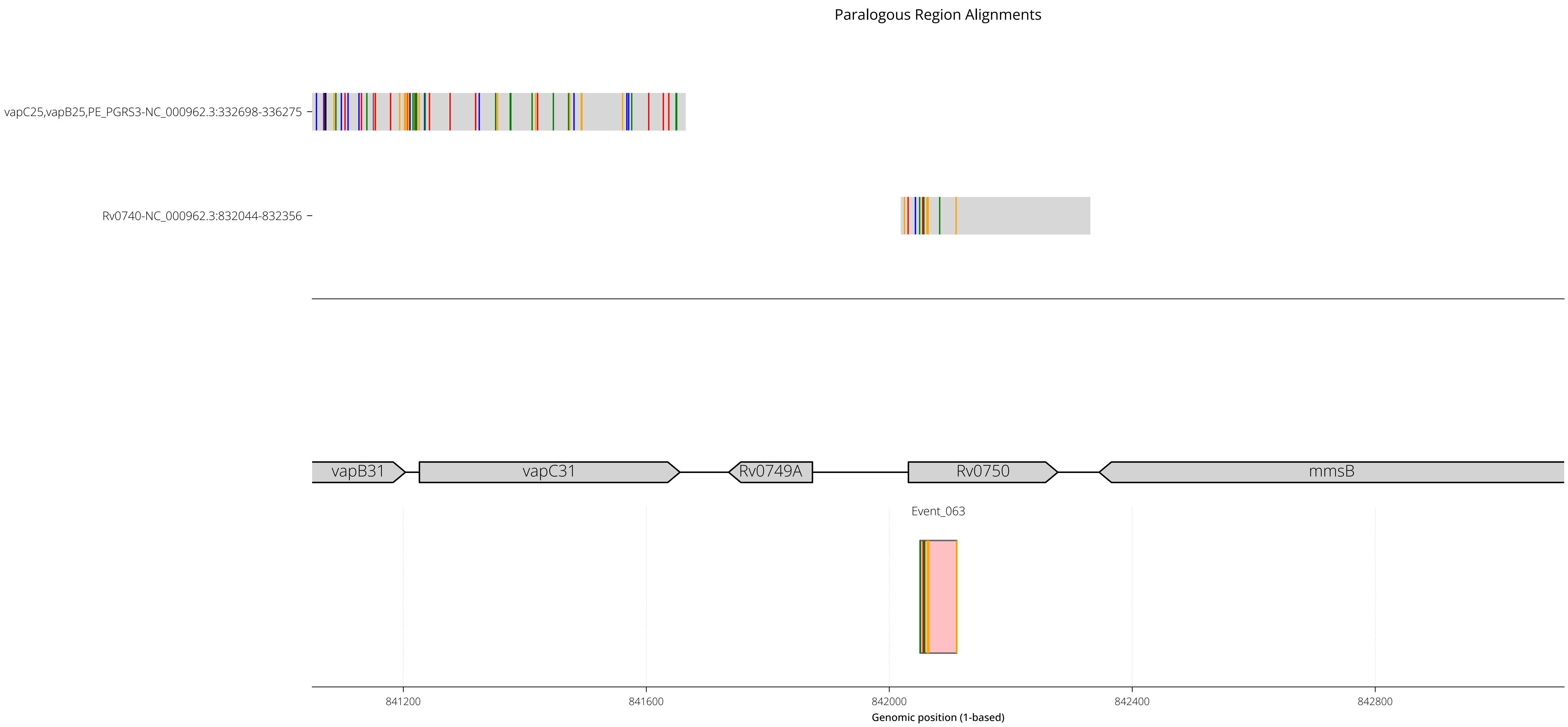

Circos Viz - GCE to Known Paralogs Locations
GCE ID: Event_063

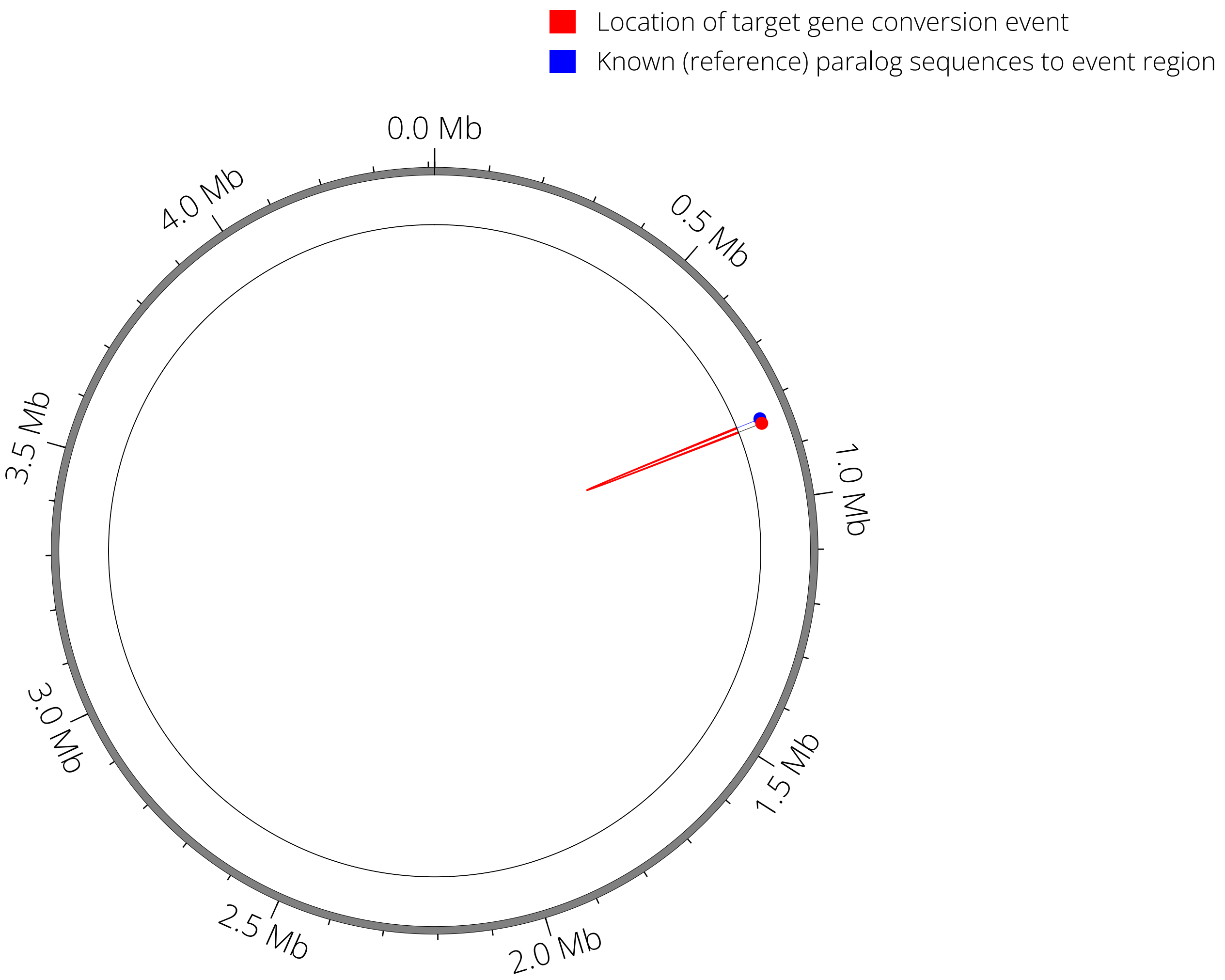

# Event_063 - 7 SNPs - k-mer analysis relative to paralogs

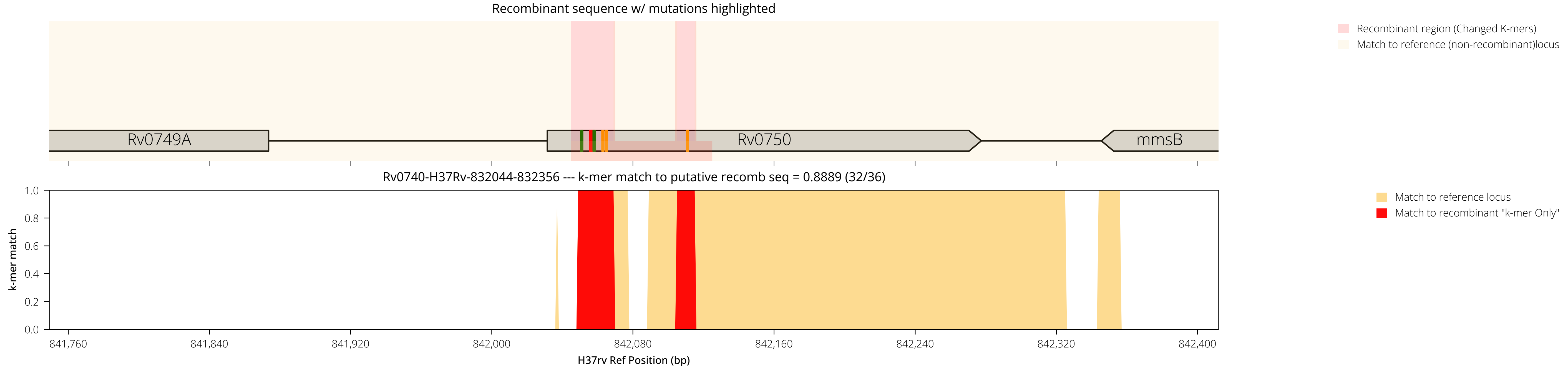

Sub-phylogeny Viz + Paralog Alignments
GCE ID: Event_063

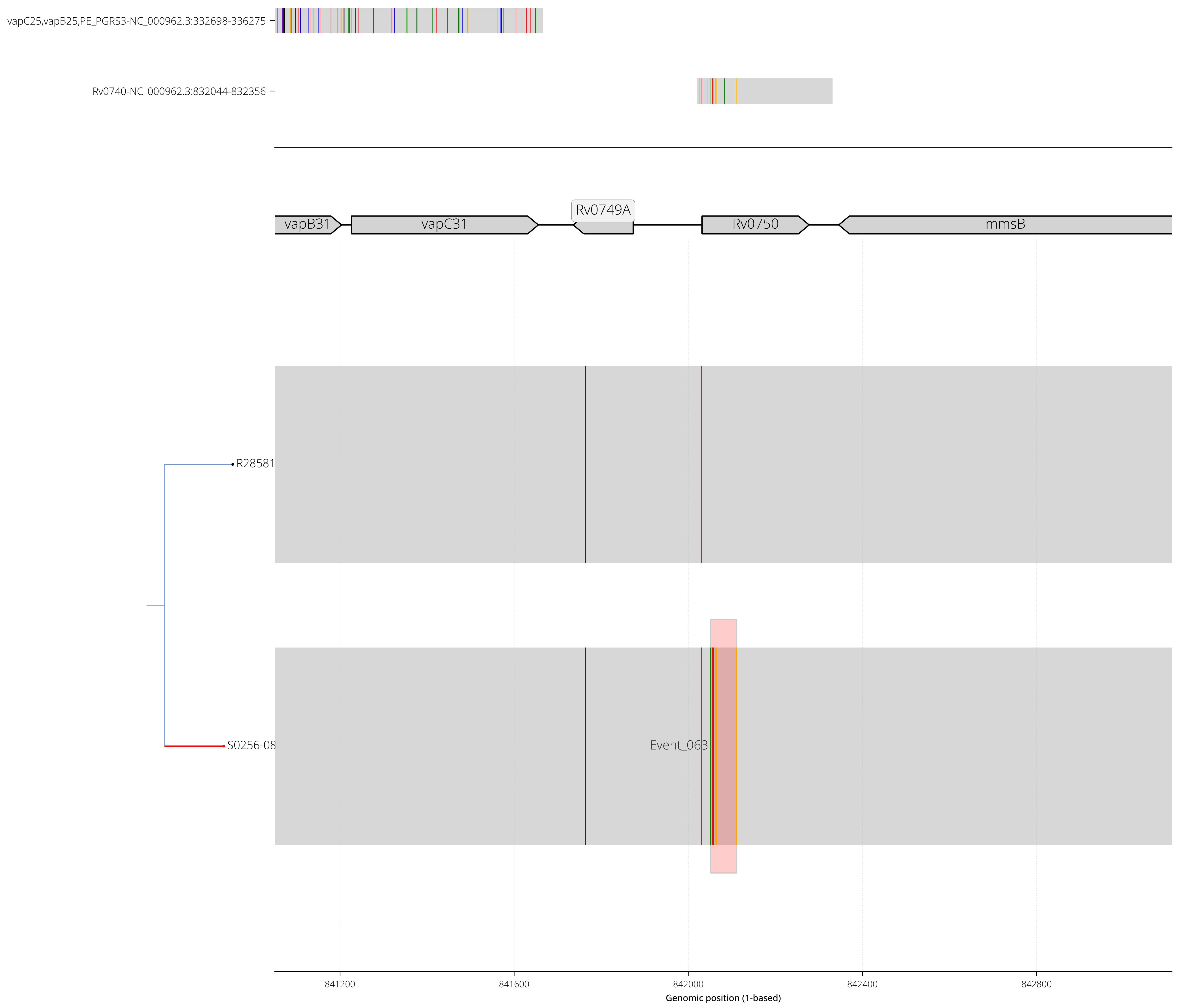

Full Phylogeny Viz + Paralog Alignments
GCE ID: Event_063

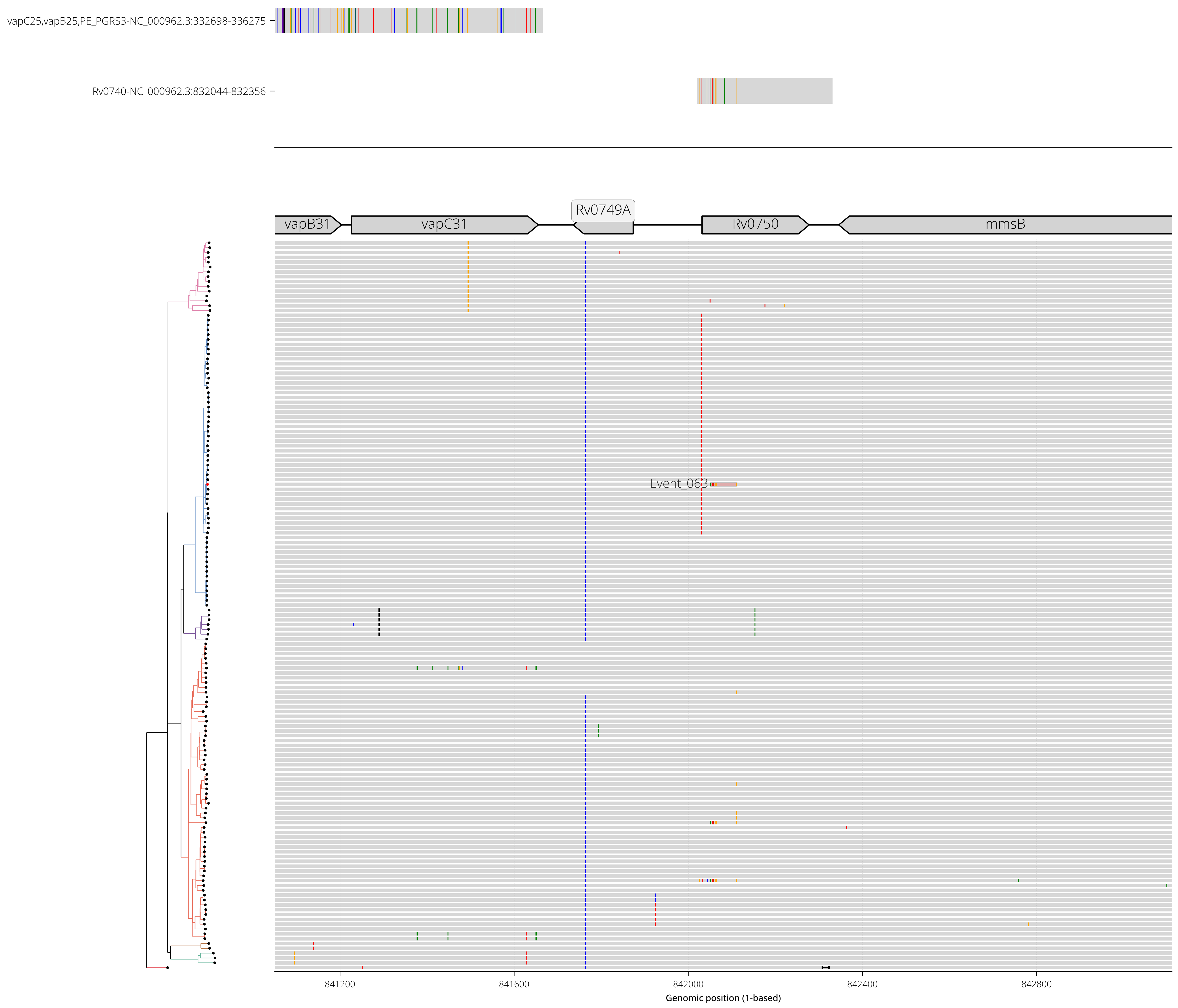

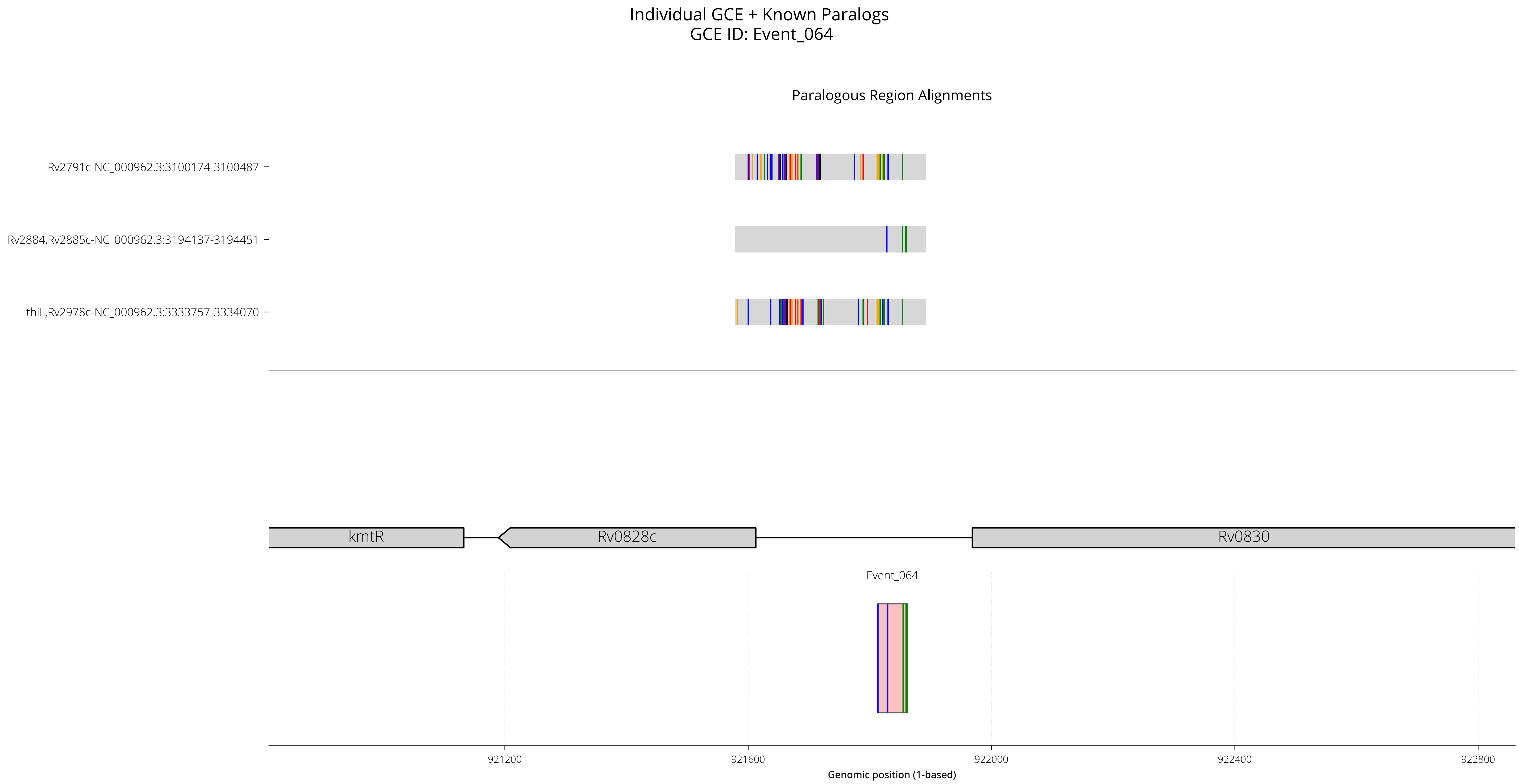

Individual GCE + Known Paralogs
GCE ID: Event_064
Paralogous Region Alignments
Rv2791c-NC_000962.3:3100174-3100487
Rv2884,Rv2885c-NC_000962.3:3194137-3194451
thiL,Rv2978c-NC_000962.3:3333757-3334070
kmtR
Rv0828c
Rv0830
Event_064
921200
921600
922000
922400
922800
Genomic position (1-based)

Circos Viz - GCE to Known Paralogs Locations
GCE ID: Event_064

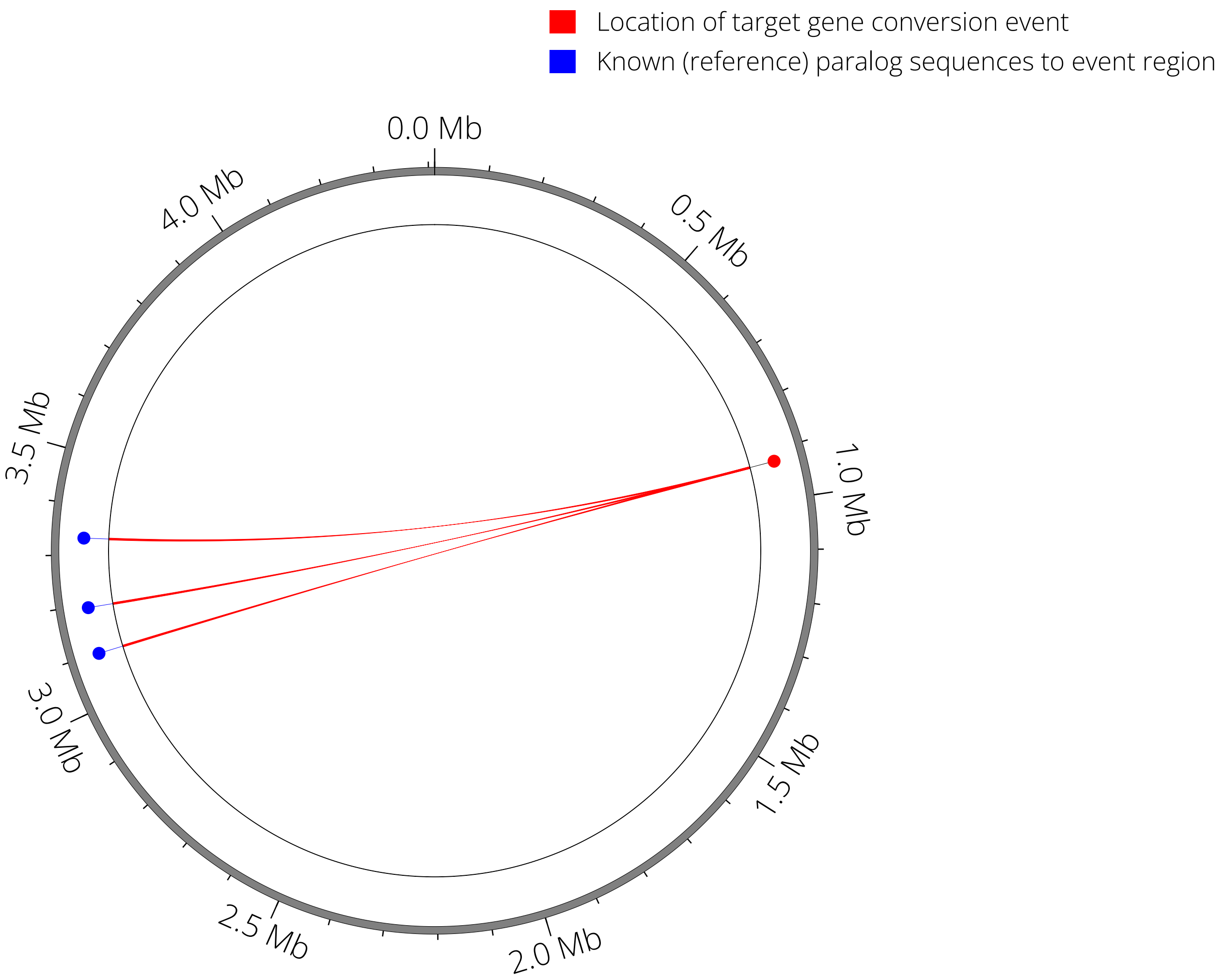

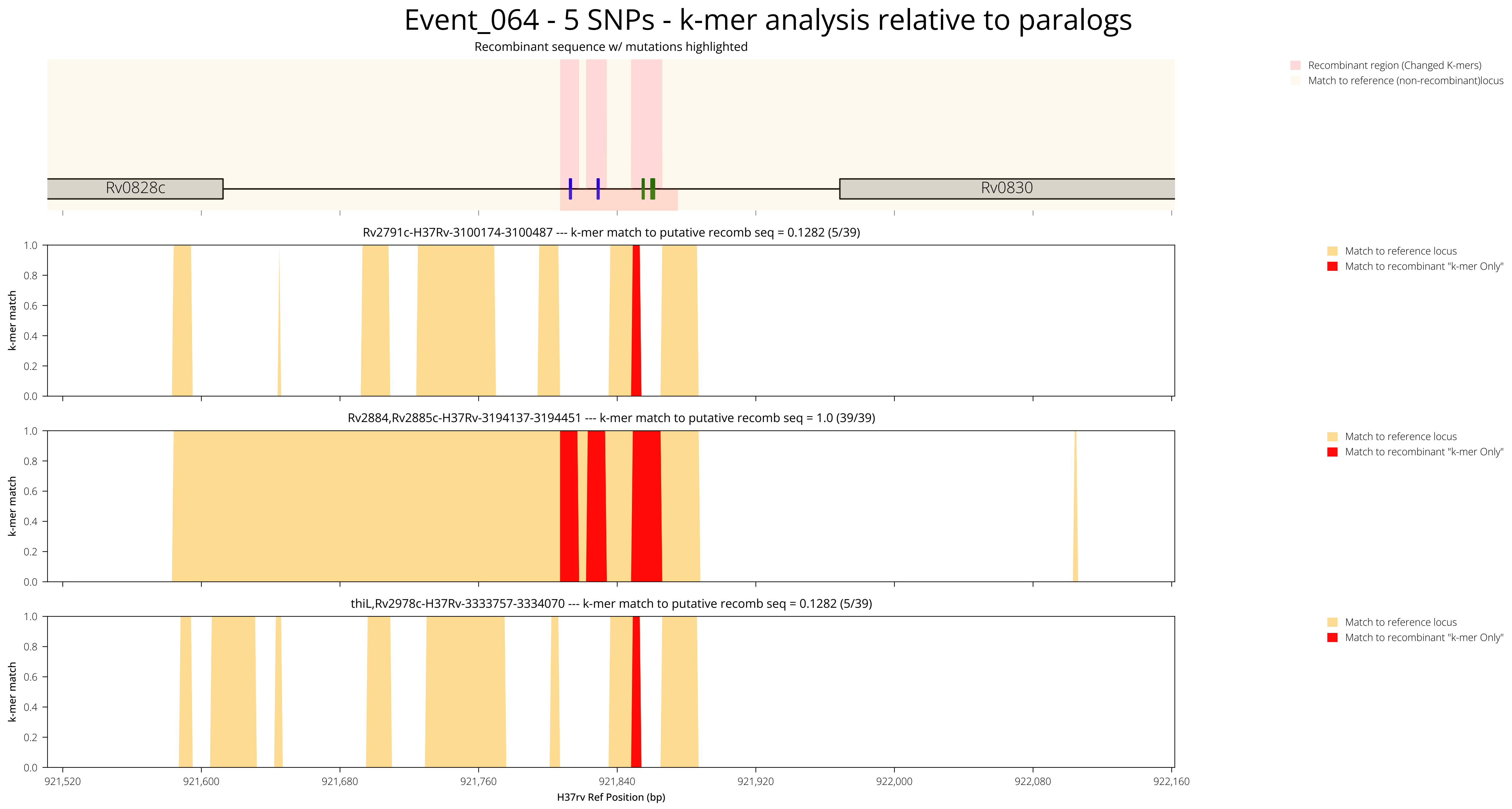
Event_064 - 5 SNPs - k-mer analysis relative to paralogs
Recombinant sequence w/ mutations highlighted
Recombinant region (Changed K-mers)
Match to reference (non-recombinant)locus
Rv0828c
Rv0830
Rv2791c-H37Rv-3100174-3100487 --- k-mer match to putative recomb seq = 0.1282 (5/39)
Rv2884,Rv2885c-H37Rv-3194137-3194451 --- k-mer match to putative recomb seq = 1.0 (39/39)
thiL,Rv2978c-H37Rv-3333757-3334070 --- k-mer match to putative recomb seq = 0.1282 (5/39)
Match to reference locus
Match to recombinant "k-mer Only"
k-mer match
1.0
0.8
0.6
0.4
0.2
0.0
921,520
921,600
921,680
921,760
921,840
921,920
922,000
922,080
922,160
H37rv Ref Position (bp)

Sub-phylogeny Viz + Paralog Alignments
GCE ID: Event_064

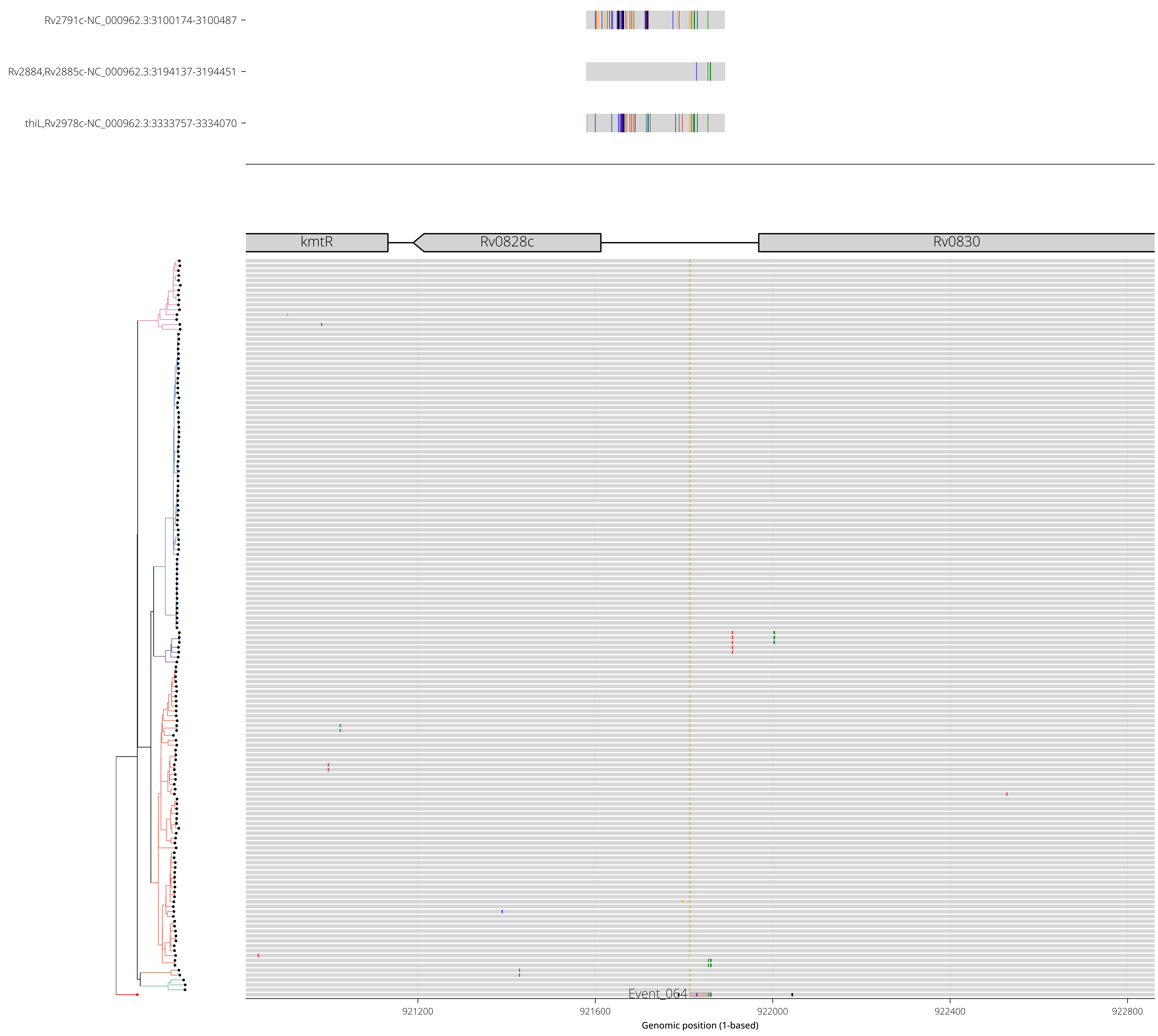

Full Phylogeny Viz + Paralog Alignments
GCE ID: Event_064

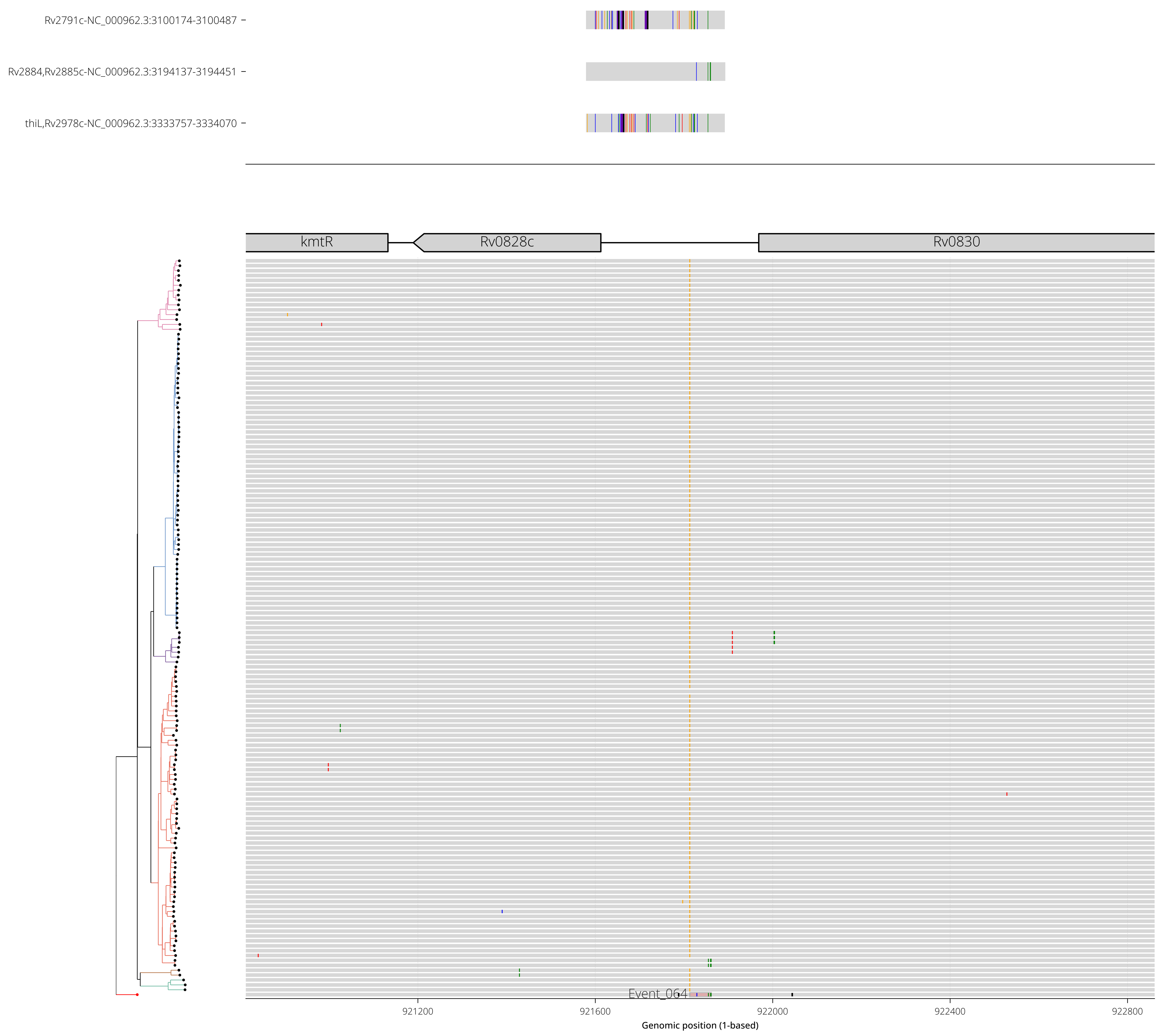

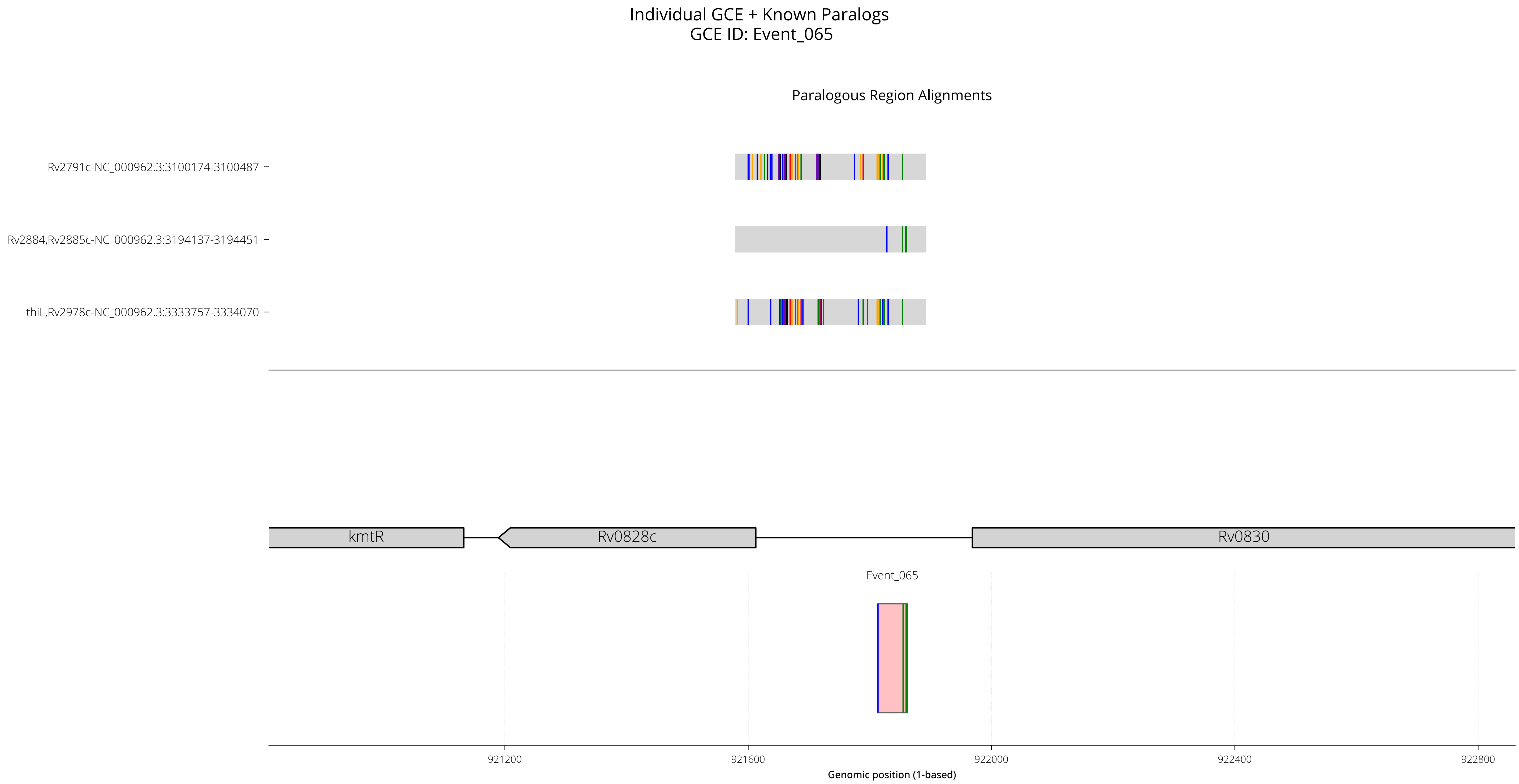

Individual GCE + Known Paralogs
GCE ID: Event_065
Paralogous Region Alignments
Rv2791c-NC_000962.3:3100174-3100487
Rv2884,Rv2885c-NC_000962.3:3194137-3194451
thiL,Rv2978c-NC_000962.3:3333757-3334070
kmtR
Rv0828c
Rv0830
Event_065
921200
921600
922000
922400
922800
Genomic position (1-based)

Circos Viz - GCE to Known Paralogs Locations
GCE ID: Event_065

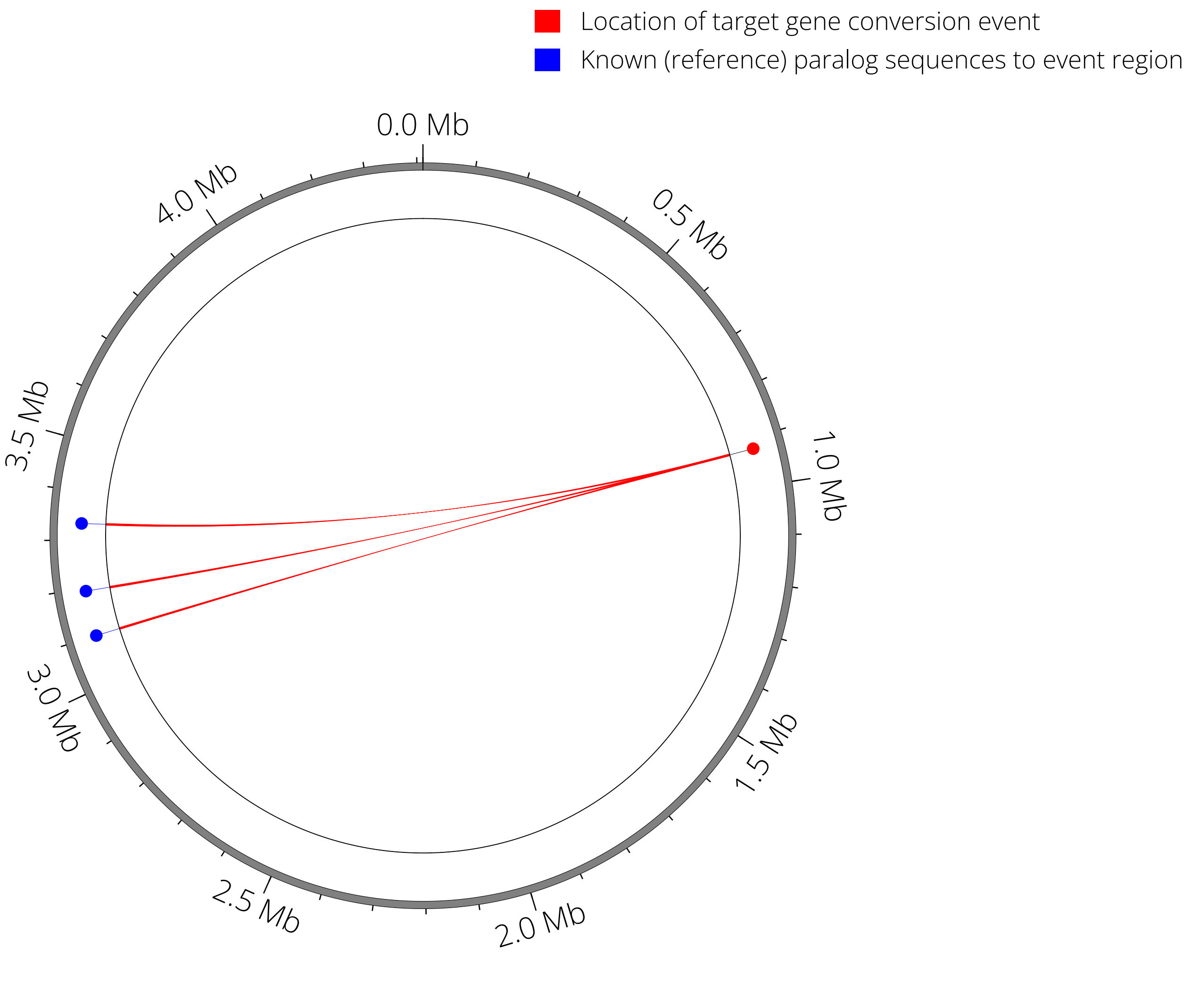

# Event_065 - 4 SNPs - k-mer analysis relative to paralogs

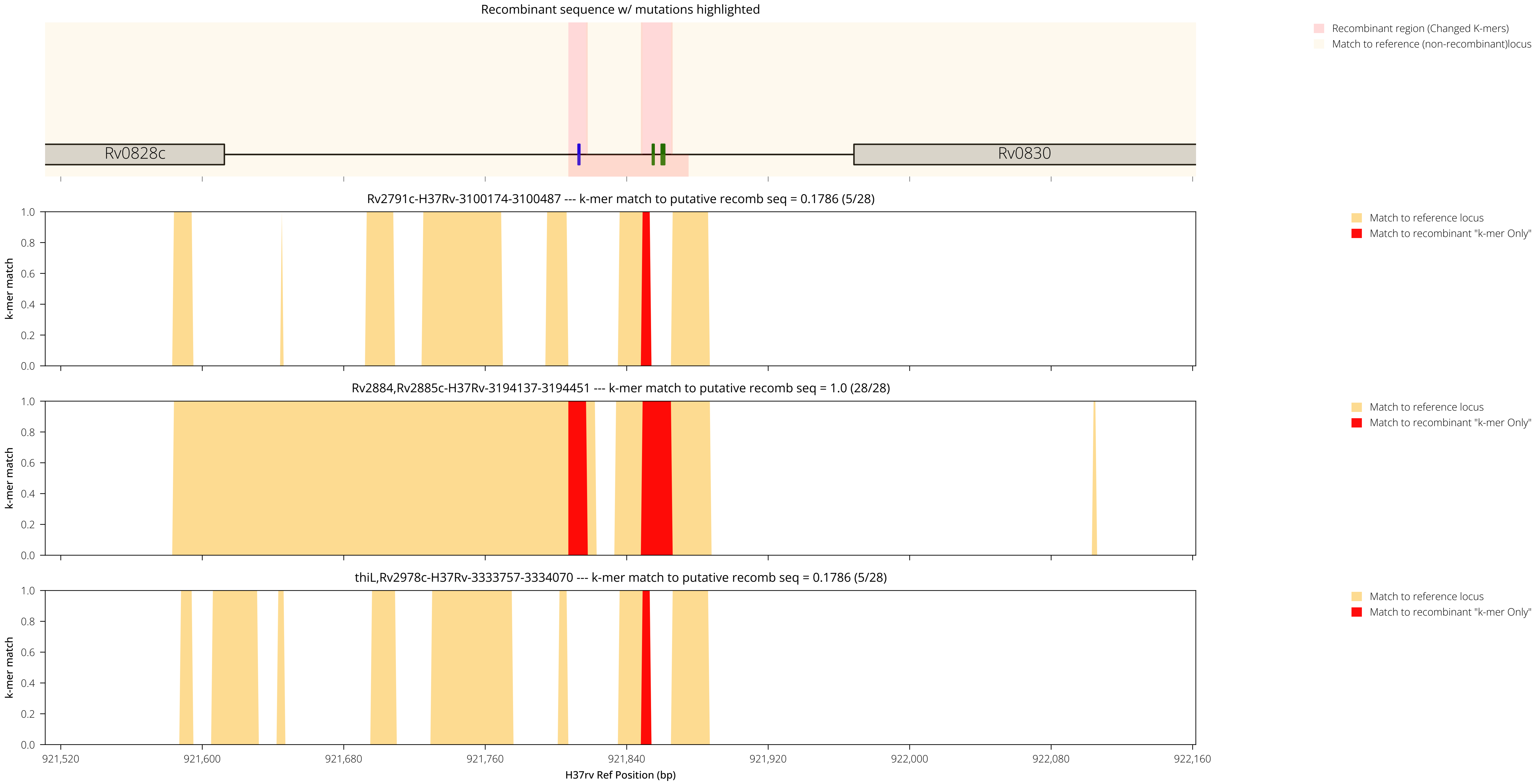

Sub-phylogeny Viz + Paralog Alignments
GCE ID: Event_065

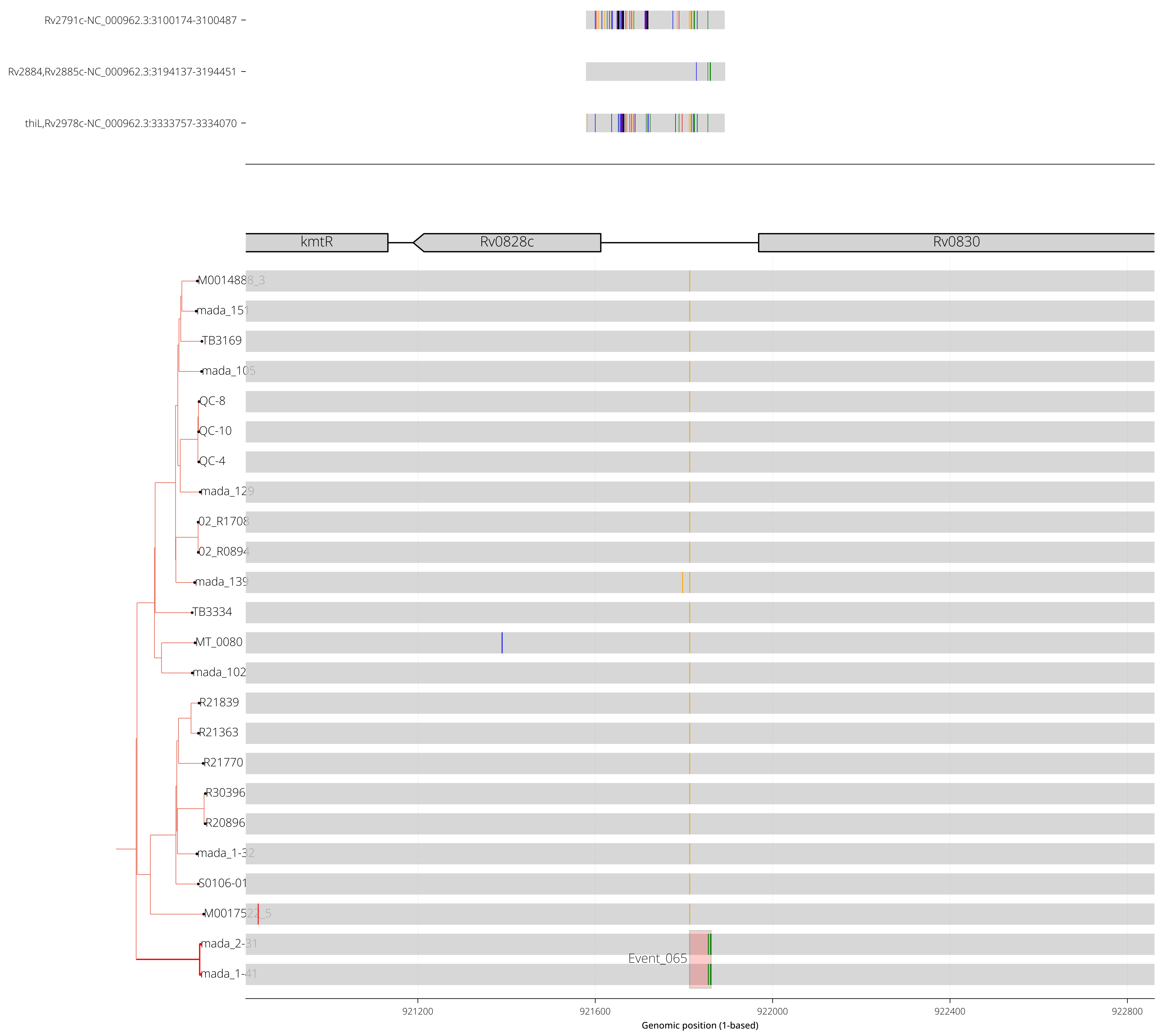

Full Phylogeny Viz + Paralog Alignments
GCE ID: Event_065

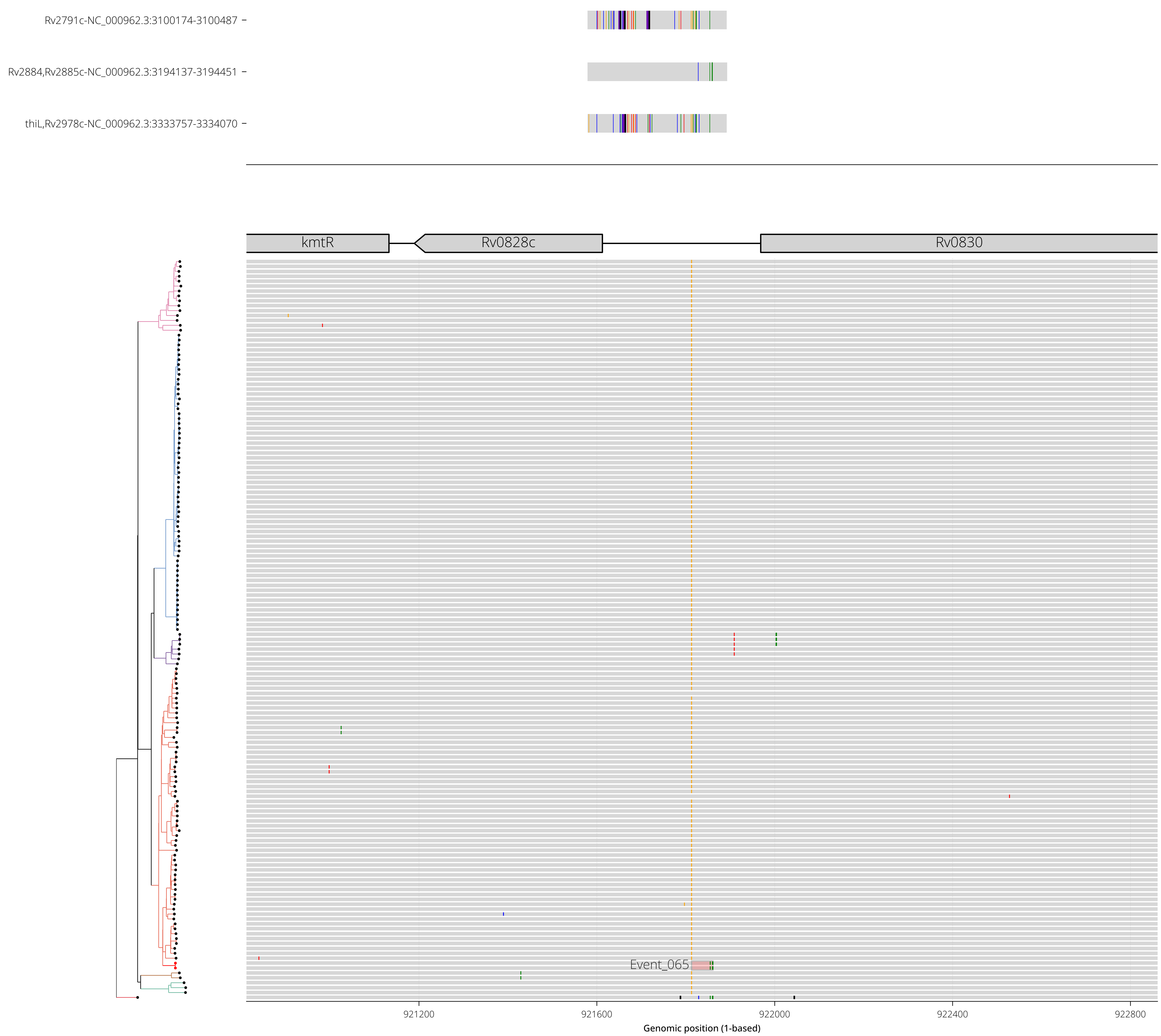

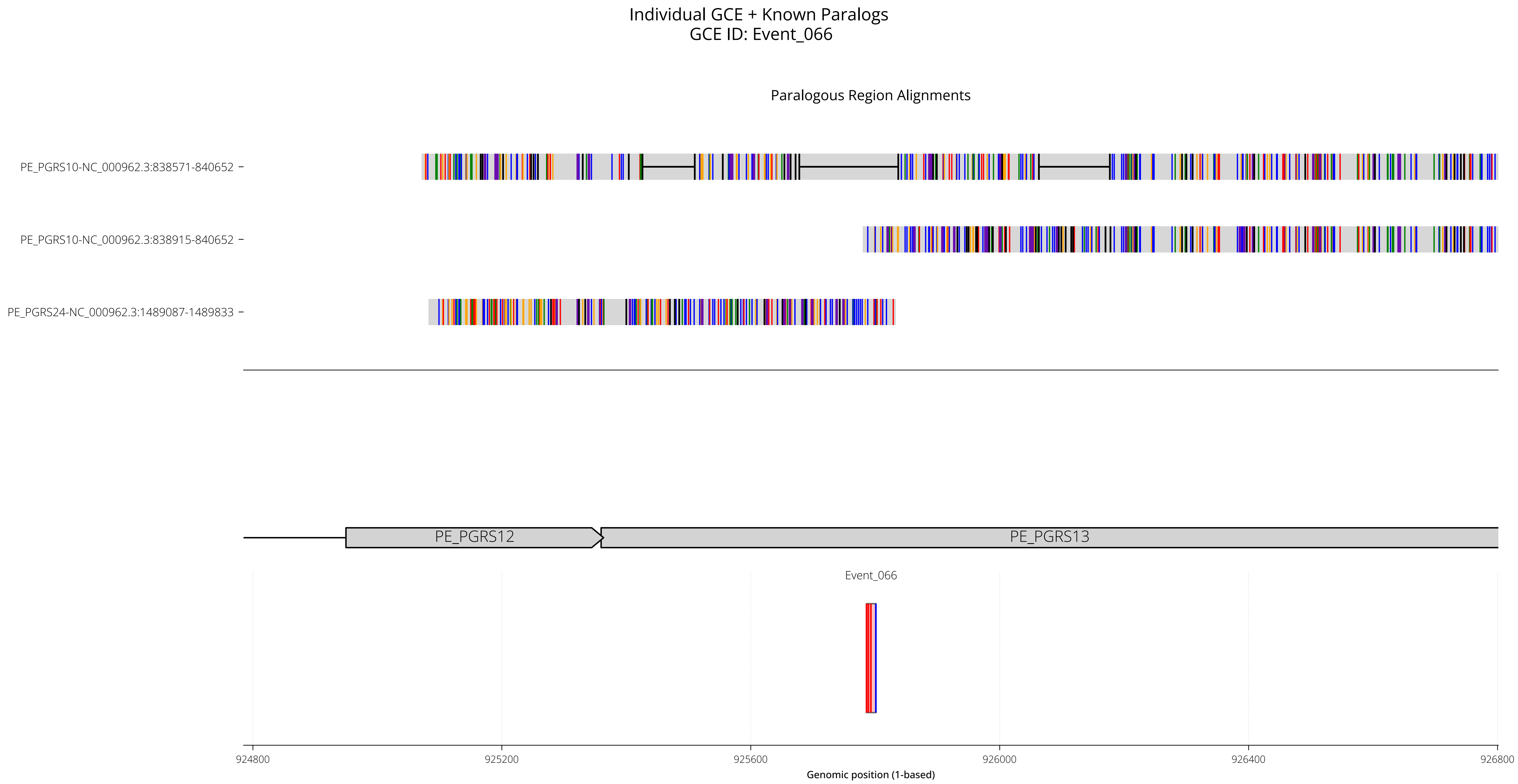
Individual GCE + Known Paralogs
GCE ID: Event_066
Paralogous Region Alignments
PE_PGRS10-NC_000962.3:838571-840652
PE_PGRS10-NC_000962.3:838915-840652
PE_PGRS24-NC_000962.3:1489087-1489833
PE_PGRS12
PE_PGRS13
Event_066
924800
925200
925600
926000
926400
926800
Genomic position (1-based)

Circos Viz - GCE to Known Paralogs Locations
GCE ID: Event_066

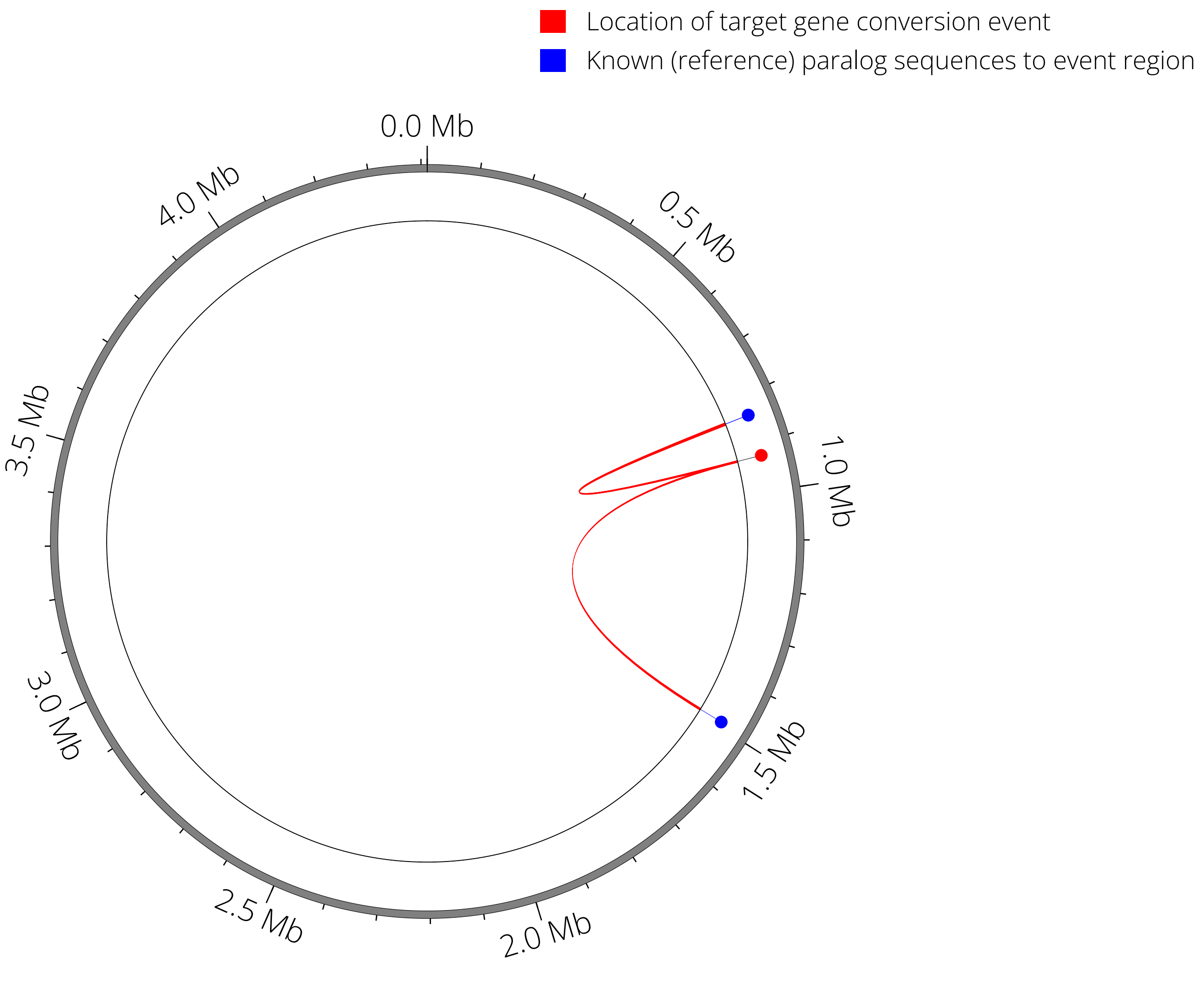

# Event_066 - 4 SNPs - k-mer analysis relative to paralogs

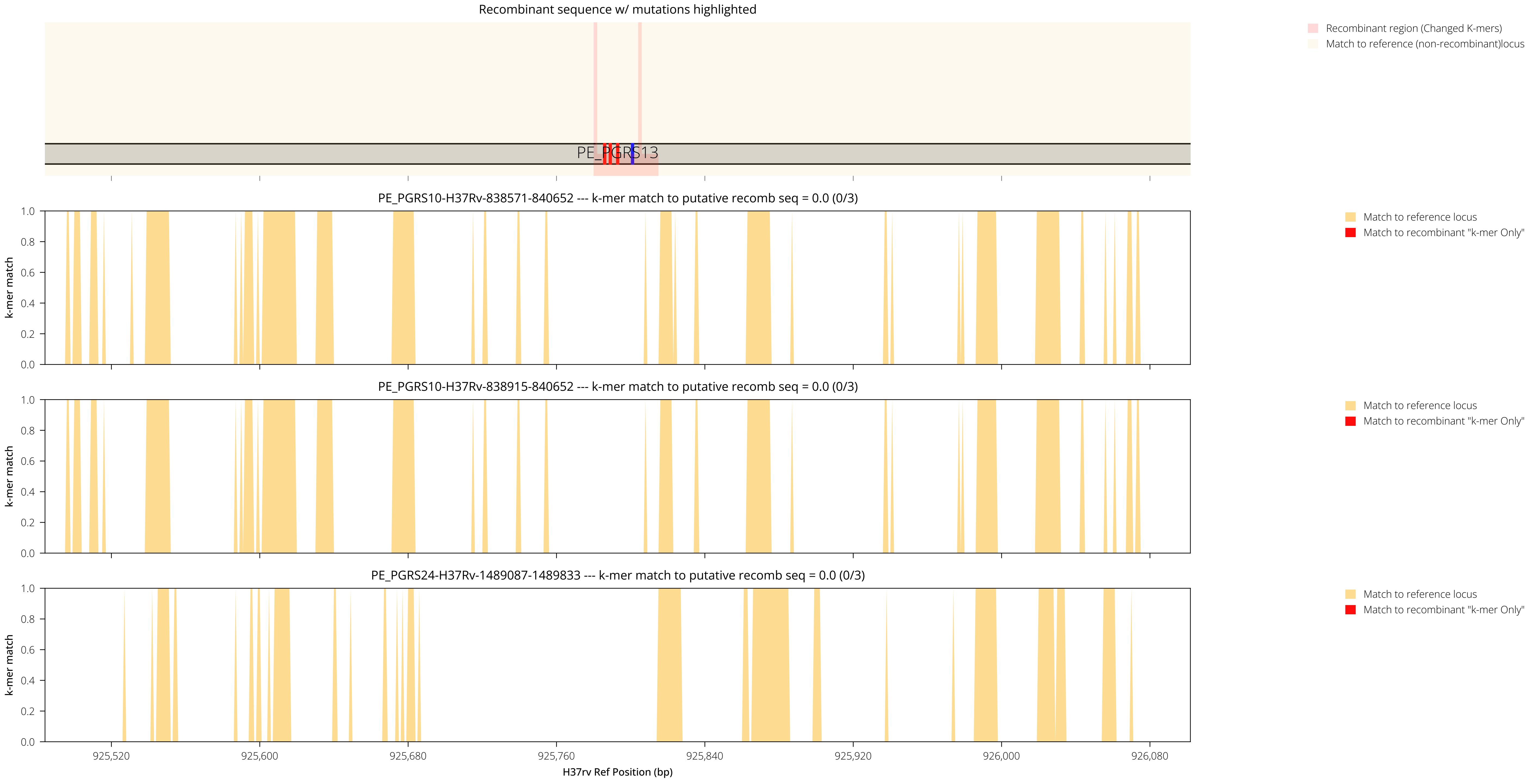

Sub-phylogeny Viz + Paralog Alignments
GCE ID: Event_066

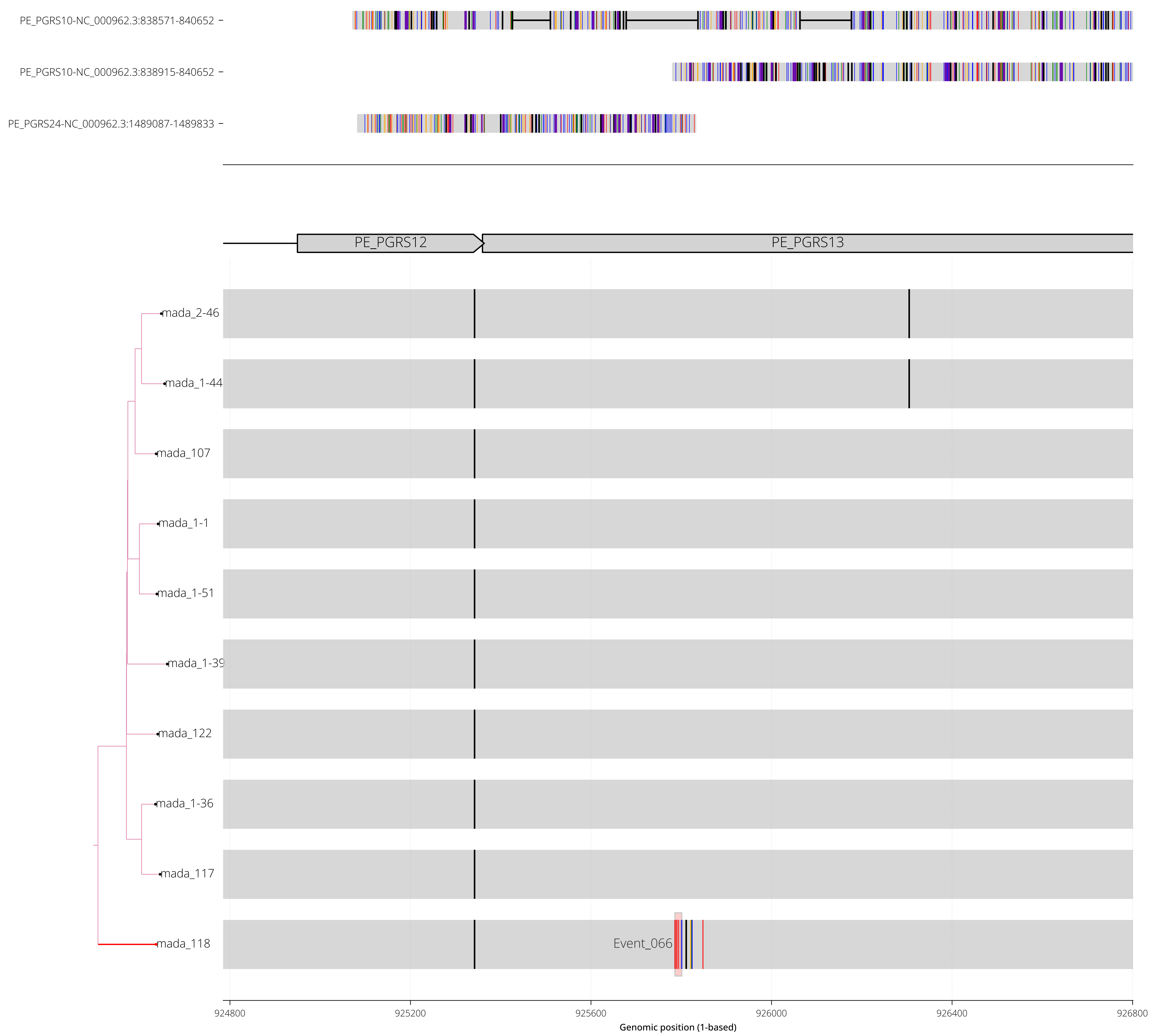

Full Phylogeny Viz + Paralog Alignments
GCE ID: Event_066

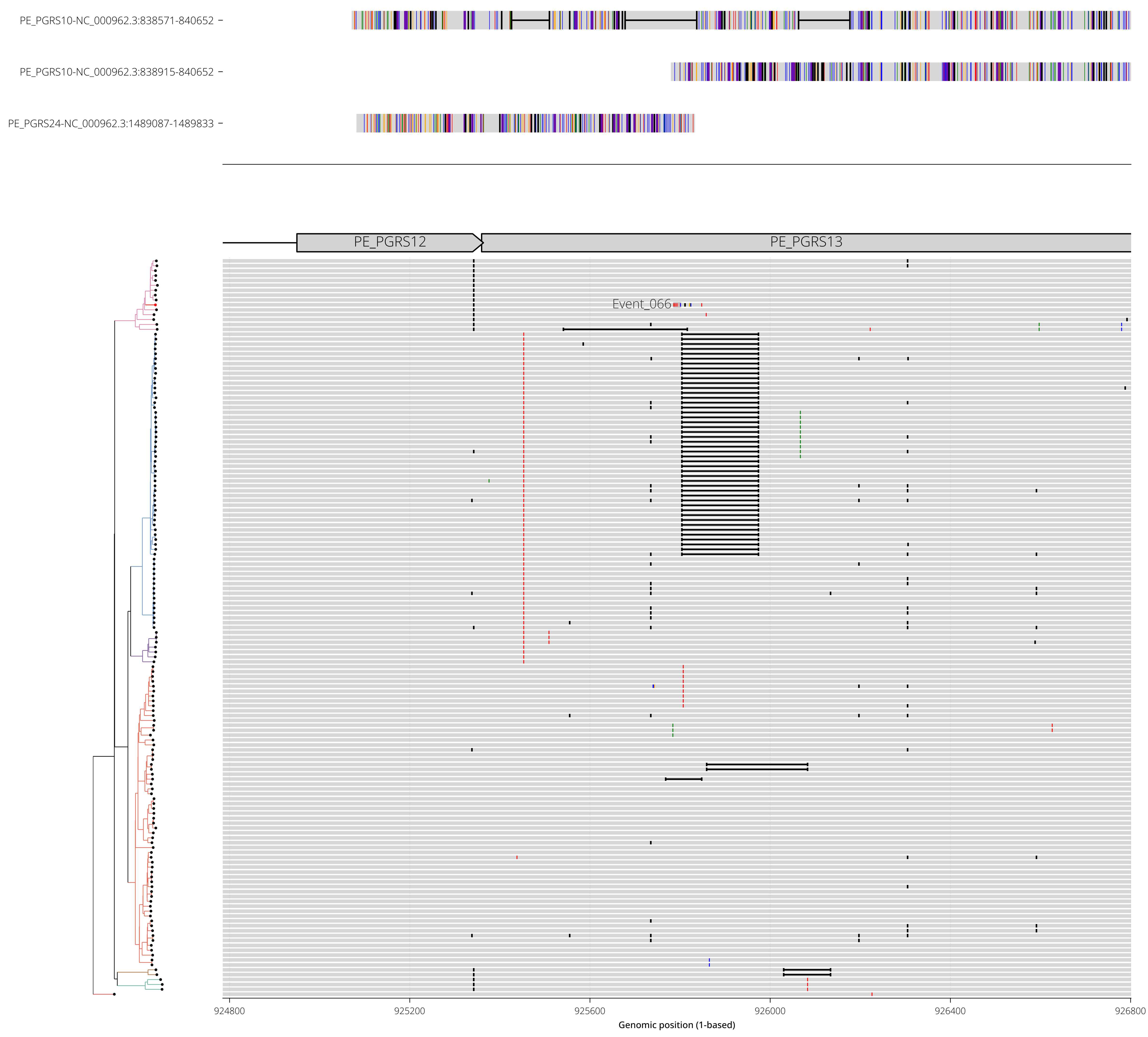

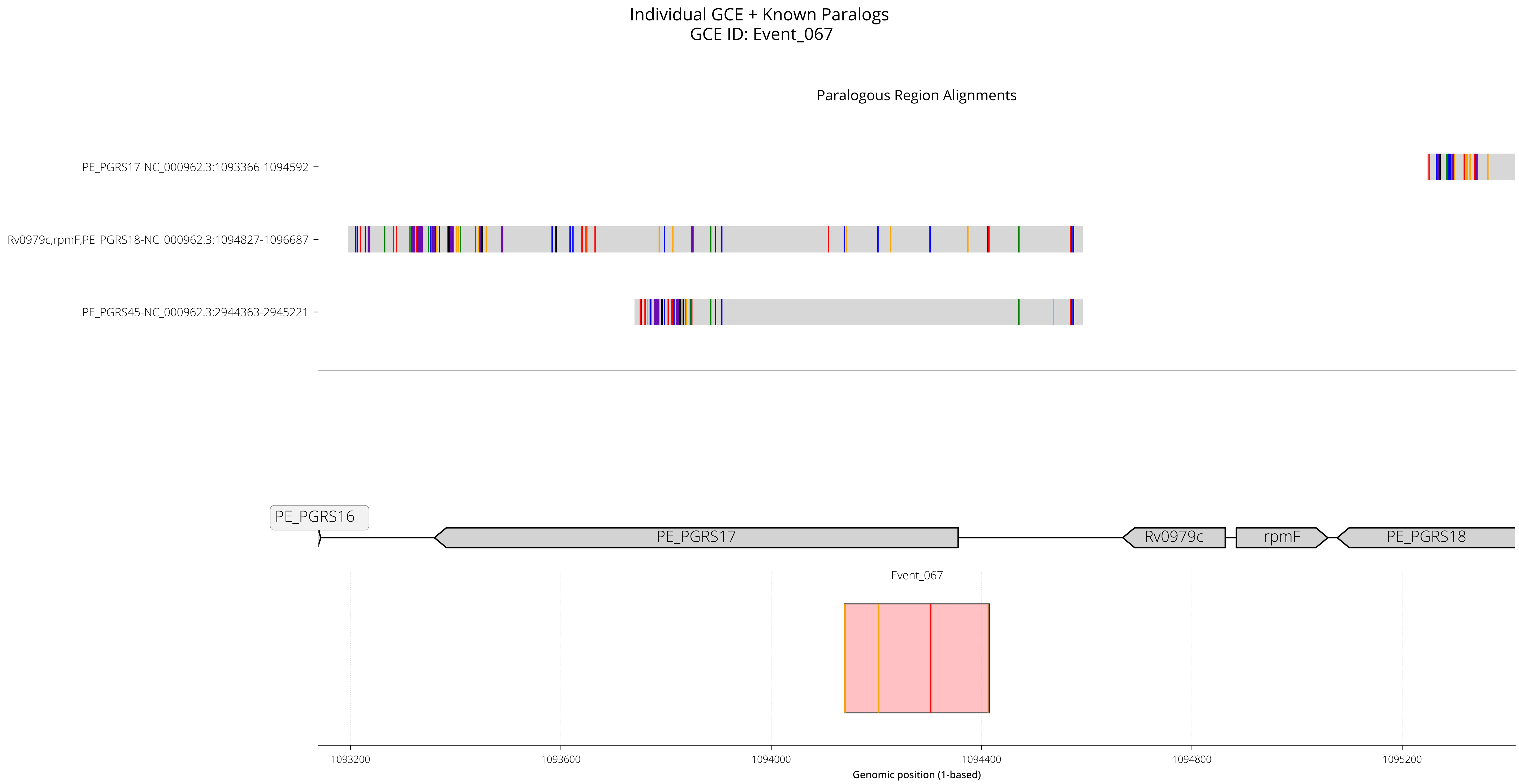
Individual GCE + Known Paralogs
GCE ID: Event_067
Paralogous Region Alignments
PE_PGRS17-NC_000962.3:1093366-1094592
Rv0979c,rpmF,PE_PGRS18-NC_000962.3:1094827-1096687
PE_PGRS45-NC_000962.3:2944363-2945221
PE_PGRS16
PE_PGRS17
Rv0979c
rpmF
PE_PGRS18
Event_067
1093200
1093600
1094000
1094400
1094800
1095200
Genomic position (1-based)

Circos Viz - GCE to Known Paralogs Locations
GCE ID: Event_067

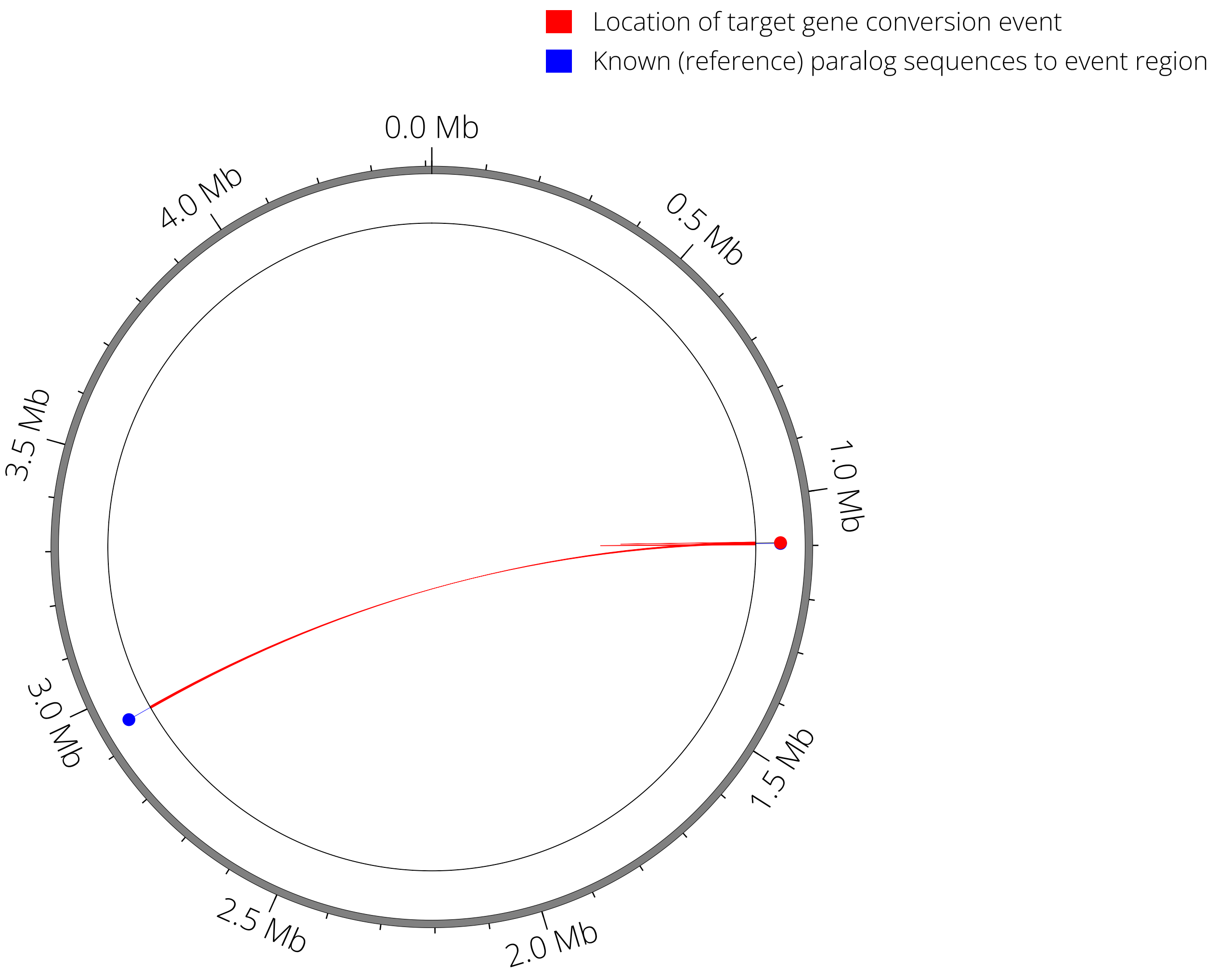

# Event_067 - 5 SNPs - k-mer analysis relative to paralogs

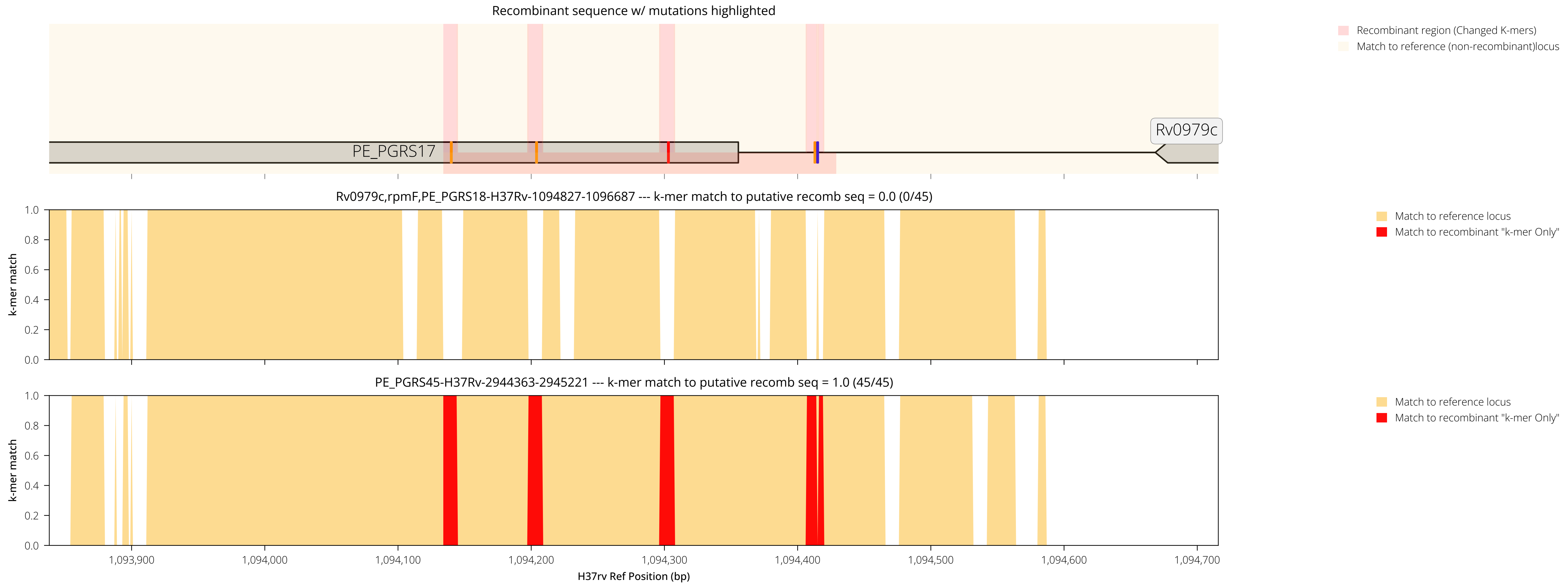

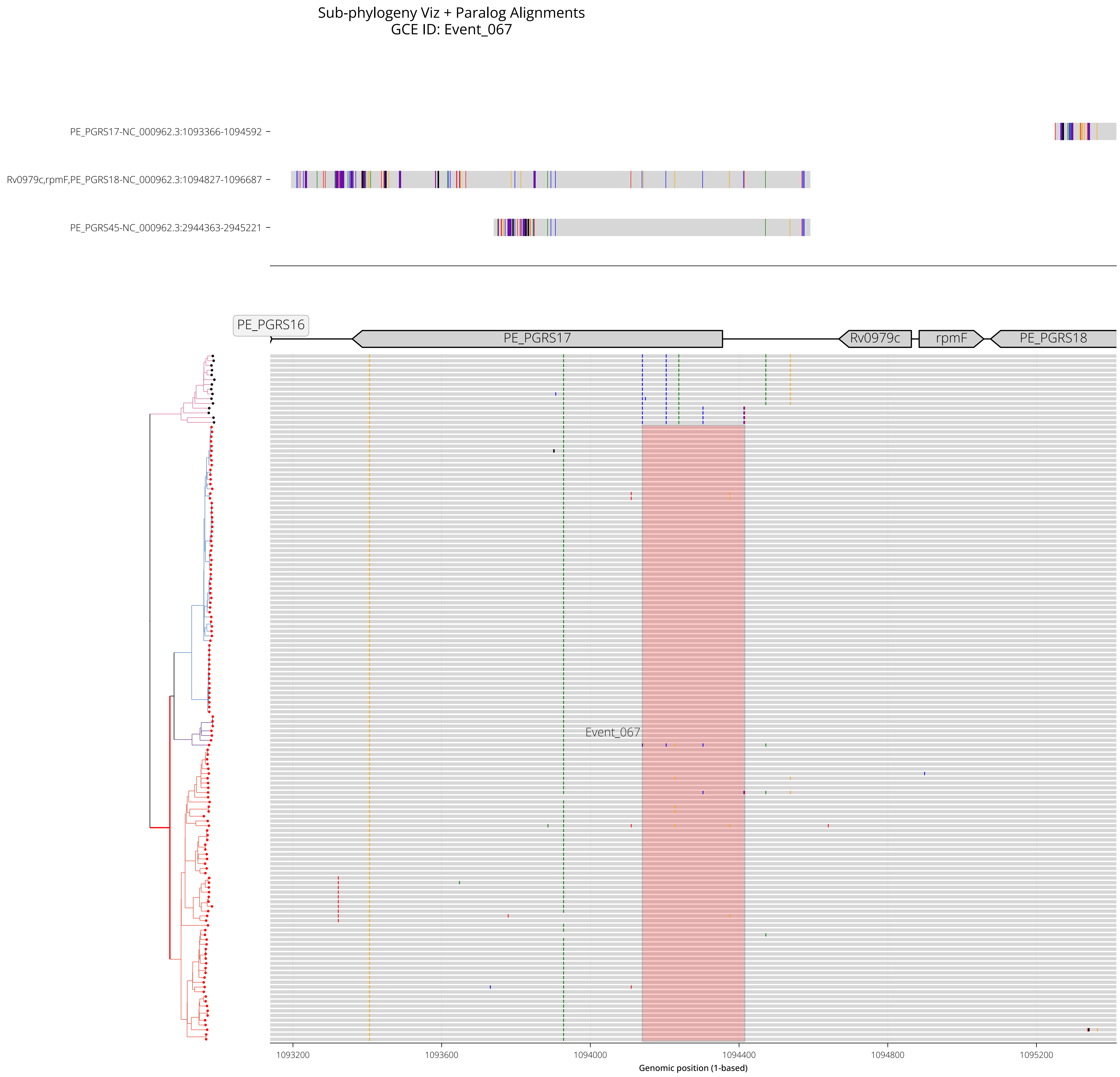

Sub-phylogeny Viz + Paralog Alignments
GCE ID: Event_067
PE_PGRS17-NC_000962.3:1093366-1094592
Rv0979c,rpmF,PE_PGRS18-NC_000962.3:1094827-1096687
PE_PGRS45-NC_000962.3:2944363-2945221
PE_PGRS16
PE_PGRS17
Rv0979c
rpmF
PE_PGRS18
Event_067
1093200
1093600
1094000
1094400
1094800
1095200
Genomic position (1-based)

Full Phylogeny Viz + Paralog Alignments
GCE ID: Event_067

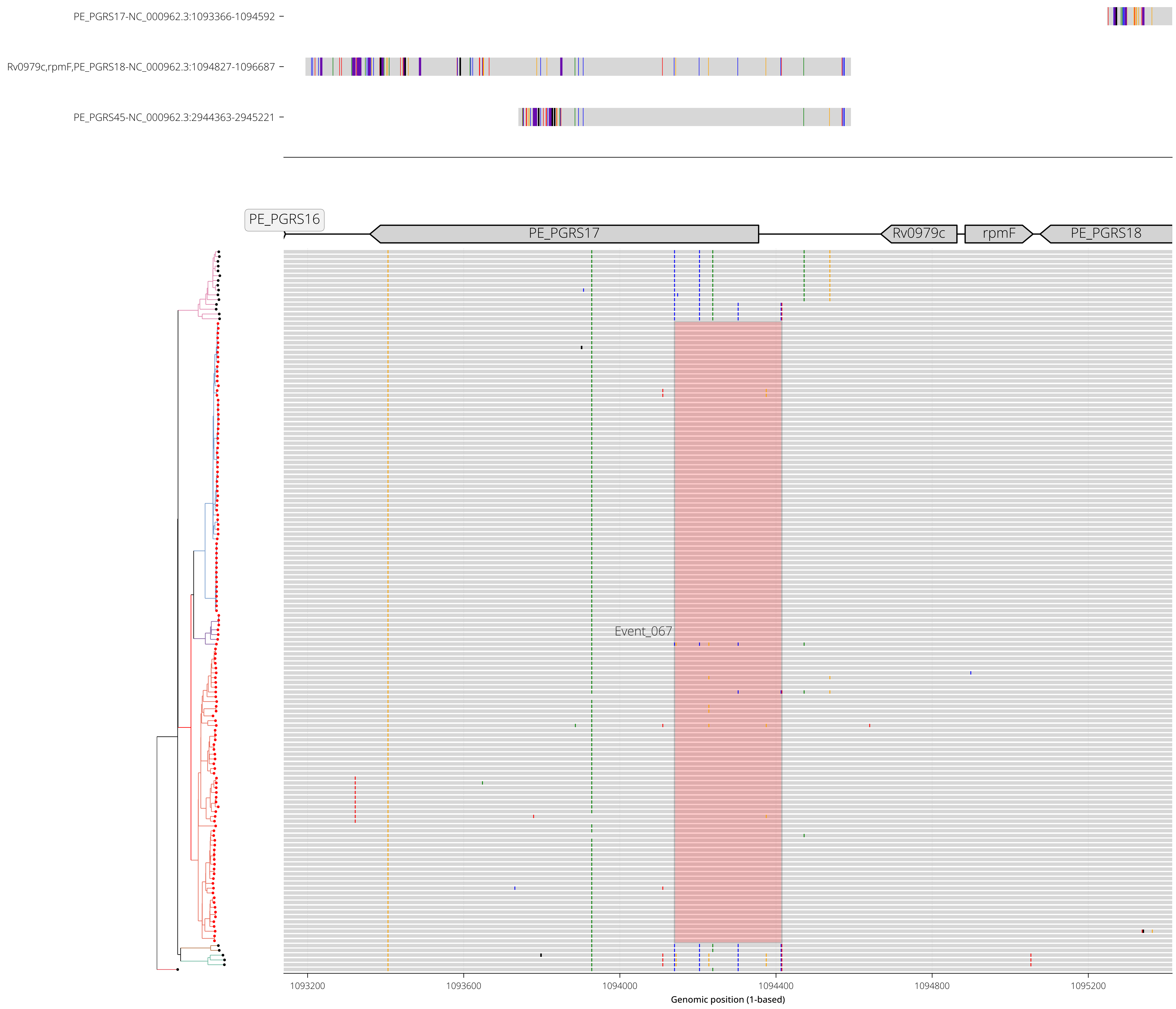

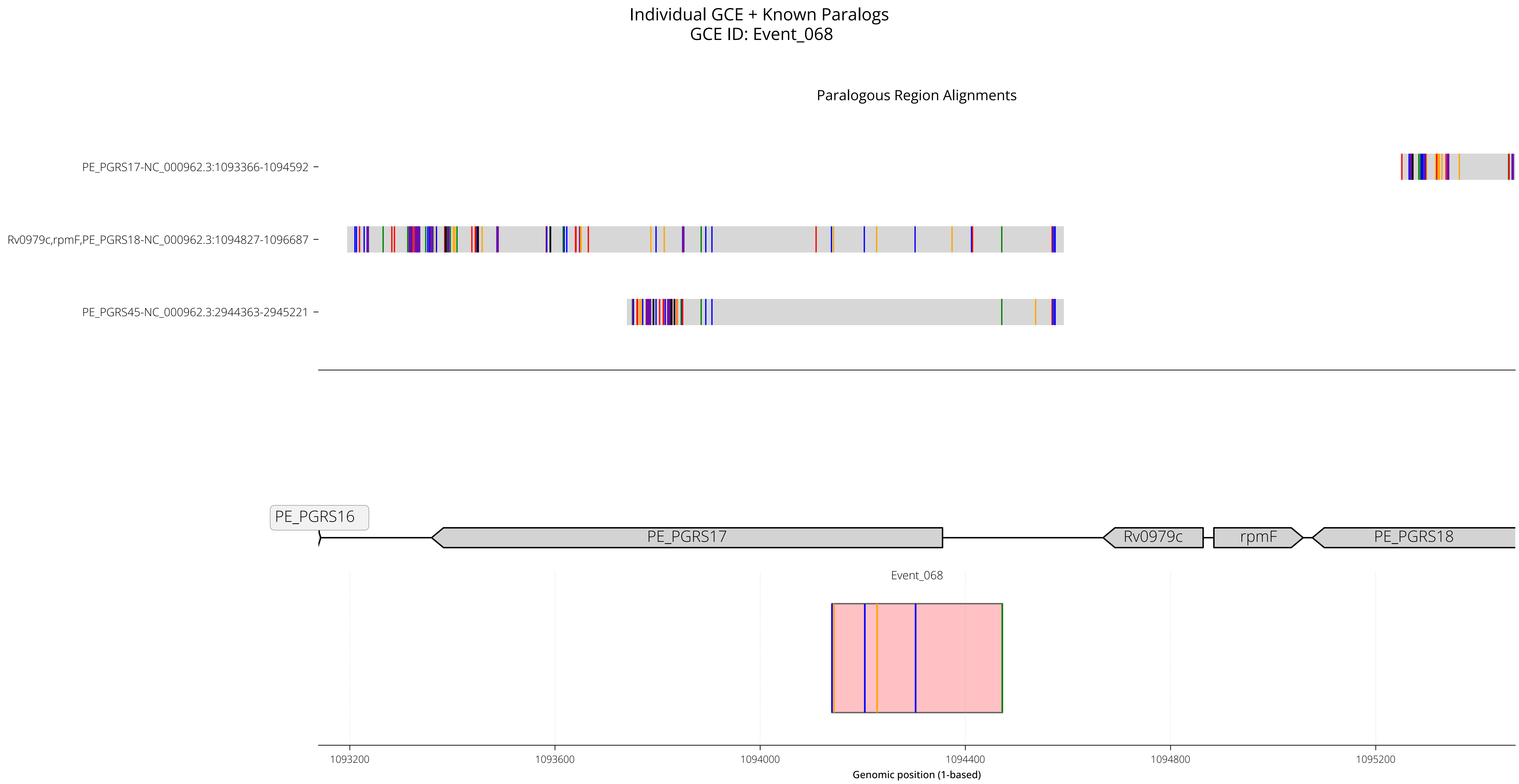
Individual GCE + Known Paralogs
GCE ID: Event_068
Paralogous Region Alignments
PE_PGRS17-NC_000962.3:1093366-1094592
Rv0979c,rpmF,PE_PGRS18-NC_000962.3:1094827-1096687
PE_PGRS45-NC_000962.3:2944363-2945221
PE_PGRS16
PE_PGRS17
Rv0979c
rpmF
PE_PGRS18
Event_068
1093200
1093600
1094000
1094400
1094800
1095200
Genomic position (1-based)

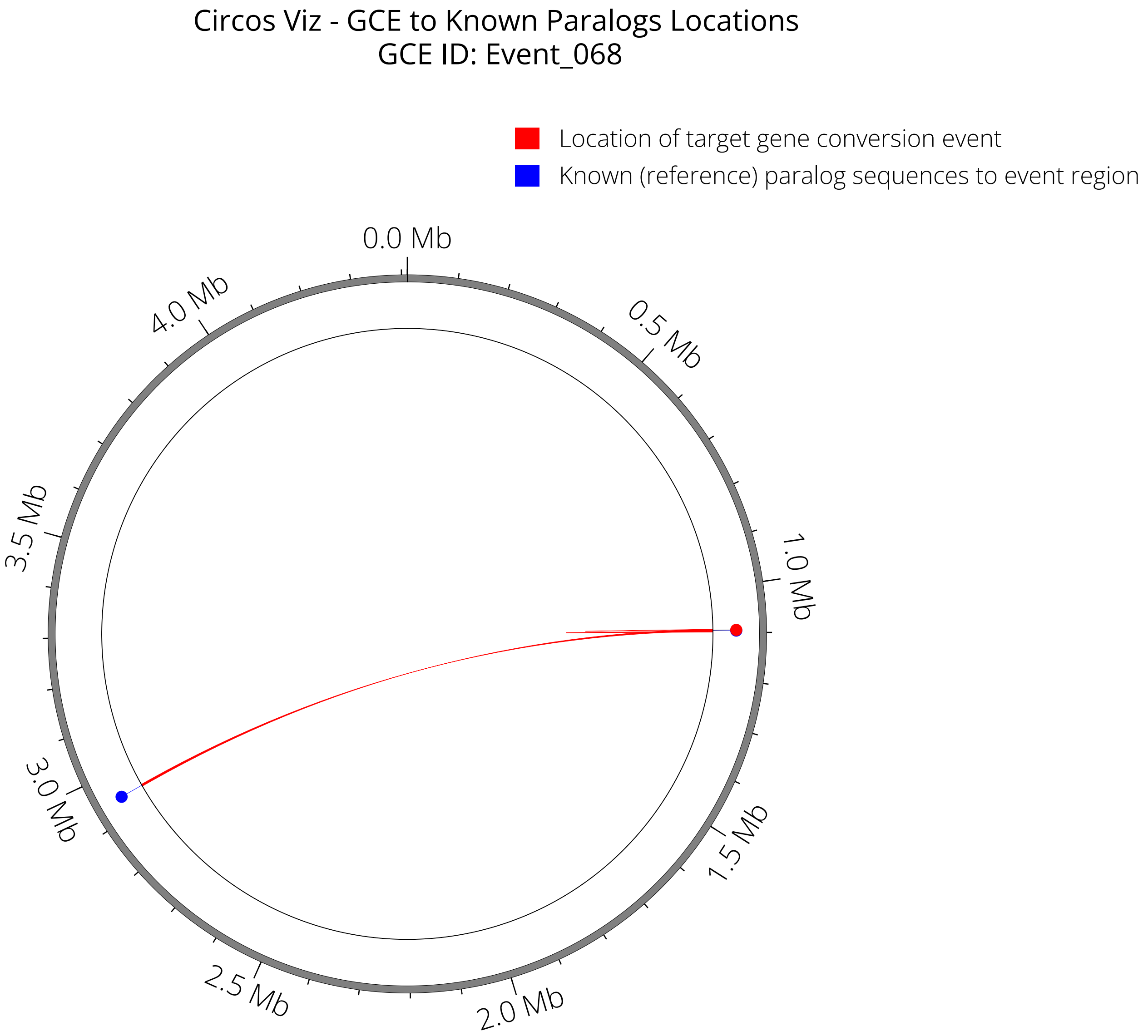
Circos Viz - GCE to Known Paralogs Locations
GCE ID: Event_068
Location of target gene conversion event
Known (reference) paralog sequences to event region
0.0 Mb
0.5 Mb
1.0 Mb
1.5 Mb
2.0 Mb
2.5 Mb
3.0 Mb
3.5 Mb
4.0 Mb

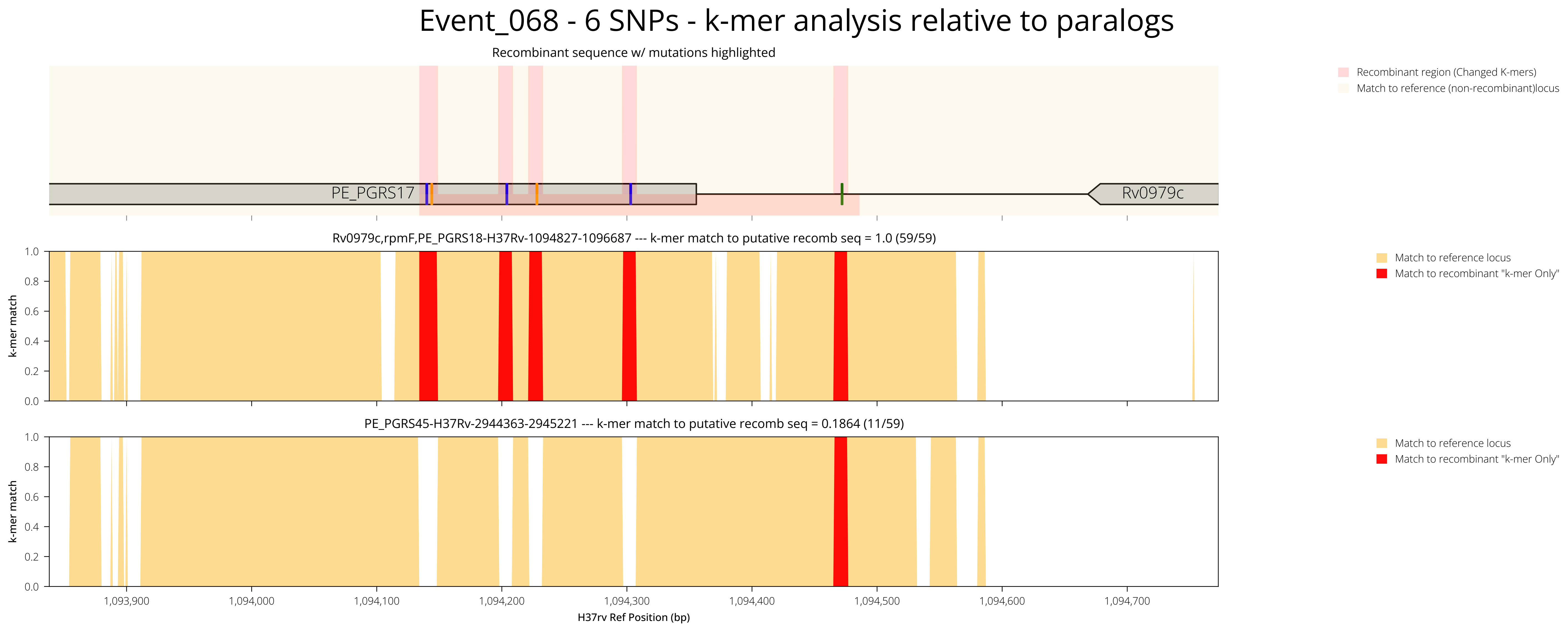

Event_068 - 6 SNPs - k-mer analysis relative to paralogs
Recombinant sequence w/ mutations highlighted
PE_PGRS17
Rv0979c
Recombinant region (Changed K-mers)
Match to reference (non-recombinant)locus
Rv0979c,rpmF,PE_PGRS18-H37Rv-1094827-1096687 --- k-mer match to putative recomb seq = 1.0 (59/59)
Match to reference locus
Match to recombinant "k-mer Only"
PE_PGRS45-H37Rv-2944363-2945221 --- k-mer match to putative recomb seq = 0.1864 (11/59)
Match to reference locus
Match to recombinant "k-mer Only"
k-mer match
1.0
0.8
0.6
0.4
0.2
0.0
1,093,900
1,094,000
1,094,100
1,094,200
1,094,300
1,094,400
1,094,500
1,094,600
1,094,700
H37rv Ref Position (bp)

Sub-phylogeny Viz + Paralog Alignments
GCE ID: Event_068

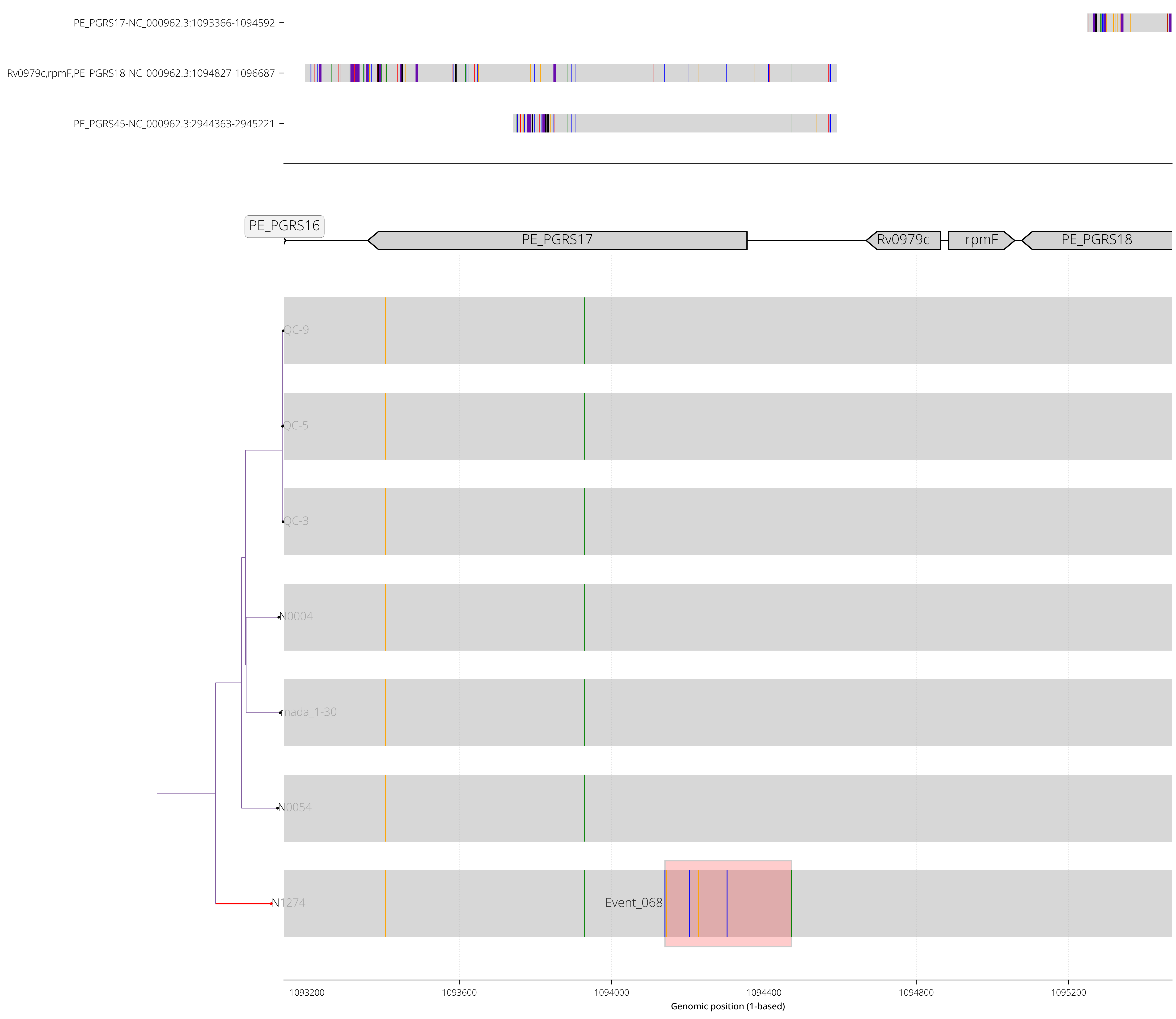

Full Phylogeny Viz + Paralog Alignments
GCE ID: Event_068

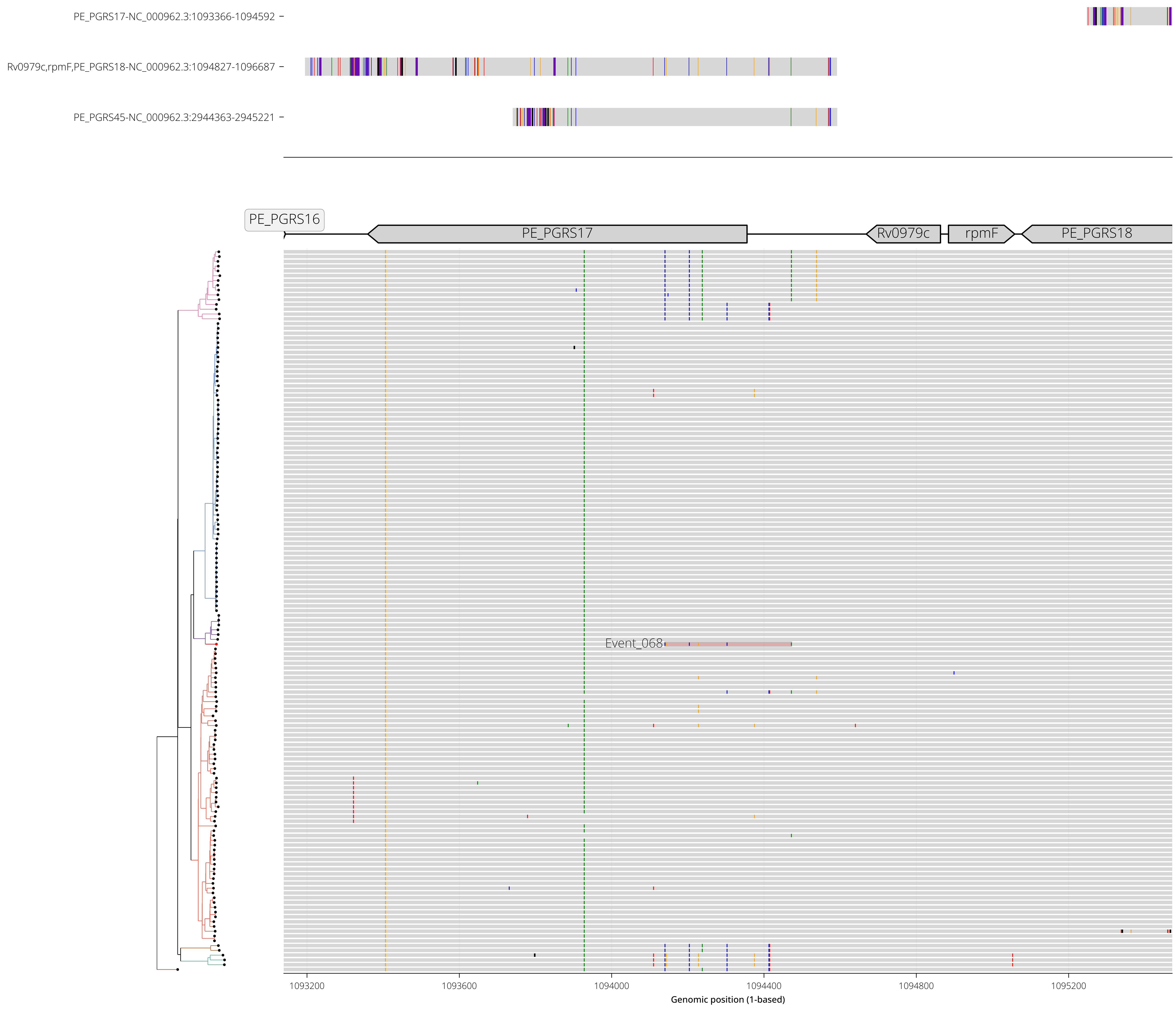

Individual GCE + Known Paralogs
GCE ID: Event_069

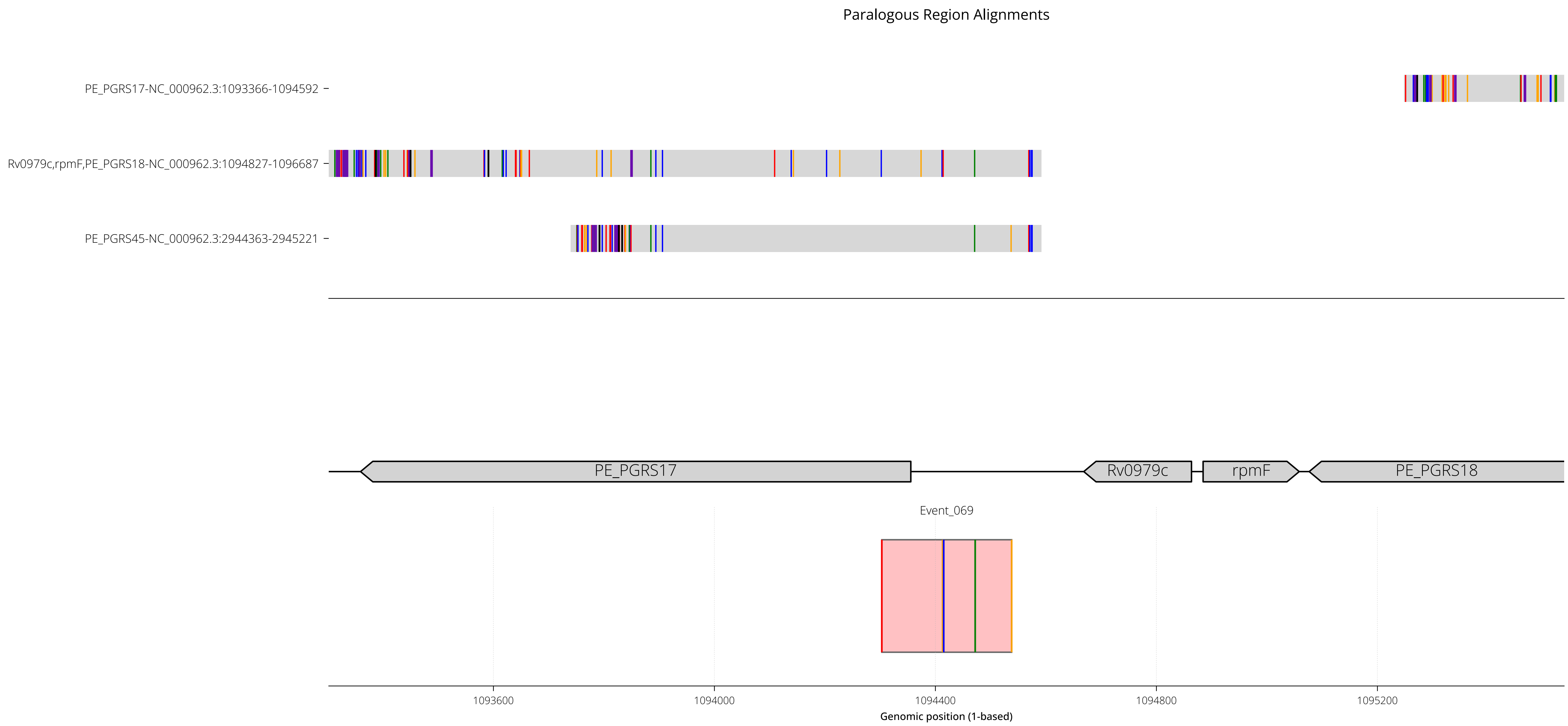

Circos Viz - GCE to Known Paralogs Locations
GCE ID: Event_069

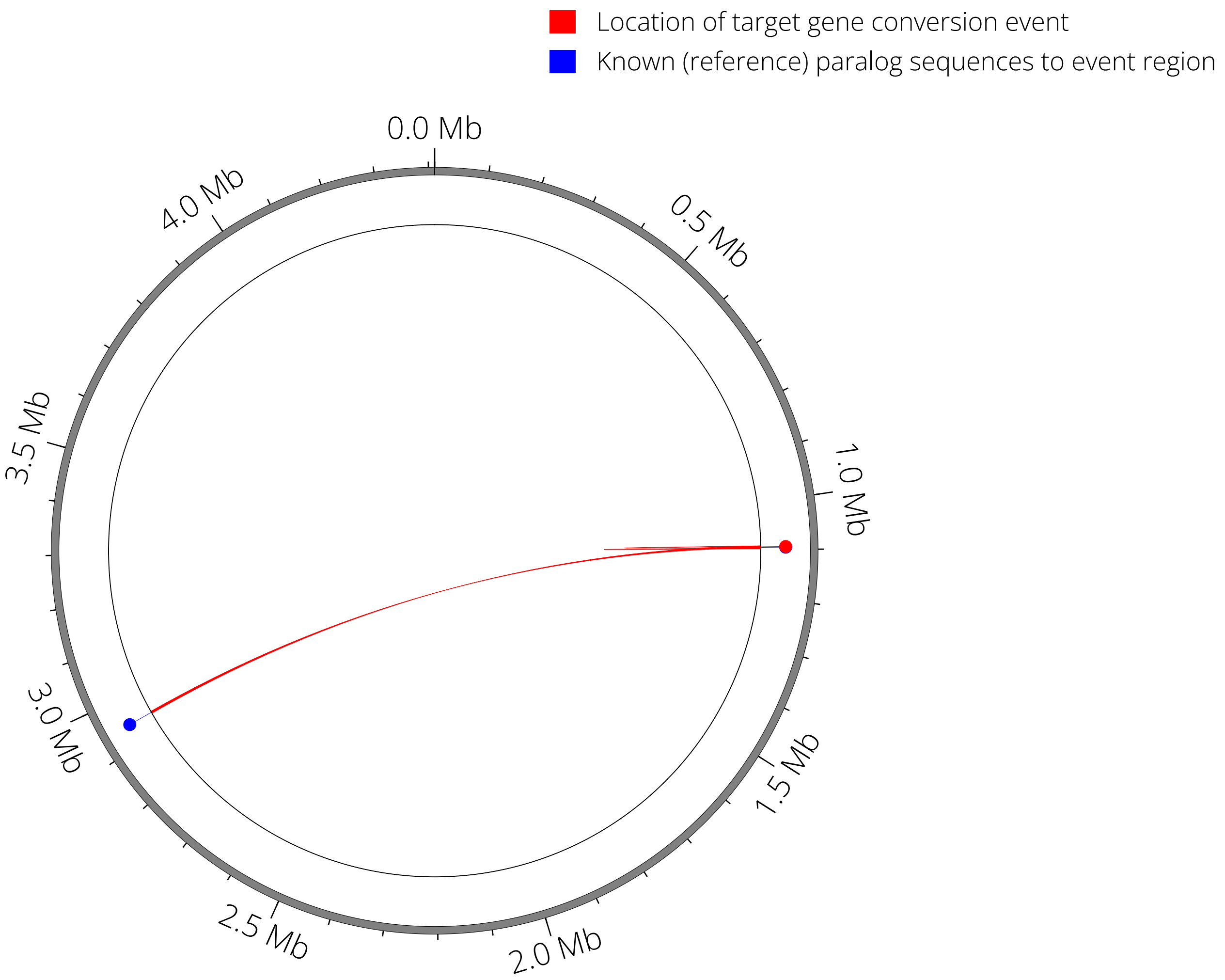

# Event_069 - 5 SNPs - k-mer analysis relative to paralogs

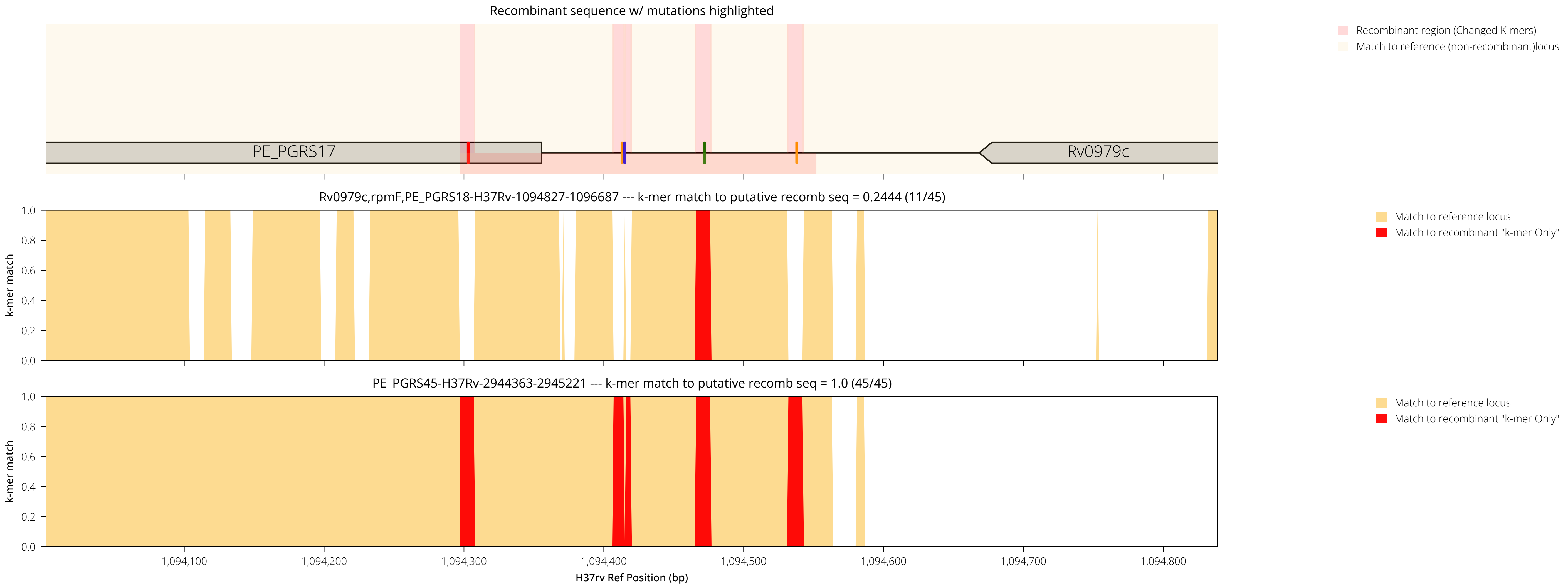

Sub-phylogeny Viz + Paralog Alignments
GCE ID: Event_069

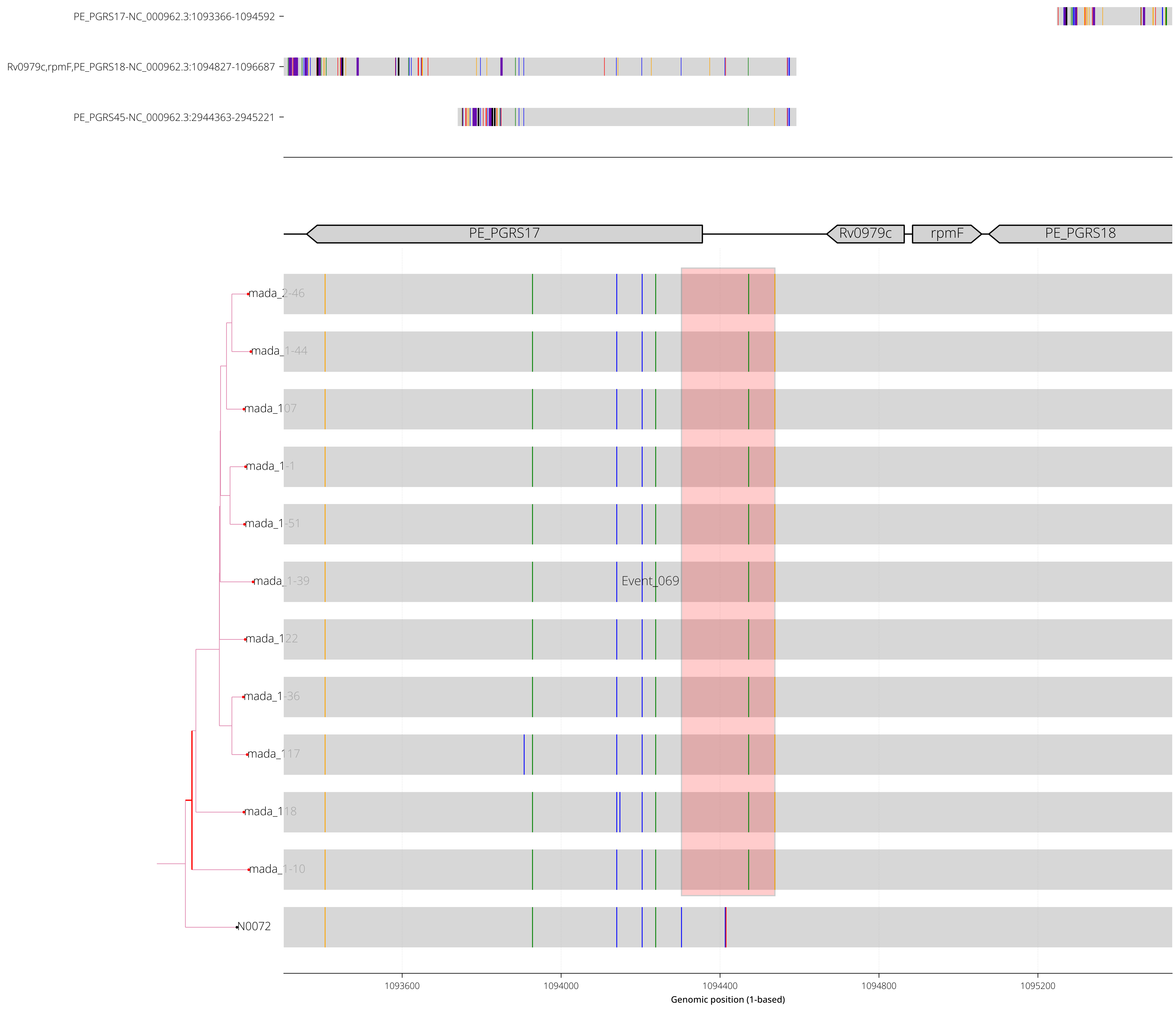

Full Phylogeny Viz + Paralog Alignments
GCE ID: Event_069

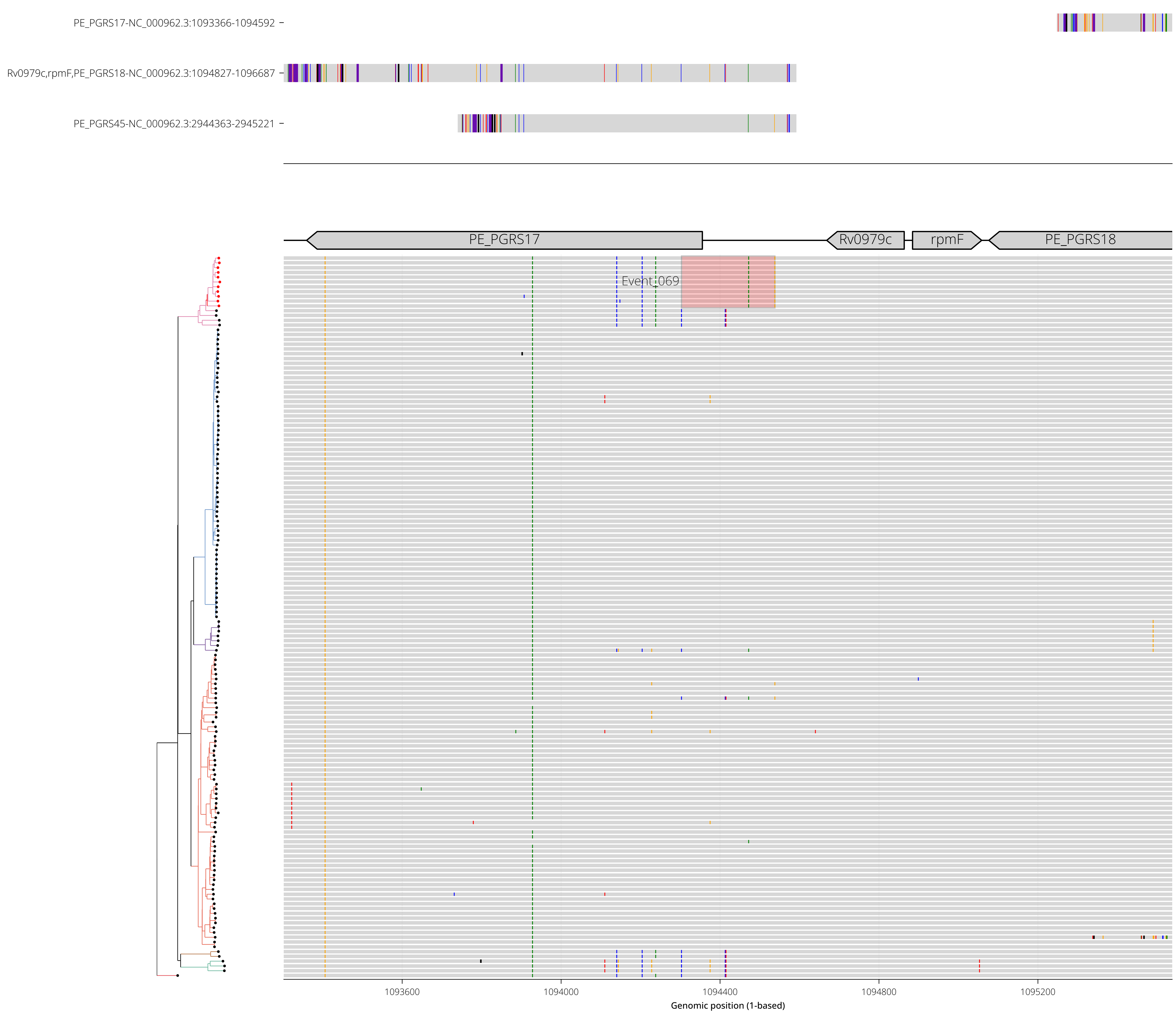

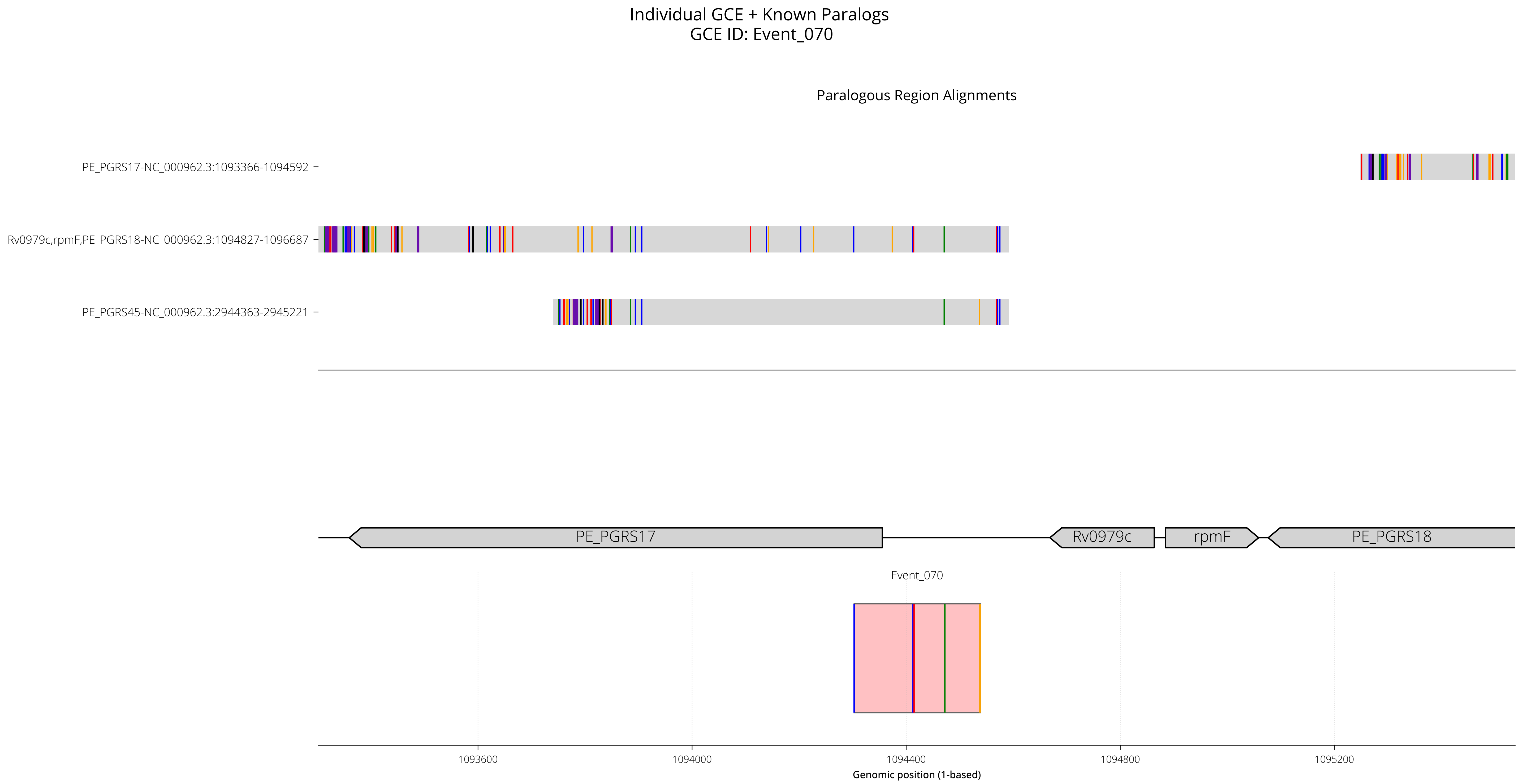
Individual GCE + Known Paralogs
GCE ID: Event_070
Paralogous Region Alignments
PE_PGRS17-NC_000962.3:1093366-1094592
Rv0979c,rpmF,PE_PGRS18-NC_000962.3:1094827-1096687
PE_PGRS45-NC_000962.3:2944363-2945221
PE_PGRS17
Rv0979c
rpmF
PE_PGRS18
Event_070
1093600
1094000
1094400
1094800
1095200
Genomic position (1-based)

Circos Viz - GCE to Known Paralogs Locations
GCE ID: Event_070

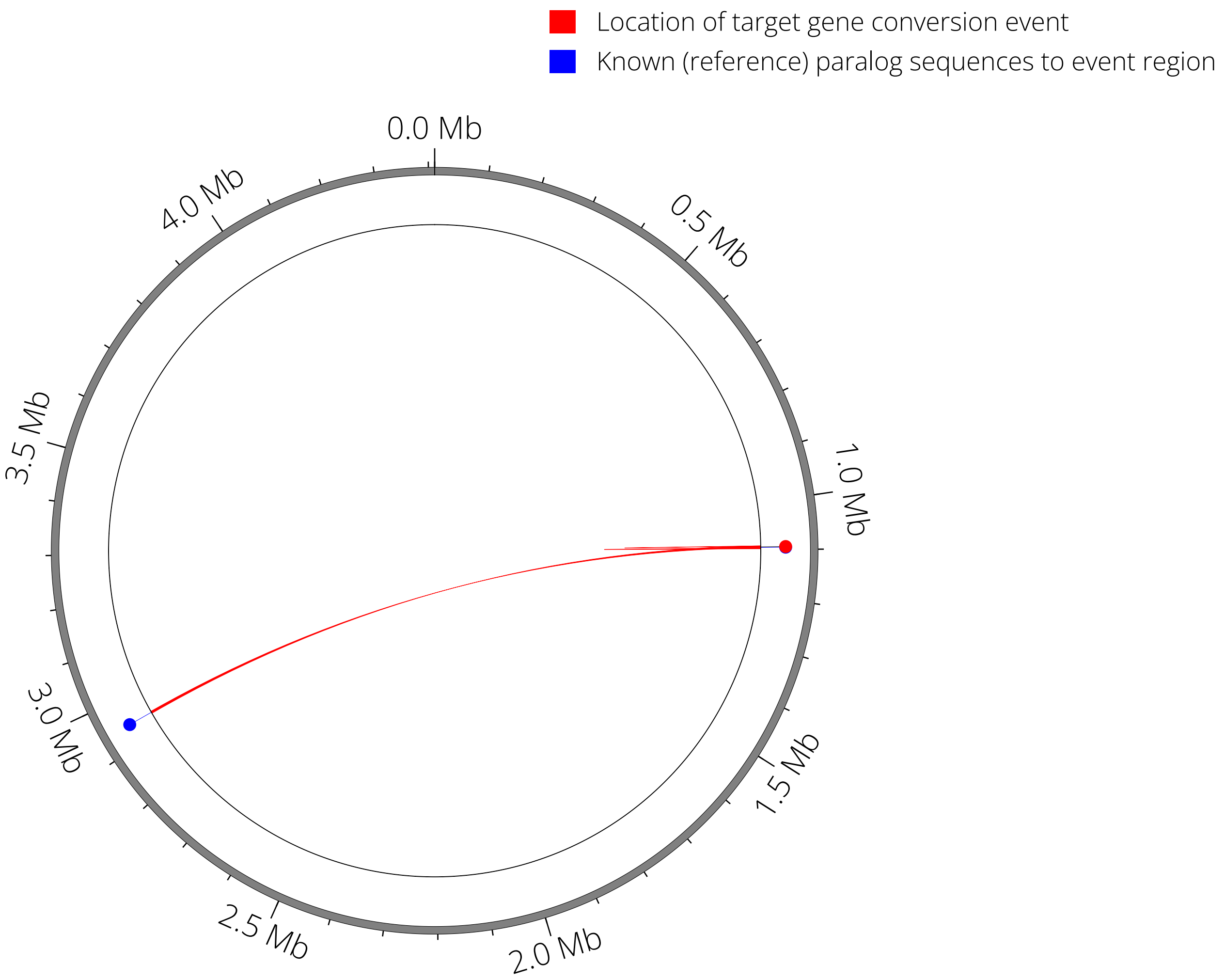

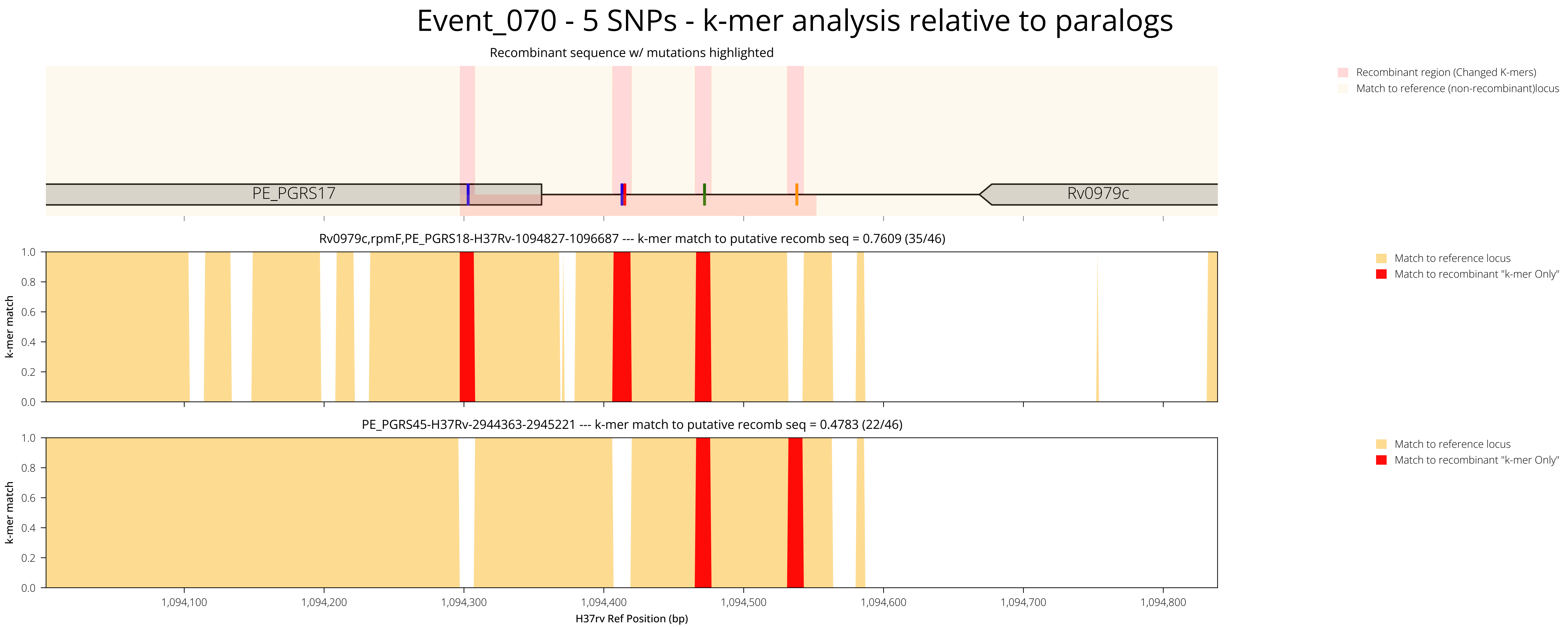
Event_070 - 5 SNPs - k-mer analysis relative to paralogs
Recombinant sequence w/ mutations highlighted
Recombinant region (Changed K-mers)
Match to reference (non-recombinant)locus
PE_PGRS17
Rv0979c
Rv0979c,rpmF,PE_PGRS18-H37Rv-1094827-1096687 --- k-mer match to putative recomb seq = 0.7609 (35/46)
Match to reference locus
Match to recombinant "k-mer Only"
k-mer match
PE_PGRS45-H37Rv-2944363-2945221 --- k-mer match to putative recomb seq = 0.4783 (22/46)
1.0
0.8
0.6
0.4
0.2
0.0
1,094,100
1,094,200
1,094,300
1,094,400
1,094,500
1,094,600
1,094,700
1,094,800
H37rv Ref Position (bp)

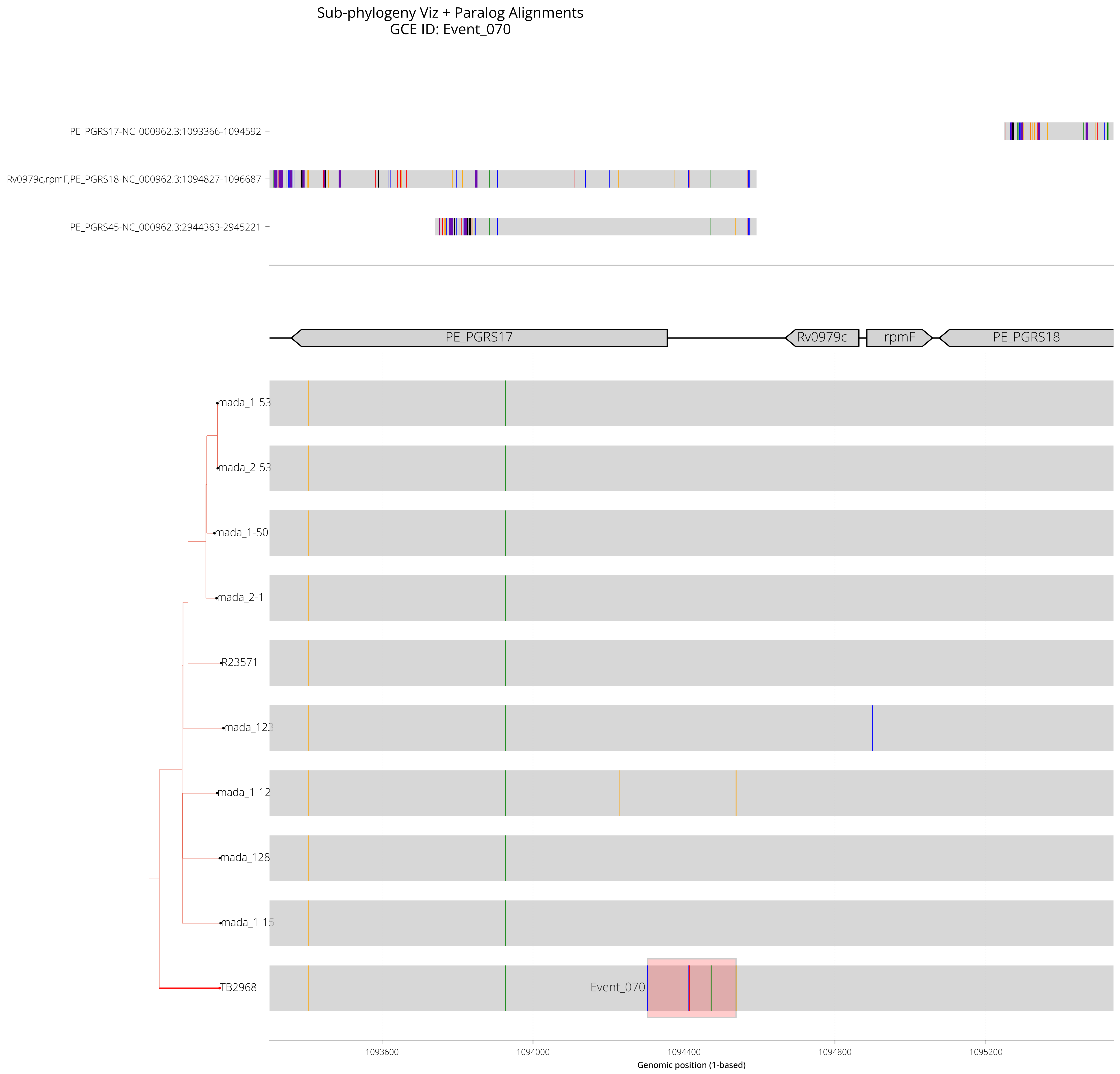

Sub-phylogeny Viz + Paralog Alignments
GCE ID: Event_070
PE_PGRS17-NC_000962.3:1093366-1094592
Rv0979c,rpmF,PE_PGRS18-NC_000962.3:1094827-1096687
PE_PGRS45-NC_000962.3:2944363-2945221
PE_PGRS17
Rv0979c
rpmF
PE_PGRS18
mada_1-53
mada_2-53
mada_1-50
mada_2-1
R23571
mada_123
mada_1-12
mada_128
mada_1-15
TB2968
Event_070
1093600
1094000
1094400
1094800
1095200
Genomic position (1-based)

Full Phylogeny Viz + Paralog Alignments
GCE ID: Event_070

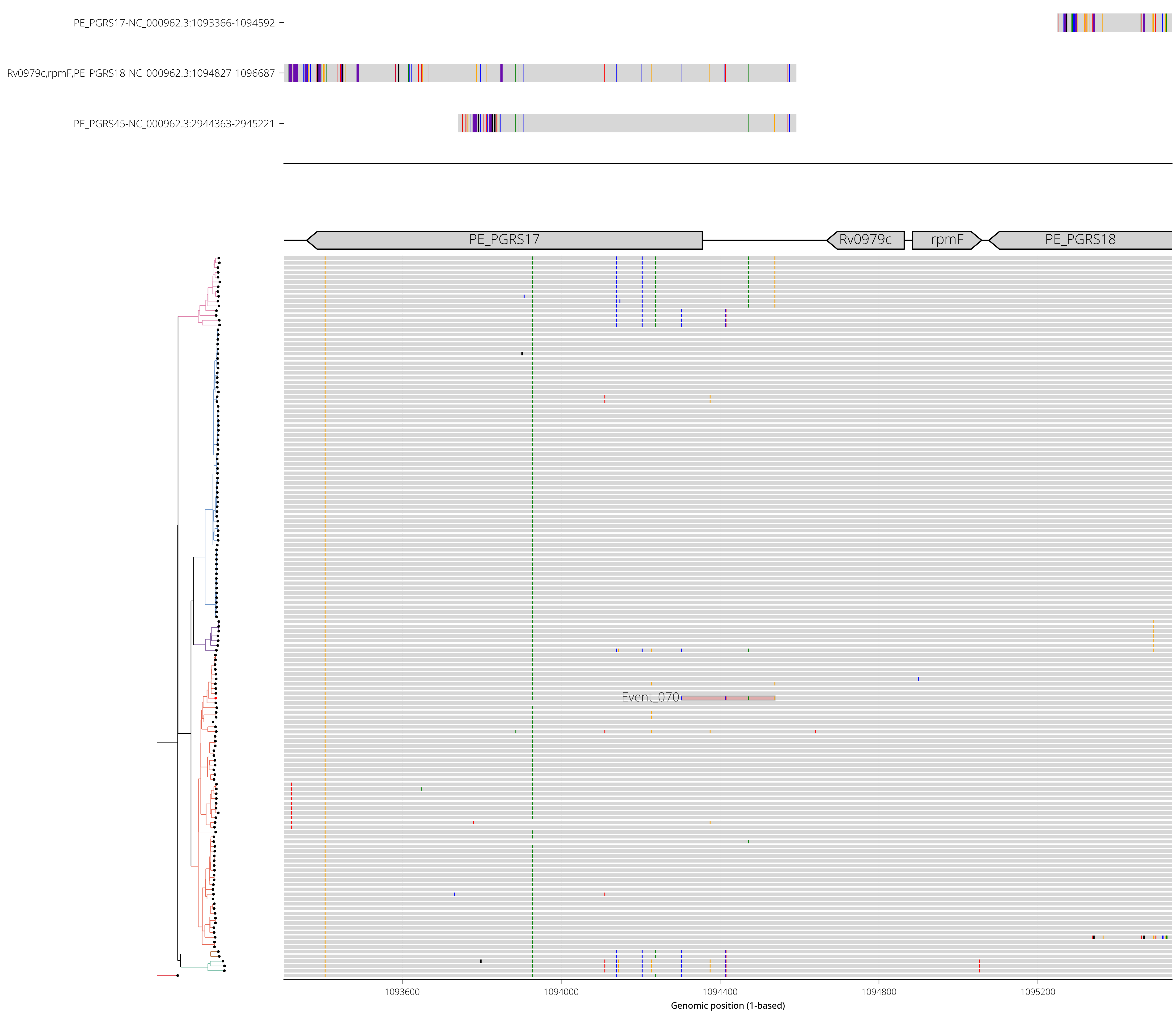

Individual GCE + Known Paralogs
GCE ID: Event_071

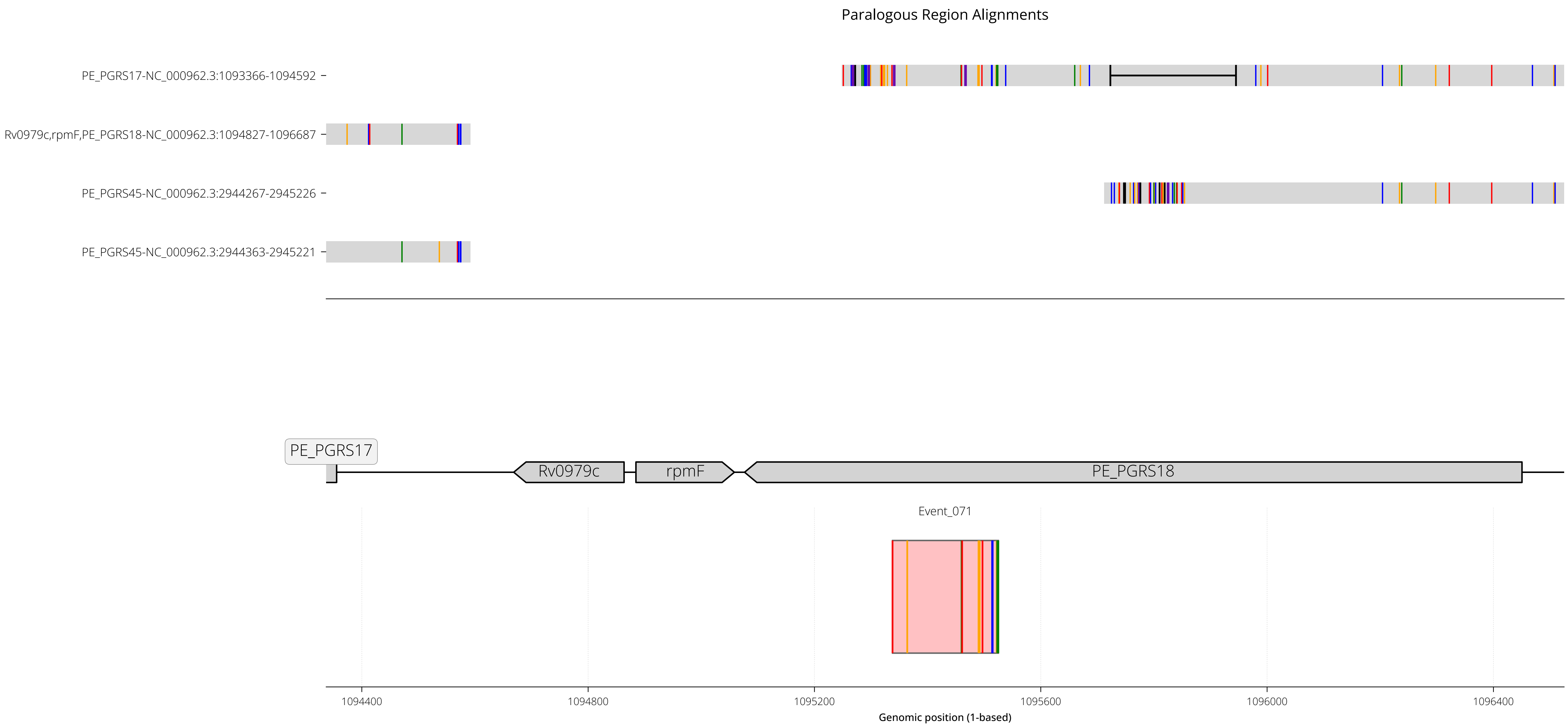

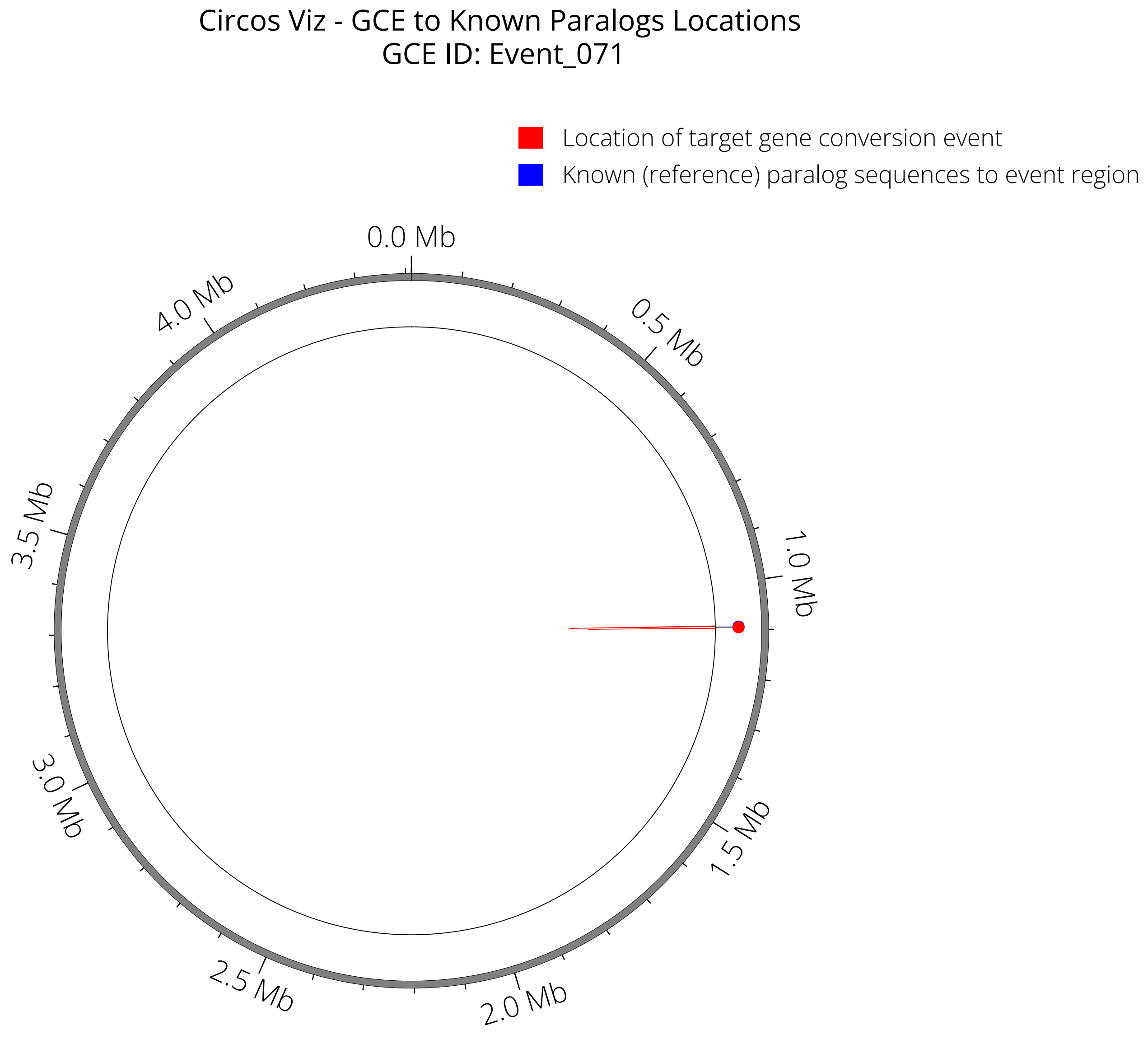
Circos Viz - GCE to Known Paralogs Locations
GCE ID: Event_071
Location of target gene conversion event
Known (reference) paralog sequences to event region
0.0 Mb
0.5 Mb
1.0 Mb
1.5 Mb
2.0 Mb
2.5 Mb
3.0 Mb
3.5 Mb
4.0 Mb

# Event_071 - 13 SNPs - k-mer analysis relative to paralogs

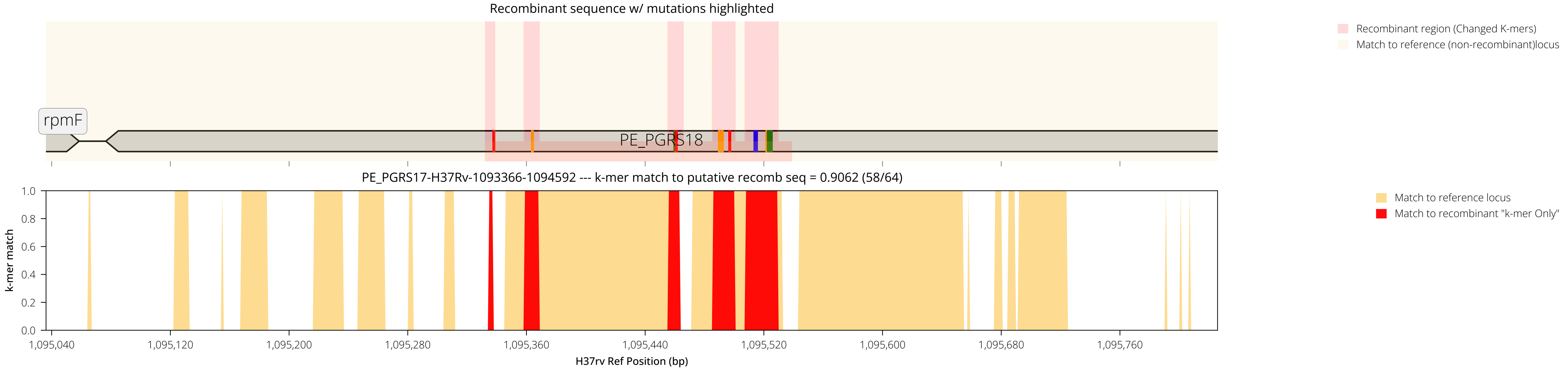

Sub-phylogeny Viz + Paralog Alignments
GCE ID: Event_071

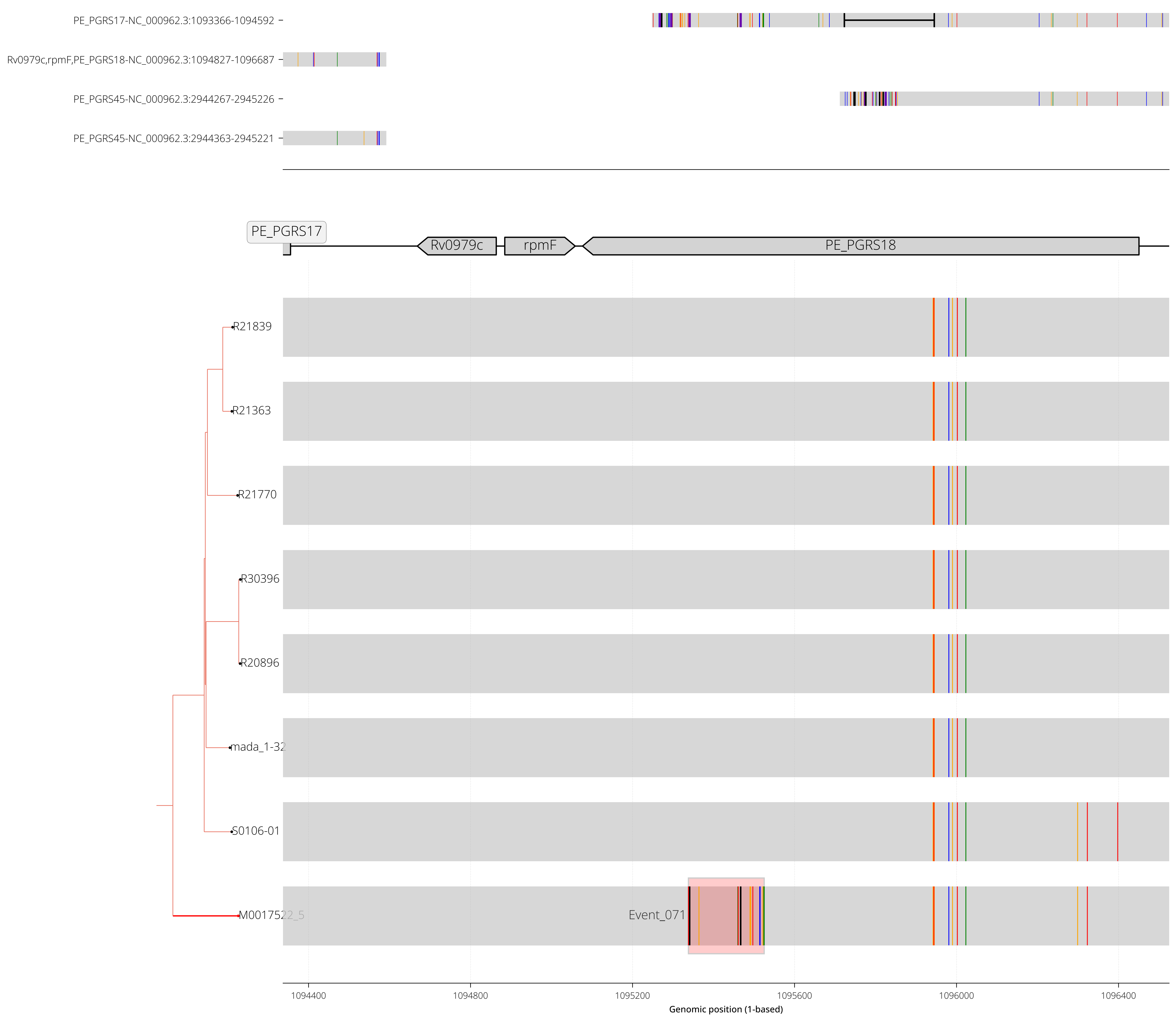

Full Phylogeny Viz + Paralog Alignments
GCE ID: Event_071

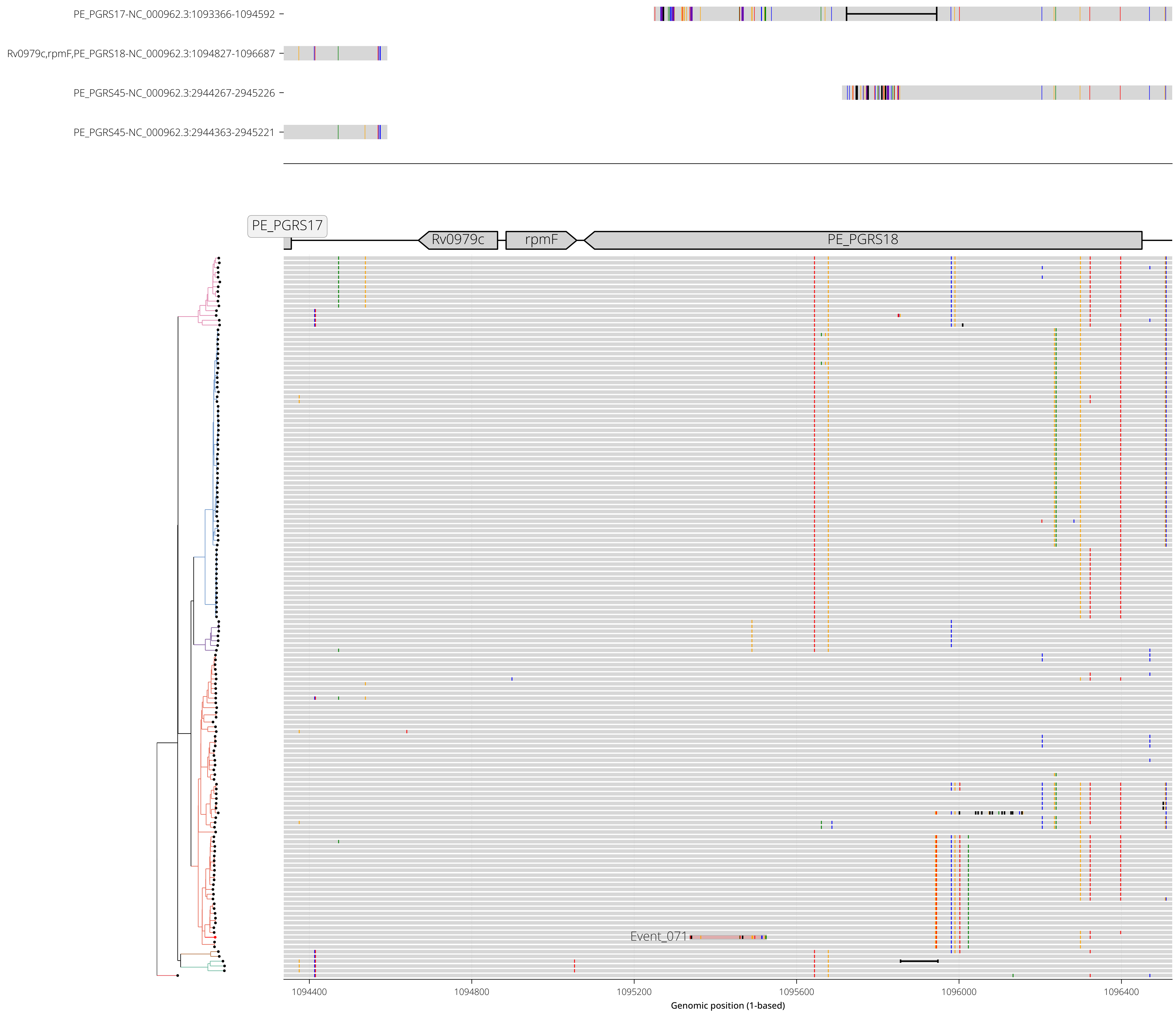

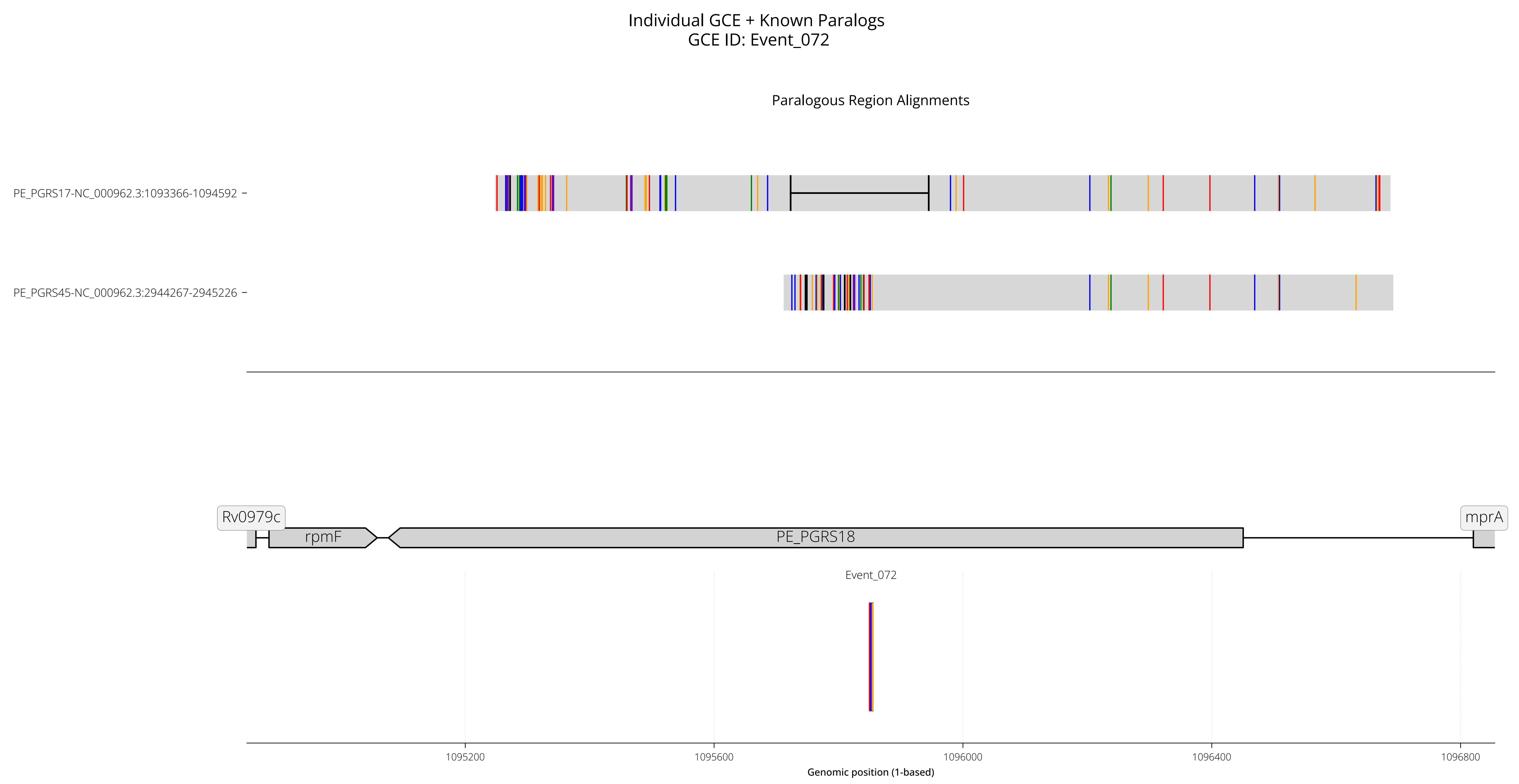
Individual GCE + Known Paralogs
GCE ID: Event_072
Paralogous Region Alignments
PE_PGRS17-NC_000962.3:1093366-1094592
PE_PGRS45-NC_000962.3:2944267-2945226
Rv0979c
rpmF
PE_PGRS18
mprA
Event_072
1095200
1095600
1096000
1096400
1096800
Genomic position (1-based)

Circos Viz - GCE to Known Paralogs Locations
GCE ID: Event_072

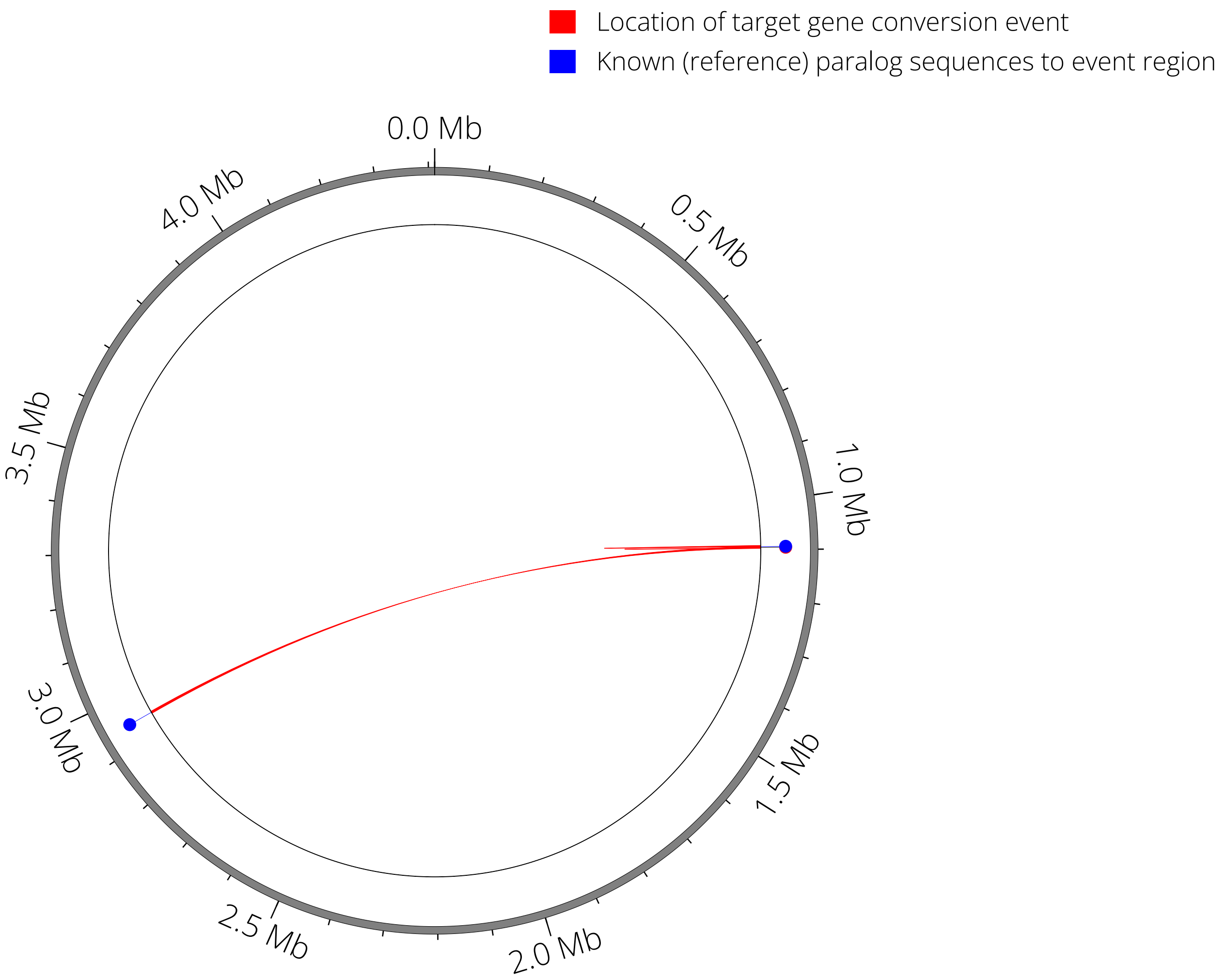

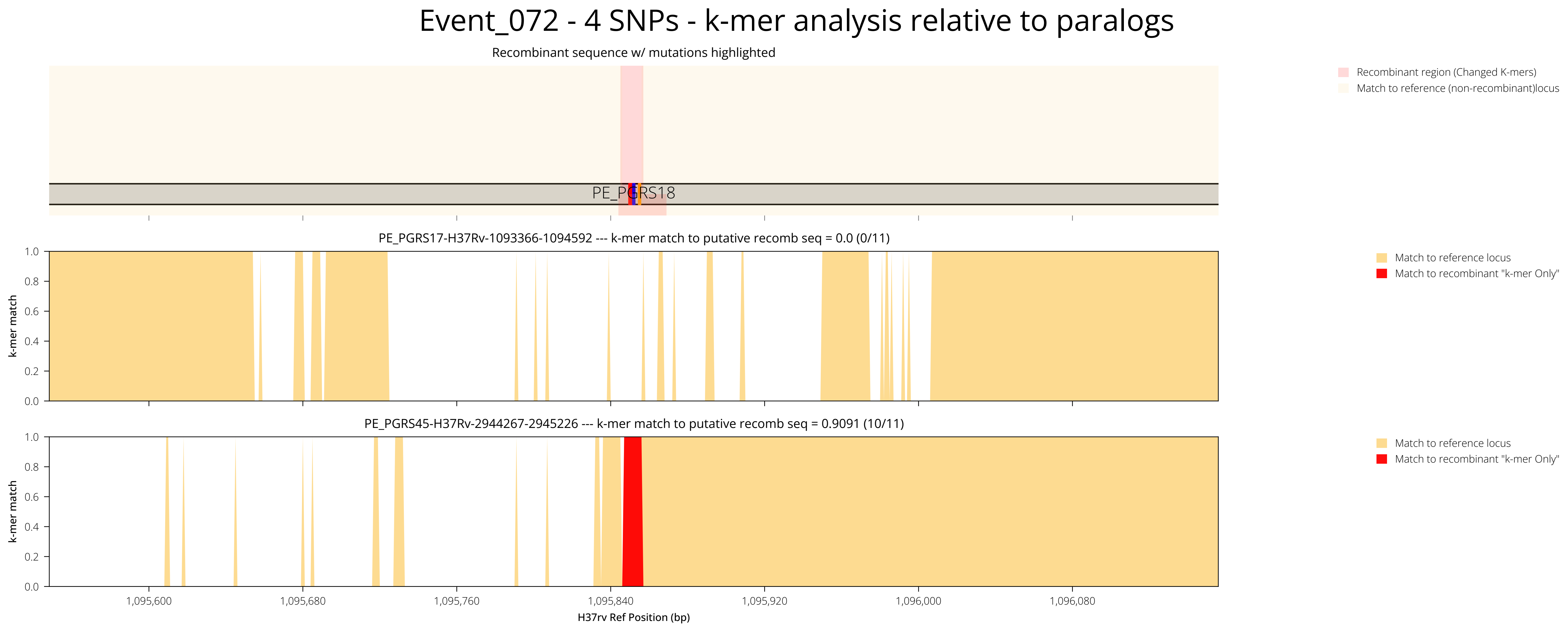
Event_072 - 4 SNPs - k-mer analysis relative to paralogs
Recombinant sequence w/ mutations highlighted
Recombinant region (Changed K-mers)
Match to reference (non-recombinant)locus
PE_PGRS18
PE_PGRS17-H37Rv-1093366-1094592 --- k-mer match to putative recomb seq = 0.0 (0/11)
Match to reference locus
Match to recombinant "k-mer Only"
k-mer match
1.0
0.8
0.6
0.4
0.2
0.0
PE_PGRS45-H37Rv-2944267-2945226 --- k-mer match to putative recomb seq = 0.9091 (10/11)
Match to reference locus
Match to recombinant "k-mer Only"
k-mer match
1,095,600
1,095,680
1,095,760
1,095,840
1,095,920
1,096,000
1,096,080
H37rv Ref Position (bp)

Sub-phylogeny Viz + Paralog Alignments
GCE ID: Event_072

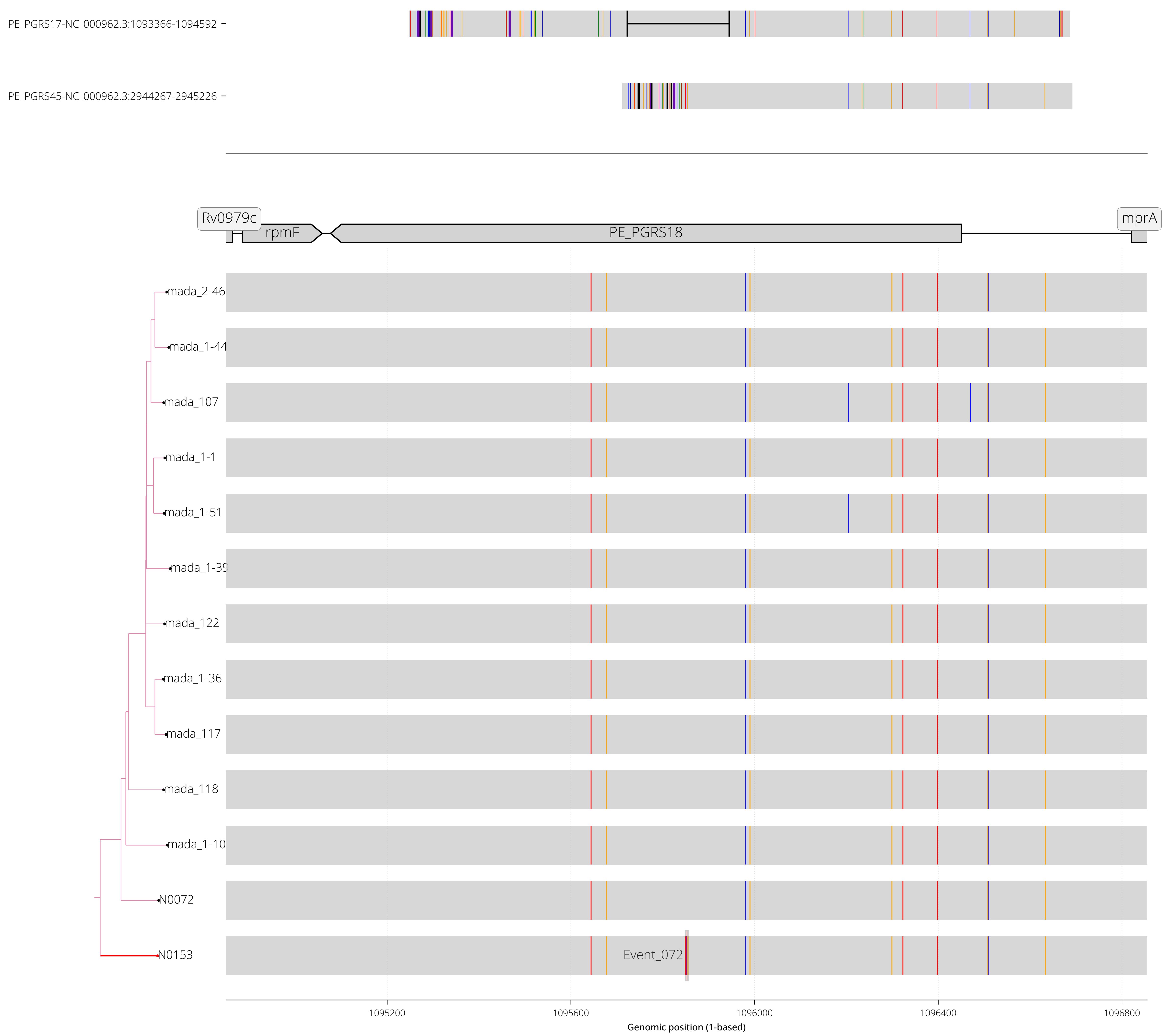

Full Phylogeny Viz + Paralog Alignments
GCE ID: Event_072

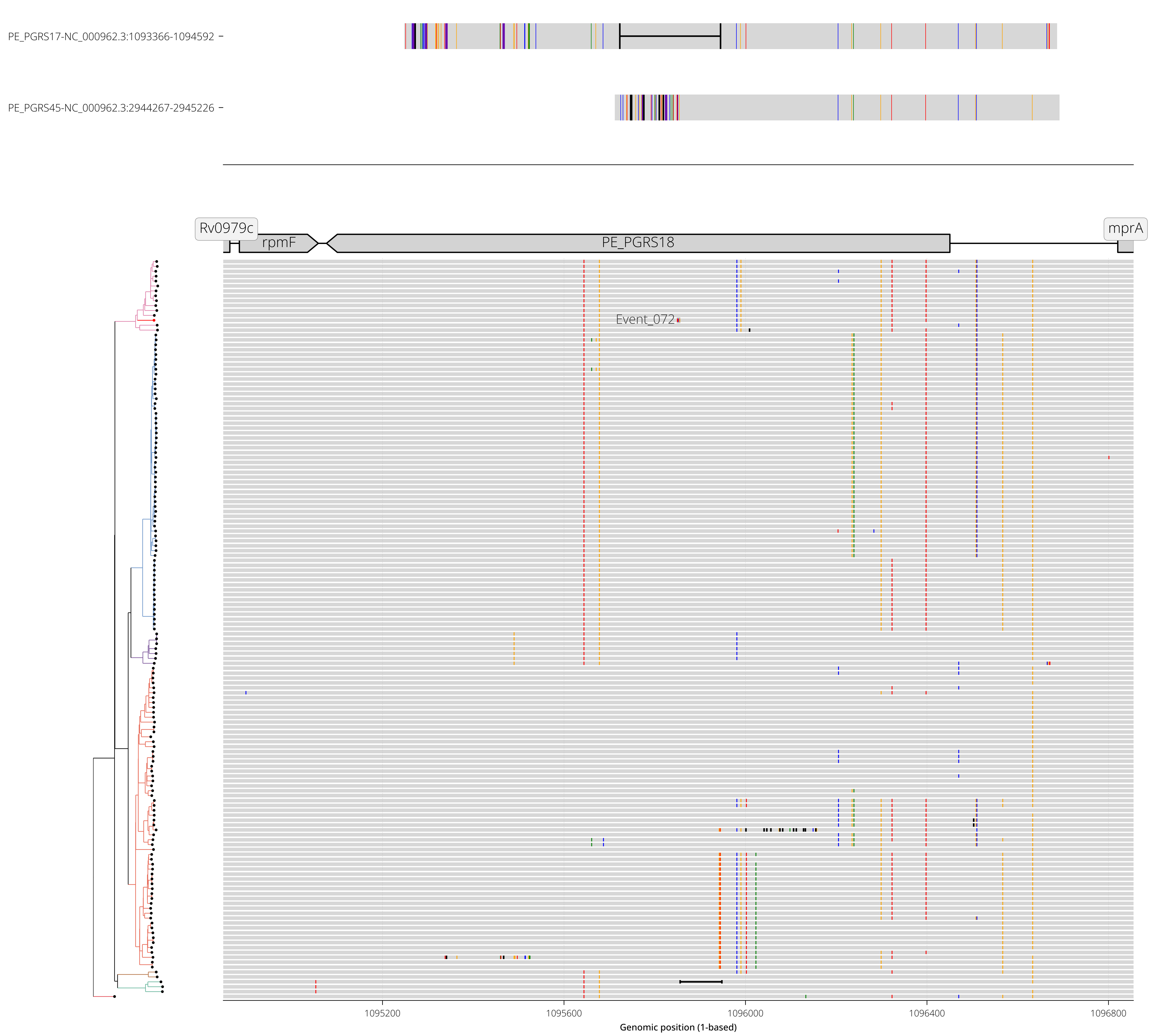

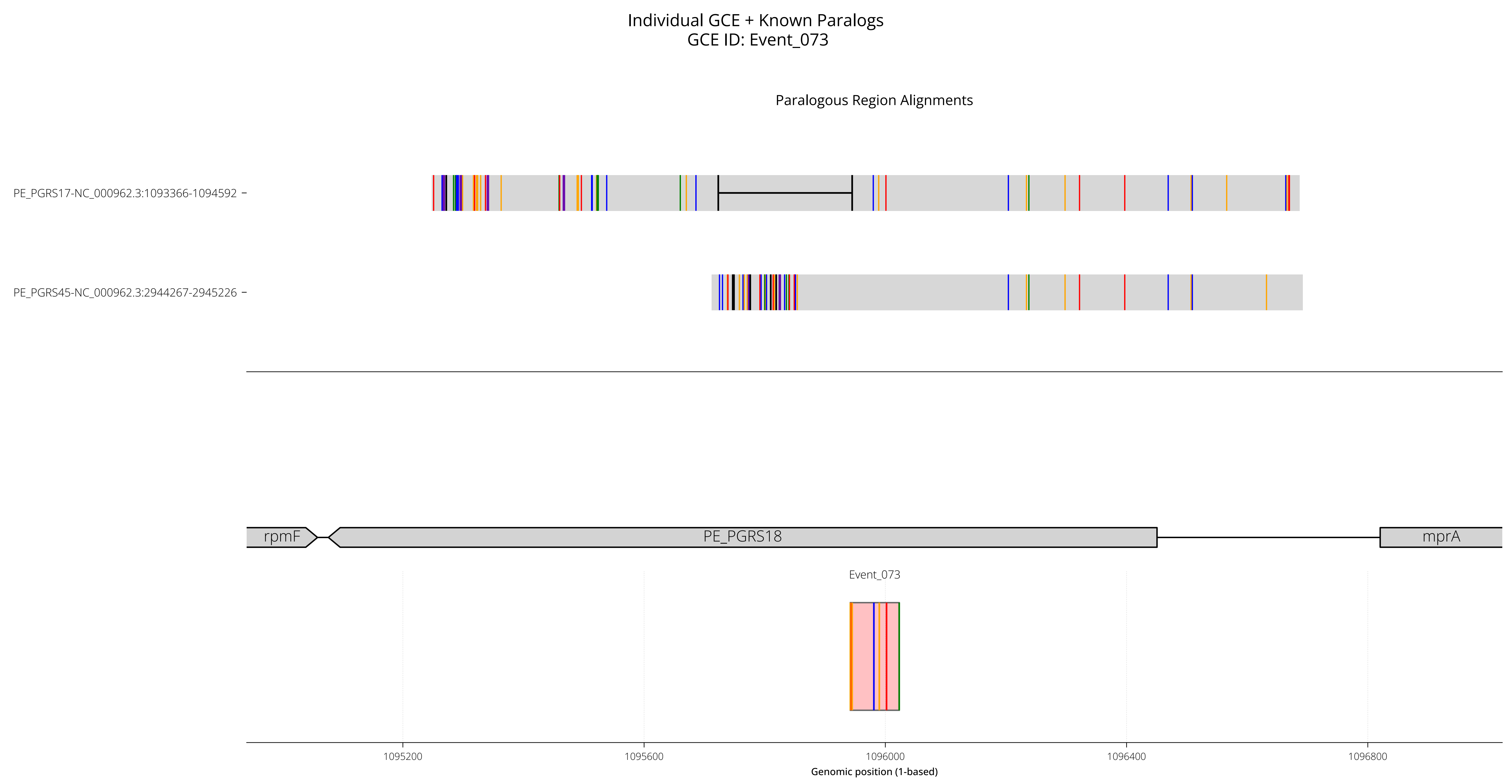
Individual GCE + Known Paralogs
GCE ID: Event_073
Paralogous Region Alignments
PE_PGRS17-NC_000962.3:1093366-1094592
PE_PGRS45-NC_000962.3:2944267-2945226
rpmF
PE_PGRS18
mprA
Event_073
1095200
1095600
1096000
1096400
1096800
Genomic position (1-based)

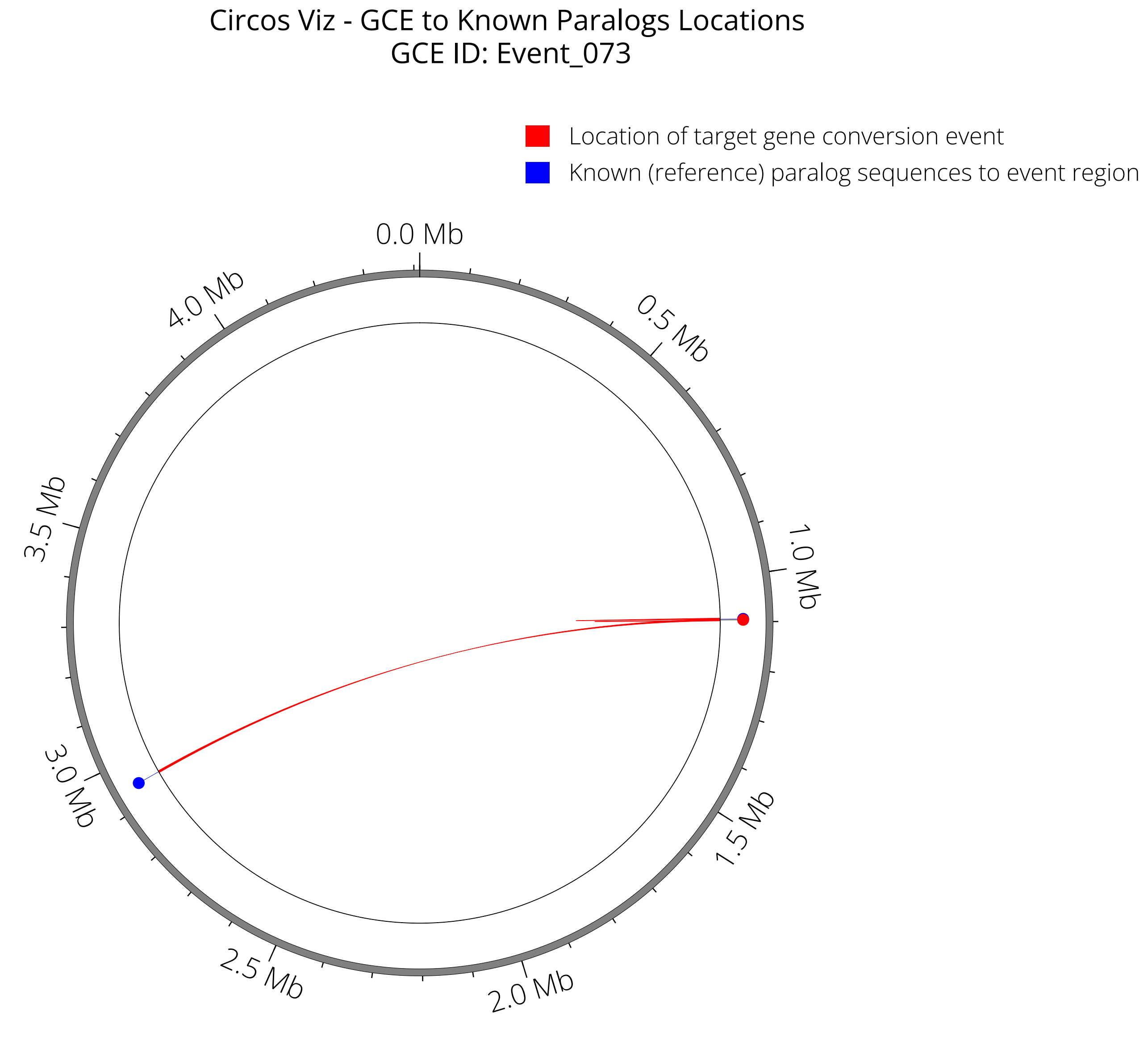

Circos Viz - GCE to Known Paralogs Locations
GCE ID: Event_073
Location of target gene conversion event
Known (reference) paralog sequences to event region
0.0 Mb
0.5 Mb
1.0 Mb
1.5 Mb
2.0 Mb
2.5 Mb
3.0 Mb
3.5 Mb
4.0 Mb

# Event_073 - 7 SNPs - k-mer analysis relative to paralogs

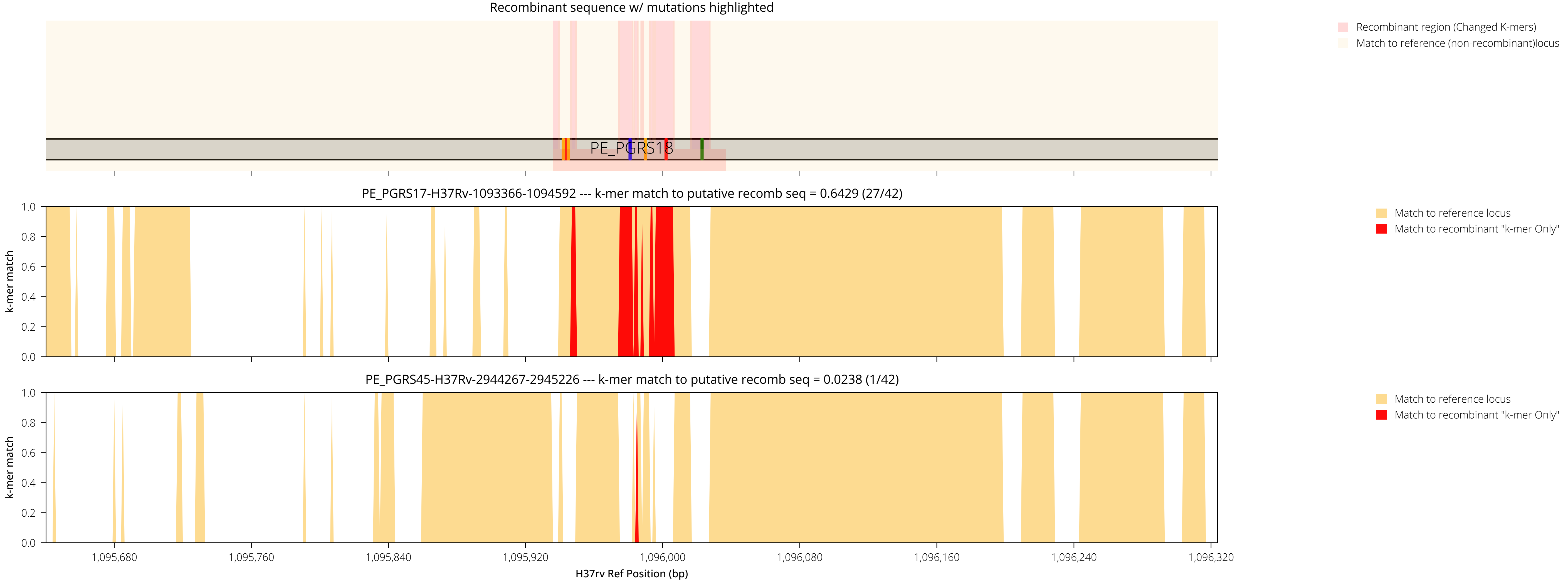

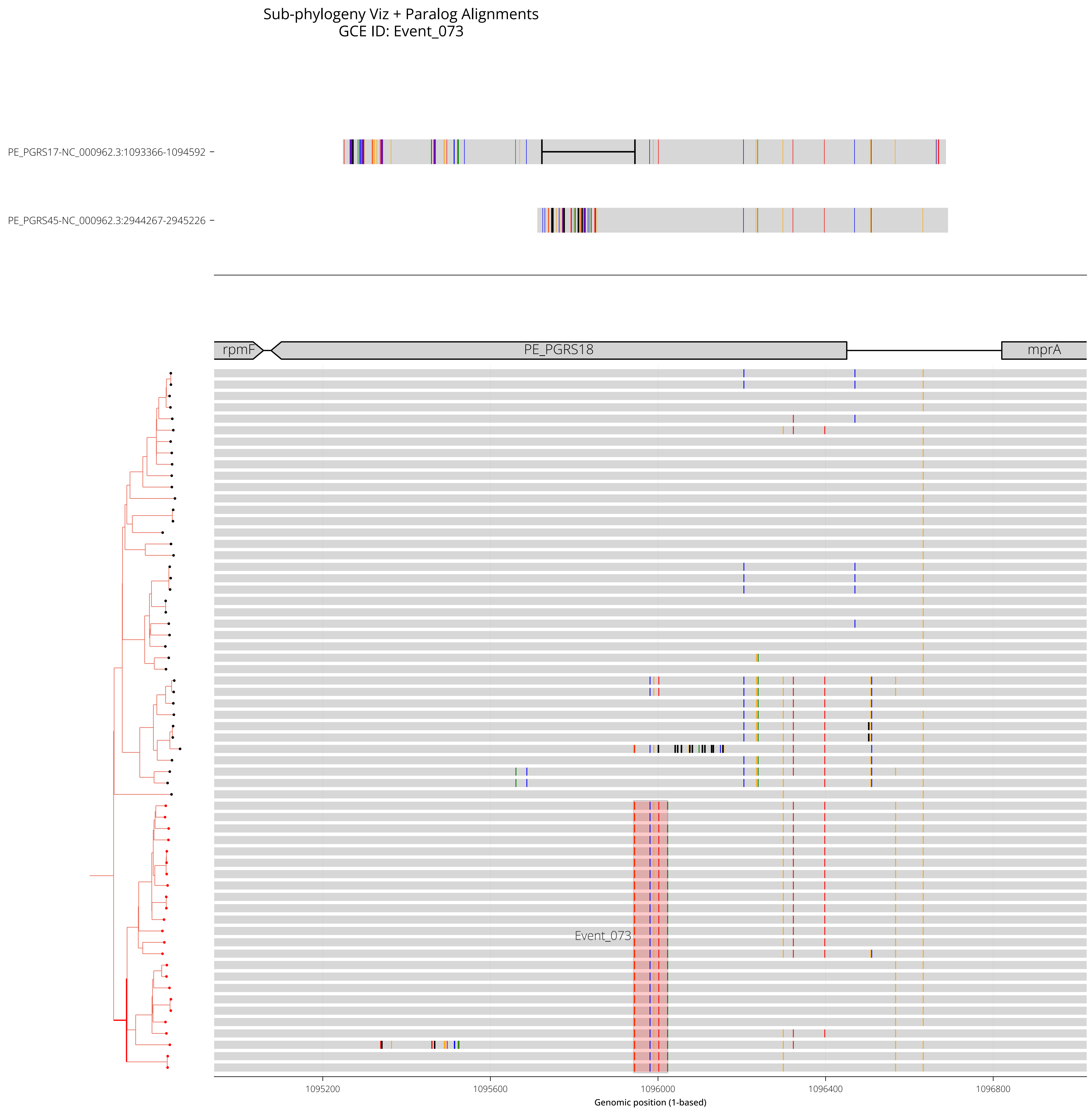

Sub-phylogeny Viz + Paralog Alignments
GCE ID: Event_073
PE_PGRS17-NC_000962.3:1093366-1094592
PE_PGRS45-NC_000962.3:2944267-2945226
rpmF
PE_PGRS18
mprA
Event_073
1095200
1095600
1096000
1096400
1096800
Genomic position (1-based)

Full Phylogeny Viz + Paralog Alignments
GCE ID: Event_073

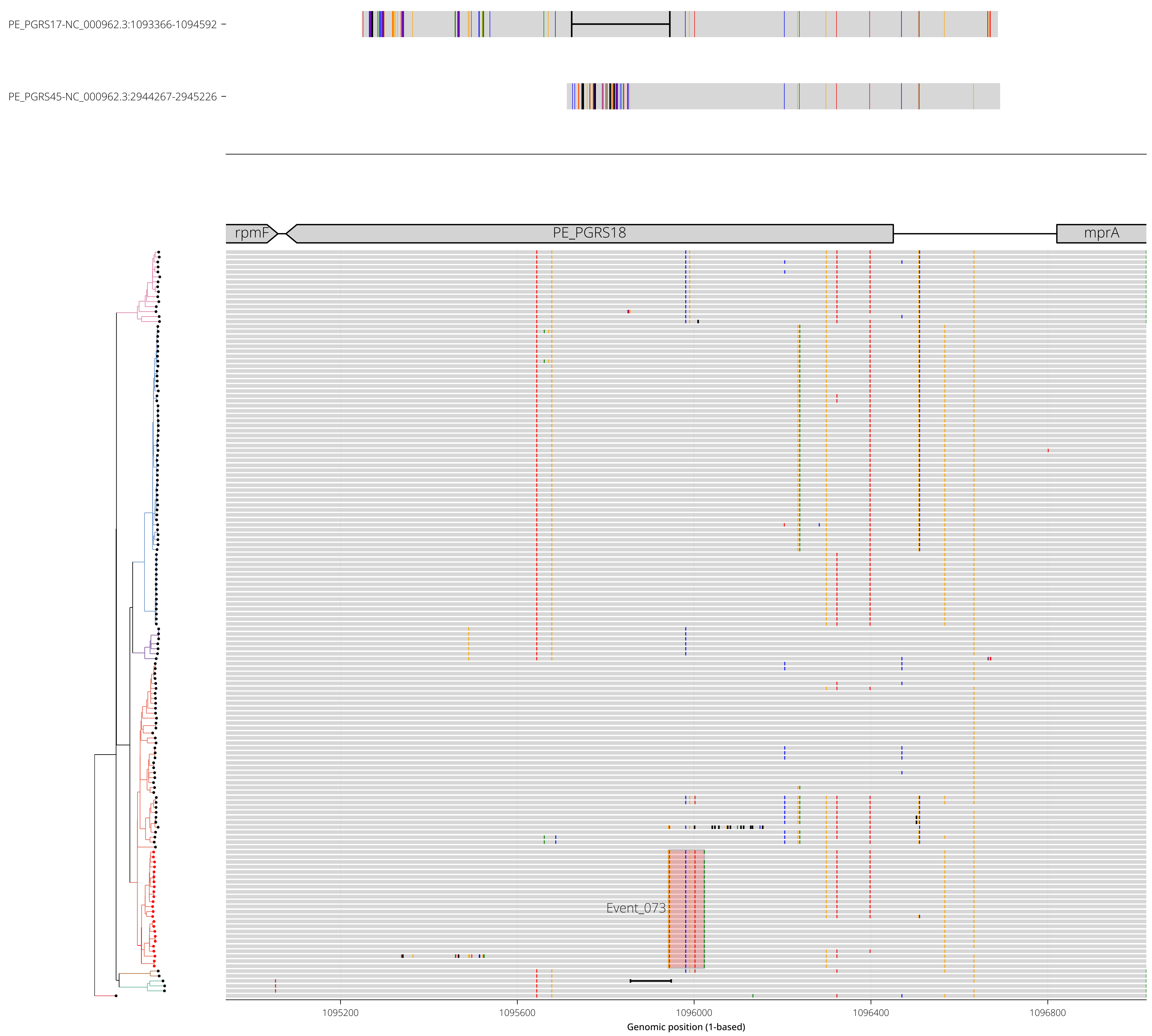

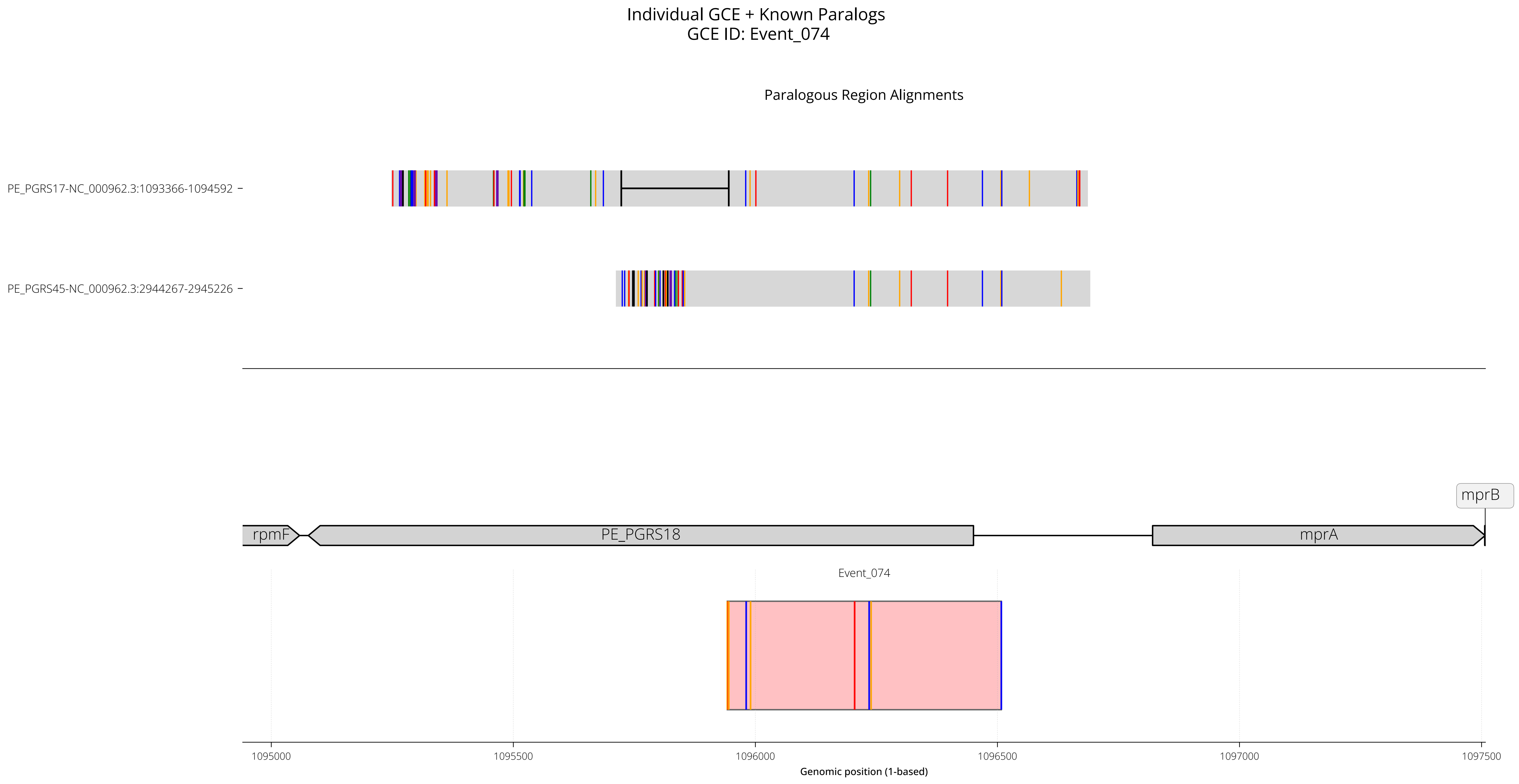

Individual GCE + Known Paralogs
GCE ID: Event_074
Paralogous Region Alignments
PE_PGRS17-NC_000962.3:1093366-1094592
PE_PGRS45-NC_000962.3:2944267-2945226
mprB
rpmF
PE_PGRS18
mprA
Event_074
1095000
1095500
1096000
1096500
1097000
1097500
Genomic position (1-based)

Circos Viz - GCE to Known Paralogs Locations
GCE ID: Event_074

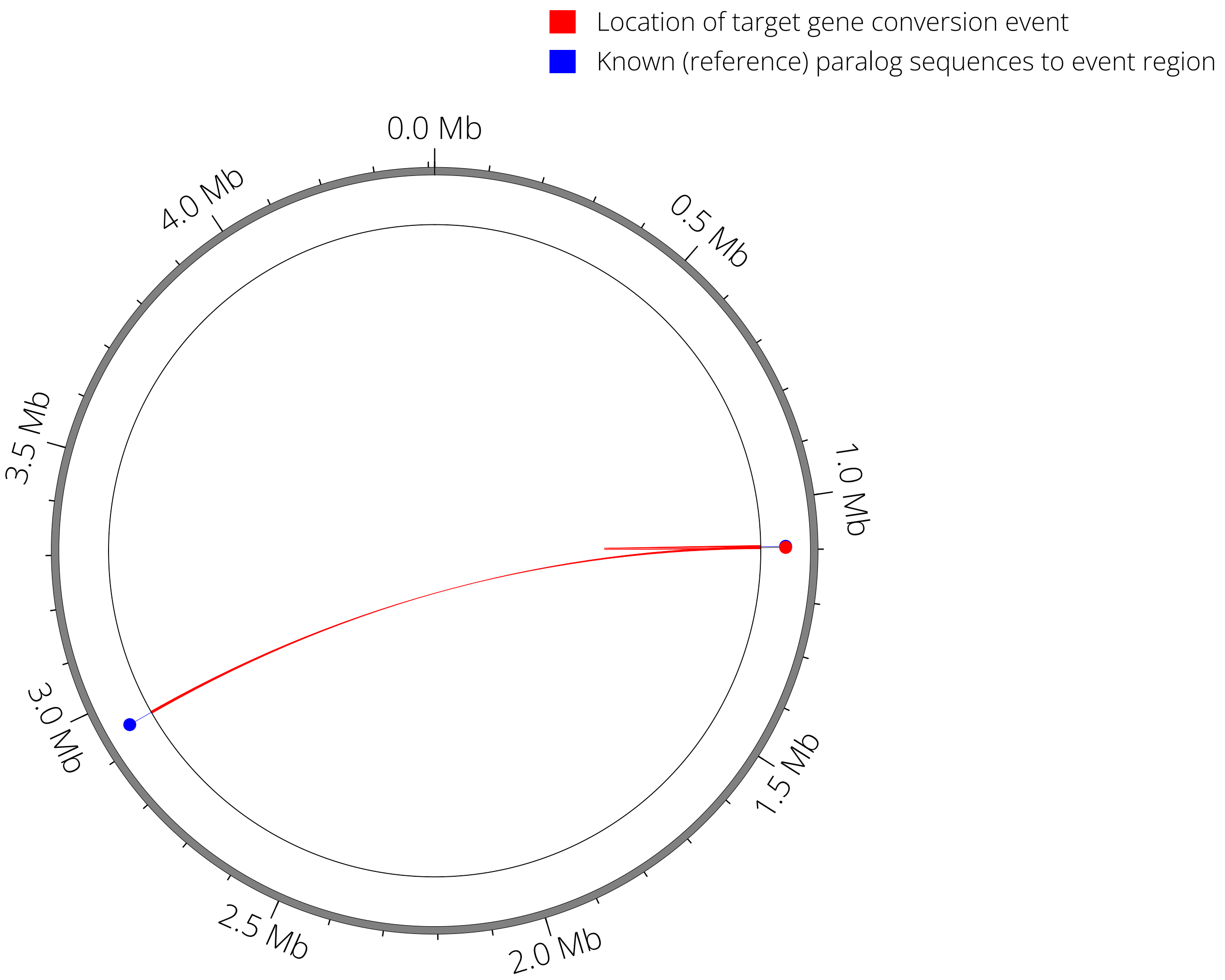

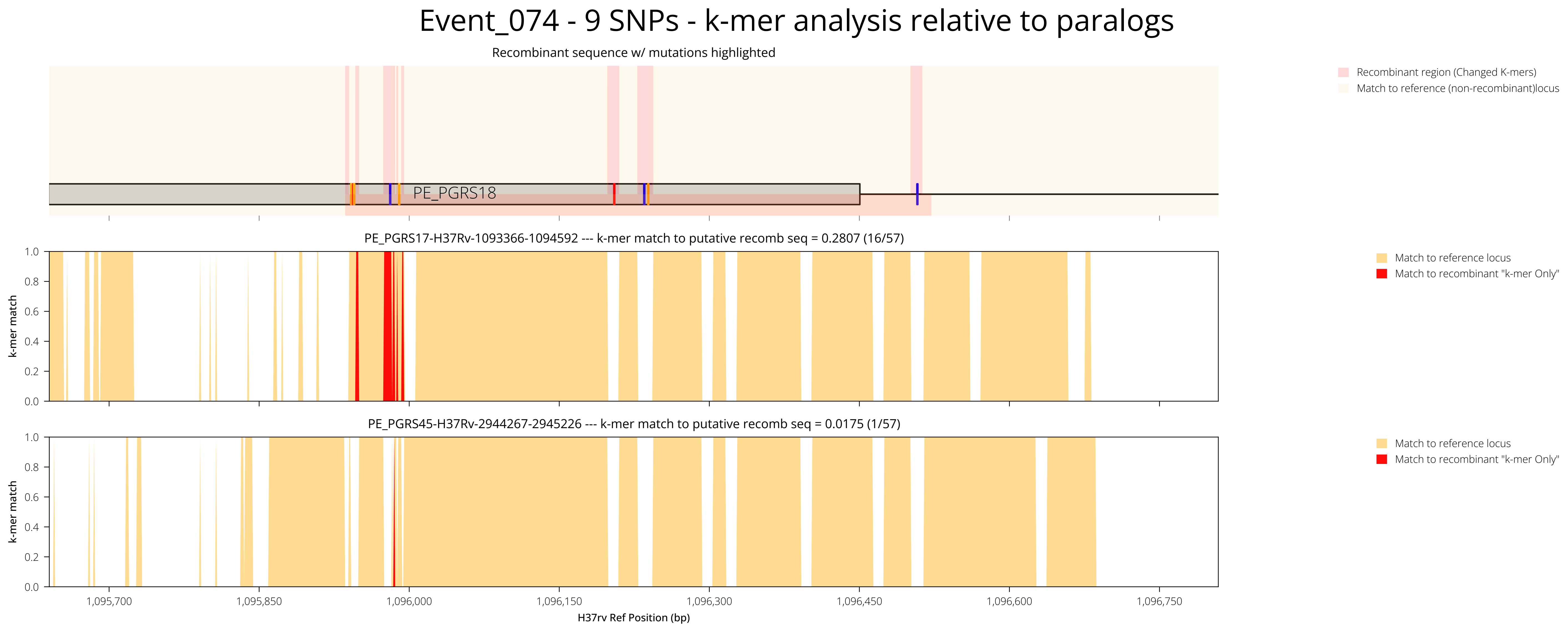

Event_074 - 9 SNPs - k-mer analysis relative to paralogs
Recombinant sequence w/ mutations highlighted
Recombinant region (Changed K-mers)
Match to reference (non-recombinant)locus
PE_PGRS18
PE_PGRS17-H37Rv-1093366-1094592 --- k-mer match to putative recomb seq = 0.2807 (16/57)
Match to reference locus
Match to recombinant "k-mer Only"
k-mer match
1.0
0.8
0.6
0.4
0.2
0.0
PE_PGRS45-H37Rv-2944267-2945226 --- k-mer match to putative recomb seq = 0.0175 (1/57)
Match to reference locus
Match to recombinant "k-mer Only"
k-mer match
1.0
0.8
0.6
0.4
0.2
0.0
1,095,700
1,095,850
1,096,000
1,096,150
1,096,300
1,096,450
1,096,600
1,096,750
H37rv Ref Position (bp)

Sub-phylogeny Viz + Paralog Alignments
GCE ID: Event_074

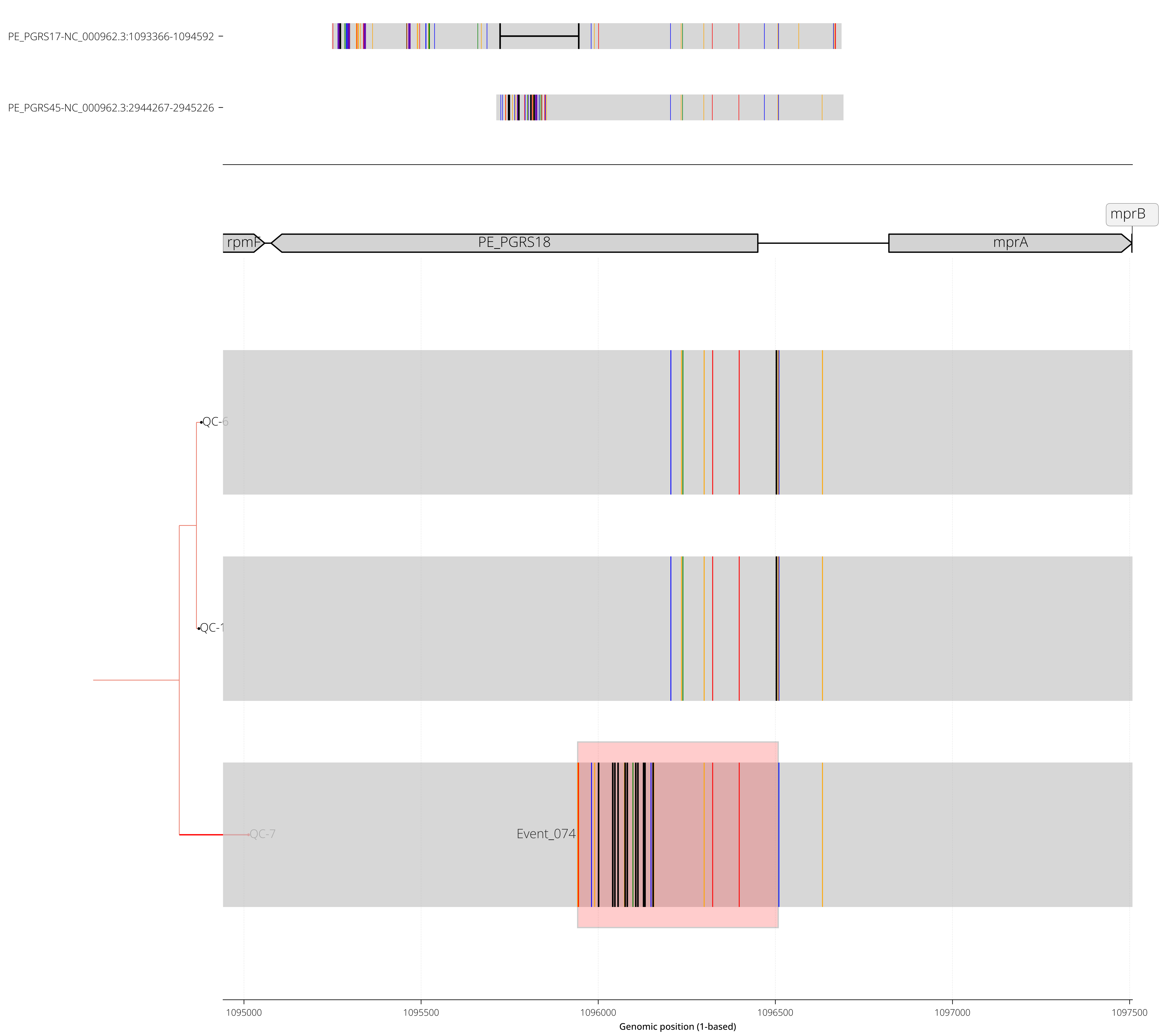

Full Phylogeny Viz + Paralog Alignments
GCE ID: Event_074

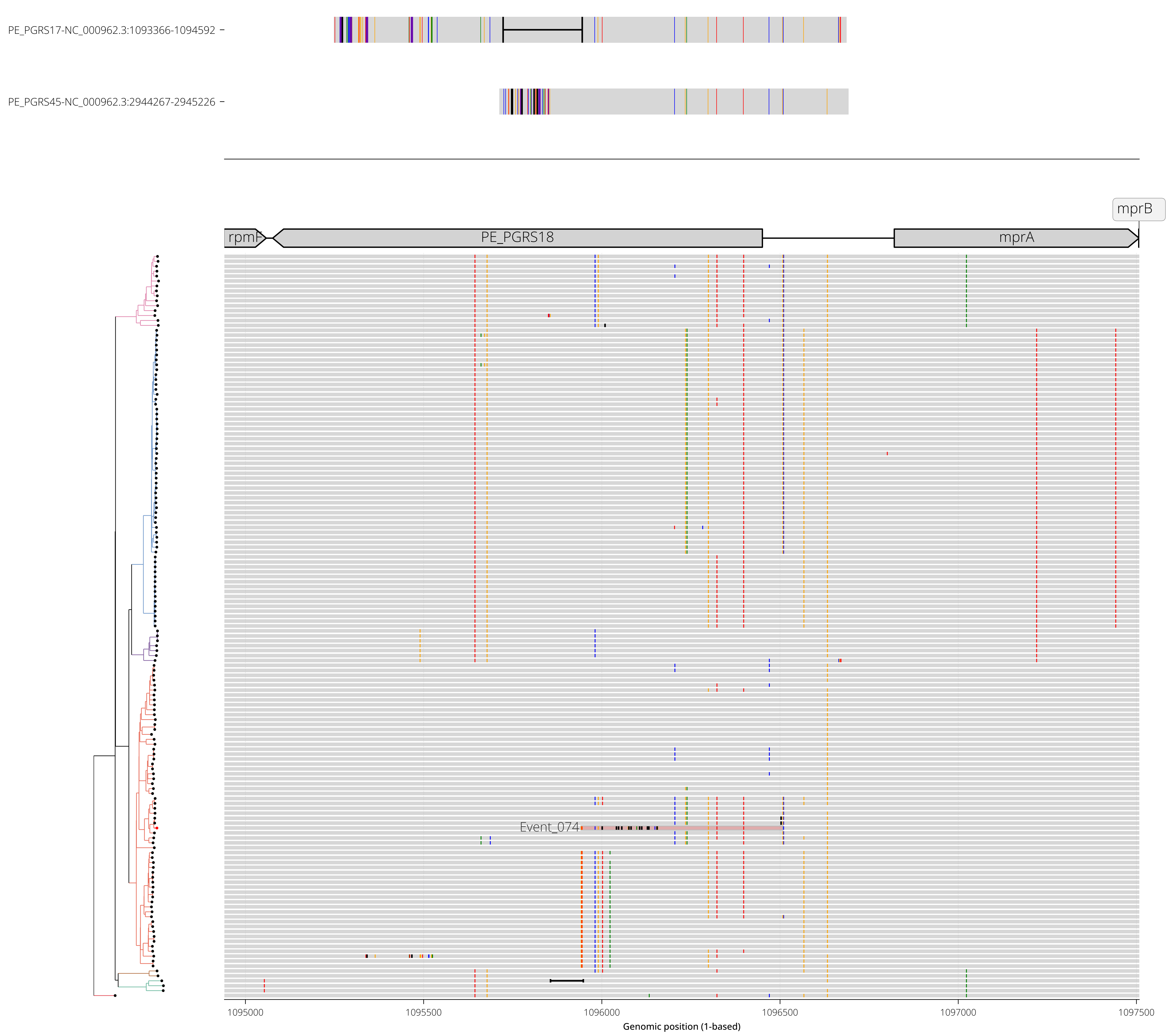

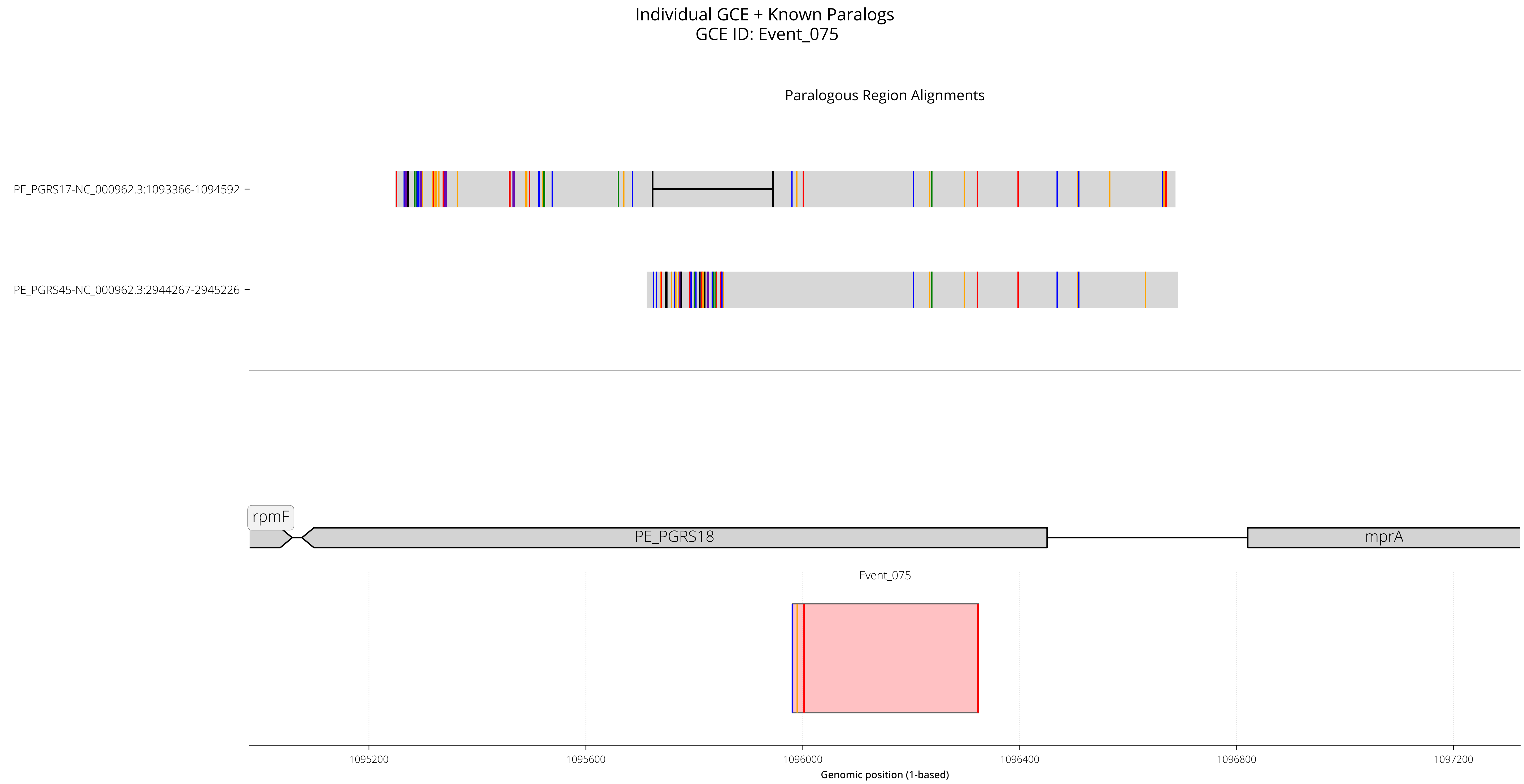

Individual GCE + Known Paralogs
GCE ID: Event_075
Paralogous Region Alignments
PE_PGRS17-NC_000962.3:1093366-1094592
PE_PGRS45-NC_000962.3:2944267-2945226
rpmF
PE_PGRS18
mprA
Event_075
1095200
1095600
1096000
1096400
1096800
1097200
Genomic position (1-based)

Circos Viz - GCE to Known Paralogs Locations
GCE ID: Event_075

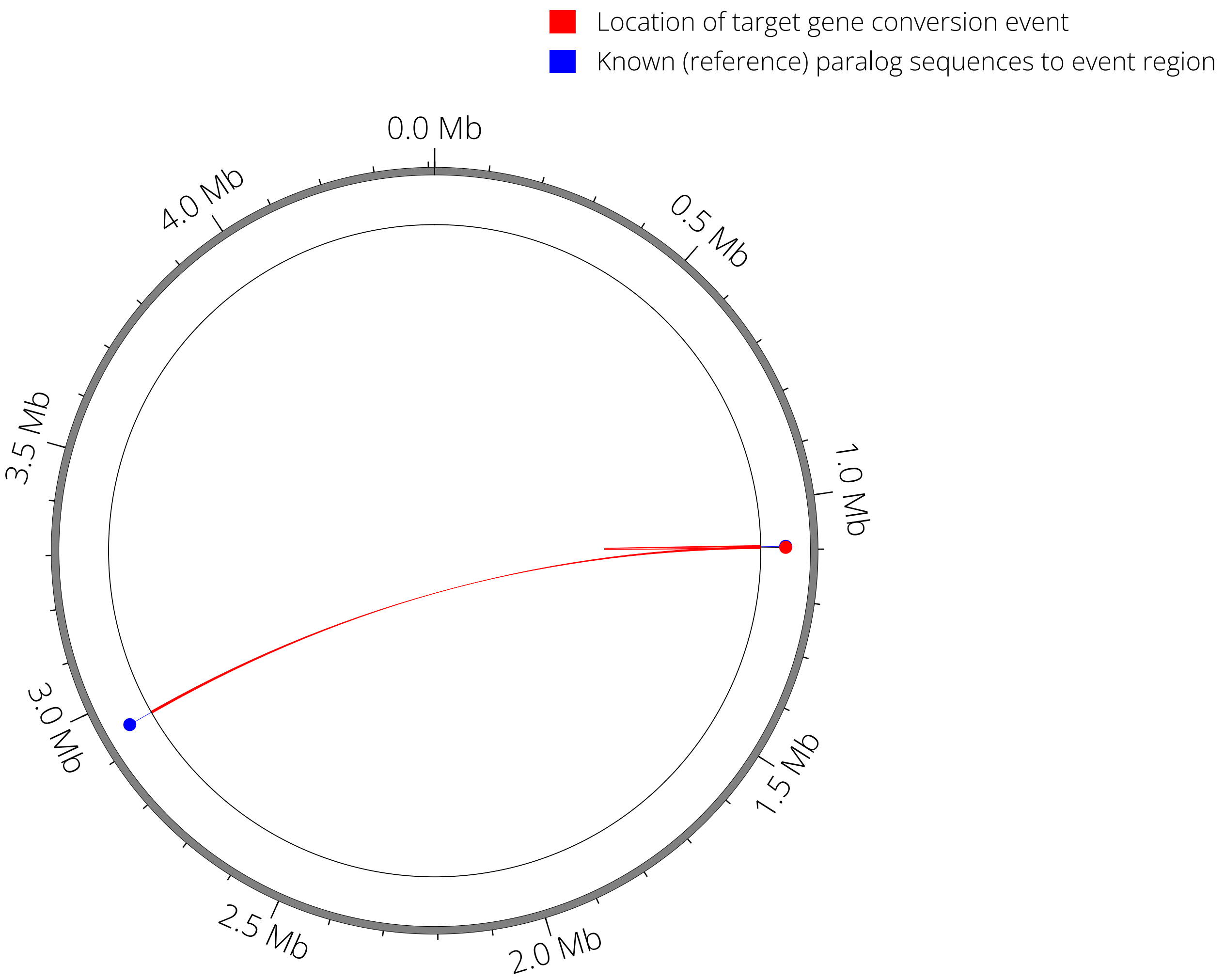

# Event_075 - 4 SNPs - k-mer analysis relative to paralogs

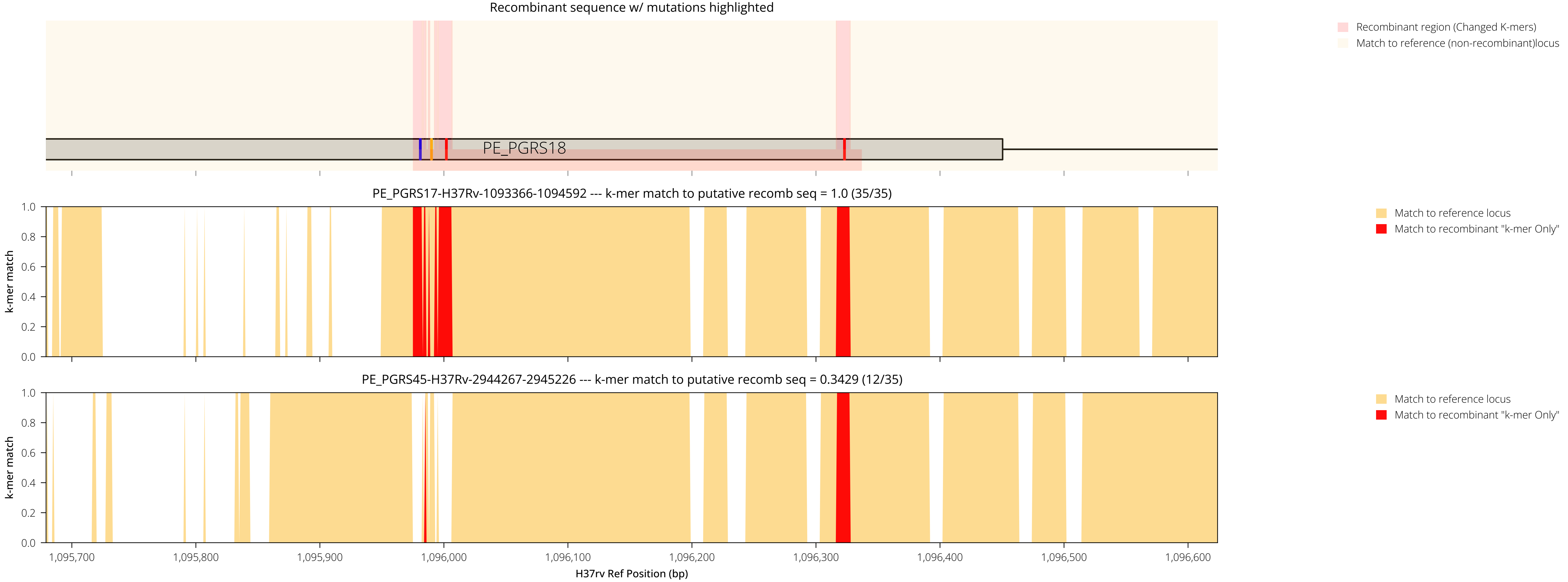

Sub-phylogeny Viz + Paralog Alignments
GCE ID: Event_075

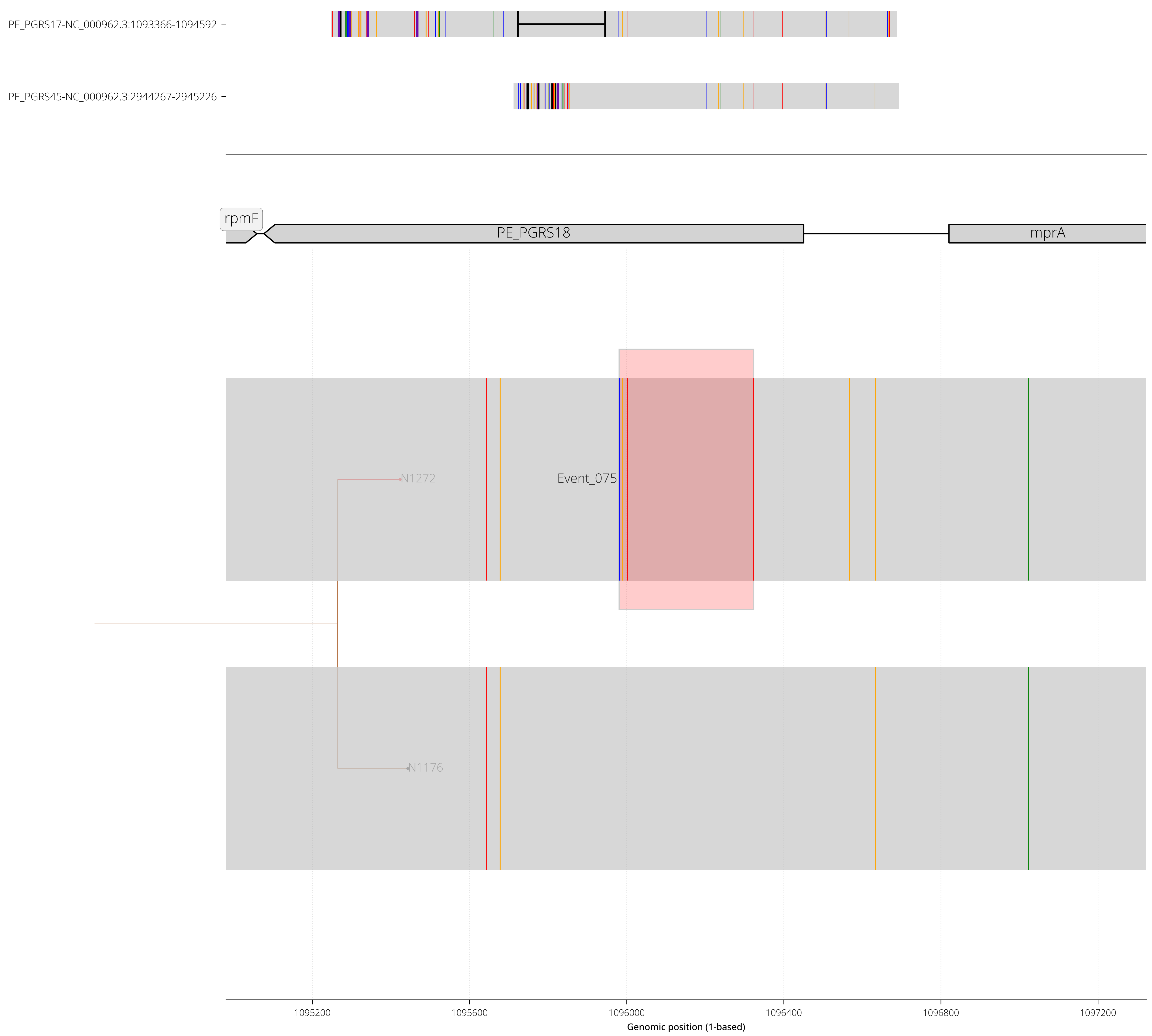

Full Phylogeny Viz + Paralog Alignments
GCE ID: Event_075

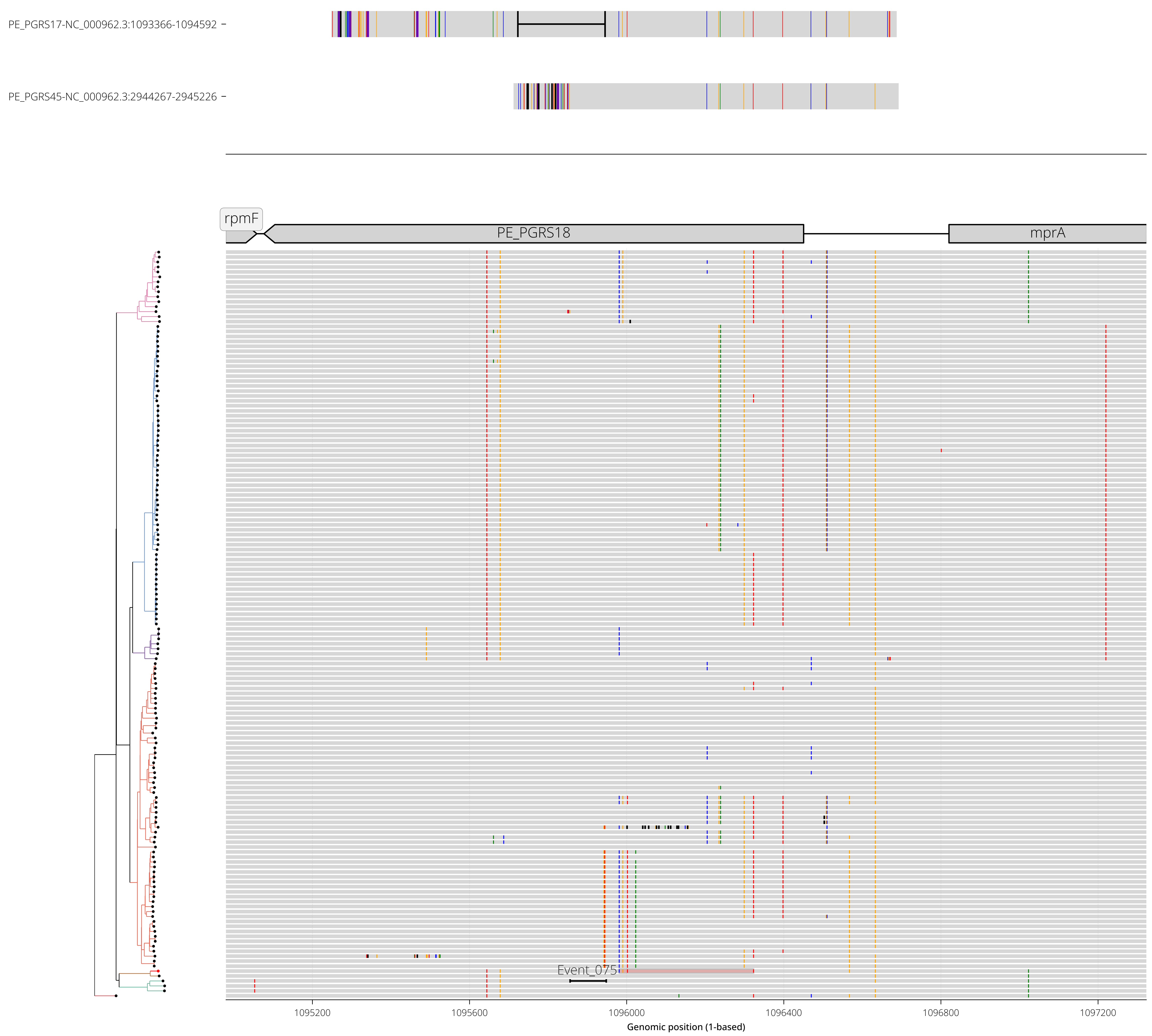

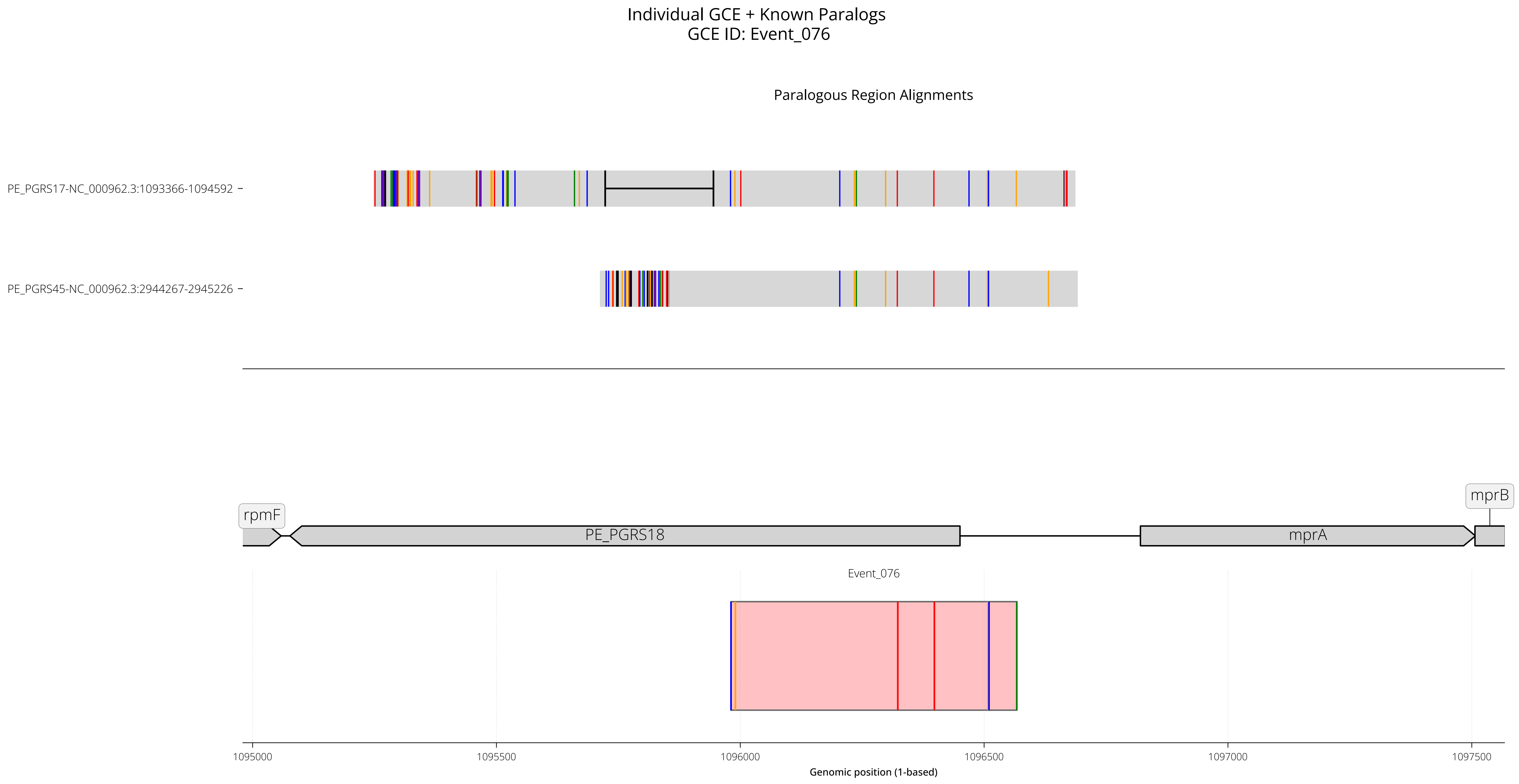
Individual GCE + Known Paralogs
GCE ID: Event_076
Paralogous Region Alignments
PE_PGRS17-NC_000962.3:1093366-1094592
PE_PGRS45-NC_000962.3:2944267-2945226
rpmF
PE_PGRS18
mprA
mprB
Event_076
1095000
1095500
1096000
1096500
1097000
1097500
Genomic position (1-based)

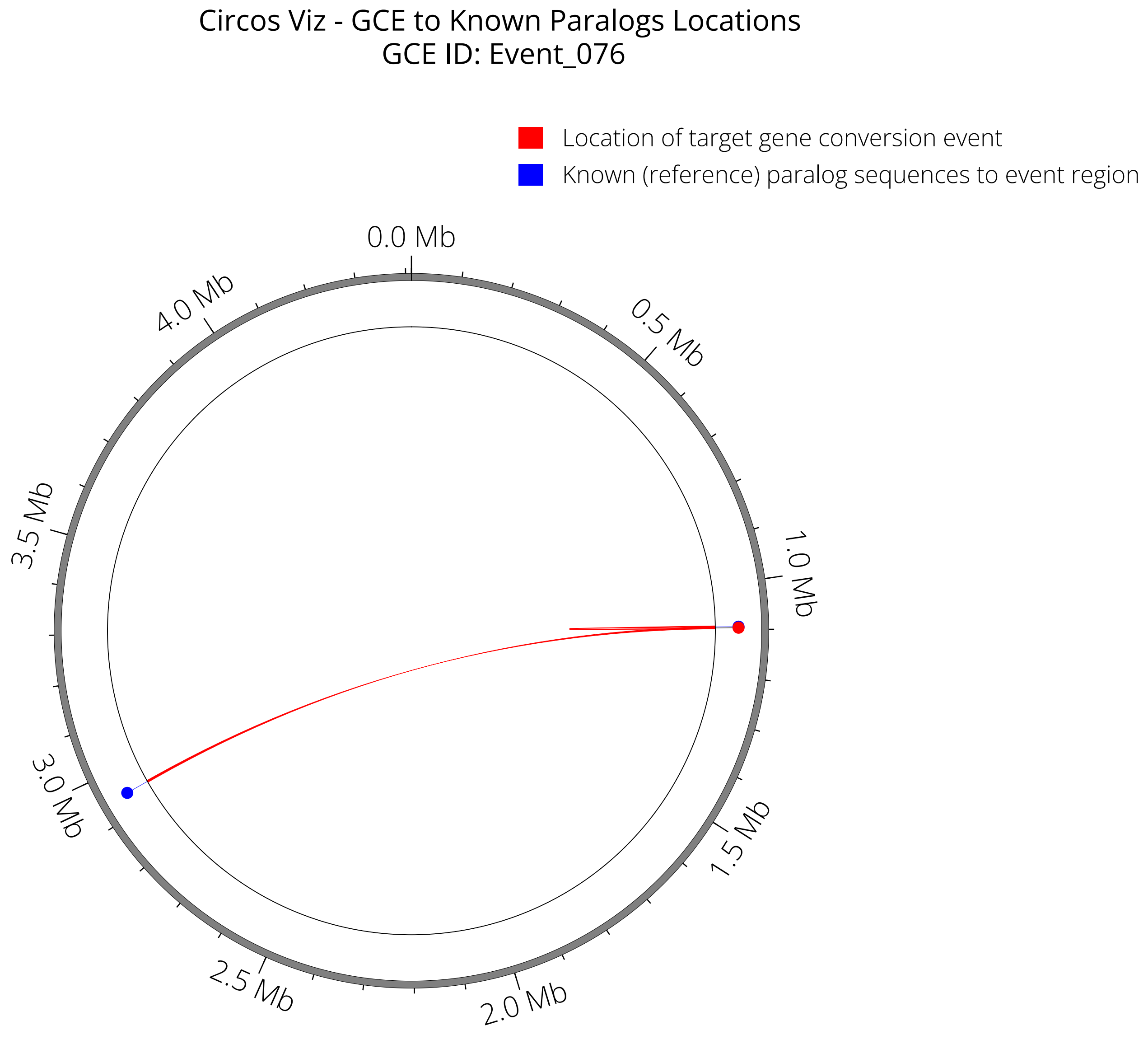

Circos Viz - GCE to Known Paralogs Locations
GCE ID: Event_076
Location of target gene conversion event
Known (reference) paralog sequences to event region
0.0 Mb
0.5 Mb
1.0 Mb
1.5 Mb
2.0 Mb
2.5 Mb
3.0 Mb
3.5 Mb
4.0 Mb

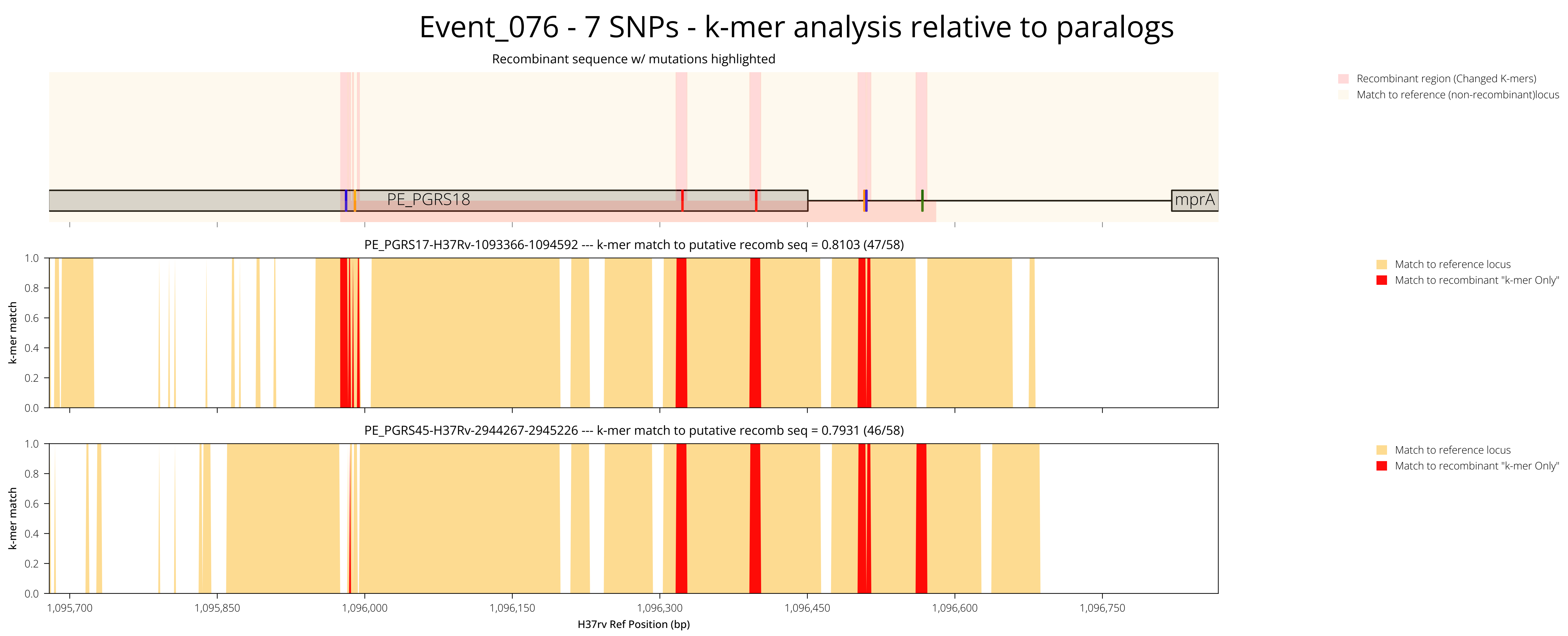

Event_076 - 7 SNPs - k-mer analysis relative to paralogs
Recombinant sequence w/ mutations highlighted
Recombinant region (Changed K-mers)
Match to reference (non-recombinant)locus
PE_PGRS18
mprA
PE_PGRS17-H37Rv-1093366-1094592 --- k-mer match to putative recomb seq = 0.8103 (47/58)
Match to reference locus
Match to recombinant "k-mer Only"
PE_PGRS45-H37Rv-2944267-2945226 --- k-mer match to putative recomb seq = 0.7931 (46/58)
Match to reference locus
Match to recombinant "k-mer Only"
k-mer match
0.0
0.2
0.4
0.6
0.8
1.0
1,095,700
1,095,850
1,096,000
1,096,150
1,096,300
1,096,450
1,096,600
1,096,750
H37rv Ref Position (bp)

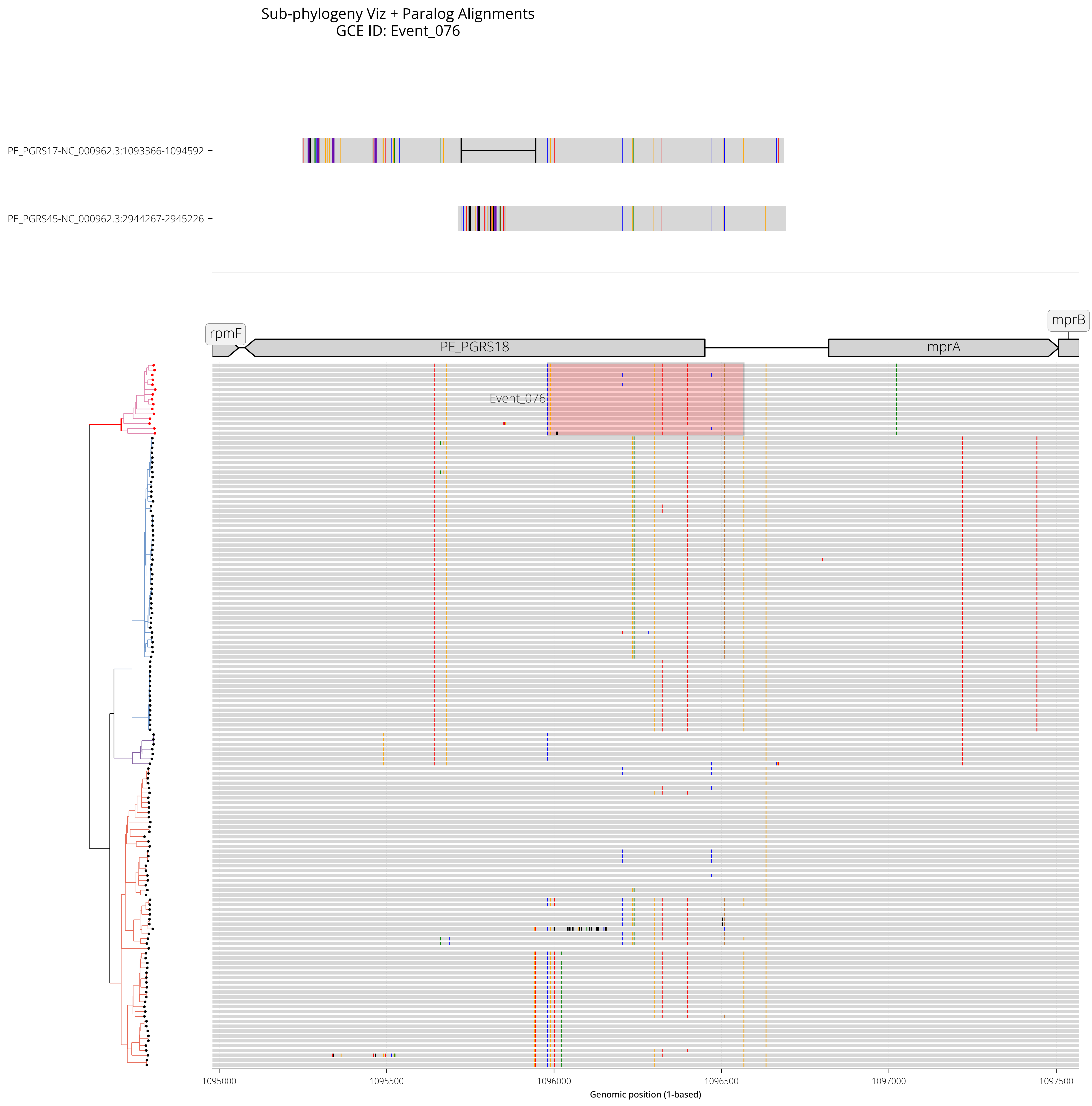

Sub-phylogeny Viz + Paralog Alignments
GCE ID: Event_076
PE_PGRS17-NC_000962.3:1093366-1094592
PE_PGRS45-NC_000962.3:2944267-2945226
rpmF
PE_PGRS18
mprA
mprB
Event_076
1095000
1095500
1096000
1096500
1097000
1097500
Genomic position (1-based)

Full Phylogeny Viz + Paralog Alignments
GCE ID: Event_076

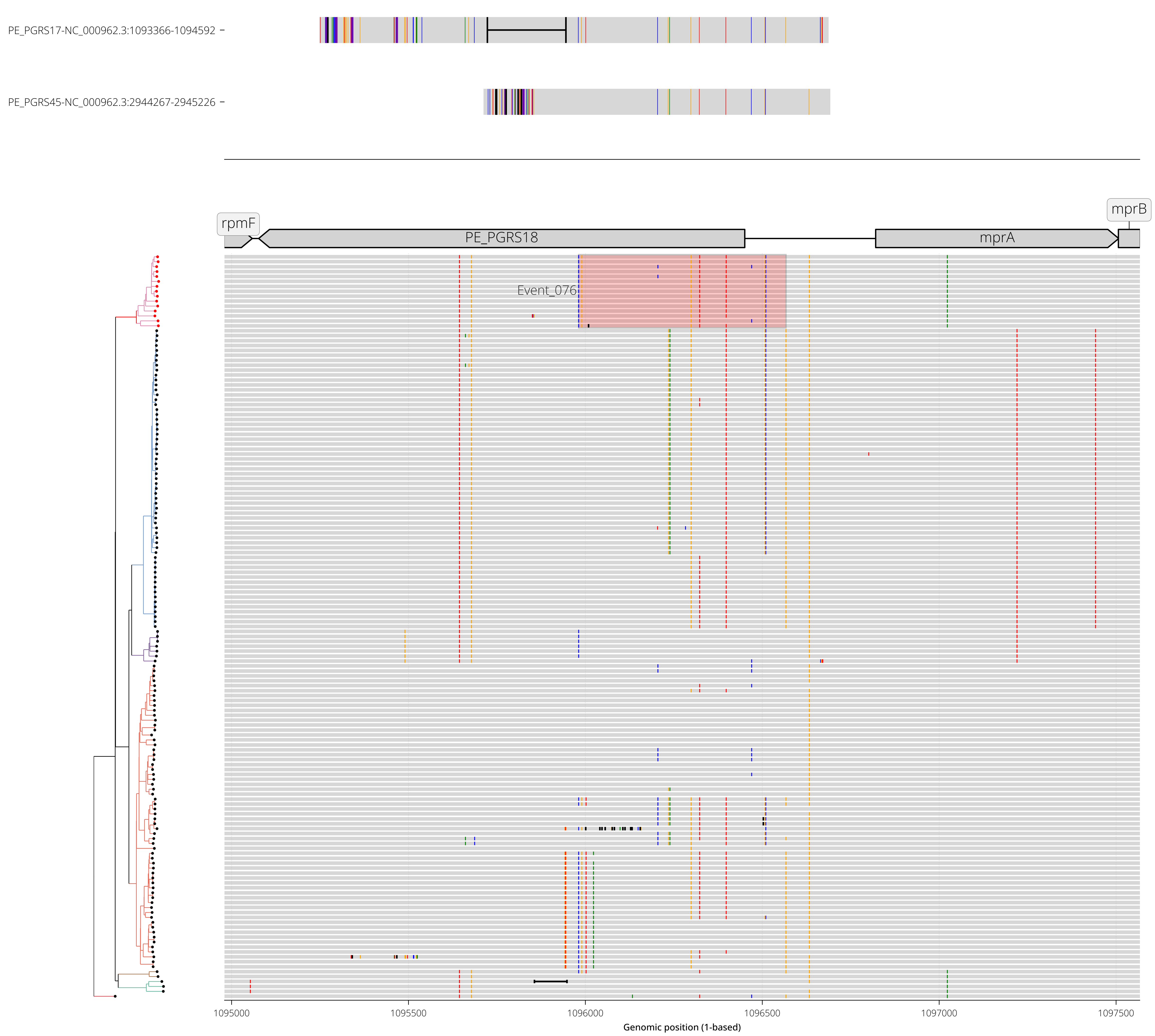

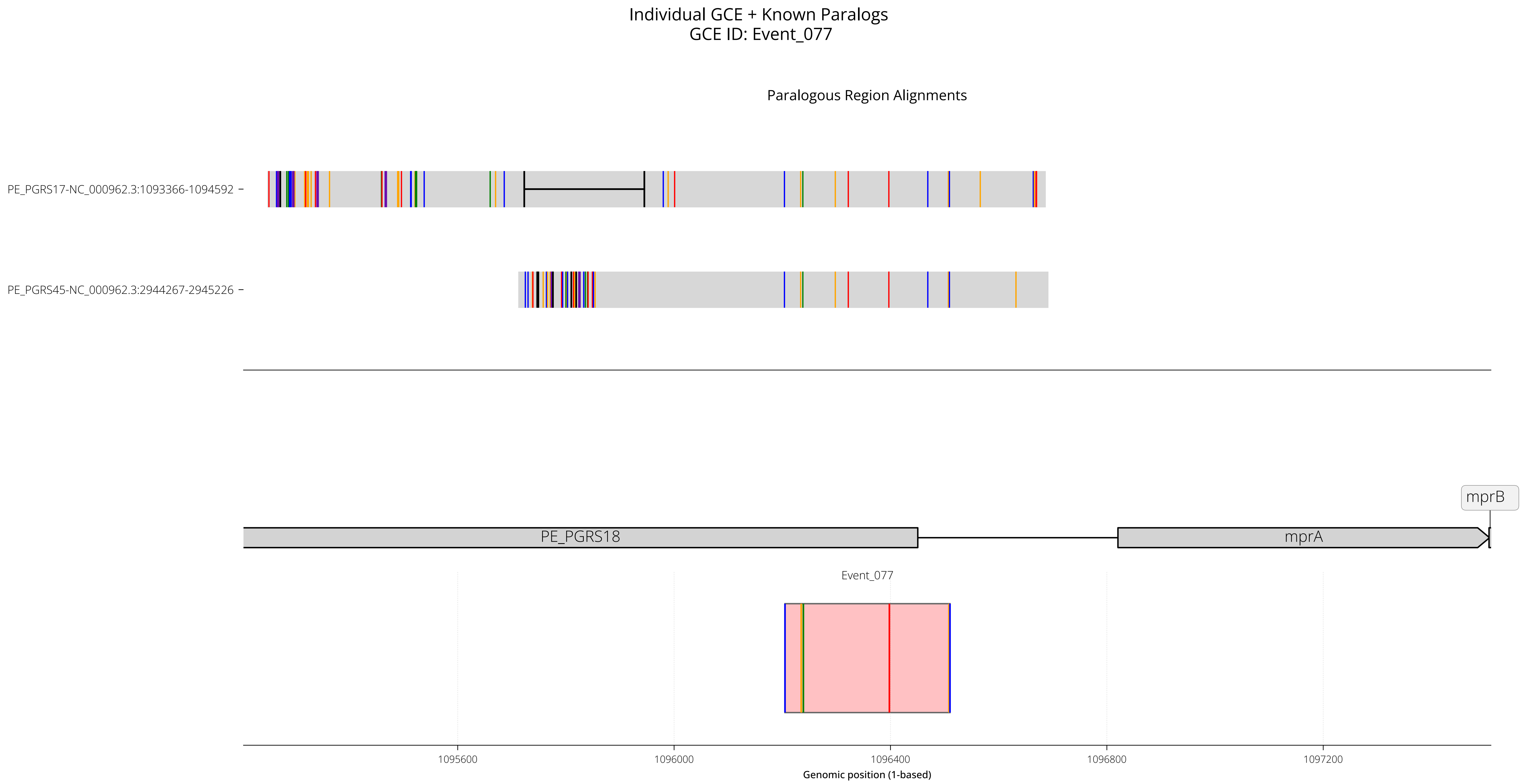

Individual GCE + Known Paralogs
GCE ID: Event_077
Paralogous Region Alignments
PE_PGRS17-NC_000962.3:1093366-1094592
PE_PGRS45-NC_000962.3:2944267-2945226
mprB
PE_PGRS18
mprA
Event_077
1095600
1096000
1096400
1096800
1097200
Genomic position (1-based)

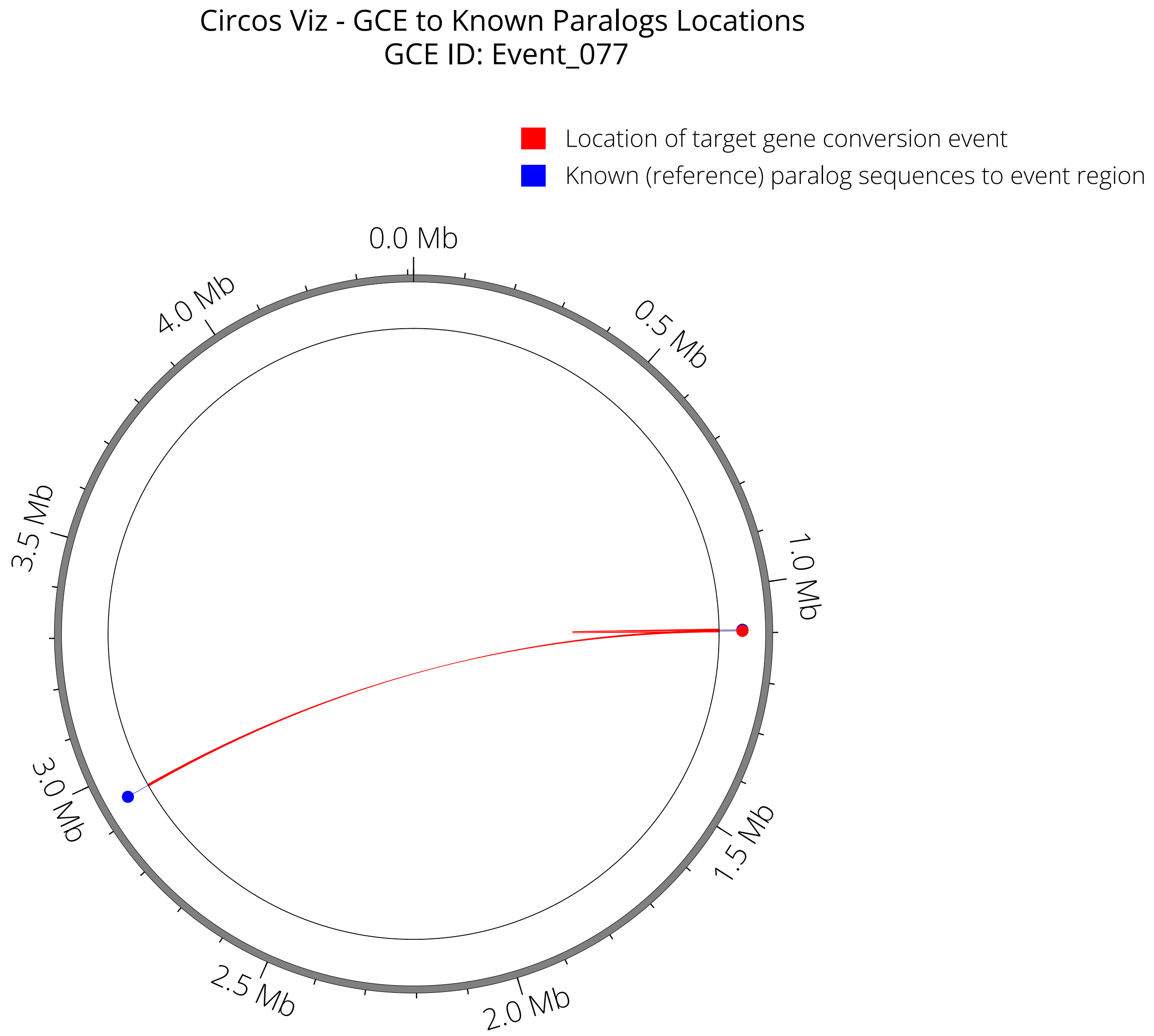
Circos Viz - GCE to Known Paralogs Locations
GCE ID: Event_077
Location of target gene conversion event
Known (reference) paralog sequences to event region
0.0 Mb
0.5 Mb
1.0 Mb
1.5 Mb
2.0 Mb
2.5 Mb
3.0 Mb
3.5 Mb
4.0 Mb

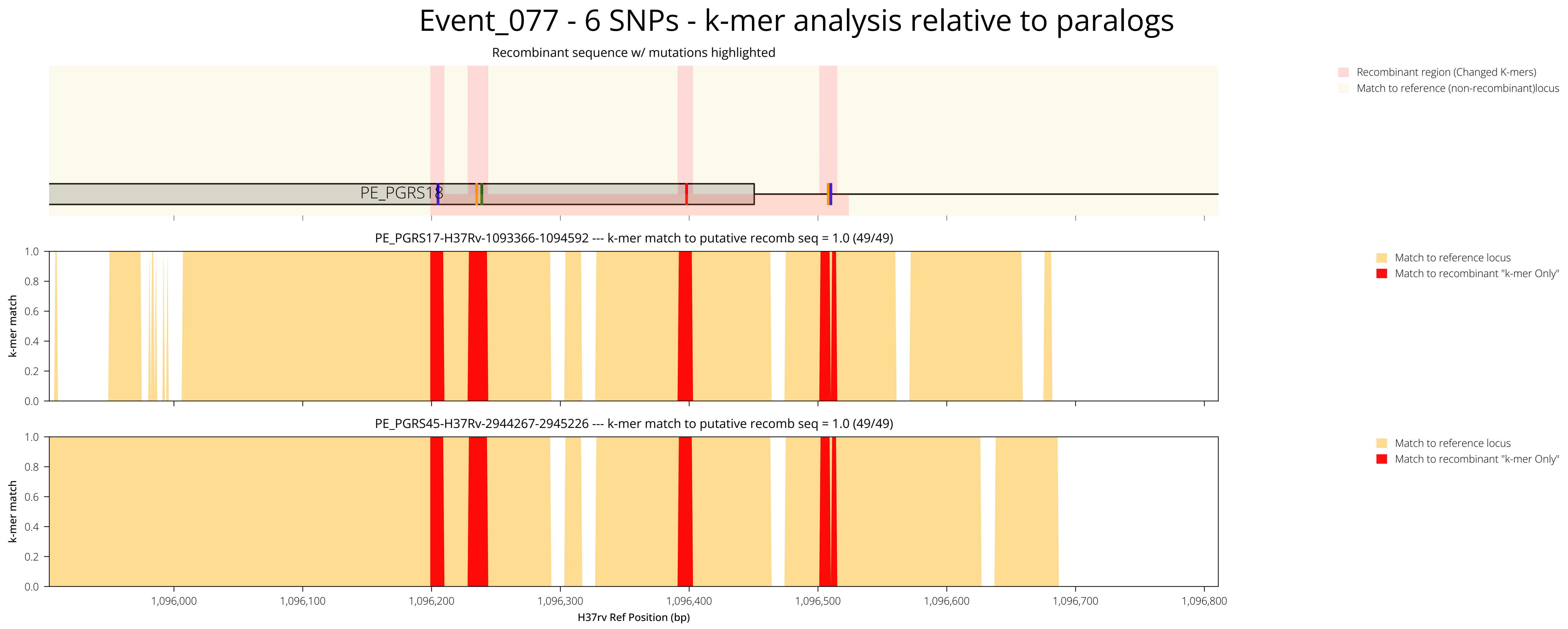
Event_077 - 6 SNPs - k-mer analysis relative to paralogs
Recombinant sequence w/ mutations highlighted
Recombinant region (Changed K-mers)
Match to reference (non-recombinant)locus
PE_PGRS18
PE_PGRS17-H37Rv-1093366-1094592 --- k-mer match to putative recomb seq = 1.0 (49/49)
Match to reference locus
Match to recombinant "k-mer Only"
PE_PGRS45-H37Rv-2944267-2945226 --- k-mer match to putative recomb seq = 1.0 (49/49)
Match to reference locus
Match to recombinant "k-mer Only"
k-mer match
1.0
0.8
0.6
0.4
0.2
0.0
1,096,000
1,096,100
1,096,200
1,096,300
1,096,400
1,096,500
1,096,600
1,096,700
1,096,800
H37rv Ref Position (bp)

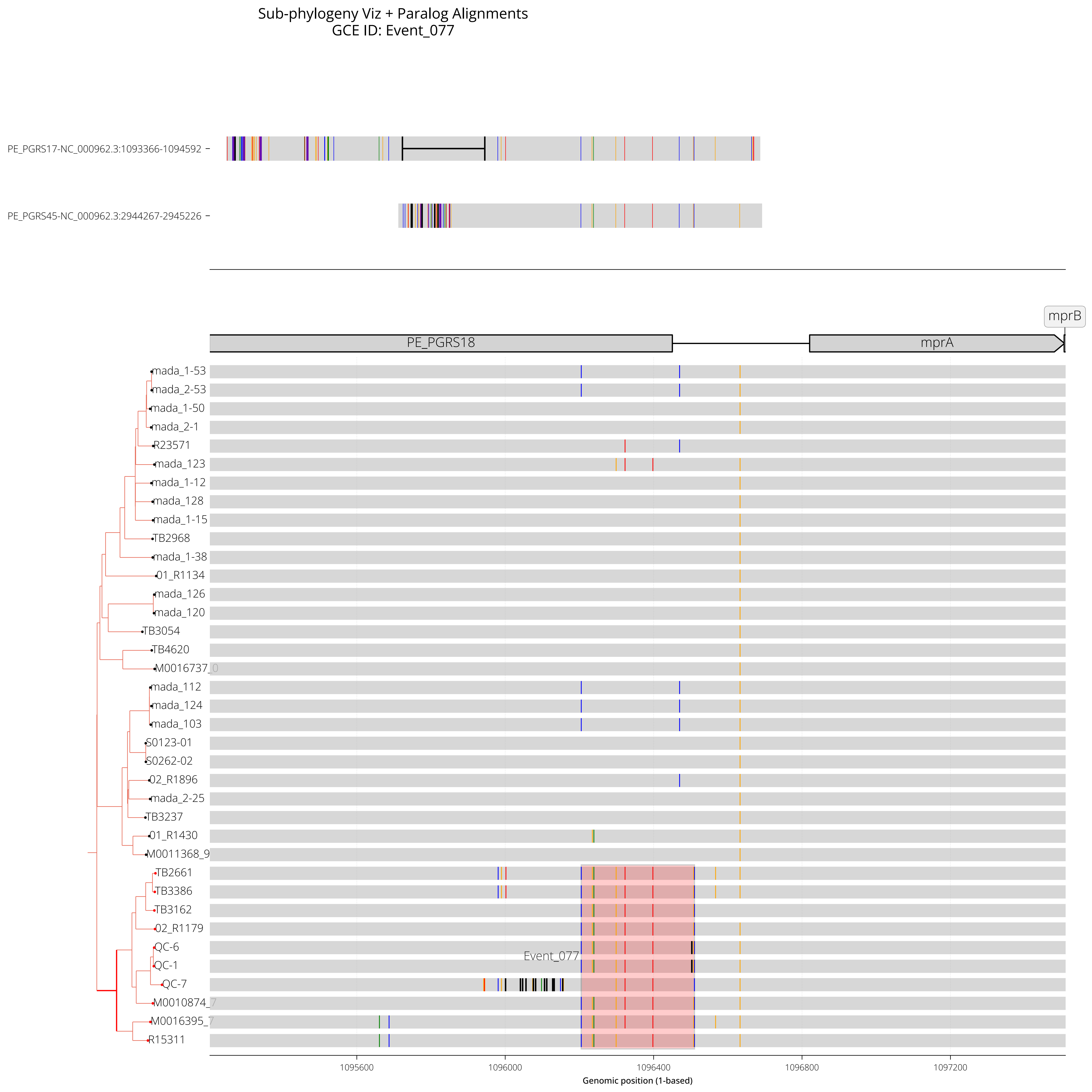

Sub-phylogeny Viz + Paralog Alignments
GCE ID: Event_077
PE_PGRS17-NC_000962.3:1093366-1094592
PE_PGRS45-NC_000962.3:2944267-2945226
mprB
PE_PGRS18
mprA
mada_1-53
mada_2-53
mada_1-50
mada_2-1
R23571
mada_123
mada_1-12
mada_128
mada_1-15
TB2968
mada_1-38
01_R1134
mada_126
mada_120
TB3054
TB4620
M0016737_0
mada_112
mada_124
mada_103
S0123-01
S0262-02
02_R1896
mada_2-25
TB3237
01_R1430
M0011368_9
TB2661
TB3386
TB3162
02_R1179
QC-6
QC-1
QC-7
M0010874_7
M0016395_7
R15311
Event_077
1095600
1096000
1096400
1096800
1097200
Genomic position (1-based)

Full Phylogeny Viz + Paralog Alignments
GCE ID: Event_077

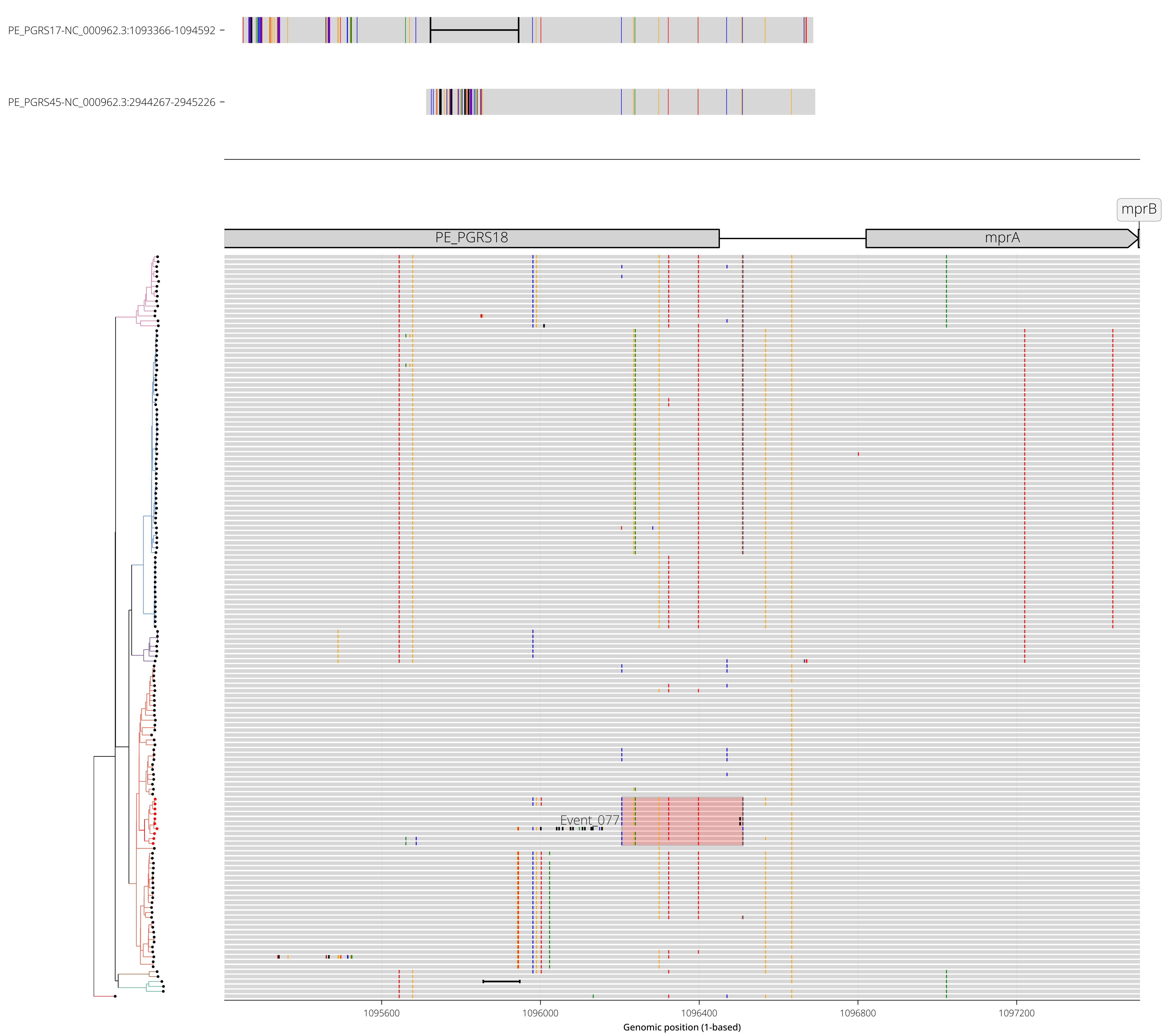

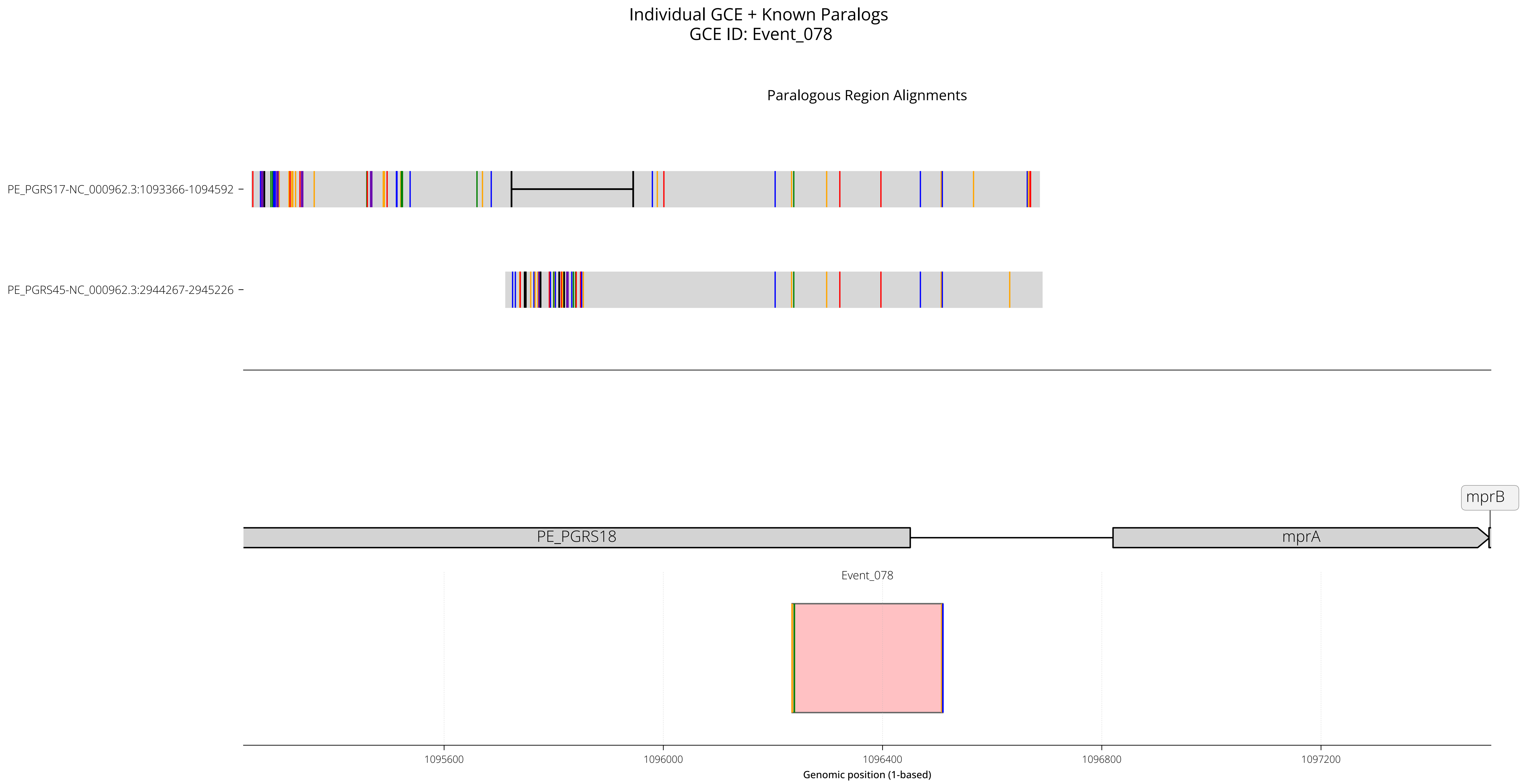
Individual GCE + Known Paralogs
GCE ID: Event_078
Paralogous Region Alignments
PE_PGRS17-NC_000962.3:1093366-1094592
PE_PGRS45-NC_000962.3:2944267-2945226
mprB
PE_PGRS18
mprA
Event_078
1095600
1096000
1096400
1096800
1097200
Genomic position (1-based)

Circos Viz - GCE to Known Paralogs Locations
GCE ID: Event_078

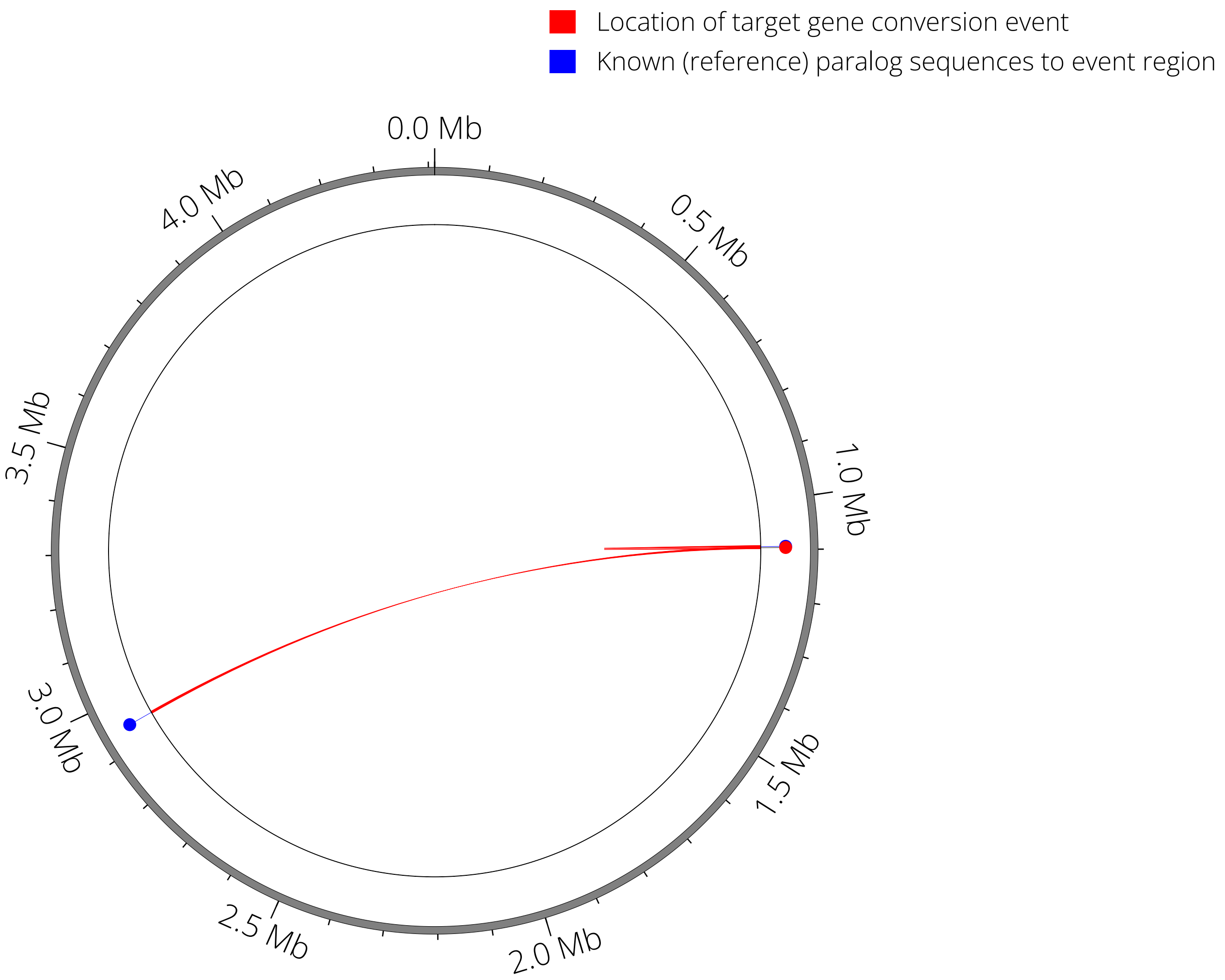

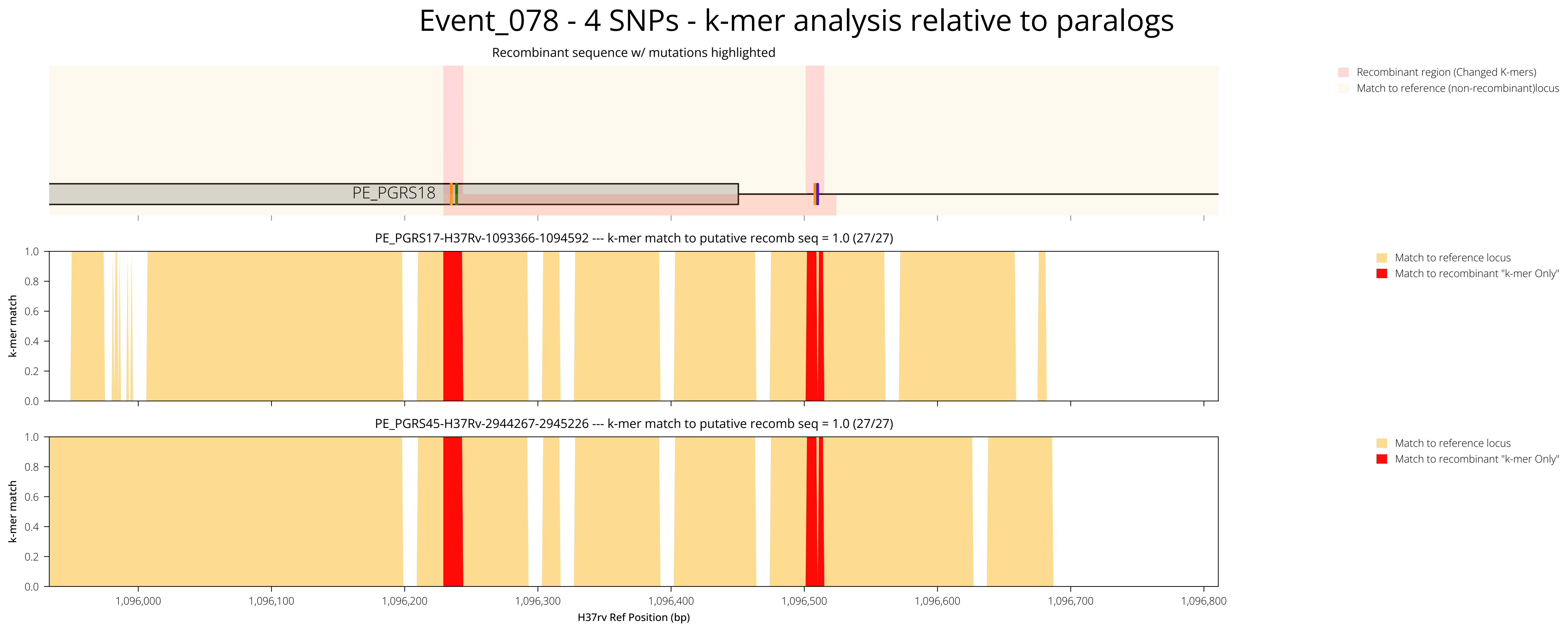

Event_078 - 4 SNPs - k-mer analysis relative to paralogs
Recombinant sequence w/ mutations highlighted
Recombinant region (Changed K-mers)
Match to reference (non-recombinant)locus
PE_PGRS18
PE_PGRS17-H37Rv-1093366-1094592 --- k-mer match to putative recomb seq = 1.0 (27/27)
Match to reference locus
Match to recombinant "k-mer Only"
k-mer match
1.0
0.8
0.6
0.4
0.2
0.0
PE_PGRS45-H37Rv-2944267-2945226 --- k-mer match to putative recomb seq = 1.0 (27/27)
Match to reference locus
Match to recombinant "k-mer Only"
k-mer match
1,096,000
1,096,100
1,096,200
1,096,300
1,096,400
1,096,500
1,096,600
1,096,700
1,096,800
H37rv Ref Position (bp)

Sub-phylogeny Viz + Paralog Alignments
GCE ID: Event_078

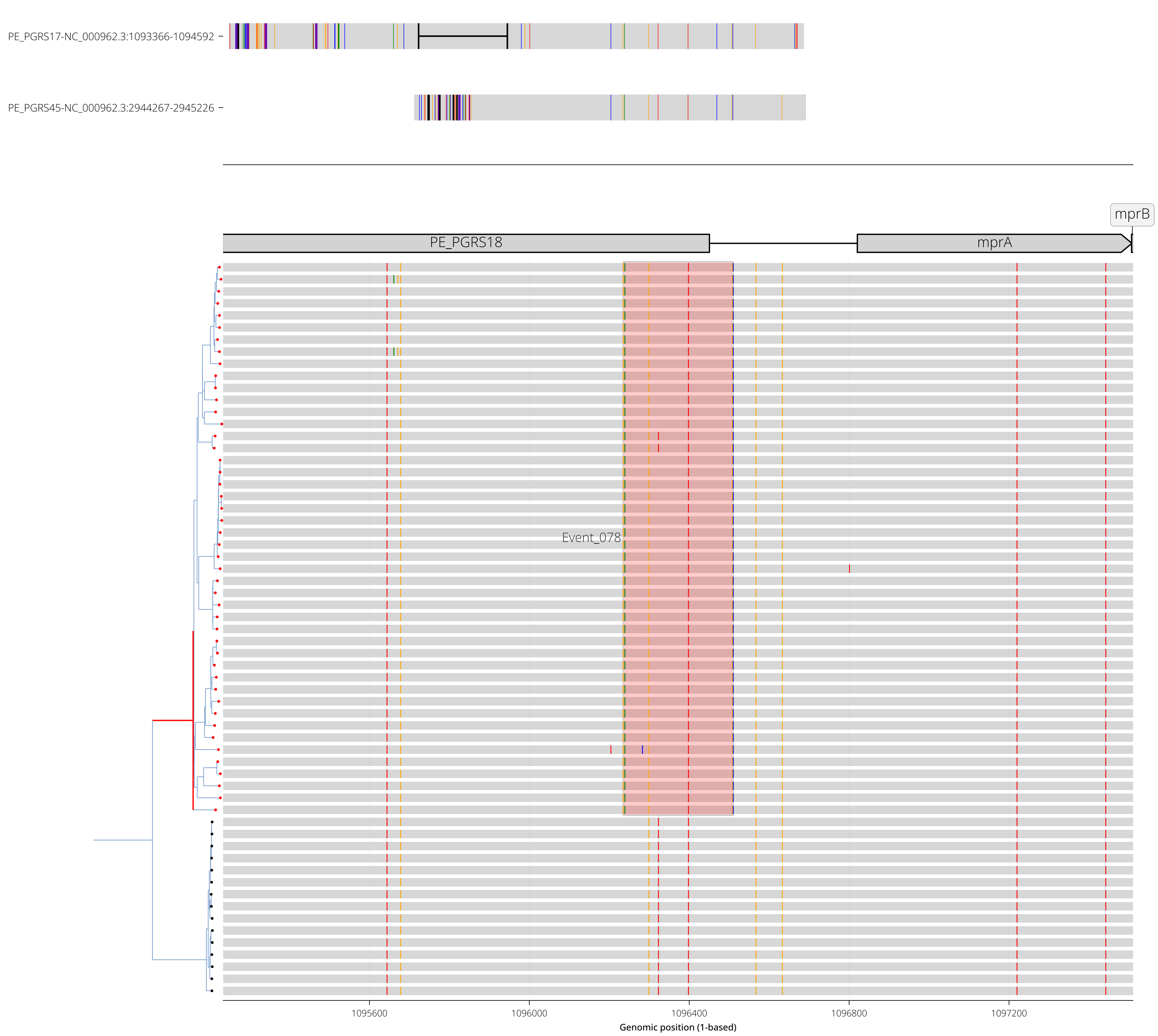

Full Phylogeny Viz + Paralog Alignments
GCE ID: Event_078

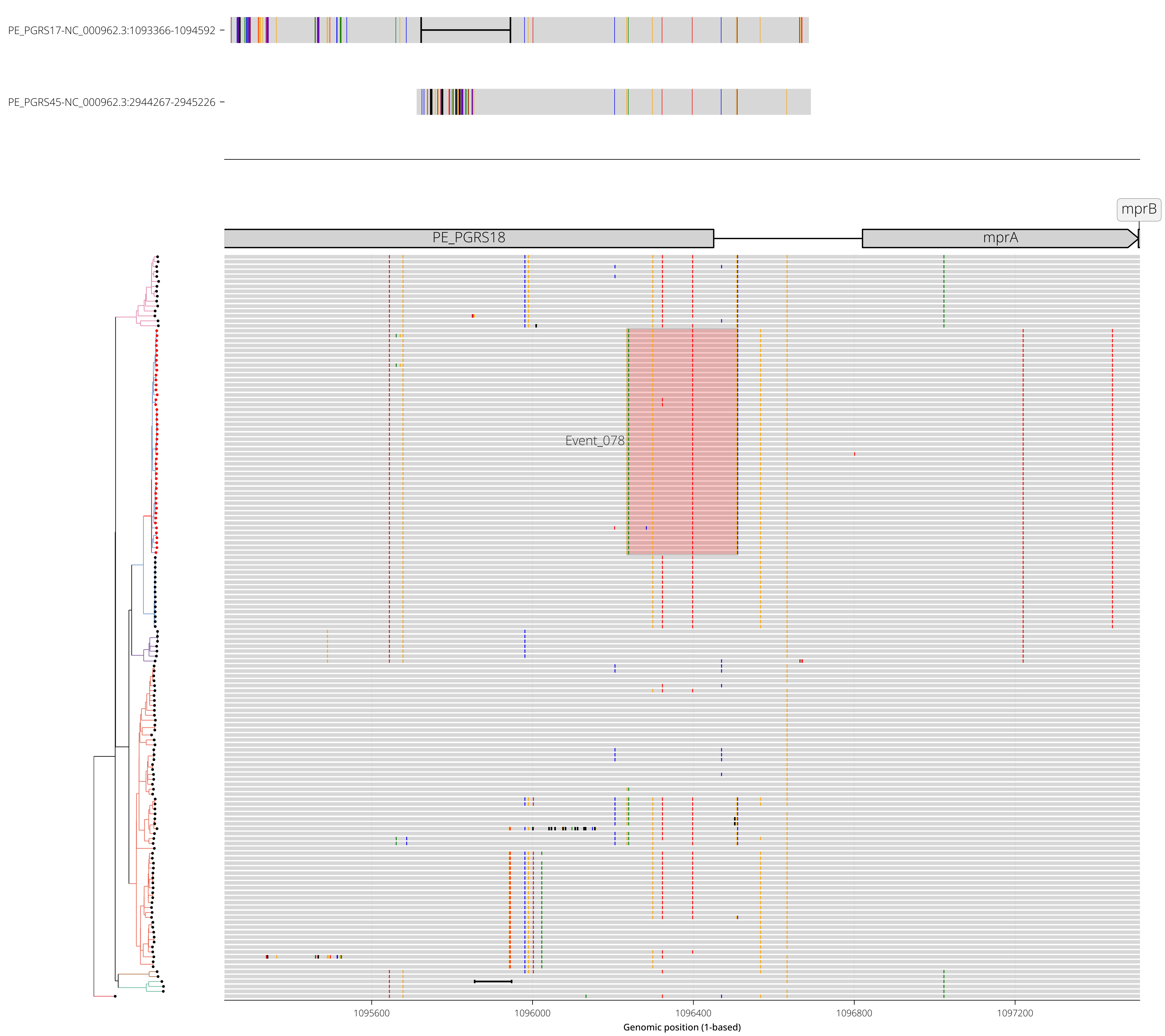

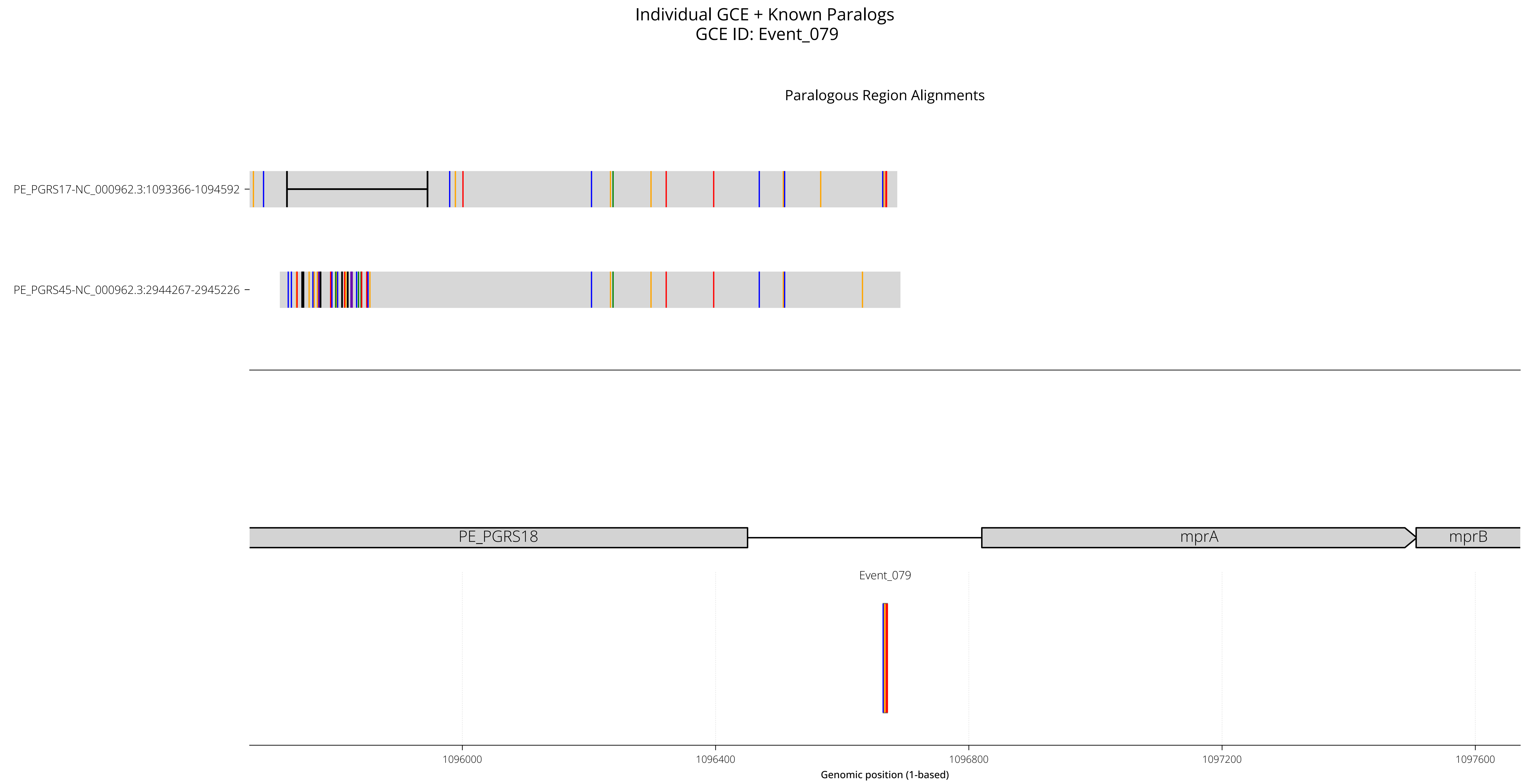
Individual GCE + Known Paralogs
GCE ID: Event_079
Paralogous Region Alignments
PE_PGRS17-NC_000962.3:1093366-1094592
PE_PGRS45-NC_000962.3:2944267-2945226
PE_PGRS18
mprA
mprB
Event_079
1096000
1096400
1096800
1097200
1097600
Genomic position (1-based)

Circos Viz - GCE to Known Paralogs Locations
GCE ID: Event_079

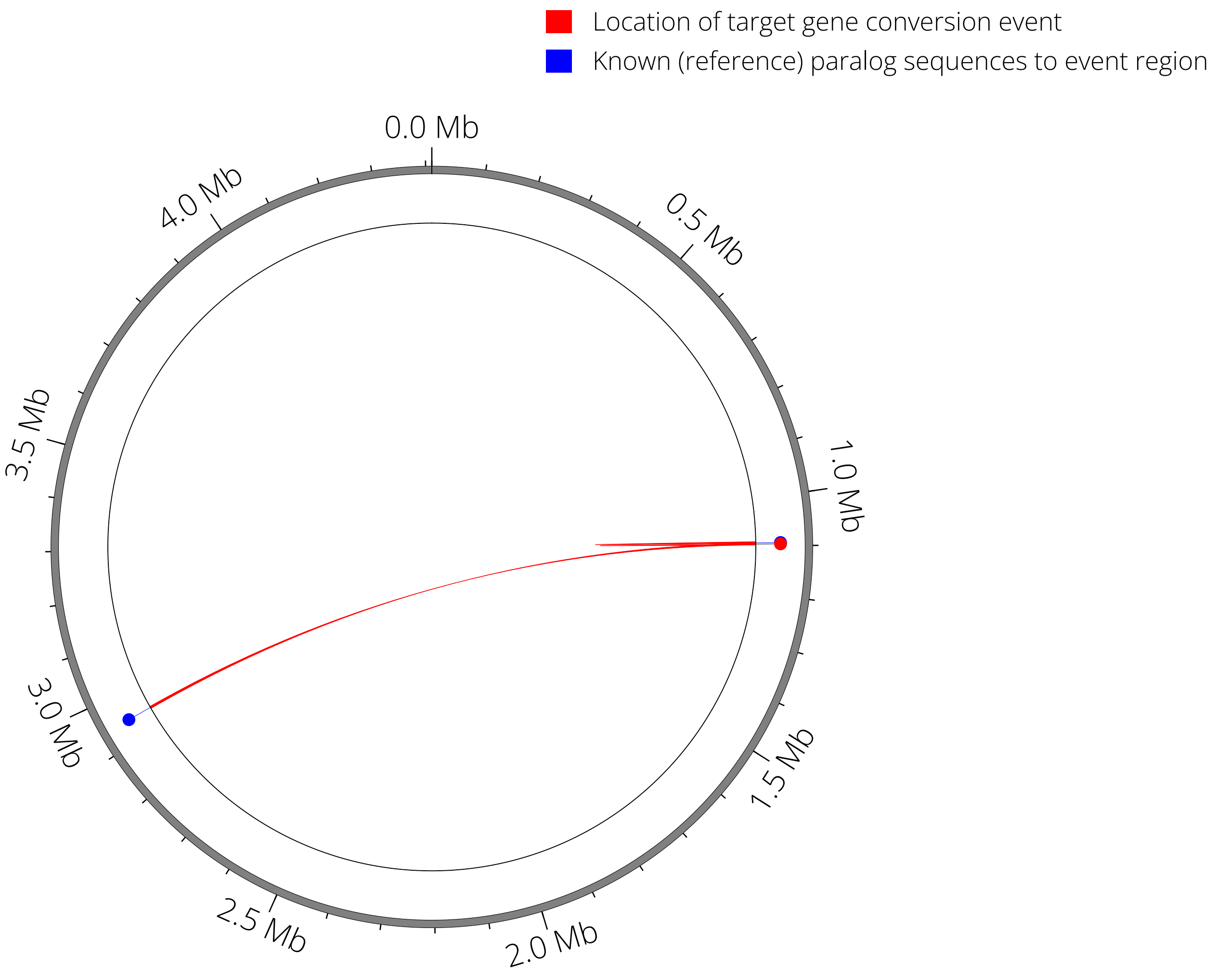

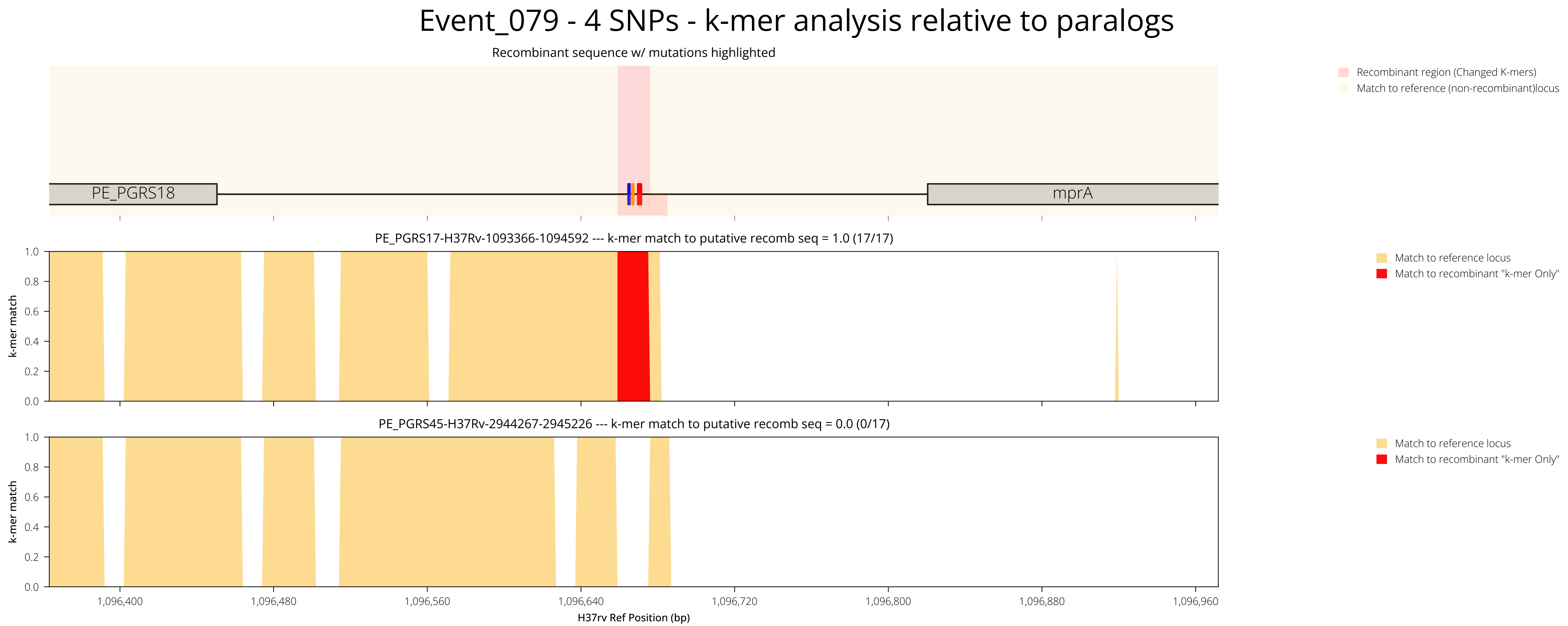

Event_079 - 4 SNPs - k-mer analysis relative to paralogs
Recombinant sequence w/ mutations highlighted
Recombinant region (Changed K-mers)
Match to reference (non-recombinant)locus
PE_PGRS18
mprA
PE_PGRS17-H37Rv-1093366-1094592 --- k-mer match to putative recomb seq = 1.0 (17/17)
Match to reference locus
Match to recombinant "k-mer Only"
k-mer match
1.0
0.8
0.6
0.4
0.2
0.0
PE_PGRS45-H37Rv-2944267-2945226 --- k-mer match to putative recomb seq = 0.0 (0/17)
Match to reference locus
Match to recombinant "k-mer Only"
k-mer match
1,096,400
1,096,480
1,096,560
1,096,640
1,096,720
1,096,800
1,096,880
1,096,960
H37rv Ref Position (bp)

Sub-phylogeny Viz + Paralog Alignments
GCE ID: Event_079

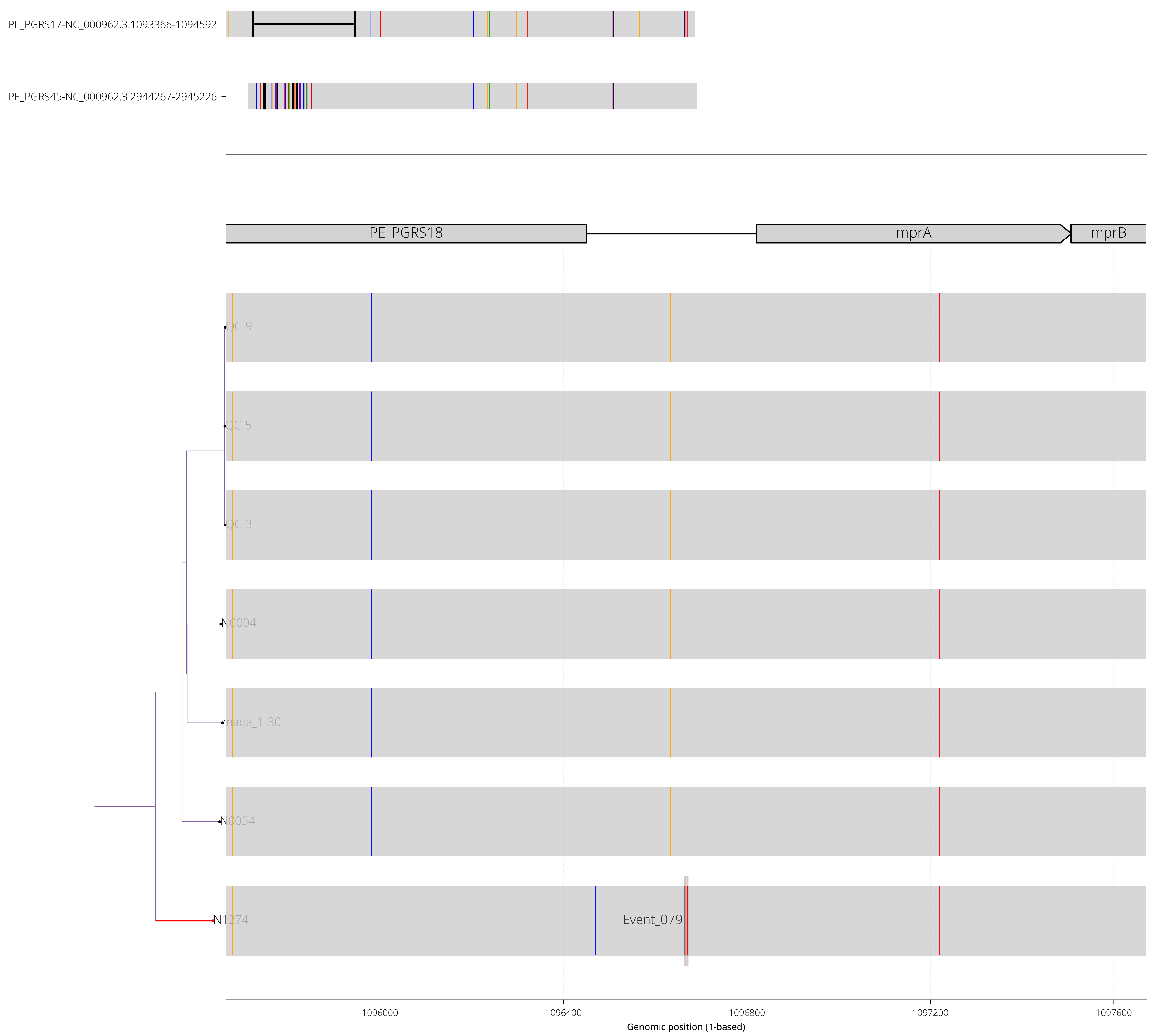

Full Phylogeny Viz + Paralog Alignments
GCE ID: Event_079

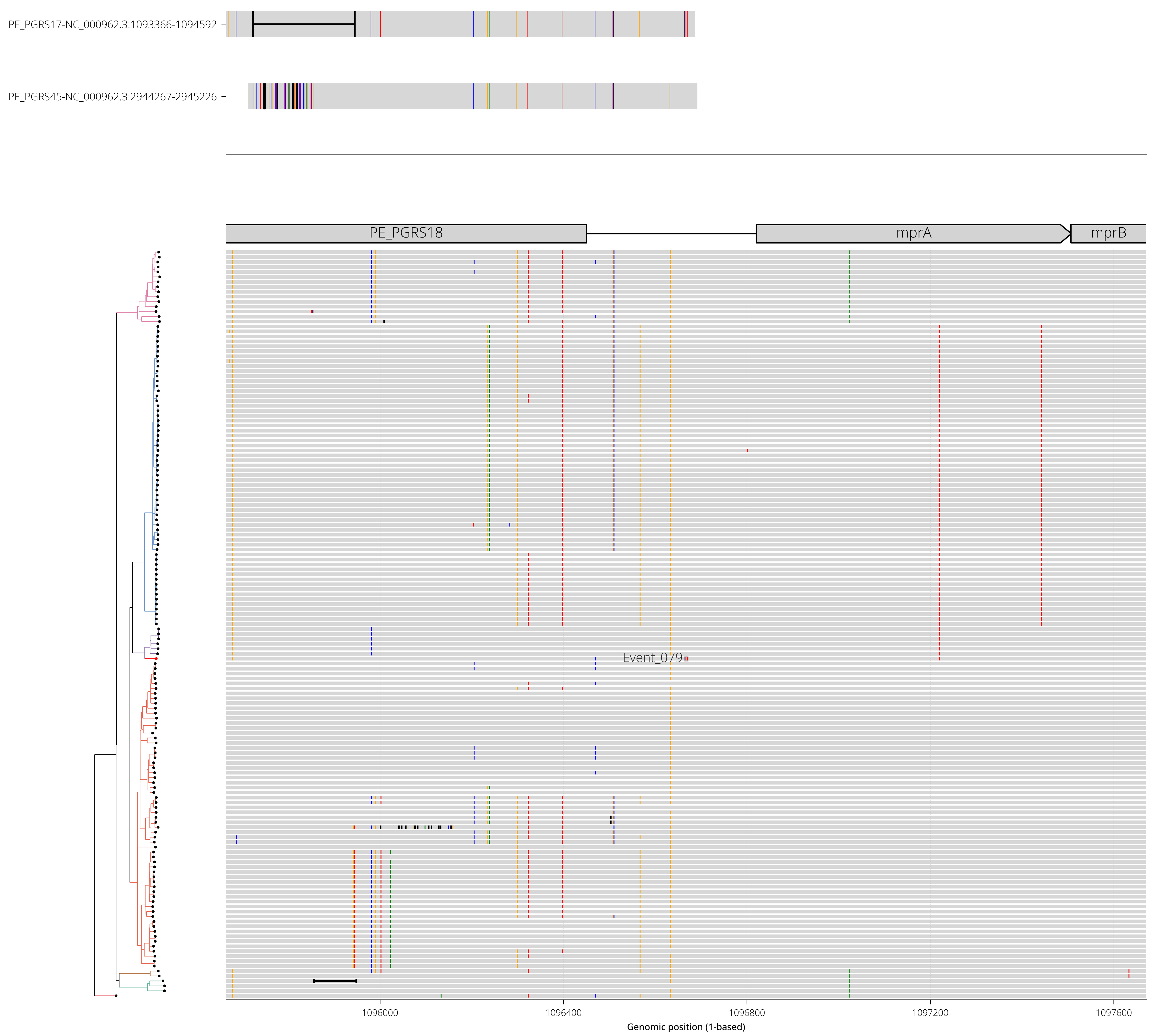

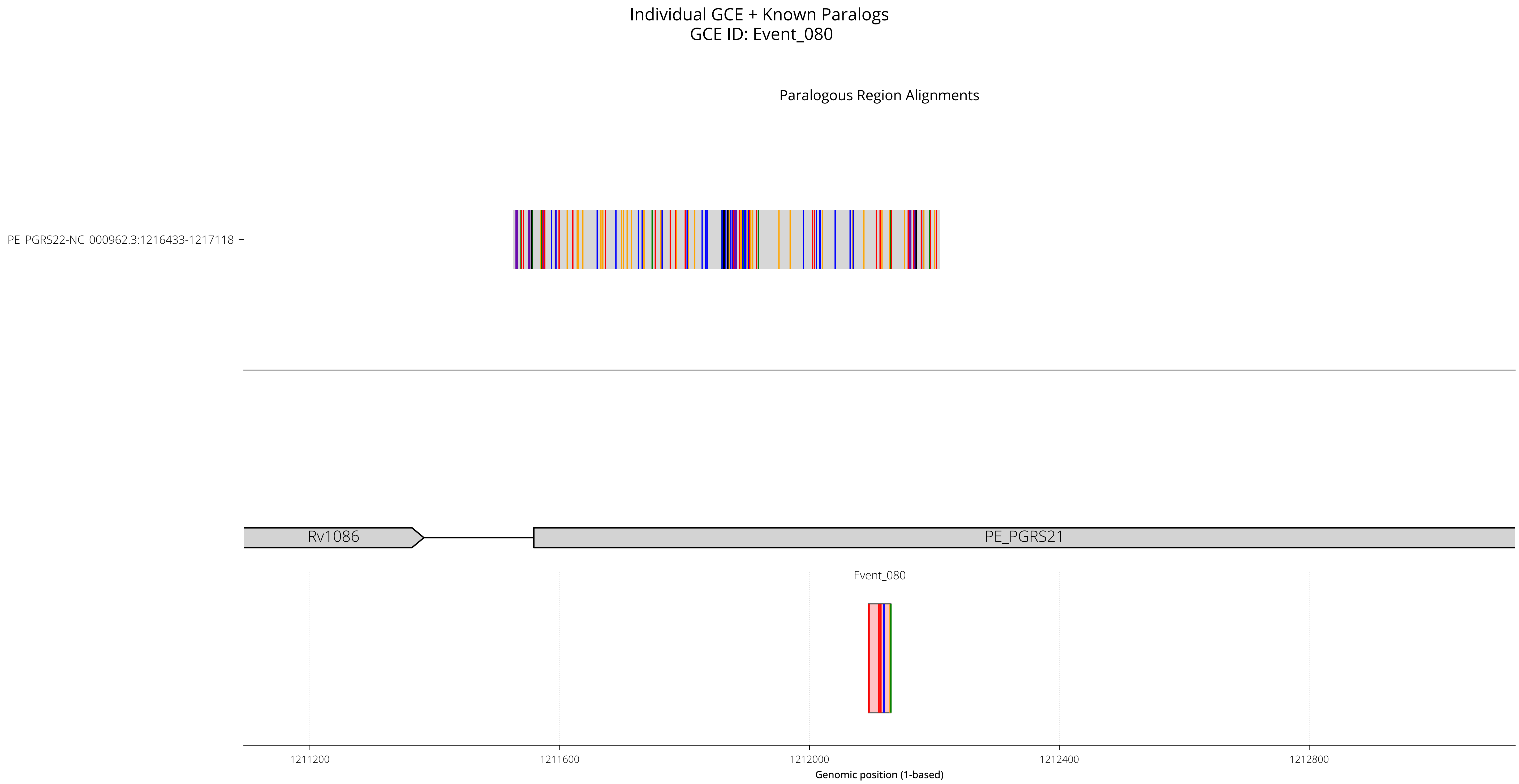
Individual GCE + Known Paralogs
GCE ID: Event_080
Paralogous Region Alignments
PE_PGRS22-NC_000962.3:1216433-1217118
Rv1086
PE_PGRS21
Event_080
1211200
1211600
1212000
1212400
1212800
Genomic position (1-based)

Circos Viz - GCE to Known Paralogs Locations
GCE ID: Event_080

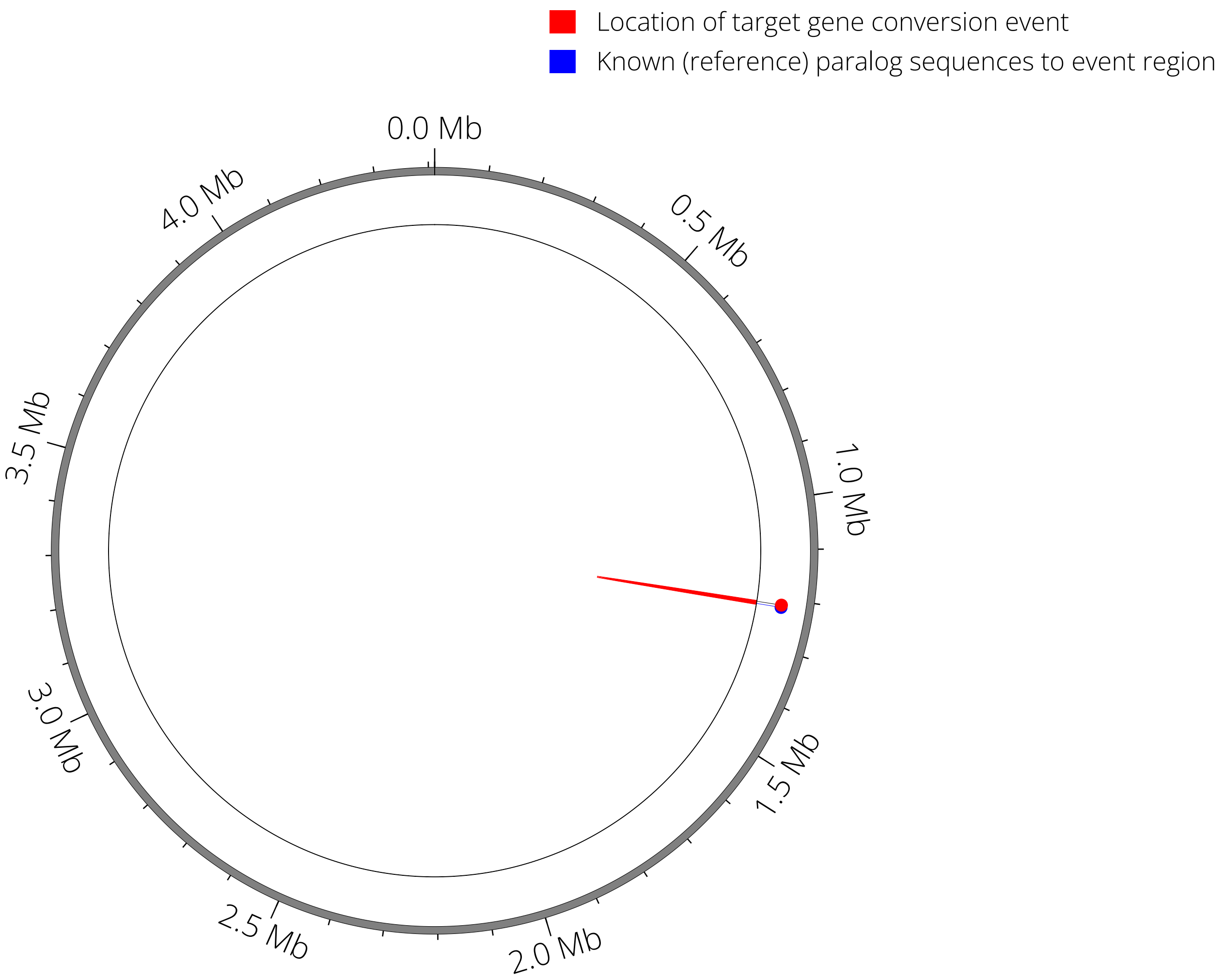

# Event_080 - 6 SNPs - k-mer analysis relative to paralogs

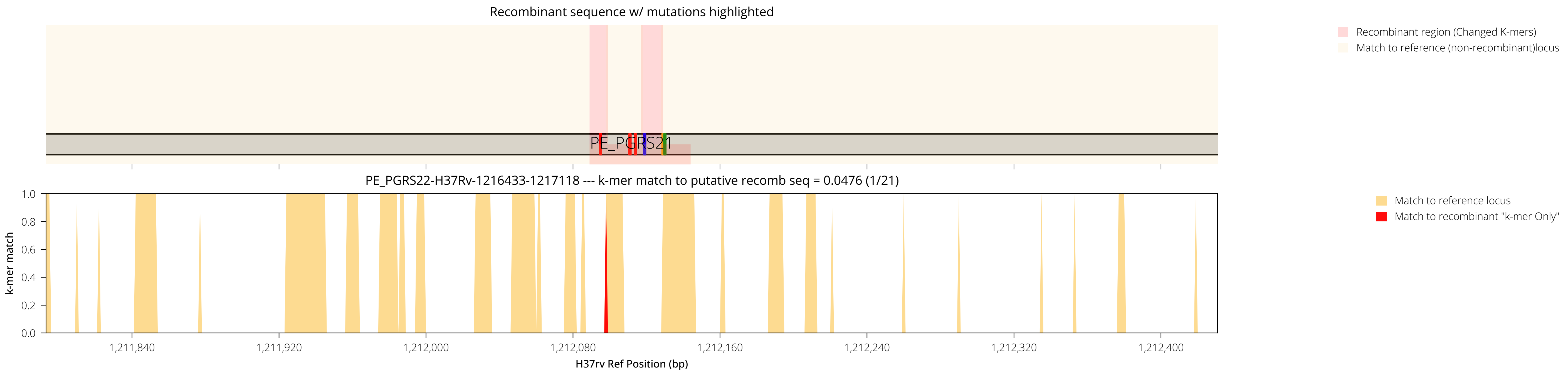

Sub-phylogeny Viz + Paralog Alignments
GCE ID: Event_080

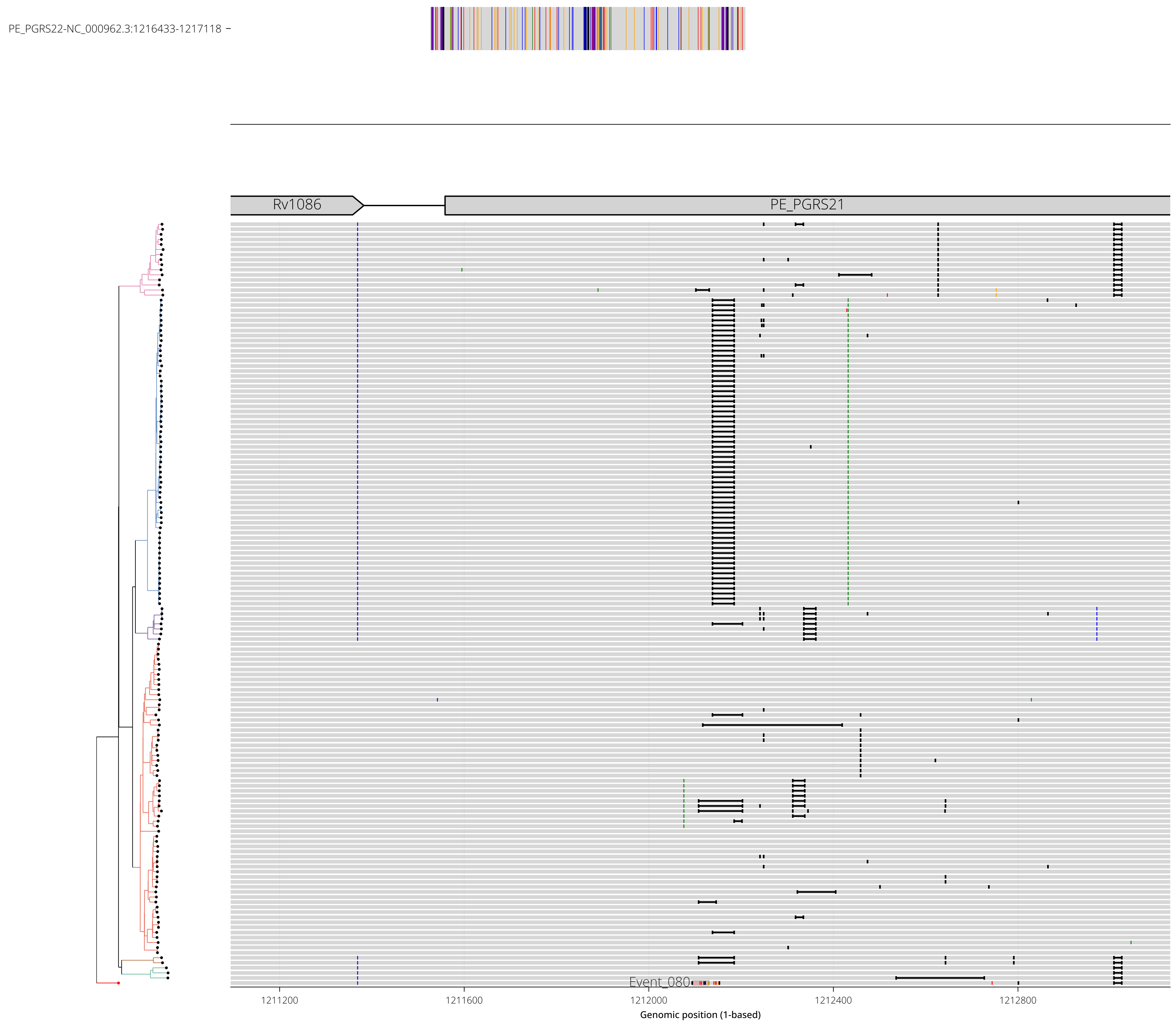

Full Phylogeny Viz + Paralog Alignments
GCE ID: Event_080

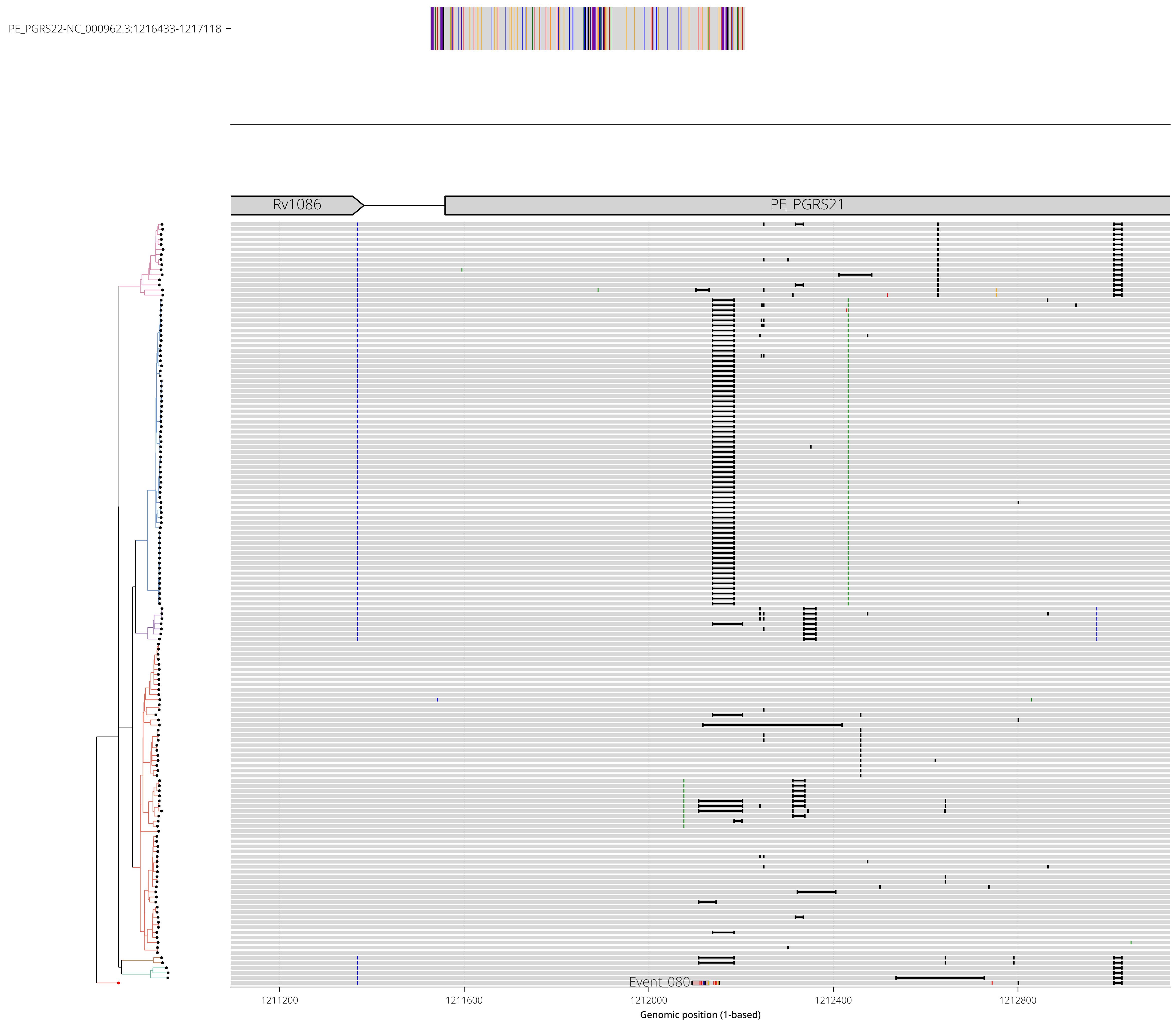

Individual GCE + Known Paralogs
GCE ID: Event_081

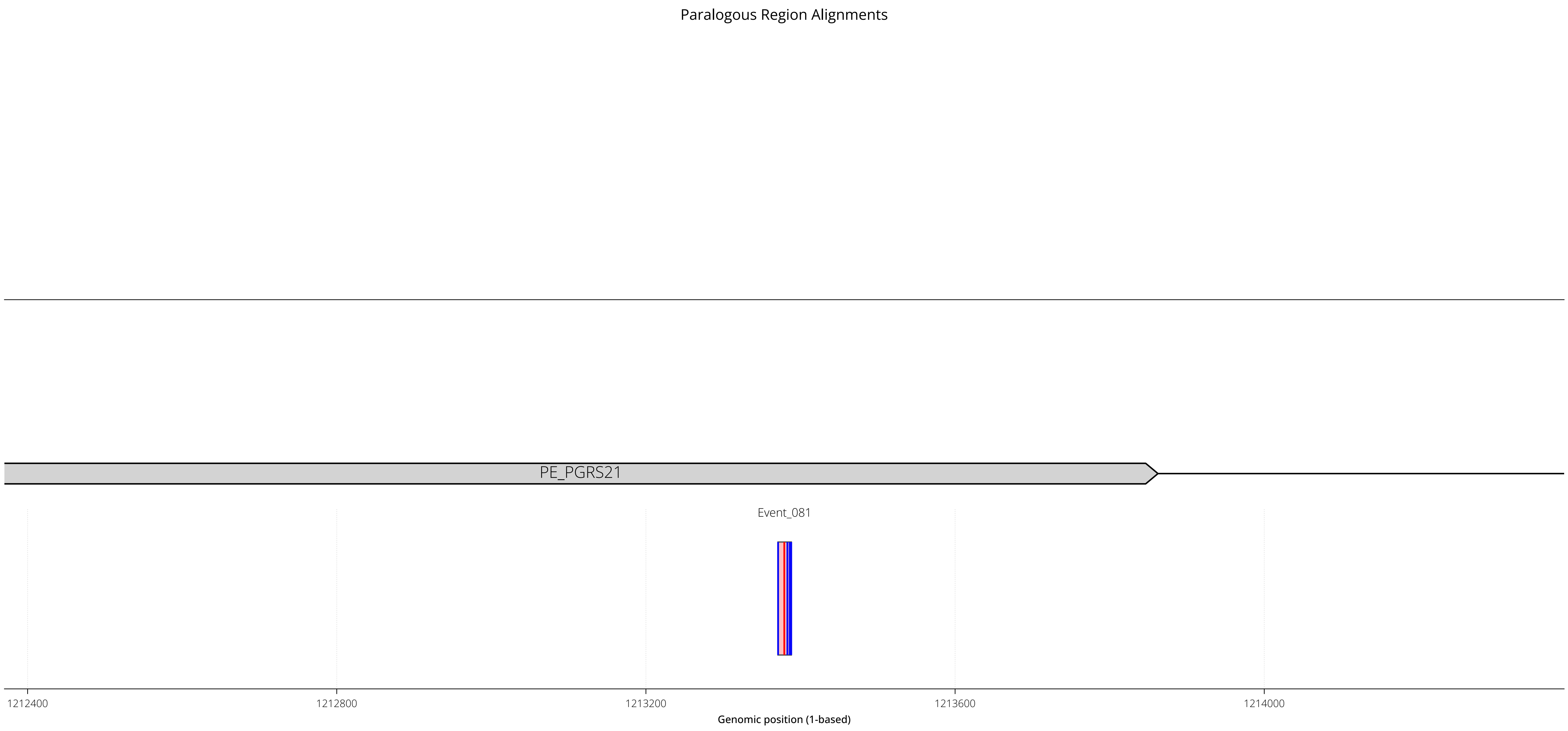

Sub-phylogeny Viz + Paralog Alignments
GCE ID: Event_081

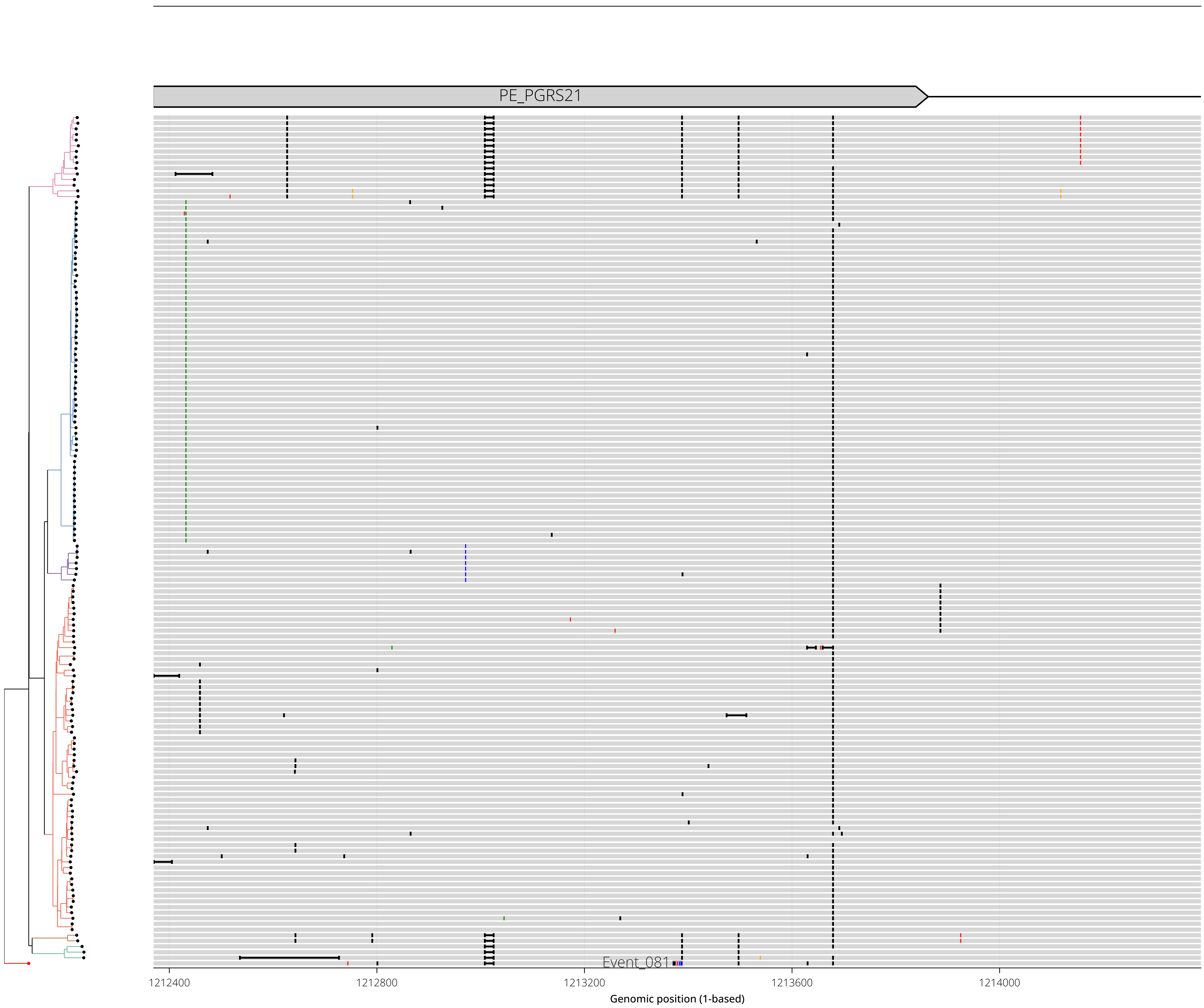

Full Phylogeny Viz + Paralog Alignments
GCE ID: Event_081

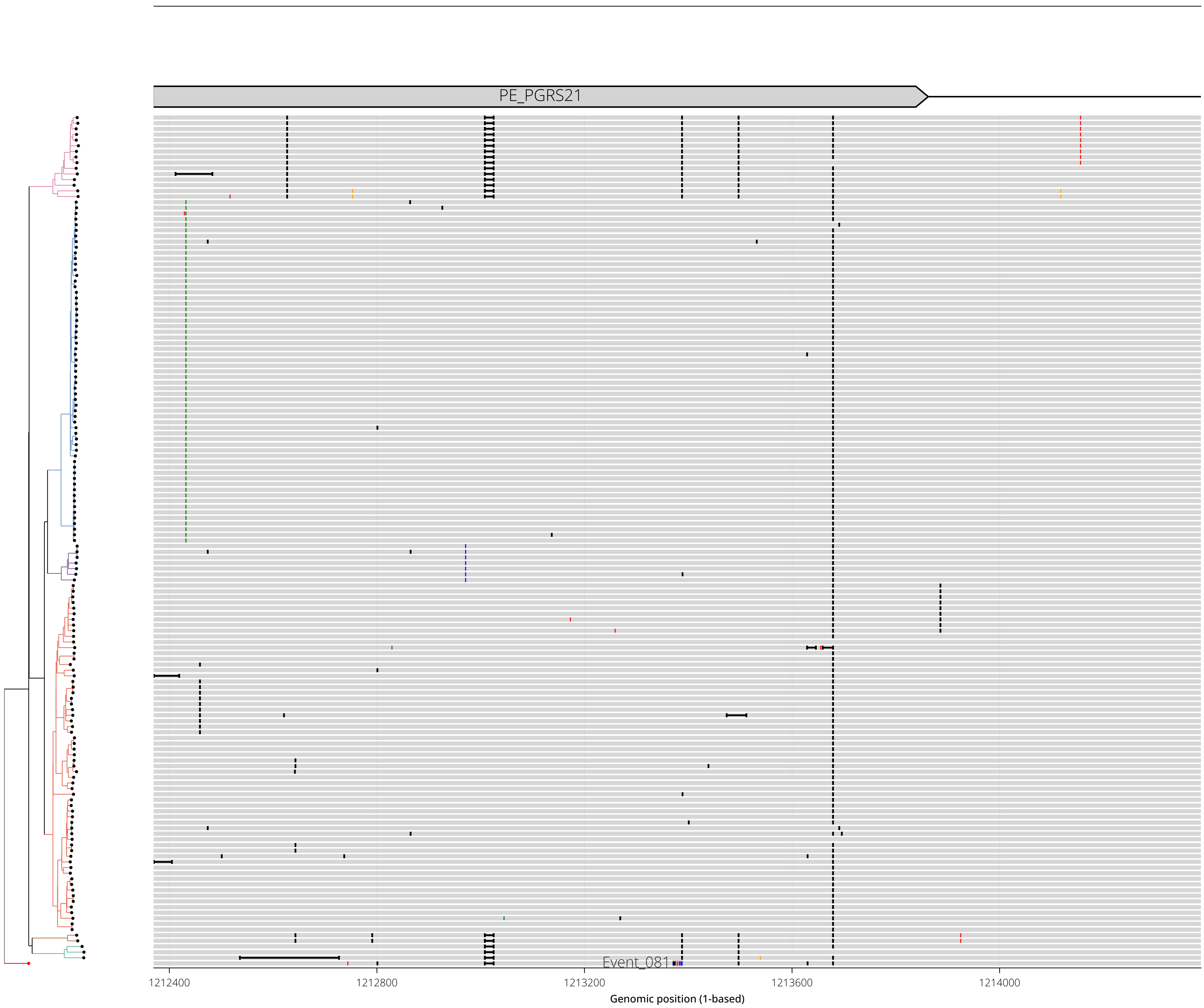

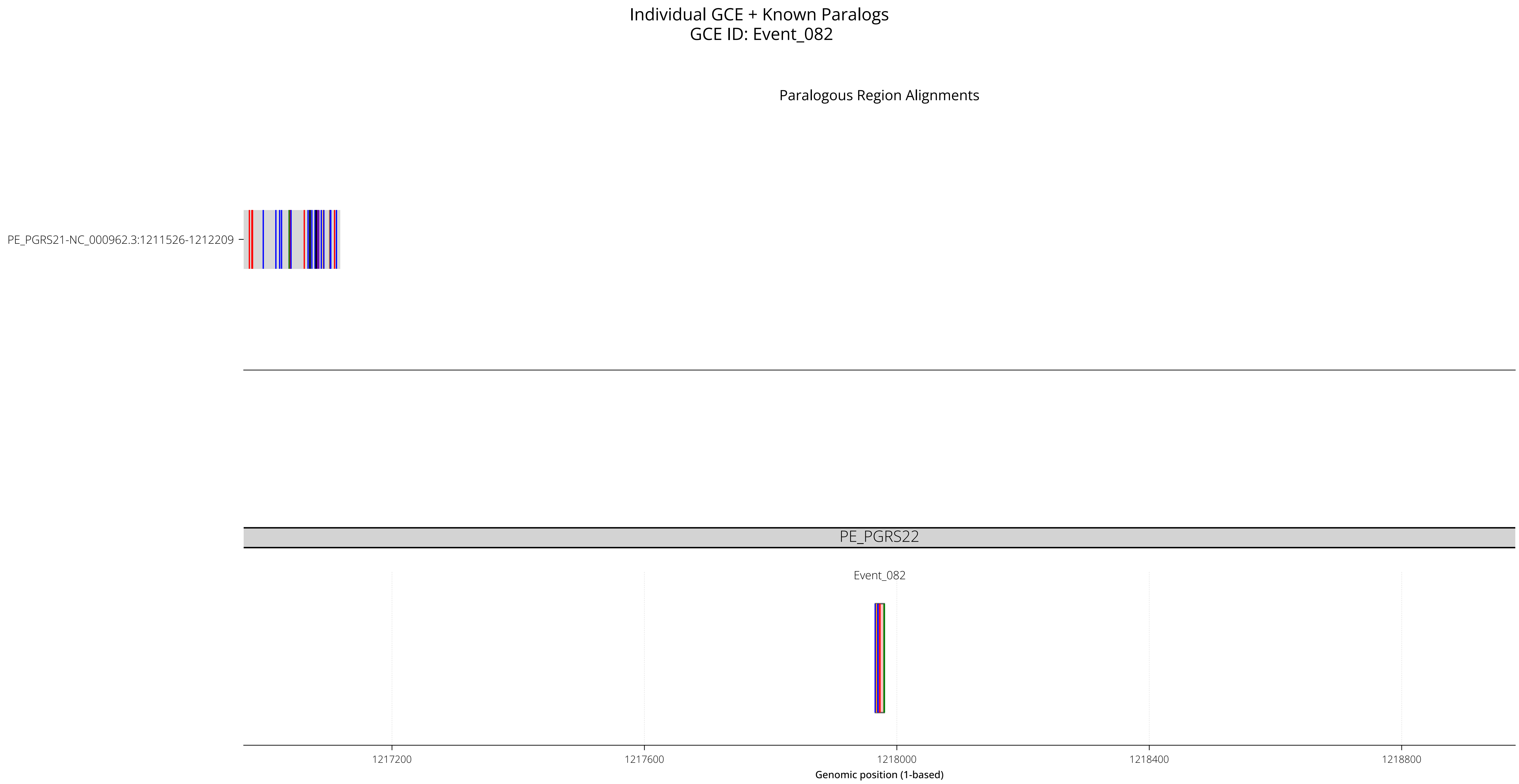

Individual GCE + Known Paralogs
GCE ID: Event_082
Paralogous Region Alignments
PE_PGRS21-NC_000962.3:1211526-1212209
PE_PGRS22
Event_082
1217200
1217600
1218000
1218400
1218800
Genomic position (1-based)

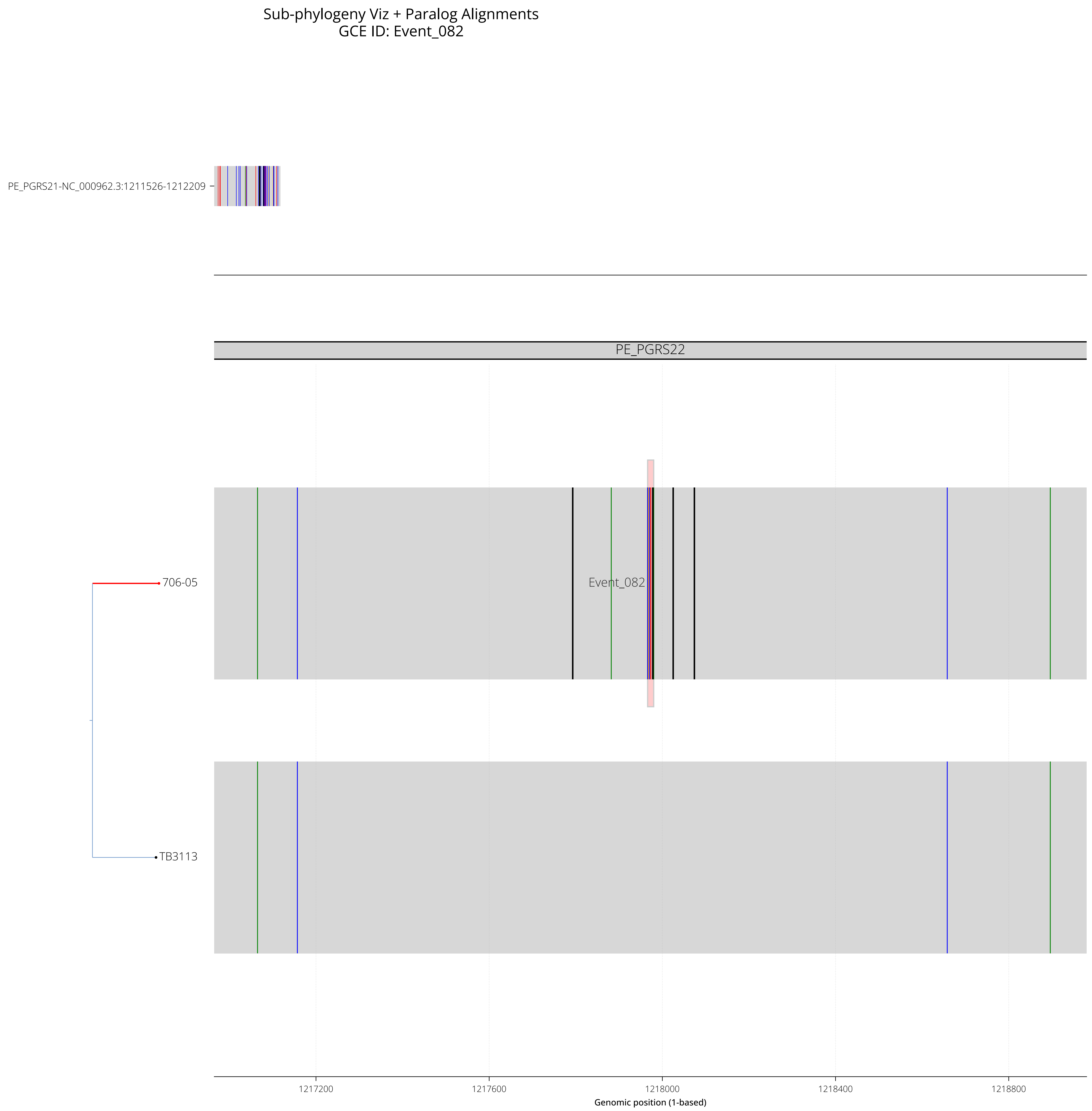

Sub-phylogeny Viz + Paralog Alignments
GCE ID: Event_082
PE_PGRS21-NC_000962.3:1211526-1212209
PE_PGRS22
706-05
Event_082
TB3113
1217200
1217600
1218000
1218400
1218800
Genomic position (1-based)

Full Phylogeny Viz + Paralog Alignments
GCE ID: Event_082

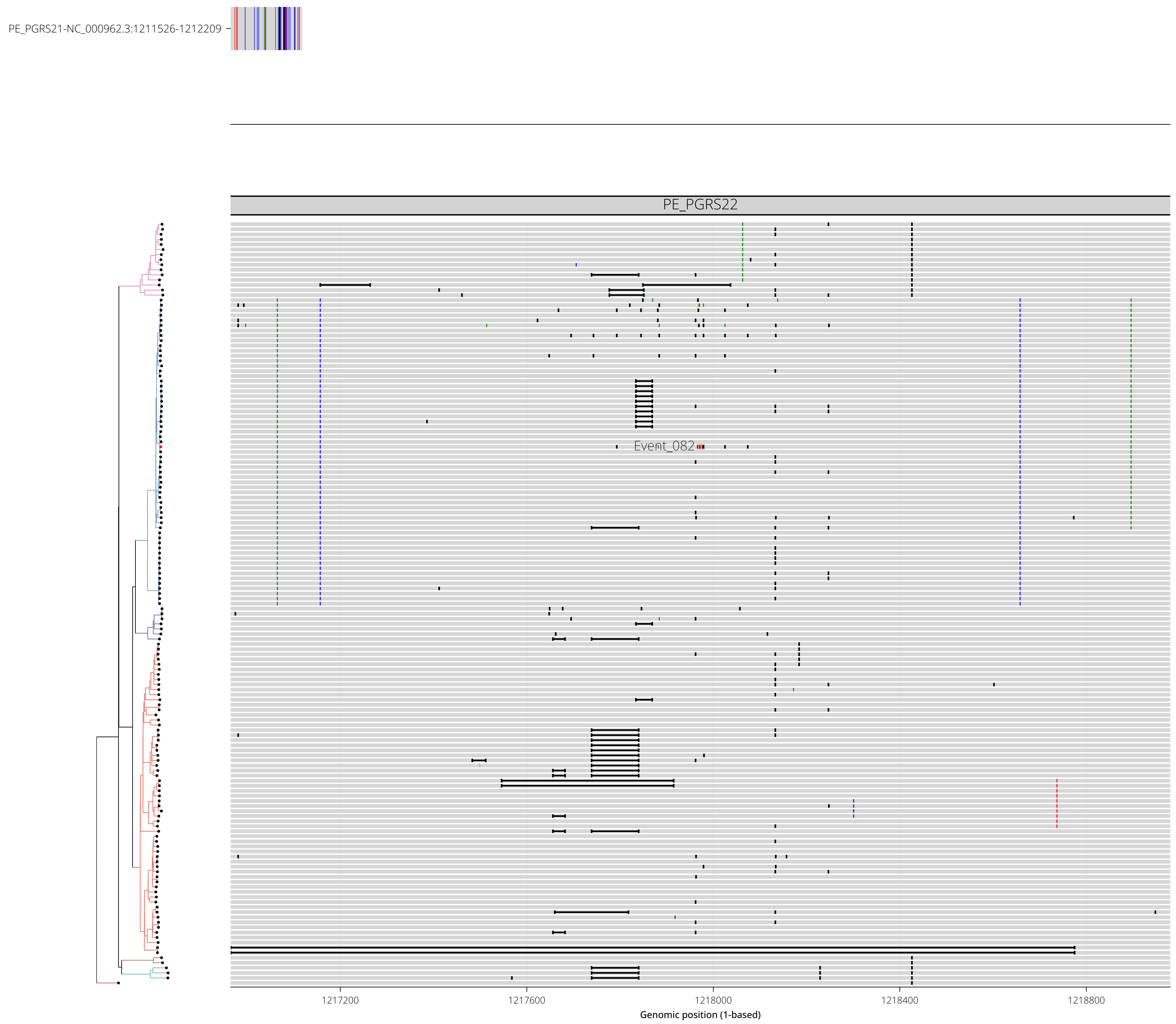

Individual GCE + Known Paralogs
GCE ID: Event_083

Paralogous Region Alignments

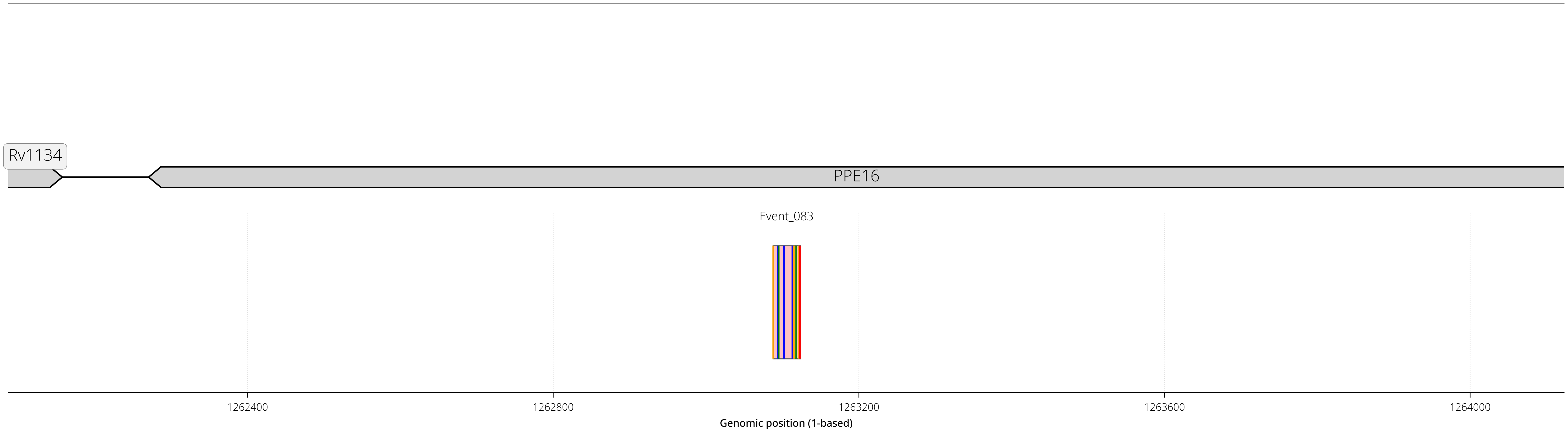

Sub-phylogeny Viz + Paralog Alignments
GCE ID: Event_083

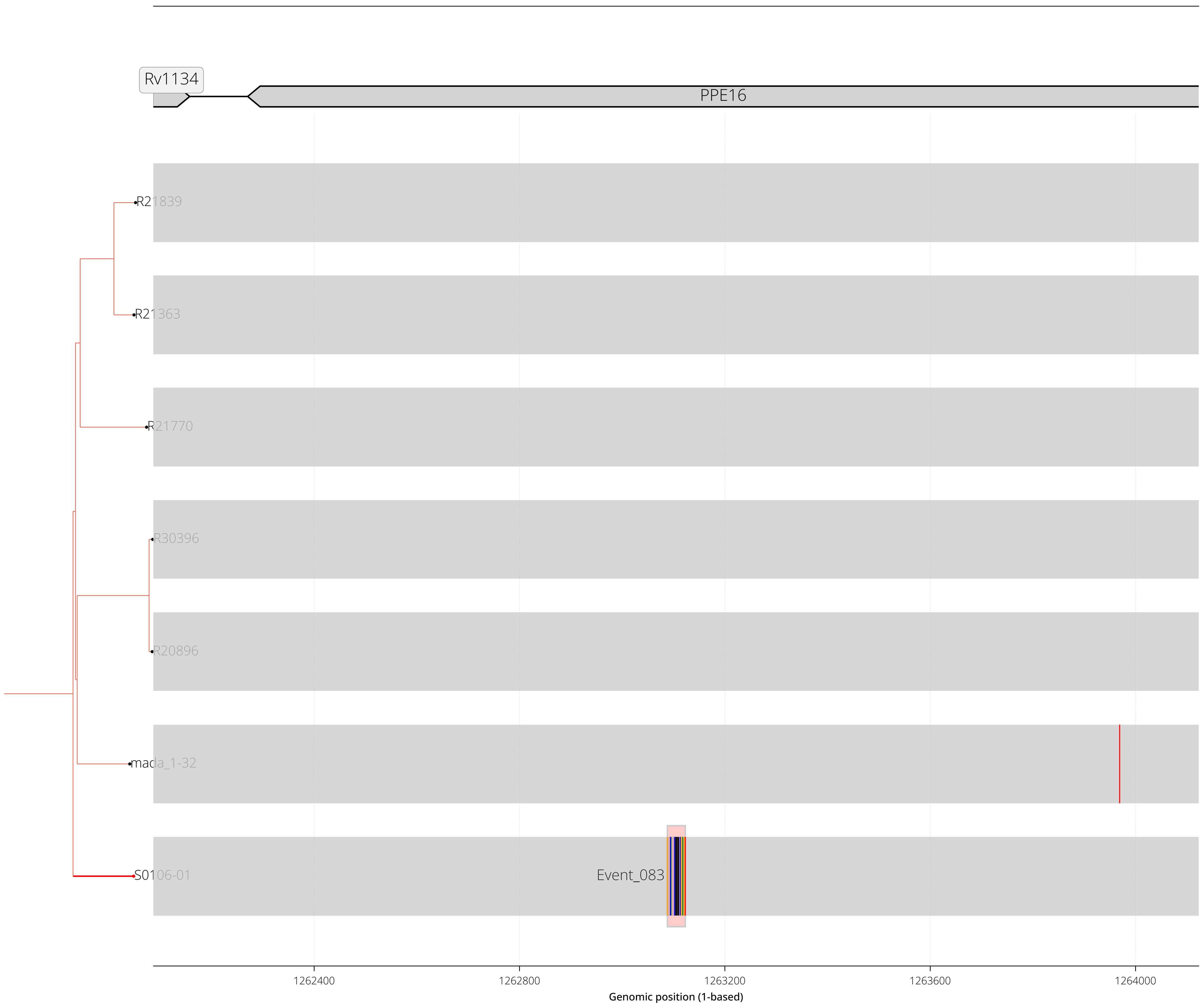

Full Phylogeny Viz + Paralog Alignments
GCE ID: Event_083

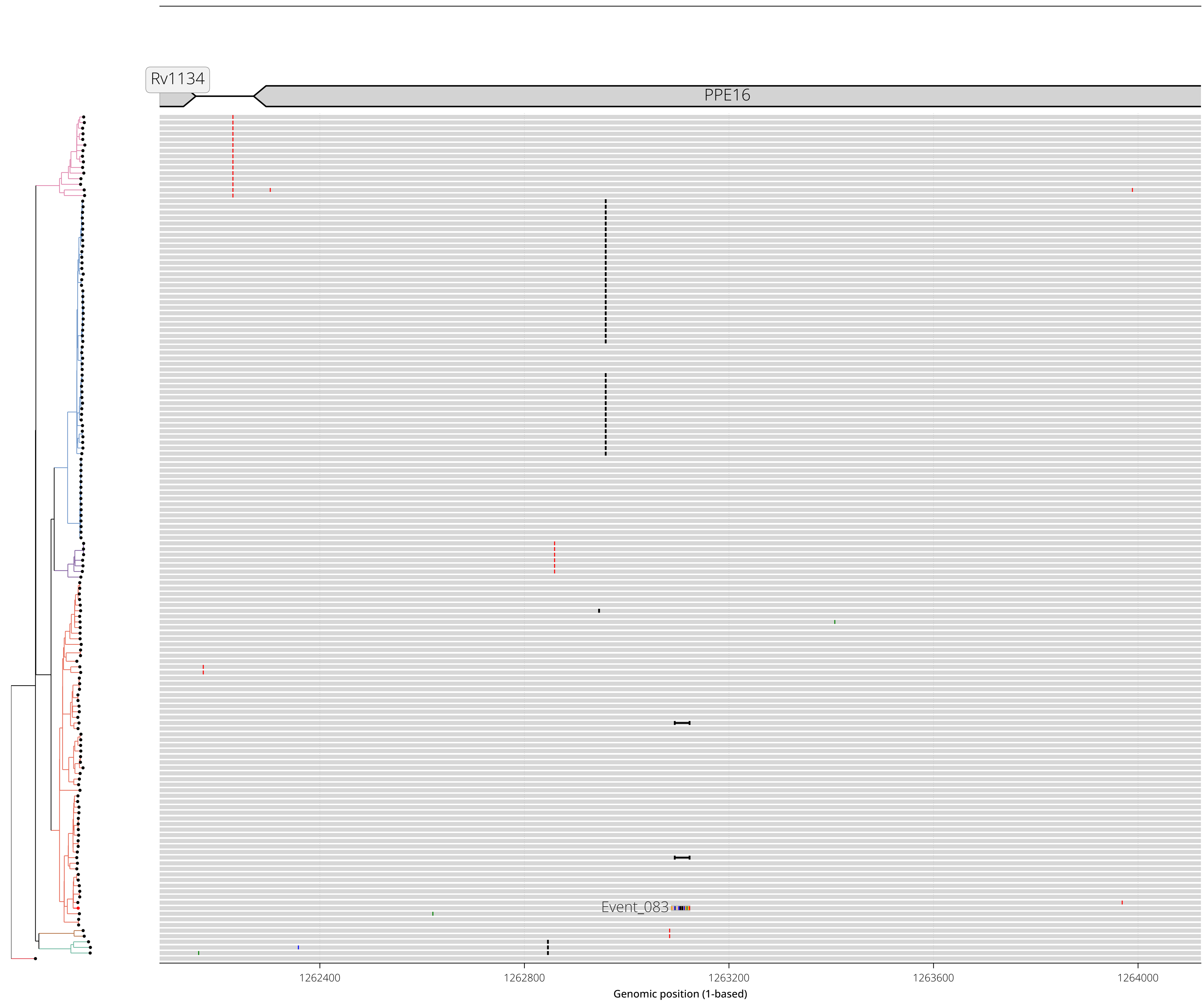

Individual GCE + Known Paralogs
GCE ID: Event_084

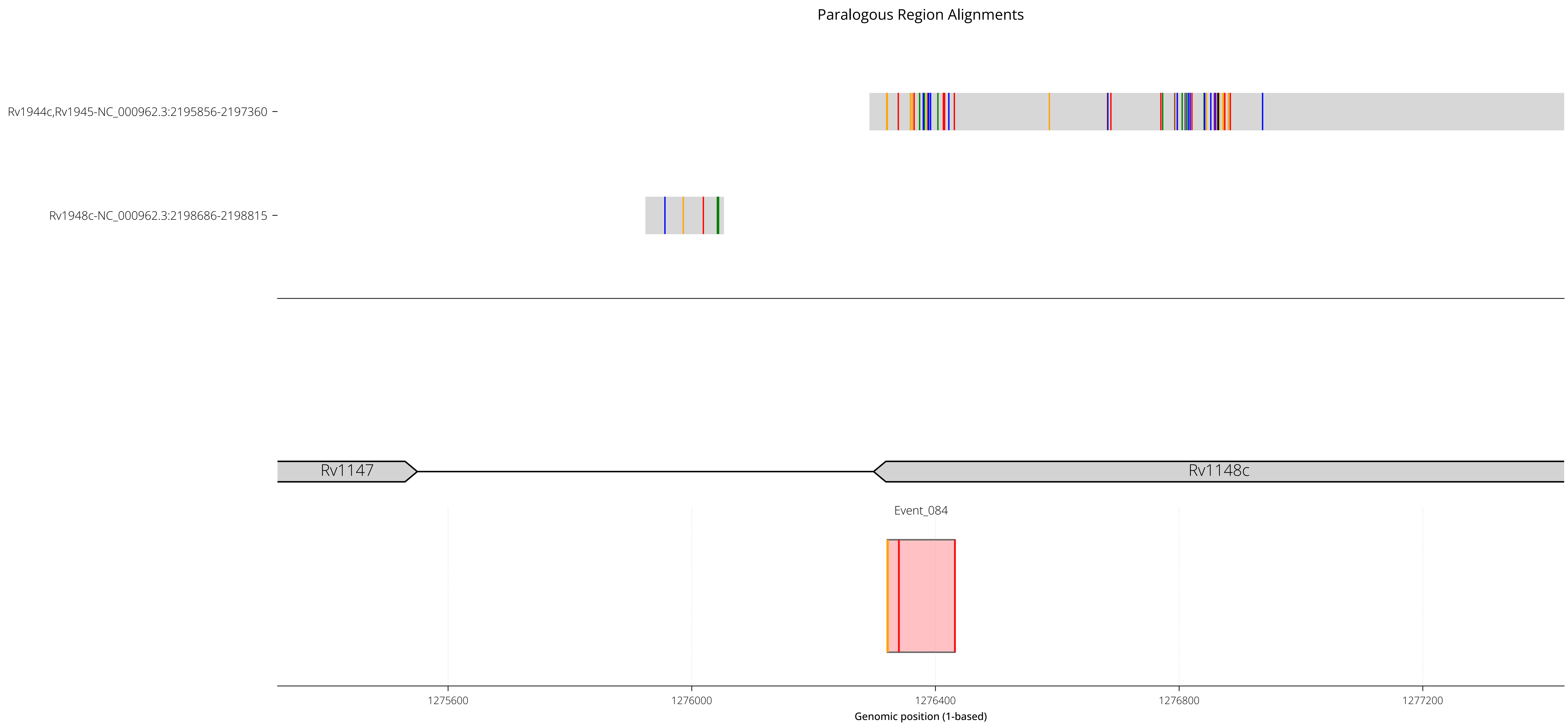

Circos Viz - GCE to Known Paralogs Locations
GCE ID: Event_084

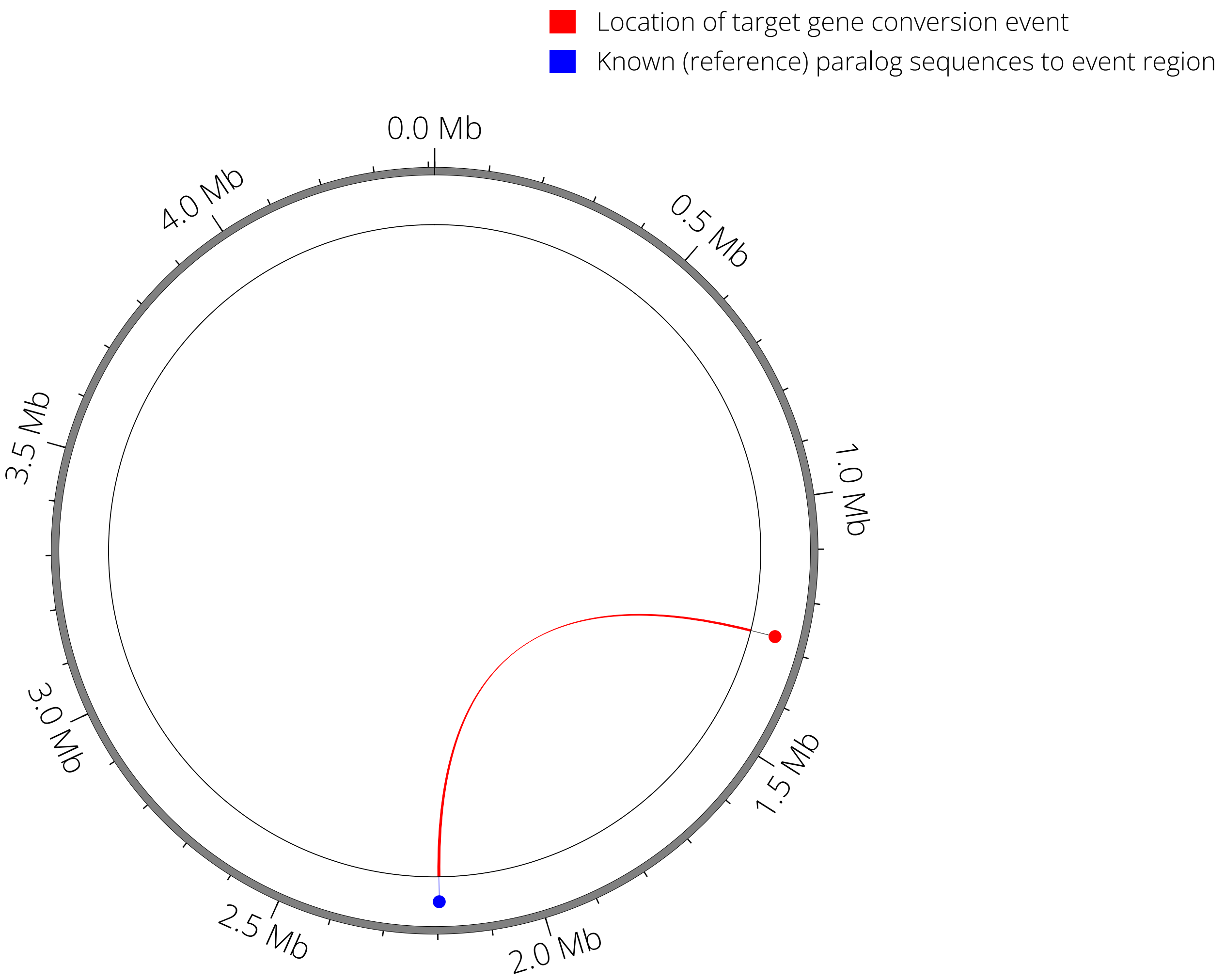

# Event_084 - 4 SNPs - k-mer analysis relative to paralogs

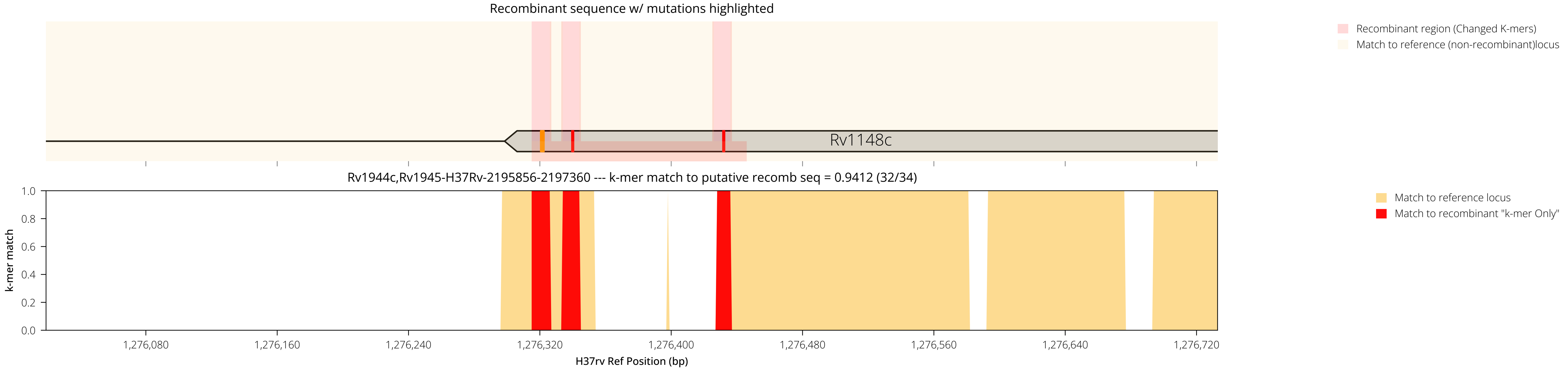

Sub-phylogeny Viz + Paralog Alignments
GCE ID: Event_084

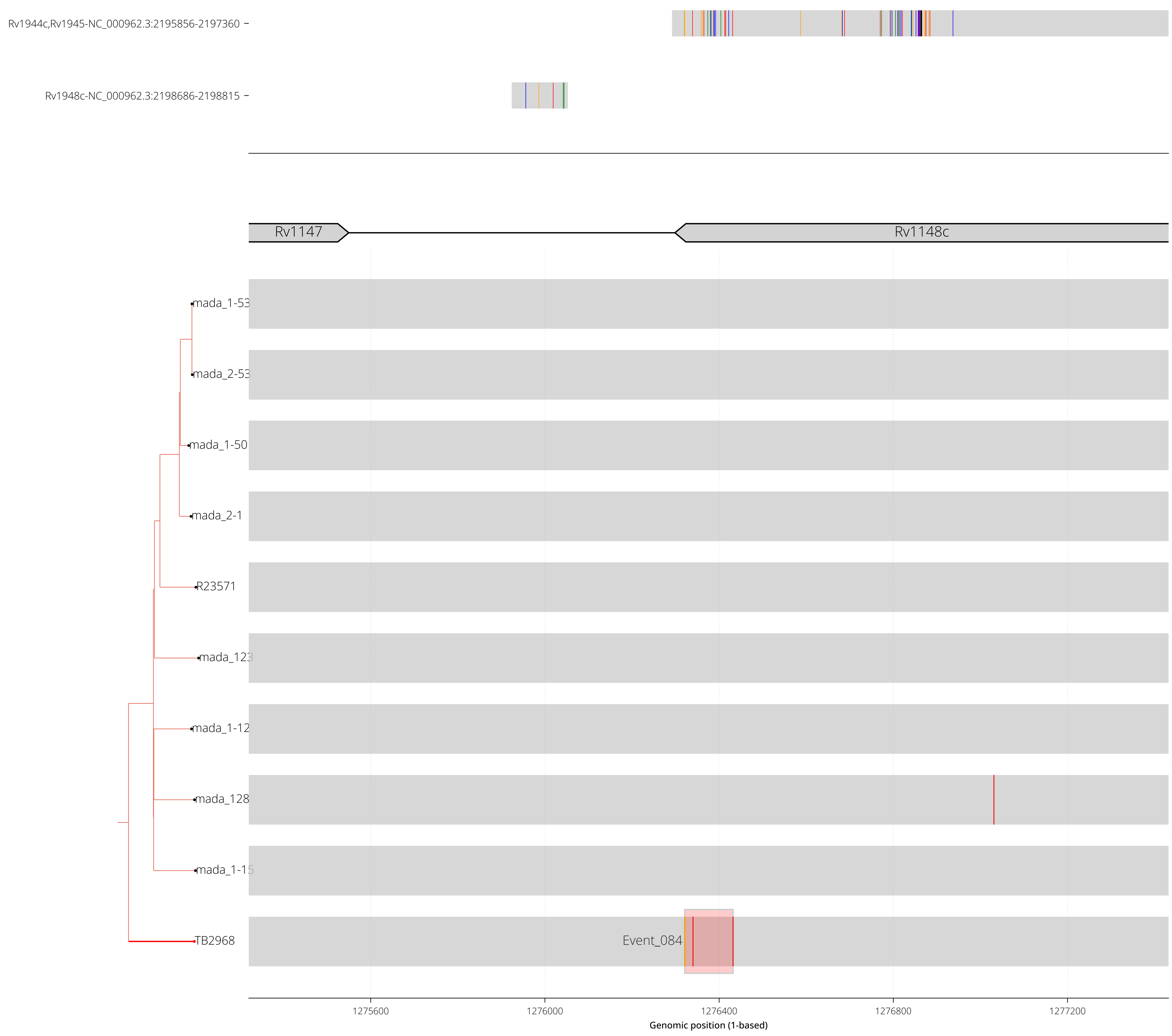

Full Phylogeny Viz + Paralog Alignments
GCE ID: Event_084

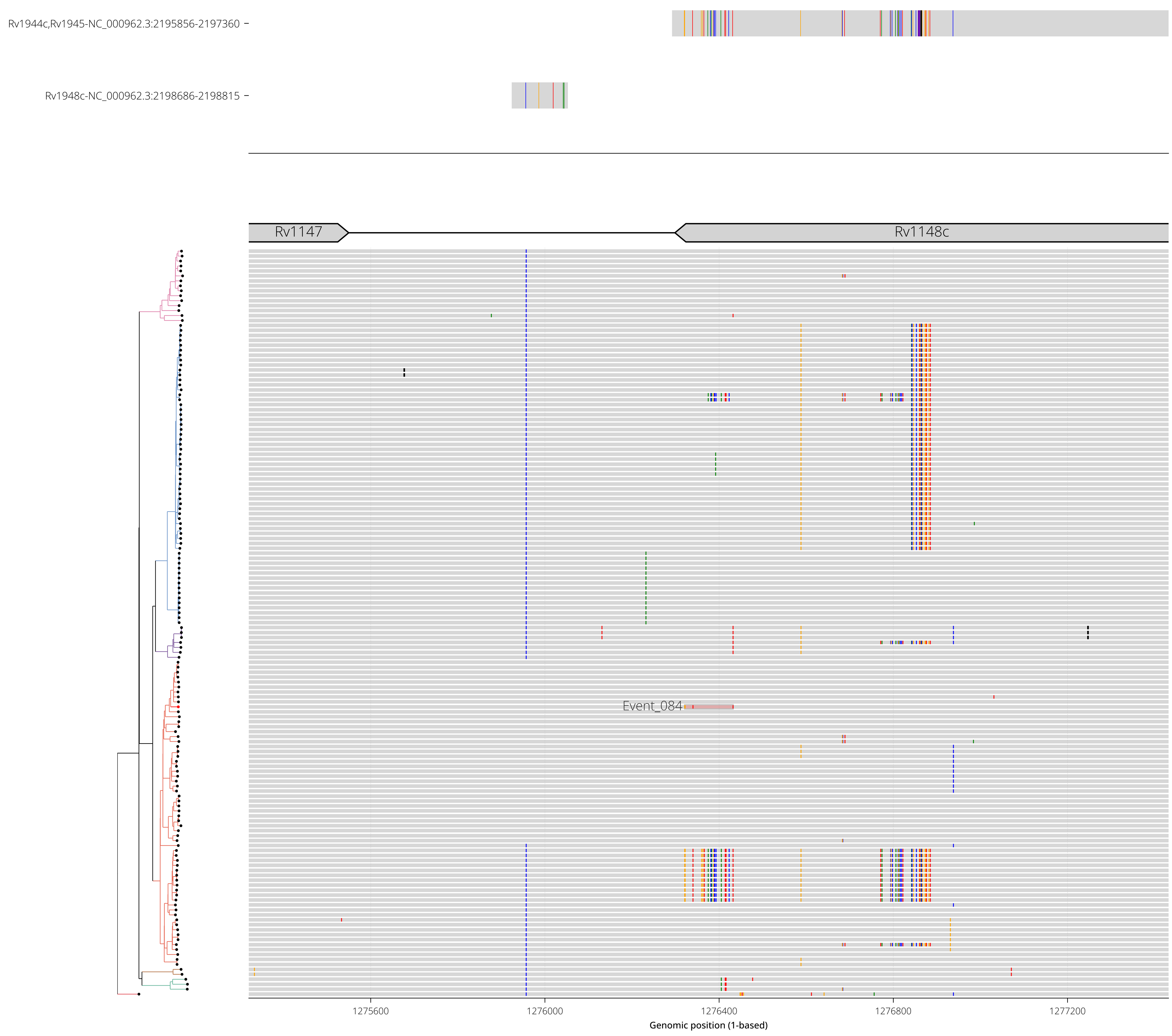

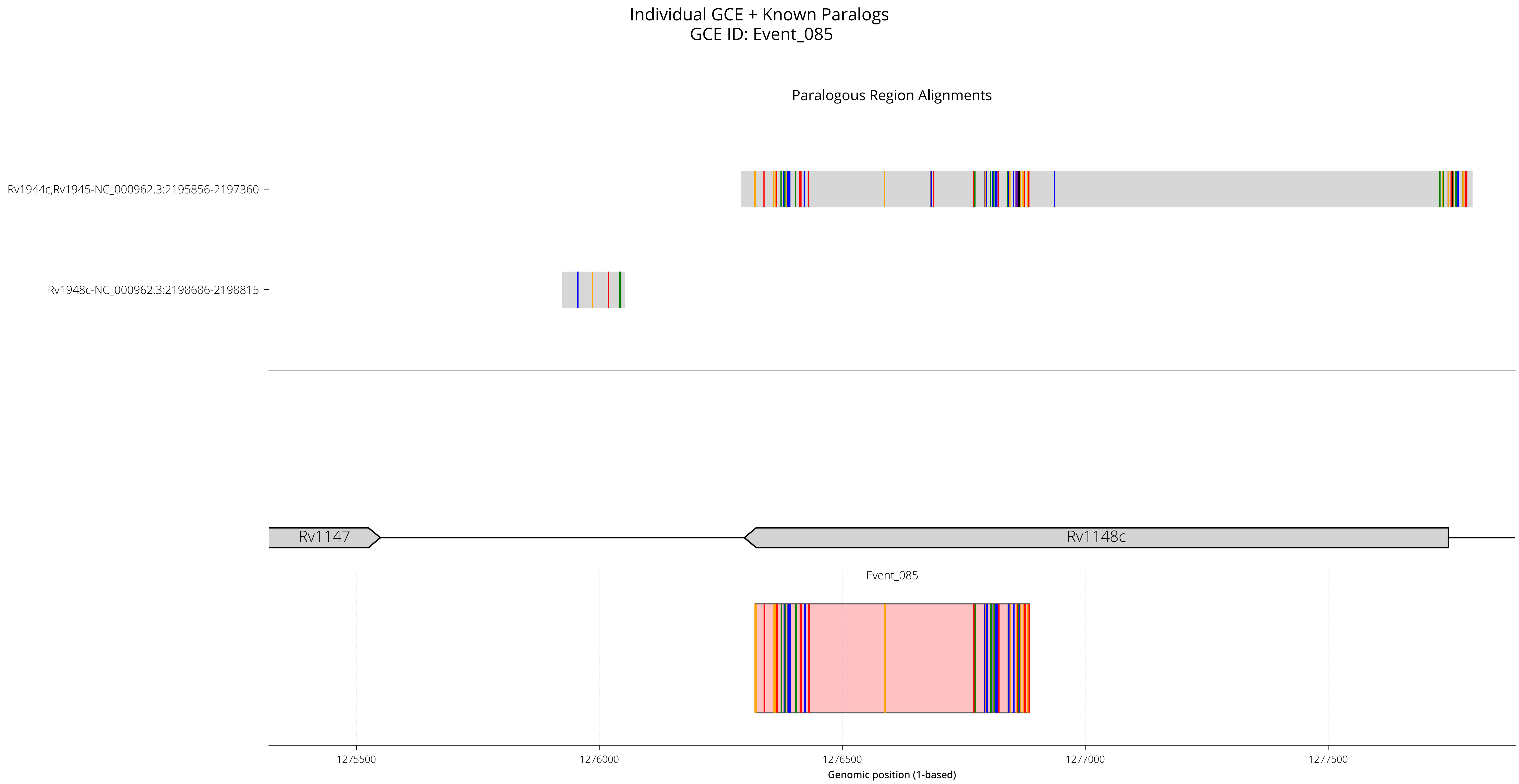
Individual GCE + Known Paralogs
GCE ID: Event_085
Paralogous Region Alignments
Rv1944c,Rv1945-NC_000962.3:2195856-2197360
Rv1948c-NC_000962.3:2198686-2198815
Rv1147
Rv1148c
Event_085
1275500
1276000
1276500
1277000
1277500
Genomic position (1-based)

Circos Viz - GCE to Known Paralogs Locations
GCE ID: Event_085

Location of target gene conversion event
Known (reference) paralog sequences to event region

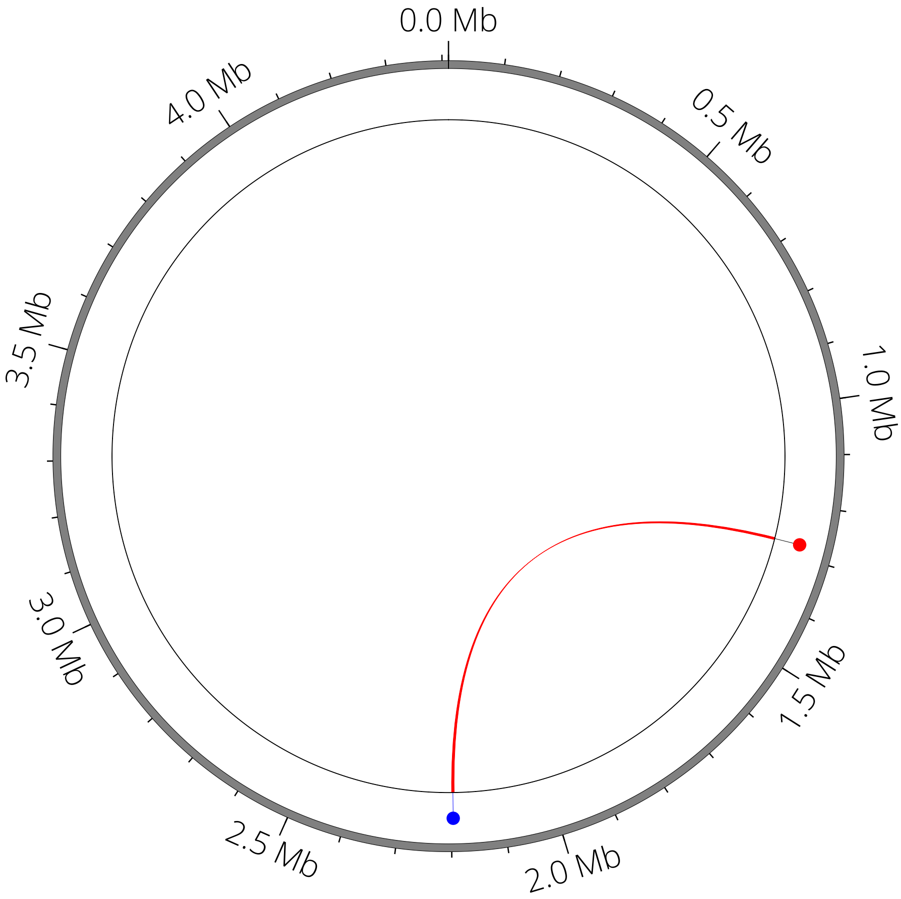

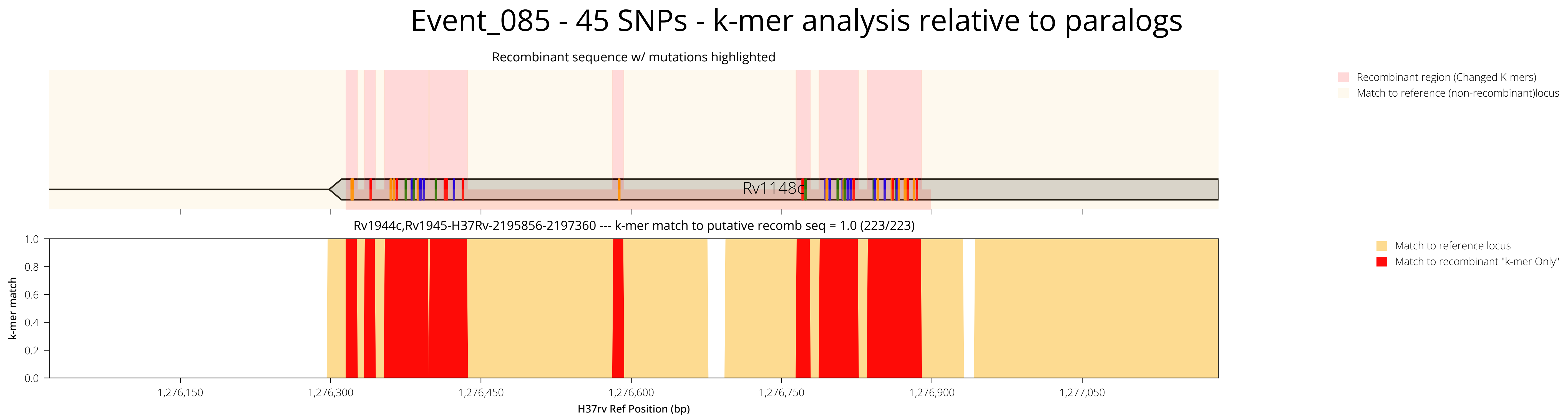

Event_085 - 45 SNPs - k-mer analysis relative to paralogs
Recombinant sequence w/ mutations highlighted
Recombinant region (Changed K-mers)
Match to reference (non-recombinant)locus
Rv1148c
Rv1944c,Rv1945-H37Rv-2195856-2197360 --- k-mer match to putative recomb seq = 1.0 (223/223)
Match to reference locus
Match to recombinant "k-mer Only"
k-mer match
1.0
0.8
0.6
0.4
0.2
0.0
1,276,150
1,276,300
1,276,450
1,276,600
1,276,750
1,276,900
1,277,050
H37rv Ref Position (bp)

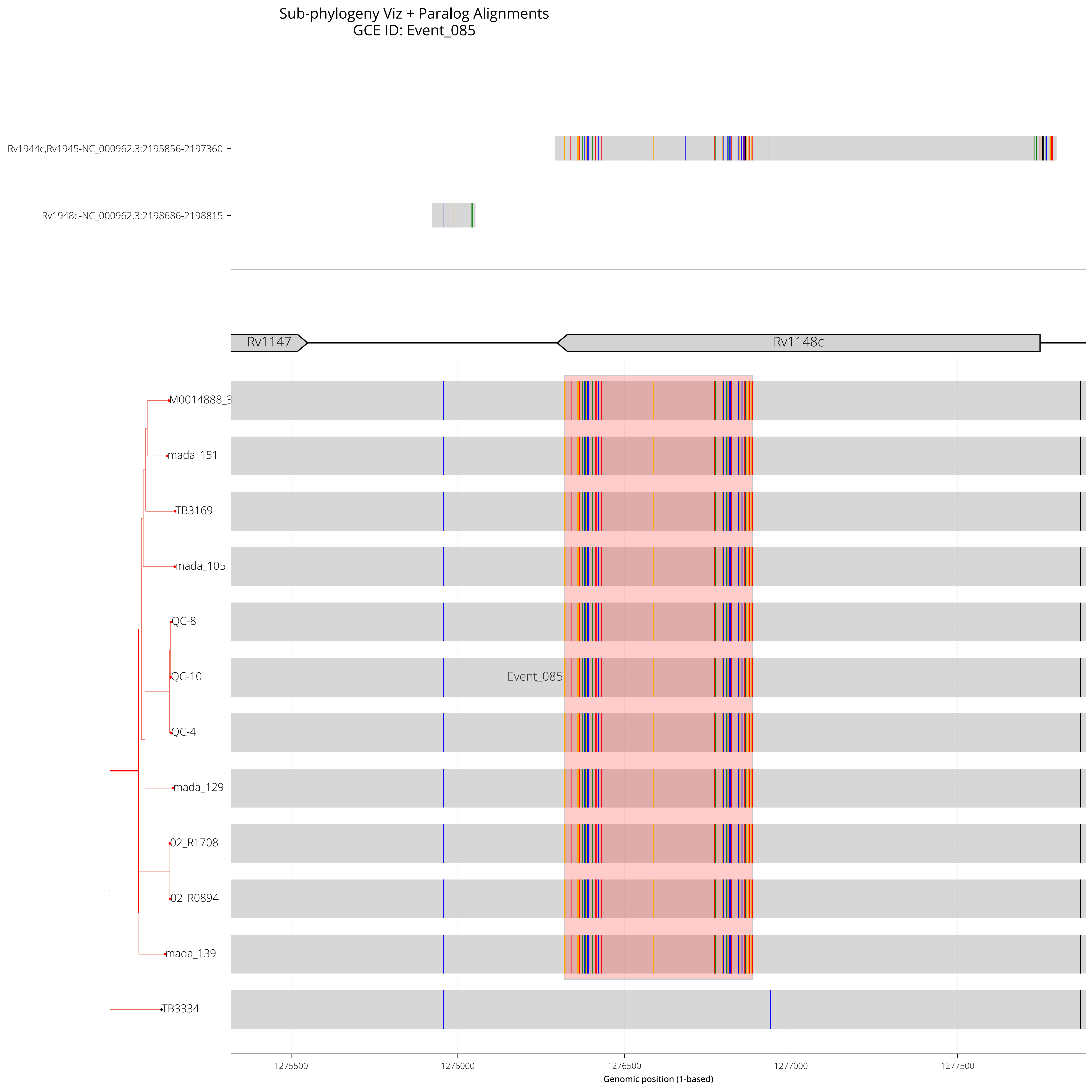

Sub-phylogeny Viz + Paralog Alignments
GCE ID: Event_085
Rv1944c,Rv1945-NC_000962.3:2195856-2197360
Rv1948c-NC_000962.3:2198686-2198815
Rv1147
Rv1148c
M0014888_3
mada_151
TB3169
mada_105
QC-8
QC-10
QC-4
mada_129
02_R1708
02_R0894
mada_139
TB3334
Event_085
1275500
1276000
1276500
1277000
1277500
Genomic position (1-based)

Full Phylogeny Viz + Paralog Alignments
GCE ID: Event_085

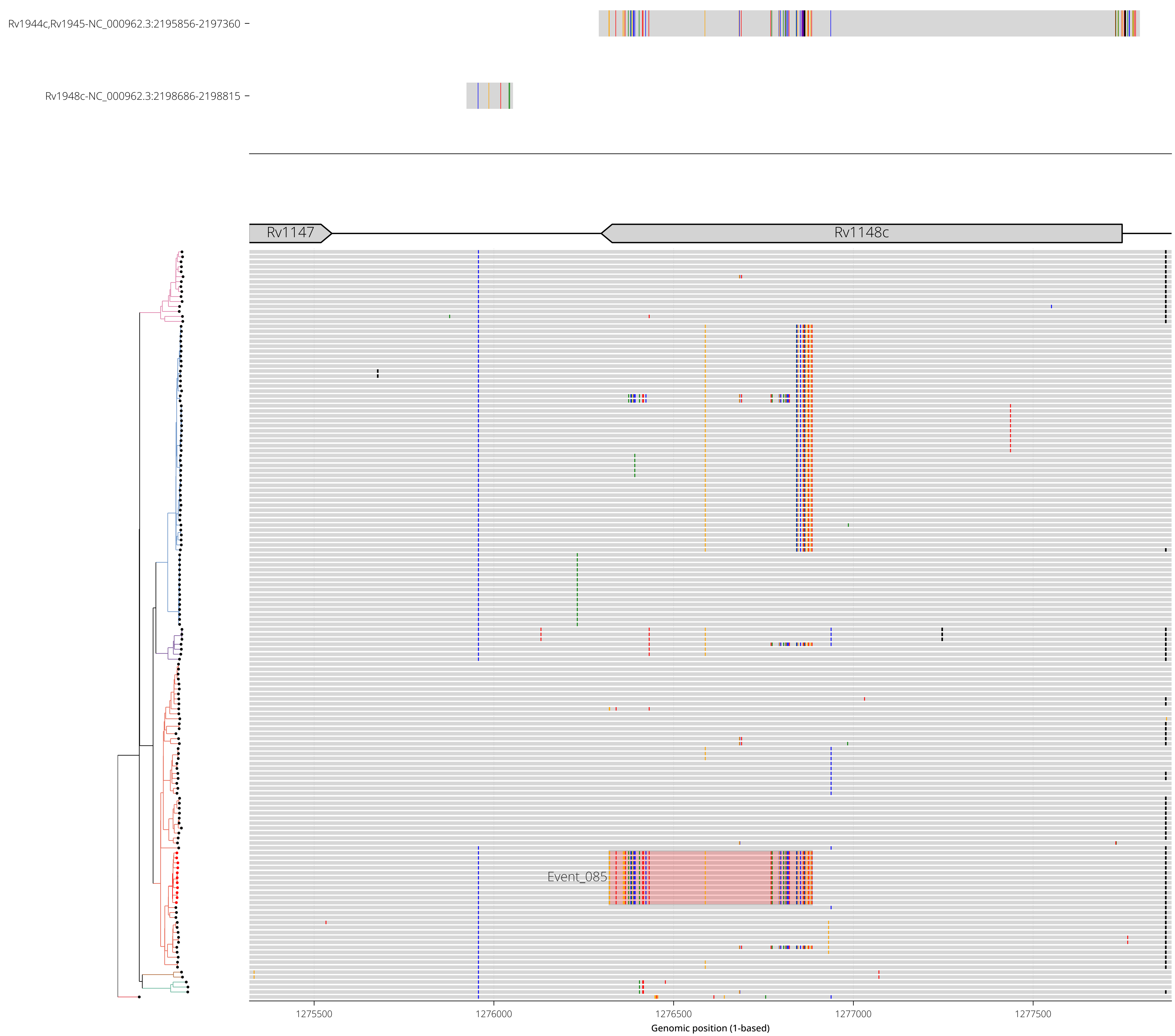

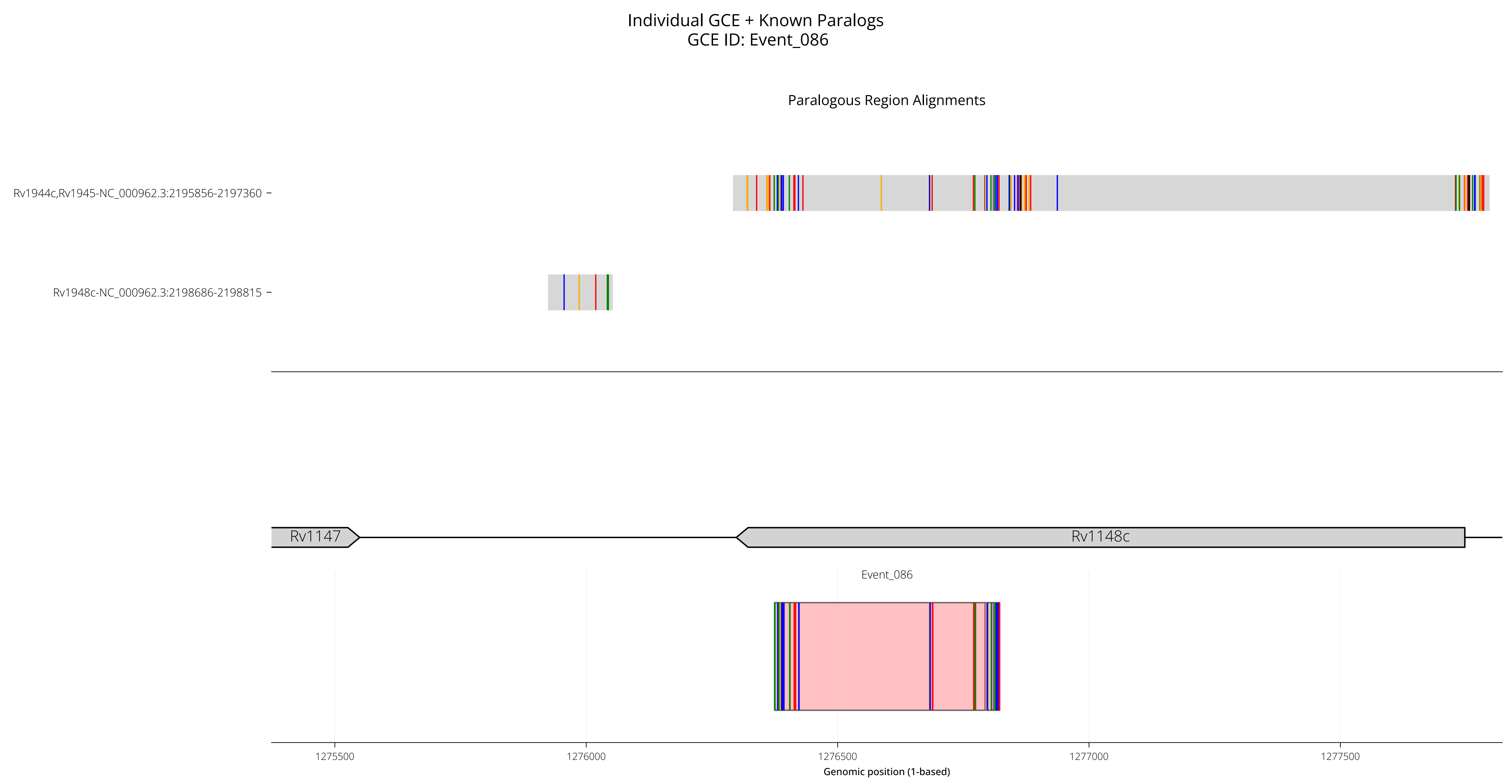
Individual GCE + Known Paralogs
GCE ID: Event_086
Paralogous Region Alignments
Rv1944c,Rv1945-NC_000962.3:2195856-2197360
Rv1948c-NC_000962.3:2198686-2198815
Rv1147
Rv1148c
Event_086
1275500
1276000
1276500
1277000
1277500
Genomic position (1-based)

Circos Viz - GCE to Known Paralogs Locations
GCE ID: Event_086

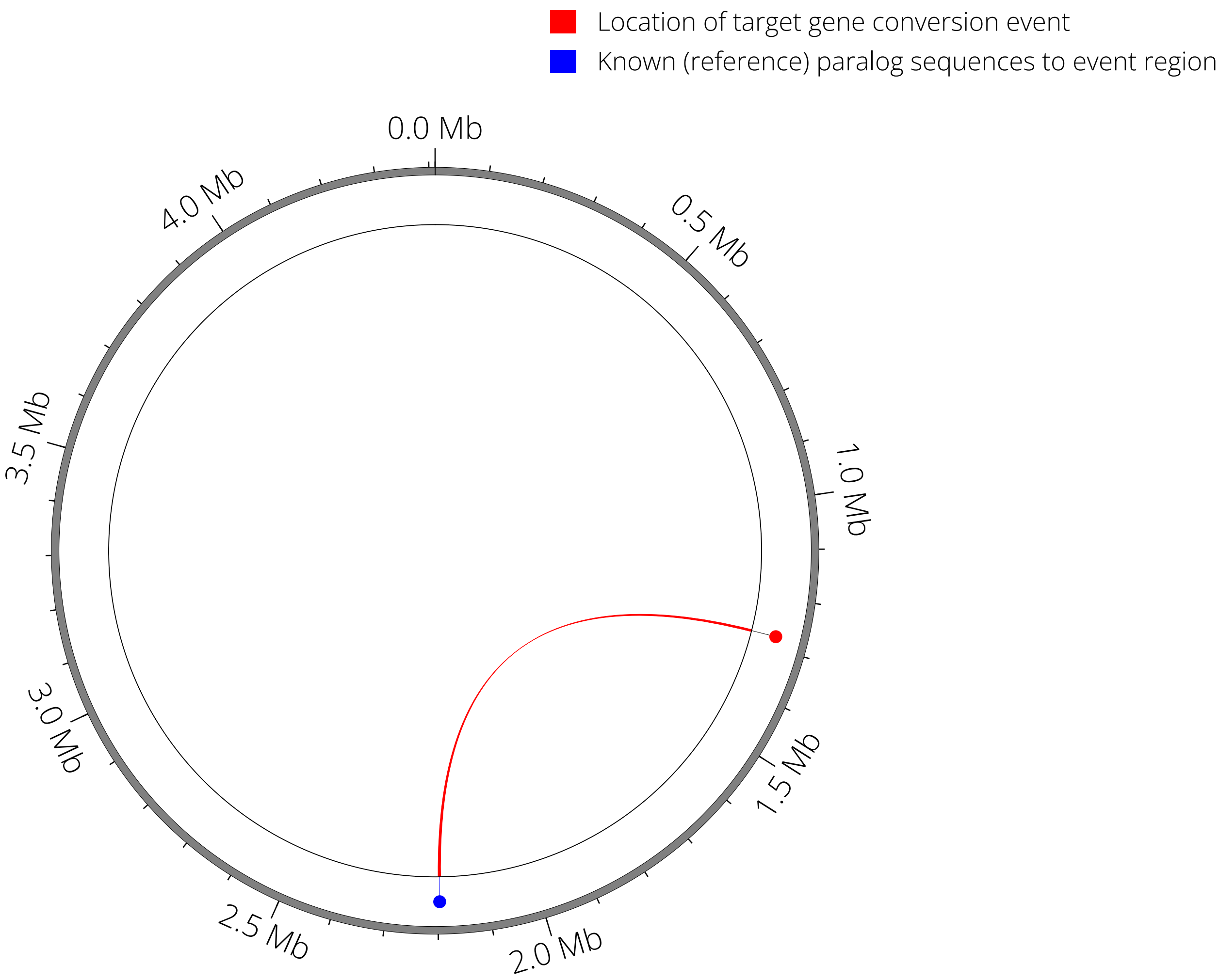

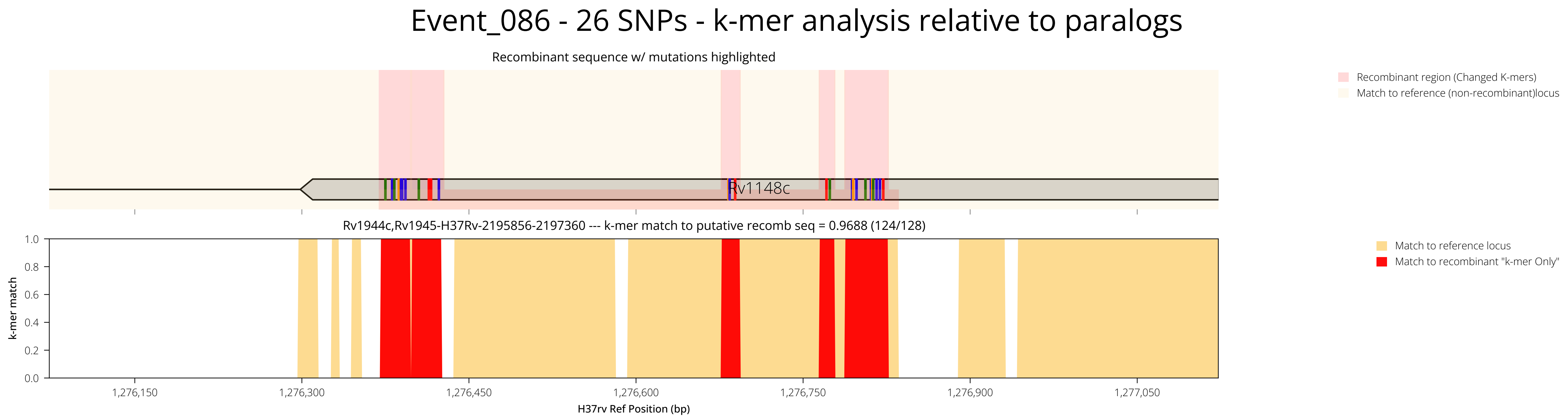

Event_086 - 26 SNPs - k-mer analysis relative to paralogs
Recombinant sequence w/ mutations highlighted
Recombinant region (Changed K-mers)
Match to reference (non-recombinant)locus
Rv1148c
Rv1944c,Rv1945-H37Rv-2195856-2197360 --- k-mer match to putative recomb seq = 0.9688 (124/128)
Match to reference locus
Match to recombinant "k-mer Only"
k-mer match
1.0
0.8
0.6
0.4
0.2
0.0
1,276,150
1,276,300
1,276,450
1,276,600
1,276,750
1,276,900
1,277,050
H37rv Ref Position (bp)

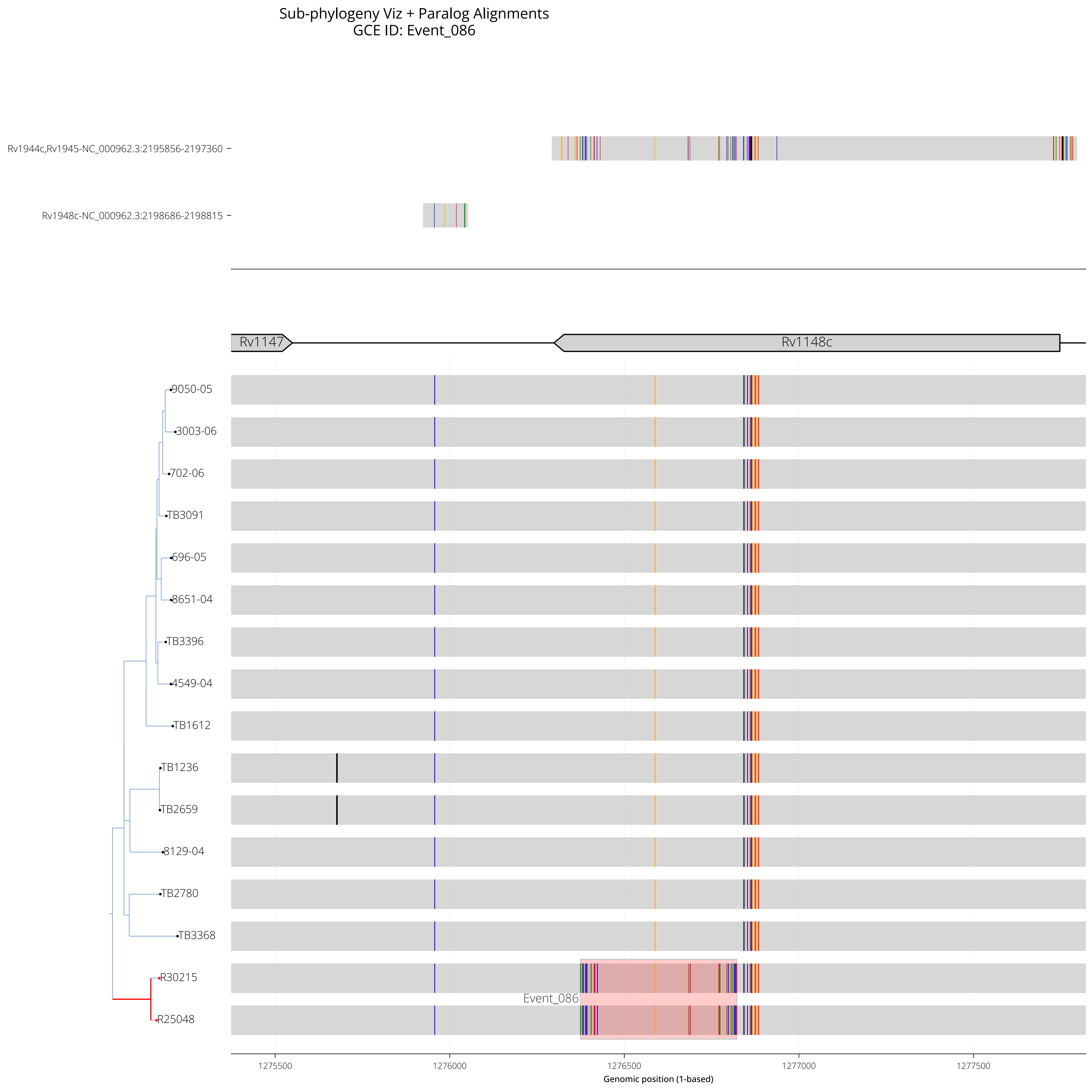

Sub-phylogeny Viz + Paralog Alignments
GCE ID: Event_086
Rv1944c,Rv1945-NC_000962.3:2195856-2197360
Rv1948c-NC_000962.3:2198686-2198815
Rv1147
Rv1148c
9050-05
3003-06
702-06
TB3091
696-05
8651-04
TB3396
4549-04
TB1612
TB1236
TB2659
8129-04
TB2780
TB3368
R30215
R25048
Event_086
1275500
1276000
1276500
1277000
1277500
Genomic position (1-based)

Full Phylogeny Viz + Paralog Alignments
GCE ID: Event_086

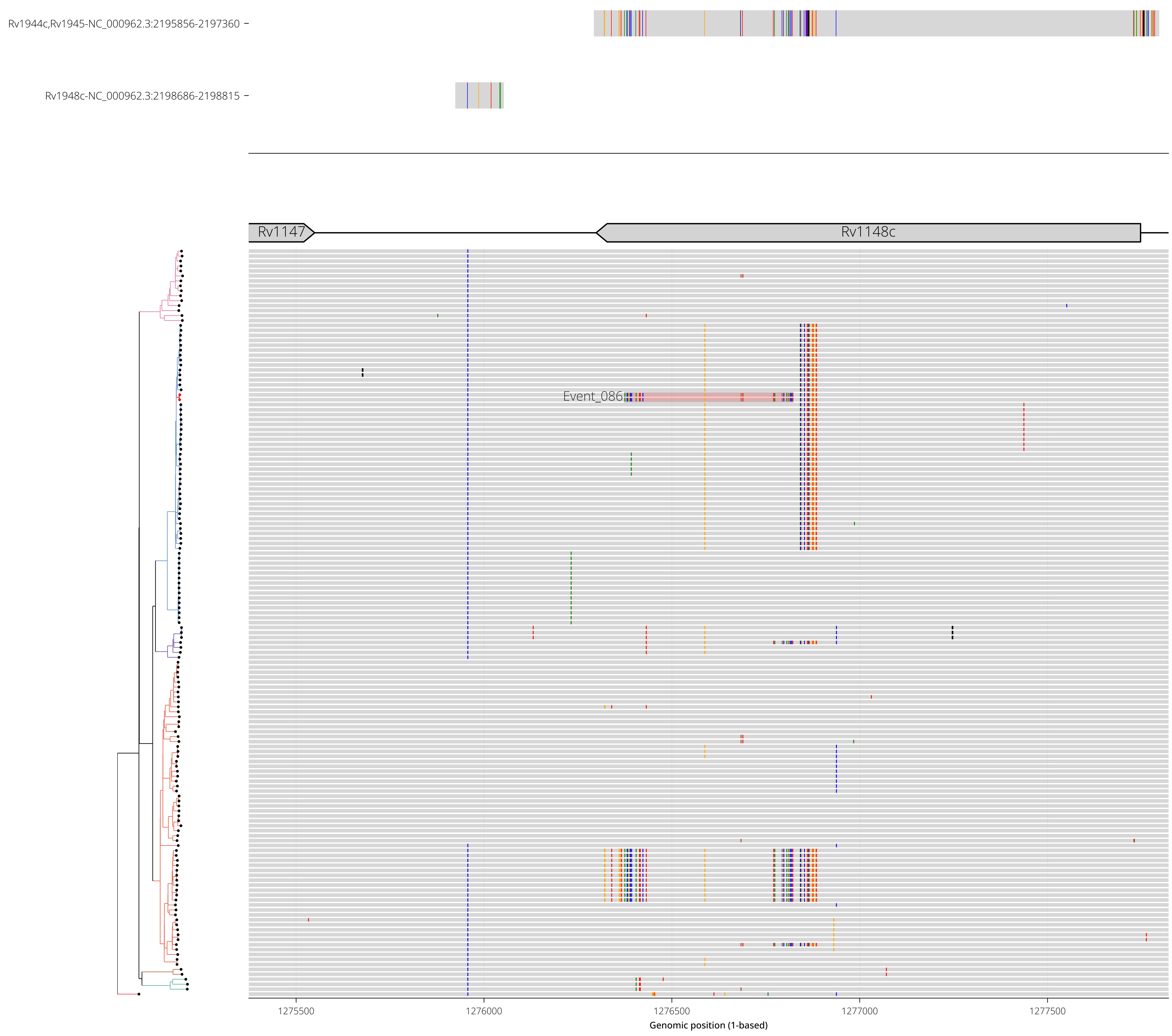

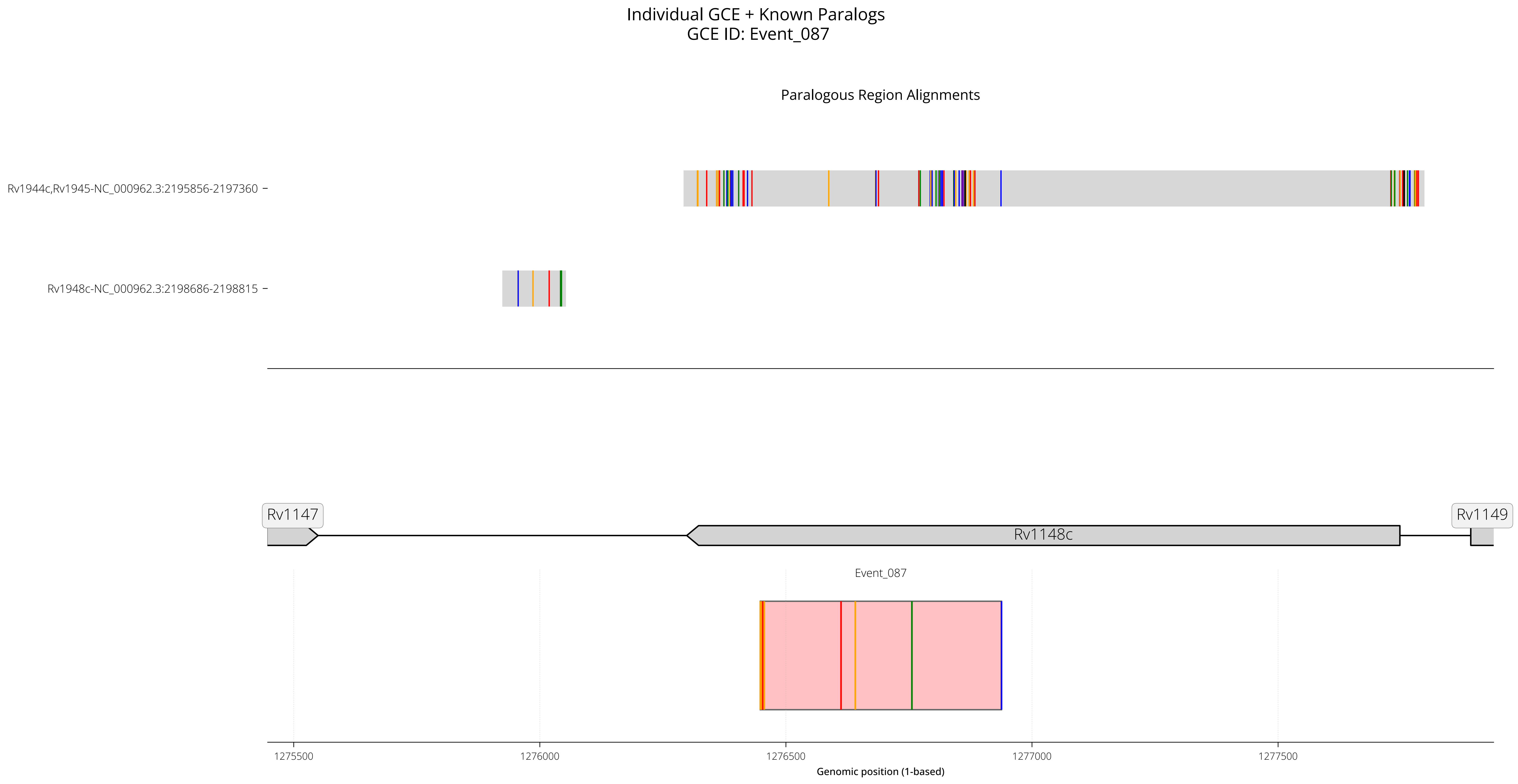

Individual GCE + Known Paralogs
GCE ID: Event_087
Paralogous Region Alignments
Rv1944c,Rv1945-NC_000962.3:2195856-2197360
Rv1948c-NC_000962.3:2198686-2198815
Rv1147
Rv1148c
Rv1149
Event_087
1275500
1276000
1276500
1277000
1277500
Genomic position (1-based)

# Circos Viz - GCE to Known Paralogs Locations
GCE ID: Event_087

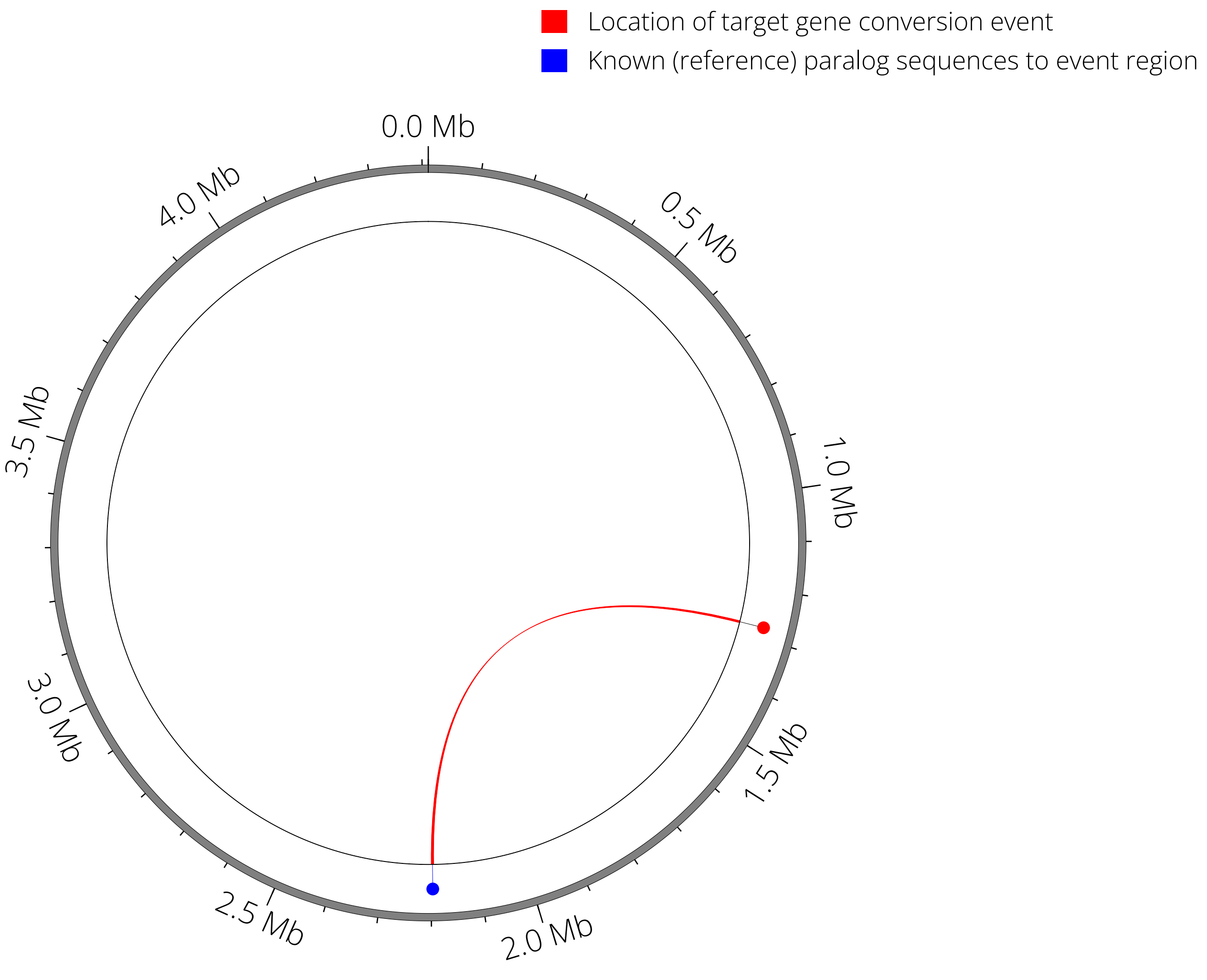

# Event_087 - 8 SNPs - k-mer analysis relative to paralogs

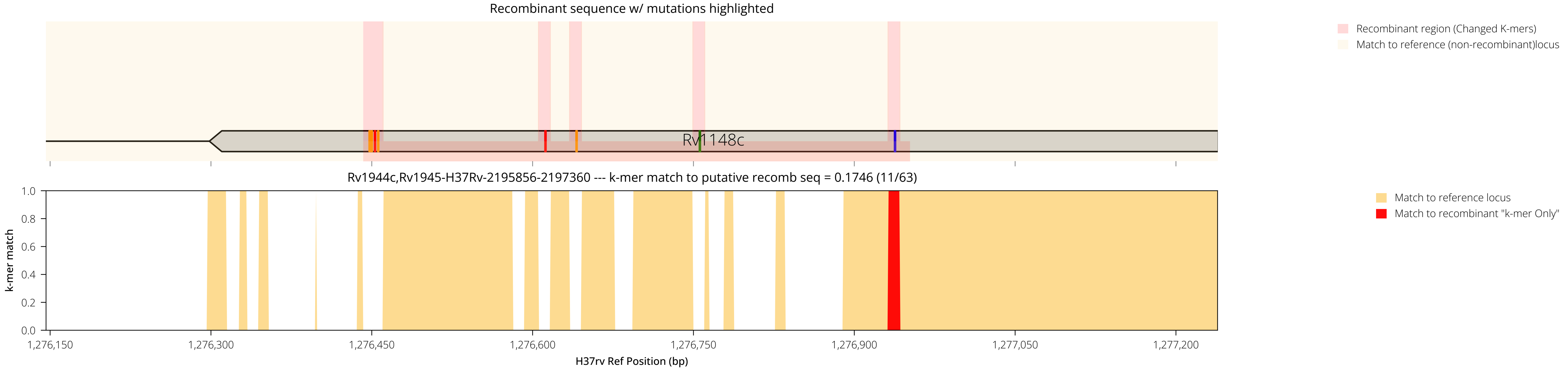

Sub-phylogeny Viz + Paralog Alignments
GCE ID: Event_087

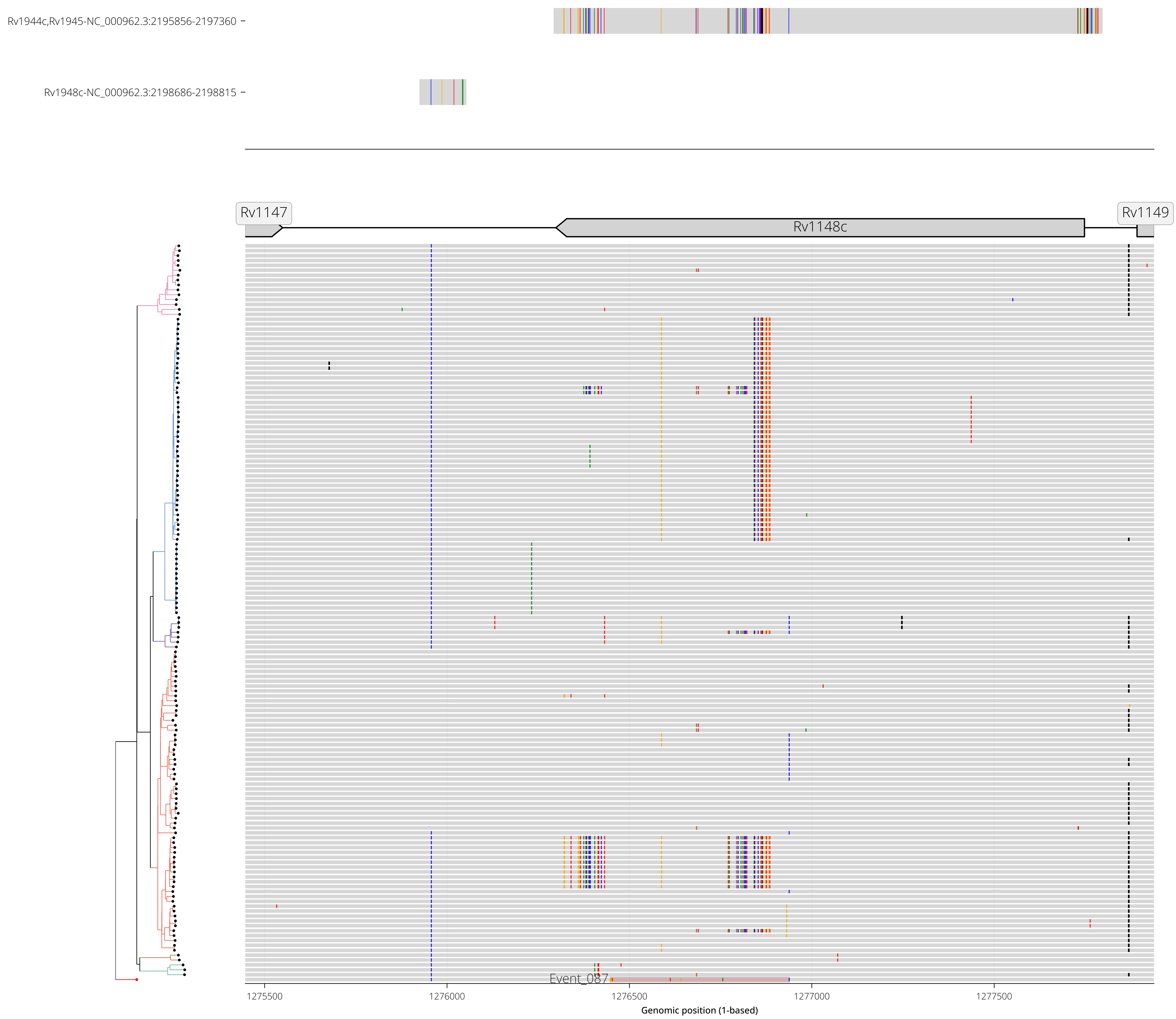

Full Phylogeny Viz + Paralog Alignments
GCE ID: Event_087

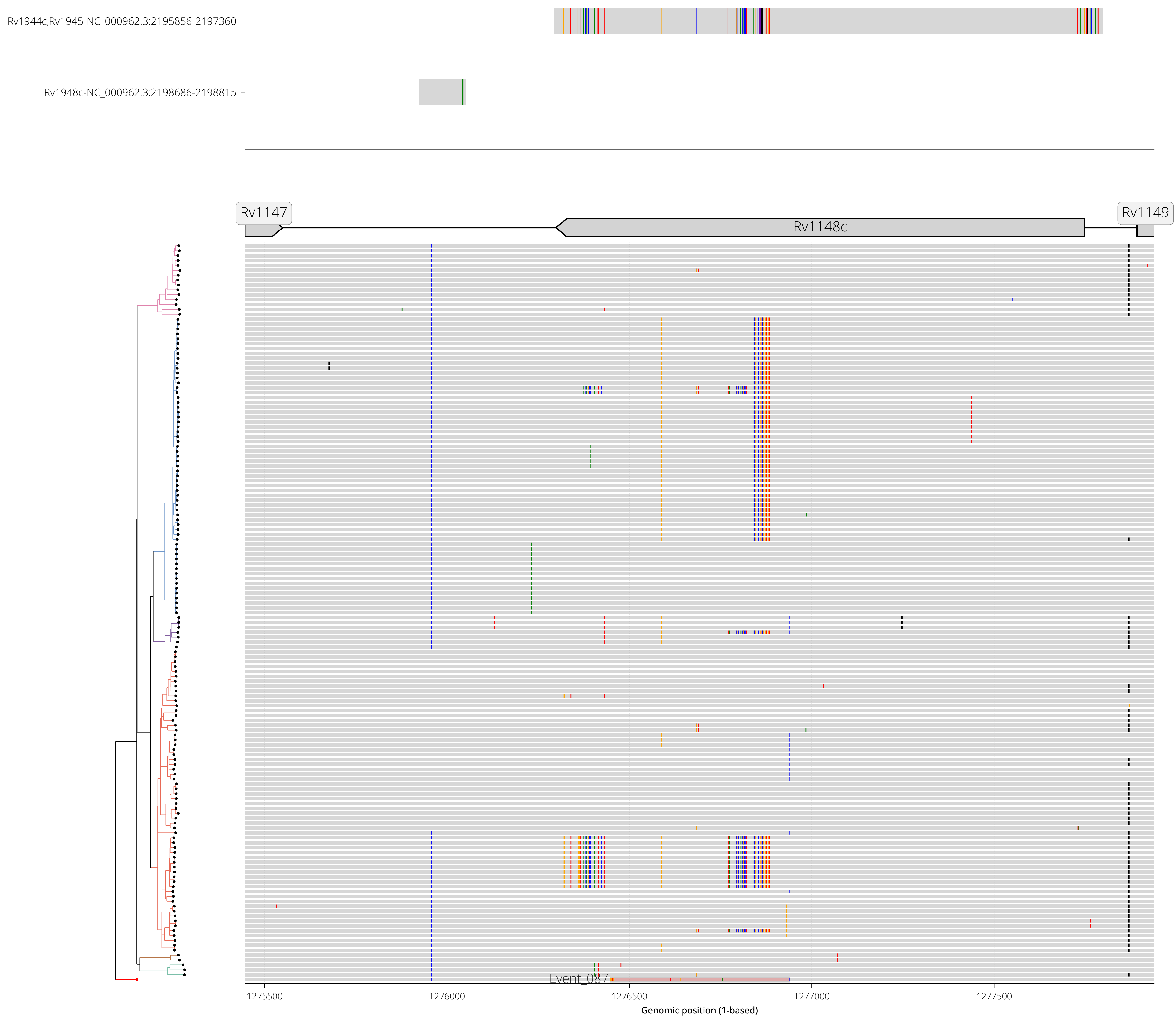

Individual GCE + Known Paralogs
GCE ID: Event_088

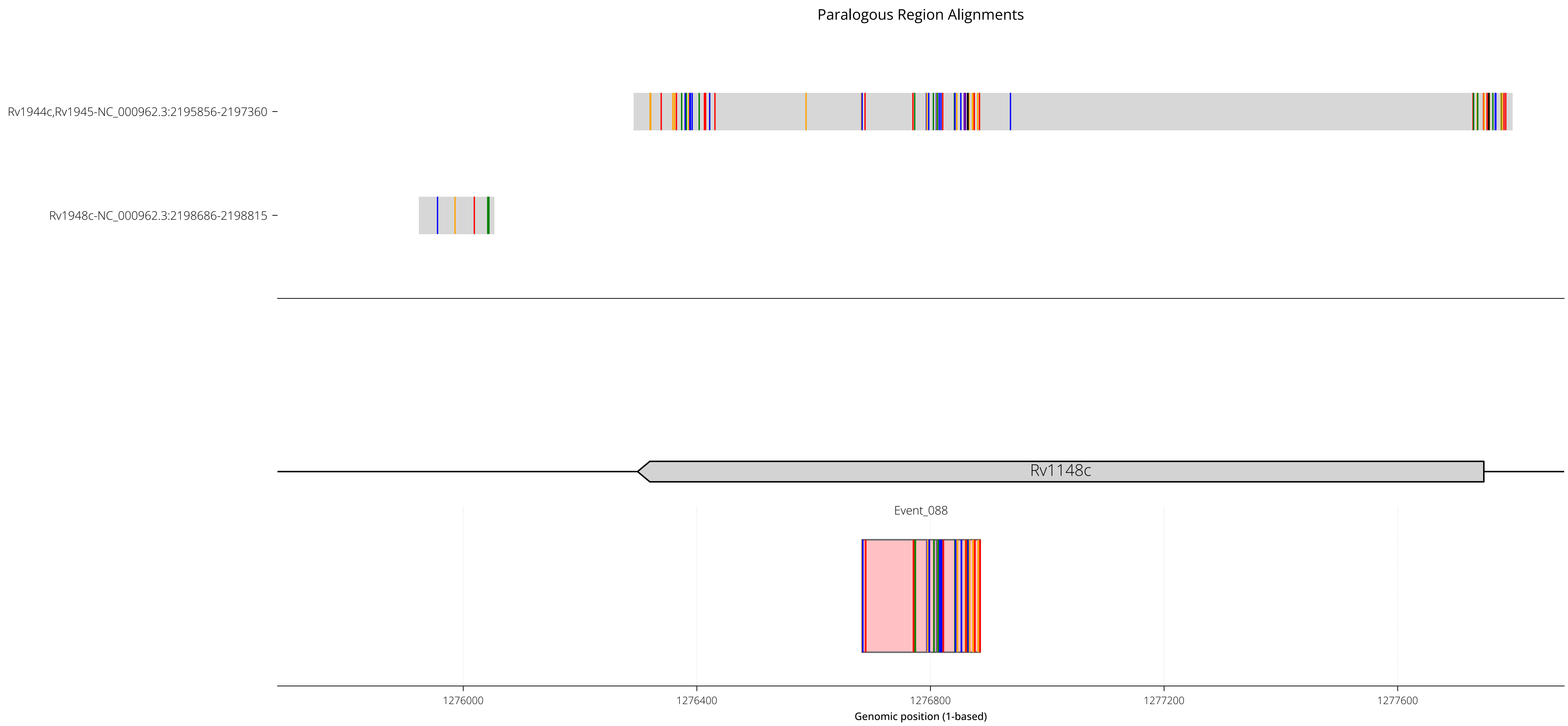

Circos Viz - GCE to Known Paralogs Locations
GCE ID: Event_088

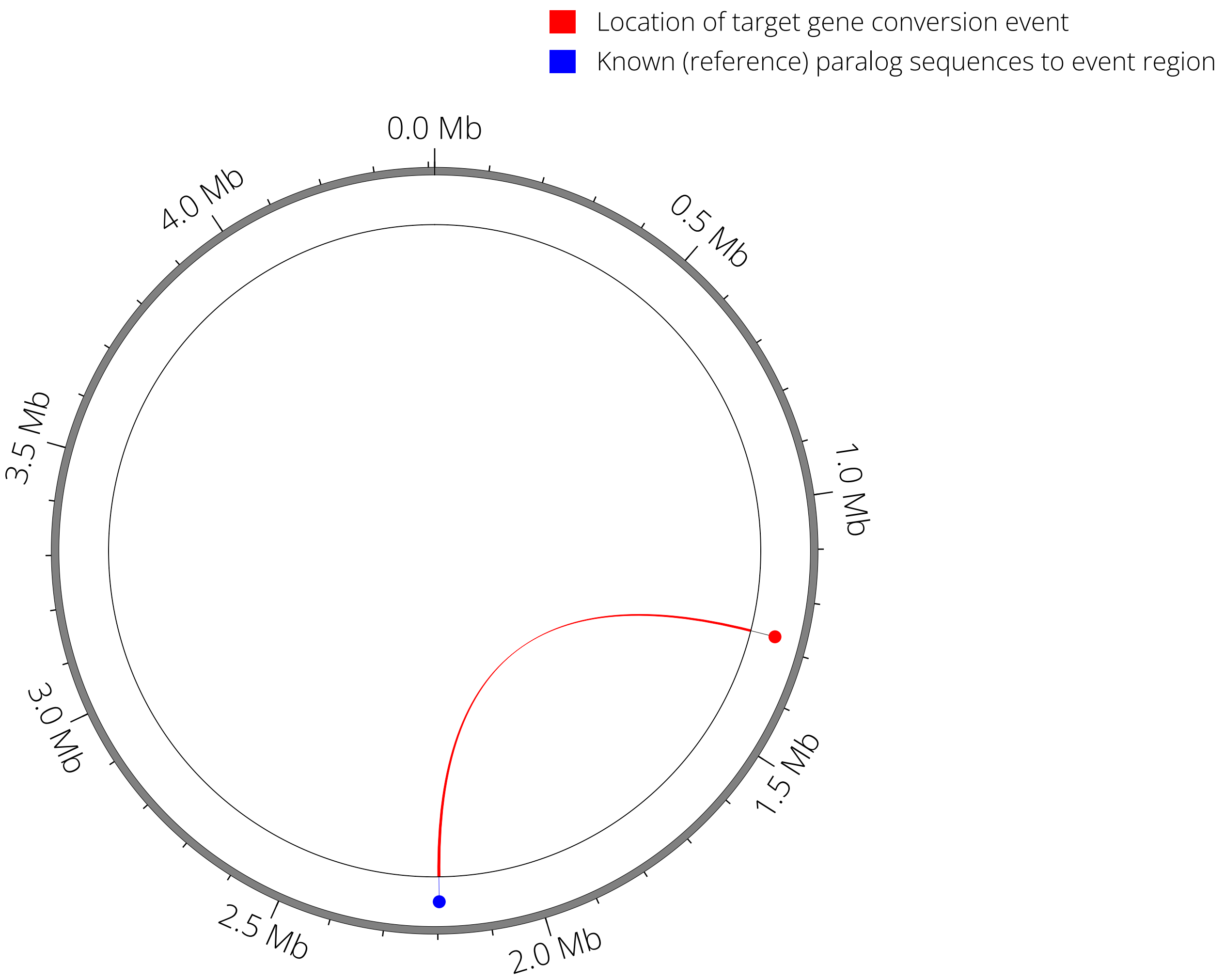

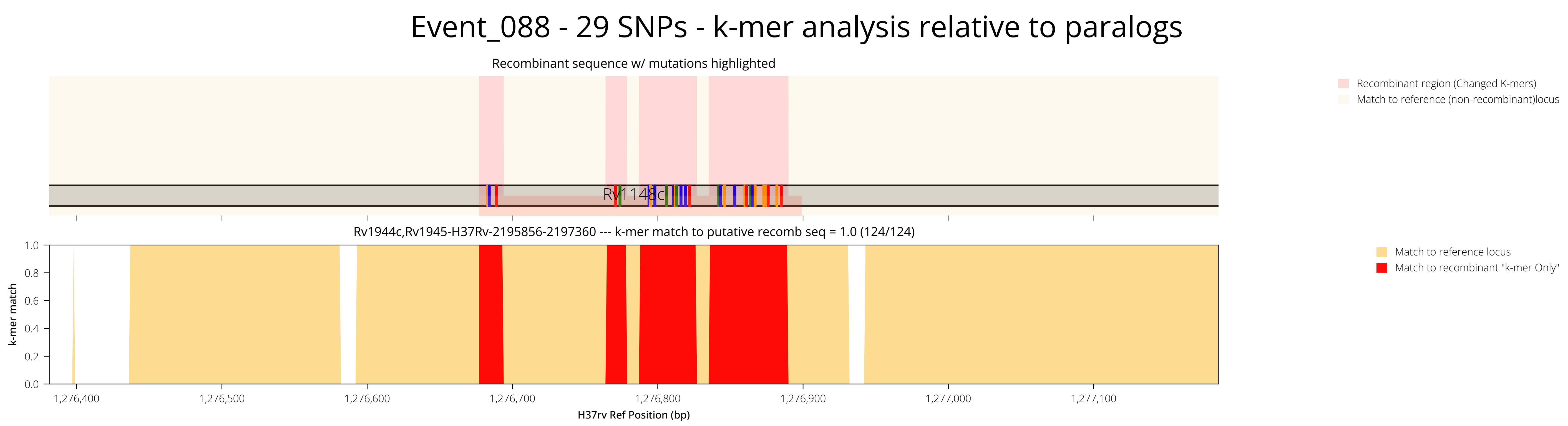

Event_088 - 29 SNPs - k-mer analysis relative to paralogs
Recombinant sequence w/ mutations highlighted
Rv1148c
Recombinant region (Changed K-mers)
Match to reference (non-recombinant)locus
Rv1944c,Rv1945-H37Rv-2195856-2197360 --- k-mer match to putative recomb seq = 1.0 (124/124)
k-mer match
1.0
0.8
0.6
0.4
0.2
0.0
1,276,400
1,276,500
1,276,600
1,276,700
1,276,800
1,276,900
1,277,000
1,277,100
H37rv Ref Position (bp)
Match to reference locus
Match to recombinant "k-mer Only"

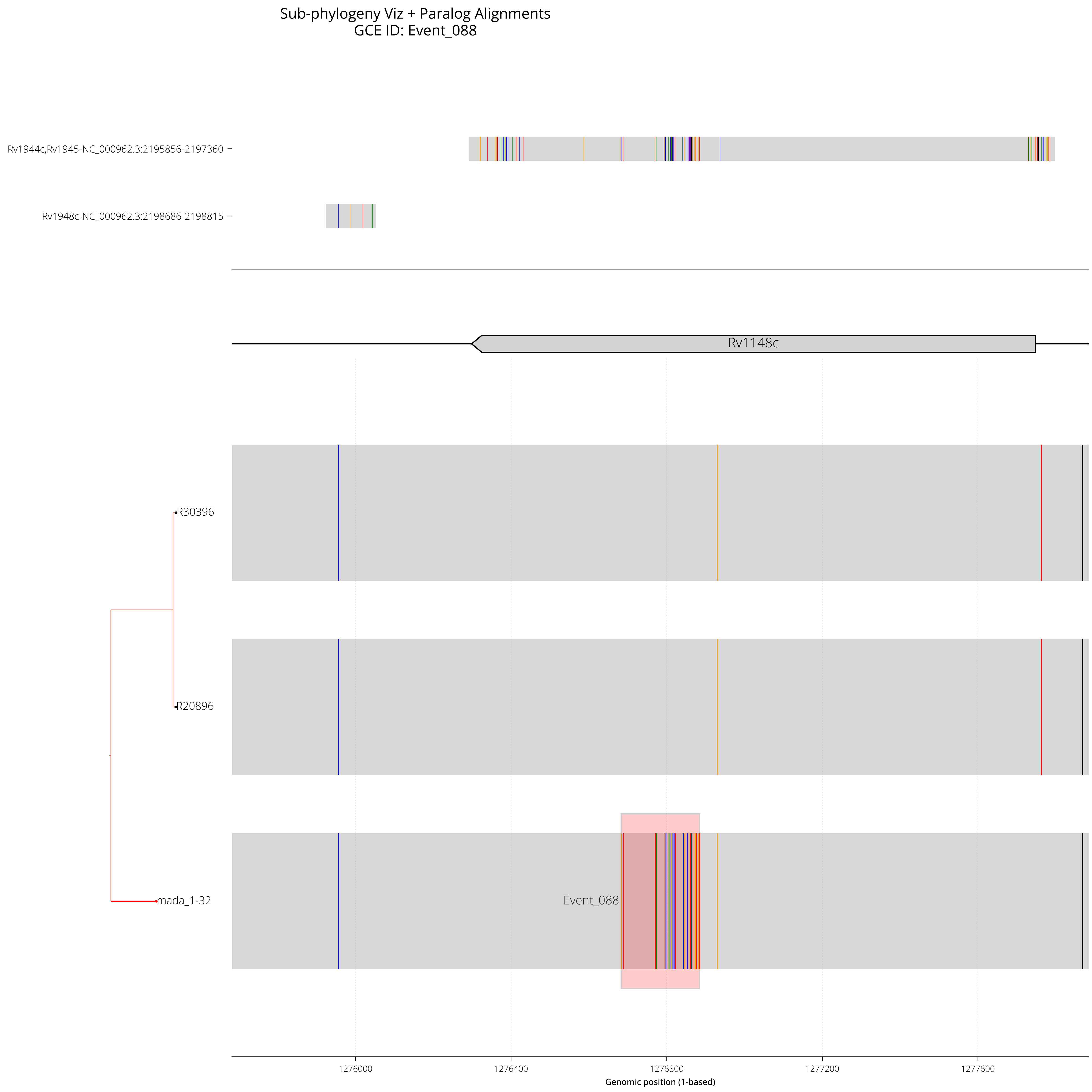

Sub-phylogeny Viz + Paralog Alignments
GCE ID: Event_088
Rv1944c,Rv1945-NC_000962.3:2195856-2197360
Rv1948c-NC_000962.3:2198686-2198815
Rv1148c
R30396
R20896
mada_1-32
Event_088
1276000
1276400
1276800
1277200
1277600
Genomic position (1-based)

Full Phylogeny Viz + Paralog Alignments
GCE ID: Event_088

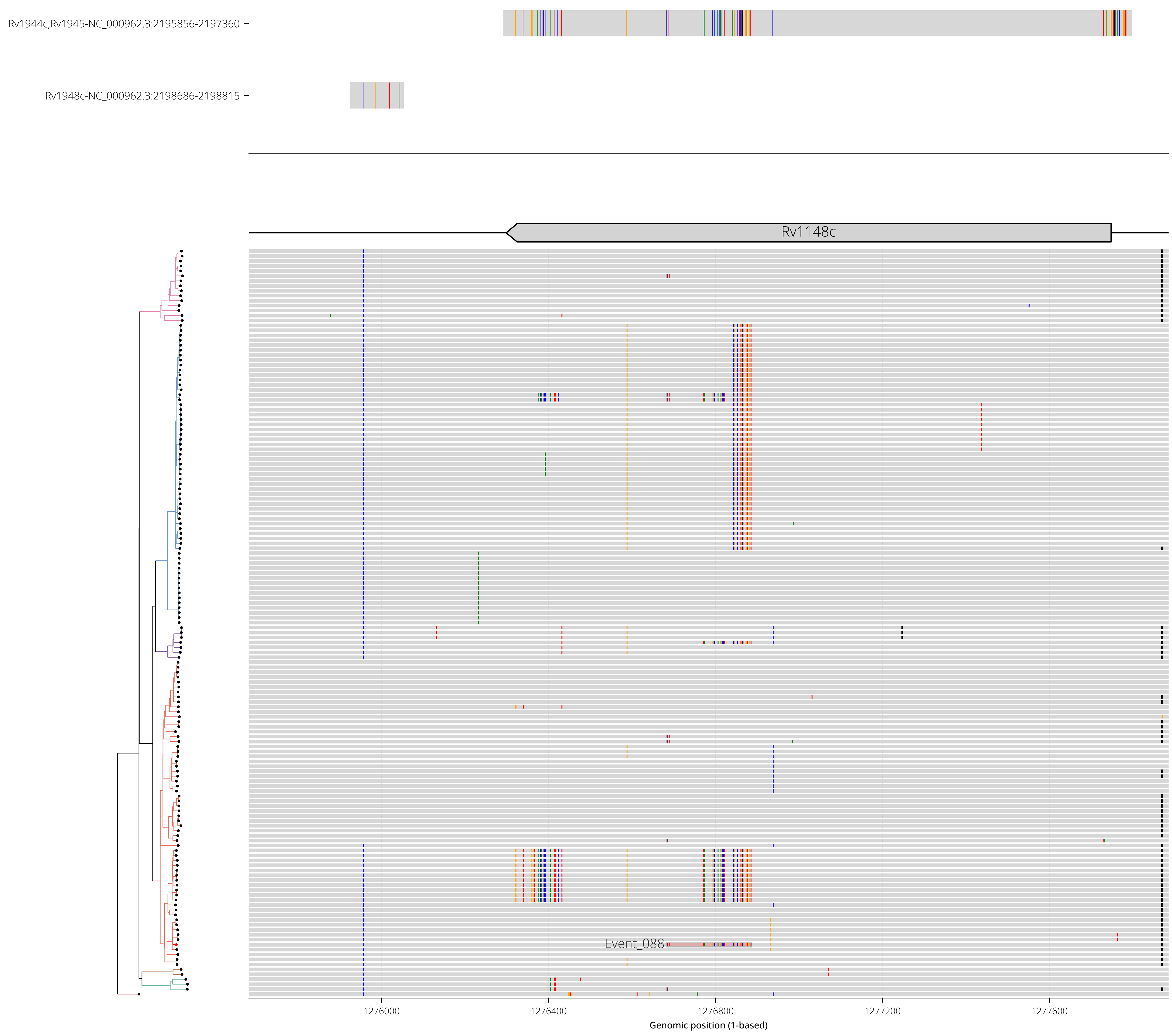

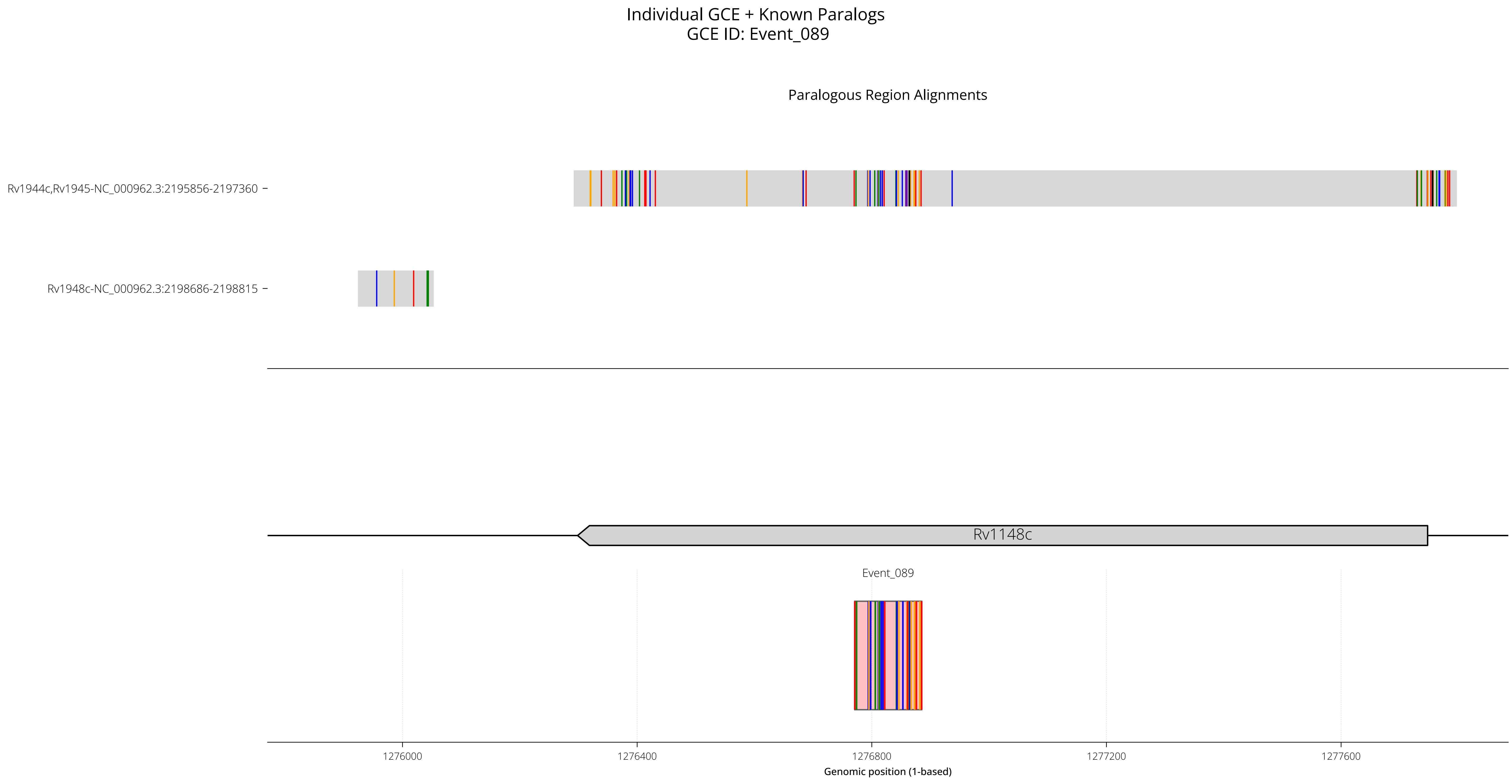
Individual GCE + Known Paralogs
GCE ID: Event_089
Paralogous Region Alignments
Rv1944c,Rv1945-NC_000962.3:2195856-2197360
Rv1948c-NC_000962.3:2198686-2198815
Rv1148c
Event_089
1276000
1276400
1276800
1277200
1277600
Genomic position (1-based)

# Circos Viz - GCE to Known Paralogs Locations
GCE ID: Event_089

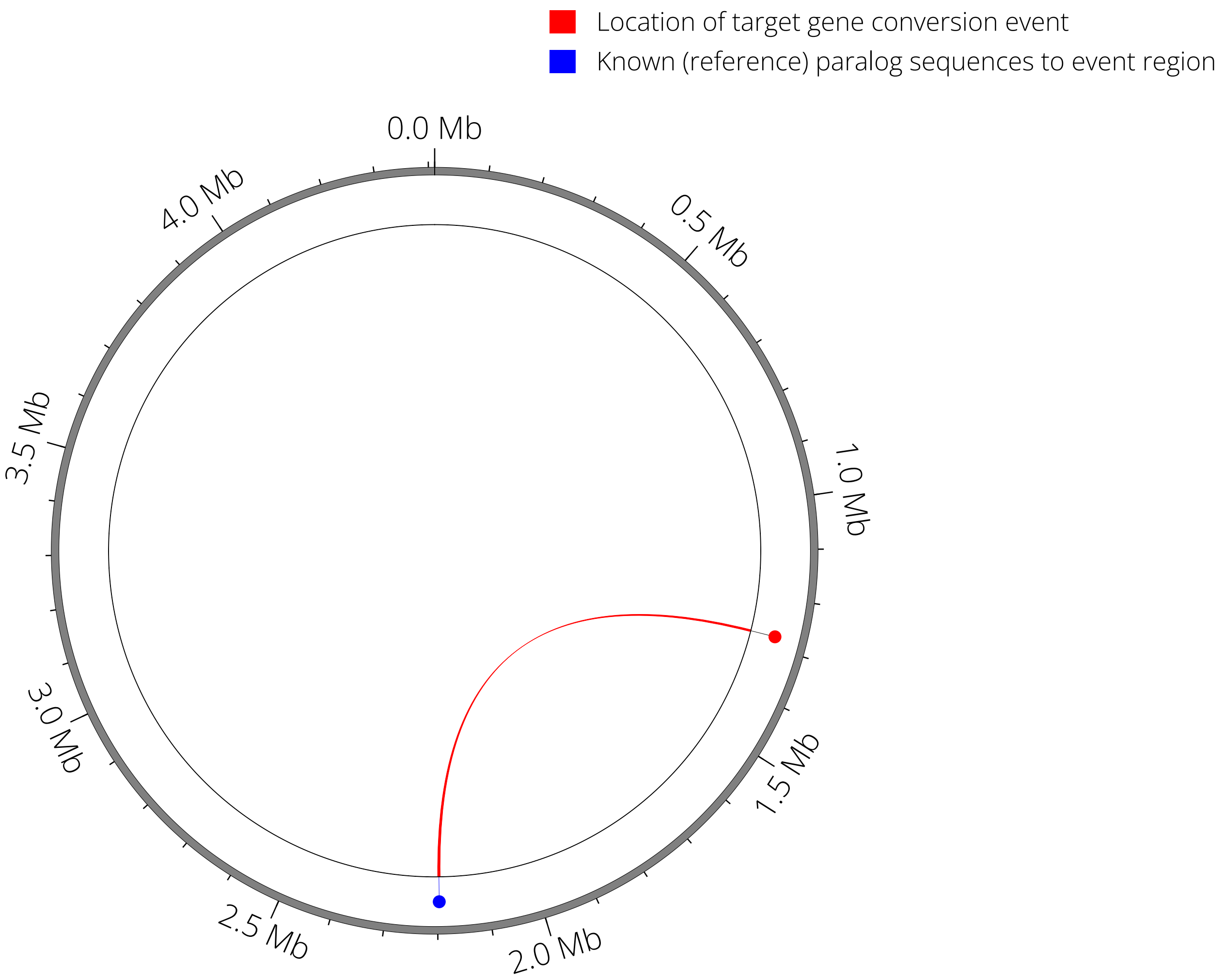

# Event_089 - 26 SNPs - k-mer analysis relative to paralogs

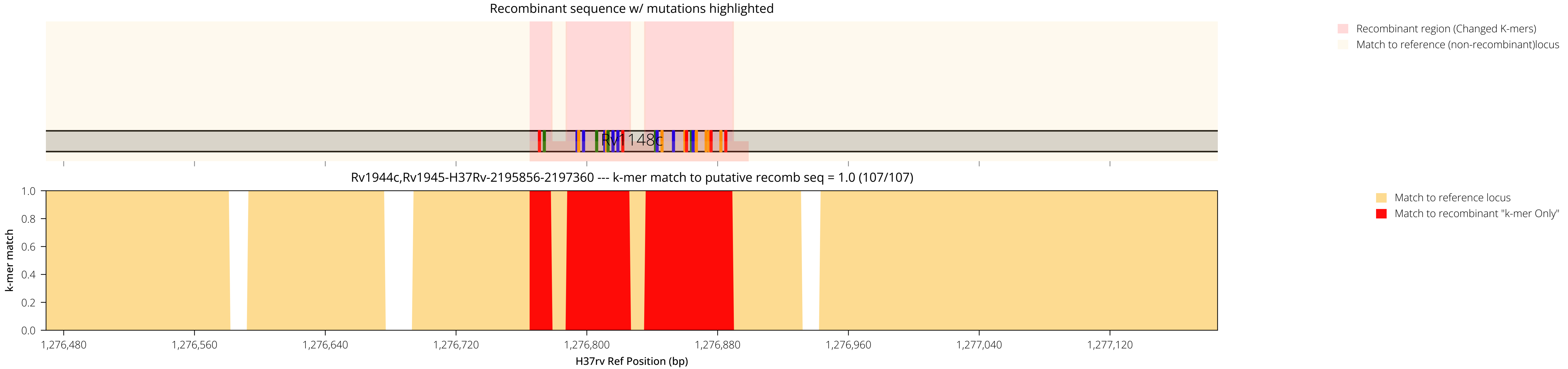

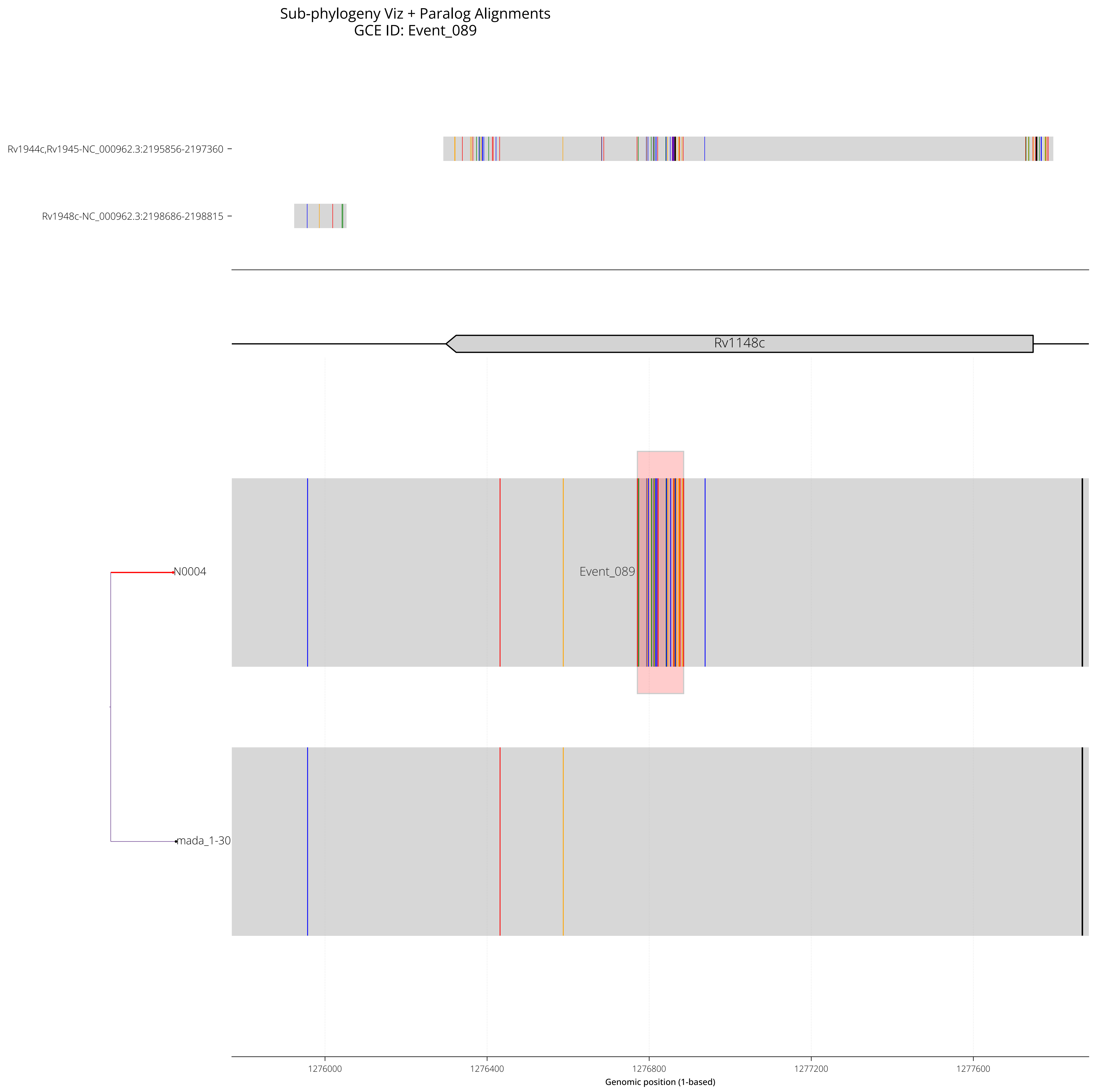

Sub-phylogeny Viz + Paralog Alignments
GCE ID: Event_089
Rv1944c,Rv1945-NC_000962.3:2195856-2197360
Rv1948c-NC_000962.3:2198686-2198815
Rv1148c
N0004
Event_089
mada_1-30
1276000
1276400
1276800
1277200
1277600
Genomic position (1-based)

Full Phylogeny Viz + Paralog Alignments
GCE ID: Event_089

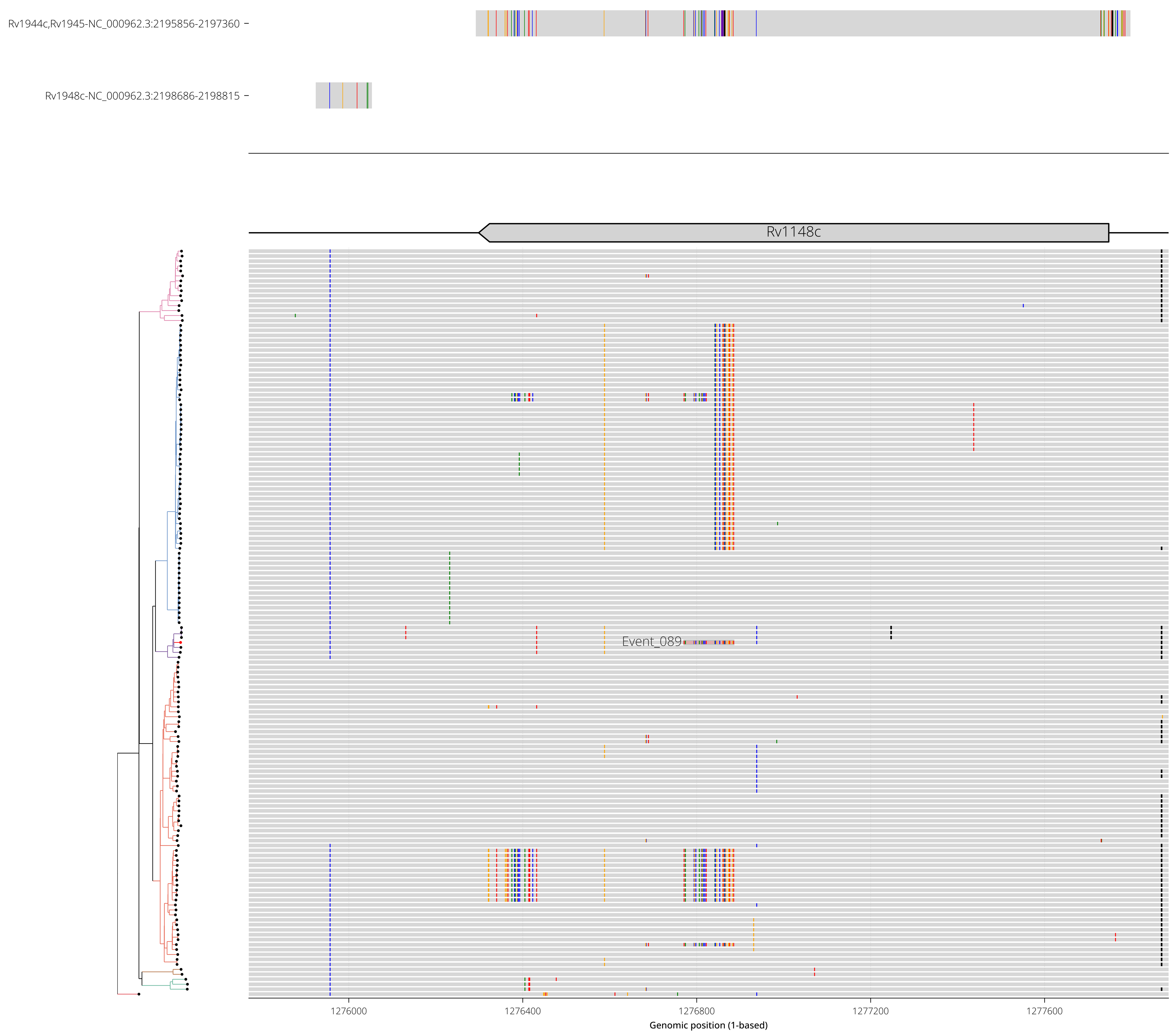

Individual GCE + Known Paralogs
GCE ID: Event_090

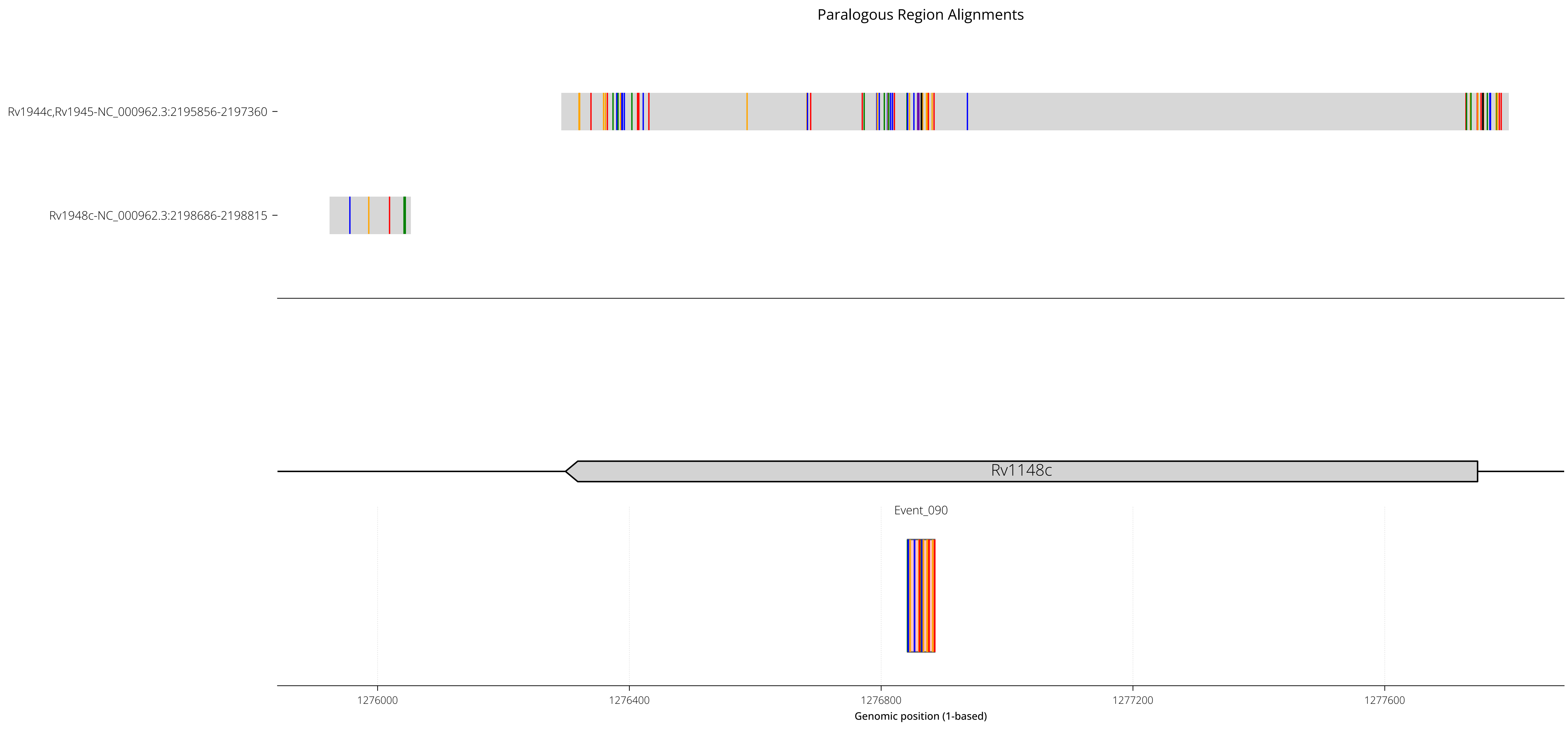

Circos Viz - GCE to Known Paralogs Locations
GCE ID: Event_090

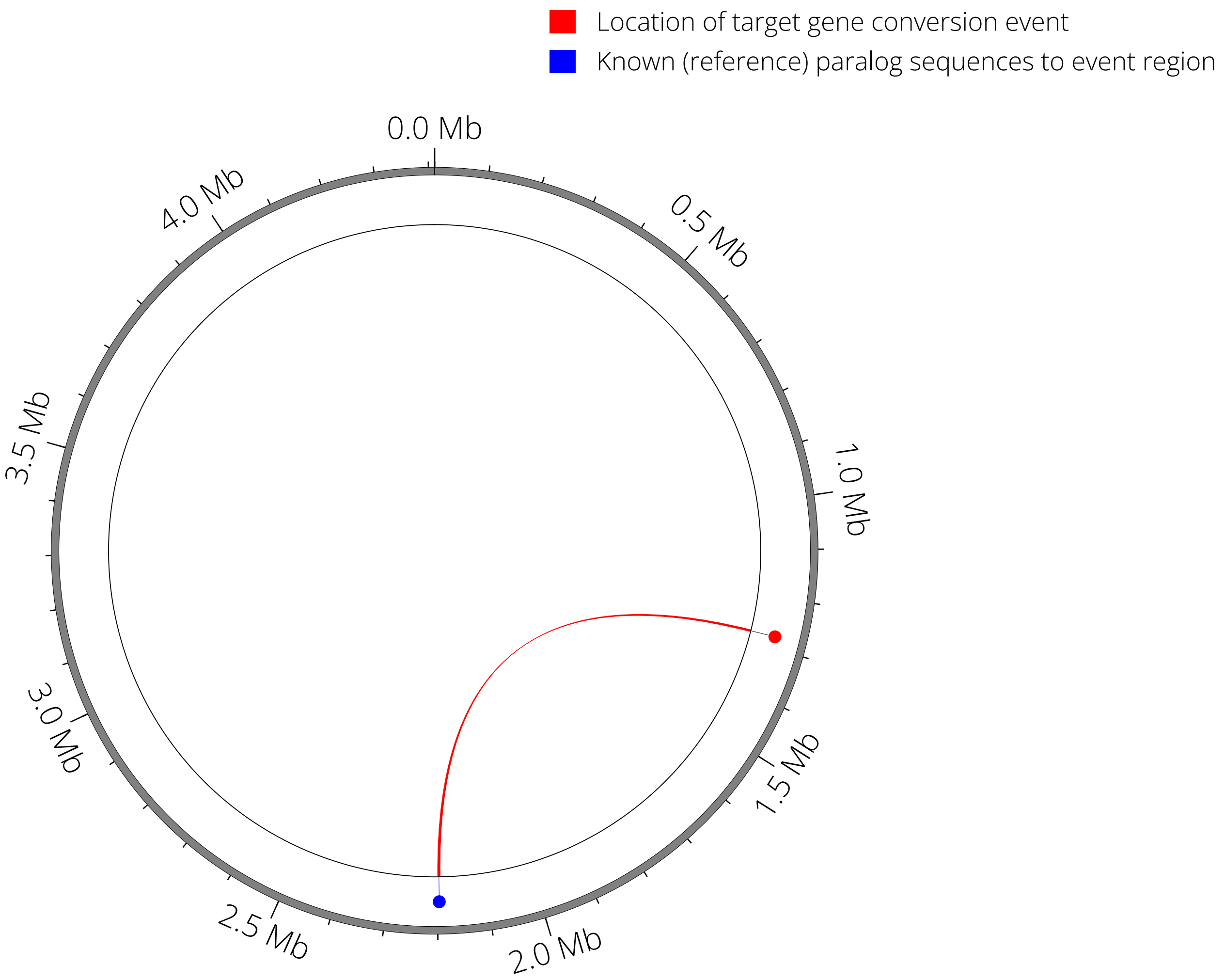

# Event_090 - 14 SNPs - k-mer analysis relative to paralogs

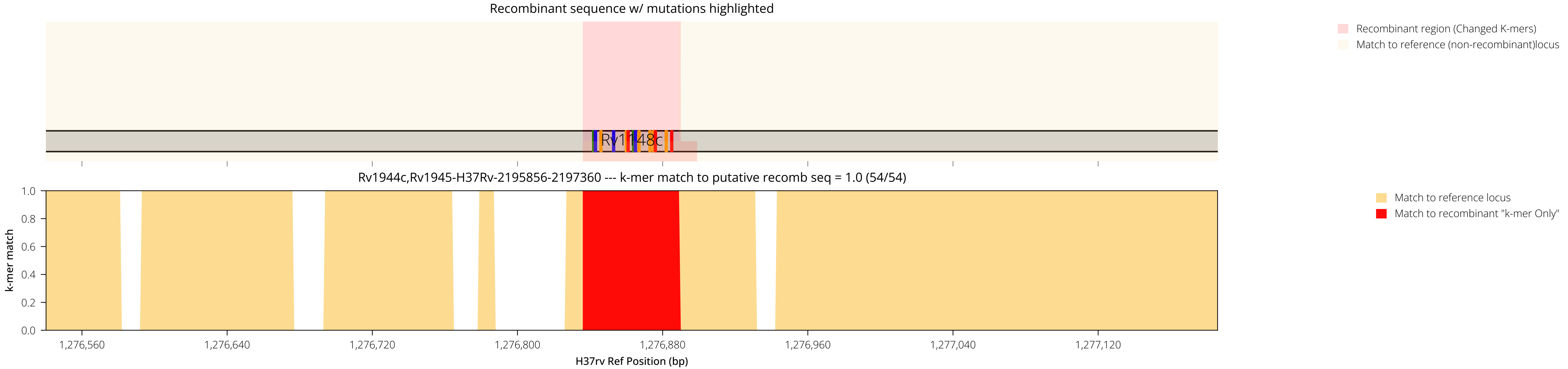

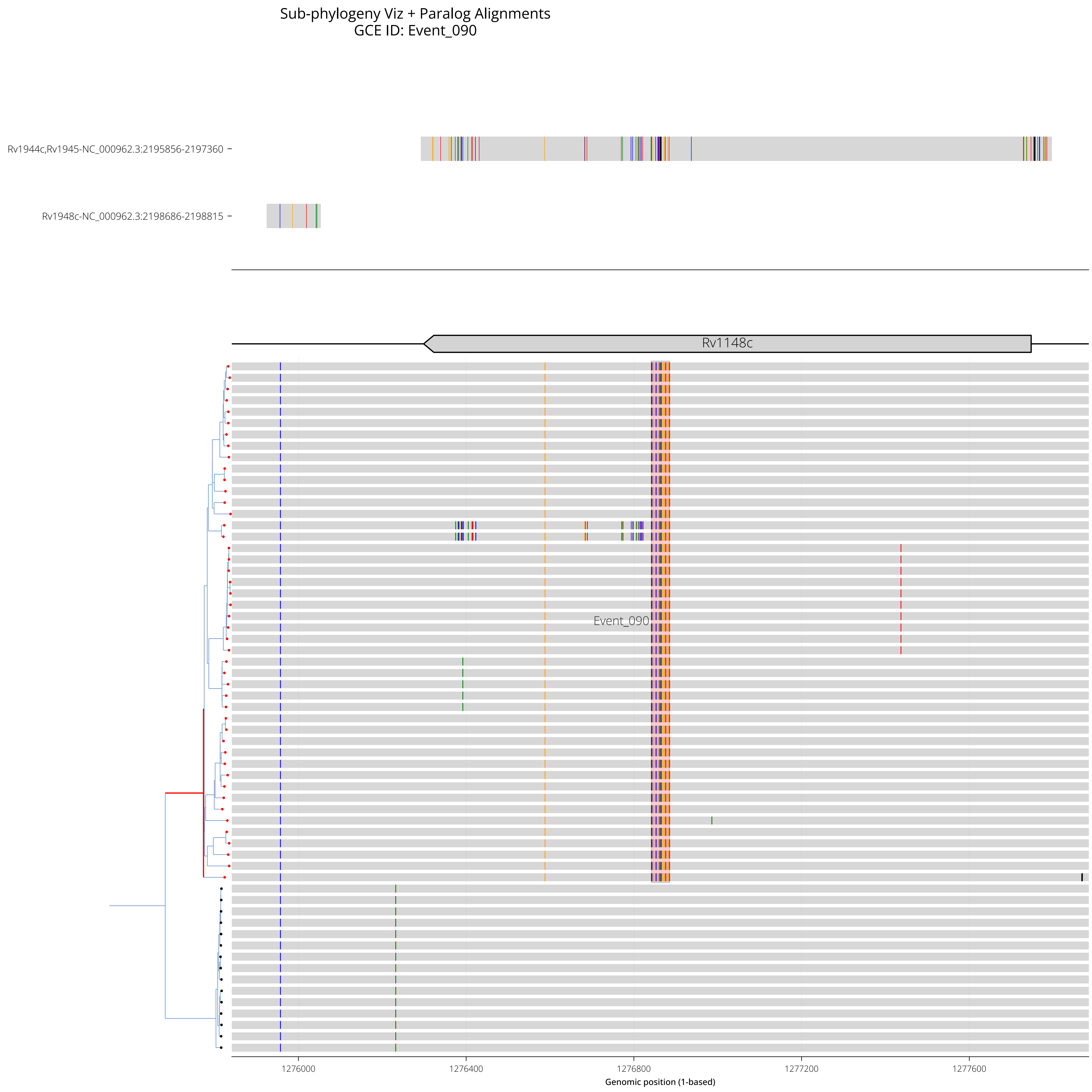

Sub-phylogeny Viz + Paralog Alignments
GCE ID: Event_090
Rv1944c,Rv1945-NC_000962.3:2195856-2197360
Rv1948c-NC_000962.3:2198686-2198815
Rv1148c
Event_090
1276000
1276400
1276800
1277200
1277600
Genomic position (1-based)

Full Phylogeny Viz + Paralog Alignments
GCE ID: Event_090

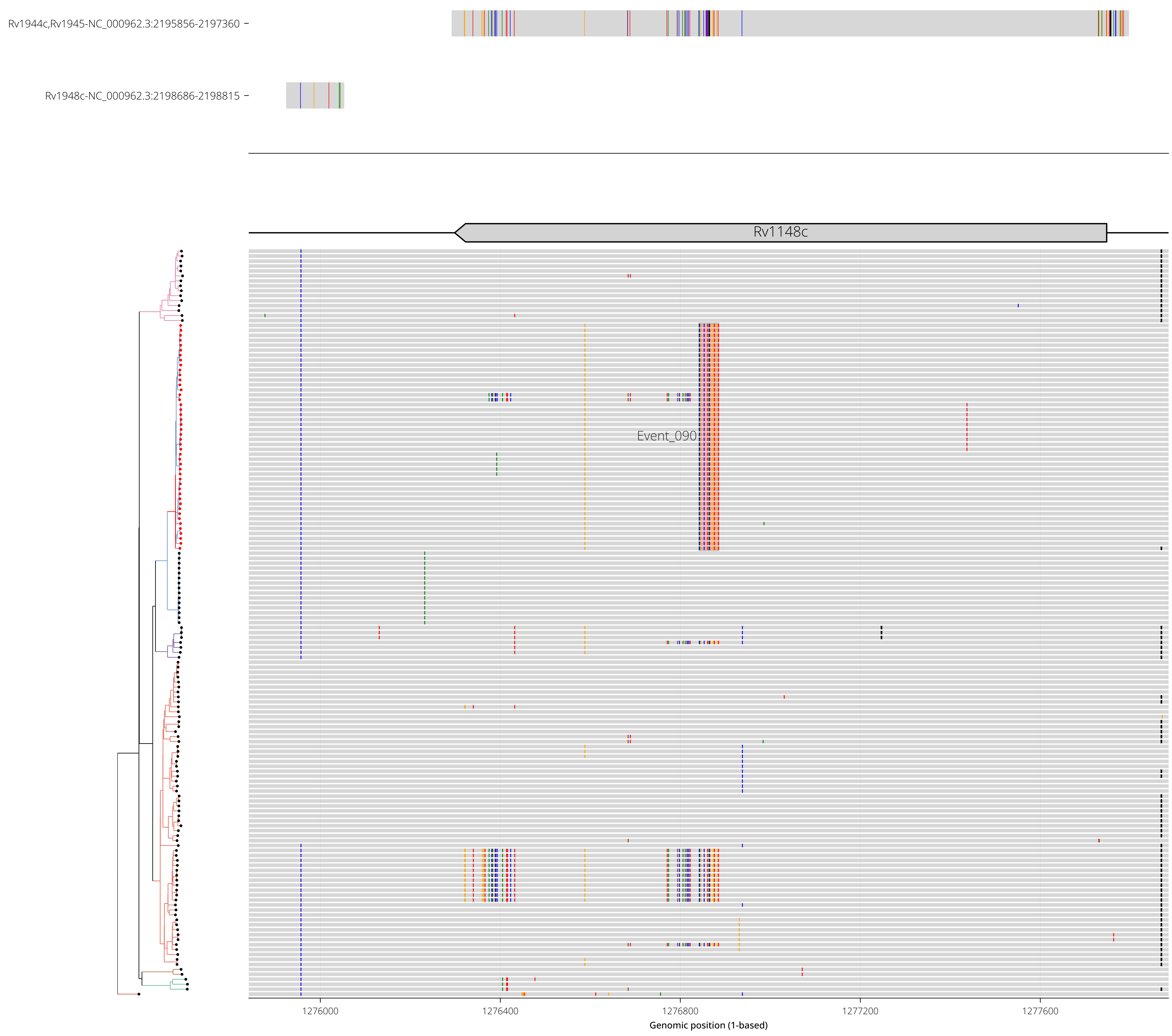

Individual GCE + Known Paralogs
GCE ID: Event_091

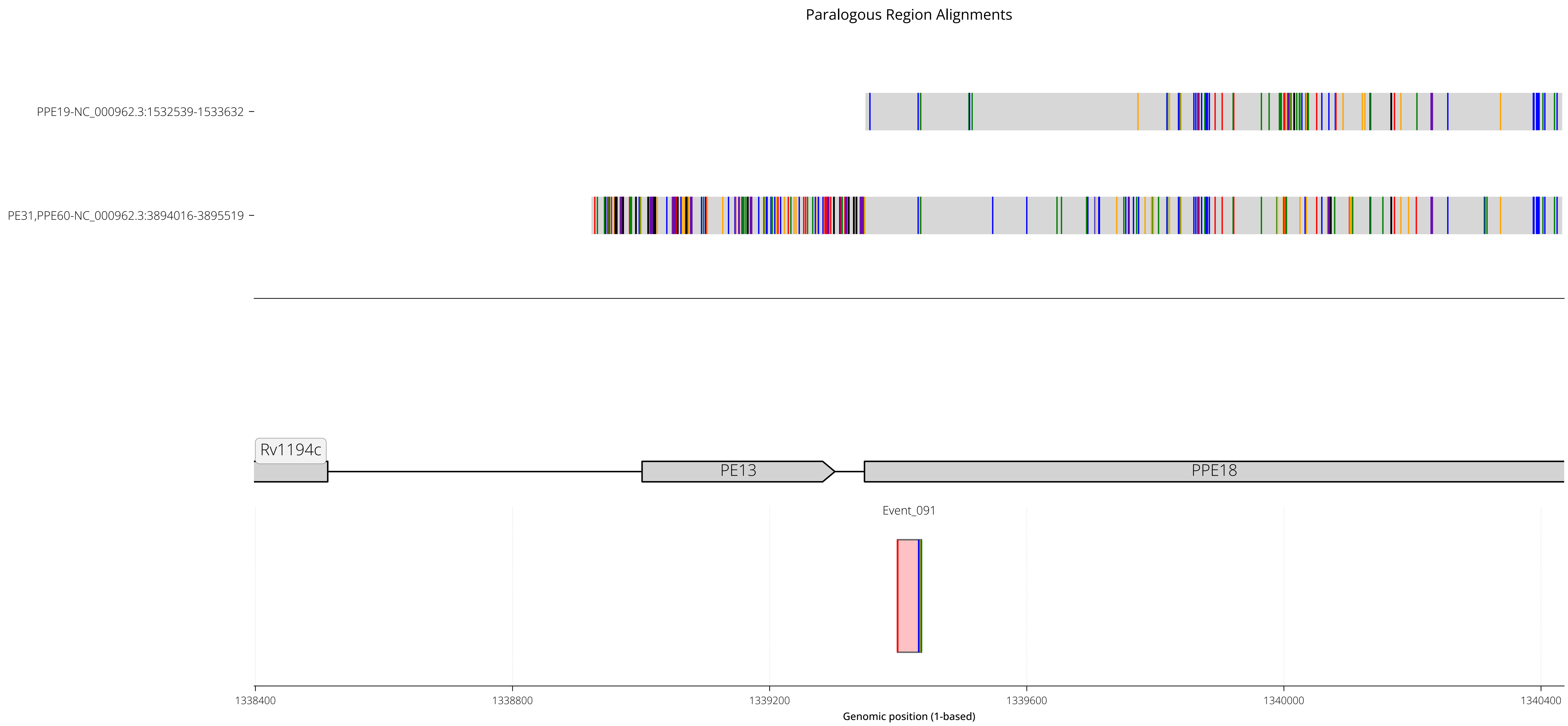

Circos Viz - GCE to Known Paralogs Locations
GCE ID: Event_091

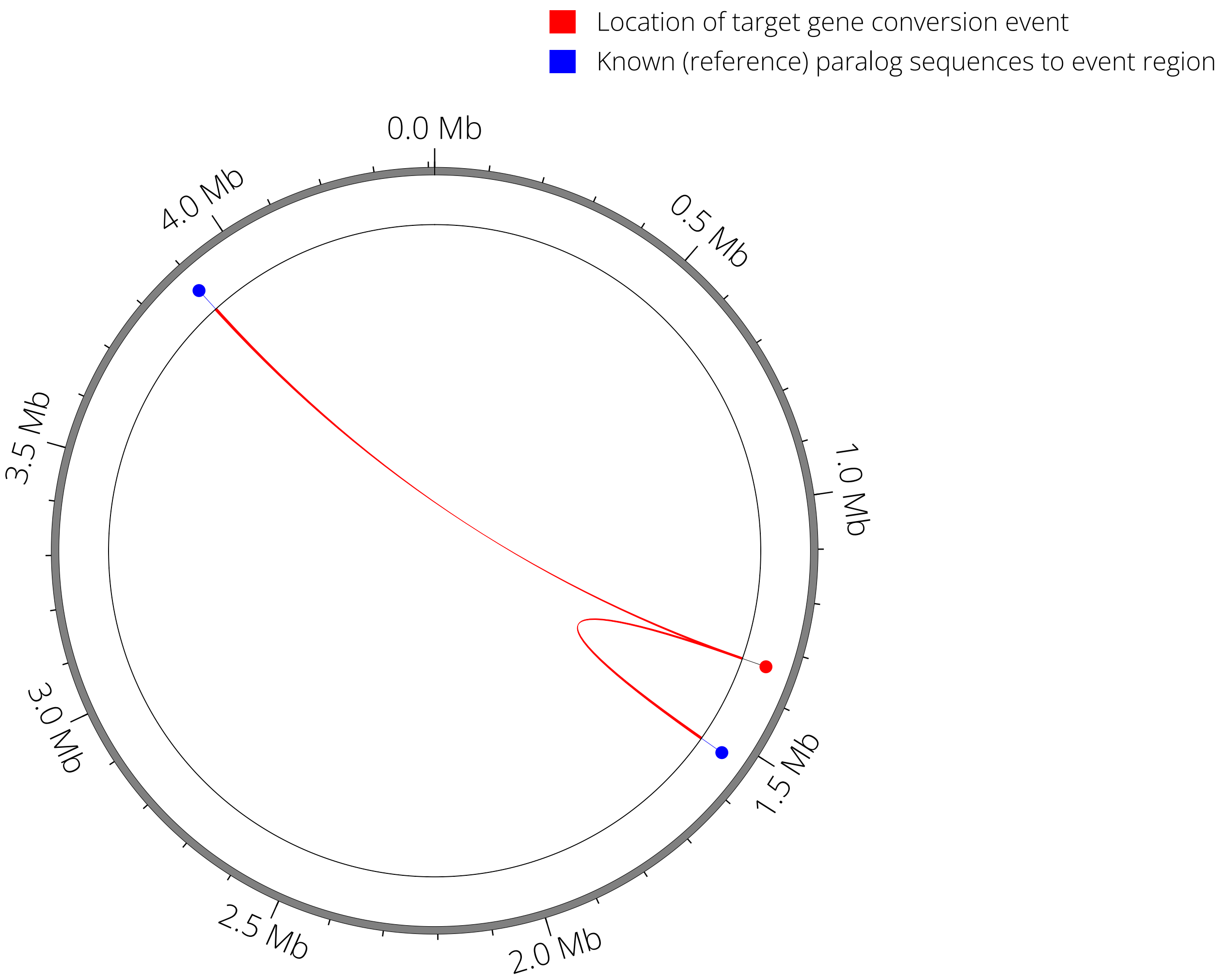

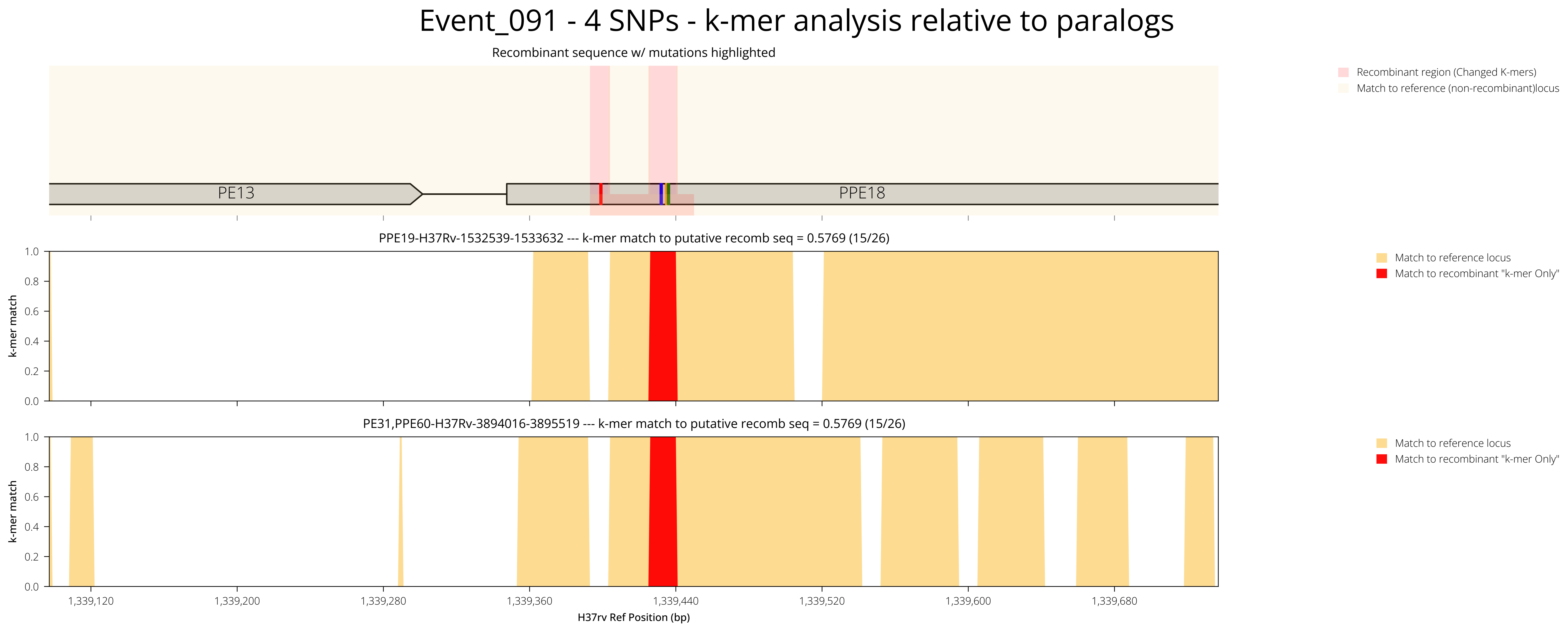

Event_091 - 4 SNPs - k-mer analysis relative to paralogs
Recombinant sequence w/ mutations highlighted
PE13
PPE18
Recombinant region (Changed K-mers)
Match to reference (non-recombinant)locus
PPE19-H37Rv-1532539-1533632 --- k-mer match to putative recomb seq = 0.5769 (15/26)
Match to reference locus
Match to recombinant "k-mer Only"
k-mer match
1.0
0.8
0.6
0.4
0.2
0.0
PE31,PPE60-H37Rv-3894016-3895519 --- k-mer match to putative recomb seq = 0.5769 (15/26)
Match to reference locus
Match to recombinant "k-mer Only"
k-mer match
1,339,120
1,339,200
1,339,280
1,339,360
1,339,440
1,339,520
1,339,600
1,339,680
H37rv Ref Position (bp)

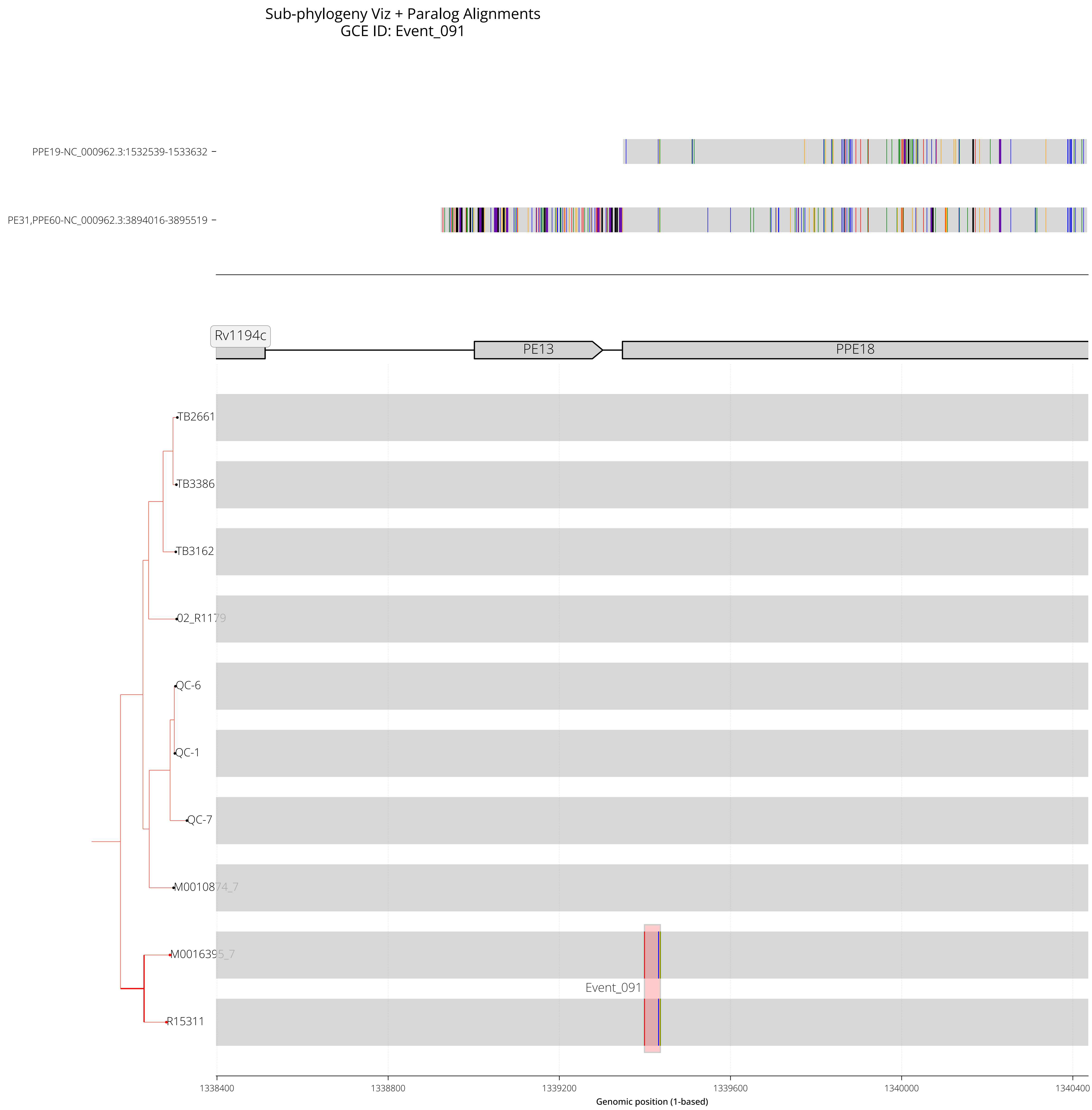

Sub-phylogeny Viz + Paralog Alignments
GCE ID: Event_091
PPE19-NC_000962.3:1532539-1533632
PE31,PPE60-NC_000962.3:3894016-3895519
Rv1194c
PE13
PPE18
TB2661
TB3386
TB3162
02_R1179
QC-6
QC-1
QC-7
M0010874_7
M0016395_7
R15311
Event_091
1338400
1338800
1339200
1339600
1340000
1340400
Genomic position (1-based)

Full Phylogeny Viz + Paralog Alignments
GCE ID: Event_091

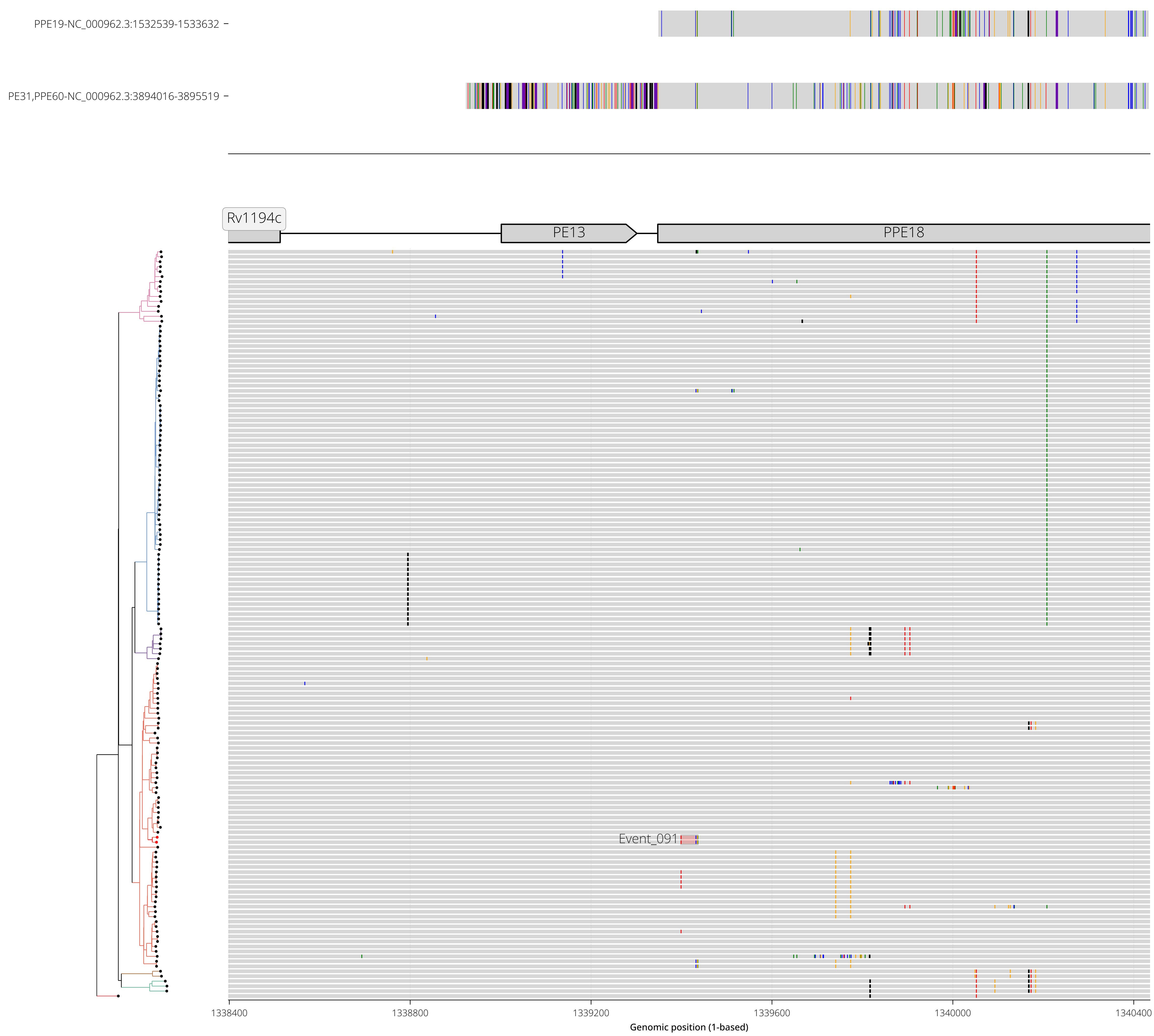

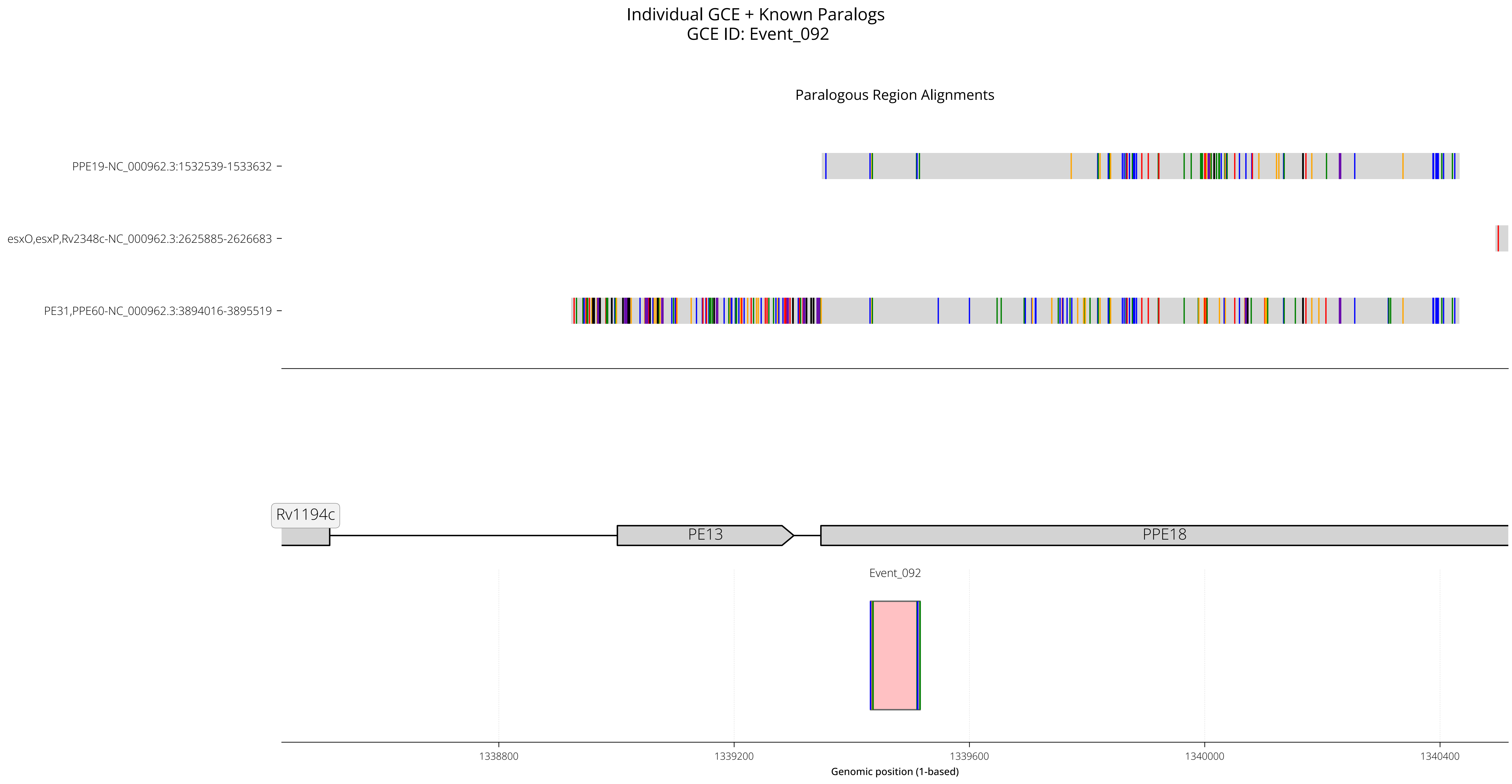
Individual GCE + Known Paralogs
GCE ID: Event_092
Paralogous Region Alignments
PPE19-NC_000962.3:1532539-1533632
esxO,esxP,Rv2348c-NC_000962.3:2625885-2626683
PE31,PPE60-NC_000962.3:3894016-3895519
Rv1194c
PE13
PPE18
Event_092
1338800
1339200
1339600
1340000
1340400
Genomic position (1-based)

Circos Viz - GCE to Known Paralogs Locations
GCE ID: Event_092

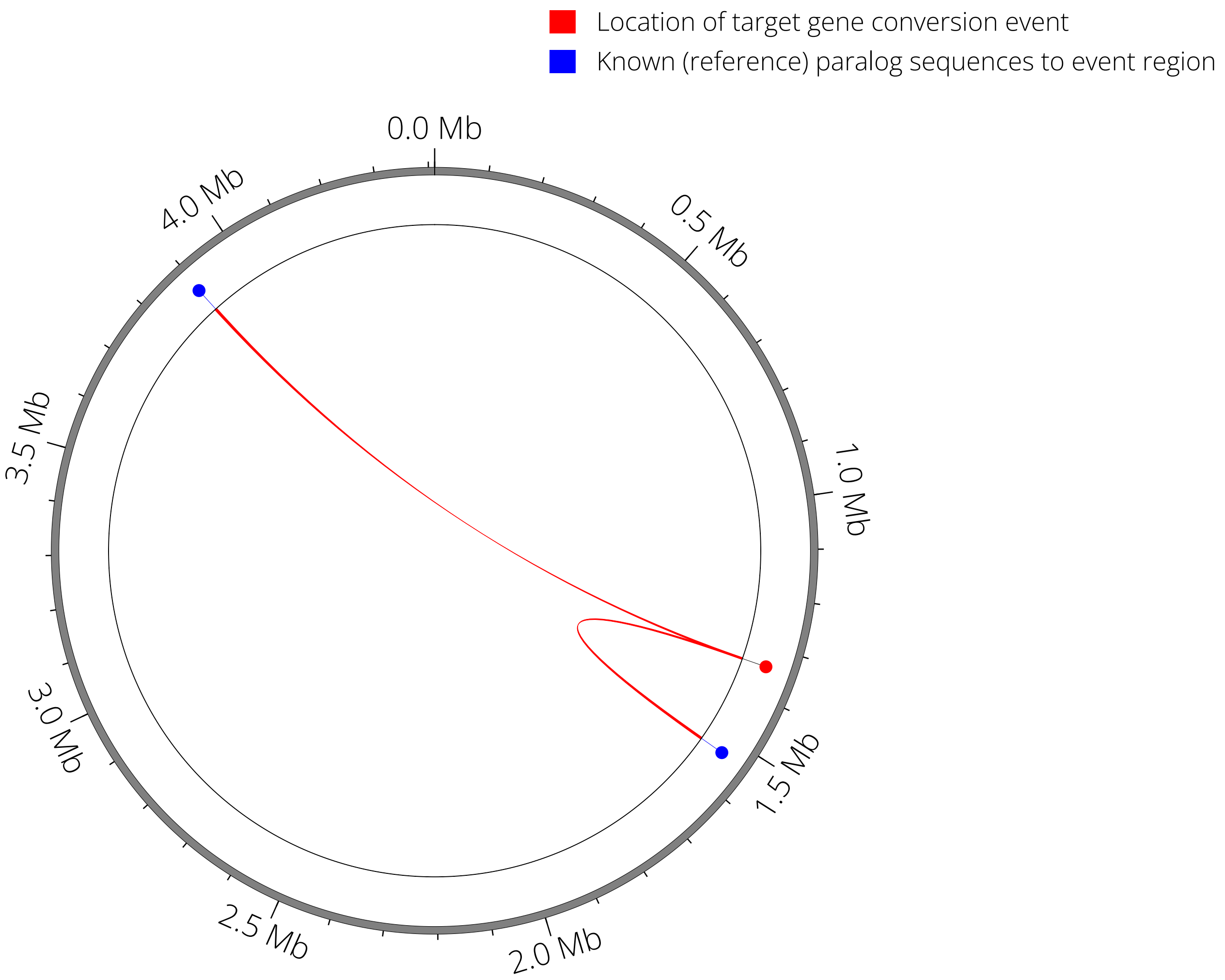

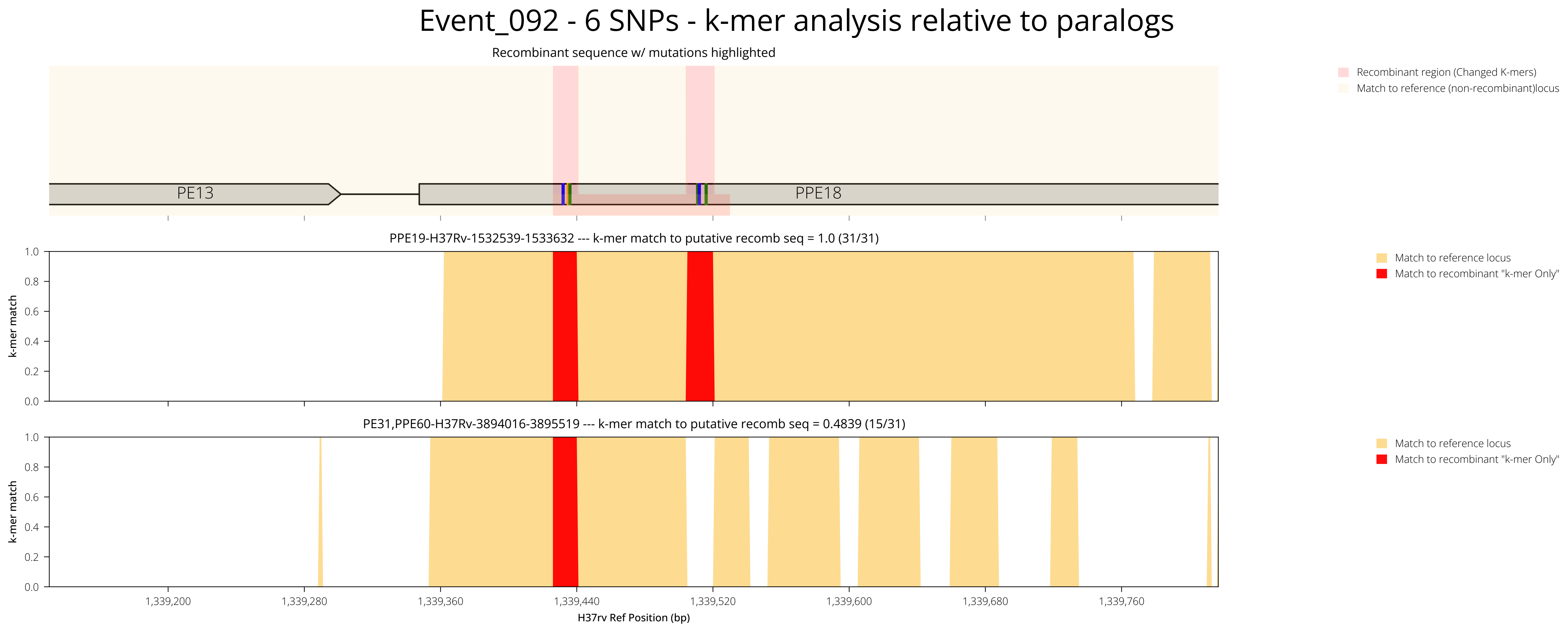
Event_092 - 6 SNPs - k-mer analysis relative to paralogs
Recombinant sequence w/ mutations highlighted
Recombinant region (Changed K-mers)
Match to reference (non-recombinant)locus
PE13
PPE18
PPE19-H37Rv-1532539-1533632 --- k-mer match to putative recomb seq = 1.0 (31/31)
Match to reference locus
Match to recombinant "k-mer Only"
k-mer match
1.0
0.8
0.6
0.4
0.2
0.0
PE31,PPE60-H37Rv-3894016-3895519 --- k-mer match to putative recomb seq = 0.4839 (15/31)
Match to reference locus
Match to recombinant "k-mer Only"
k-mer match
1.0
0.8
0.6
0.4
0.2
0.0
1,339,200
1,339,280
1,339,360
1,339,440
1,339,520
1,339,600
1,339,680
1,339,760
H37rv Ref Position (bp)

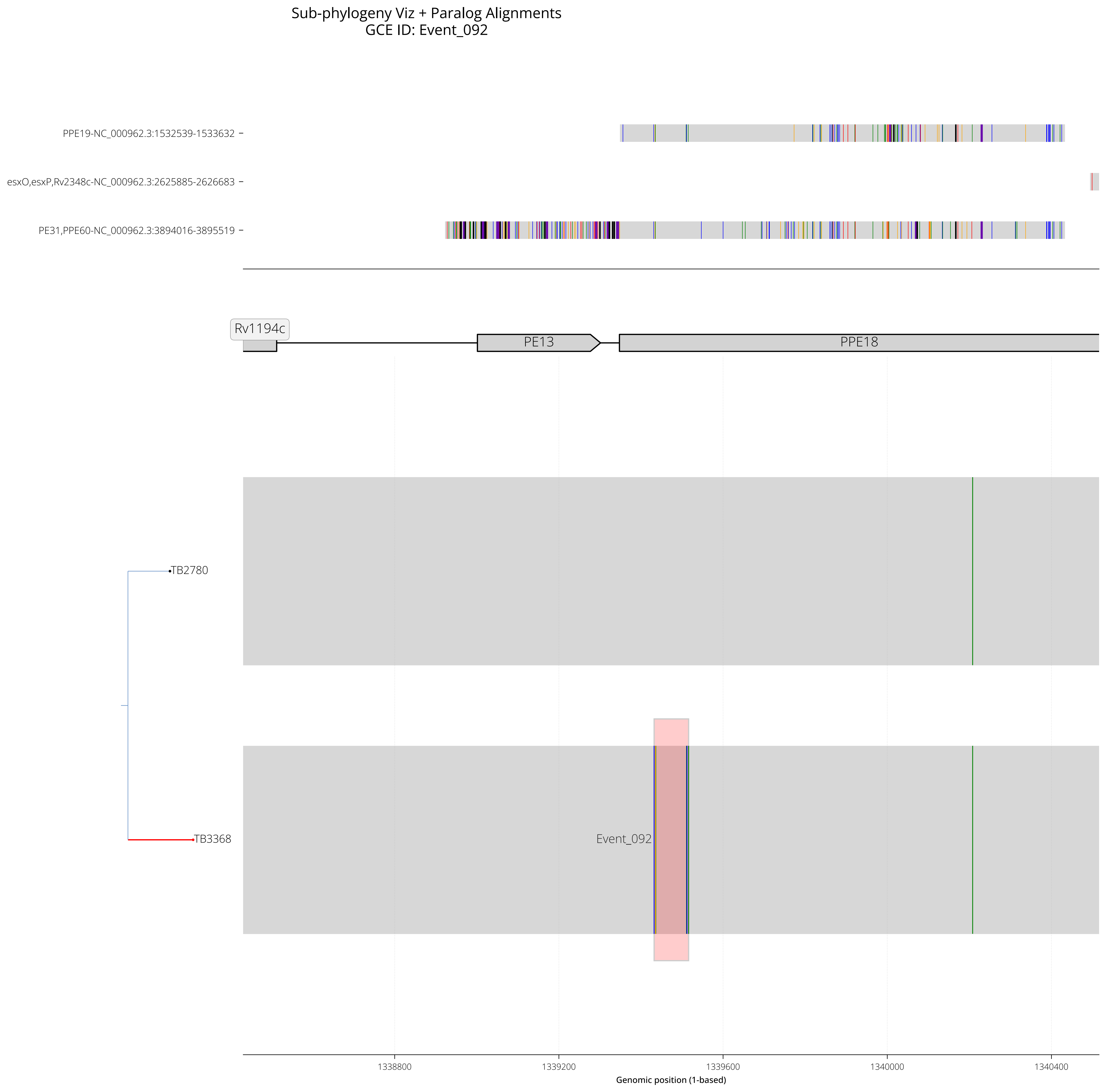

Sub-phylogeny Viz + Paralog Alignments
GCE ID: Event_092
PPE19-NC_000962.3:1532539-1533632
esxO,esxP,Rv2348c-NC_000962.3:2625885-2626683
PE31,PPE60-NC_000962.3:3894016-3895519
Rv1194c
PE13
PPE18
TB2780
TB3368
Event_092
1338800
1339200
1339600
1340000
1340400
Genomic position (1-based)

Full Phylogeny Viz + Paralog Alignments
GCE ID: Event_092

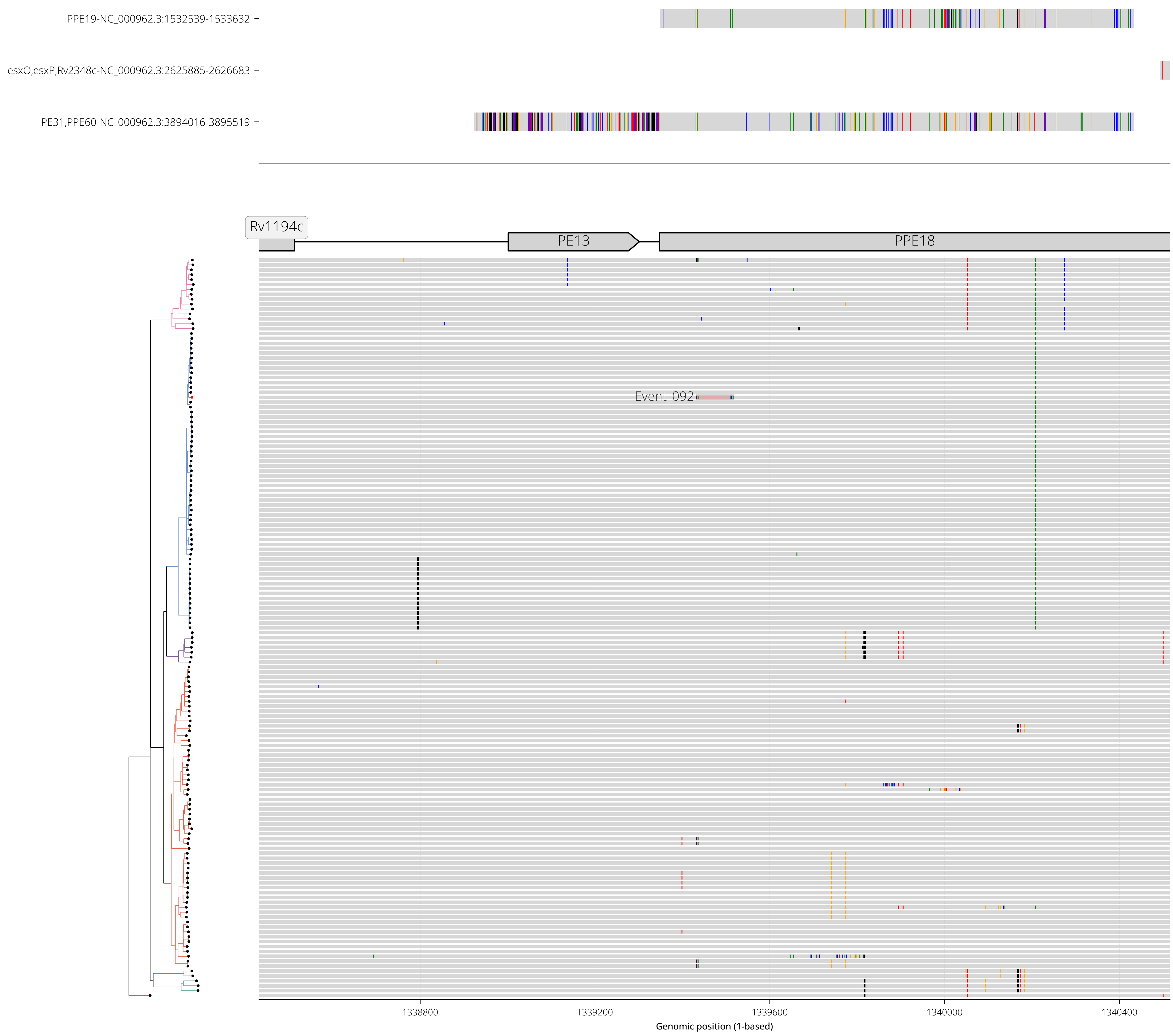

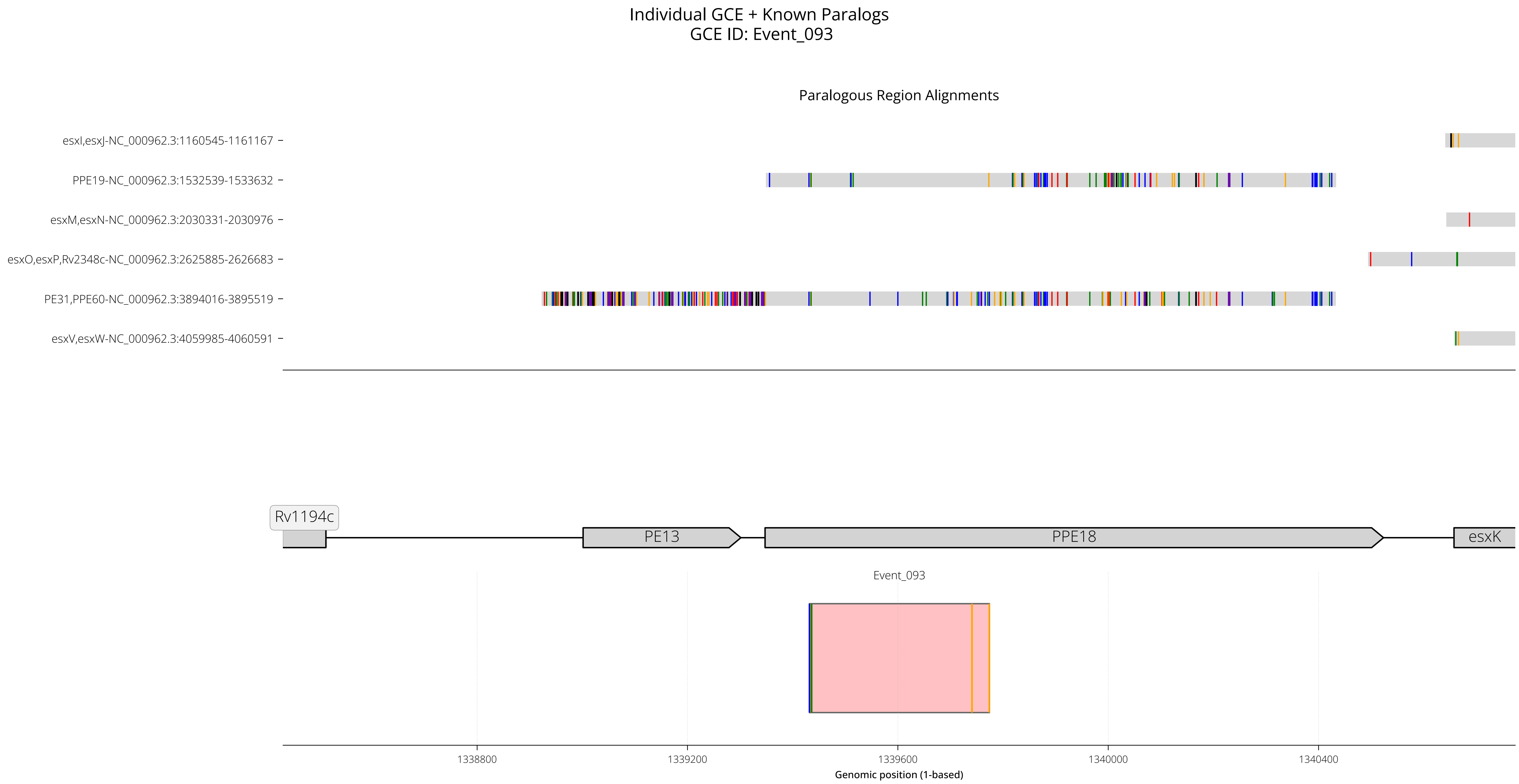

Individual GCE + Known Paralogs
GCE ID: Event_093
Paralogous Region Alignments
esxI,esxJ-NC_000962.3:1160545-1161167
PPE19-NC_000962.3:1532539-1533632
esxM,esxN-NC_000962.3:2030331-2030976
esxO,esxP,Rv2348c-NC_000962.3:2625885-2626683
PE31,PPE60-NC_000962.3:3894016-3895519
esxV,esxW-NC_000962.3:4059985-4060591
Rv1194c
PE13
PPE18
esxK
Event_093
1338800
1339200
1339600
1340000
1340400
Genomic position (1-based)

Circos Viz - GCE to Known Paralogs Locations
GCE ID: Event_093

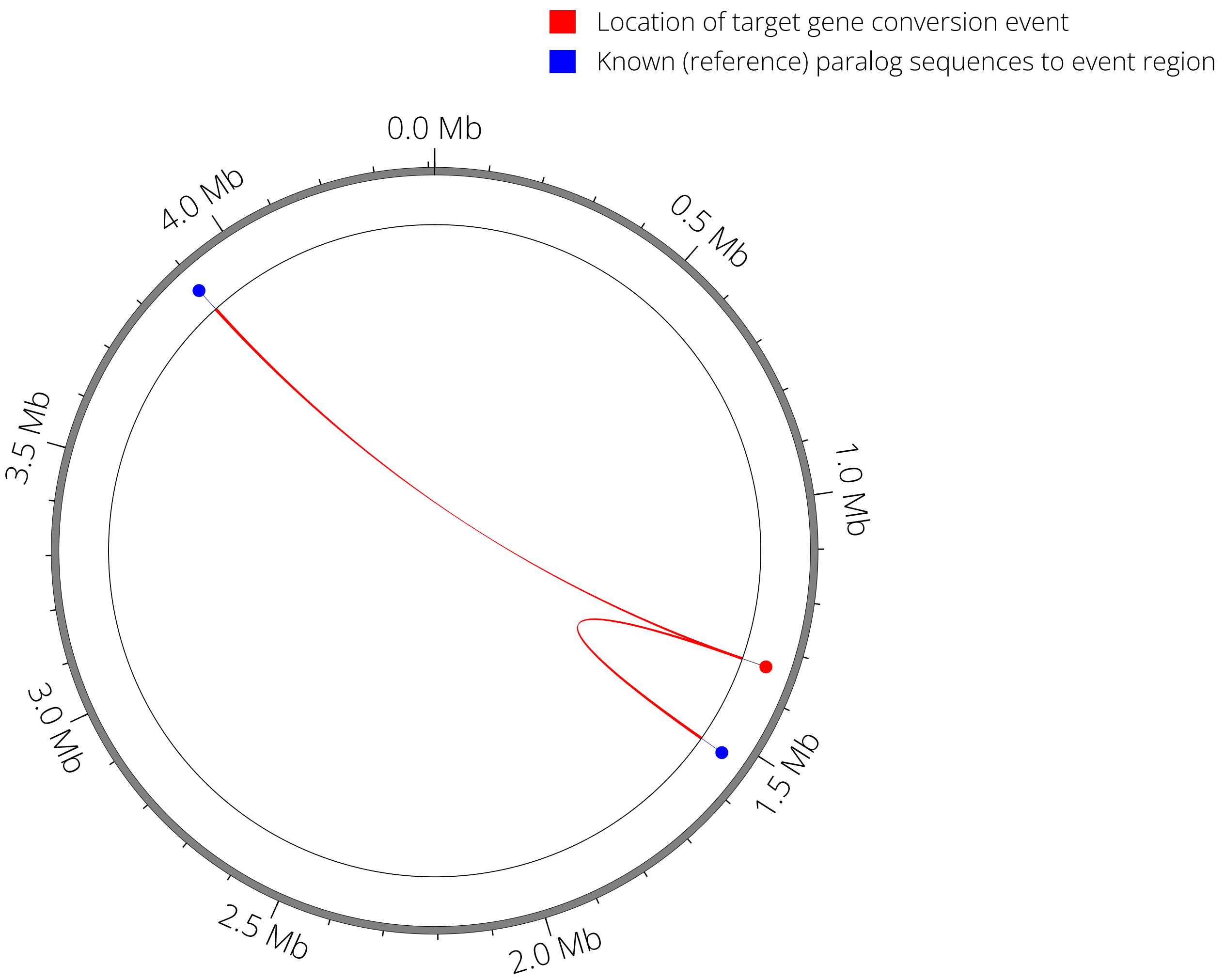

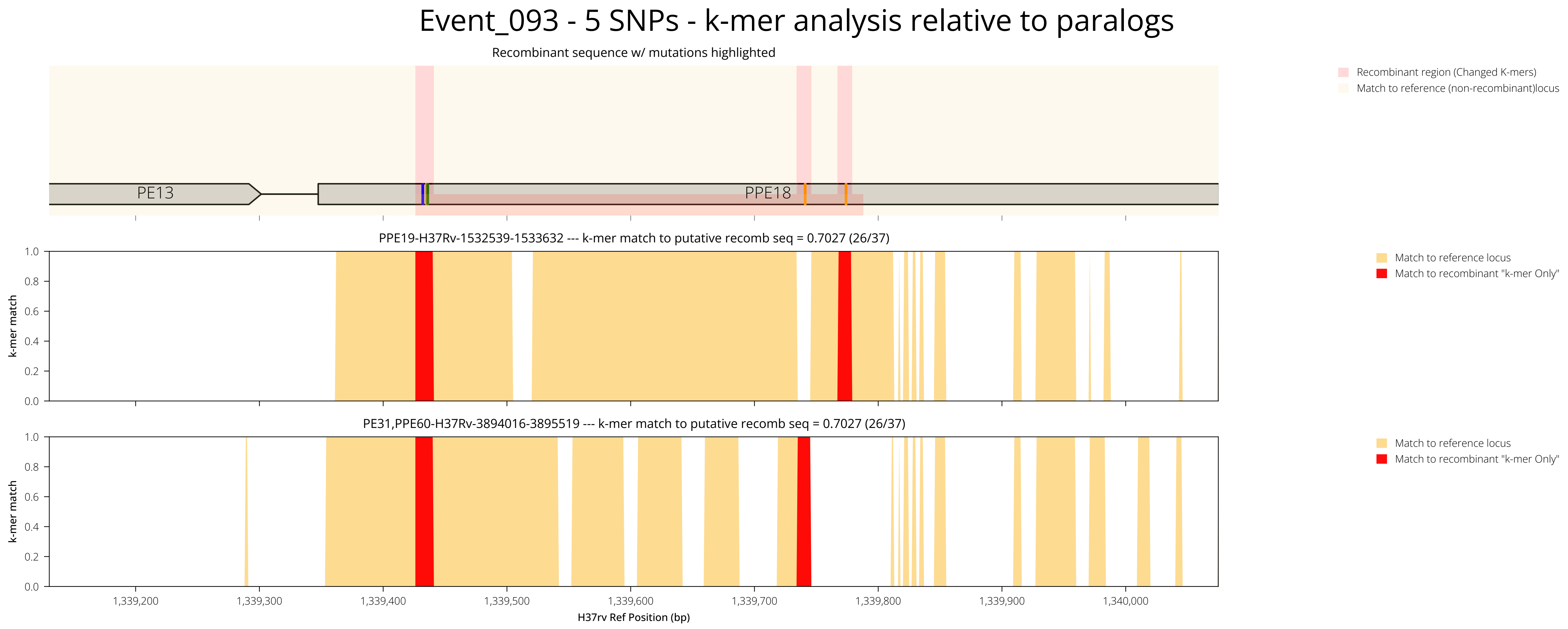
Event_093 - 5 SNPs - k-mer analysis relative to paralogs
Recombinant sequence w/ mutations highlighted
Recombinant region (Changed K-mers)
Match to reference (non-recombinant)locus
PE13
PPE18
PPE19-H37Rv-1532539-1533632 --- k-mer match to putative recomb seq = 0.7027 (26/37)
Match to reference locus
Match to recombinant "k-mer Only"
k-mer match
1.0
0.8
0.6
0.4
0.2
0.0
PE31,PPE60-H37Rv-3894016-3895519 --- k-mer match to putative recomb seq = 0.7027 (26/37)
Match to reference locus
Match to recombinant "k-mer Only"
k-mer match
1,339,200
1,339,300
1,339,400
1,339,500
1,339,600
1,339,700
1,339,800
1,339,900
1,340,000
H37rv Ref Position (bp)

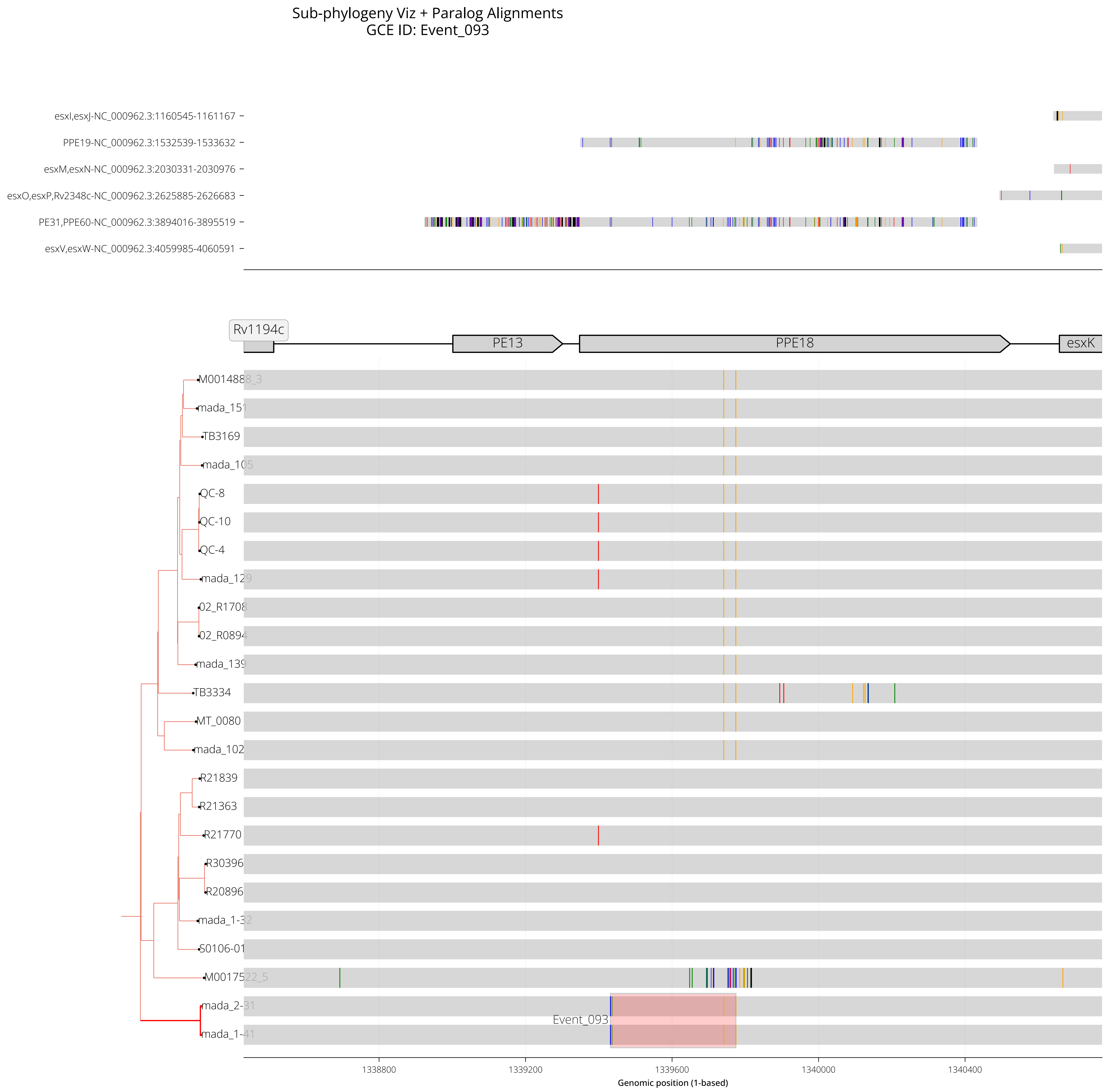

Sub-phylogeny Viz + Paralog Alignments
GCE ID: Event_093
esxI,esxJ-NC_000962.3:1160545-1161167
PPE19-NC_000962.3:1532539-1533632
esxM,esxN-NC_000962.3:2030331-2030976
esxO,esxP,Rv2348c-NC_000962.3:2625885-2626683
PE31,PPE60-NC_000962.3:3894016-3895519
esxV,esxW-NC_000962.3:4059985-4060591
Rv1194c
PE13
PPE18
esxK
M0014888_3
mada_151
TB3169
mada_105
QC-8
QC-10
QC-4
mada_129
02_R1708
02_R0894
mada_139
TB3334
MT_0080
mada_102
R21839
R21363
R21770
R30396
R20896
mada_1-32
S0106-01
M0017522_5
mada_2-31
mada_1-41
Event_093
1338800
1339200
1339600
1340000
1340400
Genomic position (1-based)

Full Phylogeny Viz + Paralog Alignments
GCE ID: Event_093

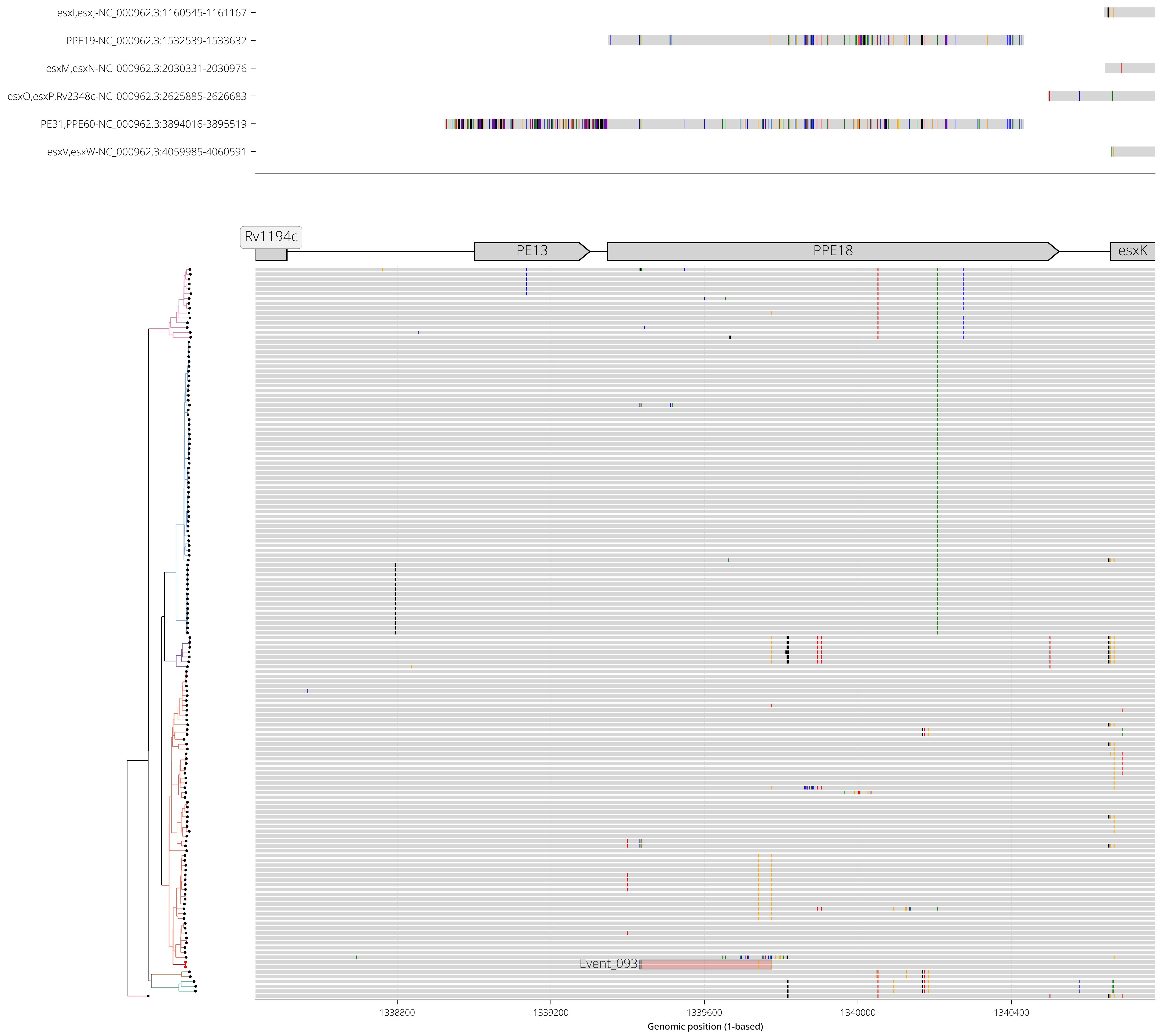

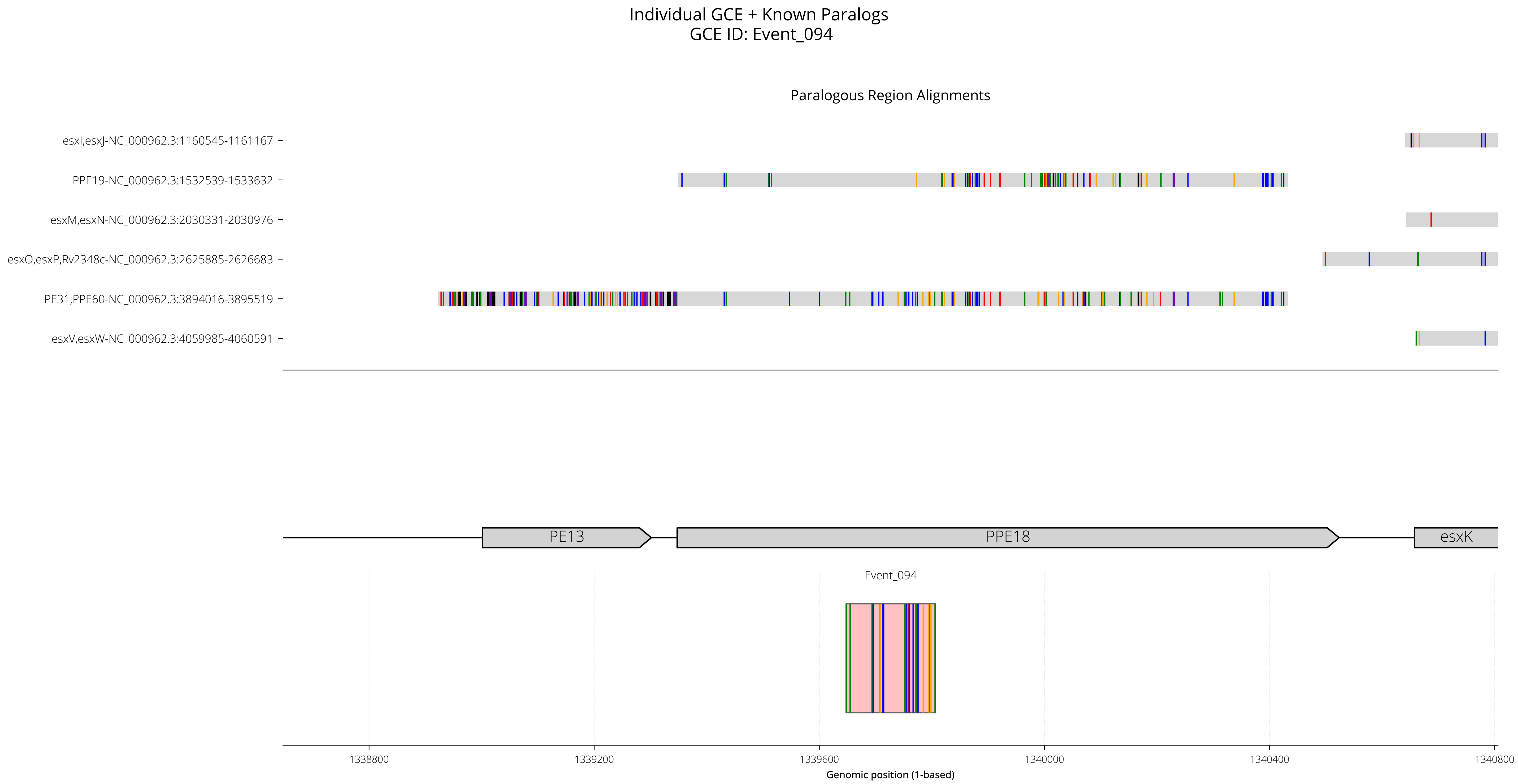

Individual GCE + Known Paralogs
GCE ID: Event_094
Paralogous Region Alignments
esxI,esxJ-NC_000962.3:1160545-1161167
PPE19-NC_000962.3:1532539-1533632
esxM,esxN-NC_000962.3:2030331-2030976
esxO,esxP,Rv2348c-NC_000962.3:2625885-2626683
PE31,PPE60-NC_000962.3:3894016-3895519
esxV,esxW-NC_000962.3:4059985-4060591
PE13
PPE18
esxK
Event_094
1338800
1339200
1339600
1340000
1340400
1340800
Genomic position (1-based)

Circos Viz - GCE to Known Paralogs Locations
GCE ID: Event_094

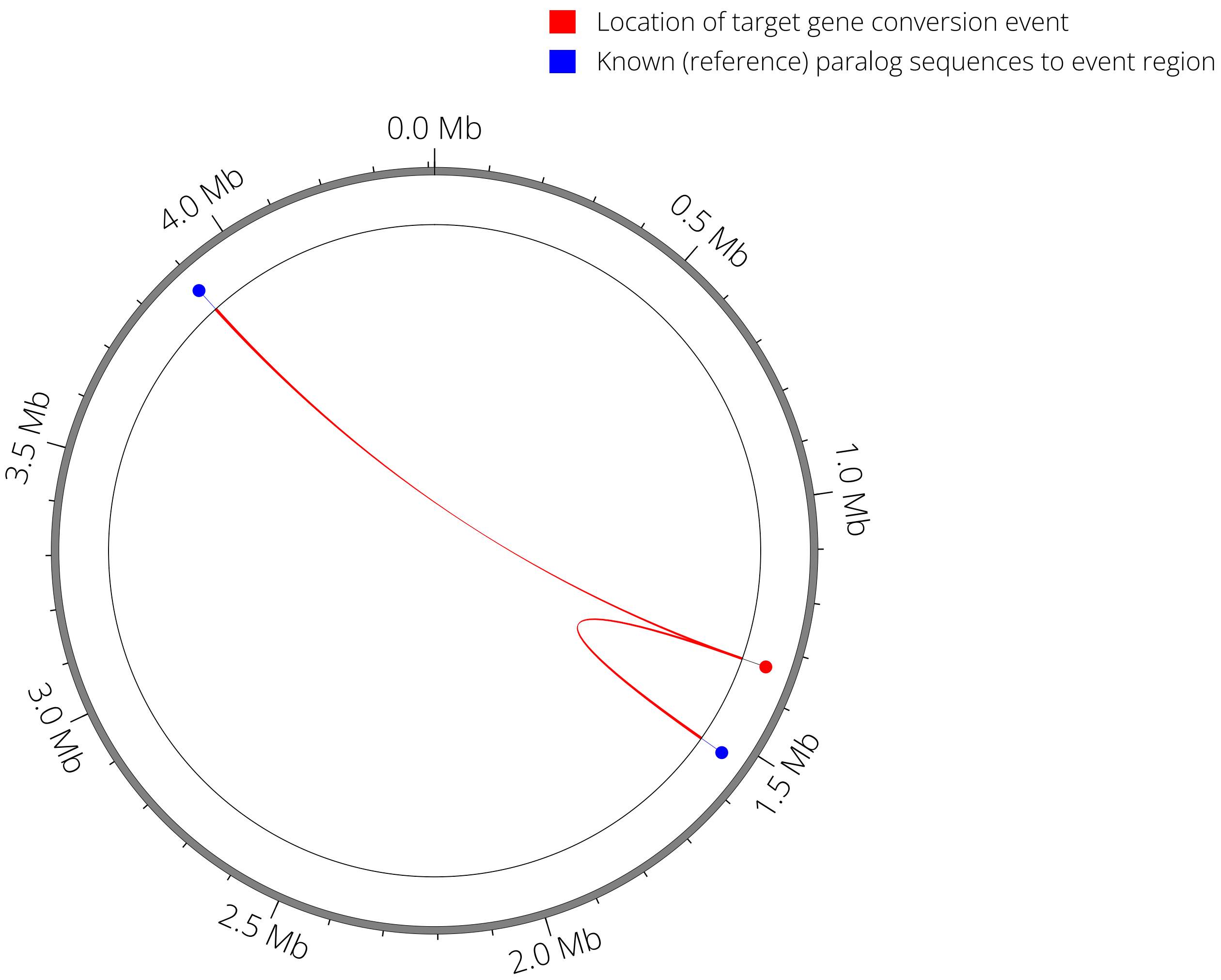

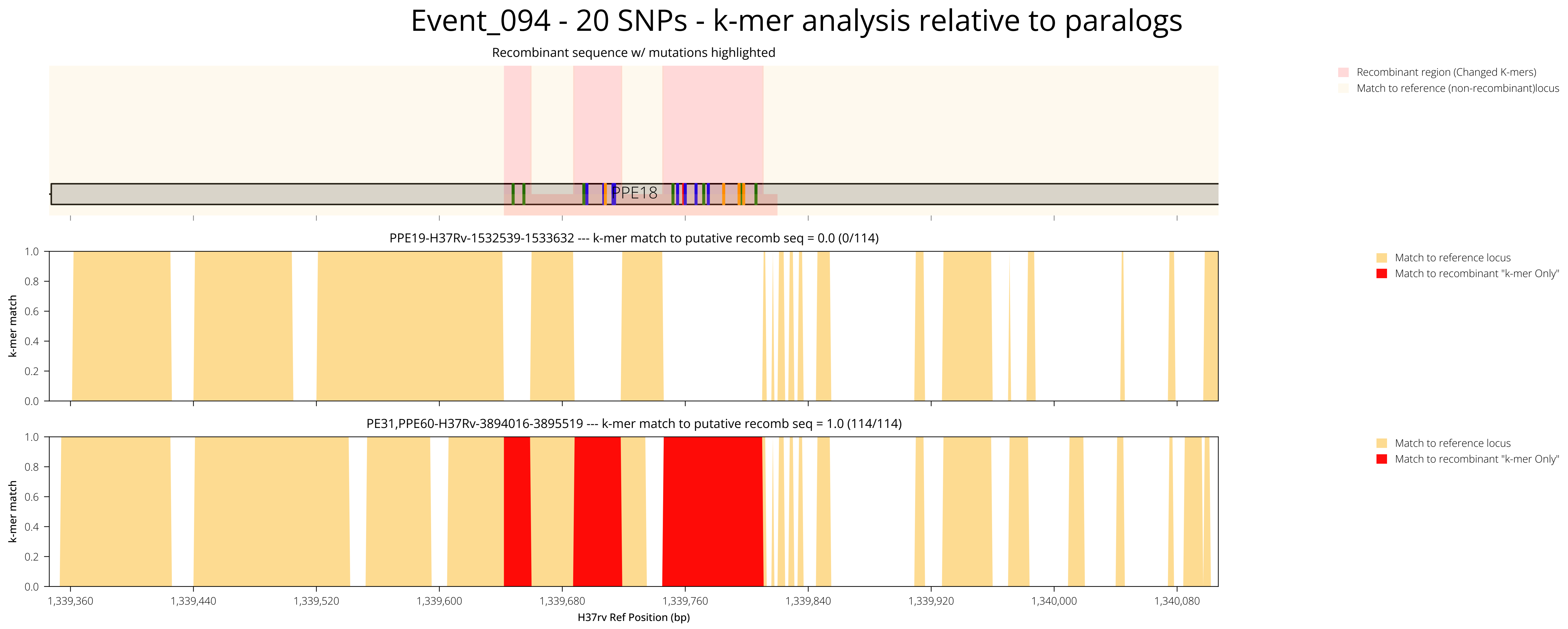

Event_094 - 20 SNPs - k-mer analysis relative to paralogs
Recombinant sequence w/ mutations highlighted
PPE18
Recombinant region (Changed K-mers)
Match to reference (non-recombinant)locus
PPE19-H37Rv-1532539-1533632 --- k-mer match to putative recomb seq = 0.0 (0/114)
Match to reference locus
Match to recombinant "k-mer Only"
PE31,PPE60-H37Rv-3894016-3895519 --- k-mer match to putative recomb seq = 1.0 (114/114)
Match to reference locus
Match to recombinant "k-mer Only"
k-mer match
1.0
0.8
0.6
0.4
0.2
0.0
1,339,360
1,339,440
1,339,520
1,339,600
1,339,680
1,339,760
1,339,840
1,339,920
1,340,000
1,340,080
H37rv Ref Position (bp)

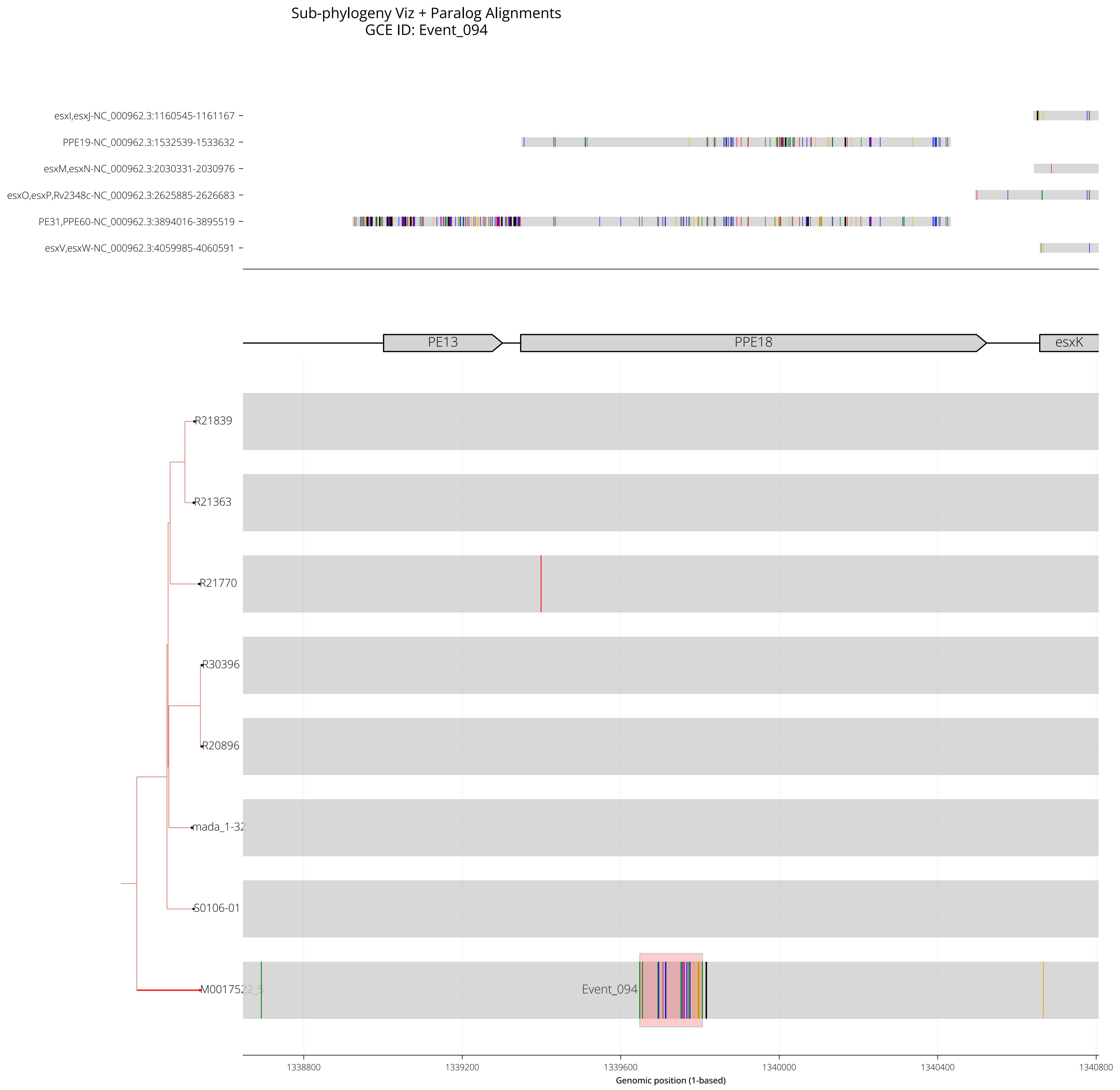

Sub-phylogeny Viz + Paralog Alignments
GCE ID: Event_094
esxI,esxJ-NC_000962.3:1160545-1161167
PPE19-NC_000962.3:1532539-1533632
esxM,esxN-NC_000962.3:2030331-2030976
esxO,esxP,Rv2348c-NC_000962.3:2625885-2626683
PE31,PPE60-NC_000962.3:3894016-3895519
esxV,esxW-NC_000962.3:4059985-4060591
PE13
PPE18
esxK
R21839
R21363
R21770
R30396
R20896
S0106-01
Event_094
1338800
1339200
1339600
1340000
1340400
1340800
Genomic position (1-based)

Full Phylogeny Viz + Paralog Alignments
GCE ID: Event_094

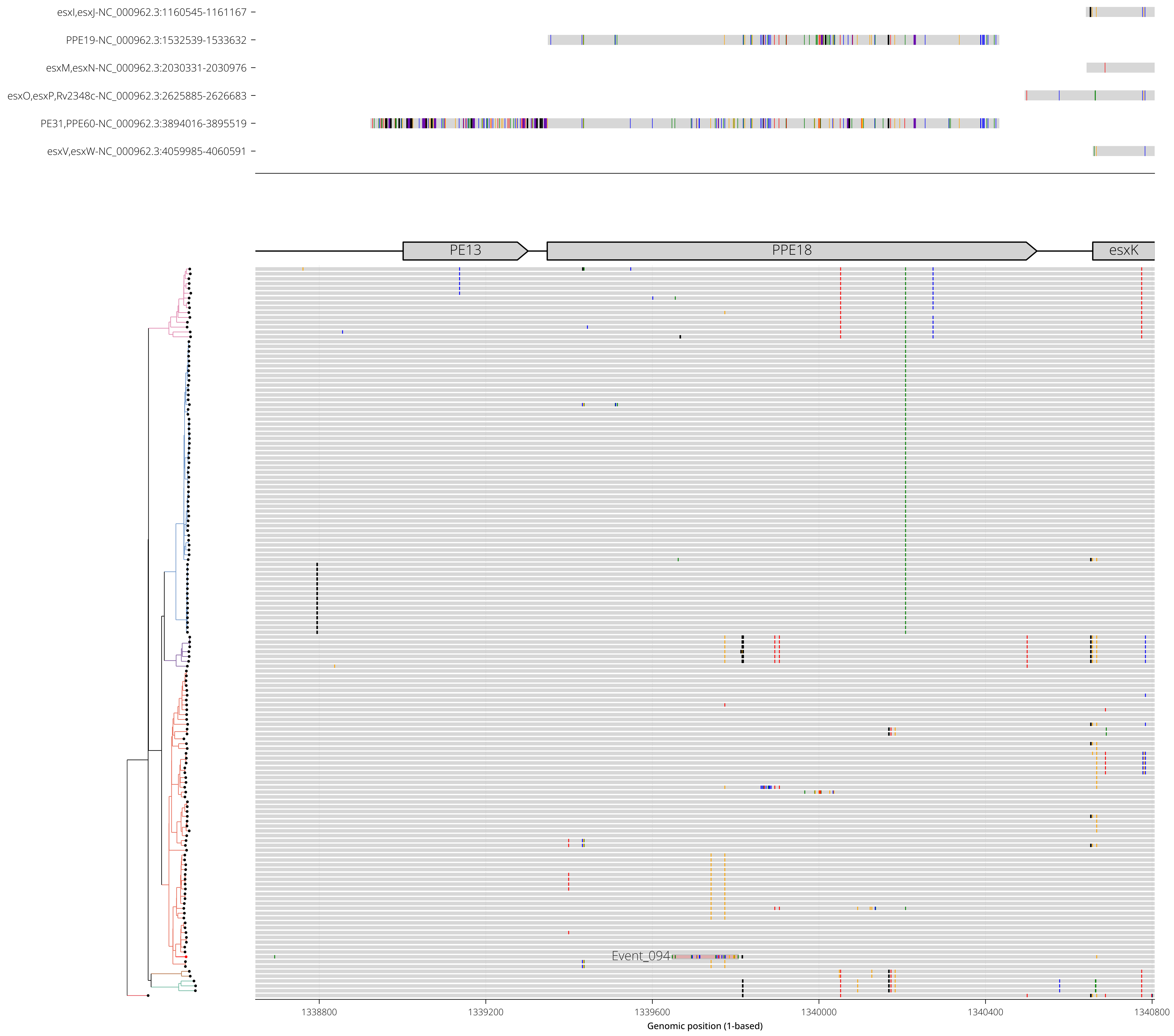

Individual GCE + Known Paralogs
GCE ID: Event_095

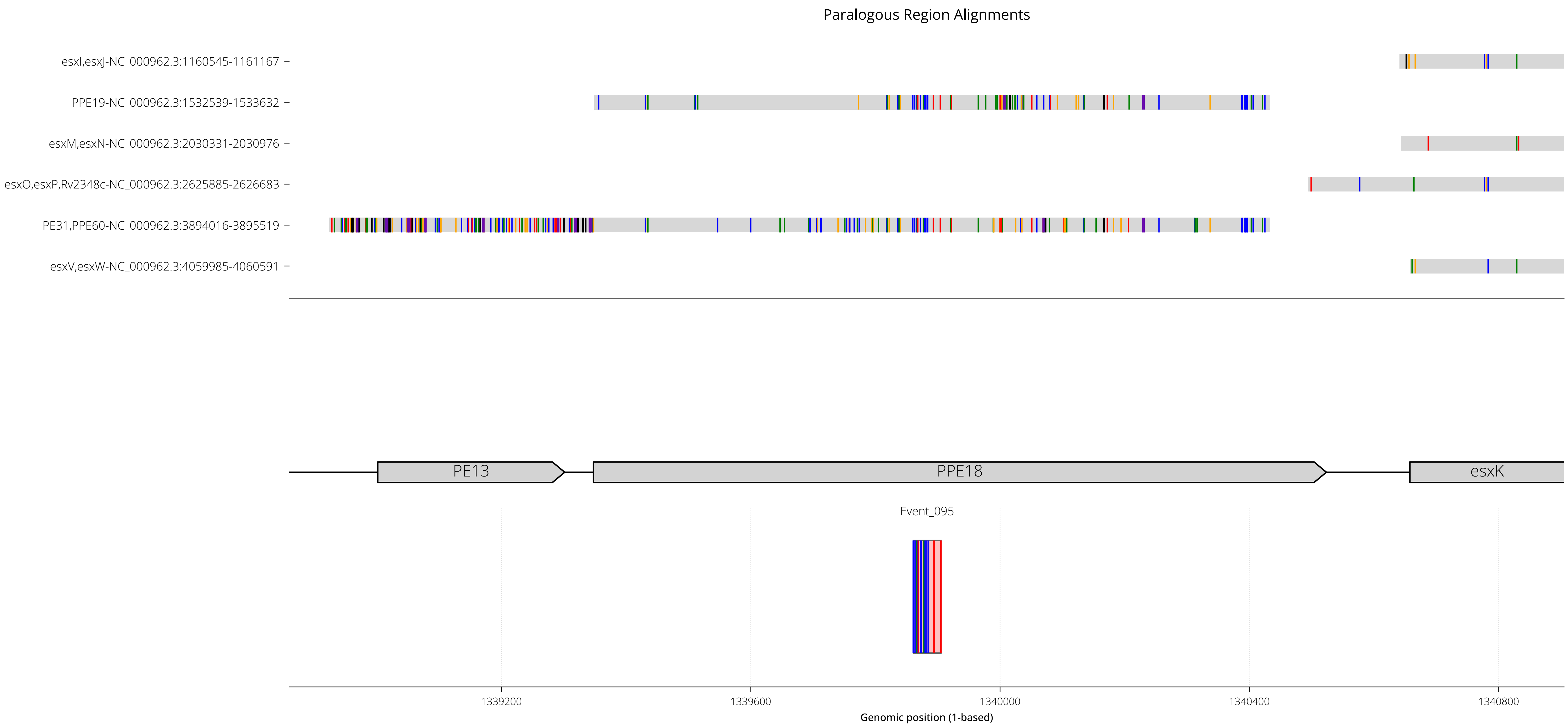

Circos Viz - GCE to Known Paralogs Locations
GCE ID: Event_095

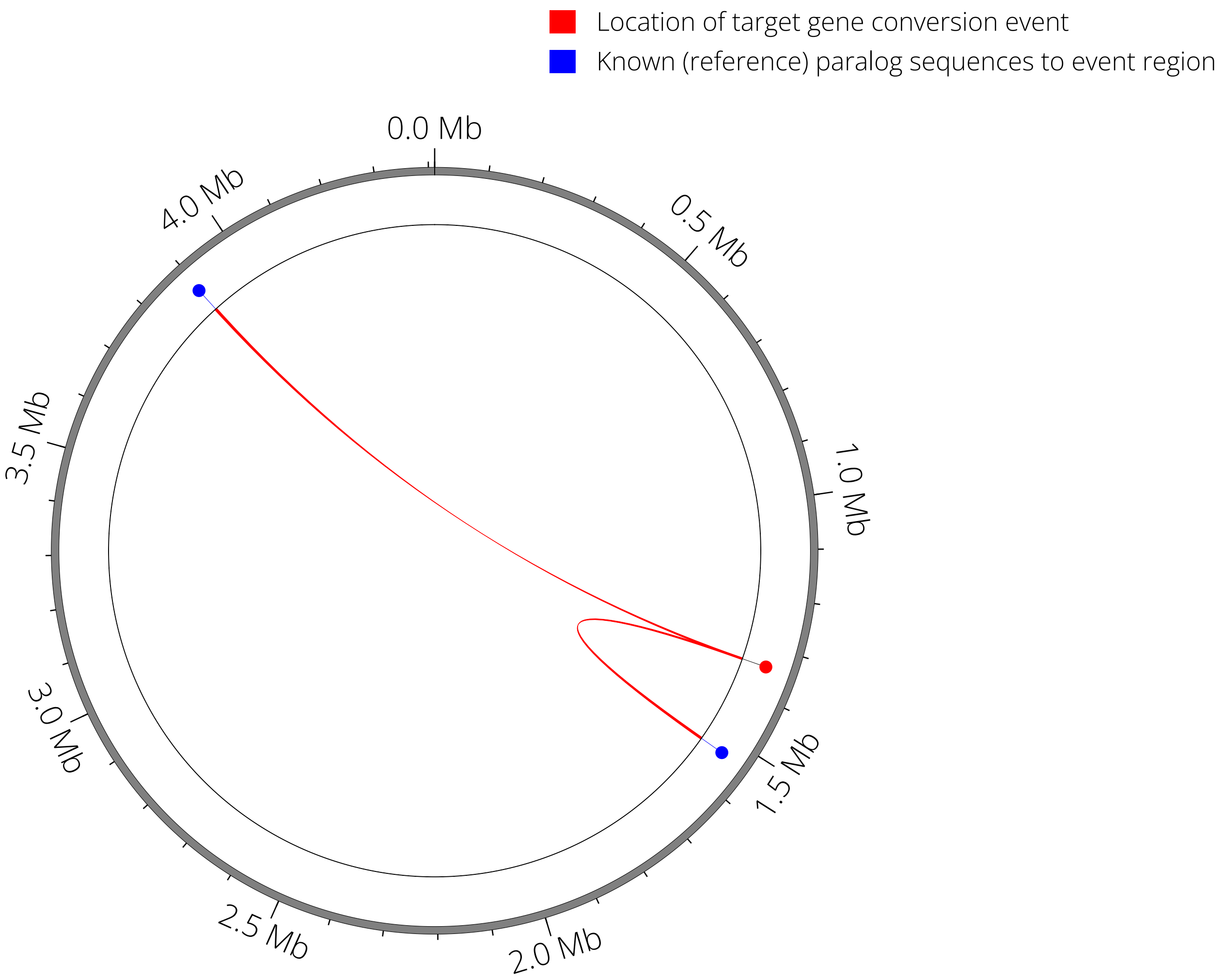

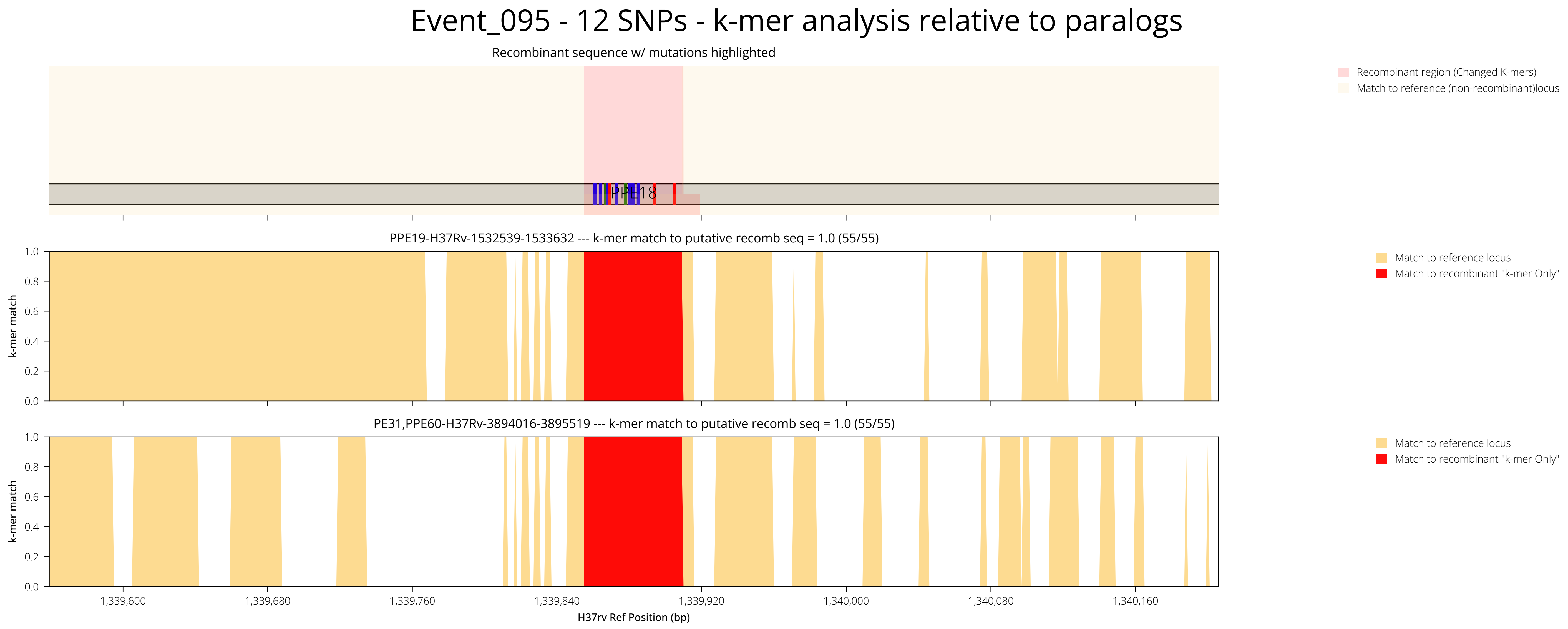
Event_095 - 12 SNPs - k-mer analysis relative to paralogs
Recombinant sequence w/ mutations highlighted
PPE18
Recombinant region (Changed K-mers)
Match to reference (non-recombinant)locus
PPE19-H37Rv-1532539-1533632 --- k-mer match to putative recomb seq = 1.0 (55/55)
k-mer match
1.0
0.8
0.6
0.4
0.2
0.0
Match to reference locus
Match to recombinant "k-mer Only"
PE31,PPE60-H37Rv-3894016-3895519 --- k-mer match to putative recomb seq = 1.0 (55/55)
k-mer match
1.0
0.8
0.6
0.4
0.2
0.0
Match to reference locus
Match to recombinant "k-mer Only"
1,339,600
1,339,680
1,339,760
1,339,840
1,339,920
1,340,000
1,340,080
1,340,160
H37rv Ref Position (bp)

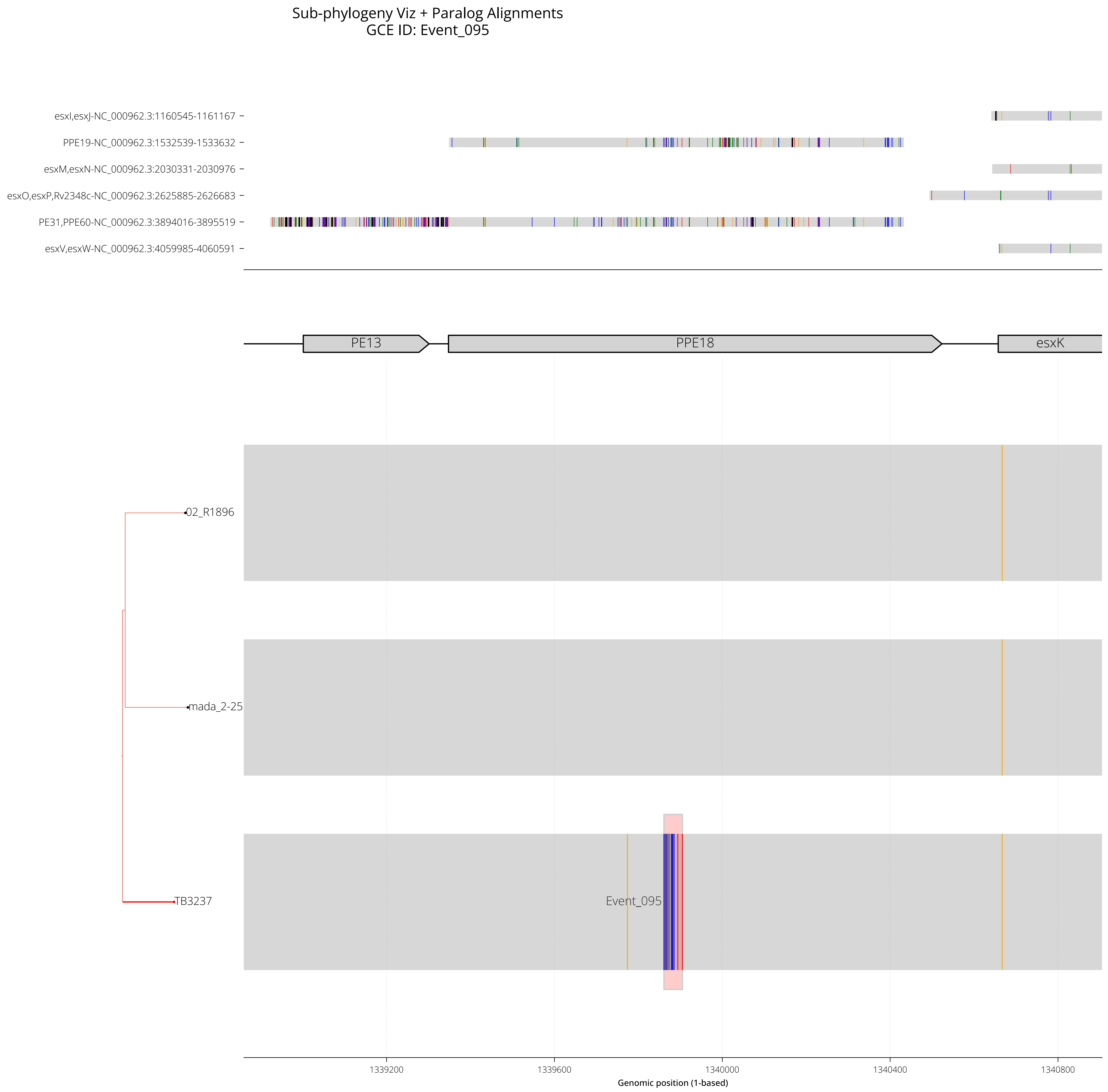

Sub-phylogeny Viz + Paralog Alignments
GCE ID: Event_095
esxI,esxJ-NC_000962.3:1160545-1161167
PPE19-NC_000962.3:1532539-1533632
esxM,esxN-NC_000962.3:2030331-2030976
esxO,esxP,Rv2348c-NC_000962.3:2625885-2626683
PE31,PPE60-NC_000962.3:3894016-3895519
esxV,esxW-NC_000962.3:4059985-4060591
PE13
PPE18
esxK
02_R1896
mada_2-25
TB3237
Event_095
1339200
1339600
1340000
1340400
1340800
Genomic position (1-based)

Full Phylogeny Viz + Paralog Alignments
GCE ID: Event_095

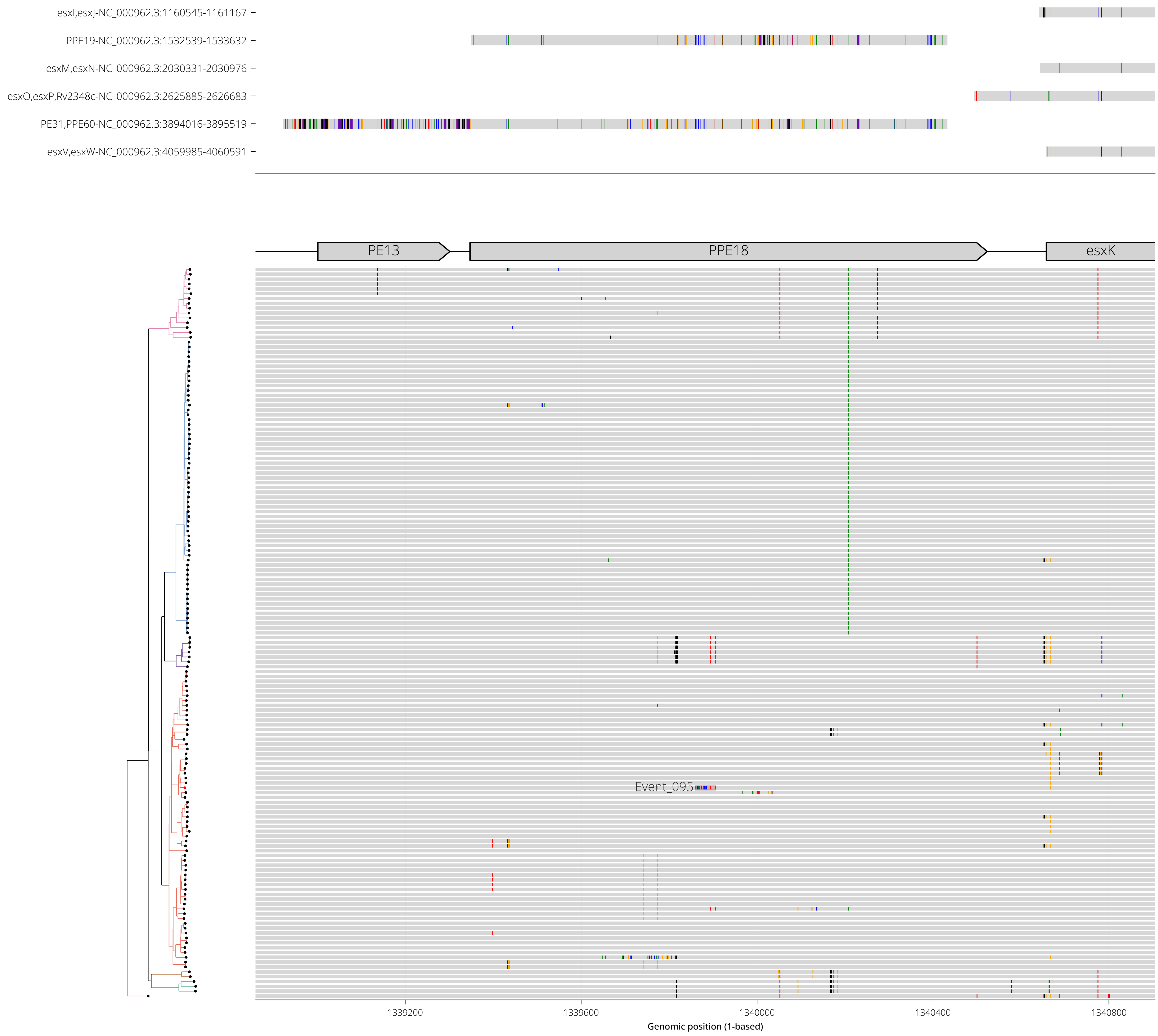

Individual GCE + Known Paralogs
GCE ID: Event_096

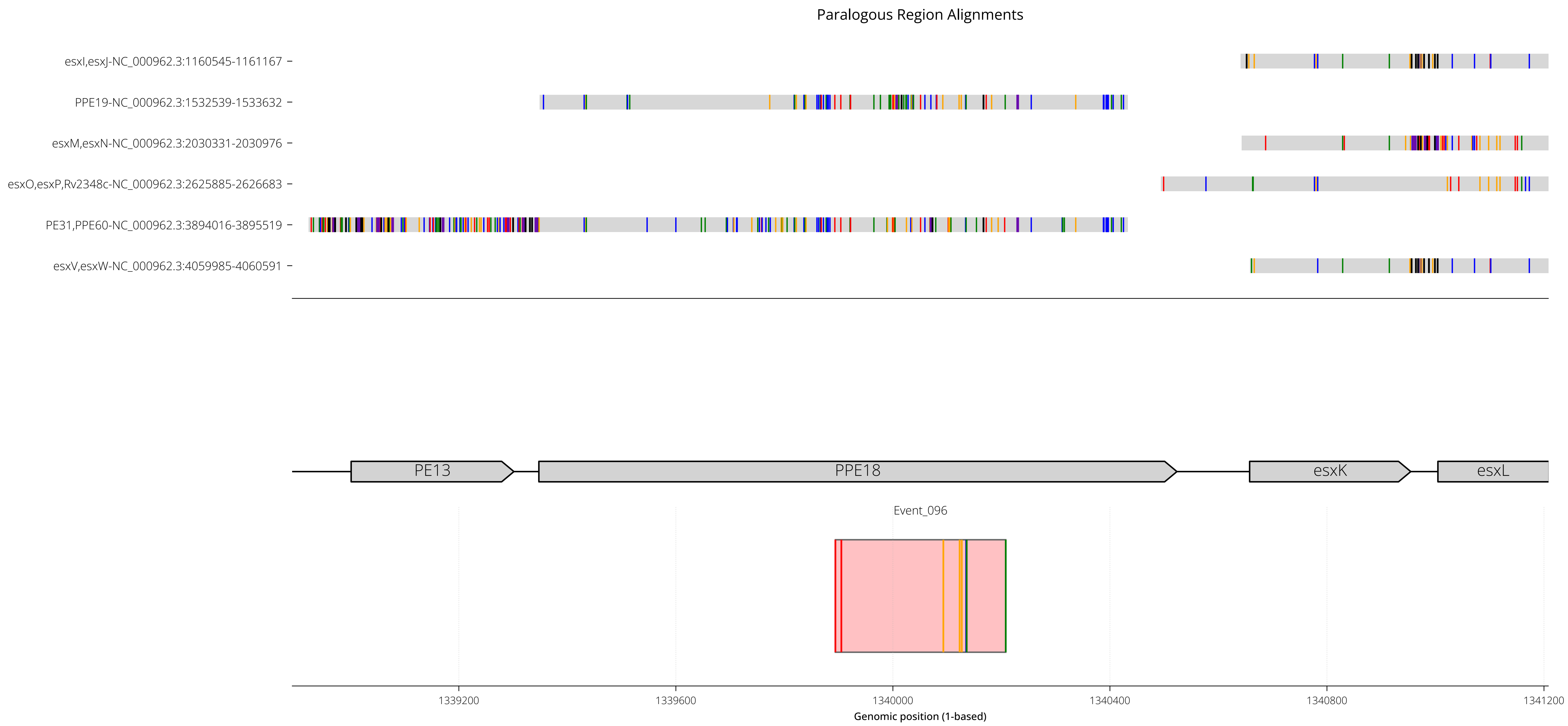

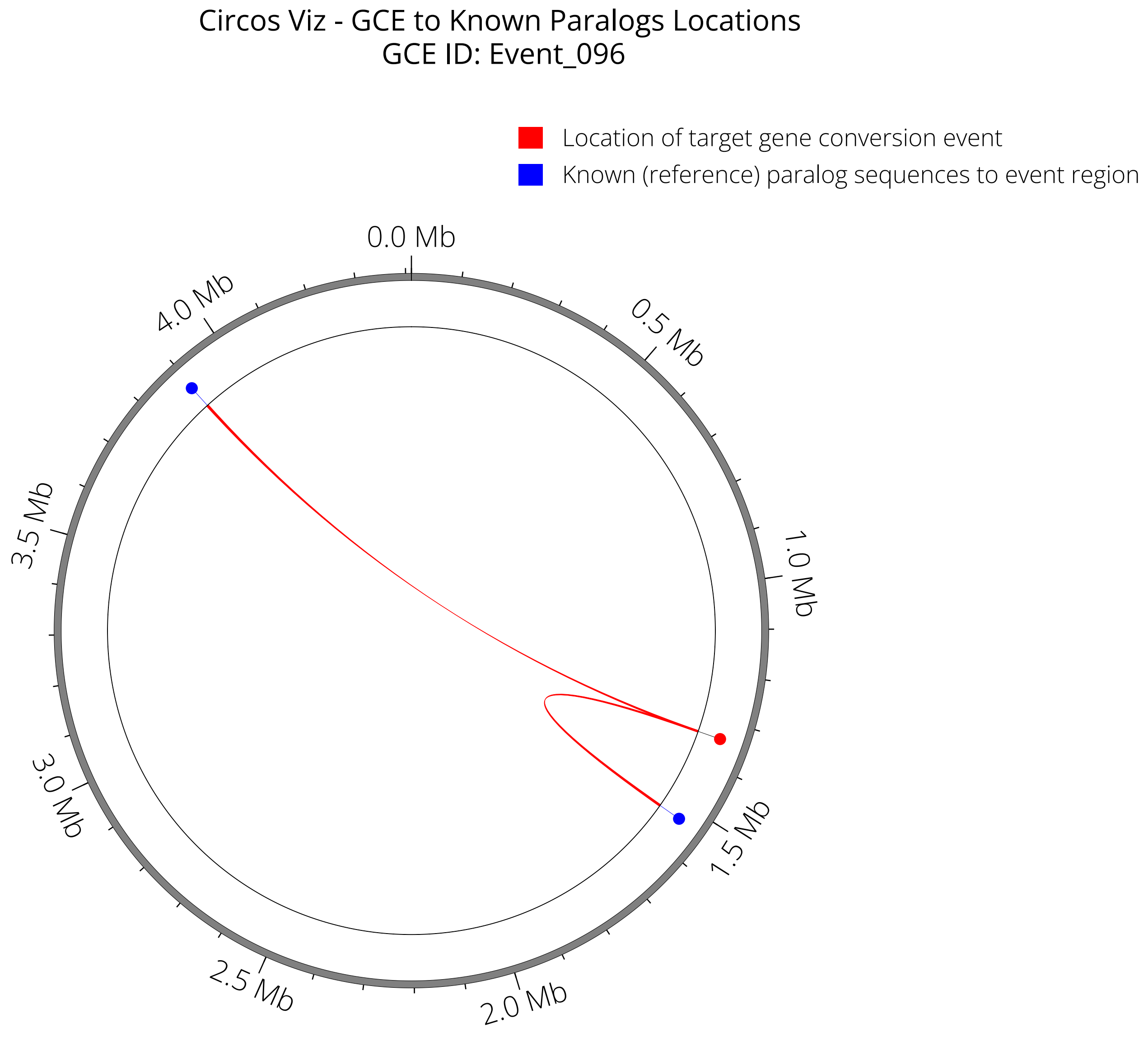

Circos Viz - GCE to Known Paralogs Locations
GCE ID: Event_096
Location of target gene conversion event
Known (reference) paralog sequences to event region
0.0 Mb
0.5 Mb
1.0 Mb
1.5 Mb
2.0 Mb
2.5 Mb
3.0 Mb
3.5 Mb
4.0 Mb

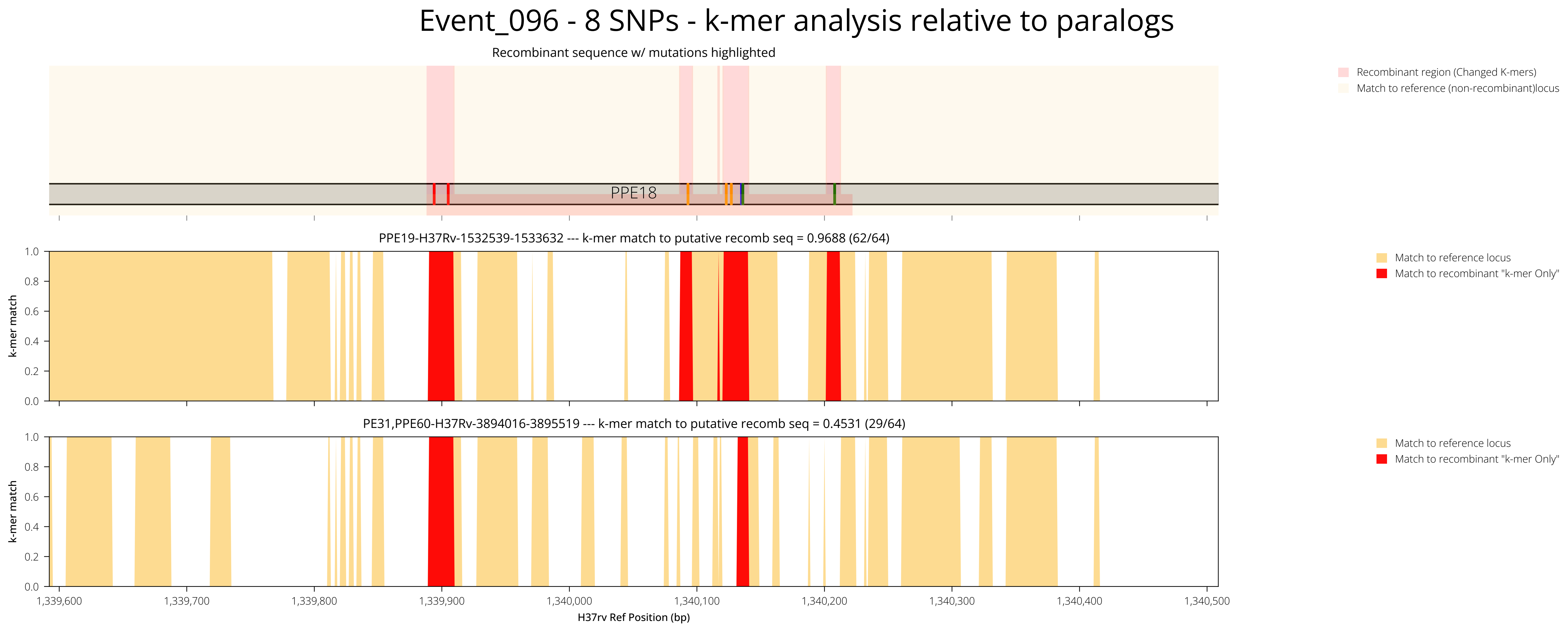

Event_096 - 8 SNPs - k-mer analysis relative to paralogs
Recombinant sequence w/ mutations highlighted
PPE18
Recombinant region (Changed K-mers)
Match to reference (non-recombinant)locus
PPE19-H37Rv-1532539-1533632 --- k-mer match to putative recomb seq = 0.9688 (62/64)
k-mer match
1.0
0.8
0.6
0.4
0.2
0.0
Match to reference locus
Match to recombinant "k-mer Only"
PE31,PPE60-H37Rv-3894016-3895519 --- k-mer match to putative recomb seq = 0.4531 (29/64)
k-mer match
1.0
0.8
0.6
0.4
0.2
0.0
Match to reference locus
Match to recombinant "k-mer Only"
1,339,600
1,339,700
1,339,800
1,339,900
1,340,000
1,340,100
1,340,200
1,340,300
1,340,400
1,340,500
H37rv Ref Position (bp)

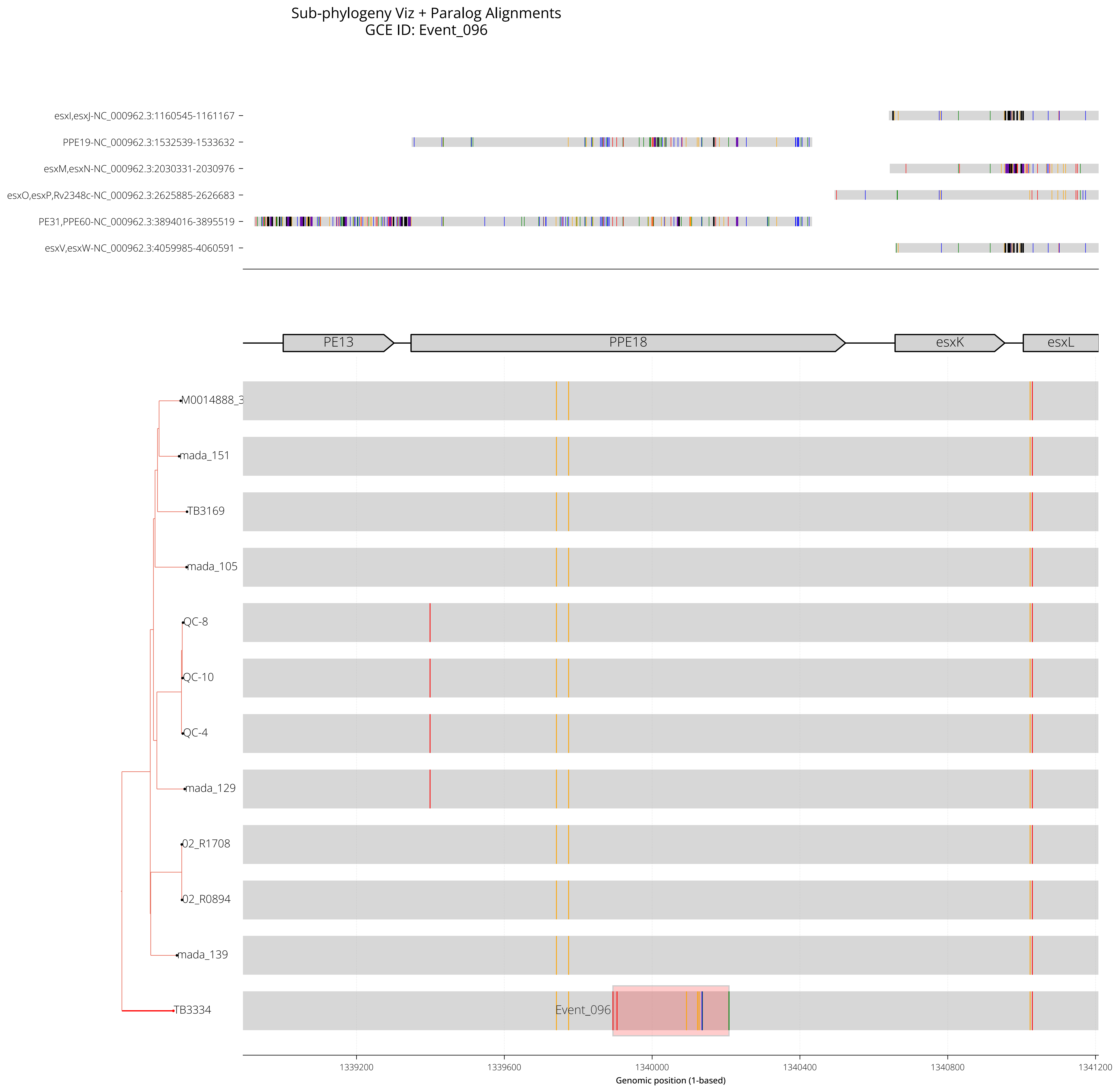

Sub-phylogeny Viz + Paralog Alignments
GCE ID: Event_096
esxI,esxJ-NC_000962.3:1160545-1161167
PPE19-NC_000962.3:1532539-1533632
esxM,esxN-NC_000962.3:2030331-2030976
esxO,esxP,Rv2348c-NC_000962.3:2625885-2626683
PE31,PPE60-NC_000962.3:3894016-3895519
esxV,esxW-NC_000962.3:4059985-4060591
PE13
PPE18
esxK
esxL
M0014888_3
mada_151
TB3169
mada_105
QC-8
QC-10
QC-4
mada_129
02_R1708
02_R0894
mada_139
TB3334
Event_096
1339200
1339600
1340000
1340400
1340800
1341200
Genomic position (1-based)

Full Phylogeny Viz + Paralog Alignments
GCE ID: Event_096

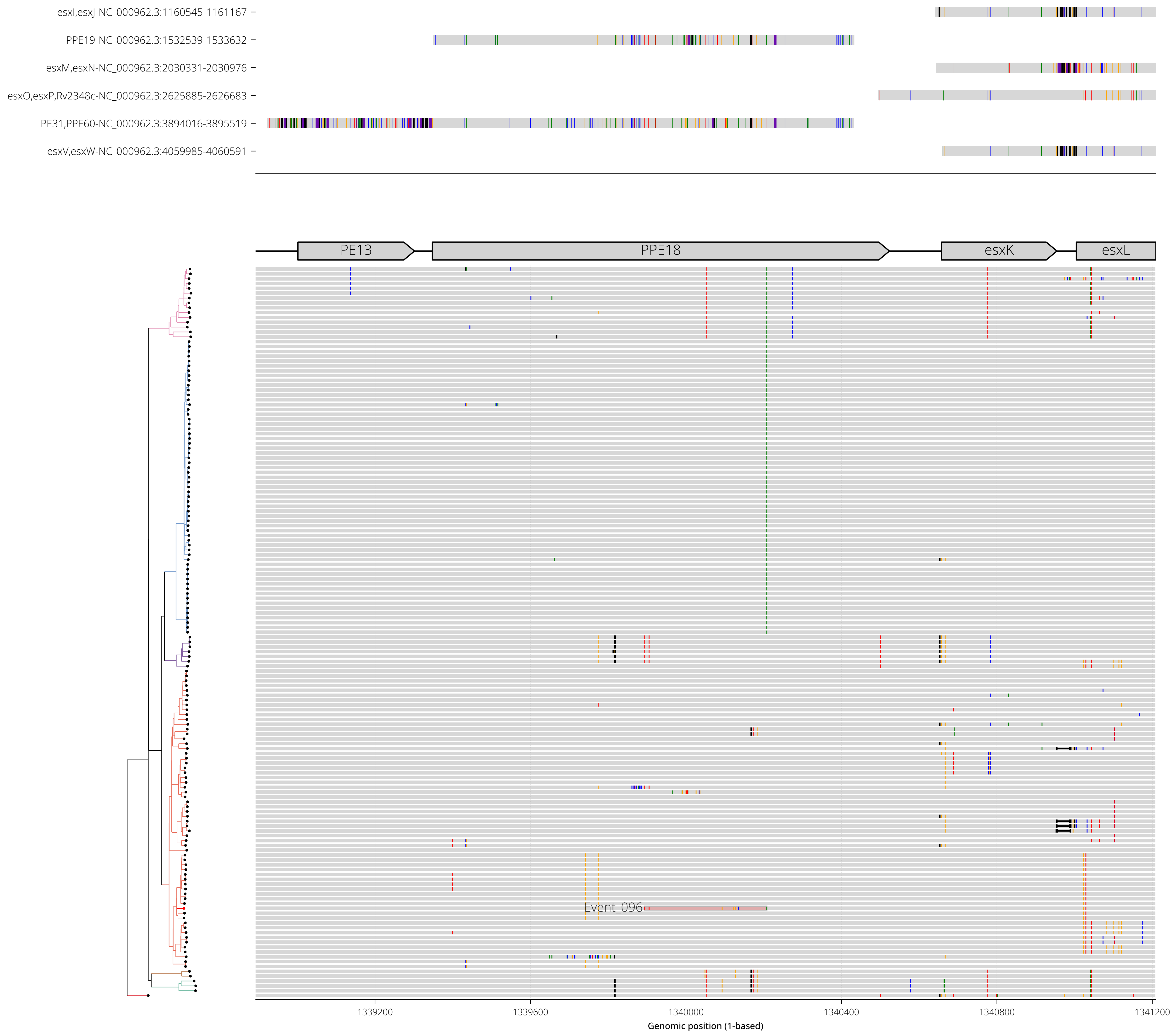

Individual GCE + Known Paralogs
GCE ID: Event_097

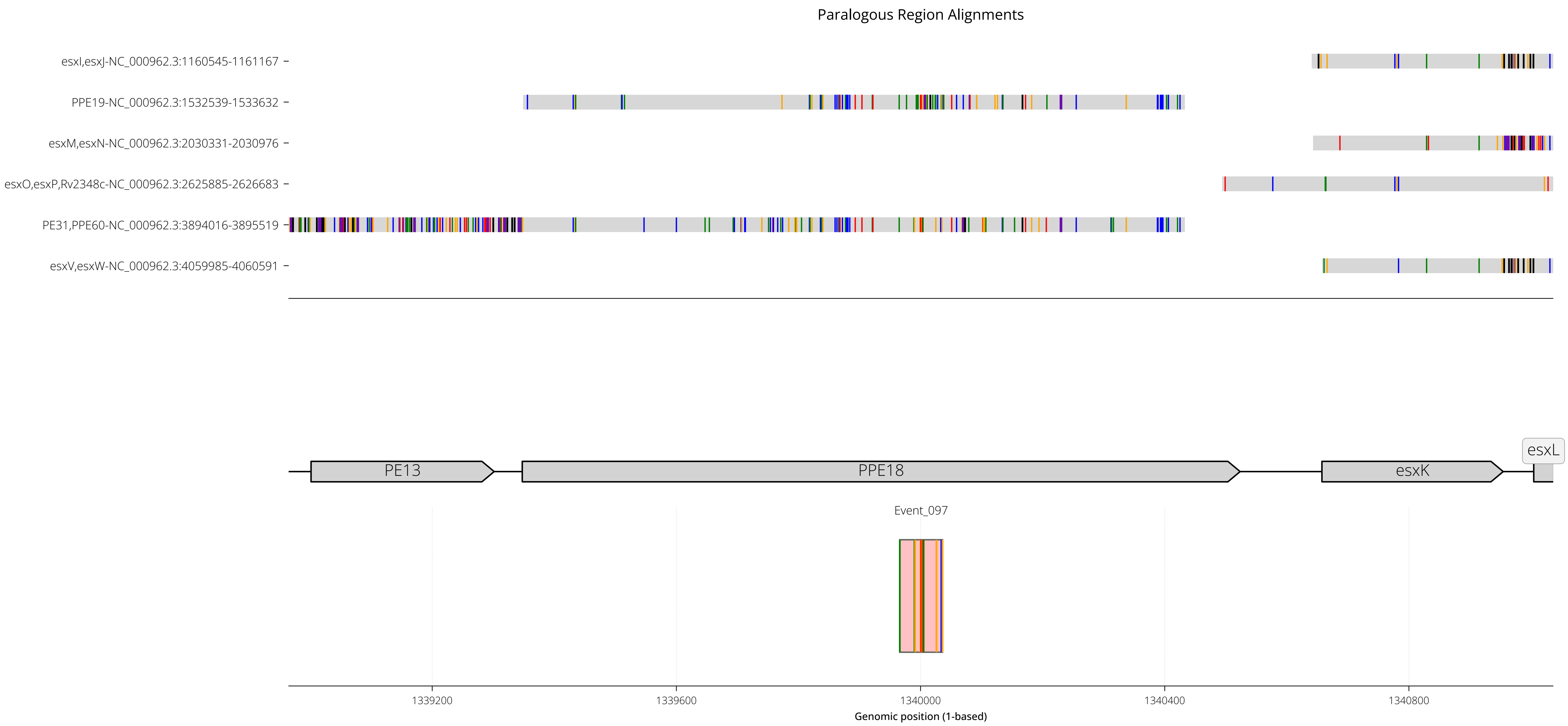

Circos Viz - GCE to Known Paralogs Locations
GCE ID: Event_097

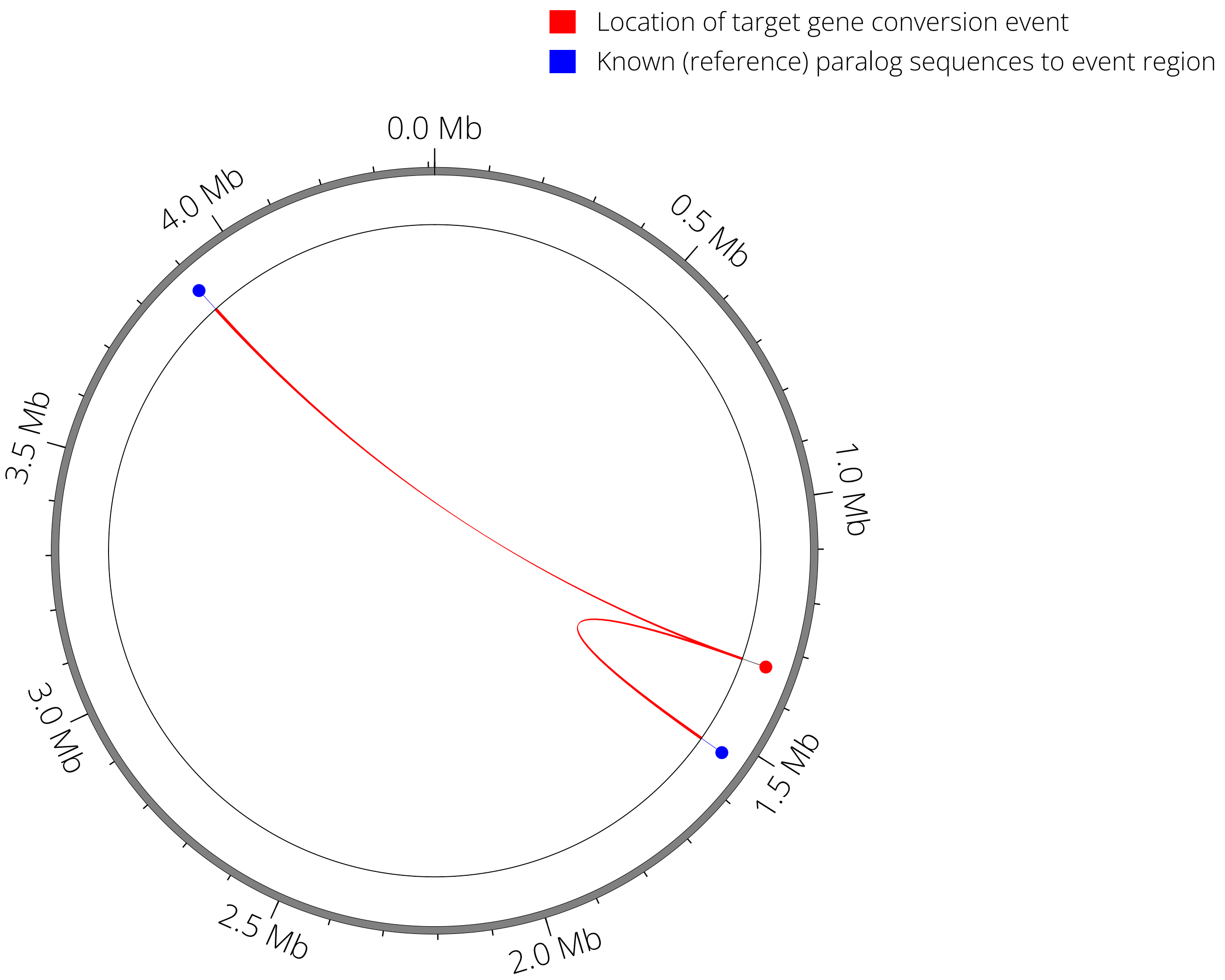

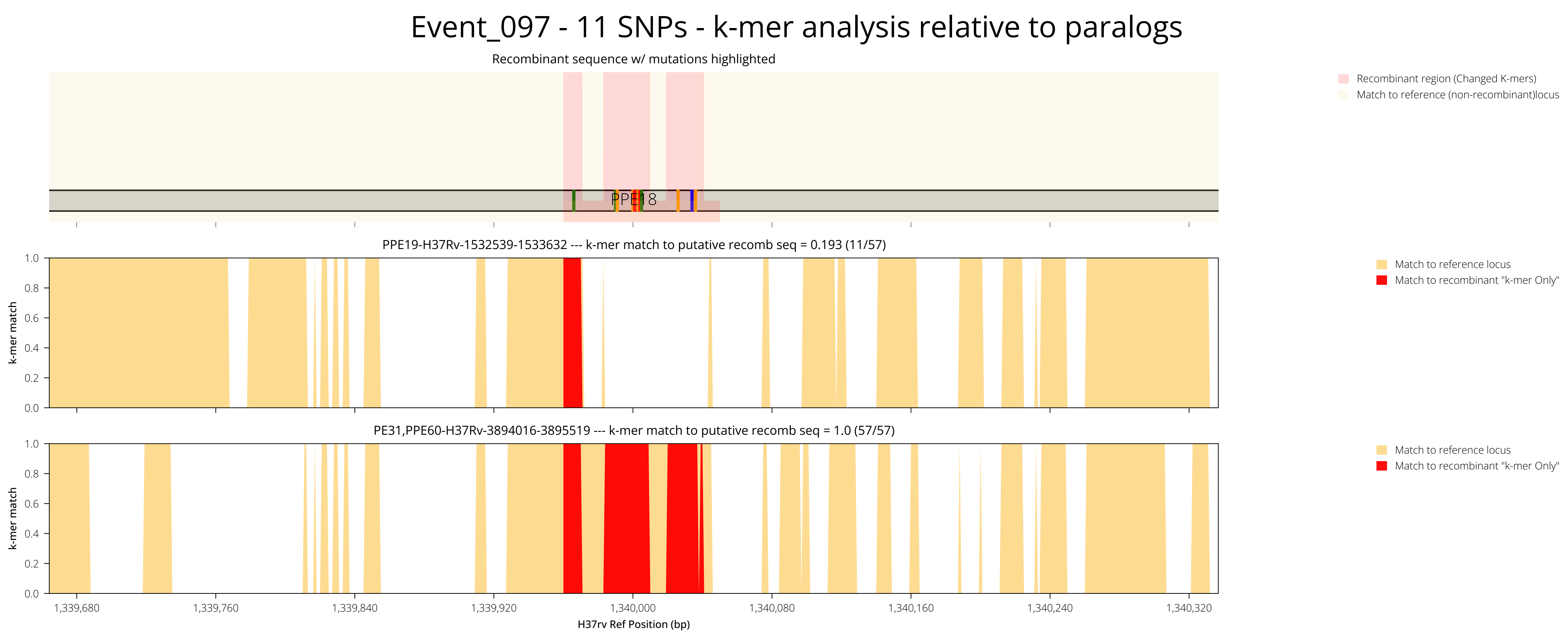

Event_097 - 11 SNPs - k-mer analysis relative to paralogs
Recombinant sequence w/ mutations highlighted
PPE18
Recombinant region (Changed K-mers)
Match to reference (non-recombinant)locus
PPE19-H37Rv-1532539-1533632 --- k-mer match to putative recomb seq = 0.193 (11/57)
Match to reference locus
Match to recombinant "k-mer Only"
PE31,PPE60-H37Rv-3894016-3895519 --- k-mer match to putative recomb seq = 1.0 (57/57)
Match to reference locus
Match to recombinant "k-mer Only"
k-mer match
1.0
0.8
0.6
0.4
0.2
0.0
1,339,680
1,339,760
1,339,840
1,339,920
1,340,000
1,340,080
1,340,160
1,340,240
1,340,320
H37rv Ref Position (bp)

Sub-phylogeny Viz + Paralog Alignments
GCE ID: Event_097

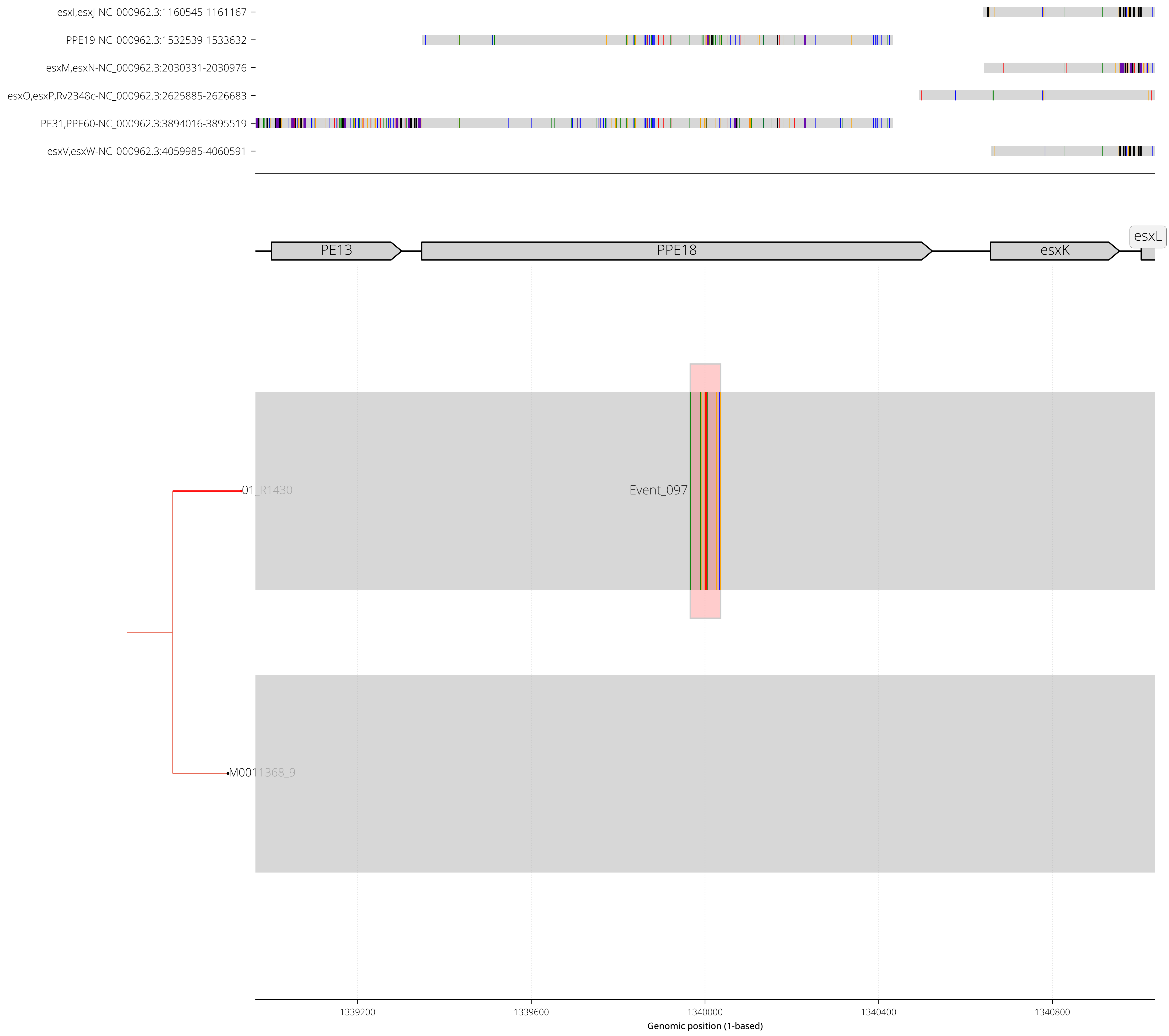

Full Phylogeny Viz + Paralog Alignments
GCE ID: Event_097

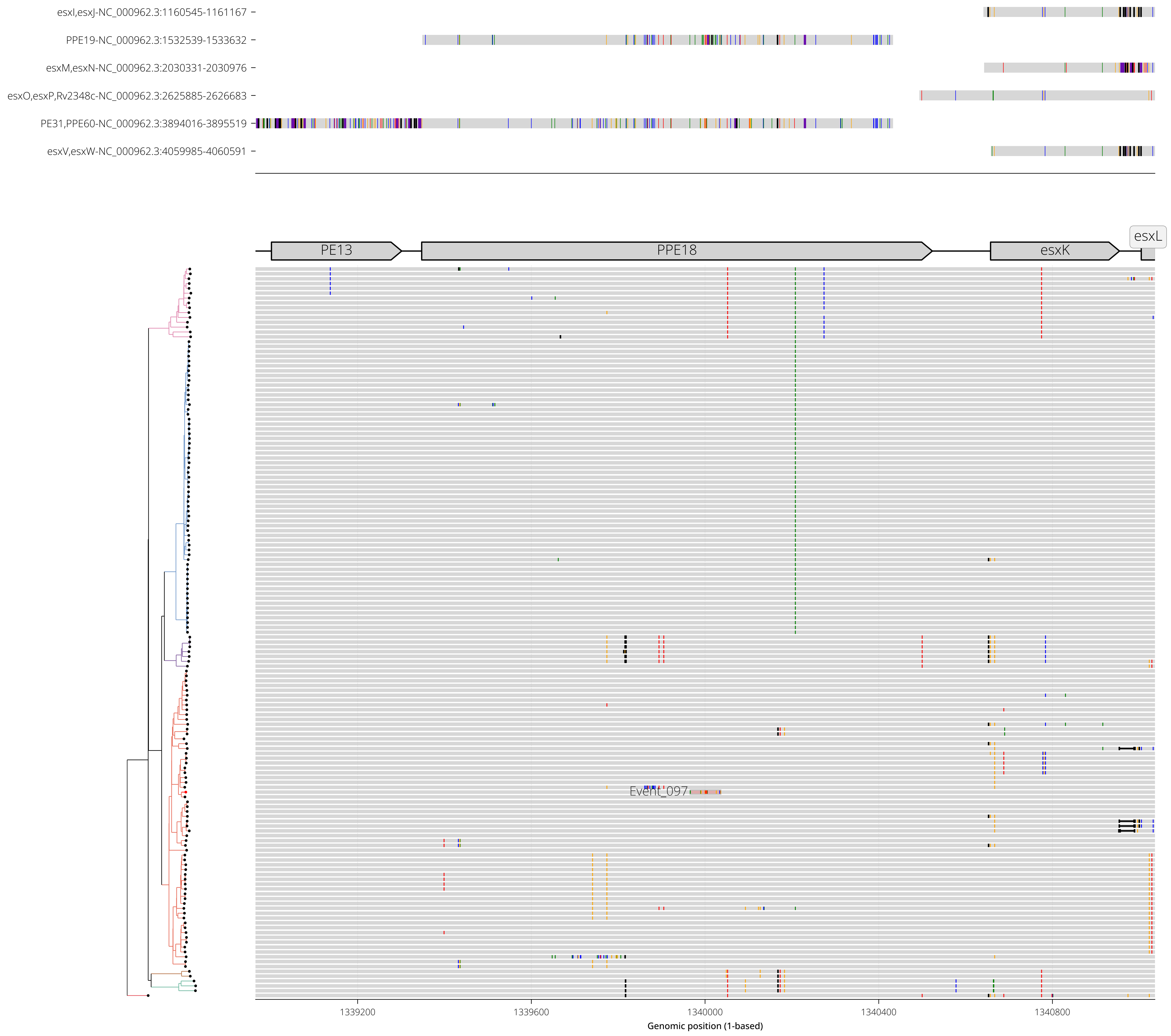

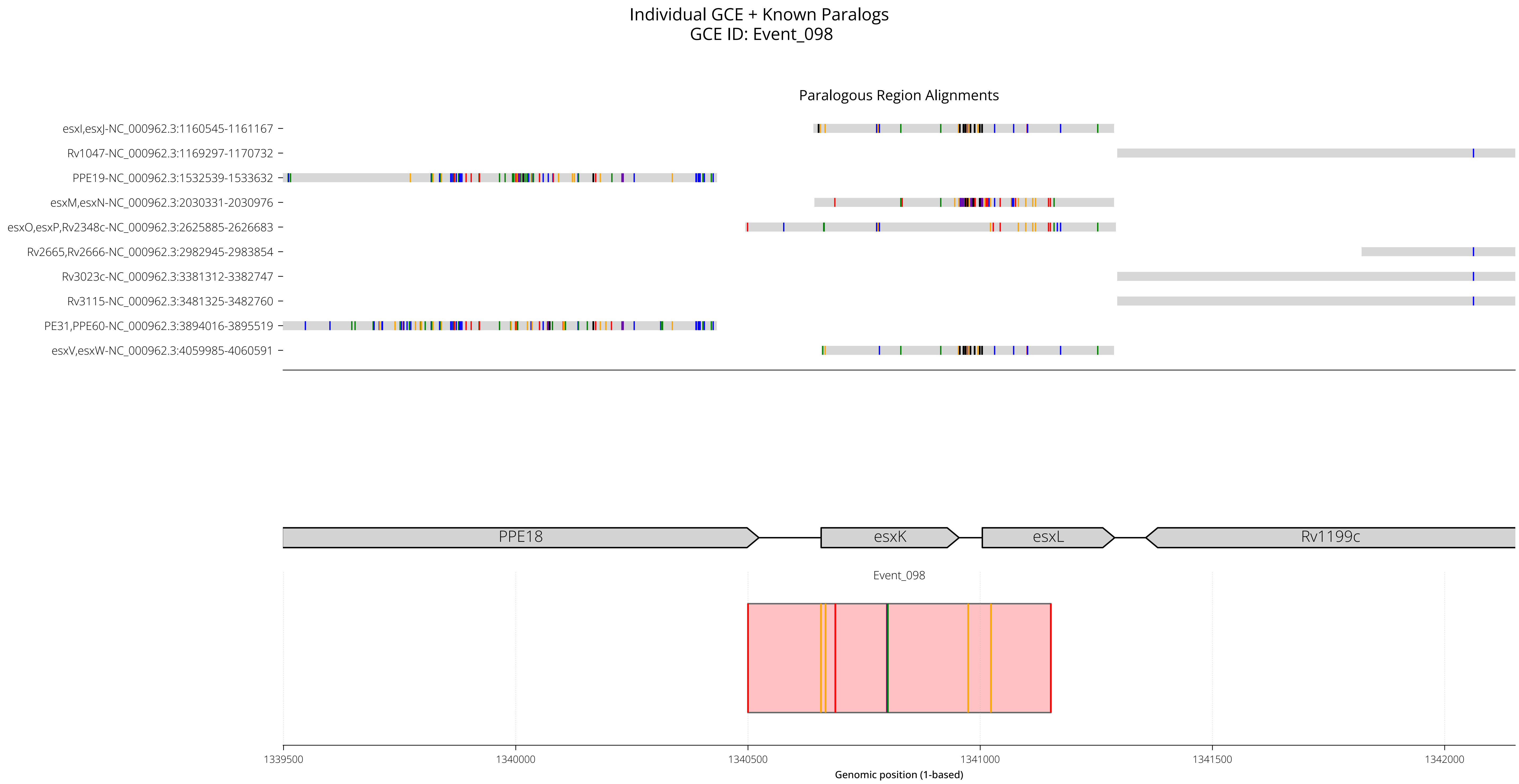
Individual GCE + Known Paralogs
GCE ID: Event_098
Paralogous Region Alignments
esxI,esxJ-NC_000962.3:1160545-1161167
Rv1047-NC_000962.3:1169297-1170732
PPE19-NC_000962.3:1532539-1533632
esxM,esxN-NC_000962.3:2030331-2030976
esxO,esxP,Rv2348c-NC_000962.3:2625885-2626683
Rv2665,Rv2666-NC_000962.3:2982945-2983854
Rv3023c-NC_000962.3:3381312-3382747
Rv3115-NC_000962.3:3481325-3482760
PE31,PPE60-NC_000962.3:3894016-3895519
esxV,esxW-NC_000962.3:4059985-4060591
PPE18
esxK
esxL
Rv1199c
Event_098
1339500
1340000
1340500
1341000
1341500
1342000
Genomic position (1-based)

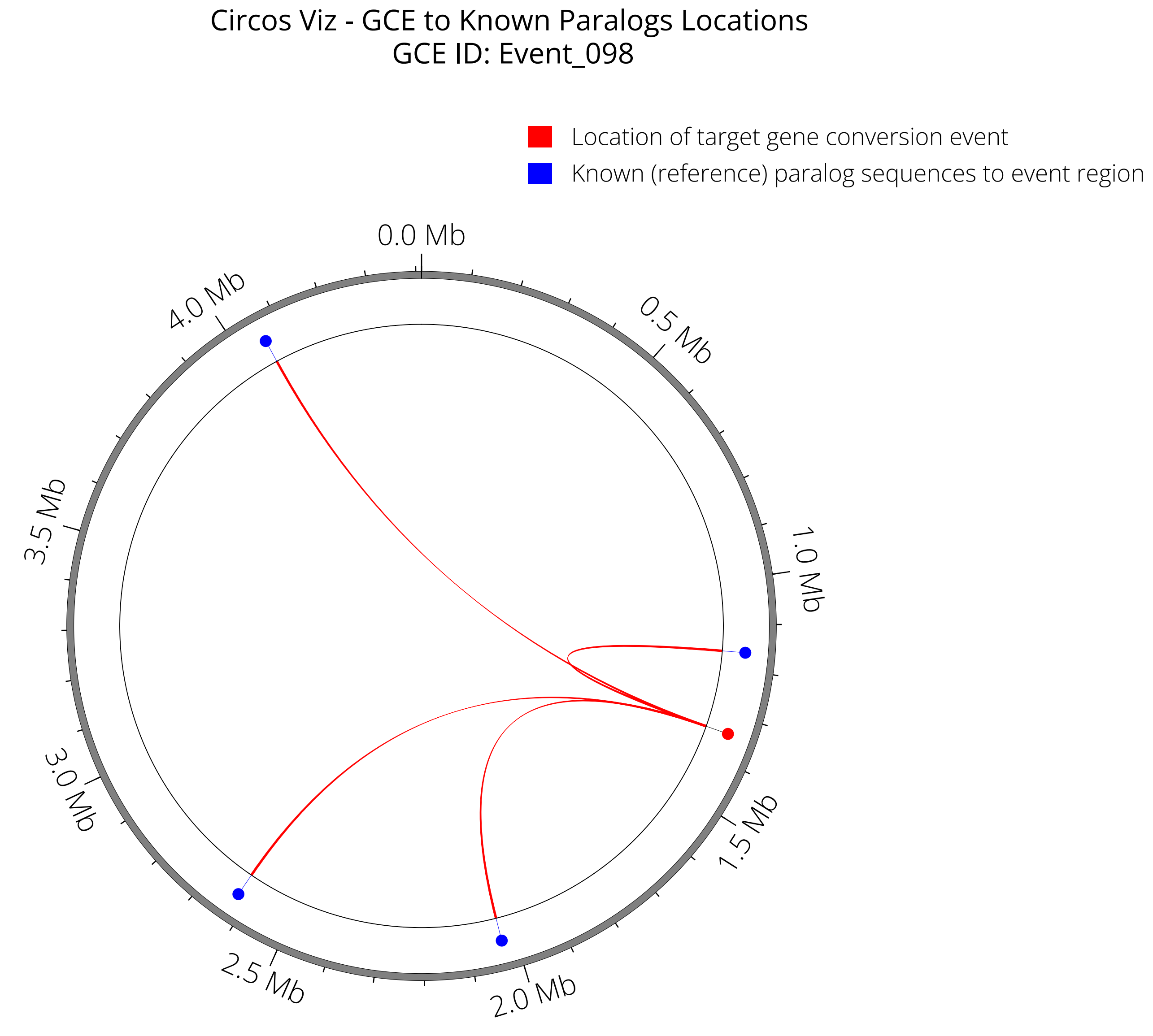
Circos Viz - GCE to Known Paralogs Locations
GCE ID: Event_098
Location of target gene conversion event
Known (reference) paralog sequences to event region
0.0 Mb
0.5 Mb
1.0 Mb
1.5 Mb
2.0 Mb
2.5 Mb
3.0 Mb
3.5 Mb
4.0 Mb

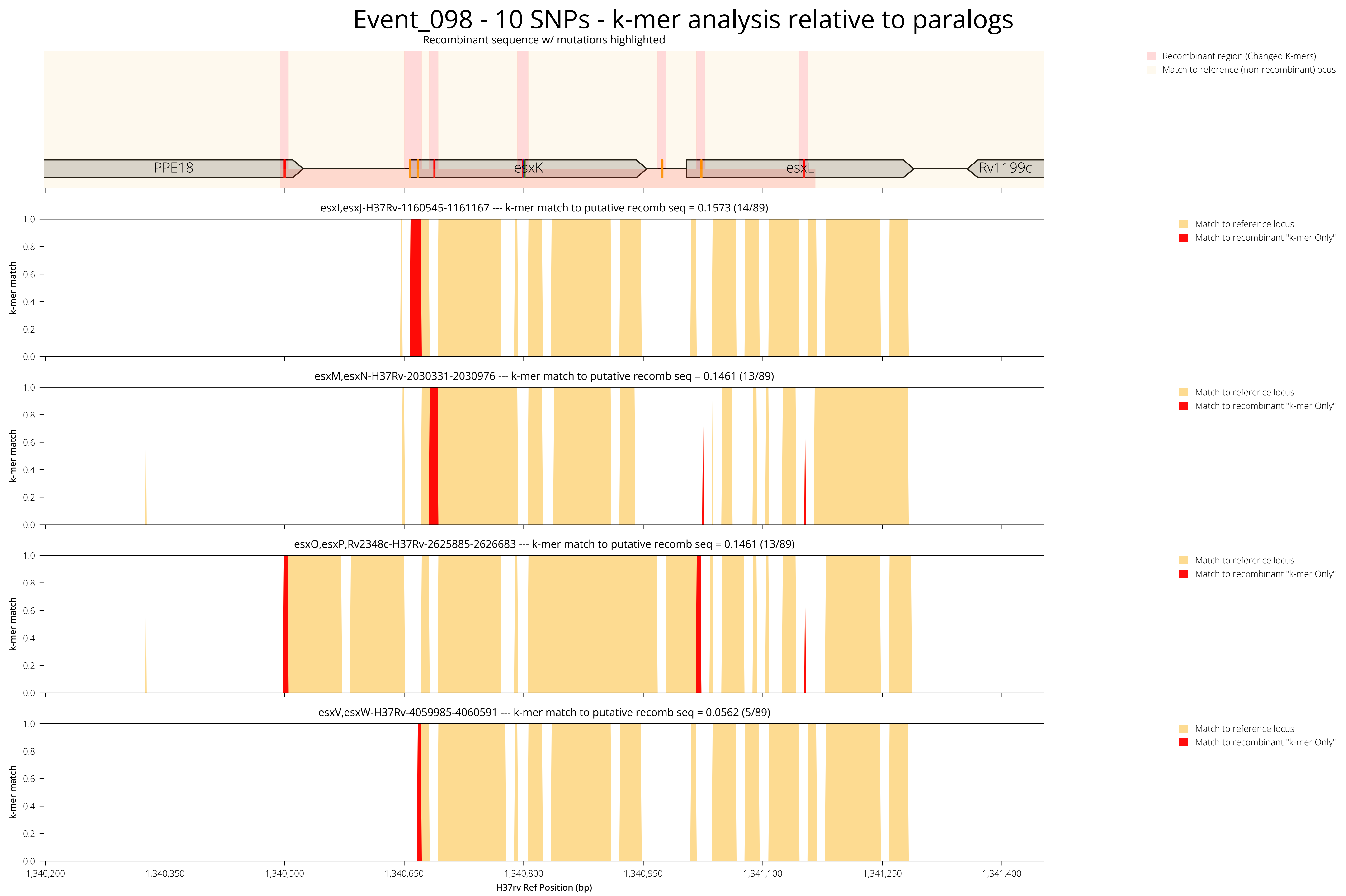

Event_098 - 10 SNPs - k-mer analysis relative to paralogs
Recombinant sequence w/ mutations highlighted
Recombinant region (Changed K-mers)
Match to reference (non-recombinant)locus
PPE18
esxK
esxL
Rv1199c
esxI,esxJ-H37Rv-1160545-1161167 --- k-mer match to putative recomb seq = 0.1573 (14/89)
esxM,esxN-H37Rv-2030331-2030976 --- k-mer match to putative recomb seq = 0.1461 (13/89)
esxO,esxP,Rv2348c-H37Rv-2625885-2626683 --- k-mer match to putative recomb seq = 0.1461 (13/89)
esxV,esxW-H37Rv-4059985-4060591 --- k-mer match to putative recomb seq = 0.0562 (5/89)
Match to reference locus
Match to recombinant "k-mer Only"
k-mer match
0.0
0.2
0.4
0.6
0.8
1.0
1,340,200
1,340,350
1,340,500
1,340,650
1,340,800
1,340,950
1,341,100
1,341,250
1,341,400
H37rv Ref Position (bp)

Sub-phylogeny Viz + Paralog Alignments
GCE ID: Event_098

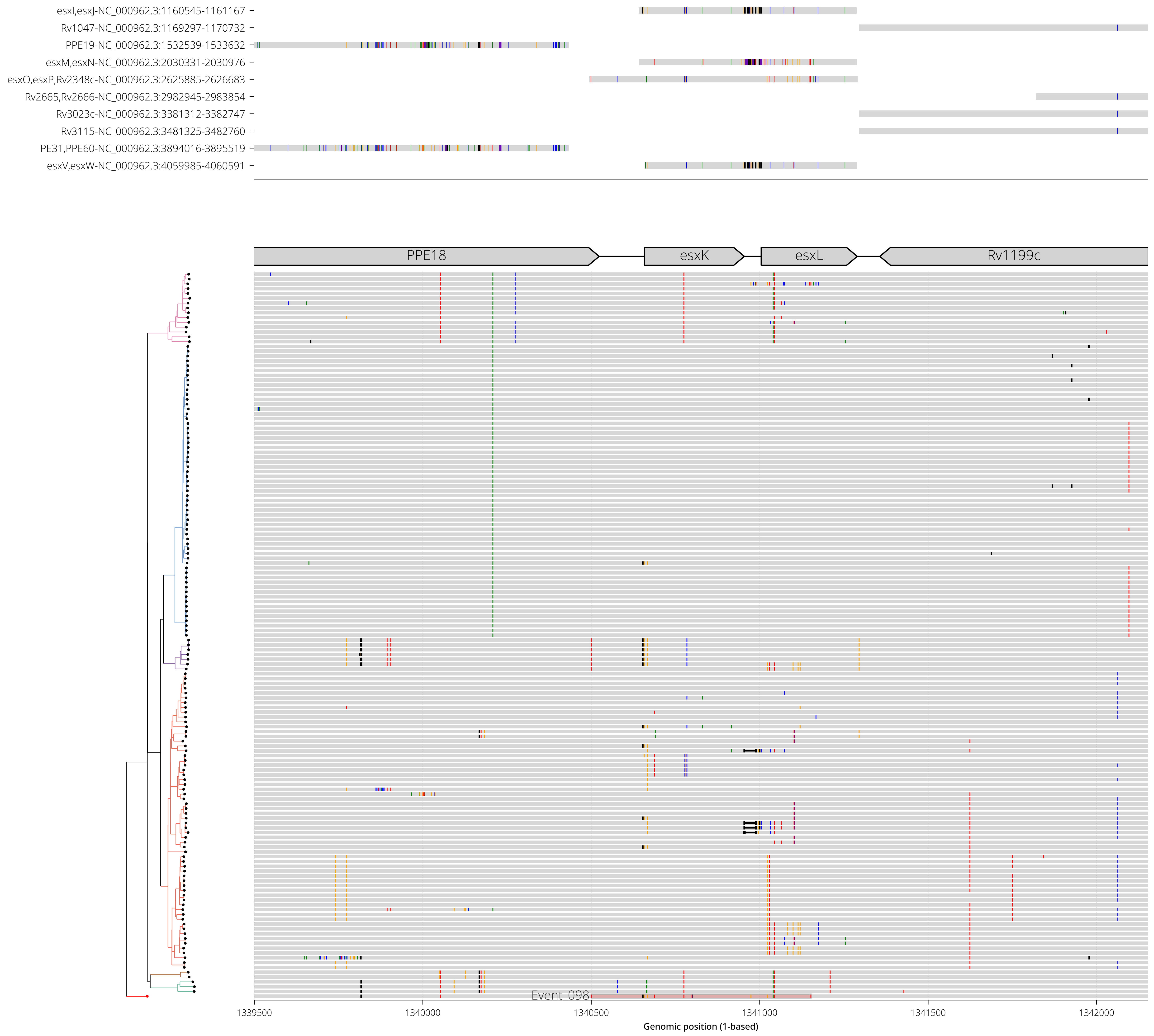

Full Phylogeny Viz + Paralog Alignments
GCE ID: Event_098

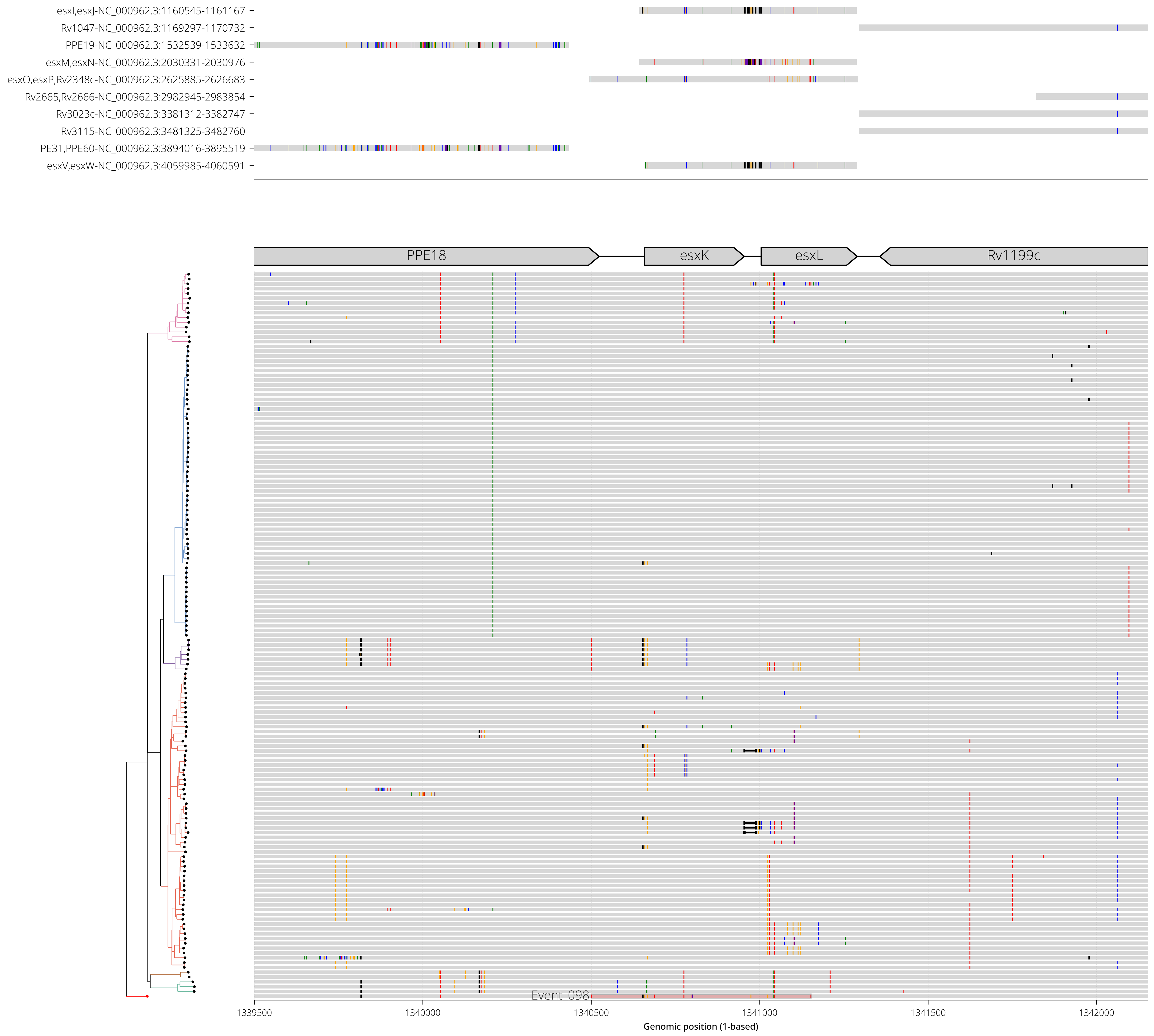

Individual GCE + Known Paralogs
GCE ID: Event_099

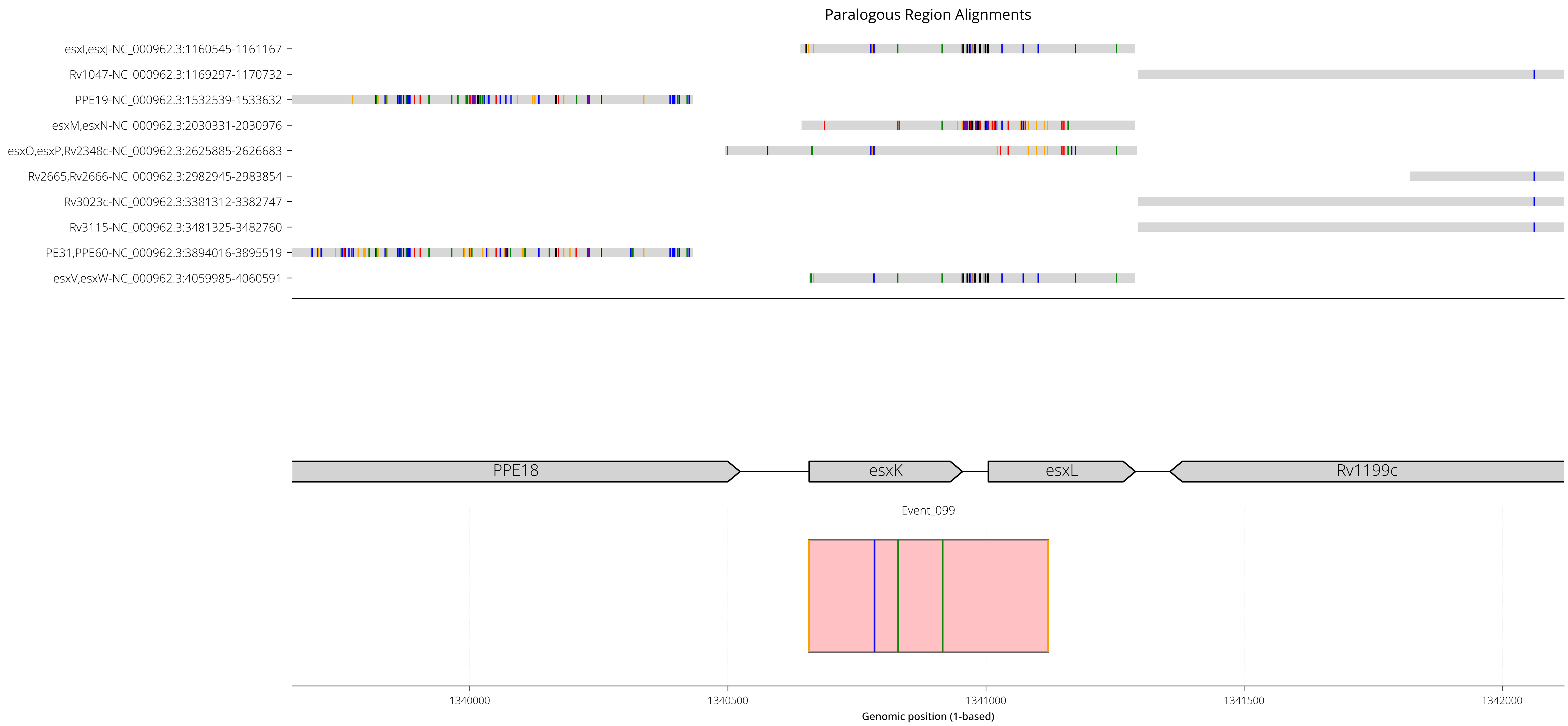

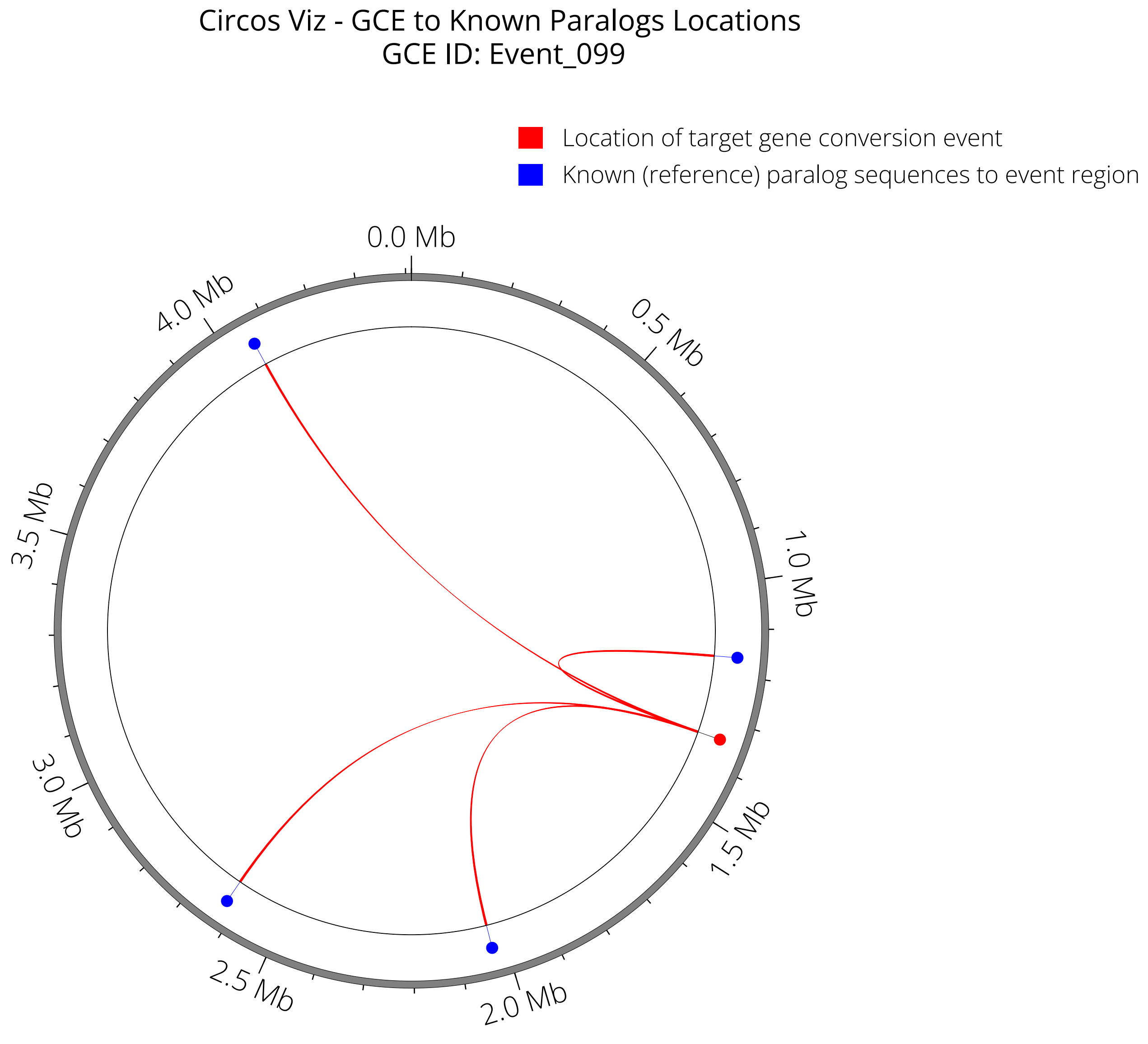
Circos Viz - GCE to Known Paralogs Locations
GCE ID: Event_099
Location of target gene conversion event
Known (reference) paralog sequences to event region
0.0 Mb
0.5 Mb
1.0 Mb
1.5 Mb
2.0 Mb
2.5 Mb
3.0 Mb
3.5 Mb
4.0 Mb

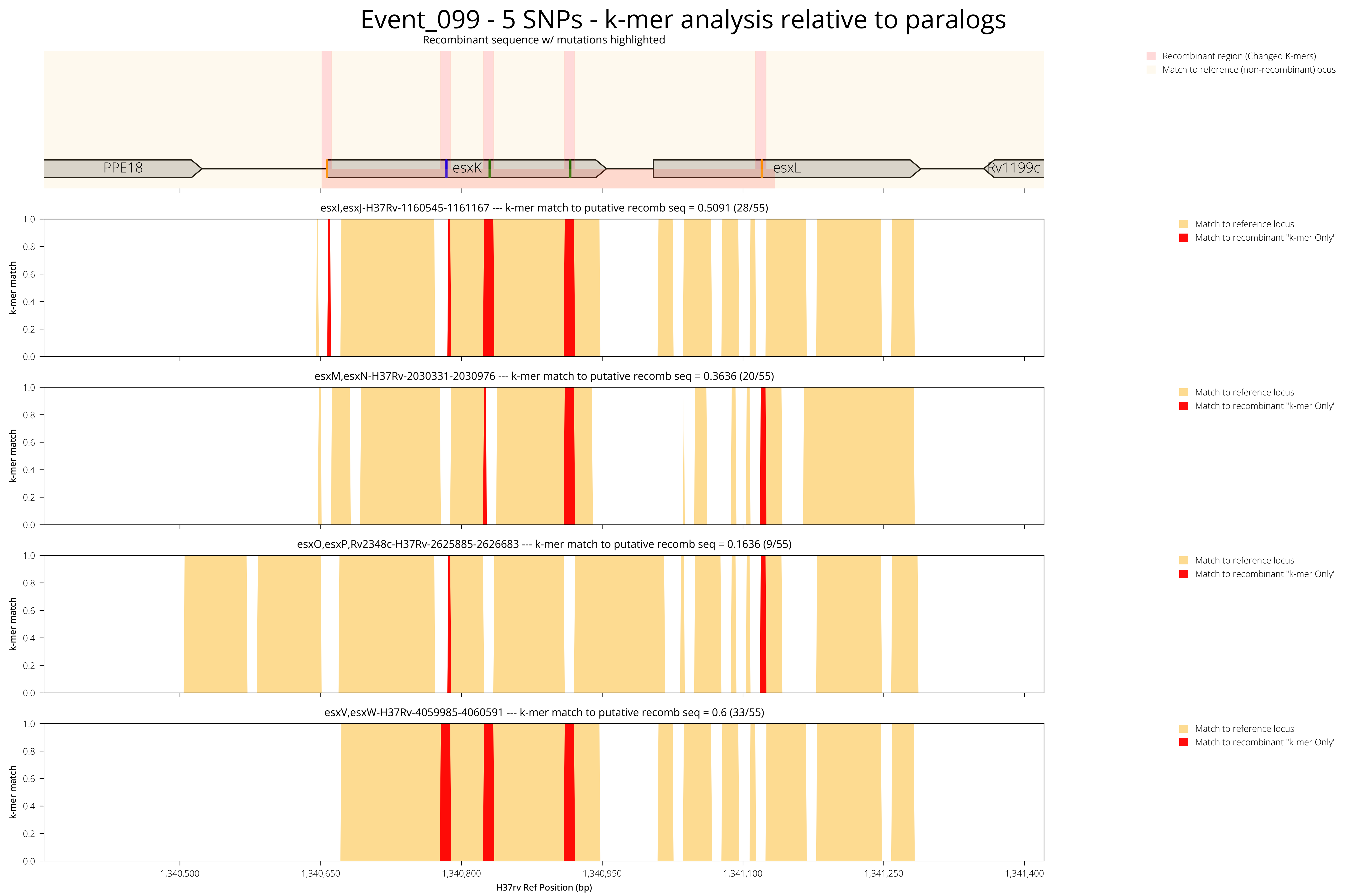
Event_099 - 5 SNPs - k-mer analysis relative to paralogs
Recombinant sequence w/ mutations highlighted
Recombinant region (Changed K-mers)
Match to reference (non-recombinant)locus
PPE18
esxK
esxL
Rv1199c
esxI,esxJ-H37Rv-1160545-1161167 --- k-mer match to putative recomb seq = 0.5091 (28/55)
esxM,esxN-H37Rv-2030331-2030976 --- k-mer match to putative recomb seq = 0.3636 (20/55)
esxO,esxP,Rv2348c-H37Rv-2625885-2626683 --- k-mer match to putative recomb seq = 0.1636 (9/55)
esxV,esxW-H37Rv-4059985-4060591 --- k-mer match to putative recomb seq = 0.6 (33/55)
Match to reference locus
Match to recombinant "k-mer Only"
k-mer match
1.0
0.8
0.6
0.4
0.2
0.0
1,340,500
1,340,650
1,340,800
1,340,950
1,341,100
1,341,250
1,341,400
H37rv Ref Position (bp)

Sub-phylogeny Viz + Paralog Alignments
GCE ID: Event_099

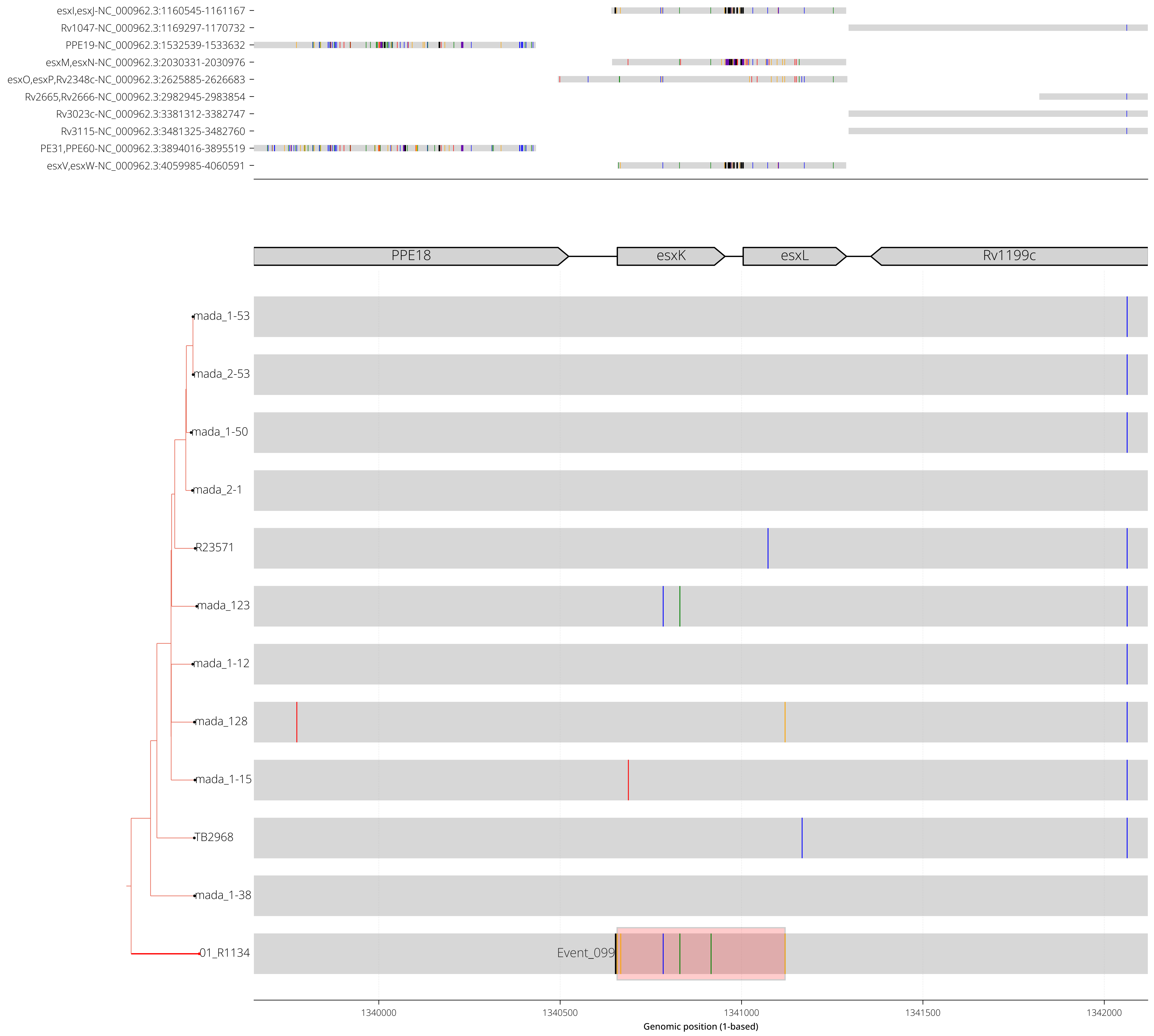

Full Phylogeny Viz + Paralog Alignments
GCE ID: Event_099

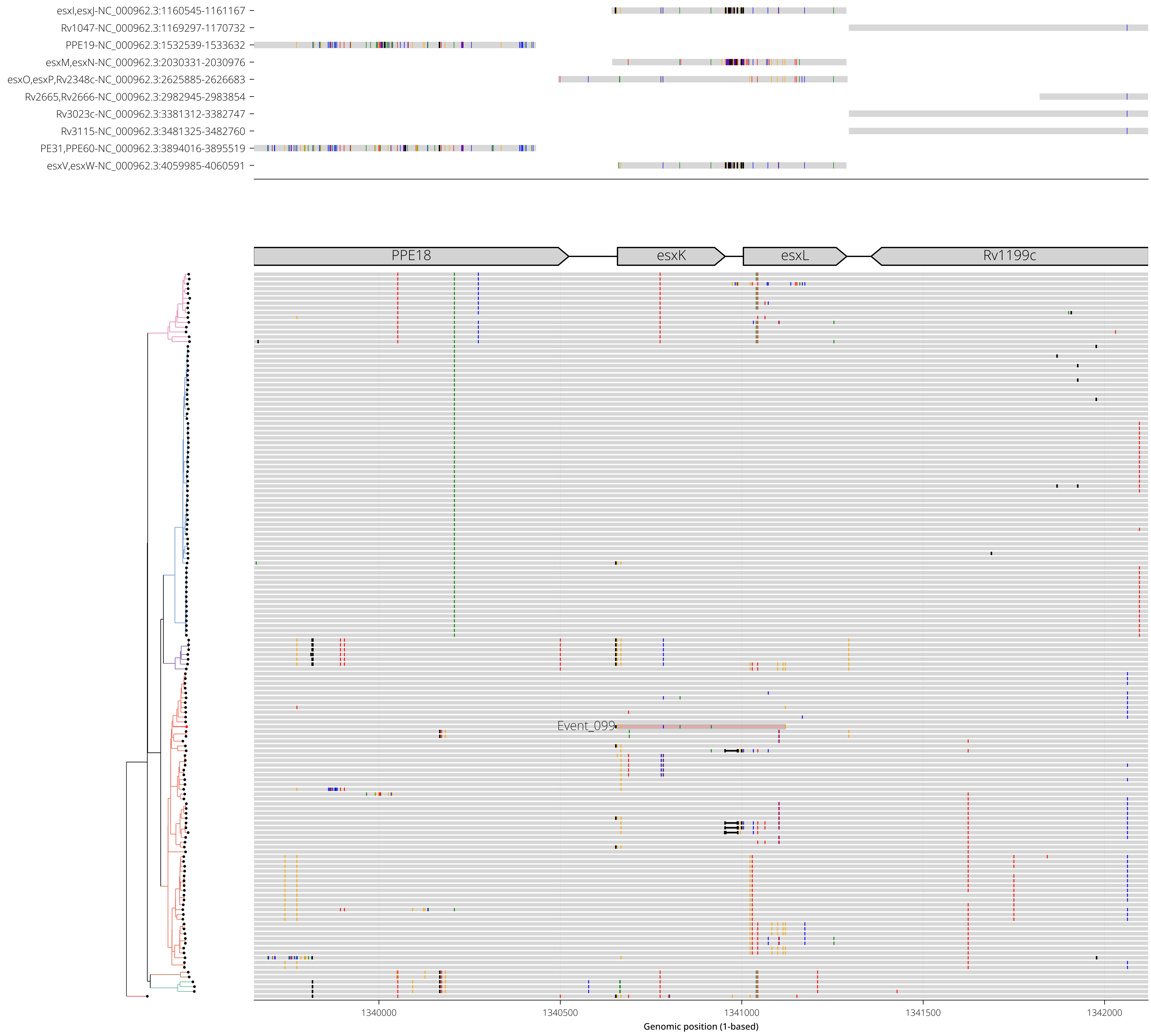

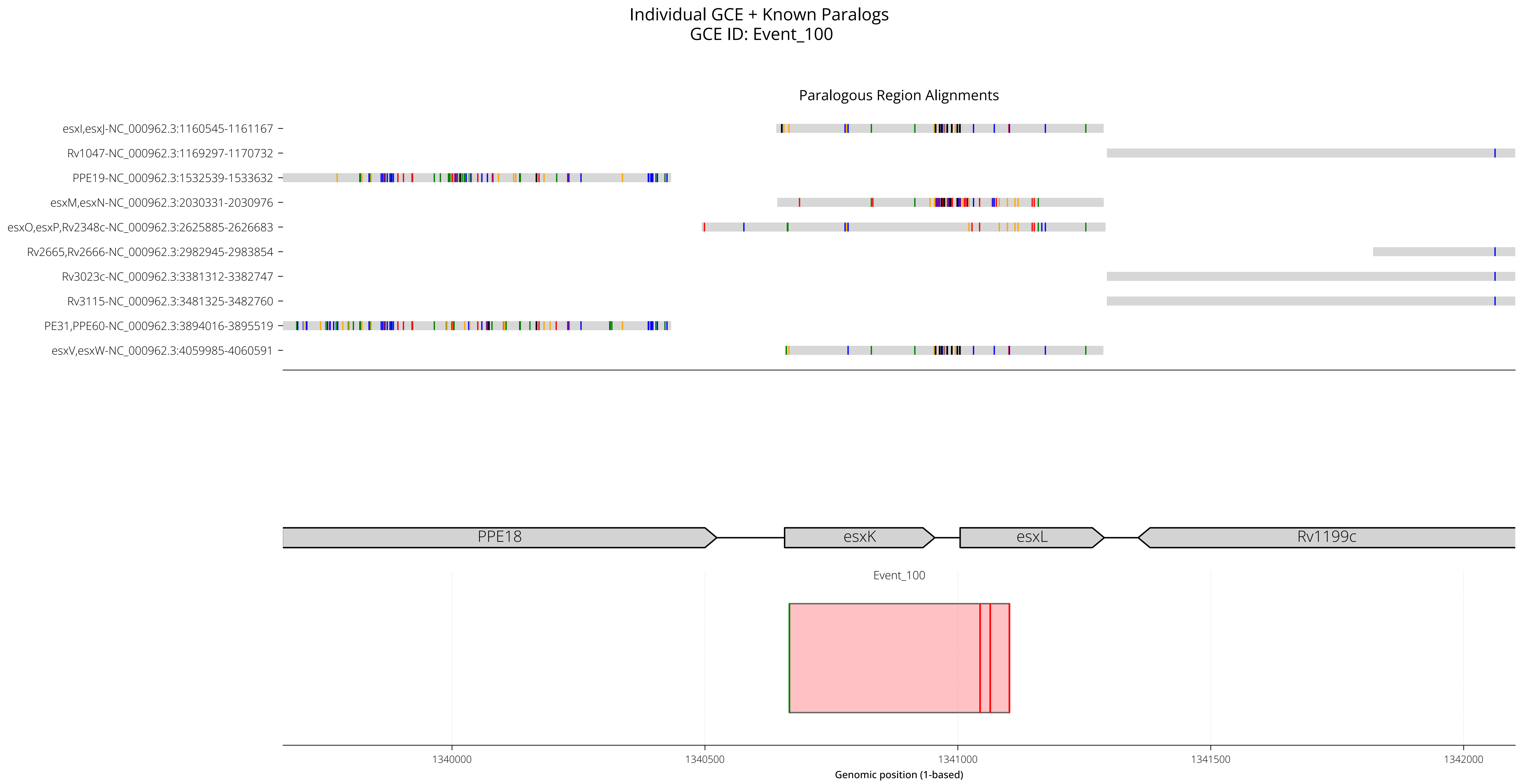
Individual GCE + Known Paralogs
GCE ID: Event_100
Paralogous Region Alignments
esxI,esxJ-NC_000962.3:1160545-1161167
Rv1047-NC_000962.3:1169297-1170732
PPE19-NC_000962.3:1532539-1533632
esxM,esxN-NC_000962.3:2030331-2030976
esxO,esxP,Rv2348c-NC_000962.3:2625885-2626683
Rv2665,Rv2666-NC_000962.3:2982945-2983854
Rv3023c-NC_000962.3:3381312-3382747
Rv3115-NC_000962.3:3481325-3482760
PE31,PPE60-NC_000962.3:3894016-3895519
esxV,esxW-NC_000962.3:4059985-4060591
PPE18
esxK
esxL
Rv1199c
Event_100
1340000
1340500
1341000
1341500
1342000
Genomic position (1-based)

Circos Viz - GCE to Known Paralogs Locations
GCE ID: Event_100

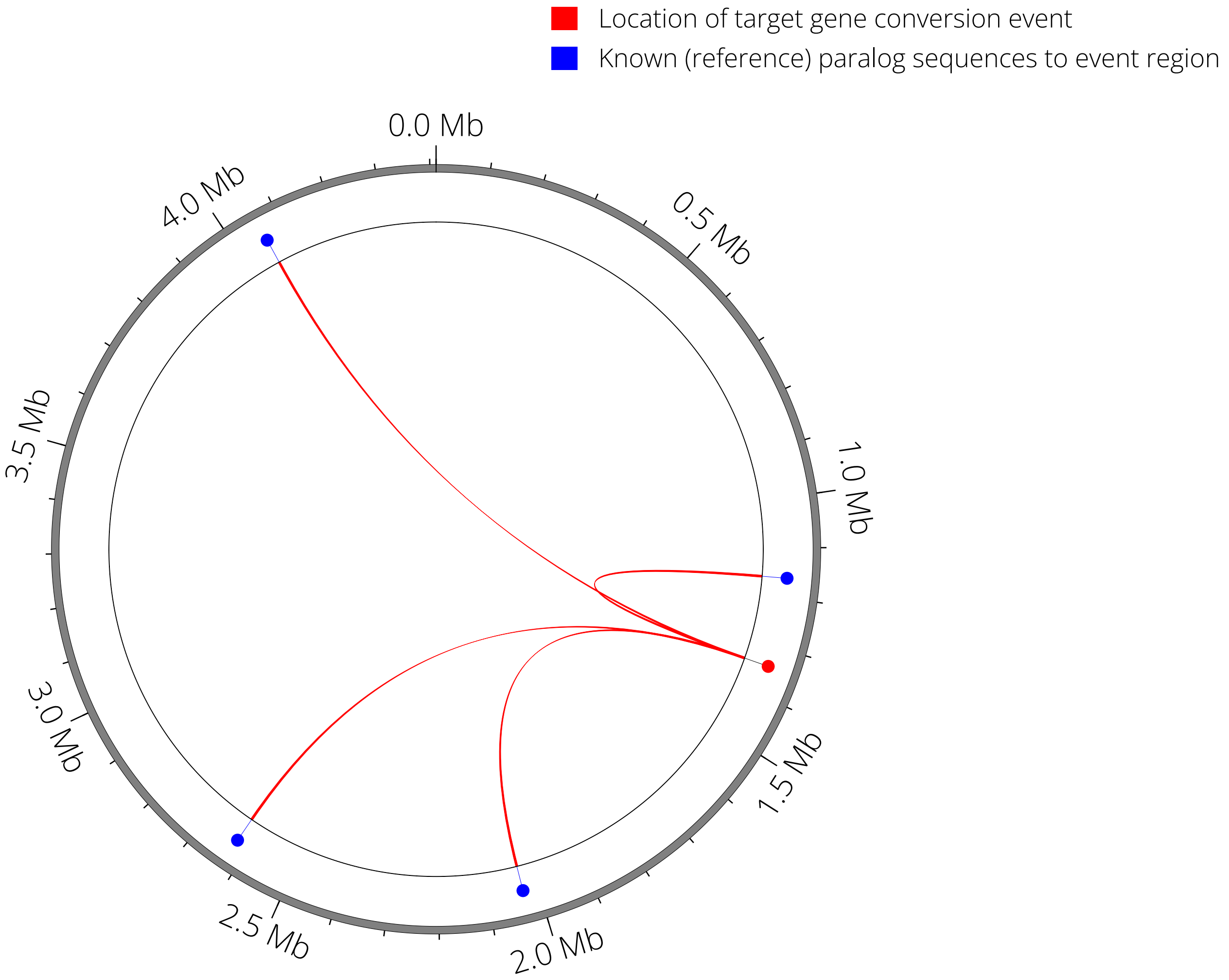

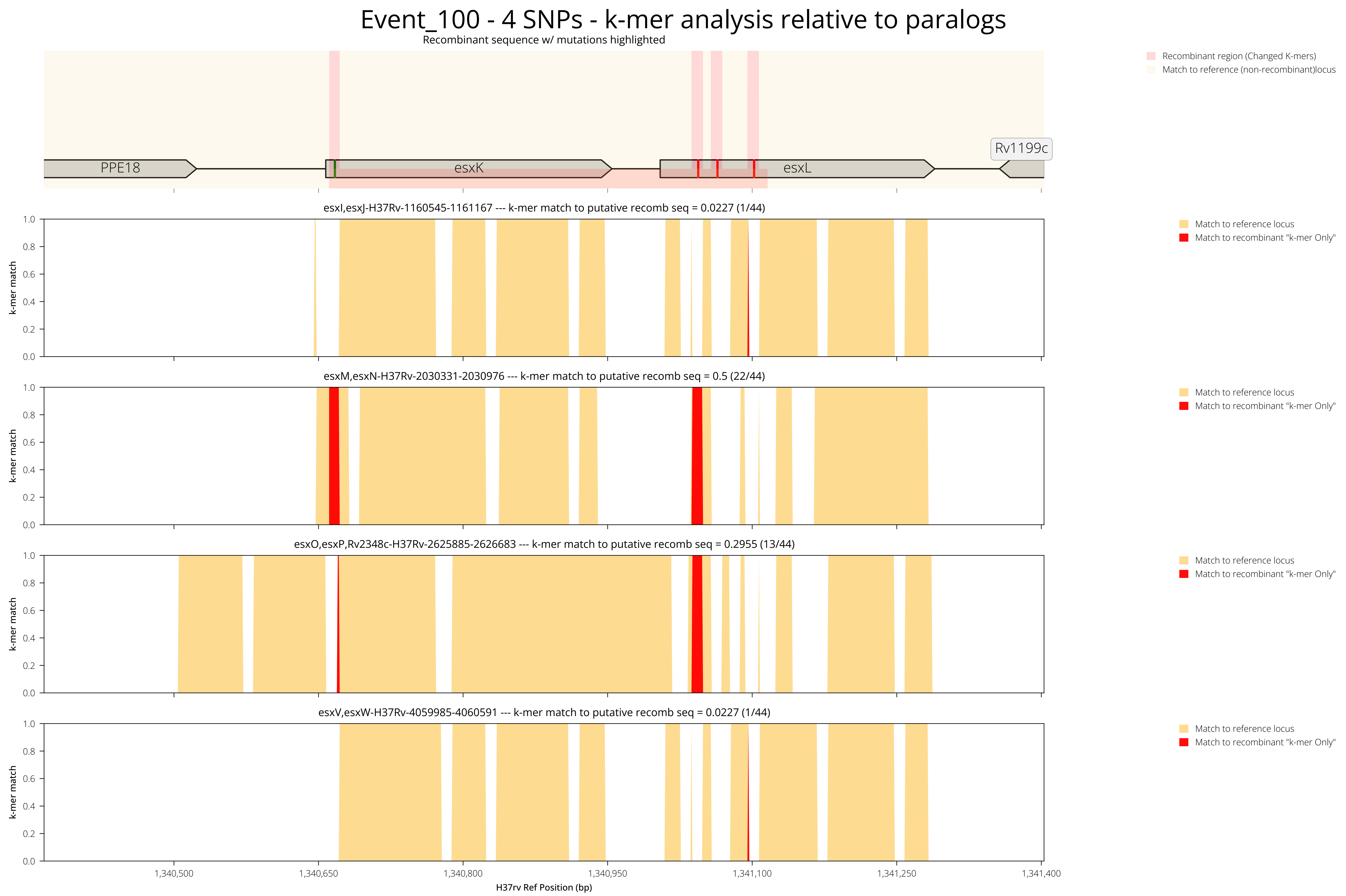
Event_100 - 4 SNPs - k-mer analysis relative to paralogs
Recombinant sequence w/ mutations highlighted
Recombinant region (Changed K-mers)
Match to reference (non-recombinant)locus
PPE18
esxK
esxL
Rv1199c
esxI,esxJ-H37Rv-1160545-1161167 --- k-mer match to putative recomb seq = 0.0227 (1/44)
esxM,esxN-H37Rv-2030331-2030976 --- k-mer match to putative recomb seq = 0.5 (22/44)
esxO,esxP,Rv2348c-H37Rv-2625885-2626683 --- k-mer match to putative recomb seq = 0.2955 (13/44)
esxV,esxW-H37Rv-4059985-4060591 --- k-mer match to putative recomb seq = 0.0227 (1/44)
Match to reference locus
Match to recombinant "k-mer Only"
k-mer match
1.0
0.8
0.6
0.4
0.2
0.0
1,340,500
1,340,650
1,340,800
1,340,950
1,341,100
1,341,250
1,341,400
H37rv Ref Position (bp)

Sub-phylogeny Viz + Paralog Alignments
GCE ID: Event_100

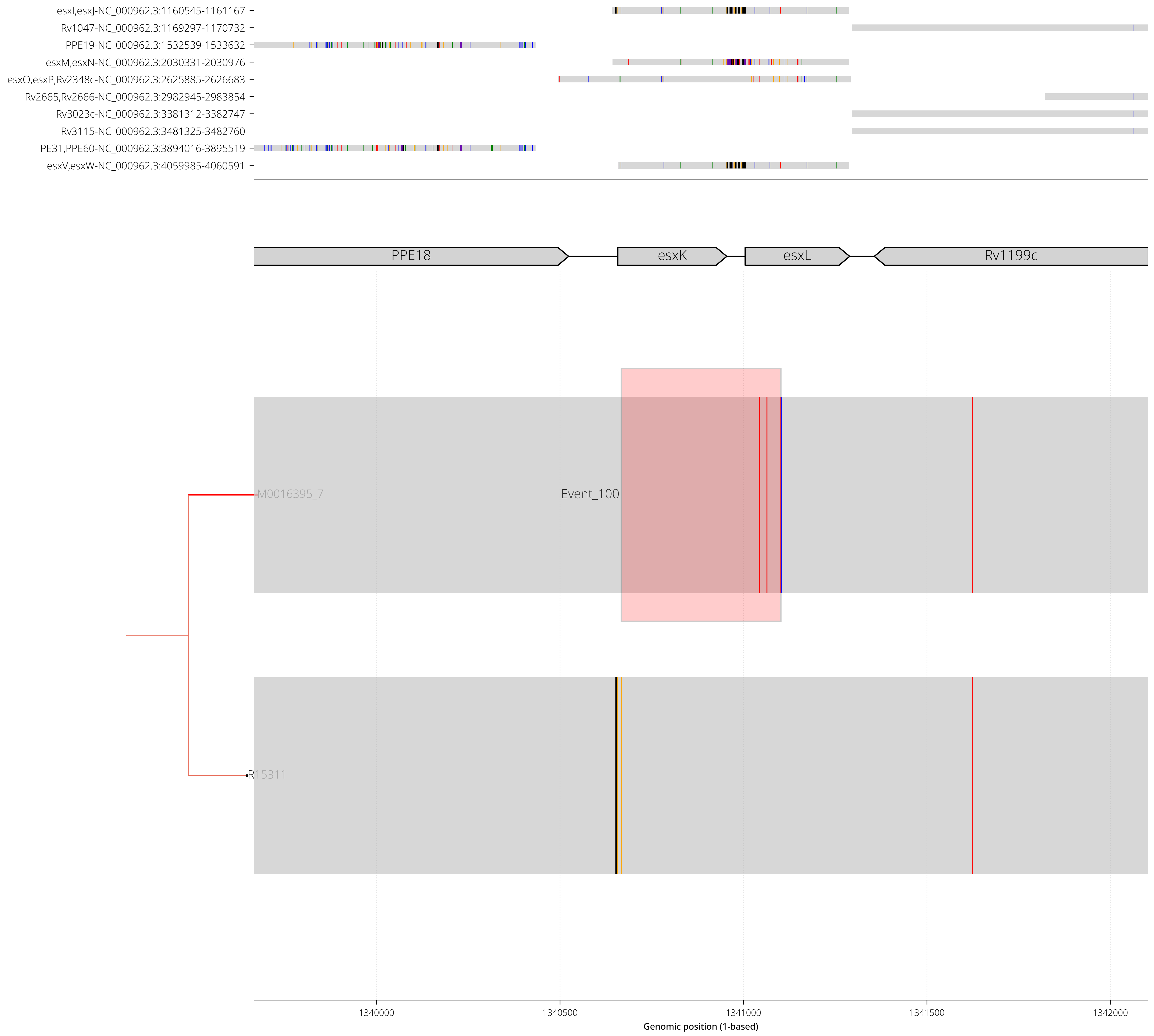

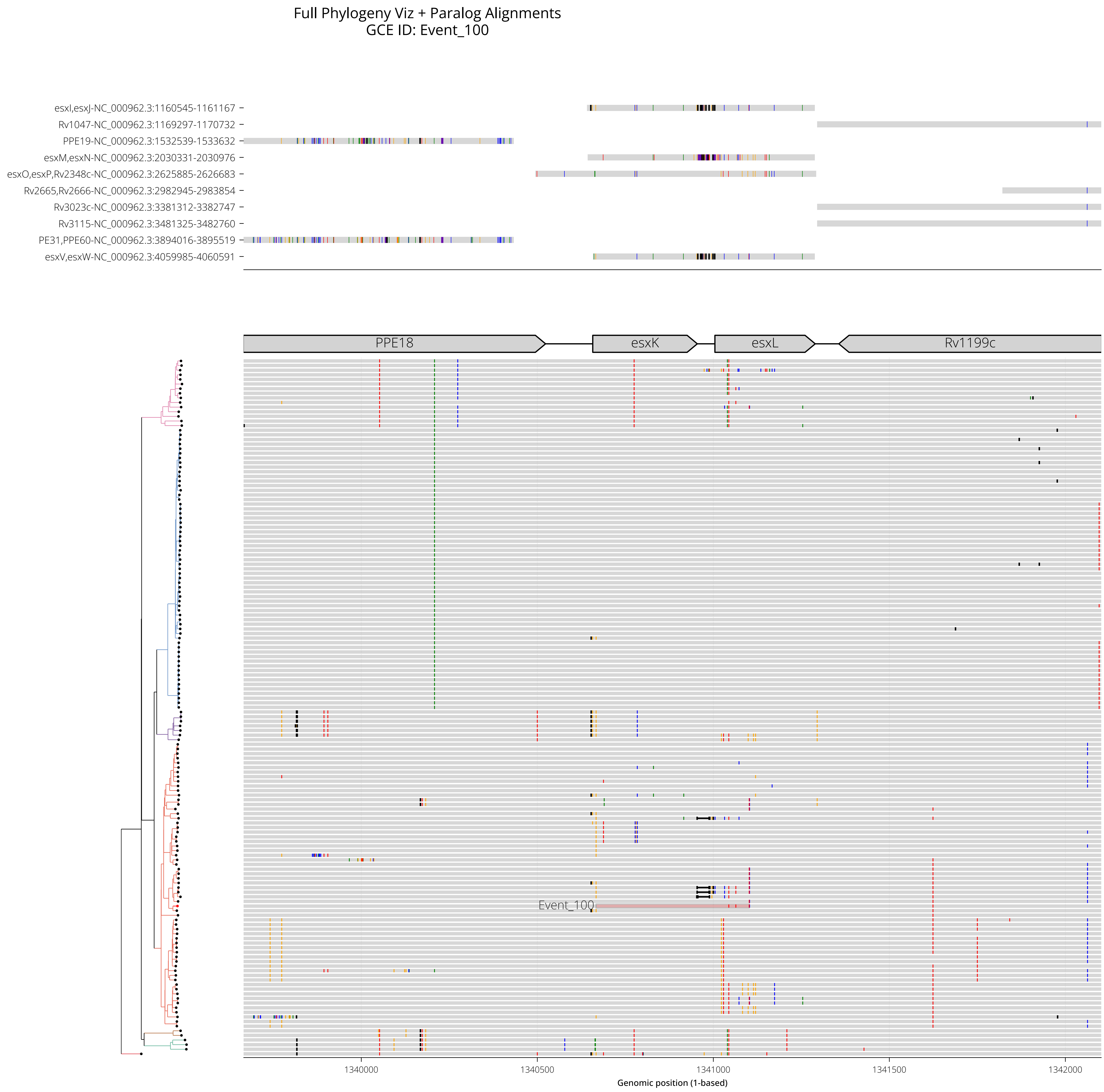

Full Phylogeny Viz + Paralog Alignments
GCE ID: Event_100
esxI,esxJ-NC_000962.3:1160545-1161167
Rv1047-NC_000962.3:1169297-1170732
PPE19-NC_000962.3:1532539-1533632
esxM,esxN-NC_000962.3:2030331-2030976
esxO,esxP,Rv2348c-NC_000962.3:2625885-2626683
Rv2665,Rv2666-NC_000962.3:2982945-2983854
Rv3023c-NC_000962.3:3381312-3382747
Rv3115-NC_000962.3:3481325-3482760
PE31,PPE60-NC_000962.3:3894016-3895519
esxV,esxW-NC_000962.3:4059985-4060591
PPE18
esxK
esxL
Rv1199c
Event_100
1340000
1340500
1341000
1341500
1342000
Genomic position (1-based)

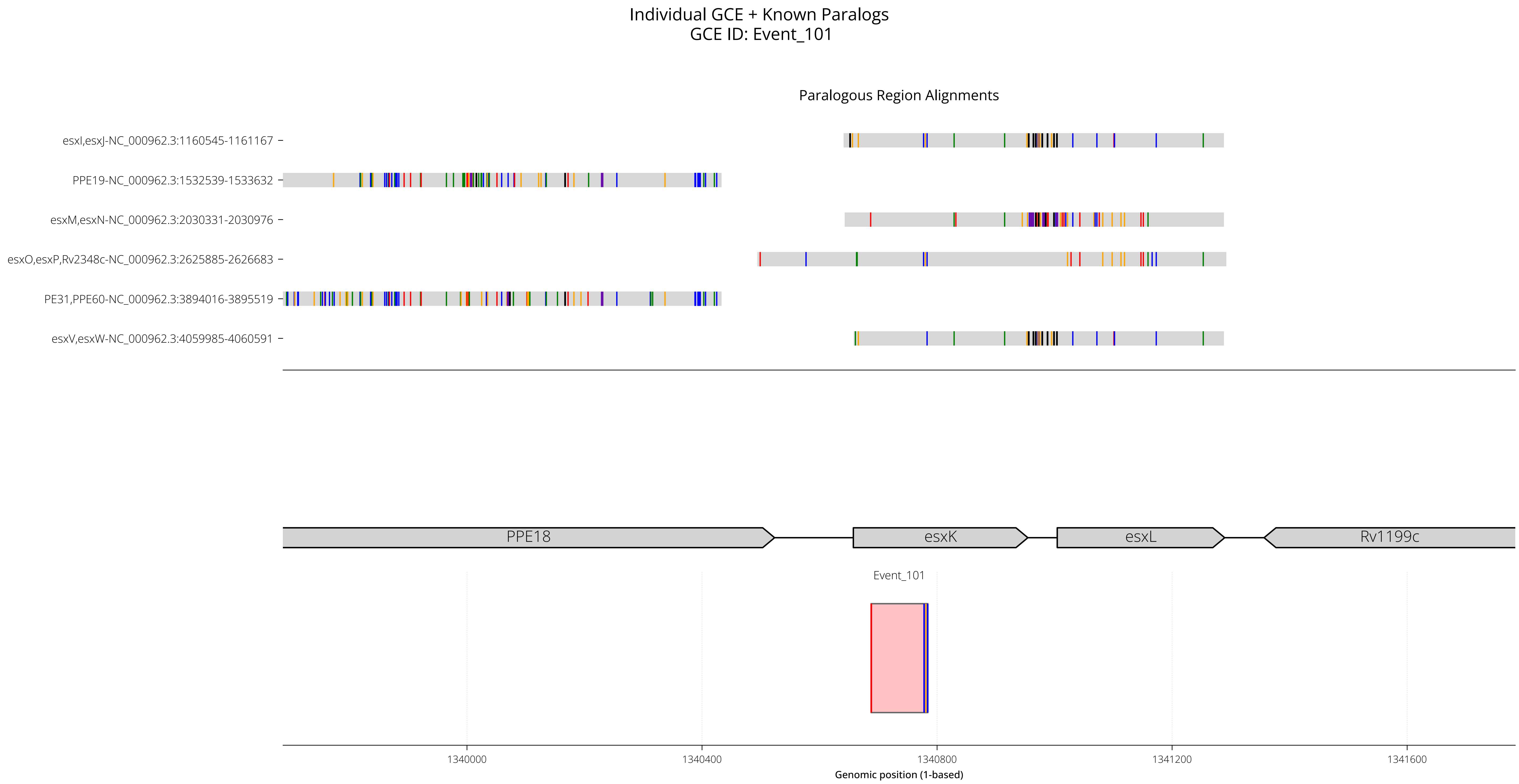
Individual GCE + Known Paralogs
GCE ID: Event_101
Paralogous Region Alignments
esxI,esxJ-NC_000962.3:1160545-1161167
PPE19-NC_000962.3:1532539-1533632
esxM,esxN-NC_000962.3:2030331-2030976
esxO,esxP,Rv2348c-NC_000962.3:2625885-2626683
PE31,PPE60-NC_000962.3:3894016-3895519
esxV,esxW-NC_000962.3:4059985-4060591
PPE18
esxK
esxL
Rv1199c
Event_101
1340000
1340400
1340800
1341200
1341600
Genomic position (1-based)

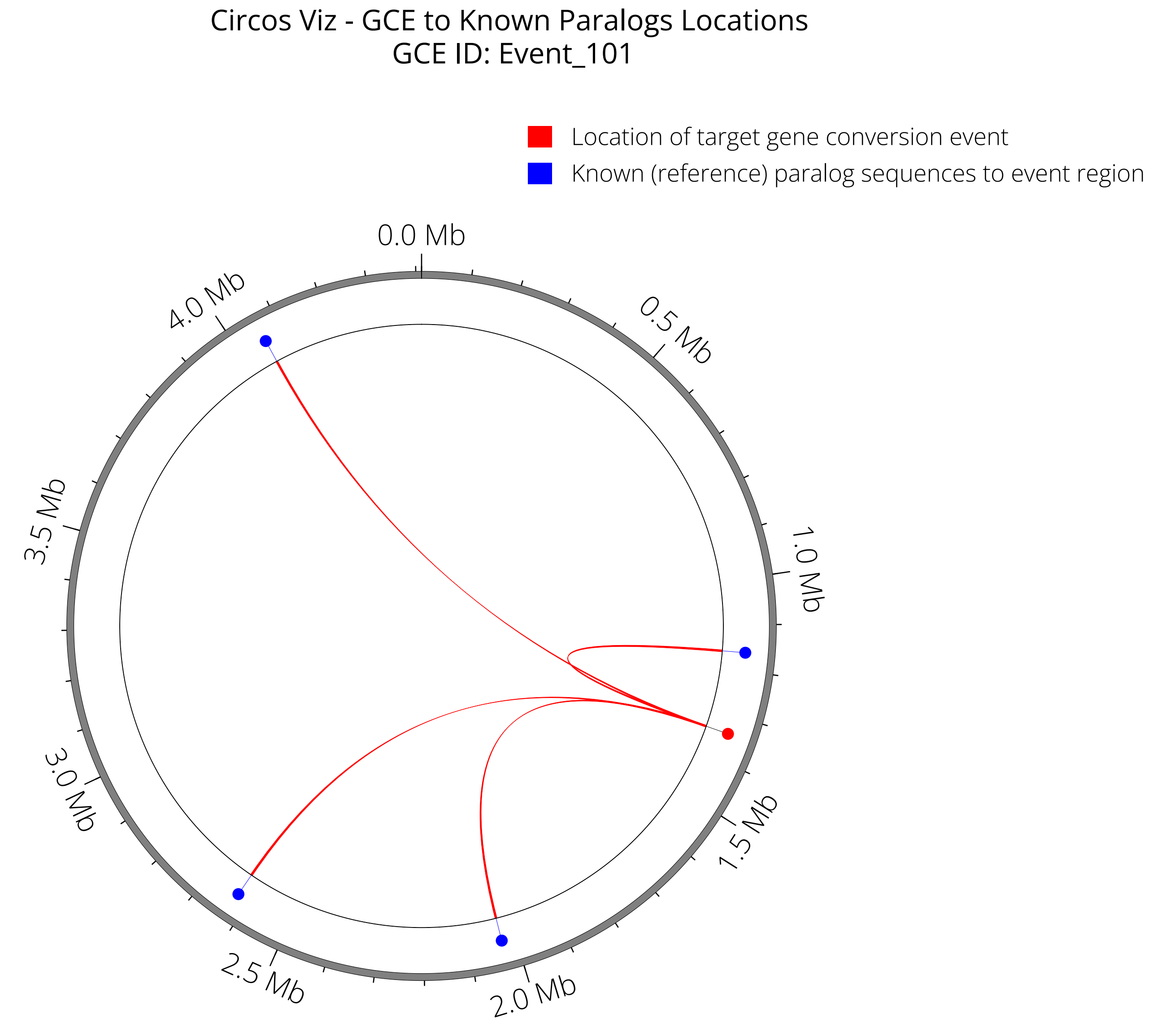
Circos Viz - GCE to Known Paralogs Locations
GCE ID: Event_101
Location of target gene conversion event
Known (reference) paralog sequences to event region
0.0 Mb
0.5 Mb
1.0 Mb
1.5 Mb
2.0 Mb
2.5 Mb
3.0 Mb
3.5 Mb
4.0 Mb

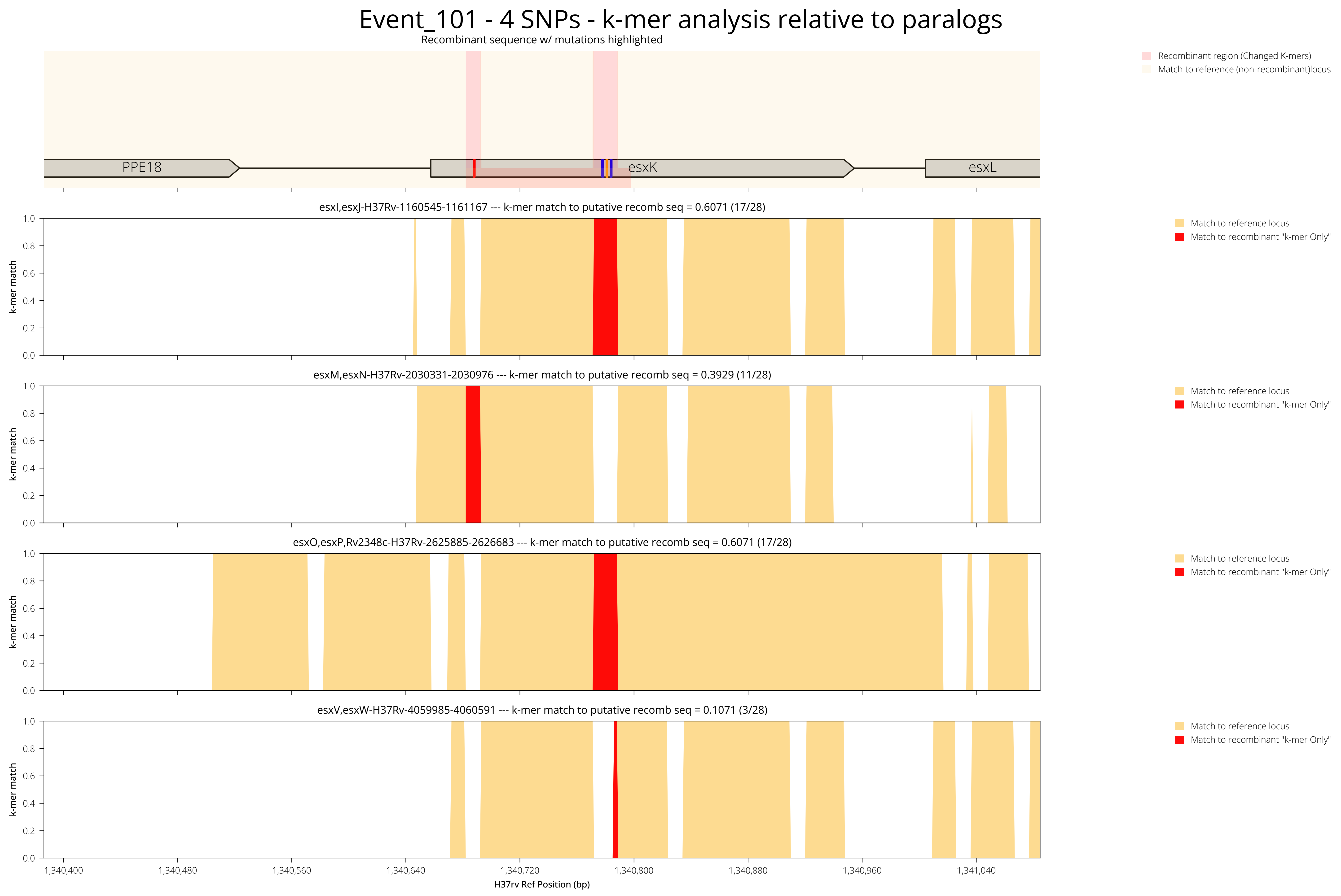

Event_101 - 4 SNPs - k-mer analysis relative to paralogs
Recombinant sequence w/ mutations highlighted
Recombinant region (Changed K-mers)
Match to reference (non-recombinant)locus
PPE18
esxK
esxL
esxI,esxJ-H37Rv-1160545-1161167 --- k-mer match to putative recomb seq = 0.6071 (17/28)
esxM,esxN-H37Rv-2030331-2030976 --- k-mer match to putative recomb seq = 0.3929 (11/28)
esxO,esxP,Rv2348c-H37Rv-2625885-2626683 --- k-mer match to putative recomb seq = 0.6071 (17/28)
esxV,esxW-H37Rv-4059985-4060591 --- k-mer match to putative recomb seq = 0.1071 (3/28)
Match to reference locus
Match to recombinant "k-mer Only"
k-mer match
1.0
0.8
0.6
0.4
0.2
0.0
1,340,400
1,340,480
1,340,560
1,340,640
1,340,720
1,340,800
1,340,880
1,340,960
1,341,040
H37rv Ref Position (bp)

Sub-phylogeny Viz + Paralog Alignments
GCE ID: Event_101

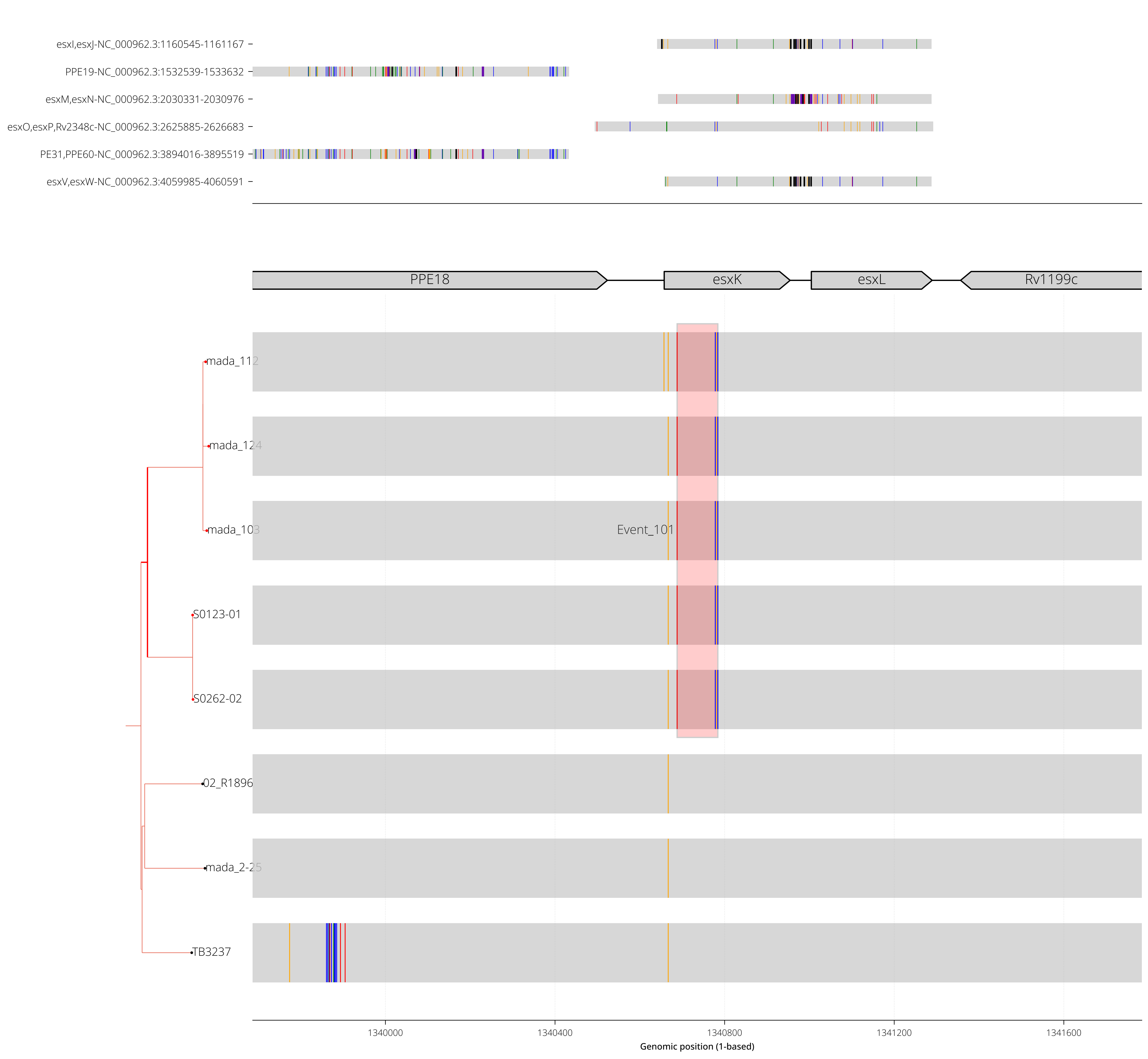

Full Phylogeny Viz + Paralog Alignments
GCE ID: Event_101

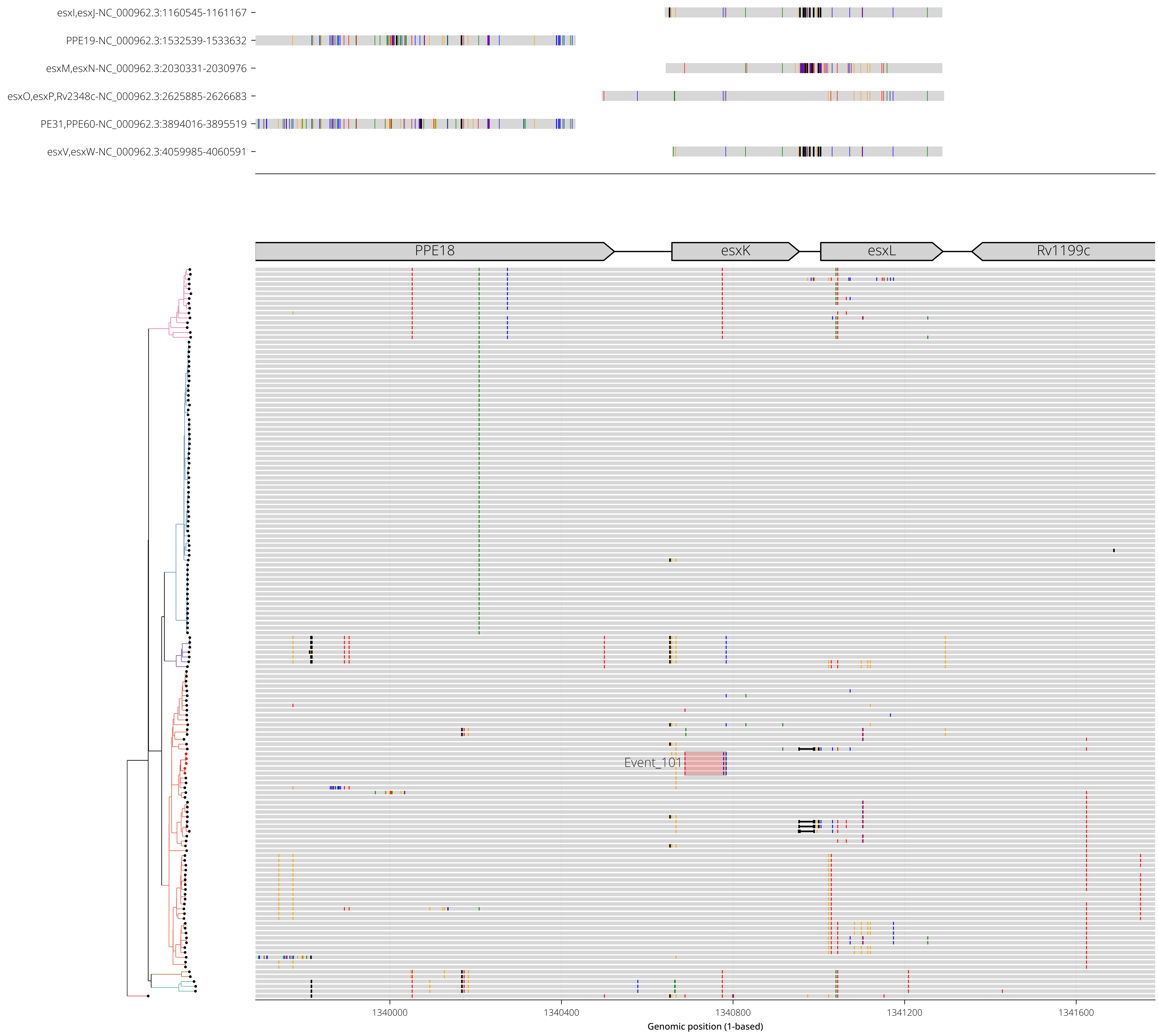

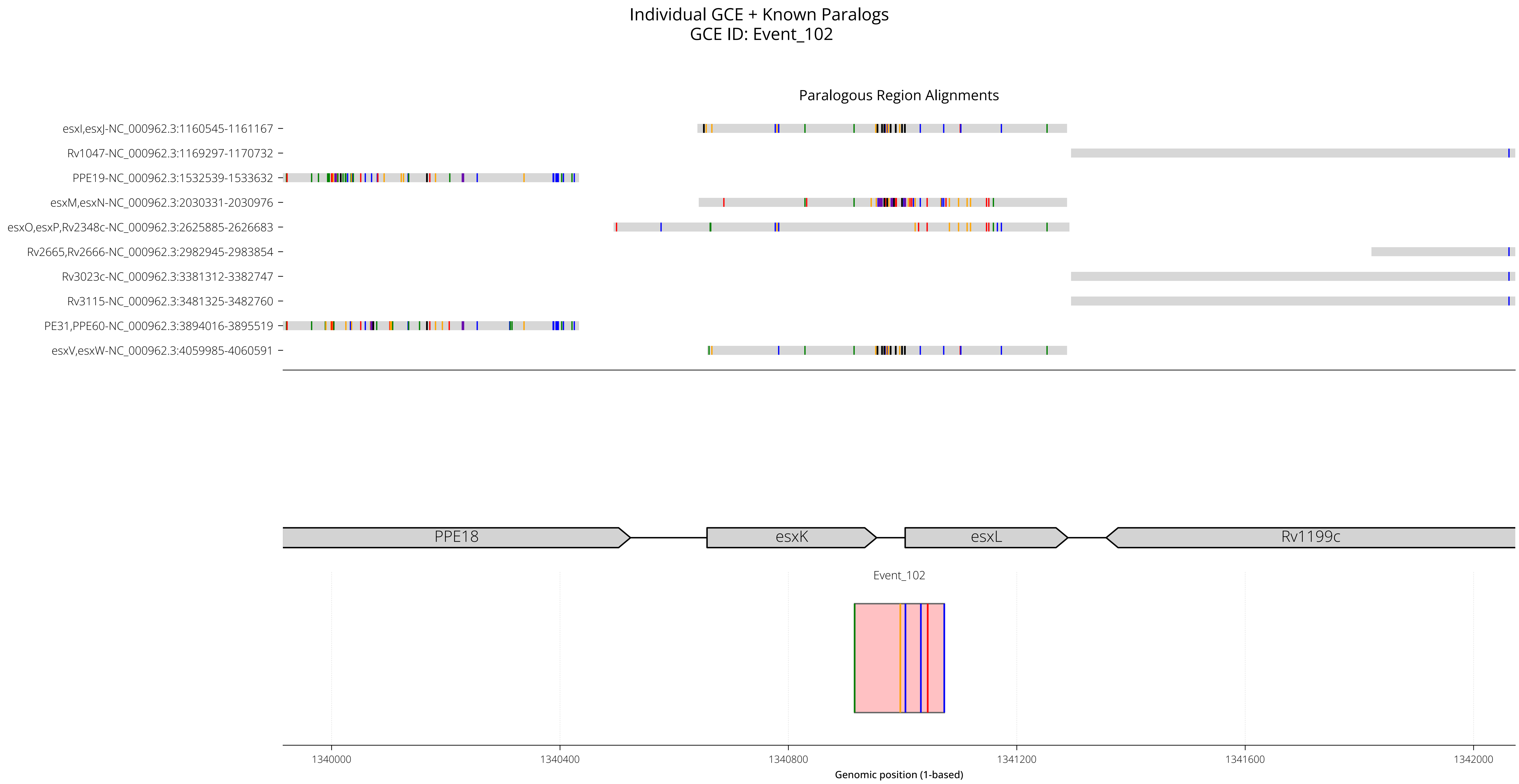
Individual GCE + Known Paralogs
GCE ID: Event_102
Paralogous Region Alignments
esxI,esxJ-NC_000962.3:1160545-1161167
Rv1047-NC_000962.3:1169297-1170732
PPE19-NC_000962.3:1532539-1533632
esxM,esxN-NC_000962.3:2030331-2030976
esxO,esxP,Rv2348c-NC_000962.3:2625885-2626683
Rv2665,Rv2666-NC_000962.3:2982945-2983854
Rv3023c-NC_000962.3:3381312-3382747
Rv3115-NC_000962.3:3481325-3482760
PE31,PPE60-NC_000962.3:3894016-3895519
esxV,esxW-NC_000962.3:4059985-4060591
PPE18
esxK
esxL
Rv1199c
Event_102
1340000
1340400
1340800
1341200
1341600
1342000
Genomic position (1-based)

Circos Viz - GCE to Known Paralogs Locations
GCE ID: Event_102

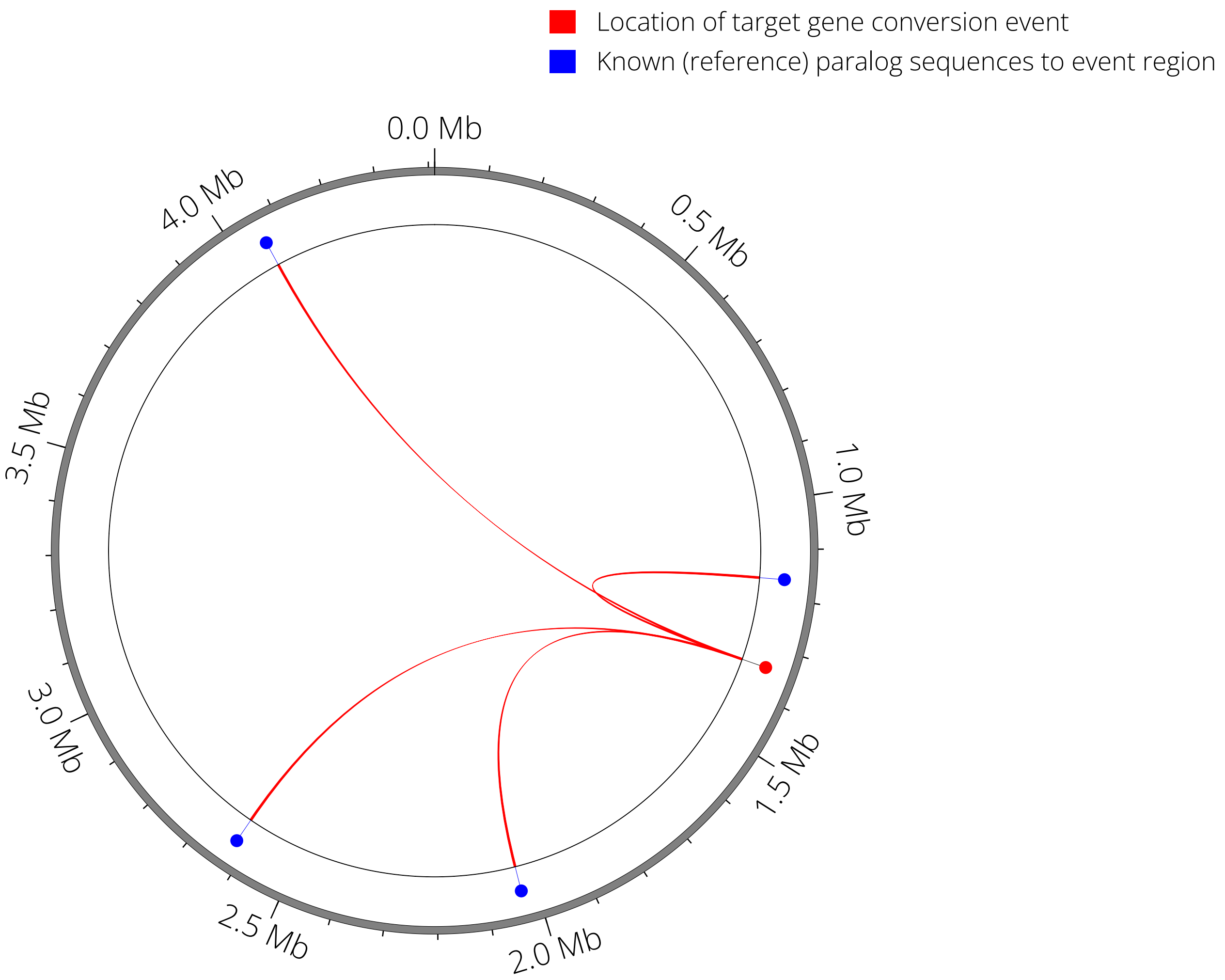

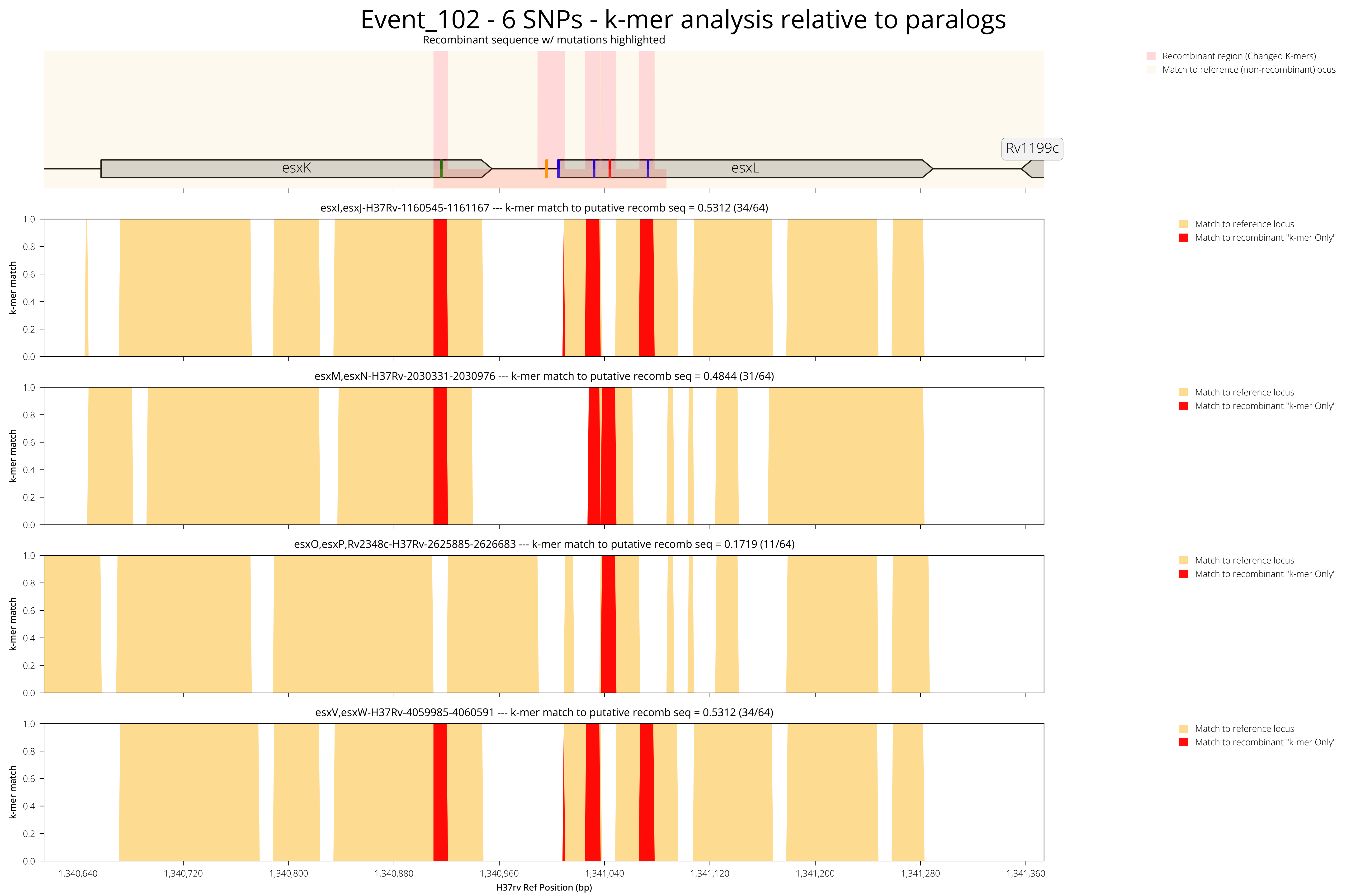

Event_102 - 6 SNPs - k-mer analysis relative to paralogs
Recombinant sequence w/ mutations highlighted
Recombinant region (Changed K-mers)
Match to reference (non-recombinant)locus
esxK
esxL
Rv1199c
esxI,esxJ-H37Rv-1160545-1161167 --- k-mer match to putative recomb seq = 0.5312 (34/64)
esxM,esxN-H37Rv-2030331-2030976 --- k-mer match to putative recomb seq = 0.4844 (31/64)
esxO,esxP,Rv2348c-H37Rv-2625885-2626683 --- k-mer match to putative recomb seq = 0.1719 (11/64)
esxV,esxW-H37Rv-4059985-4060591 --- k-mer match to putative recomb seq = 0.5312 (34/64)
Match to reference locus
Match to recombinant "k-mer Only"
k-mer match
0.0
0.2
0.4
0.6
0.8
1.0
1,340,640
1,340,720
1,340,800
1,340,880
1,340,960
1,341,040
1,341,120
1,341,200
1,341,280
1,341,360
H37rv Ref Position (bp)

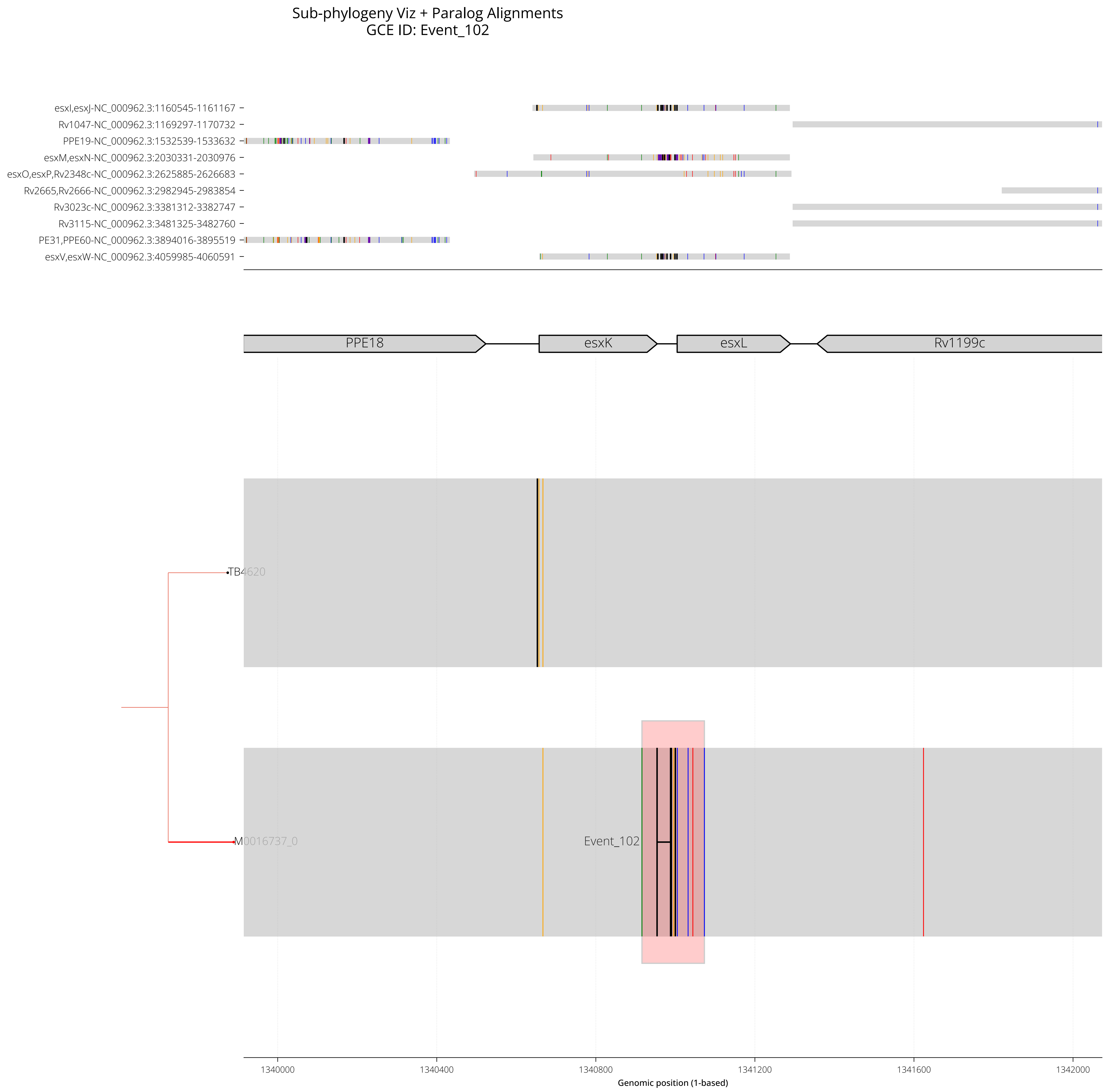

Sub-phylogeny Viz + Paralog Alignments
GCE ID: Event_102
esxI,esxJ-NC_000962.3:1160545-1161167
Rv1047-NC_000962.3:1169297-1170732
PPE19-NC_000962.3:1532539-1533632
esxM,esxN-NC_000962.3:2030331-2030976
esxO,esxP,Rv2348c-NC_000962.3:2625885-2626683
Rv2665,Rv2666-NC_000962.3:2982945-2983854
Rv3023c-NC_000962.3:3381312-3382747
Rv3115-NC_000962.3:3481325-3482760
PE31,PPE60-NC_000962.3:3894016-3895519
esxV,esxW-NC_000962.3:4059985-4060591
PPE18
esxK
esxL
Rv1199c
TB4620
M0016737_0
Event_102
1340000
1340400
1340800
1341200
1341600
1342000
Genomic position (1-based)

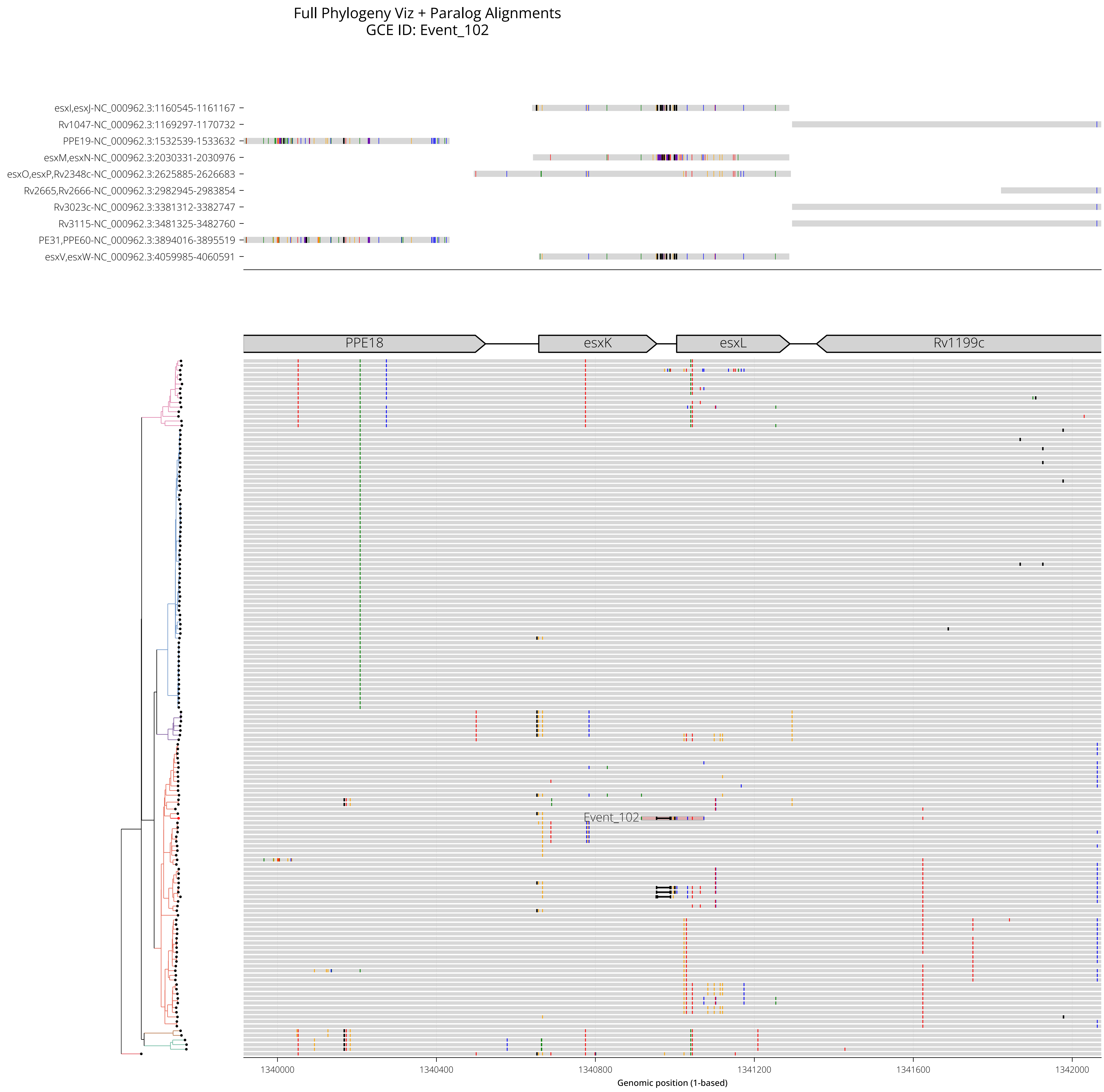

Full Phylogeny Viz + Paralog Alignments
GCE ID: Event_102
esxI,esxJ-NC_000962.3:1160545-1161167
Rv1047-NC_000962.3:1169297-1170732
PPE19-NC_000962.3:1532539-1533632
esxM,esxN-NC_000962.3:2030331-2030976
esxO,esxP,Rv2348c-NC_000962.3:2625885-2626683
Rv2665,Rv2666-NC_000962.3:2982945-2983854
Rv3023c-NC_000962.3:3381312-3382747
Rv3115-NC_000962.3:3481325-3482760
PE31,PPE60-NC_000962.3:3894016-3895519
esxV,esxW-NC_000962.3:4059985-4060591
PPE18
esxK
esxL
Rv1199c
Event_102
1340000
1340400
1340800
1341200
1341600
1342000
Genomic position (1-based)

Individual GCE + Known Paralogs
GCE ID: Event_103

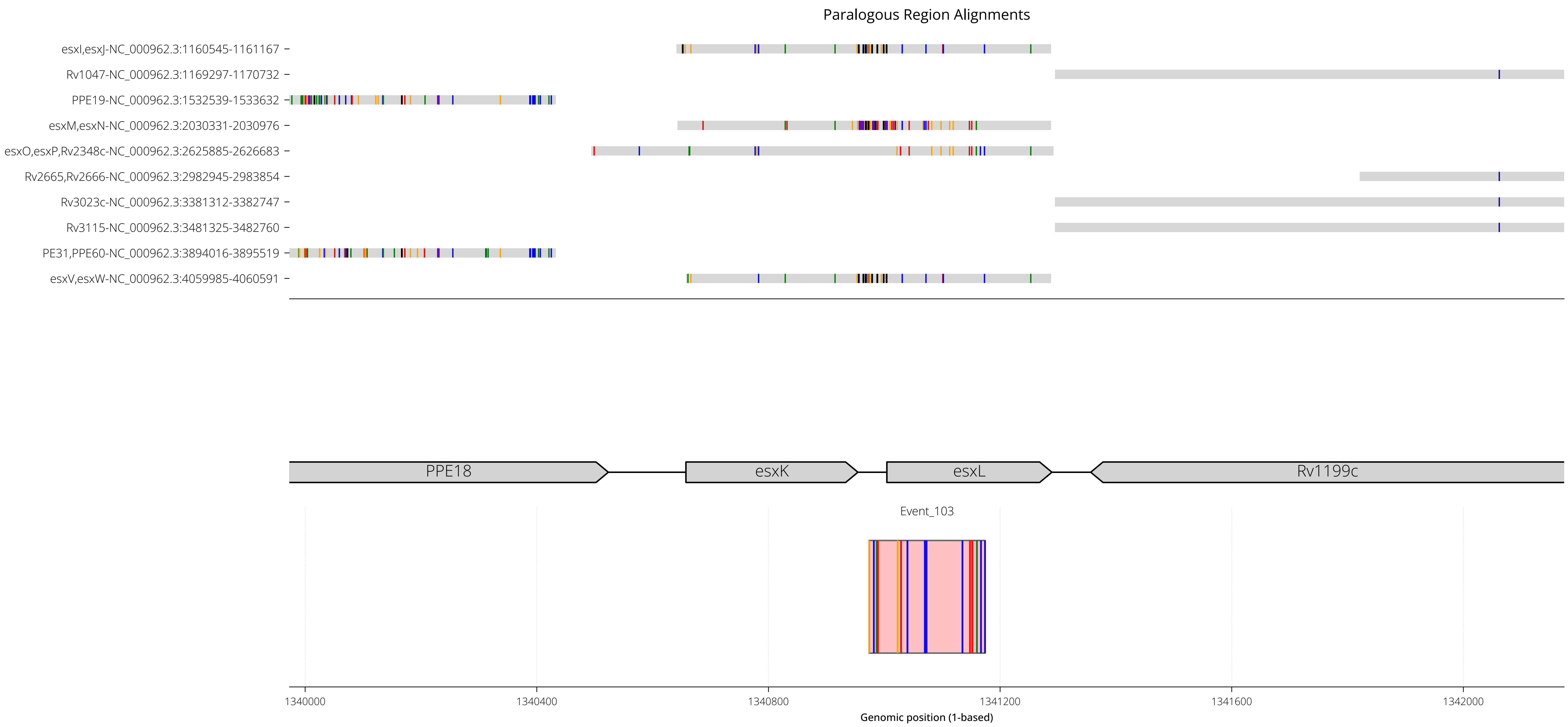

Circos Viz - GCE to Known Paralogs Locations
GCE ID: Event_103

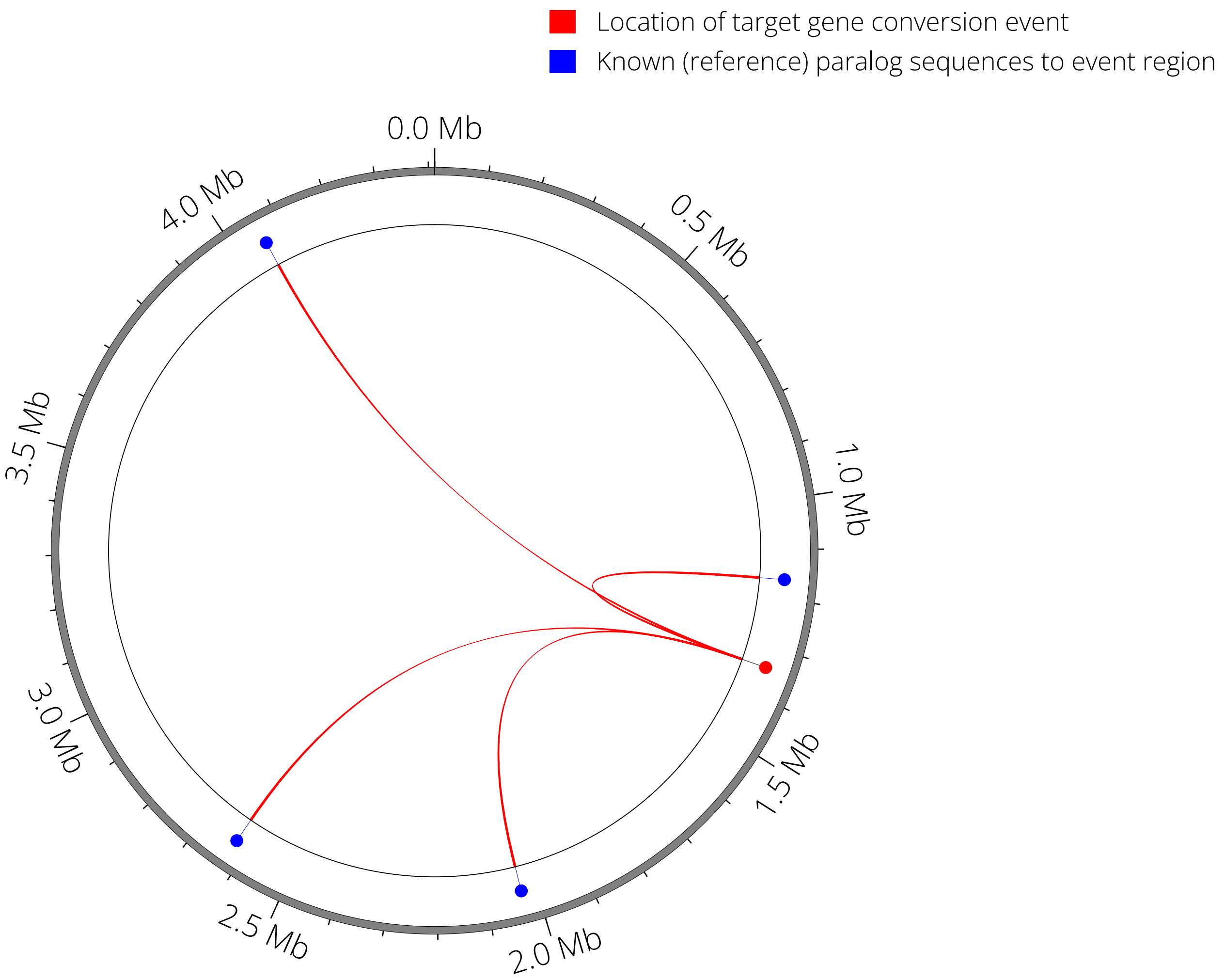

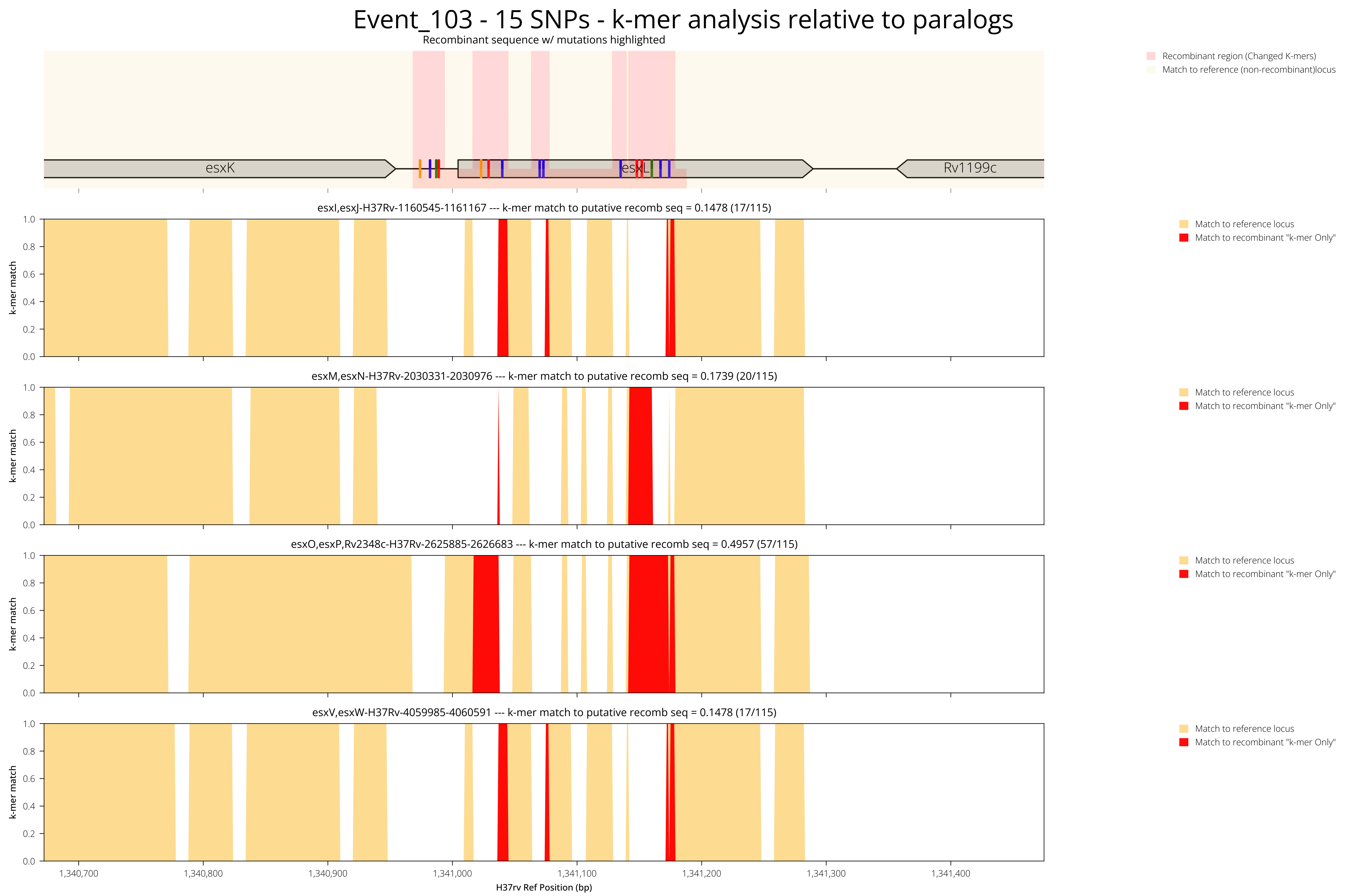
Event_103 - 15 SNPs - k-mer analysis relative to paralogs
Recombinant sequence w/ mutations highlighted
Recombinant region (Changed K-mers)
Match to reference (non-recombinant)locus
esxK
esxL
Rv1199c
esxI,esxJ-H37Rv-1160545-1161167 --- k-mer match to putative recomb seq = 0.1478 (17/115)
esxM,esxN-H37Rv-2030331-2030976 --- k-mer match to putative recomb seq = 0.1739 (20/115)
esxO,esxP,Rv2348c-H37Rv-2625885-2626683 --- k-mer match to putative recomb seq = 0.4957 (57/115)
esxV,esxW-H37Rv-4059985-4060591 --- k-mer match to putative recomb seq = 0.1478 (17/115)
Match to reference locus
Match to recombinant "k-mer Only"
k-mer match
1.0
0.8
0.6
0.4
0.2
0.0
1,340,700
1,340,800
1,340,900
1,341,000
1,341,100
1,341,200
1,341,300
1,341,400
H37rv Ref Position (bp)

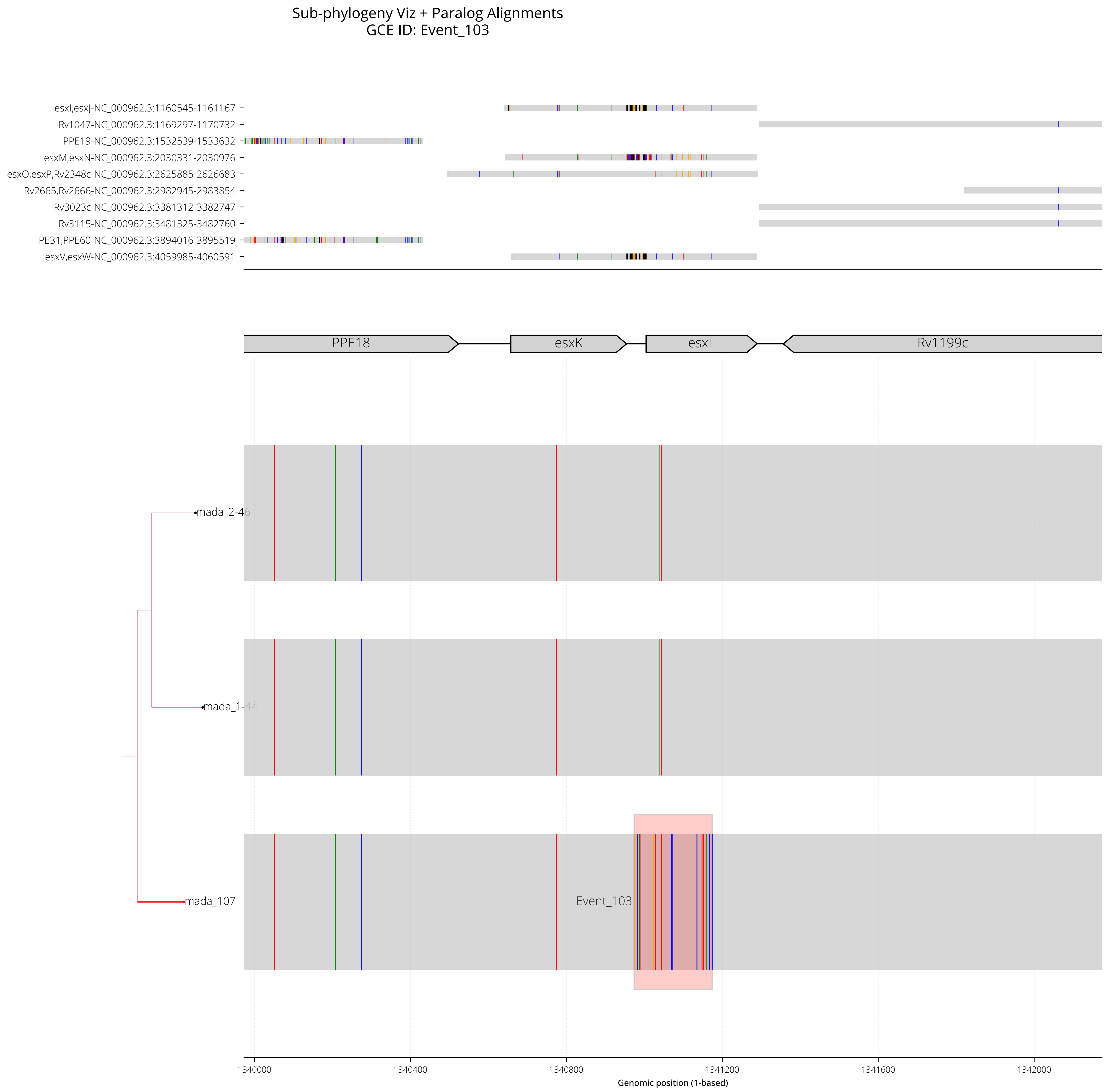

Sub-phylogeny Viz + Paralog Alignments
GCE ID: Event_103
esxI,esxJ-NC_000962.3:1160545-1161167
Rv1047-NC_000962.3:1169297-1170732
PPE19-NC_000962.3:1532539-1533632
esxM,esxN-NC_000962.3:2030331-2030976
esxO,esxP,Rv2348c-NC_000962.3:2625885-2626683
Rv2665,Rv2666-NC_000962.3:2982945-2983854
Rv3023c-NC_000962.3:3381312-3382747
Rv3115-NC_000962.3:3481325-3482760
PE31,PPE60-NC_000962.3:3894016-3895519
esxV,esxW-NC_000962.3:4059985-4060591
PPE18
esxK
esxL
Rv1199c
mada_2-46
mada_1-44
mada_107
Event_103
1340000
1340400
1340800
1341200
1341600
1342000
Genomic position (1-based)

Full Phylogeny Viz + Paralog Alignments
GCE ID: Event_103

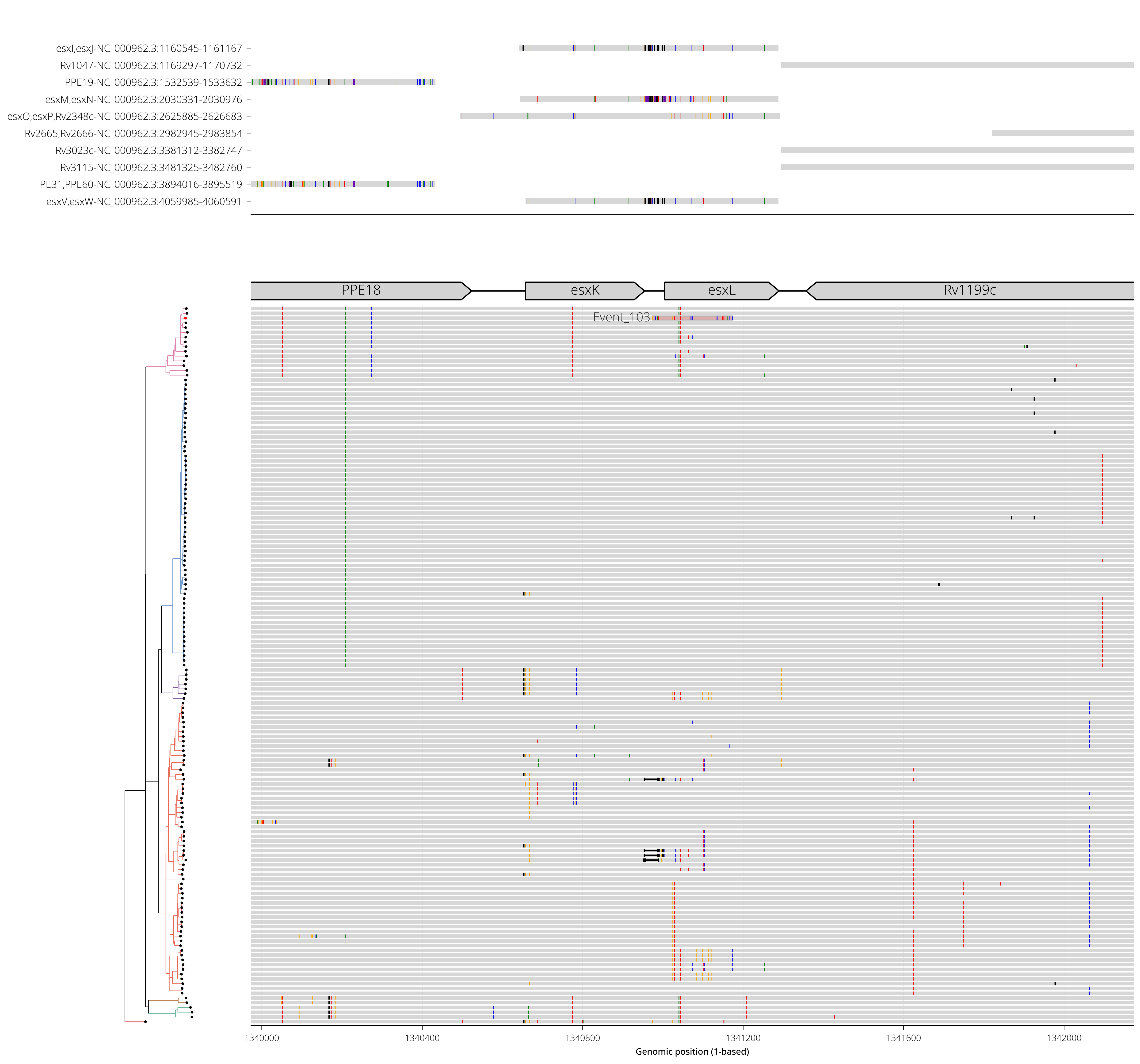

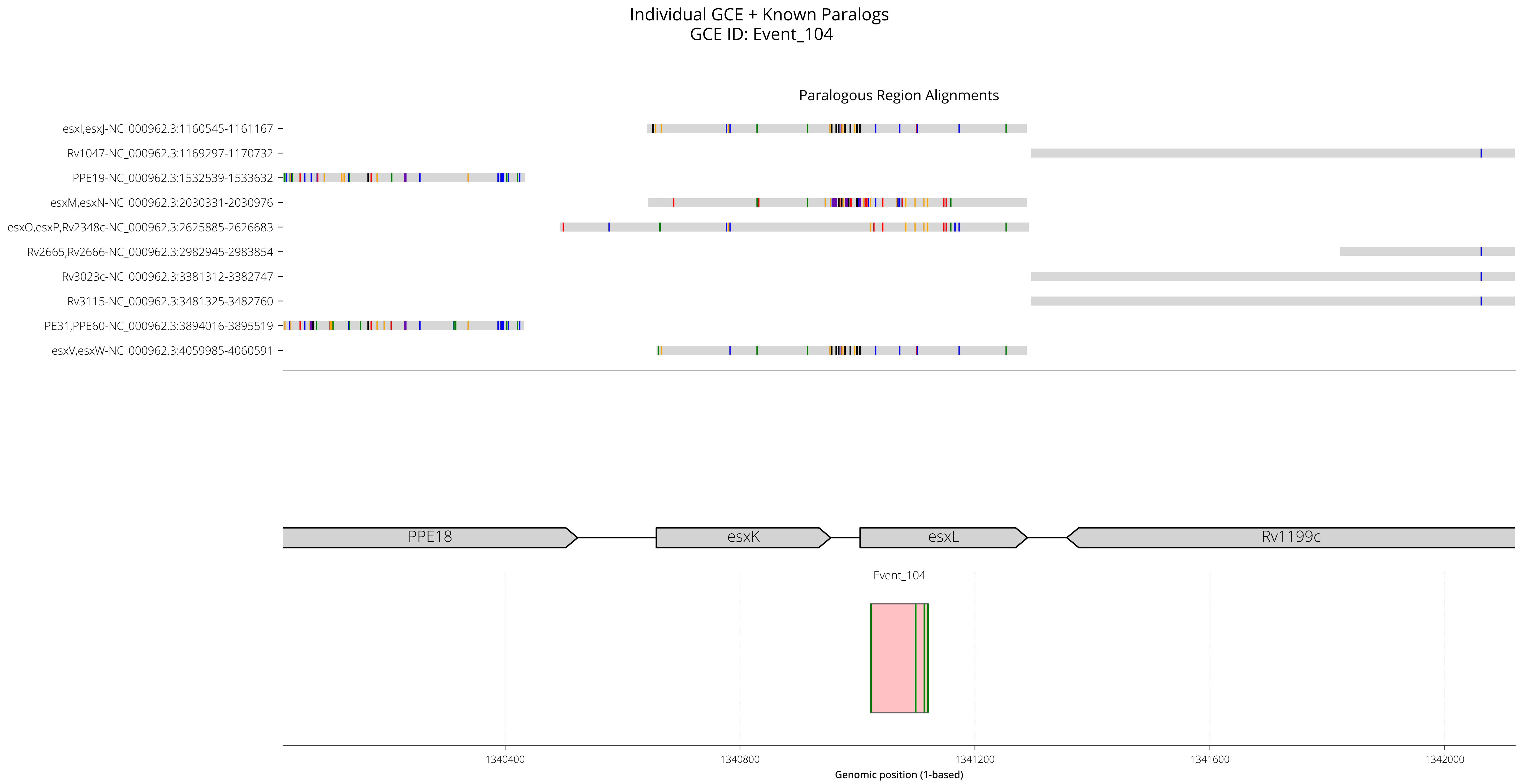
Individual GCE + Known Paralogs
GCE ID: Event_104
Paralogous Region Alignments
esxI,esxJ-NC_000962.3:1160545-1161167
Rv1047-NC_000962.3:1169297-1170732
PPE19-NC_000962.3:1532539-1533632
esxM,esxN-NC_000962.3:2030331-2030976
esxO,esxP,Rv2348c-NC_000962.3:2625885-2626683
Rv2665,Rv2666-NC_000962.3:2982945-2983854
Rv3023c-NC_000962.3:3381312-3382747
Rv3115-NC_000962.3:3481325-3482760
PE31,PPE60-NC_000962.3:3894016-3895519
esxV,esxW-NC_000962.3:4059985-4060591
PPE18
esxK
esxL
Rv1199c
Event_104
1340400
1340800
1341200
1341600
1342000
Genomic position (1-based)

Circos Viz - GCE to Known Paralogs Locations
GCE ID: Event_104

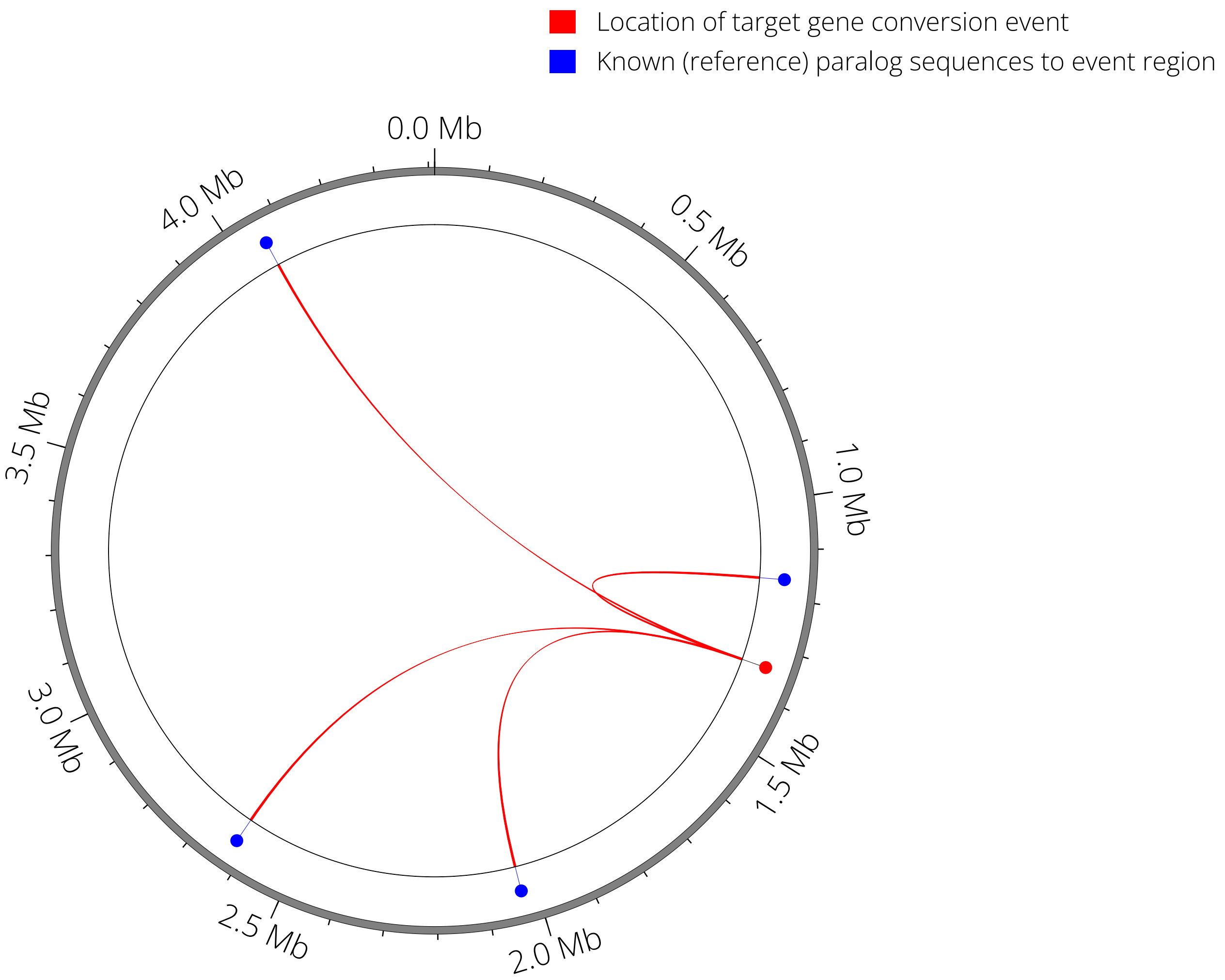

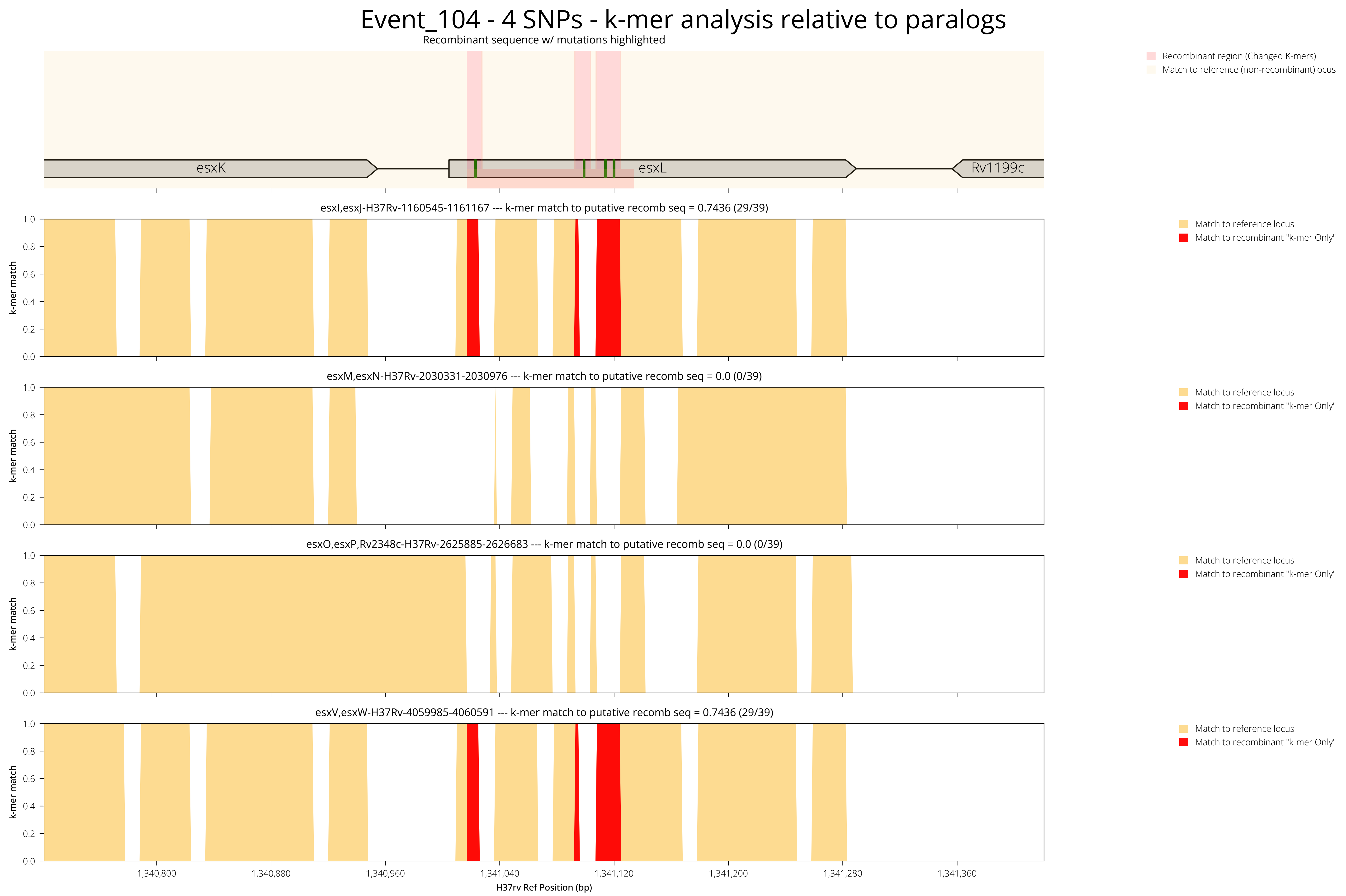

Event_104 - 4 SNPs - k-mer analysis relative to paralogs
Recombinant sequence w/ mutations highlighted
Recombinant region (Changed K-mers)
Match to reference (non-recombinant)locus
esxK
esxL
Rv1199c
esxI,esxJ-H37Rv-1160545-1161167 --- k-mer match to putative recomb seq = 0.7436 (29/39)
esxM,esxN-H37Rv-2030331-2030976 --- k-mer match to putative recomb seq = 0.0 (0/39)
esxO,esxP,Rv2348c-H37Rv-2625885-2626683 --- k-mer match to putative recomb seq = 0.0 (0/39)
esxV,esxW-H37Rv-4059985-4060591 --- k-mer match to putative recomb seq = 0.7436 (29/39)
Match to reference locus
Match to recombinant "k-mer Only"
k-mer match
1.0
0.8
0.6
0.4
0.2
0.0
1,340,800
1,340,880
1,340,960
1,341,040
1,341,120
1,341,200
1,341,280
1,341,360
H37rv Ref Position (bp)

Sub-phylogeny Viz + Paralog Alignments
GCE ID: Event_104

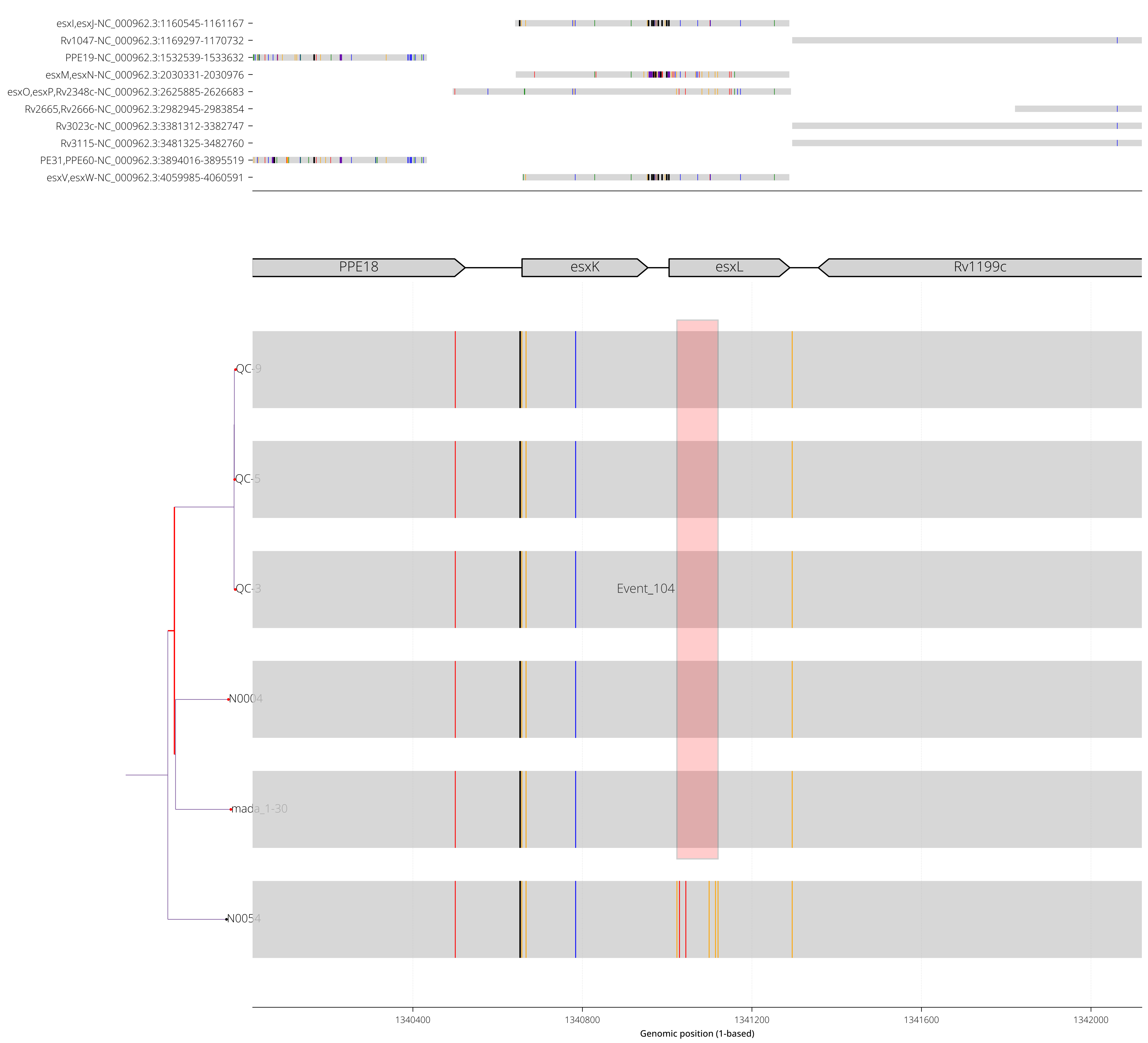

Full Phylogeny Viz + Paralog Alignments
GCE ID: Event_104

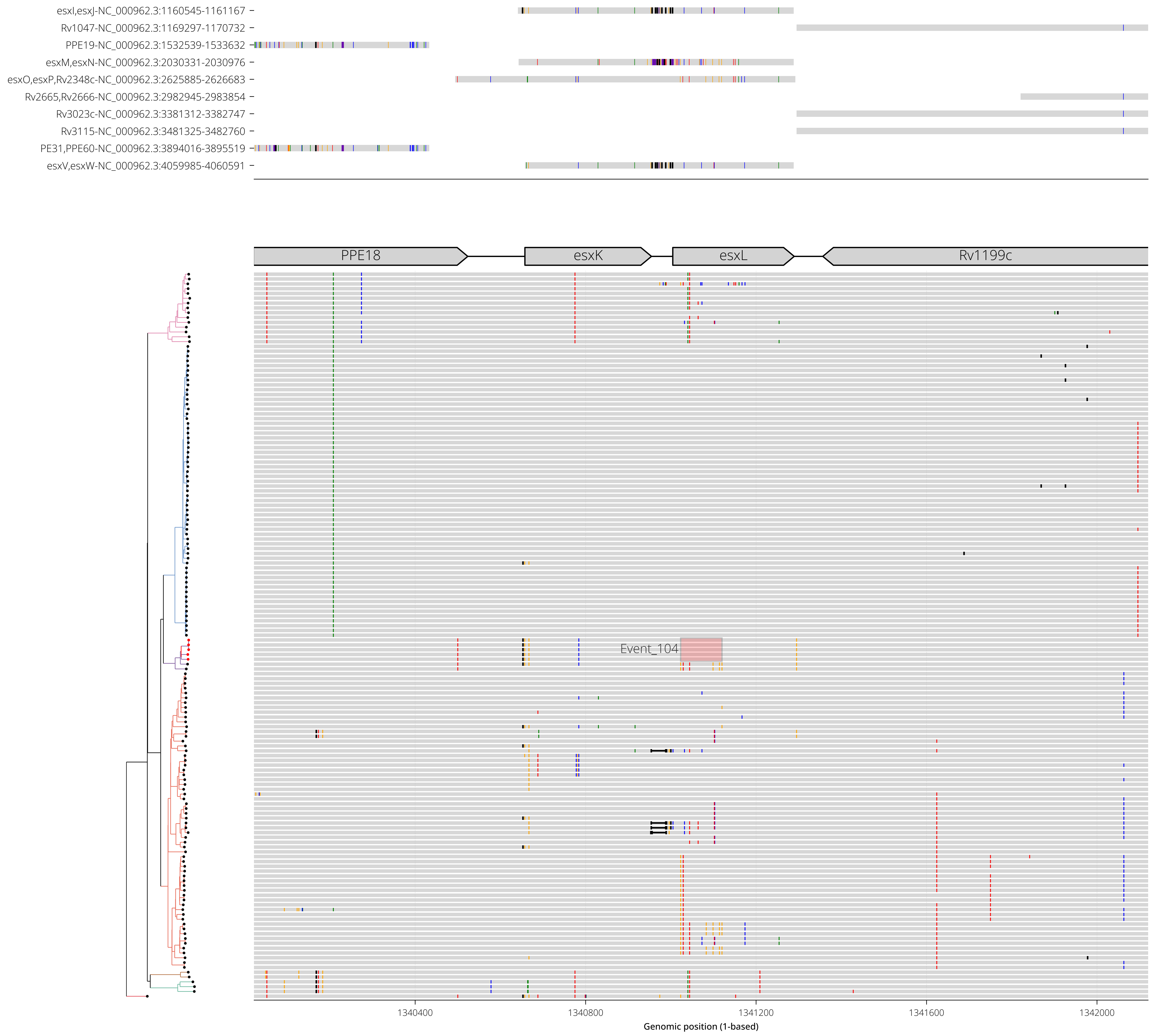

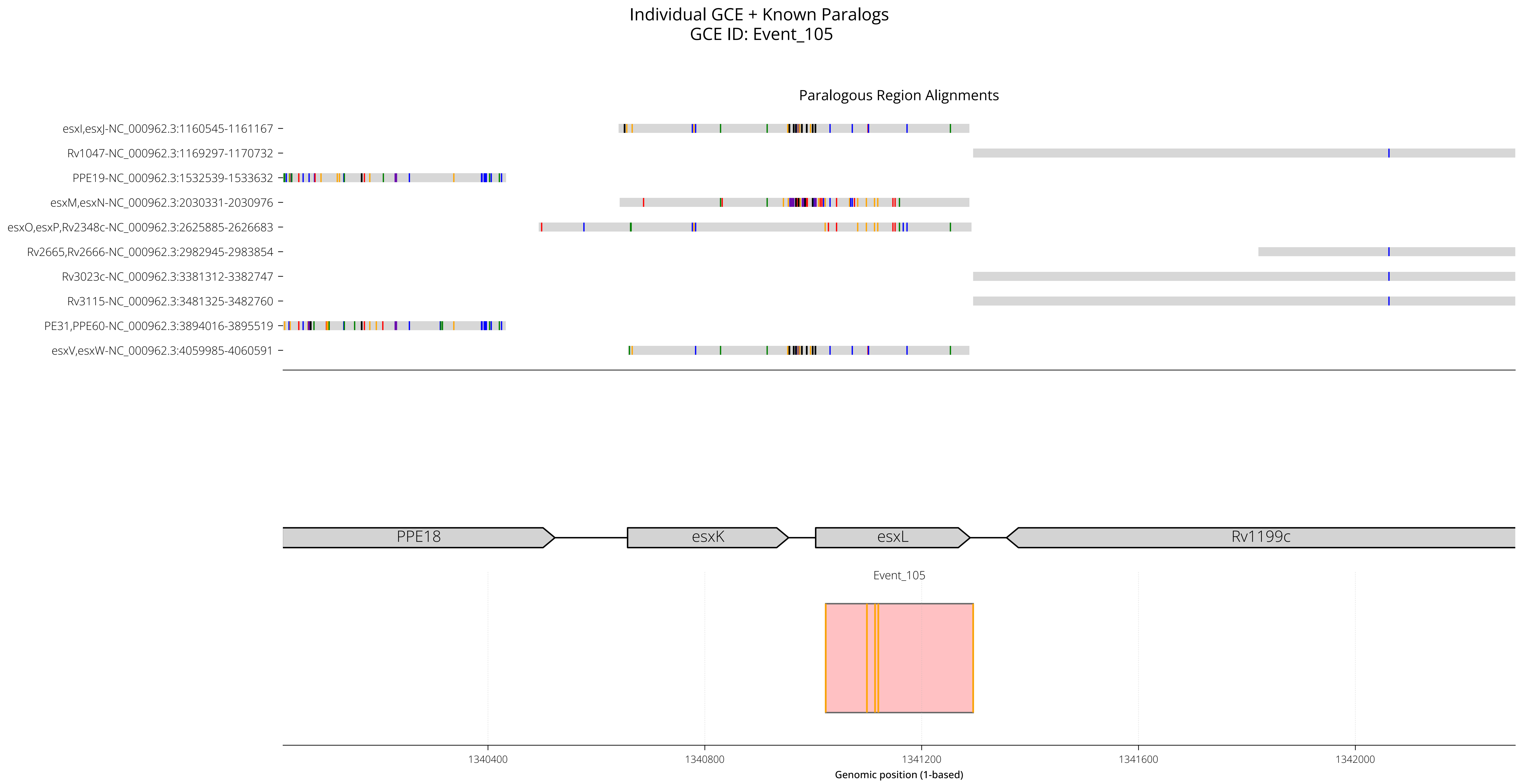

Individual GCE + Known Paralogs
GCE ID: Event_105
Paralogous Region Alignments
esxI,esxJ-NC_000962.3:1160545-1161167
Rv1047-NC_000962.3:1169297-1170732
PPE19-NC_000962.3:1532539-1533632
esxM,esxN-NC_000962.3:2030331-2030976
esxO,esxP,Rv2348c-NC_000962.3:2625885-2626683
Rv2665,Rv2666-NC_000962.3:2982945-2983854
Rv3023c-NC_000962.3:3381312-3382747
Rv3115-NC_000962.3:3481325-3482760
PE31,PPE60-NC_000962.3:3894016-3895519
esxV,esxW-NC_000962.3:4059985-4060591
PPE18
esxK
esxL
Rv1199c
Event_105
1340400
1340800
1341200
1341600
1342000
Genomic position (1-based)

Circos Viz - GCE to Known Paralogs Locations
GCE ID: Event_105

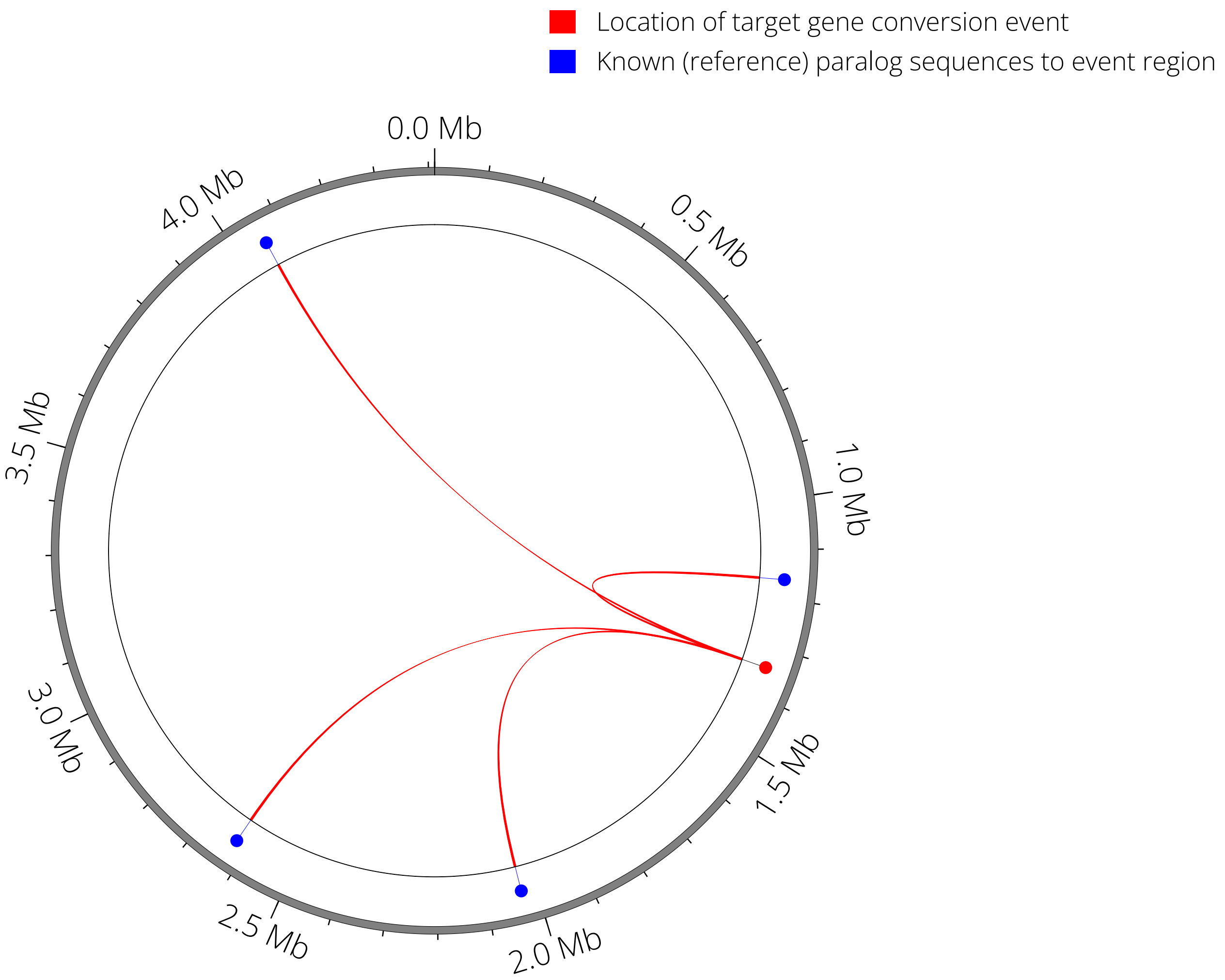

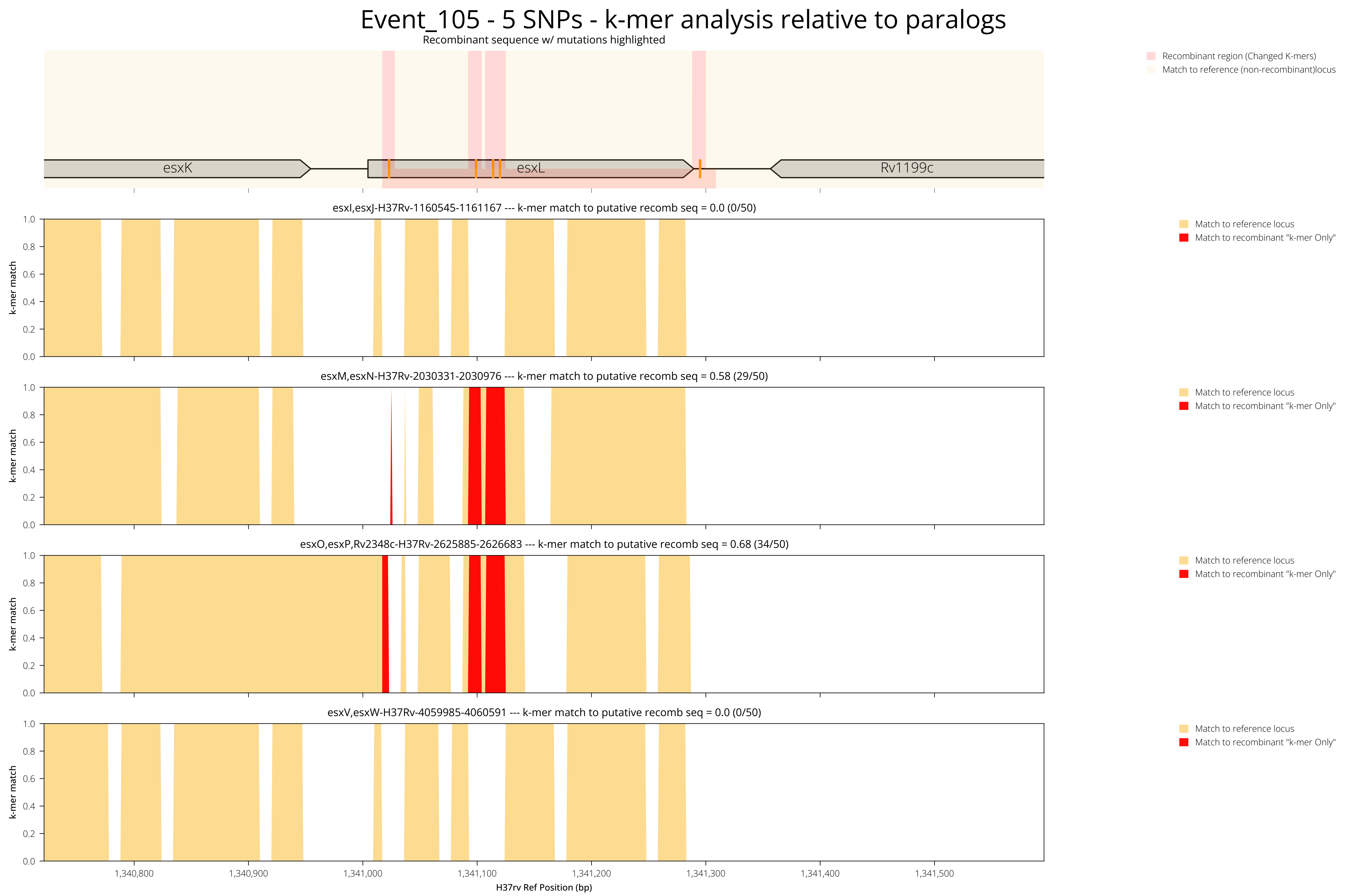

Event_105 - 5 SNPs - k-mer analysis relative to paralogs
Recombinant sequence w/ mutations highlighted
Recombinant region (Changed K-mers)
Match to reference (non-recombinant)locus
esxK
esxL
Rv1199c
esxI,esxJ-H37Rv-1160545-1161167 --- k-mer match to putative recomb seq = 0.0 (0/50)
esxM,esxN-H37Rv-2030331-2030976 --- k-mer match to putative recomb seq = 0.58 (29/50)
esxO,esxP,Rv2348c-H37Rv-2625885-2626683 --- k-mer match to putative recomb seq = 0.68 (34/50)
esxV,esxW-H37Rv-4059985-4060591 --- k-mer match to putative recomb seq = 0.0 (0/50)
Match to reference locus
Match to recombinant "k-mer Only"
k-mer match
1.0
0.8
0.6
0.4
0.2
0.0
1,340,800
1,340,900
1,341,000
1,341,100
1,341,200
1,341,300
1,341,400
1,341,500
H37rv Ref Position (bp)

Sub-phylogeny Viz + Paralog Alignments
GCE ID: Event_105

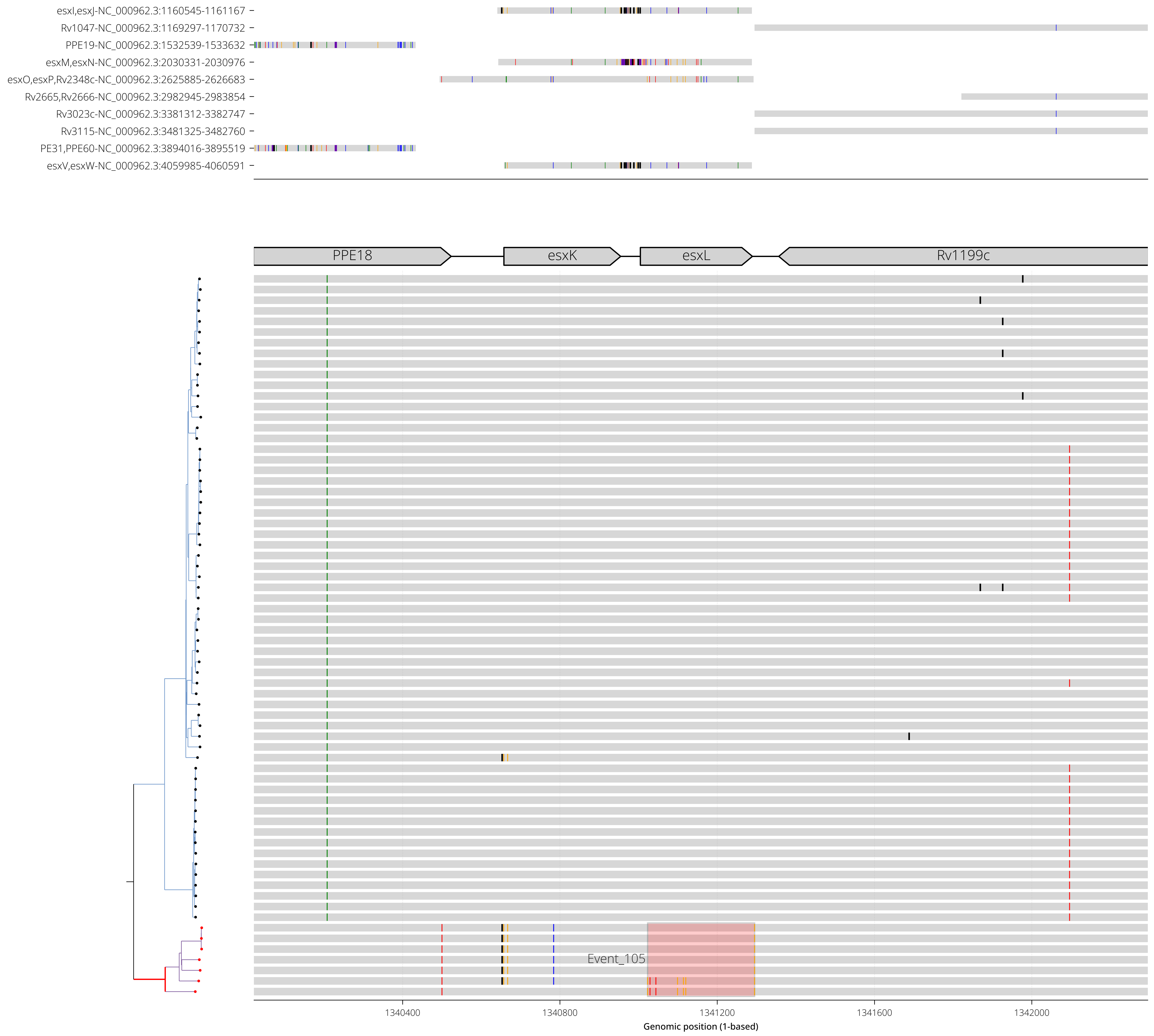

Full Phylogeny Viz + Paralog Alignments
GCE ID: Event_105

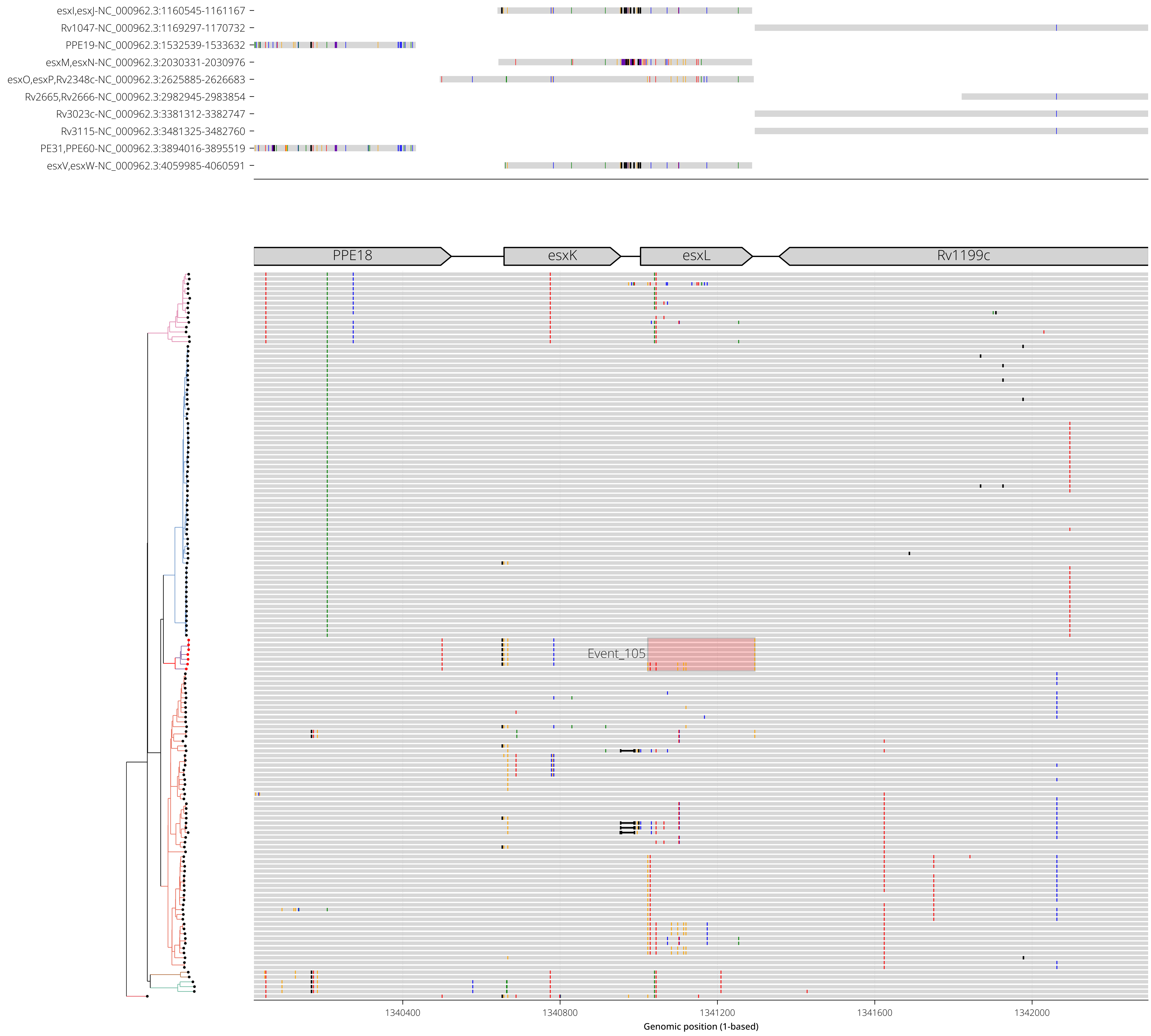

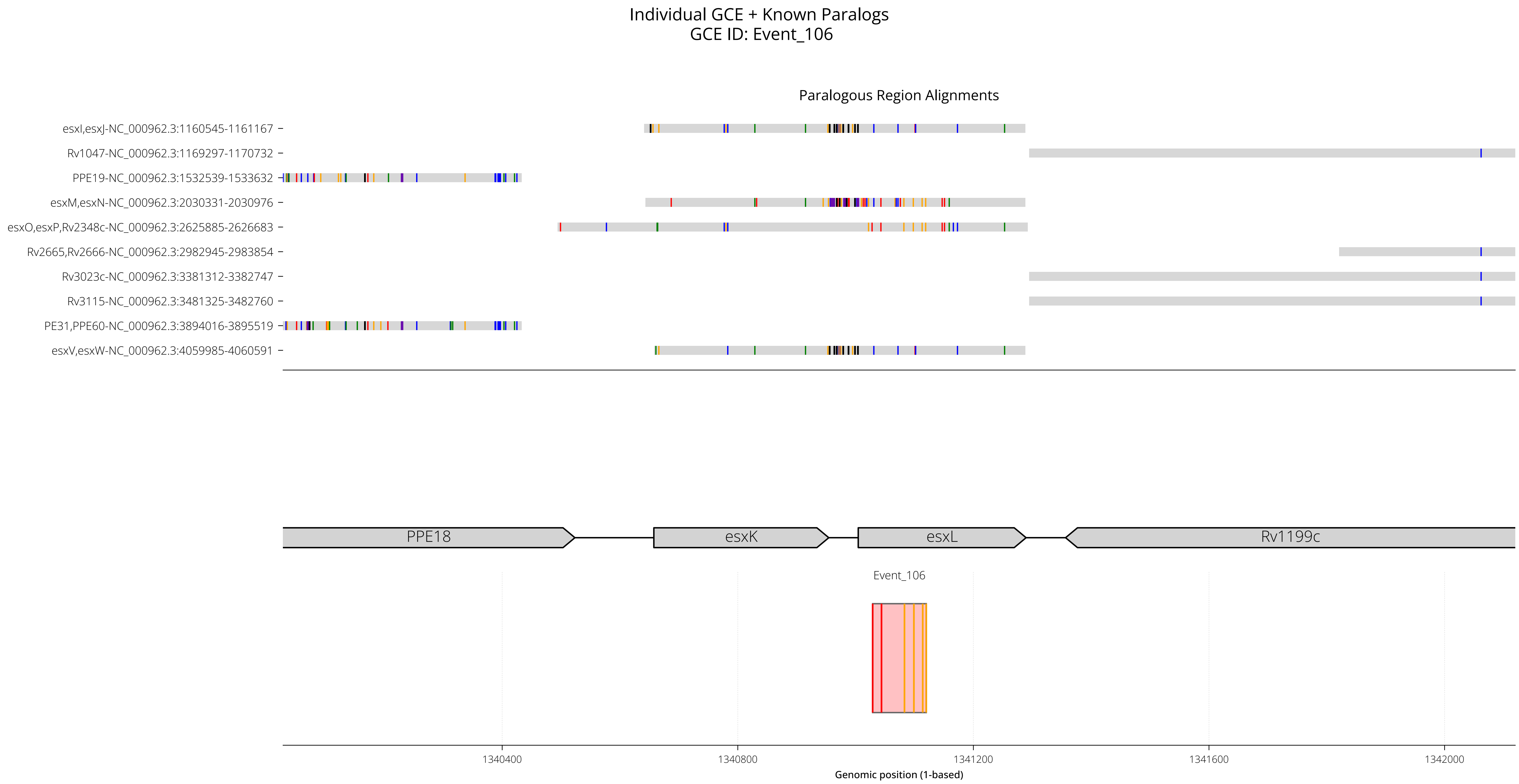

Individual GCE + Known Paralogs
GCE ID: Event_106
Paralogous Region Alignments
esxI,esxJ-NC_000962.3:1160545-1161167
Rv1047-NC_000962.3:1169297-1170732
PPE19-NC_000962.3:1532539-1533632
esxM,esxN-NC_000962.3:2030331-2030976
esxO,esxP,Rv2348c-NC_000962.3:2625885-2626683
Rv2665,Rv2666-NC_000962.3:2982945-2983854
Rv3023c-NC_000962.3:3381312-3382747
Rv3115-NC_000962.3:3481325-3482760
PE31,PPE60-NC_000962.3:3894016-3895519
esxV,esxW-NC_000962.3:4059985-4060591
PPE18
esxK
esxL
Rv1199c
Event_106
1340400
1340800
1341200
1341600
1342000
Genomic position (1-based)

Circos Viz - GCE to Known Paralogs Locations
GCE ID: Event_106

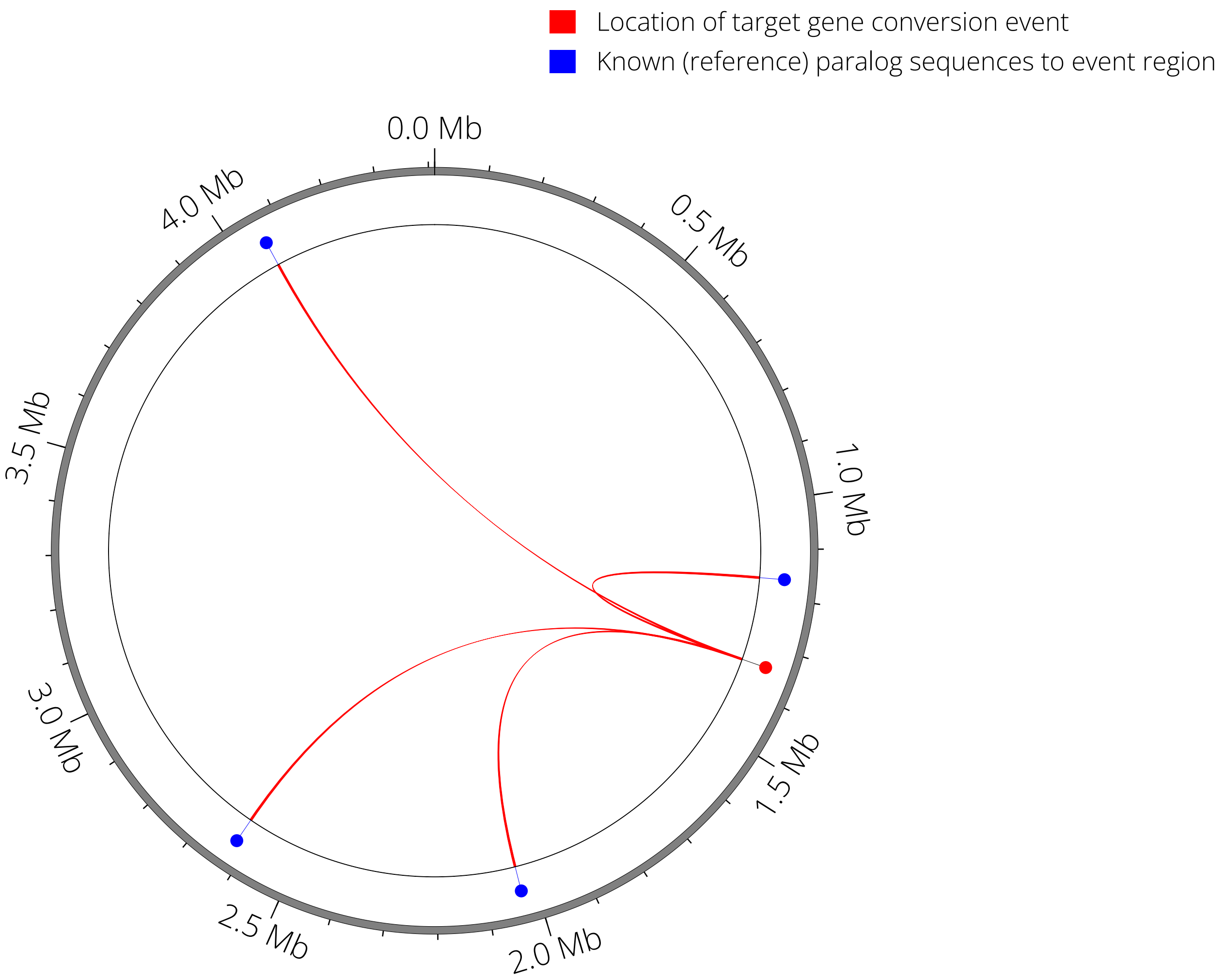

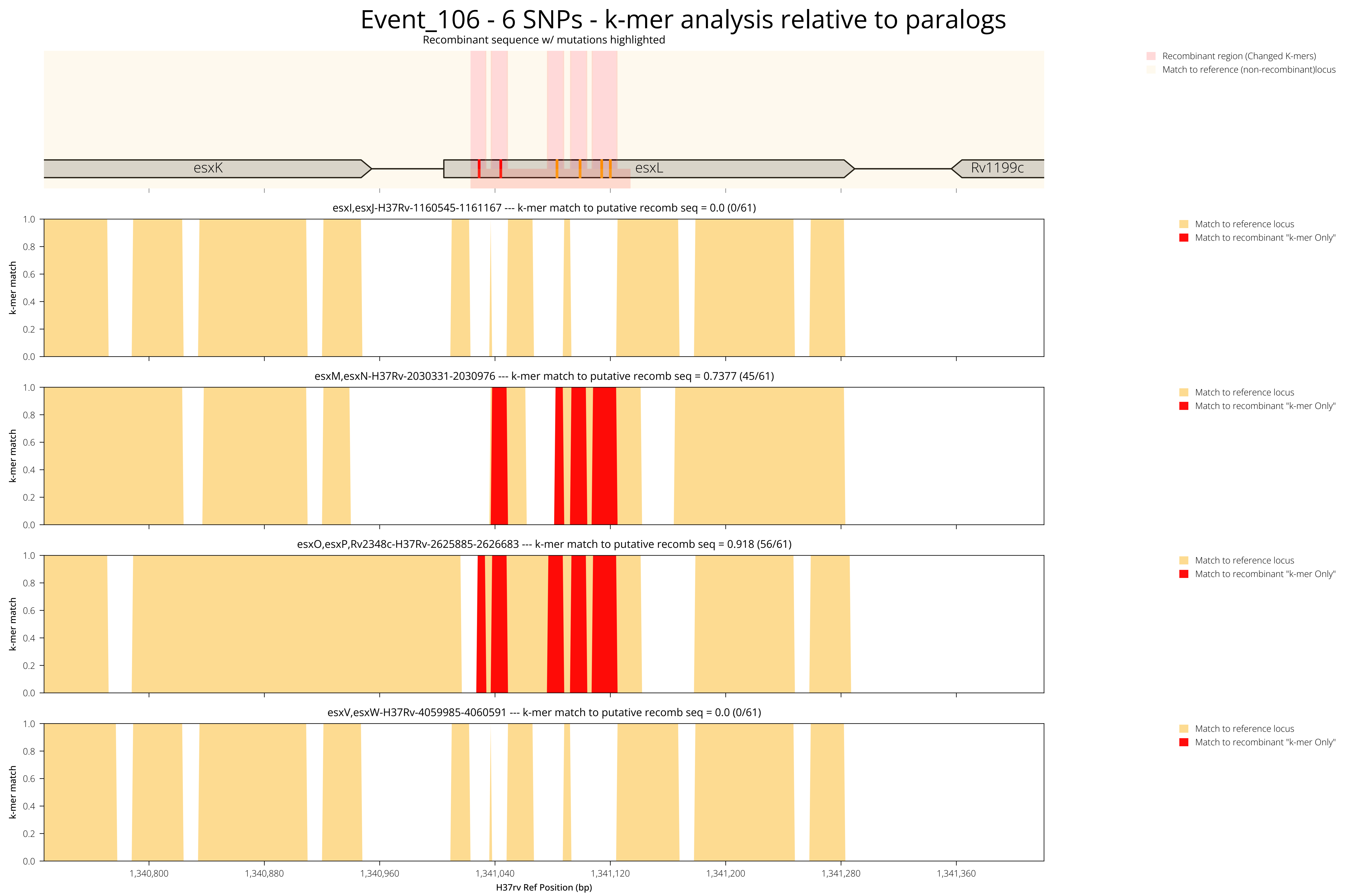
Event_106 - 6 SNPs - k-mer analysis relative to paralogs
Recombinant sequence w/ mutations highlighted
esxK
esxL
Rv1199c
Recombinant region (Changed K-mers)
Match to reference (non-recombinant)locus
esxI,esxJ-H37Rv-1160545-1161167 --- k-mer match to putative recomb seq = 0.0 (0/61)
esxM,esxN-H37Rv-2030331-2030976 --- k-mer match to putative recomb seq = 0.7377 (45/61)
esxO,esxP,Rv2348c-H37Rv-2625885-2626683 --- k-mer match to putative recomb seq = 0.918 (56/61)
esxV,esxW-H37Rv-4059985-4060591 --- k-mer match to putative recomb seq = 0.0 (0/61)
k-mer match
Match to reference locus
Match to recombinant "k-mer Only"
1,340,800
1,340,880
1,340,960
1,341,040
1,341,120
1,341,200
1,341,280
1,341,360
H37rv Ref Position (bp)

Sub-phylogeny Viz + Paralog Alignments
GCE ID: Event_106

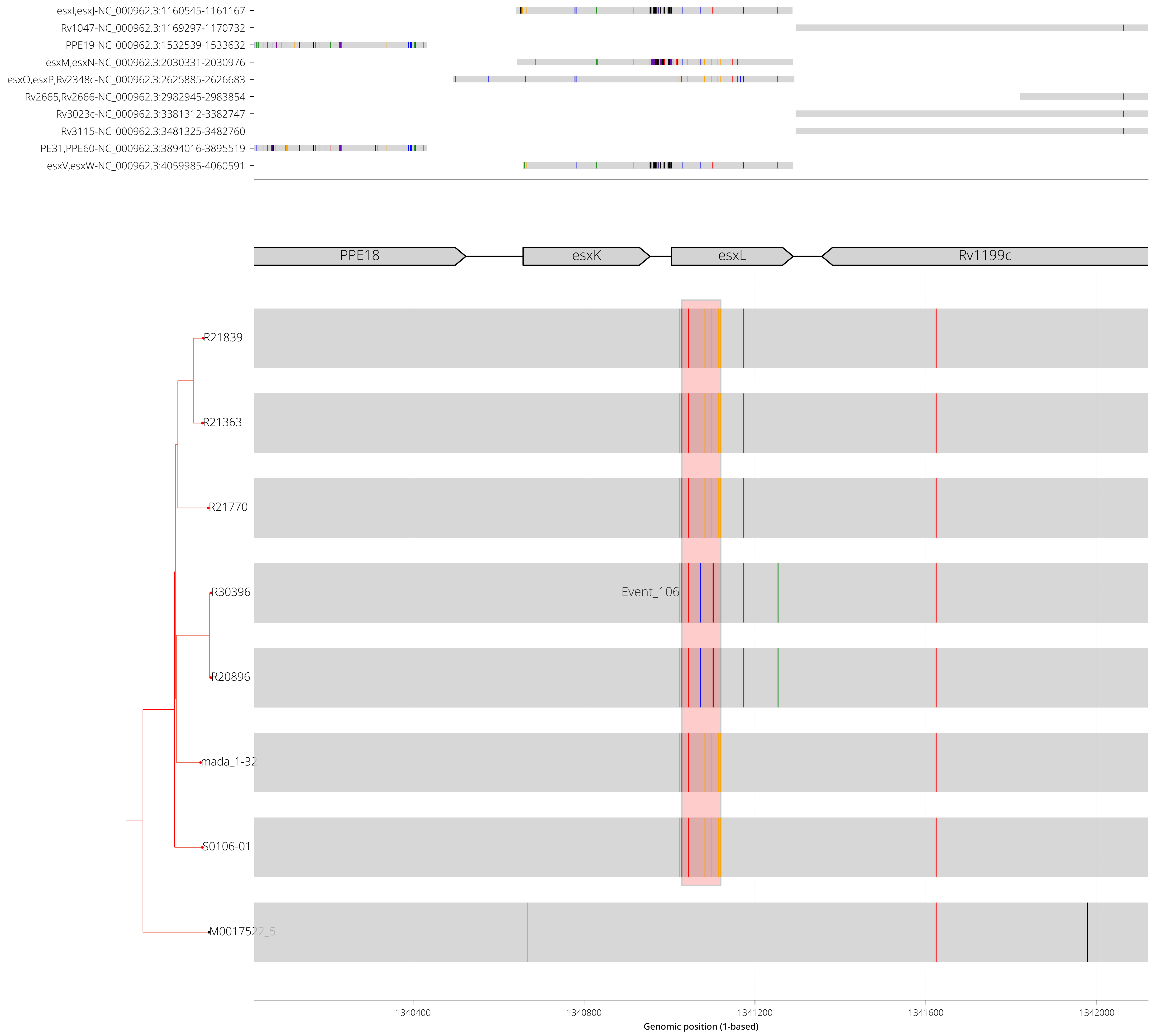

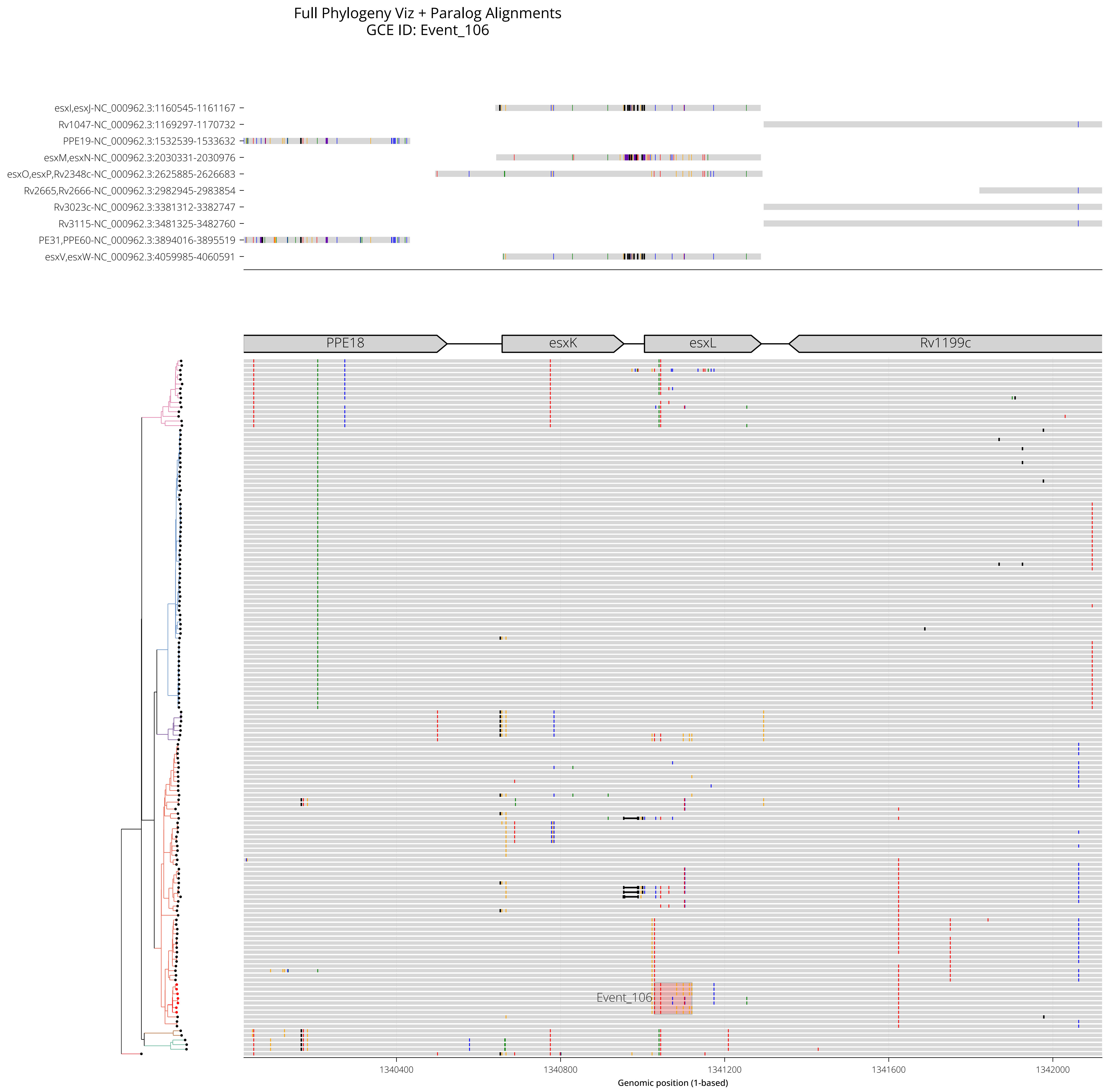

Full Phylogeny Viz + Paralog Alignments
GCE ID: Event_106
esxI,esxJ-NC_000962.3:1160545-1161167
Rv1047-NC_000962.3:1169297-1170732
PPE19-NC_000962.3:1532539-1533632
esxM,esxN-NC_000962.3:2030331-2030976
esxO,esxP,Rv2348c-NC_000962.3:2625885-2626683
Rv2665,Rv2666-NC_000962.3:2982945-2983854
Rv3023c-NC_000962.3:3381312-3382747
Rv3115-NC_000962.3:3481325-3482760
PE31,PPE60-NC_000962.3:3894016-3895519
esxV,esxW-NC_000962.3:4059985-4060591
PPE18
esxK
esxL
Rv1199c
Event_106
1340400
1340800
1341200
1341600
1342000
Genomic position (1-based)

## Individual GCE + Known Paralogs
## GCE ID: Event_107

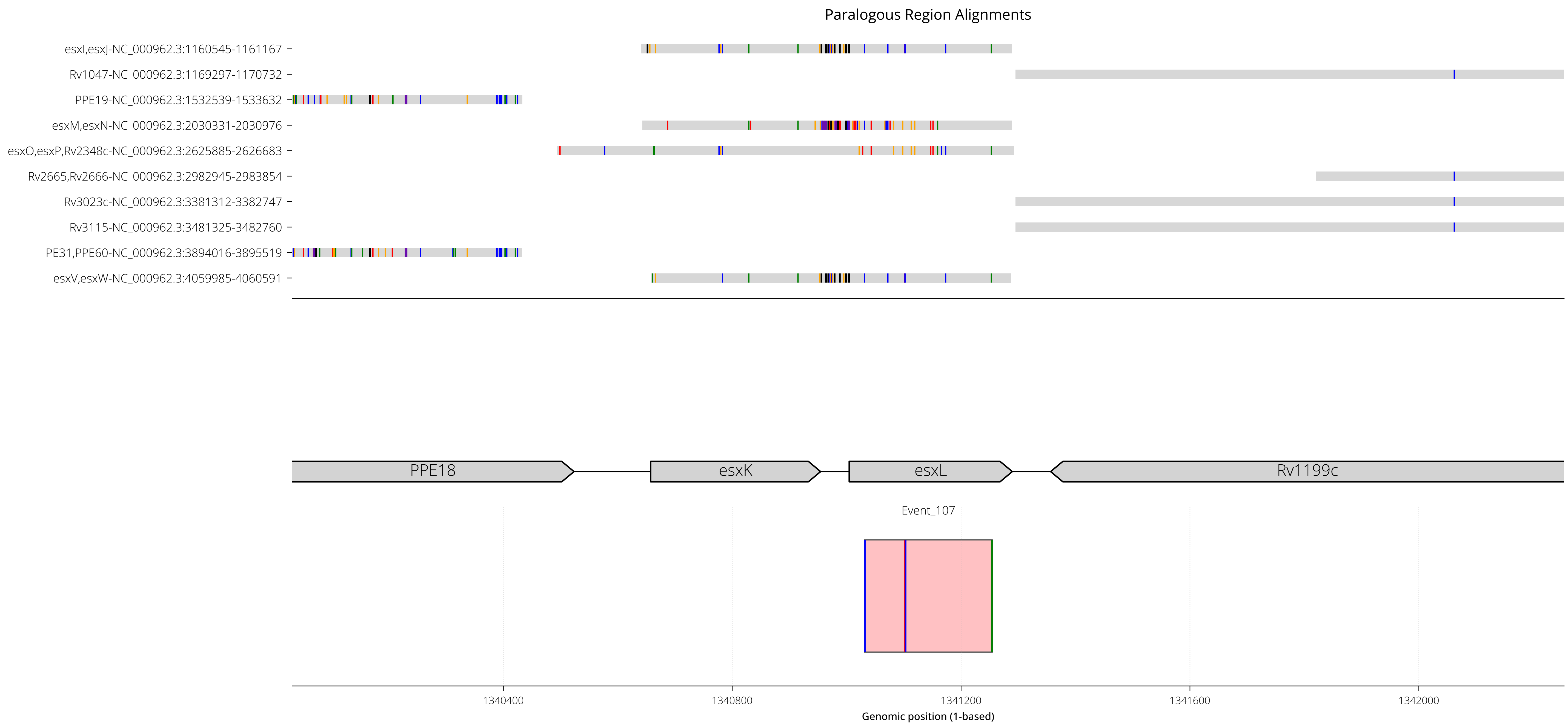

Circos Viz - GCE to Known Paralogs Locations
GCE ID: Event_107

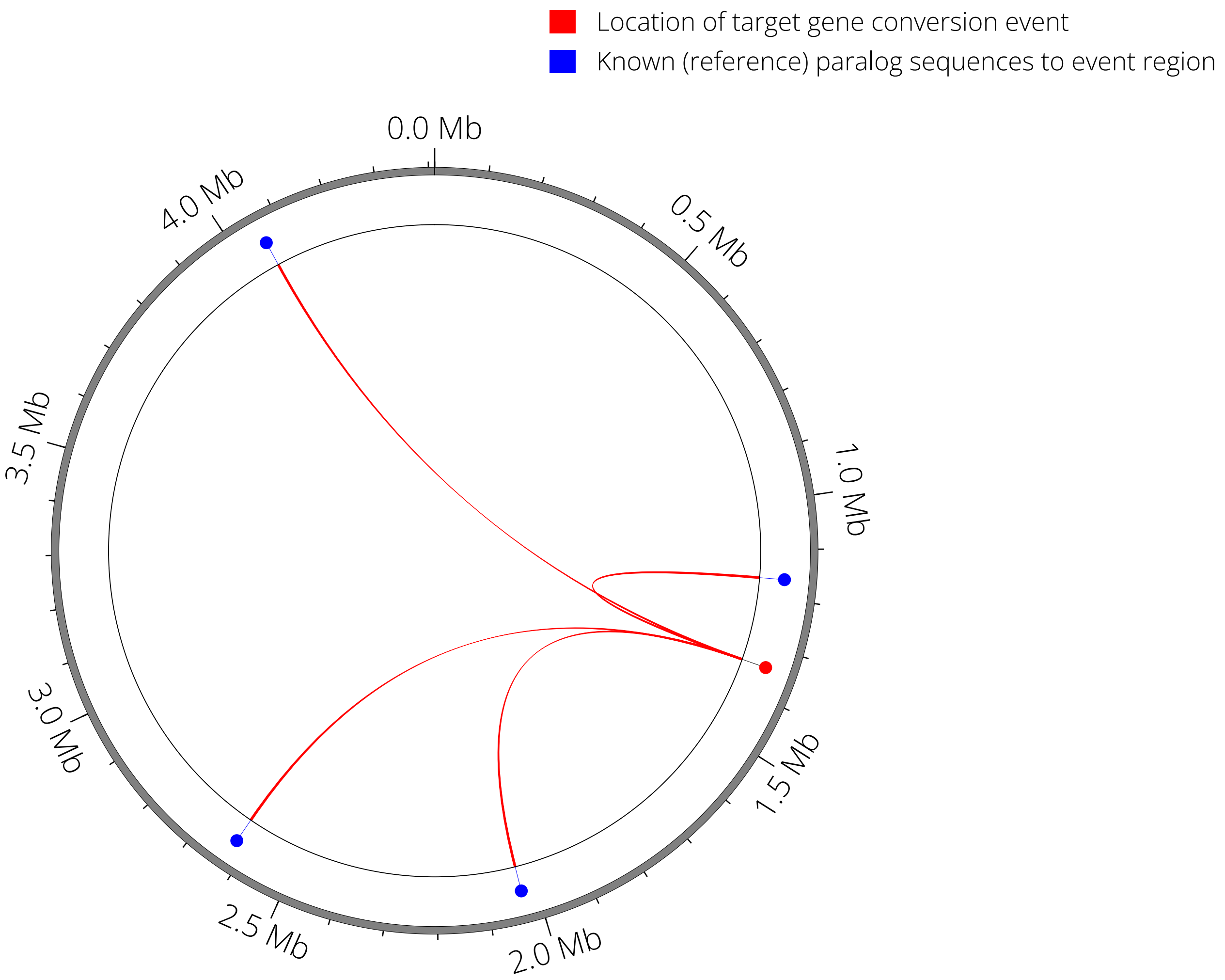

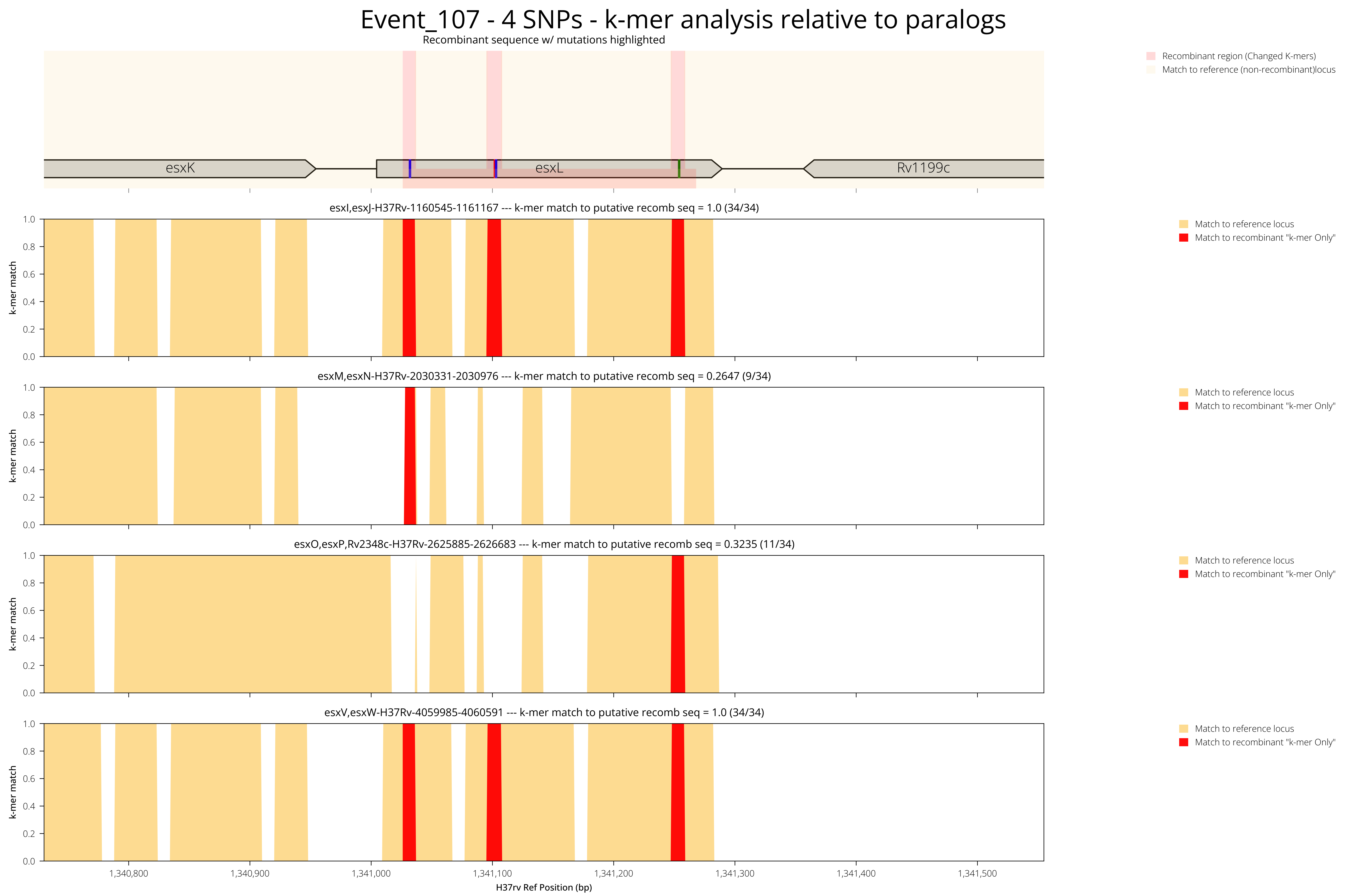

Event_107 - 4 SNPs - k-mer analysis relative to paralogs
Recombinant sequence w/ mutations highlighted
Recombinant region (Changed K-mers)
Match to reference (non-recombinant)locus
esxK
esxL
Rv1199c
esxI,esxJ-H37Rv-1160545-1161167 --- k-mer match to putative recomb seq = 1.0 (34/34)
esxM,esxN-H37Rv-2030331-2030976 --- k-mer match to putative recomb seq = 0.2647 (9/34)
esxO,esxP,Rv2348c-H37Rv-2625885-2626683 --- k-mer match to putative recomb seq = 0.3235 (11/34)
esxV,esxW-H37Rv-4059985-4060591 --- k-mer match to putative recomb seq = 1.0 (34/34)
Match to reference locus
Match to recombinant "k-mer Only"
k-mer match
1.0
0.8
0.6
0.4
0.2
0.0
1,340,800
1,340,900
1,341,000
1,341,100
1,341,200
1,341,300
1,341,400
1,341,500
H37rv Ref Position (bp)

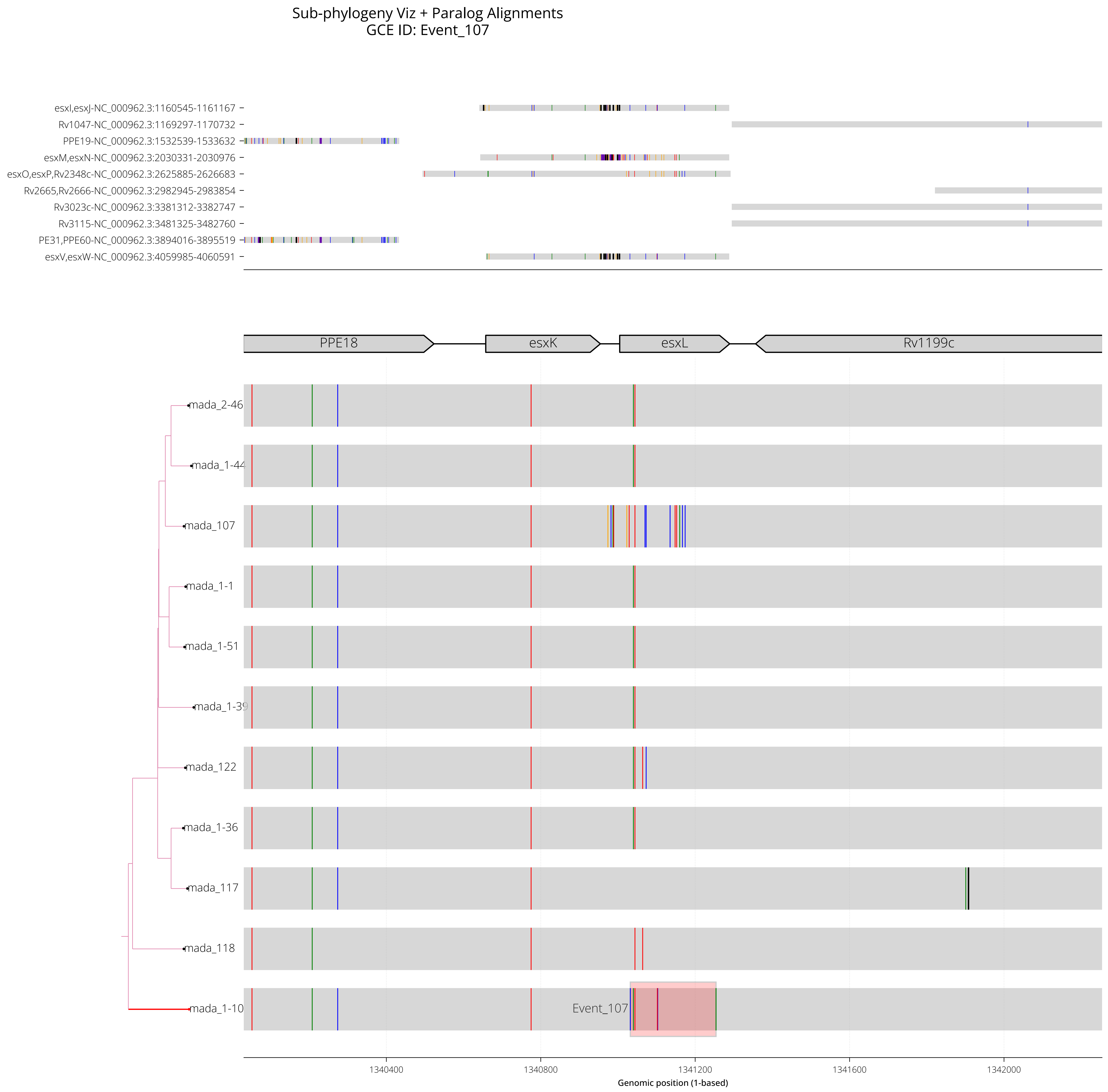

Sub-phylogeny Viz + Paralog Alignments
GCE ID: Event_107
esxI,esxJ-NC_000962.3:1160545-1161167
Rv1047-NC_000962.3:1169297-1170732
PPE19-NC_000962.3:1532539-1533632
esxM,esxN-NC_000962.3:2030331-2030976
esxO,esxP,Rv2348c-NC_000962.3:2625885-2626683
Rv2665,Rv2666-NC_000962.3:2982945-2983854
Rv3023c-NC_000962.3:3381312-3382747
Rv3115-NC_000962.3:3481325-3482760
PE31,PPE60-NC_000962.3:3894016-3895519
esxV,esxW-NC_000962.3:4059985-4060591
PPE18
esxK
esxL
Rv1199c
mada_2-46
mada_1-44
mada_107
mada_1-1
mada_1-51
mada_1-39
mada_122
mada_1-36
mada_117
mada_118
mada_1-10
Event_107
1340400
1340800
1341200
1341600
1342000
Genomic position (1-based)

# Full Phylogeny Viz + Paralog Alignments
GCE ID: Event_107

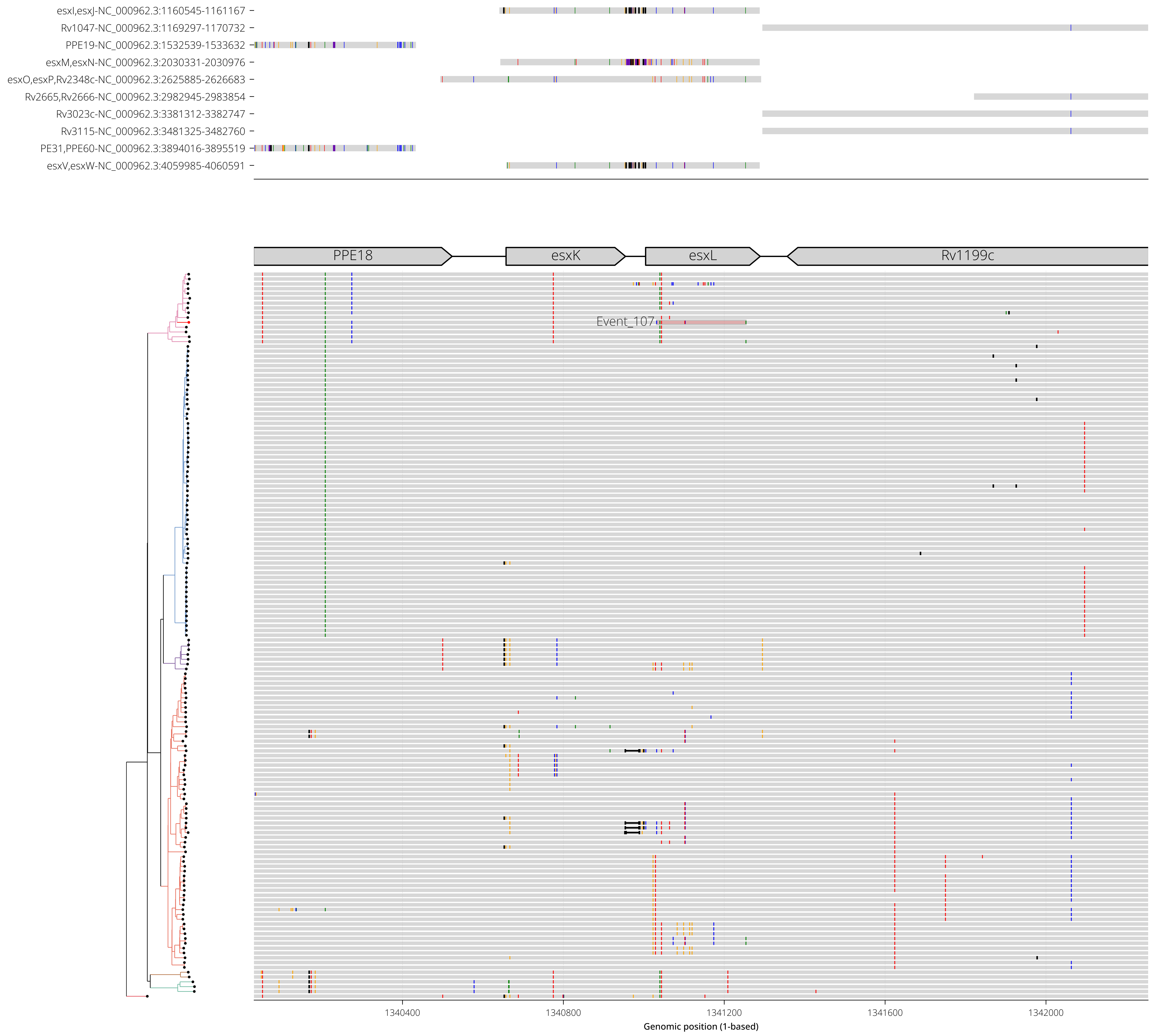

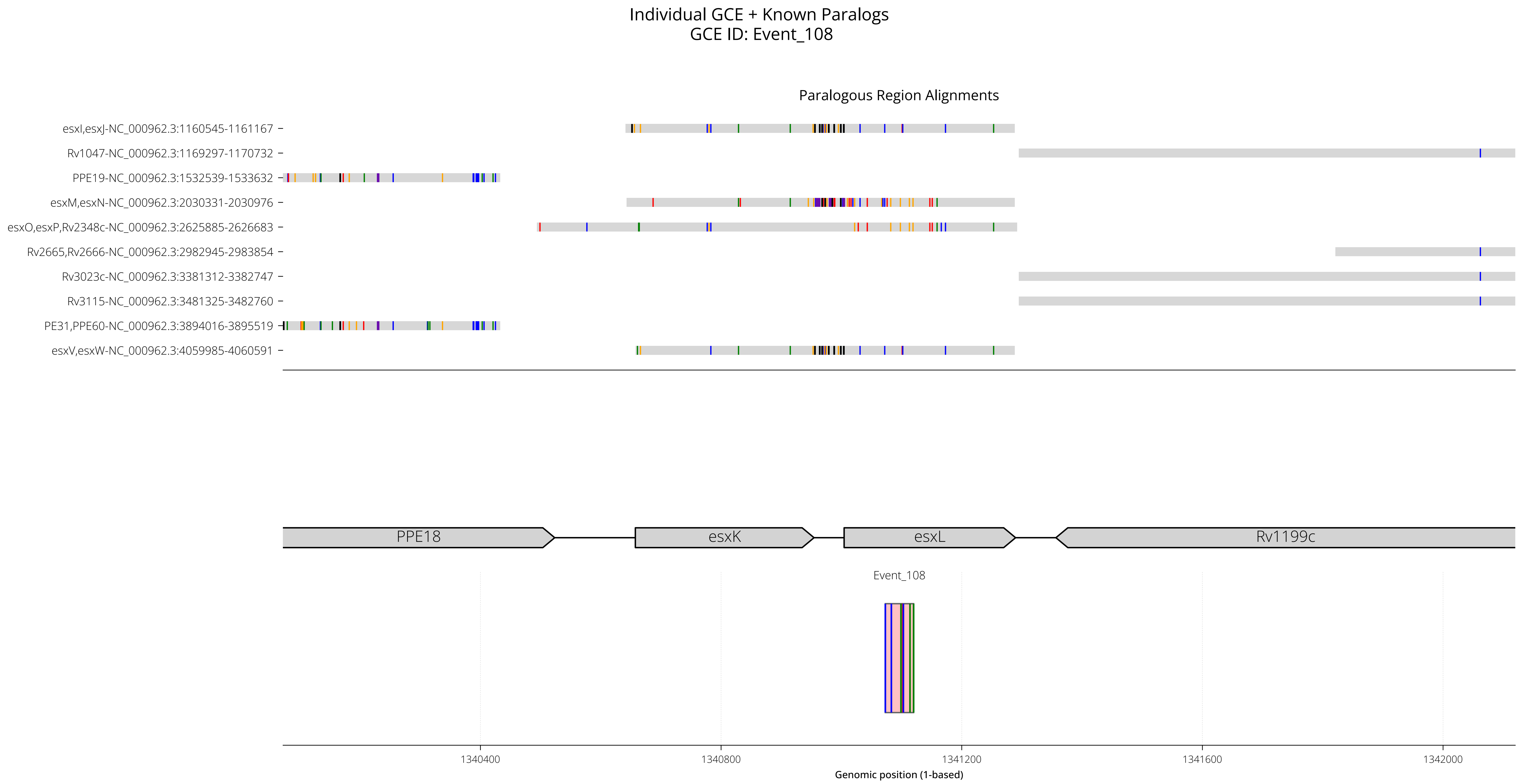

Individual GCE + Known Paralogs
GCE ID: Event_108
Paralogous Region Alignments
esxI,esxJ-NC_000962.3:1160545-1161167
Rv1047-NC_000962.3:1169297-1170732
PPE19-NC_000962.3:1532539-1533632
esxM,esxN-NC_000962.3:2030331-2030976
esxO,esxP,Rv2348c-NC_000962.3:2625885-2626683
Rv2665,Rv2666-NC_000962.3:2982945-2983854
Rv3023c-NC_000962.3:3381312-3382747
Rv3115-NC_000962.3:3481325-3482760
PE31,PPE60-NC_000962.3:3894016-3895519
esxV,esxW-NC_000962.3:4059985-4060591
PPE18
esxK
esxL
Rv1199c
Event_108
1340400
1340800
1341200
1341600
1342000
Genomic position (1-based)

Circos Viz - GCE to Known Paralogs Locations
GCE ID: Event_108

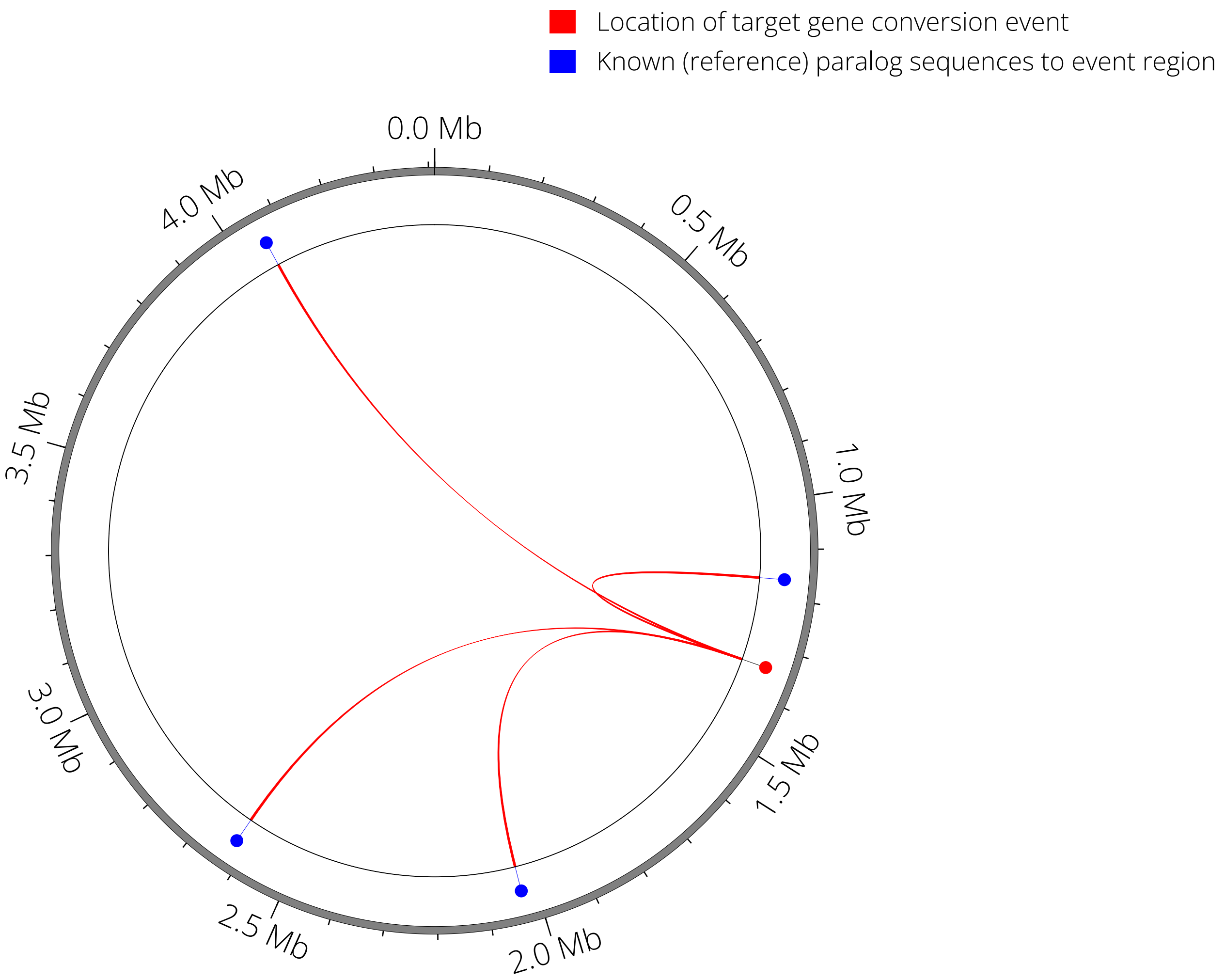

# Event_108 - 7 SNPs - k-mer analysis relative to paralogs

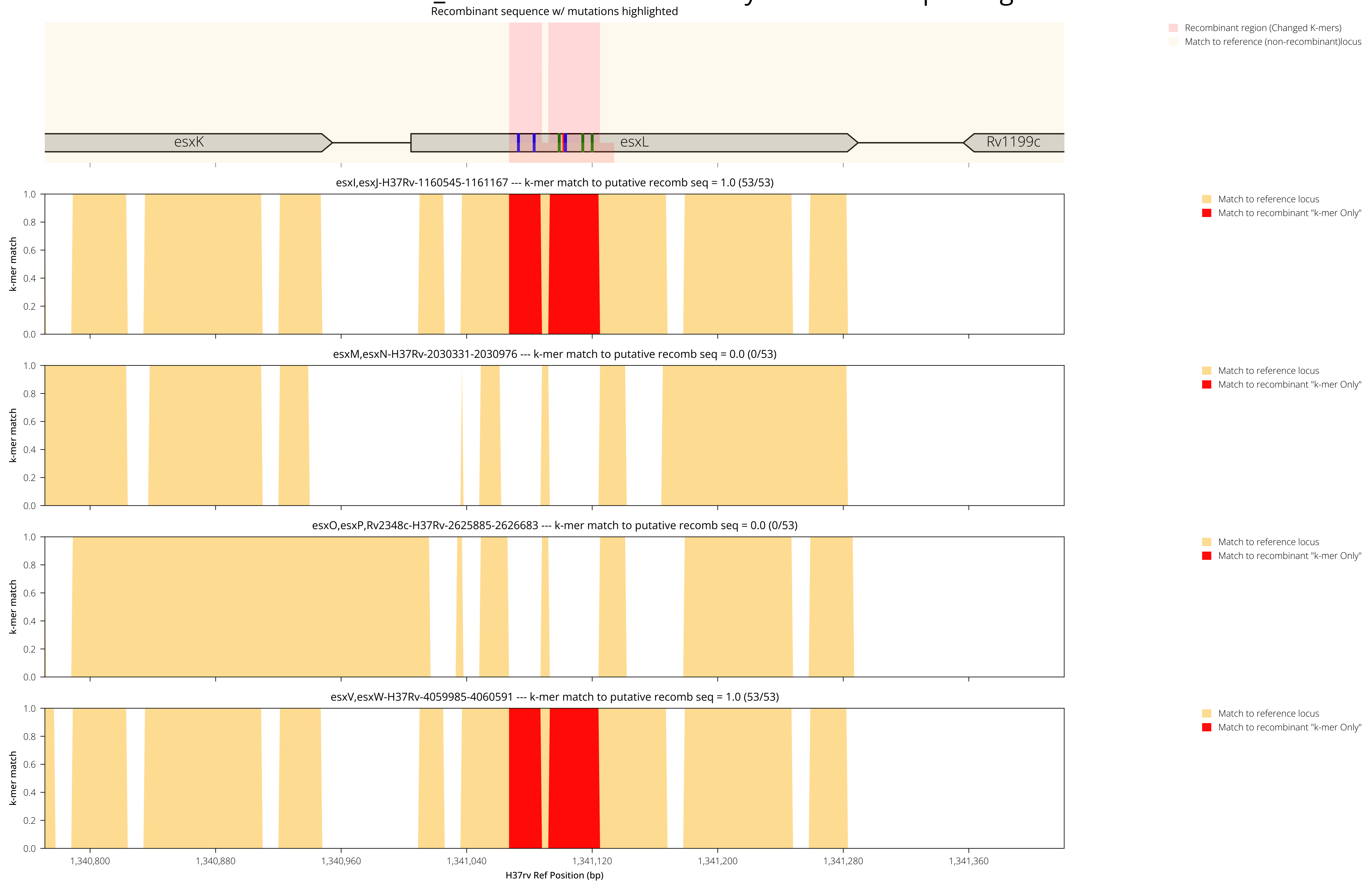

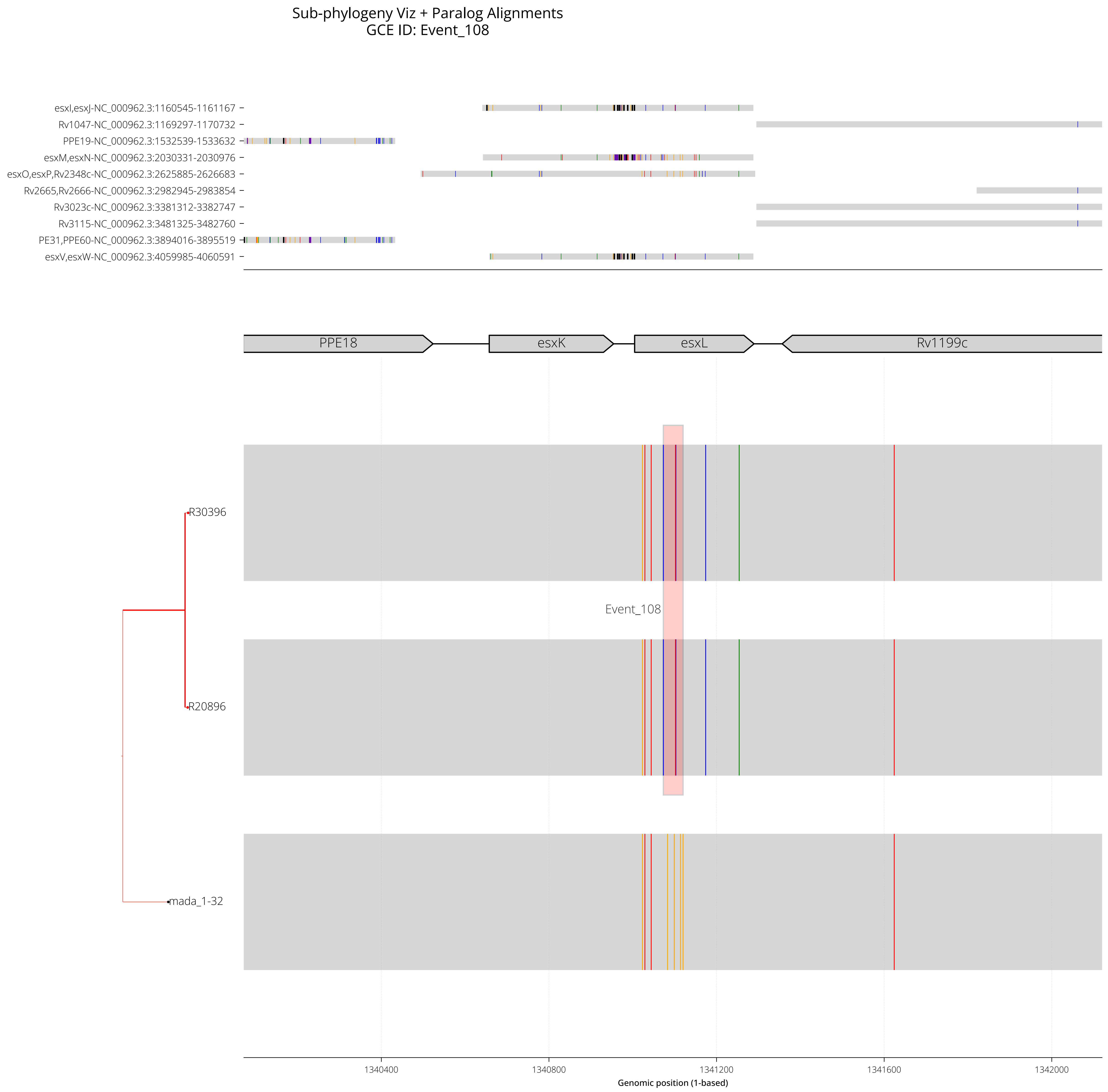
Sub-phylogeny Viz + Paralog Alignments
GCE ID: Event_108
esxI,esxJ-NC_000962.3:1160545-1161167
Rv1047-NC_000962.3:1169297-1170732
PPE19-NC_000962.3:1532539-1533632
esxM,esxN-NC_000962.3:2030331-2030976
esxO,esxP,Rv2348c-NC_000962.3:2625885-2626683
Rv2665,Rv2666-NC_000962.3:2982945-2983854
Rv3023c-NC_000962.3:3381312-3382747
Rv3115-NC_000962.3:3481325-3482760
PE31,PPE60-NC_000962.3:3894016-3895519
esxV,esxW-NC_000962.3:4059985-4060591
PPE18
esxK
esxL
Rv1199c
R30396
Event_108
R20896
mada_1-32
1340400
1340800
1341200
1341600
1342000
Genomic position (1-based)

Full Phylogeny Viz + Paralog Alignments
GCE ID: Event_108

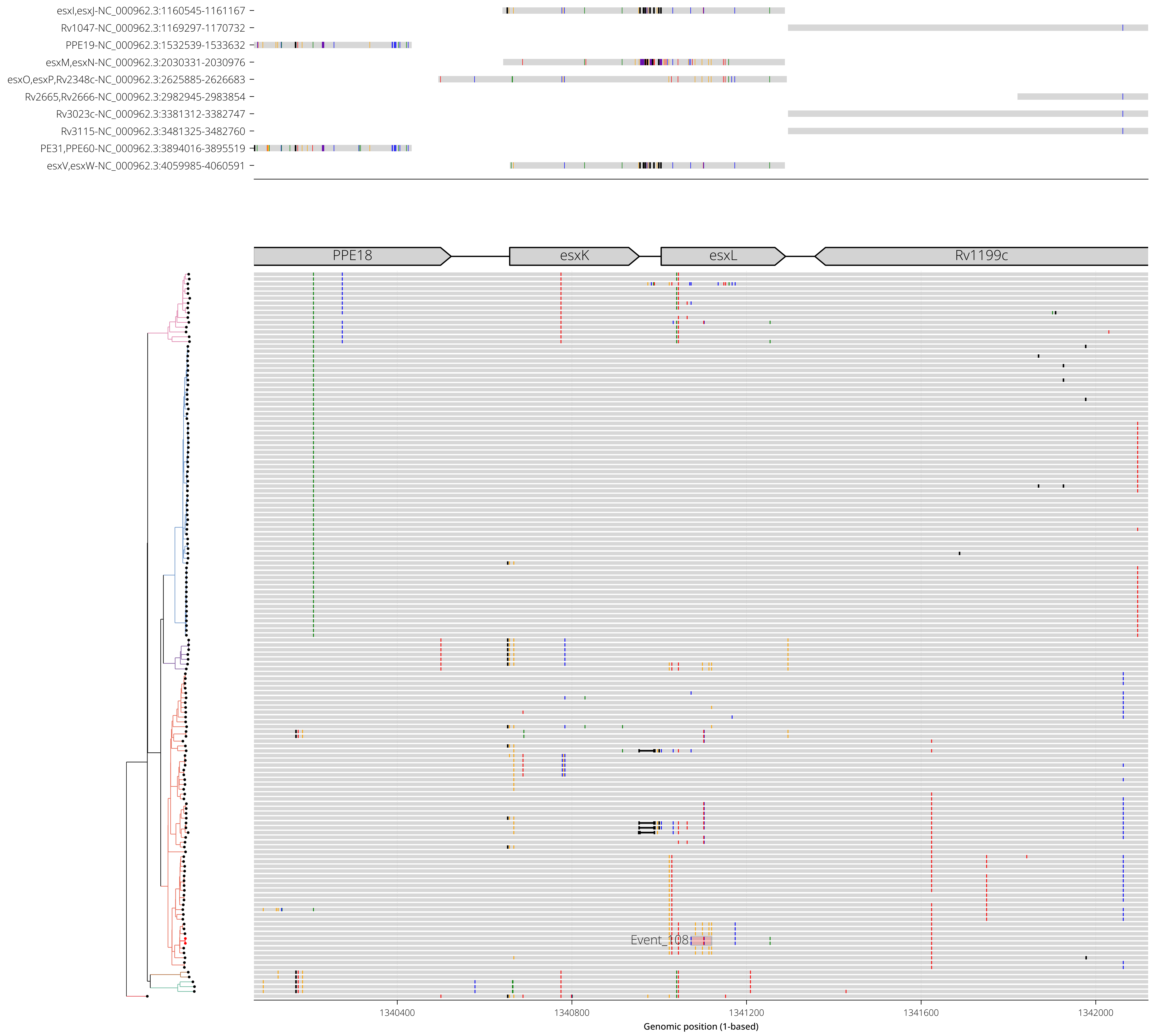

Individual GCE + Known Paralogs
GCE ID: Event_109

Paralogous Region Alignments

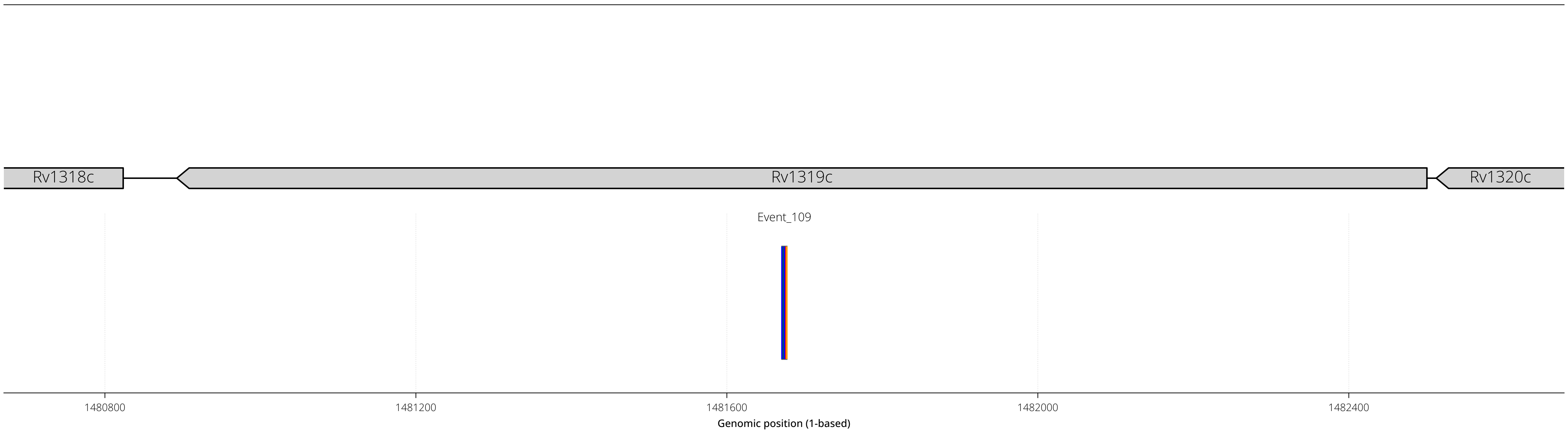

Sub-phylogeny Viz + Paralog Alignments
GCE ID: Event_109

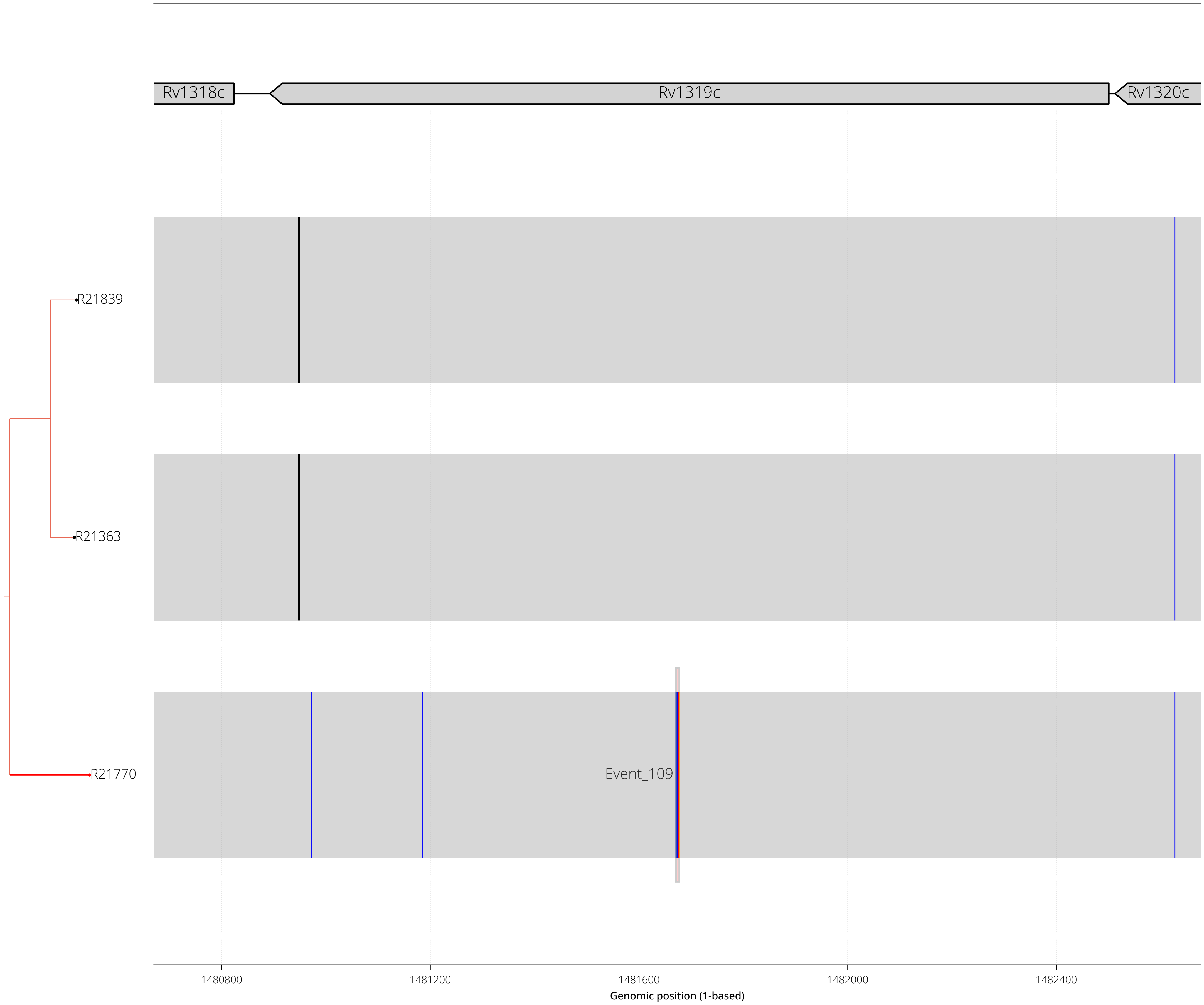

Full Phylogeny Viz + Paralog Alignments
GCE ID: Event_109

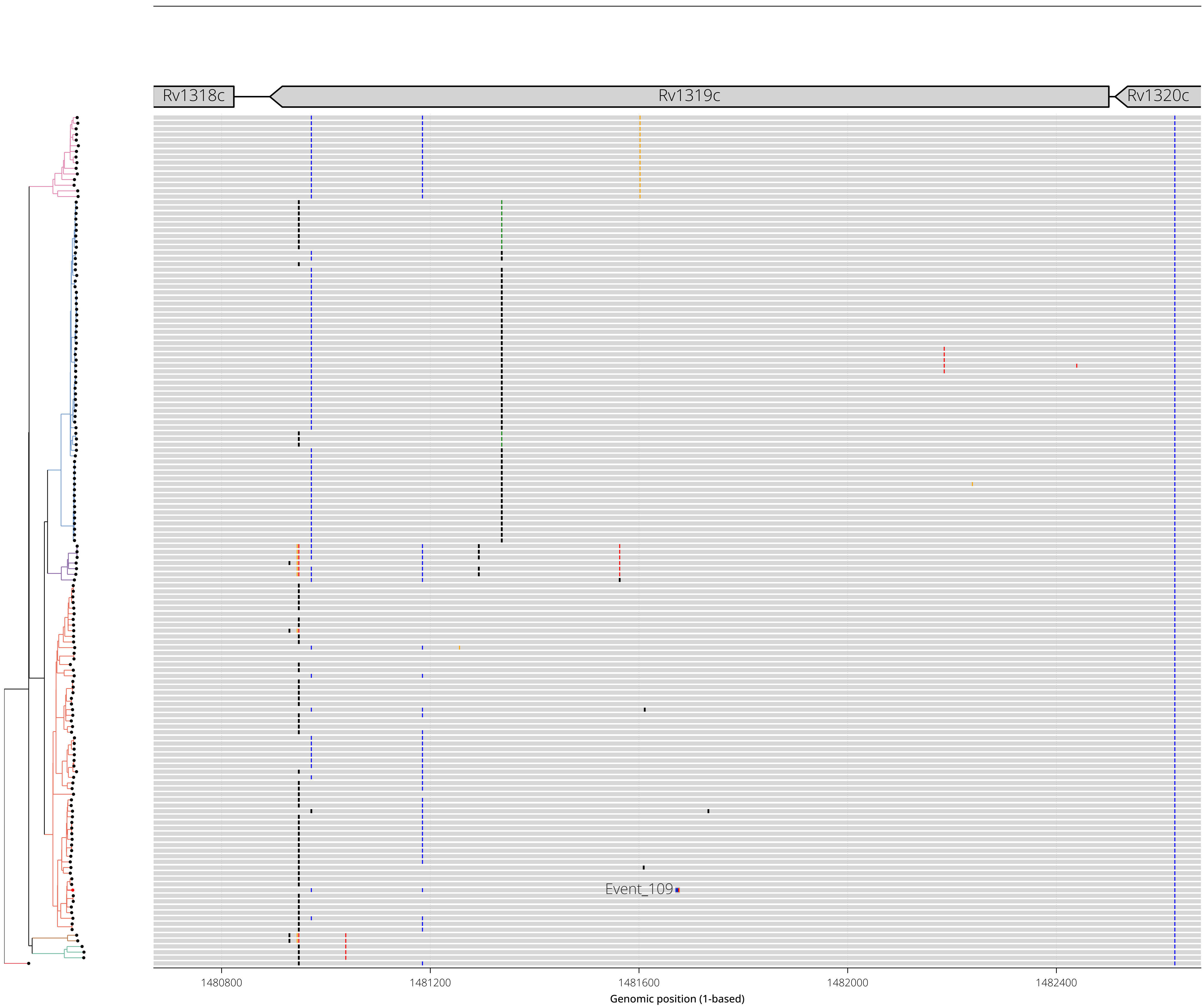

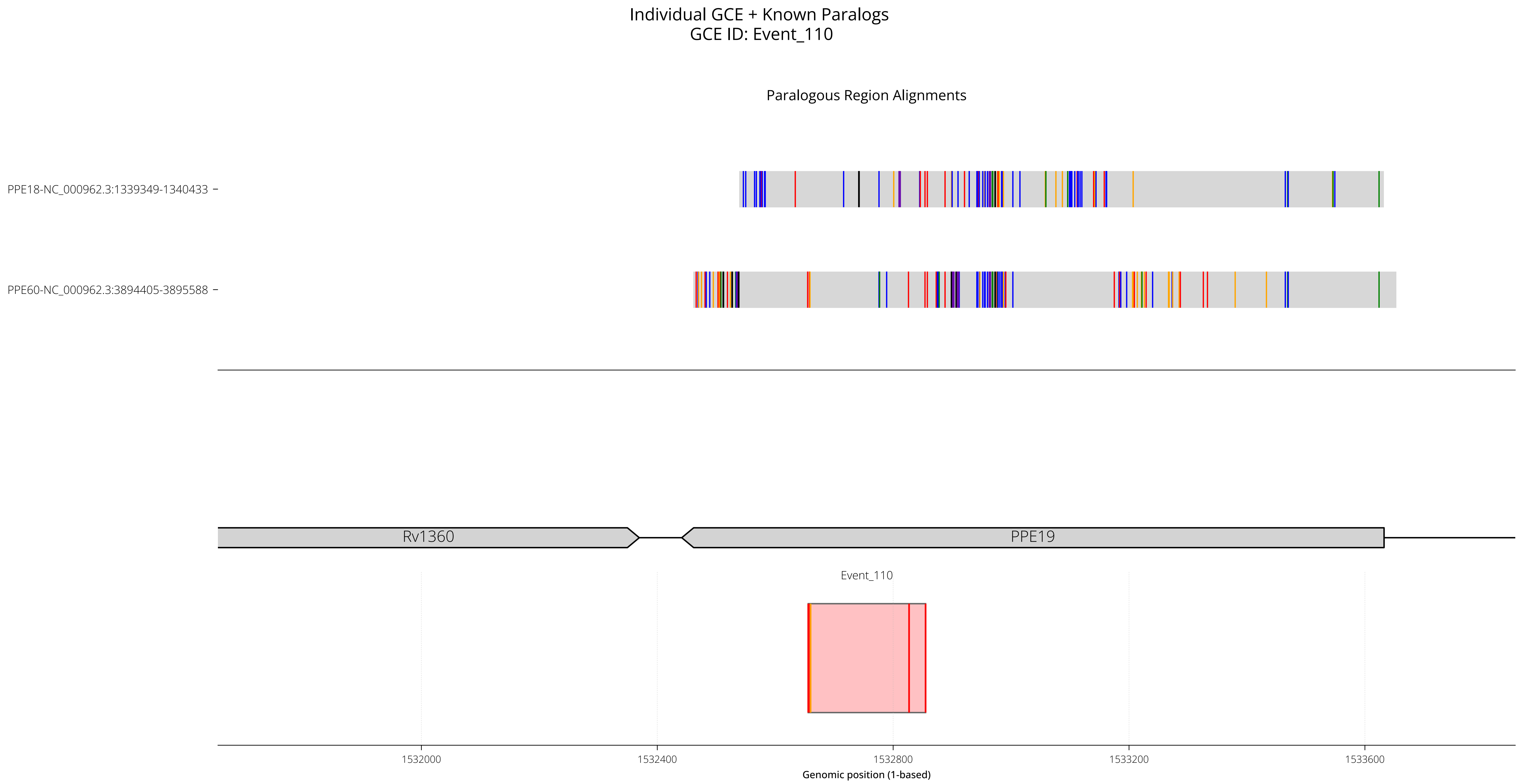

Individual GCE + Known Paralogs
GCE ID: Event_110
Paralogous Region Alignments
PPE18-NC_000962.3:1339349-1340433
PPE60-NC_000962.3:3894405-3895588
Rv1360
PPE19
Event_110
1532000
1532400
1532800
1533200
1533600
Genomic position (1-based)

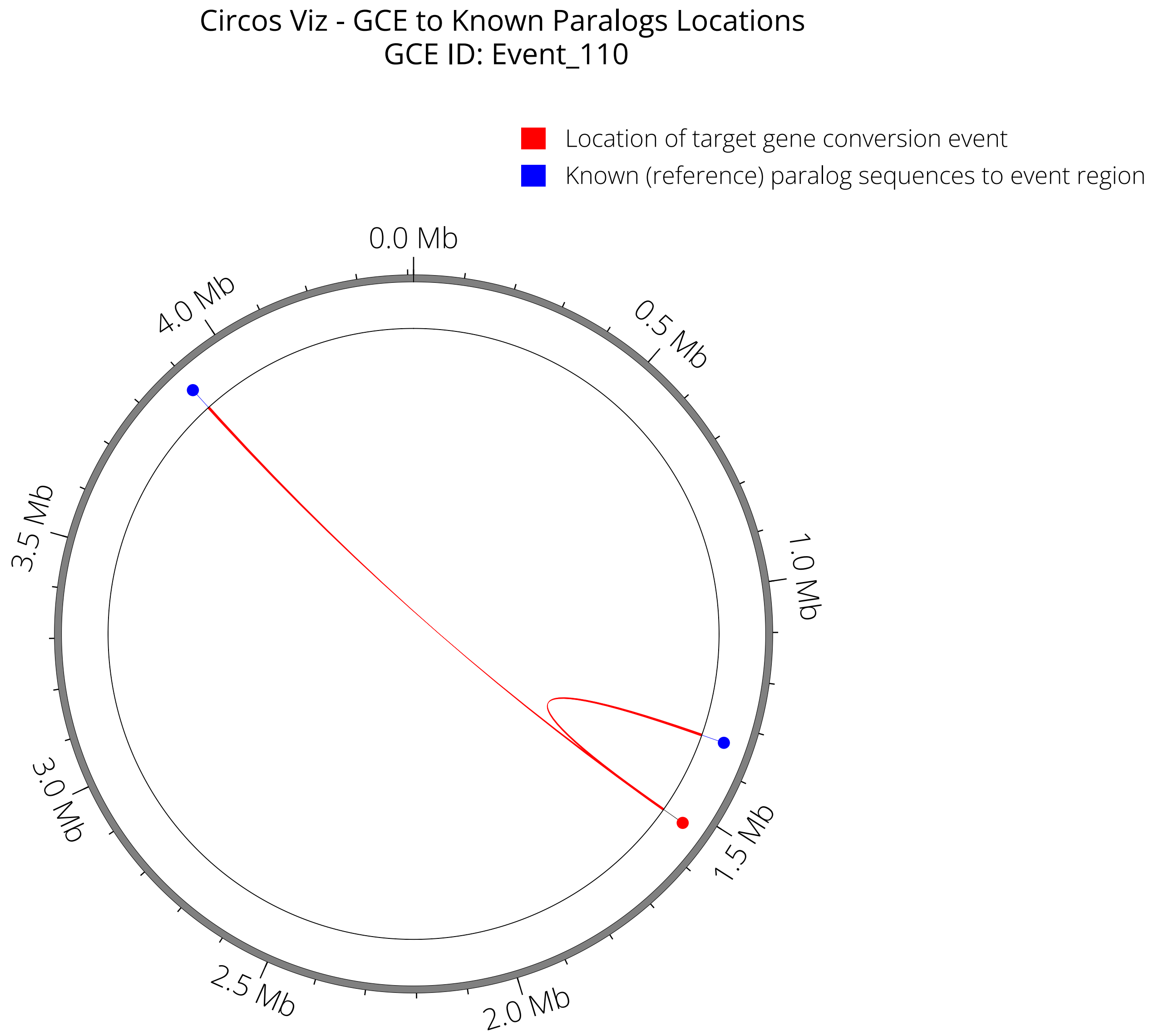
Circos Viz - GCE to Known Paralogs Locations
GCE ID: Event_110
Location of target gene conversion event
Known (reference) paralog sequences to event region
0.0 Mb
0.5 Mb
1.0 Mb
1.5 Mb
2.0 Mb
2.5 Mb
3.0 Mb
3.5 Mb
4.0 Mb

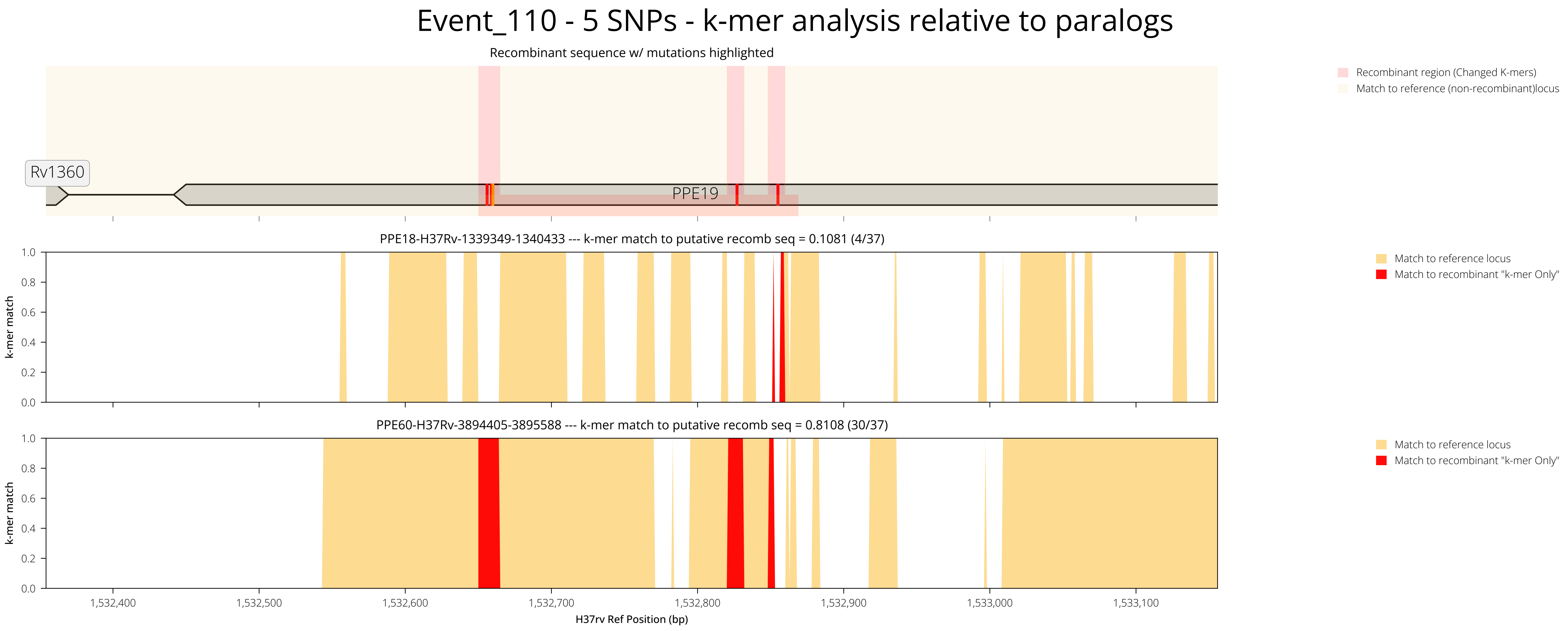
Event_110 - 5 SNPs - k-mer analysis relative to paralogs
Recombinant sequence w/ mutations highlighted
Recombinant region (Changed K-mers)
Match to reference (non-recombinant)locus
Rv1360
PPE19
PPE18-H37Rv-1339349-1340433 --- k-mer match to putative recomb seq = 0.1081 (4/37)
Match to reference locus
Match to recombinant "k-mer Only"
k-mer match
PPE60-H37Rv-3894405-3895588 --- k-mer match to putative recomb seq = 0.8108 (30/37)
Match to reference locus
Match to recombinant "k-mer Only"
k-mer match
1,532,400
1,532,500
1,532,600
1,532,700
1,532,800
1,532,900
1,533,000
1,533,100
H37rv Ref Position (bp)

Sub-phylogeny Viz + Paralog Alignments
GCE ID: Event_110

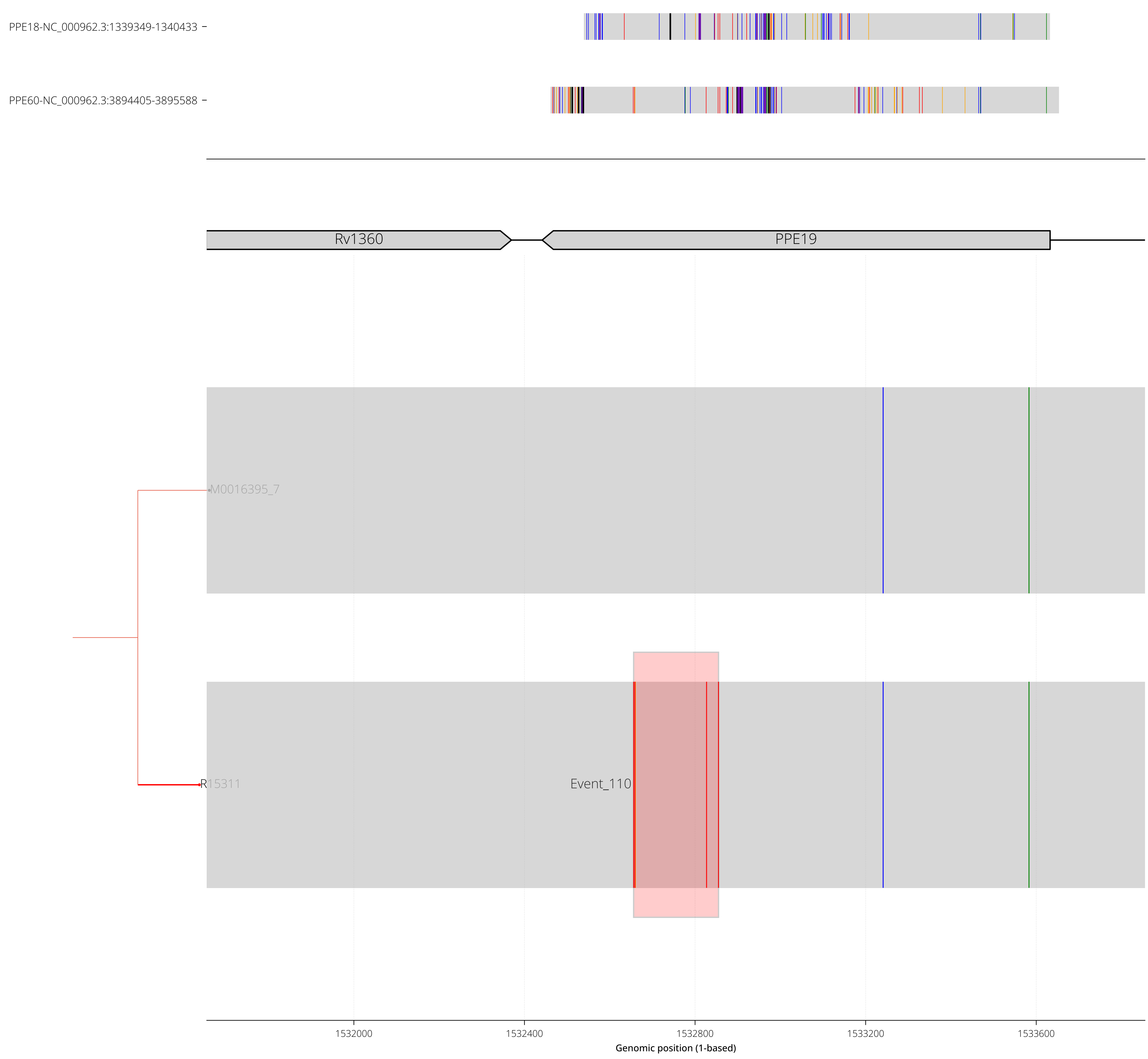

# Full Phylogeny Viz + Paralog Alignments
GCE ID: Event_110

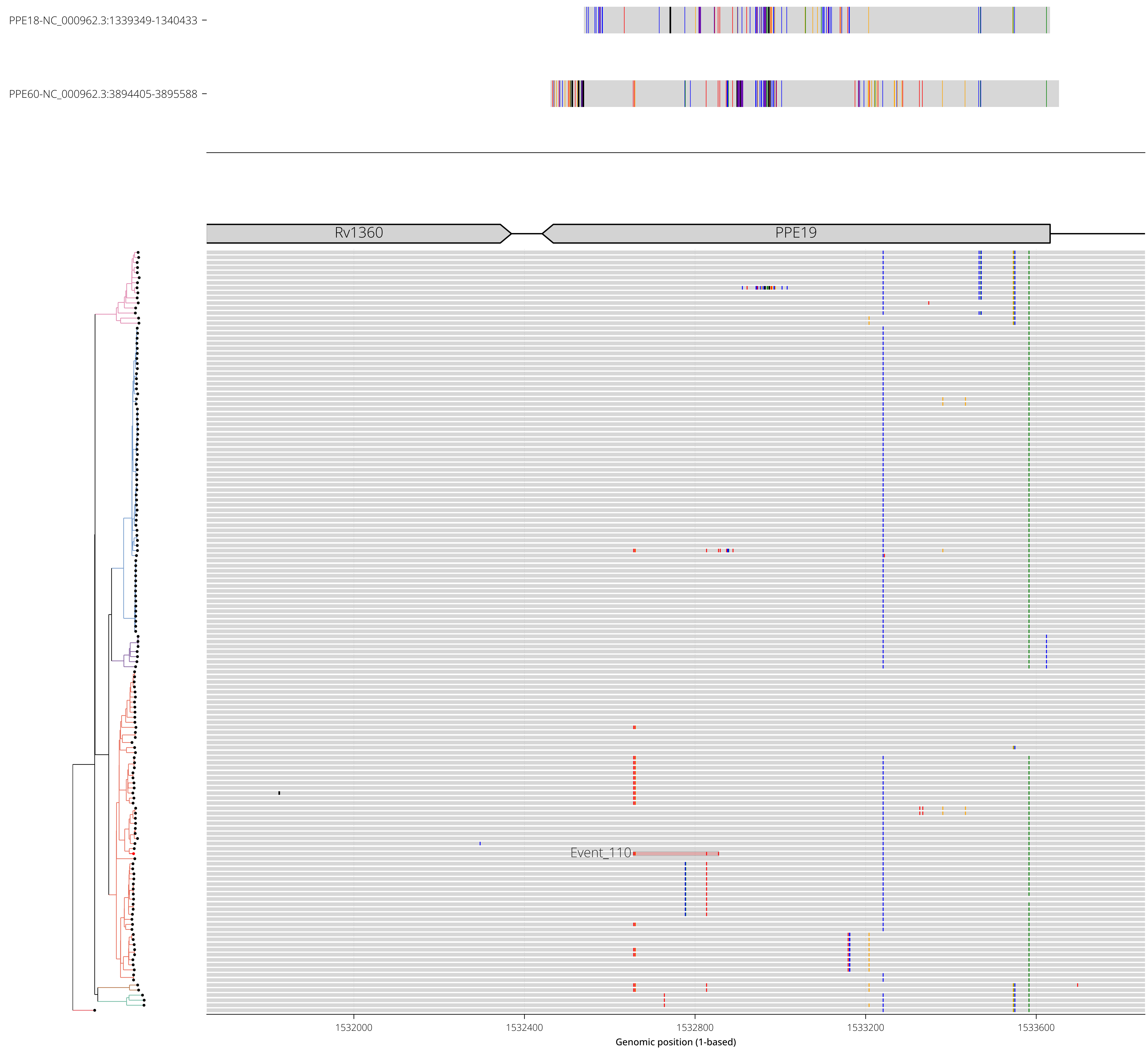

Individual GCE + Known Paralogs
GCE ID: Event_111

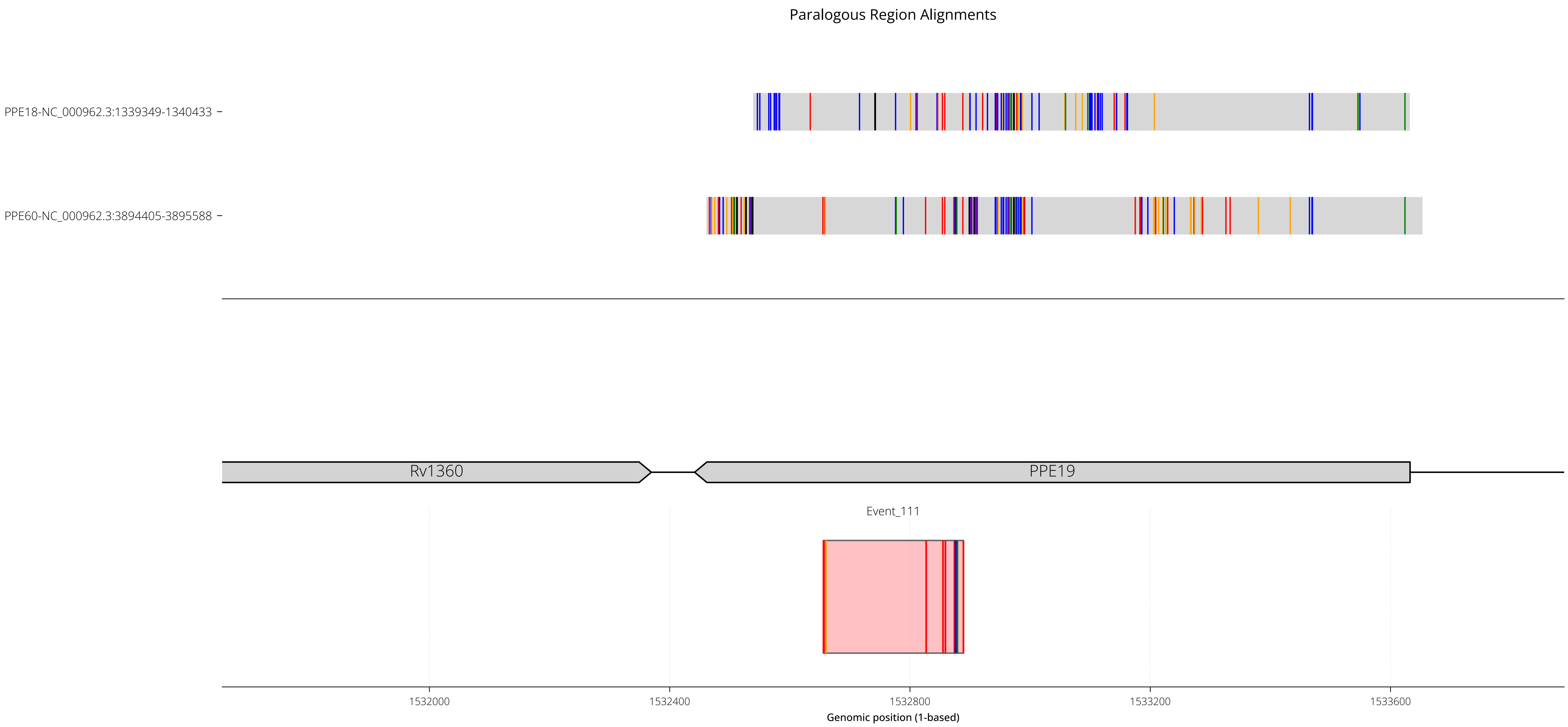

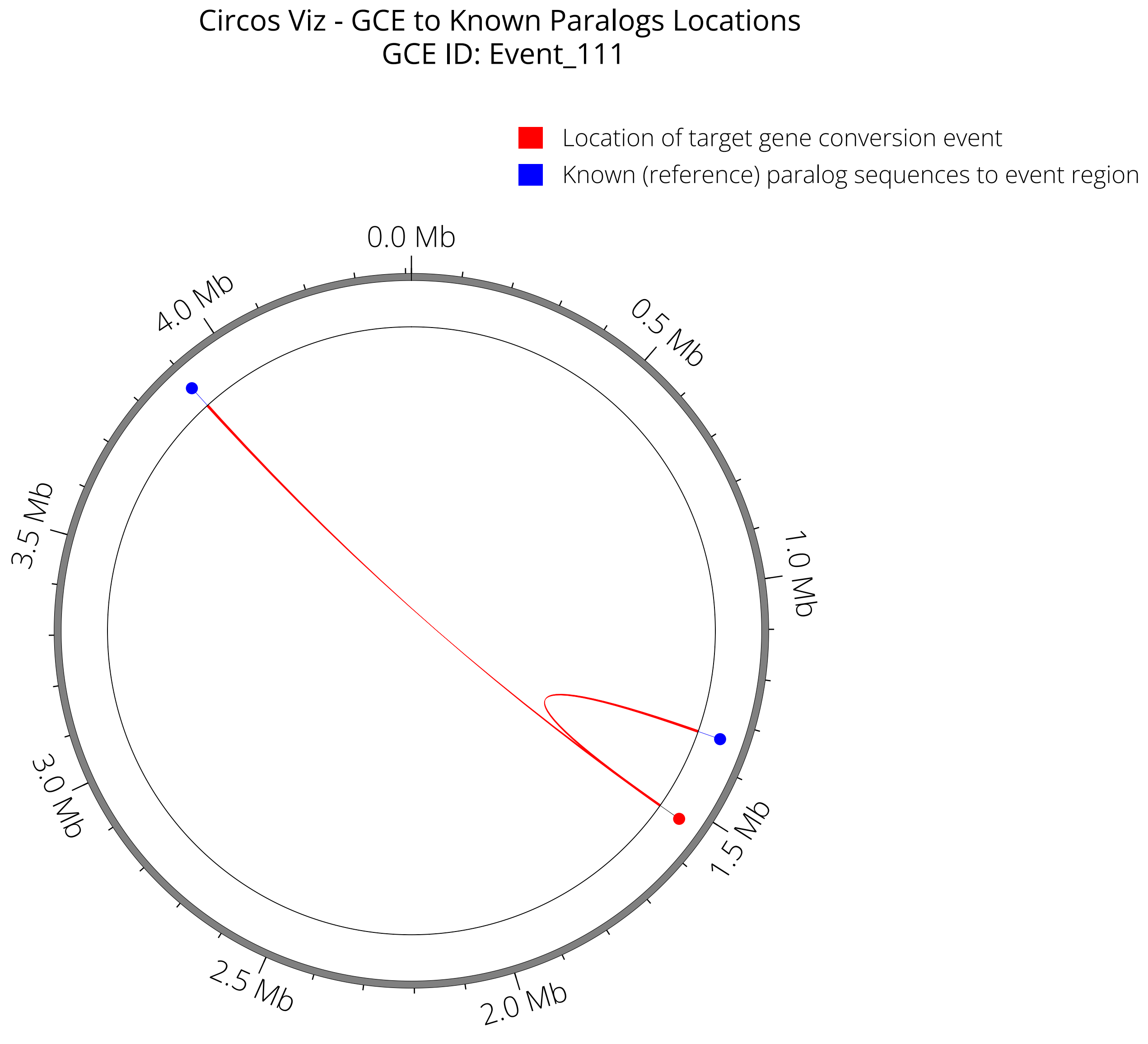
Circos Viz - GCE to Known Paralogs Locations
GCE ID: Event_111
Location of target gene conversion event
Known (reference) paralog sequences to event region
0.0 Mb
0.5 Mb
1.0 Mb
1.5 Mb
2.0 Mb
2.5 Mb
3.0 Mb
3.5 Mb
4.0 Mb

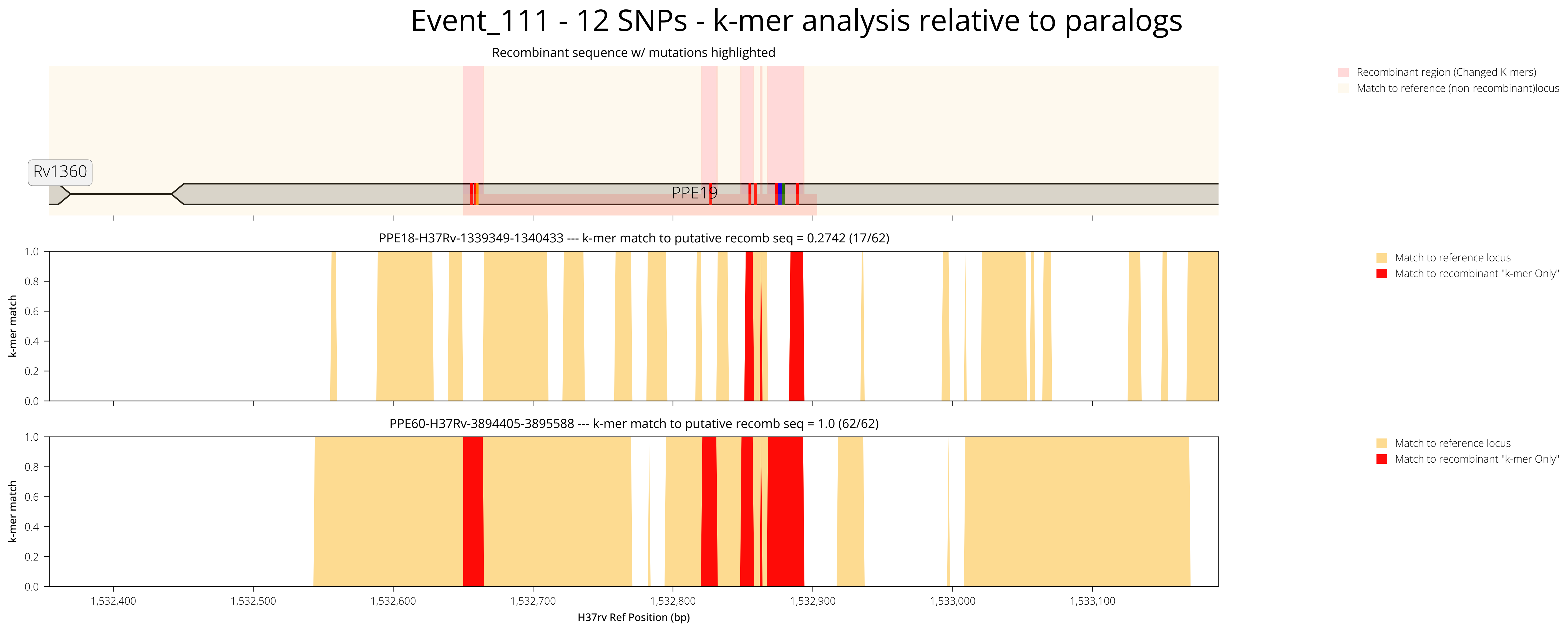

Event_111 - 12 SNPs - k-mer analysis relative to paralogs
Recombinant sequence w/ mutations highlighted
Rv1360
PPE19
Recombinant region (Changed K-mers)
Match to reference (non-recombinant)locus
PPE18-H37Rv-1339349-1340433 --- k-mer match to putative recomb seq = 0.2742 (17/62)
Match to reference locus
Match to recombinant "k-mer Only"
PPE60-H37Rv-3894405-3895588 --- k-mer match to putative recomb seq = 1.0 (62/62)
Match to reference locus
Match to recombinant "k-mer Only"
k-mer match
1.0
0.8
0.6
0.4
0.2
0.0
1,532,400
1,532,500
1,532,600
1,532,700
1,532,800
1,532,900
1,533,000
1,533,100
H37rv Ref Position (bp)

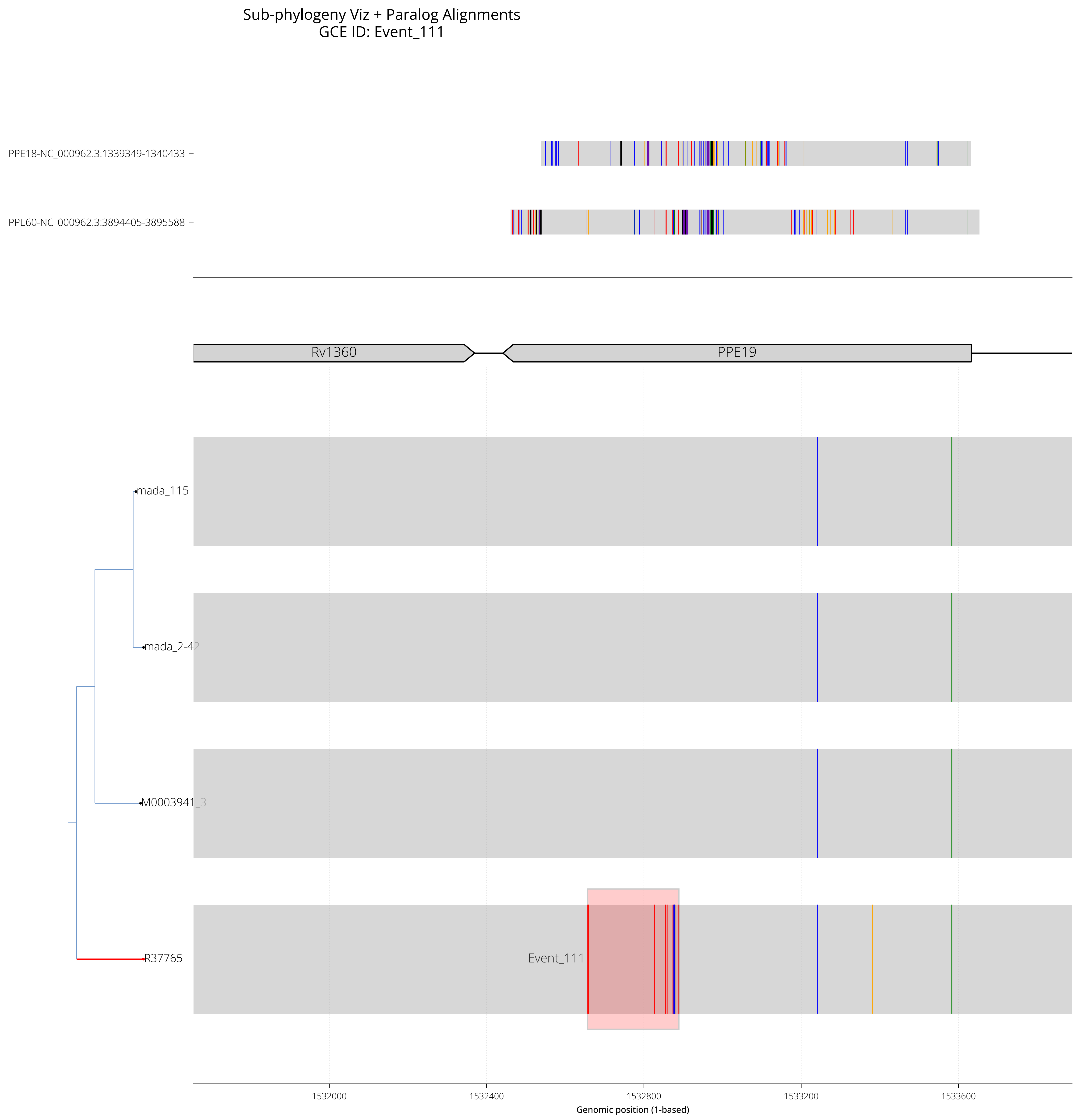
Sub-phylogeny Viz + Paralog Alignments
GCE ID: Event_111
PPE18-NC_000962.3:1339349-1340433
PPE60-NC_000962.3:3894405-3895588
Rv1360
PPE19
mada_115
mada_2-42
M0003941_3
R37765
Event_111
1532000
1532400
1532800
1533200
1533600
Genomic position (1-based)

Full Phylogeny Viz + Paralog Alignments
GCE ID: Event_111

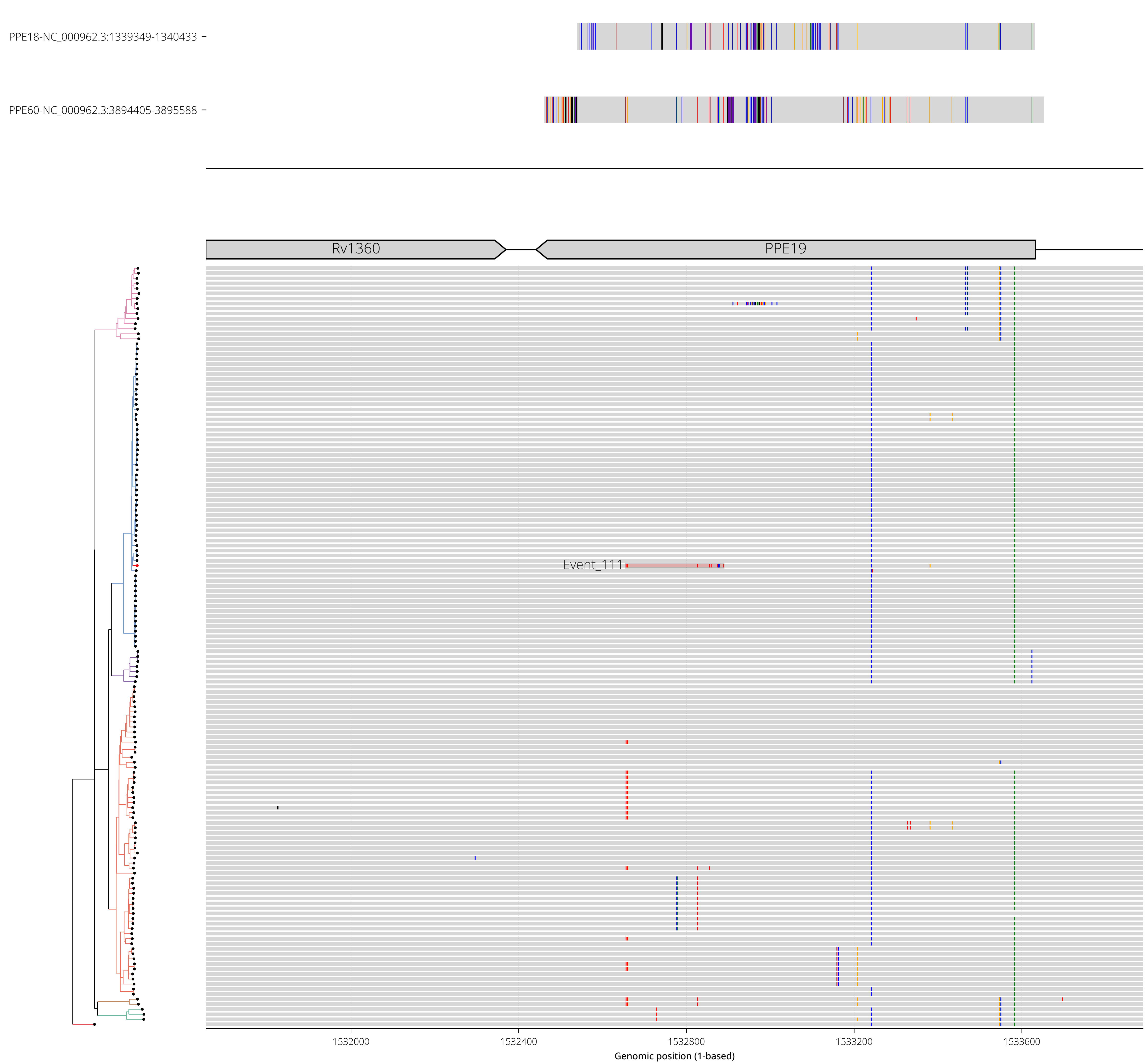

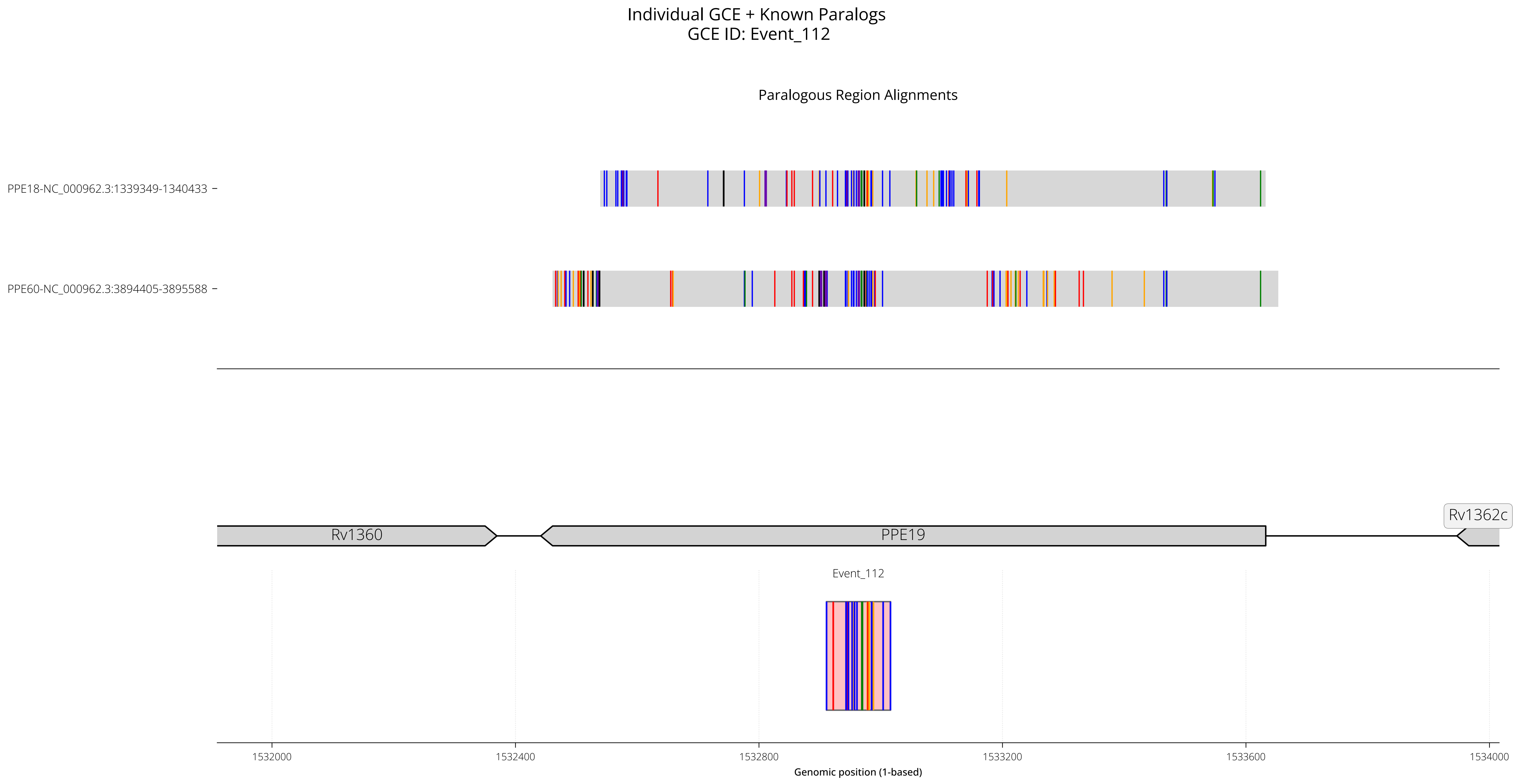
Individual GCE + Known Paralogs
GCE ID: Event_112
Paralogous Region Alignments
PPE18-NC_000962.3:1339349-1340433
PPE60-NC_000962.3:3894405-3895588
Rv1360
PPE19
Rv1362c
Event_112
1532000
1532400
1532800
1533200
1533600
1534000
Genomic position (1-based)

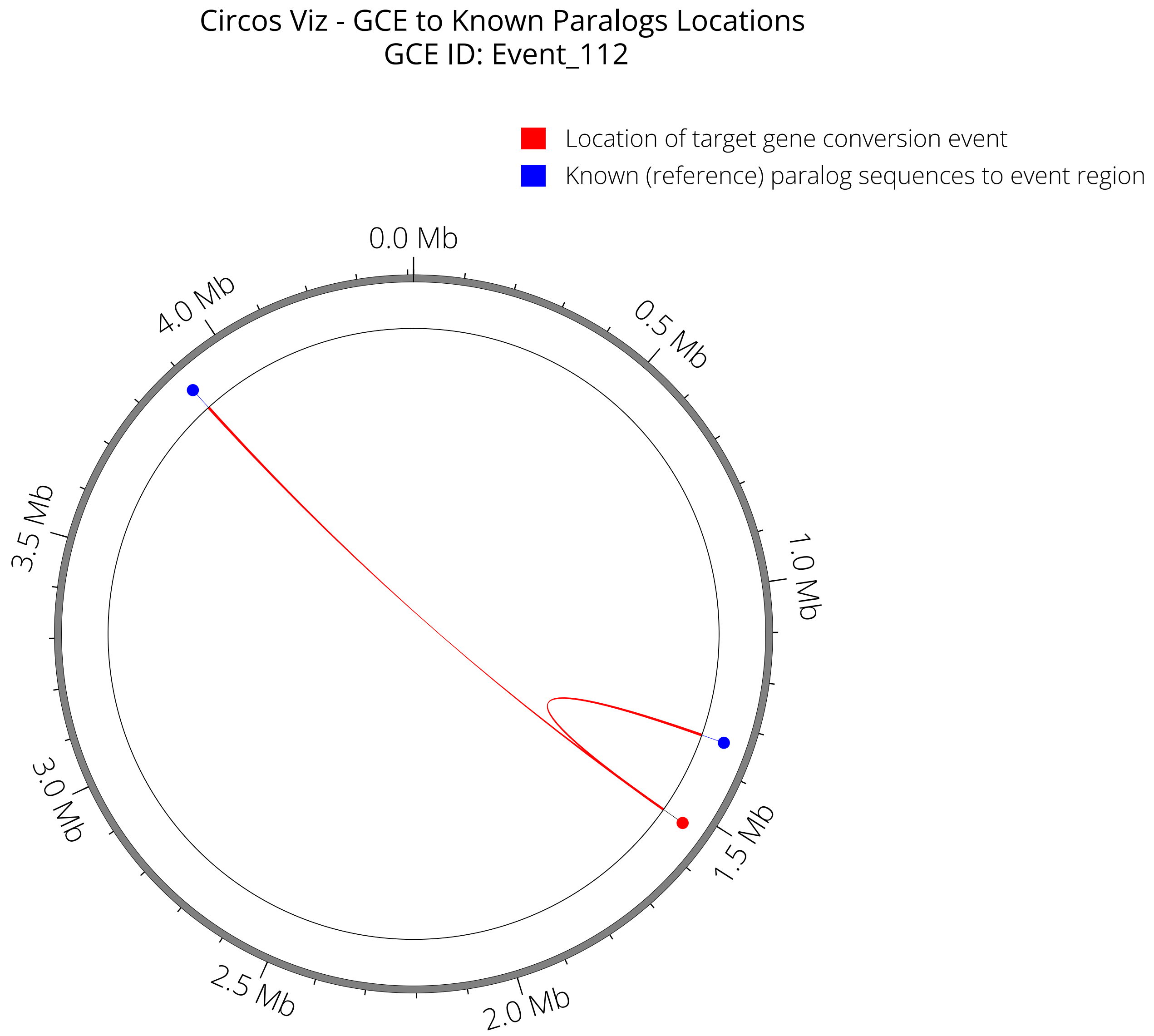
Circos Viz - GCE to Known Paralogs Locations
GCE ID: Event_112
Location of target gene conversion event
Known (reference) paralog sequences to event region
0.0 Mb
0.5 Mb
1.0 Mb
1.5 Mb
2.0 Mb
2.5 Mb
3.0 Mb
3.5 Mb
4.0 Mb

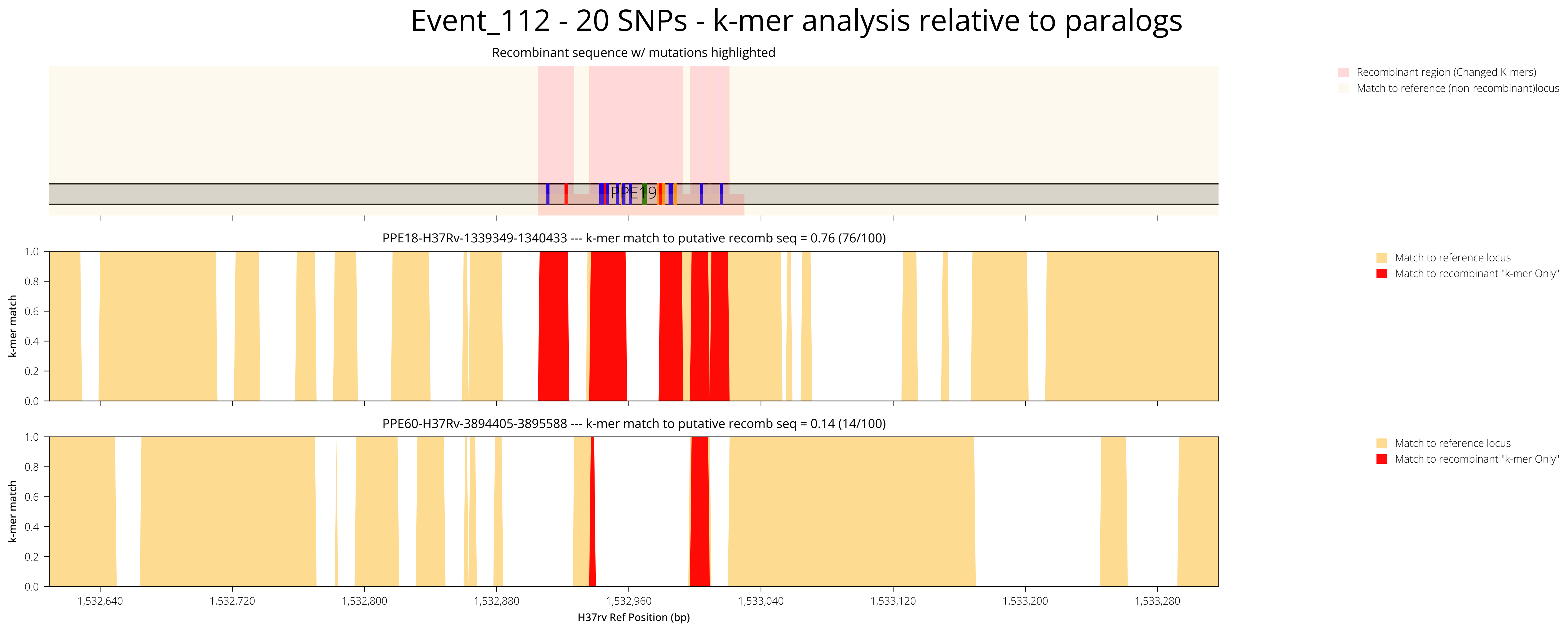

Event_112 - 20 SNPs - k-mer analysis relative to paralogs
Recombinant sequence w/ mutations highlighted
PPE19
Recombinant region (Changed K-mers)
Match to reference (non-recombinant)locus
PPE18-H37Rv-1339349-1340433 --- k-mer match to putative recomb seq = 0.76 (76/100)
Match to reference locus
Match to recombinant "k-mer Only"
k-mer match
PPE60-H37Rv-3894405-3895588 --- k-mer match to putative recomb seq = 0.14 (14/100)
Match to reference locus
Match to recombinant "k-mer Only"
k-mer match
1,532,640
1,532,720
1,532,800
1,532,880
1,532,960
1,533,040
1,533,120
1,533,200
1,533,280
H37rv Ref Position (bp)

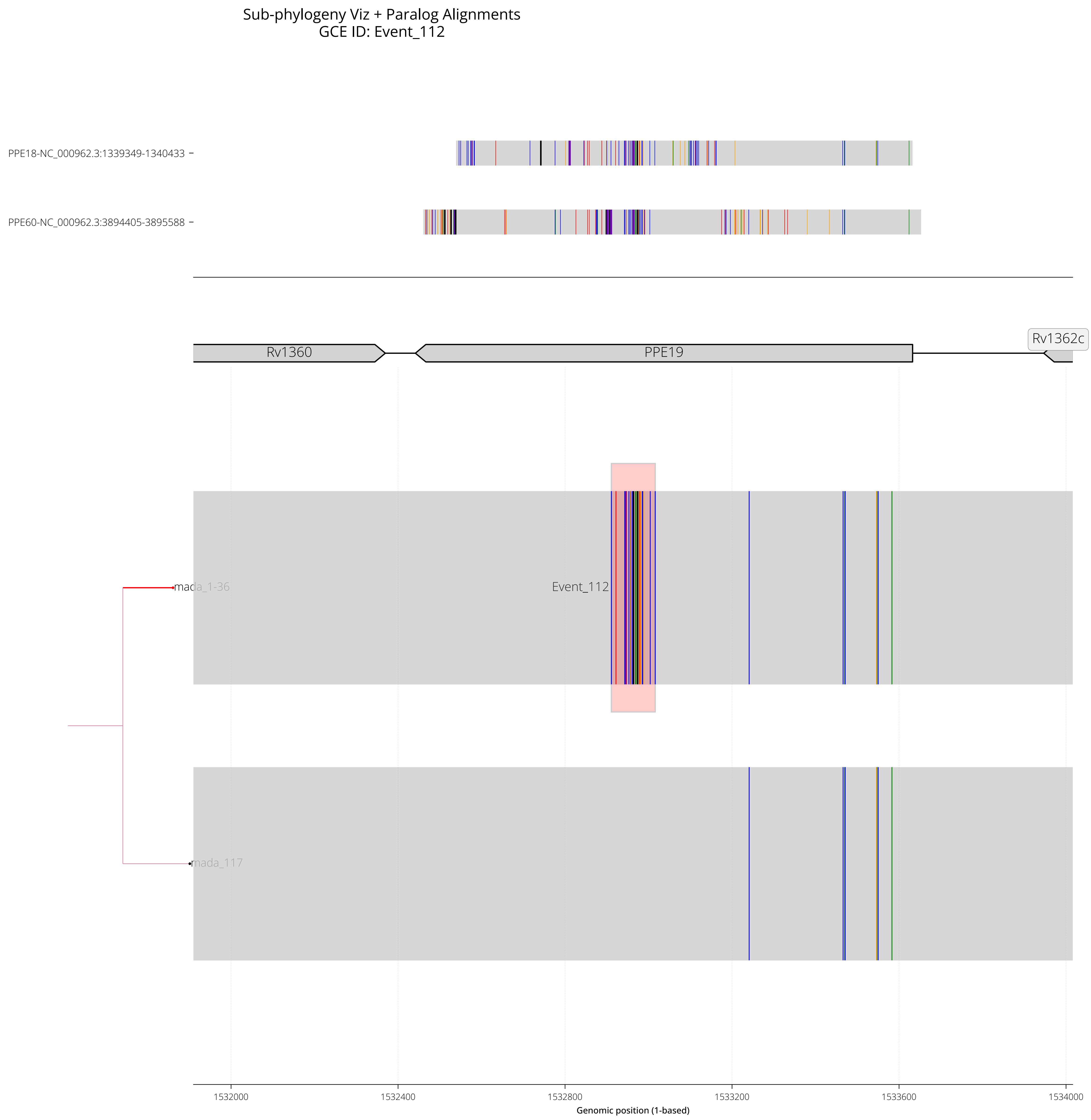
Sub-phylogeny Viz + Paralog Alignments
GCE ID: Event_112
PPE18-NC_000962.3:1339349-1340433
PPE60-NC_000962.3:3894405-3895588
Rv1360
PPE19
Rv1362c
mada_1-36
Event_112
mada_117
1532000
1532400
1532800
1533200
1533600
1534000
Genomic position (1-based)

Full Phylogeny Viz + Paralog Alignments
GCE ID: Event_112

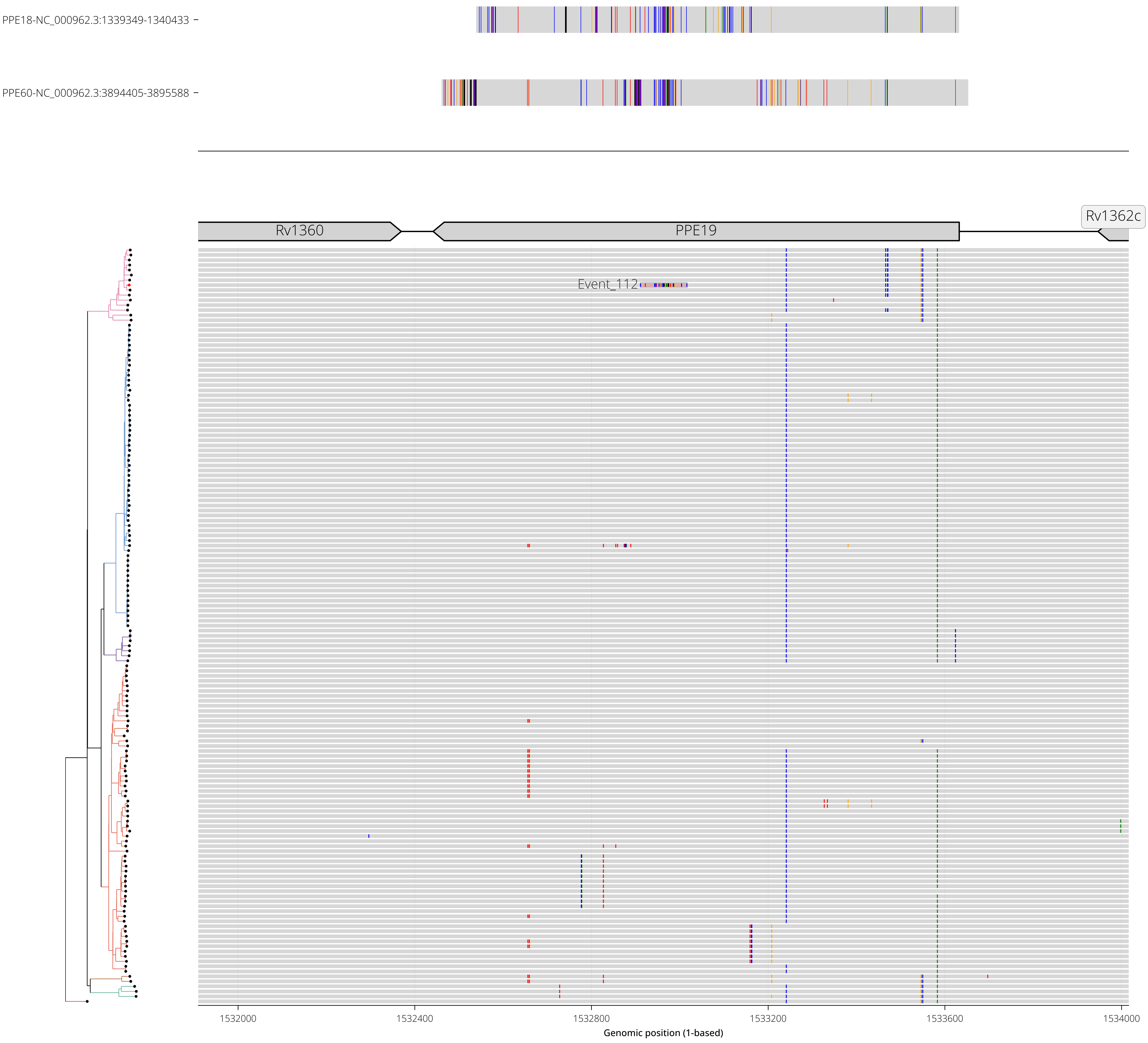

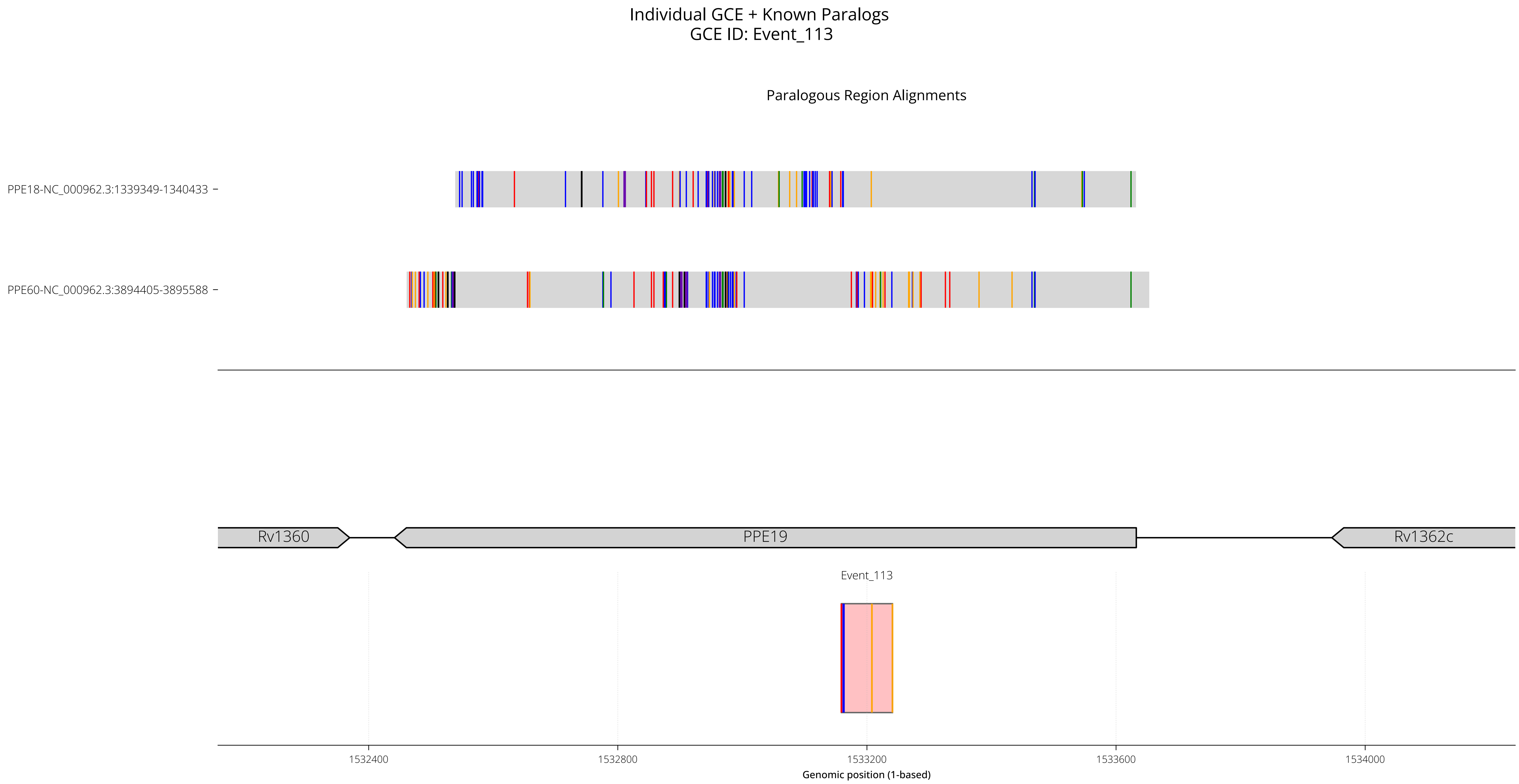
Individual GCE + Known Paralogs
GCE ID: Event_113
Paralogous Region Alignments
PPE18-NC_000962.3:1339349-1340433
PPE60-NC_000962.3:3894405-3895588
Rv1360
PPE19
Rv1362c
Event_113
1532400
1532800
1533200
1533600
1534000
Genomic position (1-based)

Circos Viz - GCE to Known Paralogs Locations
GCE ID: Event_113

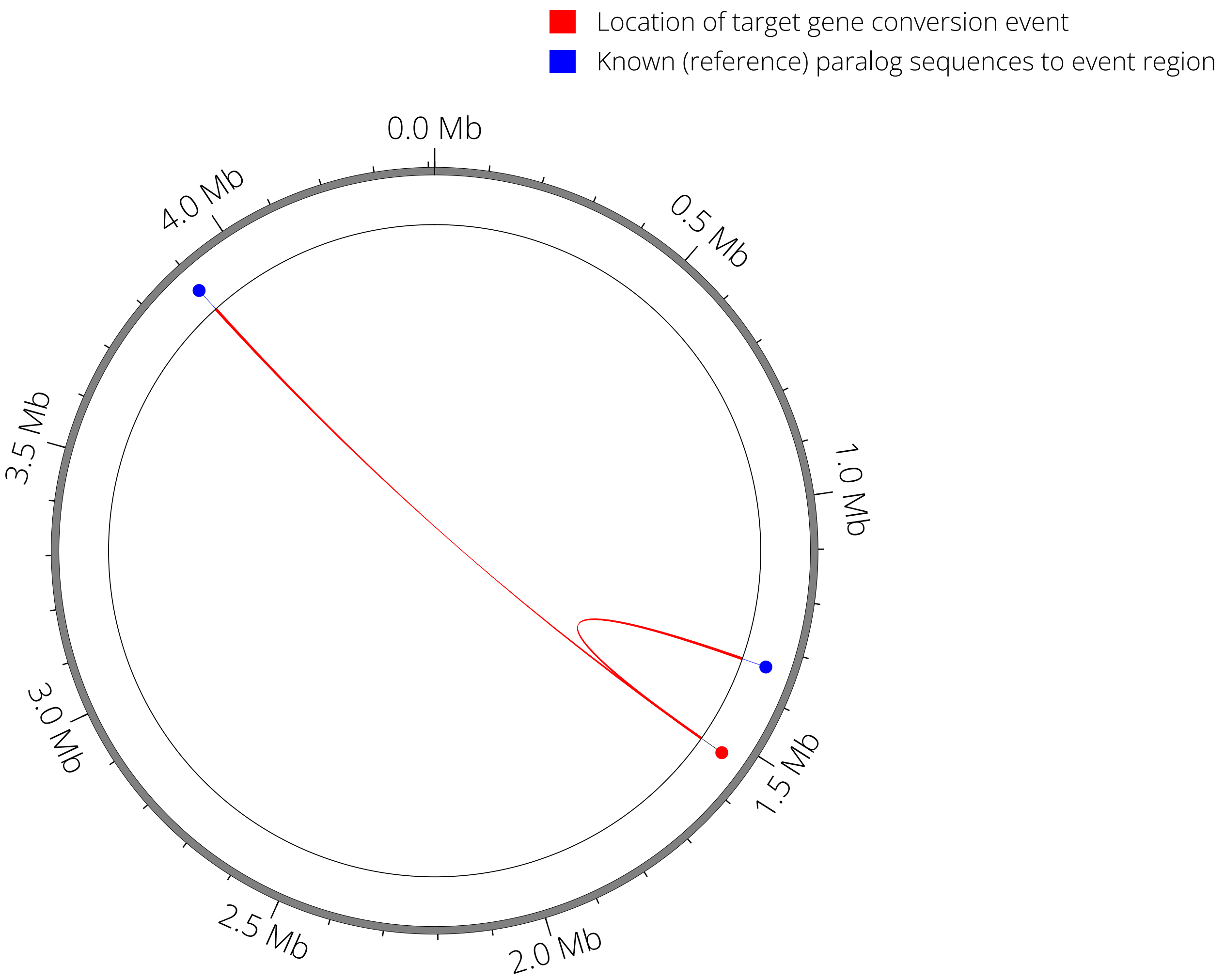

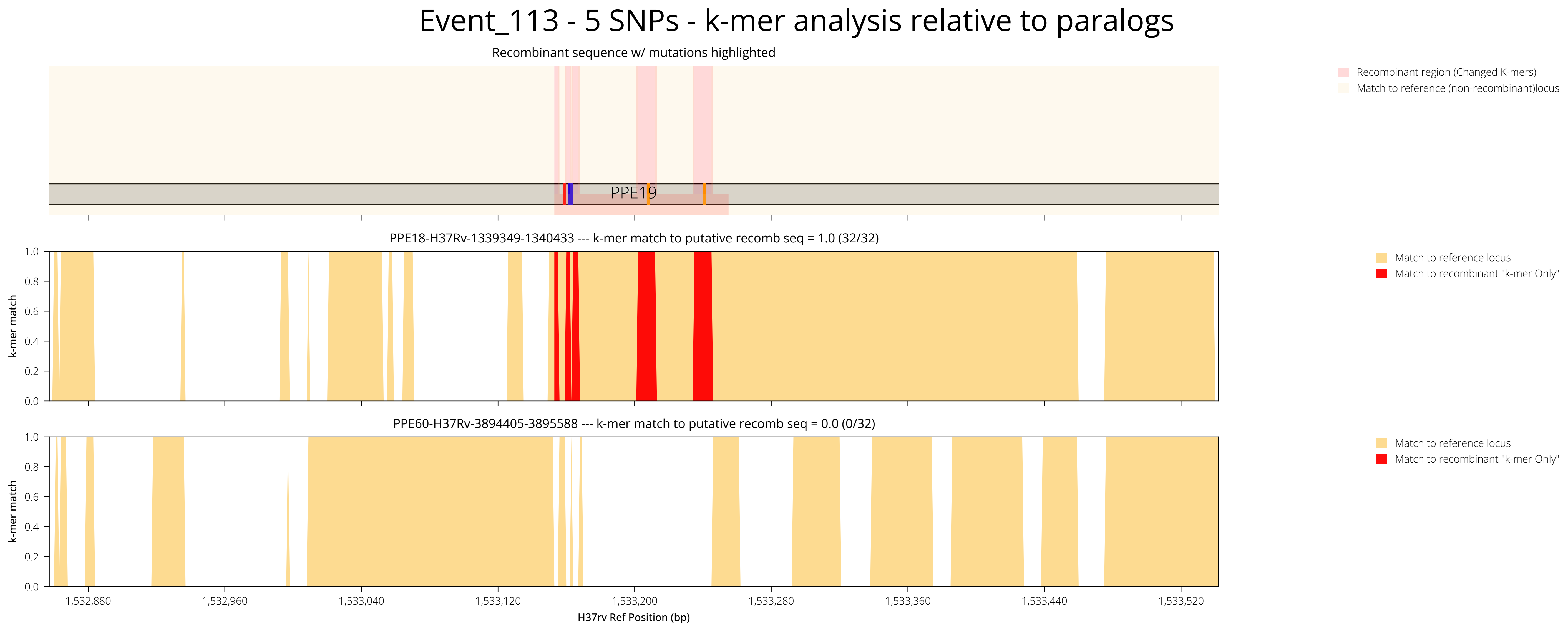
Event_113 - 5 SNPs - k-mer analysis relative to paralogs
Recombinant sequence w/ mutations highlighted
PPE19
Recombinant region (Changed K-mers)
Match to reference (non-recombinant)locus
PPE18-H37Rv-1339349-1340433 --- k-mer match to putative recomb seq = 1.0 (32/32)
Match to reference locus
Match to recombinant "k-mer Only"
PPE60-H37Rv-3894405-3895588 --- k-mer match to putative recomb seq = 0.0 (0/32)
Match to reference locus
Match to recombinant "k-mer Only"
k-mer match
1.0
0.8
0.6
0.4
0.2
0.0
1,532,880
1,532,960
1,533,040
1,533,120
1,533,200
1,533,280
1,533,360
1,533,440
1,533,520
H37rv Ref Position (bp)

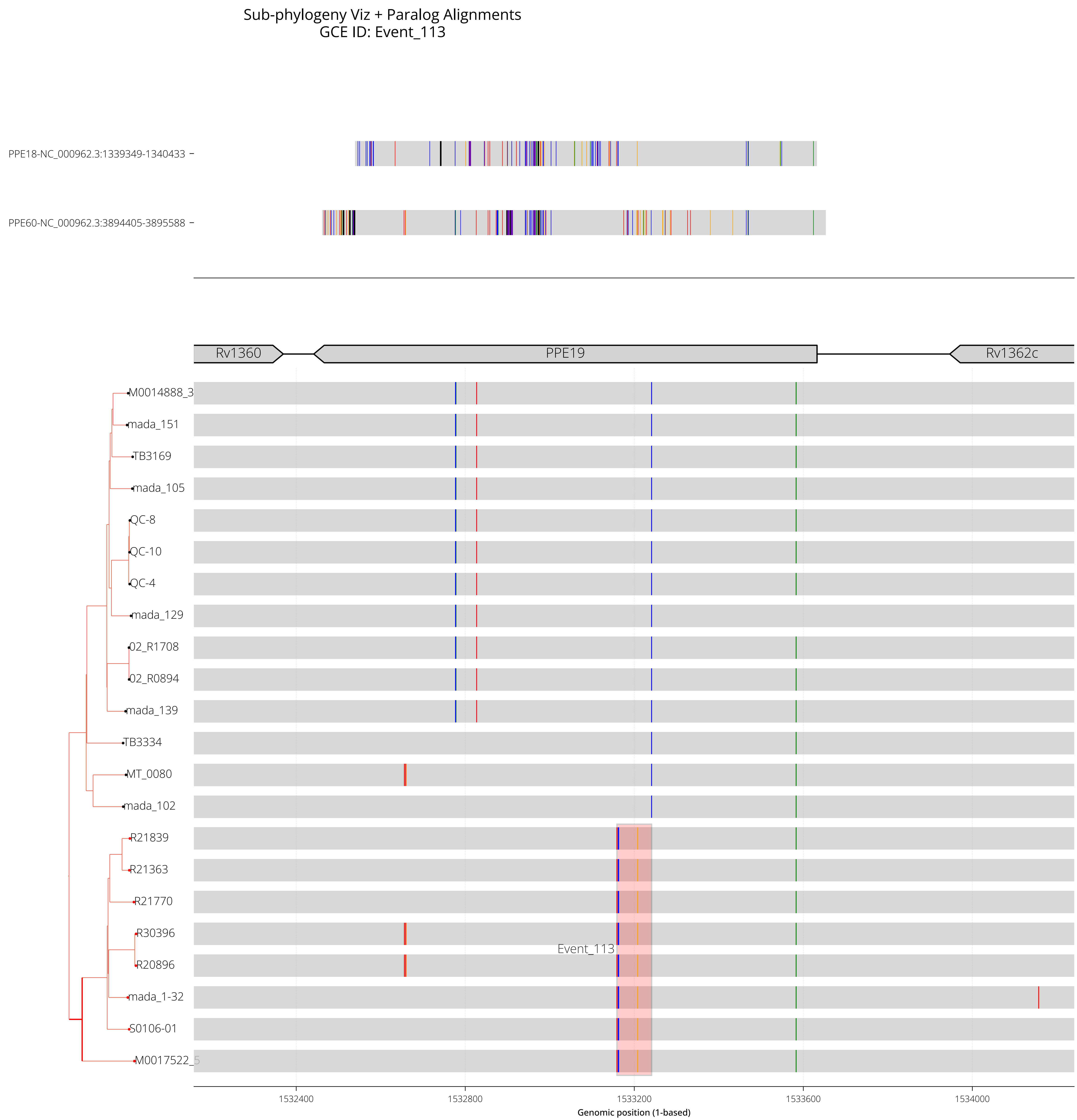

Sub-phylogeny Viz + Paralog Alignments
GCE ID: Event_113
PPE18-NC_000962.3:1339349-1340433
PPE60-NC_000962.3:3894405-3895588
Rv1360
PPE19
Rv1362c
M0014888_3
mada_151
TB3169
mada_105
QC-8
QC-10
QC-4
mada_129
02_R1708
02_R0894
mada_139
TB3334
MT_0080
mada_102
R21839
R21363
R21770
R30396
R20896
mada_1-32
S0106-01
M0017522_5
Event_113
1532400
1532800
1533200
1533600
1534000
Genomic position (1-based)

Full Phylogeny Viz + Paralog Alignments
GCE ID: Event_113

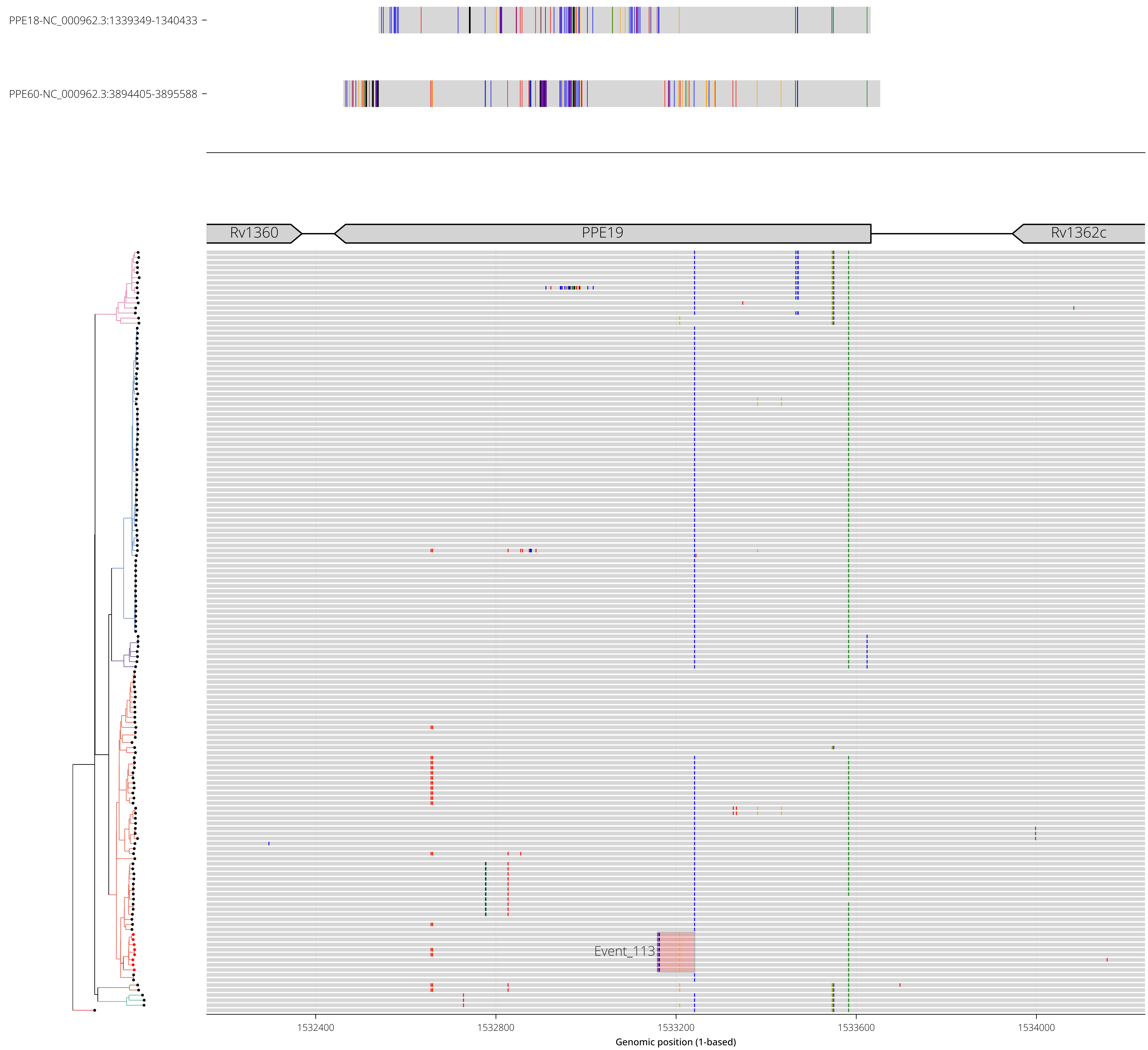

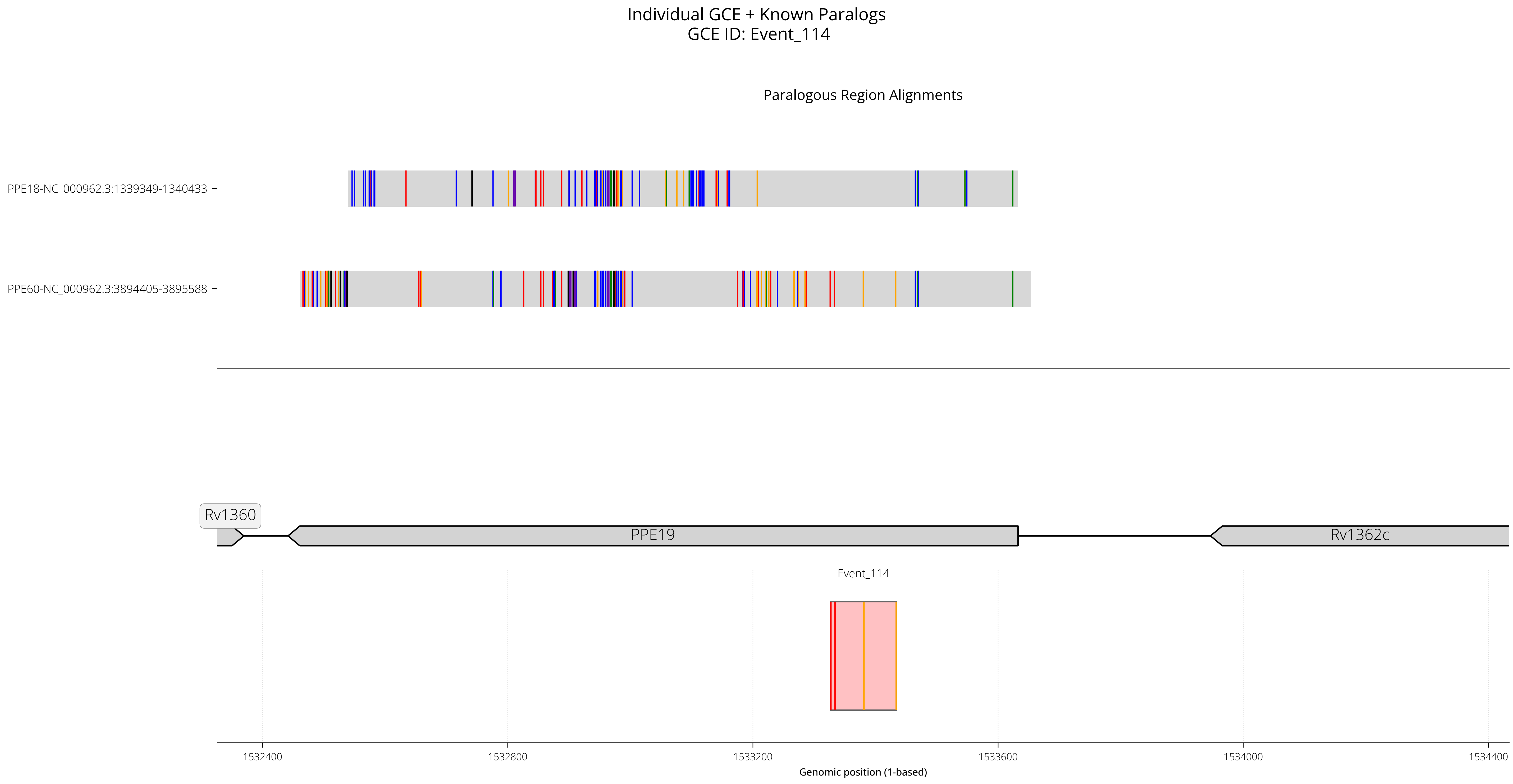

Individual GCE + Known Paralogs
GCE ID: Event_114
Paralogous Region Alignments
PPE18-NC_000962.3:1339349-1340433
PPE60-NC_000962.3:3894405-3895588
Rv1360
PPE19
Rv1362c
Event_114
1532400
1532800
1533200
1533600
1534000
1534400
Genomic position (1-based)

Circos Viz - GCE to Known Paralogs Locations
GCE ID: Event_114

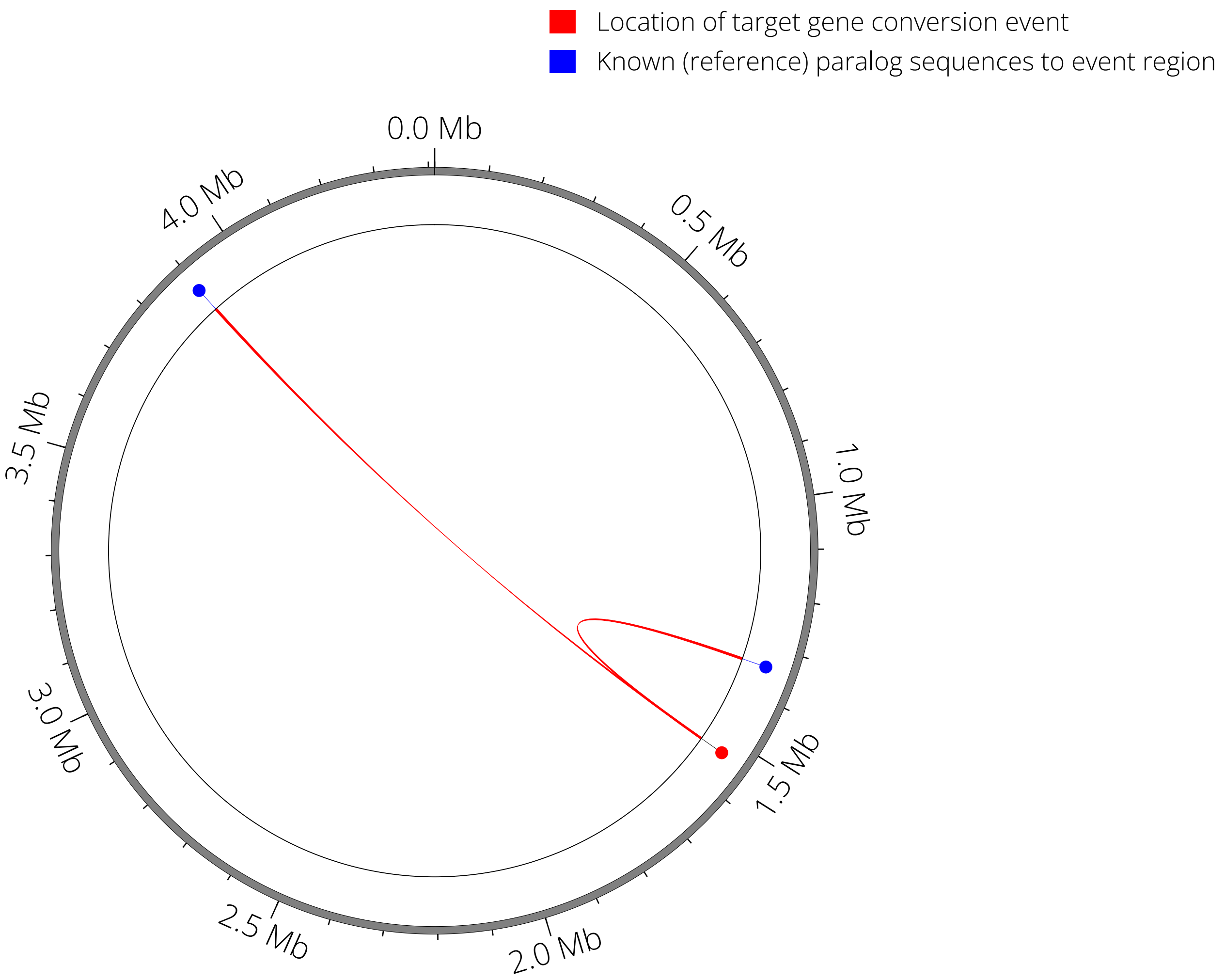

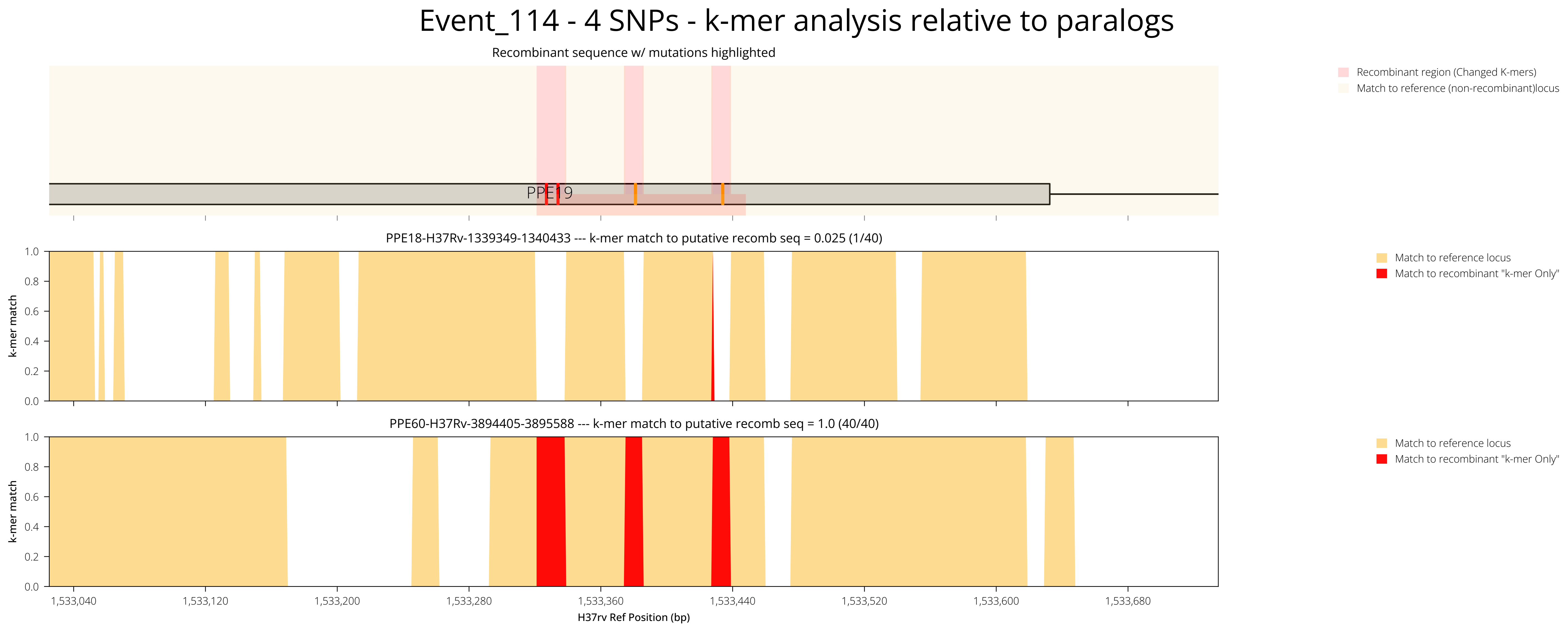
Event_114 - 4 SNPs - k-mer analysis relative to paralogs
Recombinant sequence w/ mutations highlighted
Recombinant region (Changed K-mers)
Match to reference (non-recombinant)locus
PPE19
PPE18-H37Rv-1339349-1340433 --- k-mer match to putative recomb seq = 0.025 (1/40)
Match to reference locus
Match to recombinant "k-mer Only"
k-mer match
PPE60-H37Rv-3894405-3895588 --- k-mer match to putative recomb seq = 1.0 (40/40)
Match to reference locus
Match to recombinant "k-mer Only"
k-mer match
1,533,040
1,533,120
1,533,200
1,533,280
1,533,360
1,533,440
1,533,520
1,533,600
1,533,680
H37rv Ref Position (bp)

Sub-phylogeny Viz + Paralog Alignments
GCE ID: Event_114

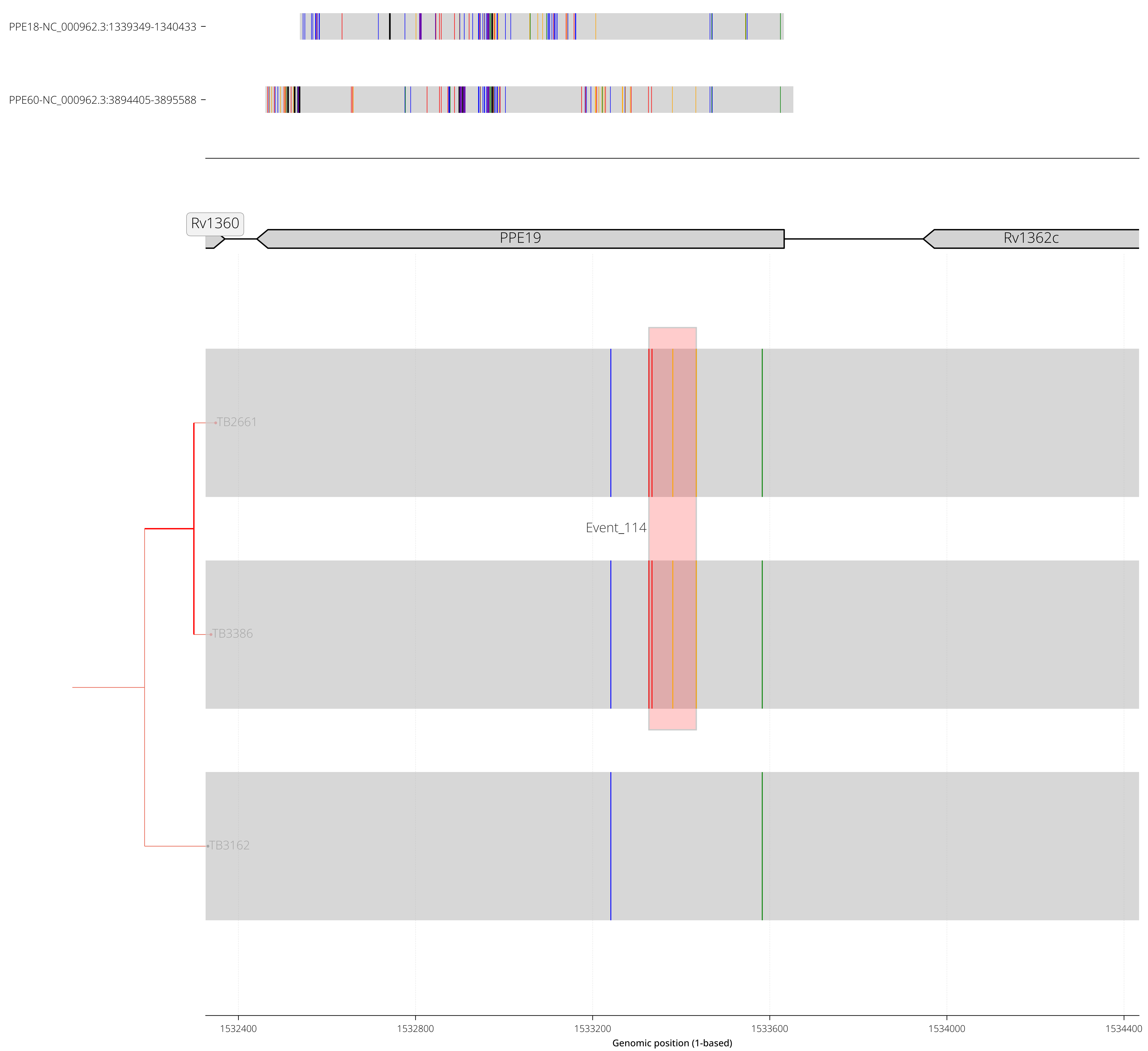

Full Phylogeny Viz + Paralog Alignments
GCE ID: Event_114

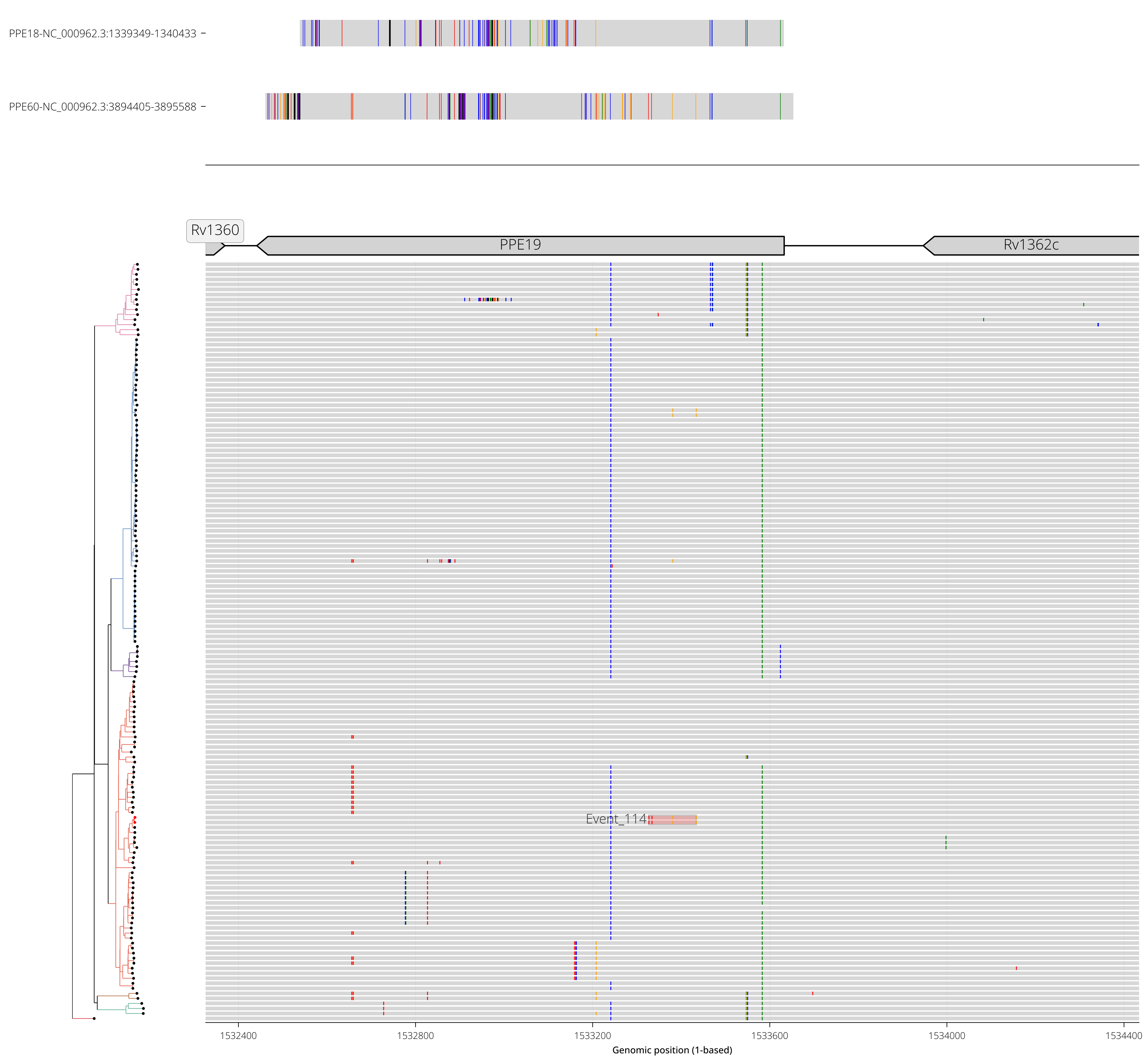

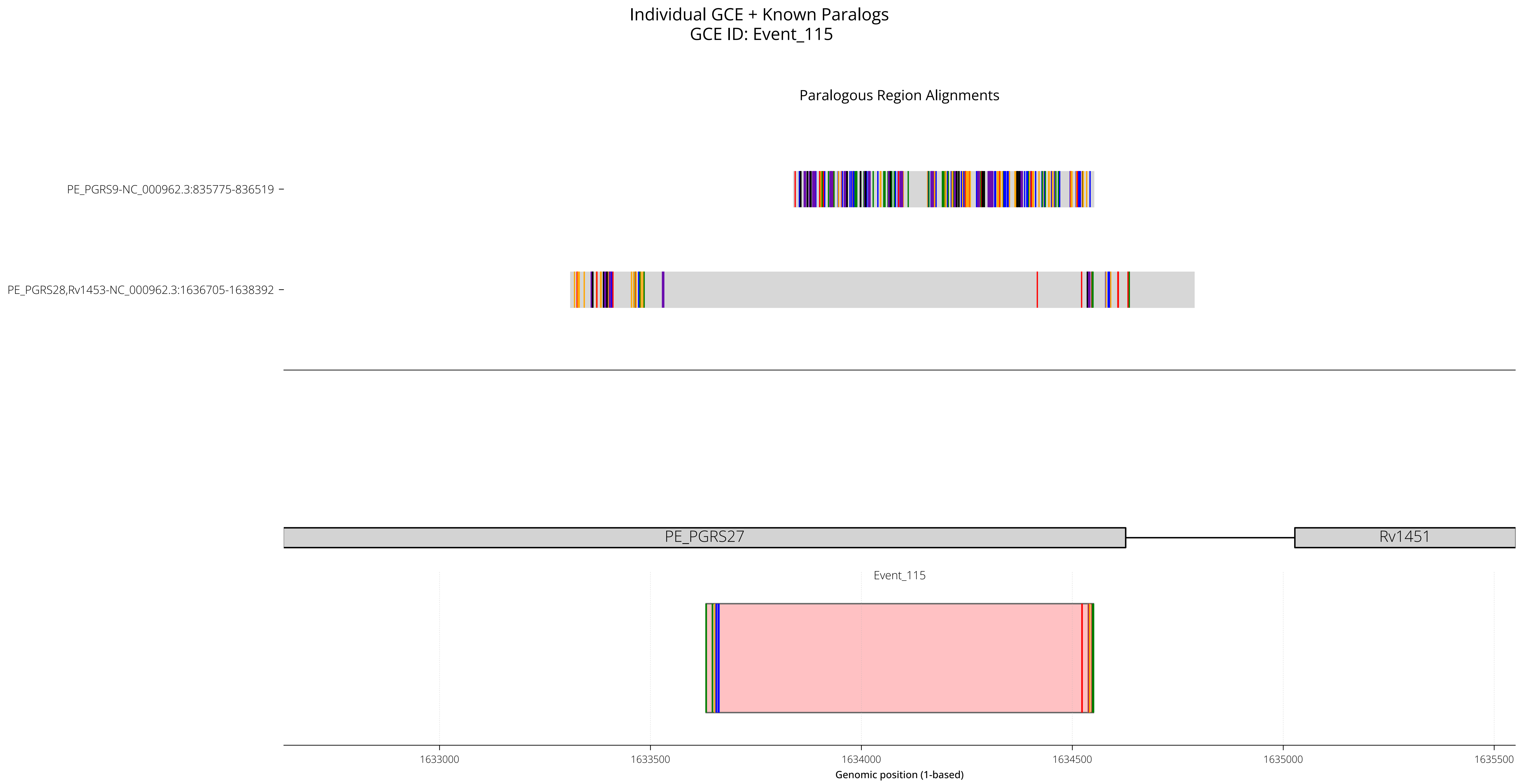

Individual GCE + Known Paralogs
GCE ID: Event_115
Paralogous Region Alignments
PE_PGRS9-NC_000962.3:835775-836519
PE_PGRS28,Rv1453-NC_000962.3:1636705-1638392
PE_PGRS27
Rv1451
Event_115
1633000
1633500
1634000
1634500
1635000
1635500
Genomic position (1-based)

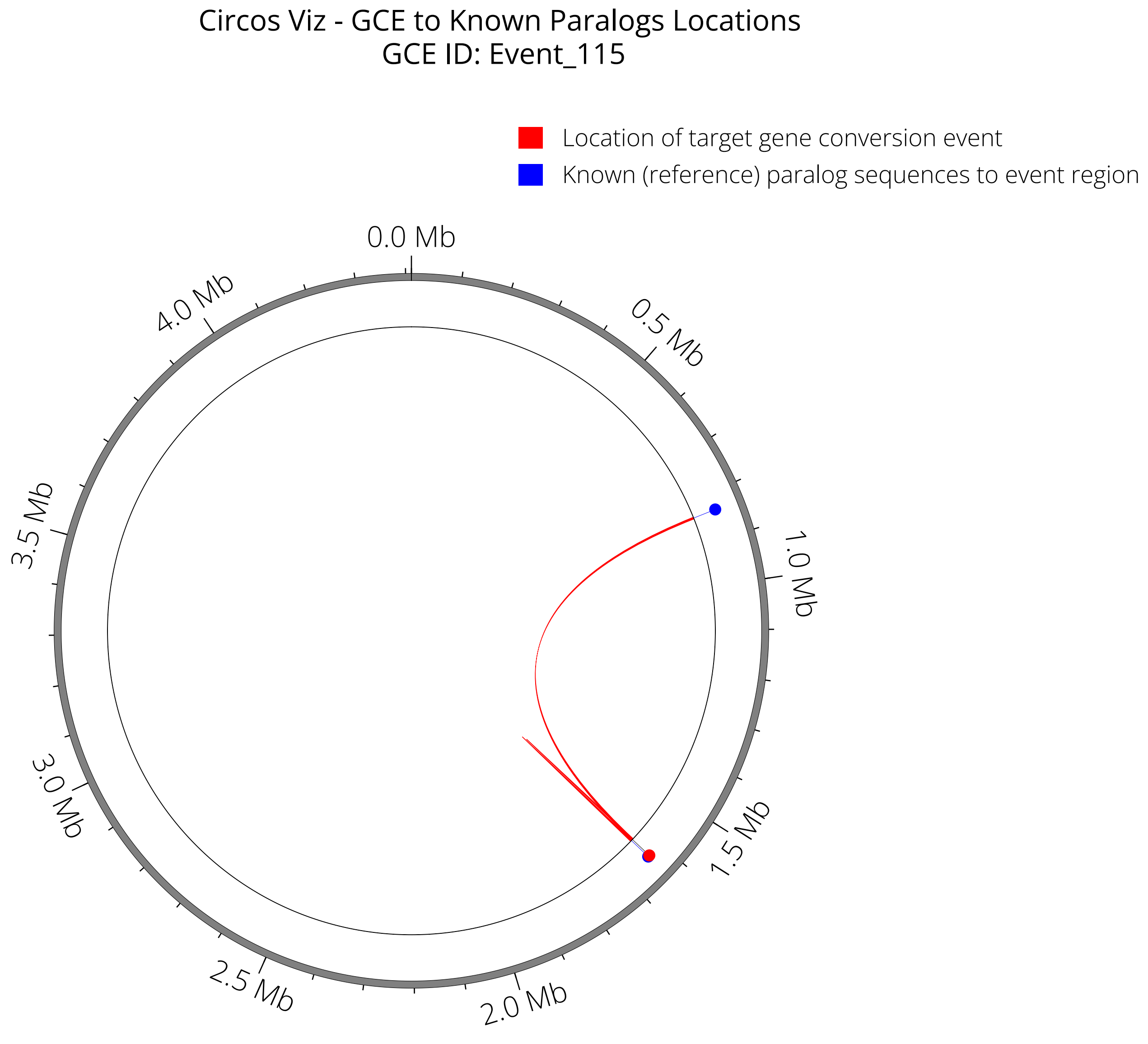
Circos Viz - GCE to Known Paralogs Locations
GCE ID: Event_115
Location of target gene conversion event
Known (reference) paralog sequences to event region
0.0 Mb
0.5 Mb
1.0 Mb
1.5 Mb
2.0 Mb
2.5 Mb
3.0 Mb
3.5 Mb
4.0 Mb

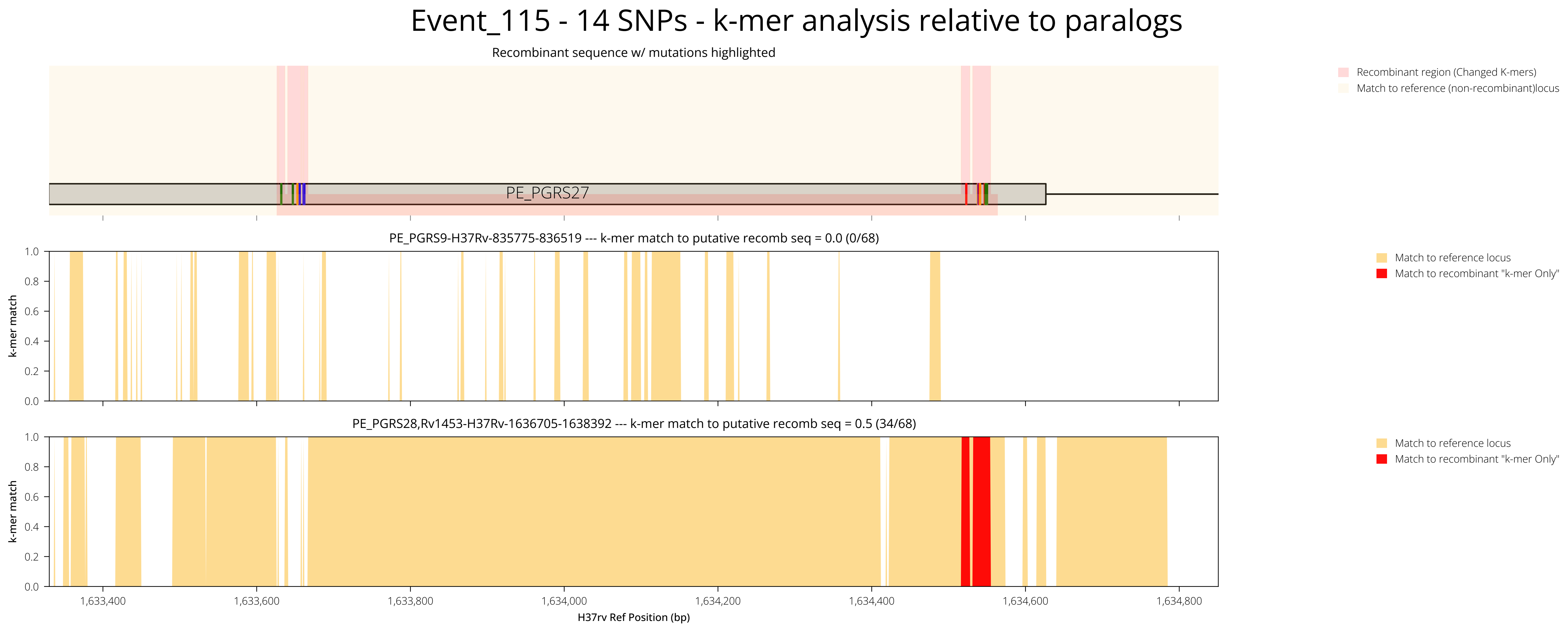

Event_115 - 14 SNPs - k-mer analysis relative to paralogs
Recombinant sequence w/ mutations highlighted
PE_PGRS27
Recombinant region (Changed K-mers)
Match to reference (non-recombinant)locus
PE_PGRS9-H37Rv-835775-836519 --- k-mer match to putative recomb seq = 0.0 (0/68)
PE_PGRS28,Rv1453-H37Rv-1636705-1638392 --- k-mer match to putative recomb seq = 0.5 (34/68)
k-mer match
1.0
0.8
0.6
0.4
0.2
0.0
Match to reference locus
Match to recombinant "k-mer Only"
1,633,400
1,633,600
1,633,800
1,634,000
1,634,200
1,634,400
1,634,600
1,634,800
H37rv Ref Position (bp)

Sub-phylogeny Viz + Paralog Alignments
GCE ID: Event_115

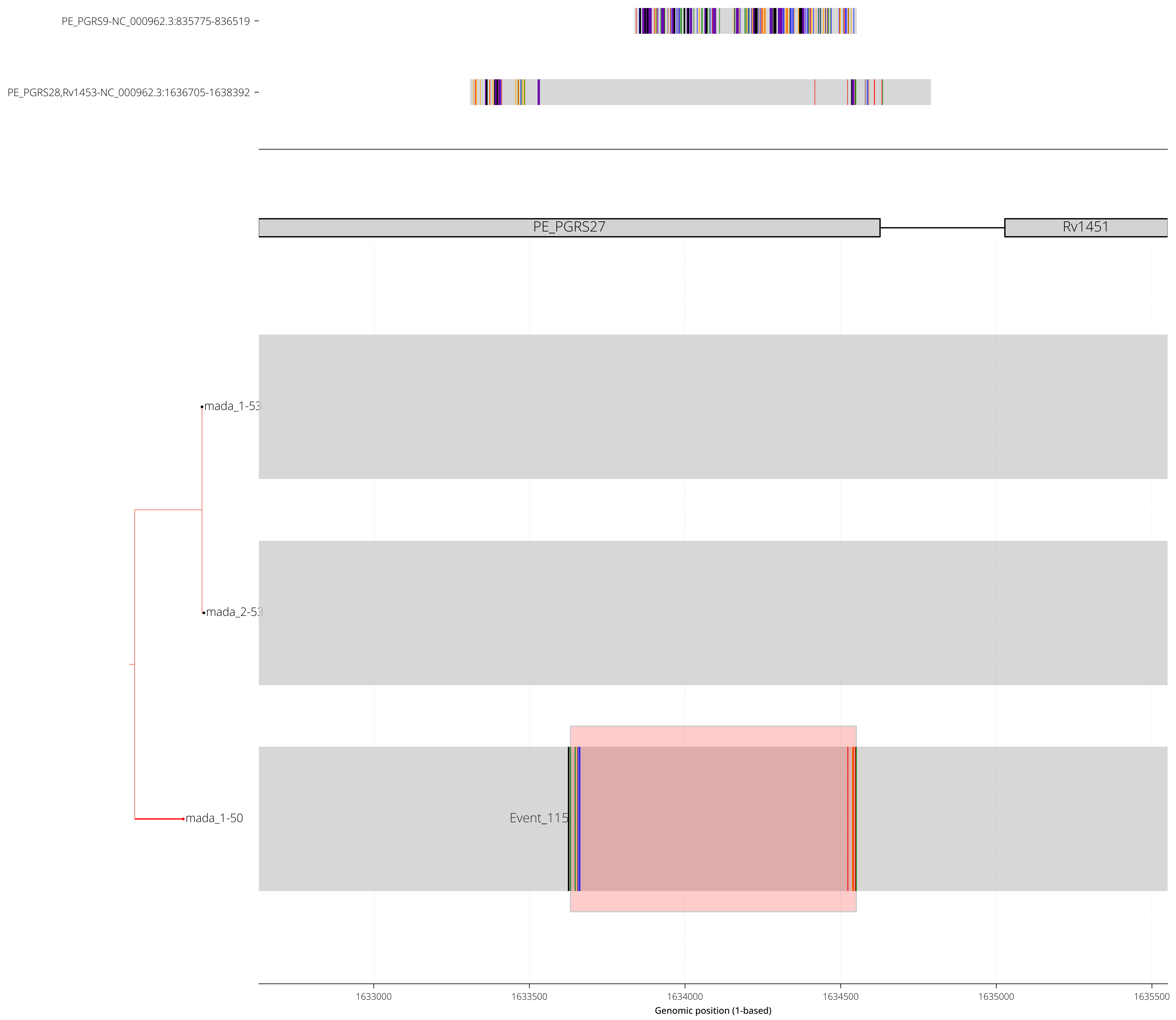

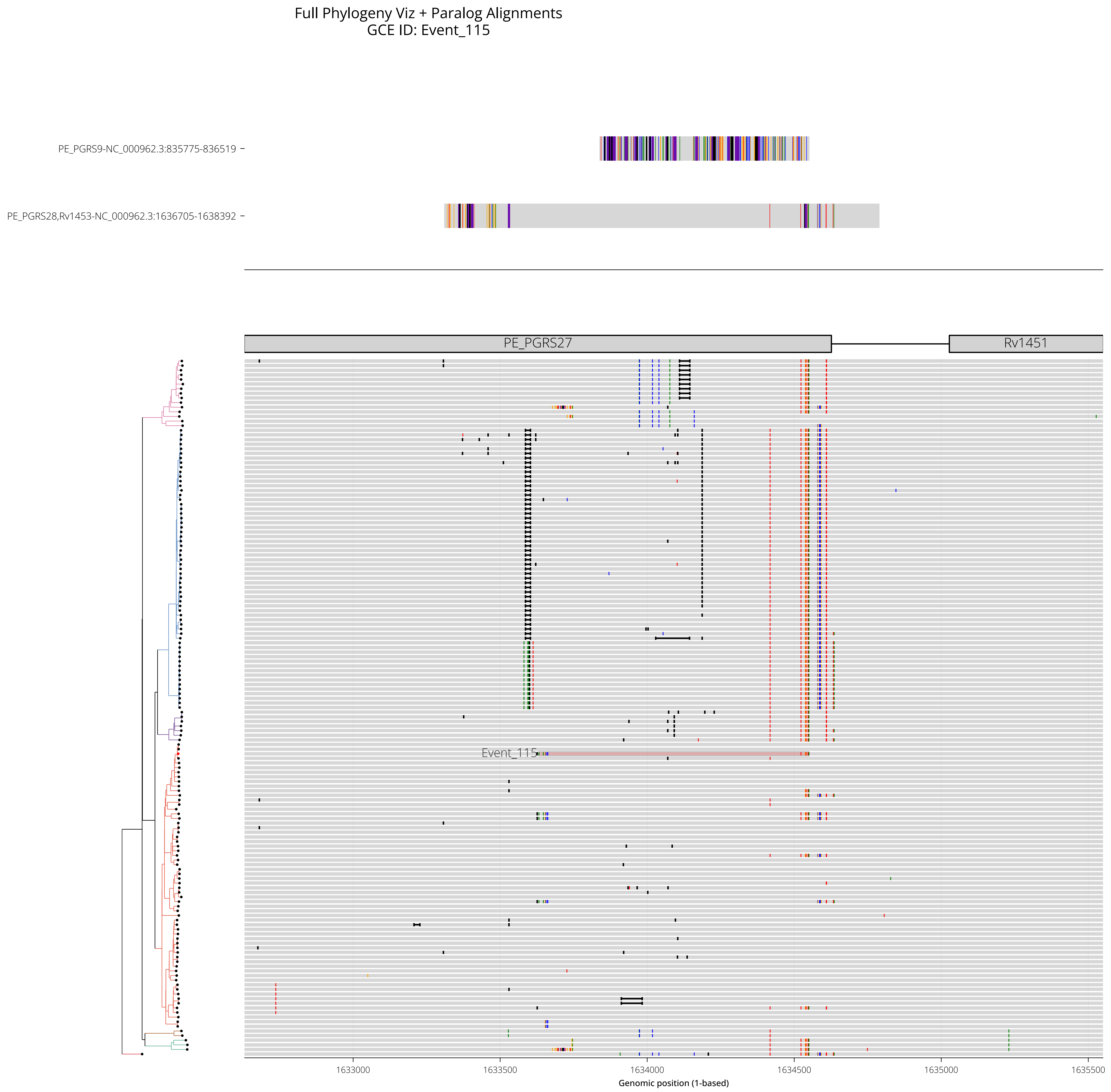

Full Phylogeny Viz + Paralog Alignments
GCE ID: Event_115
PE_PGRS9-NC_000962.3:835775-836519
PE_PGRS28,Rv1453-NC_000962.3:1636705-1638392
PE_PGRS27
Rv1451
Event_115
1633000
1633500
1634000
1634500
1635000
1635500
Genomic position (1-based)

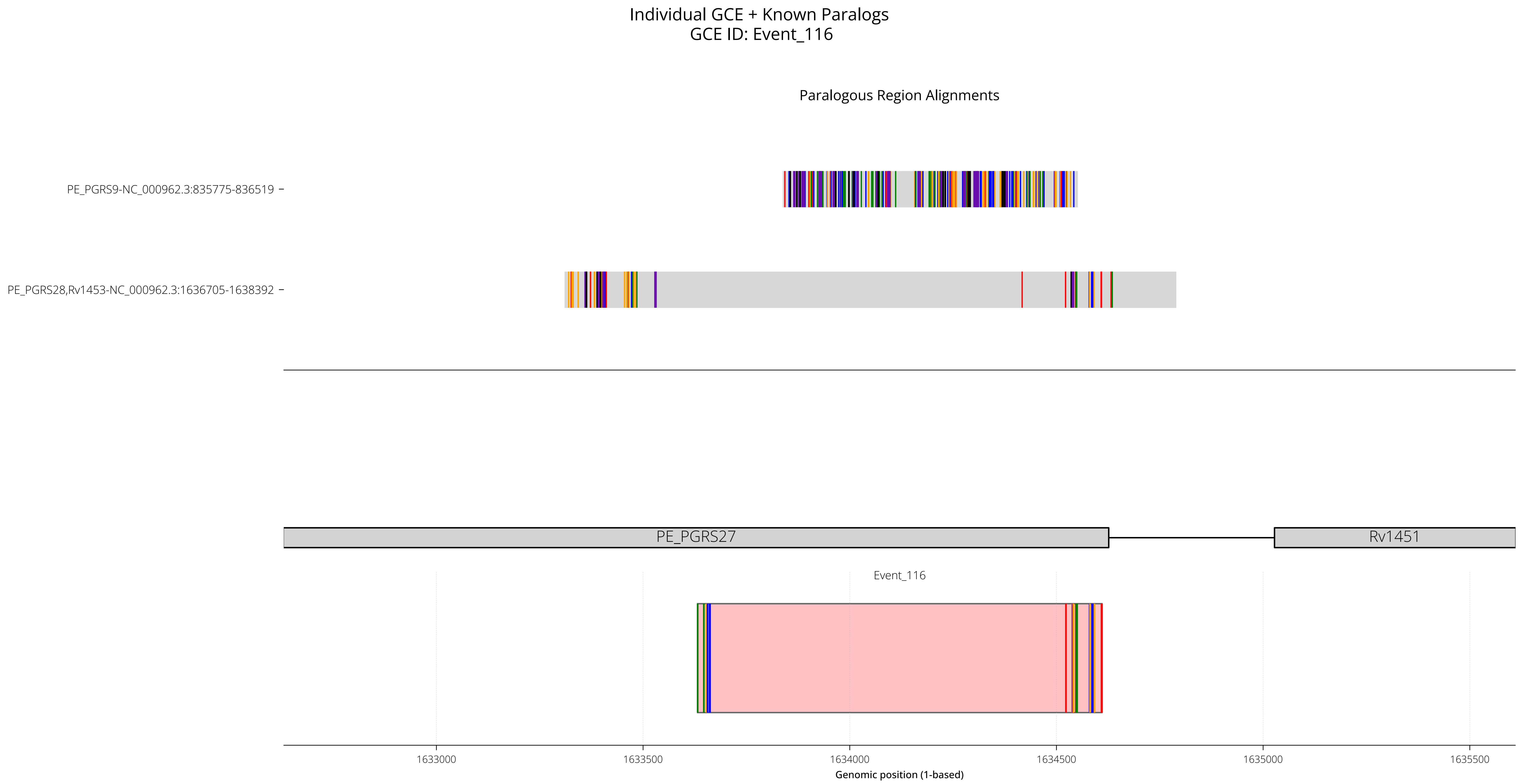

Individual GCE + Known Paralogs
GCE ID: Event_116
Paralogous Region Alignments
PE_PGRS9-NC_000962.3:835775-836519
PE_PGRS28,Rv1453-NC_000962.3:1636705-1638392
PE_PGRS27
Rv1451
Event_116
1633000
1633500
1634000
1634500
1635000
1635500
Genomic position (1-based)

Circos Viz - GCE to Known Paralogs Locations
GCE ID: Event_116

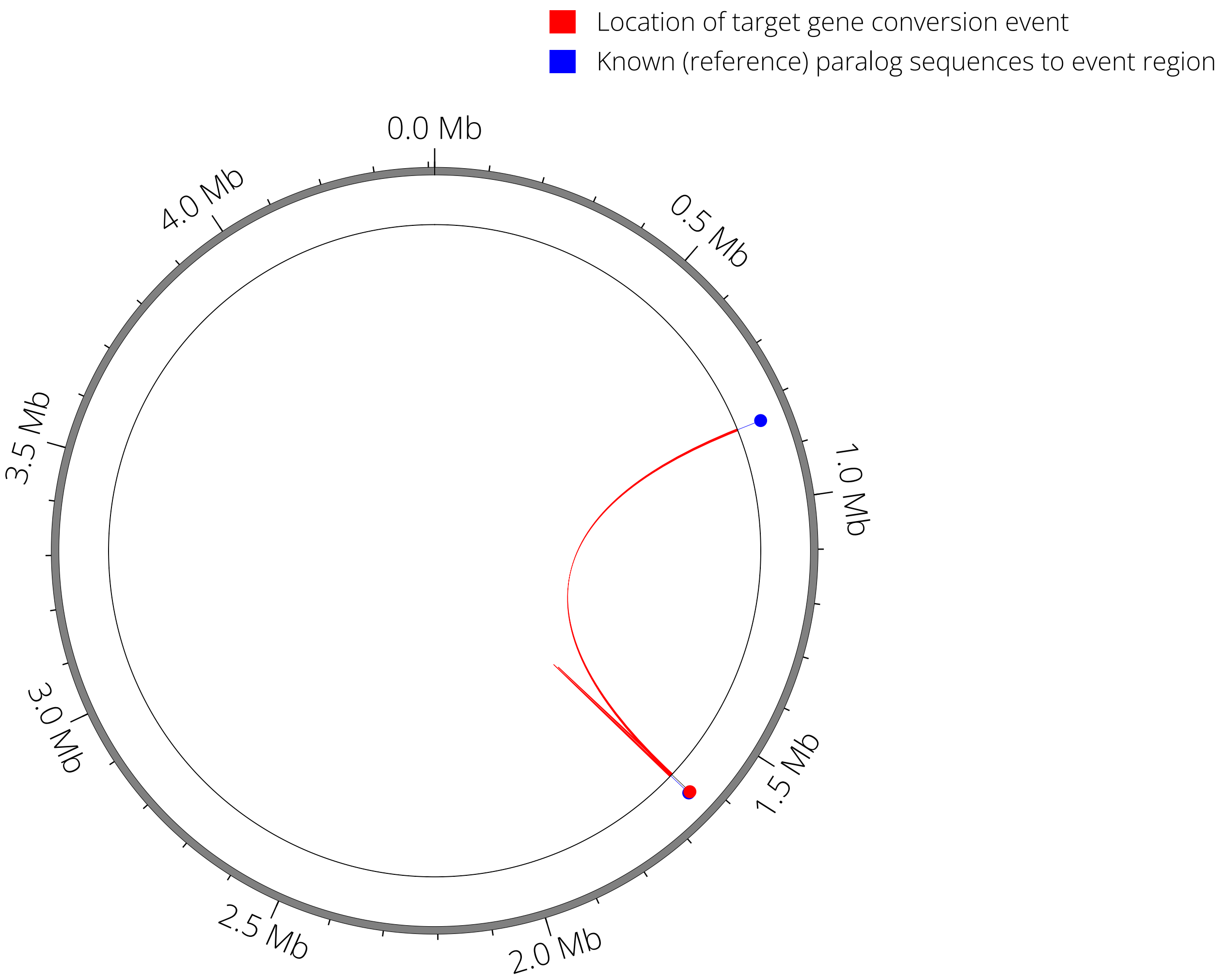

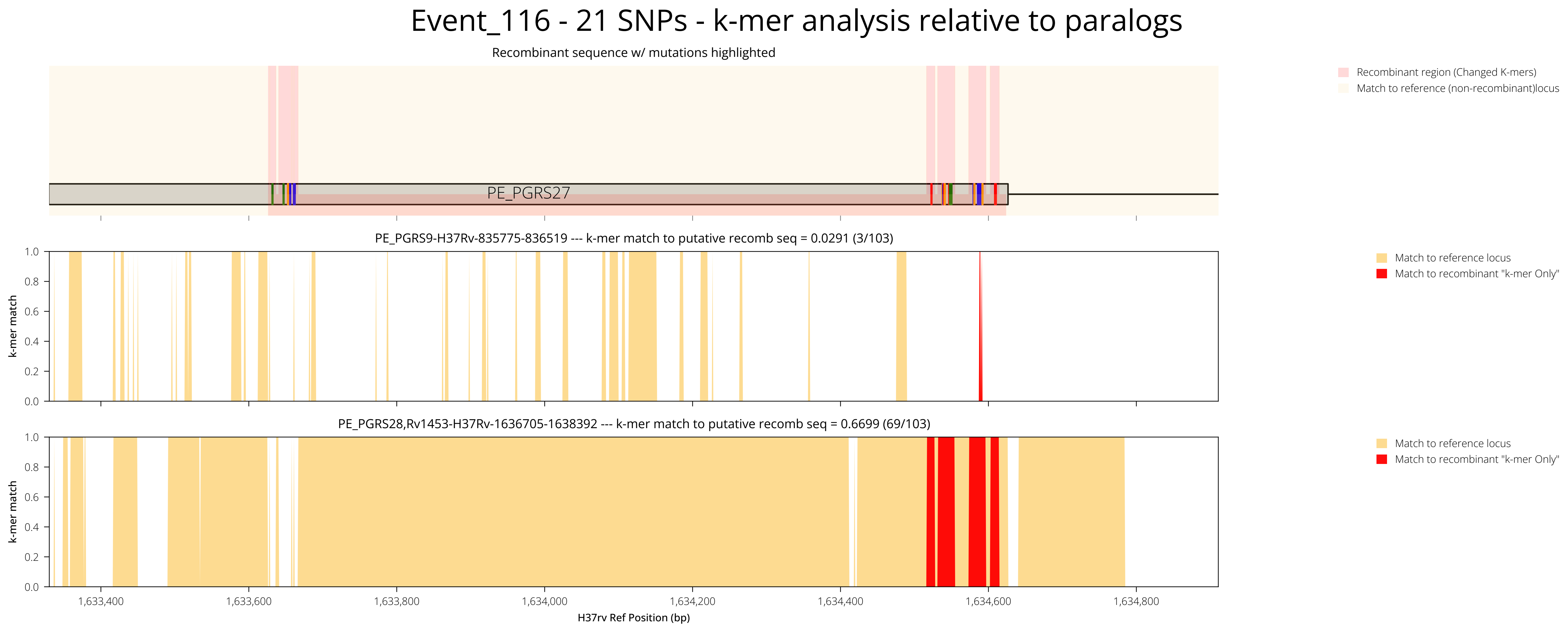

Event_116 - 21 SNPs - k-mer analysis relative to paralogs
Recombinant sequence w/ mutations highlighted
Recombinant region (Changed K-mers)
Match to reference (non-recombinant)locus
PE_PGRS27
PE_PGRS9-H37Rv-835775-836519 --- k-mer match to putative recomb seq = 0.0291 (3/103)
Match to reference locus
Match to recombinant "k-mer Only"
k-mer match
1.0
0.8
0.6
0.4
0.2
0.0
PE_PGRS28,Rv1453-H37Rv-1636705-1638392 --- k-mer match to putative recomb seq = 0.6699 (69/103)
Match to reference locus
Match to recombinant "k-mer Only"
k-mer match
1,633,400
1,633,600
1,633,800
1,634,000
1,634,200
1,634,400
1,634,600
1,634,800
H37rv Ref Position (bp)

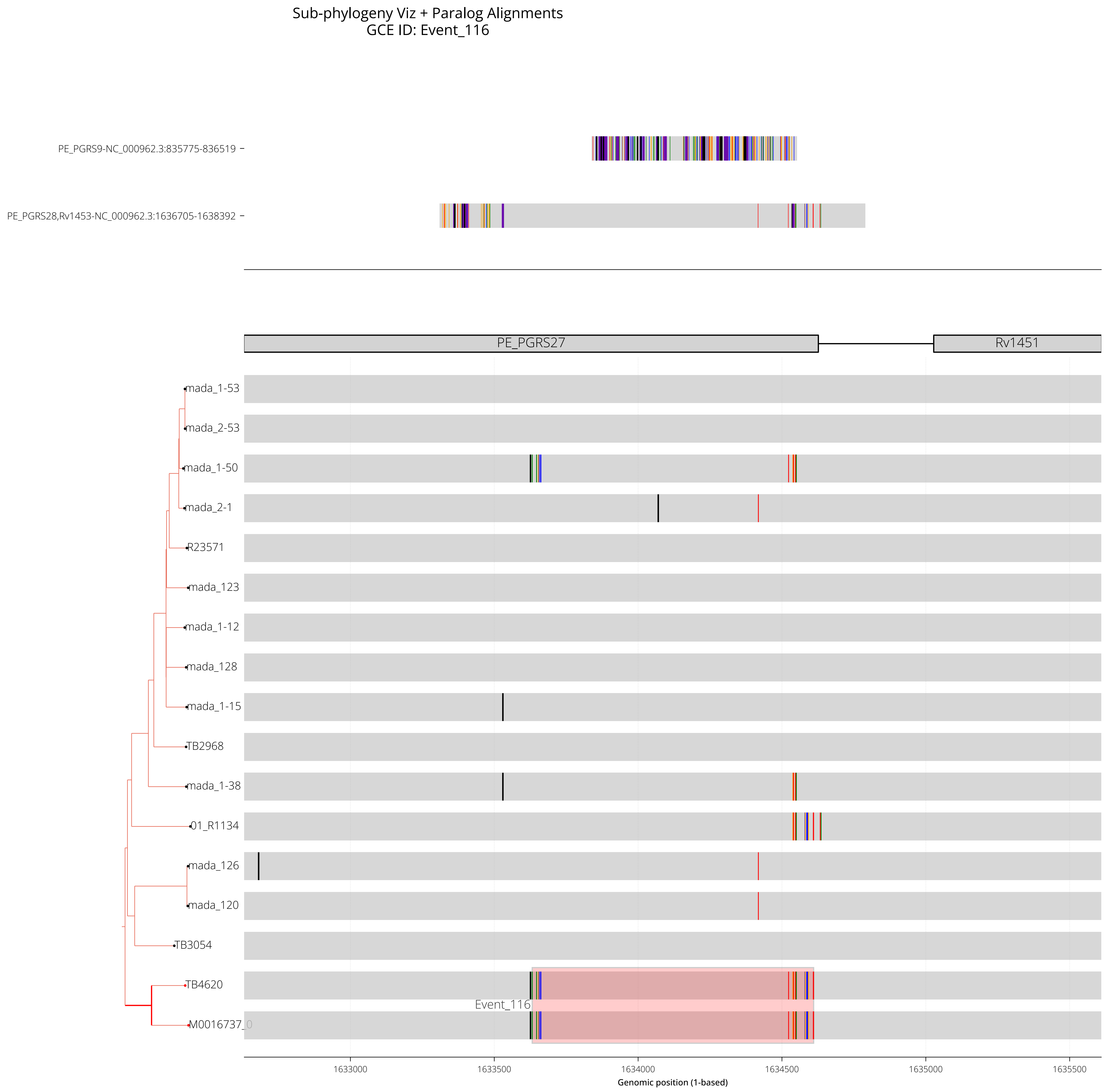

Sub-phylogeny Viz + Paralog Alignments
GCE ID: Event_116
PE_PGRS9-NC_000962.3:835775-836519
PE_PGRS28,Rv1453-NC_000962.3:1636705-1638392
PE_PGRS27
Rv1451
mada_1-53
mada_2-53
mada_1-50
mada_2-1
R23571
mada_123
mada_1-12
mada_128
mada_1-15
TB2968
mada_1-38
01_R1134
mada_126
mada_120
TB3054
TB4620
Event_116
M0016737_0
1633000
1633500
1634000
1634500
1635000
1635500
Genomic position (1-based)

Full Phylogeny Viz + Paralog Alignments
GCE ID: Event_116

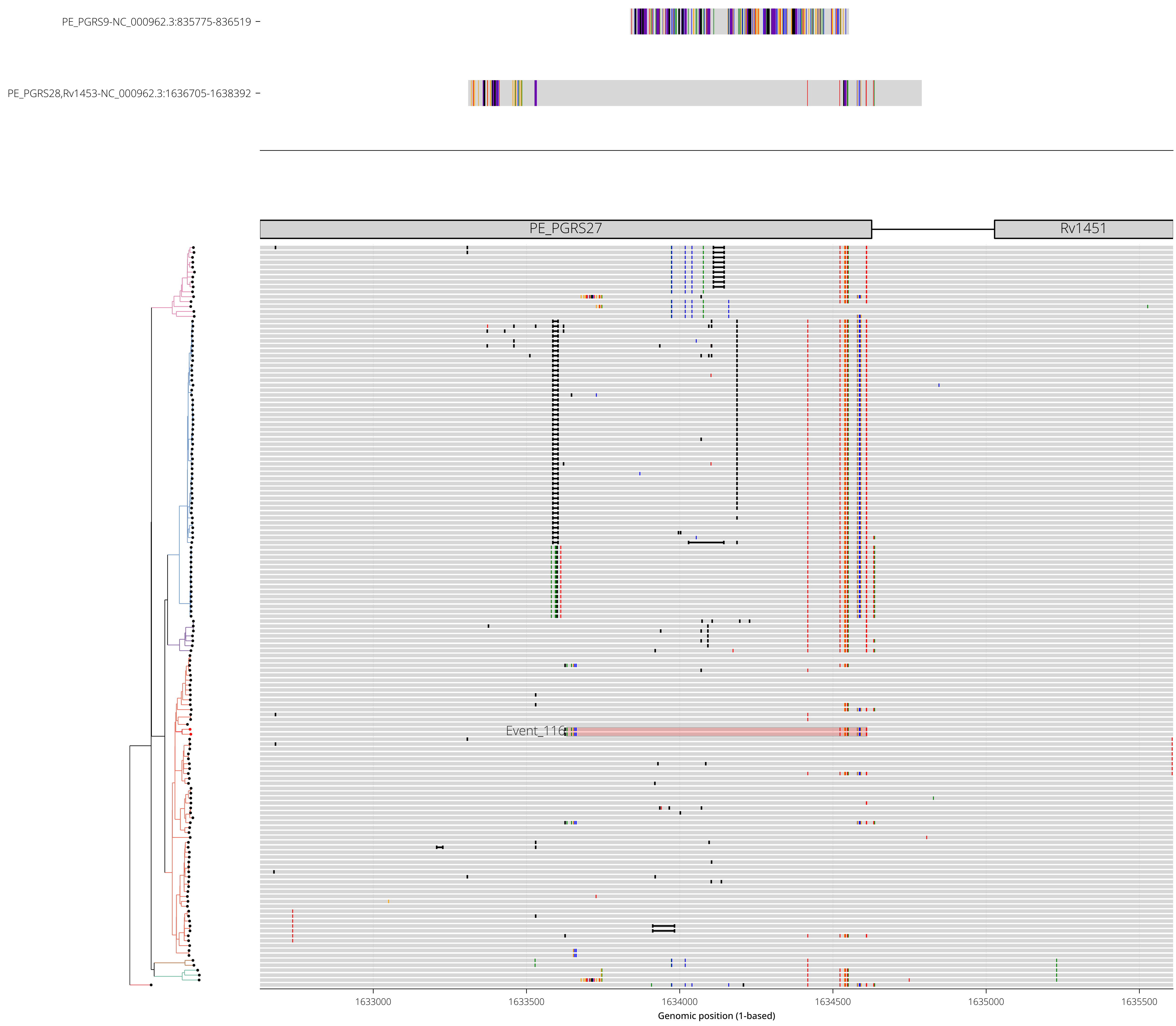

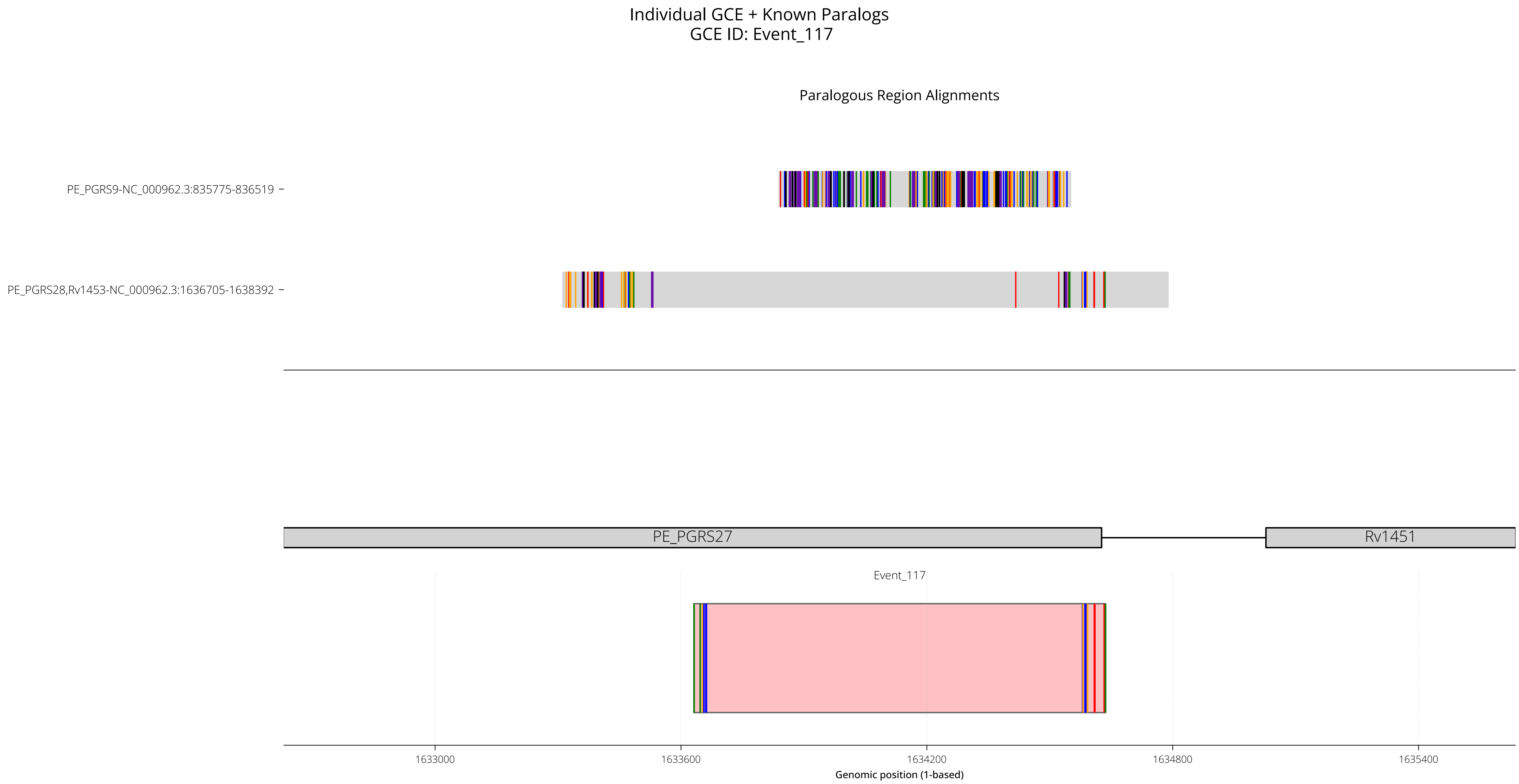

Individual GCE + Known Paralogs
GCE ID: Event_117
Paralogous Region Alignments
PE_PGRS9-NC_000962.3:835775-836519
PE_PGRS28,Rv1453-NC_000962.3:1636705-1638392
PE_PGRS27
Rv1451
Event_117
1633000
1633600
1634200
1634800
1635400
Genomic position (1-based)

Circos Viz - GCE to Known Paralogs Locations
GCE ID: Event_117

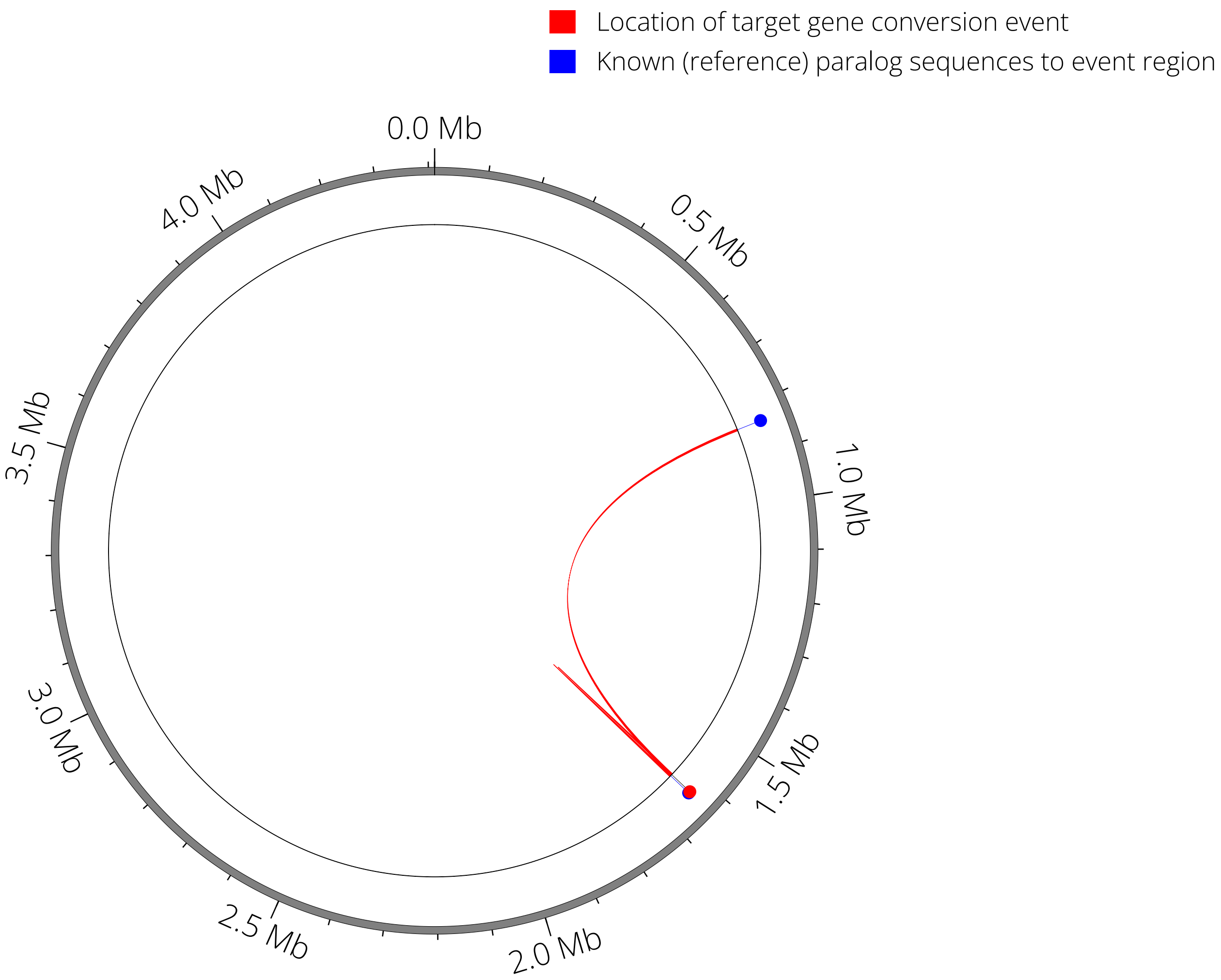

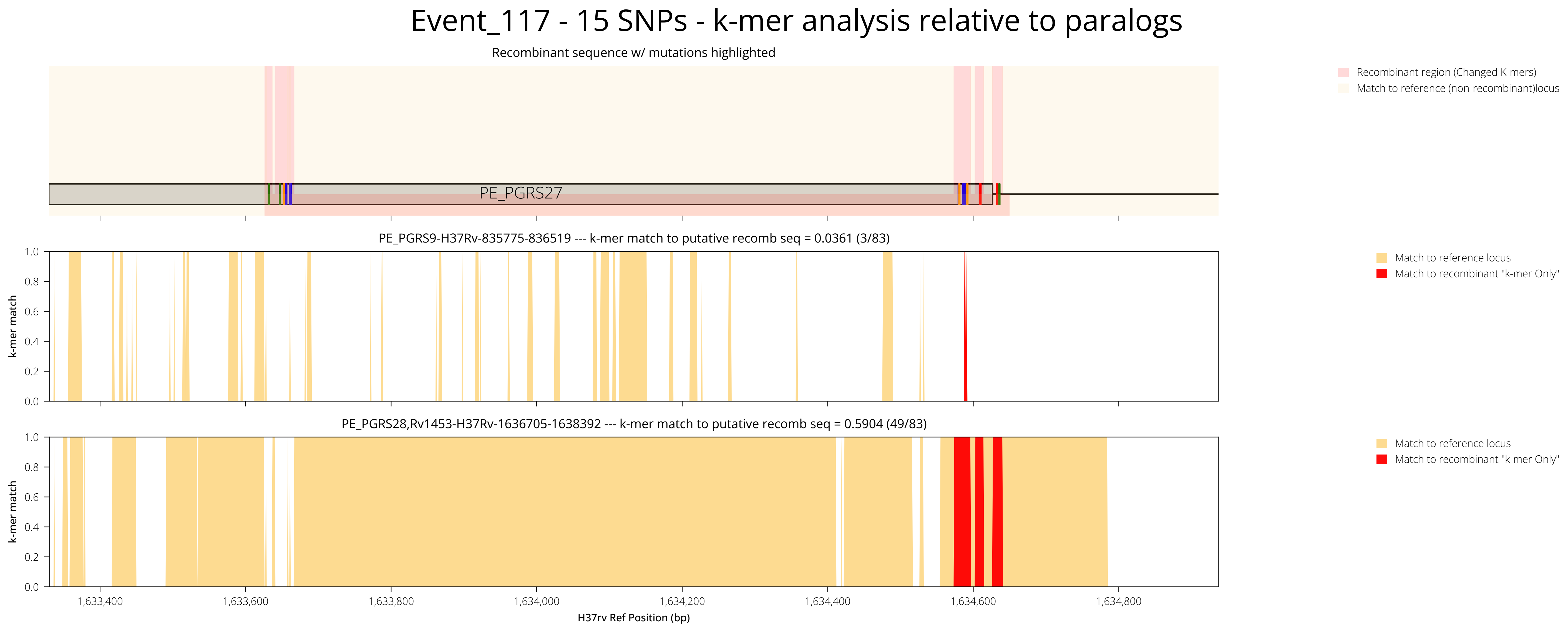
Event_117 - 15 SNPs - k-mer analysis relative to paralogs
Recombinant sequence w/ mutations highlighted
Recombinant region (Changed K-mers)
Match to reference (non-recombinant)locus
PE_PGRS27
PE_PGRS9-H37Rv-835775-836519 --- k-mer match to putative recomb seq = 0.0361 (3/83)
Match to reference locus
Match to recombinant "k-mer Only"
PE_PGRS28,Rv1453-H37Rv-1636705-1638392 --- k-mer match to putative recomb seq = 0.5904 (49/83)
Match to reference locus
Match to recombinant "k-mer Only"
k-mer match
1.0
0.8
0.6
0.4
0.2
0.0
1,633,400
1,633,600
1,633,800
1,634,000
1,634,200
1,634,400
1,634,600
1,634,800
H37rv Ref Position (bp)

Sub-phylogeny Viz + Paralog Alignments
GCE ID: Event_117

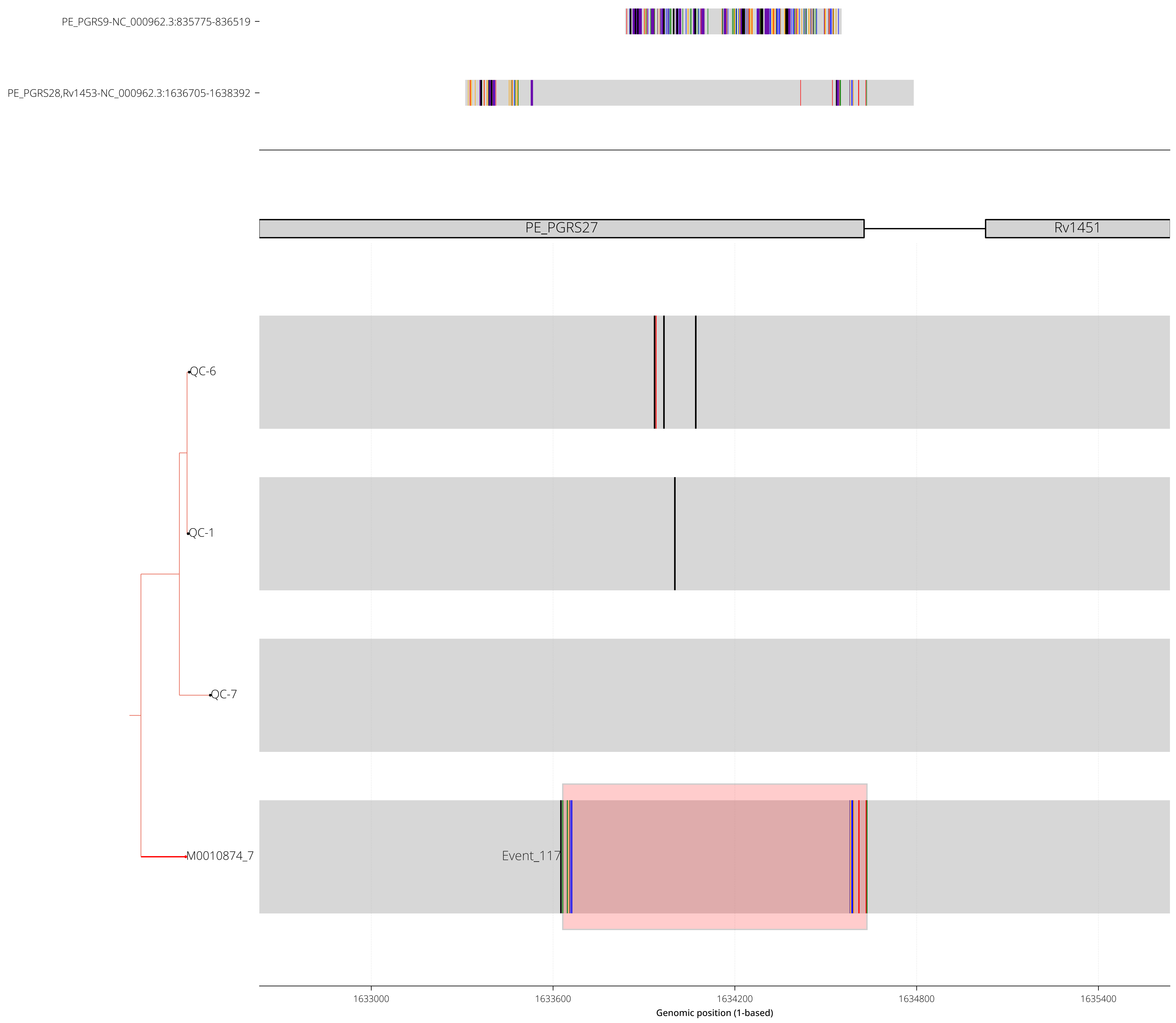

Full Phylogeny Viz + Paralog Alignments
GCE ID: Event_117

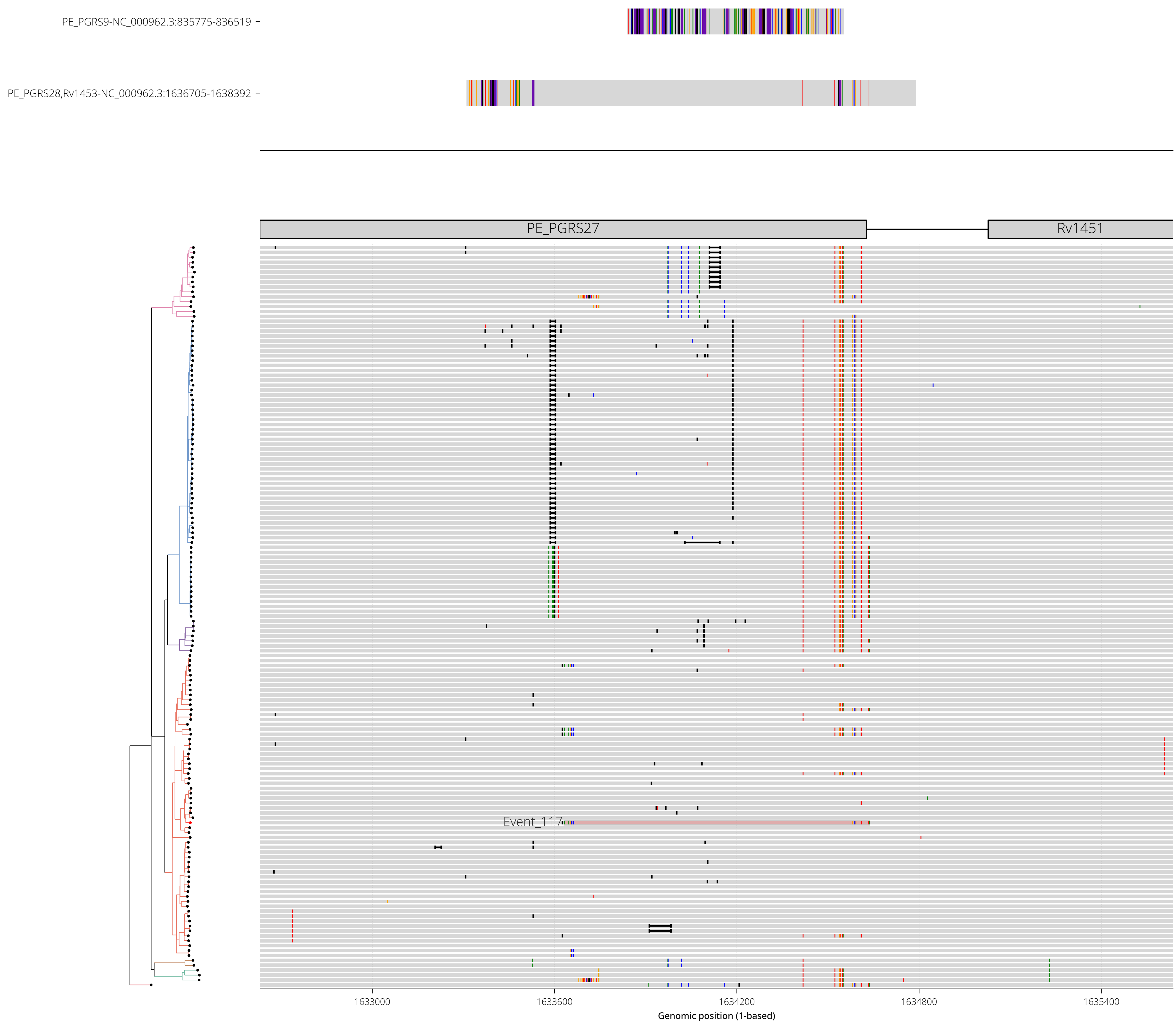

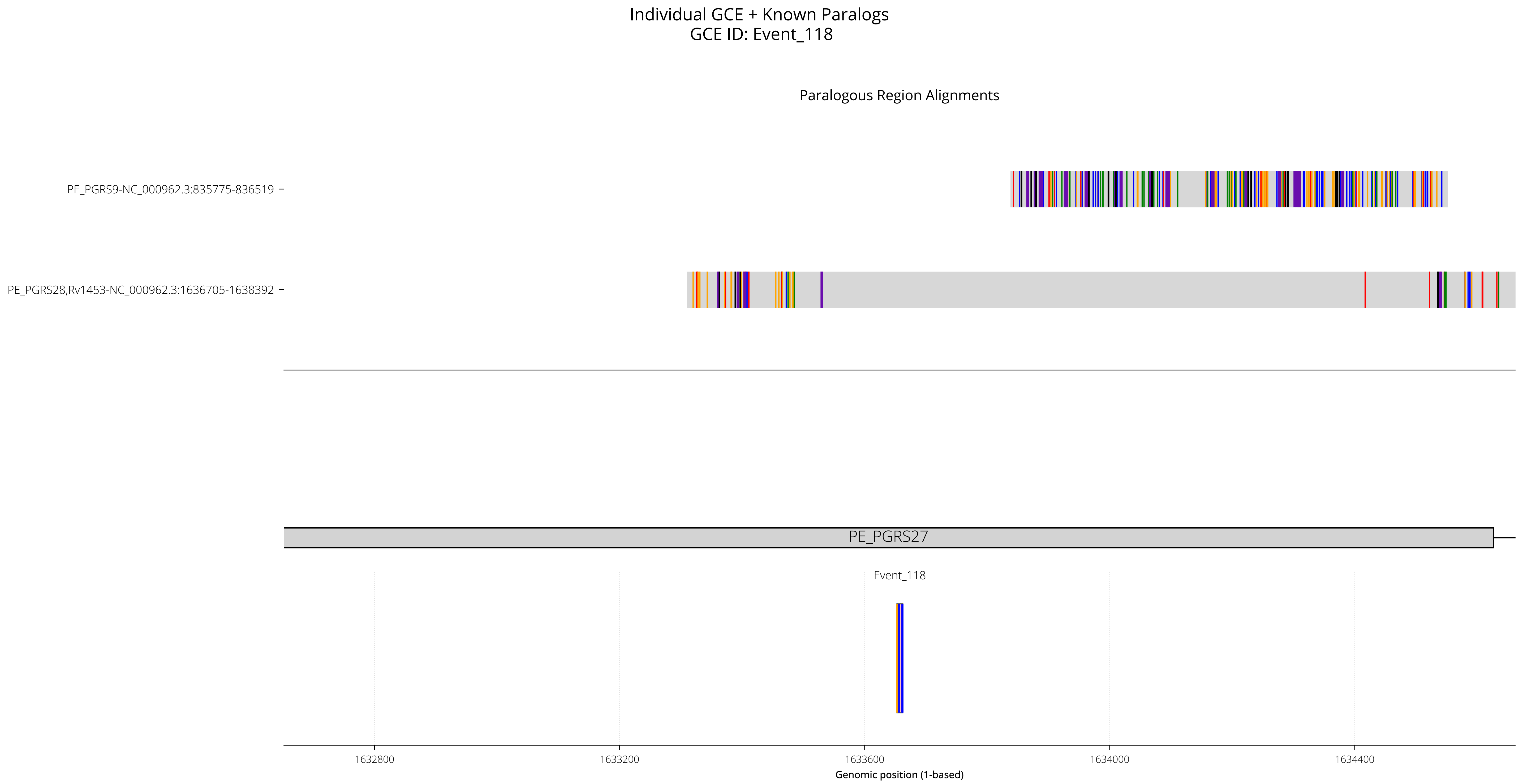
Individual GCE + Known Paralogs
GCE ID: Event_118
Paralogous Region Alignments
PE_PGRS9-NC_000962.3:835775-836519
PE_PGRS28,Rv1453-NC_000962.3:1636705-1638392
PE_PGRS27
Event_118
1632800
1633200
1633600
1634000
1634400
Genomic position (1-based)

Circos Viz - GCE to Known Paralogs Locations
GCE ID: Event_118

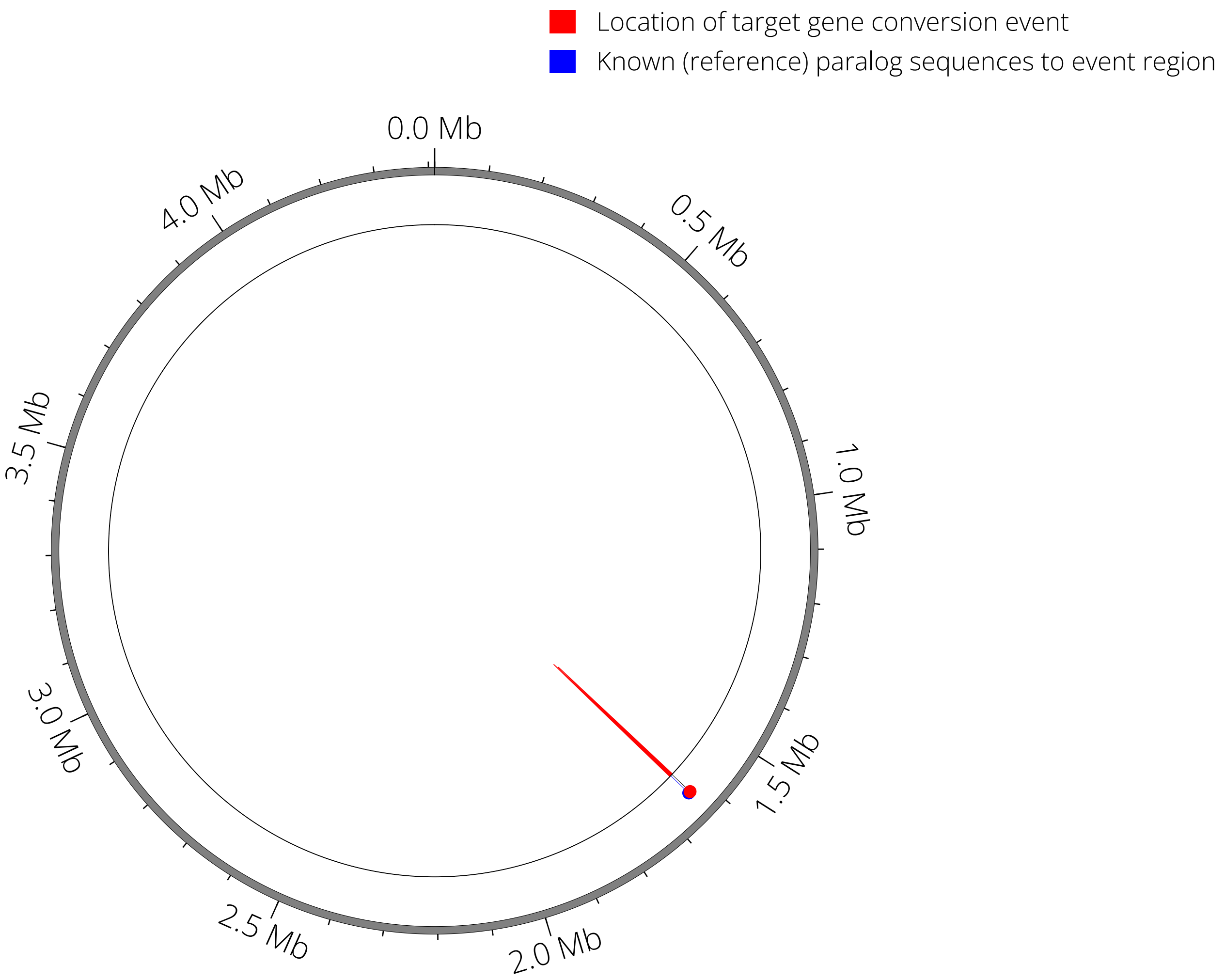

# Event_118 - 4 SNPs - k-mer analysis relative to paralogs

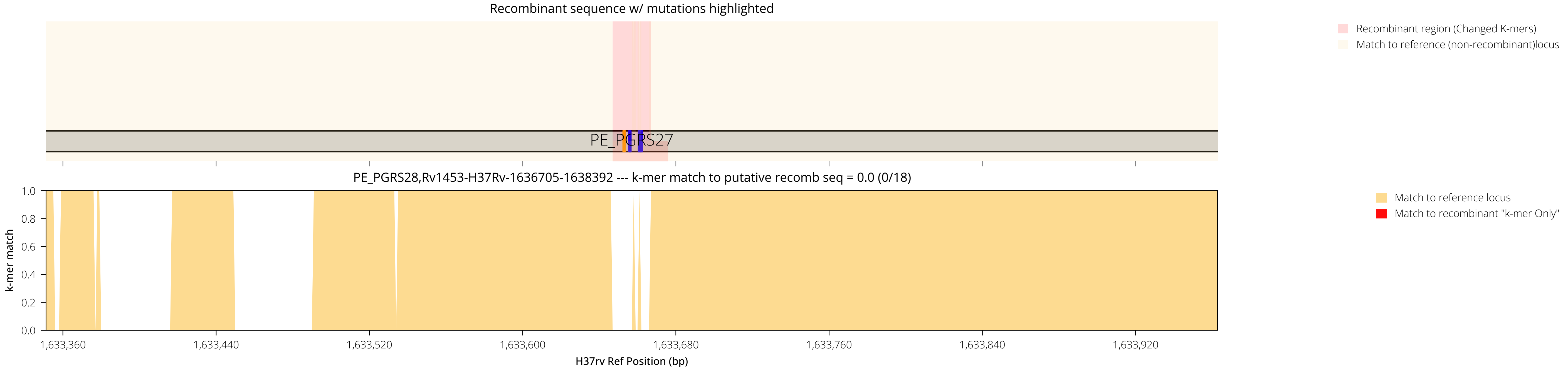

Sub-phylogeny Viz + Paralog Alignments
GCE ID: Event_118

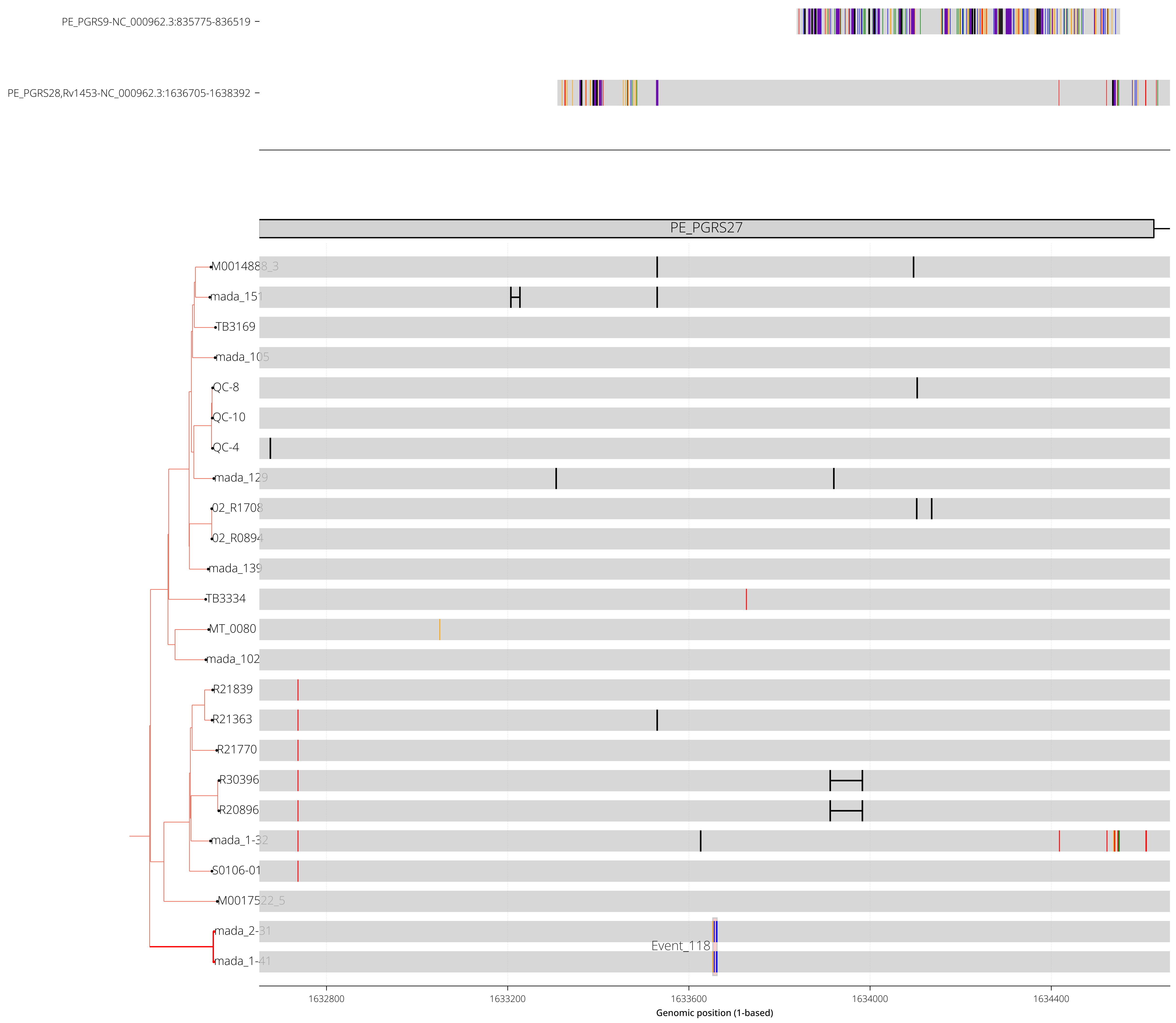

Full Phylogeny Viz + Paralog Alignments
GCE ID: Event_118

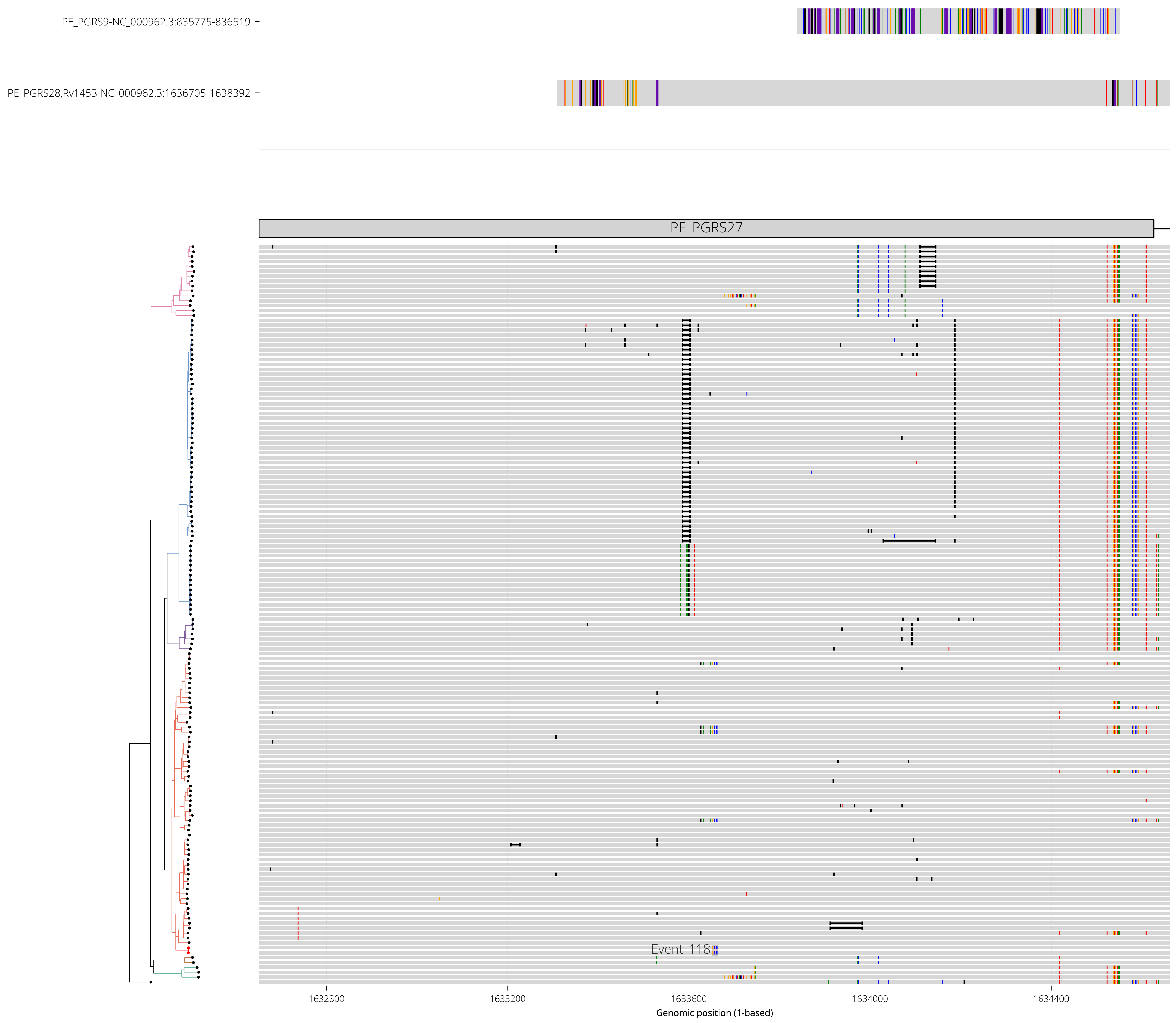

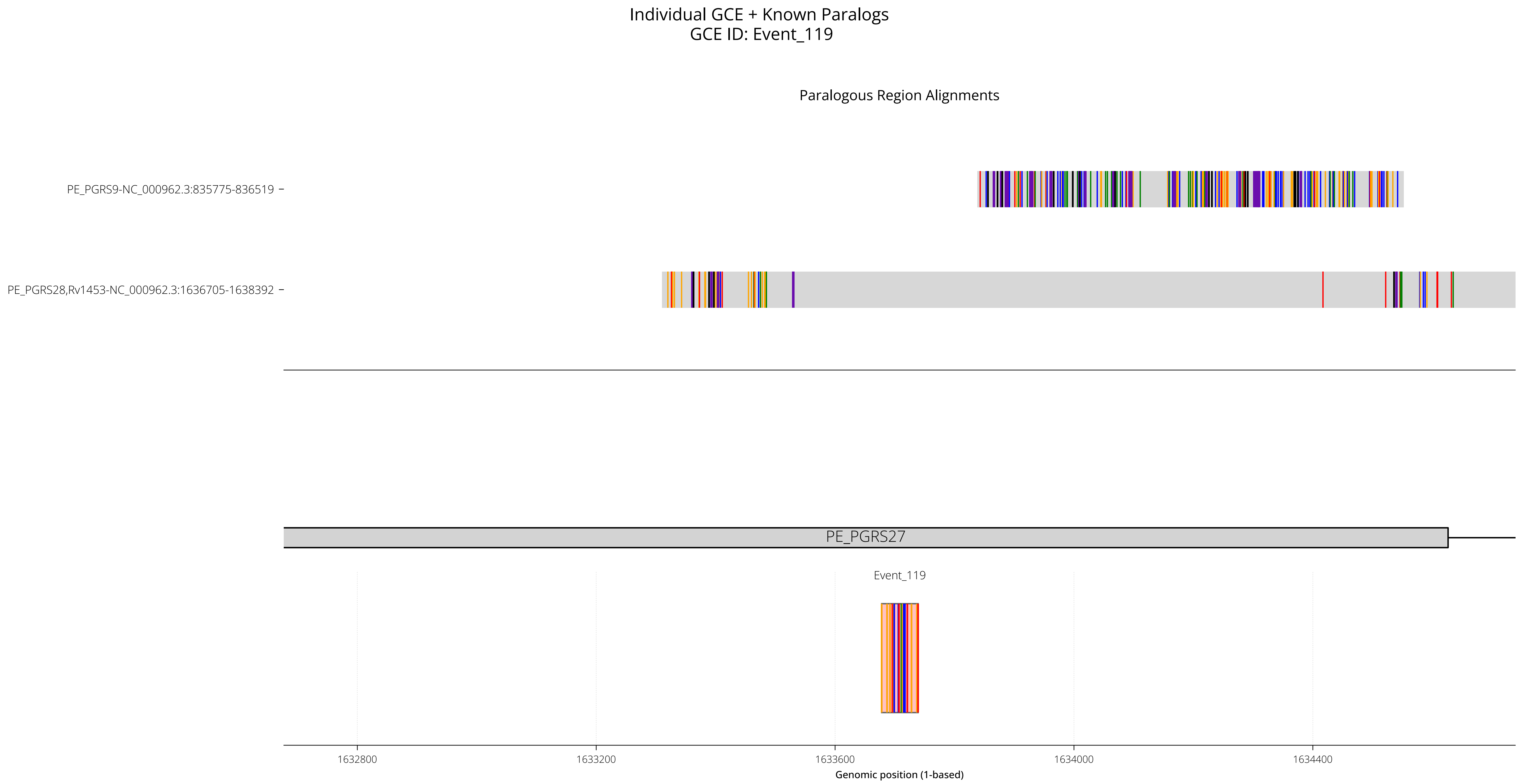
Individual GCE + Known Paralogs
GCE ID: Event_119
Paralogous Region Alignments
PE_PGRS9-NC_000962.3:835775-836519
PE_PGRS28,Rv1453-NC_000962.3:1636705-1638392
PE_PGRS27
Event_119
1632800
1633200
1633600
1634000
1634400
Genomic position (1-based)

Circos Viz - GCE to Known Paralogs Locations
GCE ID: Event_119

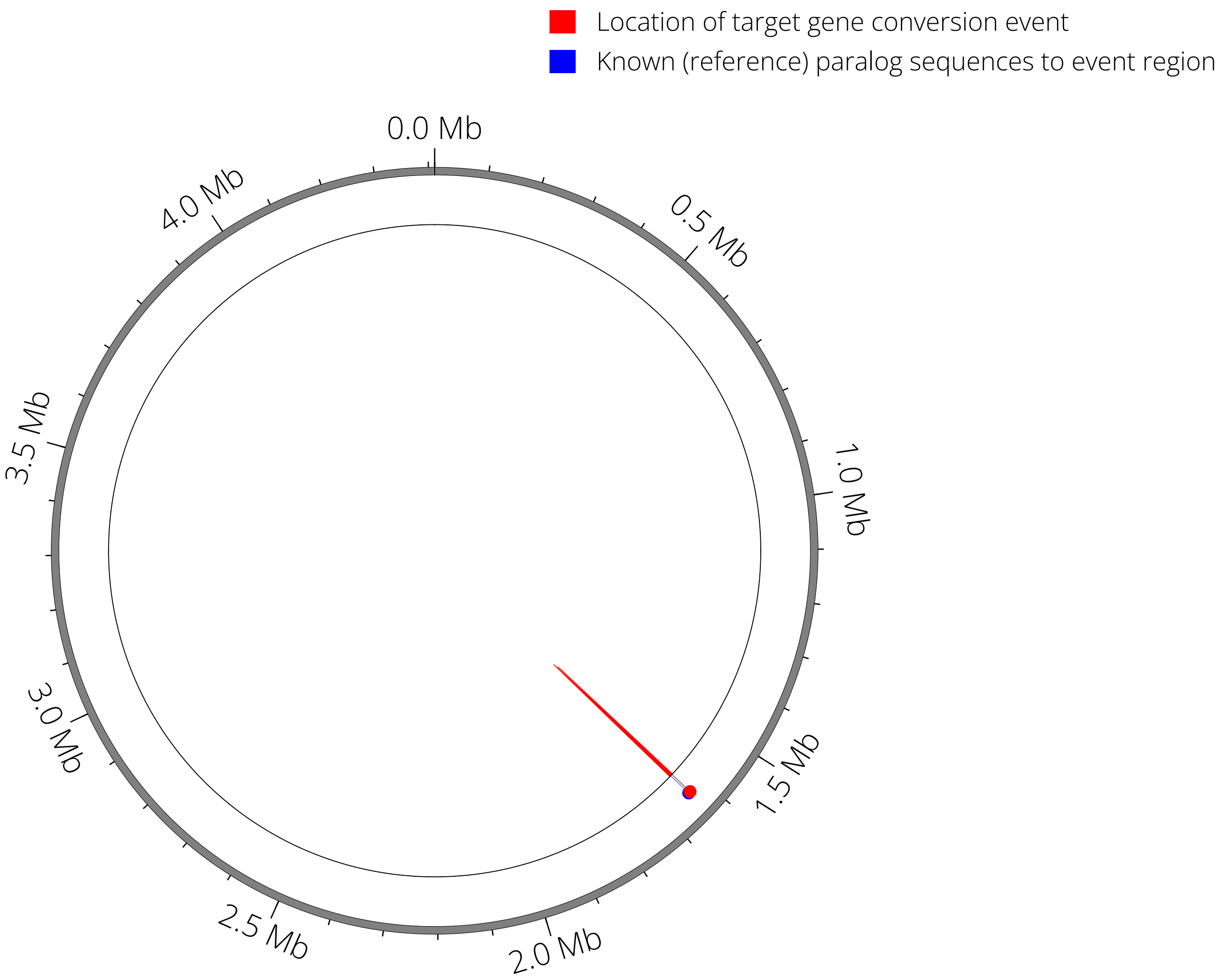

# Event_119 - 17 SNPs - k-mer analysis relative to paralogs

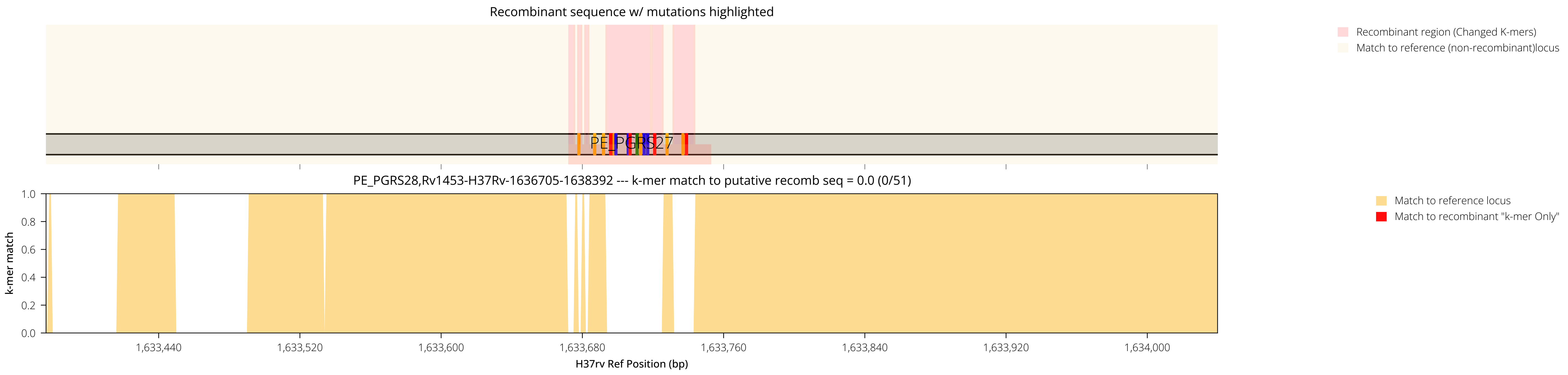

Sub-phylogeny Viz + Paralog Alignments
GCE ID: Event_119

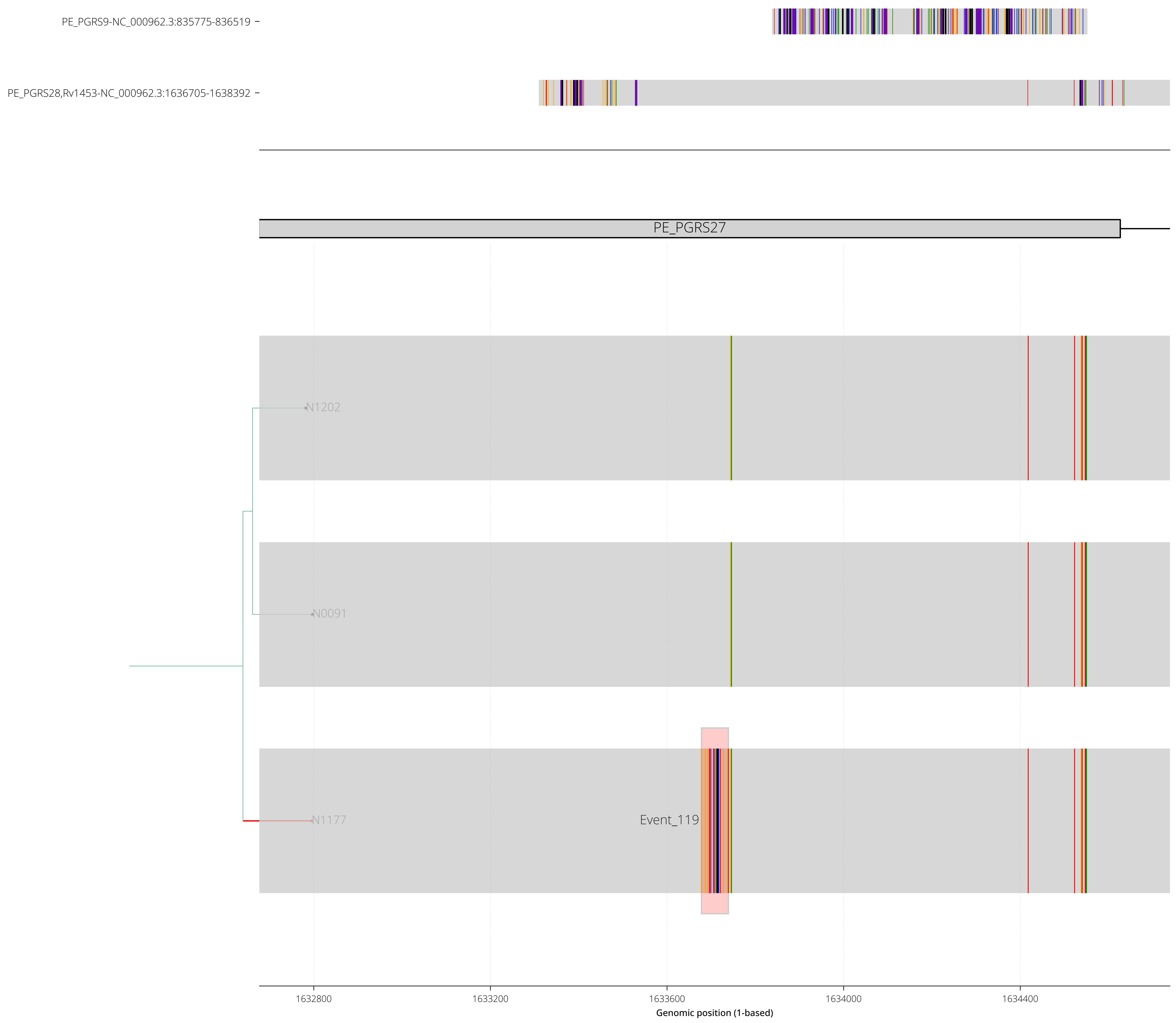

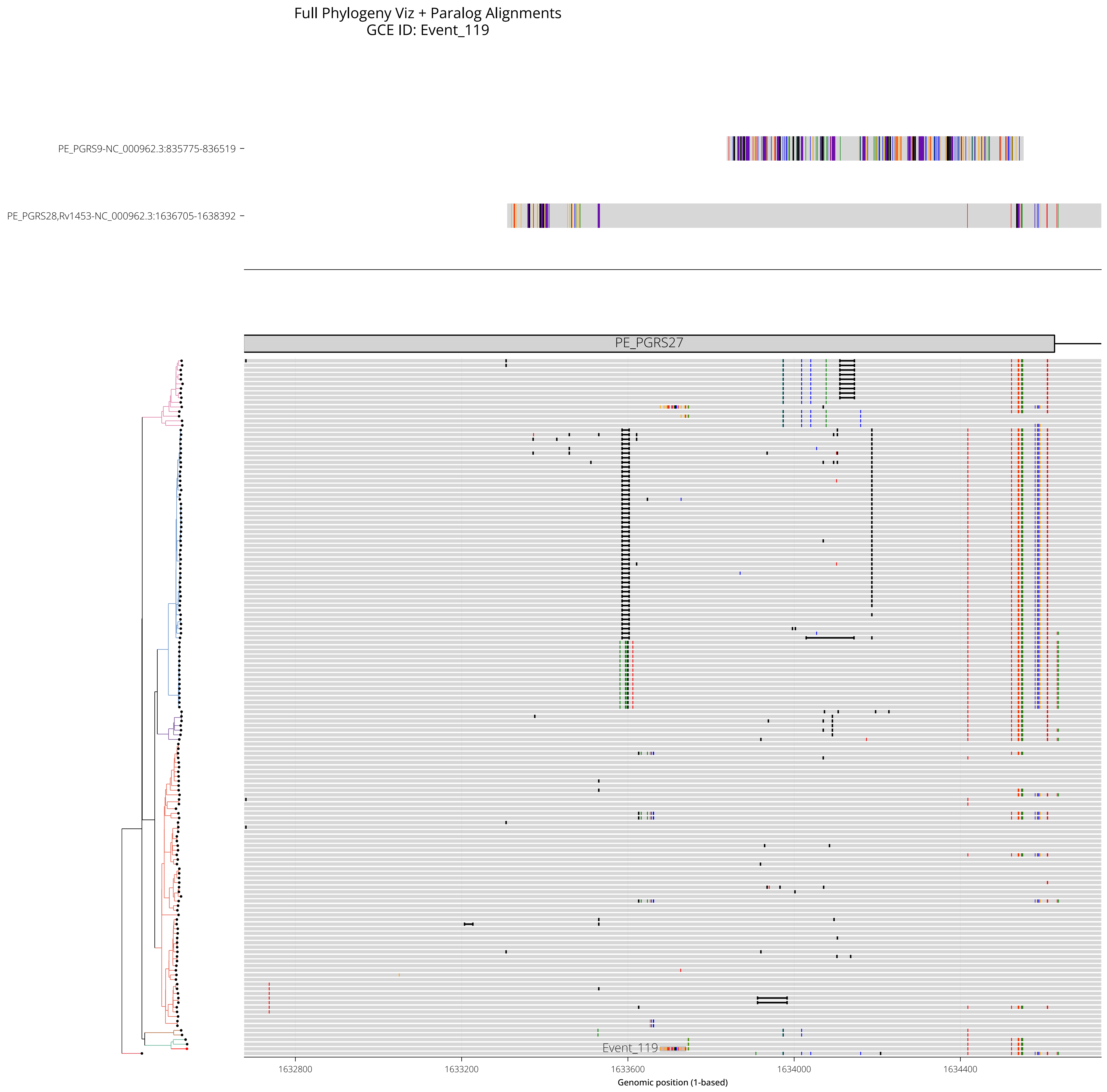

Full Phylogeny Viz + Paralog Alignments
GCE ID: Event_119
PE_PGRS9-NC_000962.3:835775-836519
PE_PGRS28,Rv1453-NC_000962.3:1636705-1638392
PE_PGRS27
Event_119
1632800
1633200
1633600
1634000
1634400
Genomic position (1-based)

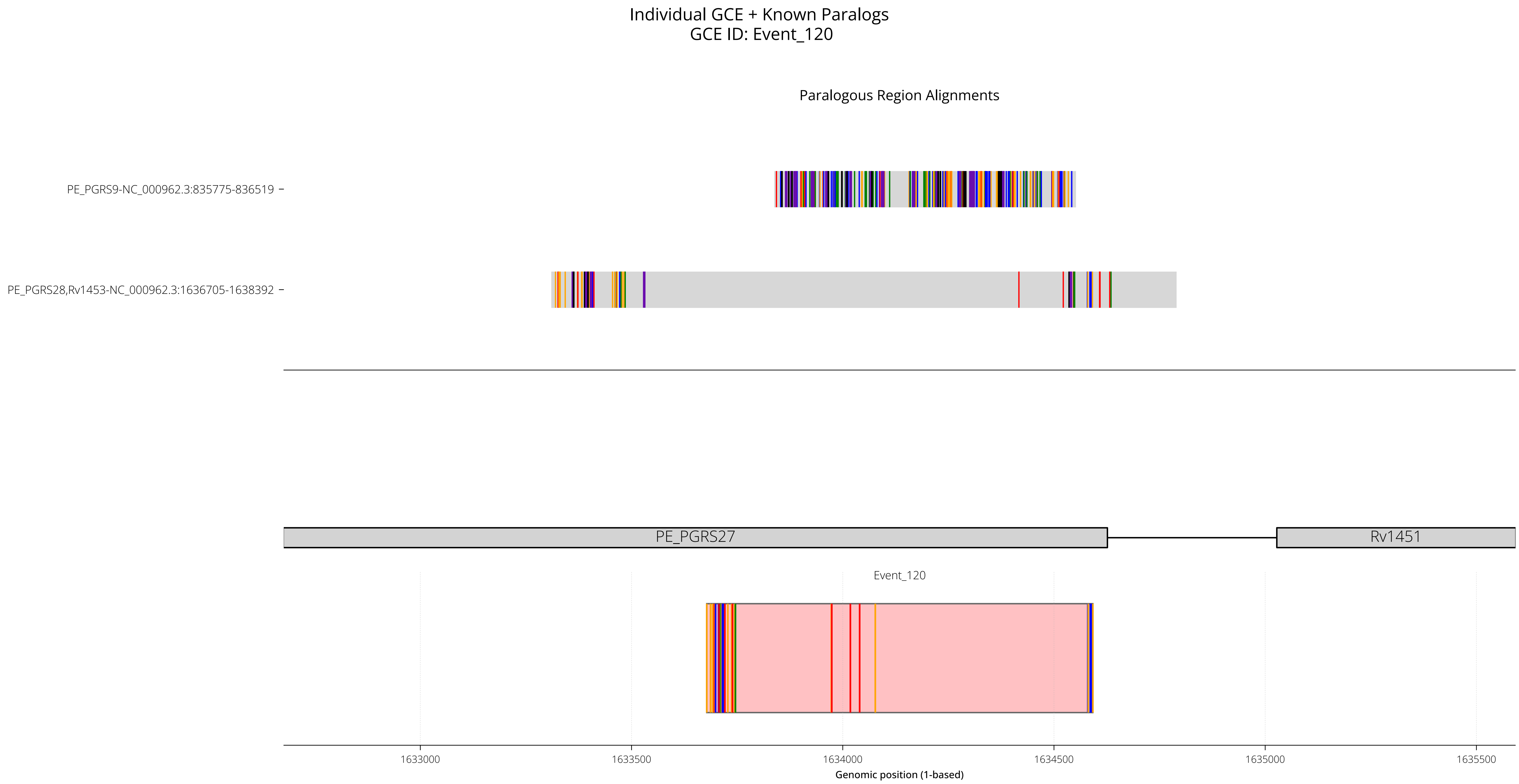
Individual GCE + Known Paralogs
GCE ID: Event_120
Paralogous Region Alignments
PE_PGRS9-NC_000962.3:835775-836519
PE_PGRS28,Rv1453-NC_000962.3:1636705-1638392
PE_PGRS27
Rv1451
Event_120
1633000
1633500
1634000
1634500
1635000
1635500
Genomic position (1-based)

Circos Viz - GCE to Known Paralogs Locations
GCE ID: Event_120

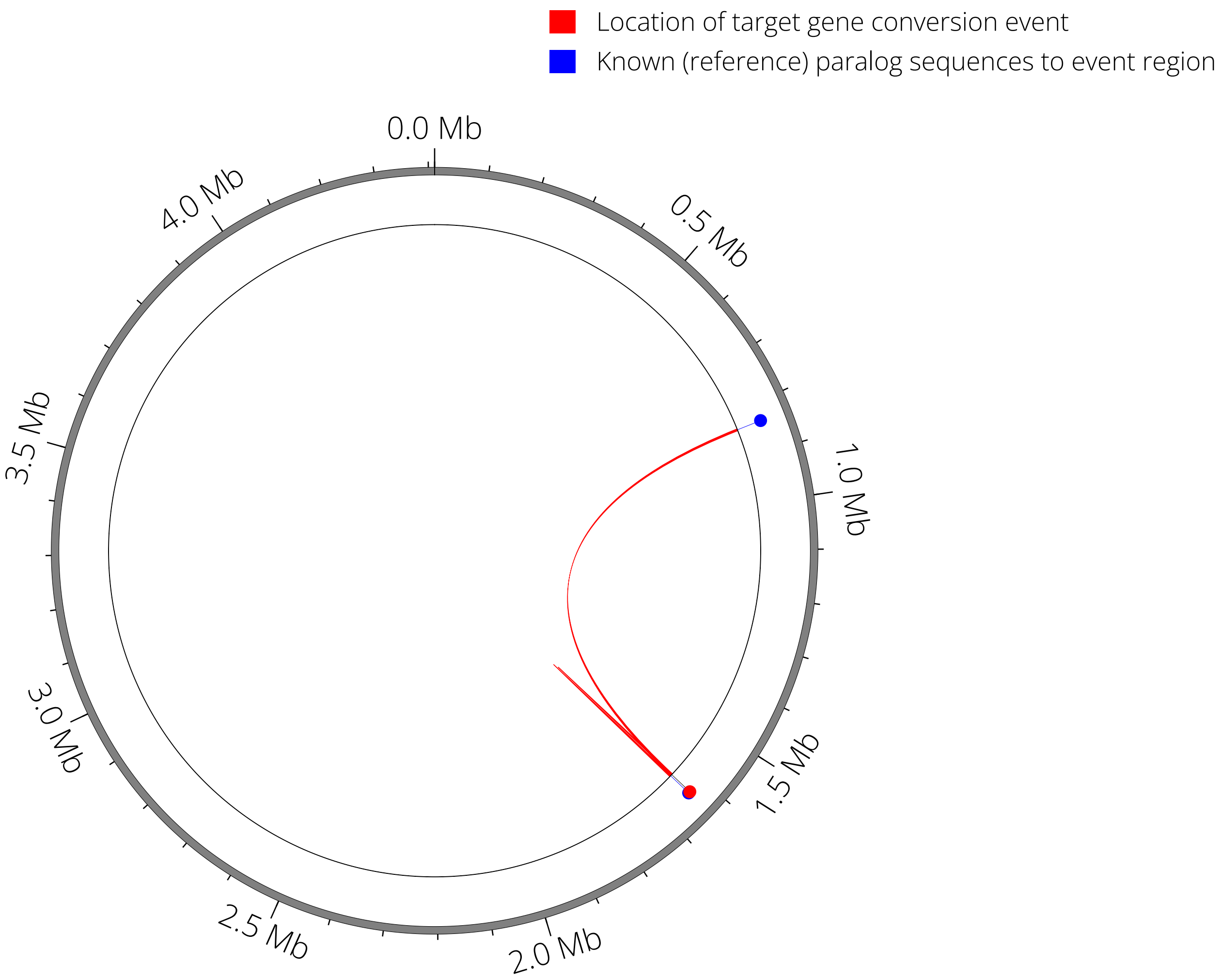

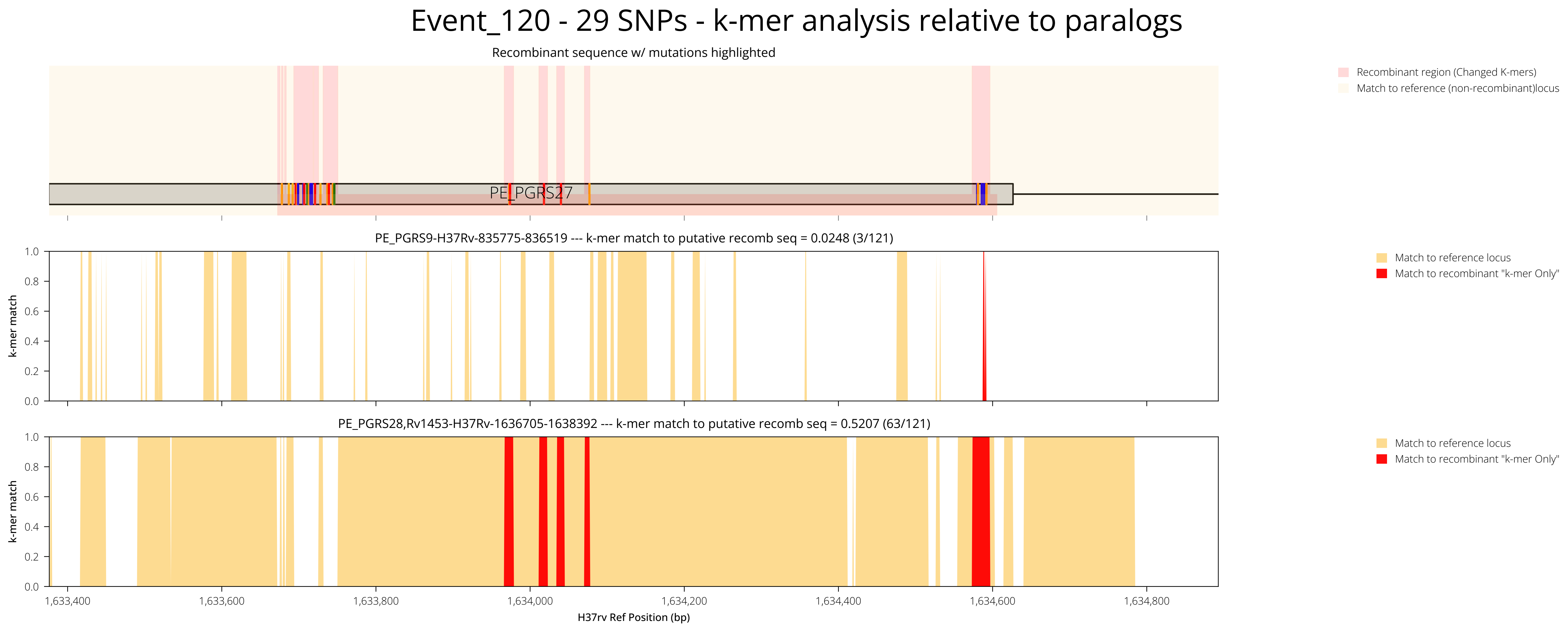

Event_120 - 29 SNPs - k-mer analysis relative to paralogs
Recombinant sequence w/ mutations highlighted
PE_PGRS27
Recombinant region (Changed K-mers)
Match to reference (non-recombinant)locus
PE_PGRS9-H37Rv-835775-836519 --- k-mer match to putative recomb seq = 0.0248 (3/121)
k-mer match
1.0
0.8
0.6
0.4
0.2
0.0
Match to reference locus
Match to recombinant "k-mer Only"
PE_PGRS28,Rv1453-H37Rv-1636705-1638392 --- k-mer match to putative recomb seq = 0.5207 (63/121)
k-mer match
1.0
0.8
0.6
0.4
0.2
0.0
Match to reference locus
Match to recombinant "k-mer Only"
1,633,400
1,633,600
1,633,800
1,634,000
1,634,200
1,634,400
1,634,600
1,634,800
H37rv Ref Position (bp)

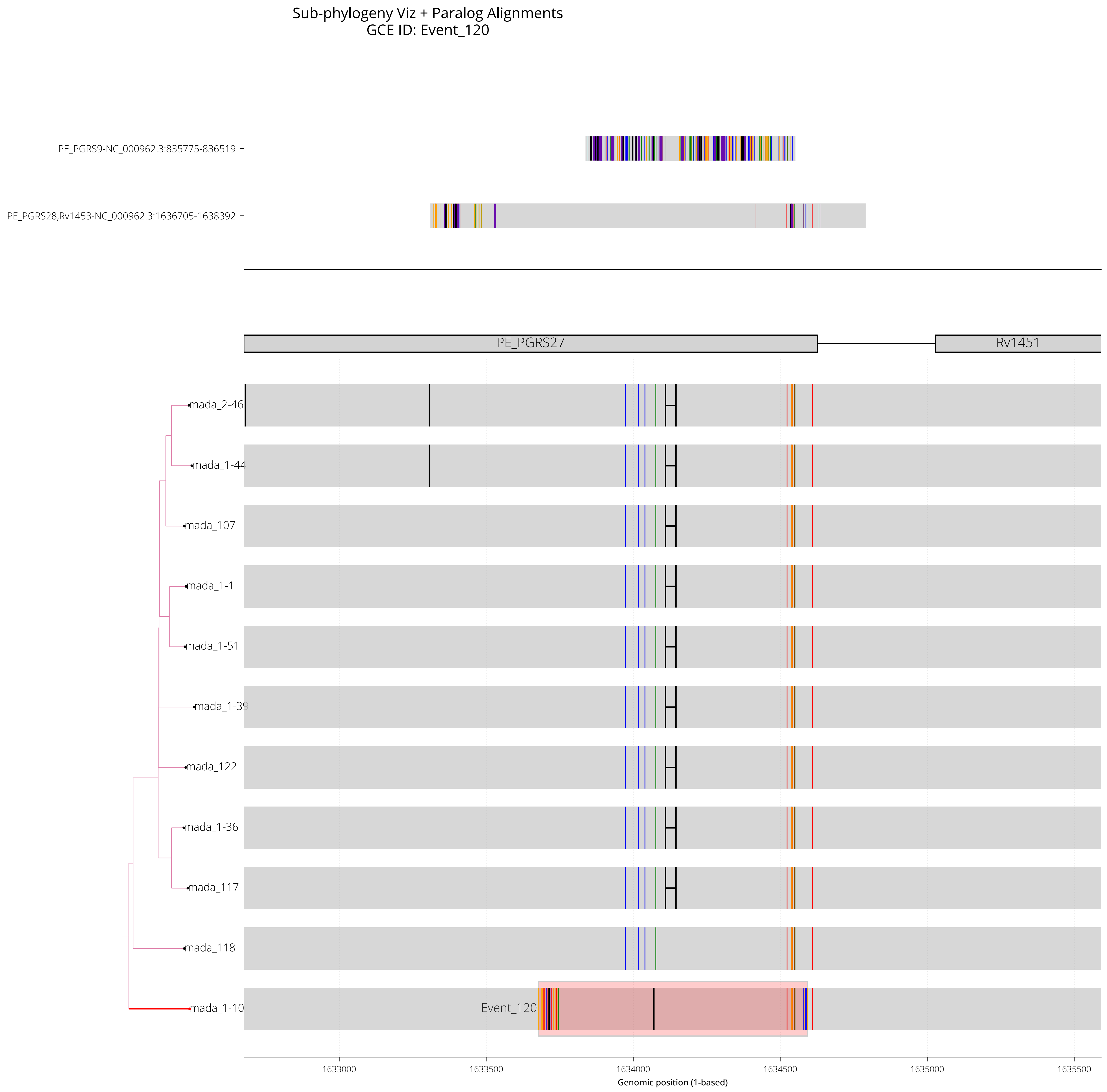

Sub-phylogeny Viz + Paralog Alignments
GCE ID: Event_120
PE_PGRS9-NC_000962.3:835775-836519
PE_PGRS28,Rv1453-NC_000962.3:1636705-1638392
PE_PGRS27
Rv1451
mada_2-46
mada_1-44
mada_107
mada_1-1
mada_1-51
mada_1-39
mada_122
mada_1-36
mada_117
mada_118
mada_1-10
Event_120
1633000
1633500
1634000
1634500
1635000
1635500
Genomic position (1-based)

Full Phylogeny Viz + Paralog Alignments
GCE ID: Event_120

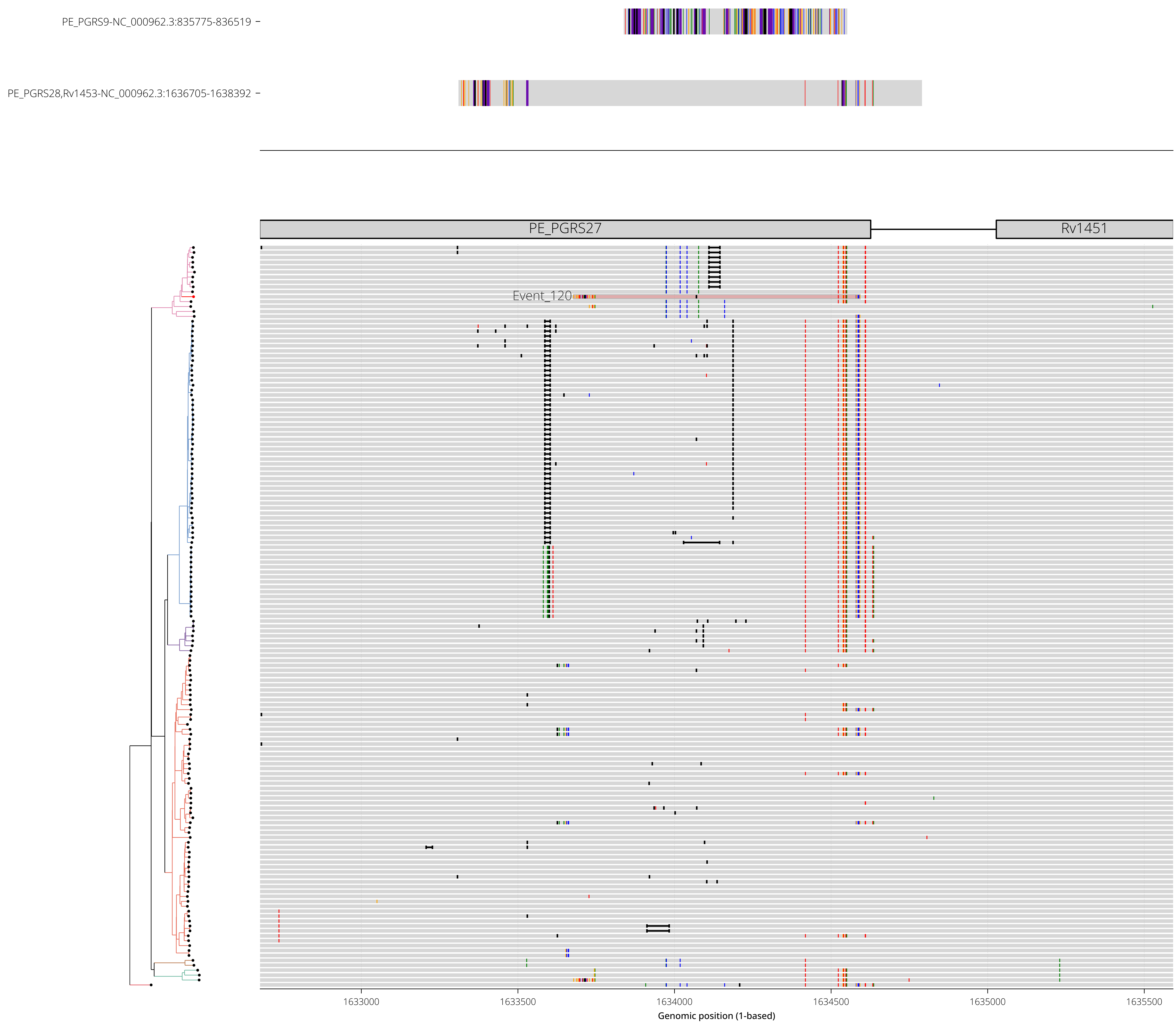

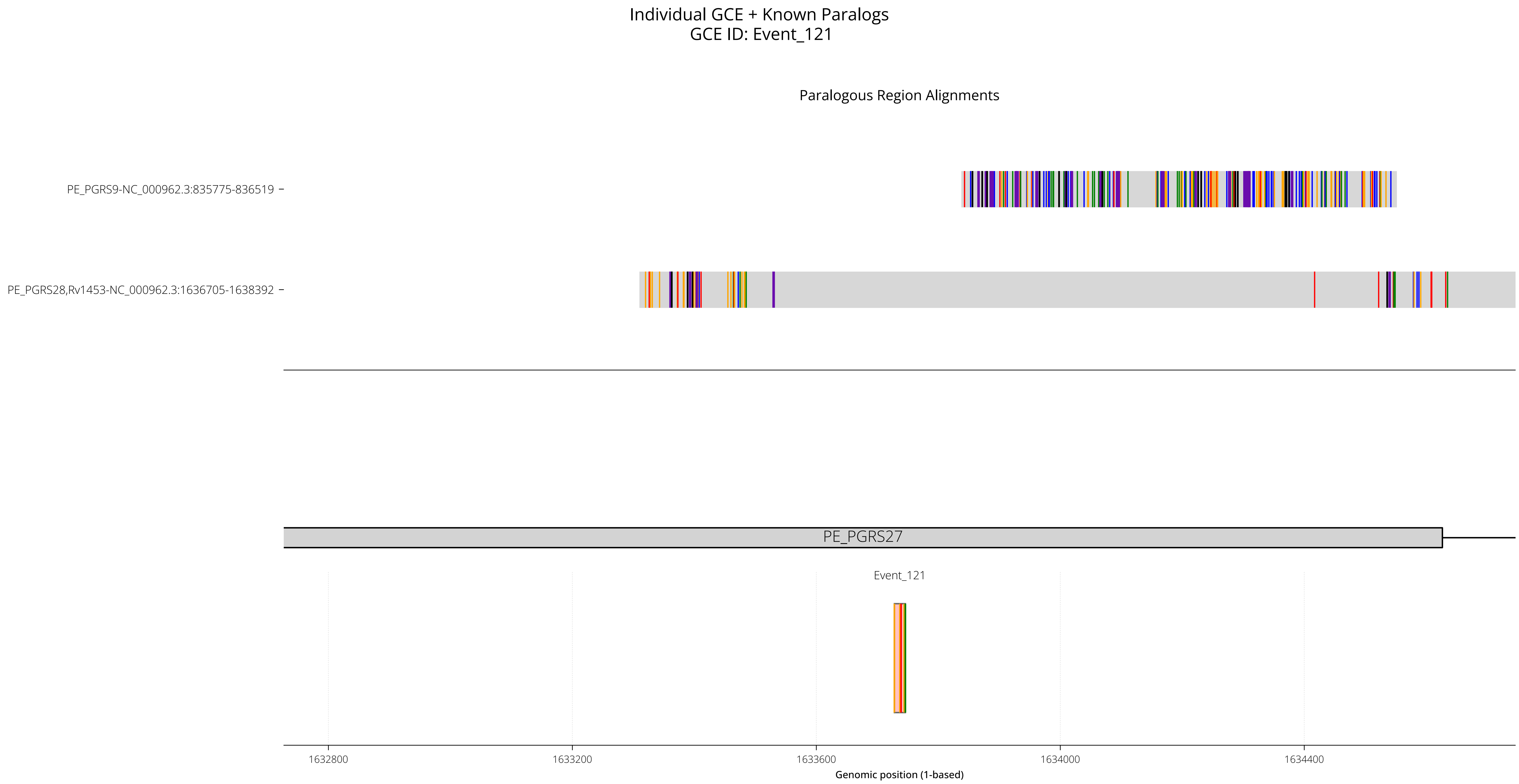

Individual GCE + Known Paralogs
GCE ID: Event_121
Paralogous Region Alignments
PE_PGRS9-NC_000962.3:835775-836519
PE_PGRS28,Rv1453-NC_000962.3:1636705-1638392
PE_PGRS27
Event_121
1632800
1633200
1633600
1634000
1634400
Genomic position (1-based)

Circos Viz - GCE to Known Paralogs Locations
GCE ID: Event_121

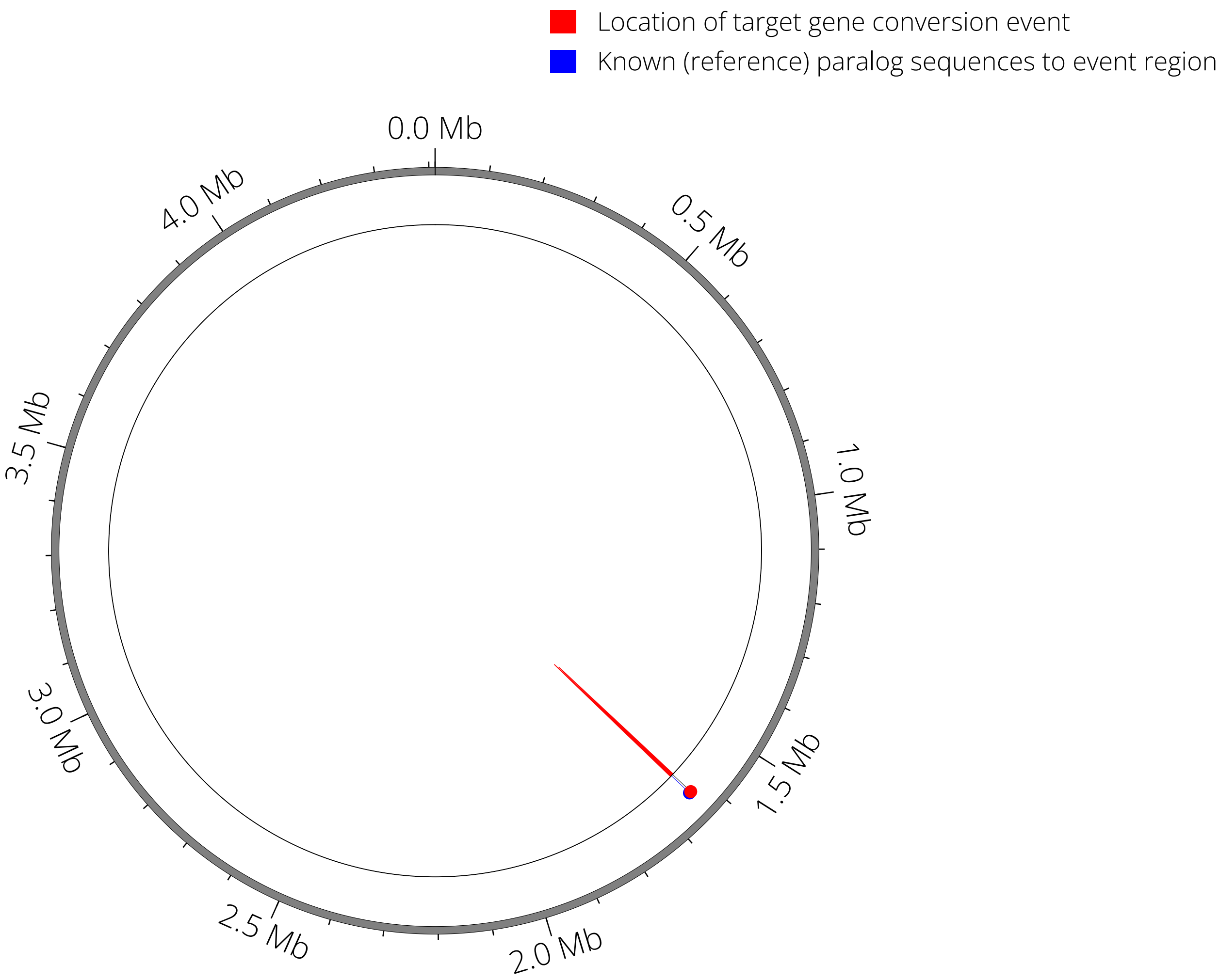

# Event_121 - 5 SNPs - k-mer analysis relative to paralogs

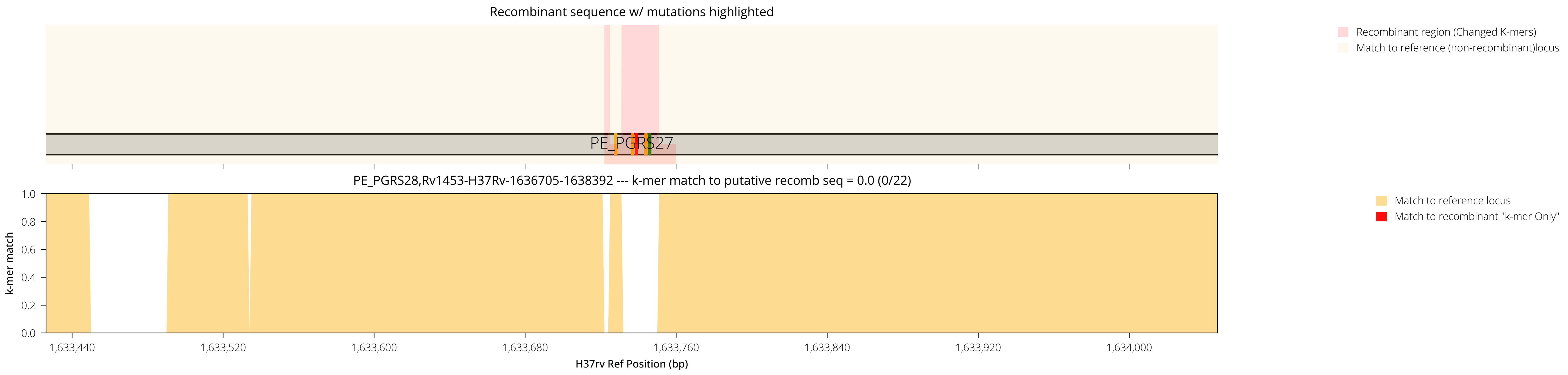

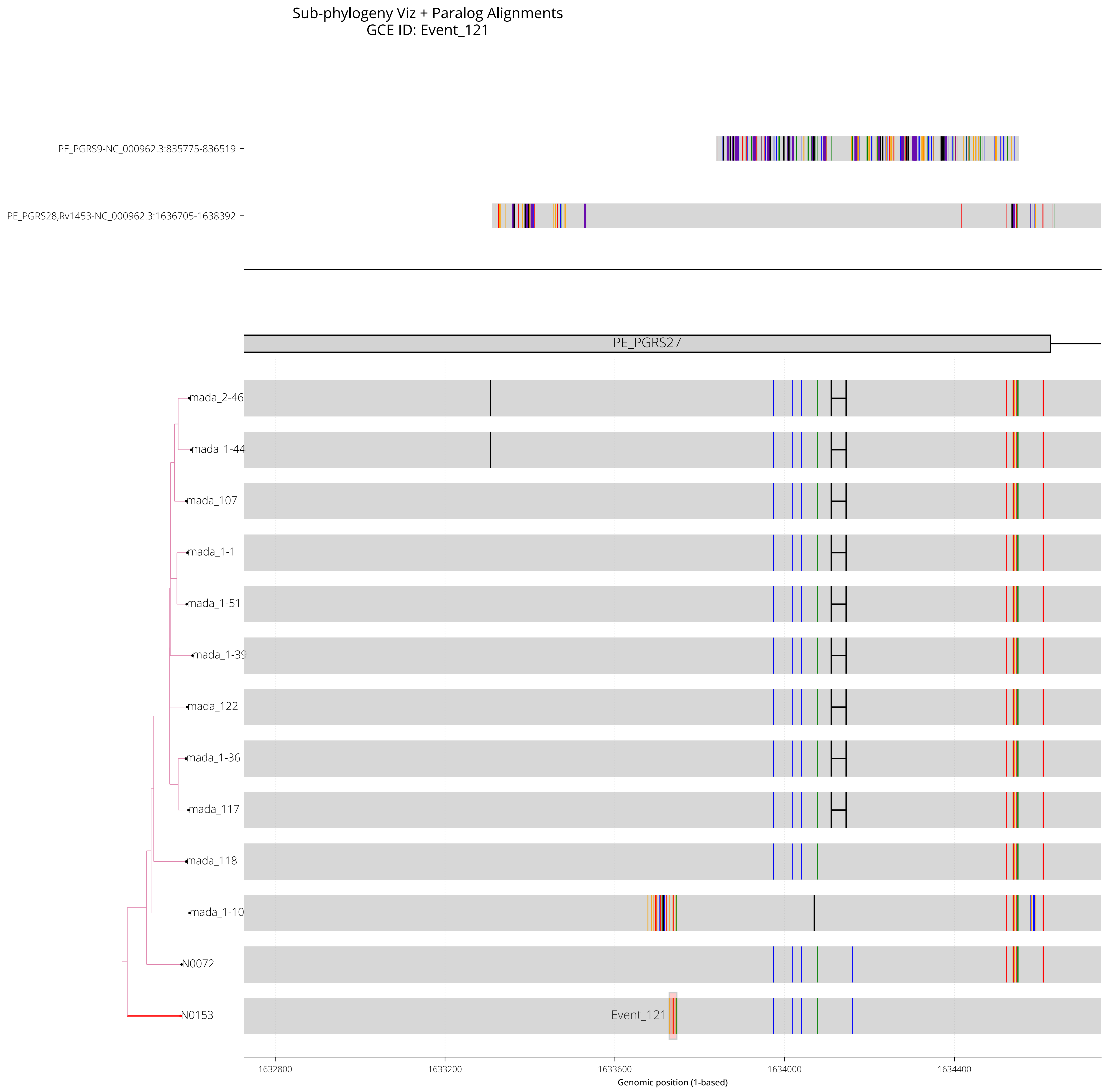

Sub-phylogeny Viz + Paralog Alignments
GCE ID: Event_121
PE_PGRS9-NC_000962.3:835775-836519
PE_PGRS28,Rv1453-NC_000962.3:1636705-1638392
PE_PGRS27
mada_2-46
mada_1-44
mada_107
mada_1-1
mada_1-51
mada_1-39
mada_122
mada_1-36
mada_117
mada_118
mada_1-10
N0072
N0153
Event_121
1632800
1633200
1633600
1634000
1634400
Genomic position (1-based)

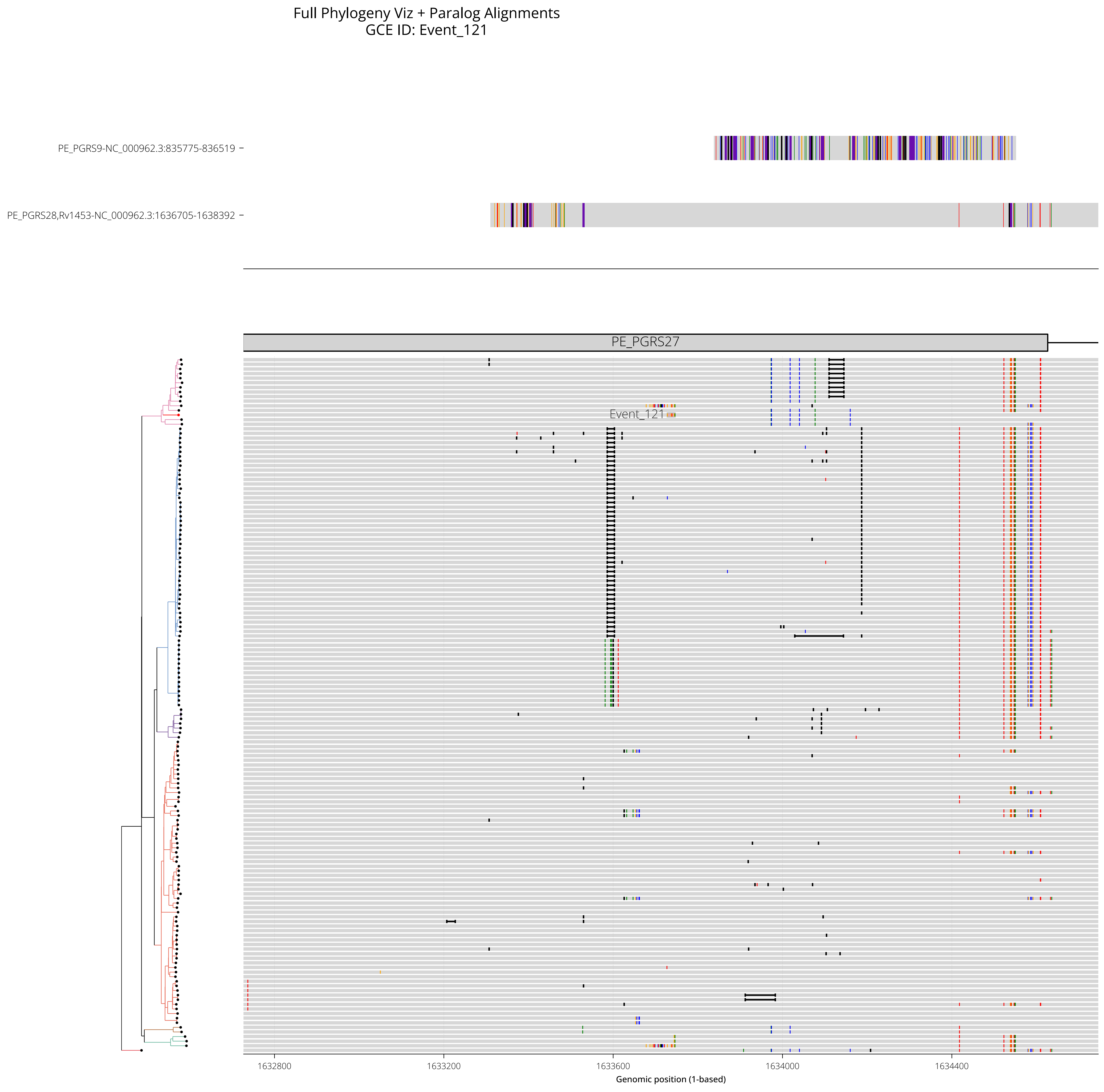

Full Phylogeny Viz + Paralog Alignments
GCE ID: Event_121
PE_PGRS9-NC_000962.3:835775-836519
PE_PGRS28,Rv1453-NC_000962.3:1636705-1638392
PE_PGRS27
Event_121
1632800
1633200
1633600
1634000
1634400
Genomic position (1-based)

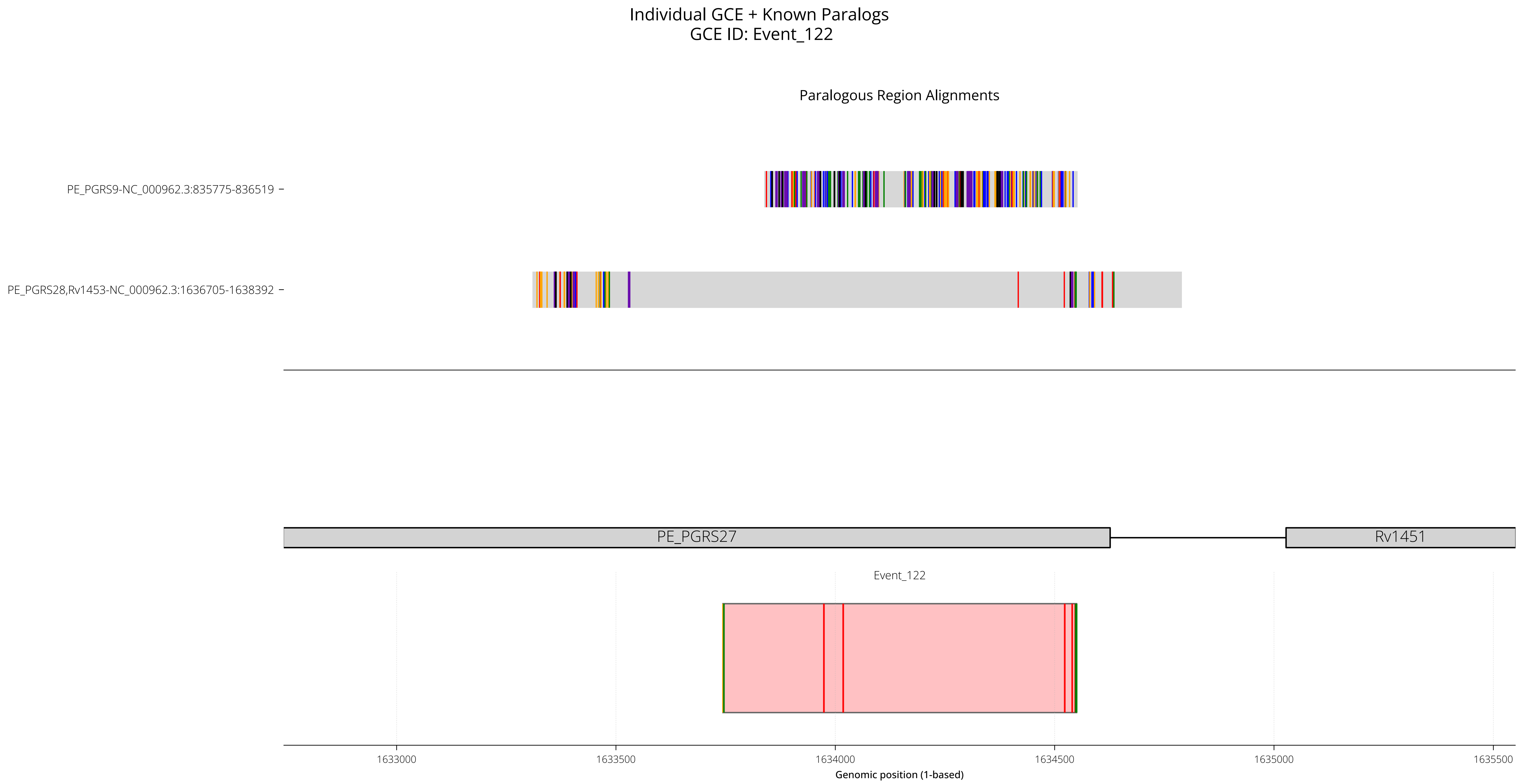

Individual GCE + Known Paralogs
GCE ID: Event_122
Paralogous Region Alignments
PE_PGRS9-NC_000962.3:835775-836519
PE_PGRS28,Rv1453-NC_000962.3:1636705-1638392
PE_PGRS27
Rv1451
Event_122
1633000
1633500
1634000
1634500
1635000
1635500
Genomic position (1-based)

Circos Viz - GCE to Known Paralogs Locations
GCE ID: Event_122

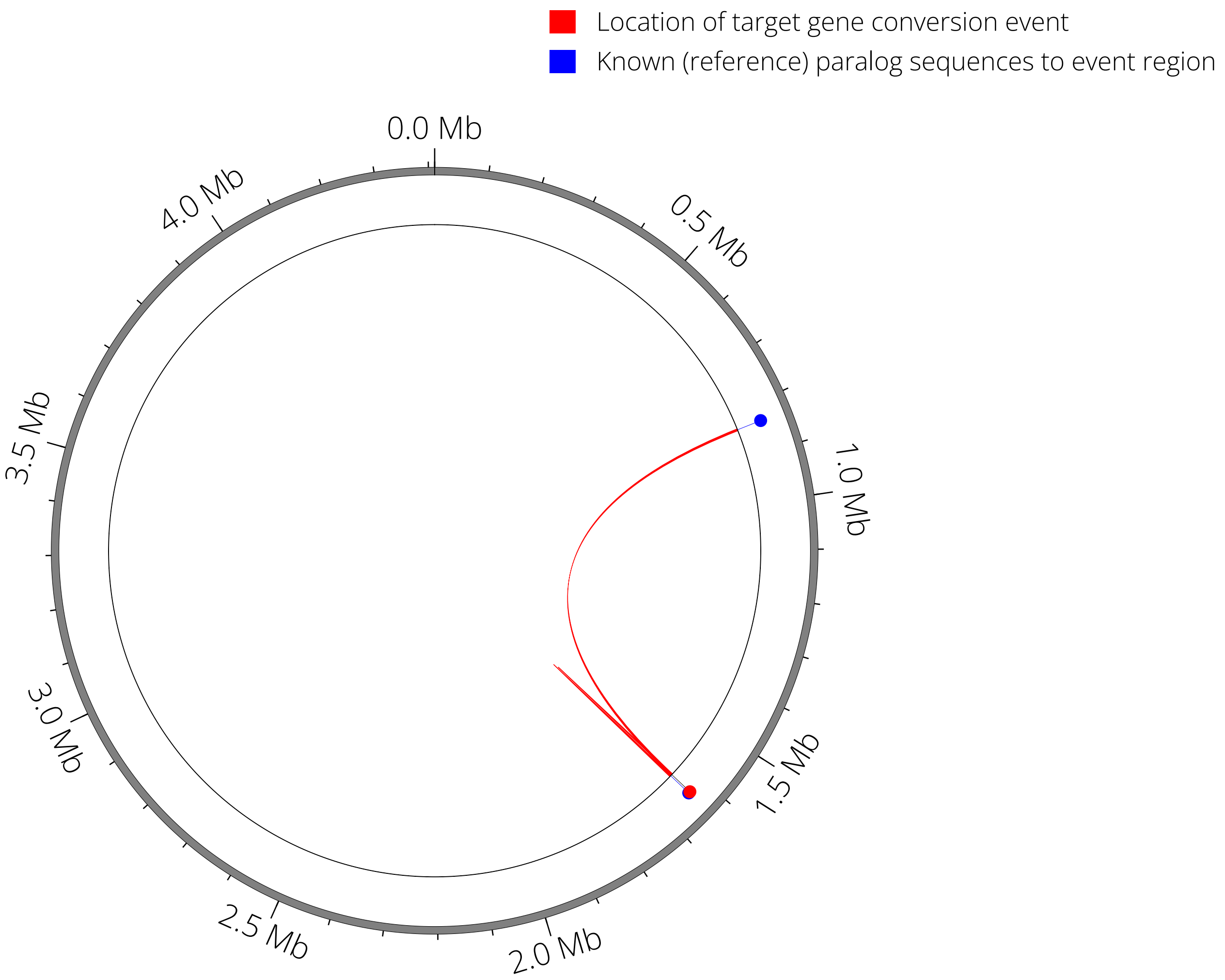

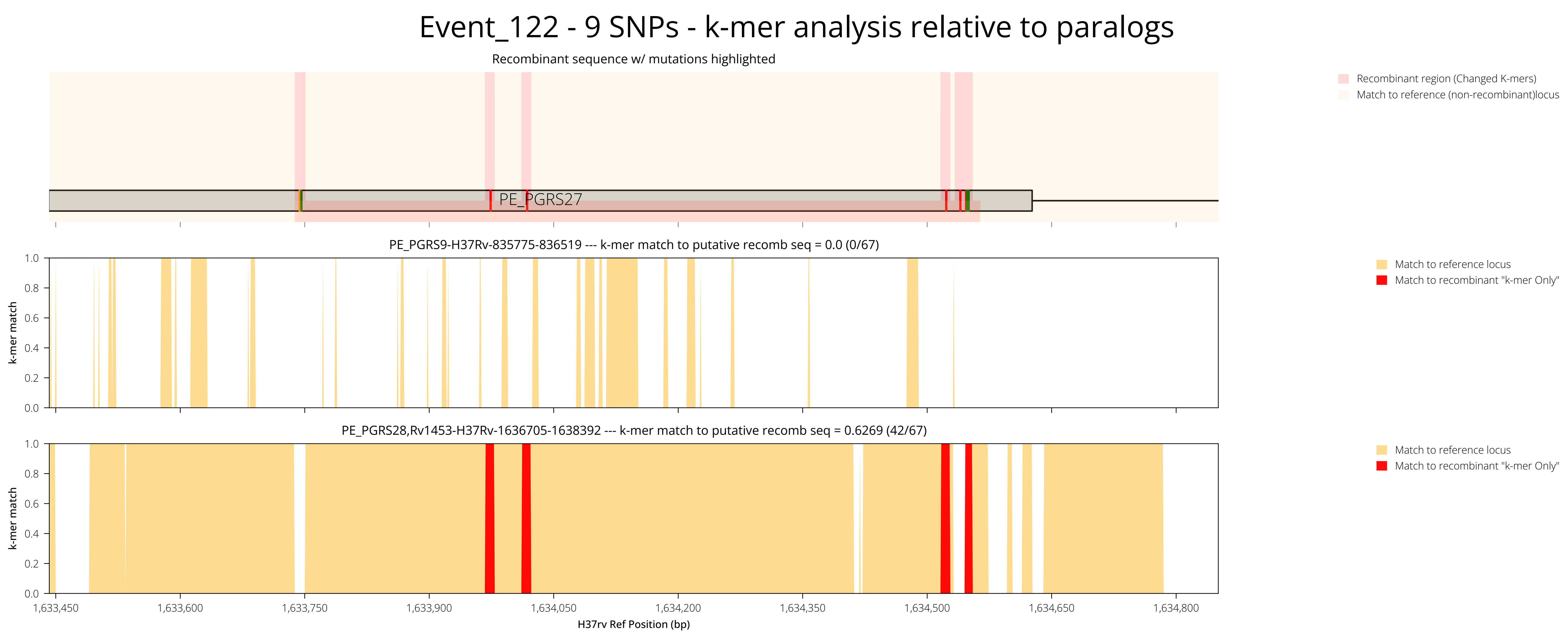
Event_122 - 9 SNPs - k-mer analysis relative to paralogs
Recombinant sequence w/ mutations highlighted
PE_PGRS27
Recombinant region (Changed K-mers)
Match to reference (non-recombinant)locus
PE_PGRS9-H37Rv-835775-836519 --- k-mer match to putative recomb seq = 0.0 (0/67)
Match to reference locus
Match to recombinant "k-mer Only"
PE_PGRS28,Rv1453-H37Rv-1636705-1638392 --- k-mer match to putative recomb seq = 0.6269 (42/67)
Match to reference locus
Match to recombinant "k-mer Only"
k-mer match
1.0
0.8
0.6
0.4
0.2
0.0
1,633,450
1,633,600
1,633,750
1,633,900
1,634,050
1,634,200
1,634,350
1,634,500
1,634,650
1,634,800
H37rv Ref Position (bp)

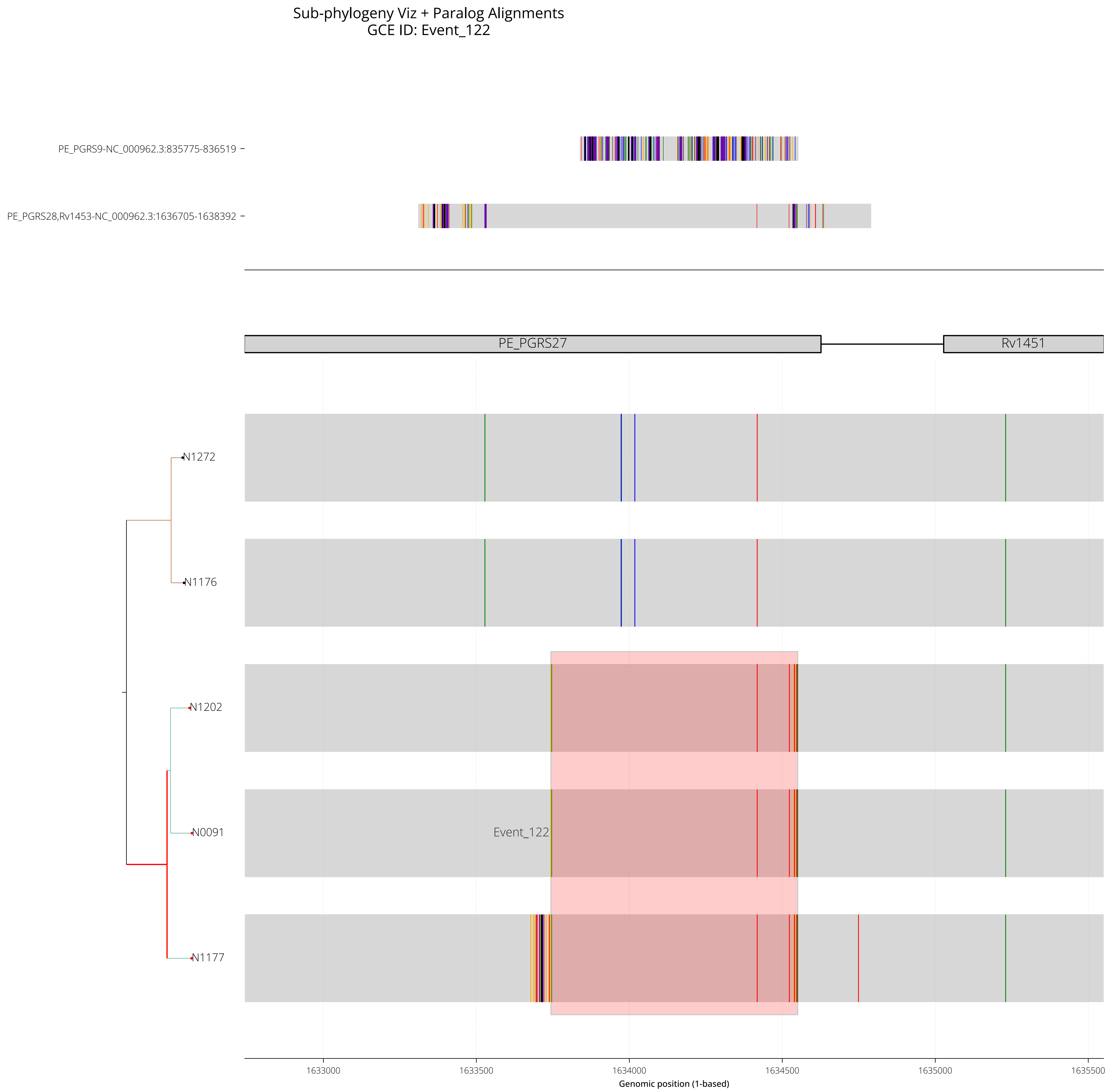

Sub-phylogeny Viz + Paralog Alignments
GCE ID: Event_122
PE_PGRS9-NC_000962.3:835775-836519
PE_PGRS28,Rv1453-NC_000962.3:1636705-1638392
PE_PGRS27
Rv1451
N1272
N1176
N1202
N0091
N1177
Event_122
1633000
1633500
1634000
1634500
1635000
1635500
Genomic position (1-based)

Full Phylogeny Viz + Paralog Alignments
GCE ID: Event_122

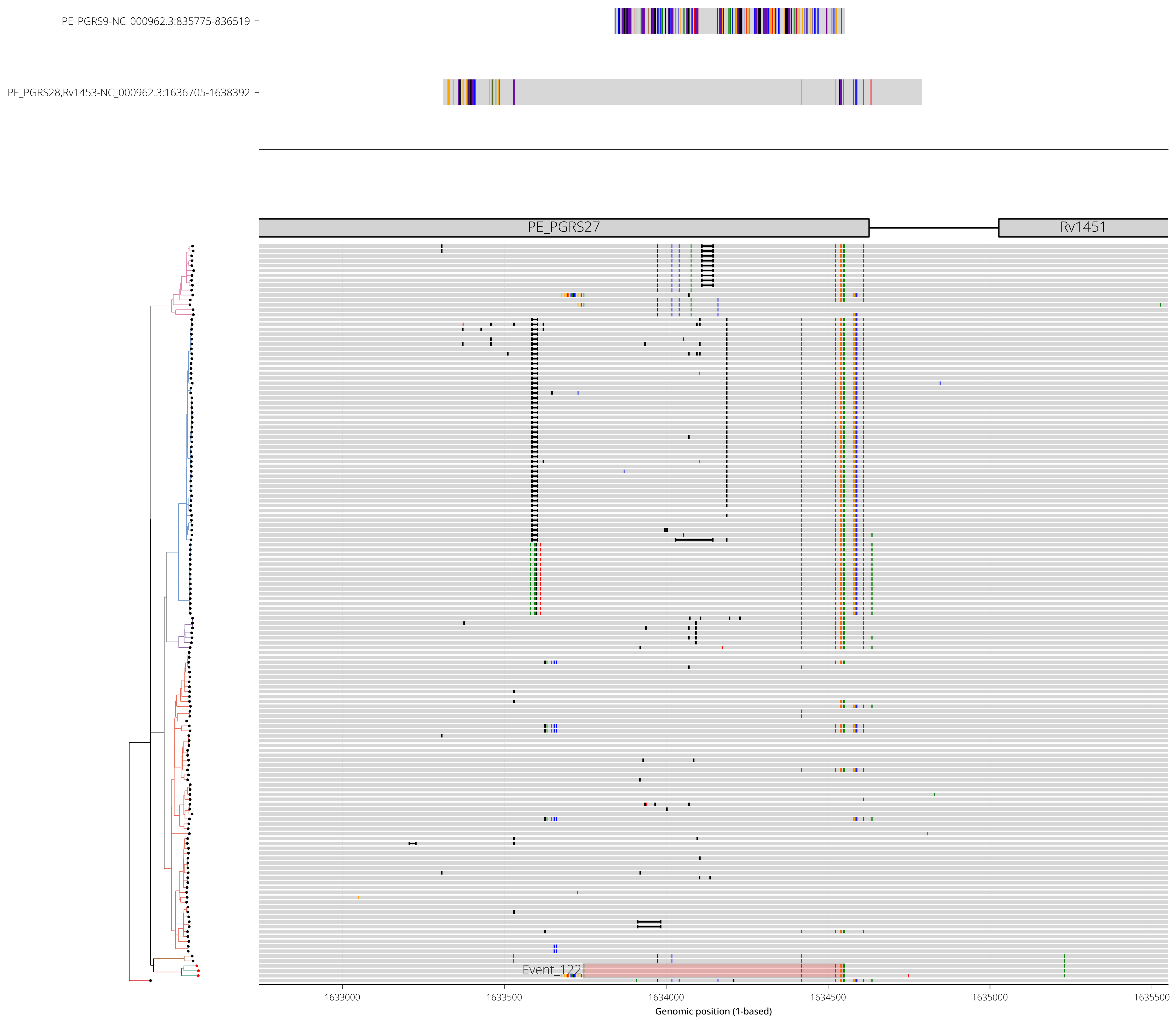

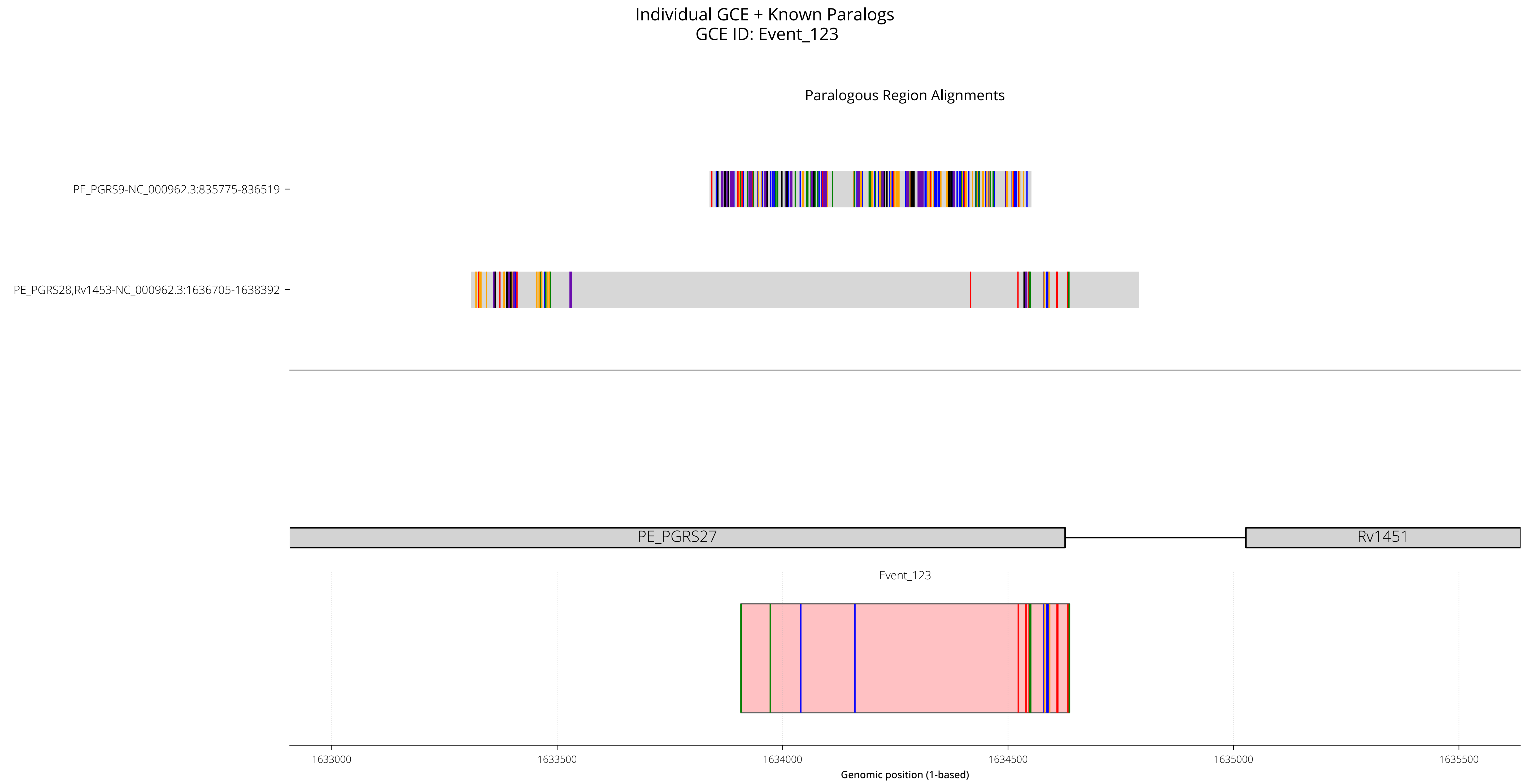

Individual GCE + Known Paralogs
GCE ID: Event_123
Paralogous Region Alignments
PE_PGRS9-NC_000962.3:835775-836519
PE_PGRS28,Rv1453-NC_000962.3:1636705-1638392
PE_PGRS27
Rv1451
Event_123
1633000
1633500
1634000
1634500
1635000
1635500
Genomic position (1-based)

# Circos Viz - GCE to Known Paralogs Locations
GCE ID: Event_123

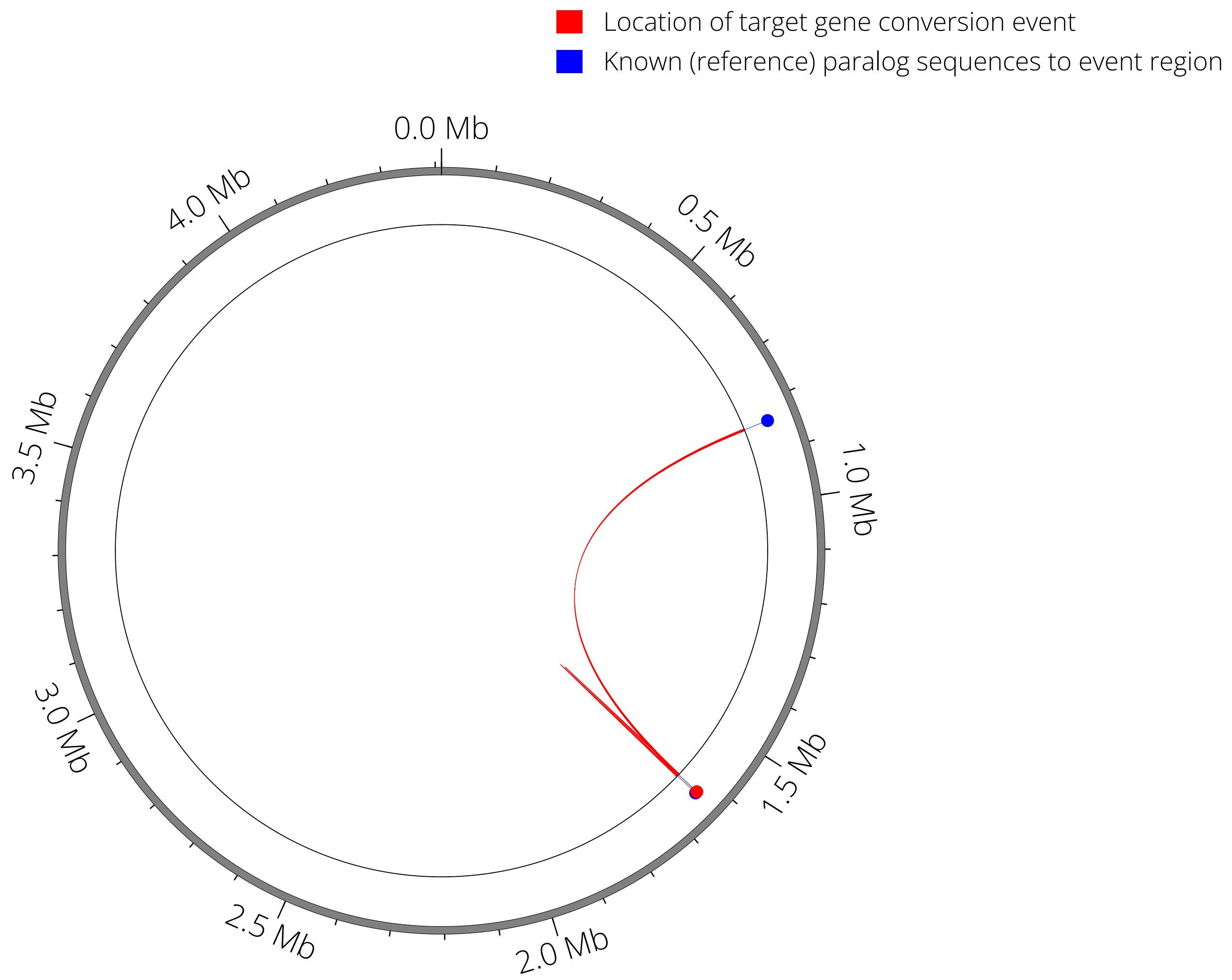

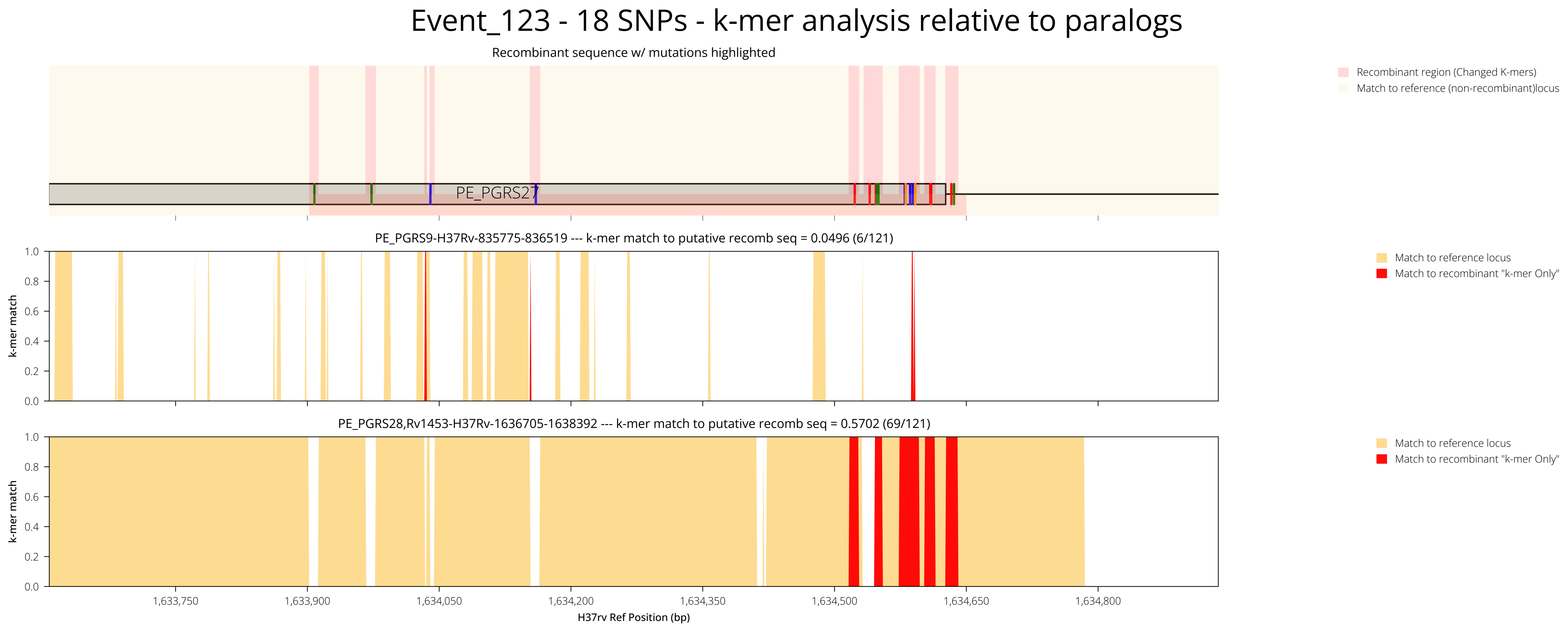
Event_123 - 18 SNPs - k-mer analysis relative to paralogs
Recombinant sequence w/ mutations highlighted
Recombinant region (Changed K-mers)
Match to reference (non-recombinant)locus
PE_PGRS27
PE_PGRS9-H37Rv-835775-836519 --- k-mer match to putative recomb seq = 0.0496 (6/121)
Match to reference locus
Match to recombinant "k-mer Only"
k-mer match
1.0
0.8
0.6
0.4
0.2
0.0
PE_PGRS28,Rv1453-H37Rv-1636705-1638392 --- k-mer match to putative recomb seq = 0.5702 (69/121)
Match to reference locus
Match to recombinant "k-mer Only"
k-mer match
1,633,750
1,633,900
1,634,050
1,634,200
1,634,350
1,634,500
1,634,650
1,634,800
H37rv Ref Position (bp)

Sub-phylogeny Viz + Paralog Alignments
GCE ID: Event_123

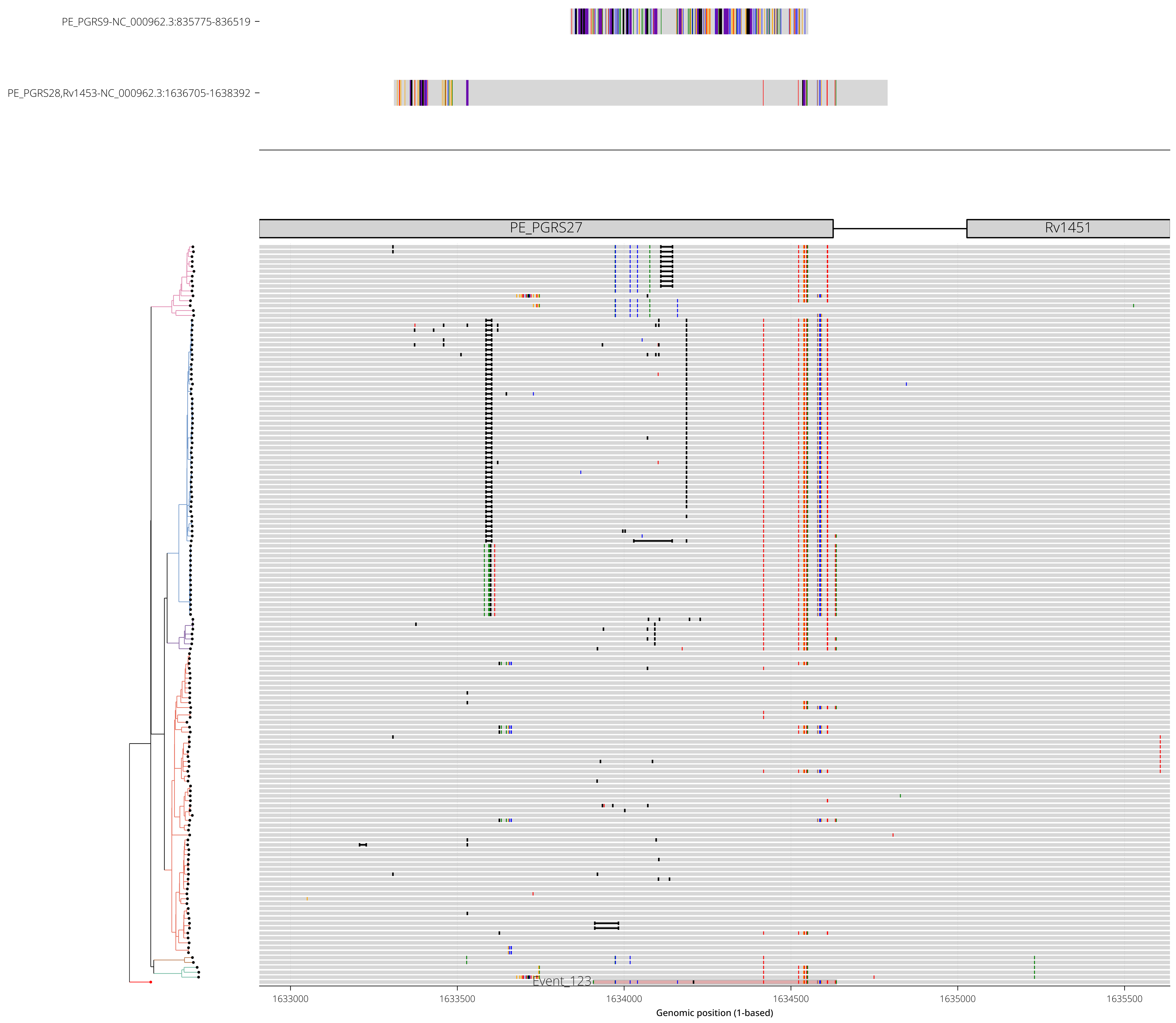

Full Phylogeny Viz + Paralog Alignments
GCE ID: Event_123

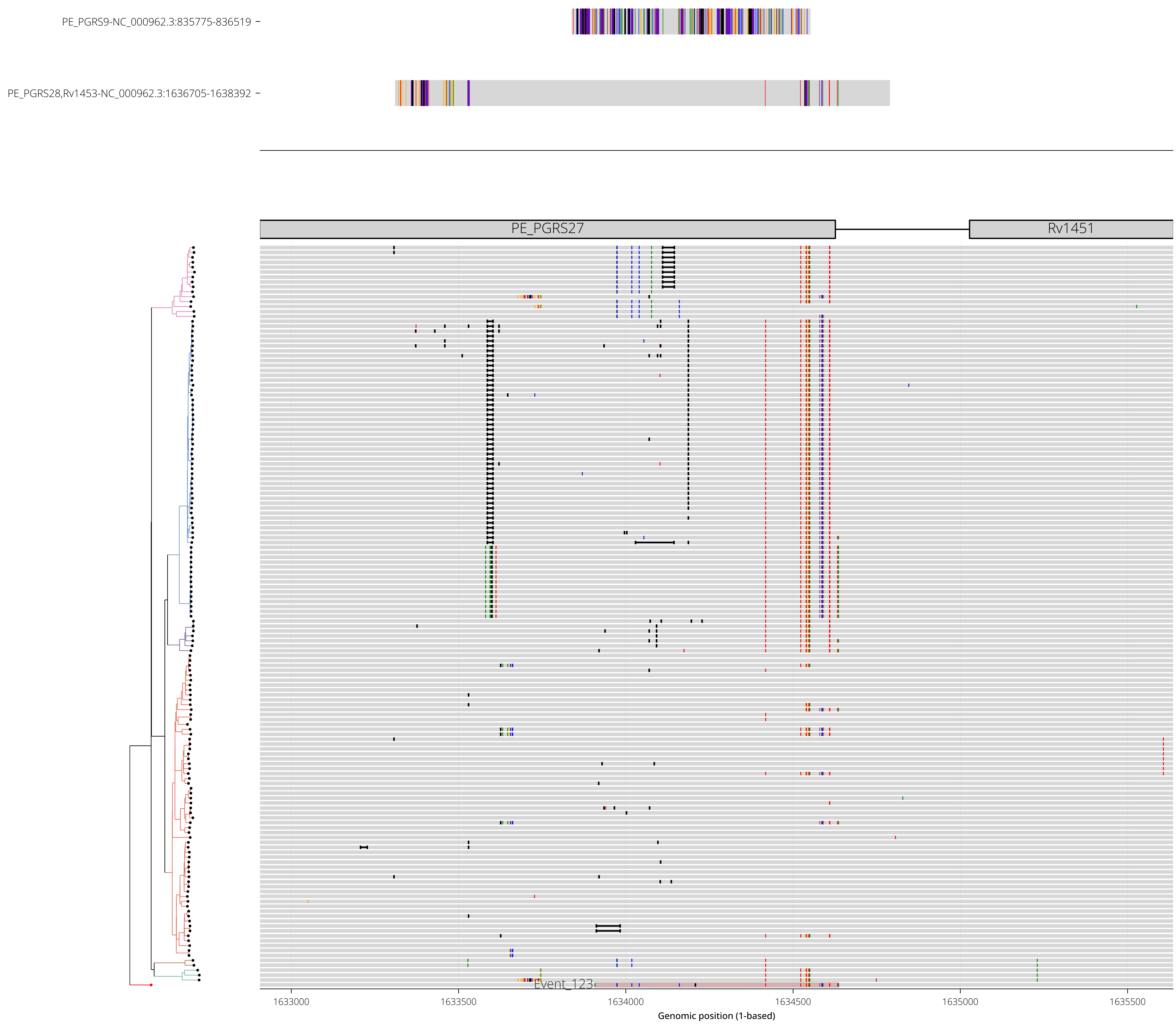

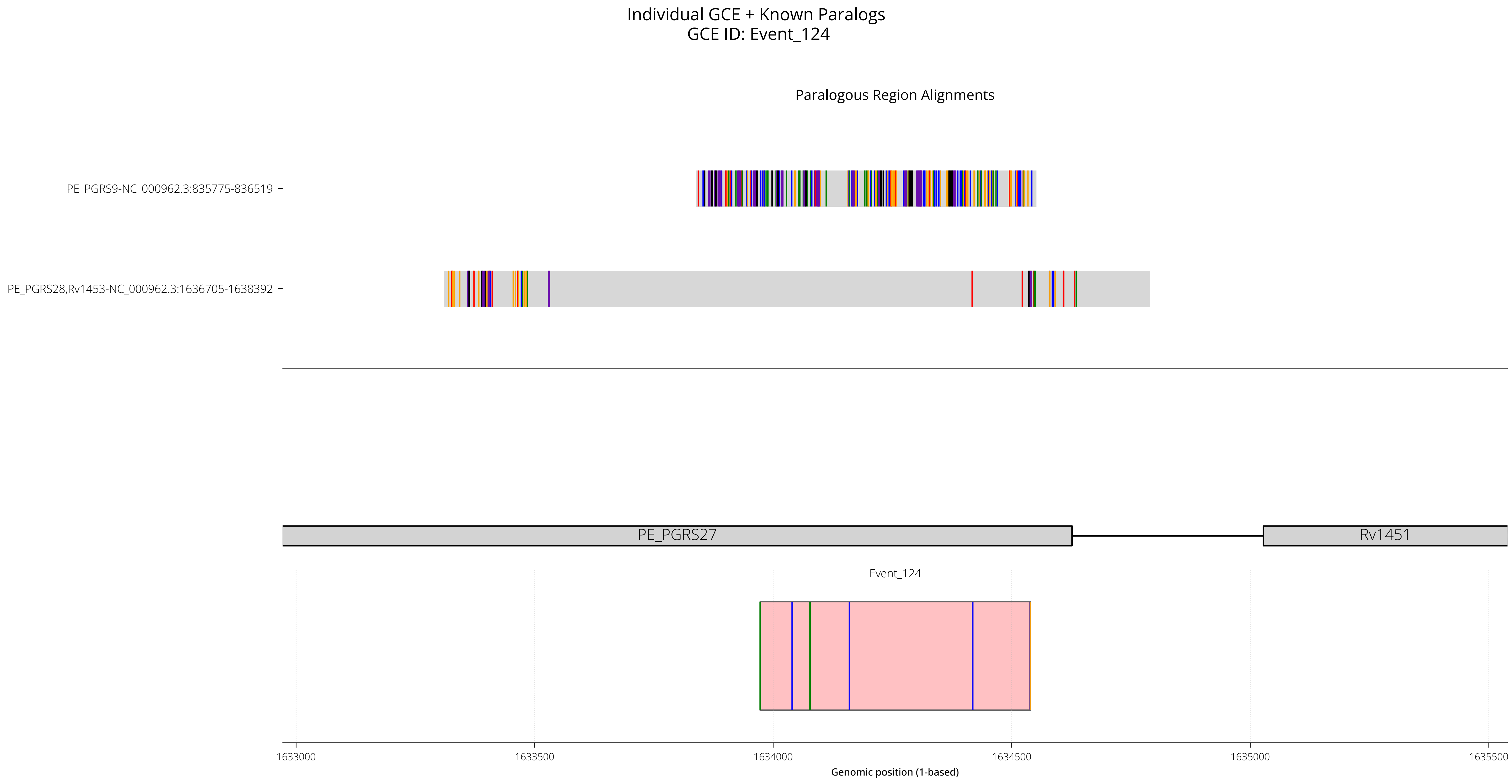
Individual GCE + Known Paralogs
GCE ID: Event_124
Paralogous Region Alignments
PE_PGRS9-NC_000962.3:835775-836519
PE_PGRS28,Rv1453-NC_000962.3:1636705-1638392
PE_PGRS27
Rv1451
Event_124
1633000
1633500
1634000
1634500
1635000
1635500
Genomic position (1-based)

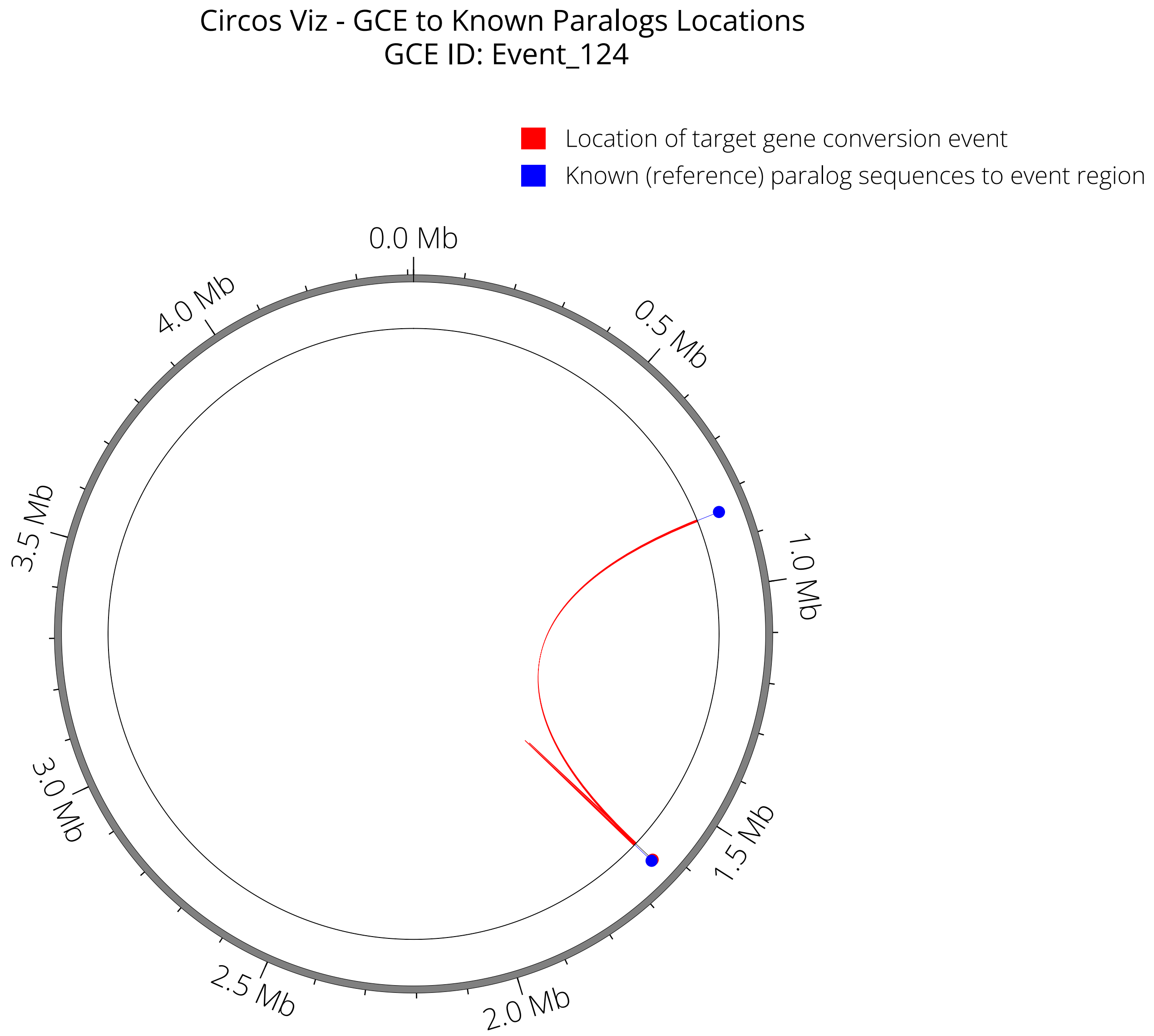
Circos Viz - GCE to Known Paralogs Locations
GCE ID: Event_124
Location of target gene conversion event
Known (reference) paralog sequences to event region
0.0 Mb
0.5 Mb
1.0 Mb
1.5 Mb
2.0 Mb
2.5 Mb
3.0 Mb
3.5 Mb
4.0 Mb

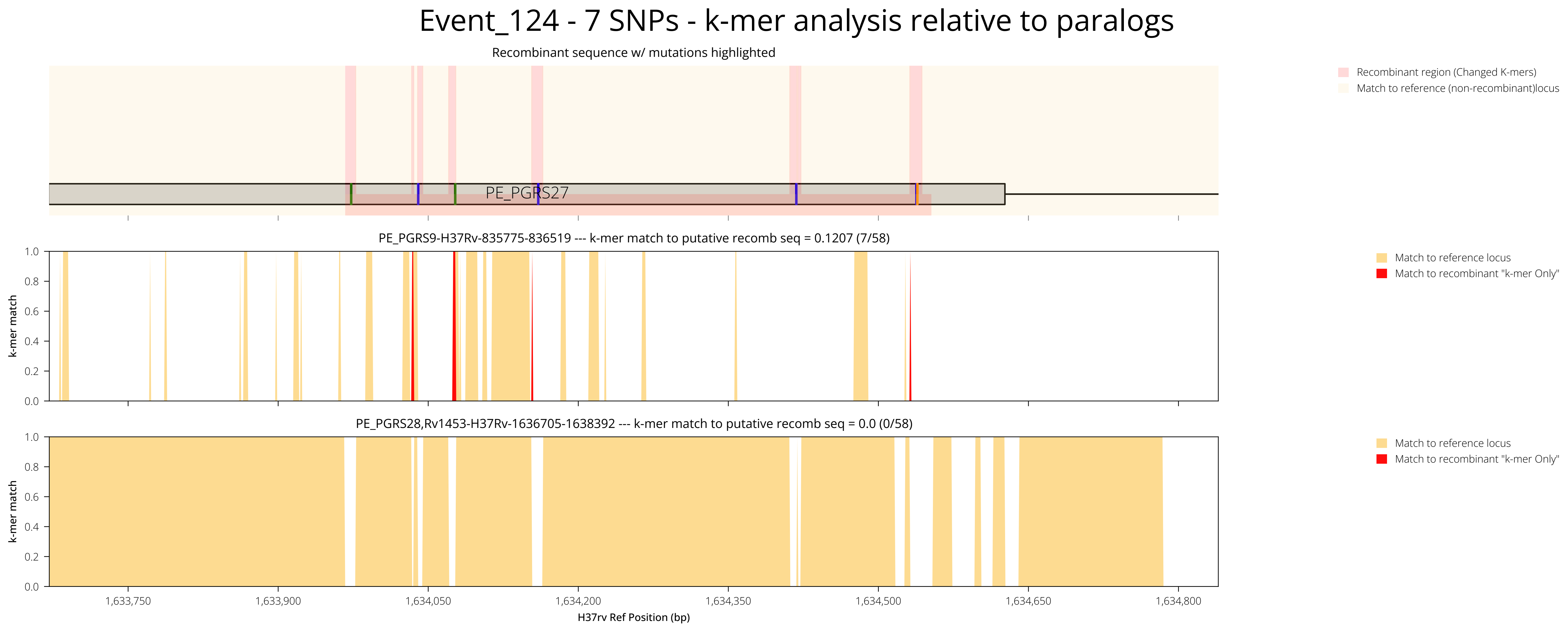

Event_124 - 7 SNPs - k-mer analysis relative to paralogs
Recombinant sequence w/ mutations highlighted
Recombinant region (Changed K-mers)
Match to reference (non-recombinant)locus
PE_PGRS27
PE_PGRS9-H37Rv-835775-836519 --- k-mer match to putative recomb seq = 0.1207 (7/58)
Match to reference locus
Match to recombinant "k-mer Only"
k-mer match
1.0
0.8
0.6
0.4
0.2
0.0
PE_PGRS28,Rv1453-H37Rv-1636705-1638392 --- k-mer match to putative recomb seq = 0.0 (0/58)
Match to reference locus
Match to recombinant "k-mer Only"
k-mer match
1.0
0.8
0.6
0.4
0.2
0.0
1,633,750
1,633,900
1,634,050
1,634,200
1,634,350
1,634,500
1,634,650
1,634,800
H37rv Ref Position (bp)

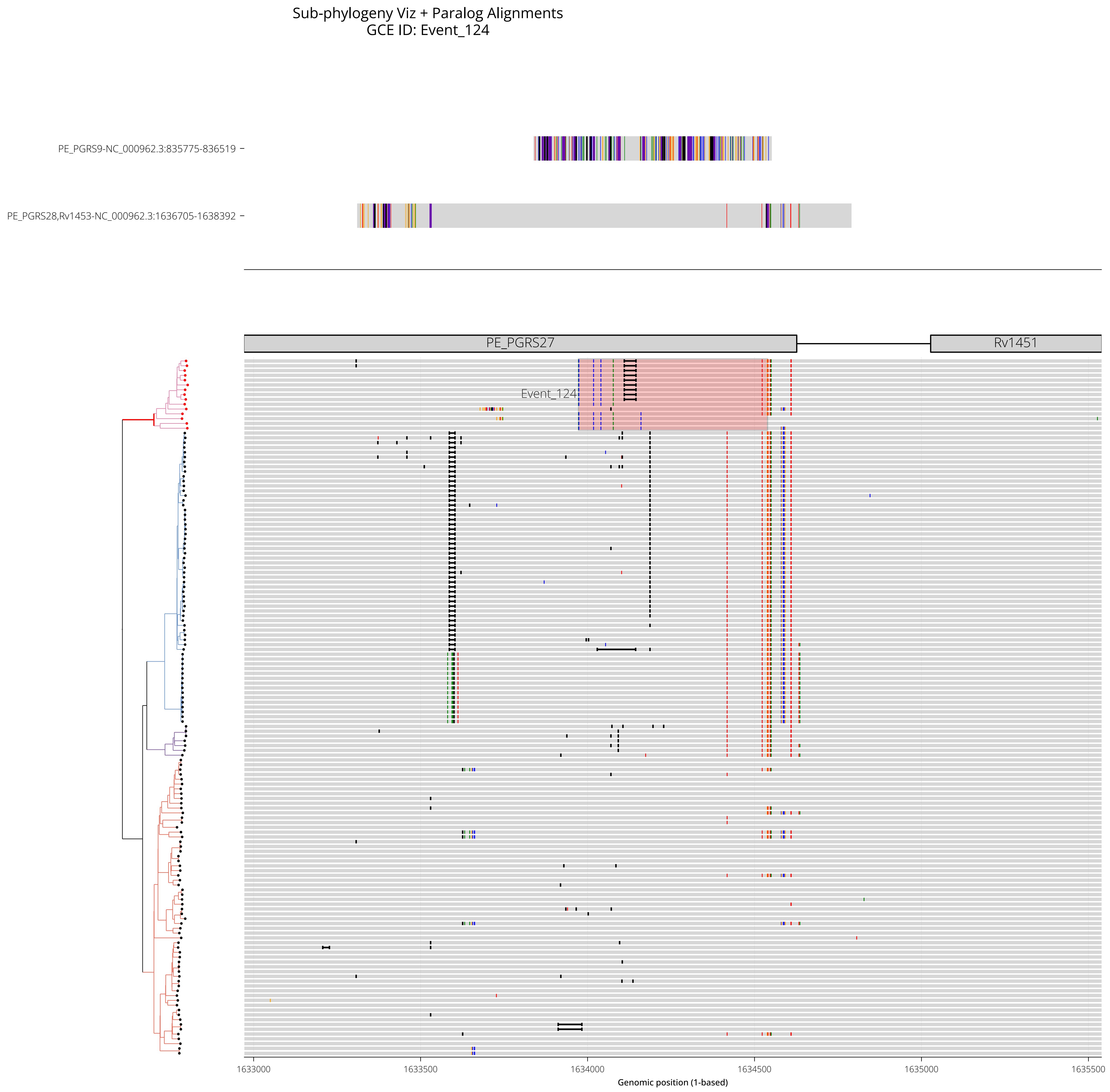

Sub-phylogeny Viz + Paralog Alignments
GCE ID: Event_124
PE_PGRS9-NC_000962.3:835775-836519
PE_PGRS28,Rv1453-NC_000962.3:1636705-1638392
PE_PGRS27
Rv1451
Event_124
1633000
1633500
1634000
1634500
1635000
1635500
Genomic position (1-based)

Full Phylogeny Viz + Paralog Alignments
GCE ID: Event_124

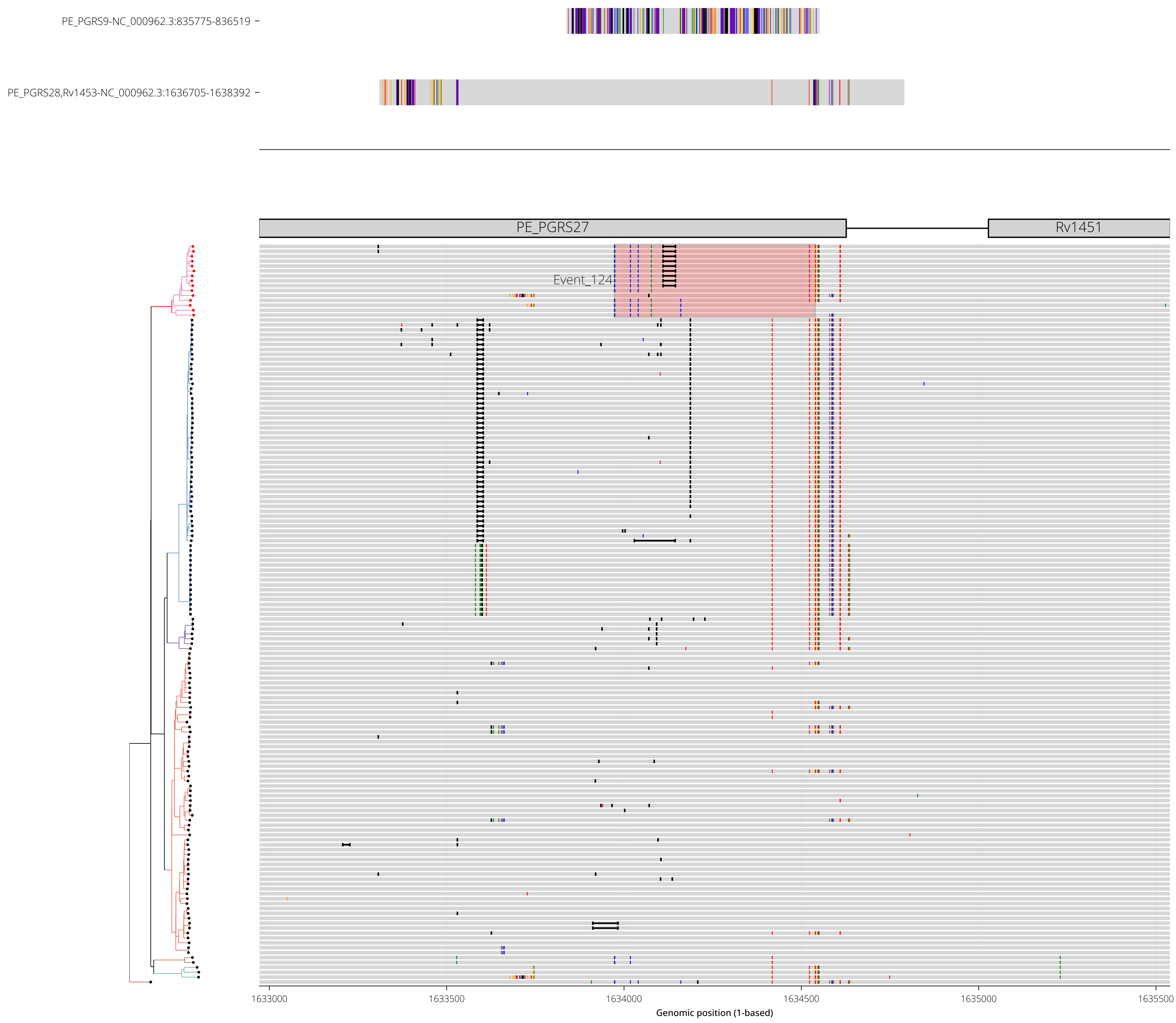

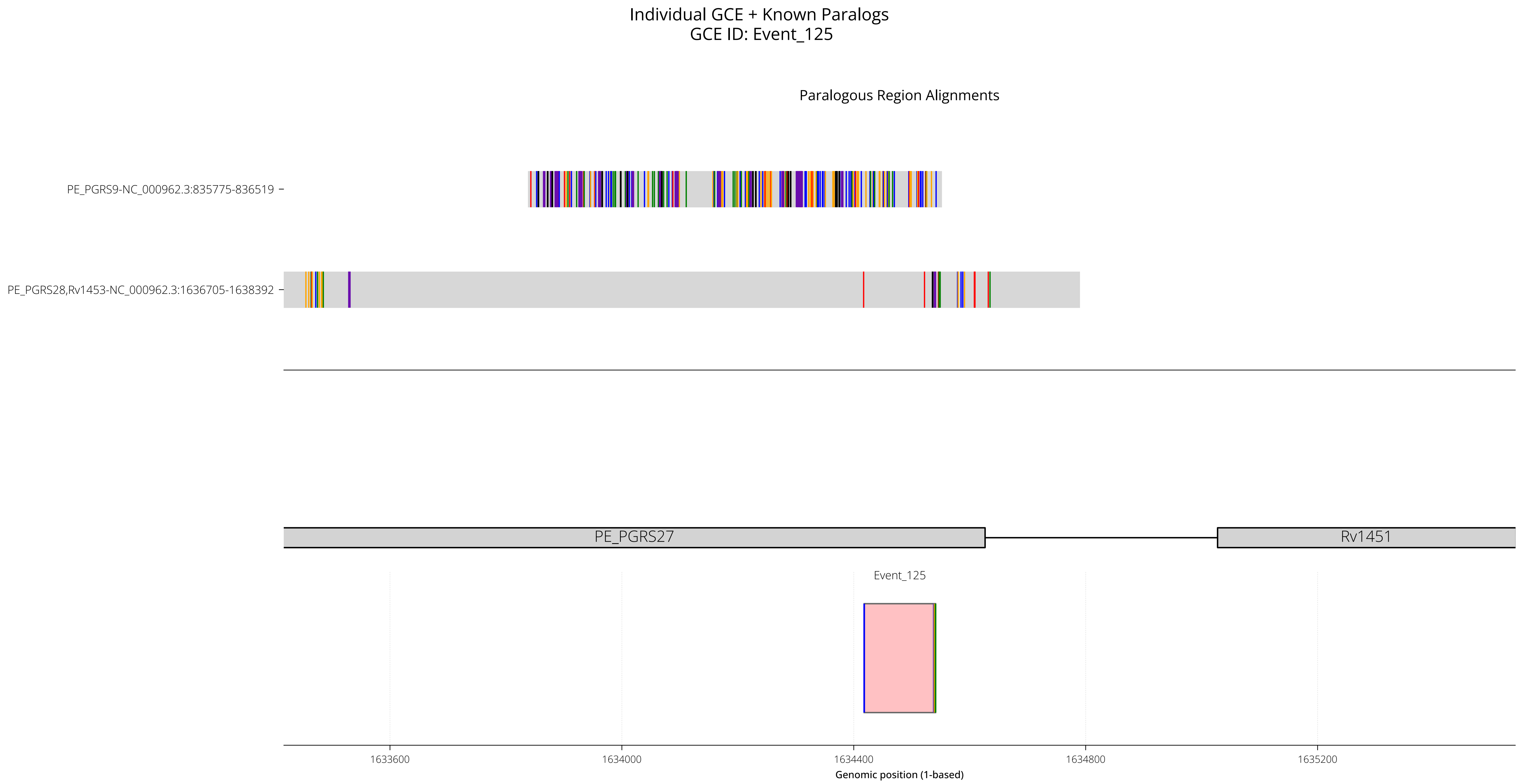
Individual GCE + Known Paralogs
GCE ID: Event_125
Paralogous Region Alignments
PE_PGRS9-NC_000962.3:835775-836519
PE_PGRS28,Rv1453-NC_000962.3:1636705-1638392
PE_PGRS27
Rv1451
Event_125
1633600
1634000
1634400
1634800
1635200
Genomic position (1-based)

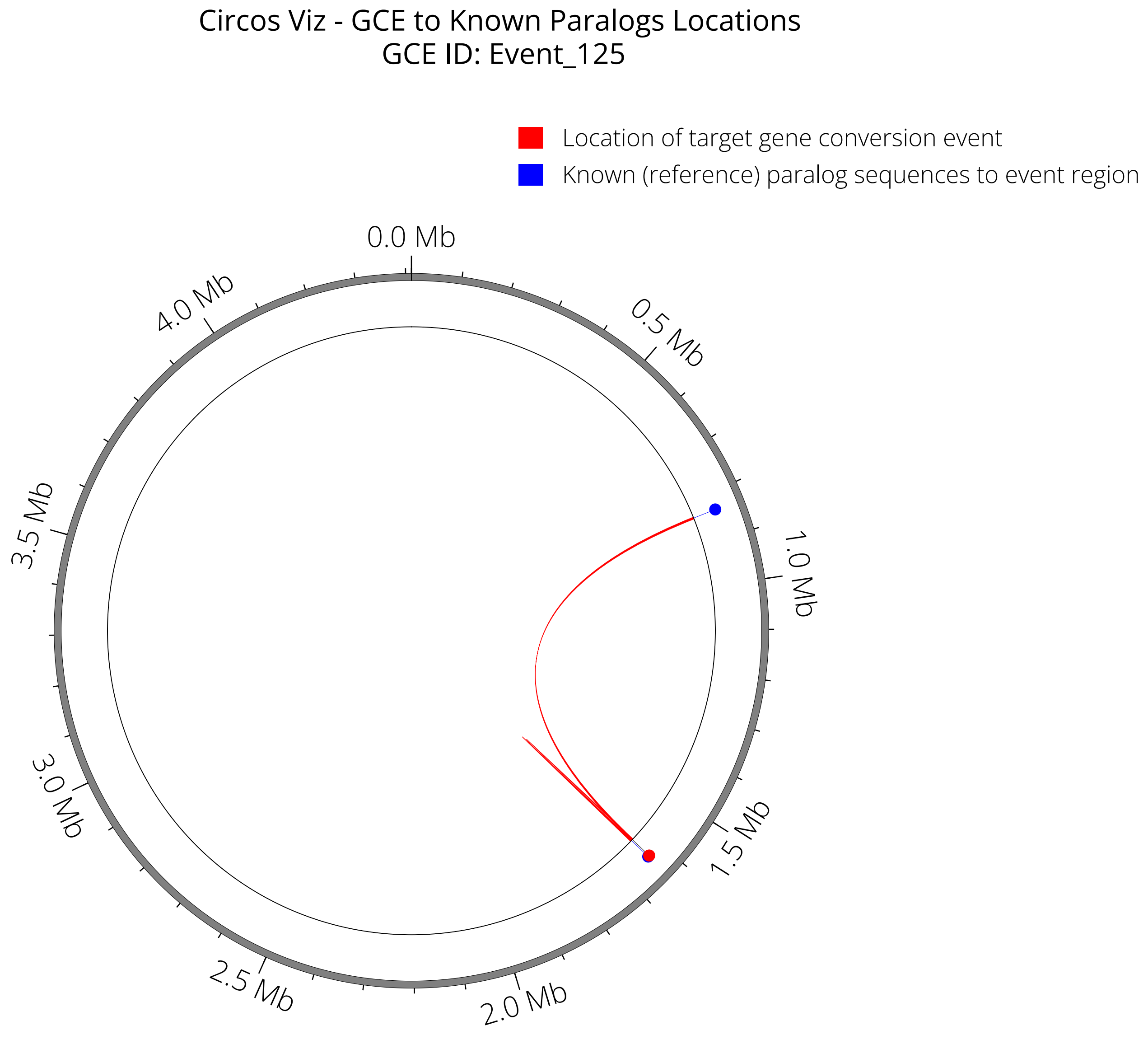

Circos Viz - GCE to Known Paralogs Locations
GCE ID: Event_125
Location of target gene conversion event
Known (reference) paralog sequences to event region
0.0 Mb
0.5 Mb
1.0 Mb
1.5 Mb
2.0 Mb
2.5 Mb
3.0 Mb
3.5 Mb
4.0 Mb

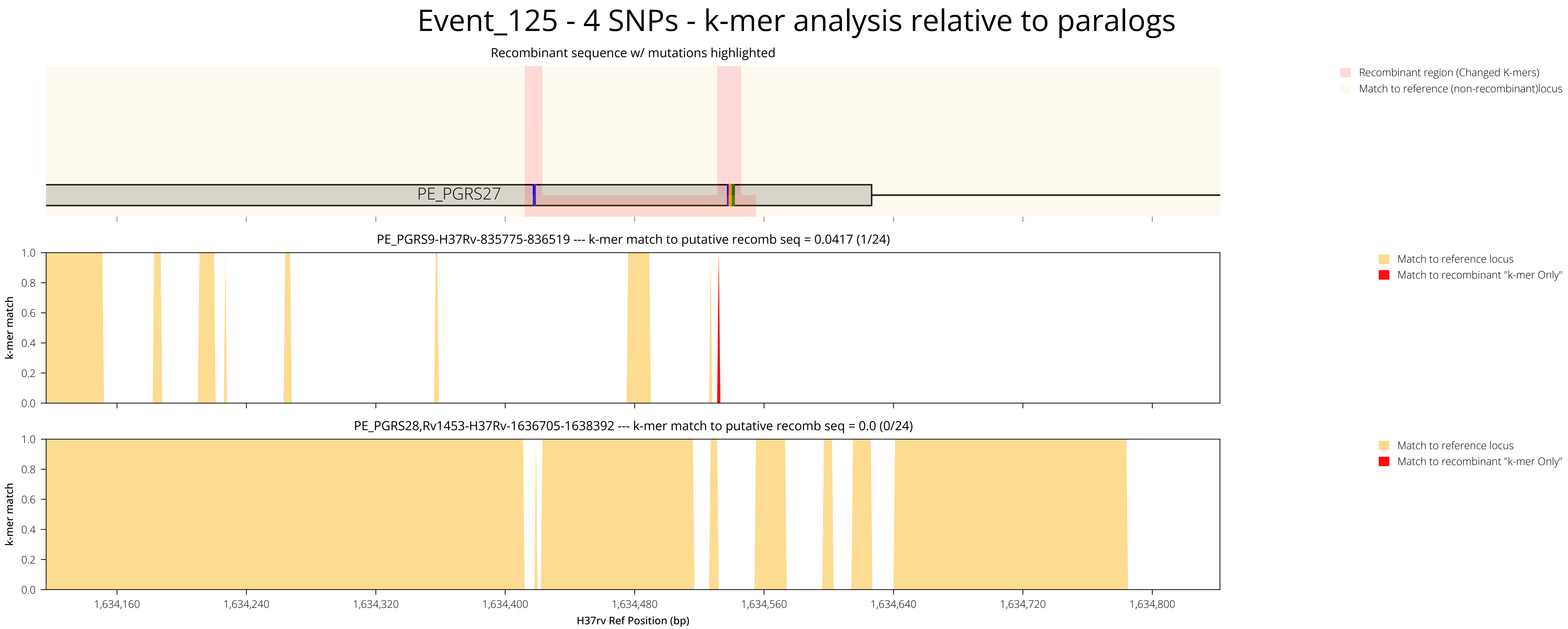

Event_125 - 4 SNPs - k-mer analysis relative to paralogs
Recombinant sequence w/ mutations highlighted
Recombinant region (Changed K-mers)
Match to reference (non-recombinant)locus
PE_PGRS27
PE_PGRS9-H37Rv-835775-836519 --- k-mer match to putative recomb seq = 0.0417 (1/24)
Match to reference locus
Match to recombinant "k-mer Only"
k-mer match
1.0
0.8
0.6
0.4
0.2
0.0
PE_PGRS28,Rv1453-H37Rv-1636705-1638392 --- k-mer match to putative recomb seq = 0.0 (0/24)
Match to reference locus
Match to recombinant "k-mer Only"
k-mer match
1,634,160
1,634,240
1,634,320
1,634,400
1,634,480
1,634,560
1,634,640
1,634,720
1,634,800
H37rv Ref Position (bp)

Sub-phylogeny Viz + Paralog Alignments
GCE ID: Event_125

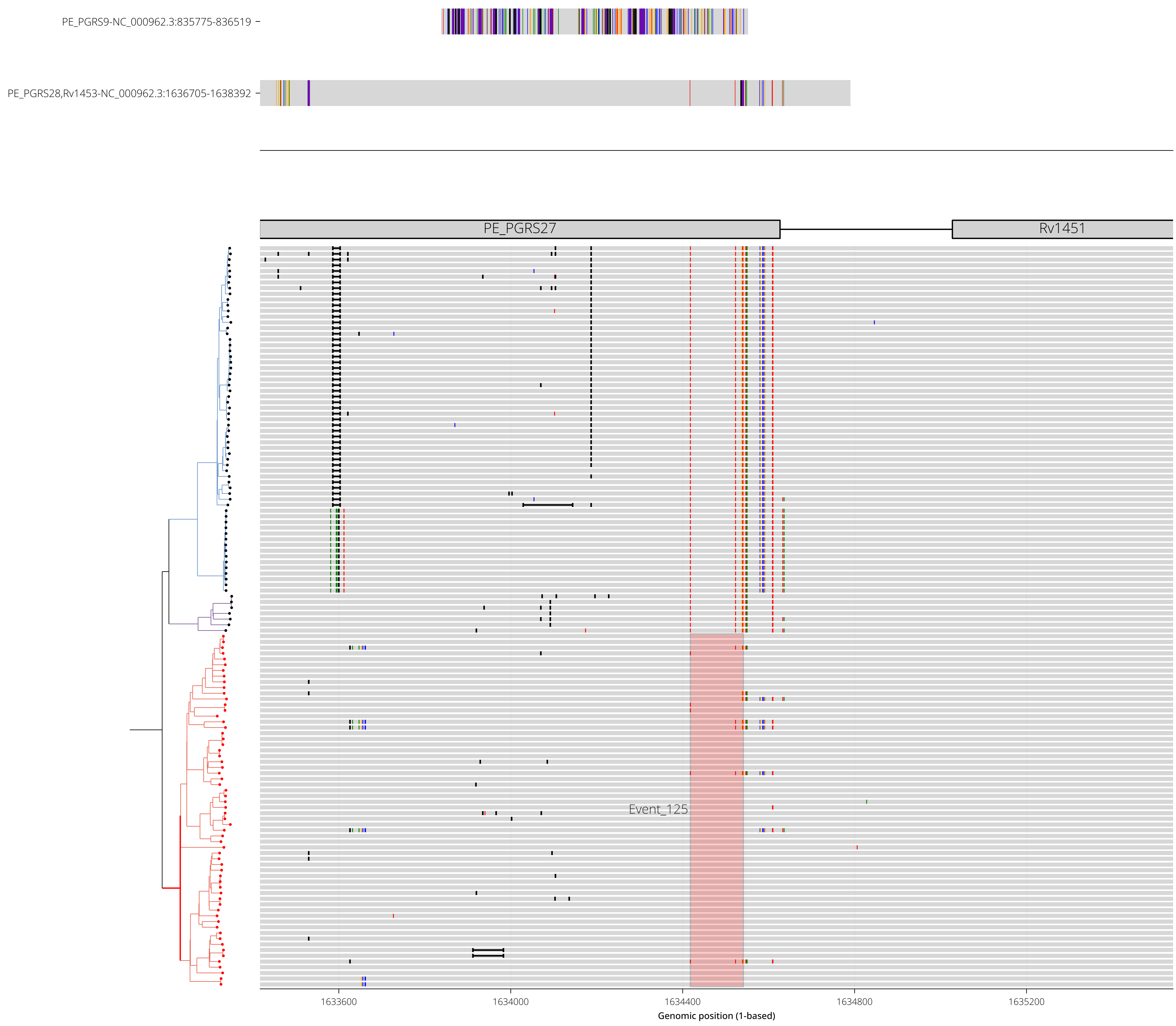

Full Phylogeny Viz + Paralog Alignments
GCE ID: Event_125

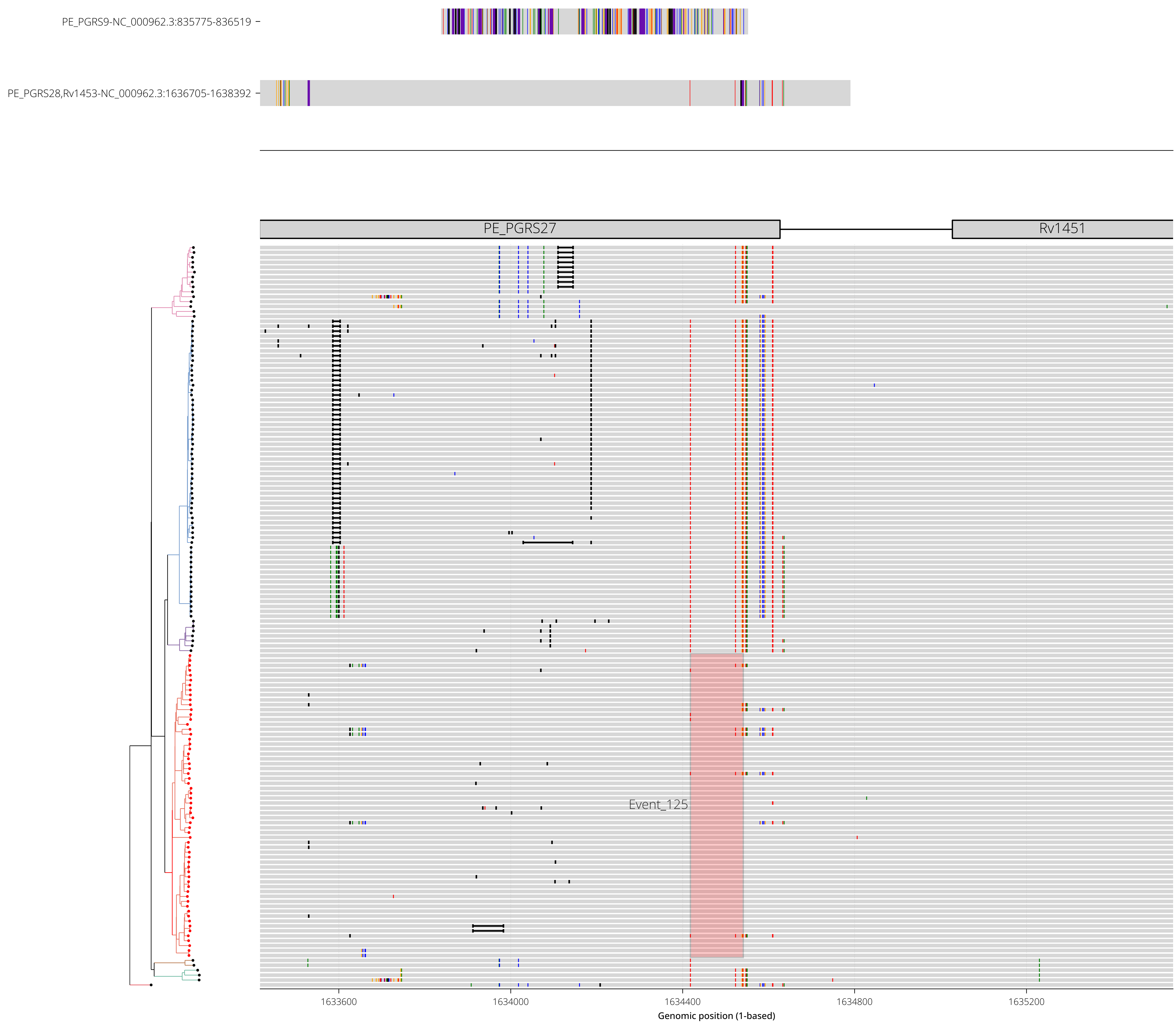

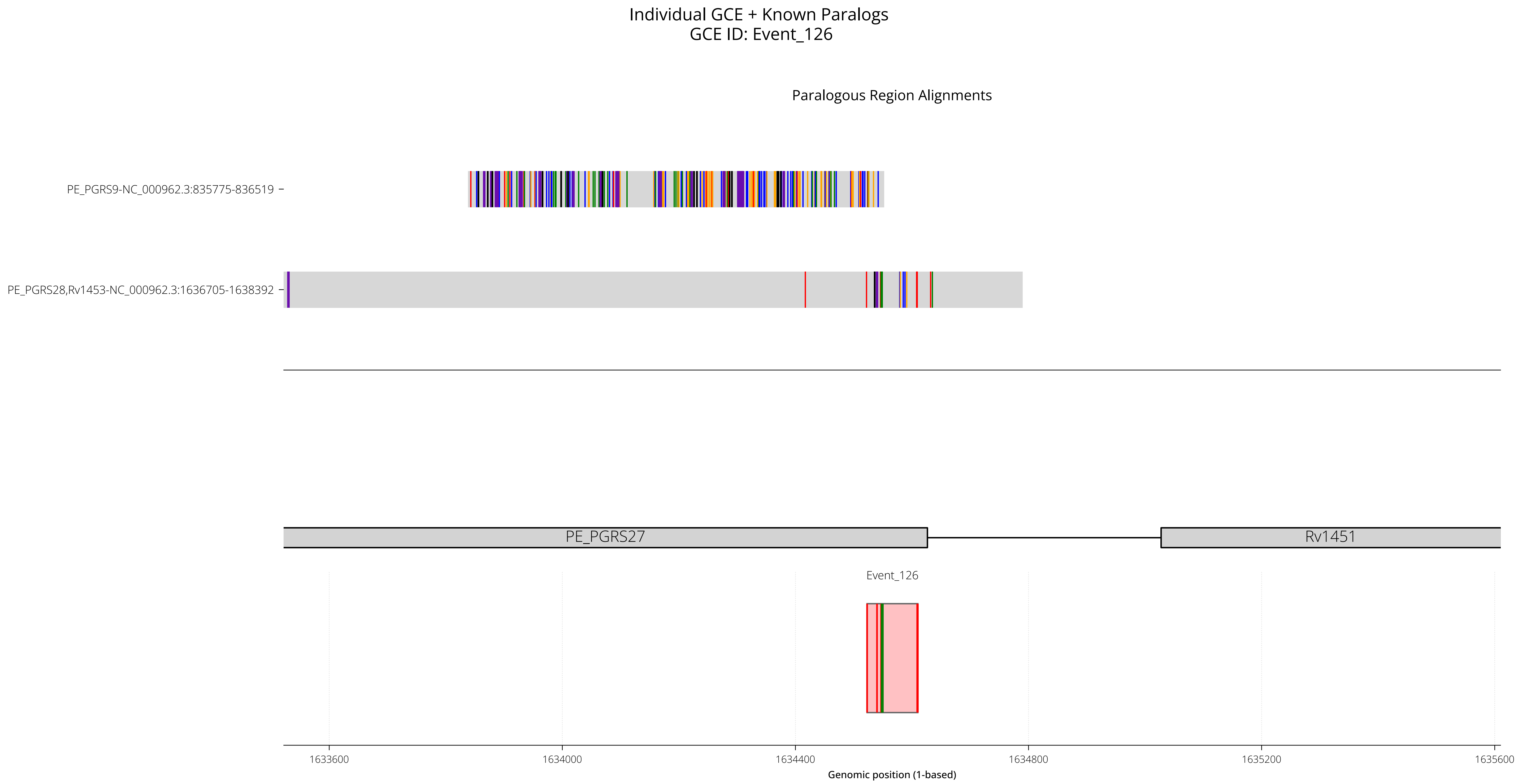

Individual GCE + Known Paralogs
GCE ID: Event_126
Paralogous Region Alignments
PE_PGRS9-NC_000962.3:835775-836519
PE_PGRS28,Rv1453-NC_000962.3:1636705-1638392
PE_PGRS27
Rv1451
Event_126
1633600
1634000
1634400
1634800
1635200
1635600
Genomic position (1-based)

Circos Viz - GCE to Known Paralogs Locations
GCE ID: Event_126

Location of target gene conversion event
Known (reference) paralog sequences to event region

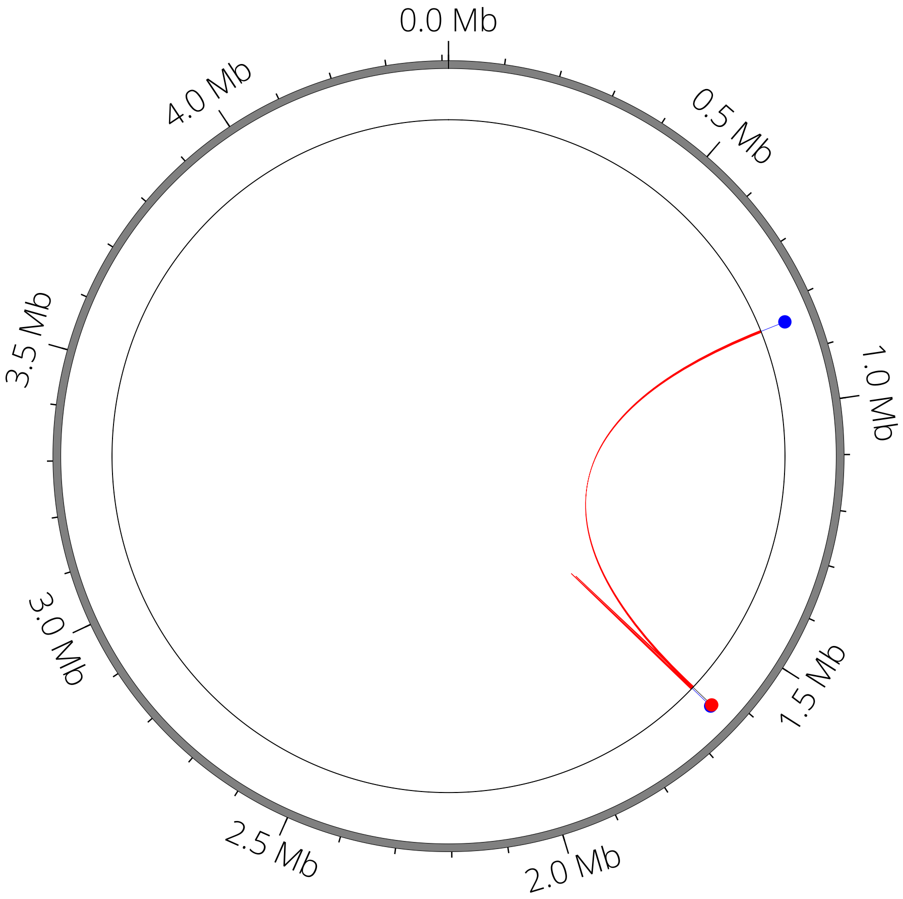

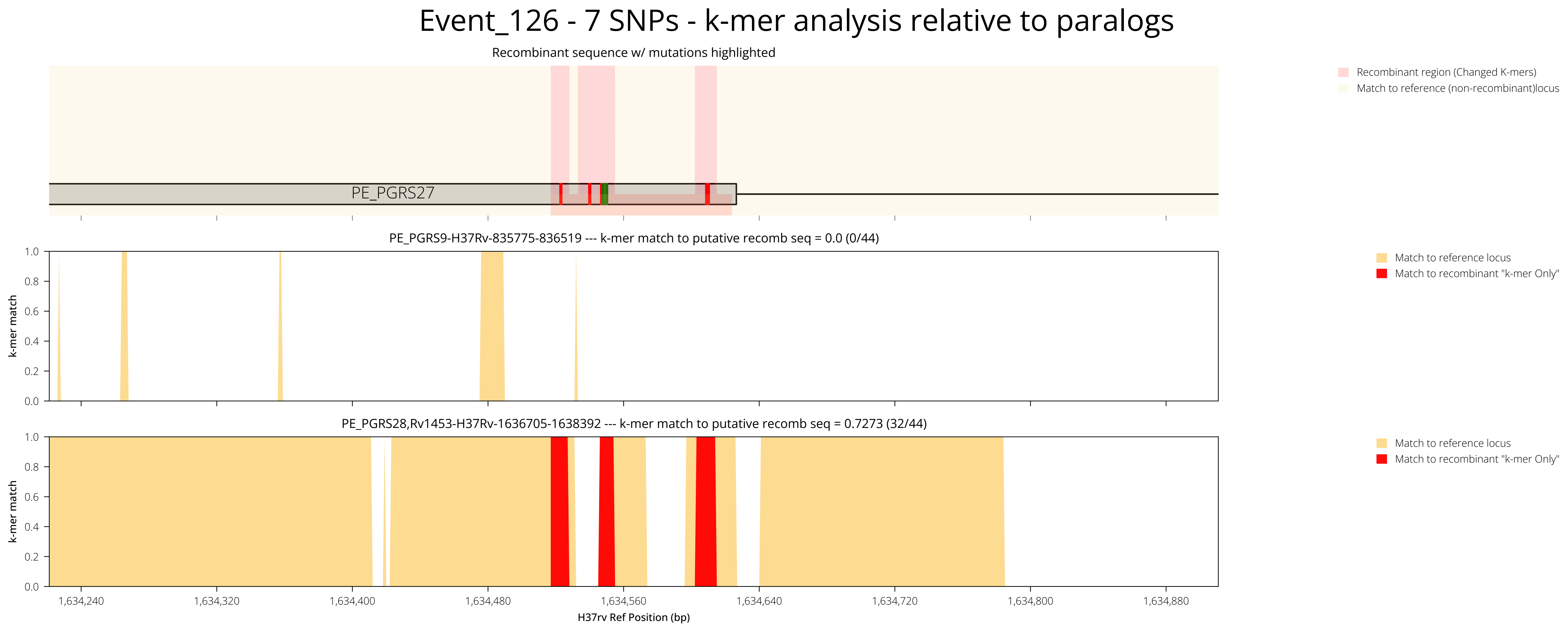

Event_126 - 7 SNPs - k-mer analysis relative to paralogs
Recombinant sequence w/ mutations highlighted
PE_PGRS27
Recombinant region (Changed K-mers)
Match to reference (non-recombinant)locus
PE_PGRS9-H37Rv-835775-836519 --- k-mer match to putative recomb seq = 0.0 (0/44)
k-mer match
1.0
0.8
0.6
0.4
0.2
0.0
Match to reference locus
Match to recombinant "k-mer Only"
PE_PGRS28,Rv1453-H37Rv-1636705-1638392 --- k-mer match to putative recomb seq = 0.7273 (32/44)
k-mer match
Match to reference locus
Match to recombinant "k-mer Only"
1,634,240
1,634,320
1,634,400
1,634,480
1,634,560
1,634,640
1,634,720
1,634,800
1,634,880
H37rv Ref Position (bp)

Sub-phylogeny Viz + Paralog Alignments
GCE ID: Event_126

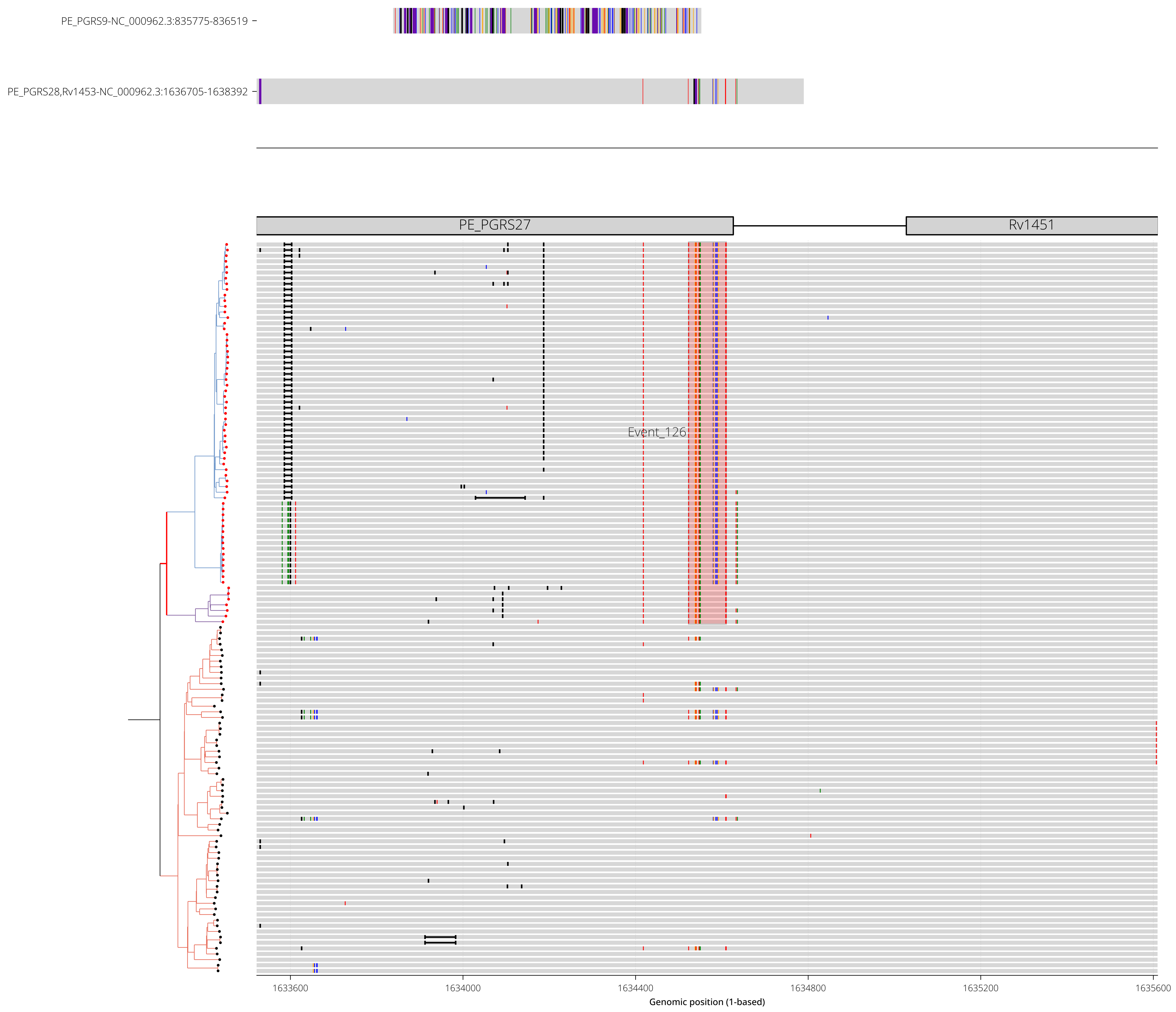

Full Phylogeny Viz + Paralog Alignments
GCE ID: Event_126

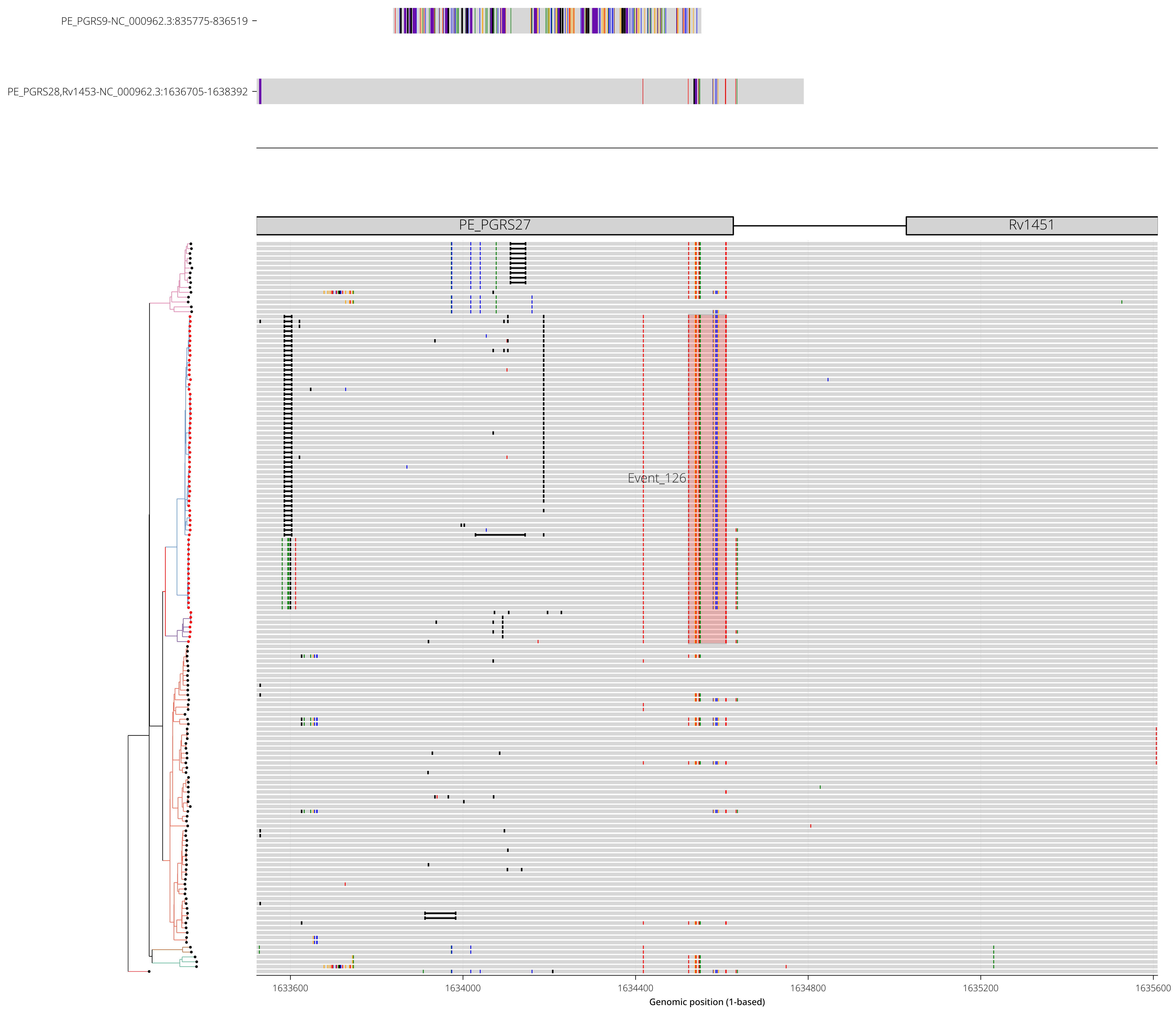

Individual GCE + Known Paralogs
GCE ID: Event_127

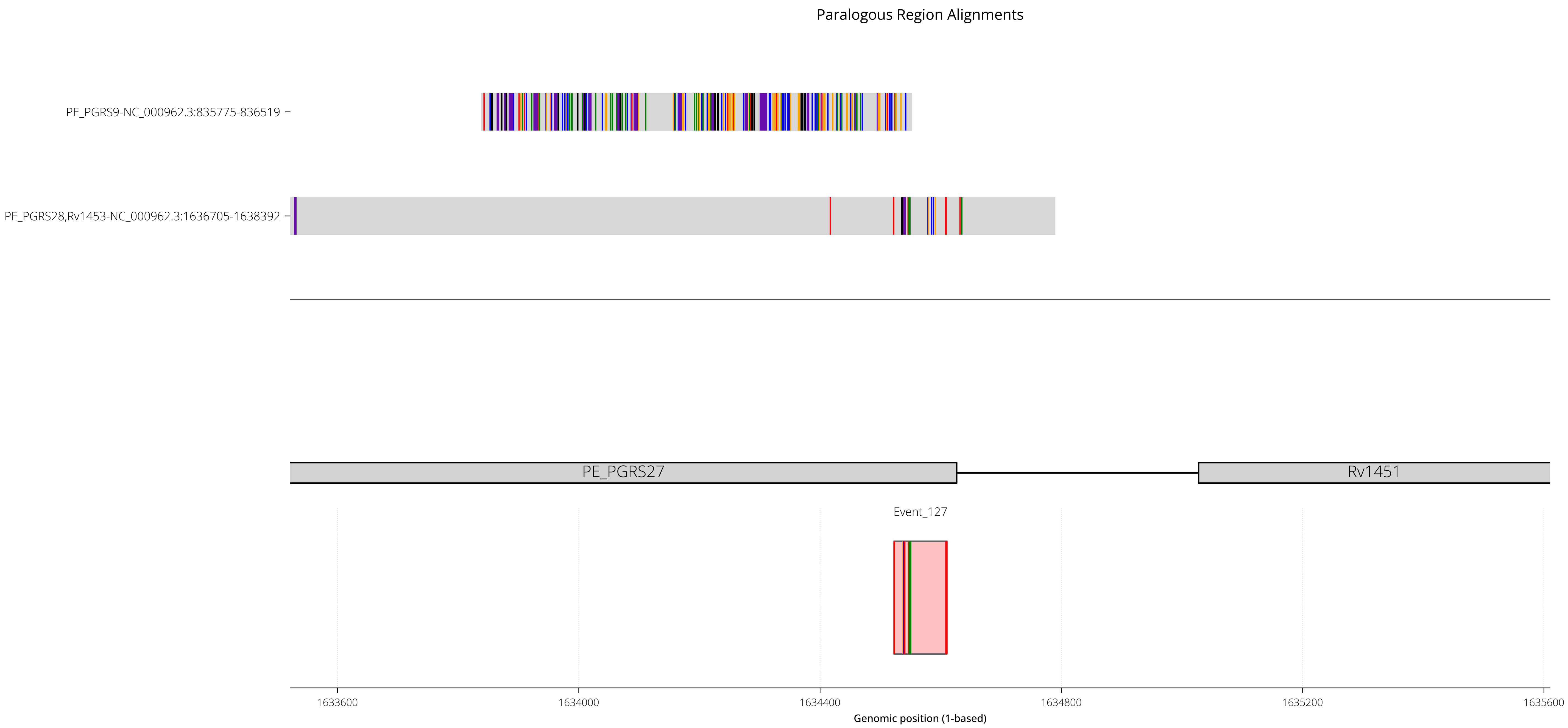

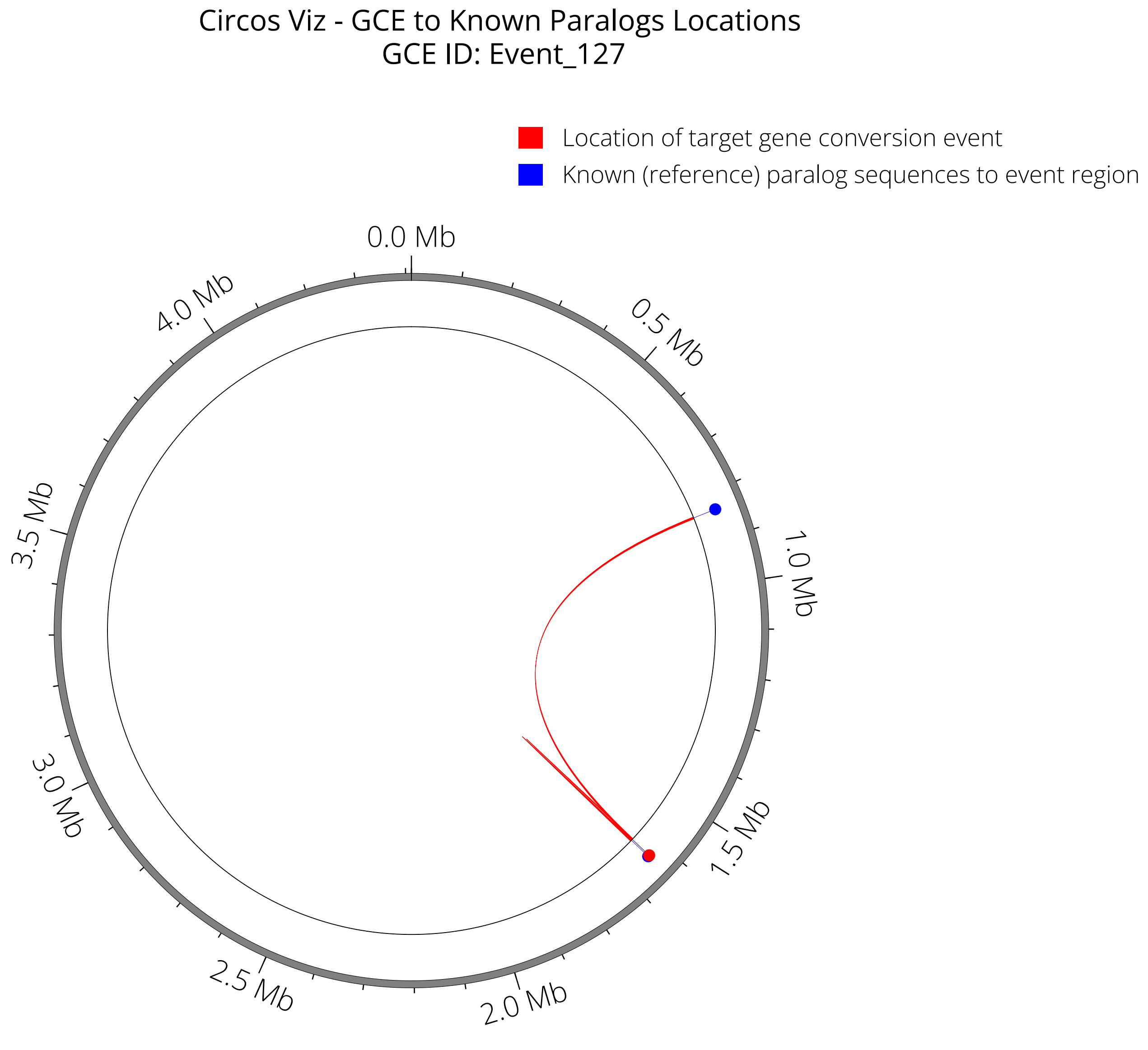
Circos Viz - GCE to Known Paralogs Locations
GCE ID: Event_127
Location of target gene conversion event
Known (reference) paralog sequences to event region
0.0 Mb
0.5 Mb
1.0 Mb
1.5 Mb
2.0 Mb
2.5 Mb
3.0 Mb
3.5 Mb
4.0 Mb

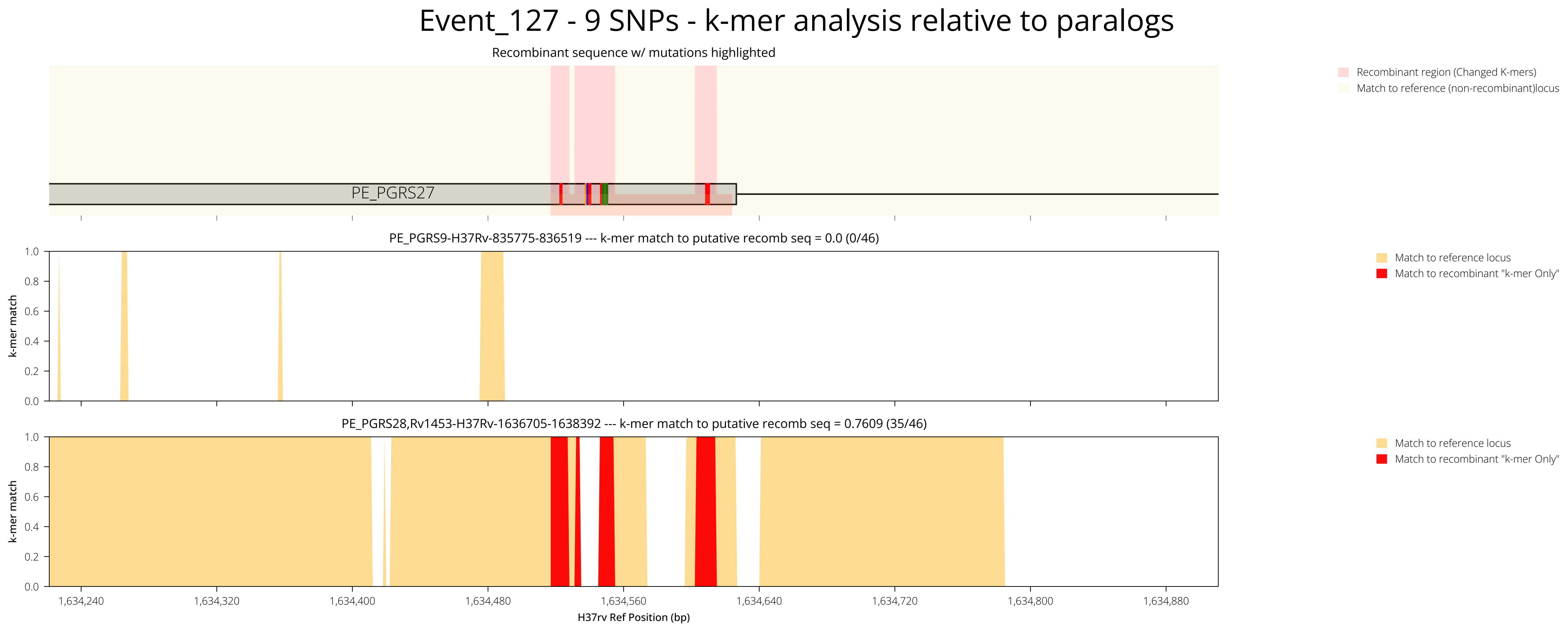
Event_127 - 9 SNPs - k-mer analysis relative to paralogs
Recombinant sequence w/ mutations highlighted
Recombinant region (Changed K-mers)
Match to reference (non-recombinant)locus
PE_PGRS27
PE_PGRS9-H37Rv-835775-836519 --- k-mer match to putative recomb seq = 0.0 (0/46)
Match to reference locus
Match to recombinant "k-mer Only"
k-mer match
1.0
0.8
0.6
0.4
0.2
0.0
PE_PGRS28,Rv1453-H37Rv-1636705-1638392 --- k-mer match to putative recomb seq = 0.7609 (35/46)
1,634,240
1,634,320
1,634,400
1,634,480
1,634,560
1,634,640
1,634,720
1,634,800
1,634,880
H37rv Ref Position (bp)

Sub-phylogeny Viz + Paralog Alignments
GCE ID: Event_127

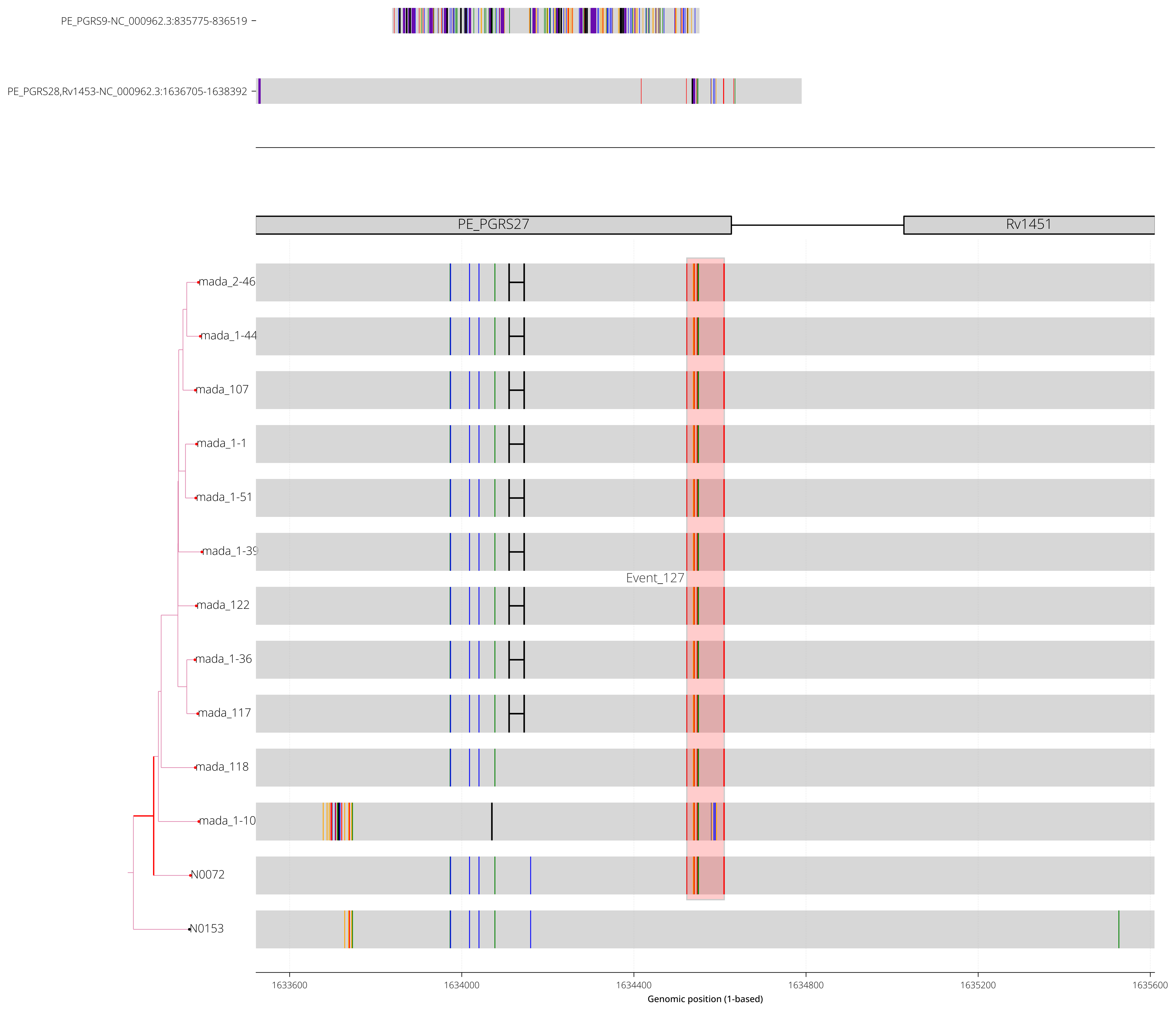

Full Phylogeny Viz + Paralog Alignments
GCE ID: Event_127

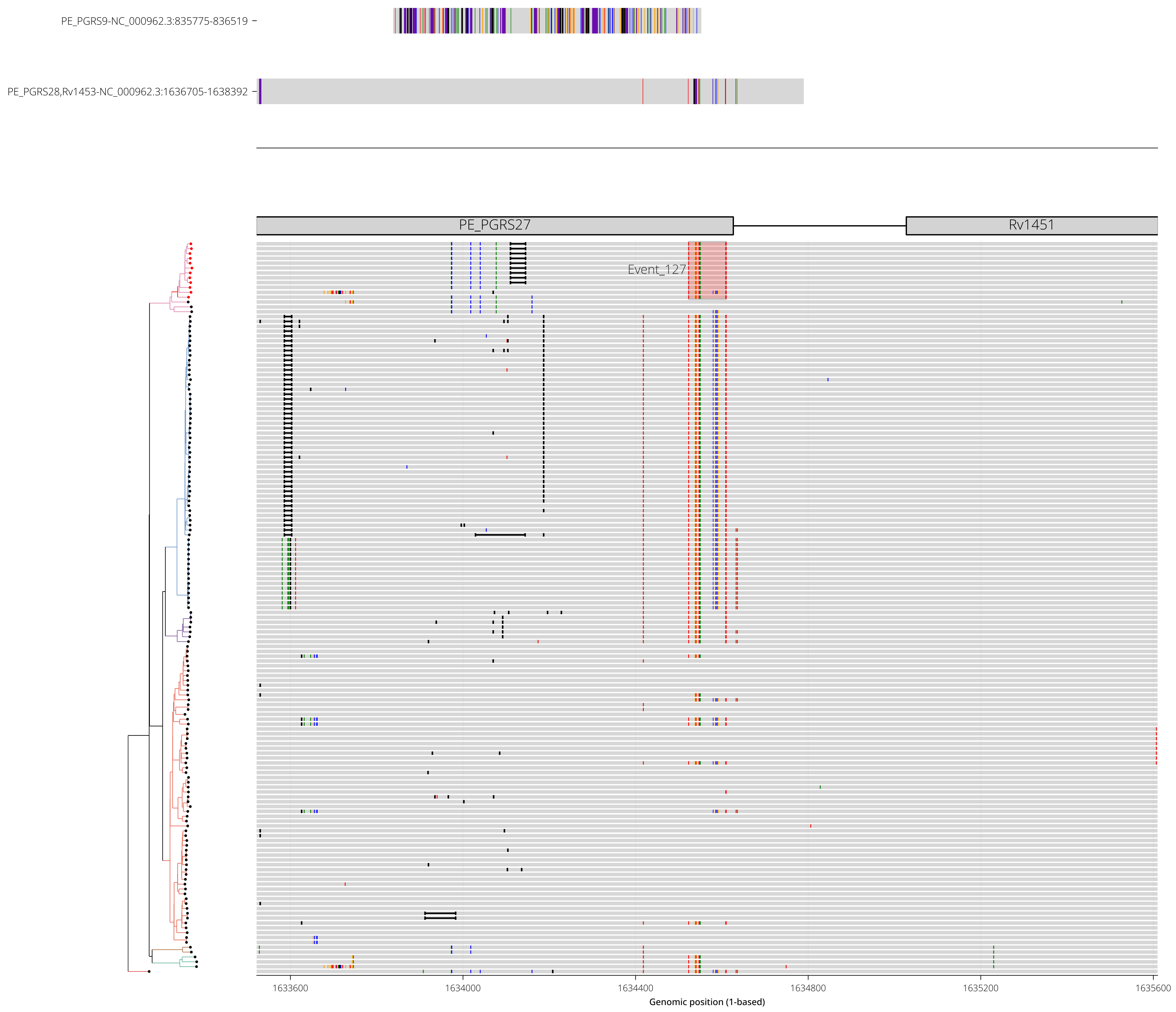

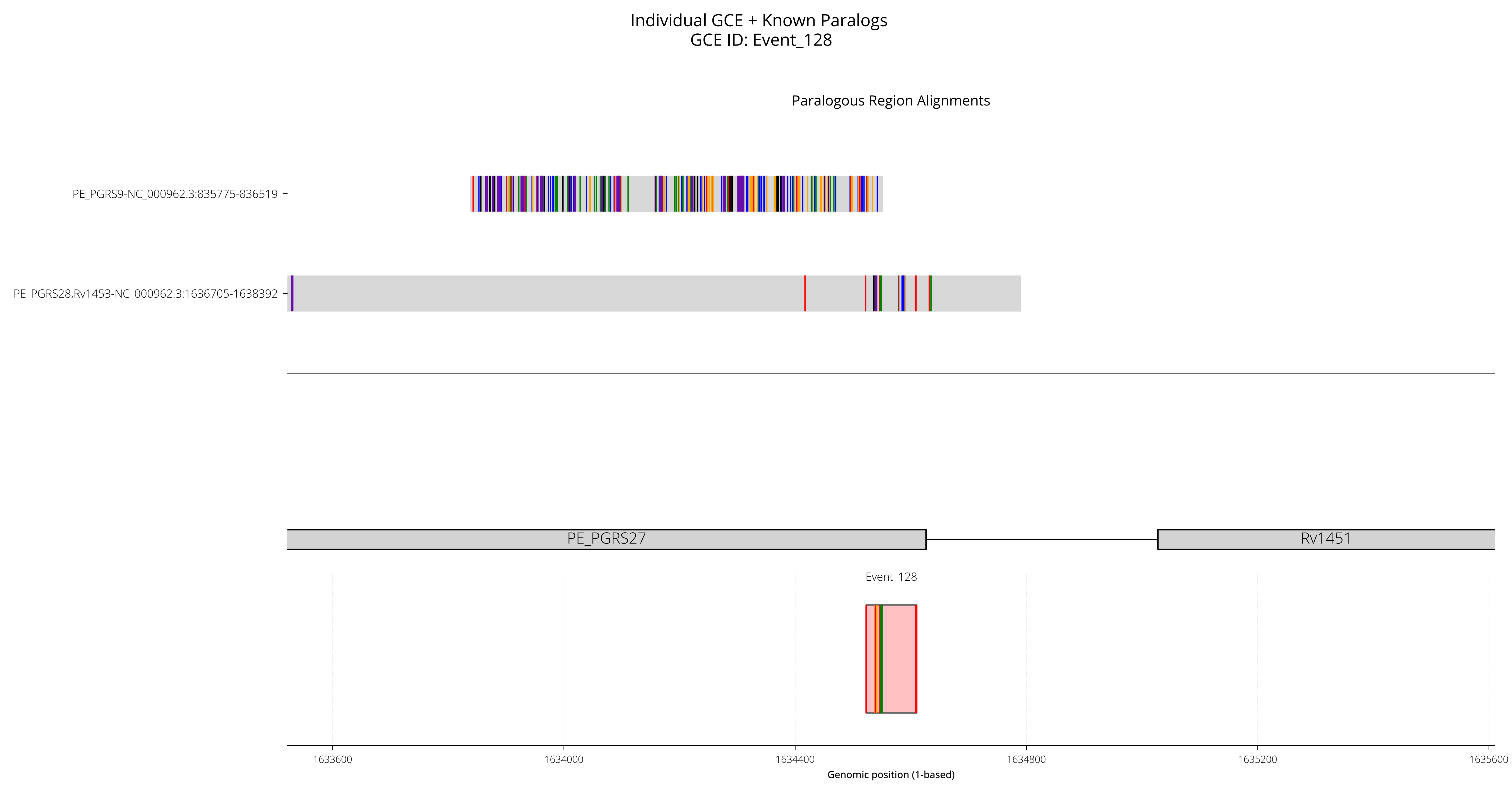
Individual GCE + Known Paralogs
GCE ID: Event_128
Paralogous Region Alignments
PE_PGRS9-NC_000962.3:835775-836519
PE_PGRS28,Rv1453-NC_000962.3:1636705-1638392
PE_PGRS27
Rv1451
Event_128
1633600
1634000
1634400
1634800
1635200
1635600
Genomic position (1-based)

Circos Viz - GCE to Known Paralogs Locations
GCE ID: Event_128

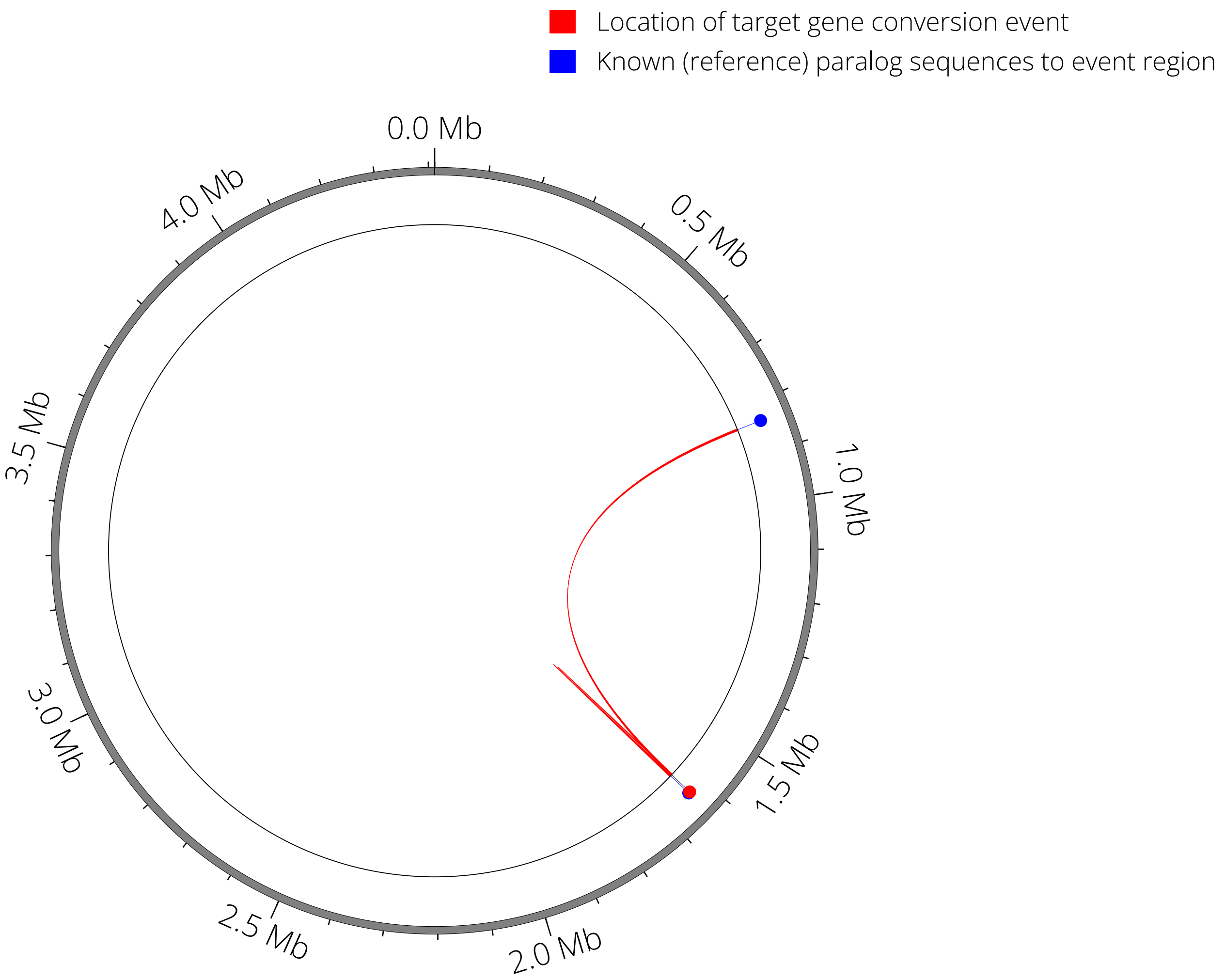

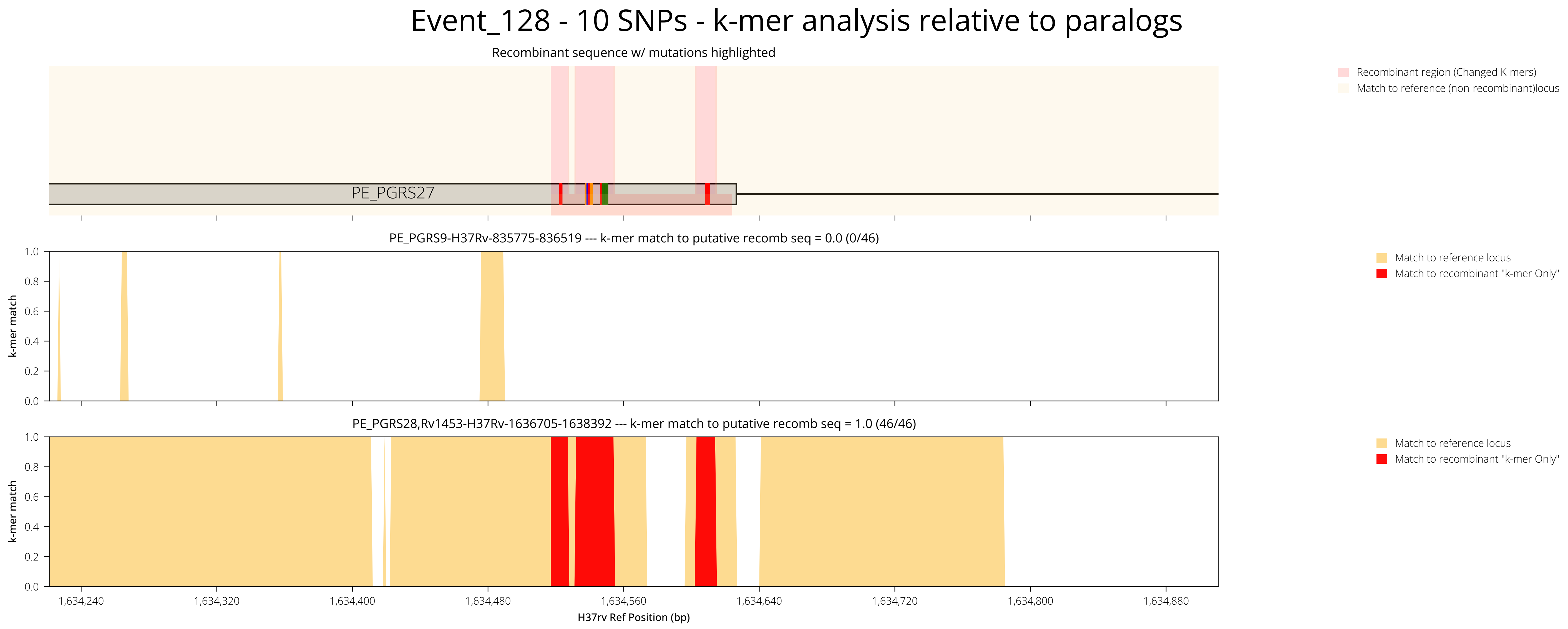
Event_128 - 10 SNPs - k-mer analysis relative to paralogs
Recombinant sequence w/ mutations highlighted
Recombinant region (Changed K-mers)
Match to reference (non-recombinant)locus
PE_PGRS27
PE_PGRS9-H37Rv-835775-836519 --- k-mer match to putative recomb seq = 0.0 (0/46)
Match to reference locus
Match to recombinant "k-mer Only"
k-mer match
1.0
0.8
0.6
0.4
0.2
0.0
PE_PGRS28,Rv1453-H37Rv-1636705-1638392 --- k-mer match to putative recomb seq = 1.0 (46/46)
Match to reference locus
Match to recombinant "k-mer Only"
k-mer match
1,634,240
1,634,320
1,634,400
1,634,480
1,634,560
1,634,640
1,634,720
1,634,800
1,634,880
H37rv Ref Position (bp)

Sub-phylogeny Viz + Paralog Alignments
GCE ID: Event_128

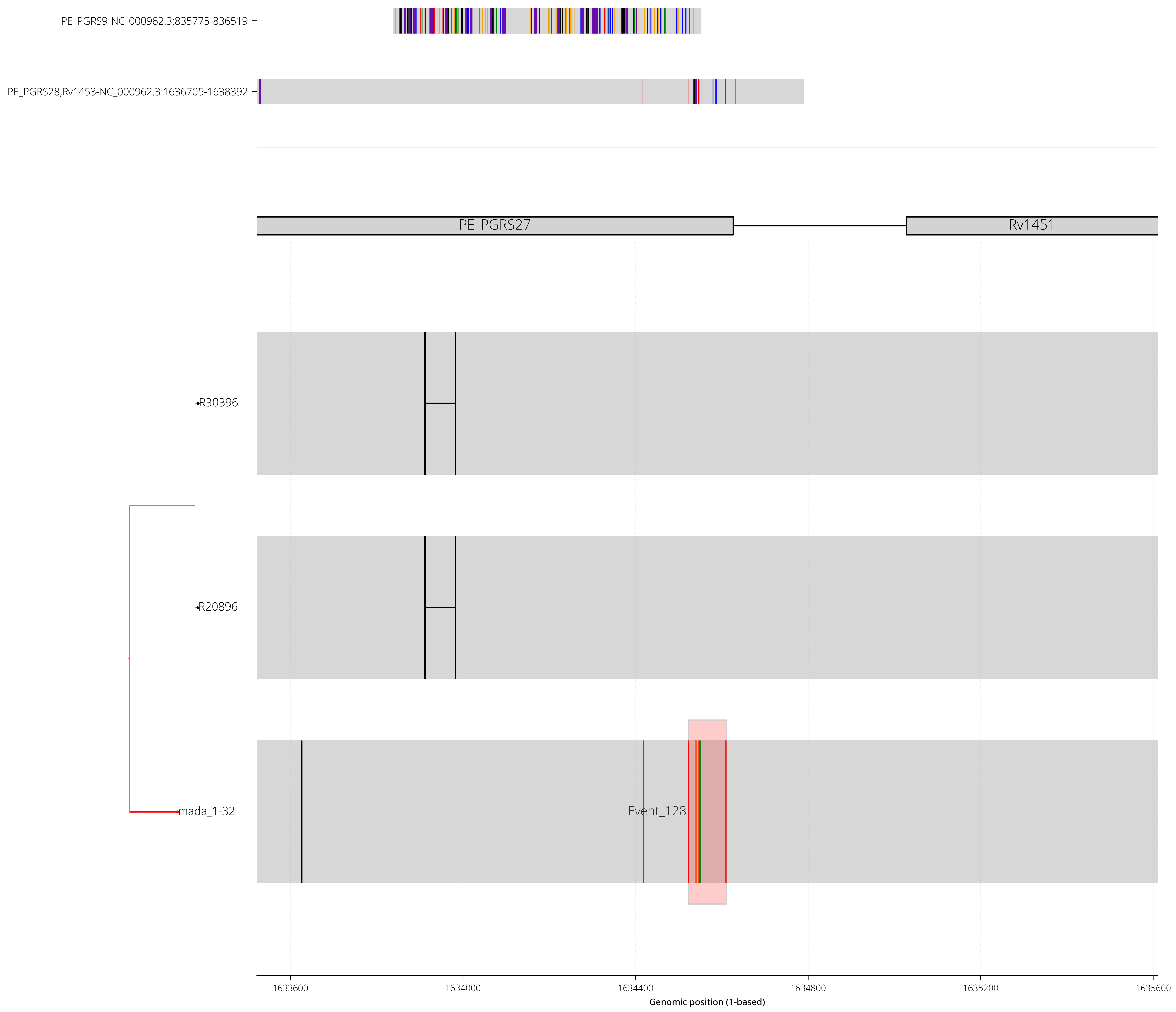

Full Phylogeny Viz + Paralog Alignments
GCE ID: Event_128

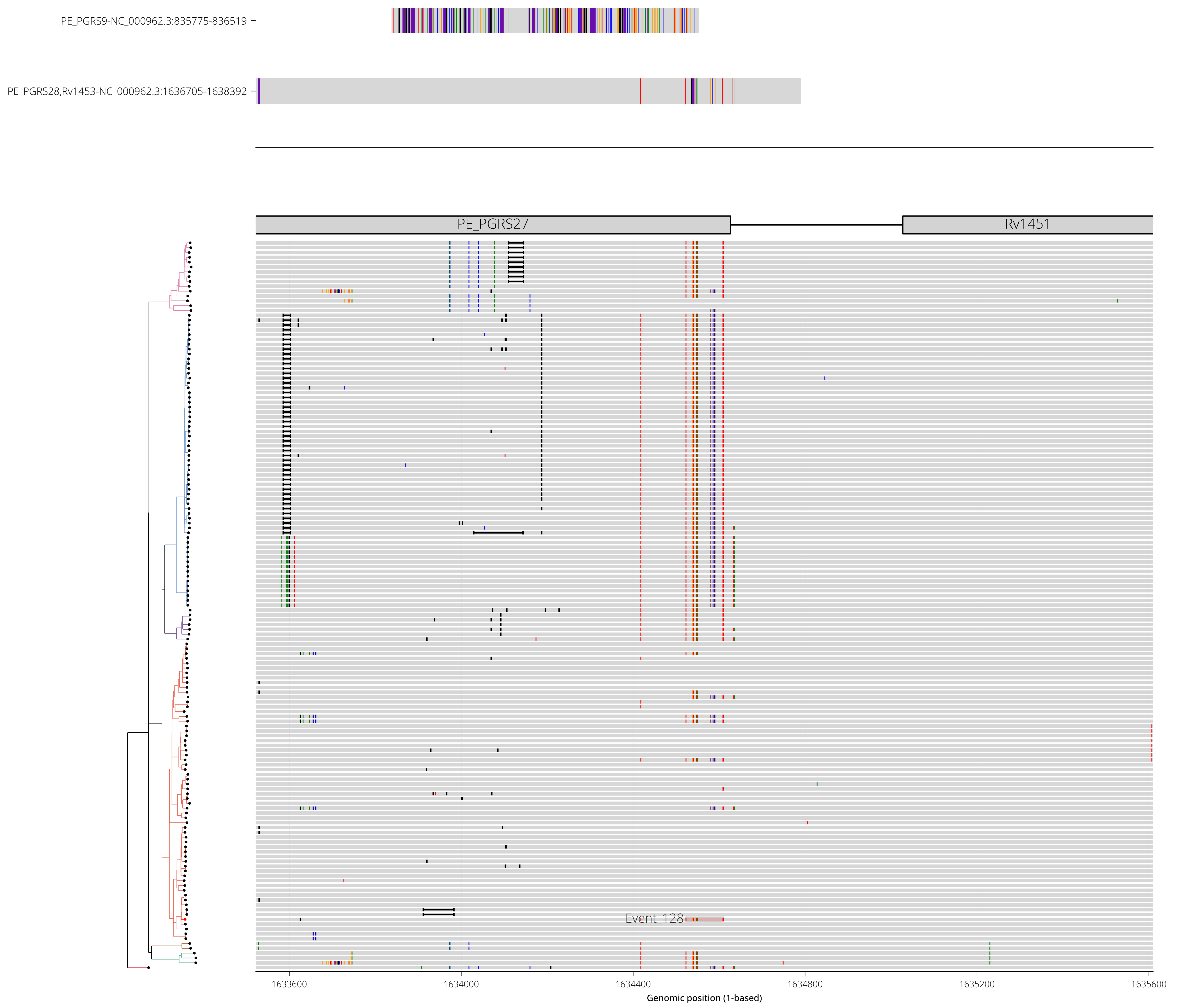

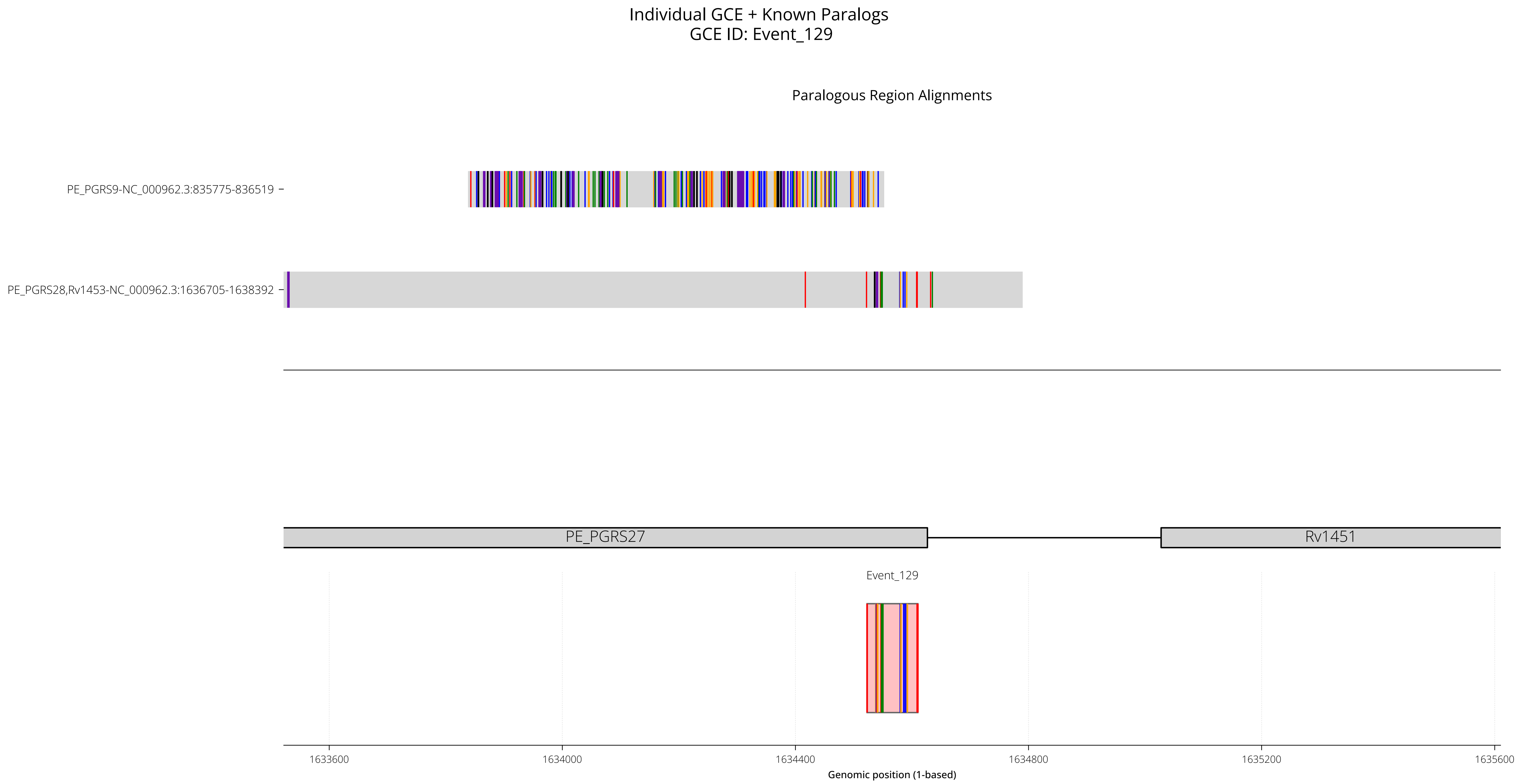
Individual GCE + Known Paralogs
GCE ID: Event_129
Paralogous Region Alignments
PE_PGRS9-NC_000962.3:835775-836519
PE_PGRS28,Rv1453-NC_000962.3:1636705-1638392
PE_PGRS27
Rv1451
Event_129
1633600
1634000
1634400
1634800
1635200
1635600
Genomic position (1-based)

Circos Viz - GCE to Known Paralogs Locations
GCE ID: Event_129

Location of target gene conversion event
Known (reference) paralog sequences to event region

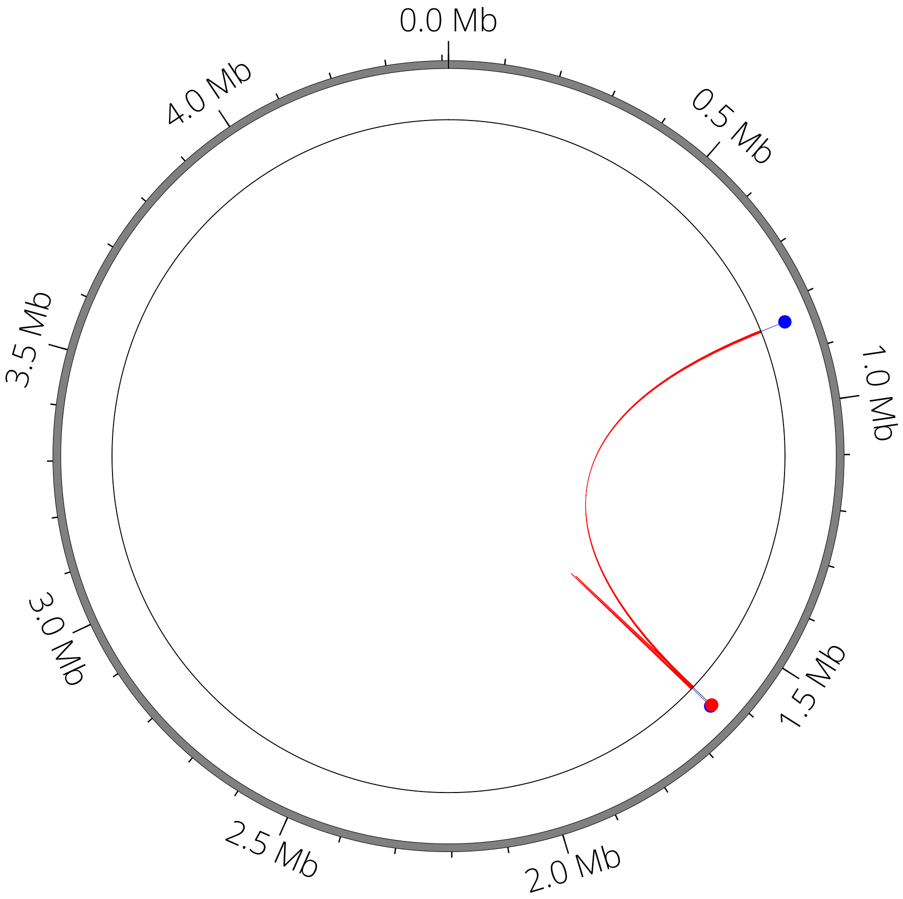

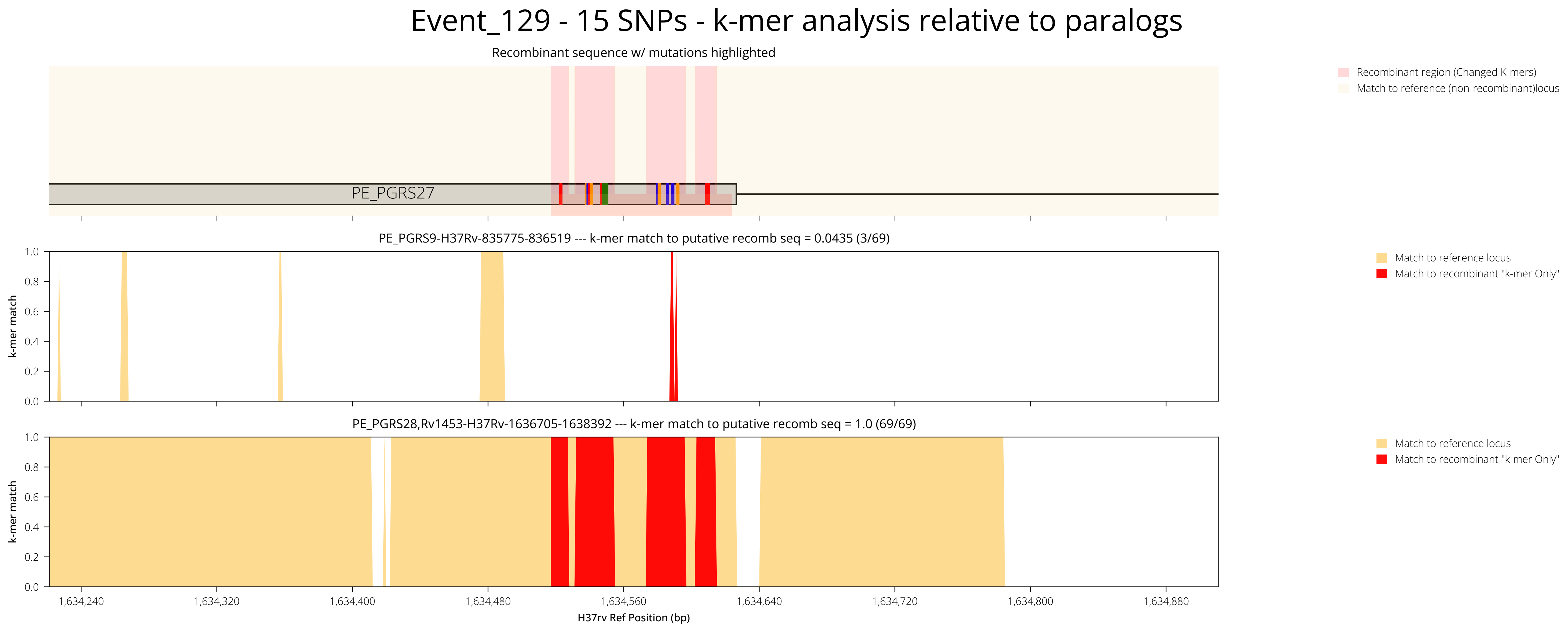

Event_129 - 15 SNPs - k-mer analysis relative to paralogs
Recombinant sequence w/ mutations highlighted
PE_PGRS27
Recombinant region (Changed K-mers)
Match to reference (non-recombinant)locus
PE_PGRS9-H37Rv-835775-836519 --- k-mer match to putative recomb seq = 0.0435 (3/69)
Match to reference locus
Match to recombinant "k-mer Only"
PE_PGRS28,Rv1453-H37Rv-1636705-1638392 --- k-mer match to putative recomb seq = 1.0 (69/69)
Match to reference locus
Match to recombinant "k-mer Only"
k-mer match
1.0
0.8
0.6
0.4
0.2
0.0
1,634,240
1,634,320
1,634,400
1,634,480
1,634,560
1,634,640
1,634,720
1,634,800
1,634,880
H37rv Ref Position (bp)

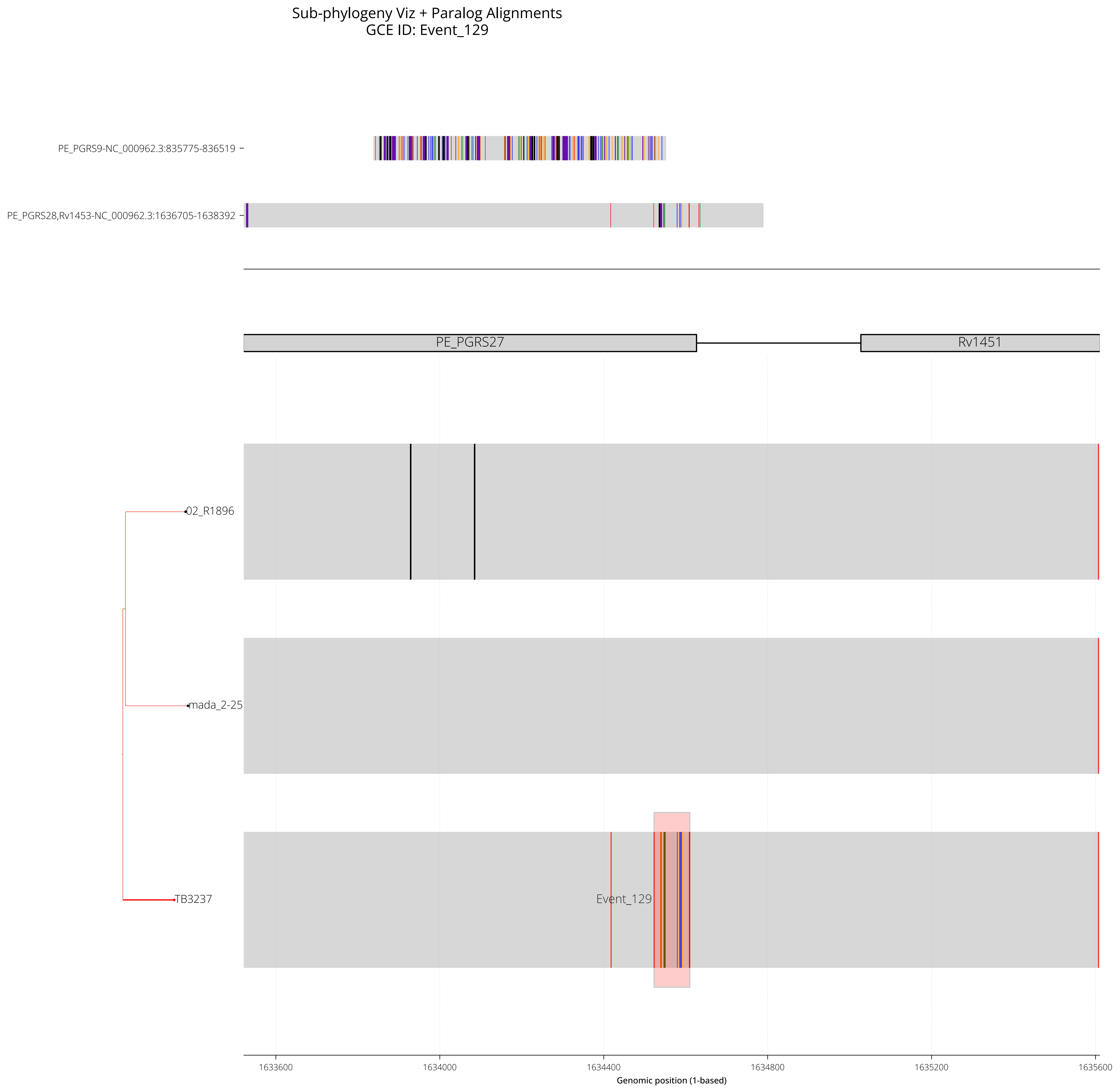

Sub-phylogeny Viz + Paralog Alignments
GCE ID: Event_129
PE_PGRS9-NC_000962.3:835775-836519
PE_PGRS28,Rv1453-NC_000962.3:1636705-1638392
PE_PGRS27
Rv1451
02_R1896
mada_2-25
TB3237
Event_129
1633600
1634000
1634400
1634800
1635200
1635600
Genomic position (1-based)

Full Phylogeny Viz + Paralog Alignments
GCE ID: Event_129

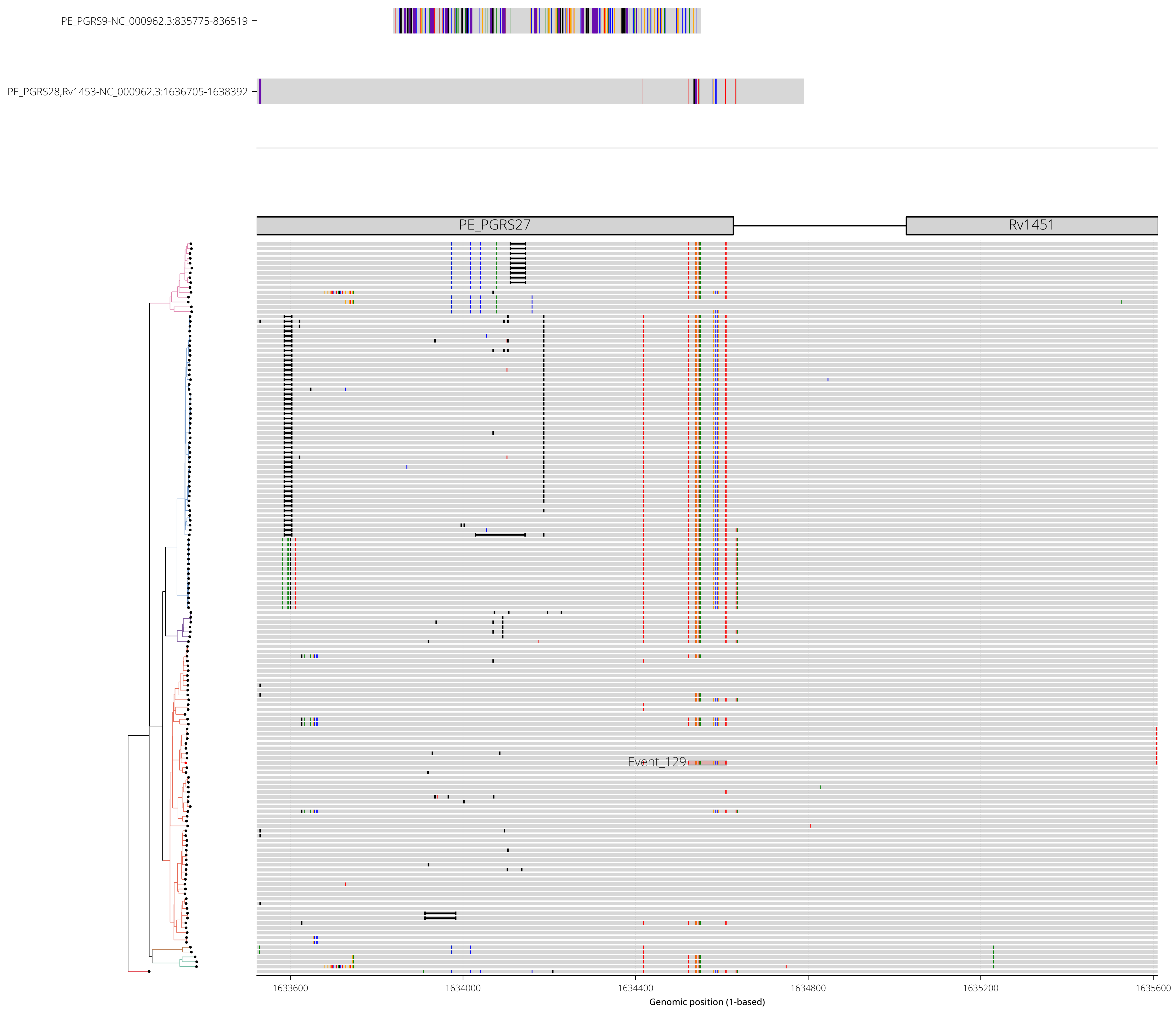

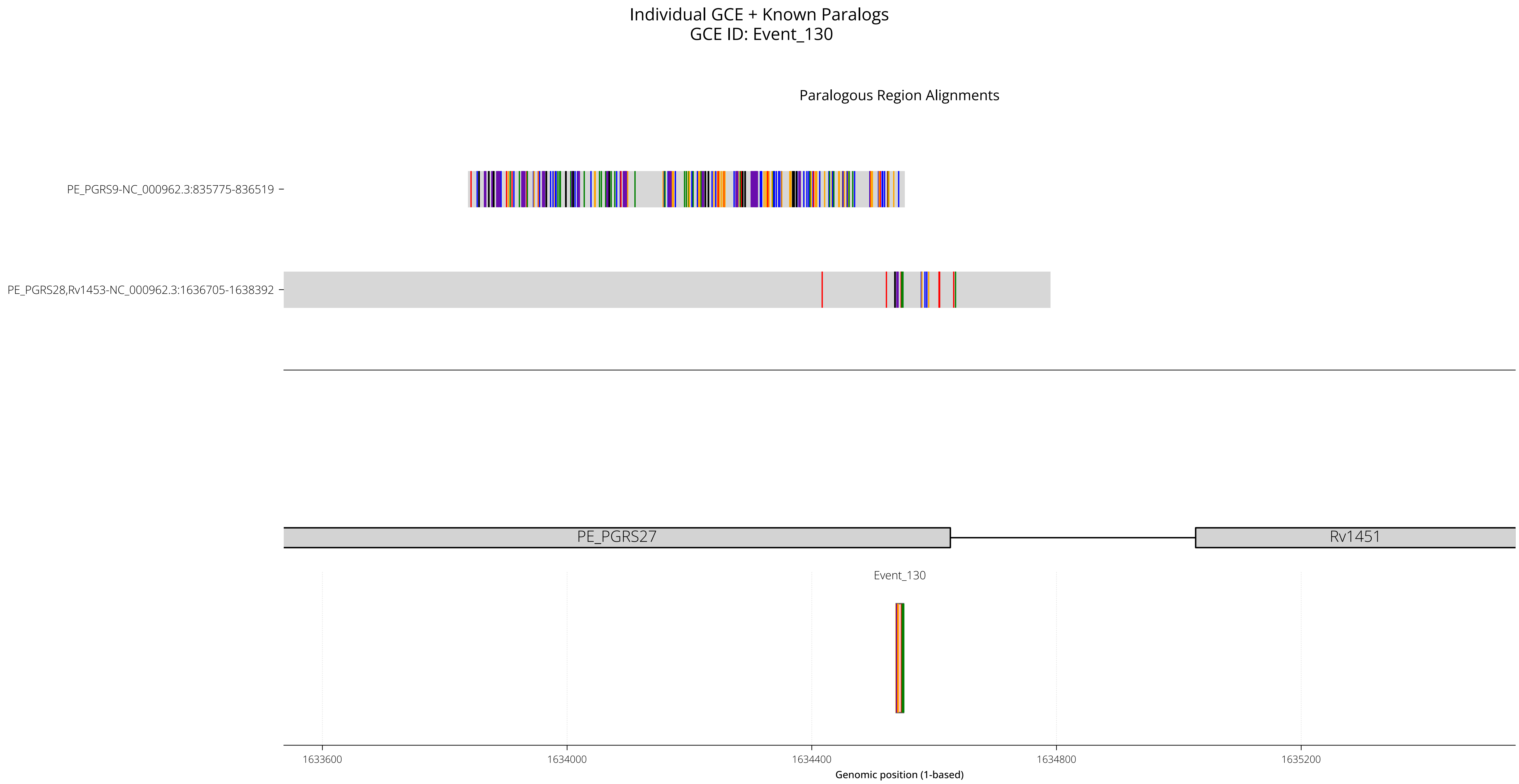

Individual GCE + Known Paralogs
GCE ID: Event_130
Paralogous Region Alignments
PE_PGRS9-NC_000962.3:835775-836519
PE_PGRS28,Rv1453-NC_000962.3:1636705-1638392
PE_PGRS27
Rv1451
Event_130
1633600
1634000
1634400
1634800
1635200
Genomic position (1-based)

Circos Viz - GCE to Known Paralogs Locations
GCE ID: Event_130

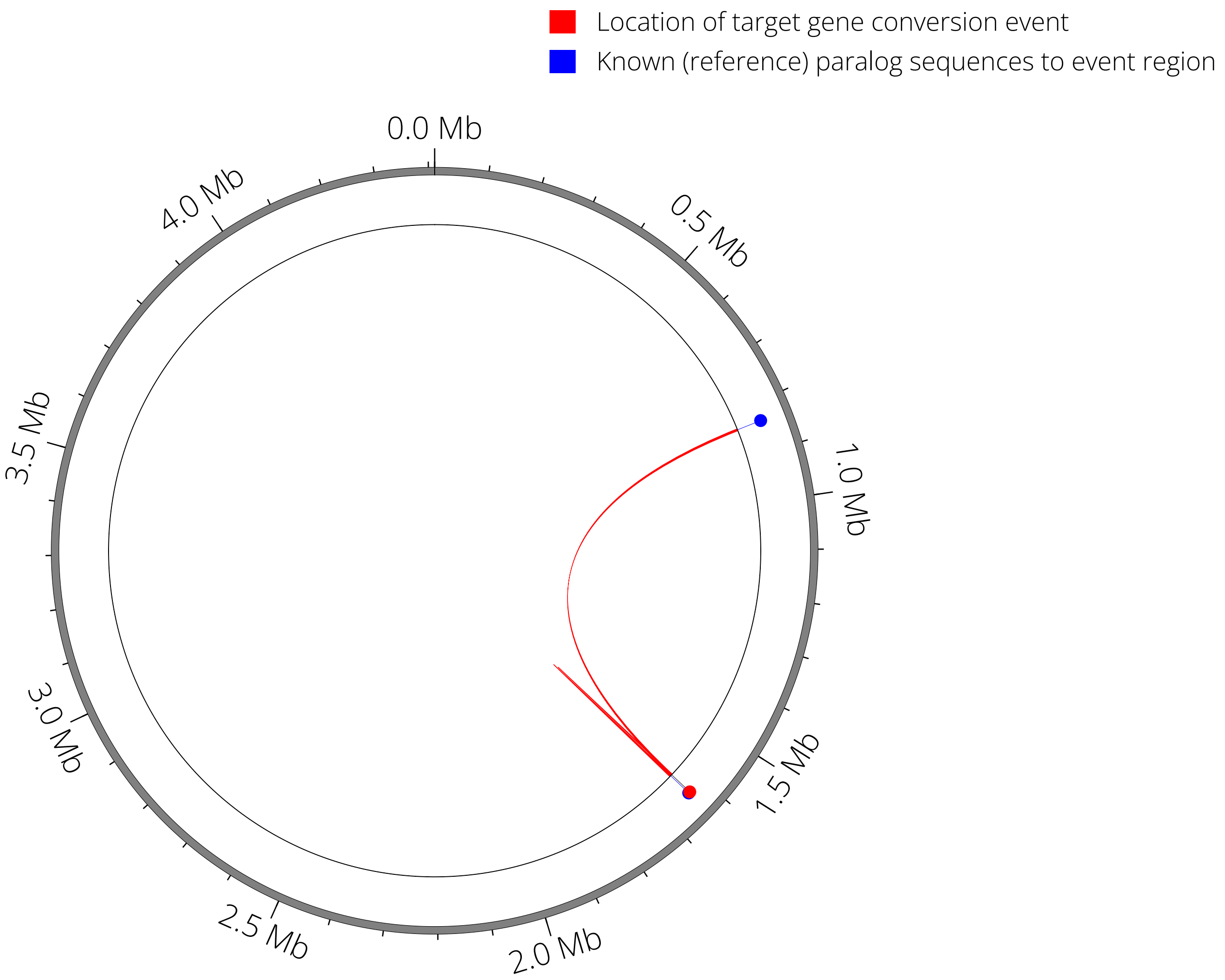

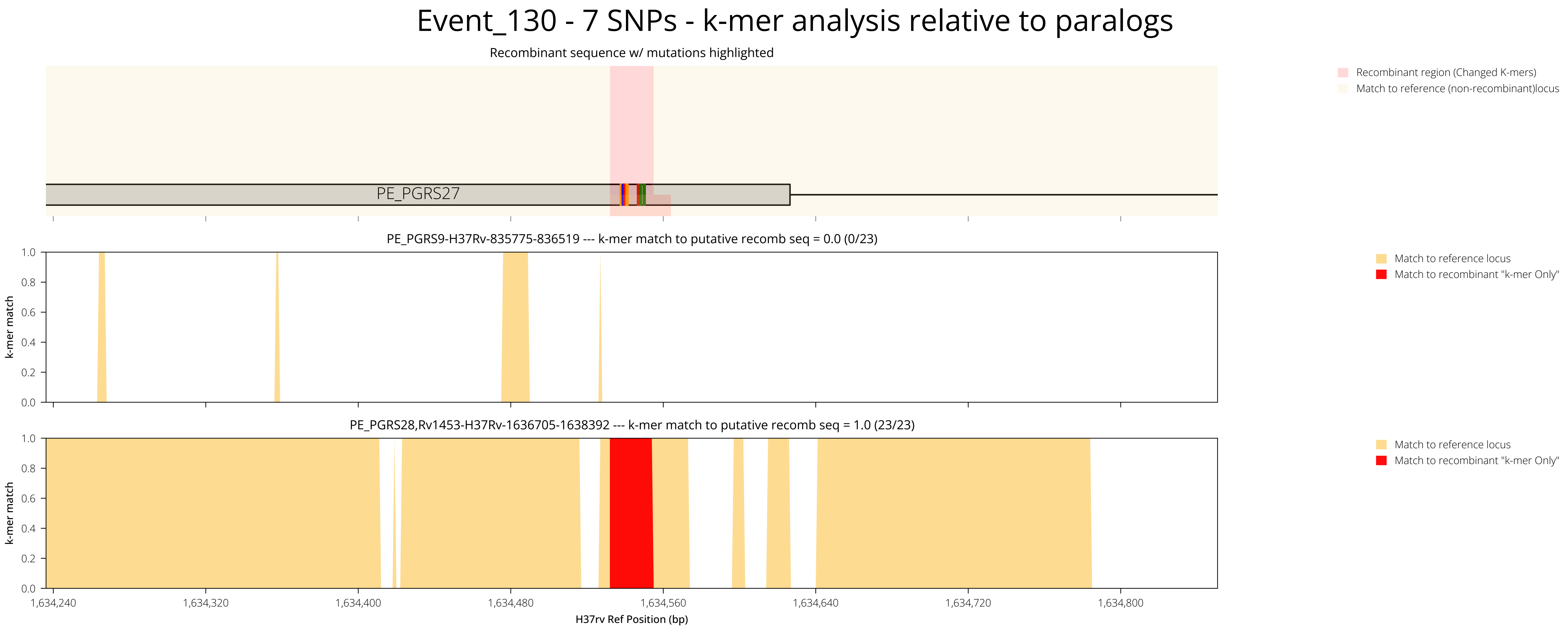

Event_130 - 7 SNPs - k-mer analysis relative to paralogs
Recombinant sequence w/ mutations highlighted
Recombinant region (Changed K-mers)
Match to reference (non-recombinant)locus
PE_PGRS27
PE_PGRS9-H37Rv-835775-836519 --- k-mer match to putative recomb seq = 0.0 (0/23)
Match to reference locus
Match to recombinant "k-mer Only"
PE_PGRS28,Rv1453-H37Rv-1636705-1638392 --- k-mer match to putative recomb seq = 1.0 (23/23)
Match to reference locus
Match to recombinant "k-mer Only"
k-mer match
1.0
0.8
0.6
0.4
0.2
0.0
1,634,240
1,634,320
1,634,400
1,634,480
1,634,560
1,634,640
1,634,720
1,634,800
H37rv Ref Position (bp)

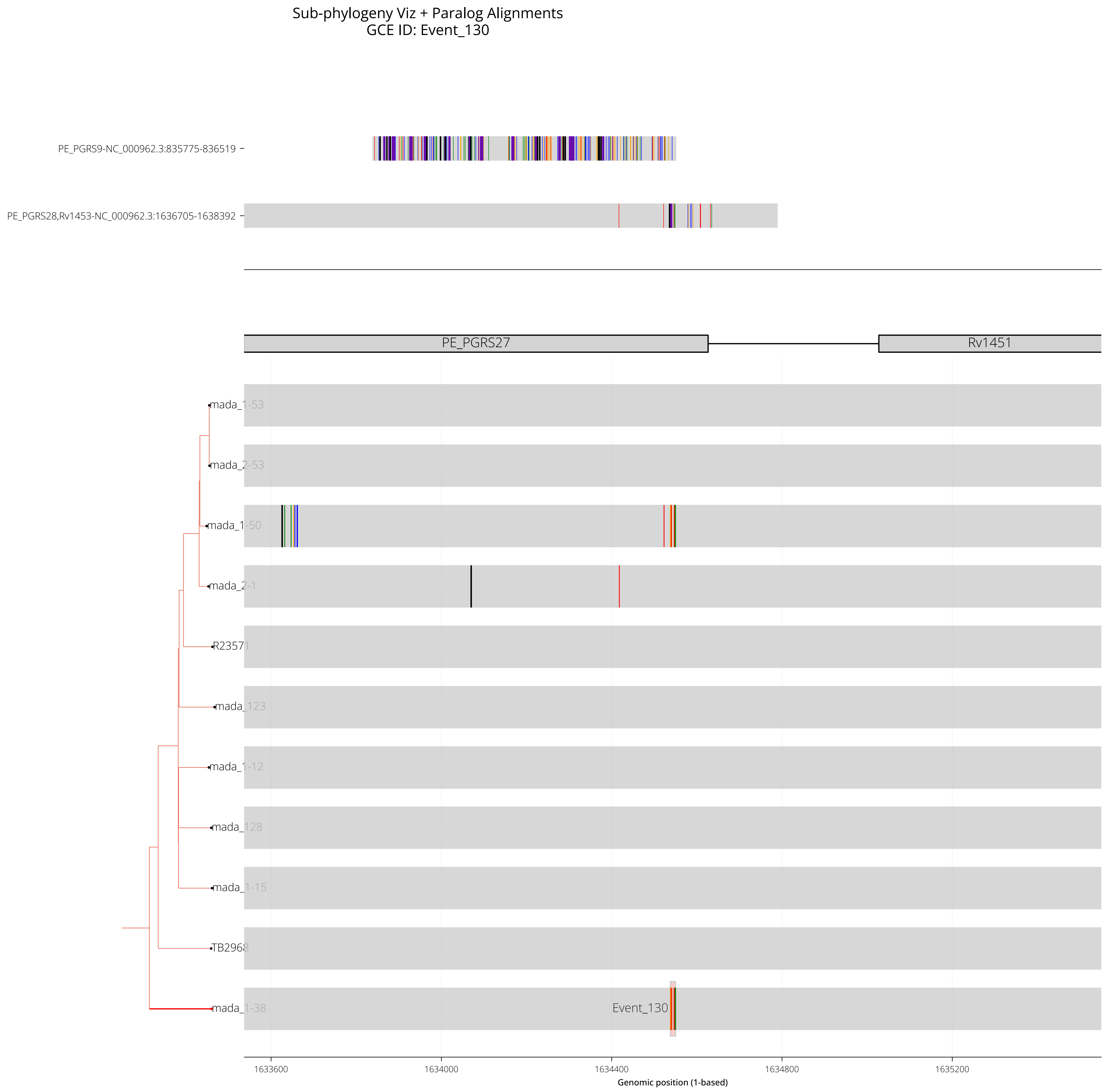

Sub-phylogeny Viz + Paralog Alignments
GCE ID: Event_130
PE_PGRS9-NC_000962.3:835775-836519
PE_PGRS28,Rv1453-NC_000962.3:1636705-1638392
PE_PGRS27
Rv1451
mada_1-53
mada_2-53
mada_1-50
mada_2-1
R23571
mada_123
mada_1-12
mada_128
mada_1-15
TB2968
mada_1-38
Event_130
1633600
1634000
1634400
1634800
1635200
Genomic position (1-based)

Full Phylogeny Viz + Paralog Alignments
GCE ID: Event_130

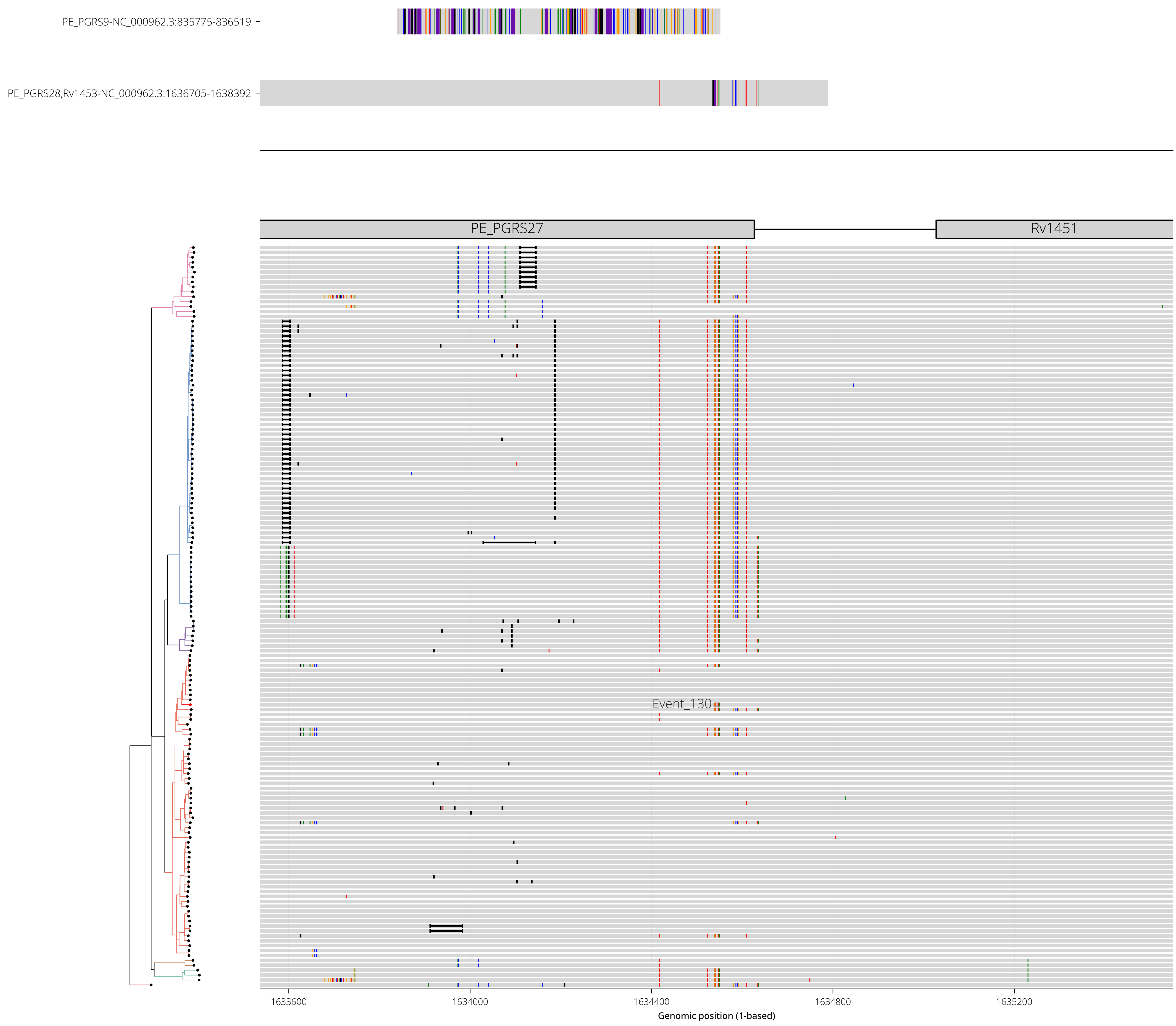

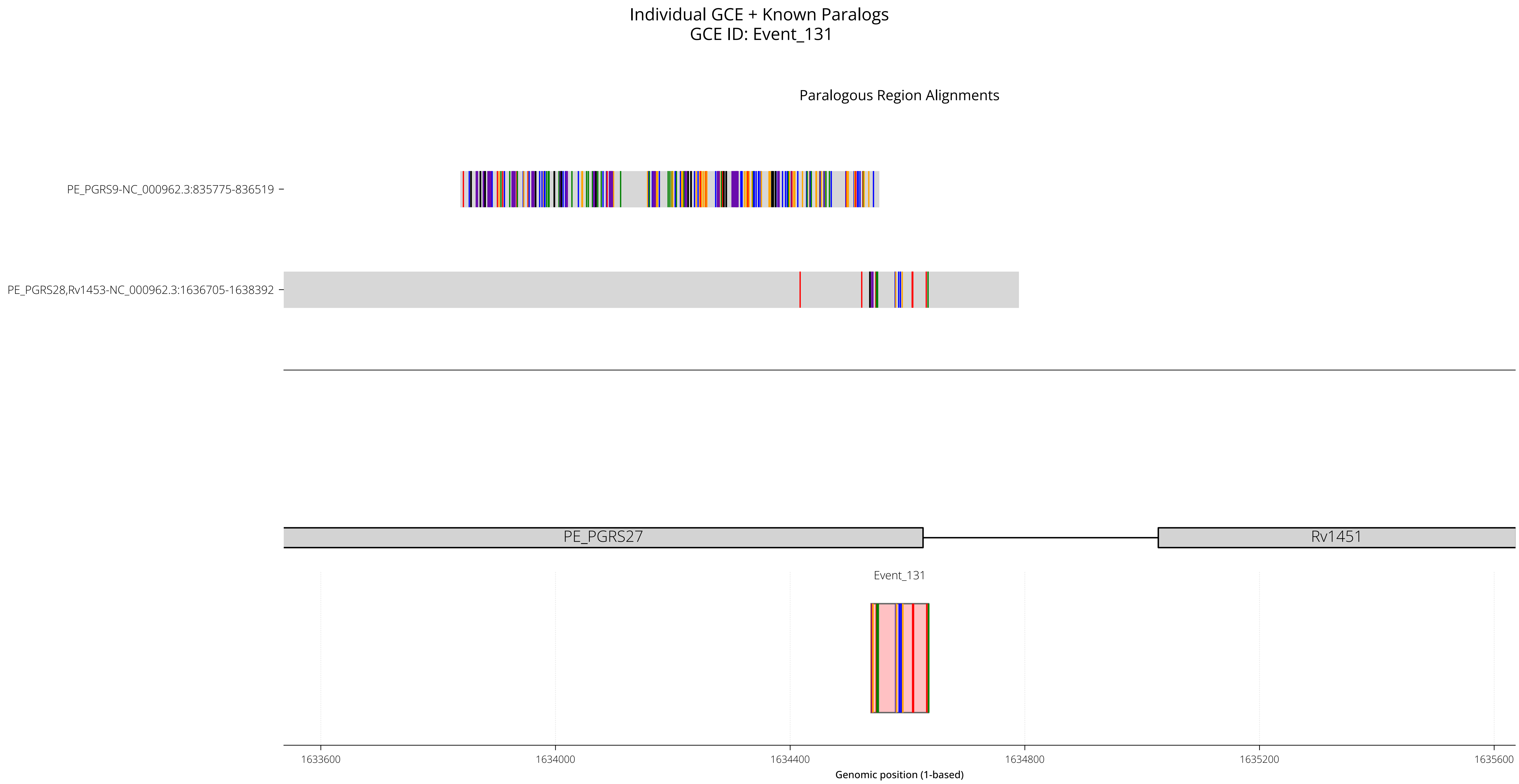
Individual GCE + Known Paralogs
GCE ID: Event_131
Paralogous Region Alignments
PE_PGRS9-NC_000962.3:835775-836519
PE_PGRS28,Rv1453-NC_000962.3:1636705-1638392
PE_PGRS27
Rv1451
Event_131
1633600
1634000
1634400
1634800
1635200
1635600
Genomic position (1-based)

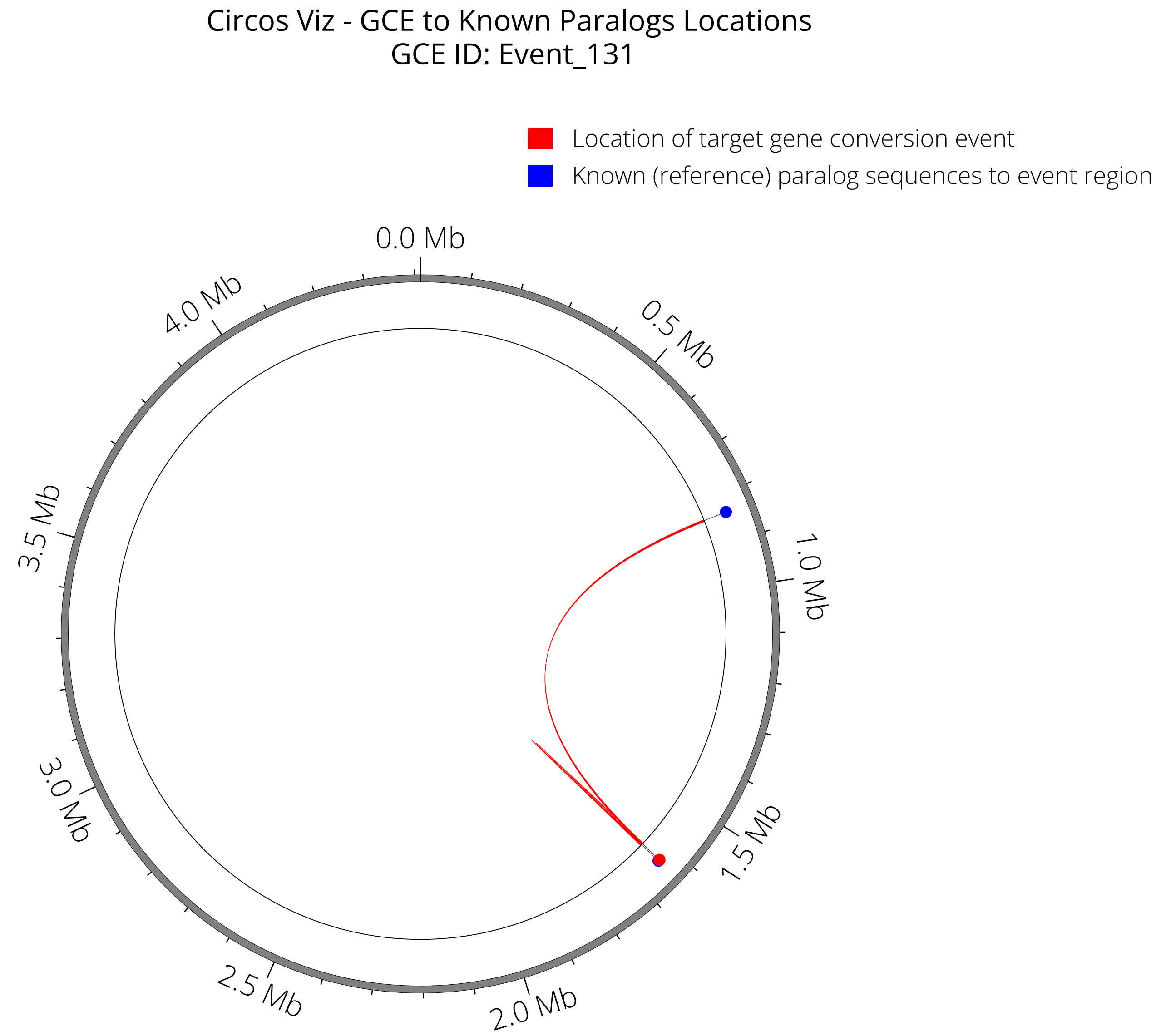
Circos Viz - GCE to Known Paralogs Locations
GCE ID: Event_131
Location of target gene conversion event
Known (reference) paralog sequences to event region
0.0 Mb
0.5 Mb
1.0 Mb
1.5 Mb
2.0 Mb
2.5 Mb
3.0 Mb
3.5 Mb
4.0 Mb

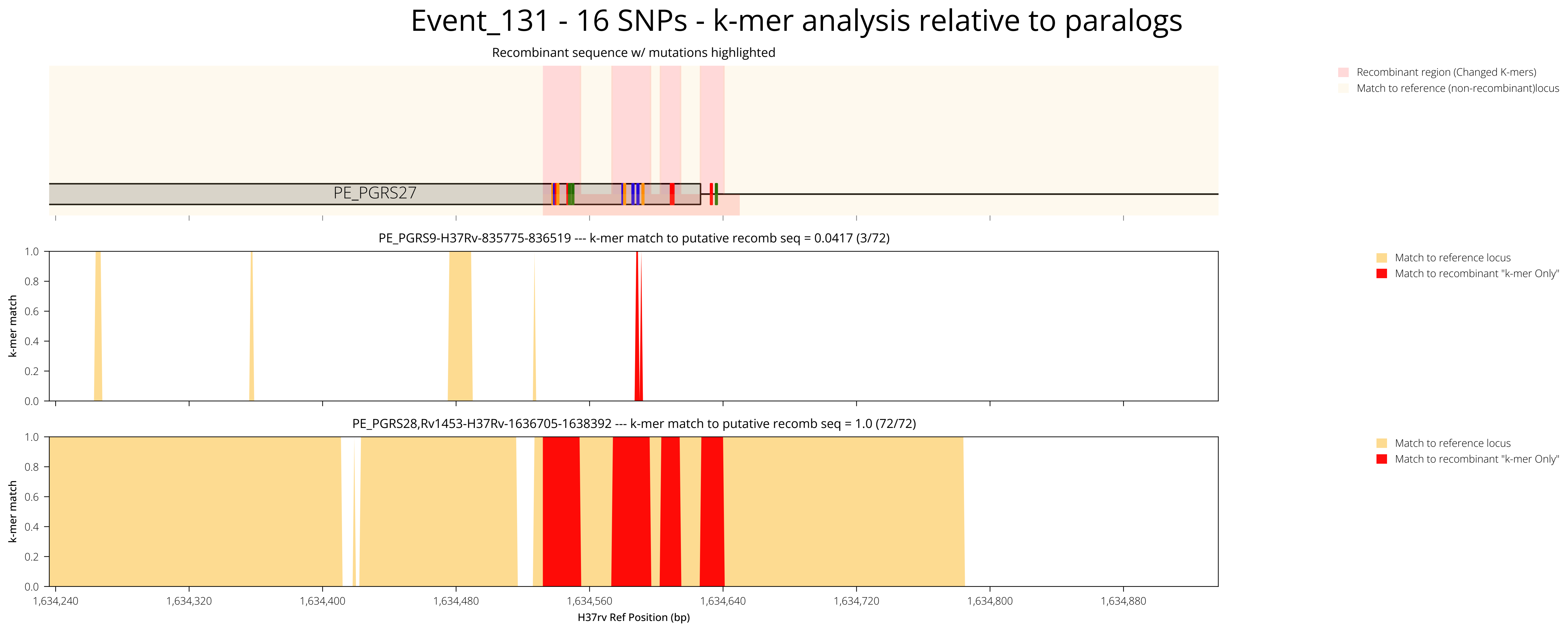

Event_131 - 16 SNPs - k-mer analysis relative to paralogs
Recombinant sequence w/ mutations highlighted
Recombinant region (Changed K-mers)
Match to reference (non-recombinant)locus
PE_PGRS27
PE_PGRS9-H37Rv-835775-836519 --- k-mer match to putative recomb seq = 0.0417 (3/72)
Match to reference locus
Match to recombinant "k-mer Only"
k-mer match
1.0
0.8
0.6
0.4
0.2
0.0
PE_PGRS28,Rv1453-H37Rv-1636705-1638392 --- k-mer match to putative recomb seq = 1.0 (72/72)
Match to reference locus
Match to recombinant "k-mer Only"
k-mer match
1,634,240
1,634,320
1,634,400
1,634,480
1,634,560
1,634,640
1,634,720
1,634,800
1,634,880
H37rv Ref Position (bp)

Sub-phylogeny Viz + Paralog Alignments
GCE ID: Event_131

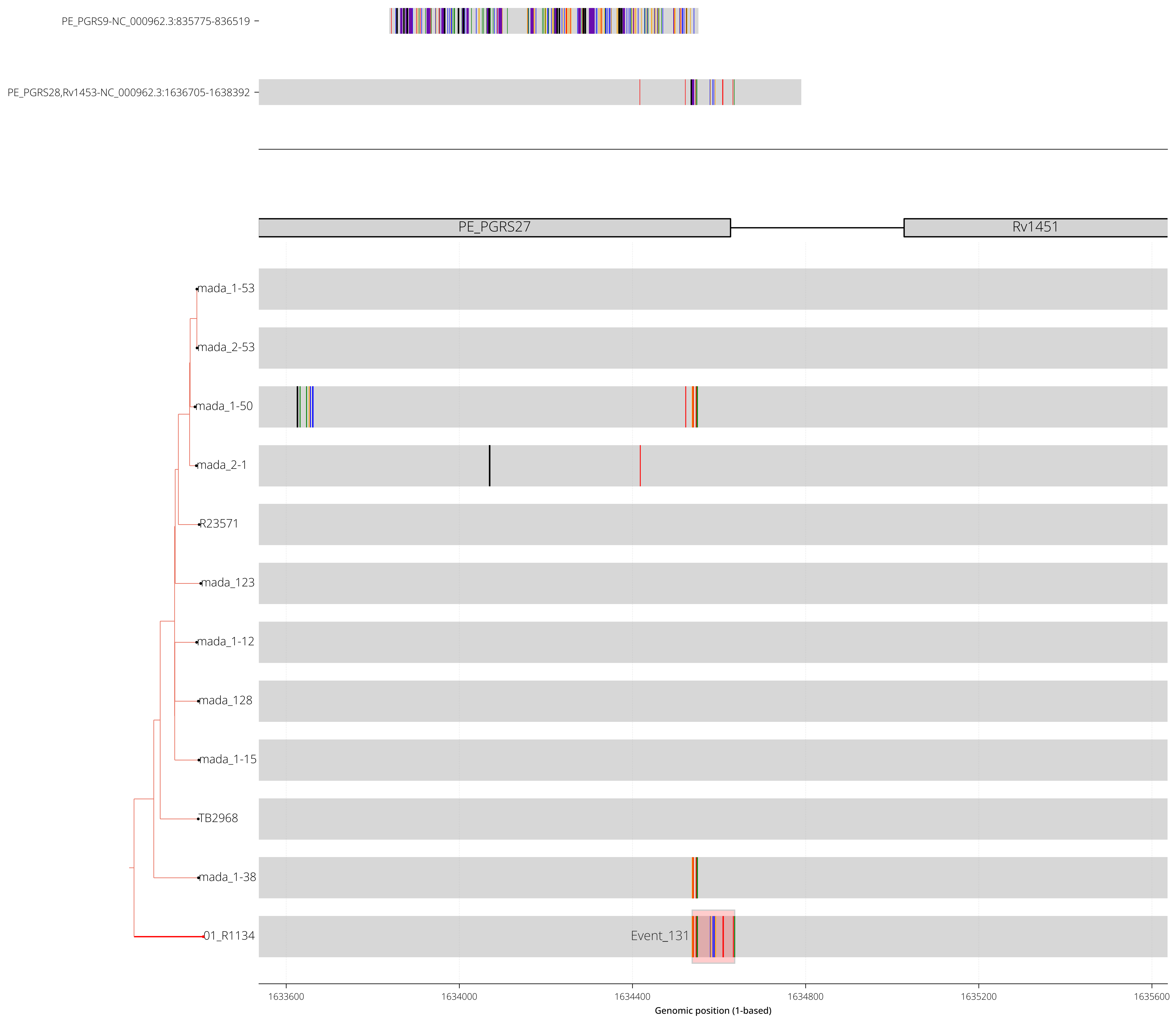

Full Phylogeny Viz + Paralog Alignments
GCE ID: Event_131

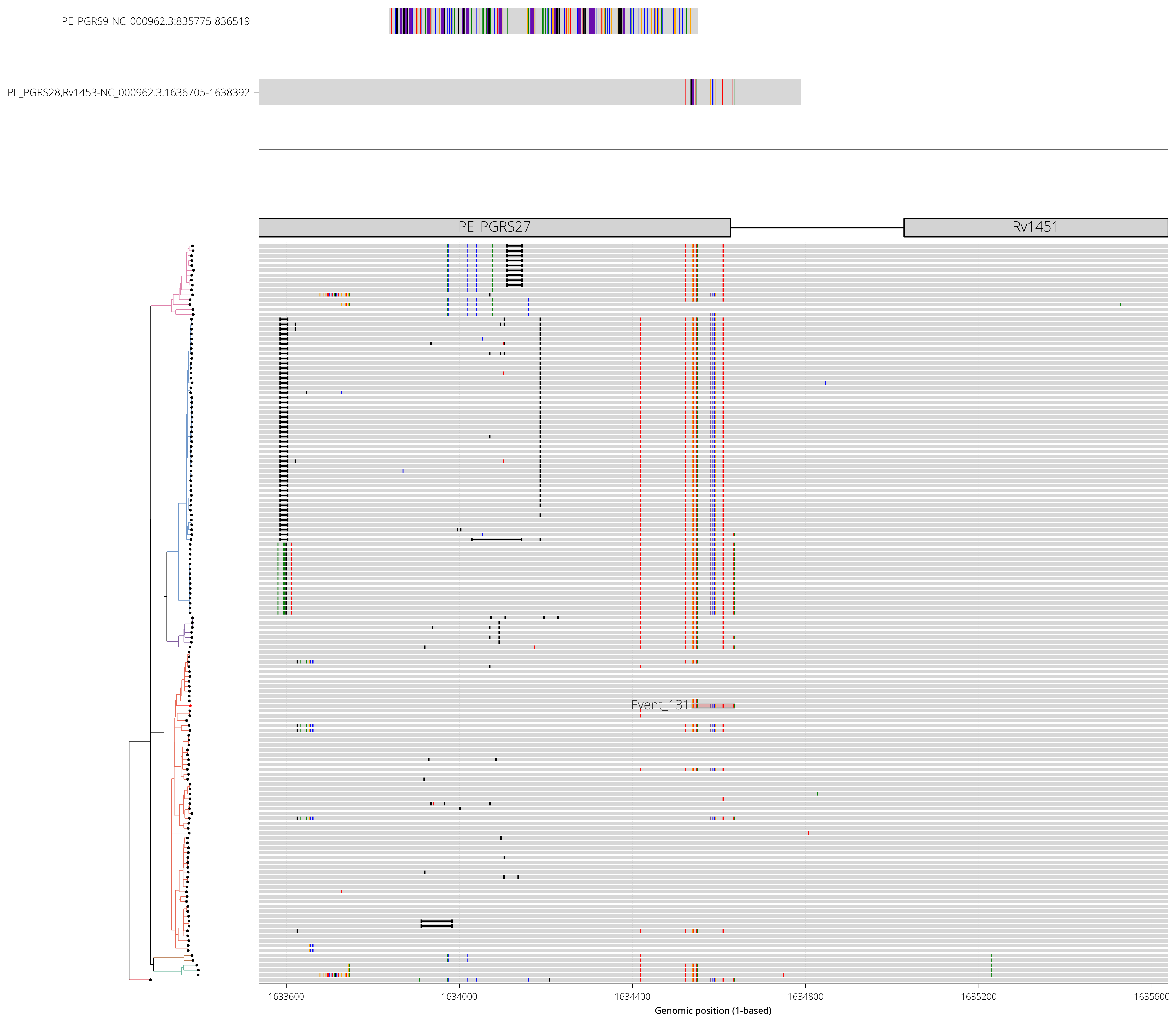

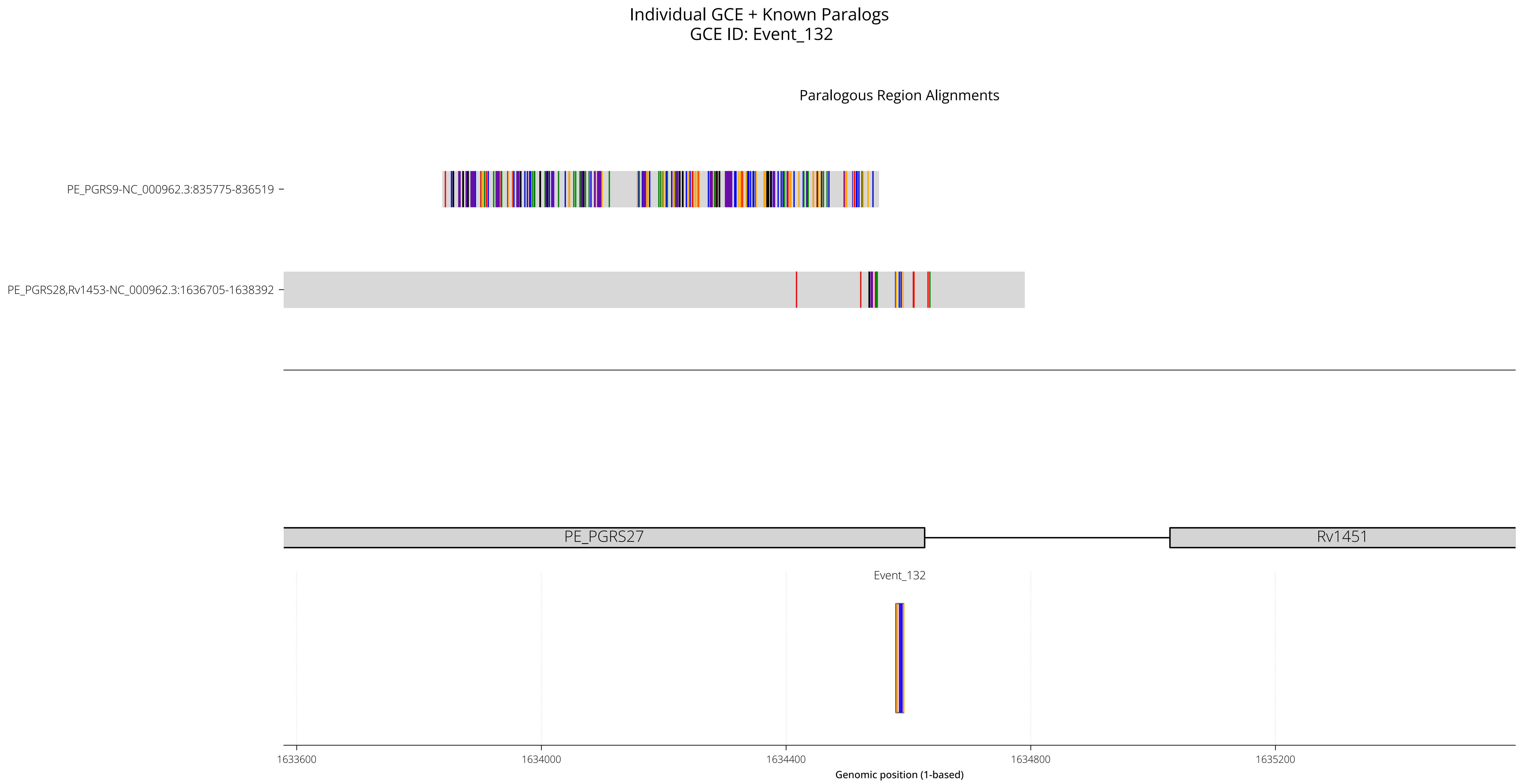

Individual GCE + Known Paralogs
GCE ID: Event_132
Paralogous Region Alignments
PE_PGRS9-NC_000962.3:835775-836519
PE_PGRS28,Rv1453-NC_000962.3:1636705-1638392
PE_PGRS27
Rv1451
Event_132
1633600
1634000
1634400
1634800
1635200
Genomic position (1-based)

Circos Viz - GCE to Known Paralogs Locations
GCE ID: Event_132

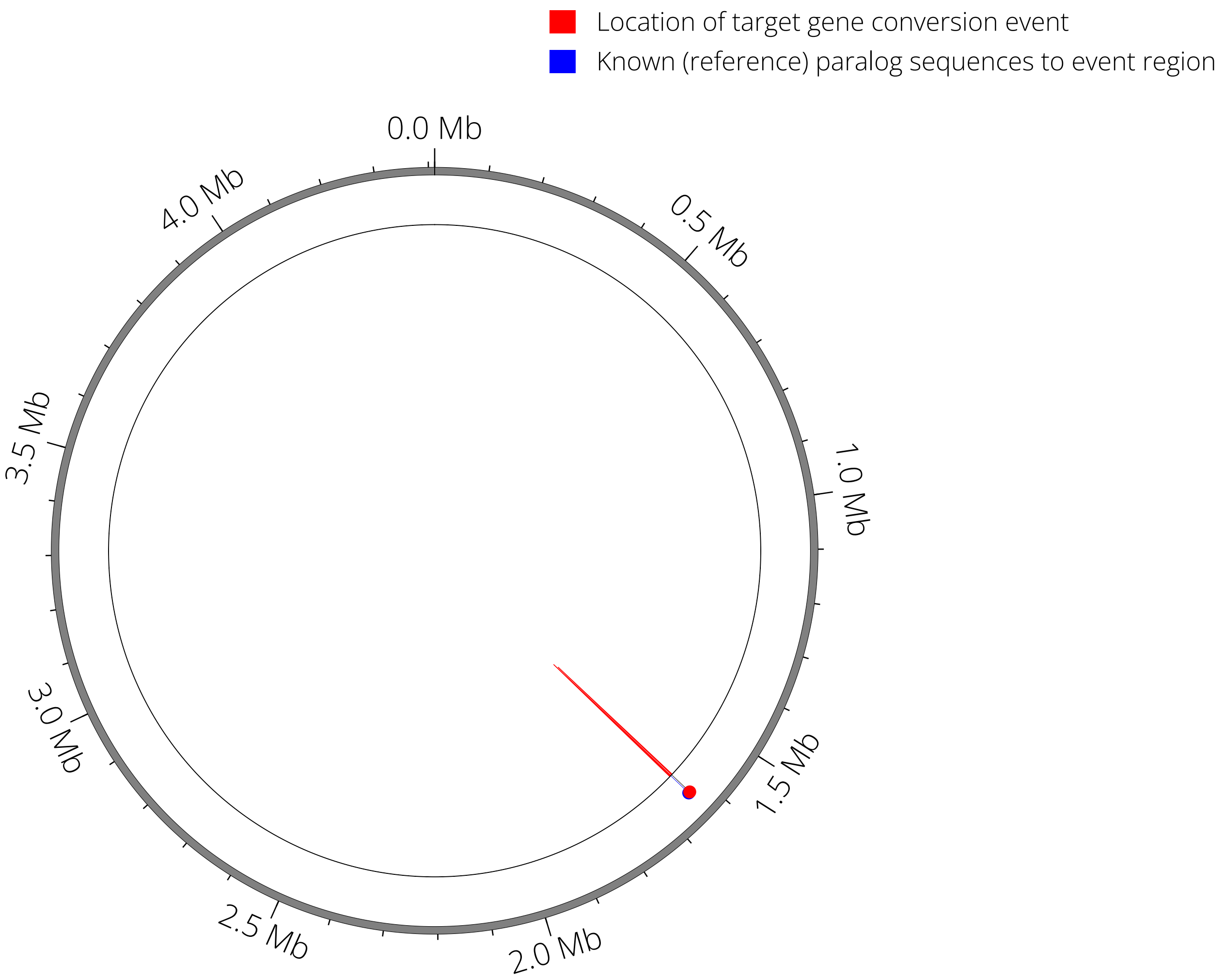

# Event_132 - 5 SNPs - k-mer analysis relative to paralogs

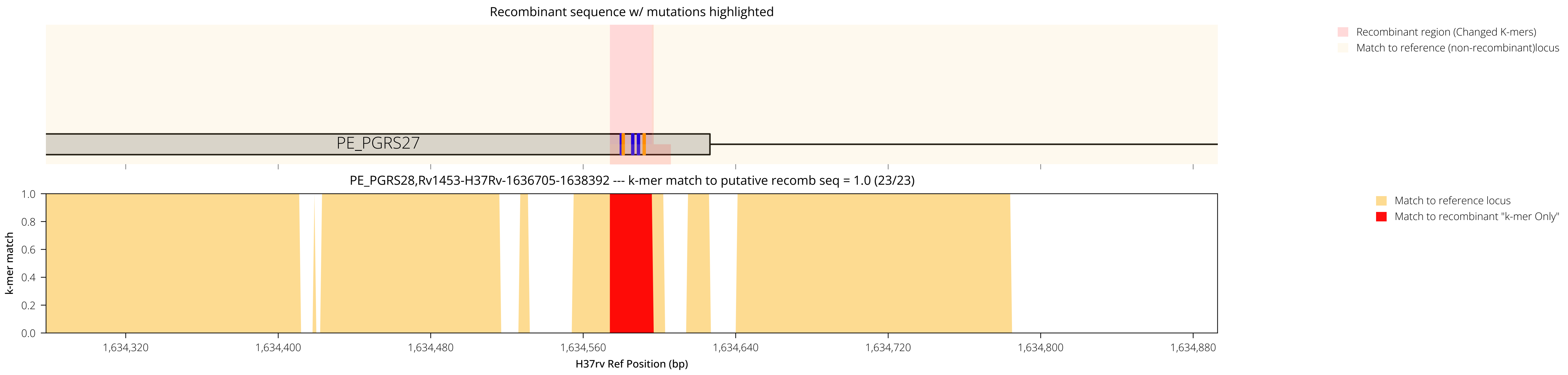

Sub-phylogeny Viz + Paralog Alignments
GCE ID: Event_132

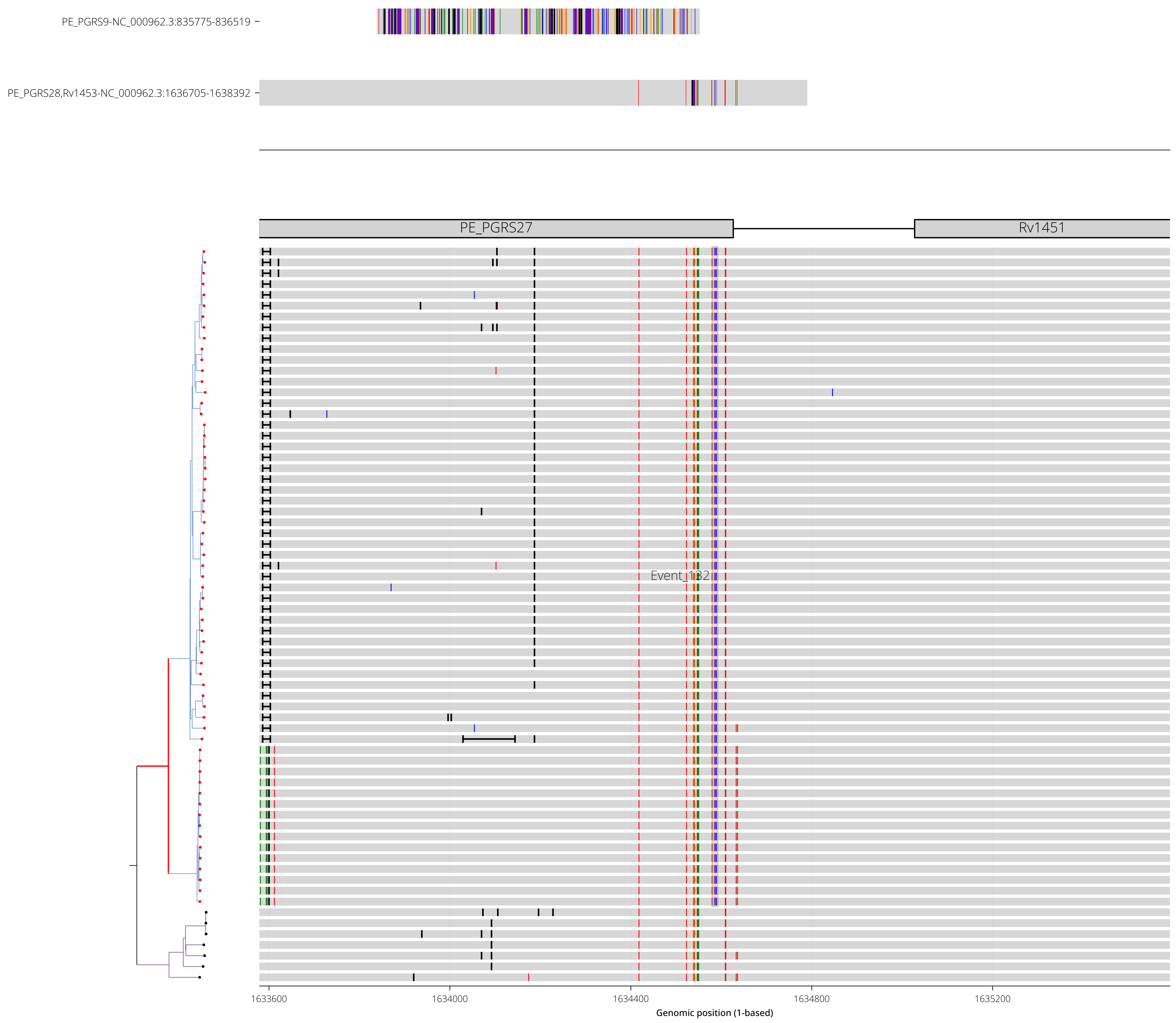

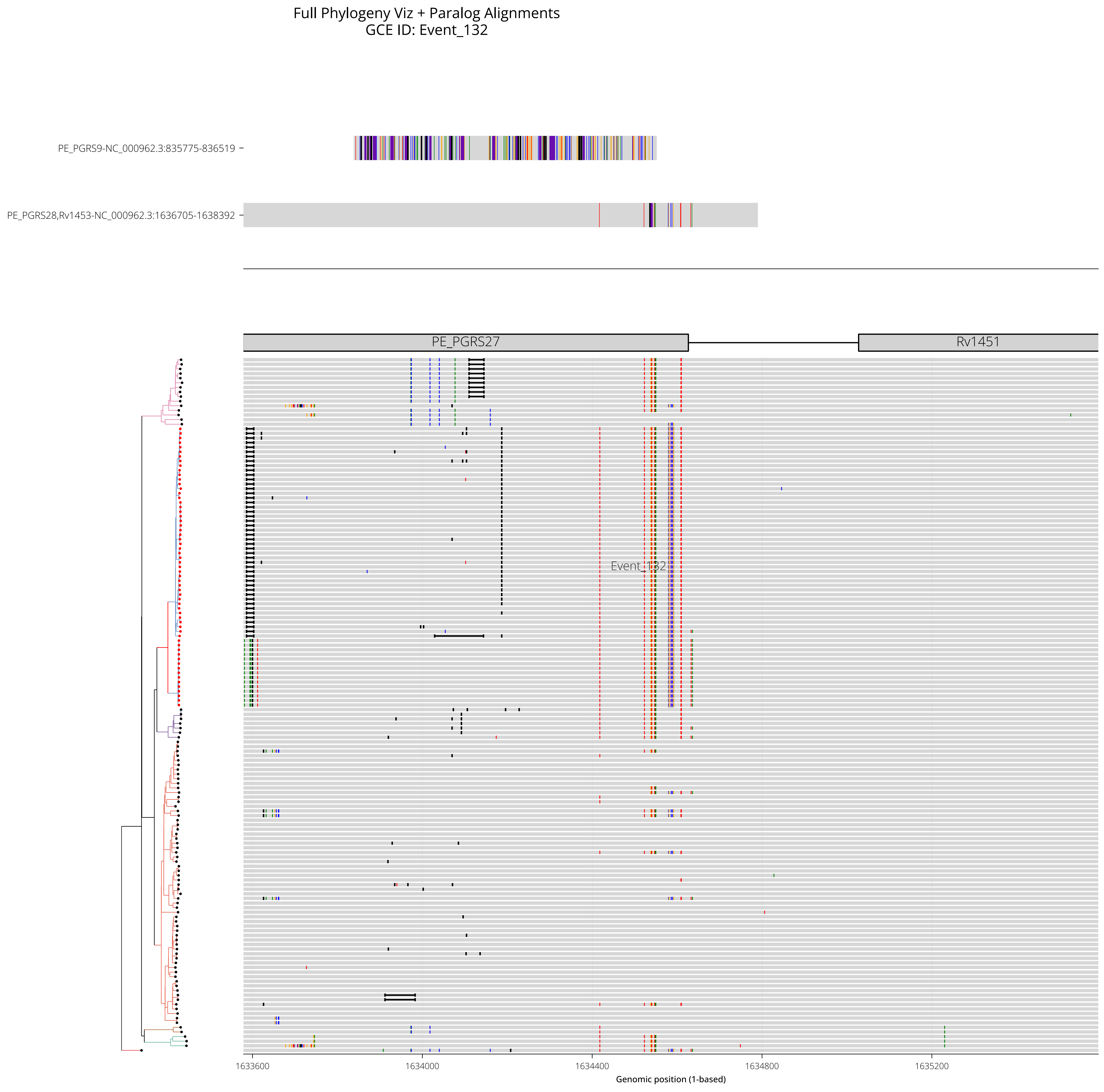

Full Phylogeny Viz + Paralog Alignments
GCE ID: Event_132
PE_PGRS9-NC_000962.3:835775-836519
PE_PGRS28,Rv1453-NC_000962.3:1636705-1638392
PE_PGRS27
Rv1451
Event_132
1633600
1634000
1634400
1634800
1635200
Genomic position (1-based)

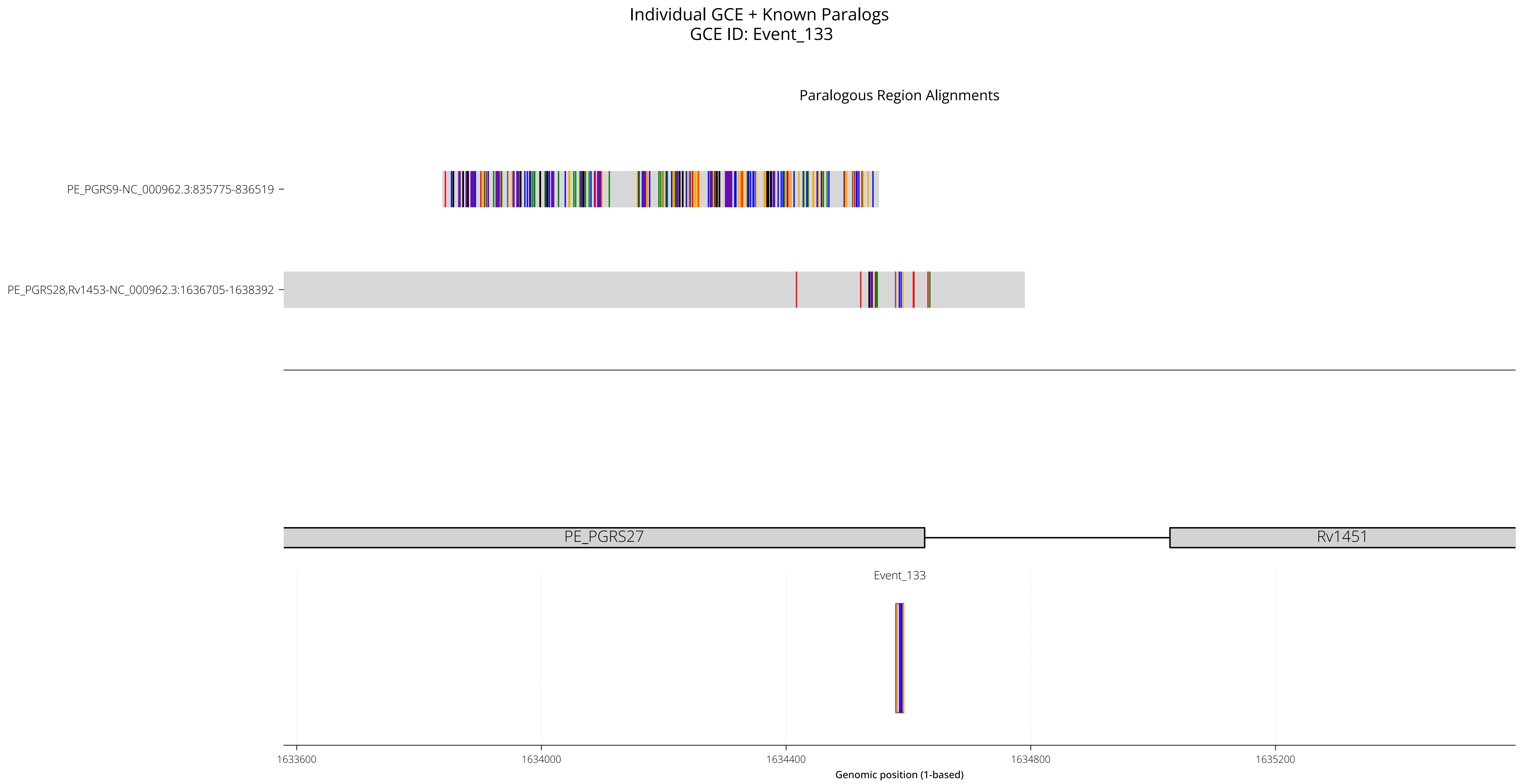

Individual GCE + Known Paralogs
GCE ID: Event_133
Paralogous Region Alignments
PE_PGRS9-NC_000962.3:835775-836519
PE_PGRS28,Rv1453-NC_000962.3:1636705-1638392
PE_PGRS27
Rv1451
Event_133
1633600
1634000
1634400
1634800
1635200
Genomic position (1-based)

# Circos Viz - GCE to Known Paralogs Locations
GCE ID: Event_133

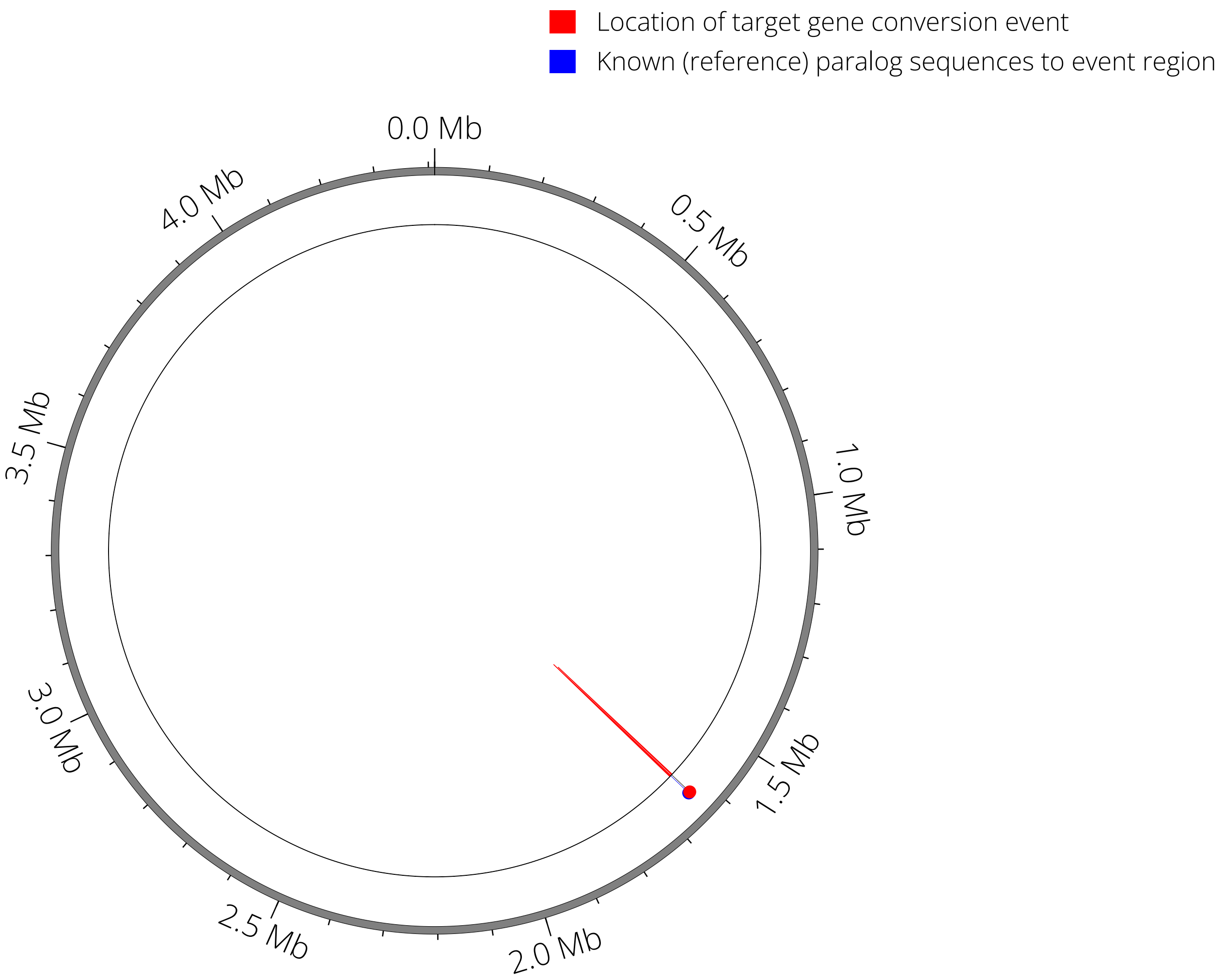

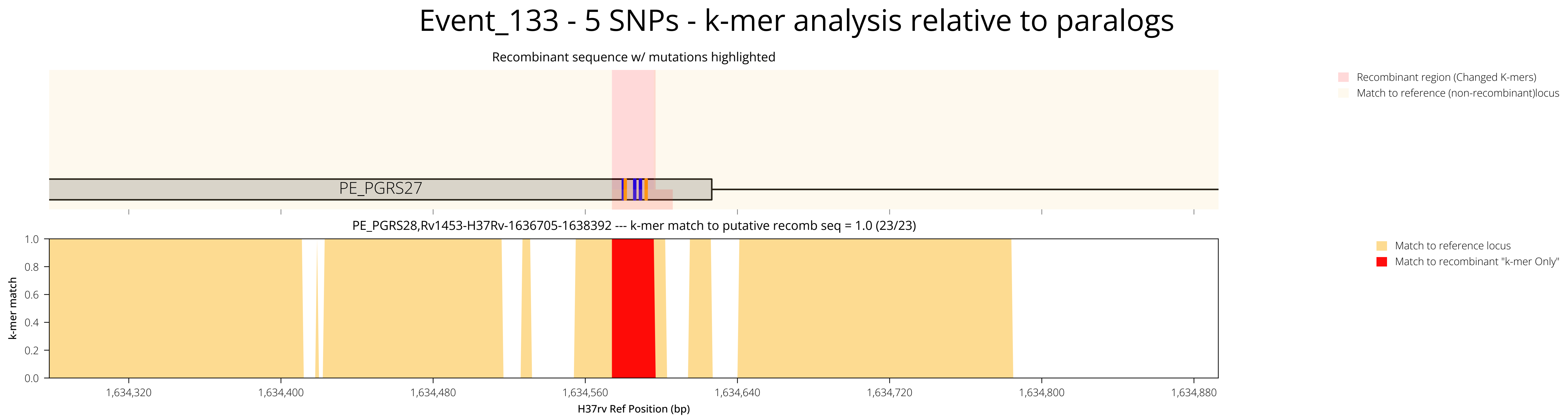
Event_133 - 5 SNPs - k-mer analysis relative to paralogs
Recombinant sequence w/ mutations highlighted
PE_PGRS27
Recombinant region (Changed K-mers)
Match to reference (non-recombinant)locus
PE_PGRS28,Rv1453-H37Rv-1636705-1638392 --- k-mer match to putative recomb seq = 1.0 (23/23)
Match to reference locus
Match to recombinant "k-mer Only"
k-mer match
1.0
0.8
0.6
0.4
0.2
0.0
1,634,320
1,634,400
1,634,480
1,634,560
1,634,640
1,634,720
1,634,800
1,634,880
H37rv Ref Position (bp)

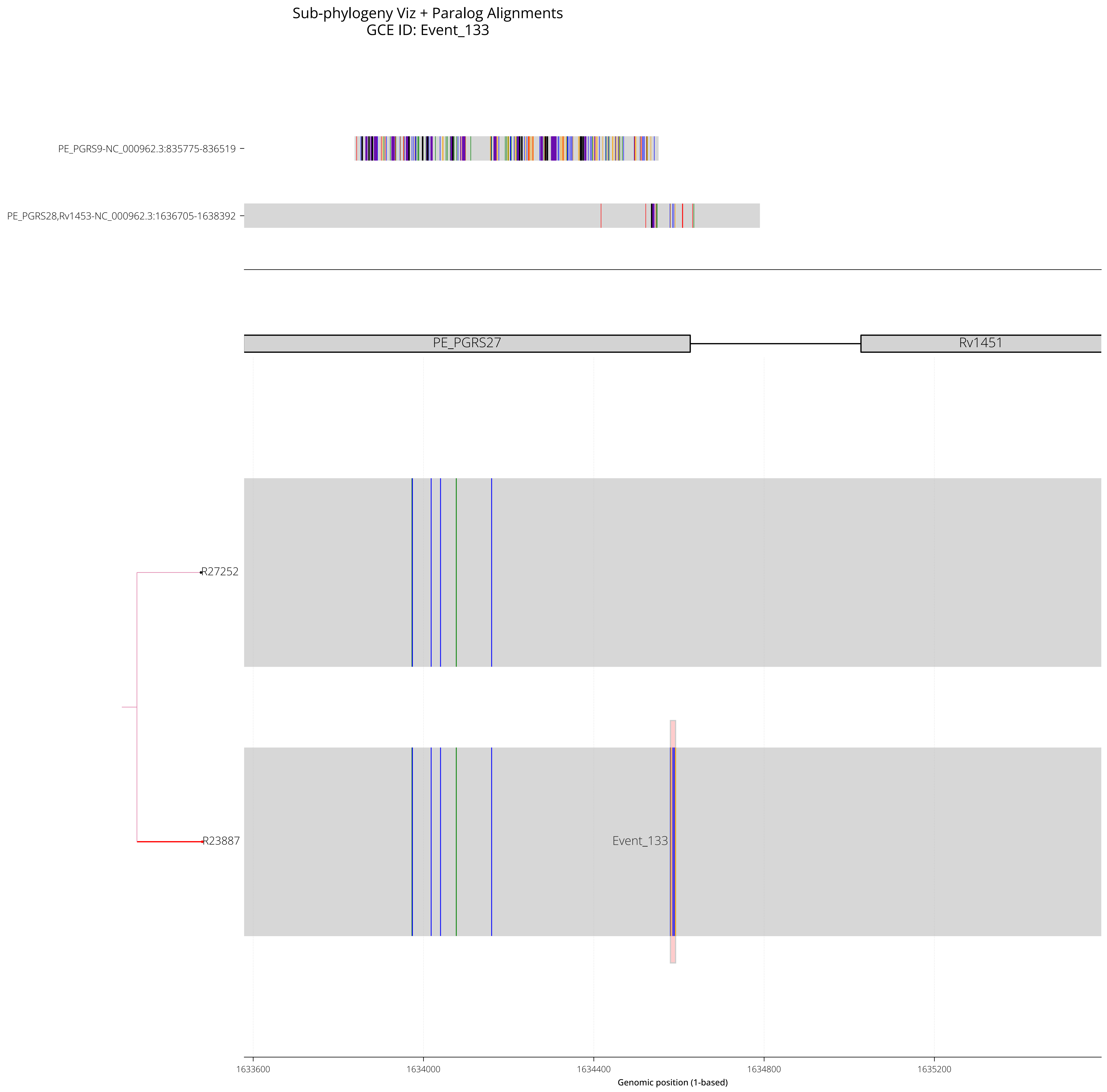
Sub-phylogeny Viz + Paralog Alignments
GCE ID: Event_133
PE_PGRS9-NC_000962.3:835775-836519
PE_PGRS28,Rv1453-NC_000962.3:1636705-1638392
PE_PGRS27
Rv1451
R27252
R23887
Event_133
1633600
1634000
1634400
1634800
1635200
Genomic position (1-based)

Full Phylogeny Viz + Paralog Alignments
GCE ID: Event_133

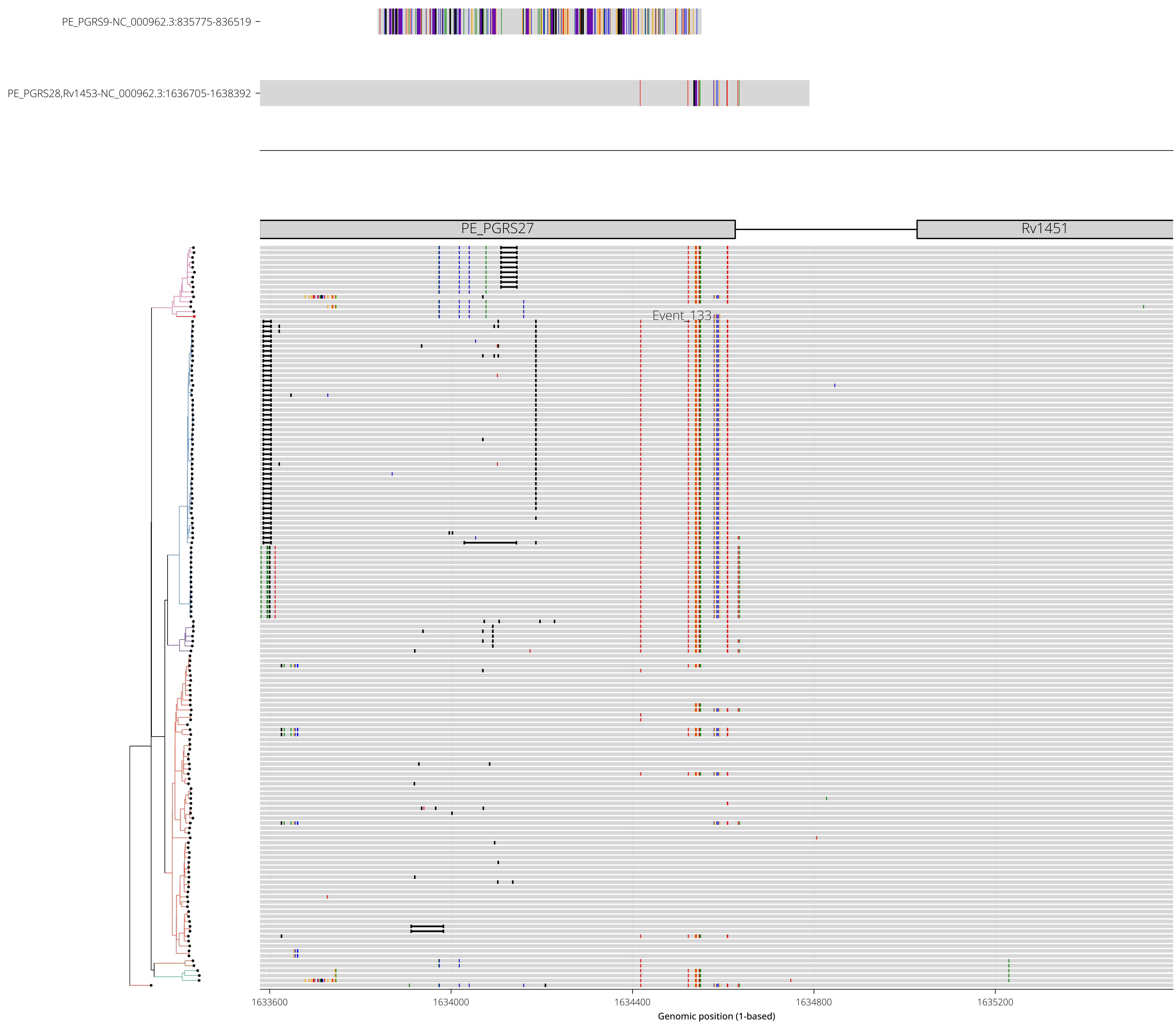

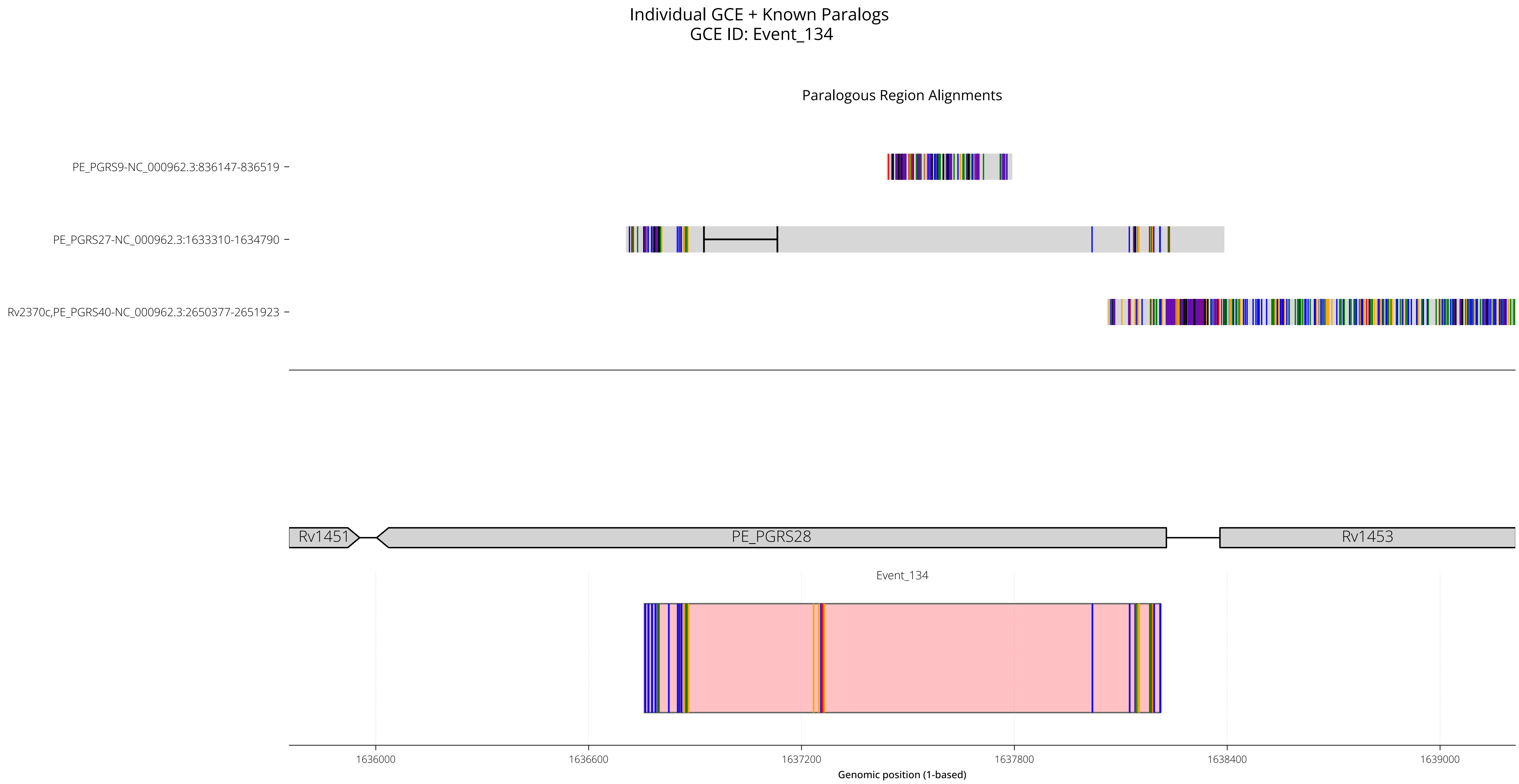

Individual GCE + Known Paralogs
GCE ID: Event_134
Paralogous Region Alignments
PE_PGRS9-NC_000962.3:836147-836519
PE_PGRS27-NC_000962.3:1633310-1634790
Rv2370c,PE_PGRS40-NC_000962.3:2650377-2651923
Rv1451
PE_PGRS28
Rv1453
Event_134
1636000
1636600
1637200
1637800
1638400
1639000
Genomic position (1-based)

Circos Viz - GCE to Known Paralogs Locations
GCE ID: Event_134

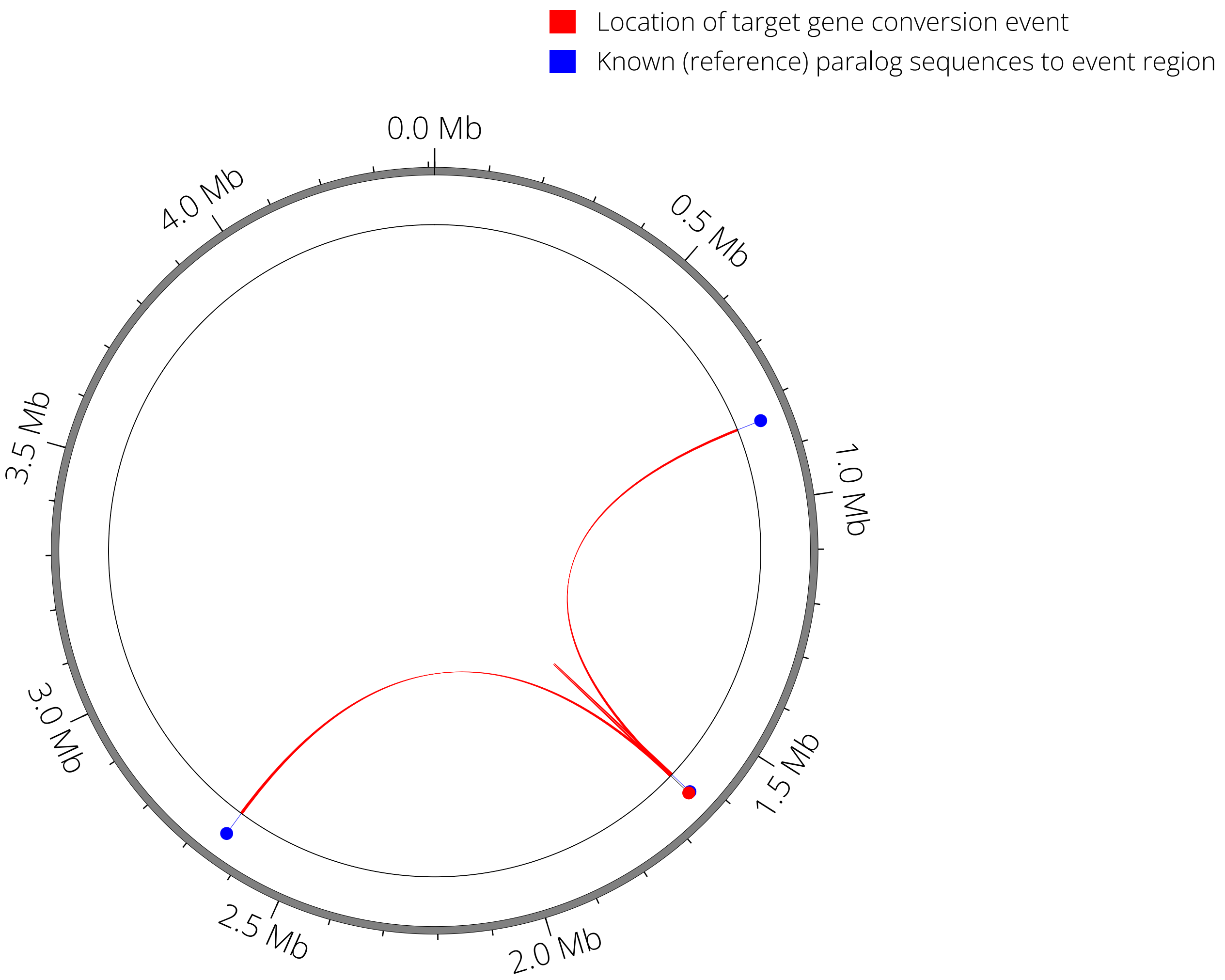

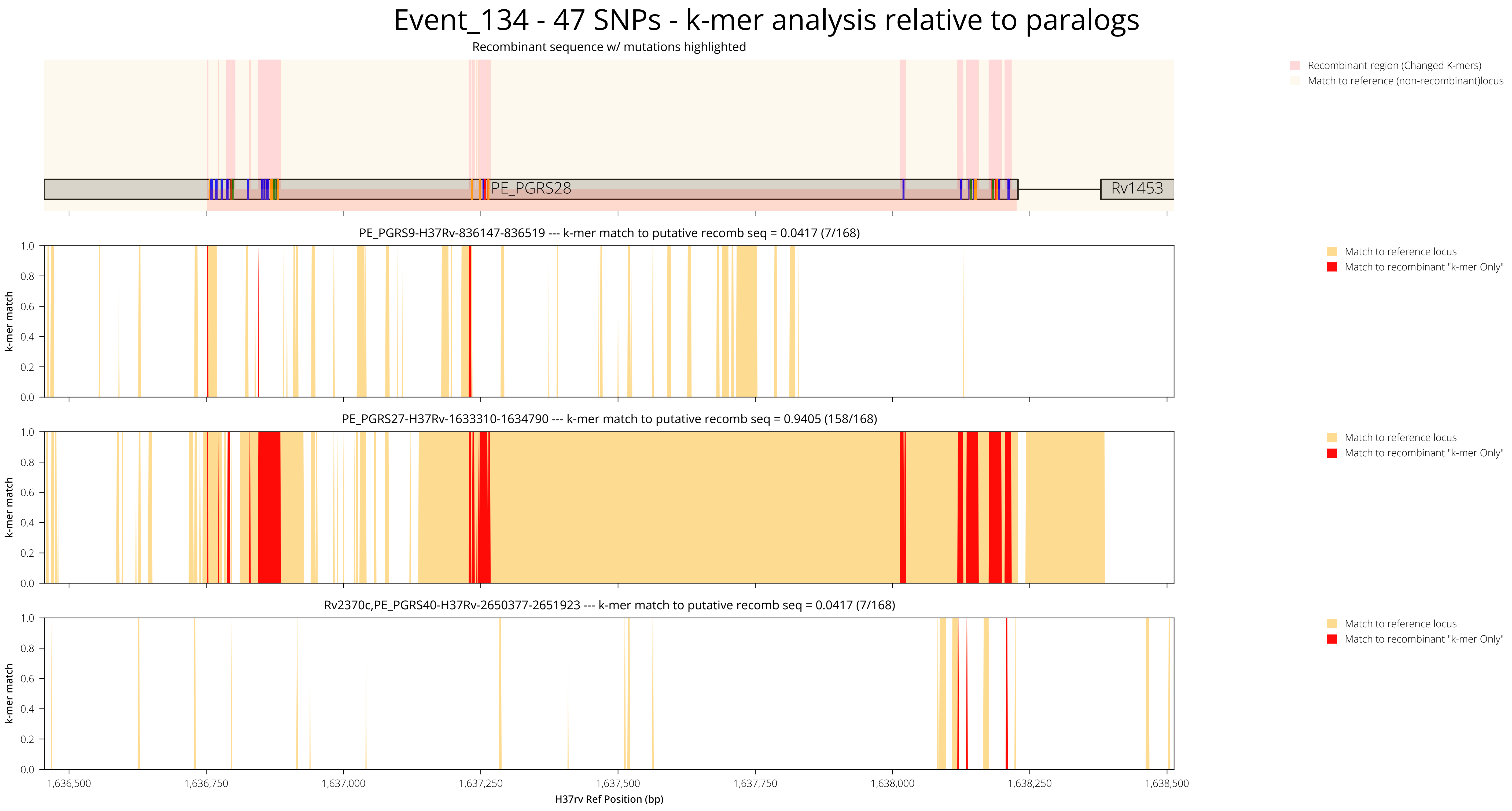

Event_134 - 47 SNPs - k-mer analysis relative to paralogs
Recombinant sequence w/ mutations highlighted
Recombinant region (Changed K-mers)
Match to reference (non-recombinant)locus
PE_PGRS28
Rv1453
PE_PGRS9-H37Rv-836147-836519 --- k-mer match to putative recomb seq = 0.0417 (7/168)
PE_PGRS27-H37Rv-1633310-1634790 --- k-mer match to putative recomb seq = 0.9405 (158/168)
Rv2370c,PE_PGRS40-H37Rv-2650377-2651923 --- k-mer match to putative recomb seq = 0.0417 (7/168)
Match to reference locus
Match to recombinant "k-mer Only"
k-mer match
1.0
0.8
0.6
0.4
0.2
0.0
1,636,500
1,636,750
1,637,000
1,637,250
1,637,500
1,637,750
1,638,000
1,638,250
1,638,500
H37rv Ref Position (bp)

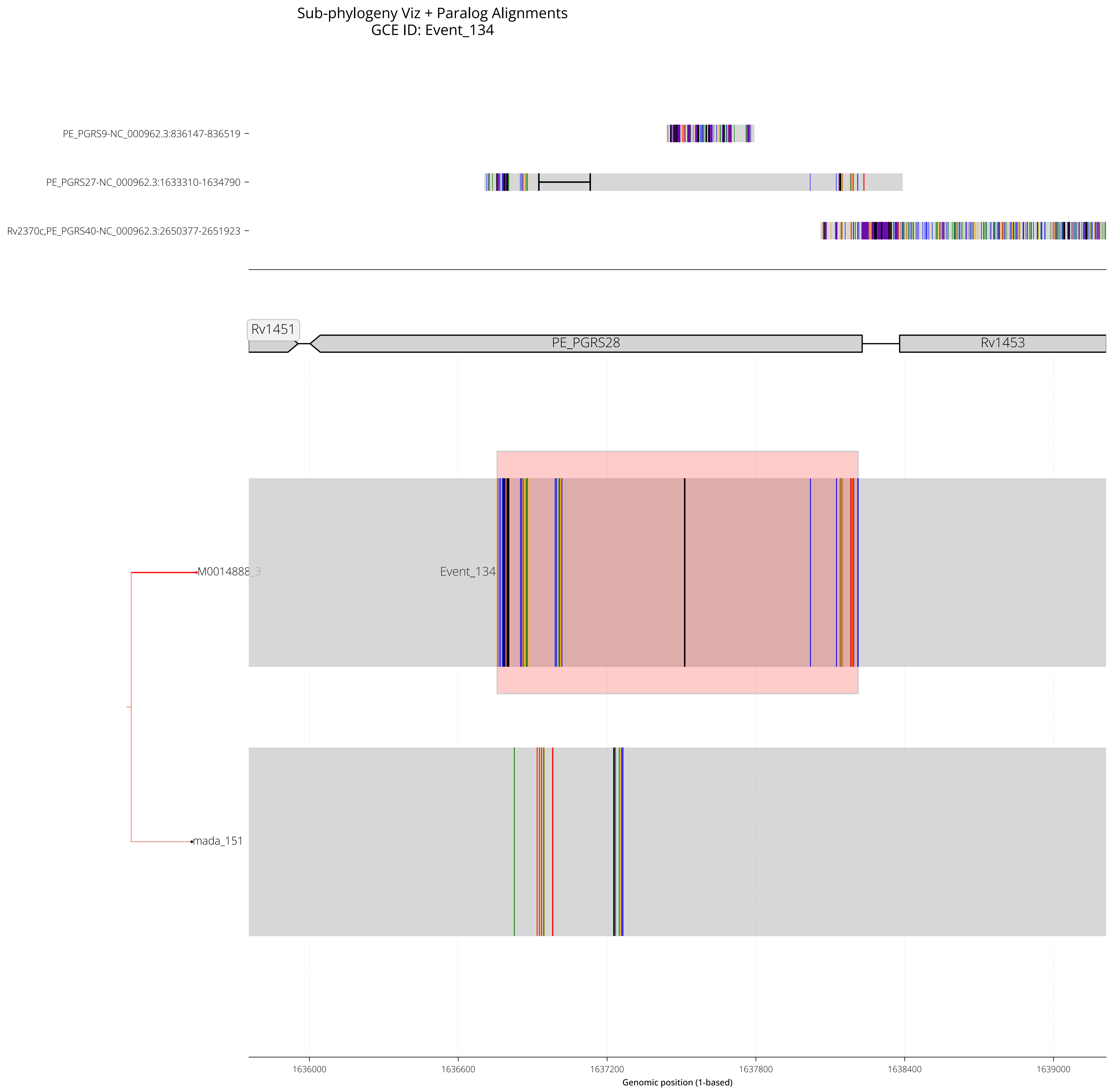

Sub-phylogeny Viz + Paralog Alignments
GCE ID: Event_134
PE_PGRS9-NC_000962.3:836147-836519
PE_PGRS27-NC_000962.3:1633310-1634790
Rv2370c,PE_PGRS40-NC_000962.3:2650377-2651923
Rv1451
PE_PGRS28
Rv1453
M0014888_3
Event_134
mada_151
1636000
1636600
1637200
1637800
1638400
1639000
Genomic position (1-based)

Full Phylogeny Viz + Paralog Alignments
GCE ID: Event_134

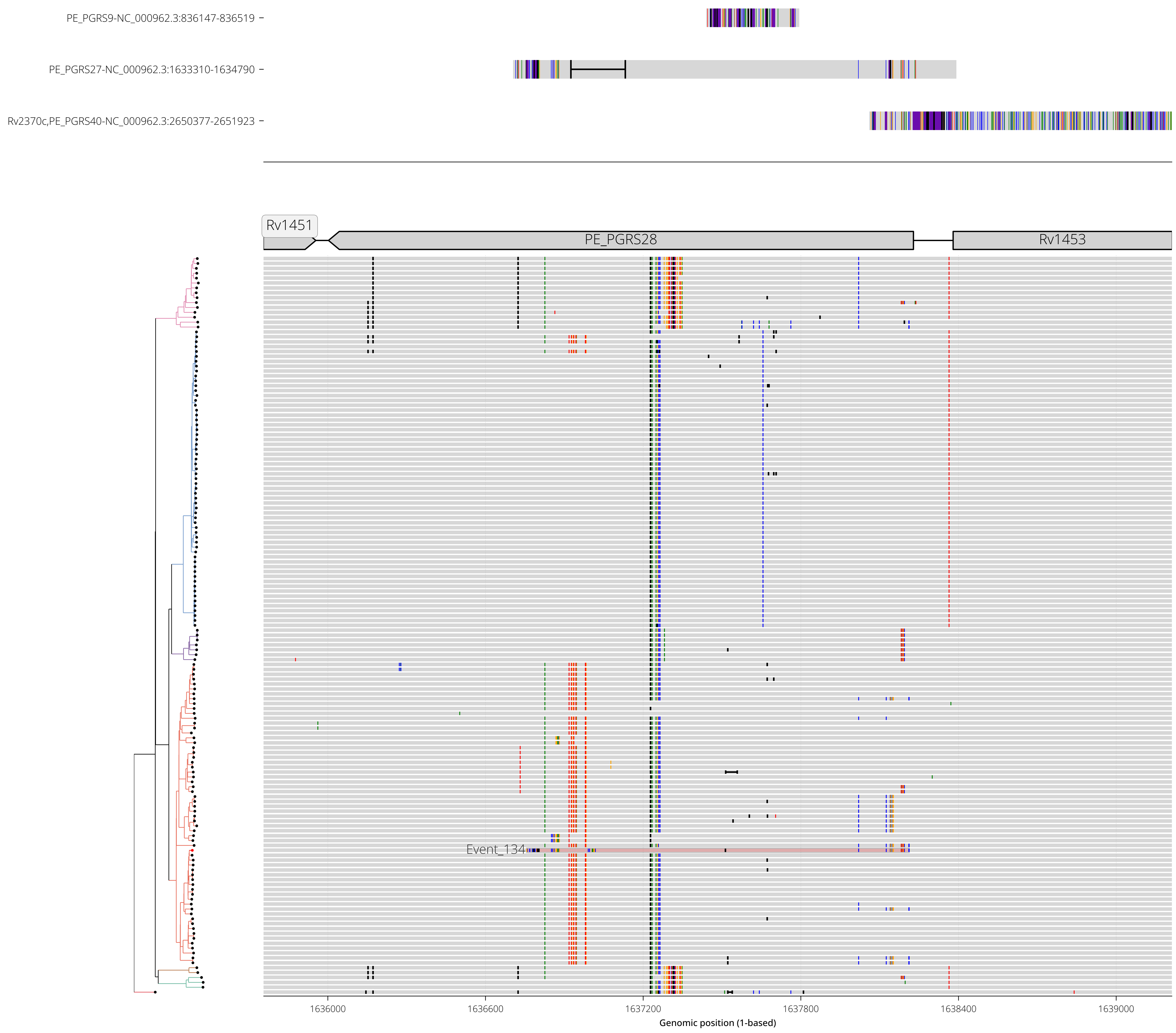

Individual GCE + Known Paralogs
GCE ID: Event_135

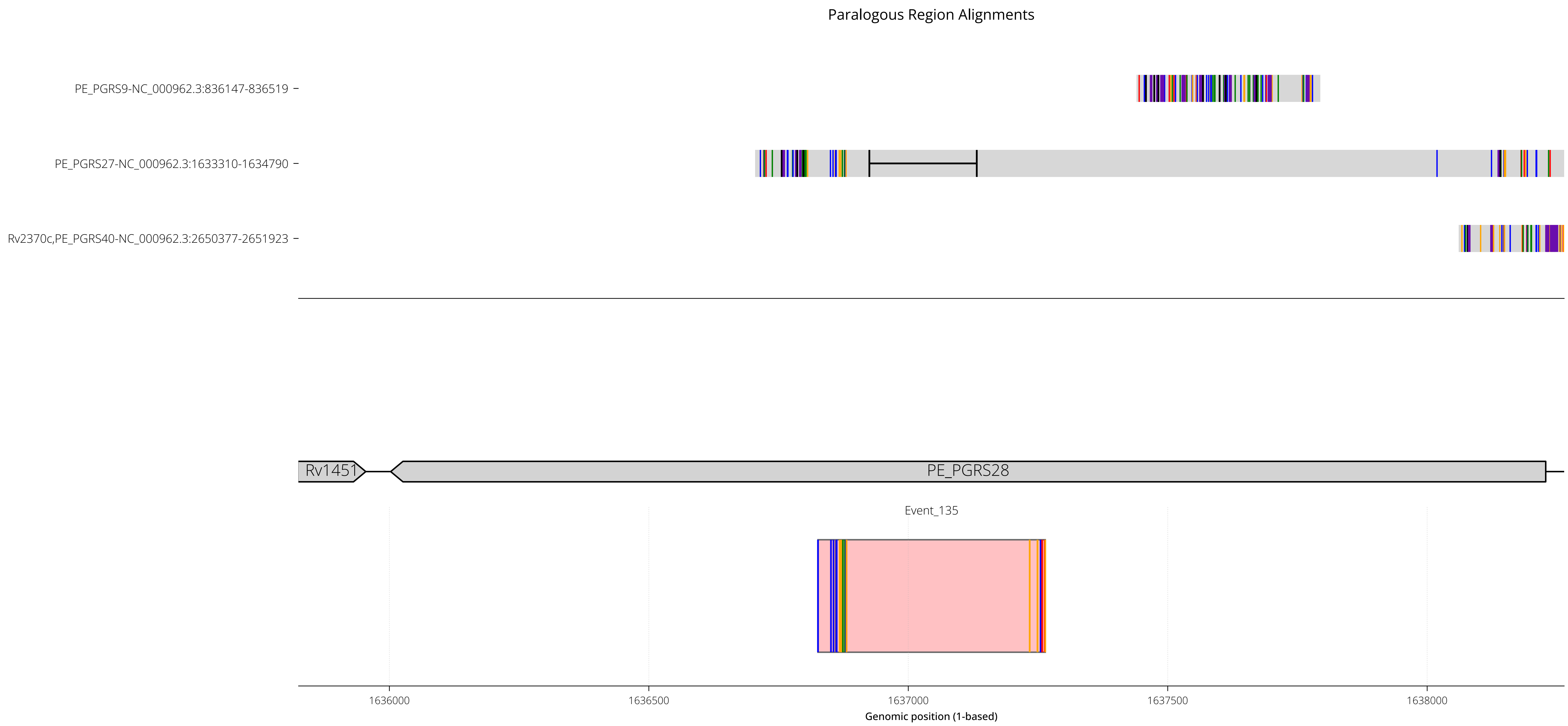

Circos Viz - GCE to Known Paralogs Locations
GCE ID: Event_135

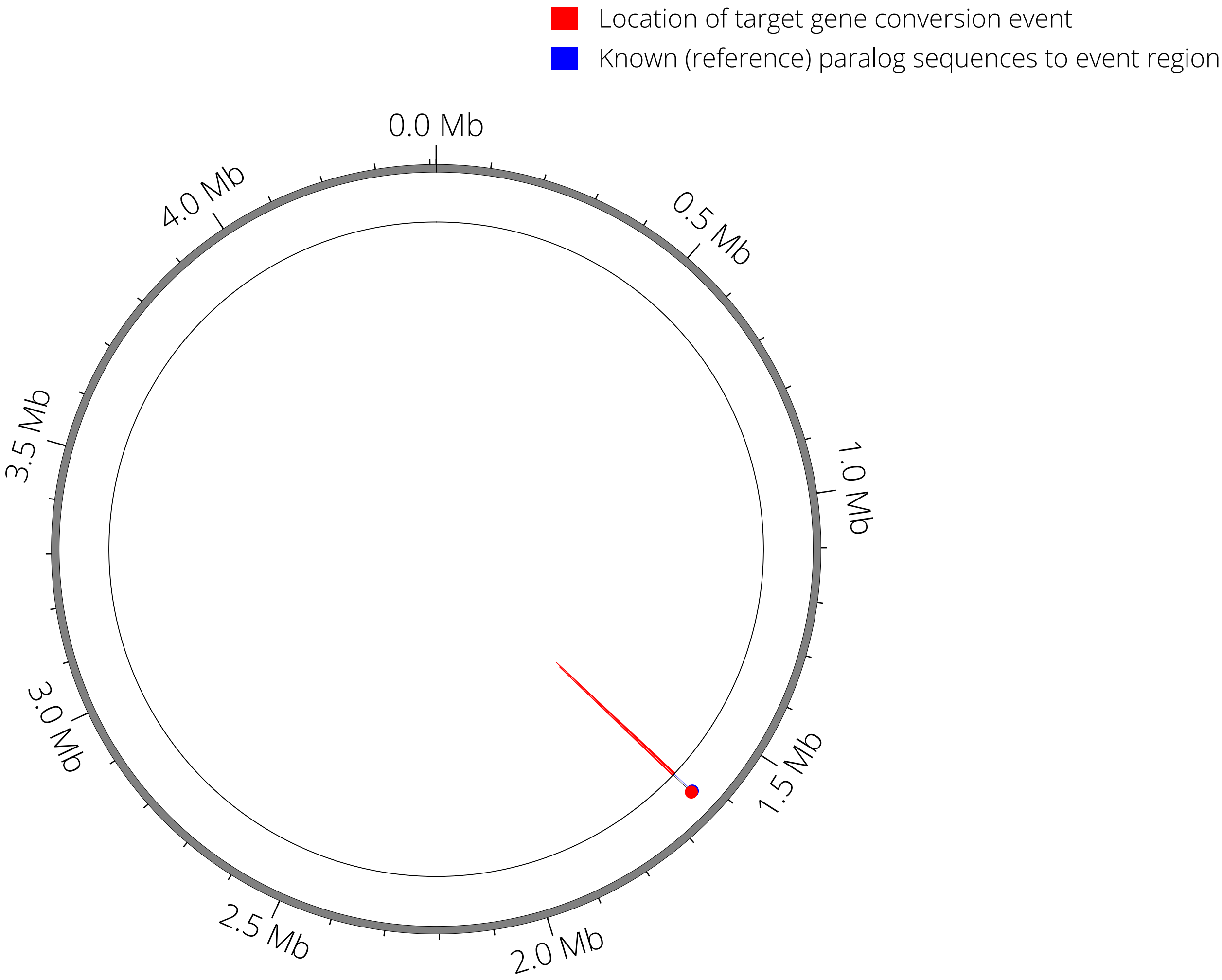

# Event_135 - 18 SNPs - k-mer analysis relative to paralogs

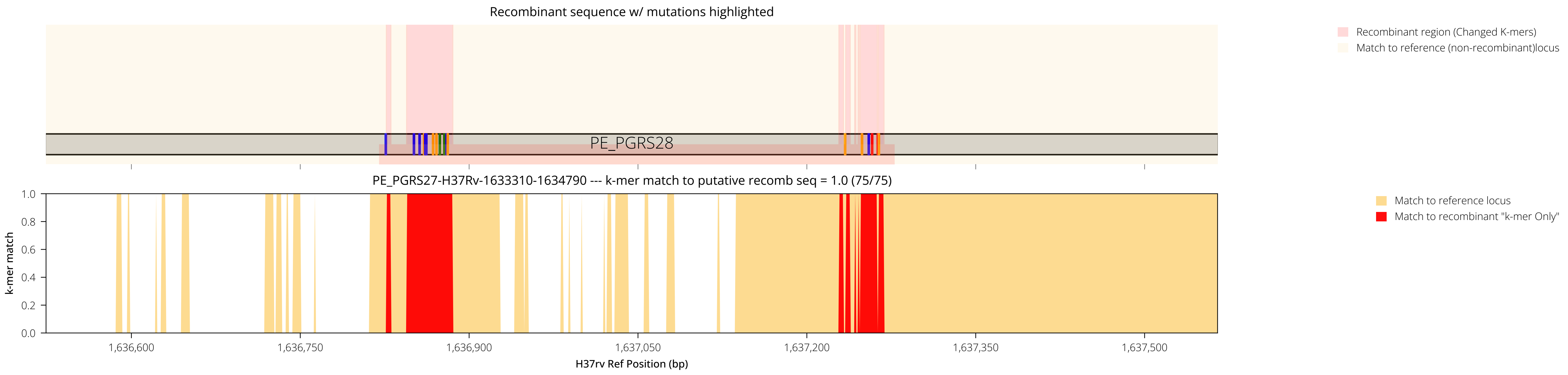

Sub-phylogeny Viz + Paralog Alignments
GCE ID: Event_135

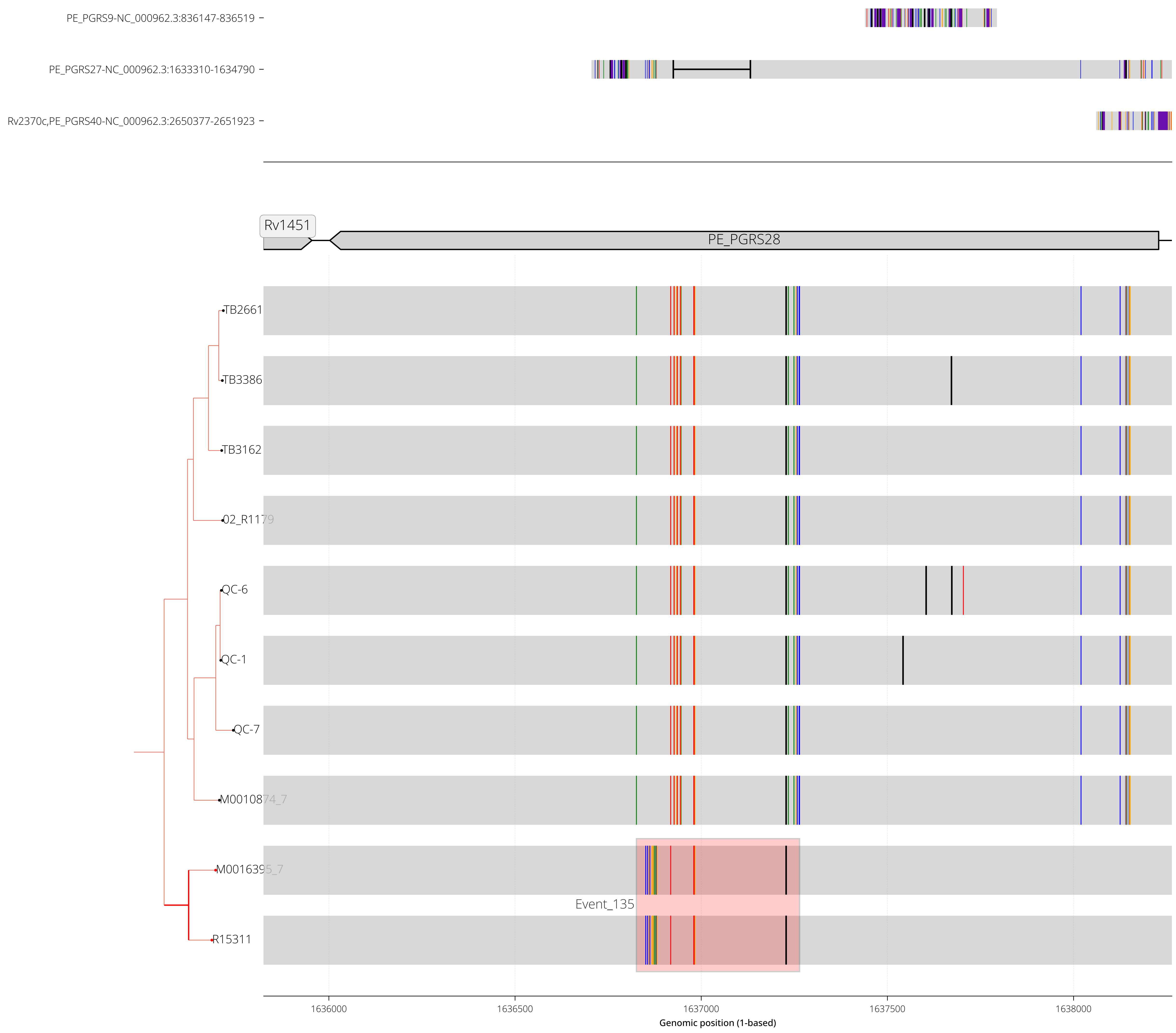

Full Phylogeny Viz + Paralog Alignments
GCE ID: Event_135

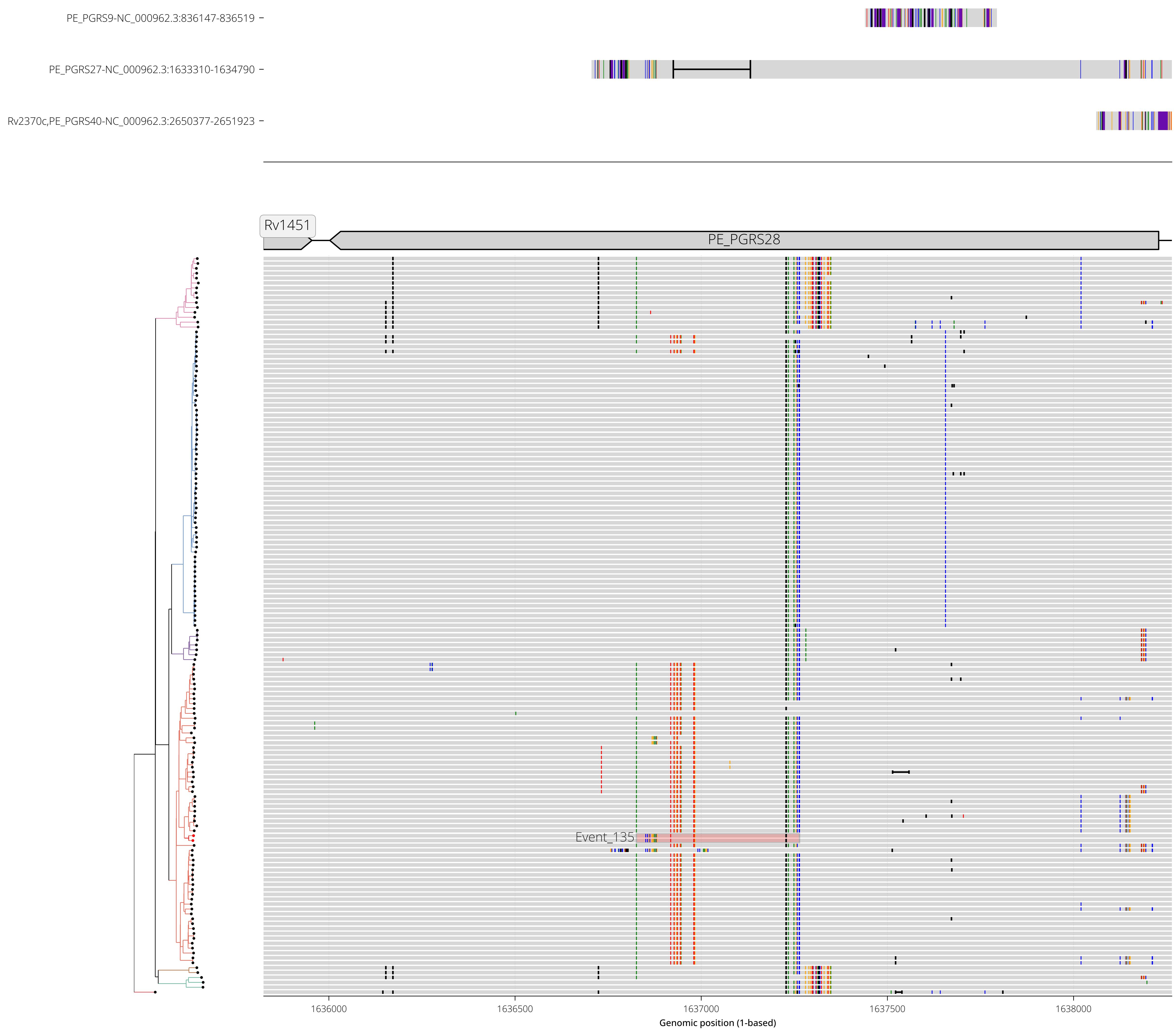

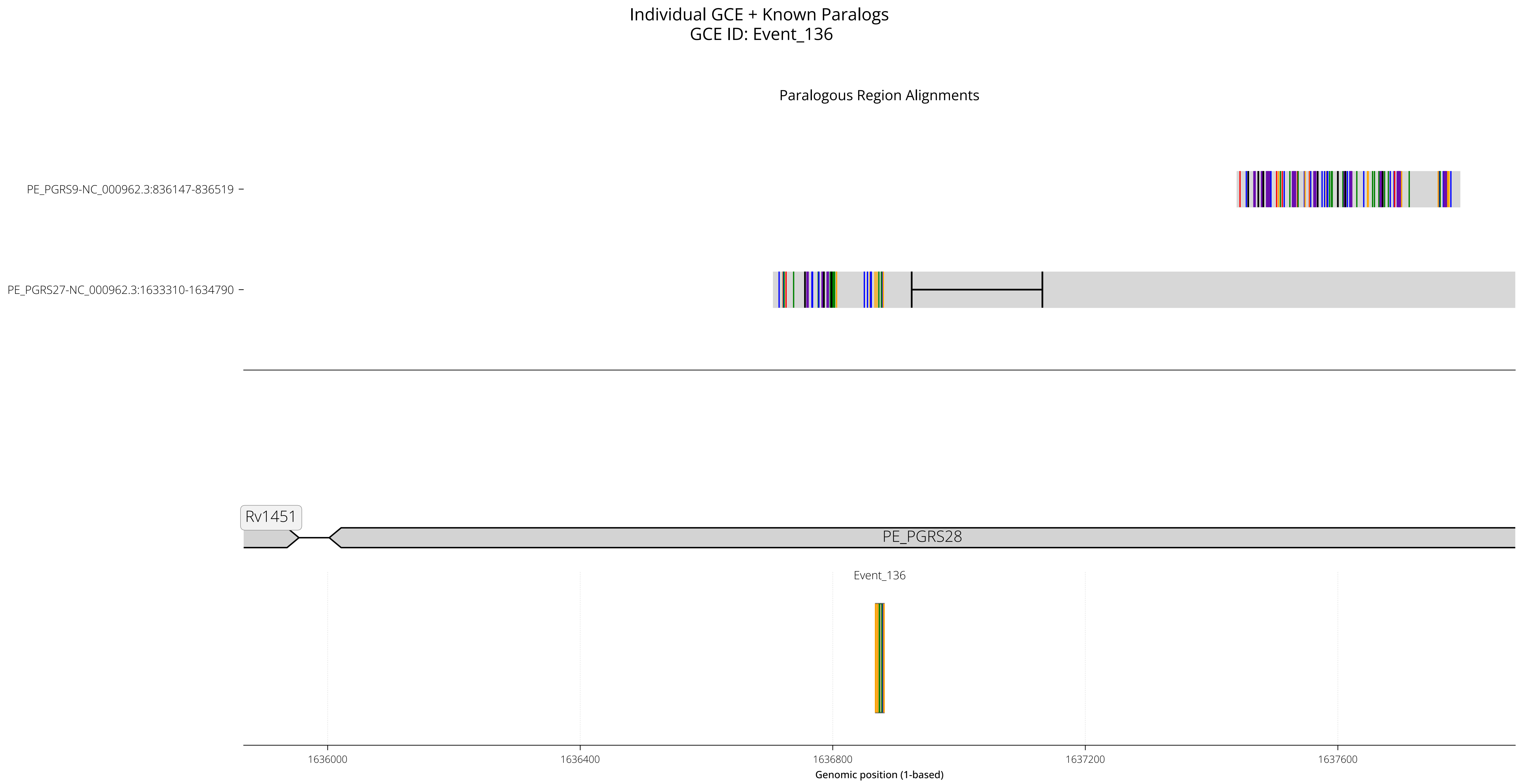

Individual GCE + Known Paralogs
GCE ID: Event_136
Paralogous Region Alignments
PE_PGRS9-NC_000962.3:836147-836519
PE_PGRS27-NC_000962.3:1633310-1634790
Rv1451
PE_PGRS28
Event_136
1636000
1636400
1636800
1637200
1637600
Genomic position (1-based)

Circos Viz - GCE to Known Paralogs Locations
GCE ID: Event_136

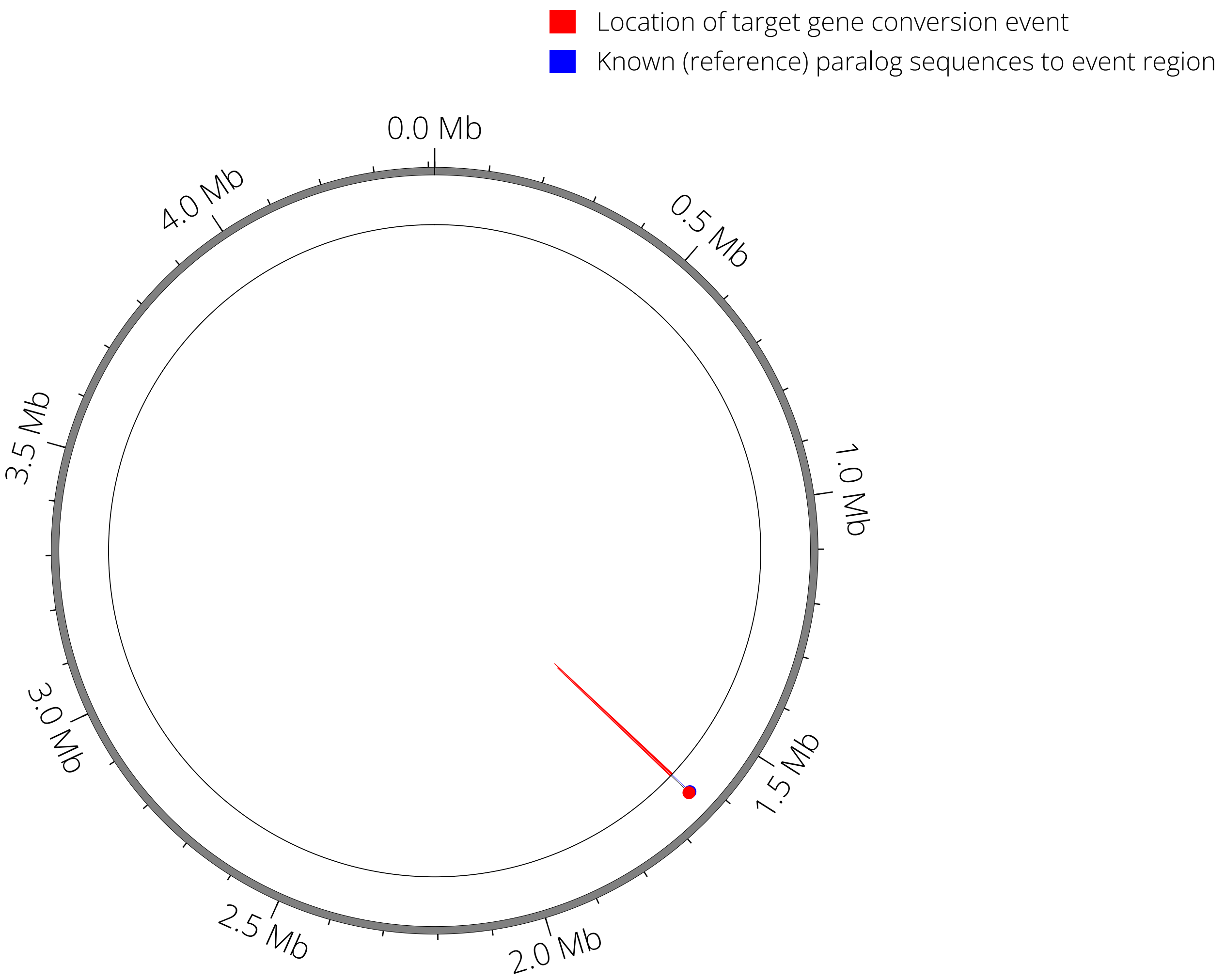

# Event_136 - 6 SNPs - k-mer analysis relative to paralogs

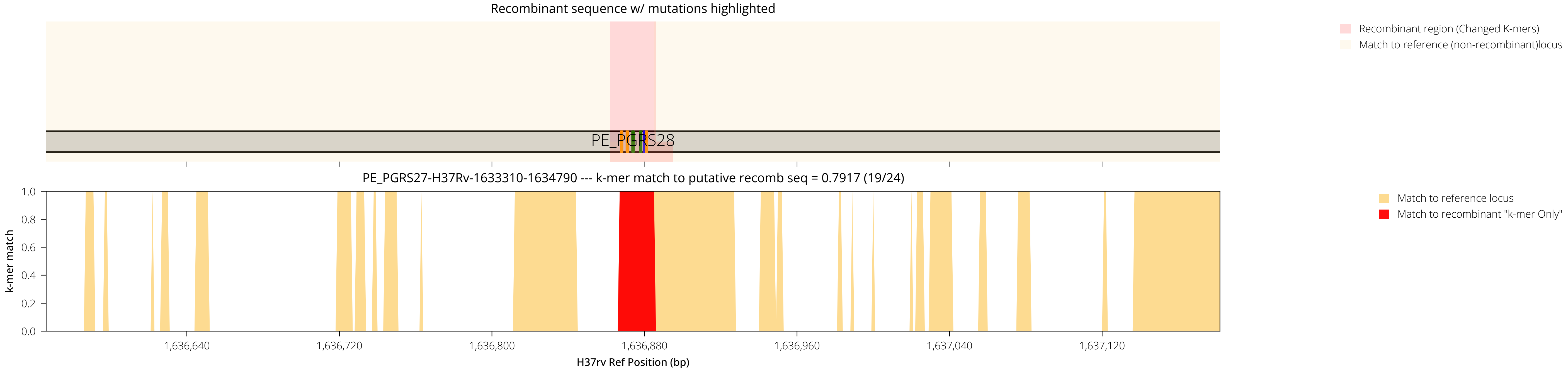

Sub-phylogeny Viz + Paralog Alignments
GCE ID: Event_136

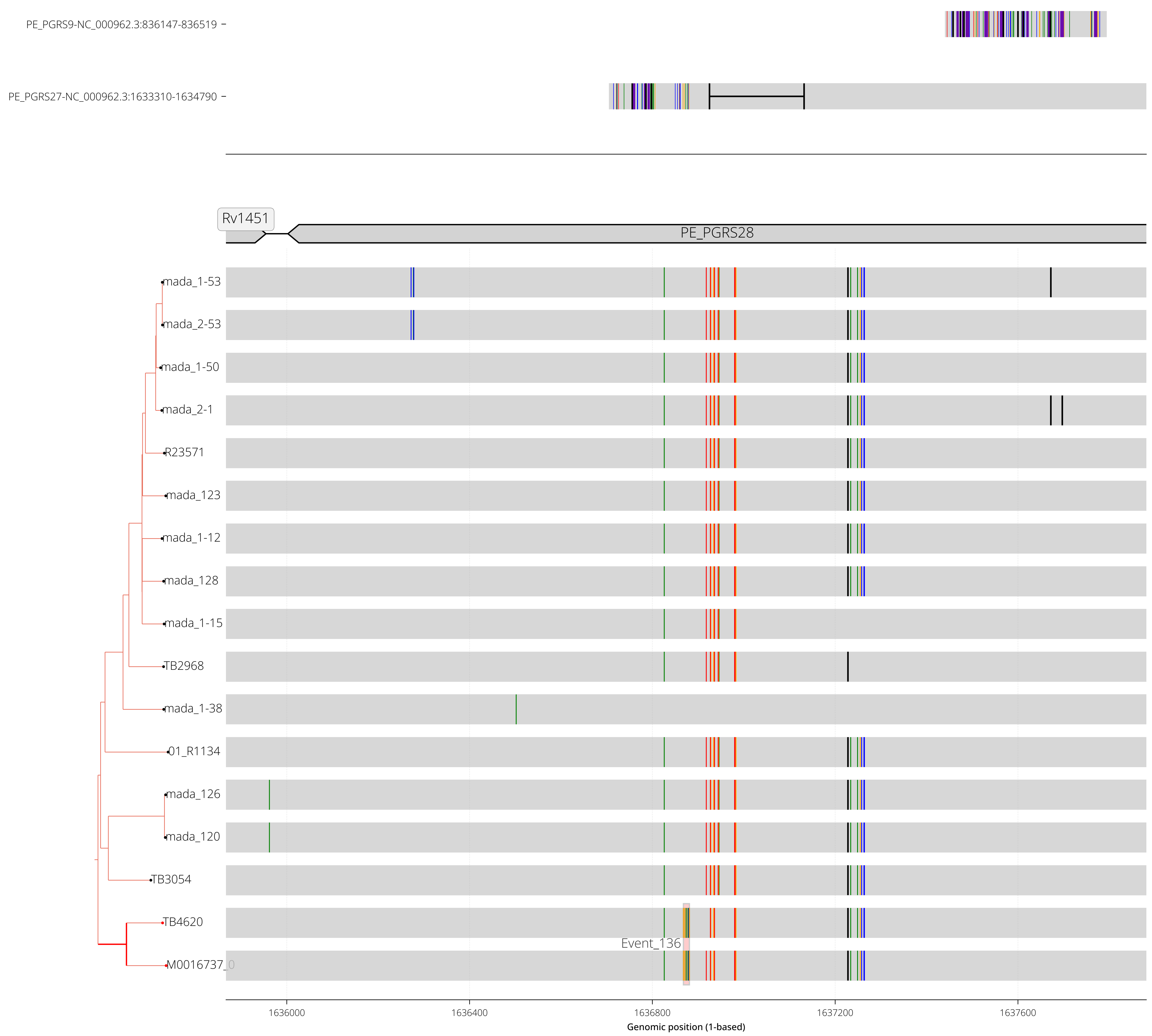

Full Phylogeny Viz + Paralog Alignments
GCE ID: Event_136

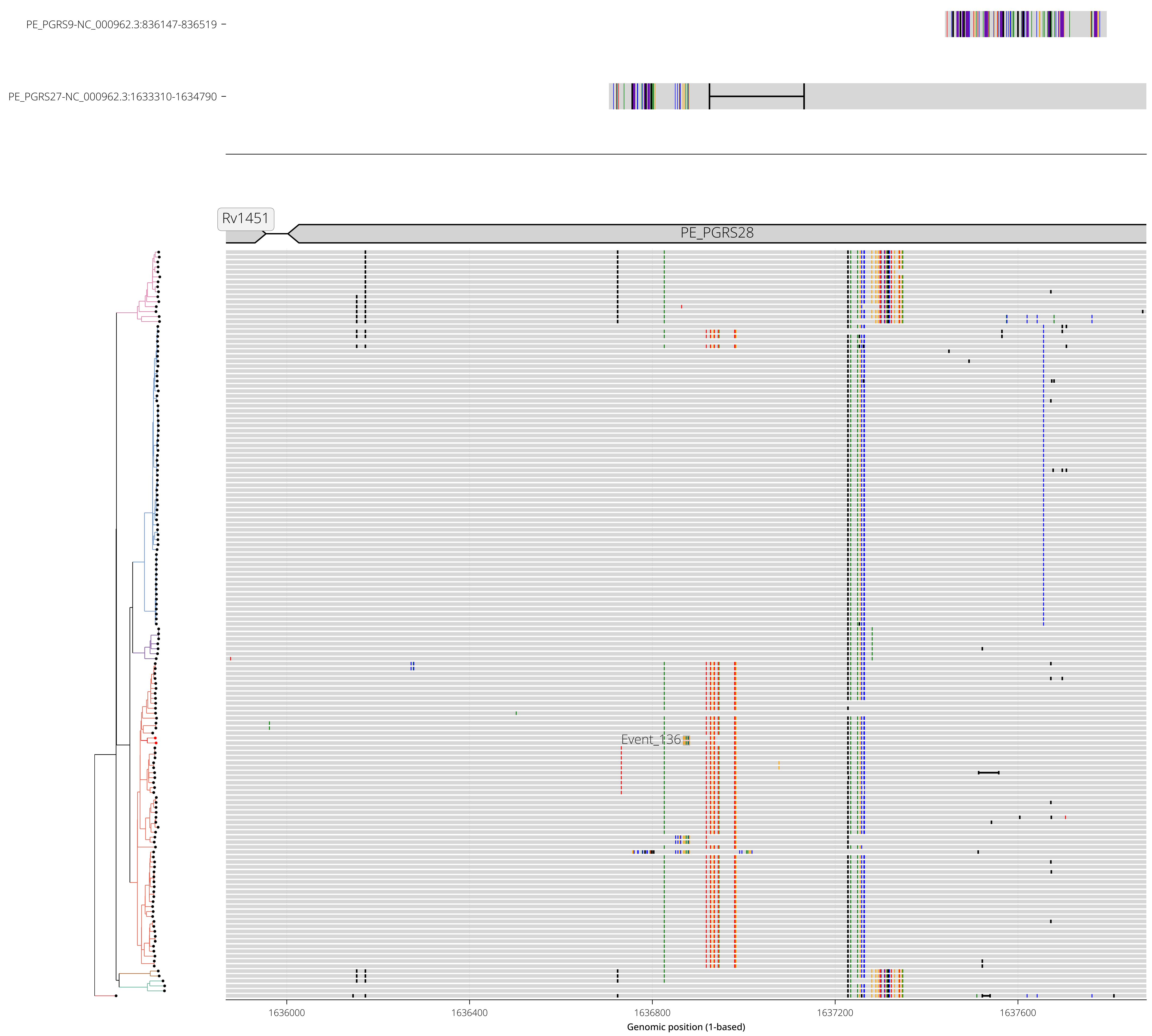

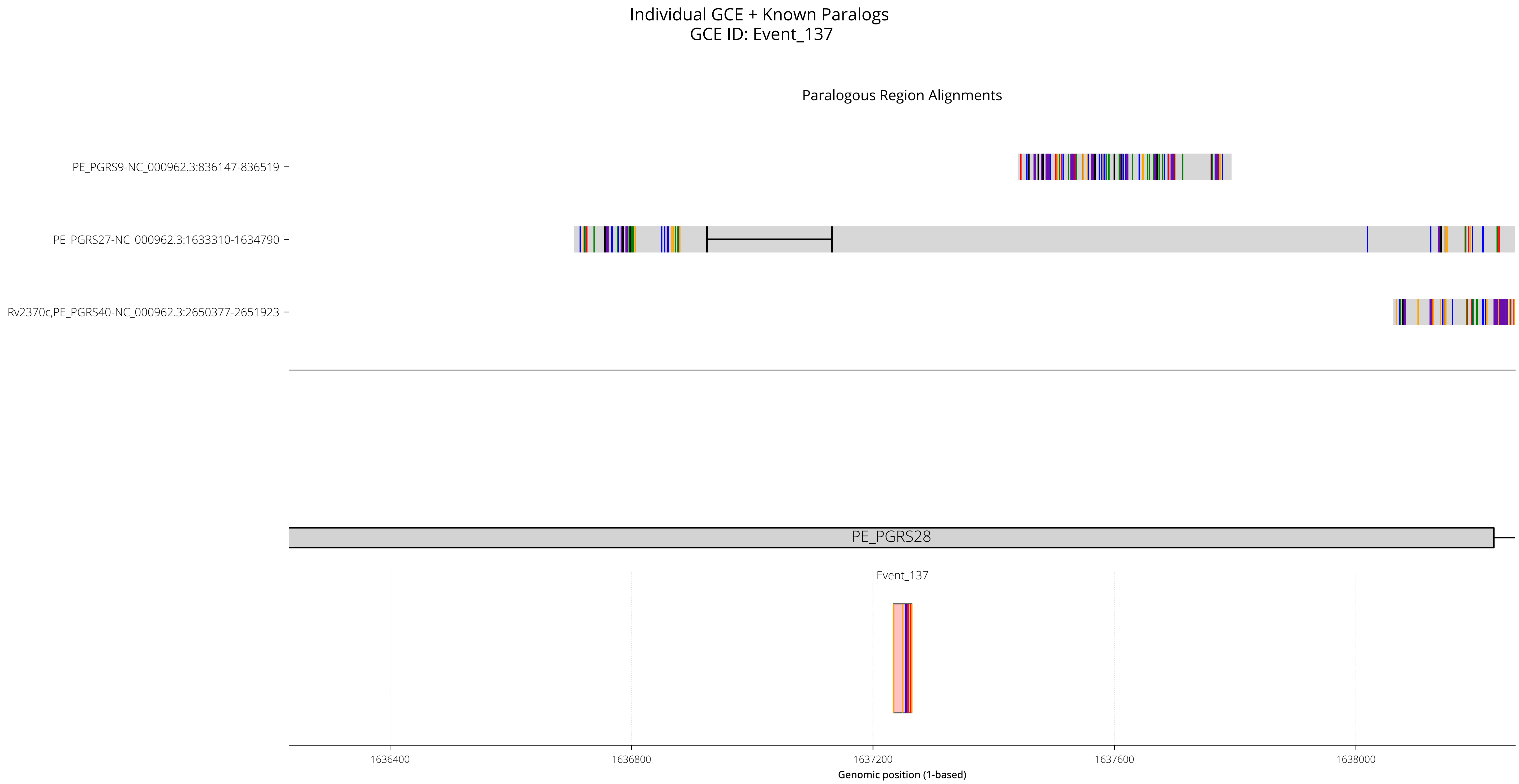

Individual GCE + Known Paralogs
GCE ID: Event_137
Paralogous Region Alignments
PE_PGRS9-NC_000962.3:836147-836519
PE_PGRS27-NC_000962.3:1633310-1634790
Rv2370c,PE_PGRS40-NC_000962.3:2650377-2651923
PE_PGRS28
Event_137
1636400
1636800
1637200
1637600
1638000
Genomic position (1-based)

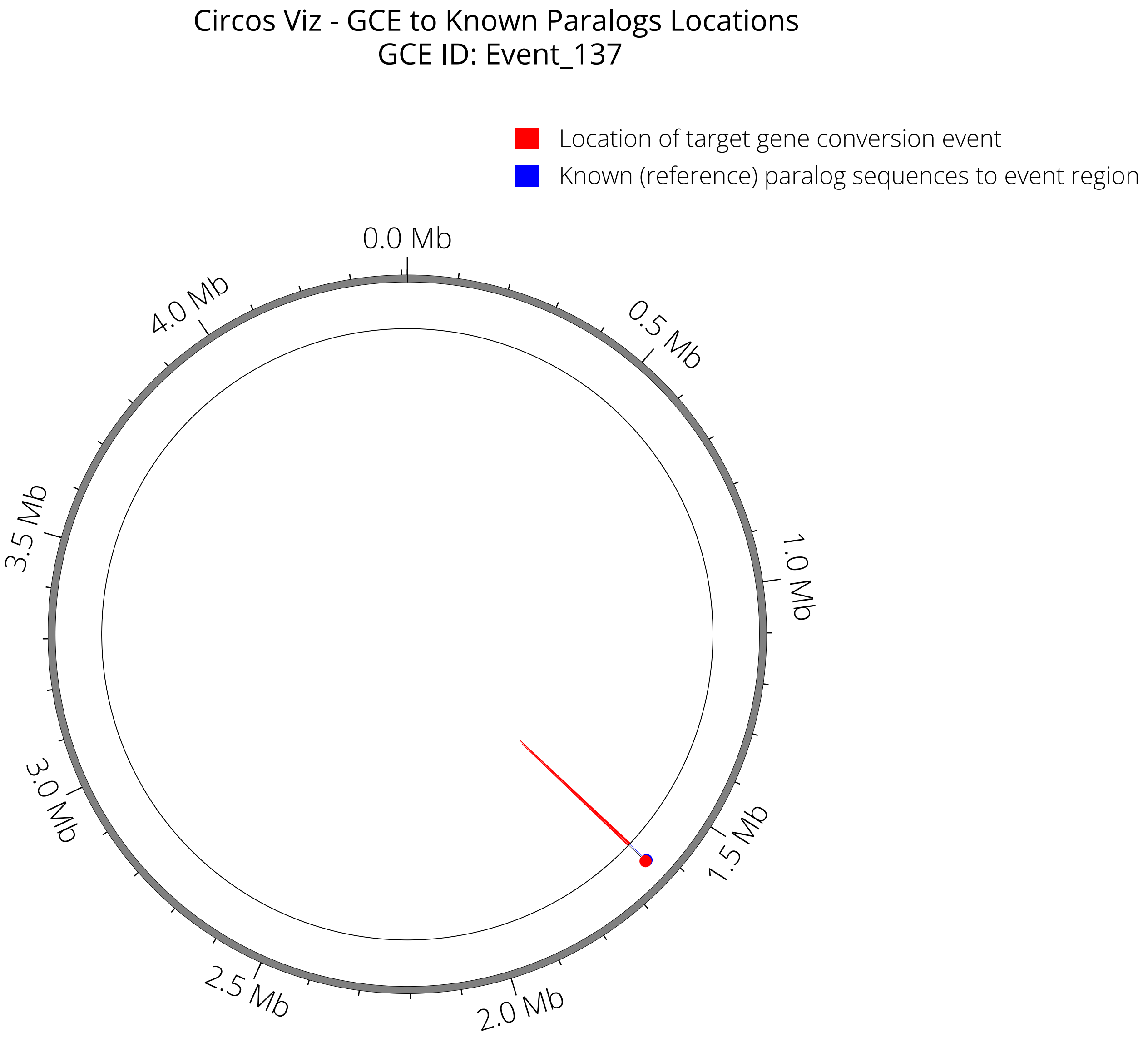
Circos Viz - GCE to Known Paralogs Locations
GCE ID: Event_137
Location of target gene conversion event
Known (reference) paralog sequences to event region
0.0 Mb
0.5 Mb
1.0 Mb
1.5 Mb
2.0 Mb
2.5 Mb
3.0 Mb
3.5 Mb
4.0 Mb

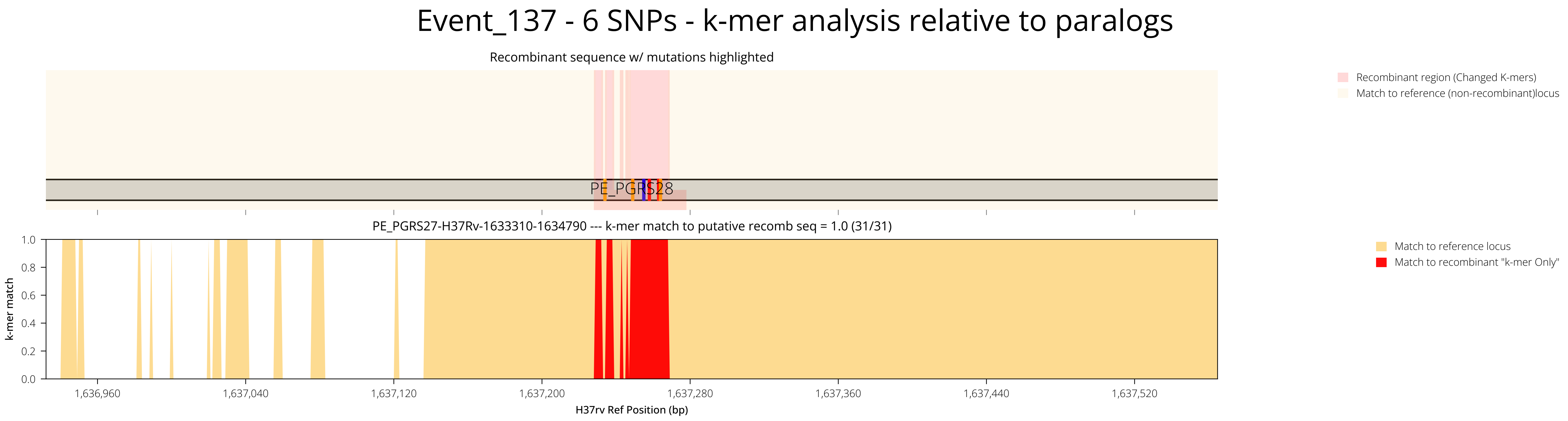

Event_137 - 6 SNPs - k-mer analysis relative to paralogs
Recombinant sequence w/ mutations highlighted
Recombinant region (Changed K-mers)
Match to reference (non-recombinant)locus
PE_PGRS28
PE_PGRS27-H37Rv-1633310-1634790 --- k-mer match to putative recomb seq = 1.0 (31/31)
Match to reference locus
Match to recombinant "k-mer Only"
k-mer match
1.0
0.8
0.6
0.4
0.2
0.0
1,636,960
1,637,040
1,637,120
1,637,200
1,637,280
1,637,360
1,637,440
1,637,520
H37rv Ref Position (bp)

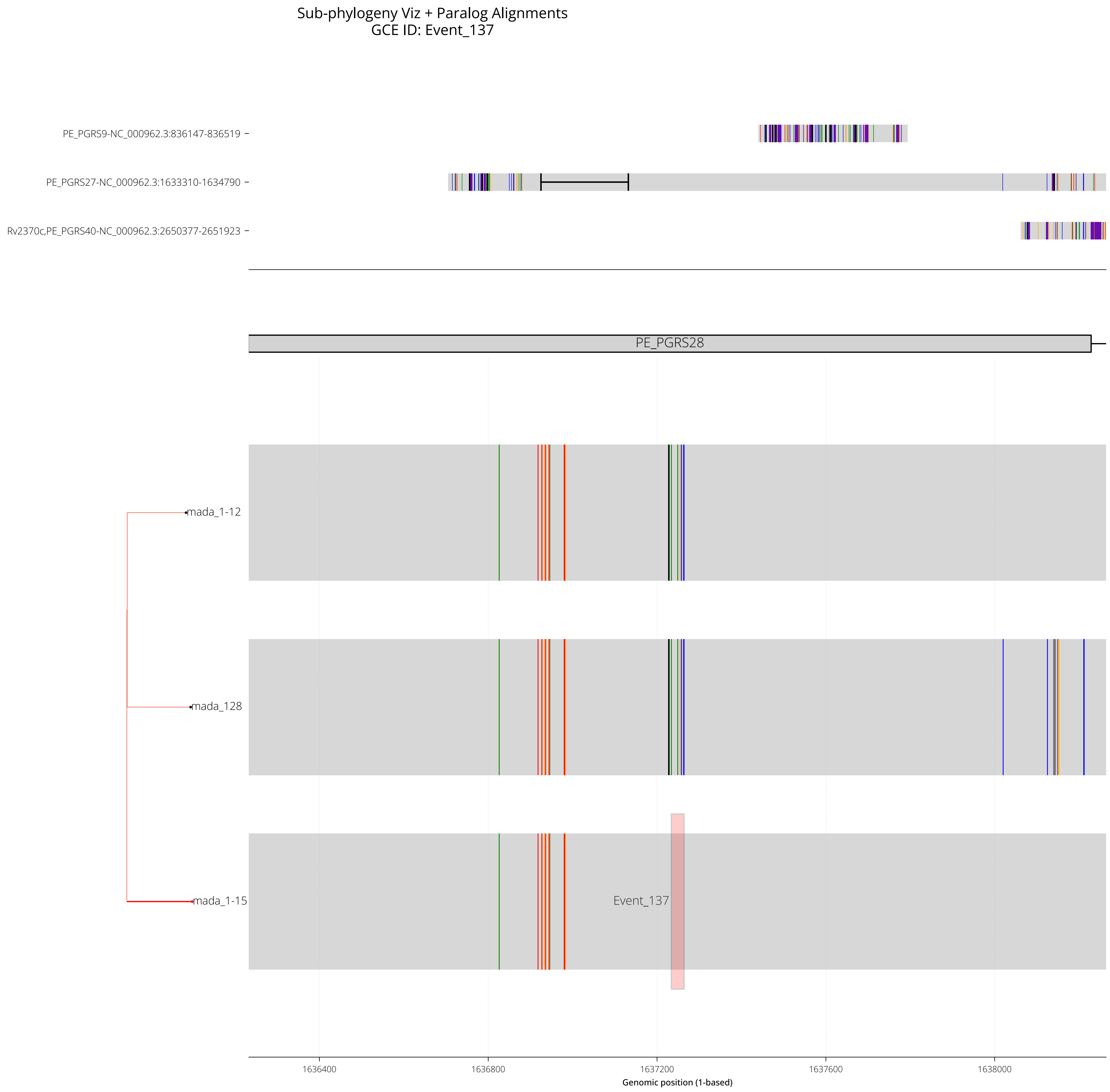

Sub-phylogeny Viz + Paralog Alignments
GCE ID: Event_137
PE_PGRS9-NC_000962.3:836147-836519
PE_PGRS27-NC_000962.3:1633310-1634790
Rv2370c,PE_PGRS40-NC_000962.3:2650377-2651923
PE_PGRS28
mada_1-12
mada_128
mada_1-15
Event_137
1636400
1636800
1637200
1637600
1638000
Genomic position (1-based)

Full Phylogeny Viz + Paralog Alignments
GCE ID: Event_137

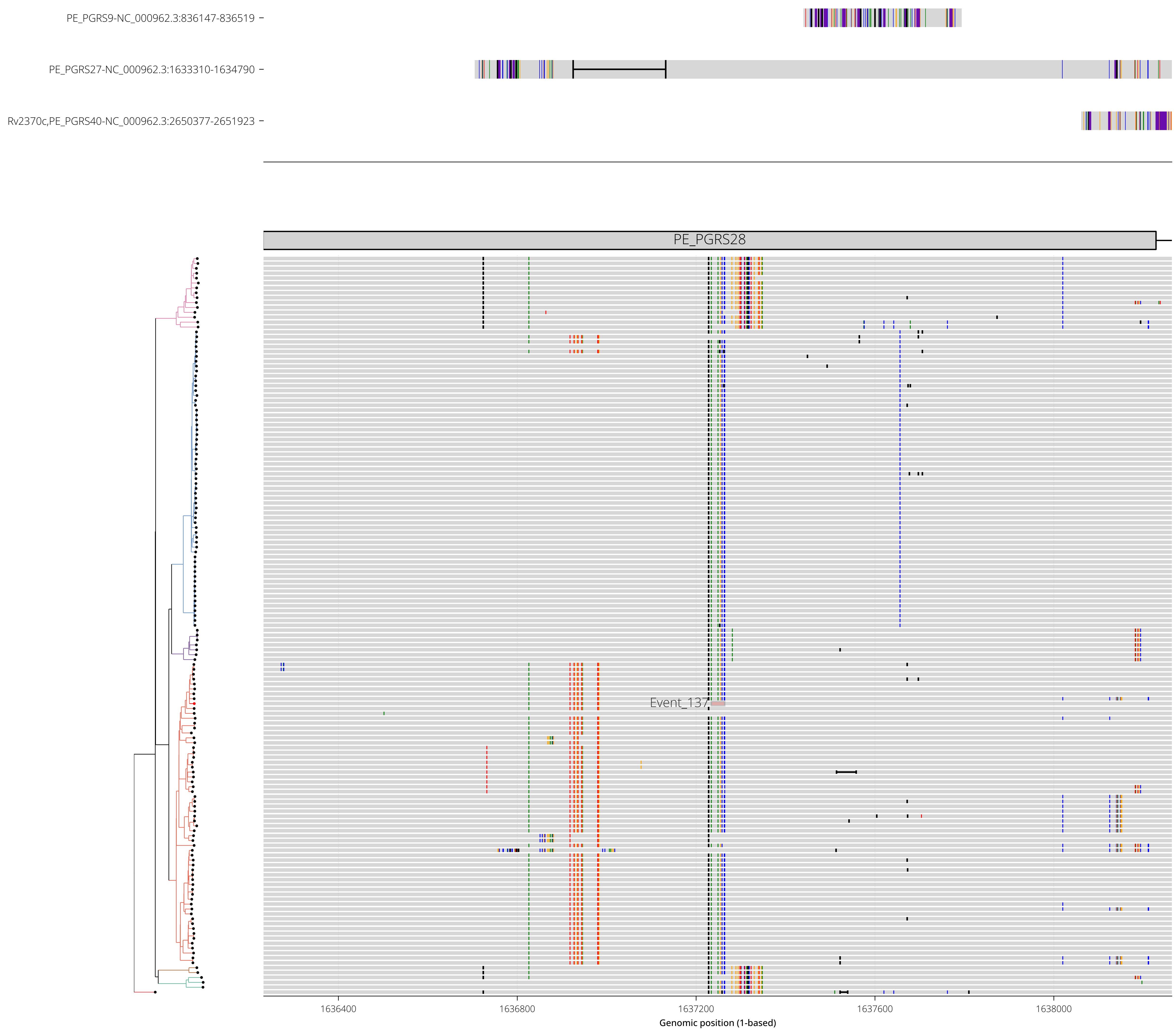

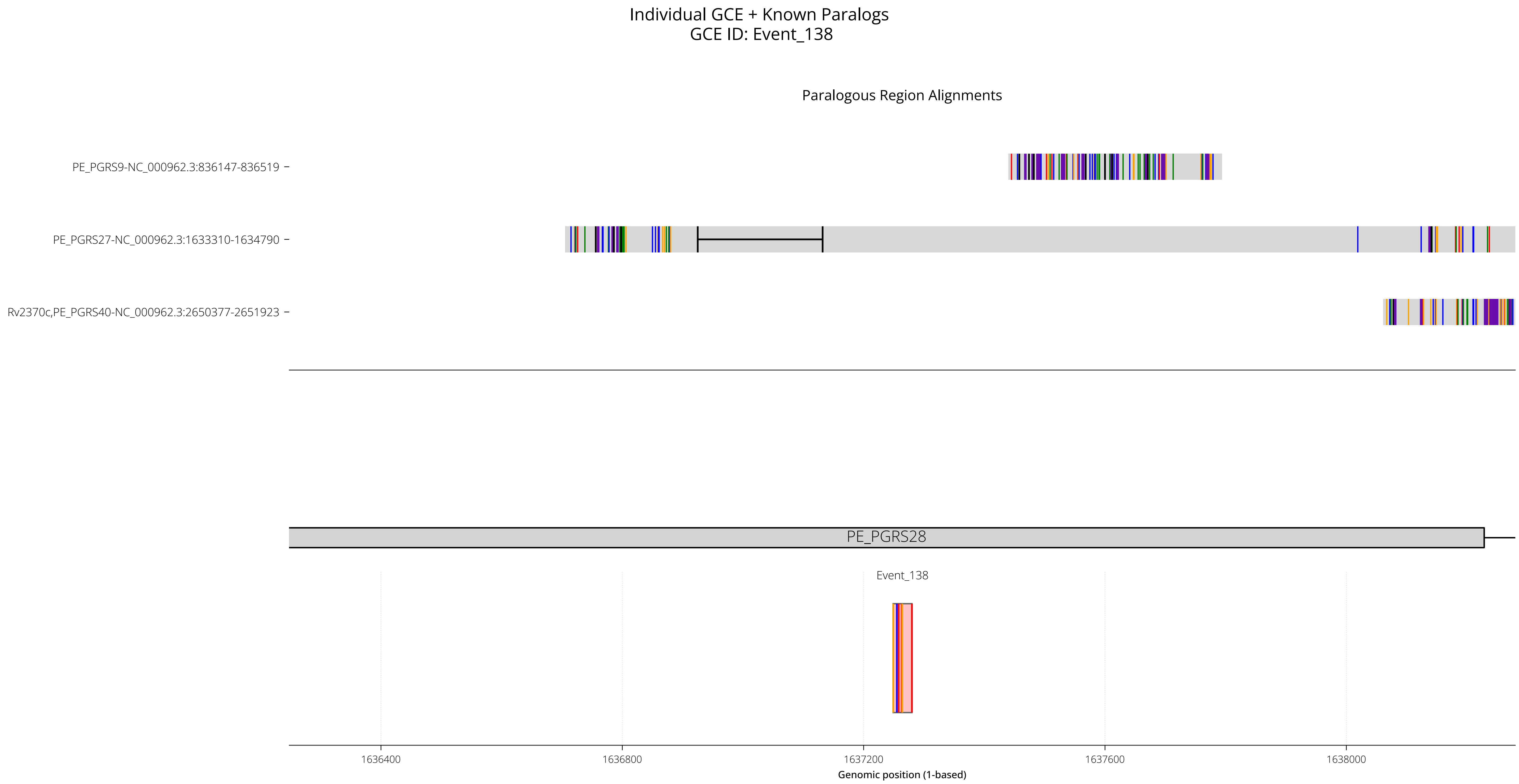
Individual GCE + Known Paralogs
GCE ID: Event_138
Paralogous Region Alignments
PE_PGRS9-NC_000962.3:836147-836519
PE_PGRS27-NC_000962.3:1633310-1634790
Rv2370c,PE_PGRS40-NC_000962.3:2650377-2651923
PE_PGRS28
Event_138
1636400
1636800
1637200
1637600
1638000
Genomic position (1-based)

Circos Viz - GCE to Known Paralogs Locations
GCE ID: Event_138

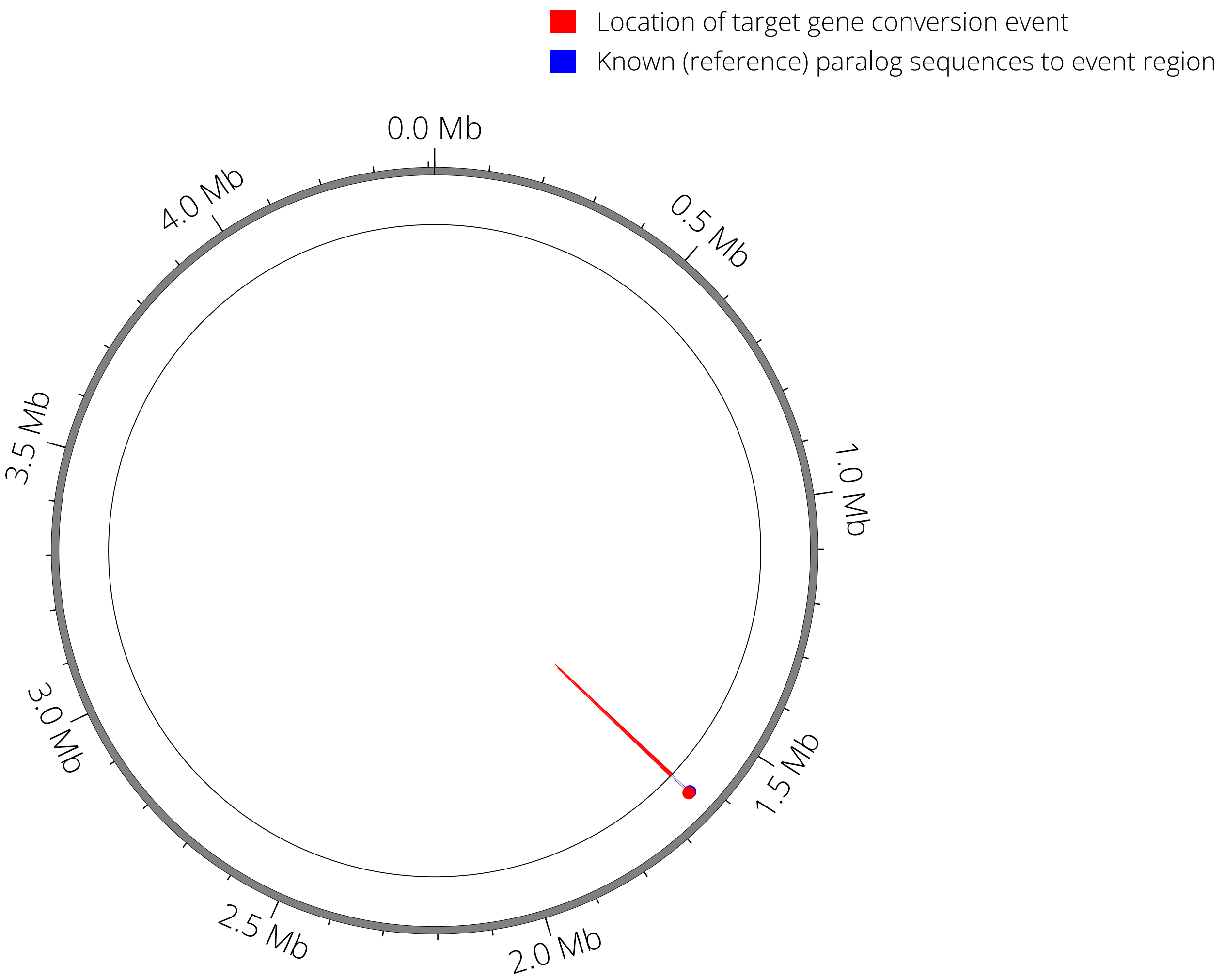

# Event_138 - 6 SNPs - k-mer analysis relative to paralogs

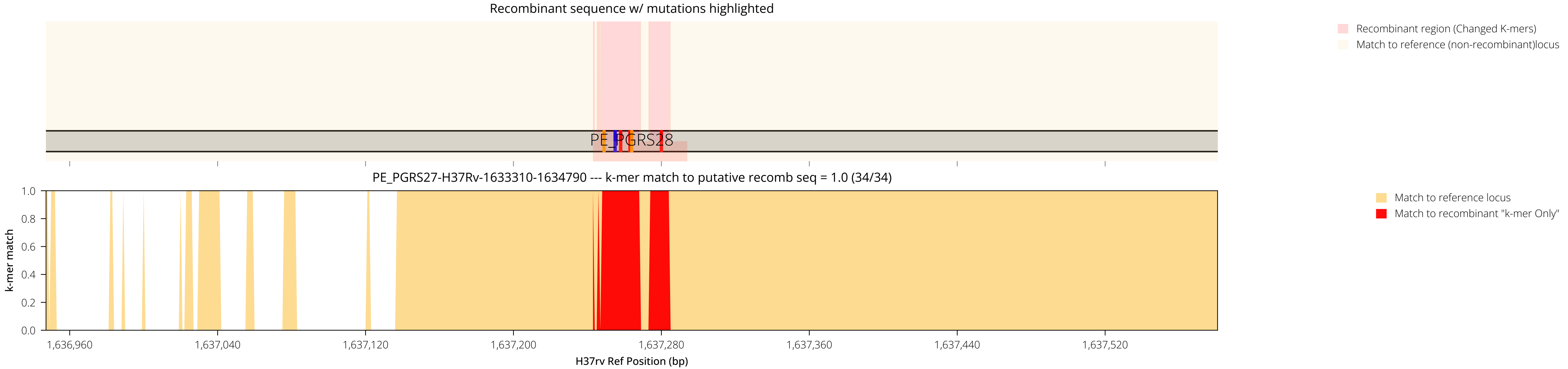

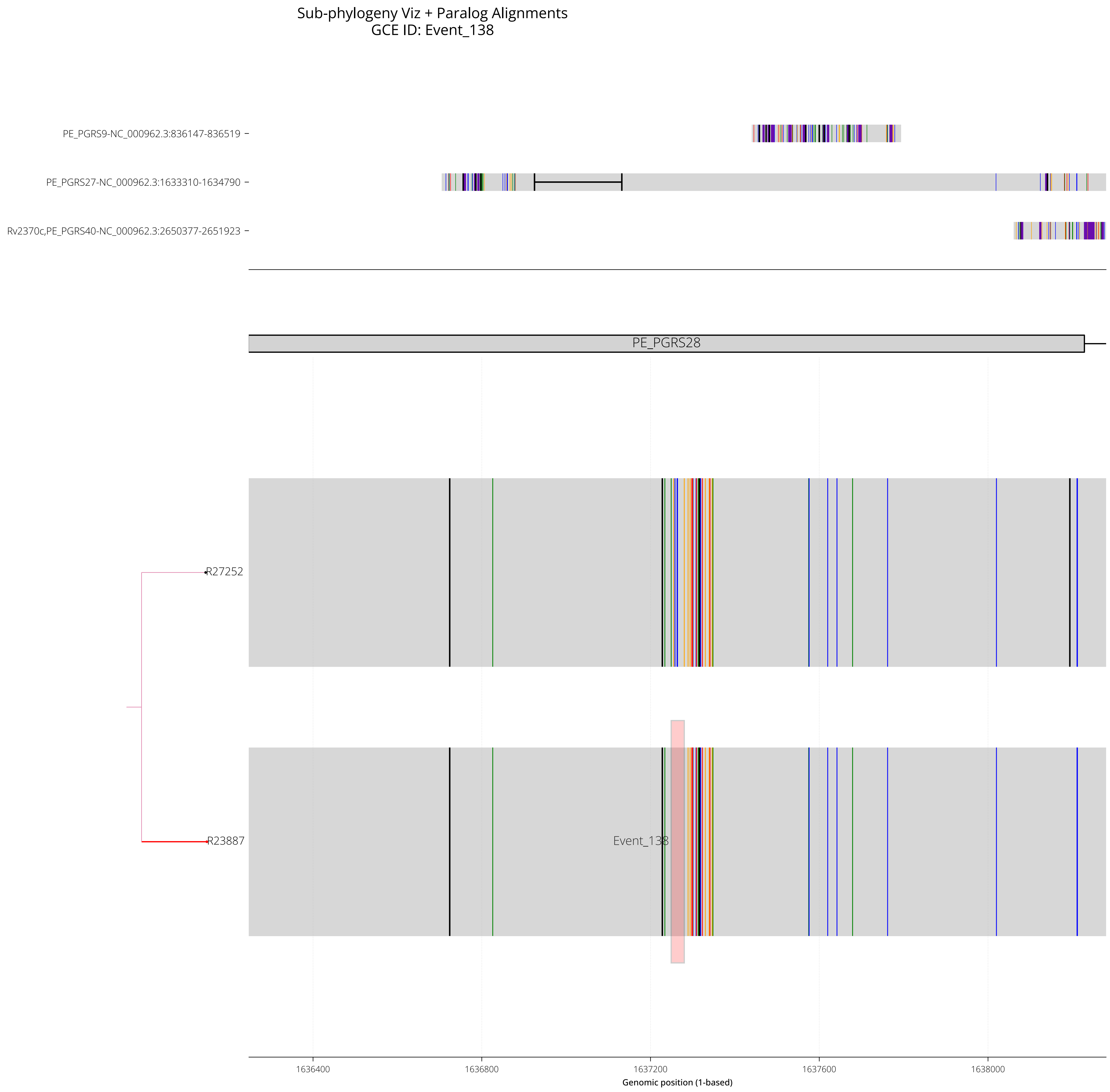

Sub-phylogeny Viz + Paralog Alignments
GCE ID: Event_138
PE_PGRS9-NC_000962.3:836147-836519
PE_PGRS27-NC_000962.3:1633310-1634790
Rv2370c,PE_PGRS40-NC_000962.3:2650377-2651923
PE_PGRS28
R27252
R23887
Event_138
1636400
1636800
1637200
1637600
1638000
Genomic position (1-based)

Full Phylogeny Viz + Paralog Alignments
GCE ID: Event_138

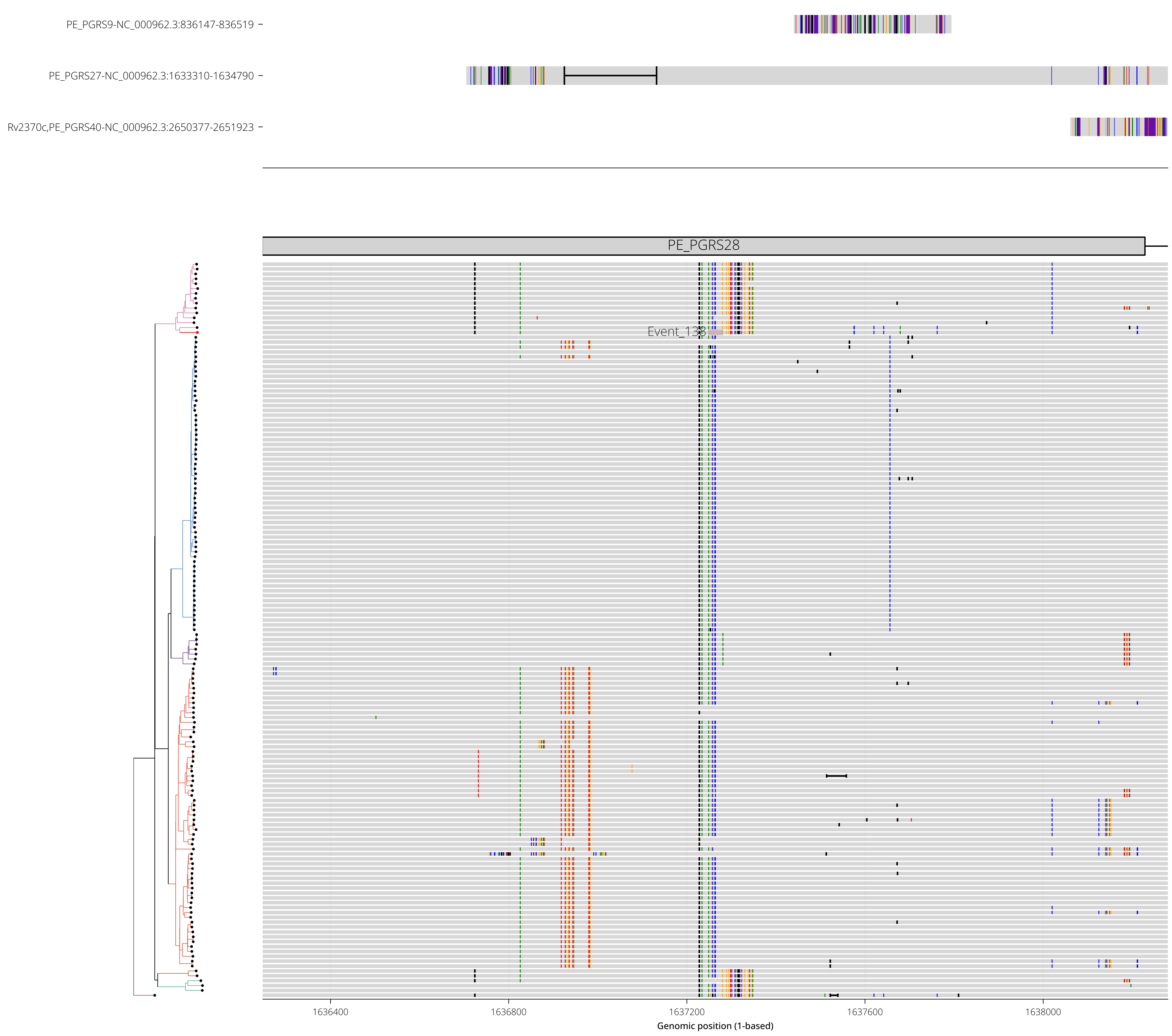

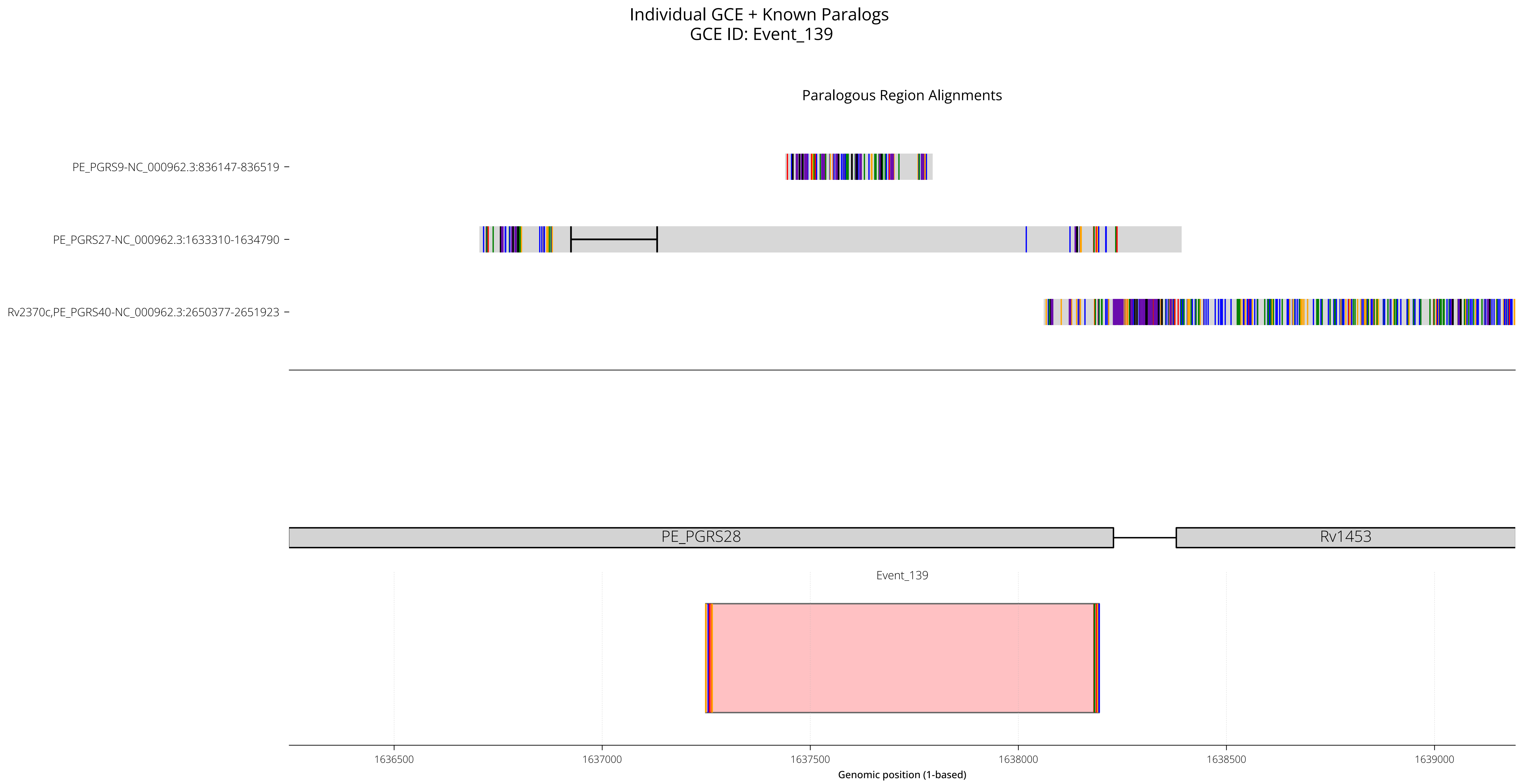
Individual GCE + Known Paralogs
GCE ID: Event_139
Paralogous Region Alignments
PE_PGRS9-NC_000962.3:836147-836519
PE_PGRS27-NC_000962.3:1633310-1634790
Rv2370c,PE_PGRS40-NC_000962.3:2650377-2651923
PE_PGRS28
Rv1453
Event_139
1636500
1637000
1637500
1638000
1638500
1639000
Genomic position (1-based)

Circos Viz - GCE to Known Paralogs Locations
GCE ID: Event_139

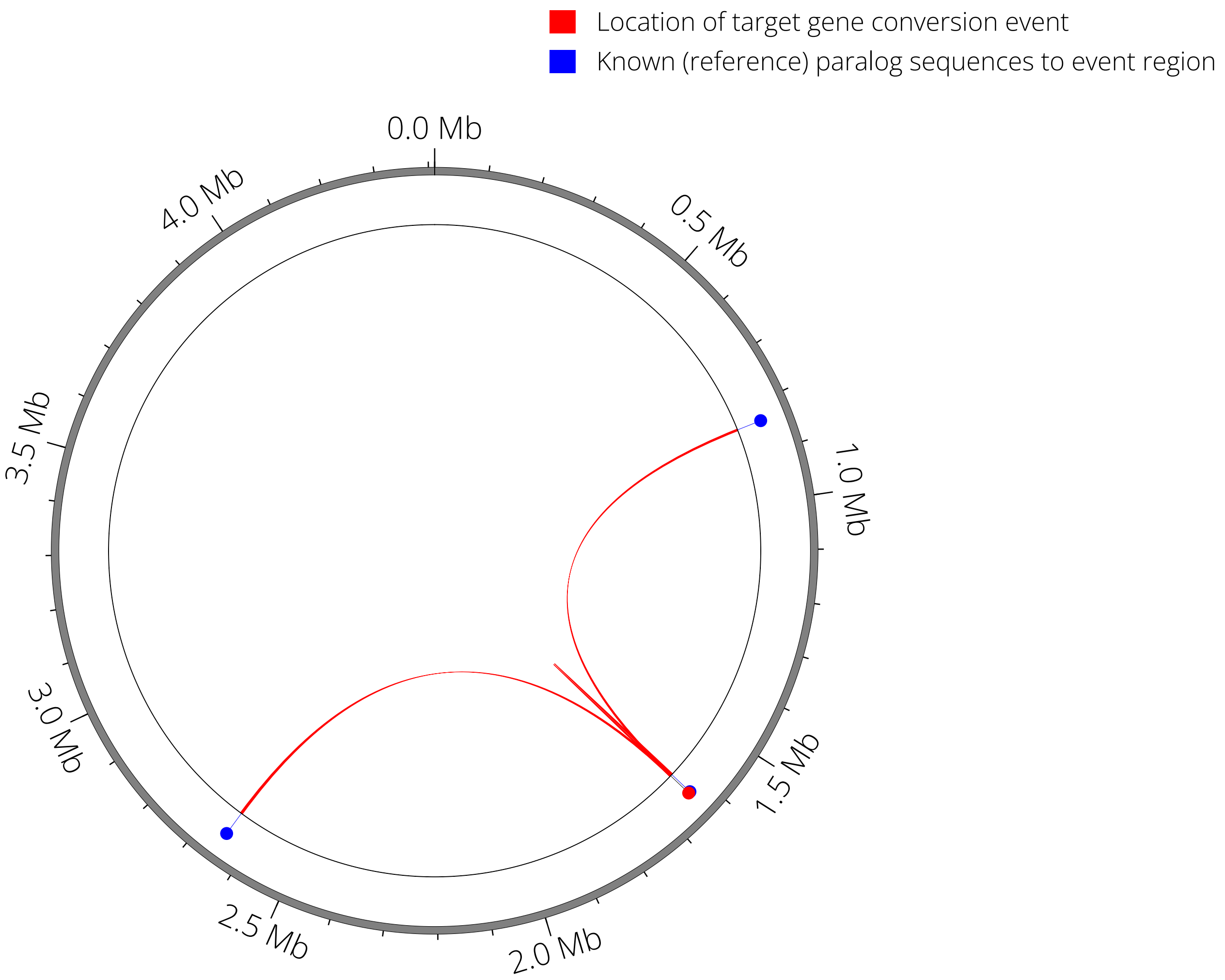

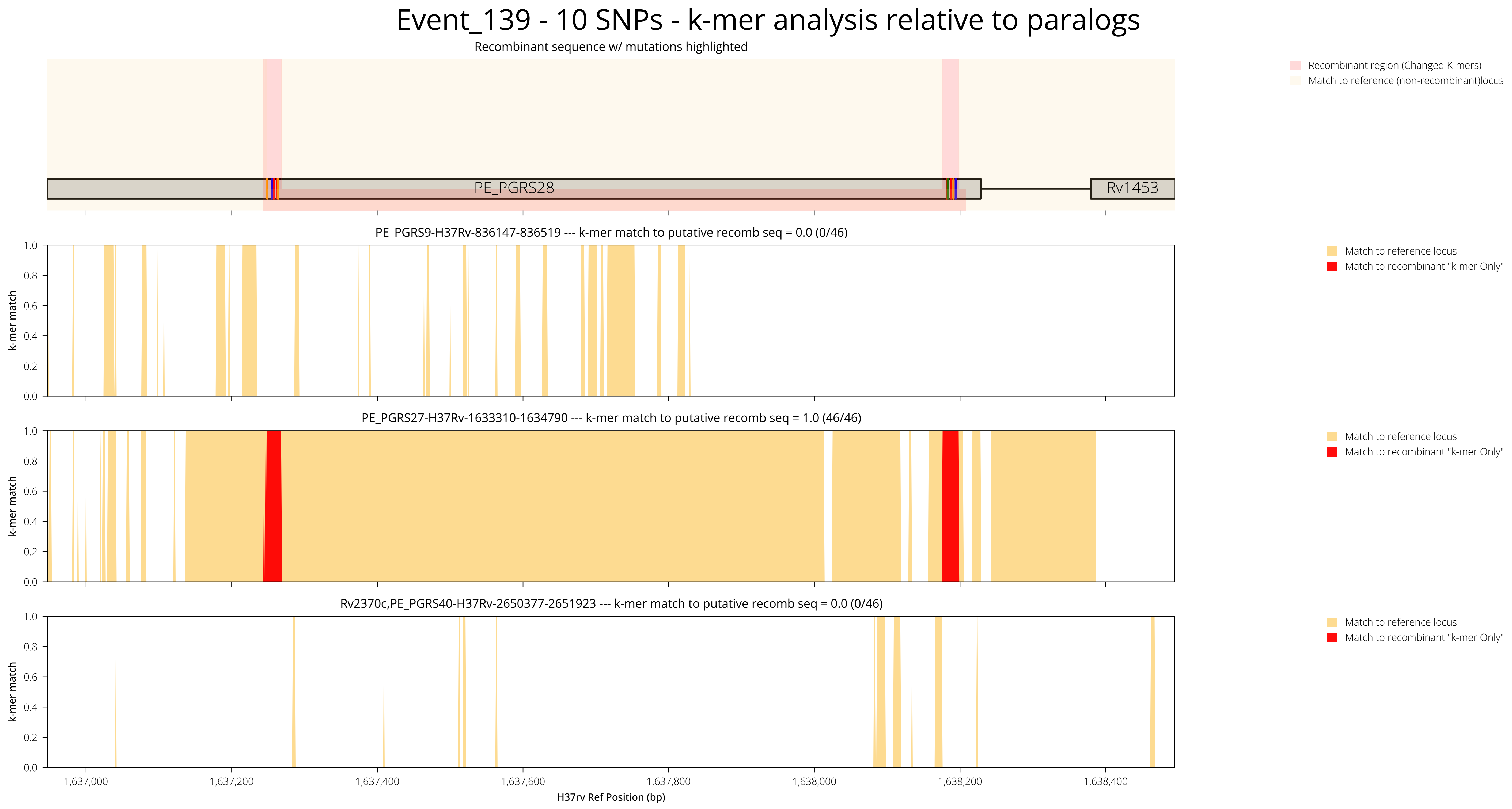
Event_139 - 10 SNPs - k-mer analysis relative to paralogs
Recombinant sequence w/ mutations highlighted
Recombinant region (Changed K-mers)
Match to reference (non-recombinant)locus
PE_PGRS28
Rv1453
PE_PGRS9-H37Rv-836147-836519 --- k-mer match to putative recomb seq = 0.0 (0/46)
PE_PGRS27-H37Rv-1633310-1634790 --- k-mer match to putative recomb seq = 1.0 (46/46)
Rv2370c,PE_PGRS40-H37Rv-2650377-2651923 --- k-mer match to putative recomb seq = 0.0 (0/46)
Match to reference locus
Match to recombinant "k-mer Only"
k-mer match
0.0
0.2
0.4
0.6
0.8
1.0
1,637,000
1,637,200
1,637,400
1,637,600
1,637,800
1,638,000
1,638,200
1,638,400
H37rv Ref Position (bp)

Sub-phylogeny Viz + Paralog Alignments
GCE ID: Event_139

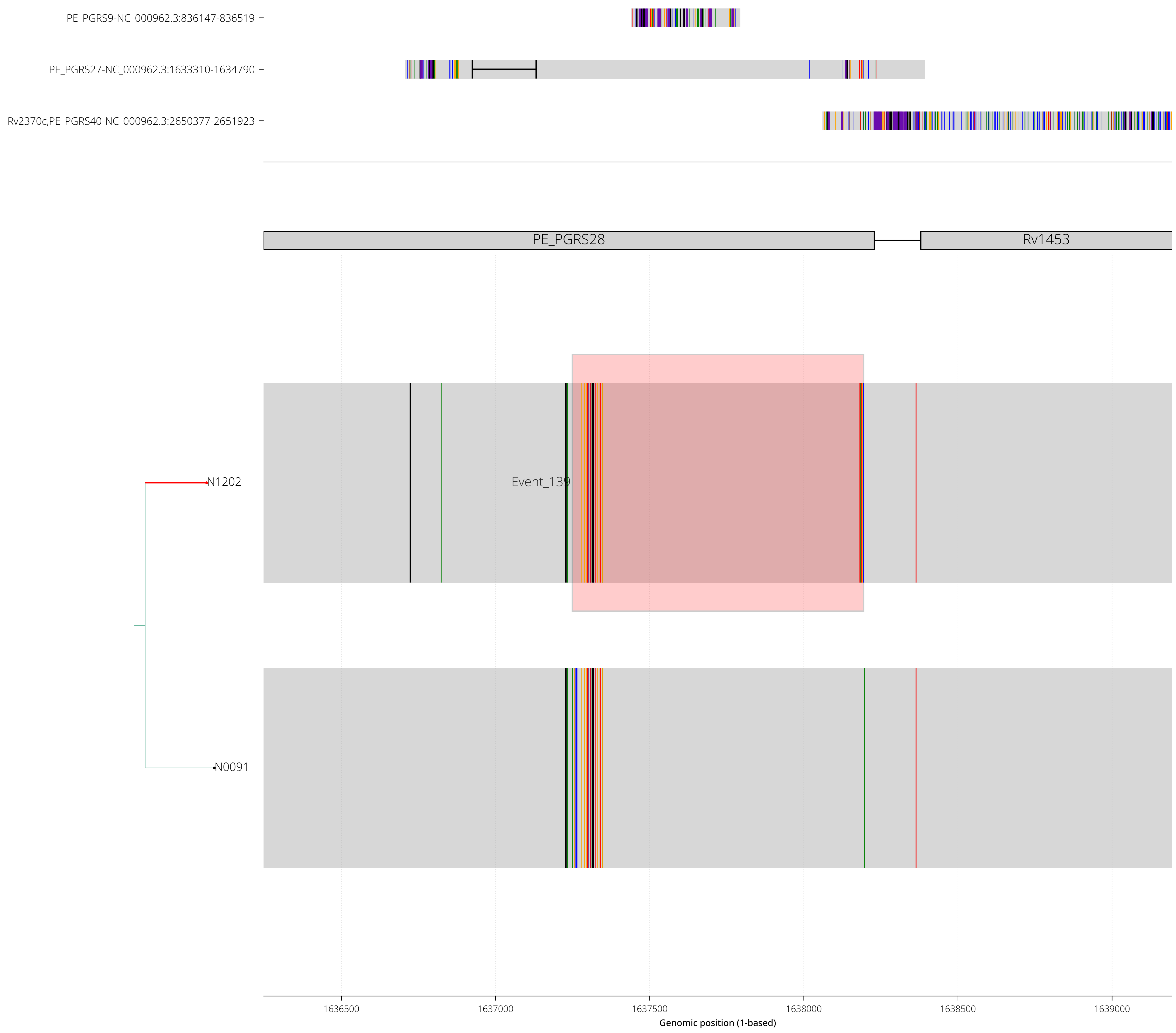

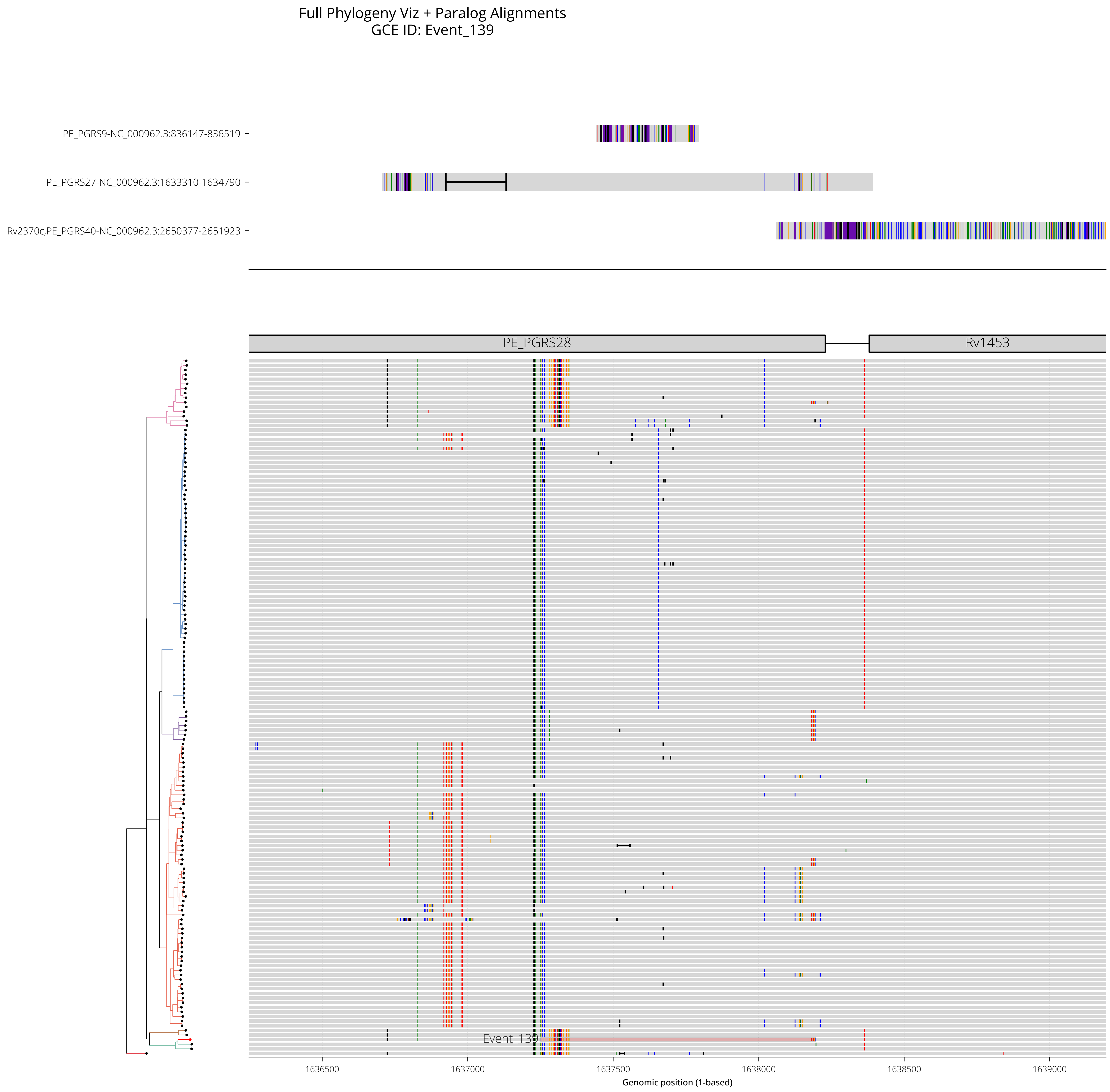

Full Phylogeny Viz + Paralog Alignments
GCE ID: Event_139
PE_PGRS9-NC_000962.3:836147-836519
PE_PGRS27-NC_000962.3:1633310-1634790
Rv2370c,PE_PGRS40-NC_000962.3:2650377-2651923
PE_PGRS28
Rv1453
Event_139
1636500
1637000
1637500
1638000
1638500
1639000
Genomic position (1-based)

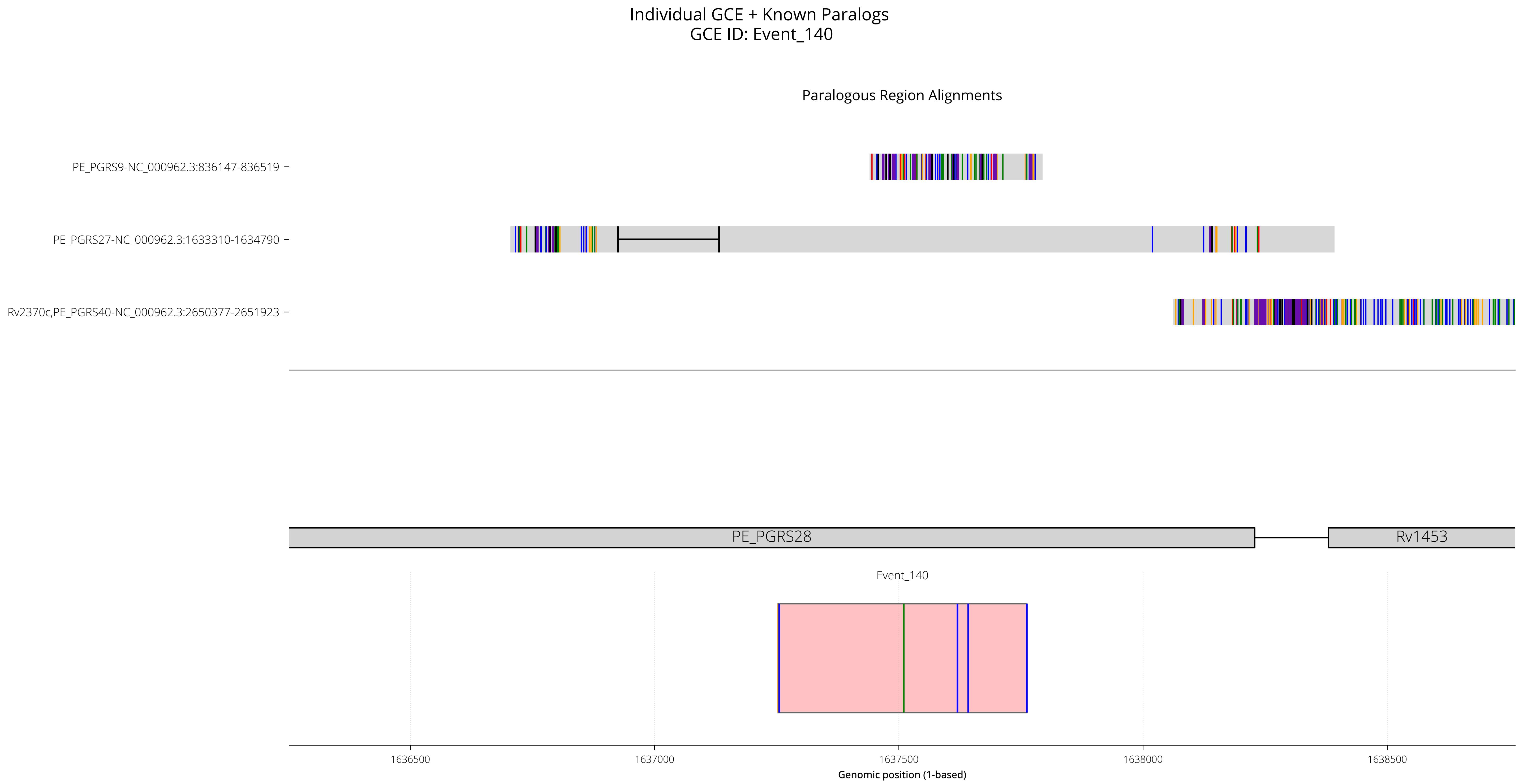
Individual GCE + Known Paralogs
GCE ID: Event_140
Paralogous Region Alignments
PE_PGRS9-NC_000962.3:836147-836519
PE_PGRS27-NC_000962.3:1633310-1634790
Rv2370c,PE_PGRS40-NC_000962.3:2650377-2651923
PE_PGRS28
Rv1453
Event_140
1636500
1637000
1637500
1638000
1638500
Genomic position (1-based)

Circos Viz - GCE to Known Paralogs Locations
GCE ID: Event_140

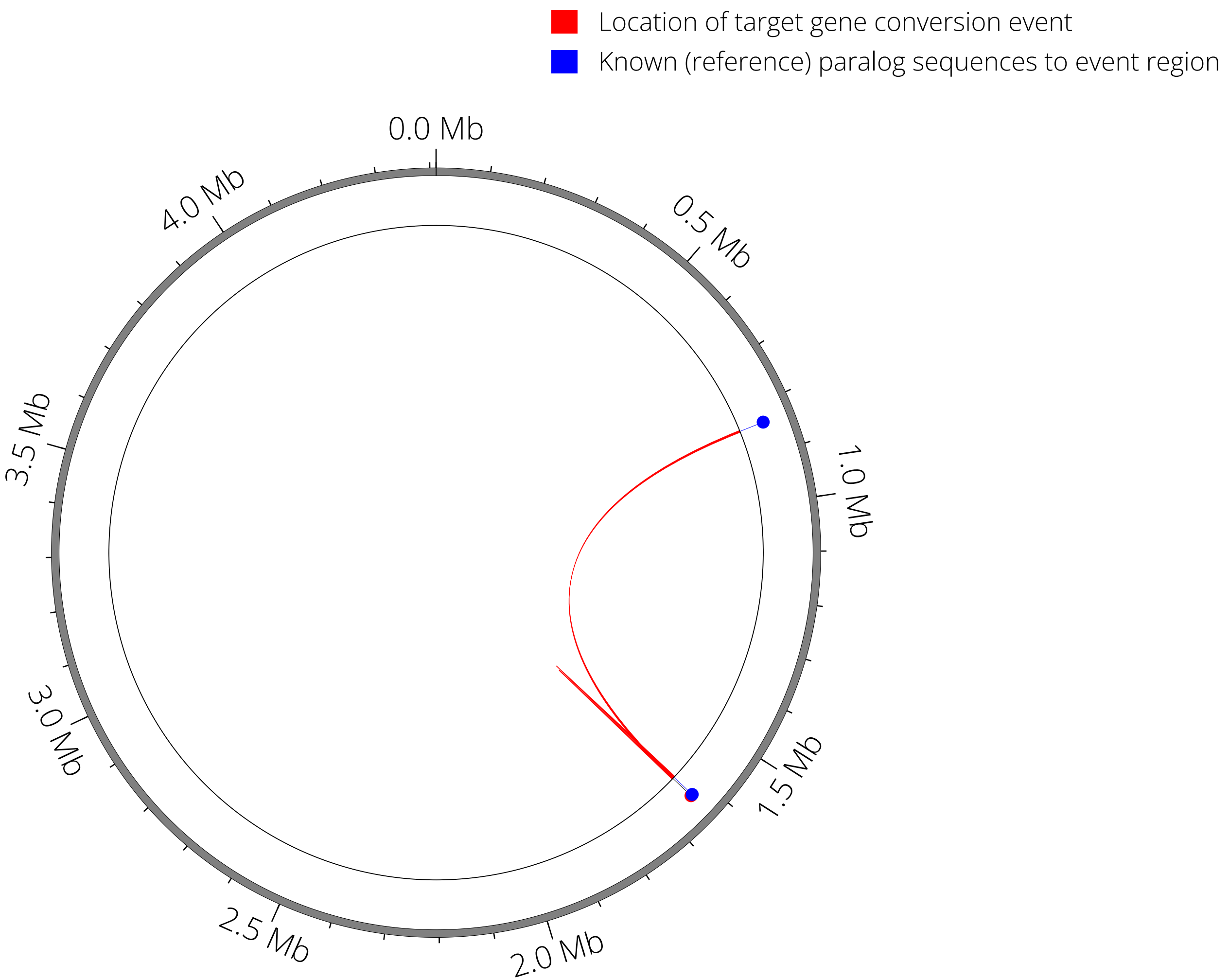

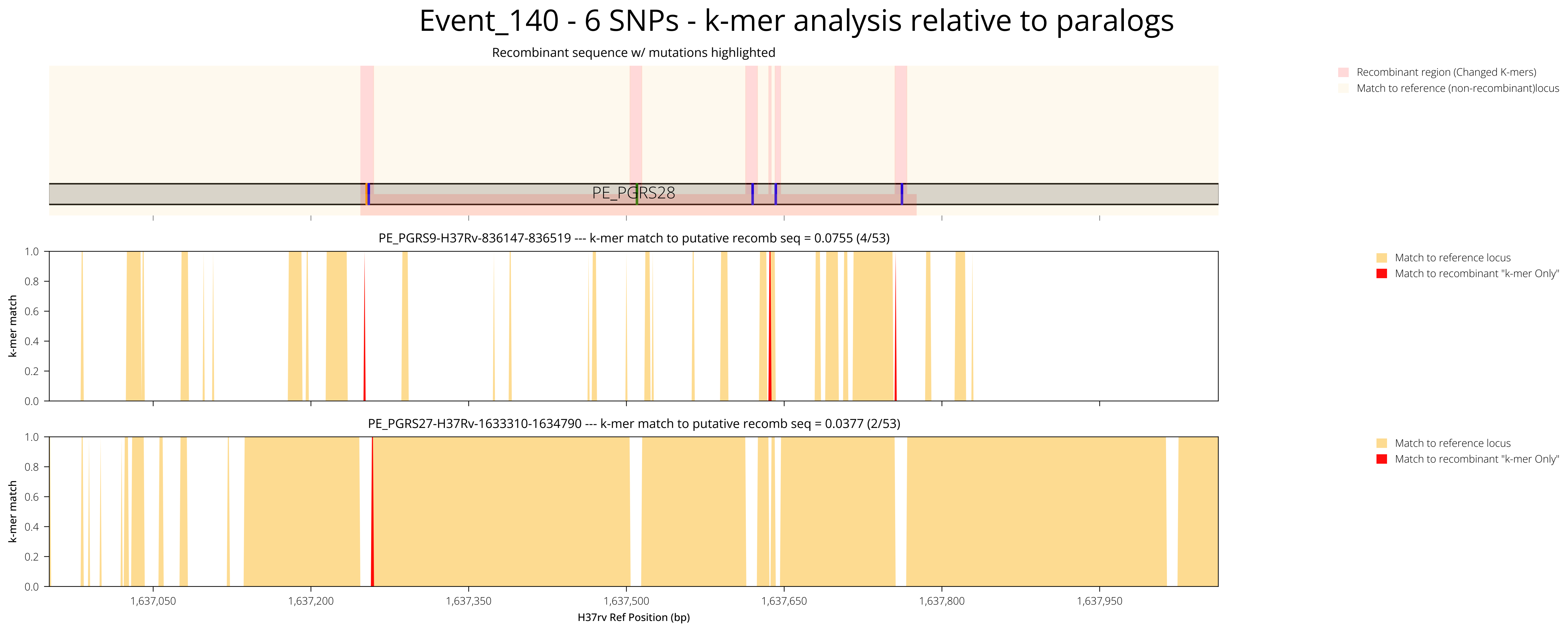

Event_140 - 6 SNPs - k-mer analysis relative to paralogs
Recombinant sequence w/ mutations highlighted
Recombinant region (Changed K-mers)
Match to reference (non-recombinant)locus
PE_PGRS28
PE_PGRS9-H37Rv-836147-836519 --- k-mer match to putative recomb seq = 0.0755 (4/53)
Match to reference locus
Match to recombinant "k-mer Only"
PE_PGRS27-H37Rv-1633310-1634790 --- k-mer match to putative recomb seq = 0.0377 (2/53)
Match to reference locus
Match to recombinant "k-mer Only"
k-mer match
1.0
0.8
0.6
0.4
0.2
0.0
1,637,050
1,637,200
1,637,350
1,637,500
1,637,650
1,637,800
1,637,950
H37rv Ref Position (bp)

Sub-phylogeny Viz + Paralog Alignments
GCE ID: Event_140

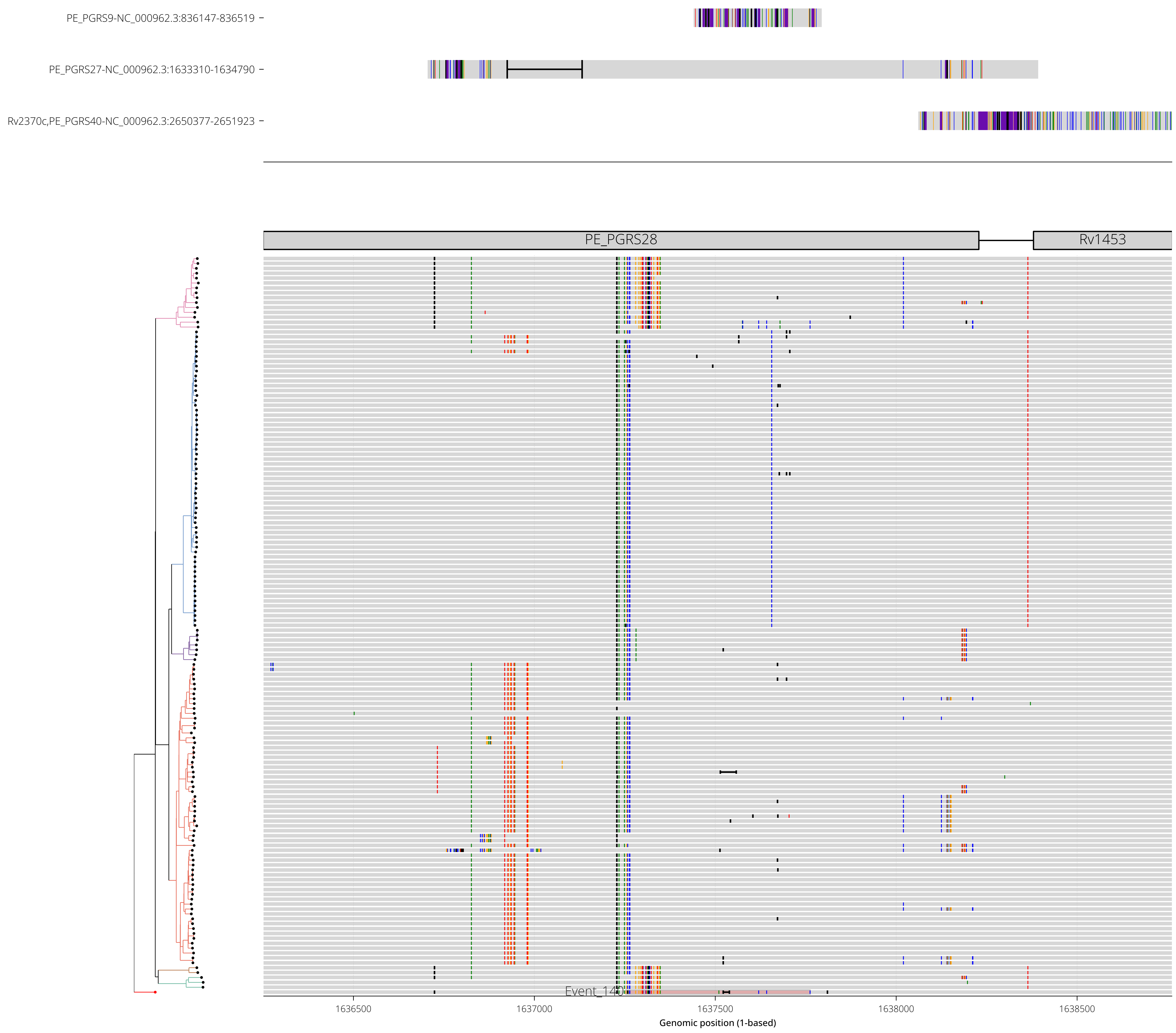

Full Phylogeny Viz + Paralog Alignments
GCE ID: Event_140

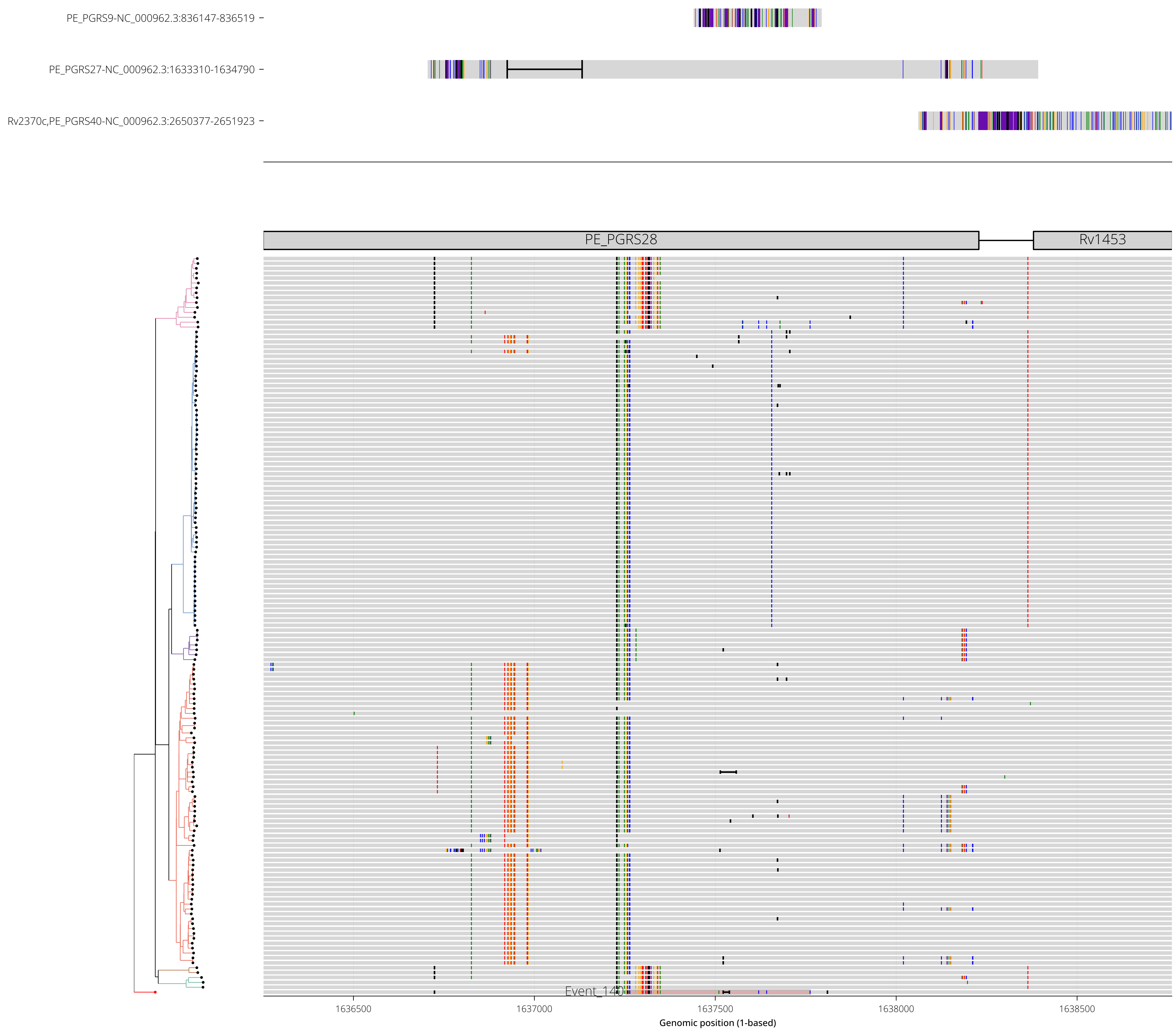

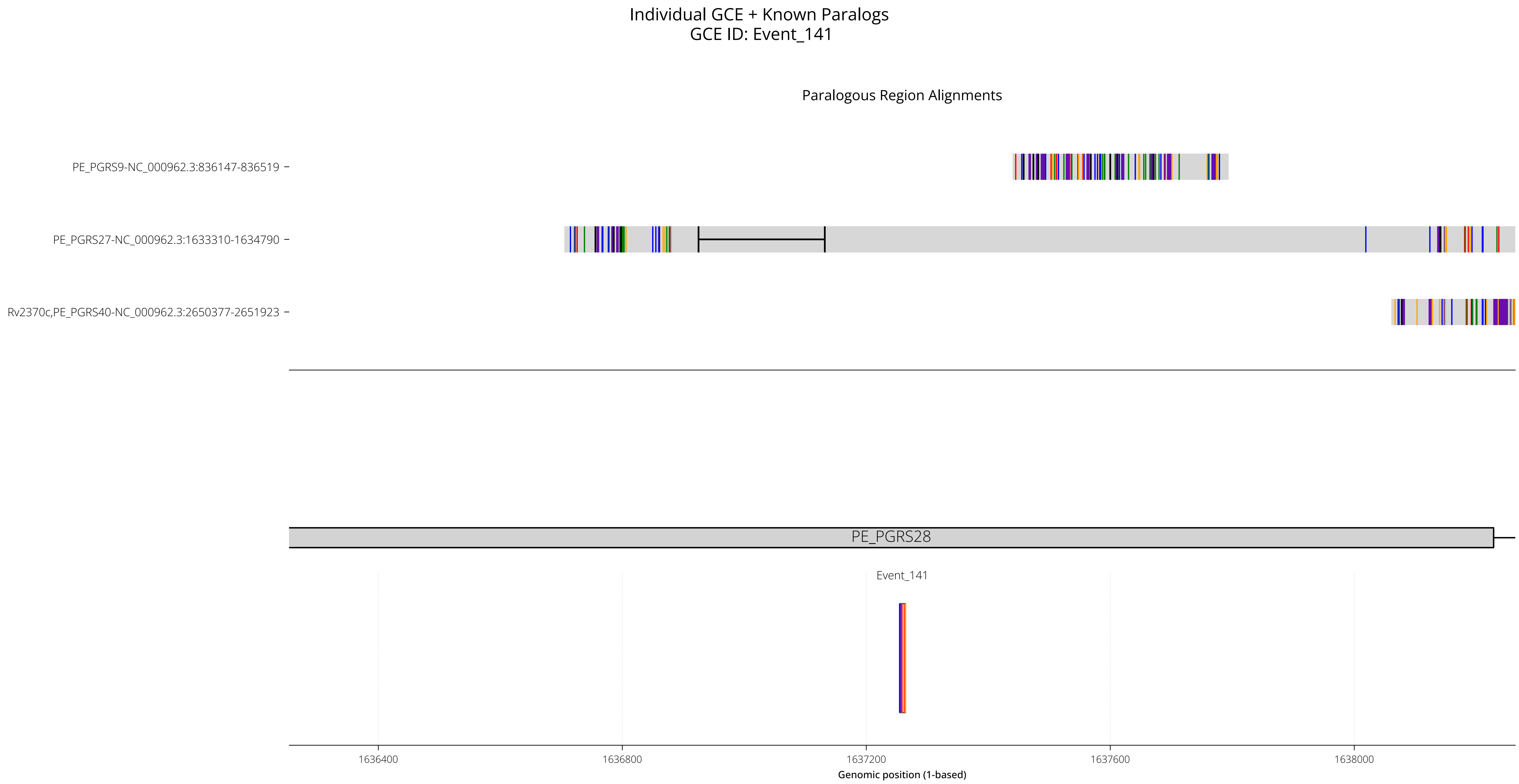

Individual GCE + Known Paralogs
GCE ID: Event_141
Paralogous Region Alignments
PE_PGRS9-NC_000962.3:836147-836519
PE_PGRS27-NC_000962.3:1633310-1634790
Rv2370c,PE_PGRS40-NC_000962.3:2650377-2651923
PE_PGRS28
Event_141
1636400
1636800
1637200
1637600
1638000
Genomic position (1-based)

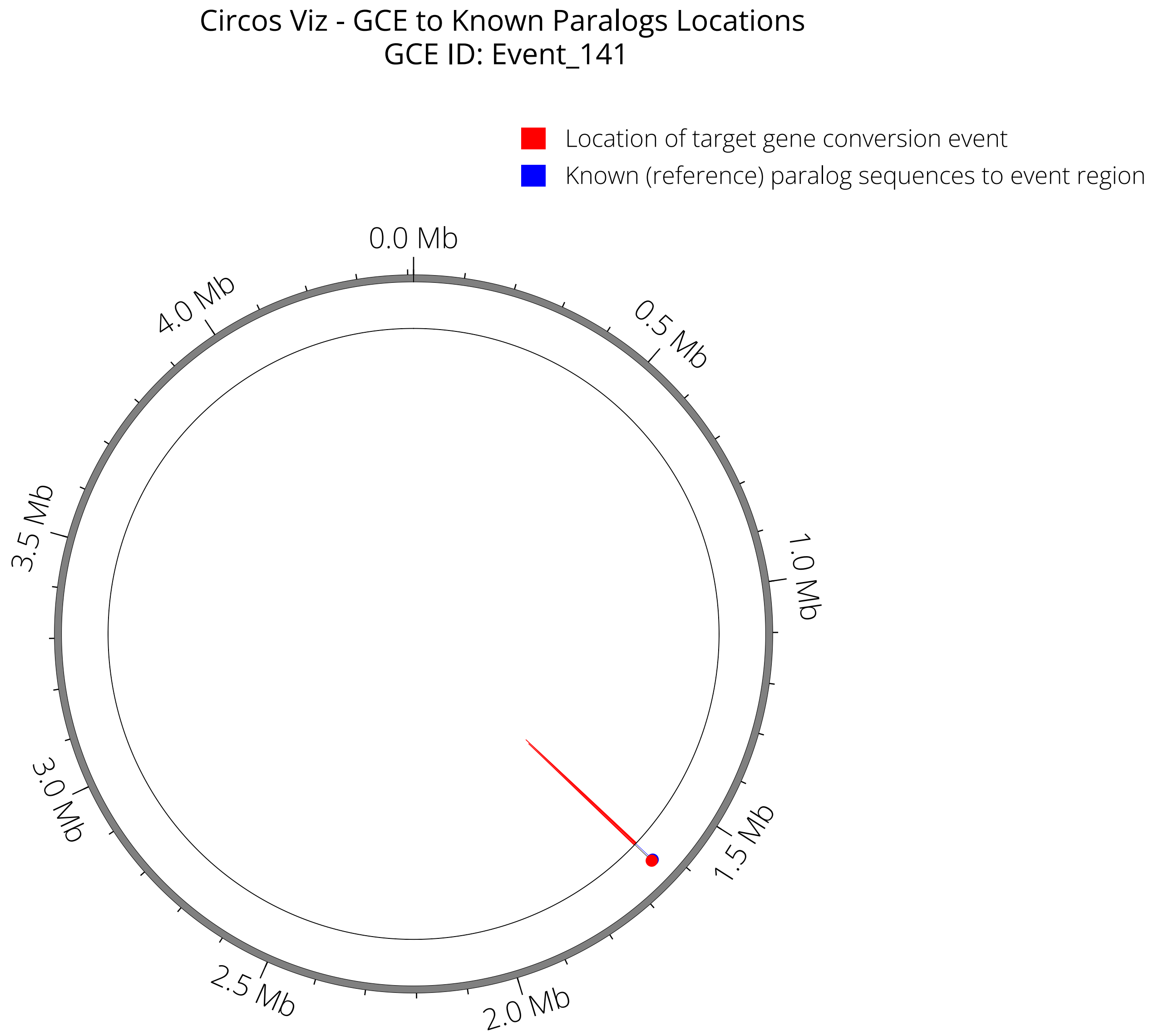

Circos Viz - GCE to Known Paralogs Locations
GCE ID: Event_141
Location of target gene conversion event
Known (reference) paralog sequences to event region
0.0 Mb
0.5 Mb
1.0 Mb
1.5 Mb
2.0 Mb
2.5 Mb
3.0 Mb
3.5 Mb
4.0 Mb

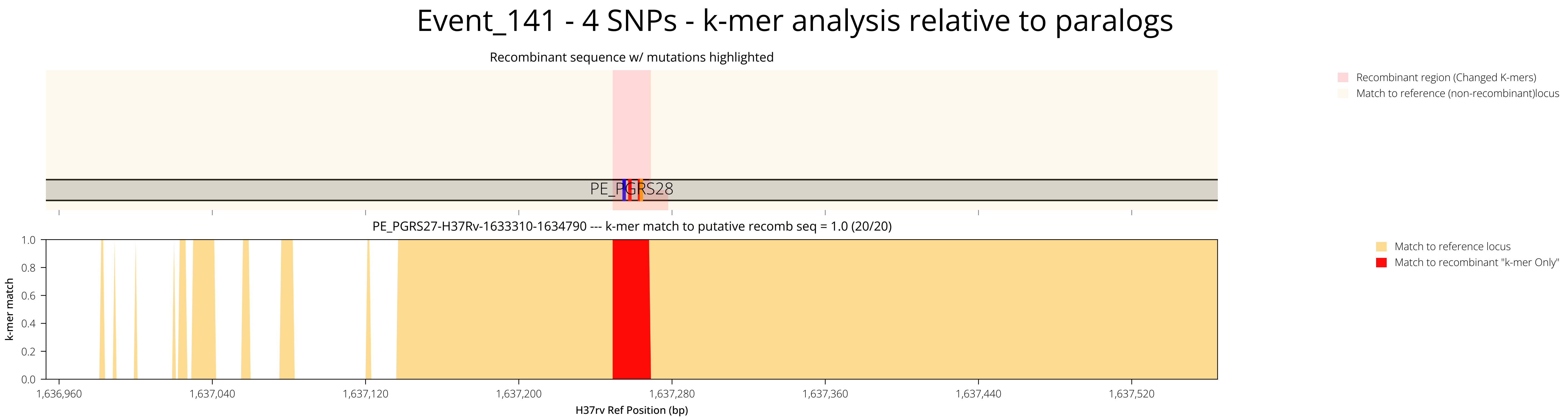
Event_141 - 4 SNPs - k-mer analysis relative to paralogs
Recombinant sequence w/ mutations highlighted
PE_PGRS28
Recombinant region (Changed K-mers)
Match to reference (non-recombinant)locus
PE_PGRS27-H37Rv-1633310-1634790 --- k-mer match to putative recomb seq = 1.0 (20/20)
Match to reference locus
Match to recombinant "k-mer Only"
k-mer match
1.0
0.8
0.6
0.4
0.2
0.0
1,636,960
1,637,040
1,637,120
1,637,200
1,637,280
1,637,360
1,637,440
1,637,520
H37rv Ref Position (bp)

Sub-phylogeny Viz + Paralog Alignments
GCE ID: Event_141

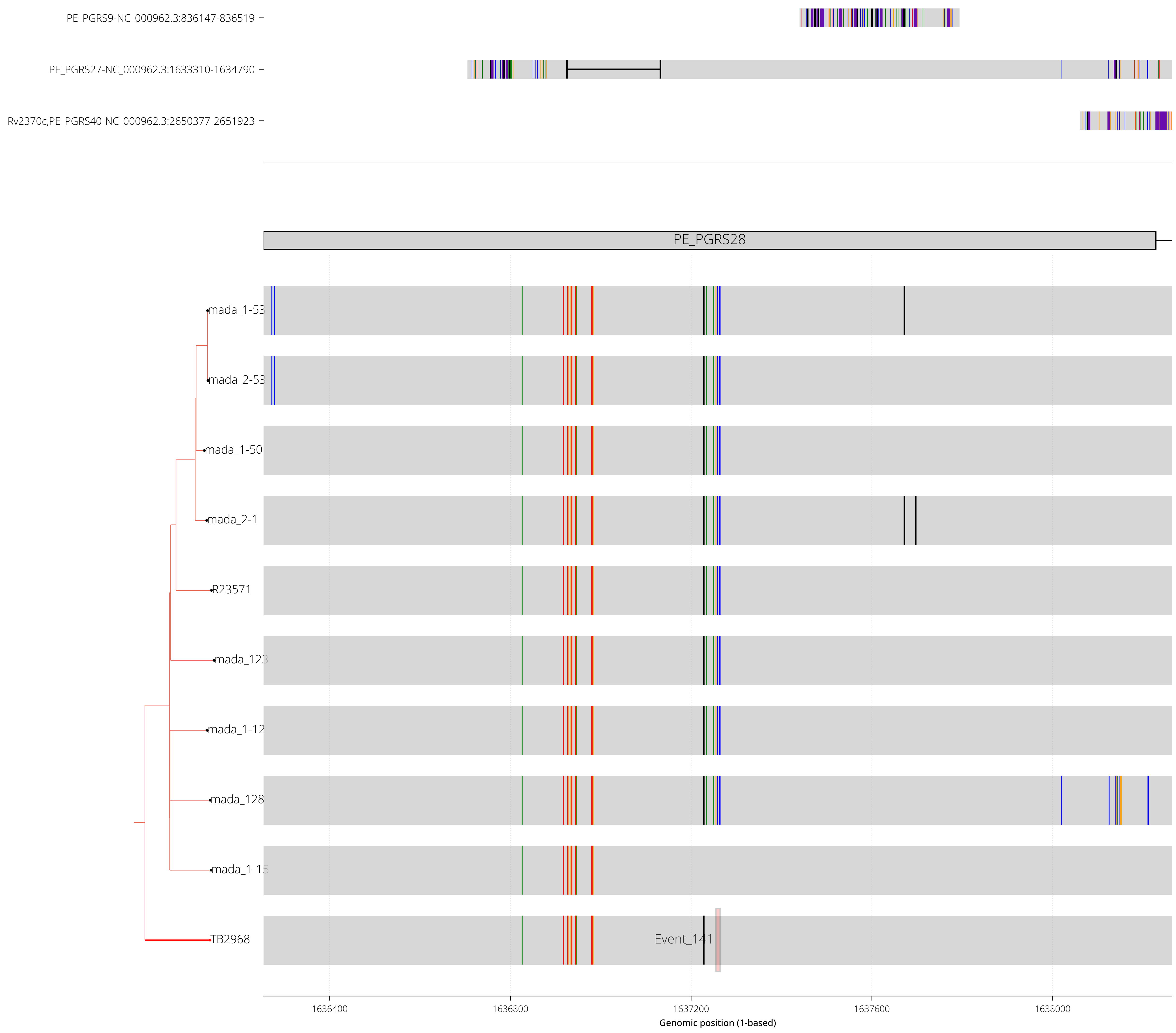

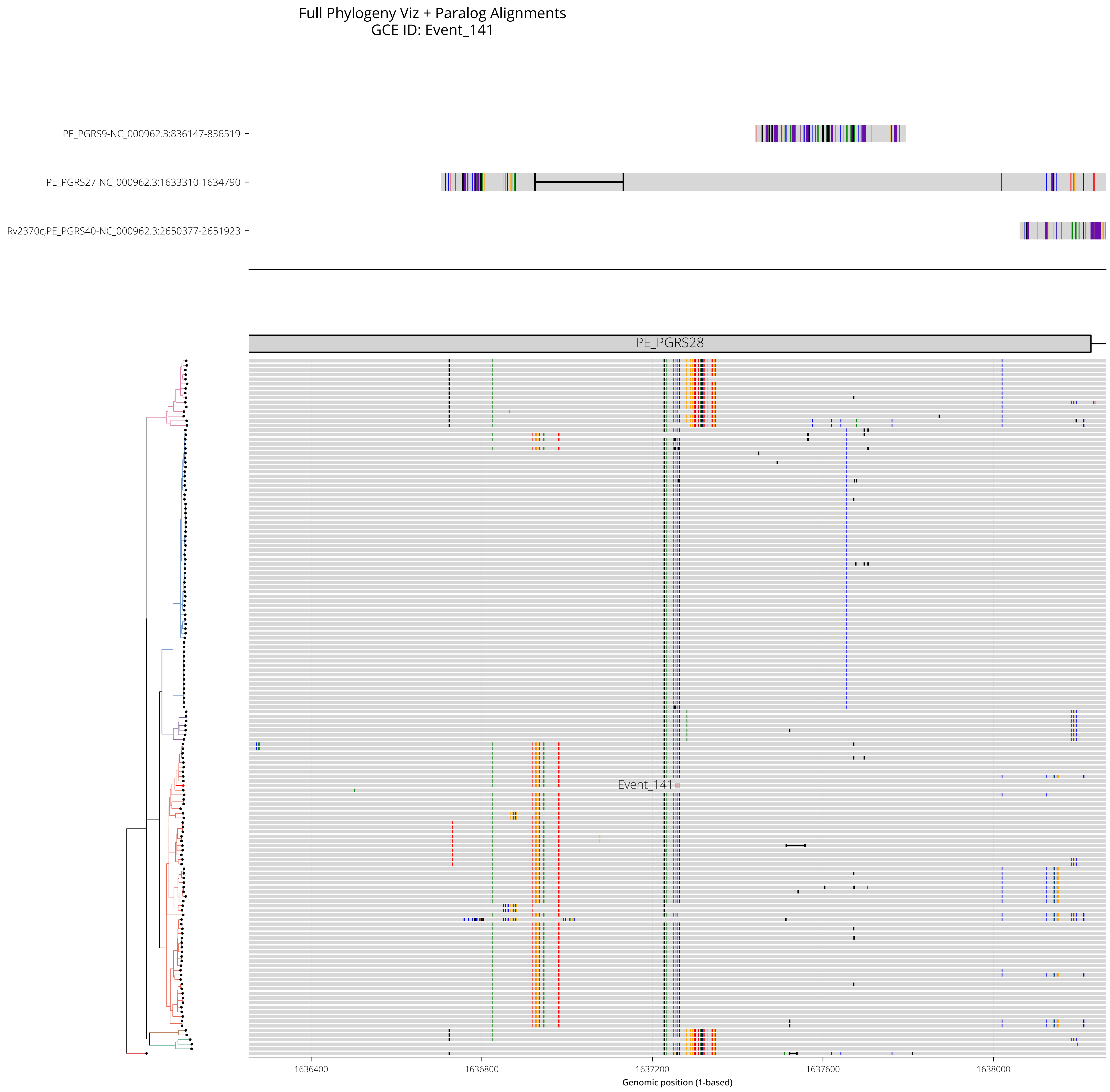

Full Phylogeny Viz + Paralog Alignments
GCE ID: Event_141
PE_PGRS9-NC_000962.3:836147-836519
PE_PGRS27-NC_000962.3:1633310-1634790
Rv2370c,PE_PGRS40-NC_000962.3:2650377-2651923
PE_PGRS28
Event_141
1636400
1636800
1637200
1637600
1638000
Genomic position (1-based)

Individual GCE + Known Paralogs
GCE ID: Event_142

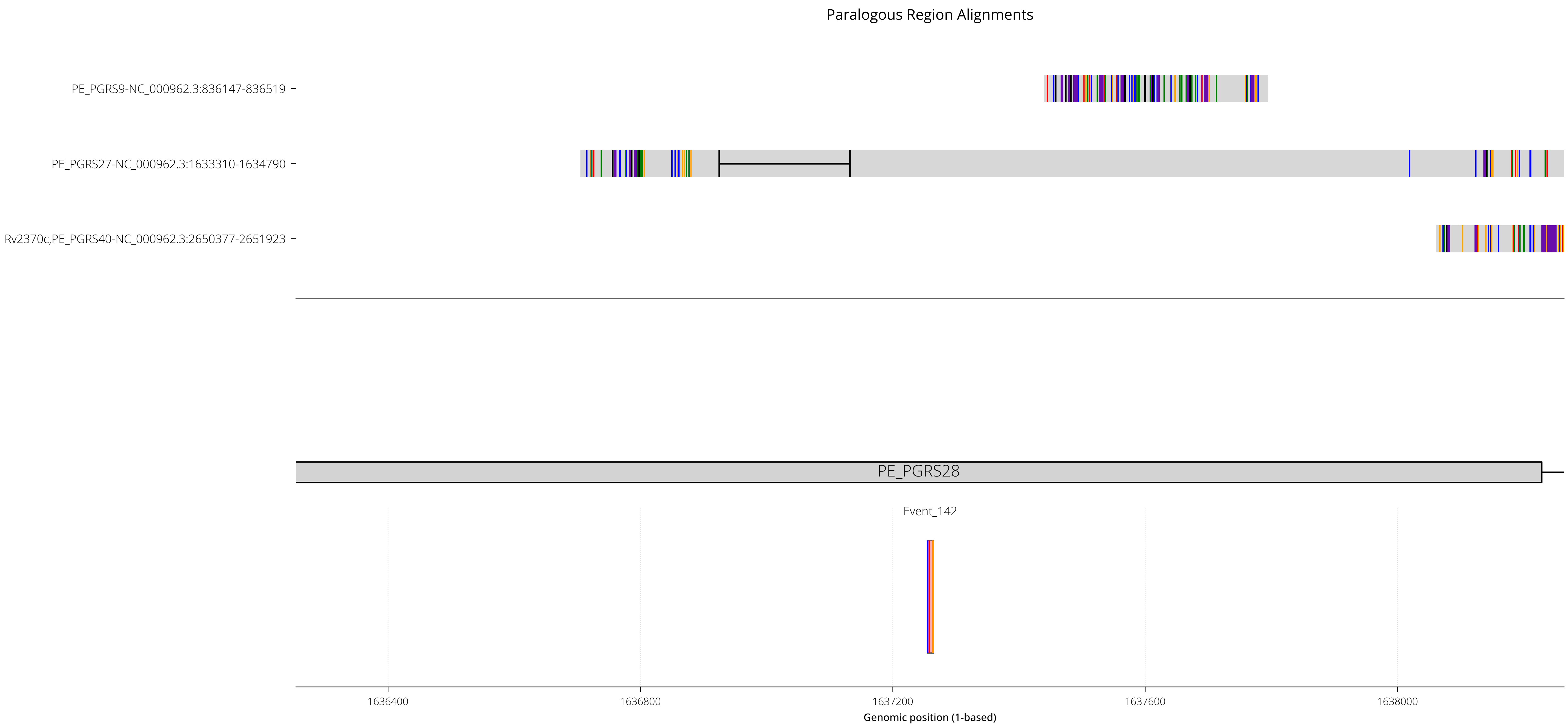

Circos Viz - GCE to Known Paralogs Locations
GCE ID: Event_142

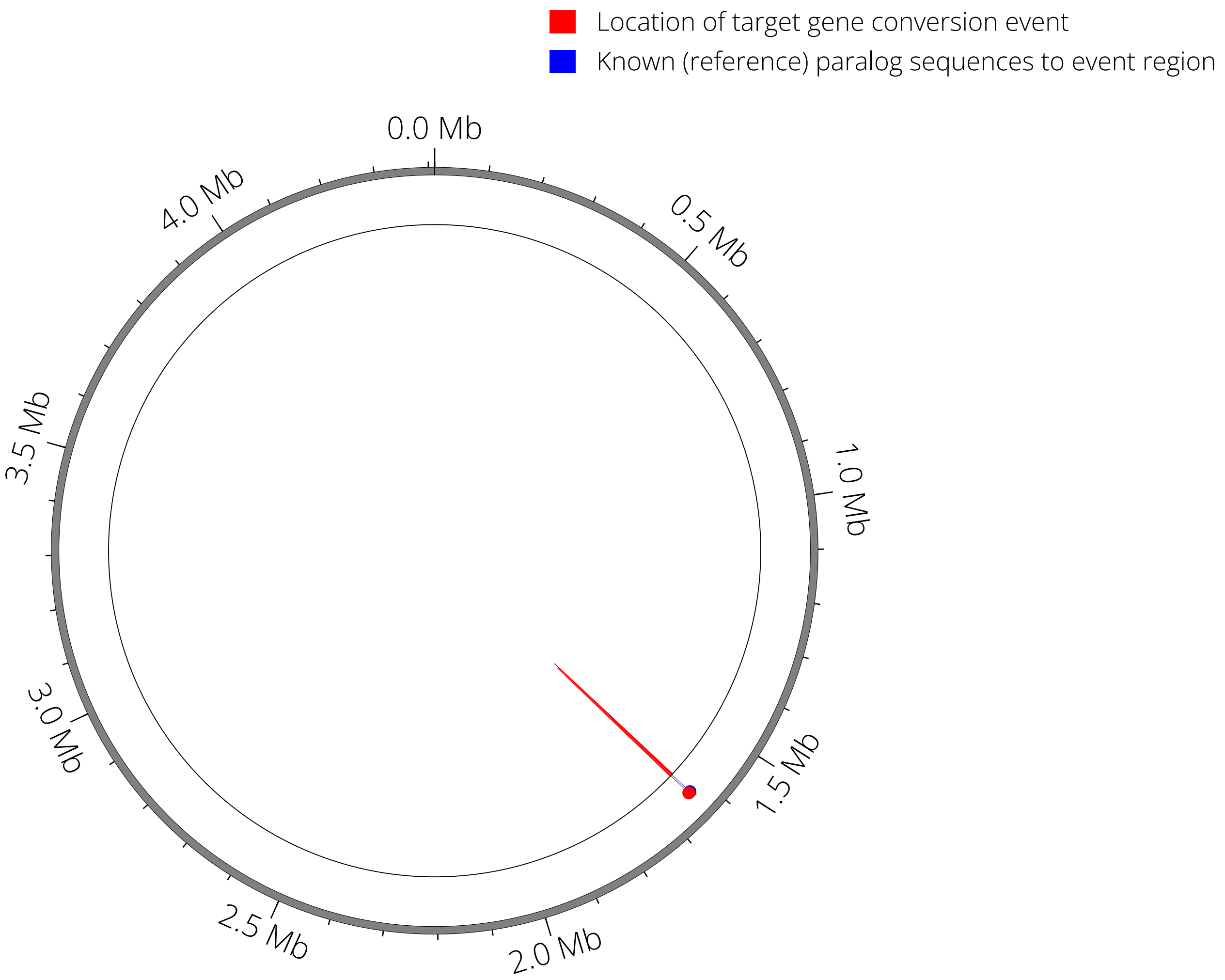

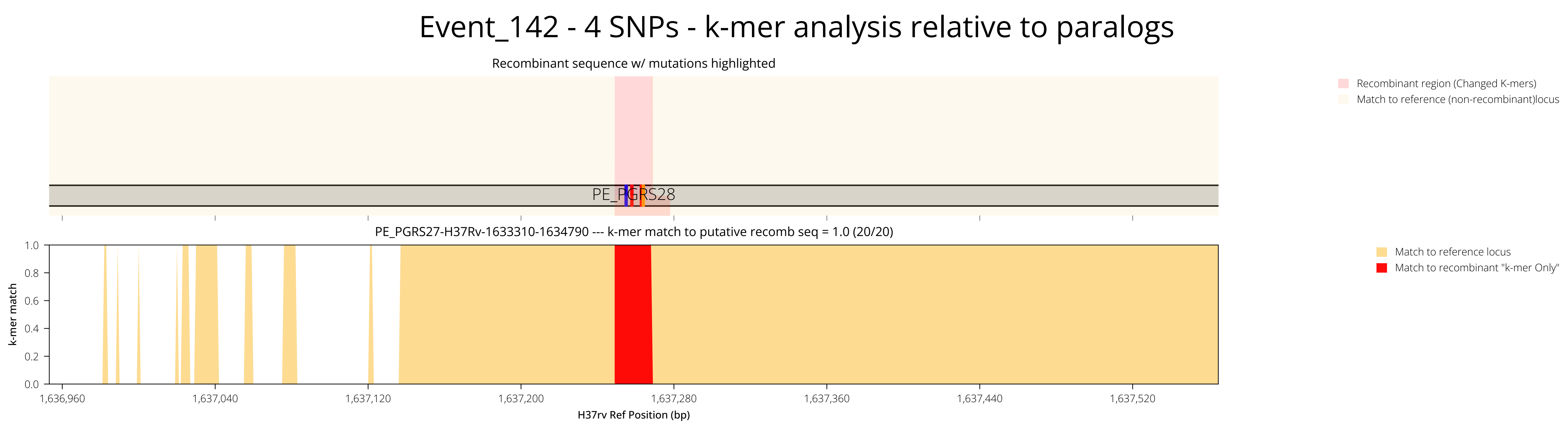
Event_142 - 4 SNPs - k-mer analysis relative to paralogs
Recombinant sequence w/ mutations highlighted
Recombinant region (Changed K-mers)
Match to reference (non-recombinant)locus
PE_PGRS28
PE_PGRS27-H37Rv-1633310-1634790 --- k-mer match to putative recomb seq = 1.0 (20/20)
k-mer match
1.0
0.8
0.6
0.4
0.2
0.0
Match to reference locus
Match to recombinant "k-mer Only"
1,636,960
1,637,040
1,637,120
1,637,200
1,637,280
1,637,360
1,637,440
1,637,520
H37rv Ref Position (bp)

Sub-phylogeny Viz + Paralog Alignments
GCE ID: Event_142

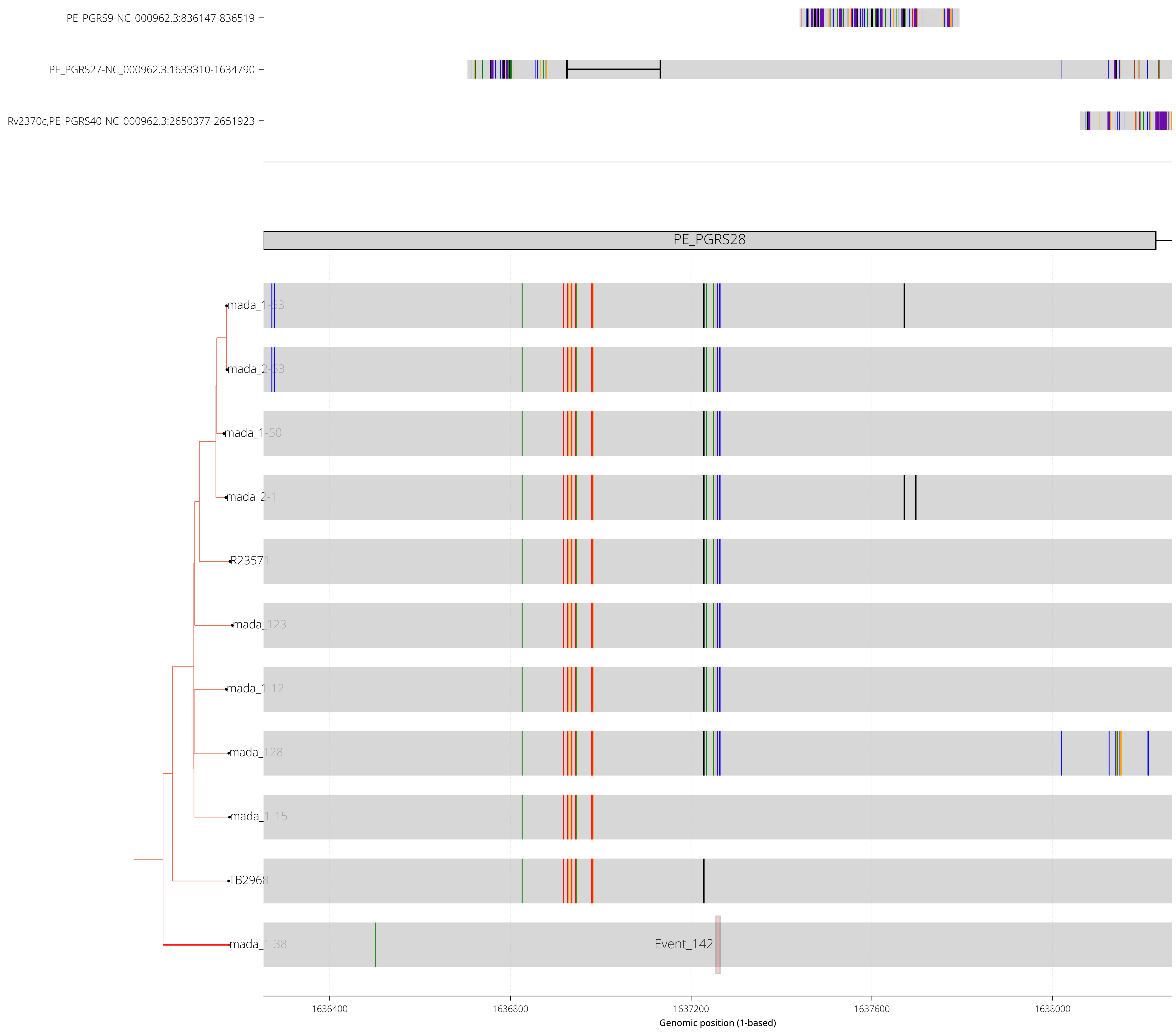

Full Phylogeny Viz + Paralog Alignments
GCE ID: Event_142

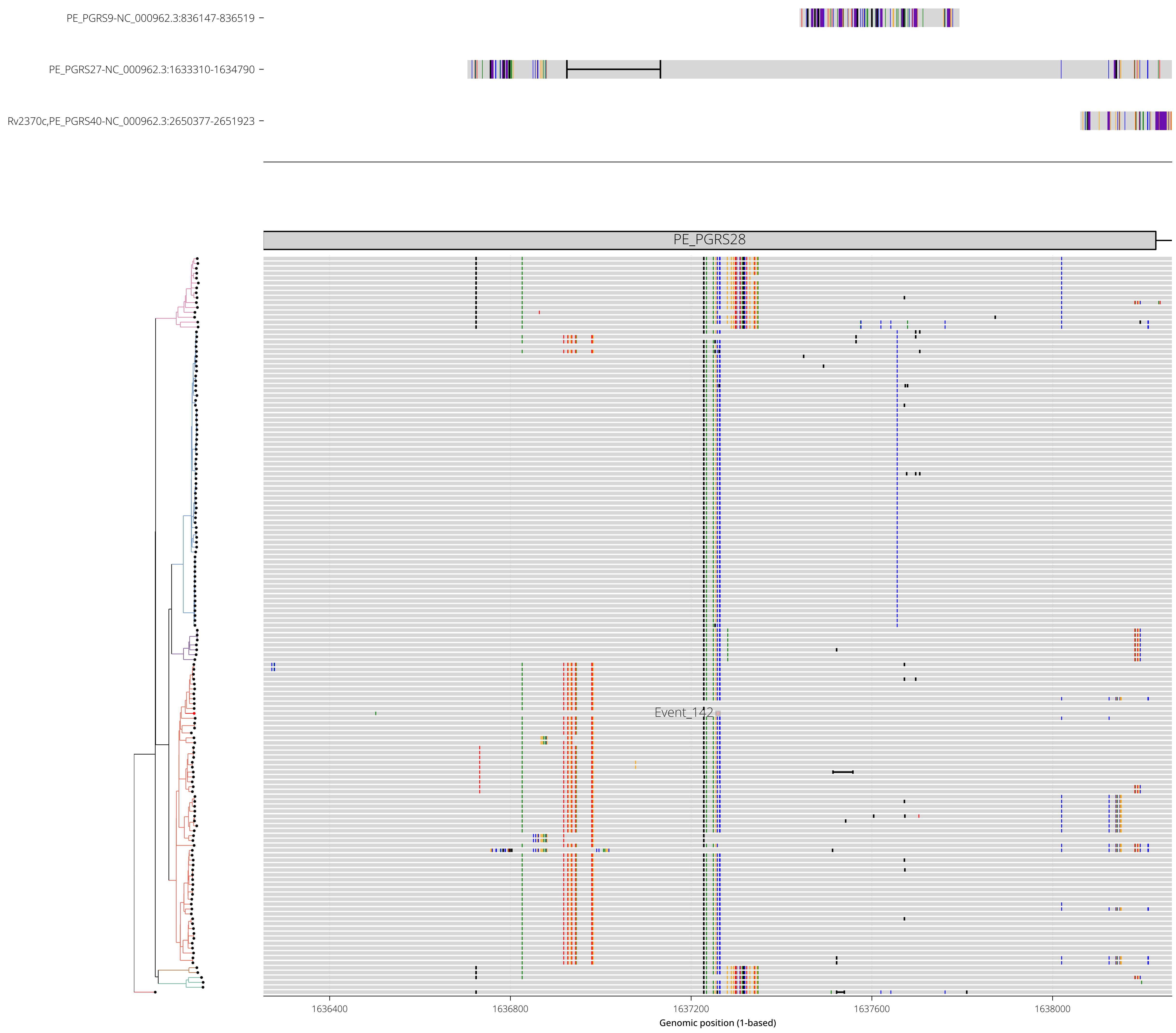

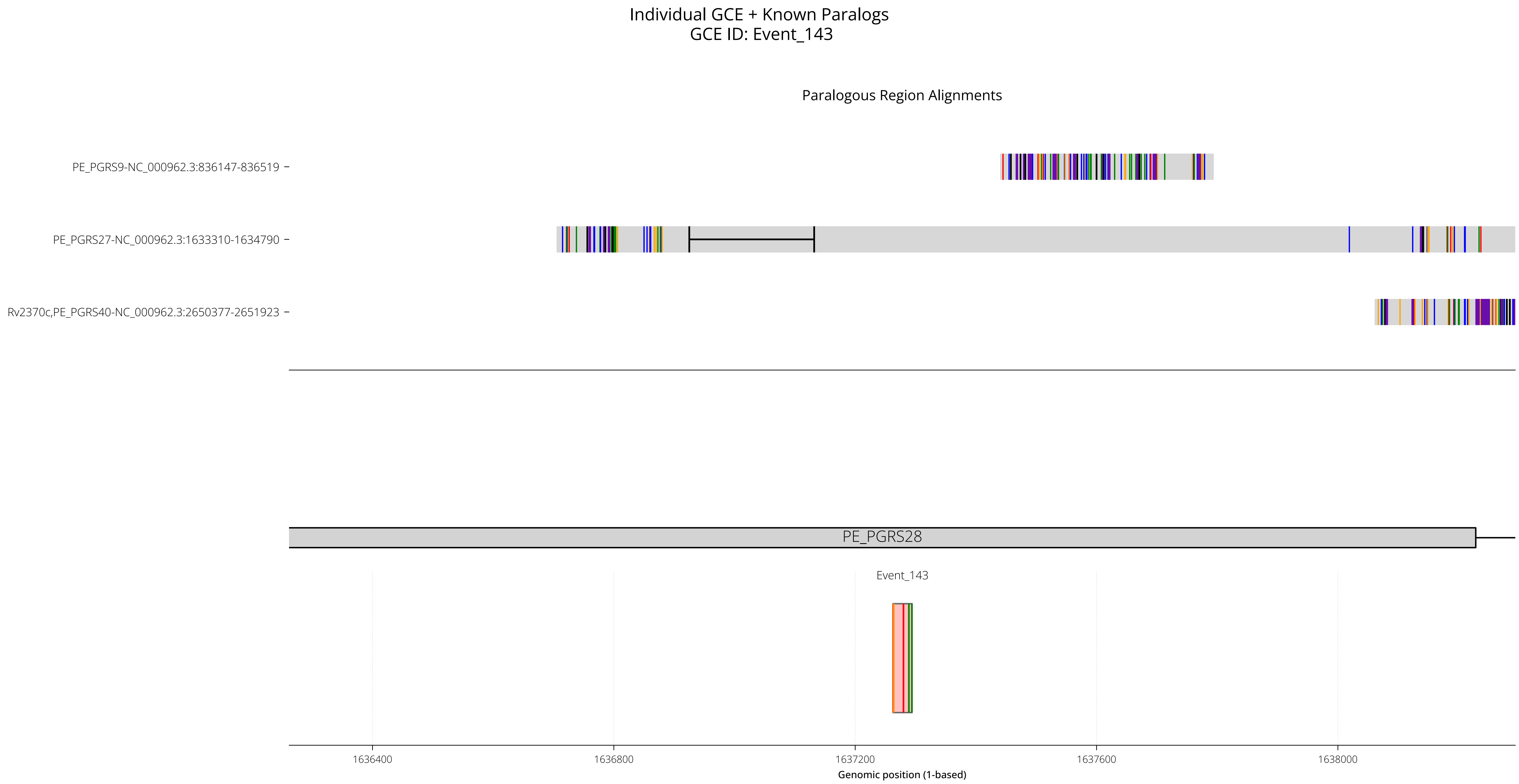

Individual GCE + Known Paralogs
GCE ID: Event_143
Paralogous Region Alignments
PE_PGRS9-NC_000962.3:836147-836519
PE_PGRS27-NC_000962.3:1633310-1634790
Rv2370c,PE_PGRS40-NC_000962.3:2650377-2651923
PE_PGRS28
Event_143
1636400
1636800
1637200
1637600
1638000
Genomic position (1-based)

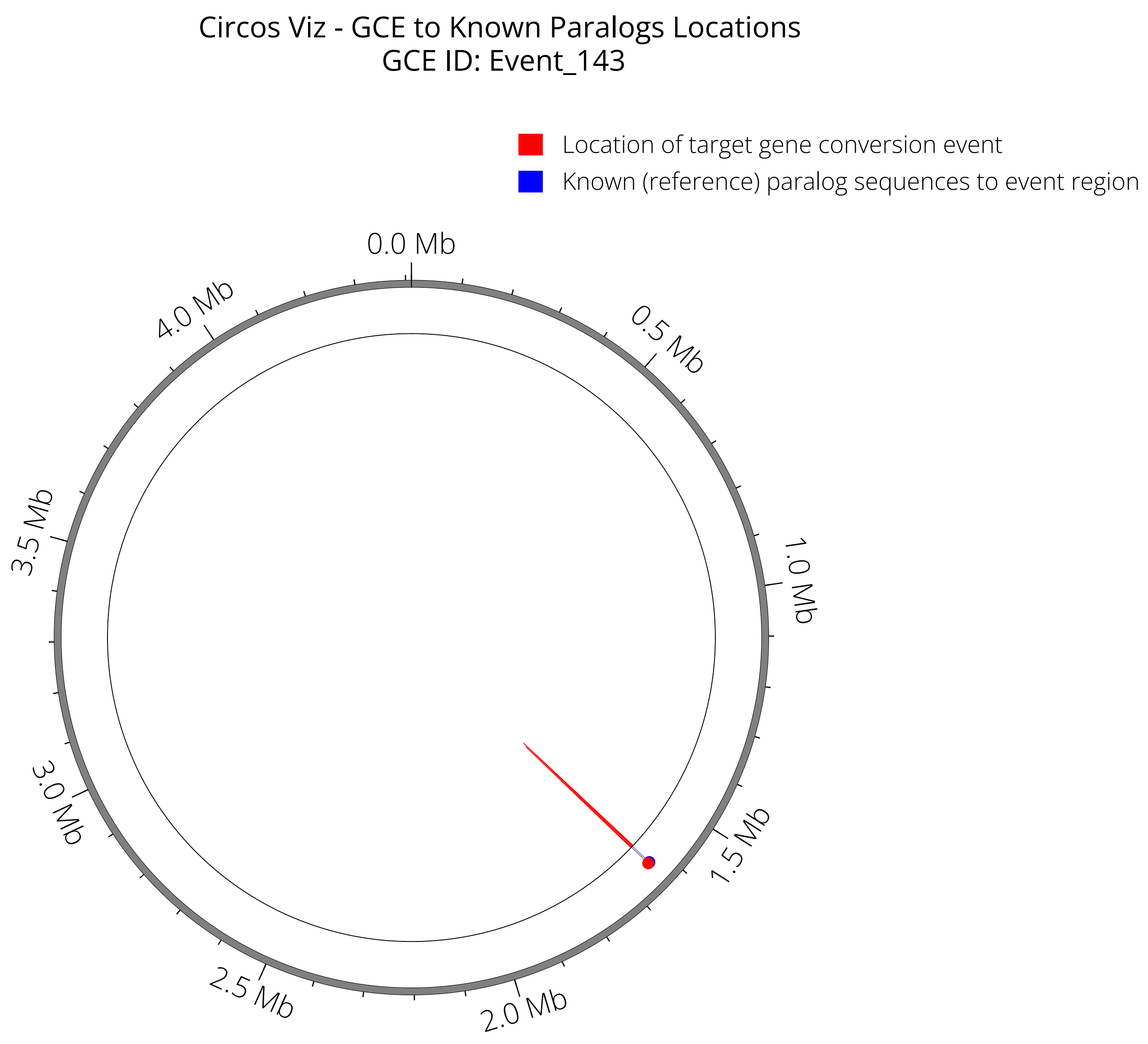
Circos Viz - GCE to Known Paralogs Locations
GCE ID: Event_143
Location of target gene conversion event
Known (reference) paralog sequences to event region
0.0 Mb
0.5 Mb
1.0 Mb
1.5 Mb
2.0 Mb
2.5 Mb
3.0 Mb
3.5 Mb
4.0 Mb

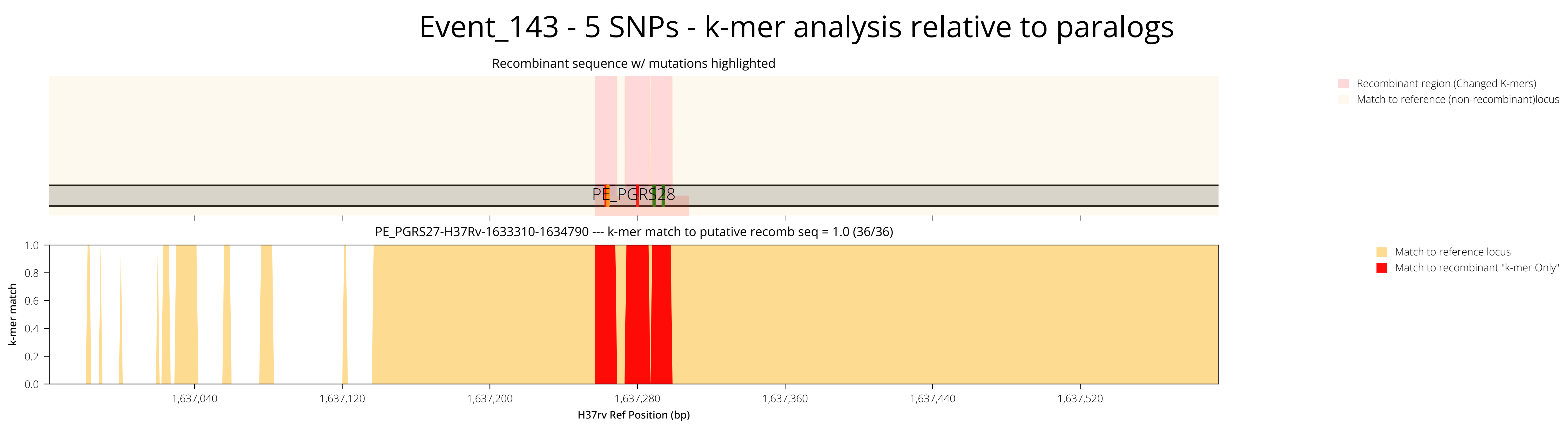
Event_143 - 5 SNPs - k-mer analysis relative to paralogs
Recombinant sequence w/ mutations highlighted
Recombinant region (Changed K-mers)
Match to reference (non-recombinant)locus
PE_PGRS28
PE_PGRS27-H37Rv-1633310-1634790 --- k-mer match to putative recomb seq = 1.0 (36/36)
Match to reference locus
Match to recombinant "k-mer Only"
k-mer match
1.0
0.8
0.6
0.4
0.2
0.0
1,637,040
1,637,120
1,637,200
1,637,280
1,637,360
1,637,440
1,637,520
H37rv Ref Position (bp)

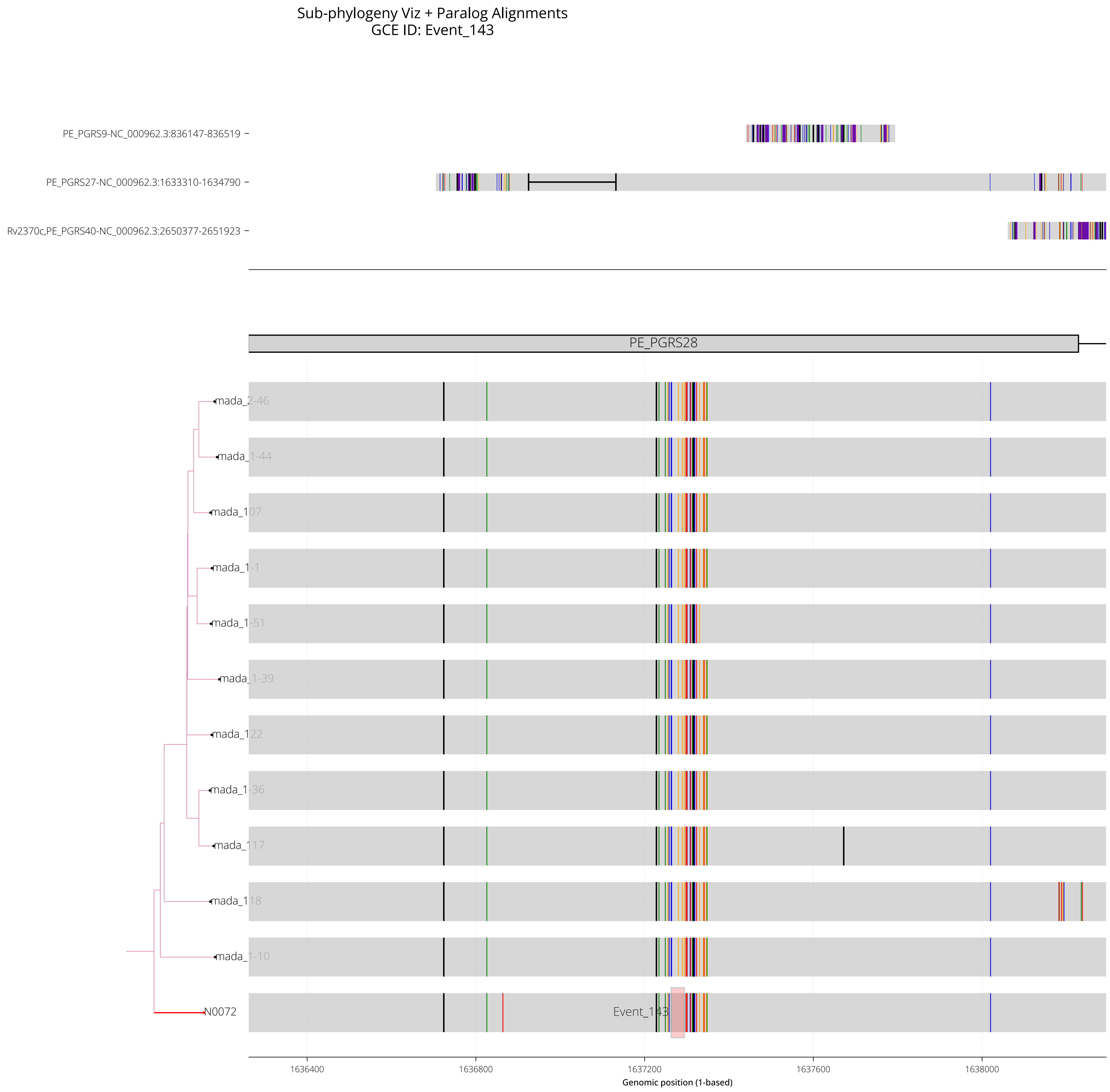
Sub-phylogeny Viz + Paralog Alignments
GCE ID: Event_143
PE_PGRS9-NC_000962.3:836147-836519
PE_PGRS27-NC_000962.3:1633310-1634790
Rv2370c,PE_PGRS40-NC_000962.3:2650377-2651923
PE_PGRS28
mada_2-46
mada_1-44
mada_107
mada_1-1
mada_1-51
mada_1-39
mada_122
mada_1-36
mada_117
mada_118
mada_1-10
N0072
Event_143
1636400
1636800
1637200
1637600
1638000
Genomic position (1-based)

Full Phylogeny Viz + Paralog Alignments
GCE ID: Event_143

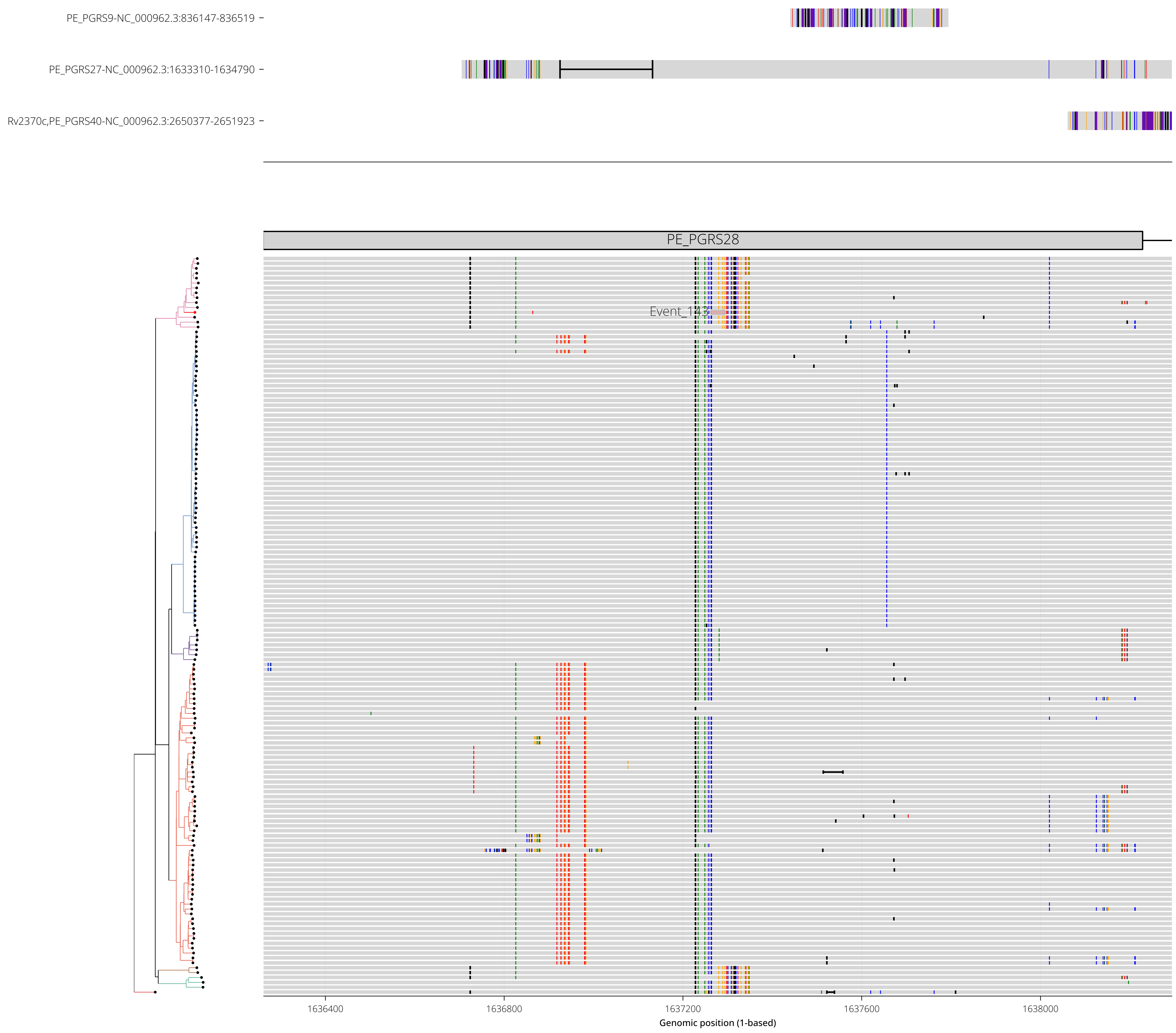

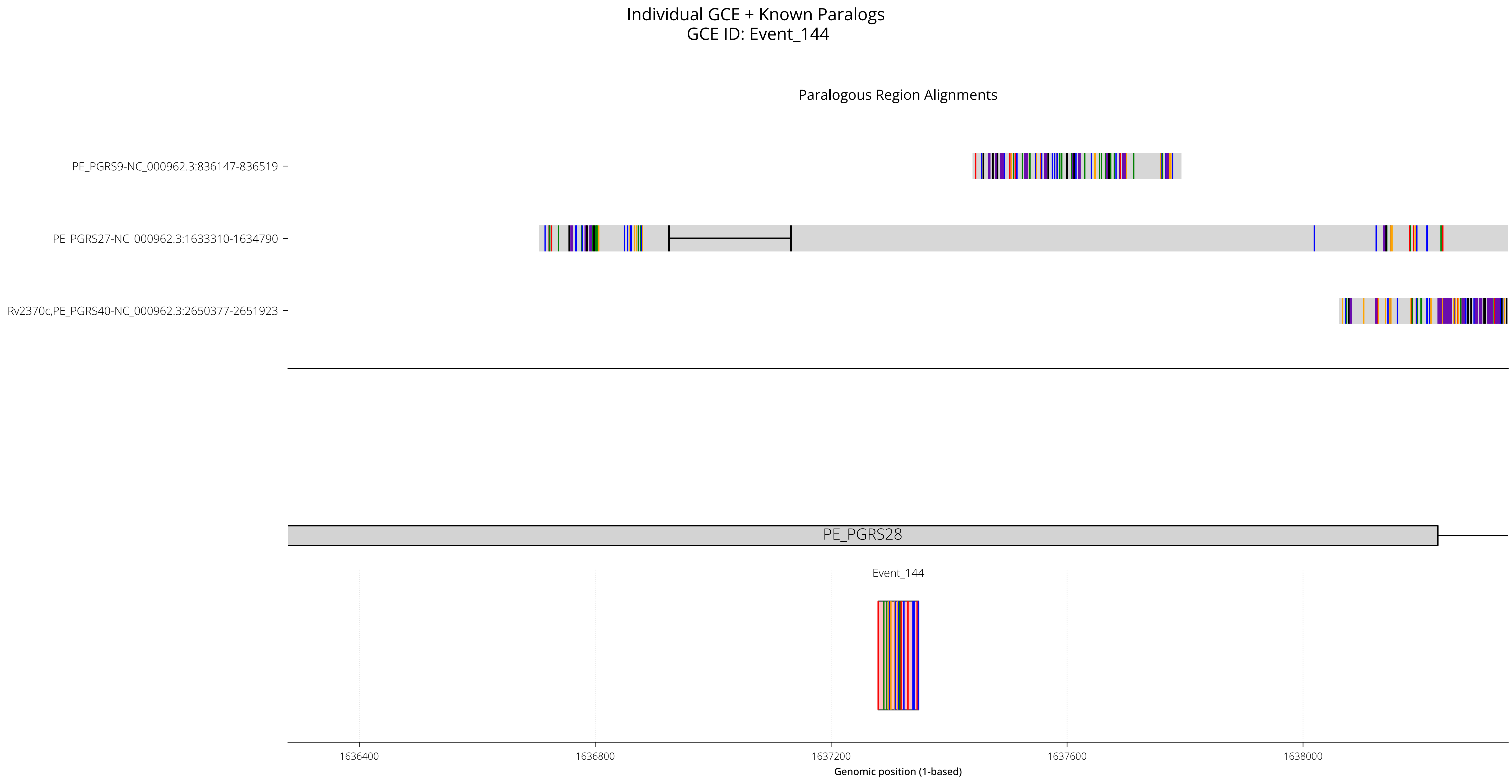

Individual GCE + Known Paralogs
GCE ID: Event_144
Paralogous Region Alignments
PE_PGRS9-NC_000962.3:836147-836519
PE_PGRS27-NC_000962.3:1633310-1634790
Rv2370c,PE_PGRS40-NC_000962.3:2650377-2651923
PE_PGRS28
Event_144
1636400
1636800
1637200
1637600
1638000
Genomic position (1-based)

Circos Viz - GCE to Known Paralogs Locations
GCE ID: Event_144

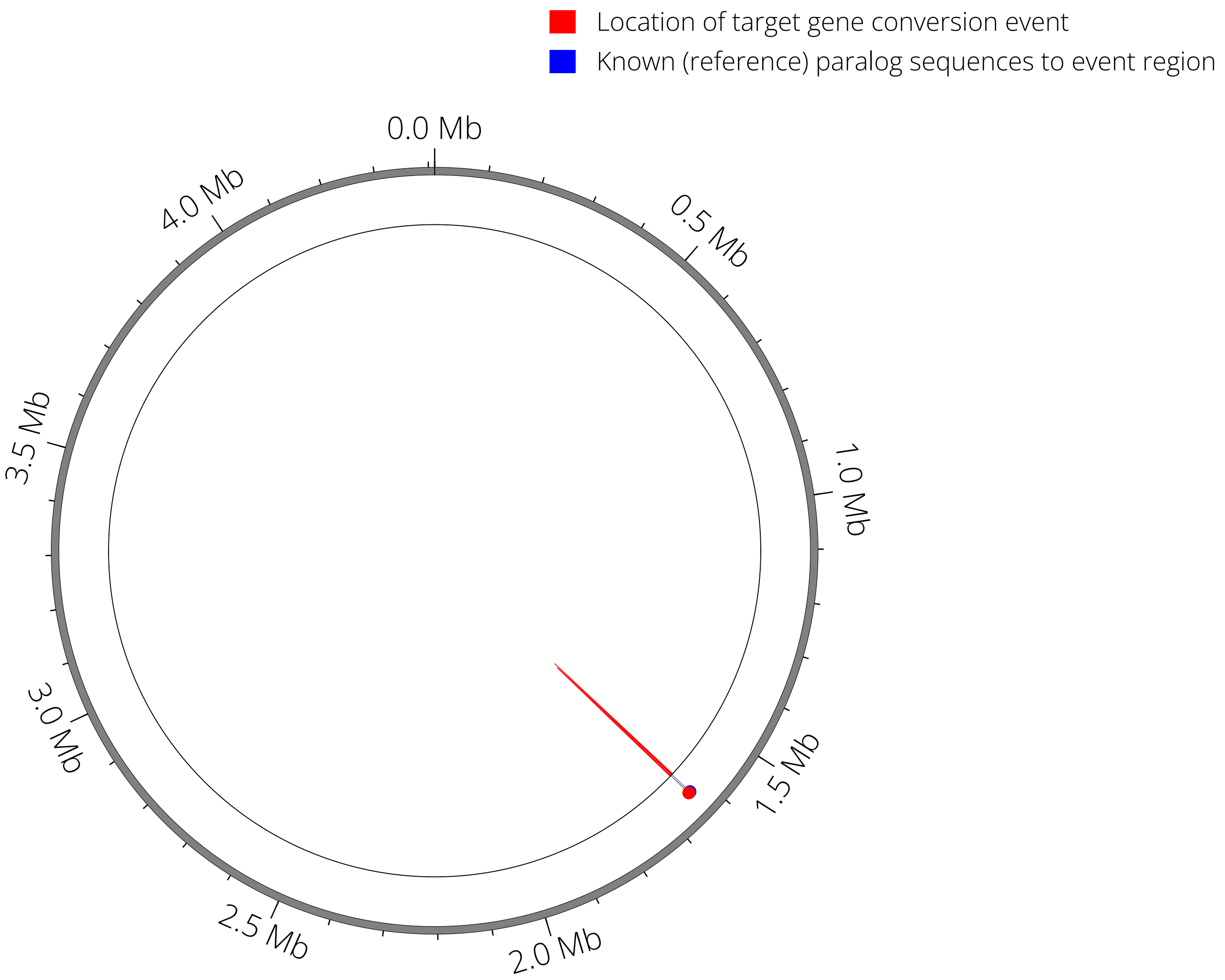

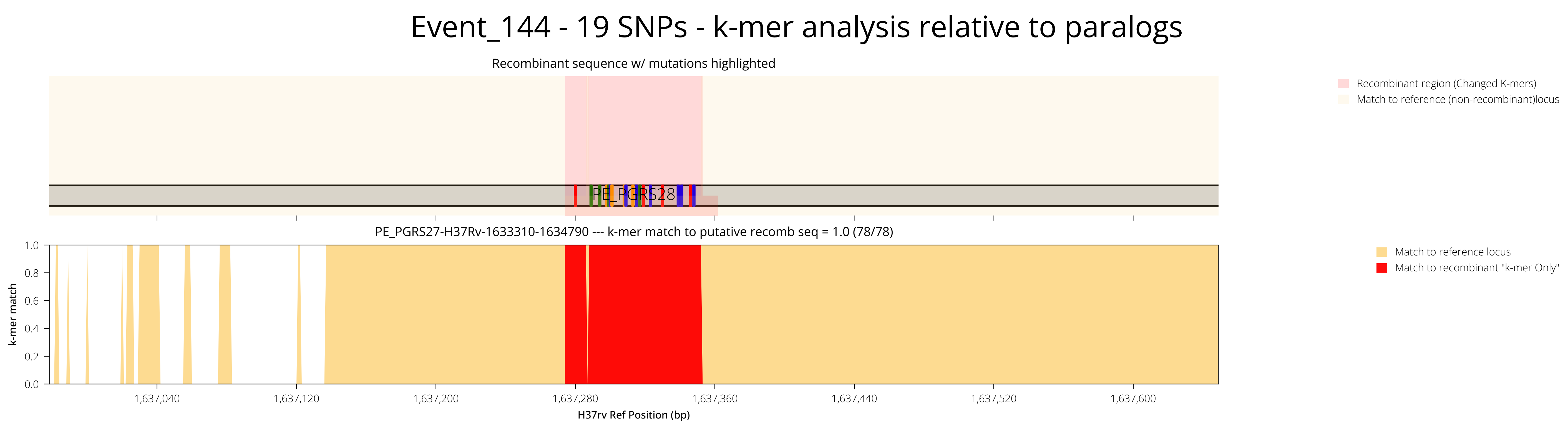
Event_144 - 19 SNPs - k-mer analysis relative to paralogs
Recombinant sequence w/ mutations highlighted
Recombinant region (Changed K-mers)
Match to reference (non-recombinant)locus
PE_PGRS28
PE_PGRS27-H37Rv-1633310-1634790 --- k-mer match to putative recomb seq = 1.0 (78/78)
Match to reference locus
Match to recombinant "k-mer Only"
k-mer match
1.0
0.8
0.6
0.4
0.2
0.0
1,637,040
1,637,120
1,637,200
1,637,280
1,637,360
1,637,440
1,637,520
1,637,600
H37rv Ref Position (bp)

Sub-phylogeny Viz + Paralog Alignments
GCE ID: Event_144

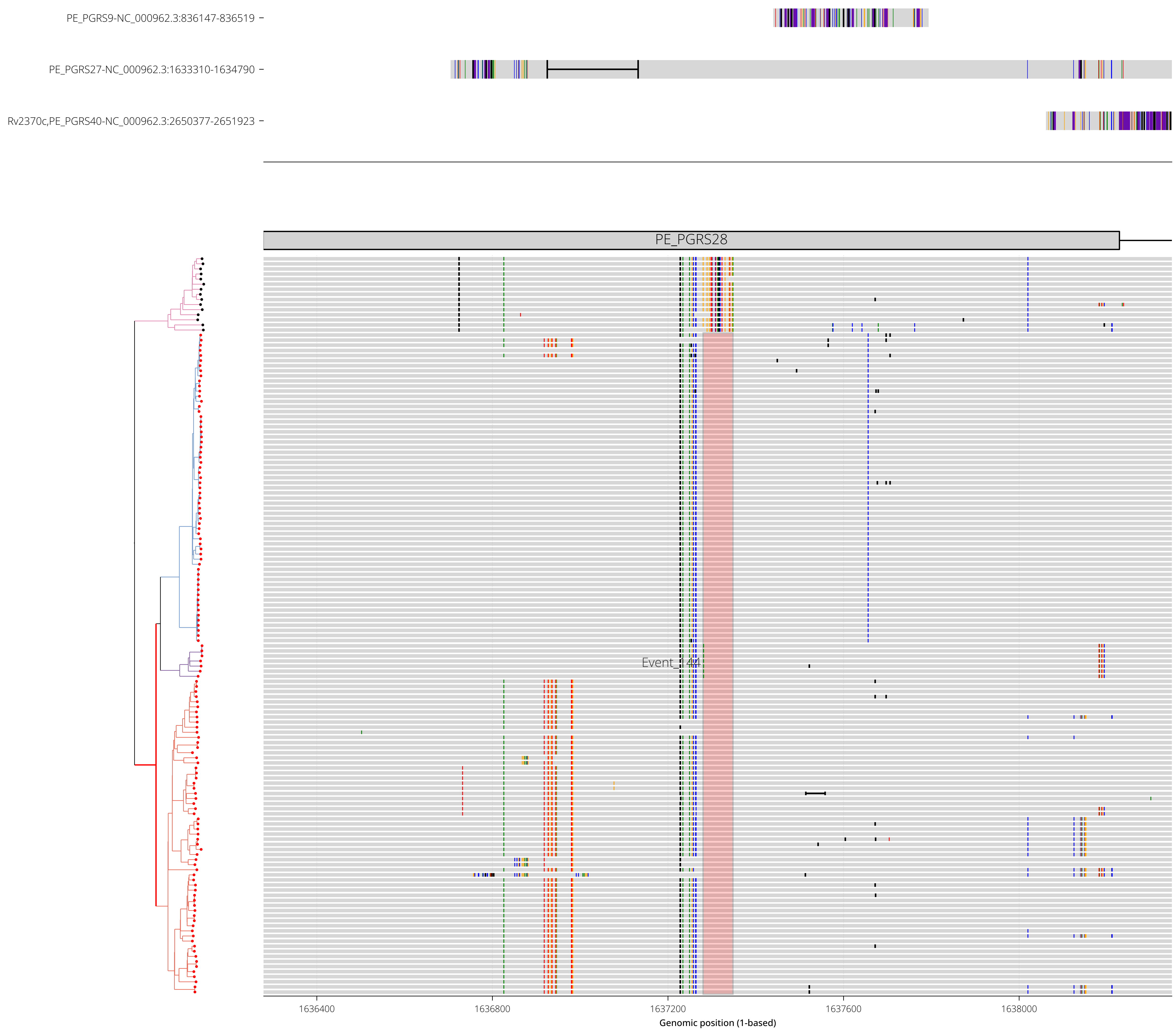

Full Phylogeny Viz + Paralog Alignments
GCE ID: Event_144

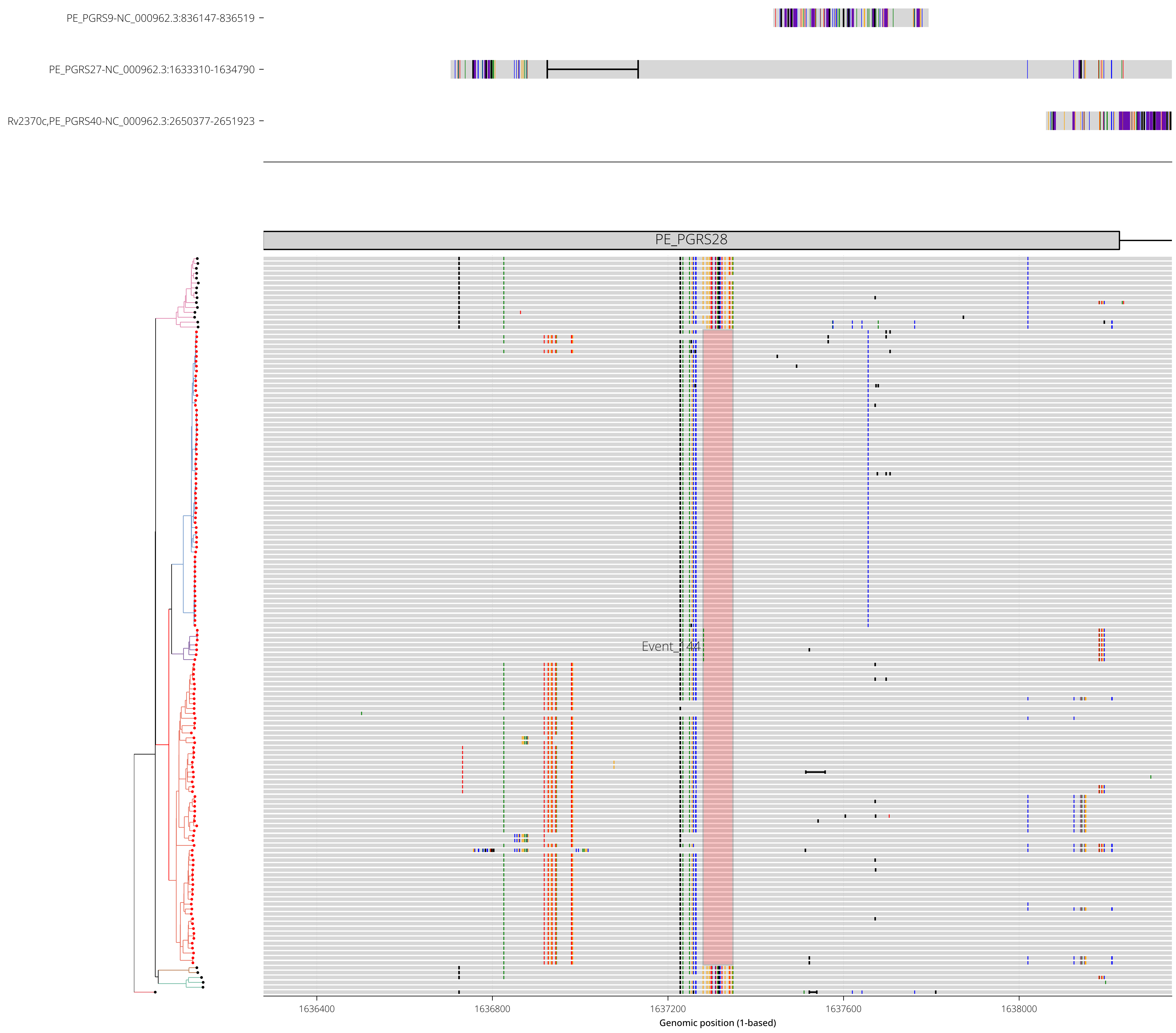

Individual GCE + Known Paralogs
GCE ID: Event_145

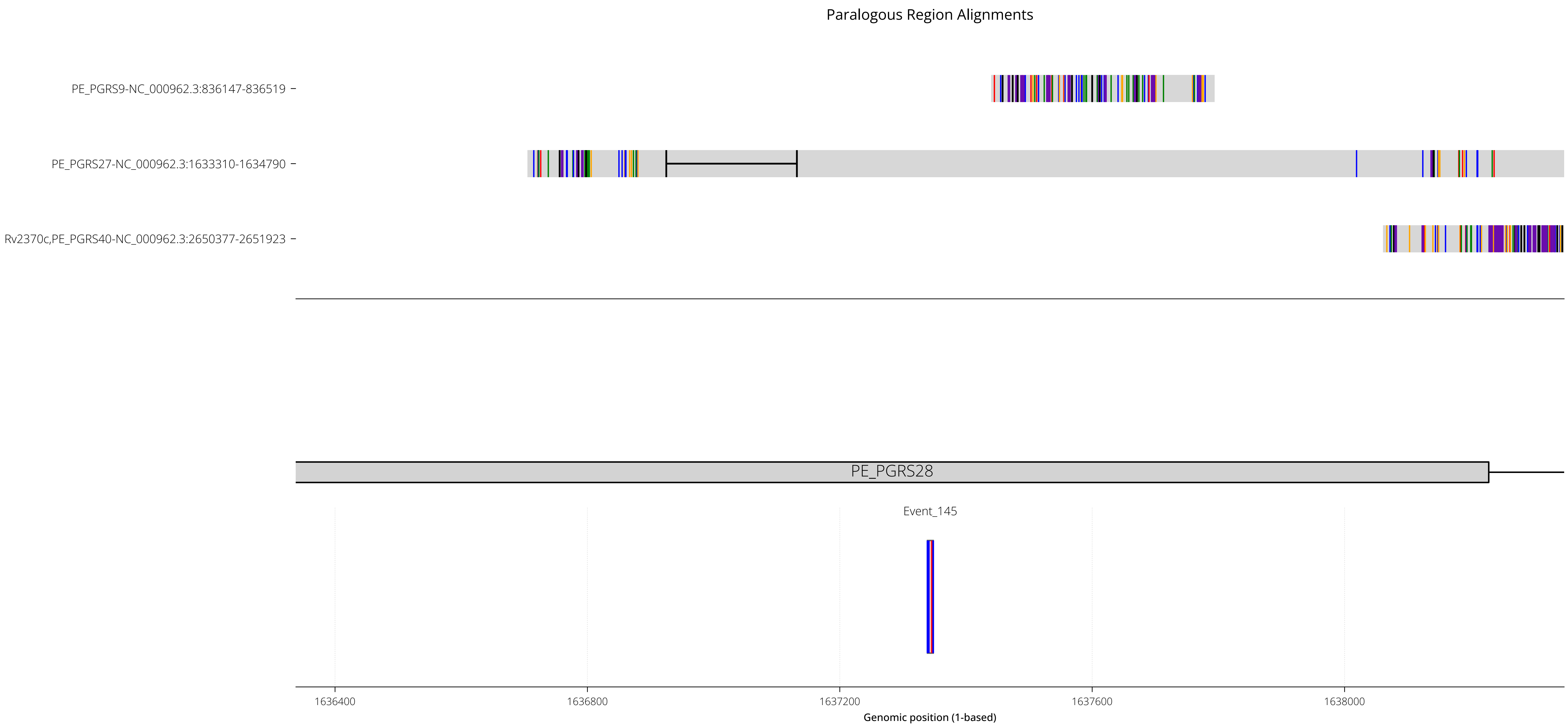

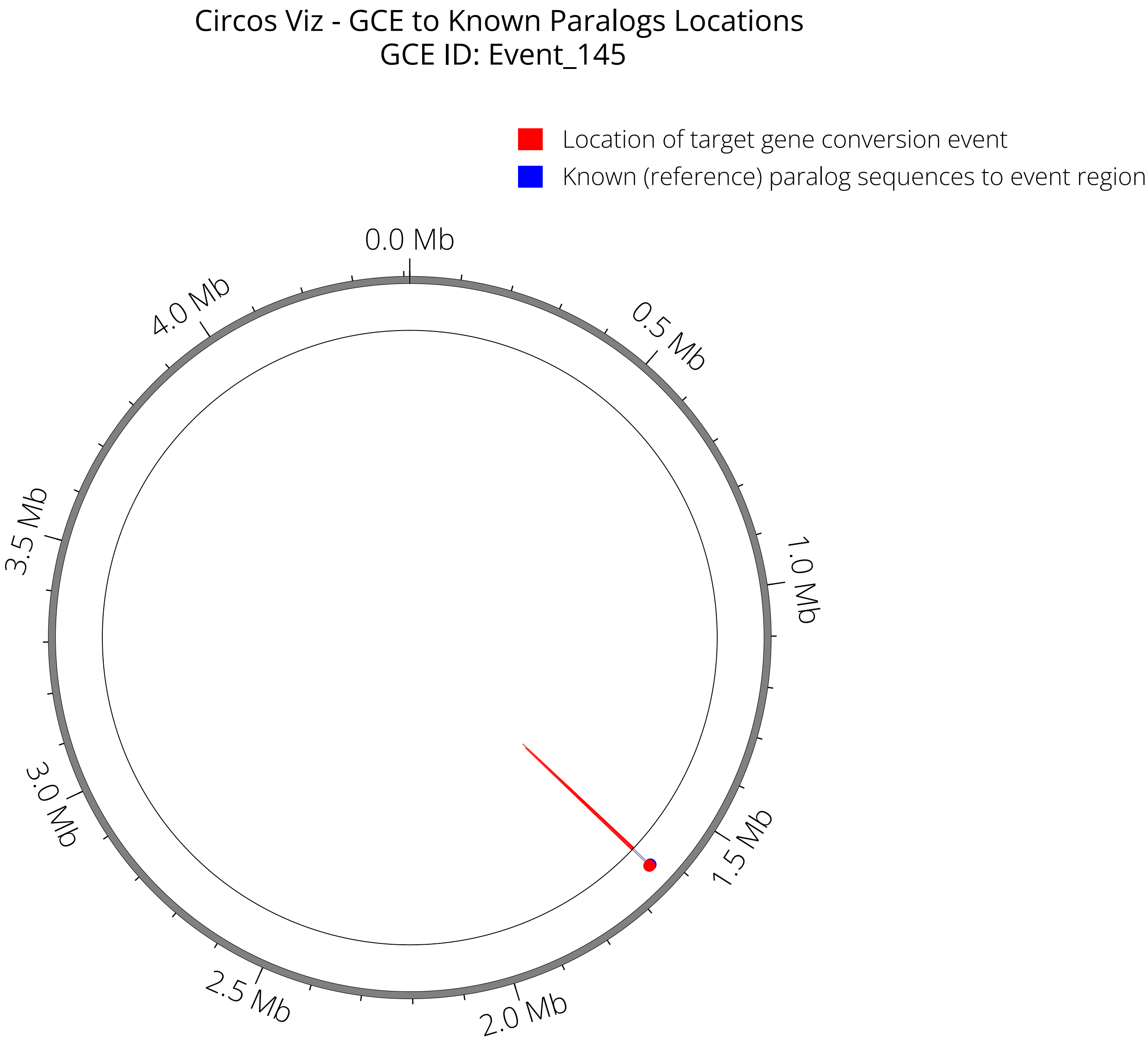
Circos Viz - GCE to Known Paralogs Locations
GCE ID: Event_145
Location of target gene conversion event
Known (reference) paralog sequences to event region
0.0 Mb
0.5 Mb
1.0 Mb
1.5 Mb
2.0 Mb
2.5 Mb
3.0 Mb
3.5 Mb
4.0 Mb

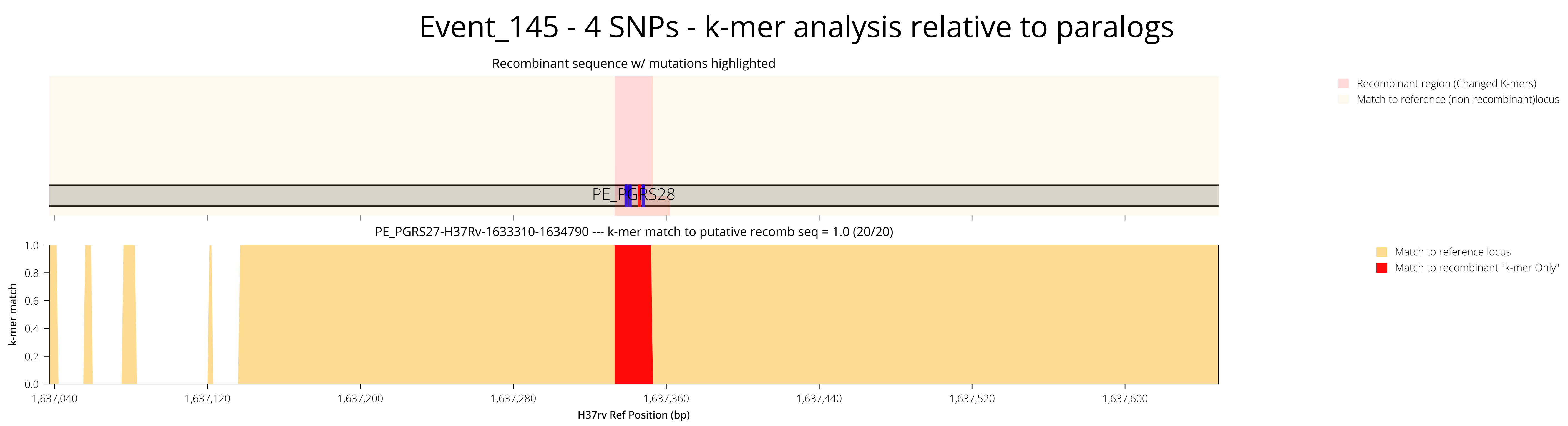
Event_145 - 4 SNPs - k-mer analysis relative to paralogs
Recombinant sequence w/ mutations highlighted
PE_PGRS28
Recombinant region (Changed K-mers)
Match to reference (non-recombinant)locus
PE_PGRS27-H37Rv-1633310-1634790 --- k-mer match to putative recomb seq = 1.0 (20/20)
Match to reference locus
Match to recombinant "k-mer Only"
k-mer match
1.0
0.8
0.6
0.4
0.2
0.0
1,637,040
1,637,120
1,637,200
1,637,280
1,637,360
1,637,440
1,637,520
1,637,600
H37rv Ref Position (bp)

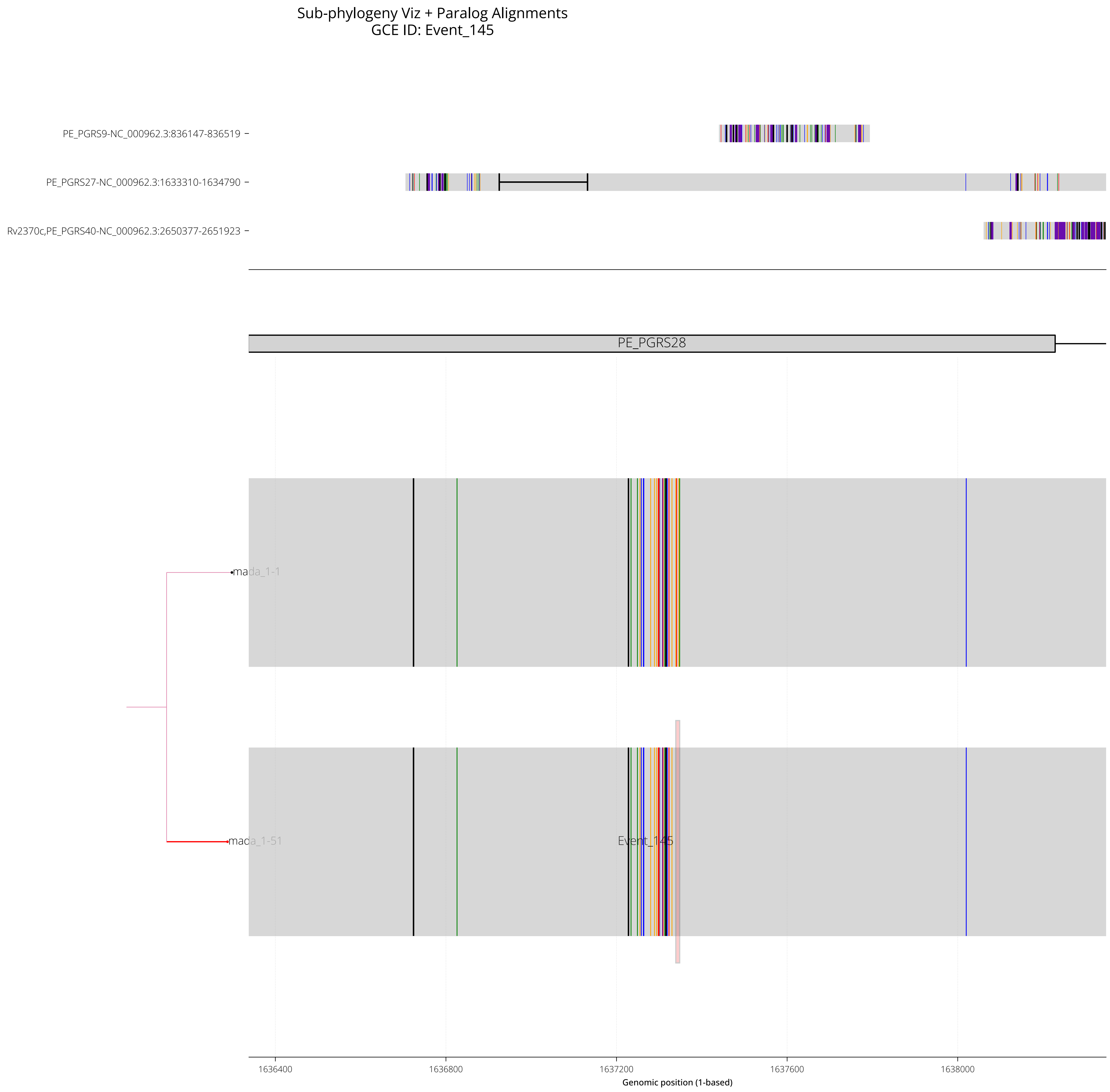
Sub-phylogeny Viz + Paralog Alignments
GCE ID: Event_145
PE_PGRS9-NC_000962.3:836147-836519
PE_PGRS27-NC_000962.3:1633310-1634790
Rv2370c,PE_PGRS40-NC_000962.3:2650377-2651923
PE_PGRS28
mada_1-1
mada_1-51
Event_145
1636400
1636800
1637200
1637600
1638000
Genomic position (1-based)

Full Phylogeny Viz + Paralog Alignments
GCE ID: Event_145

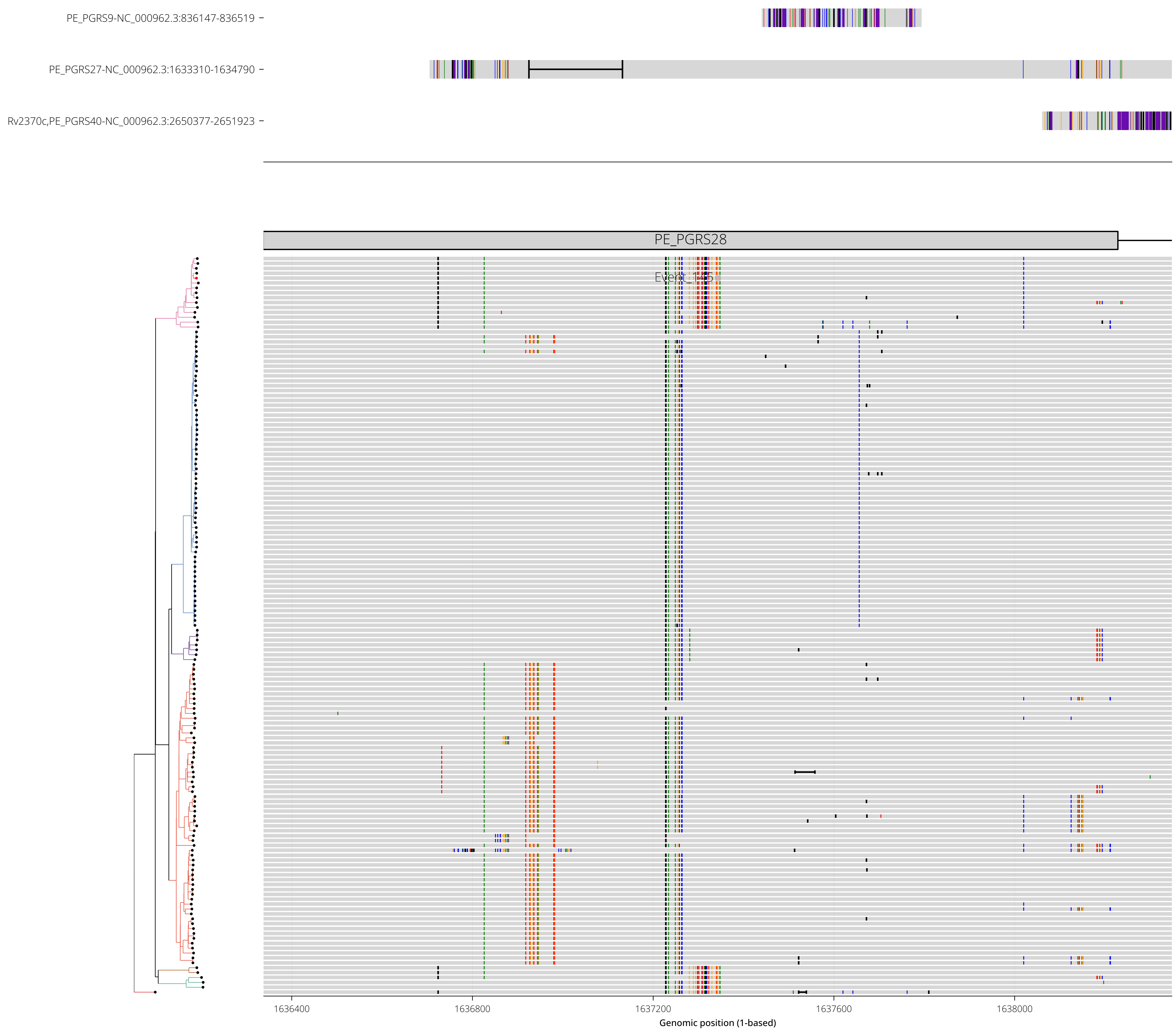

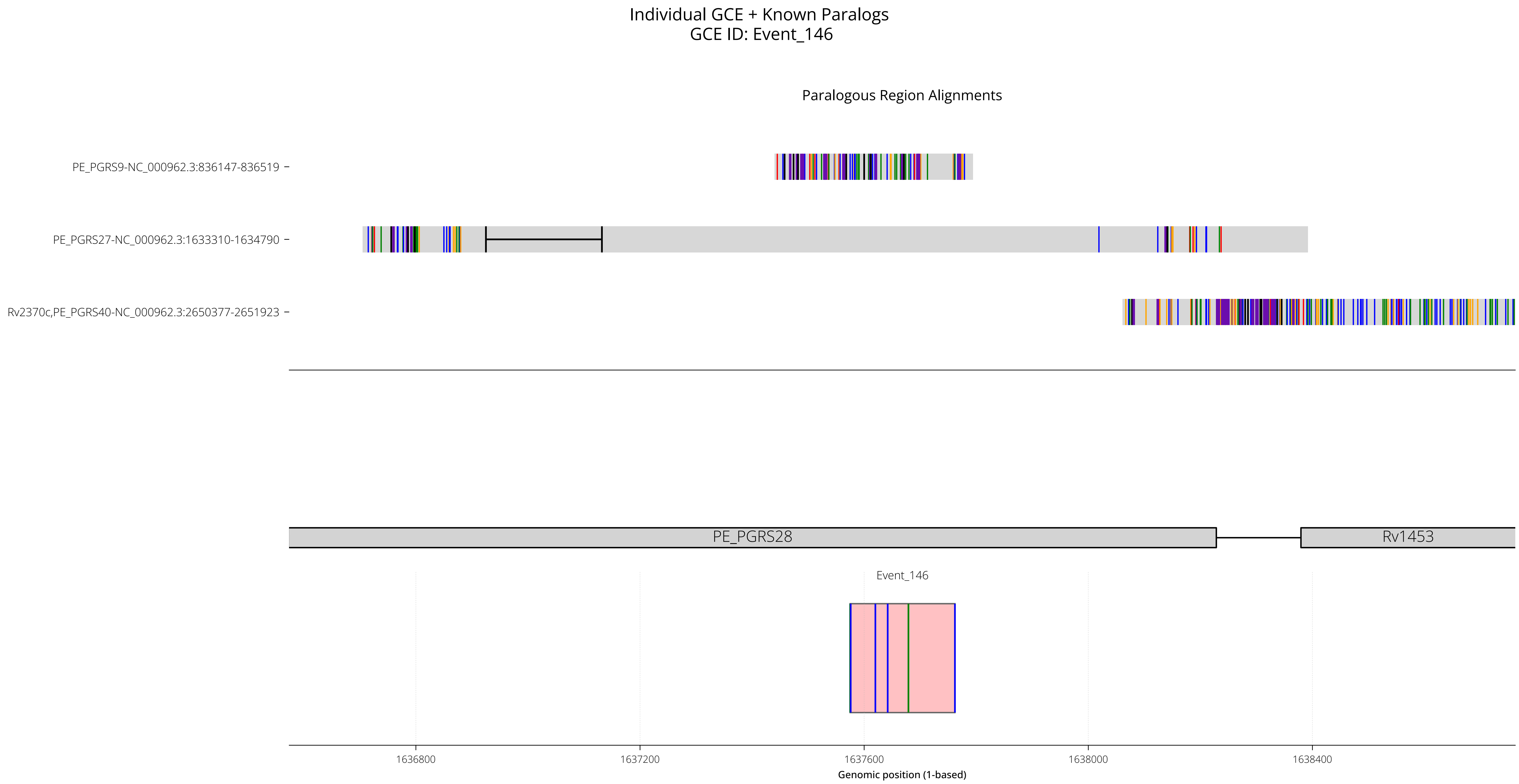
Individual GCE + Known Paralogs
GCE ID: Event_146
Paralogous Region Alignments
PE_PGRS9-NC_000962.3:836147-836519
PE_PGRS27-NC_000962.3:1633310-1634790
Rv2370c,PE_PGRS40-NC_000962.3:2650377-2651923
PE_PGRS28
Rv1453
Event_146
1636800
1637200
1637600
1638000
1638400
Genomic position (1-based)

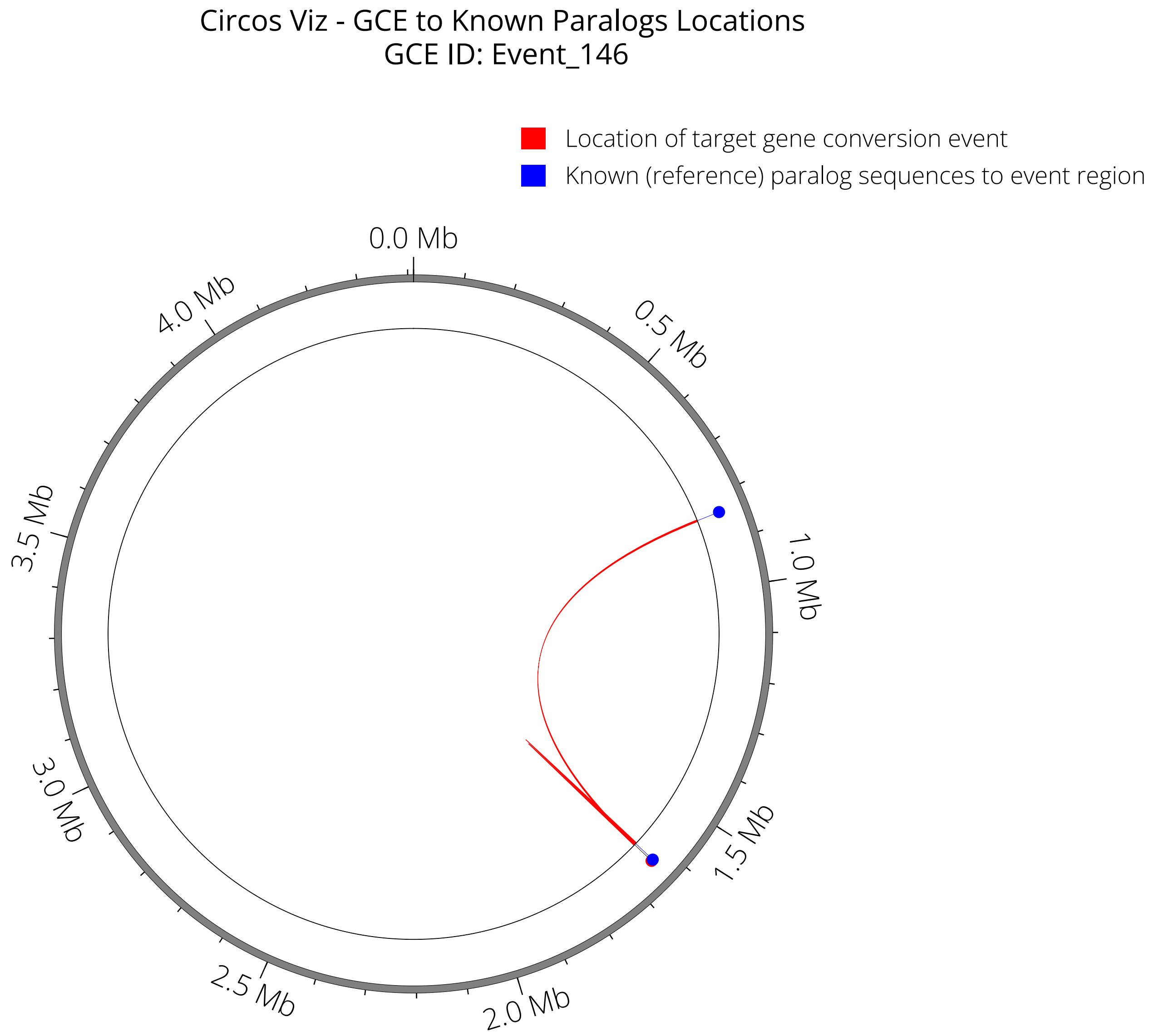

Circos Viz - GCE to Known Paralogs Locations
GCE ID: Event_146
Location of target gene conversion event
Known (reference) paralog sequences to event region
0.0 Mb
0.5 Mb
1.0 Mb
1.5 Mb
2.0 Mb
2.5 Mb
3.0 Mb
3.5 Mb
4.0 Mb

# Event_146 - 6 SNPs - k-mer analysis relative to paralogs

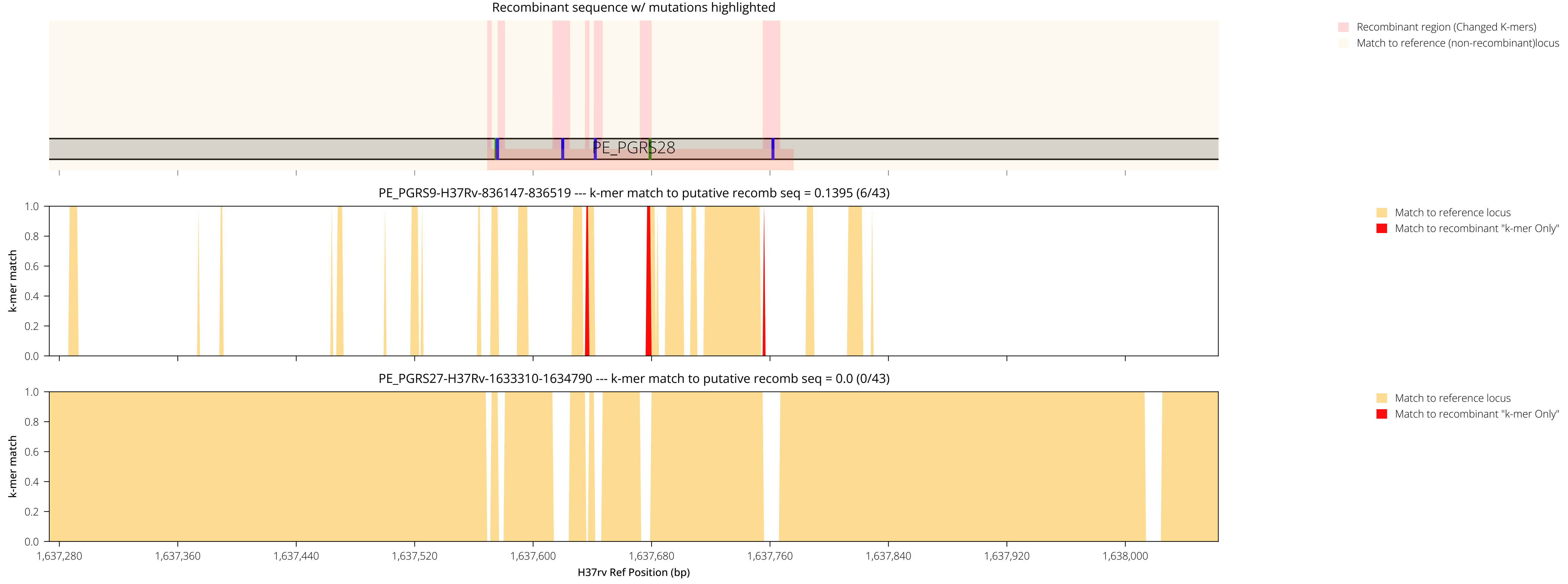

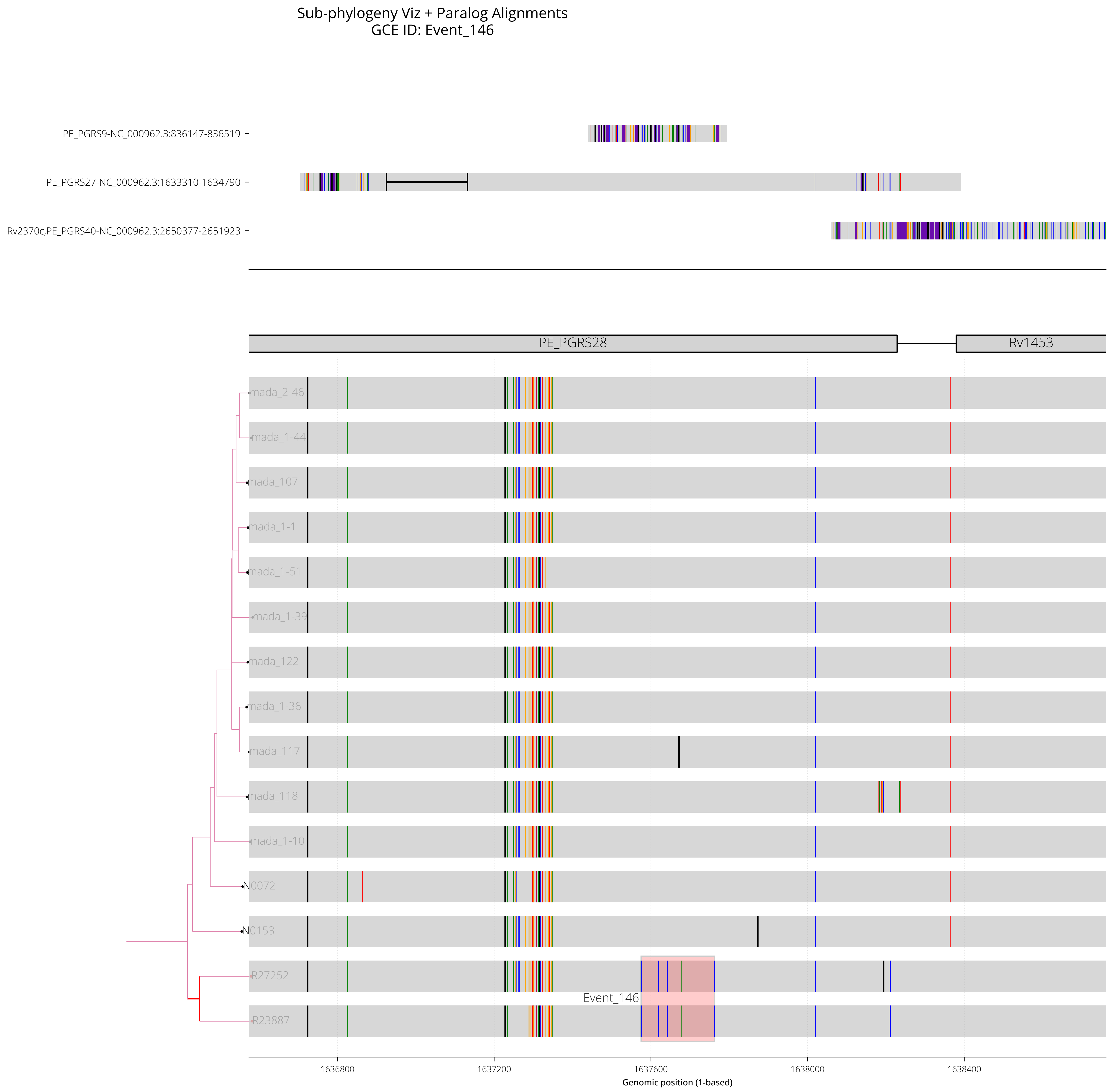

Sub-phylogeny Viz + Paralog Alignments
GCE ID: Event_146
PE_PGRS9-NC_000962.3:836147-836519
PE_PGRS27-NC_000962.3:1633310-1634790
Rv2370c,PE_PGRS40-NC_000962.3:2650377-2651923
PE_PGRS28
Rv1453
mada_2-46
mada_1-44
mada_107
mada_1-1
mada_1-51
mada_1-39
mada_122
mada_1-36
mada_117
mada_118
mada_1-10
N0072
N0153
R27252
Event_146
R23887
1636800
1637200
1637600
1638000
1638400
Genomic position (1-based)

Full Phylogeny Viz + Paralog Alignments
GCE ID: Event_146

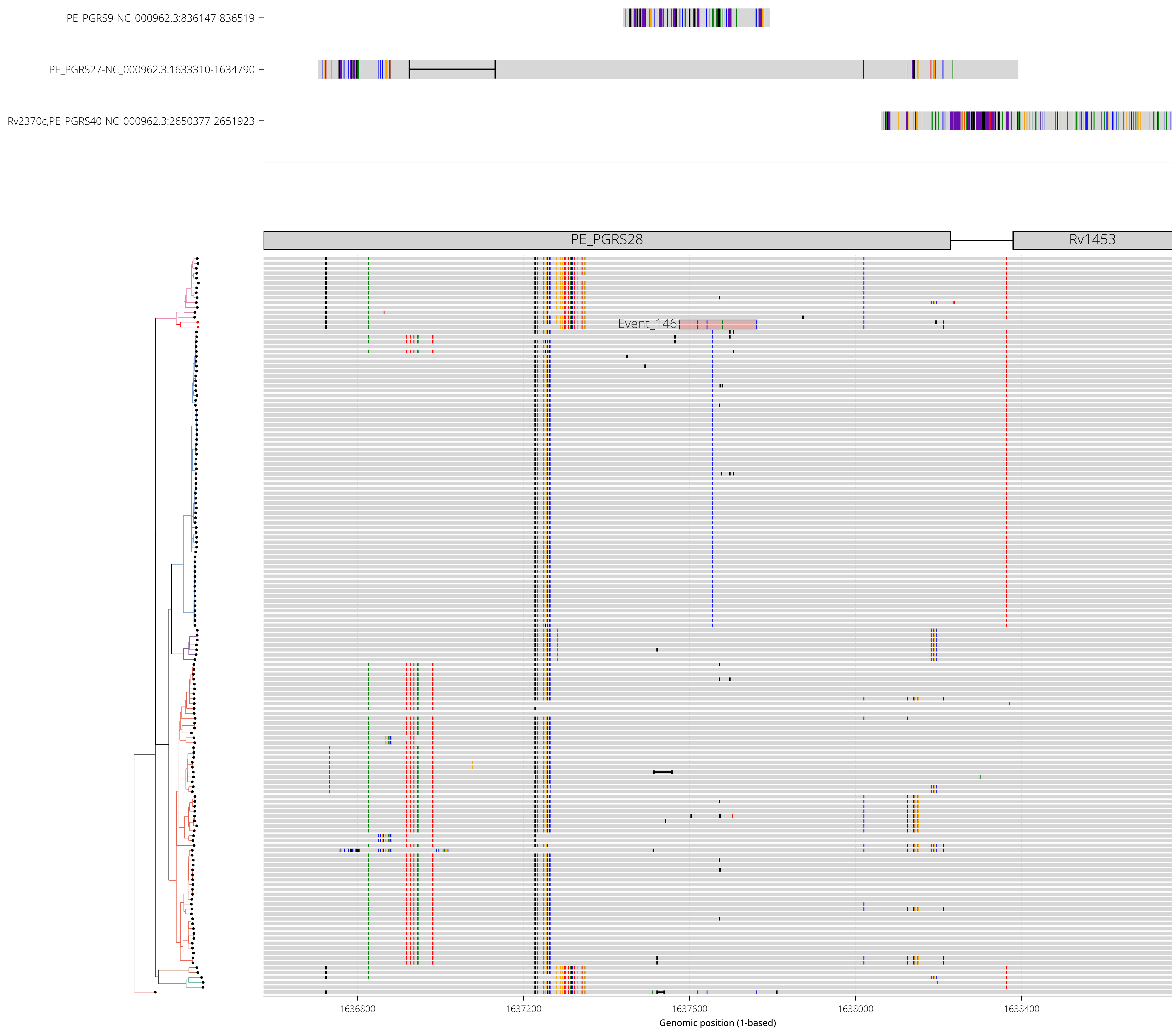

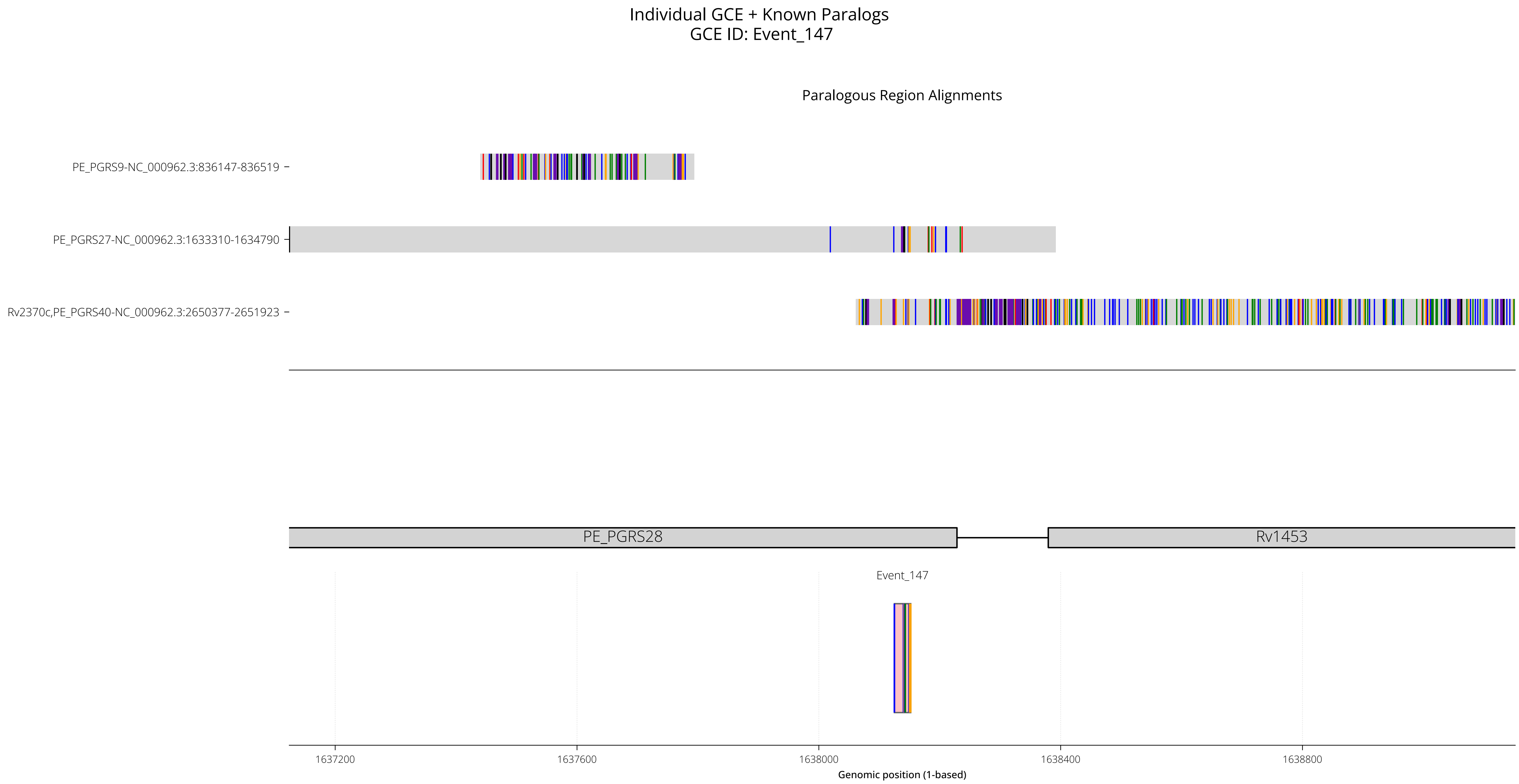
Individual GCE + Known Paralogs
GCE ID: Event_147
Paralogous Region Alignments
PE_PGRS9-NC_000962.3:836147-836519
PE_PGRS27-NC_000962.3:1633310-1634790
Rv2370c,PE_PGRS40-NC_000962.3:2650377-2651923
PE_PGRS28
Rv1453
Event_147
1637200
1637600
1638000
1638400
1638800
Genomic position (1-based)

Circos Viz - GCE to Known Paralogs Locations
GCE ID: Event_147

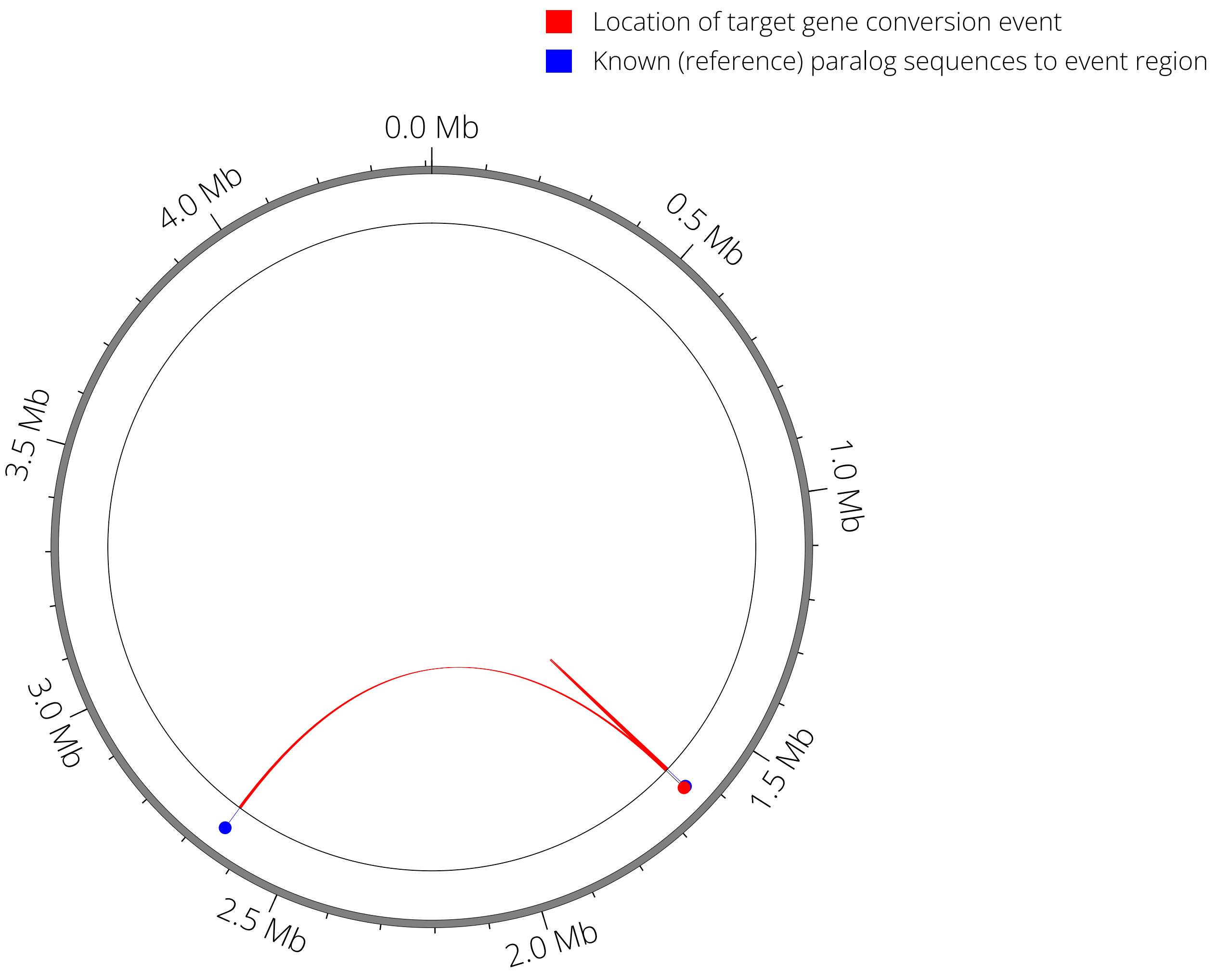

# Event_147 - 8 SNPs - k-mer analysis relative to paralogs

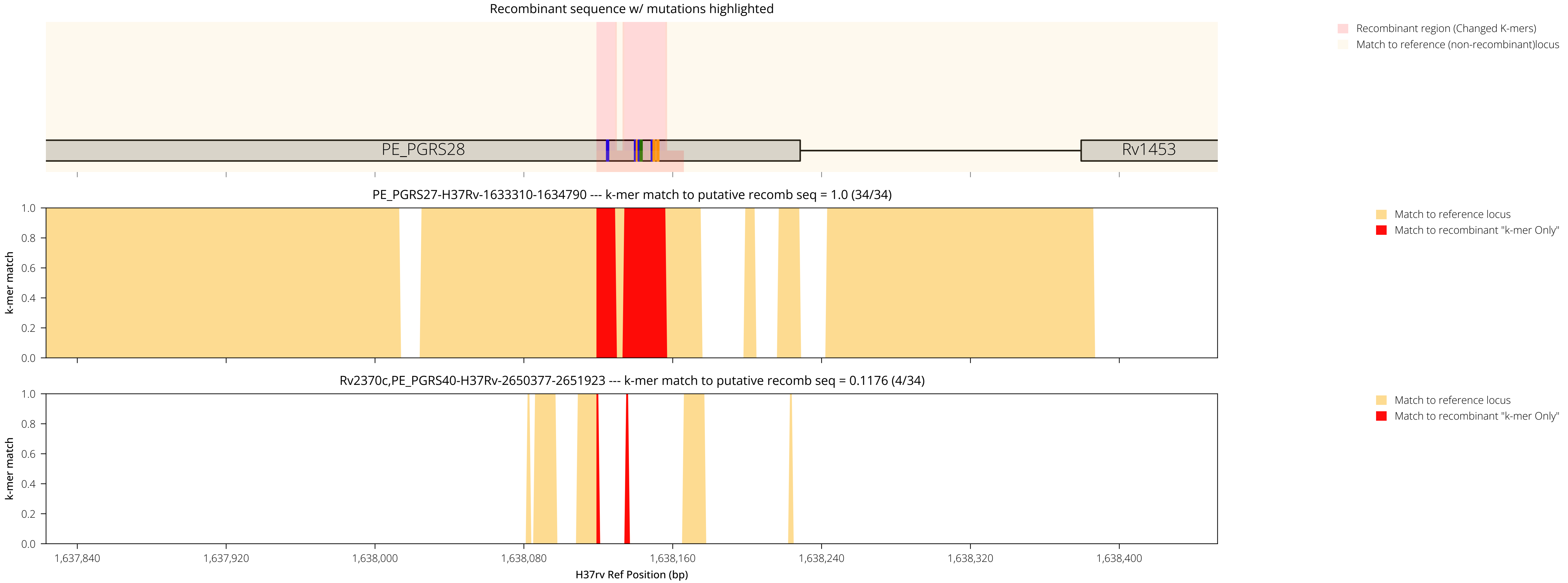

Sub-phylogeny Viz + Paralog Alignments
GCE ID: Event_147

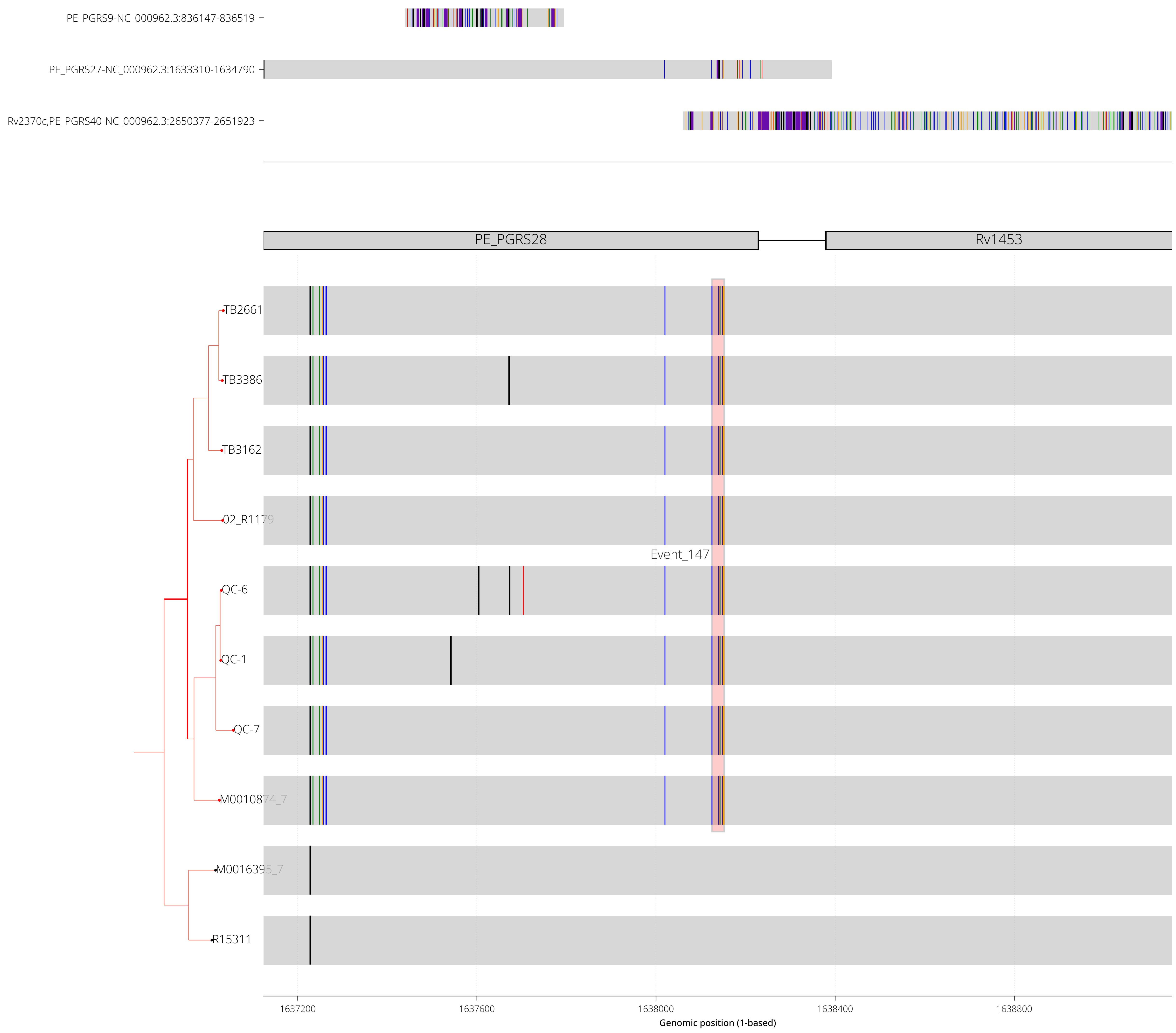

Full Phylogeny Viz + Paralog Alignments
GCE ID: Event_147

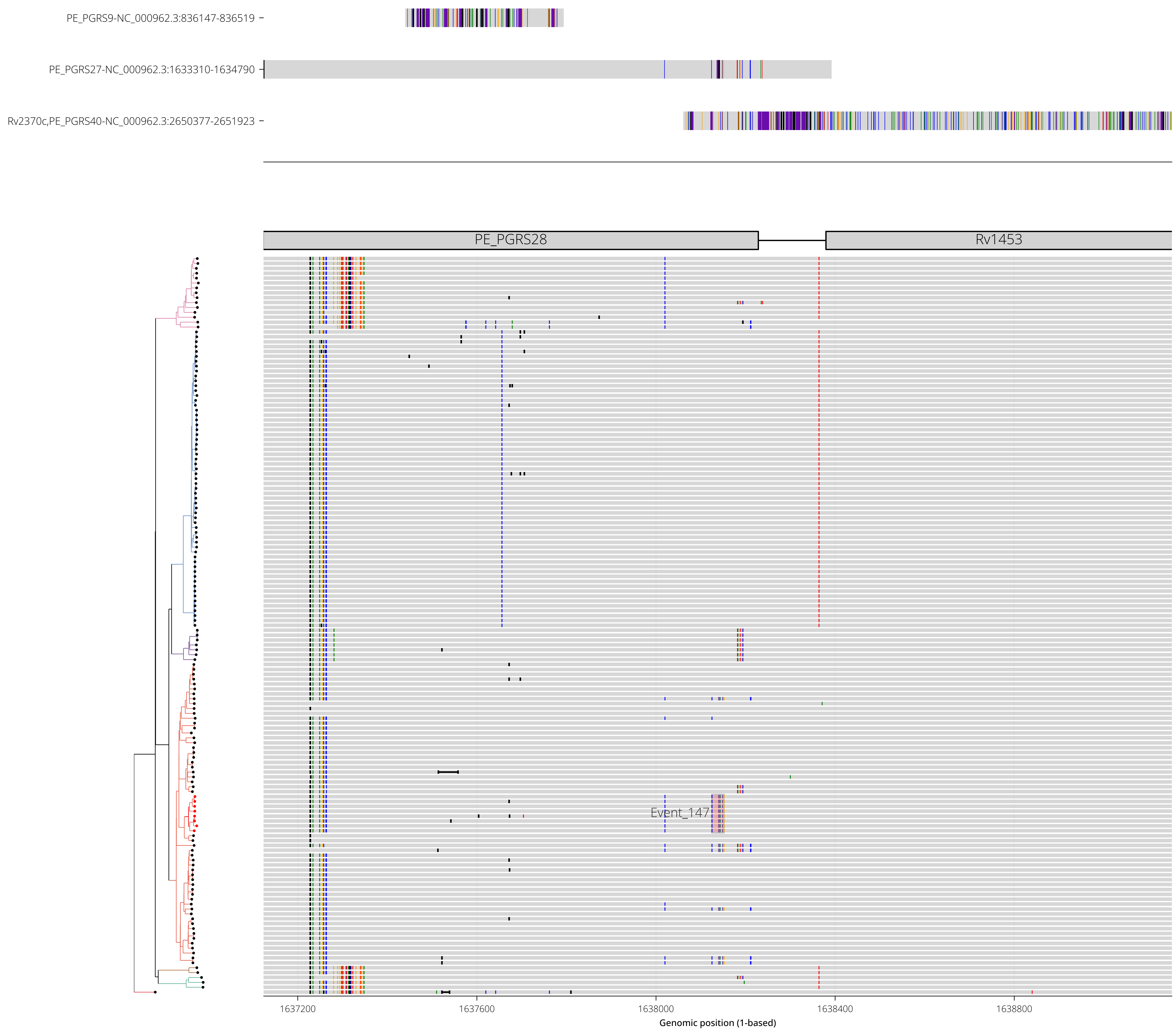

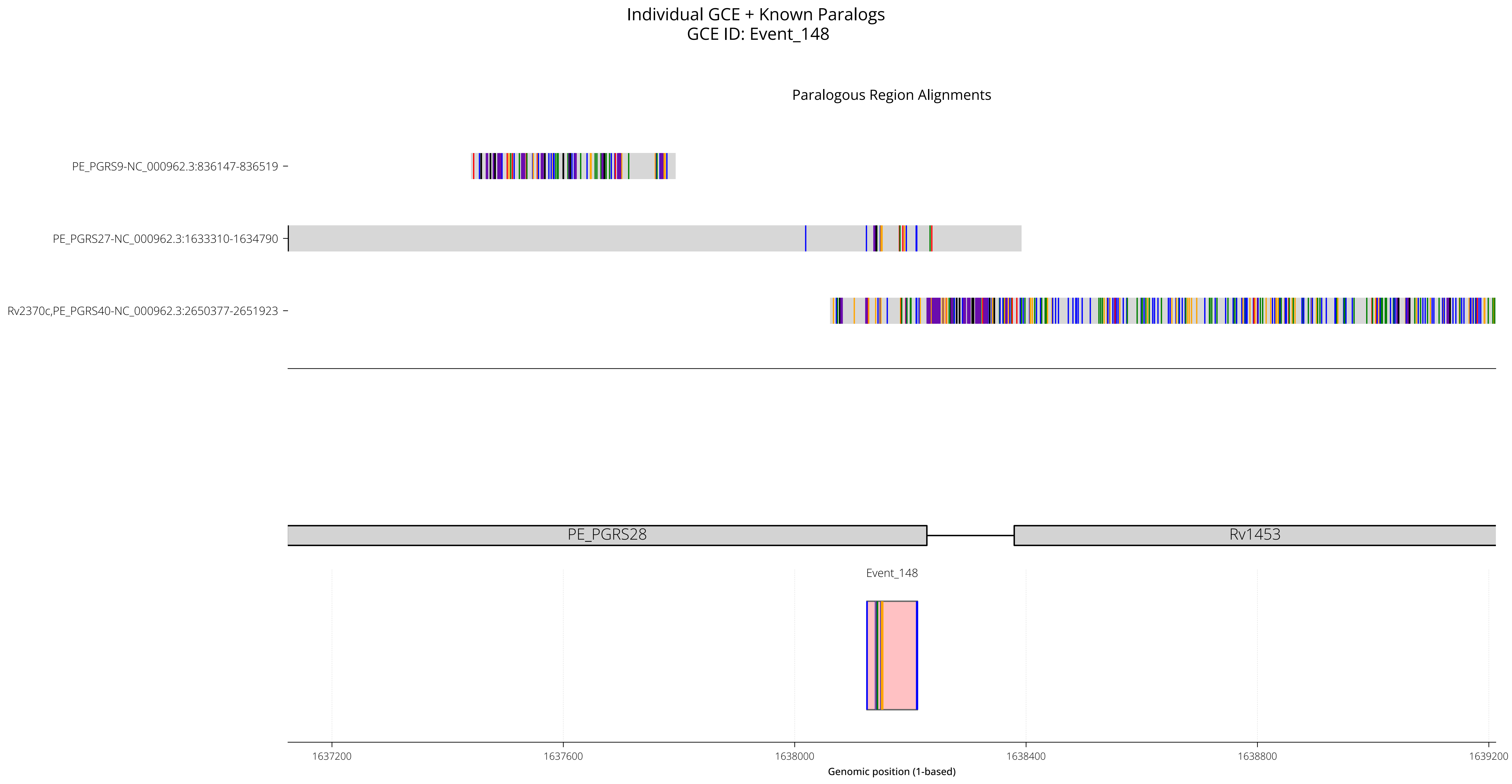

Individual GCE + Known Paralogs
GCE ID: Event_148
Paralogous Region Alignments
PE_PGRS9-NC_000962.3:836147-836519
PE_PGRS27-NC_000962.3:1633310-1634790
Rv2370c,PE_PGRS40-NC_000962.3:2650377-2651923
PE_PGRS28
Rv1453
Event_148
1637200
1637600
1638000
1638400
1638800
1639200
Genomic position (1-based)

Circos Viz - GCE to Known Paralogs Locations
GCE ID: Event_148

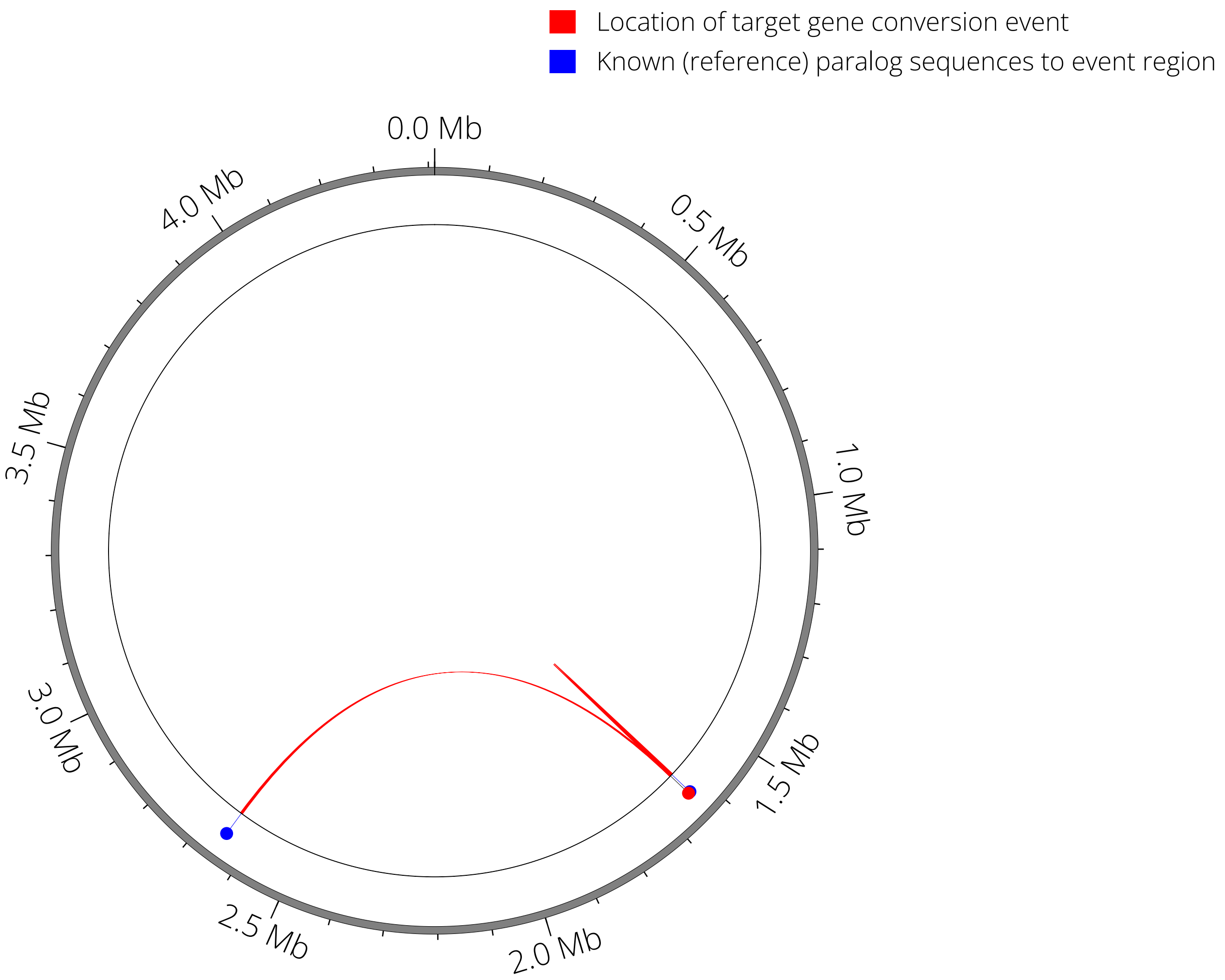

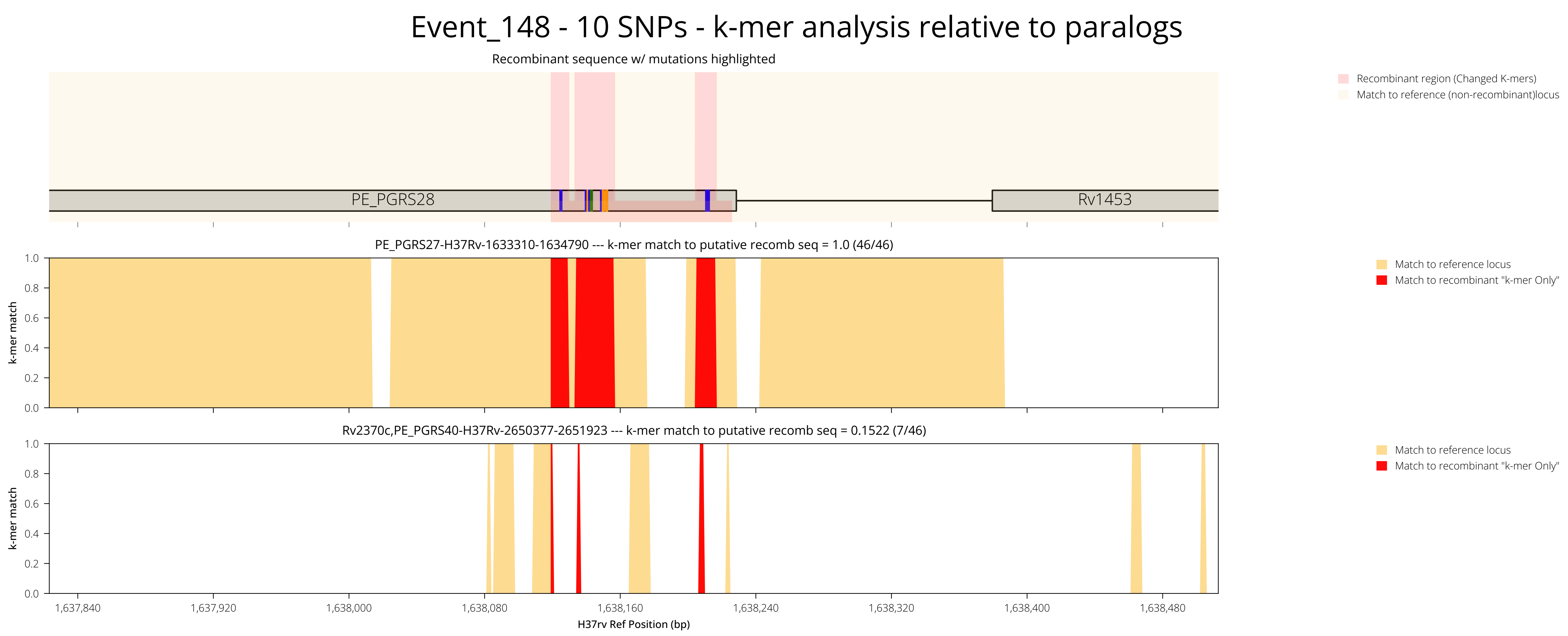

Event_148 - 10 SNPs - k-mer analysis relative to paralogs
Recombinant sequence w/ mutations highlighted
Recombinant region (Changed K-mers)
Match to reference (non-recombinant)locus
PE_PGRS28
Rv1453
PE_PGRS27-H37Rv-1633310-1634790 --- k-mer match to putative recomb seq = 1.0 (46/46)
Match to reference locus
Match to recombinant "k-mer Only"
k-mer match
1.0
0.8
0.6
0.4
0.2
0.0
Rv2370c,PE_PGRS40-H37Rv-2650377-2651923 --- k-mer match to putative recomb seq = 0.1522 (7/46)
Match to reference locus
Match to recombinant "k-mer Only"
k-mer match
1.0
0.8
0.6
0.4
0.2
0.0
1,637,840
1,637,920
1,638,000
1,638,080
1,638,160
1,638,240
1,638,320
1,638,400
1,638,480
H37rv Ref Position (bp)

Sub-phylogeny Viz + Paralog Alignments
GCE ID: Event_148

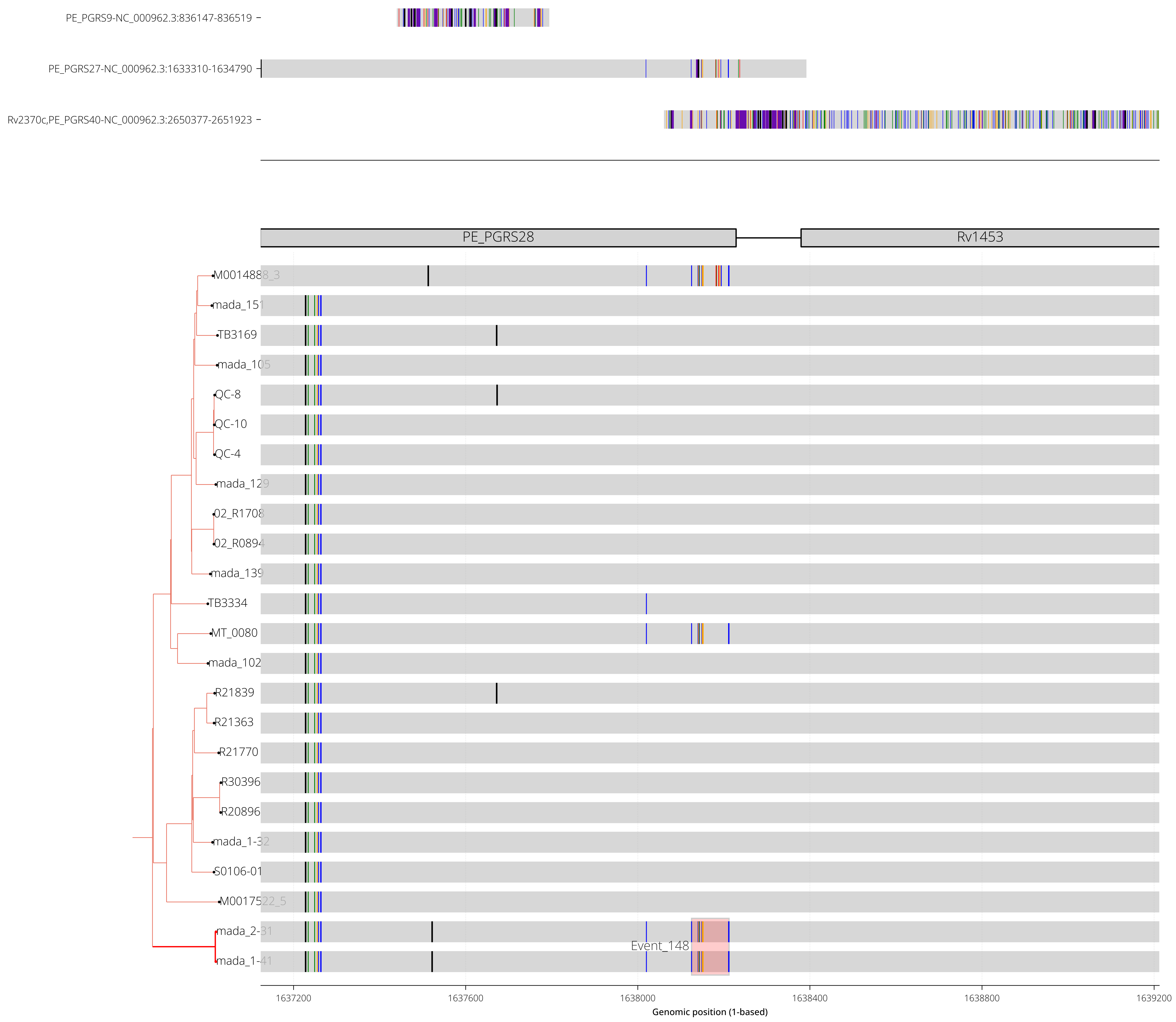

Full Phylogeny Viz + Paralog Alignments
GCE ID: Event_148

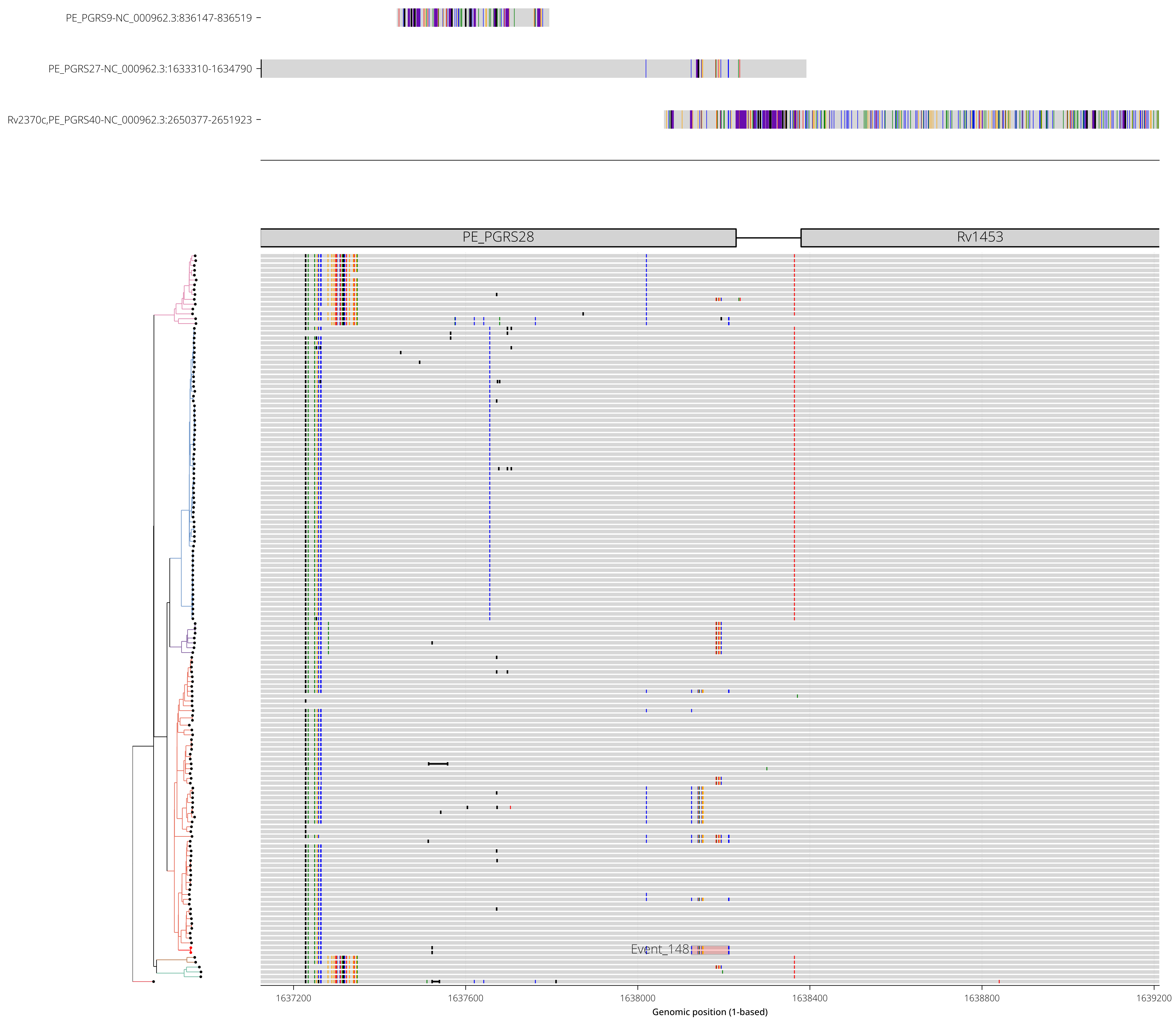

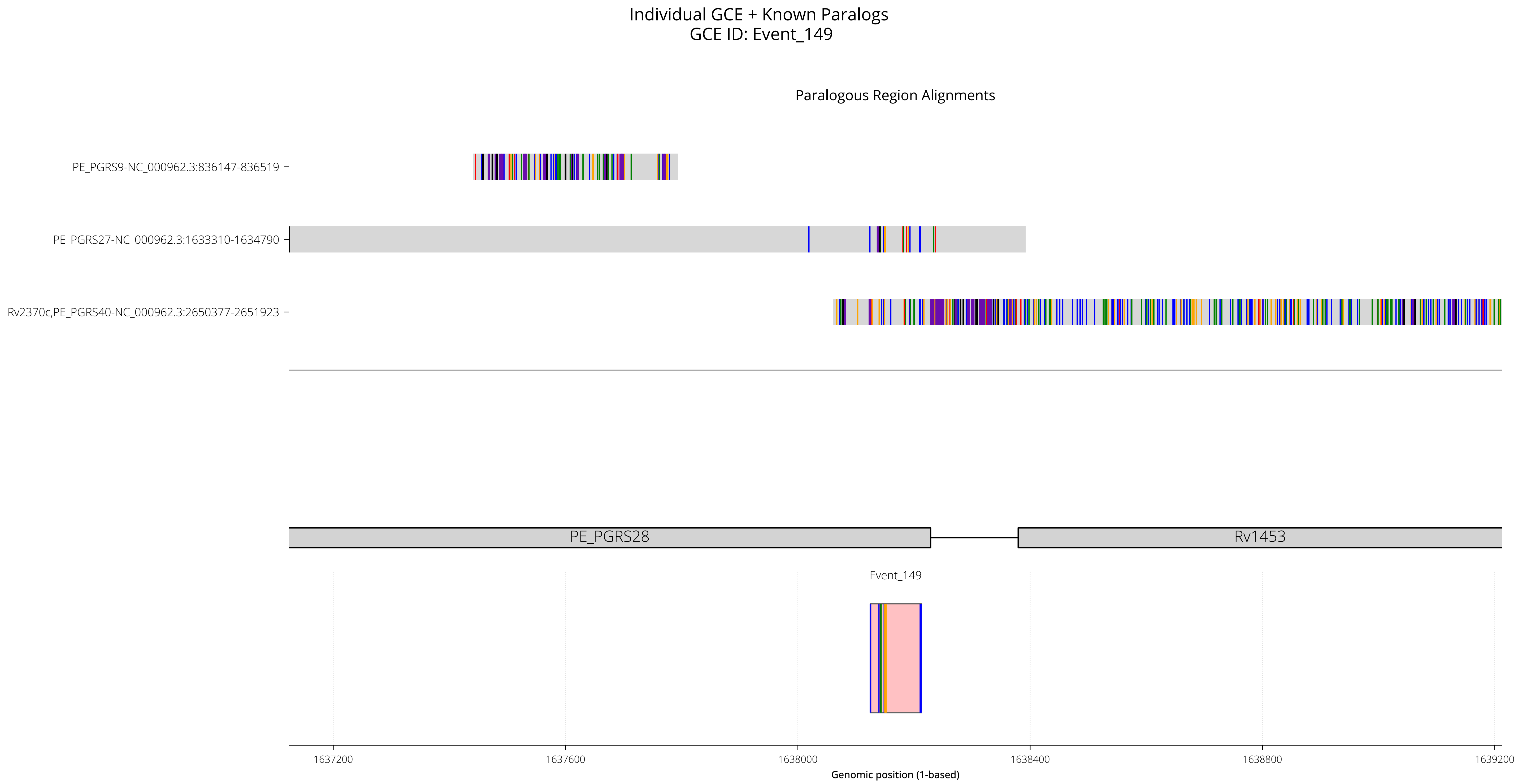
Individual GCE + Known Paralogs
GCE ID: Event_149
Paralogous Region Alignments
PE_PGRS9-NC_000962.3:836147-836519
PE_PGRS27-NC_000962.3:1633310-1634790
Rv2370c,PE_PGRS40-NC_000962.3:2650377-2651923
PE_PGRS28
Rv1453
Event_149
1637200
1637600
1638000
1638400
1638800
1639200
Genomic position (1-based)

Circos Viz - GCE to Known Paralogs Locations
GCE ID: Event_149

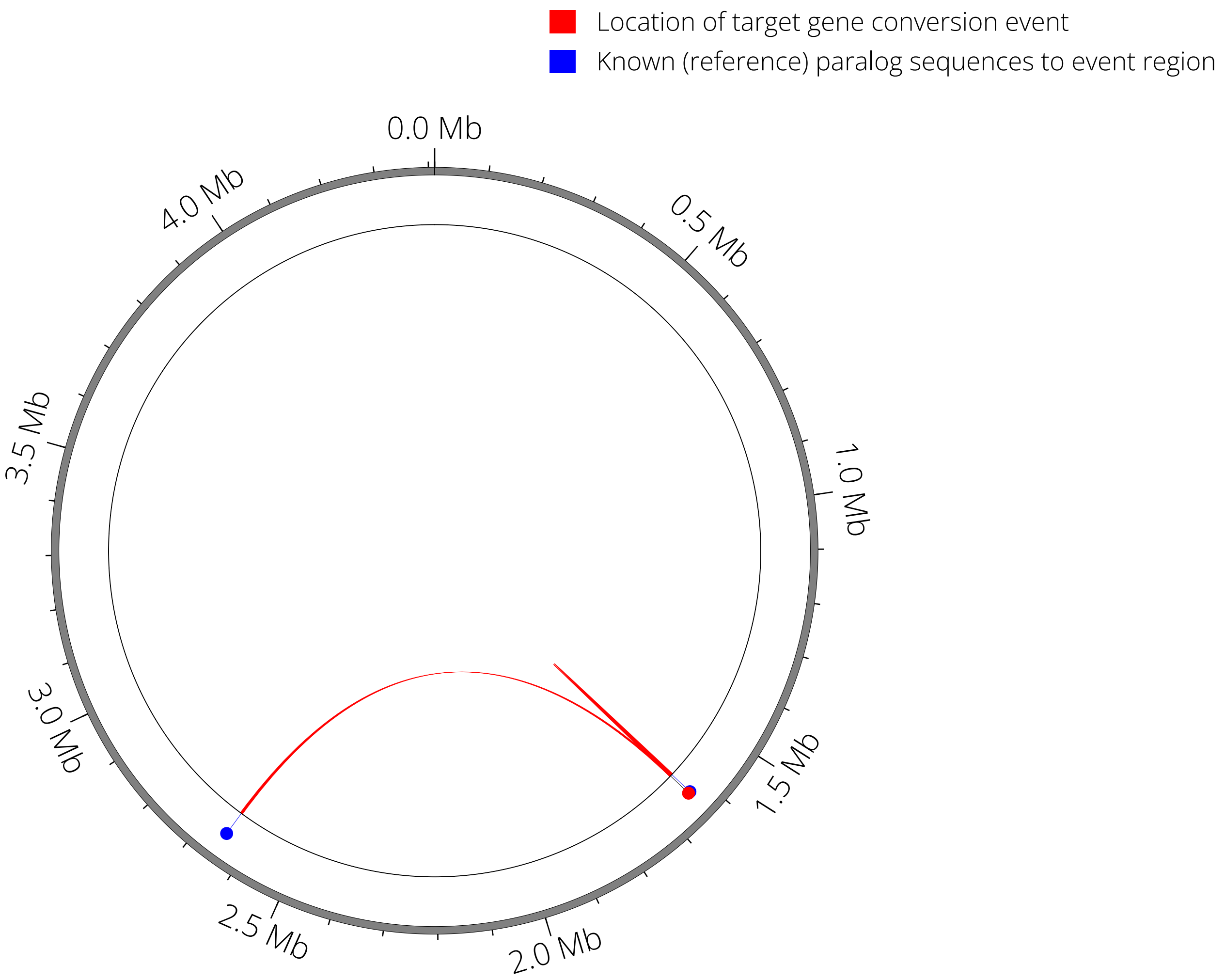

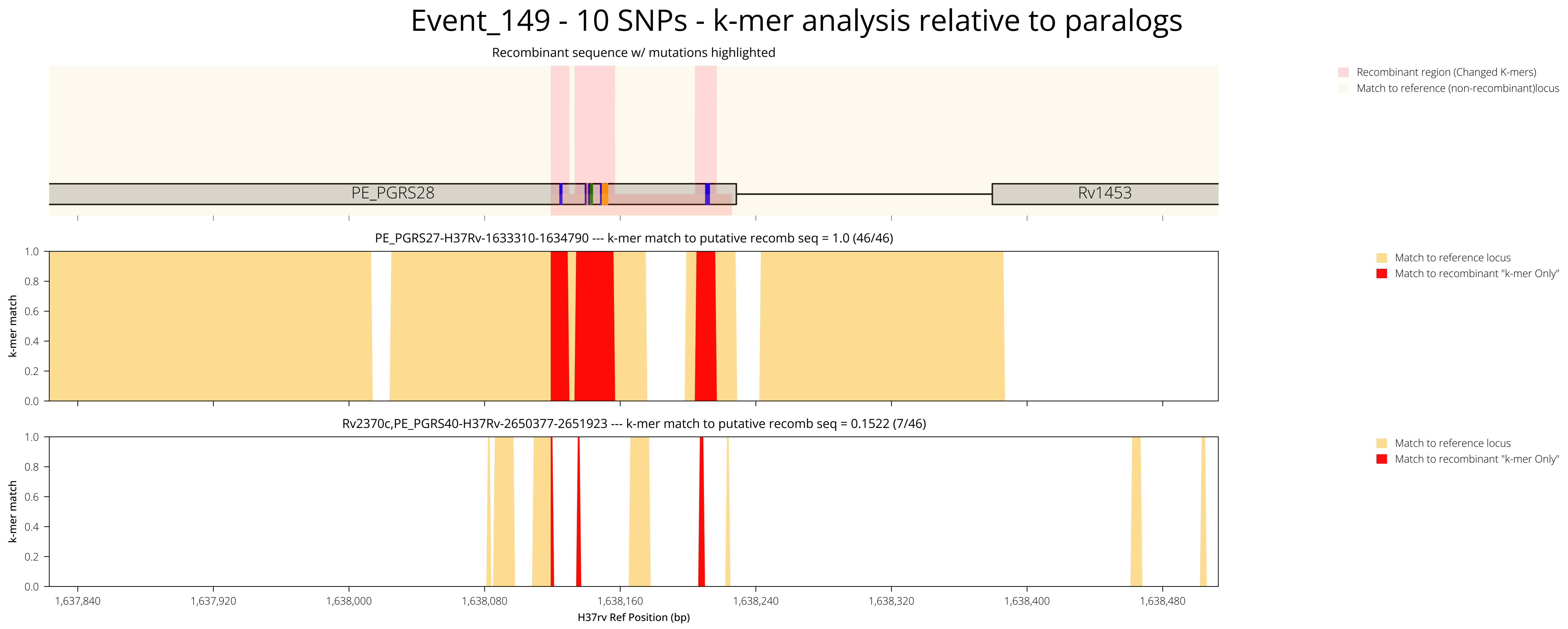

Event_149 - 10 SNPs - k-mer analysis relative to paralogs
Recombinant sequence w/ mutations highlighted
PE_PGRS28
Rv1453
Recombinant region (Changed K-mers)
Match to reference (non-recombinant)locus
PE_PGRS27-H37Rv-1633310-1634790 --- k-mer match to putative recomb seq = 1.0 (46/46)
Match to reference locus
Match to recombinant "k-mer Only"
Rv2370c,PE_PGRS40-H37Rv-2650377-2651923 --- k-mer match to putative recomb seq = 0.1522 (7/46)
Match to reference locus
Match to recombinant "k-mer Only"
k-mer match
1.0
0.8
0.6
0.4
0.2
0.0
1,637,840
1,637,920
1,638,000
1,638,080
1,638,160
1,638,240
1,638,320
1,638,400
1,638,480
H37rv Ref Position (bp)

Sub-phylogeny Viz + Paralog Alignments
GCE ID: Event_149

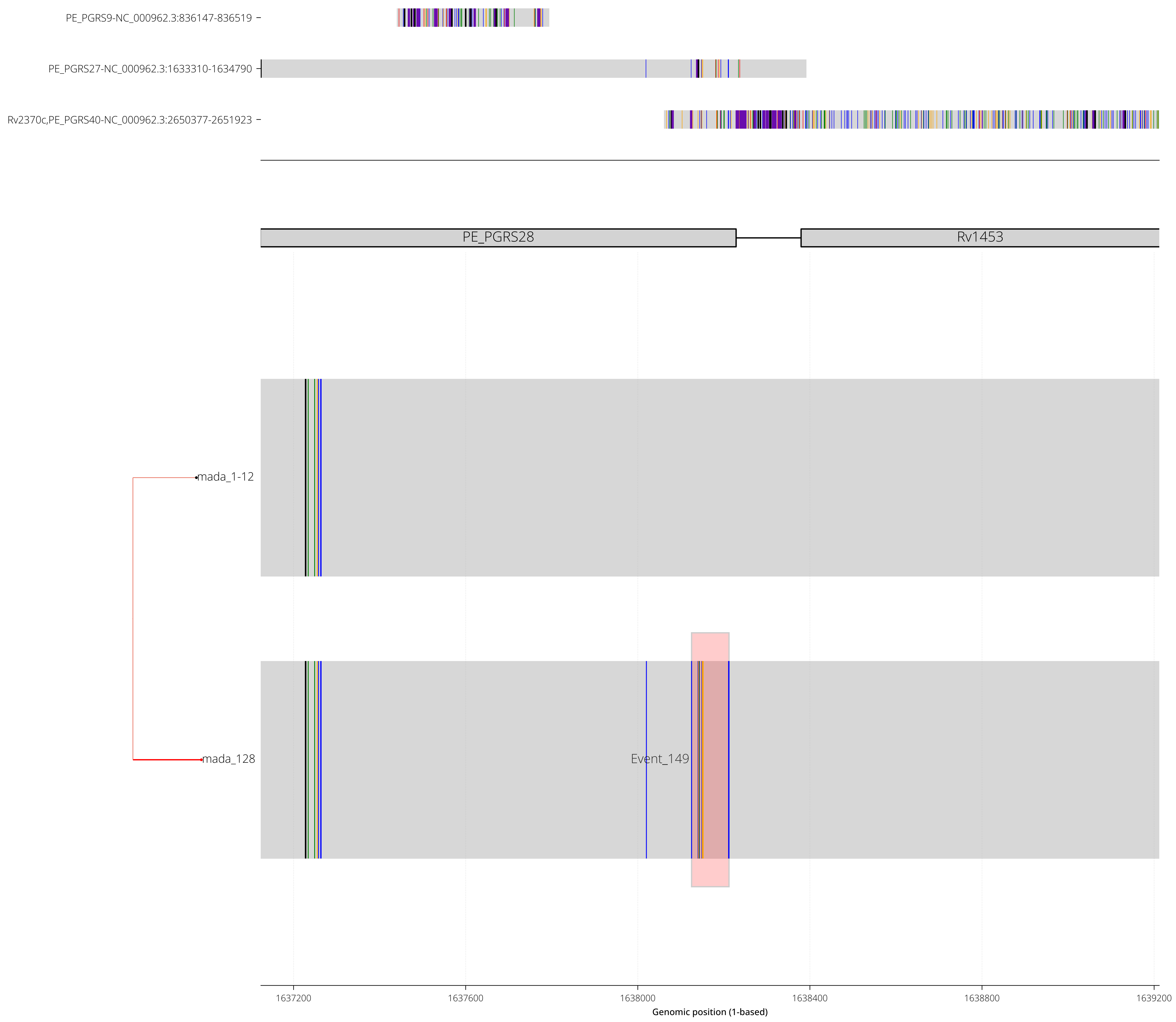

Full Phylogeny Viz + Paralog Alignments
GCE ID: Event_149

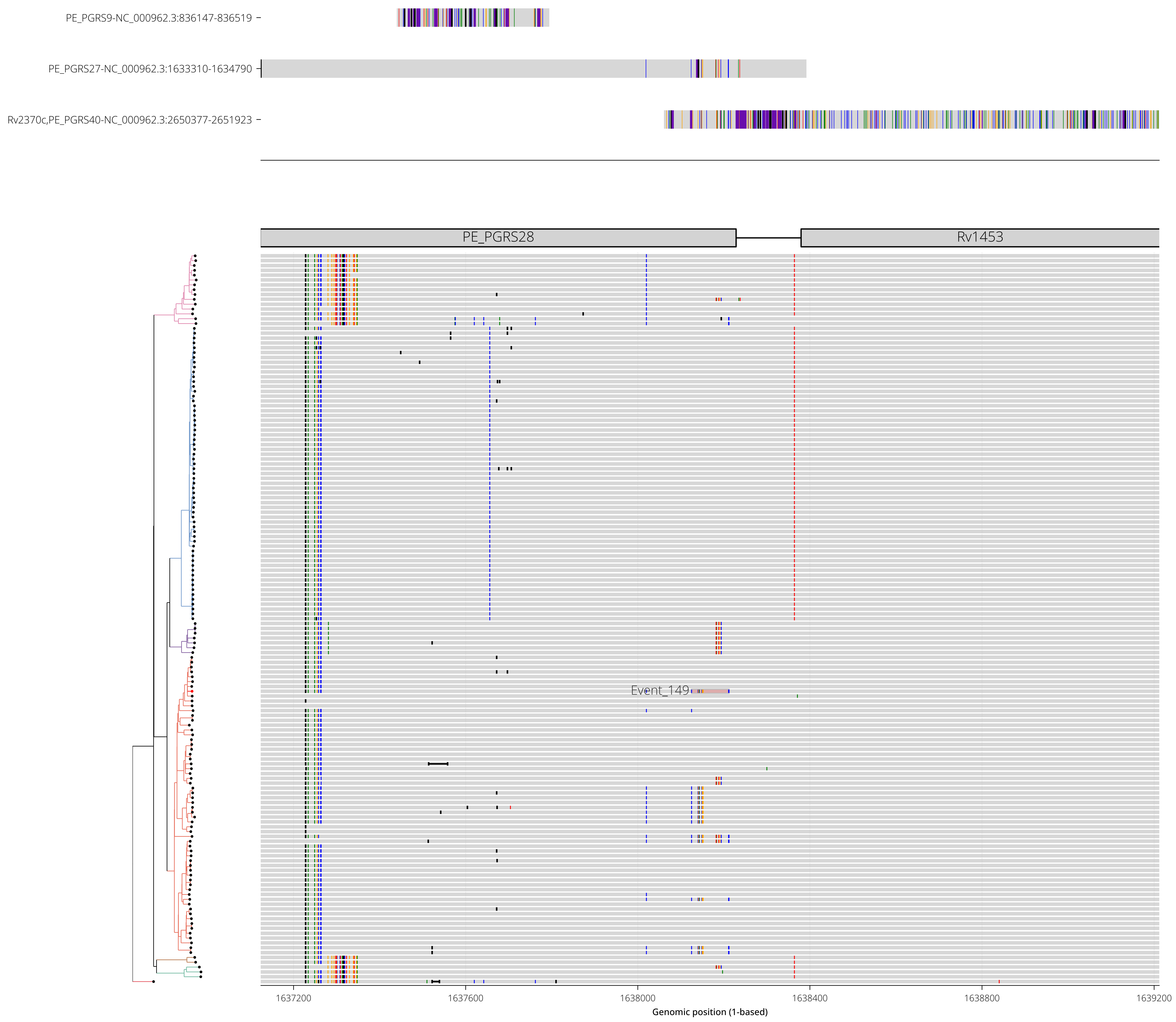

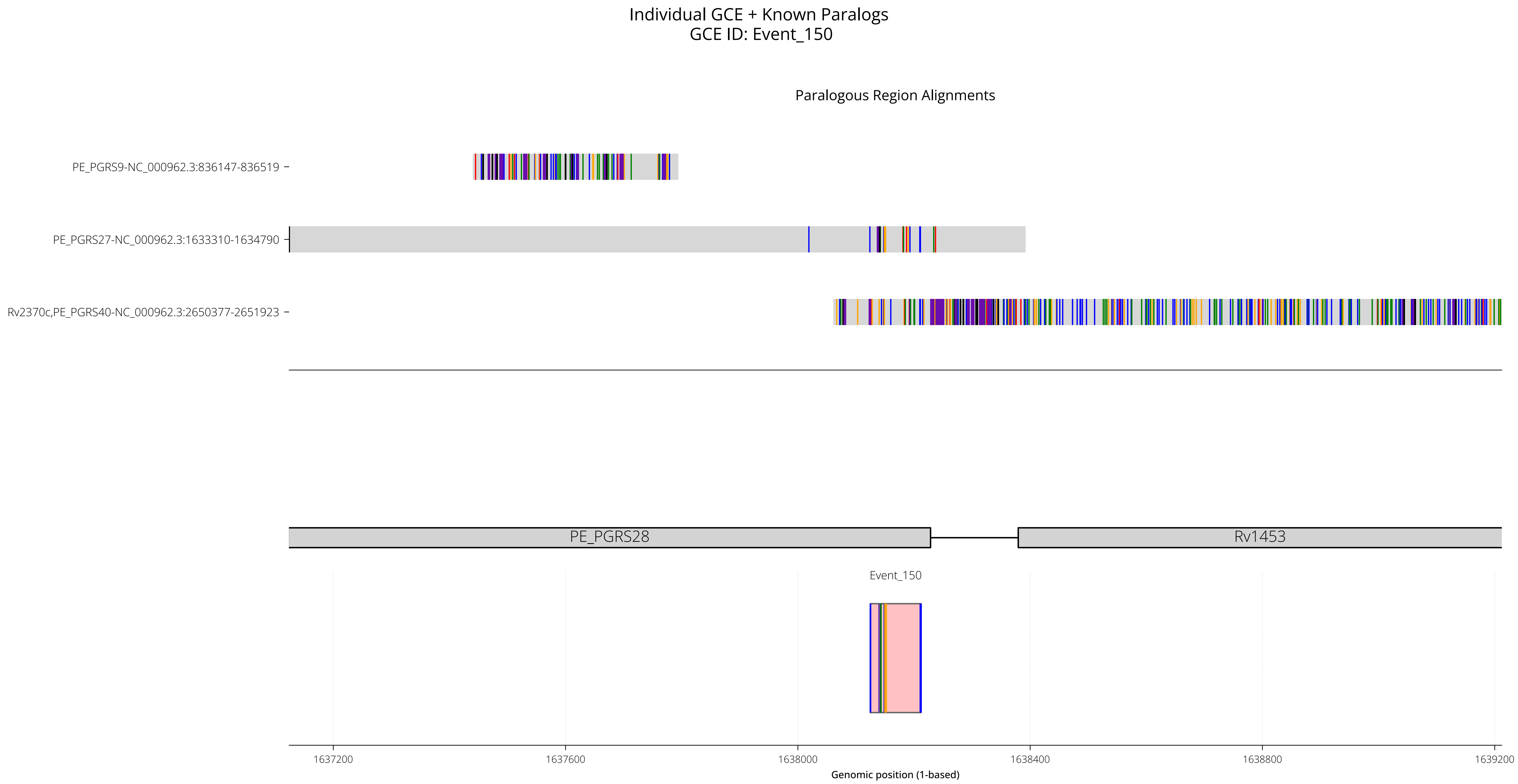

Individual GCE + Known Paralogs
GCE ID: Event_150
Paralogous Region Alignments
PE_PGRS9-NC_000962.3:836147-836519
PE_PGRS27-NC_000962.3:1633310-1634790
Rv2370c,PE_PGRS40-NC_000962.3:2650377-2651923
PE_PGRS28
Rv1453
Event_150
1637200
1637600
1638000
1638400
1638800
1639200
Genomic position (1-based)

# Circos Viz - GCE to Known Paralogs Locations
GCE ID: Event_150

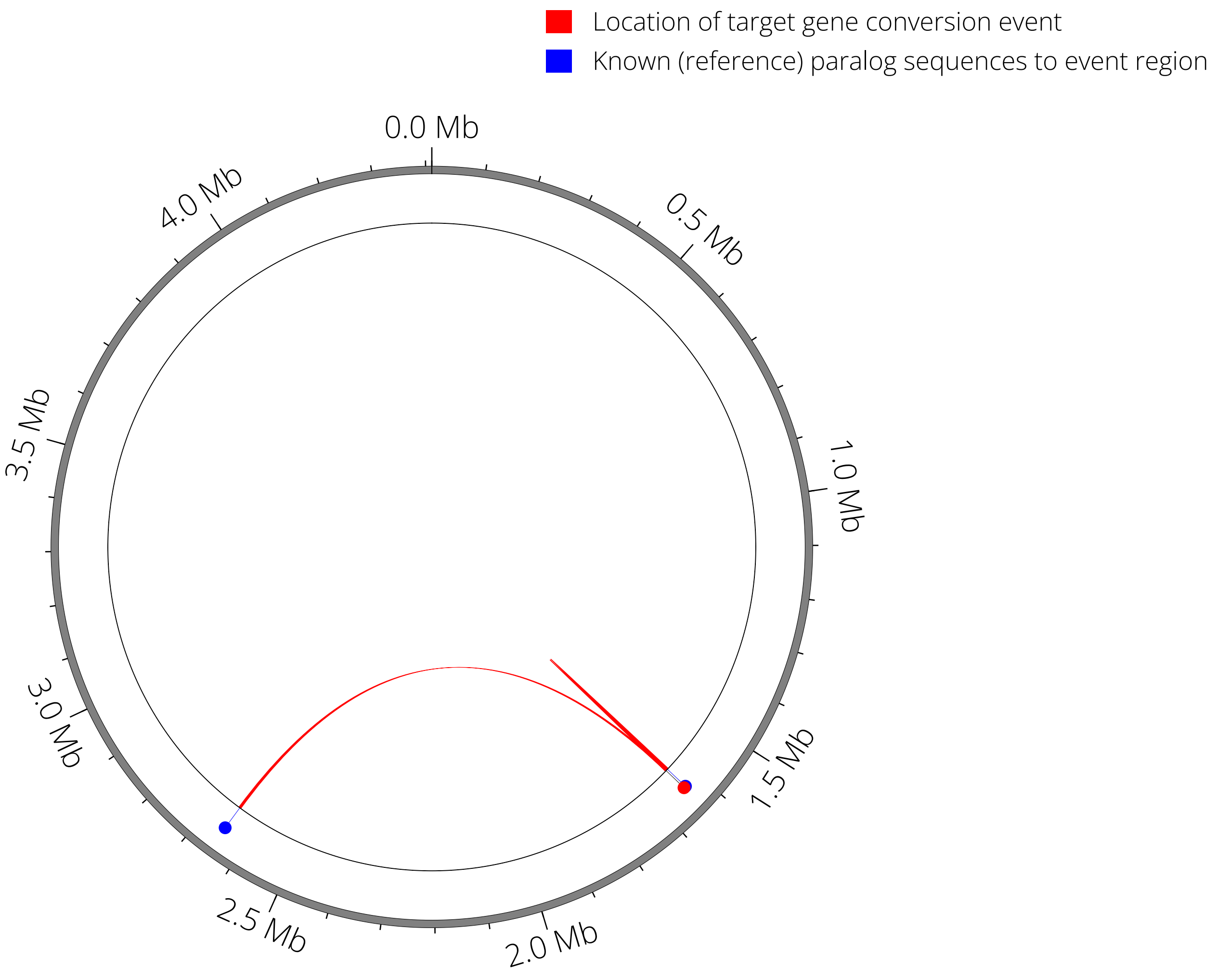

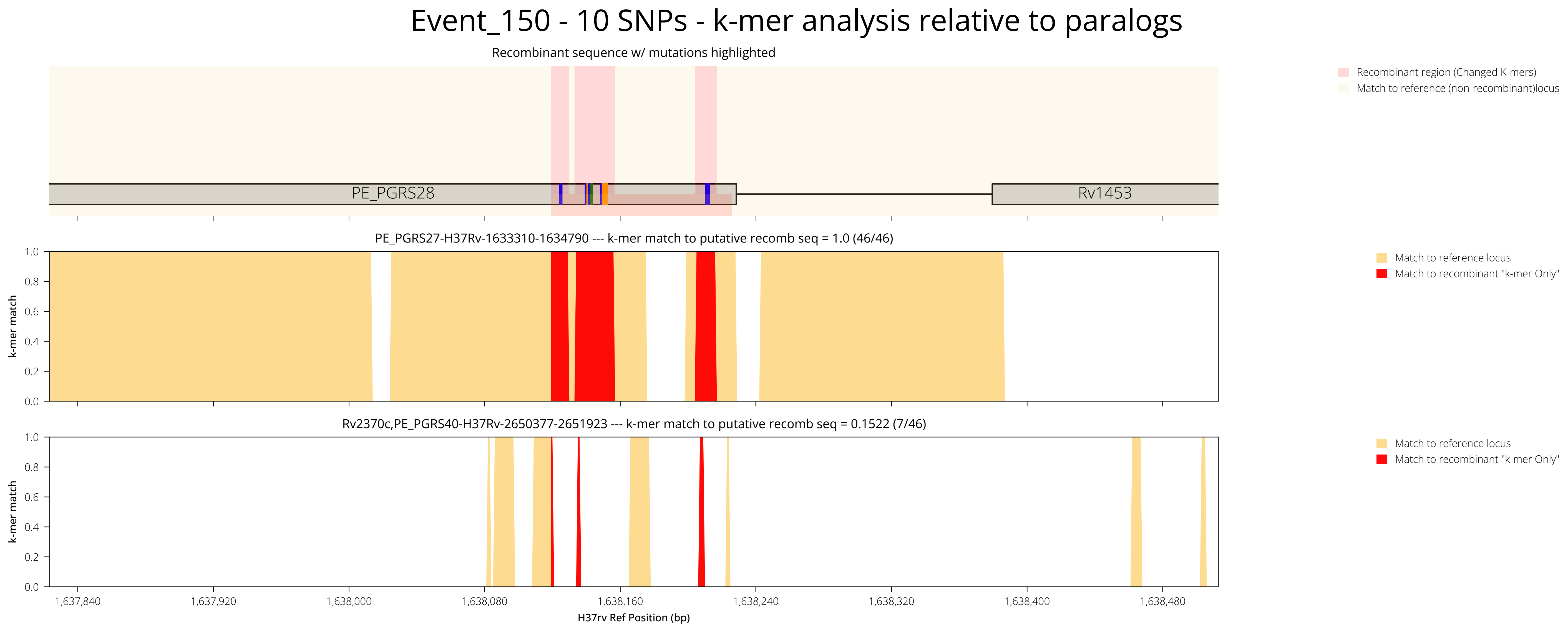
Event_150 - 10 SNPs - k-mer analysis relative to paralogs
Recombinant sequence w/ mutations highlighted
PE_PGRS28
Rv1453
Recombinant region (Changed K-mers)
Match to reference (non-recombinant)locus
PE_PGRS27-H37Rv-1633310-1634790 --- k-mer match to putative recomb seq = 1.0 (46/46)
k-mer match
1.0
0.8
0.6
0.4
0.2
0.0
Match to reference locus
Match to recombinant "k-mer Only"
Rv2370c,PE_PGRS40-H37Rv-2650377-2651923 --- k-mer match to putative recomb seq = 0.1522 (7/46)
k-mer match
1.0
0.8
0.6
0.4
0.2
0.0
Match to reference locus
Match to recombinant "k-mer Only"
1,637,840
1,637,920
1,638,000
1,638,080
1,638,160
1,638,240
1,638,320
1,638,400
1,638,480
H37rv Ref Position (bp)

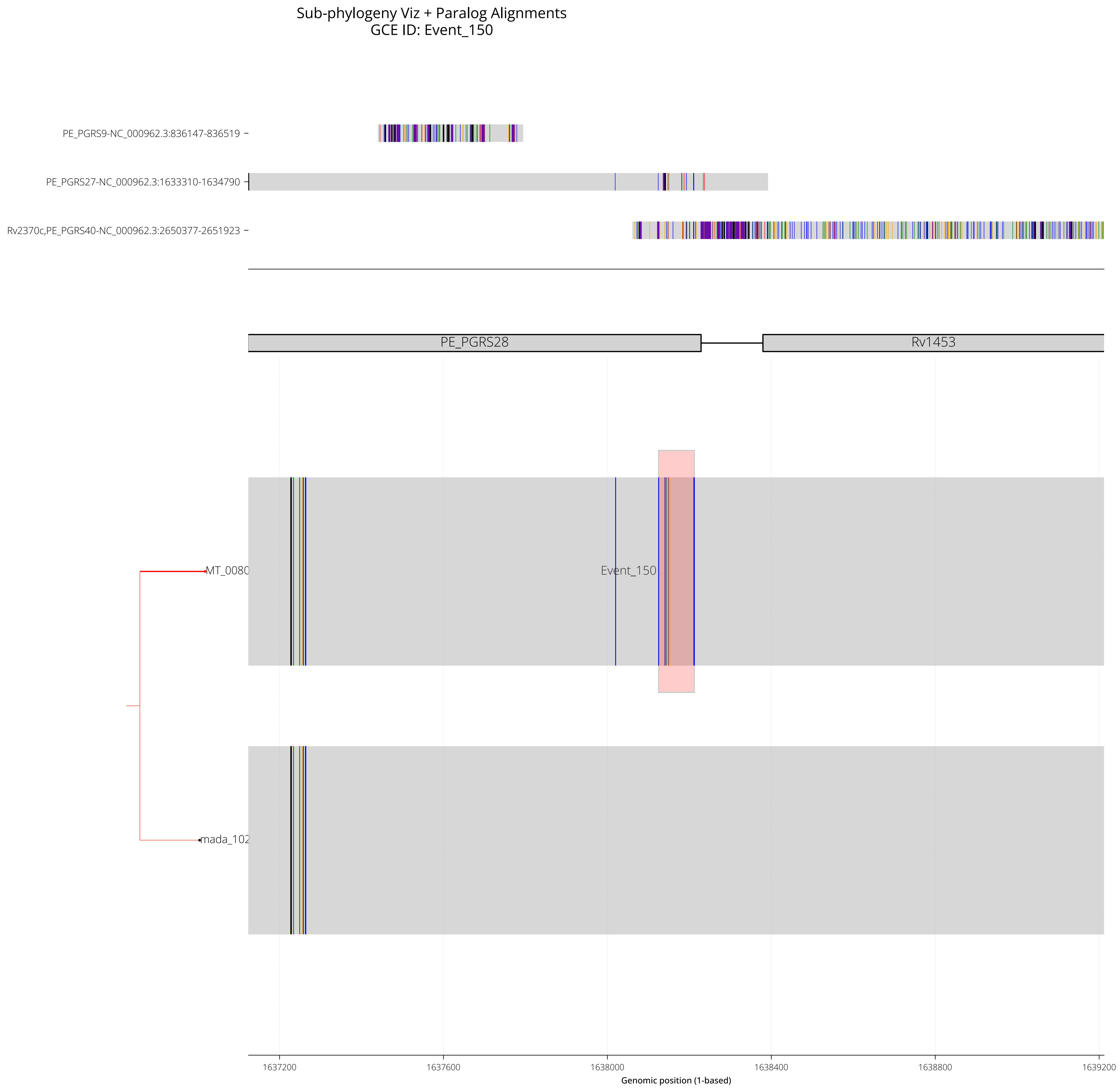
Sub-phylogeny Viz + Paralog Alignments
GCE ID: Event_150
PE_PGRS9-NC_000962.3:836147-836519
PE_PGRS27-NC_000962.3:1633310-1634790
Rv2370c,PE_PGRS40-NC_000962.3:2650377-2651923
PE_PGRS28
Rv1453
MT_0080
Event_150
mada_102
1637200
1637600
1638000
1638400
1638800
1639200
Genomic position (1-based)

Full Phylogeny Viz + Paralog Alignments
GCE ID: Event_150

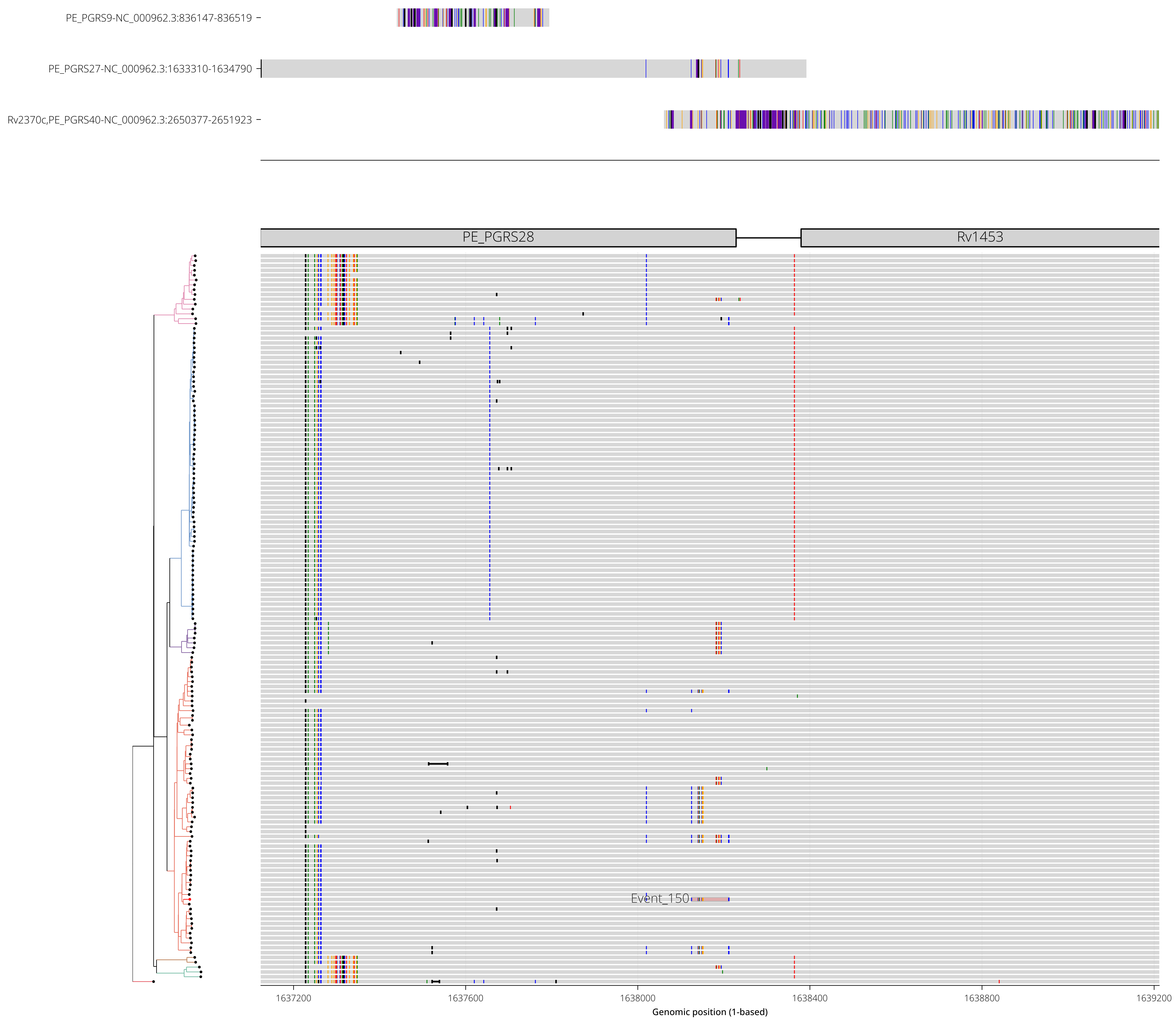

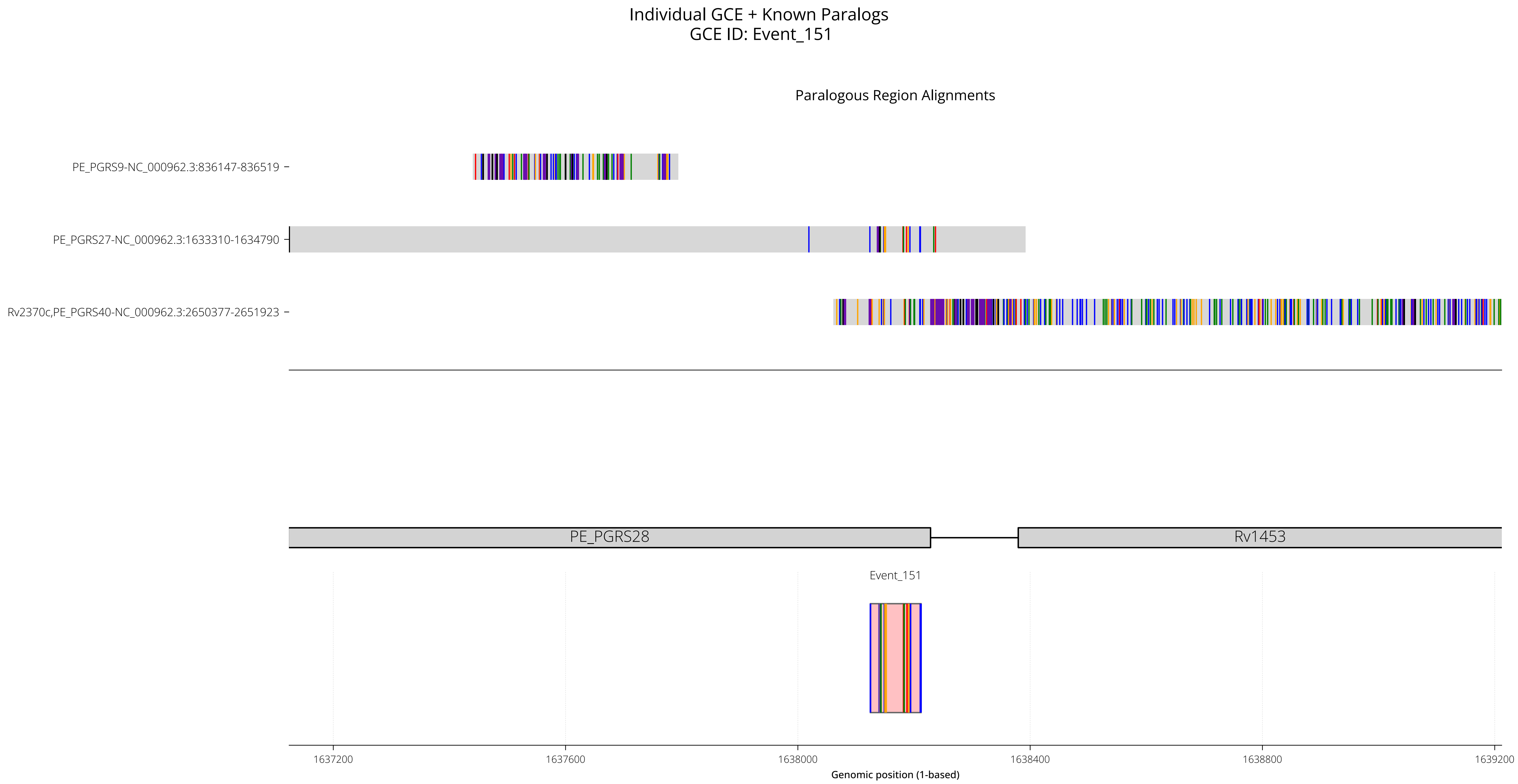
Individual GCE + Known Paralogs
GCE ID: Event_151
Paralogous Region Alignments
PE_PGRS9-NC_000962.3:836147-836519
PE_PGRS27-NC_000962.3:1633310-1634790
Rv2370c,PE_PGRS40-NC_000962.3:2650377-2651923
PE_PGRS28
Rv1453
Event_151
1637200
1637600
1638000
1638400
1638800
1639200
Genomic position (1-based)

Circos Viz - GCE to Known Paralogs Locations
GCE ID: Event_151

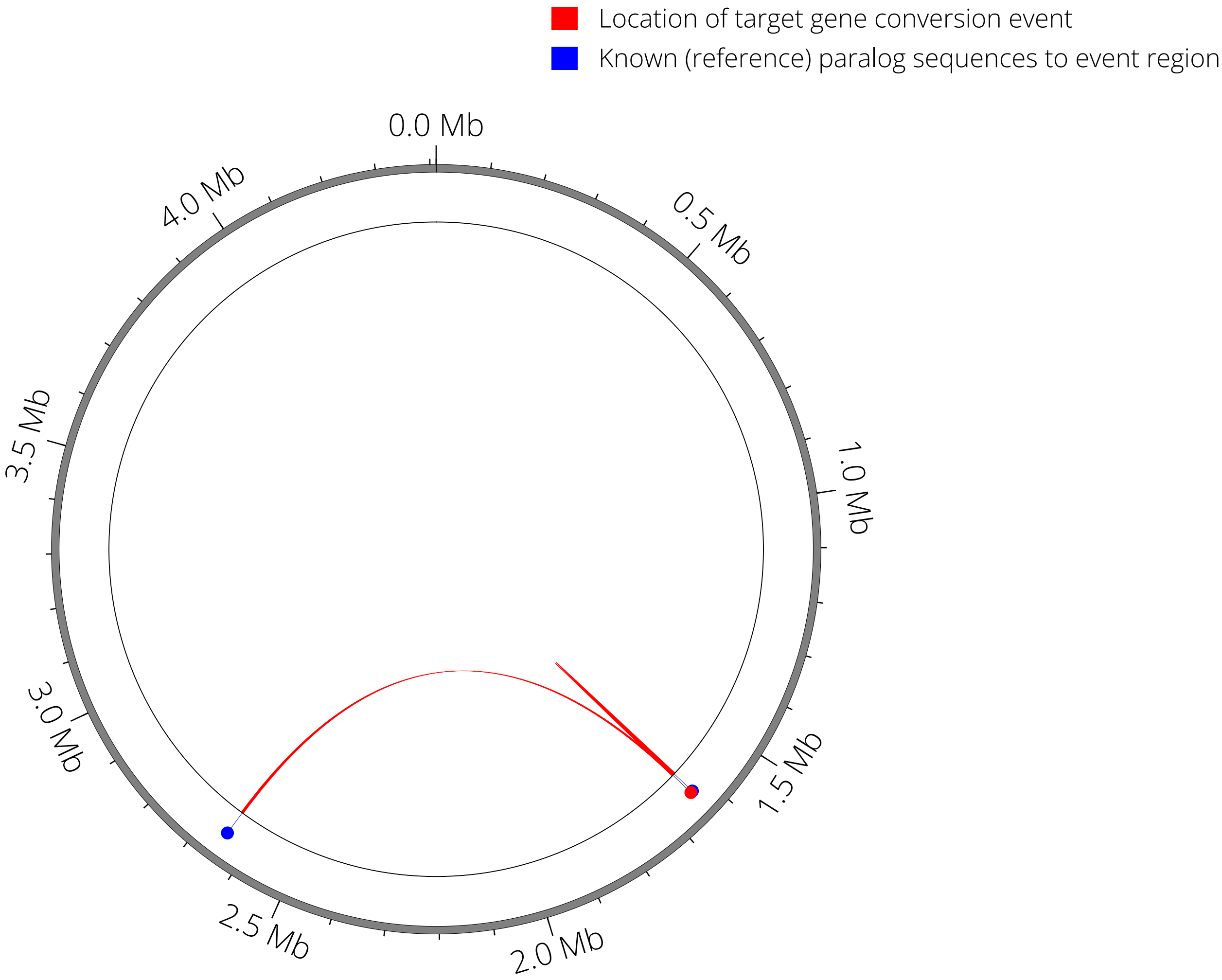

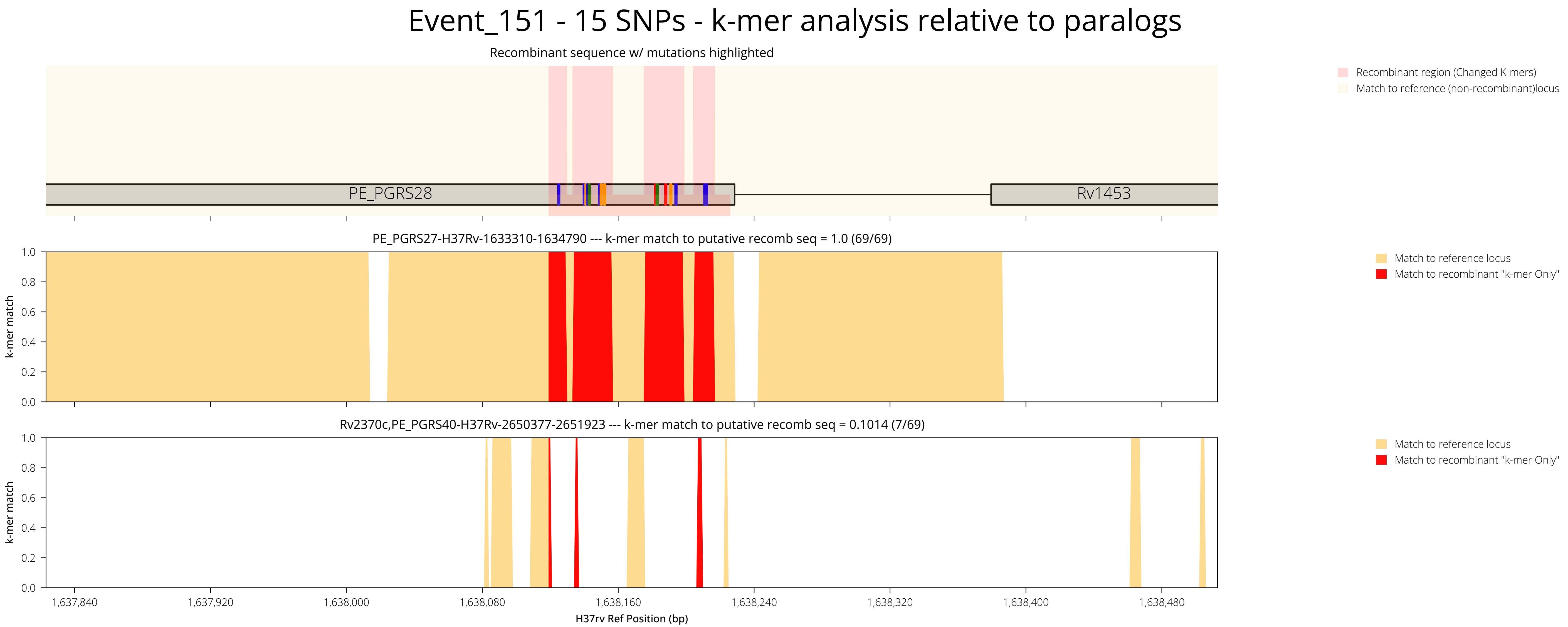

Event_151 - 15 SNPs - k-mer analysis relative to paralogs
Recombinant sequence w/ mutations highlighted
Recombinant region (Changed K-mers)
Match to reference (non-recombinant)locus
PE_PGRS28
Rv1453
PE_PGRS27-H37Rv-1633310-1634790 --- k-mer match to putative recomb seq = 1.0 (69/69)
Match to reference locus
Match to recombinant "k-mer Only"
k-mer match
1.0
0.8
0.6
0.4
0.2
0.0
Rv2370c,PE_PGRS40-H37Rv-2650377-2651923 --- k-mer match to putative recomb seq = 0.1014 (7/69)
Match to reference locus
Match to recombinant "k-mer Only"
k-mer match
1.0
0.8
0.6
0.4
0.2
0.0
1,637,840
1,637,920
1,638,000
1,638,080
1,638,160
1,638,240
1,638,320
1,638,400
1,638,480
H37rv Ref Position (bp)

Sub-phylogeny Viz + Paralog Alignments
GCE ID: Event_151

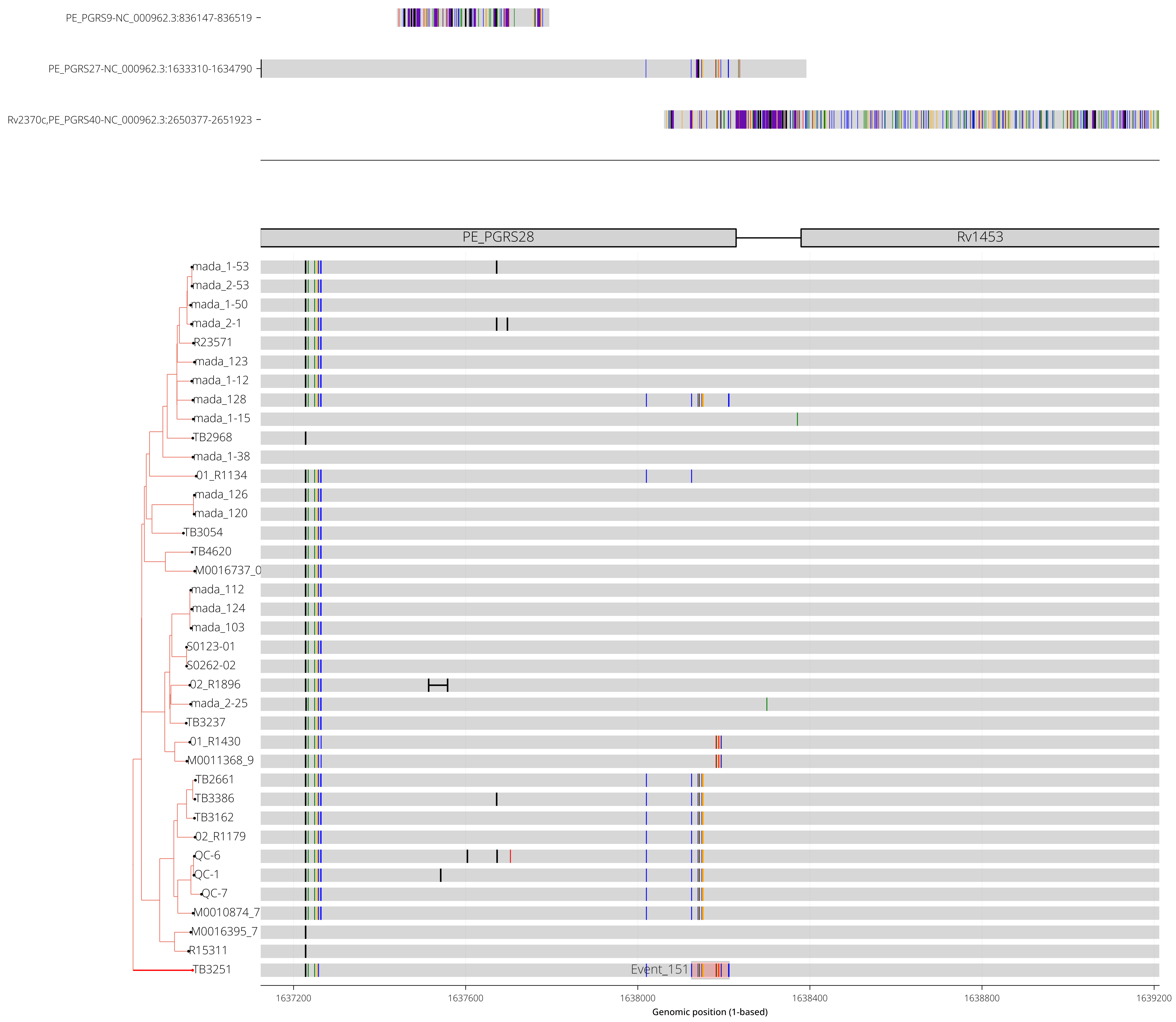

Full Phylogeny Viz + Paralog Alignments
GCE ID: Event_151

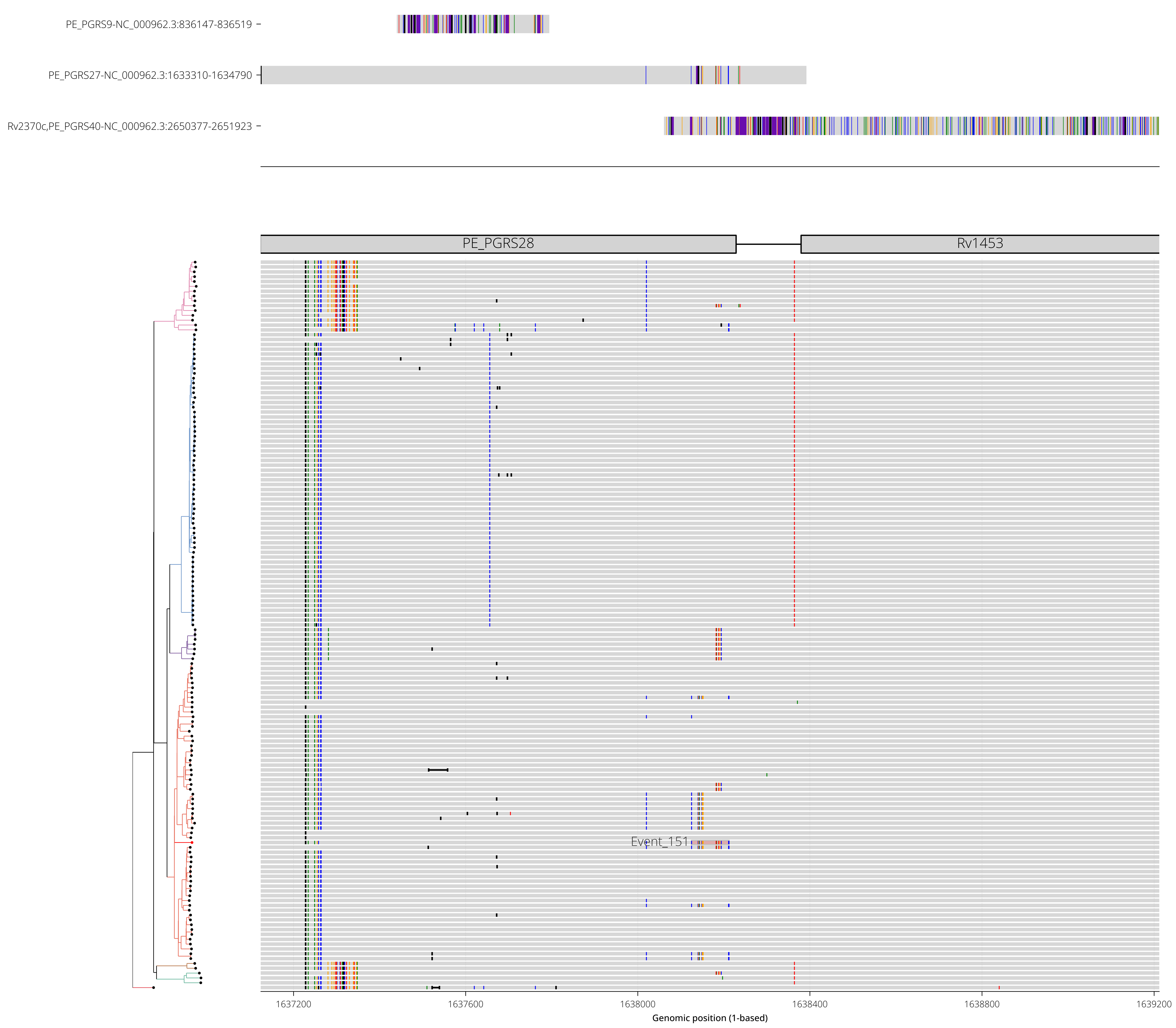

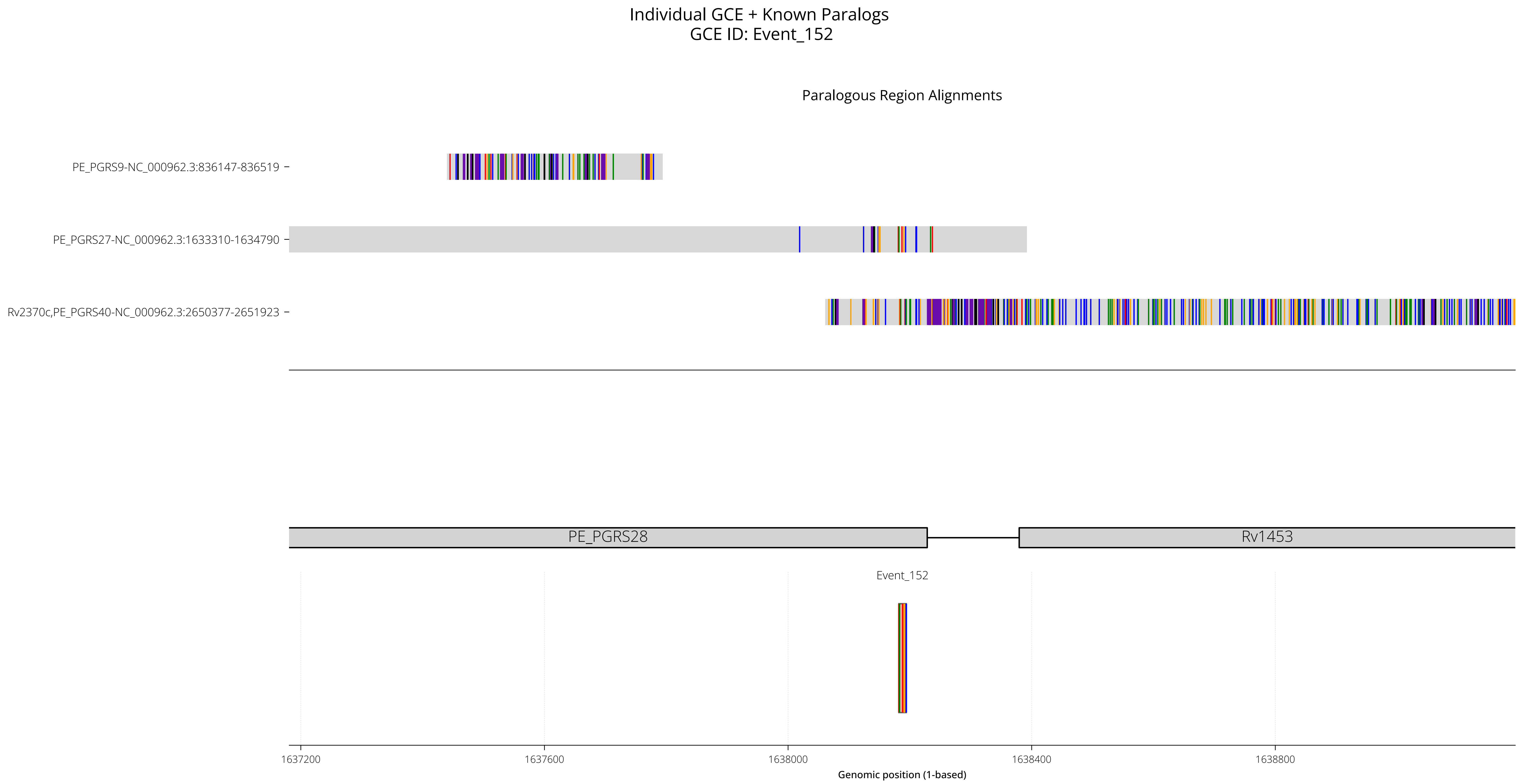

Individual GCE + Known Paralogs
GCE ID: Event_152
Paralogous Region Alignments
PE_PGRS9-NC_000962.3:836147-836519
PE_PGRS27-NC_000962.3:1633310-1634790
Rv2370c,PE_PGRS40-NC_000962.3:2650377-2651923
PE_PGRS28
Rv1453
Event_152
1637200
1637600
1638000
1638400
1638800
Genomic position (1-based)

Circos Viz - GCE to Known Paralogs Locations
GCE ID: Event_152

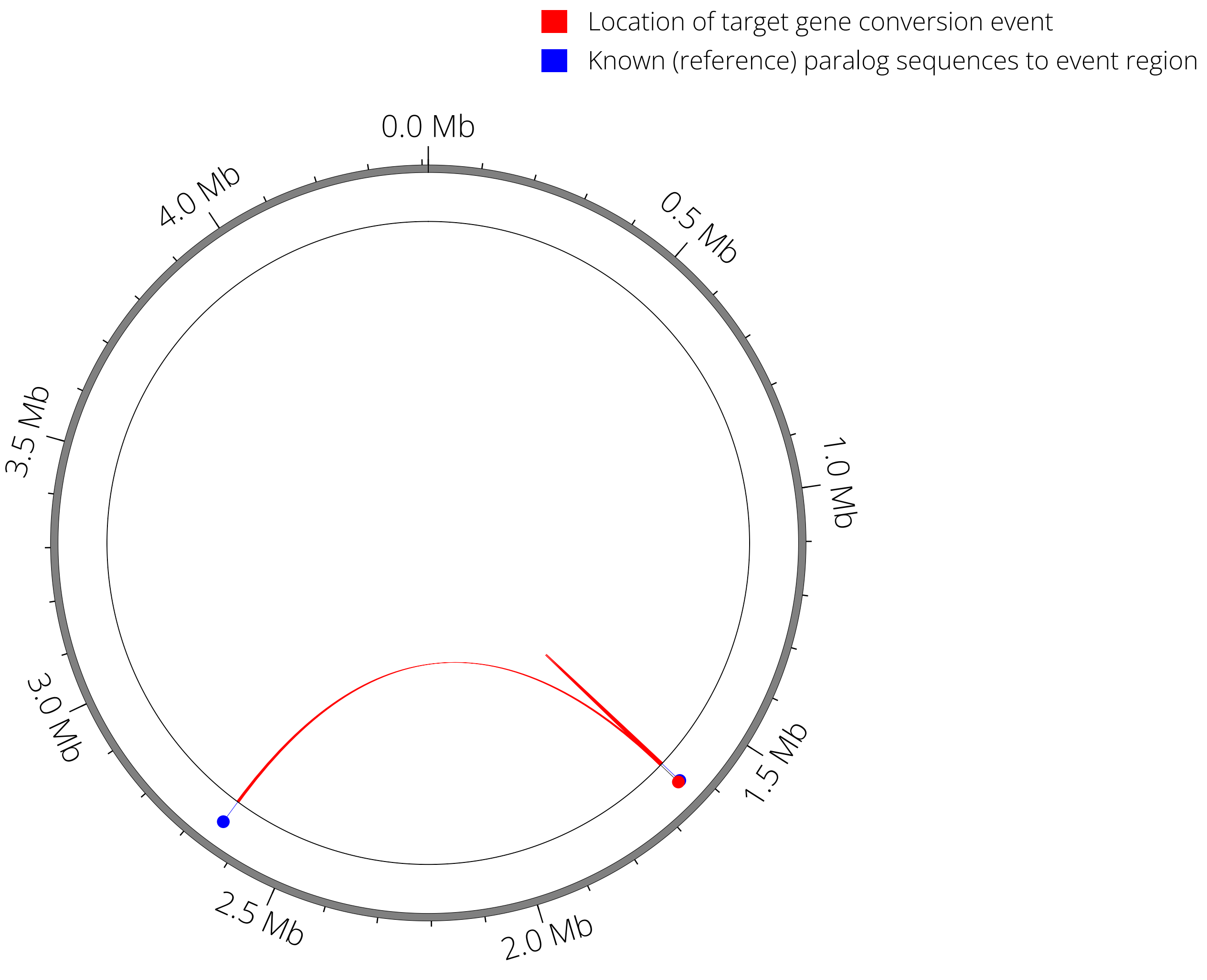

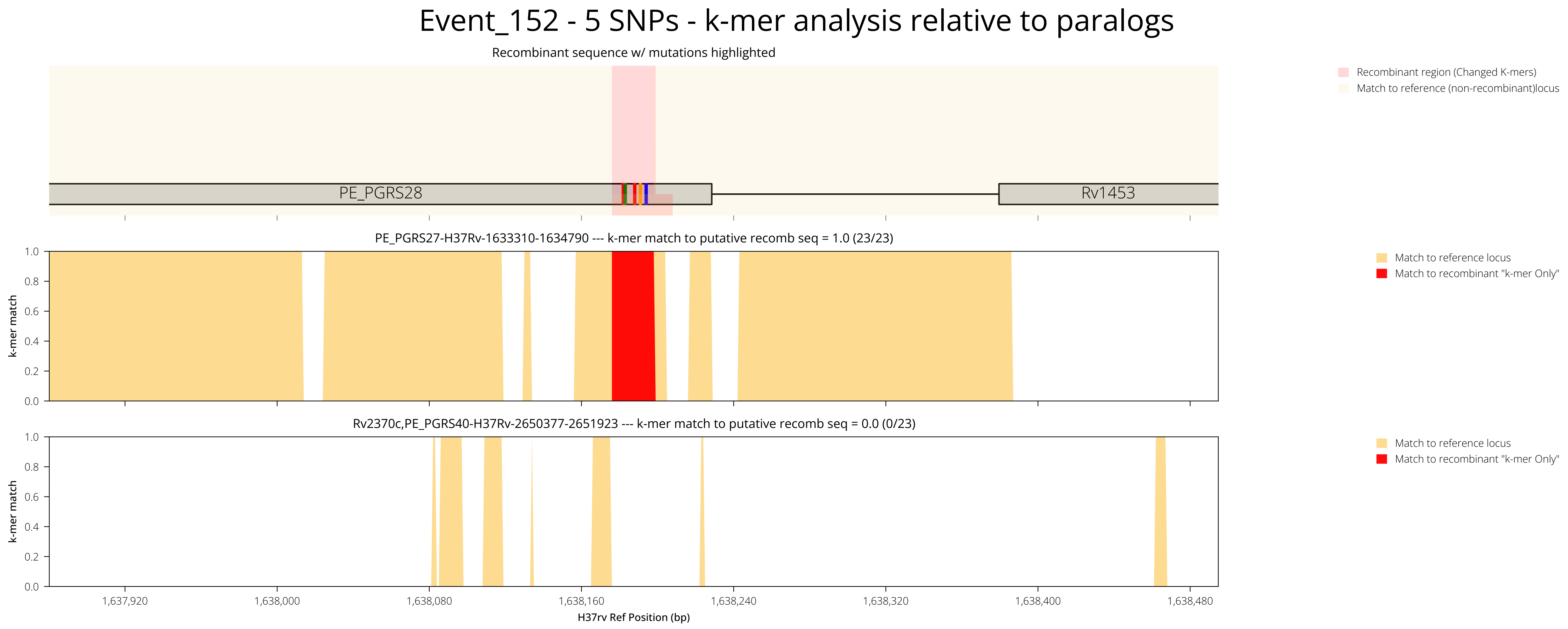
Event_152 - 5 SNPs - k-mer analysis relative to paralogs
Recombinant sequence w/ mutations highlighted
Recombinant region (Changed K-mers)
Match to reference (non-recombinant)locus
PE_PGRS28
Rv1453
PE_PGRS27-H37Rv-1633310-1634790 --- k-mer match to putative recomb seq = 1.0 (23/23)
Match to reference locus
Match to recombinant "k-mer Only"
k-mer match
1.0
0.8
0.6
0.4
0.2
0.0
Rv2370c,PE_PGRS40-H37Rv-2650377-2651923 --- k-mer match to putative recomb seq = 0.0 (0/23)
Match to reference locus
Match to recombinant "k-mer Only"
k-mer match
1,637,920
1,638,000
1,638,080
1,638,160
1,638,240
1,638,320
1,638,400
1,638,480
H37rv Ref Position (bp)

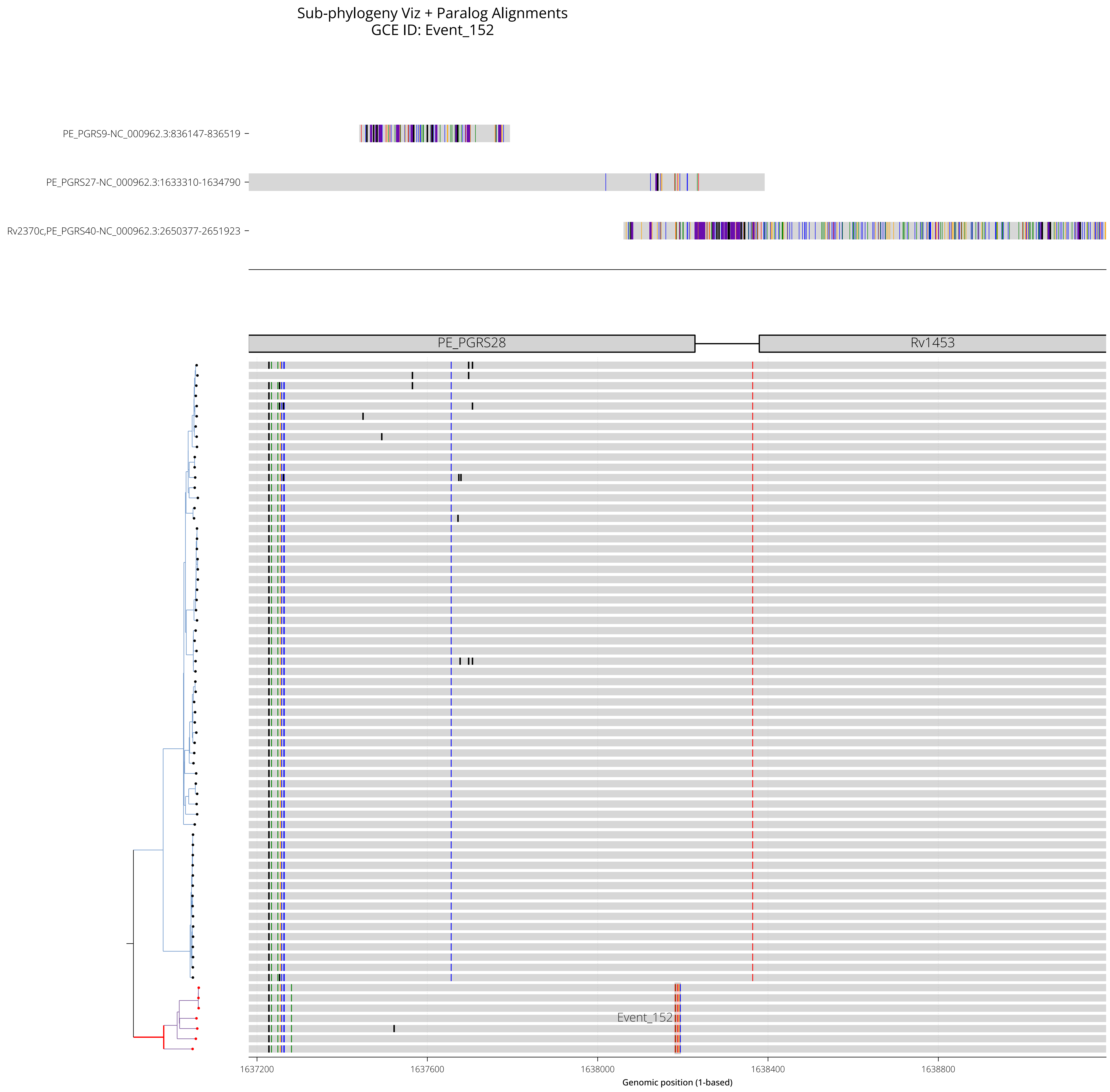
Sub-phylogeny Viz + Paralog Alignments
GCE ID: Event_152
PE_PGRS9-NC_000962.3:836147-836519
PE_PGRS27-NC_000962.3:1633310-1634790
Rv2370c,PE_PGRS40-NC_000962.3:2650377-2651923
PE_PGRS28
Rv1453
Event_152
1637200
1637600
1638000
1638400
1638800
Genomic position (1-based)

Full Phylogeny Viz + Paralog Alignments
GCE ID: Event_152

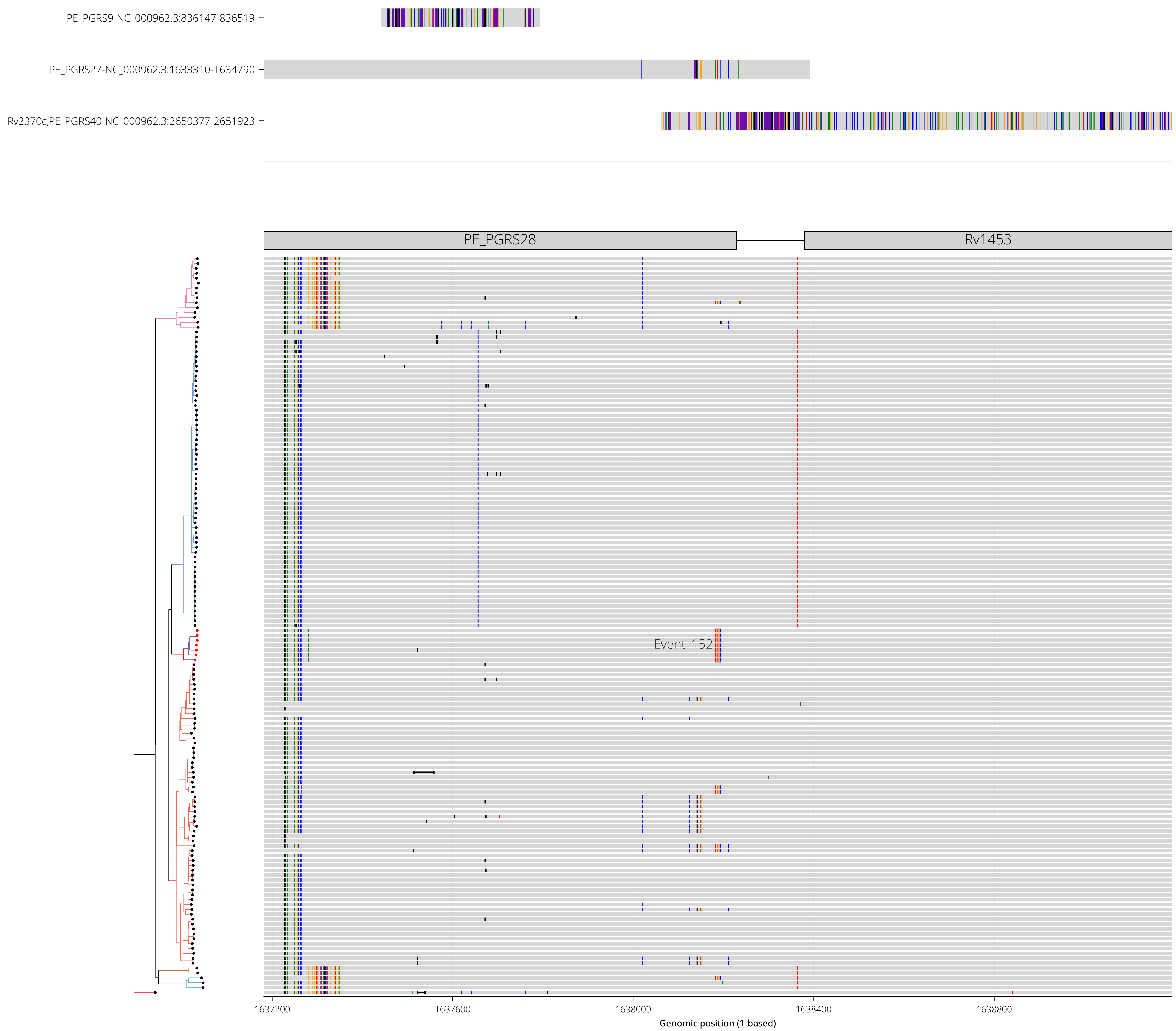

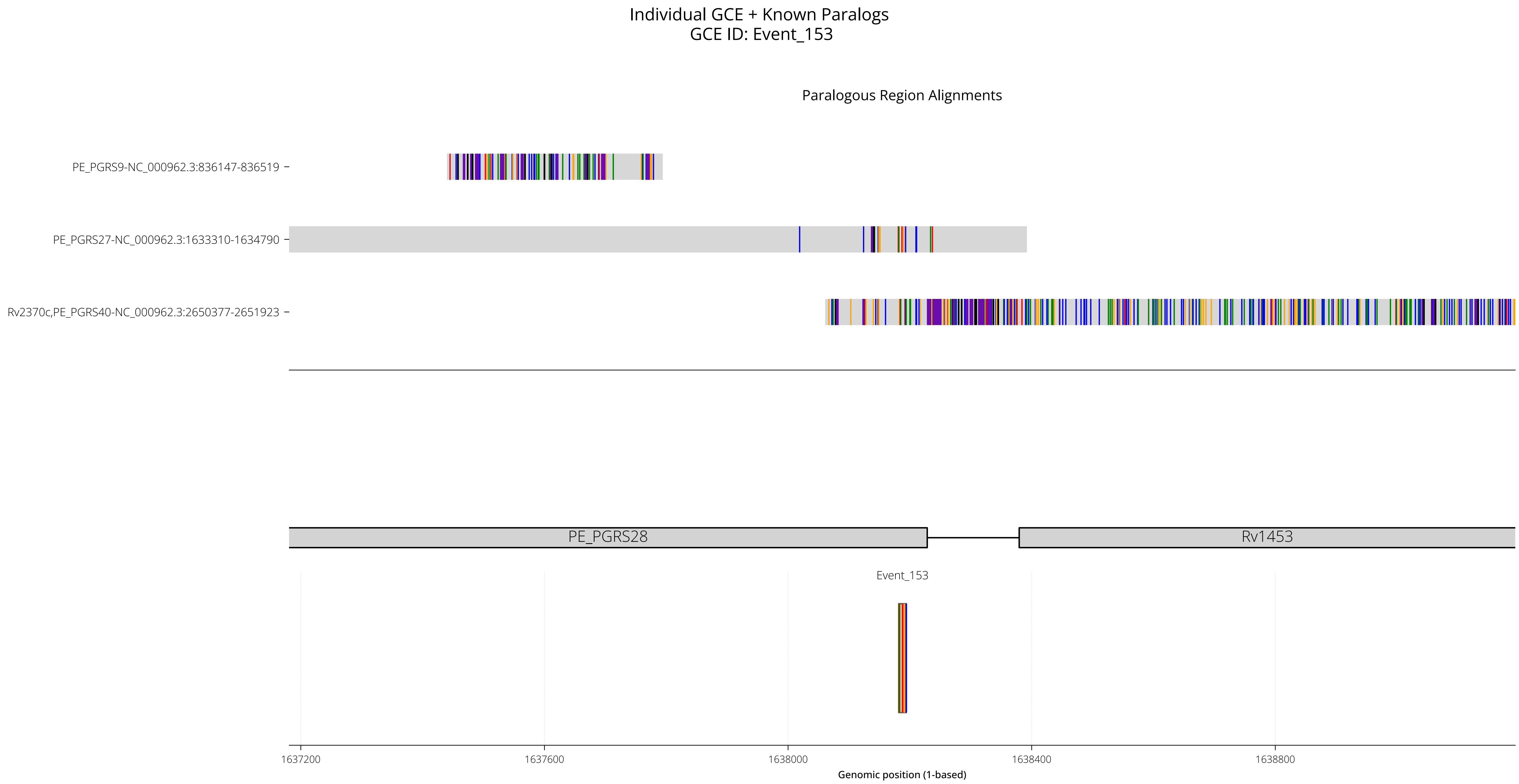
Individual GCE + Known Paralogs
GCE ID: Event_153
Paralogous Region Alignments
PE_PGRS9-NC_000962.3:836147-836519
PE_PGRS27-NC_000962.3:1633310-1634790
Rv2370c,PE_PGRS40-NC_000962.3:2650377-2651923
PE_PGRS28
Rv1453
Event_153
1637200
1637600
1638000
1638400
1638800
Genomic position (1-based)

Circos Viz - GCE to Known Paralogs Locations
GCE ID: Event_153

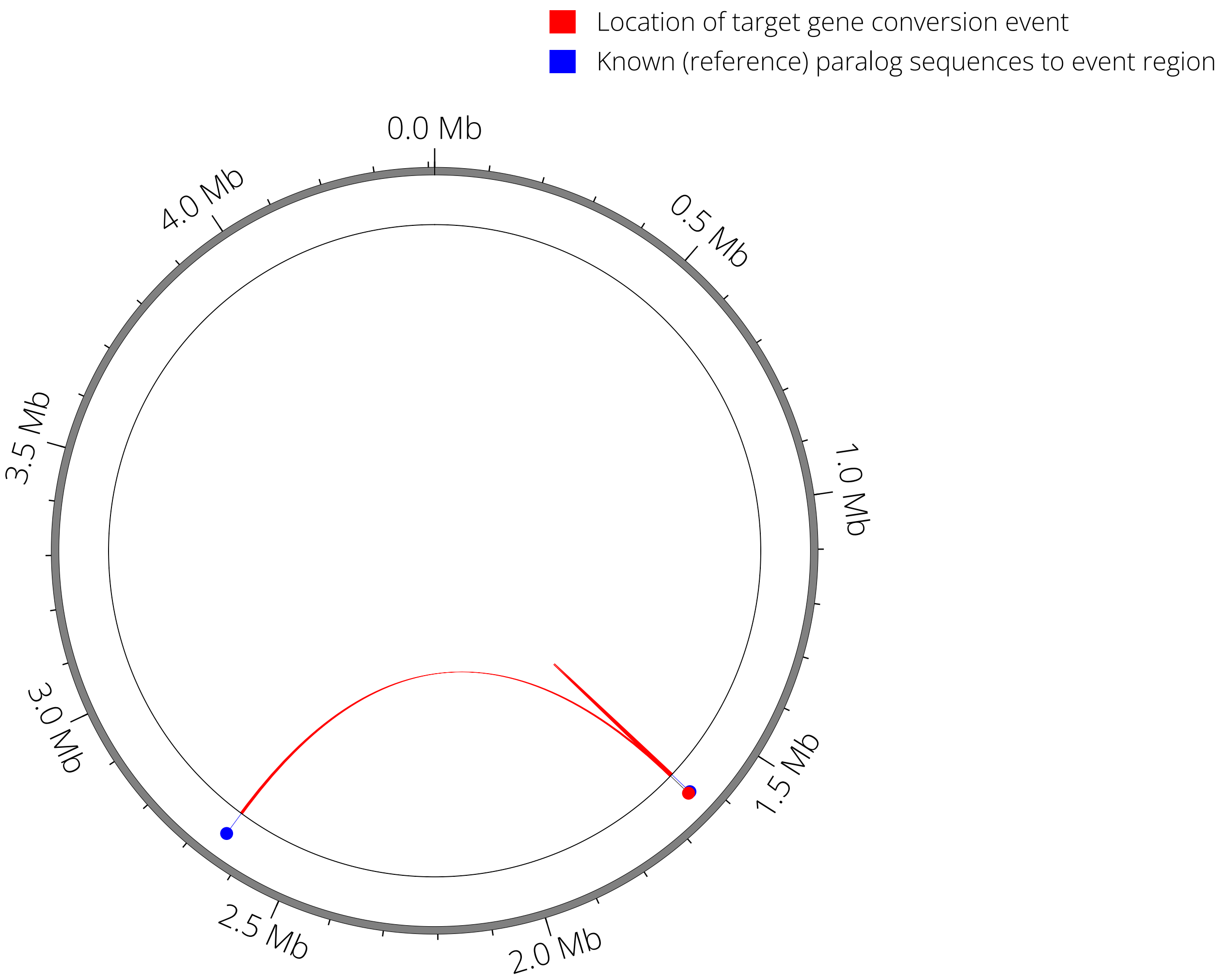

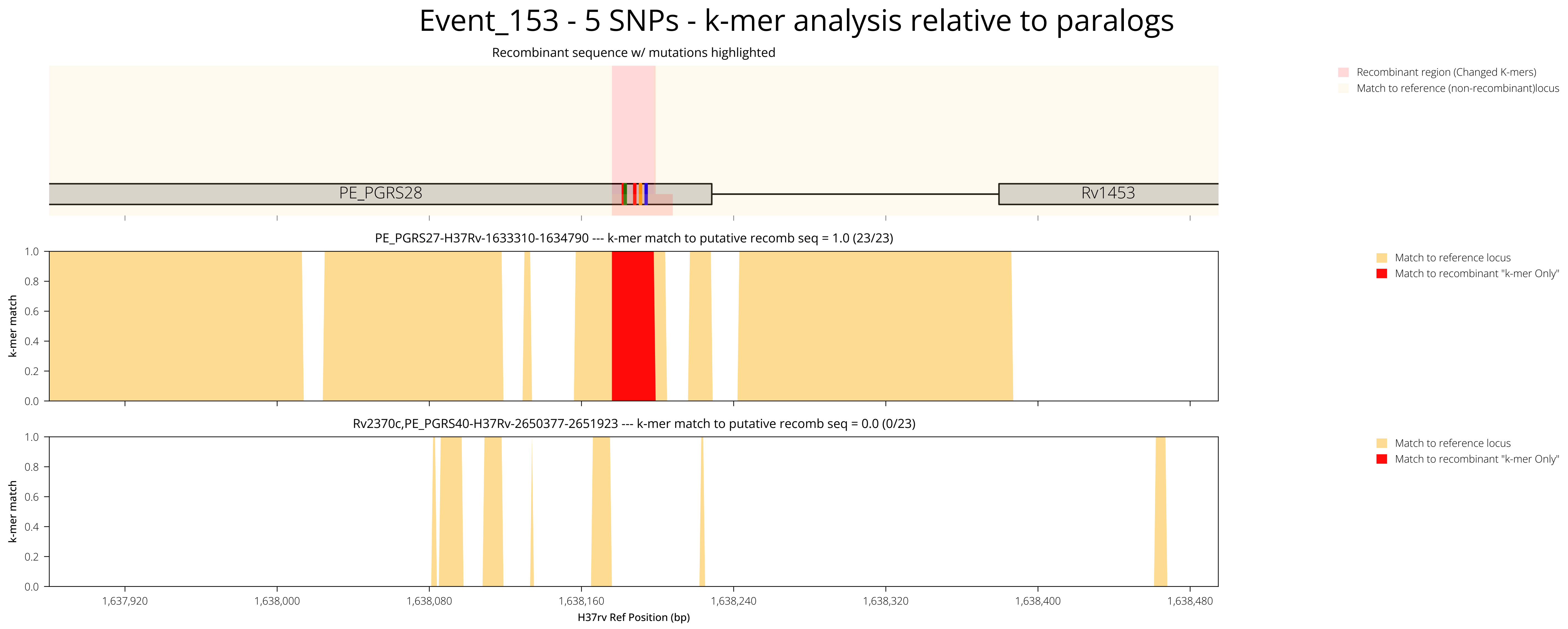
Event_153 - 5 SNPs - k-mer analysis relative to paralogs
Recombinant sequence w/ mutations highlighted
PE_PGRS28
Rv1453
Recombinant region (Changed K-mers)
Match to reference (non-recombinant)locus
PE_PGRS27-H37Rv-1633310-1634790 --- k-mer match to putative recomb seq = 1.0 (23/23)
k-mer match
1.0
0.8
0.6
0.4
0.2
0.0
Match to reference locus
Match to recombinant "k-mer Only"
Rv2370c,PE_PGRS40-H37Rv-2650377-2651923 --- k-mer match to putative recomb seq = 0.0 (0/23)
k-mer match
1.0
0.8
0.6
0.4
0.2
0.0
Match to reference locus
Match to recombinant "k-mer Only"
1,637,920
1,638,000
1,638,080
1,638,160
1,638,240
1,638,320
1,638,400
1,638,480
H37rv Ref Position (bp)

Sub-phylogeny Viz + Paralog Alignments
GCE ID: Event_153

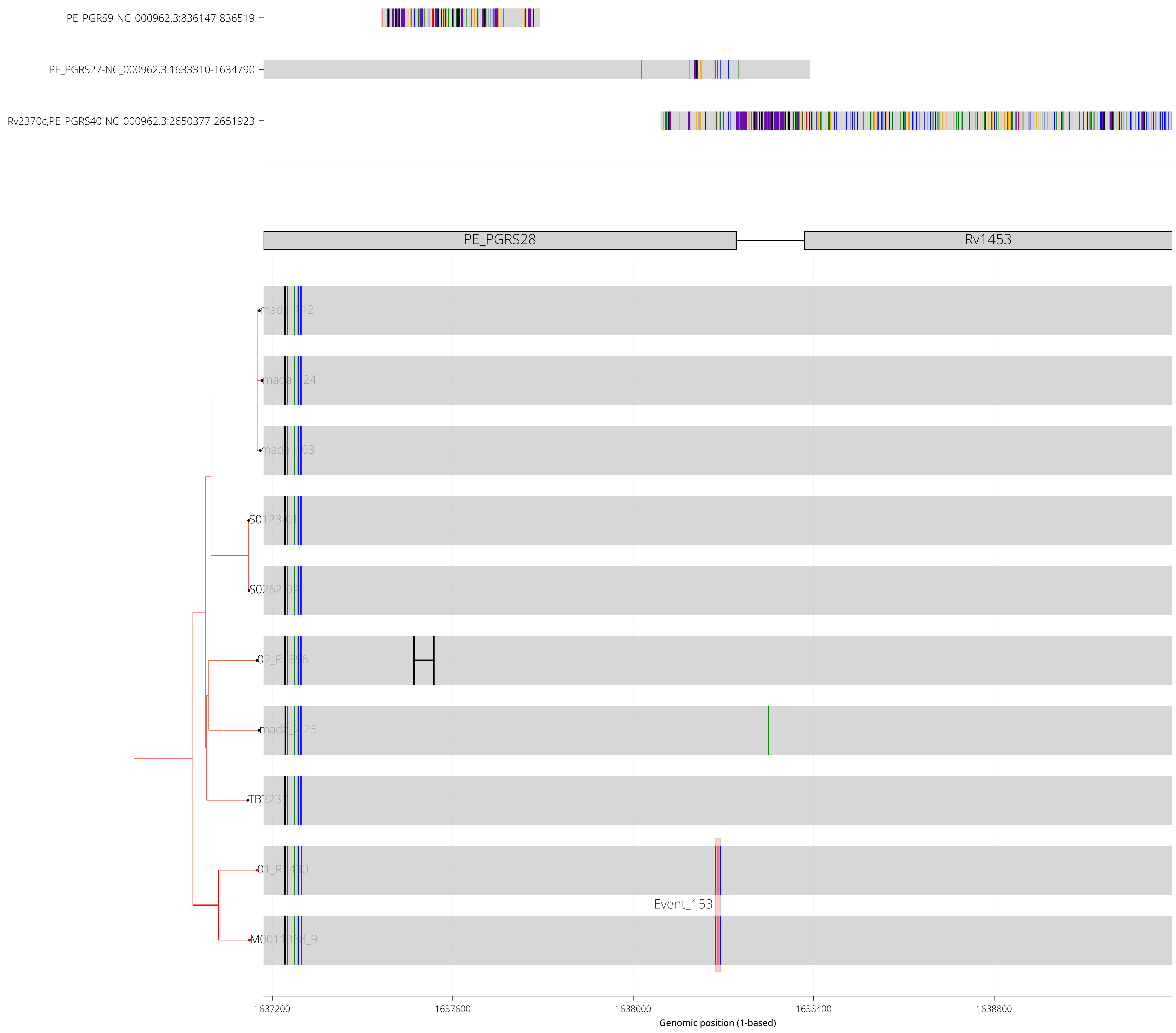

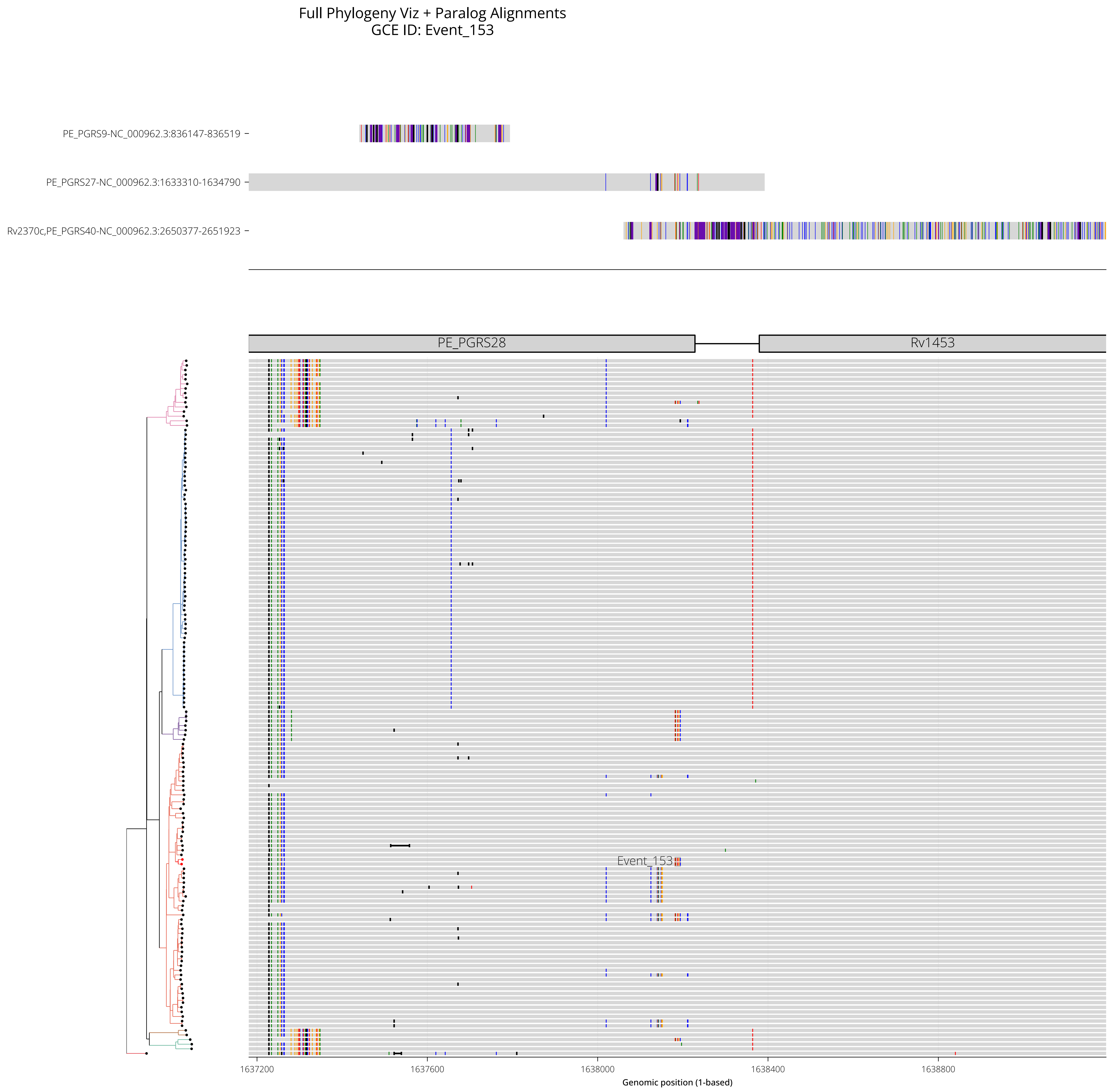

Full Phylogeny Viz + Paralog Alignments
GCE ID: Event_153
PE_PGRS9-NC_000962.3:836147-836519
PE_PGRS27-NC_000962.3:1633310-1634790
Rv2370c,PE_PGRS40-NC_000962.3:2650377-2651923
PE_PGRS28
Rv1453
Event_153
1637200
1637600
1638000
1638400
1638800
Genomic position (1-based)

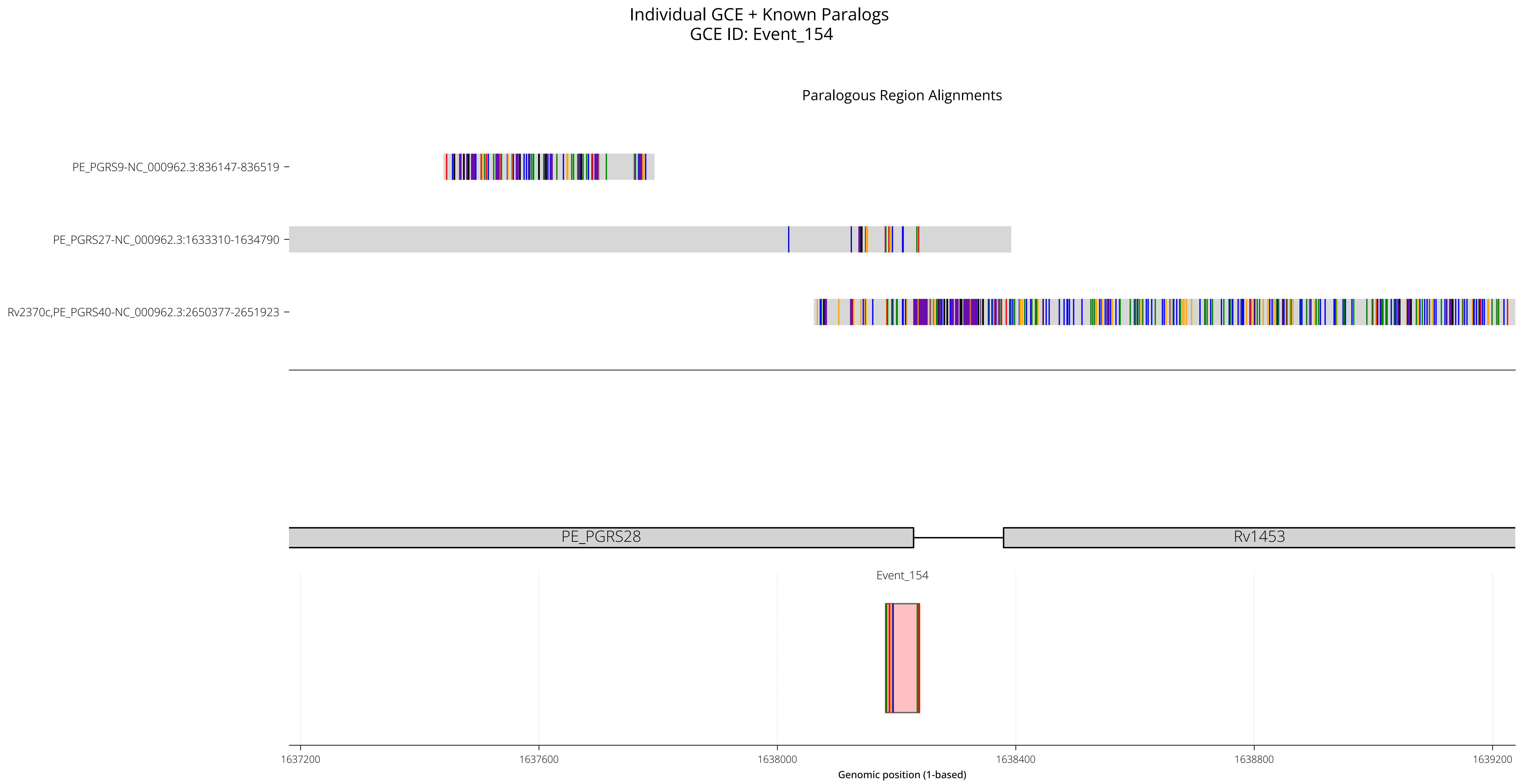

Individual GCE + Known Paralogs
GCE ID: Event_154
Paralogous Region Alignments
PE_PGRS9-NC_000962.3:836147-836519
PE_PGRS27-NC_000962.3:1633310-1634790
Rv2370c,PE_PGRS40-NC_000962.3:2650377-2651923
PE_PGRS28
Rv1453
Event_154
1637200
1637600
1638000
1638400
1638800
1639200
Genomic position (1-based)

Circos Viz - GCE to Known Paralogs Locations
GCE ID: Event_154

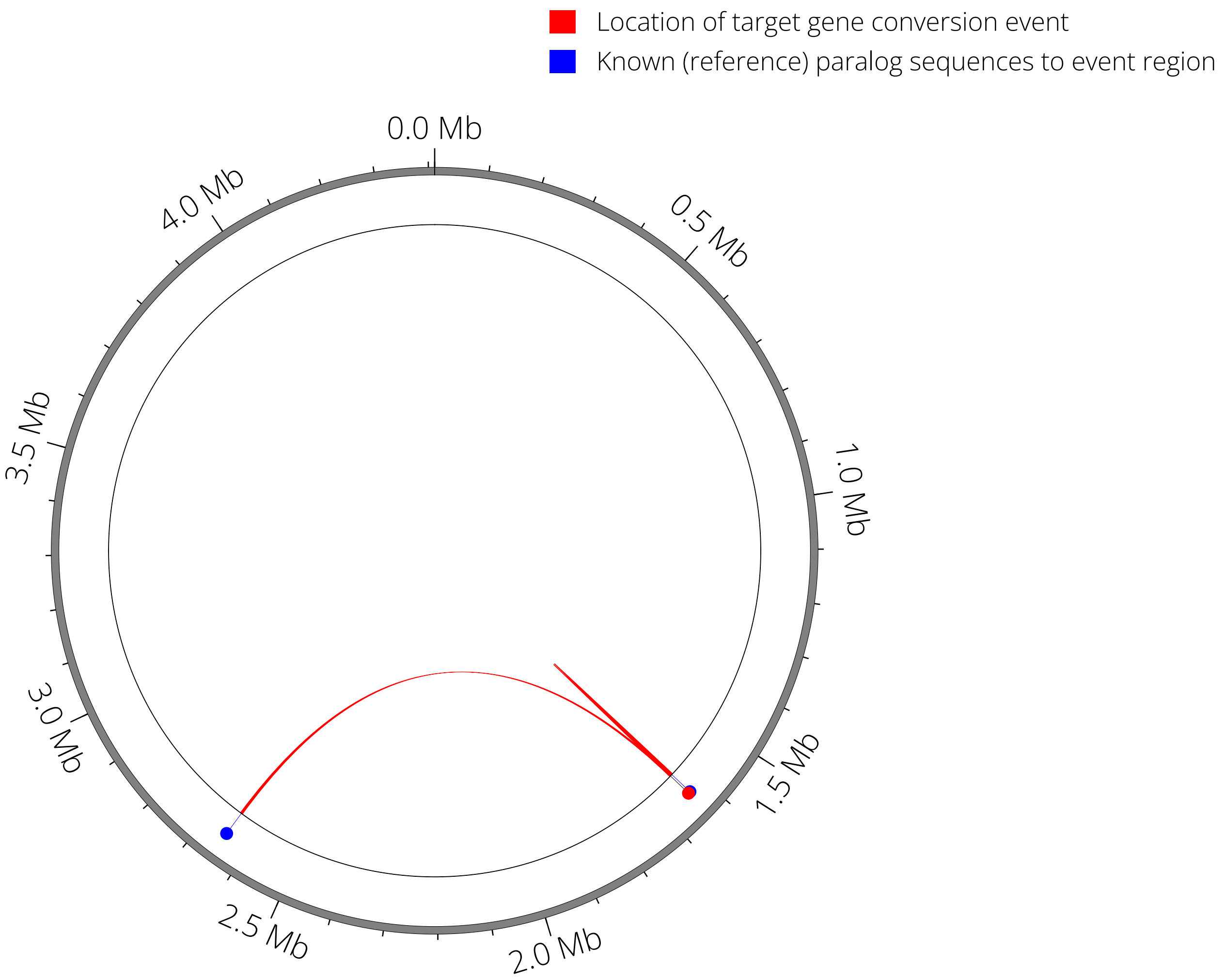

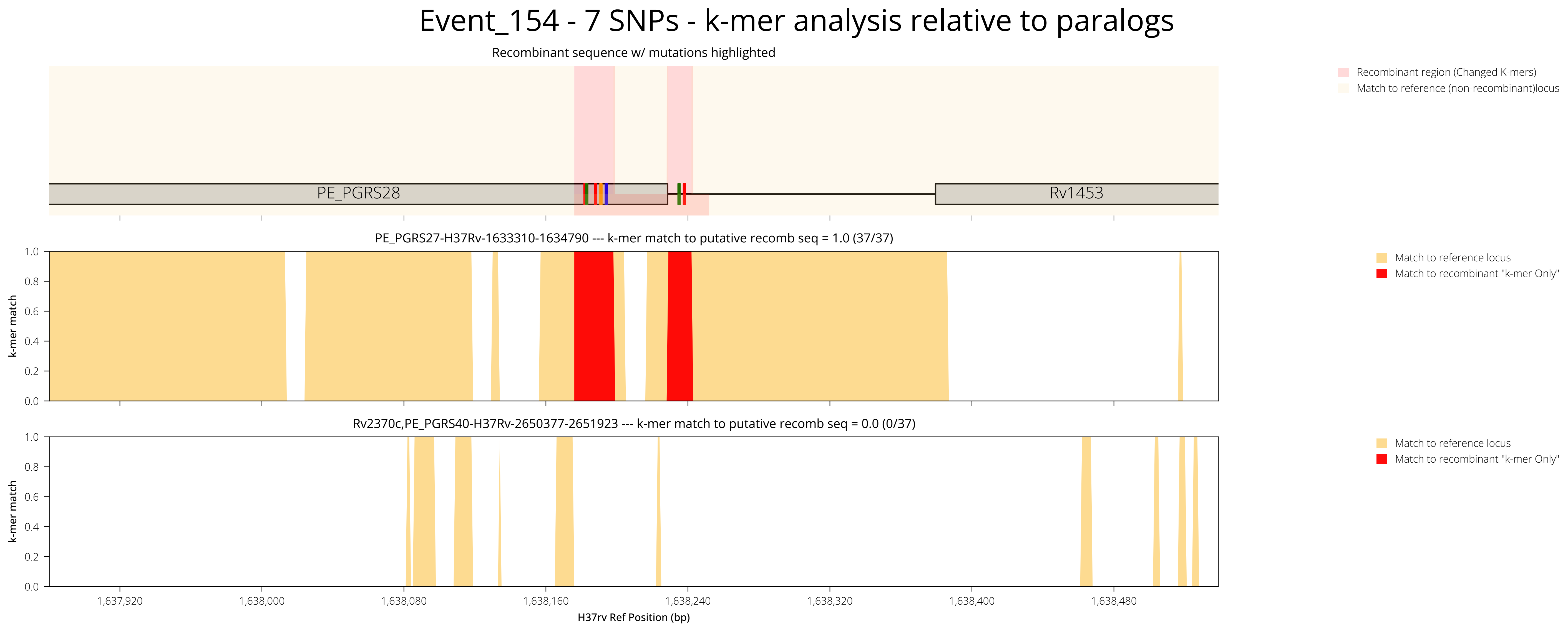

Event_154 - 7 SNPs - k-mer analysis relative to paralogs
Recombinant sequence w/ mutations highlighted
PE_PGRS28
Rv1453
Recombinant region (Changed K-mers)
Match to reference (non-recombinant)locus
PE_PGRS27-H37Rv-1633310-1634790 --- k-mer match to putative recomb seq = 1.0 (37/37)
k-mer match
1.0
0.8
0.6
0.4
0.2
0.0
Match to reference locus
Match to recombinant "k-mer Only"
Rv2370c,PE_PGRS40-H37Rv-2650377-2651923 --- k-mer match to putative recomb seq = 0.0 (0/37)
k-mer match
1,637,920
1,638,000
1,638,080
1,638,160
1,638,240
1,638,320
1,638,400
1,638,480
H37rv Ref Position (bp)

Sub-phylogeny Viz + Paralog Alignments
GCE ID: Event_154

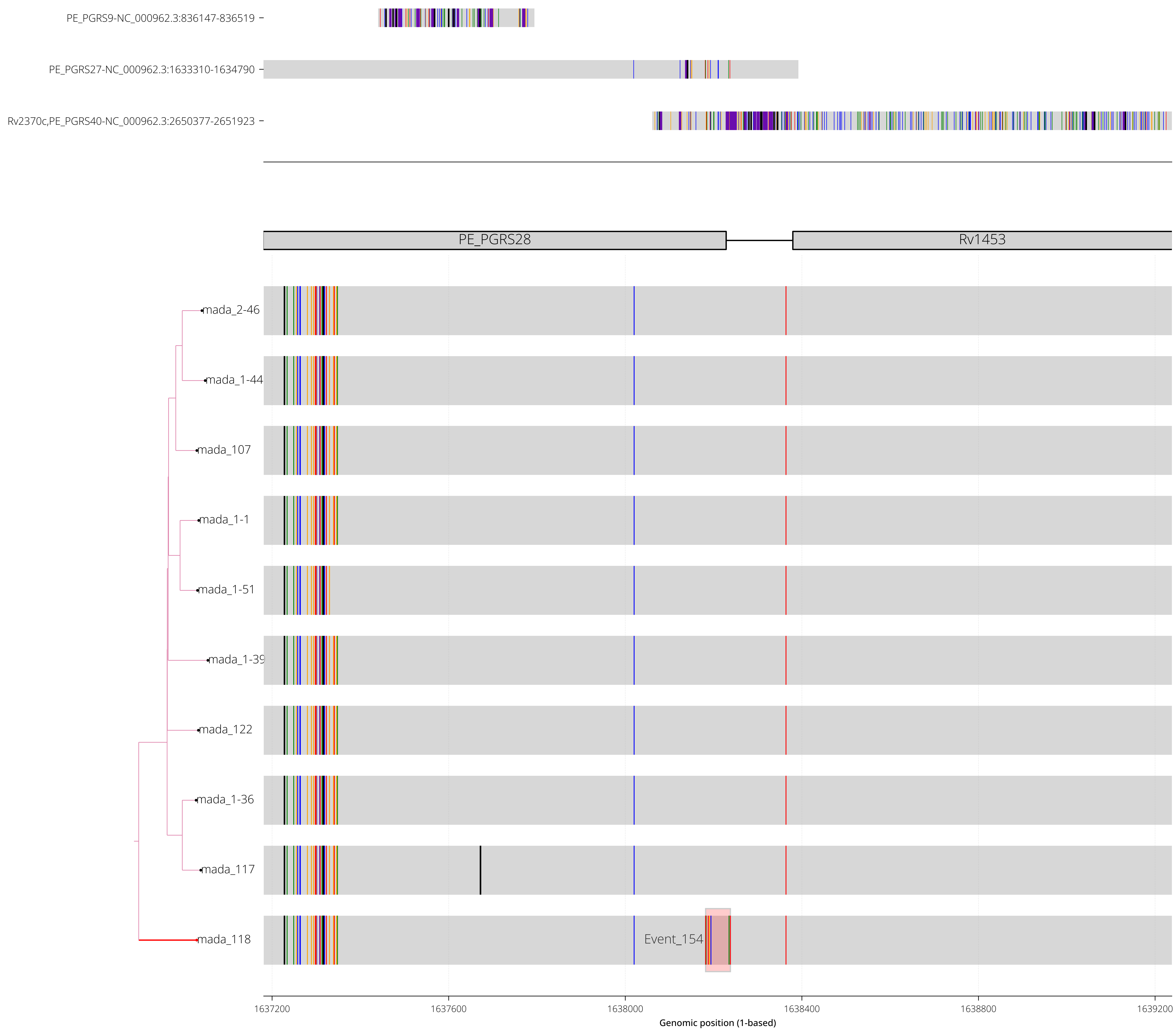

Full Phylogeny Viz + Paralog Alignments
GCE ID: Event_154

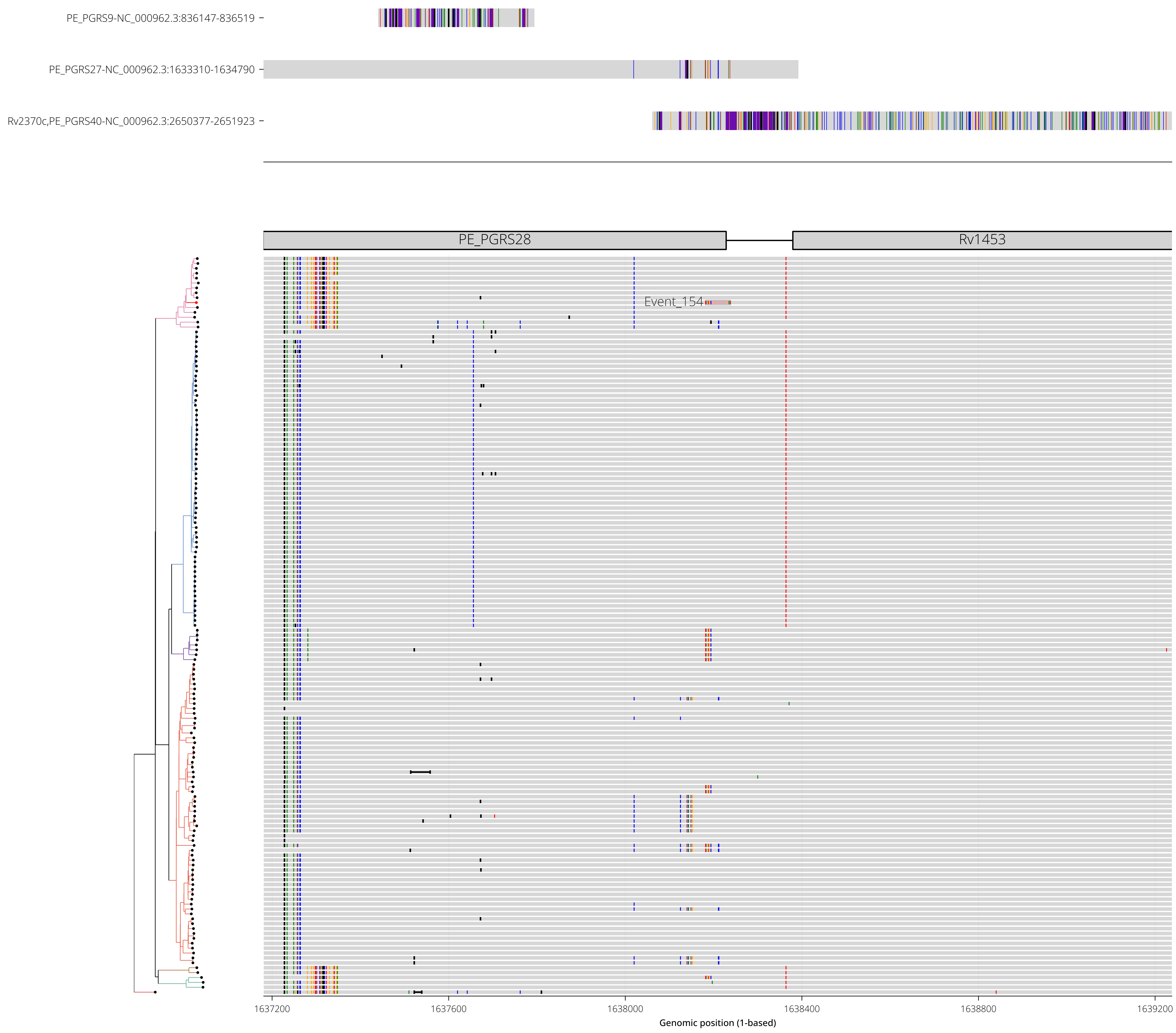

Individual GCE + Known Paralogs
GCE ID: Event_155

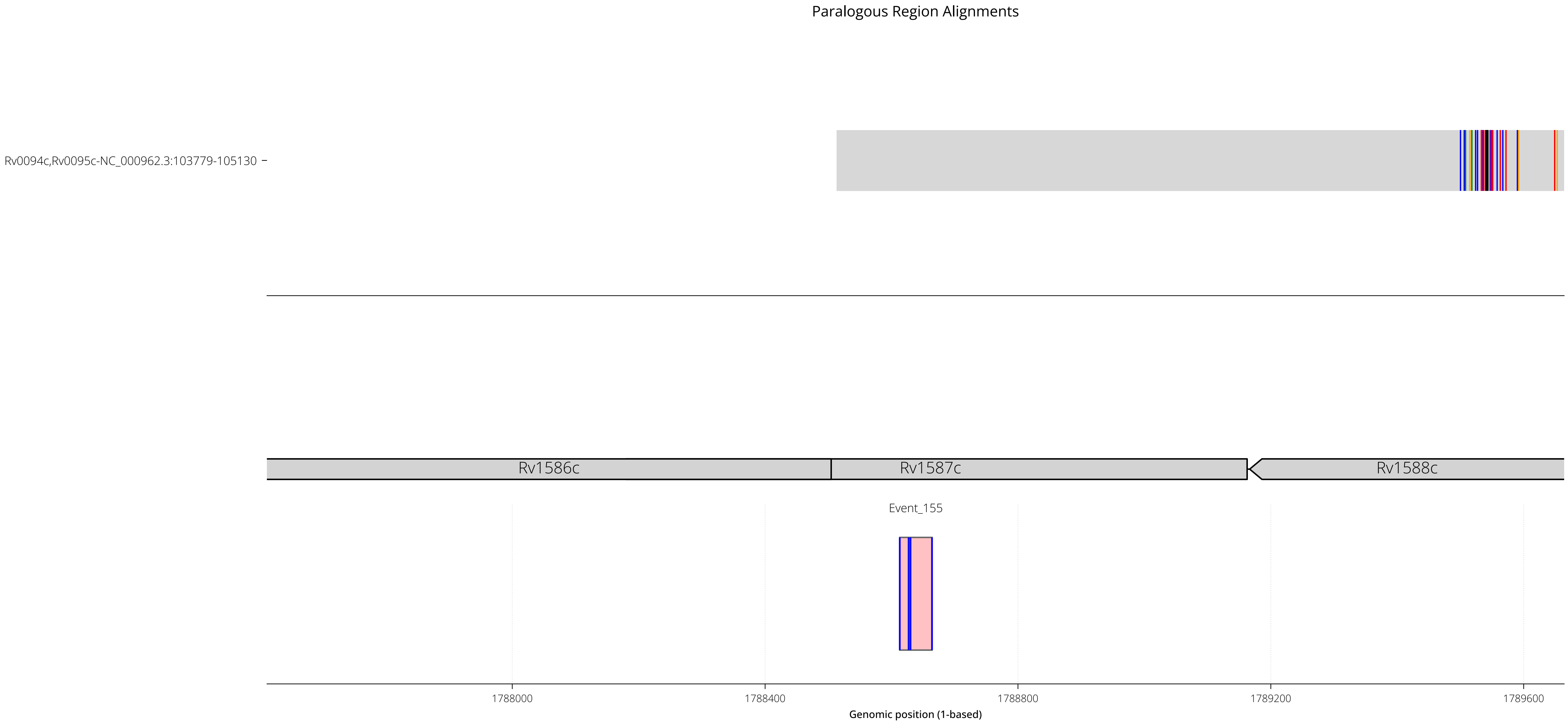

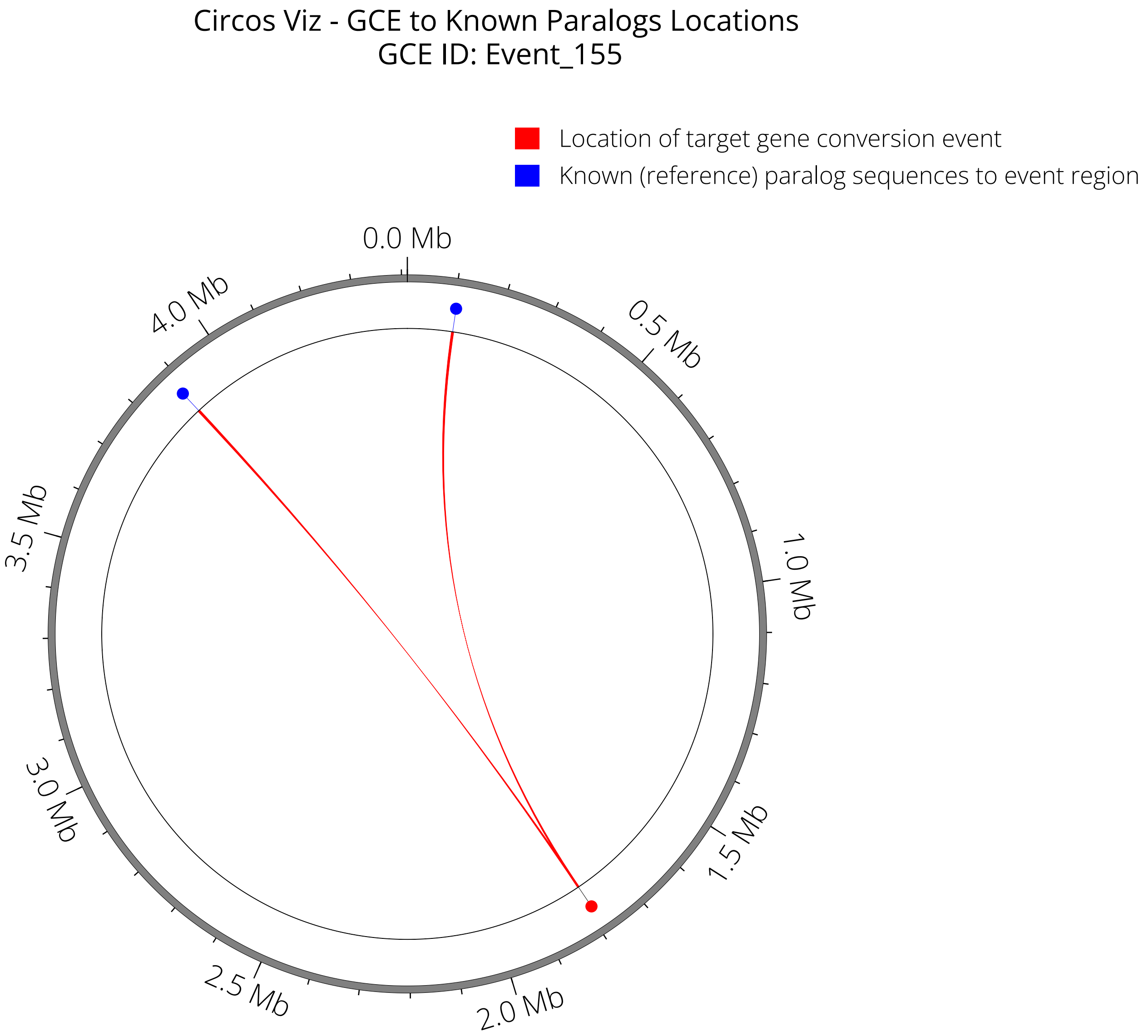
Circos Viz - GCE to Known Paralogs Locations
GCE ID: Event_155
Location of target gene conversion event
Known (reference) paralog sequences to event region
0.0 Mb
0.5 Mb
1.0 Mb
1.5 Mb
2.0 Mb
2.5 Mb
3.0 Mb
3.5 Mb
4.0 Mb

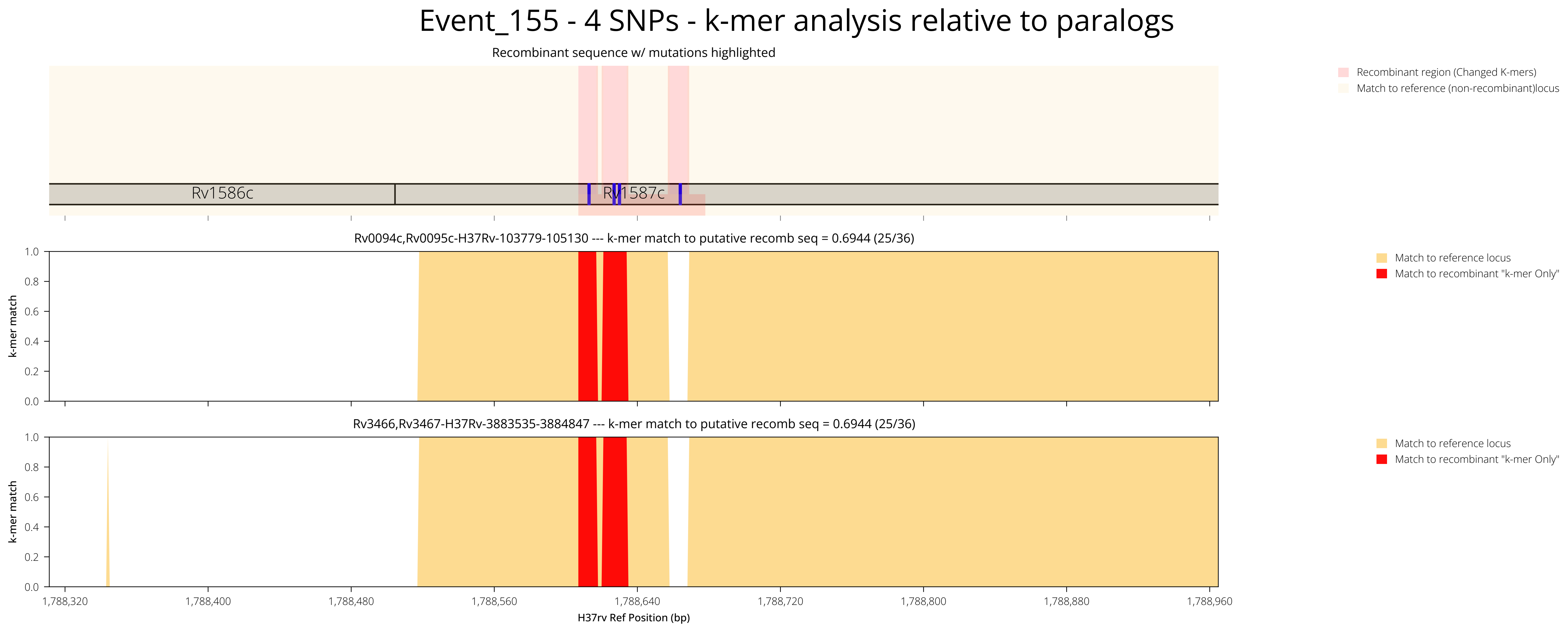

Event_155 - 4 SNPs - k-mer analysis relative to paralogs
Recombinant sequence w/ mutations highlighted
Recombinant region (Changed K-mers)
Match to reference (non-recombinant)locus
Rv1586c
Rv1587c
Rv0094c,Rv0095c-H37Rv-103779-105130 --- k-mer match to putative recomb seq = 0.6944 (25/36)
Match to reference locus
Match to recombinant "k-mer Only"
Rv3466,Rv3467-H37Rv-3883535-3884847 --- k-mer match to putative recomb seq = 0.6944 (25/36)
k-mer match
1.0
0.8
0.6
0.4
0.2
0.0
1,788,320
1,788,400
1,788,480
1,788,560
1,788,640
1,788,720
1,788,800
1,788,880
1,788,960
H37rv Ref Position (bp)

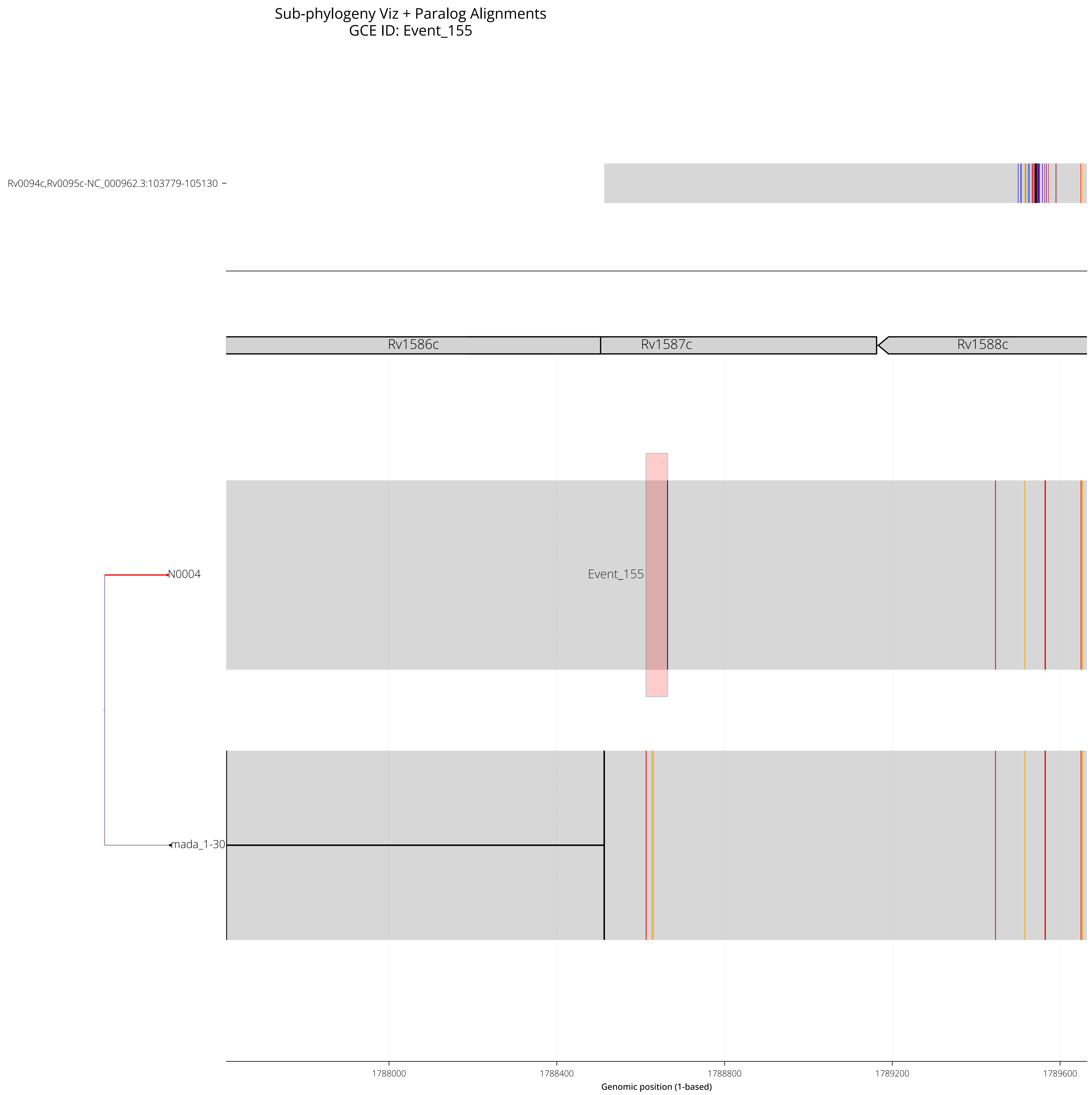

Sub-phylogeny Viz + Paralog Alignments
GCE ID: Event_155
Rv0094c,Rv0095c-NC_000962.3:103779-105130
Rv1586c
Rv1587c
Rv1588c
N0004
Event_155
mada_1-30
1788000
1788400
1788800
1789200
1789600
Genomic position (1-based)

Full Phylogeny Viz + Paralog Alignments
GCE ID: Event_155

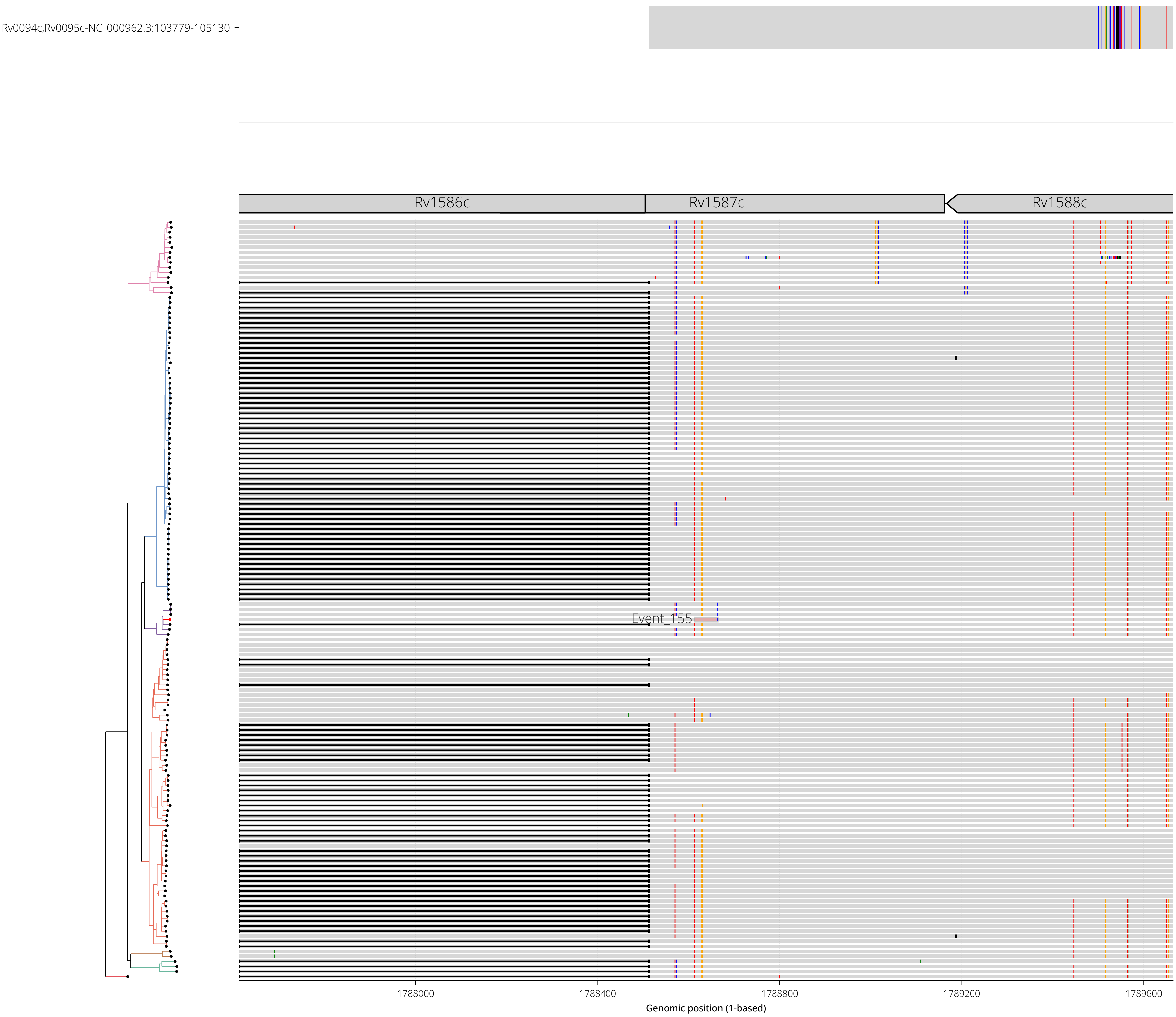

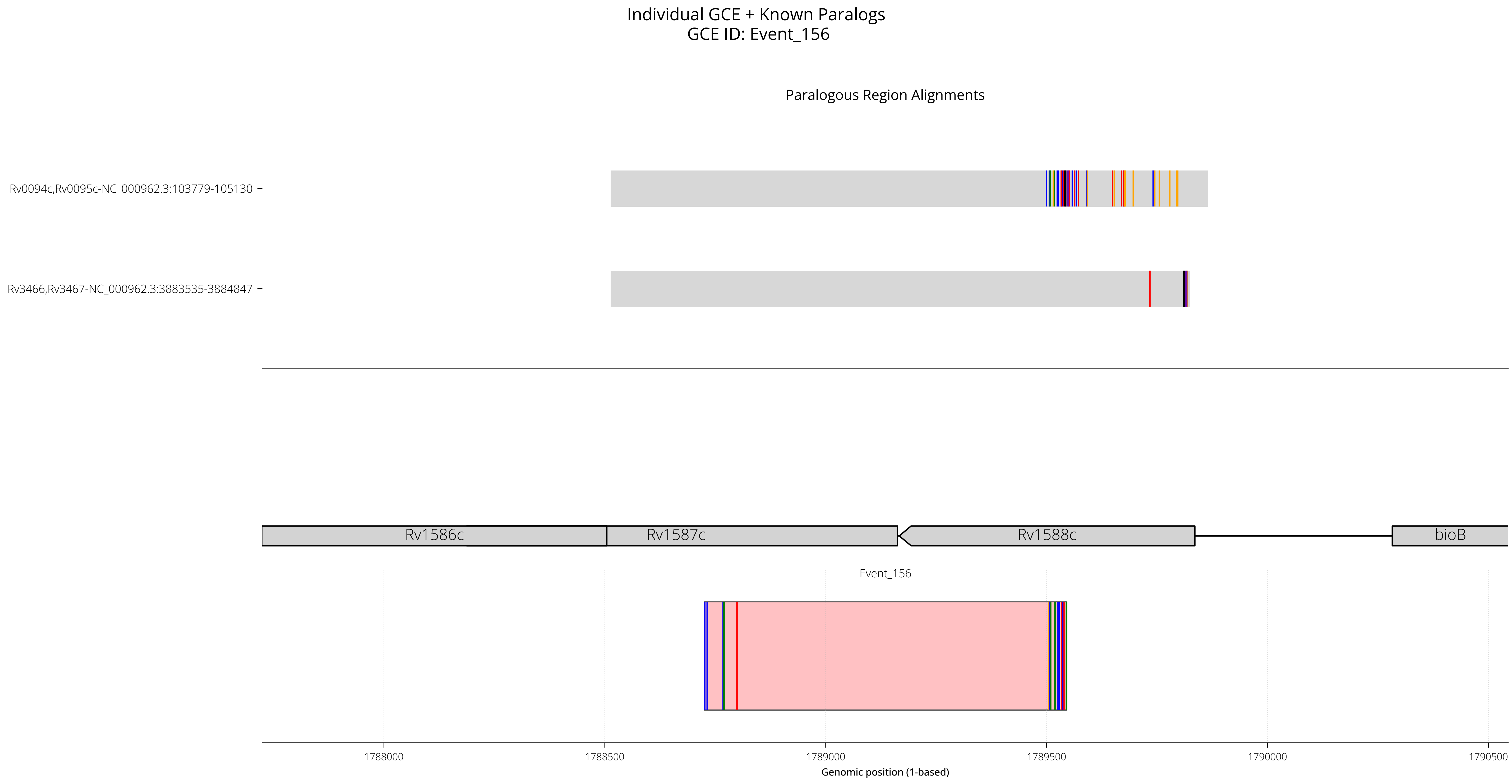
Individual GCE + Known Paralogs
GCE ID: Event_156
Paralogous Region Alignments
Rv0094c,Rv0095c-NC_000962.3:103779-105130
Rv3466,Rv3467-NC_000962.3:3883535-3884847
Rv1586c
Rv1587c
Rv1588c
bioB
Event_156
1788000
1788500
1789000
1789500
1790000
1790500
Genomic position (1-based)

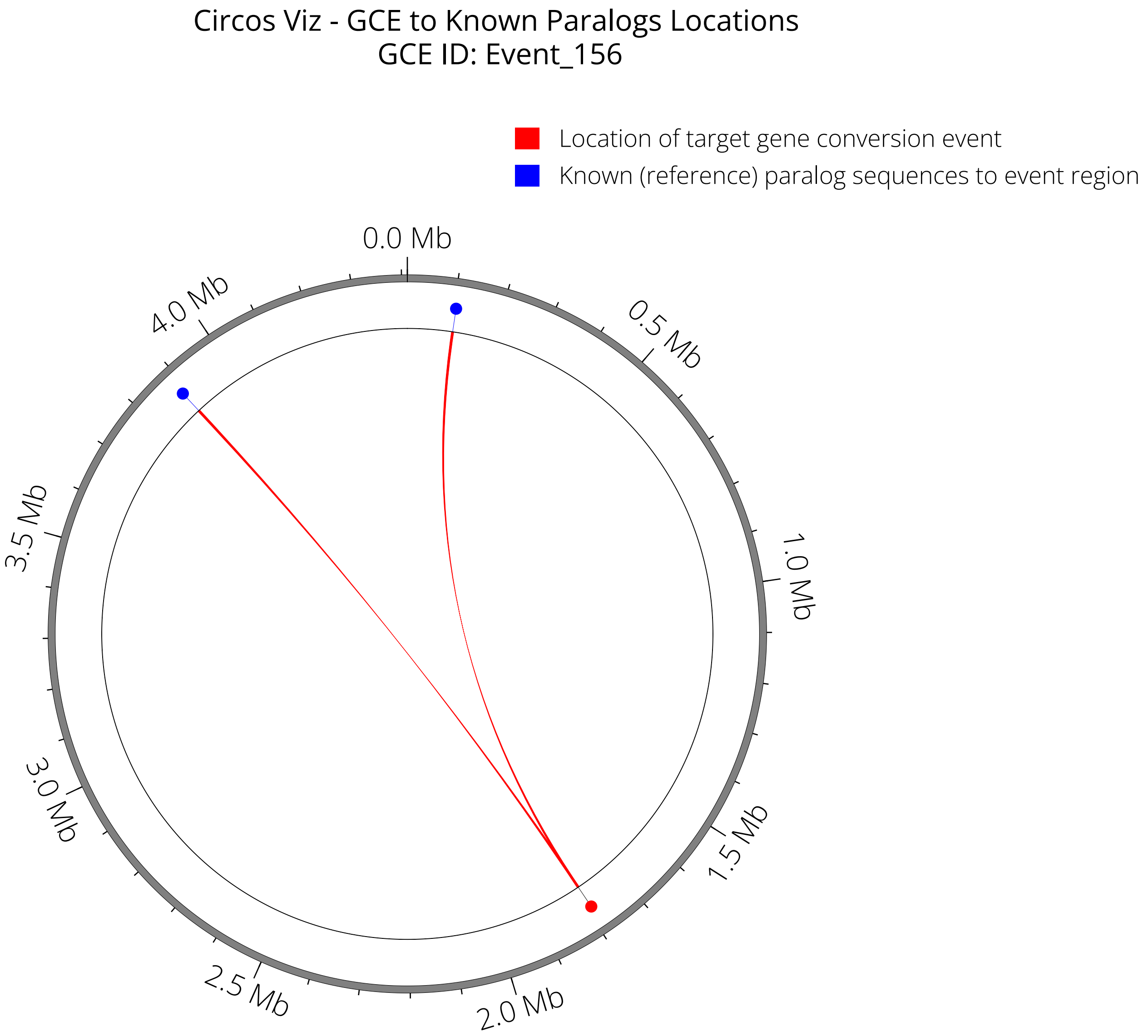
Circos Viz - GCE to Known Paralogs Locations
GCE ID: Event_156
Location of target gene conversion event
Known (reference) paralog sequences to event region
0.0 Mb
0.5 Mb
1.0 Mb
1.5 Mb
2.0 Mb
2.5 Mb
3.0 Mb
3.5 Mb
4.0 Mb

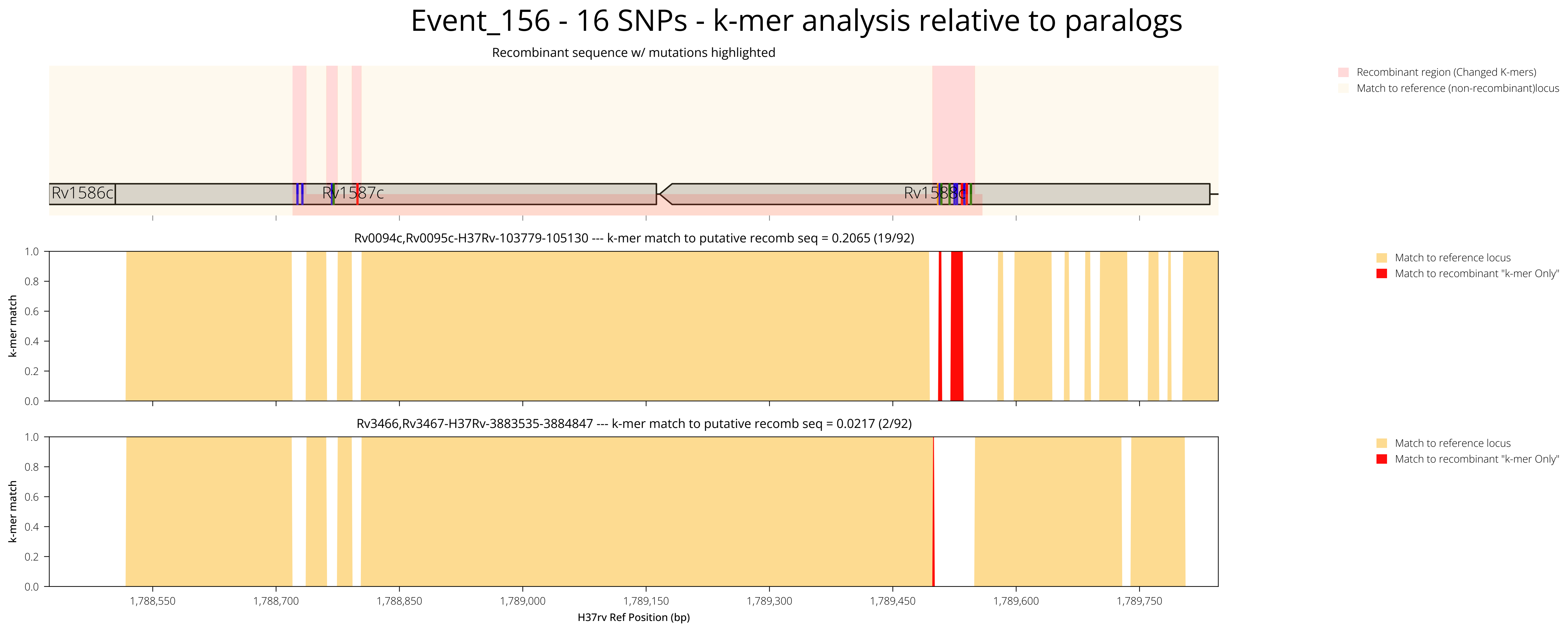

Event_156 - 16 SNPs - k-mer analysis relative to paralogs
Recombinant sequence w/ mutations highlighted
Recombinant region (Changed K-mers)
Match to reference (non-recombinant)locus
Rv1586c
Rv1587c
Rv1588c
Rv0094c,Rv0095c-H37Rv-103779-105130 --- k-mer match to putative recomb seq = 0.2065 (19/92)
Match to reference locus
Match to recombinant "k-mer Only"
k-mer match
1.0
0.8
0.6
0.4
0.2
0.0
Rv3466,Rv3467-H37Rv-3883535-3884847 --- k-mer match to putative recomb seq = 0.0217 (2/92)
Match to reference locus
Match to recombinant "k-mer Only"
k-mer match
1.0
0.8
0.6
0.4
0.2
0.0
1,788,550
1,788,700
1,788,850
1,789,000
1,789,150
1,789,300
1,789,450
1,789,600
1,789,750
H37rv Ref Position (bp)

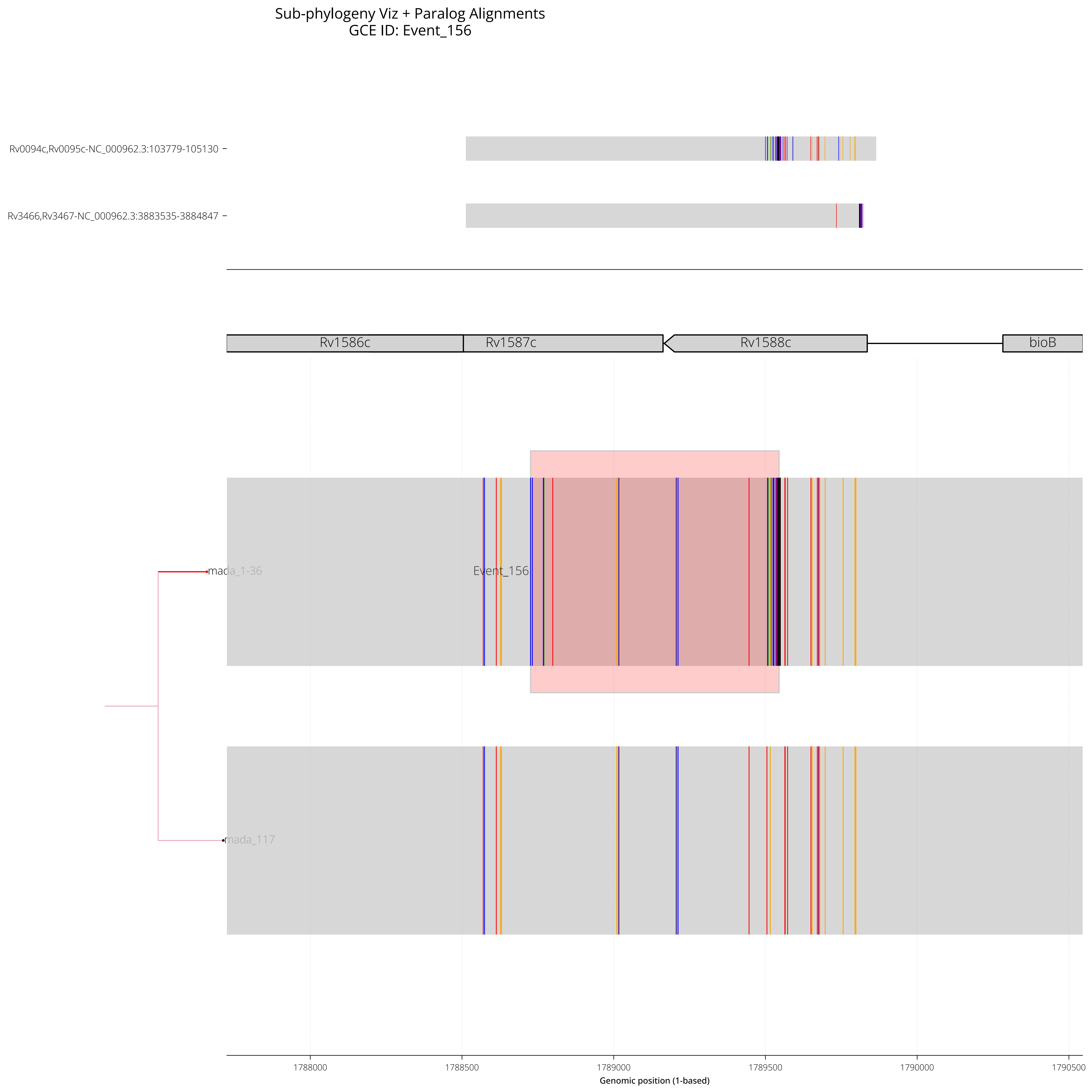

Sub-phylogeny Viz + Paralog Alignments
GCE ID: Event_156
Rv0094c,Rv0095c-NC_000962.3:103779-105130
Rv3466,Rv3467-NC_000962.3:3883535-3884847
Rv1586c
Rv1587c
Rv1588c
bioB
mada_1-36
Event_156
mada_117
1788000
1788500
1789000
1789500
1790000
1790500
Genomic position (1-based)

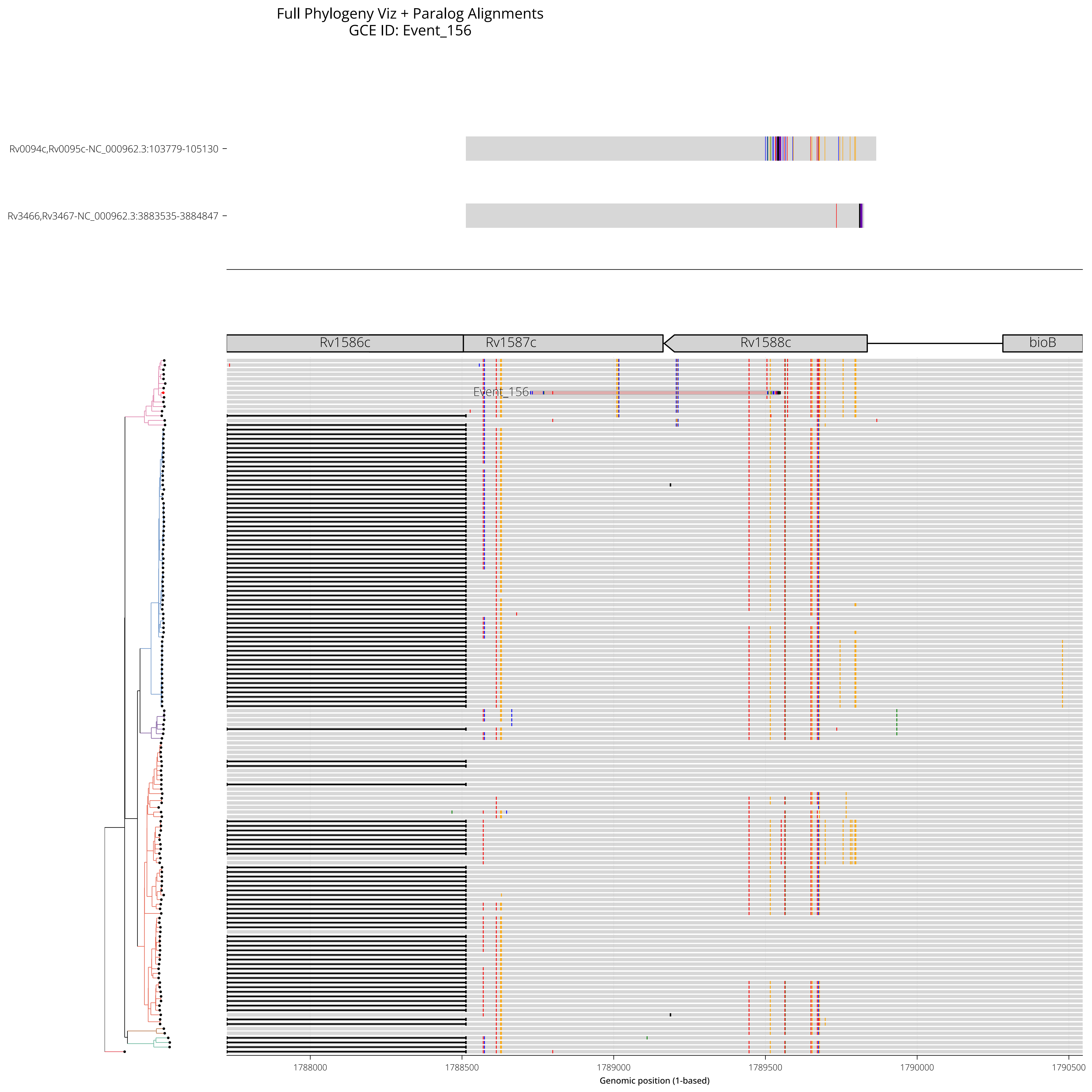
Full Phylogeny Viz + Paralog Alignments
GCE ID: Event_156
Rv0094c,Rv0095c-NC_000962.3:103779-105130
Rv3466,Rv3467-NC_000962.3:3883535-3884847
Rv1586c
Rv1587c
Rv1588c
bioB
Event_156
1788000
1788500
1789000
1789500
1790000
1790500
Genomic position (1-based)

Individual GCE + Known Paralogs
GCE ID: Event_157

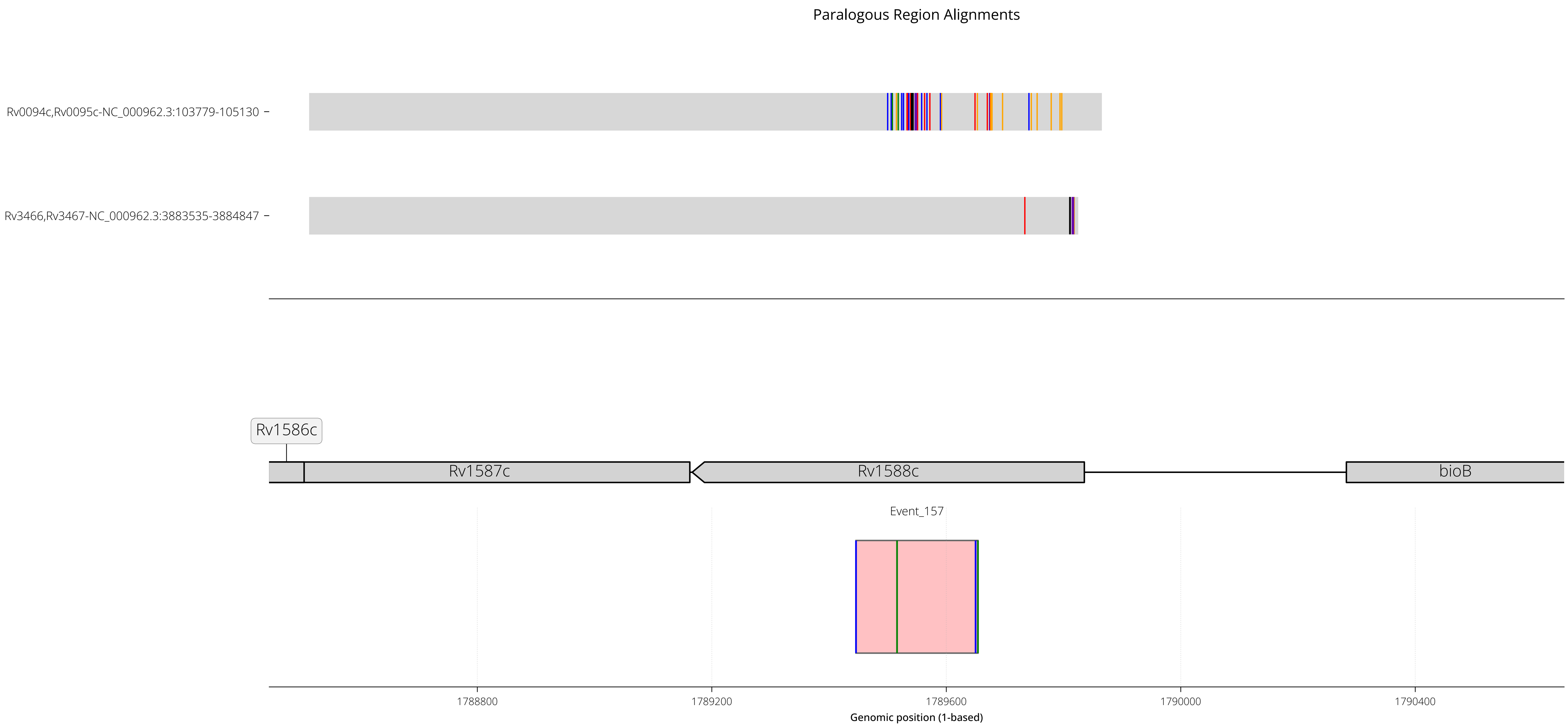

Circos Viz - GCE to Known Paralogs Locations
GCE ID: Event_157

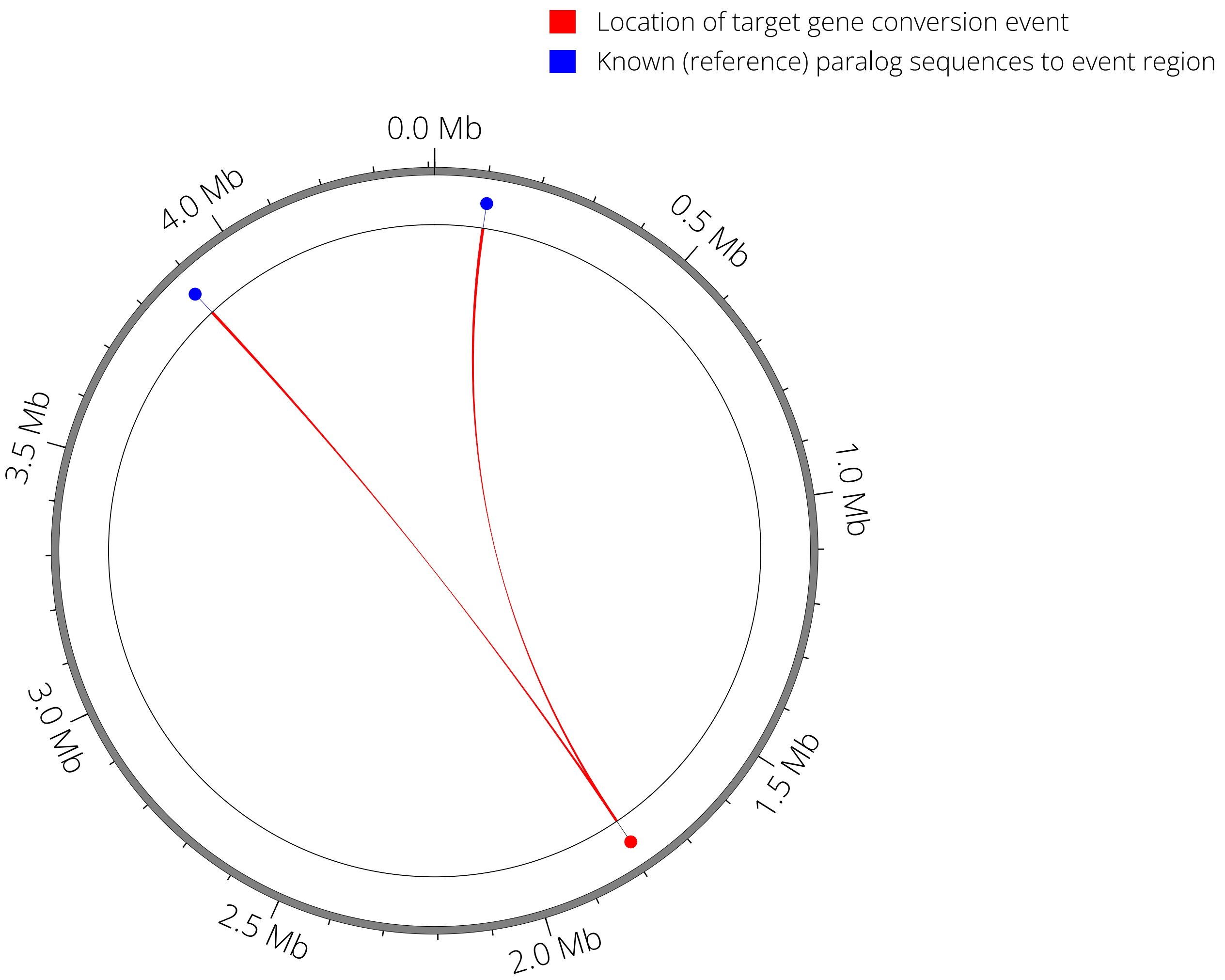

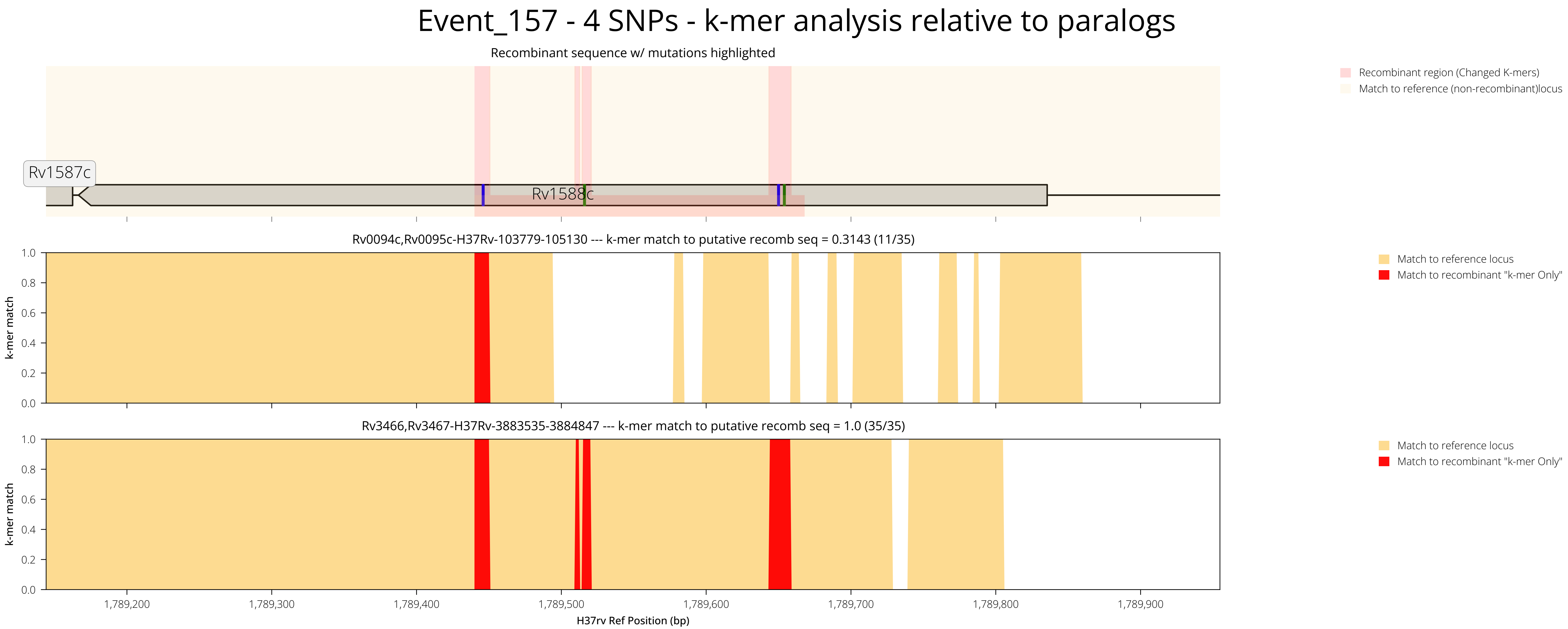

Event_157 - 4 SNPs - k-mer analysis relative to paralogs
Recombinant sequence w/ mutations highlighted
Recombinant region (Changed K-mers)
Match to reference (non-recombinant)locus
Rv1587c
Rv1588c
Rv0094c,Rv0095c-H37Rv-103779-105130 --- k-mer match to putative recomb seq = 0.3143 (11/35)
Match to reference locus
Match to recombinant "k-mer Only"
k-mer match
1.0
0.8
0.6
0.4
0.2
0.0
Rv3466,Rv3467-H37Rv-3883535-3884847 --- k-mer match to putative recomb seq = 1.0 (35/35)
Match to reference locus
Match to recombinant "k-mer Only"
k-mer match
1.0
0.8
0.6
0.4
0.2
0.0
1,789,200
1,789,300
1,789,400
1,789,500
1,789,600
1,789,700
1,789,800
1,789,900
H37rv Ref Position (bp)

Sub-phylogeny Viz + Paralog Alignments
GCE ID: Event_157

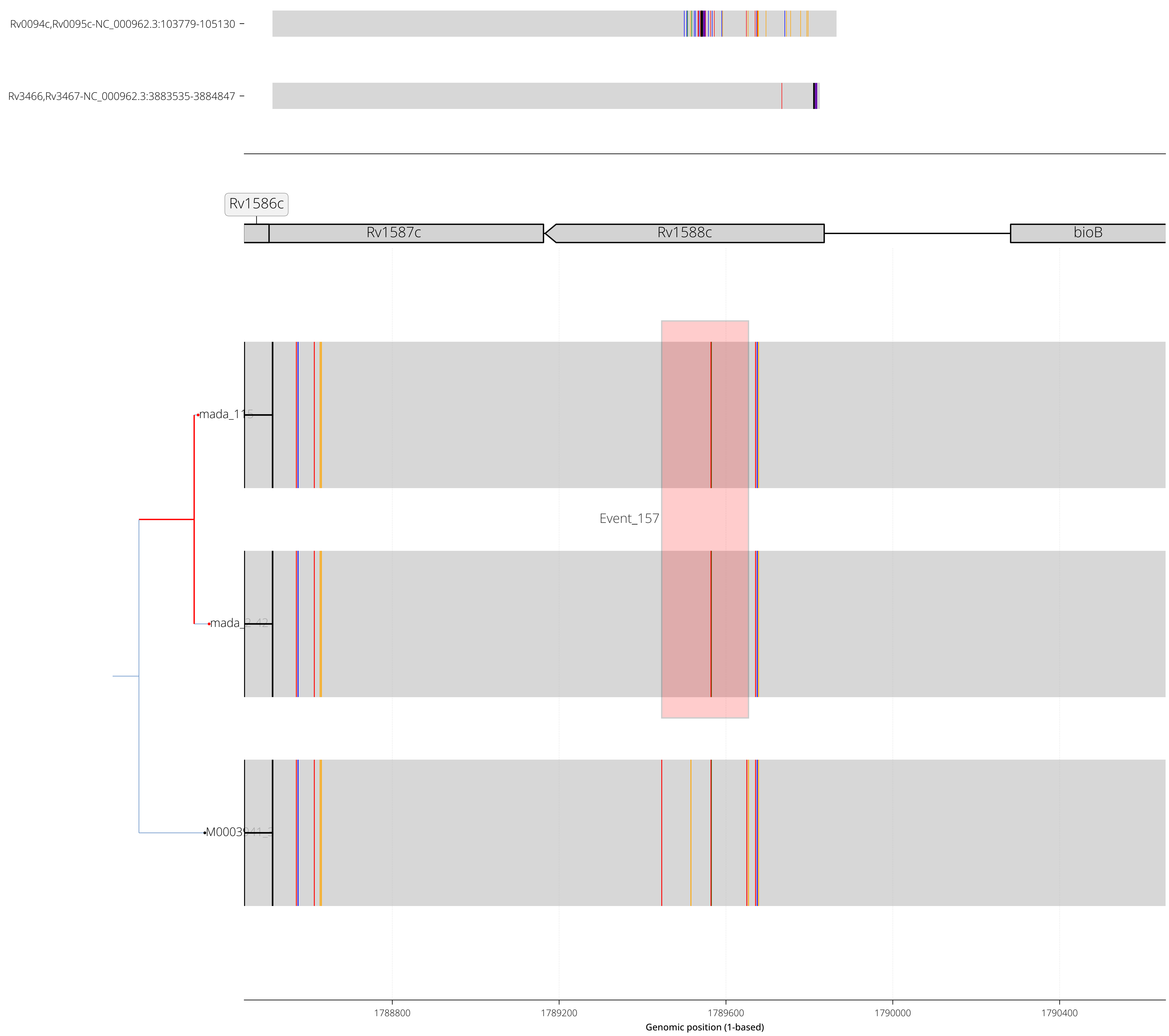

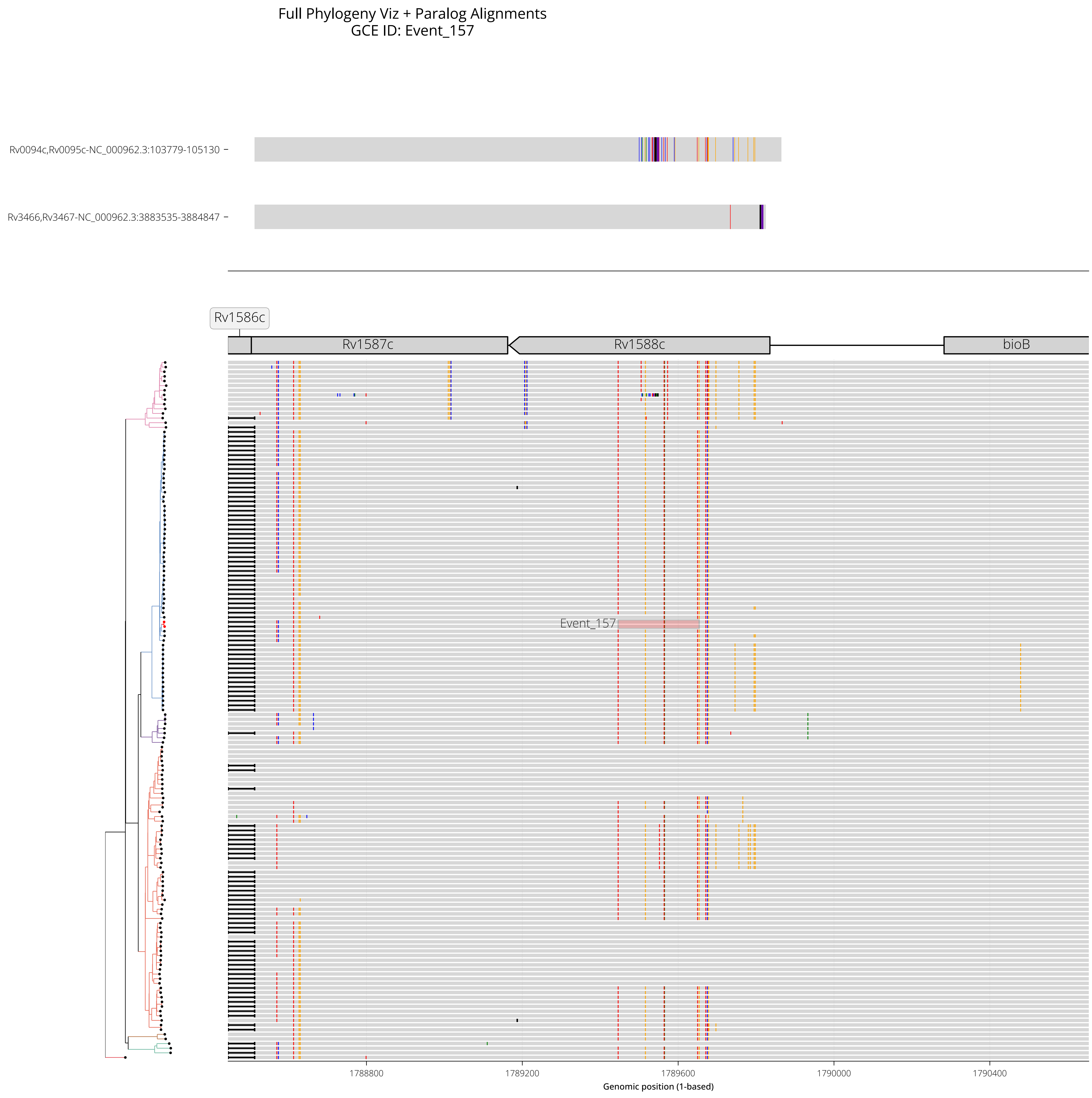

Full Phylogeny Viz + Paralog Alignments
GCE ID: Event_157
Rv0094c,Rv0095c-NC_000962.3:103779-105130
Rv3466,Rv3467-NC_000962.3:3883535-3884847
Rv1586c
Rv1587c
Rv1588c
bioB
Event_157
1788800
1789200
1789600
1790000
1790400
Genomic position (1-based)

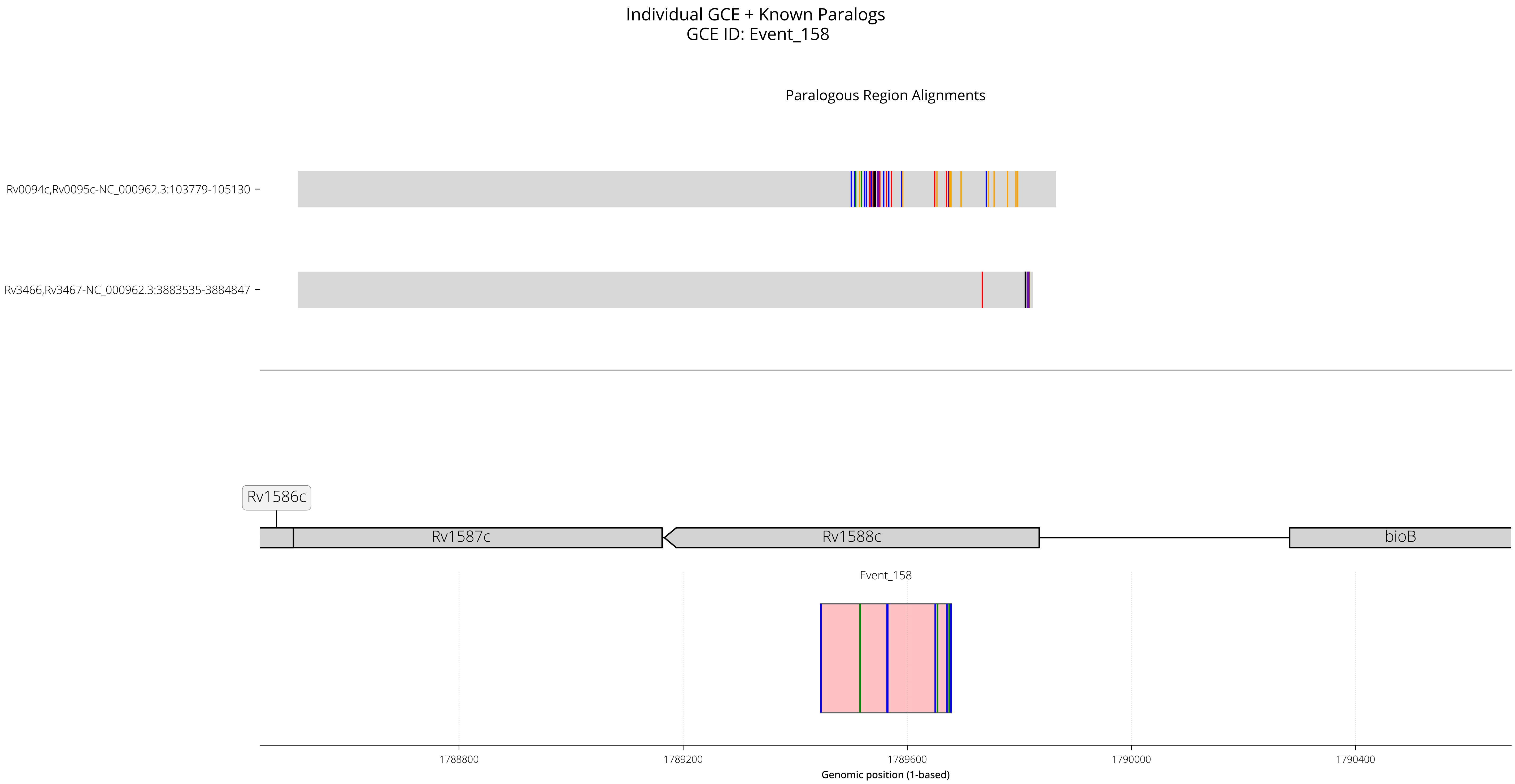

Individual GCE + Known Paralogs
GCE ID: Event_158
Paralogous Region Alignments
Rv0094c,Rv0095c-NC_000962.3:103779-105130
Rv3466,Rv3467-NC_000962.3:3883535-3884847
Rv1586c
Rv1587c
Rv1588c
bioB
Event_158
1788800
1789200
1789600
1790000
1790400
Genomic position (1-based)

Circos Viz - GCE to Known Paralogs Locations
GCE ID: Event_158

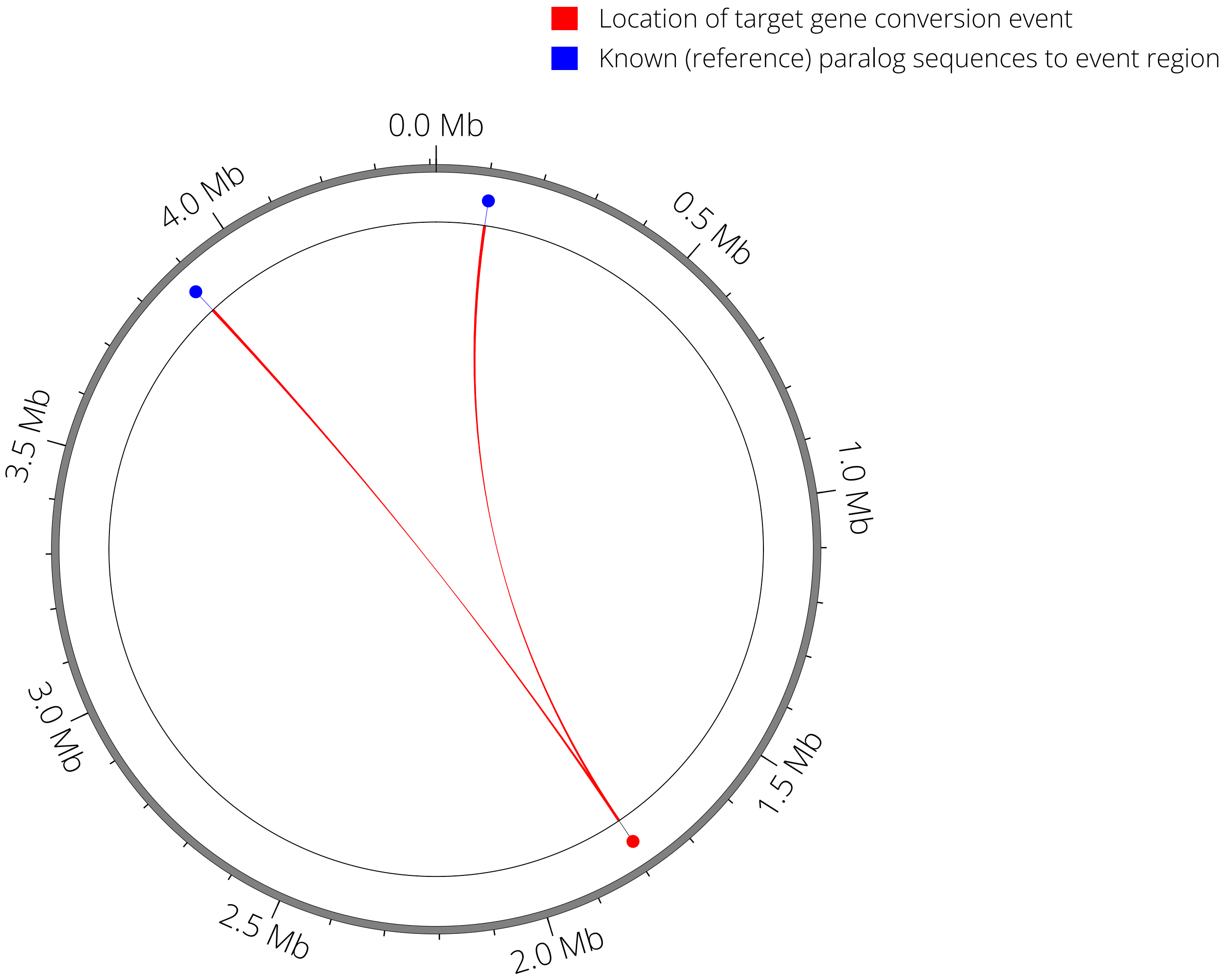

# Event_158 - 9 SNPs - k-mer analysis relative to paralogs

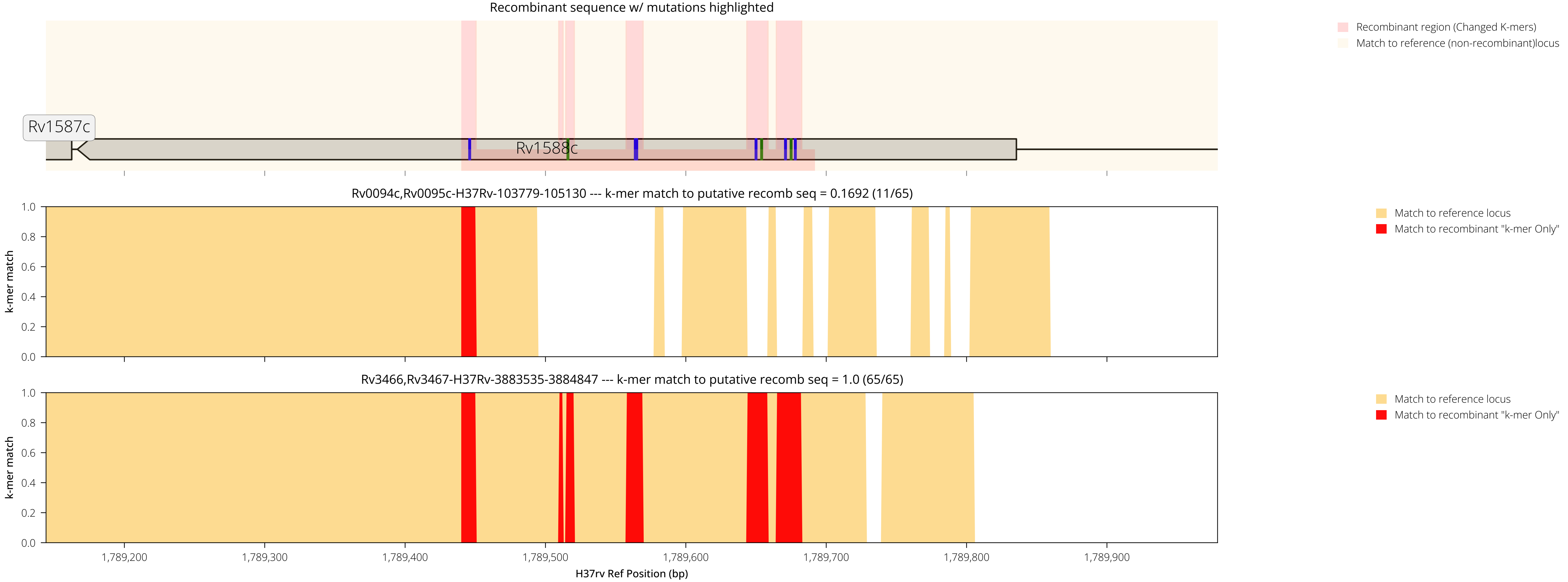

Sub-phylogeny Viz + Paralog Alignments
GCE ID: Event_158

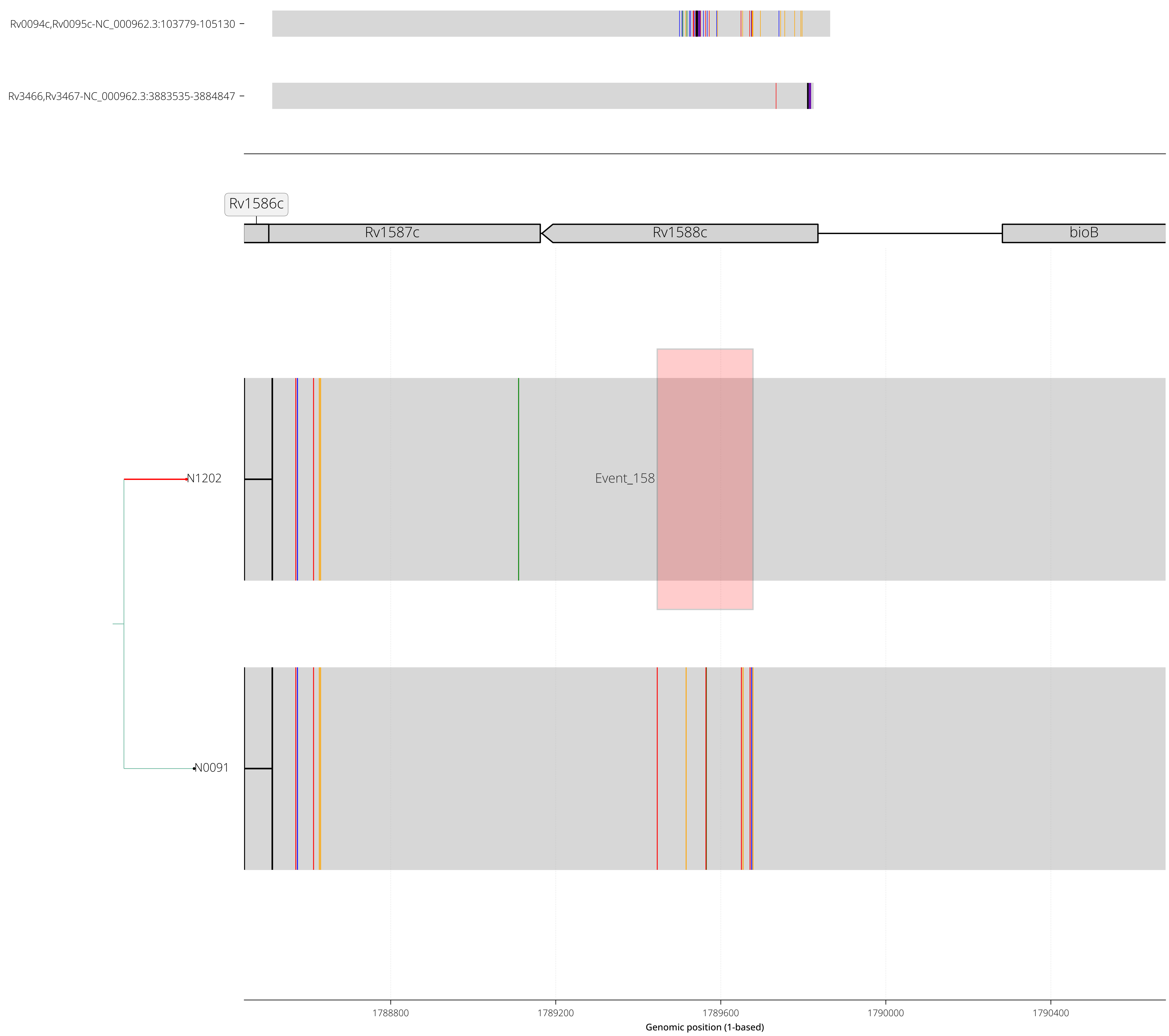

Full Phylogeny Viz + Paralog Alignments
GCE ID: Event_158

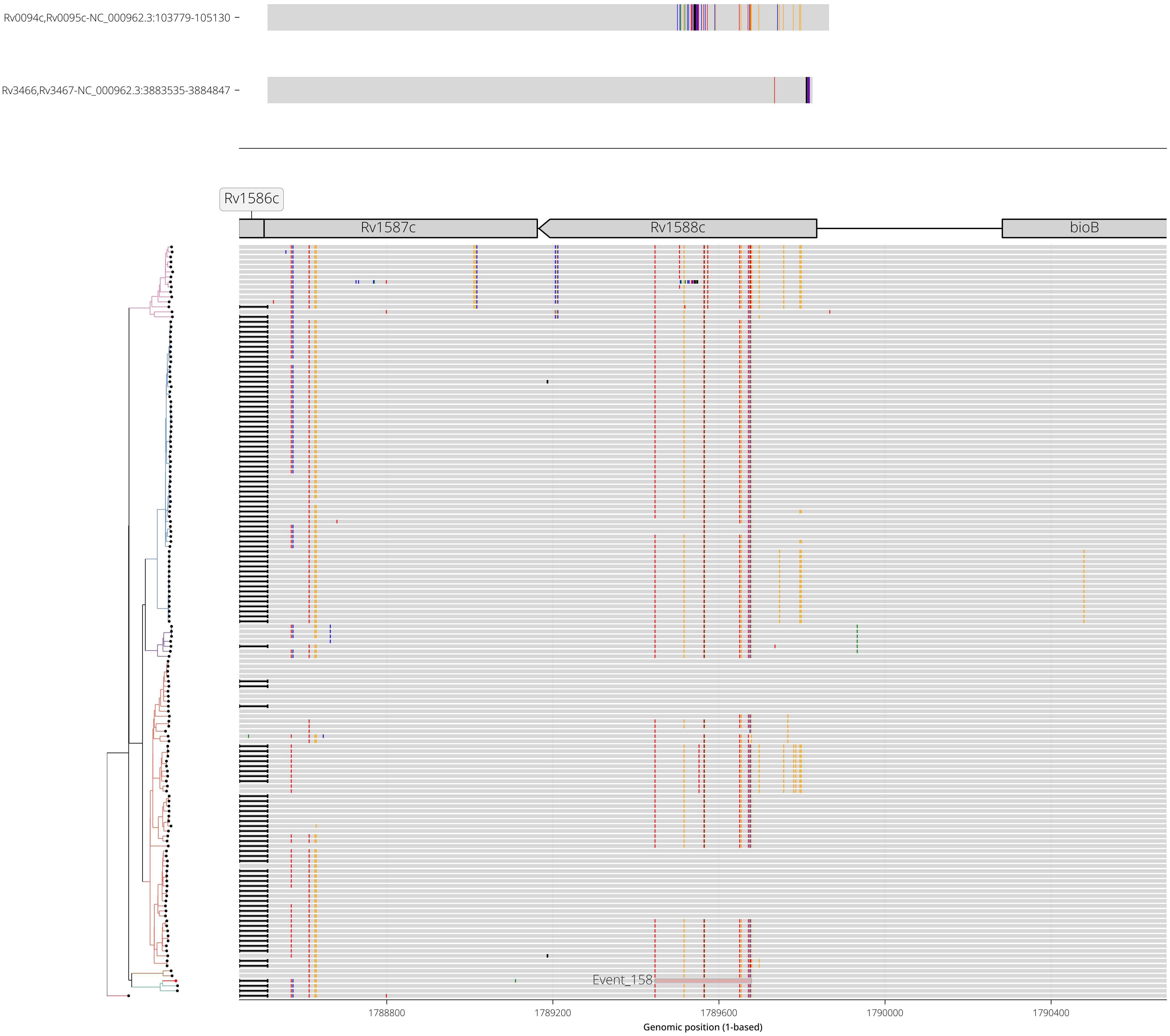

Individual GCE + Known Paralogs
GCE ID: Event_159

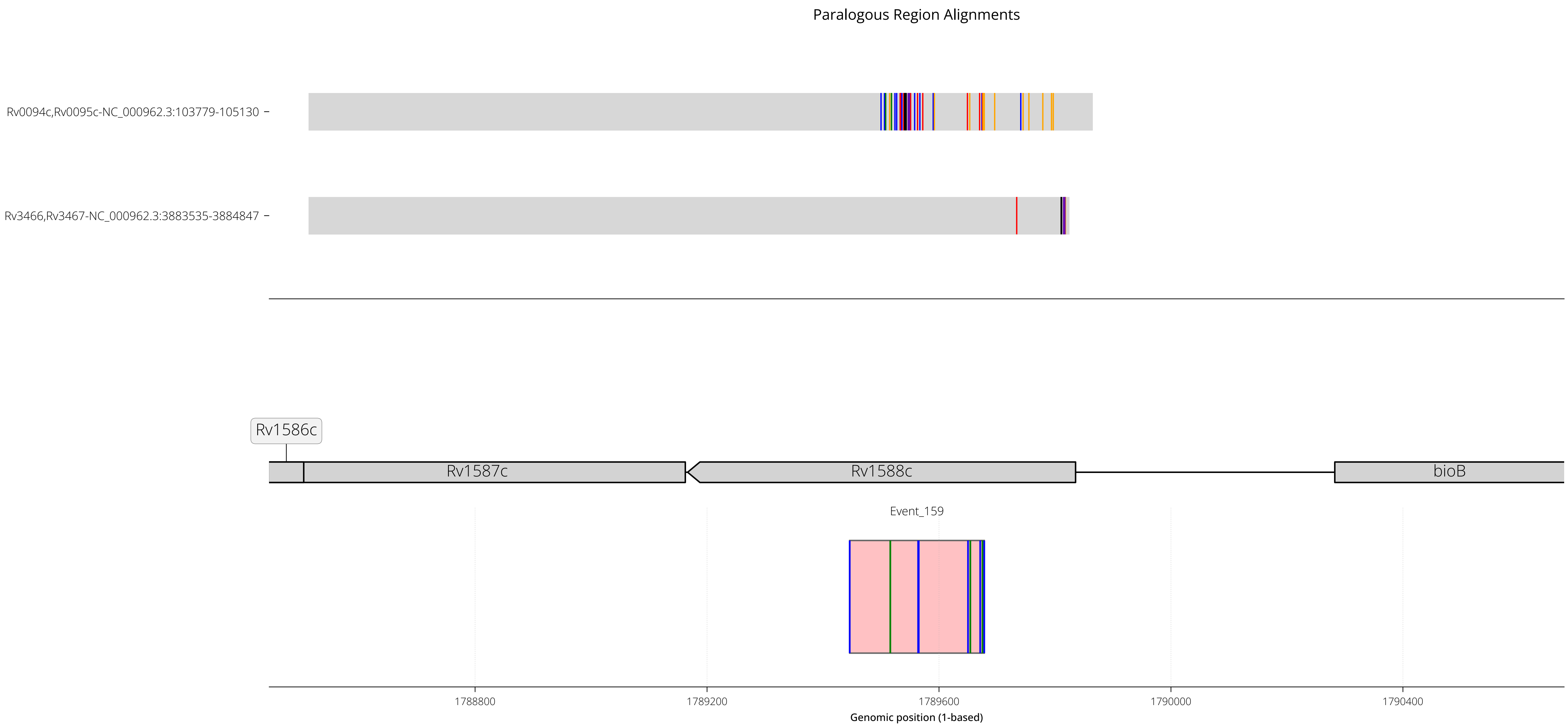

Circos Viz - GCE to Known Paralogs Locations
GCE ID: Event_159

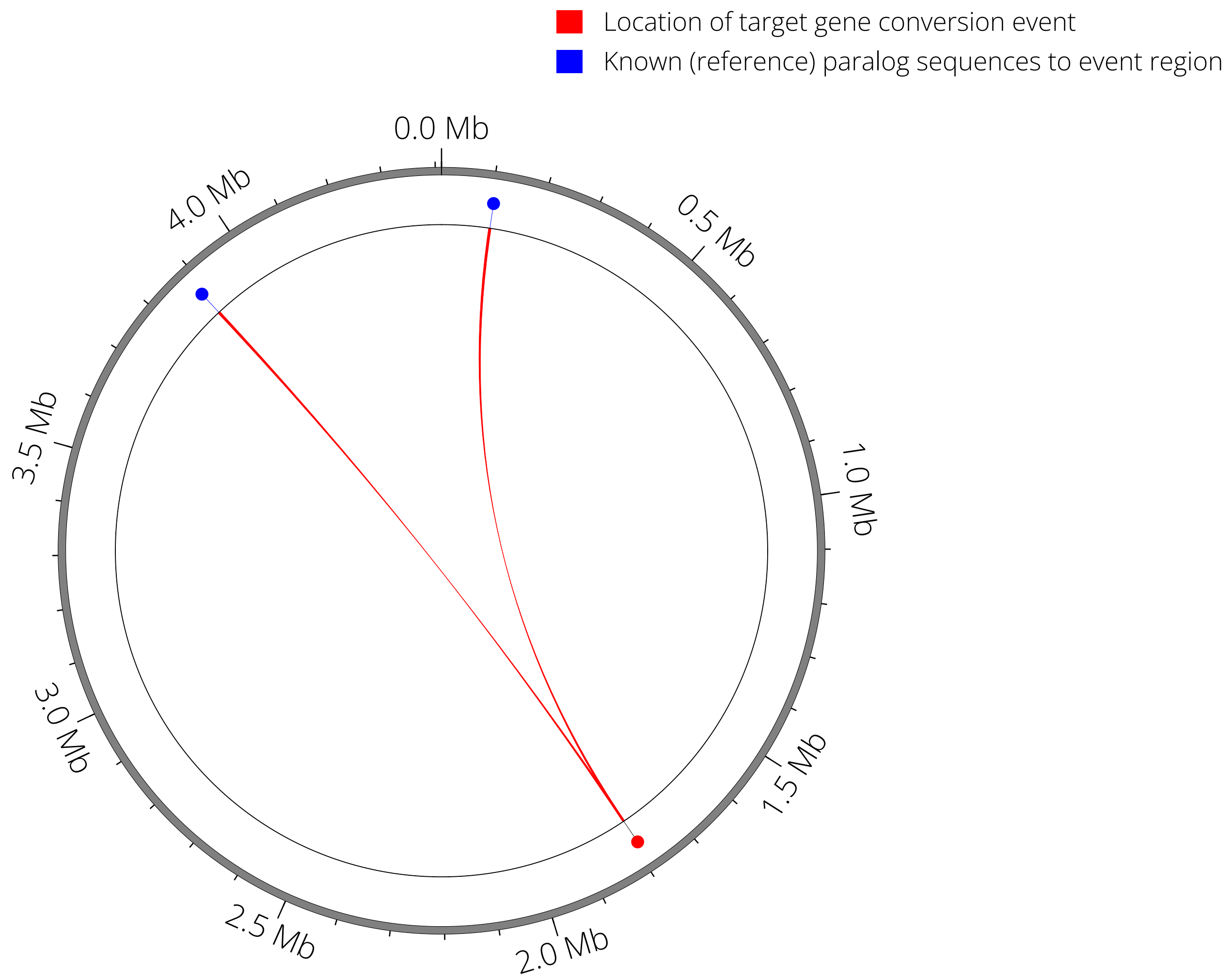

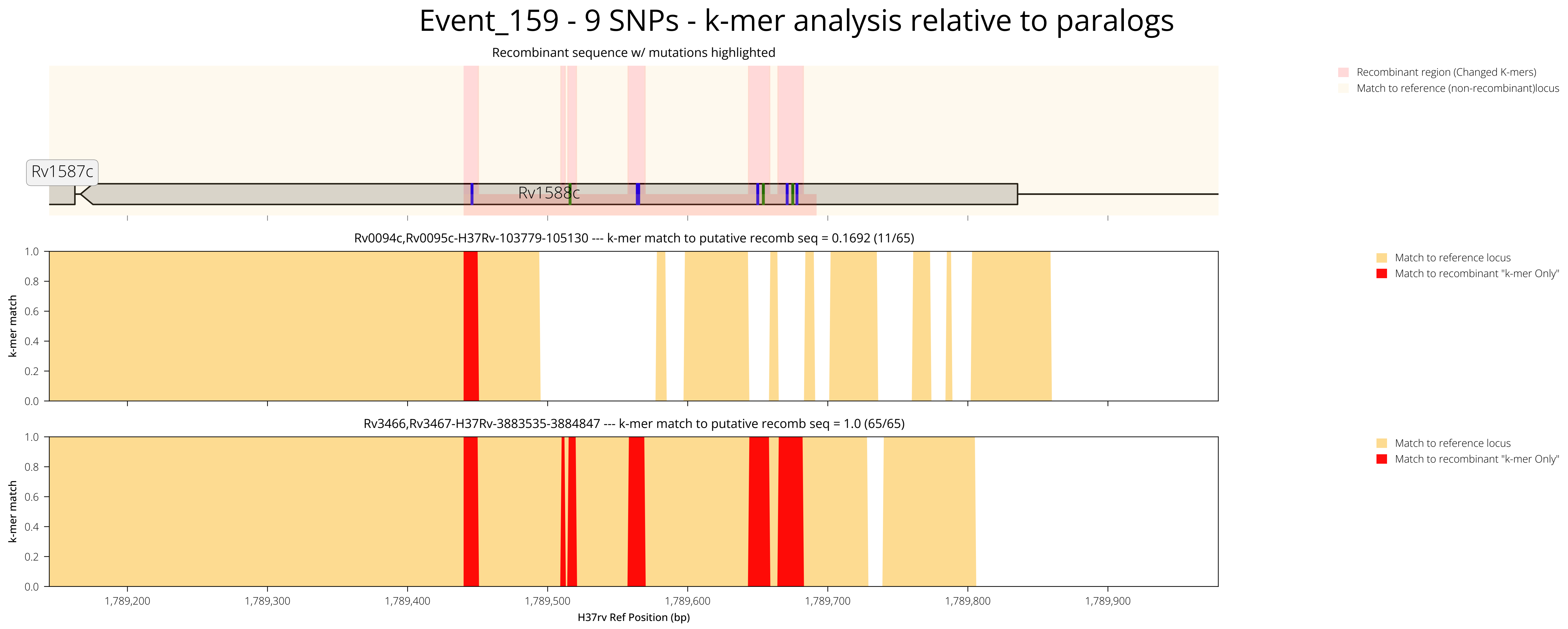
Event_159 - 9 SNPs - k-mer analysis relative to paralogs
Recombinant sequence w/ mutations highlighted
Recombinant region (Changed K-mers)
Match to reference (non-recombinant)locus
Rv1587c
Rv1588c
Rv0094c,Rv0095c-H37Rv-103779-105130 --- k-mer match to putative recomb seq = 0.1692 (11/65)
Match to reference locus
Match to recombinant "k-mer Only"
k-mer match
1.0
0.8
0.6
0.4
0.2
0.0
Rv3466,Rv3467-H37Rv-3883535-3884847 --- k-mer match to putative recomb seq = 1.0 (65/65)
1,789,200
1,789,300
1,789,400
1,789,500
1,789,600
1,789,700
1,789,800
1,789,900
H37rv Ref Position (bp)

Sub-phylogeny Viz + Paralog Alignments
GCE ID: Event_159

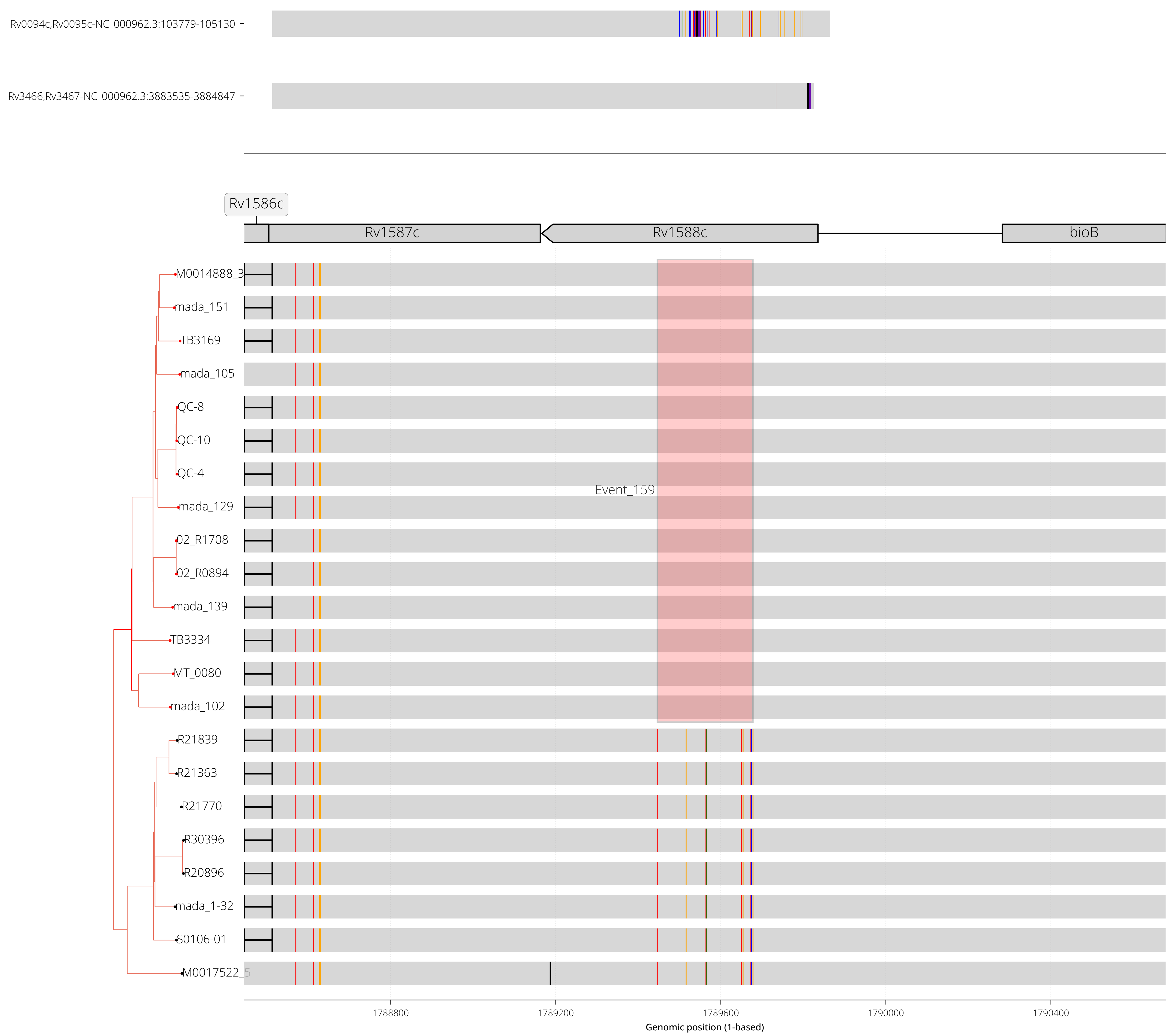

Full Phylogeny Viz + Paralog Alignments
GCE ID: Event_159

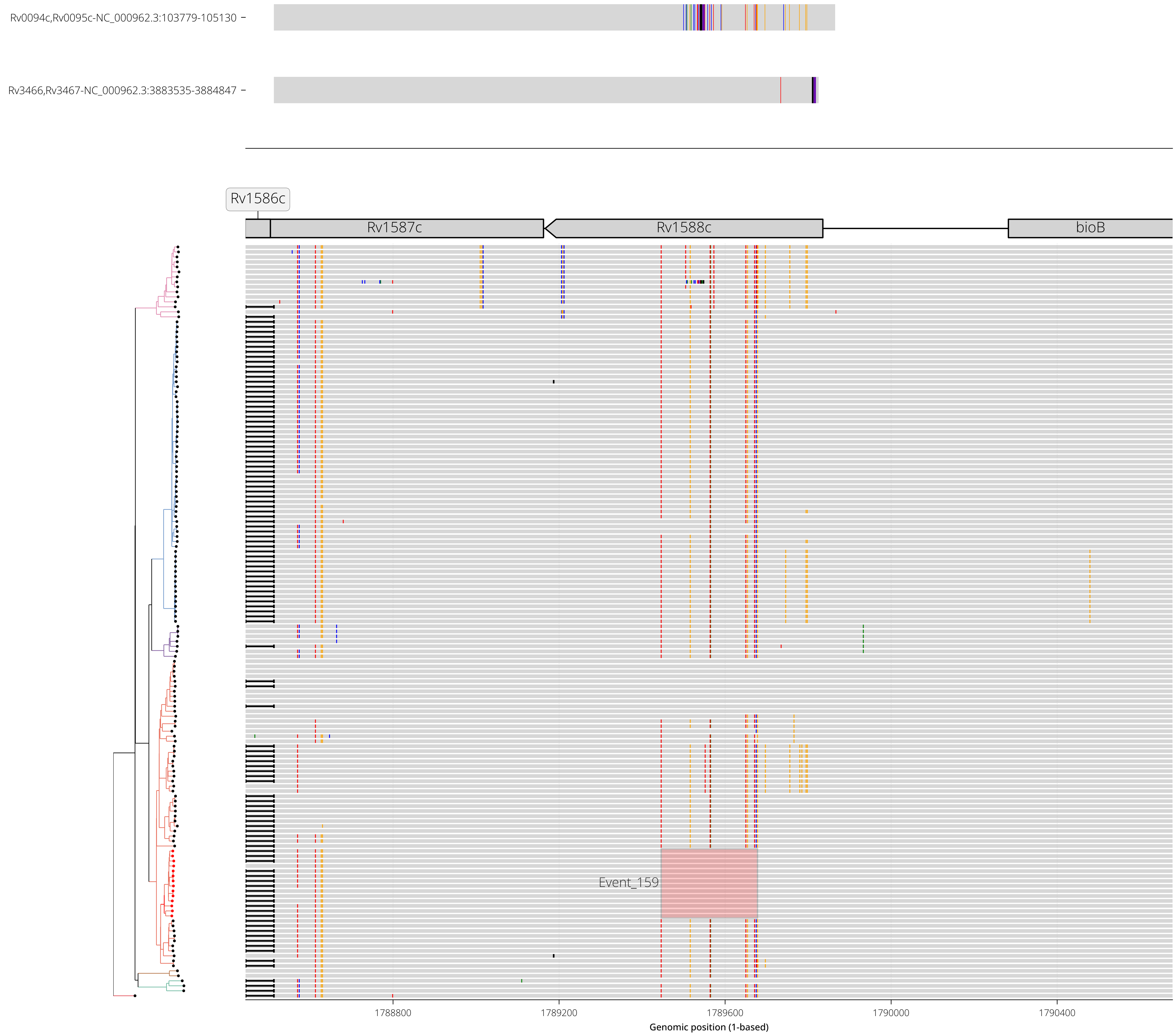

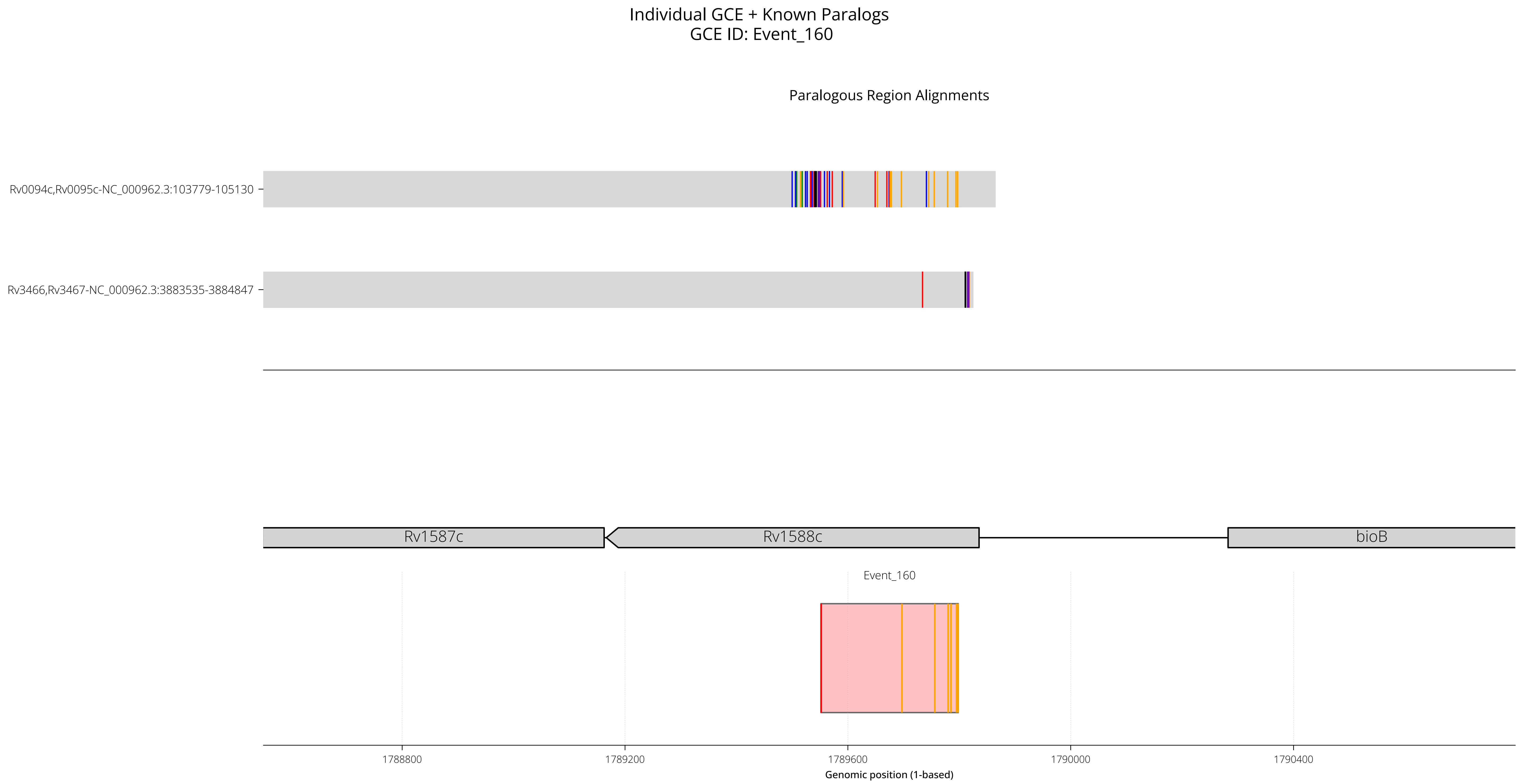
Individual GCE + Known Paralogs
GCE ID: Event_160
Paralogous Region Alignments
Rv0094c,Rv0095c-NC_000962.3:103779-105130
Rv3466,Rv3467-NC_000962.3:3883535-3884847
Rv1587c
Rv1588c
bioB
Event_160
1788800
1789200
1789600
1790000
1790400
Genomic position (1-based)

Circos Viz - GCE to Known Paralogs Locations
GCE ID: Event_160

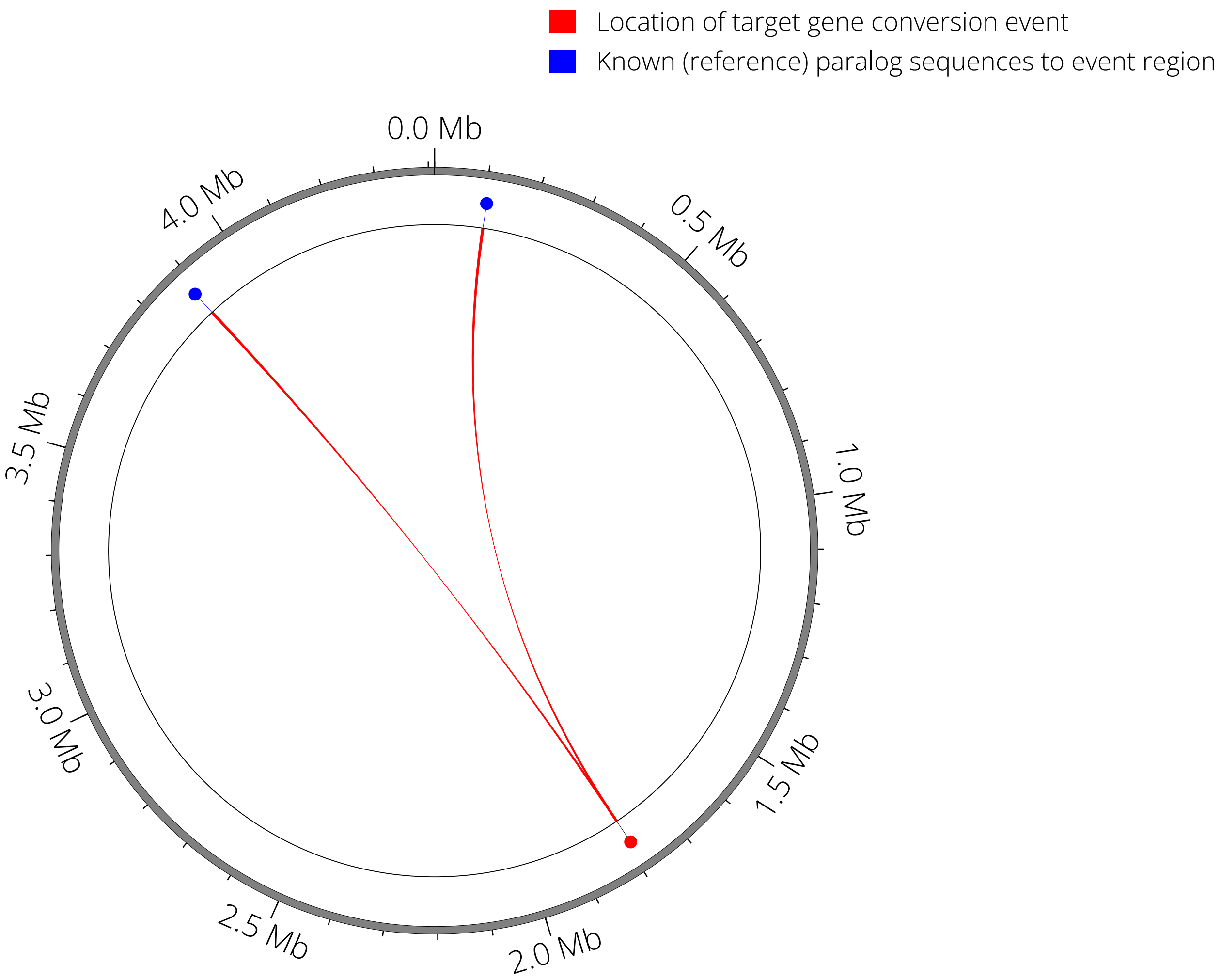

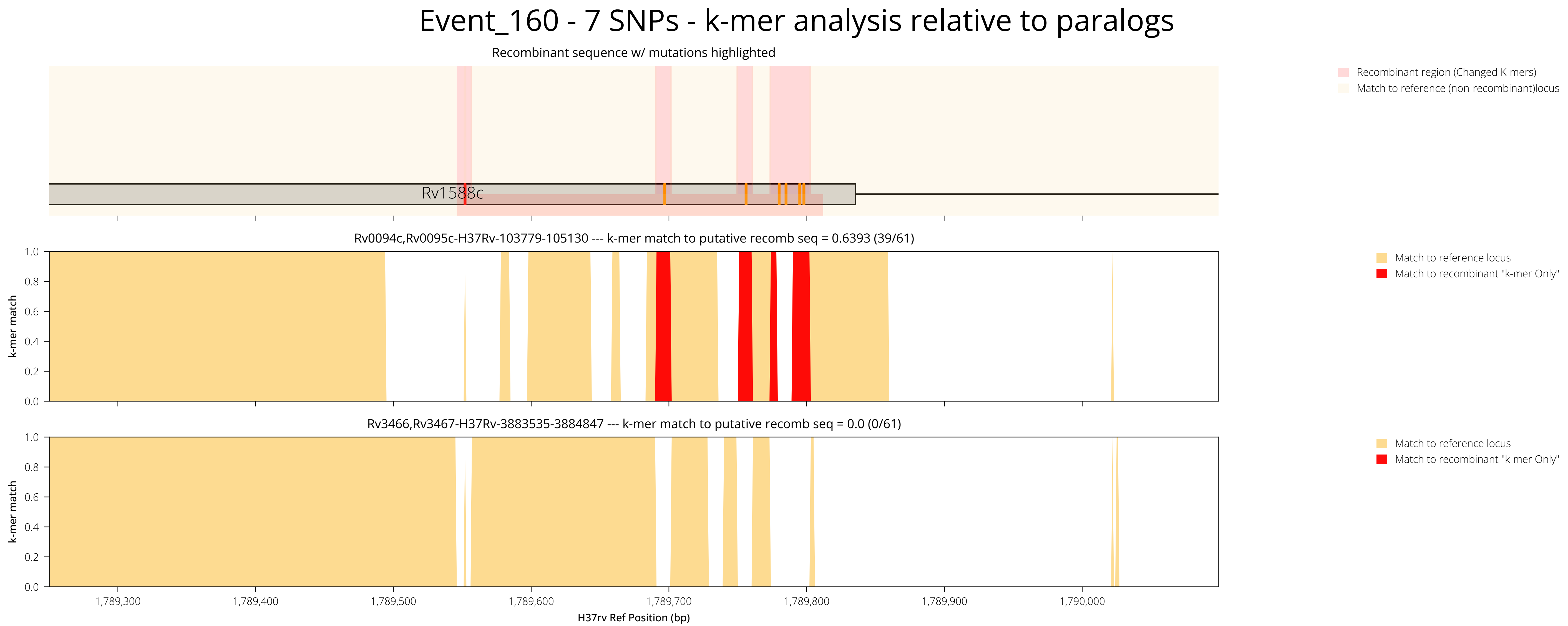
Event_160 - 7 SNPs - k-mer analysis relative to paralogs
Recombinant sequence w/ mutations highlighted
Rv1588c
Recombinant region (Changed K-mers)
Match to reference (non-recombinant)locus
Rv0094c,Rv0095c-H37Rv-103779-105130 --- k-mer match to putative recomb seq = 0.6393 (39/61)
Match to reference locus
Match to recombinant "k-mer Only"
Rv3466,Rv3467-H37Rv-3883535-3884847 --- k-mer match to putative recomb seq = 0.0 (0/61)
Match to reference locus
Match to recombinant "k-mer Only"
k-mer match
1.0
0.8
0.6
0.4
0.2
0.0
1,789,300
1,789,400
1,789,500
1,789,600
1,789,700
1,789,800
1,789,900
1,790,000
H37rv Ref Position (bp)

Sub-phylogeny Viz + Paralog Alignments
GCE ID: Event_160

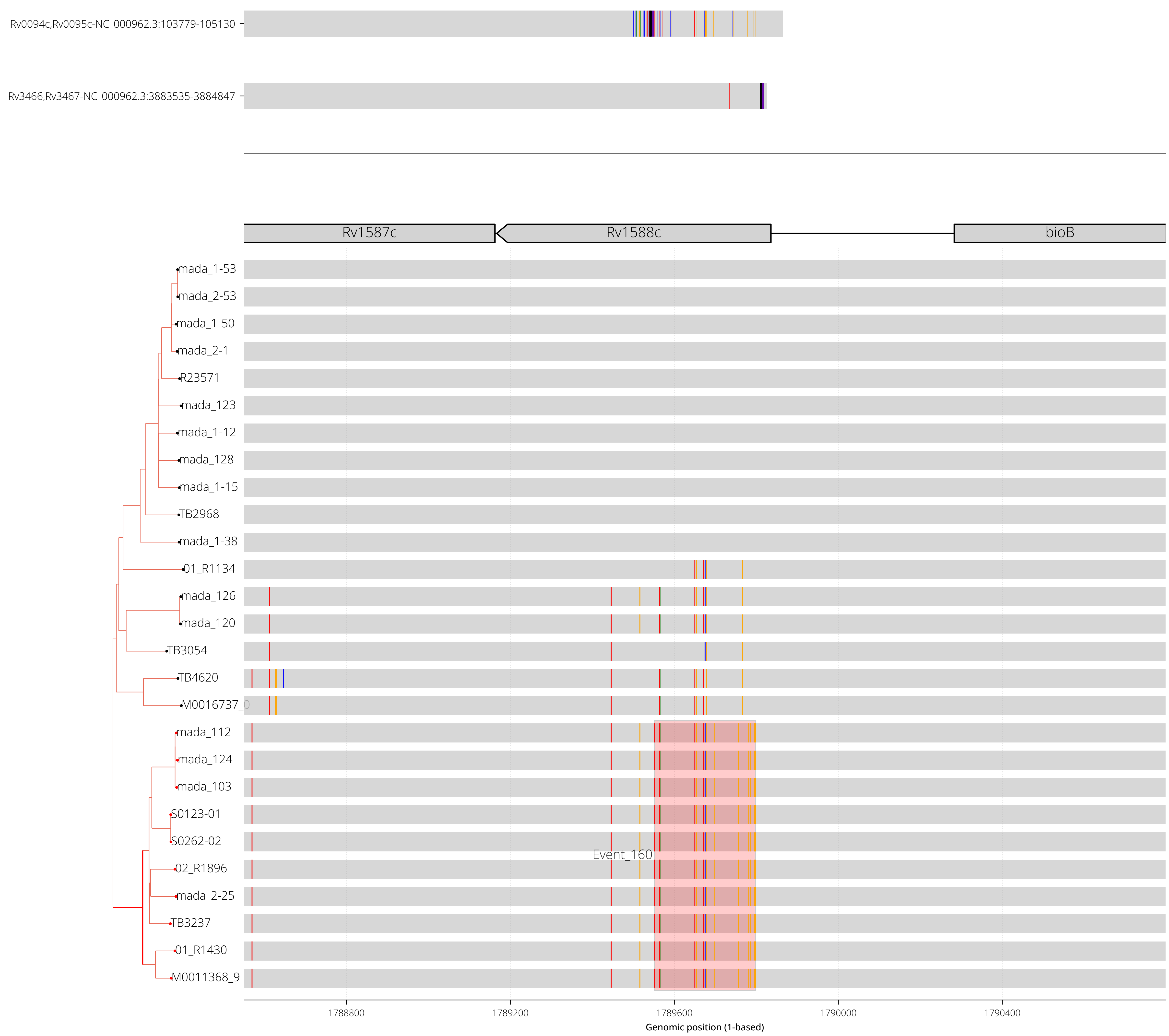

# Full Phylogeny Viz + Paralog Alignments
GCE ID: Event_160

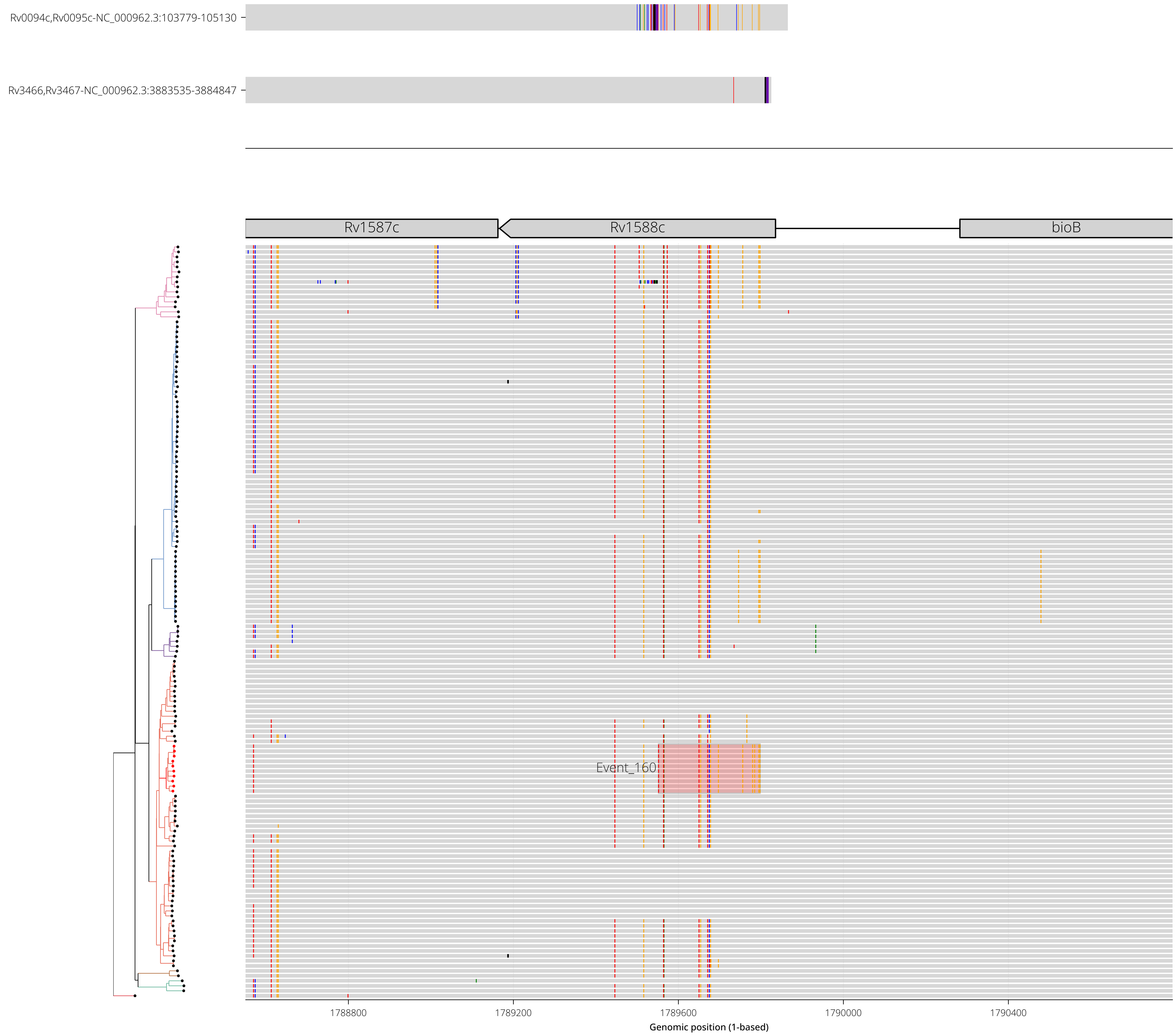

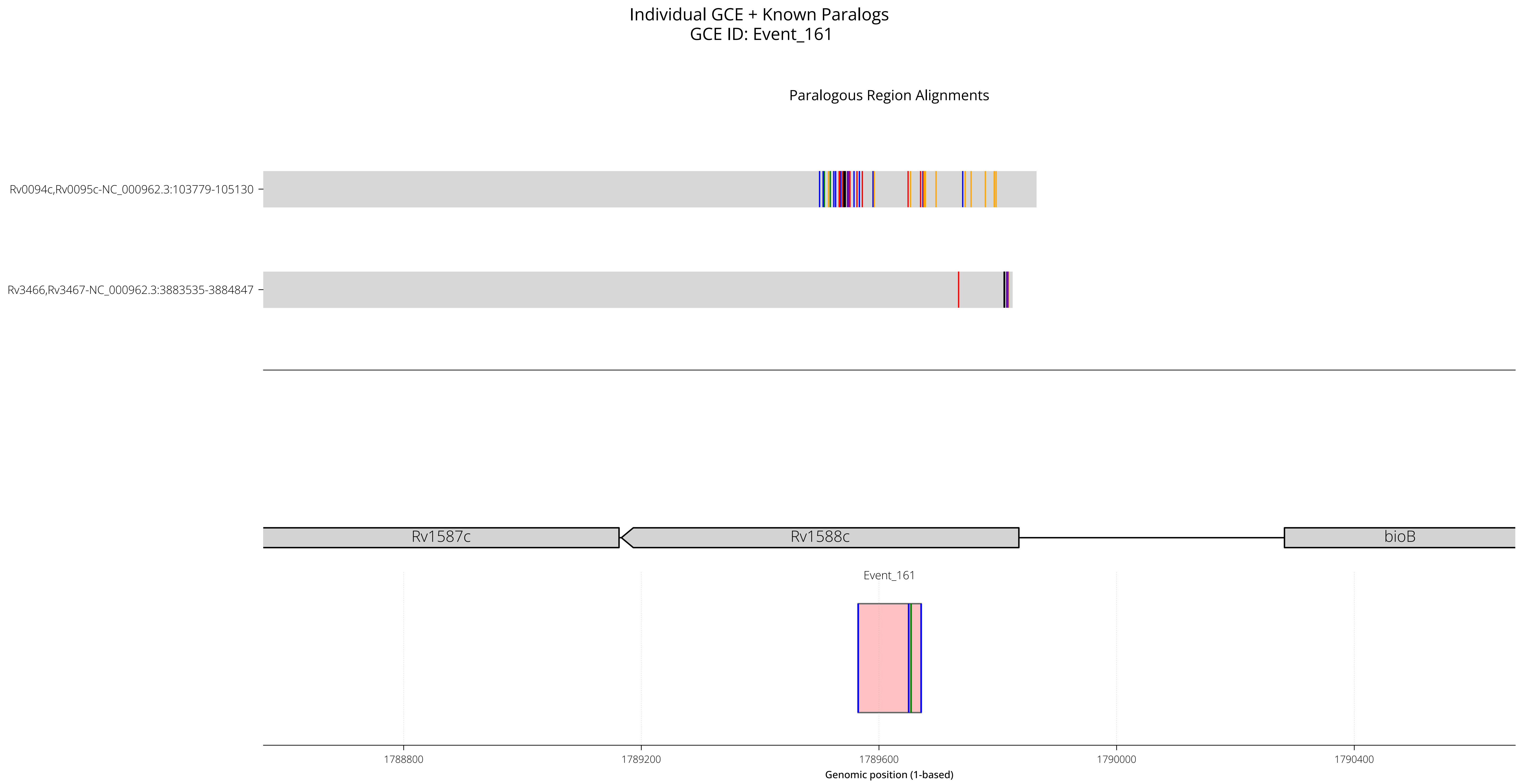

Individual GCE + Known Paralogs
GCE ID: Event_161
Paralogous Region Alignments
Rv0094c,Rv0095c-NC_000962.3:103779-105130
Rv3466,Rv3467-NC_000962.3:3883535-3884847
Rv1587c
Rv1588c
bioB
Event_161
1788800
1789200
1789600
1790000
1790400
Genomic position (1-based)

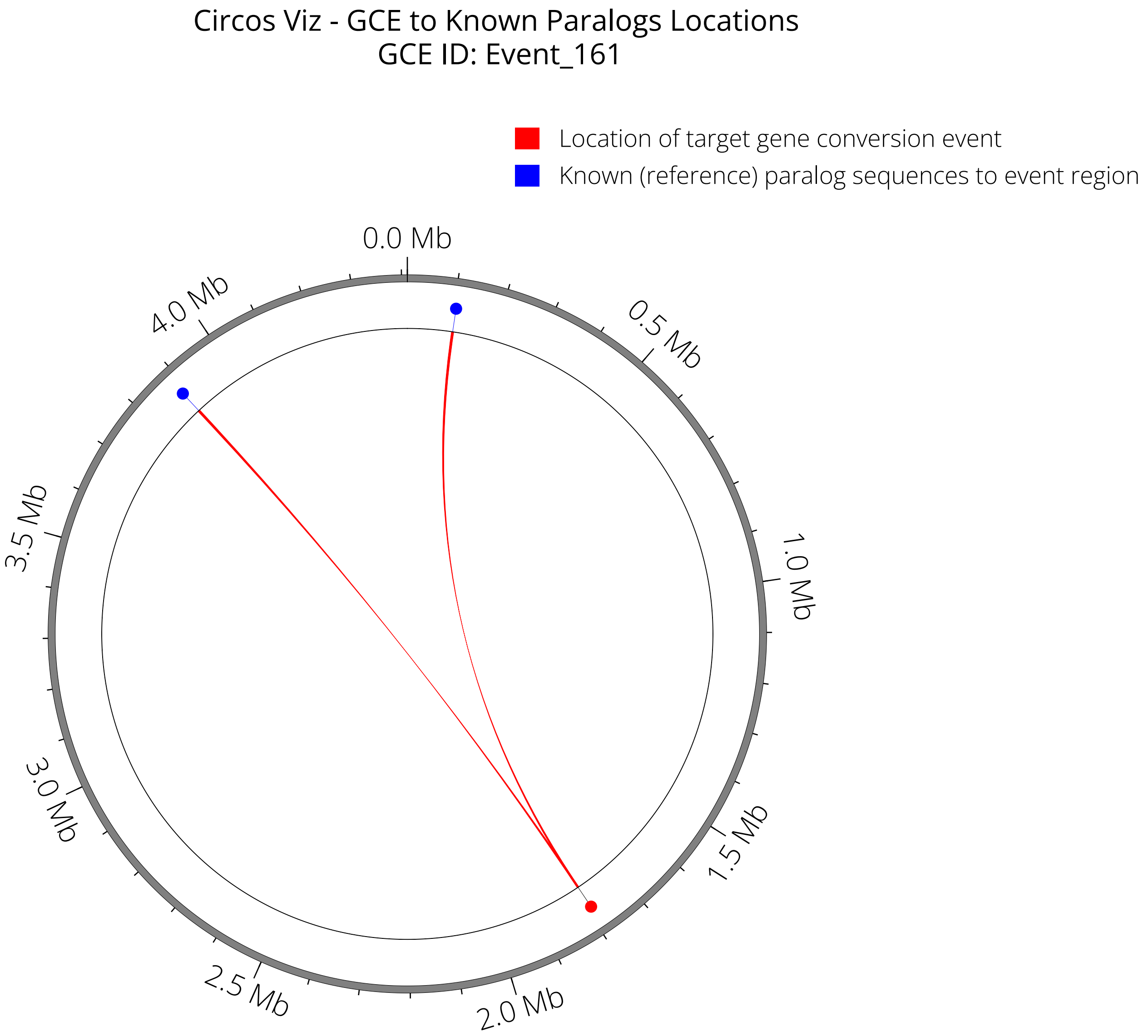

Circos Viz - GCE to Known Paralogs Locations
GCE ID: Event_161
Location of target gene conversion event
Known (reference) paralog sequences to event region
0.0 Mb
0.5 Mb
1.0 Mb
1.5 Mb
2.0 Mb
2.5 Mb
3.0 Mb
3.5 Mb
4.0 Mb

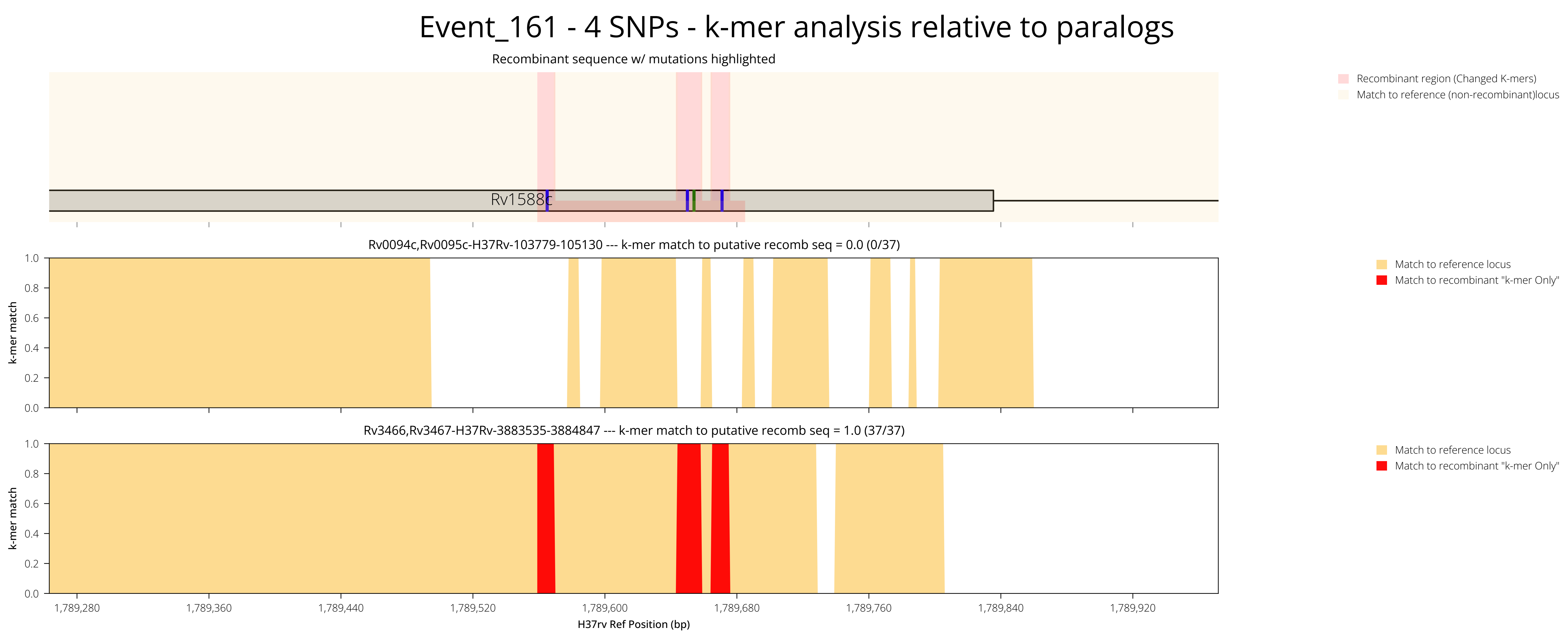

Event_161 - 4 SNPs - k-mer analysis relative to paralogs
Recombinant sequence w/ mutations highlighted
Rv1588c
Recombinant region (Changed K-mers)
Match to reference (non-recombinant)locus
Rv0094c,Rv0095c-H37Rv-103779-105130 --- k-mer match to putative recomb seq = 0.0 (0/37)
Match to reference locus
Match to recombinant "k-mer Only"
k-mer match
1.0
0.8
0.6
0.4
0.2
0.0
Rv3466,Rv3467-H37Rv-3883535-3884847 --- k-mer match to putative recomb seq = 1.0 (37/37)
Match to reference locus
Match to recombinant "k-mer Only"
k-mer match
1,789,280
1,789,360
1,789,440
1,789,520
1,789,600
1,789,680
1,789,760
1,789,840
1,789,920
H37rv Ref Position (bp)

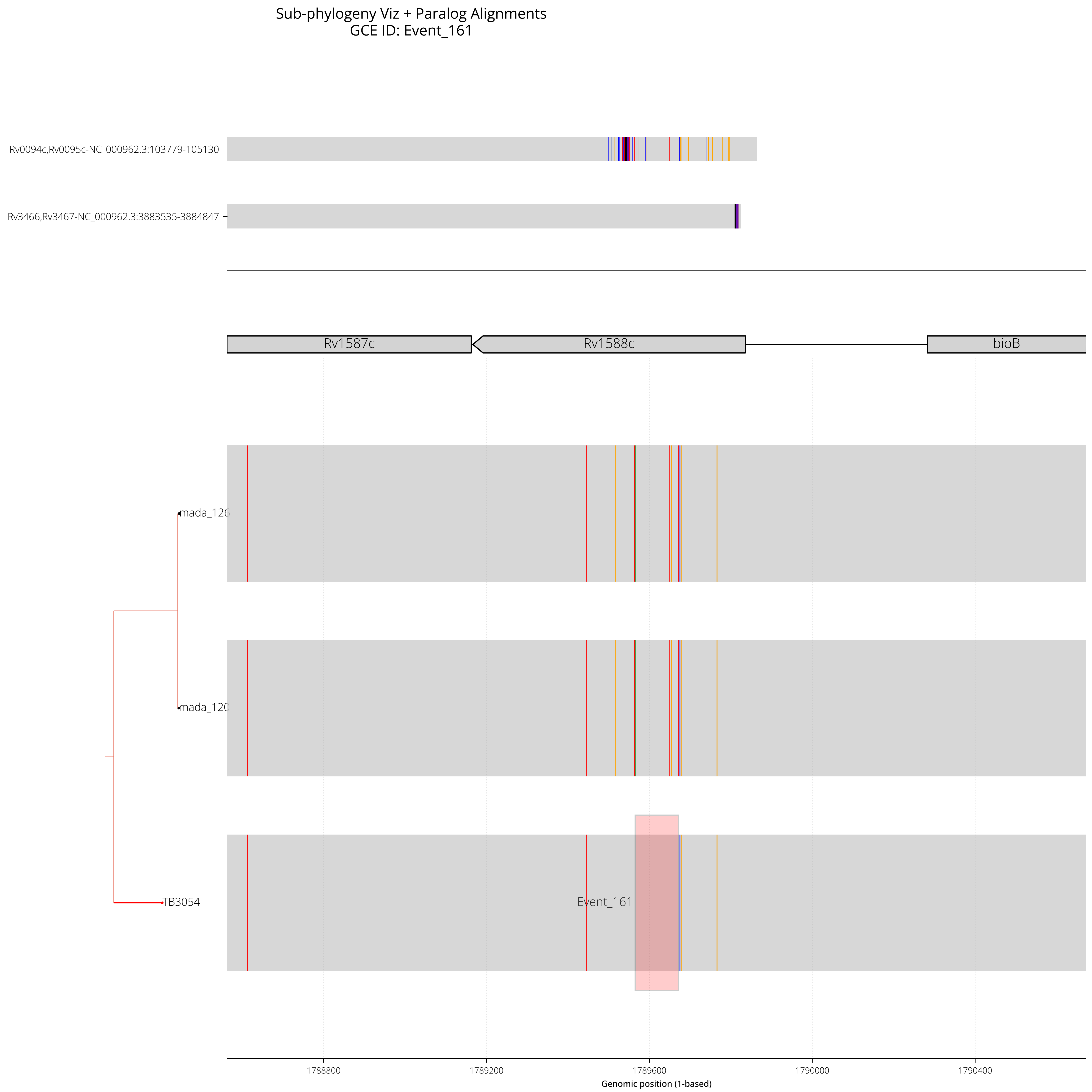

Sub-phylogeny Viz + Paralog Alignments
GCE ID: Event_161
Rv0094c,Rv0095c-NC_000962.3:103779-105130
Rv3466,Rv3467-NC_000962.3:3883535-3884847
Rv1587c
Rv1588c
bioB
mada_126
mada_120
TB3054
Event_161
1788800
1789200
1789600
1790000
1790400
Genomic position (1-based)

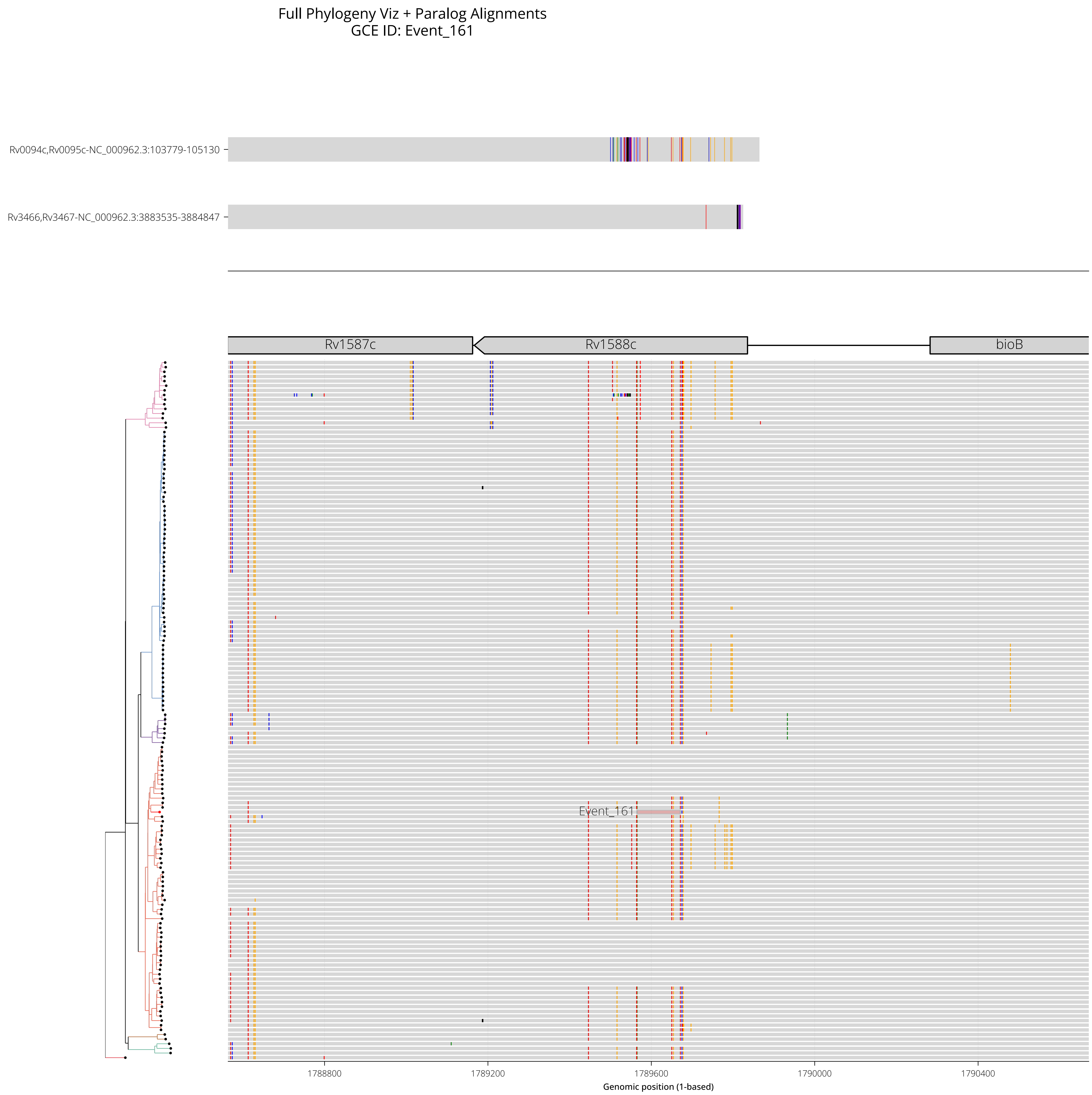
Full Phylogeny Viz + Paralog Alignments
GCE ID: Event_161
Rv0094c,Rv0095c-NC_000962.3:103779-105130
Rv3466,Rv3467-NC_000962.3:3883535-3884847
Rv1587c
Rv1588c
bioB
Event_161
1788800
1789200
1789600
1790000
1790400
Genomic position (1-based)

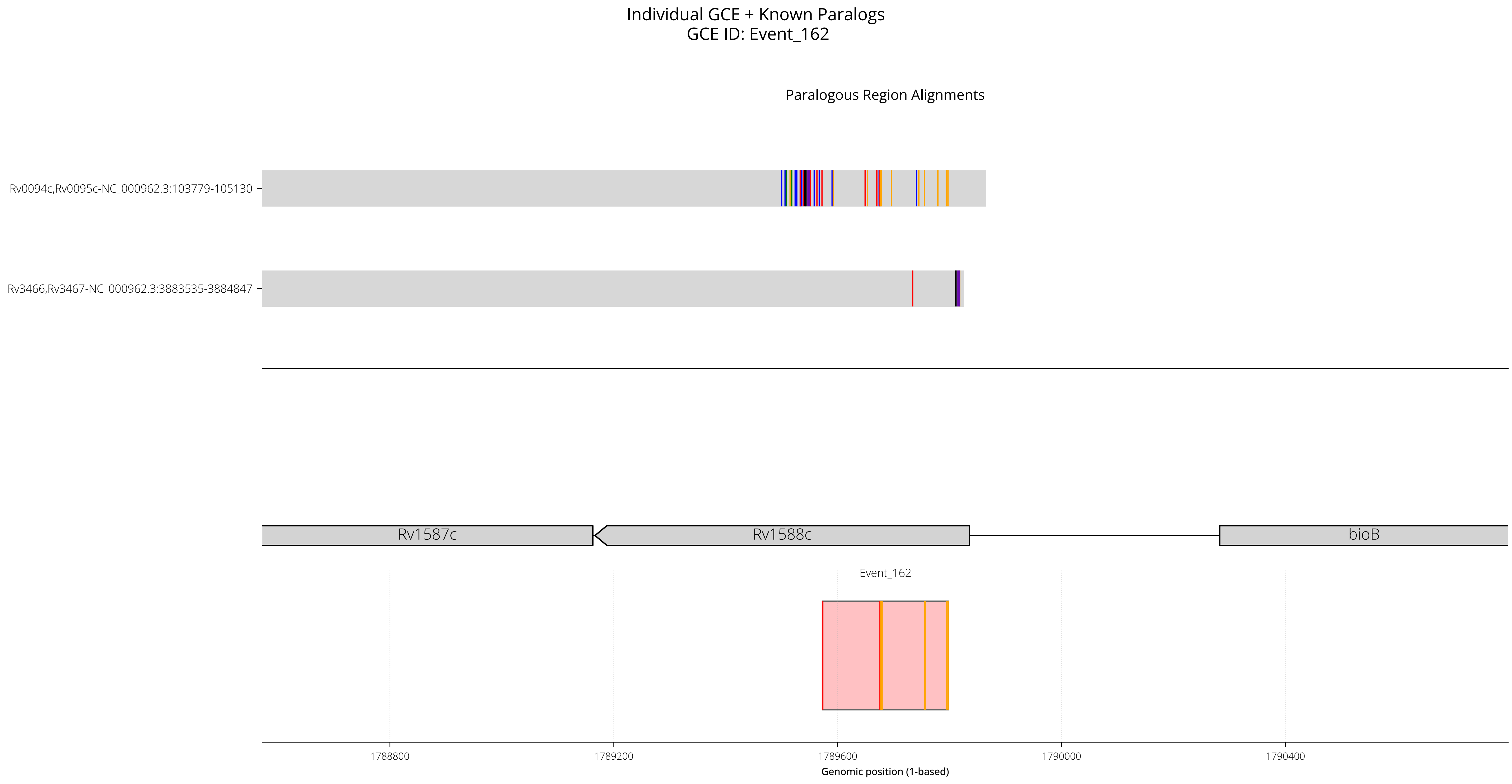

Individual GCE + Known Paralogs
GCE ID: Event_162
Paralogous Region Alignments
Rv0094c,Rv0095c-NC_000962.3:103779-105130
Rv3466,Rv3467-NC_000962.3:3883535-3884847
Rv1587c
Rv1588c
bioB
Event_162
1788800
1789200
1789600
1790000
1790400
Genomic position (1-based)

Circos Viz - GCE to Known Paralogs Locations
GCE ID: Event_162

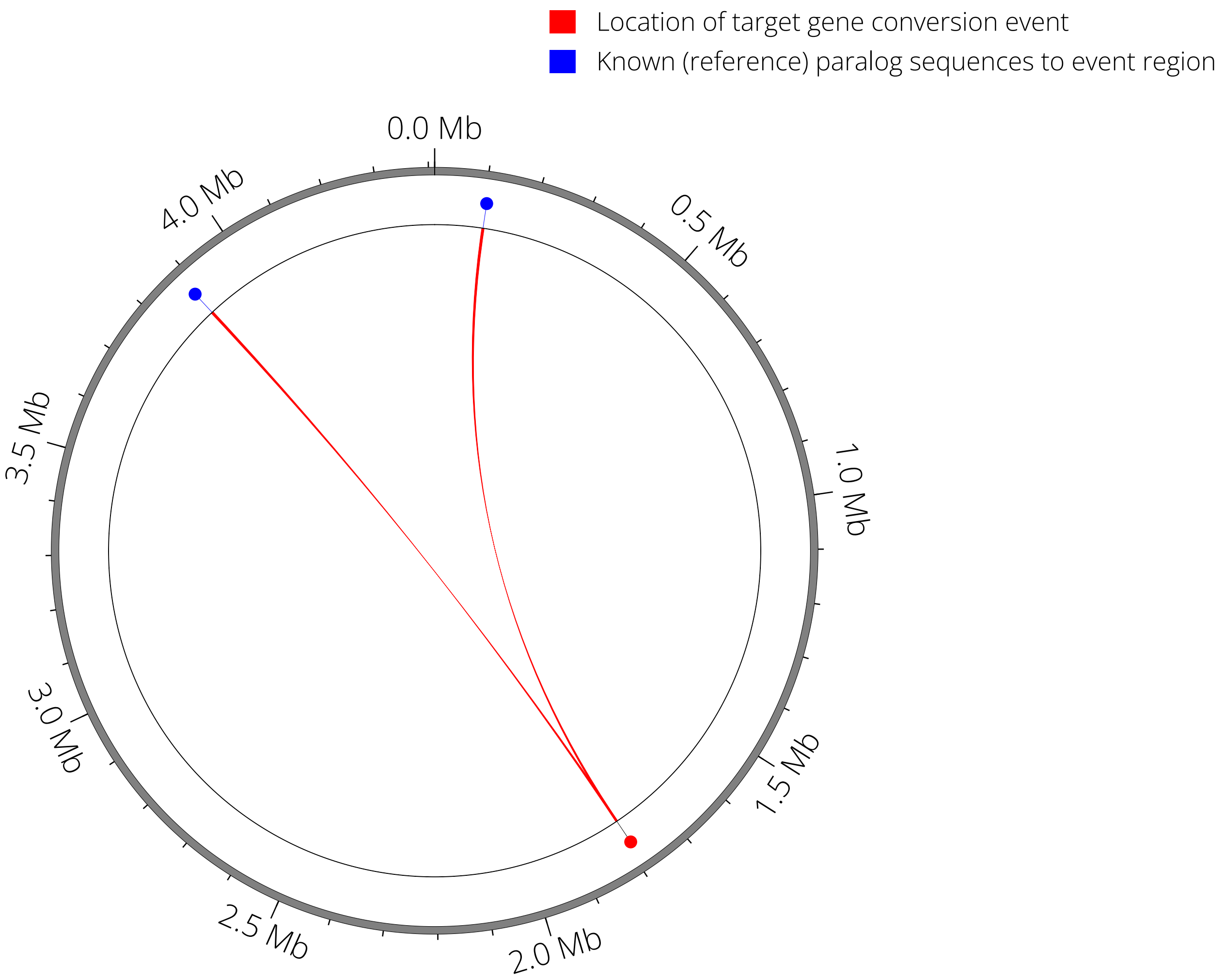

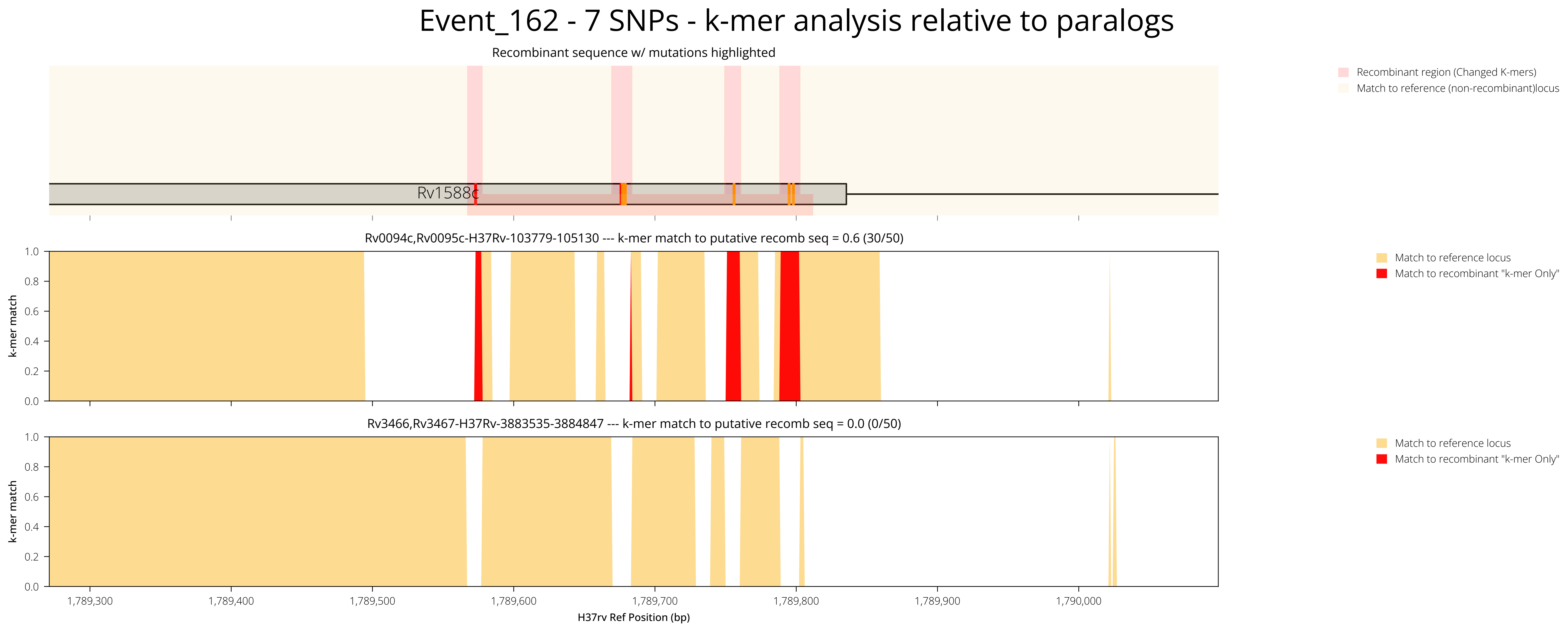
Event_162 - 7 SNPs - k-mer analysis relative to paralogs
Recombinant sequence w/ mutations highlighted
Rv1588c
Recombinant region (Changed K-mers)
Match to reference (non-recombinant)locus
Rv0094c,Rv0095c-H37Rv-103779-105130 --- k-mer match to putative recomb seq = 0.6 (30/50)
Match to reference locus
Match to recombinant "k-mer Only"
Rv3466,Rv3467-H37Rv-3883535-3884847 --- k-mer match to putative recomb seq = 0.0 (0/50)
k-mer match
1.0
0.8
0.6
0.4
0.2
0.0
1,789,300
1,789,400
1,789,500
1,789,600
1,789,700
1,789,800
1,789,900
1,790,000
H37rv Ref Position (bp)

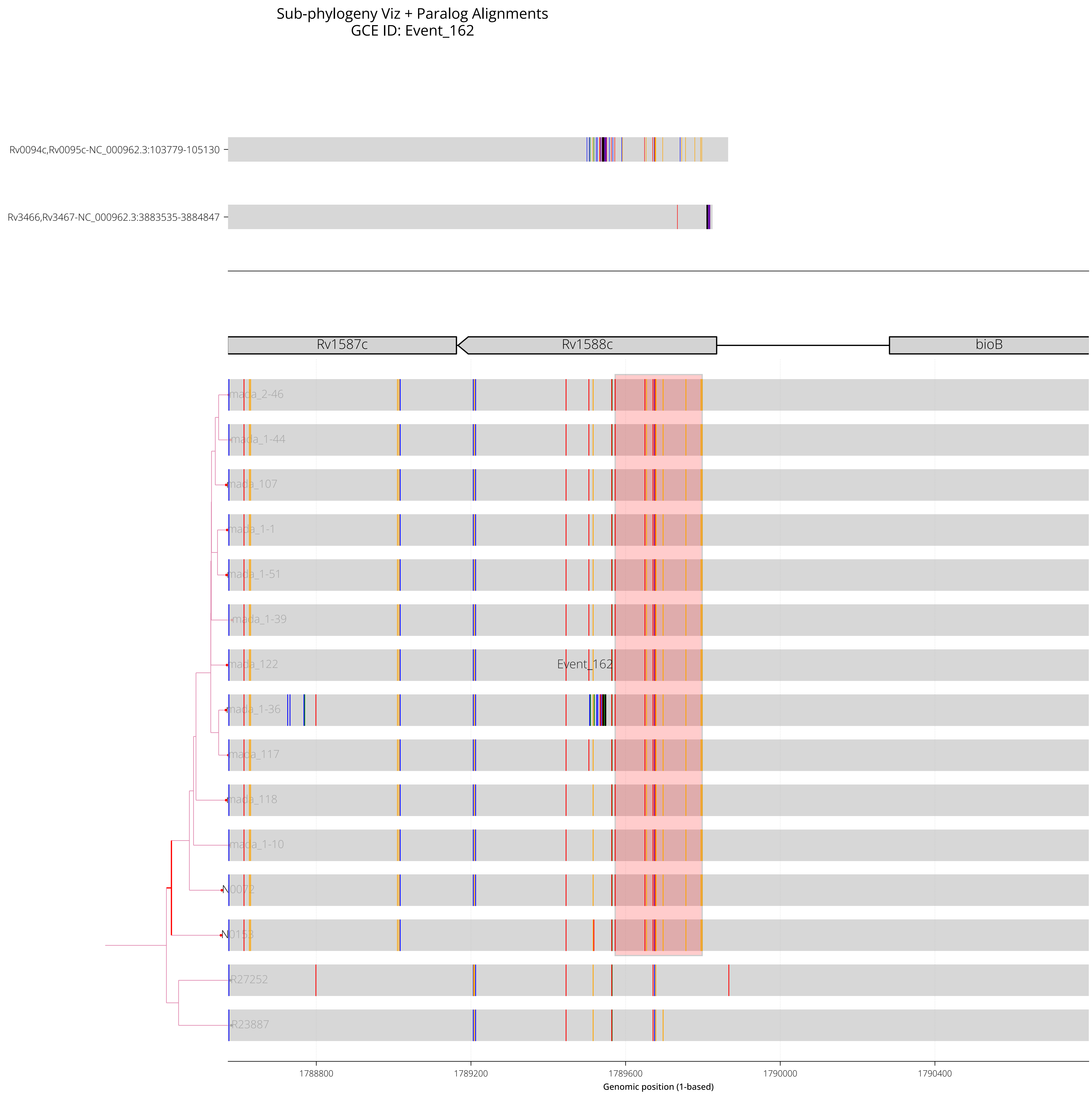
Sub-phylogeny Viz + Paralog Alignments
GCE ID: Event_162
Rv0094c,Rv0095c-NC_000962.3:103779-105130
Rv3466,Rv3467-NC_000962.3:3883535-3884847
Rv1587c
Rv1588c
bioB
mada_2-46
mada_1-44
mada_107
mada_1-1
mada_1-51
mada_1-39
mada_122
Event_162
mada_1-36
mada_117
mada_118
mada_1-10
N0072
N0153
R27252
R23887
1788800
1789200
1789600
1790000
1790400
Genomic position (1-based)

Full Phylogeny Viz + Paralog Alignments
GCE ID: Event_162

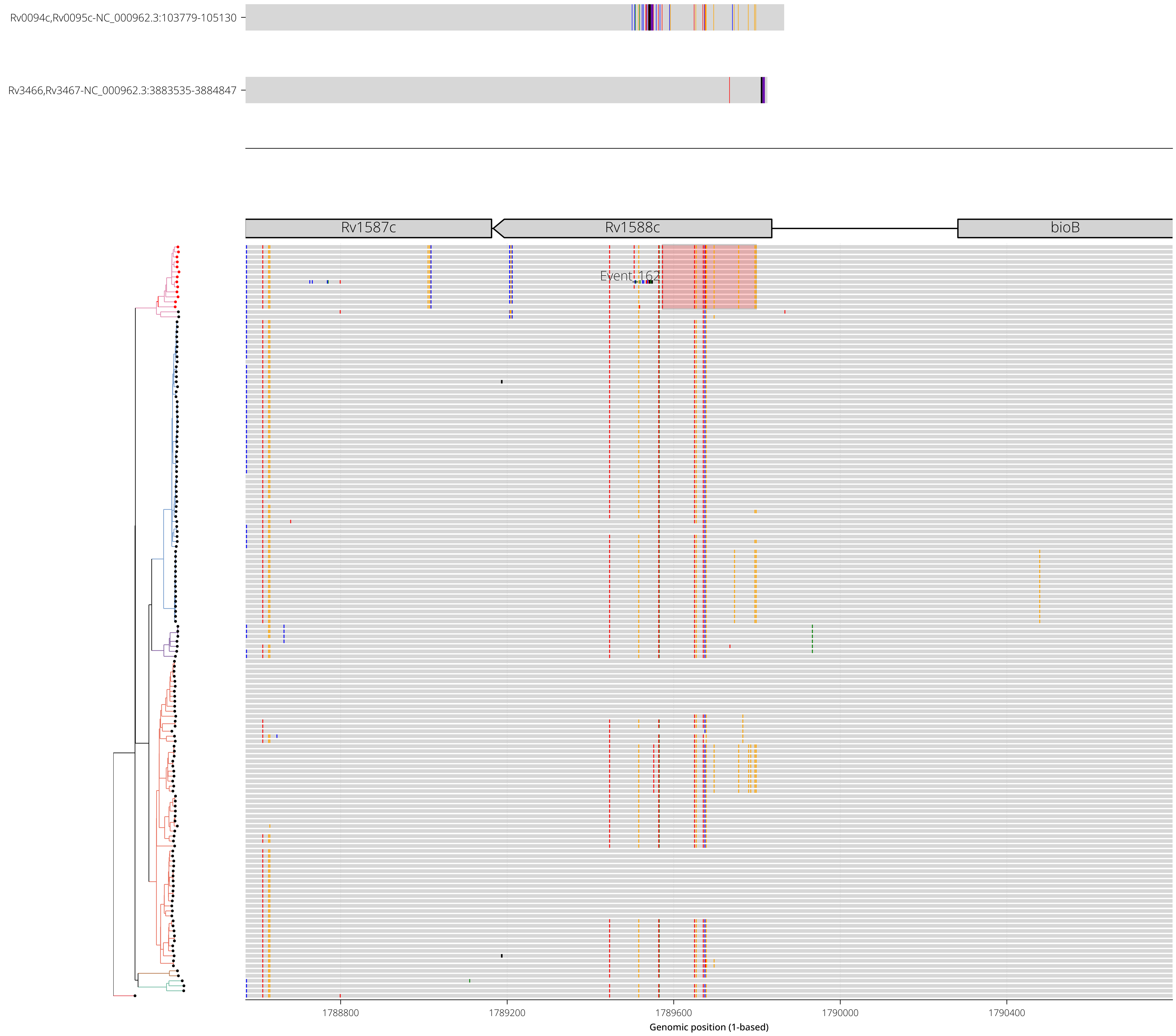

Individual GCE + Known Paralogs
GCE ID: Event_163

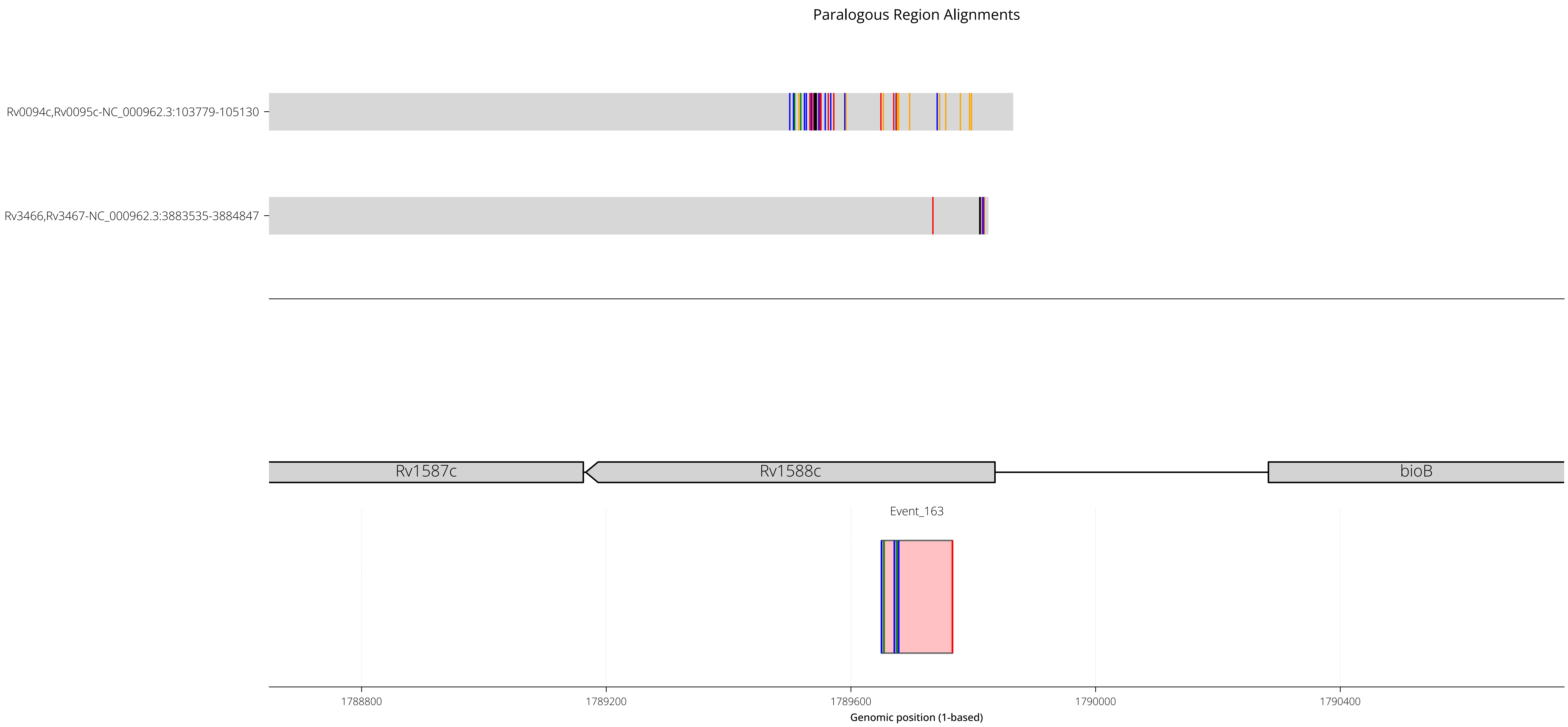

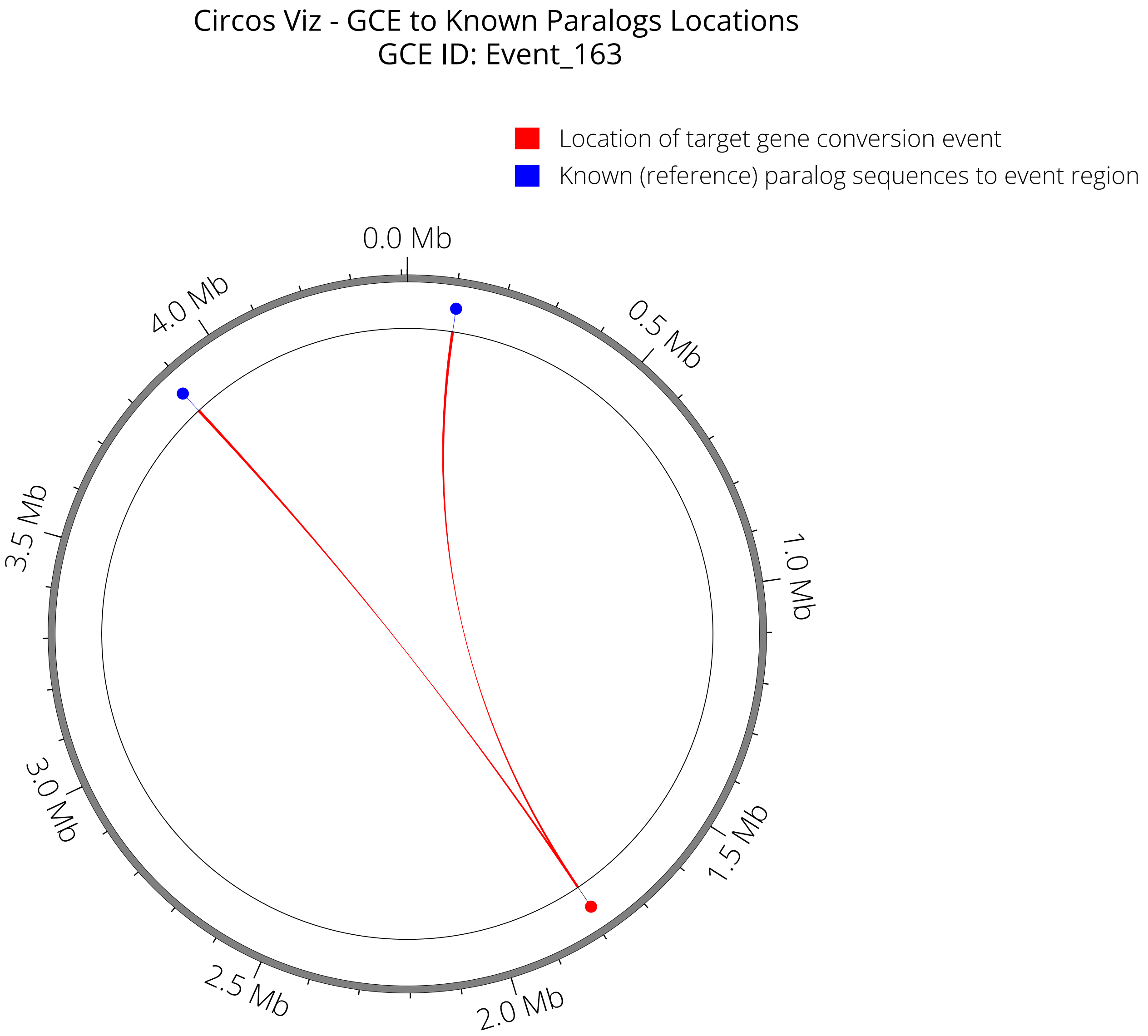
Circos Viz - GCE to Known Paralogs Locations
GCE ID: Event_163
Location of target gene conversion event
Known (reference) paralog sequences to event region
0.0 Mb
0.5 Mb
1.0 Mb
1.5 Mb
2.0 Mb
2.5 Mb
3.0 Mb
3.5 Mb
4.0 Mb

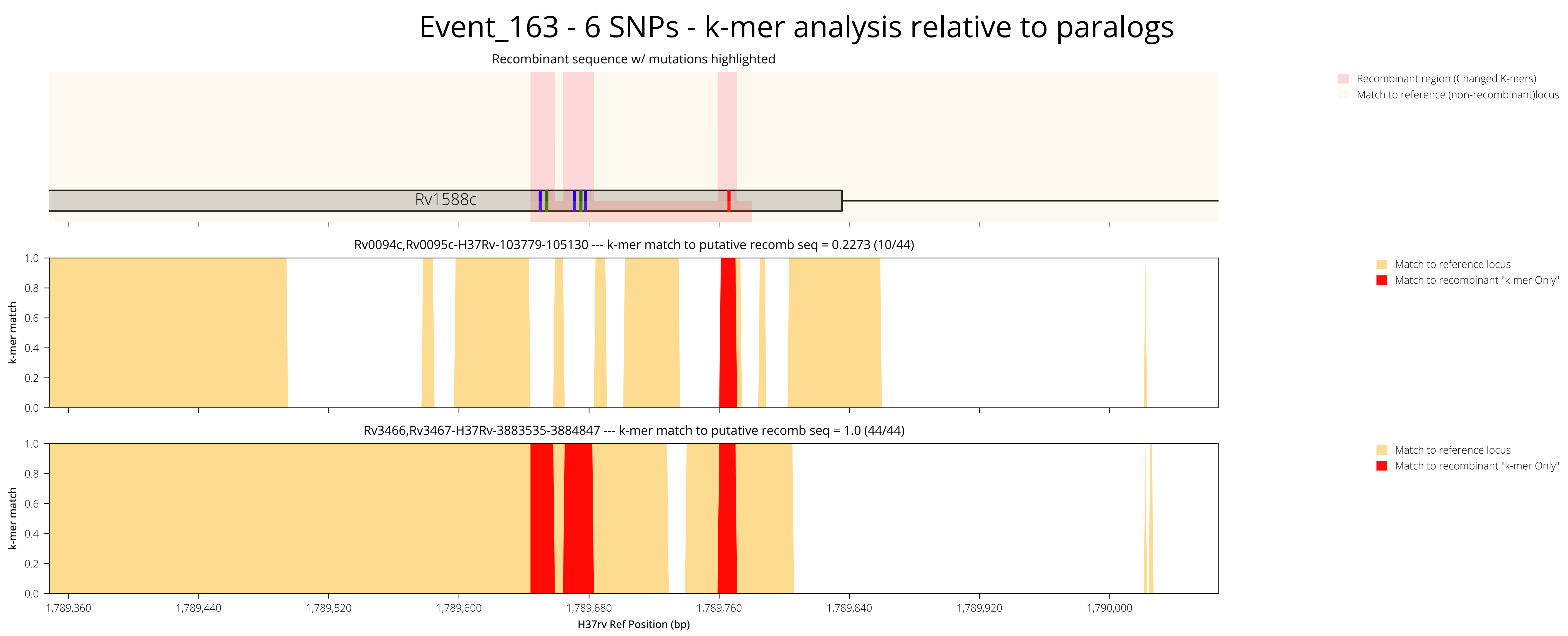

Event_163 - 6 SNPs - k-mer analysis relative to paralogs
Recombinant sequence w/ mutations highlighted
Recombinant region (Changed K-mers)
Match to reference (non-recombinant)locus
Rv1588c
Rv0094c,Rv0095c-H37Rv-103779-105130 --- k-mer match to putative recomb seq = 0.2273 (10/44)
Match to reference locus
Match to recombinant "k-mer Only"
Rv3466,Rv3467-H37Rv-3883535-3884847 --- k-mer match to putative recomb seq = 1.0 (44/44)
Match to reference locus
Match to recombinant "k-mer Only"
k-mer match
1.0
0.8
0.6
0.4
0.2
0.0
1,789,360
1,789,440
1,789,520
1,789,600
1,789,680
1,789,760
1,789,840
1,789,920
1,790,000
H37rv Ref Position (bp)

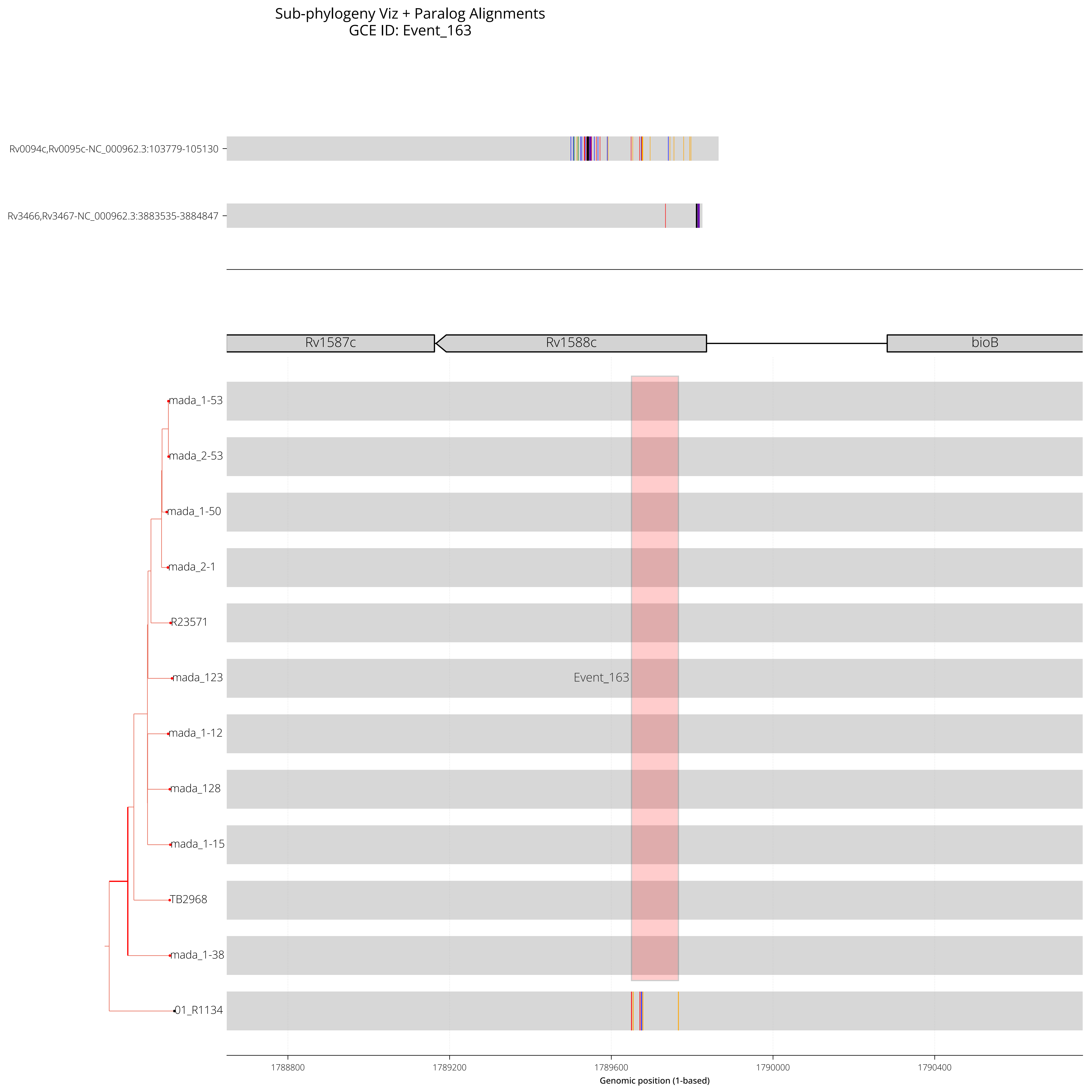

Sub-phylogeny Viz + Paralog Alignments
GCE ID: Event_163
Rv0094c,Rv0095c-NC_000962.3:103779-105130
Rv3466,Rv3467-NC_000962.3:3883535-3884847
Rv1587c
Rv1588c
bioB
mada_1-53
mada_2-53
mada_1-50
mada_2-1
R23571
mada_123
Event_163
mada_1-12
mada_128
mada_1-15
TB2968
mada_1-38
01_R1134
1788800
1789200
1789600
1790000
1790400
Genomic position (1-based)

Full Phylogeny Viz + Paralog Alignments
GCE ID: Event_163

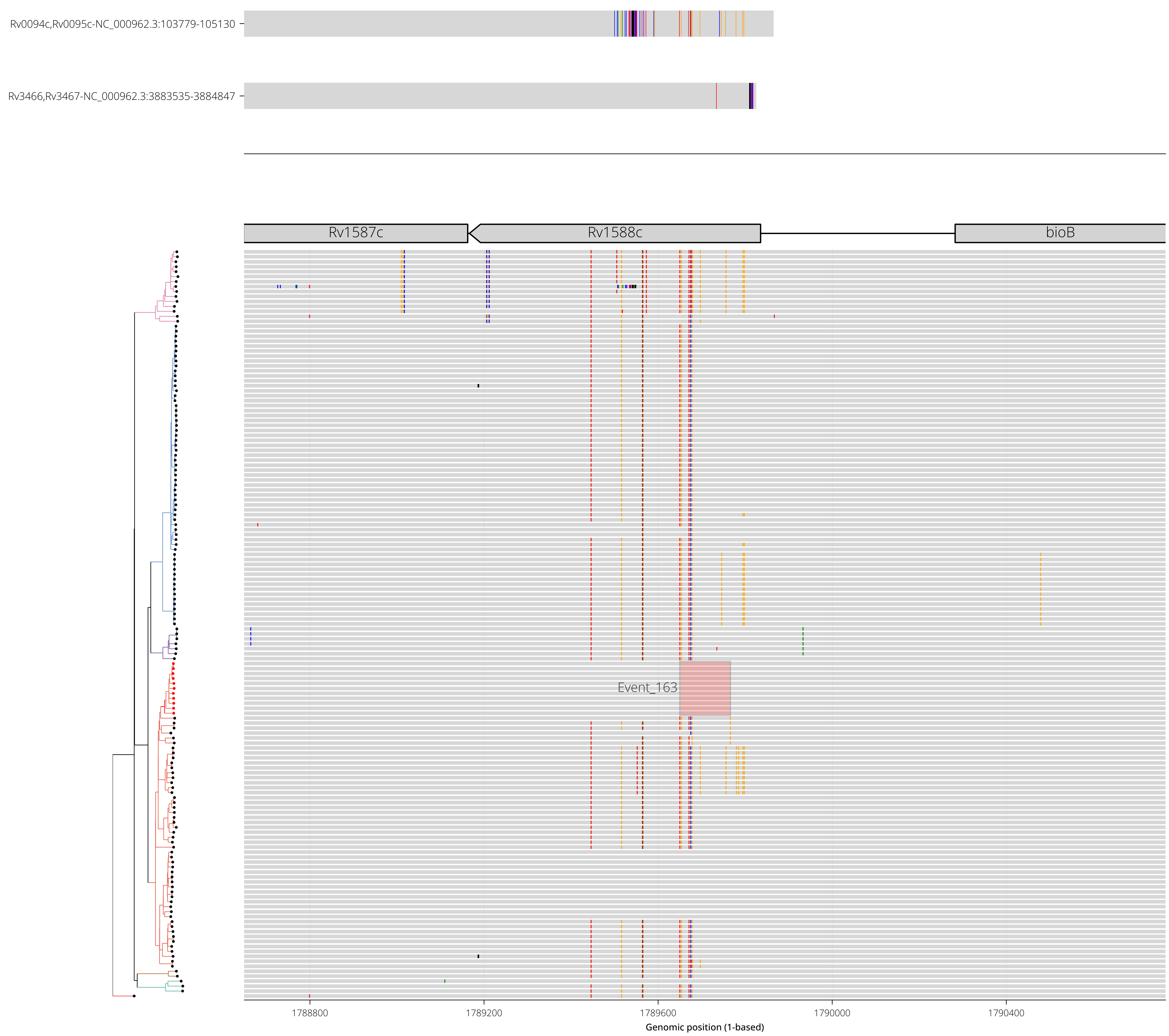

Individual GCE + Known Paralogs
GCE ID: Event_164

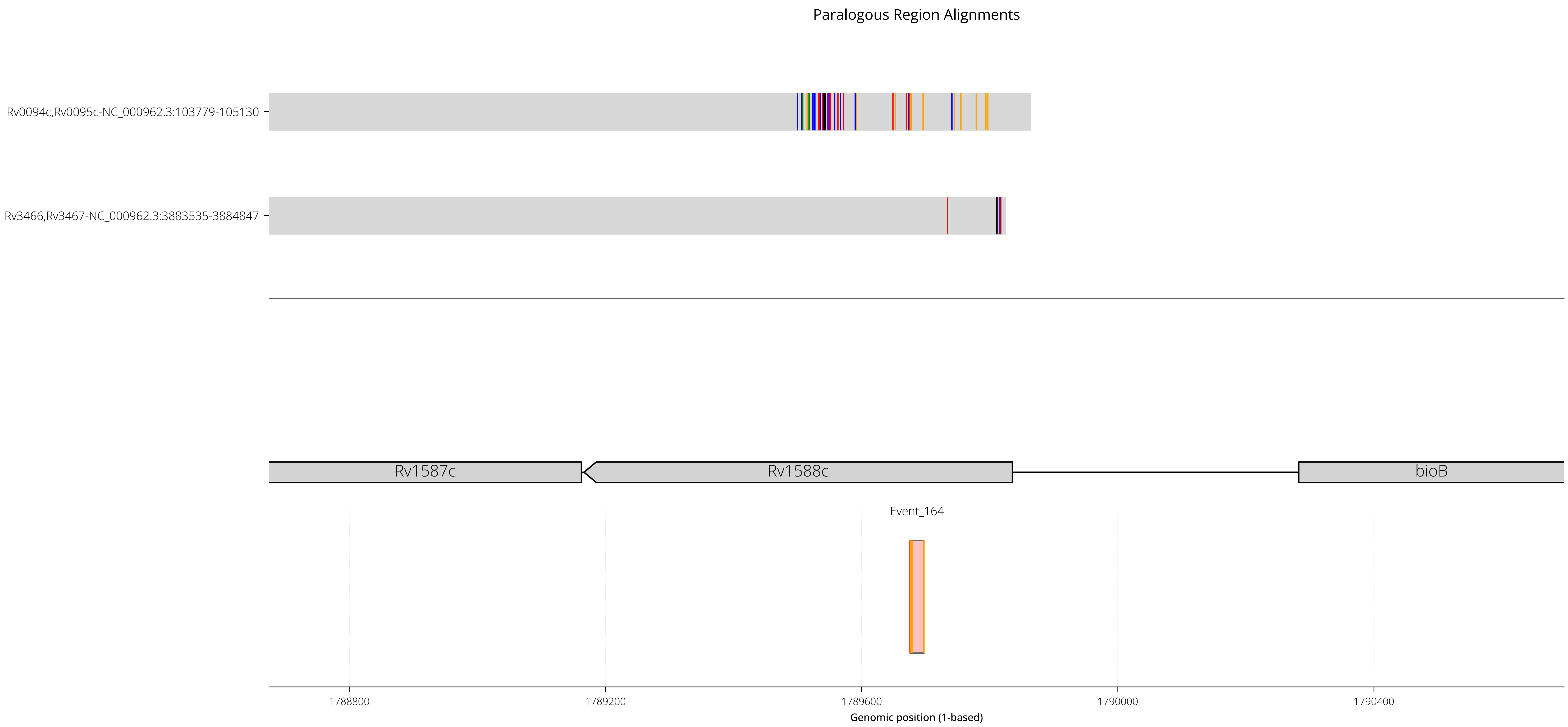

Circos Viz - GCE to Known Paralogs Locations
GCE ID: Event_164

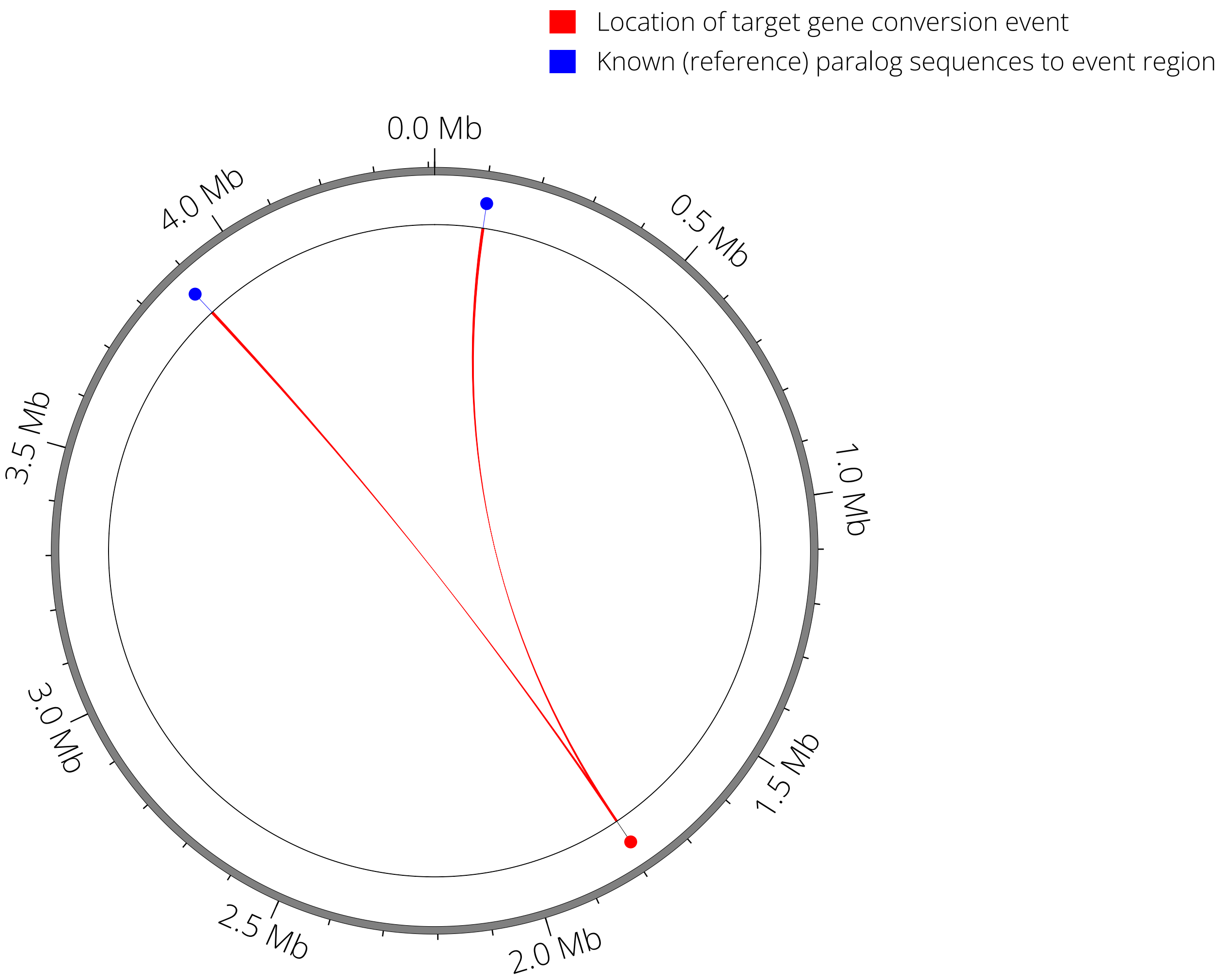

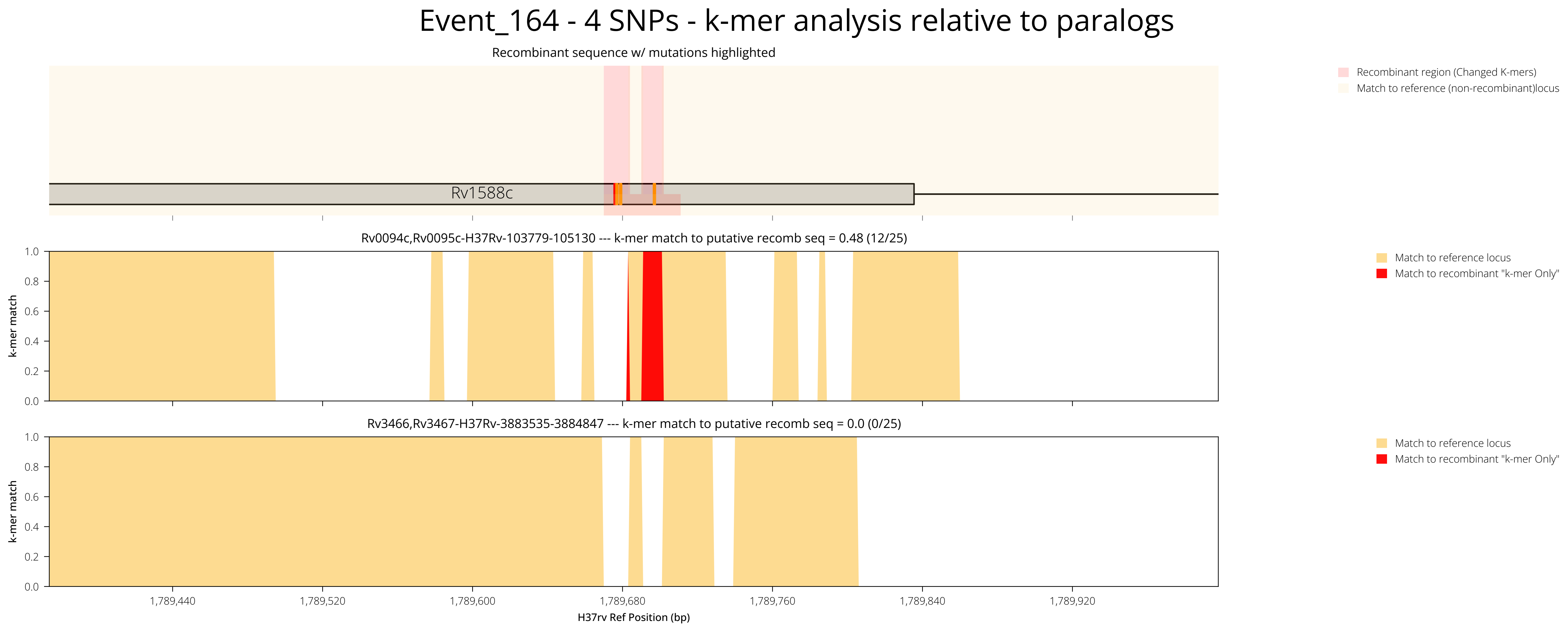

Event_164 - 4 SNPs - k-mer analysis relative to paralogs
Recombinant sequence w/ mutations highlighted
Recombinant region (Changed K-mers)
Match to reference (non-recombinant)locus
Rv1588c
Rv0094c,Rv0095c-H37Rv-103779-105130 --- k-mer match to putative recomb seq = 0.48 (12/25)
Match to reference locus
Match to recombinant "k-mer Only"
k-mer match
1.0
0.8
0.6
0.4
0.2
0.0
Rv3466,Rv3467-H37Rv-3883535-3884847 --- k-mer match to putative recomb seq = 0.0 (0/25)
Match to reference locus
Match to recombinant "k-mer Only"
k-mer match
1,789,440
1,789,520
1,789,600
1,789,680
1,789,760
1,789,840
1,789,920
H37rv Ref Position (bp)

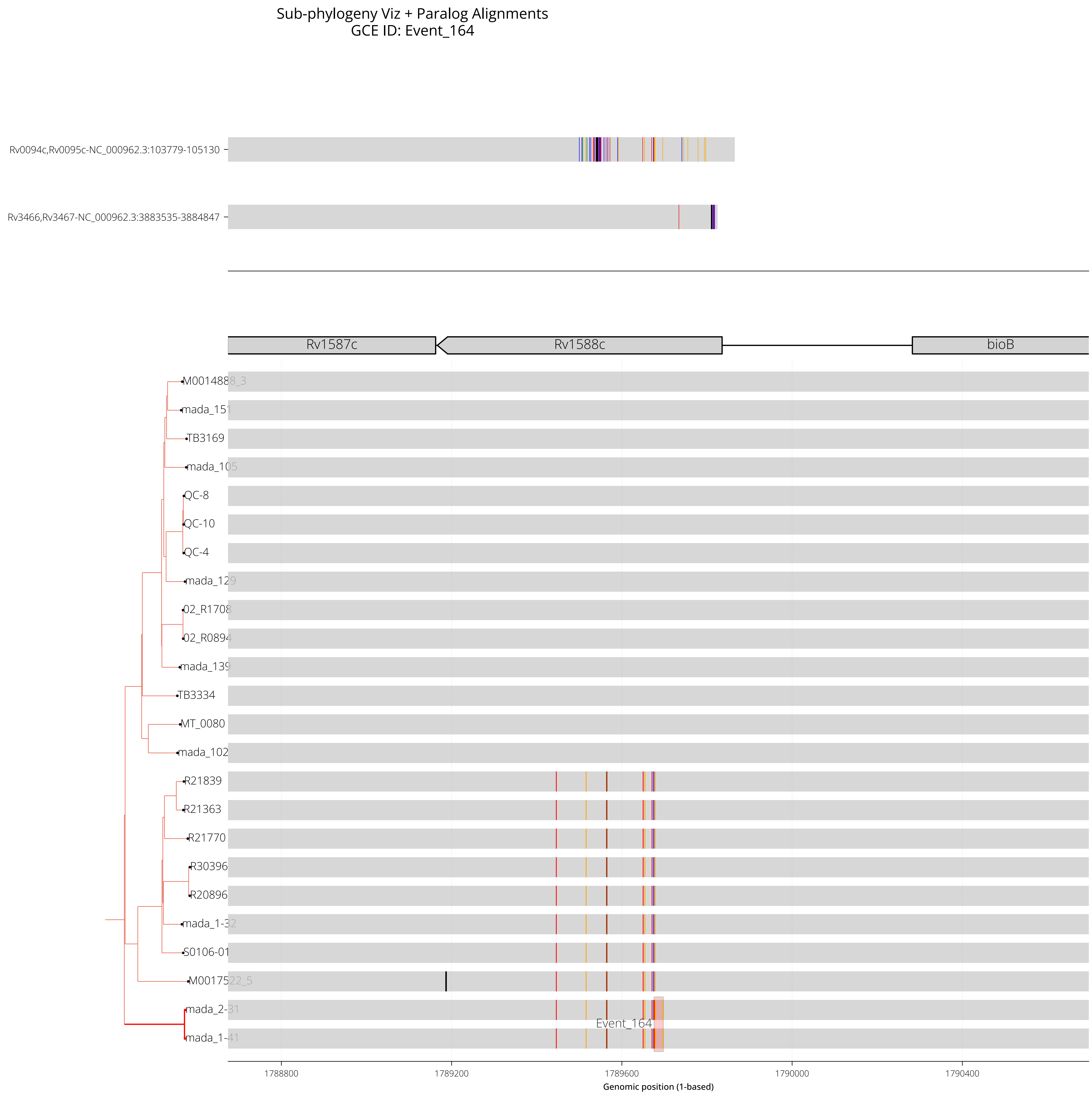

Sub-phylogeny Viz + Paralog Alignments
GCE ID: Event_164
Rv0094c,Rv0095c-NC_000962.3:103779-105130
Rv3466,Rv3467-NC_000962.3:3883535-3884847
Rv1587c
Rv1588c
bioB
M0014888_3
mada_151
TB3169
mada_105
QC-8
QC-10
QC-4
mada_129
02_R1708
02_R0894
mada_139
TB3334
MT_0080
mada_102
R21839
R21363
R21770
R30396
R20896
mada_1-32
S0106-01
M0017522_5
mada_2-31
Event_164
mada_1-41
1788800
1789200
1789600
1790000
1790400
Genomic position (1-based)

Full Phylogeny Viz + Paralog Alignments
GCE ID: Event_164

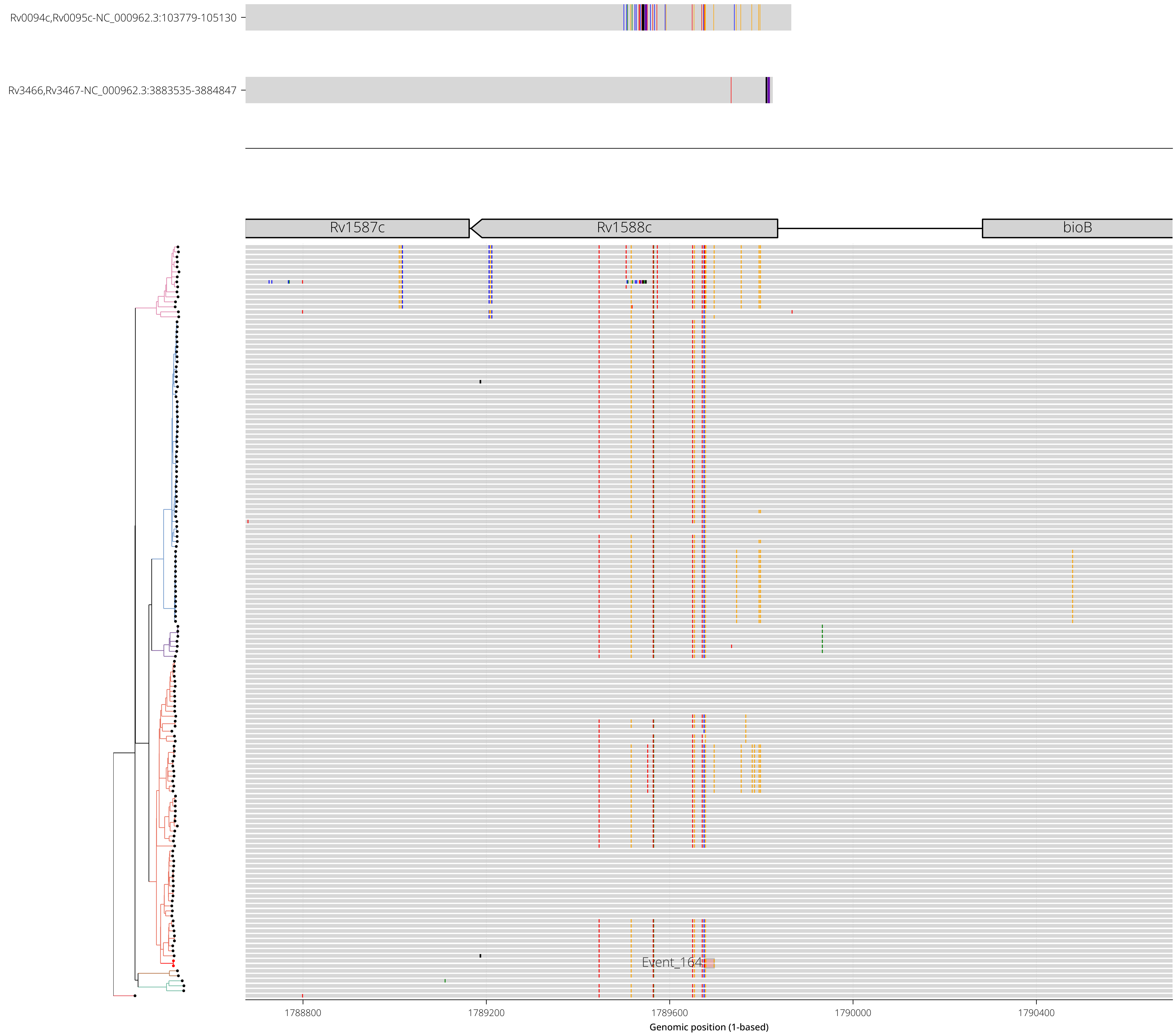

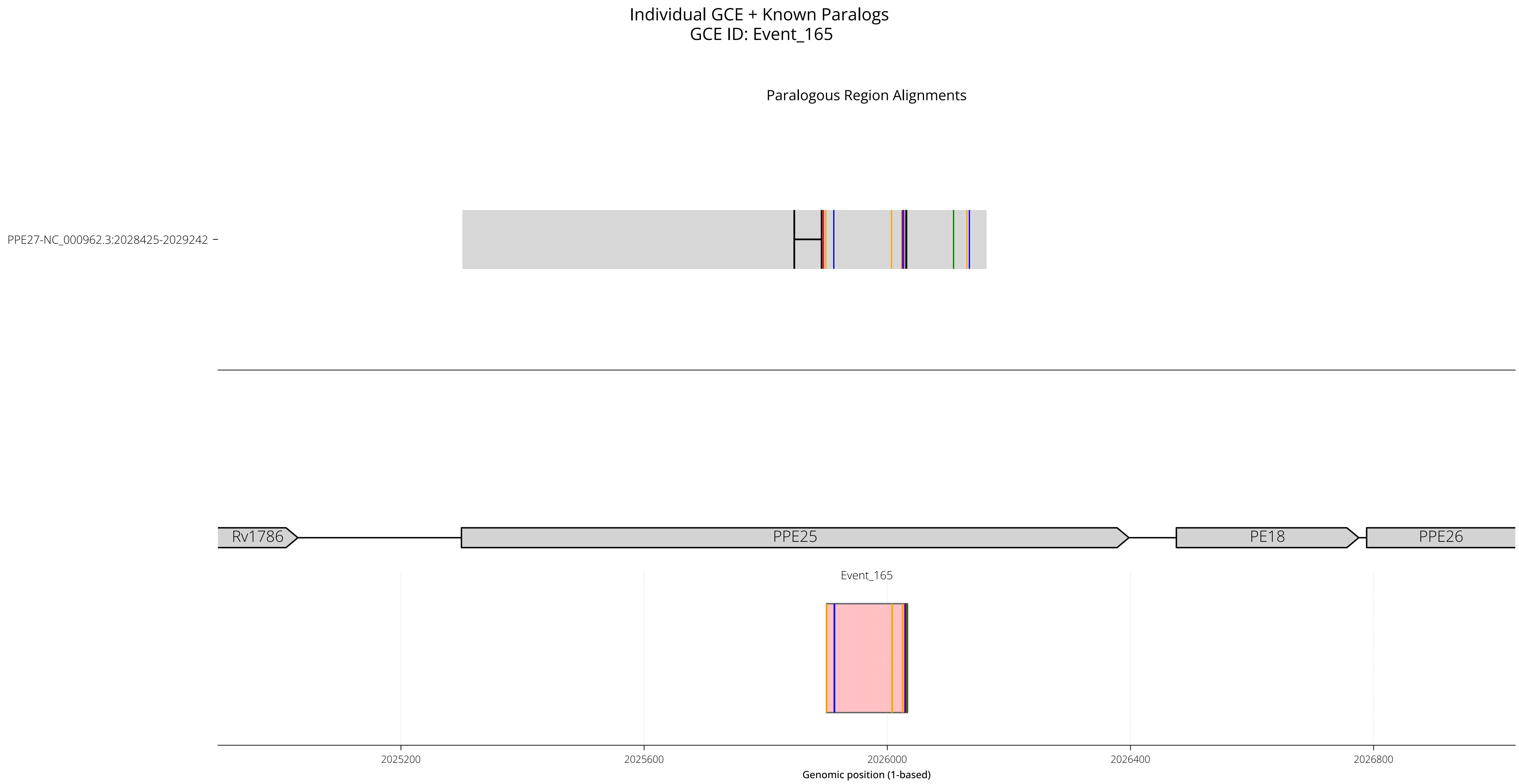

Individual GCE + Known Paralogs
GCE ID: Event_165
Paralogous Region Alignments
PPE27-NC_000962.3:2028425-2029242
Rv1786
PPE25
PE18
PPE26
Event_165
2025200
2025600
2026000
2026400
2026800
Genomic position (1-based)

Circos Viz - GCE to Known Paralogs Locations
GCE ID: Event_165

Location of target gene conversion event
Known (reference) paralog sequences to event region

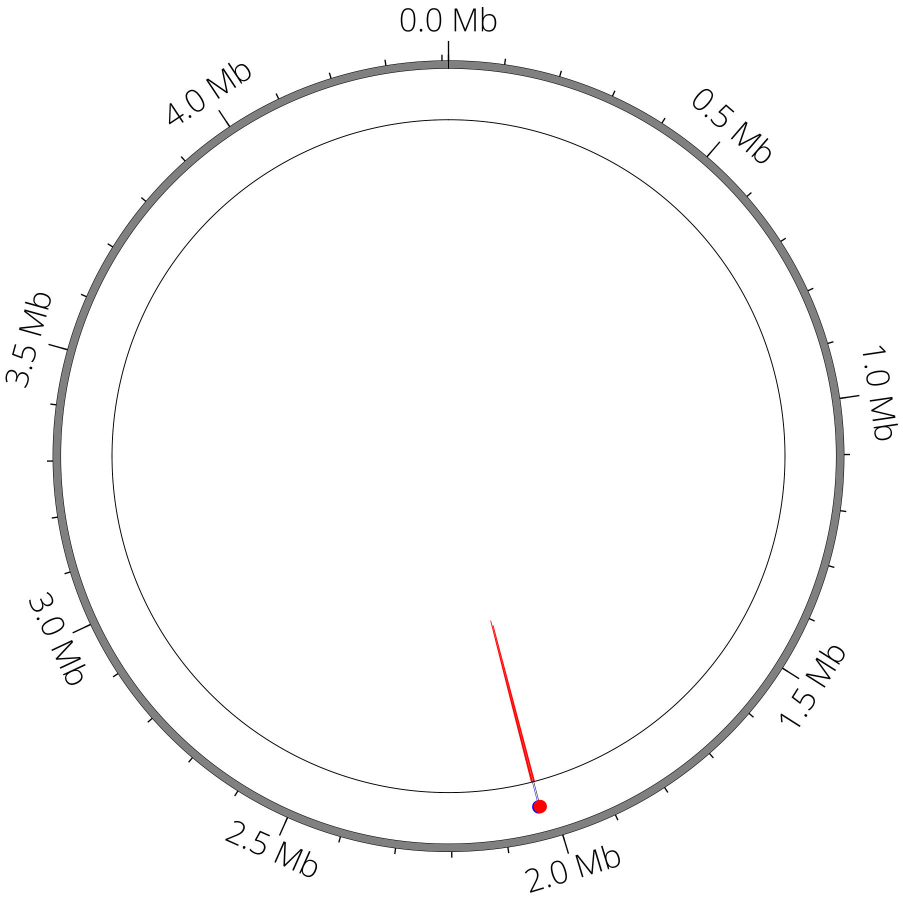

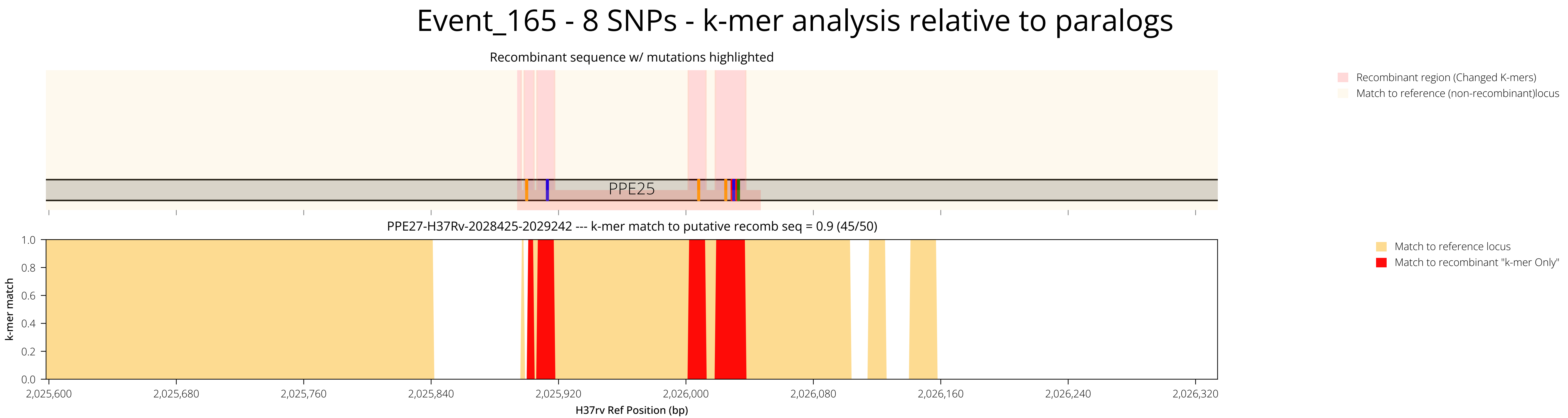
Event_165 - 8 SNPs - k-mer analysis relative to paralogs
Recombinant sequence w/ mutations highlighted
Recombinant region (Changed K-mers)
Match to reference (non-recombinant)locus
PPE25
PPE27-H37Rv-2028425-2029242 --- k-mer match to putative recomb seq = 0.9 (45/50)
Match to reference locus
Match to recombinant "k-mer Only"
k-mer match
1.0
0.8
0.6
0.4
0.2
0.0
2,025,600
2,025,680
2,025,760
2,025,840
2,025,920
2,026,000
2,026,080
2,026,160
2,026,240
2,026,320
H37rv Ref Position (bp)

Sub-phylogeny Viz + Paralog Alignments
GCE ID: Event_165

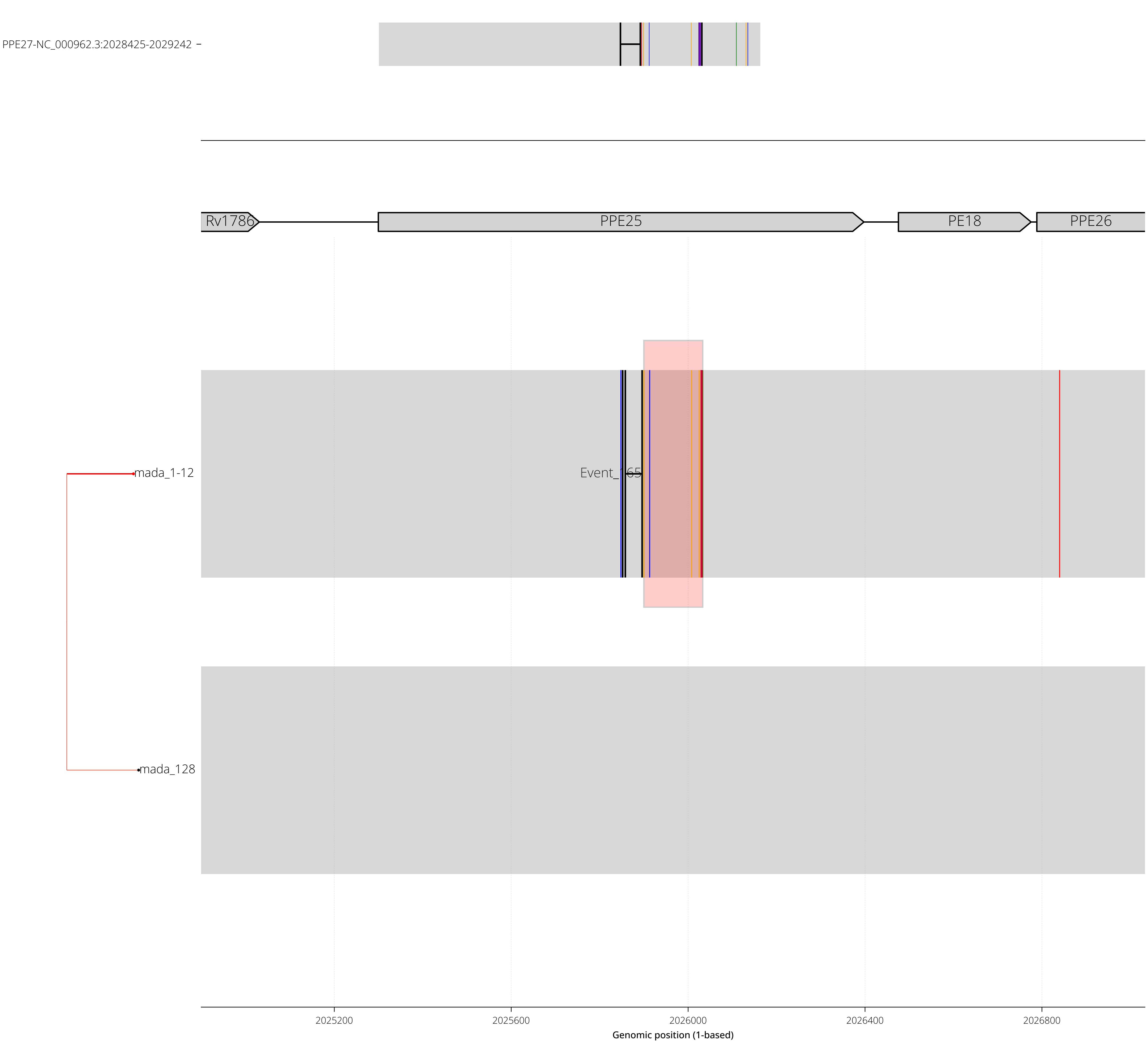

Full Phylogeny Viz + Paralog Alignments
GCE ID: Event_165

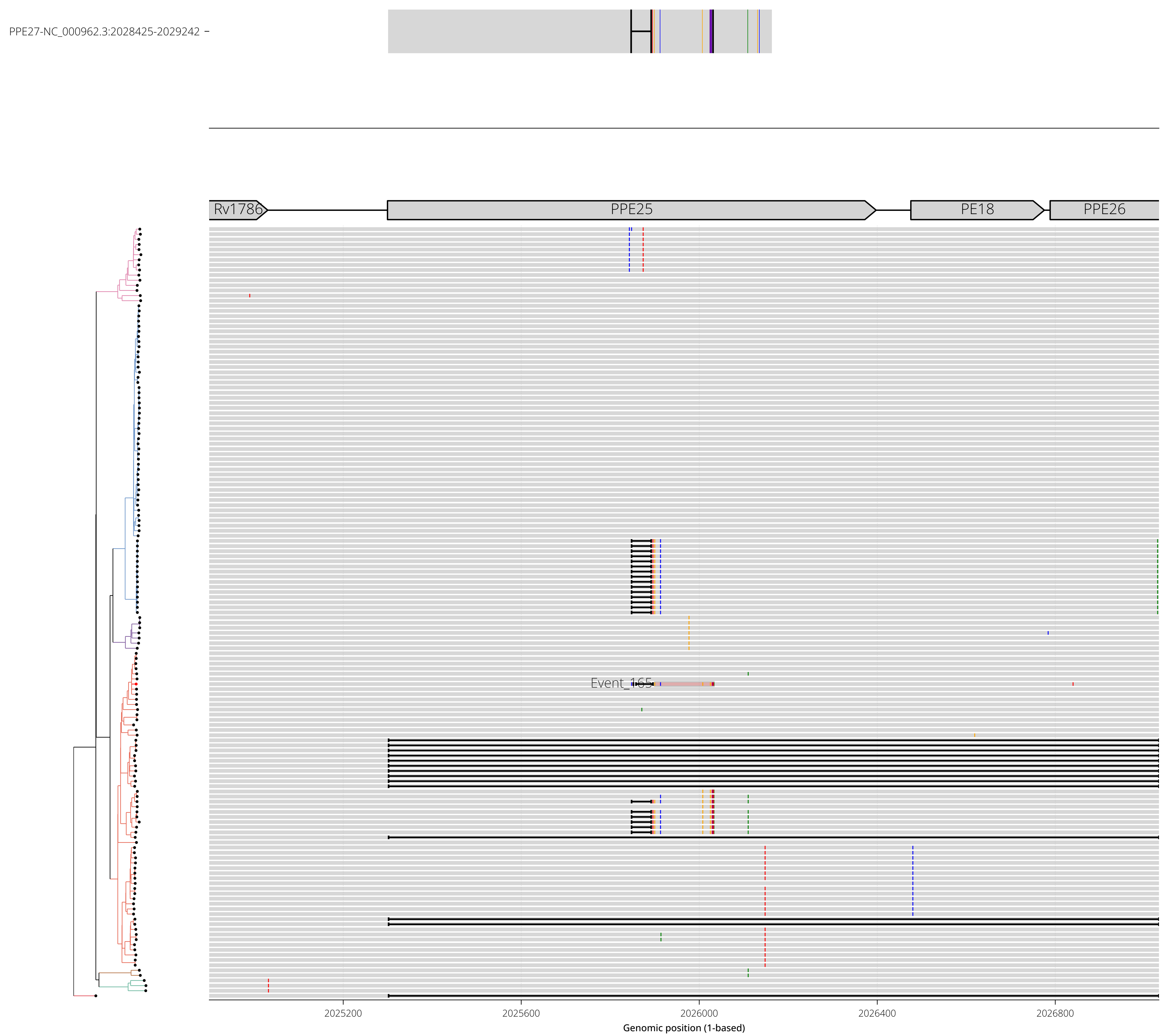

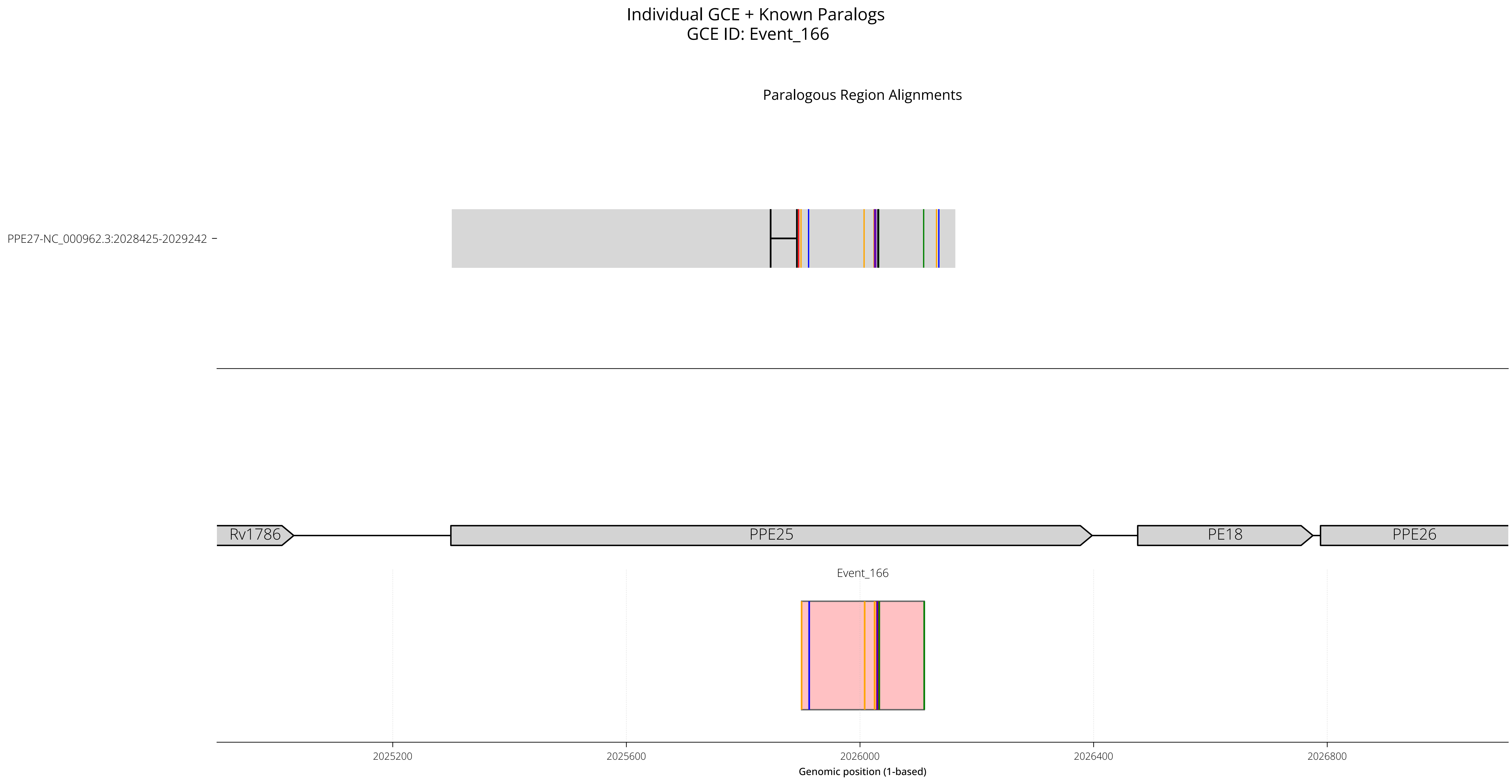
Individual GCE + Known Paralogs
GCE ID: Event_166
Paralogous Region Alignments
PPE27-NC_000962.3:2028425-2029242
Rv1786
PPE25
PE18
PPE26
Event_166
2025200
2025600
2026000
2026400
2026800
Genomic position (1-based)

Circos Viz - GCE to Known Paralogs Locations
GCE ID: Event_166

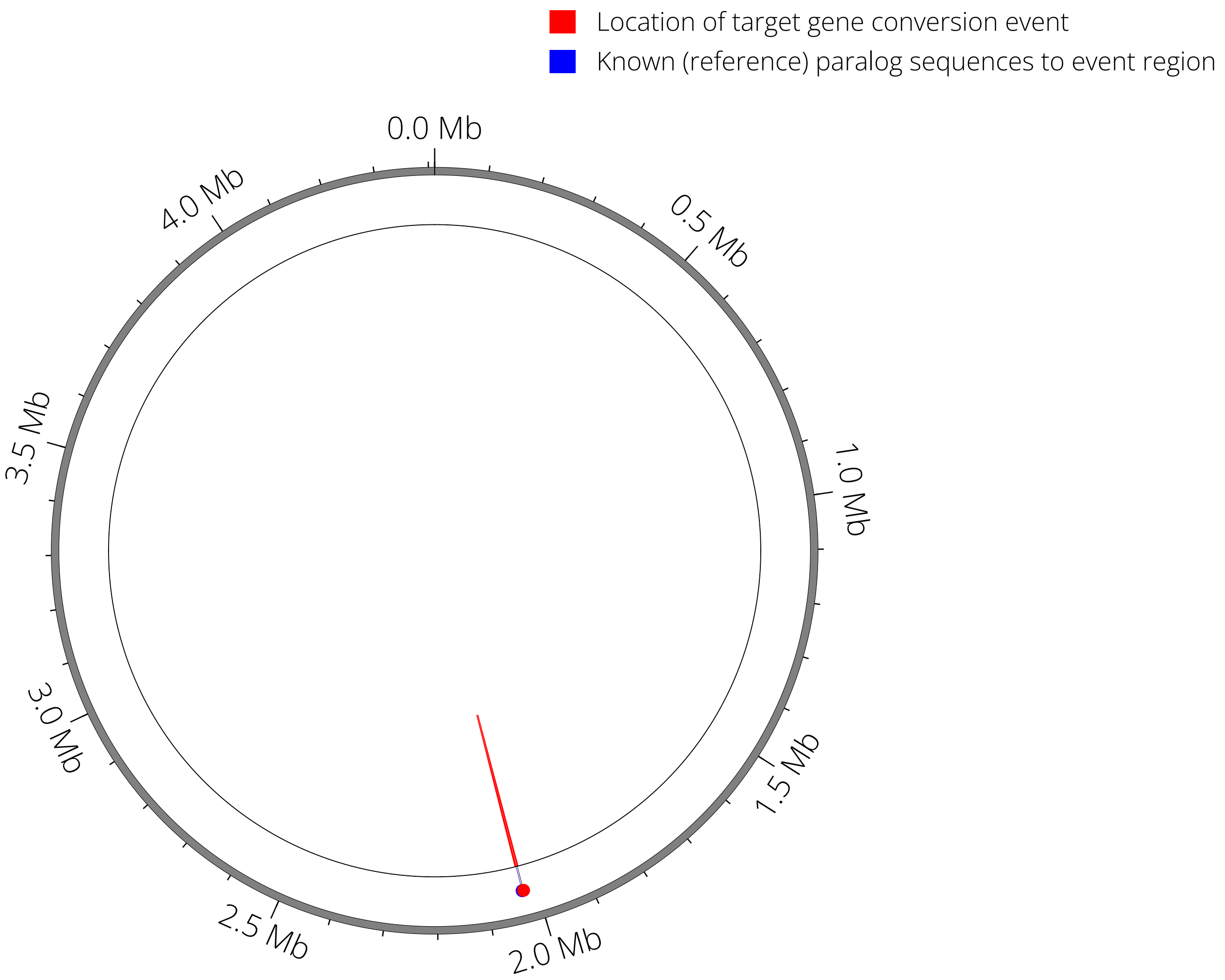

# Event_166 - 9 SNPs - k-mer analysis relative to paralogs

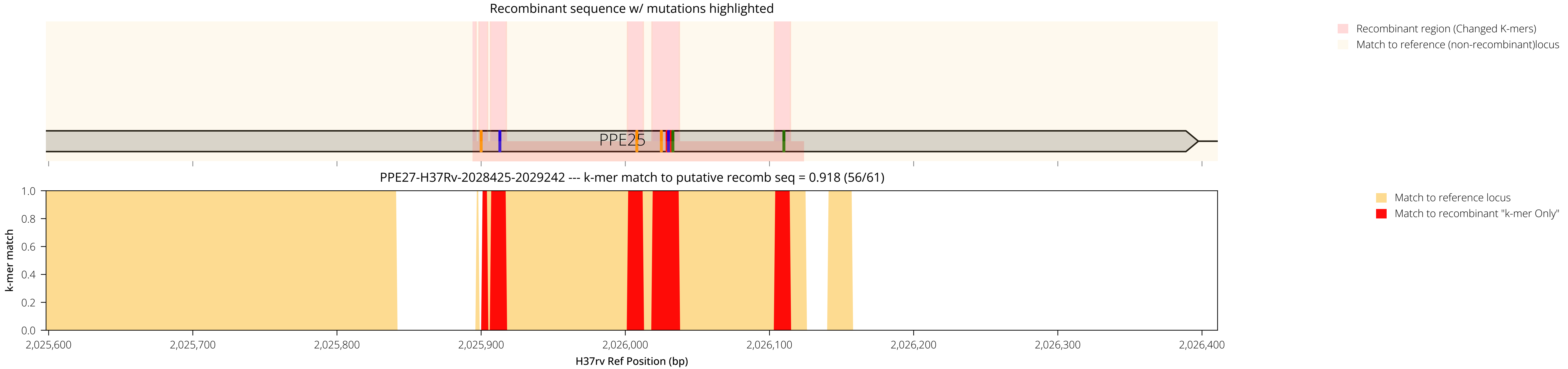

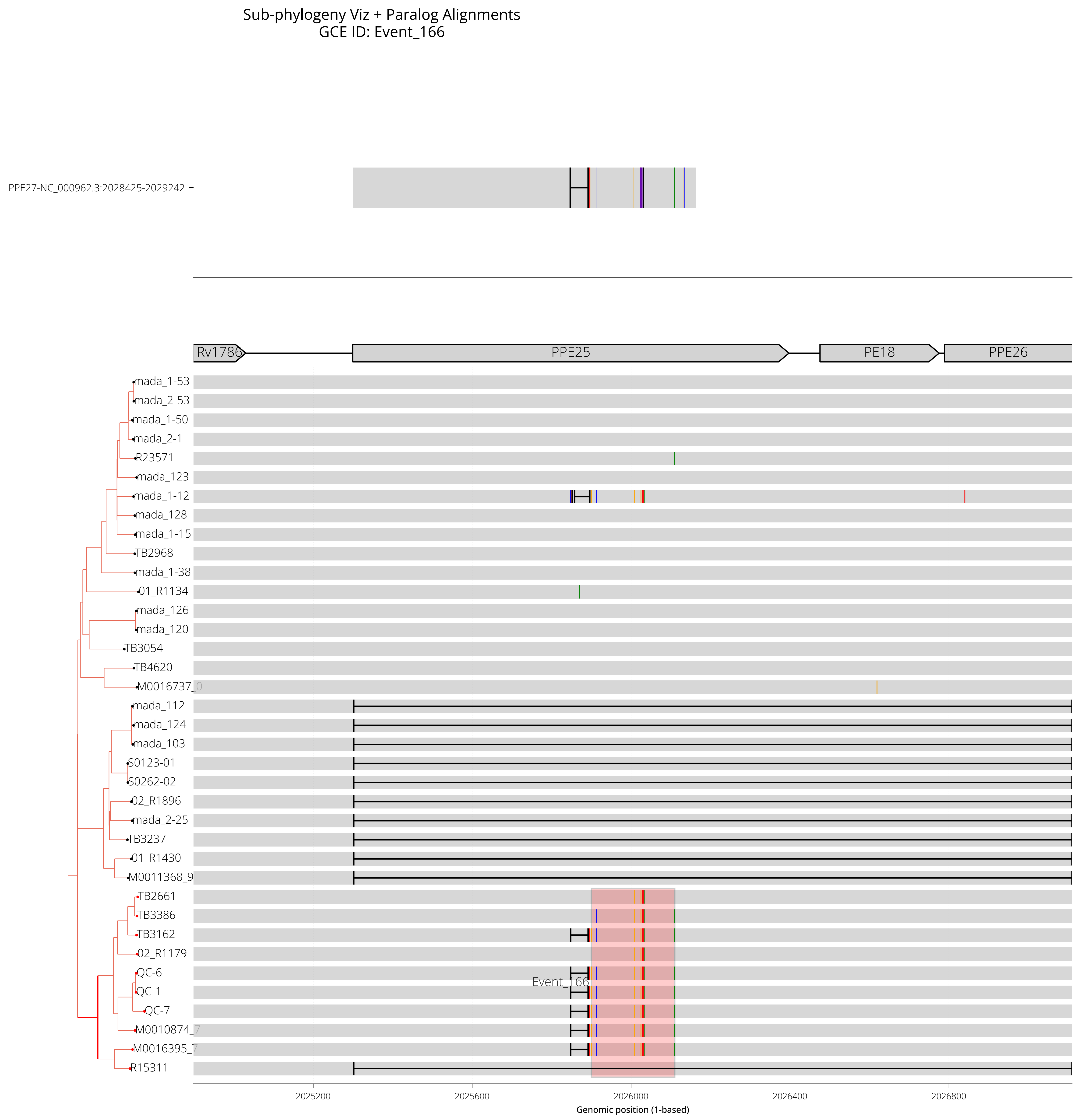

Sub-phylogeny Viz + Paralog Alignments
GCE ID: Event_166
PPE27-NC_000962.3:2028425-2029242
Rv1786
PPE25
PE18
PPE26
mada_1-53
mada_2-53
mada_1-50
mada_2-1
R23571
mada_123
mada_1-12
mada_128
mada_1-15
TB2968
mada_1-38
01_R1134
mada_126
mada_120
TB3054
TB4620
M0016737_0
mada_112
mada_124
mada_103
S0123-01
S0262-02
02_R1896
mada_2-25
TB3237
01_R1430
M0011368_9
TB2661
TB3386
TB3162
02_R1179
QC-6
QC-1
QC-7
M0010874_7
M0016395_7
R15311
Event_166
2025200
2025600
2026000
2026400
2026800
Genomic position (1-based)

Full Phylogeny Viz + Paralog Alignments
GCE ID: Event_166

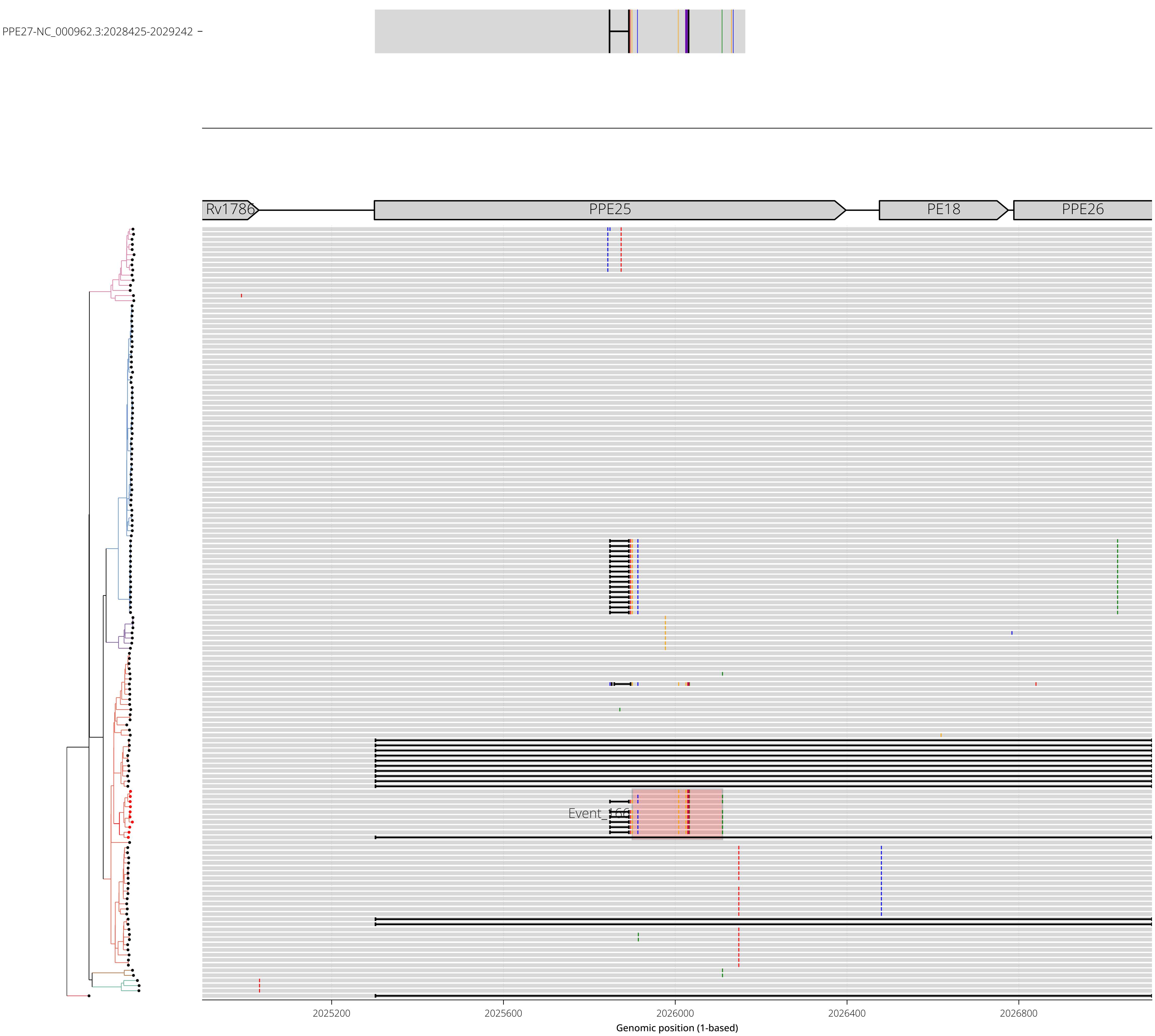

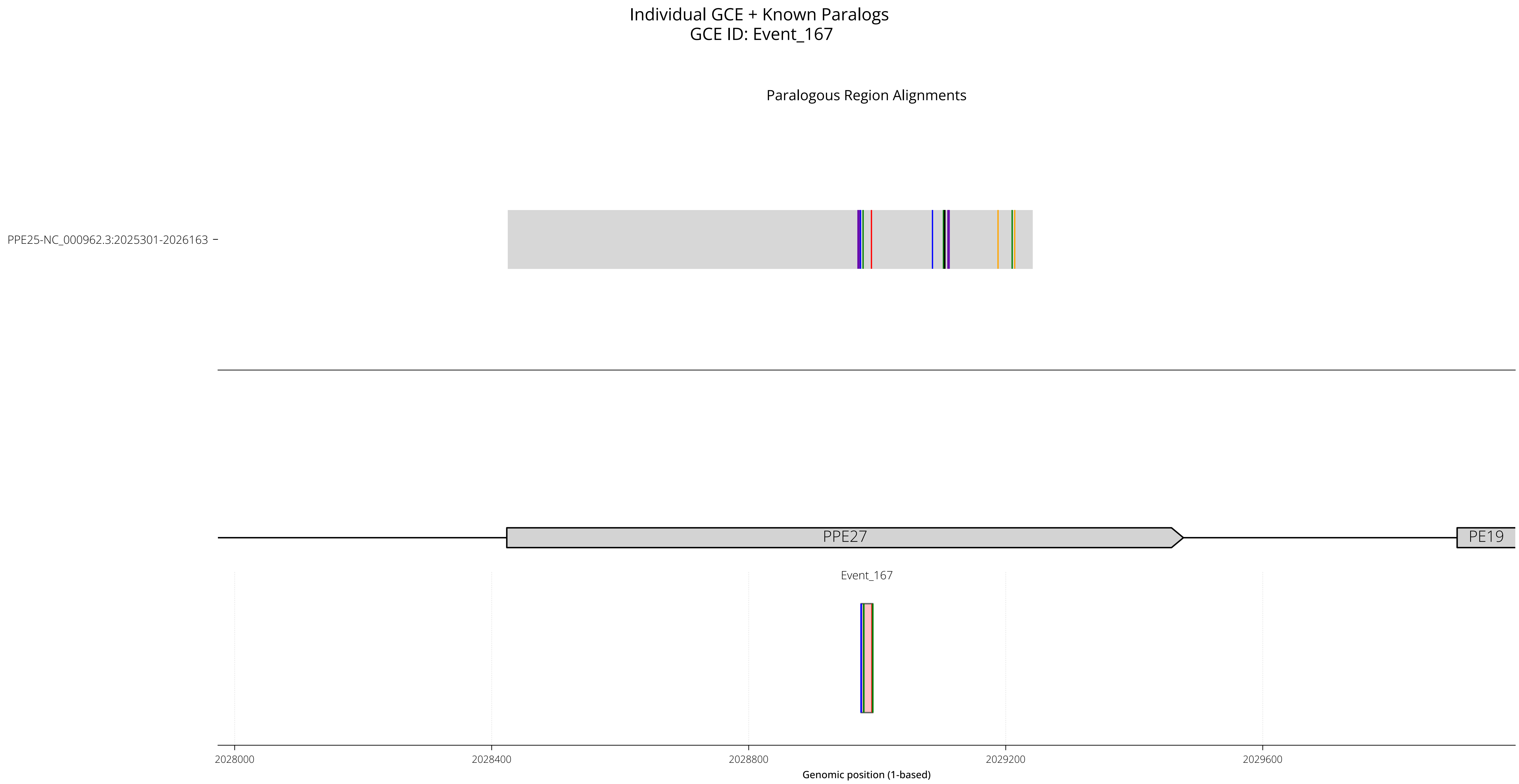

Individual GCE + Known Paralogs
GCE ID: Event_167
Paralogous Region Alignments
PPE25-NC_000962.3:2025301-2026163
PPE27
PE19
Event_167
2028000
2028400
2028800
2029200
2029600
Genomic position (1-based)

Circos Viz - GCE to Known Paralogs Locations
GCE ID: Event_167

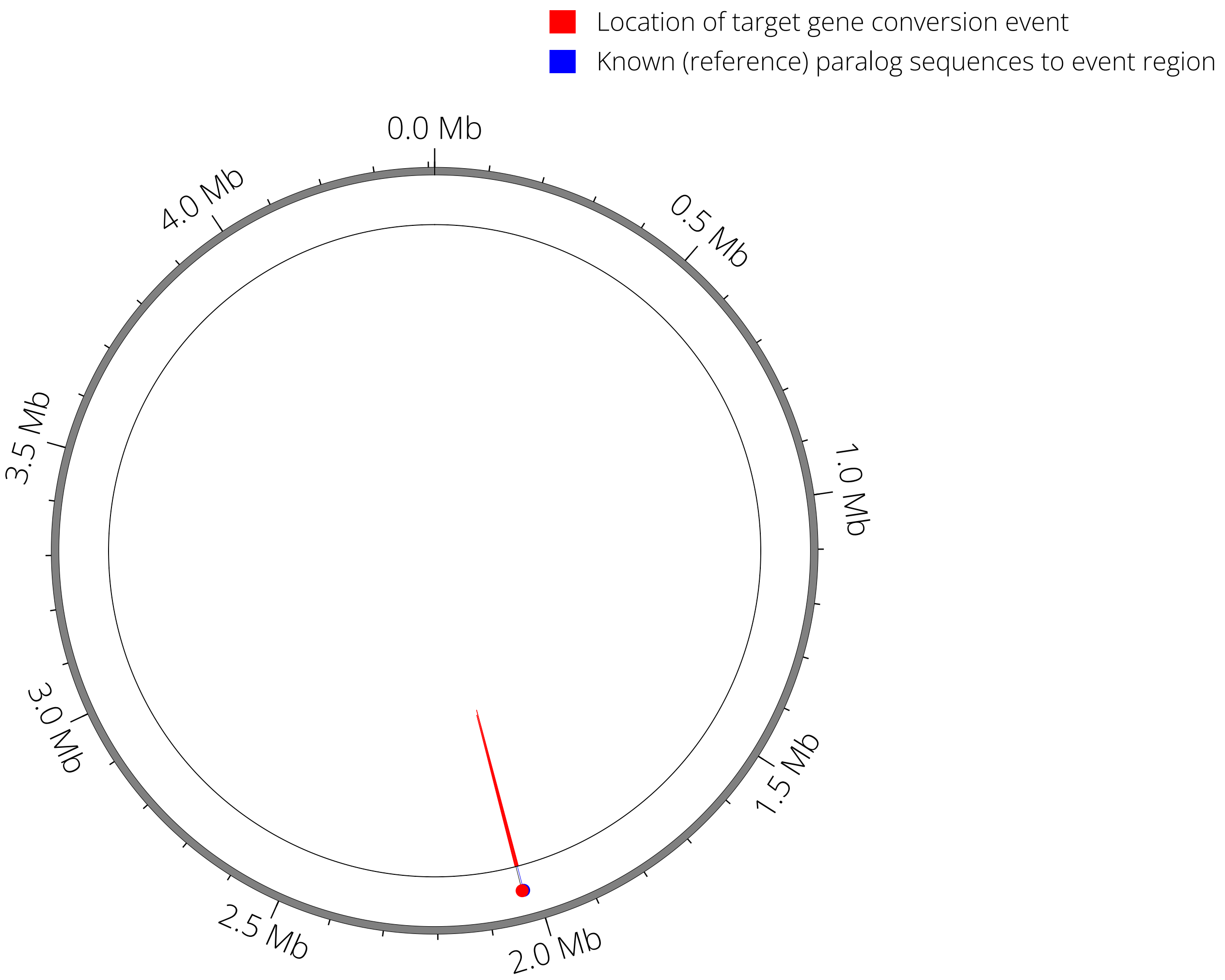

# Event_167 - 4 SNPs - k-mer analysis relative to paralogs

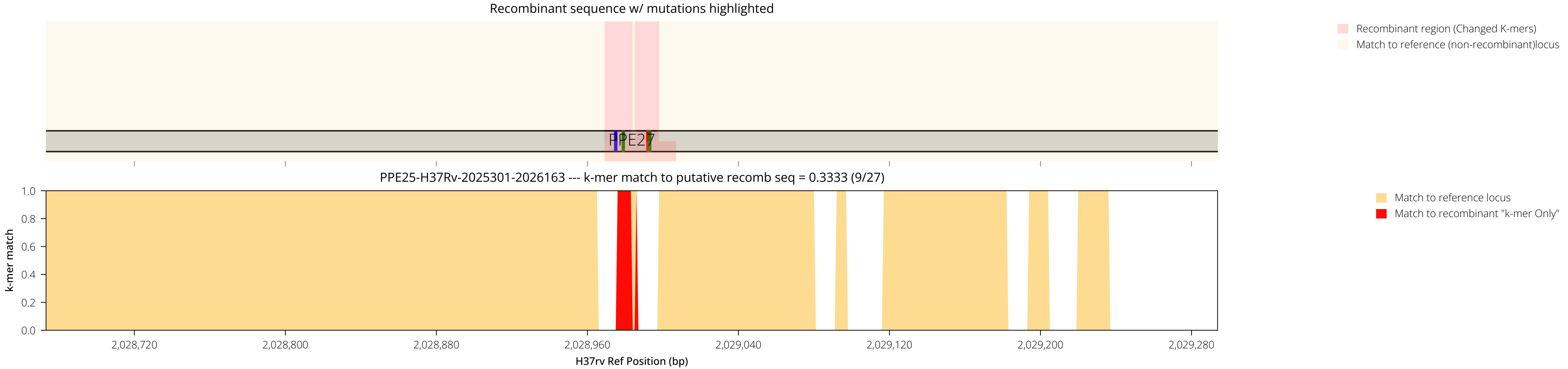

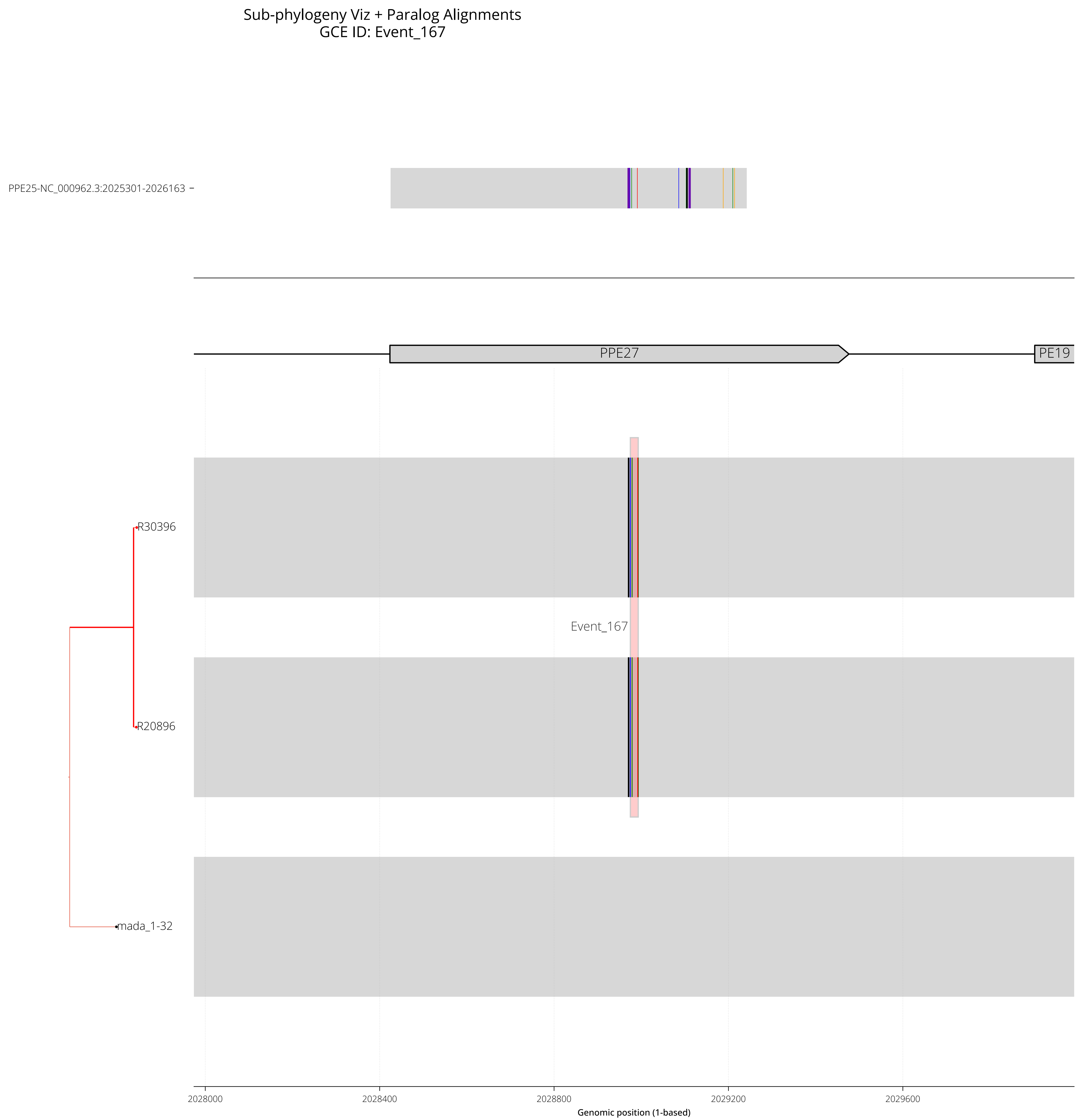
Sub-phylogeny Viz + Paralog Alignments
GCE ID: Event_167
PPE25-NC_000962.3:2025301-2026163
PPE27
PE19
R30396
Event_167
R20896
mada_1-32
2028000
2028400
2028800
2029200
2029600
Genomic position (1-based)

Full Phylogeny Viz + Paralog Alignments
GCE ID: Event_167

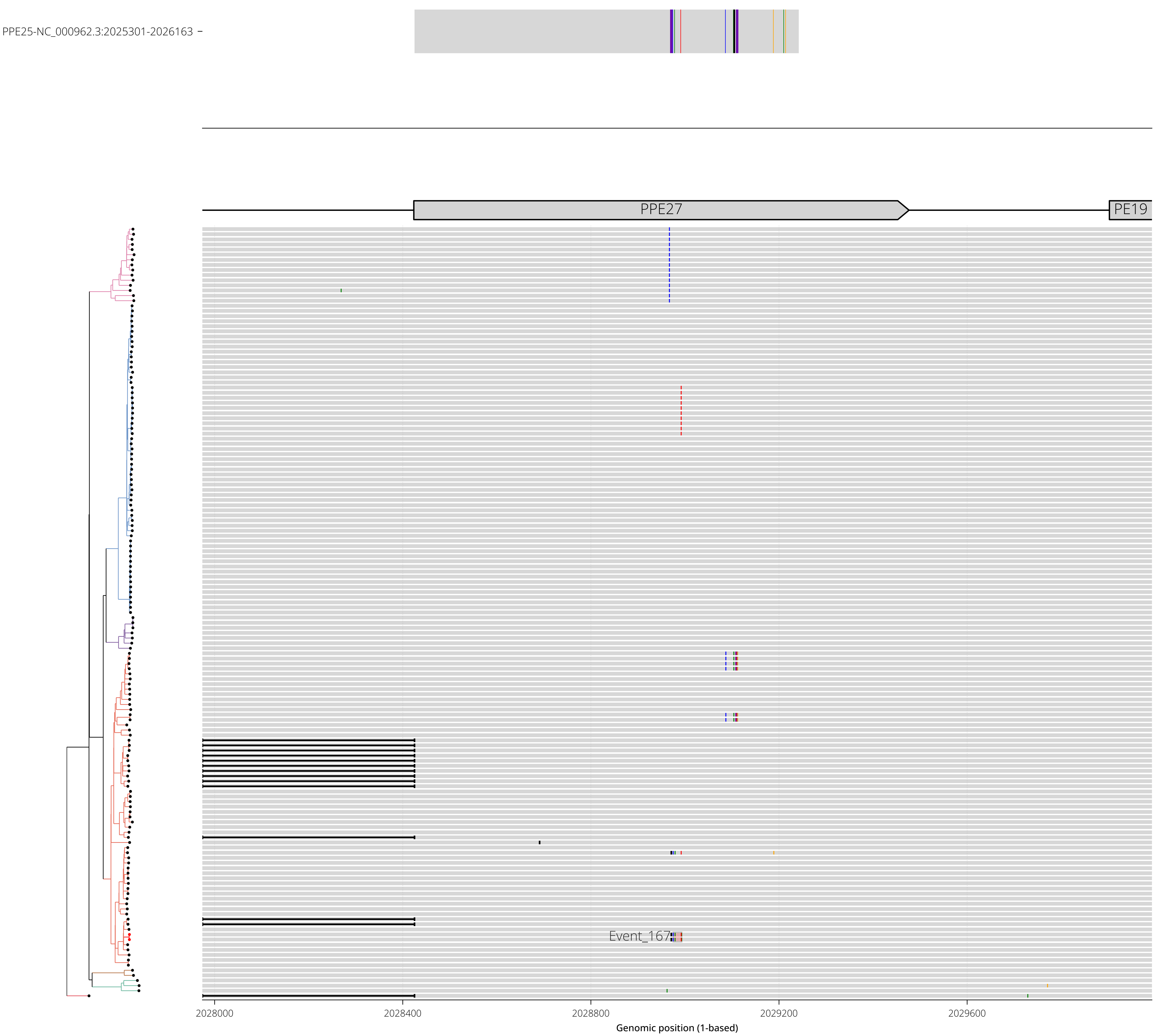

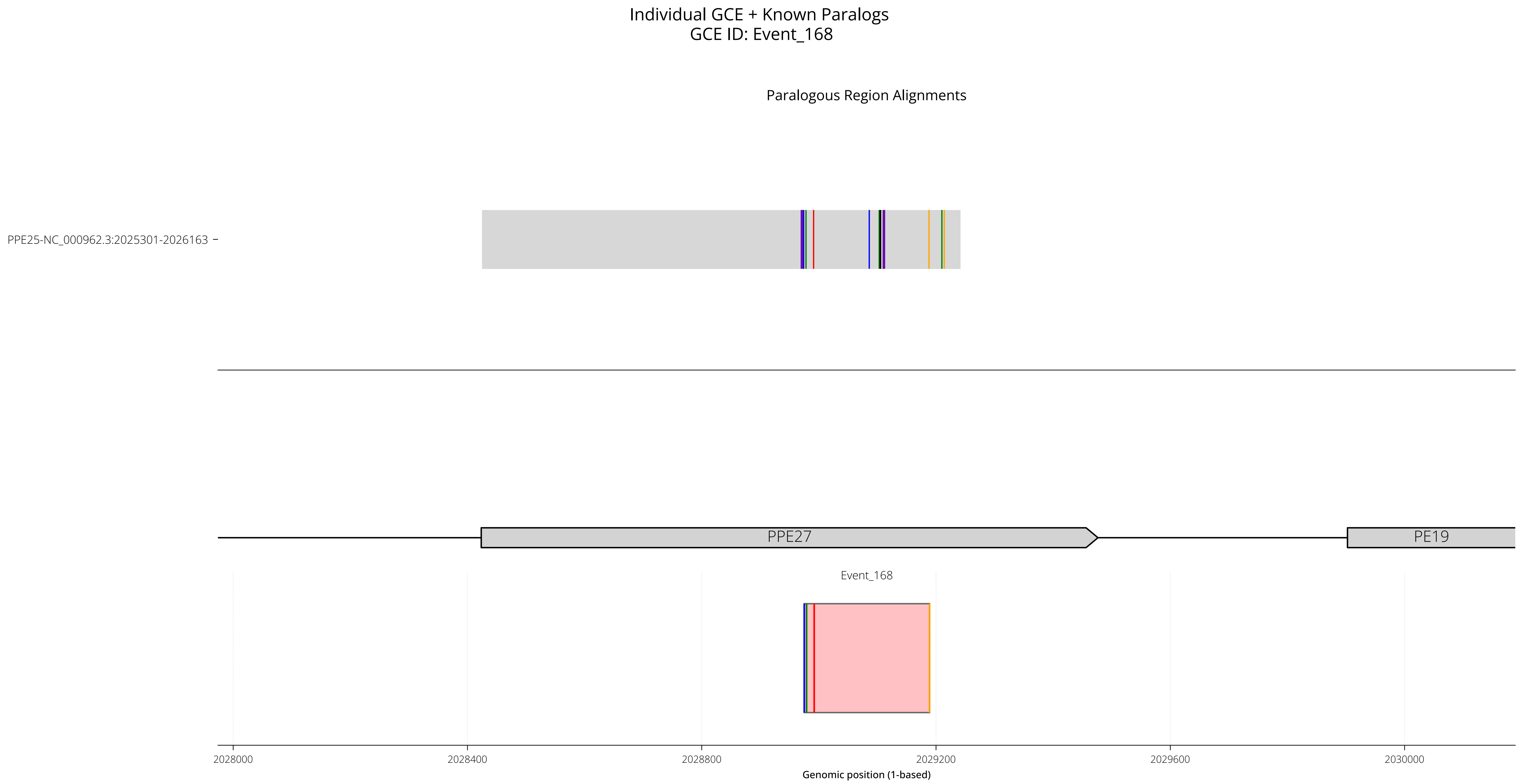

Individual GCE + Known Paralogs
GCE ID: Event_168
Paralogous Region Alignments
PPE25-NC_000962.3:2025301-2026163
PPE27
PE19
Event_168
2028000
2028400
2028800
2029200
2029600
2030000
Genomic position (1-based)

Circos Viz - GCE to Known Paralogs Locations
GCE ID: Event_168

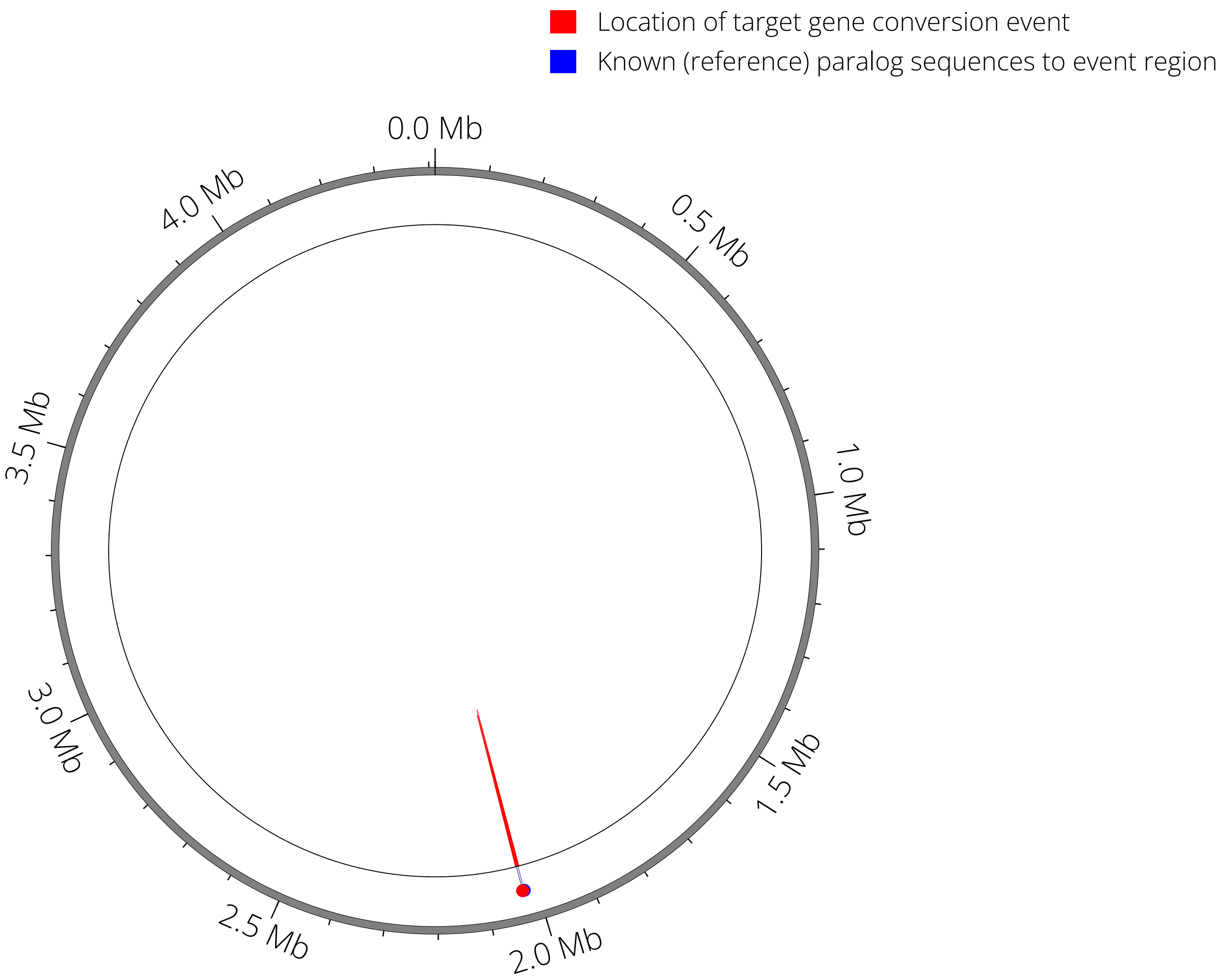

# Event_168 - 4 SNPs - k-mer analysis relative to paralogs

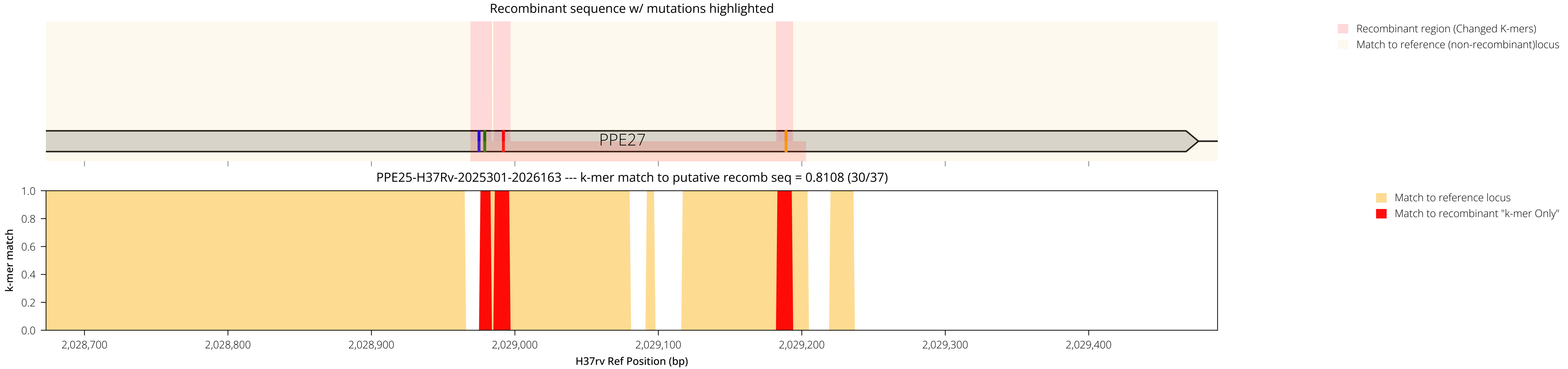

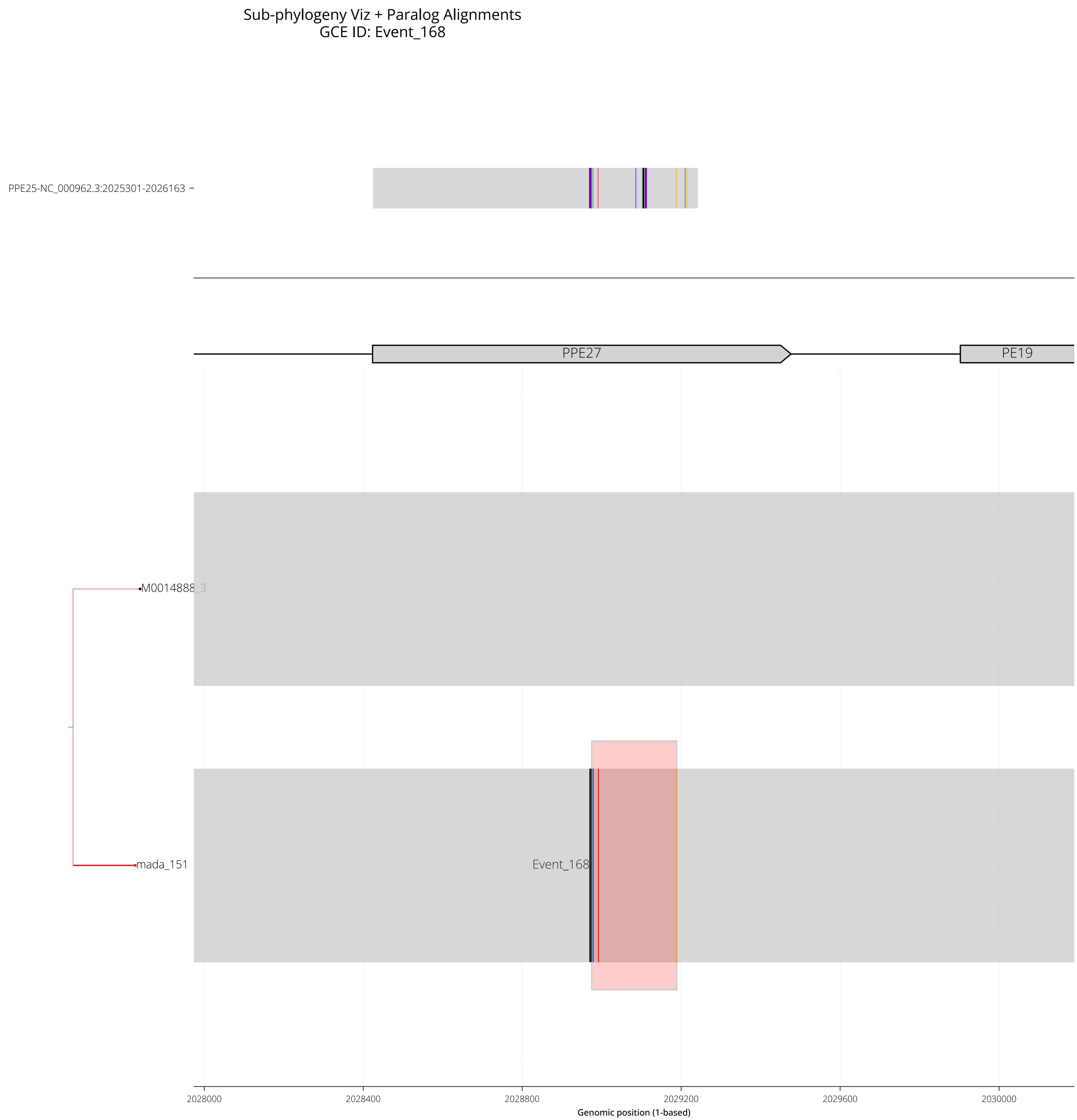

Sub-phylogeny Viz + Paralog Alignments
GCE ID: Event_168
PPE25-NC_000962.3:2025301-2026163
PPE27
PE19
M0014888_3
mada_151
Event_168
2028000
2028400
2028800
2029200
2029600
2030000
Genomic position (1-based)

Full Phylogeny Viz + Paralog Alignments
GCE ID: Event_168

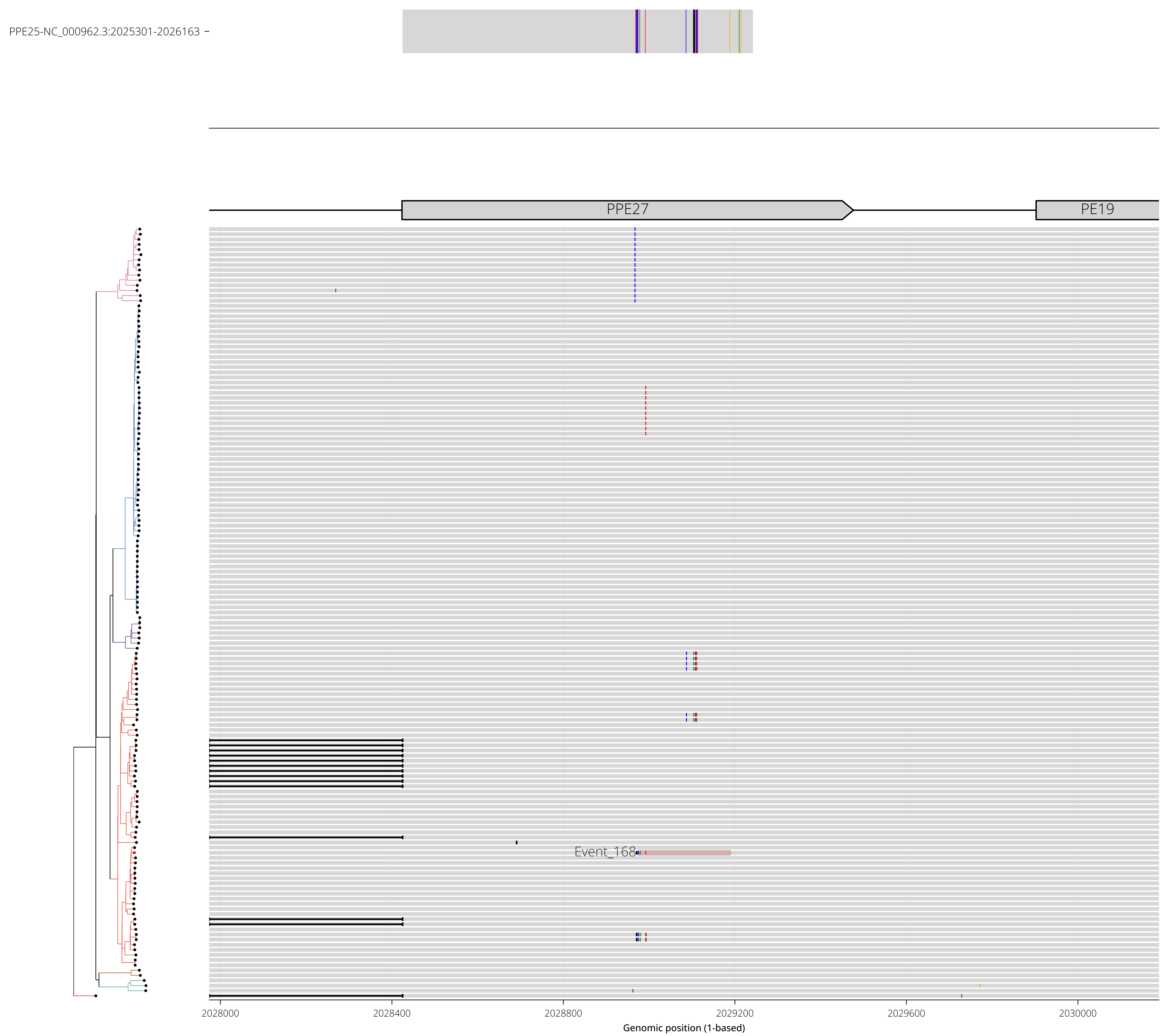

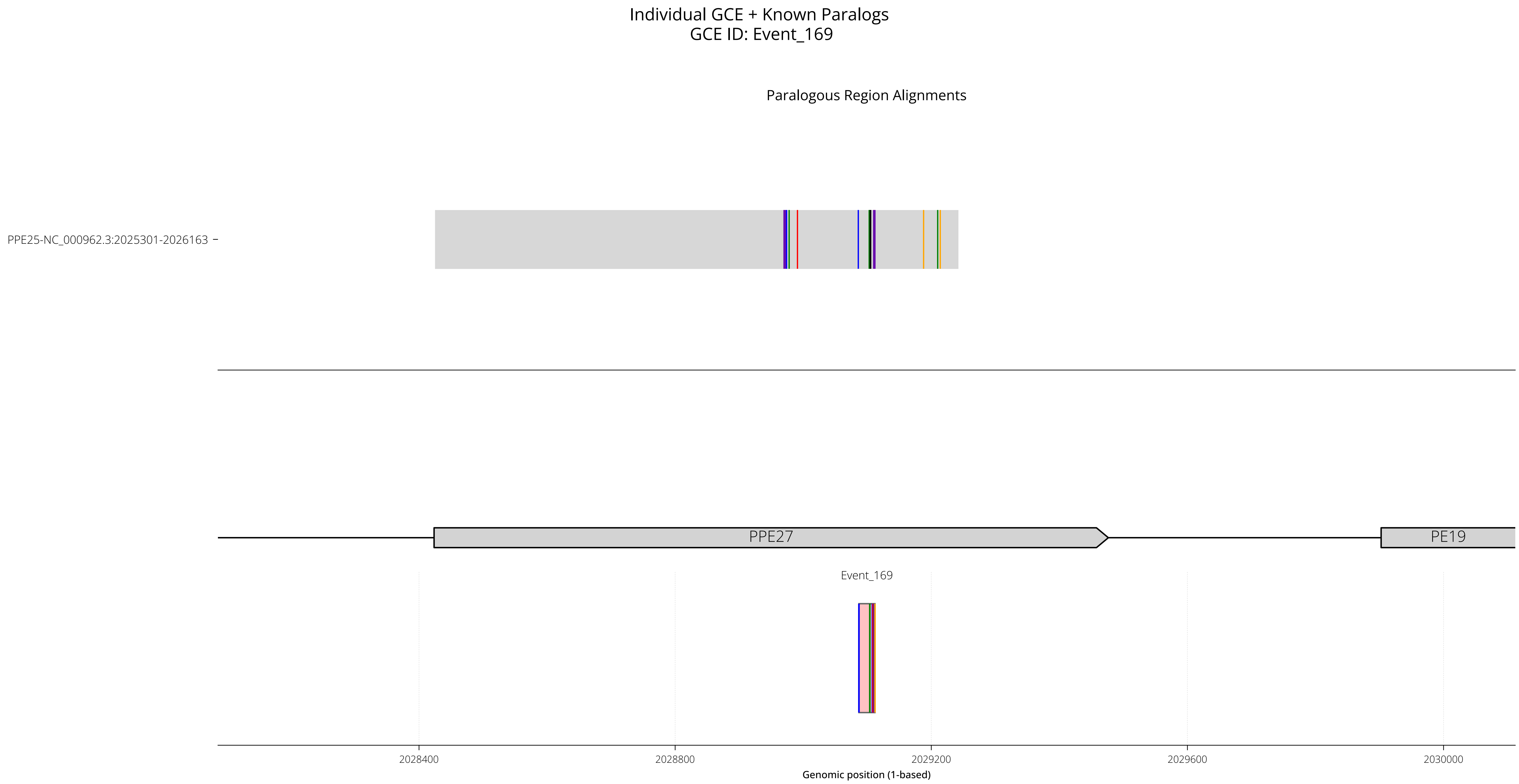
Individual GCE + Known Paralogs
GCE ID: Event_169
Paralogous Region Alignments
PPE25-NC_000962.3:2025301-2026163
PPE27
PE19
Event_169
2028400
2028800
2029200
2029600
2030000
Genomic position (1-based)

Circos Viz - GCE to Known Paralogs Locations
GCE ID: Event_169

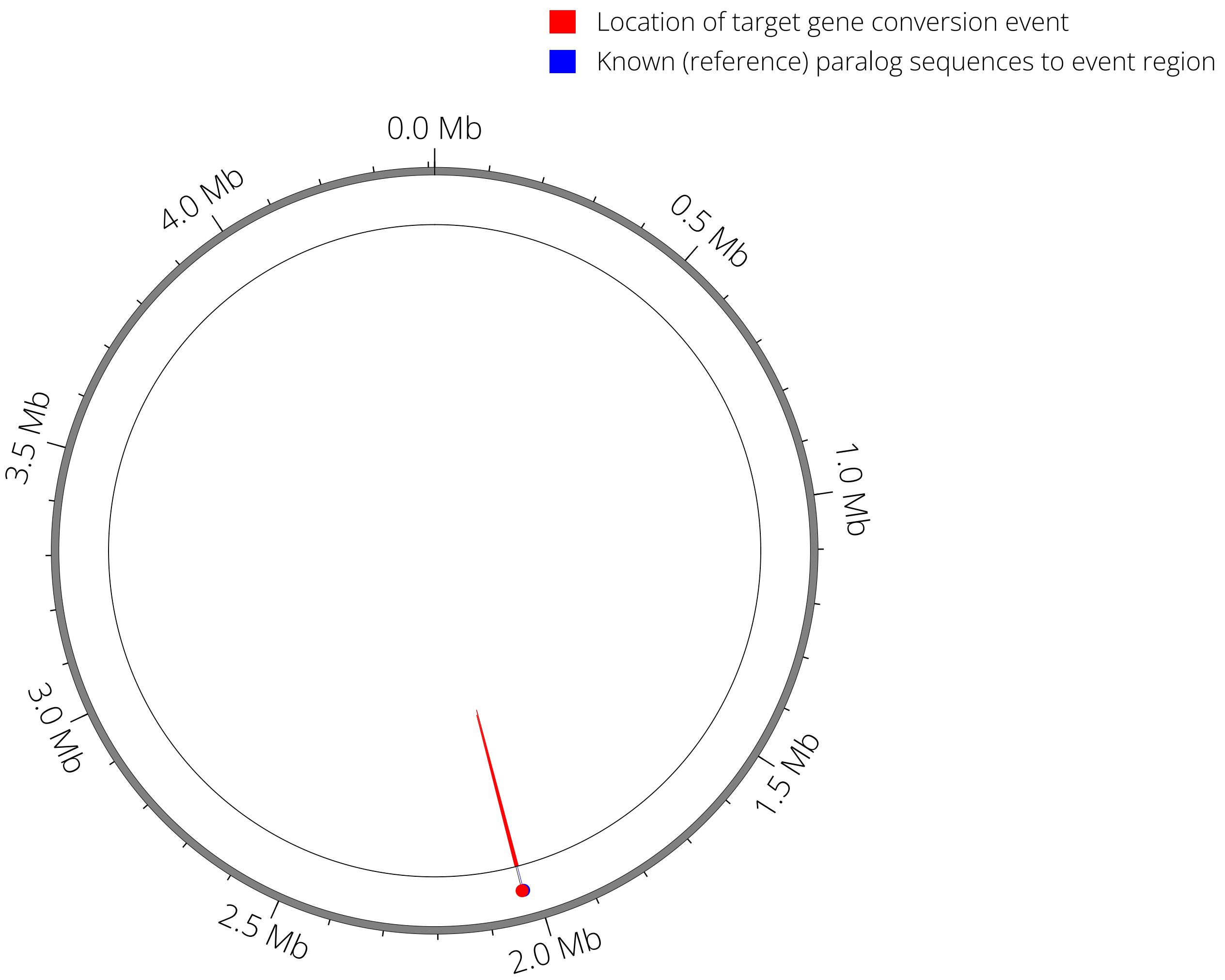

# Event_169 - 6 SNPs - k-mer analysis relative to paralogs

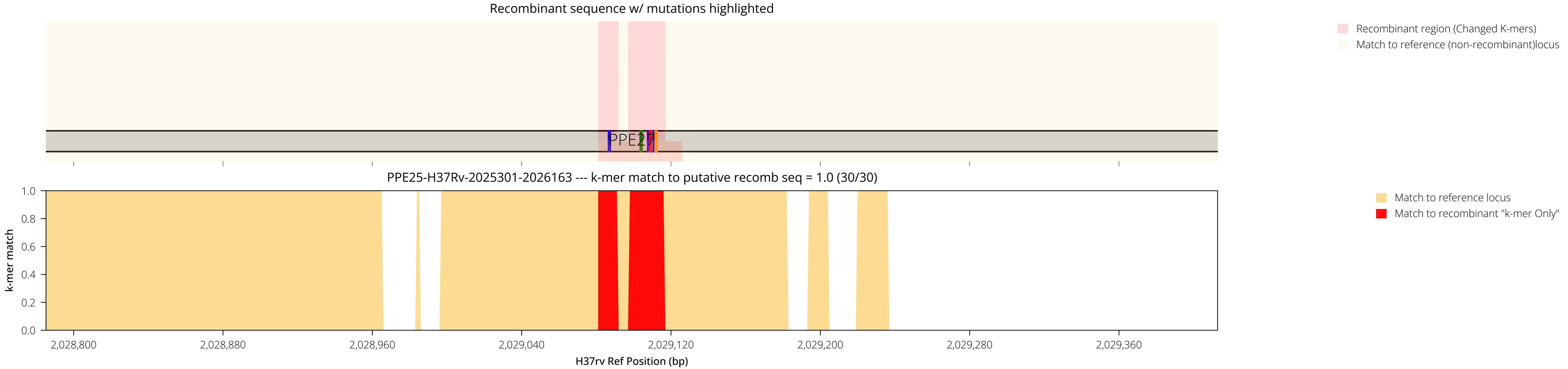

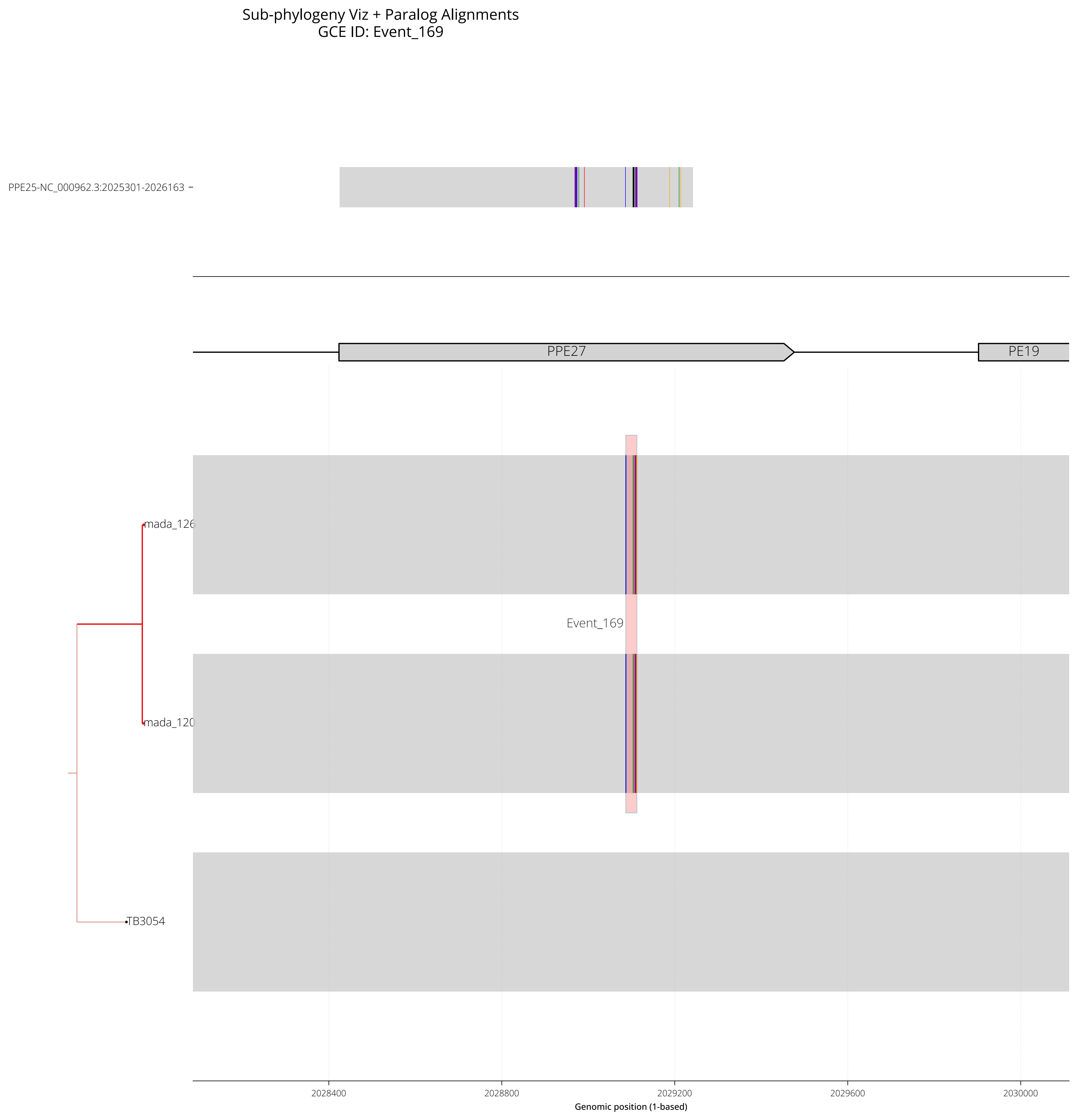

Sub-phylogeny Viz + Paralog Alignments
GCE ID: Event_169
PPE25-NC_000962.3:2025301-2026163
PPE27
PE19
mada_126
mada_120
TB3054
Event_169
2028400
2028800
2029200
2029600
2030000
Genomic position (1-based)

Full Phylogeny Viz + Paralog Alignments
GCE ID: Event_169

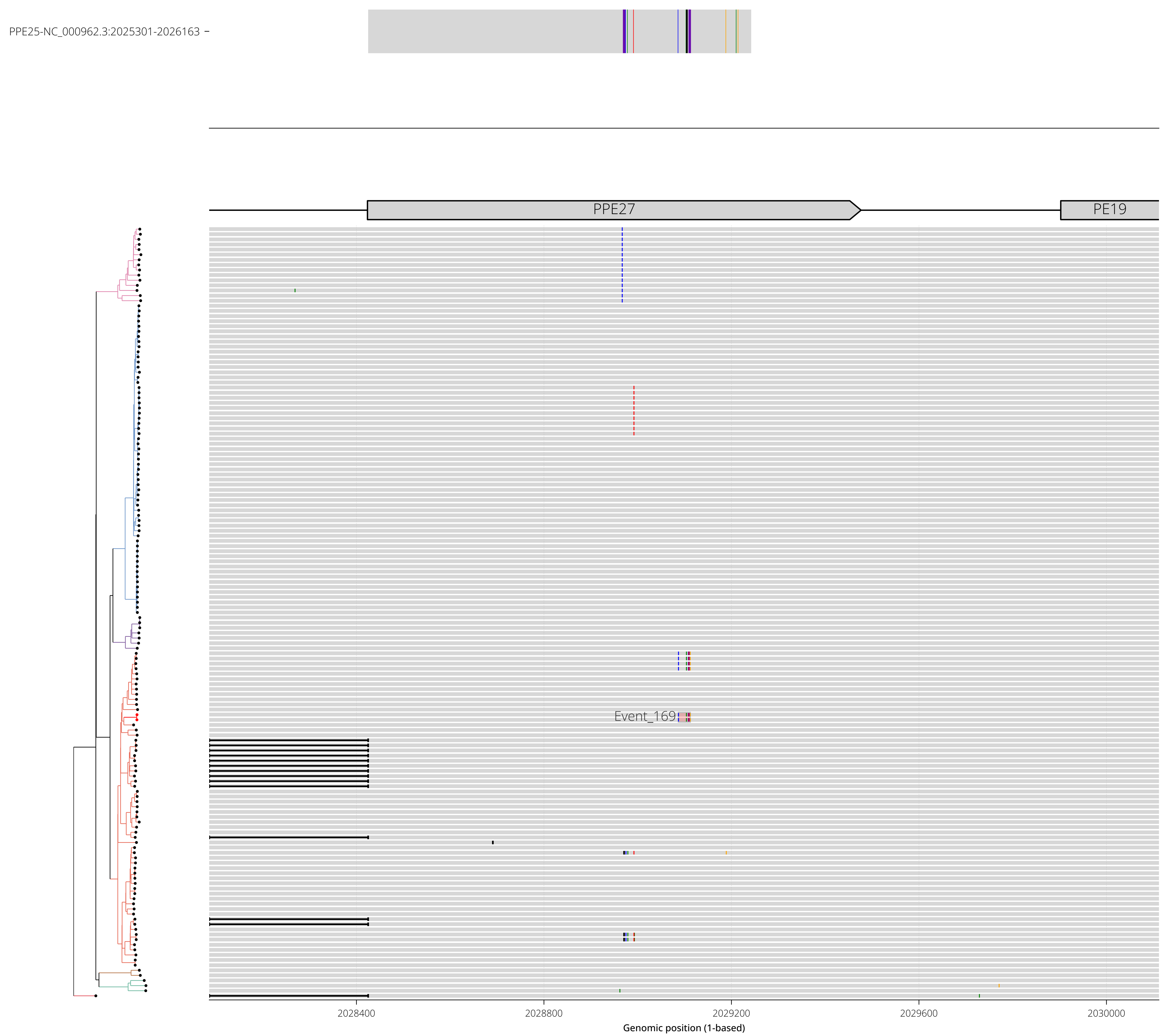

Individual GCE + Known Paralogs
GCE ID: Event_170

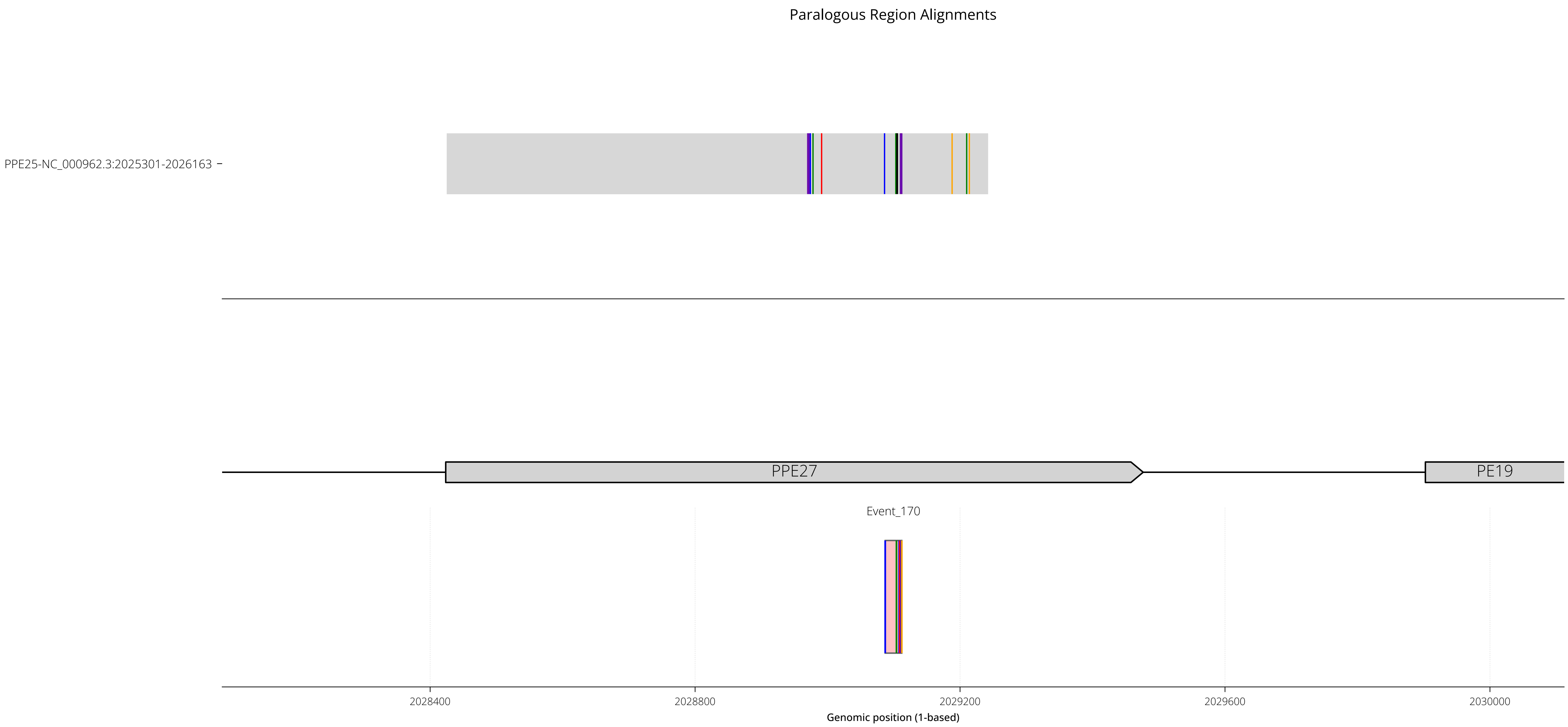

Circos Viz - GCE to Known Paralogs Locations
GCE ID: Event_170

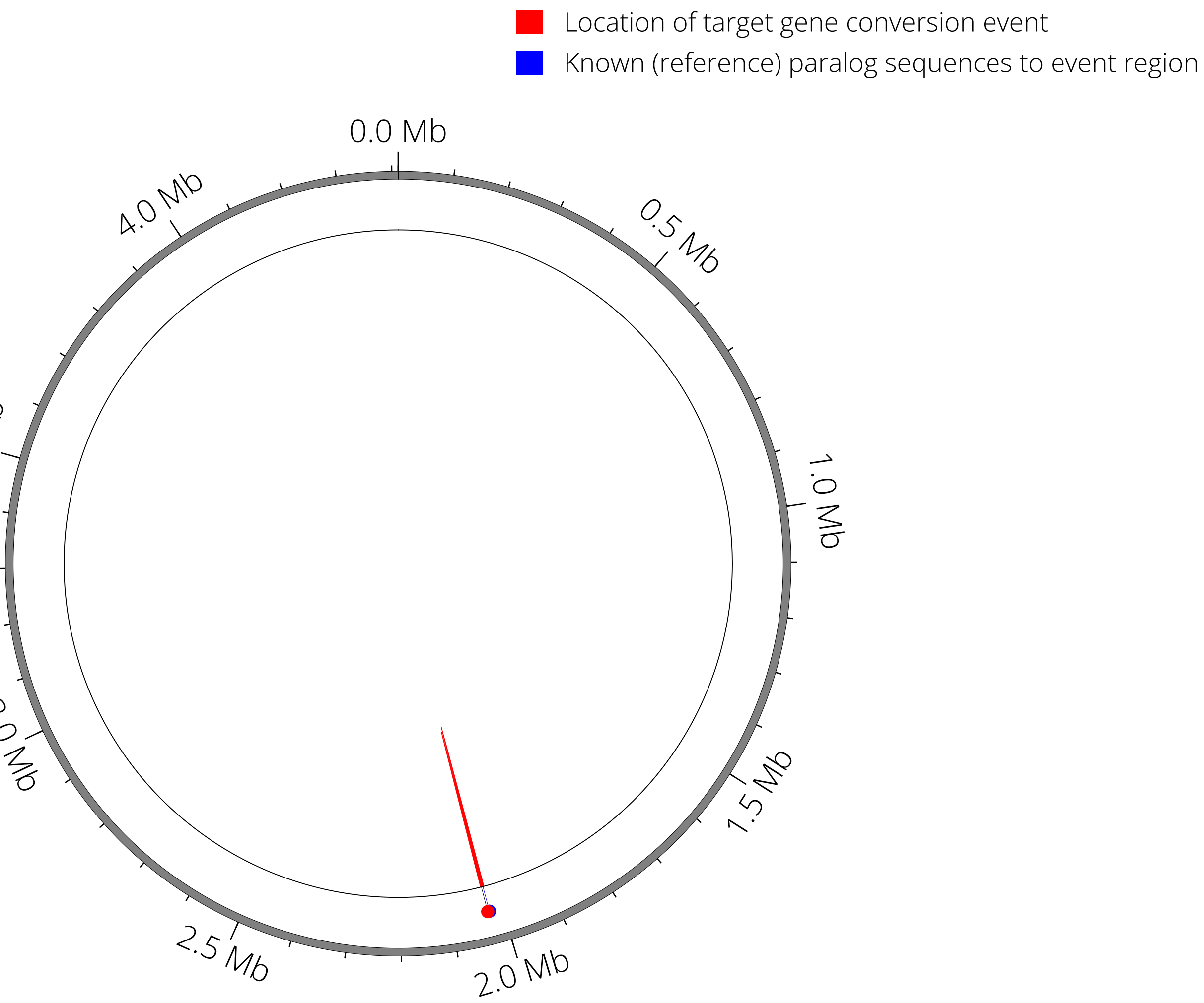

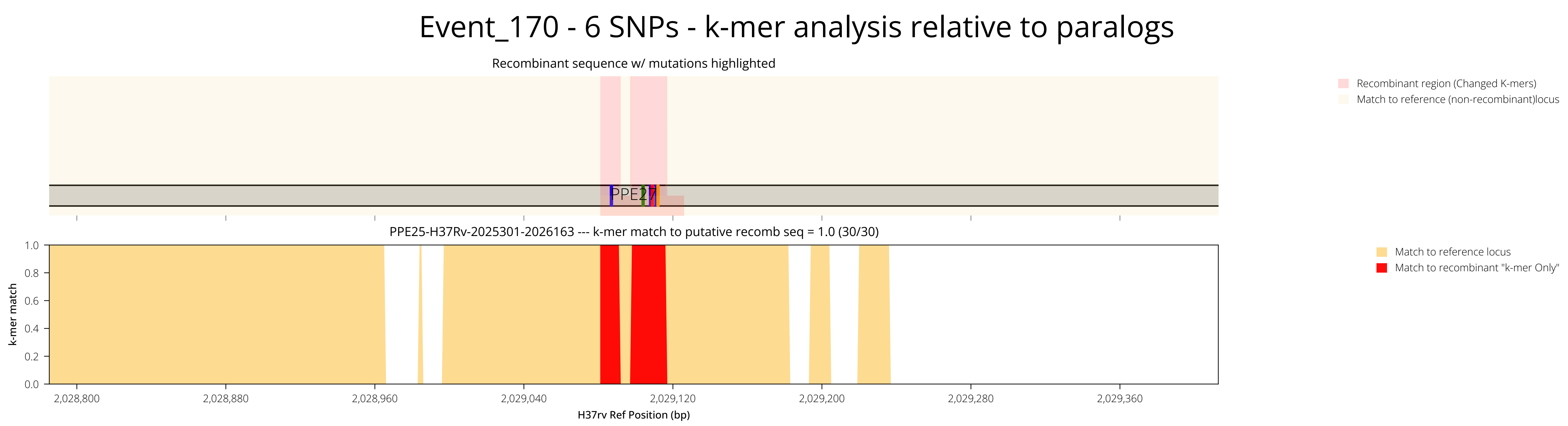

Event_170 - 6 SNPs - k-mer analysis relative to paralogs
Recombinant sequence w/ mutations highlighted
PPE27
Recombinant region (Changed K-mers)
Match to reference (non-recombinant)locus
PPE25-H37Rv-2025301-2026163 --- k-mer match to putative recomb seq = 1.0 (30/30)
k-mer match
1.0
0.8
0.6
0.4
0.2
0.0
2,028,800
2,028,880
2,028,960
2,029,040
2,029,120
2,029,200
2,029,280
2,029,360
H37rv Ref Position (bp)
Match to reference locus
Match to recombinant "k-mer Only"

Sub-phylogeny Viz + Paralog Alignments
GCE ID: Event_170

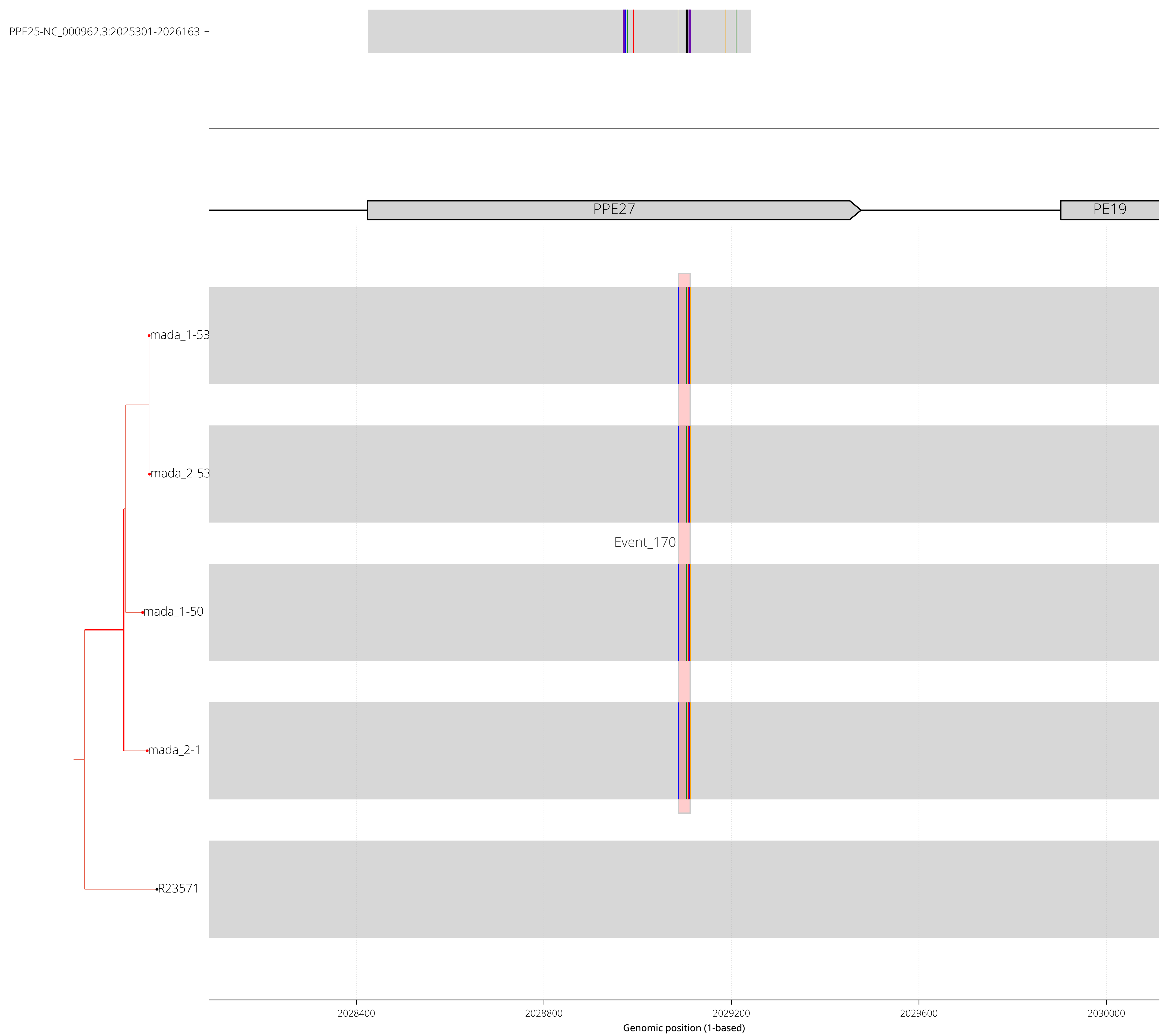

Full Phylogeny Viz + Paralog Alignments
GCE ID: Event_170

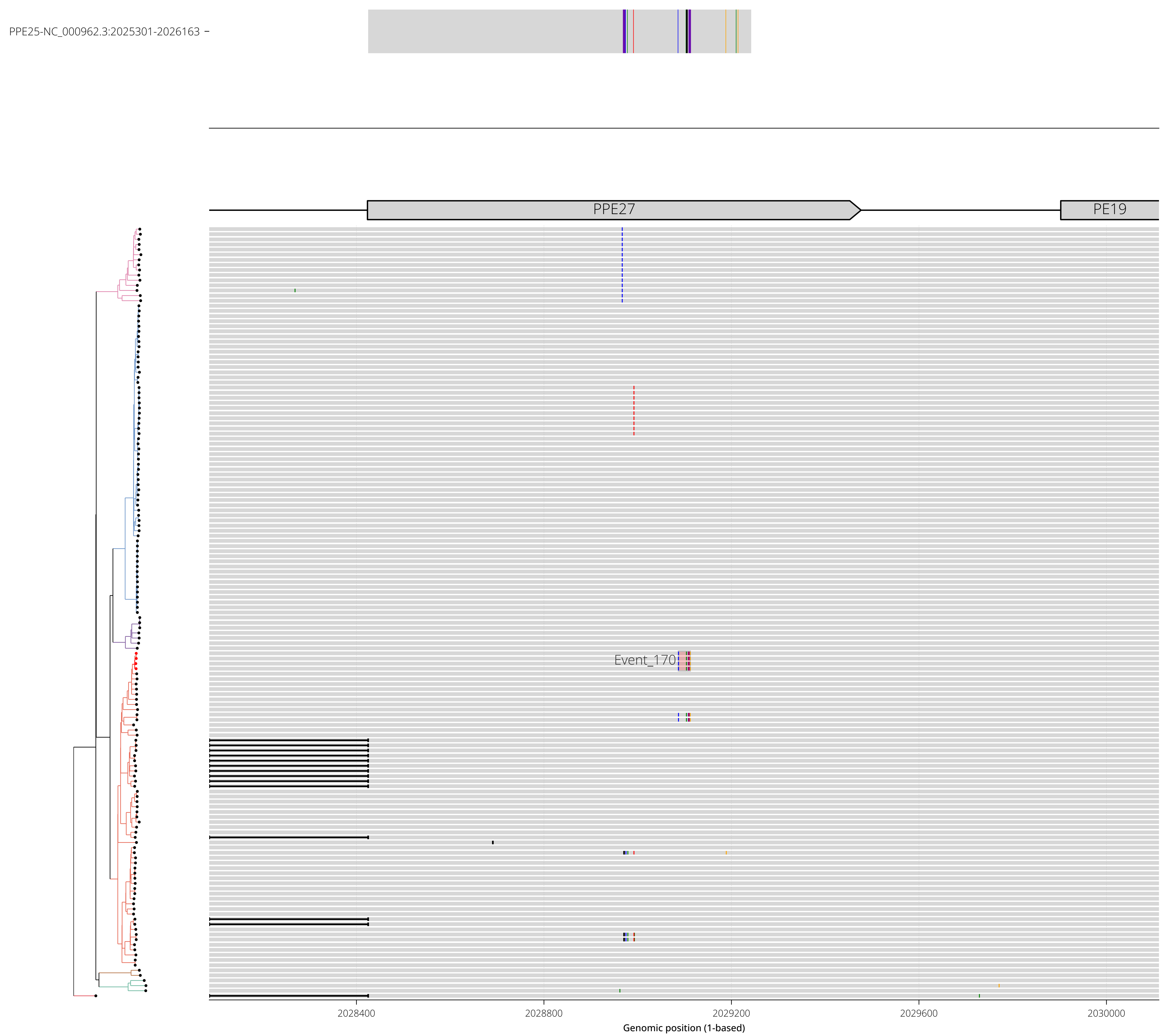

Individual GCE + Known Paralogs
GCE ID: Event_171

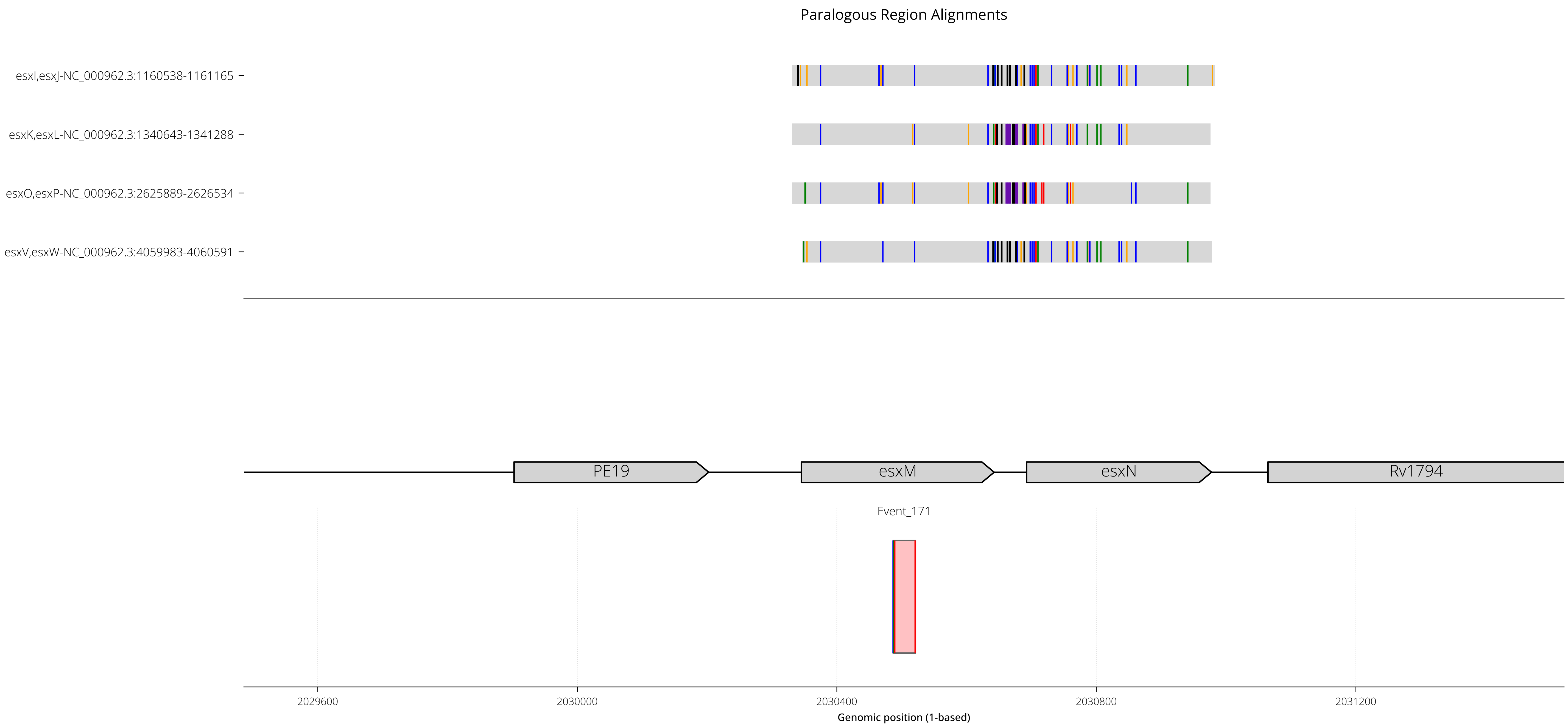

Circos Viz - GCE to Known Paralogs Locations
GCE ID: Event_171

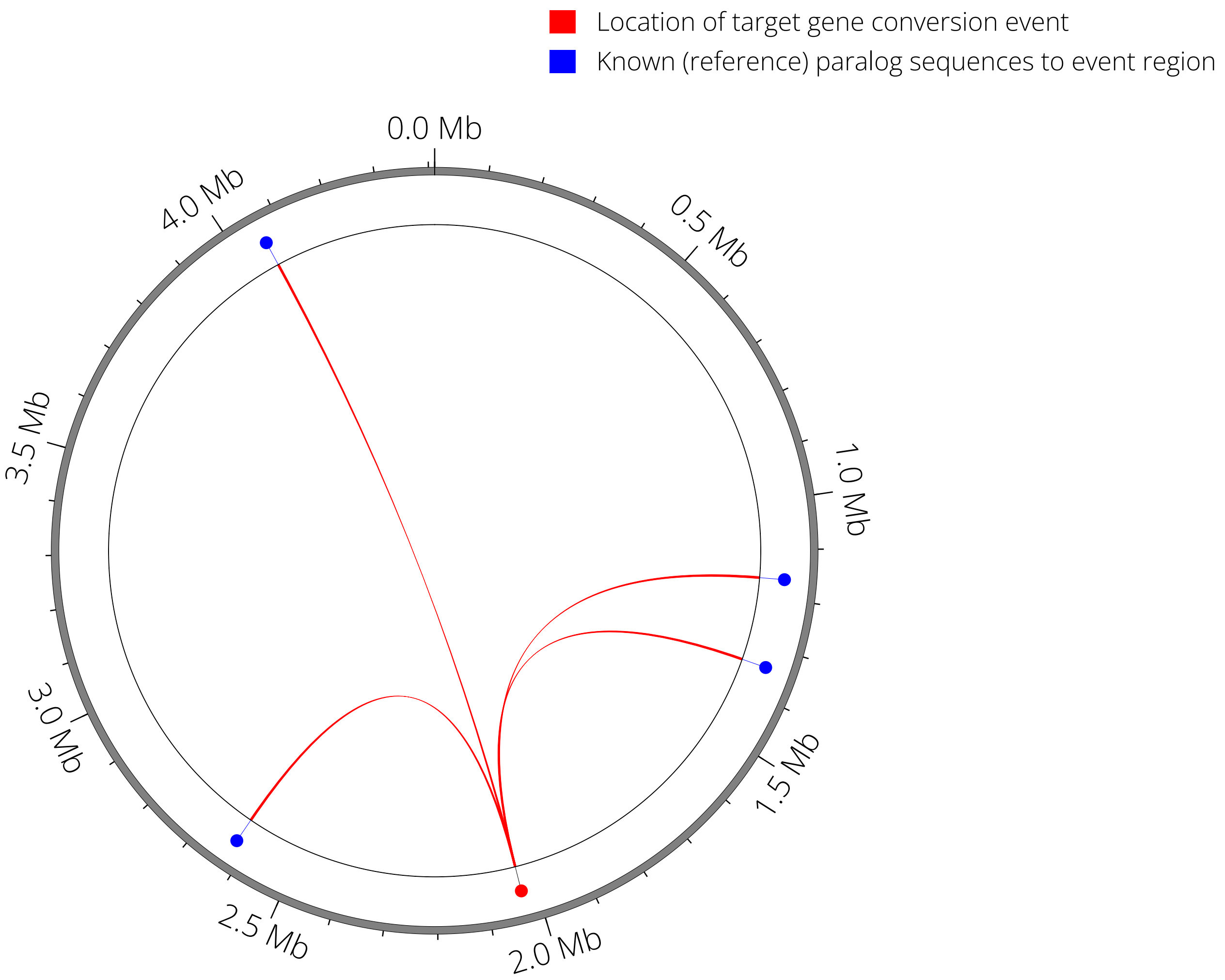

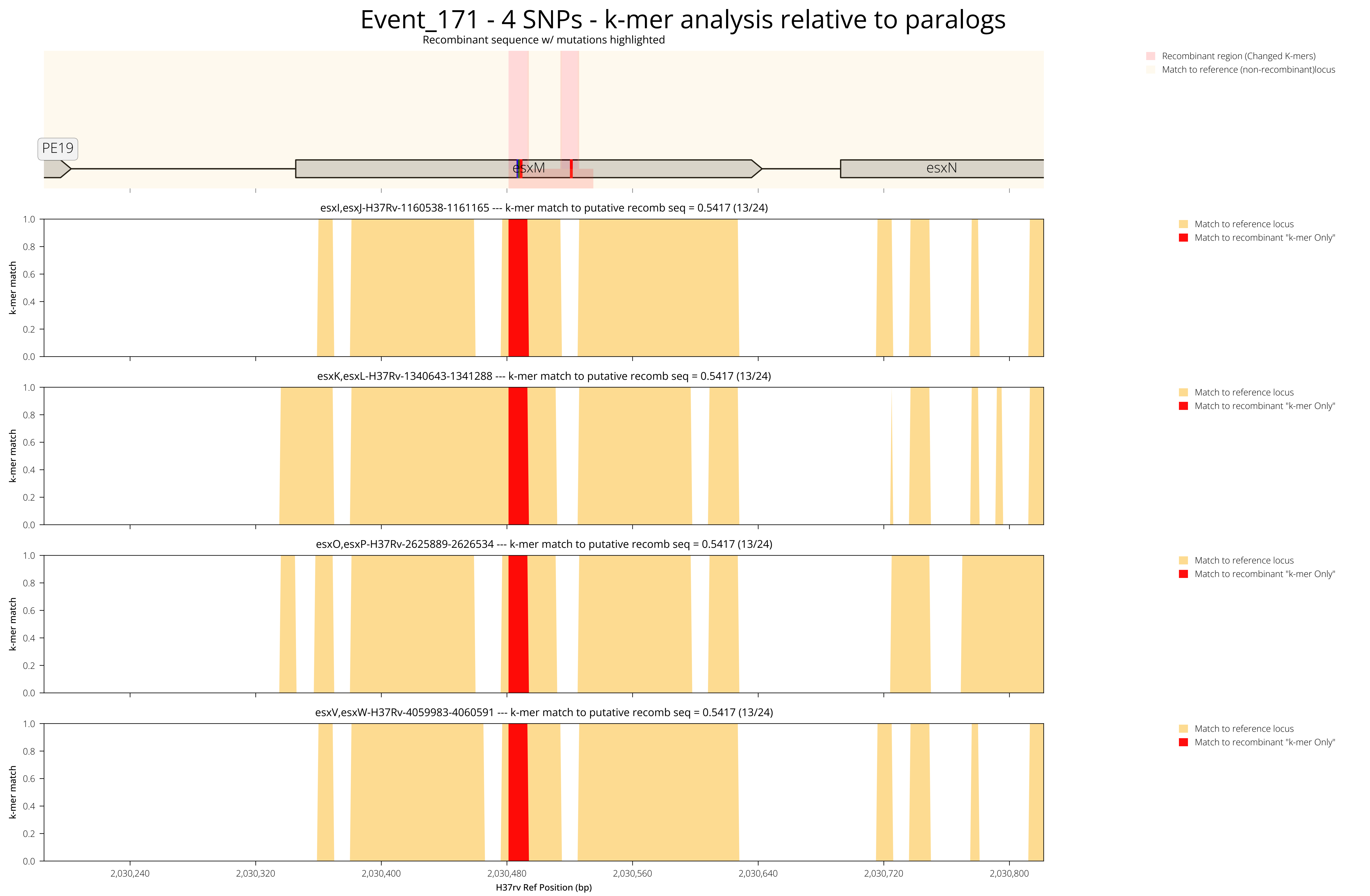

Event_171 - 4 SNPs - k-mer analysis relative to paralogs
Recombinant sequence w/ mutations highlighted
Recombinant region (Changed K-mers)
Match to reference (non-recombinant)locus
PE19
esxM
esxN
esxI,esxJ-H37Rv-1160538-1161165 --- k-mer match to putative recomb seq = 0.5417 (13/24)
esxK,esxL-H37Rv-1340643-1341288 --- k-mer match to putative recomb seq = 0.5417 (13/24)
esxO,esxP-H37Rv-2625889-2626534 --- k-mer match to putative recomb seq = 0.5417 (13/24)
esxV,esxW-H37Rv-4059983-4060591 --- k-mer match to putative recomb seq = 0.5417 (13/24)
Match to reference locus
Match to recombinant "k-mer Only"
k-mer match
1.0
0.8
0.6
0.4
0.2
0.0
2,030,240
2,030,320
2,030,400
2,030,480
2,030,560
2,030,640
2,030,720
2,030,800
H37rv Ref Position (bp)

Sub-phylogeny Viz + Paralog Alignments
GCE ID: Event_171

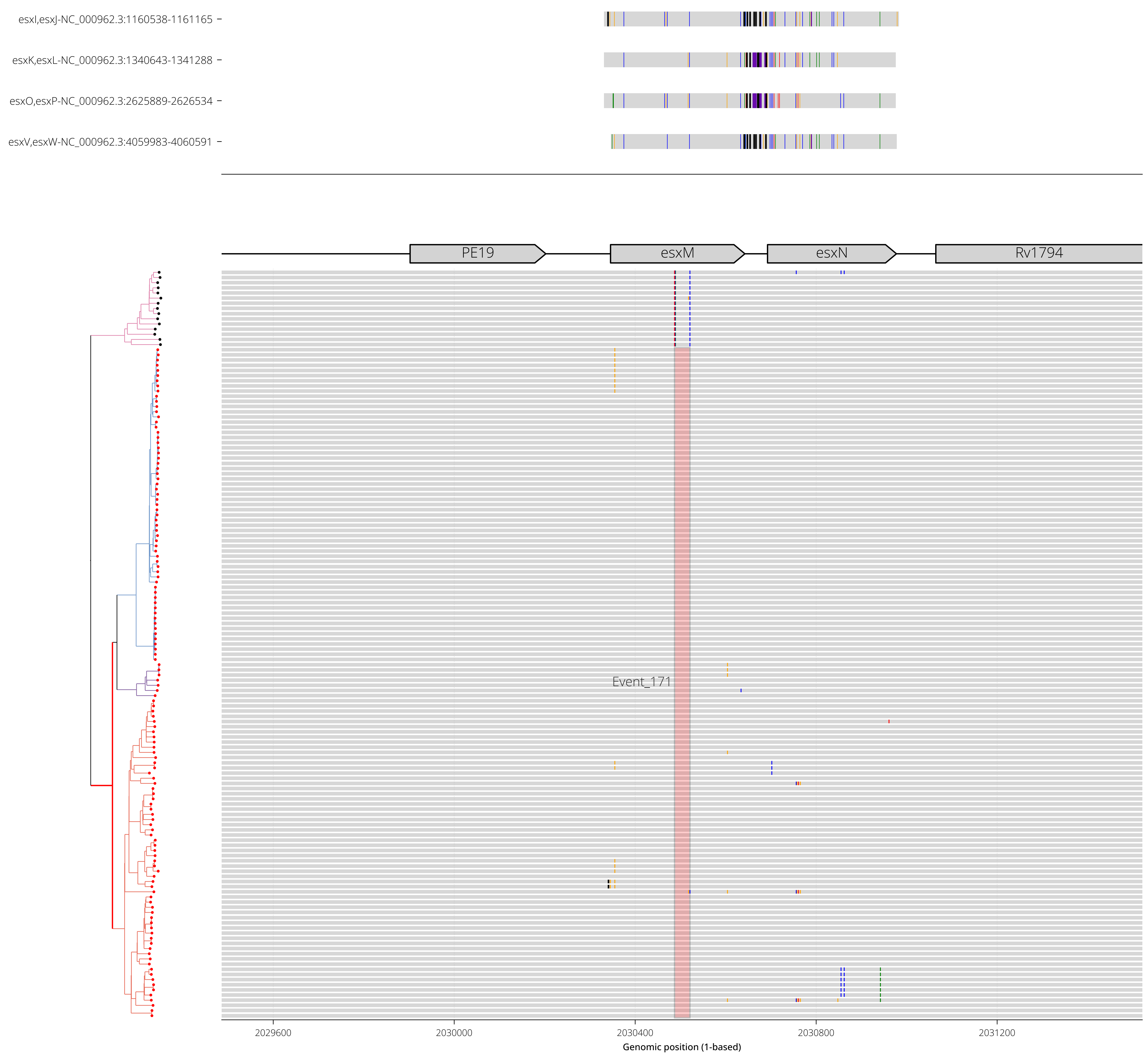

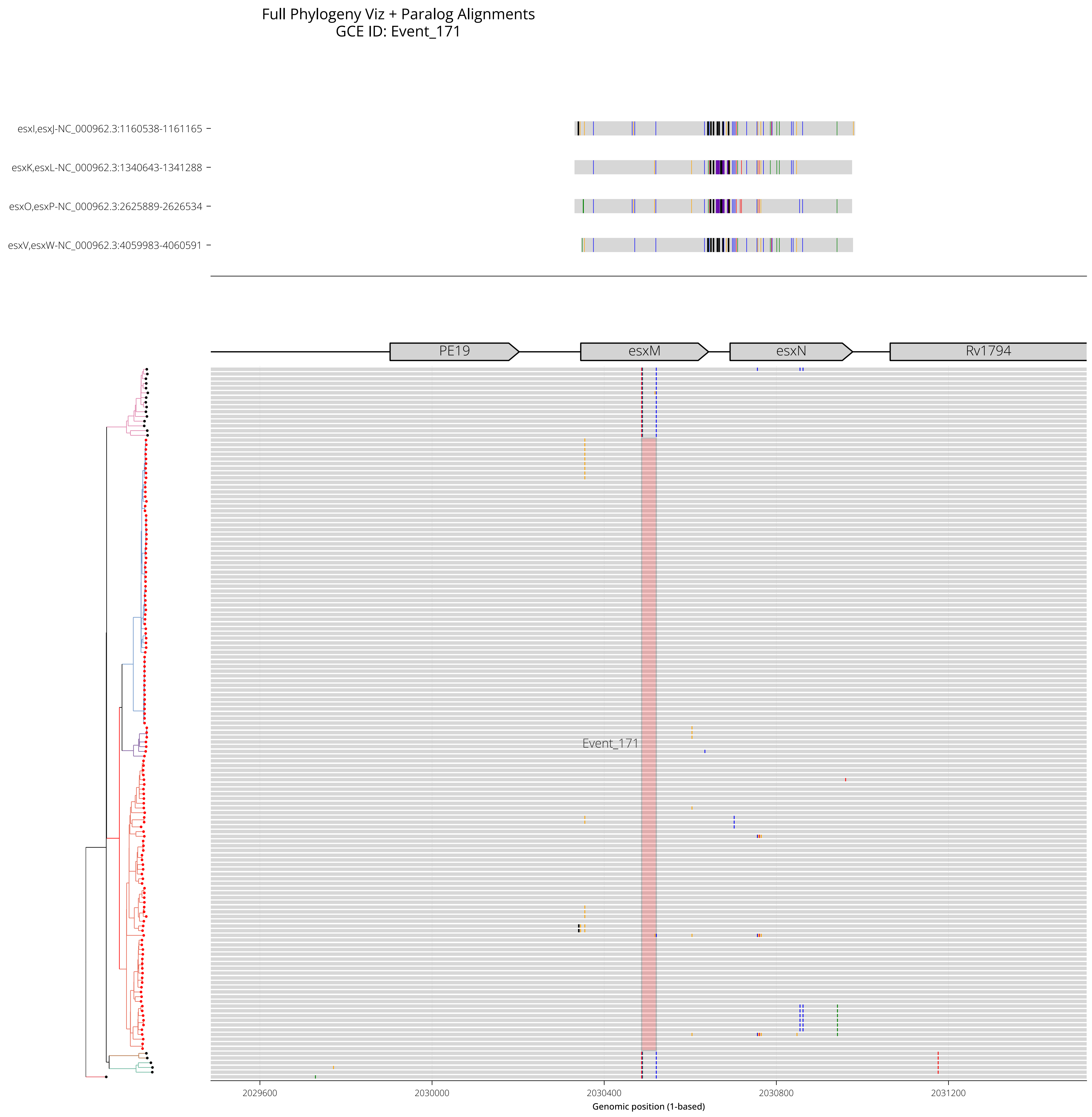

Full Phylogeny Viz + Paralog Alignments
GCE ID: Event_171
esxI,esxJ-NC_000962.3:1160538-1161165
esxK,esxL-NC_000962.3:1340643-1341288
esxO,esxP-NC_000962.3:2625889-2626534
esxV,esxW-NC_000962.3:4059983-4060591
PE19
esxM
esxN
Rv1794
Event_171
2029600
2030000
2030400
2030800
2031200
Genomic position (1-based)

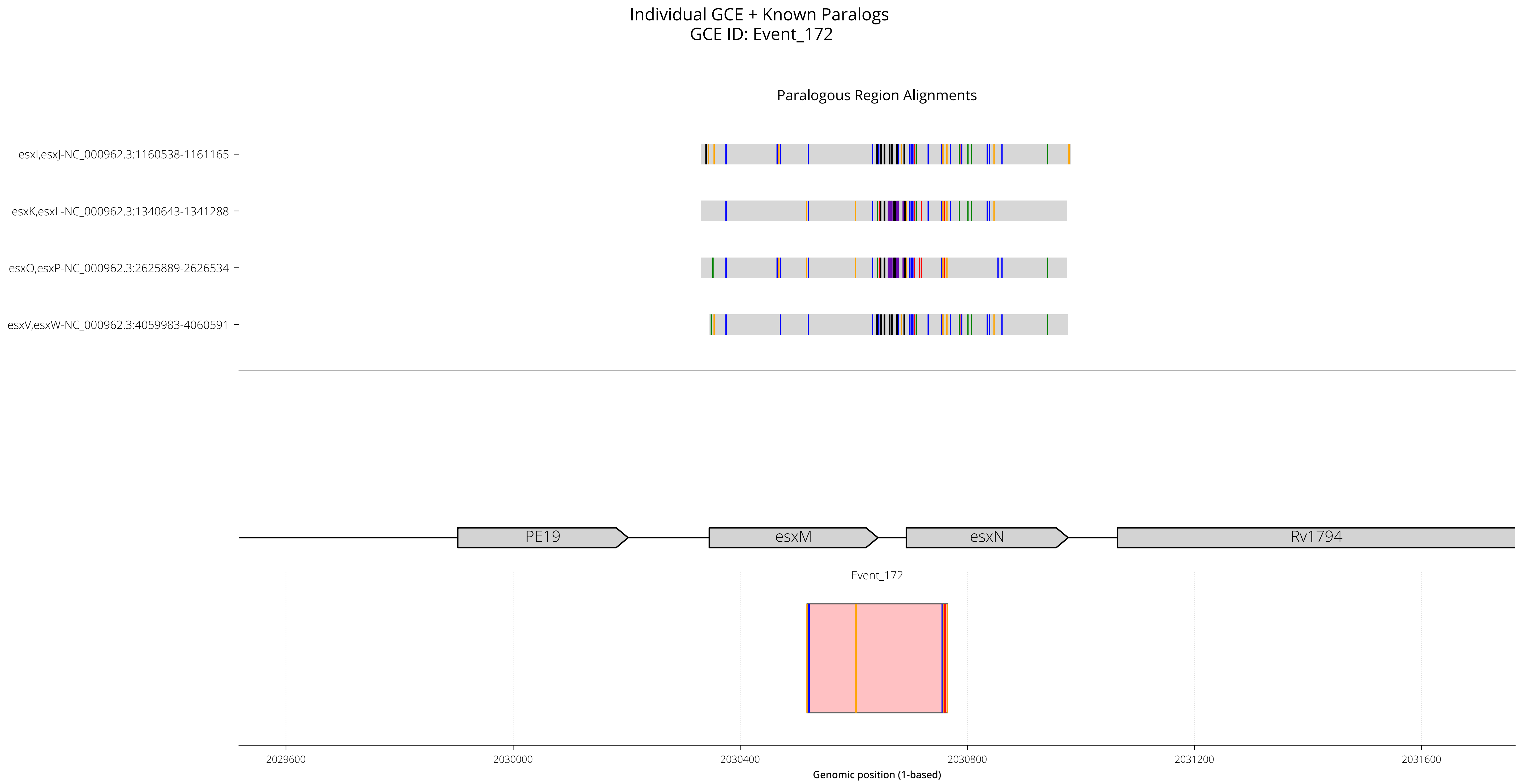

Individual GCE + Known Paralogs
GCE ID: Event_172
Paralogous Region Alignments
esxI,esxJ-NC_000962.3:1160538-1161165
esxK,esxL-NC_000962.3:1340643-1341288
esxO,esxP-NC_000962.3:2625889-2626534
esxV,esxW-NC_000962.3:4059983-4060591
PE19
esxM
esxN
Rv1794
Event_172
2029600
2030000
2030400
2030800
2031200
2031600
Genomic position (1-based)

Circos Viz - GCE to Known Paralogs Locations
GCE ID: Event_172

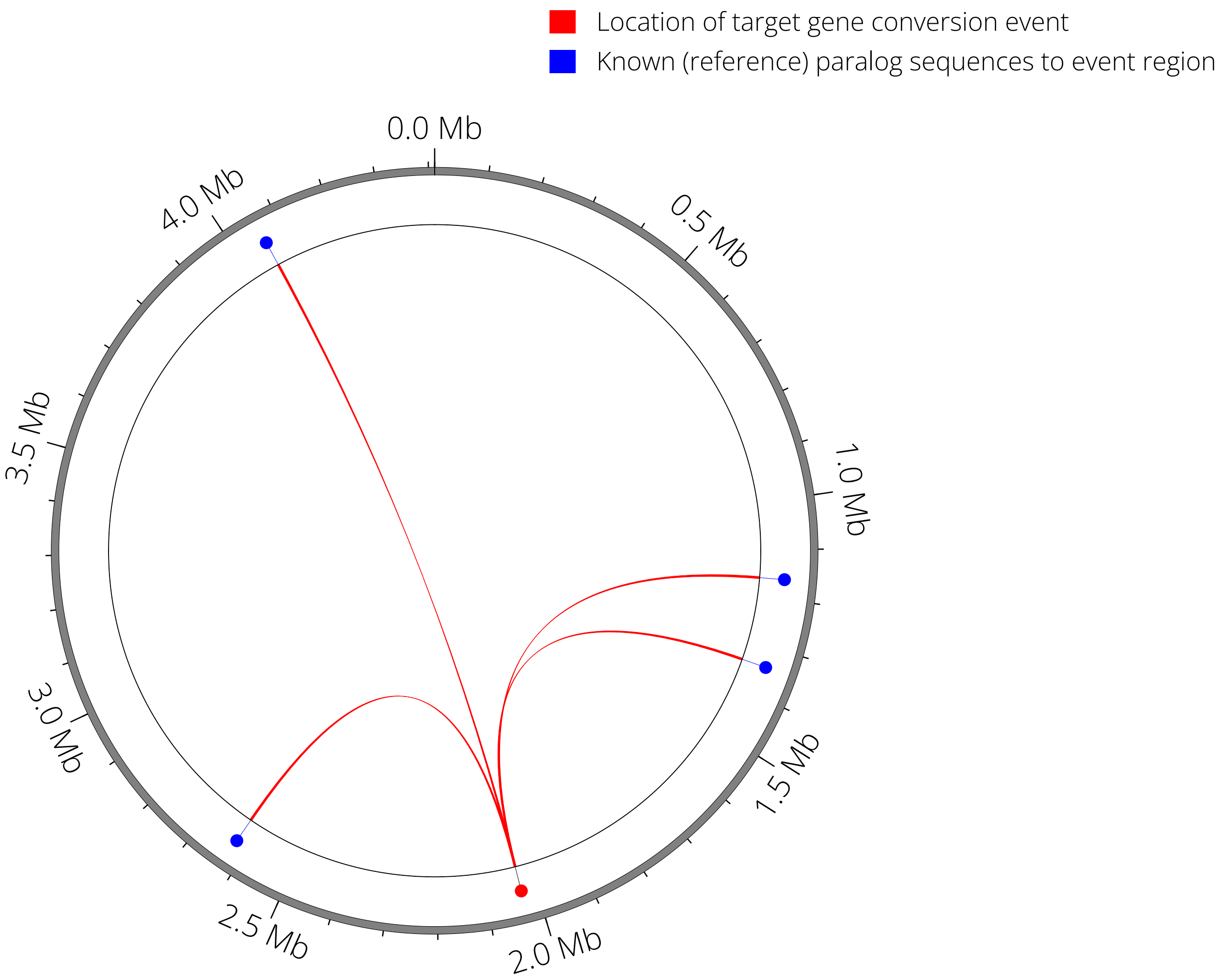

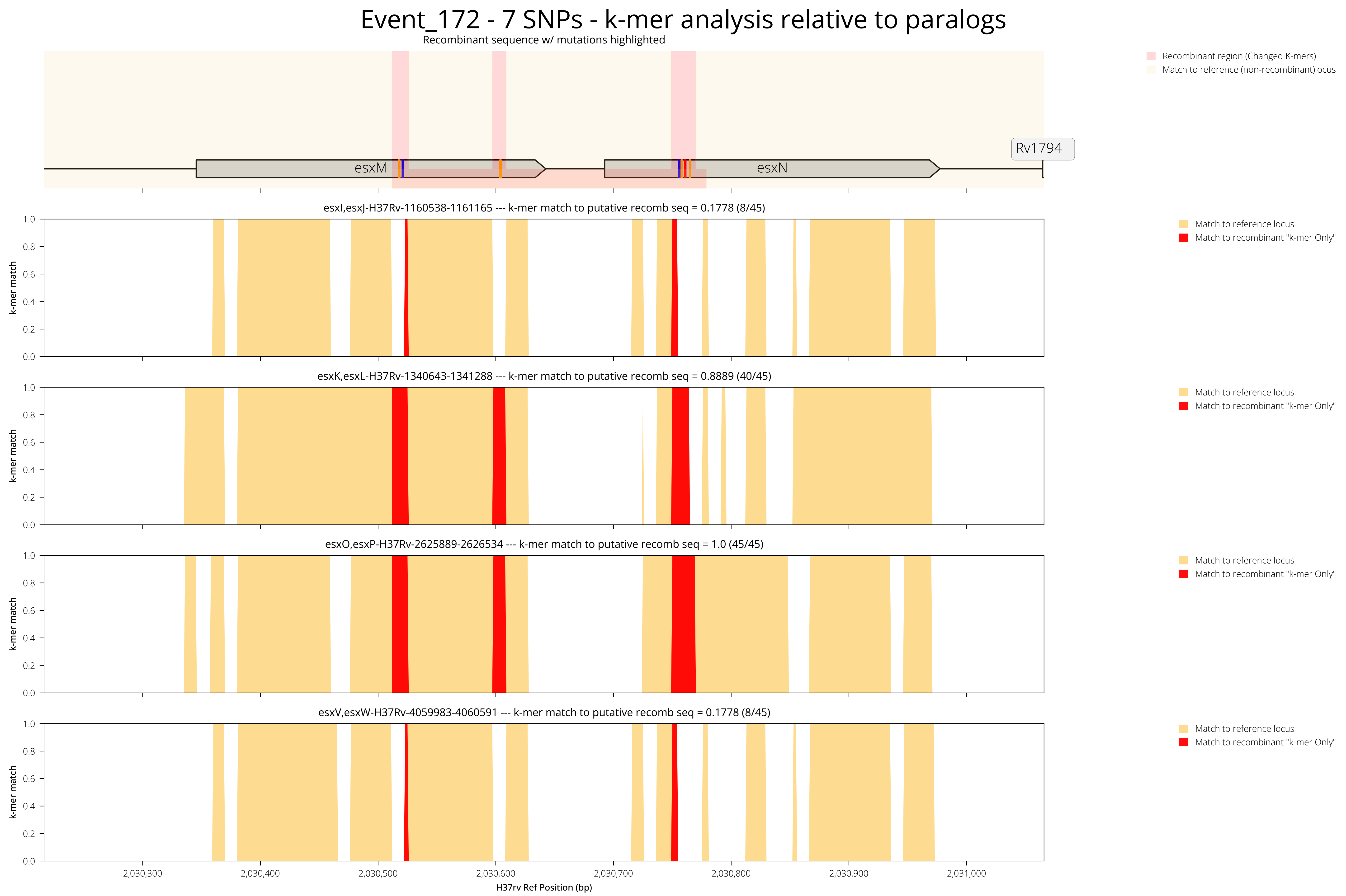

Event_172 - 7 SNPs - k-mer analysis relative to paralogs
Recombinant sequence w/ mutations highlighted
Recombinant region (Changed K-mers)
Match to reference (non-recombinant)locus
esxM
esxN
Rv1794
esxI,esxJ-H37Rv-1160538-1161165 --- k-mer match to putative recomb seq = 0.1778 (8/45)
esxK,esxL-H37Rv-1340643-1341288 --- k-mer match to putative recomb seq = 0.8889 (40/45)
esxO,esxP-H37Rv-2625889-2626534 --- k-mer match to putative recomb seq = 1.0 (45/45)
esxV,esxW-H37Rv-4059983-4060591 --- k-mer match to putative recomb seq = 0.1778 (8/45)
Match to reference locus
Match to recombinant "k-mer Only"
k-mer match
1.0
0.8
0.6
0.4
0.2
0.0
2,030,300
2,030,400
2,030,500
2,030,600
2,030,700
2,030,800
2,030,900
2,031,000
H37rv Ref Position (bp)

Sub-phylogeny Viz + Paralog Alignments
GCE ID: Event_172

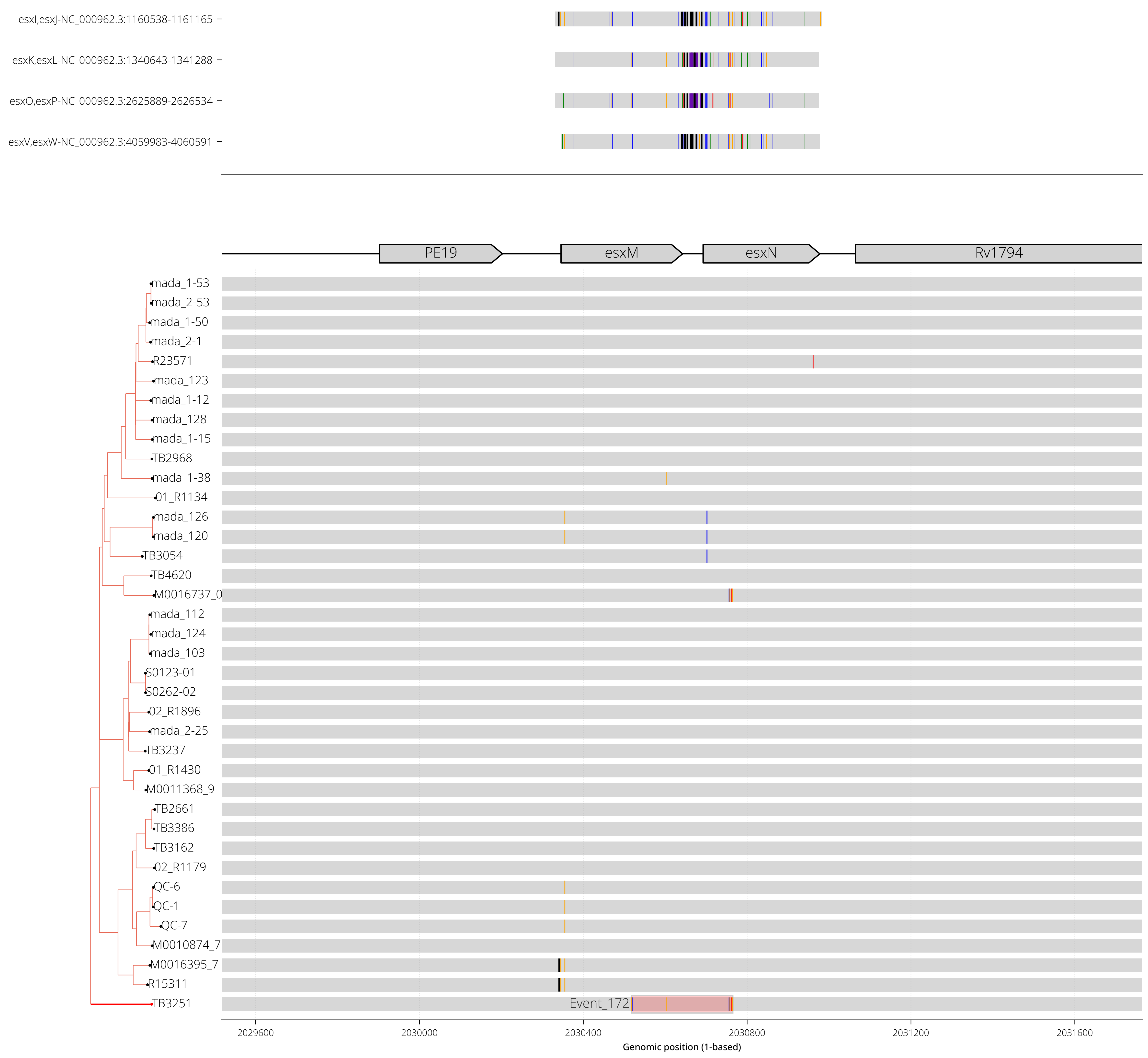

Full Phylogeny Viz + Paralog Alignments
GCE ID: Event_172

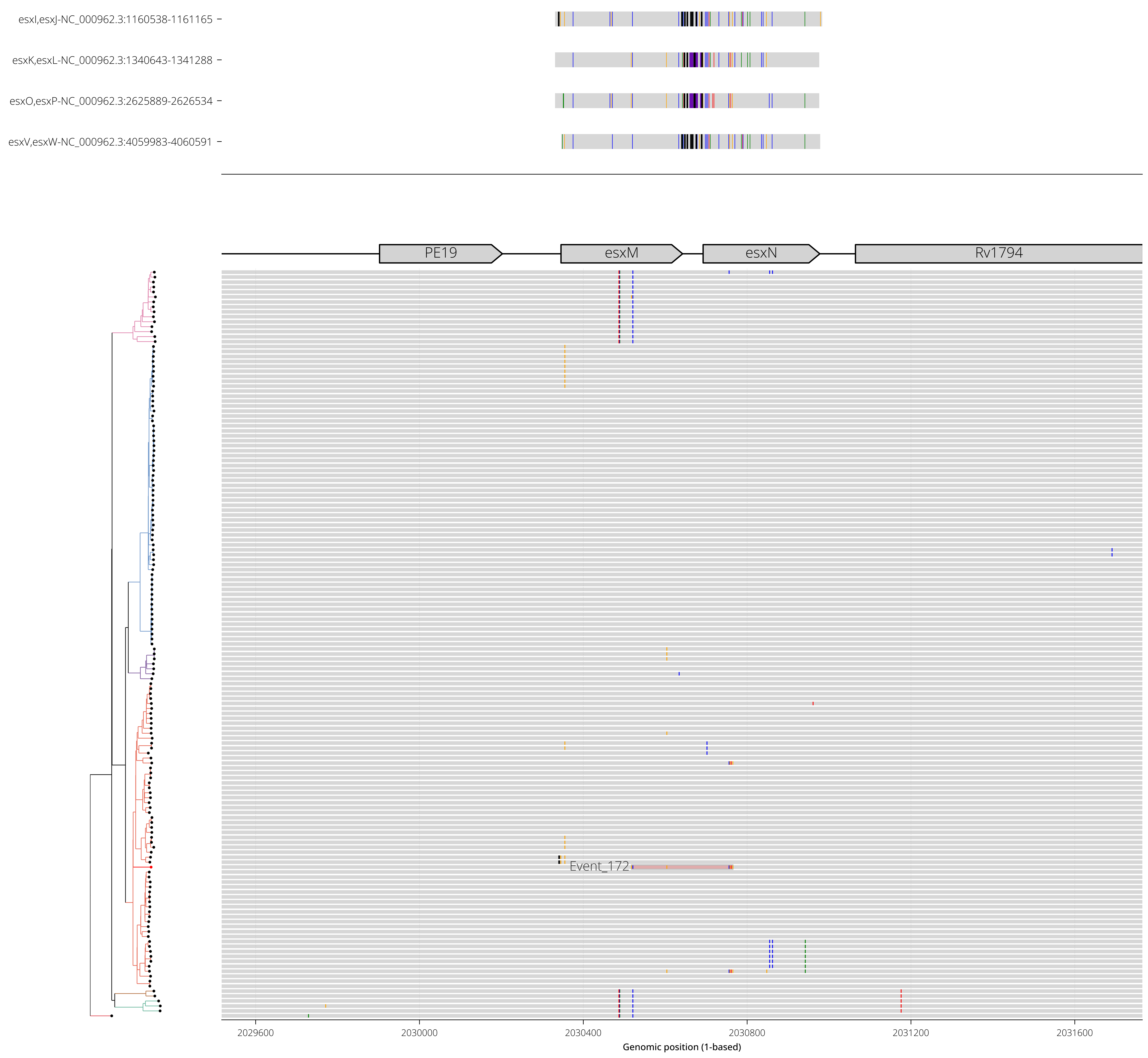

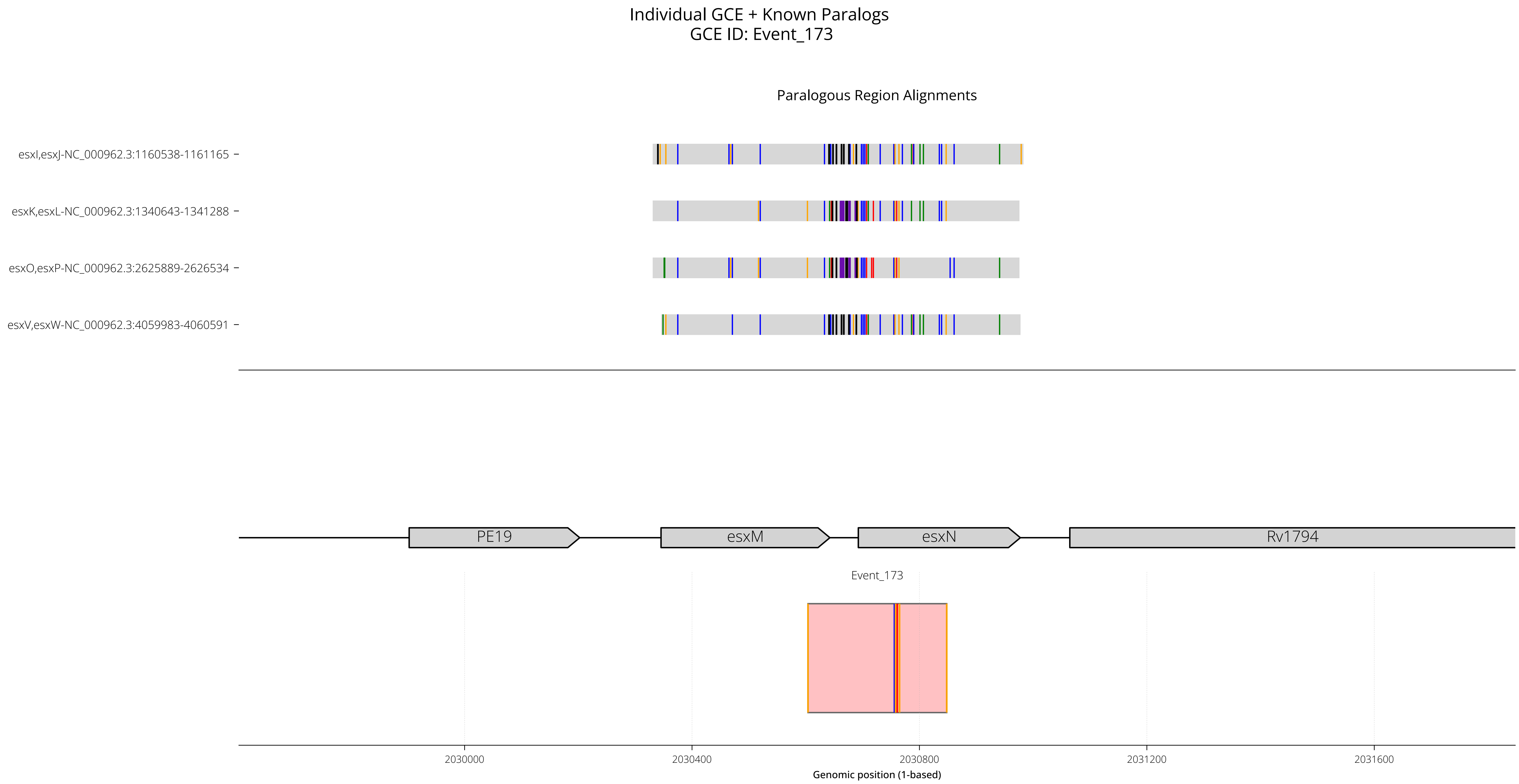
Individual GCE + Known Paralogs
GCE ID: Event_173
Paralogous Region Alignments
esxI,esxJ-NC_000962.3:1160538-1161165
esxK,esxL-NC_000962.3:1340643-1341288
esxO,esxP-NC_000962.3:2625889-2626534
esxV,esxW-NC_000962.3:4059983-4060591
PE19
esxM
esxN
Rv1794
Event_173
2030000
2030400
2030800
2031200
2031600
Genomic position (1-based)

Circos Viz - GCE to Known Paralogs Locations
GCE ID: Event_173

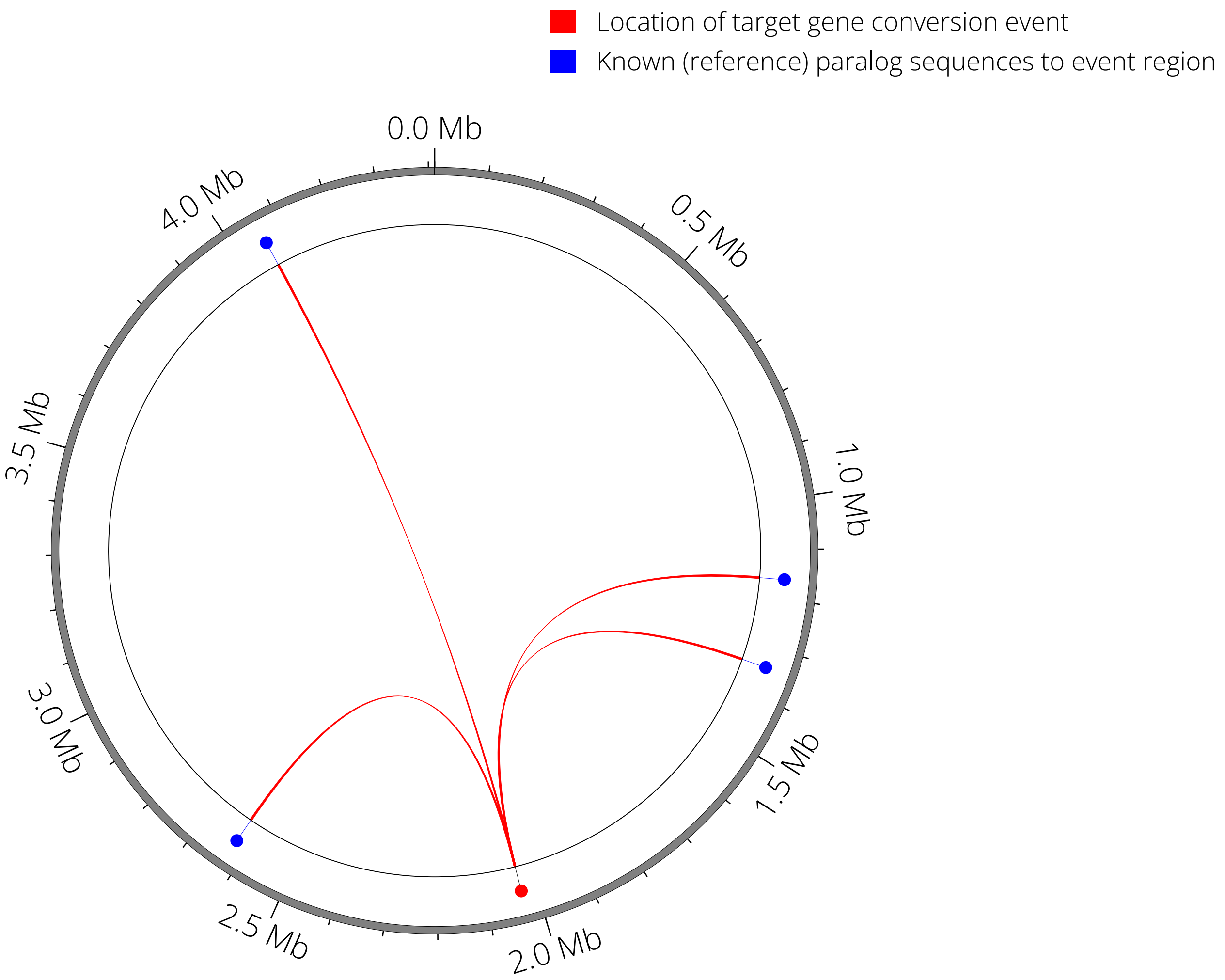

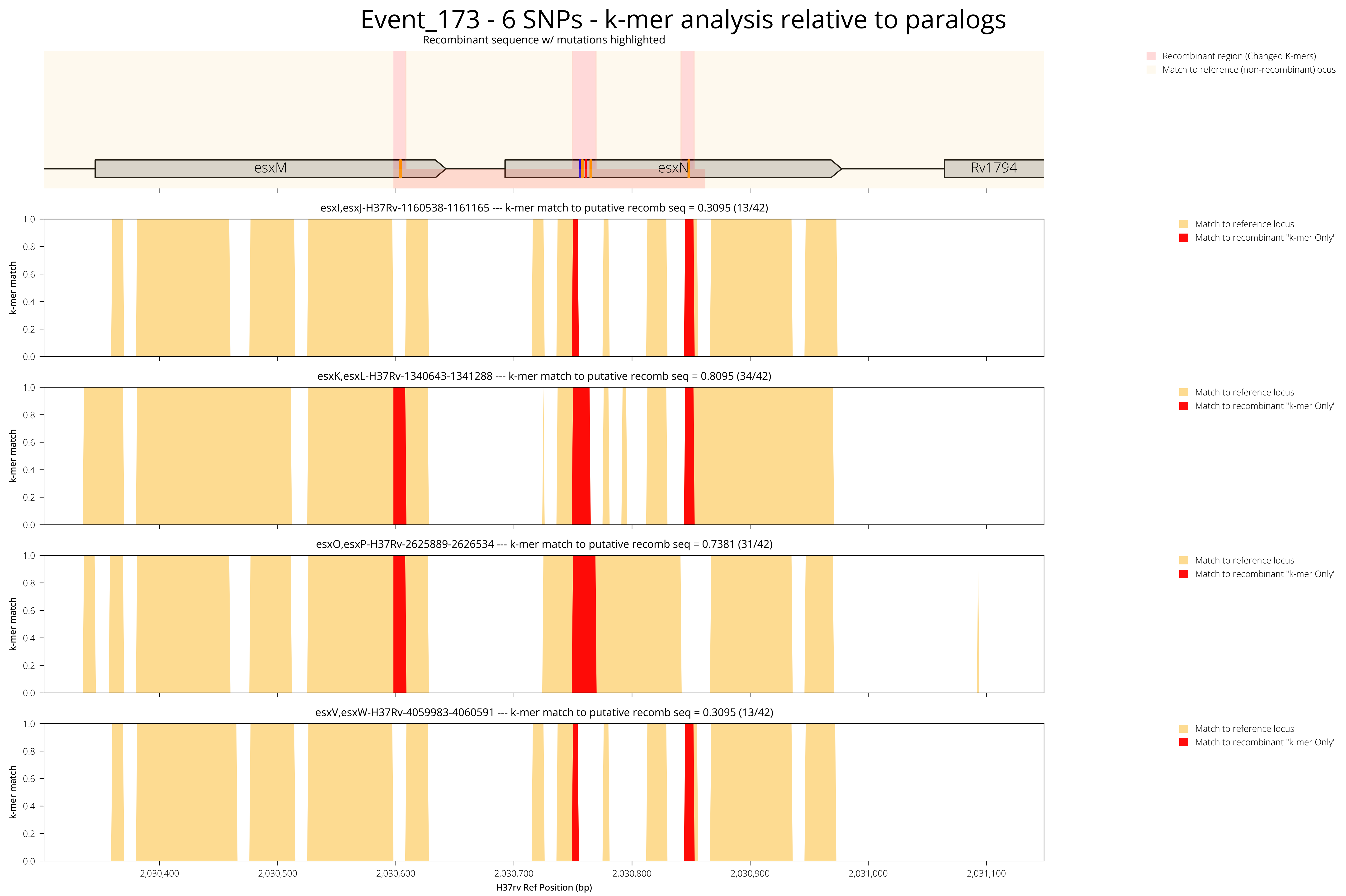
Event_173 - 6 SNPs - k-mer analysis relative to paralogs
Recombinant sequence w/ mutations highlighted
esxM
esxN
Rv1794
Recombinant region (Changed K-mers)
Match to reference (non-recombinant)locus
esxI,esxJ-H37Rv-1160538-1161165 --- k-mer match to putative recomb seq = 0.3095 (13/42)
esxK,esxL-H37Rv-1340643-1341288 --- k-mer match to putative recomb seq = 0.8095 (34/42)
esxO,esxP-H37Rv-2625889-2626534 --- k-mer match to putative recomb seq = 0.7381 (31/42)
esxV,esxW-H37Rv-4059983-4060591 --- k-mer match to putative recomb seq = 0.3095 (13/42)
Match to reference locus
Match to recombinant "k-mer Only"
k-mer match
0.0
0.2
0.4
0.6
0.8
1.0
2,030,400
2,030,500
2,030,600
2,030,700
2,030,800
2,030,900
2,031,000
2,031,100
H37rv Ref Position (bp)

Sub-phylogeny Viz + Paralog Alignments
GCE ID: Event_173

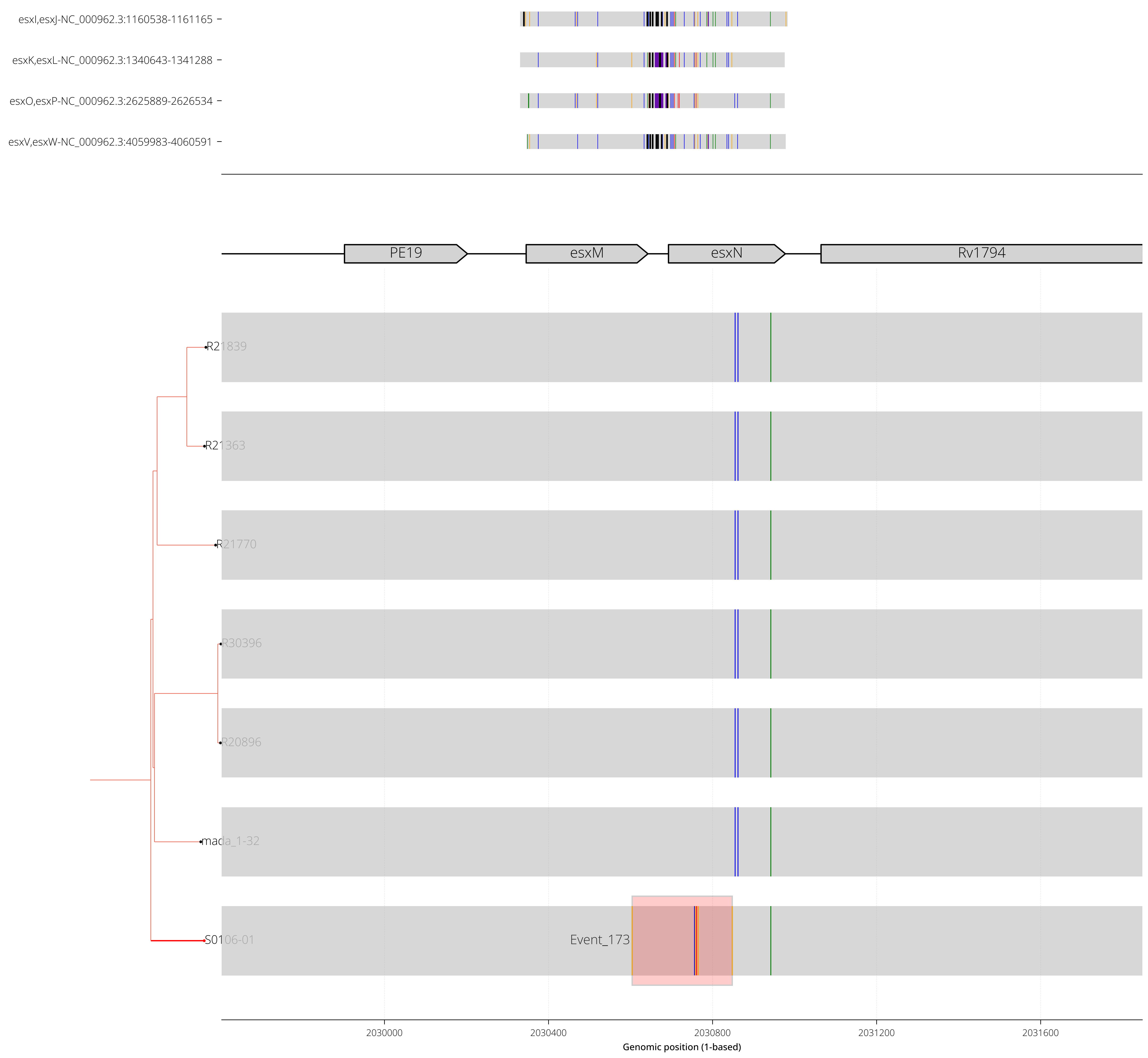

## Full Phylogeny Viz + Paralog Alignments
## GCE ID: Event_173

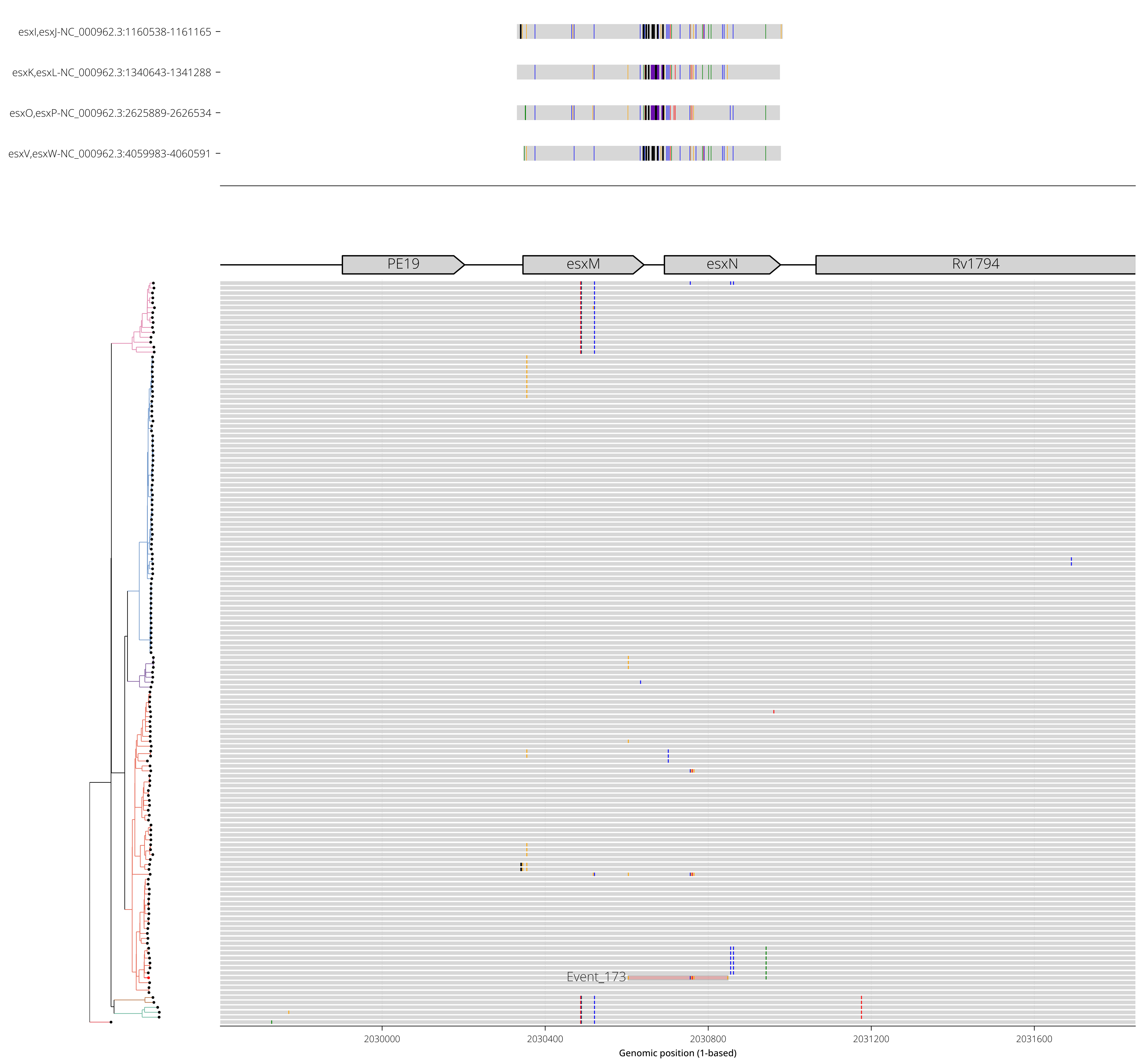

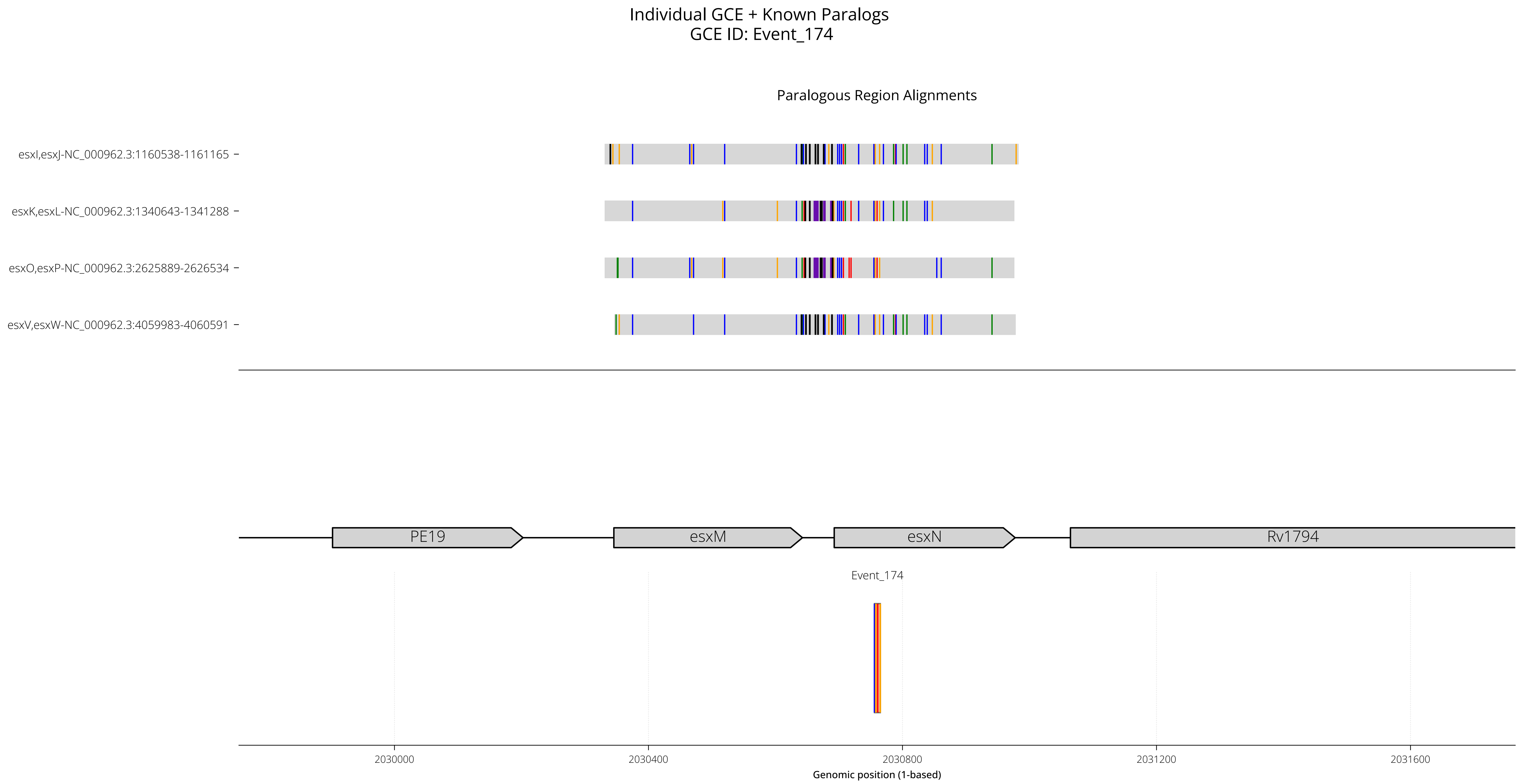
Individual GCE + Known Paralogs
GCE ID: Event_174
Paralogous Region Alignments
esxI,esxJ-NC_000962.3:1160538-1161165
esxK,esxL-NC_000962.3:1340643-1341288
esxO,esxP-NC_000962.3:2625889-2626534
esxV,esxW-NC_000962.3:4059983-4060591
PE19
esxM
esxN
Rv1794
Event_174
2030000
2030400
2030800
2031200
2031600
Genomic position (1-based)

Circos Viz - GCE to Known Paralogs Locations
GCE ID: Event_174

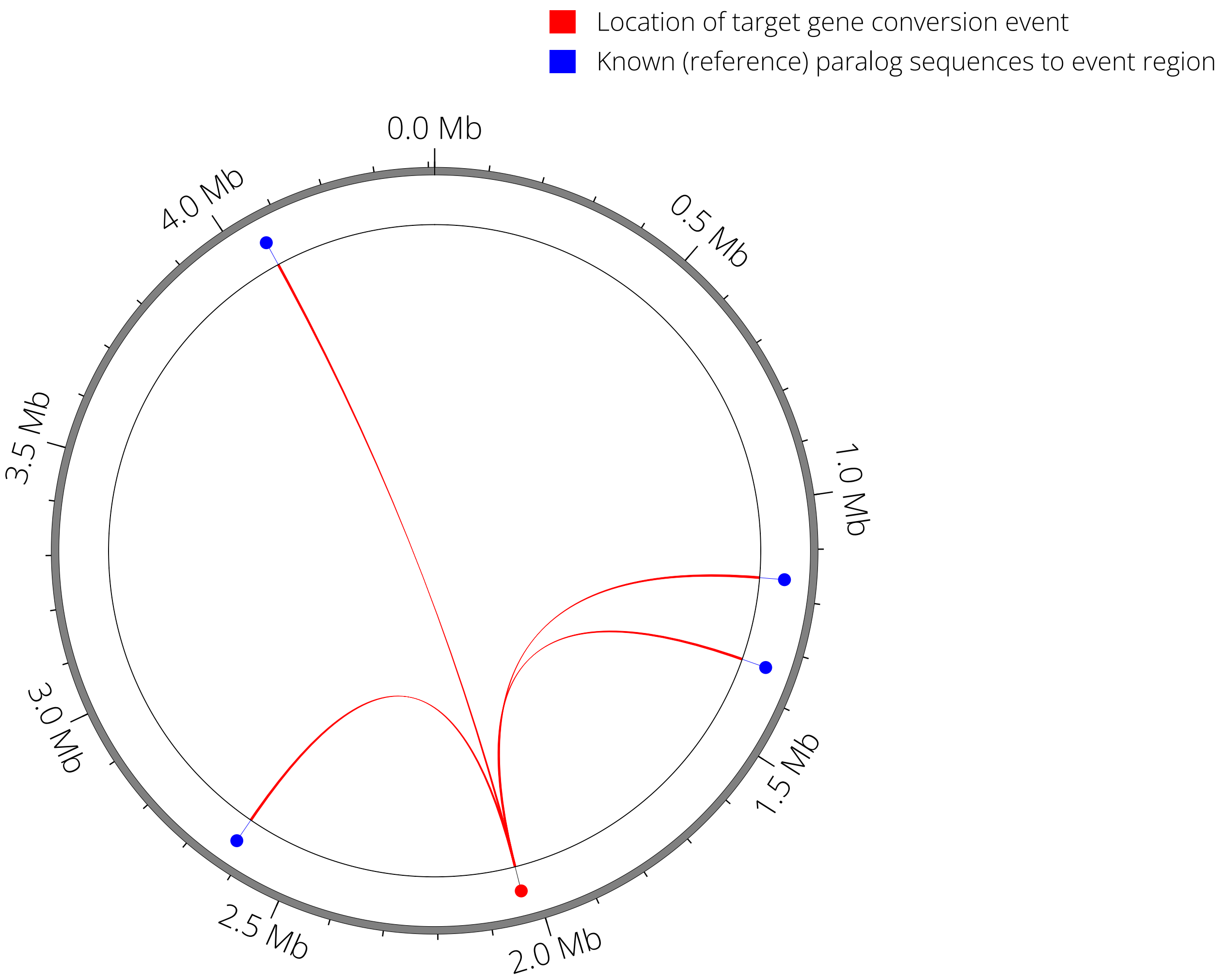

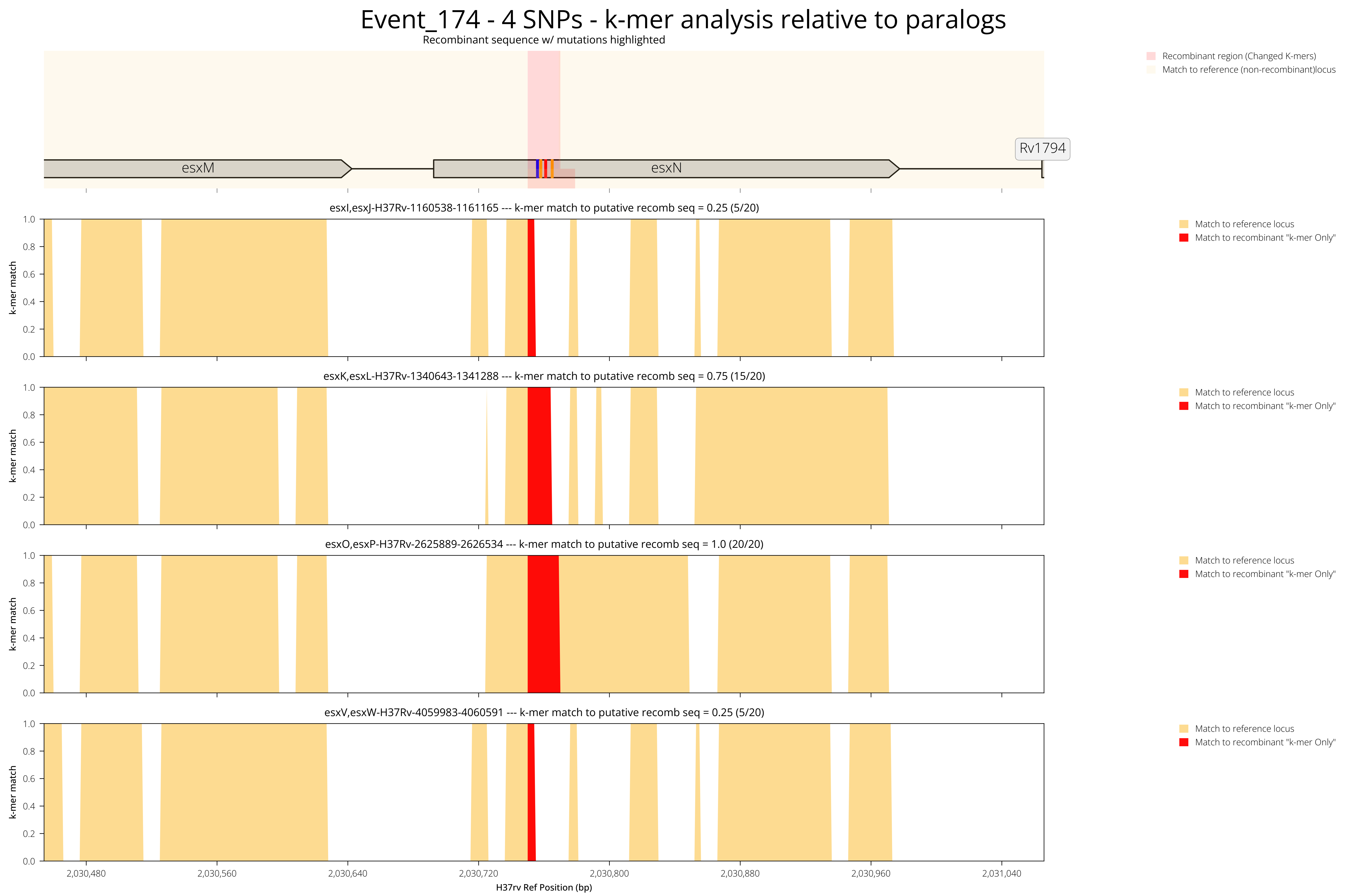
Event_174 - 4 SNPs - k-mer analysis relative to paralogs
Recombinant sequence w/ mutations highlighted
Recombinant region (Changed K-mers)
Match to reference (non-recombinant)locus
esxM
esxN
Rv1794
esxI,esxJ-H37Rv-1160538-1161165 --- k-mer match to putative recomb seq = 0.25 (5/20)
esxK,esxL-H37Rv-1340643-1341288 --- k-mer match to putative recomb seq = 0.75 (15/20)
esxO,esxP-H37Rv-2625889-2626534 --- k-mer match to putative recomb seq = 1.0 (20/20)
esxV,esxW-H37Rv-4059983-4060591 --- k-mer match to putative recomb seq = 0.25 (5/20)
Match to reference locus
Match to recombinant "k-mer Only"
k-mer match
1.0
0.8
0.6
0.4
0.2
0.0
2,030,480
2,030,560
2,030,640
2,030,720
2,030,800
2,030,880
2,030,960
2,031,040
H37rv Ref Position (bp)

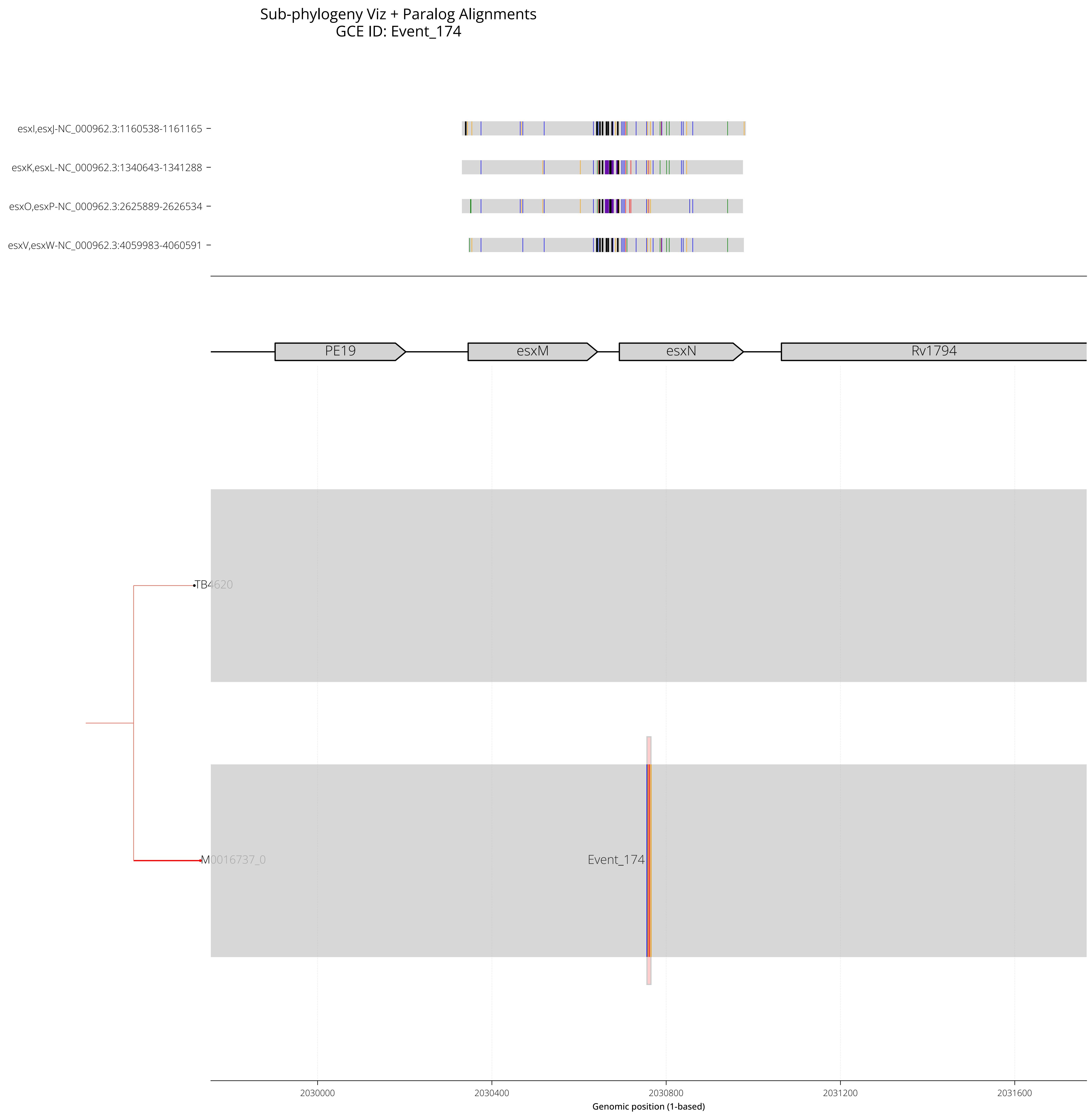

Sub-phylogeny Viz + Paralog Alignments
GCE ID: Event_174
esxI,esxJ-NC_000962.3:1160538-1161165
esxK,esxL-NC_000962.3:1340643-1341288
esxO,esxP-NC_000962.3:2625889-2626534
esxV,esxW-NC_000962.3:4059983-4060591
PE19
esxM
esxN
Rv1794
TB4620
M0016737_0
Event_174
2030000
2030400
2030800
2031200
2031600
Genomic position (1-based)

Full Phylogeny Viz + Paralog Alignments
GCE ID: Event_174

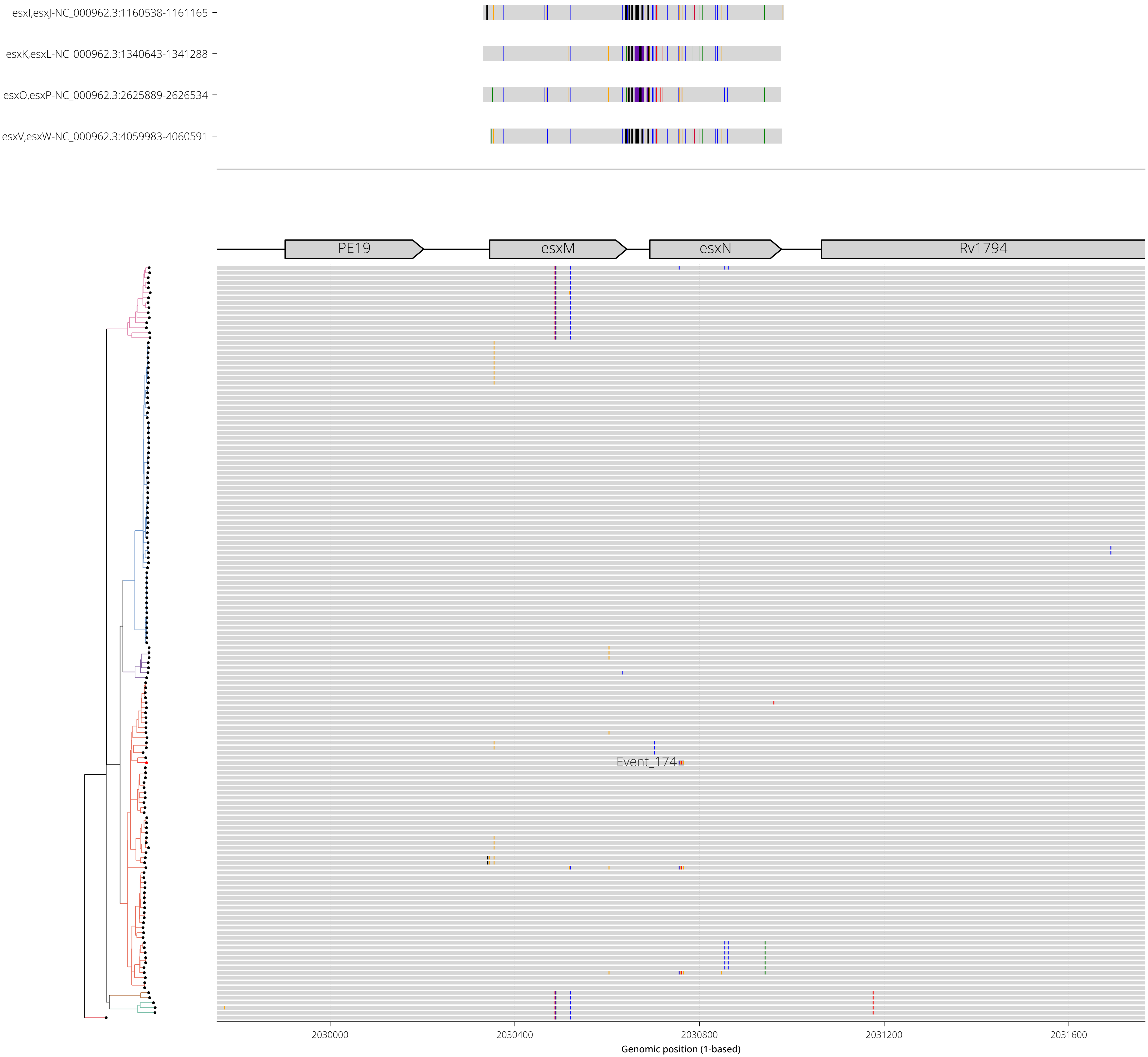

# Individual GCE + Known Paralogs
# GCE ID: Event_175

Paralogous Region Alignments

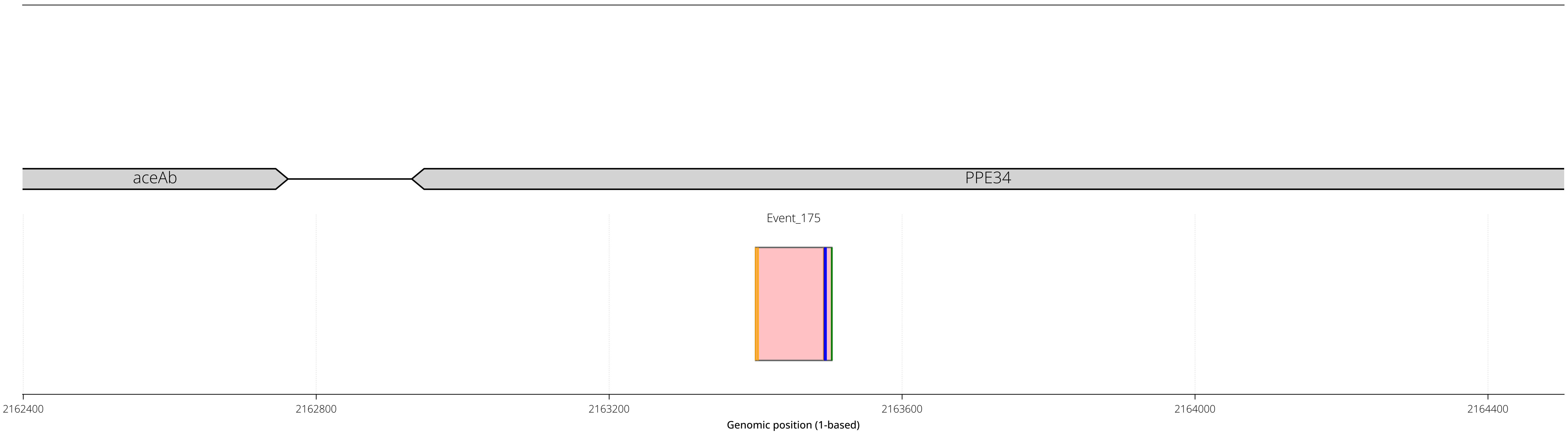

Sub-phylogeny Viz + Paralog Alignments
GCE ID: Event_175

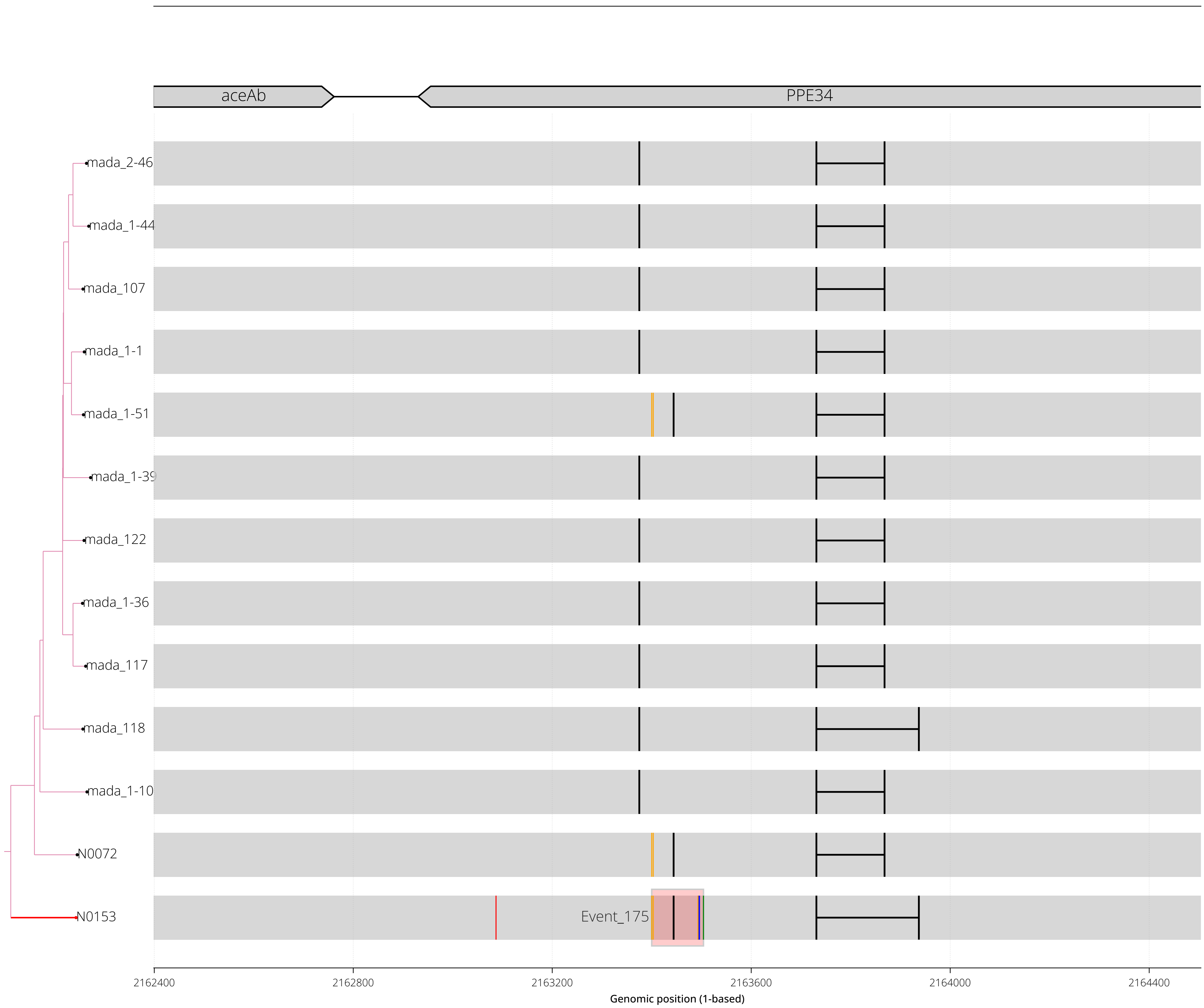

Full Phylogeny Viz + Paralog Alignments
GCE ID: Event_175

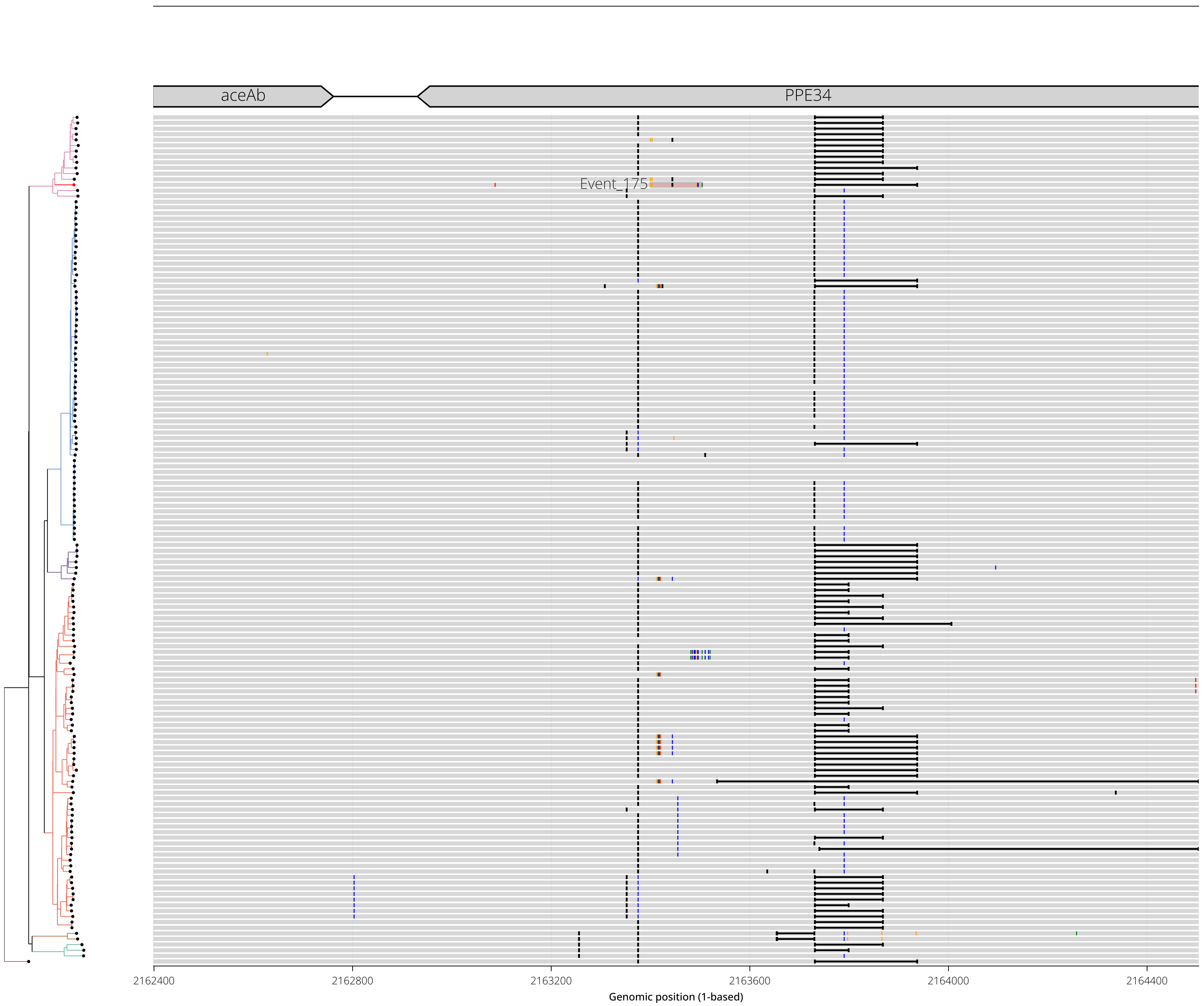

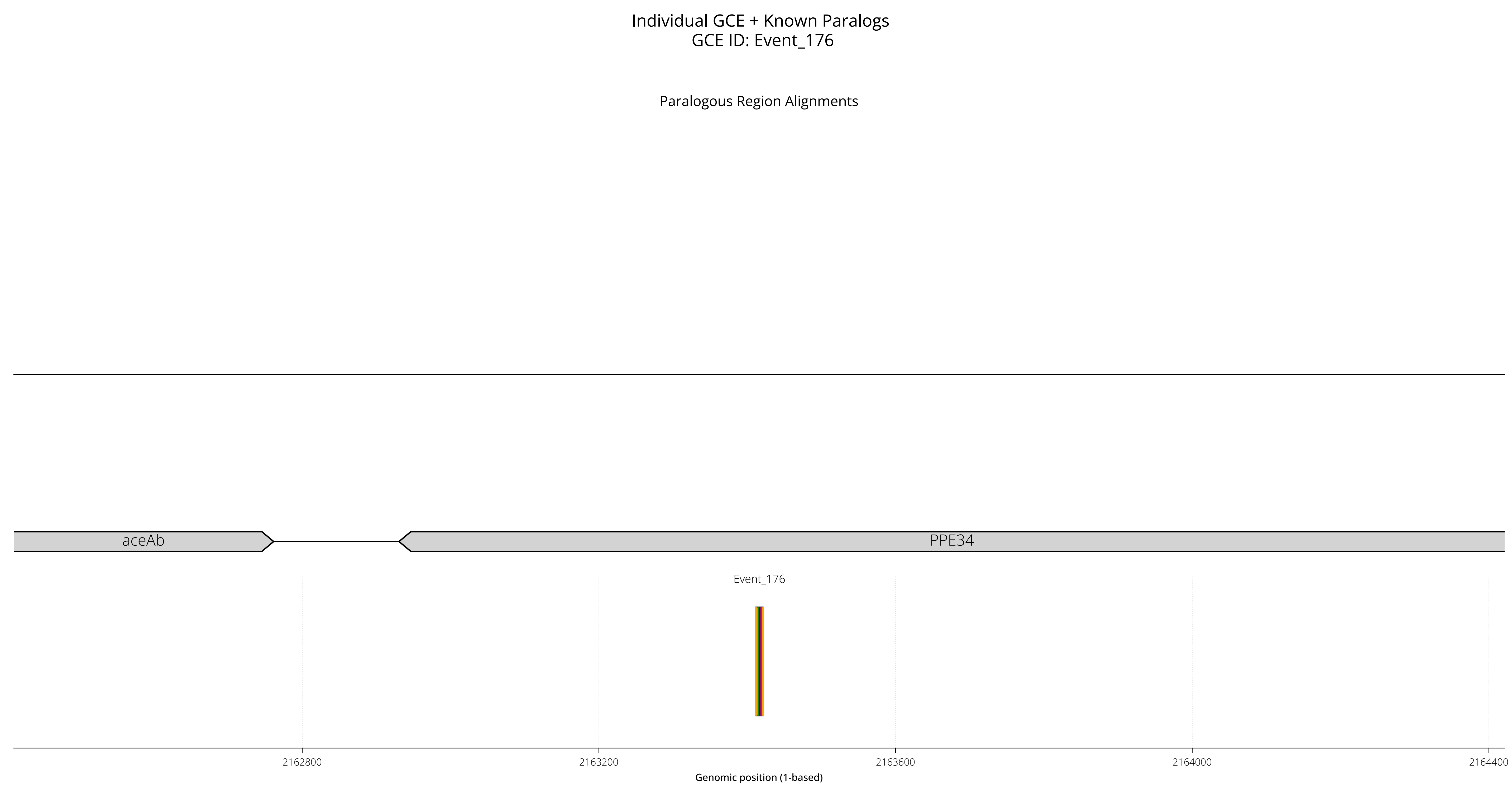
Individual GCE + Known Paralogs
GCE ID: Event_176
Paralogous Region Alignments
aceAb
PPE34
Event_176
2162800
2163200
2163600
2164000
2164400
Genomic position (1-based)

Sub-phylogeny Viz + Paralog Alignments
GCE ID: Event_176

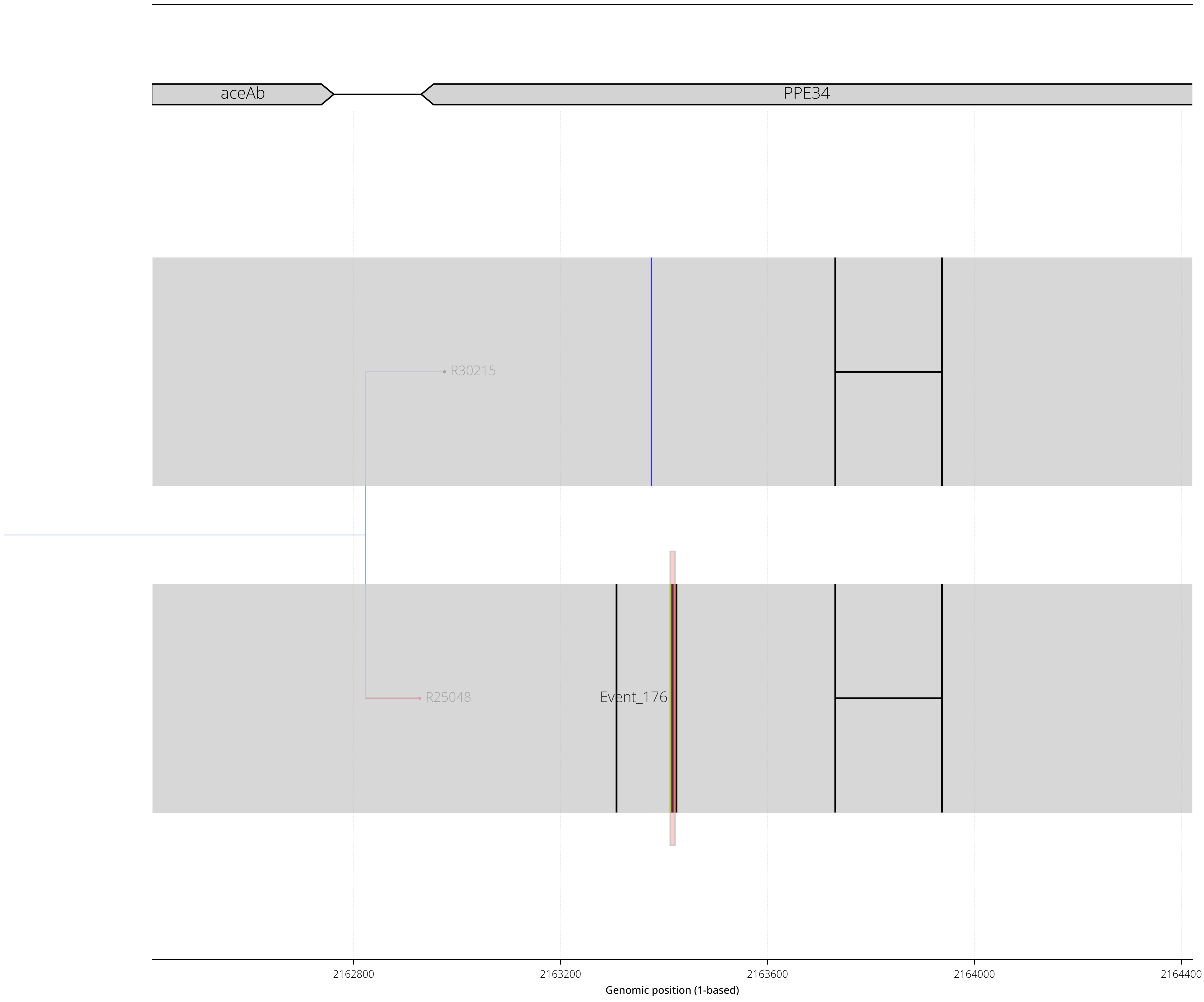

Full Phylogeny Viz + Paralog Alignments
GCE ID: Event_176

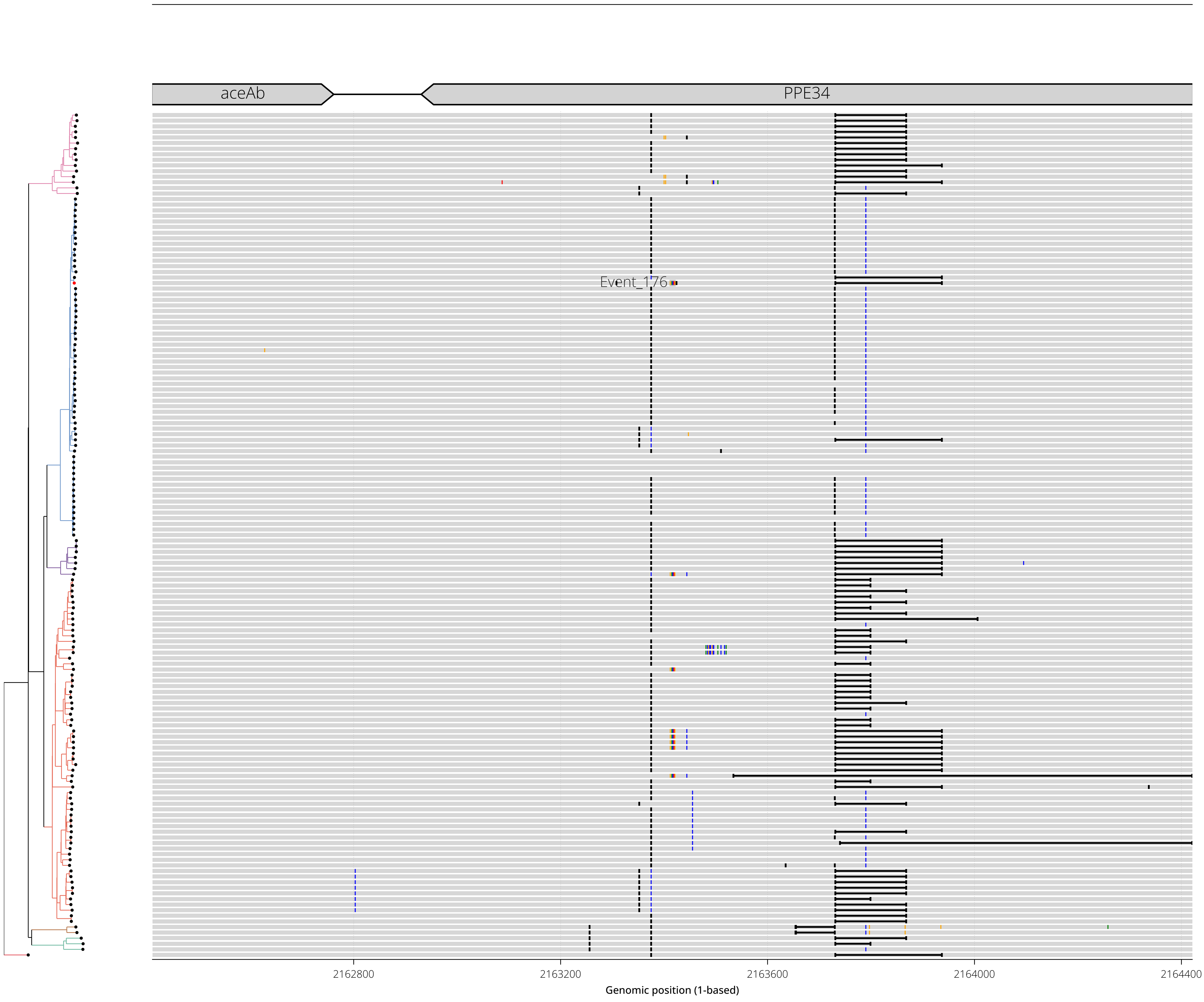

Individual GCE + Known Paralogs
GCE ID: Event_177

Paralogous Region Alignments

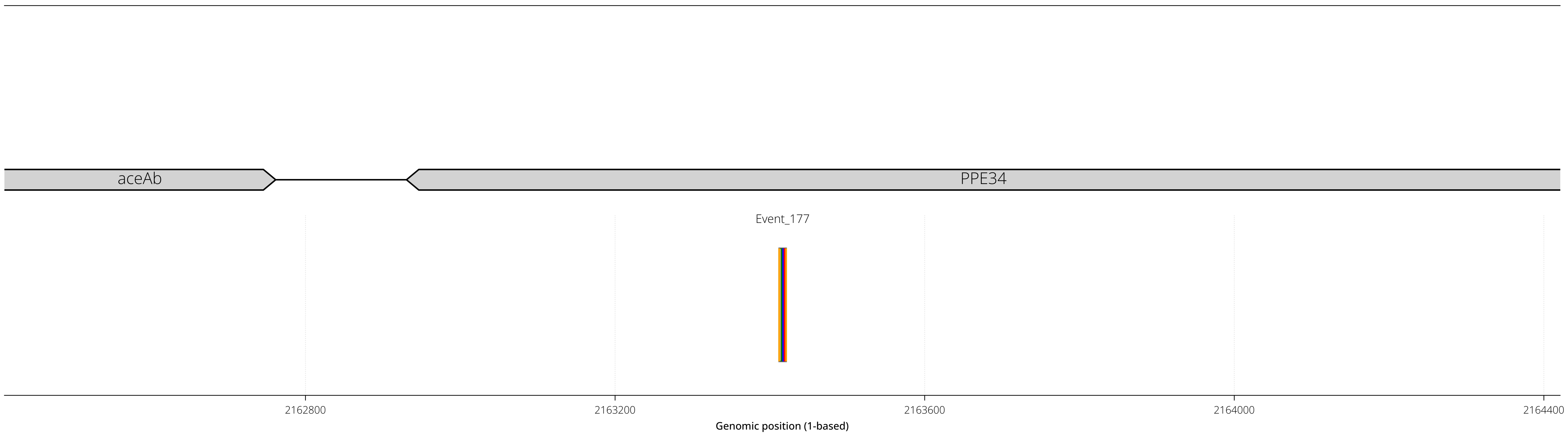

Sub-phylogeny Viz + Paralog Alignments
GCE ID: Event_177

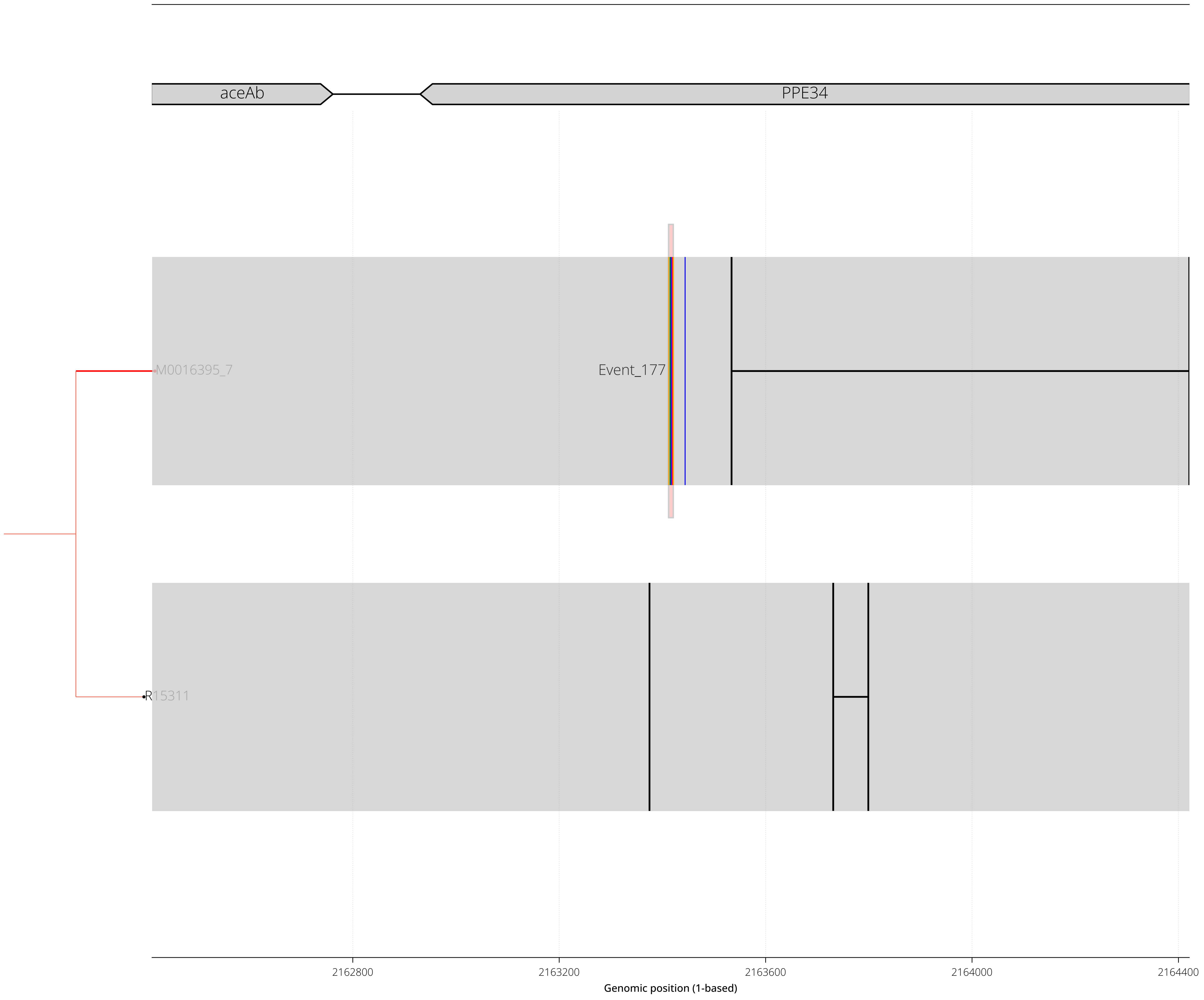

Full Phylogeny Viz + Paralog Alignments
GCE ID: Event_177

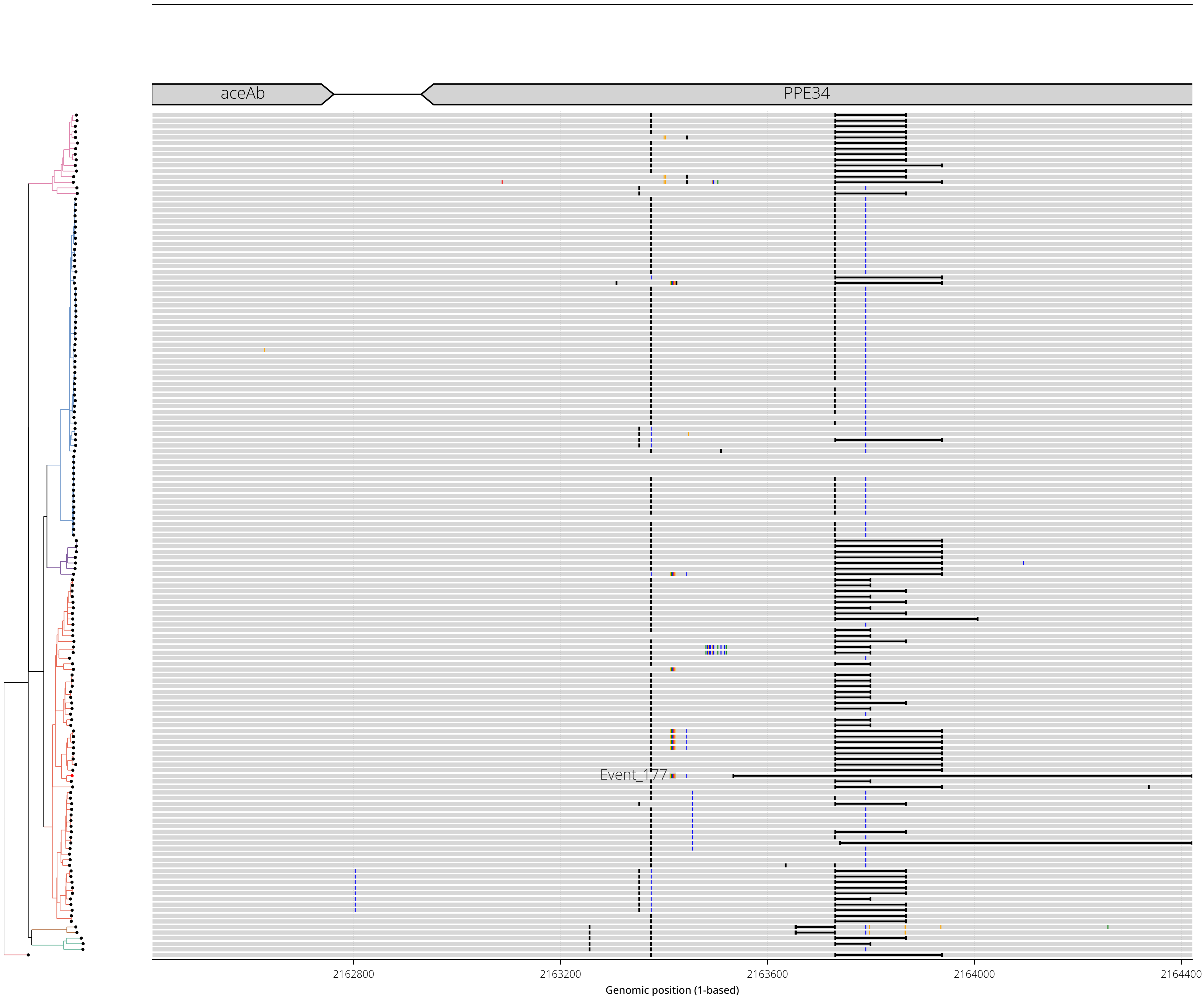

Individual GCE + Known Paralogs
GCE ID: Event_178

Paralogous Region Alignments

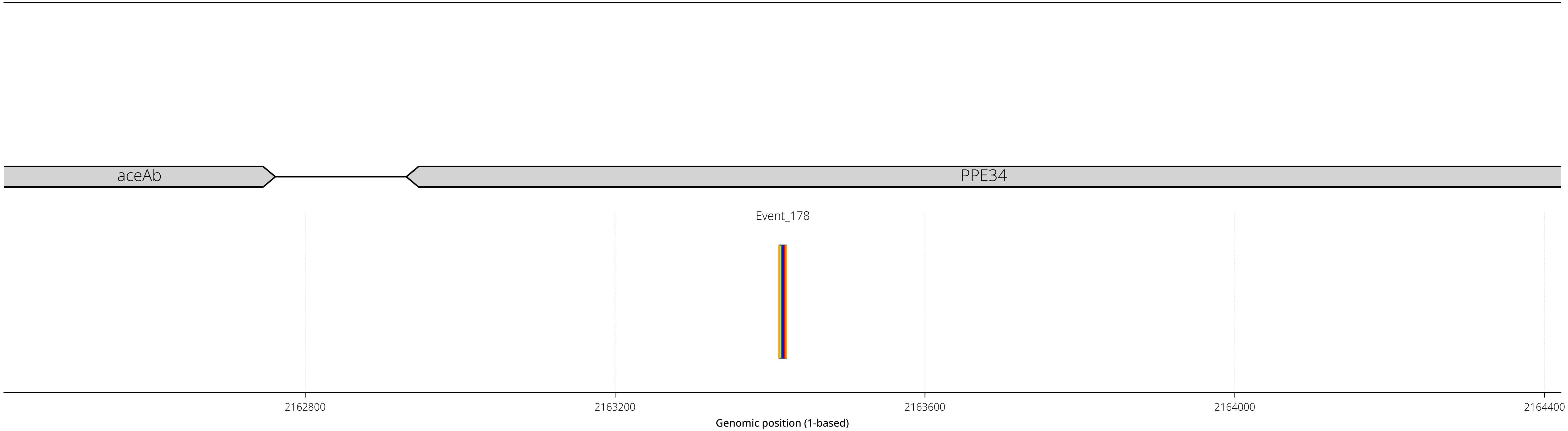

Sub-phylogeny Viz + Paralog Alignments
GCE ID: Event_178

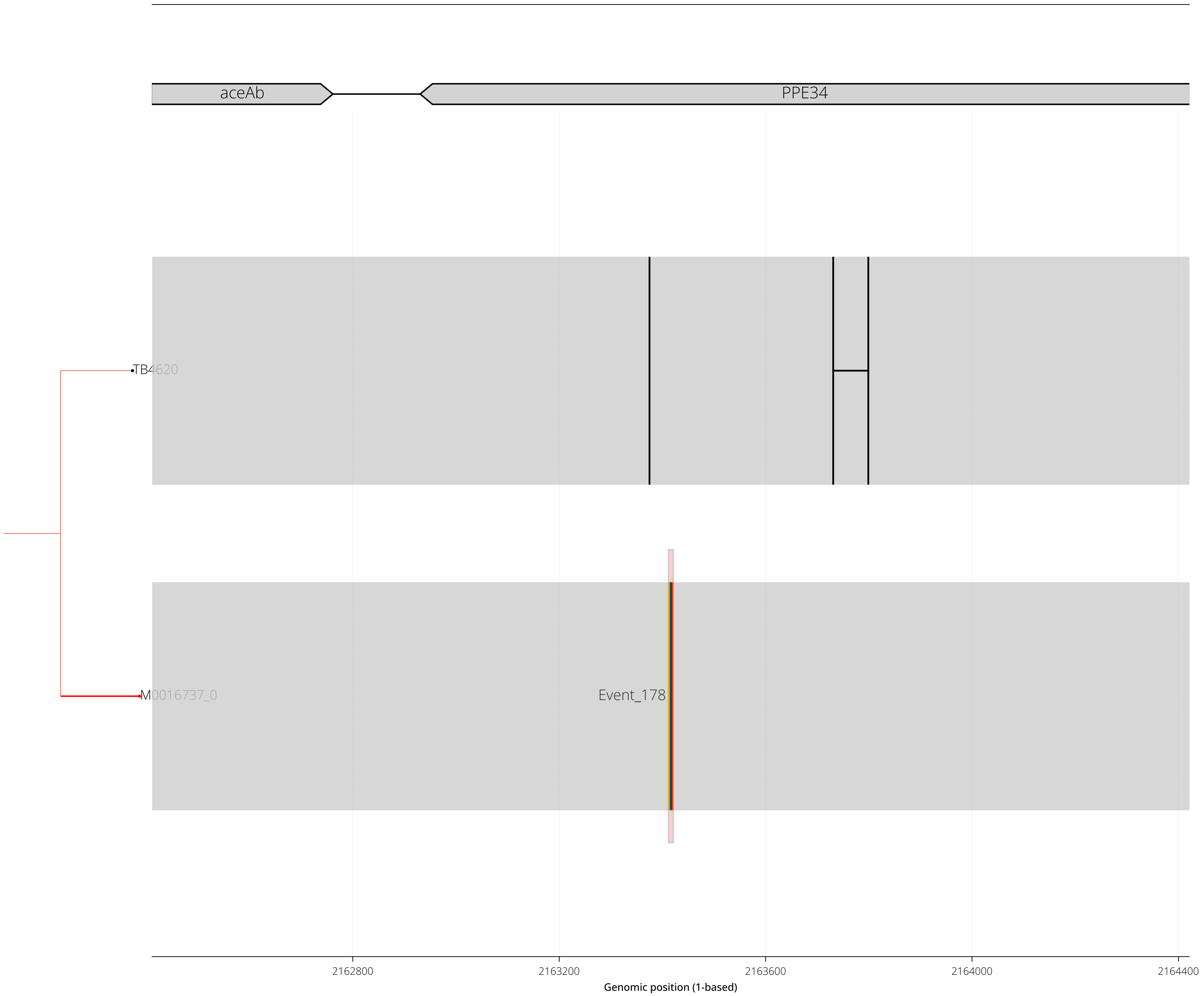

Full Phylogeny Viz + Paralog Alignments
GCE ID: Event_178

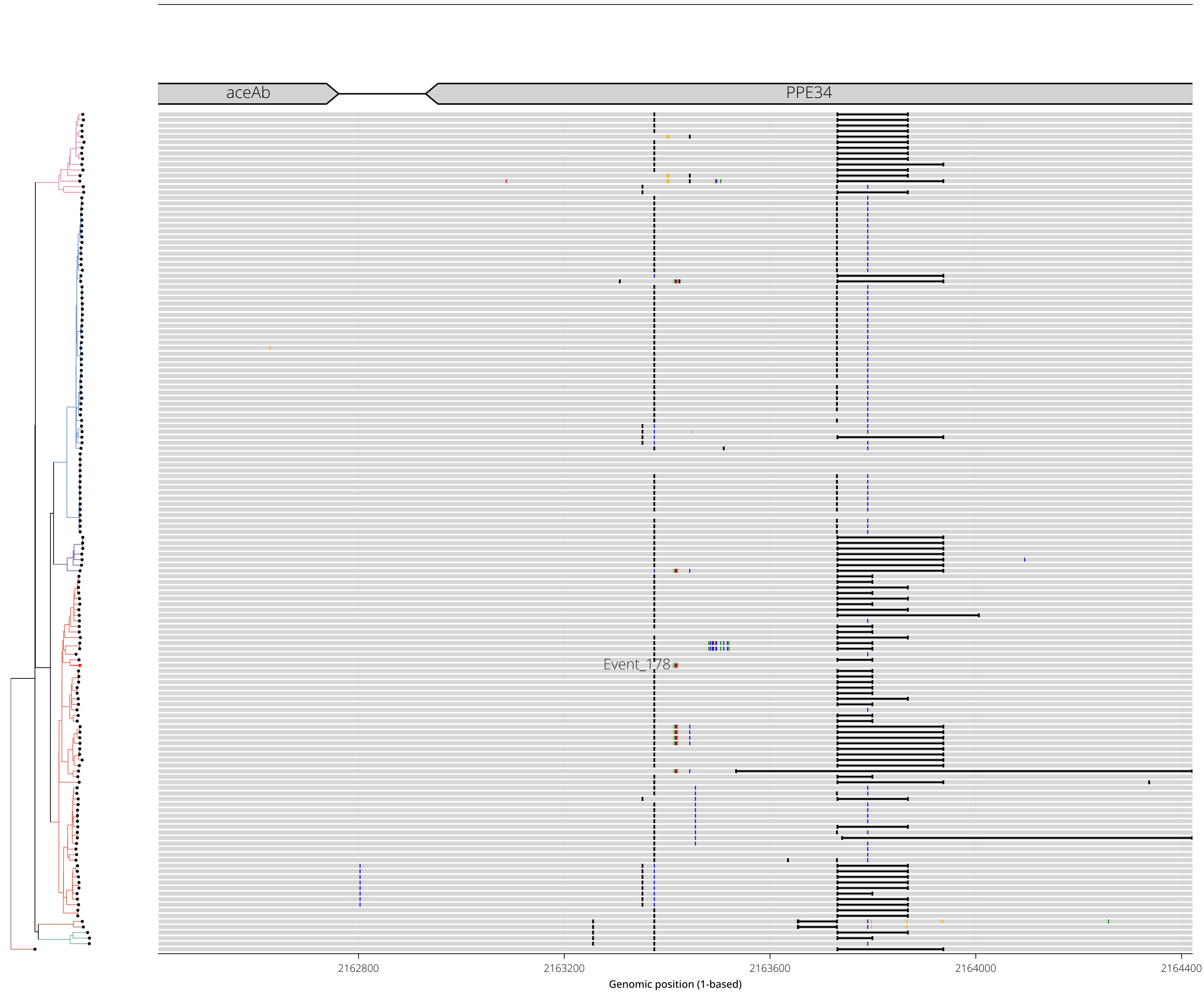

Individual GCE + Known Paralogs
GCE ID: Event_179

Paralogous Region Alignments

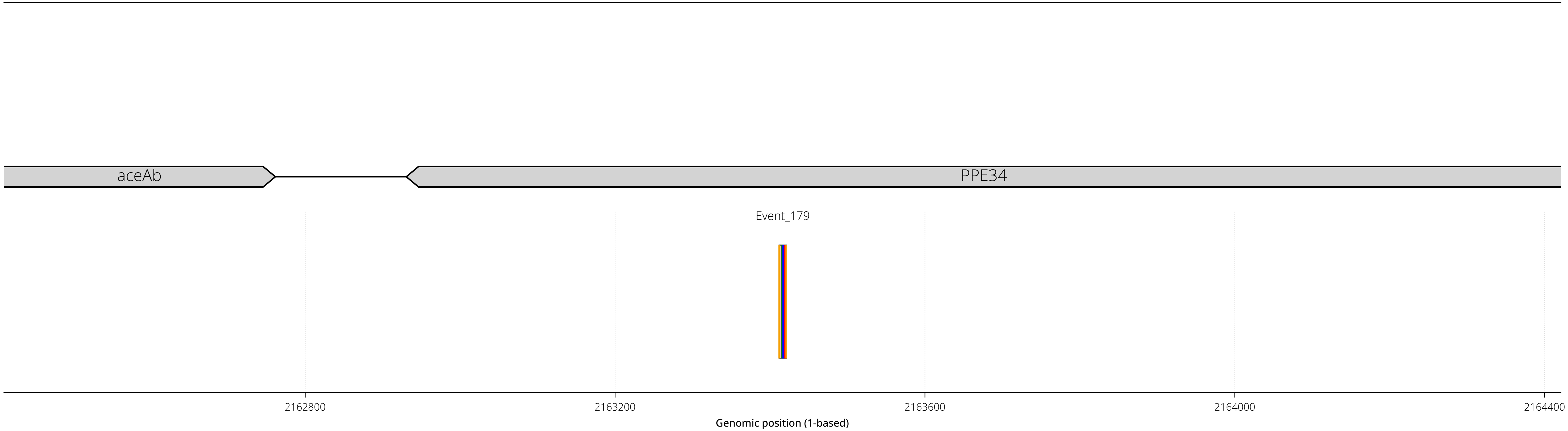

Sub-phylogeny Viz + Paralog Alignments
GCE ID: Event_179

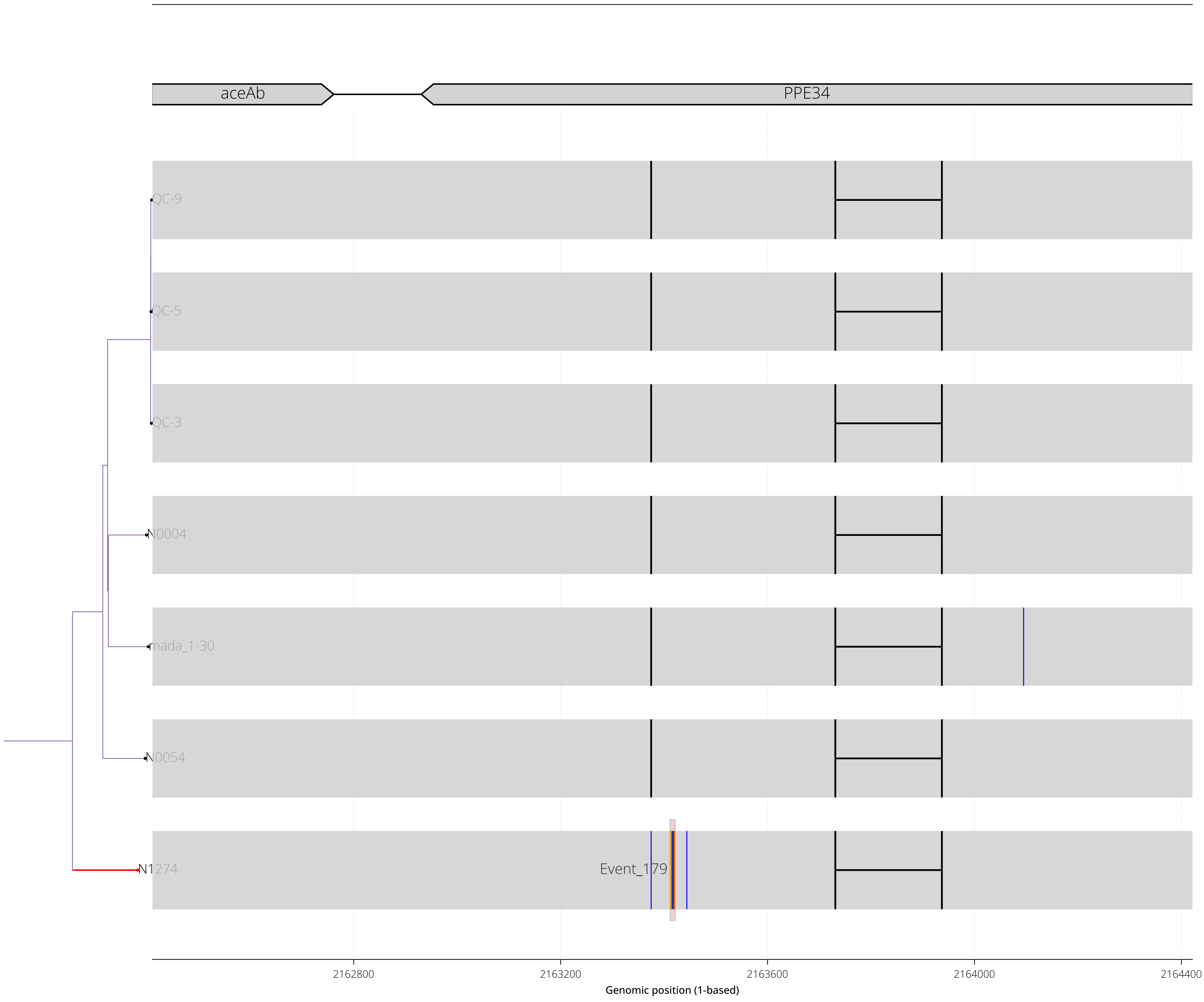

Full Phylogeny Viz + Paralog Alignments
GCE ID: Event_179

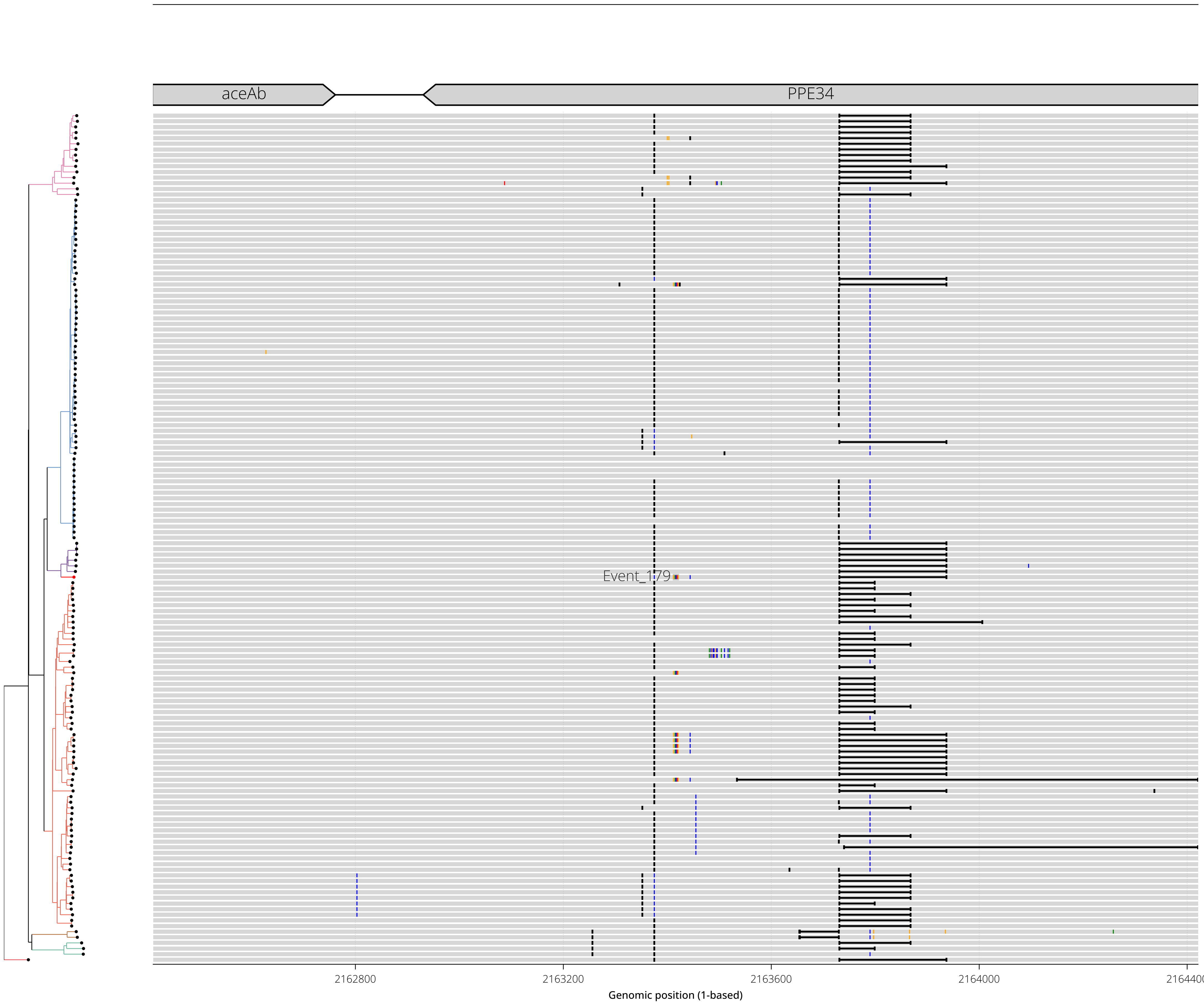

Individual GCE + Known Paralogs
GCE ID: Event_180

Paralogous Region Alignments

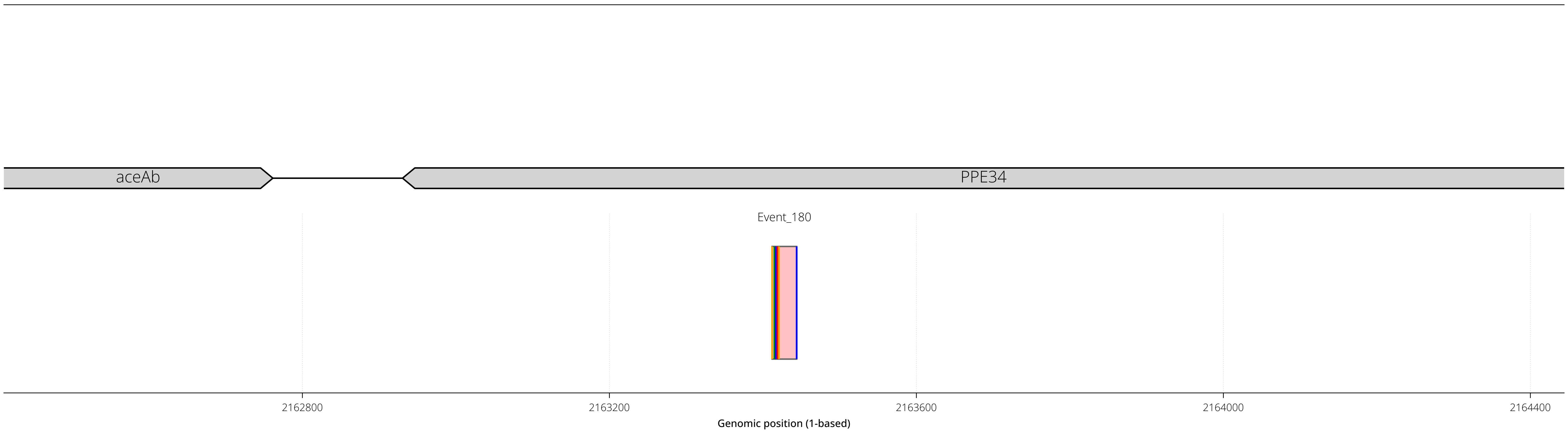

Sub-phylogeny Viz + Paralog Alignments
GCE ID: Event_180

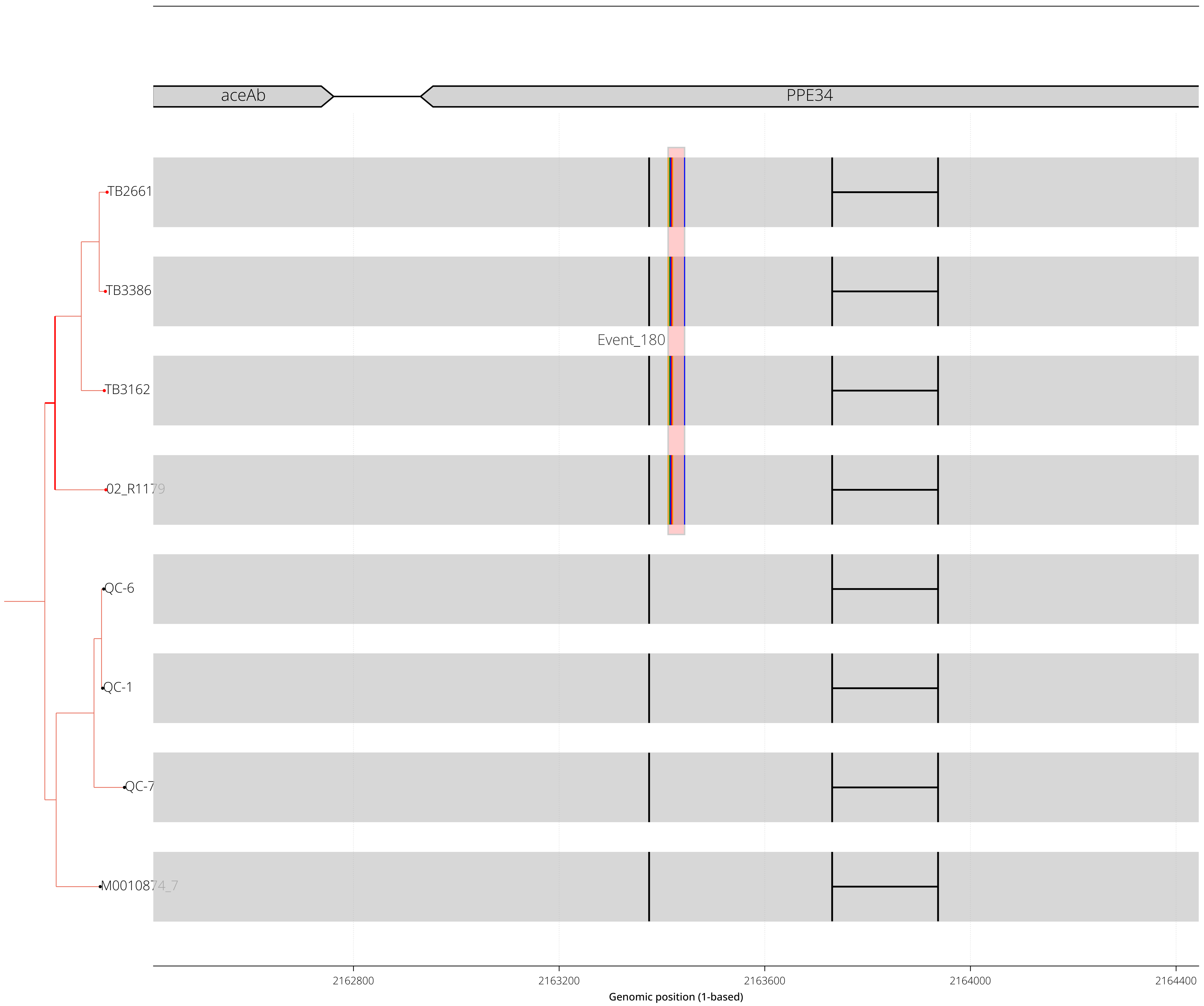

Full Phylogeny Viz + Paralog Alignments
GCE ID: Event_180

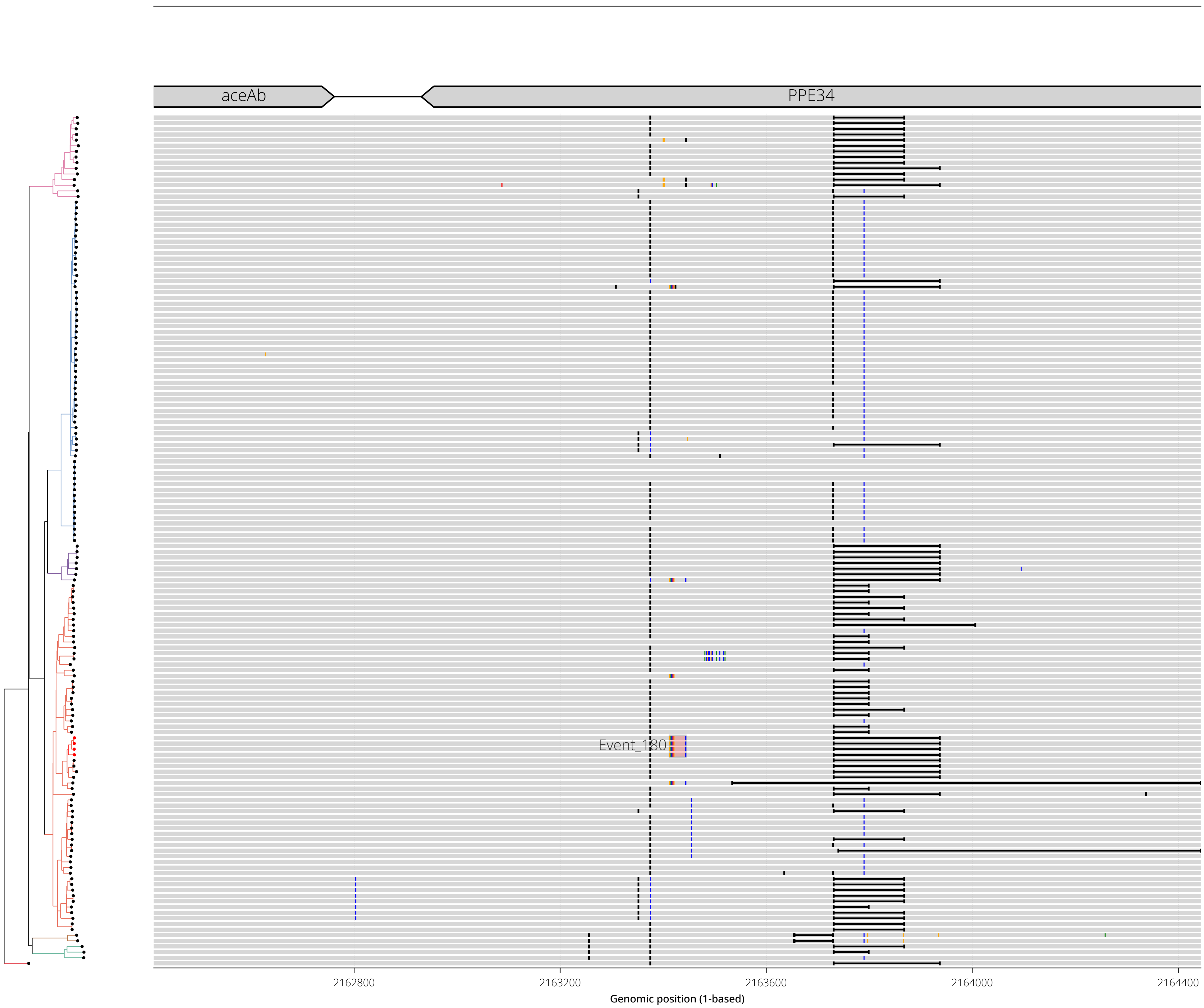

Individual GCE + Known Paralogs
GCE ID: Event_181

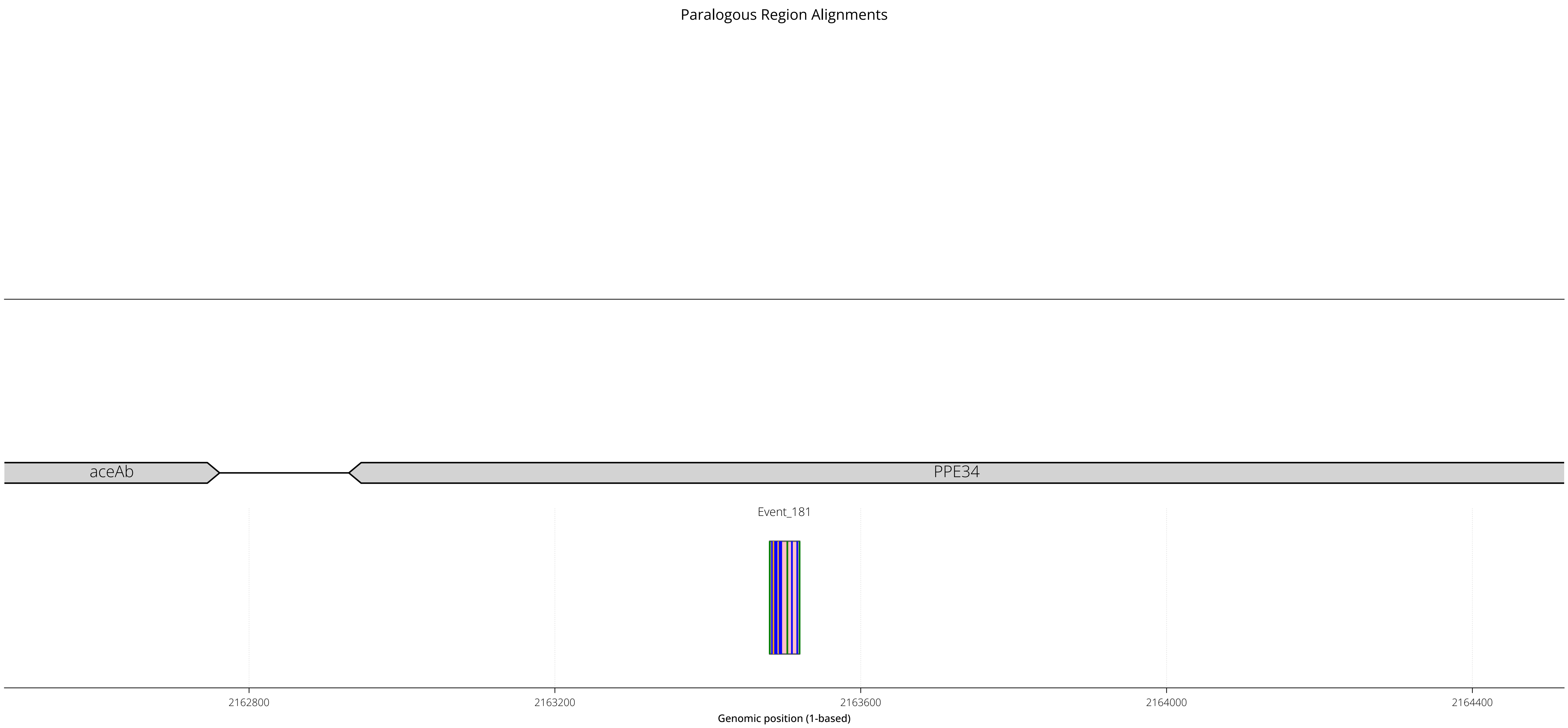

Sub-phylogeny Viz + Paralog Alignments
GCE ID: Event_181

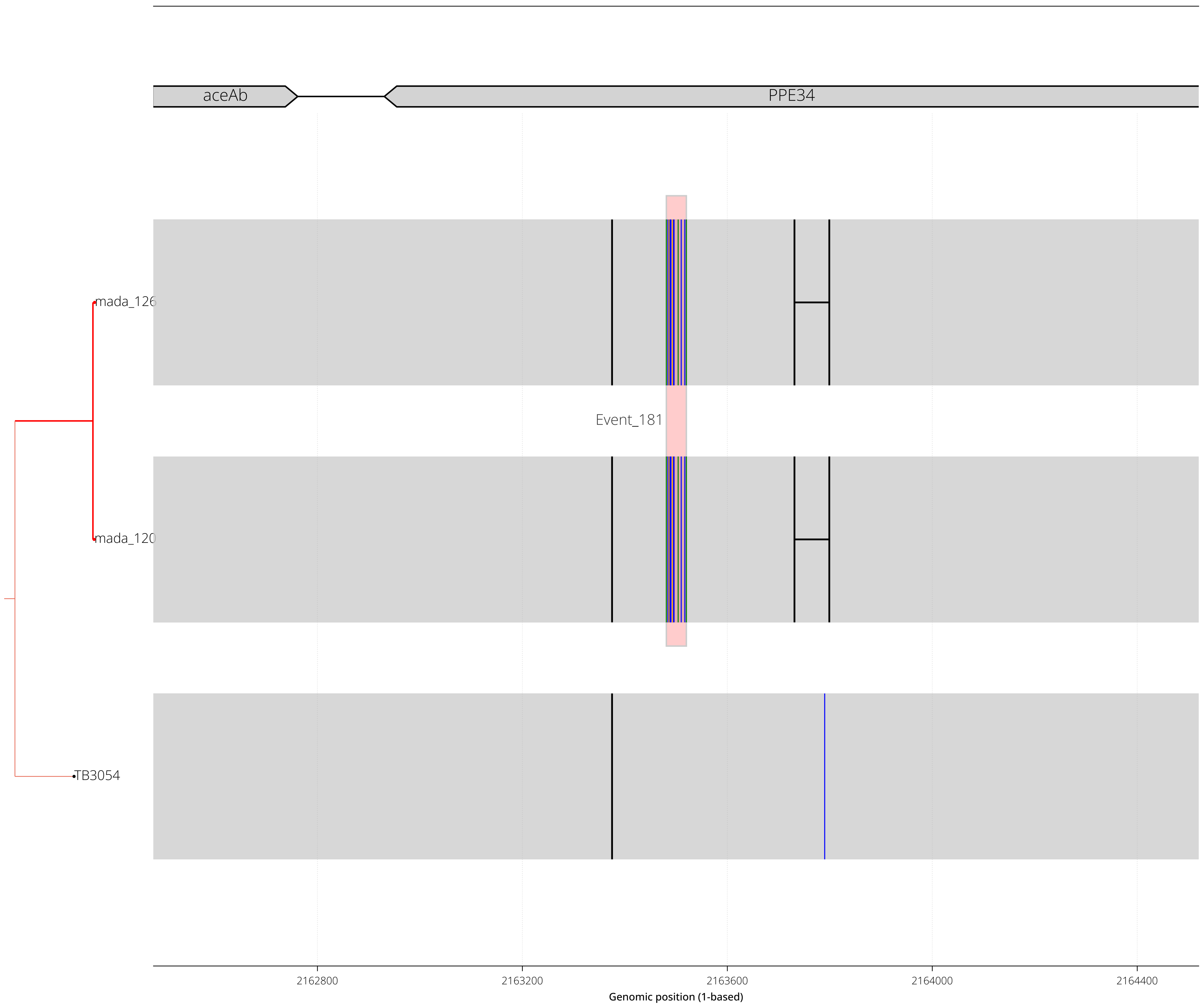

Full Phylogeny Viz + Paralog Alignments
GCE ID: Event_181

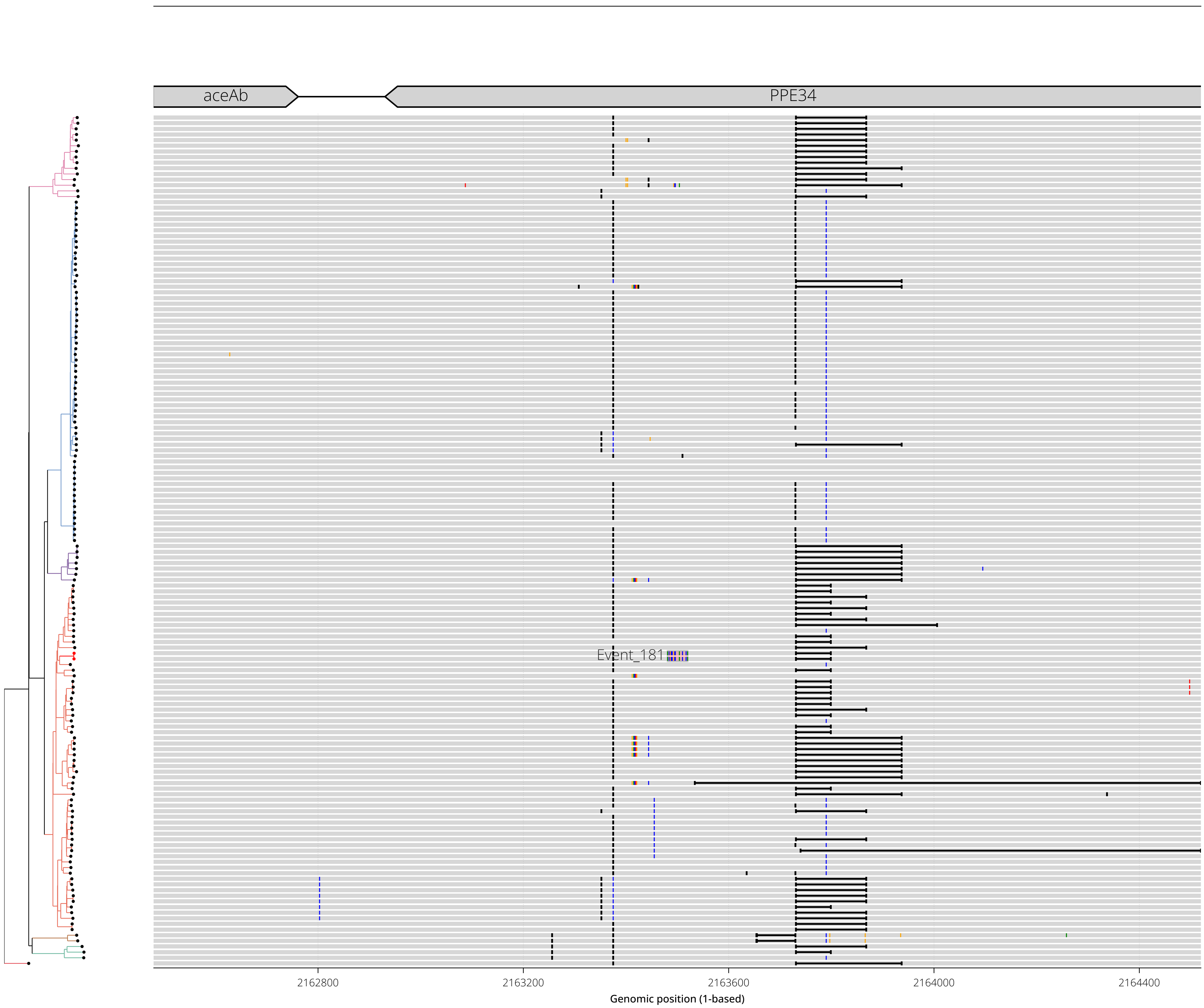

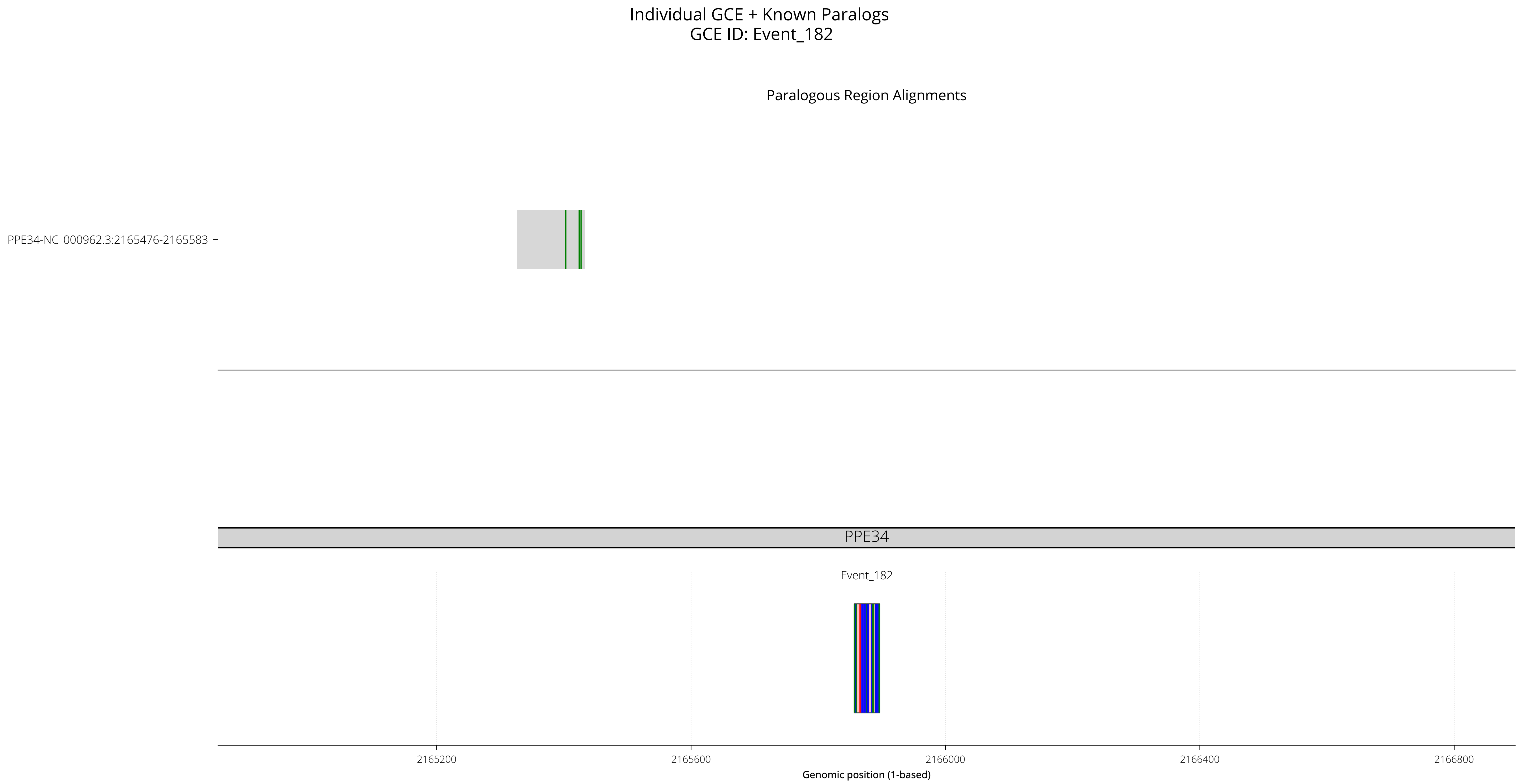

Individual GCE + Known Paralogs
GCE ID: Event_182
Paralogous Region Alignments
PPE34-NC_000962.3:2165476-2165583
PPE34
Event_182
2165200
2165600
2166000
2166400
2166800
Genomic position (1-based)

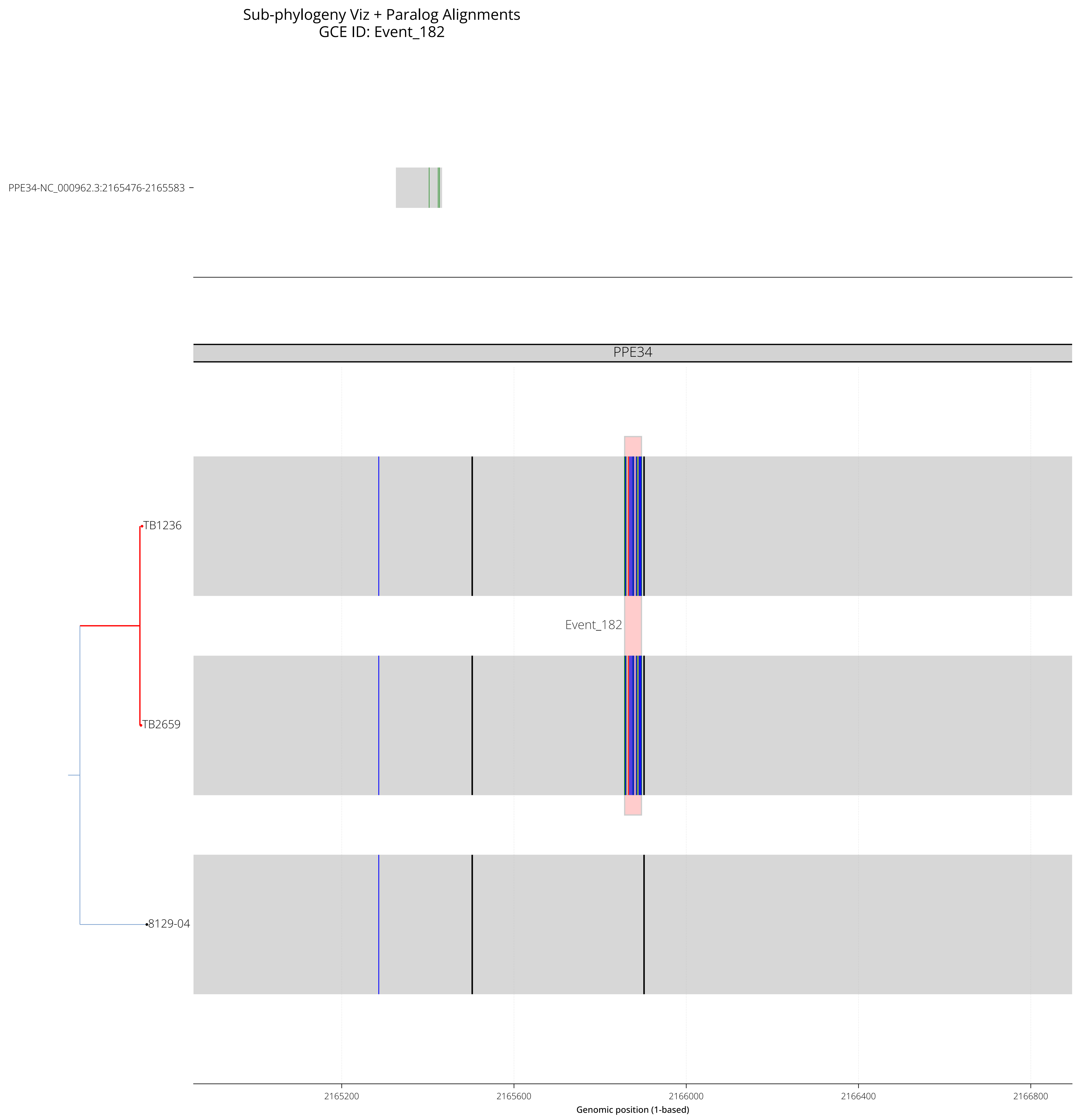

Sub-phylogeny Viz + Paralog Alignments
GCE ID: Event_182
PPE34-NC_000962.3:2165476-2165583
PPE34
TB1236
Event_182
TB2659
8129-04
2165200
2165600
2166000
2166400
2166800
Genomic position (1-based)

Full Phylogeny Viz + Paralog Alignments
GCE ID: Event_182

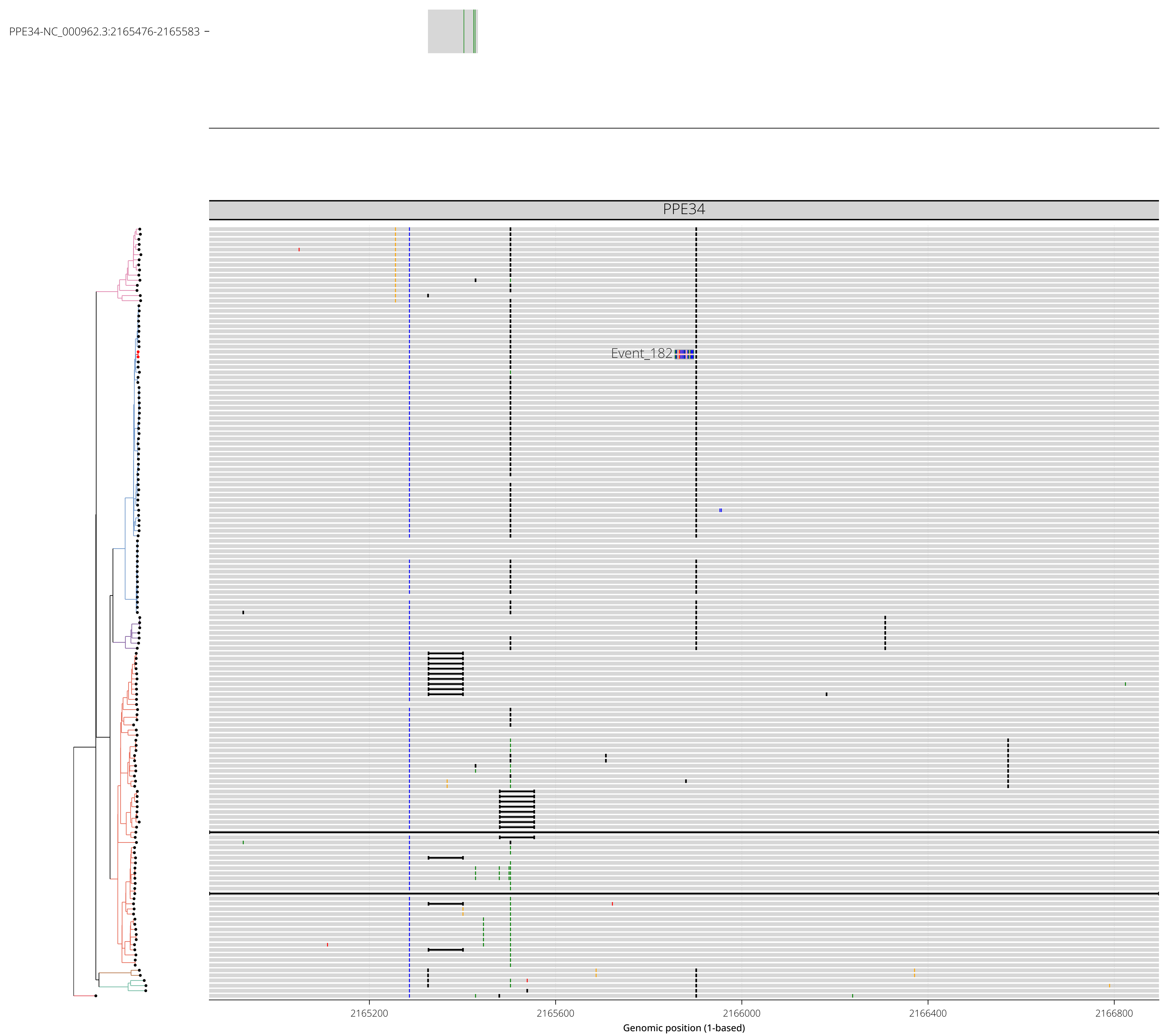

Individual GCE + Known Paralogs
GCE ID: Event_183

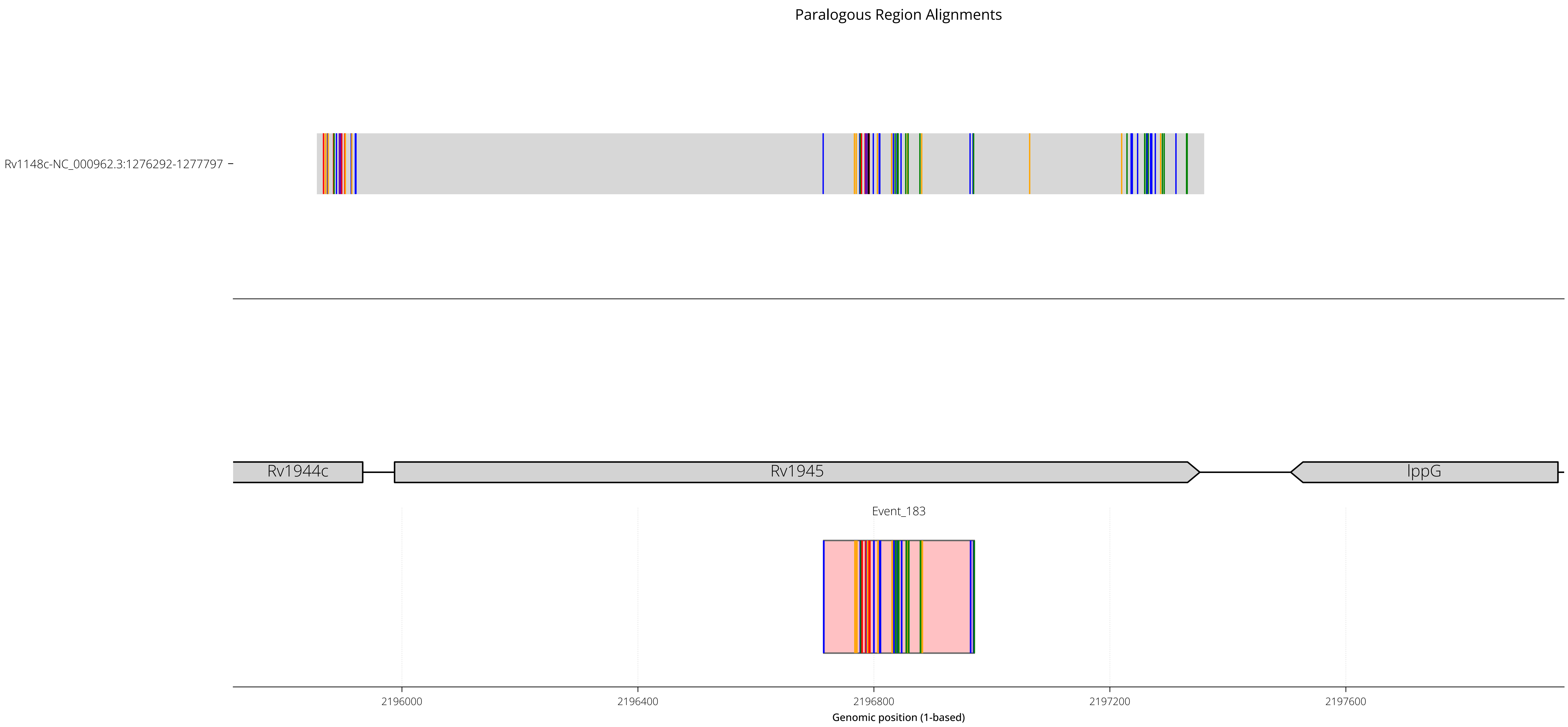

Circos Viz - GCE to Known Paralogs Locations
GCE ID: Event_183

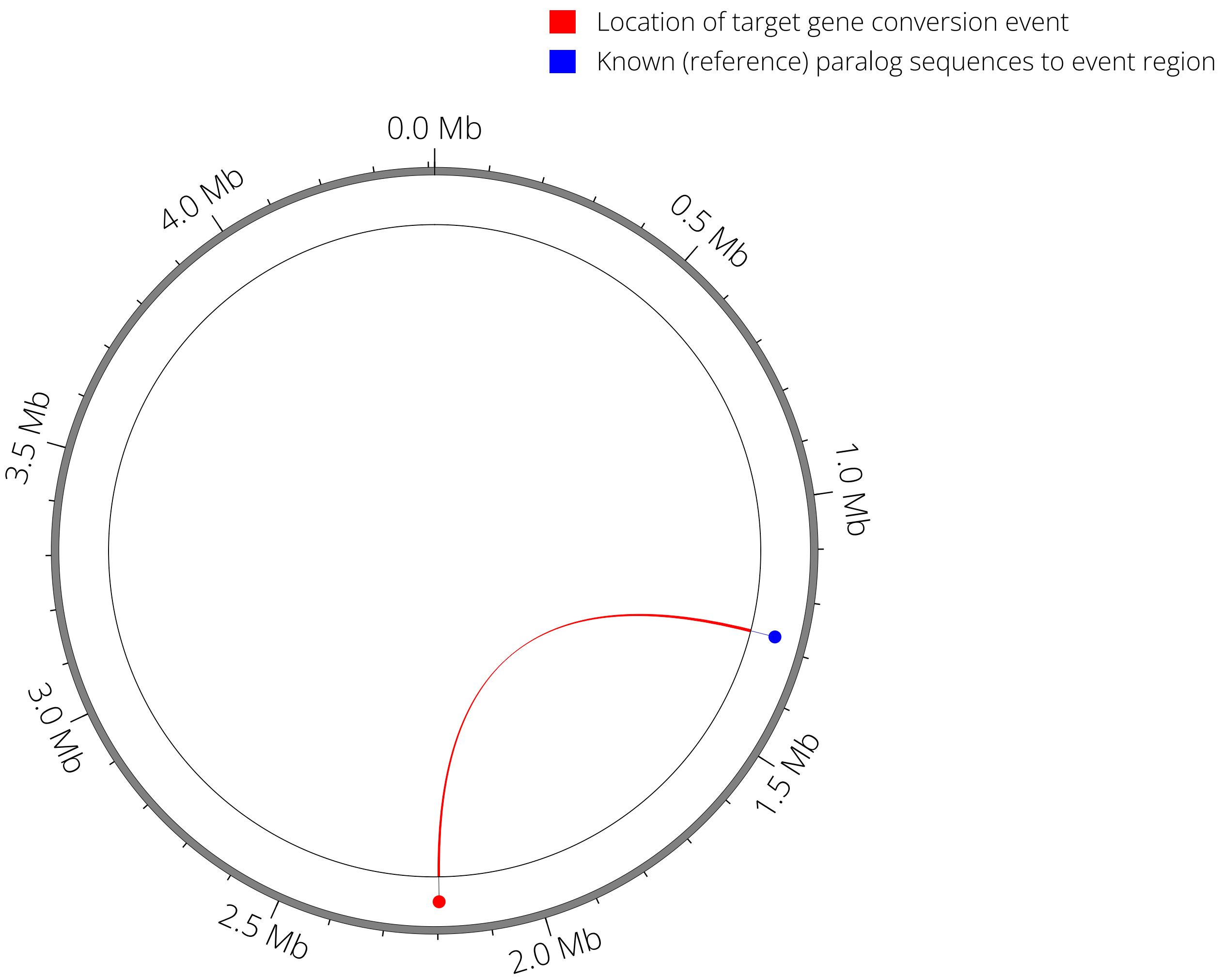

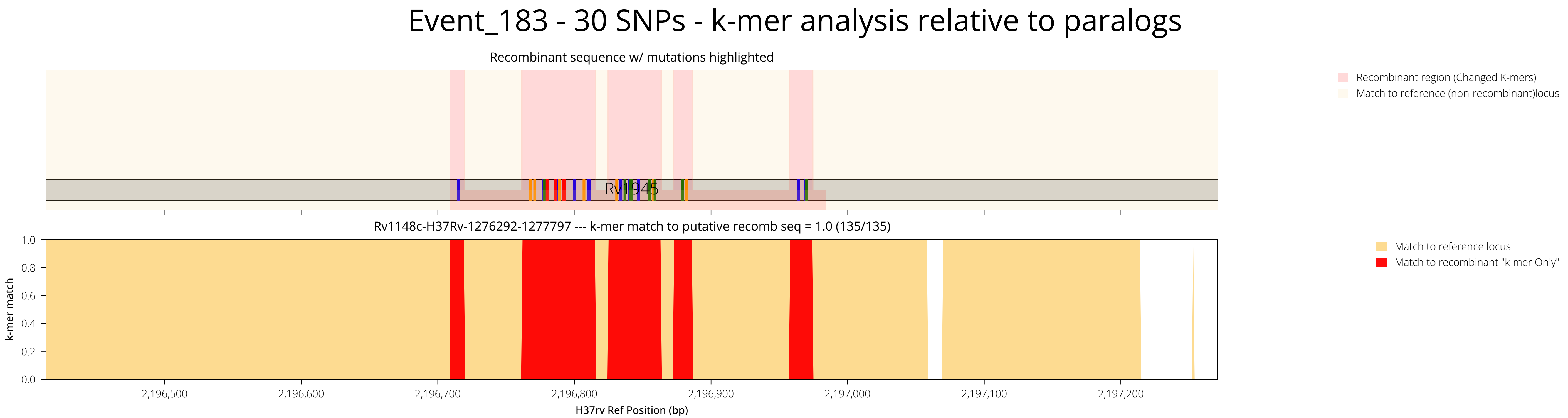
Event_183 - 30 SNPs - k-mer analysis relative to paralogs
Recombinant sequence w/ mutations highlighted
Recombinant region (Changed K-mers)
Match to reference (non-recombinant)locus
Rv1945
Rv1148c-H37Rv-1276292-1277797 --- k-mer match to putative recomb seq = 1.0 (135/135)
Match to reference locus
Match to recombinant "k-mer Only"
k-mer match
1.0
0.8
0.6
0.4
0.2
0.0
2,196,500
2,196,600
2,196,700
2,196,800
2,196,900
2,197,000
2,197,100
2,197,200
H37rv Ref Position (bp)

Sub-phylogeny Viz + Paralog Alignments
GCE ID: Event_183

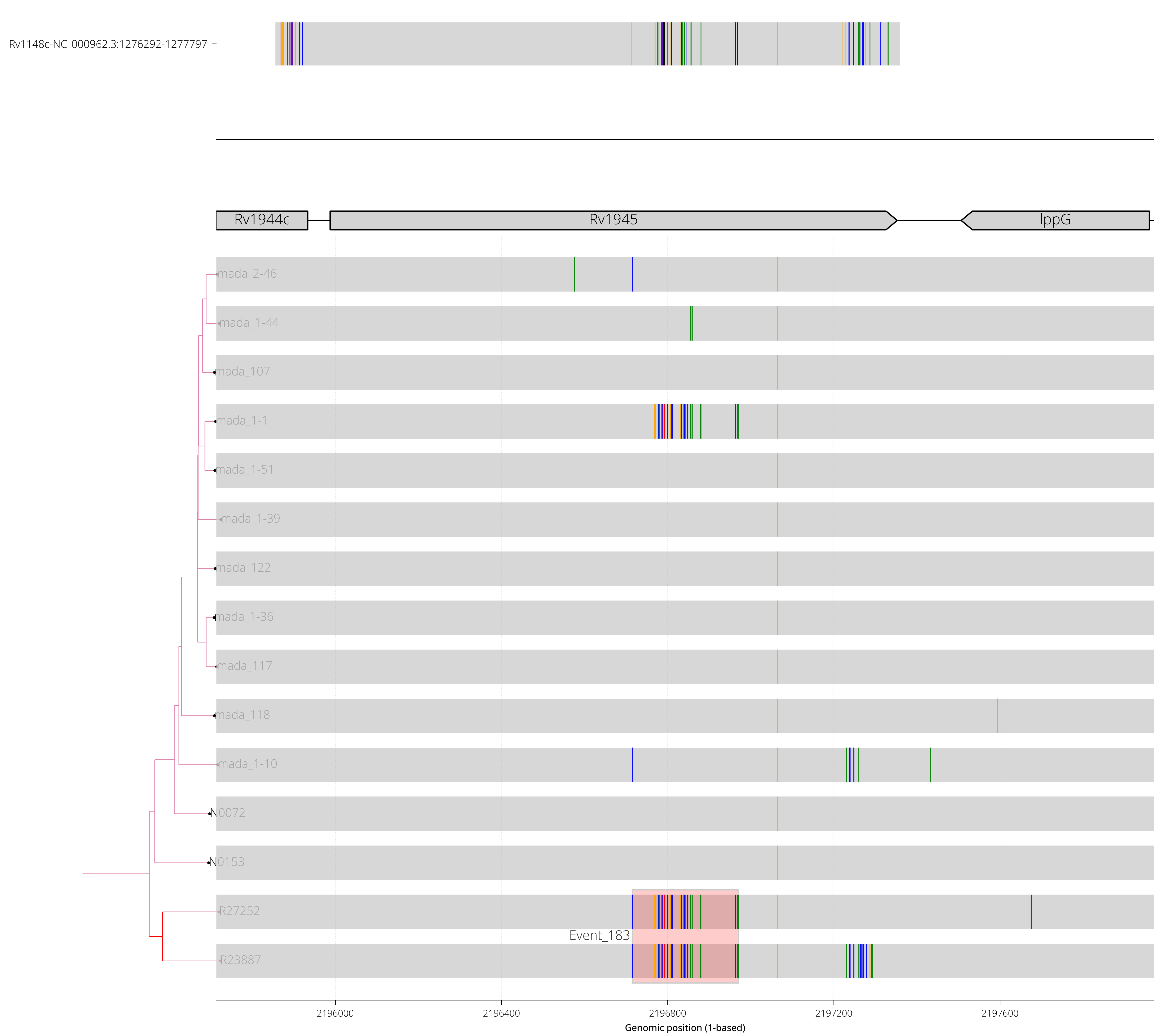

Full Phylogeny Viz + Paralog Alignments
GCE ID: Event_183

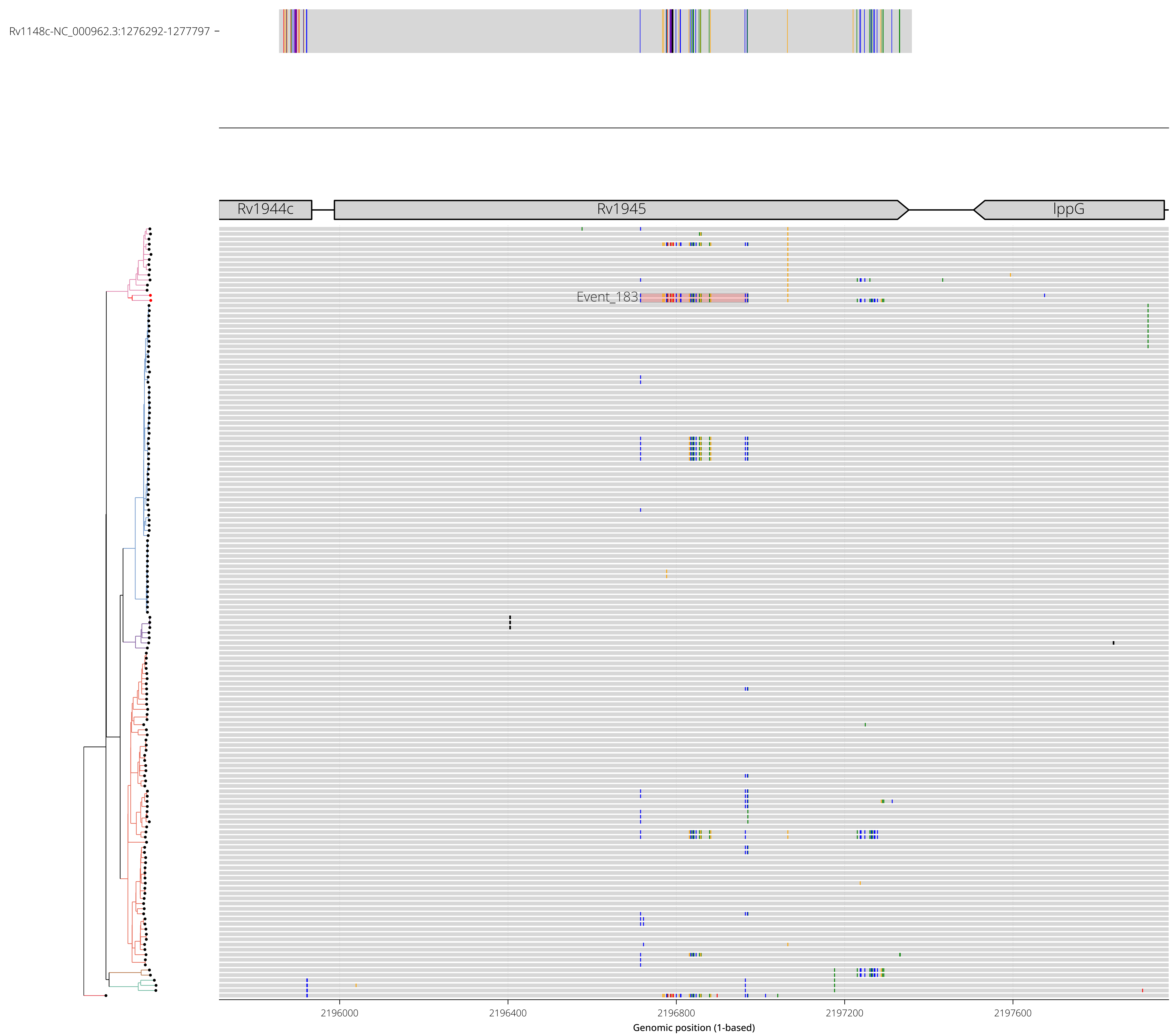

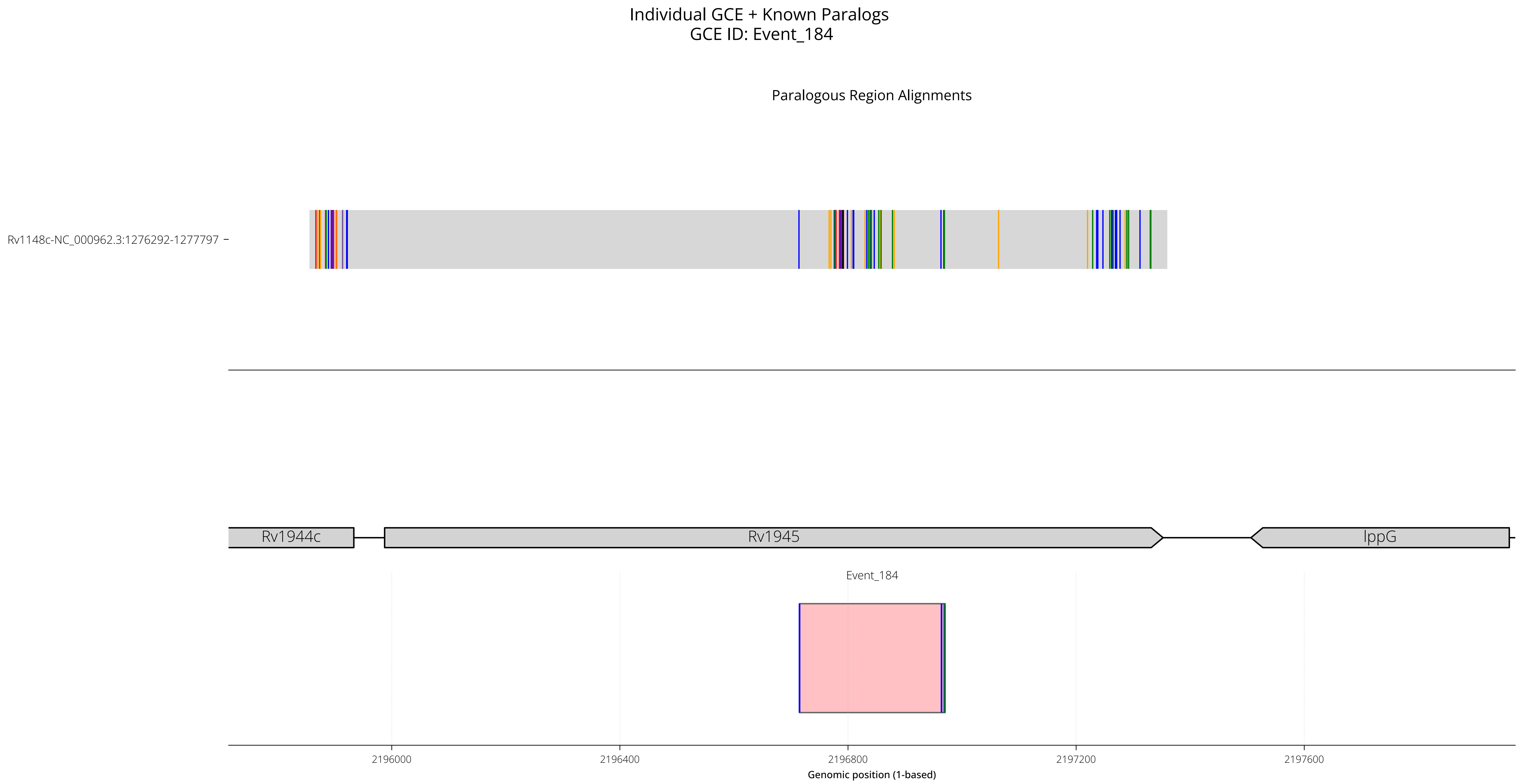

Individual GCE + Known Paralogs
GCE ID: Event_184
Paralogous Region Alignments
Rv1148c-NC_000962.3:1276292-1277797
Rv1944c
Rv1945
lppG
Event_184
2196000
2196400
2196800
2197200
2197600
Genomic position (1-based)

Circos Viz - GCE to Known Paralogs Locations
GCE ID: Event_184

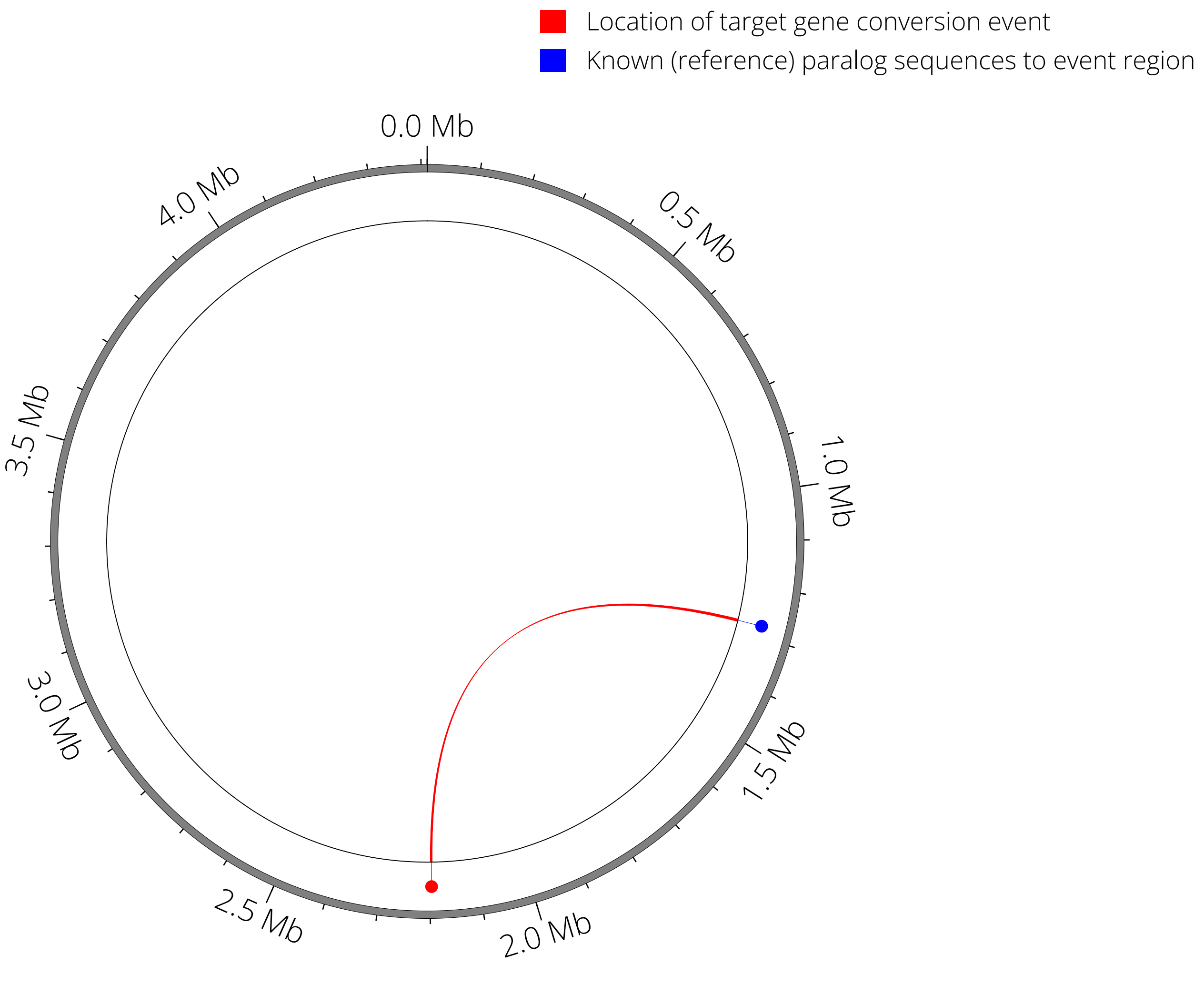

# Event_184 - 4 SNPs - k-mer analysis relative to paralogs

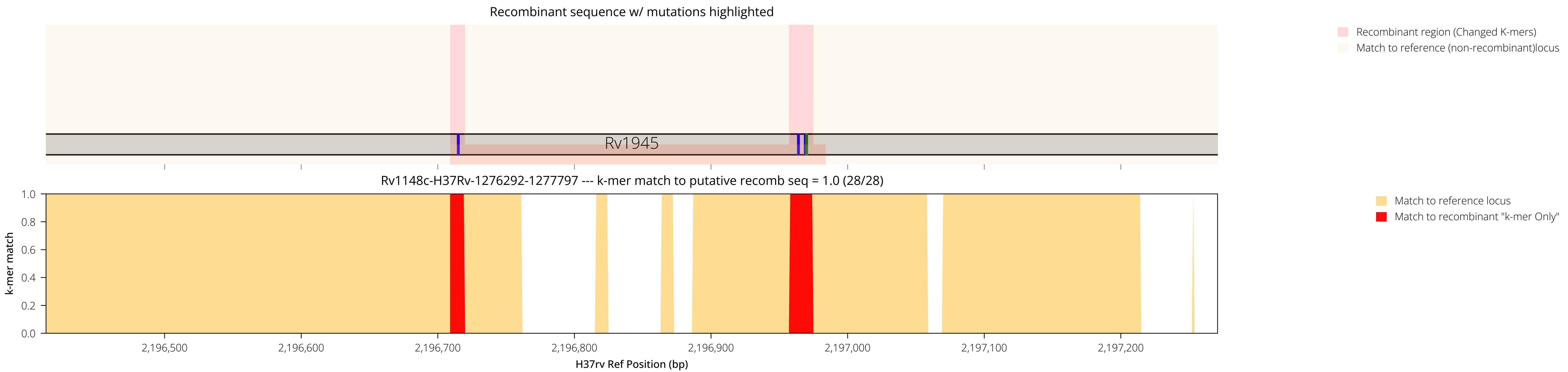

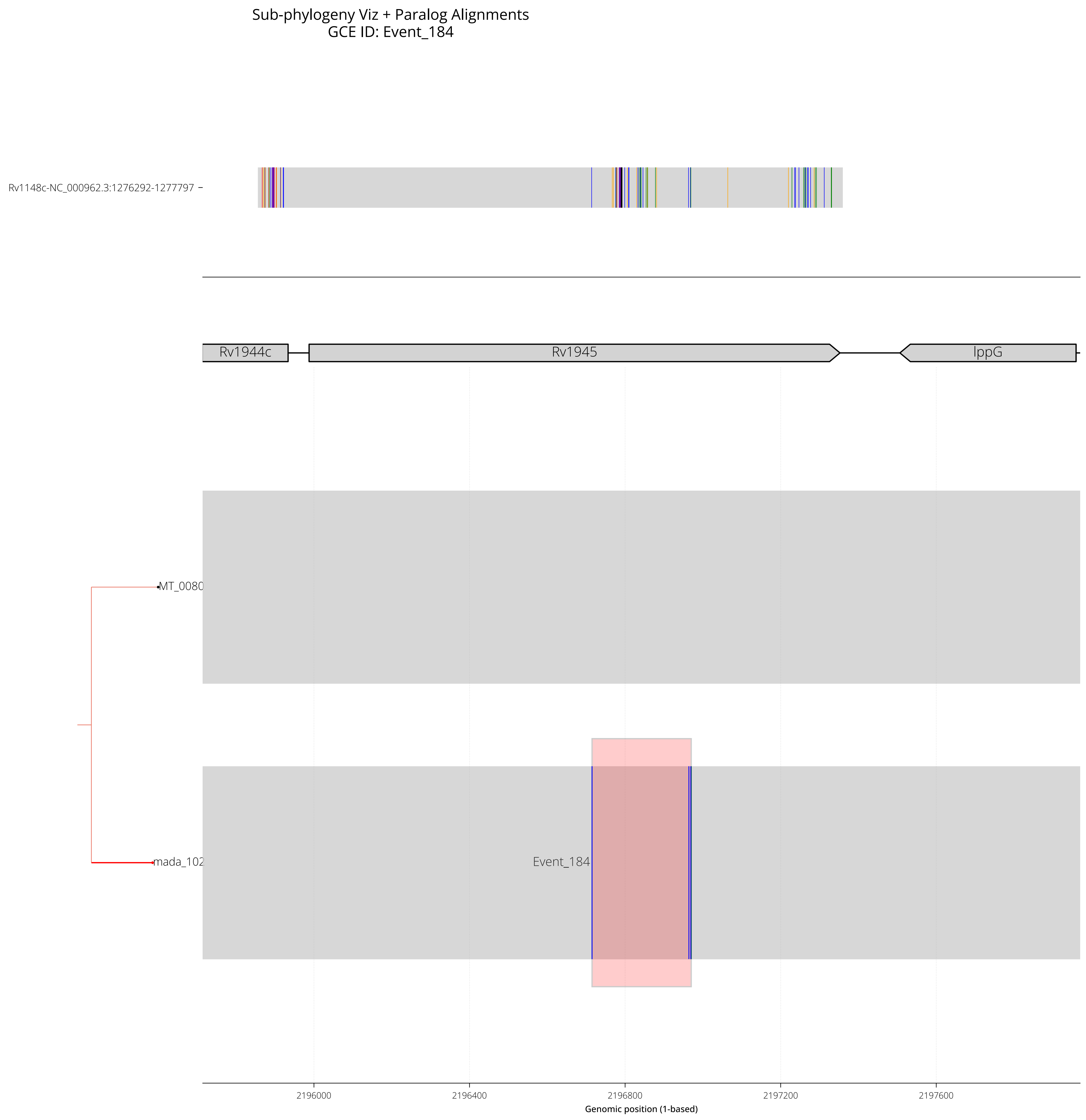
Sub-phylogeny Viz + Paralog Alignments
GCE ID: Event_184
Rv1148c-NC_000962.3:1276292-1277797
Rv1944c
Rv1945
lppG
MT_0080
mada_102
Event_184
2196000
2196400
2196800
2197200
2197600
Genomic position (1-based)

Full Phylogeny Viz + Paralog Alignments
GCE ID: Event_184

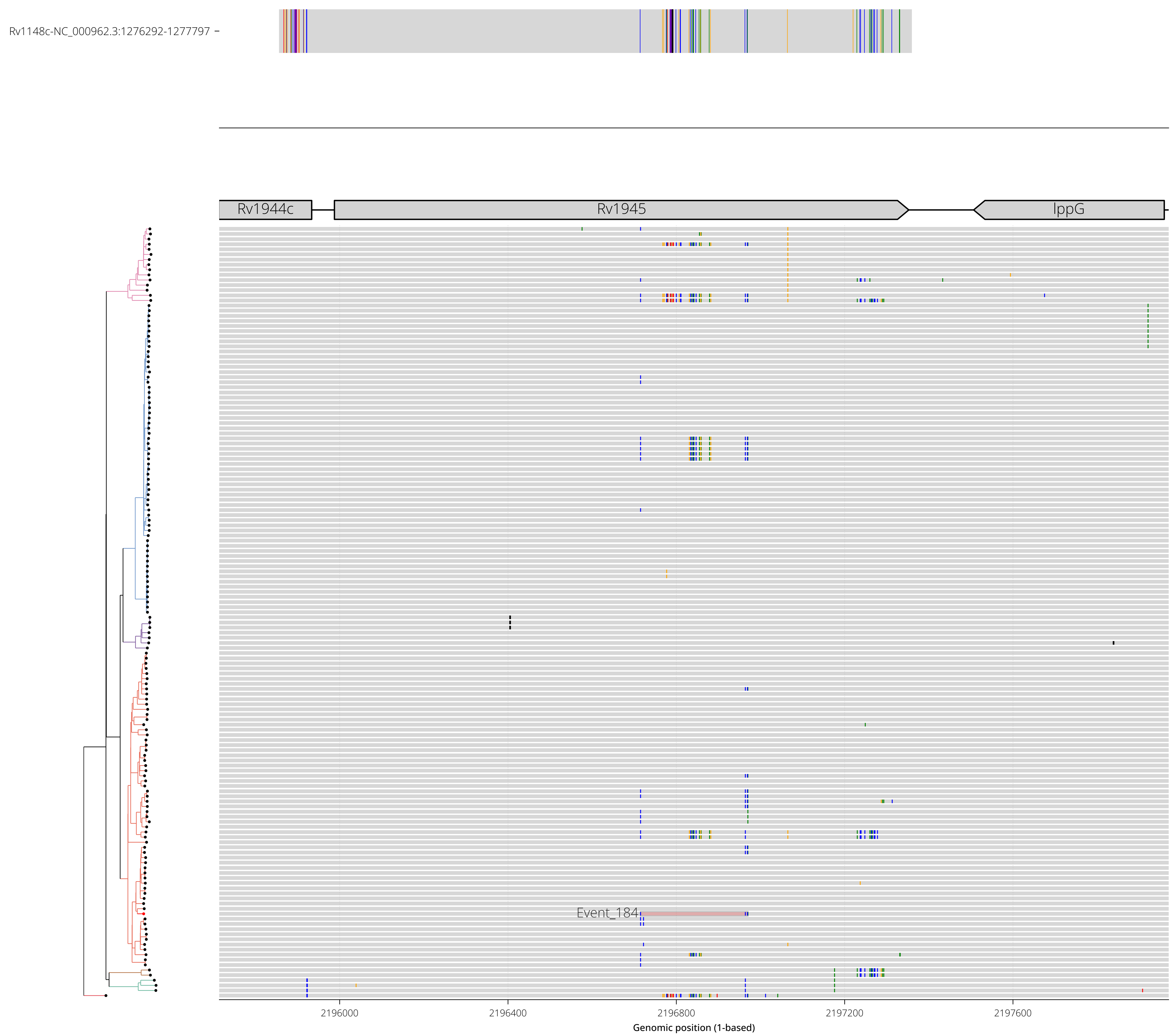

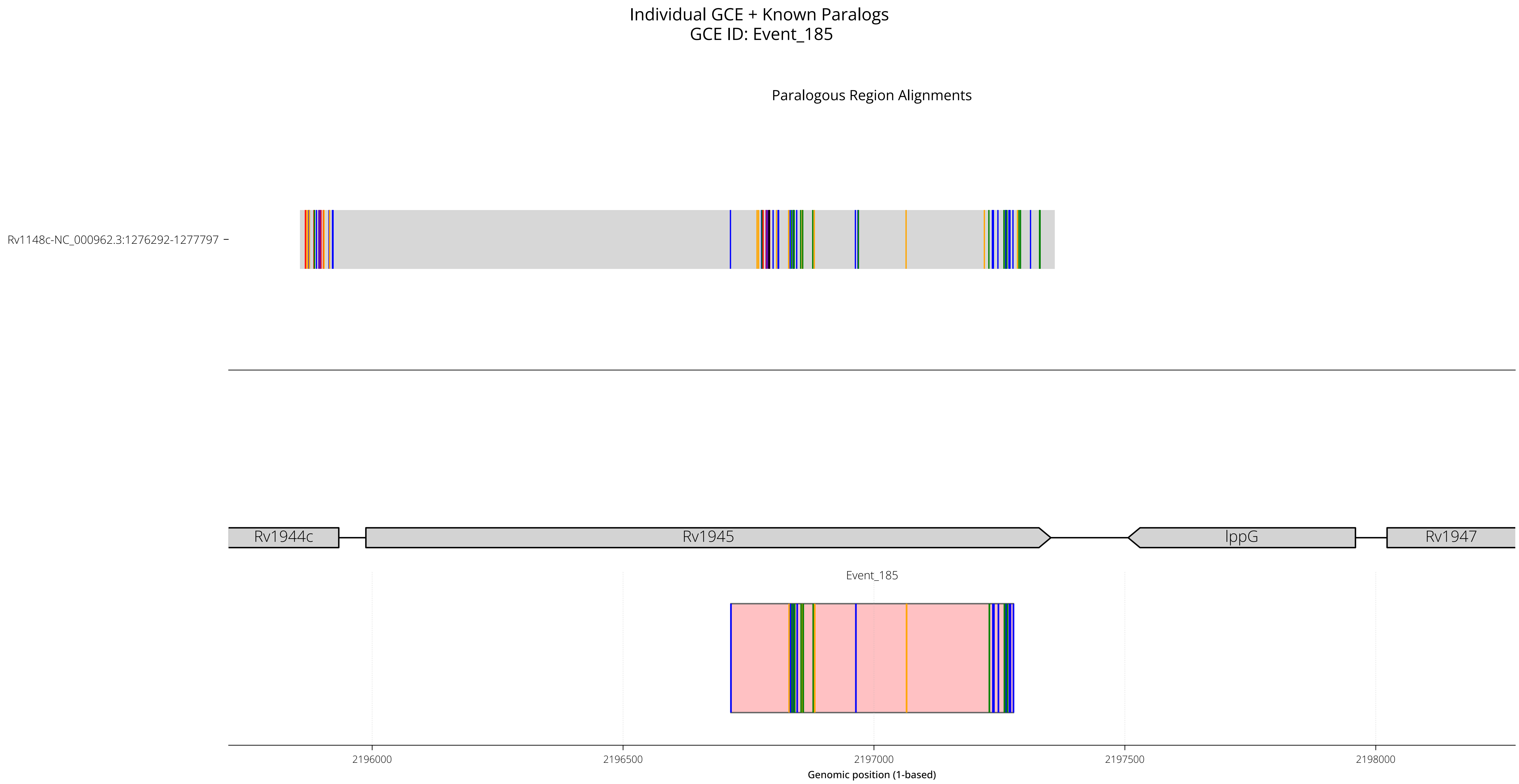

Individual GCE + Known Paralogs
GCE ID: Event_185
Paralogous Region Alignments
Rv1148c-NC_000962.3:1276292-1277797
Rv1944c
Rv1945
lppG
Rv1947
Event_185
2196000
2196500
2197000
2197500
2198000
Genomic position (1-based)

Circos Viz - GCE to Known Paralogs Locations
GCE ID: Event_185

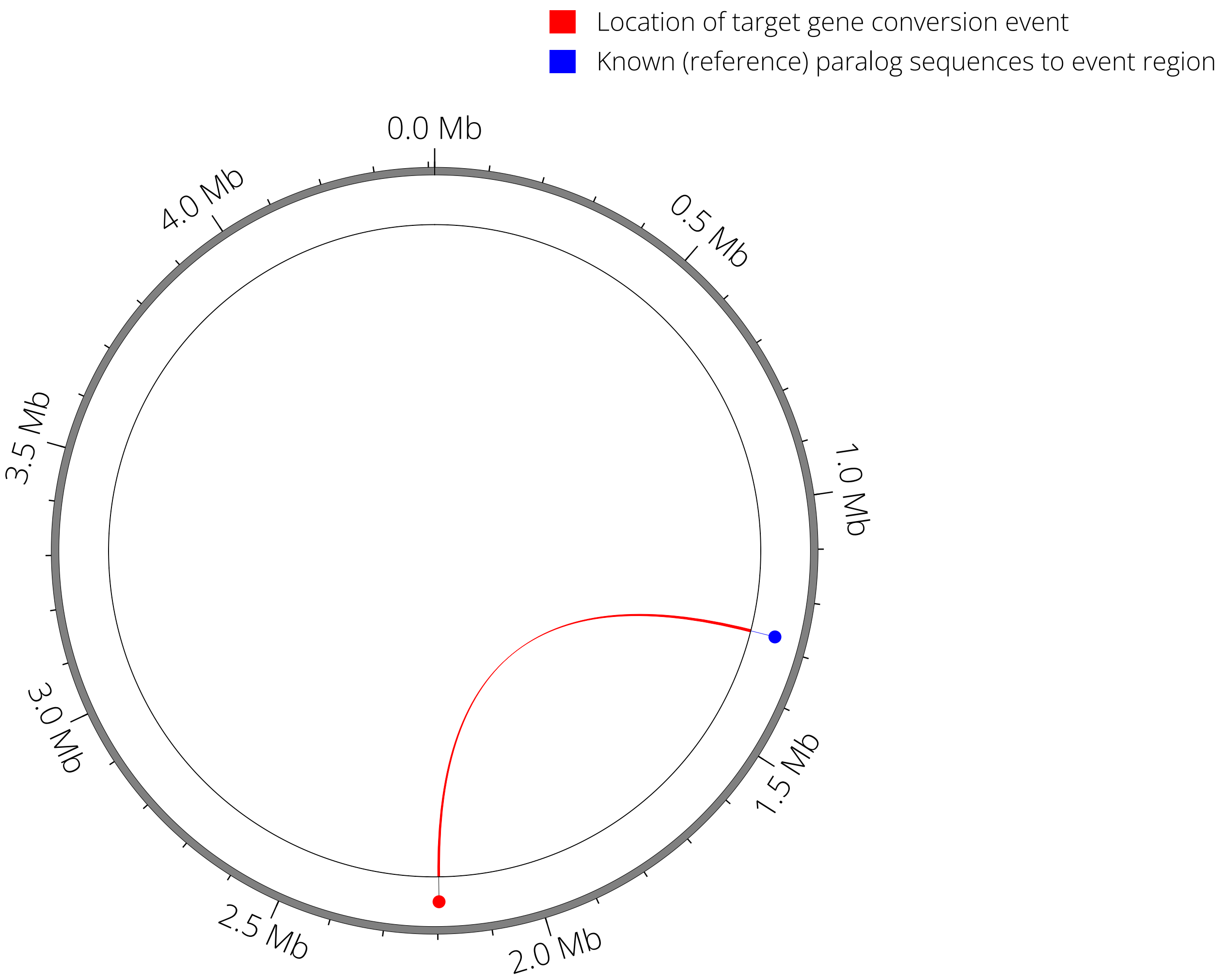

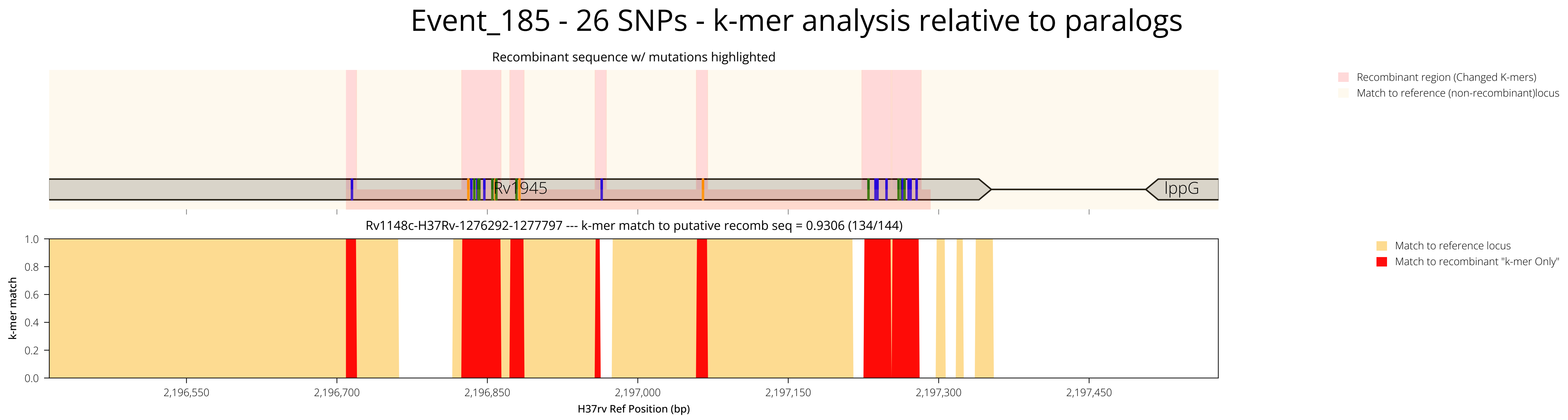

Event_185 - 26 SNPs - k-mer analysis relative to paralogs
Recombinant sequence w/ mutations highlighted
Recombinant region (Changed K-mers)
Match to reference (non-recombinant)locus
Rv1945
lppG
Rv1148c-H37Rv-1276292-1277797 --- k-mer match to putative recomb seq = 0.9306 (134/144)
Match to reference locus
Match to recombinant "k-mer Only"
k-mer match
1.0
0.8
0.6
0.4
0.2
0.0
2,196,550
2,196,700
2,196,850
2,197,000
2,197,150
2,197,300
2,197,450
H37rv Ref Position (bp)

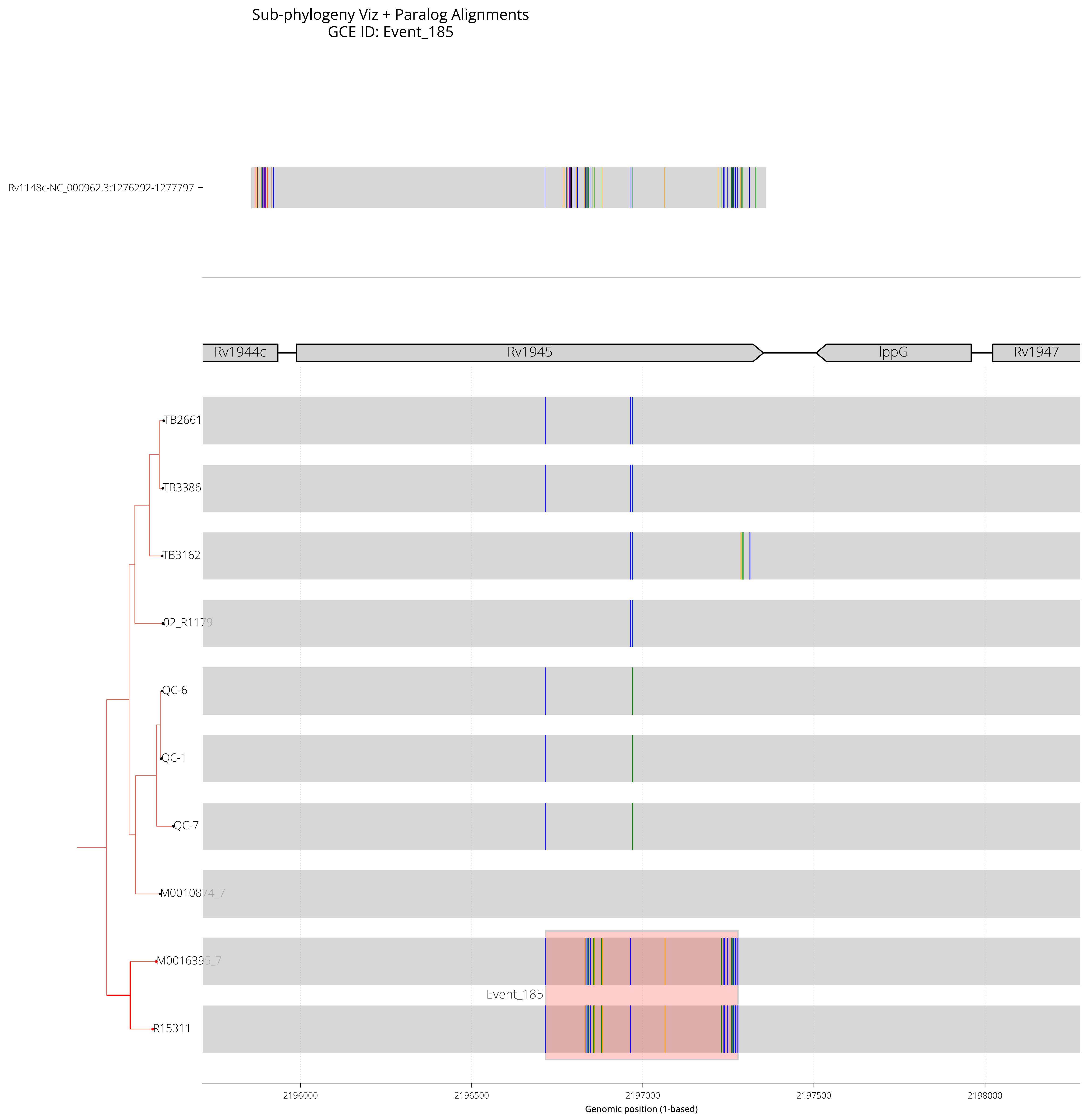

Sub-phylogeny Viz + Paralog Alignments
GCE ID: Event_185
Rv1148c-NC_000962.3:1276292-1277797
Rv1944c
Rv1945
lppG
Rv1947
TB2661
TB3386
TB3162
02_R1179
QC-6
QC-1
QC-7
M0010874_7
M0016395_7
Event_185
R15311
2196000
2196500
2197000
2197500
2198000
Genomic position (1-based)

Full Phylogeny Viz + Paralog Alignments
GCE ID: Event_185

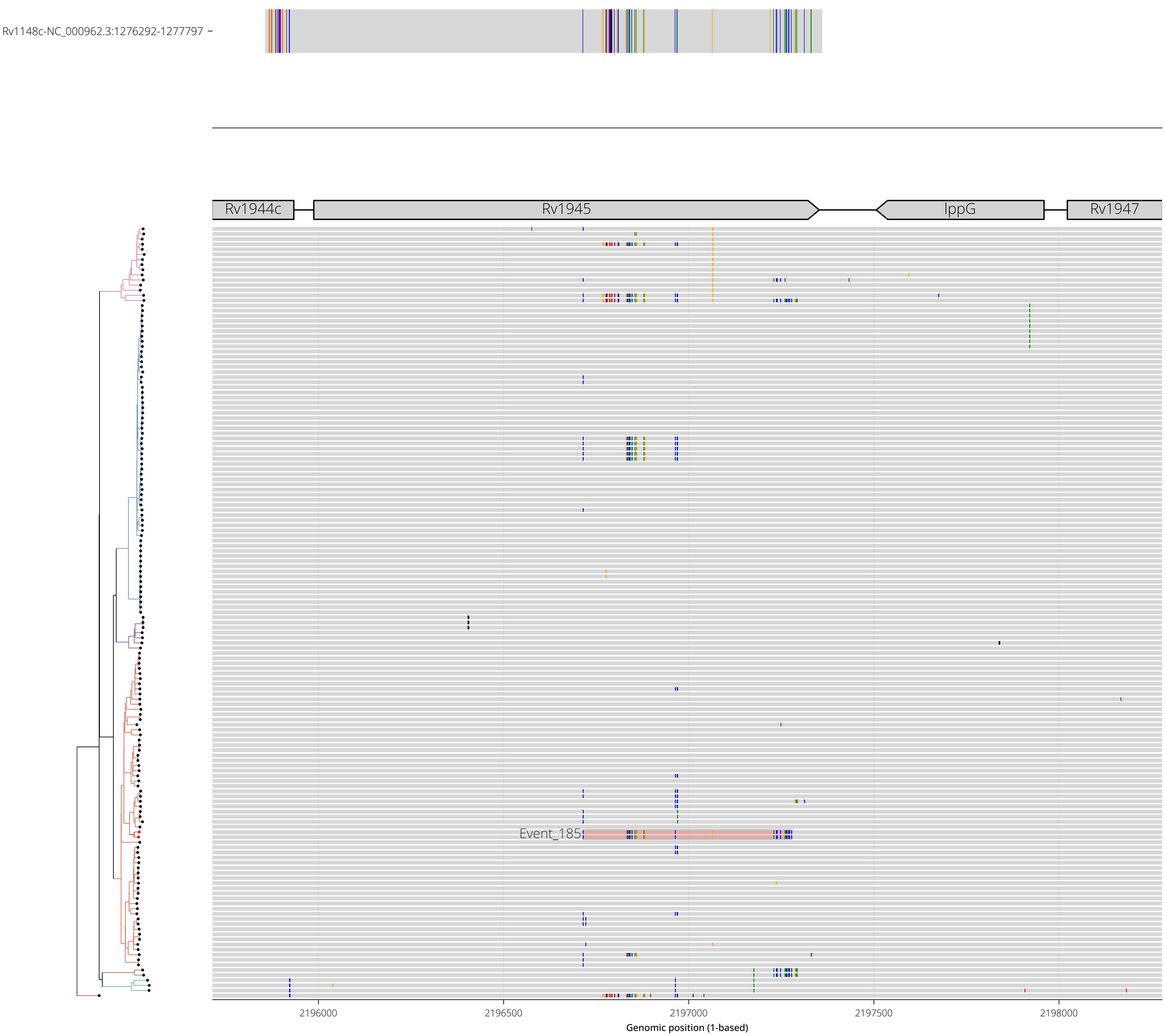

Individual GCE + Known Paralogs
GCE ID: Event_186

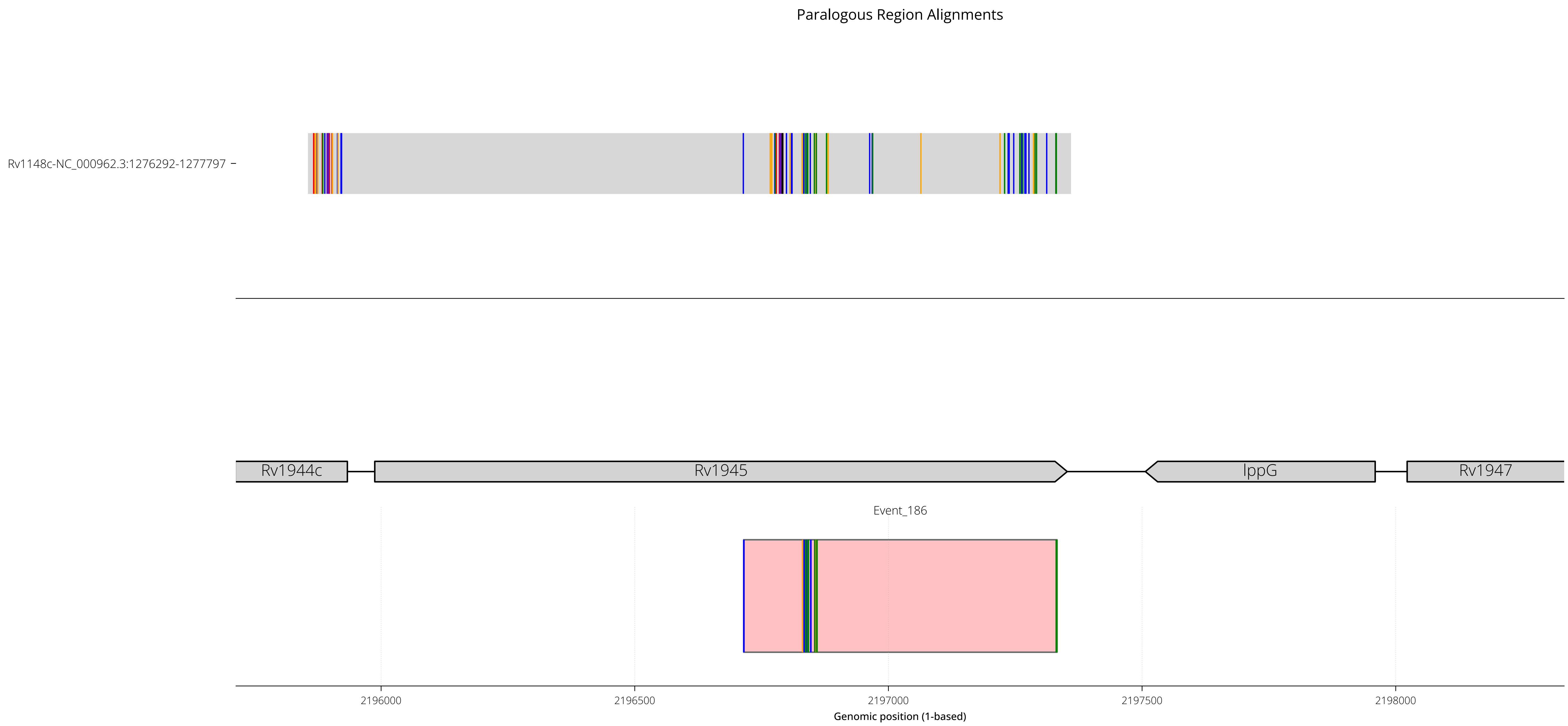

Circos Viz - GCE to Known Paralogs Locations
GCE ID: Event_186

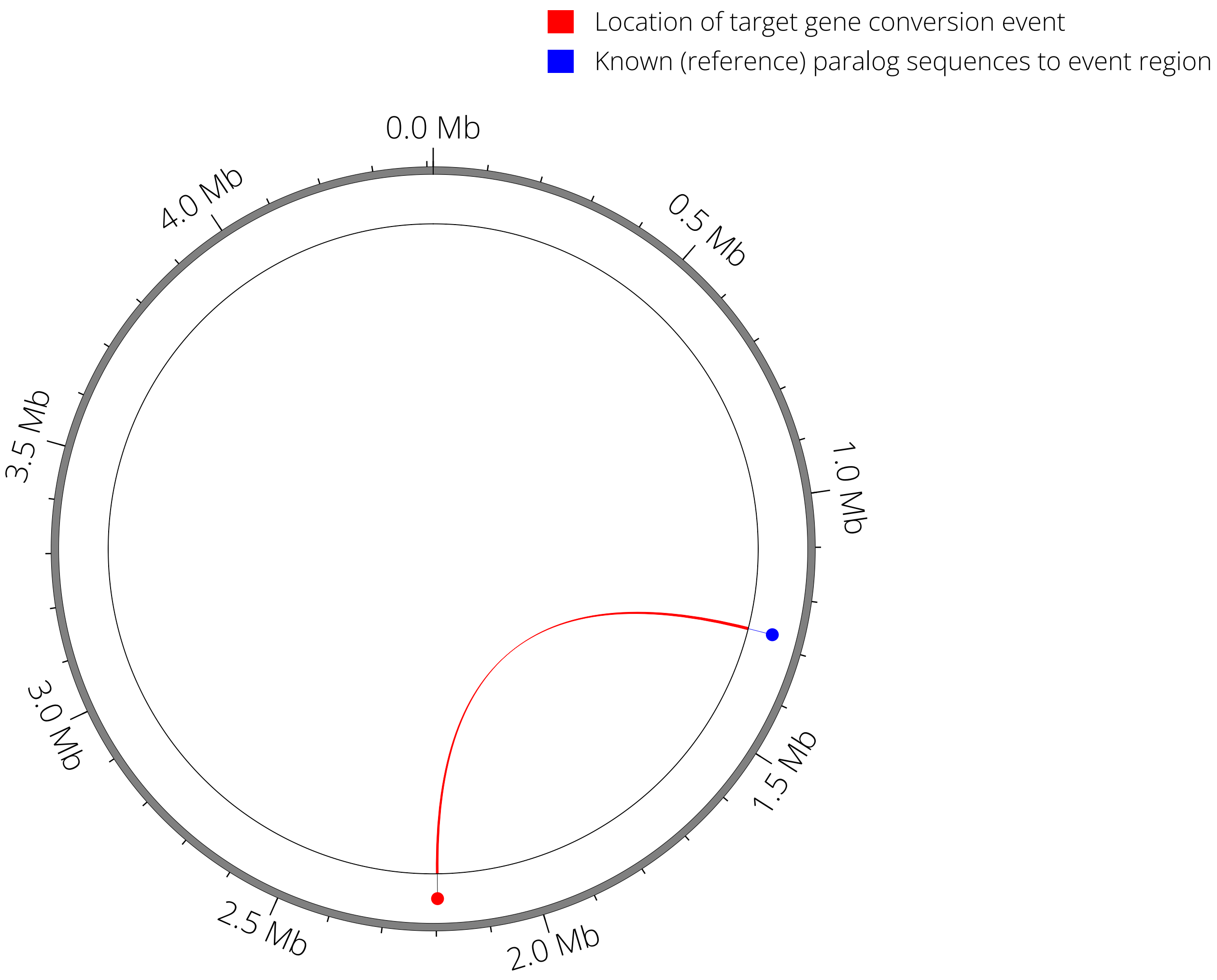

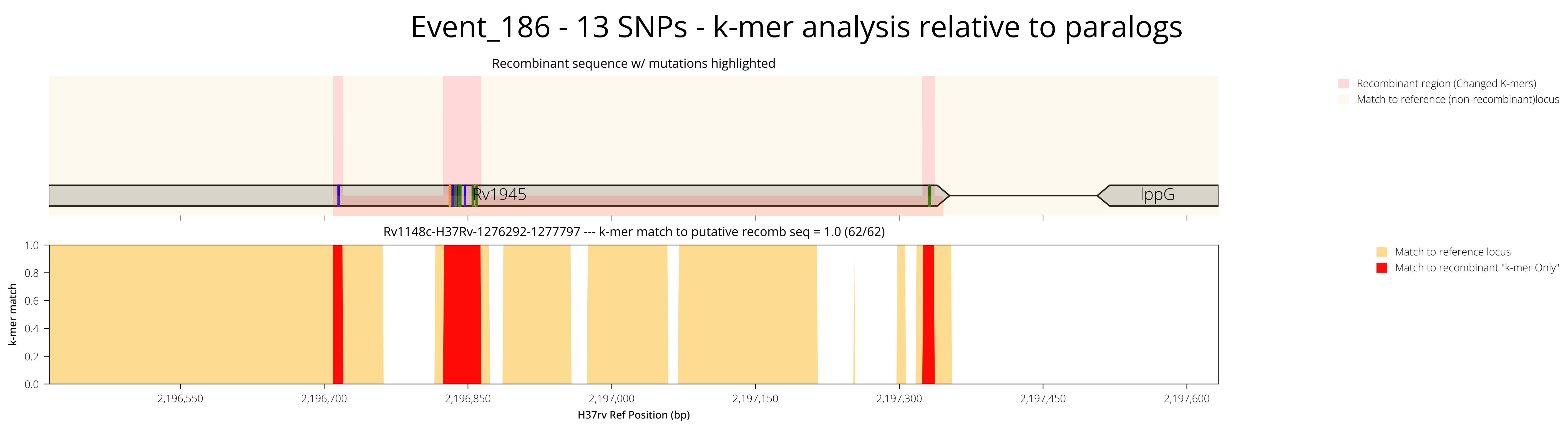
Event_186 - 13 SNPs - k-mer analysis relative to paralogs
Recombinant sequence w/ mutations highlighted
Rv1945
lppG
Recombinant region (Changed K-mers)
Match to reference (non-recombinant)locus
Rv1148c-H37Rv-1276292-1277797 --- k-mer match to putative recomb seq = 1.0 (62/62)
k-mer match
1.0
0.8
0.6
0.4
0.2
0.0
2,196,550
2,196,700
2,196,850
2,197,000
2,197,150
2,197,300
2,197,450
2,197,600
H37rv Ref Position (bp)
Match to reference locus
Match to recombinant "k-mer Only"

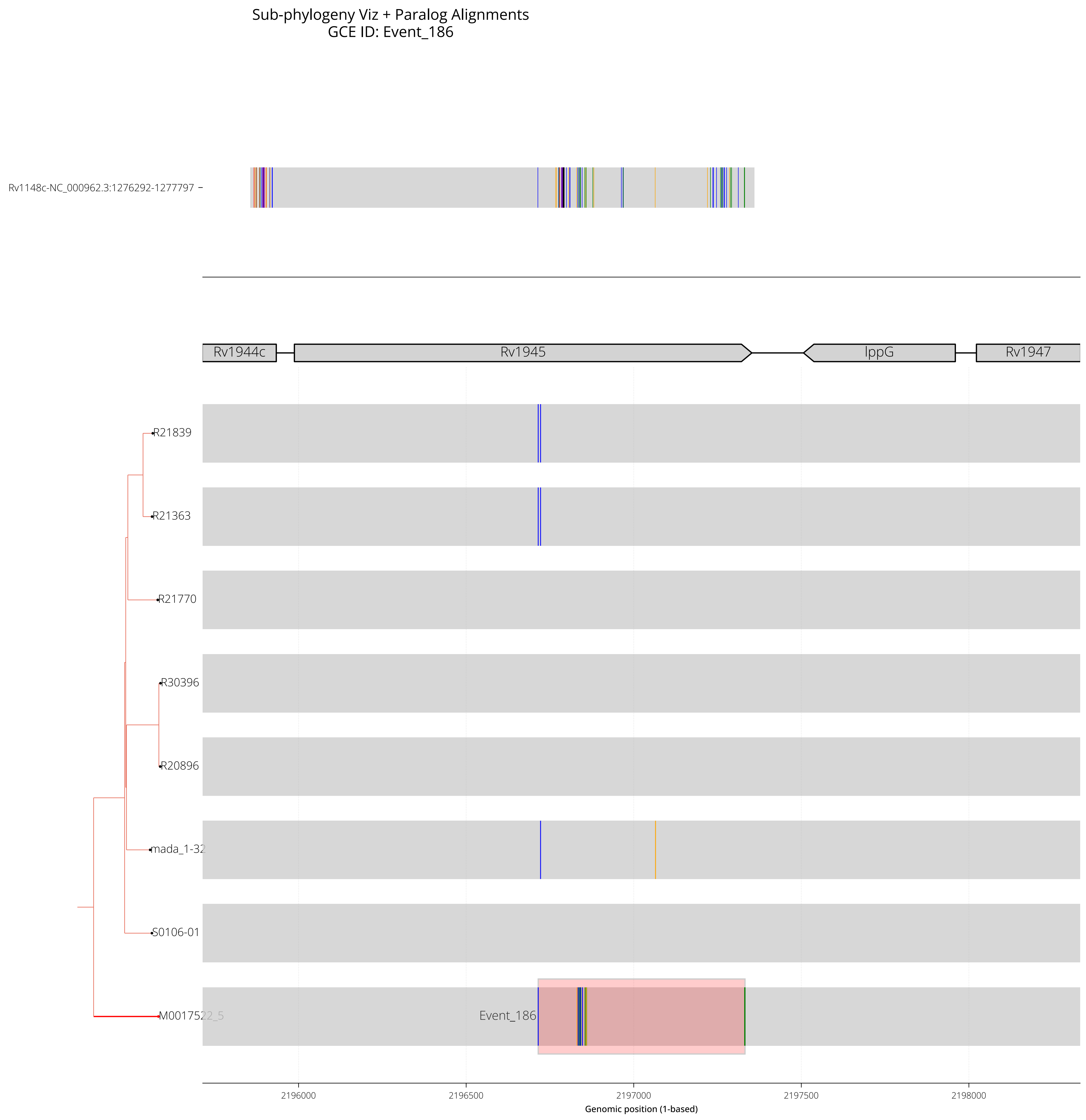

Sub-phylogeny Viz + Paralog Alignments
GCE ID: Event_186
Rv1148c-NC_000962.3:1276292-1277797
Rv1944c
Rv1945
lppG
Rv1947
R21839
R21363
R21770
R30396
R20896
mada_1-32
S0106-01
M0017522_5
Event_186
2196000
2196500
2197000
2197500
2198000
Genomic position (1-based)

Full Phylogeny Viz + Paralog Alignments
GCE ID: Event_186

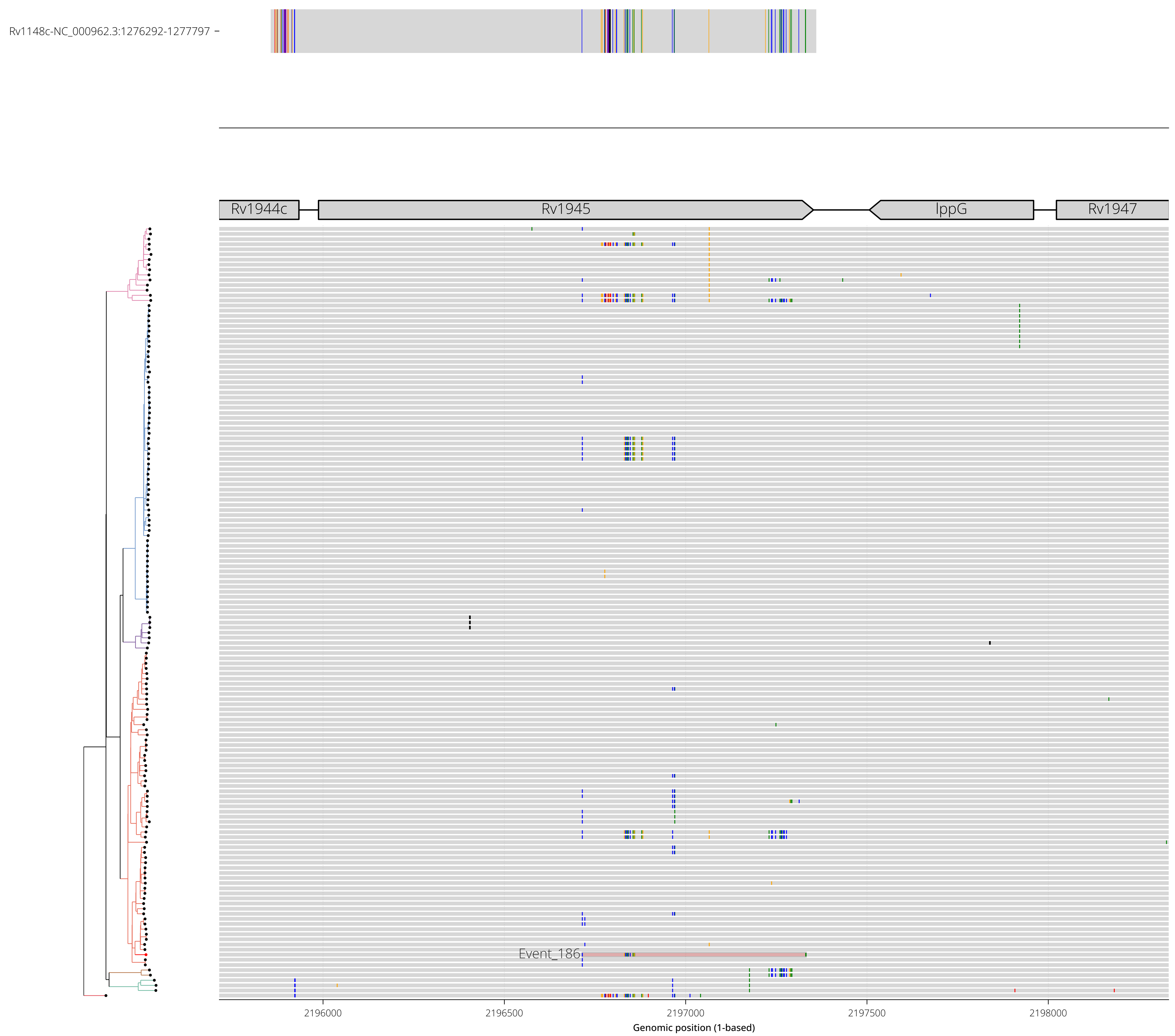

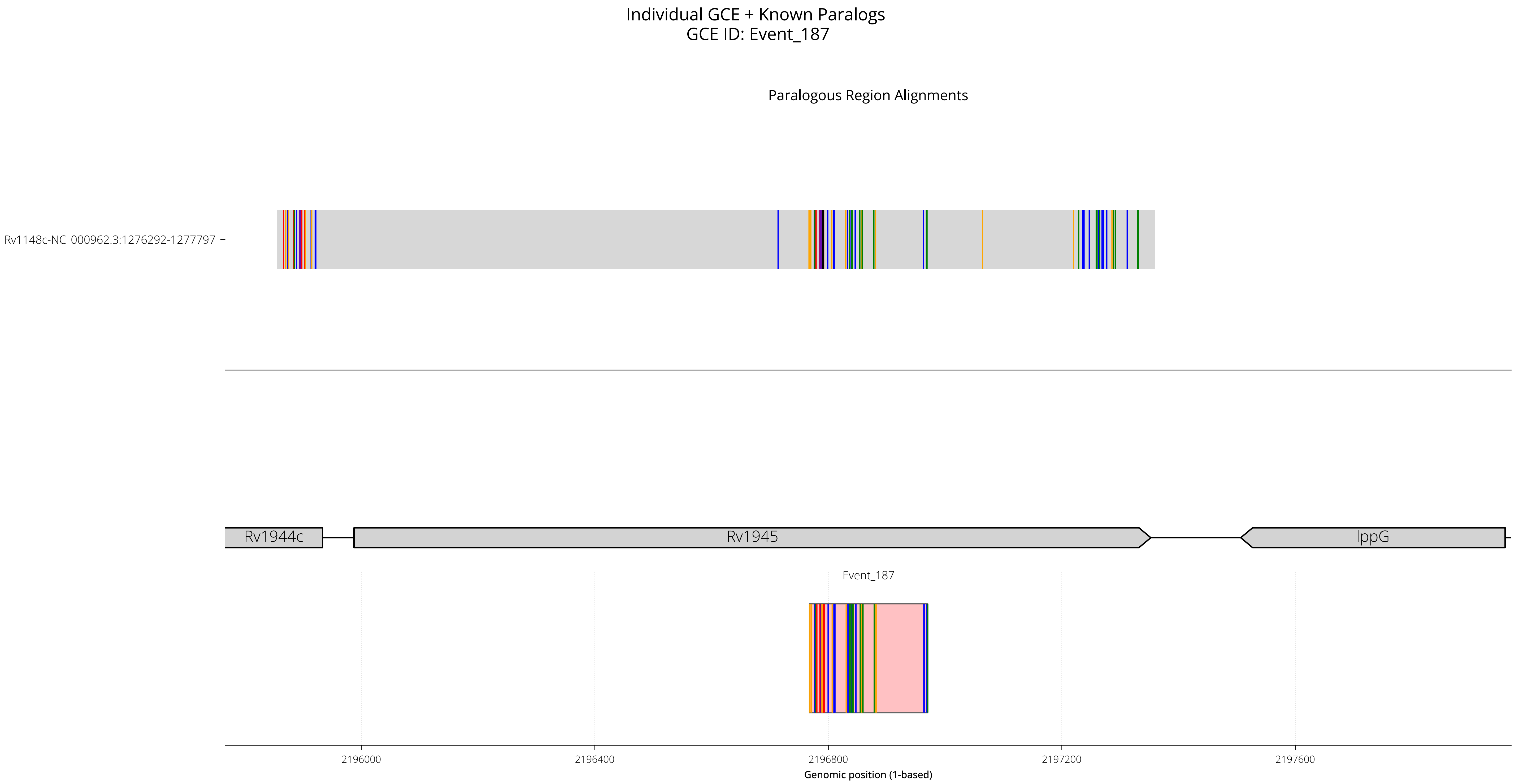
Individual GCE + Known Paralogs
GCE ID: Event_187
Paralogous Region Alignments
Rv1148c-NC_000962.3:1276292-1277797
Rv1944c
Rv1945
lppG
Event_187
2196000
2196400
2196800
2197200
2197600
Genomic position (1-based)

Circos Viz - GCE to Known Paralogs Locations
GCE ID: Event_187

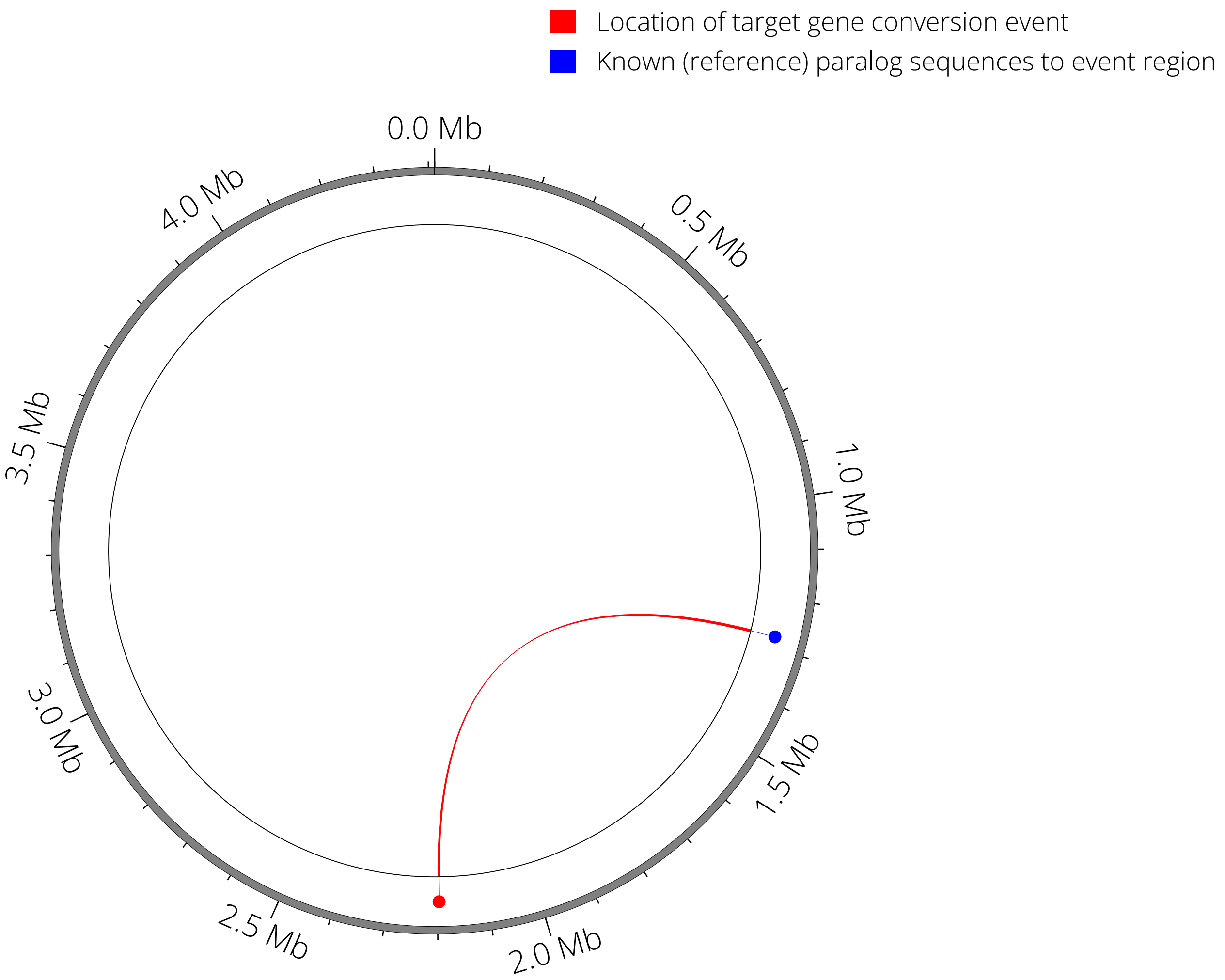

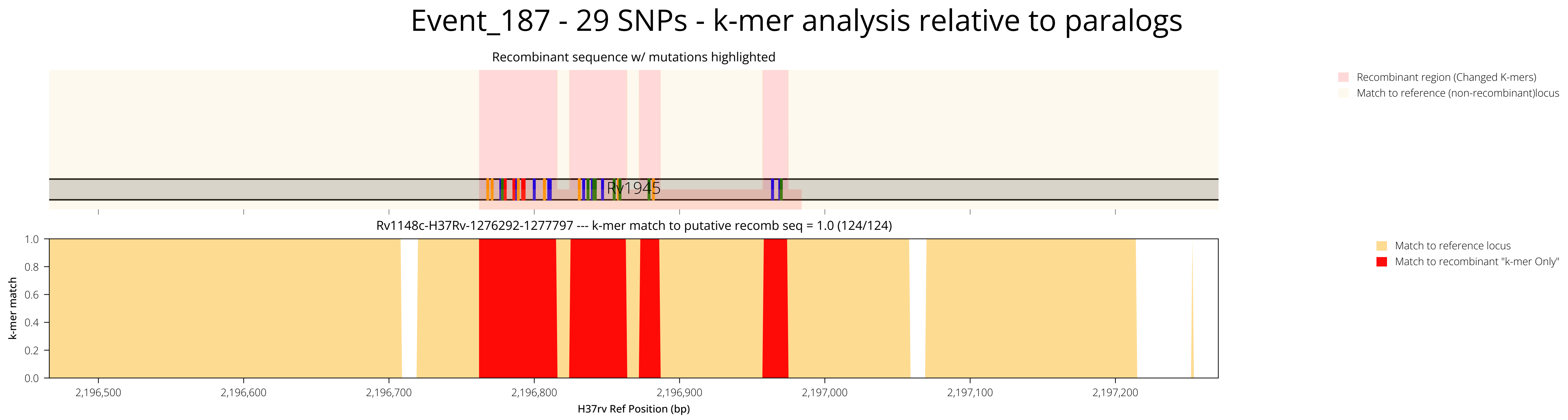

Event_187 - 29 SNPs - k-mer analysis relative to paralogs
Recombinant sequence w/ mutations highlighted
Rv1945
Recombinant region (Changed K-mers)
Match to reference (non-recombinant)locus
Rv1148c-H37Rv-1276292-1277797 --- k-mer match to putative recomb seq = 1.0 (124/124)
k-mer match
1.0
0.8
0.6
0.4
0.2
0.0
Match to reference locus
Match to recombinant "k-mer Only"
2,196,500
2,196,600
2,196,700
2,196,800
2,196,900
2,197,000
2,197,100
2,197,200
H37rv Ref Position (bp)

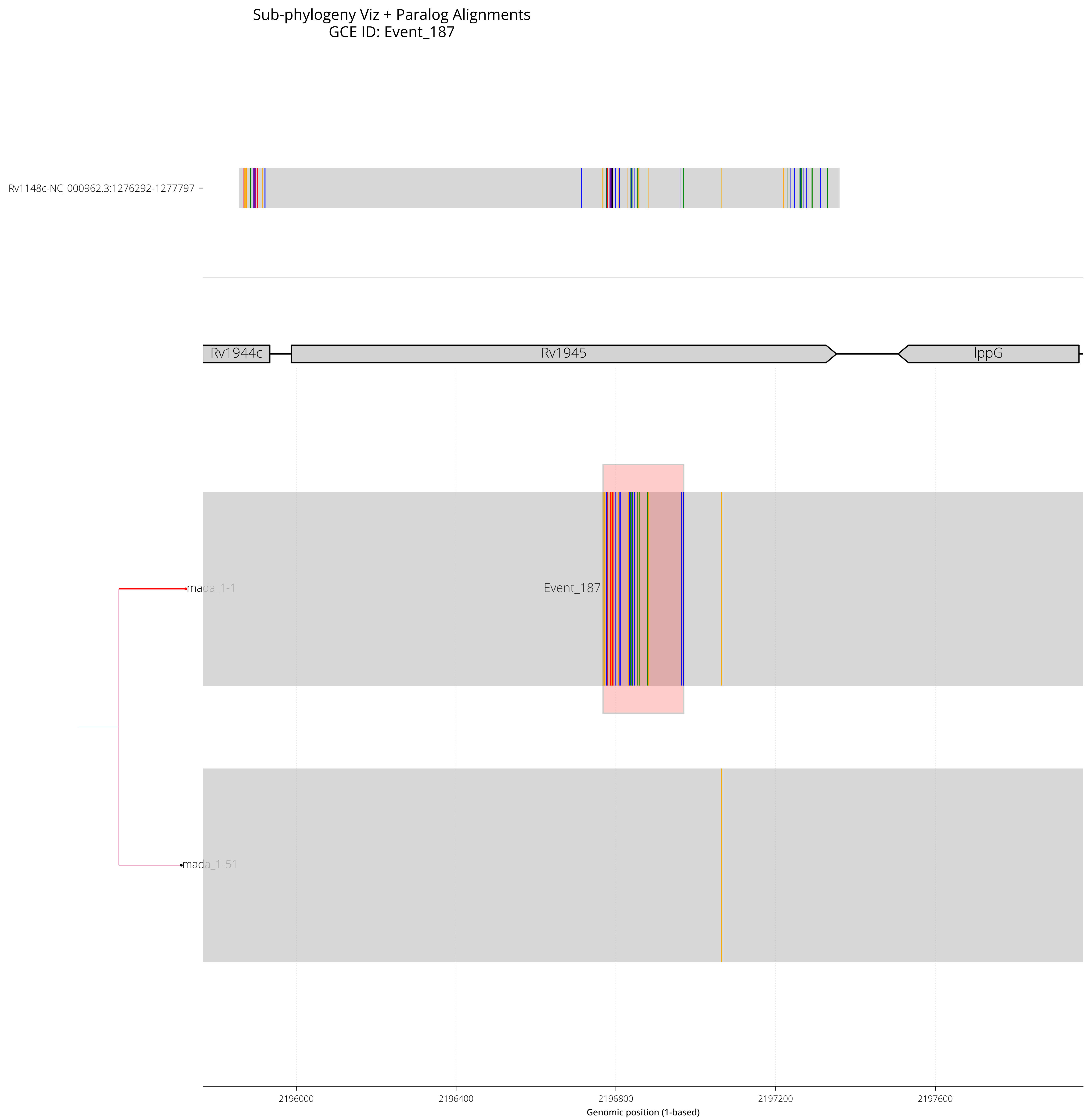
Sub-phylogeny Viz + Paralog Alignments
GCE ID: Event_187
Rv1148c-NC_000962.3:1276292-1277797
Rv1944c
Rv1945
lppG
mada_1-1
Event_187
mada_1-51
2196000
2196400
2196800
2197200
2197600
Genomic position (1-based)

Full Phylogeny Viz + Paralog Alignments
GCE ID: Event_187

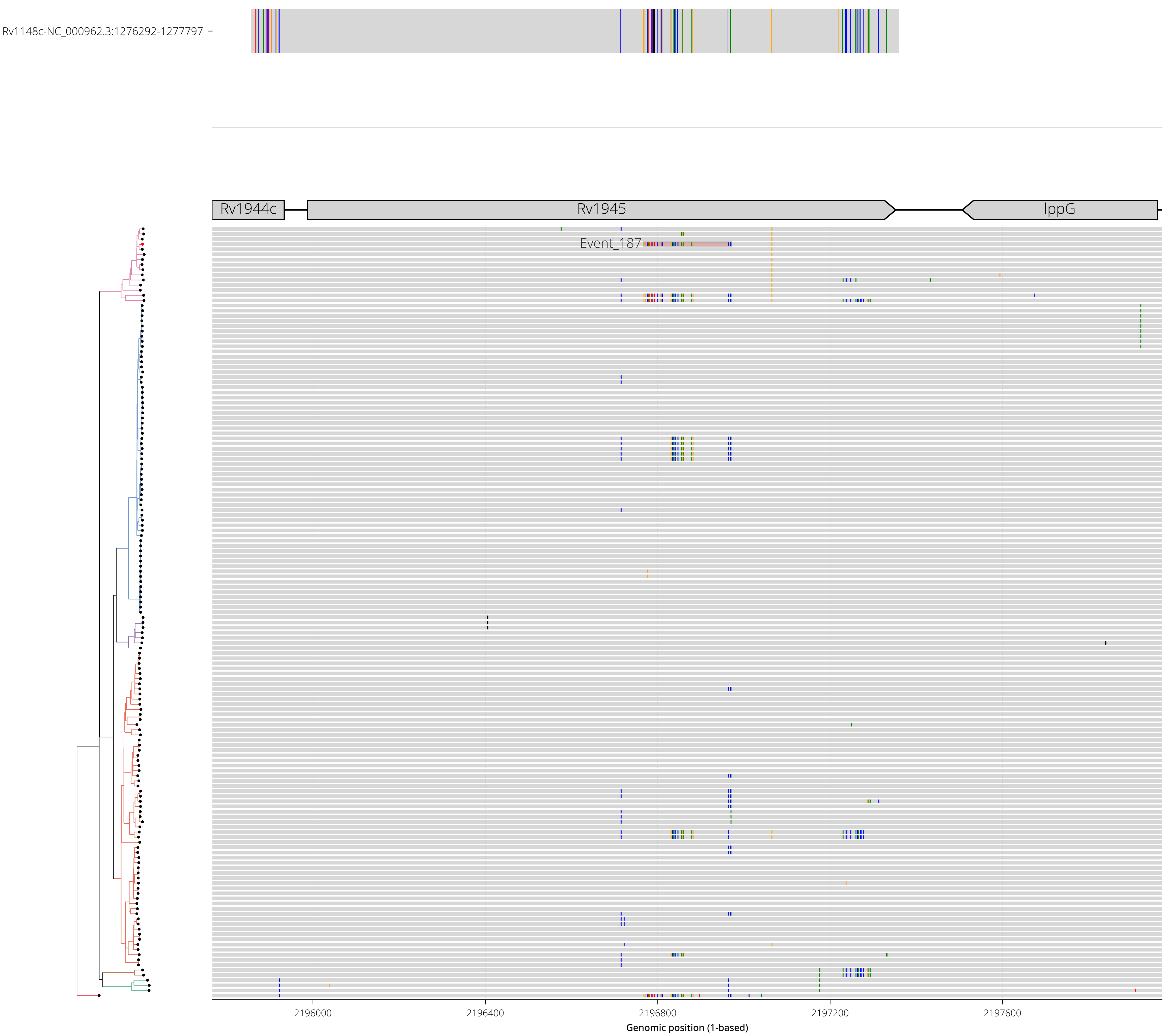

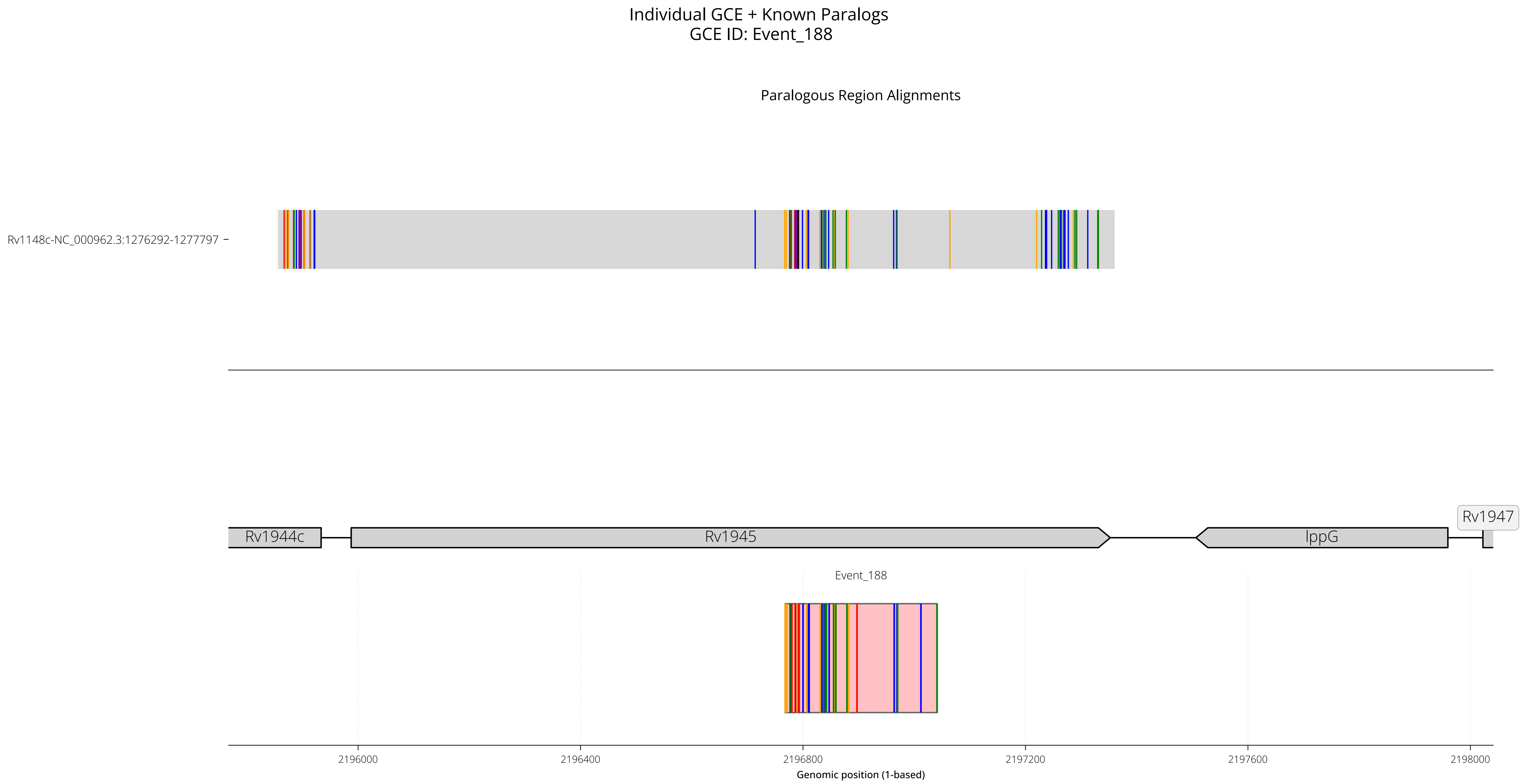
Individual GCE + Known Paralogs
GCE ID: Event_188
Paralogous Region Alignments
Rv1148c-NC_000962.3:1276292-1277797
Rv1944c
Rv1945
lppG
Rv1947
Event_188
2196000
2196400
2196800
2197200
2197600
2198000
Genomic position (1-based)

Circos Viz - GCE to Known Paralogs Locations
GCE ID: Event_188

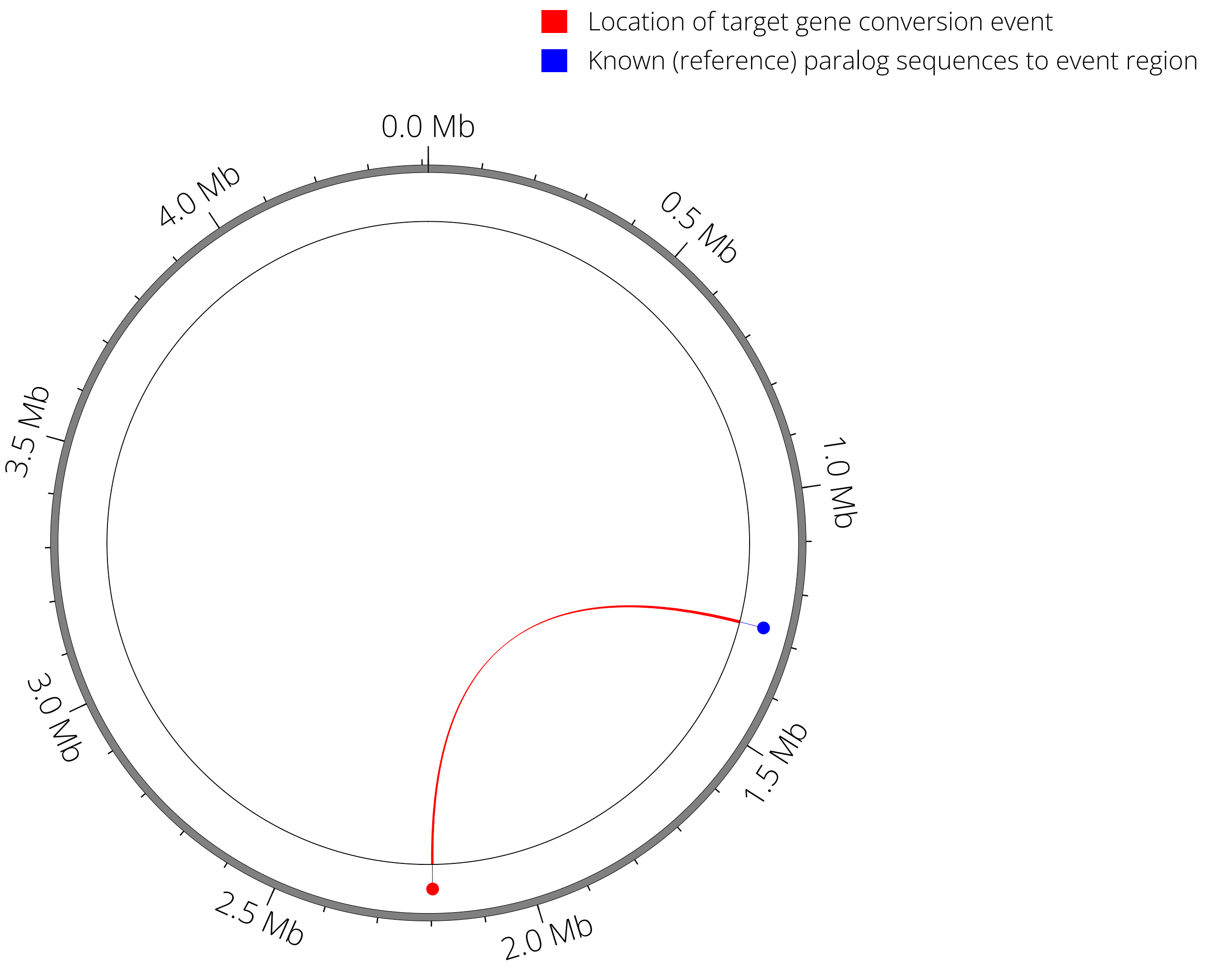

# Event_188 - 32 SNPs - k-mer analysis relative to paralogs

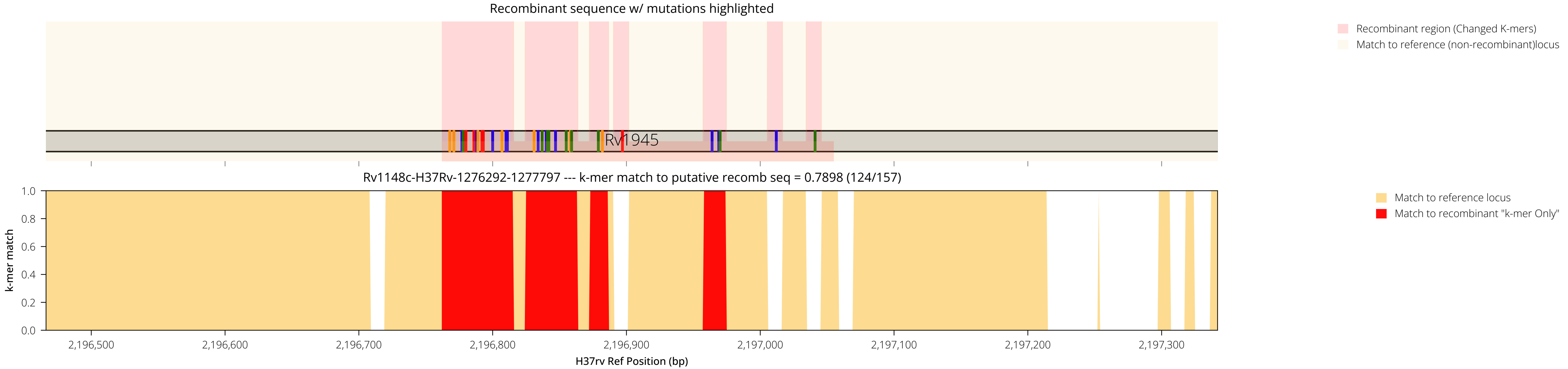

Sub-phylogeny Viz + Paralog Alignments
GCE ID: Event_188

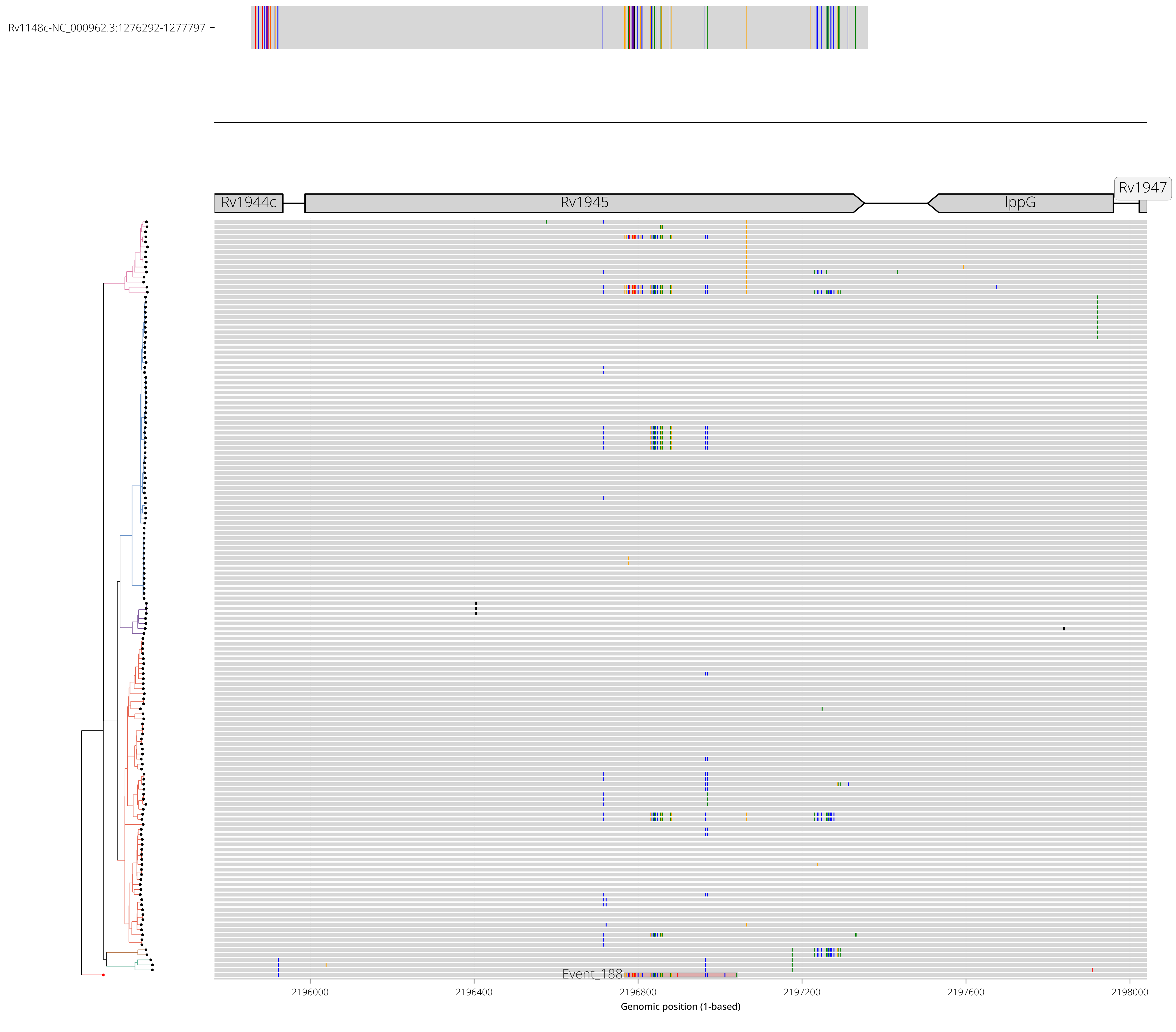

Full Phylogeny Viz + Paralog Alignments
GCE ID: Event_188

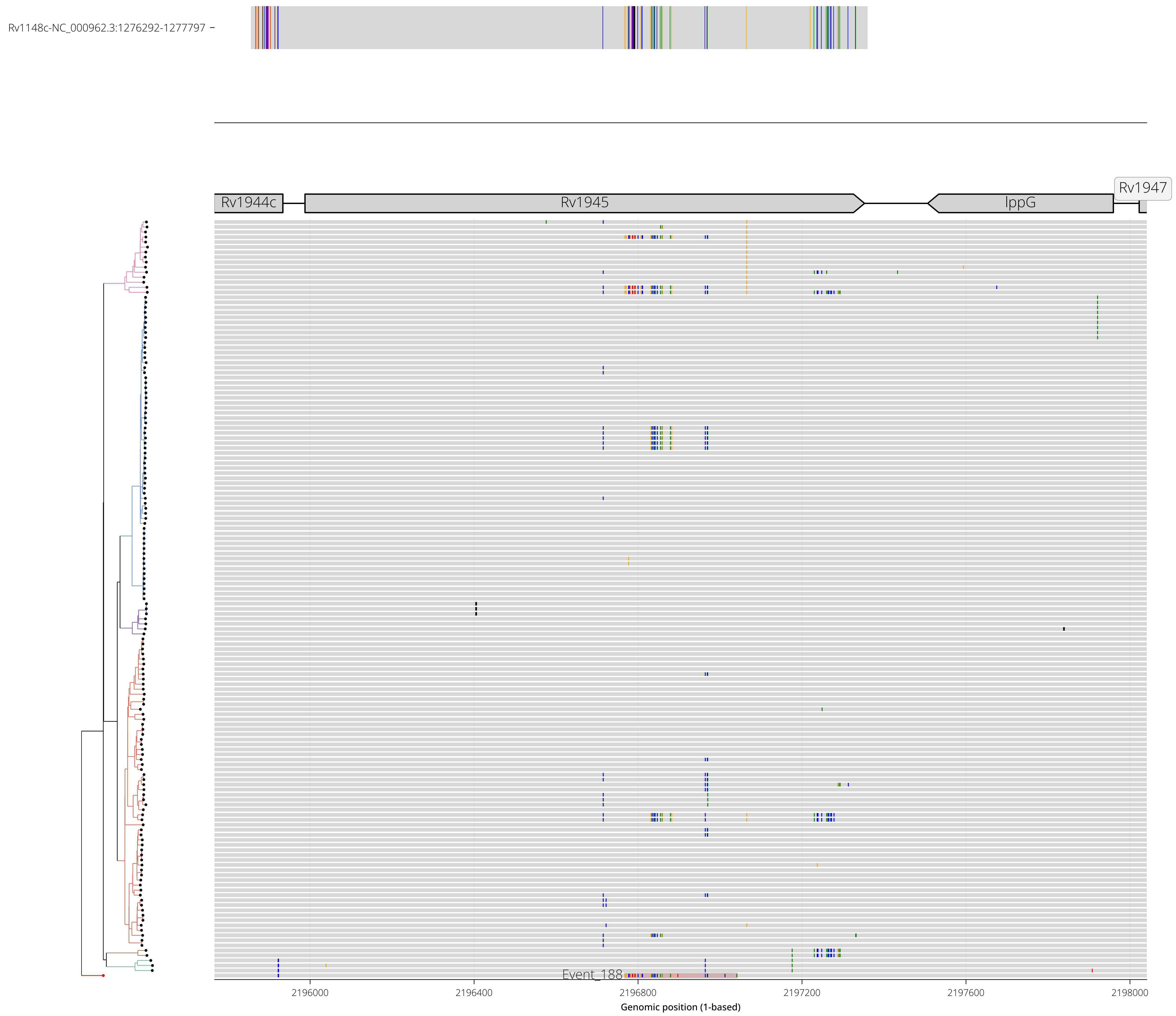

Individual GCE + Known Paralogs
GCE ID: Event_189

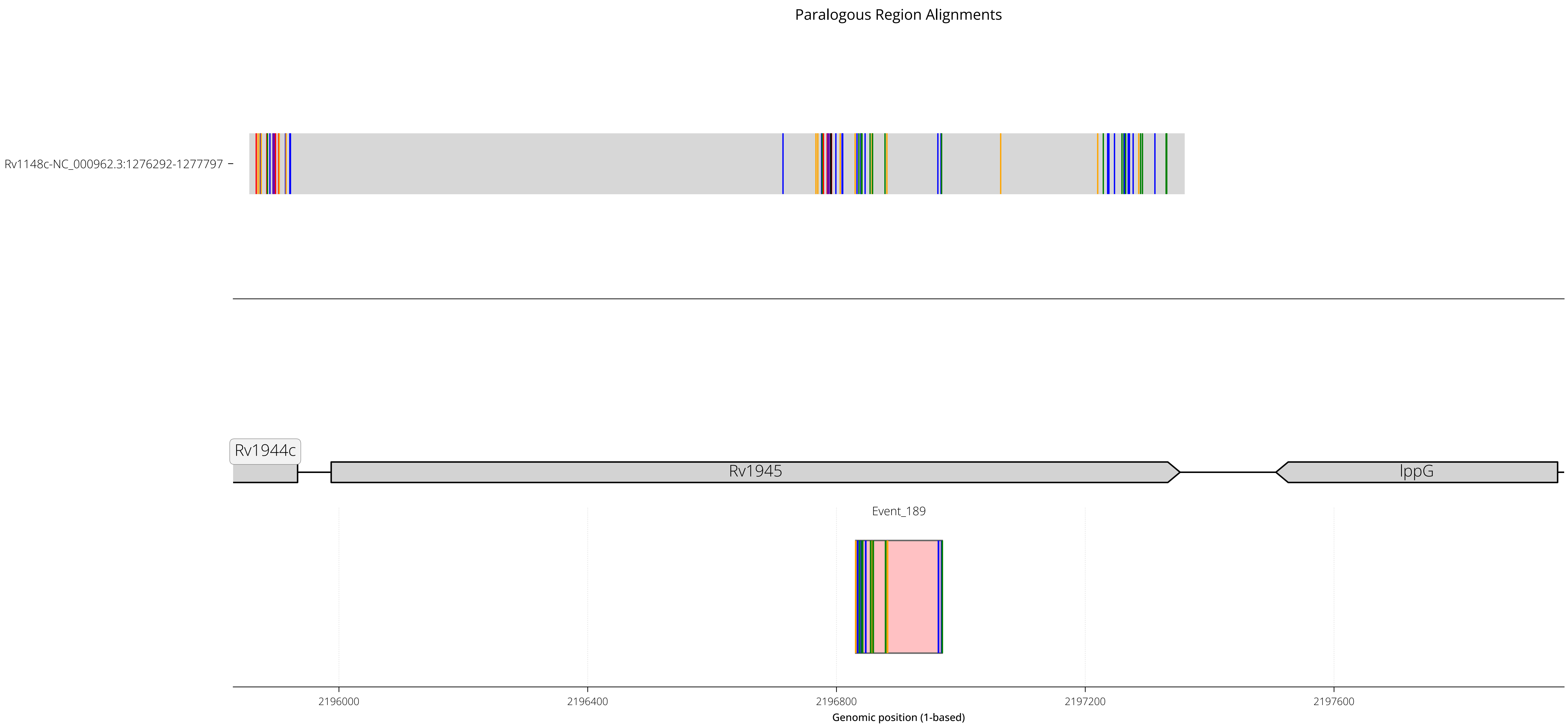

Circos Viz - GCE to Known Paralogs Locations
GCE ID: Event_189

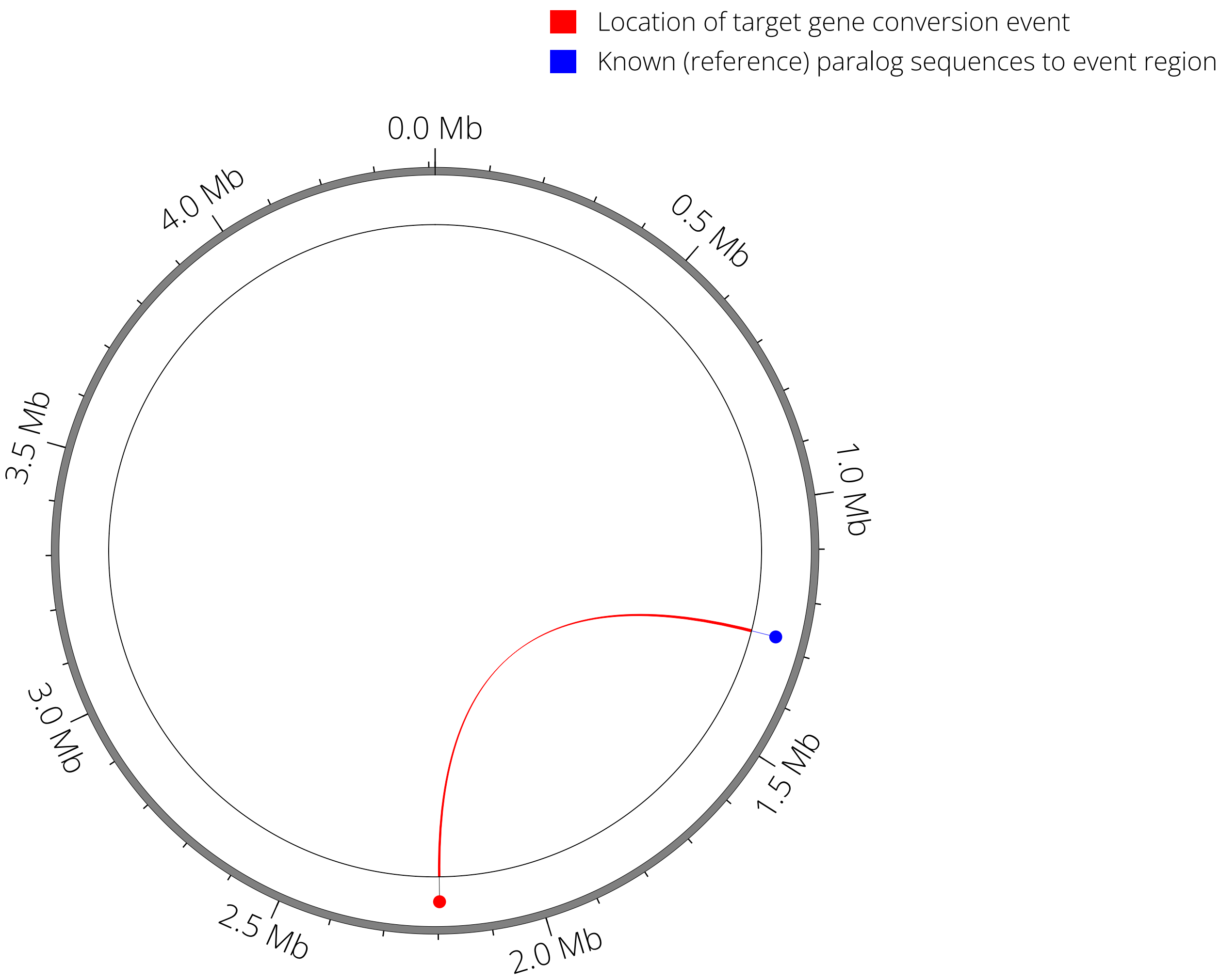

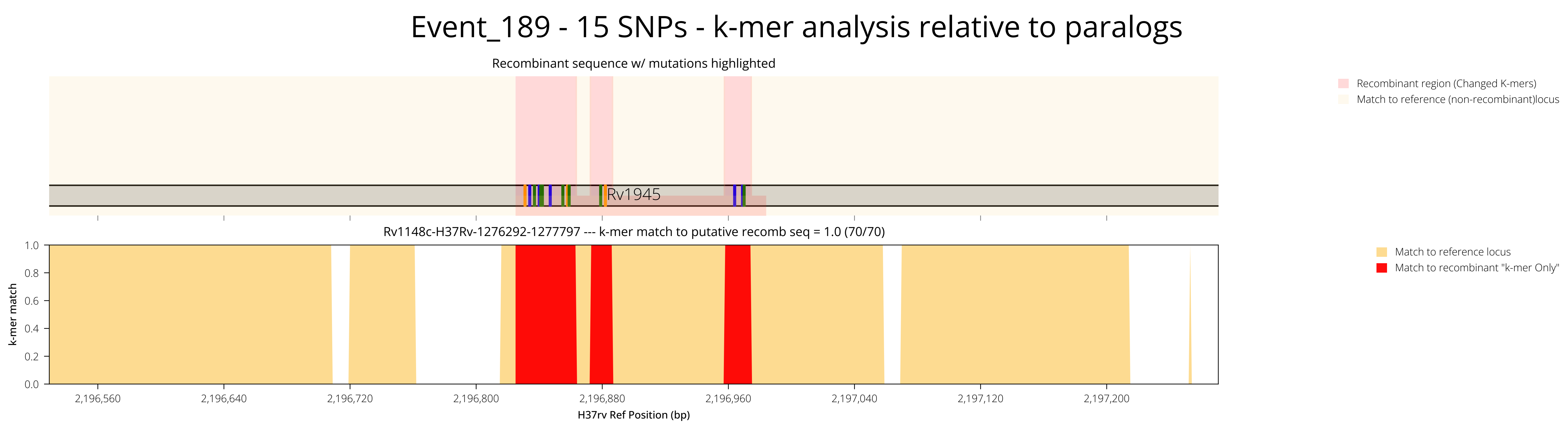

Event_189 - 15 SNPs - k-mer analysis relative to paralogs
Recombinant sequence w/ mutations highlighted
Recombinant region (Changed K-mers)
Match to reference (non-recombinant)locus
Rv1945
Rv1148c-H37Rv-1276292-1277797 --- k-mer match to putative recomb seq = 1.0 (70/70)
Match to reference locus
Match to recombinant "k-mer Only"
k-mer match
1.0
0.8
0.6
0.4
0.2
0.0
2,196,560
2,196,640
2,196,720
2,196,800
2,196,880
2,196,960
2,197,040
2,197,120
2,197,200
H37rv Ref Position (bp)

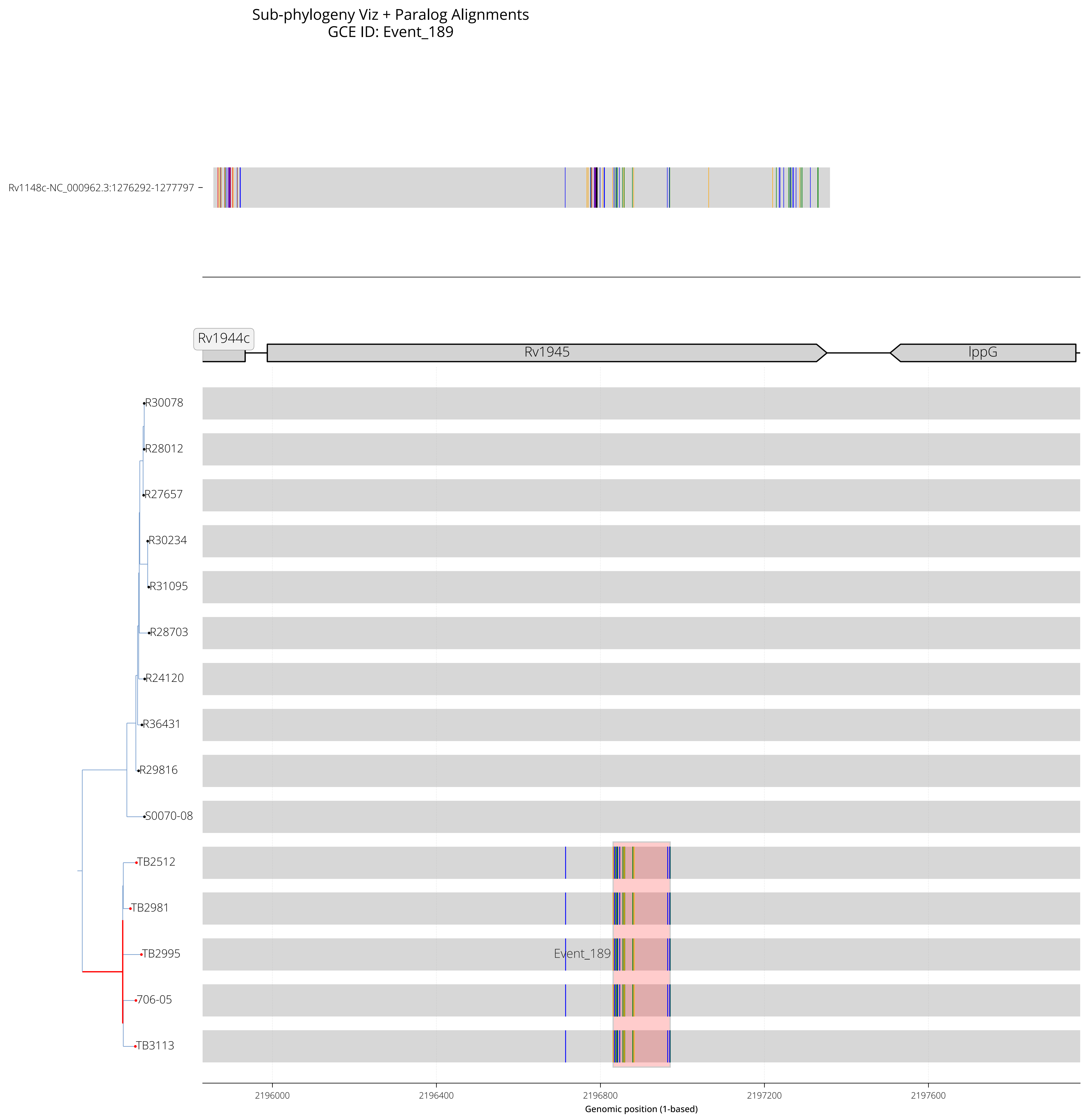

Sub-phylogeny Viz + Paralog Alignments
GCE ID: Event_189
Rv1148c-NC_000962.3:1276292-1277797
Rv1944c
Rv1945
lppG
R30078
R28012
R27657
R30234
R31095
R28703
R24120
R36431
R29816
S0070-08
TB2512
TB2981
TB2995
706-05
TB3113
Event_189
2196000
2196400
2196800
2197200
2197600
Genomic position (1-based)

Full Phylogeny Viz + Paralog Alignments
GCE ID: Event_189

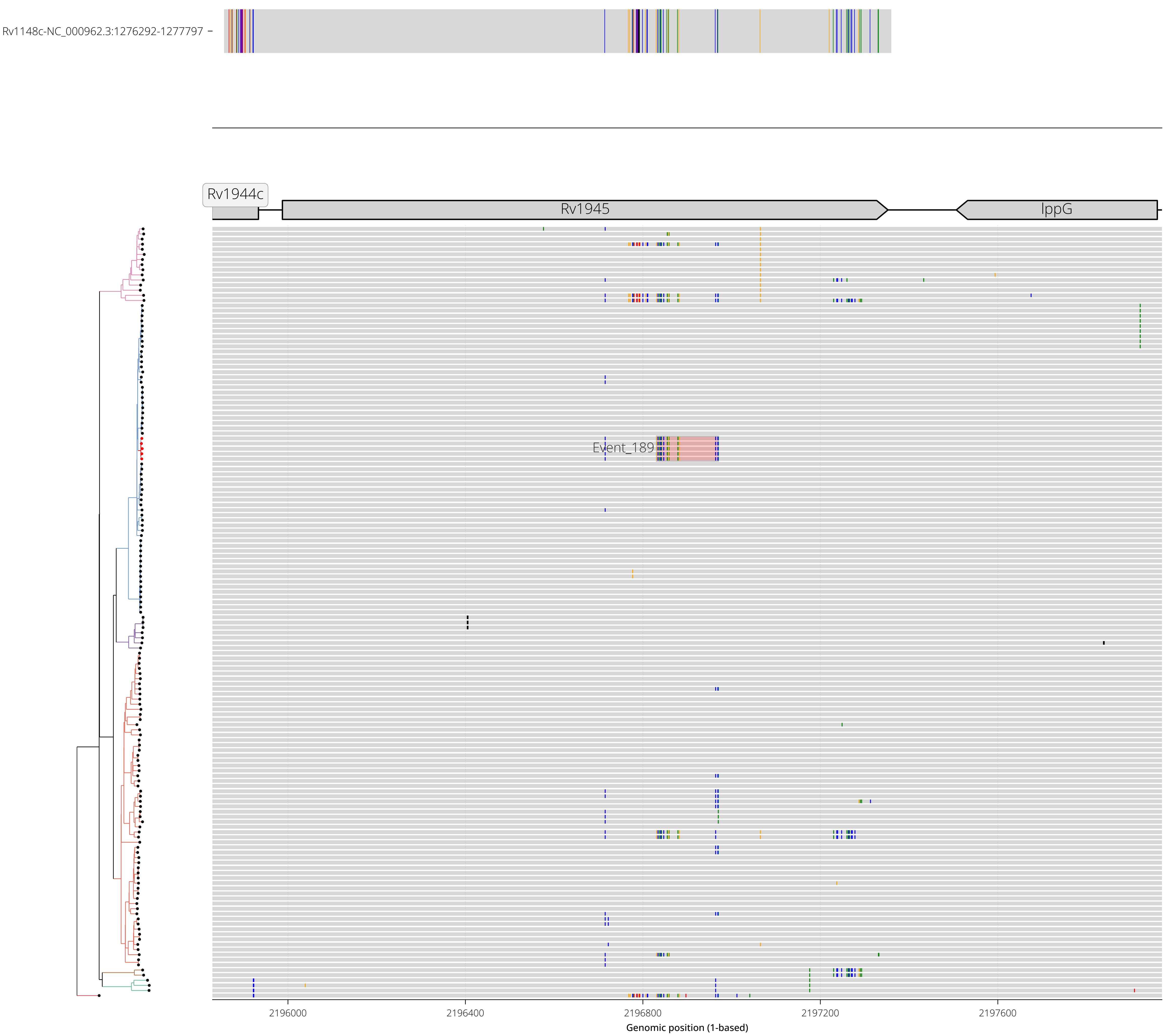

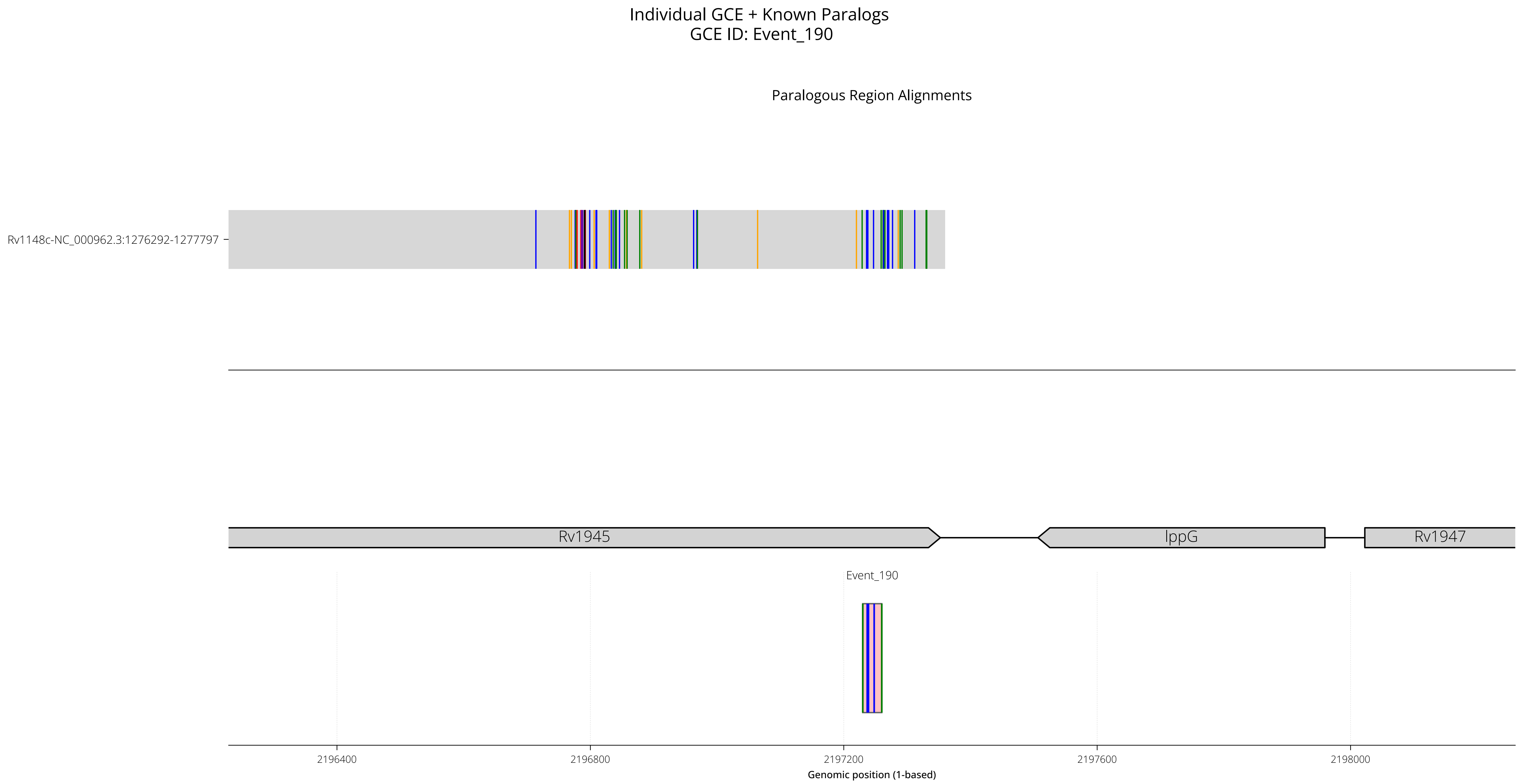
Individual GCE + Known Paralogs
GCE ID: Event_190
Paralogous Region Alignments
Rv1148c-NC_000962.3:1276292-1277797
Rv1945
lppG
Rv1947
Event_190
2196400
2196800
2197200
2197600
2198000
Genomic position (1-based)

Circos Viz - GCE to Known Paralogs Locations
GCE ID: Event_190

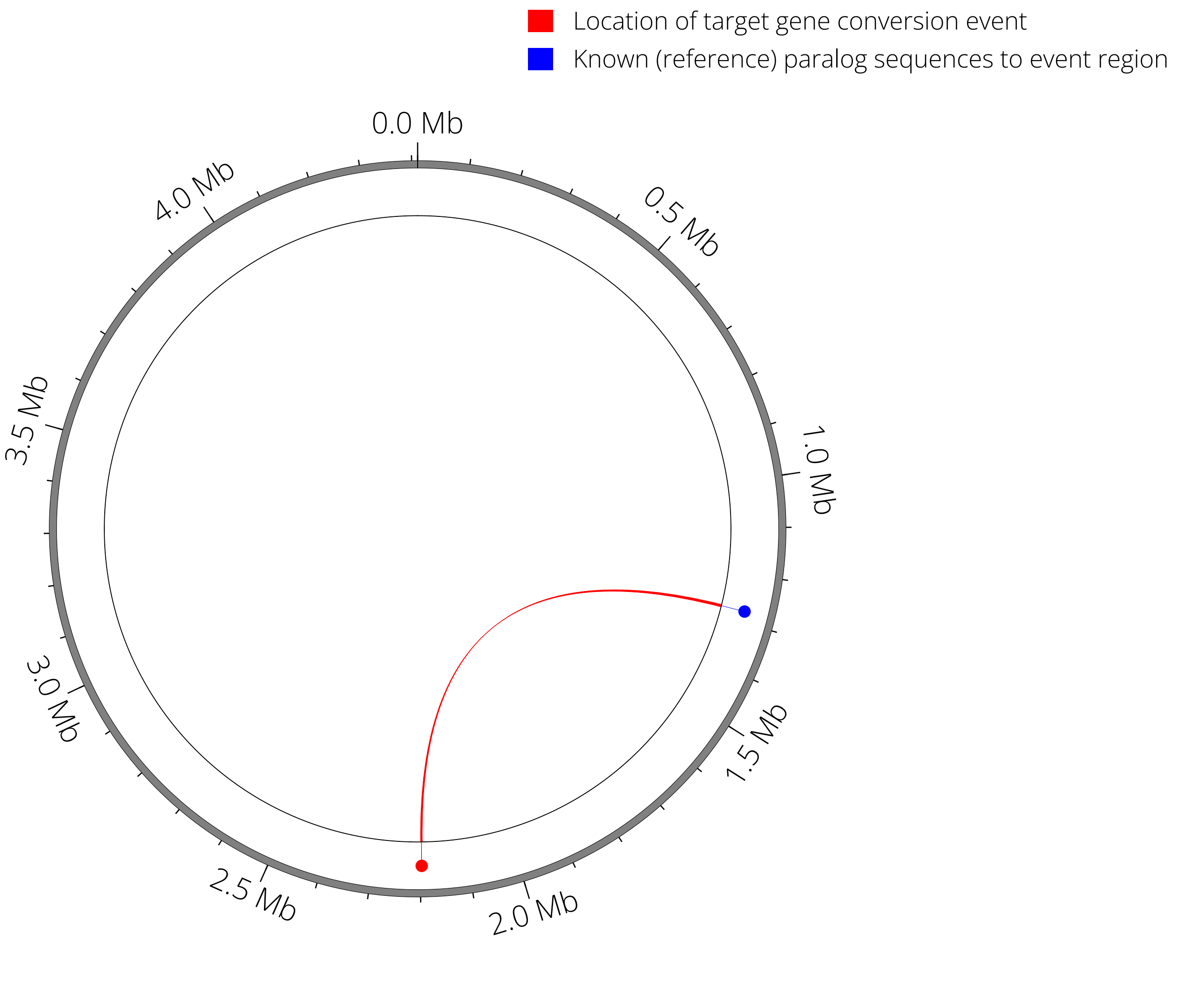

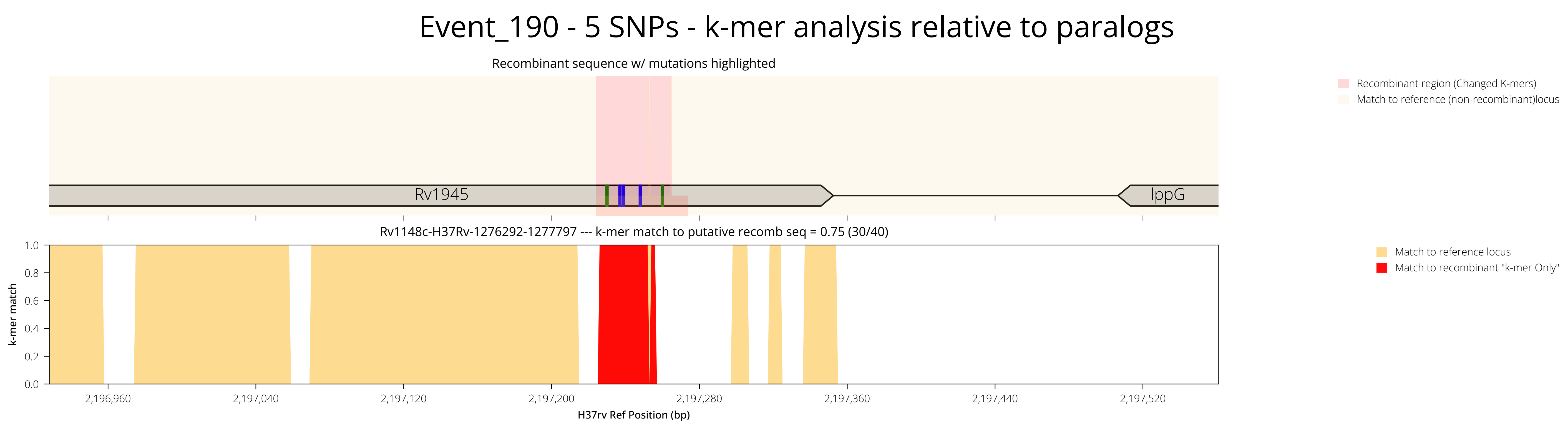

Event_190 - 5 SNPs - k-mer analysis relative to paralogs
Recombinant sequence w/ mutations highlighted
Recombinant region (Changed K-mers)
Match to reference (non-recombinant)locus
Rv1945
lppG
Rv1148c-H37Rv-1276292-1277797 --- k-mer match to putative recomb seq = 0.75 (30/40)
Match to reference locus
Match to recombinant "k-mer Only"
k-mer match
1.0
0.8
0.6
0.4
0.2
0.0
2,196,960
2,197,040
2,197,120
2,197,200
2,197,280
2,197,360
2,197,440
2,197,520
H37rv Ref Position (bp)

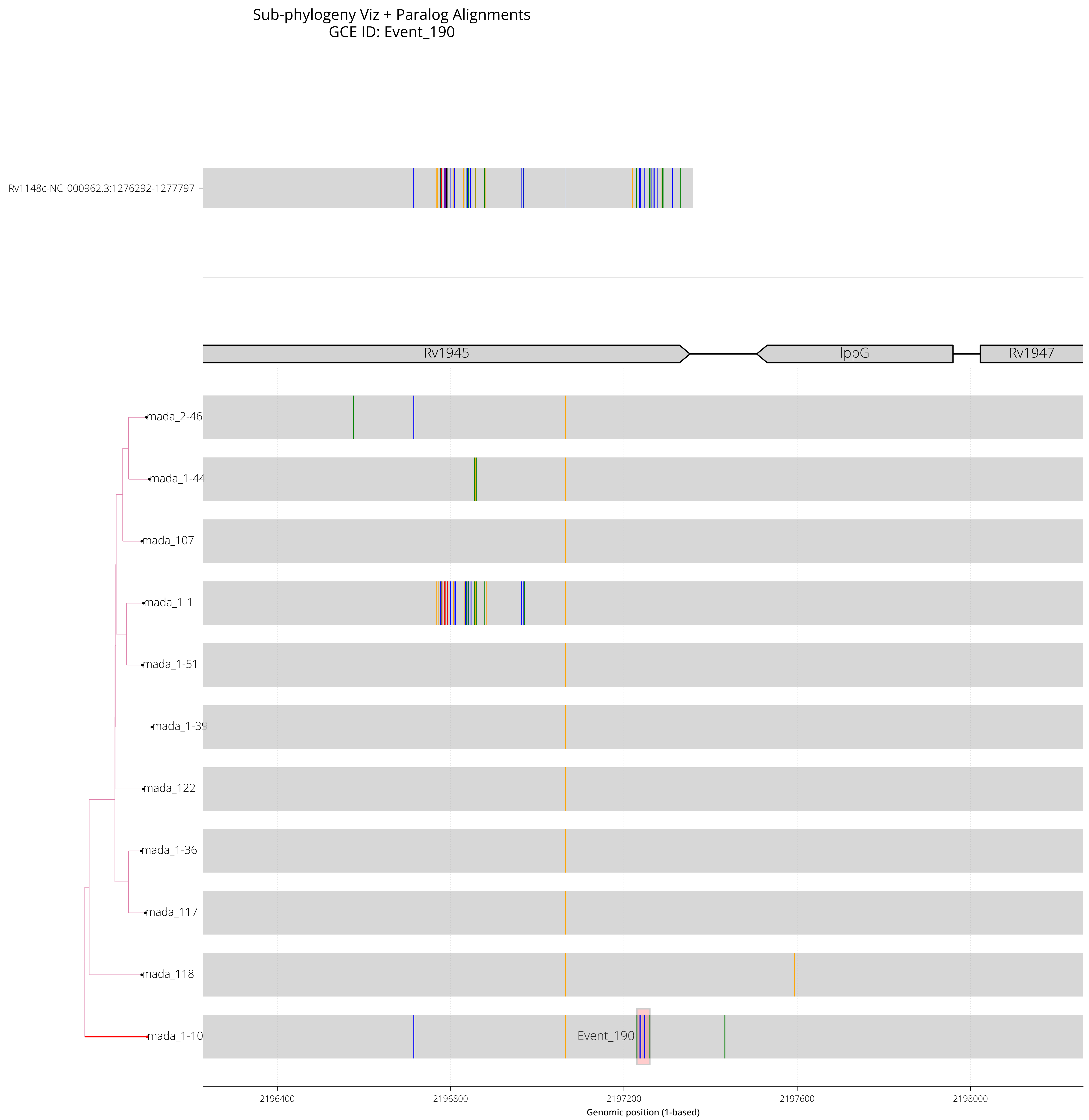
Sub-phylogeny Viz + Paralog Alignments
GCE ID: Event_190
Rv1148c-NC_000962.3:1276292-1277797
Rv1945
lppG
Rv1947
mada_2-46
mada_1-44
mada_107
mada_1-1
mada_1-51
mada_1-39
mada_122
mada_1-36
mada_117
mada_118
mada_1-10
Event_190
2196400
2196800
2197200
2197600
2198000
Genomic position (1-based)

# Full Phylogeny Viz + Paralog Alignments
GCE ID: Event_190

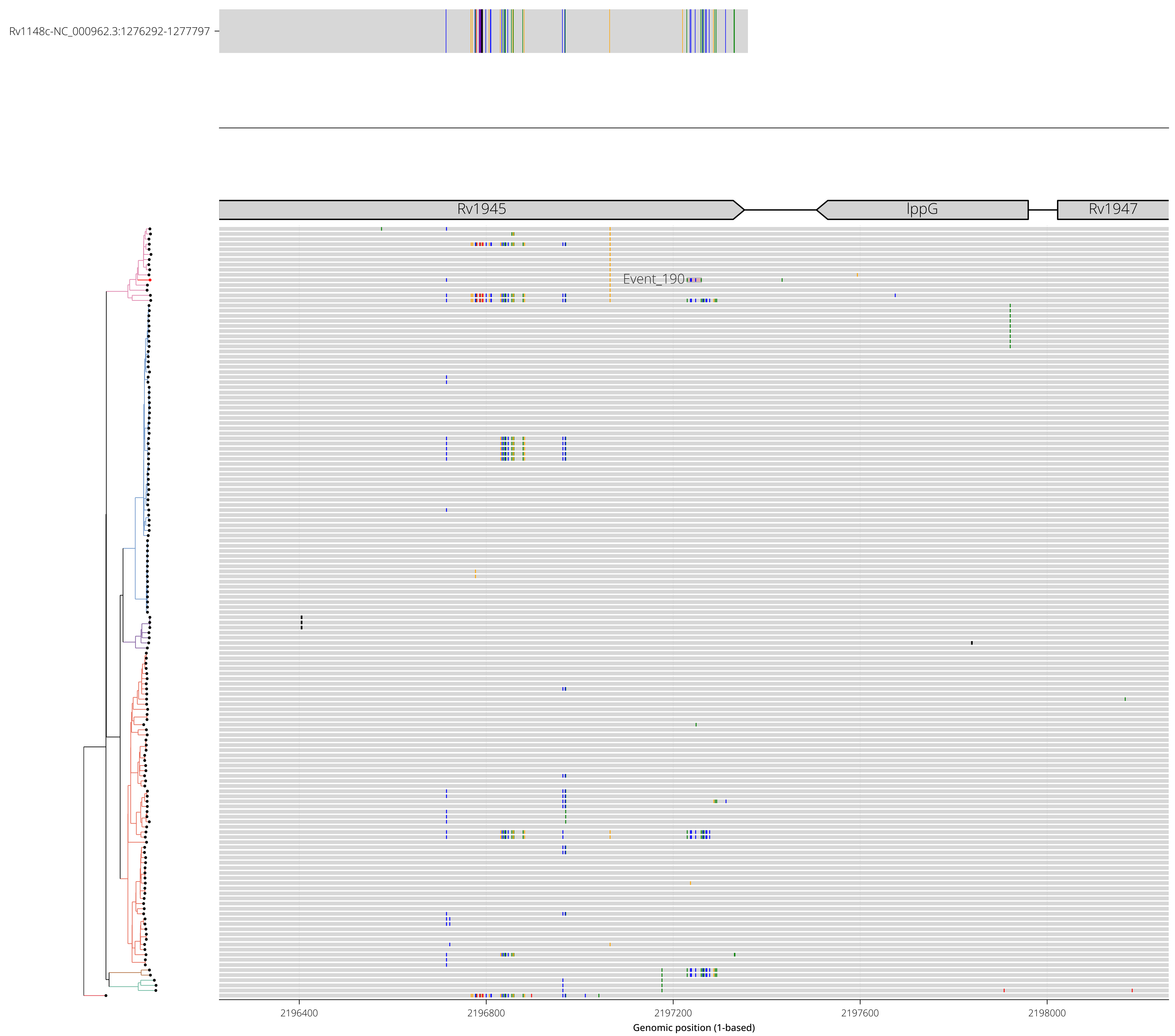

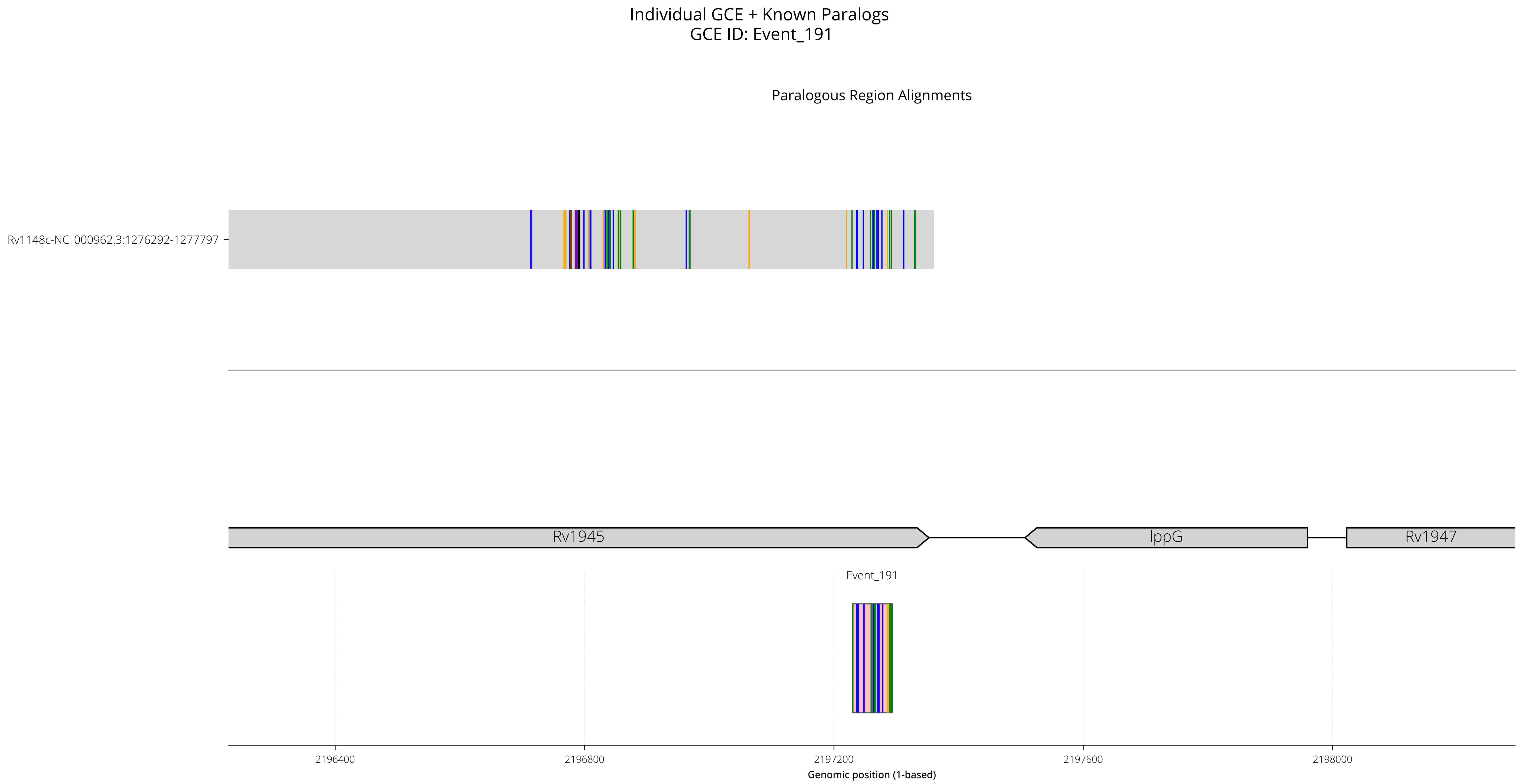
Individual GCE + Known Paralogs
GCE ID: Event_191
Paralogous Region Alignments
Rv1148c-NC_000962.3:1276292-1277797
Rv1945
lppG
Rv1947
Event_191
2196400
2196800
2197200
2197600
2198000
Genomic position (1-based)

# Circos Viz - GCE to Known Paralogs Locations
GCE ID: Event_191

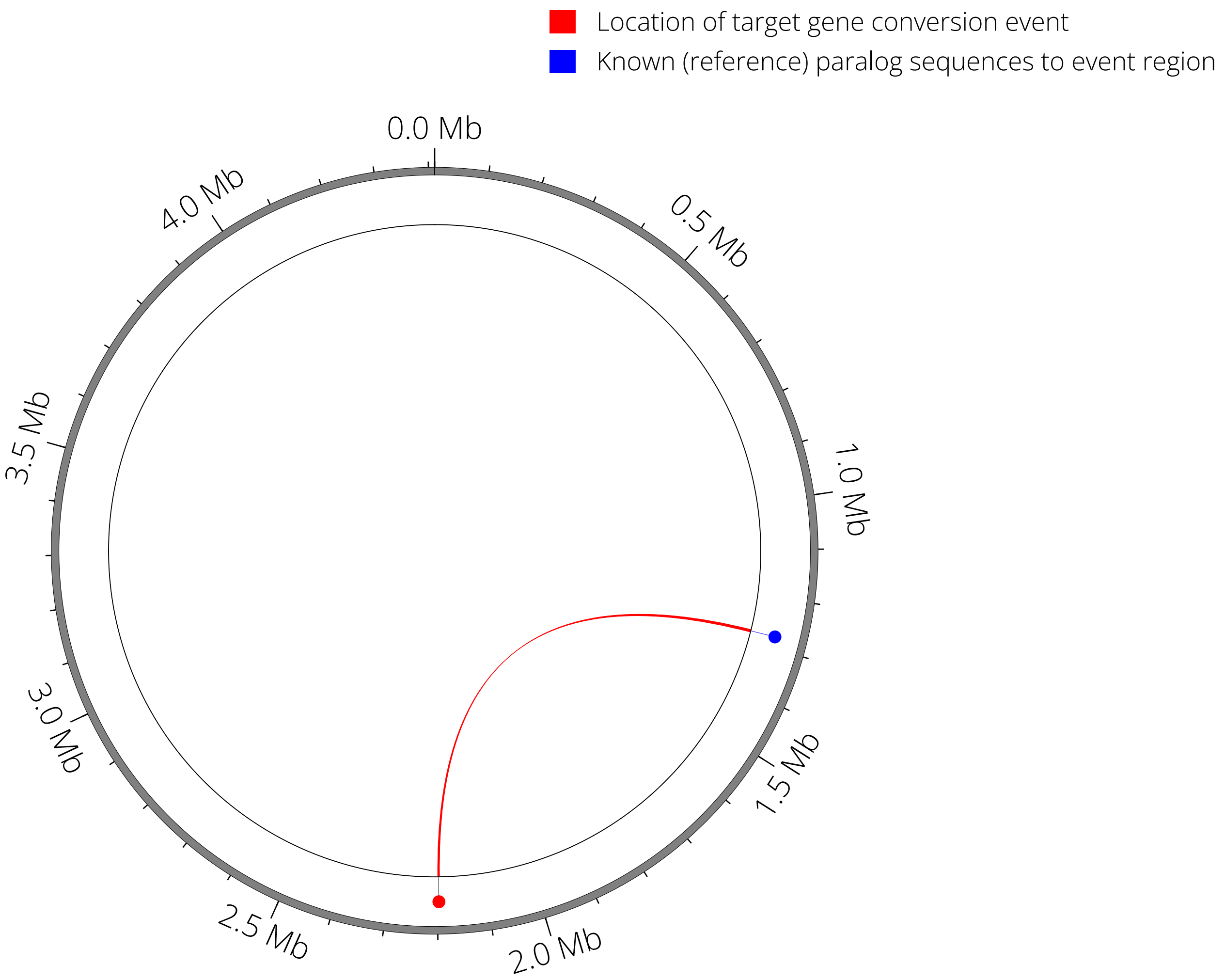

# Event_191 - 14 SNPs - k-mer analysis relative to paralogs

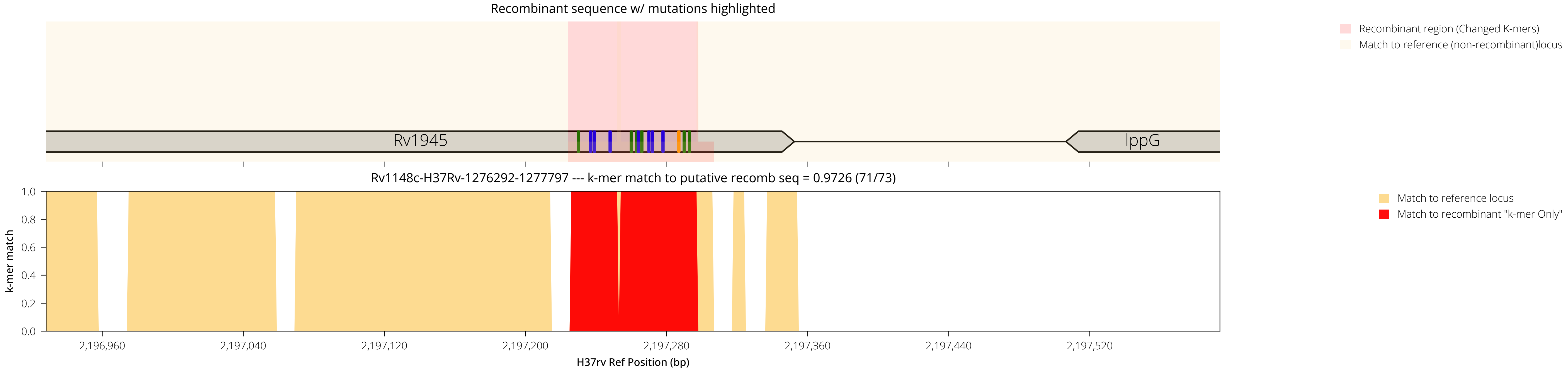

Sub-phylogeny Viz + Paralog Alignments
GCE ID: Event_191

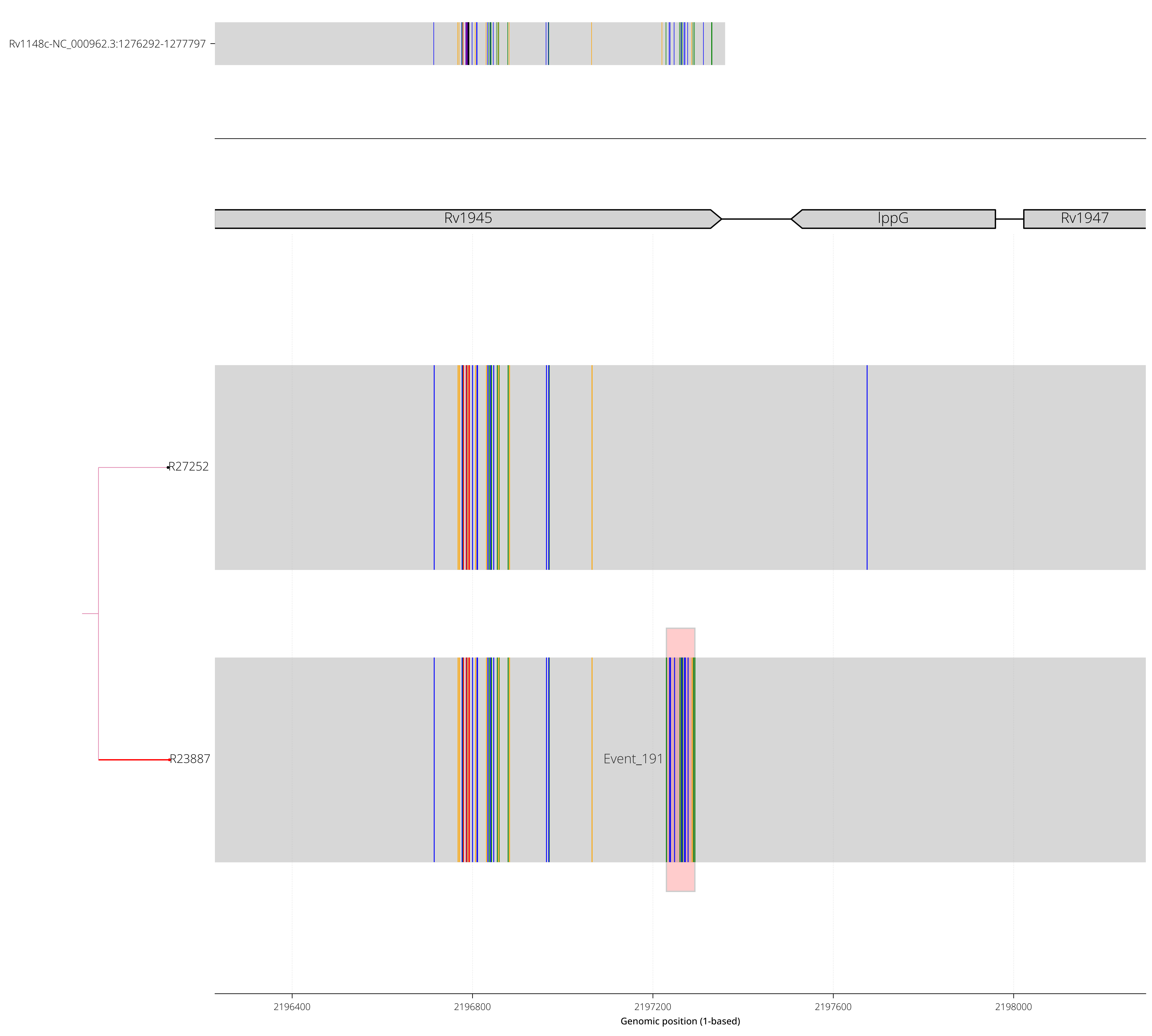

Full Phylogeny Viz + Paralog Alignments
GCE ID: Event_191

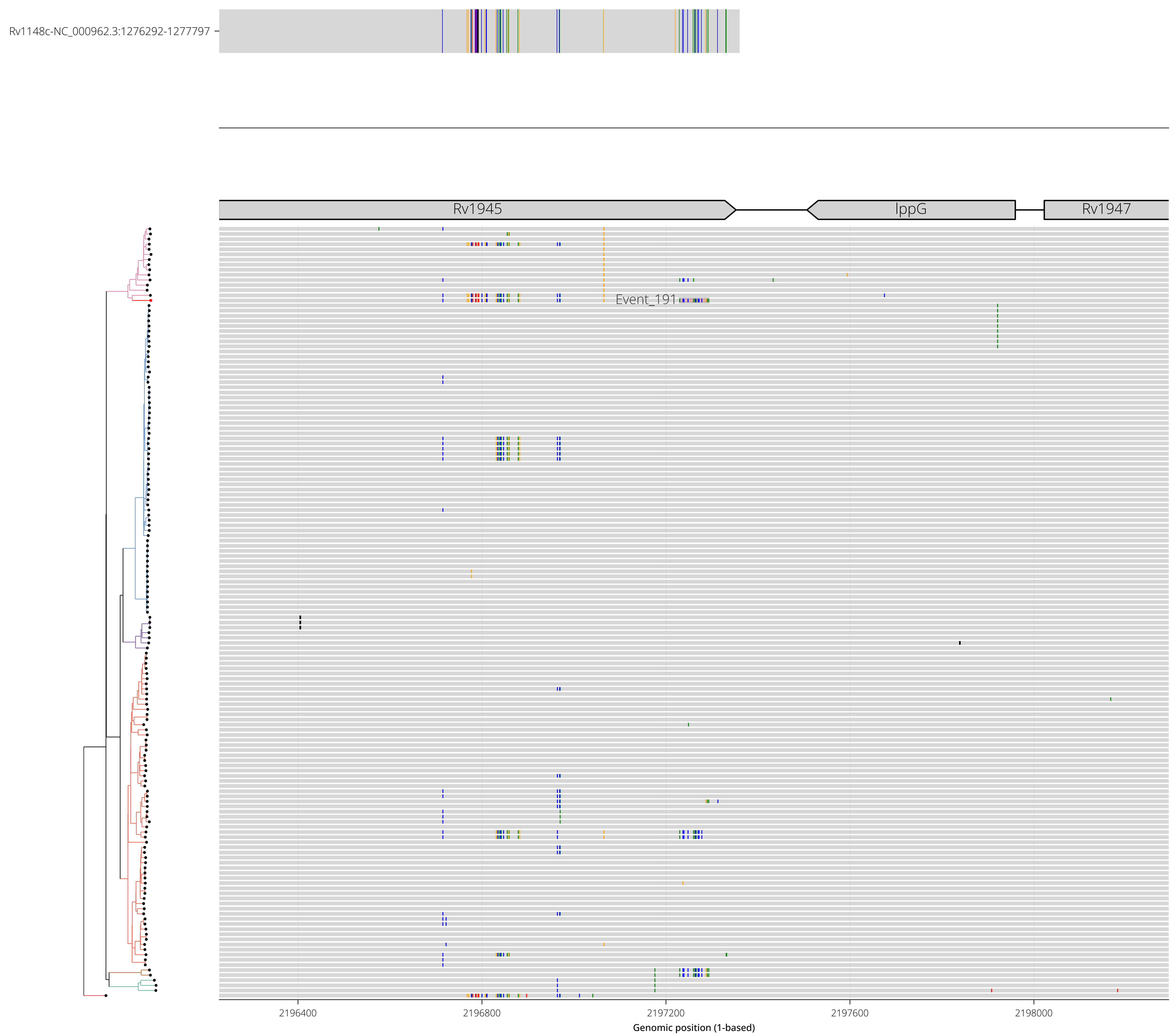

Individual GCE + Known Paralogs
GCE ID: Event_192

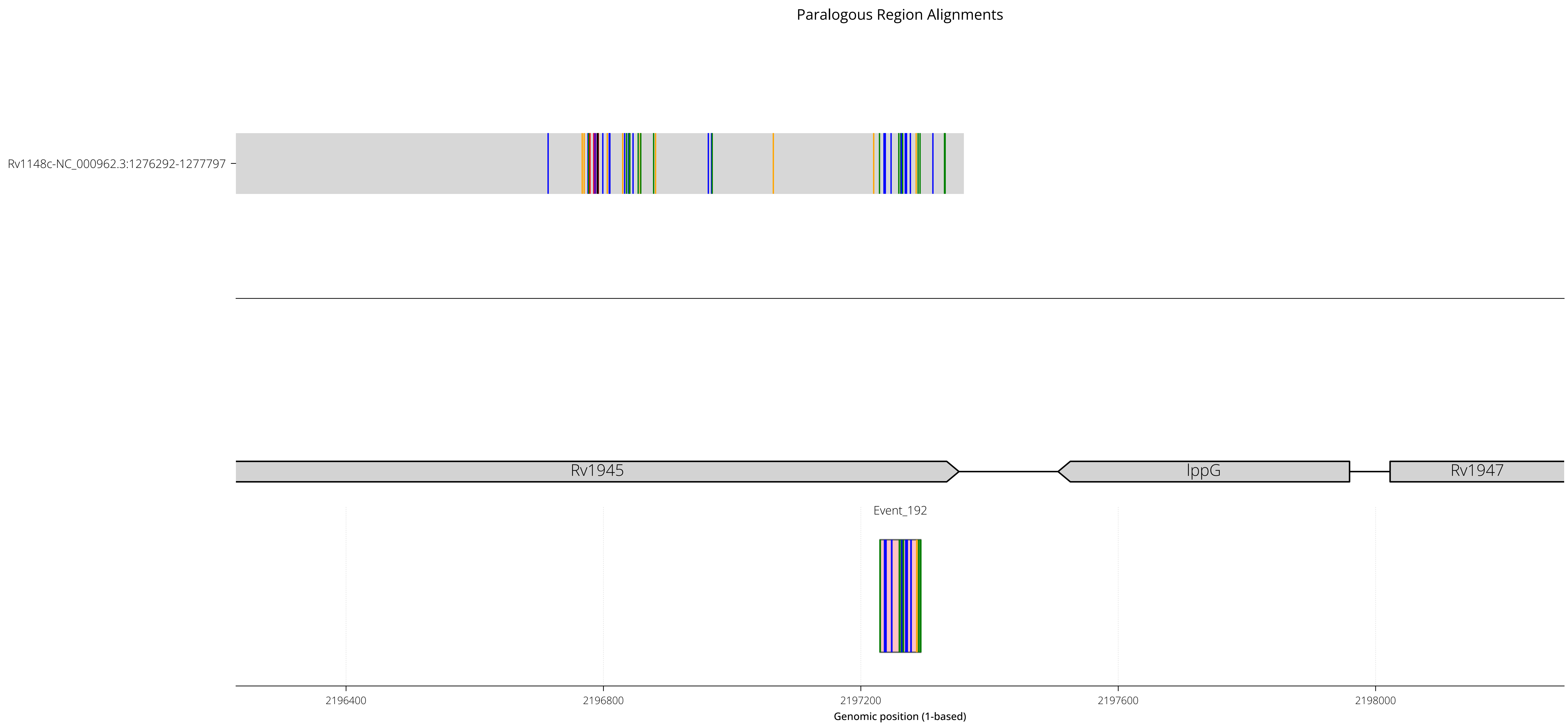

Circos Viz - GCE to Known Paralogs Locations
GCE ID: Event_192

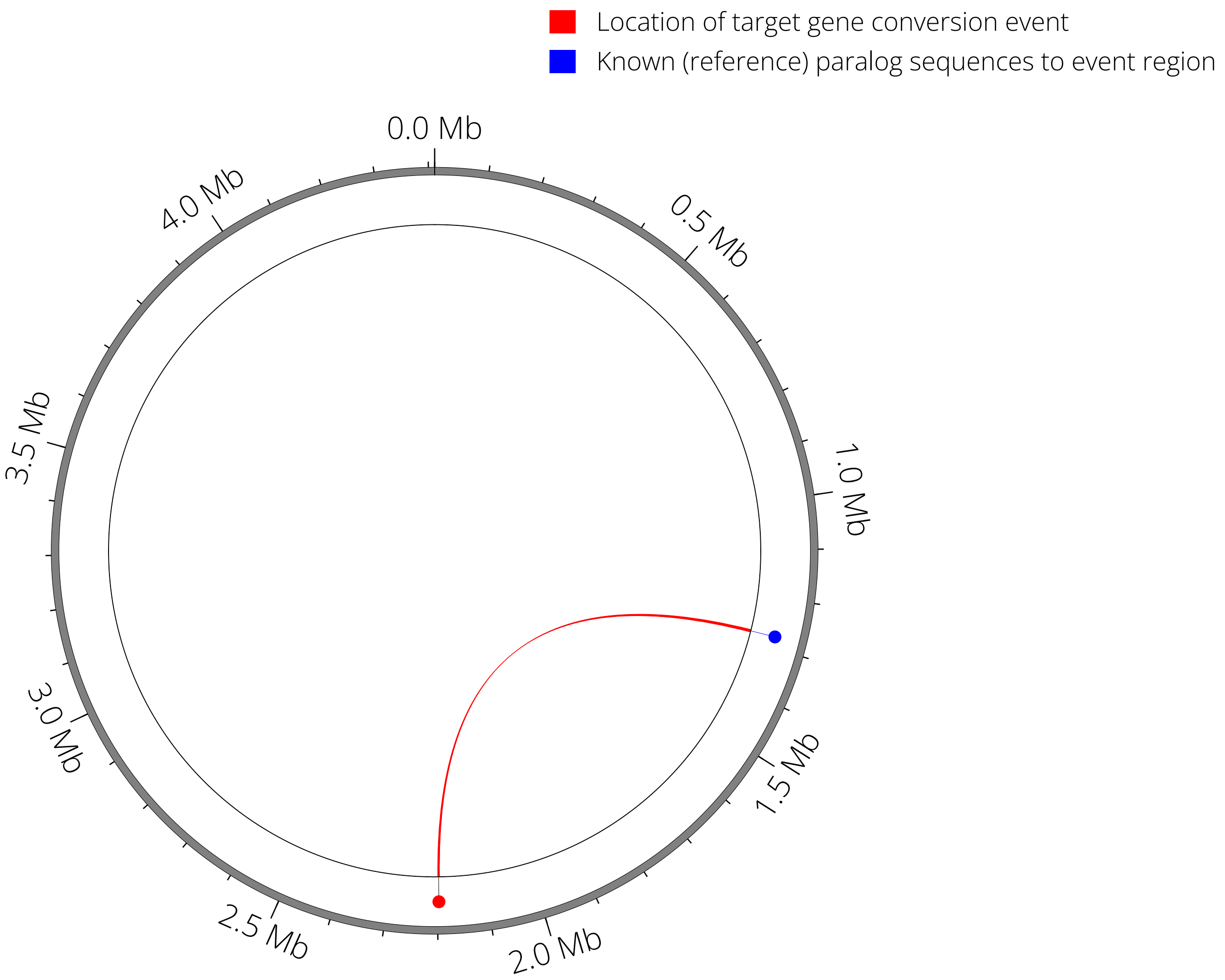

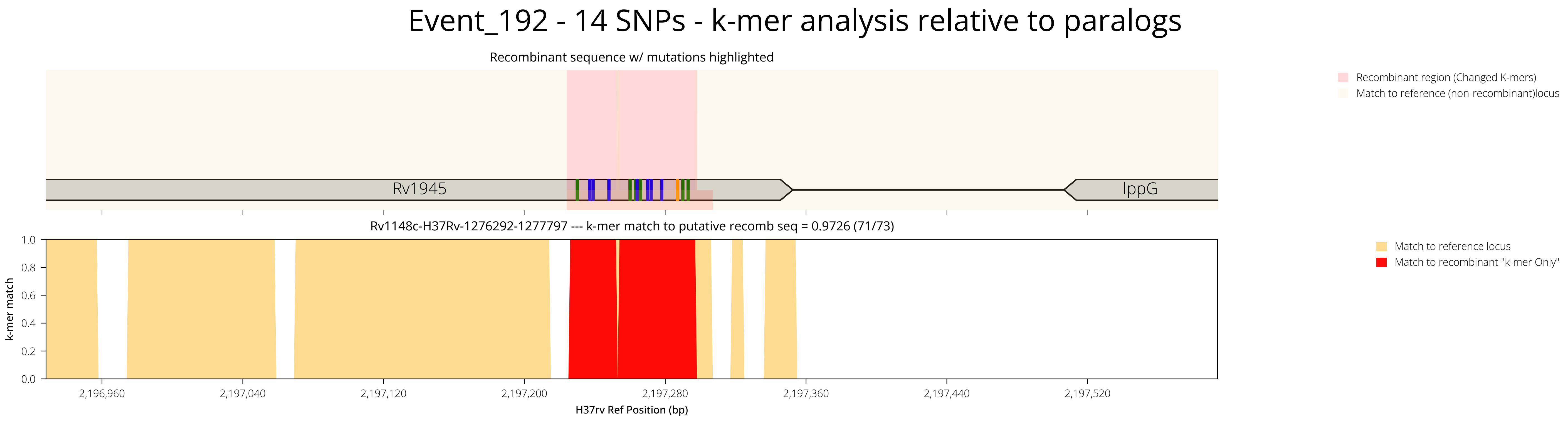

Event_192 - 14 SNPs - k-mer analysis relative to paralogs
Recombinant sequence w/ mutations highlighted
Recombinant region (Changed K-mers)
Match to reference (non-recombinant)locus
Rv1945
lppG
Rv1148c-H37Rv-1276292-1277797 --- k-mer match to putative recomb seq = 0.9726 (71/73)
Match to reference locus
Match to recombinant "k-mer Only"
k-mer match
1.0
0.8
0.6
0.4
0.2
0.0
2,196,960
2,197,040
2,197,120
2,197,200
2,197,280
2,197,360
2,197,440
2,197,520
H37rv Ref Position (bp)

Sub-phylogeny Viz + Paralog Alignments
GCE ID: Event_192

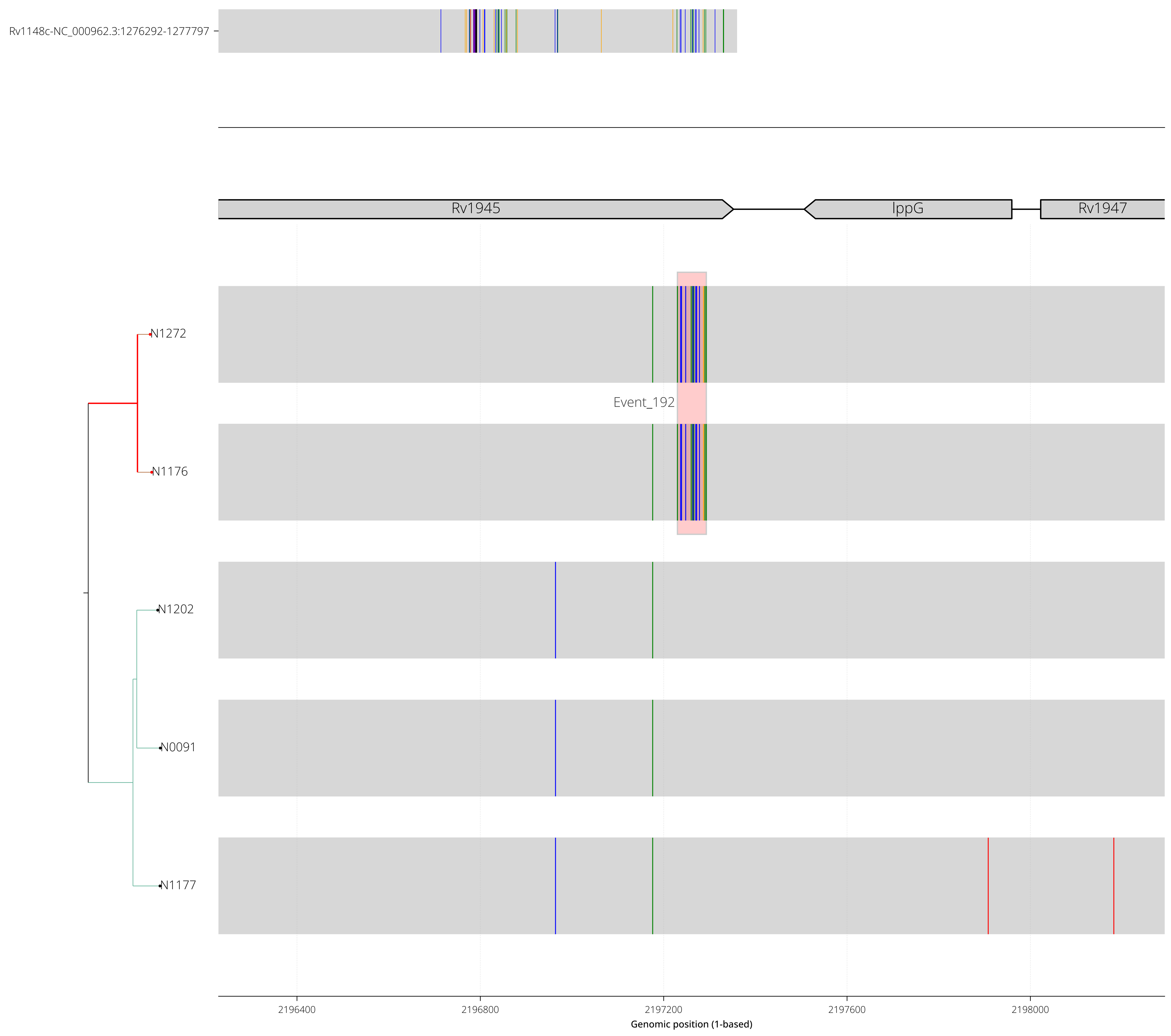

Full Phylogeny Viz + Paralog Alignments
GCE ID: Event_192

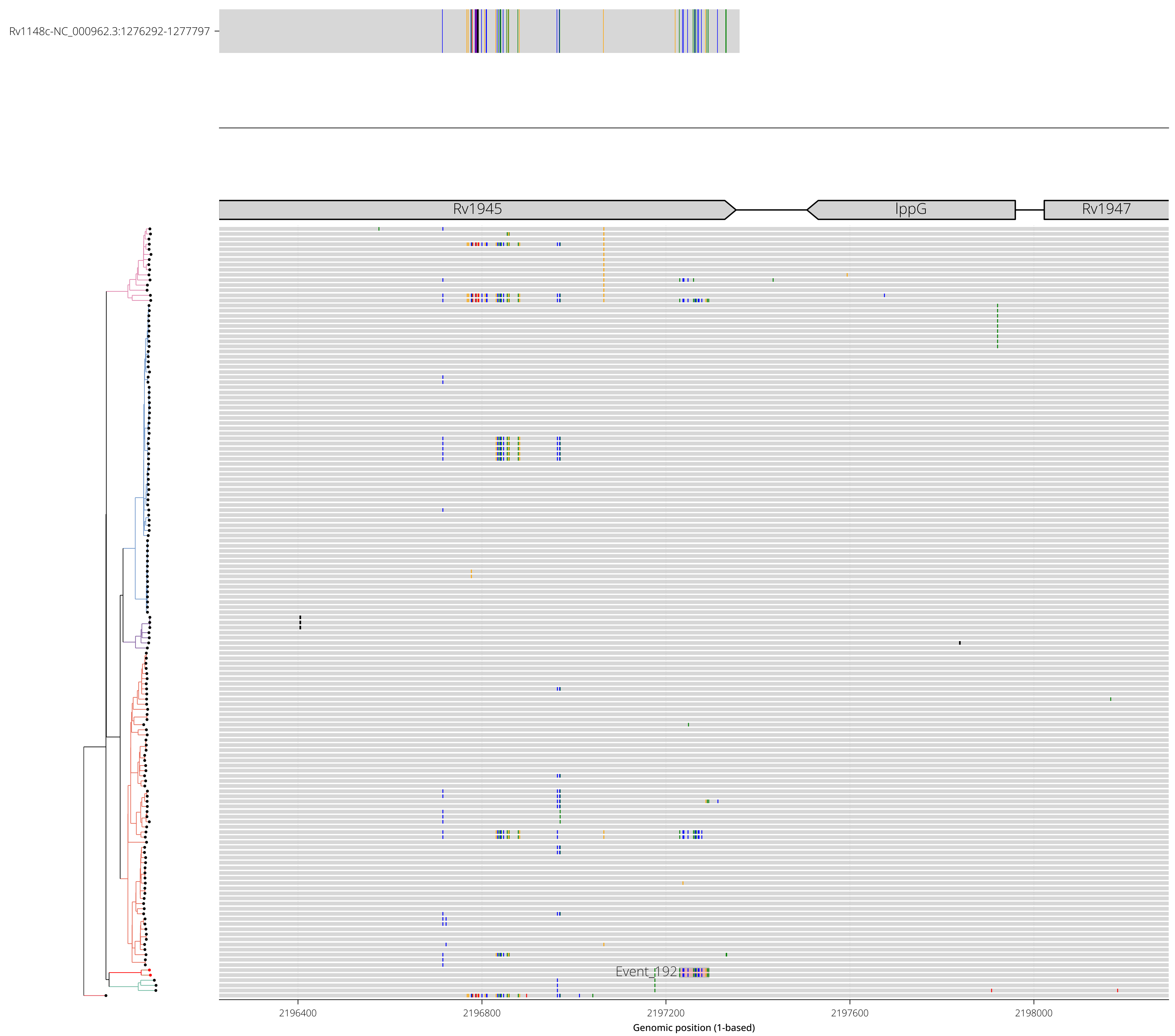

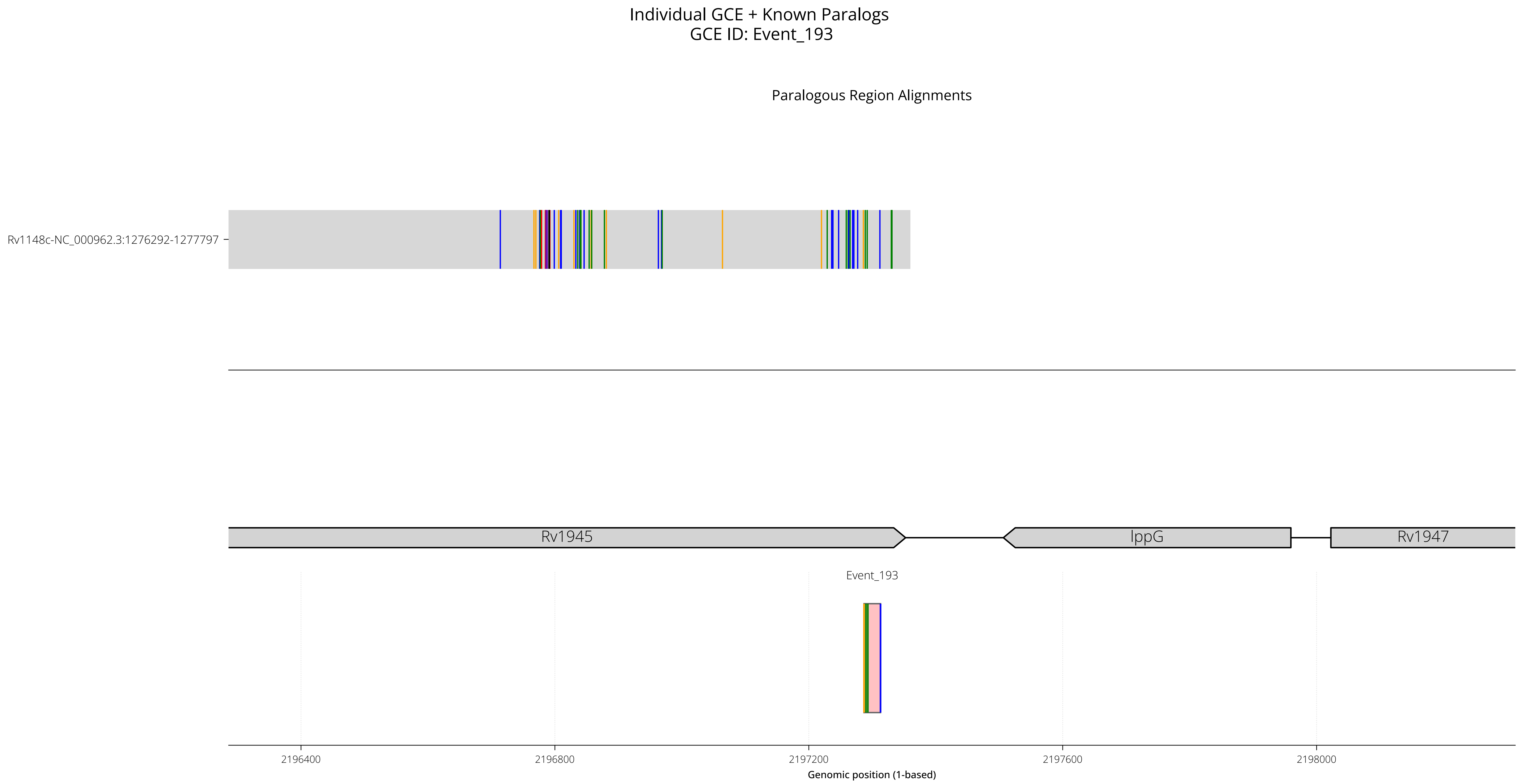
Individual GCE + Known Paralogs
GCE ID: Event_193
Paralogous Region Alignments
Rv1148c-NC_000962.3:1276292-1277797
Rv1945
lppG
Rv1947
Event_193
2196400
2196800
2197200
2197600
2198000
Genomic position (1-based)

Circos Viz - GCE to Known Paralogs Locations
GCE ID: Event_193

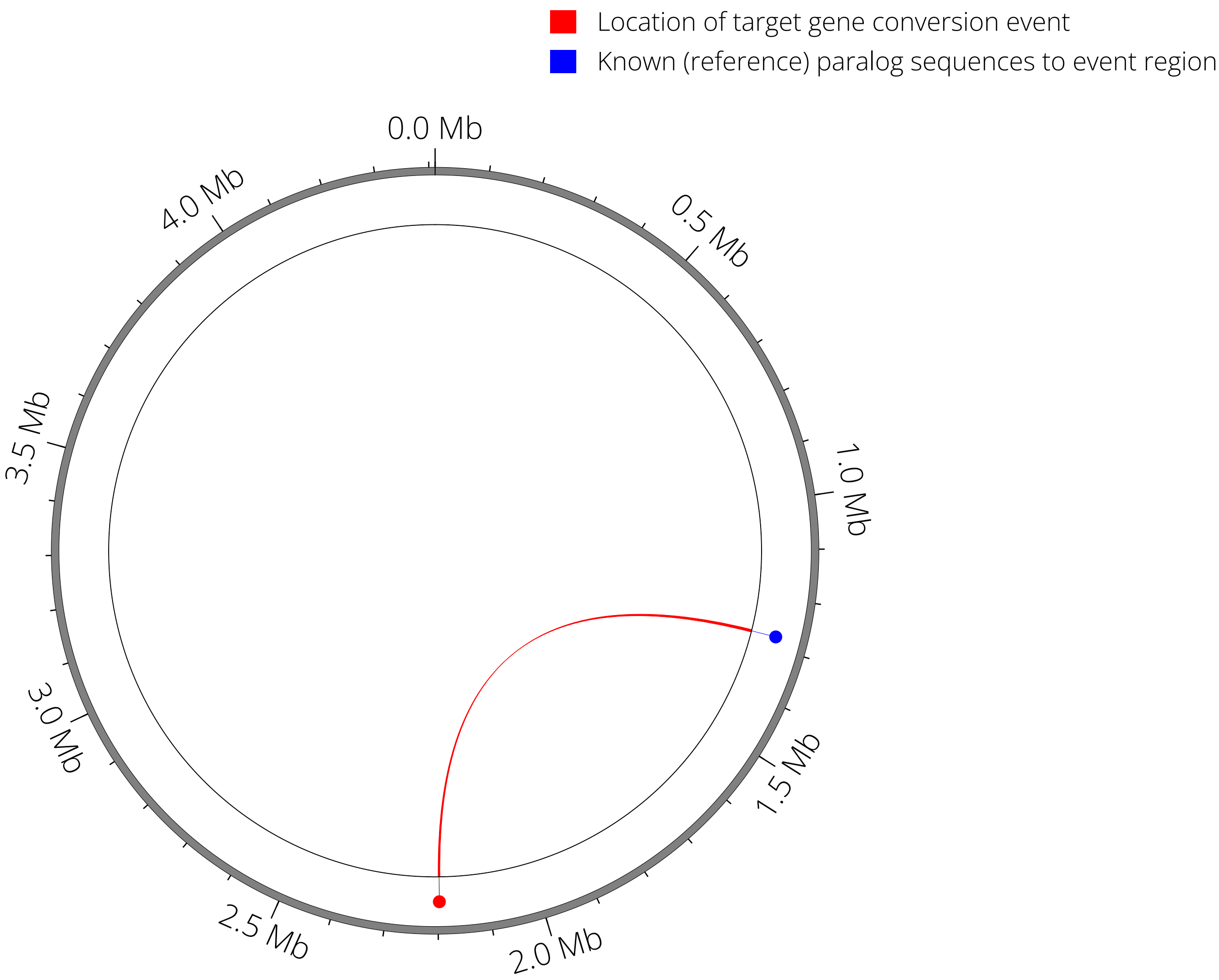

# Event_193 - 4 SNPs - k-mer analysis relative to paralogs

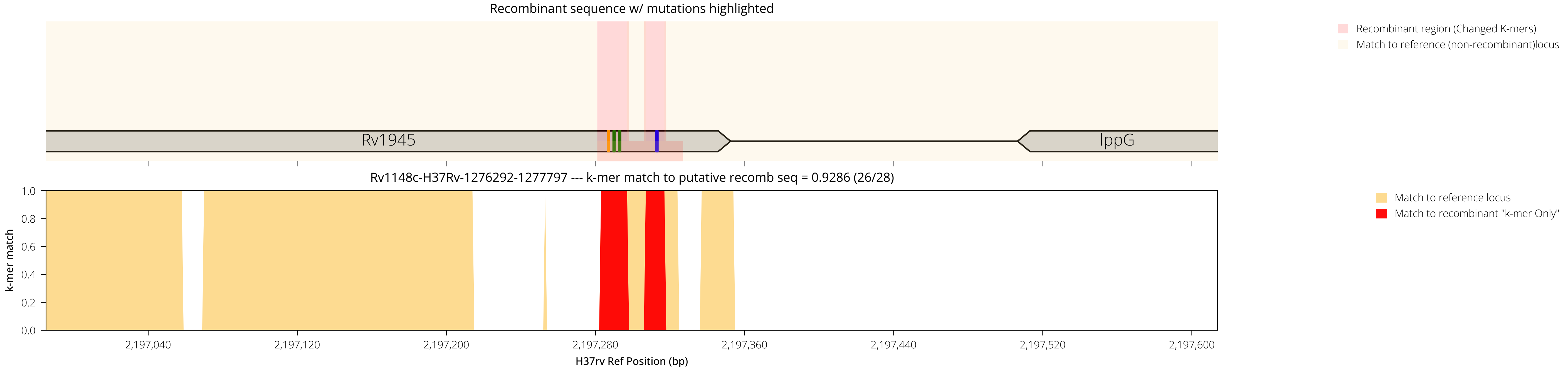

Sub-phylogeny Viz + Paralog Alignments
GCE ID: Event_193

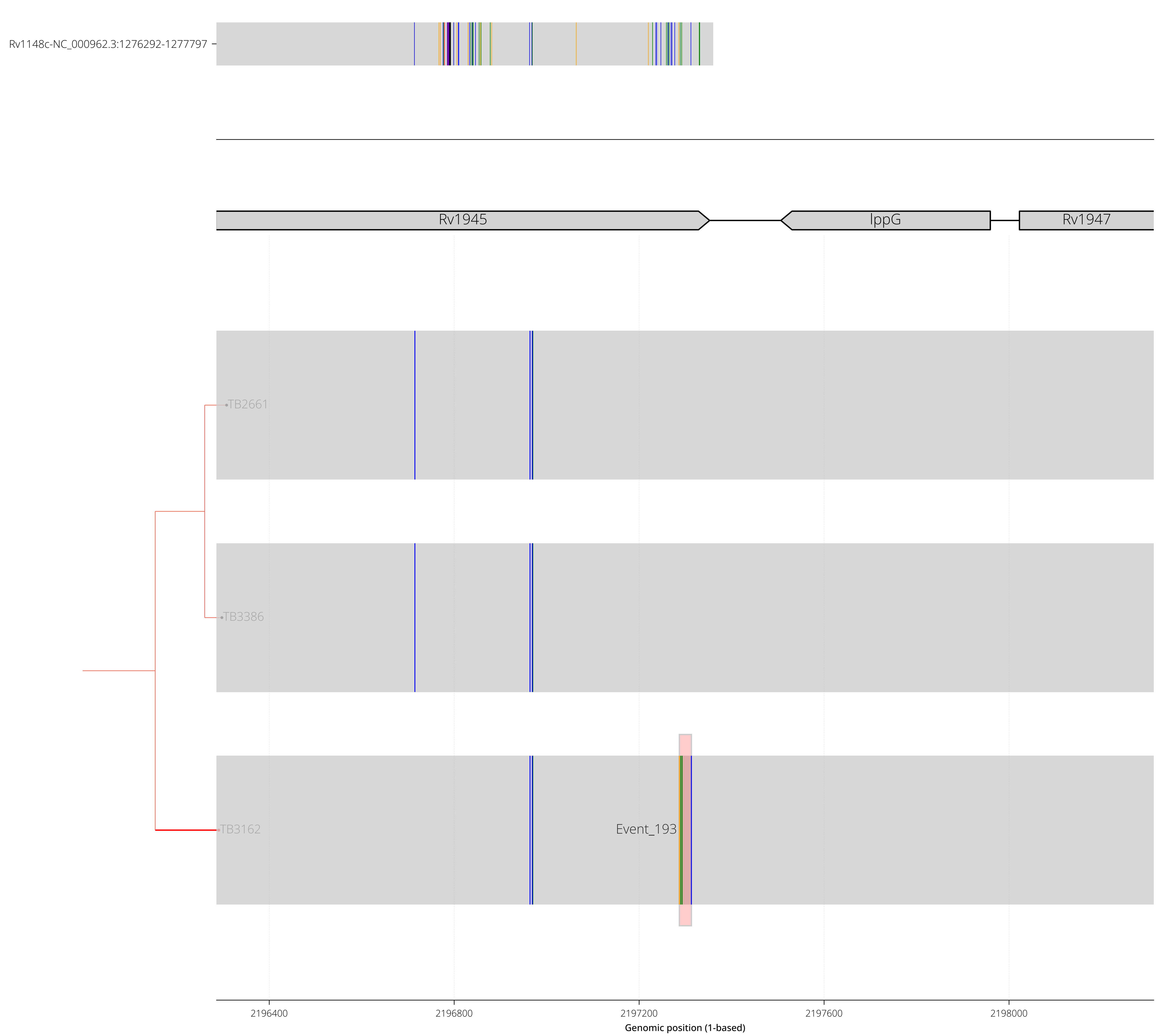

Full Phylogeny Viz + Paralog Alignments
GCE ID: Event_193

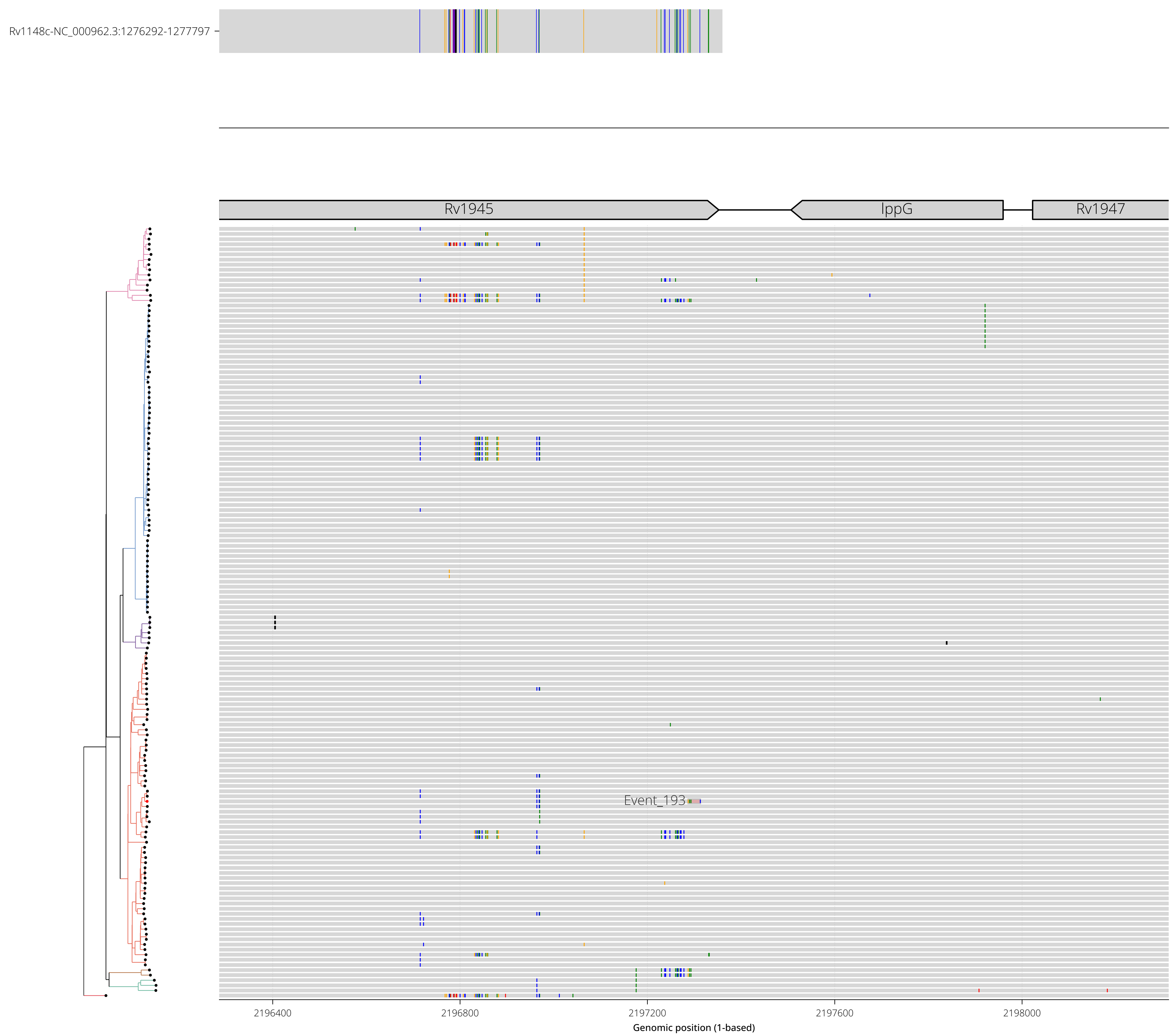

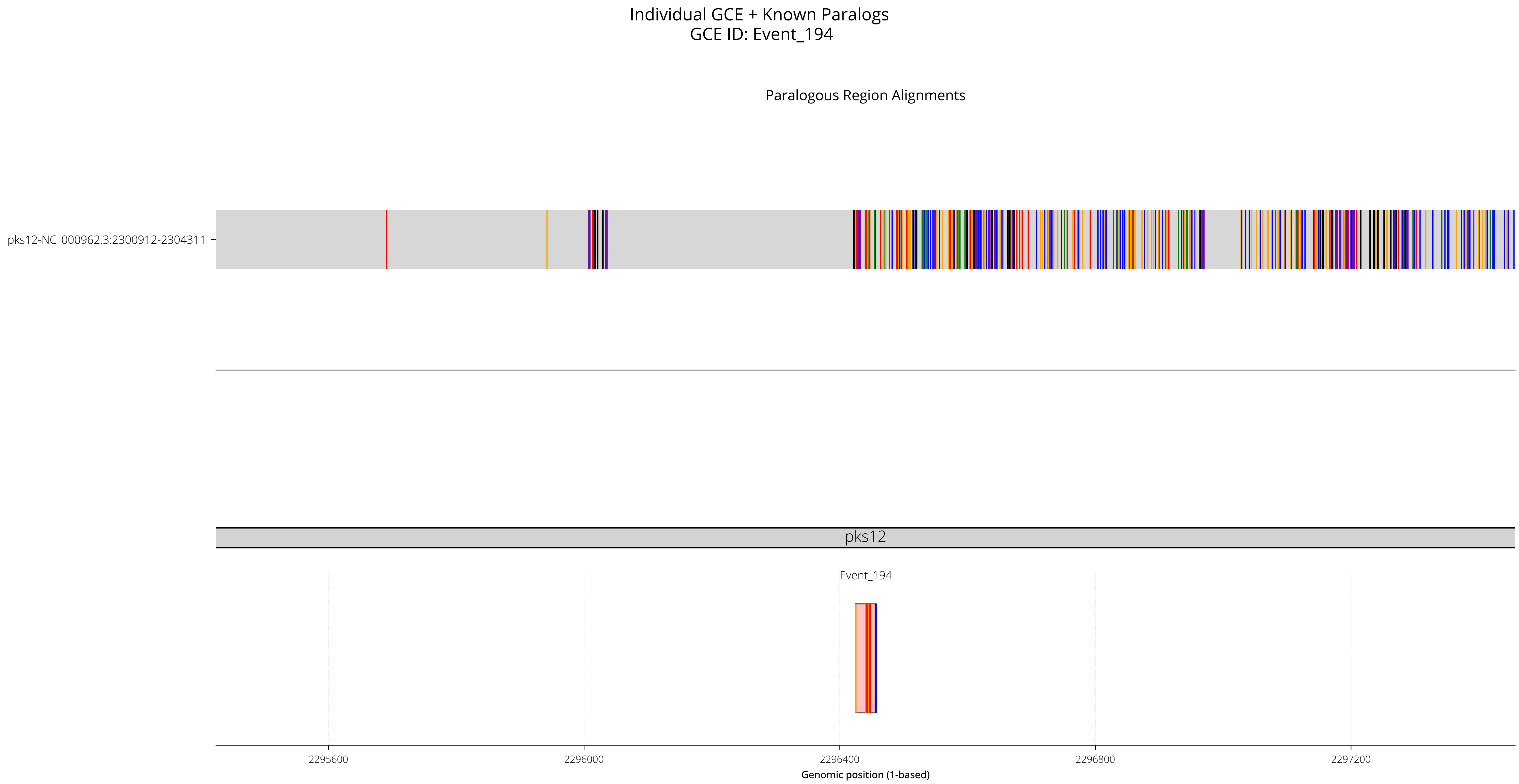
Individual GCE + Known Paralogs
GCE ID: Event_194
Paralogous Region Alignments
pks12-NC_000962.3:2300912-2304311
pks12
Event_194
2295600
2296000
2296400
2296800
2297200
Genomic position (1-based)

Circos Viz - GCE to Known Paralogs Locations
GCE ID: Event_194

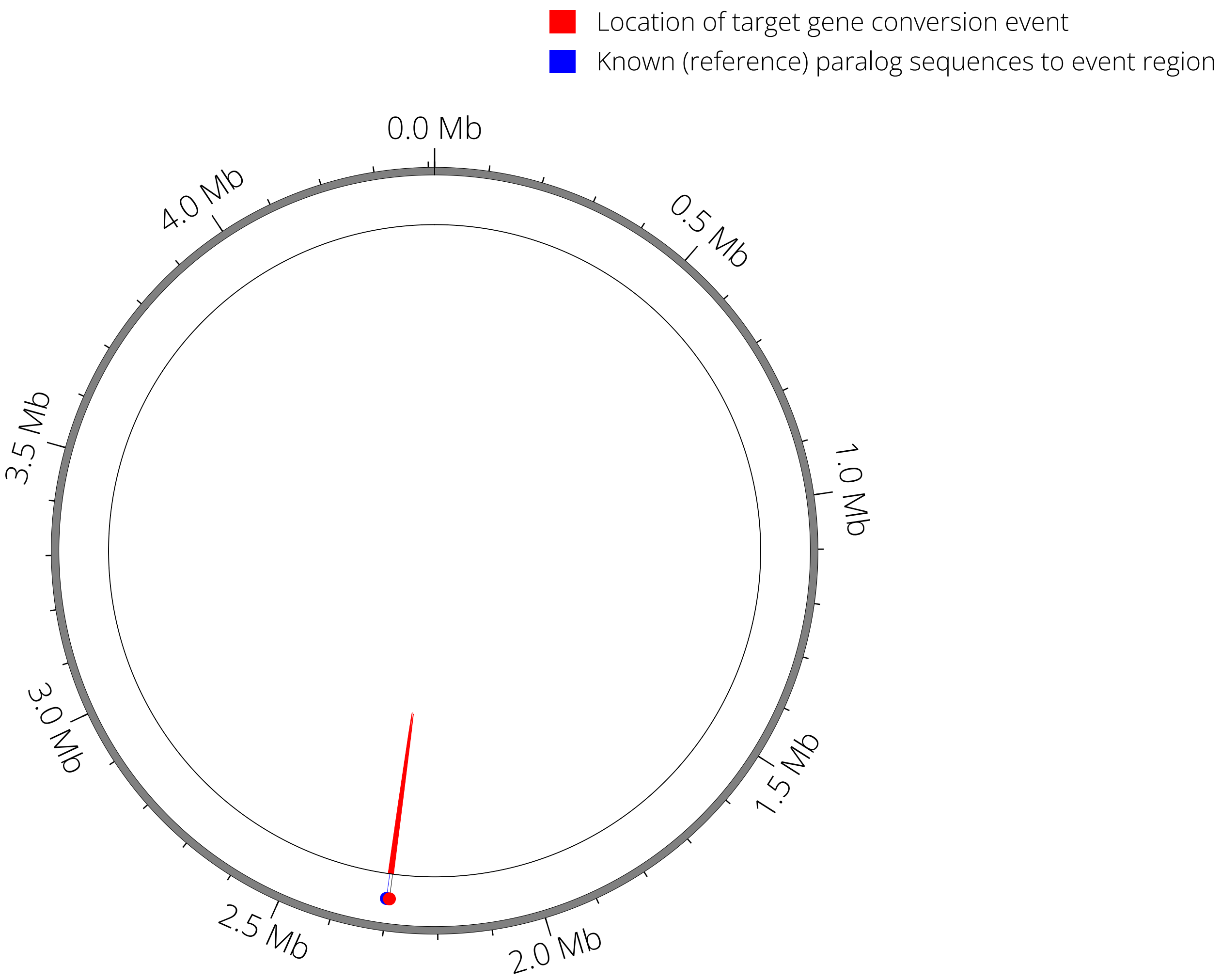

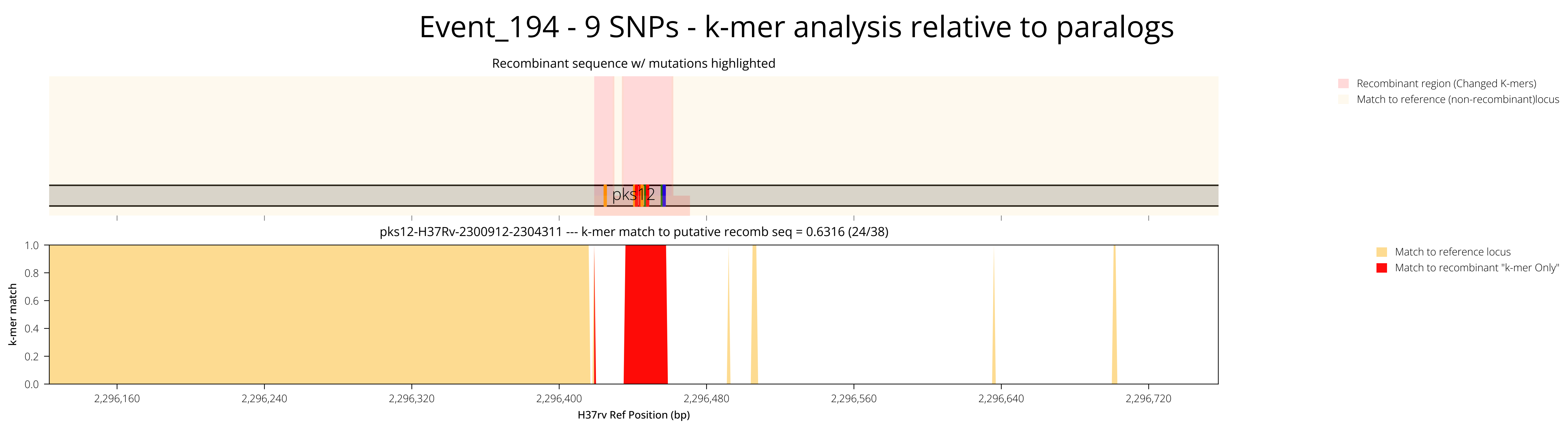
Event_194 - 9 SNPs - k-mer analysis relative to paralogs
Recombinant sequence w/ mutations highlighted
Recombinant region (Changed K-mers)
Match to reference (non-recombinant)locus
pks12
pks12-H37Rv-2300912-2304311 --- k-mer match to putative recomb seq = 0.6316 (24/38)
Match to reference locus
Match to recombinant "k-mer Only"
k-mer match
1.0
0.8
0.6
0.4
0.2
0.0
2,296,160
2,296,240
2,296,320
2,296,400
2,296,480
2,296,560
2,296,640
2,296,720
H37rv Ref Position (bp)

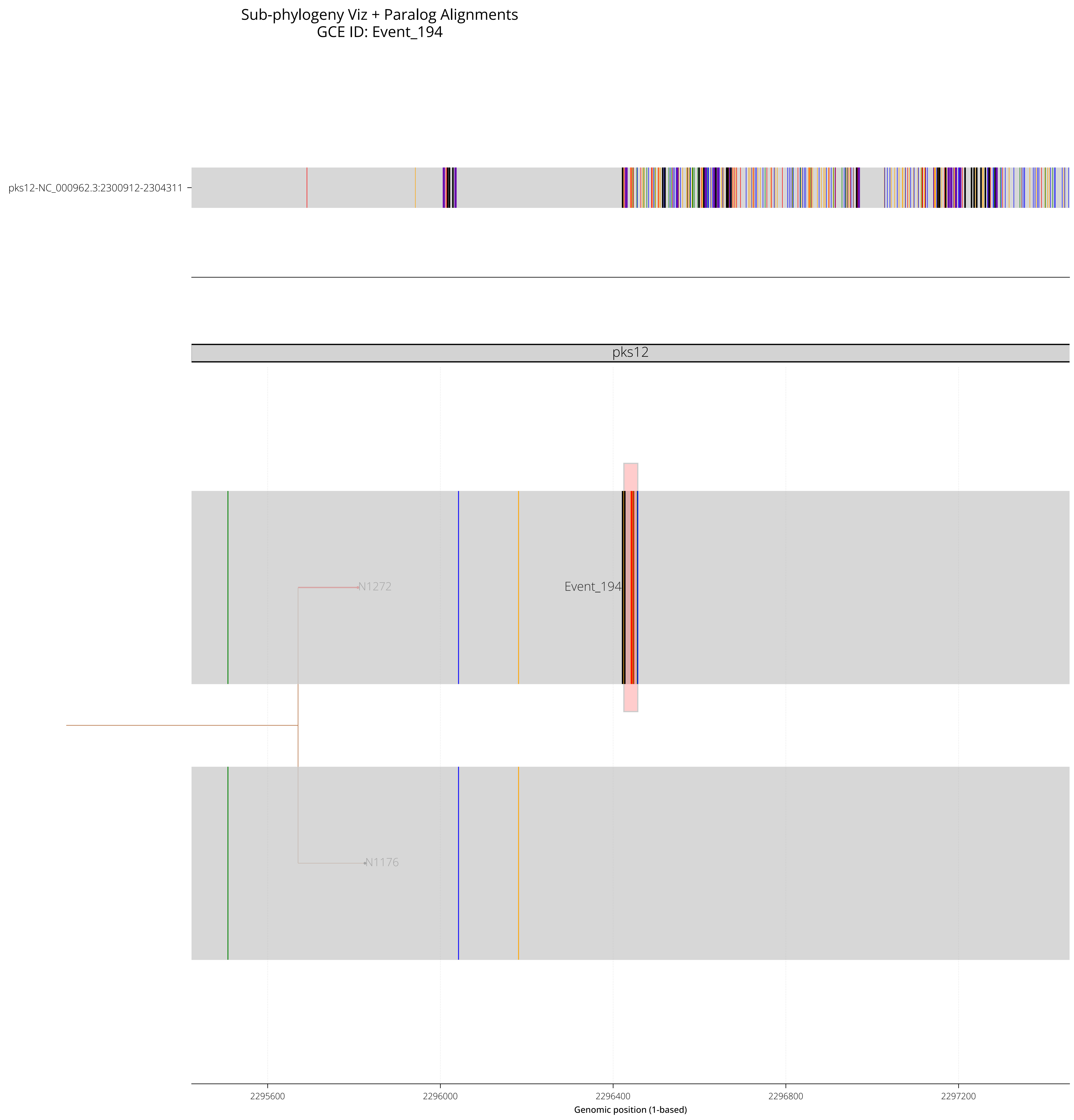

Sub-phylogeny Viz + Paralog Alignments
GCE ID: Event_194
pks12-NC_000962.3:2300912-2304311
pks12
N1272
Event_194
N1176
2295600
2296000
2296400
2296800
2297200
Genomic position (1-based)

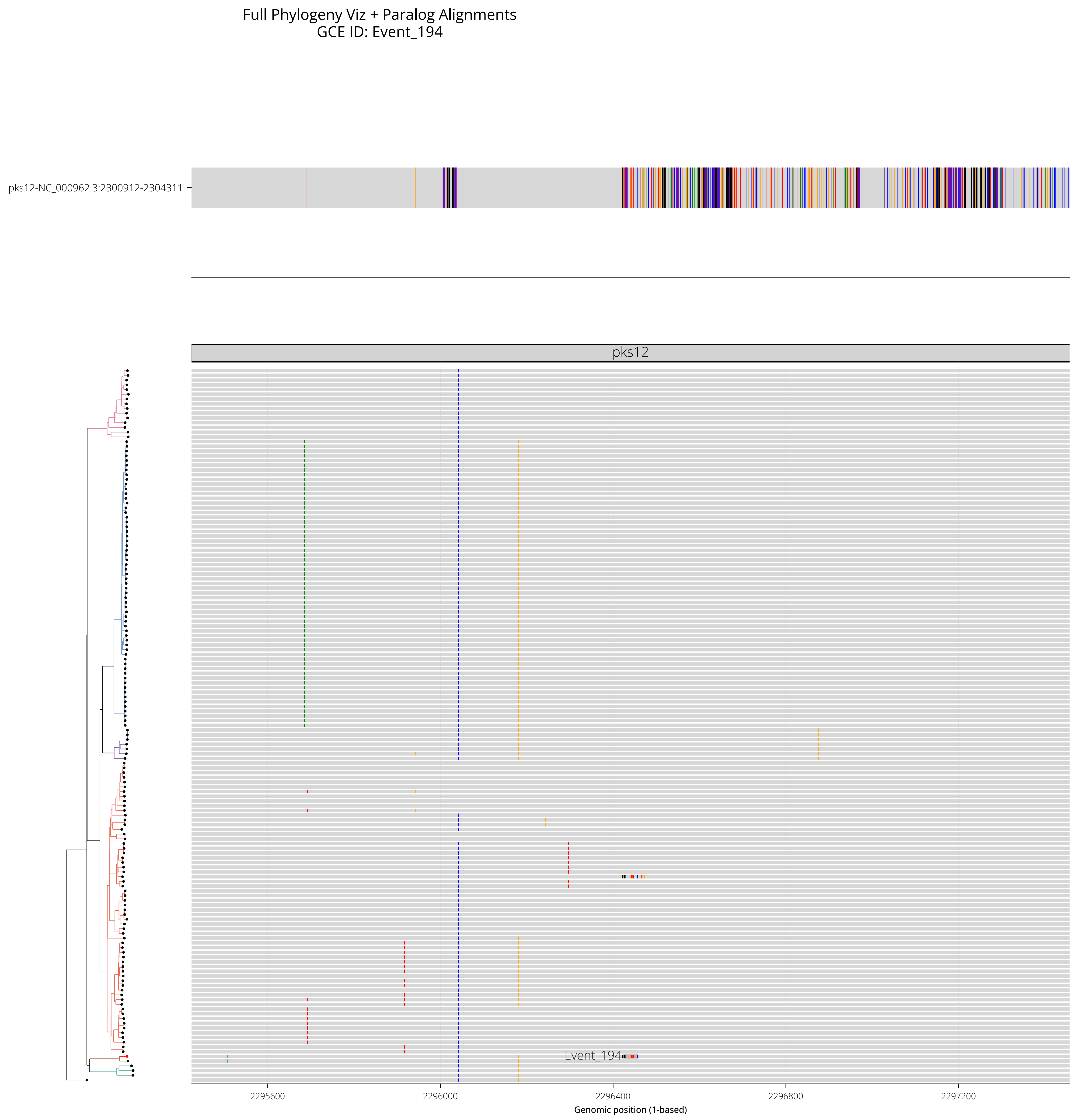

Full Phylogeny Viz + Paralog Alignments
GCE ID: Event_194
pks12-NC_000962.3:2300912-2304311
pks12
Event_194
2295600
2296000
2296400
2296800
2297200
Genomic position (1-based)

Individual GCE + Known Paralogs
GCE ID: Event_195

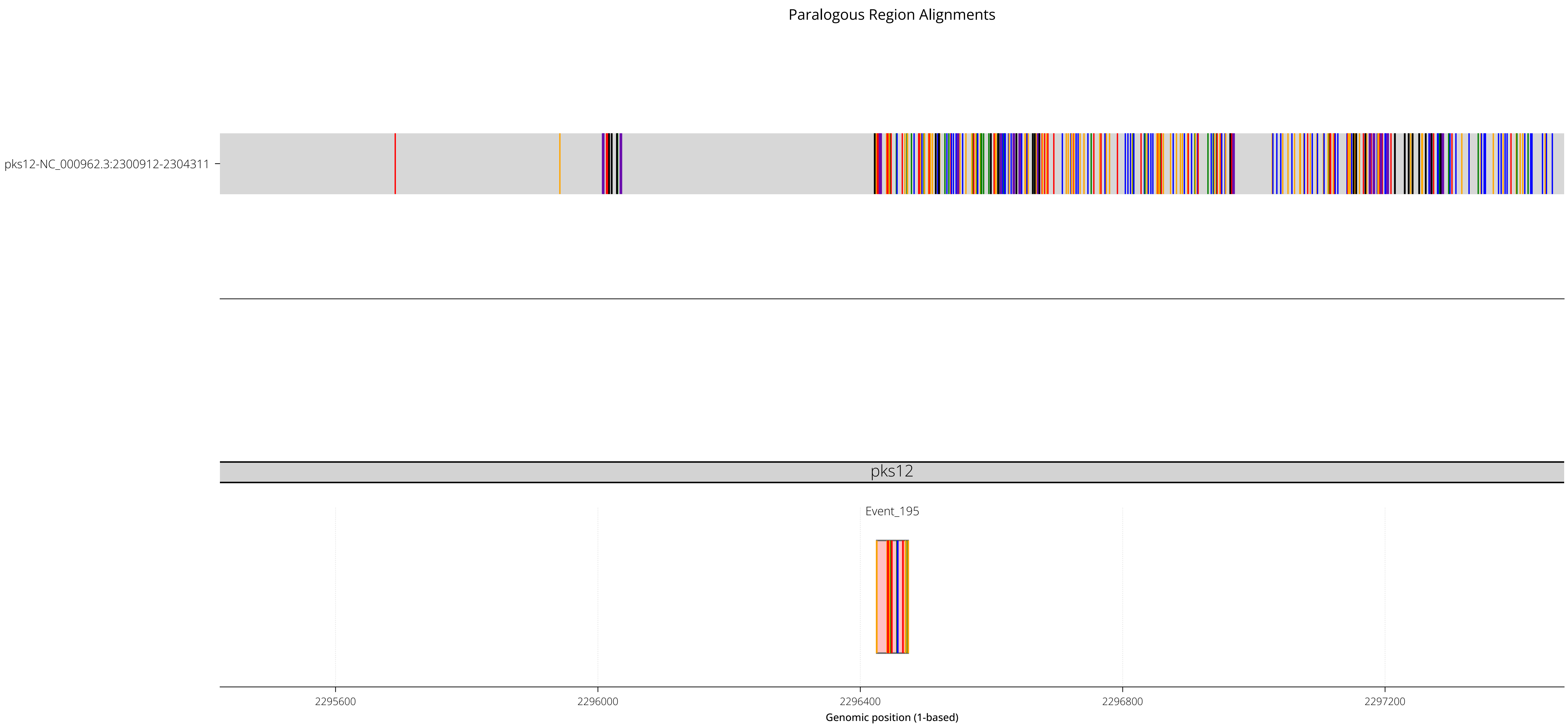

Circos Viz - GCE to Known Paralogs Locations
GCE ID: Event_195

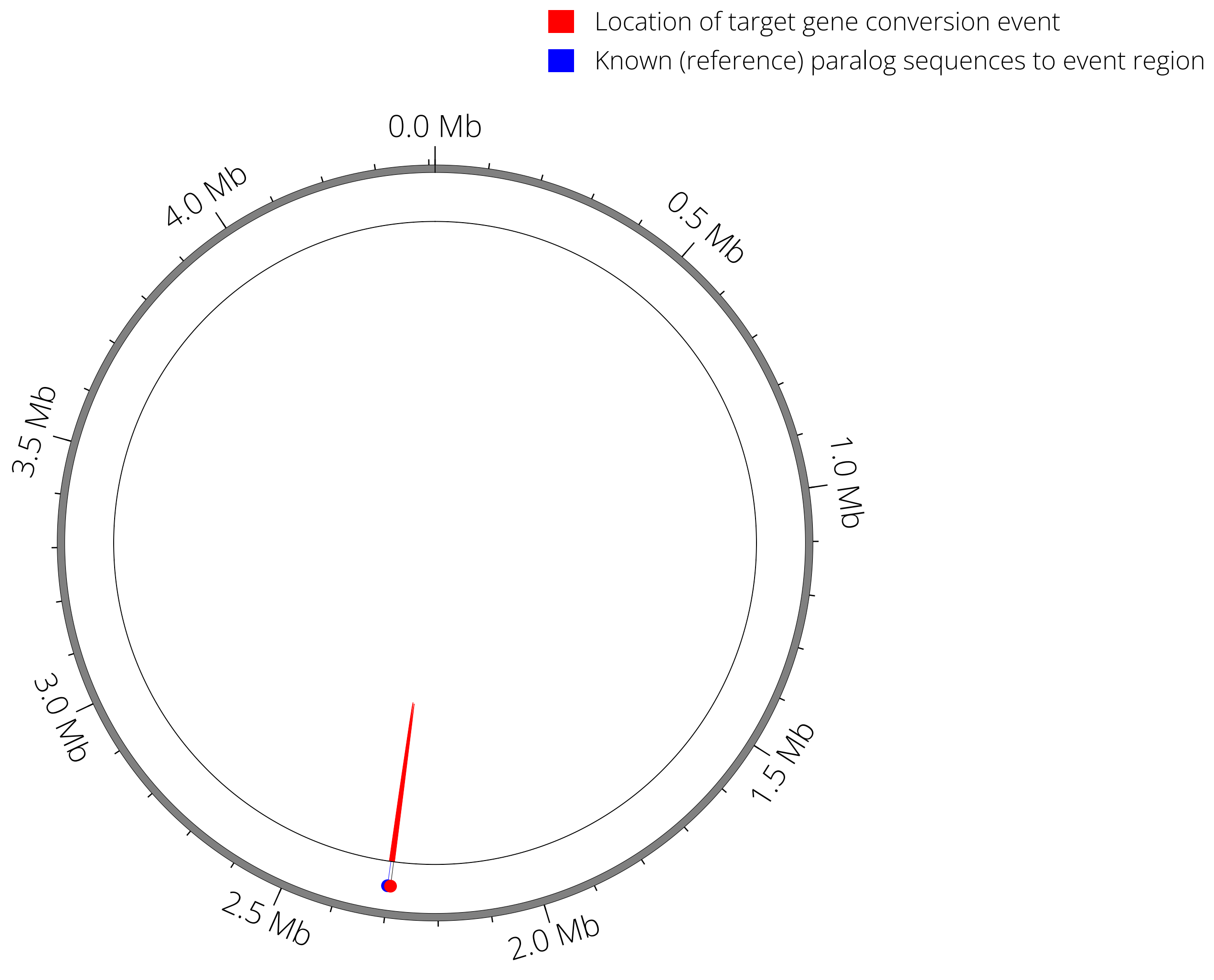

# Event_195 - 13 SNPs - k-mer analysis relative to paralogs

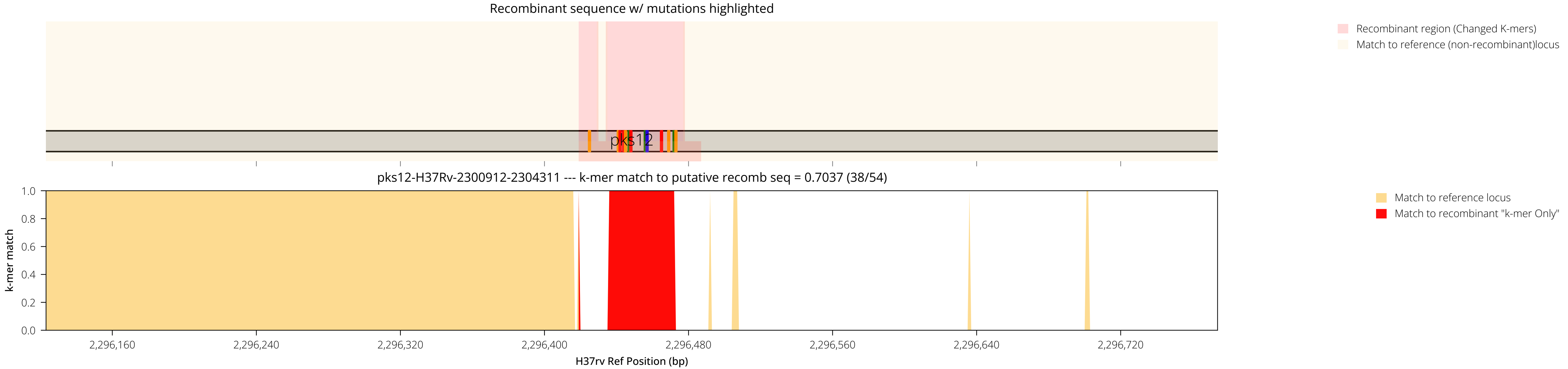

Sub-phylogeny Viz + Paralog Alignments
GCE ID: Event_195

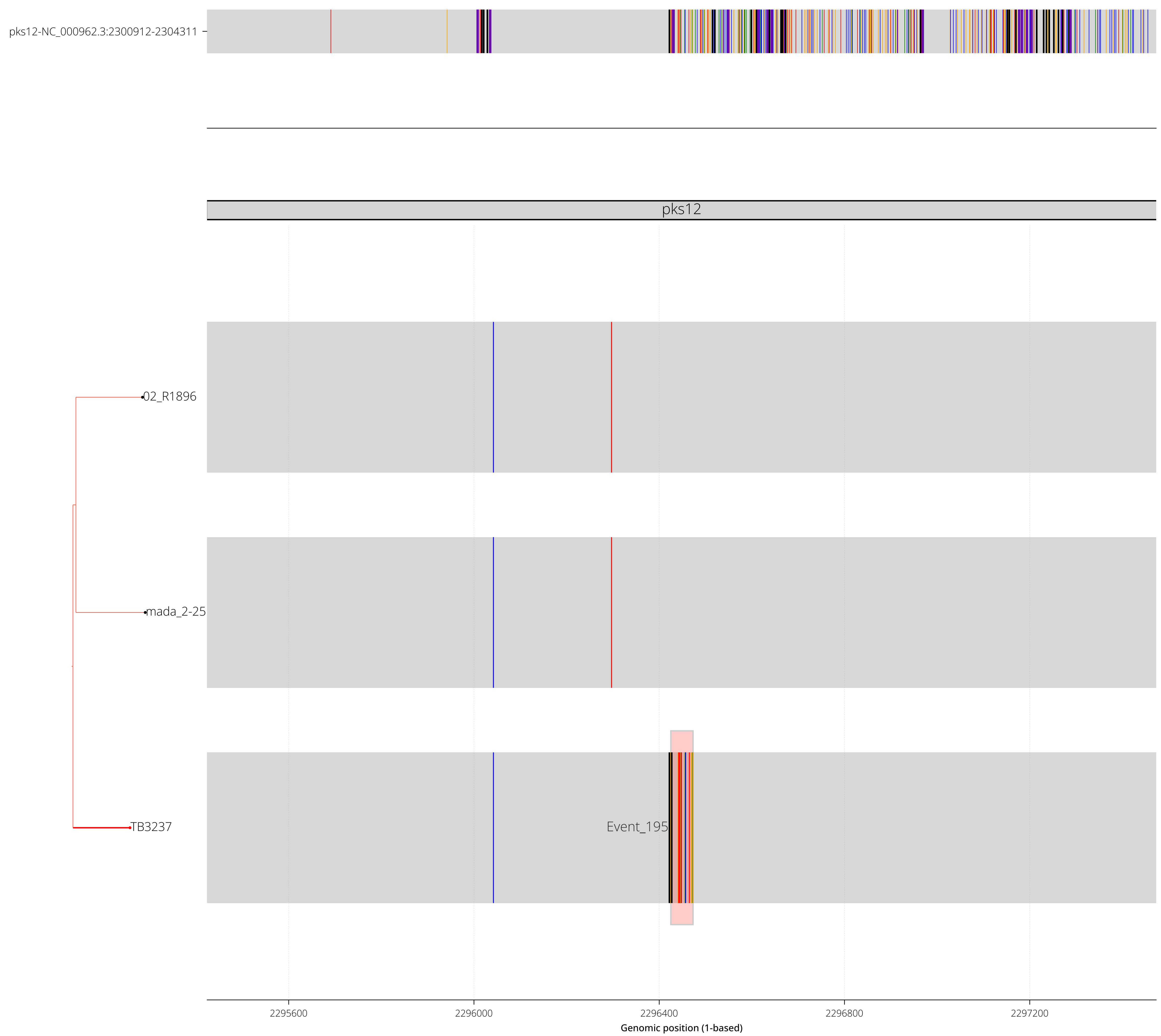

Full Phylogeny Viz + Paralog Alignments
GCE ID: Event_195

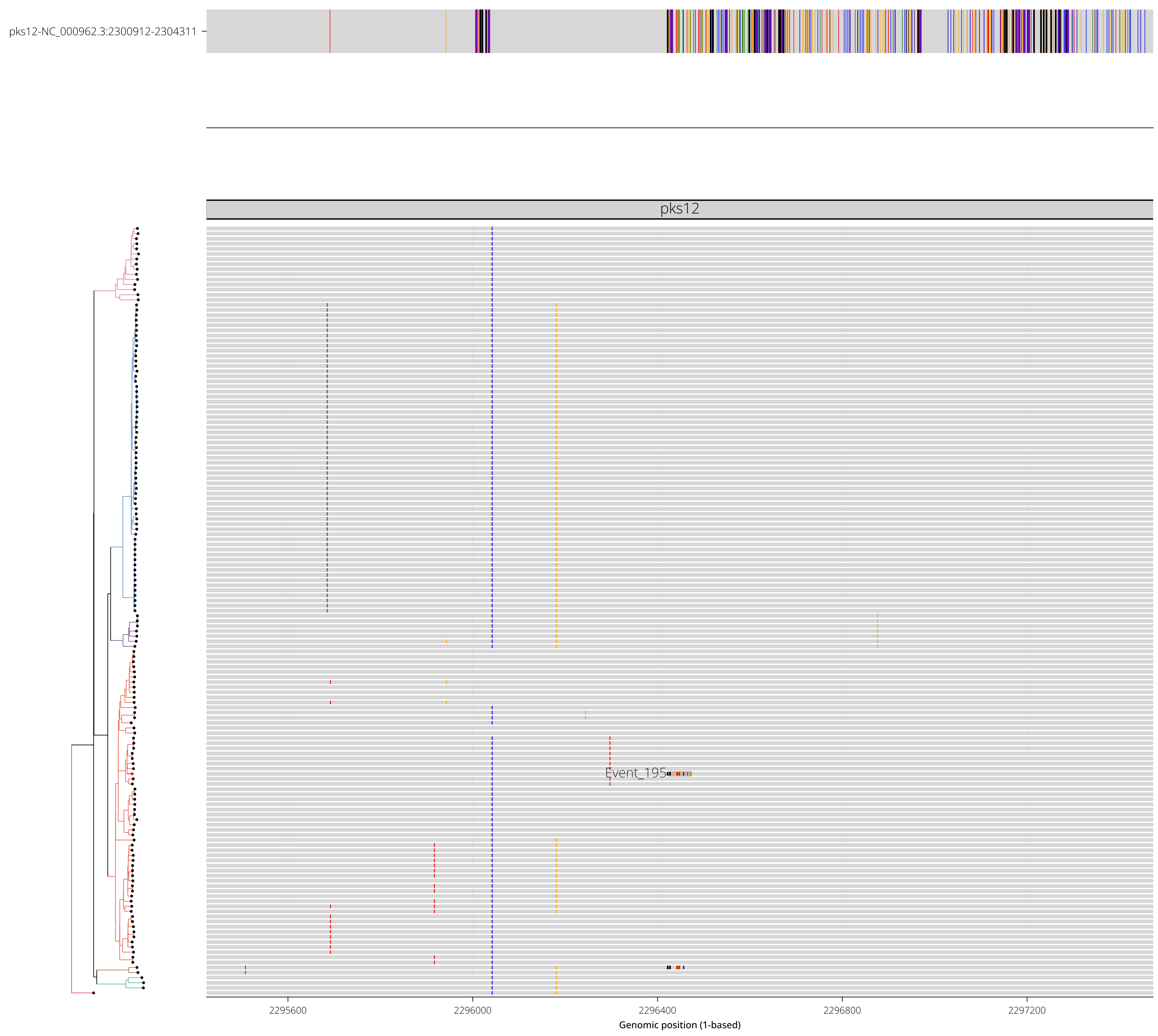

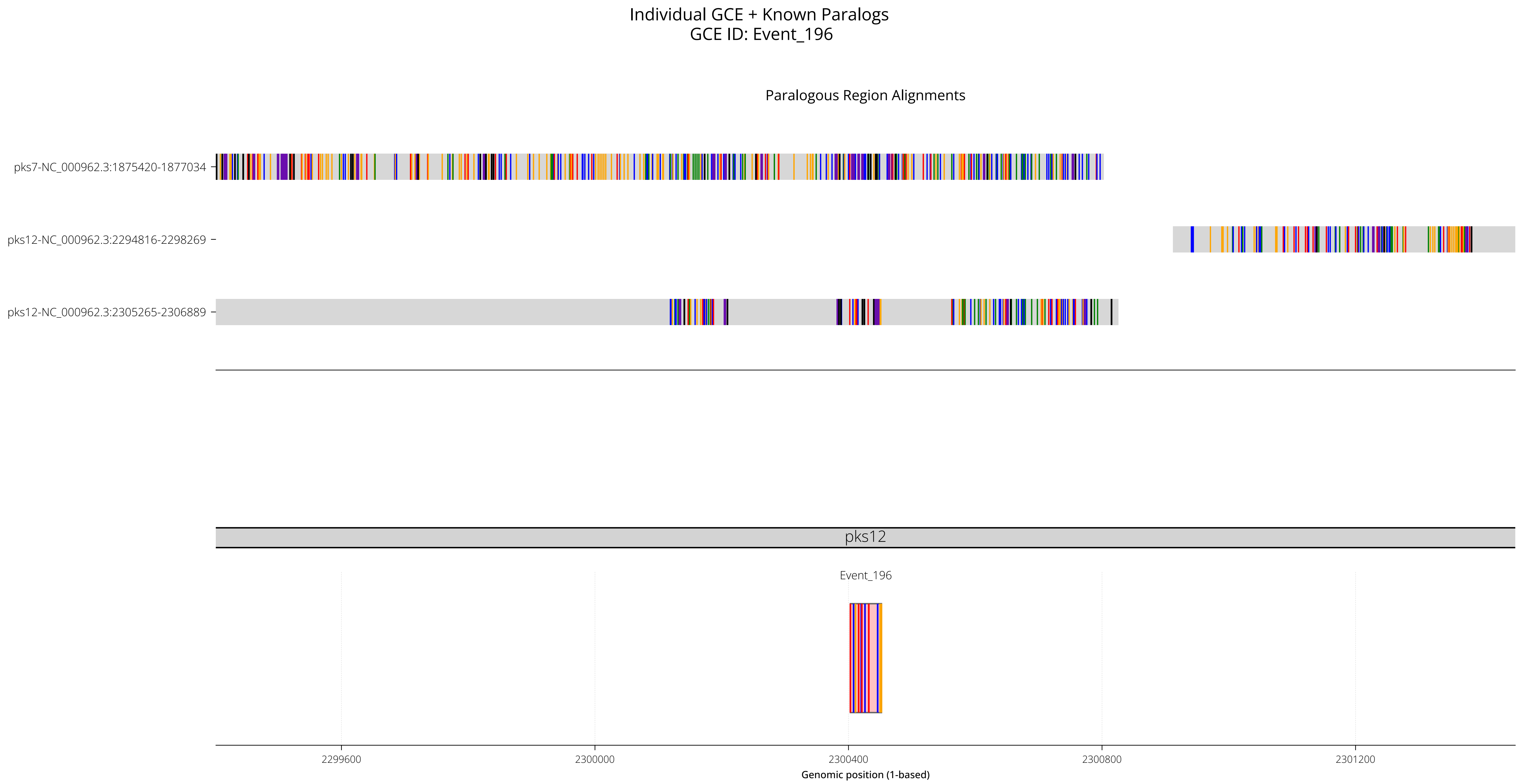
Individual GCE + Known Paralogs
GCE ID: Event_196
Paralogous Region Alignments
pks7-NC_000962.3:1875420-1877034
pks12-NC_000962.3:2294816-2298269
pks12-NC_000962.3:2305265-2306889
pks12
Event_196
2299600
2300000
2300400
2300800
2301200
Genomic position (1-based)

Circos Viz - GCE to Known Paralogs Locations
GCE ID: Event_196

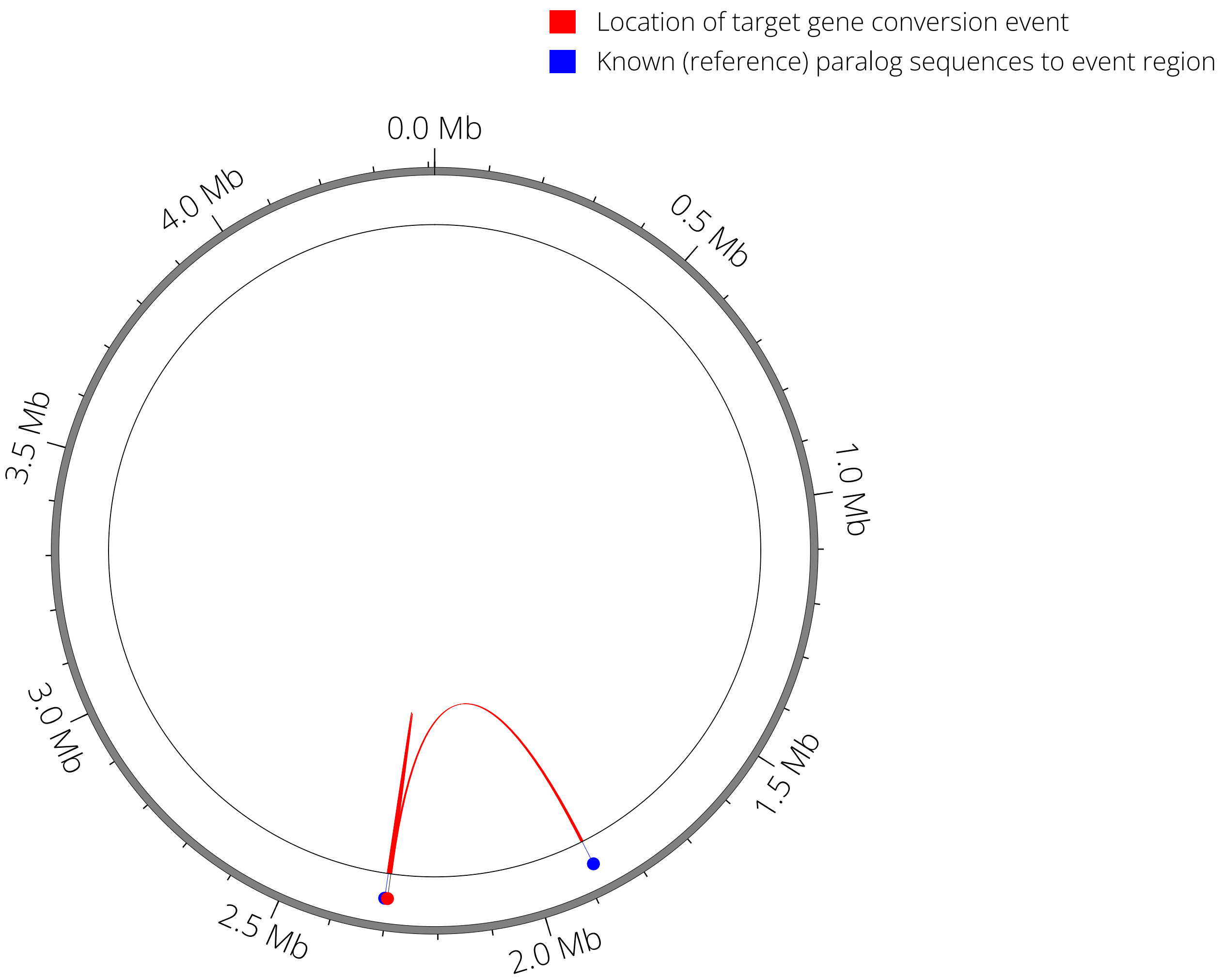

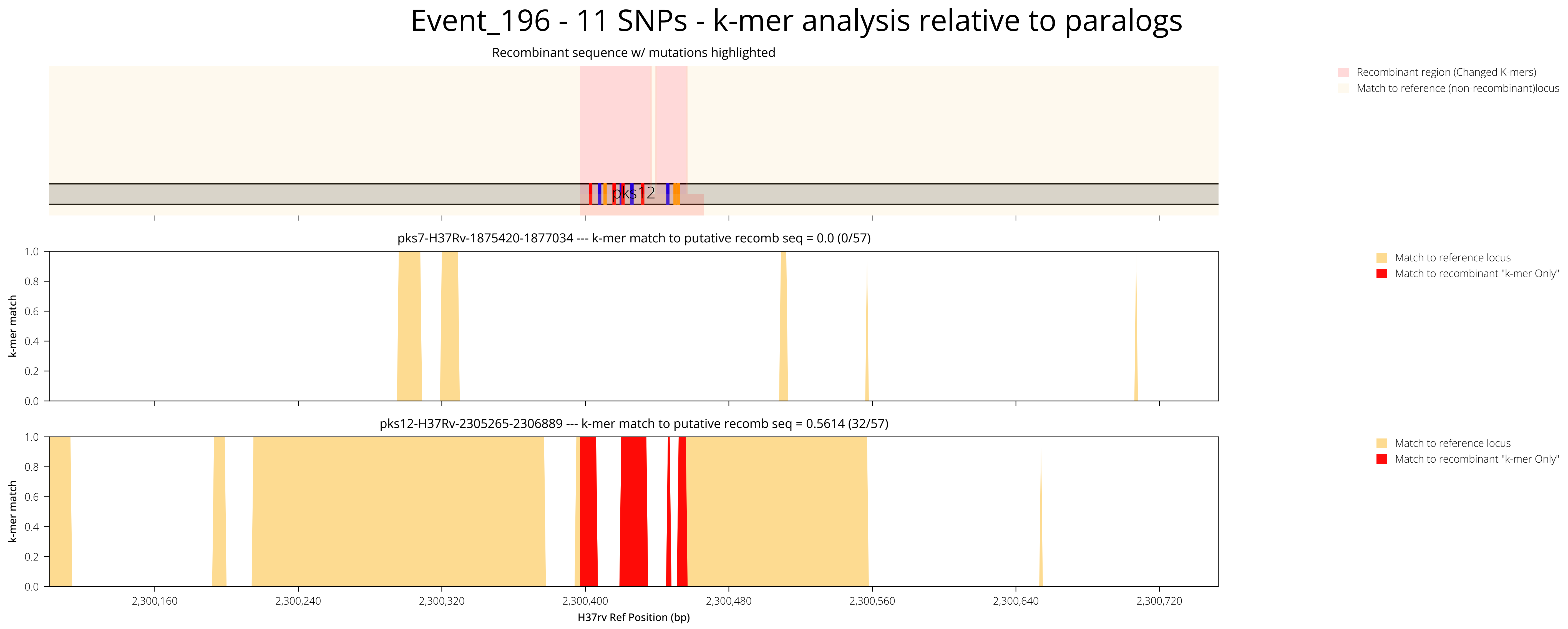

Event_196 - 11 SNPs - k-mer analysis relative to paralogs
Recombinant sequence w/ mutations highlighted
pks12
Recombinant region (Changed K-mers)
Match to reference (non-recombinant)locus
pks7-H37Rv-1875420-1877034 --- k-mer match to putative recomb seq = 0.0 (0/57)
Match to reference locus
Match to recombinant "k-mer Only"
pks12-H37Rv-2305265-2306889 --- k-mer match to putative recomb seq = 0.5614 (32/57)
Match to reference locus
Match to recombinant "k-mer Only"
k-mer match
1.0
0.8
0.6
0.4
0.2
0.0
2,300,160
2,300,240
2,300,320
2,300,400
2,300,480
2,300,560
2,300,640
2,300,720
H37rv Ref Position (bp)

Sub-phylogeny Viz + Paralog Alignments
GCE ID: Event_196

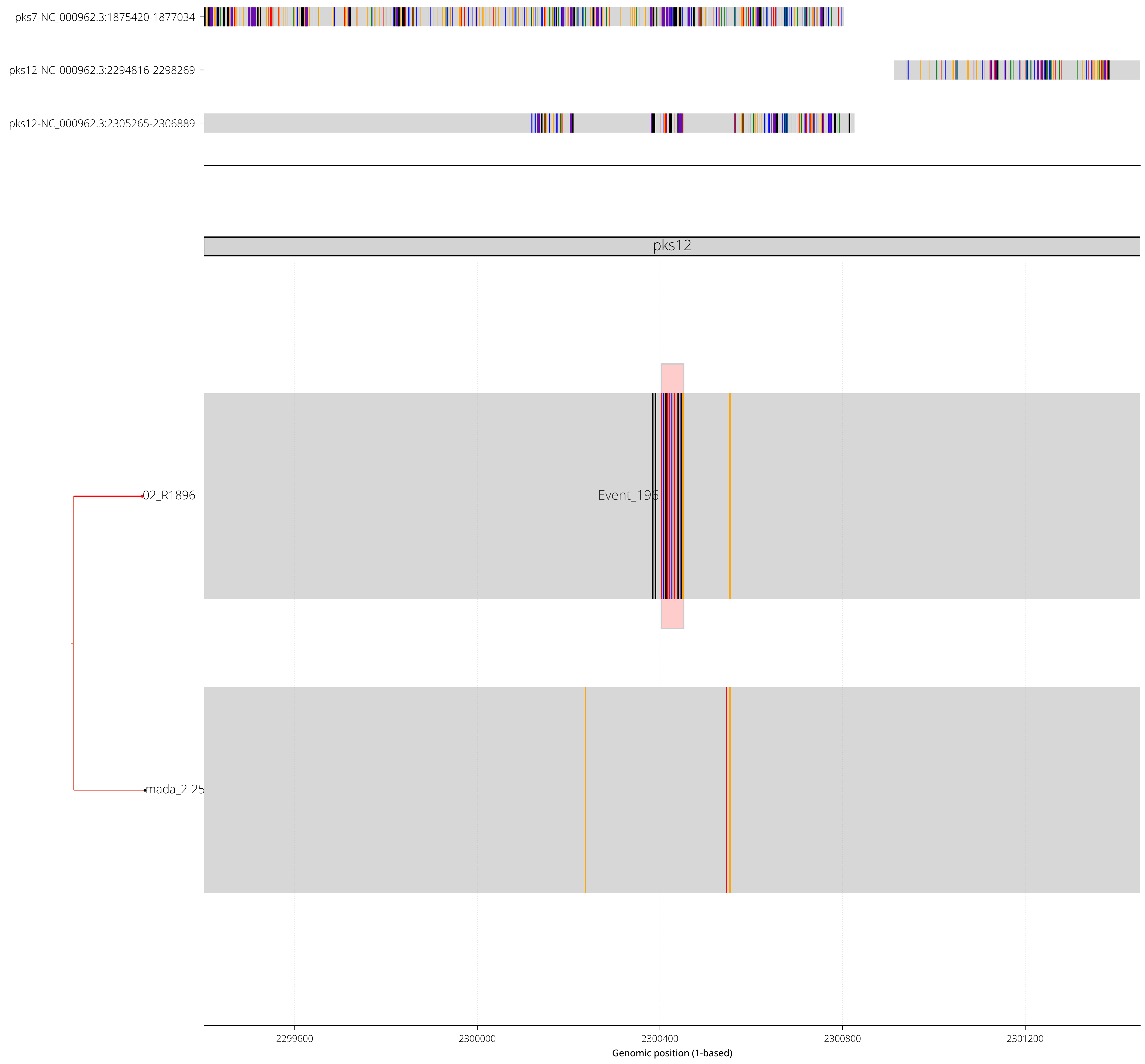

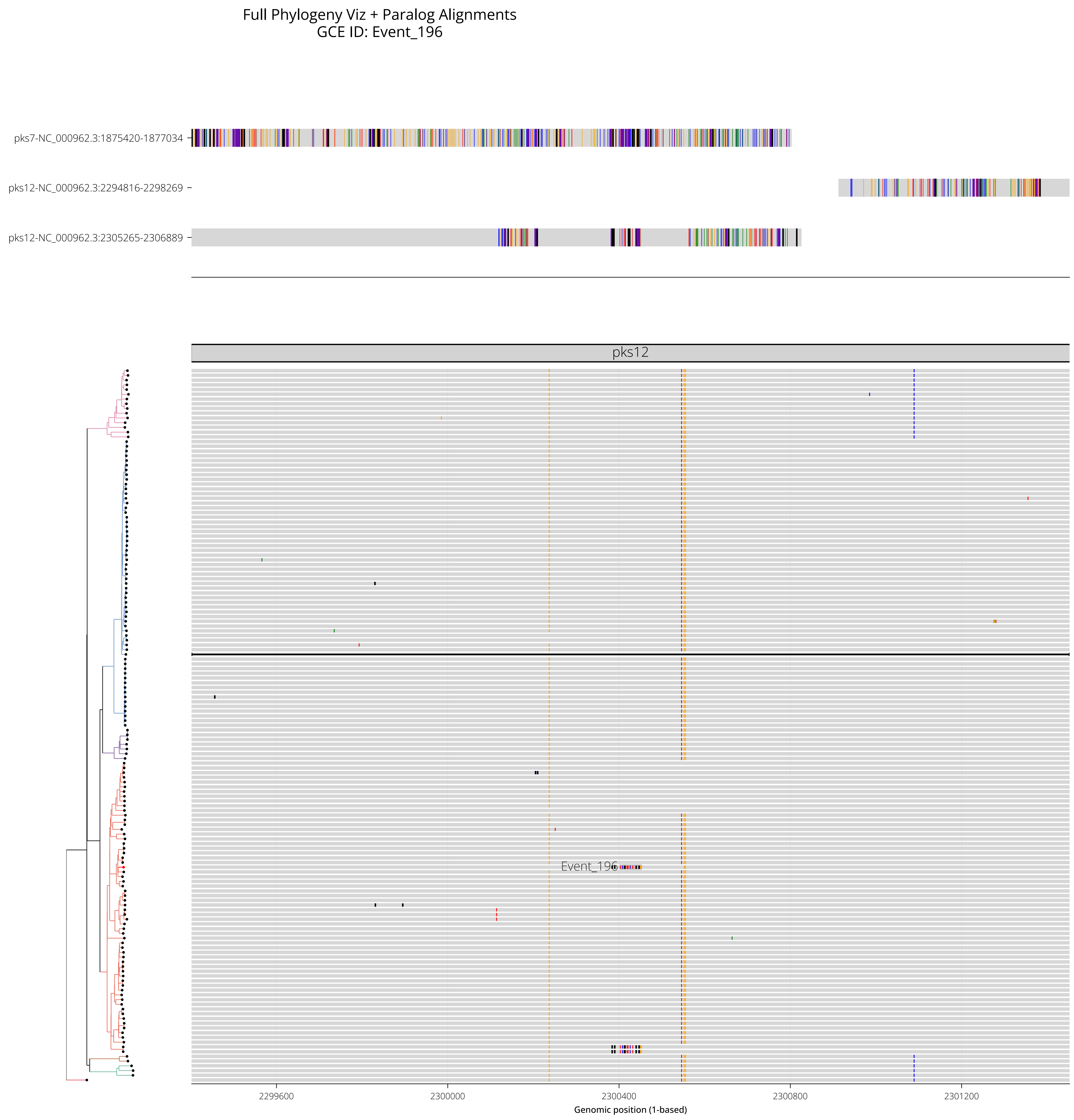

Full Phylogeny Viz + Paralog Alignments
GCE ID: Event_196
pks7-NC_000962.3:1875420-1877034
pks12-NC_000962.3:2294816-2298269
pks12-NC_000962.3:2305265-2306889
pks12
Event_196
2299600
2300000
2300400
2300800
2301200
Genomic position (1-based)

## Individual GCE + Known Paralogs
## GCE ID: Event_197

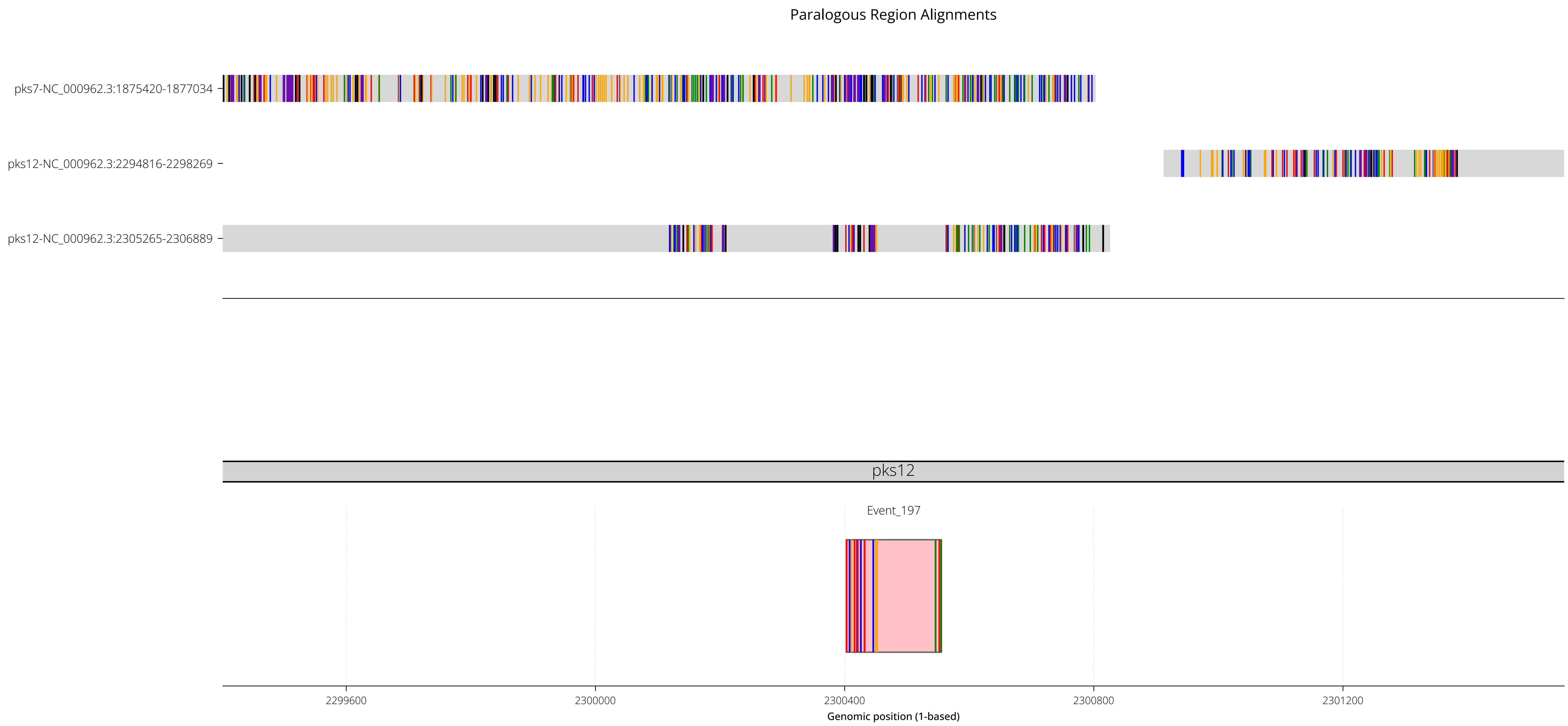

Circos Viz - GCE to Known Paralogs Locations
GCE ID: Event_197

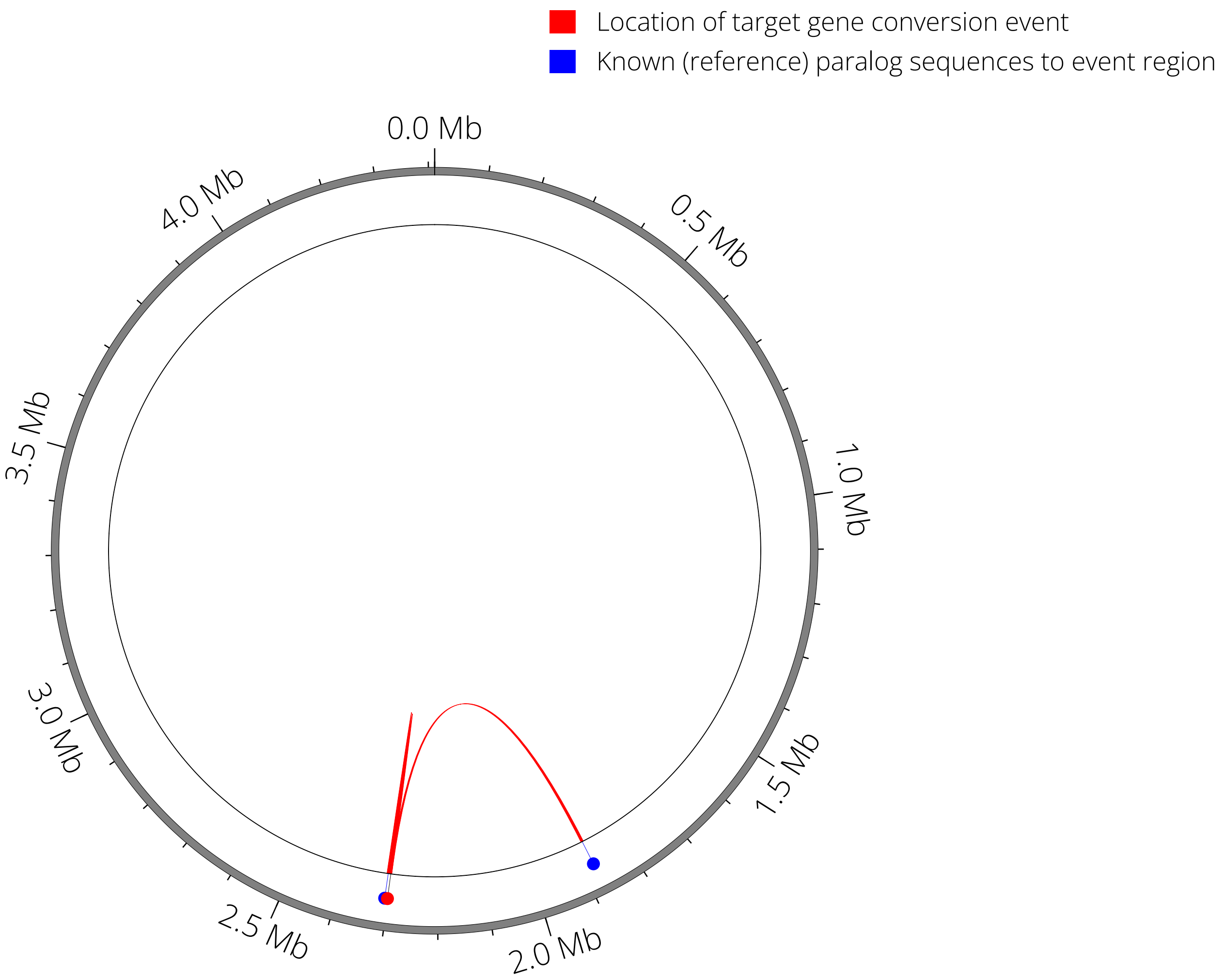

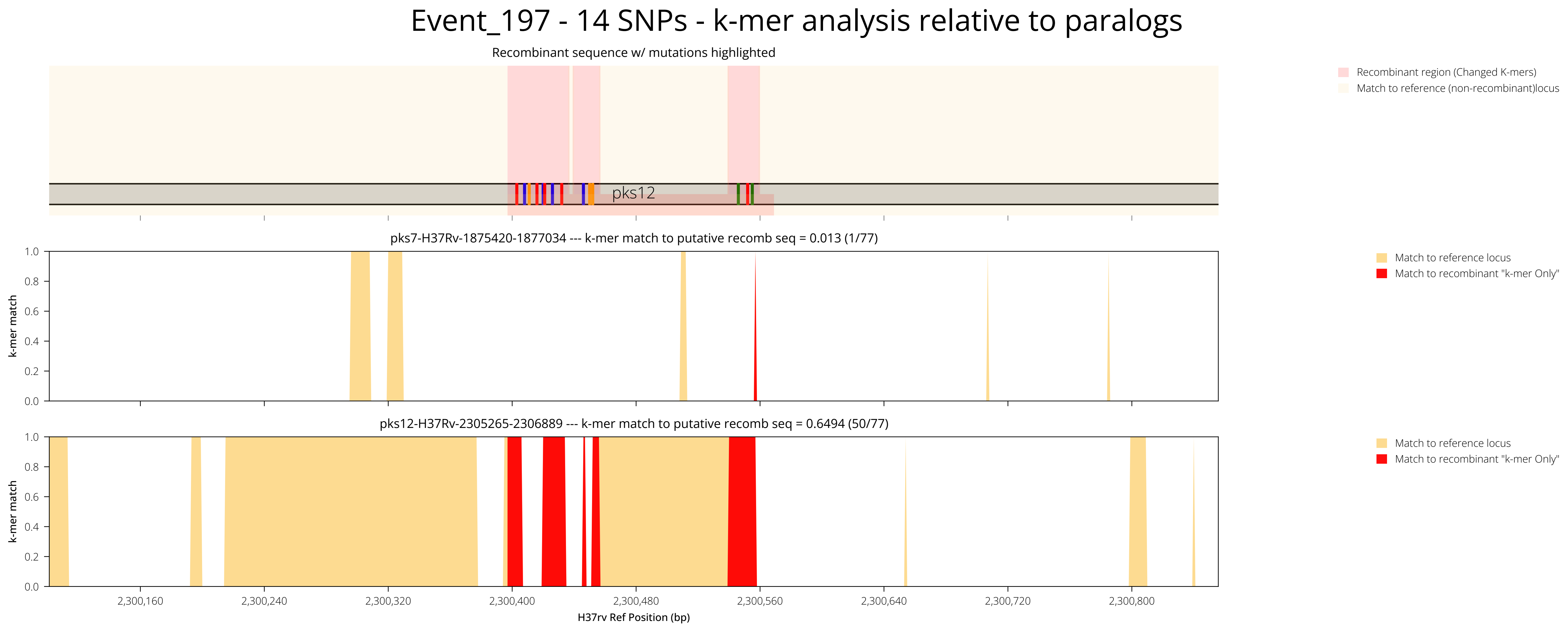

Event_197 - 14 SNPs - k-mer analysis relative to paralogs
Recombinant sequence w/ mutations highlighted
Recombinant region (Changed K-mers)
Match to reference (non-recombinant)locus
pks12
pks7-H37Rv-1875420-1877034 --- k-mer match to putative recomb seq = 0.013 (1/77)
Match to reference locus
Match to recombinant "k-mer Only"
k-mer match
1.0
0.8
0.6
0.4
0.2
0.0
pks12-H37Rv-2305265-2306889 --- k-mer match to putative recomb seq = 0.6494 (50/77)
Match to reference locus
Match to recombinant "k-mer Only"
k-mer match
1.0
0.8
0.6
0.4
0.2
0.0
2,300,160
2,300,240
2,300,320
2,300,400
2,300,480
2,300,560
2,300,640
2,300,720
2,300,800
H37rv Ref Position (bp)

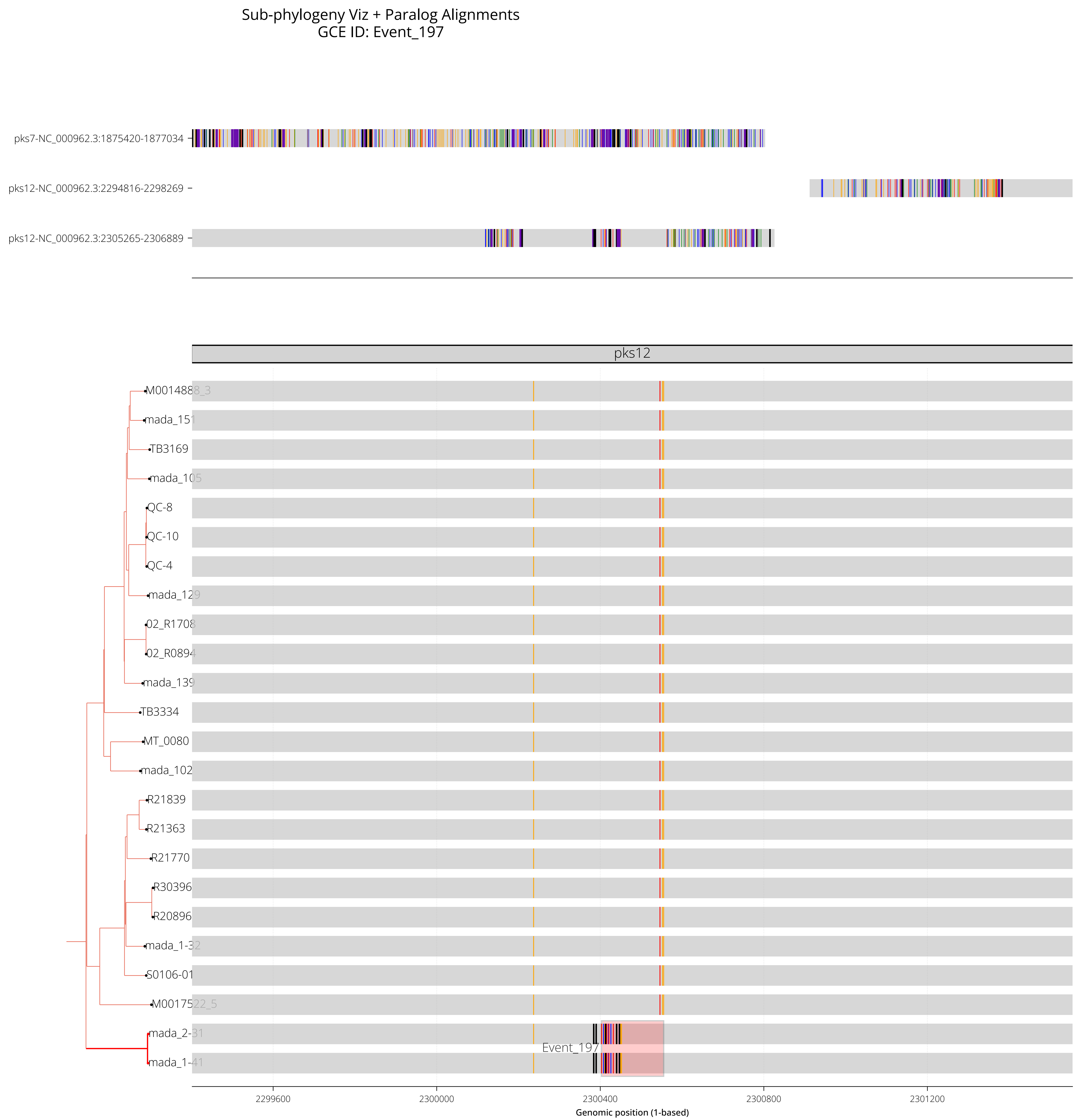

Sub-phylogeny Viz + Paralog Alignments
GCE ID: Event_197
pks7-NC_000962.3:1875420-1877034
pks12-NC_000962.3:2294816-2298269
pks12-NC_000962.3:2305265-2306889
pks12
M0014888_3
mada_151
TB3169
mada_105
QC-8
QC-10
QC-4
mada_129
02_R1708
02_R0894
mada_139
TB3334
MT_0080
mada_102
R21839
R21363
R21770
R30396
R20896
mada_1-32
S0106-01
M0017522_5
mada_2-31
mada_1-41
Event_197
2299600
2300000
2300400
2300800
2301200
Genomic position (1-based)

Full Phylogeny Viz + Paralog Alignments
GCE ID: Event_197

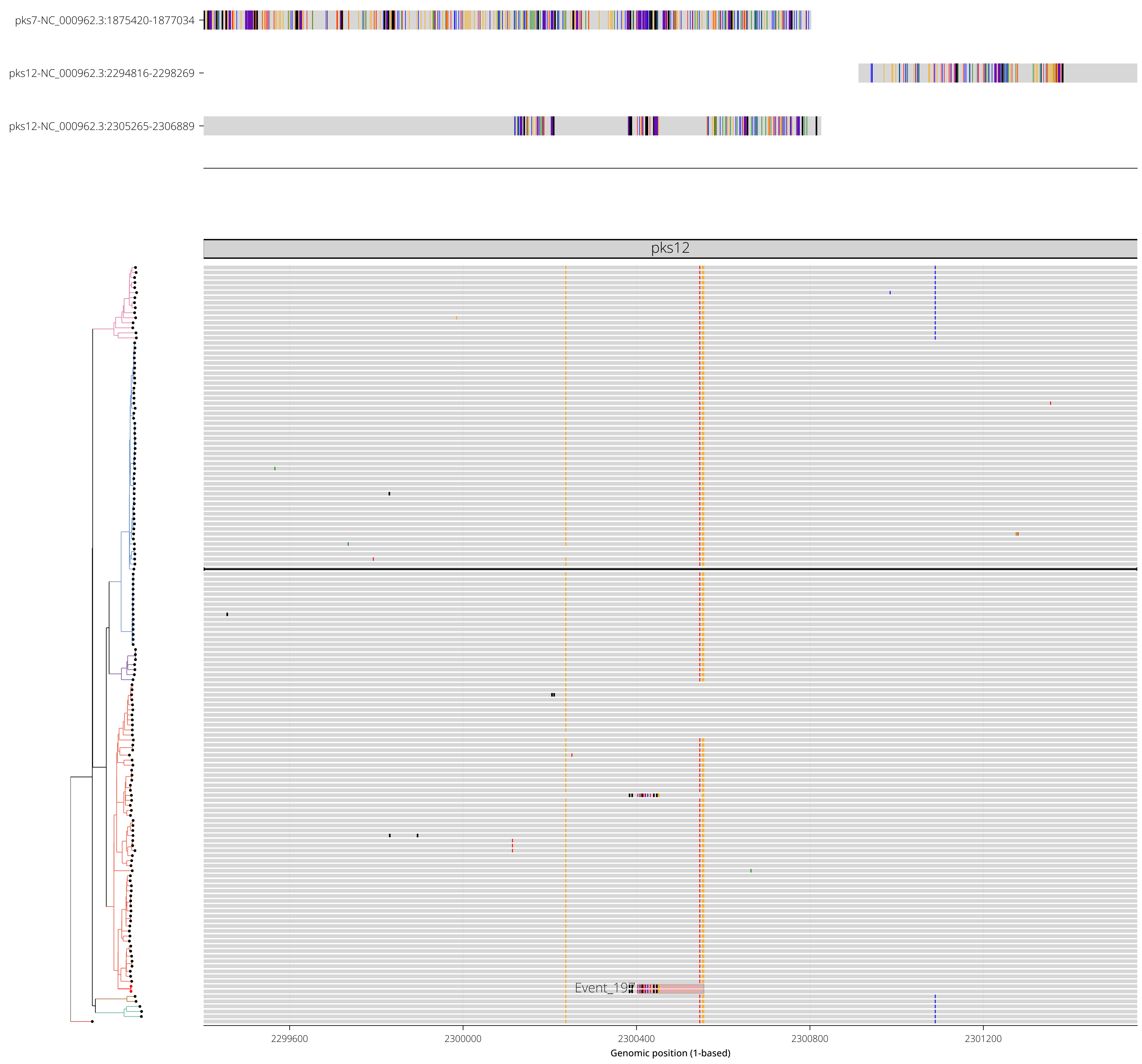

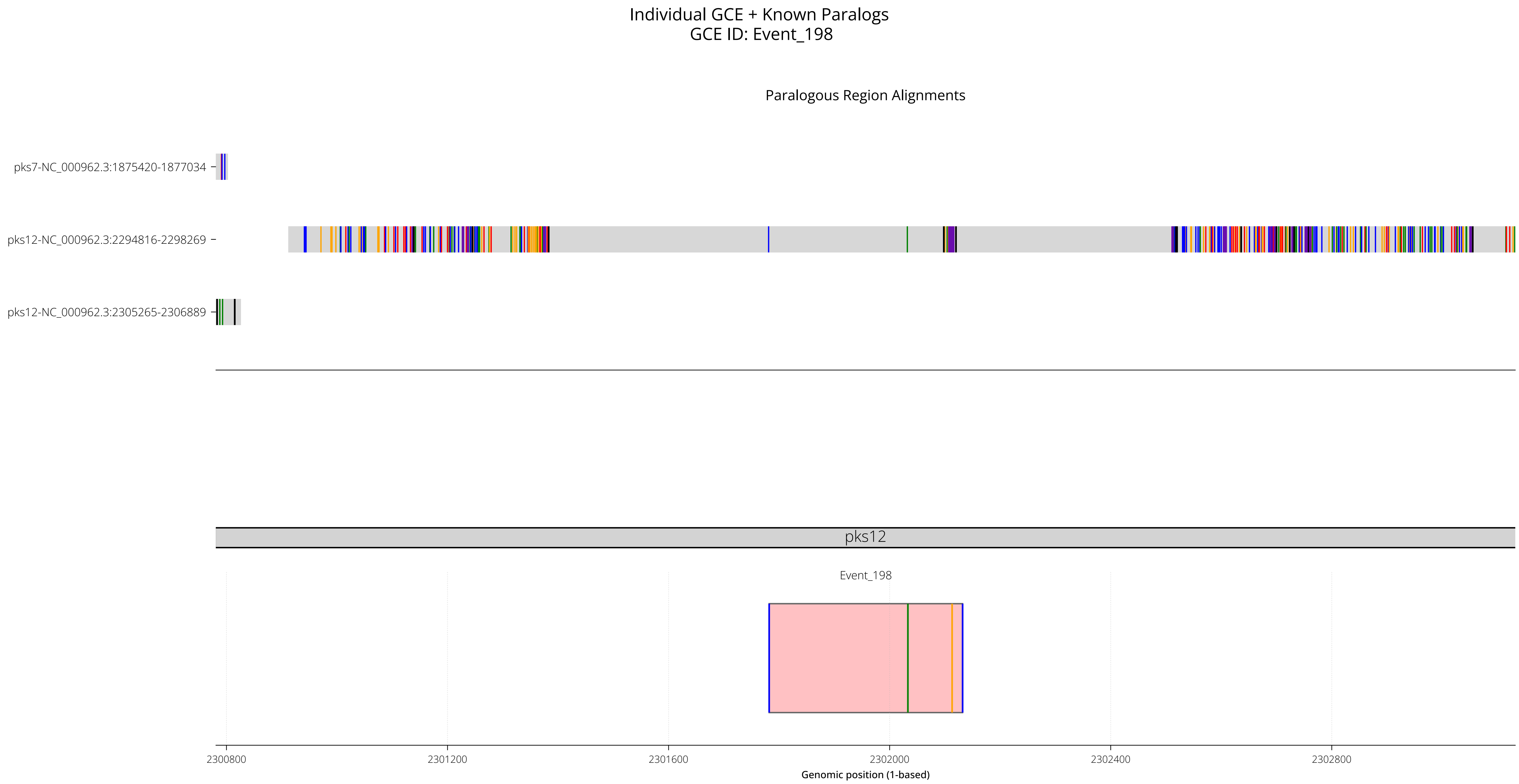
Individual GCE + Known Paralogs
GCE ID: Event_198
Paralogous Region Alignments
pks7-NC_000962.3:1875420-1877034
pks12-NC_000962.3:2294816-2298269
pks12-NC_000962.3:2305265-2306889
pks12
Event_198
2300800
2301200
2301600
2302000
2302400
2302800
Genomic position (1-based)

Circos Viz - GCE to Known Paralogs Locations
GCE ID: Event_198

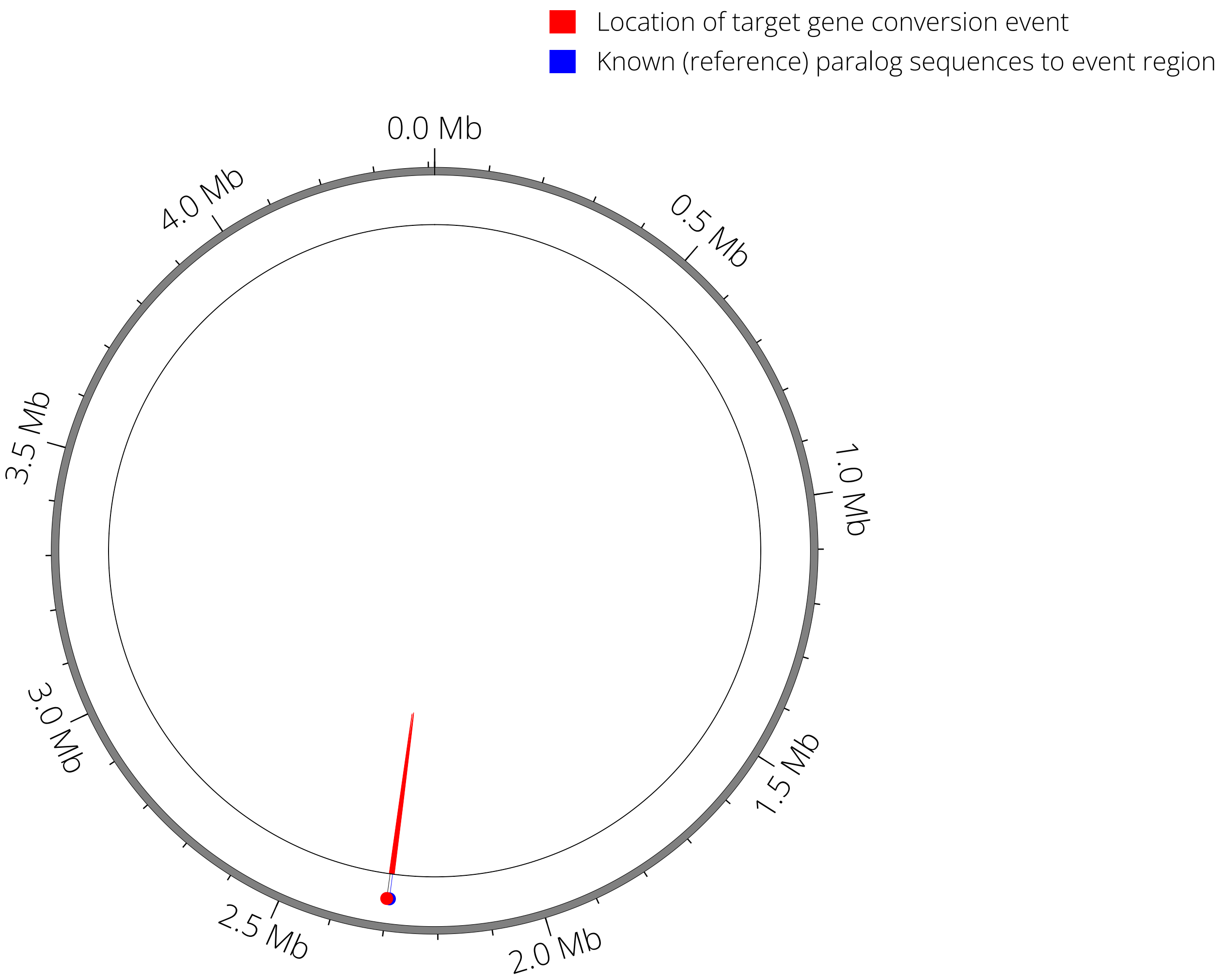

# Event_198 - 4 SNPs - k-mer analysis relative to paralogs

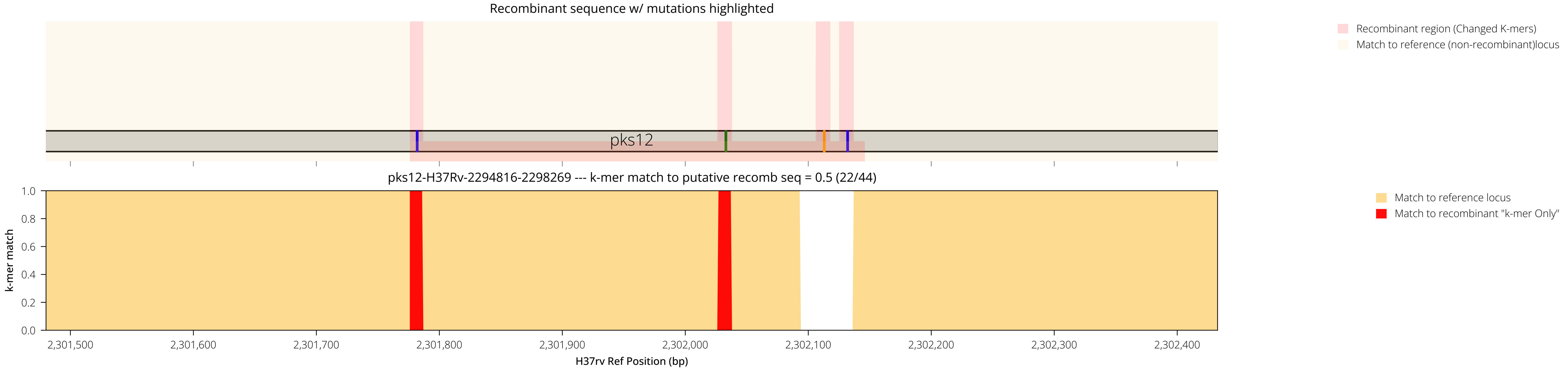

Sub-phylogeny Viz + Paralog Alignments
GCE ID: Event_198

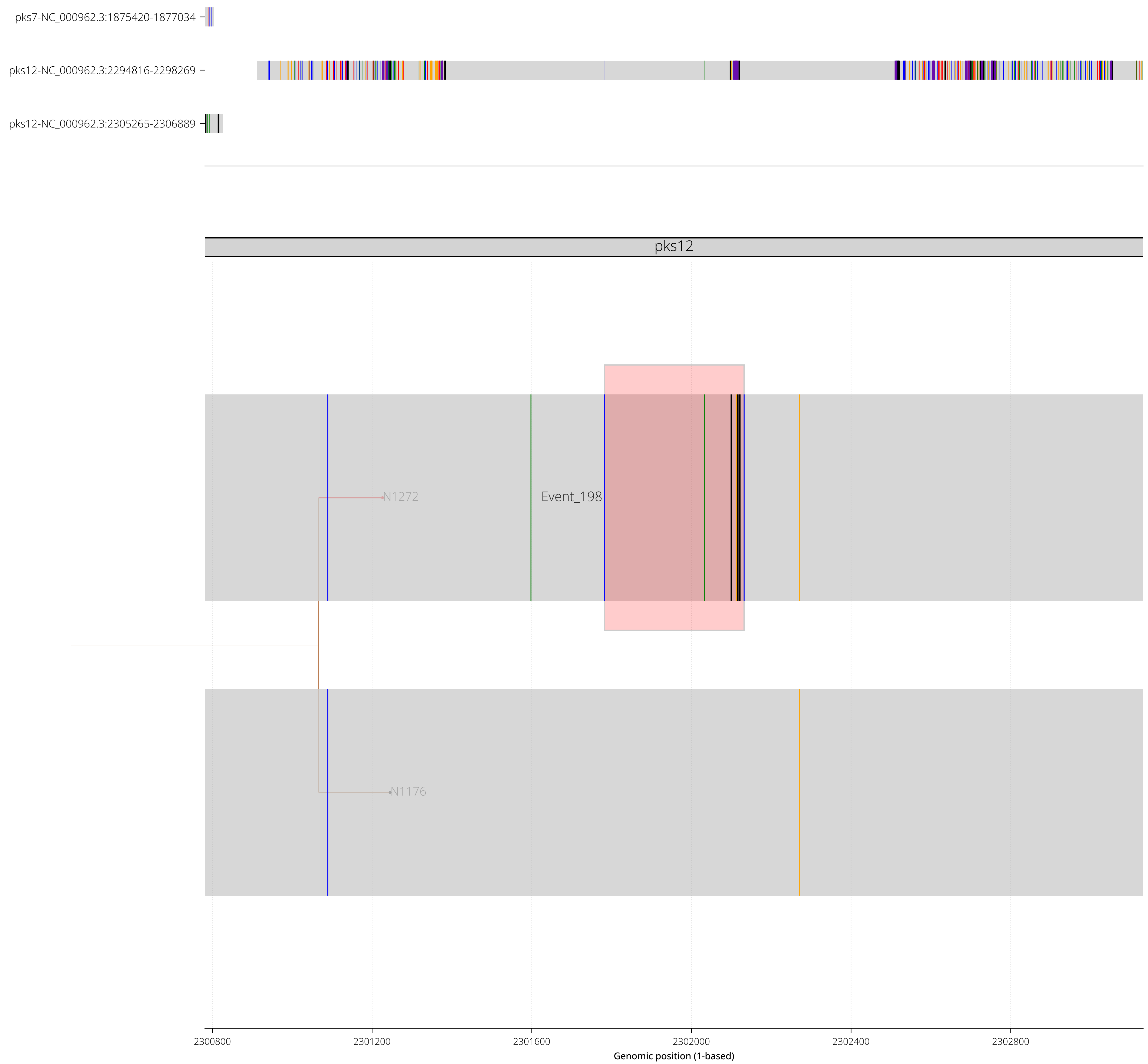

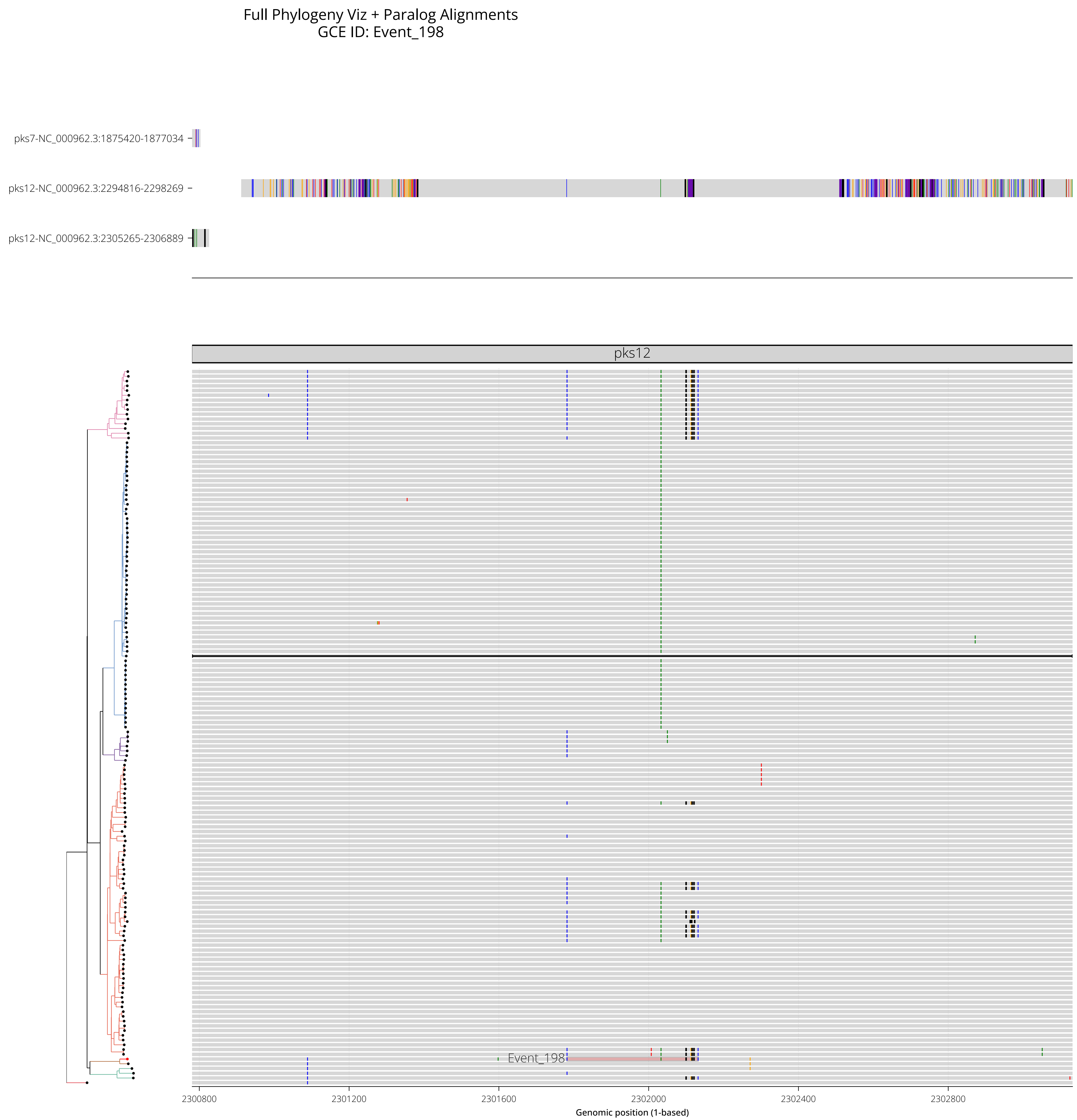
Full Phylogeny Viz + Paralog Alignments
GCE ID: Event_198
pks7-NC_000962.3:1875420-1877034
pks12-NC_000962.3:2294816-2298269
pks12-NC_000962.3:2305265-2306889
pks12
Event_198
2300800
2301200
2301600
2302000
2302400
2302800
Genomic position (1-based)

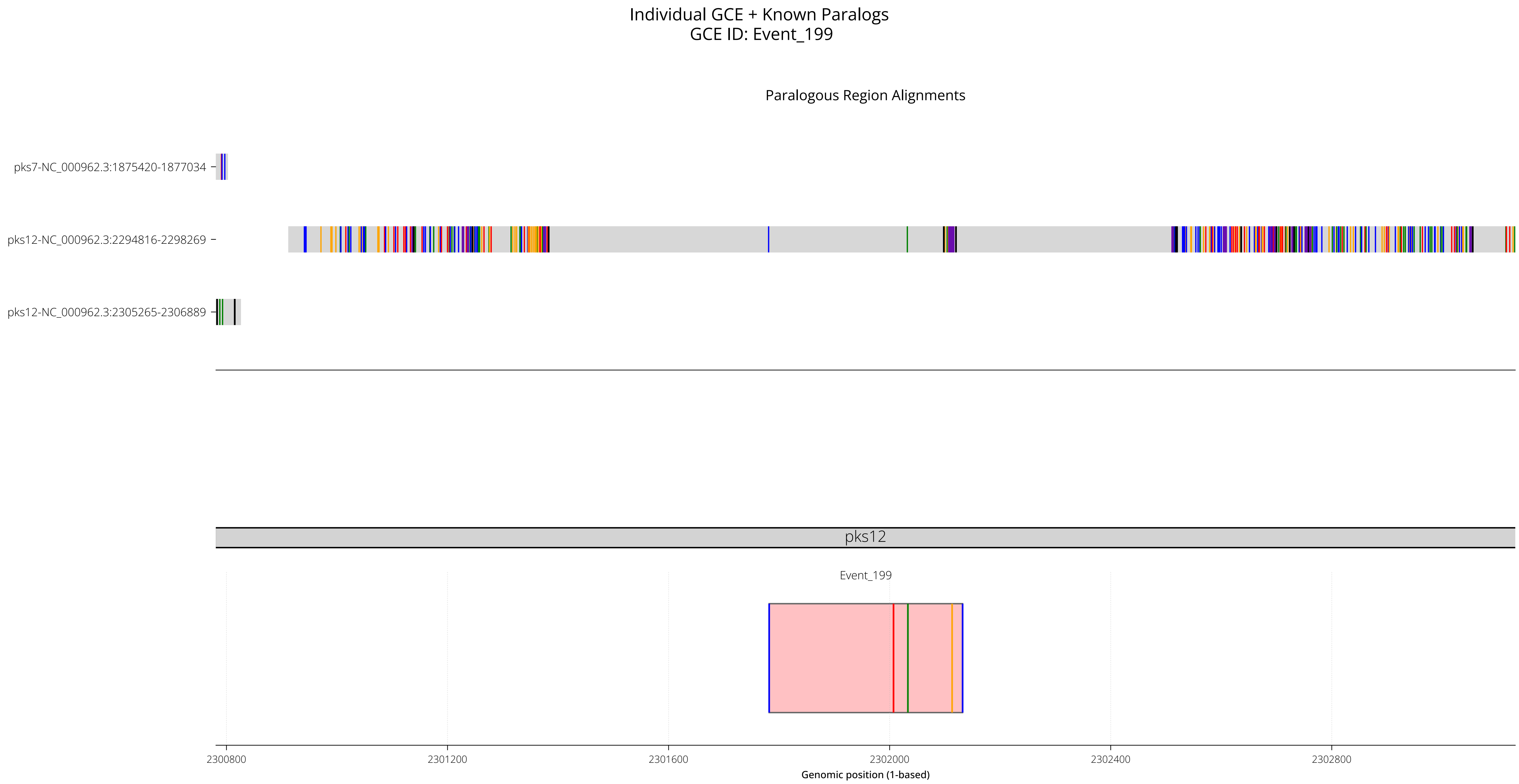

Individual GCE + Known Paralogs
GCE ID: Event_199
Paralogous Region Alignments
pks7-NC_000962.3:1875420-1877034
pks12-NC_000962.3:2294816-2298269
pks12-NC_000962.3:2305265-2306889
pks12
Event_199
2300800
2301200
2301600
2302000
2302400
2302800
Genomic position (1-based)

Circos Viz - GCE to Known Paralogs Locations
GCE ID: Event_199

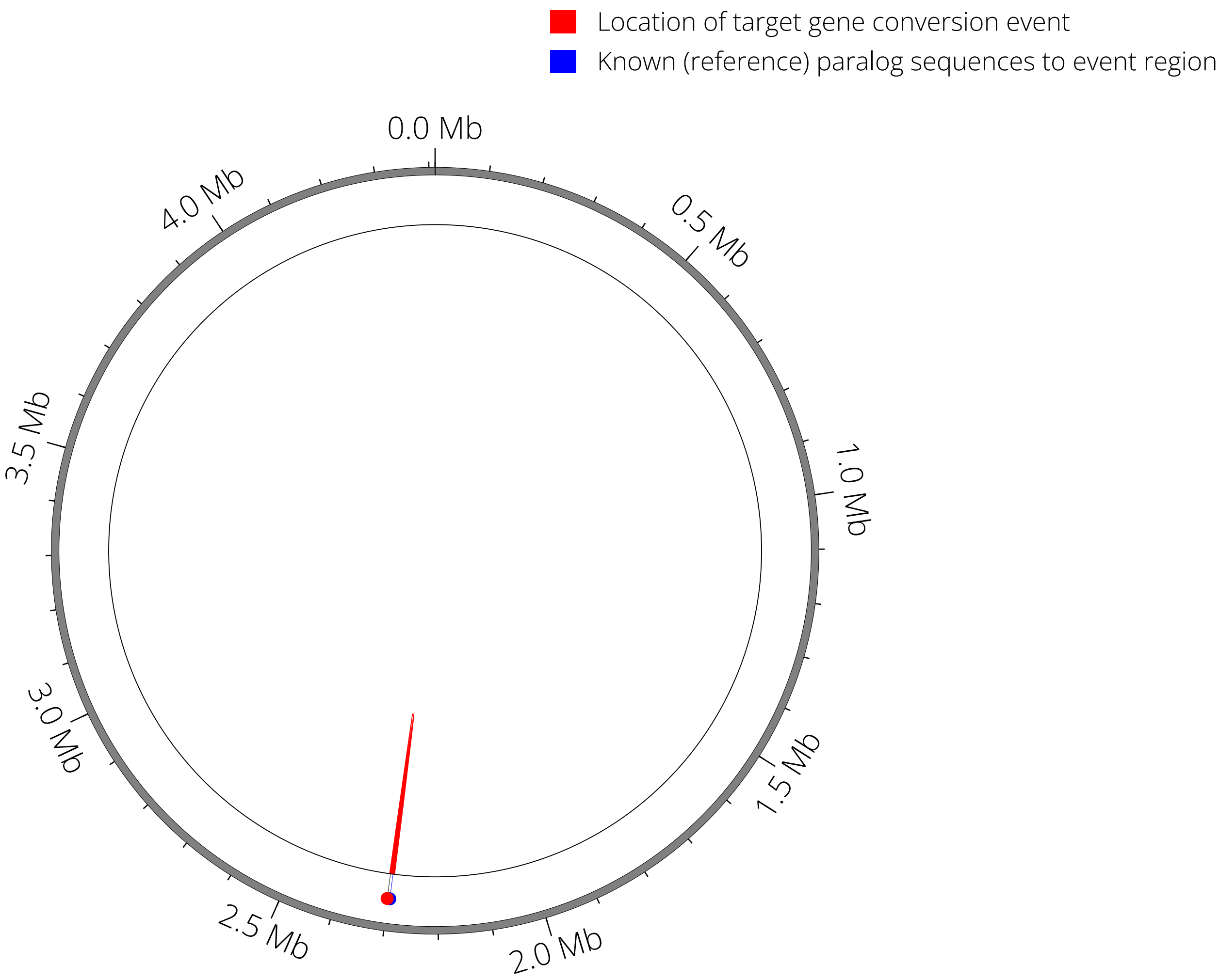

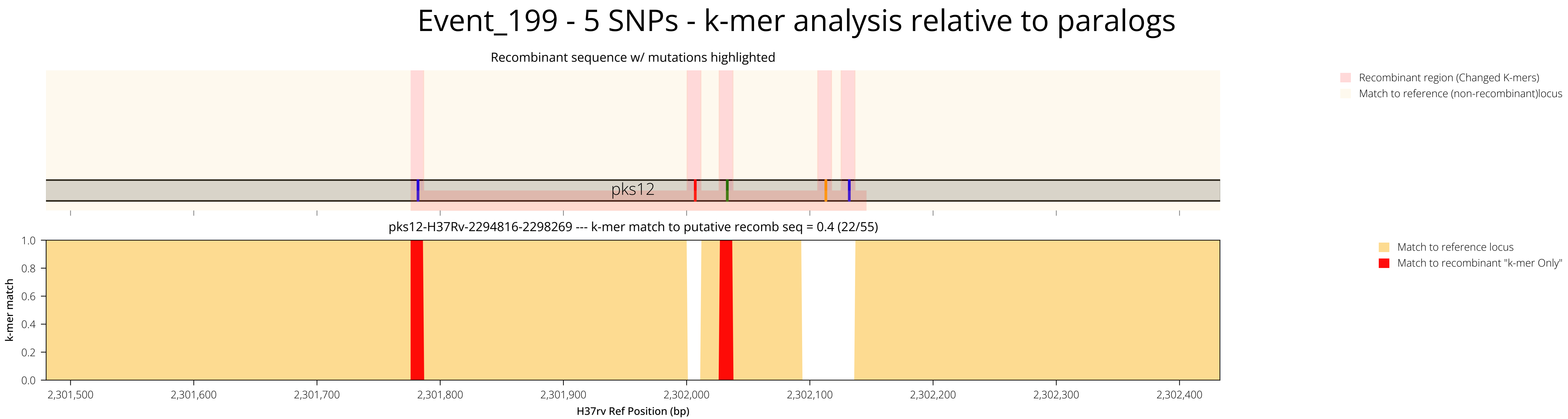

Event_199 - 5 SNPs - k-mer analysis relative to paralogs
Recombinant sequence w/ mutations highlighted
pks12
Recombinant region (Changed K-mers)
Match to reference (non-recombinant)locus
pks12-H37Rv-2294816-2298269 --- k-mer match to putative recomb seq = 0.4 (22/55)
k-mer match
1.0
0.8
0.6
0.4
0.2
0.0
2,301,500
2,301,600
2,301,700
2,301,800
2,301,900
2,302,000
2,302,100
2,302,200
2,302,300
2,302,400
H37rv Ref Position (bp)
Match to reference locus
Match to recombinant "k-mer Only"

Sub-phylogeny Viz + Paralog Alignments
GCE ID: Event_199

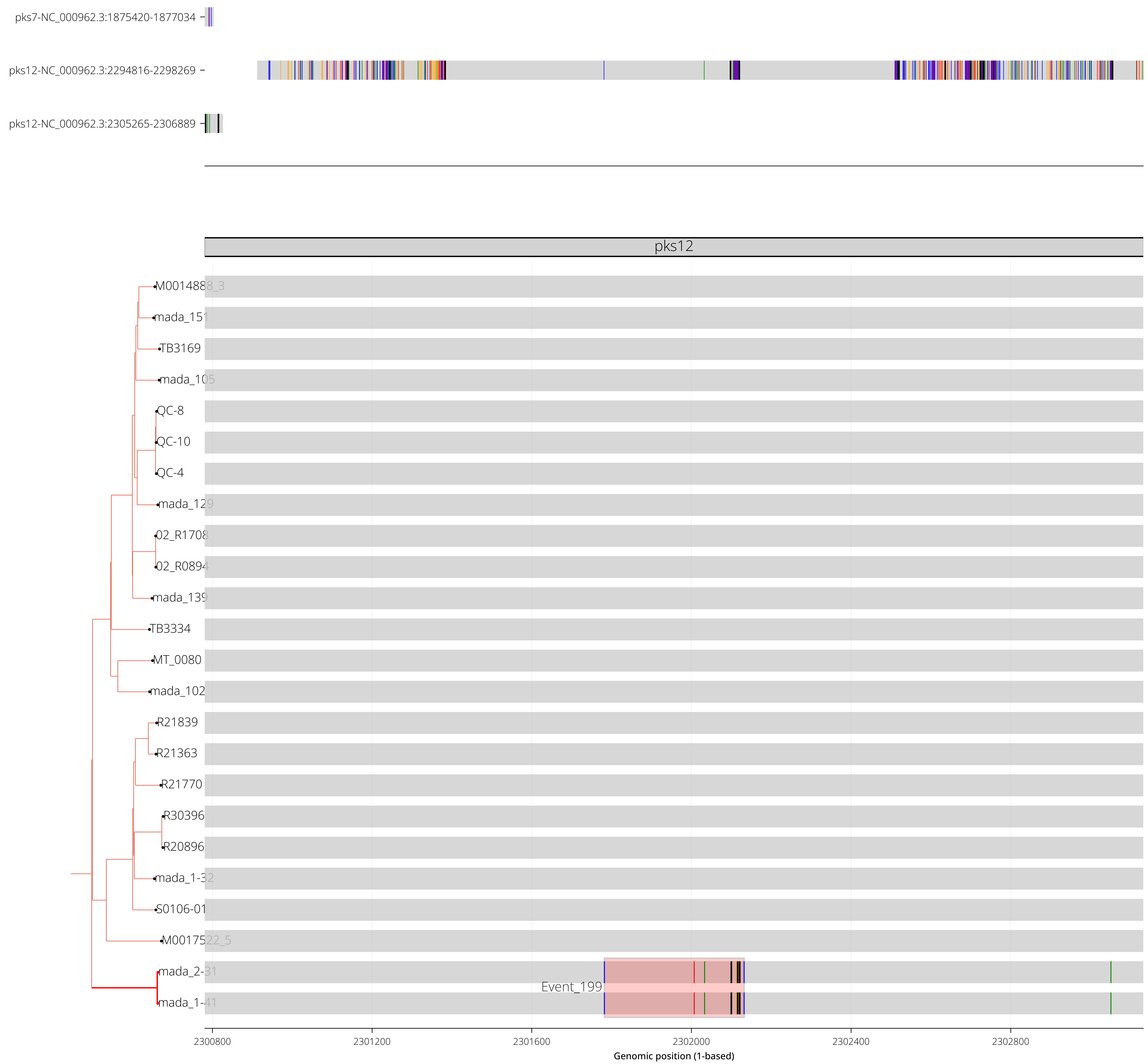

# Full Phylogeny Viz + Paralog Alignments
GCE ID: Event_199

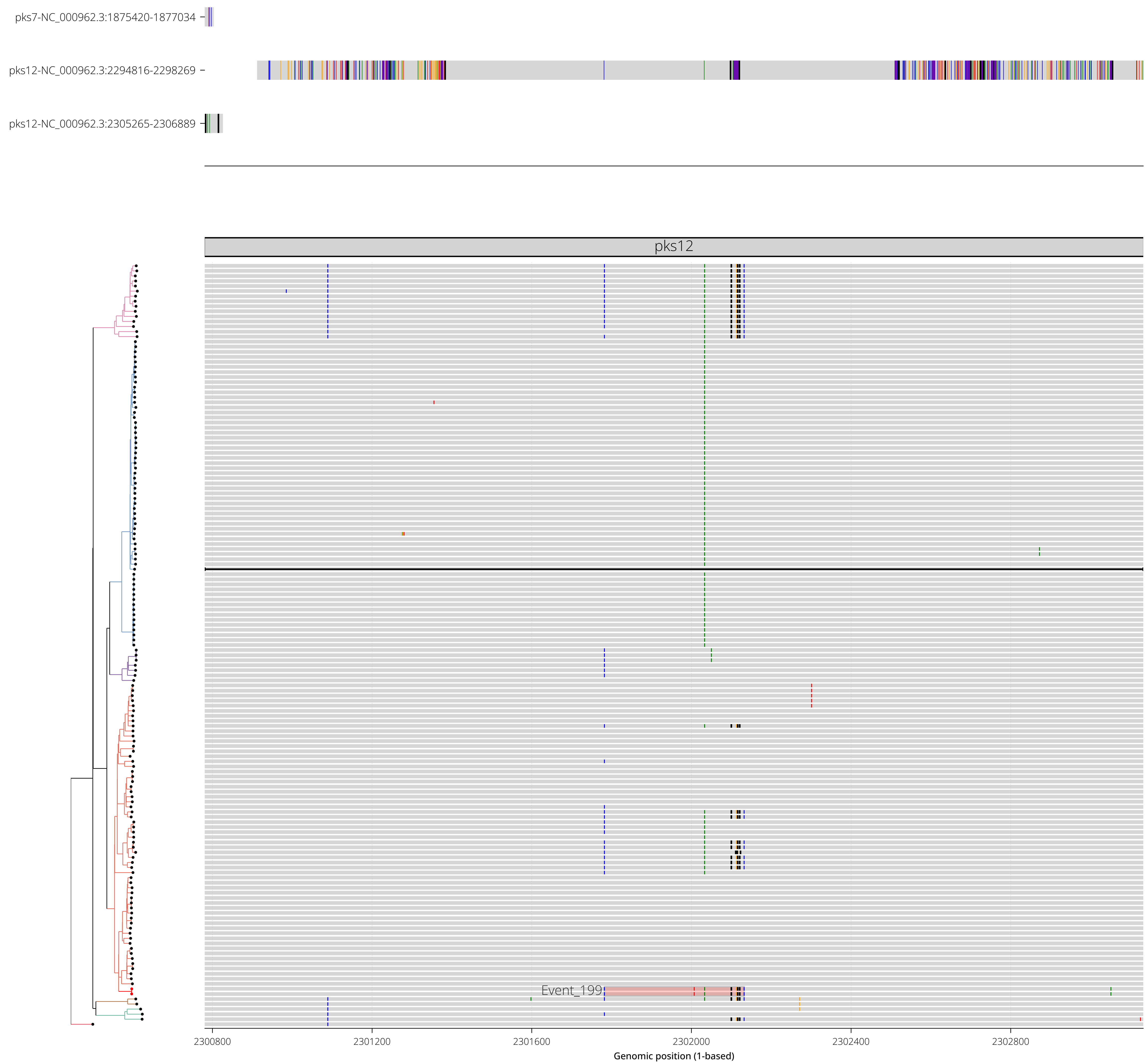

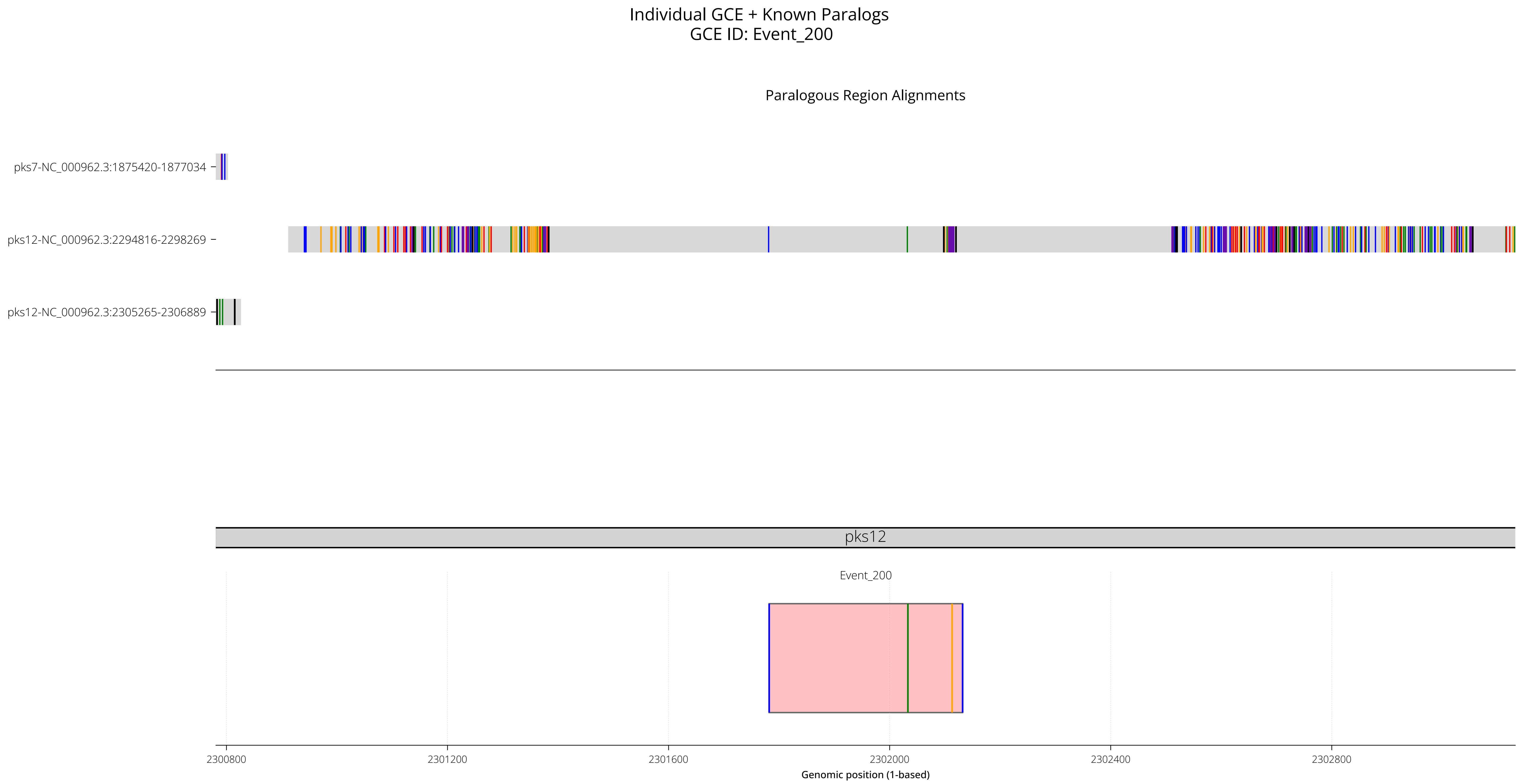

Individual GCE + Known Paralogs
GCE ID: Event_200
Paralogous Region Alignments
pks7-NC_000962.3:1875420-1877034
pks12-NC_000962.3:2294816-2298269
pks12-NC_000962.3:2305265-2306889
pks12
Event_200
2300800
2301200
2301600
2302000
2302400
2302800
Genomic position (1-based)

Circos Viz - GCE to Known Paralogs Locations
GCE ID: Event_200

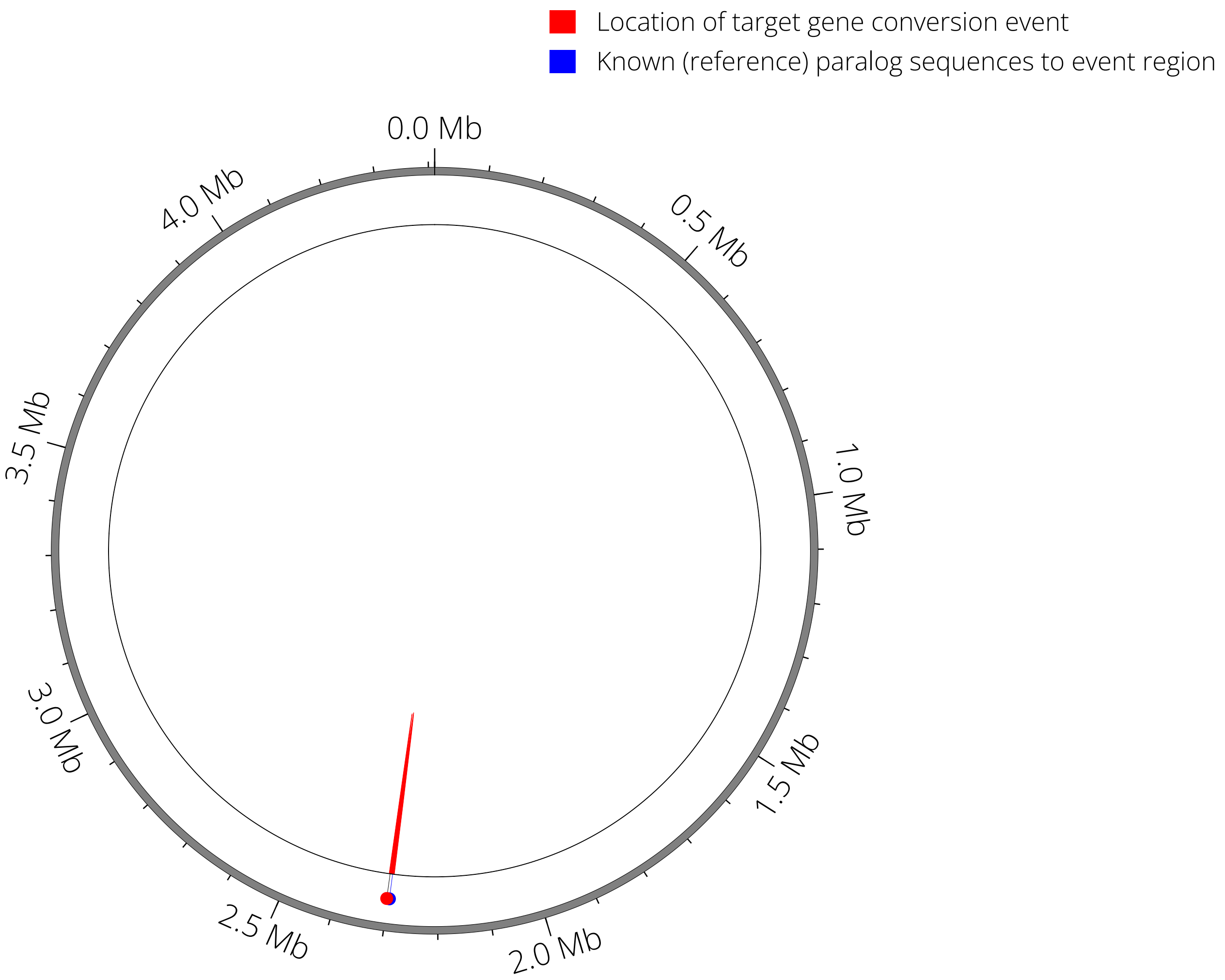

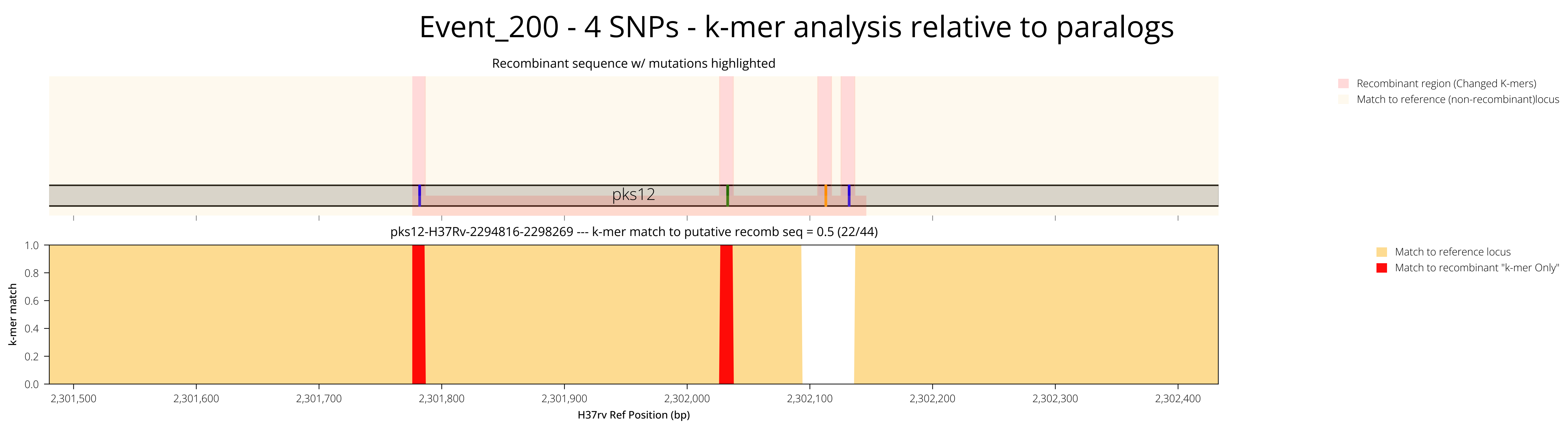
Event_200 - 4 SNPs - k-mer analysis relative to paralogs
Recombinant sequence w/ mutations highlighted
pks12
Recombinant region (Changed K-mers)
Match to reference (non-recombinant)locus
pks12-H37Rv-2294816-2298269 --- k-mer match to putative recomb seq = 0.5 (22/44)
1.0
0.8
0.6
0.4
0.2
0.0
k-mer match
Match to reference locus
Match to recombinant "k-mer Only"
2,301,500
2,301,600
2,301,700
2,301,800
2,301,900
2,302,000
2,302,100
2,302,200
2,302,300
2,302,400
H37rv Ref Position (bp)

Sub-phylogeny Viz + Paralog Alignments
GCE ID: Event_200

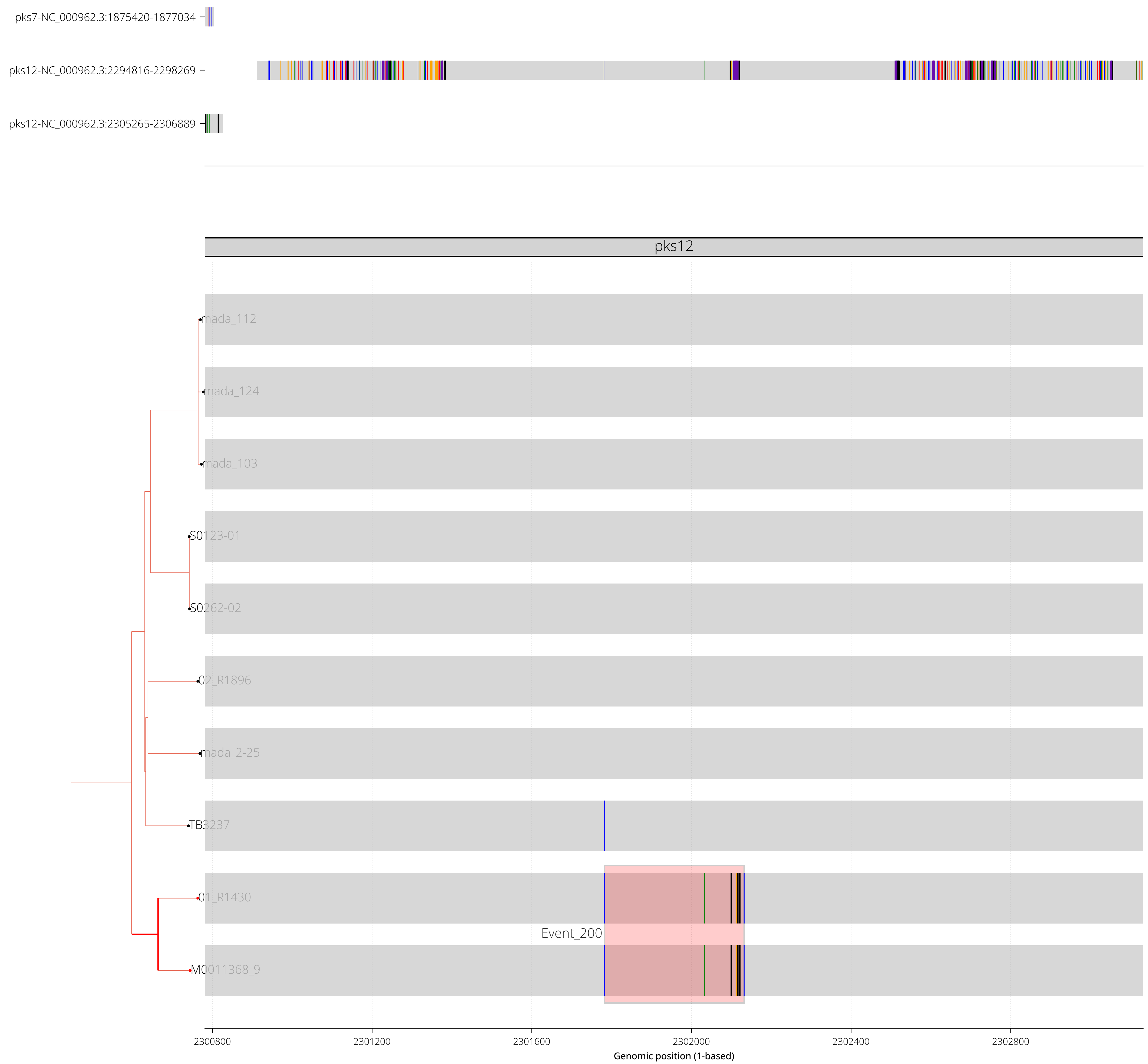

# Full Phylogeny Viz + Paralog Alignments
GCE ID: Event_200

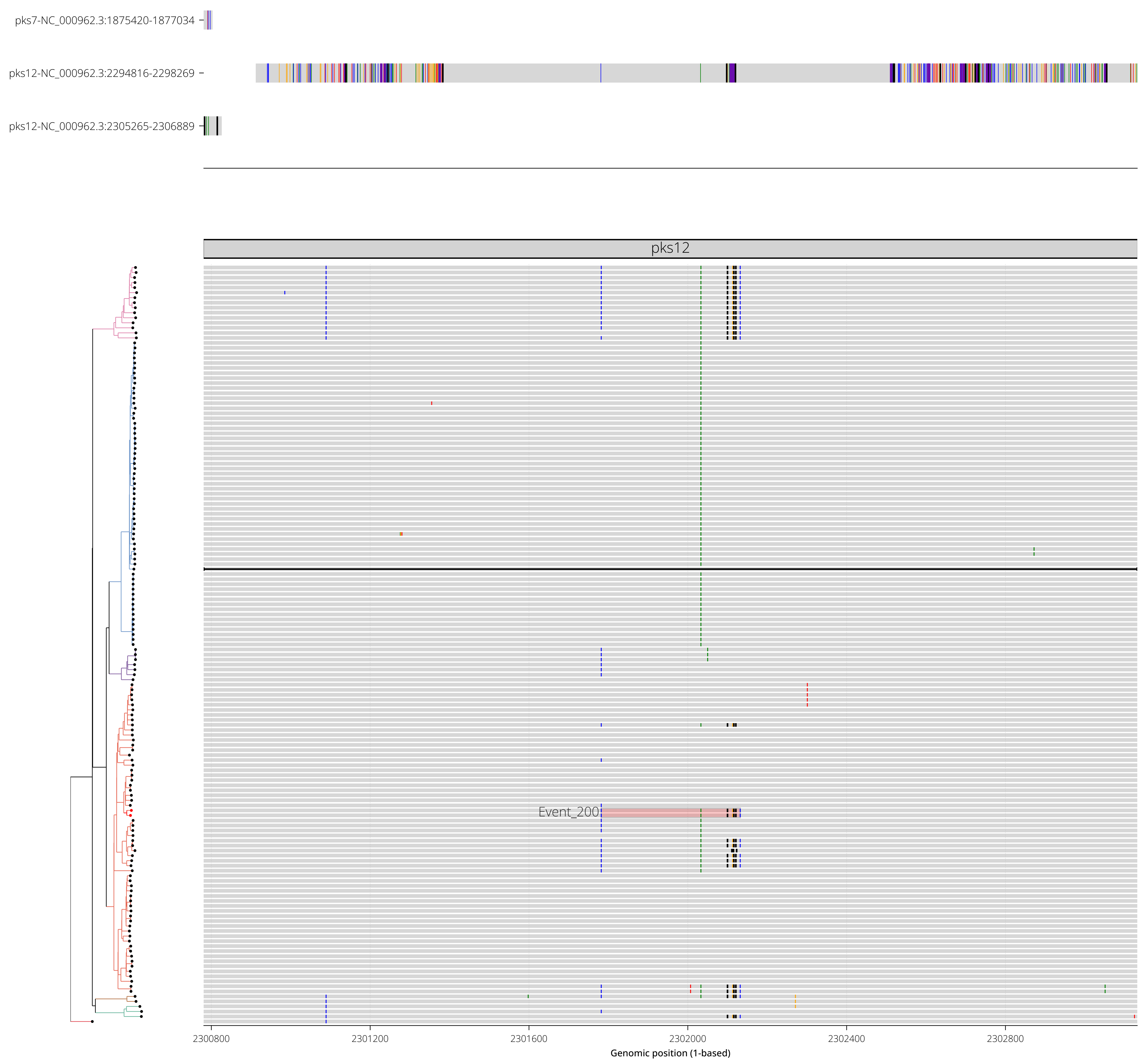

Individual GCE + Known Paralogs
GCE ID: Event_201

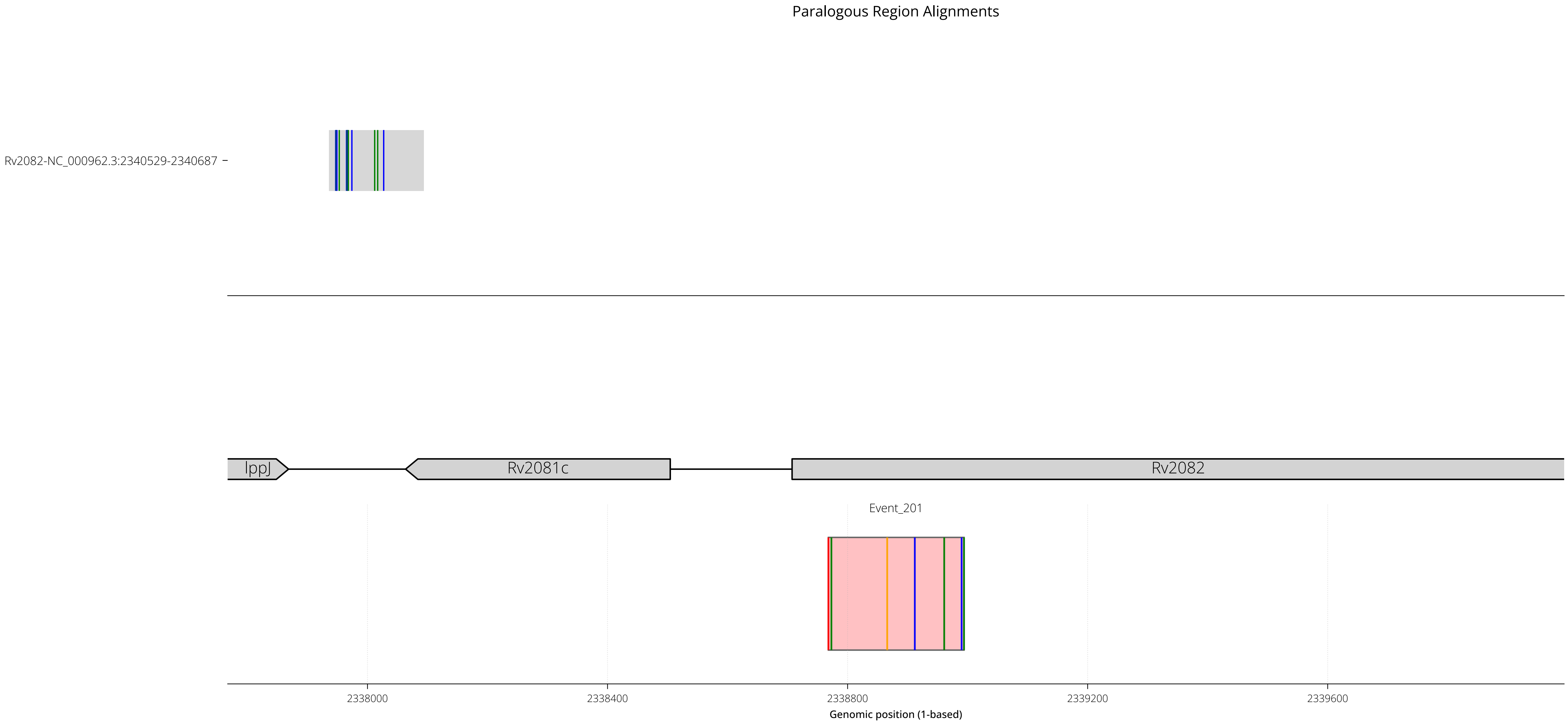

Sub-phylogeny Viz + Paralog Alignments
GCE ID: Event_201

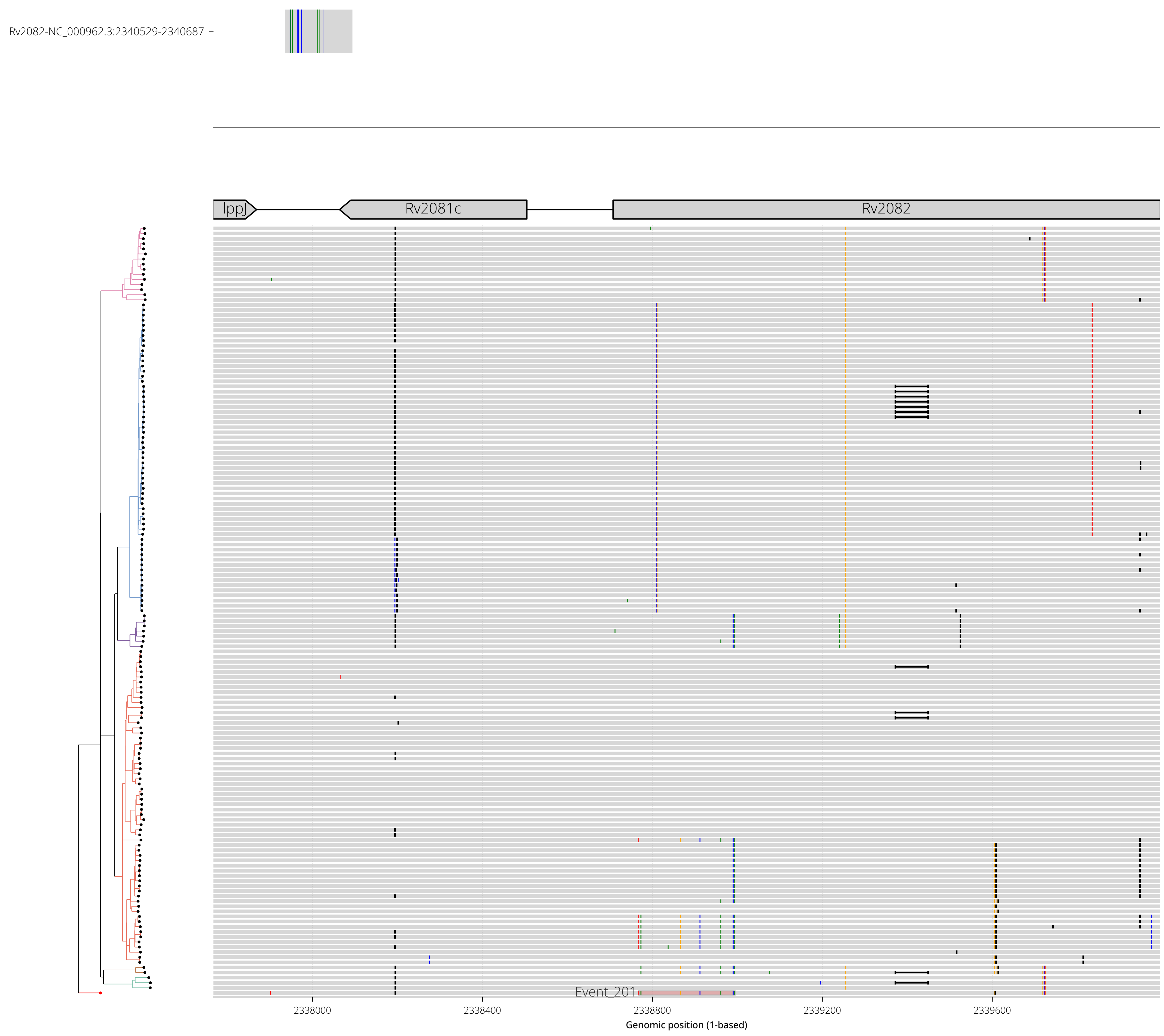

Full Phylogeny Viz + Paralog Alignments
GCE ID: Event_201

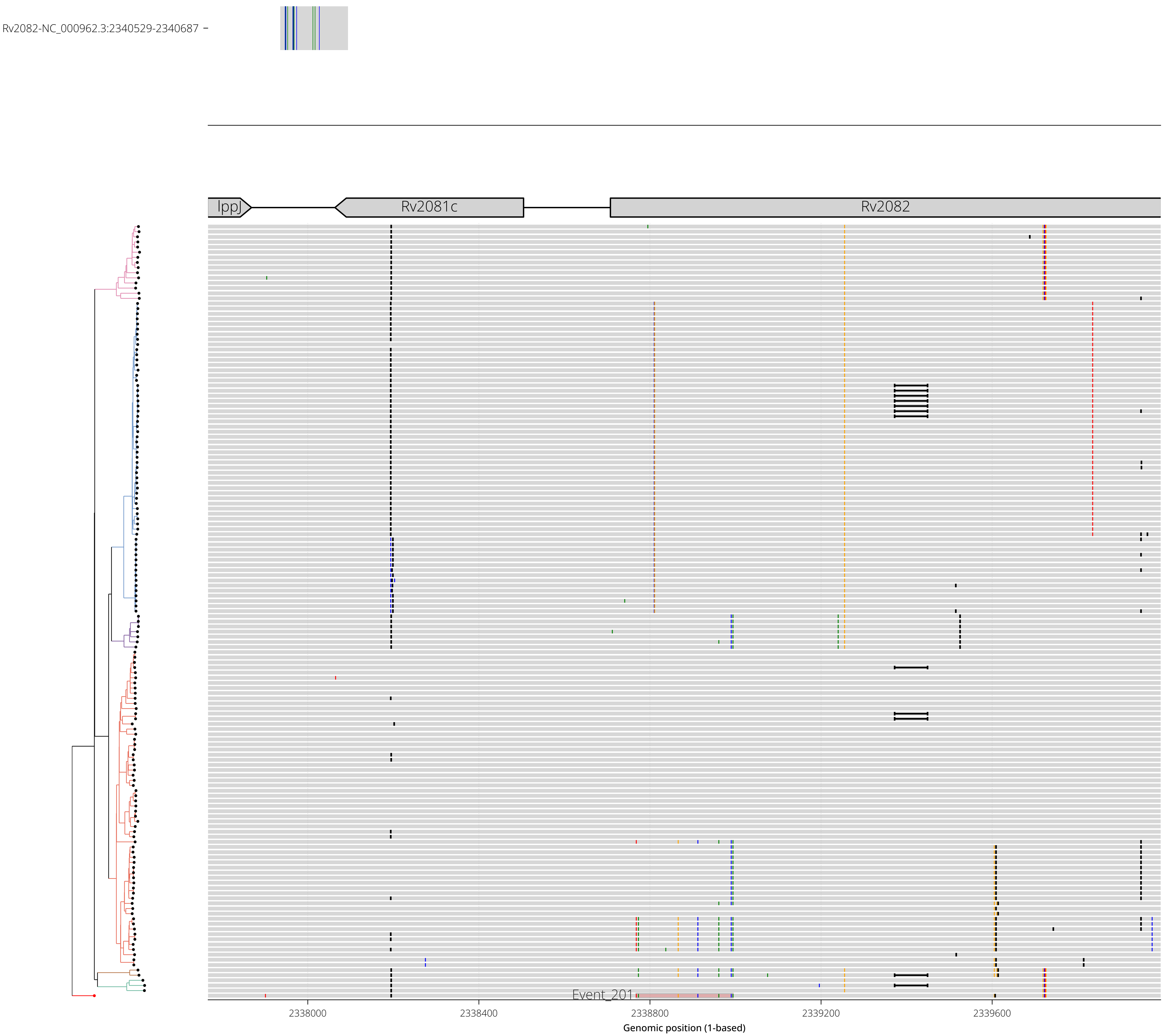

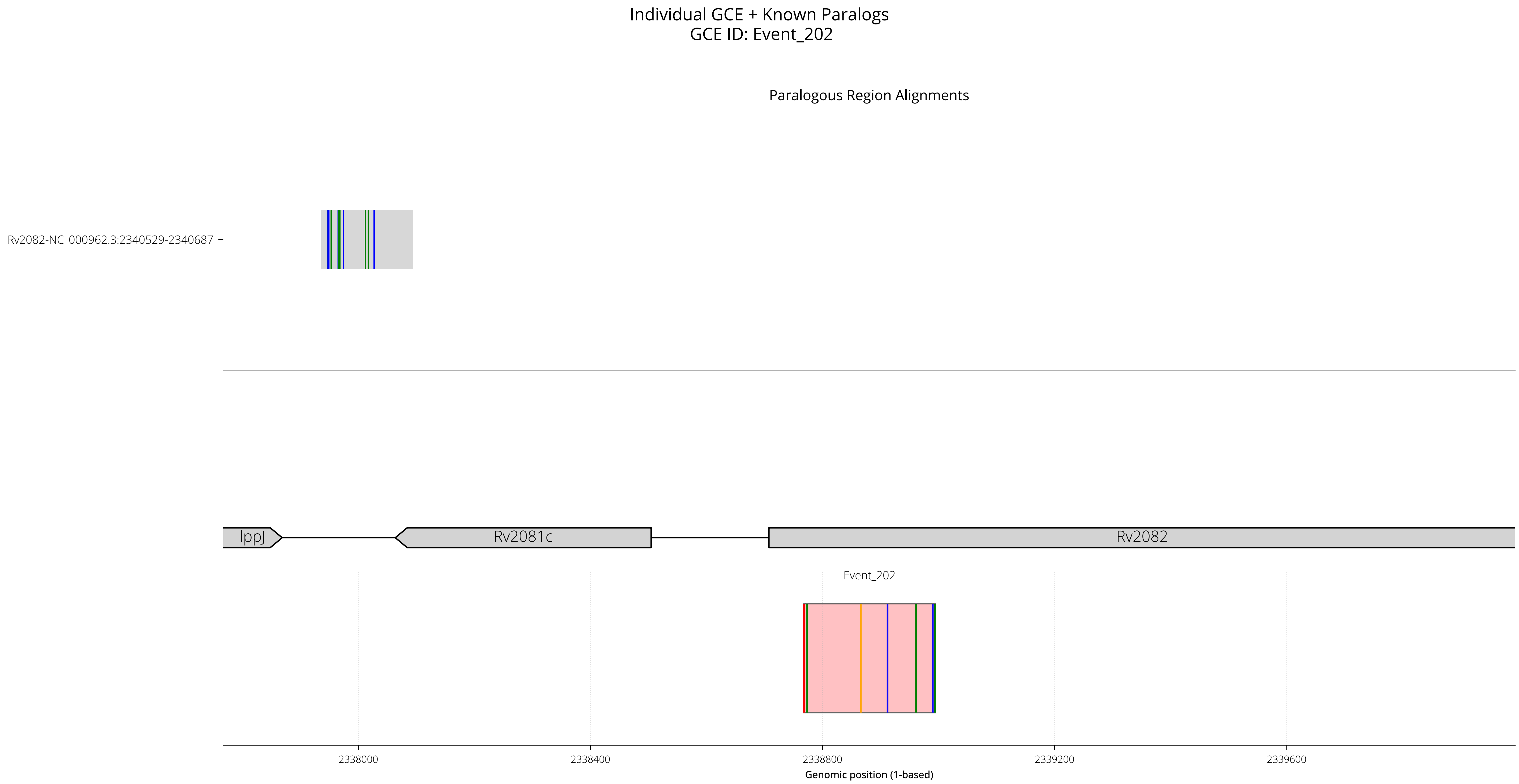

Individual GCE + Known Paralogs
GCE ID: Event_202
Paralogous Region Alignments
Rv2082-NC_000962.3:2340529-2340687
lppJ
Rv2081c
Rv2082
Event_202
2338000
2338400
2338800
2339200
2339600
Genomic position (1-based)

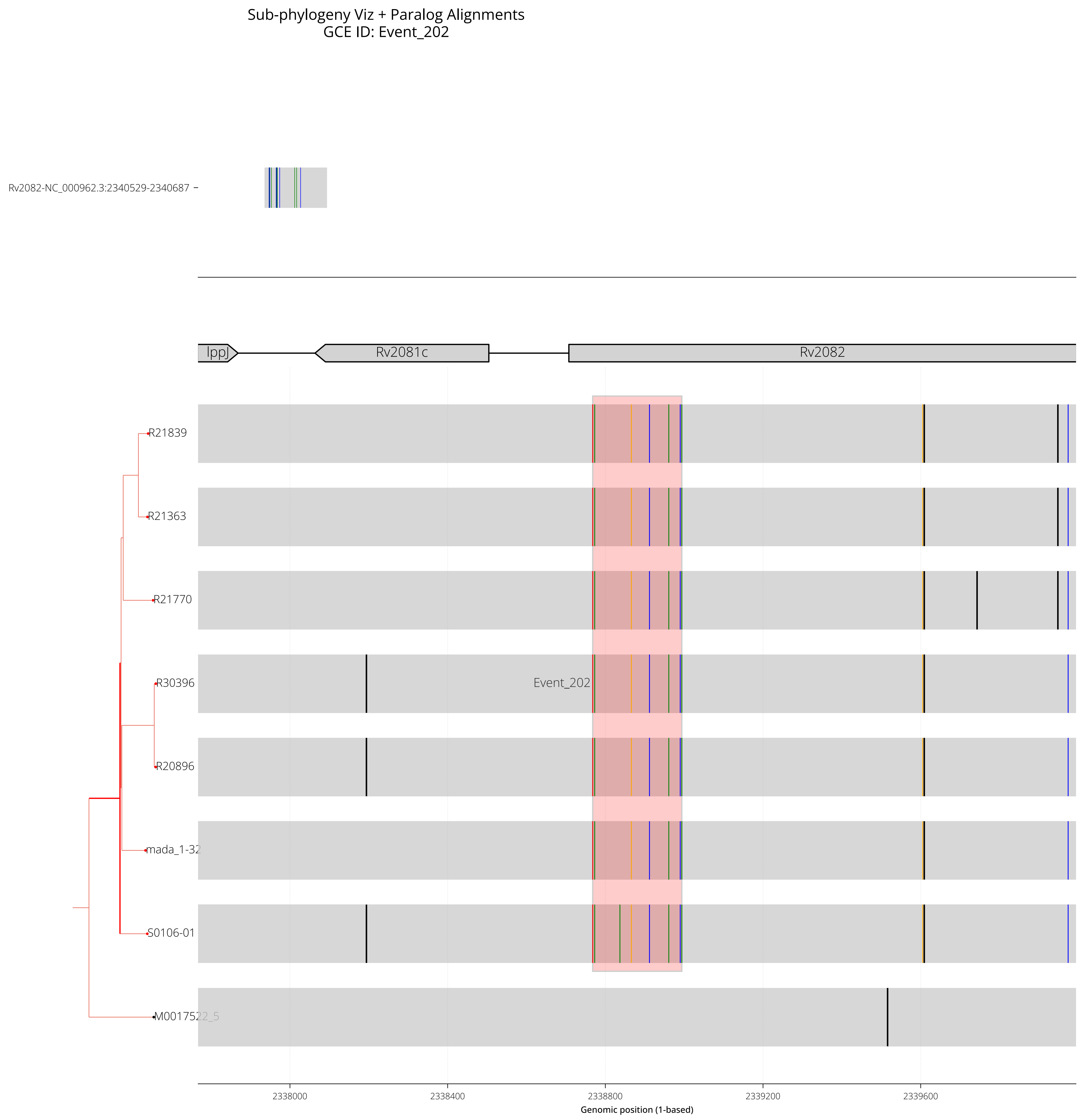

Sub-phylogeny Viz + Paralog Alignments
GCE ID: Event_202
Rv2082-NC_000962.3:2340529-2340687
lppJ
Rv2081c
Rv2082
R21839
R21363
R21770
R30396
R20896
mada_1-32
S0106-01
M0017522_5
Event_202
2338000
2338400
2338800
2339200
2339600
Genomic position (1-based)

Full Phylogeny Viz + Paralog Alignments
GCE ID: Event_202

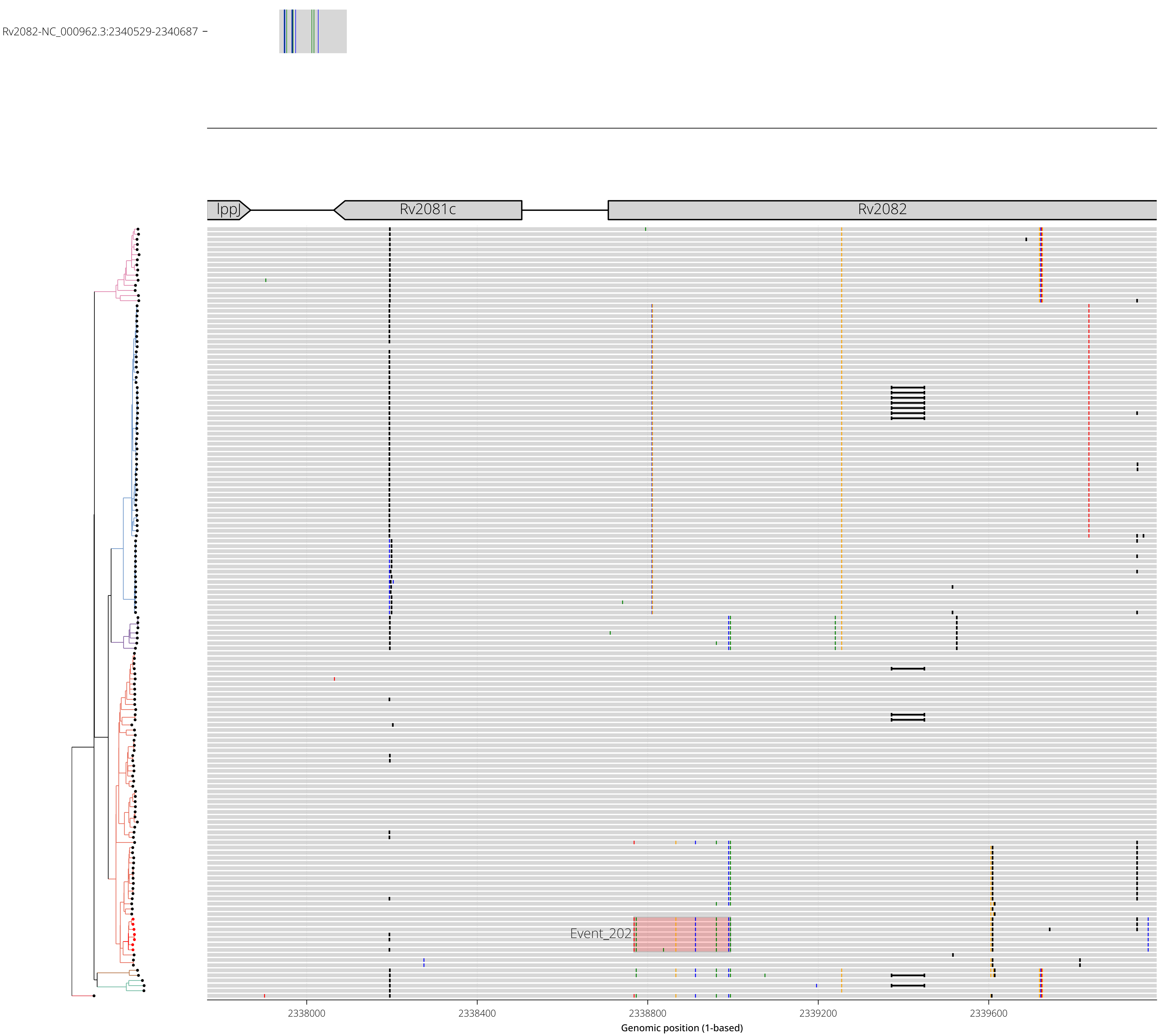

Individual GCE + Known Paralogs
GCE ID: Event_203

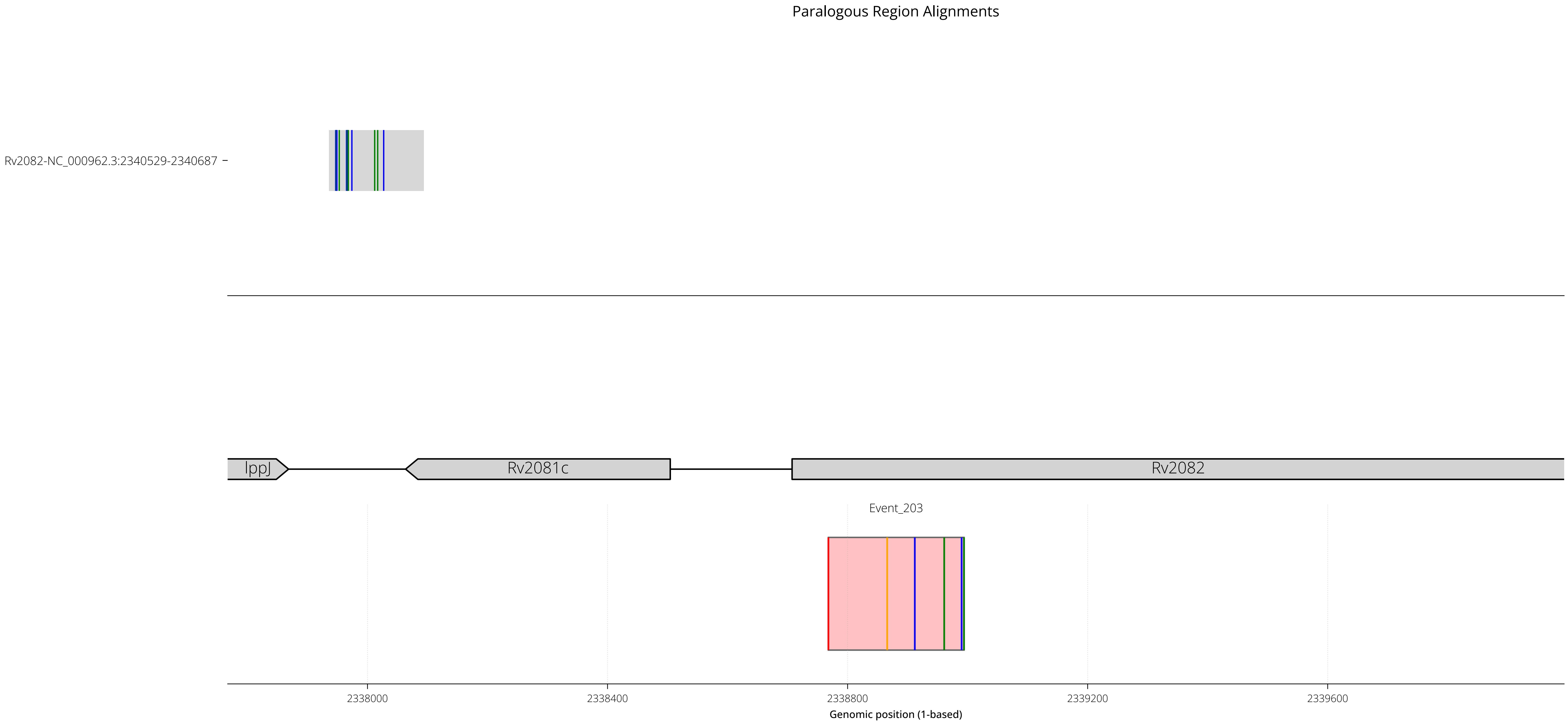

Sub-phylogeny Viz + Paralog Alignments
GCE ID: Event_203

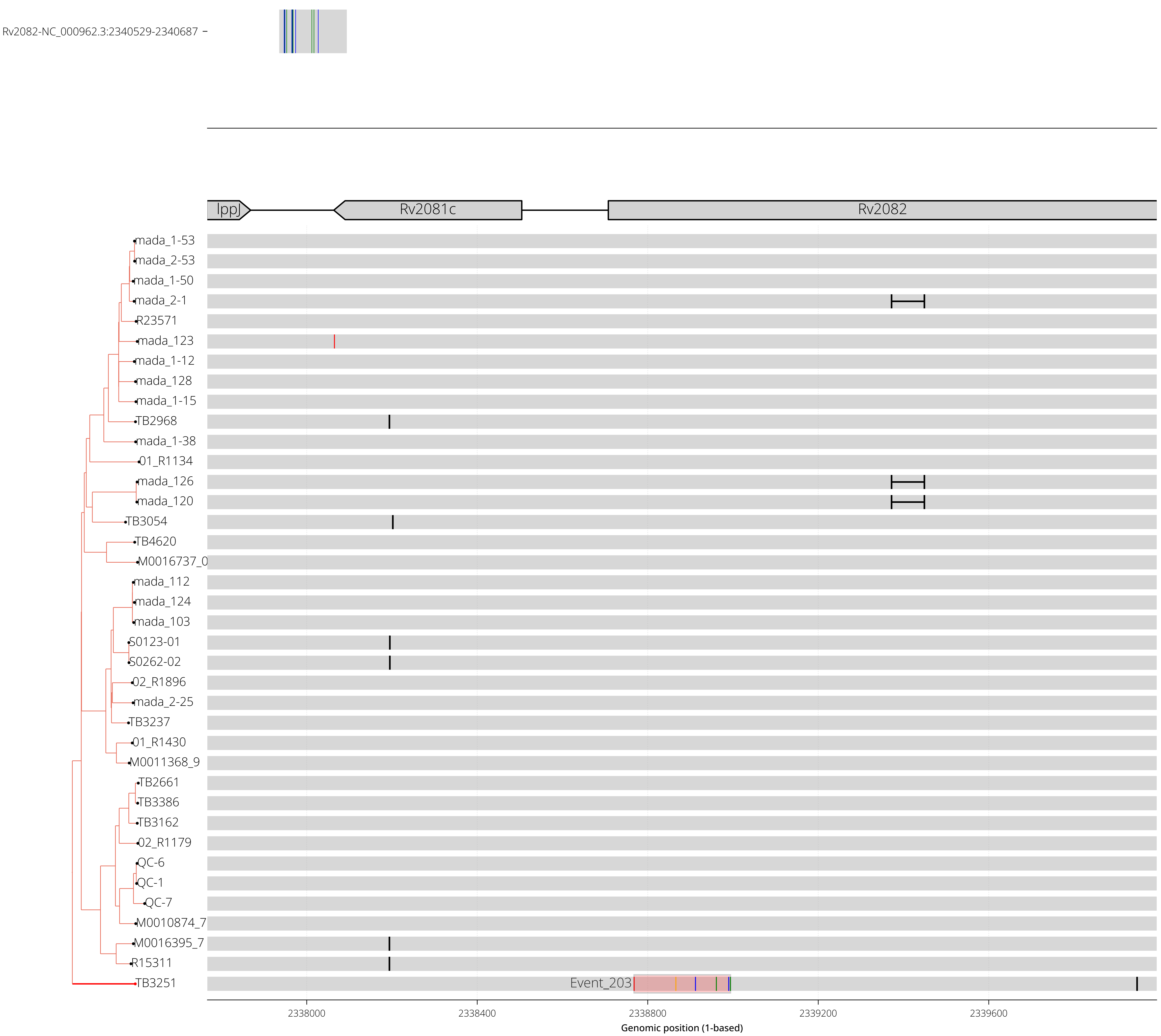

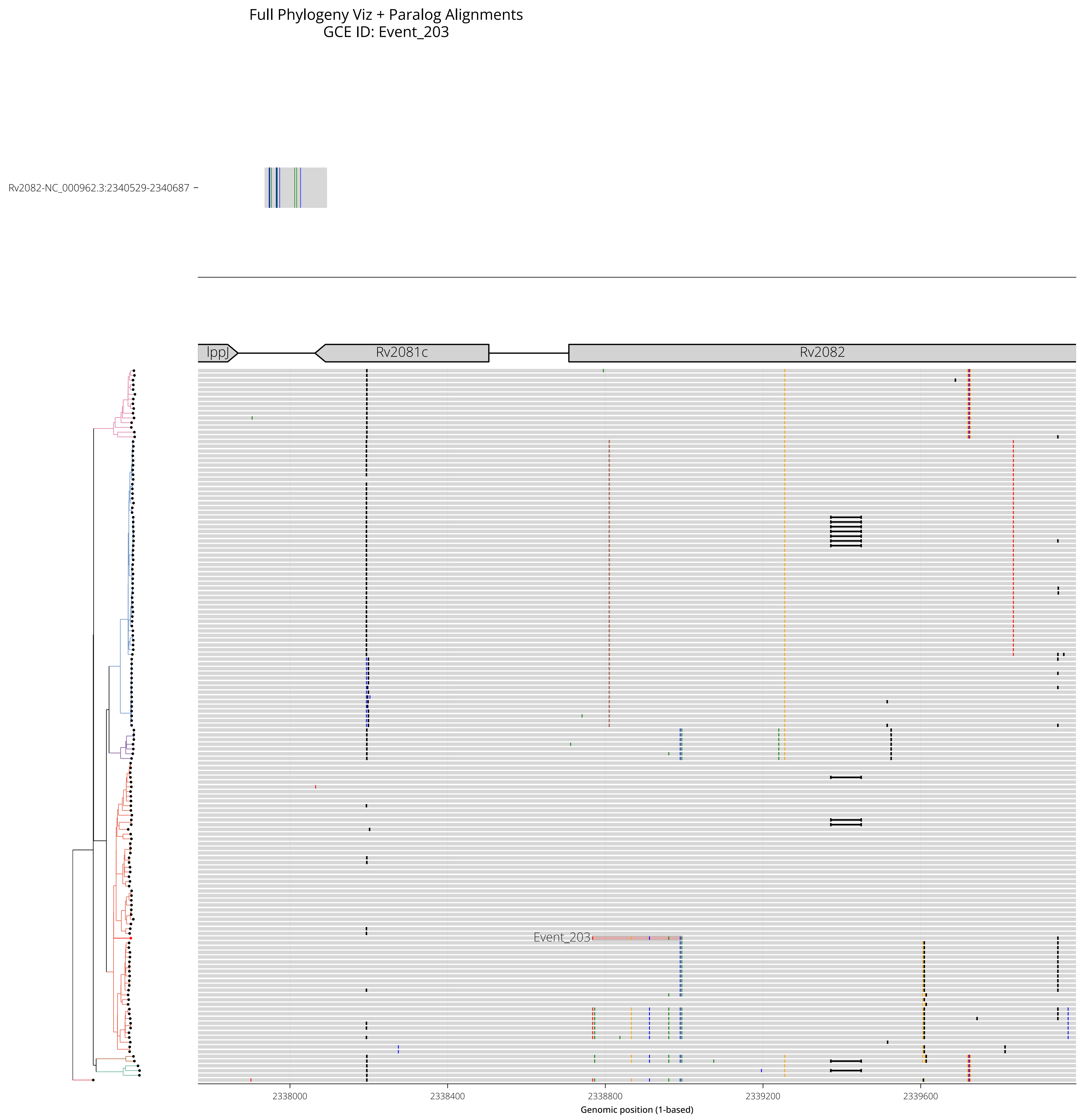

Full Phylogeny Viz + Paralog Alignments
GCE ID: Event_203
Rv2082-NC_000962.3:2340529-2340687
lppJ
Rv2081c
Rv2082
Event_203
2338000
2338400
2338800
2339200
2339600
Genomic position (1-based)

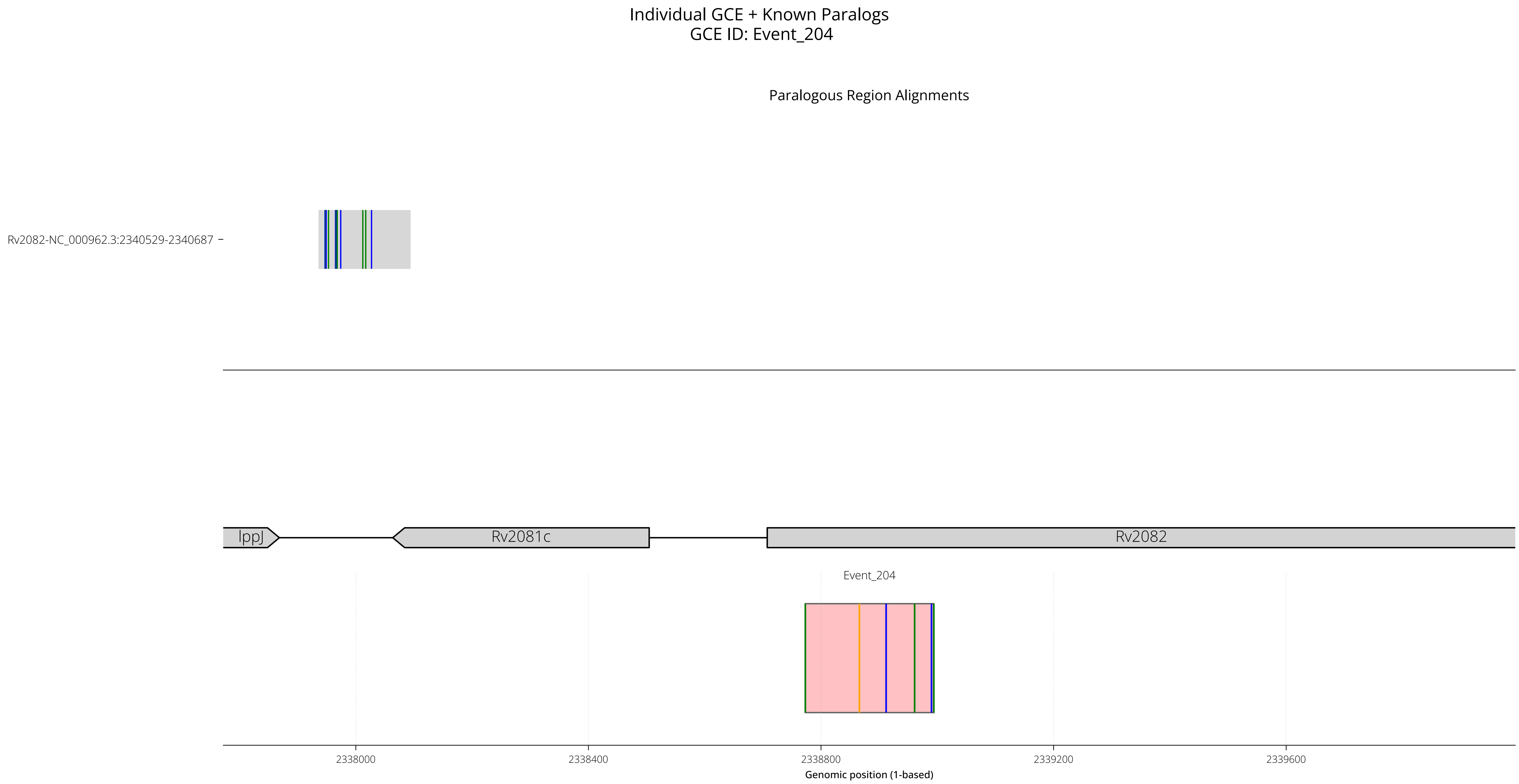
Individual GCE + Known Paralogs
GCE ID: Event_204
Paralogous Region Alignments
Rv2082-NC_000962.3:2340529-2340687
lppJ
Rv2081c
Rv2082
Event_204
2338000
2338400
2338800
2339200
2339600
Genomic position (1-based)

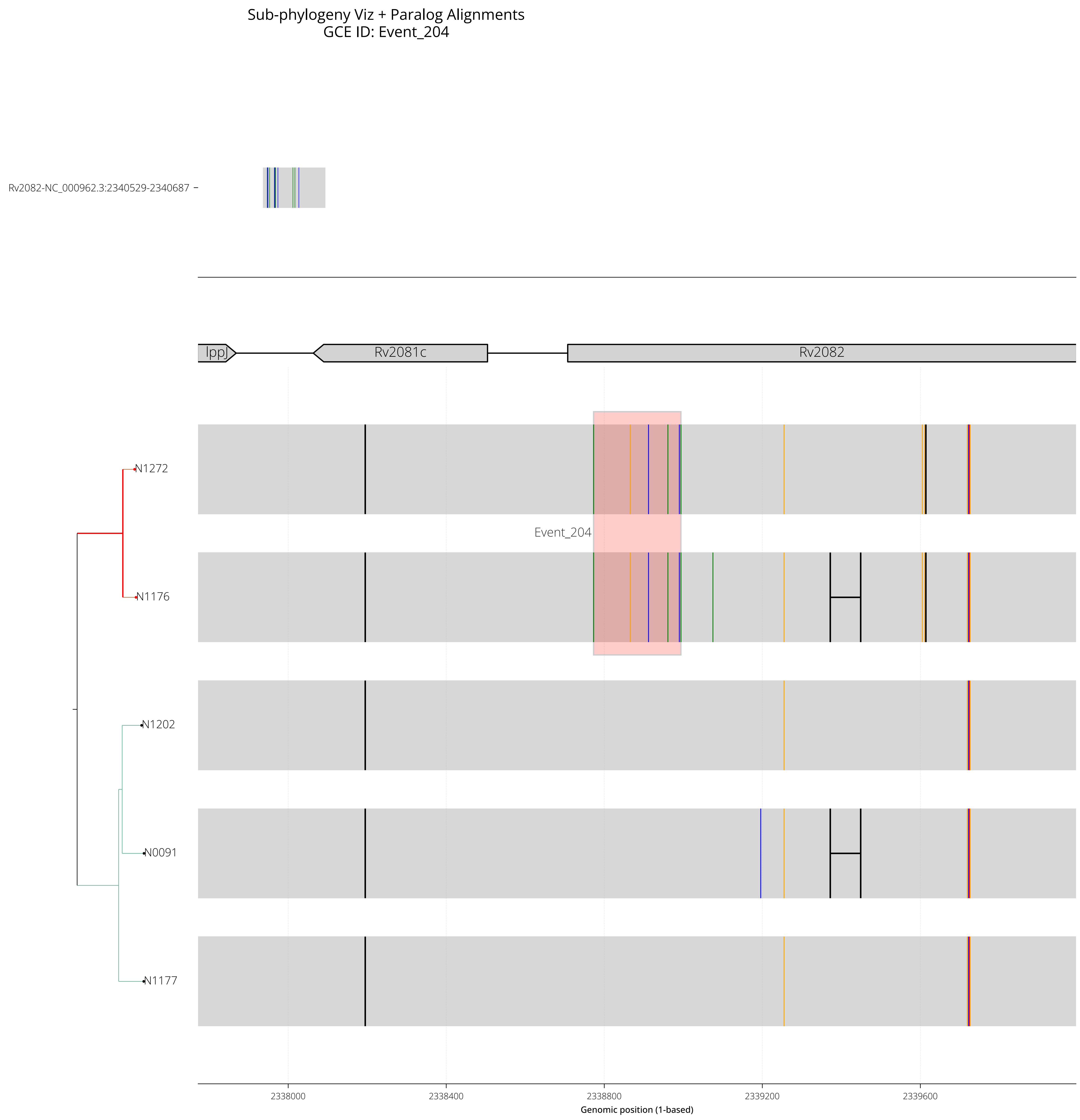
Sub-phylogeny Viz + Paralog Alignments
GCE ID: Event_204
Rv2082-NC_000962.3:2340529-2340687
lppJ
Rv2081c
Rv2082
N1272
Event_204
N1176
N1202
N0091
N1177
2338000
2338400
2338800
2339200
2339600
Genomic position (1-based)

Full Phylogeny Viz + Paralog Alignments
GCE ID: Event_204

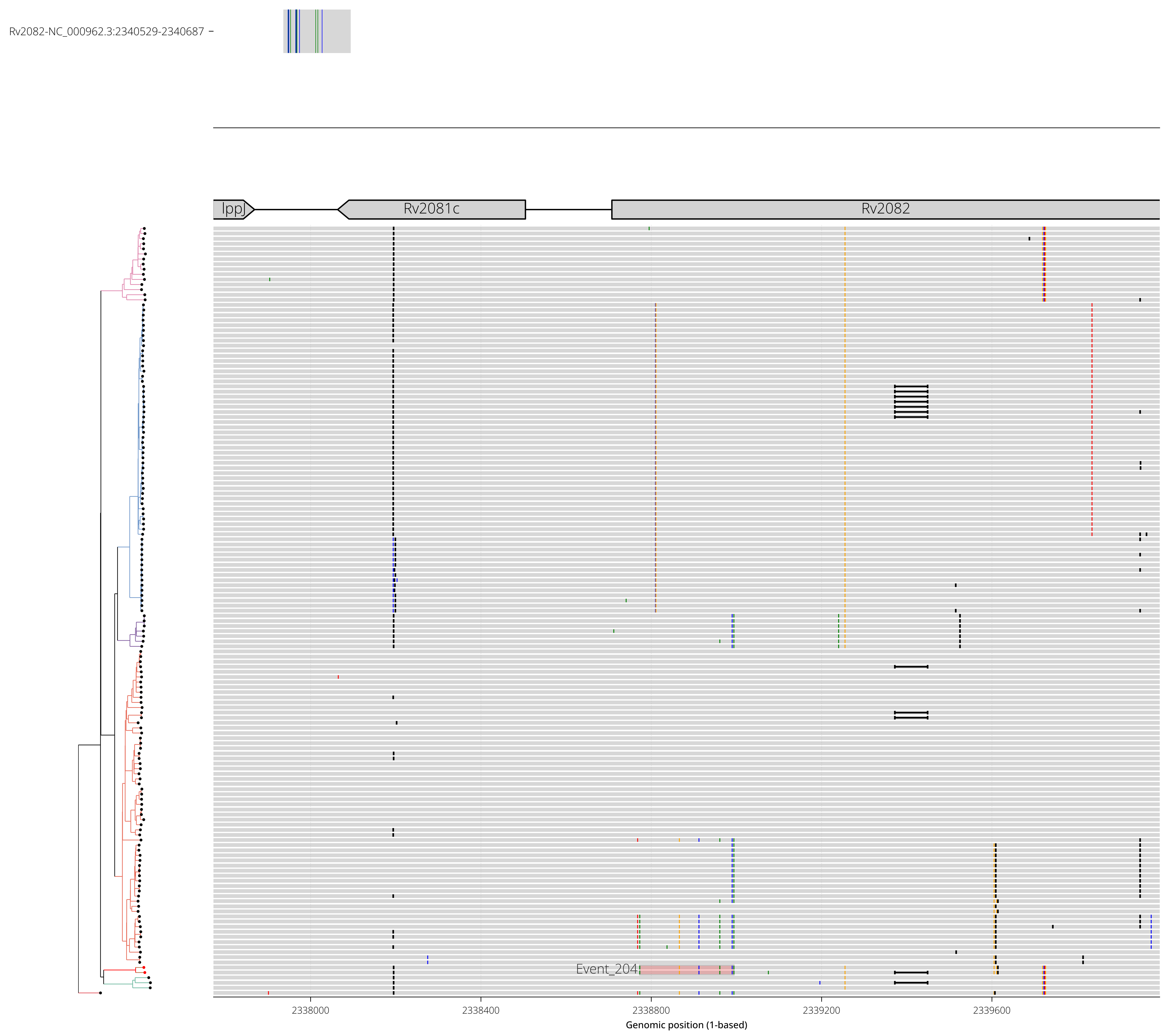

Individual GCE + Known Paralogs
GCE ID: Event_205

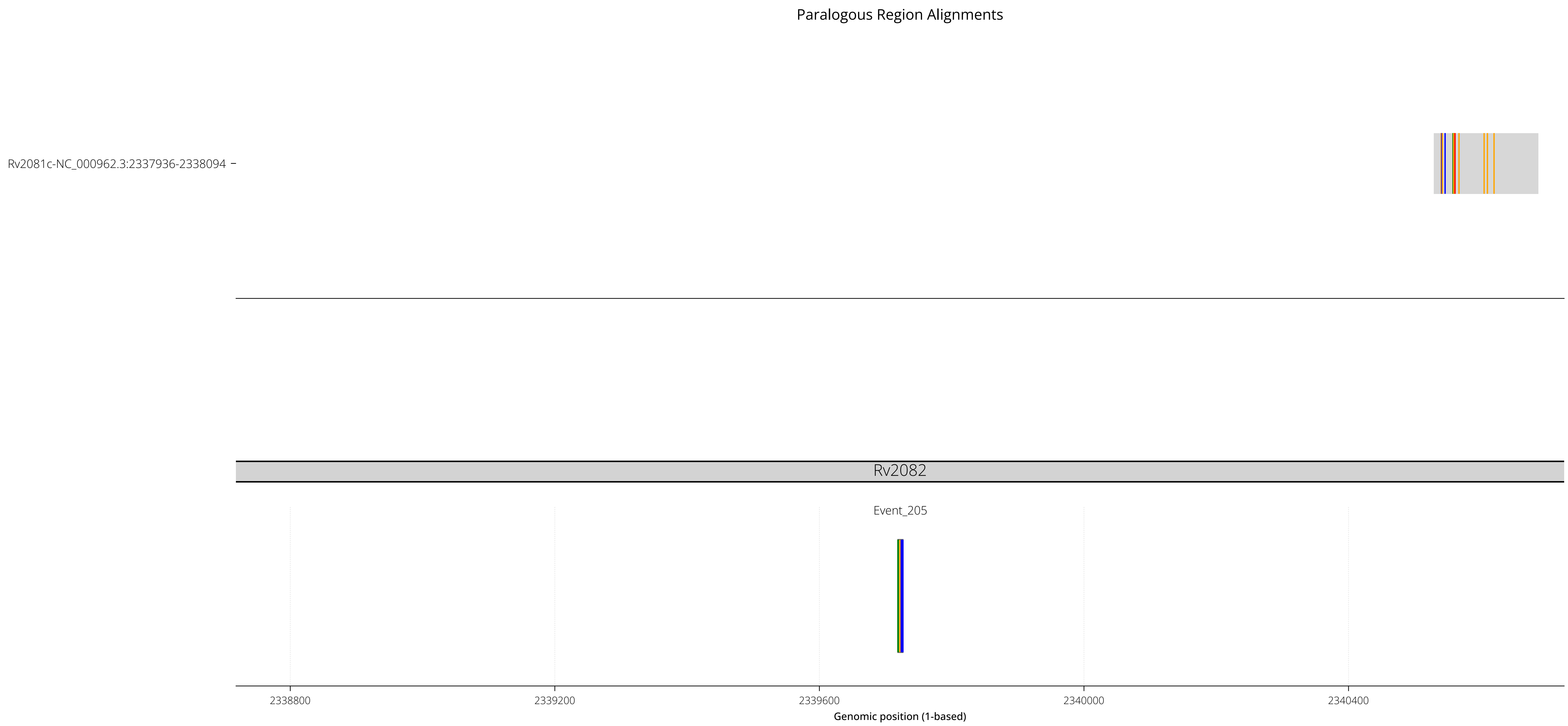

Sub-phylogeny Viz + Paralog Alignments
GCE ID: Event_205

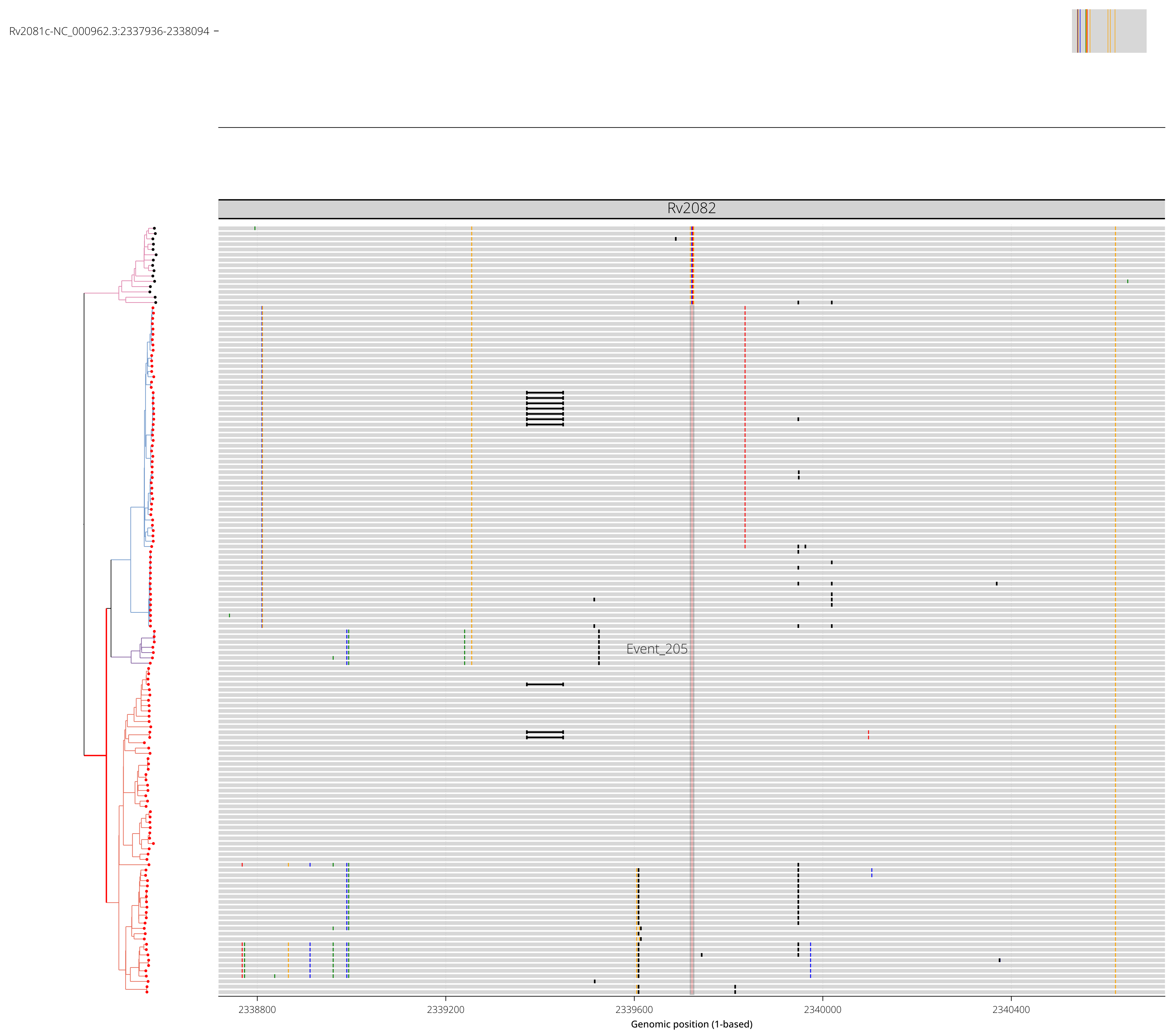

Full Phylogeny Viz + Paralog Alignments
GCE ID: Event_205

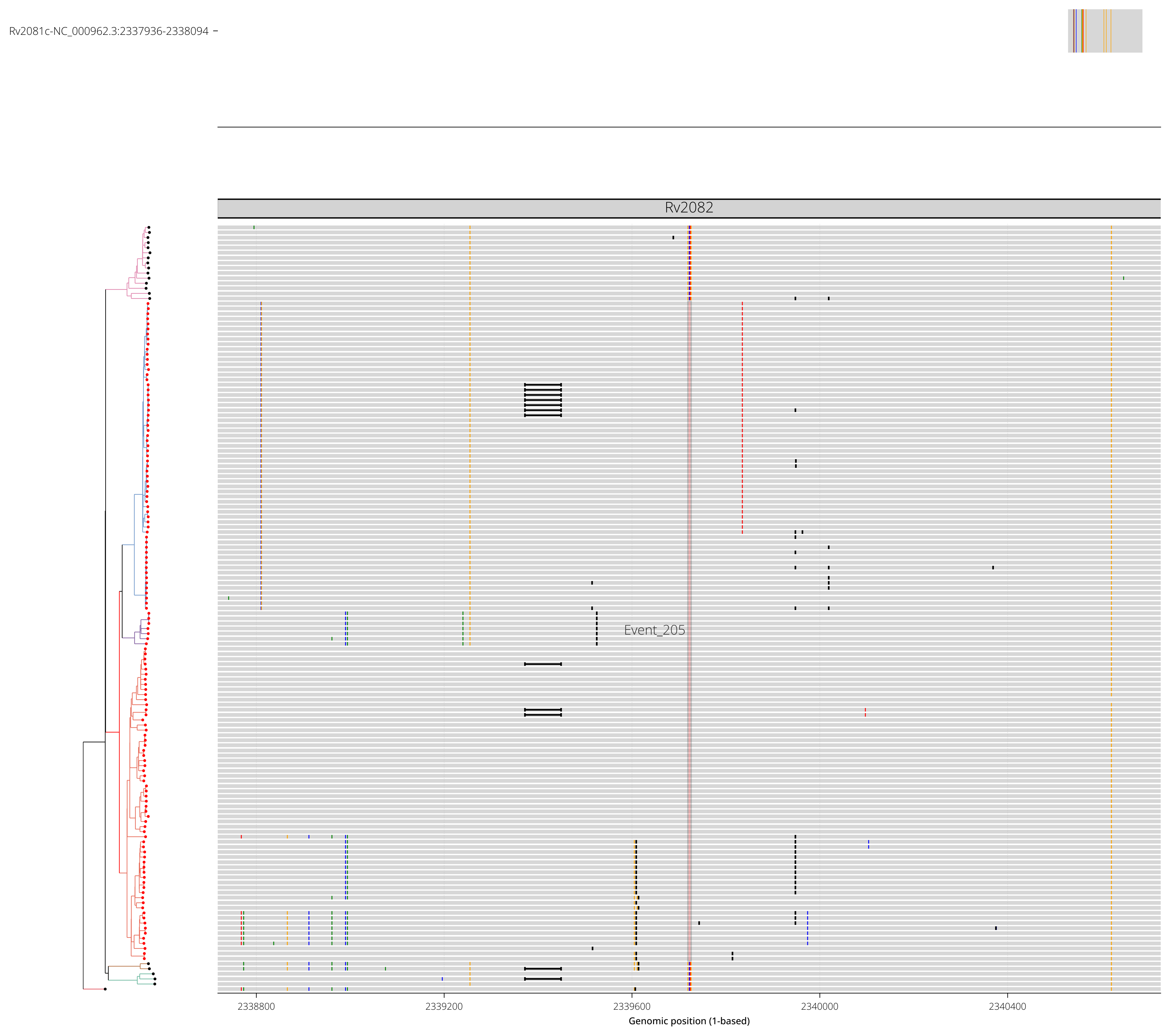

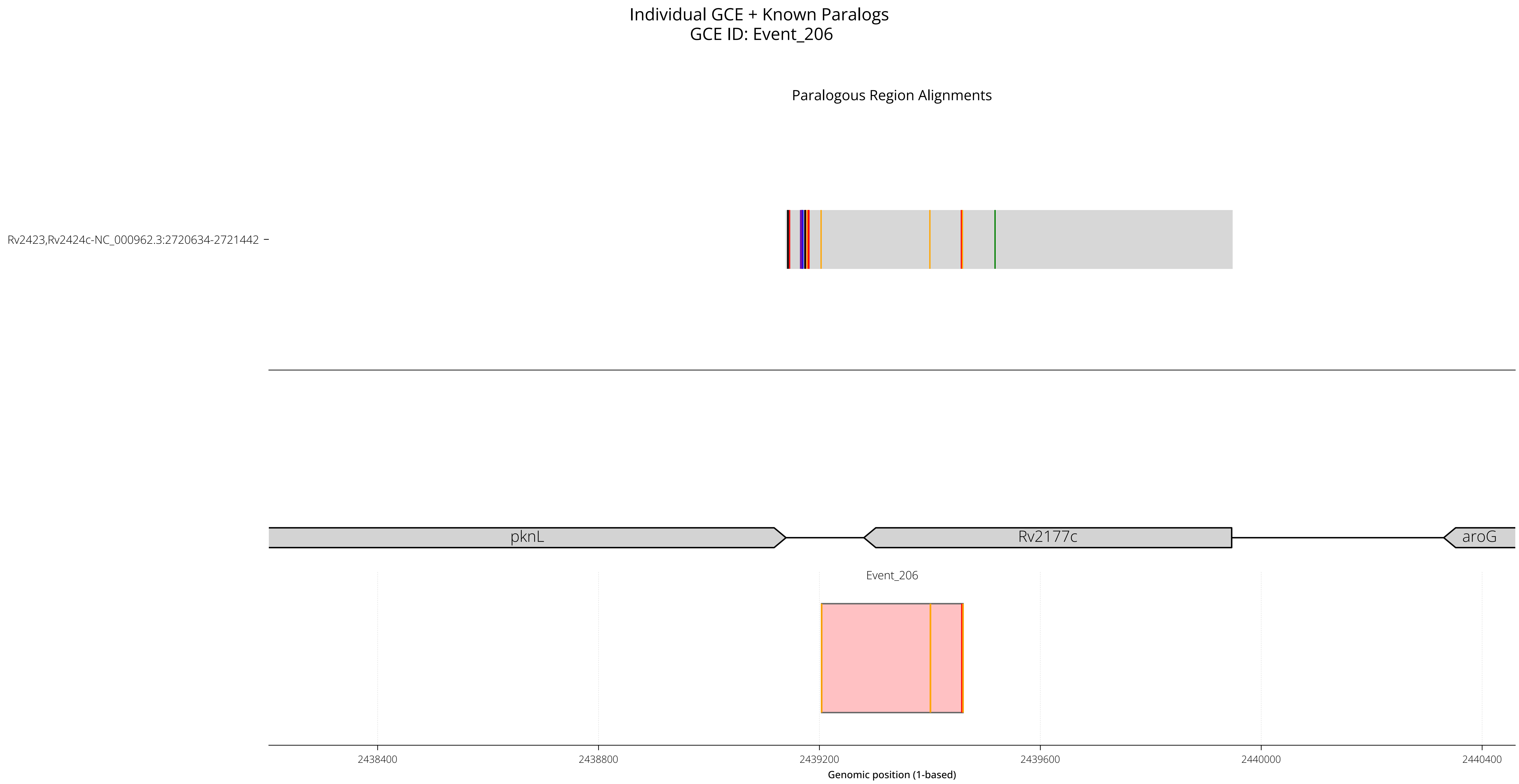

Individual GCE + Known Paralogs
GCE ID: Event_206
Paralogous Region Alignments
Rv2423,Rv2424c-NC_000962.3:2720634-2721442
pknL
Rv2177c
aroG
Event_206
2438400
2438800
2439200
2439600
2440000
2440400
Genomic position (1-based)

Circos Viz - GCE to Known Paralogs Locations
GCE ID: Event_206

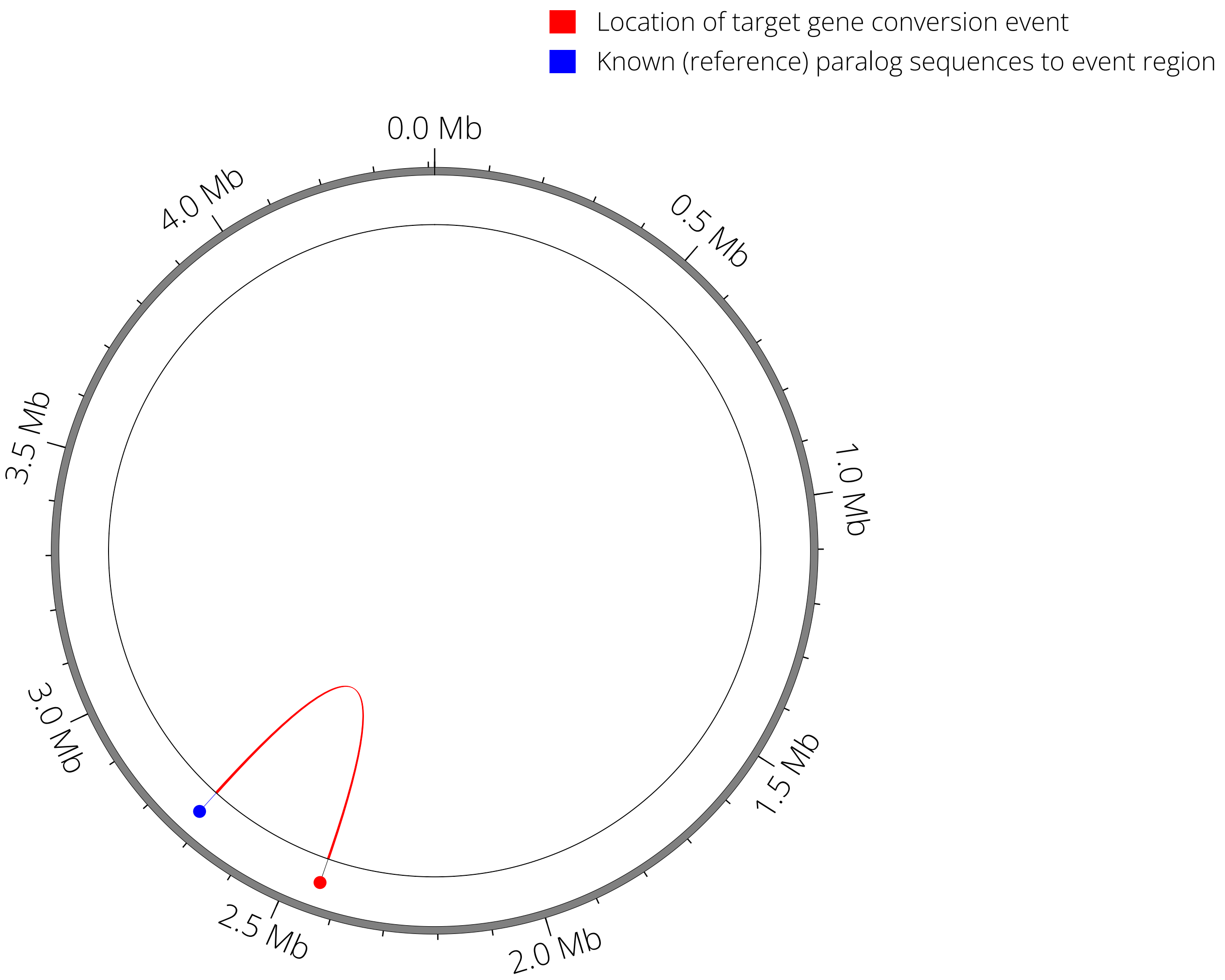

# Event_206 - 4 SNPs - k-mer analysis relative to paralogs

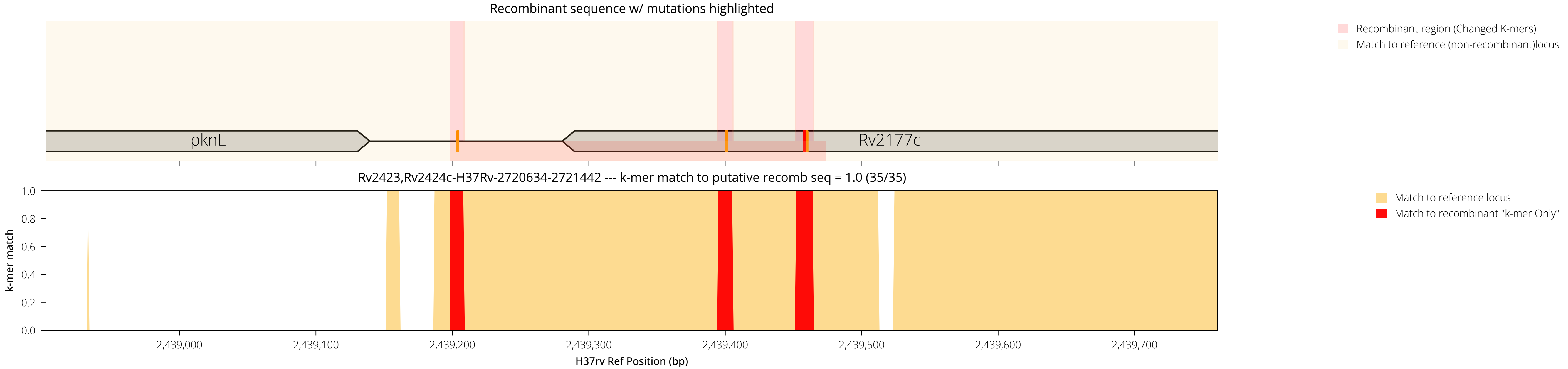

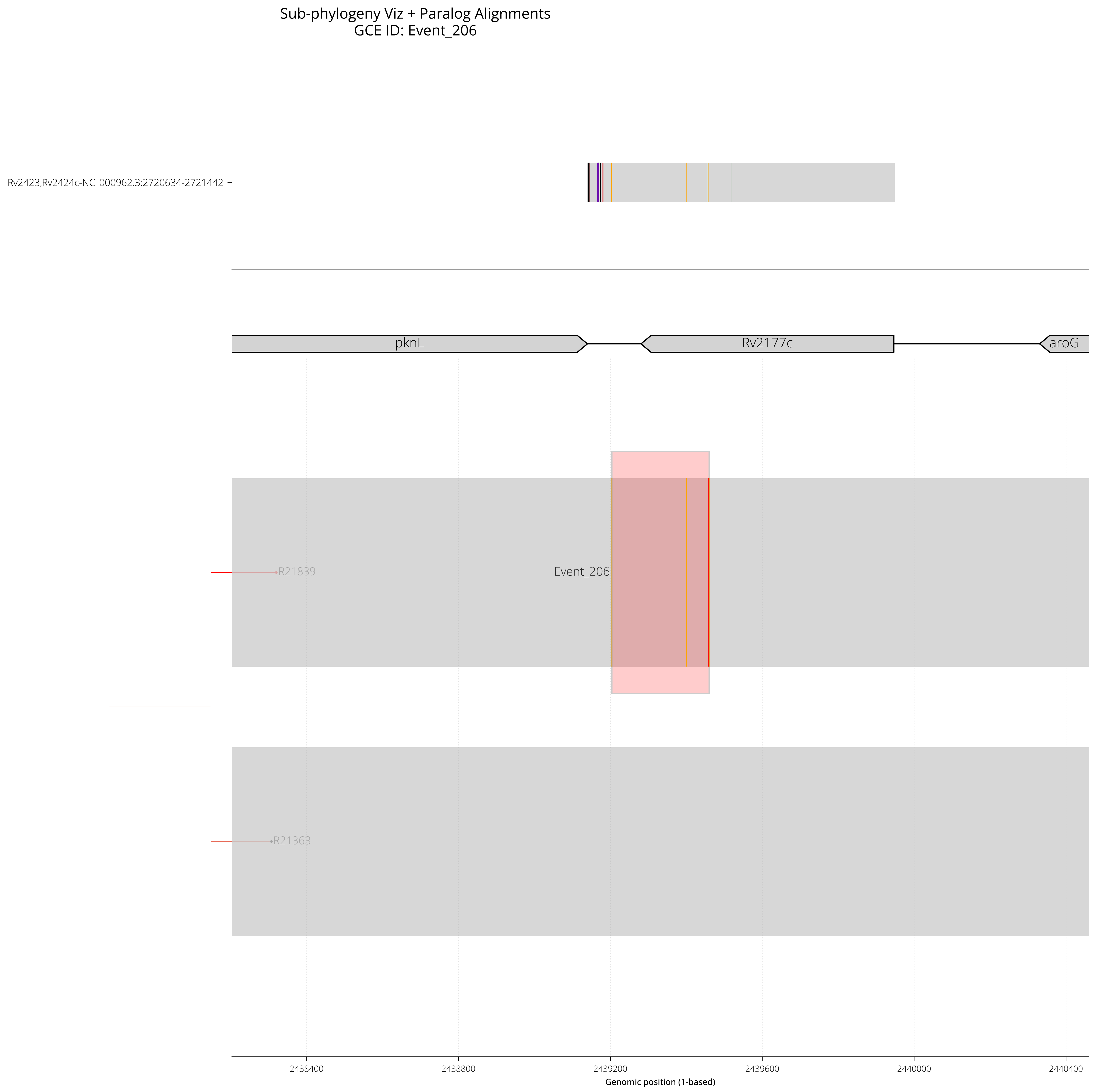

Sub-phylogeny Viz + Paralog Alignments
GCE ID: Event_206
Rv2423,Rv2424c-NC_000962.3:2720634-2721442
pknL
Rv2177c
aroG
R21839
Event_206
R21363
2438400
2438800
2439200
2439600
2440000
2440400
Genomic position (1-based)

Full Phylogeny Viz + Paralog Alignments
GCE ID: Event_206

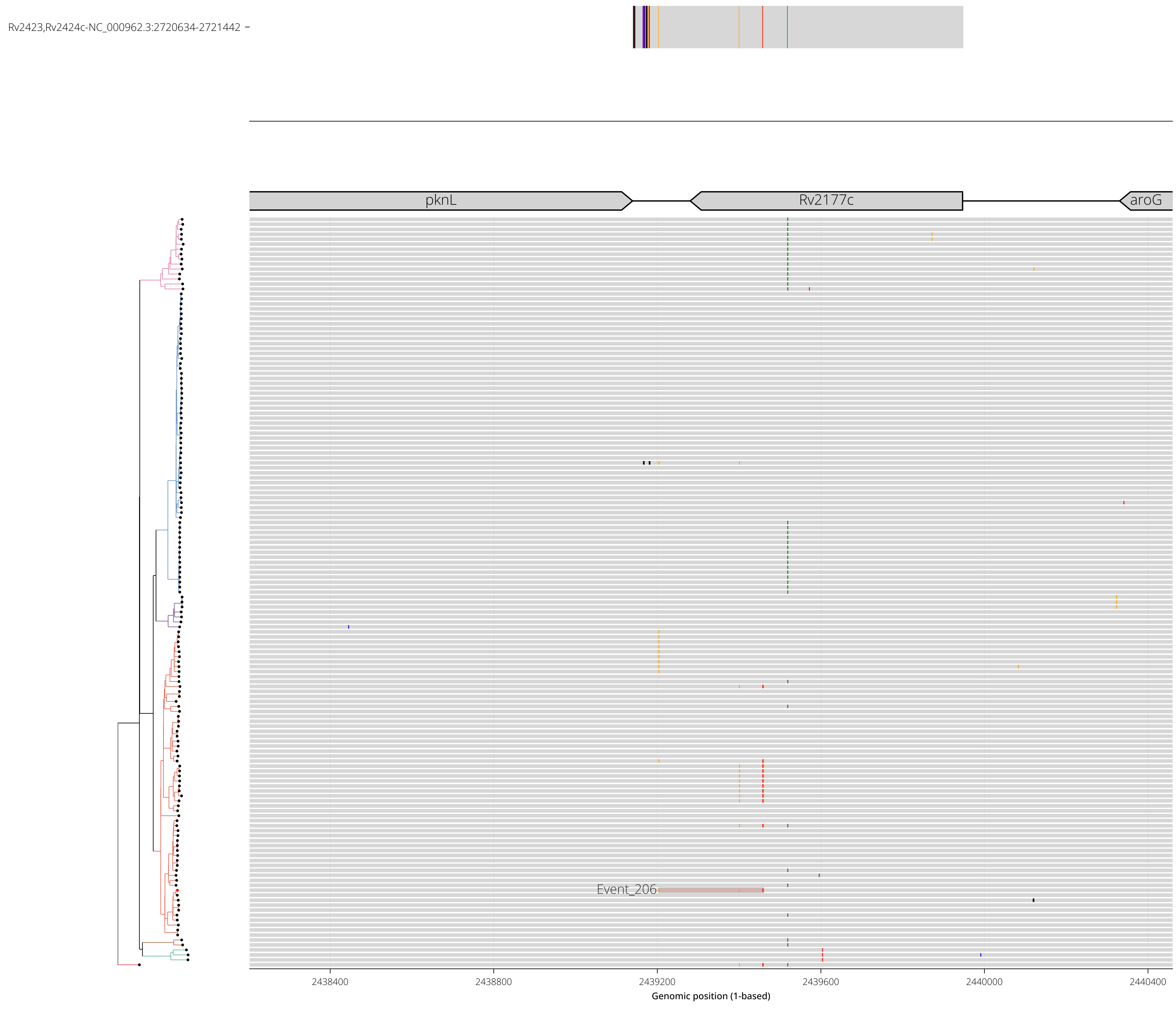

Individual GCE + Known Paralogs
GCE ID: Event_207

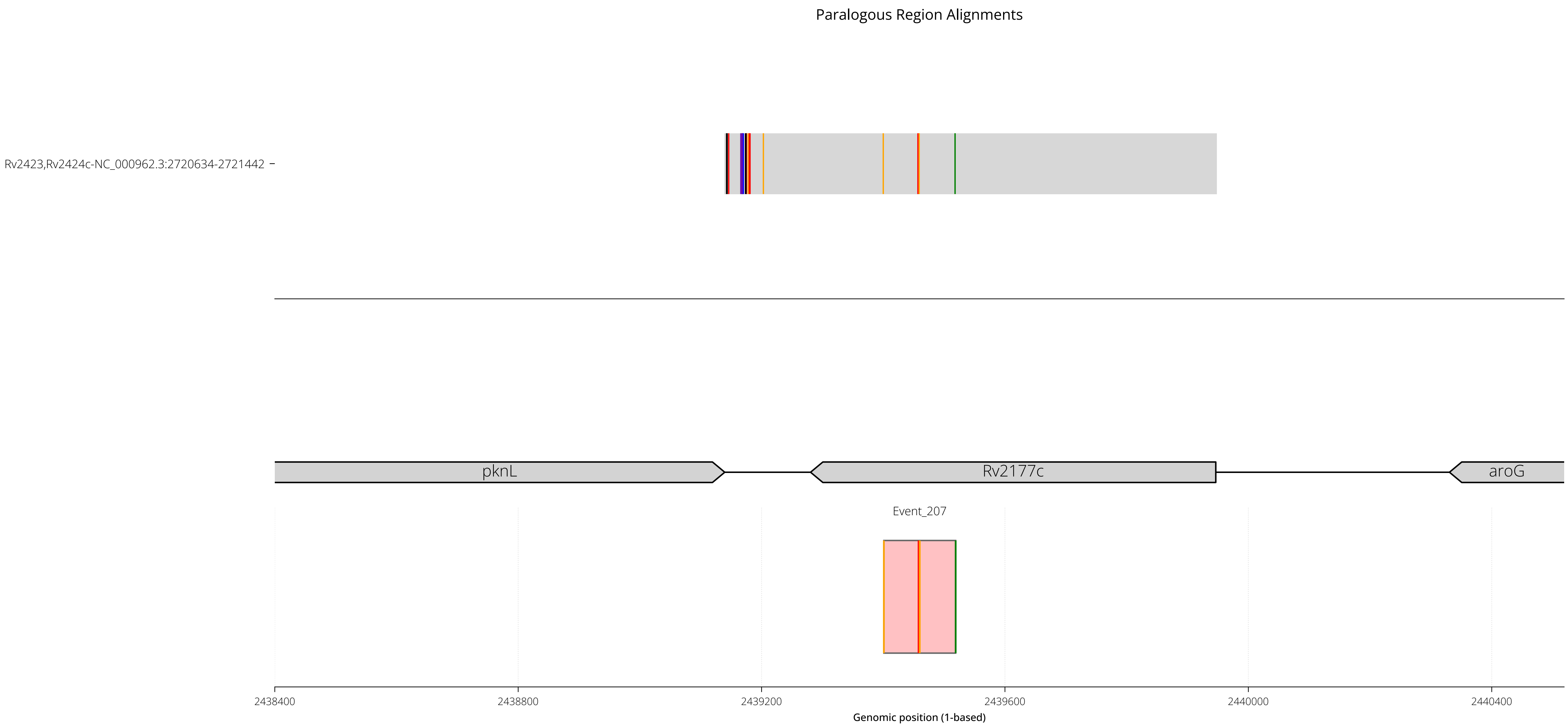

Circos Viz - GCE to Known Paralogs Locations
GCE ID: Event_207

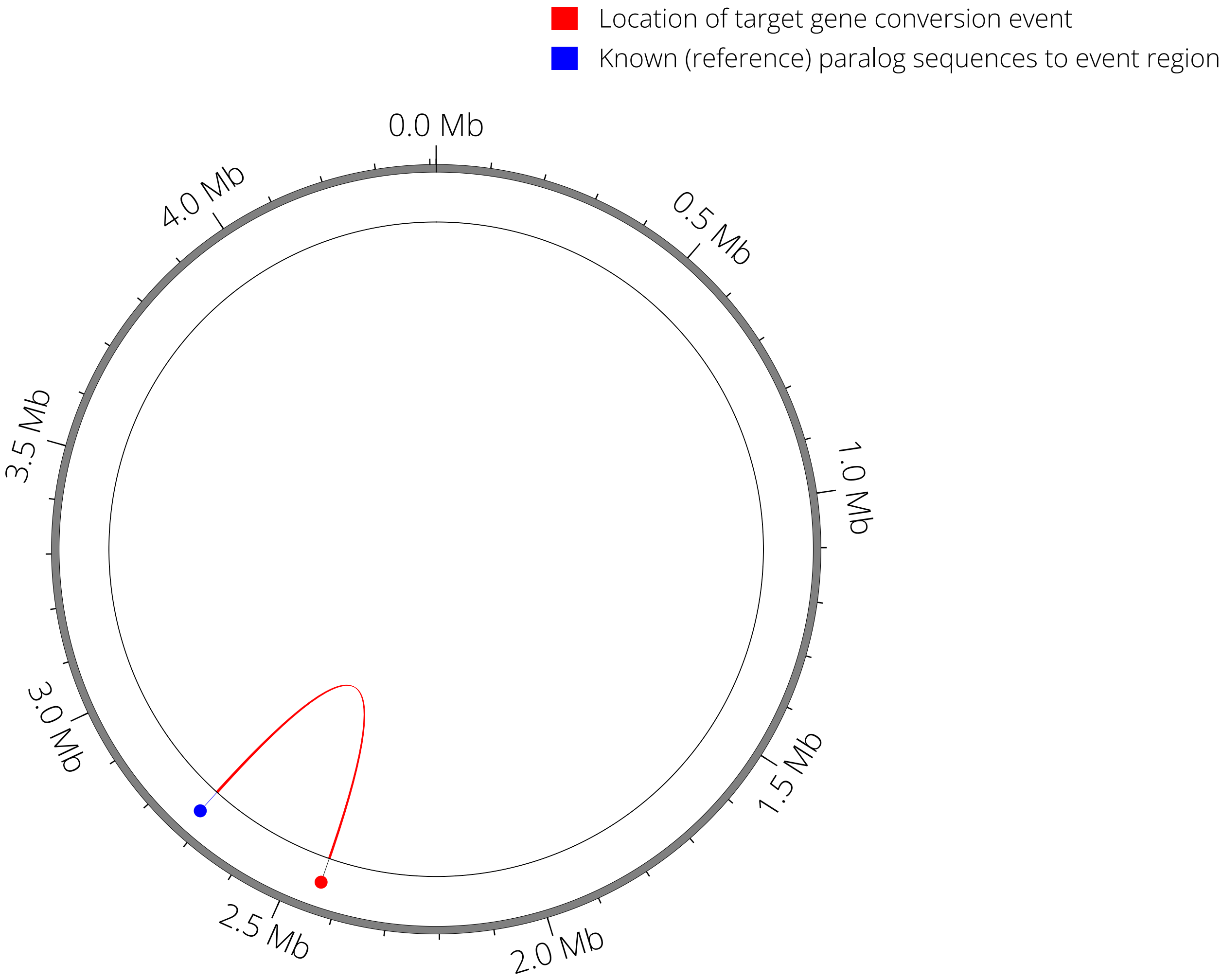

# Event_207 - 4 SNPs - k-mer analysis relative to paralogs

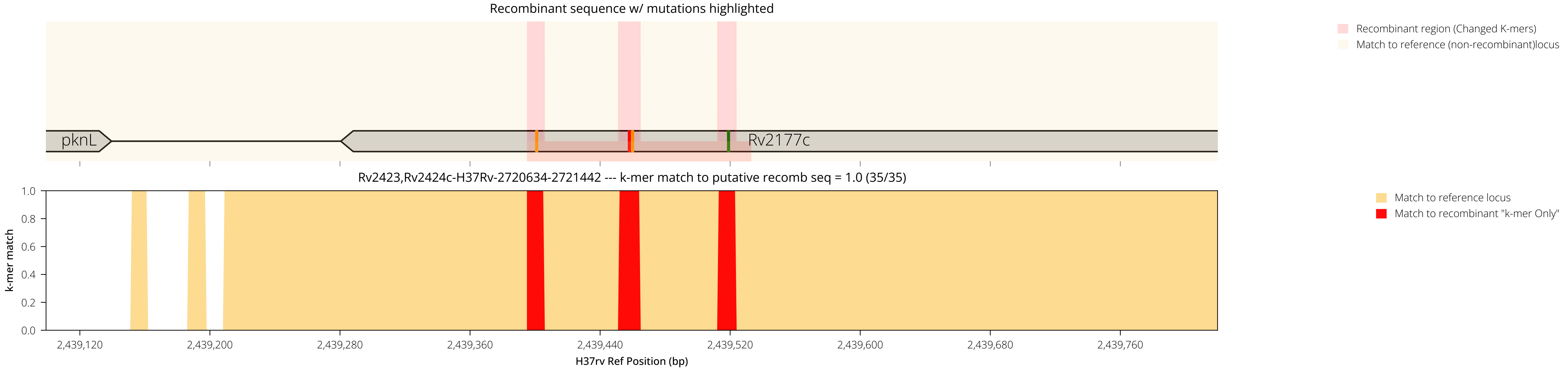

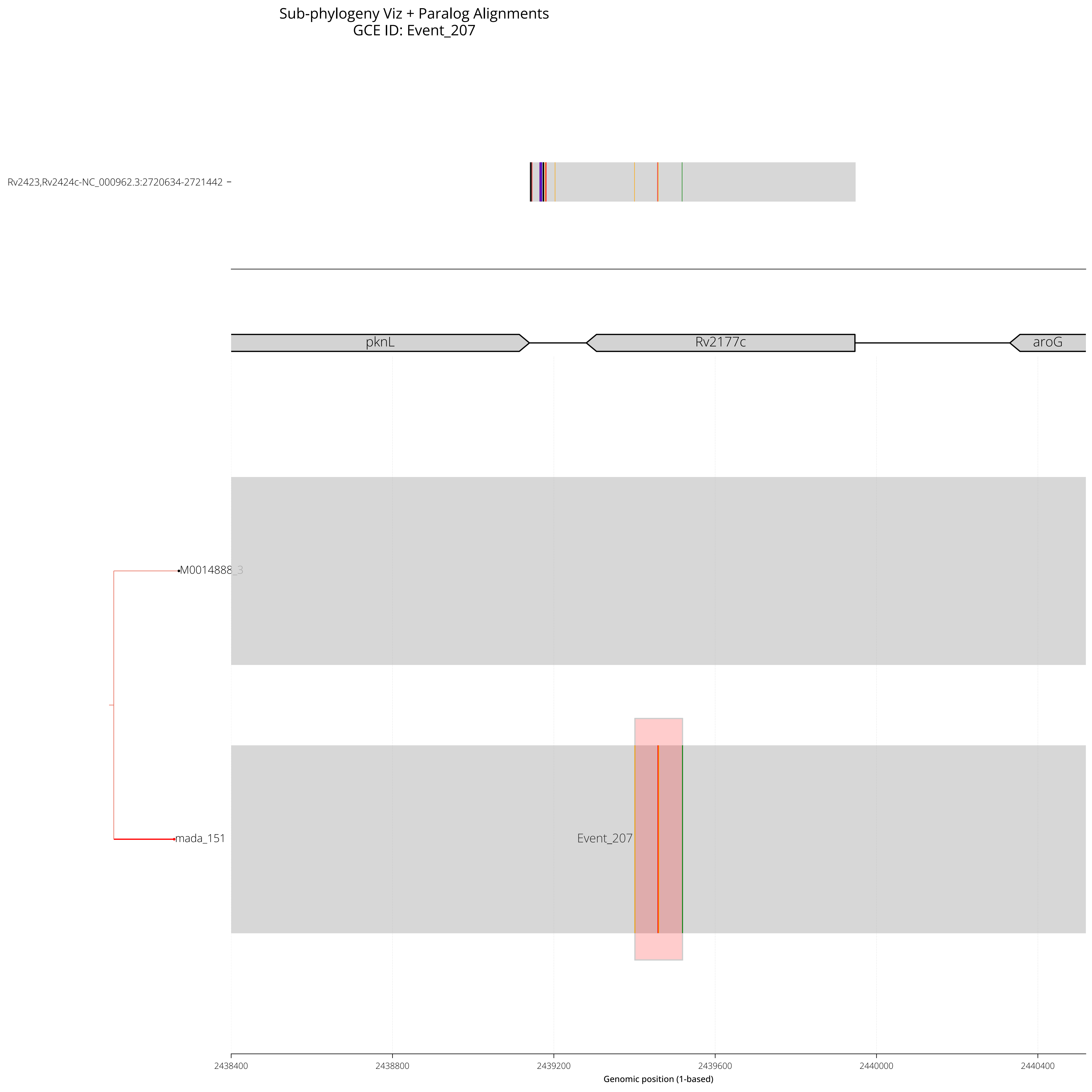

Sub-phylogeny Viz + Paralog Alignments
GCE ID: Event_207
Rv2423,Rv2424c-NC_000962.3:2720634-2721442
pknL
Rv2177c
aroG
M0014888_3
mada_151
Event_207
2438400
2438800
2439200
2439600
2440000
2440400
Genomic position (1-based)

Full Phylogeny Viz + Paralog Alignments
GCE ID: Event_207

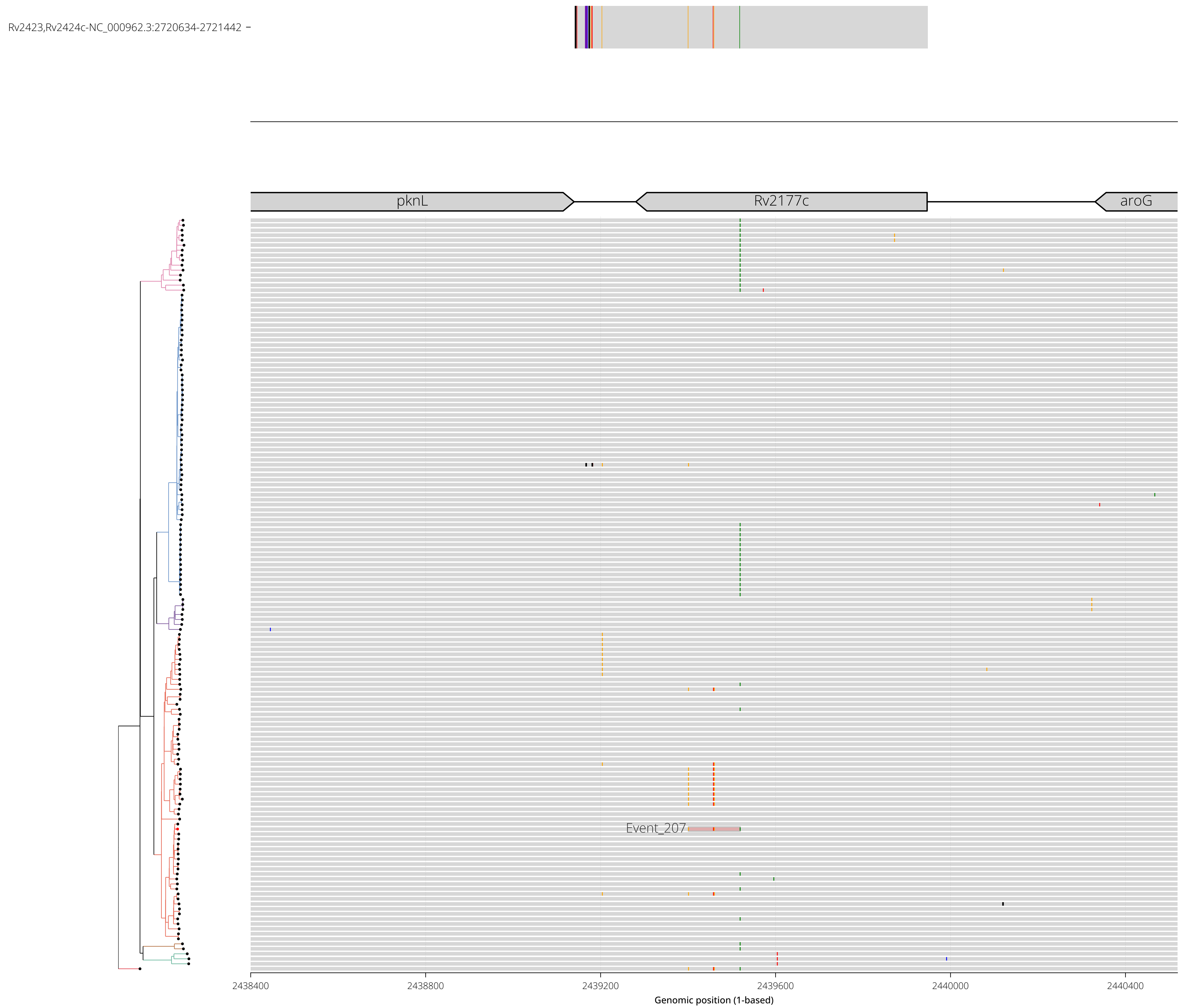

Individual GCE + Known Paralogs
GCE ID: Event_208

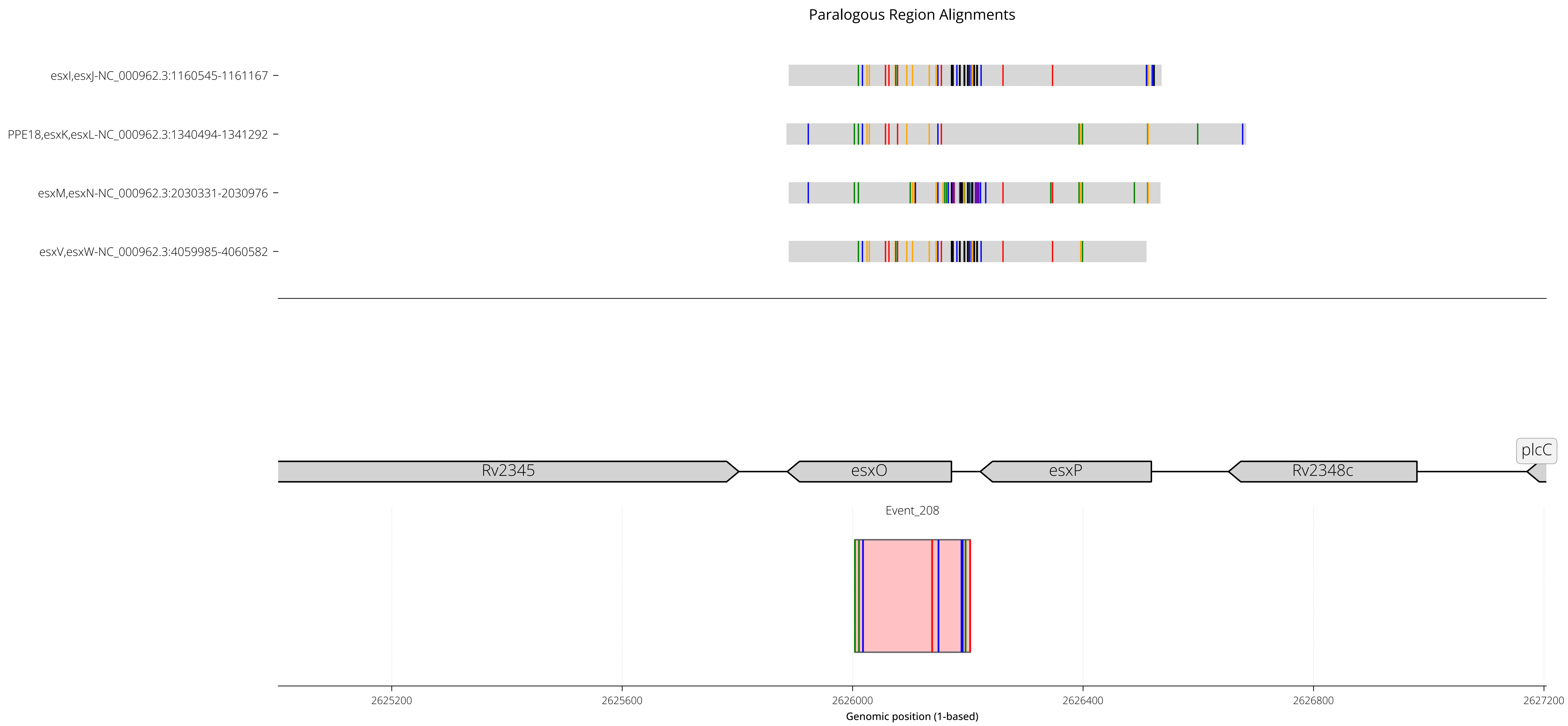

Circos Viz - GCE to Known Paralogs Locations
GCE ID: Event_208

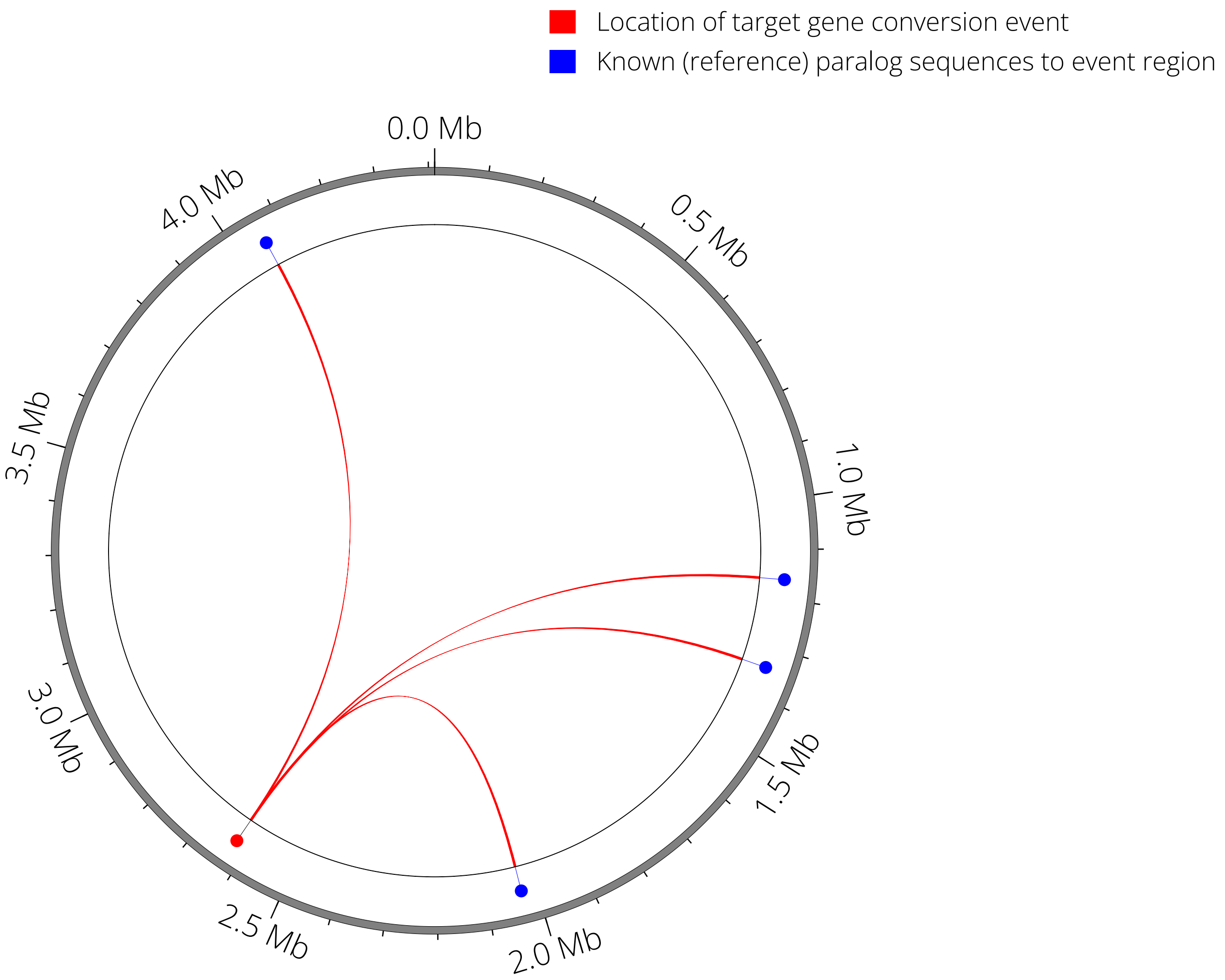

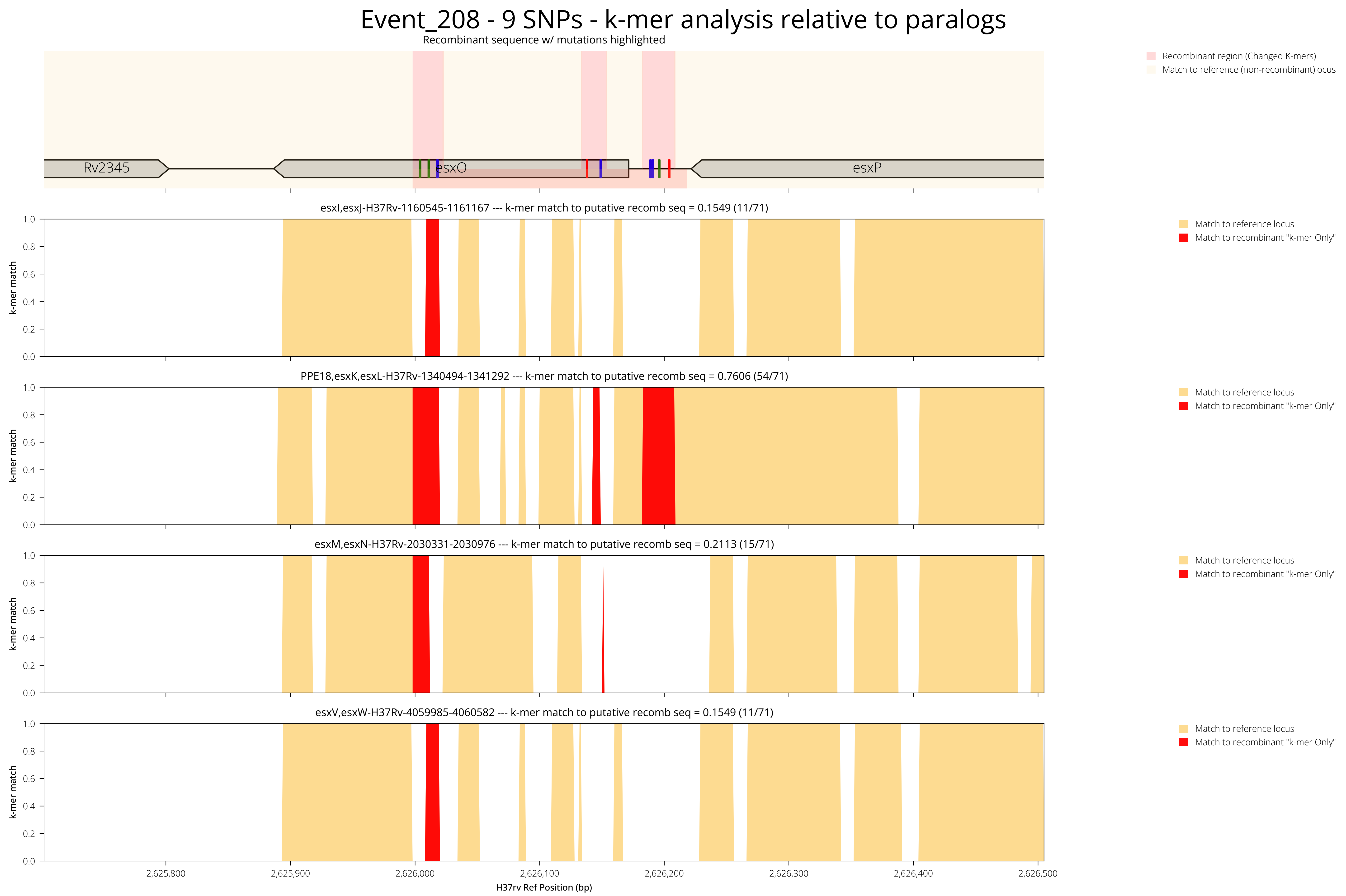

Event_208 - 9 SNPs - k-mer analysis relative to paralogs
Recombinant sequence w/ mutations highlighted
Recombinant region (Changed K-mers)
Match to reference (non-recombinant)locus
Rv2345
esxO
esxP
esxI,esxJ-H37Rv-1160545-1161167 --- k-mer match to putative recomb seq = 0.1549 (11/71)
PPE18,esxK,esxL-H37Rv-1340494-1341292 --- k-mer match to putative recomb seq = 0.7606 (54/71)
esxM,esxN-H37Rv-2030331-2030976 --- k-mer match to putative recomb seq = 0.2113 (15/71)
esxV,esxW-H37Rv-4059985-4060582 --- k-mer match to putative recomb seq = 0.1549 (11/71)
k-mer match
Match to reference locus
Match to recombinant "k-mer Only"
1.0
0.8
0.6
0.4
0.2
0.0
2,625,800
2,625,900
2,626,000
2,626,100
2,626,200
2,626,300
2,626,400
2,626,500
H37rv Ref Position (bp)

Sub-phylogeny Viz + Paralog Alignments
GCE ID: Event_208

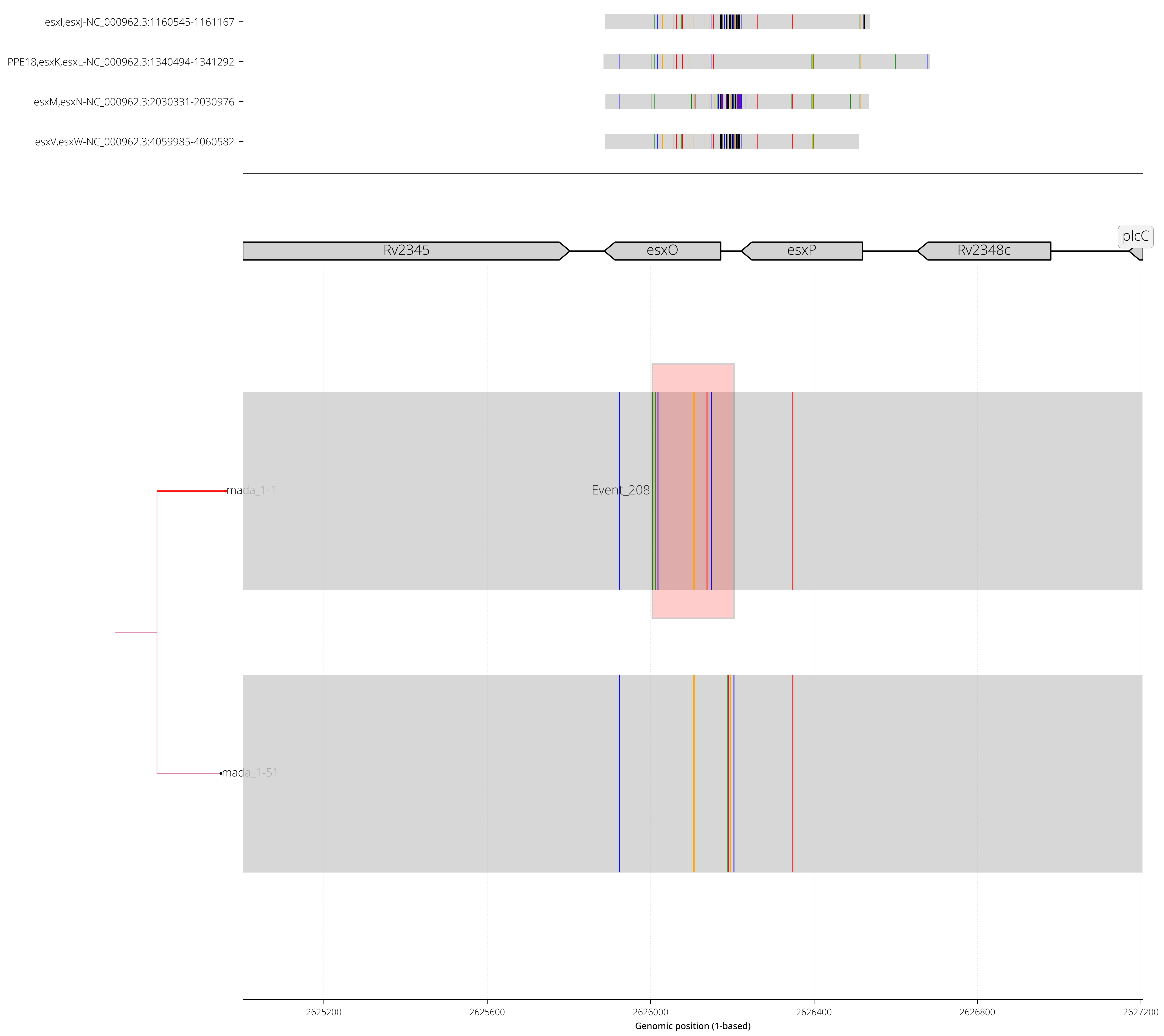

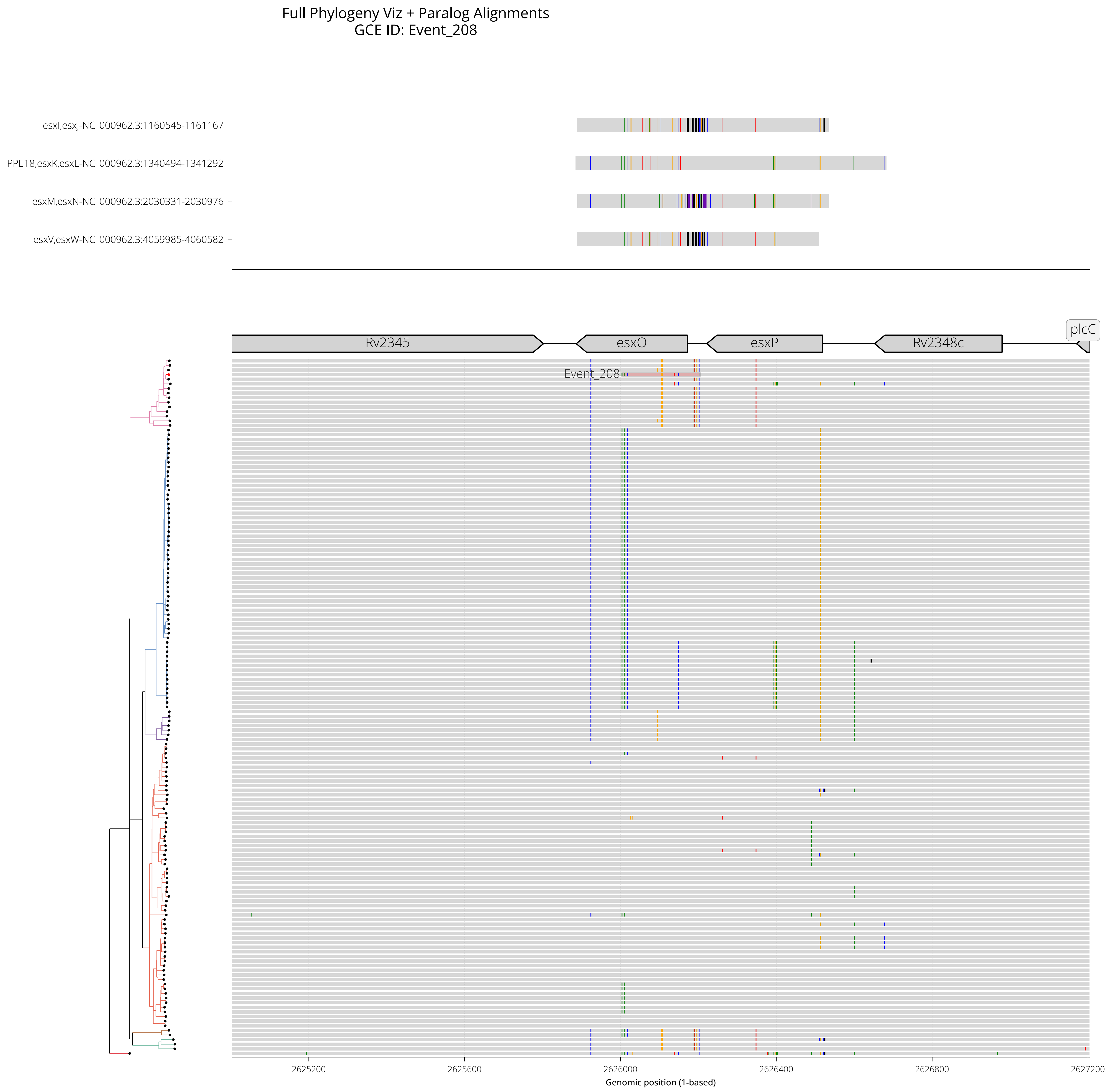

Full Phylogeny Viz + Paralog Alignments
GCE ID: Event_208
esxI,esxJ-NC_000962.3:1160545-1161167
PPE18,esxK,esxL-NC_000962.3:1340494-1341292
esxM,esxN-NC_000962.3:2030331-2030976
esxV,esxW-NC_000962.3:4059985-4060582
Rv2345
esxO
esxP
Rv2348c
plcC
Event_208
2625200
2625600
2626000
2626400
2626800
2627200
Genomic position (1-based)

Individual GCE + Known Paralogs
GCE ID: Event_209

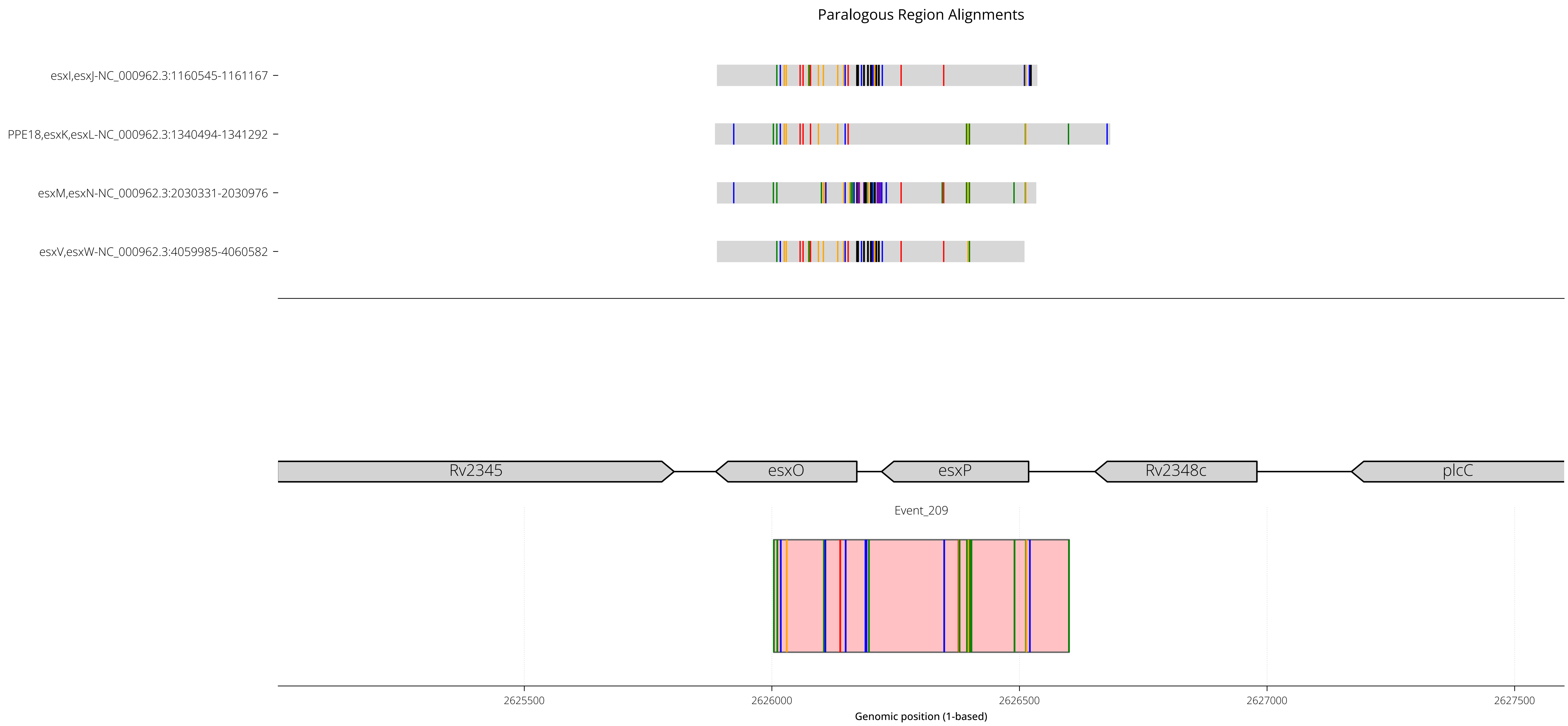

Circos Viz - GCE to Known Paralogs Locations
GCE ID: Event_209

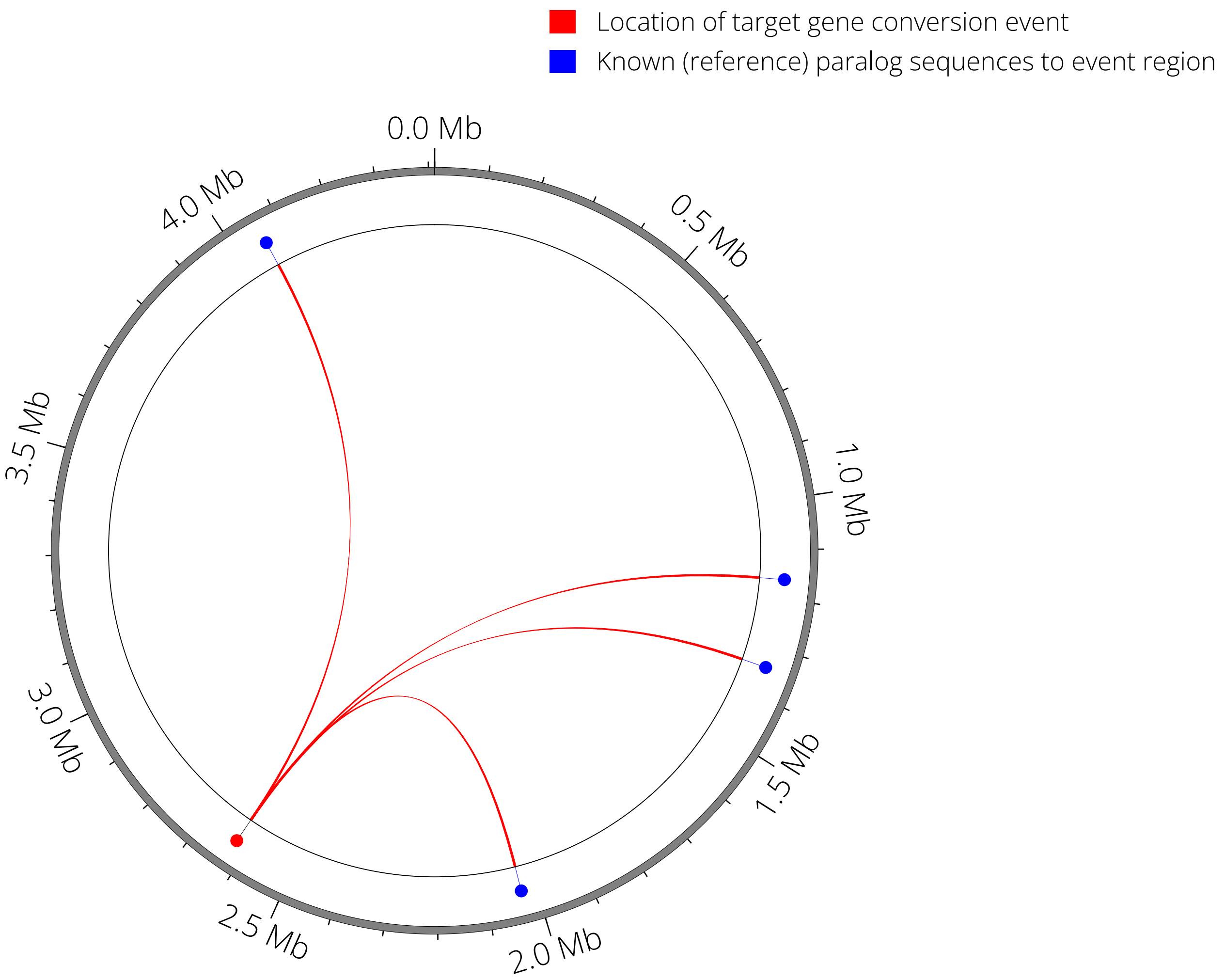

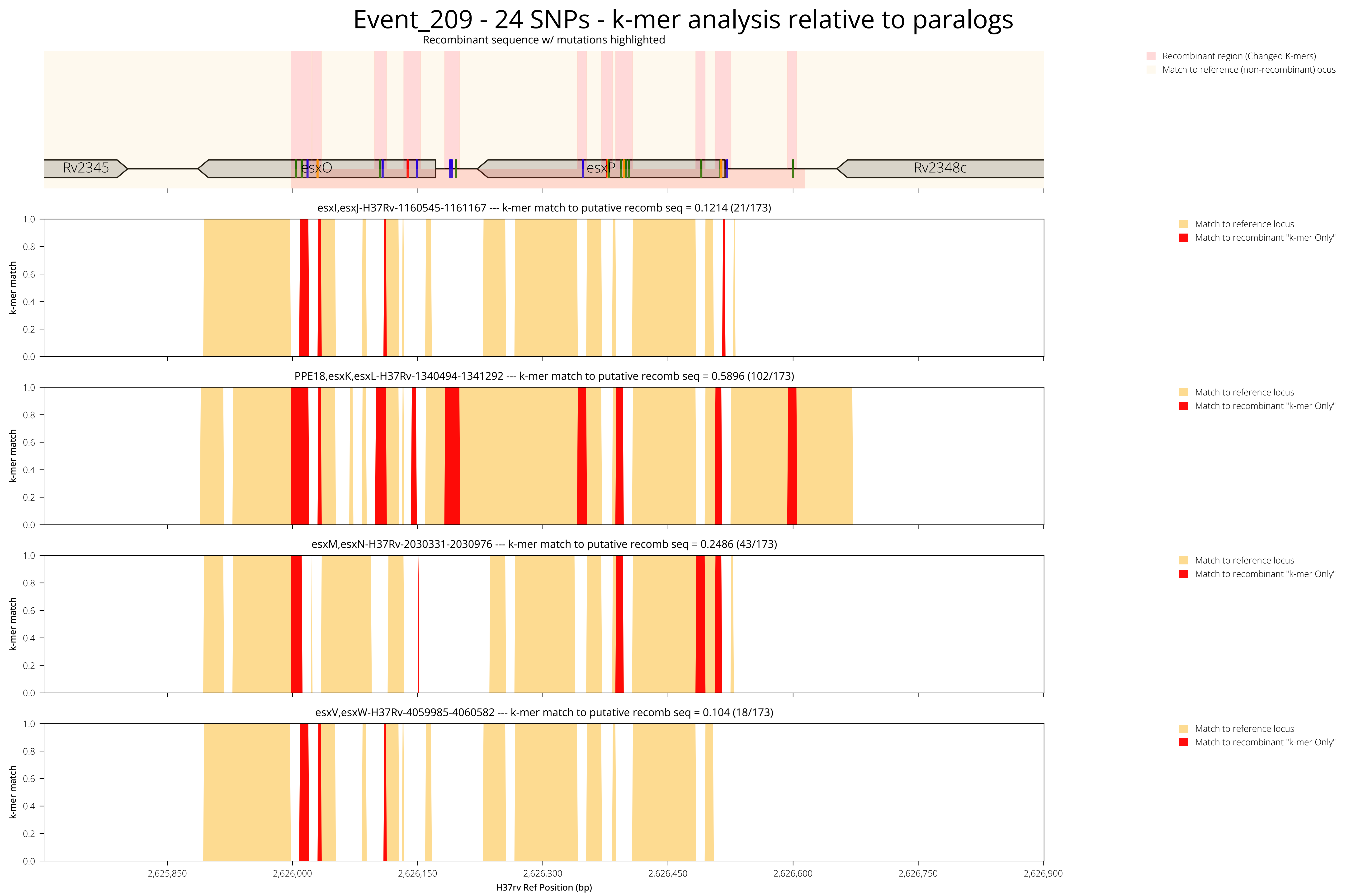

Event_209 - 24 SNPs - k-mer analysis relative to paralogs
Recombinant sequence w/ mutations highlighted
Recombinant region (Changed K-mers)
Match to reference (non-recombinant)locus
Rv2345
esxO
esxP
Rv2348c
esxI,esxJ-H37Rv-1160545-1161167 --- k-mer match to putative recomb seq = 0.1214 (21/173)
PPE18,esxK,esxL-H37Rv-1340494-1341292 --- k-mer match to putative recomb seq = 0.5896 (102/173)
esxM,esxN-H37Rv-2030331-2030976 --- k-mer match to putative recomb seq = 0.2486 (43/173)
esxV,esxW-H37Rv-4059985-4060582 --- k-mer match to putative recomb seq = 0.104 (18/173)
Match to reference locus
Match to recombinant "k-mer Only"
k-mer match
1.0
0.8
0.6
0.4
0.2
0.0
2,625,850
2,626,000
2,626,150
2,626,300
2,626,450
2,626,600
2,626,750
2,626,900
H37rv Ref Position (bp)

Sub-phylogeny Viz + Paralog Alignments
GCE ID: Event_209

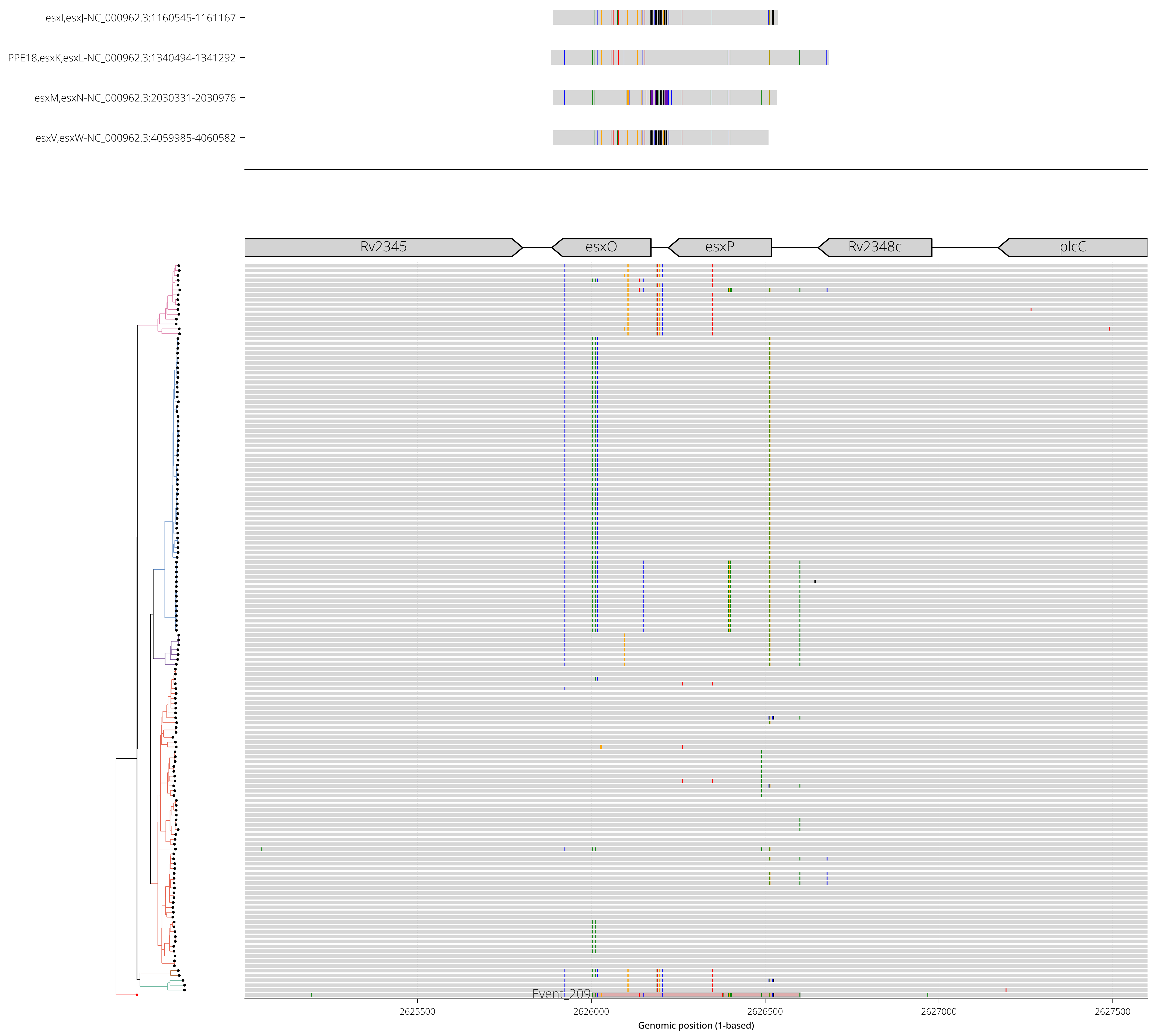

Full Phylogeny Viz + Paralog Alignments
GCE ID: Event_209

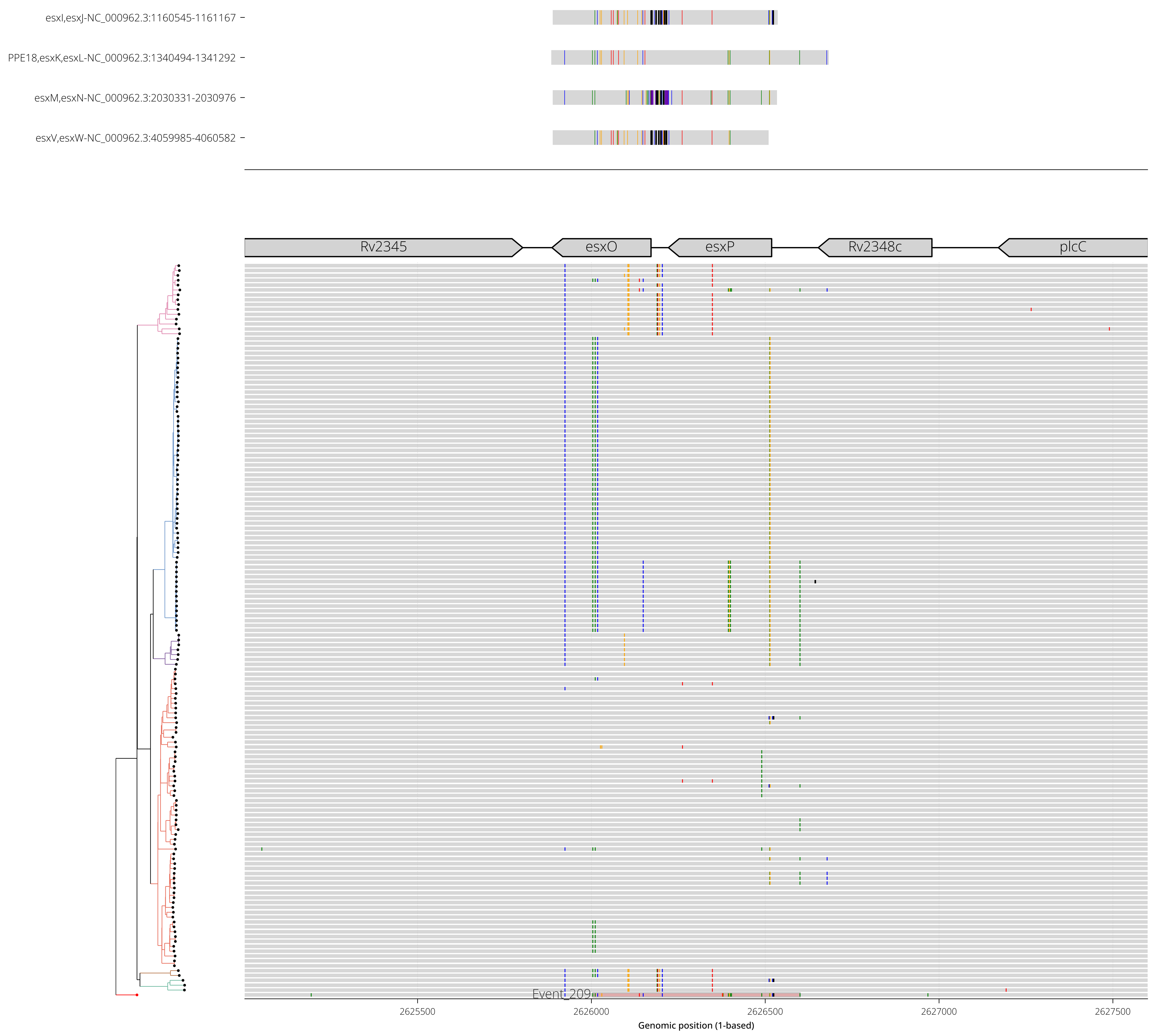

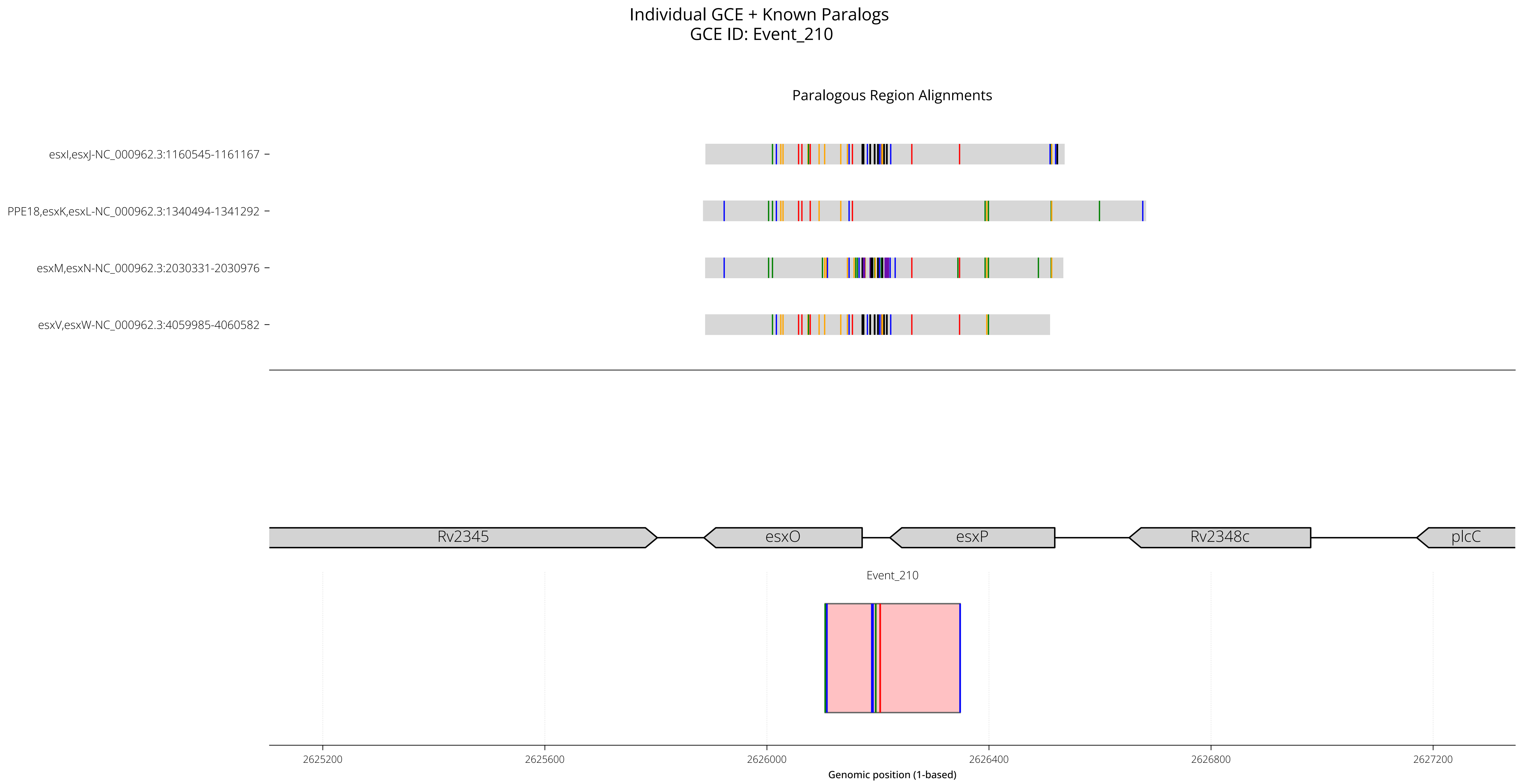

Individual GCE + Known Paralogs
GCE ID: Event_210
Paralogous Region Alignments
esxI,esxJ-NC_000962.3:1160545-1161167
PPE18,esxK,esxL-NC_000962.3:1340494-1341292
esxM,esxN-NC_000962.3:2030331-2030976
esxV,esxW-NC_000962.3:4059985-4060582
Rv2345
esxO
esxP
Rv2348c
plcC
Event_210
2625200
2625600
2626000
2626400
2626800
2627200
Genomic position (1-based)

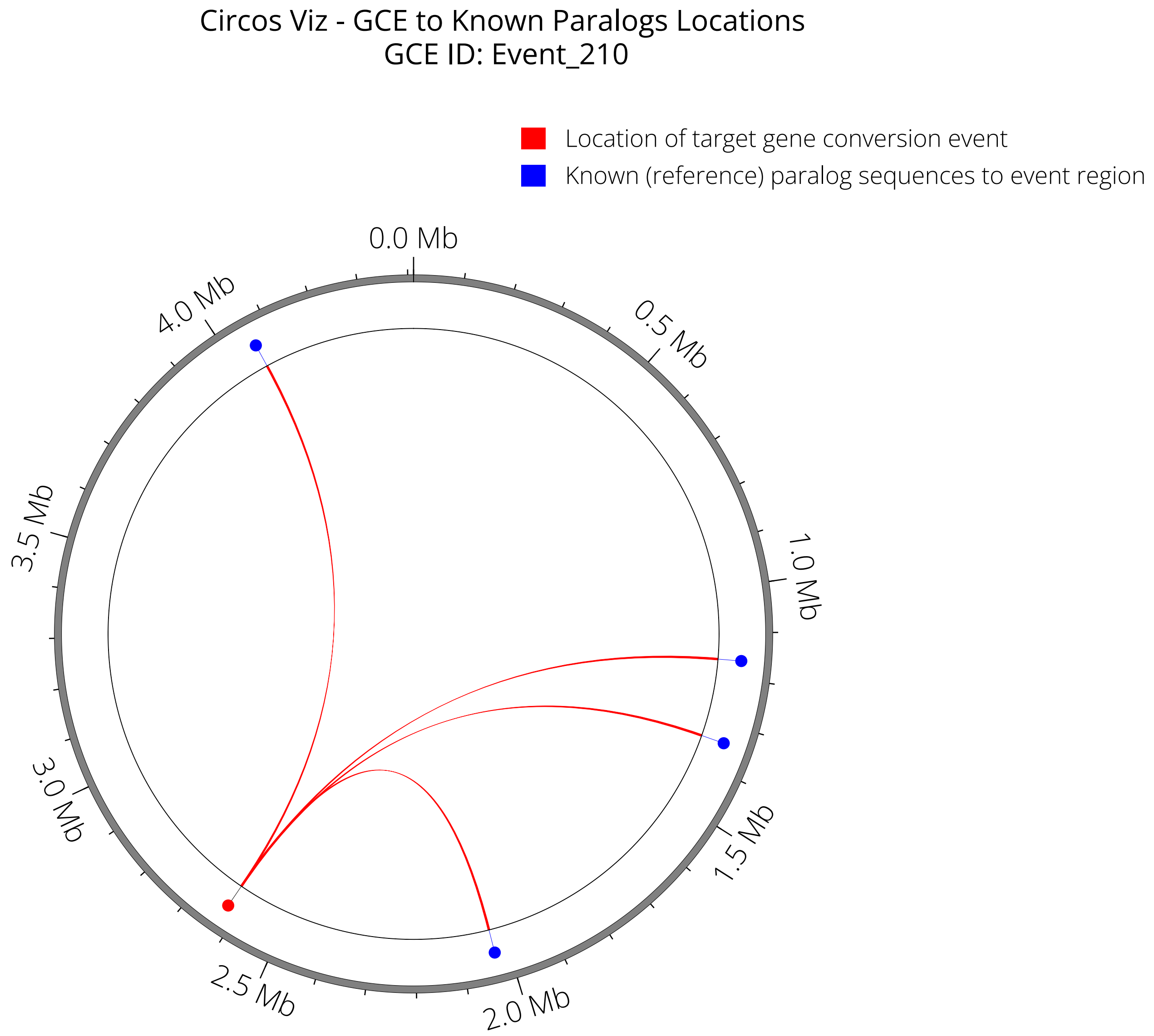
Circos Viz - GCE to Known Paralogs Locations
GCE ID: Event_210
Location of target gene conversion event
Known (reference) paralog sequences to event region
0.0 Mb
0.5 Mb
1.0 Mb
1.5 Mb
2.0 Mb
2.5 Mb
3.0 Mb
3.5 Mb
4.0 Mb

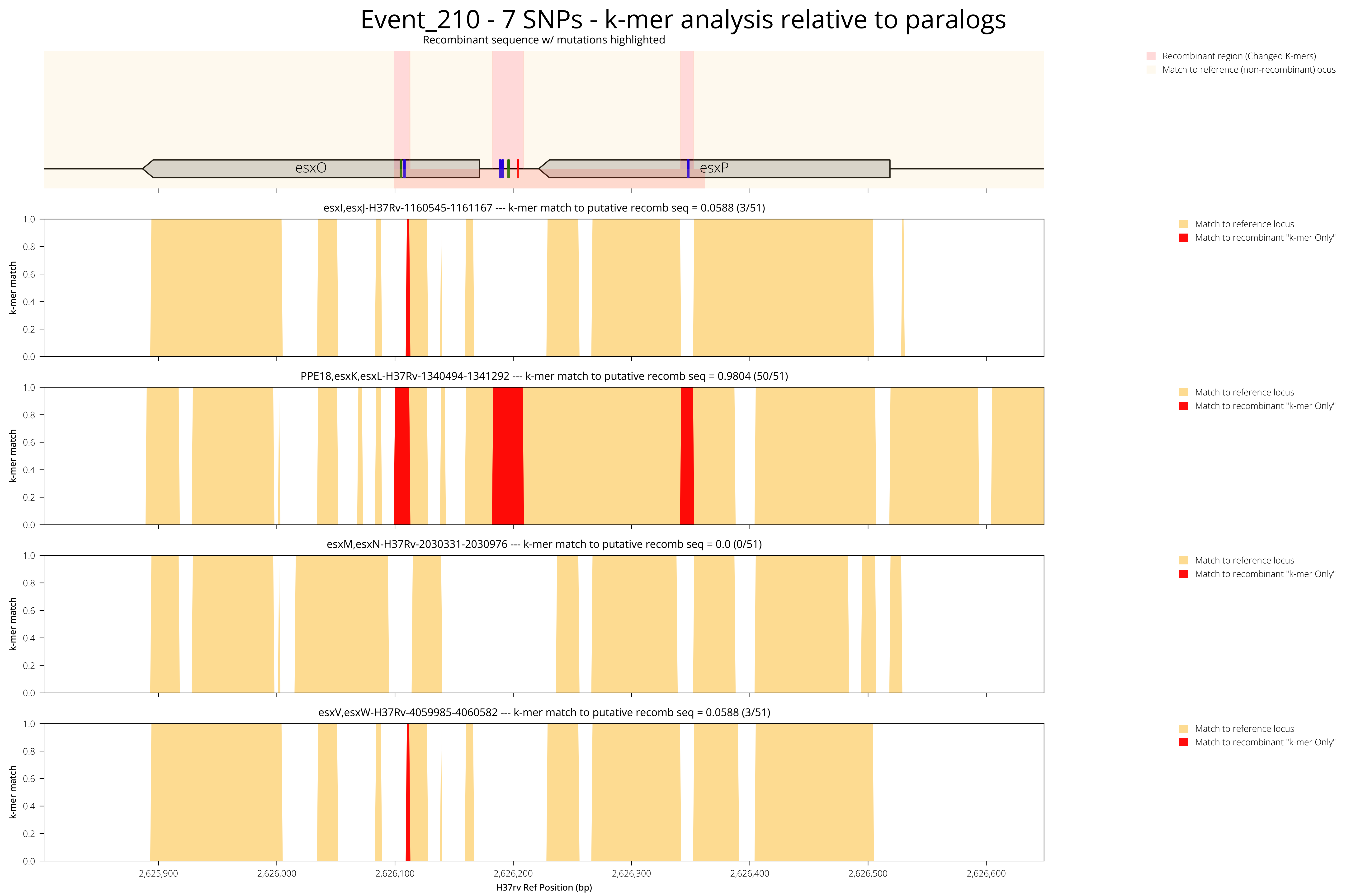
Event_210 - 7 SNPs - k-mer analysis relative to paralogs
Recombinant sequence w/ mutations highlighted
Recombinant region (Changed K-mers)
Match to reference (non-recombinant)locus
esxO
esxP
esxI,esxJ-H37Rv-1160545-1161167 --- k-mer match to putative recomb seq = 0.0588 (3/51)
PPE18,esxK,esxL-H37Rv-1340494-1341292 --- k-mer match to putative recomb seq = 0.9804 (50/51)
esxM,esxN-H37Rv-2030331-2030976 --- k-mer match to putative recomb seq = 0.0 (0/51)
esxV,esxW-H37Rv-4059985-4060582 --- k-mer match to putative recomb seq = 0.0588 (3/51)
Match to reference locus
Match to recombinant "k-mer Only"
k-mer match
1.0
0.8
0.6
0.4
0.2
0.0
2,625,900
2,626,000
2,626,100
2,626,200
2,626,300
2,626,400
2,626,500
2,626,600
H37rv Ref Position (bp)

Sub-phylogeny Viz + Paralog Alignments
GCE ID: Event_210

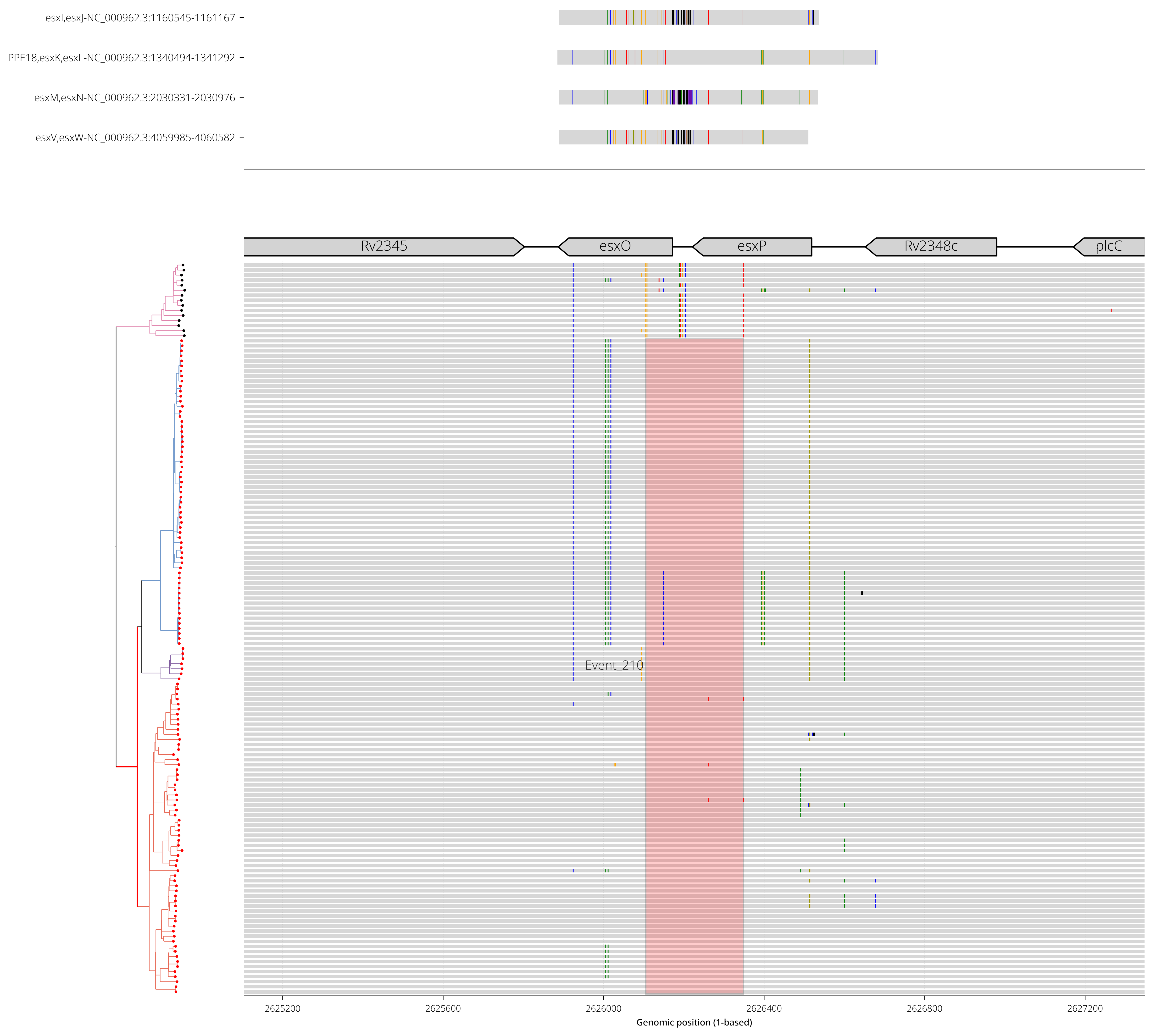

Full Phylogeny Viz + Paralog Alignments
GCE ID: Event_210

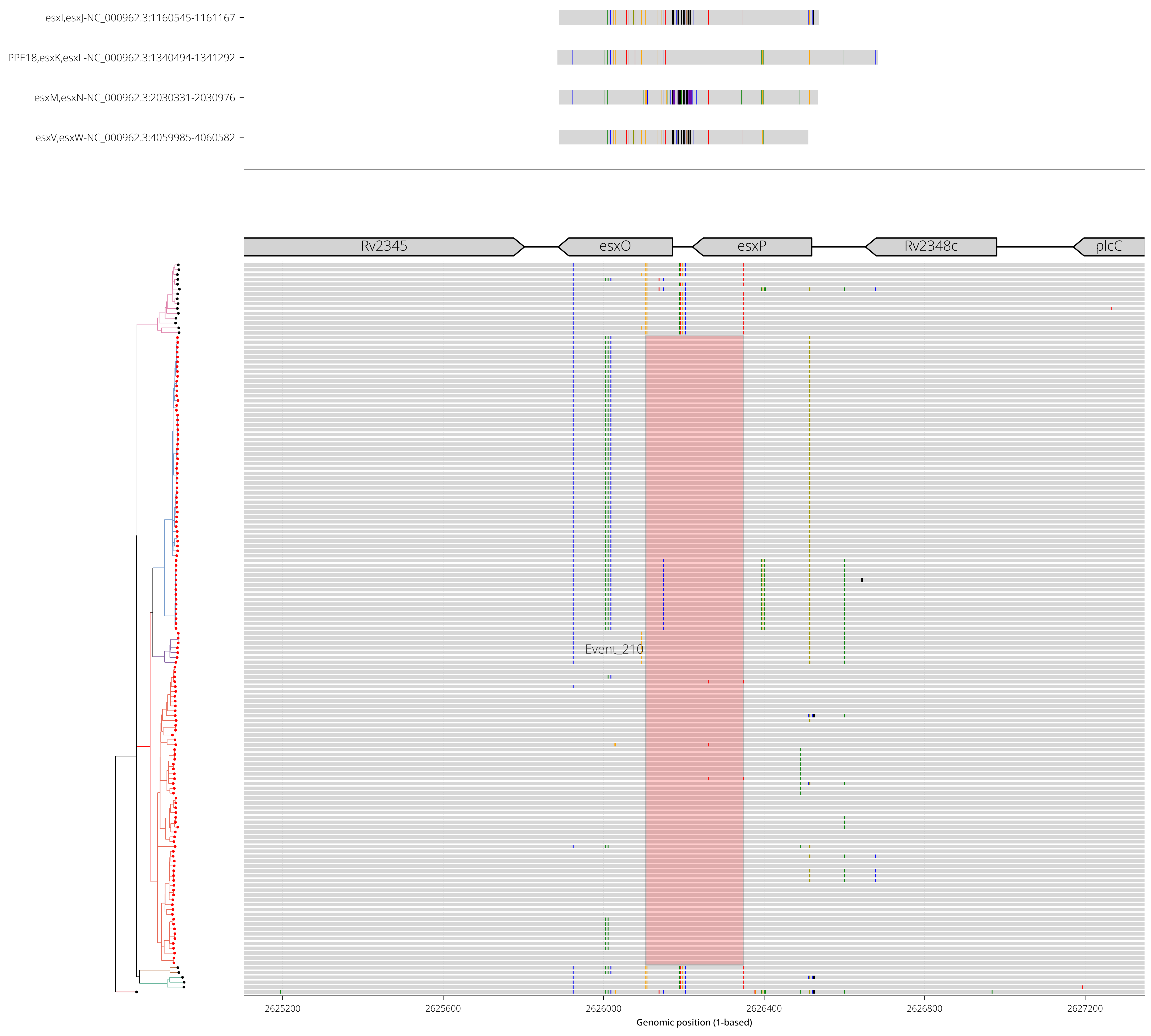

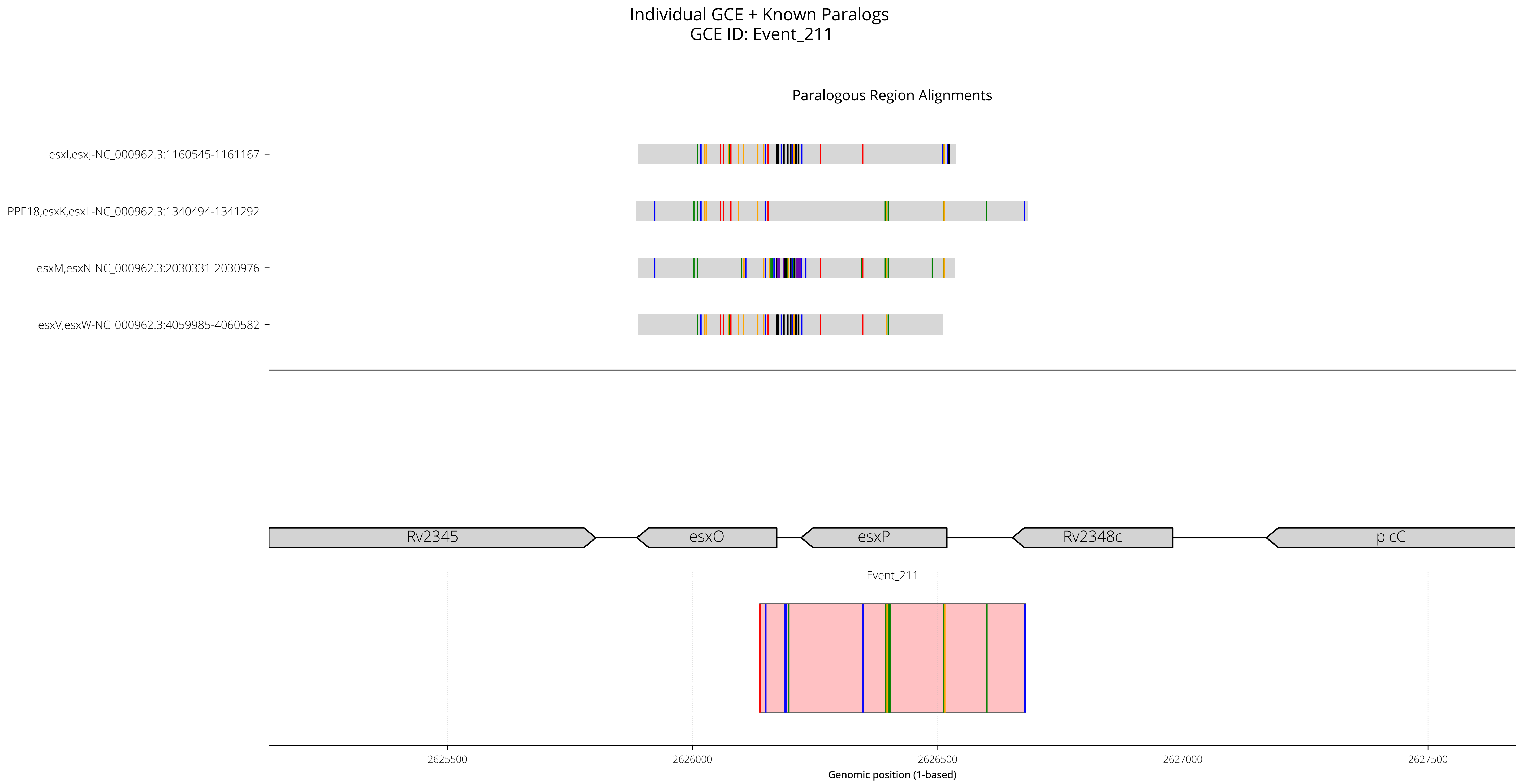

Individual GCE + Known Paralogs
GCE ID: Event_211
Paralogous Region Alignments
esxI,esxJ-NC_000962.3:1160545-1161167
PPE18,esxK,esxL-NC_000962.3:1340494-1341292
esxM,esxN-NC_000962.3:2030331-2030976
esxV,esxW-NC_000962.3:4059985-4060582
Rv2345
esxO
esxP
Rv2348c
plcC
Event_211
2625500
2626000
2626500
2627000
2627500
Genomic position (1-based)

Circos Viz - GCE to Known Paralogs Locations
GCE ID: Event_211

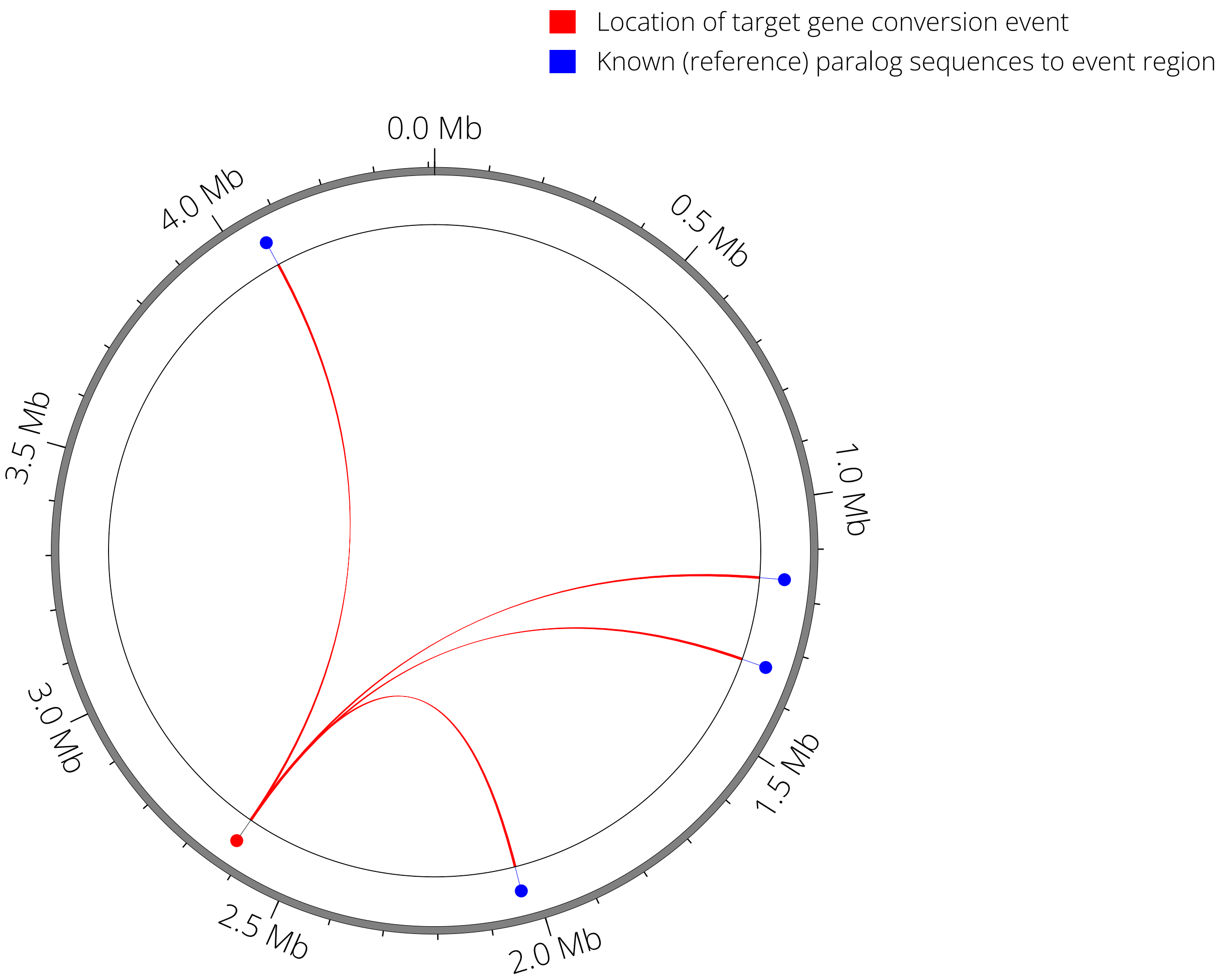

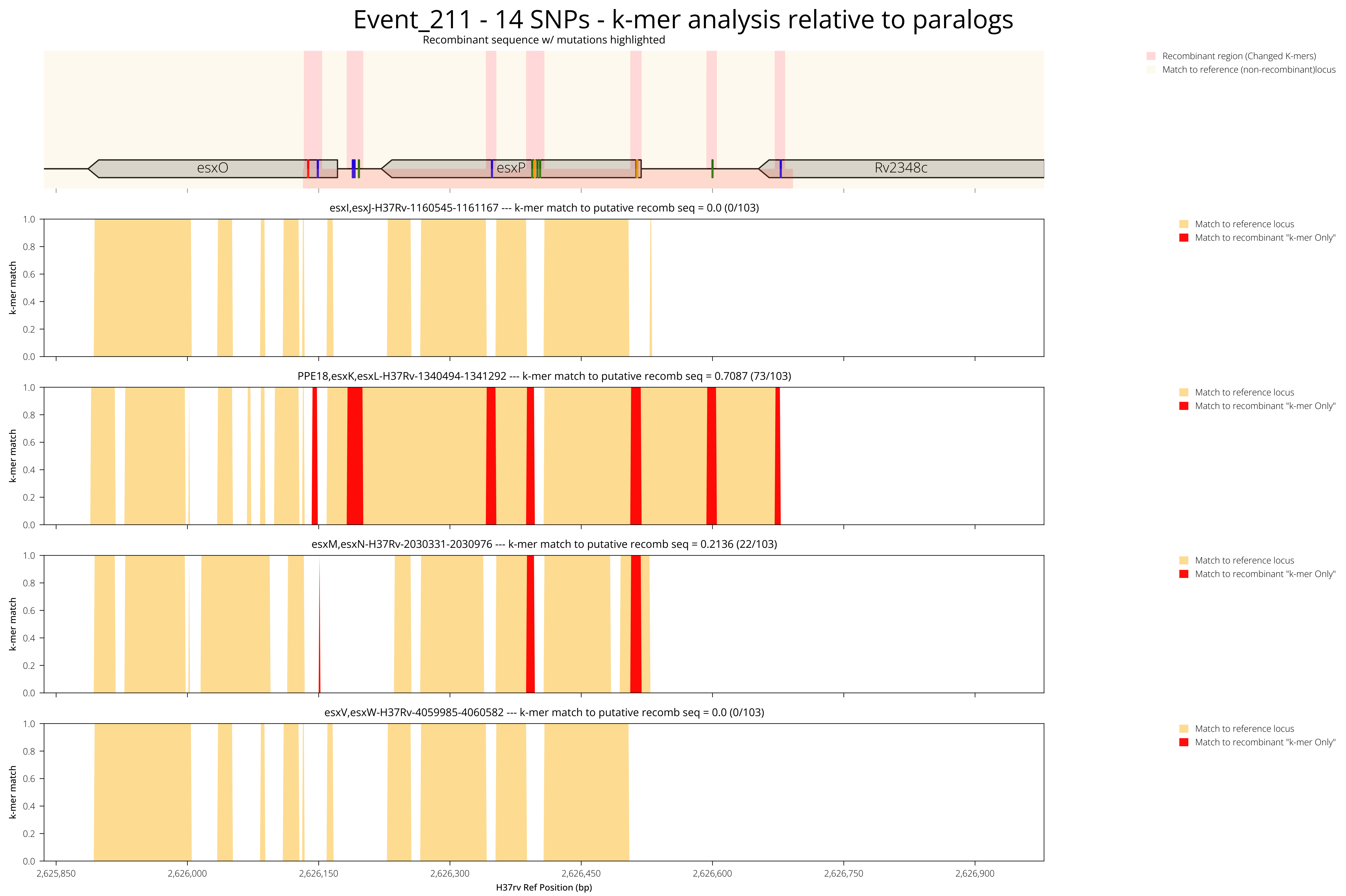
Event_211 - 14 SNPs - k-mer analysis relative to paralogs
Recombinant sequence w/ mutations highlighted
Recombinant region (Changed K-mers)
Match to reference (non-recombinant)locus
esxO
esxP
Rv2348c
esxI,esxJ-H37Rv-1160545-1161167 --- k-mer match to putative recomb seq = 0.0 (0/103)
PPE18,esxK,esxL-H37Rv-1340494-1341292 --- k-mer match to putative recomb seq = 0.7087 (73/103)
esxM,esxN-H37Rv-2030331-2030976 --- k-mer match to putative recomb seq = 0.2136 (22/103)
esxV,esxW-H37Rv-4059985-4060582 --- k-mer match to putative recomb seq = 0.0 (0/103)
Match to reference locus
Match to recombinant "k-mer Only"
k-mer match
1.0
0.8
0.6
0.4
0.2
0.0
2,625,850
2,626,000
2,626,150
2,626,300
2,626,450
2,626,600
2,626,750
2,626,900
H37rv Ref Position (bp)

# Sub-phylogeny Viz + Paralog Alignments
GCE ID: Event_211

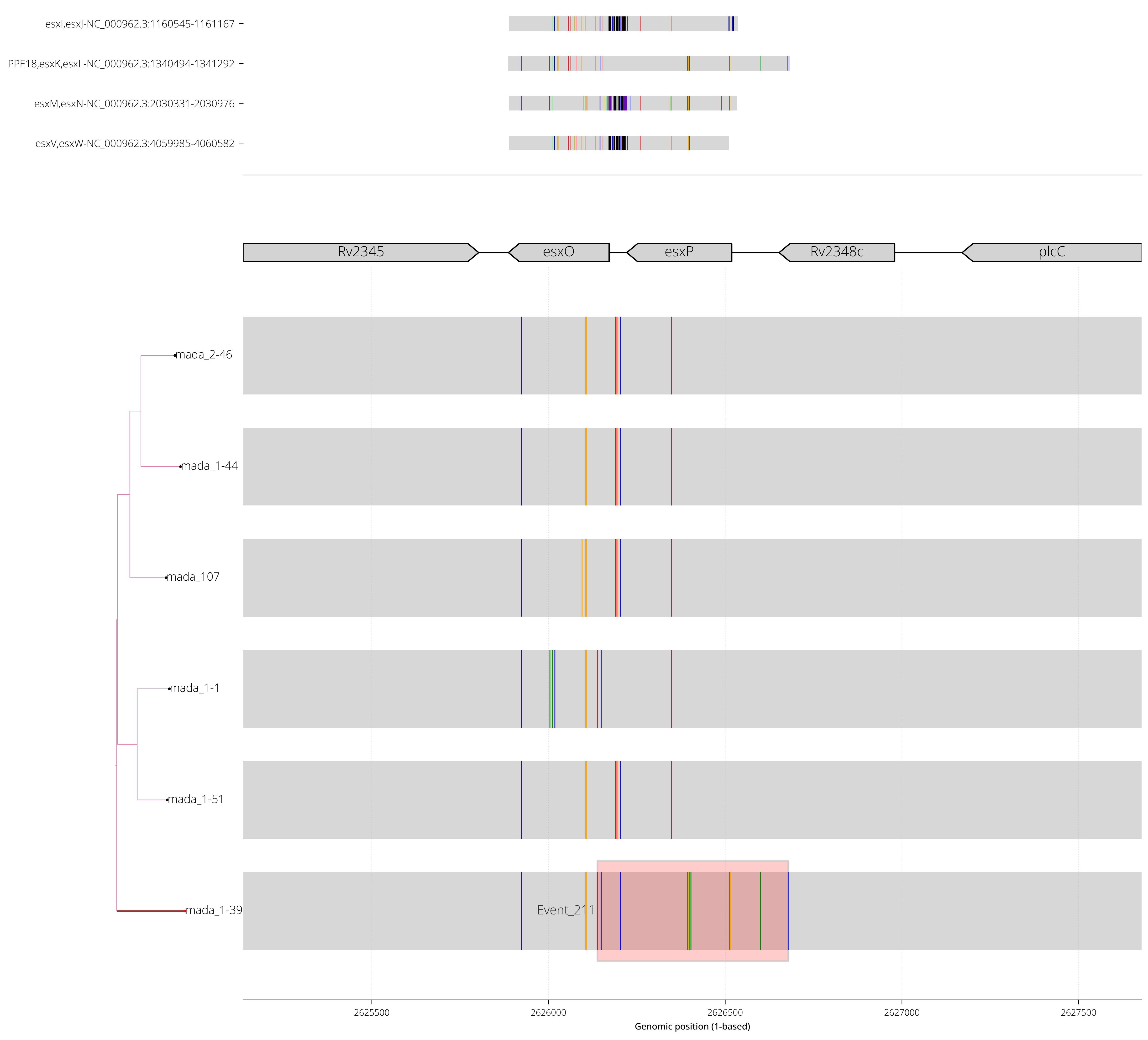

Full Phylogeny Viz + Paralog Alignments
GCE ID: Event_211

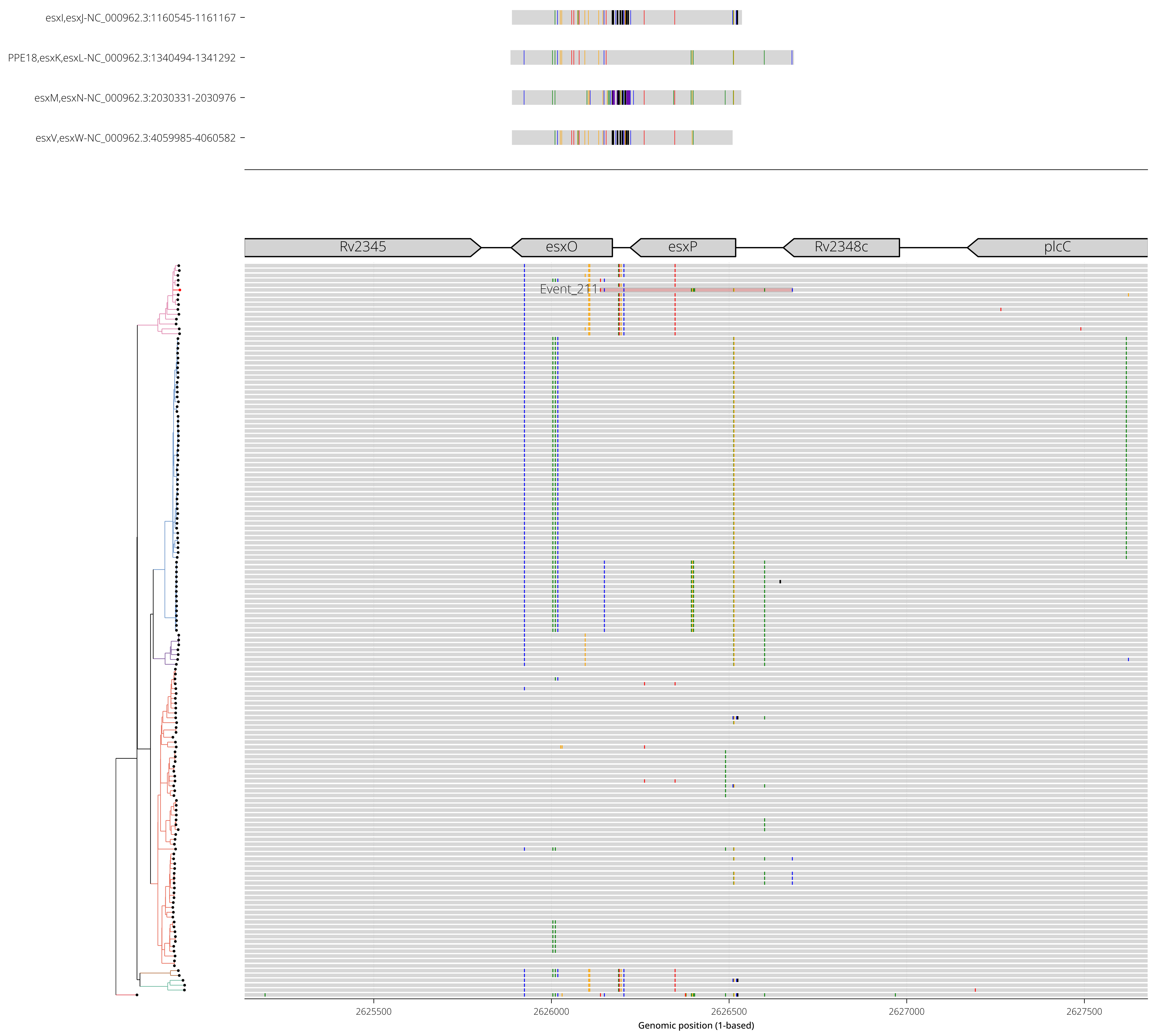

Individual GCE + Known Paralogs
GCE ID: Event_212

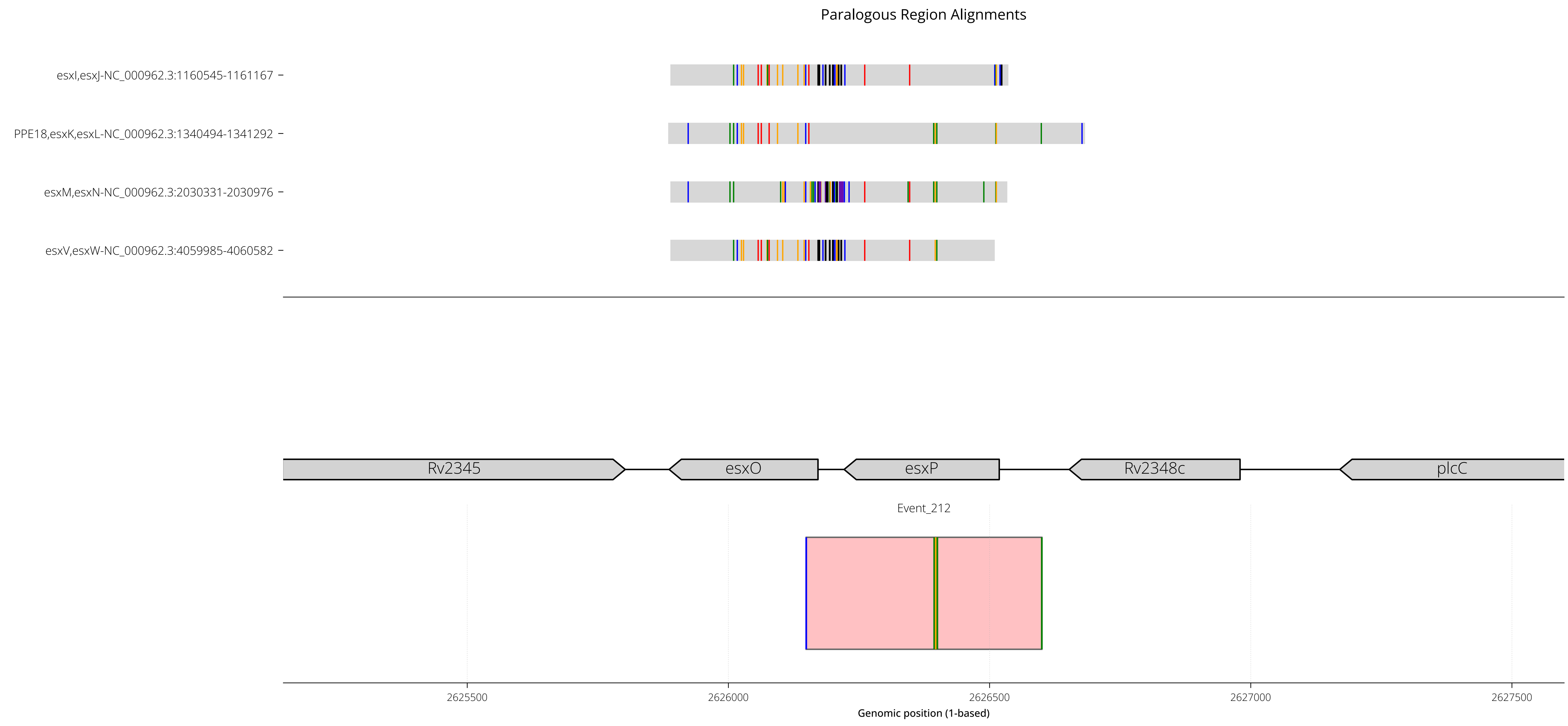

Circos Viz - GCE to Known Paralogs Locations
GCE ID: Event_212

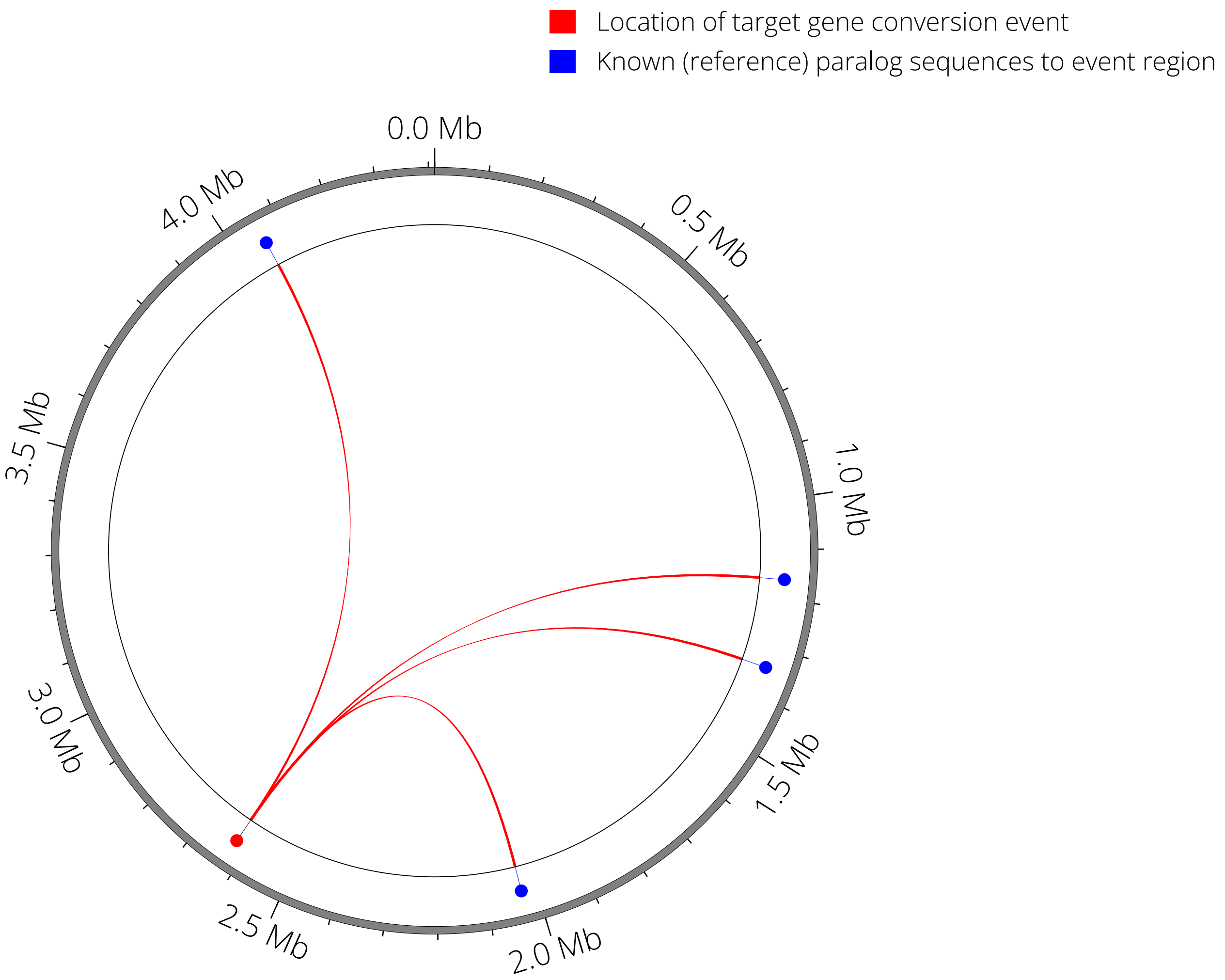

# Event_212 - 5 SNPs - k-mer analysis relative to paralogs

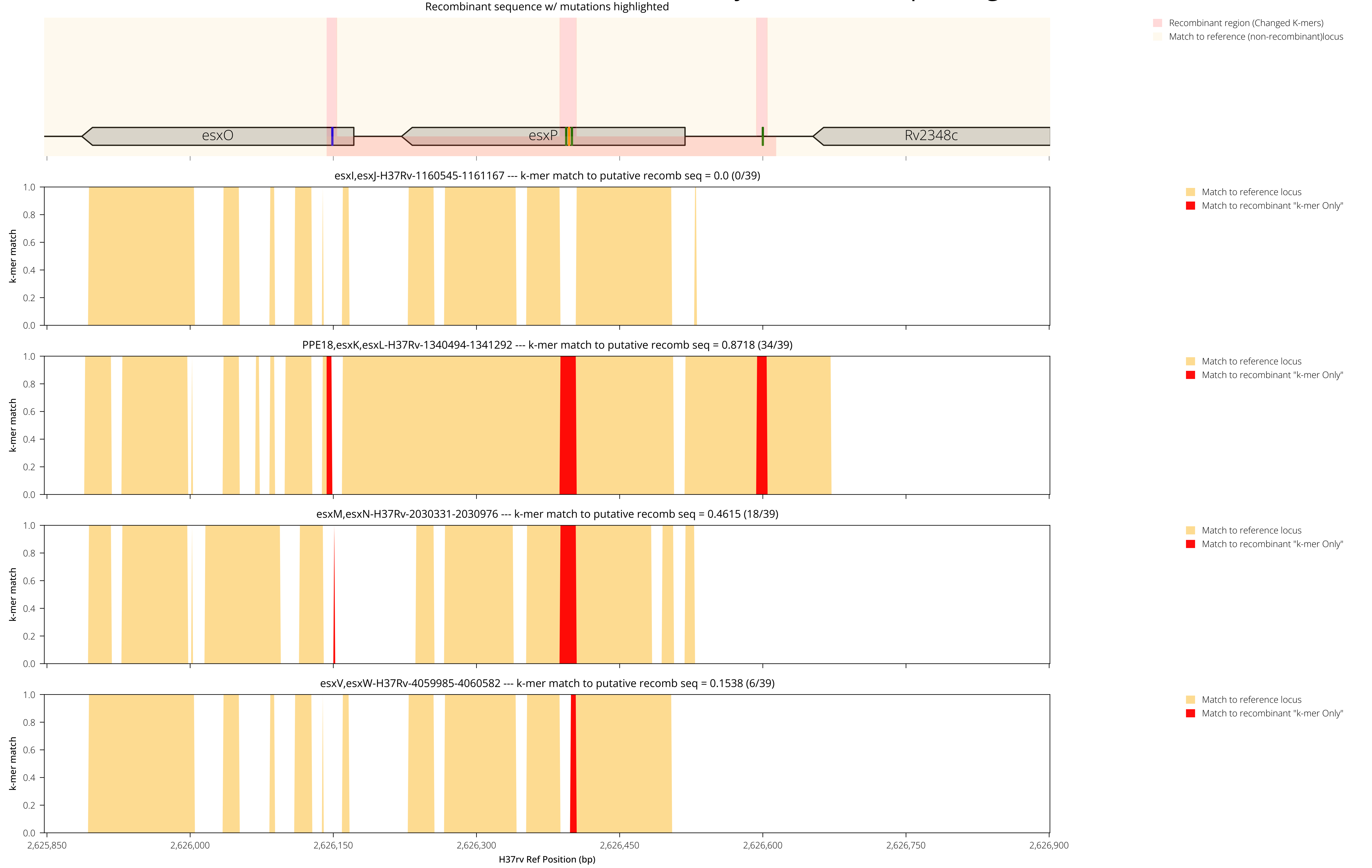

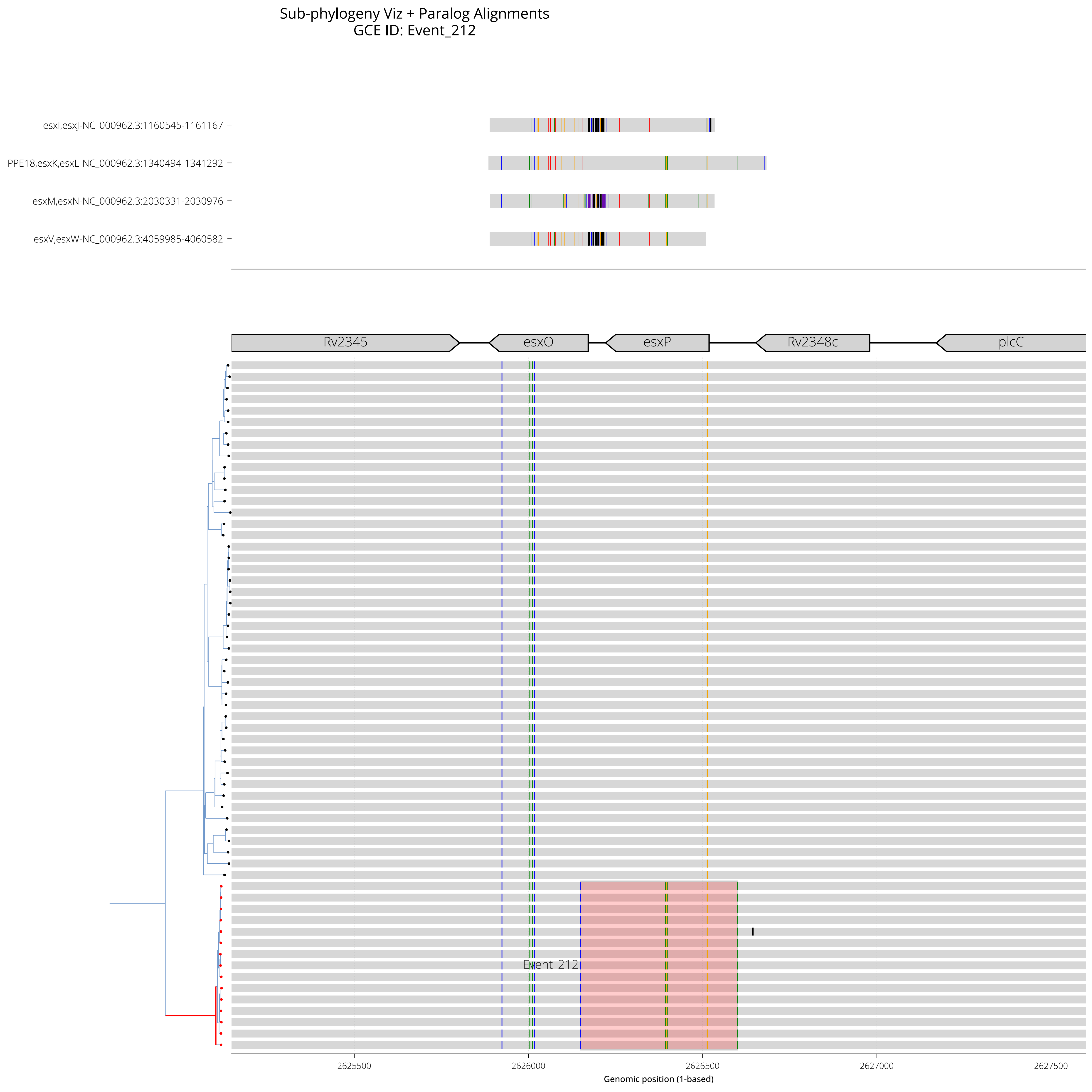
Sub-phylogeny Viz + Paralog Alignments
GCE ID: Event_212
esxI,esxJ-NC_000962.3:1160545-1161167
PPE18,esxK,esxL-NC_000962.3:1340494-1341292
esxM,esxN-NC_000962.3:2030331-2030976
esxV,esxW-NC_000962.3:4059985-4060582
Rv2345
esxO
esxP
Rv2348c
plcC
Event_212
2625500
2626000
2626500
2627000
2627500
Genomic position (1-based)

Full Phylogeny Viz + Paralog Alignments
GCE ID: Event_212

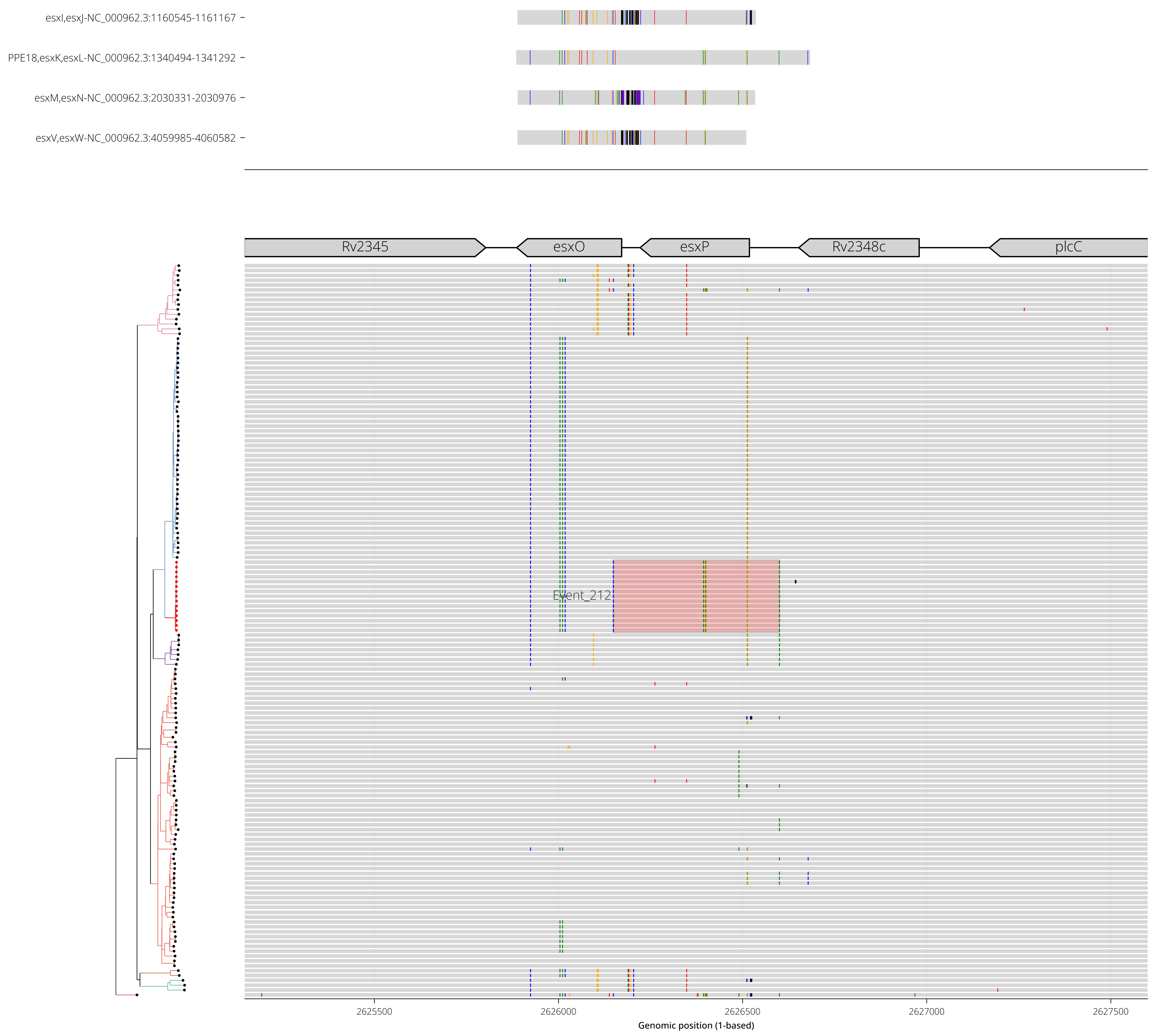

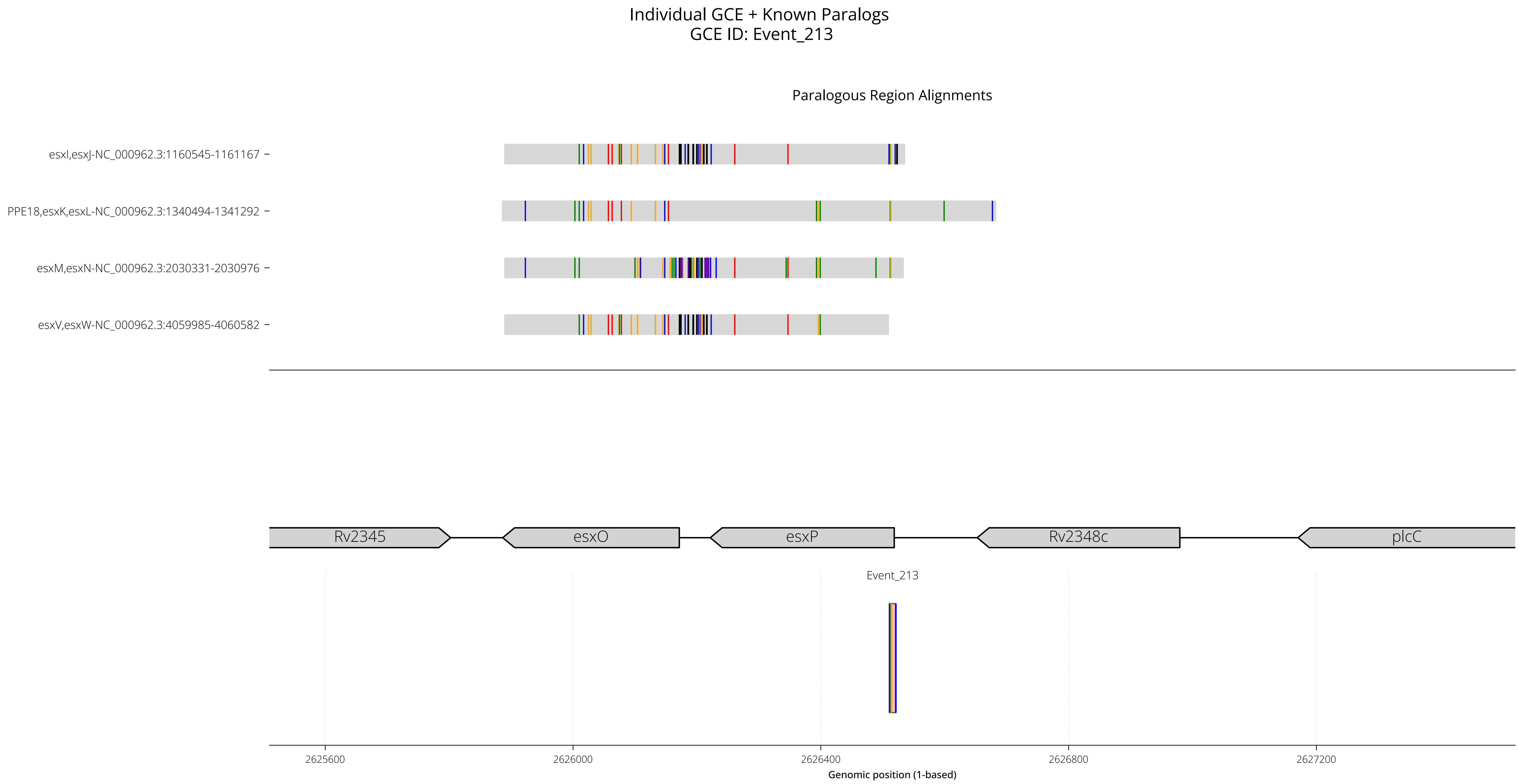
Individual GCE + Known Paralogs
GCE ID: Event_213
Paralogous Region Alignments
esxI,esxJ-NC_000962.3:1160545-1161167
PPE18,esxK,esxL-NC_000962.3:1340494-1341292
esxM,esxN-NC_000962.3:2030331-2030976
esxV,esxW-NC_000962.3:4059985-4060582
Rv2345
esxO
esxP
Rv2348c
plcC
Event_213
2625600
2626000
2626400
2626800
2627200
Genomic position (1-based)

Circos Viz - GCE to Known Paralogs Locations
GCE ID: Event_213

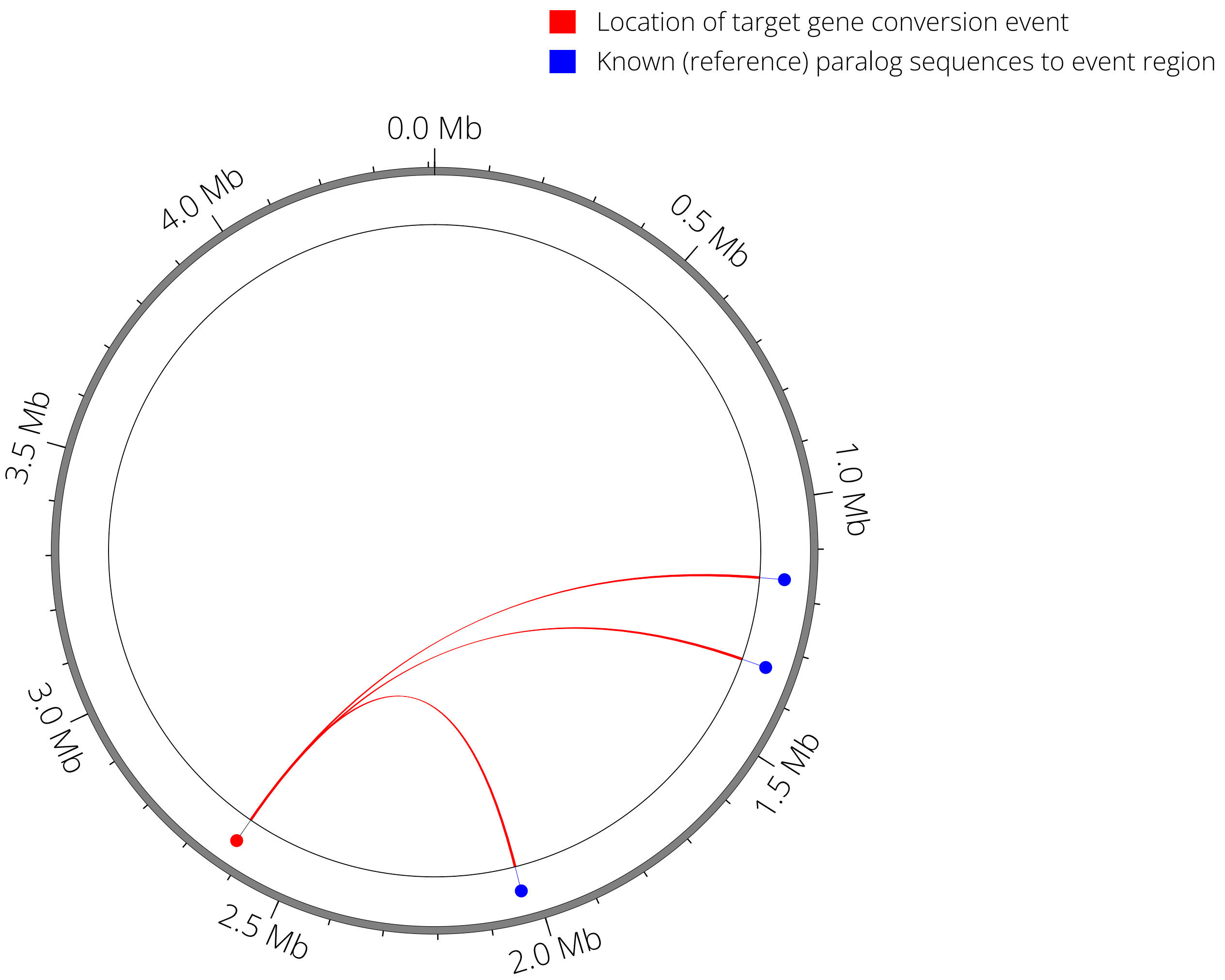

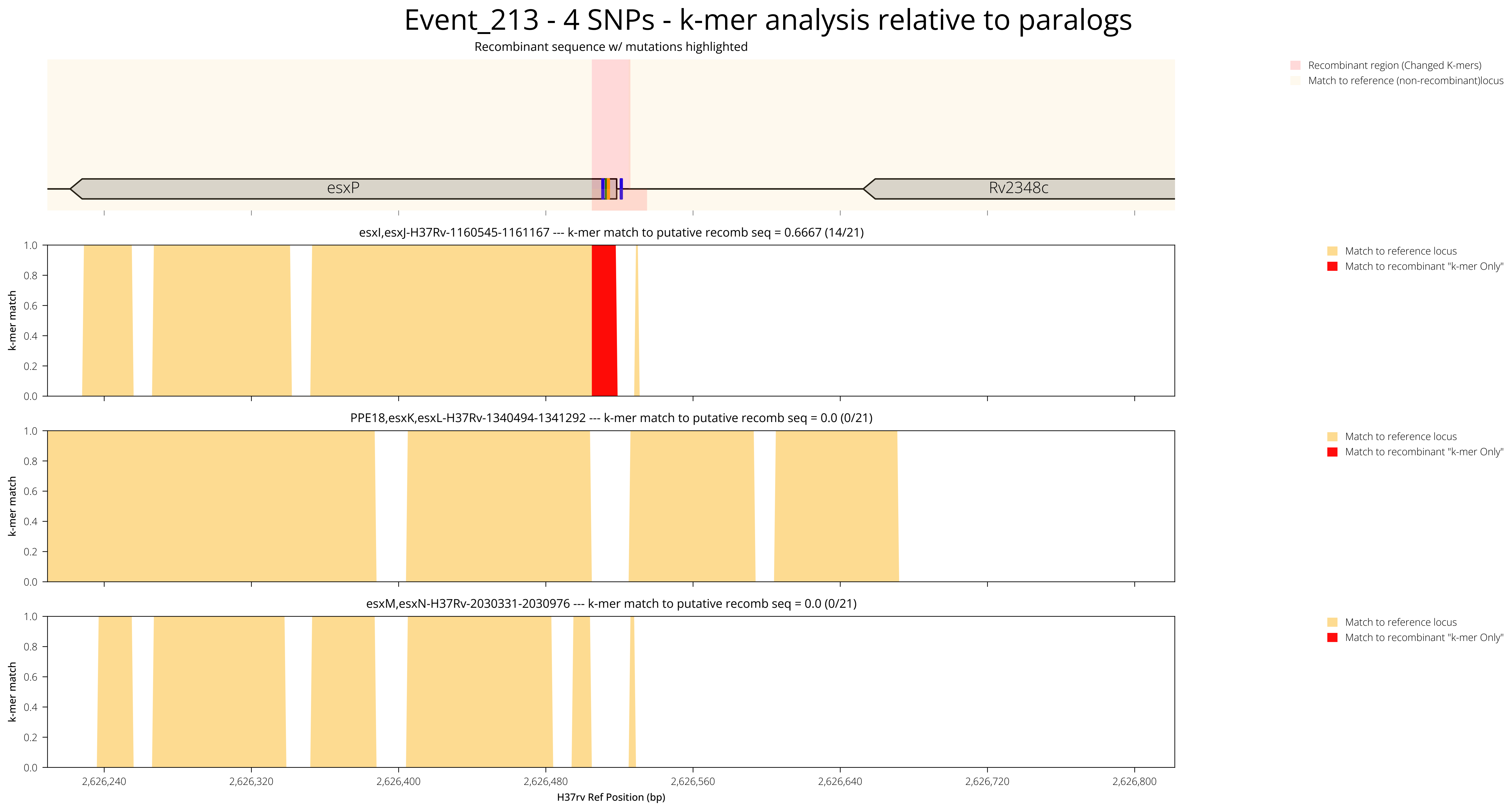
Event_213 - 4 SNPs - k-mer analysis relative to paralogs
Recombinant sequence w/ mutations highlighted
Recombinant region (Changed K-mers)
Match to reference (non-recombinant)locus
esxP
Rv2348c
esxI,esxJ-H37Rv-1160545-1161167 --- k-mer match to putative recomb seq = 0.6667 (14/21)
PPE18,esxK,esxL-H37Rv-1340494-1341292 --- k-mer match to putative recomb seq = 0.0 (0/21)
esxM,esxN-H37Rv-2030331-2030976 --- k-mer match to putative recomb seq = 0.0 (0/21)
Match to reference locus
Match to recombinant "k-mer Only"
k-mer match
1.0
0.8
0.6
0.4
0.2
0.0
2,626,240
2,626,320
2,626,400
2,626,480
2,626,560
2,626,640
2,626,720
2,626,800
H37rv Ref Position (bp)

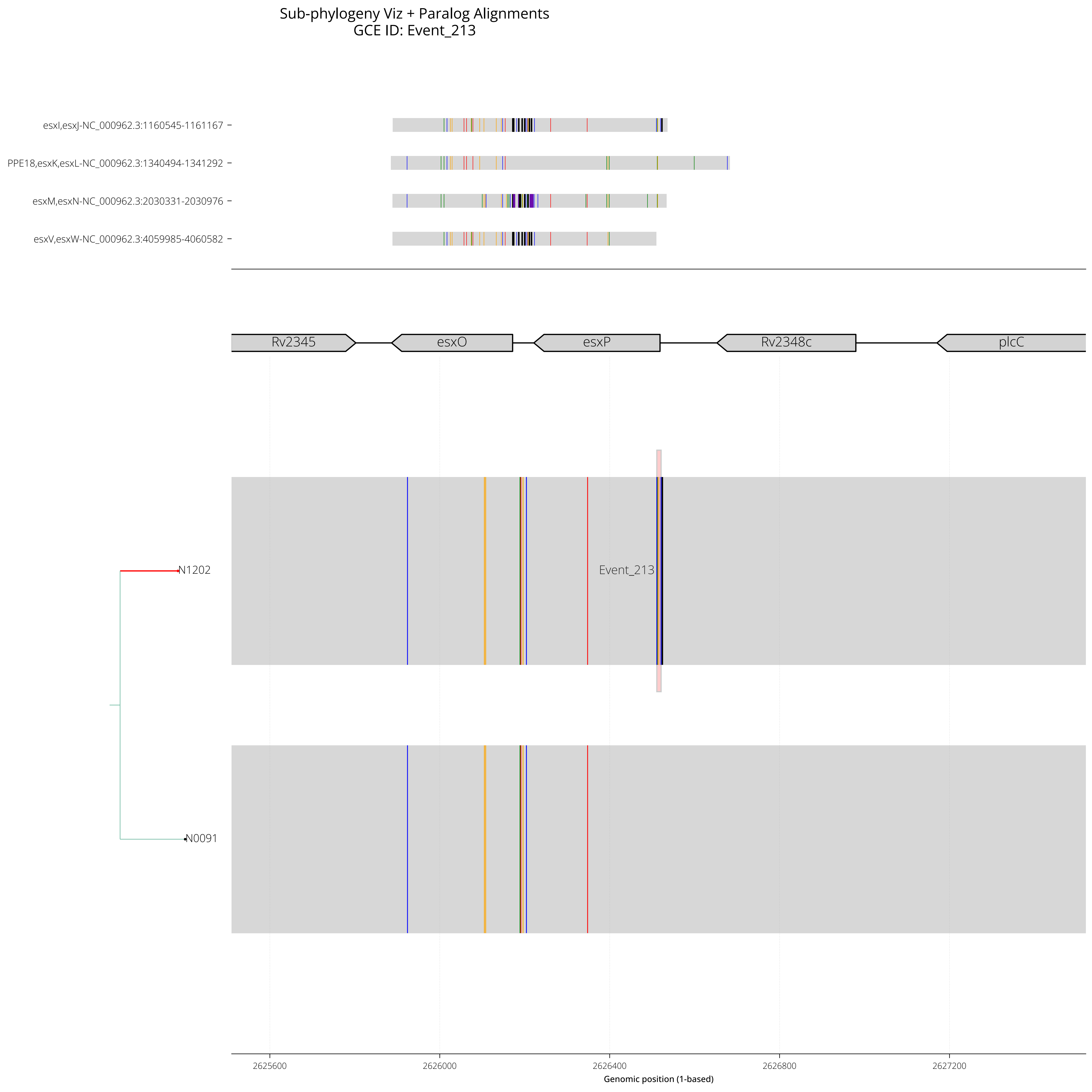

Sub-phylogeny Viz + Paralog Alignments
GCE ID: Event_213
esxI,esxJ-NC_000962.3:1160545-1161167
PPE18,esxK,esxL-NC_000962.3:1340494-1341292
esxM,esxN-NC_000962.3:2030331-2030976
esxV,esxW-NC_000962.3:4059985-4060582
Rv2345
esxO
esxP
Rv2348c
plcC
N1202
Event_213
N0091
2625600
2626000
2626400
2626800
2627200
Genomic position (1-based)

# Full Phylogeny Viz + Paralog Alignments
GCE ID: Event_213

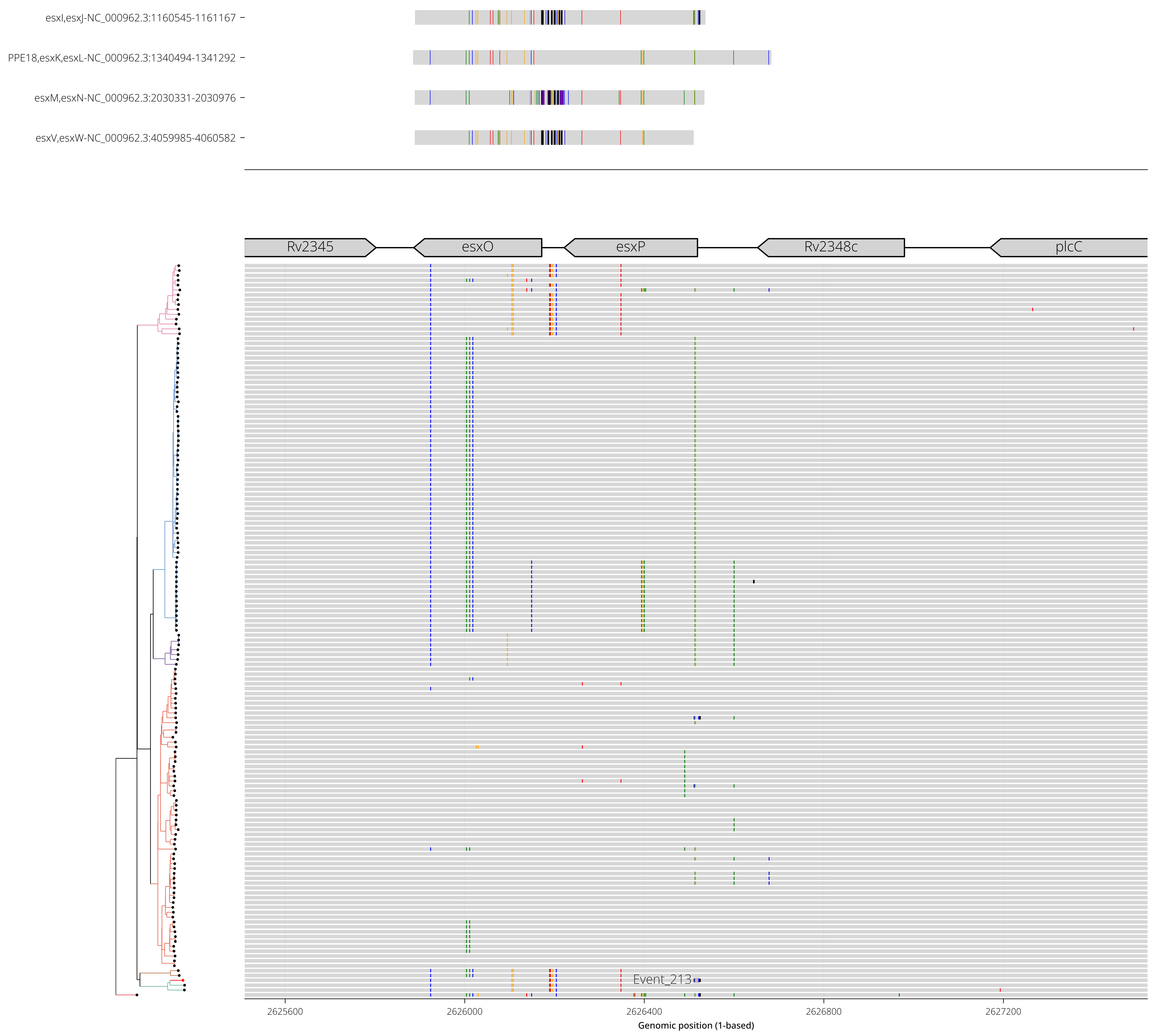

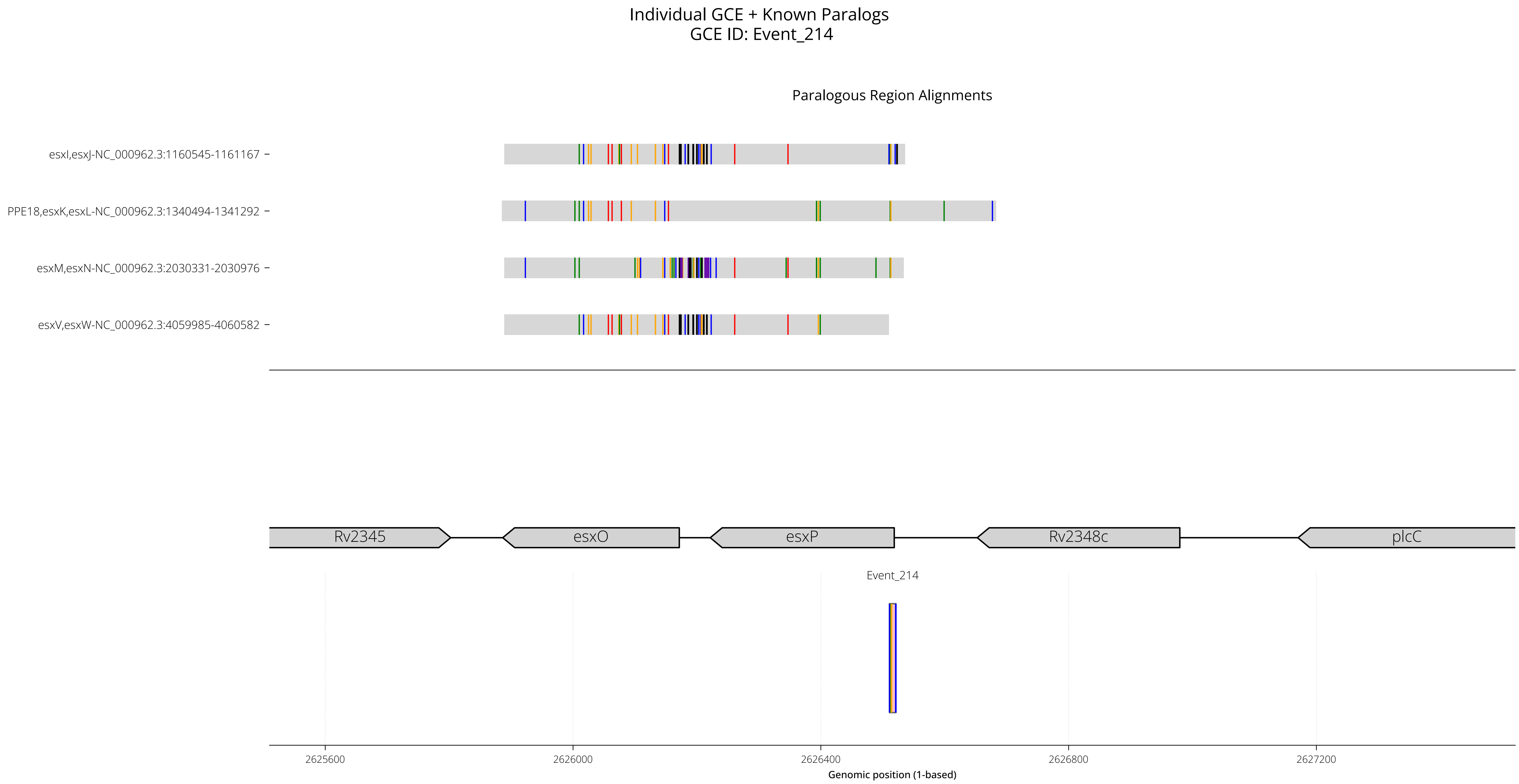

Individual GCE + Known Paralogs
GCE ID: Event_214
Paralogous Region Alignments
esxI,esxJ-NC_000962.3:1160545-1161167
PPE18,esxK,esxL-NC_000962.3:1340494-1341292
esxM,esxN-NC_000962.3:2030331-2030976
esxV,esxW-NC_000962.3:4059985-4060582
Rv2345
esxO
esxP
Rv2348c
plcC
Event_214
2625600
2626000
2626400
2626800
2627200
Genomic position (1-based)

# Circos Viz - GCE to Known Paralogs Locations
GCE ID: Event_214

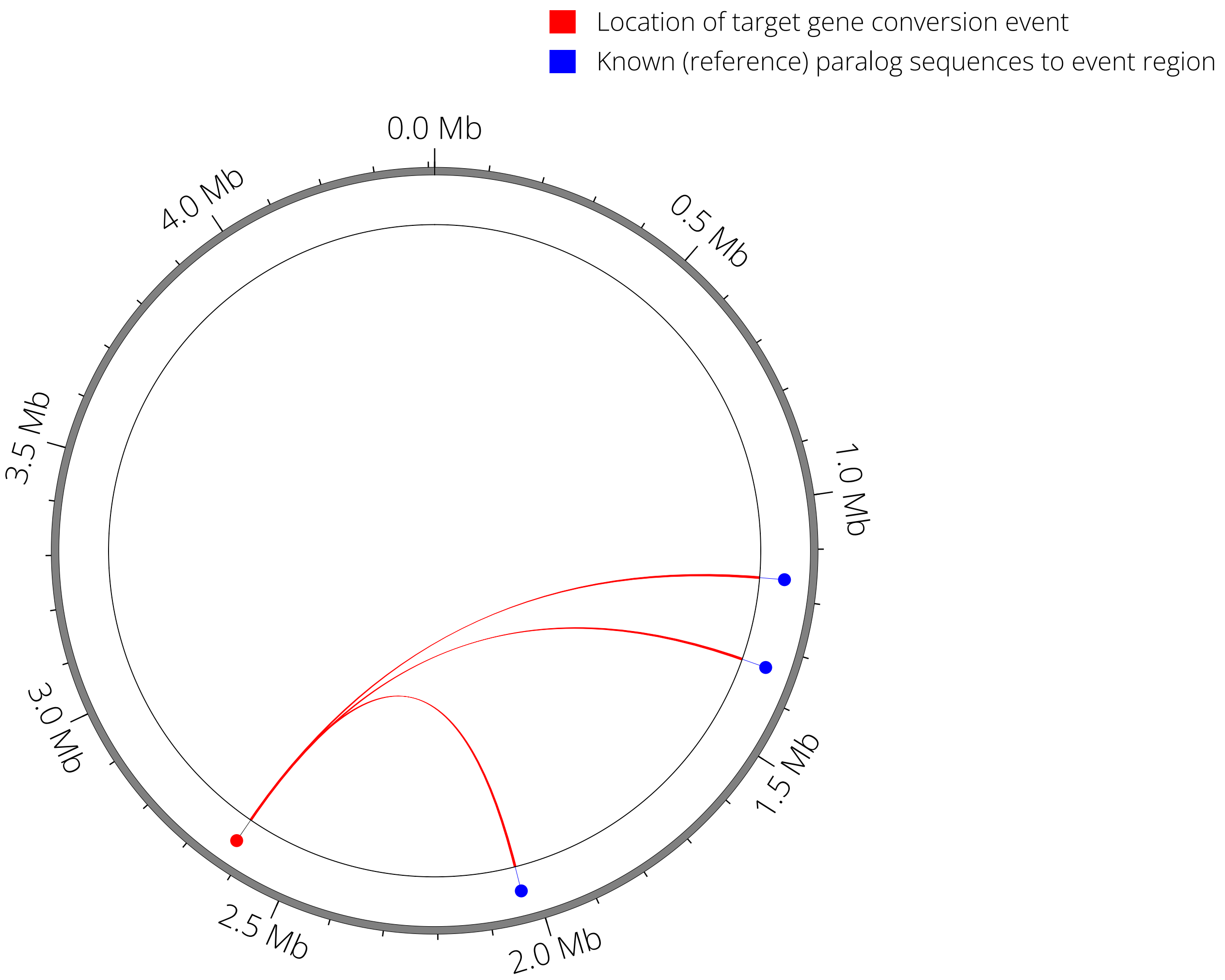

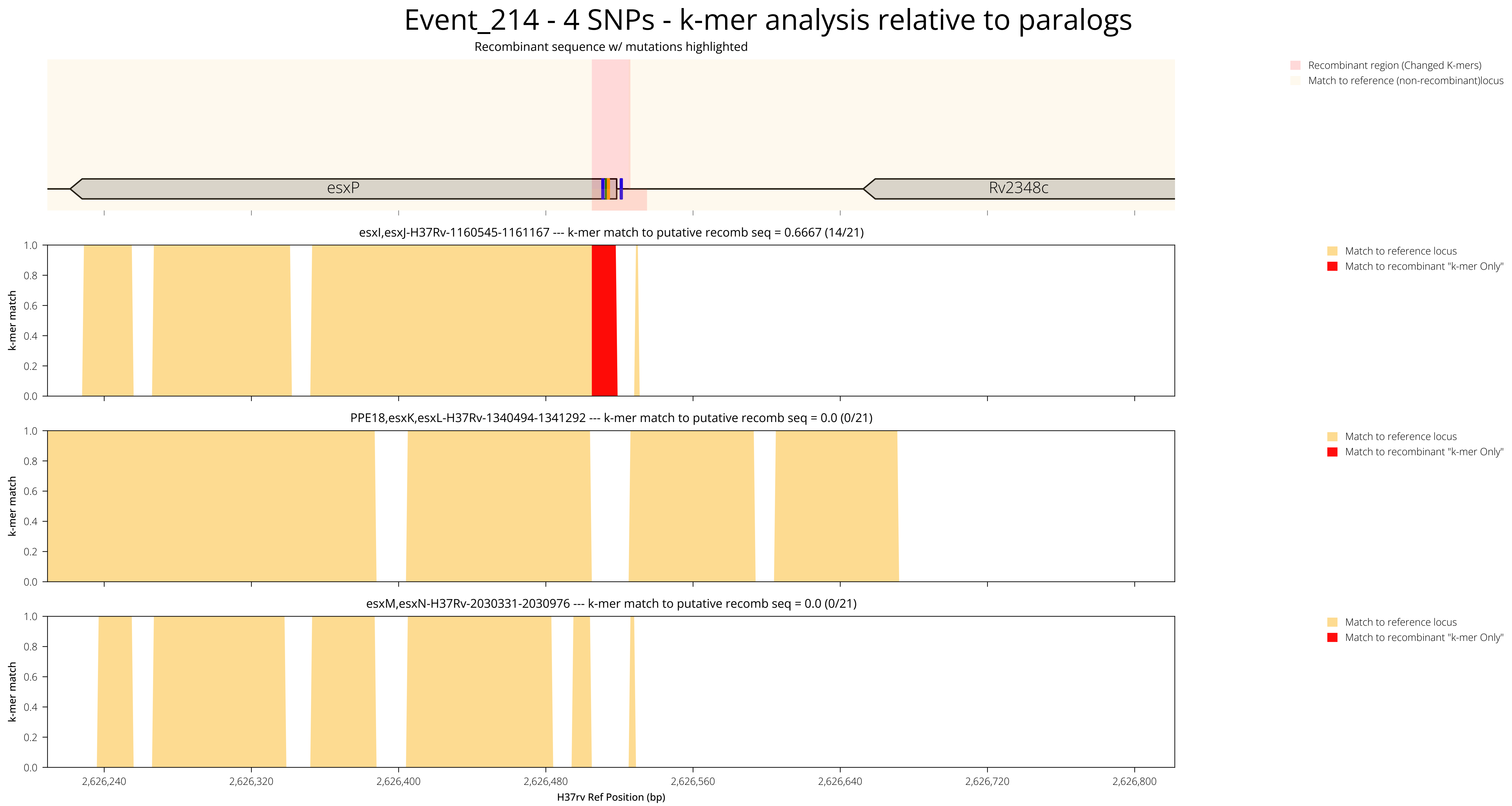

Event_214 - 4 SNPs - k-mer analysis relative to paralogs
Recombinant sequence w/ mutations highlighted
Recombinant region (Changed K-mers)
Match to reference (non-recombinant)locus
esxP
Rv2348c
esxI,esxJ-H37Rv-1160545-1161167 --- k-mer match to putative recomb seq = 0.6667 (14/21)
Match to reference locus
Match to recombinant "k-mer Only"
PPE18,esxK,esxL-H37Rv-1340494-1341292 --- k-mer match to putative recomb seq = 0.0 (0/21)
Match to reference locus
Match to recombinant "k-mer Only"
esxM,esxN-H37Rv-2030331-2030976 --- k-mer match to putative recomb seq = 0.0 (0/21)
Match to reference locus
Match to recombinant "k-mer Only"
k-mer match
1.0
0.8
0.6
0.4
0.2
0.0
2,626,240
2,626,320
2,626,400
2,626,480
2,626,560
2,626,640
2,626,720
2,626,800
H37rv Ref Position (bp)

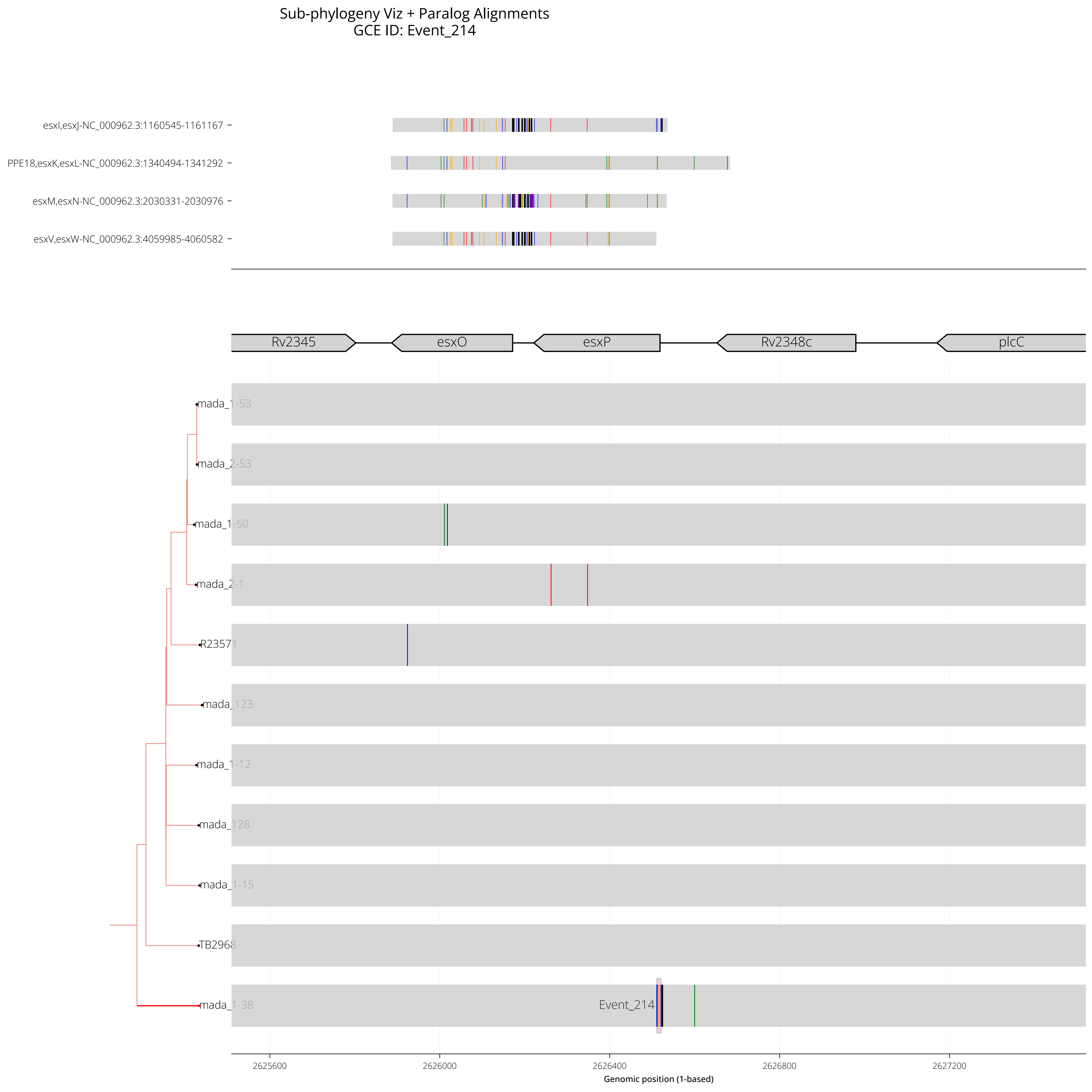

Sub-phylogeny Viz + Paralog Alignments
GCE ID: Event_214
esxI,esxJ-NC_000962.3:1160545-1161167
PPE18,esxK,esxL-NC_000962.3:1340494-1341292
esxM,esxN-NC_000962.3:2030331-2030976
esxV,esxW-NC_000962.3:4059985-4060582
Rv2345
esxO
esxP
Rv2348c
plcC
mada_1-53
mada_2-53
mada_1-50
mada_2-1
R23571
mada_123
mada_1-12
mada_128
mada_1-15
TB2968
mada_1-38
Event_214
2625600
2626000
2626400
2626800
2627200
Genomic position (1-based)

Full Phylogeny Viz + Paralog Alignments
GCE ID: Event_214

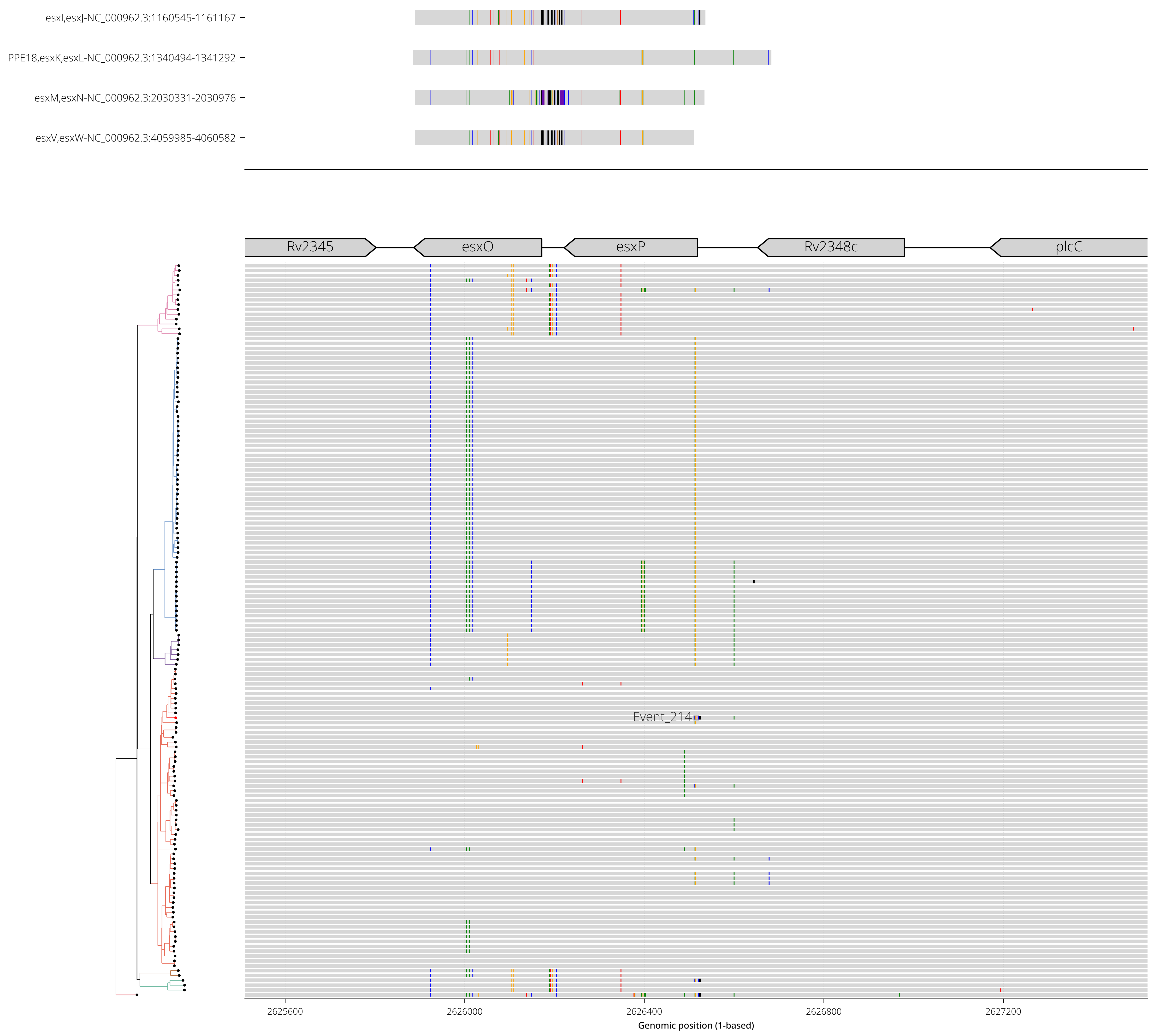

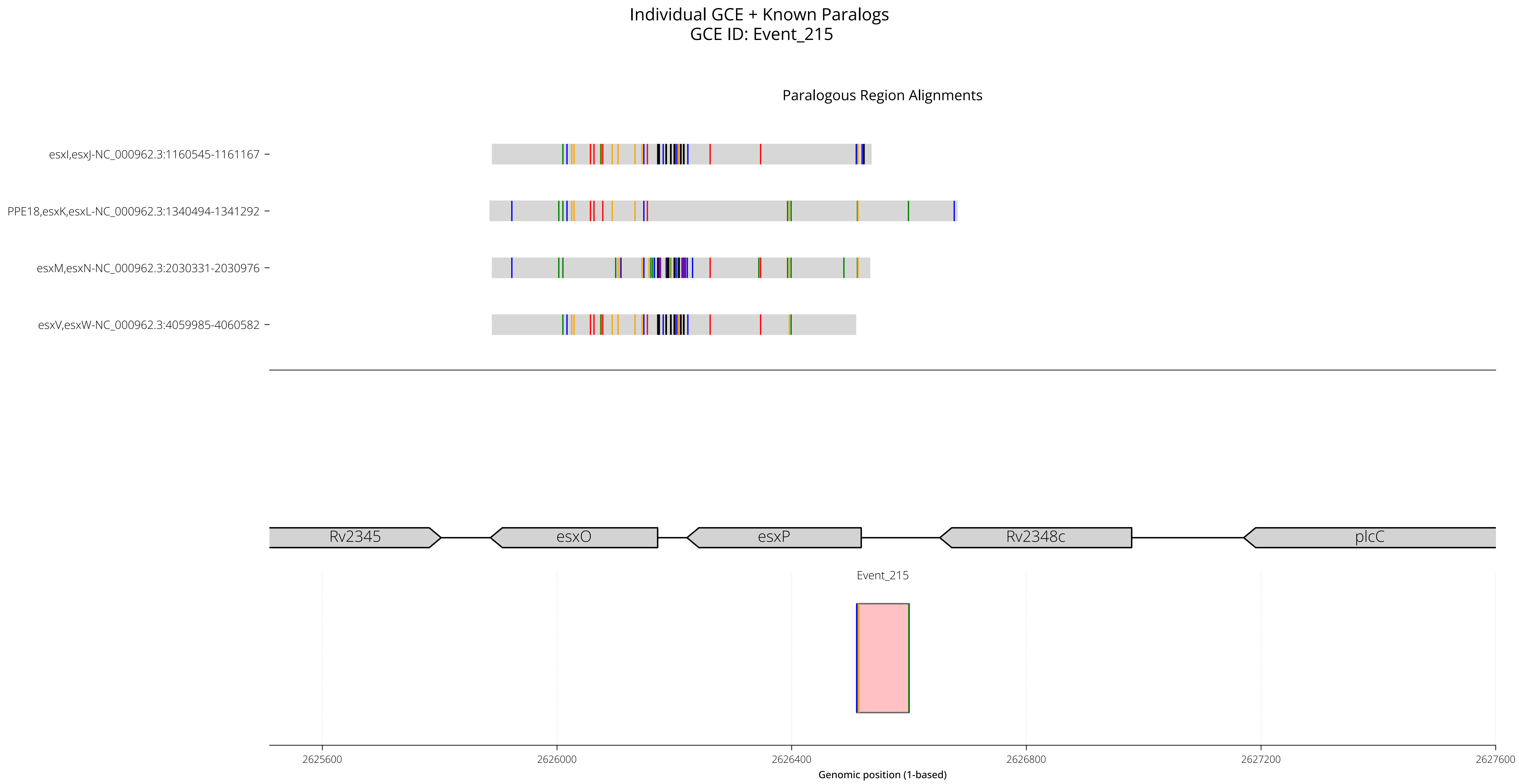
Individual GCE + Known Paralogs
GCE ID: Event_215
Paralogous Region Alignments
esxI,esxJ-NC_000962.3:1160545-1161167
PPE18,esxK,esxL-NC_000962.3:1340494-1341292
esxM,esxN-NC_000962.3:2030331-2030976
esxV,esxW-NC_000962.3:4059985-4060582
Rv2345
esxO
esxP
Rv2348c
plcC
Event_215
2625600
2626000
2626400
2626800
2627200
2627600
Genomic position (1-based)

Circos Viz - GCE to Known Paralogs Locations
GCE ID: Event_215

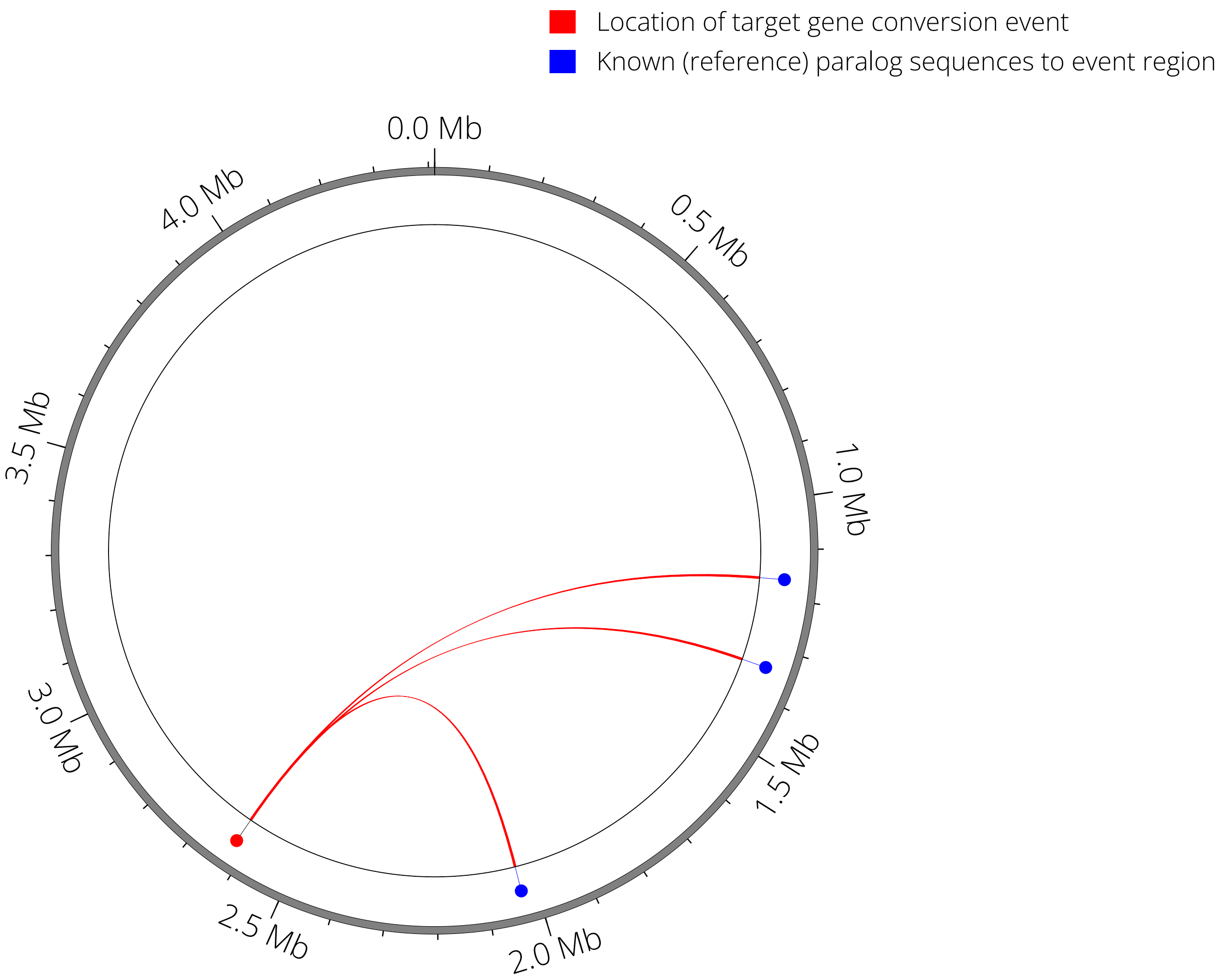

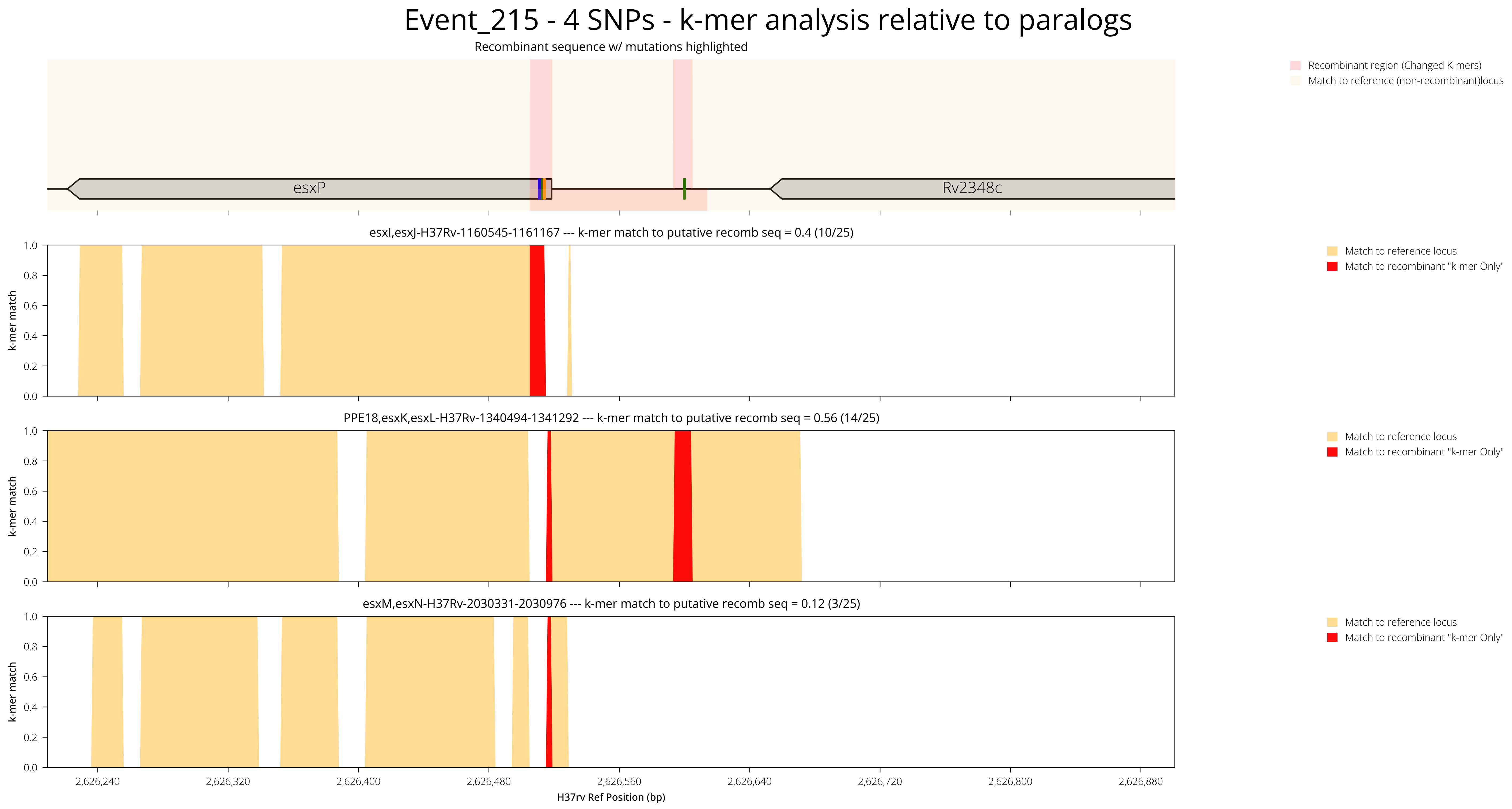

Event_215 - 4 SNPs - k-mer analysis relative to paralogs
Recombinant sequence w/ mutations highlighted
Recombinant region (Changed K-mers)
Match to reference (non-recombinant)locus
esxP
Rv2348c
esxI,esxJ-H37Rv-1160545-1161167 --- k-mer match to putative recomb seq = 0.4 (10/25)
PPE18,esxK,esxL-H37Rv-1340494-1341292 --- k-mer match to putative recomb seq = 0.56 (14/25)
esxM,esxN-H37Rv-2030331-2030976 --- k-mer match to putative recomb seq = 0.12 (3/25)
Match to reference locus
Match to recombinant "k-mer Only"
k-mer match
0.0
0.2
0.4
0.6
0.8
1.0
2,626,240
2,626,320
2,626,400
2,626,480
2,626,560
2,626,640
2,626,720
2,626,800
2,626,880
H37rv Ref Position (bp)

Sub-phylogeny Viz + Paralog Alignments
GCE ID: Event_215

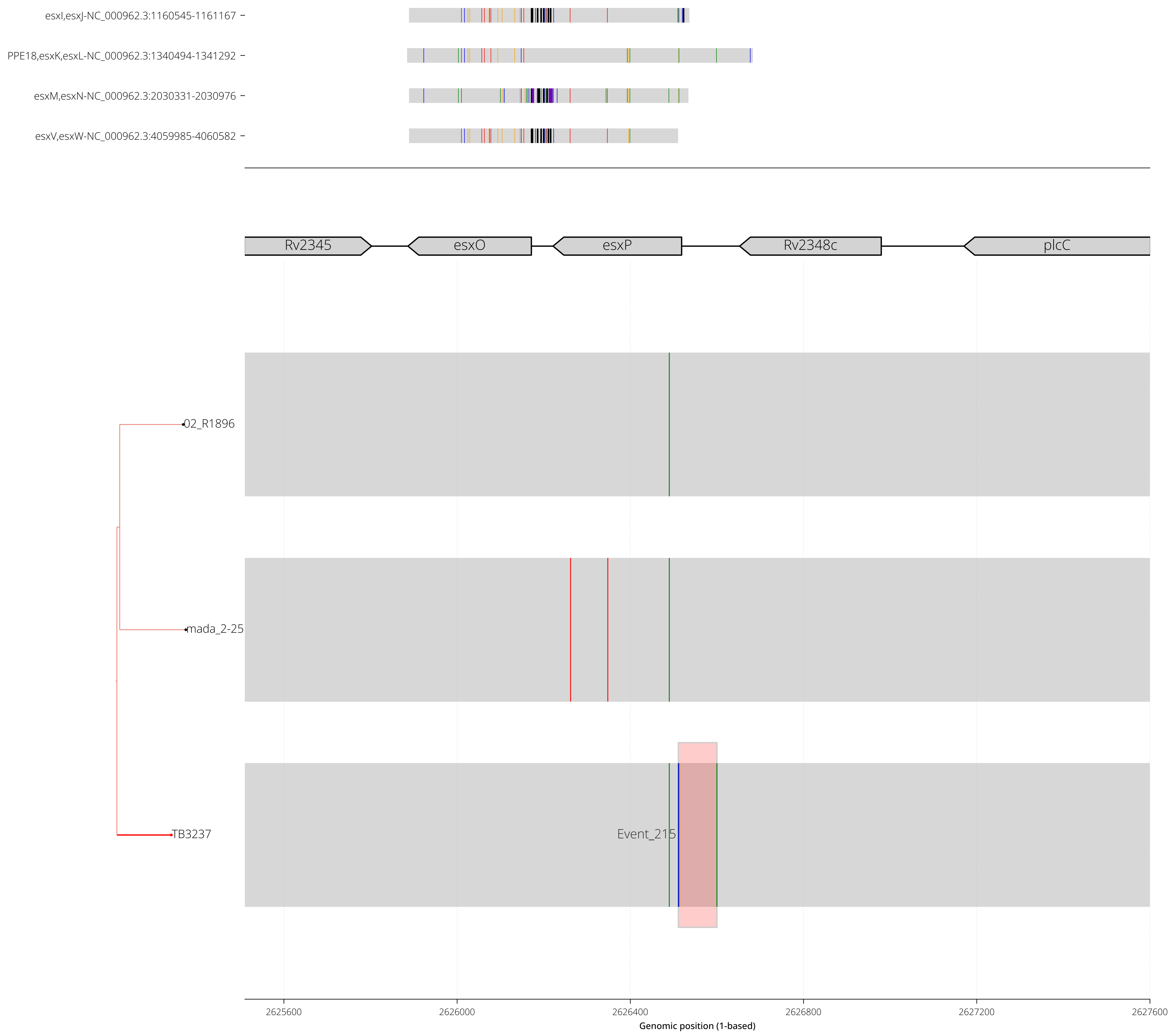

Full Phylogeny Viz + Paralog Alignments
GCE ID: Event_215

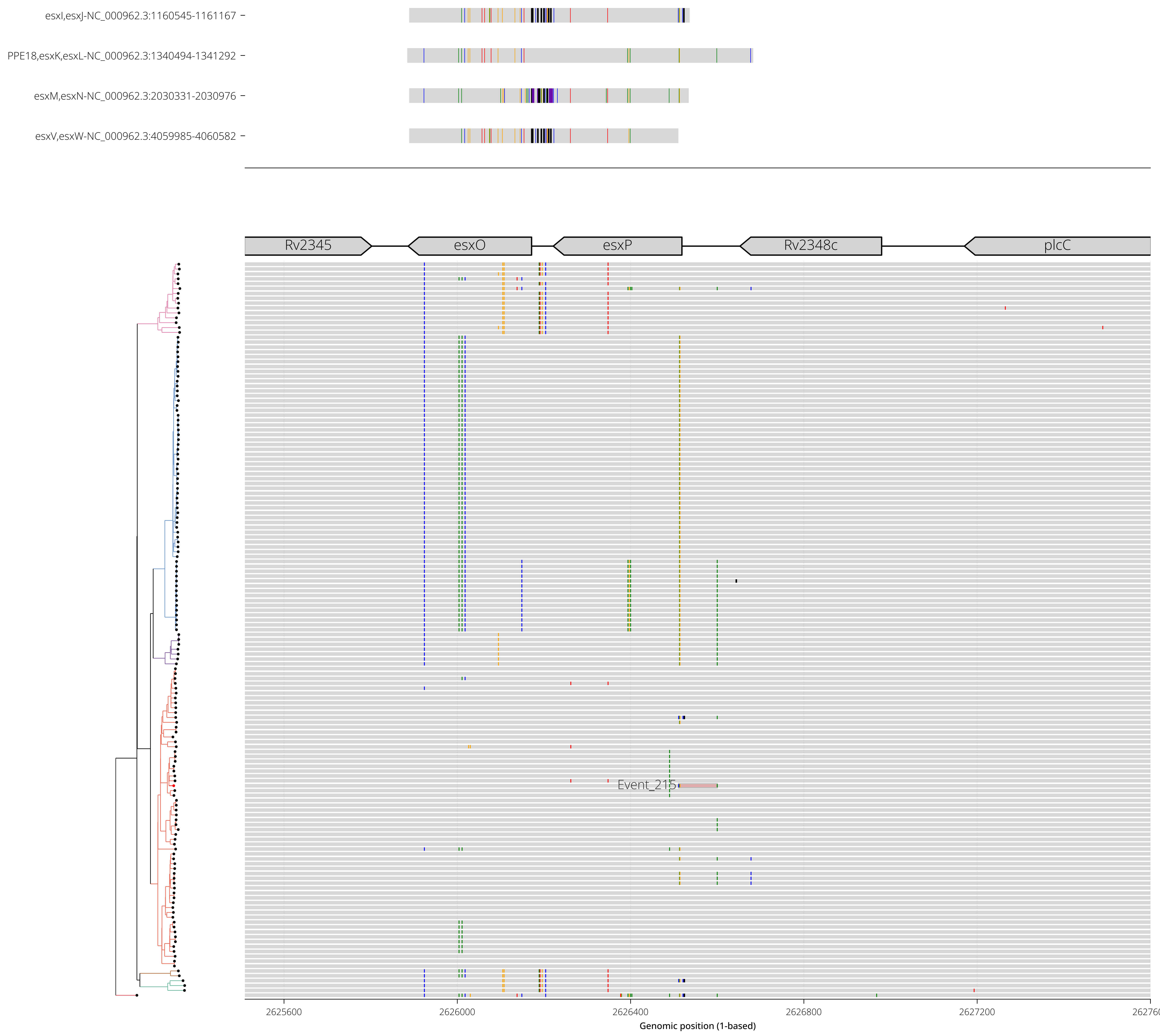

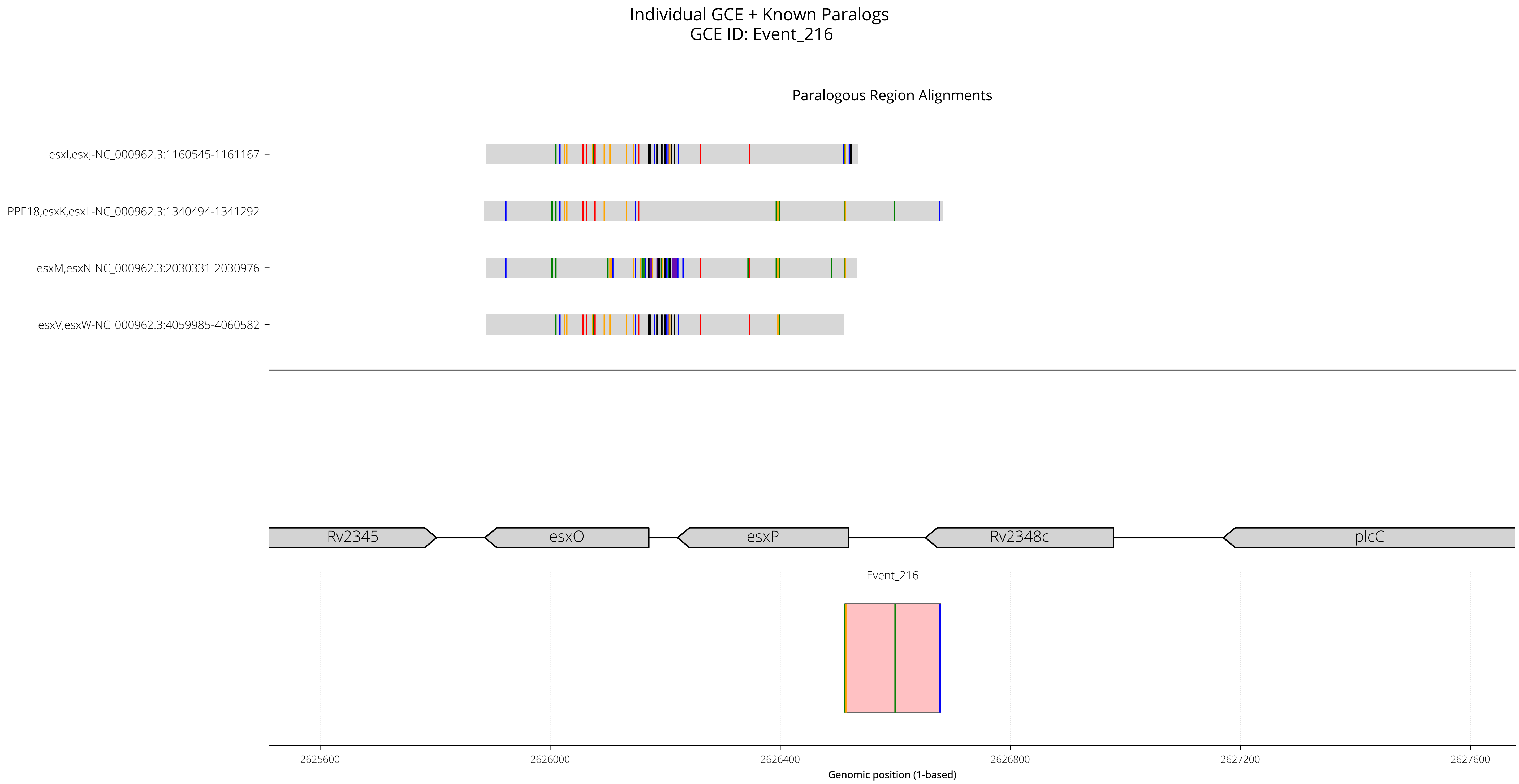
Individual GCE + Known Paralogs
GCE ID: Event_216
Paralogous Region Alignments
esxI,esxJ-NC_000962.3:1160545-1161167
PPE18,esxK,esxL-NC_000962.3:1340494-1341292
esxM,esxN-NC_000962.3:2030331-2030976
esxV,esxW-NC_000962.3:4059985-4060582
Rv2345
esxO
esxP
Rv2348c
plcC
Event_216
2625600
2626000
2626400
2626800
2627200
2627600
Genomic position (1-based)

Circos Viz - GCE to Known Paralogs Locations
GCE ID: Event_216

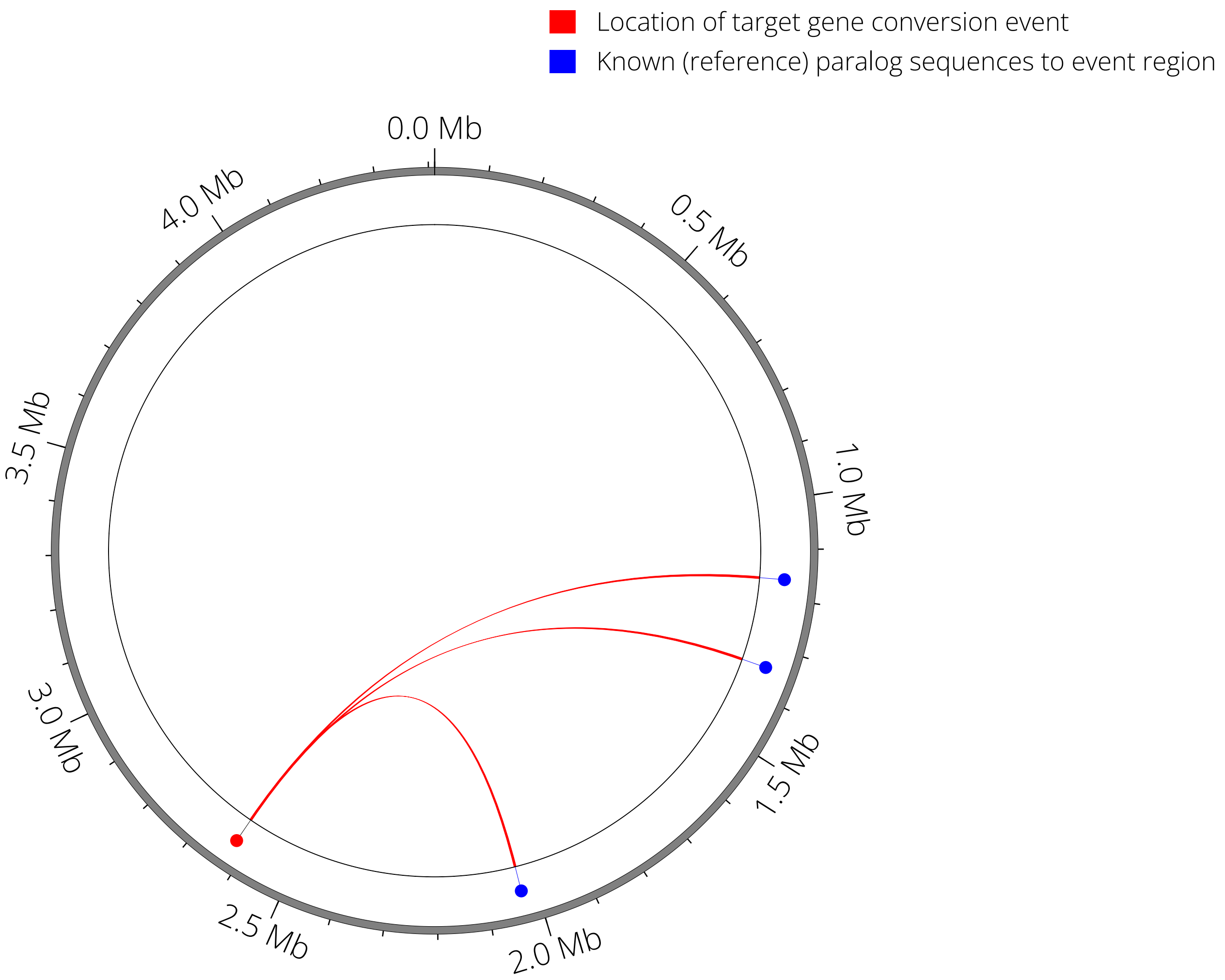

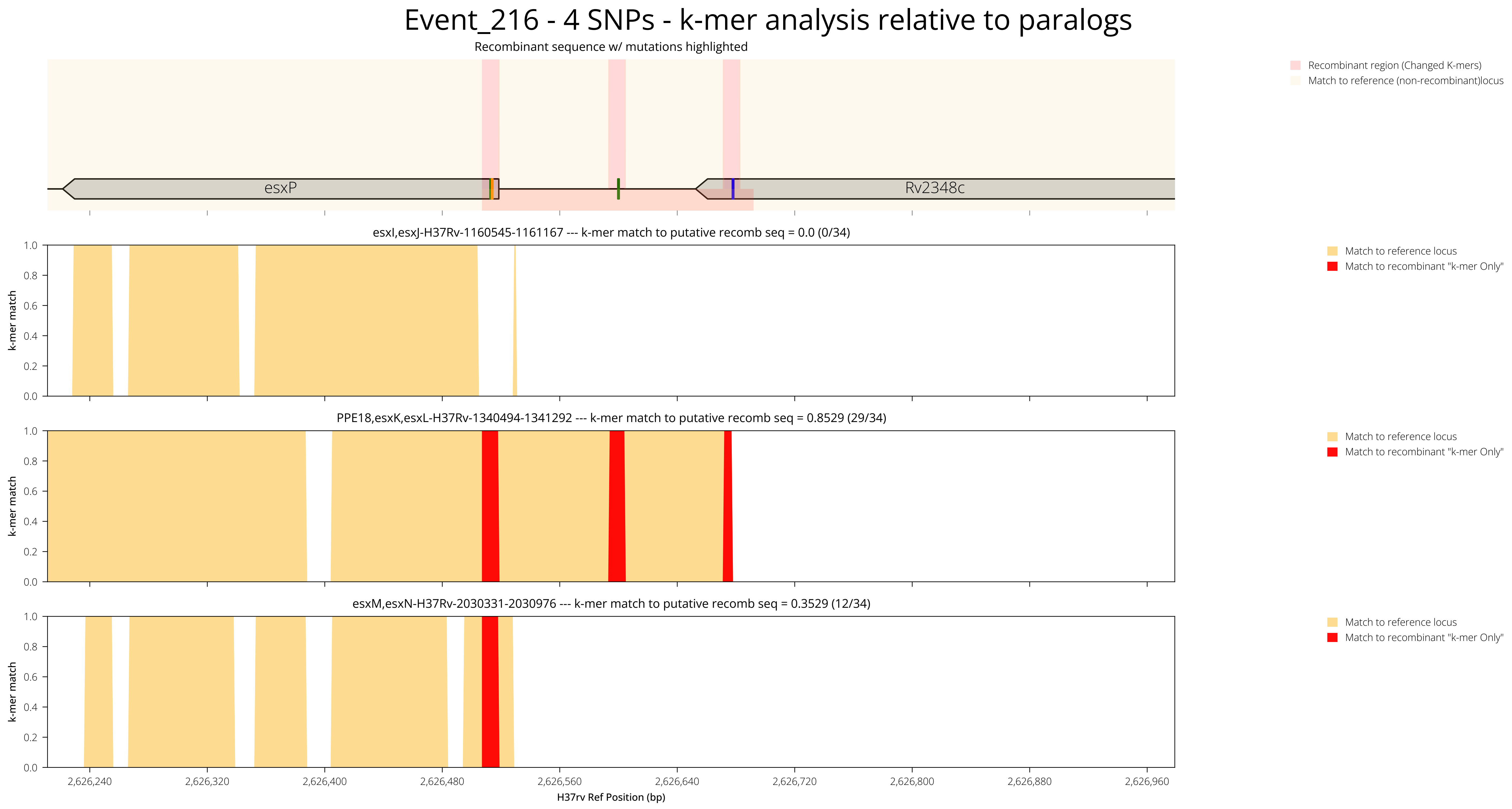

Event_216 - 4 SNPs - k-mer analysis relative to paralogs
Recombinant sequence w/ mutations highlighted
Recombinant region (Changed K-mers)
Match to reference (non-recombinant)locus
esxP
Rv2348c
esxI,esxJ-H37Rv-1160545-1161167 --- k-mer match to putative recomb seq = 0.0 (0/34)
PPE18,esxK,esxL-H37Rv-1340494-1341292 --- k-mer match to putative recomb seq = 0.8529 (29/34)
esxM,esxN-H37Rv-2030331-2030976 --- k-mer match to putative recomb seq = 0.3529 (12/34)
Match to reference locus
Match to recombinant "k-mer Only"
k-mer match
1.0
0.8
0.6
0.4
0.2
0.0
2,626,240
2,626,320
2,626,400
2,626,480
2,626,560
2,626,640
2,626,720
2,626,800
2,626,880
2,626,960
H37rv Ref Position (bp)

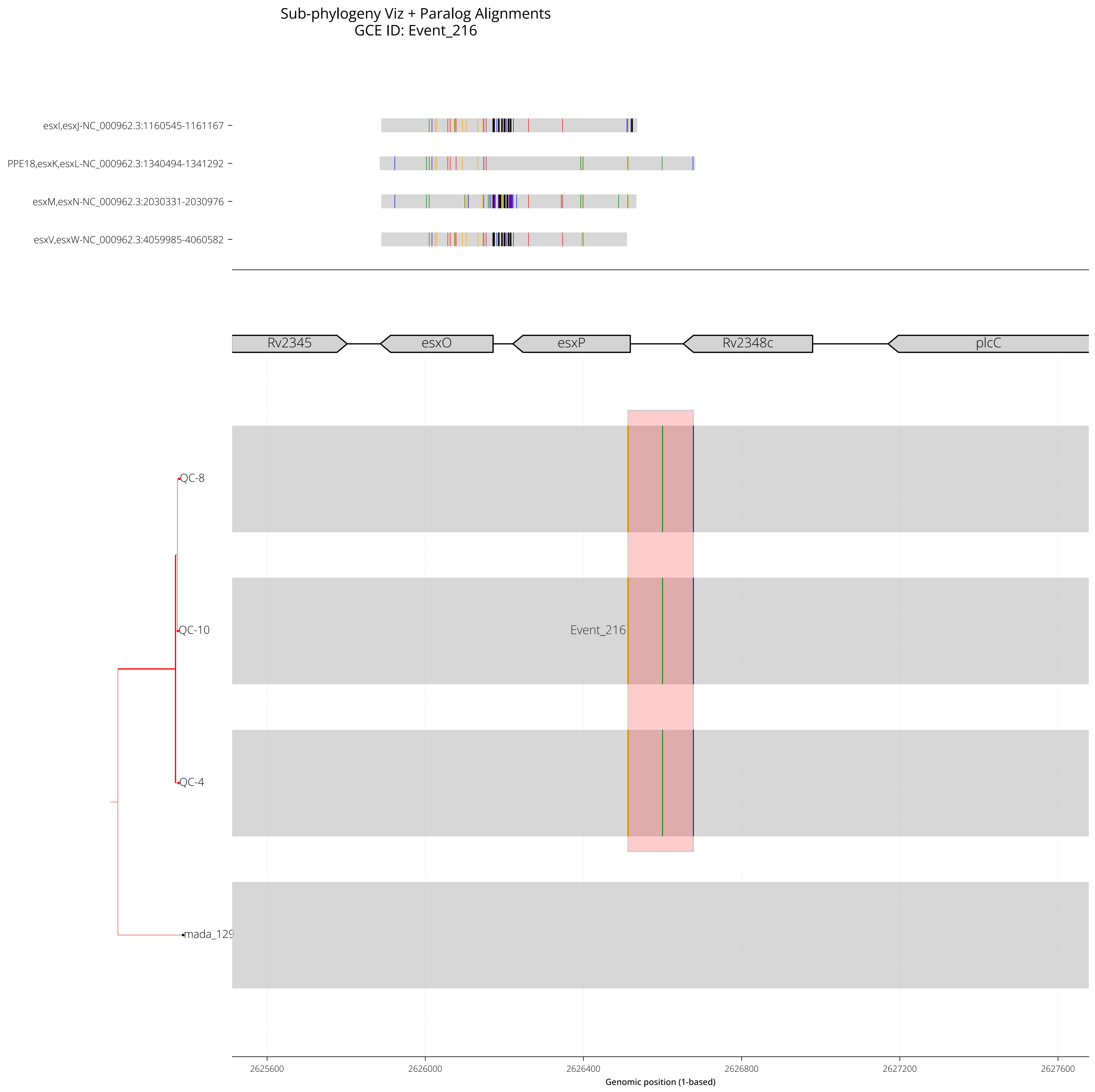
Sub-phylogeny Viz + Paralog Alignments
GCE ID: Event_216
esxI,esxJ-NC_000962.3:1160545-1161167
PPE18,esxK,esxL-NC_000962.3:1340494-1341292
esxM,esxN-NC_000962.3:2030331-2030976
esxV,esxW-NC_000962.3:4059985-4060582
Rv2345
esxO
esxP
Rv2348c
plcC
QC-8
QC-10
Event_216
QC-4
mada_129
2625600
2626000
2626400
2626800
2627200
2627600
Genomic position (1-based)

Full Phylogeny Viz + Paralog Alignments
GCE ID: Event_216

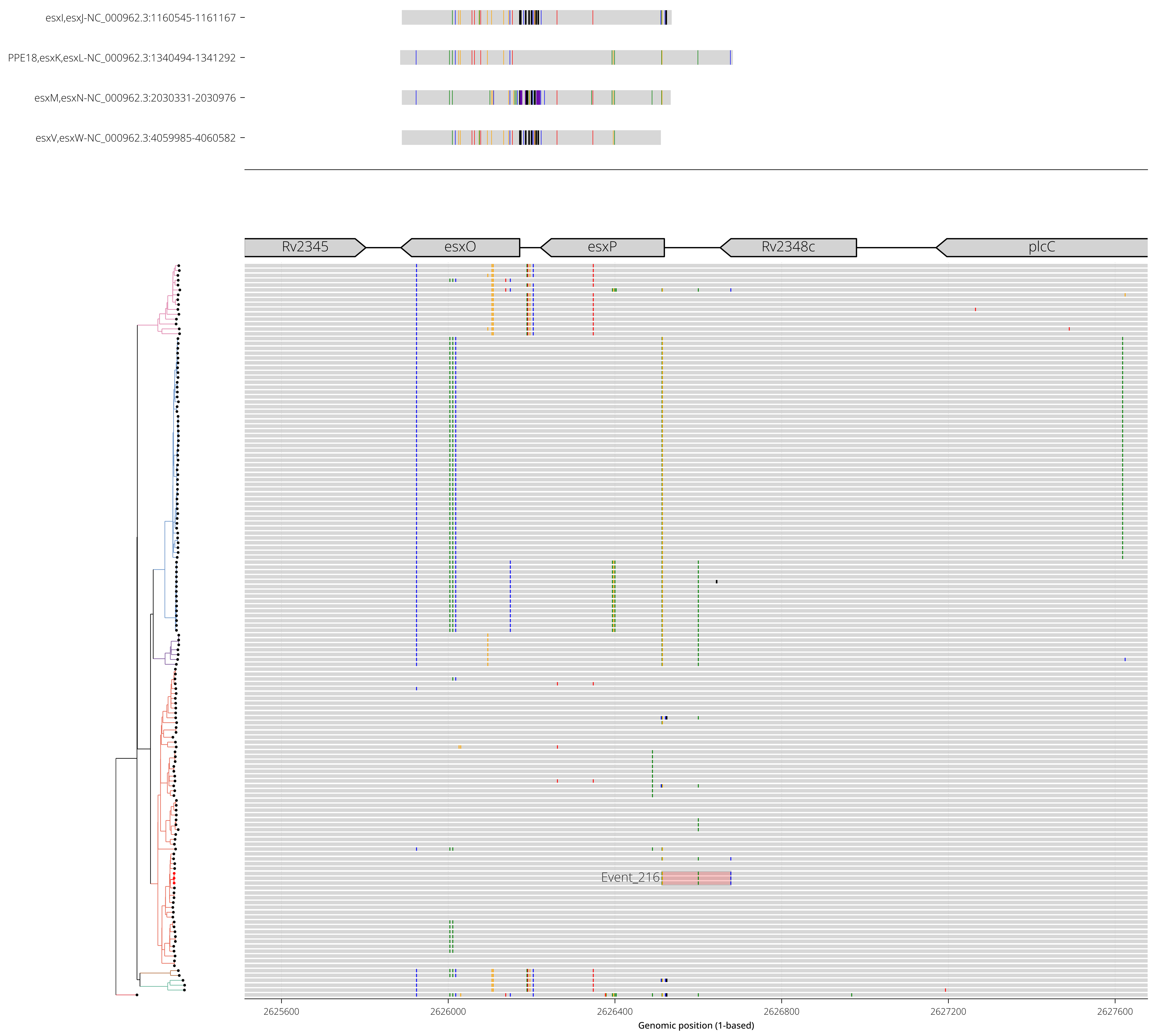

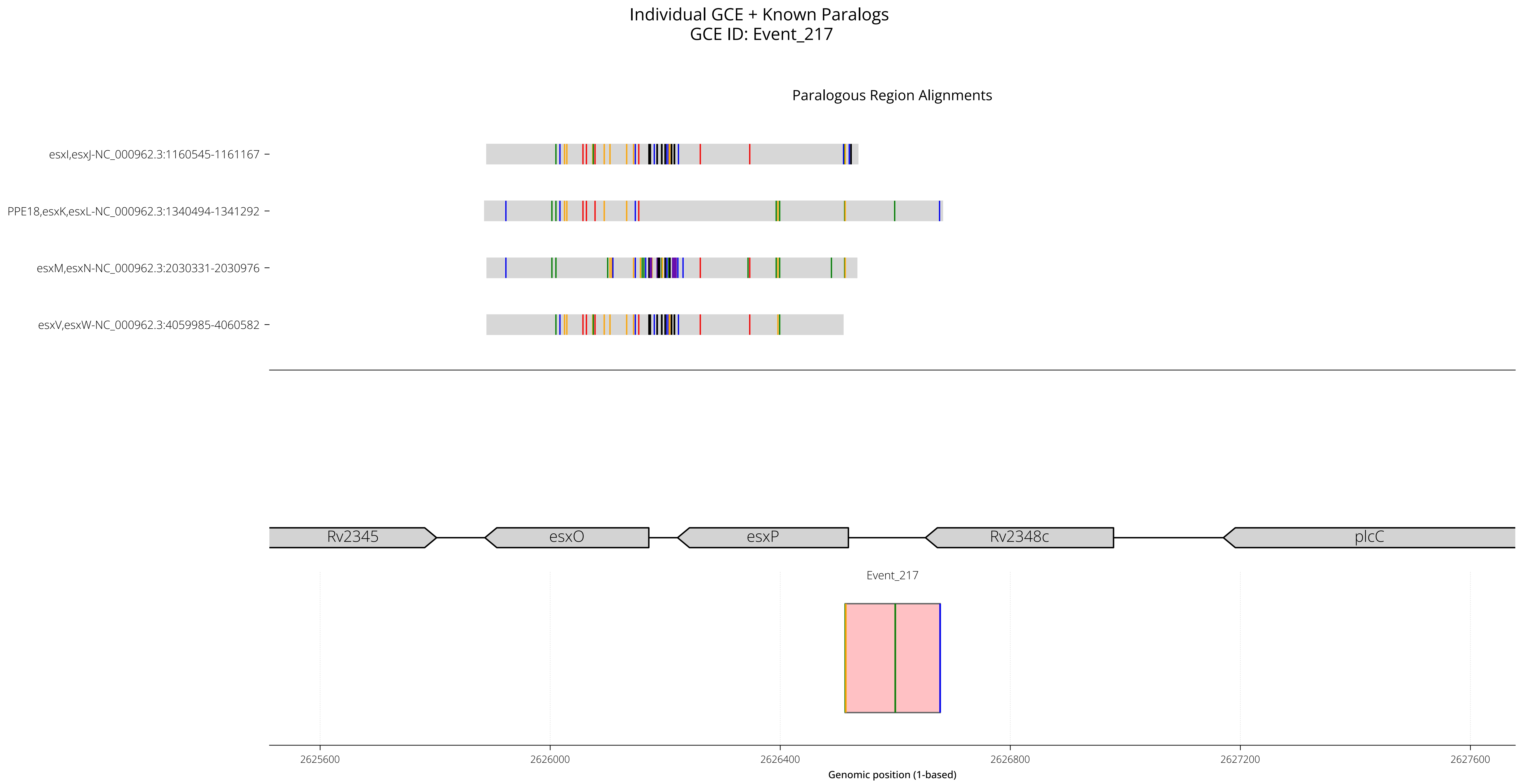

Individual GCE + Known Paralogs
GCE ID: Event_217
Paralogous Region Alignments
esxI,esxJ-NC_000962.3:1160545-1161167
PPE18,esxK,esxL-NC_000962.3:1340494-1341292
esxM,esxN-NC_000962.3:2030331-2030976
esxV,esxW-NC_000962.3:4059985-4060582
Rv2345
esxO
esxP
Rv2348c
plcC
Event_217
2625600
2626000
2626400
2626800
2627200
2627600
Genomic position (1-based)

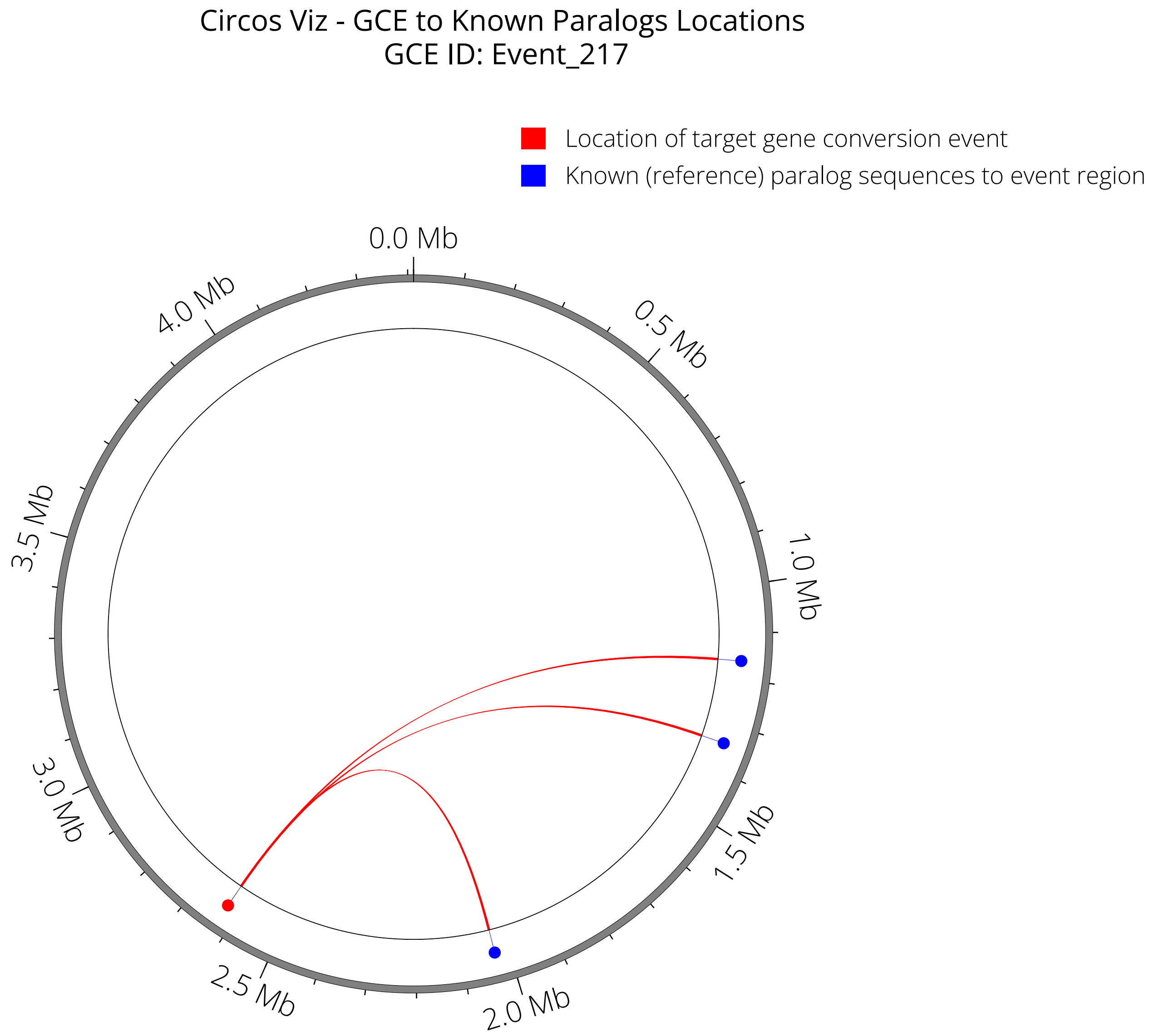
Circos Viz - GCE to Known Paralogs Locations
GCE ID: Event_217
Location of target gene conversion event
Known (reference) paralog sequences to event region
0.0 Mb
0.5 Mb
1.0 Mb
1.5 Mb
2.0 Mb
2.5 Mb
3.0 Mb
3.5 Mb
4.0 Mb

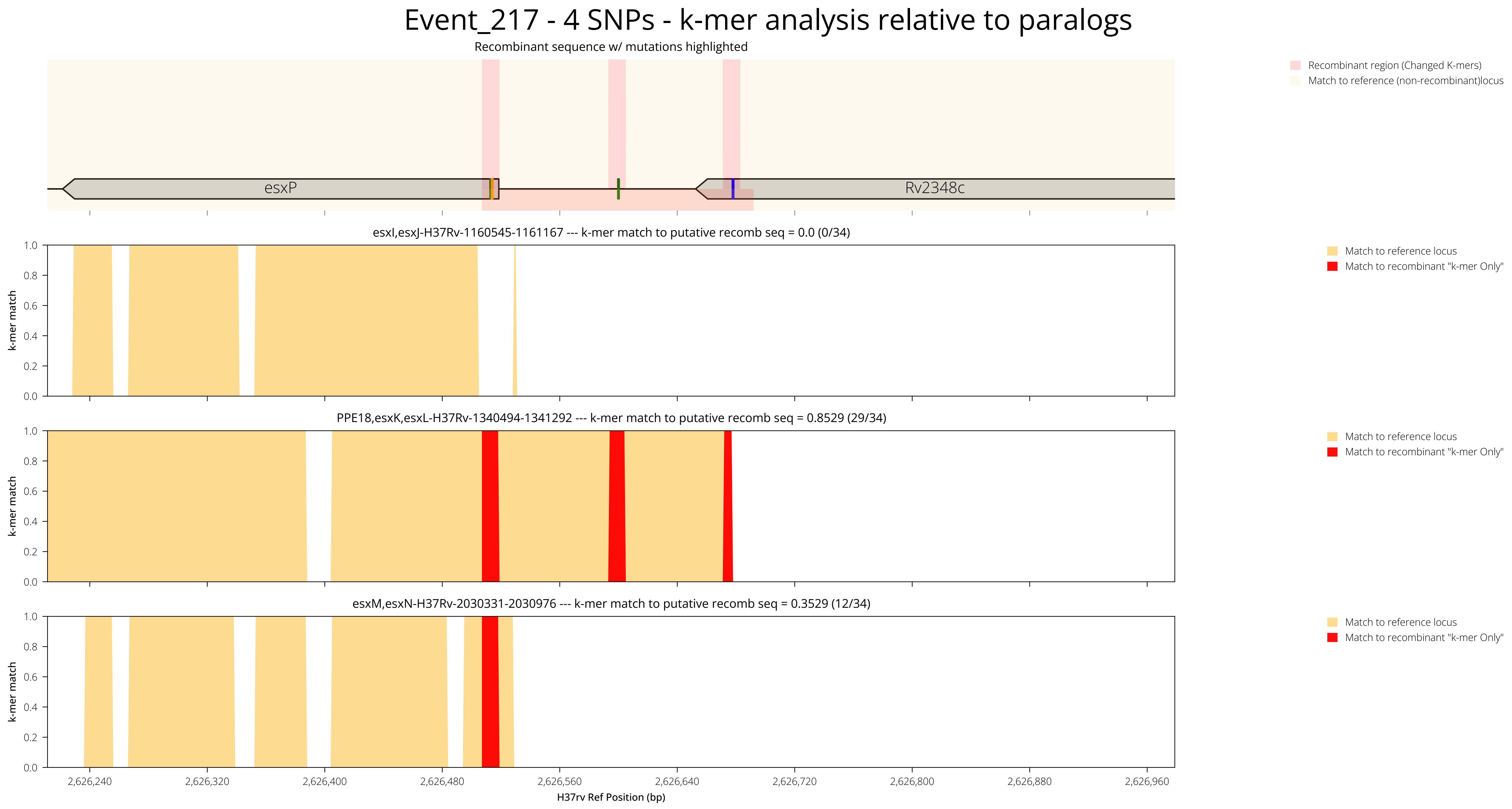
Event_217 - 4 SNPs - k-mer analysis relative to paralogs
Recombinant sequence w/ mutations highlighted
Recombinant region (Changed K-mers)
Match to reference (non-recombinant)locus
esxP
Rv2348c
esxI,esxJ-H37Rv-1160545-1161167 --- k-mer match to putative recomb seq = 0.0 (0/34)
PPE18,esxK,esxL-H37Rv-1340494-1341292 --- k-mer match to putative recomb seq = 0.8529 (29/34)
esxM,esxN-H37Rv-2030331-2030976 --- k-mer match to putative recomb seq = 0.3529 (12/34)
Match to reference locus
Match to recombinant "k-mer Only"
k-mer match
0.0
0.2
0.4
0.6
0.8
1.0
2,626,240
2,626,320
2,626,400
2,626,480
2,626,560
2,626,640
2,626,720
2,626,800
2,626,880
2,626,960
H37rv Ref Position (bp)

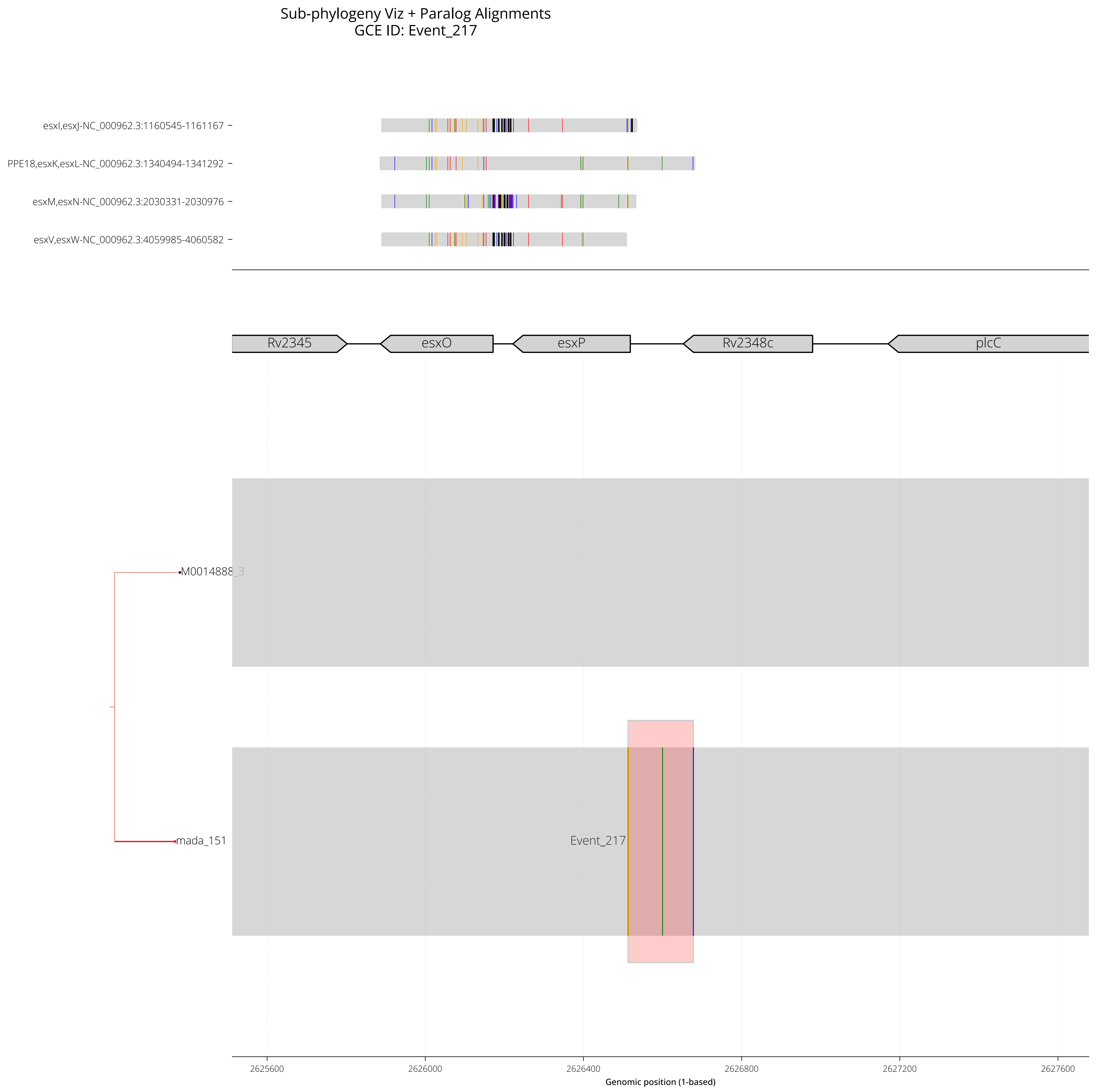
Sub-phylogeny Viz + Paralog Alignments
GCE ID: Event_217
esxI,esxJ-NC_000962.3:1160545-1161167
PPE18,esxK,esxL-NC_000962.3:1340494-1341292
esxM,esxN-NC_000962.3:2030331-2030976
esxV,esxW-NC_000962.3:4059985-4060582
Rv2345
esxO
esxP
Rv2348c
plcC
M0014888_3
mada_151
Event_217
2625600
2626000
2626400
2626800
2627200
2627600
Genomic position (1-based)

Full Phylogeny Viz + Paralog Alignments
GCE ID: Event_217

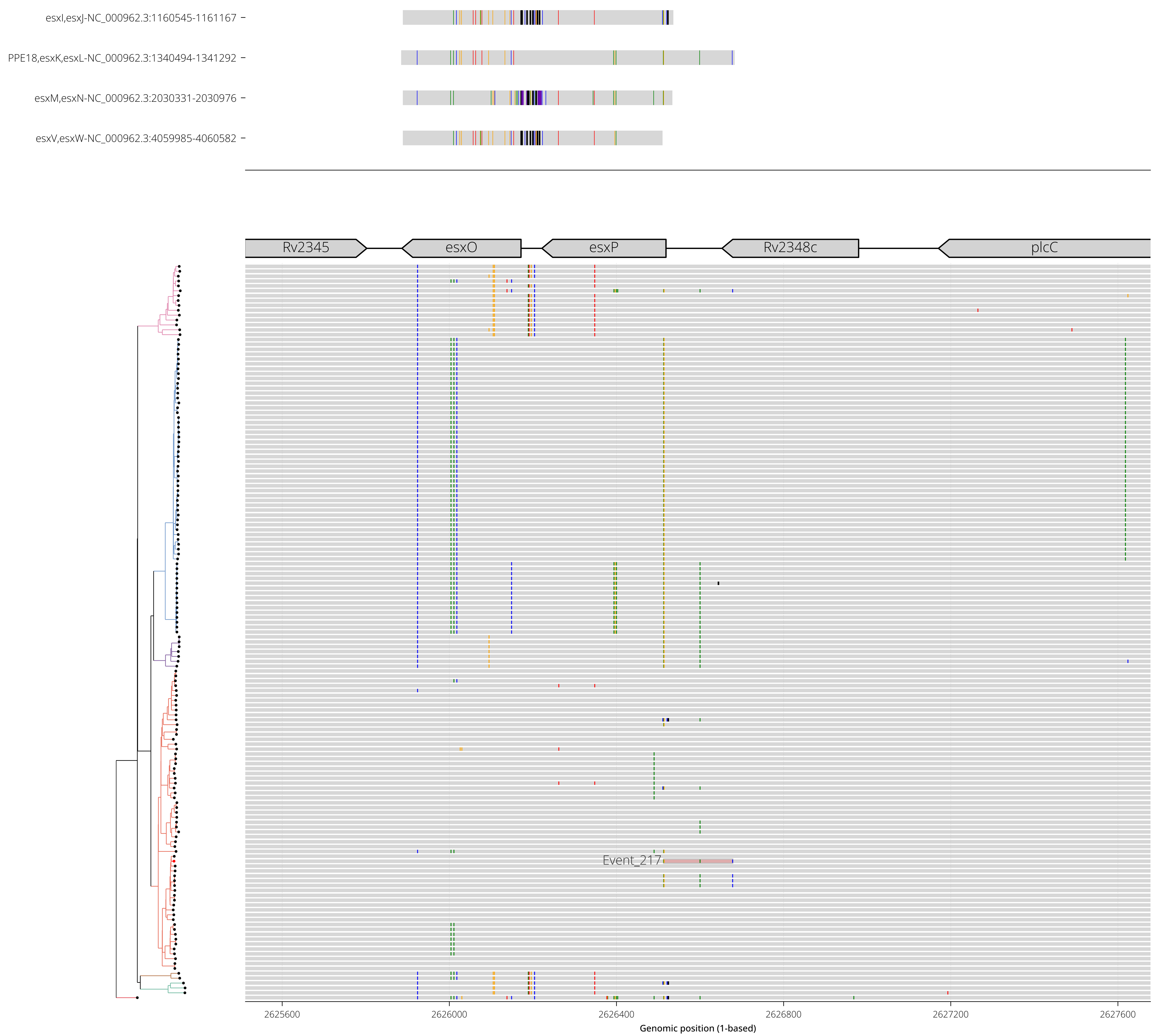

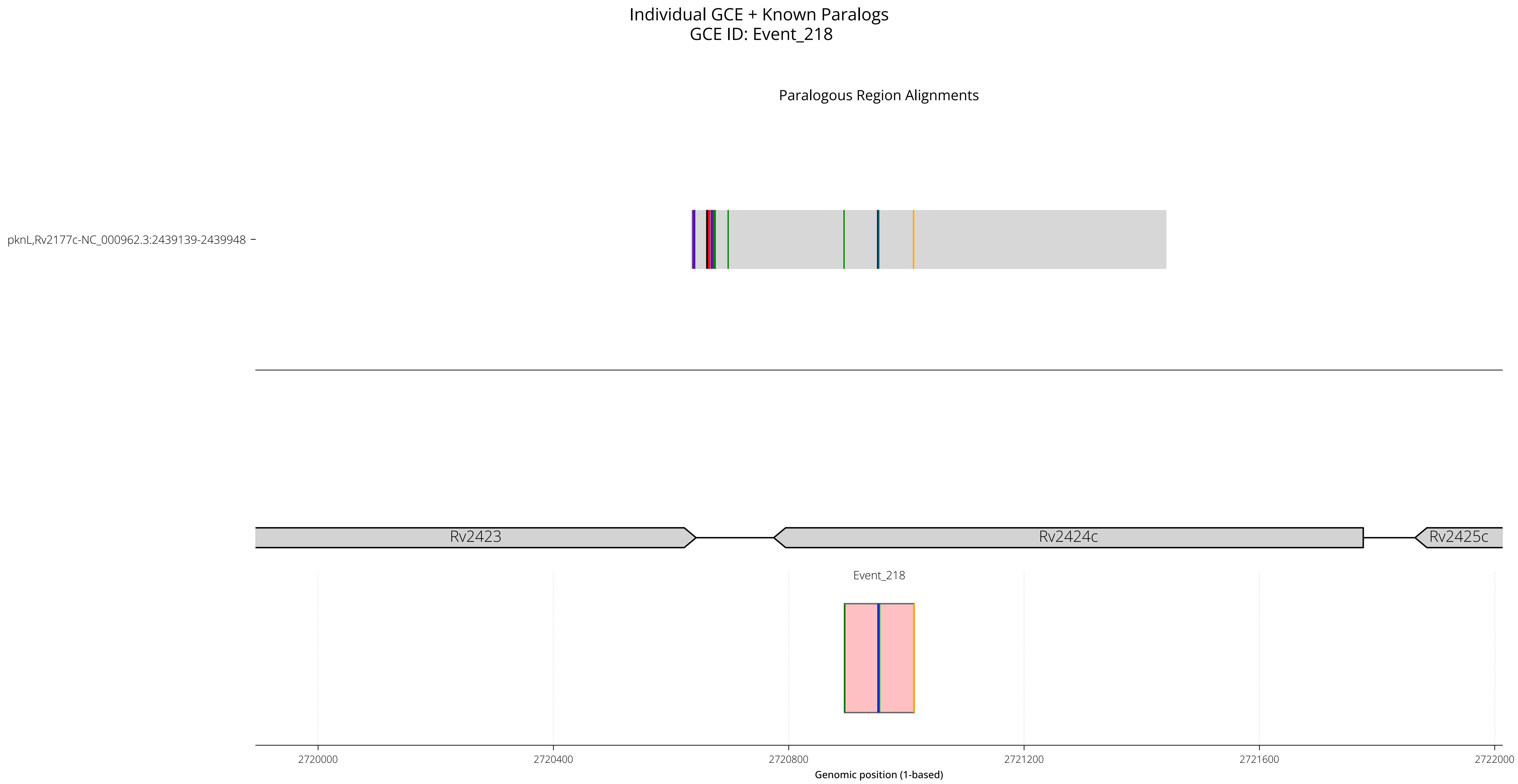

Individual GCE + Known Paralogs
GCE ID: Event_218
Paralogous Region Alignments
pknL,Rv2177c-NC_000962.3:2439139-2439948
Rv2423
Rv2424c
Rv2425c
Event_218
2720000
2720400
2720800
2721200
2721600
2722000
Genomic position (1-based)

Circos Viz - GCE to Known Paralogs Locations
GCE ID: Event_218

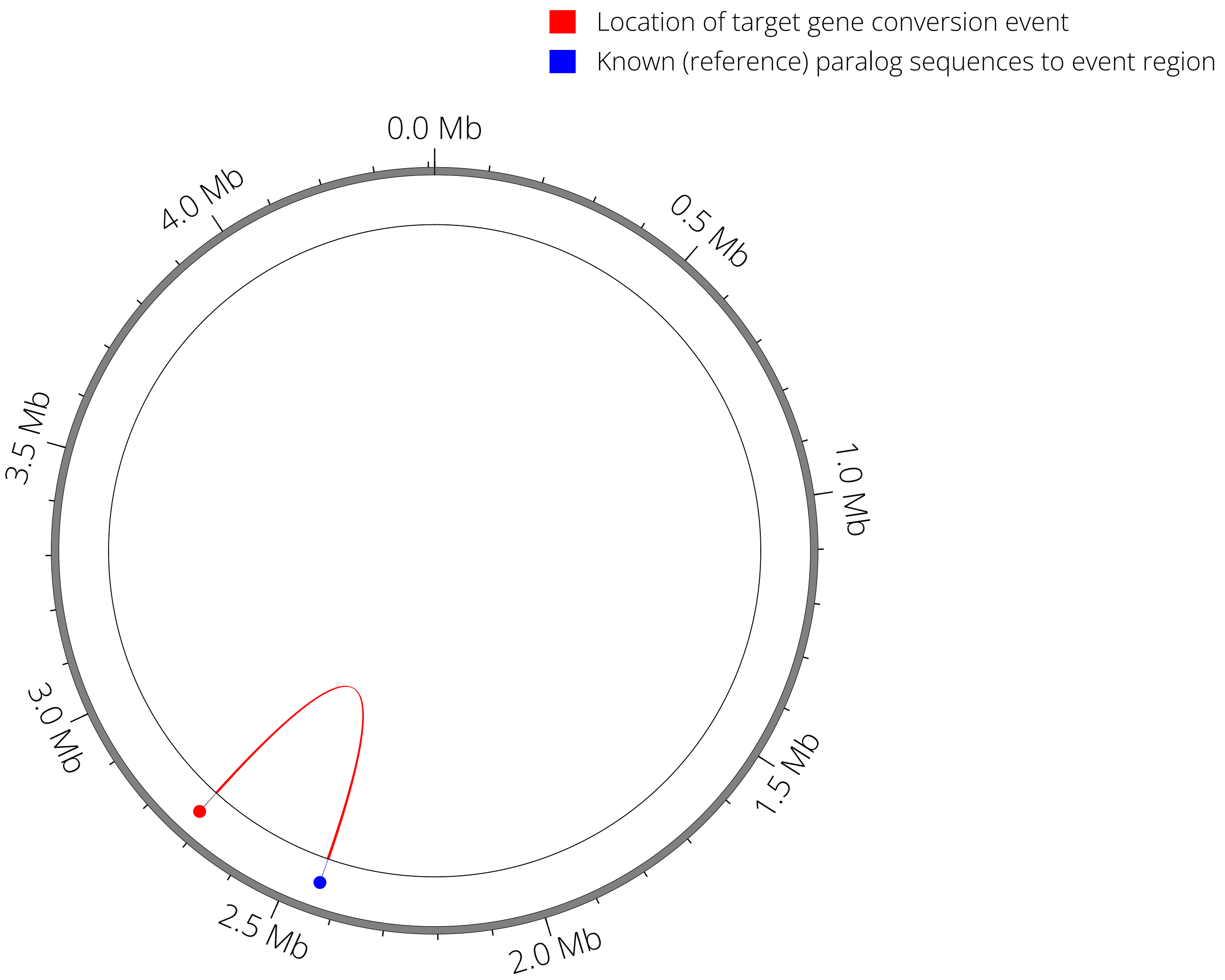

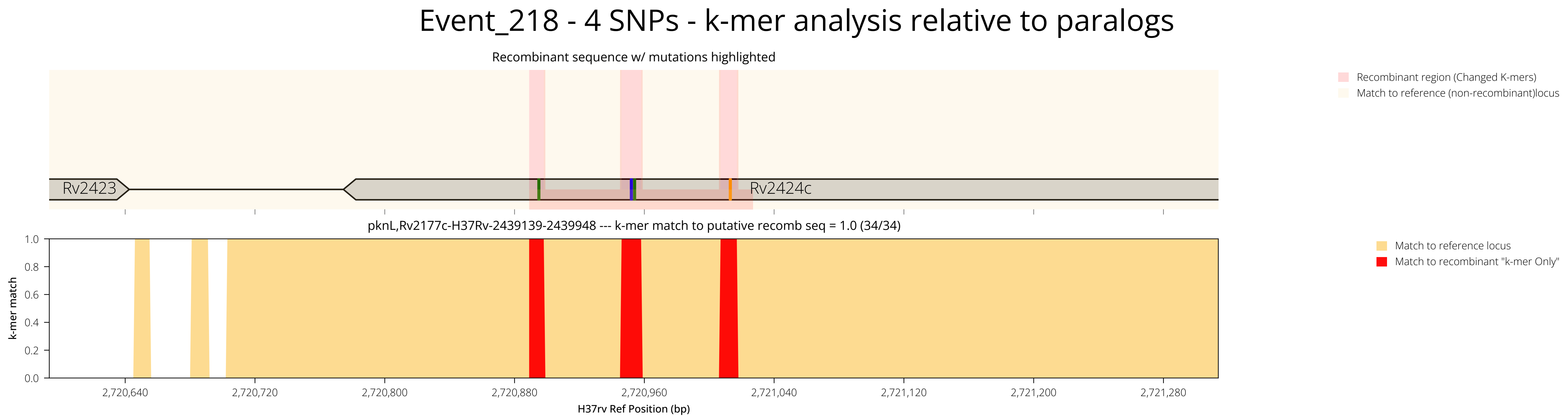

Event_218 - 4 SNPs - k-mer analysis relative to paralogs
Recombinant sequence w/ mutations highlighted
Rv2423
Rv2424c
pknL,Rv2177c-H37Rv-2439139-2439948 --- k-mer match to putative recomb seq = 1.0 (34/34)
k-mer match
1.0
0.8
0.6
0.4
0.2
0.0
2,720,640
2,720,720
2,720,800
2,720,880
2,720,960
2,721,040
2,721,120
2,721,200
2,721,280
H37rv Ref Position (bp)
Recombinant region (Changed K-mers)
Match to reference (non-recombinant)locus
Match to reference locus
Match to recombinant "k-mer Only"

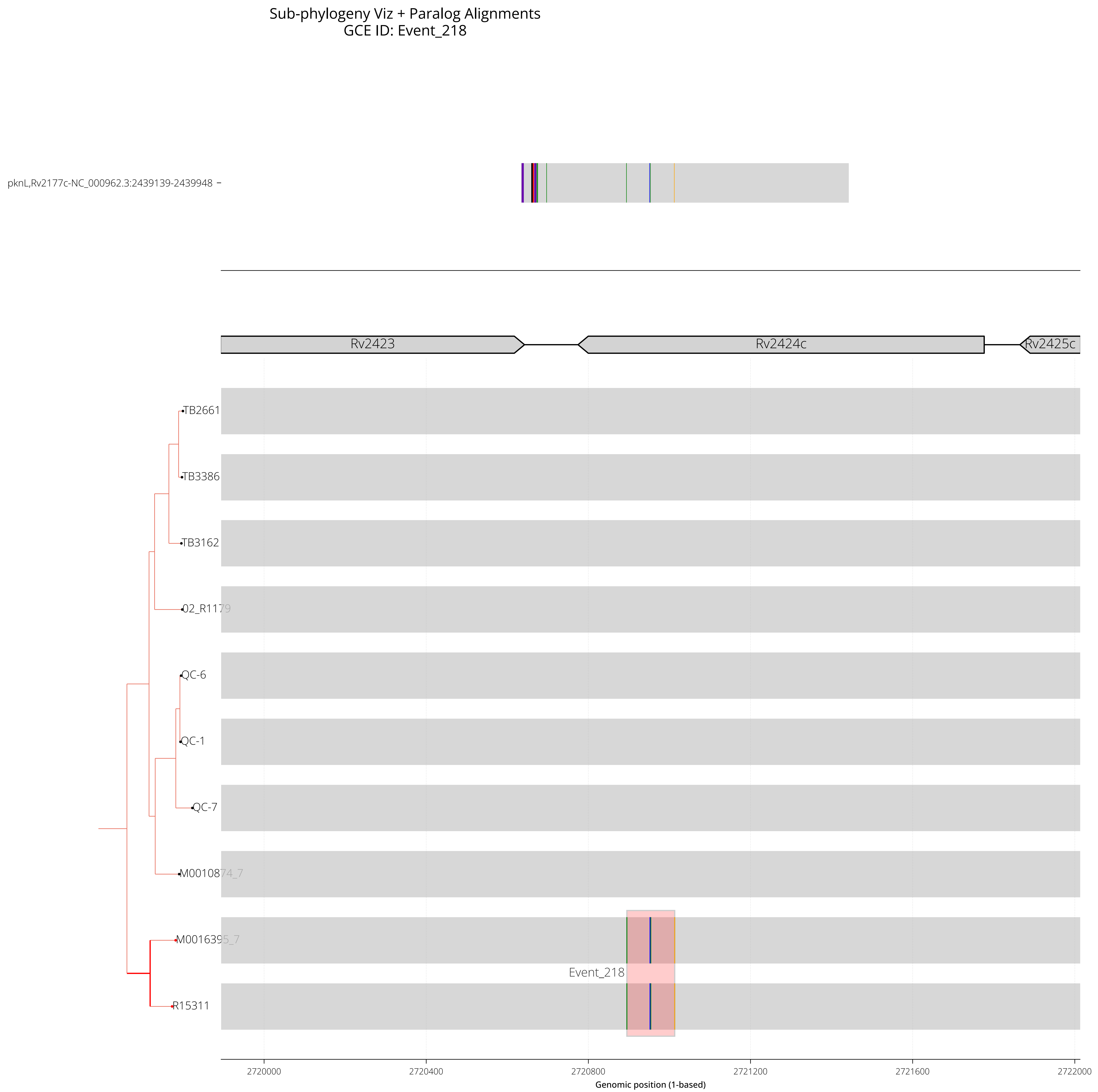
Sub-phylogeny Viz + Paralog Alignments
GCE ID: Event_218
pknL,Rv2177c-NC_000962.3:2439139-2439948
Rv2423
Rv2424c
Rv2425c
TB2661
TB3386
TB3162
02_R1179
QC-6
QC-1
QC-7
M0010874_7
M0016395_7
R15311
Event_218
2720000
2720400
2720800
2721200
2721600
2722000
Genomic position (1-based)

Full Phylogeny Viz + Paralog Alignments
GCE ID: Event_218

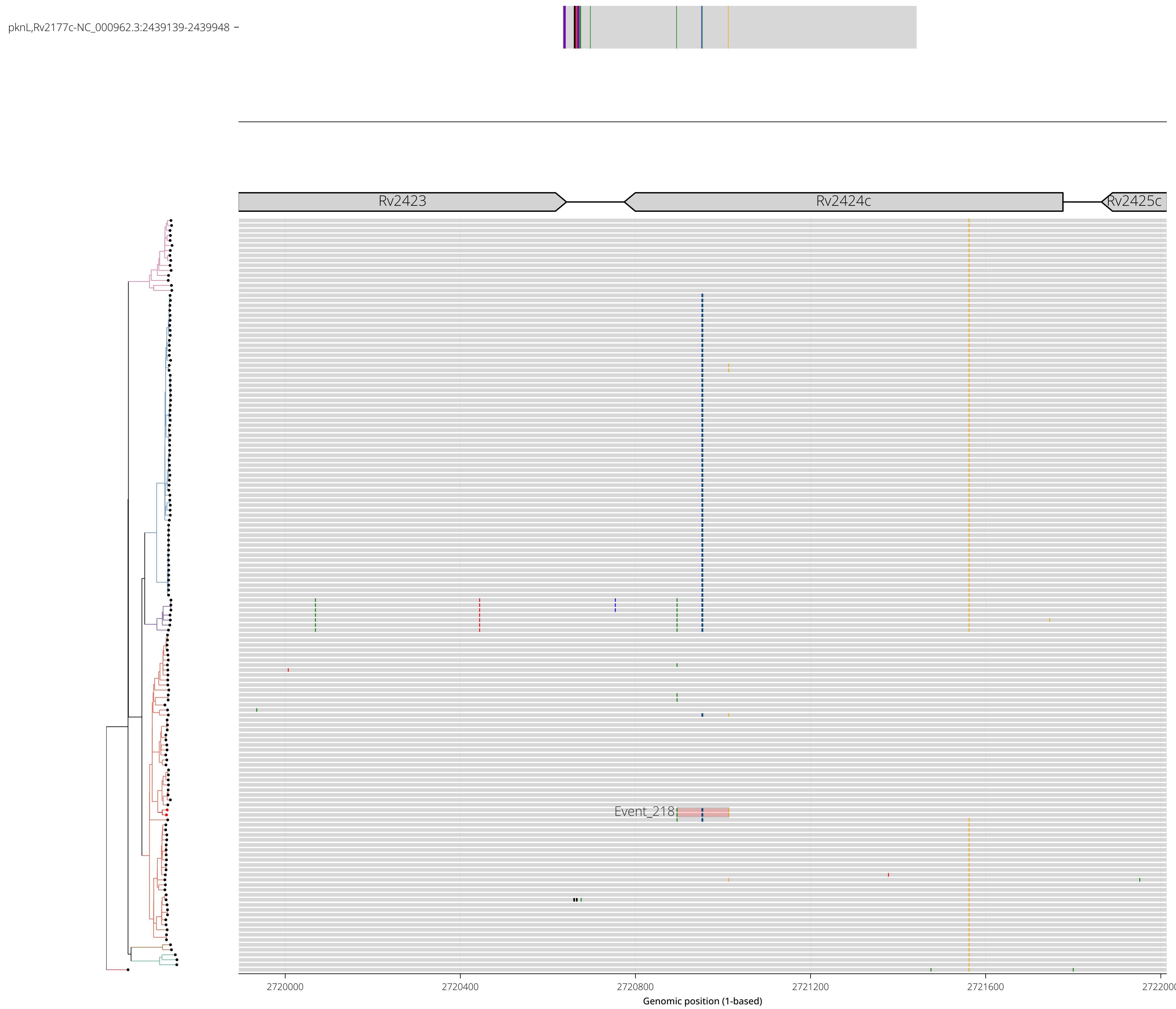

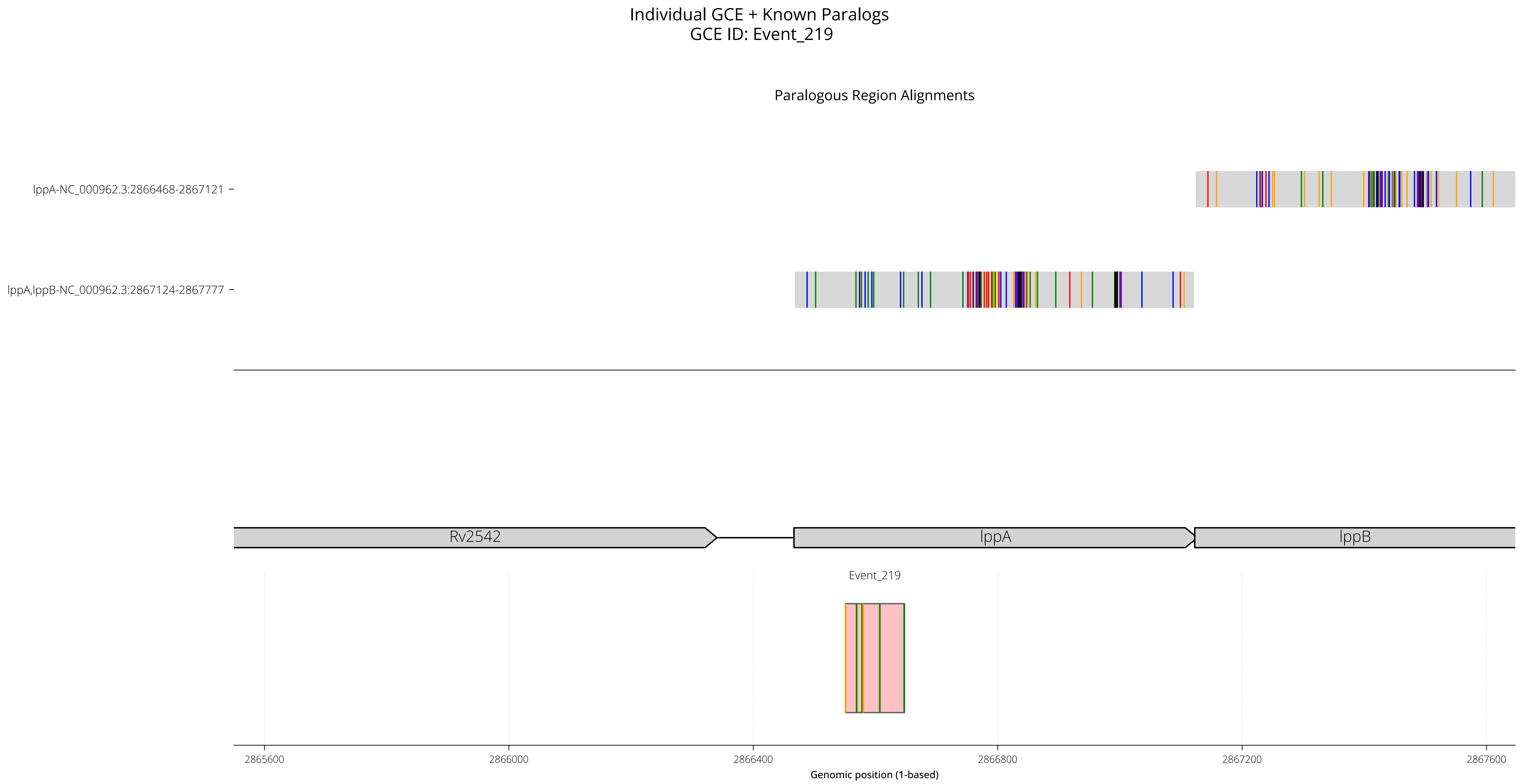

Individual GCE + Known Paralogs
GCE ID: Event_219
Paralogous Region Alignments
lppA-NC_000962.3:2866468-2867121
lppA,lppB-NC_000962.3:2867124-2867777
Rv2542
lppA
lppB
Event_219
2865600
2866000
2866400
2866800
2867200
2867600
Genomic position (1-based)

Circos Viz - GCE to Known Paralogs Locations
GCE ID: Event_219

Location of target gene conversion event
Known (reference) paralog sequences to event region

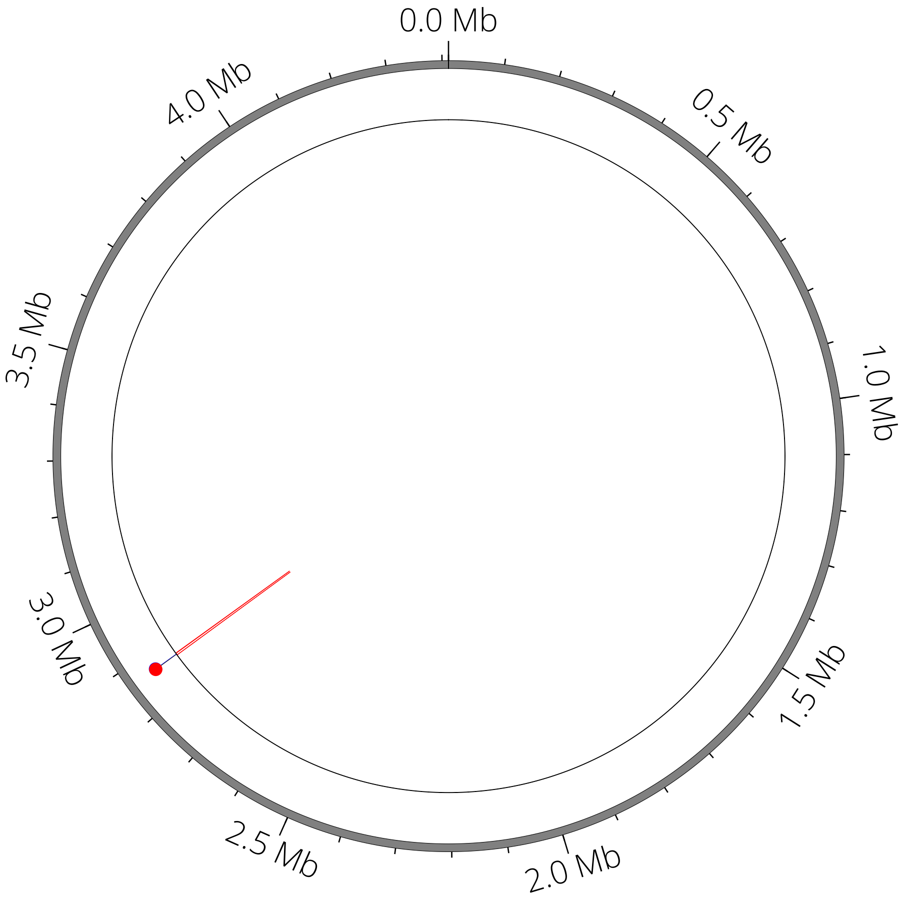

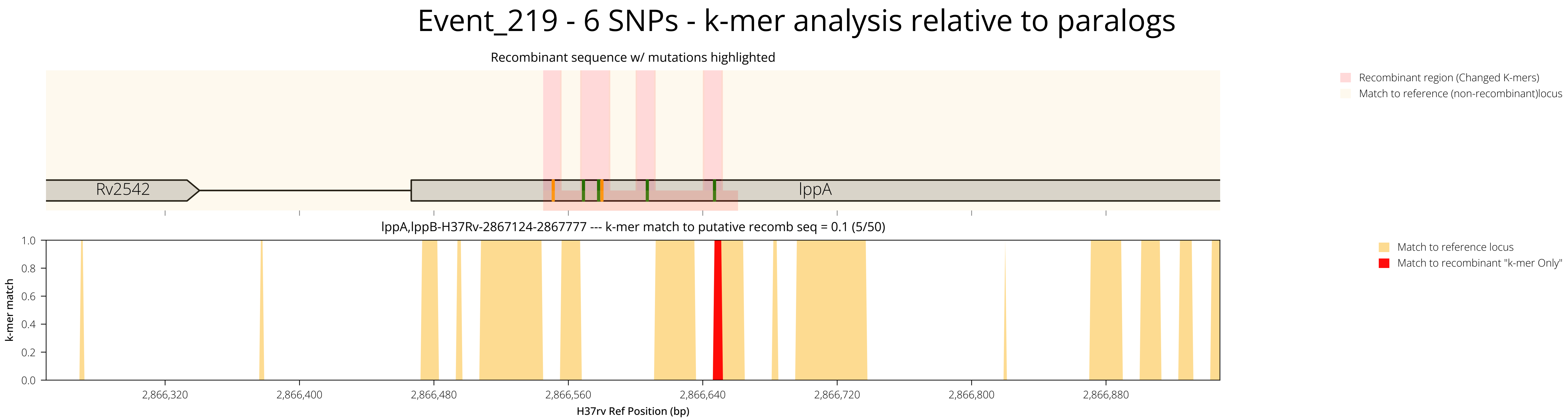
Event_219 - 6 SNPs - k-mer analysis relative to paralogs
Recombinant sequence w/ mutations highlighted
Recombinant region (Changed K-mers)
Match to reference (non-recombinant)locus
Rv2542
lppA
lppA,lppB-H37Rv-2867124-2867777 --- k-mer match to putative recomb seq = 0.1 (5/50)
Match to reference locus
Match to recombinant "k-mer Only"
k-mer match
1.0
0.8
0.6
0.4
0.2
0.0
2,866,320
2,866,400
2,866,480
2,866,560
2,866,640
2,866,720
2,866,800
2,866,880
H37rv Ref Position (bp)

Sub-phylogeny Viz + Paralog Alignments
GCE ID: Event_219

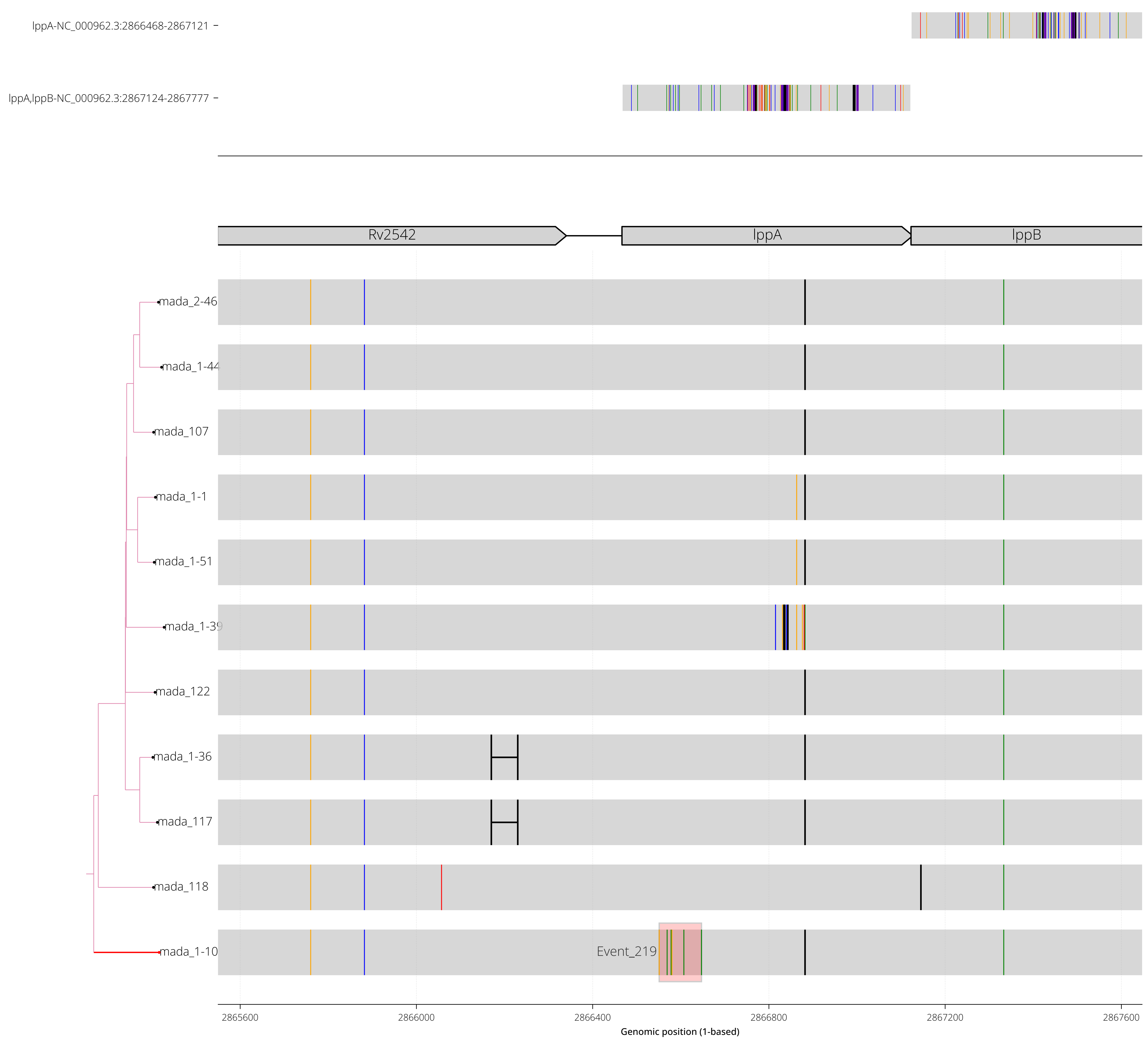

Full Phylogeny Viz + Paralog Alignments
GCE ID: Event_219

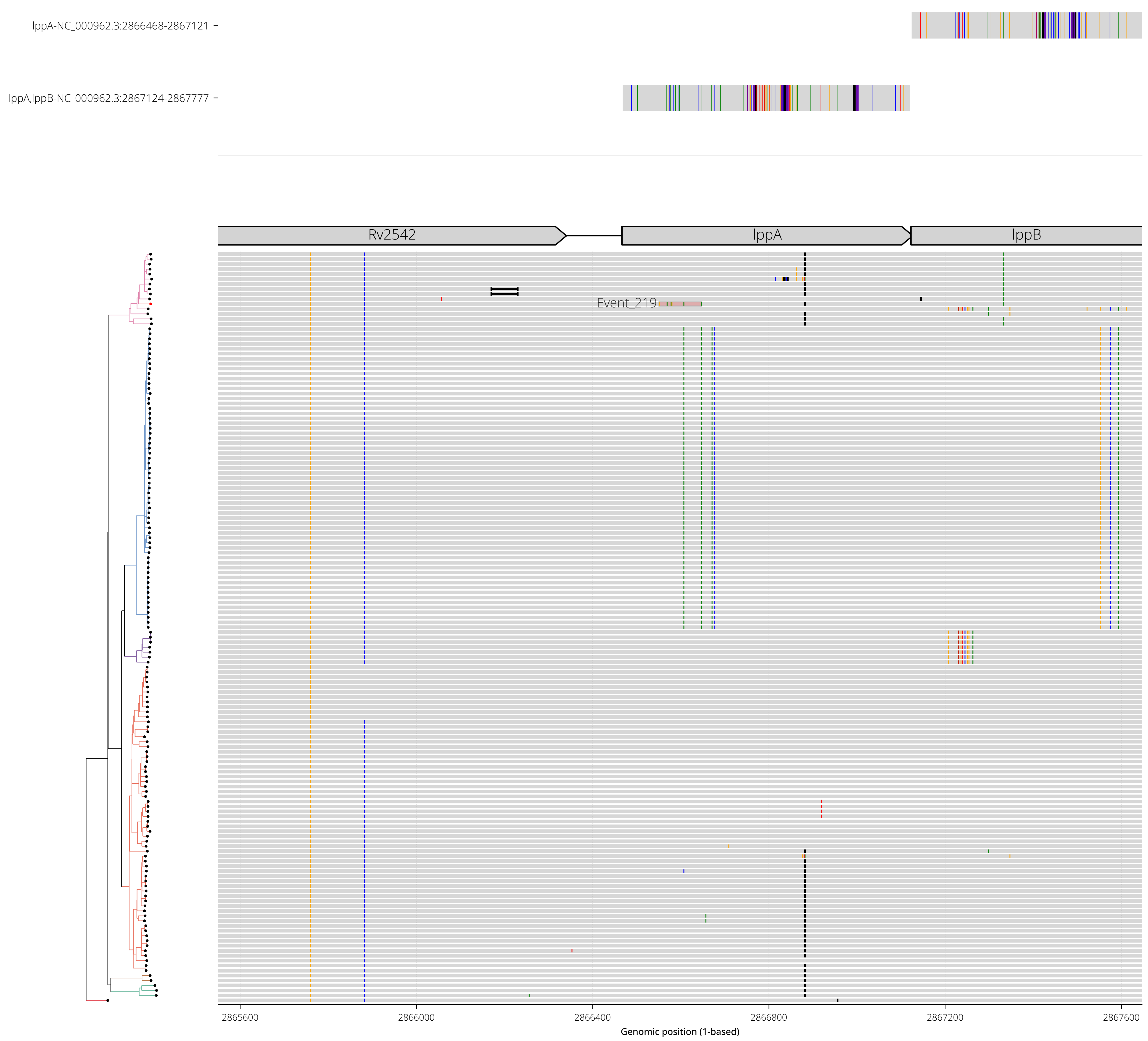

Individual GCE + Known Paralogs
GCE ID: Event_220

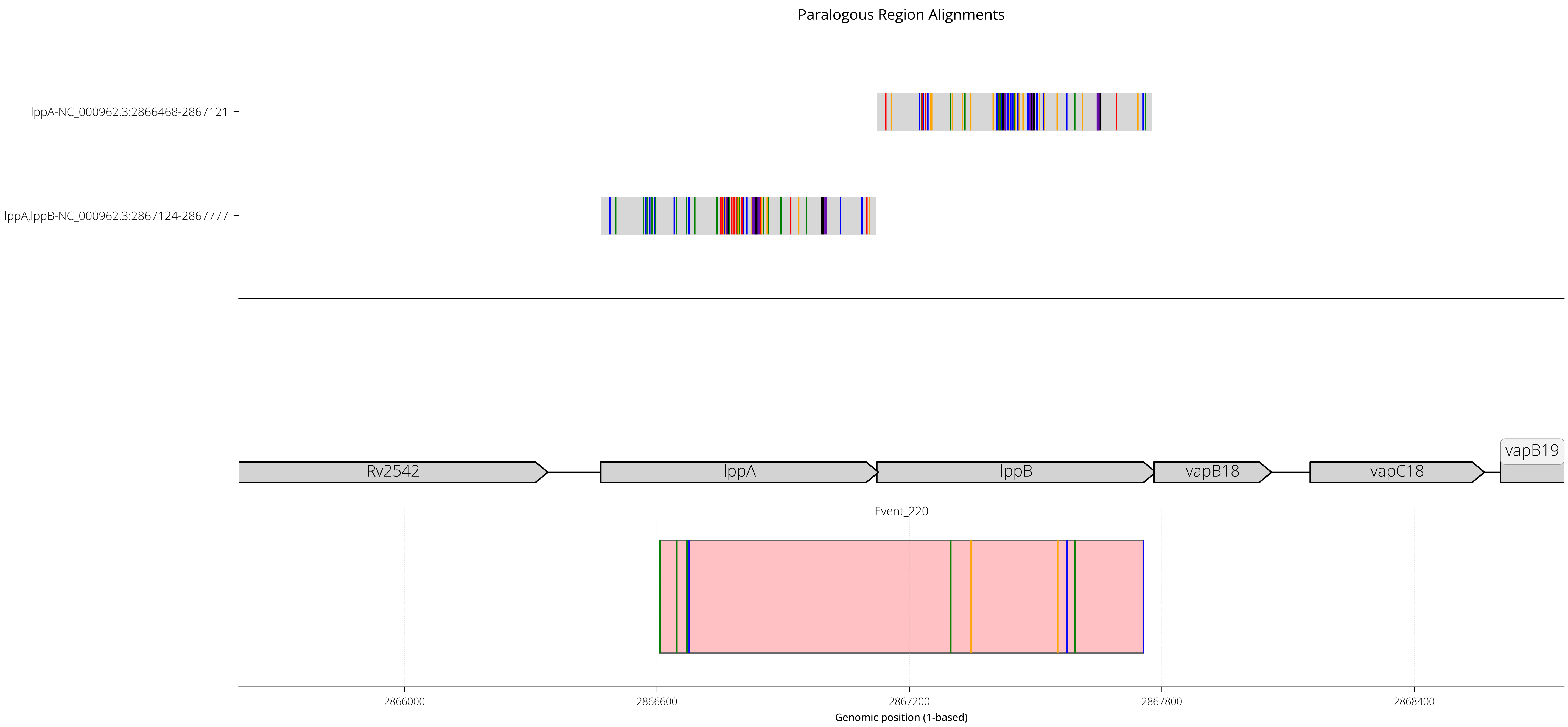

Circos Viz - GCE to Known Paralogs Locations
GCE ID: Event_220

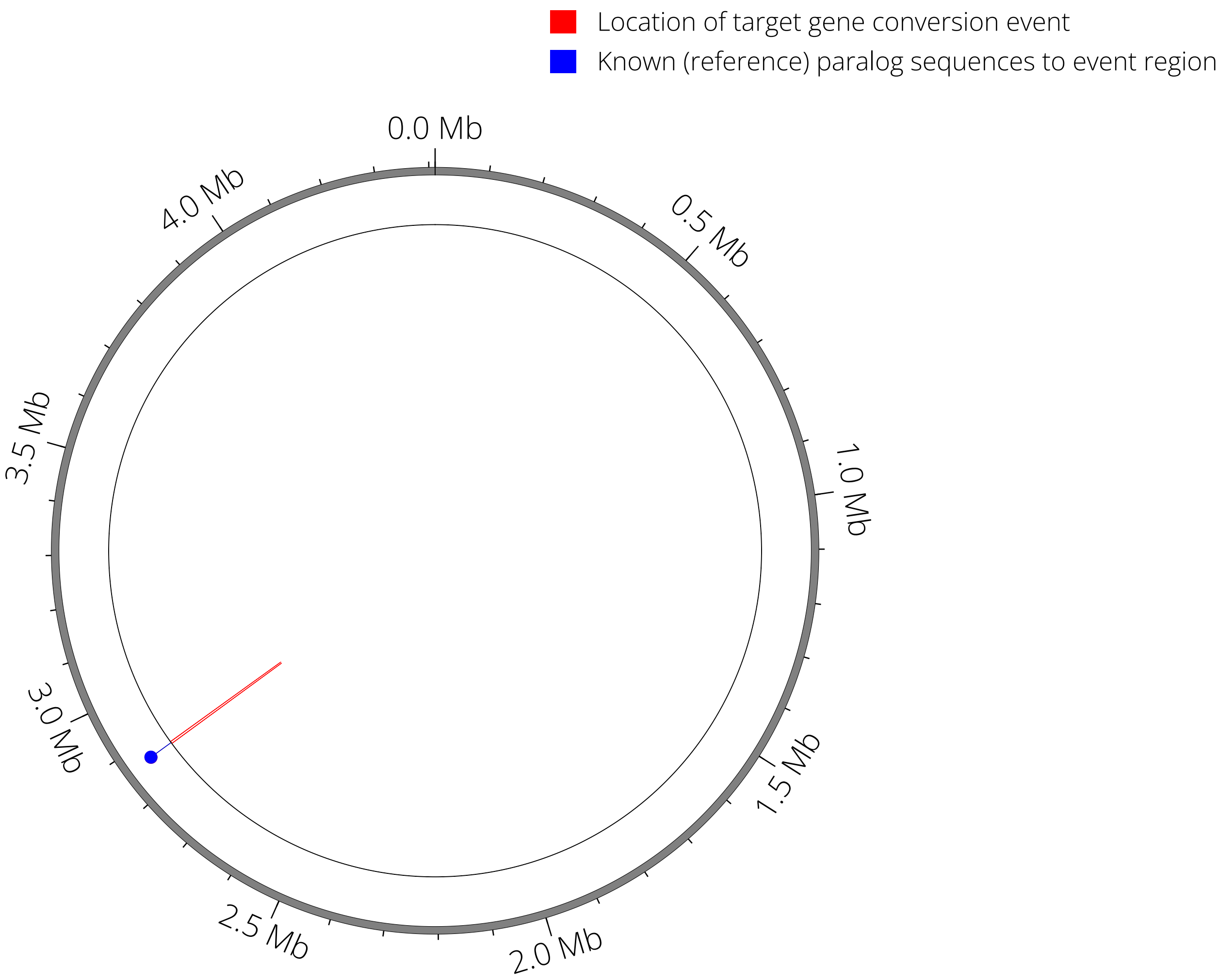

# Event_220 - 10 SNPs - k-mer analysis relative to paralogs

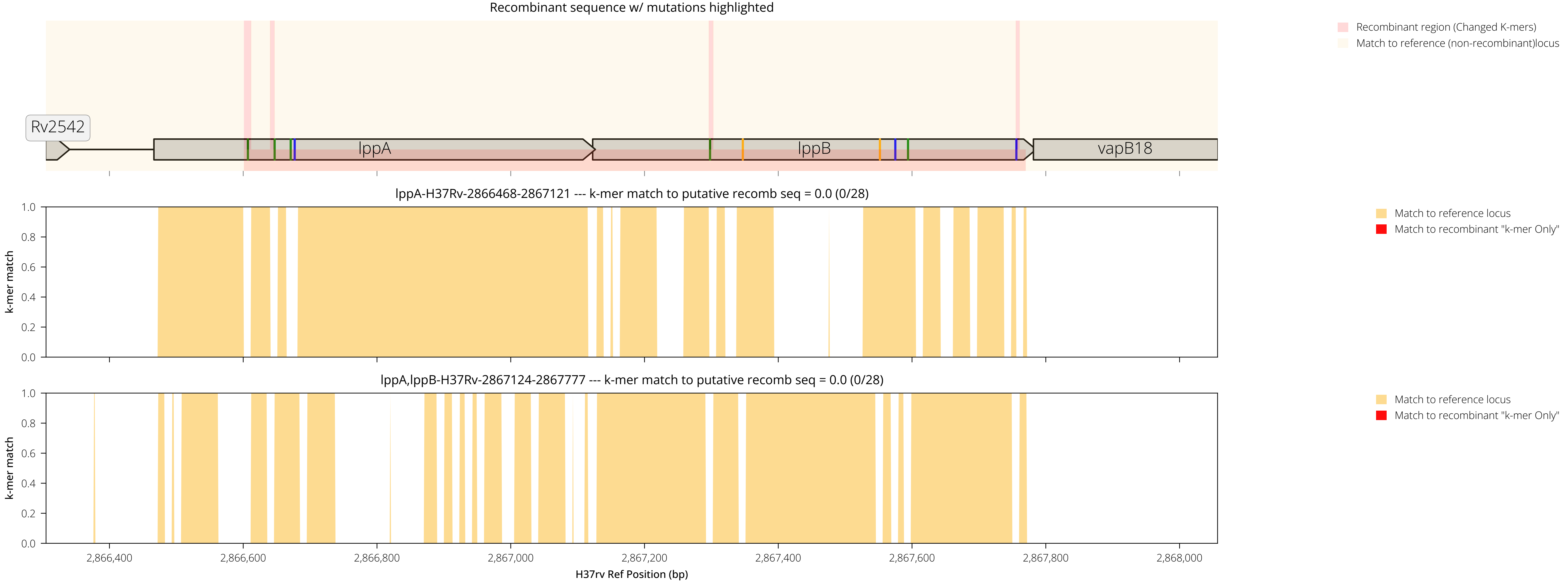

Sub-phylogeny Viz + Paralog Alignments
GCE ID: Event_220

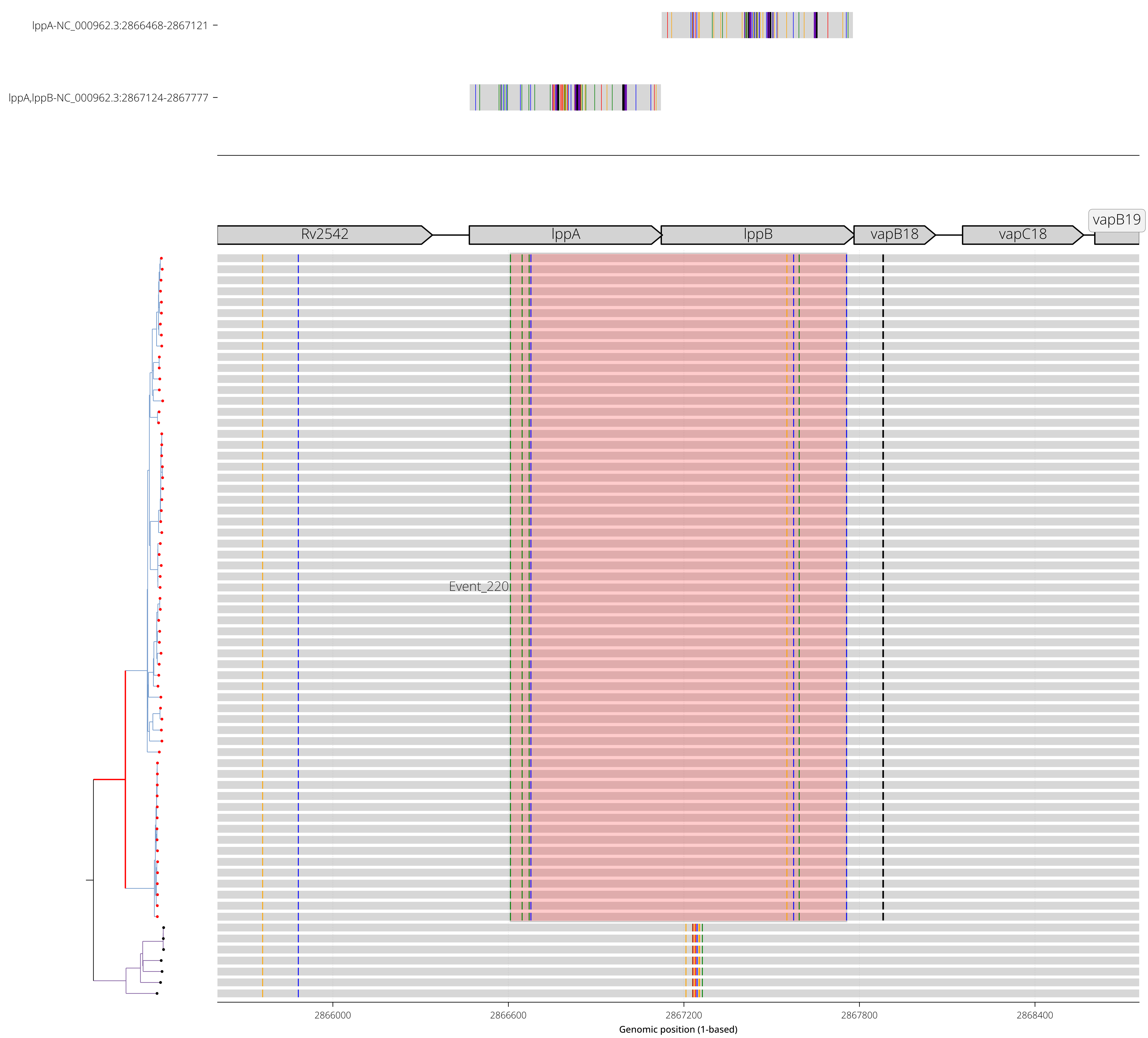

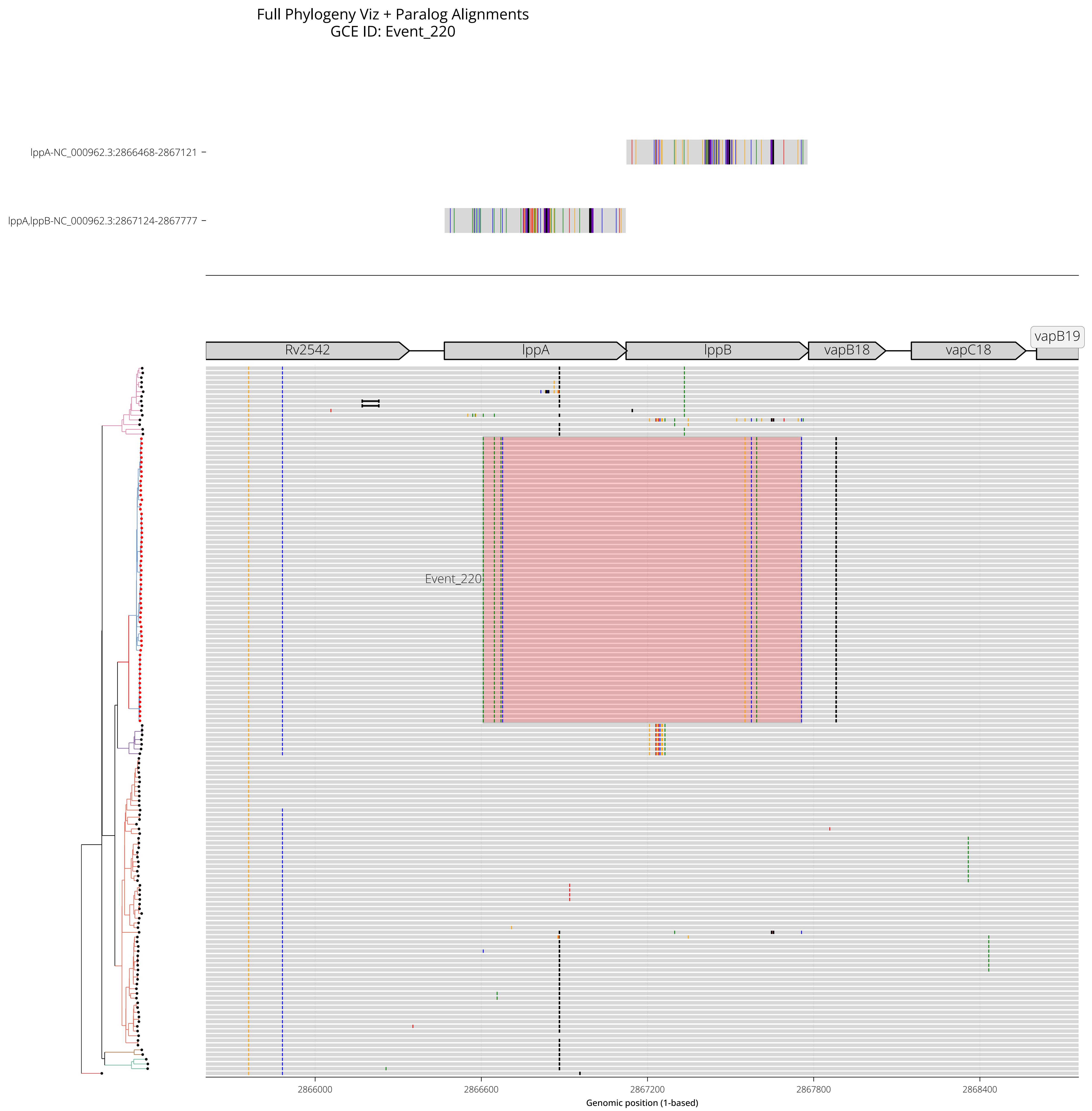

Full Phylogeny Viz + Paralog Alignments
GCE ID: Event_220
lppA-NC_000962.3:2866468-2867121
lppA,lppB-NC_000962.3:2867124-2867777
Rv2542
lppA
lppB
vapB18
vapC18
vapB19
Event_220
2866000
2866600
2867200
2867800
2868400
Genomic position (1-based)

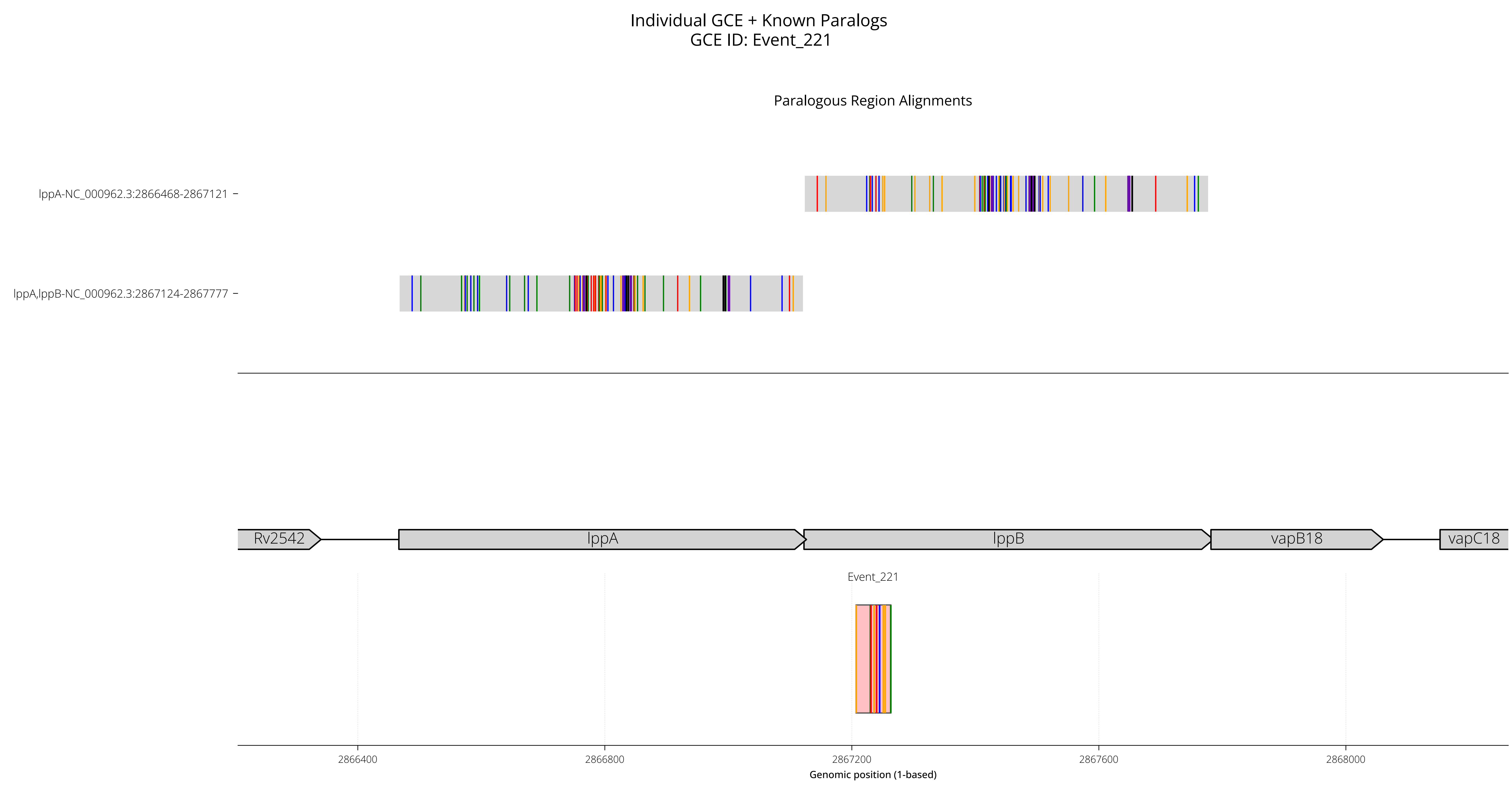

Individual GCE + Known Paralogs
GCE ID: Event_221
Paralogous Region Alignments
lppA-NC_000962.3:2866468-2867121
lppA,lppB-NC_000962.3:2867124-2867777
Rv2542
lppA
lppB
vapB18
vapC18
Event_221
2866400
2866800
2867200
2867600
2868000
Genomic position (1-based)

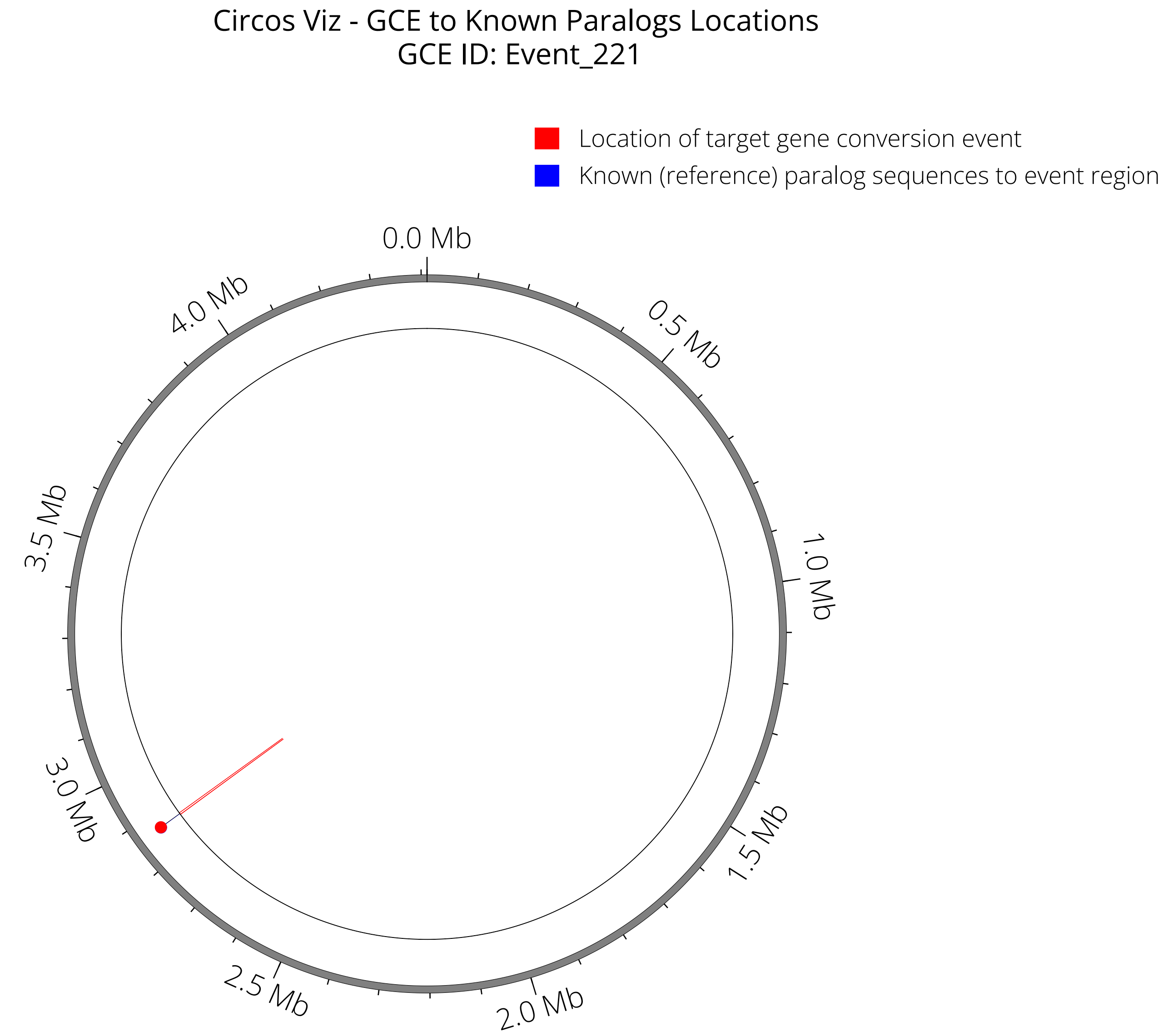
Circos Viz - GCE to Known Paralogs Locations
GCE ID: Event_221
Location of target gene conversion event
Known (reference) paralog sequences to event region
0.0 Mb
0.5 Mb
1.0 Mb
1.5 Mb
2.0 Mb
2.5 Mb
3.0 Mb
3.5 Mb
4.0 Mb

# Event_221 - 9 SNPs - k-mer analysis relative to paralogs

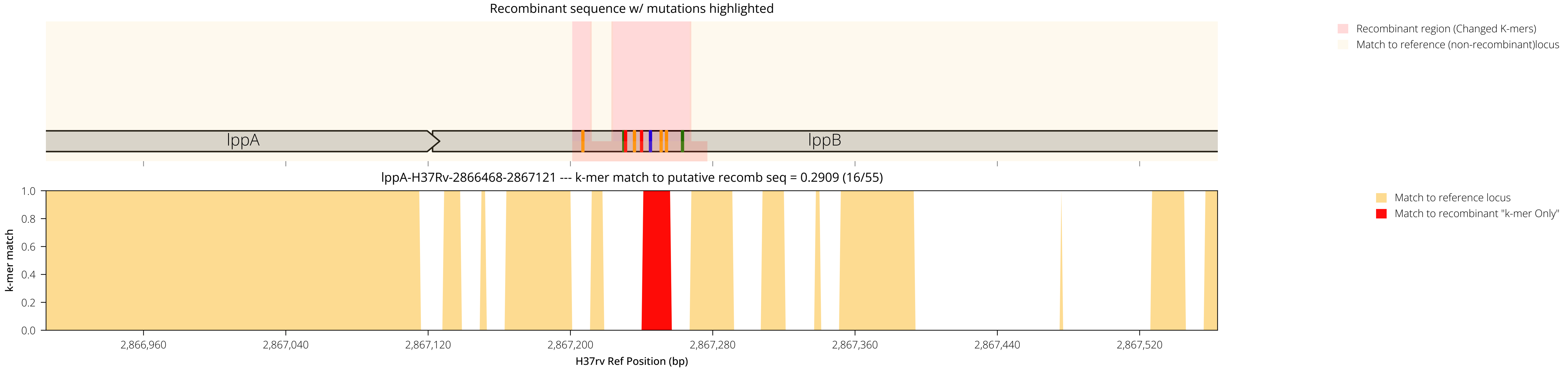

Sub-phylogeny Viz + Paralog Alignments
GCE ID: Event_221

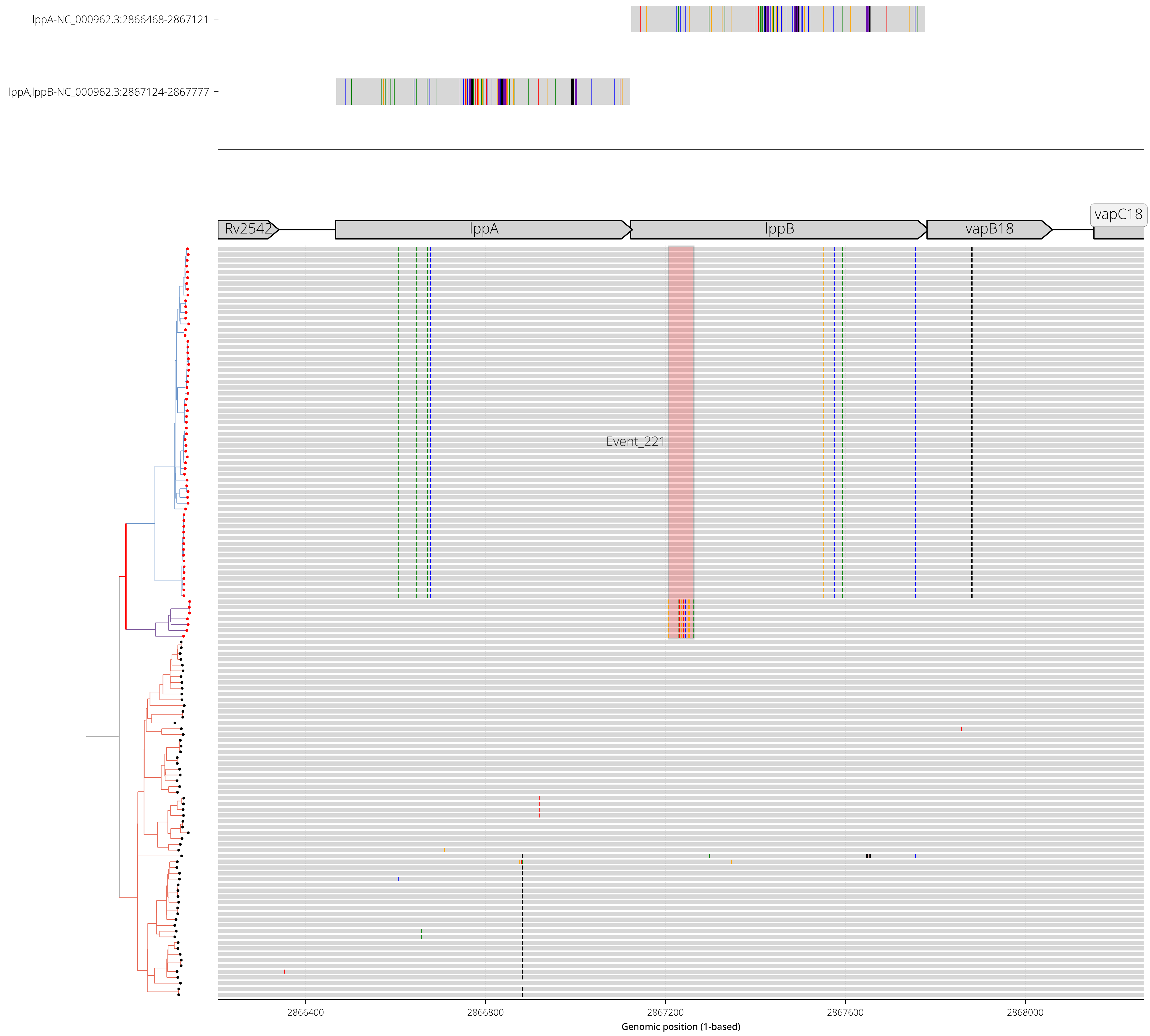

Full Phylogeny Viz + Paralog Alignments
GCE ID: Event_221

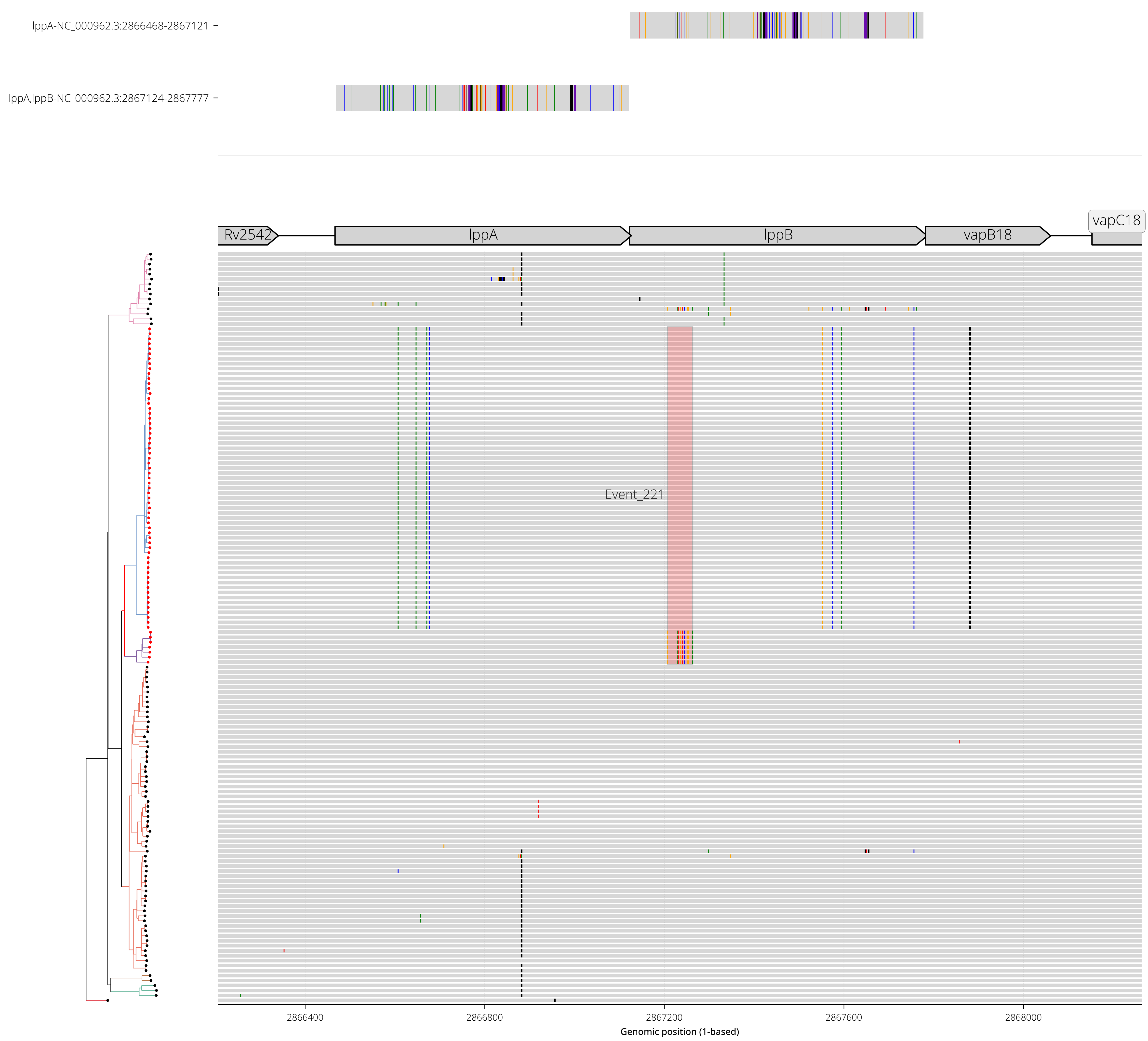

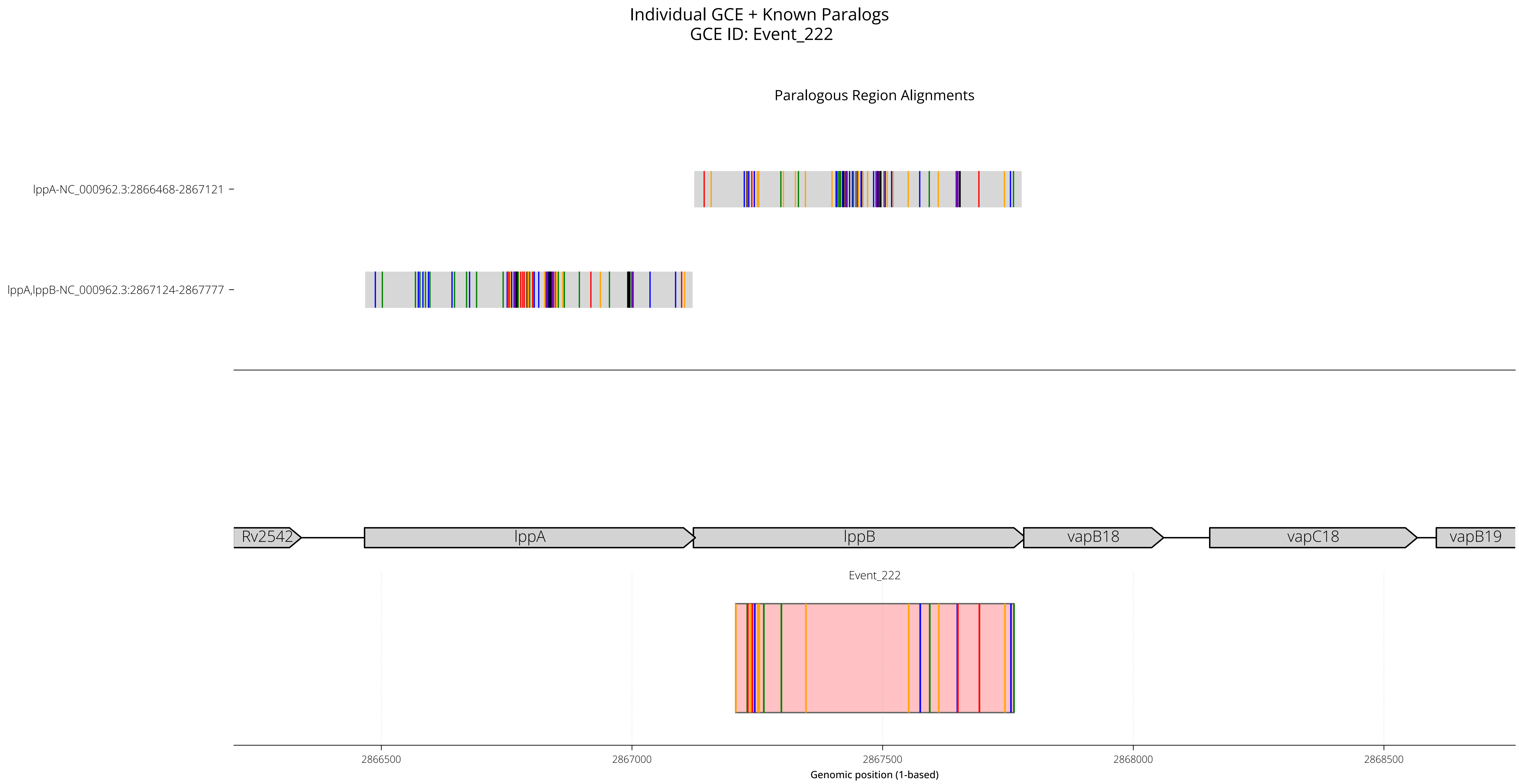

Individual GCE + Known Paralogs
GCE ID: Event_222
Paralogous Region Alignments
lppA-NC_000962.3:2866468-2867121
lppA,lppB-NC_000962.3:2867124-2867777
Rv2542
lppA
lppB
vapB18
vapC18
vapB19
Event_222
2866500
2867000
2867500
2868000
2868500
Genomic position (1-based)

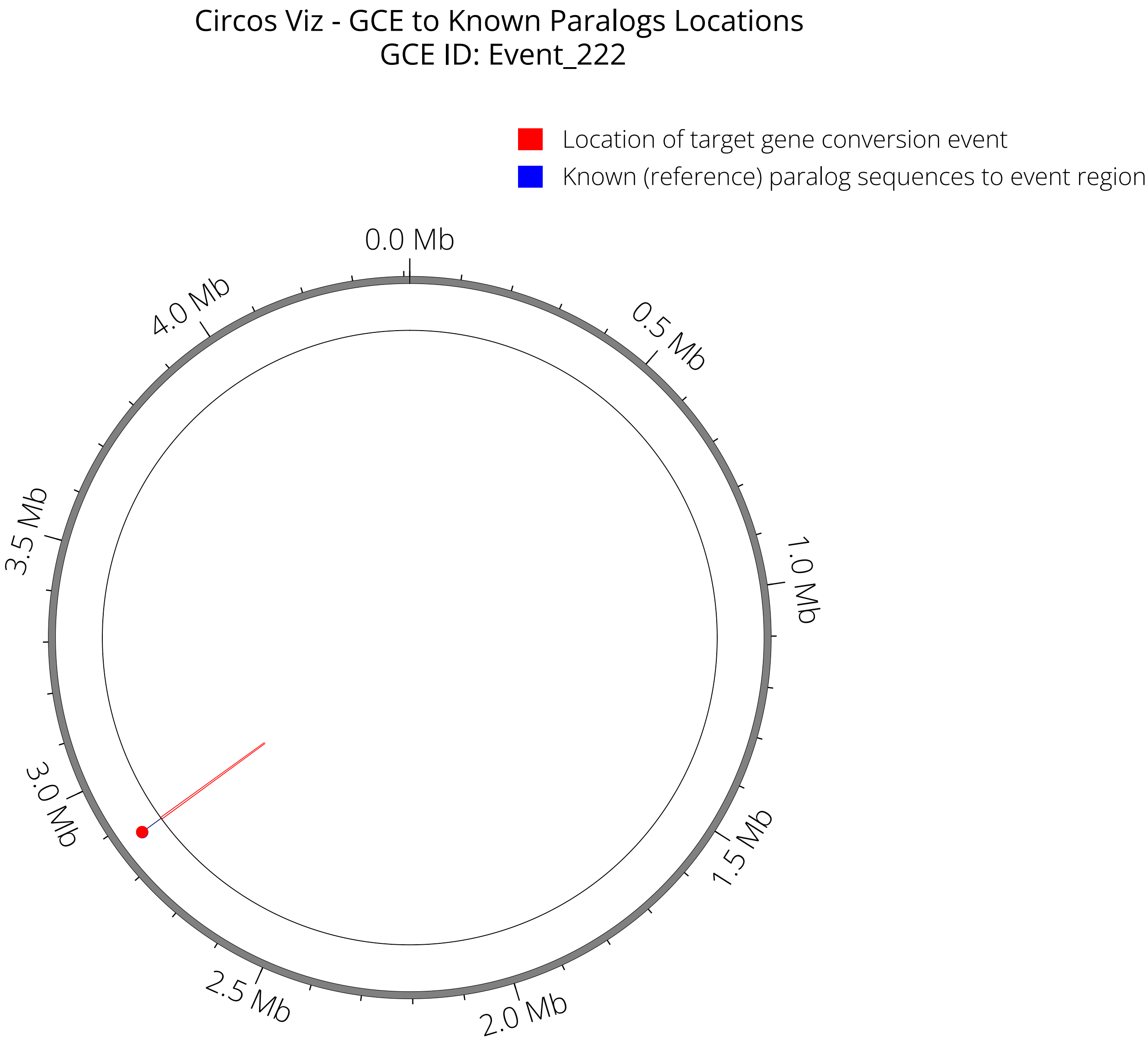
Circos Viz - GCE to Known Paralogs Locations
GCE ID: Event_222
Location of target gene conversion event
Known (reference) paralog sequences to event region
0.0 Mb
0.5 Mb
1.0 Mb
1.5 Mb
2.0 Mb
2.5 Mb
3.0 Mb
3.5 Mb
4.0 Mb

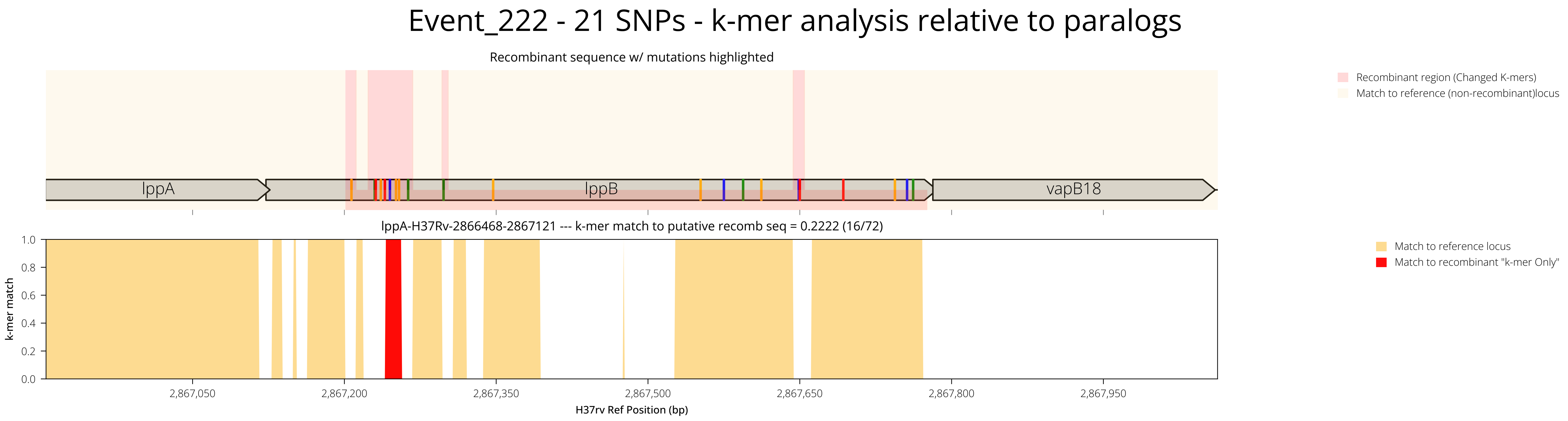

Event_222 - 21 SNPs - k-mer analysis relative to paralogs
Recombinant sequence w/ mutations highlighted
lppA
lppB
vapB18
Recombinant region (Changed K-mers)
Match to reference (non-recombinant)locus
lppA-H37Rv-2866468-2867121 --- k-mer match to putative recomb seq = 0.2222 (16/72)
k-mer match
1.0
0.8
0.6
0.4
0.2
0.0
2,867,050
2,867,200
2,867,350
2,867,500
2,867,650
2,867,800
2,867,950
H37rv Ref Position (bp)
Match to reference locus
Match to recombinant "k-mer Only"

Sub-phylogeny Viz + Paralog Alignments
GCE ID: Event_222

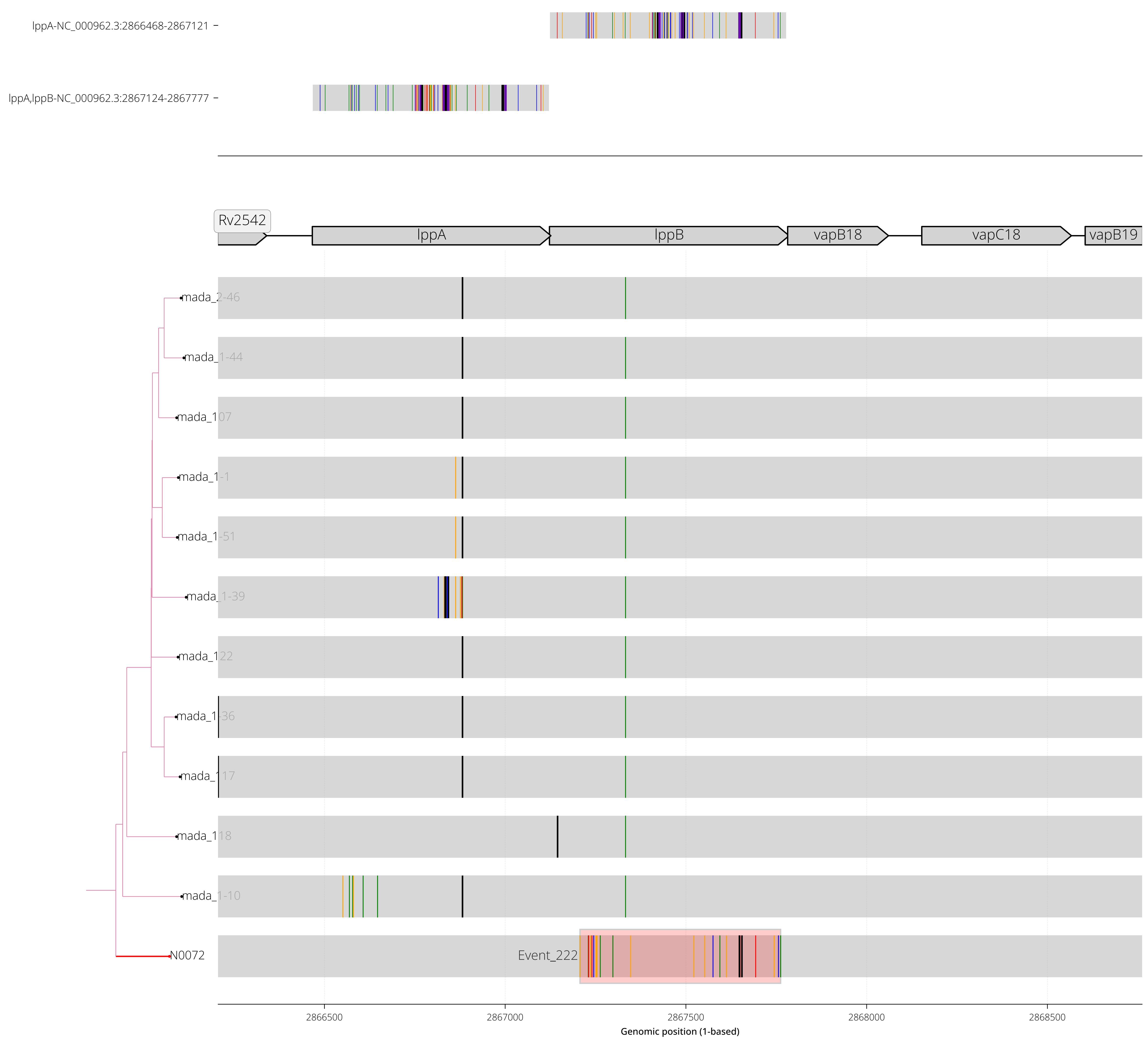

Full Phylogeny Viz + Paralog Alignments
GCE ID: Event_222

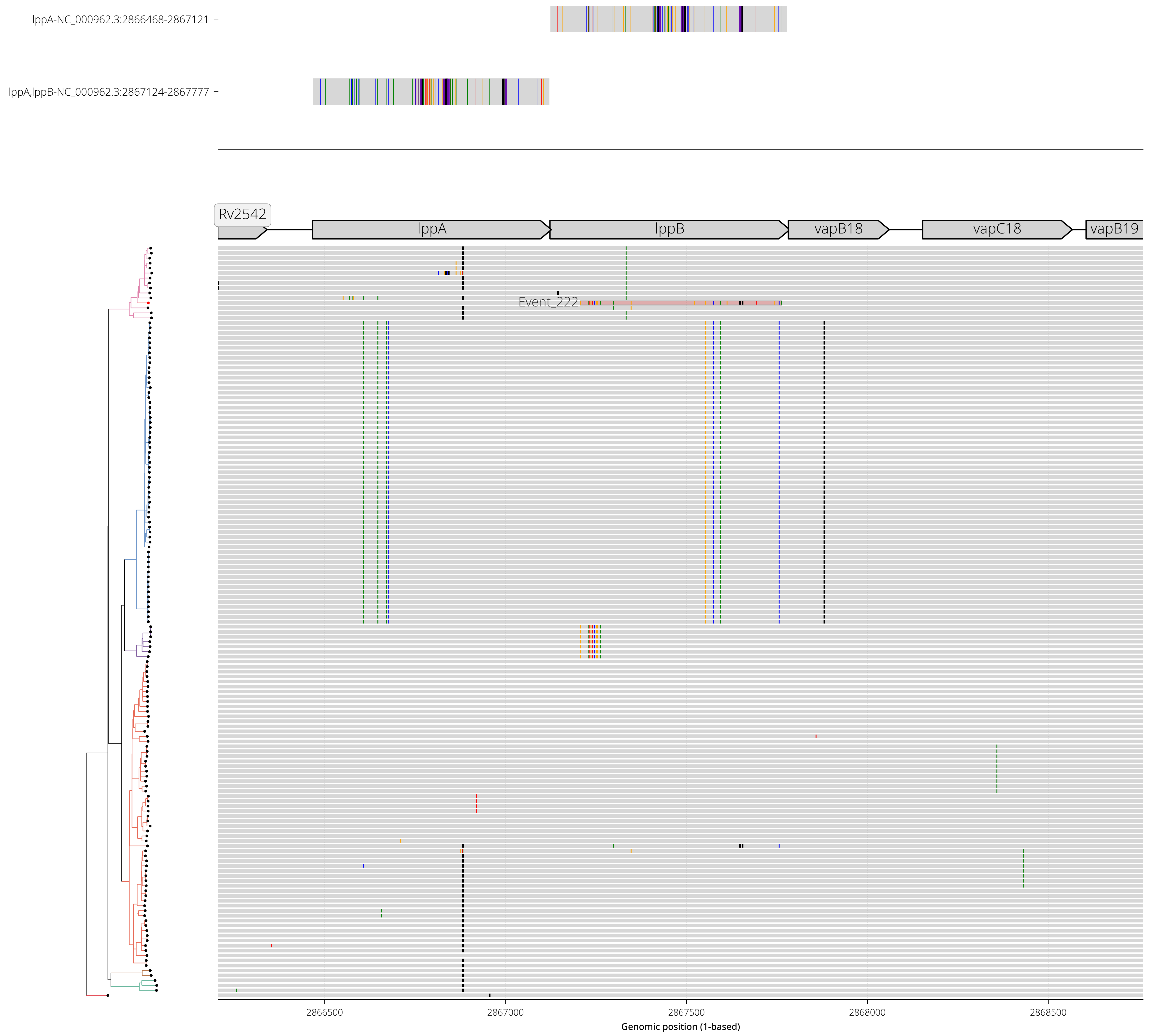

Individual GCE + Known Paralogs
GCE ID: Event_223

Paralogous Region Alignments

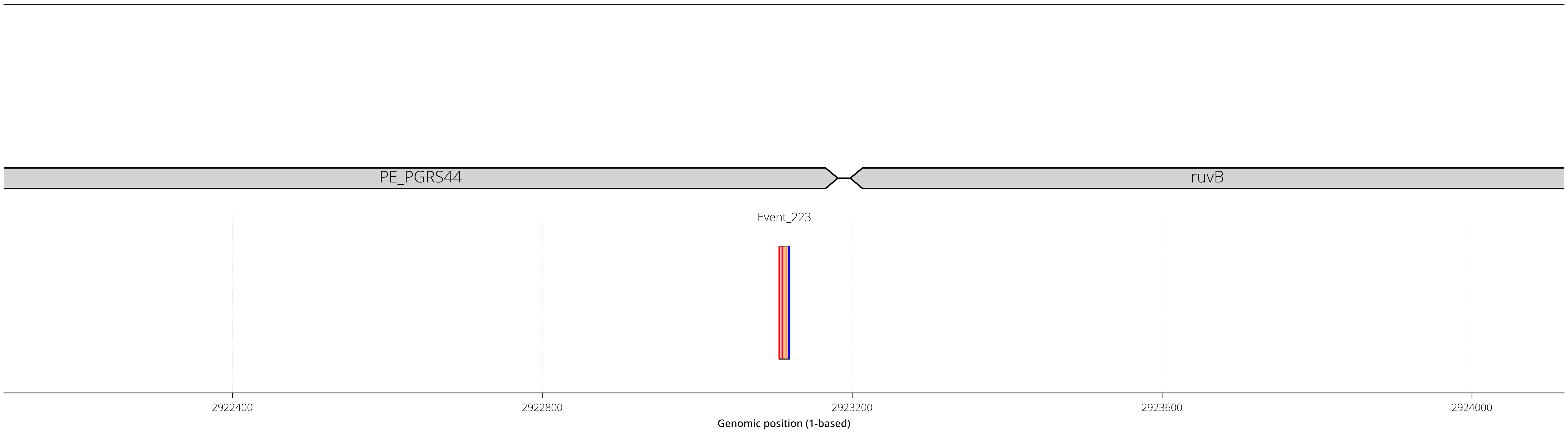

Sub-phylogeny Viz + Paralog Alignments
GCE ID: Event_223

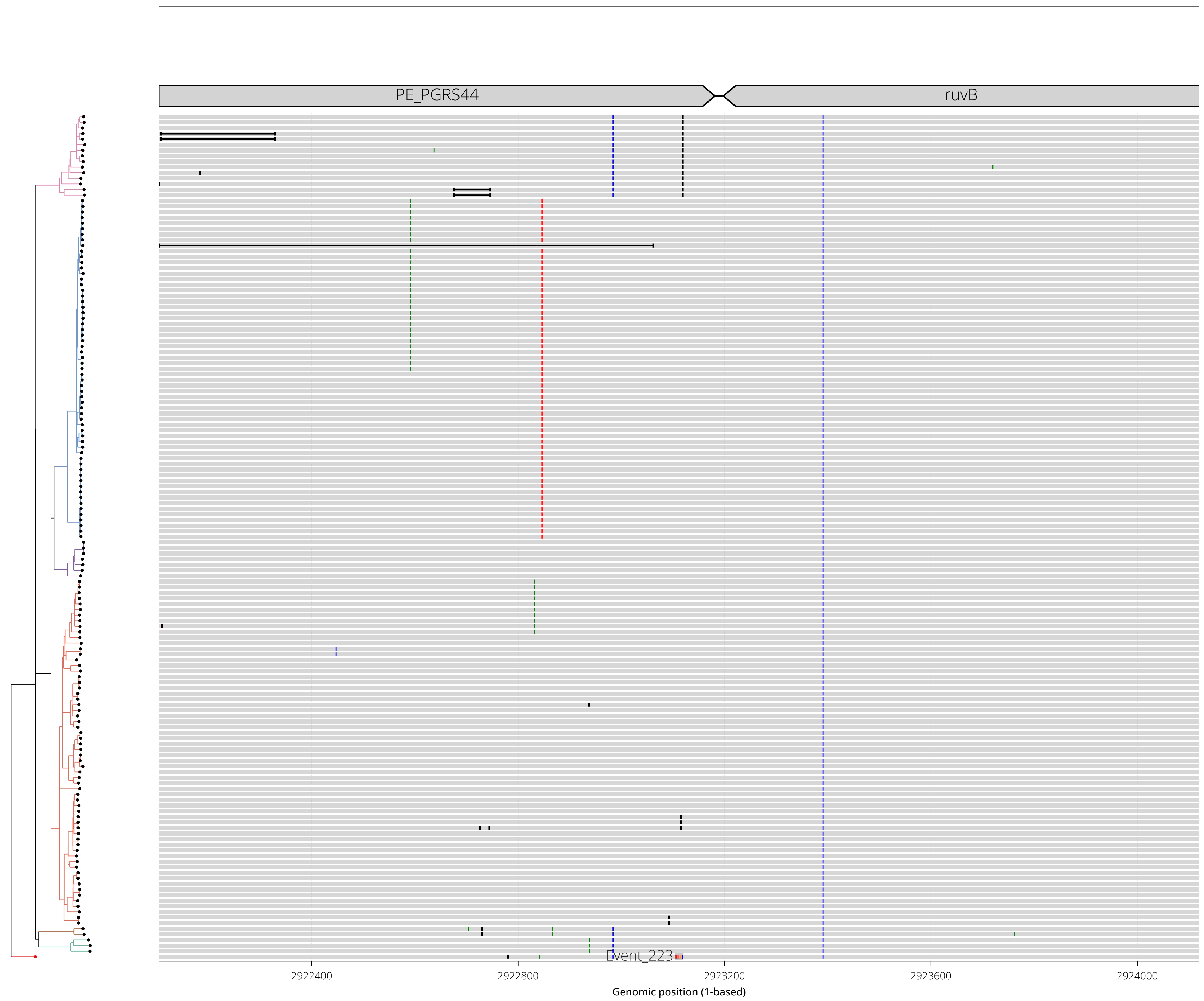

Full Phylogeny Viz + Paralog Alignments
GCE ID: Event_223

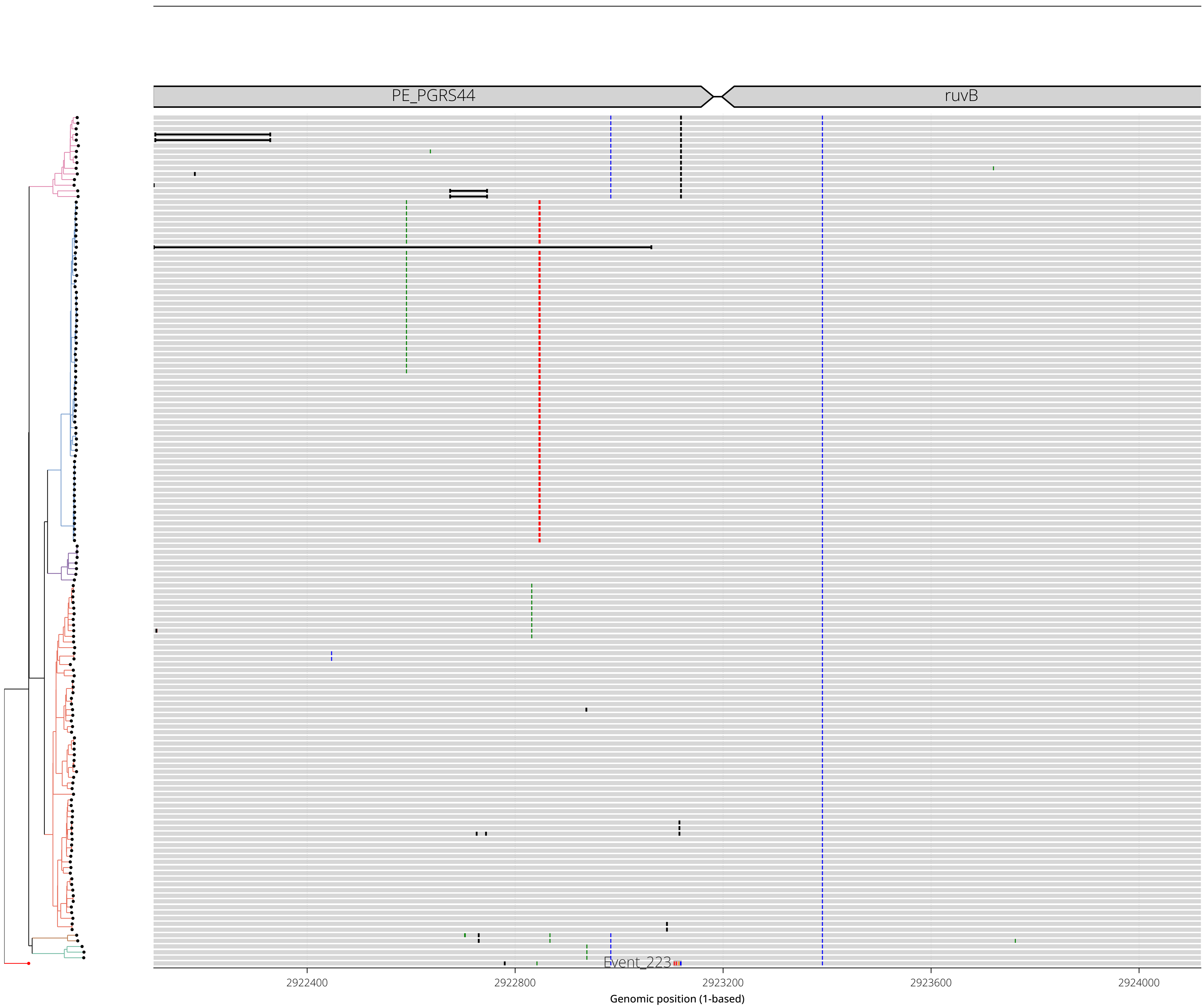

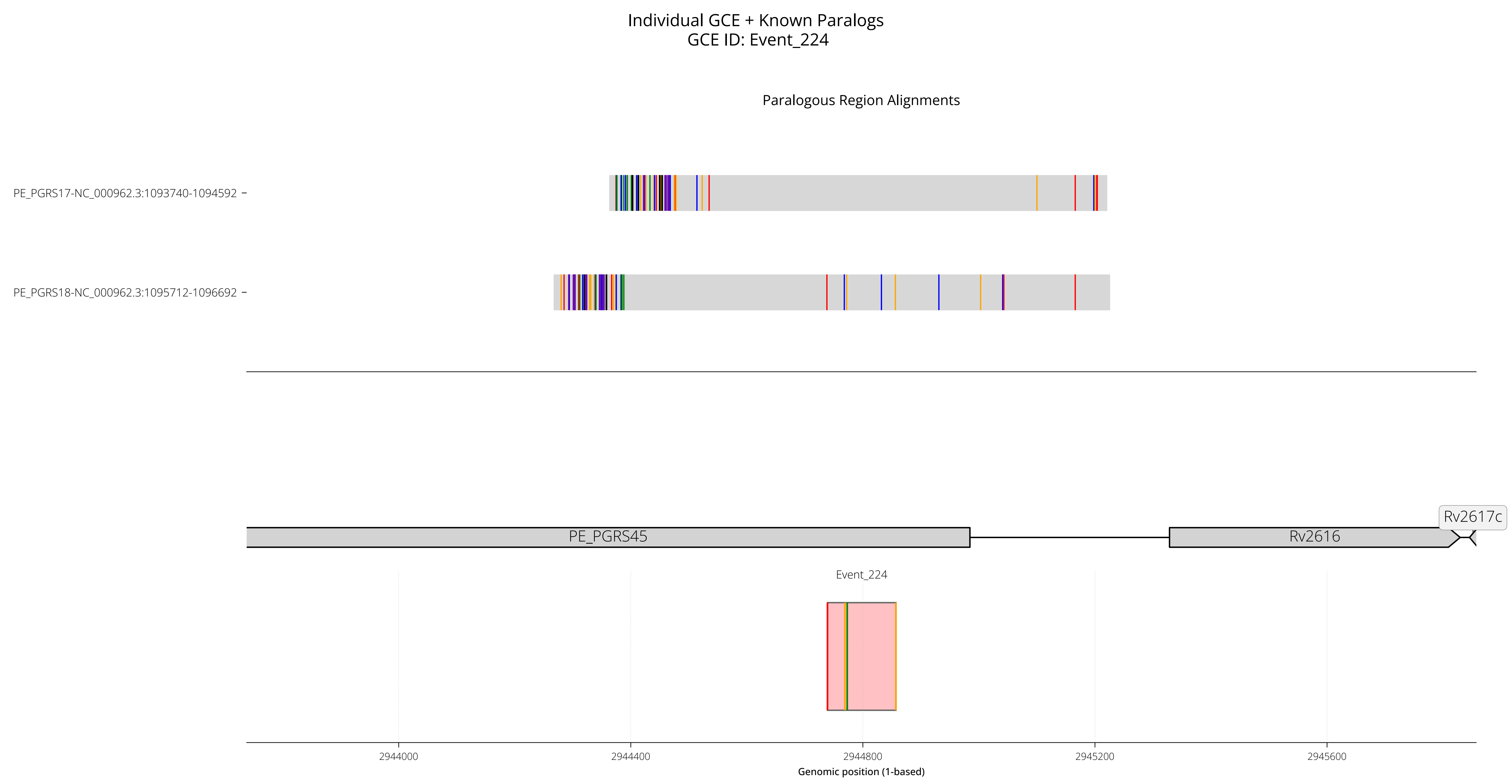

Individual GCE + Known Paralogs
GCE ID: Event_224
Paralogous Region Alignments
PE_PGRS17-NC_000962.3:1093740-1094592
PE_PGRS18-NC_000962.3:1095712-1096692
PE_PGRS45
Rv2616
Rv2617c
Event_224
2944000
2944400
2944800
2945200
2945600
Genomic position (1-based)

Circos Viz - GCE to Known Paralogs Locations
GCE ID: Event_224

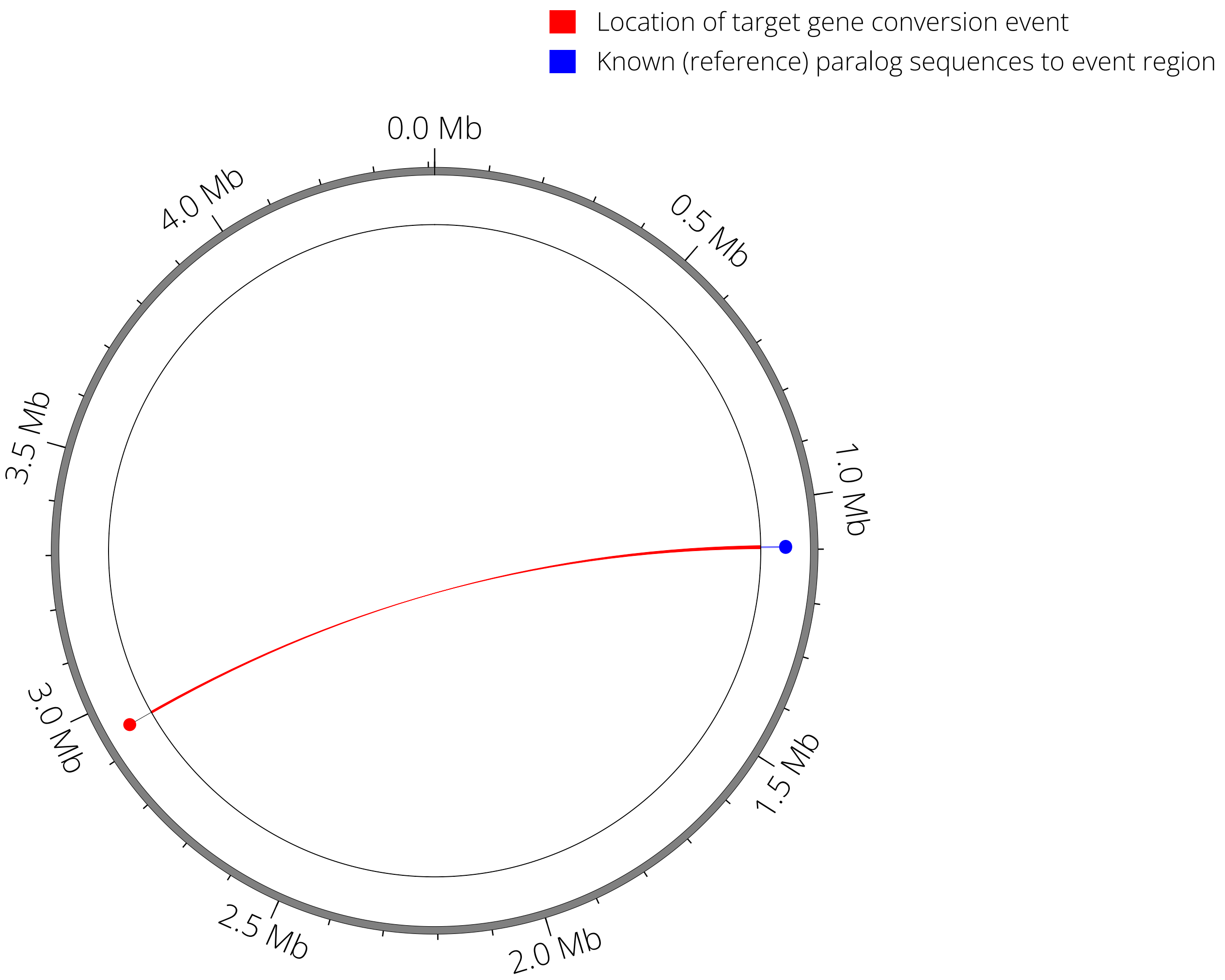

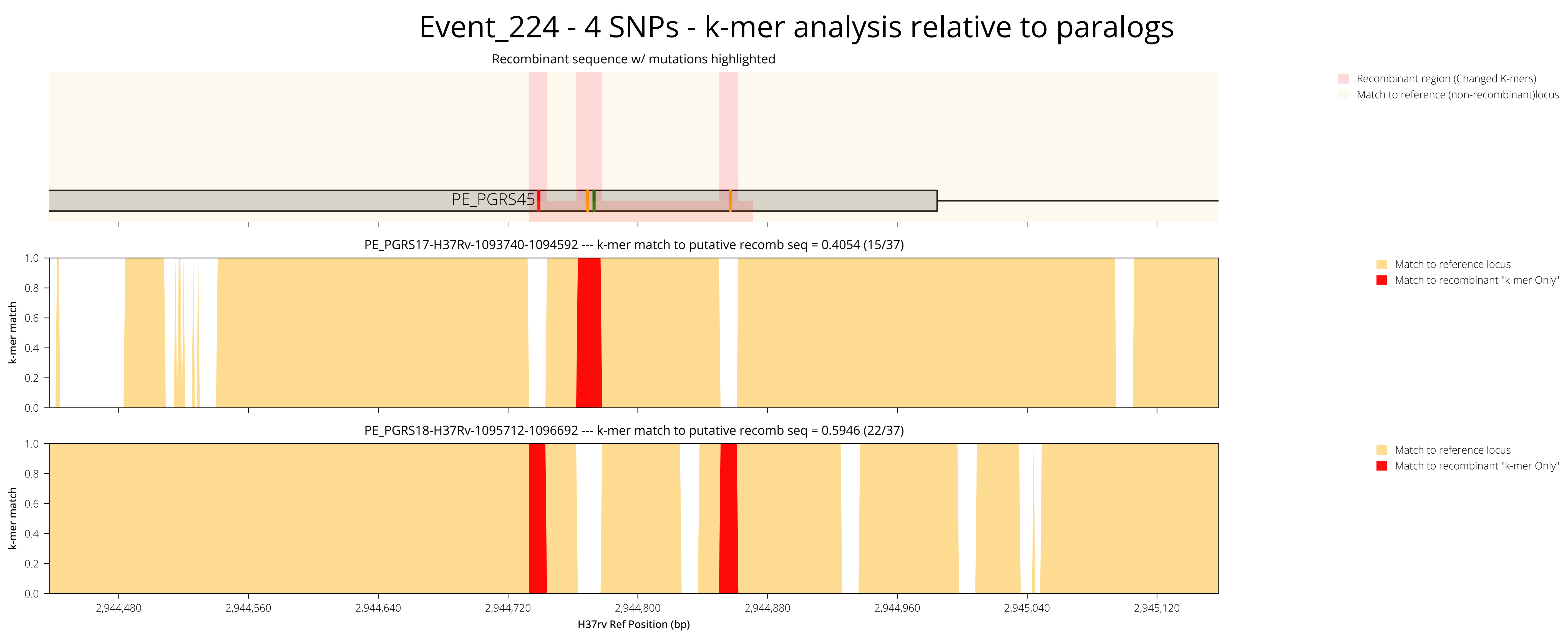
Event_224 - 4 SNPs - k-mer analysis relative to paralogs
Recombinant sequence w/ mutations highlighted
PE_PGRS45
Recombinant region (Changed K-mers)
Match to reference (non-recombinant)locus
PE_PGRS17-H37Rv-1093740-1094592 --- k-mer match to putative recomb seq = 0.4054 (15/37)
Match to reference locus
Match to recombinant "k-mer Only"
k-mer match
1.0
0.8
0.6
0.4
0.2
0.0
PE_PGRS18-H37Rv-1095712-1096692 --- k-mer match to putative recomb seq = 0.5946 (22/37)
Match to reference locus
Match to recombinant "k-mer Only"
k-mer match
2,944,480
2,944,560
2,944,640
2,944,720
2,944,800
2,944,880
2,944,960
2,945,040
2,945,120
H37rv Ref Position (bp)

Sub-phylogeny Viz + Paralog Alignments
GCE ID: Event_224

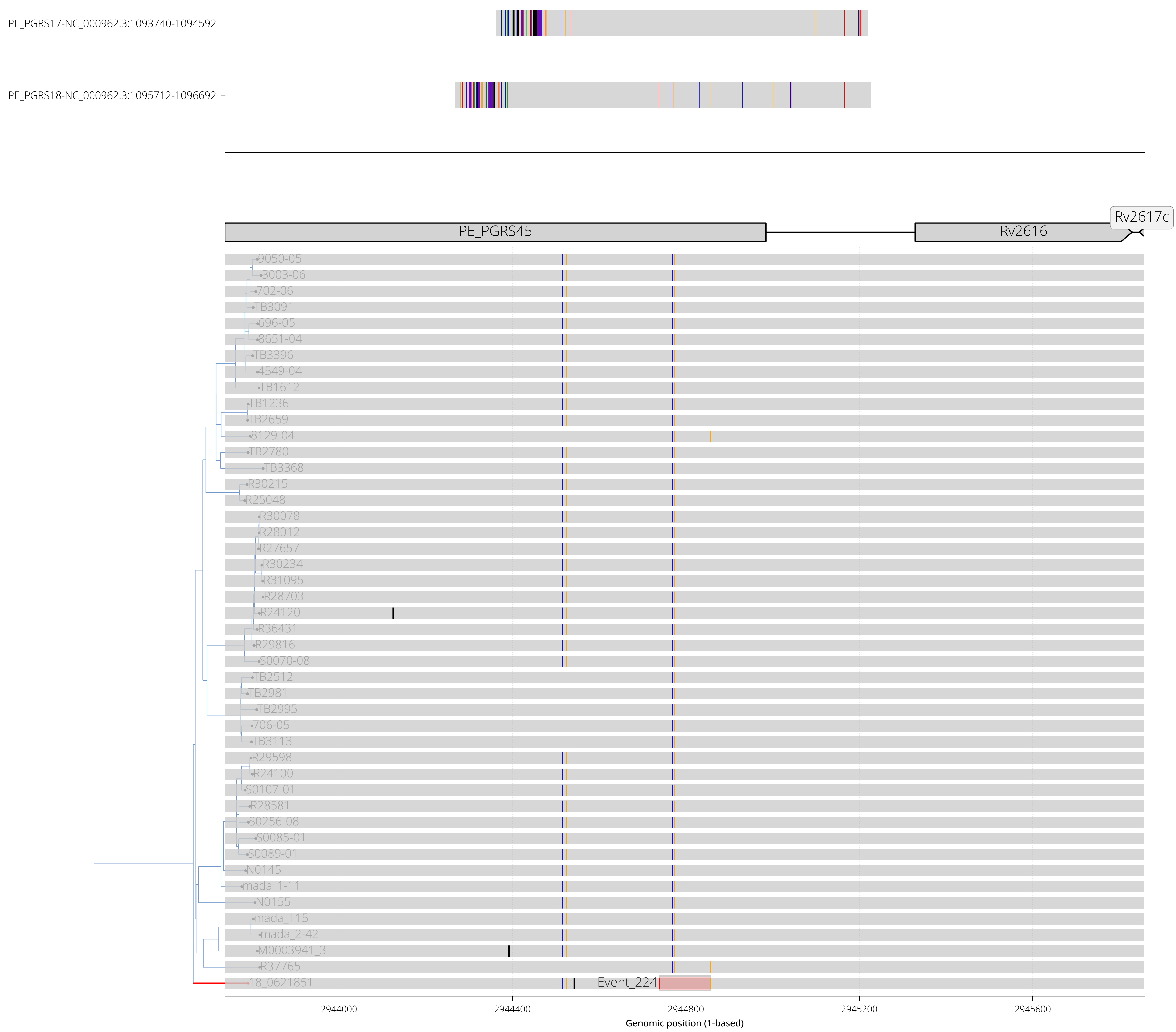

Full Phylogeny Viz + Paralog Alignments
GCE ID: Event_224

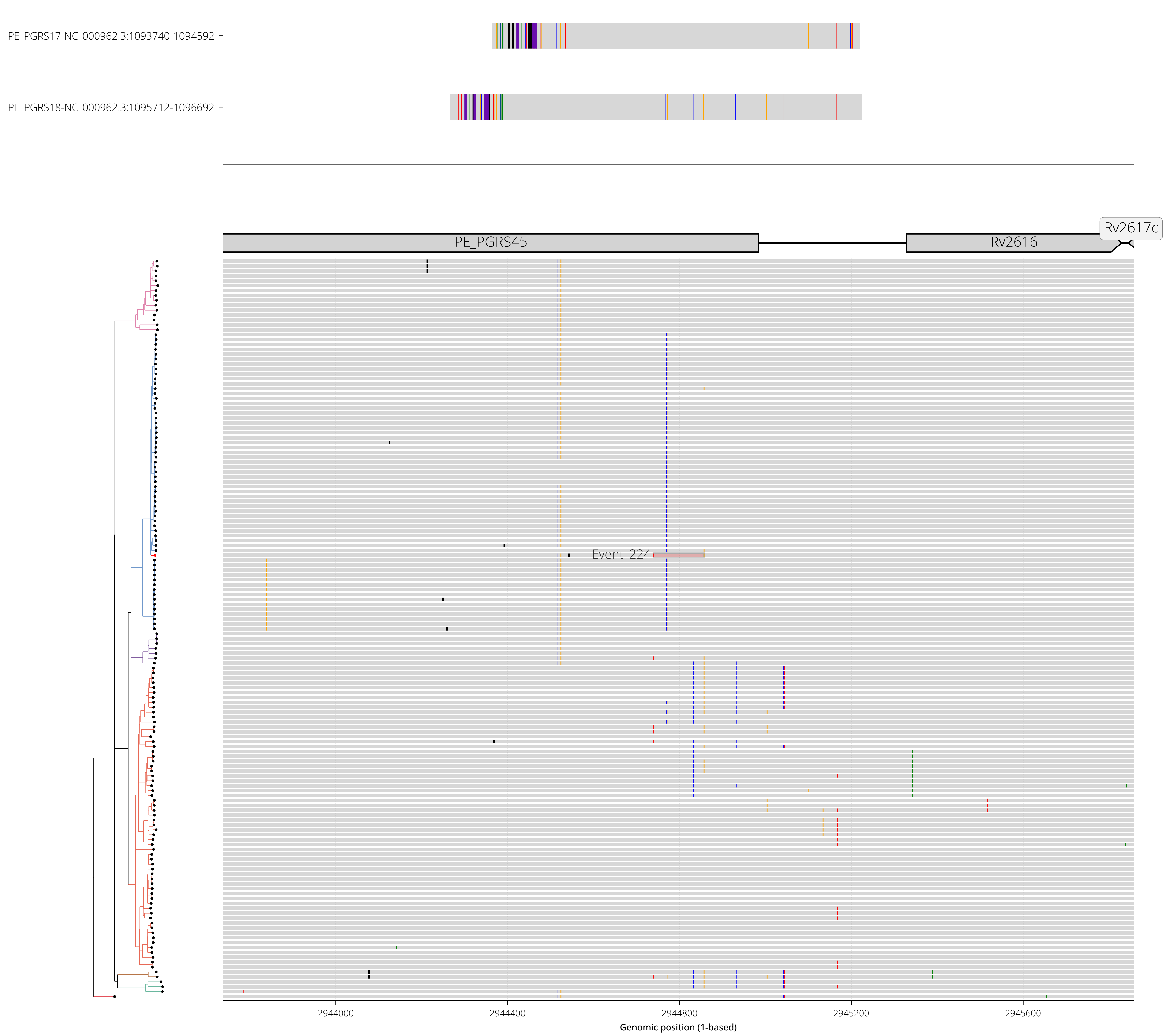

Individual GCE + Known Paralogs
GCE ID: Event_225

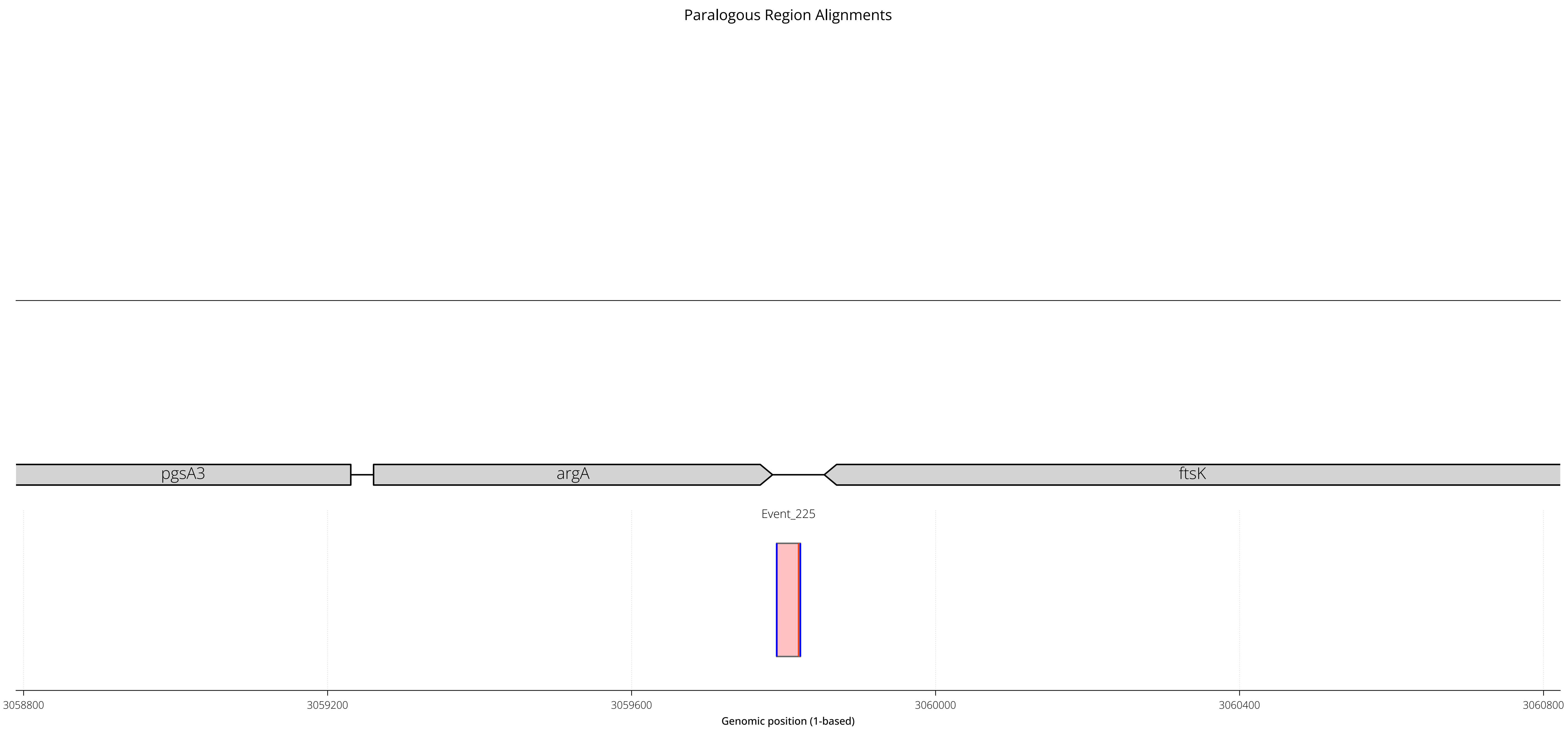

Sub-phylogeny Viz + Paralog Alignments
GCE ID: Event_225

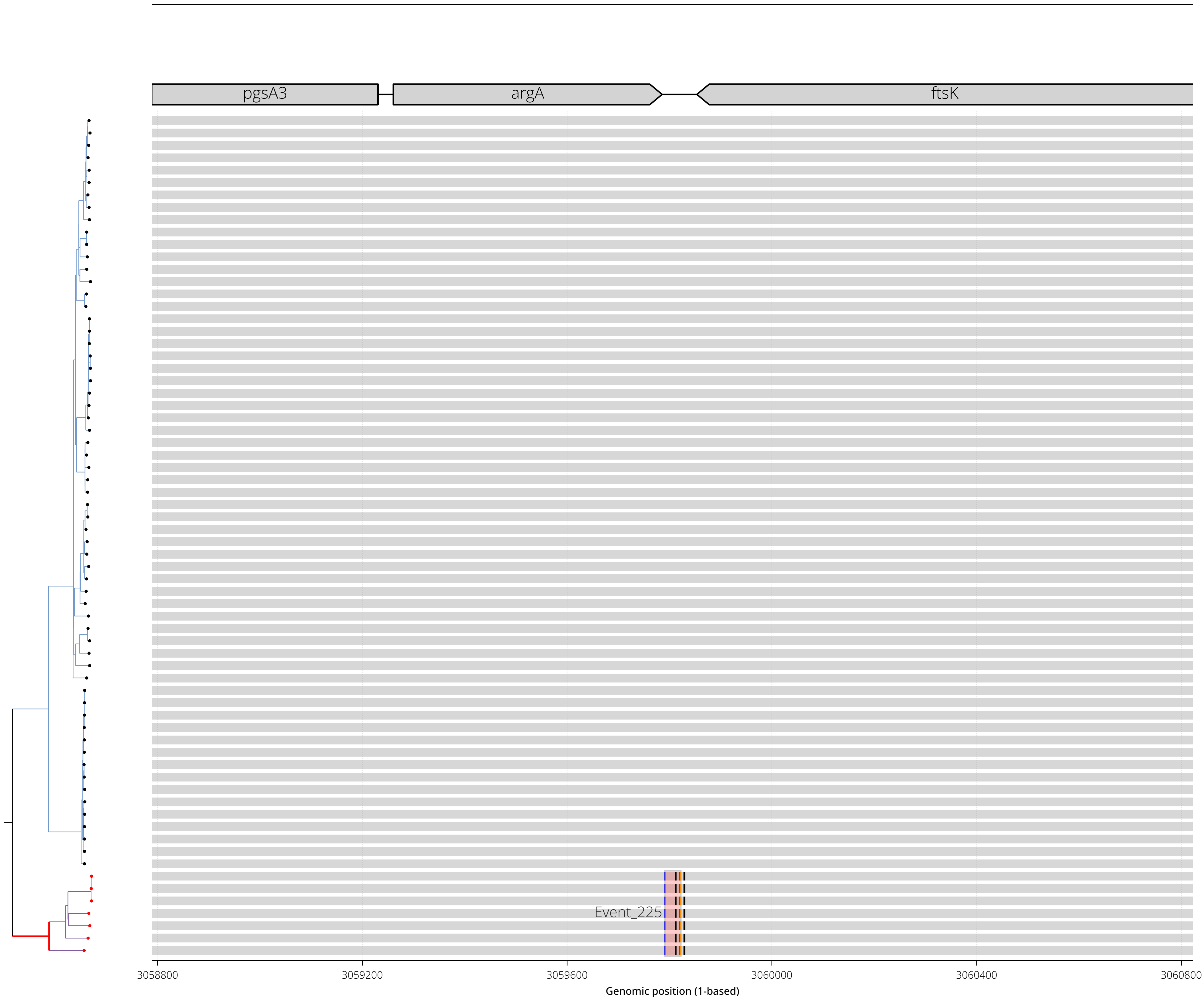

Full Phylogeny Viz + Paralog Alignments
GCE ID: Event_225

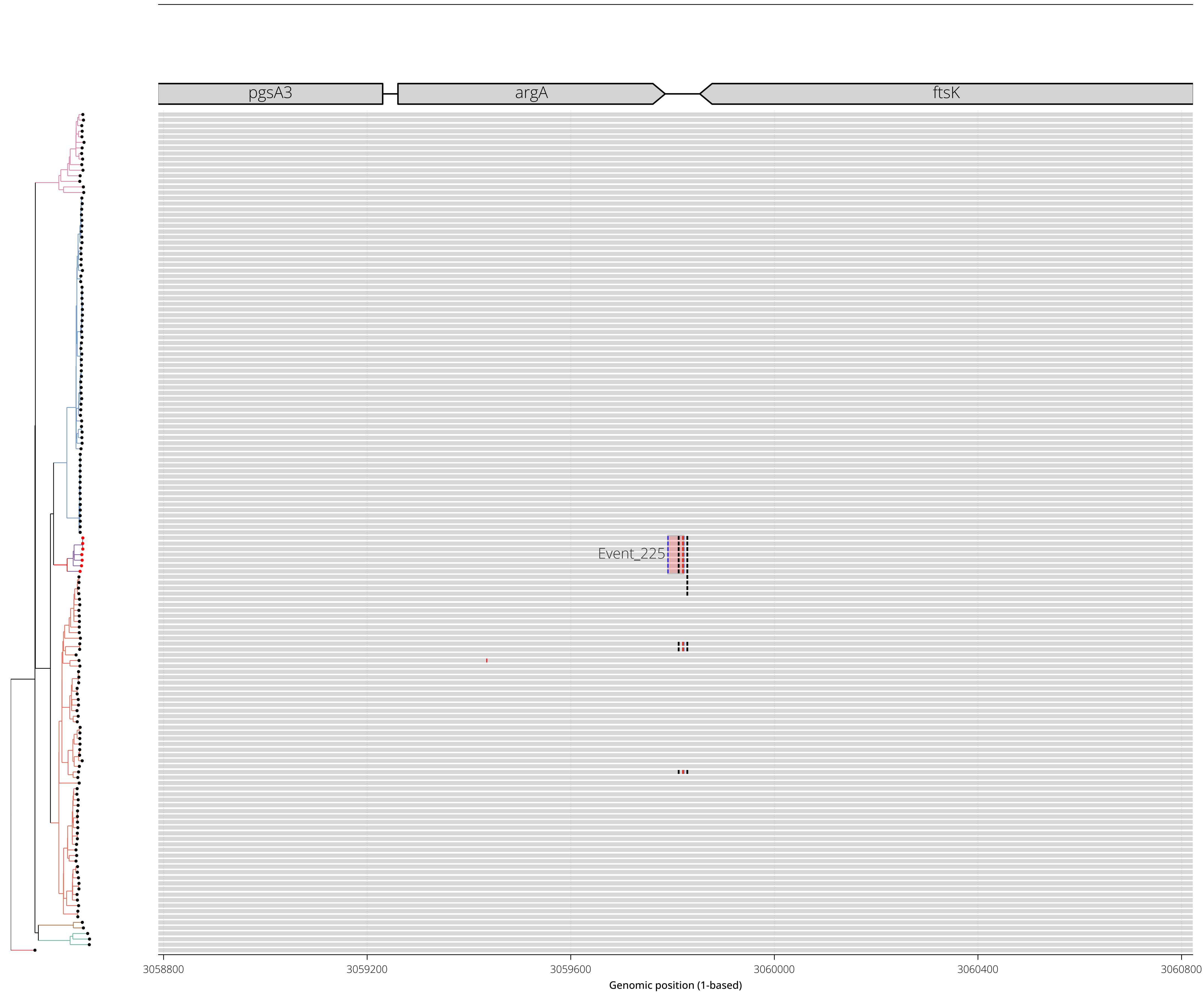

Individual GCE + Known Paralogs
GCE ID: Event_226

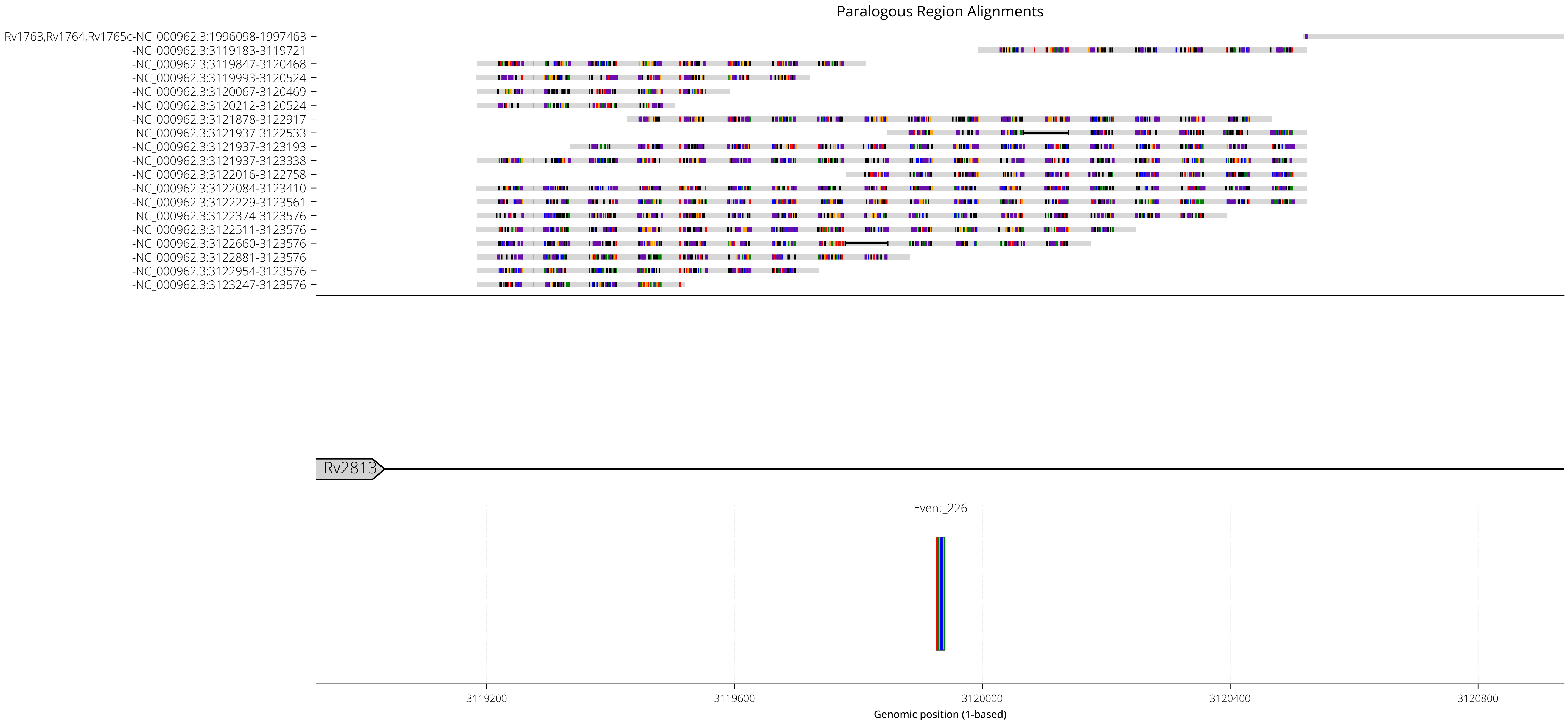

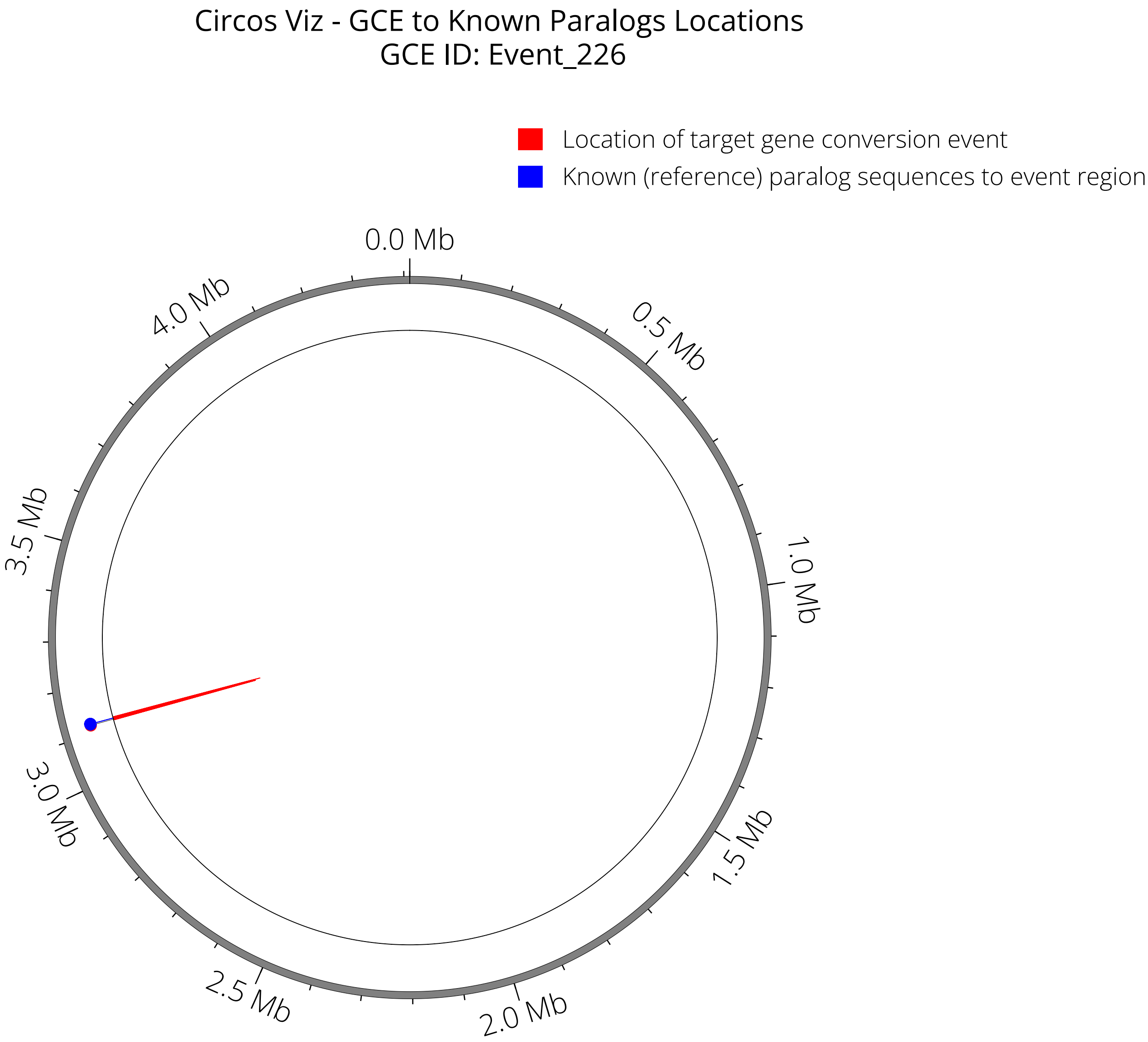
Circos Viz - GCE to Known Paralogs Locations
GCE ID: Event_226
Location of target gene conversion event
Known (reference) paralog sequences to event region
0.0 Mb
0.5 Mb
1.0 Mb
1.5 Mb
2.0 Mb
2.5 Mb
3.0 Mb
3.5 Mb
4.0 Mb

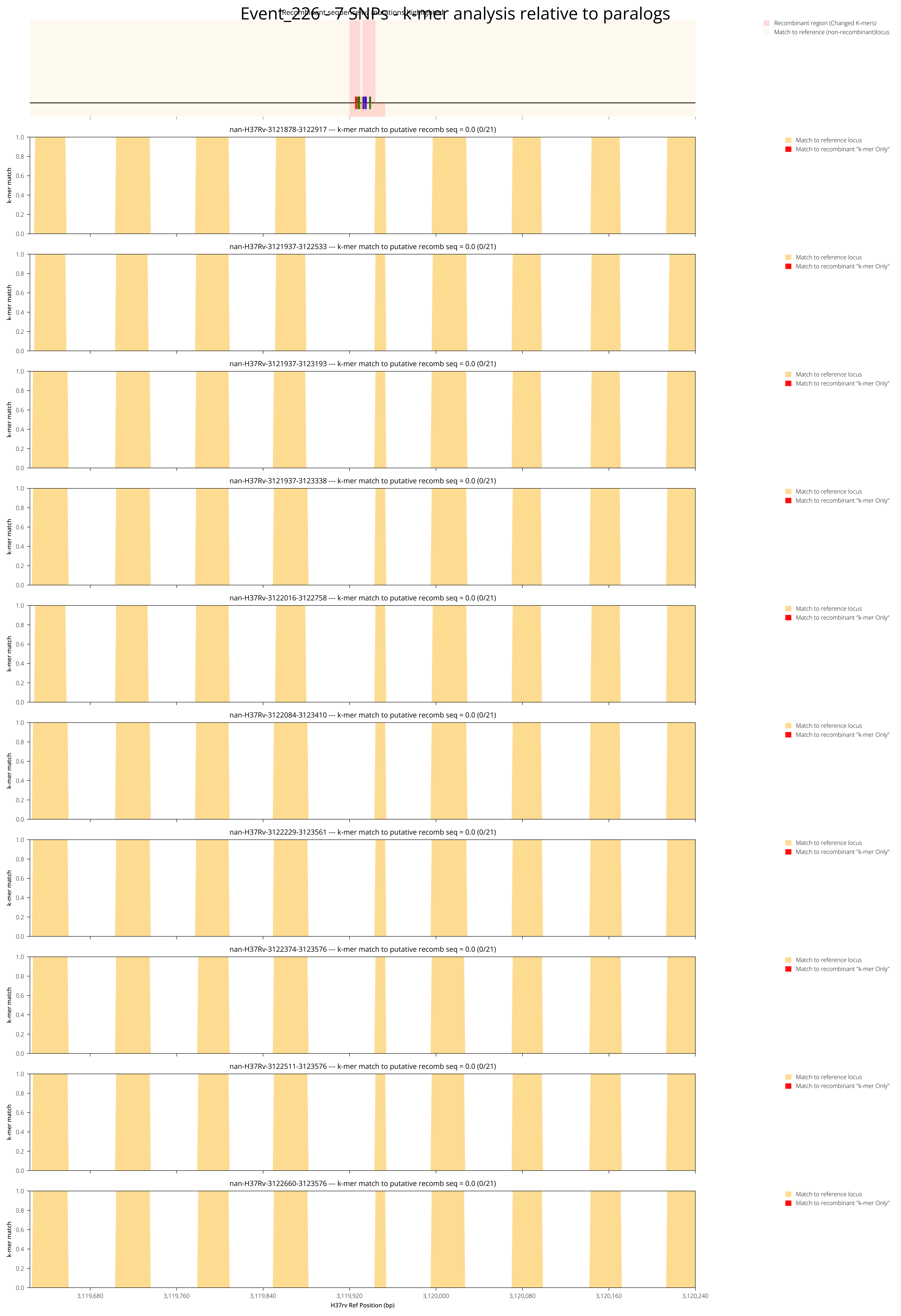

Event_226 - 7 SNPs - k-mer analysis relative to paralogs
Recombinant sequence w/ mutations highlighted
Recombinant region (Changed K-mers)
Match to reference (non-recombinant)locus
nan-H37Rv-3121878-3122917 --- k-mer match to putative recomb seq = 0.0 (0/21)
nan-H37Rv-3121937-3122533 --- k-mer match to putative recomb seq = 0.0 (0/21)
nan-H37Rv-3121937-3123193 --- k-mer match to putative recomb seq = 0.0 (0/21)
nan-H37Rv-3121937-3123338 --- k-mer match to putative recomb seq = 0.0 (0/21)
nan-H37Rv-3122016-3122758 --- k-mer match to putative recomb seq = 0.0 (0/21)
nan-H37Rv-3122084-3123410 --- k-mer match to putative recomb seq = 0.0 (0/21)
nan-H37Rv-3122229-3123561 --- k-mer match to putative recomb seq = 0.0 (0/21)
nan-H37Rv-3122374-3123576 --- k-mer match to putative recomb seq = 0.0 (0/21)
nan-H37Rv-3122511-3123576 --- k-mer match to putative recomb seq = 0.0 (0/21)
nan-H37Rv-3122660-3123576 --- k-mer match to putative recomb seq = 0.0 (0/21)
Match to reference locus
Match to recombinant "k-mer Only"
k-mer match
0.0
0.2
0.4
0.6
0.8
1.0
3,119,680
3,119,760
3,119,840
3,119,920
3,120,000
3,120,080
3,120,160
3,120,240
H37rv Ref Position (bp)

# Sub-phylogeny Viz + Paralog Alignments
## GCE ID: Event_226

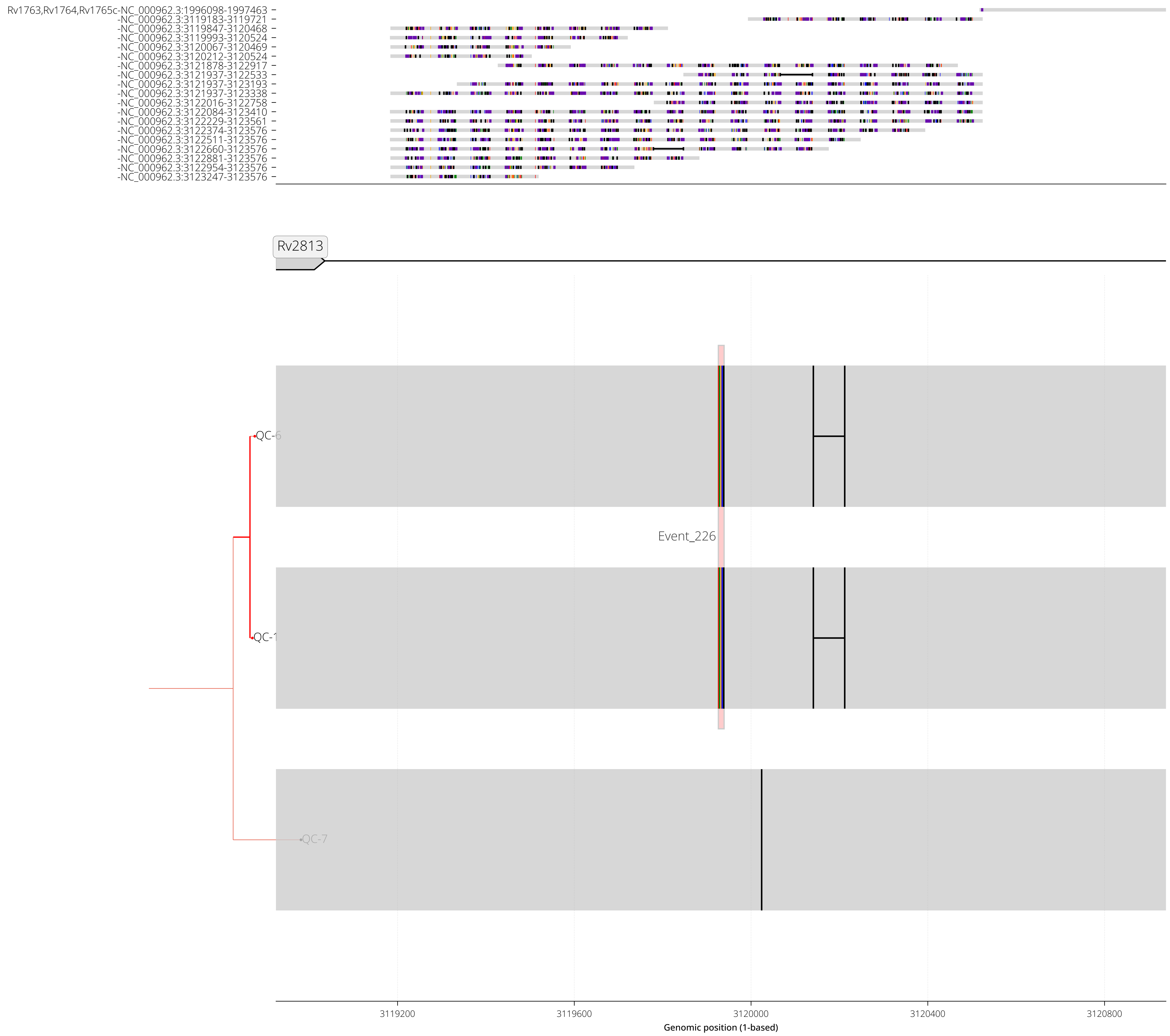

Full Phylogeny Viz + Paralog Alignments
GCE ID: Event_226

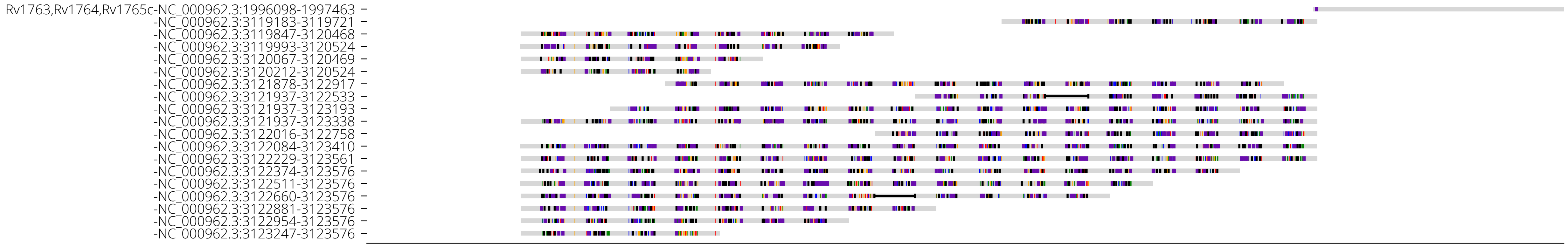


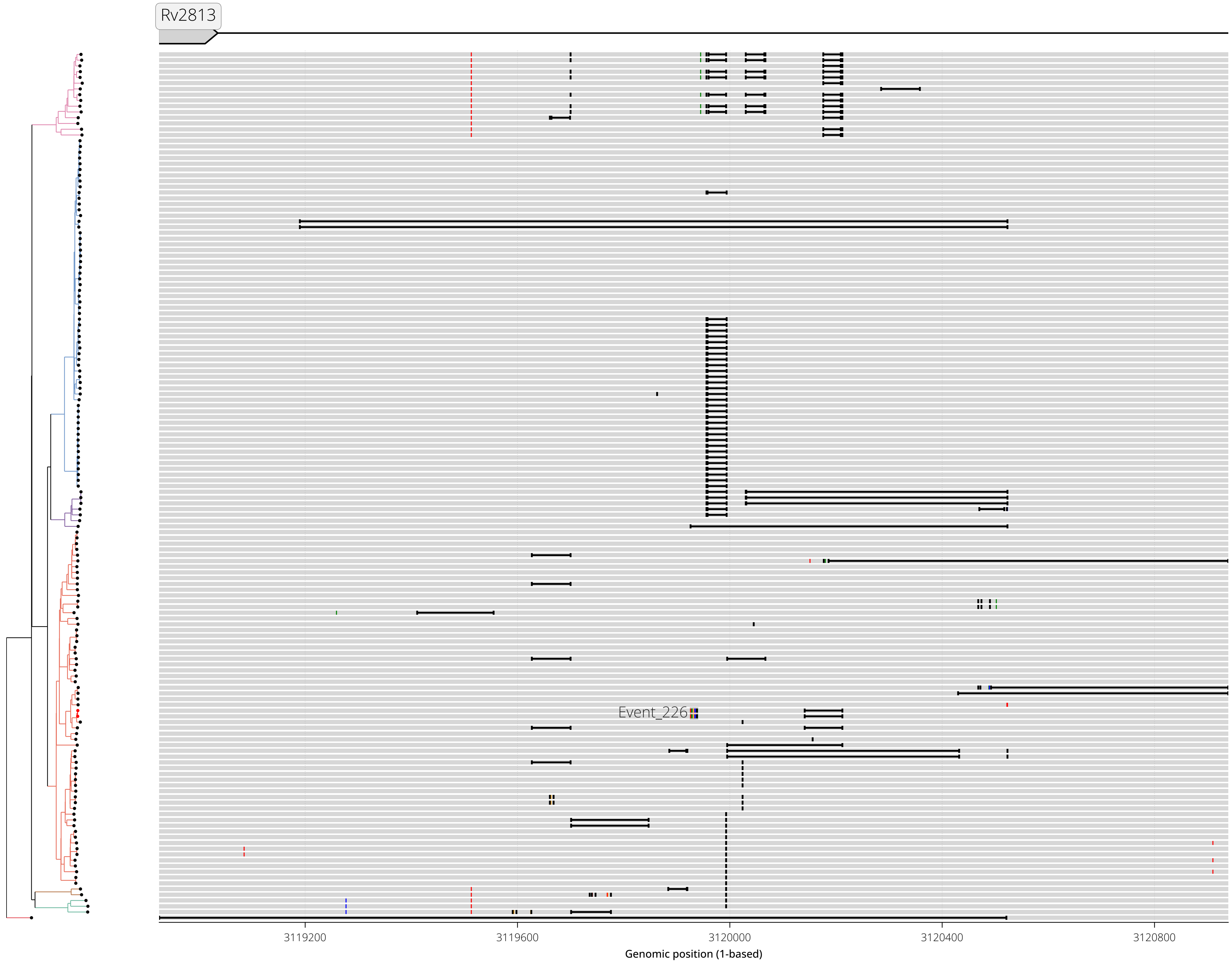

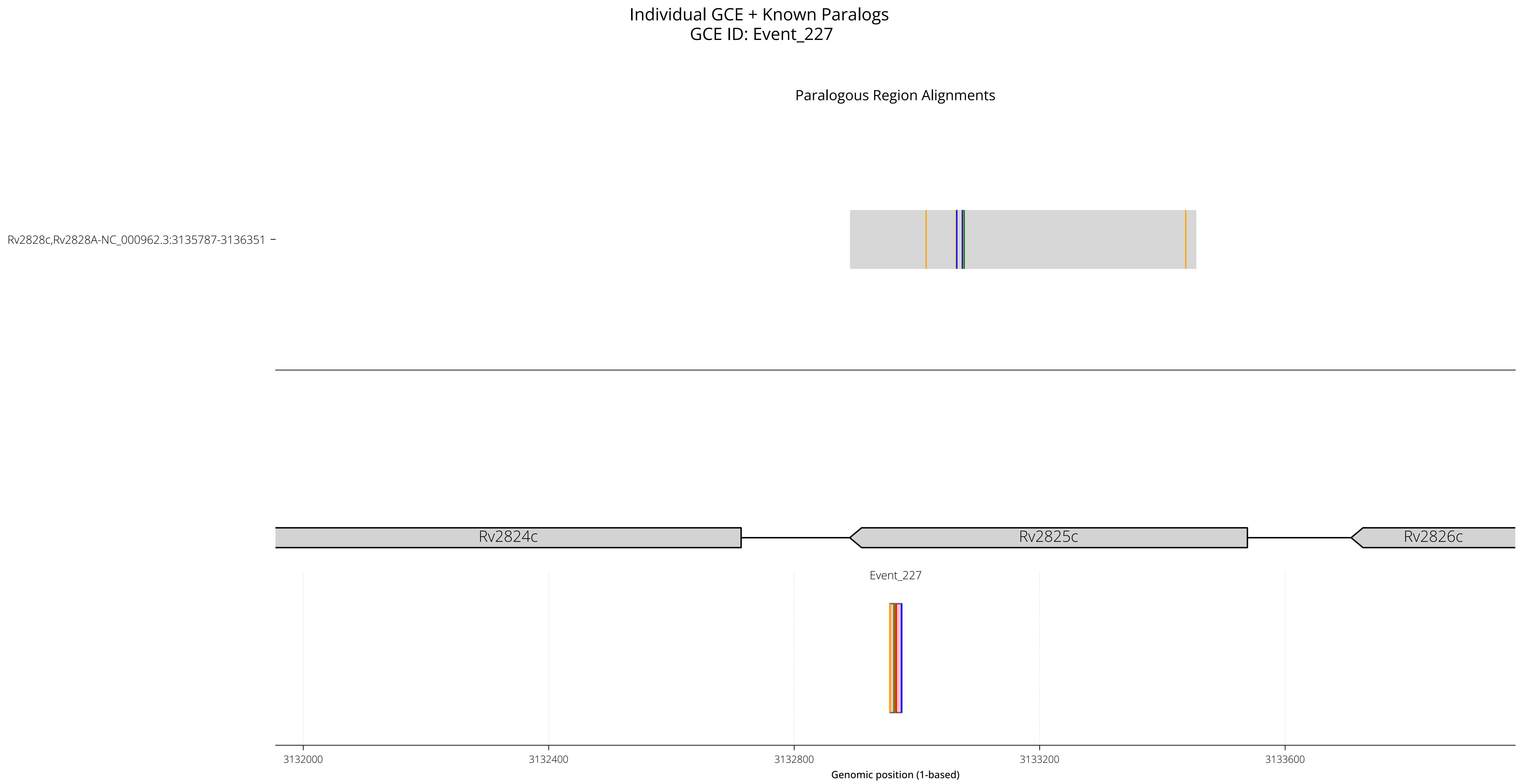

Individual GCE + Known Paralogs
GCE ID: Event_227
Paralogous Region Alignments
Rv2828c,Rv2828A-NC_000962.3:3135787-3136351
Rv2824c
Rv2825c
Rv2826c
Event_227
3132000
3132400
3132800
3133200
3133600
Genomic position (1-based)

Circos Viz - GCE to Known Paralogs Locations
GCE ID: Event_227

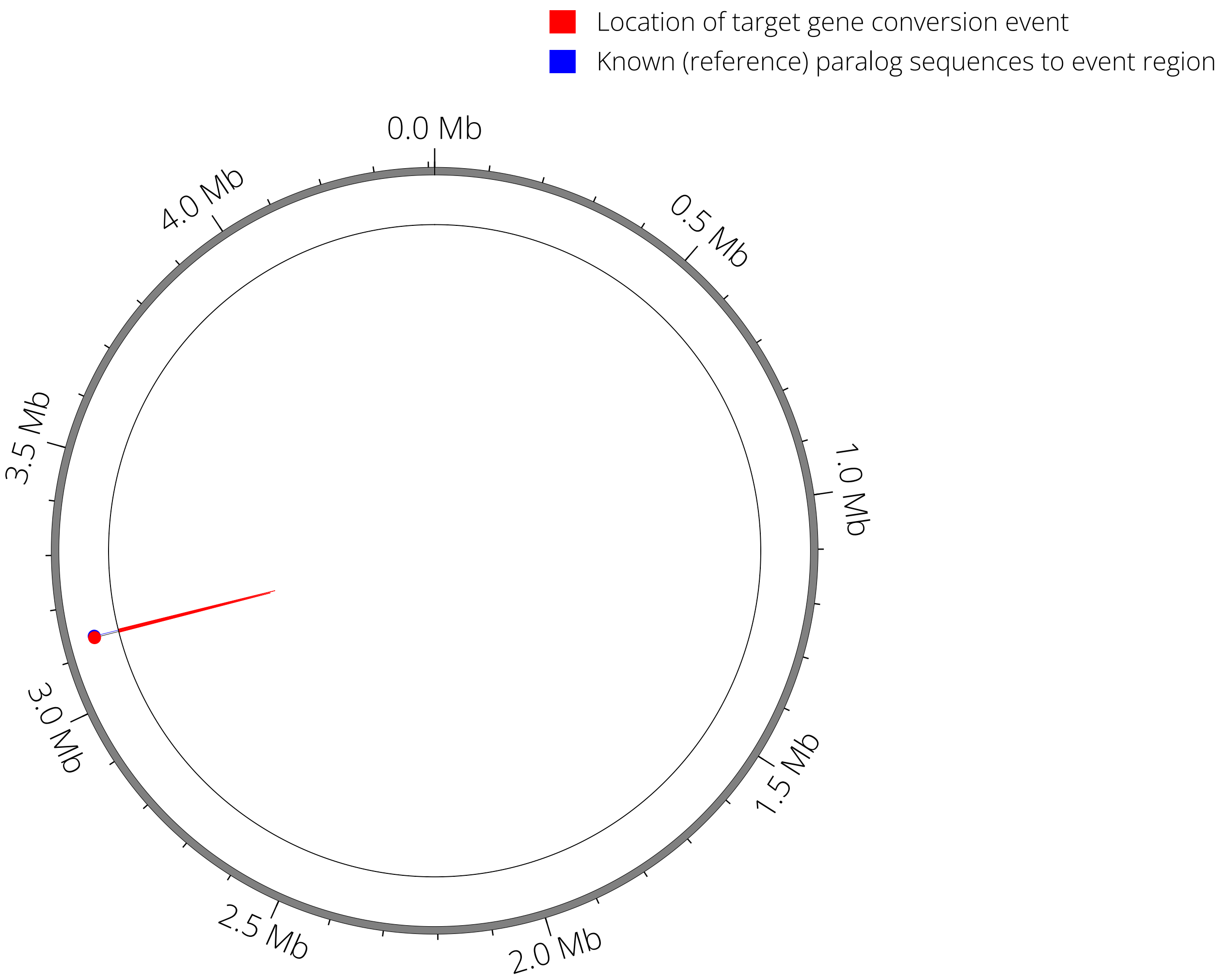

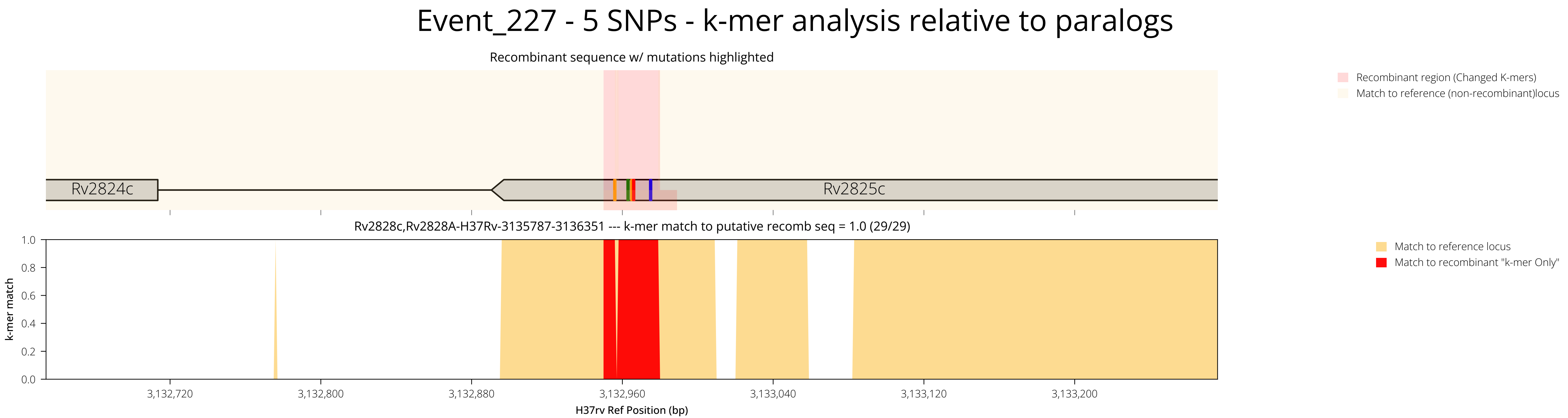

Event_227 - 5 SNPs - k-mer analysis relative to paralogs
Recombinant sequence w/ mutations highlighted
Rv2824c
Rv2825c
Recombinant region (Changed K-mers)
Match to reference (non-recombinant)locus
Rv2828c,Rv2828A-H37Rv-3135787-3136351 --- k-mer match to putative recomb seq = 1.0 (29/29)
1.0
0.8
0.6
0.4
0.2
0.0
k-mer match
3,132,720
3,132,800
3,132,880
3,132,960
3,133,040
3,133,120
3,133,200
H37rv Ref Position (bp)
Match to reference locus
Match to recombinant "k-mer Only"

Sub-phylogeny Viz + Paralog Alignments
GCE ID: Event_227

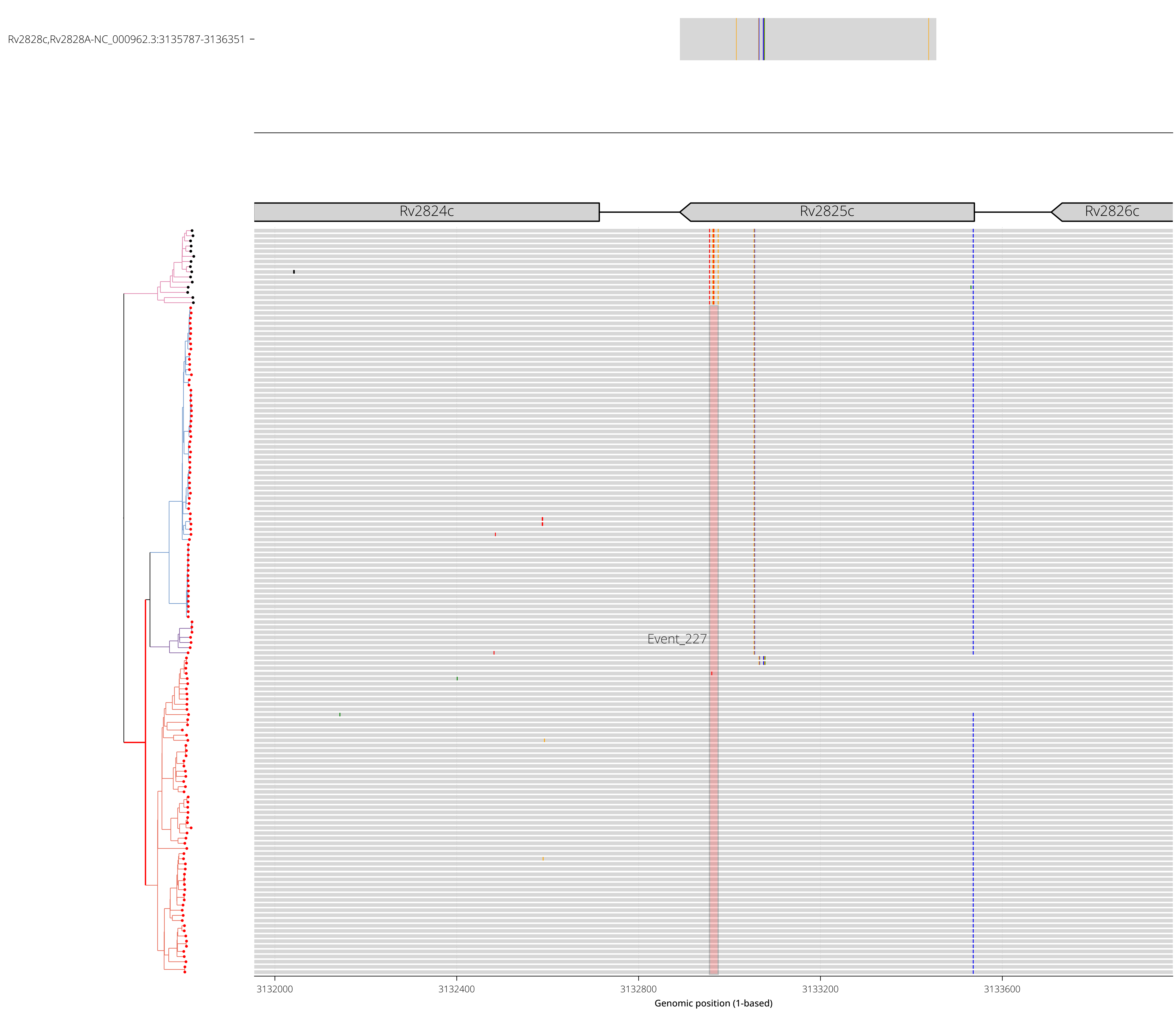

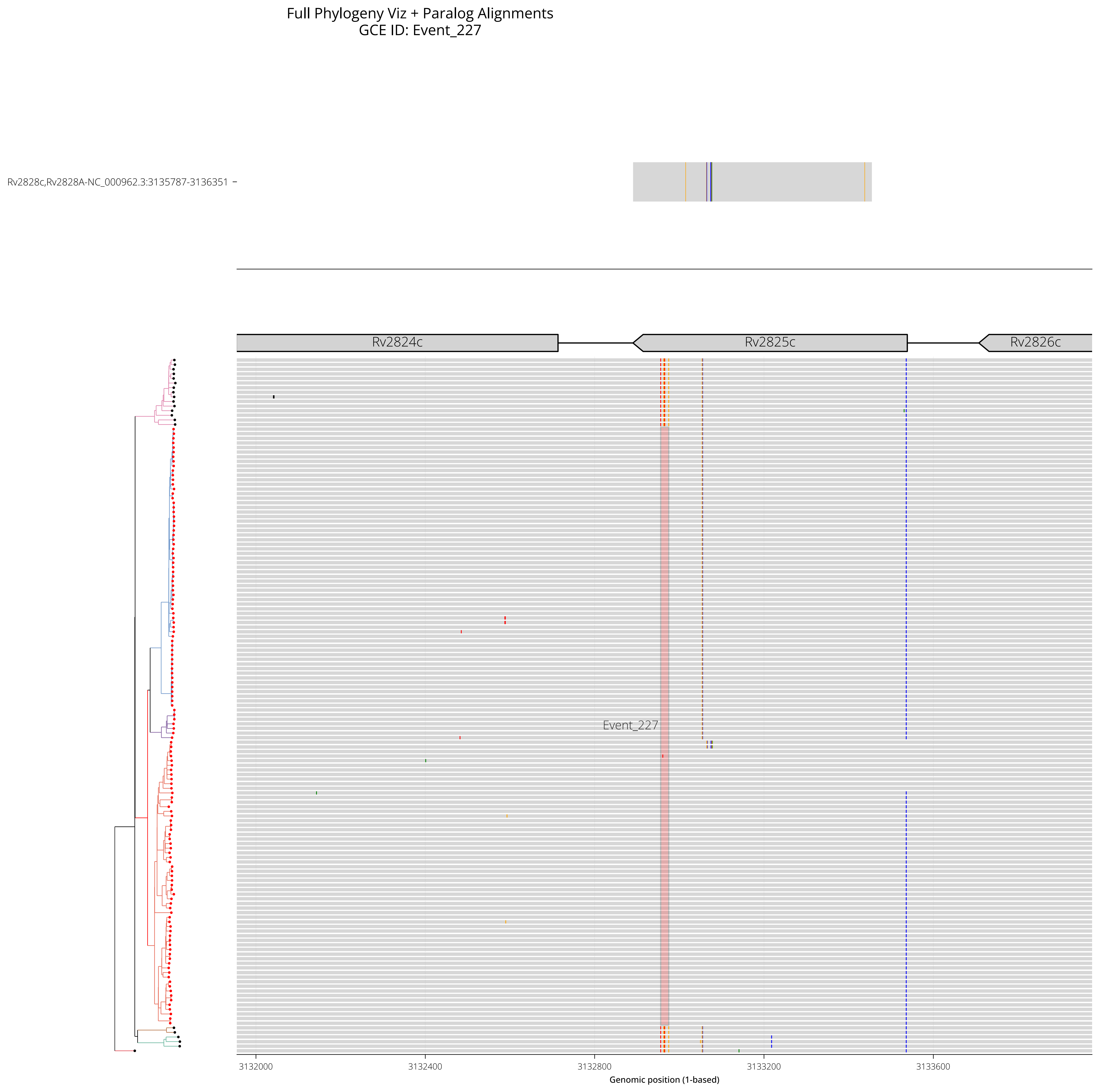

Full Phylogeny Viz + Paralog Alignments
GCE ID: Event_227
Rv2828c,Rv2828A-NC_000962.3:3135787-3136351
Rv2824c
Rv2825c
Rv2826c
Event_227
3132000
3132400
3132800
3133200
3133600
Genomic position (1-based)

Individual GCE + Known Paralogs
GCE ID: Event_228

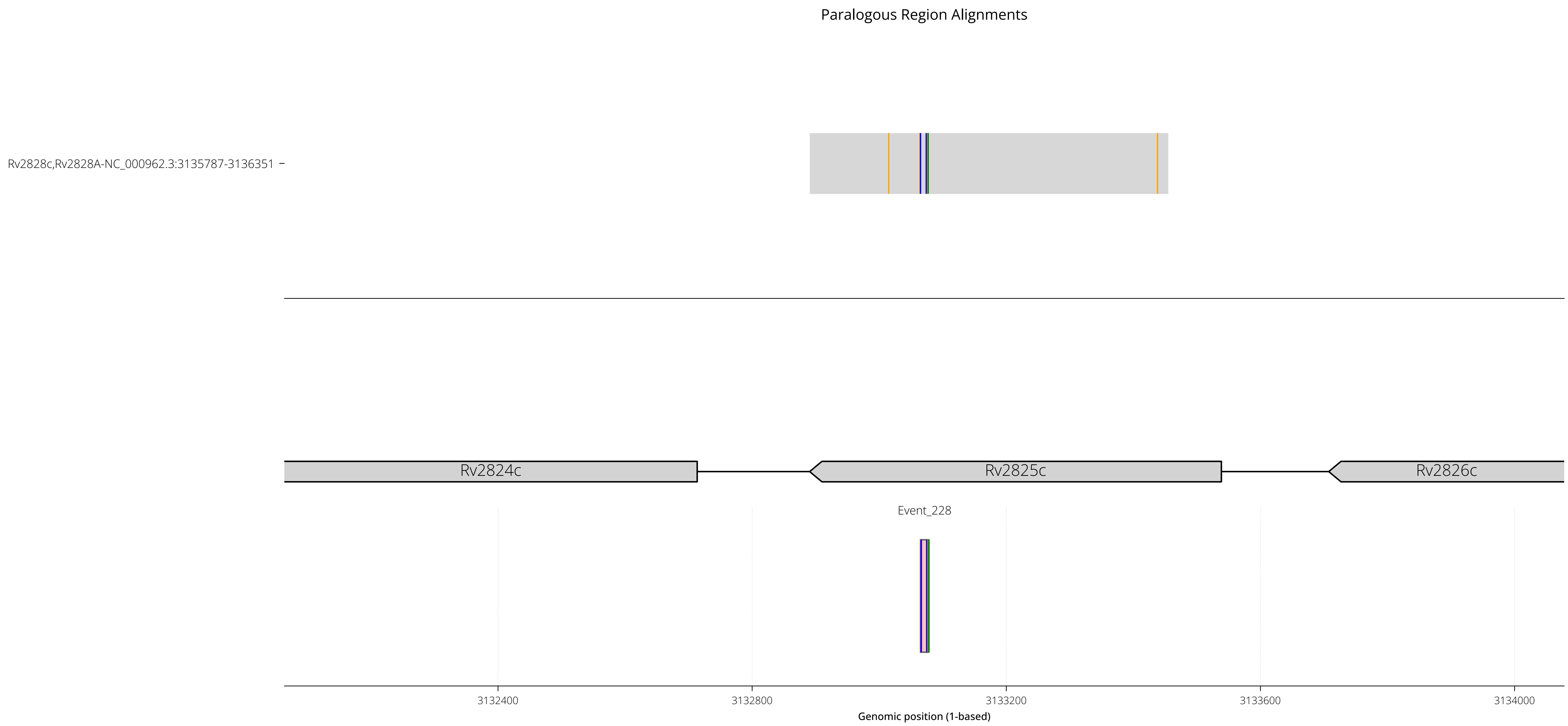

Circos Viz - GCE to Known Paralogs Locations
GCE ID: Event_228

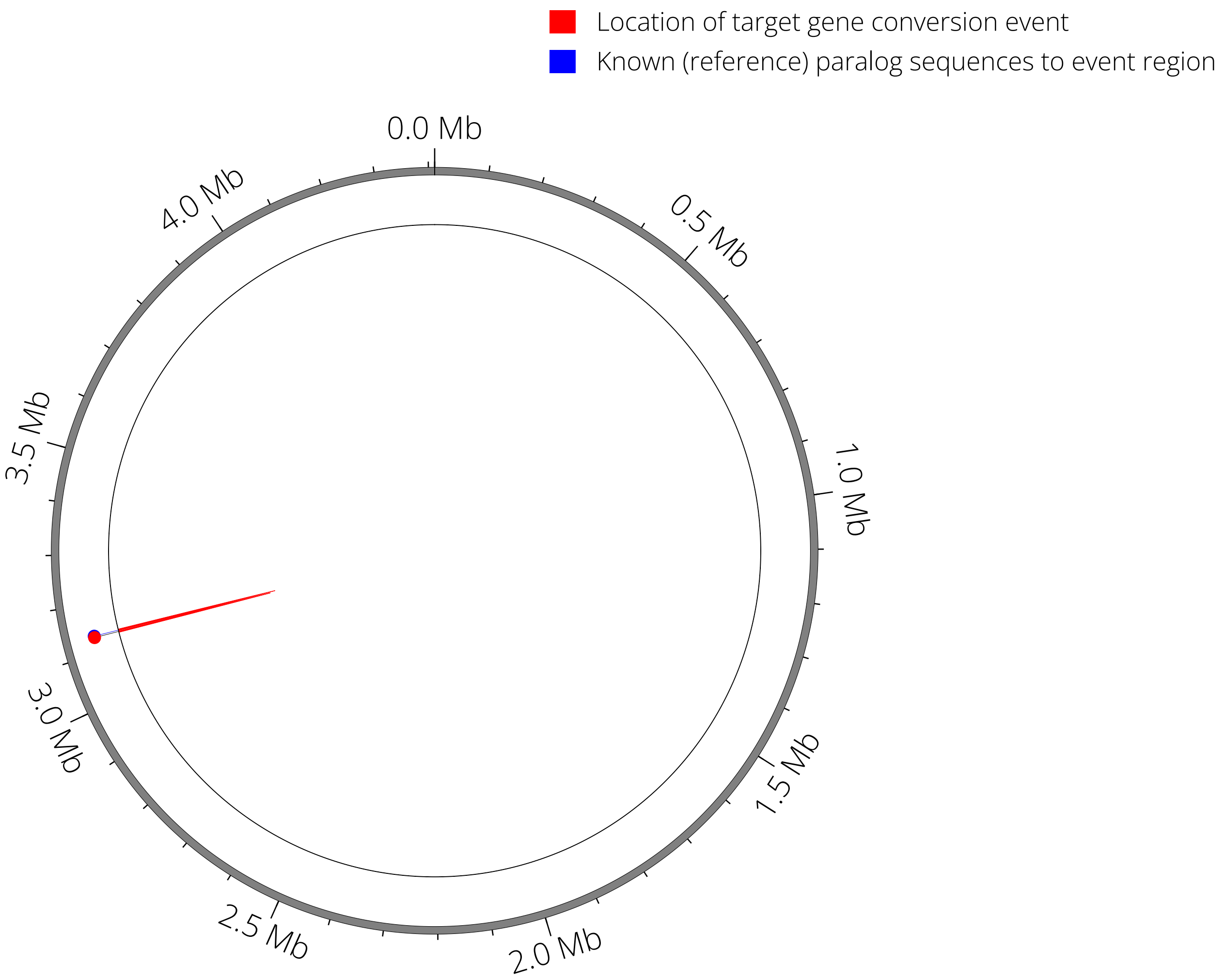

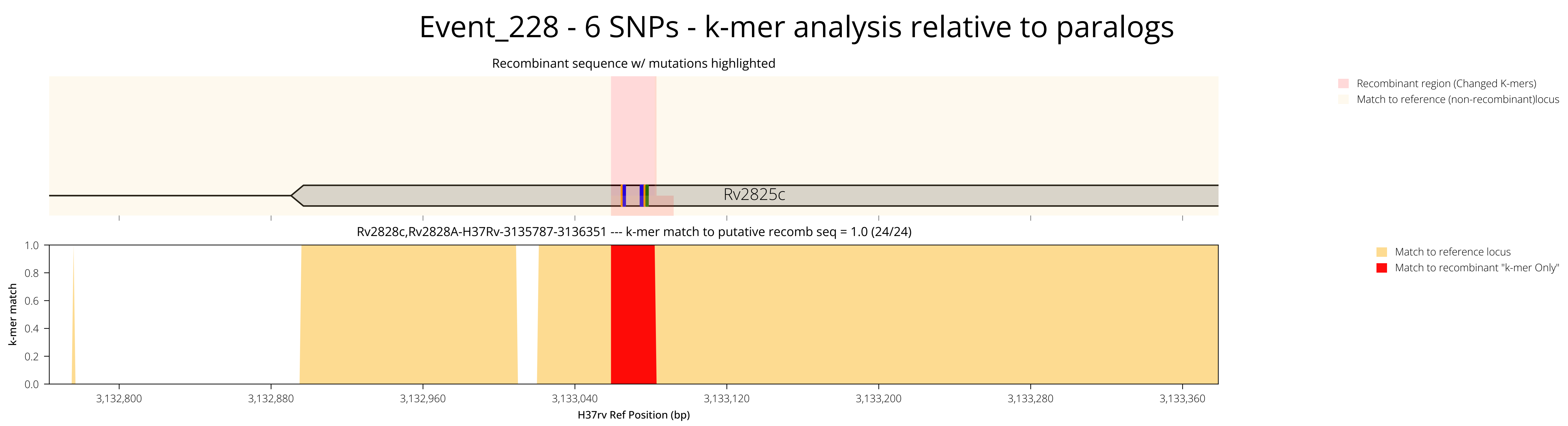

Event_228 - 6 SNPs - k-mer analysis relative to paralogs
Recombinant sequence w/ mutations highlighted
Recombinant region (Changed K-mers)
Match to reference (non-recombinant)locus
Rv2825c
Rv2828c,Rv2828A-H37Rv-3135787-3136351 --- k-mer match to putative recomb seq = 1.0 (24/24)
Match to reference locus
Match to recombinant "k-mer Only"
k-mer match
1.0
0.8
0.6
0.4
0.2
0.0
3,132,800
3,132,880
3,132,960
3,133,040
3,133,120
3,133,200
3,133,280
3,133,360
H37rv Ref Position (bp)

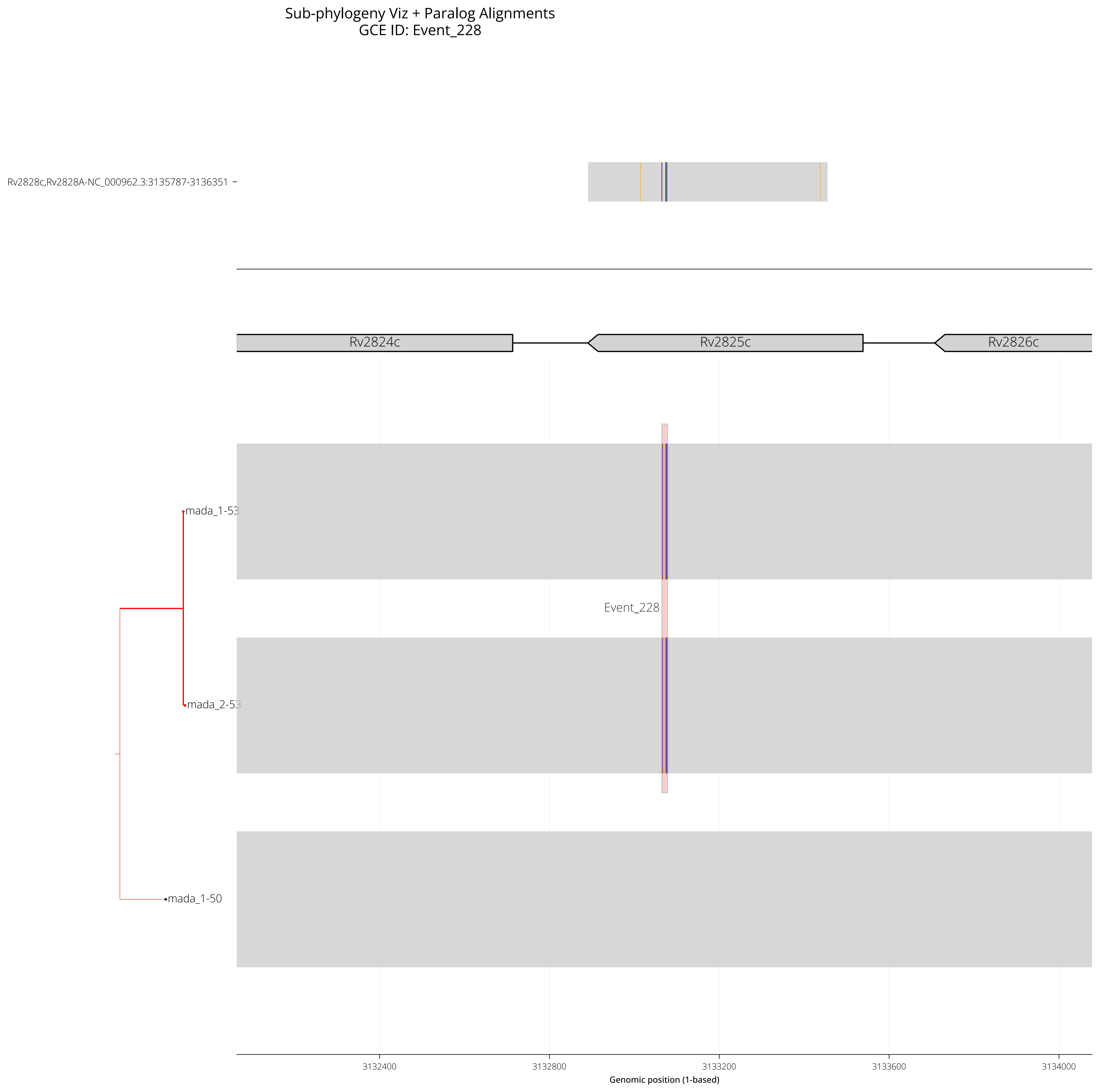

Sub-phylogeny Viz + Paralog Alignments
GCE ID: Event_228
Rv2828c,Rv2828A-NC_000962.3:3135787-3136351
Rv2824c
Rv2825c
Rv2826c
mada_1-53
Event_228
mada_2-53
mada_1-50
3132400
3132800
3133200
3133600
3134000
Genomic position (1-based)

Full Phylogeny Viz + Paralog Alignments
GCE ID: Event_228

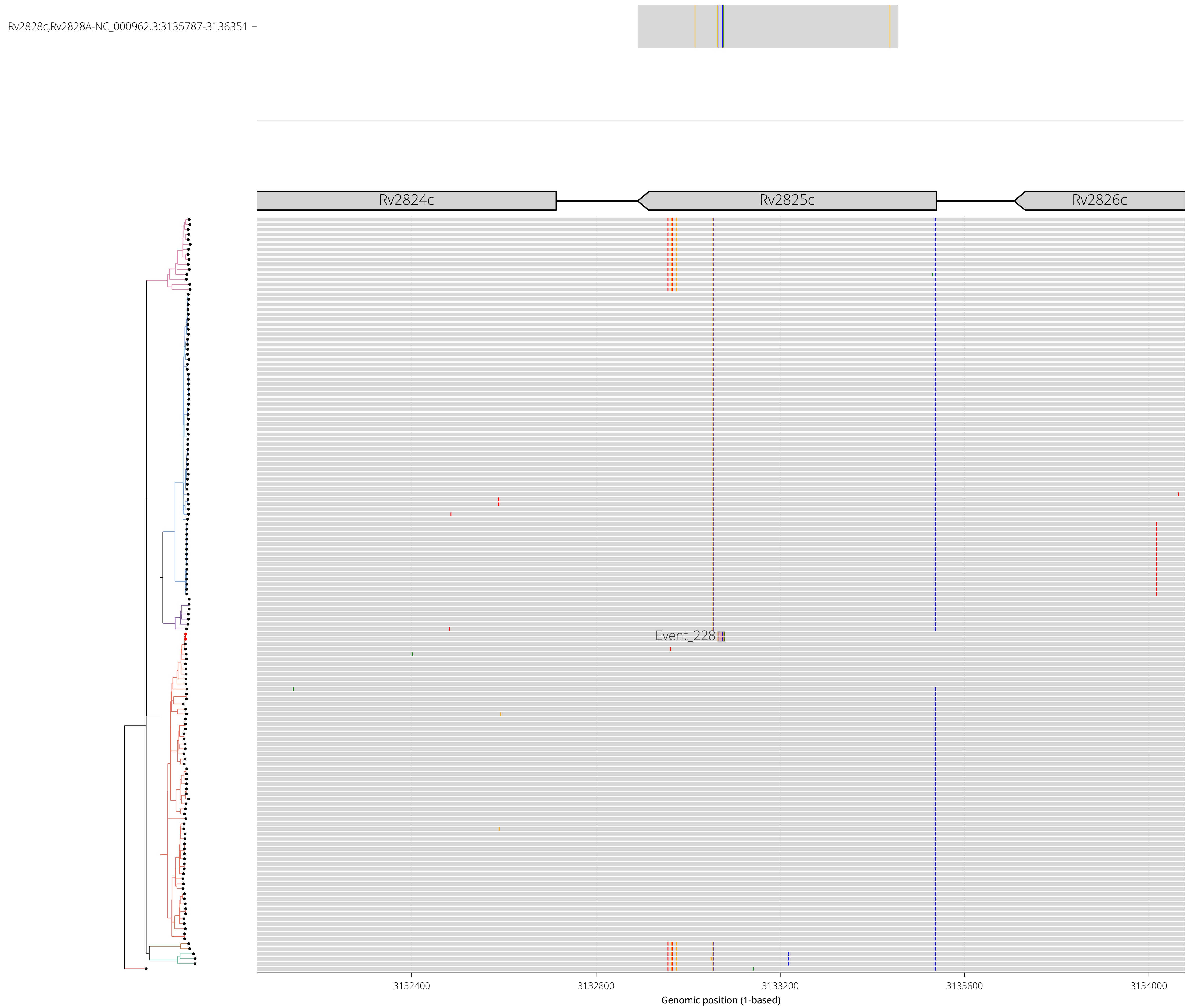

Individual GCE + Known Paralogs
GCE ID: Event_229

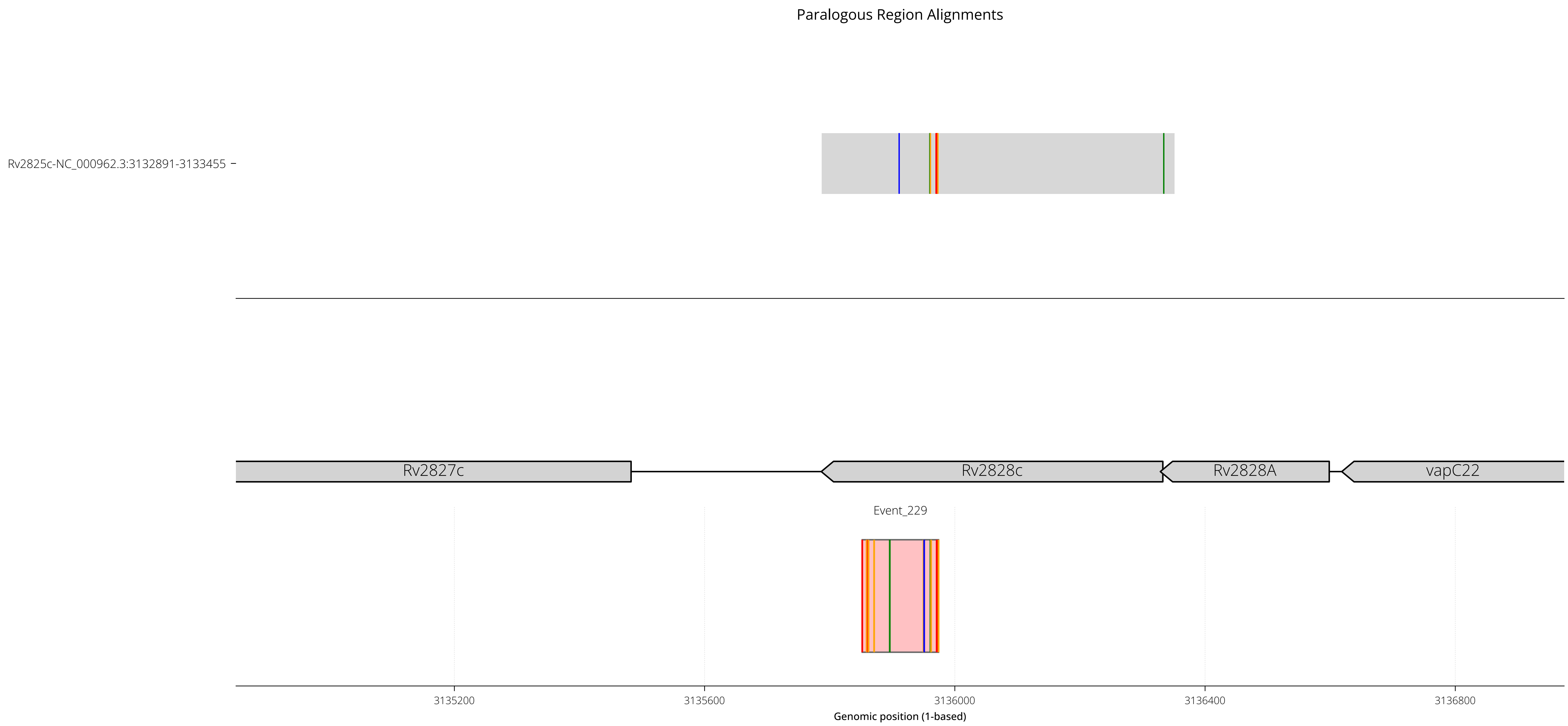

Circos Viz - GCE to Known Paralogs Locations
GCE ID: Event_229

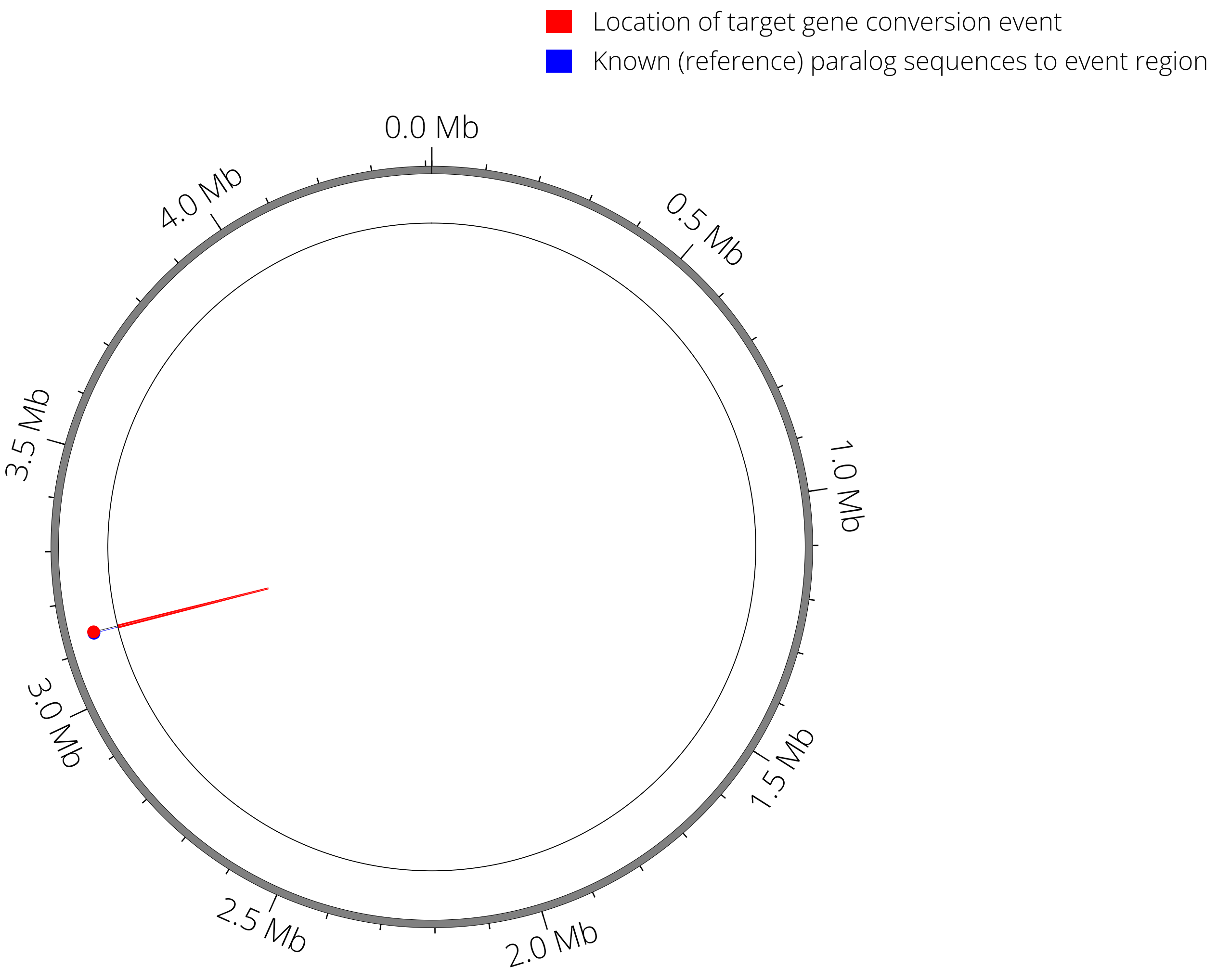

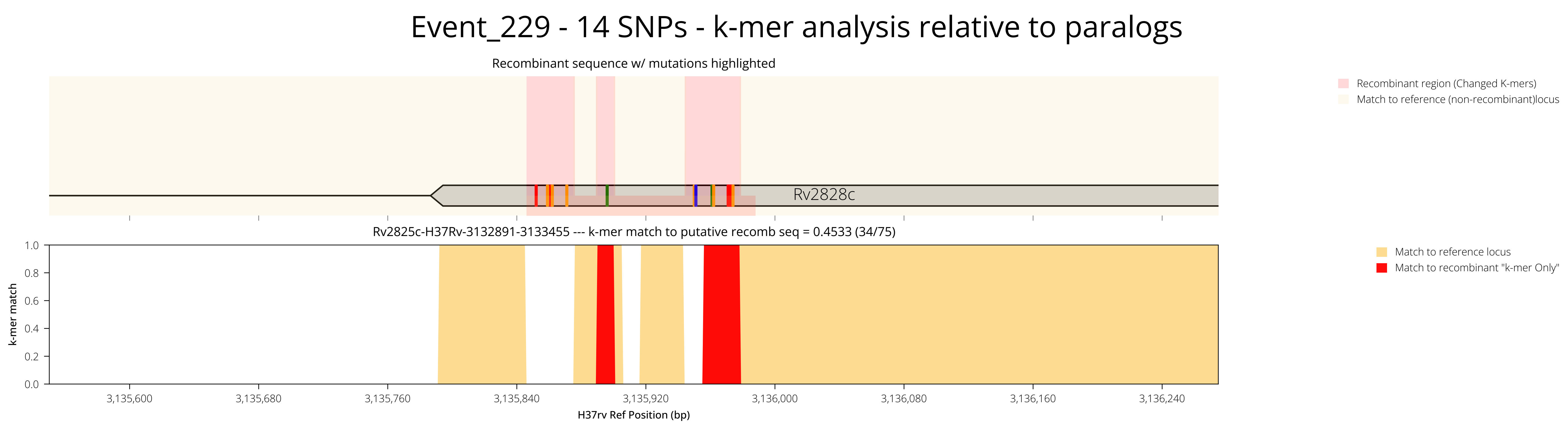

Event_229 - 14 SNPs - k-mer analysis relative to paralogs
Recombinant sequence w/ mutations highlighted
Recombinant region (Changed K-mers)
Match to reference (non-recombinant)locus
Rv2828c
Rv2825c-H37Rv-3132891-3133455 --- k-mer match to putative recomb seq = 0.4533 (34/75)
Match to reference locus
Match to recombinant "k-mer Only"
k-mer match
1.0
0.8
0.6
0.4
0.2
0.0
3,135,600
3,135,680
3,135,760
3,135,840
3,135,920
3,136,000
3,136,080
3,136,160
3,136,240
H37rv Ref Position (bp)

Sub-phylogeny Viz + Paralog Alignments
GCE ID: Event_229

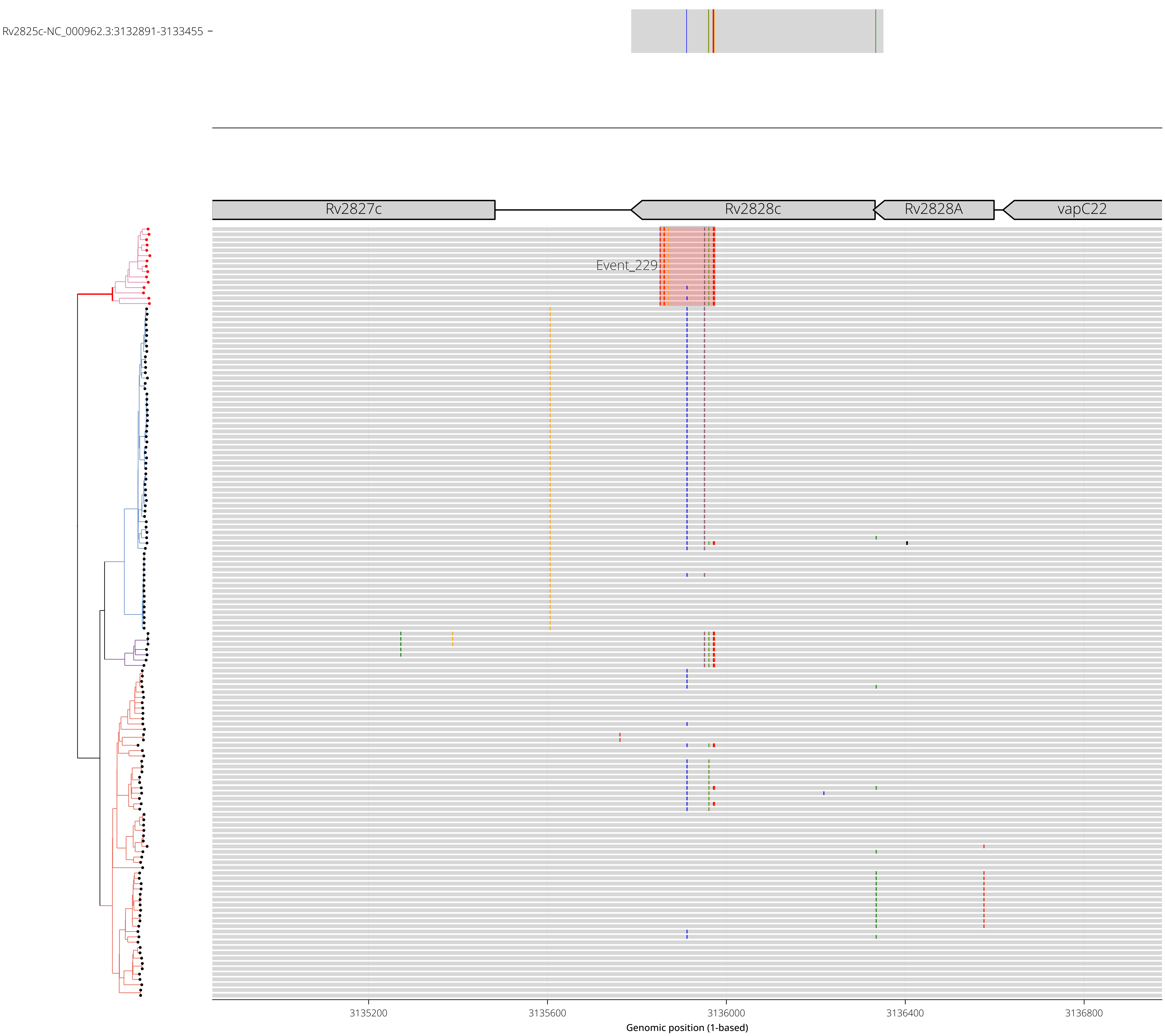

Full Phylogeny Viz + Paralog Alignments
GCE ID: Event_229

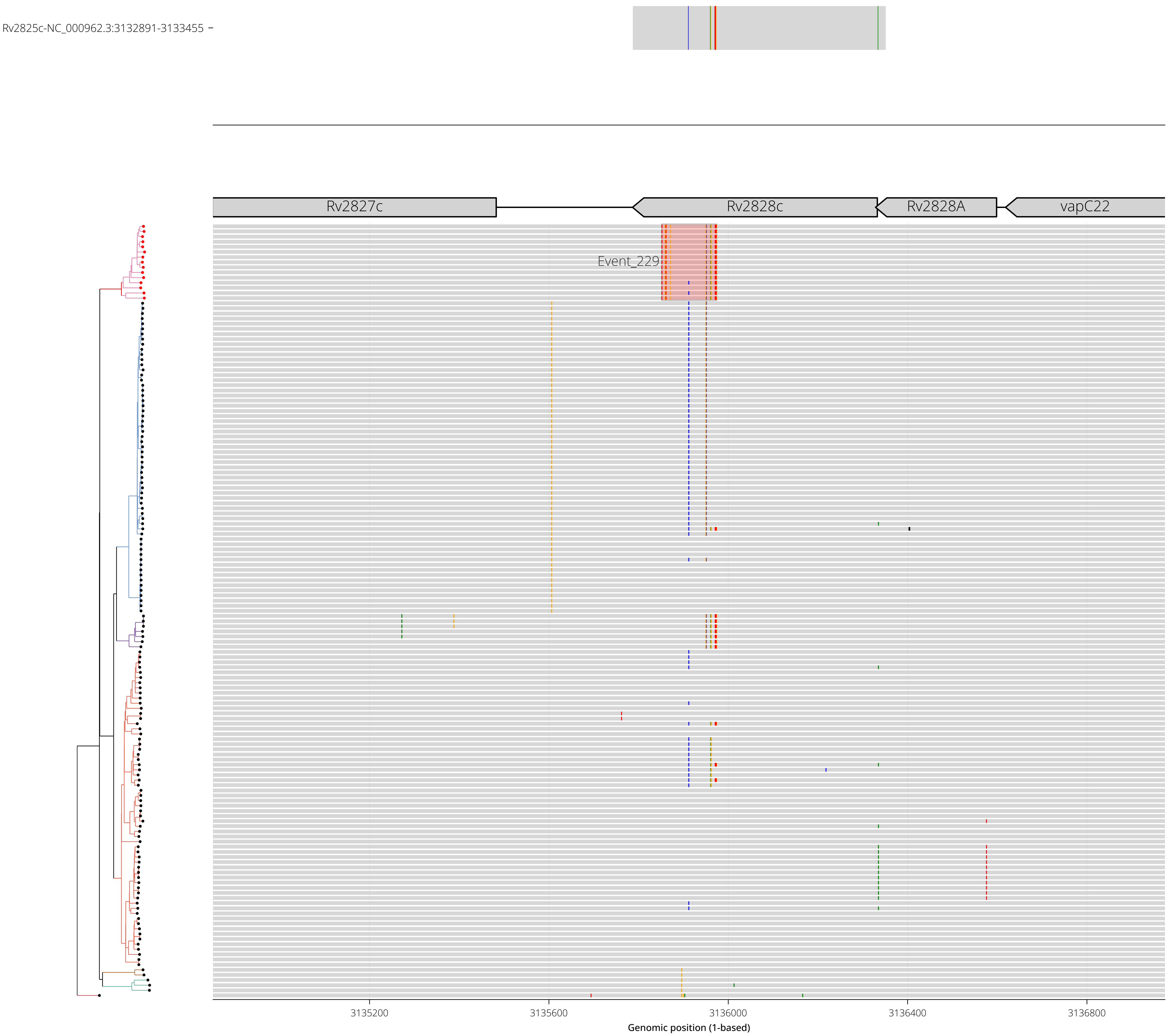

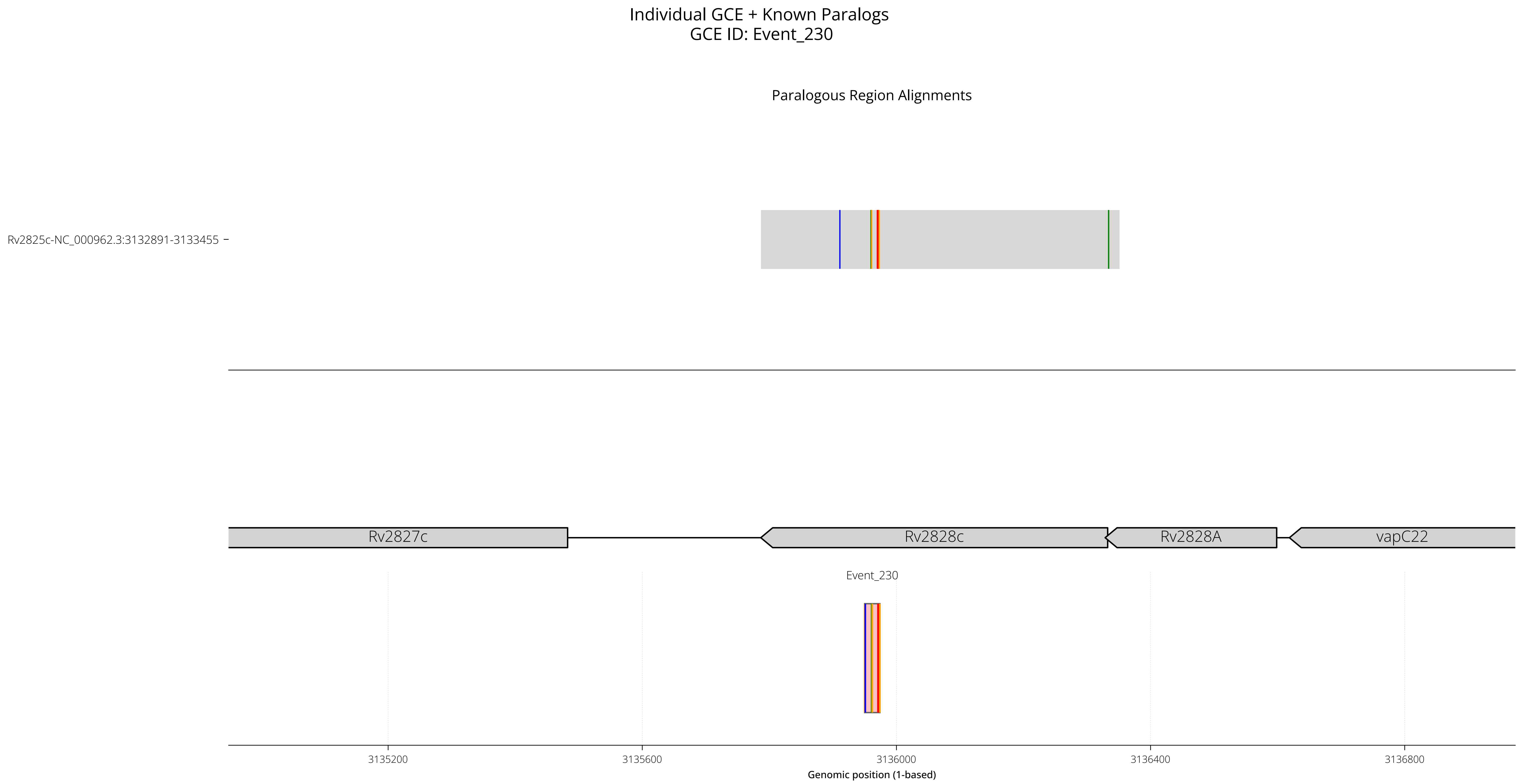

Individual GCE + Known Paralogs
GCE ID: Event_230
Paralogous Region Alignments
Rv2825c-NC_000962.3:3132891-3133455
Rv2827c
Rv2828c
Rv2828A
vapC22
Event_230
3135200
3135600
3136000
3136400
3136800
Genomic position (1-based)

Circos Viz - GCE to Known Paralogs Locations
GCE ID: Event_230

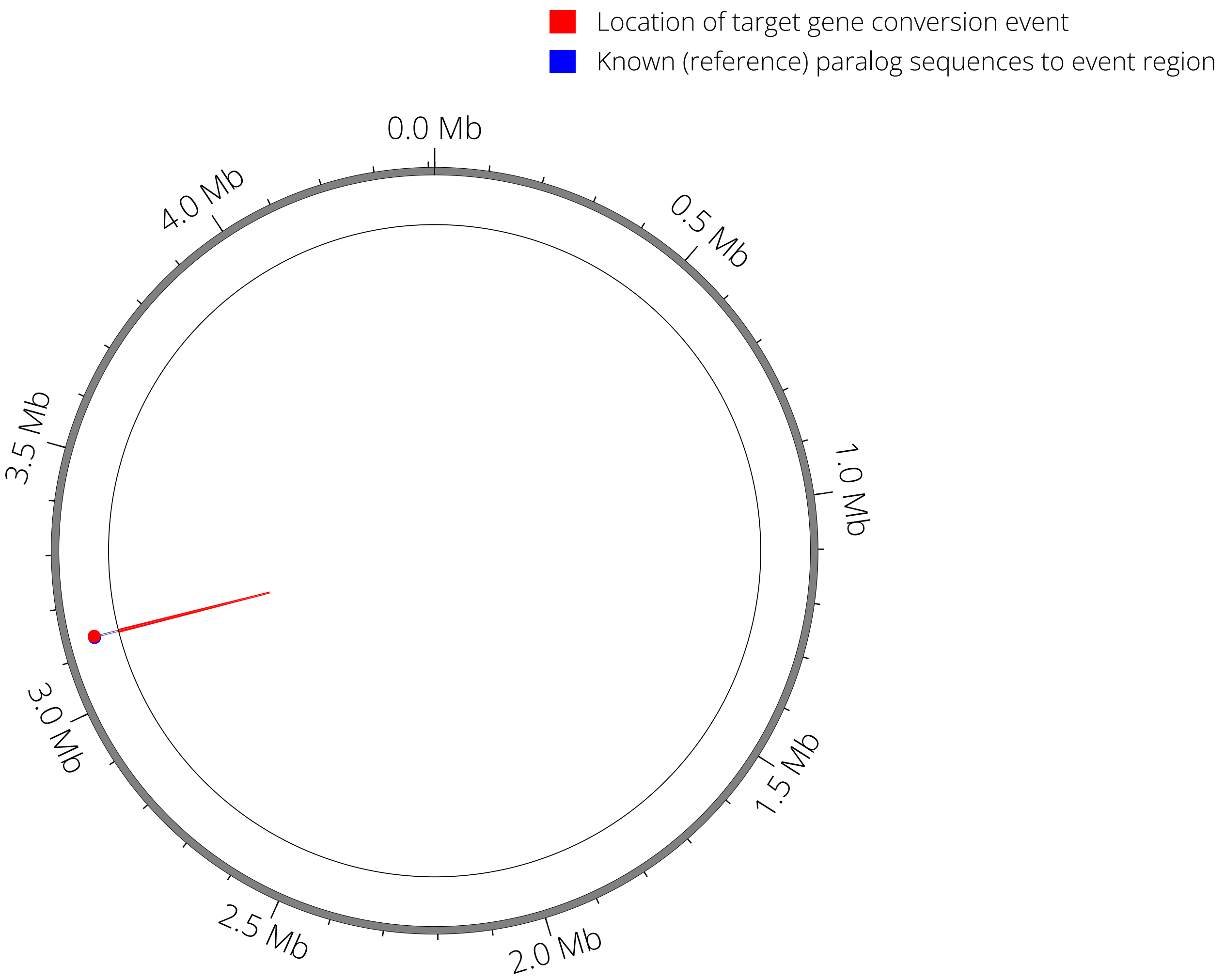

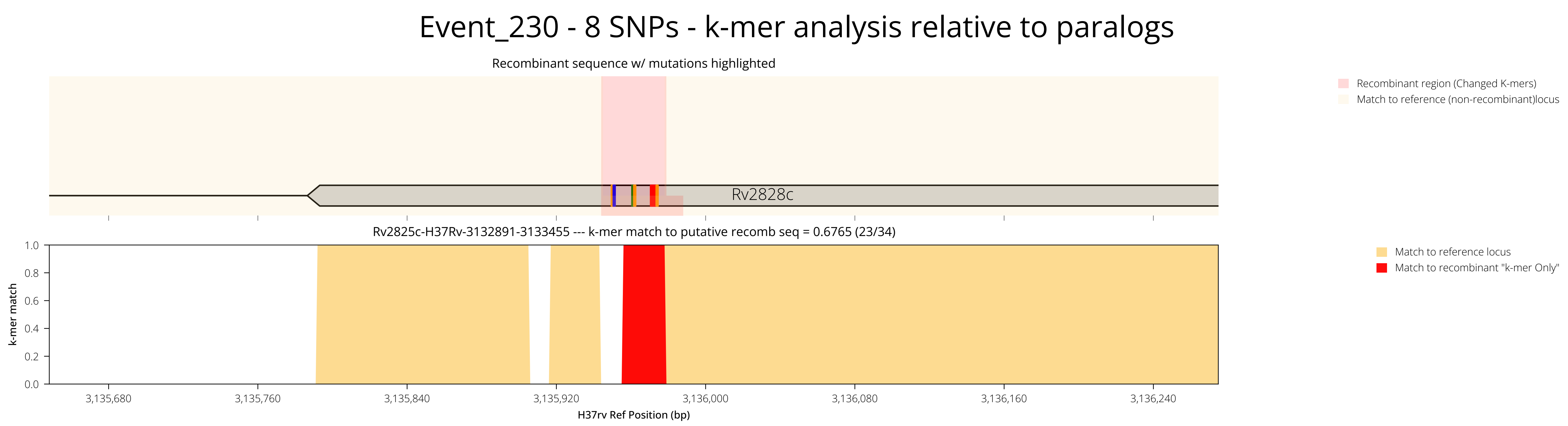

Event_230 - 8 SNPs - k-mer analysis relative to paralogs
Recombinant sequence w/ mutations highlighted
Rv2828c
Recombinant region (Changed K-mers)
Match to reference (non-recombinant)locus
Rv2825c-H37Rv-3132891-3133455 --- k-mer match to putative recomb seq = 0.6765 (23/34)
Match to reference locus
Match to recombinant "k-mer Only"
k-mer match
1.0
0.8
0.6
0.4
0.2
0.0
3,135,680
3,135,760
3,135,840
3,135,920
3,136,000
3,136,080
3,136,160
3,136,240
H37rv Ref Position (bp)

Sub-phylogeny Viz + Paralog Alignments
GCE ID: Event_230

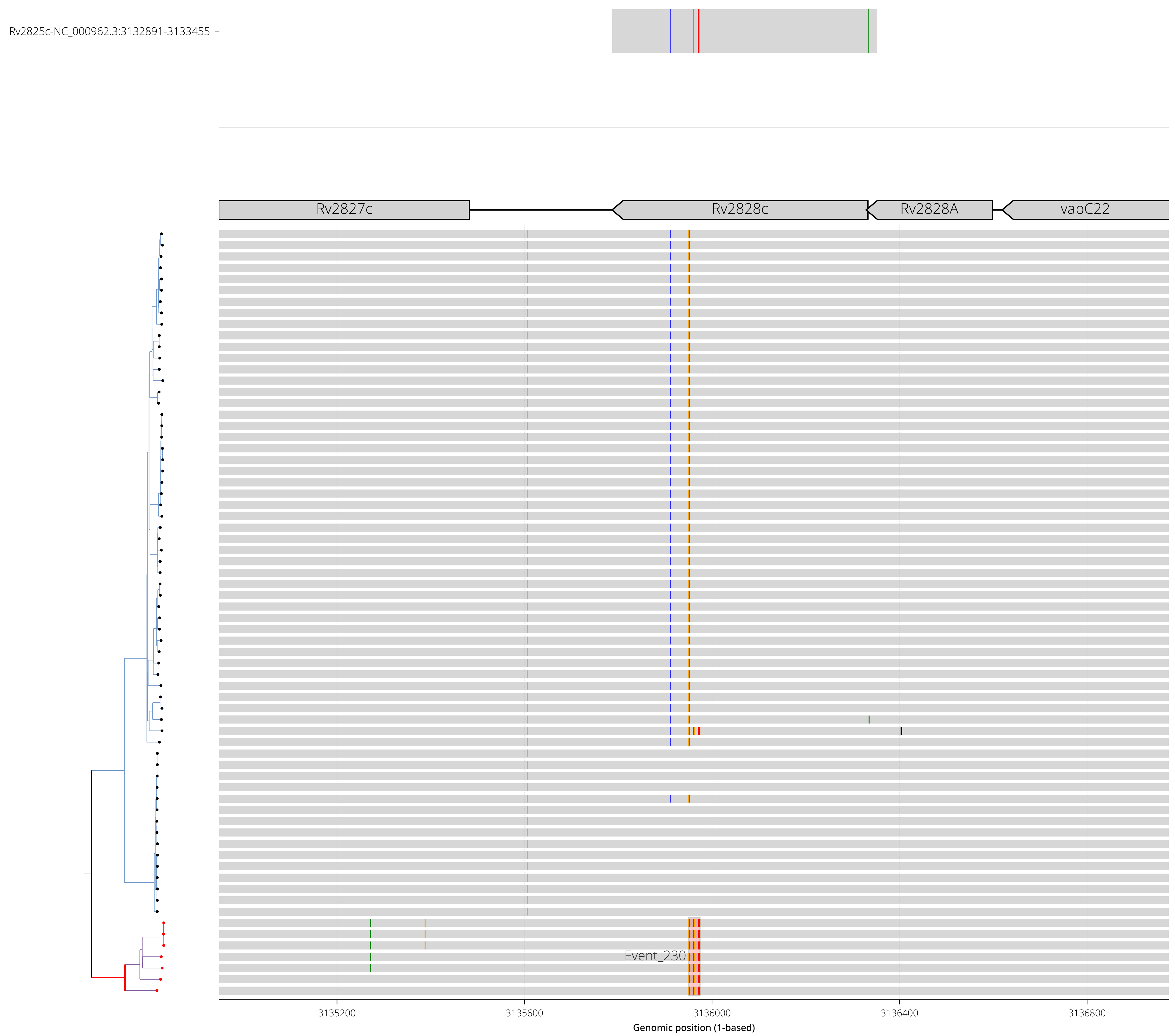

Full Phylogeny Viz + Paralog Alignments
GCE ID: Event_230

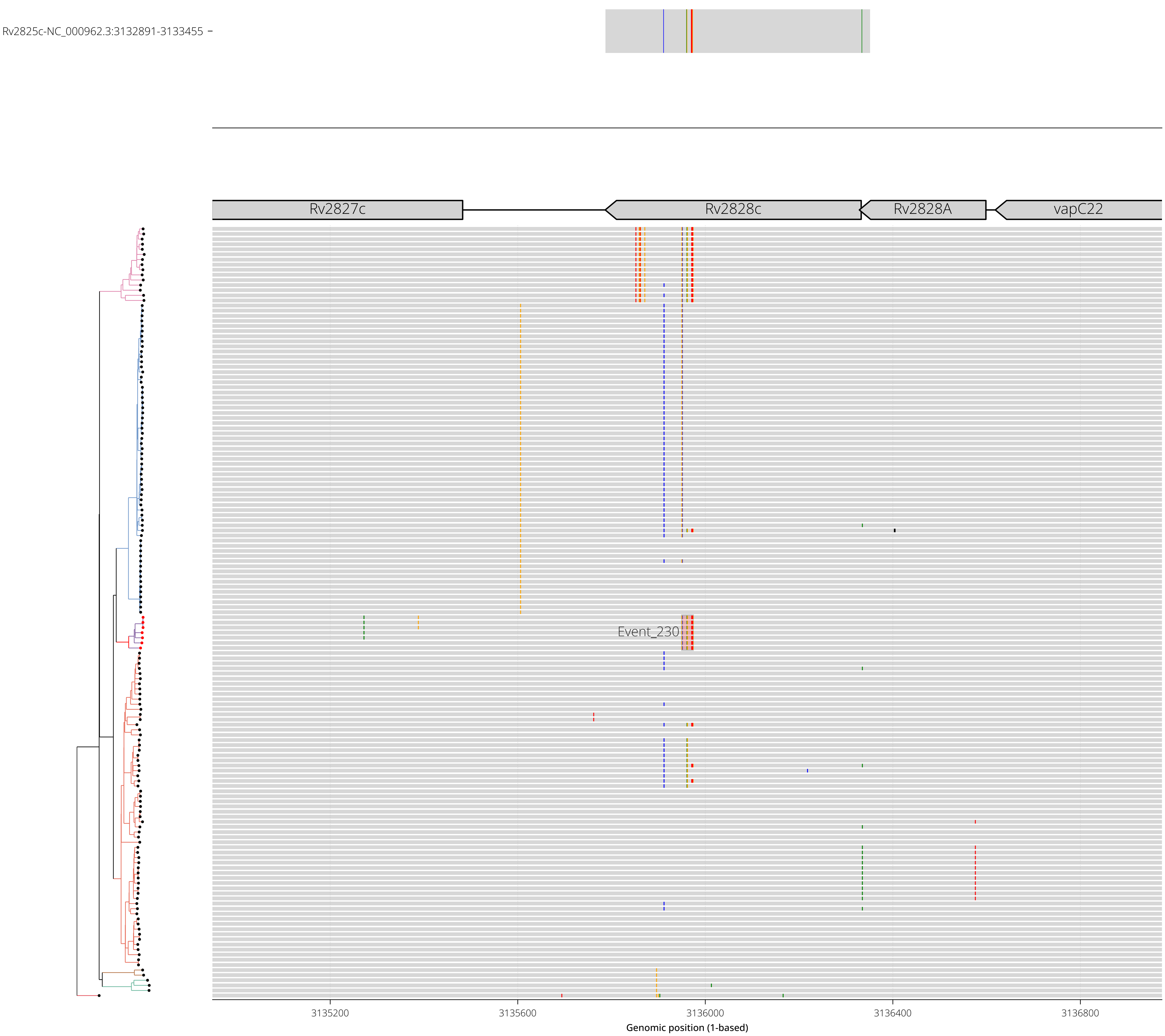

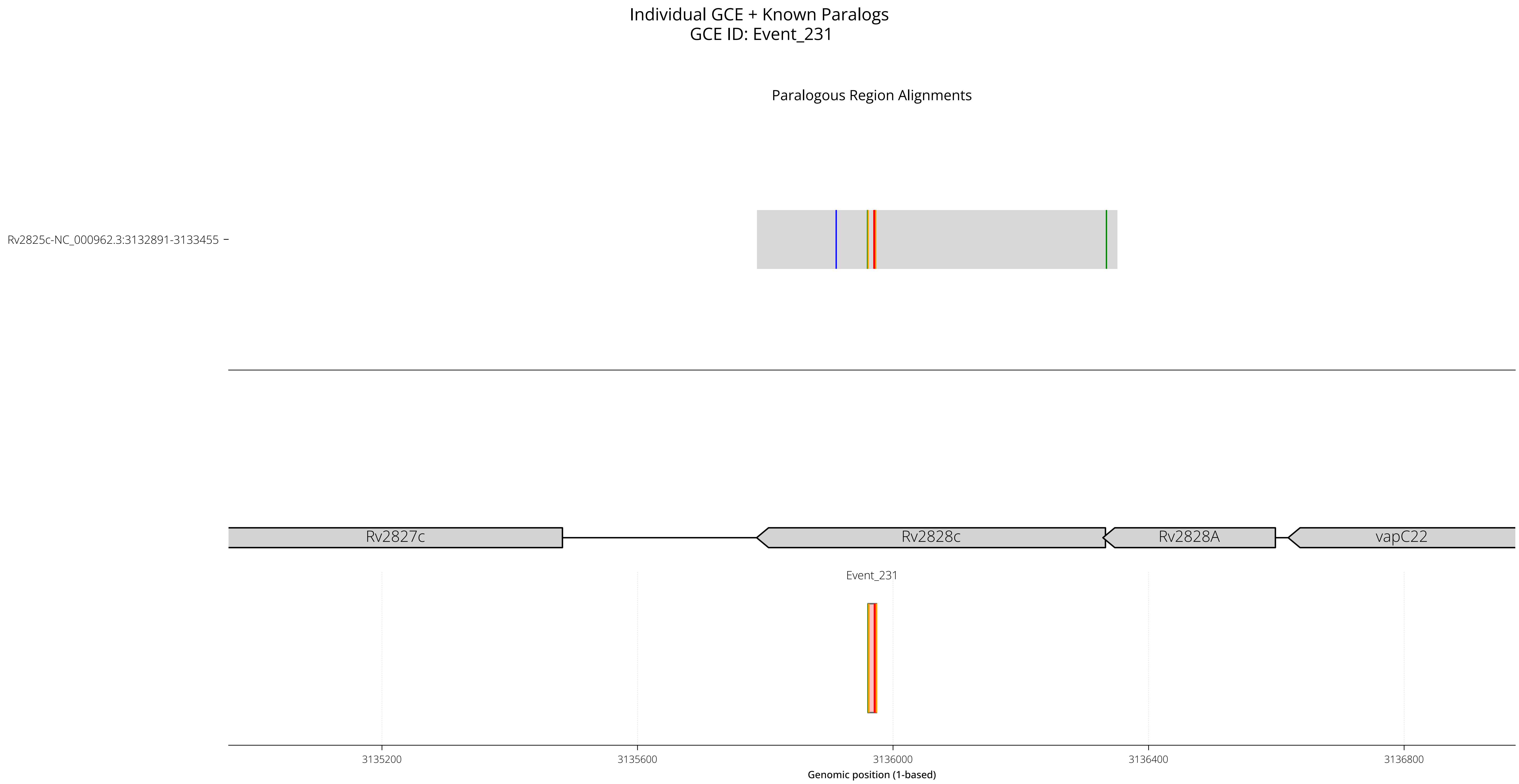
Individual GCE + Known Paralogs
GCE ID: Event_231
Paralogous Region Alignments
Rv2825c-NC_000962.3:3132891-3133455
Rv2827c
Rv2828c
Rv2828A
vapC22
Event_231
3135200
3135600
3136000
3136400
3136800
Genomic position (1-based)

Circos Viz - GCE to Known Paralogs Locations
GCE ID: Event_231

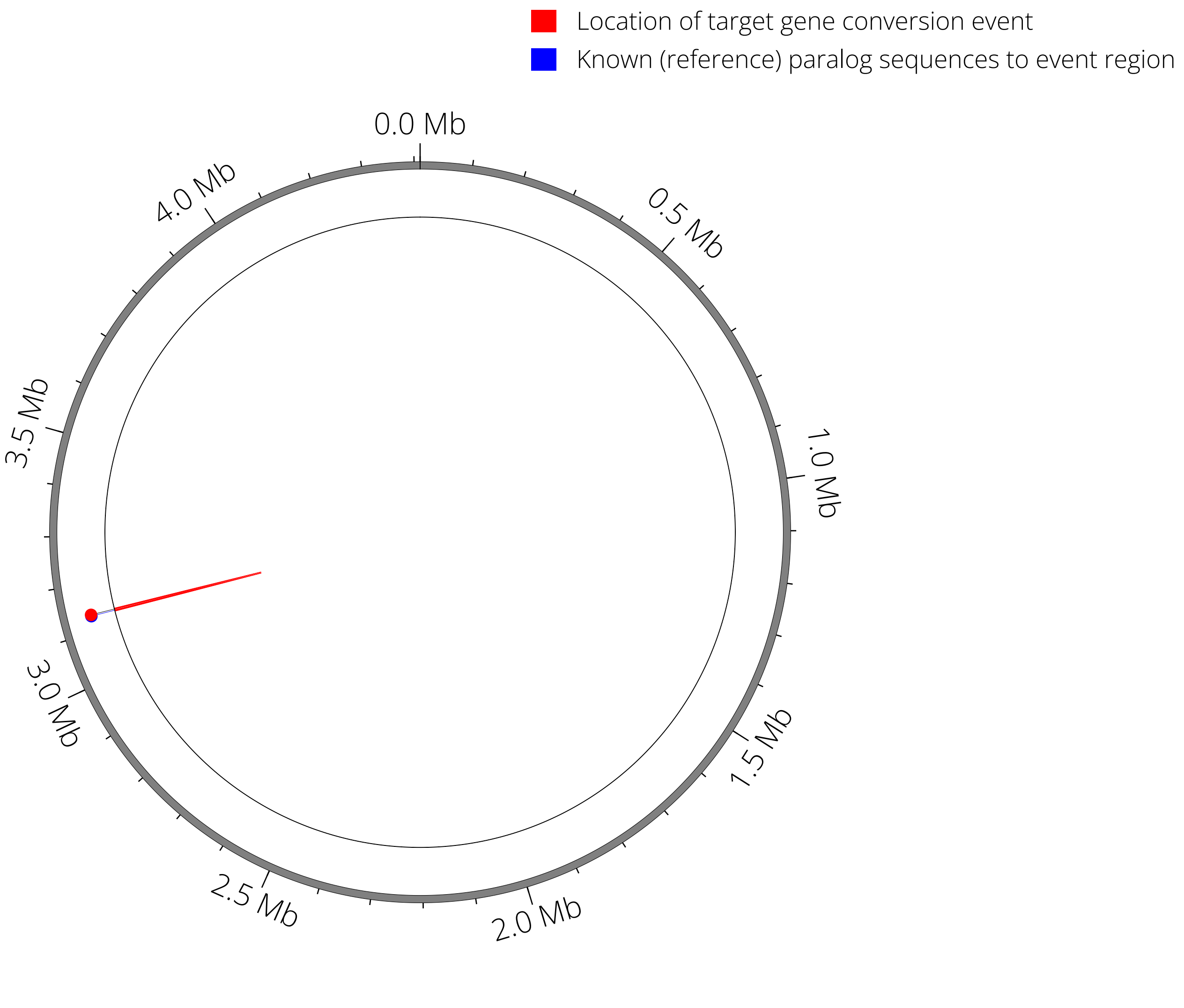

# Event_231 - 6 SNPs - k-mer analysis relative to paralogs

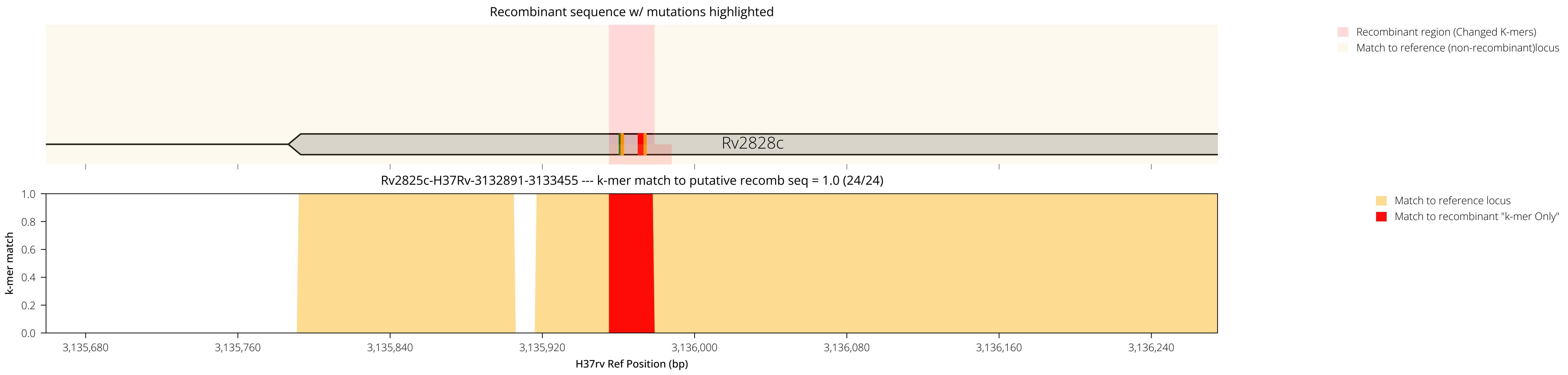

Sub-phylogeny Viz + Paralog Alignments
GCE ID: Event_231

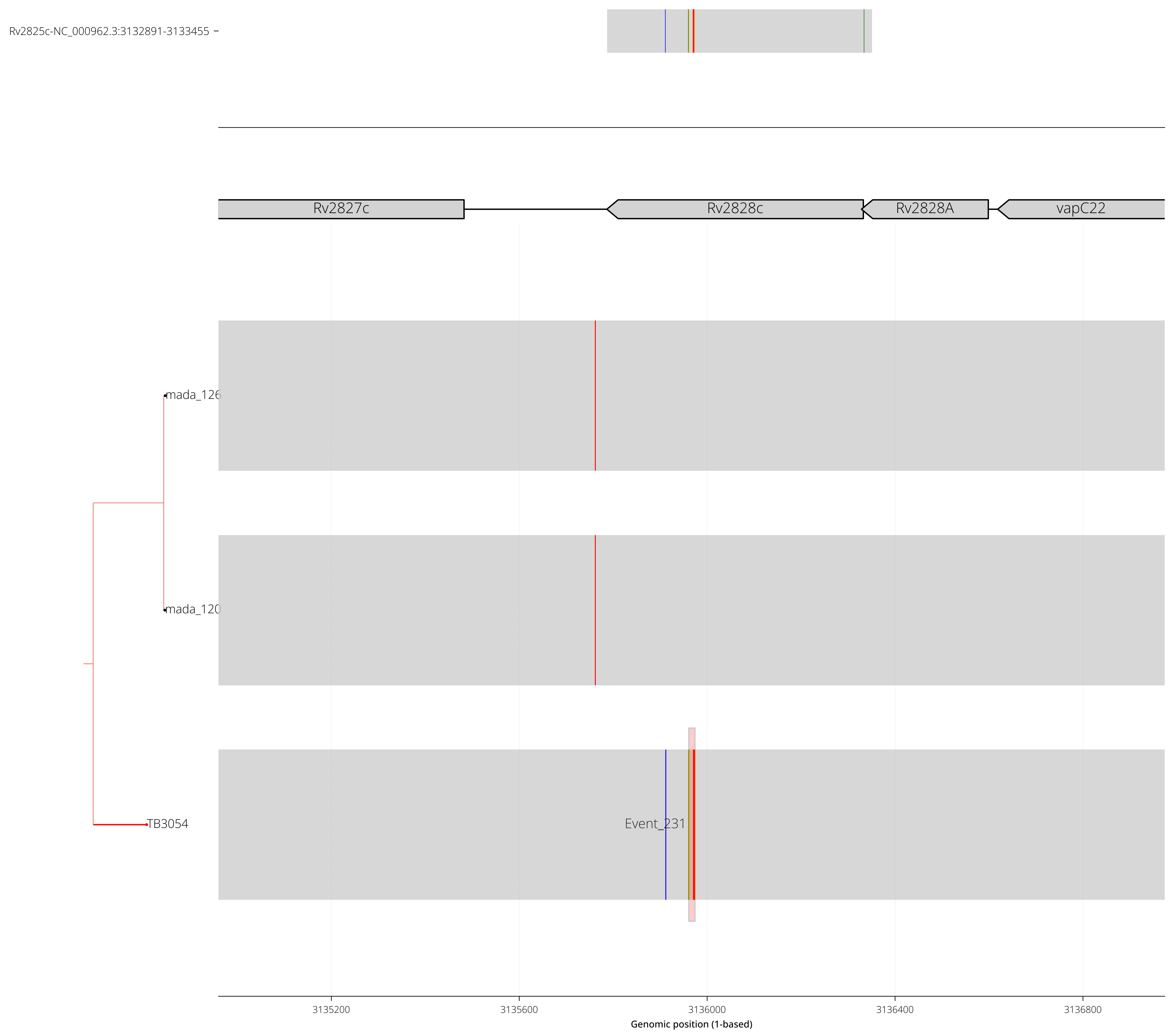

Full Phylogeny Viz + Paralog Alignments
GCE ID: Event_231

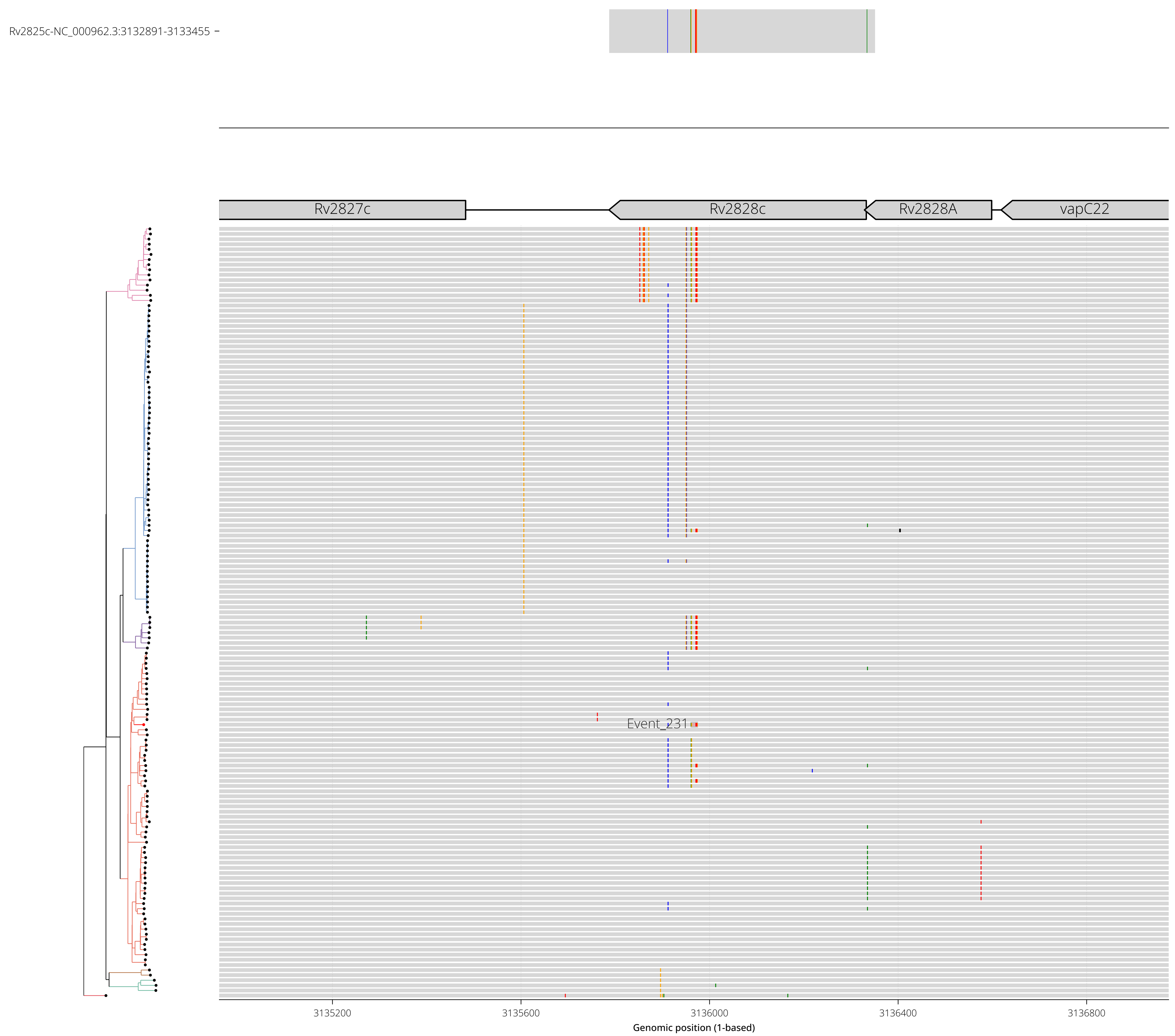

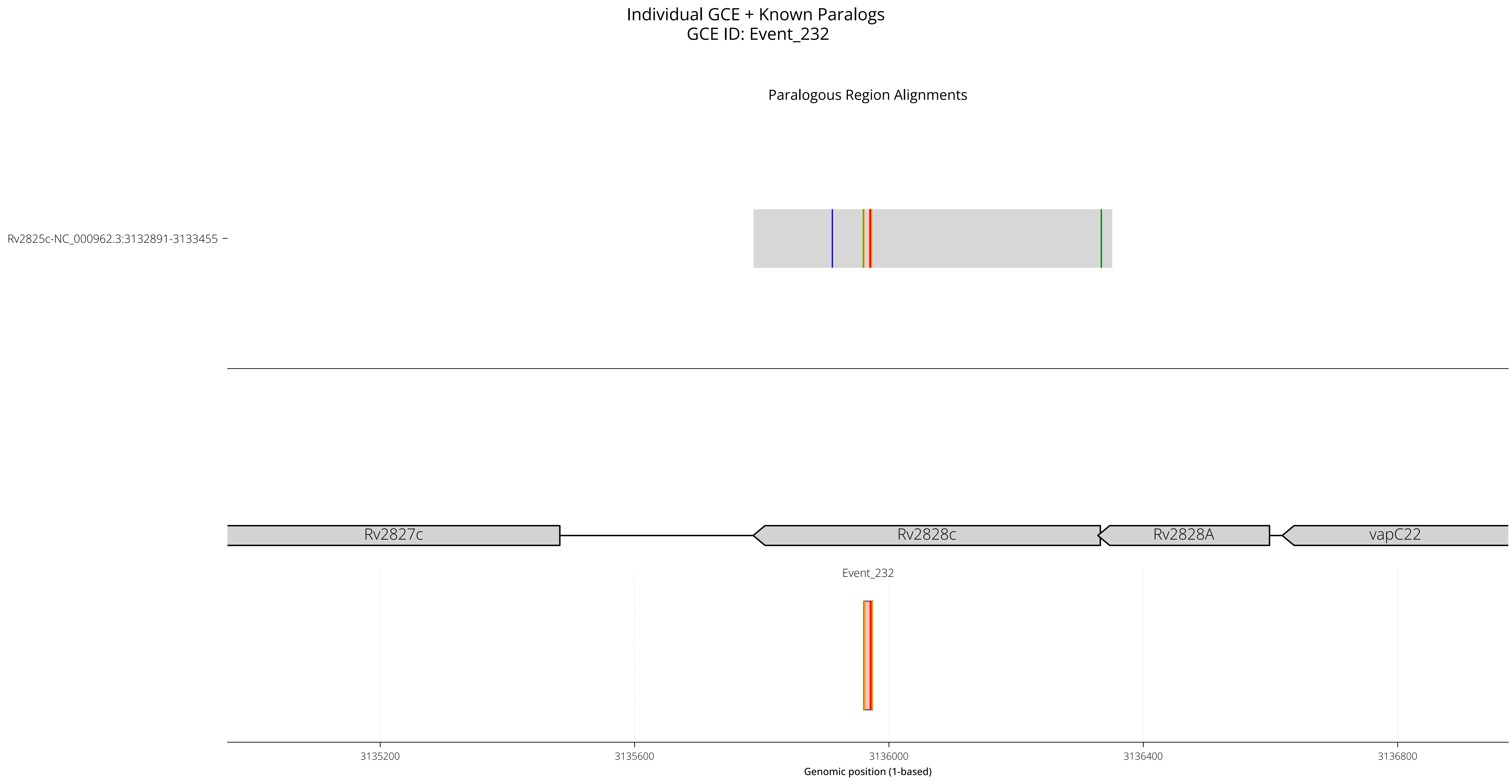
Individual GCE + Known Paralogs
GCE ID: Event_232
Paralogous Region Alignments
Rv2825c-NC_000962.3:3132891-3133455
Rv2827c
Rv2828c
Rv2828A
vapC22
Event_232
3135200
3135600
3136000
3136400
3136800
Genomic position (1-based)

Circos Viz - GCE to Known Paralogs Locations
GCE ID: Event_232

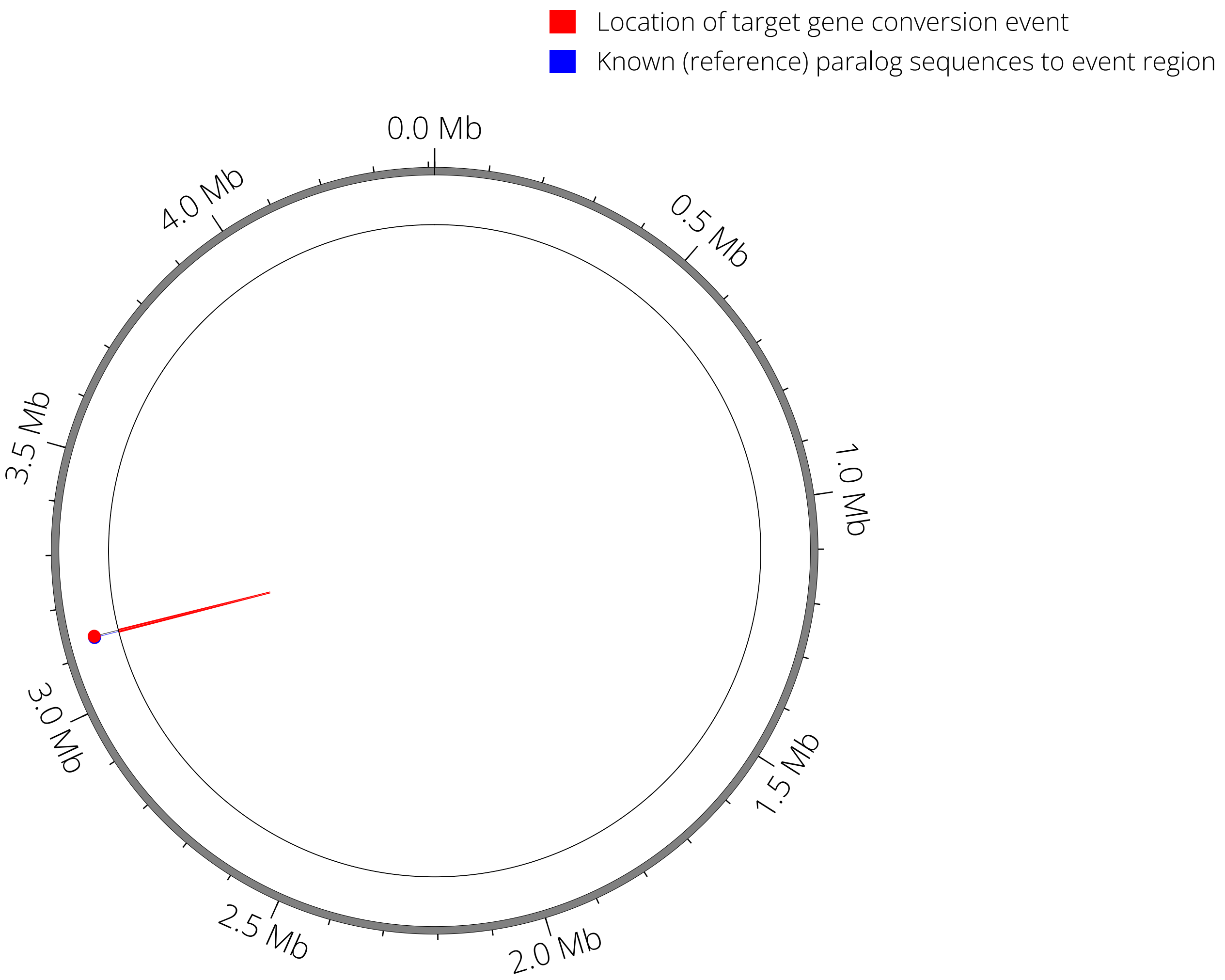

# Event_232 - 6 SNPs - k-mer analysis relative to paralogs

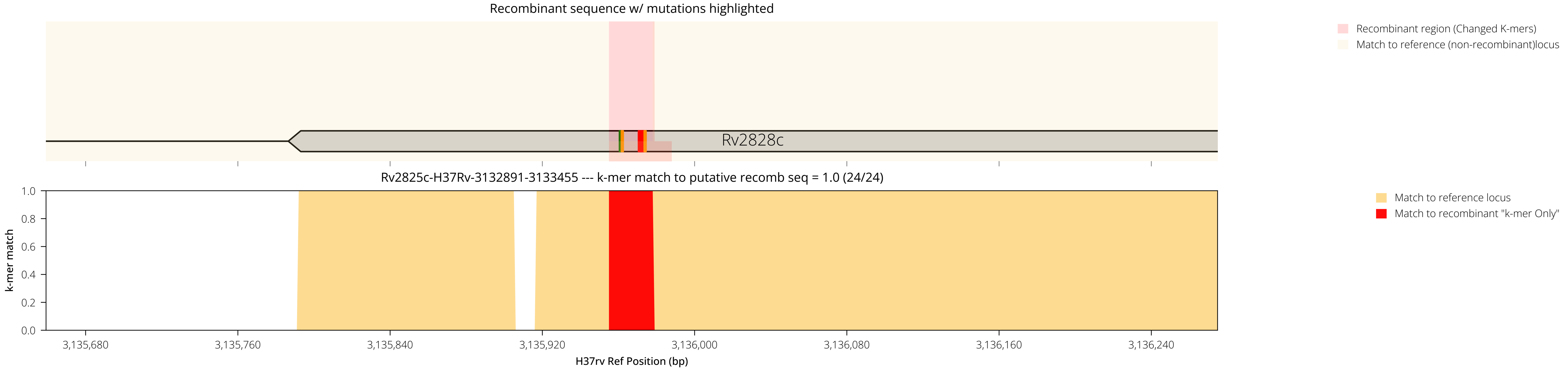

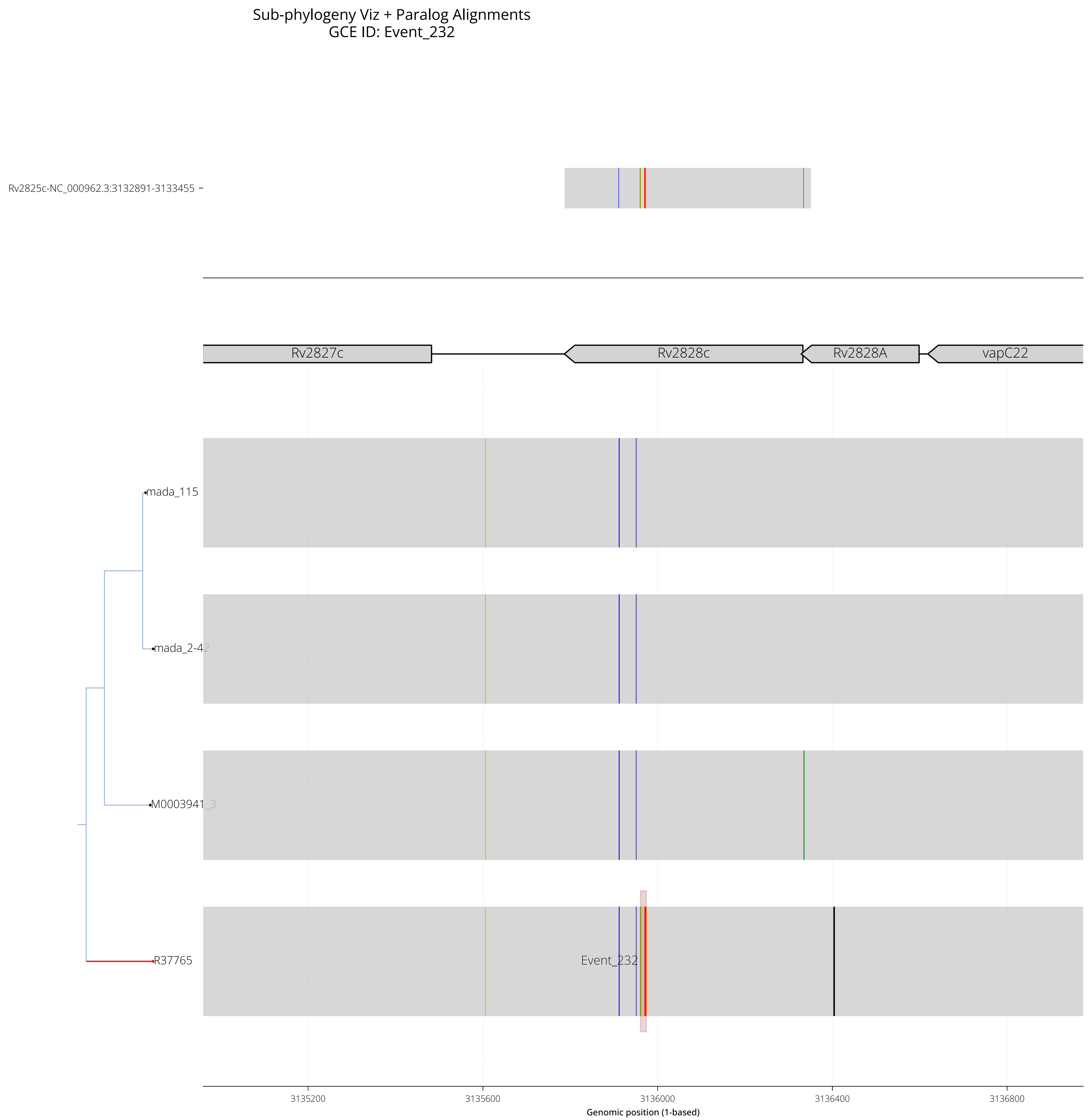

Sub-phylogeny Viz + Paralog Alignments
GCE ID: Event_232
Rv2825c-NC_000962.3:3132891-3133455
Rv2827c
Rv2828c
Rv2828A
vapC22
mada_115
mada_2-42
M0003941_3
R37765
Event_232
3135200
3135600
3136000
3136400
3136800
Genomic position (1-based)

Full Phylogeny Viz + Paralog Alignments
GCE ID: Event_232

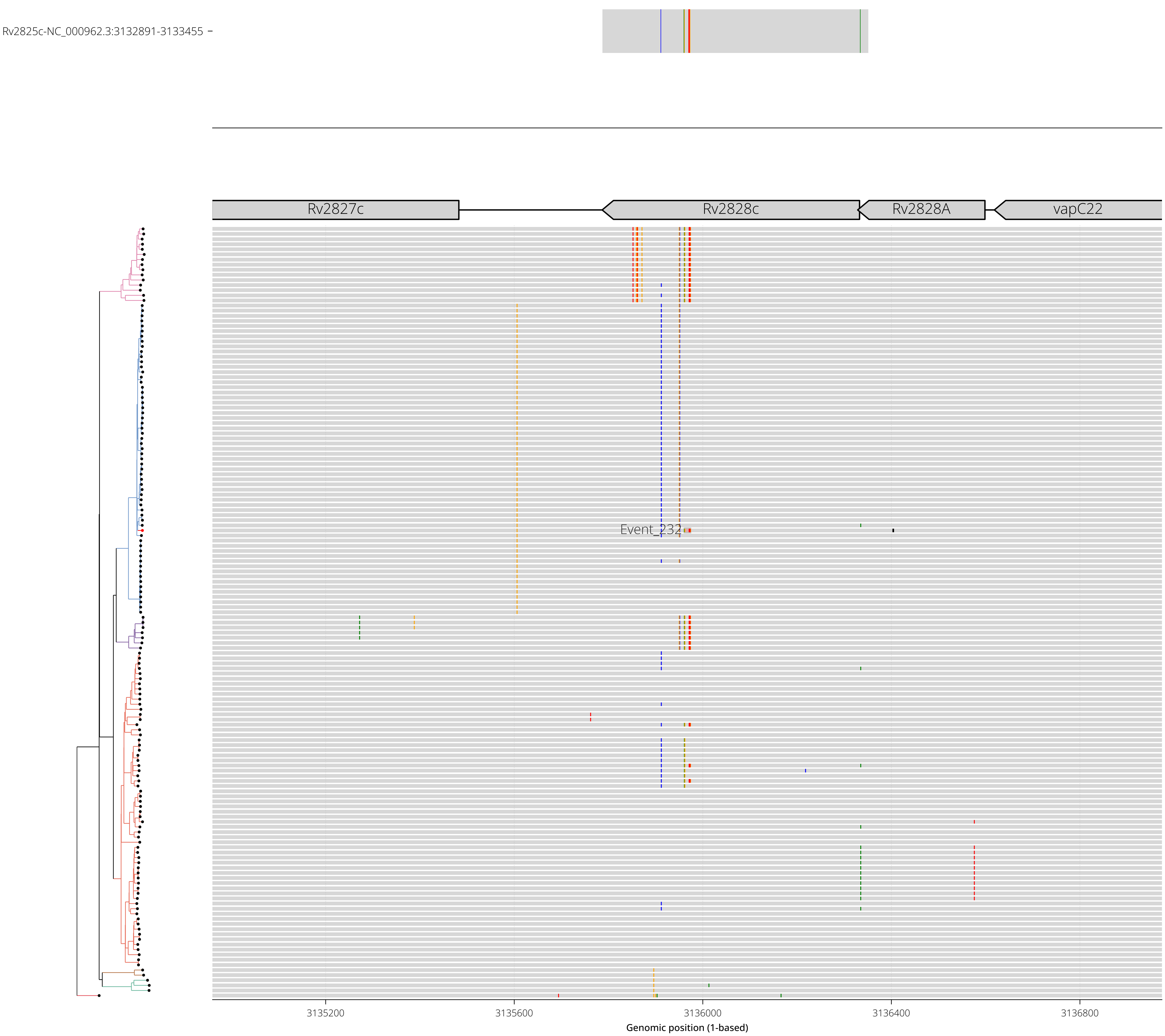

# Individual GCE + Known Paralogs
GCE ID: Event_233

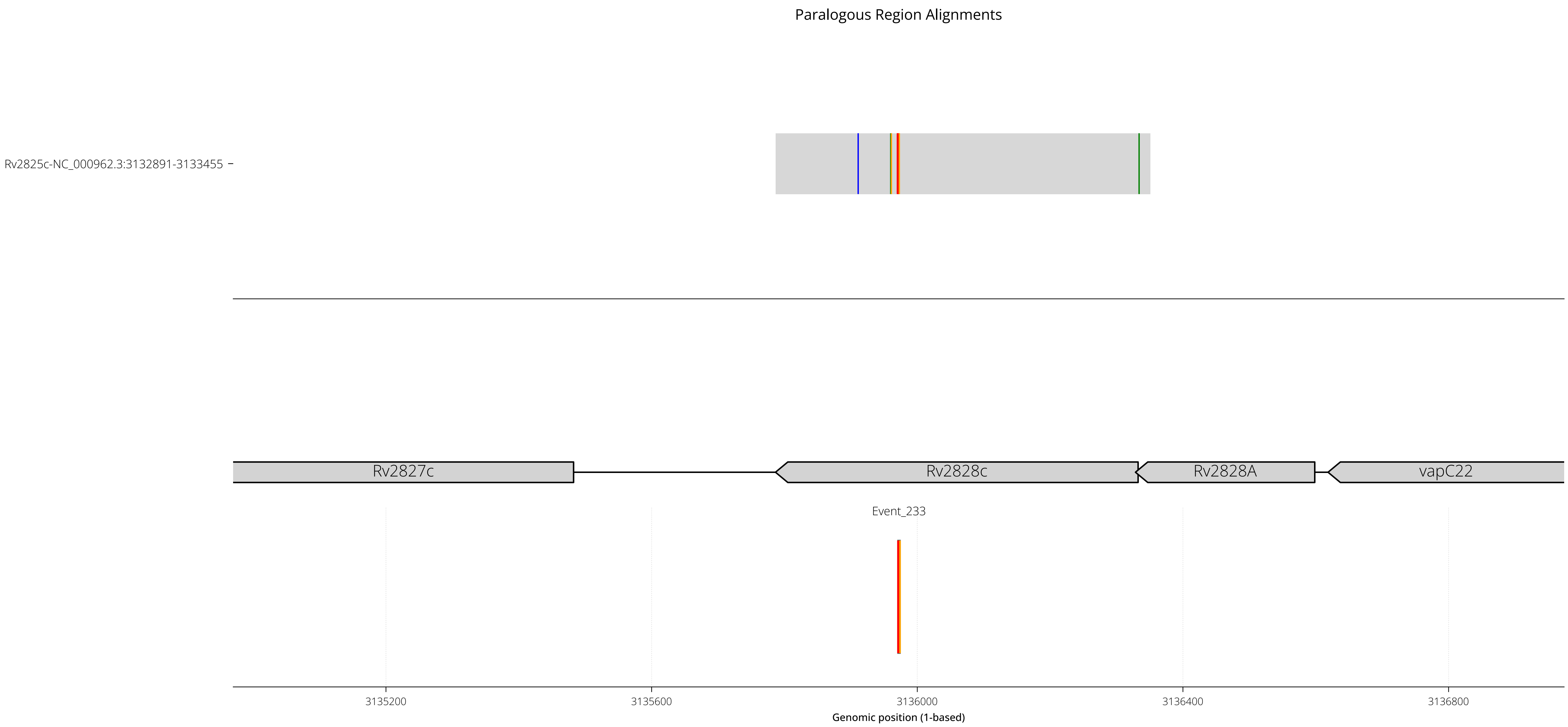

Circos Viz - GCE to Known Paralogs Locations
GCE ID: Event_233

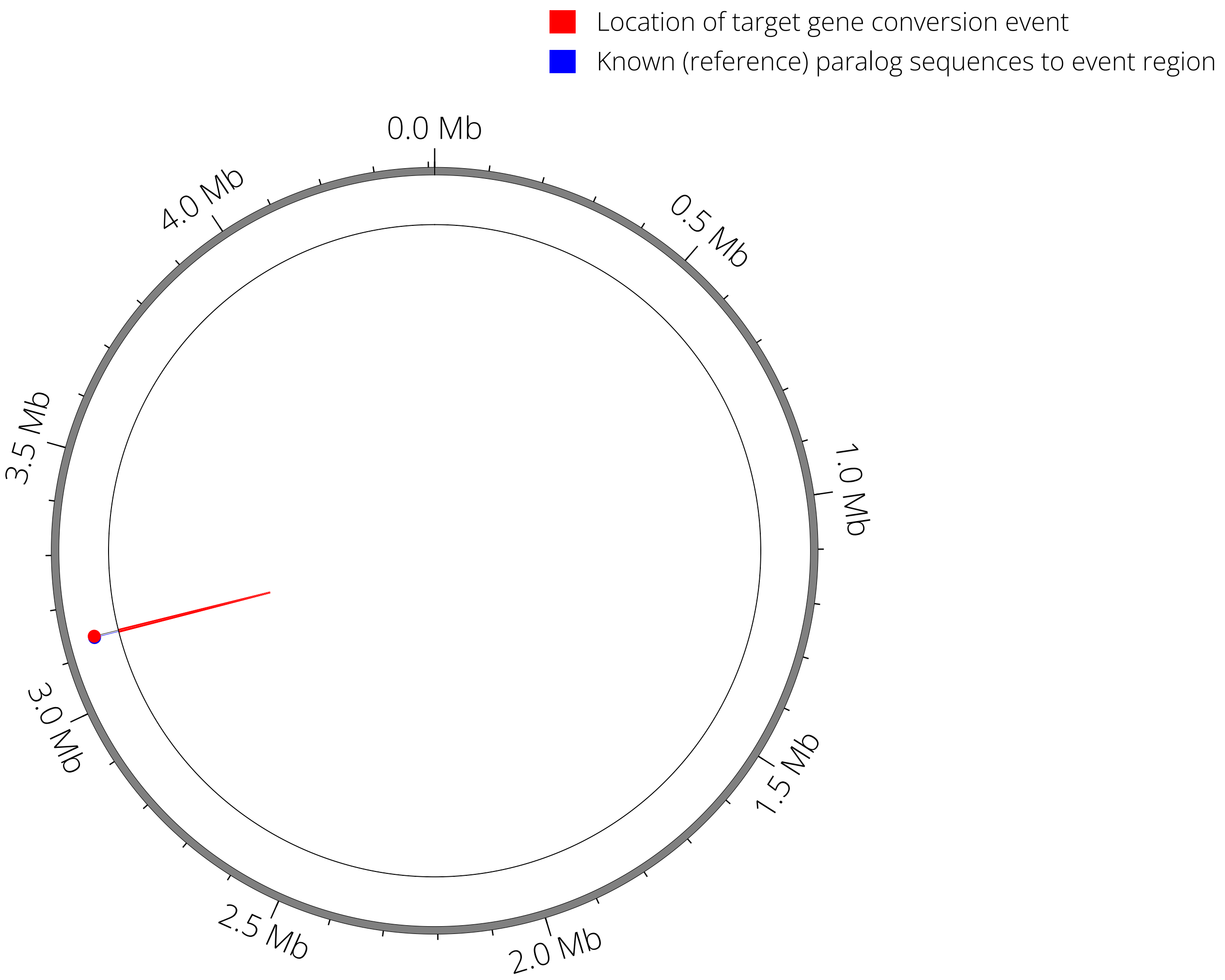

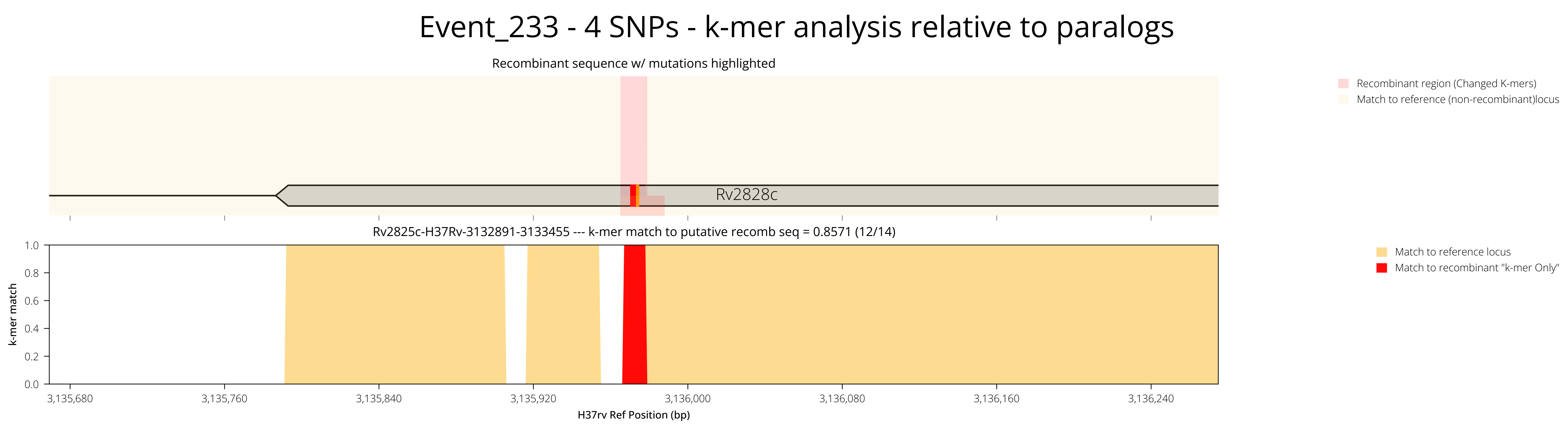

Event_233 - 4 SNPs - k-mer analysis relative to paralogs
Recombinant sequence w/ mutations highlighted
Rv2828c
Recombinant region (Changed K-mers)
Match to reference (non-recombinant)locus
Rv2825c-H37Rv-3132891-3133455 --- k-mer match to putative recomb seq = 0.8571 (12/14)
k-mer match
1.0
0.8
0.6
0.4
0.2
0.0
3,135,680
3,135,760
3,135,840
3,135,920
3,136,000
3,136,080
3,136,160
3,136,240
H37rv Ref Position (bp)
Match to reference locus
Match to recombinant "k-mer Only"

Sub-phylogeny Viz + Paralog Alignments
GCE ID: Event_233

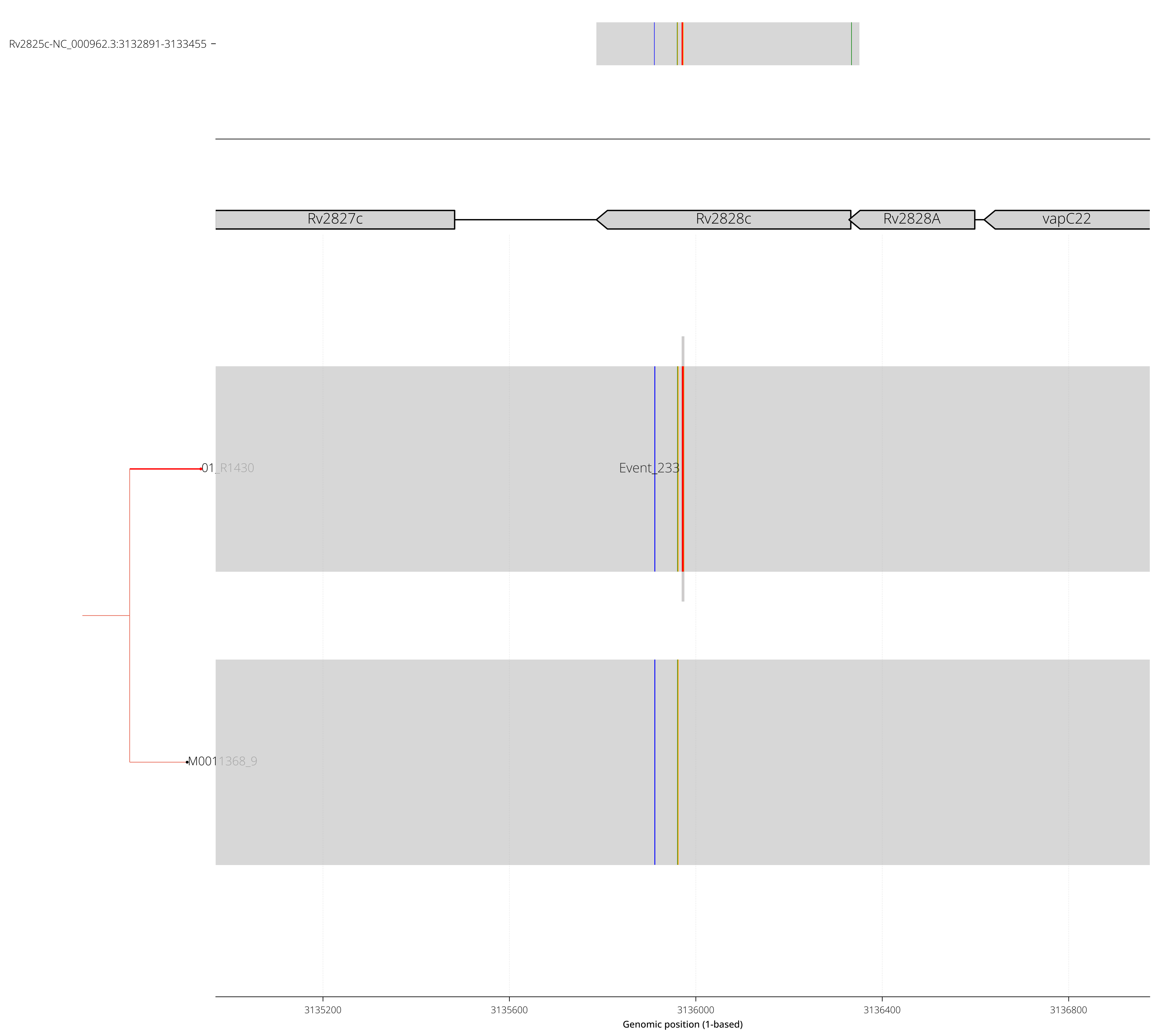

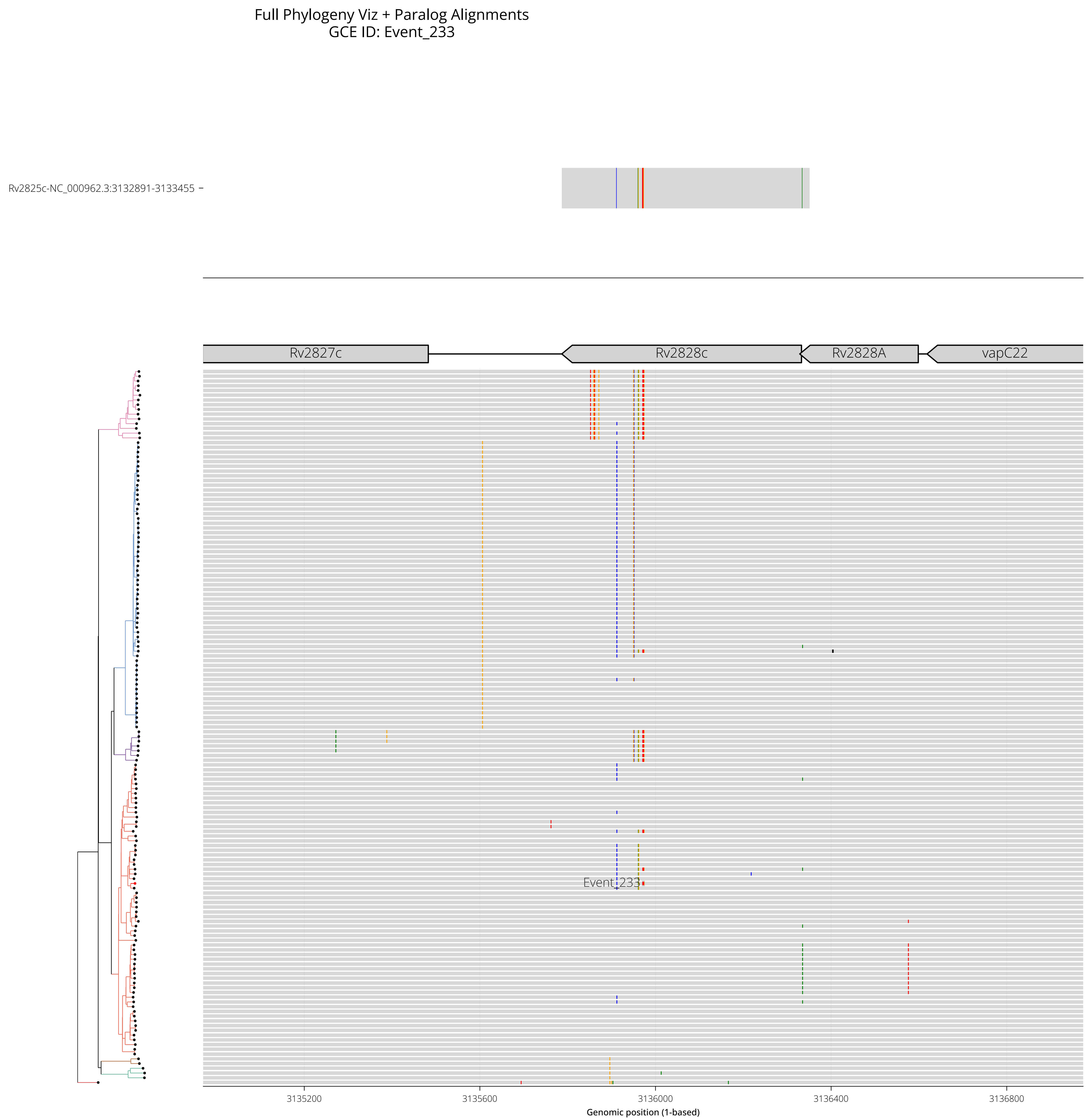

Full Phylogeny Viz + Paralog Alignments
GCE ID: Event_233
Rv2825c-NC_000962.3:3132891-3133455
Rv2827c
Rv2828c
Rv2828A
vapC22
Event_233
3135200
3135600
3136000
3136400
3136800
Genomic position (1-based)

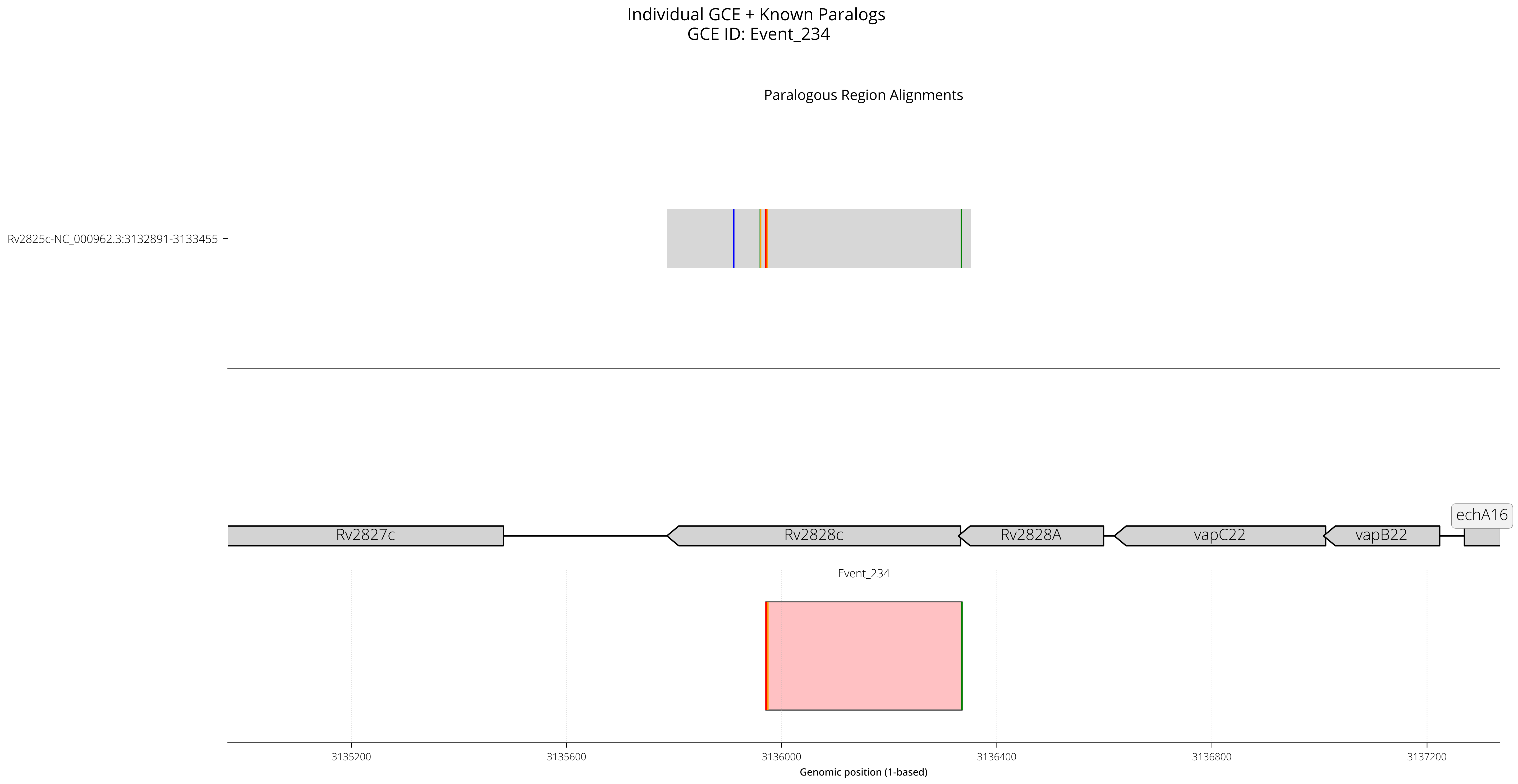
Individual GCE + Known Paralogs
GCE ID: Event_234
Paralogous Region Alignments
Rv2825c-NC_000962.3:3132891-3133455
Rv2827c
Rv2828c
Rv2828A
vapC22
vapB22
echA16
Event_234
3135200
3135600
3136000
3136400
3136800
3137200
Genomic position (1-based)

Circos Viz - GCE to Known Paralogs Locations
GCE ID: Event_234

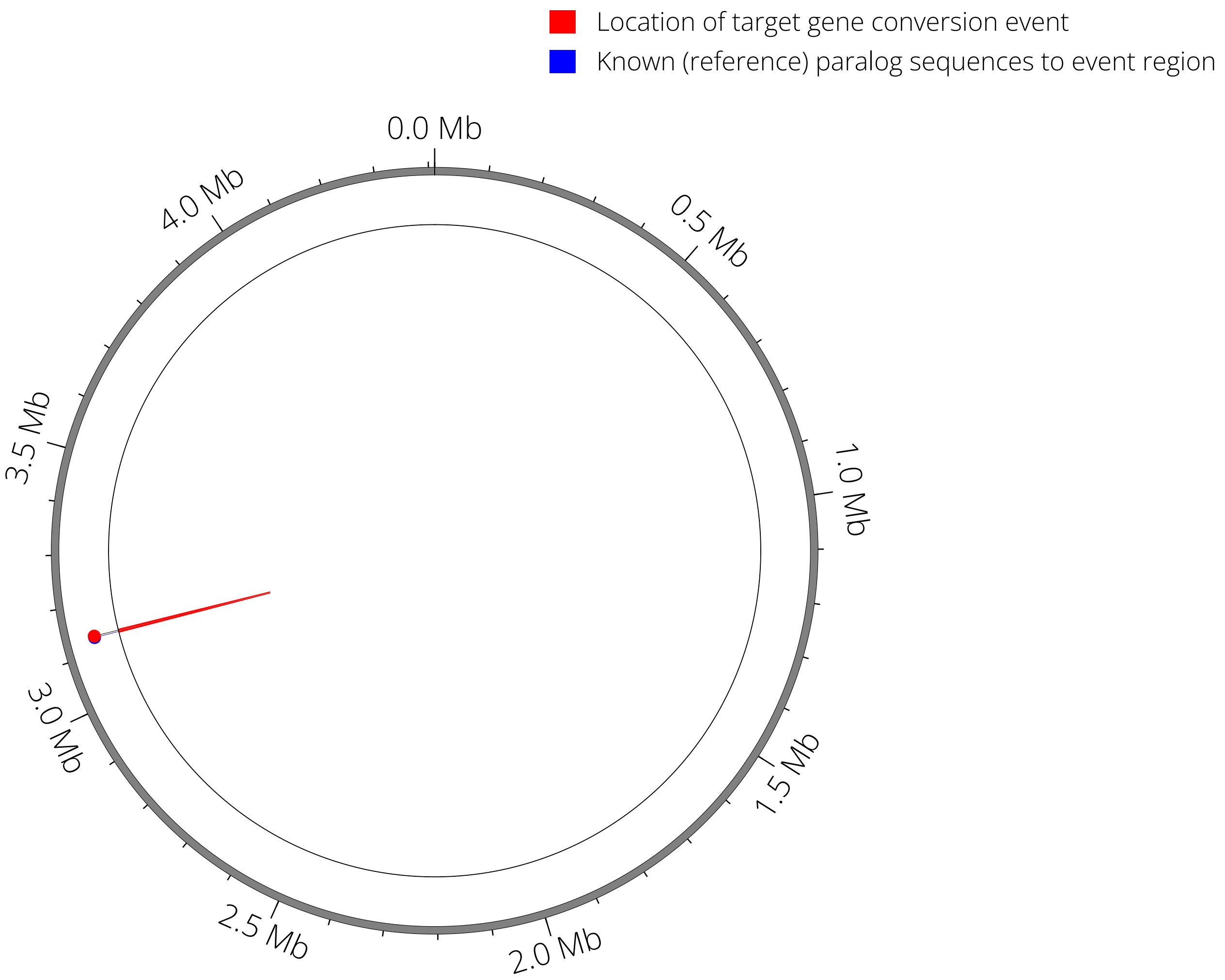

# Event_234 - 5 SNPs - k-mer analysis relative to paralogs

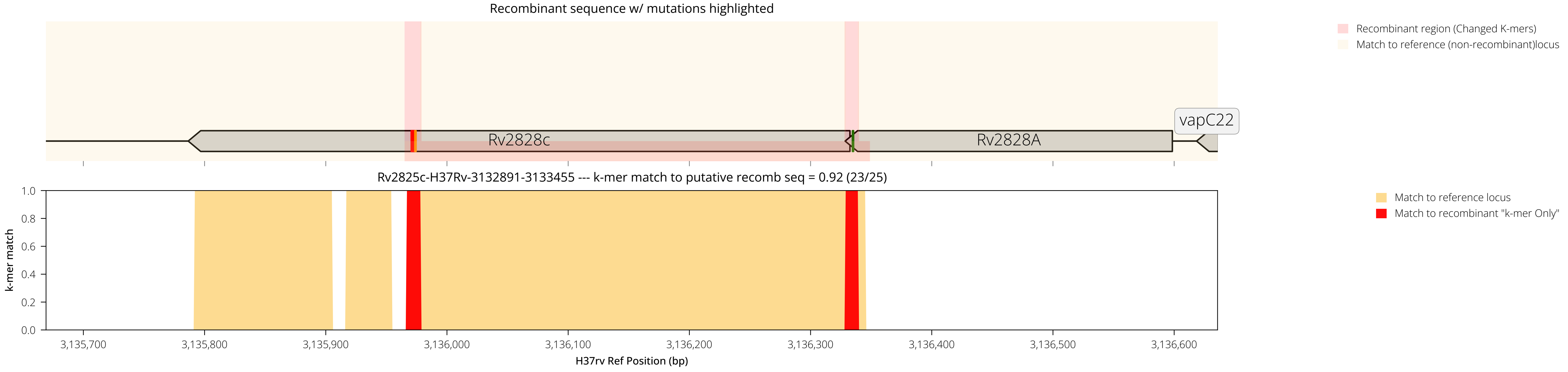

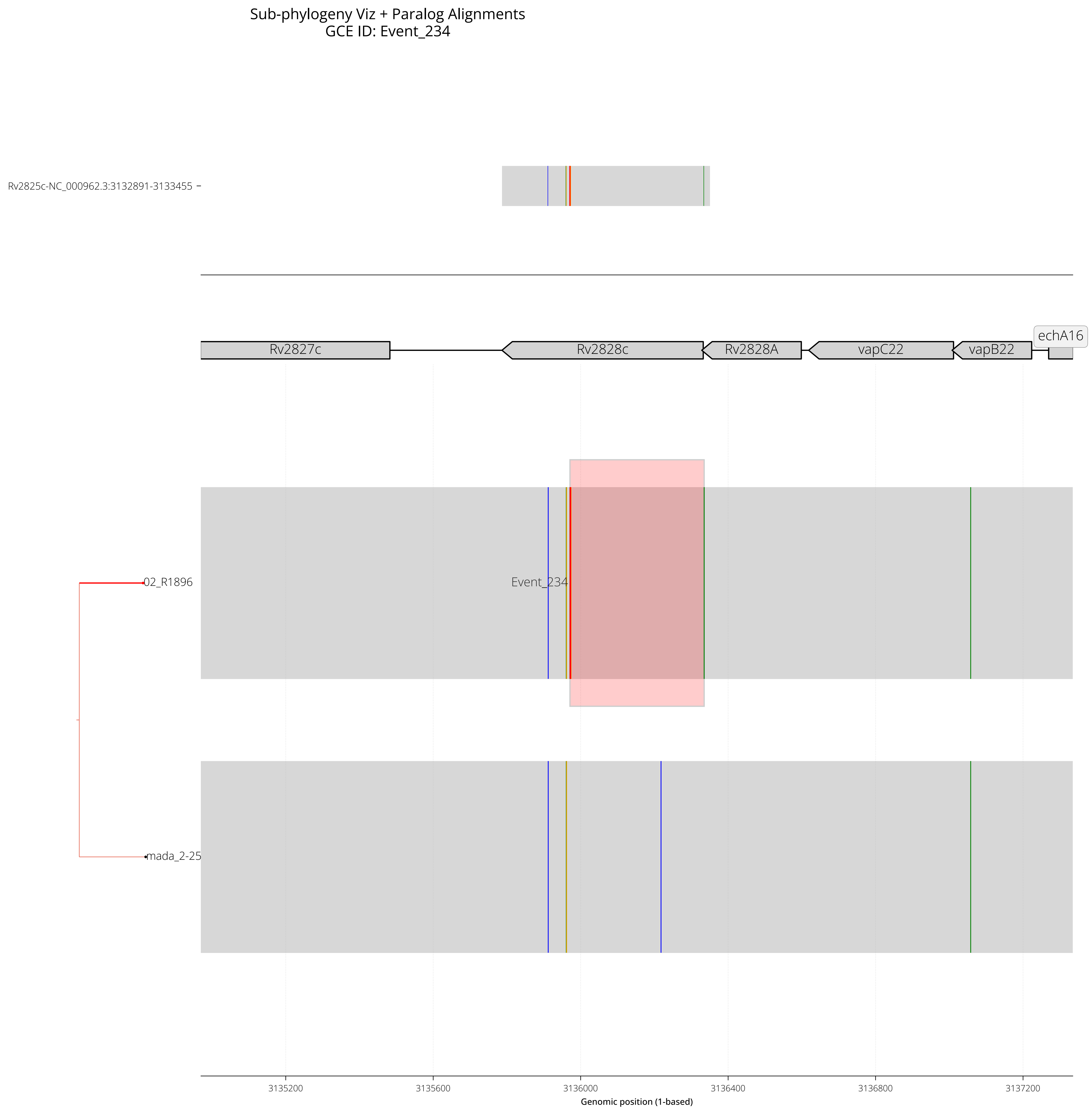

Sub-phylogeny Viz + Paralog Alignments
GCE ID: Event_234
Rv2825c-NC_000962.3:3132891-3133455
Rv2827c
Rv2828c
Rv2828A
vapC22
vapB22
echA16
02_R1896
Event_234
mada_2-25
3135200
3135600
3136000
3136400
3136800
3137200
Genomic position (1-based)

Full Phylogeny Viz + Paralog Alignments
GCE ID: Event_234

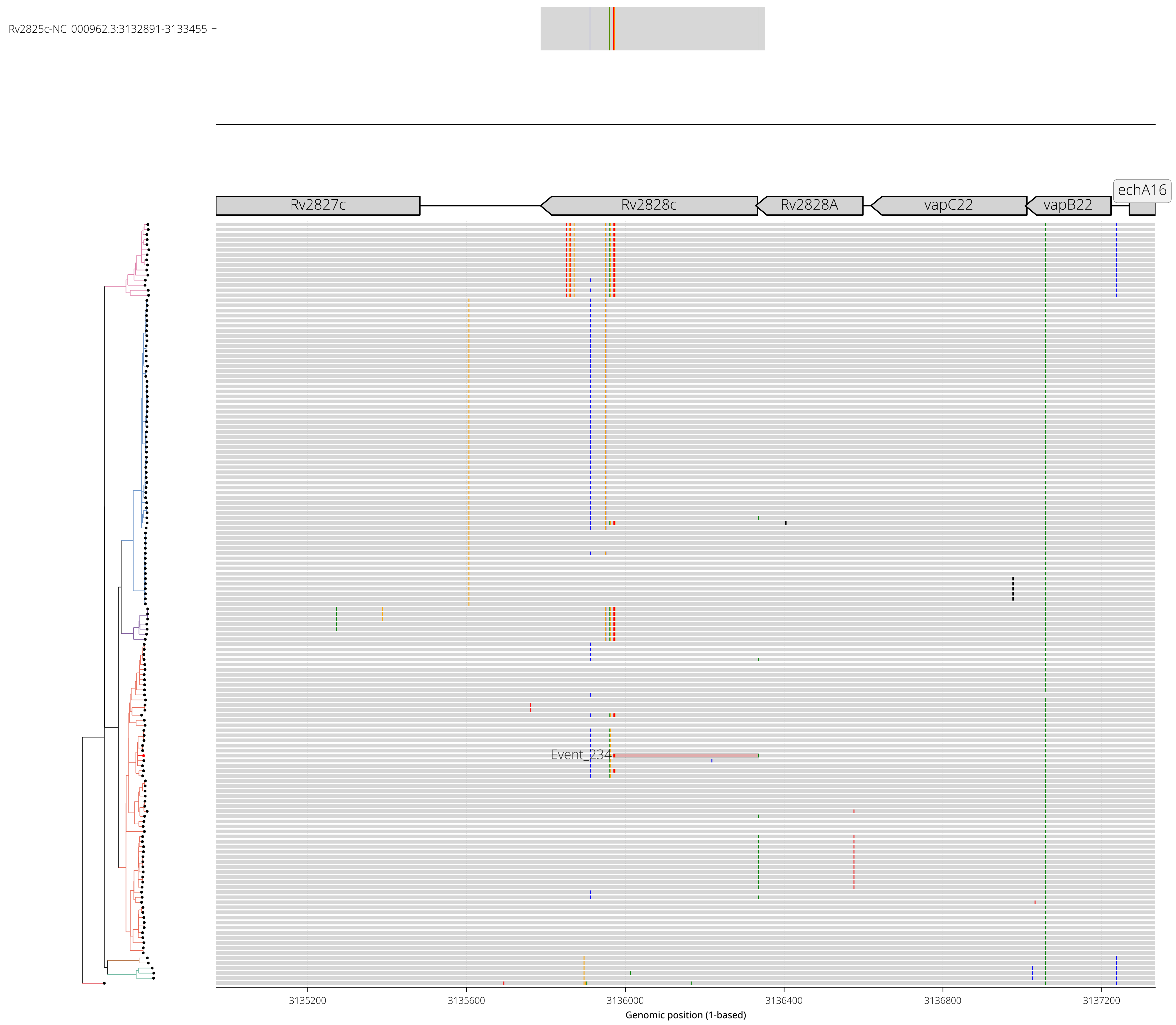

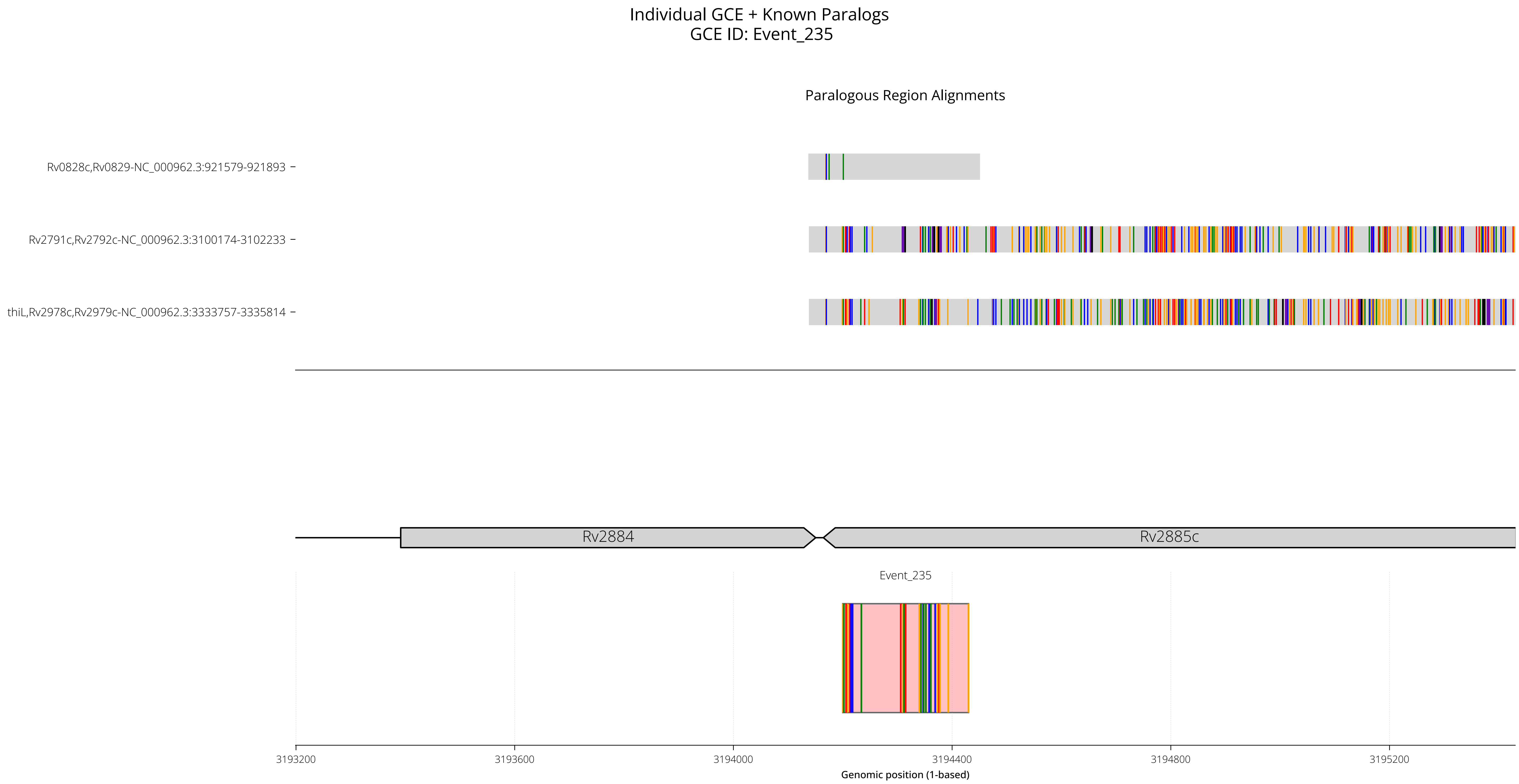

Individual GCE + Known Paralogs
GCE ID: Event_235
Paralogous Region Alignments
Rv0828c,Rv0829-NC_000962.3:921579-921893
Rv2791c,Rv2792c-NC_000962.3:3100174-3102233
thiL,Rv2978c,Rv2979c-NC_000962.3:3333757-3335814
Rv2884
Rv2885c
Event_235
3193200
3193600
3194000
3194400
3194800
3195200
Genomic position (1-based)

Circos Viz - GCE to Known Paralogs Locations
GCE ID: Event_235

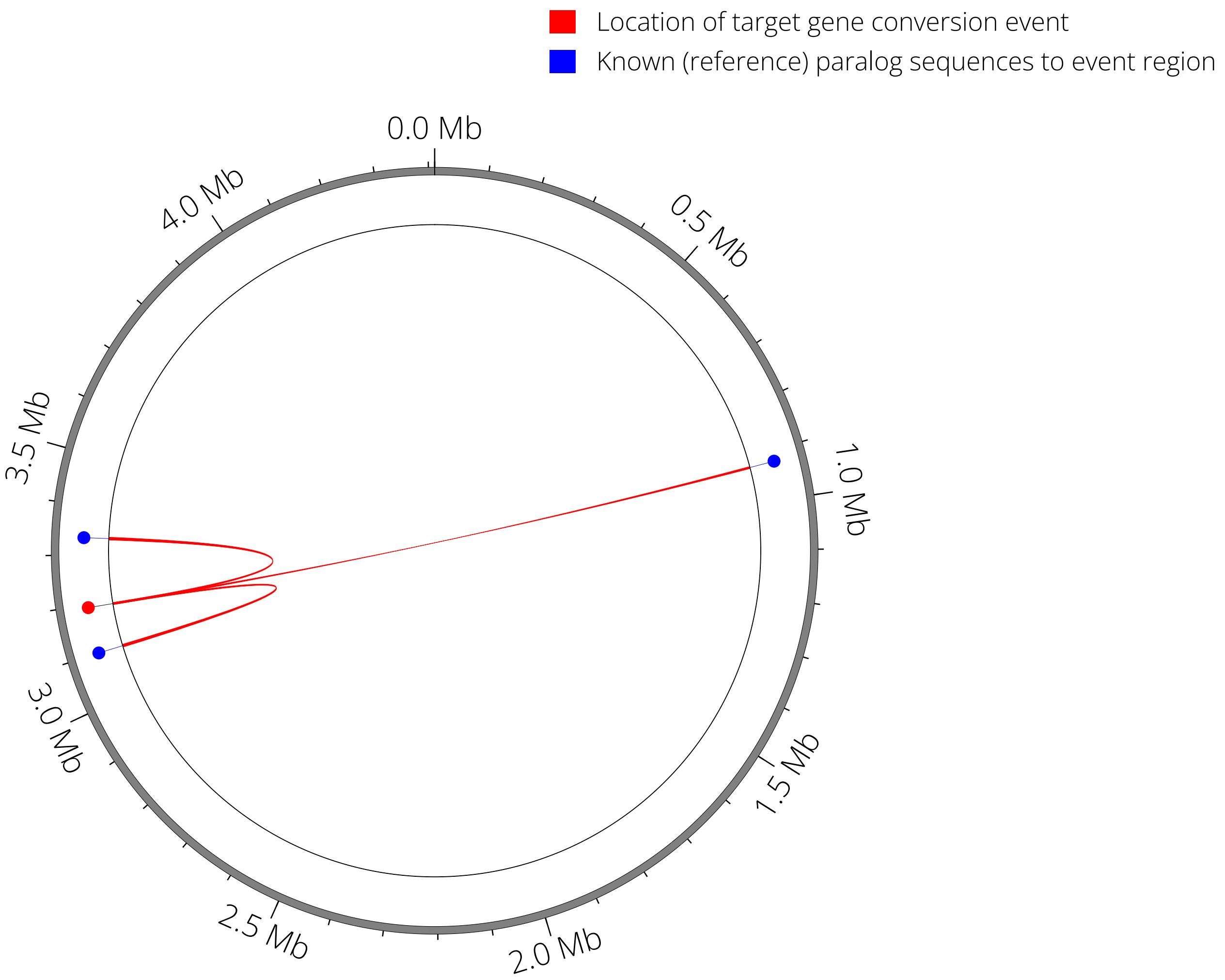

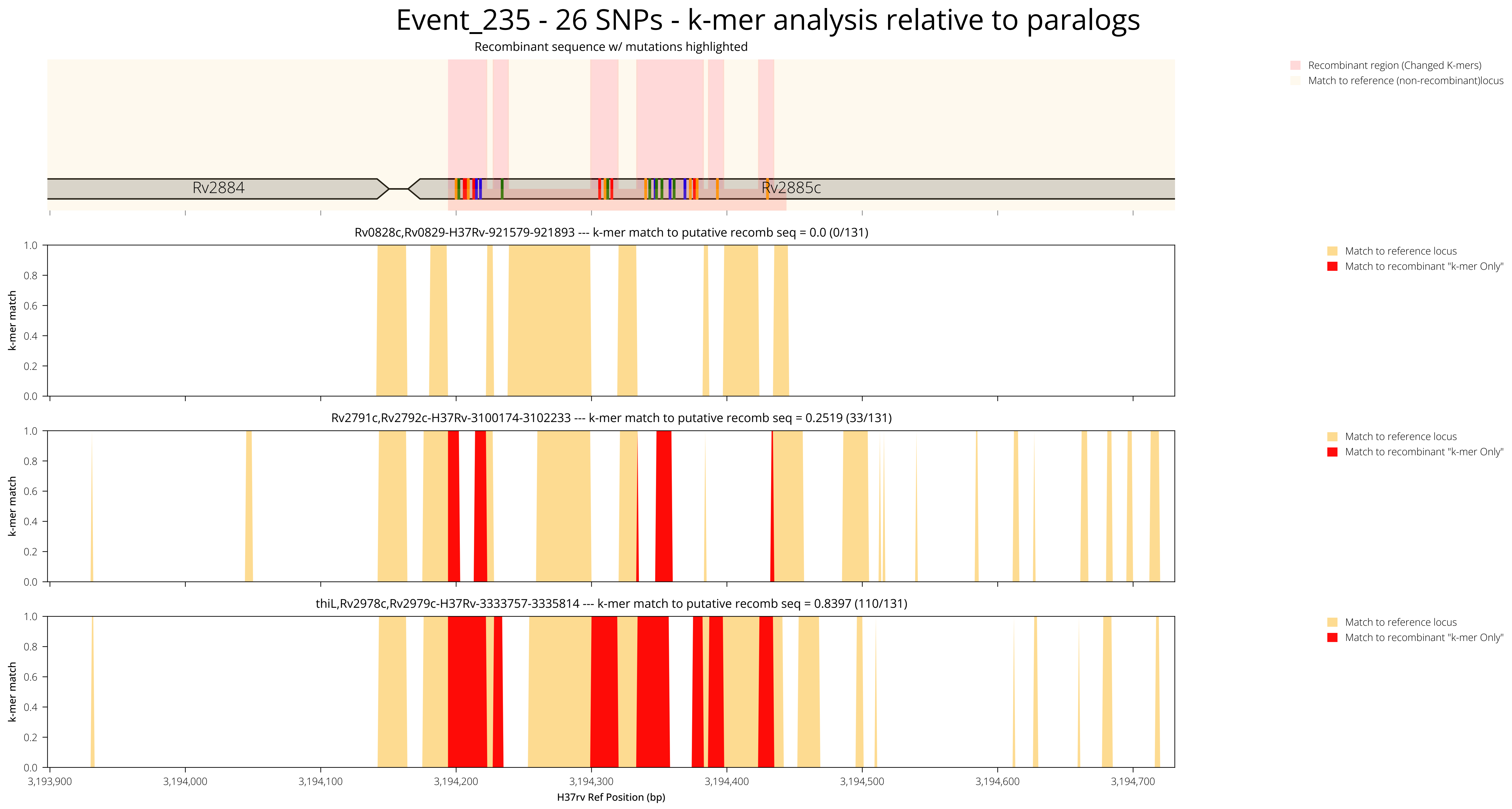
Event_235 - 26 SNPs - k-mer analysis relative to paralogs
Recombinant sequence w/ mutations highlighted
Recombinant region (Changed K-mers)
Match to reference (non-recombinant)locus
Rv2884
Rv2885c
Rv0828c,Rv0829-H37Rv-921579-921893 --- k-mer match to putative recomb seq = 0.0 (0/131)
Match to reference locus
Match to recombinant "k-mer Only"
Rv2791c,Rv2792c-H37Rv-3100174-3102233 --- k-mer match to putative recomb seq = 0.2519 (33/131)
Match to reference locus
Match to recombinant "k-mer Only"
thiL,Rv2978c,Rv2979c-H37Rv-3333757-3335814 --- k-mer match to putative recomb seq = 0.8397 (110/131)
Match to reference locus
Match to recombinant "k-mer Only"
k-mer match
0.0
0.2
0.4
0.6
0.8
1.0
3,193,900
3,194,000
3,194,100
3,194,200
3,194,300
3,194,400
3,194,500
3,194,600
3,194,700
H37rv Ref Position (bp)

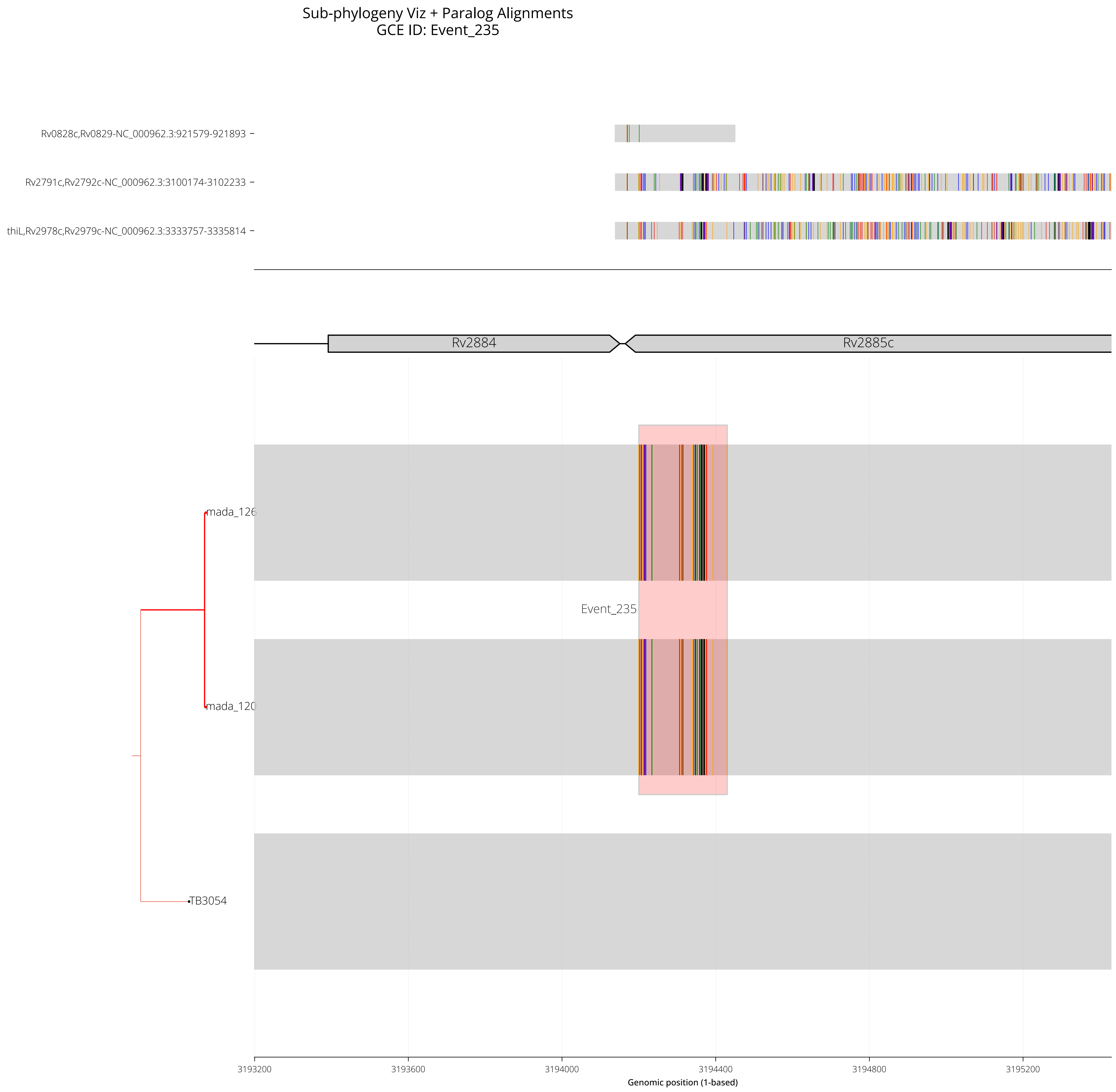
Sub-phylogeny Viz + Paralog Alignments
GCE ID: Event_235
Rv0828c,Rv0829-NC_000962.3:921579-921893
Rv2791c,Rv2792c-NC_000962.3:3100174-3102233
thiL,Rv2978c,Rv2979c-NC_000962.3:3333757-3335814
Rv2884
Rv2885c
mada_126
mada_120
TB3054
Event_235
3193200
3193600
3194000
3194400
3194800
3195200
Genomic position (1-based)

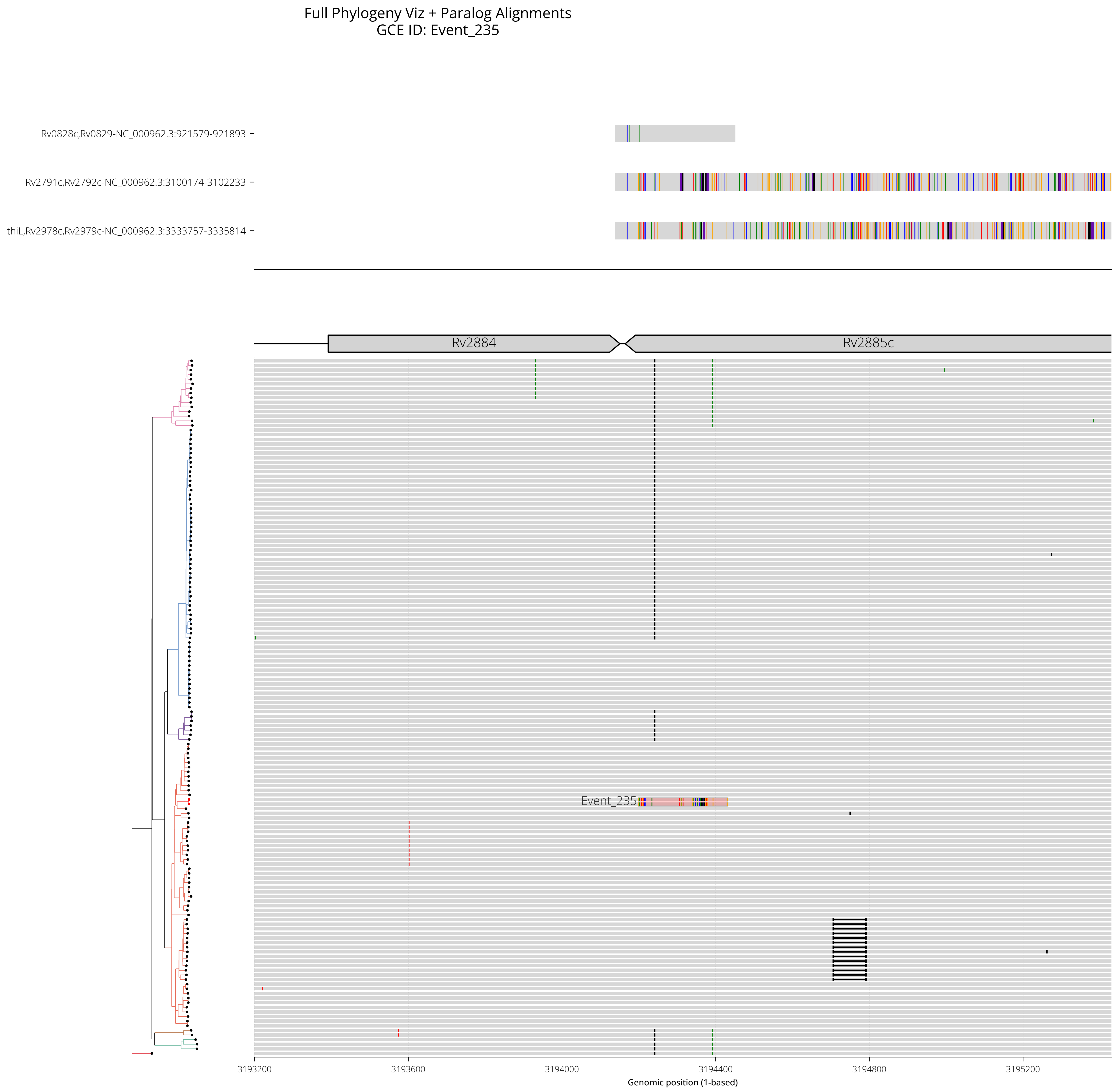

Full Phylogeny Viz + Paralog Alignments
GCE ID: Event_235
Rv0828c,Rv0829-NC_000962.3:921579-921893
Rv2791c,Rv2792c-NC_000962.3:3100174-3102233
thiL,Rv2978c,Rv2979c-NC_000962.3:3333757-3335814
Rv2884
Rv2885c
Event_235
3193200
3193600
3194000
3194400
3194800
3195200
Genomic position (1-based)

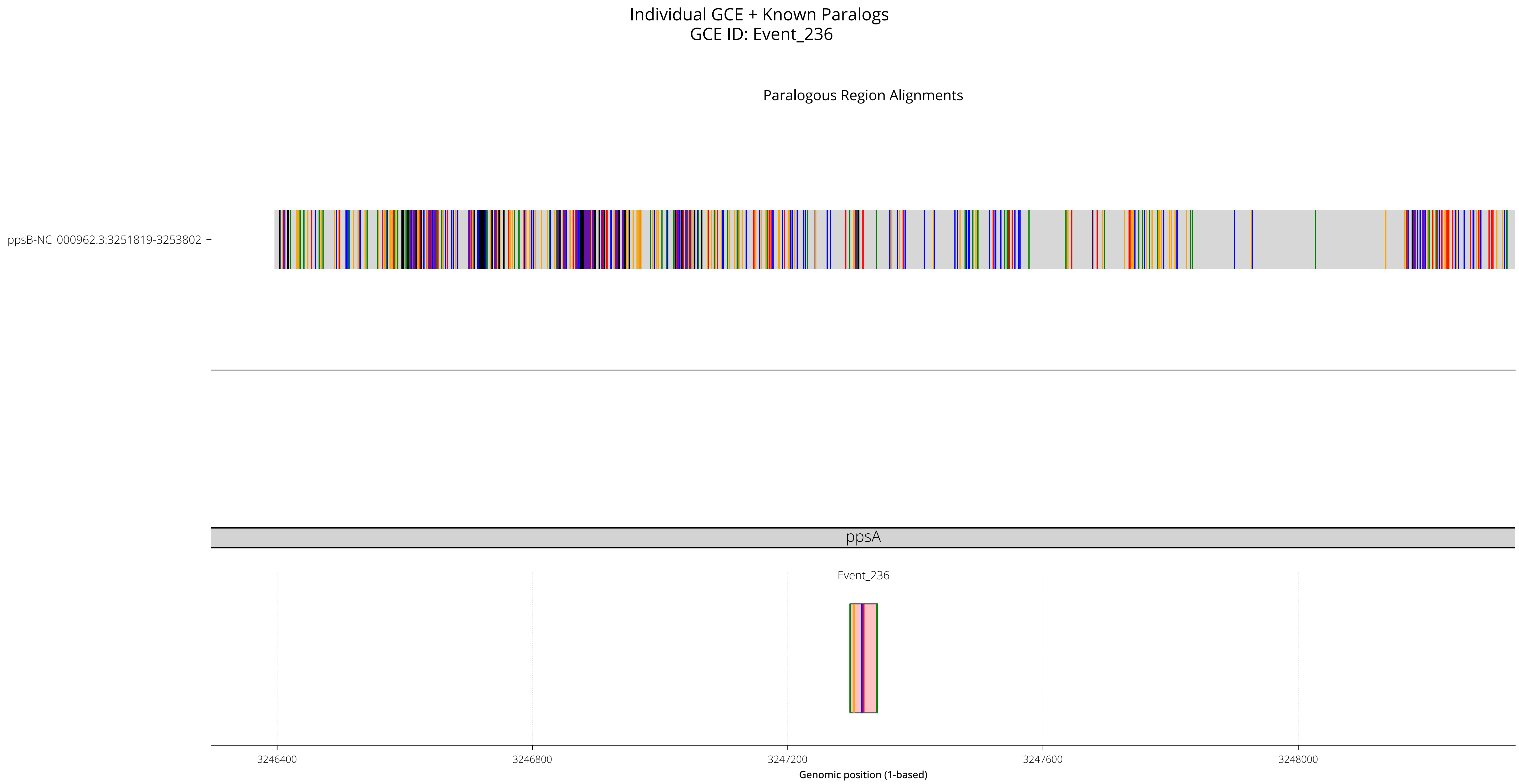

Individual GCE + Known Paralogs
GCE ID: Event_236
Paralogous Region Alignments
ppsB-NC_000962.3:3251819-3253802
ppsA
Event_236
3246400
3246800
3247200
3247600
3248000
Genomic position (1-based)

Circos Viz - GCE to Known Paralogs Locations
GCE ID: Event_236

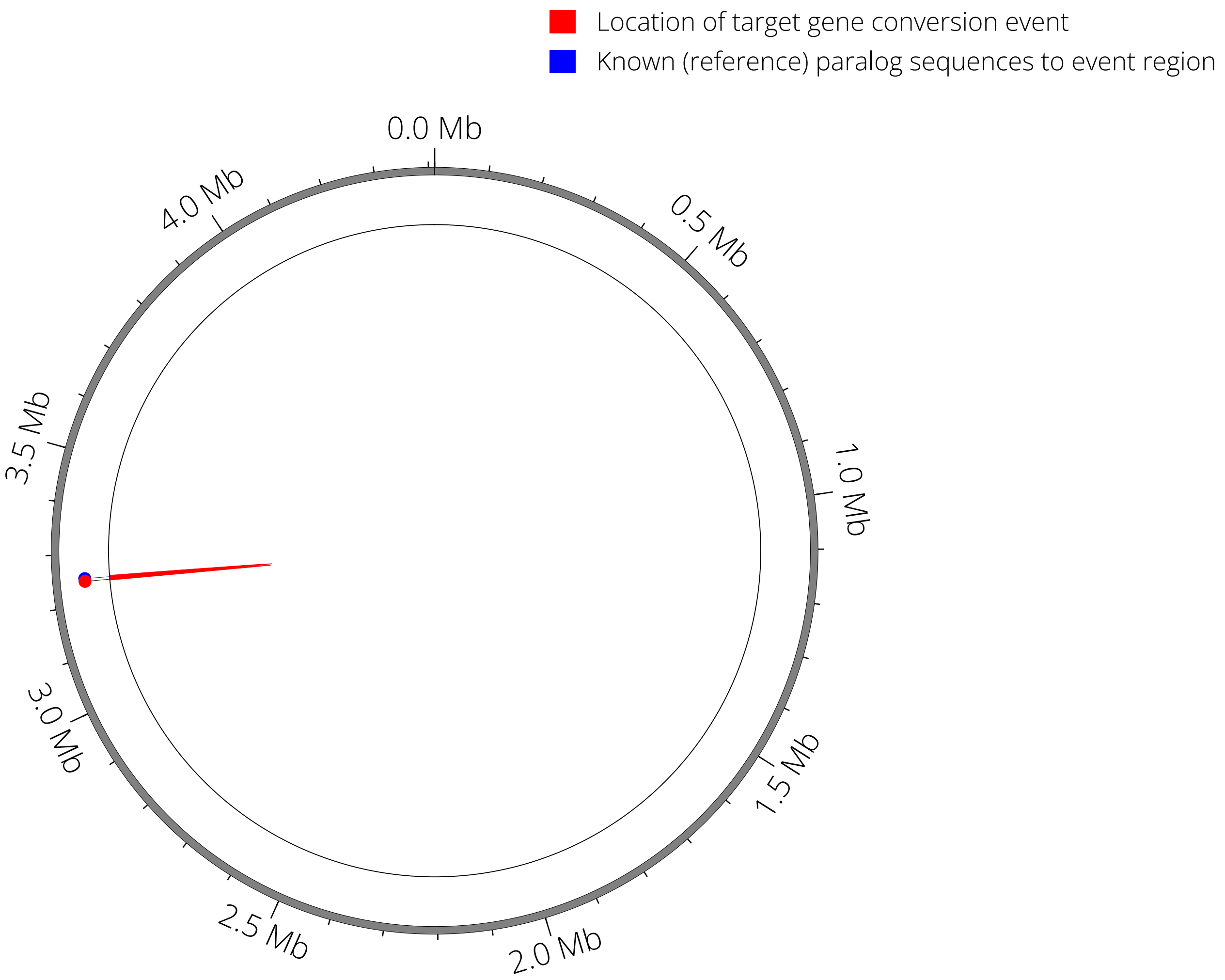

# Event_236 - 5 SNPs - k-mer analysis relative to paralogs

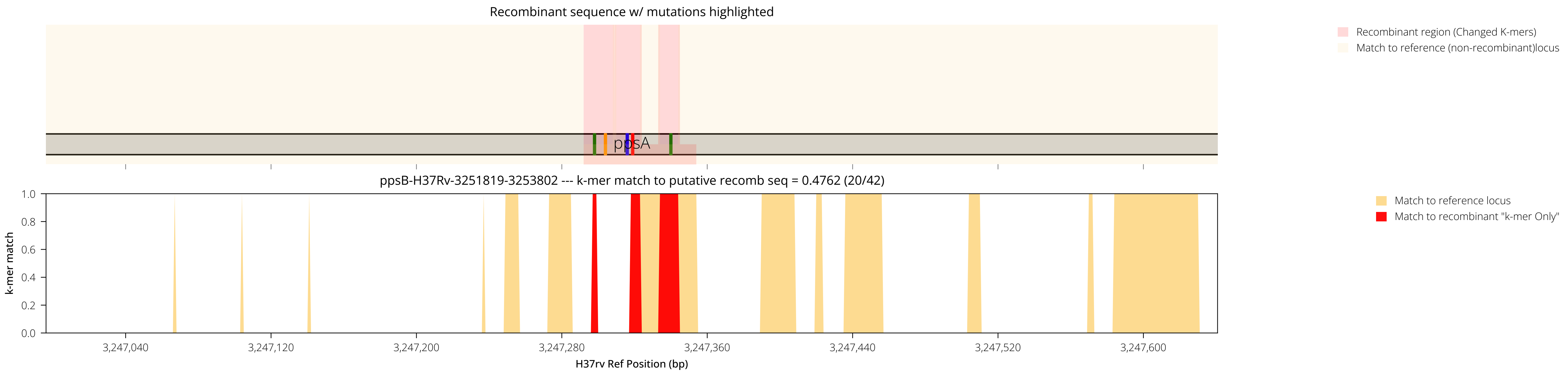

Sub-phylogeny Viz + Paralog Alignments
GCE ID: Event_236

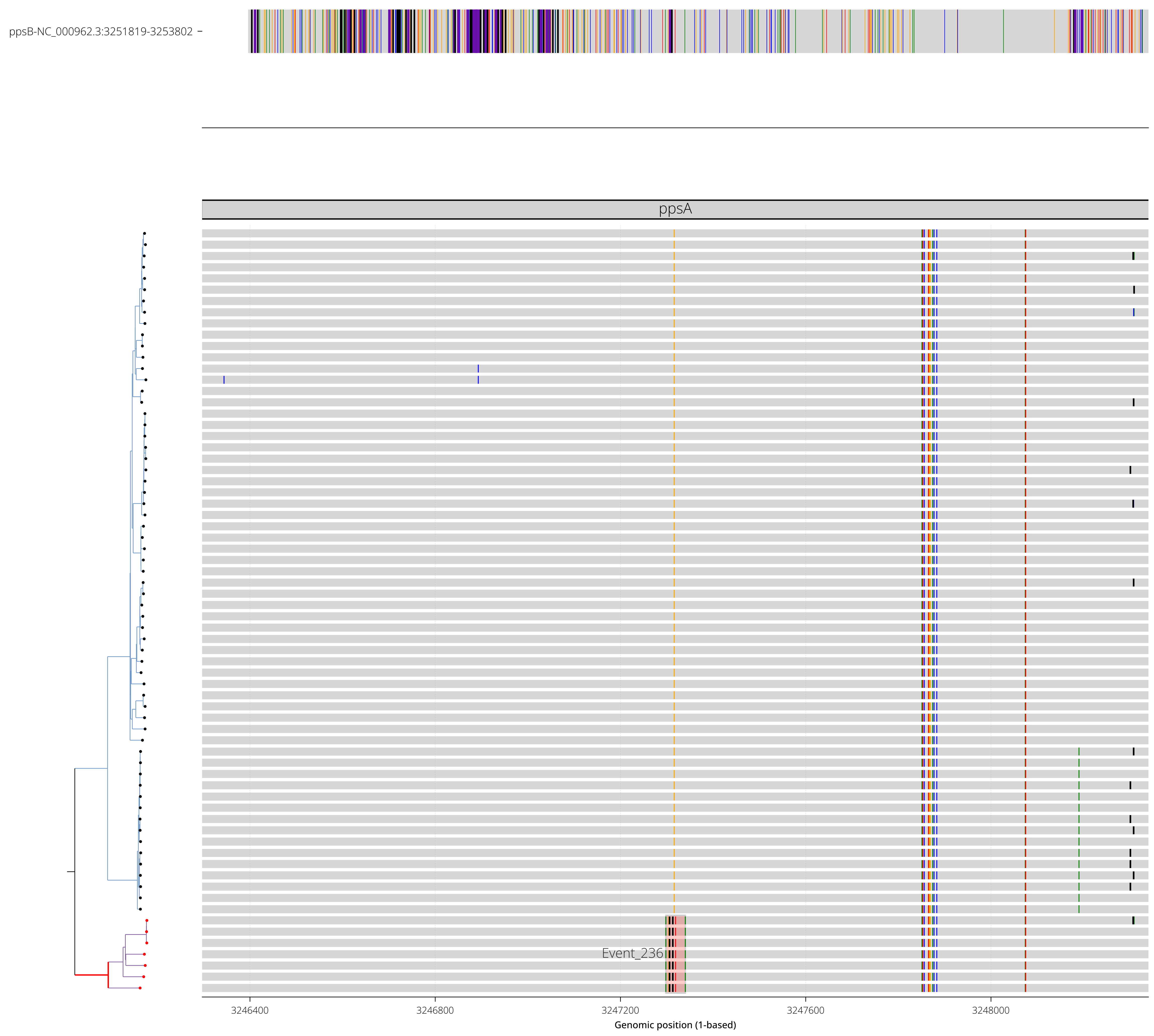

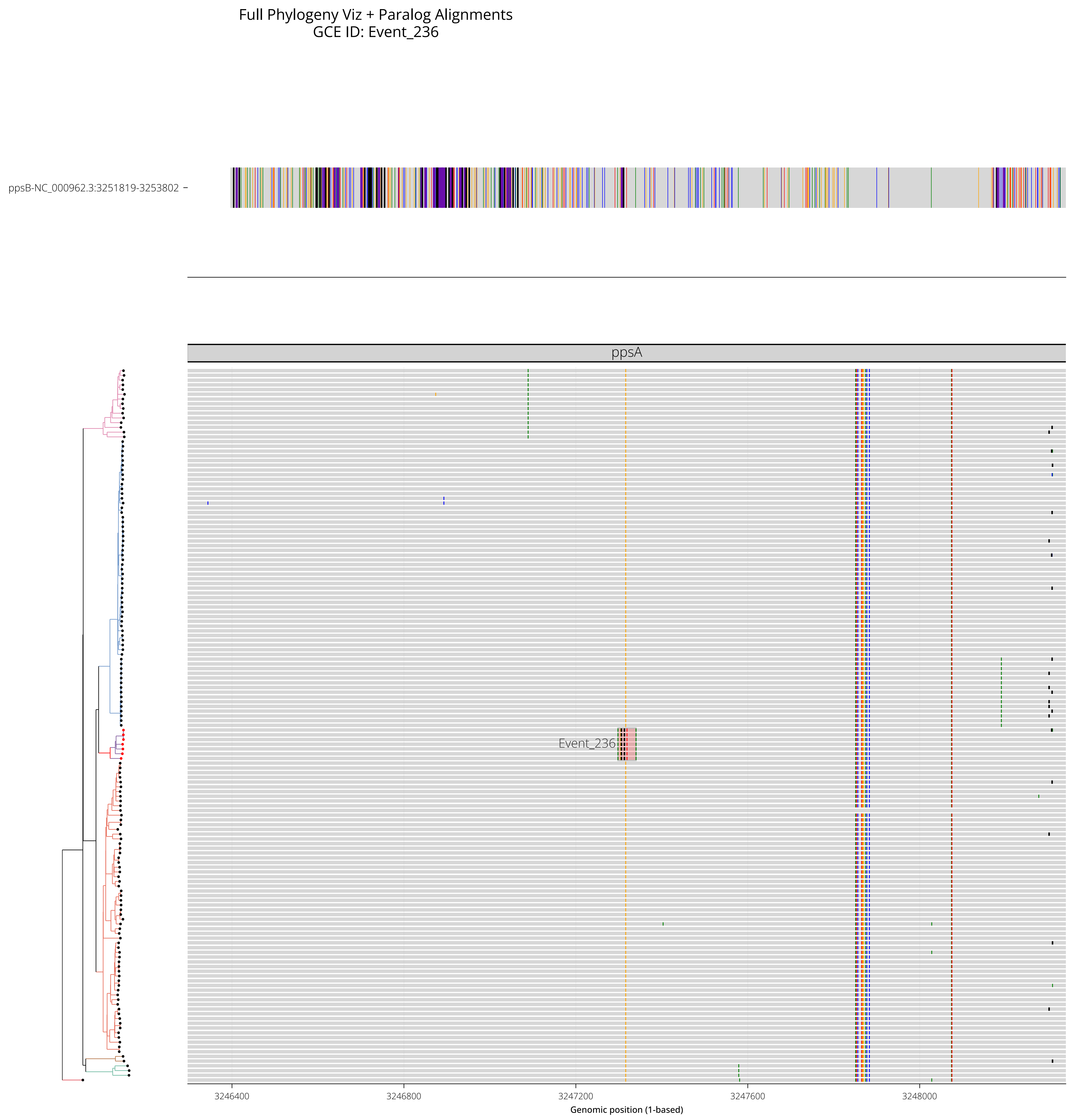

Full Phylogeny Viz + Paralog Alignments
GCE ID: Event_236
ppsB-NC_000962.3:3251819-3253802
ppsA
Event_236
3246400
3246800
3247200
3247600
3248000
Genomic position (1-based)

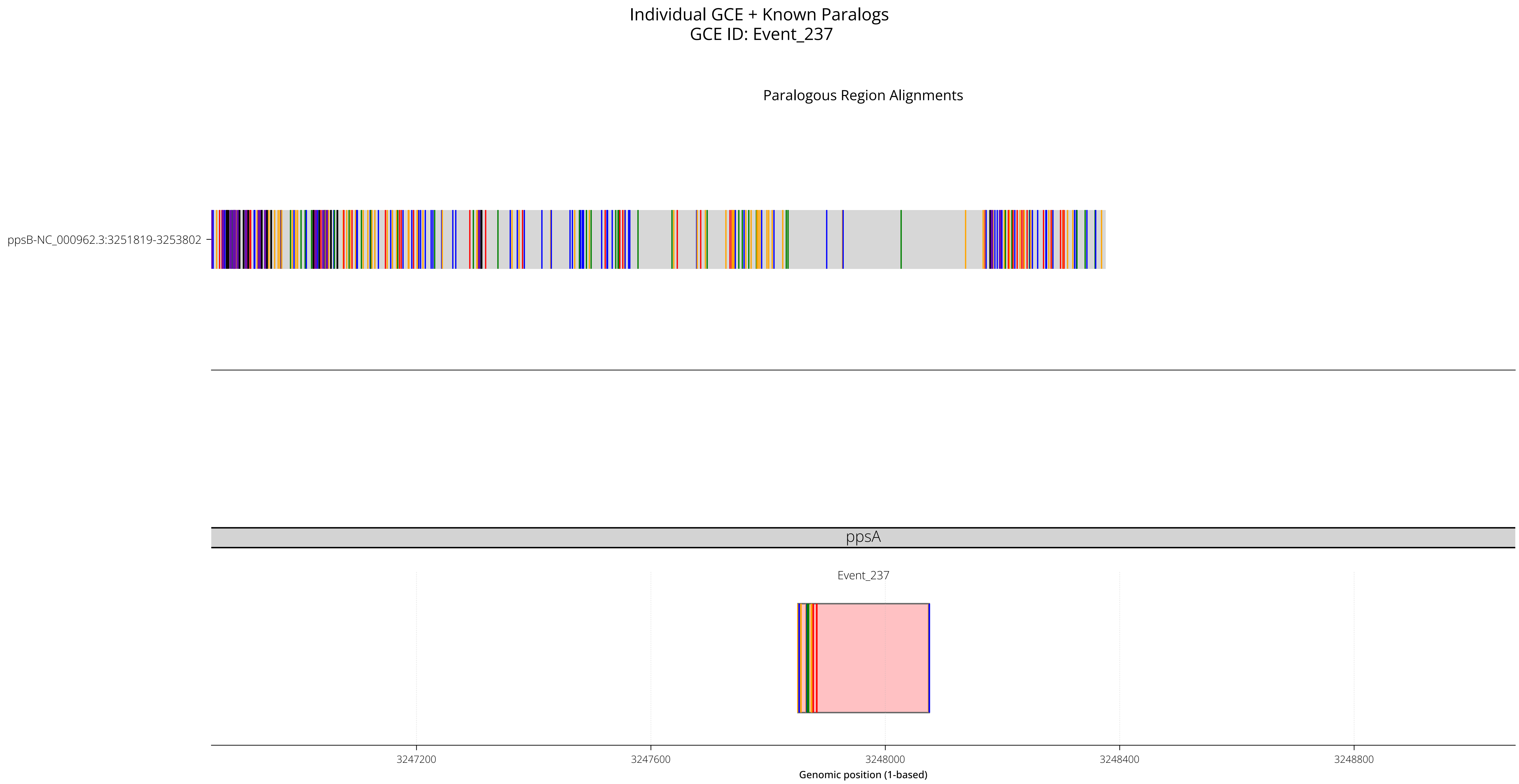

Individual GCE + Known Paralogs
GCE ID: Event_237
Paralogous Region Alignments
ppsB-NC_000962.3:3251819-3253802
ppsA
Event_237
3247200
3247600
3248000
3248400
3248800
Genomic position (1-based)

Circos Viz - GCE to Known Paralogs Locations
GCE ID: Event_237

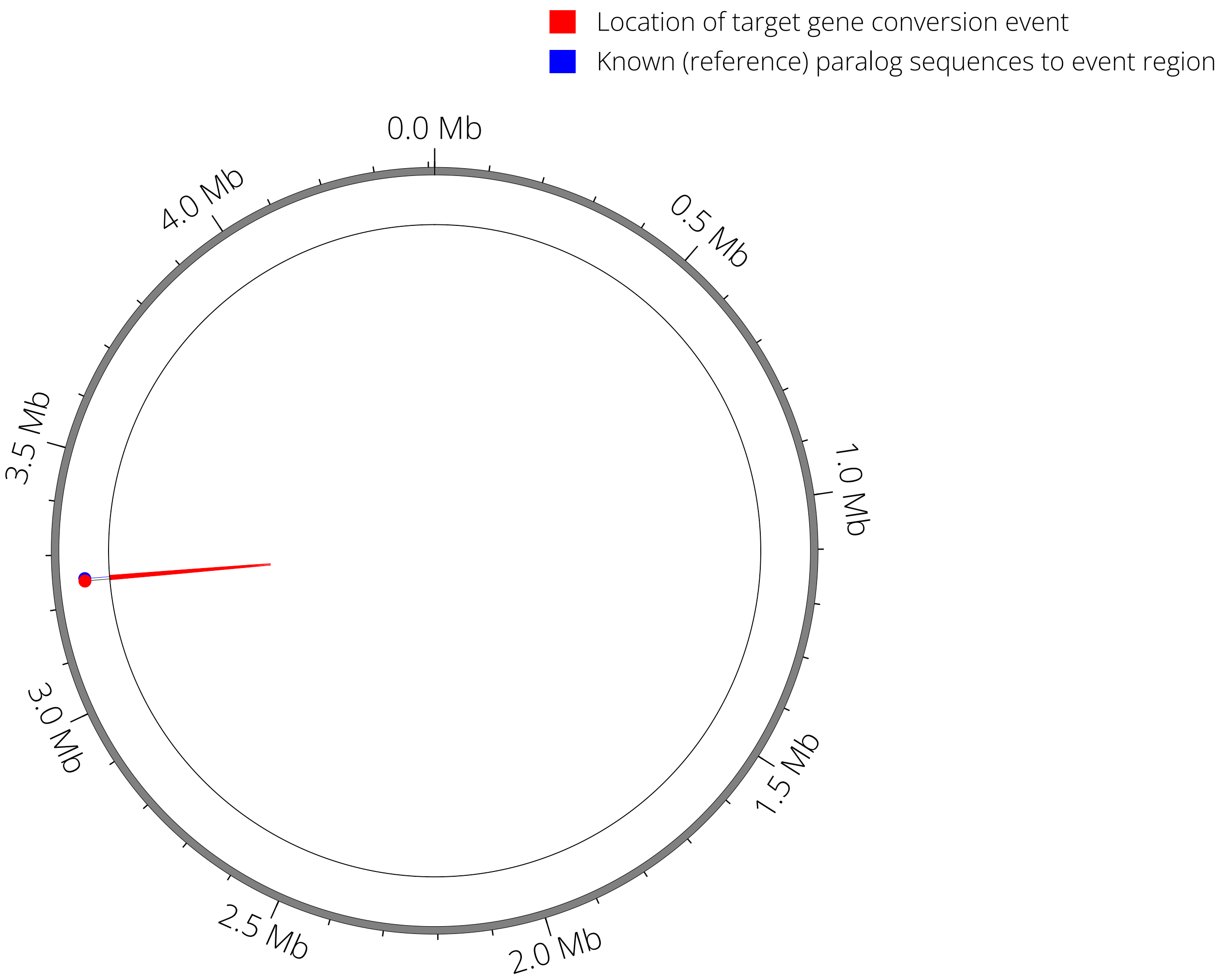

# Event_237 - 13 SNPs - k-mer analysis relative to paralogs

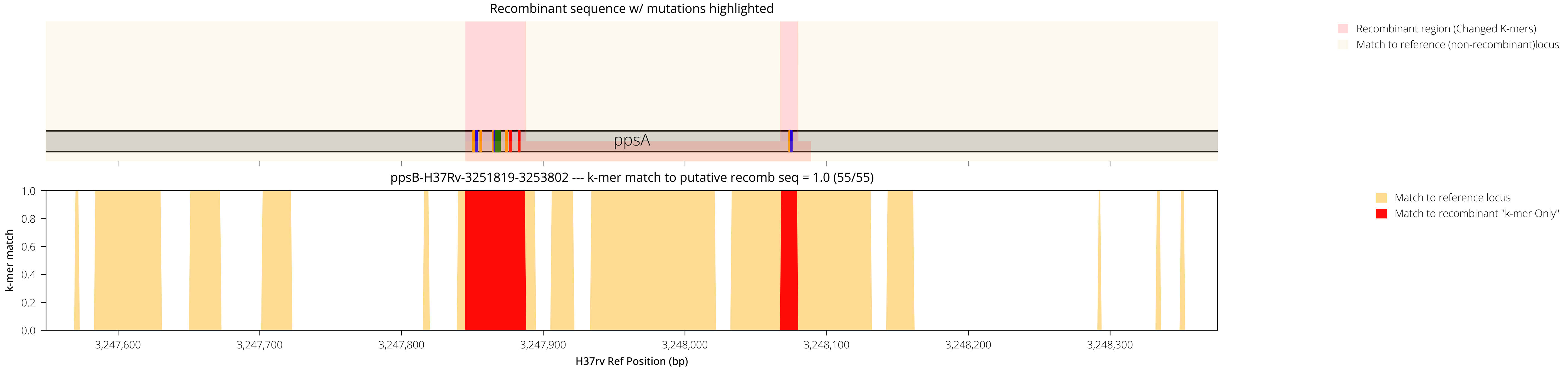

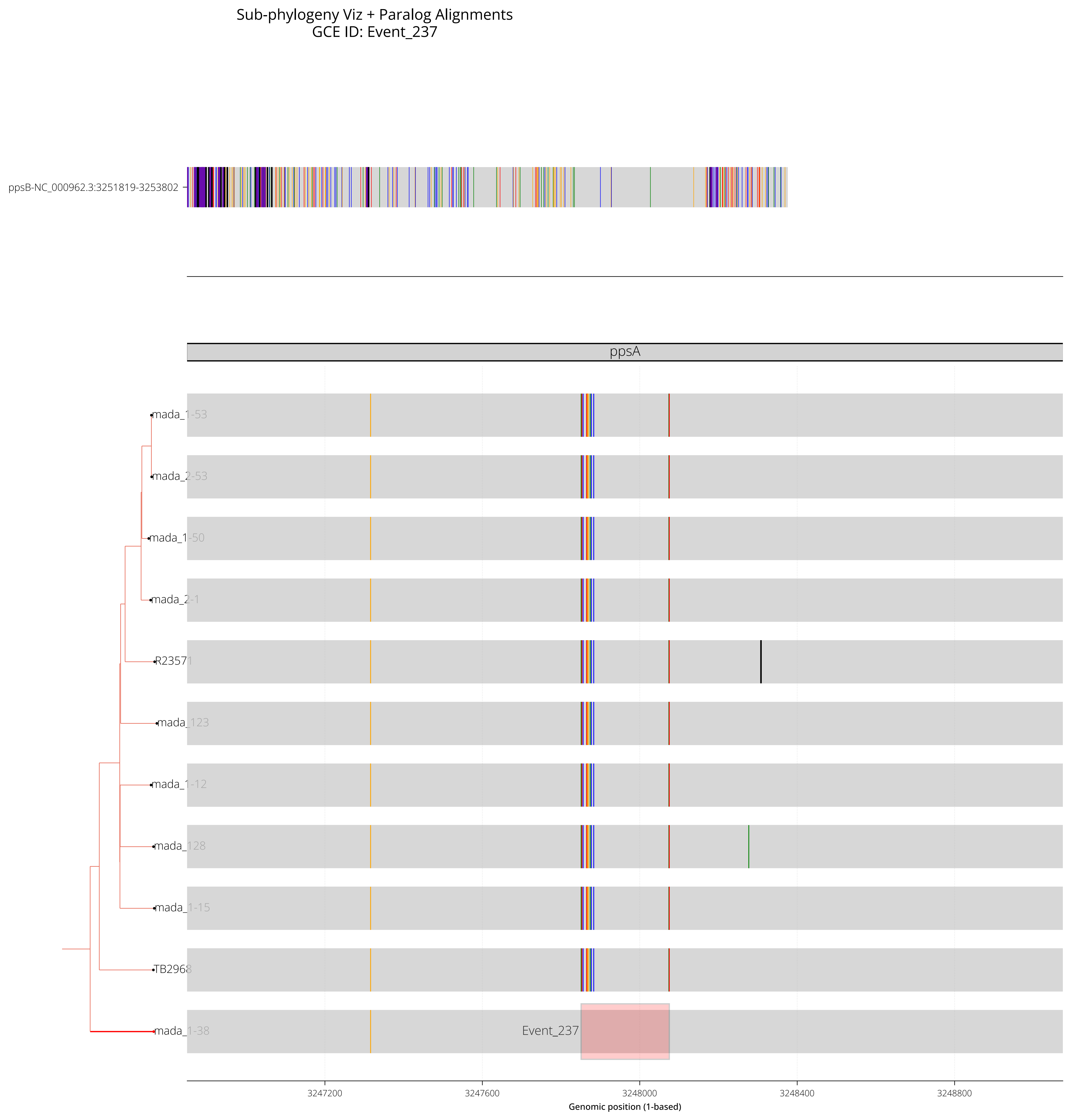

Sub-phylogeny Viz + Paralog Alignments
GCE ID: Event_237
ppsB-NC_000962.3:3251819-3253802
ppsA
mada_1-53
mada_2-53
mada_1-50
mada_2-1
R23571
mada_123
mada_1-12
mada_128
mada_1-15
TB2968
mada_1-38
Event_237
3247200
3247600
3248000
3248400
3248800
Genomic position (1-based)

Full Phylogeny Viz + Paralog Alignments
GCE ID: Event_237

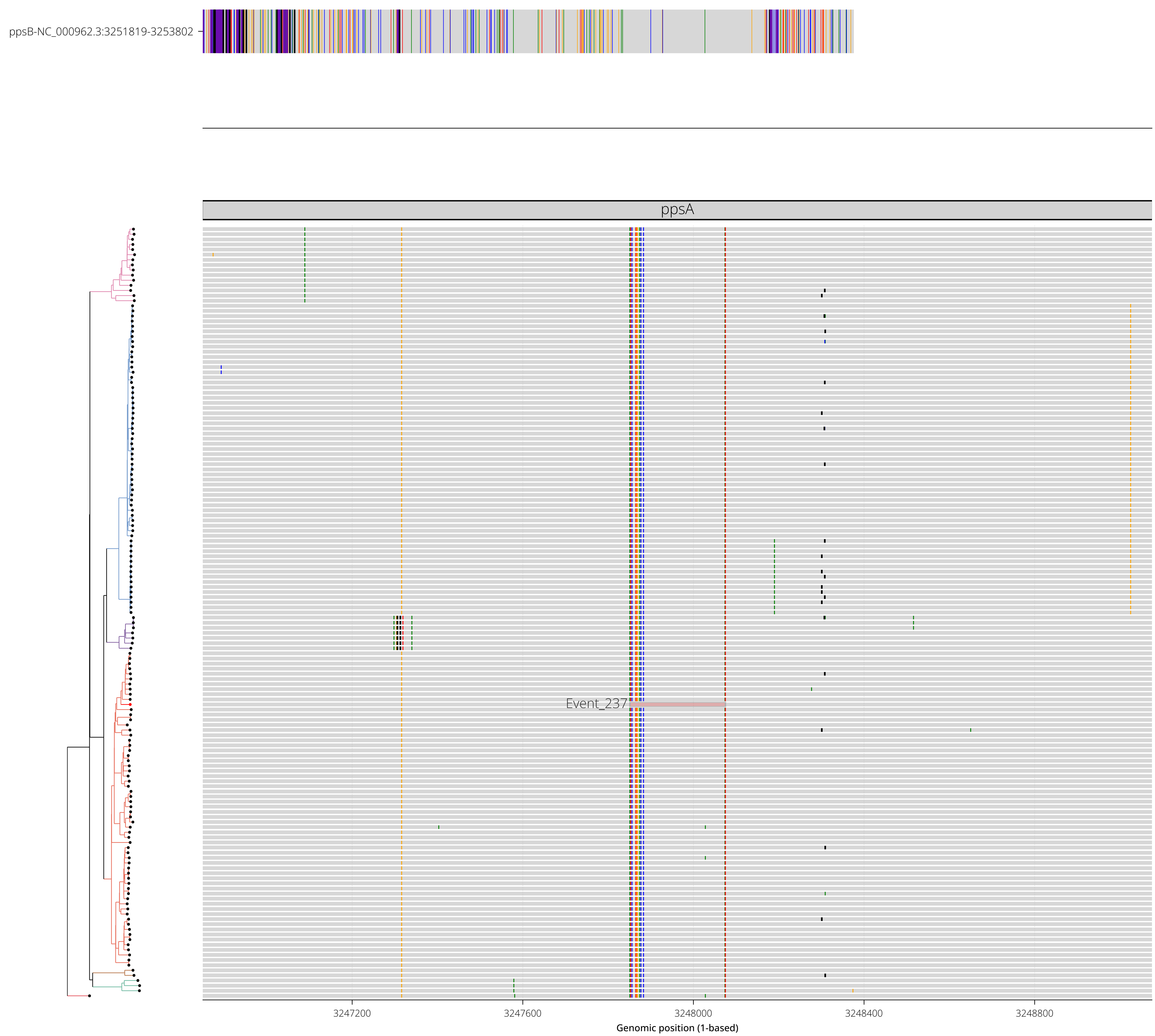

# Individual GCE + Known Paralogs
GCE ID: Event_238

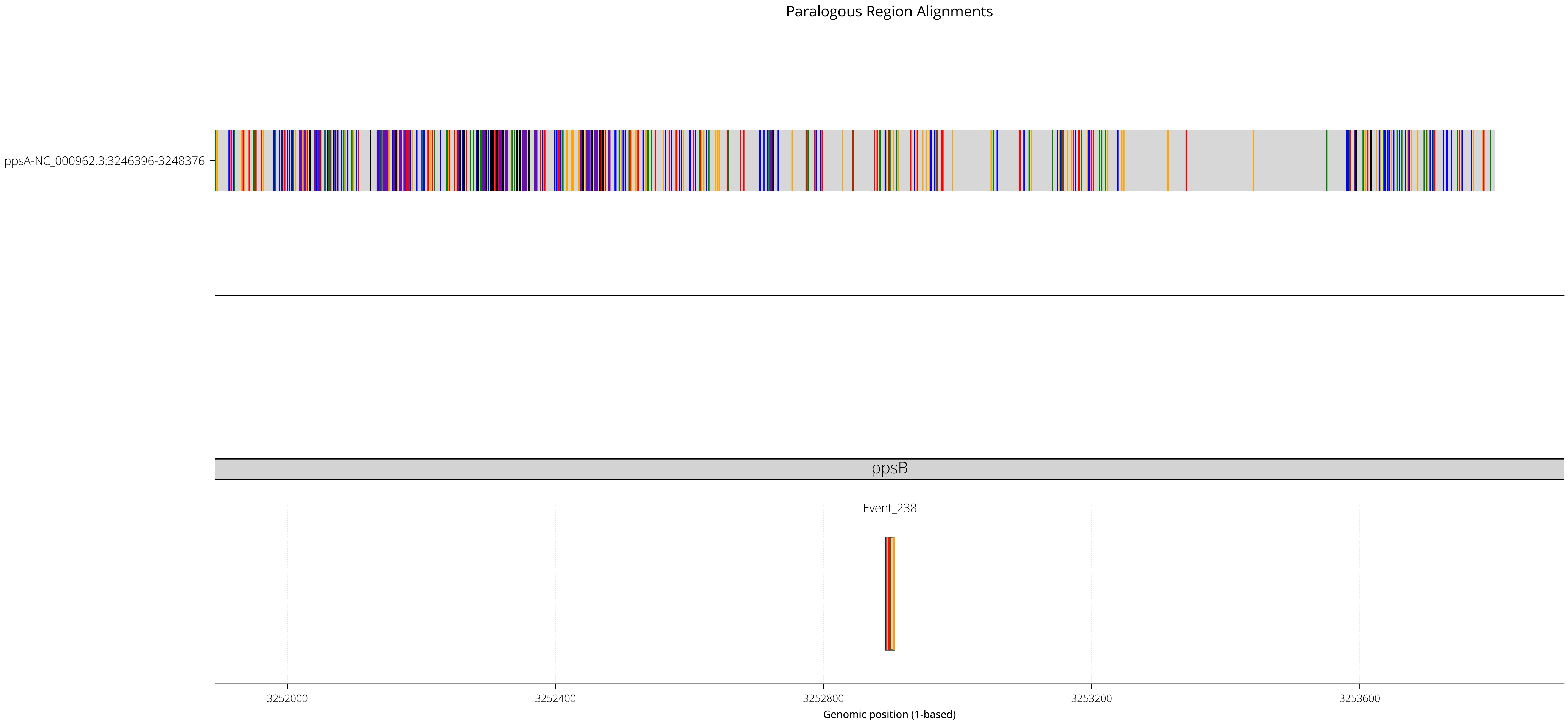

Circos Viz - GCE to Known Paralogs Locations
GCE ID: Event_238

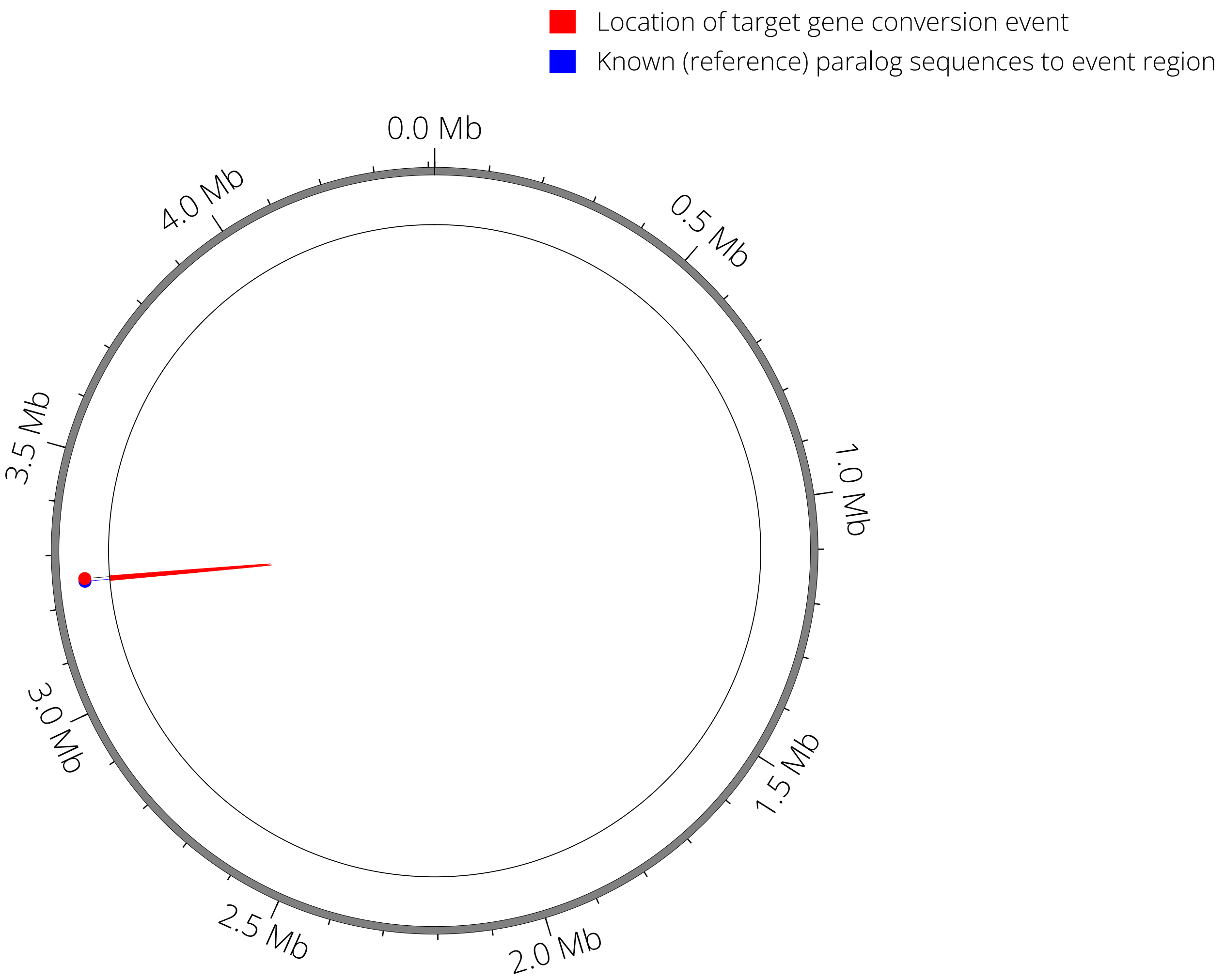

# Event_238 - 5 SNPs - k-mer analysis relative to paralogs

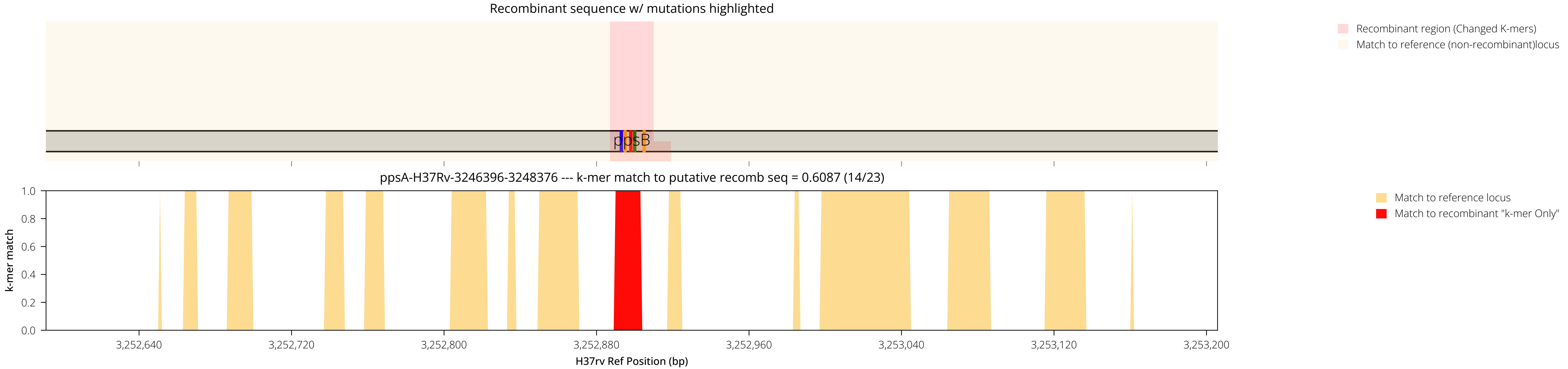

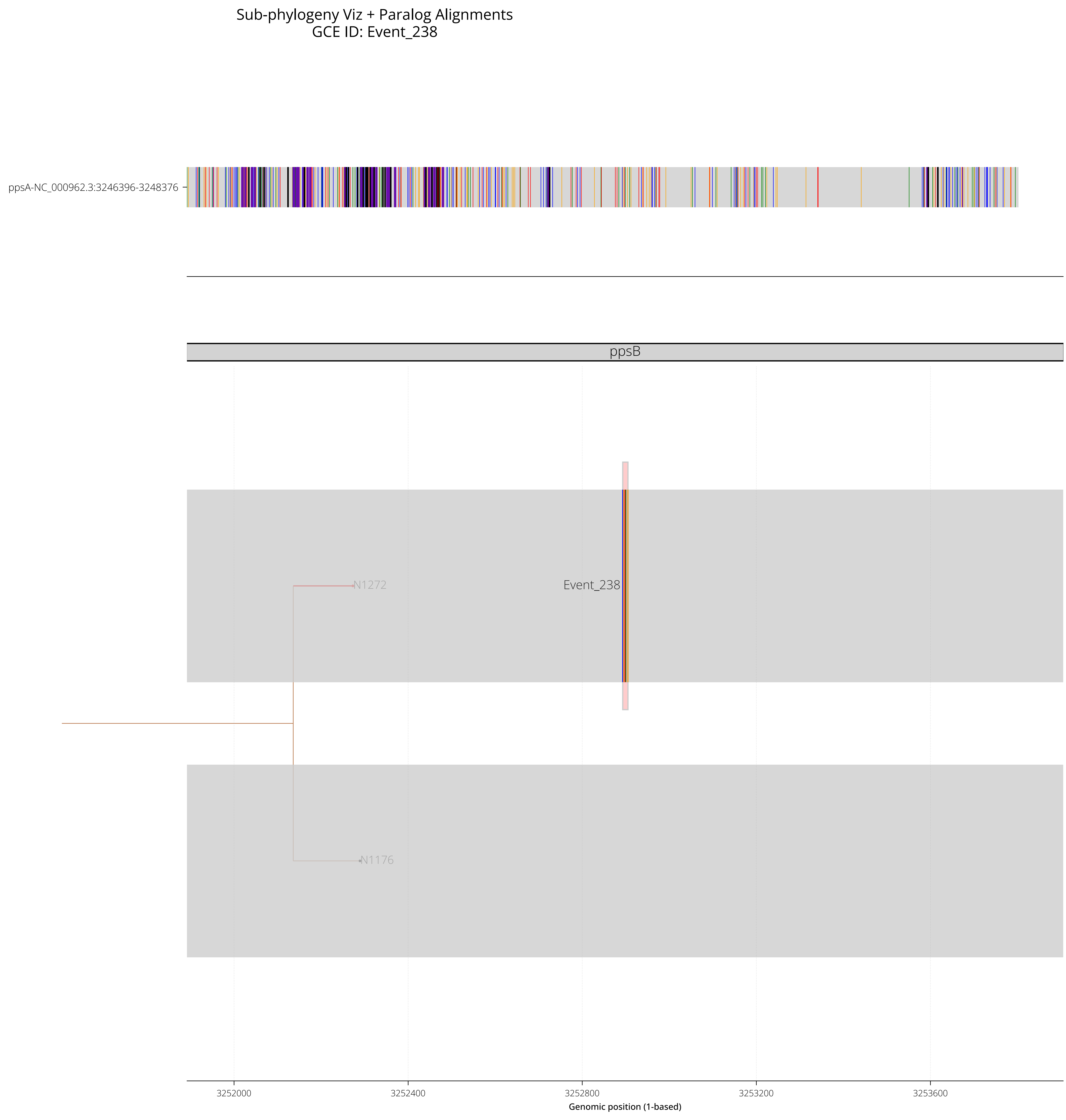

Sub-phylogeny Viz + Paralog Alignments
GCE ID: Event_238
ppsA-NC_000962.3:3246396-3248376
ppsB
N1272
Event_238
N1176
3252000
3252400
3252800
3253200
3253600
Genomic position (1-based)

Full Phylogeny Viz + Paralog Alignments
GCE ID: Event_238

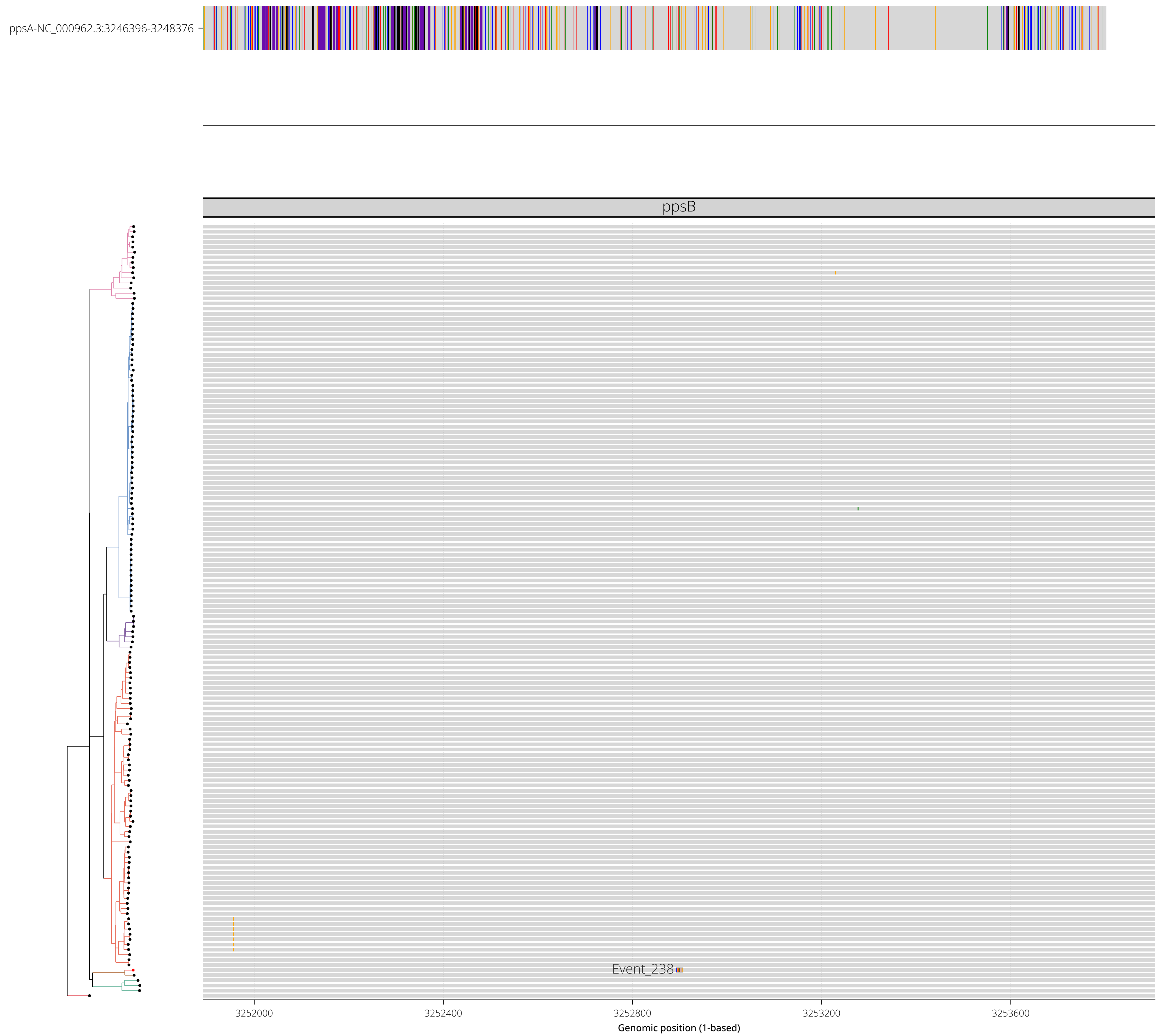

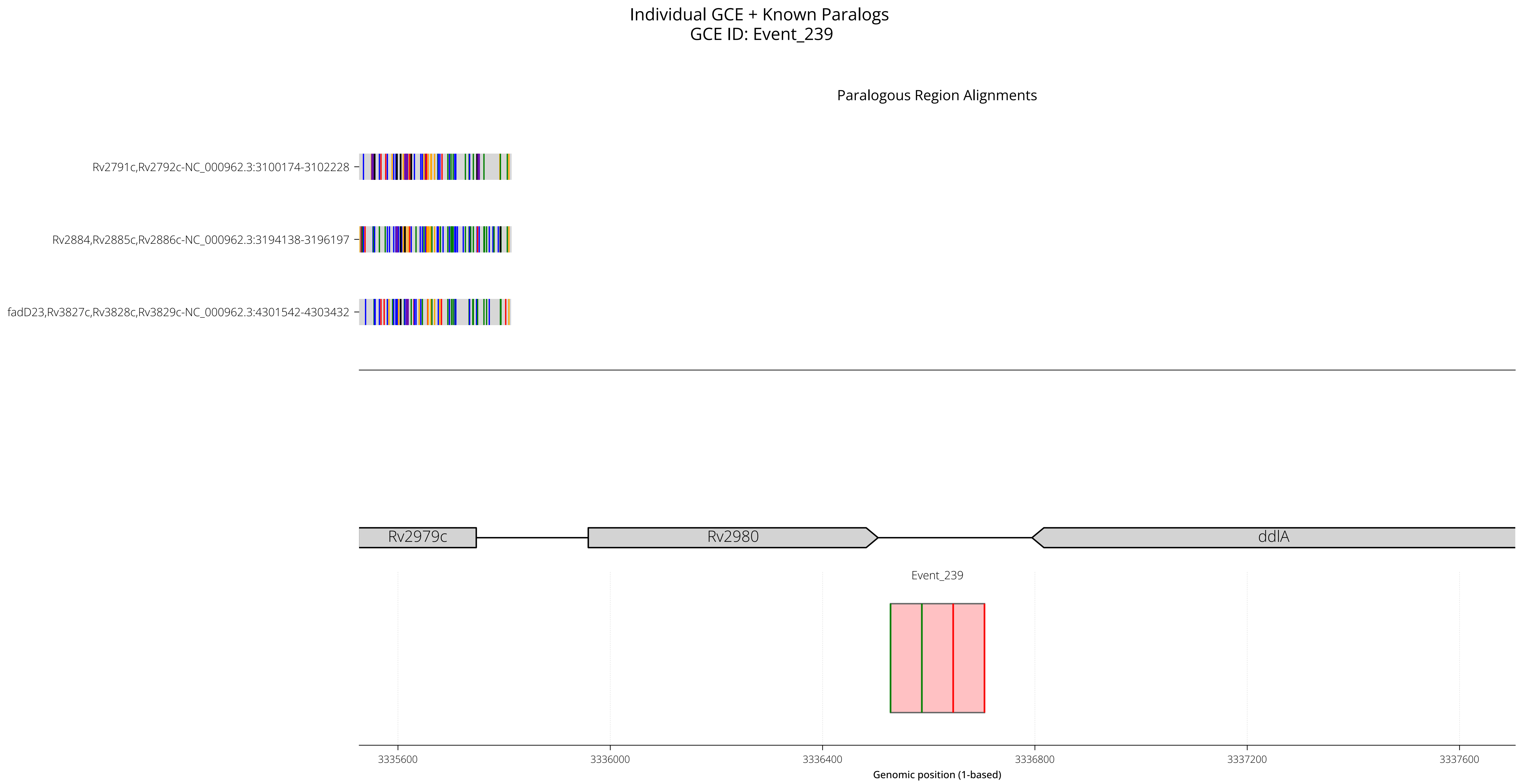

Individual GCE + Known Paralogs
GCE ID: Event_239
Paralogous Region Alignments
Rv2791c,Rv2792c-NC_000962.3:3100174-3102228
Rv2884,Rv2885c,Rv2886c-NC_000962.3:3194138-3196197
fadD23,Rv3827c,Rv3828c,Rv3829c-NC_000962.3:4301542-4303432
Rv2979c
Rv2980
ddlA
Event_239
3335600
3336000
3336400
3336800
3337200
3337600
Genomic position (1-based)

Sub-phylogeny Viz + Paralog Alignments
GCE ID: Event_239

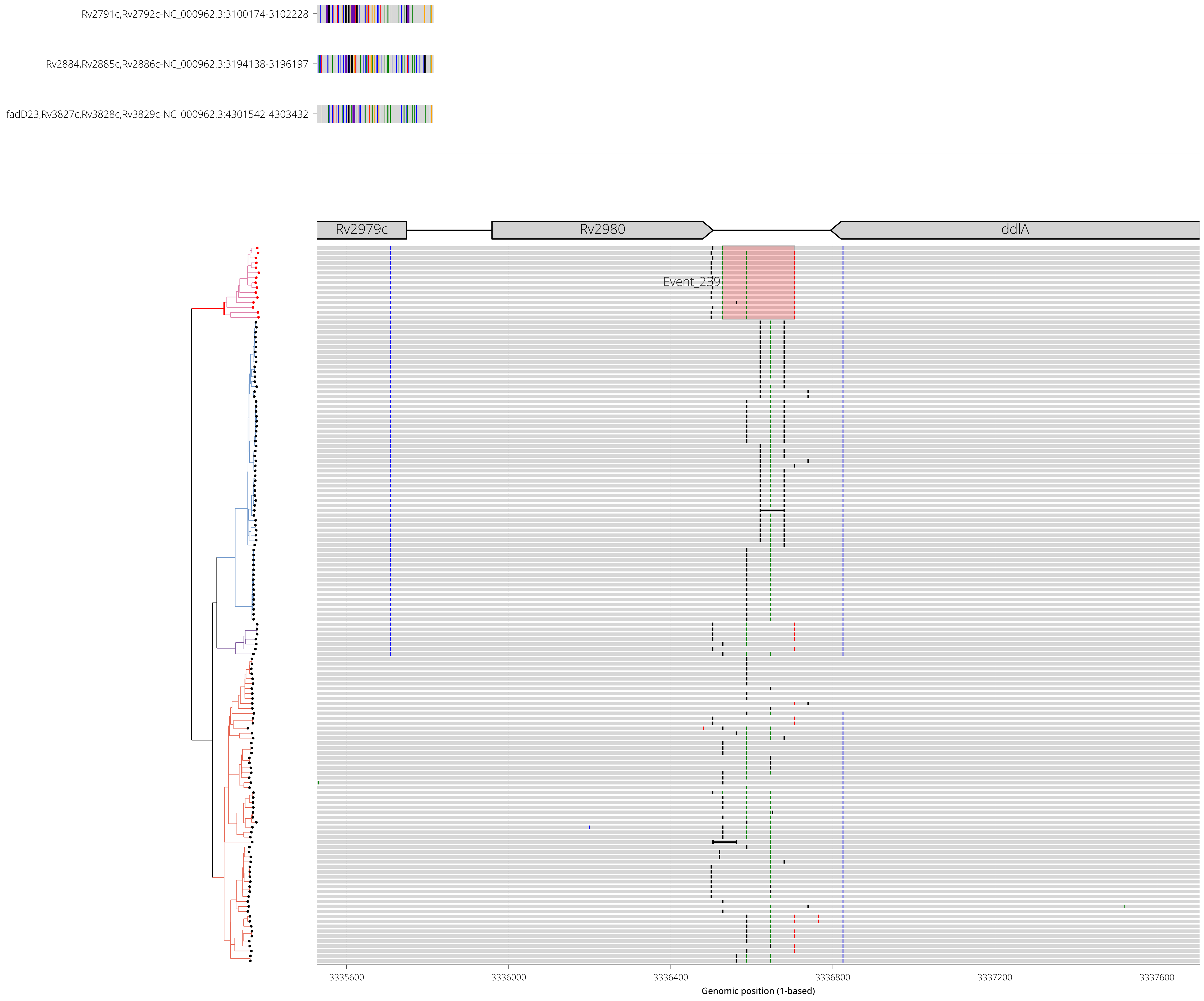

Full Phylogeny Viz + Paralog Alignments
GCE ID: Event_239

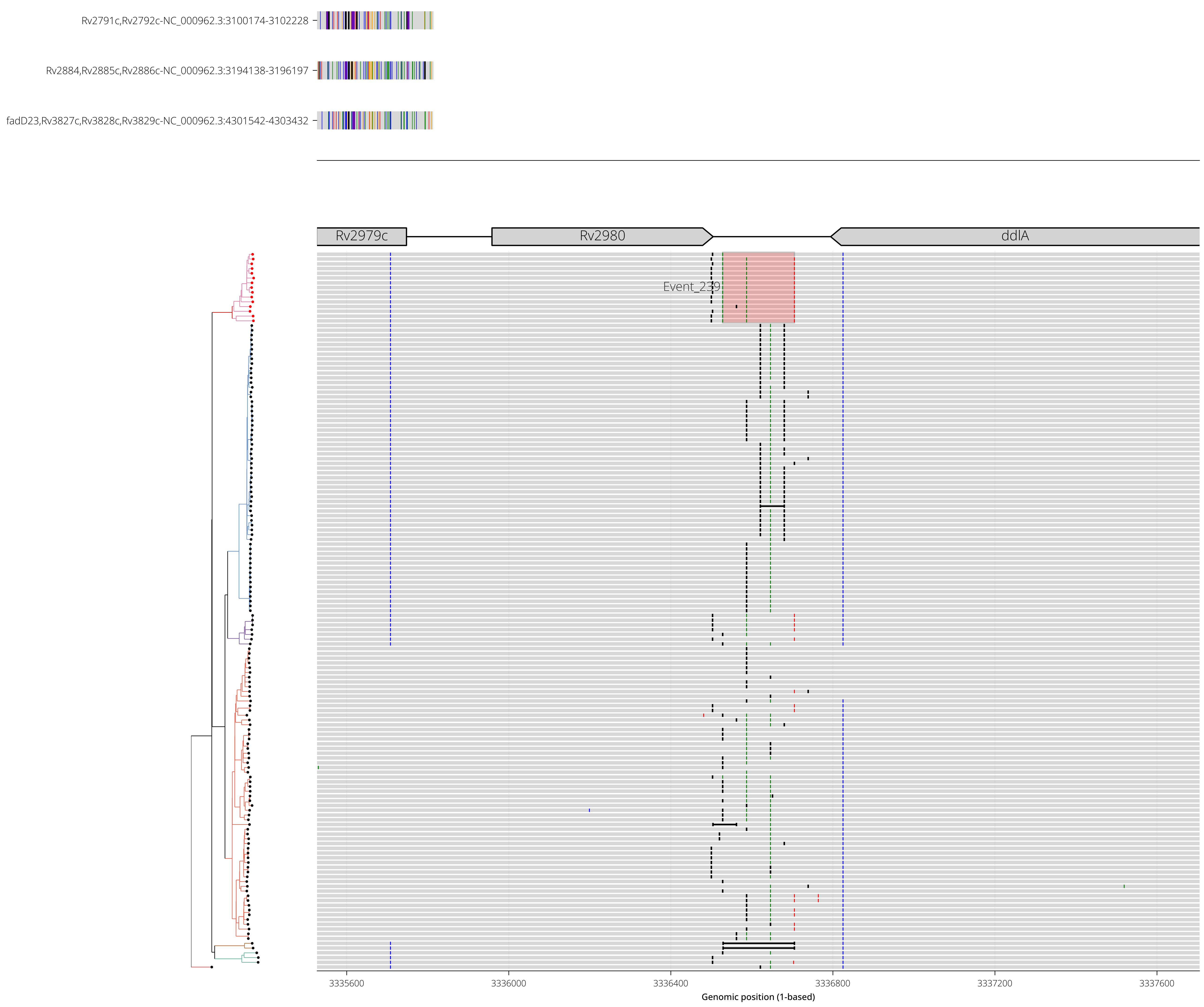

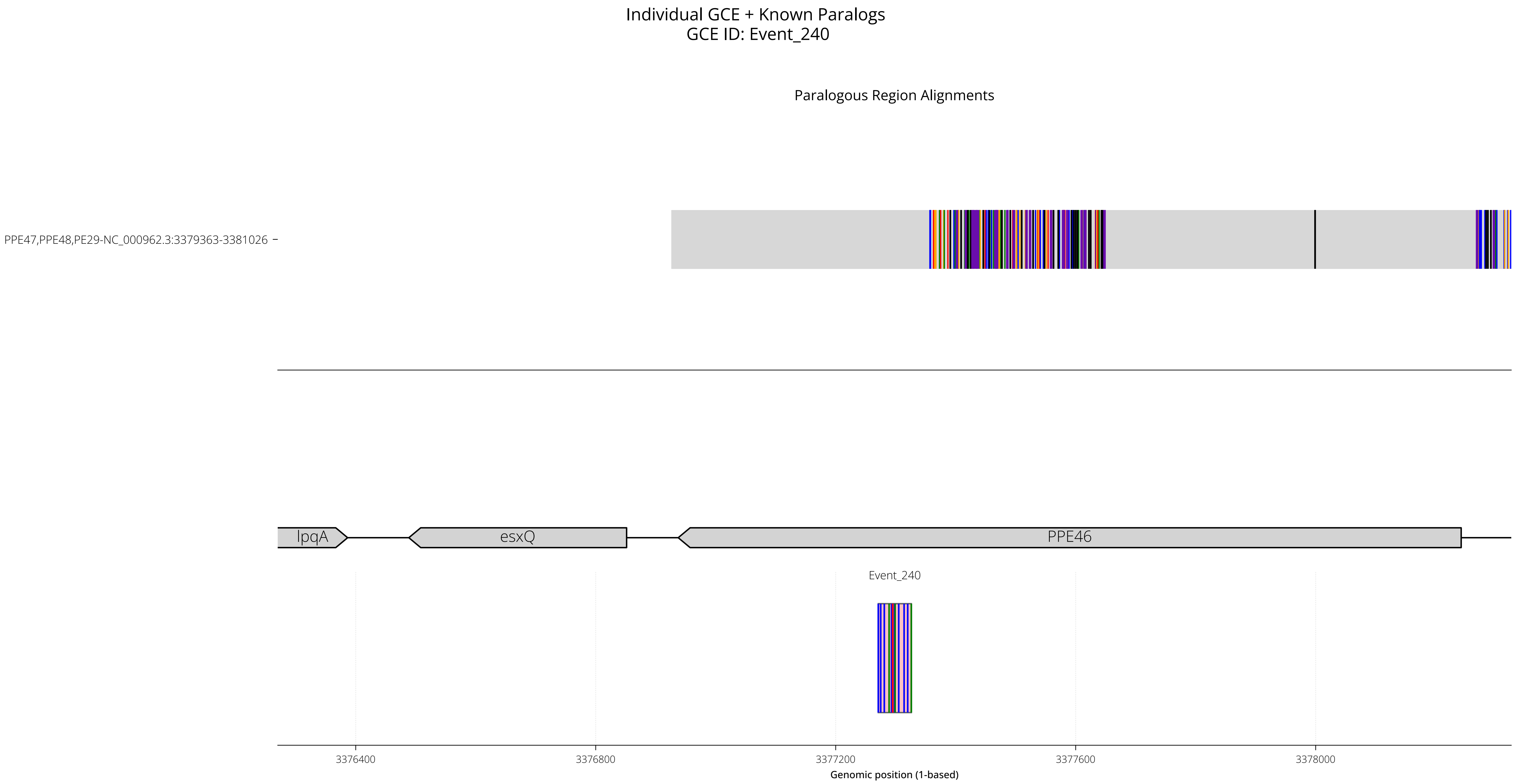
Individual GCE + Known Paralogs
GCE ID: Event_240
Paralogous Region Alignments
PPE47,PPE48,PE29-NC_000962.3:3379363-3381026
lpqA
esxQ
PPE46
Event_240
3376400
3376800
3377200
3377600
3378000
Genomic position (1-based)

Circos Viz - GCE to Known Paralogs Locations
GCE ID: Event_240

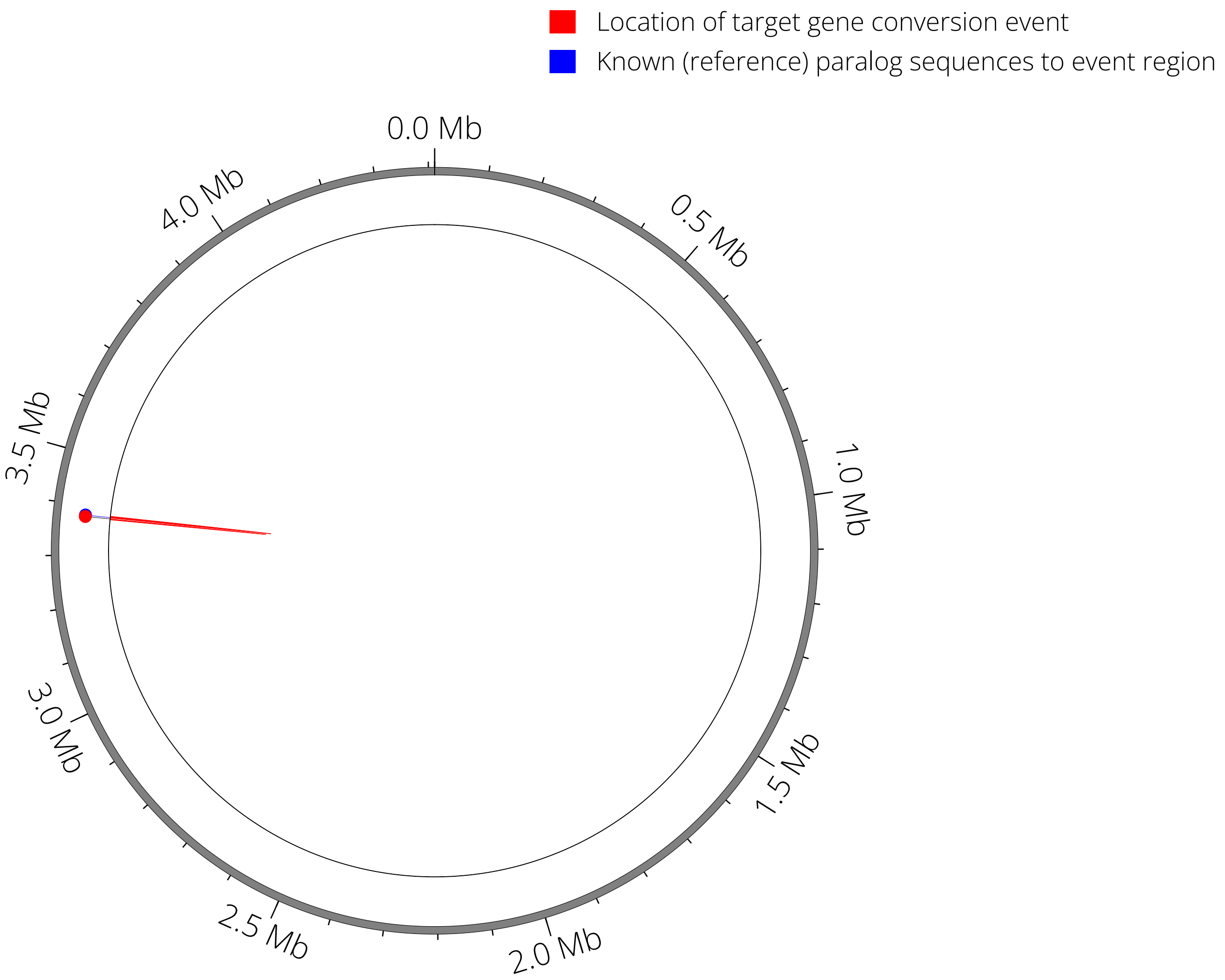

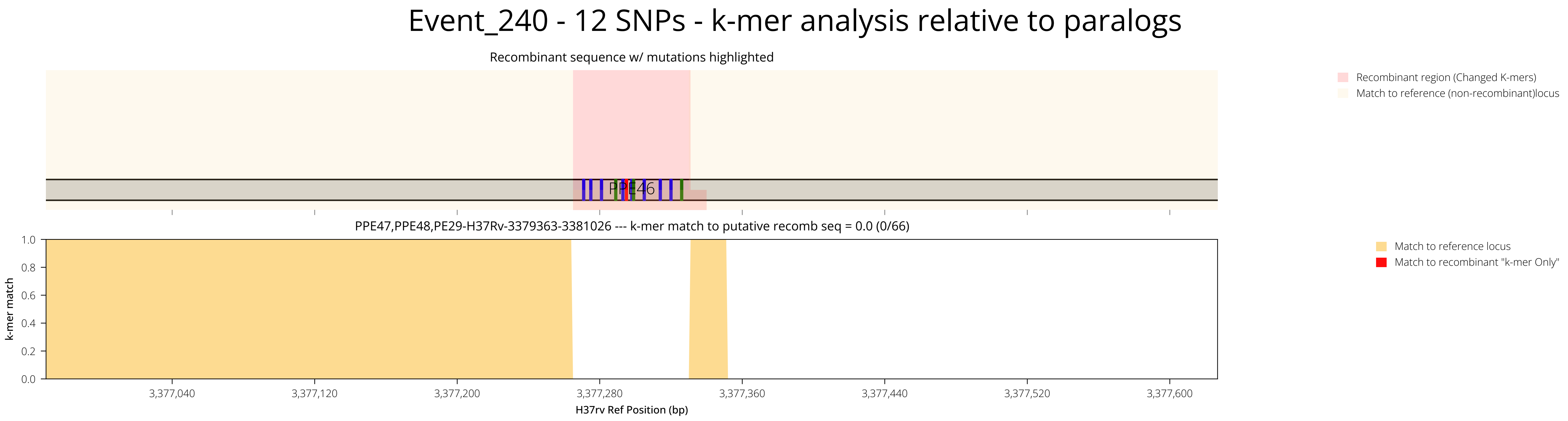

Event_240 - 12 SNPs - k-mer analysis relative to paralogs
Recombinant sequence w/ mutations highlighted
Recombinant region (Changed K-mers)
Match to reference (non-recombinant)locus
PPE46
PPE47,PPE48,PE29-H37Rv-3379363-3381026 --- k-mer match to putative recomb seq = 0.0 (0/66)
Match to reference locus
Match to recombinant "k-mer Only"
k-mer match
1.0
0.8
0.6
0.4
0.2
0.0
3,377,040
3,377,120
3,377,200
3,377,280
3,377,360
3,377,440
3,377,520
3,377,600
H37rv Ref Position (bp)

Sub-phylogeny Viz + Paralog Alignments
GCE ID: Event_240

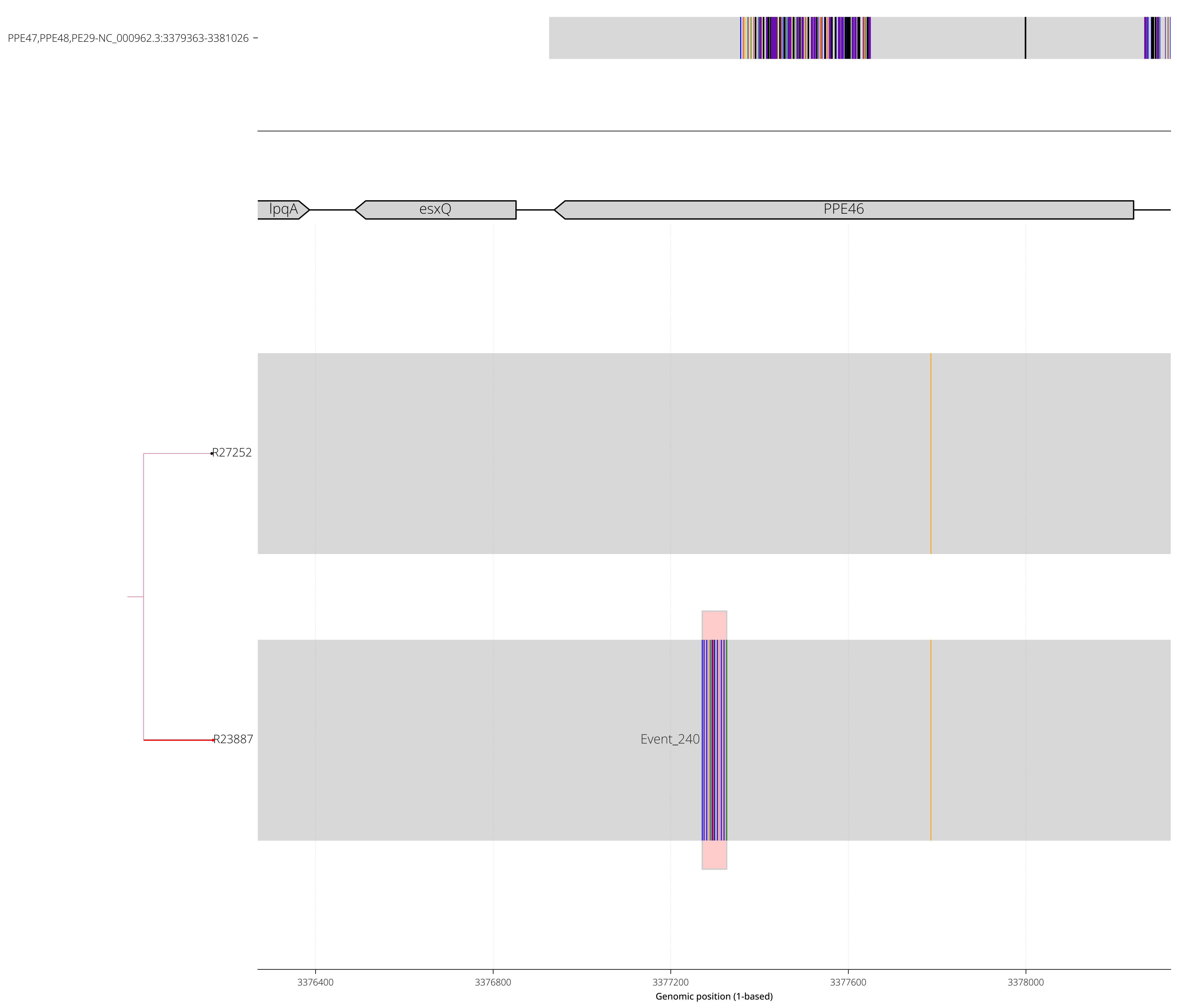

Full Phylogeny Viz + Paralog Alignments
GCE ID: Event_240

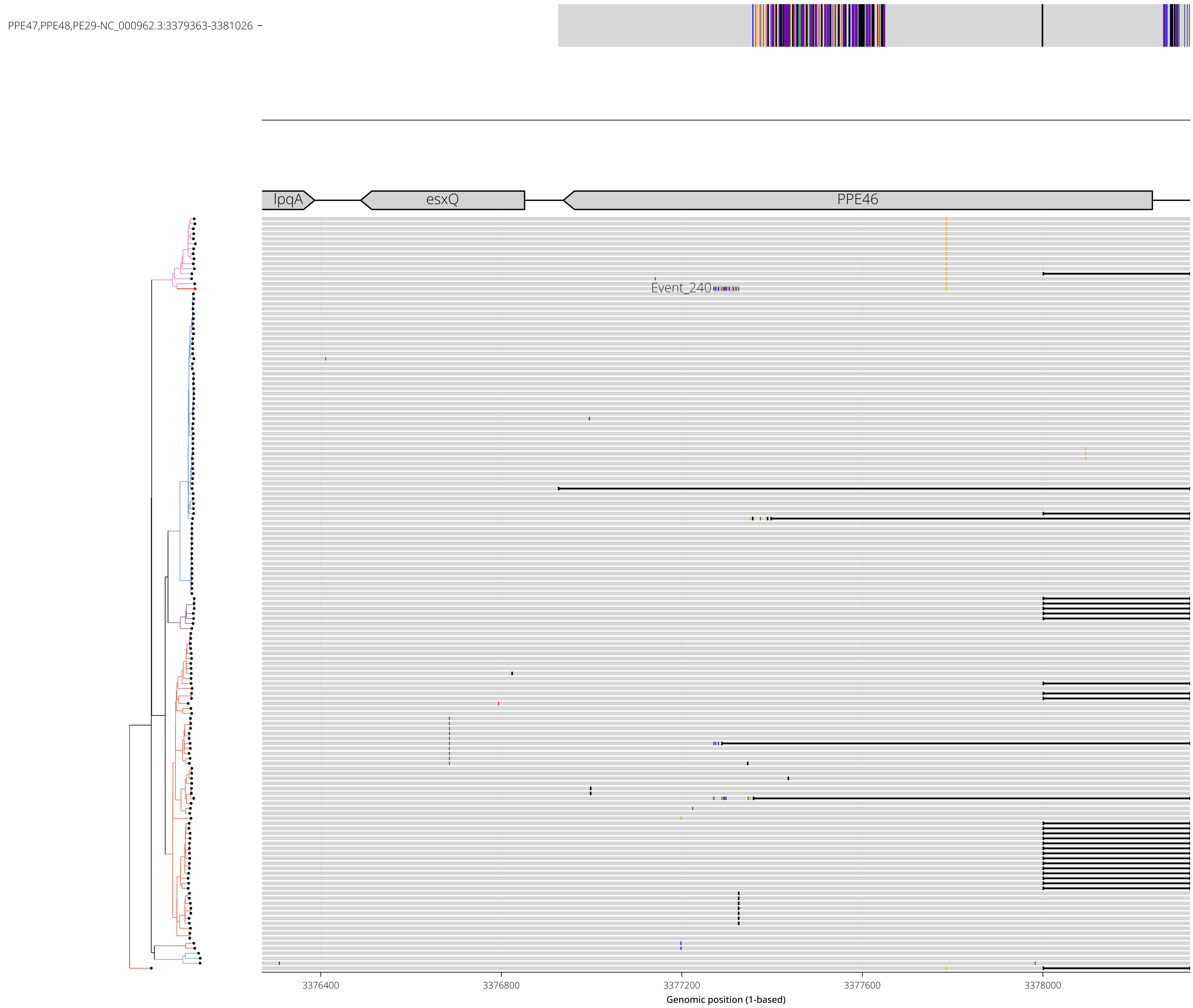

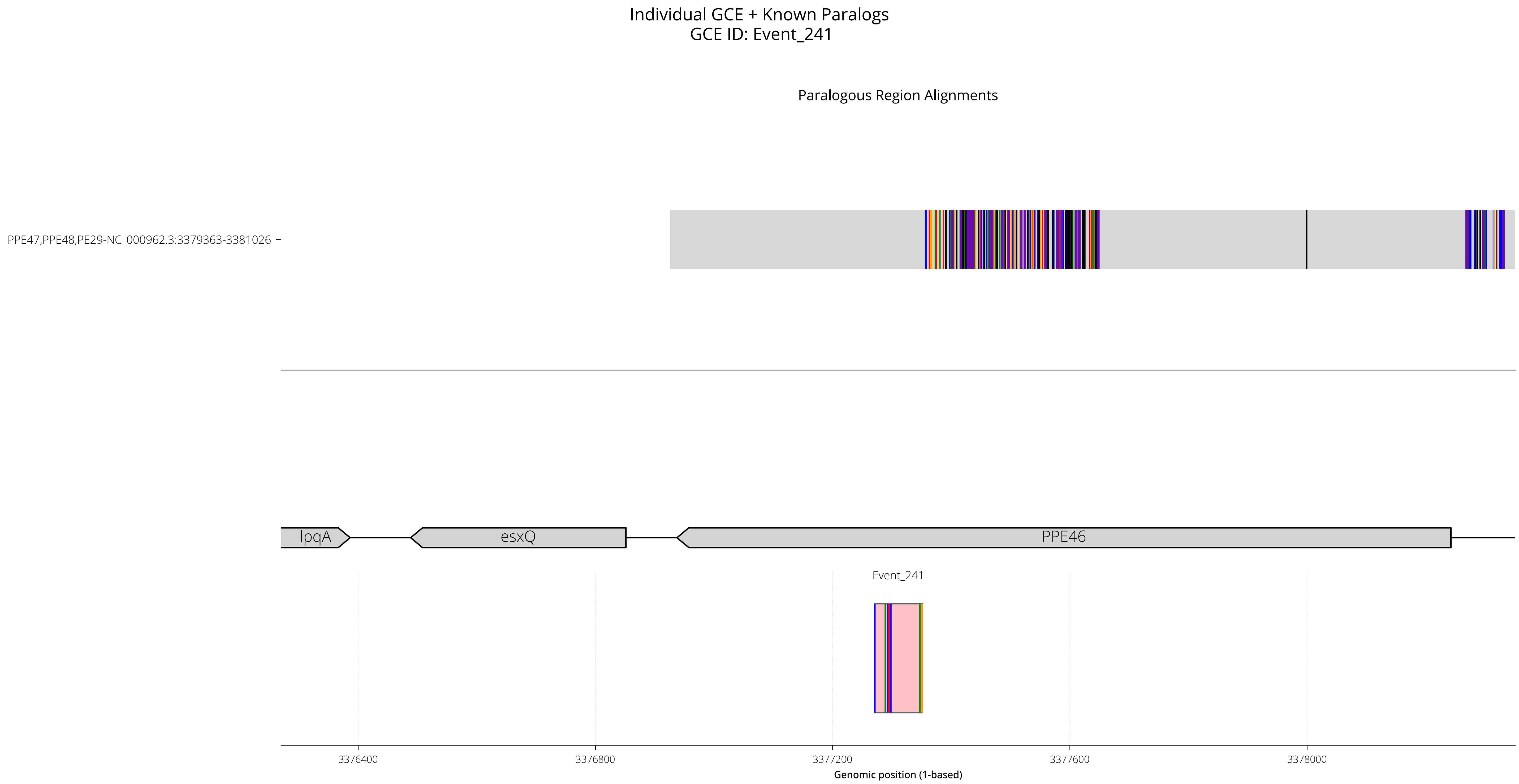
Individual GCE + Known Paralogs
GCE ID: Event_241
Paralogous Region Alignments
PPE47,PPE48,PE29-NC_000962.3:3379363-3381026
lpqA
esxQ
PPE46
Event_241
3376400
3376800
3377200
3377600
3378000
Genomic position (1-based)

Circos Viz - GCE to Known Paralogs Locations
GCE ID: Event_241

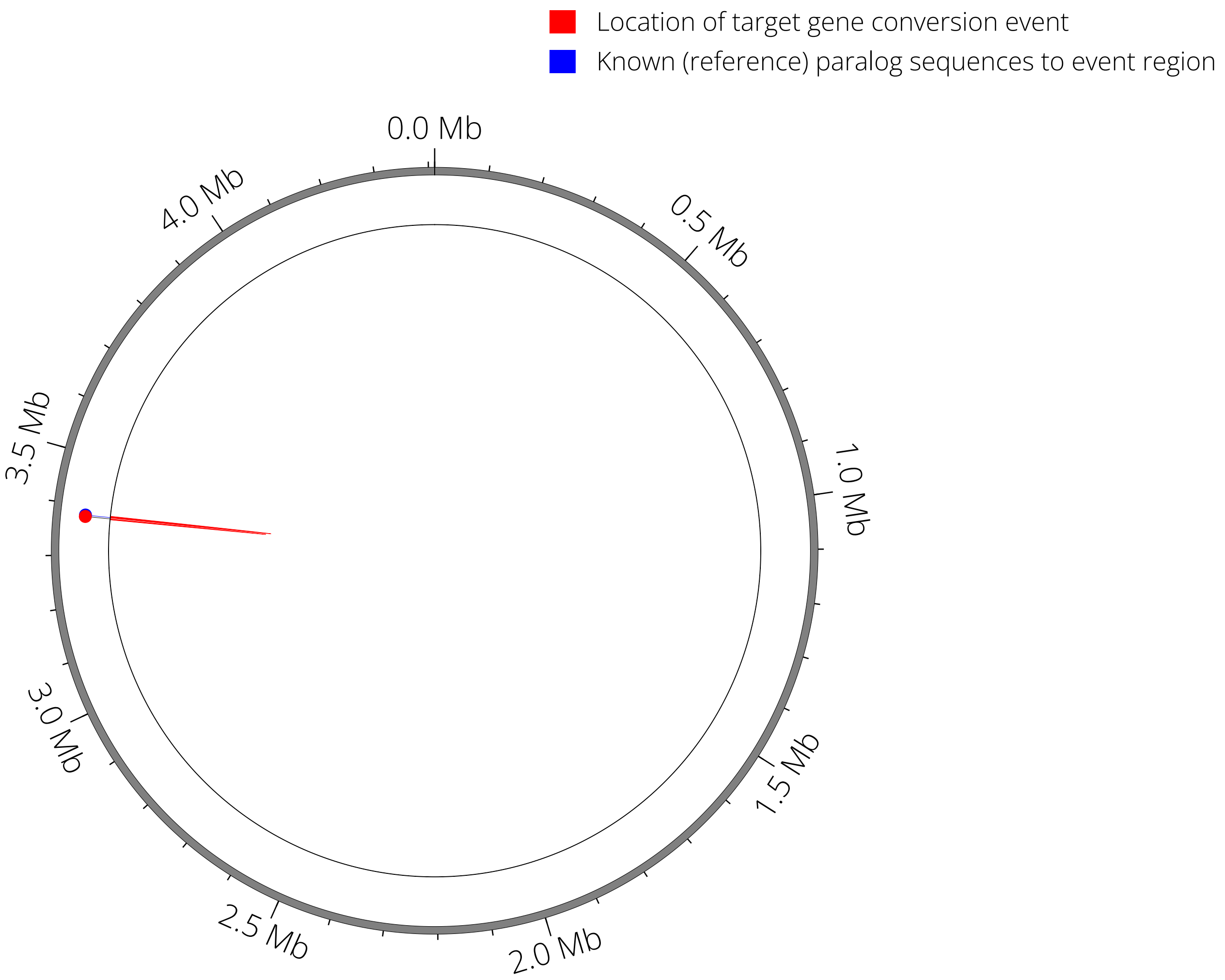

# Event_241 - 7 SNPs - k-mer analysis relative to paralogs

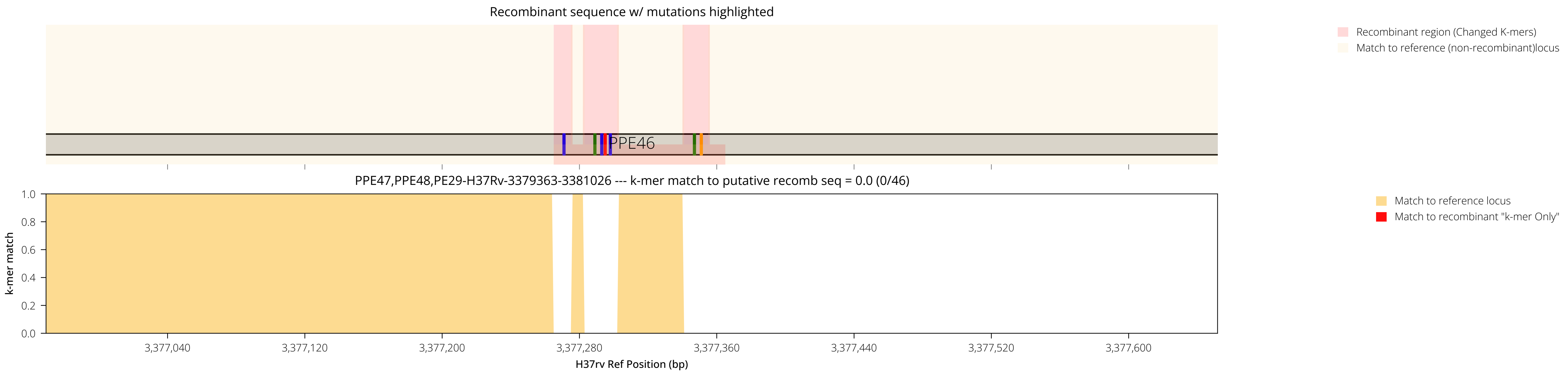

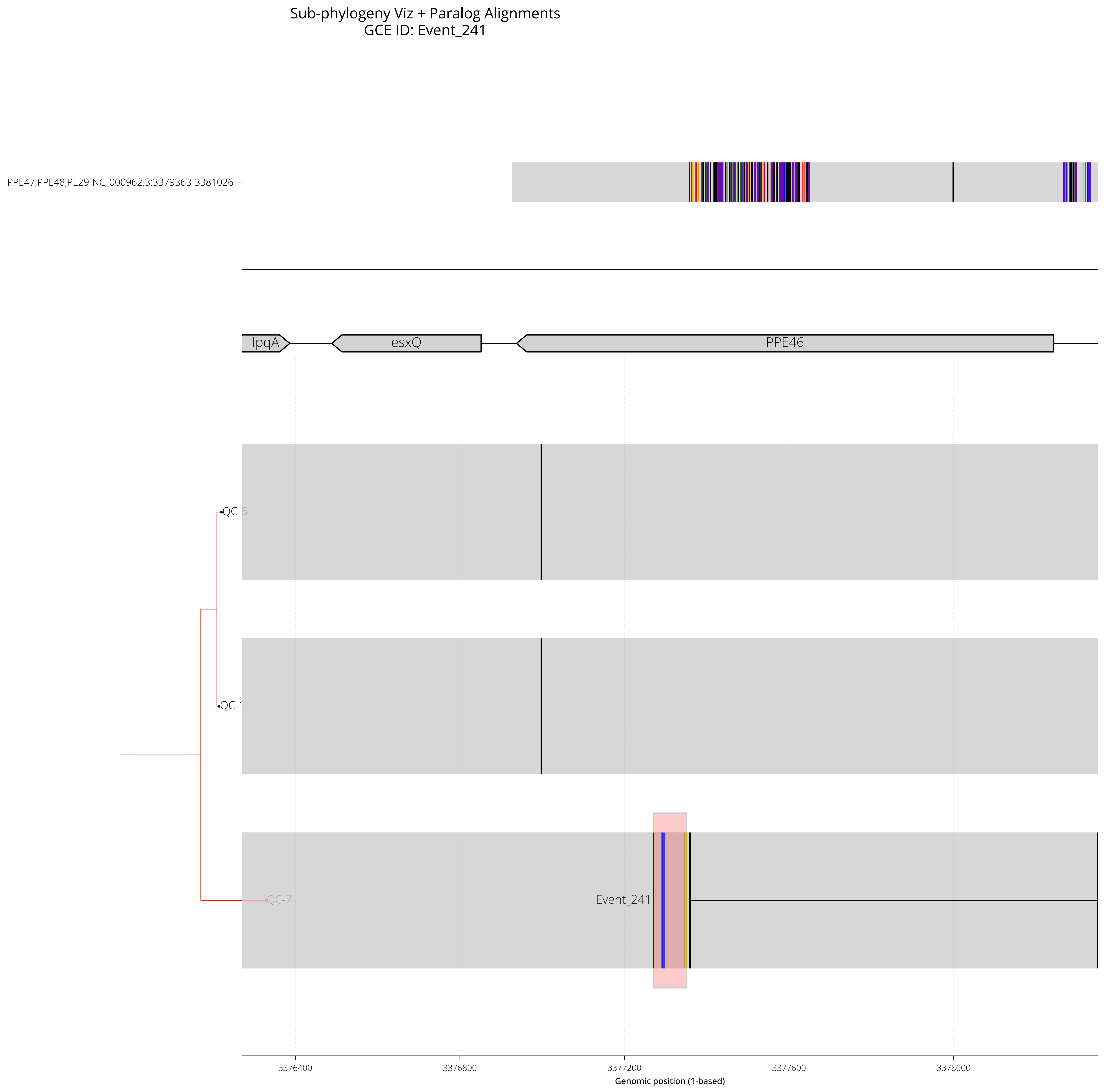

Sub-phylogeny Viz + Paralog Alignments
GCE ID: Event_241
PPE47,PPE48,PE29-NC_000962.3:3379363-3381026
lpqA
esxQ
PPE46
QC-6
QC-1
QC-7
Event_241
3376400
3376800
3377200
3377600
3378000
Genomic position (1-based)

Full Phylogeny Viz + Paralog Alignments
GCE ID: Event_241

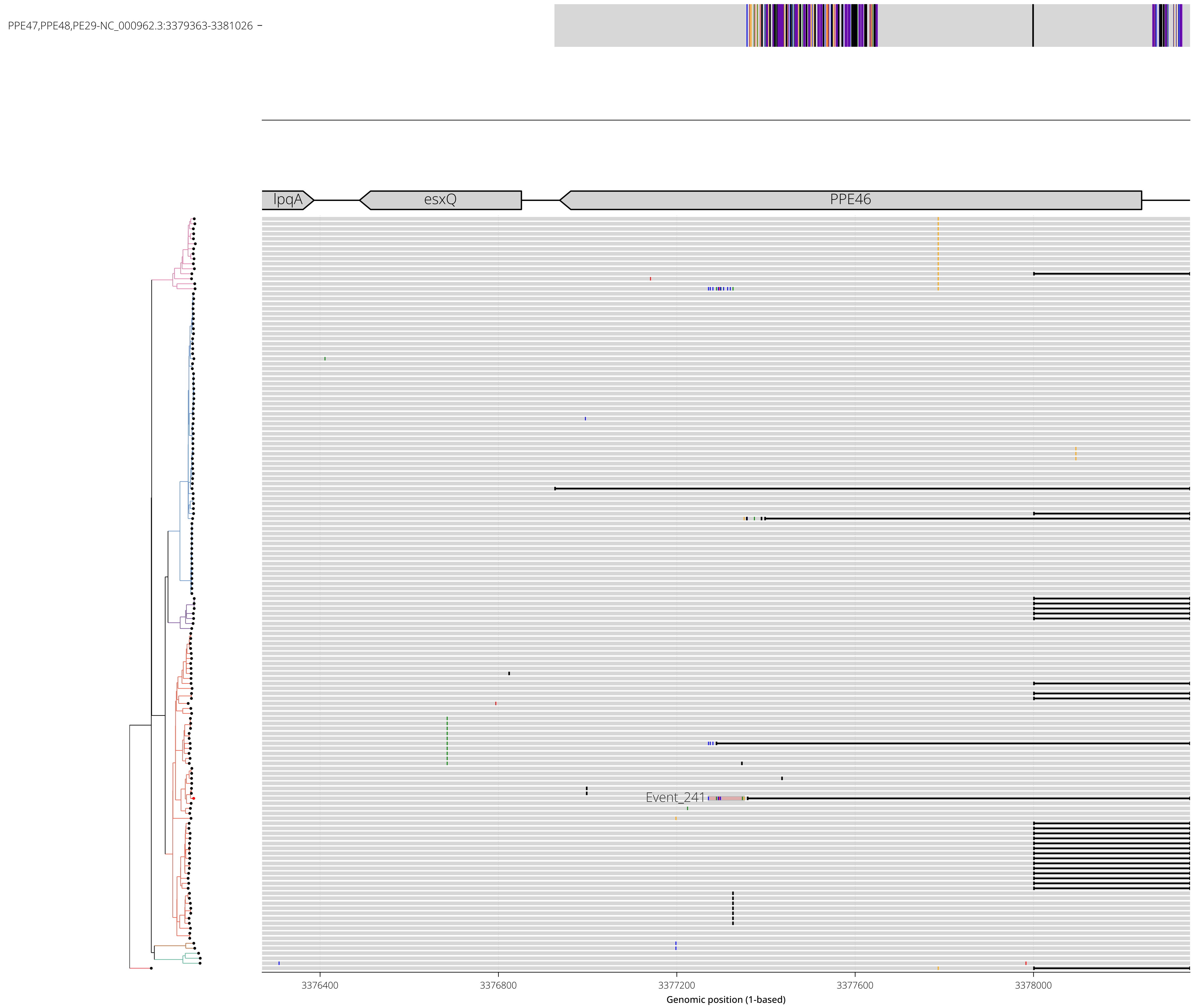

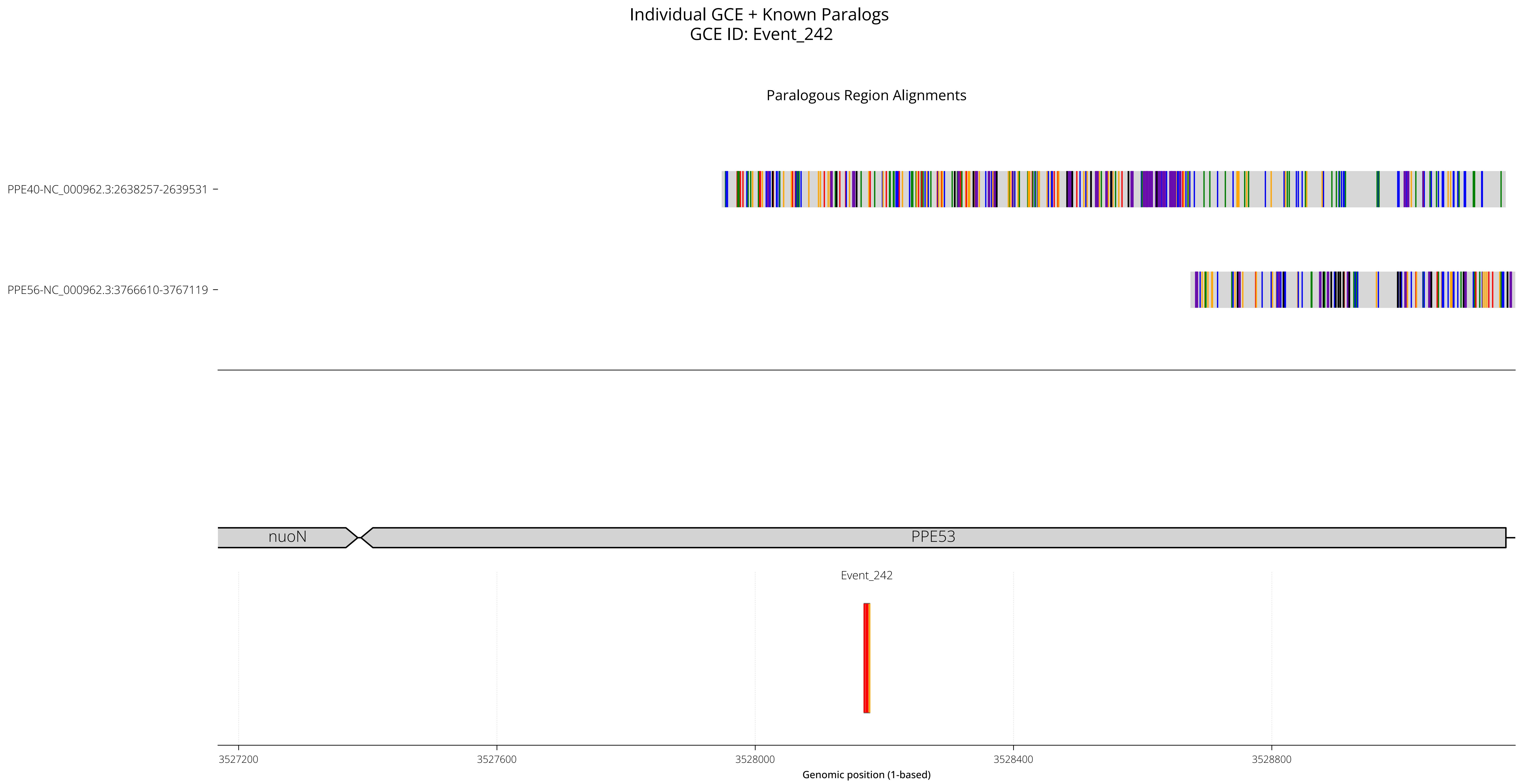
Individual GCE + Known Paralogs
GCE ID: Event_242
Paralogous Region Alignments
PPE40-NC_000962.3:2638257-2639531
PPE56-NC_000962.3:3766610-3767119
nuoN
PPE53
Event_242
3527200
3527600
3528000
3528400
3528800
Genomic position (1-based)

Circos Viz - GCE to Known Paralogs Locations
GCE ID: Event_242

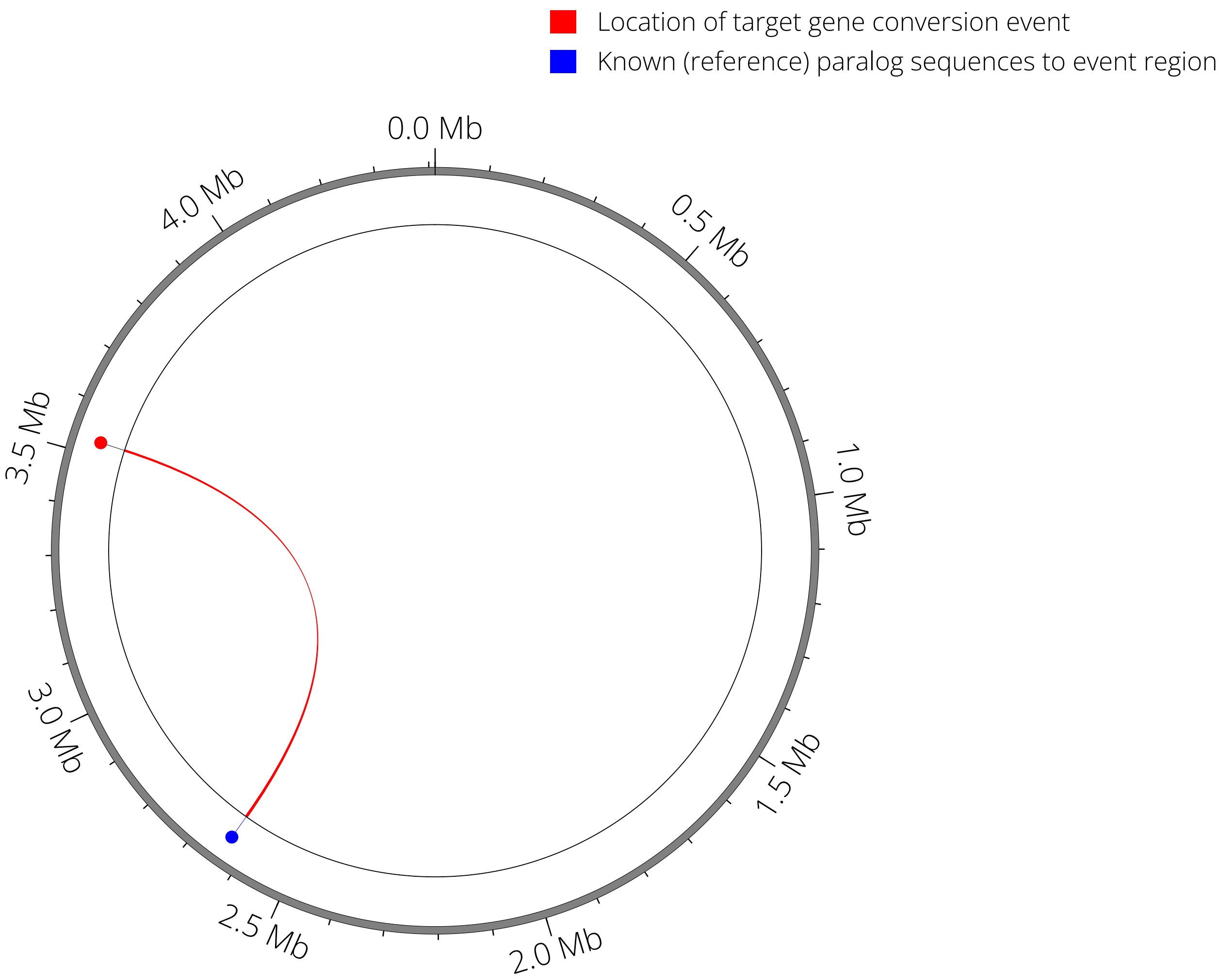

# Event_242 - 5 SNPs - k-mer analysis relative to paralogs

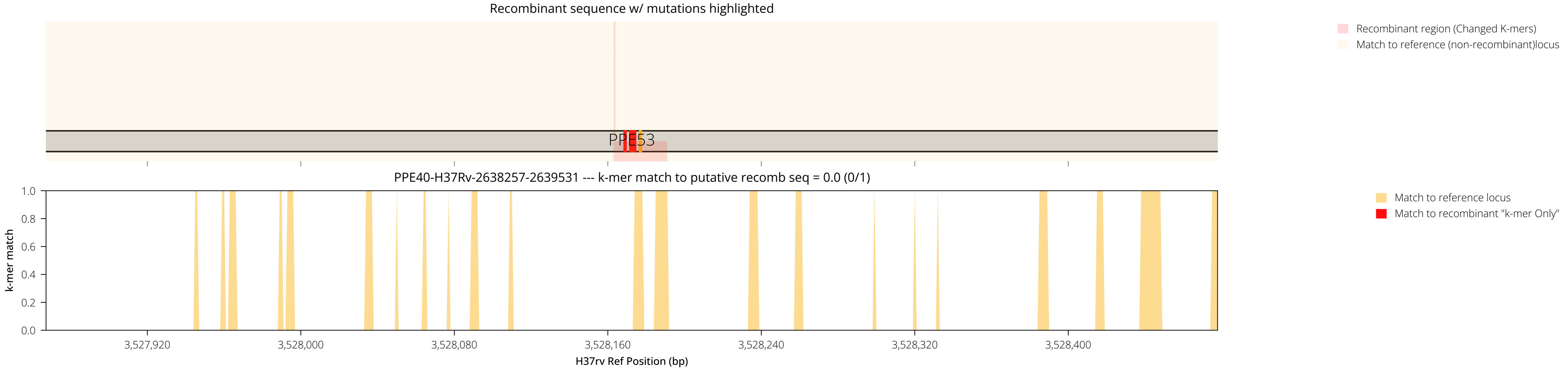

Sub-phylogeny Viz + Paralog Alignments
GCE ID: Event_242

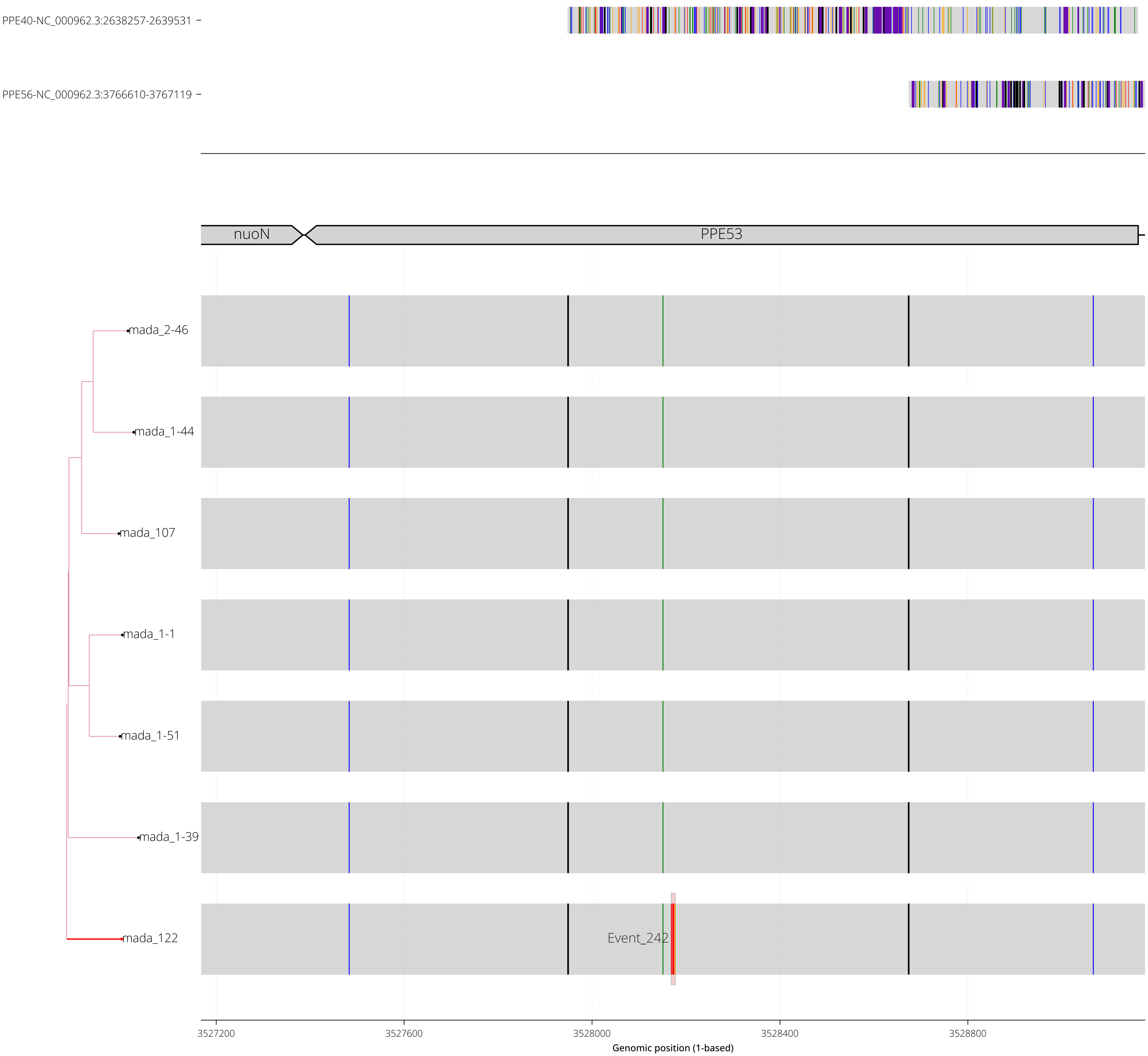

Full Phylogeny Viz + Paralog Alignments
GCE ID: Event_242

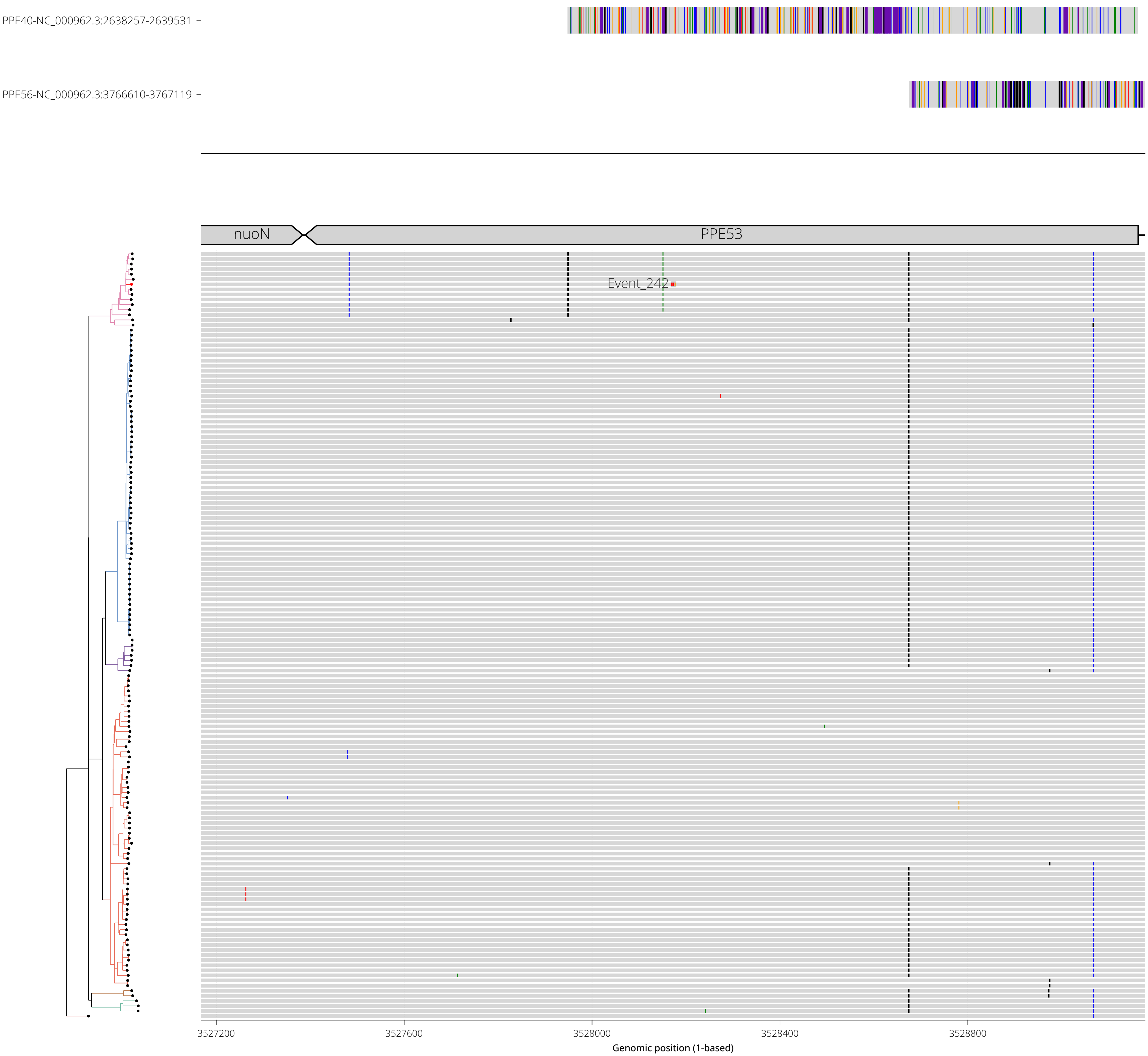

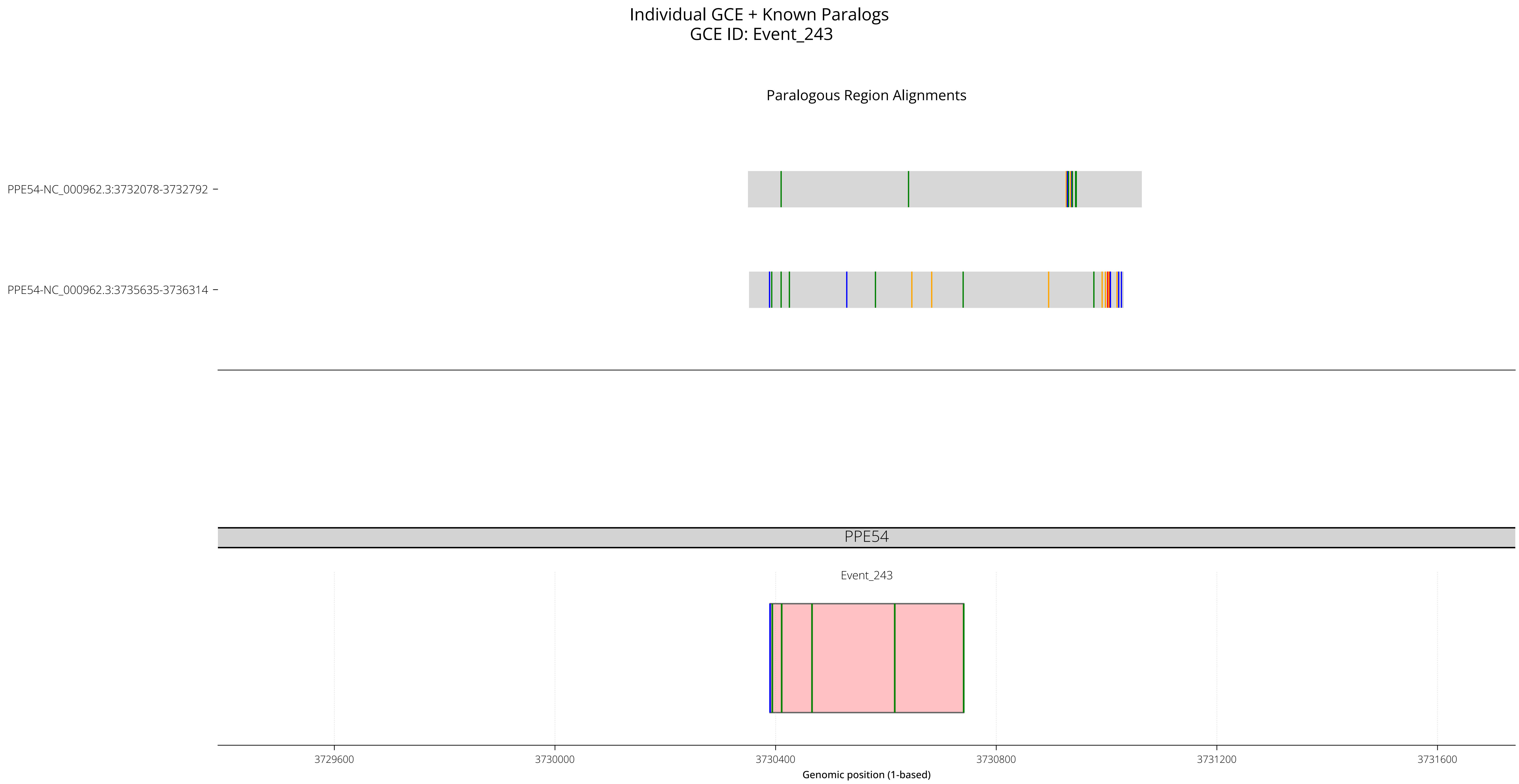
Individual GCE + Known Paralogs
GCE ID: Event_243
Paralogous Region Alignments
PPE54-NC_000962.3:3732078-3732792
PPE54-NC_000962.3:3735635-3736314
PPE54
Event_243
3729600
3730000
3730400
3730800
3731200
3731600
Genomic position (1-based)

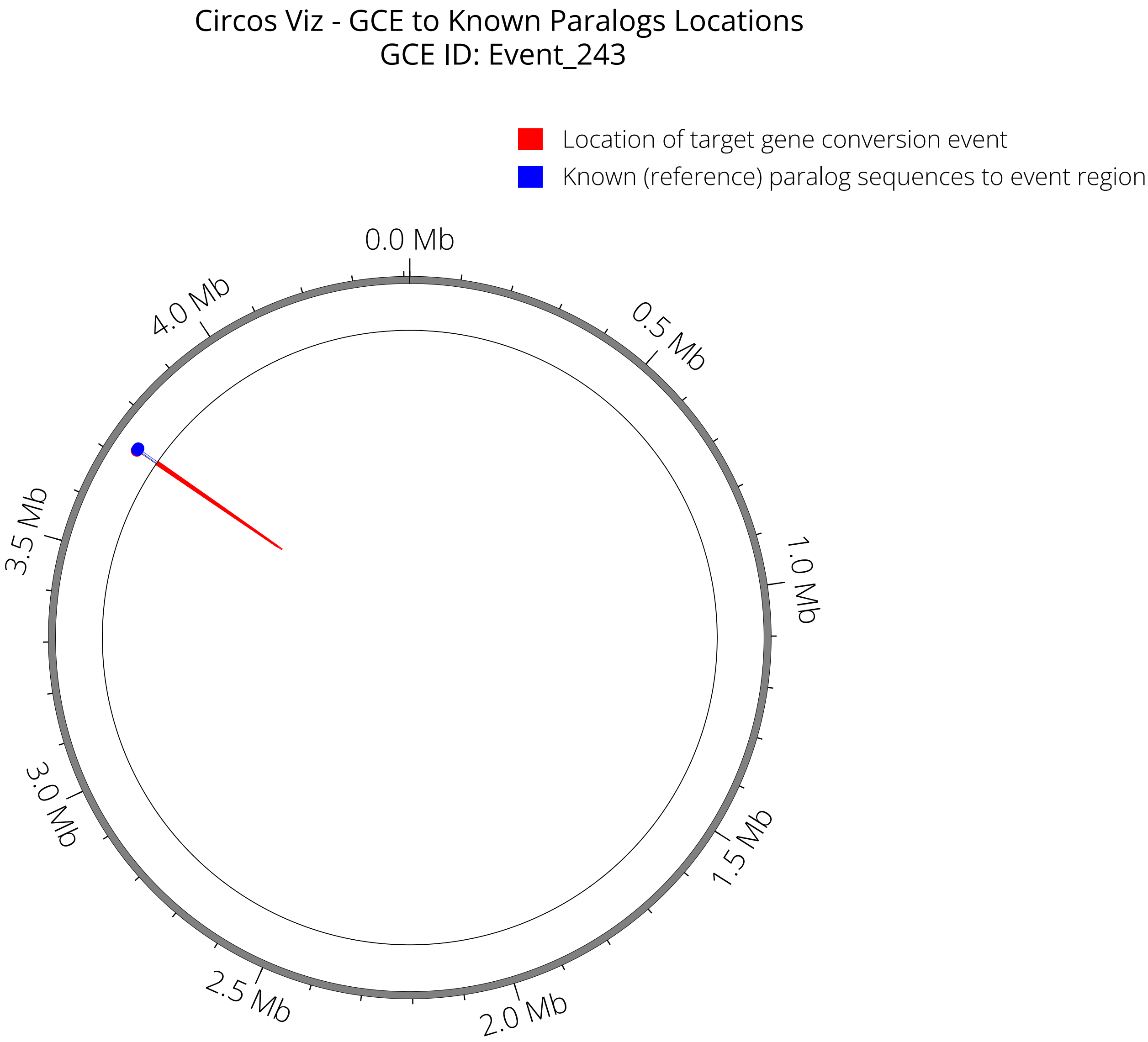
Circos Viz - GCE to Known Paralogs Locations
GCE ID: Event_243
Location of target gene conversion event
Known (reference) paralog sequences to event region
0.0 Mb
0.5 Mb
1.0 Mb
1.5 Mb
2.0 Mb
2.5 Mb
3.0 Mb
3.5 Mb
4.0 Mb

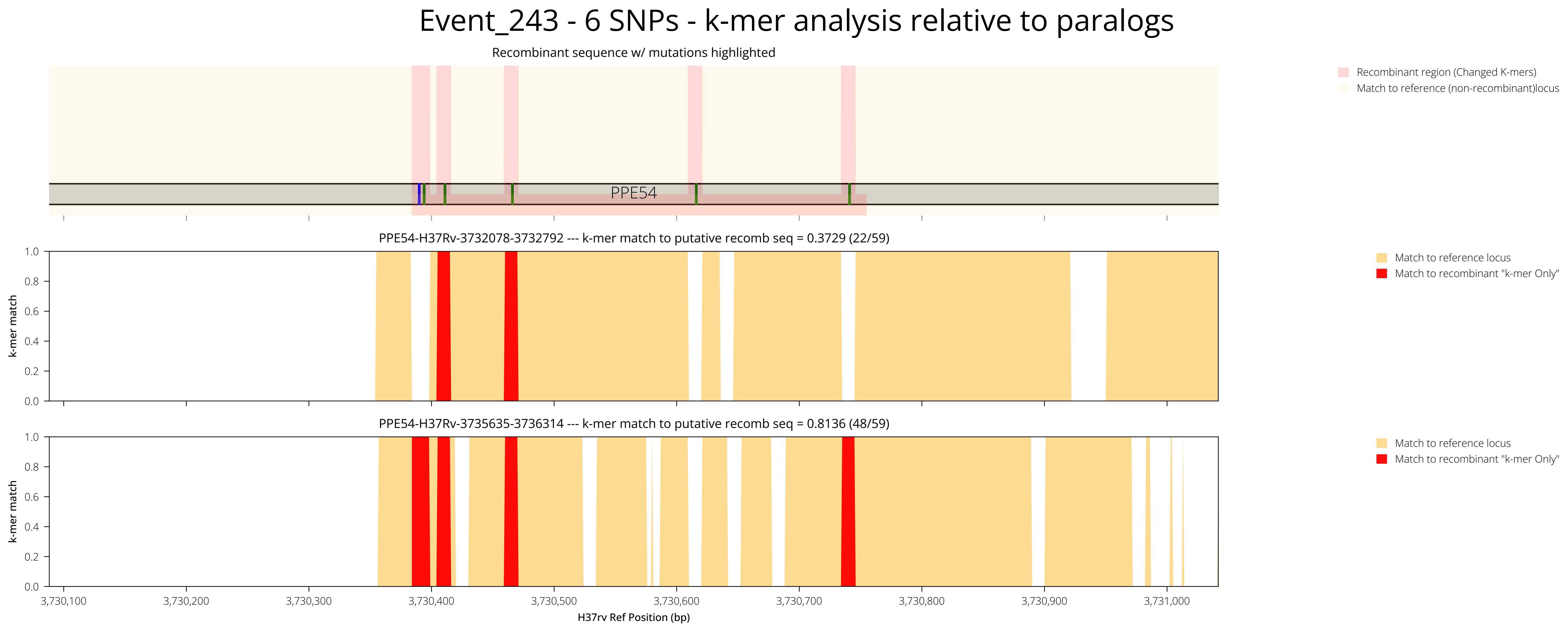

Event_243 - 6 SNPs - k-mer analysis relative to paralogs
Recombinant sequence w/ mutations highlighted
Recombinant region (Changed K-mers)
Match to reference (non-recombinant)locus
PPE54
PPE54-H37Rv-3732078-3732792 --- k-mer match to putative recomb seq = 0.3729 (22/59)
Match to reference locus
Match to recombinant "k-mer Only"
k-mer match
1.0
0.8
0.6
0.4
0.2
0.0
PPE54-H37Rv-3735635-3736314 --- k-mer match to putative recomb seq = 0.8136 (48/59)
Match to reference locus
Match to recombinant "k-mer Only"
k-mer match
1.0
0.8
0.6
0.4
0.2
0.0
3,730,100
3,730,200
3,730,300
3,730,400
3,730,500
3,730,600
3,730,700
3,730,800
3,730,900
3,731,000
H37rv Ref Position (bp)

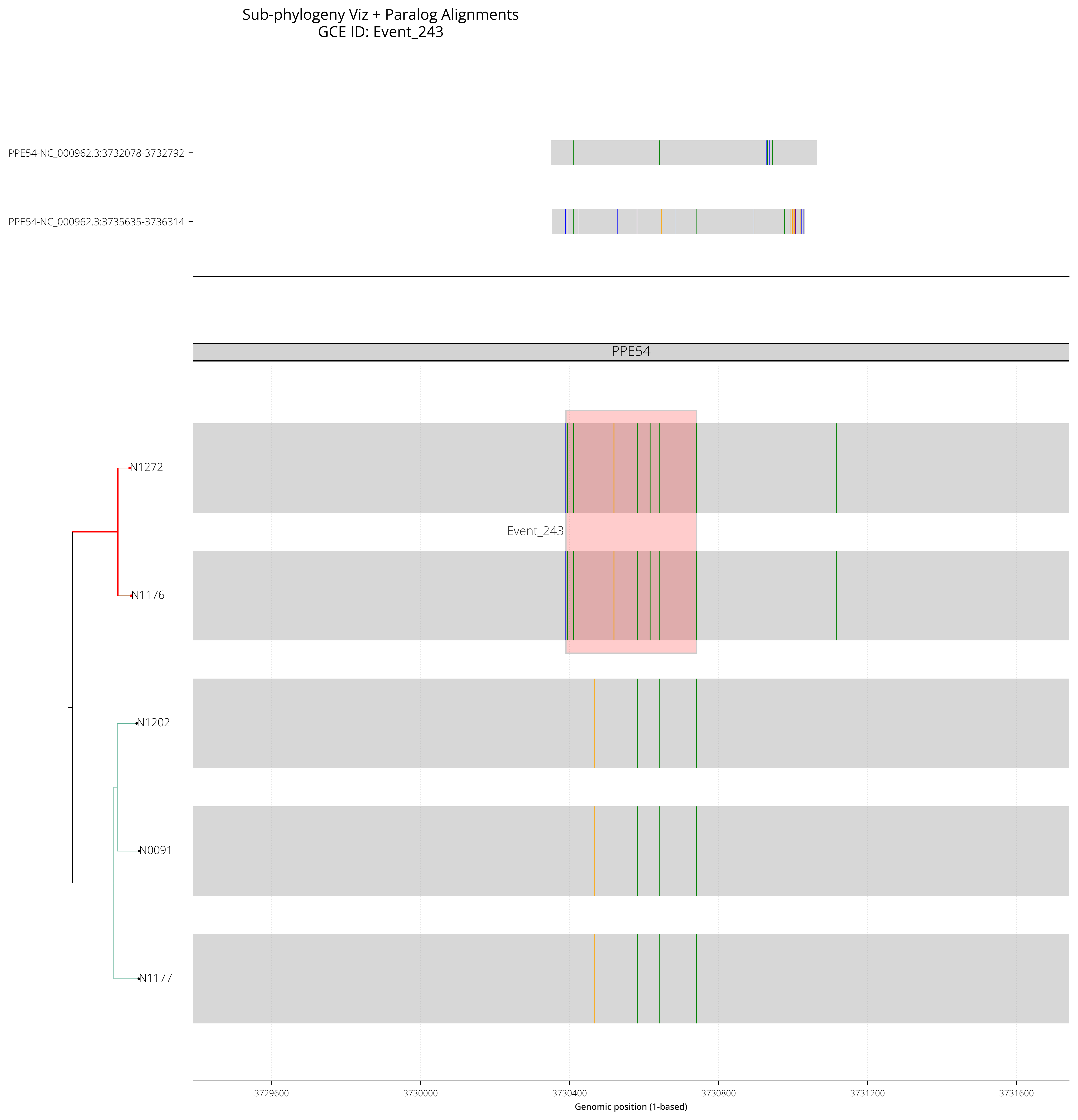

Sub-phylogeny Viz + Paralog Alignments
GCE ID: Event_243
PPE54-NC_000962.3:3732078-3732792
PPE54-NC_000962.3:3735635-3736314
PPE54
N1272
Event_243
N1176
N1202
N0091
N1177
3729600
3730000
3730400
3730800
3731200
3731600
Genomic position (1-based)

Full Phylogeny Viz + Paralog Alignments
GCE ID: Event_243

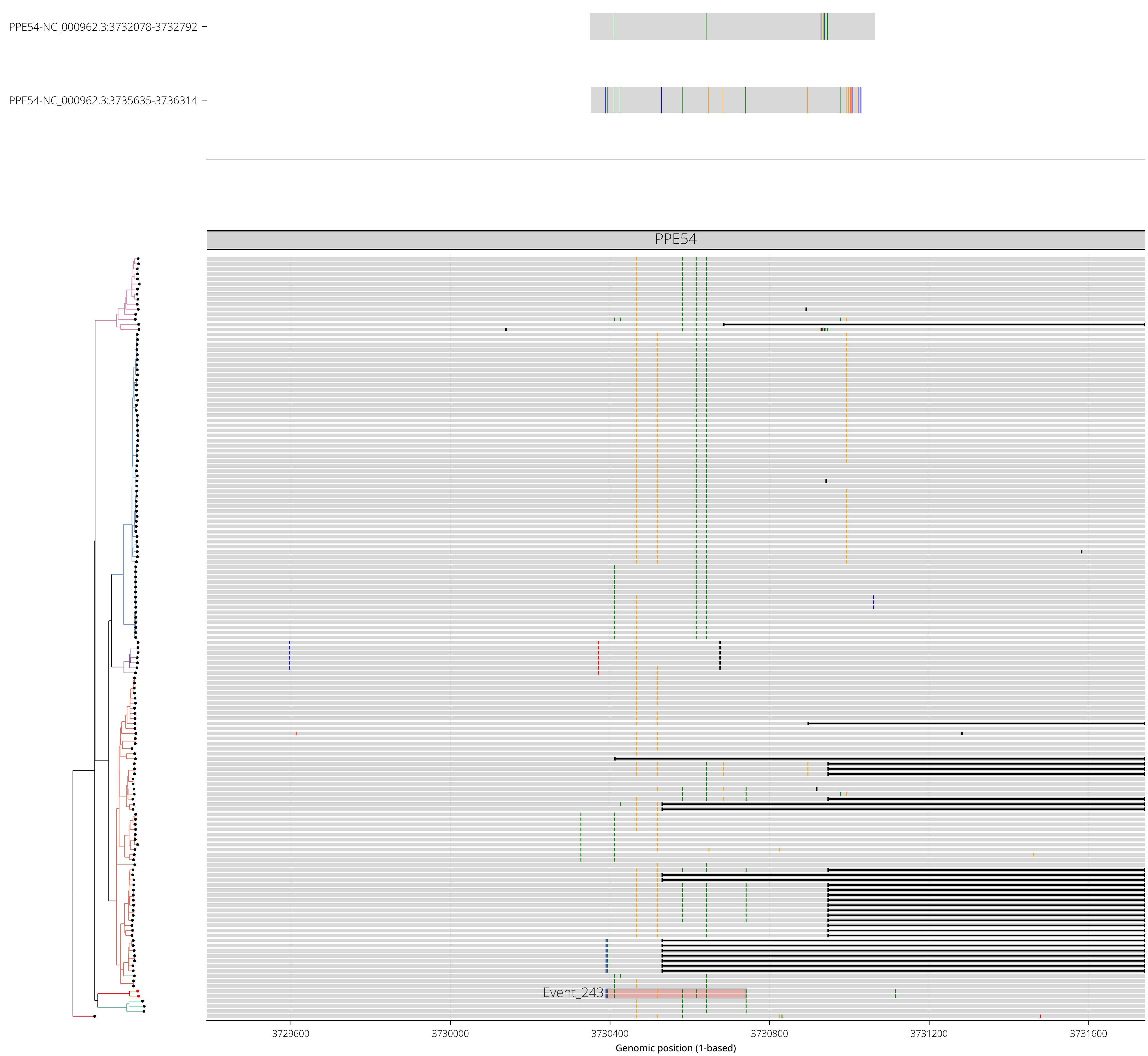

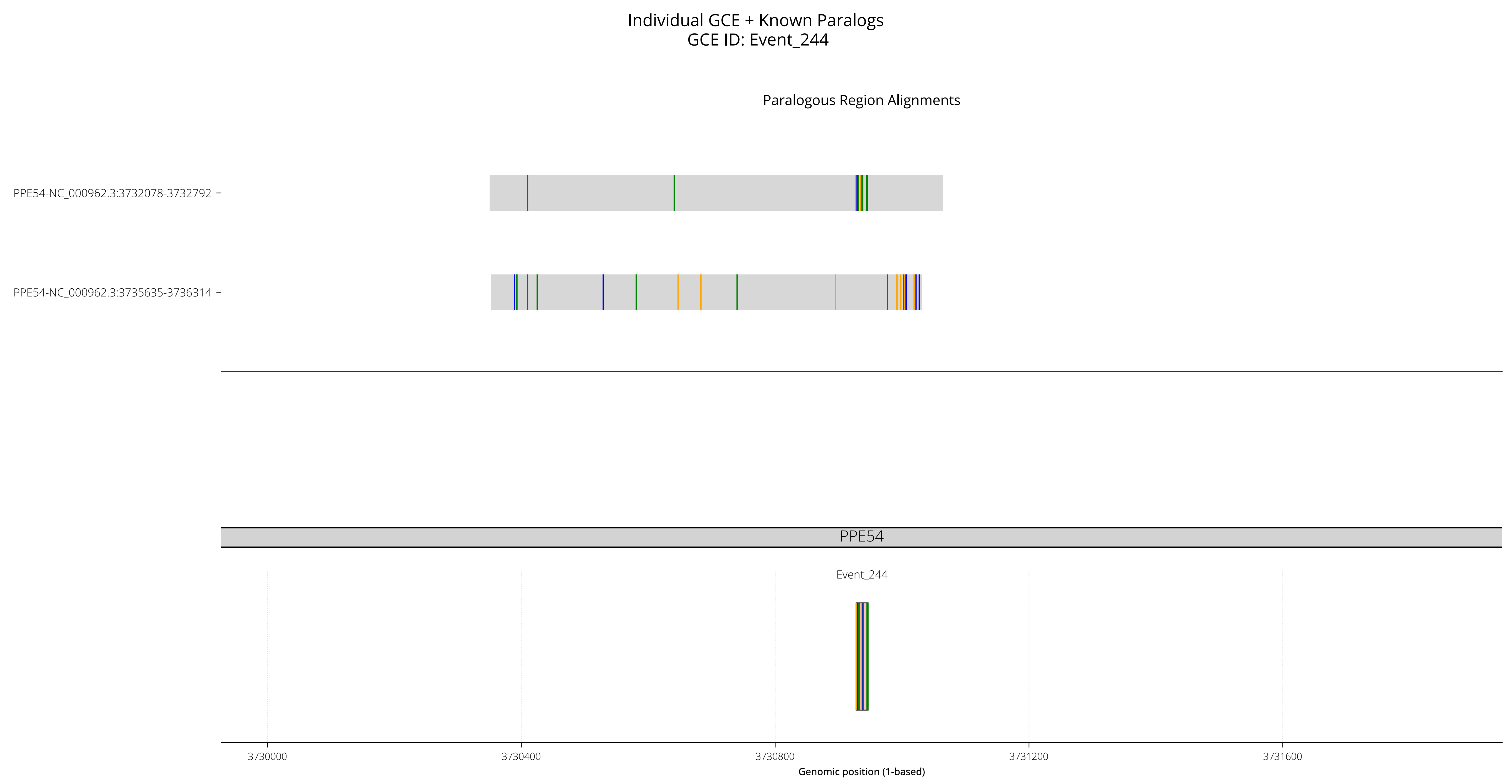

Individual GCE + Known Paralogs
GCE ID: Event_244
Paralogous Region Alignments
PPE54-NC_000962.3:3732078-3732792
PPE54-NC_000962.3:3735635-3736314
PPE54
Event_244
3730000
3730400
3730800
3731200
3731600
Genomic position (1-based)

Circos Viz - GCE to Known Paralogs Locations
GCE ID: Event_244

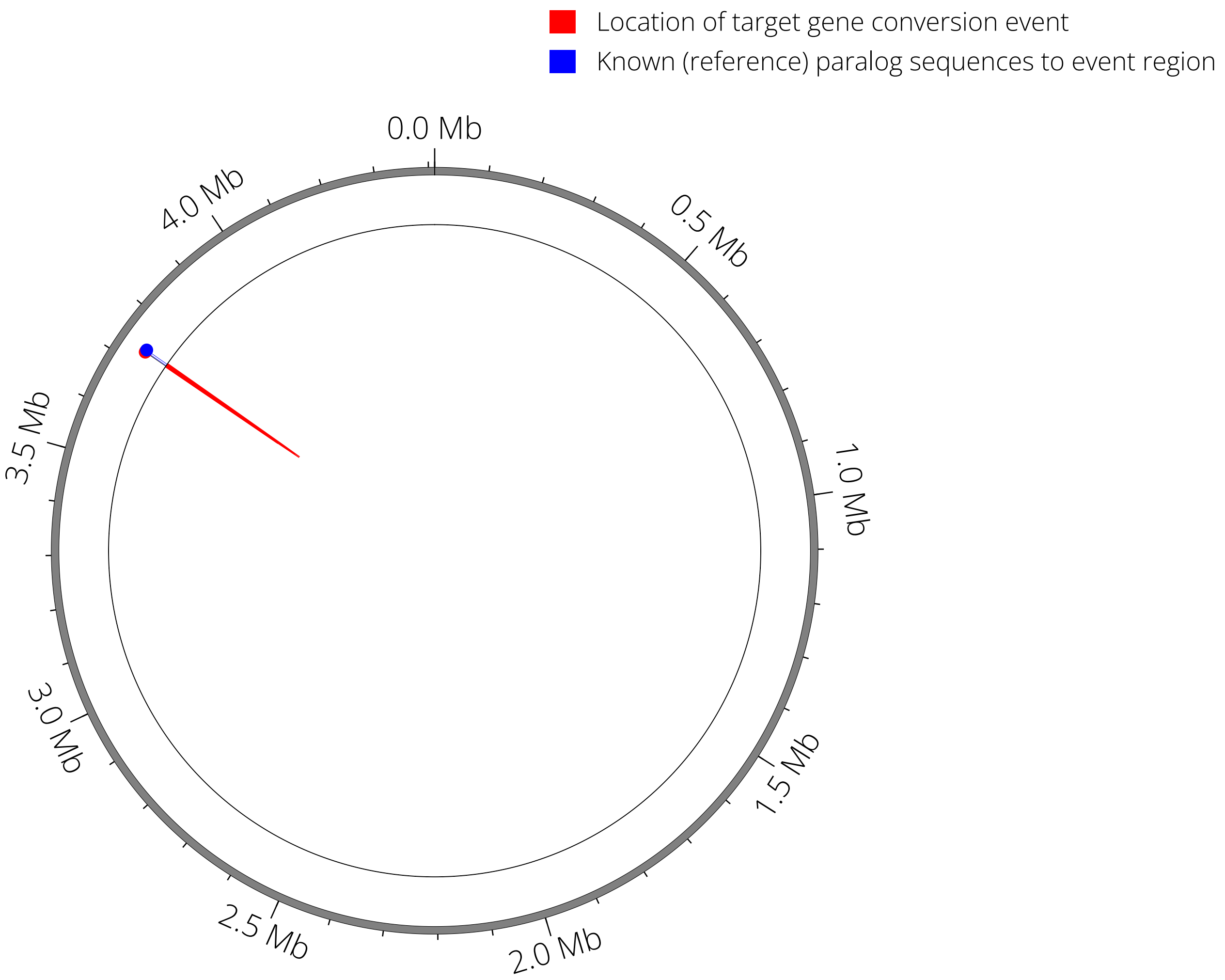

# Event_244 - 8 SNPs - k-mer analysis relative to paralogs

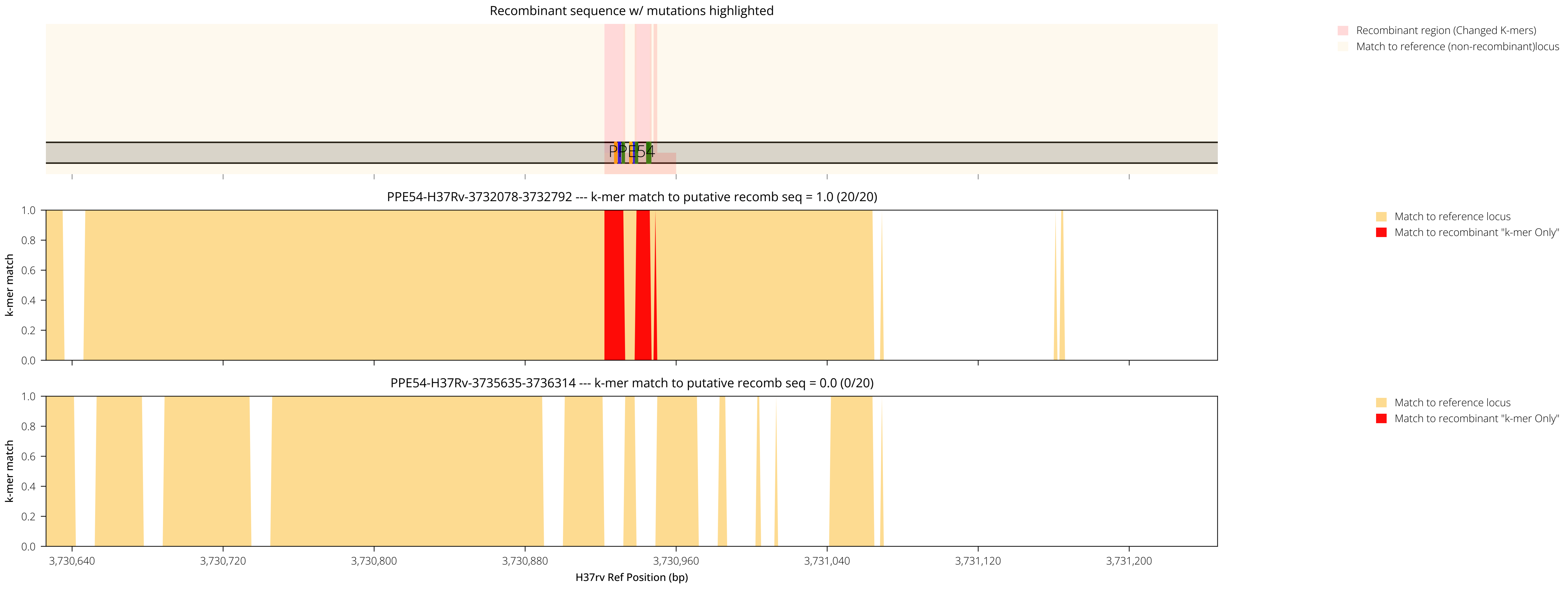

Sub-phylogeny Viz + Paralog Alignments
GCE ID: Event_244

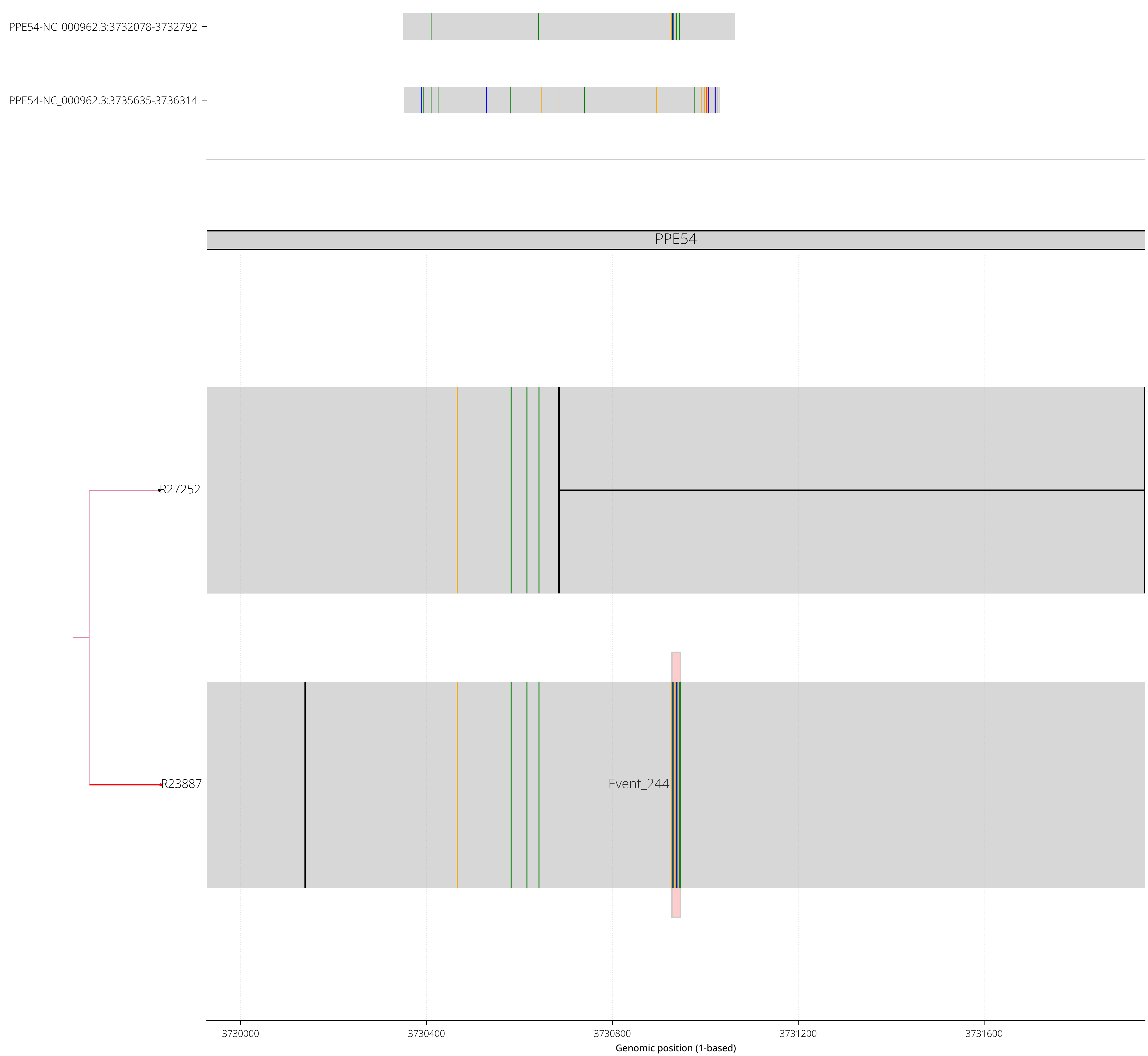

Full Phylogeny Viz + Paralog Alignments
GCE ID: Event_244

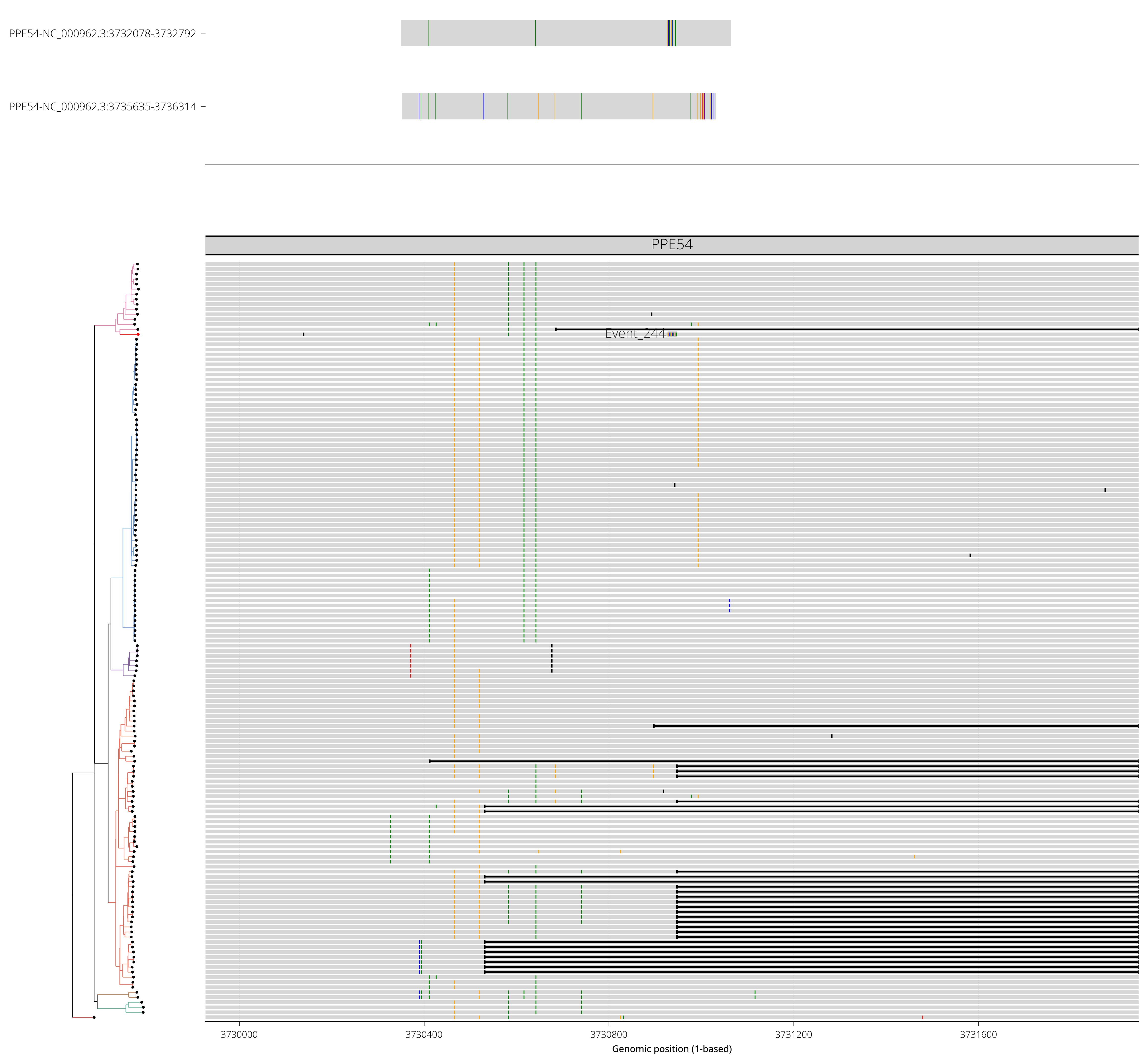

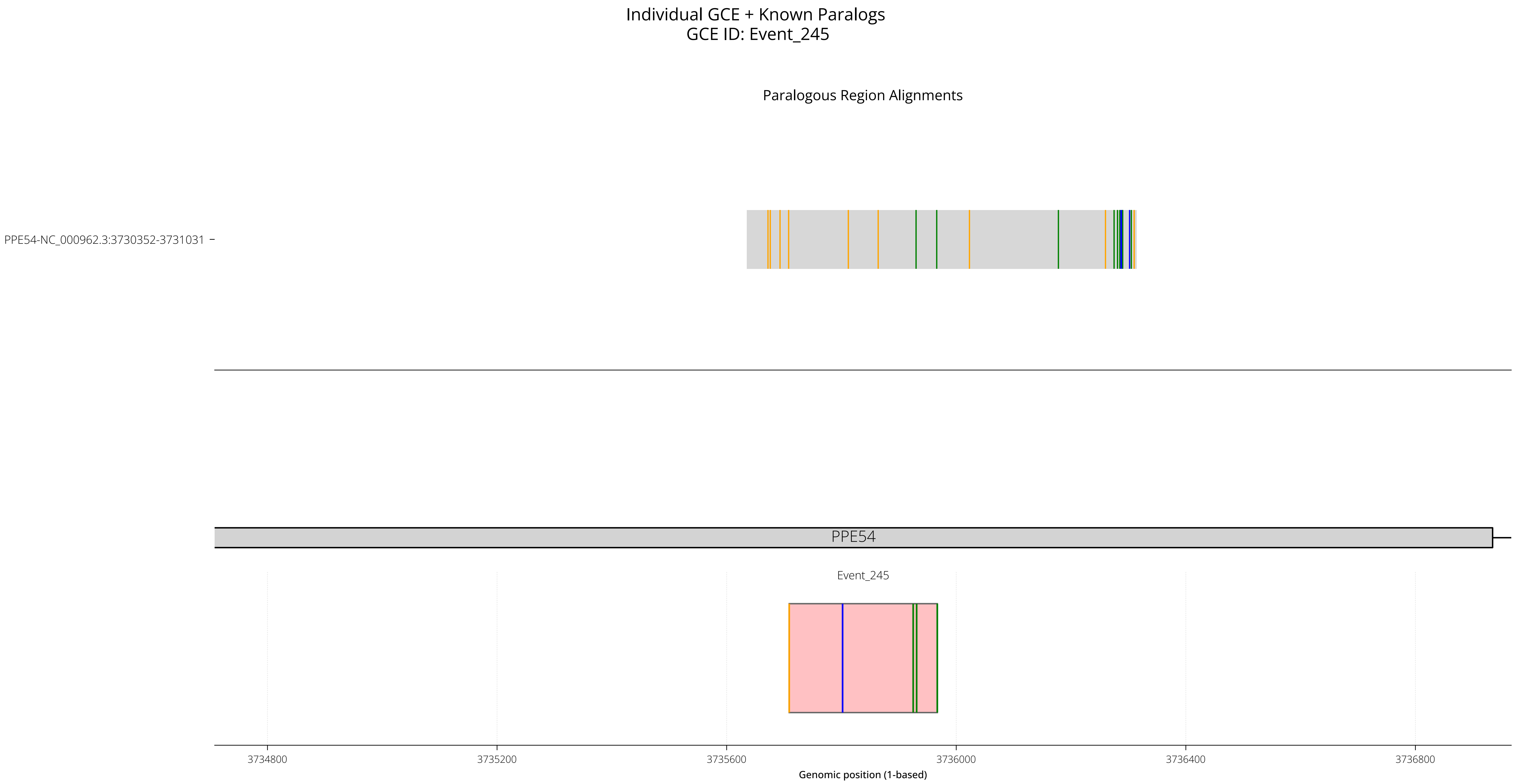

Individual GCE + Known Paralogs
GCE ID: Event_245
Paralogous Region Alignments
PPE54-NC_000962.3:3730352-3731031
PPE54
Event_245
3734800
3735200
3735600
3736000
3736400
3736800
Genomic position (1-based)

Circos Viz - GCE to Known Paralogs Locations
GCE ID: Event_245

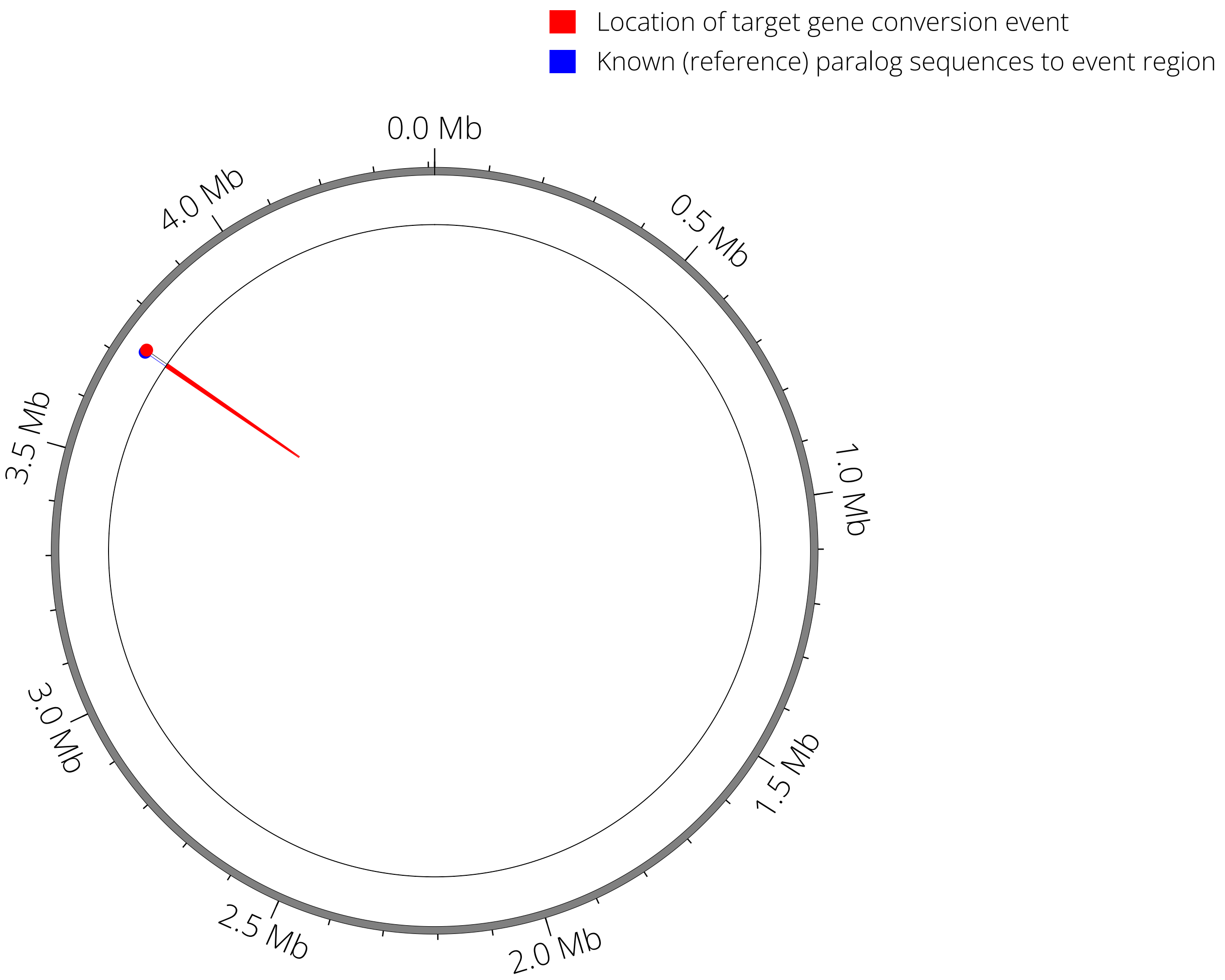

# Event_245 - 5 SNPs - k-mer analysis relative to paralogs

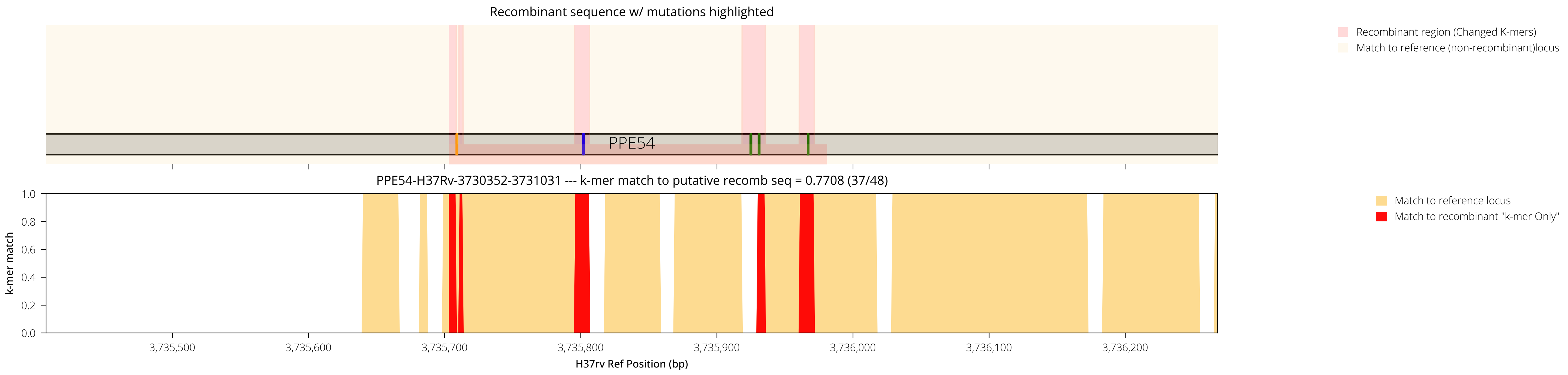

Sub-phylogeny Viz + Paralog Alignments
GCE ID: Event_245

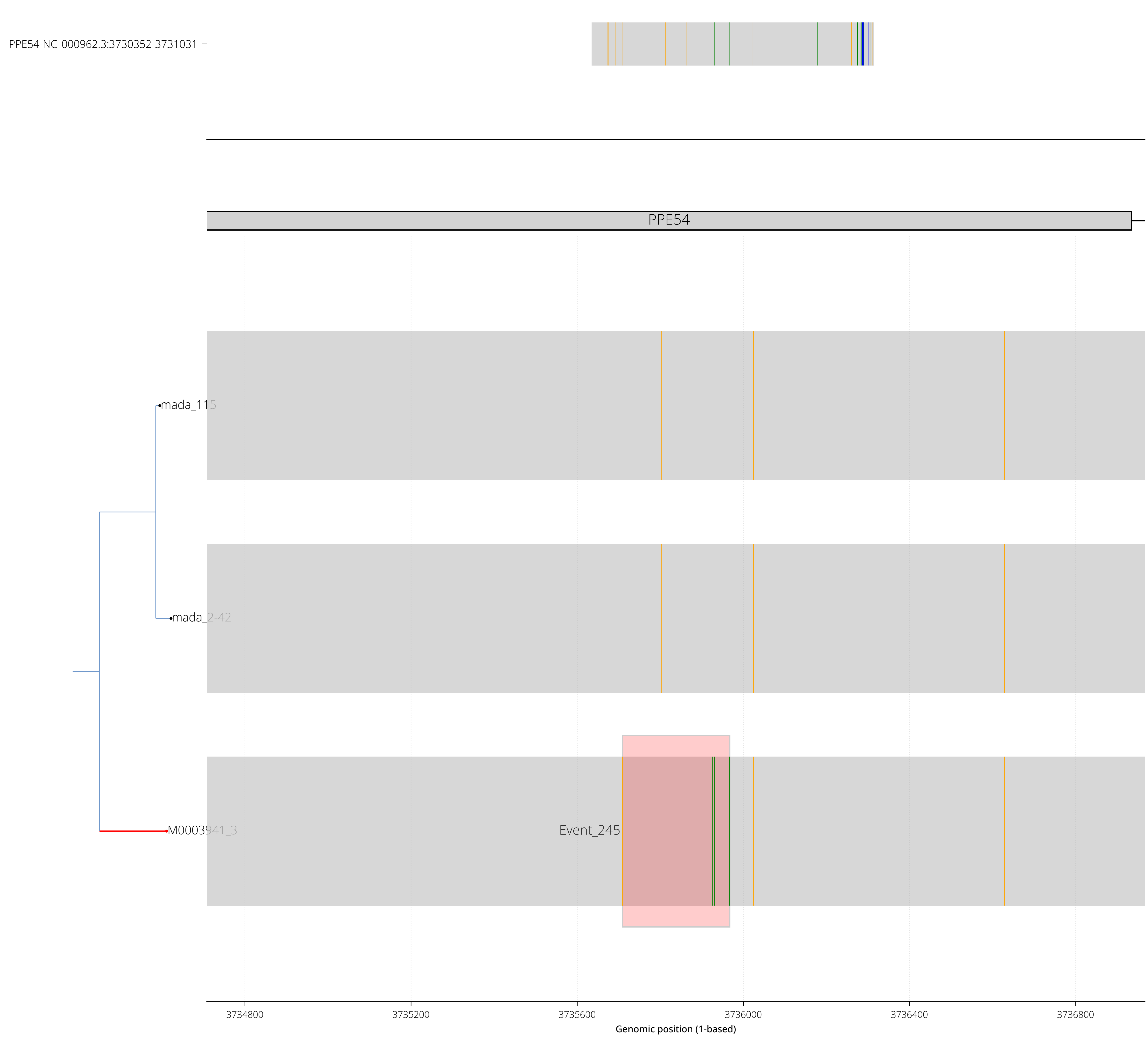

Full Phylogeny Viz + Paralog Alignments
GCE ID: Event_245

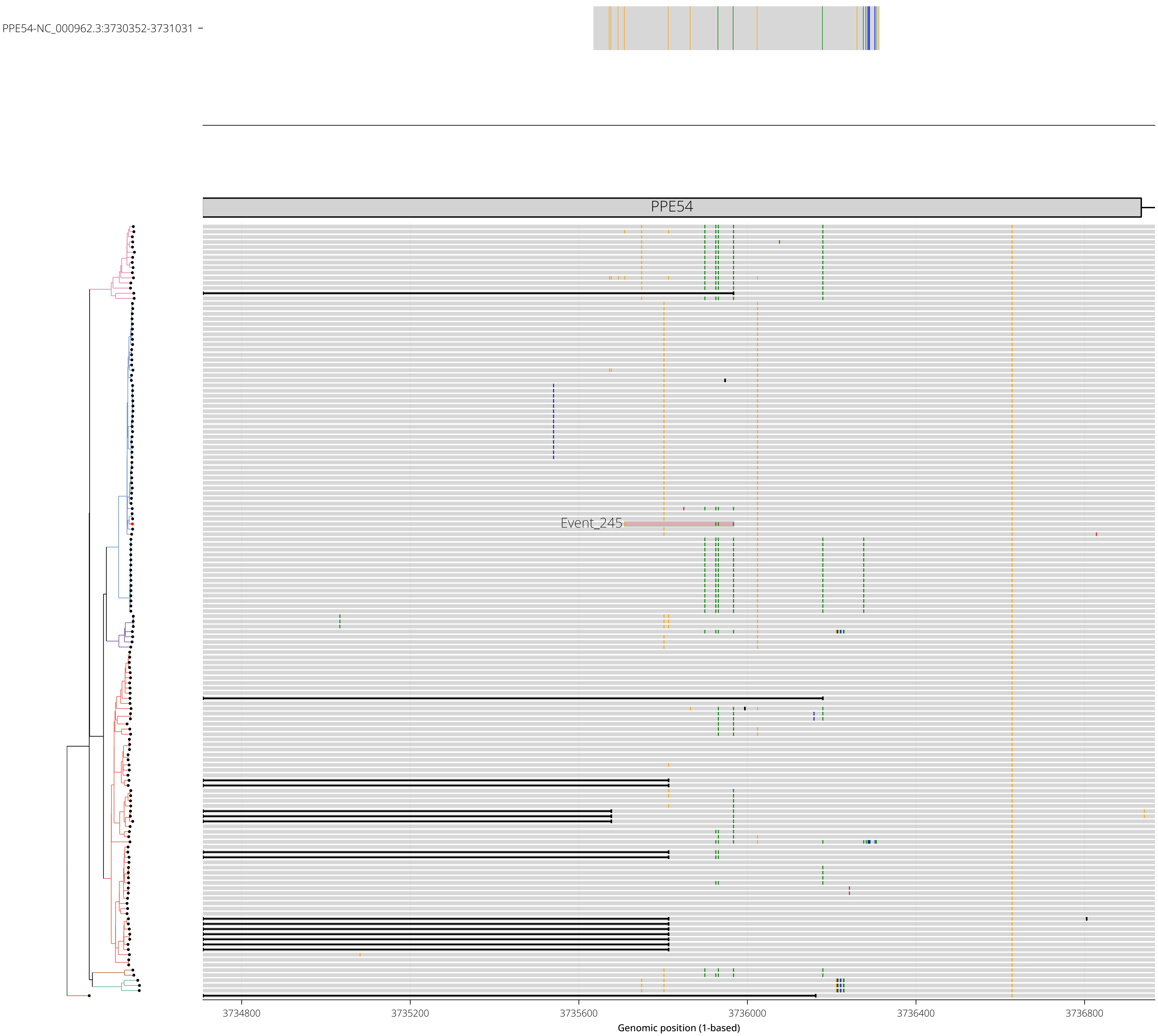

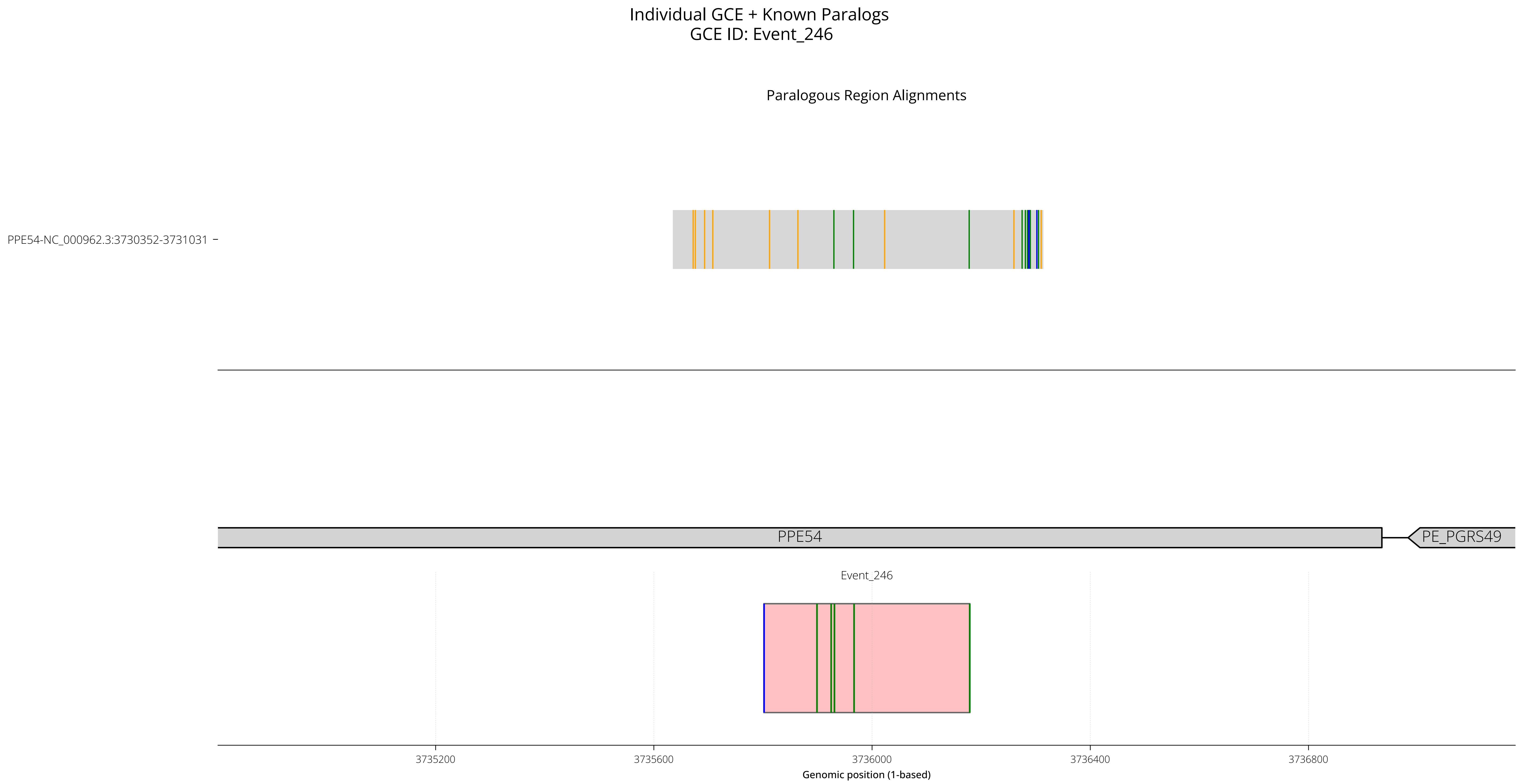

Individual GCE + Known Paralogs
GCE ID: Event_246
Paralogous Region Alignments
PPE54-NC_000962.3:3730352-3731031
PPE54
PE_PGRS49
Event_246
3735200
3735600
3736000
3736400
3736800
Genomic position (1-based)

Circos Viz - GCE to Known Paralogs Locations
GCE ID: Event_246

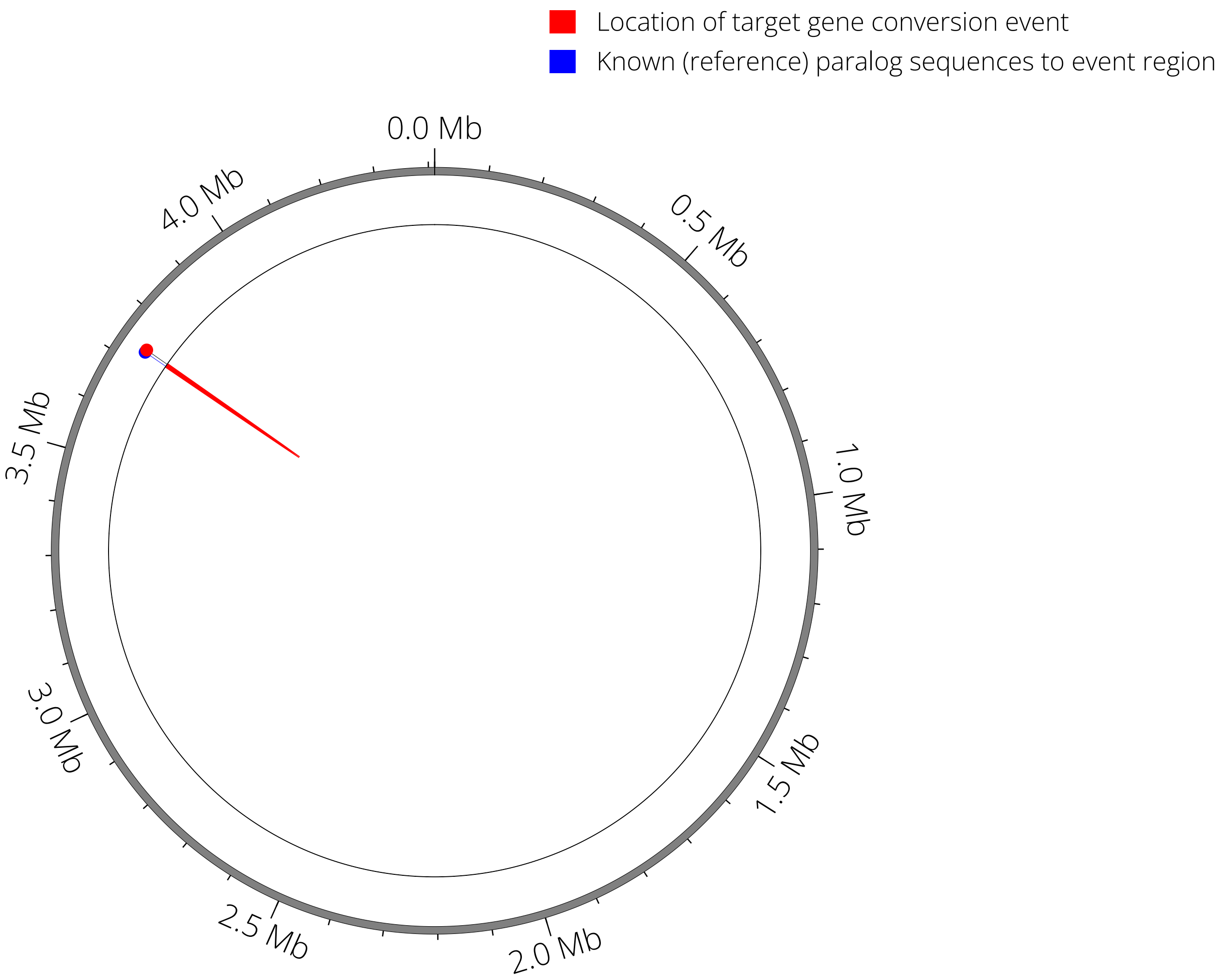

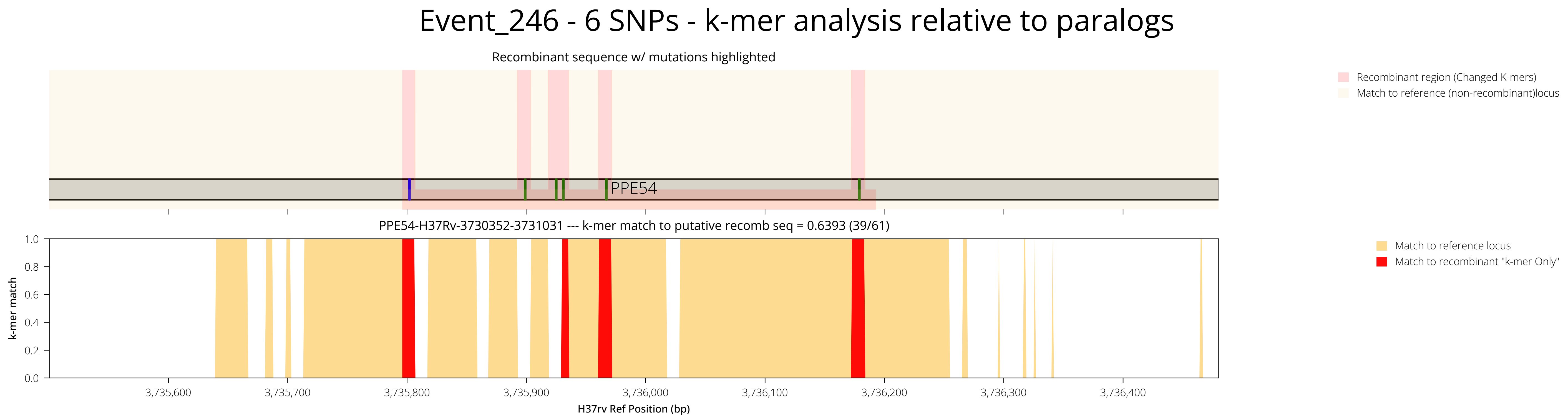
Event_246 - 6 SNPs - k-mer analysis relative to paralogs
Recombinant sequence w/ mutations highlighted
Recombinant region (Changed K-mers)
Match to reference (non-recombinant)locus
PPE54
PPE54-H37Rv-3730352-3731031 --- k-mer match to putative recomb seq = 0.6393 (39/61)
Match to reference locus
Match to recombinant "k-mer Only"
k-mer match
1.0
0.8
0.6
0.4
0.2
0.0
3,735,600
3,735,700
3,735,800
3,735,900
3,736,000
3,736,100
3,736,200
3,736,300
3,736,400
H37rv Ref Position (bp)

Sub-phylogeny Viz + Paralog Alignments
GCE ID: Event_246

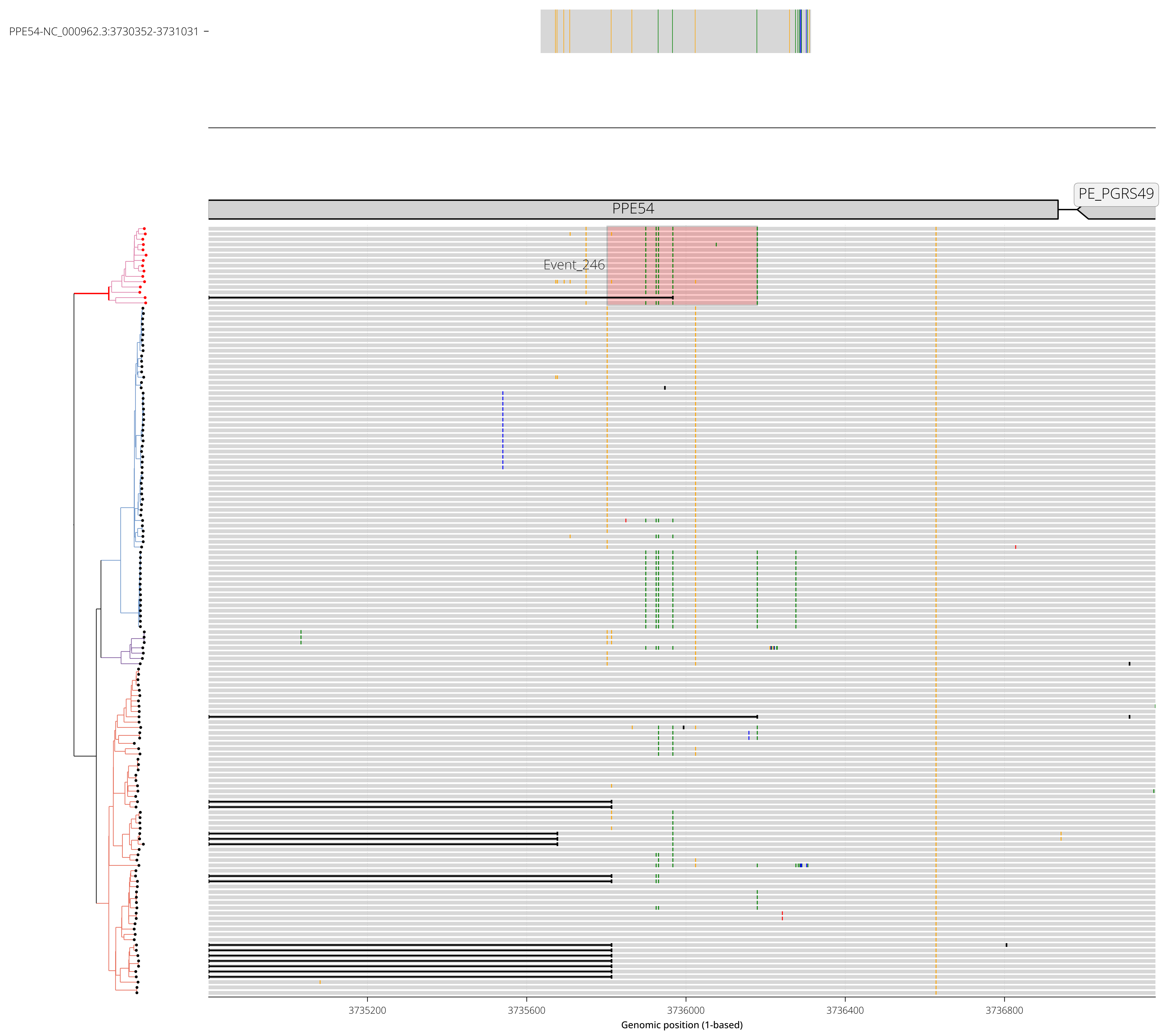

Full Phylogeny Viz + Paralog Alignments
GCE ID: Event_246

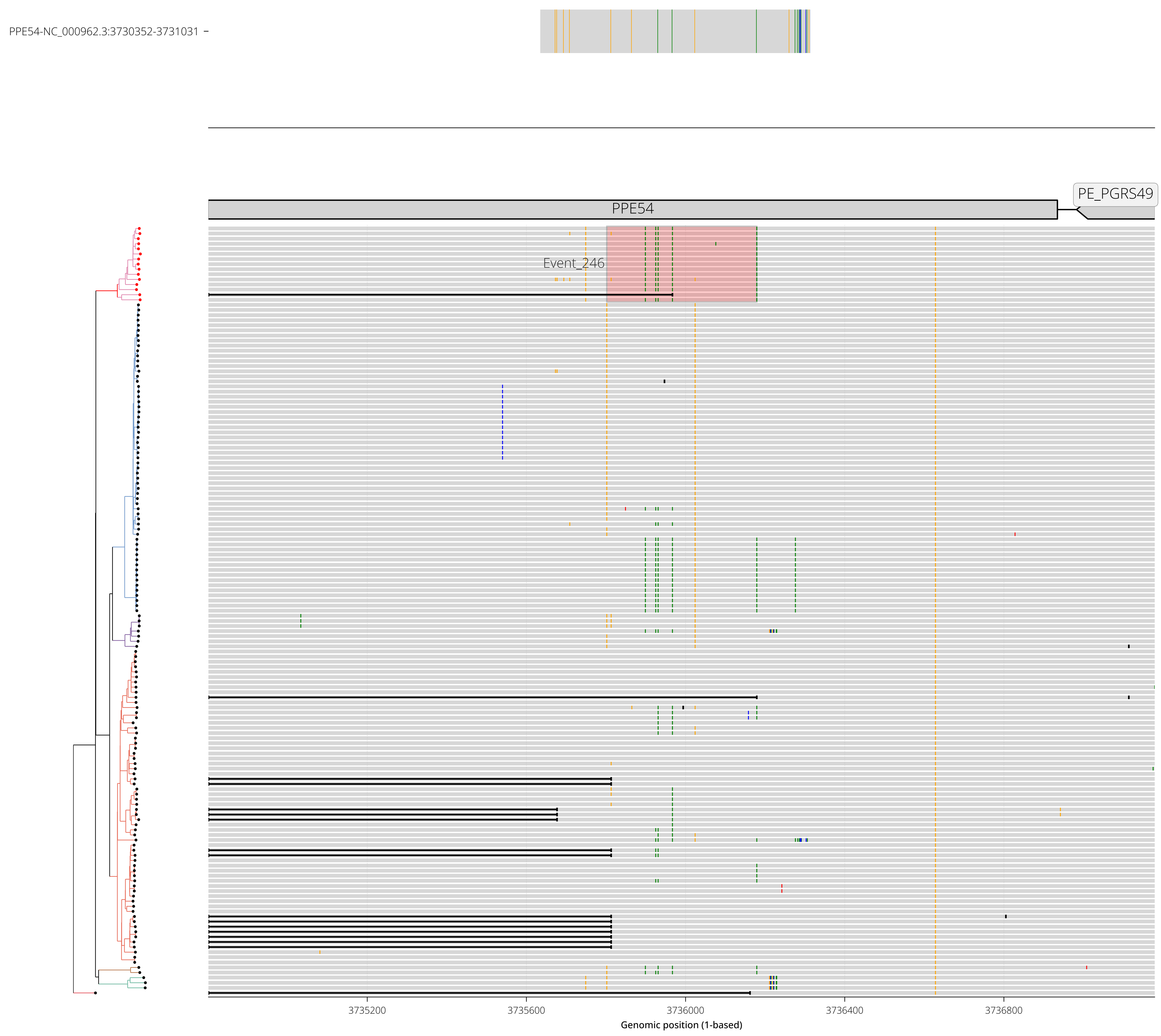

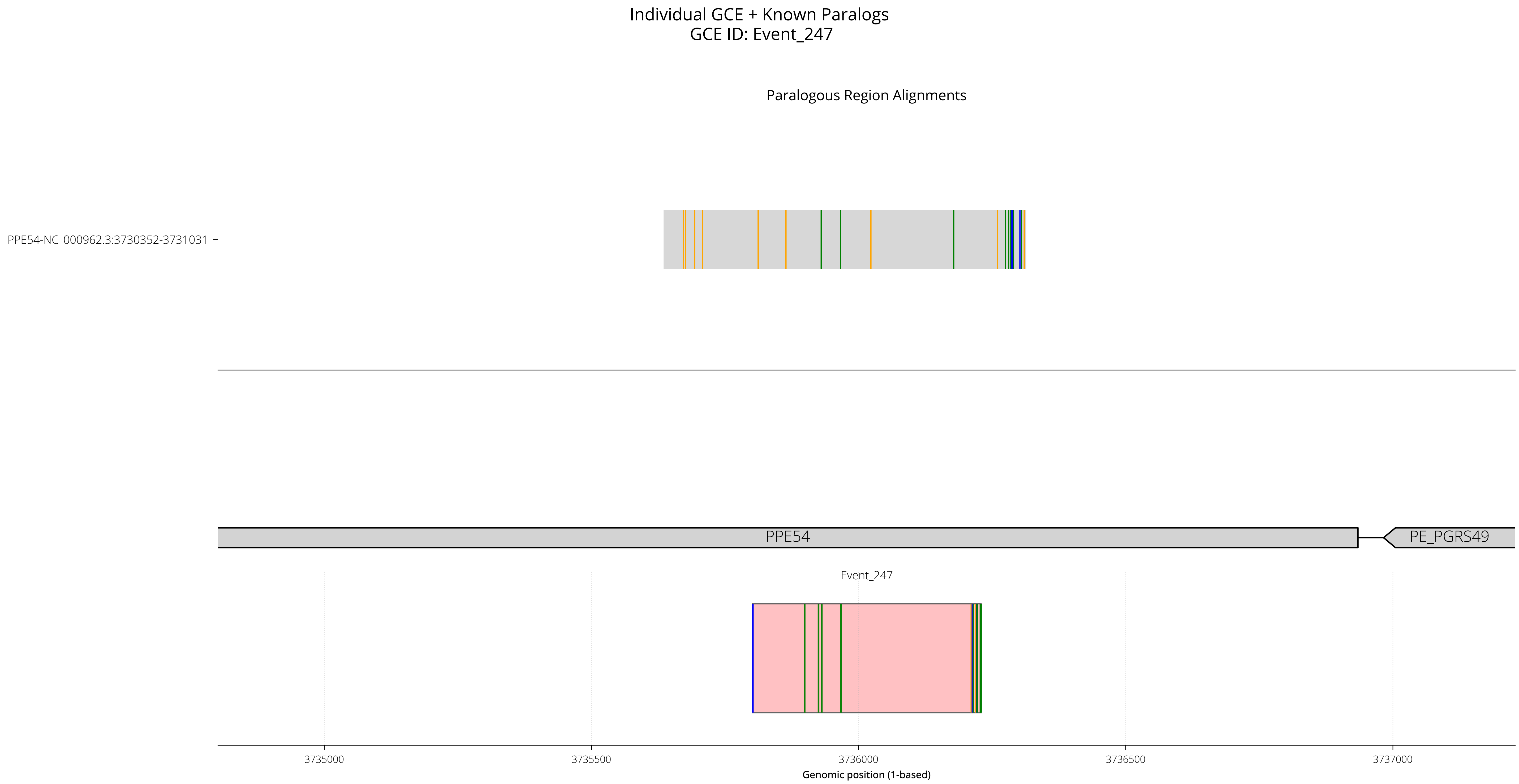

Individual GCE + Known Paralogs
GCE ID: Event_247
Paralogous Region Alignments
PPE54-NC_000962.3:3730352-3731031
PPE54
PE_PGRS49
Event_247
3735000
3735500
3736000
3736500
3737000
Genomic position (1-based)

# Circos Viz - GCE to Known Paralogs Locations
GCE ID: Event_247

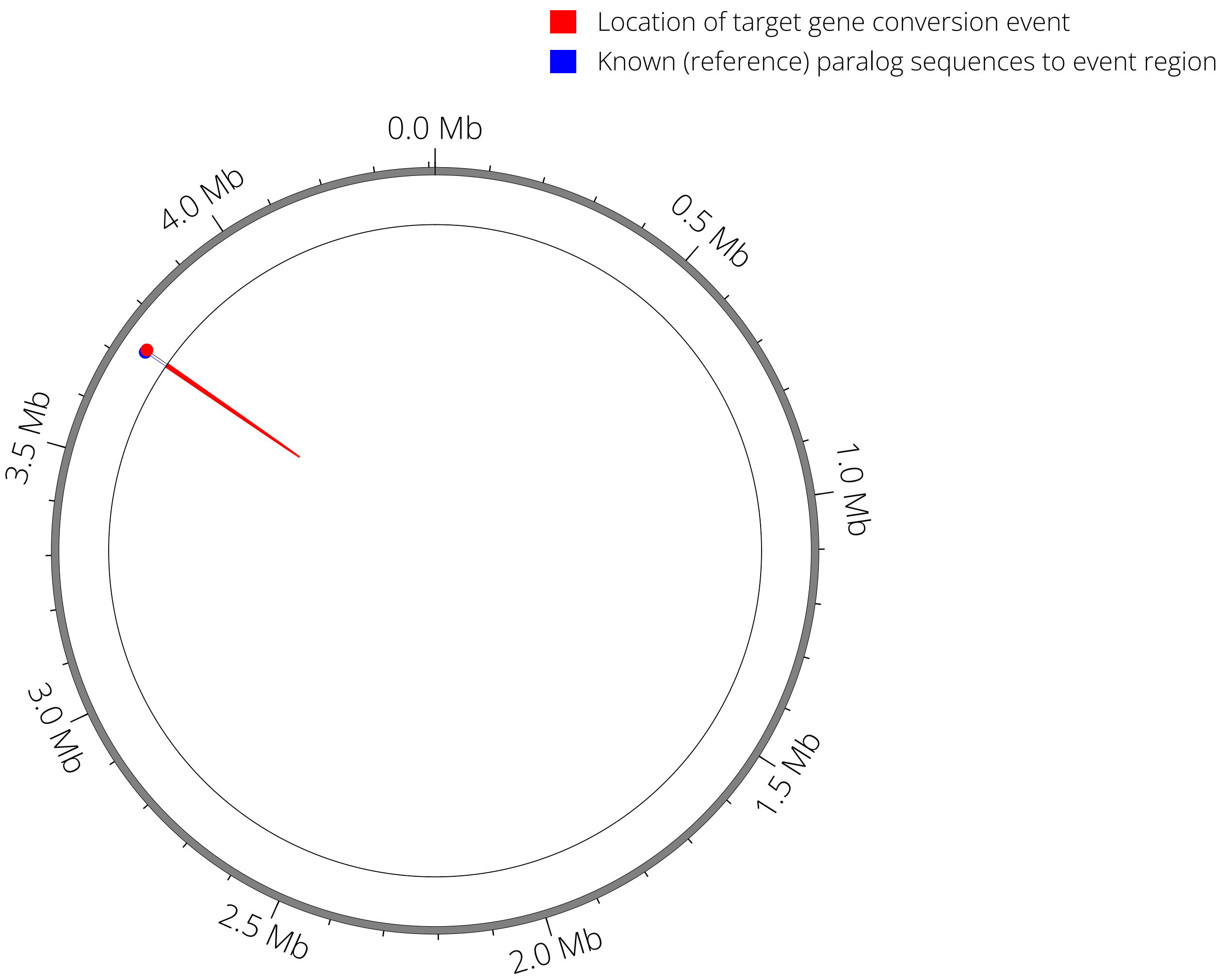

# Event_247 - 13 SNPs - k-mer analysis relative to paralogs

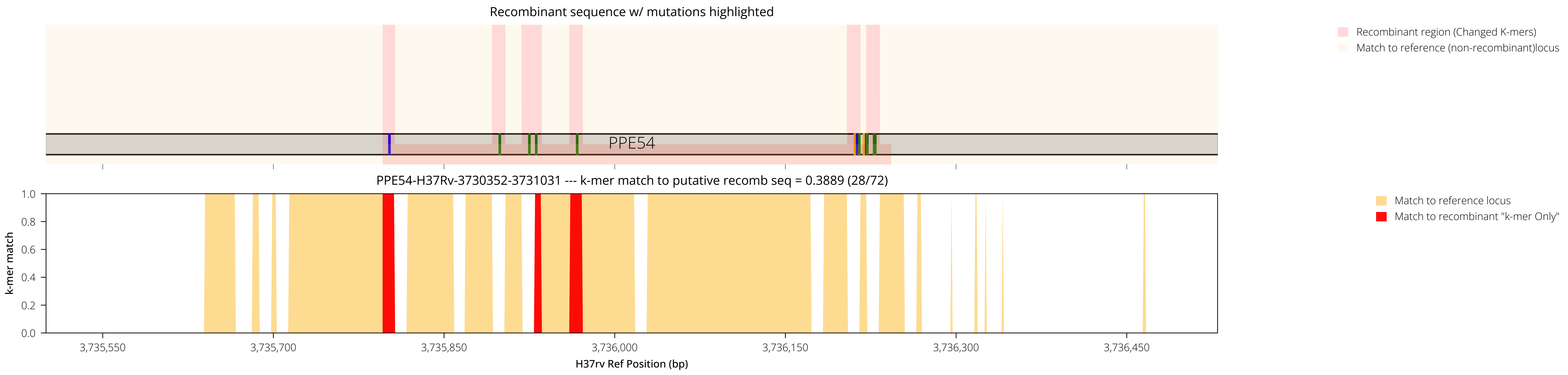

Sub-phylogeny Viz + Paralog Alignments
GCE ID: Event_247

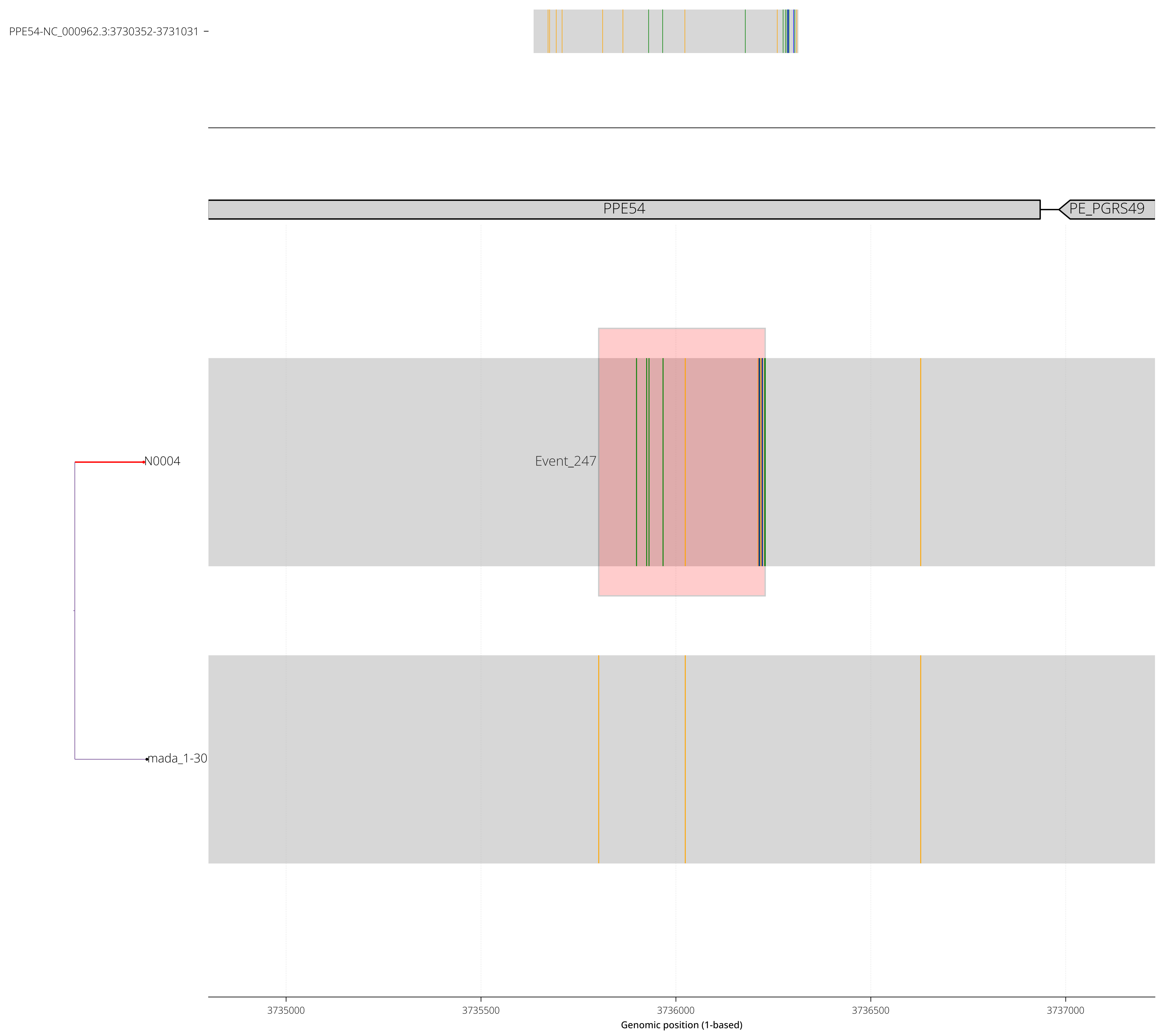

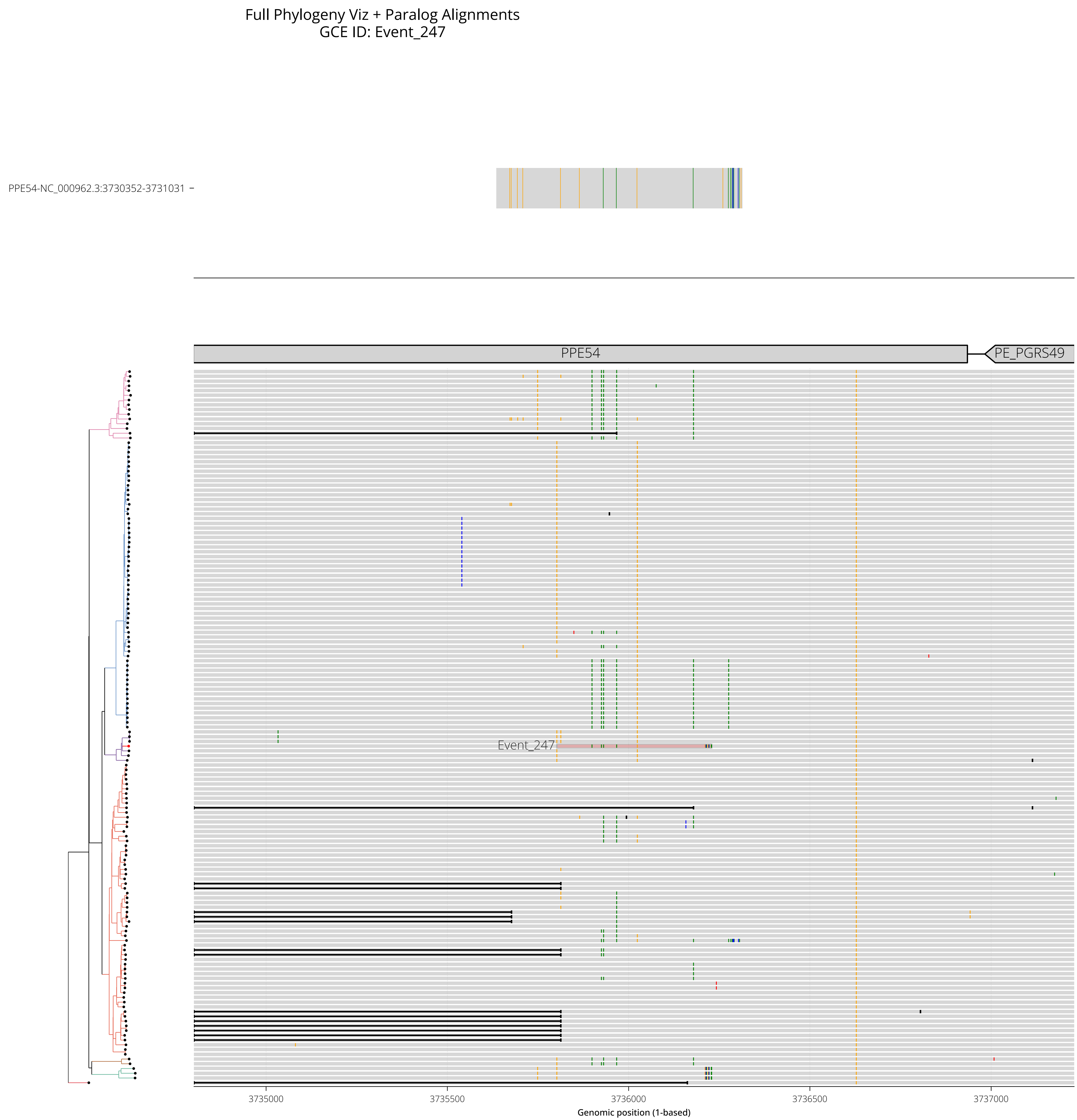
Full Phylogeny Viz + Paralog Alignments
GCE ID: Event_247
PPE54-NC_000962.3:3730352-3731031
PPE54
PE_PGRS49
Event_247
3735000
3735500
3736000
3736500
3737000
Genomic position (1-based)

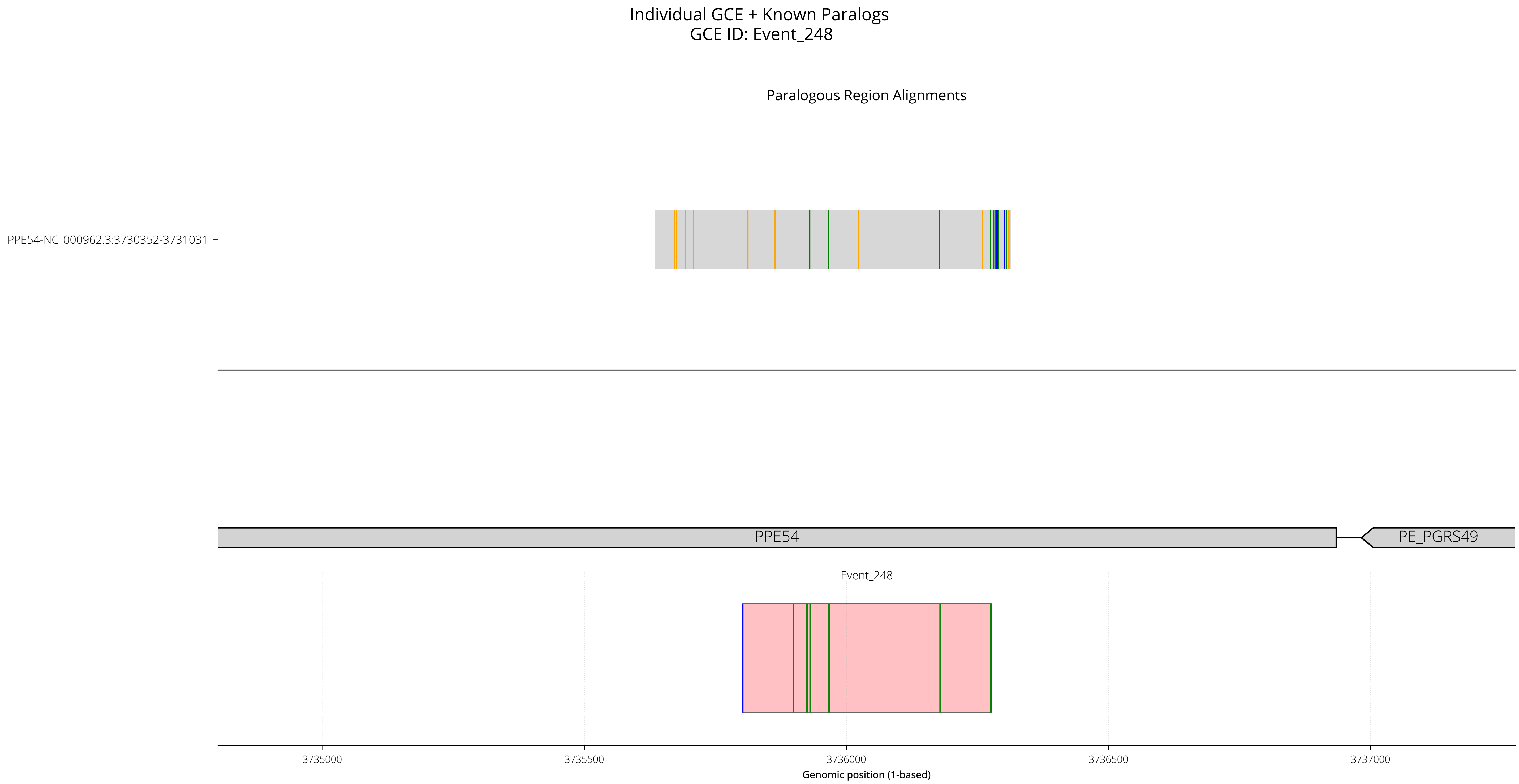
Individual GCE + Known Paralogs
GCE ID: Event_248
Paralogous Region Alignments
PPE54-NC_000962.3:3730352-3731031
PPE54
PE_PGRS49
Event_248
3735000
3735500
3736000
3736500
3737000
Genomic position (1-based)

Circos Viz - GCE to Known Paralogs Locations
GCE ID: Event_248

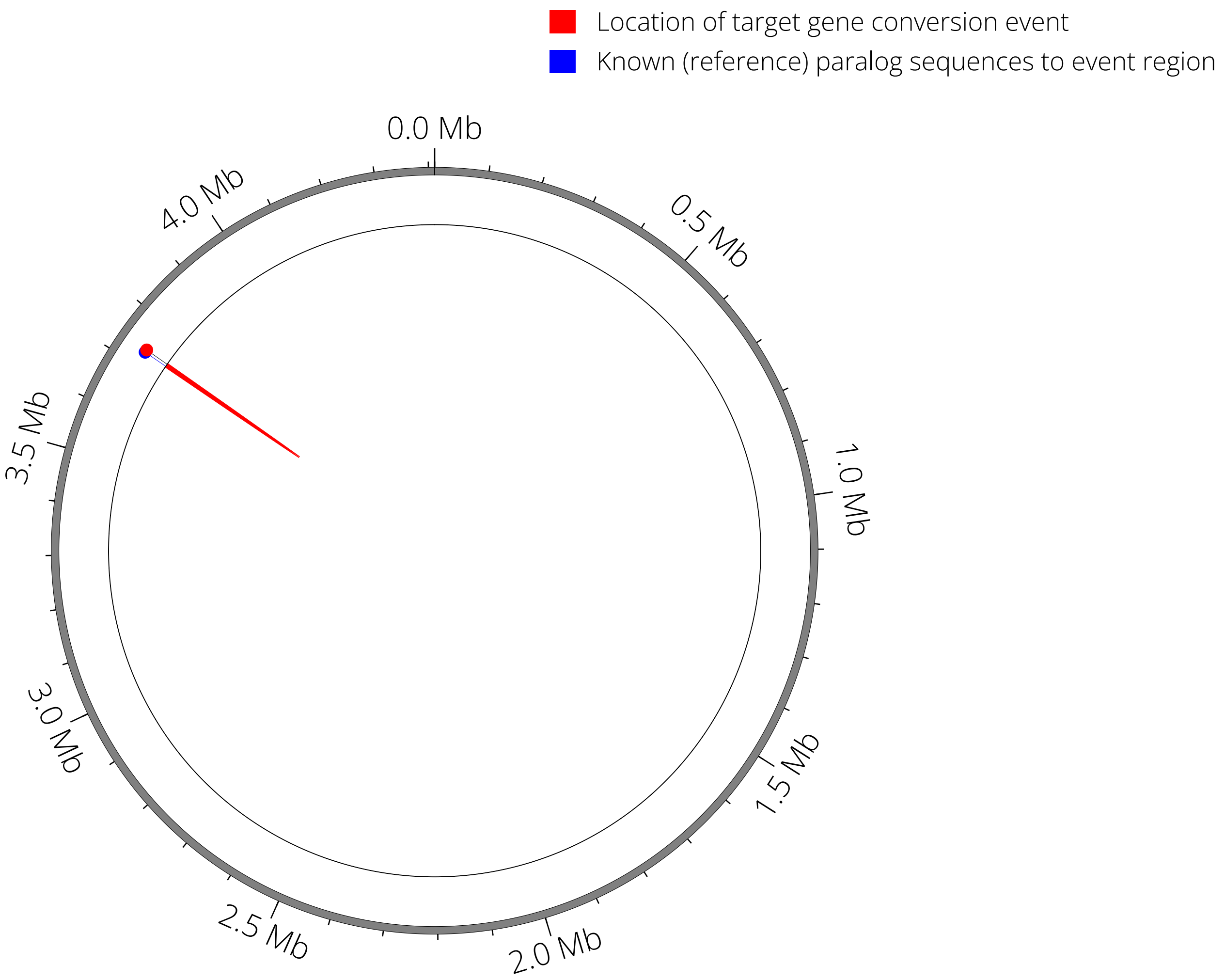

# Event_248 - 7 SNPs - k-mer analysis relative to paralogs

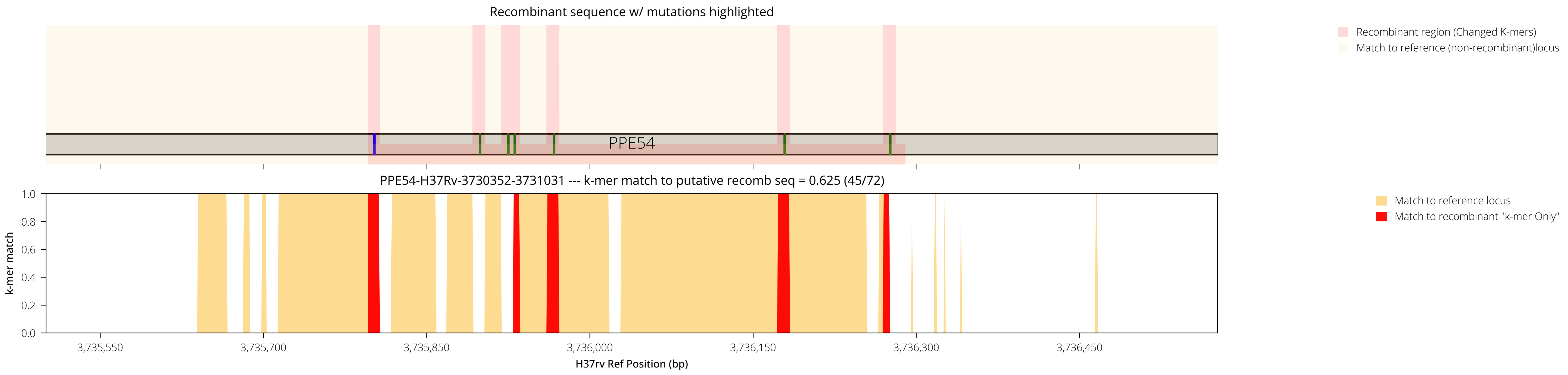

Sub-phylogeny Viz + Paralog Alignments
GCE ID: Event_248

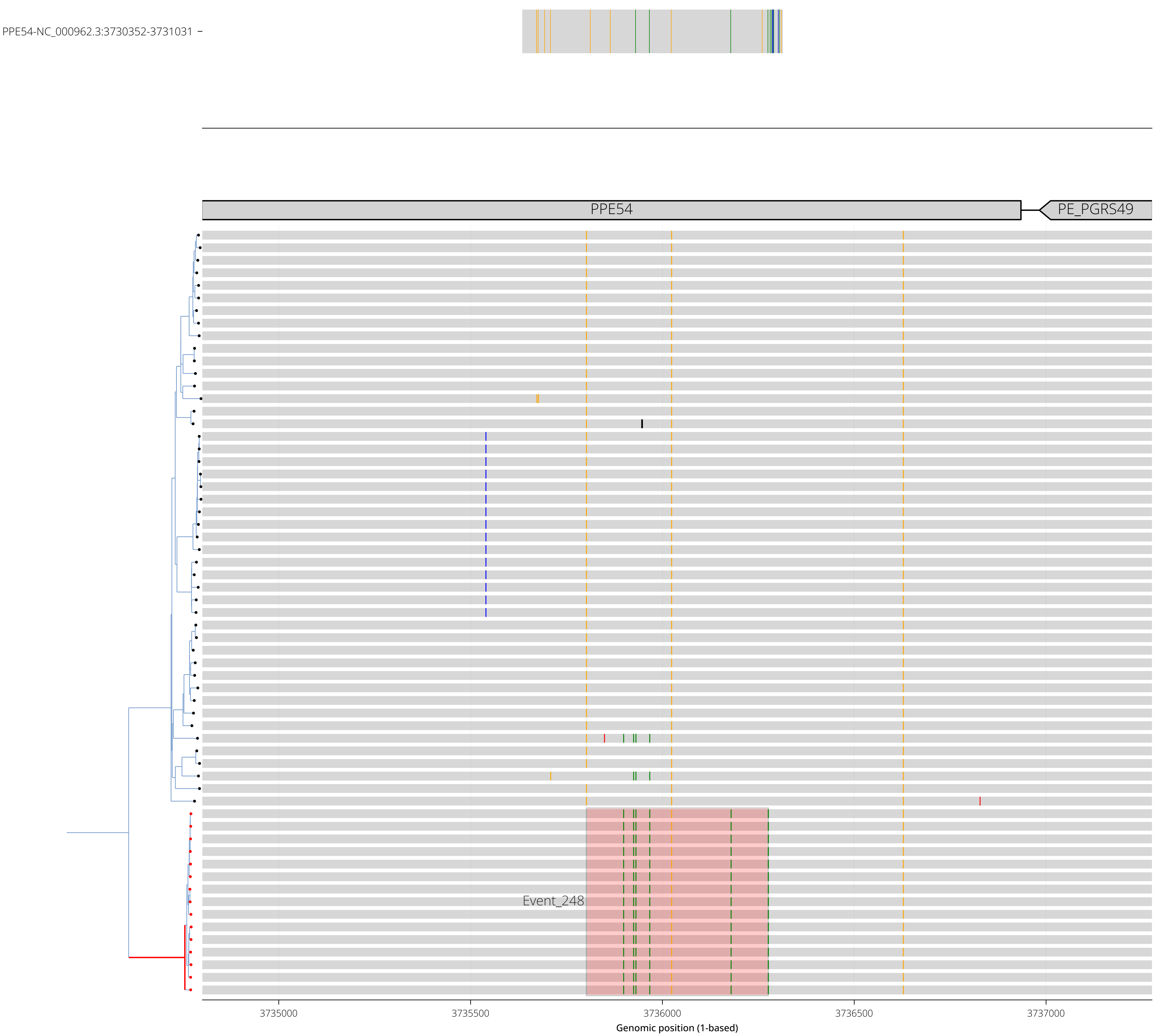

Full Phylogeny Viz + Paralog Alignments
GCE ID: Event_248

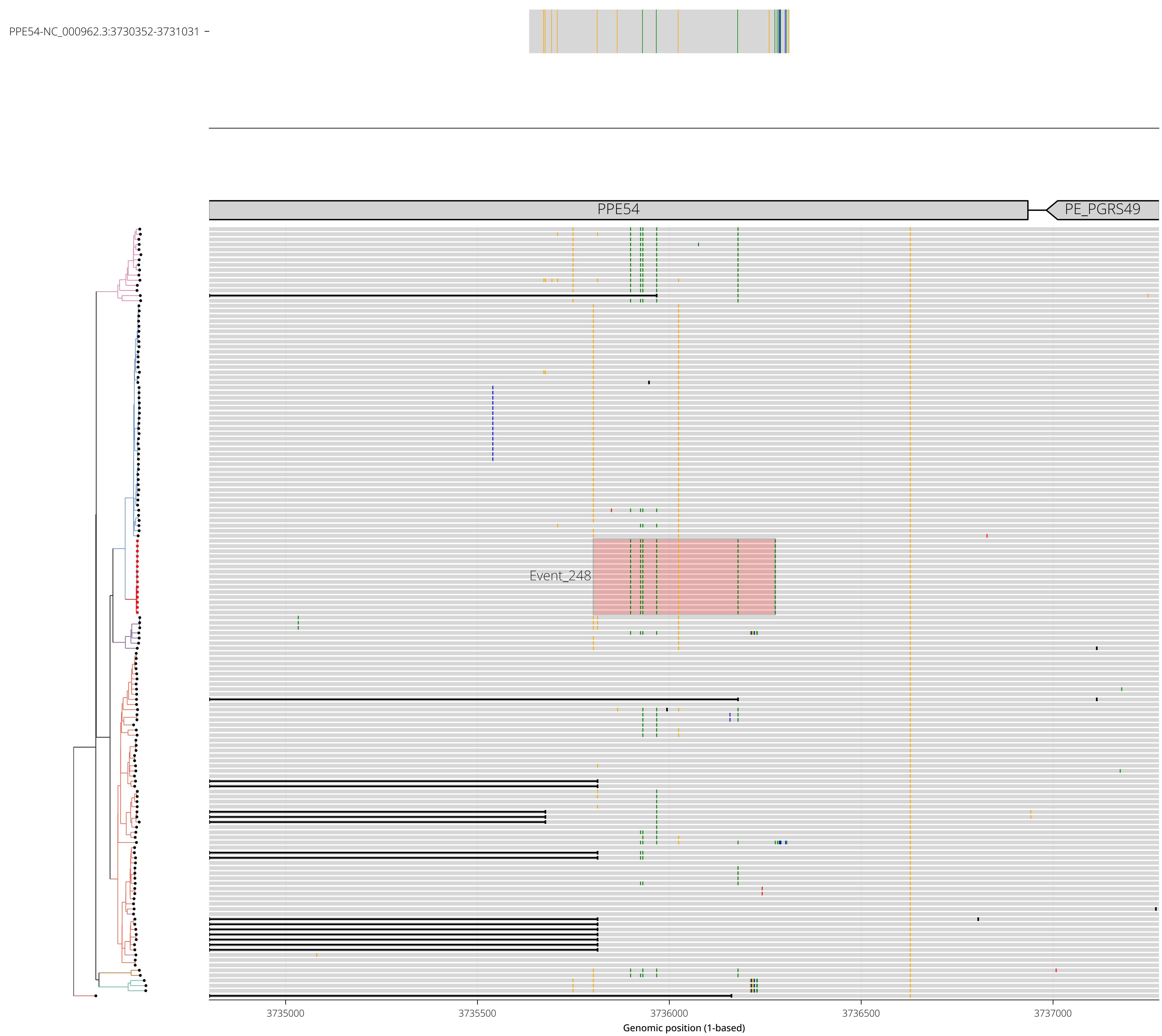

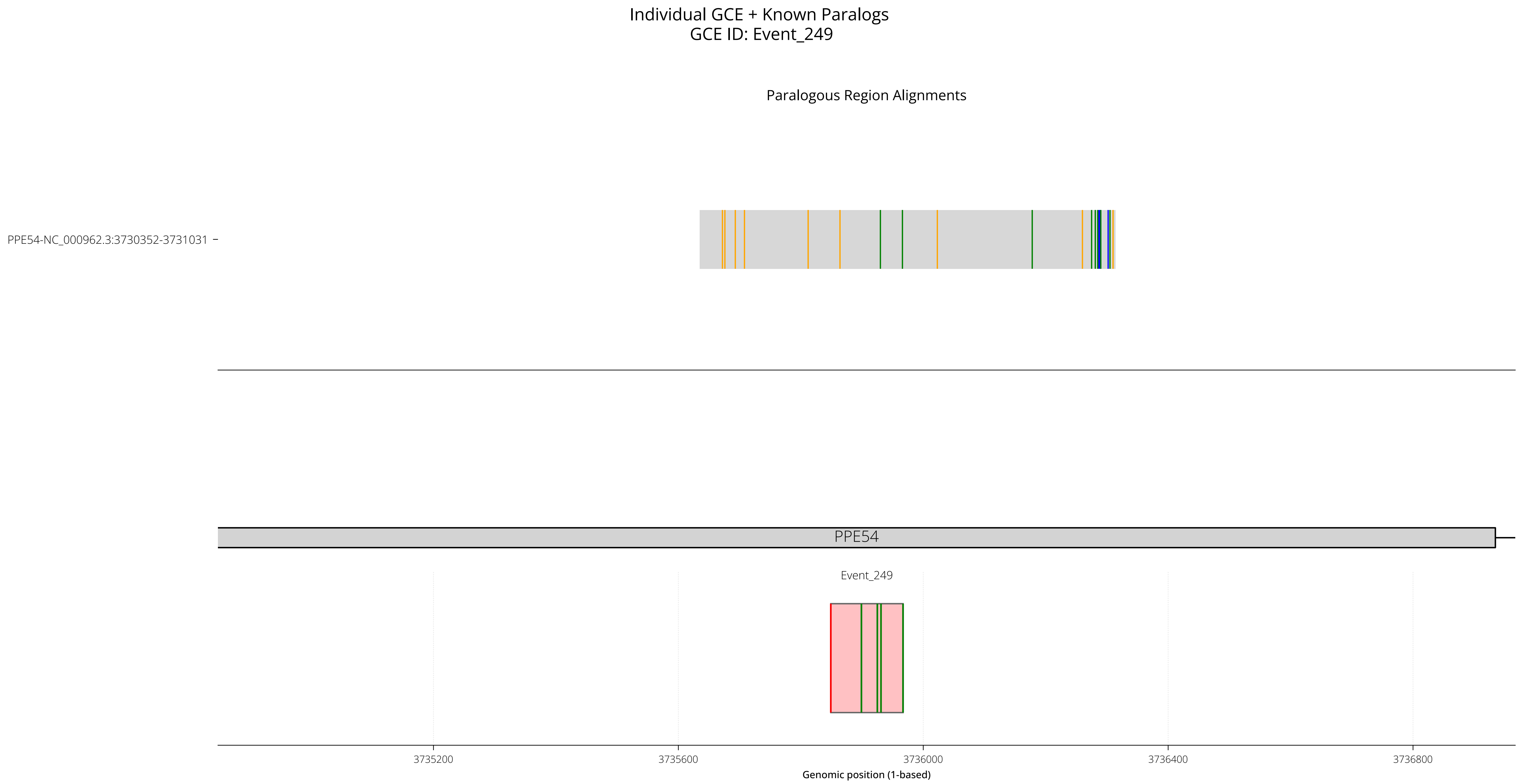
Individual GCE + Known Paralogs
GCE ID: Event_249
Paralogous Region Alignments
PPE54-NC_000962.3:3730352-3731031
PPE54
Event_249
3735200
3735600
3736000
3736400
3736800
Genomic position (1-based)

Circos Viz - GCE to Known Paralogs Locations
GCE ID: Event_249

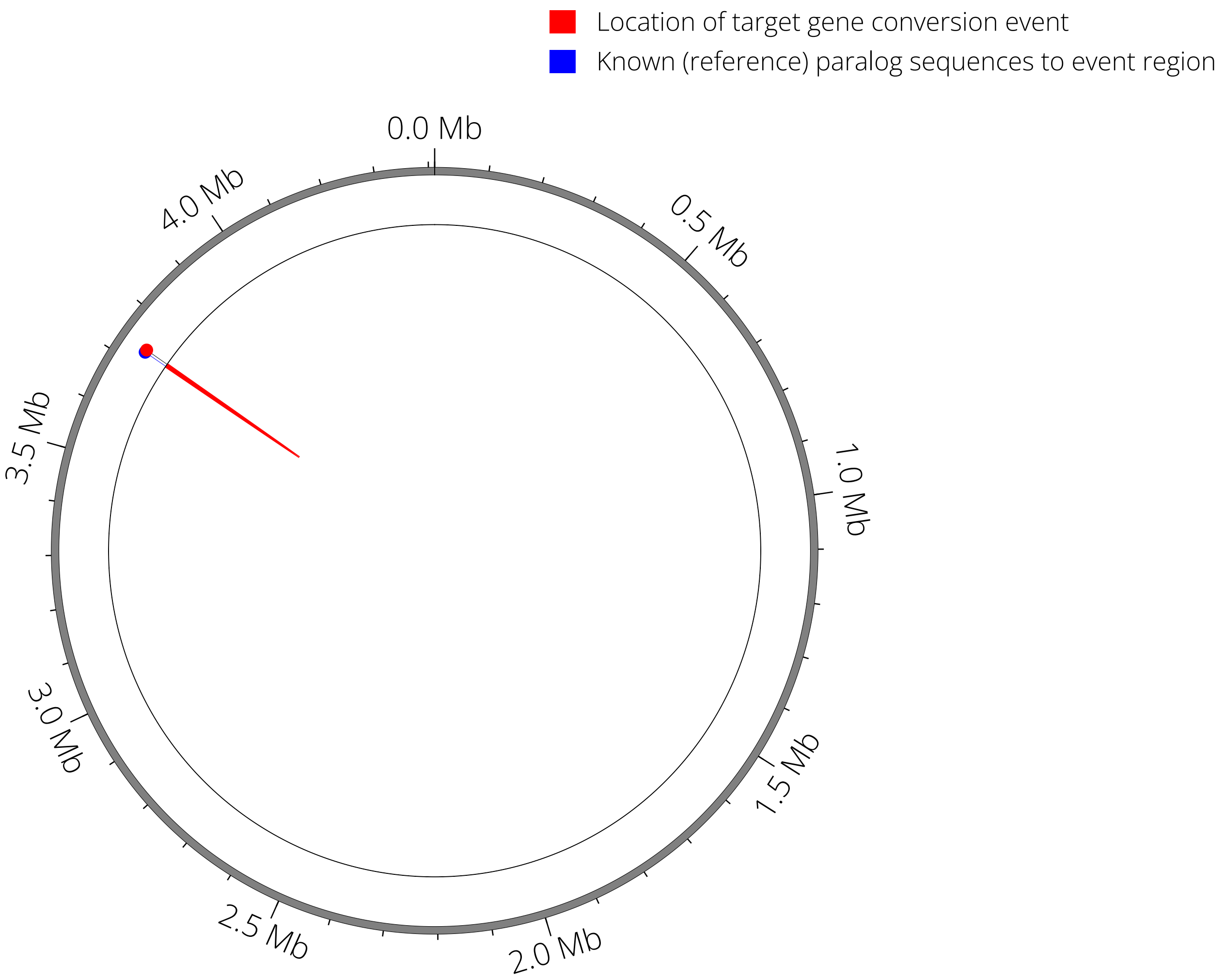

# Event_249 - 5 SNPs - k-mer analysis relative to paralogs

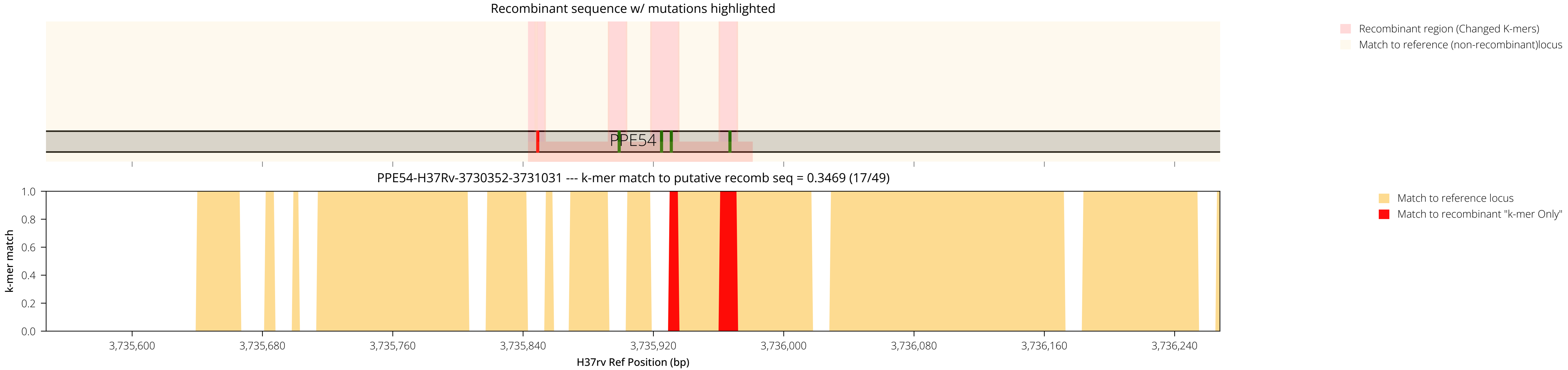

Sub-phylogeny Viz + Paralog Alignments
GCE ID: Event_249

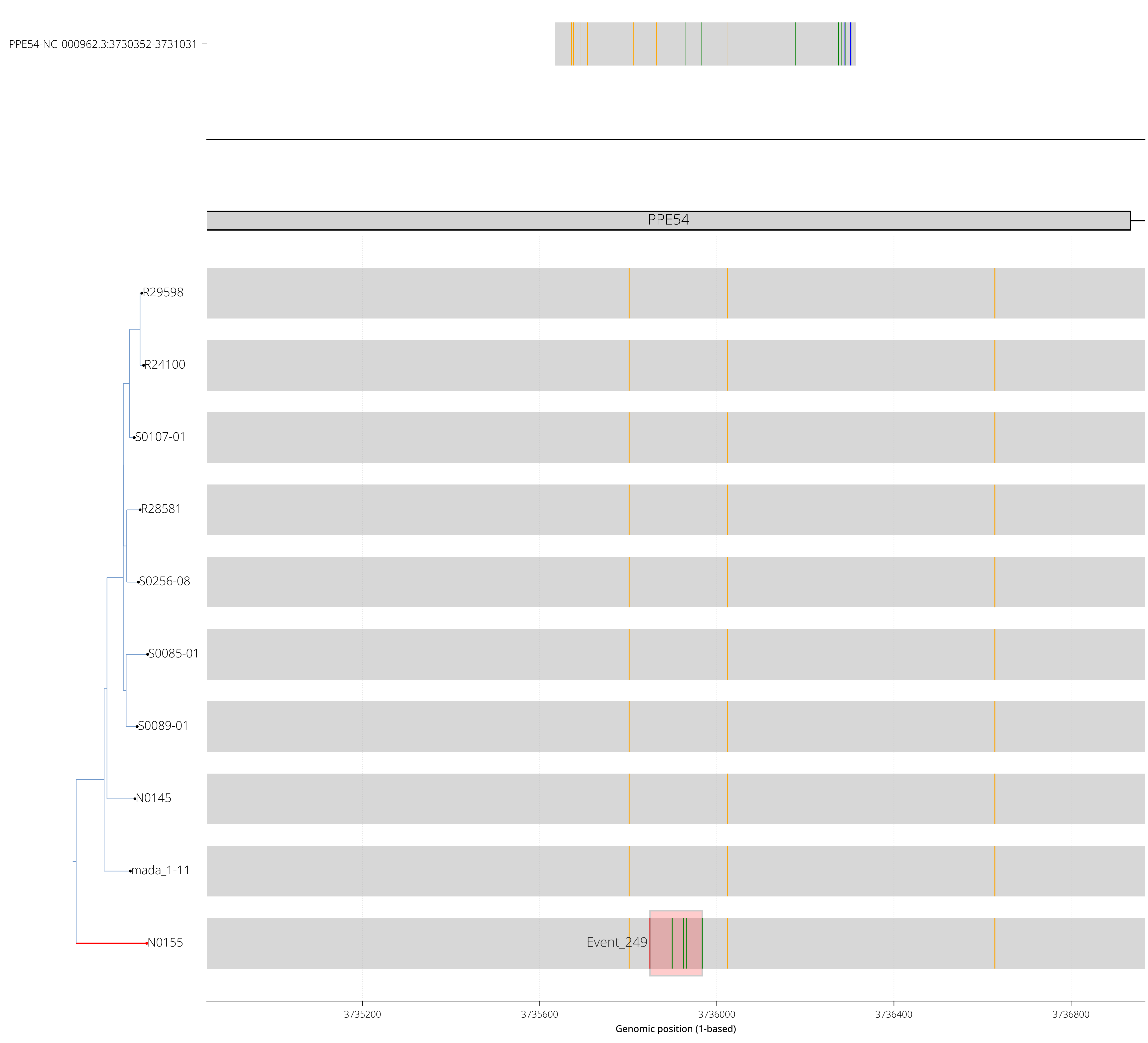

Full Phylogeny Viz + Paralog Alignments
GCE ID: Event_249

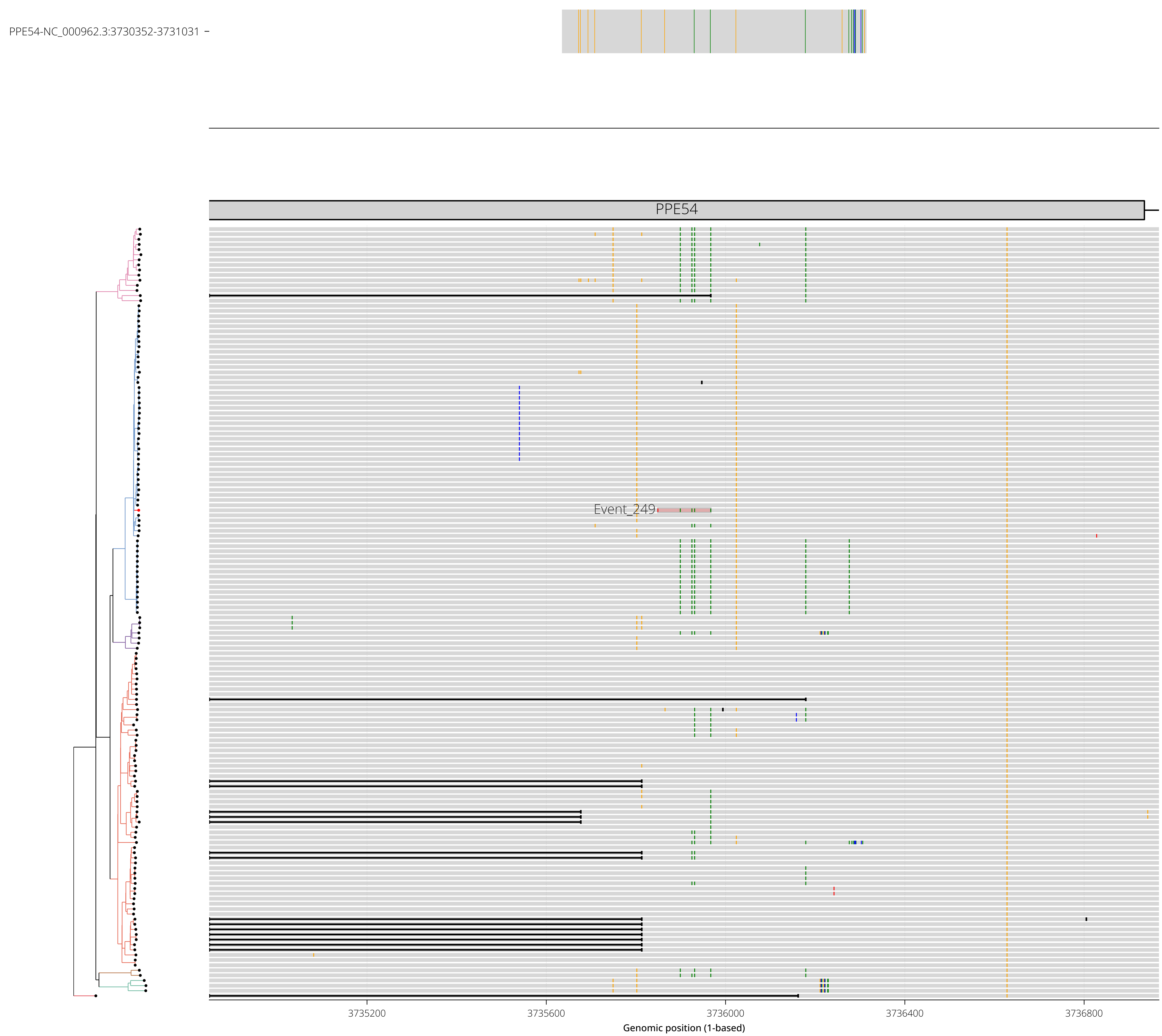

Individual GCE + Known Paralogs
GCE ID: Event_250

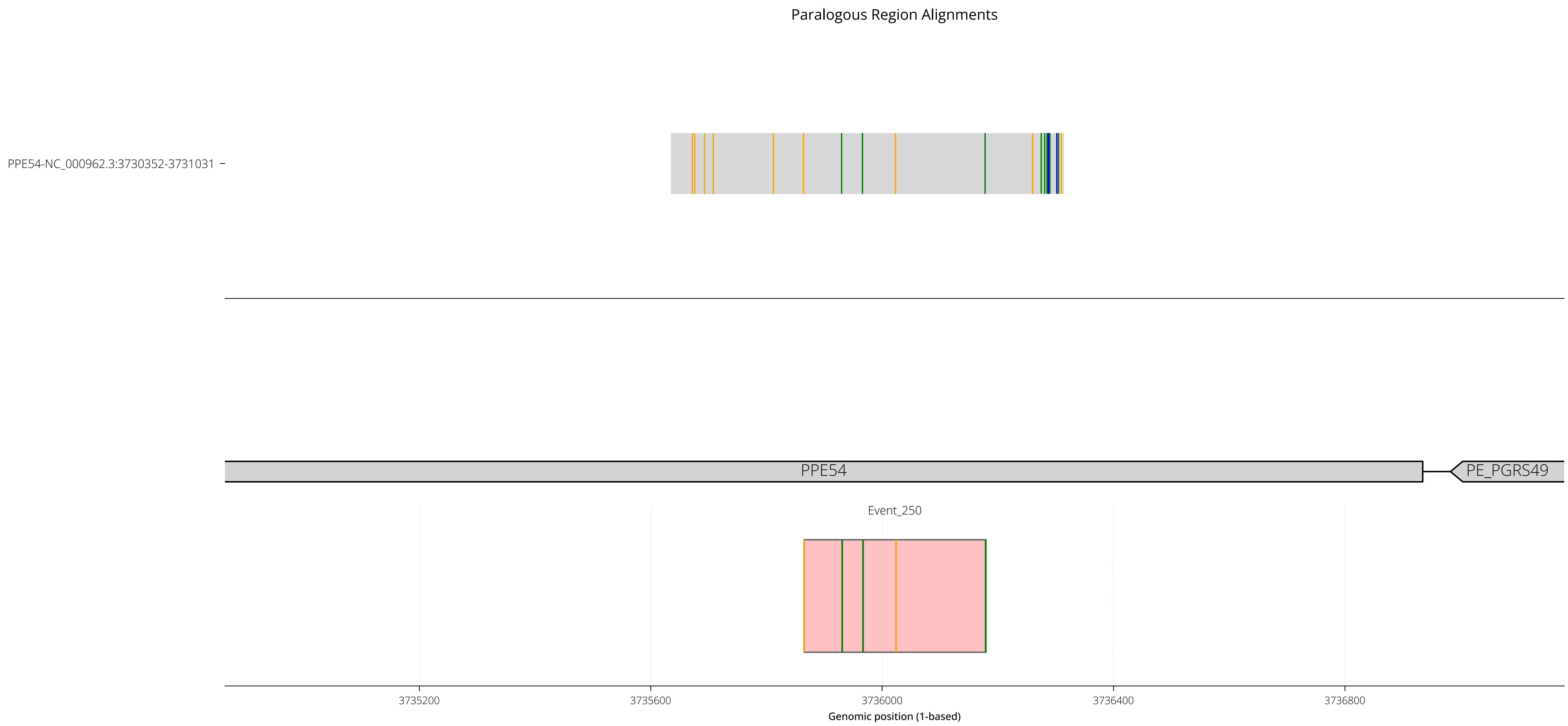

Circos Viz - GCE to Known Paralogs Locations
GCE ID: Event_250

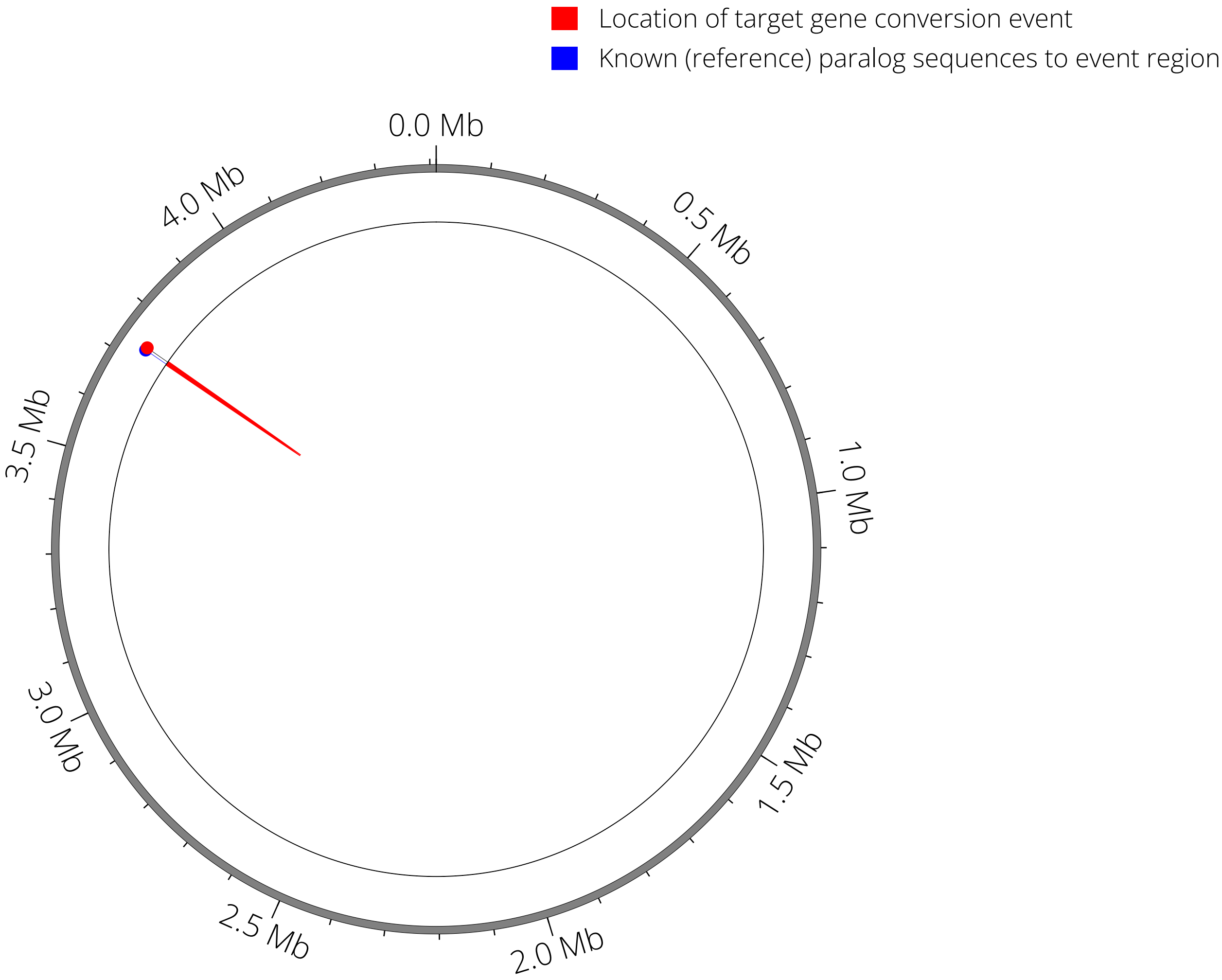

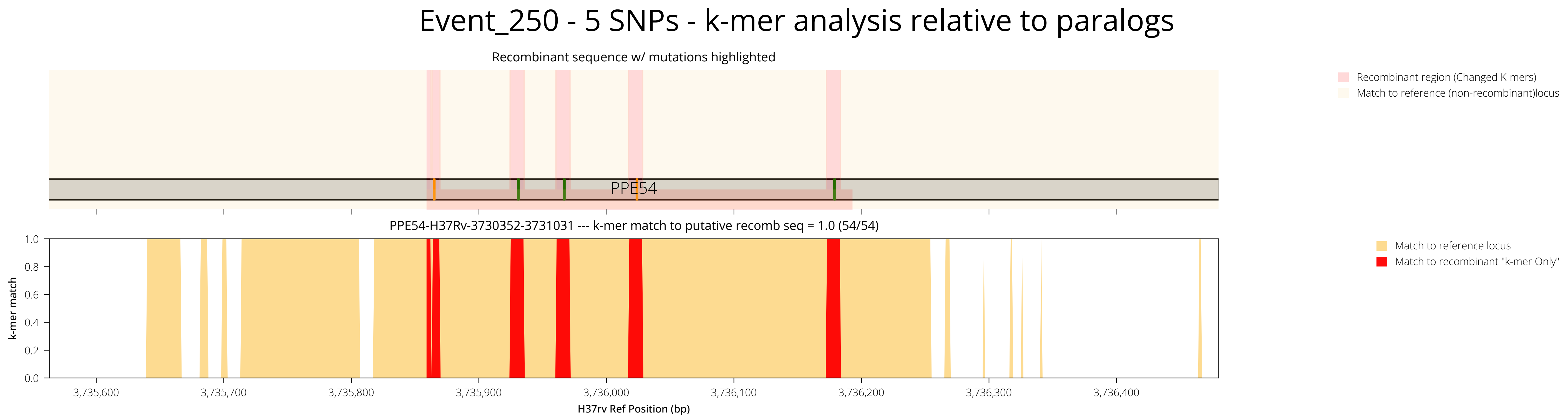

Event_250 - 5 SNPs - k-mer analysis relative to paralogs
Recombinant sequence w/ mutations highlighted
PPE54
Recombinant region (Changed K-mers)
Match to reference (non-recombinant)locus
PPE54-H37Rv-3730352-3731031 --- k-mer match to putative recomb seq = 1.0 (54/54)
k-mer match
1.0
0.8
0.6
0.4
0.2
0.0
3,735,600
3,735,700
3,735,800
3,735,900
3,736,000
3,736,100
3,736,200
3,736,300
3,736,400
H37rv Ref Position (bp)
Match to reference locus
Match to recombinant "k-mer Only"

Sub-phylogeny Viz + Paralog Alignments
GCE ID: Event_250

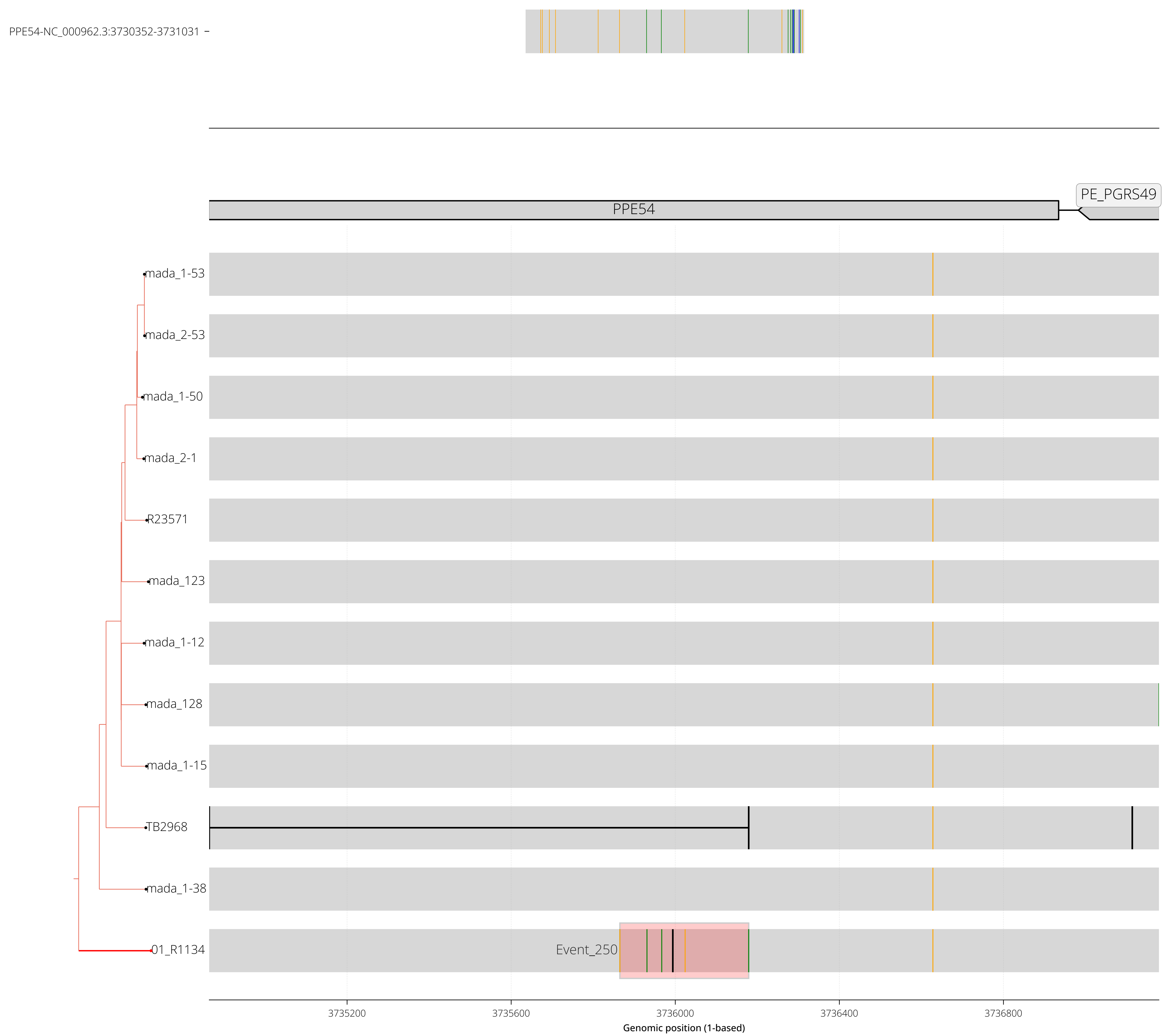

Full Phylogeny Viz + Paralog Alignments
GCE ID: Event_250

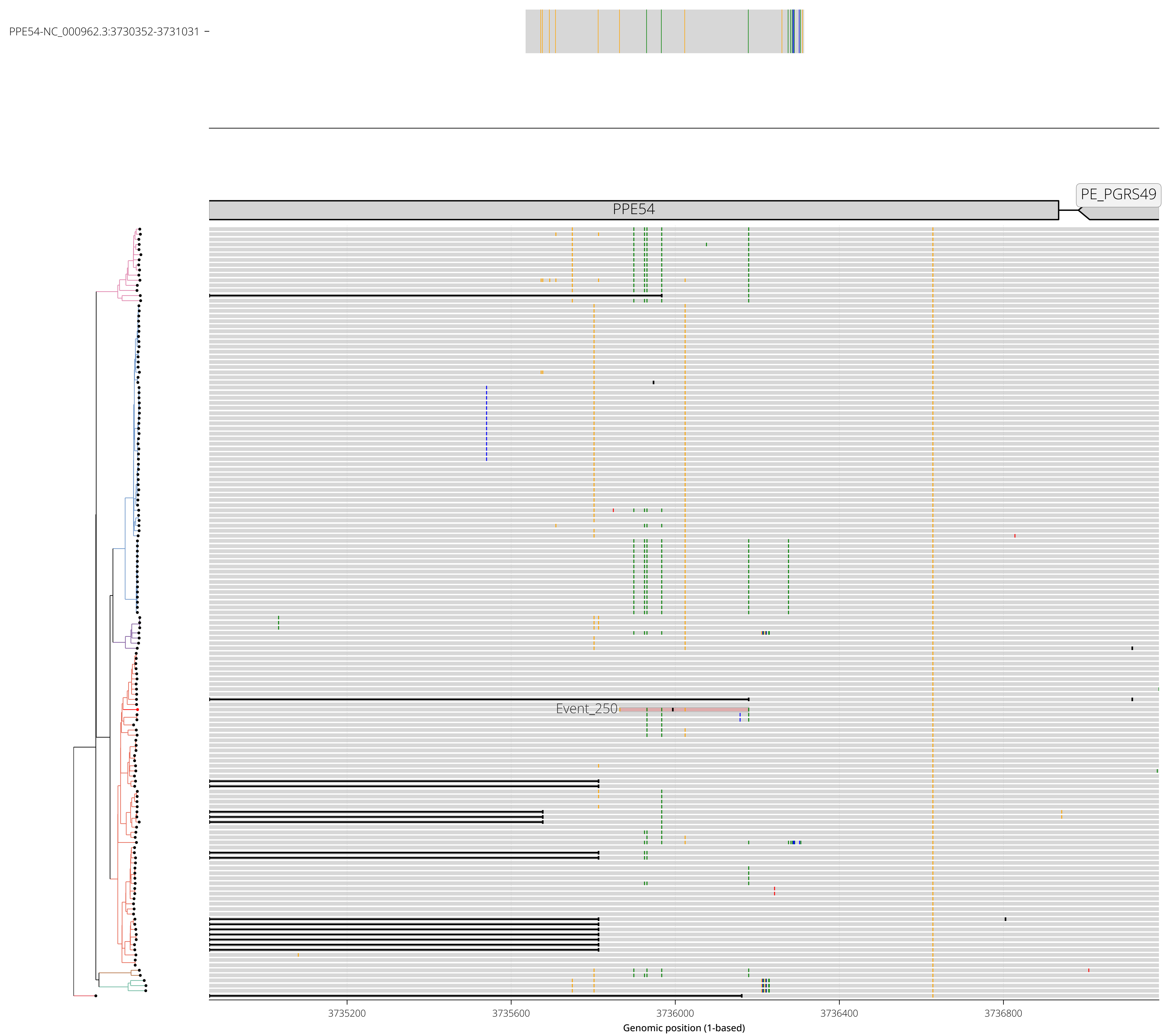

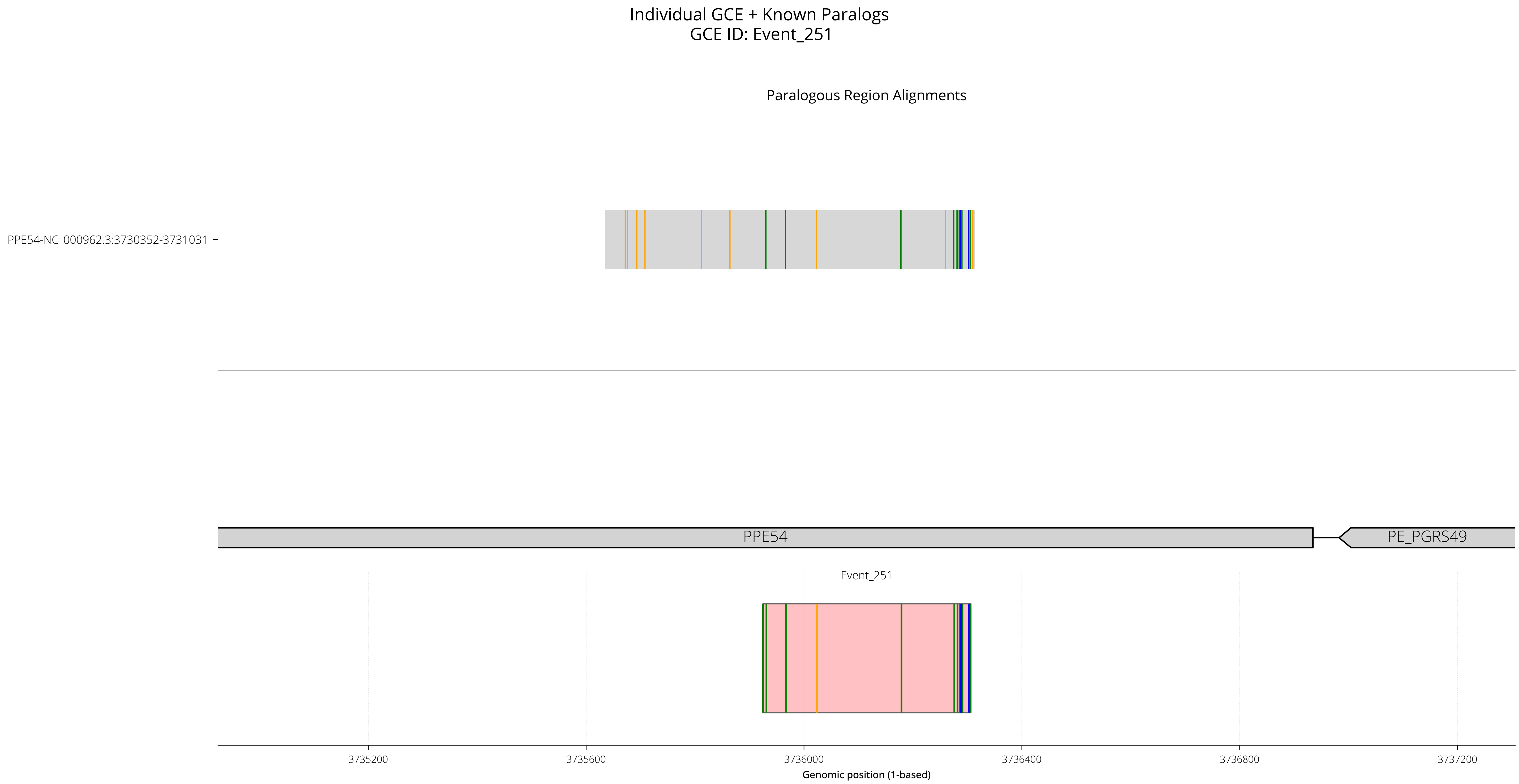

Individual GCE + Known Paralogs
GCE ID: Event_251
Paralogous Region Alignments
PPE54-NC_000962.3:3730352-3731031
PPE54
PE_PGRS49
Event_251
3735200
3735600
3736000
3736400
3736800
3737200
Genomic position (1-based)

Circos Viz - GCE to Known Paralogs Locations
GCE ID: Event_251

Location of target gene conversion event
Known (reference) paralog sequences to event region

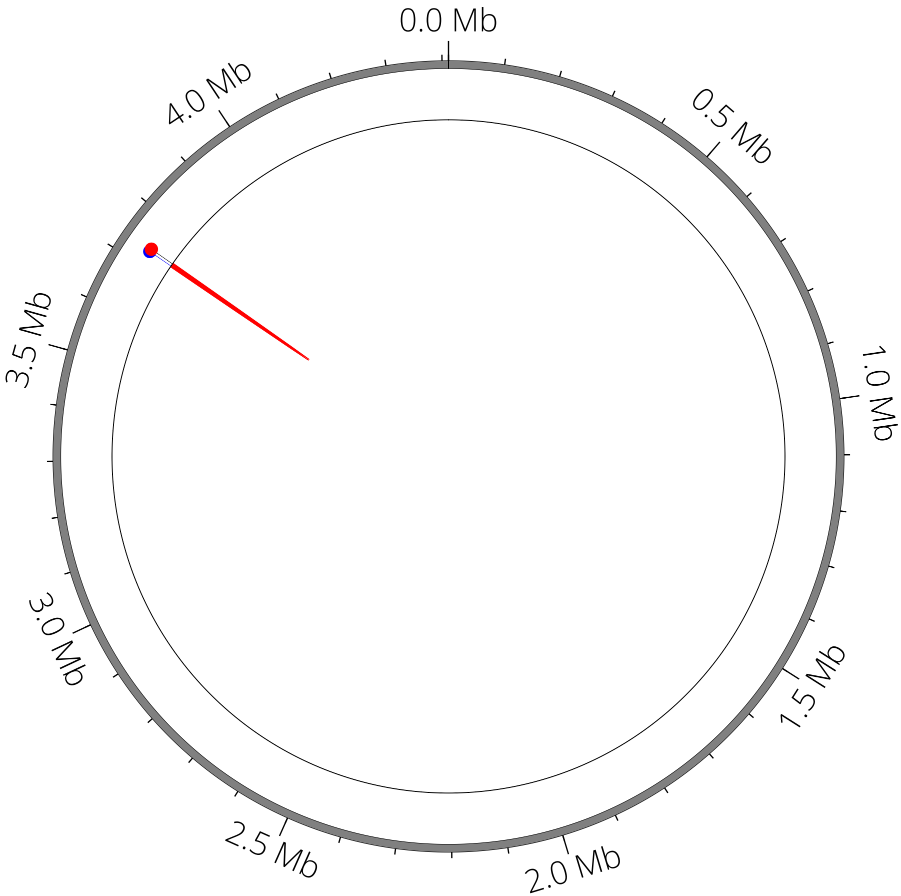

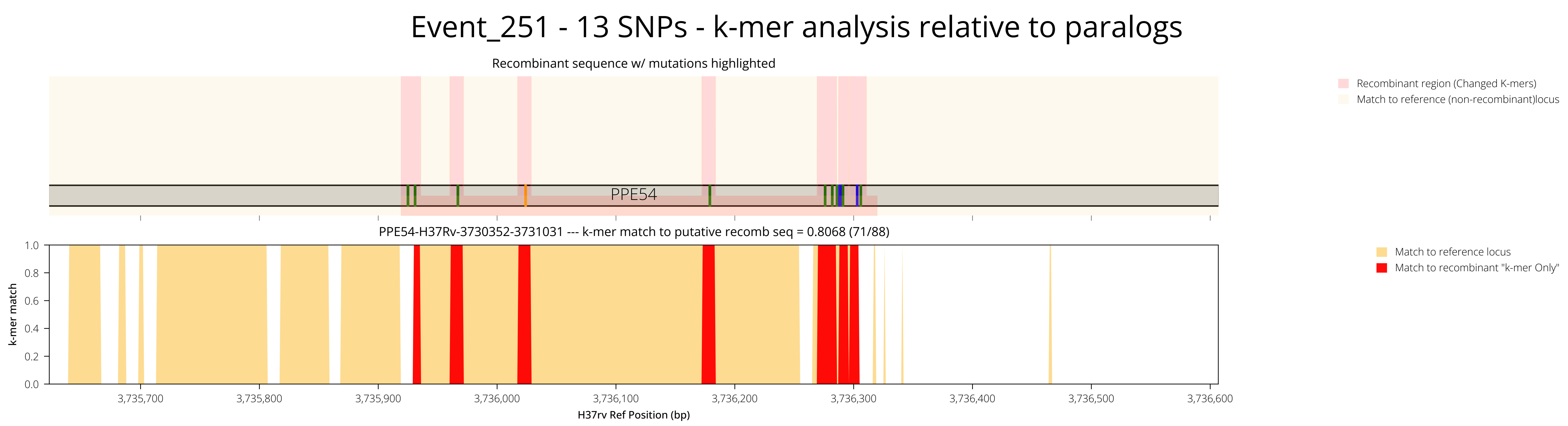

Event_251 - 13 SNPs - k-mer analysis relative to paralogs
Recombinant sequence w/ mutations highlighted
Recombinant region (Changed K-mers)
Match to reference (non-recombinant)locus
PPE54
PPE54-H37Rv-3730352-3731031 --- k-mer match to putative recomb seq = 0.8068 (71/88)
Match to reference locus
Match to recombinant "k-mer Only"
k-mer match
1.0
0.8
0.6
0.4
0.2
0.0
3,735,700
3,735,800
3,735,900
3,736,000
3,736,100
3,736,200
3,736,300
3,736,400
3,736,500
3,736,600
H37rv Ref Position (bp)

Sub-phylogeny Viz + Paralog Alignments
GCE ID: Event_251

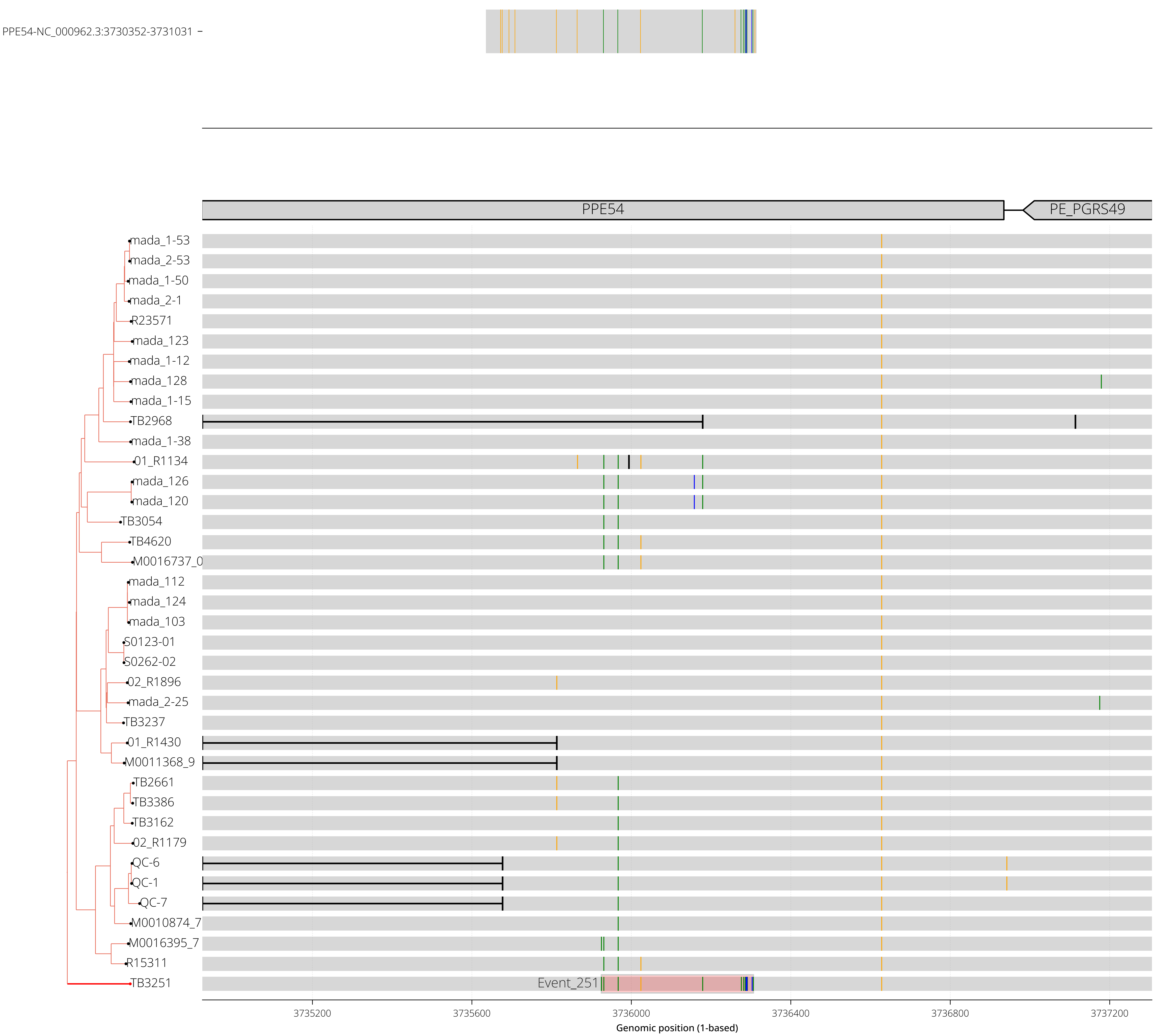

Full Phylogeny Viz + Paralog Alignments
GCE ID: Event_251

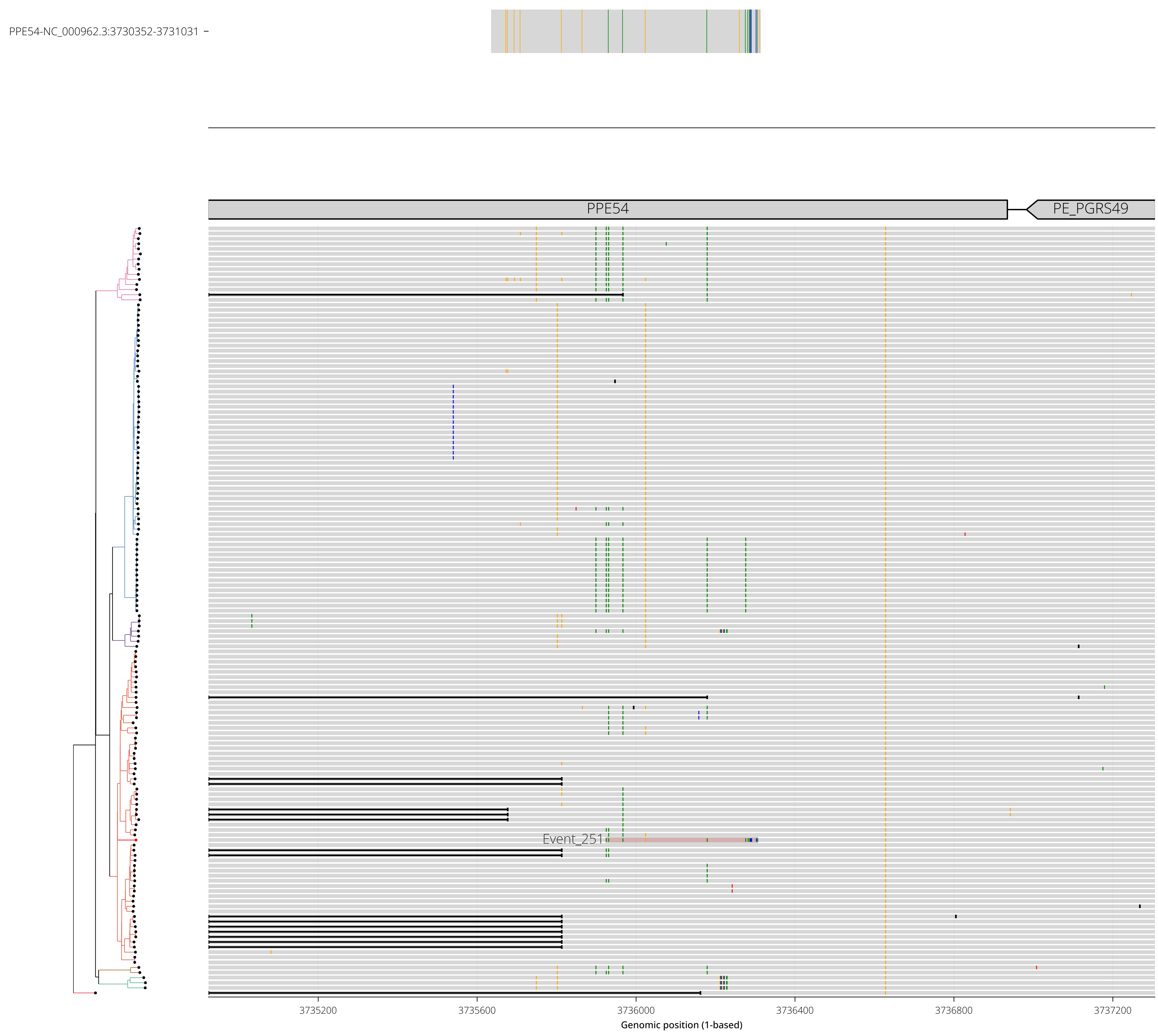

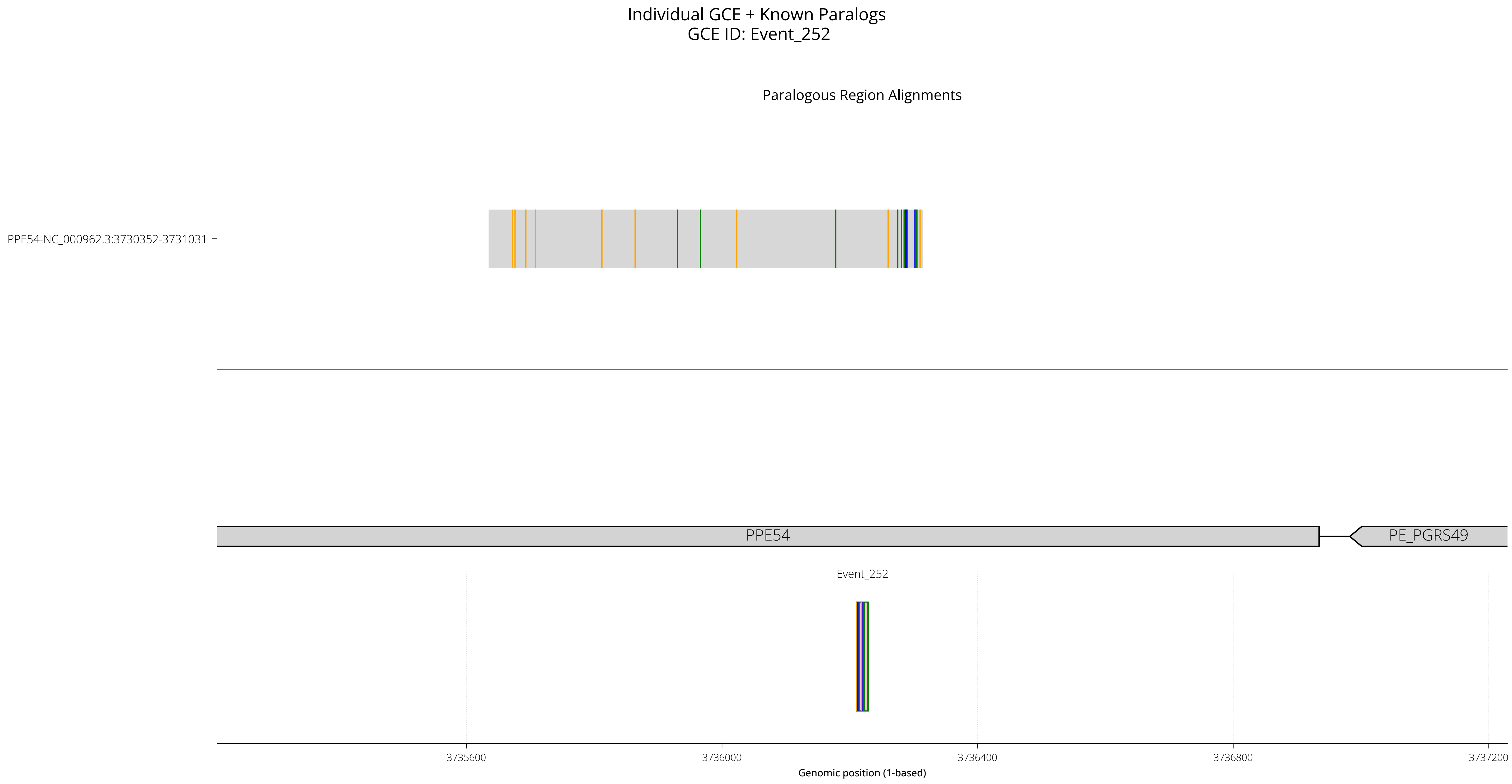

Individual GCE + Known Paralogs
GCE ID: Event_252
Paralogous Region Alignments
PPE54-NC_000962.3:3730352-3731031
PPE54
PE_PGRS49
Event_252
3735600
3736000
3736400
3736800
3737200
Genomic position (1-based)

Circos Viz - GCE to Known Paralogs Locations
GCE ID: Event_252

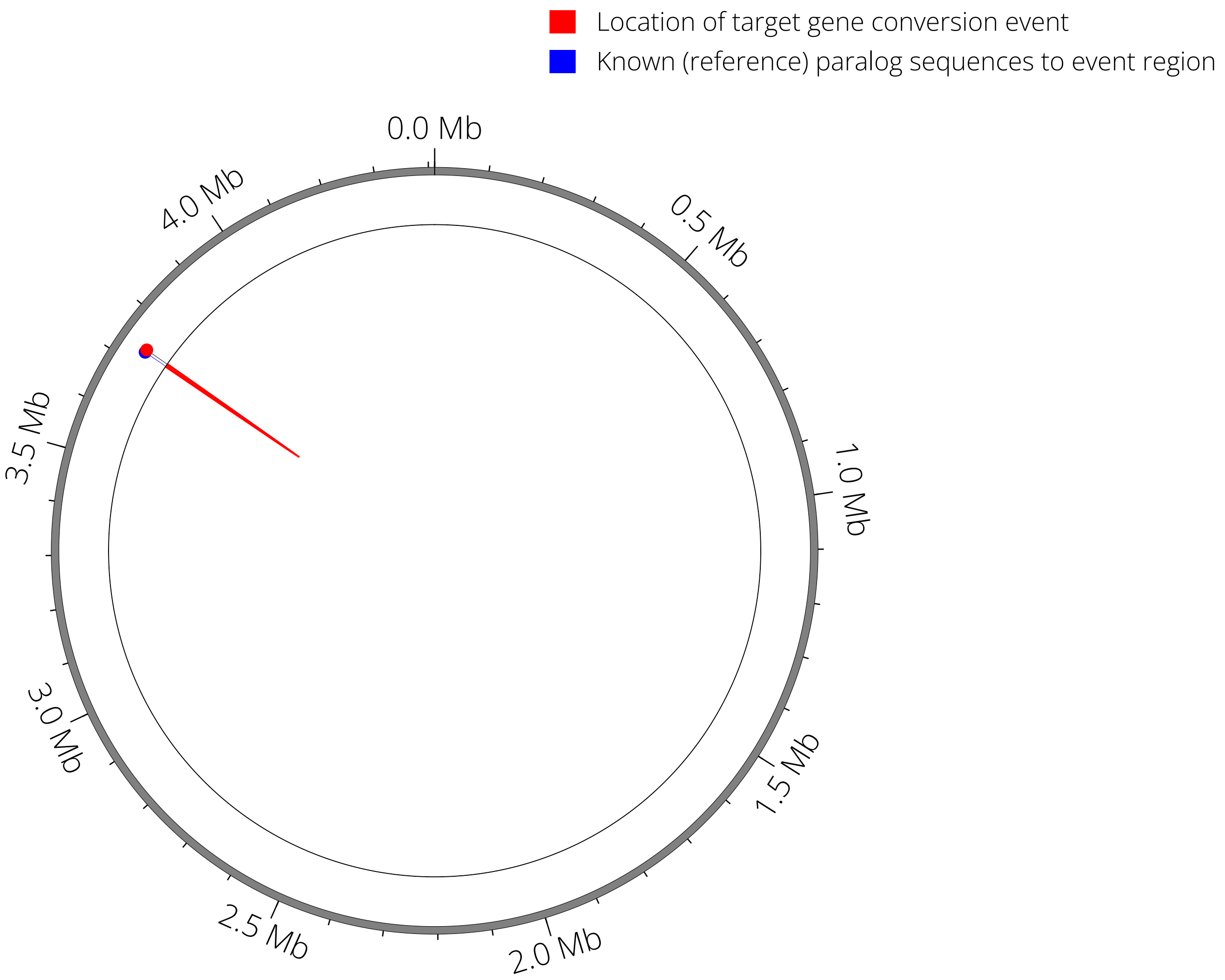

# Event_252 - 8 SNPs - k-mer analysis relative to paralogs

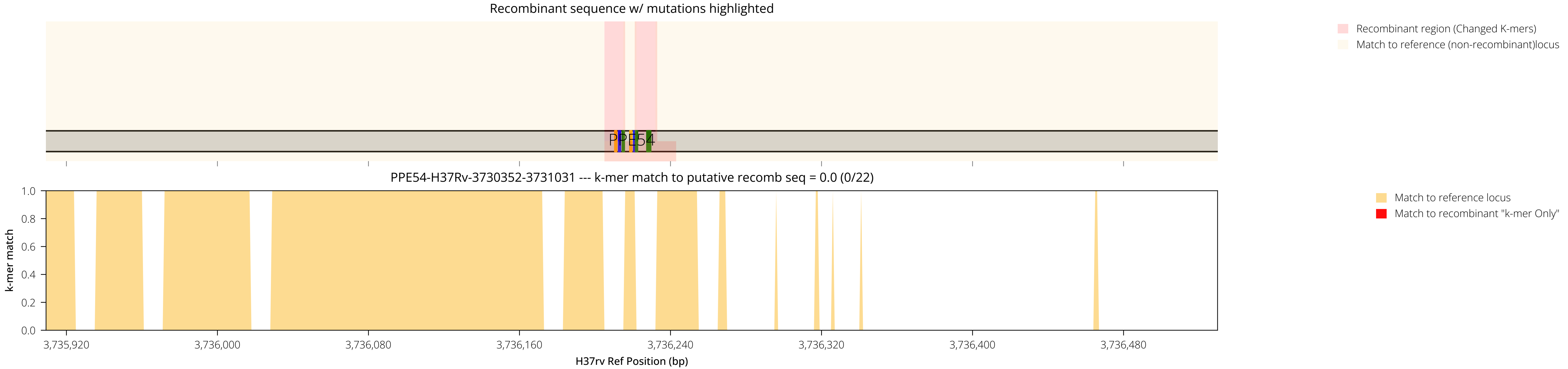

Sub-phylogeny Viz + Paralog Alignments
GCE ID: Event_252

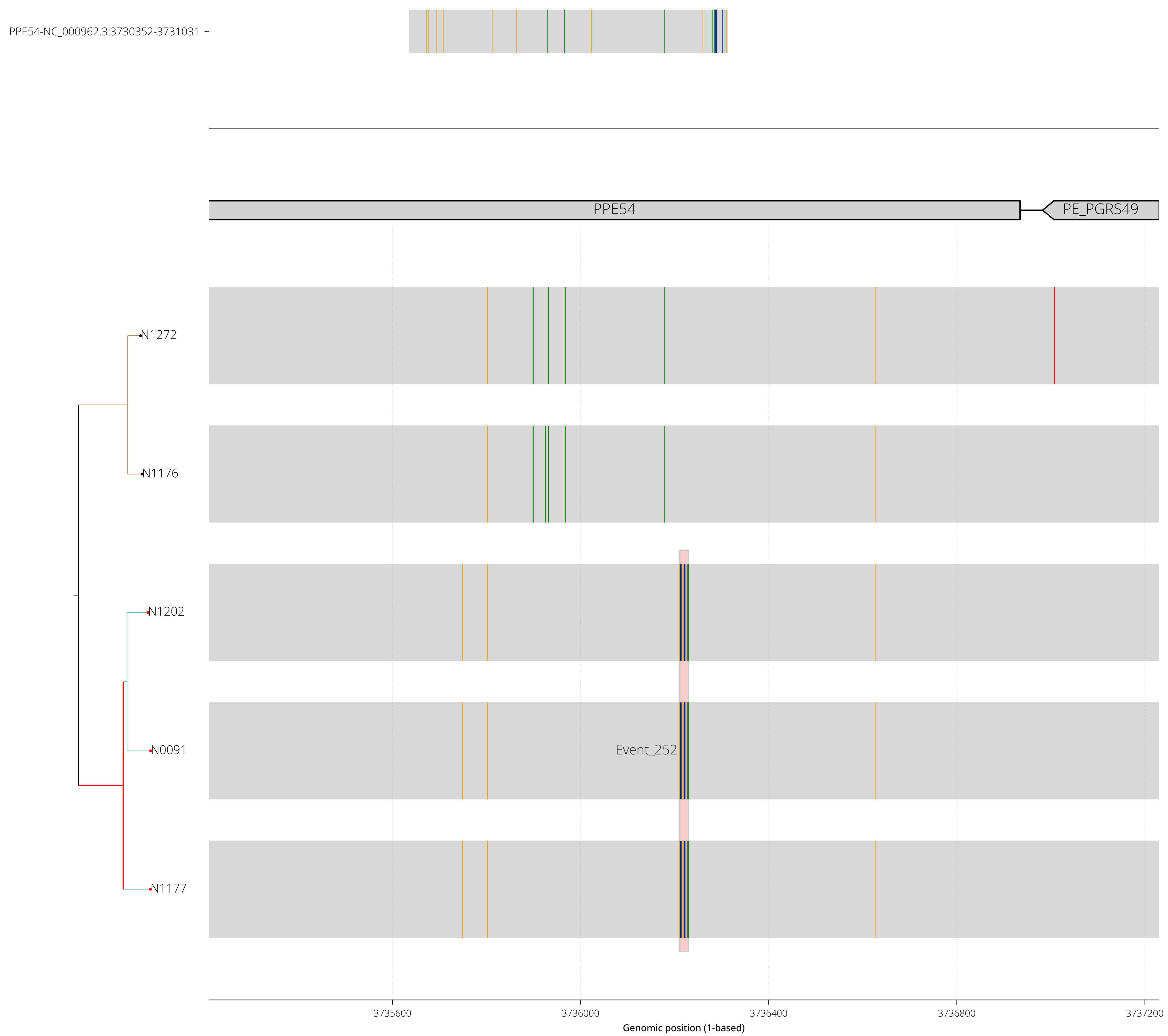

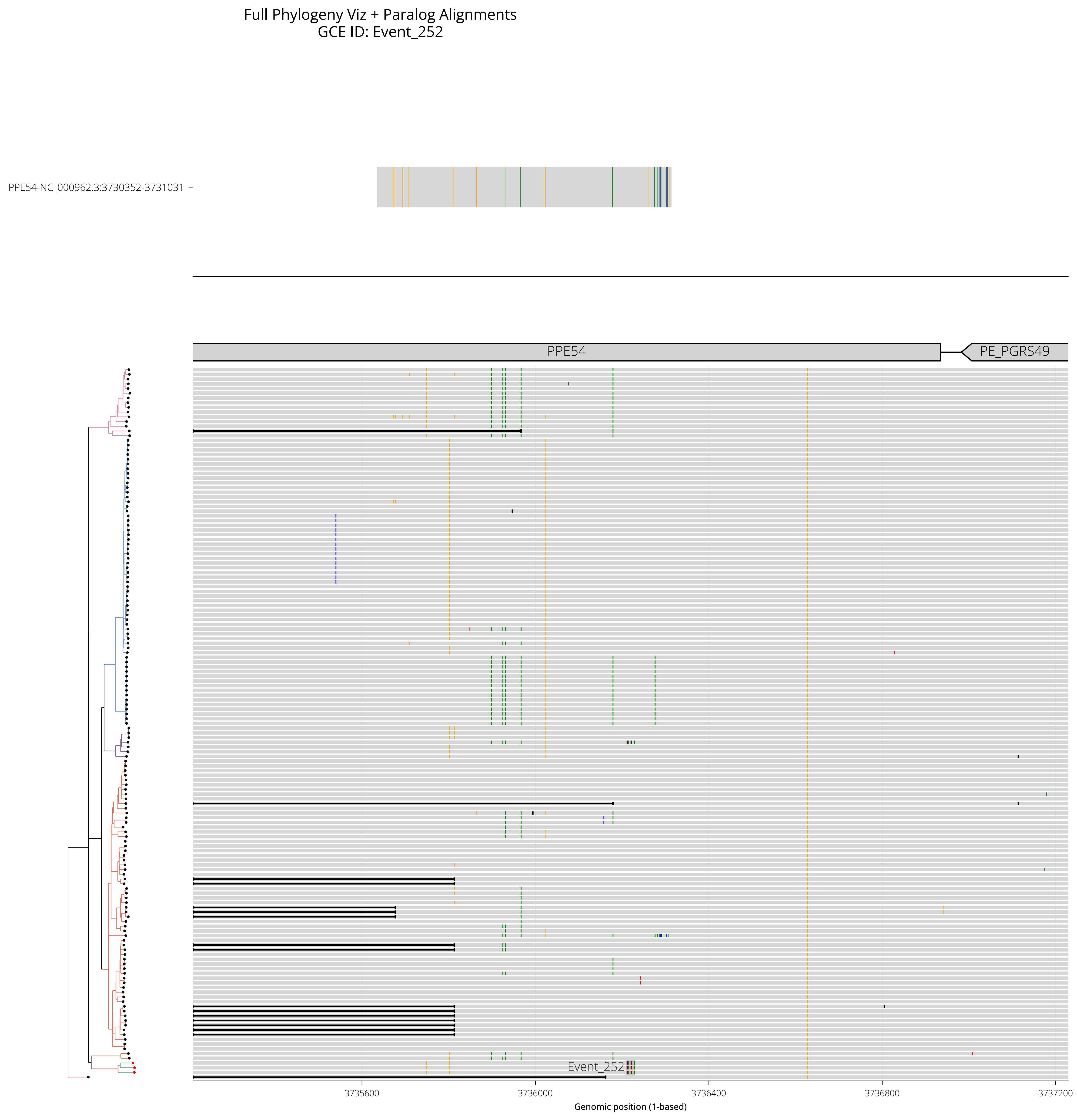

Full Phylogeny Viz + Paralog Alignments
GCE ID: Event_252
PPE54-NC_000962.3:3730352-3731031
PPE54
PE_PGRS49
Event_252
3735600
3736000
3736400
3736800
3737200
Genomic position (1-based)

Individual GCE + Known Paralogs
GCE ID: Event_253

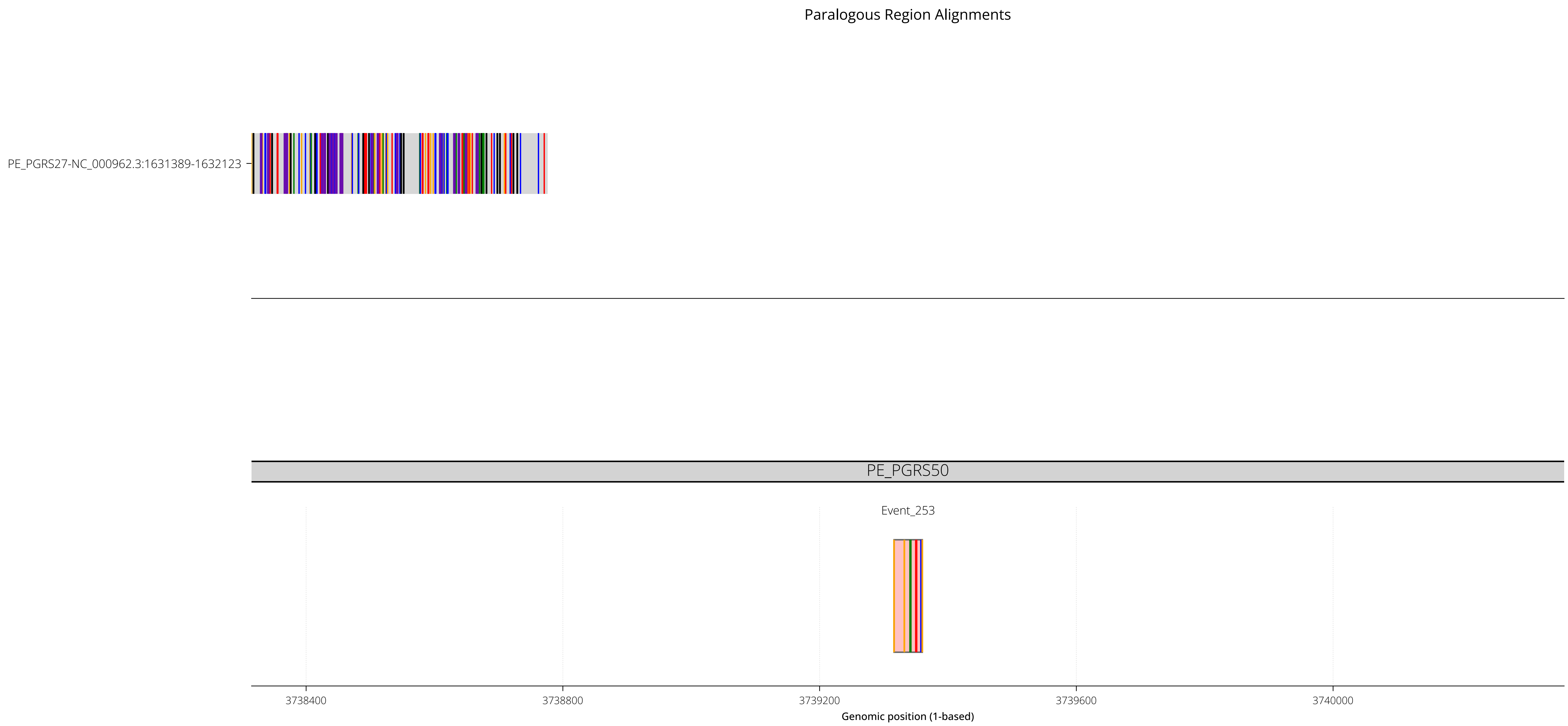

Sub-phylogeny Viz + Paralog Alignments
GCE ID: Event_253

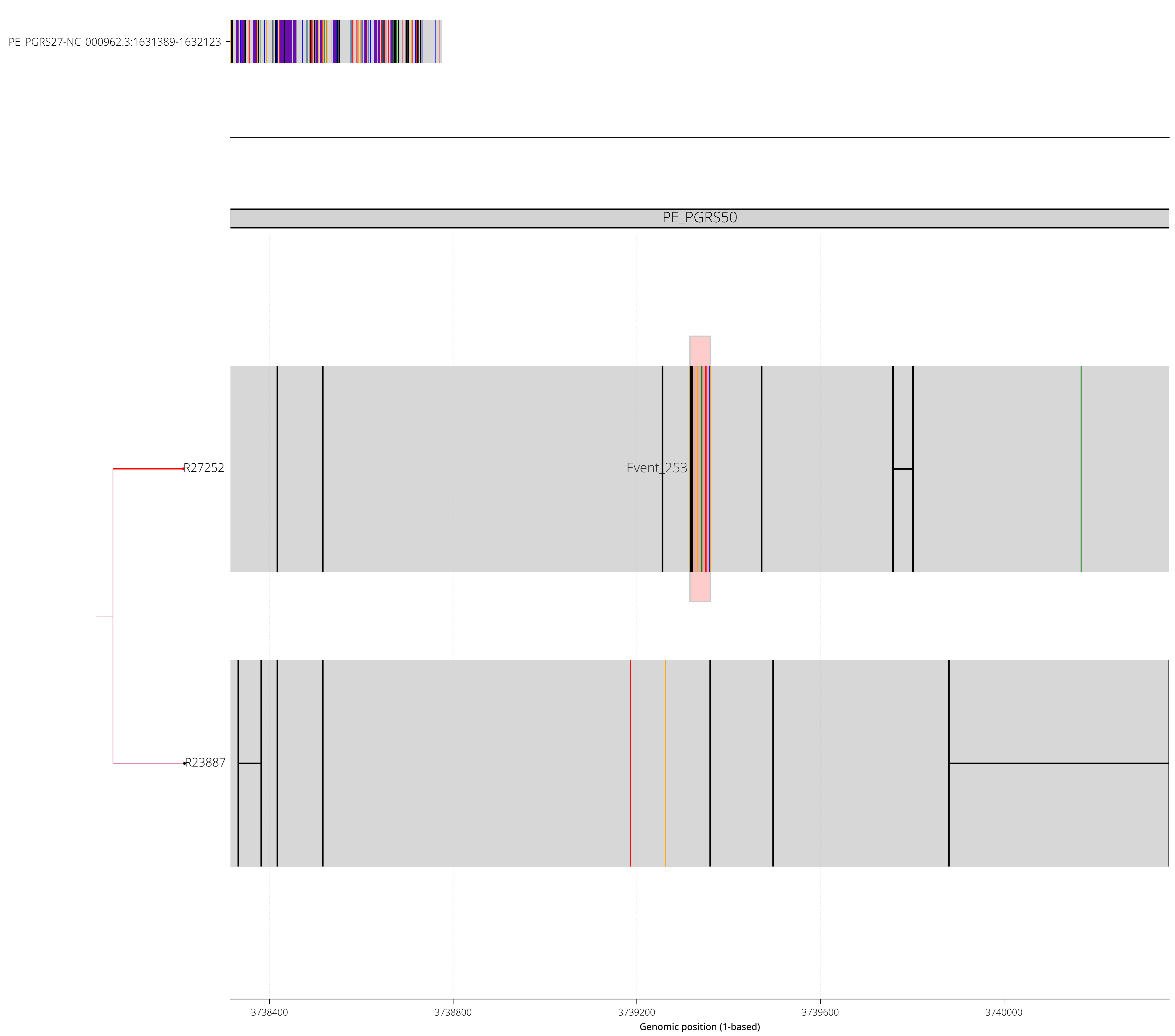

Full Phylogeny Viz + Paralog Alignments
GCE ID: Event_253

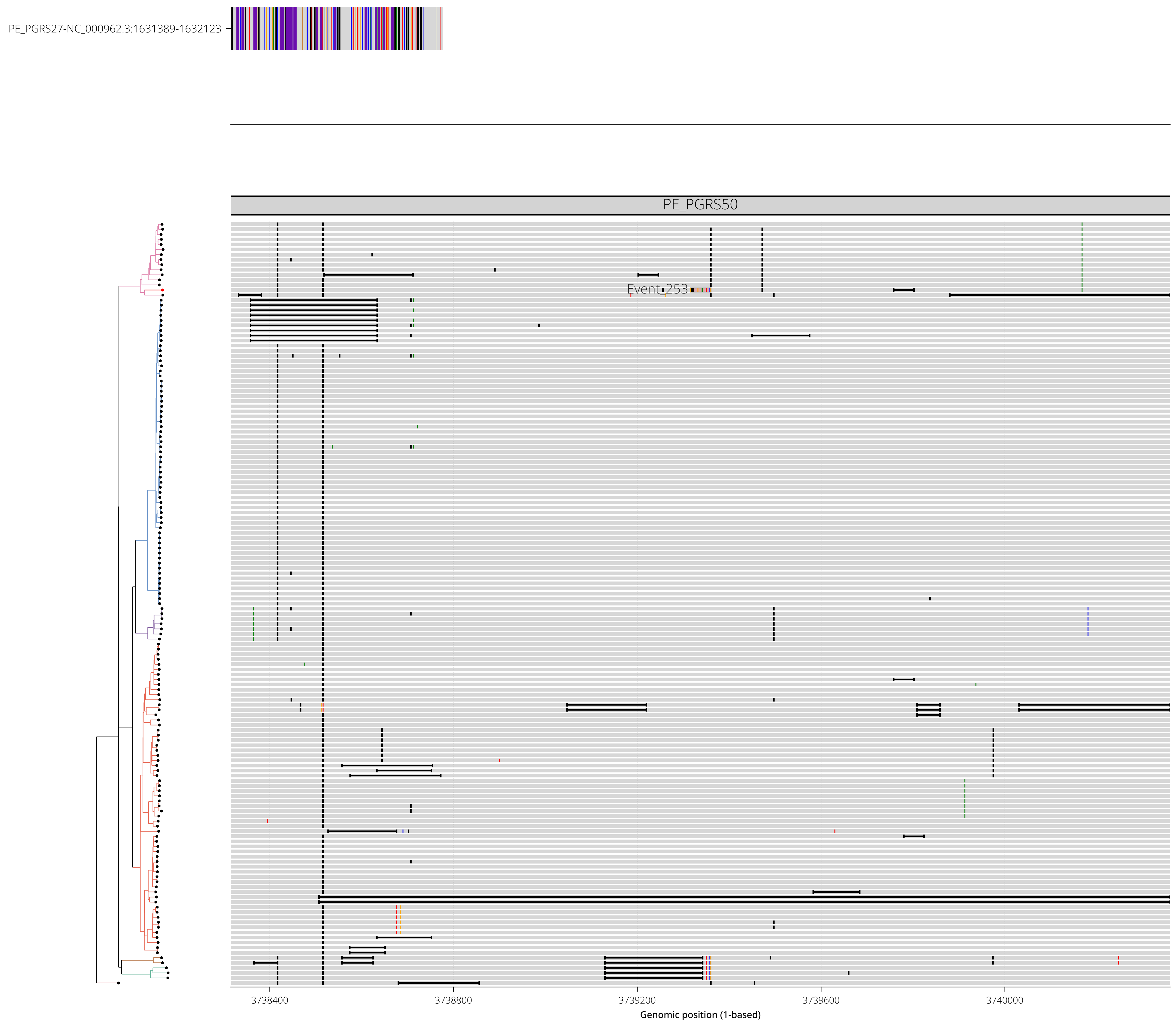

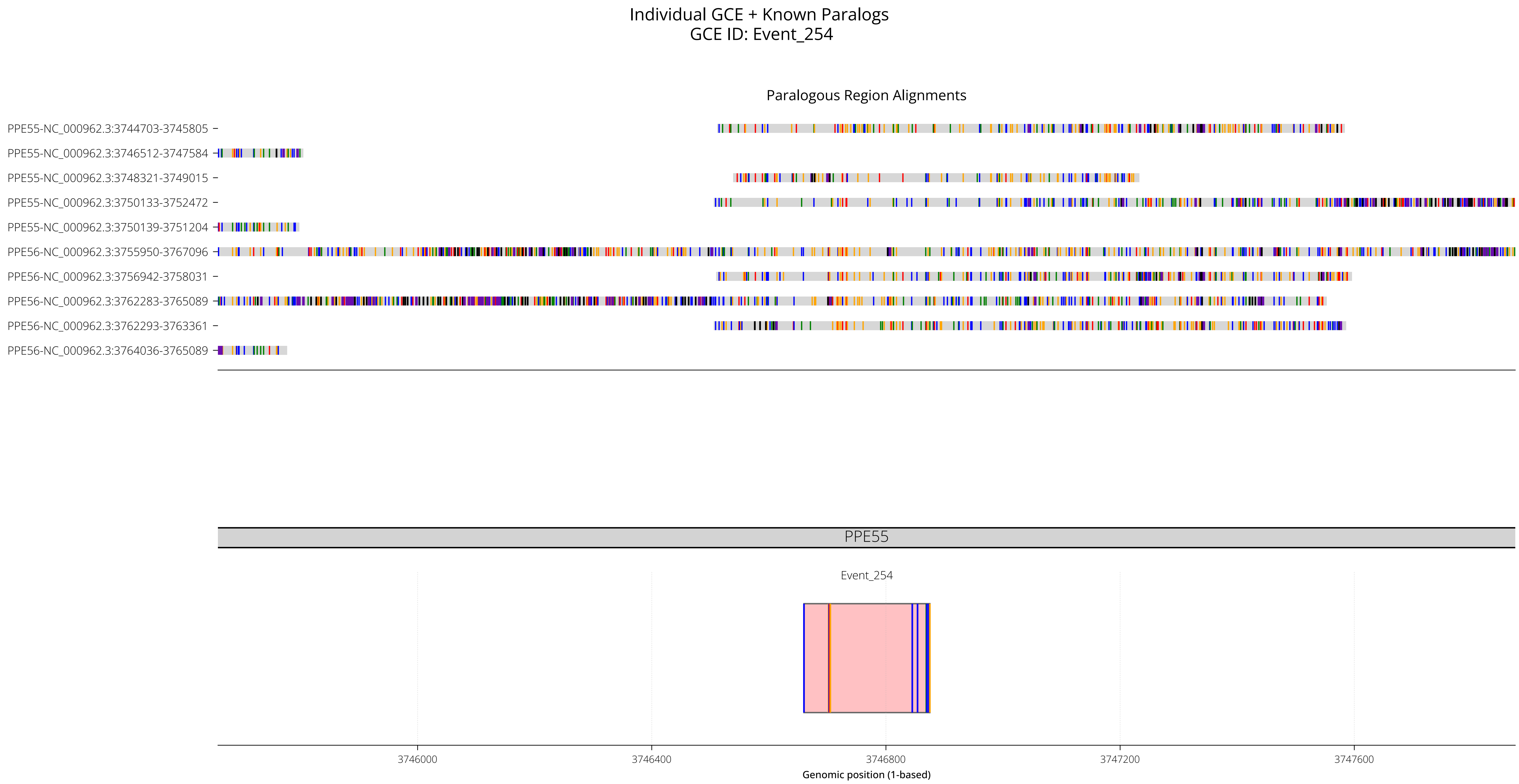
Individual GCE + Known Paralogs
GCE ID: Event_254
Paralogous Region Alignments
PPE55-NC_000962.3:3744703-3745805
PPE55-NC_000962.3:3746512-3747584
PPE55-NC_000962.3:3748321-3749015
PPE55-NC_000962.3:3750133-3752472
PPE55-NC_000962.3:3750139-3751204
PPE56-NC_000962.3:3755950-3767096
PPE56-NC_000962.3:3756942-3758031
PPE56-NC_000962.3:3762283-3765089
PPE56-NC_000962.3:3762293-3763361
PPE56-NC_000962.3:3764036-3765089
PPE55
Event_254
3746000
3746400
3746800
3747200
3747600
Genomic position (1-based)

Circos Viz - GCE to Known Paralogs Locations
GCE ID: Event_254

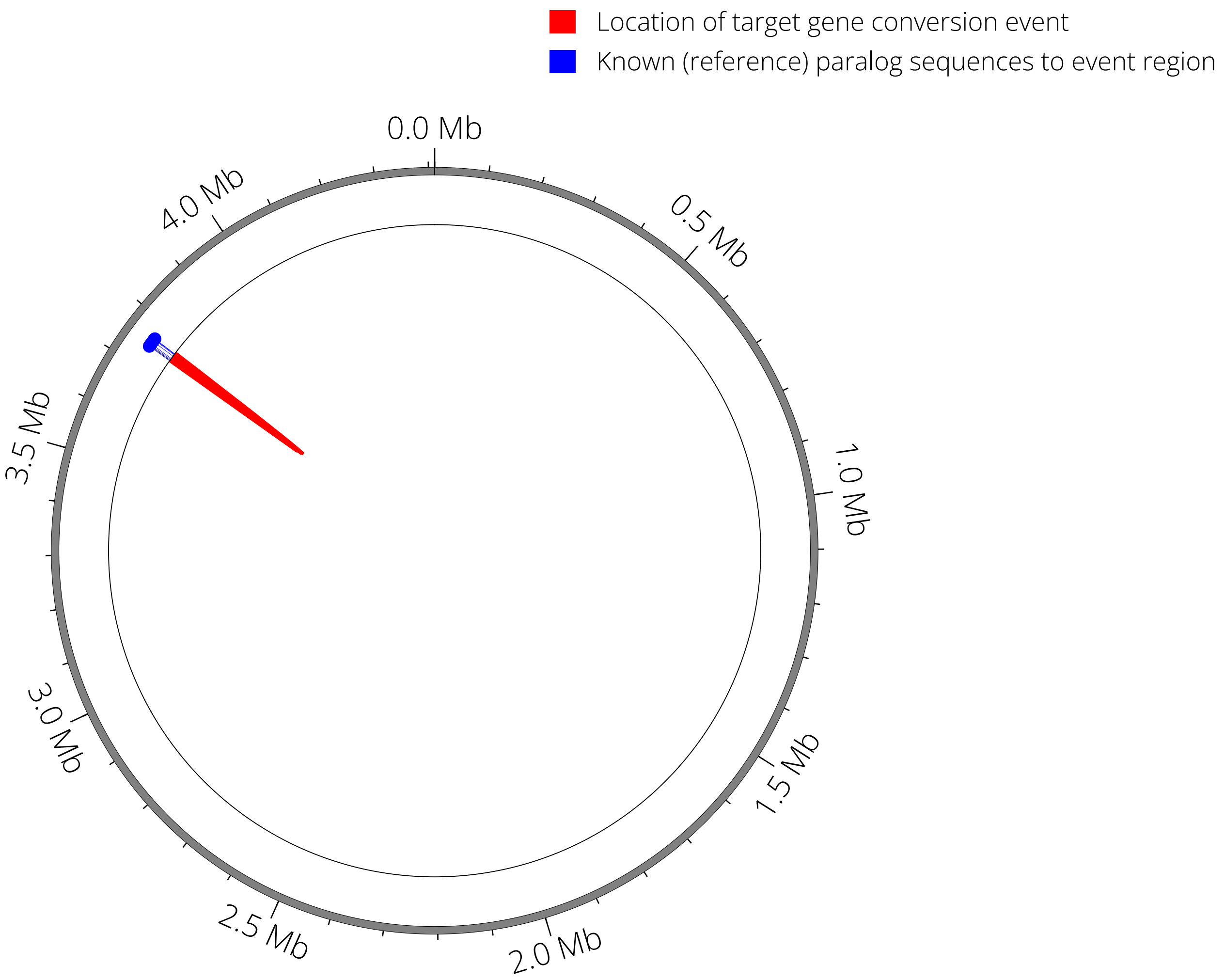

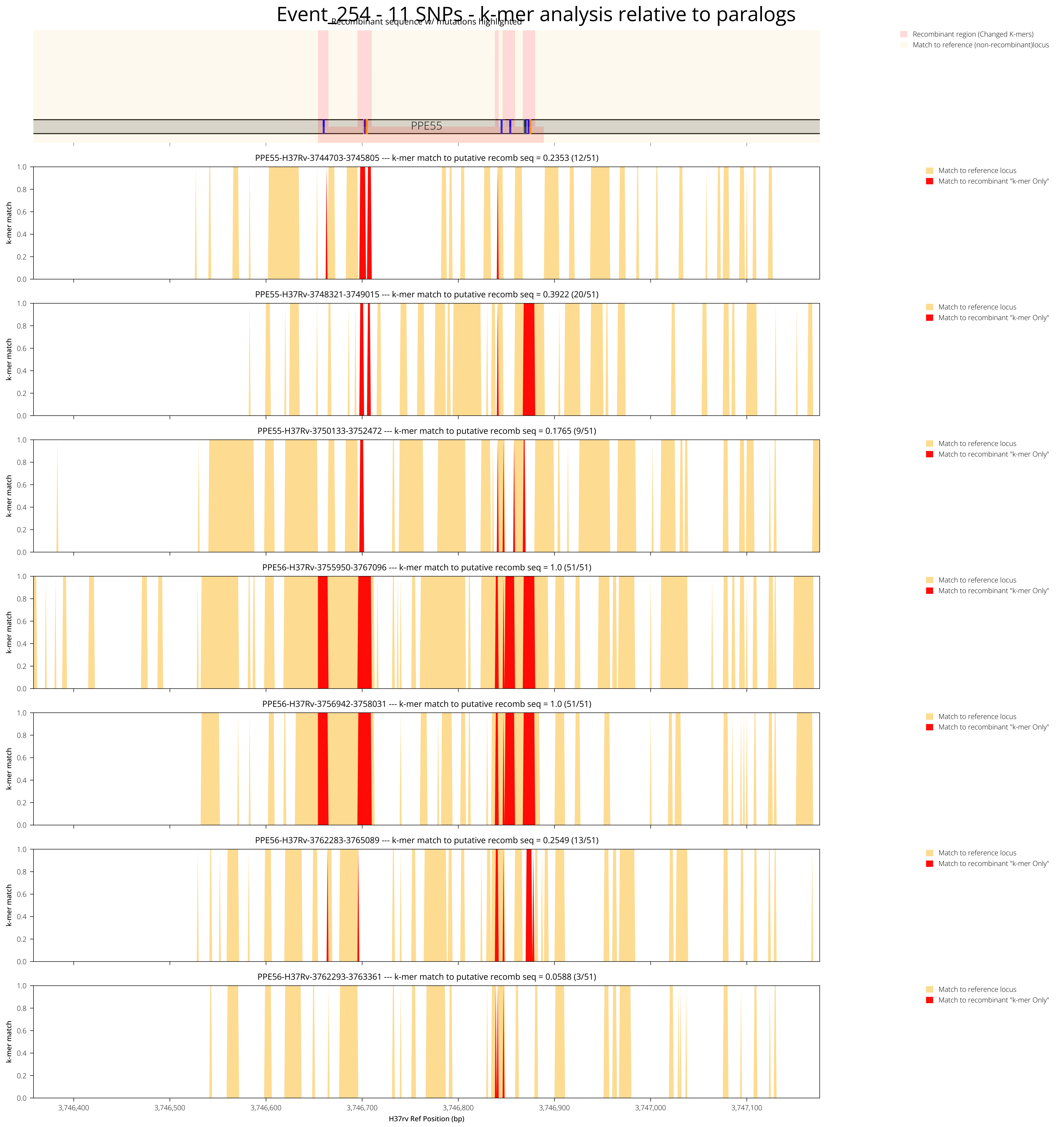

Event_254 - 11 SNPs - k-mer analysis relative to paralogs
Recombinant sequence w/ mutations highlighted
Recombinant region (Changed K-mers)
Match to reference (non-recombinant)locus
PPE55
PPE55-H37Rv-3744703-3745805 --- k-mer match to putative recomb seq = 0.2353 (12/51)
PPE55-H37Rv-3748321-3749015 --- k-mer match to putative recomb seq = 0.3922 (20/51)
PPE55-H37Rv-3750133-3752472 --- k-mer match to putative recomb seq = 0.1765 (9/51)
PPE56-H37Rv-3755950-3767096 --- k-mer match to putative recomb seq = 1.0 (51/51)
PPE56-H37Rv-3756942-3758031 --- k-mer match to putative recomb seq = 1.0 (51/51)
PPE56-H37Rv-3762283-3765089 --- k-mer match to putative recomb seq = 0.2549 (13/51)
PPE56-H37Rv-3762293-3763361 --- k-mer match to putative recomb seq = 0.0588 (3/51)
Match to reference locus
Match to recombinant "k-mer Only"
k-mer match
0.0
0.2
0.4
0.6
0.8
1.0
3,746,400
3,746,500
3,746,600
3,746,700
3,746,800
3,746,900
3,747,000
3,747,100
H37rv Ref Position (bp)

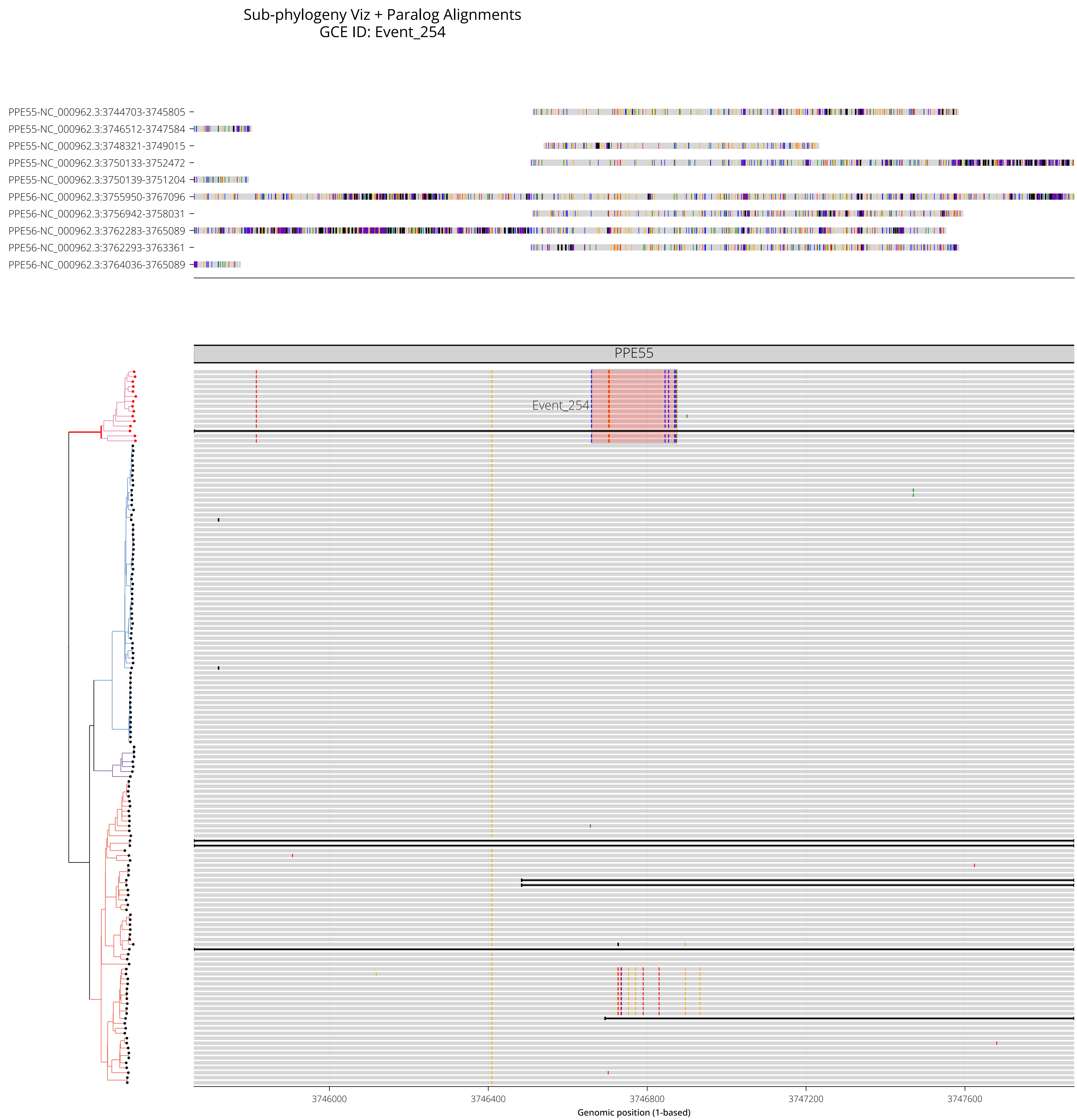

Sub-phylogeny Viz + Paralog Alignments
GCE ID: Event_254
PPE55-NC_000962.3:3744703-3745805
PPE55-NC_000962.3:3746512-3747584
PPE55-NC_000962.3:3748321-3749015
PPE55-NC_000962.3:3750133-3752472
PPE55-NC_000962.3:3750139-3751204
PPE56-NC_000962.3:3755950-3767096
PPE56-NC_000962.3:3756942-3758031
PPE56-NC_000962.3:3762283-3765089
PPE56-NC_000962.3:3762293-3763361
PPE56-NC_000962.3:3764036-3765089
PPE55
Event_254
3746000
3746400
3746800
3747200
3747600
Genomic position (1-based)

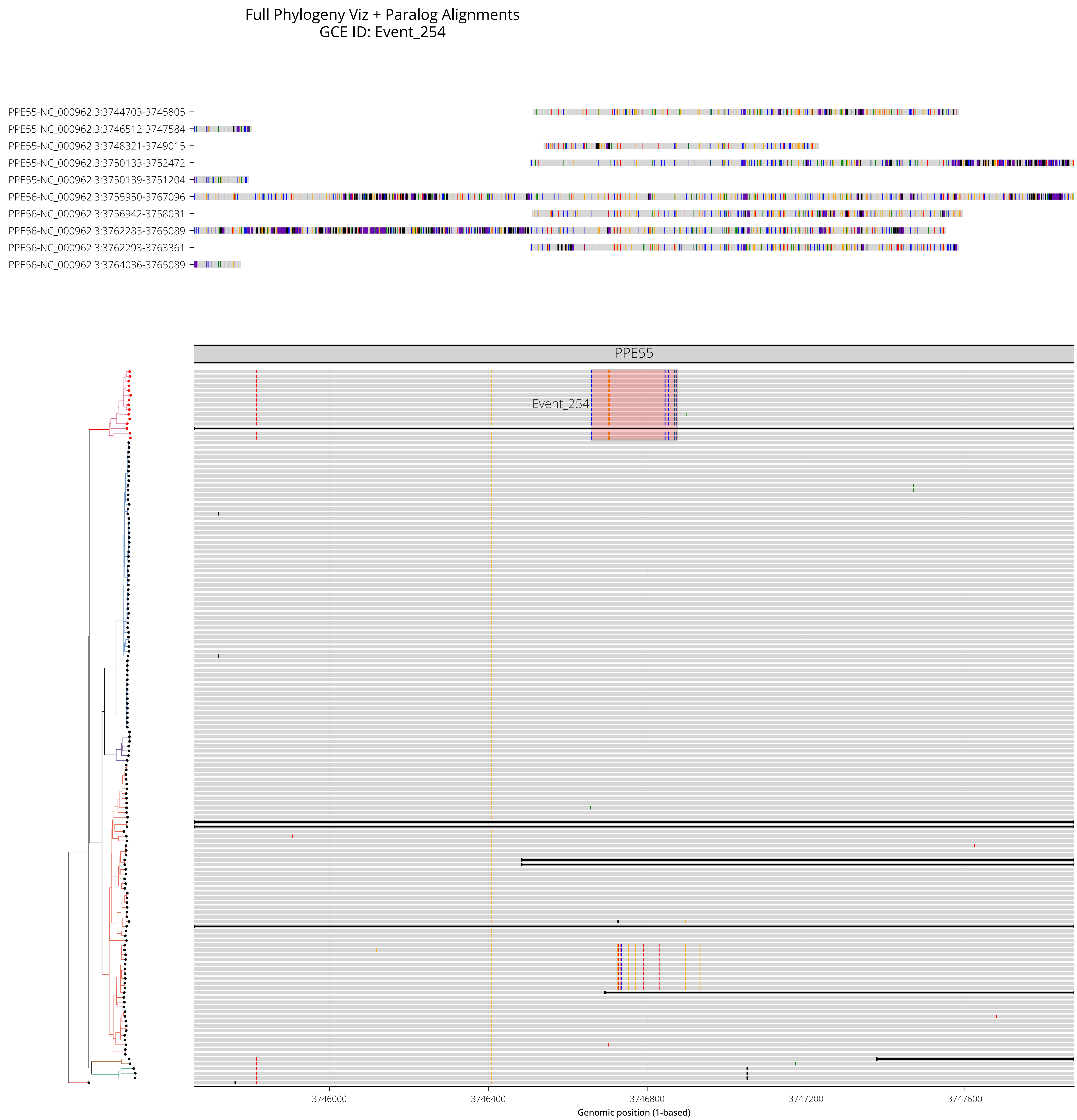

Full Phylogeny Viz + Paralog Alignments
GCE ID: Event_254
PPE55-NC_000962.3:3744703-3745805
PPE55-NC_000962.3:3746512-3747584
PPE55-NC_000962.3:3748321-3749015
PPE55-NC_000962.3:3750133-3752472
PPE55-NC_000962.3:3750139-3751204
PPE56-NC_000962.3:3755950-3767096
PPE56-NC_000962.3:3756942-3758031
PPE56-NC_000962.3:3762283-3765089
PPE56-NC_000962.3:3762293-3763361
PPE56-NC_000962.3:3764036-3765089
PPE55
Event_254
3746000
3746400
3746800
3747200
3747600
Genomic position (1-based)

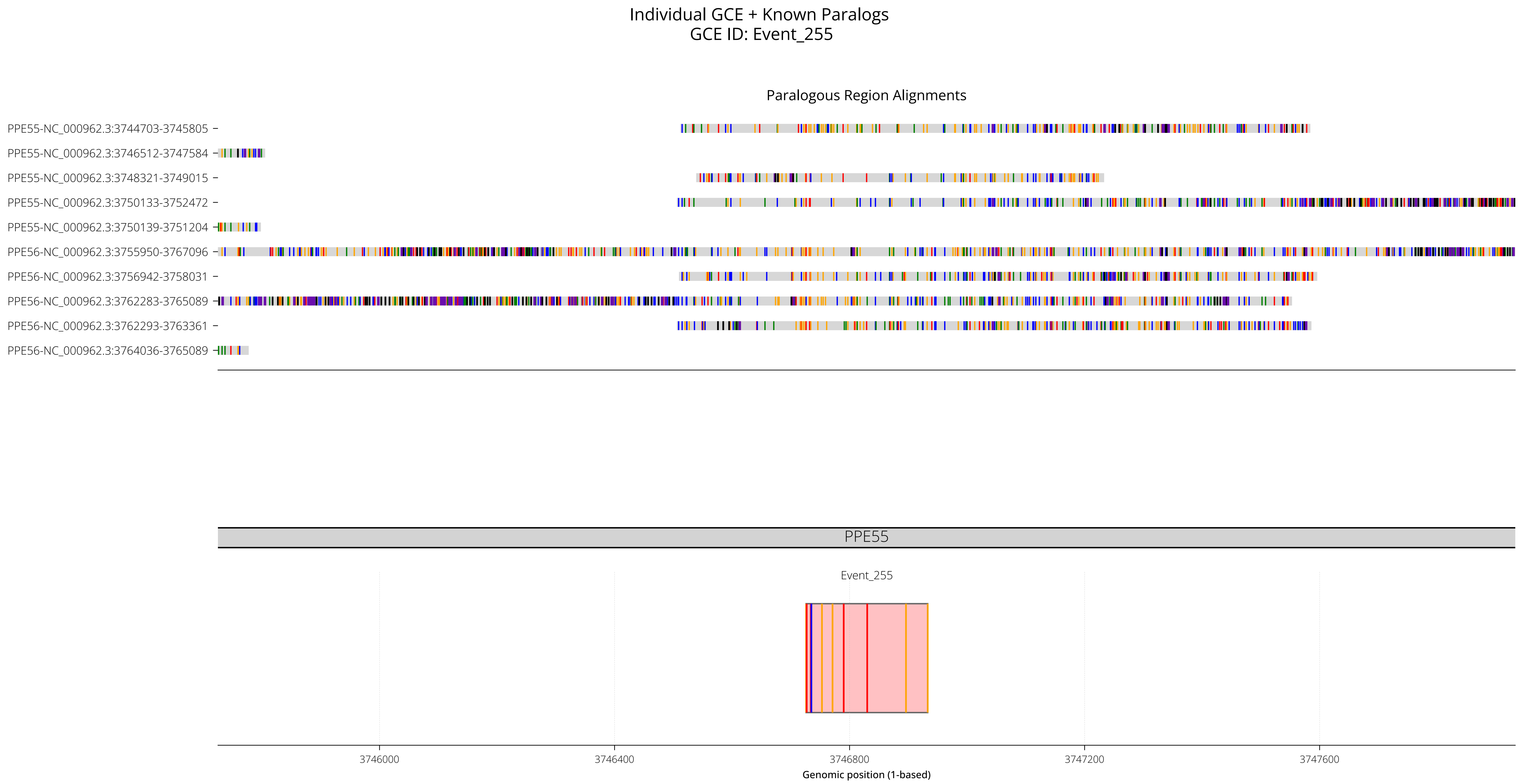

Individual GCE + Known Paralogs
GCE ID: Event_255
Paralogous Region Alignments
PPE55-NC_000962.3:3744703-3745805
PPE55-NC_000962.3:3746512-3747584
PPE55-NC_000962.3:3748321-3749015
PPE55-NC_000962.3:3750133-3752472
PPE55-NC_000962.3:3750139-3751204
PPE56-NC_000962.3:3755950-3767096
PPE56-NC_000962.3:3756942-3758031
PPE56-NC_000962.3:3762283-3765089
PPE56-NC_000962.3:3762293-3763361
PPE56-NC_000962.3:3764036-3765089
PPE55
Event_255
3746000
3746400
3746800
3747200
3747600
Genomic position (1-based)

Circos Viz - GCE to Known Paralogs Locations
GCE ID: Event_255

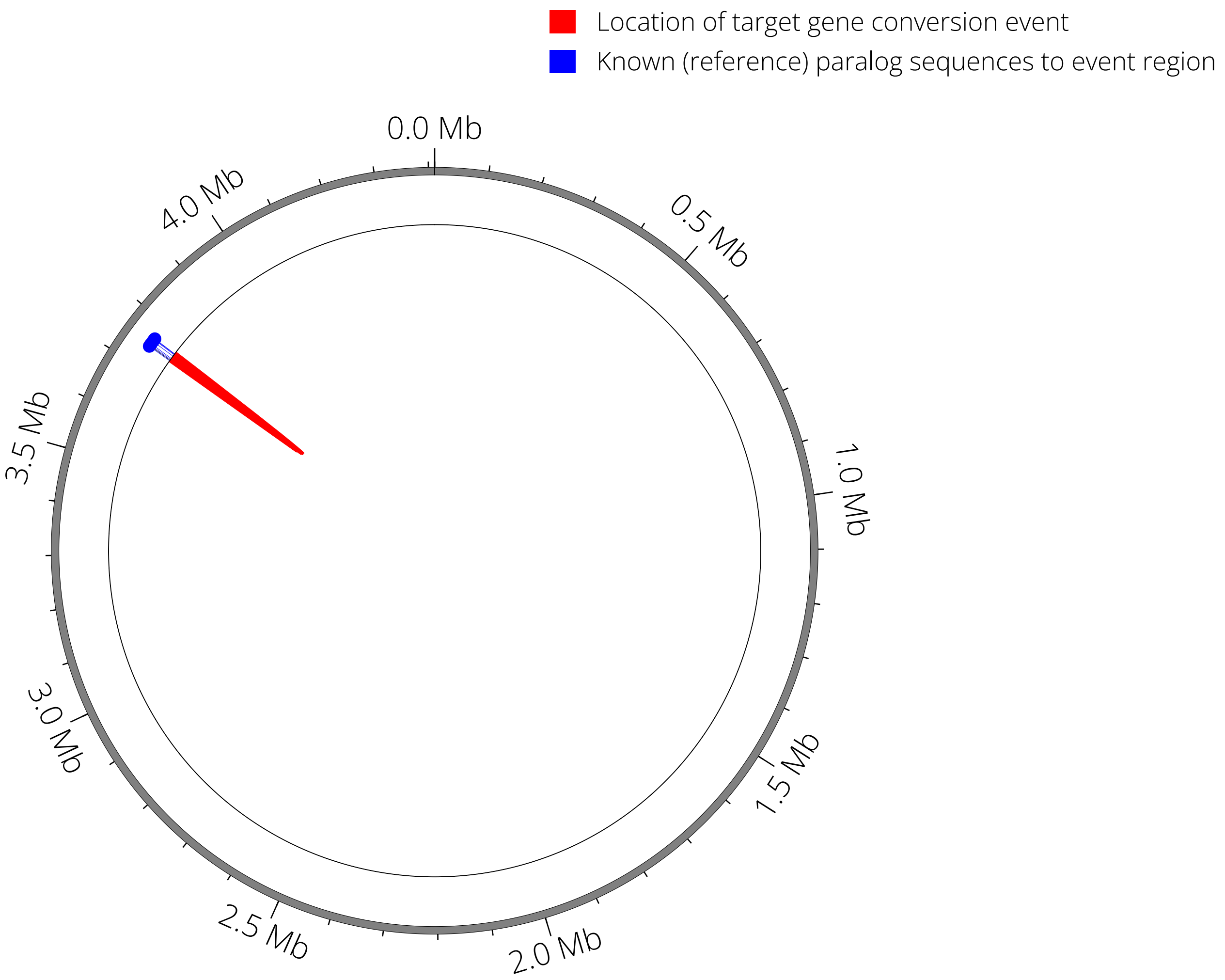

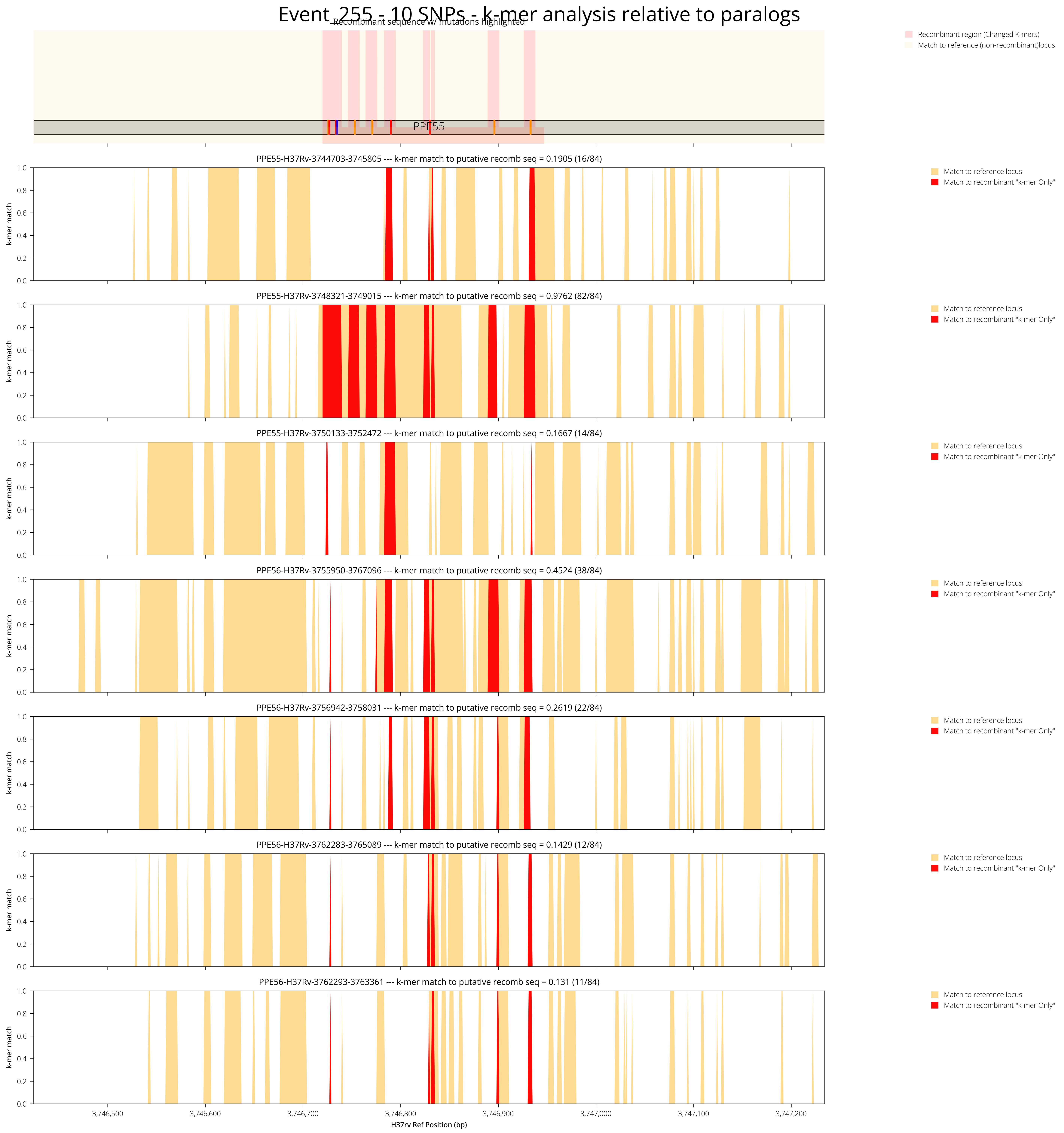

Event_255 - 10 SNPs - k-mer analysis relative to paralogs
Recombinant sequence w/ mutations highlighted
Recombinant region (Changed K-mers)
Match to reference (non-recombinant)locus
PPE55
PPE55-H37Rv-3744703-3745805 --- k-mer match to putative recomb seq = 0.1905 (16/84)
PPE55-H37Rv-3748321-3749015 --- k-mer match to putative recomb seq = 0.9762 (82/84)
PPE55-H37Rv-3750133-3752472 --- k-mer match to putative recomb seq = 0.1667 (14/84)
PPE56-H37Rv-3755950-3767096 --- k-mer match to putative recomb seq = 0.4524 (38/84)
PPE56-H37Rv-3756942-3758031 --- k-mer match to putative recomb seq = 0.2619 (22/84)
PPE56-H37Rv-3762283-3765089 --- k-mer match to putative recomb seq = 0.1429 (12/84)
PPE56-H37Rv-3762293-3763361 --- k-mer match to putative recomb seq = 0.131 (11/84)
Match to reference locus
Match to recombinant "k-mer Only"
k-mer match
1.0
0.8
0.6
0.4
0.2
0.0
3,746,500
3,746,600
3,746,700
3,746,800
3,746,900
3,747,000
3,747,100
3,747,200
H37rv Ref Position (bp)

Sub-phylogeny Viz + Paralog Alignments
GCE ID: Event_255

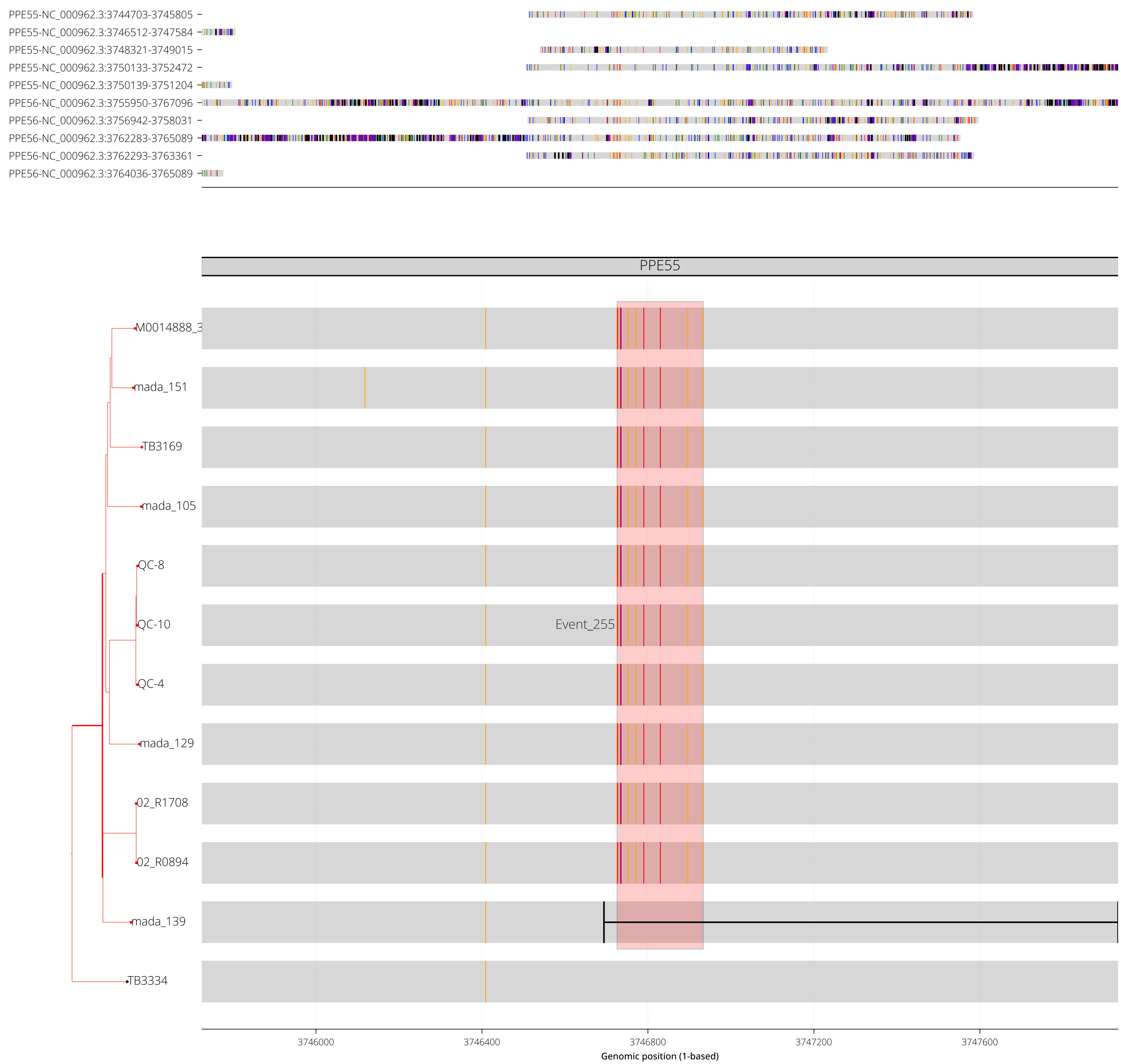

Full Phylogeny Viz + Paralog Alignments
GCE ID: Event_255

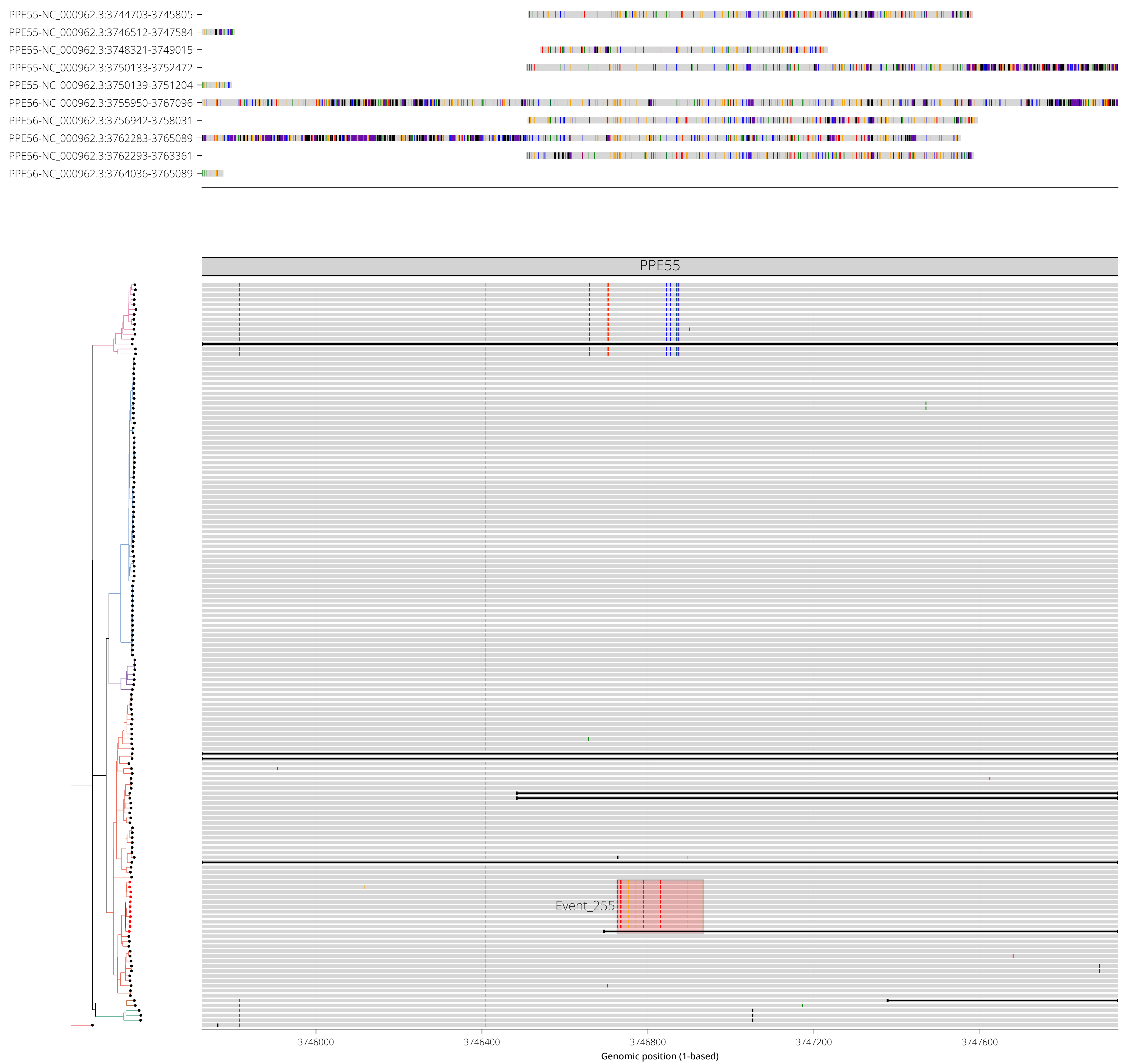

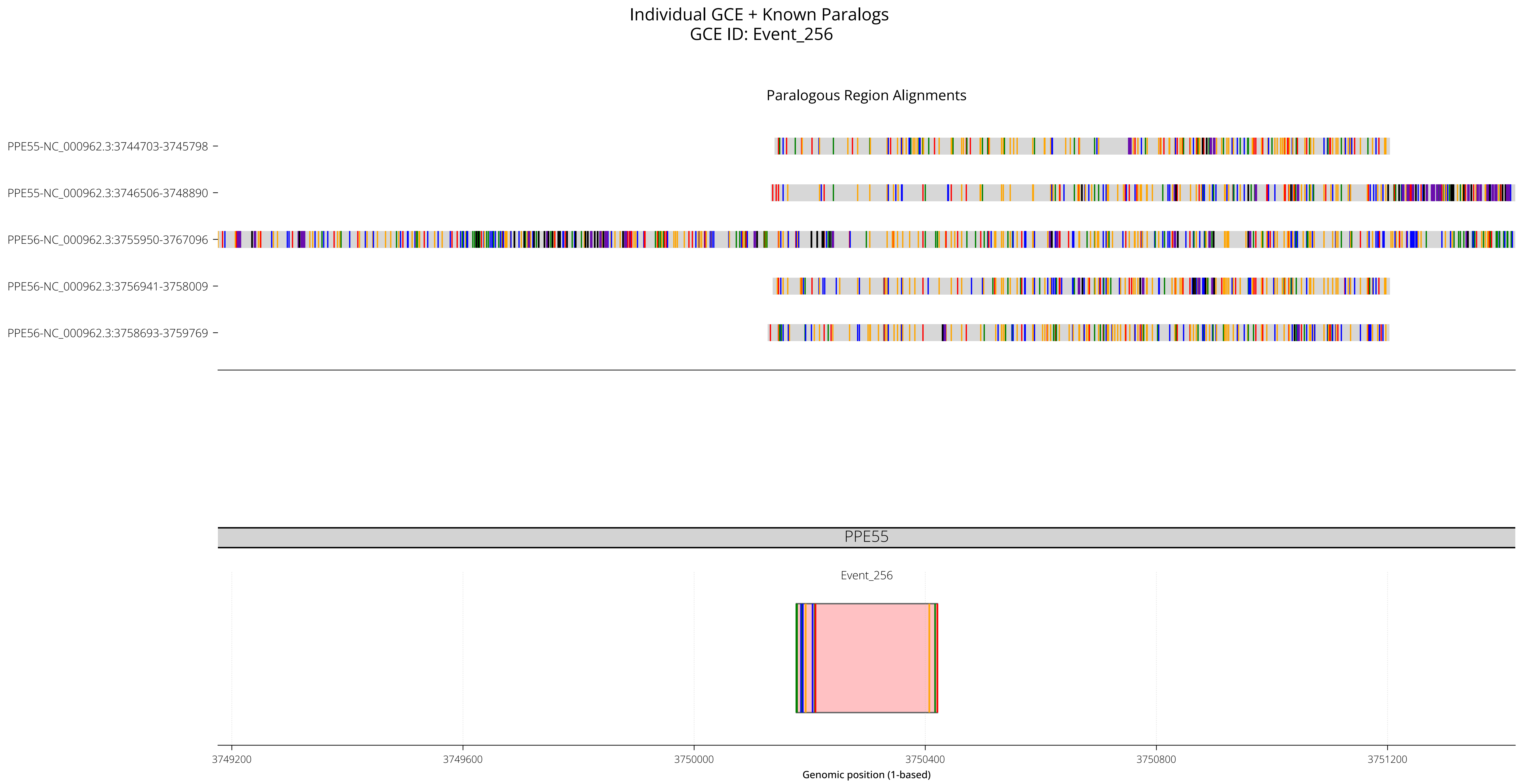
Individual GCE + Known Paralogs
GCE ID: Event_256
Paralogous Region Alignments
PPE55-NC_000962.3:3744703-3745798
PPE55-NC_000962.3:3746506-3748890
PPE56-NC_000962.3:3755950-3767096
PPE56-NC_000962.3:3756941-3758009
PPE56-NC_000962.3:3758693-3759769
PPE55
Event_256
3749200
3749600
3750000
3750400
3750800
3751200
Genomic position (1-based)

Circos Viz - GCE to Known Paralogs Locations
GCE ID: Event_256

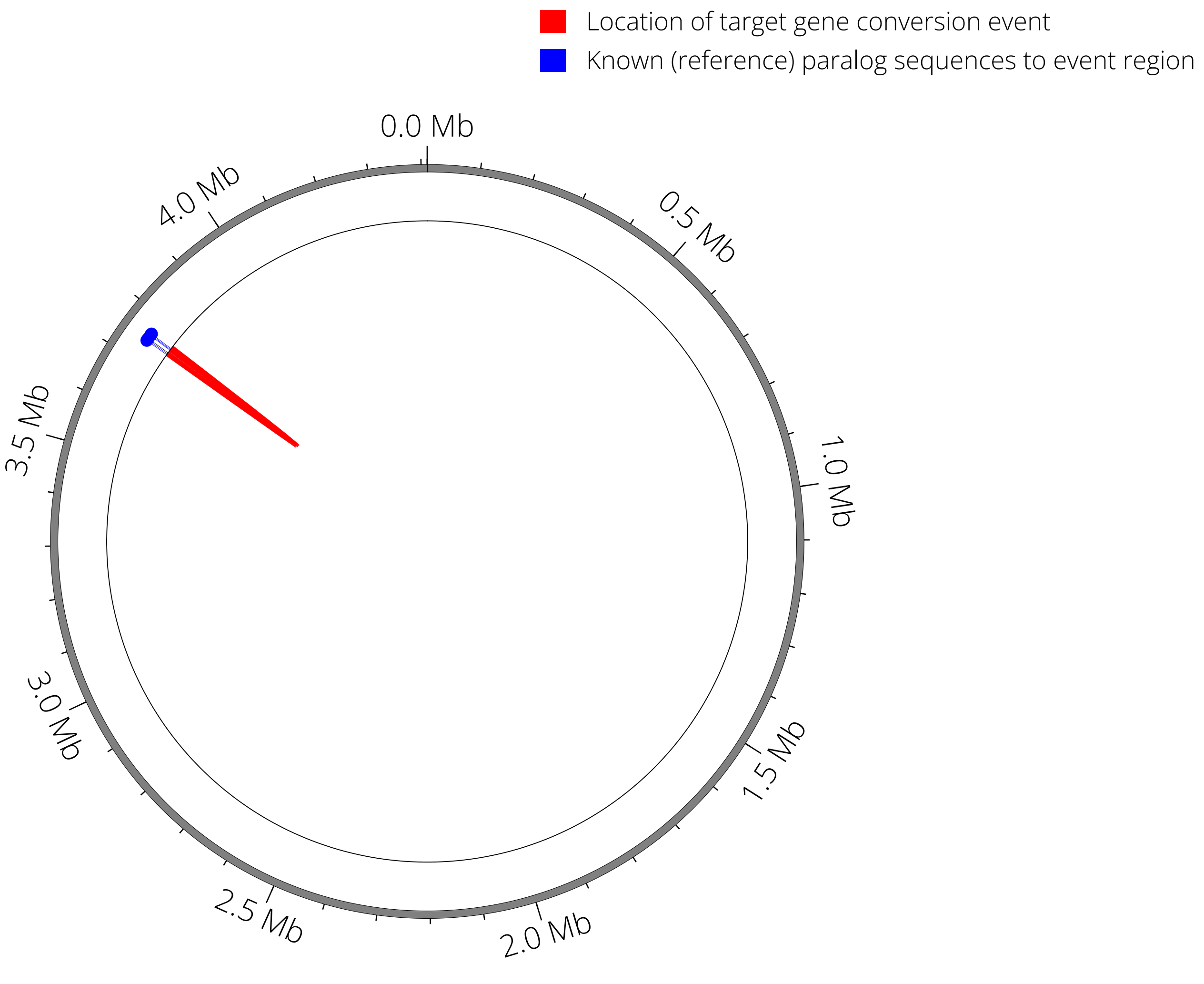

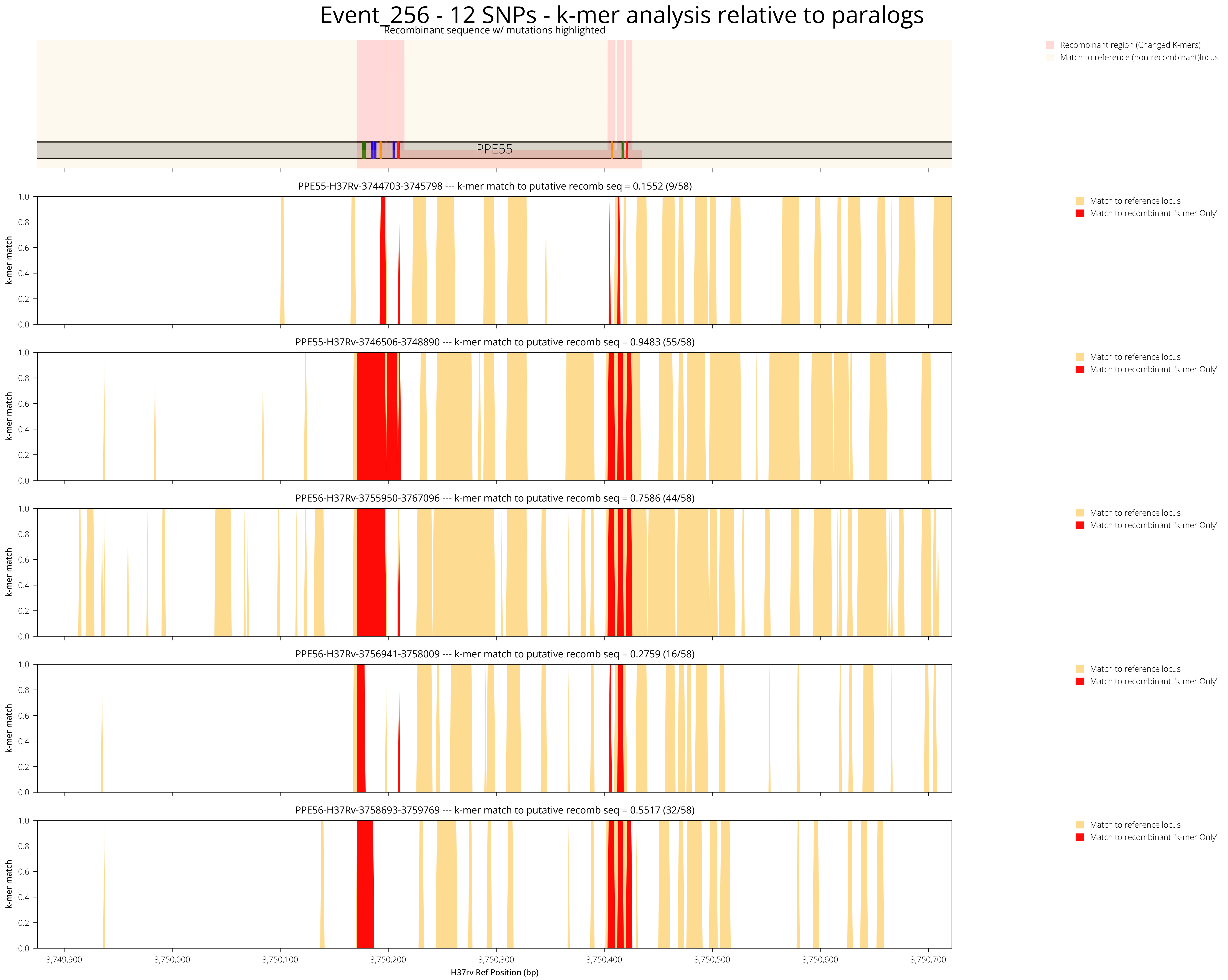

Event_256 - 12 SNPs - k-mer analysis relative to paralogs
Recombinant sequence w/ mutations highlighted
Recombinant region (Changed K-mers)
Match to reference (non-recombinant)locus
PPE55
PPE55-H37Rv-3744703-3745798 --- k-mer match to putative recomb seq = 0.1552 (9/58)
PPE55-H37Rv-3746506-3748890 --- k-mer match to putative recomb seq = 0.9483 (55/58)
PPE56-H37Rv-3755950-3767096 --- k-mer match to putative recomb seq = 0.7586 (44/58)
PPE56-H37Rv-3756941-3758009 --- k-mer match to putative recomb seq = 0.2759 (16/58)
PPE56-H37Rv-3758693-3759769 --- k-mer match to putative recomb seq = 0.5517 (32/58)
Match to reference locus
Match to recombinant "k-mer Only"
k-mer match
0.0
0.2
0.4
0.6
0.8
1.0
3,749,900
3,750,000
3,750,100
3,750,200
3,750,300
3,750,400
3,750,500
3,750,600
3,750,700
H37rv Ref Position (bp)

Sub-phylogeny Viz + Paralog Alignments
GCE ID: Event_256

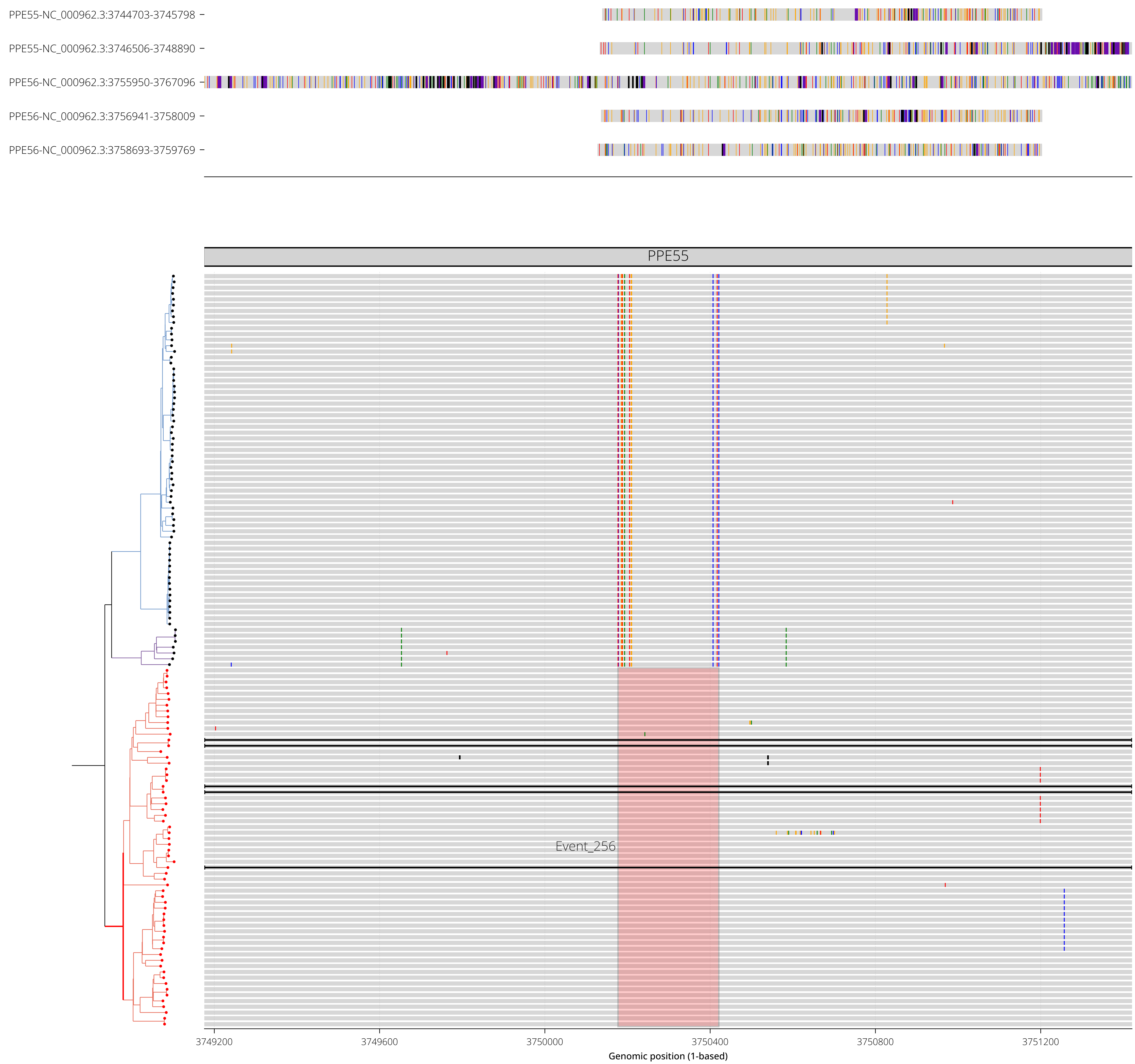

Full Phylogeny Viz + Paralog Alignments
GCE ID: Event_256

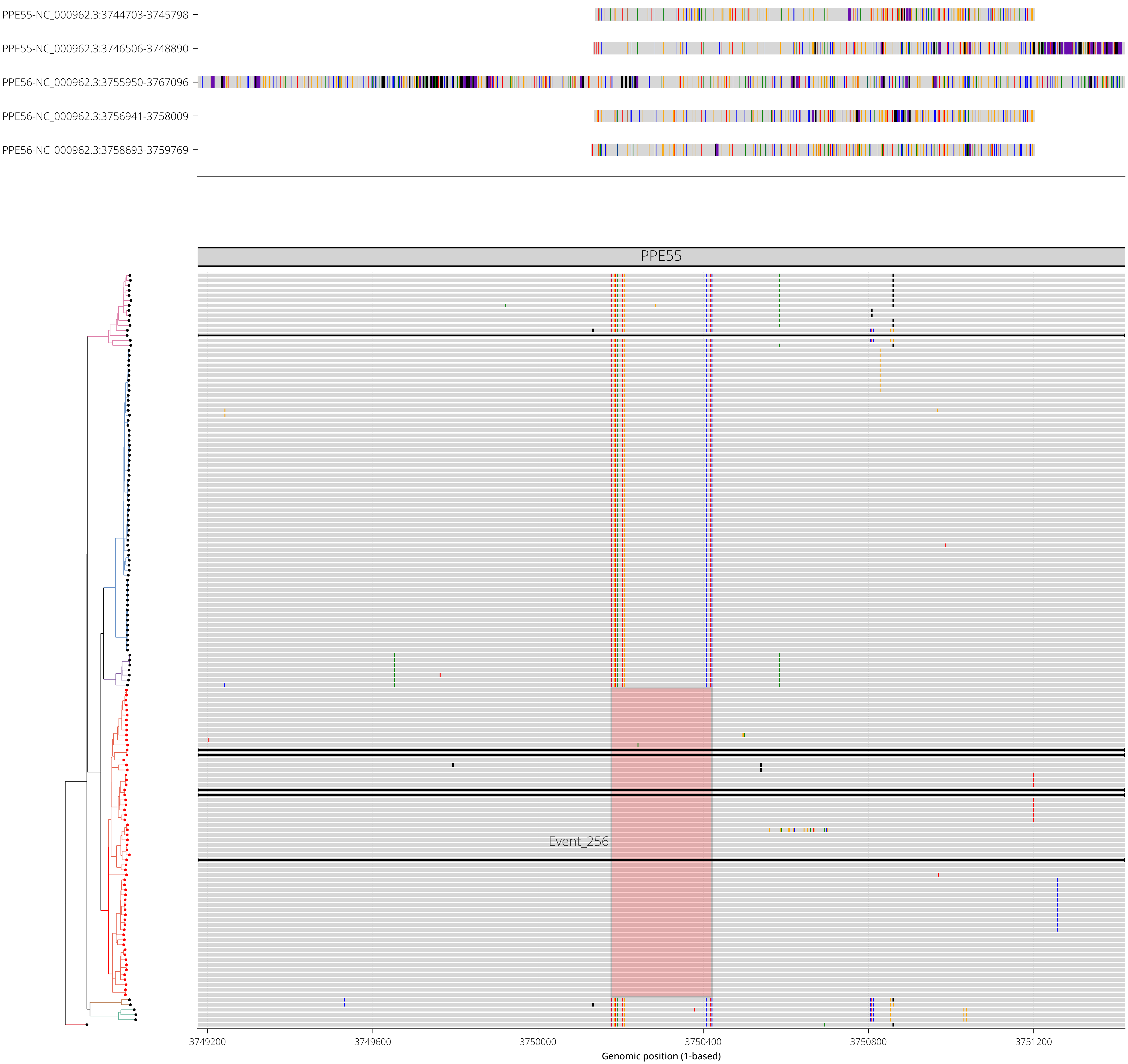

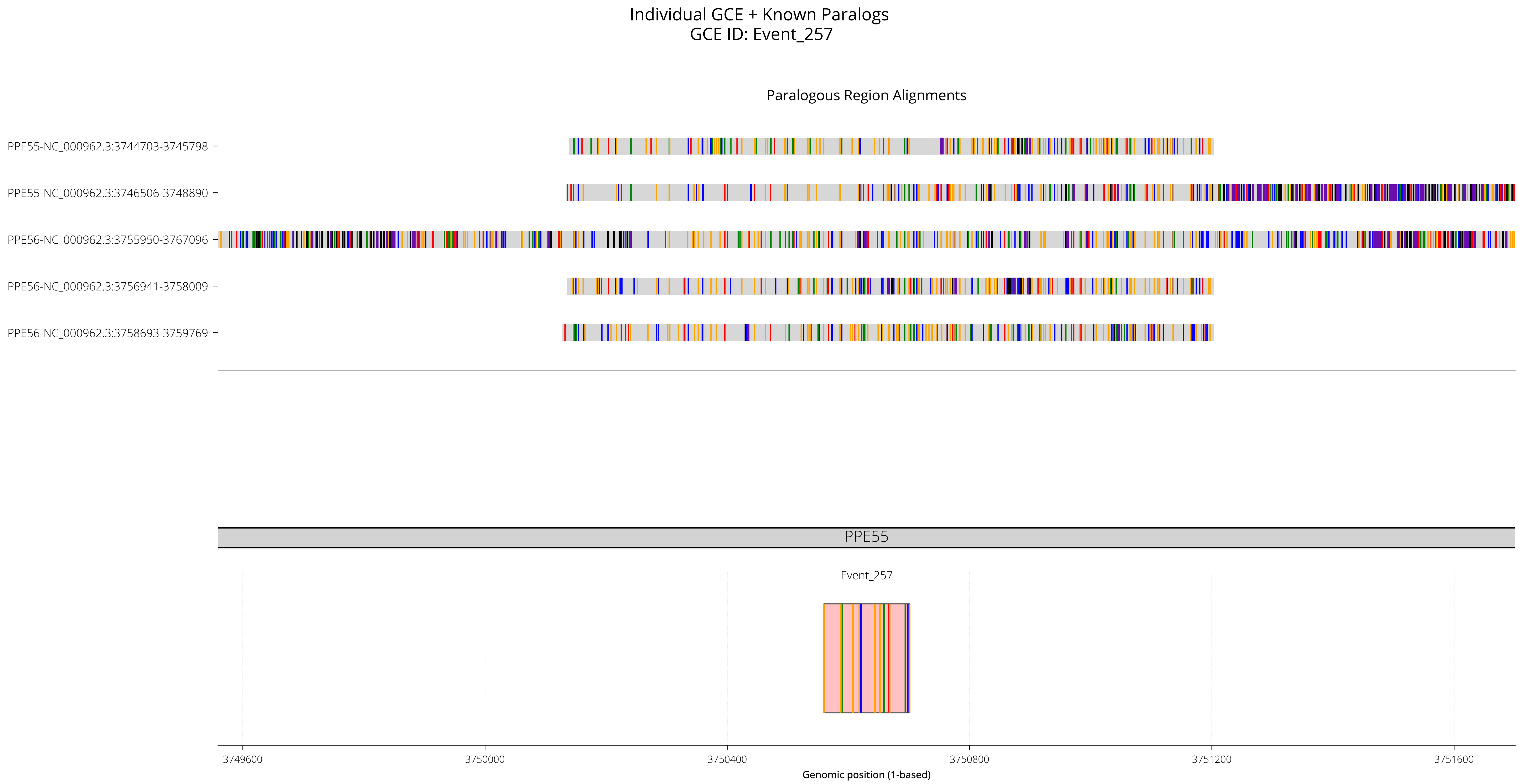
Individual GCE + Known Paralogs
GCE ID: Event_257
Paralogous Region Alignments
PPE55-NC_000962.3:3744703-3745798
PPE55-NC_000962.3:3746506-3748890
PPE56-NC_000962.3:3755950-3767096
PPE56-NC_000962.3:3756941-3758009
PPE56-NC_000962.3:3758693-3759769
PPE55
Event_257
3749600
3750000
3750400
3750800
3751200
3751600
Genomic position (1-based)

Circos Viz - GCE to Known Paralogs Locations
GCE ID: Event_257

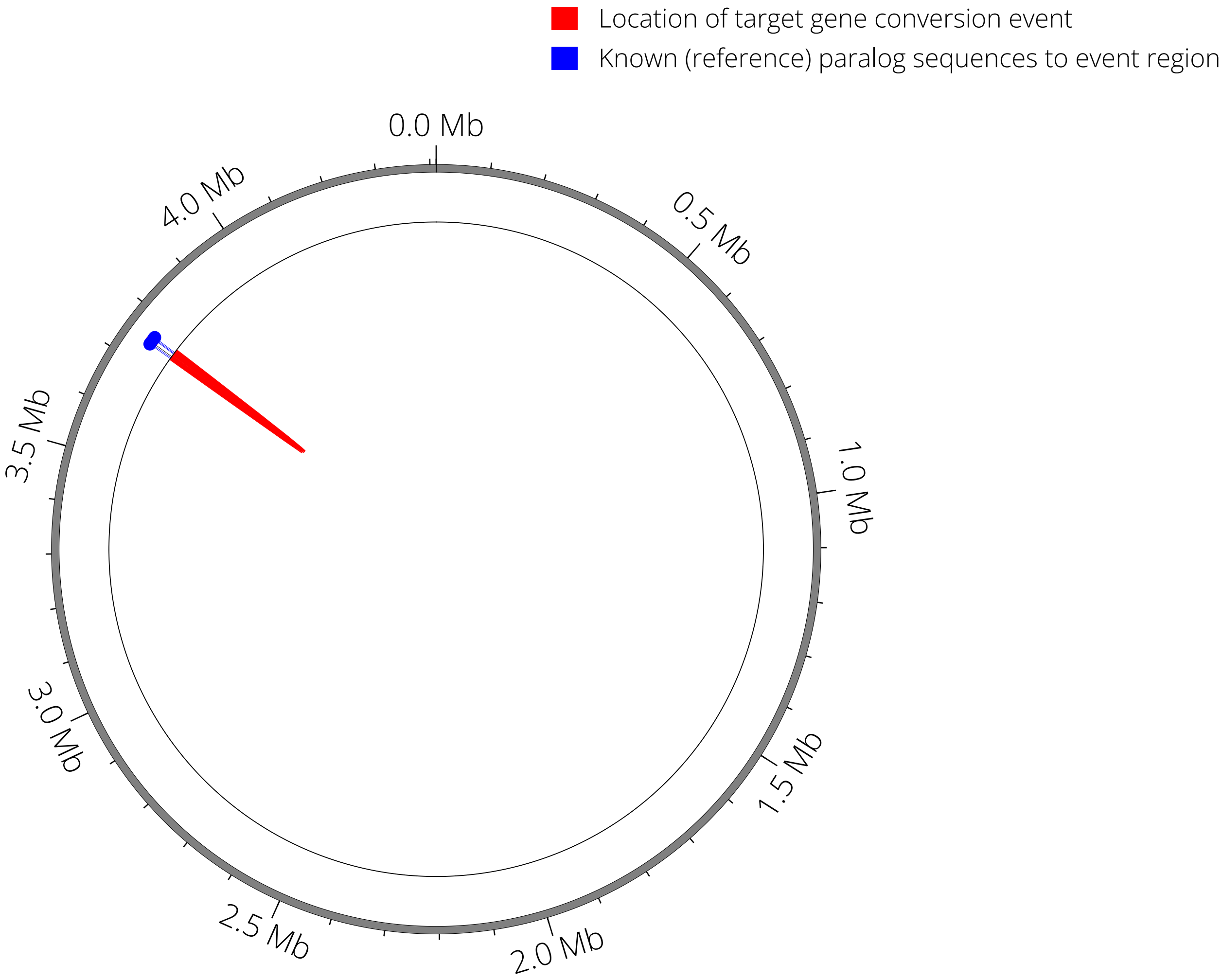

# Event_257 - 16 SNPs - k-mer analysis relative to paralogs

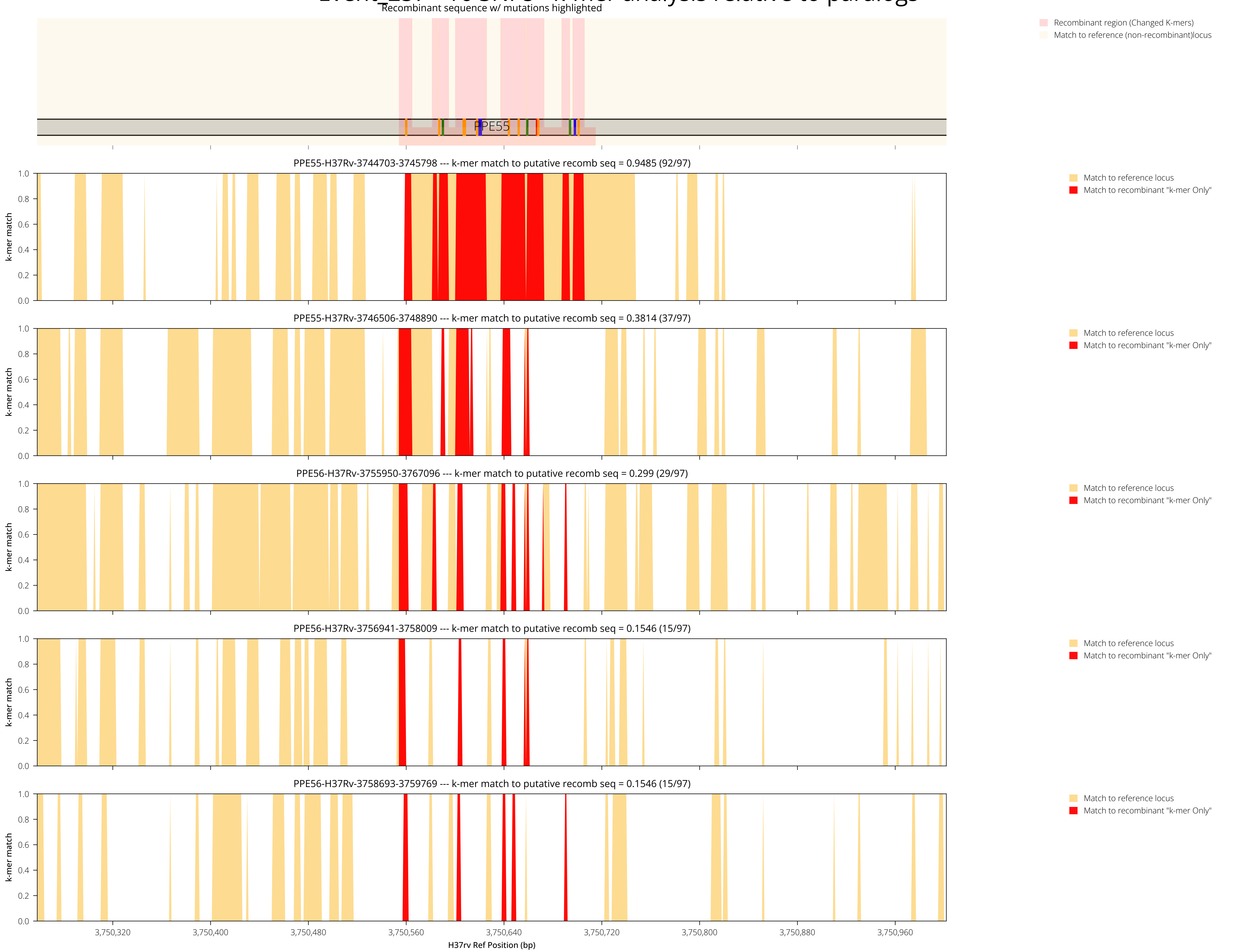

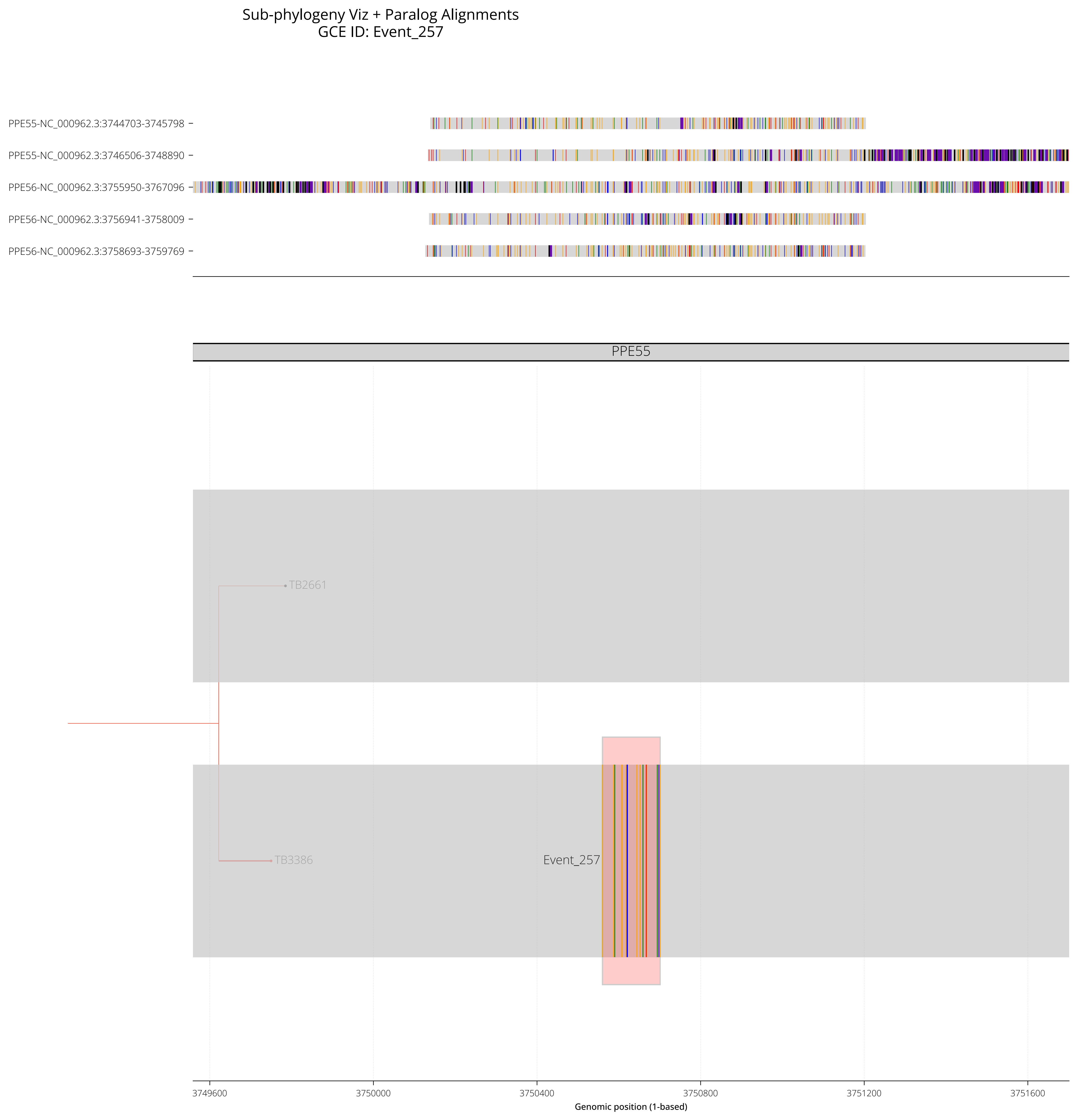

Sub-phylogeny Viz + Paralog Alignments
GCE ID: Event_257
PPE55-NC_000962.3:3744703-3745798
PPE55-NC_000962.3:3746506-3748890
PPE56-NC_000962.3:3755950-3767096
PPE56-NC_000962.3:3756941-3758009
PPE56-NC_000962.3:3758693-3759769
PPE55
TB2661
TB3386
Event_257
3749600
3750000
3750400
3750800
3751200
3751600
Genomic position (1-based)

Full Phylogeny Viz + Paralog Alignments
GCE ID: Event_257

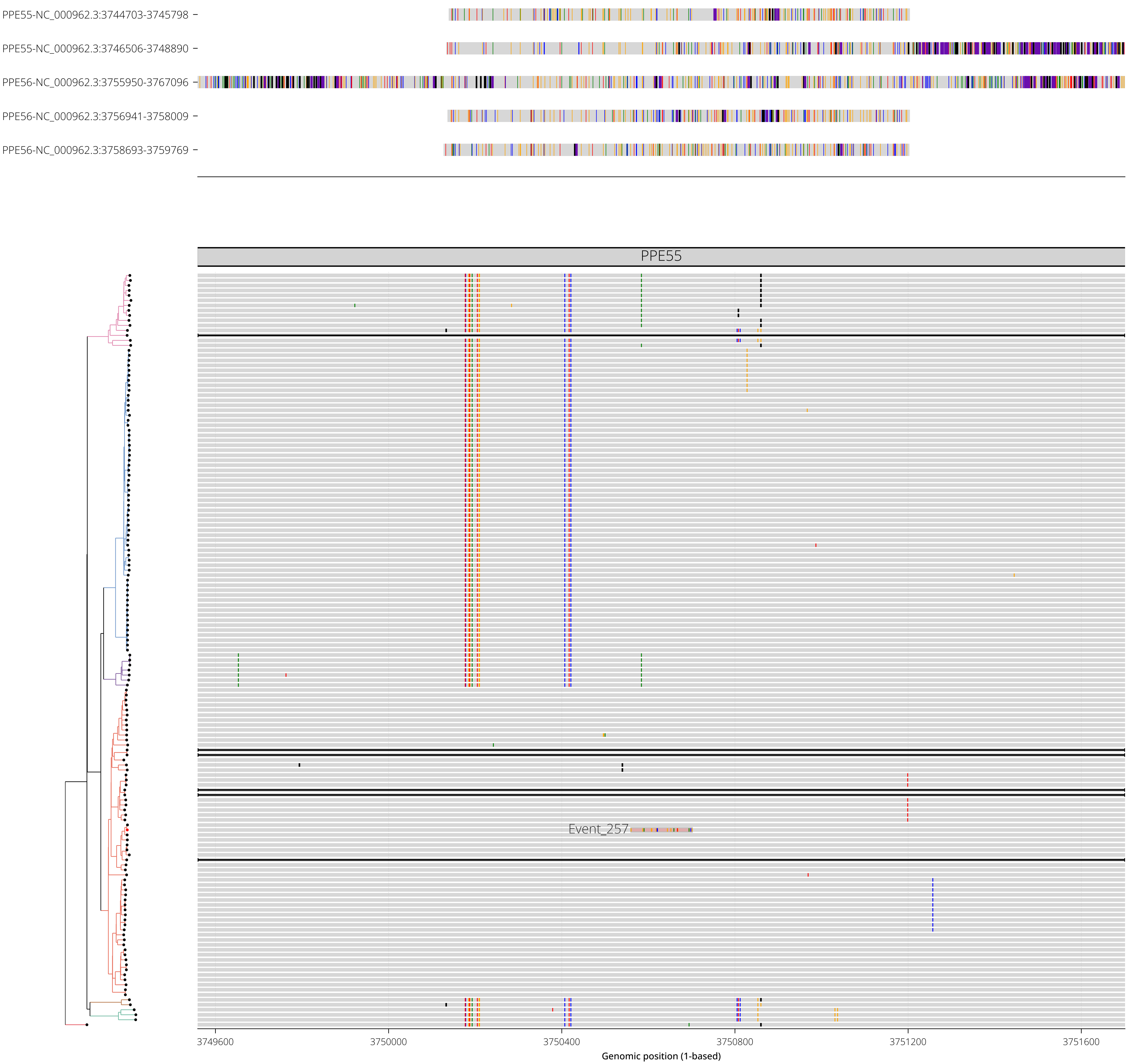

Individual GCE + Known Paralogs
GCE ID: Event_258

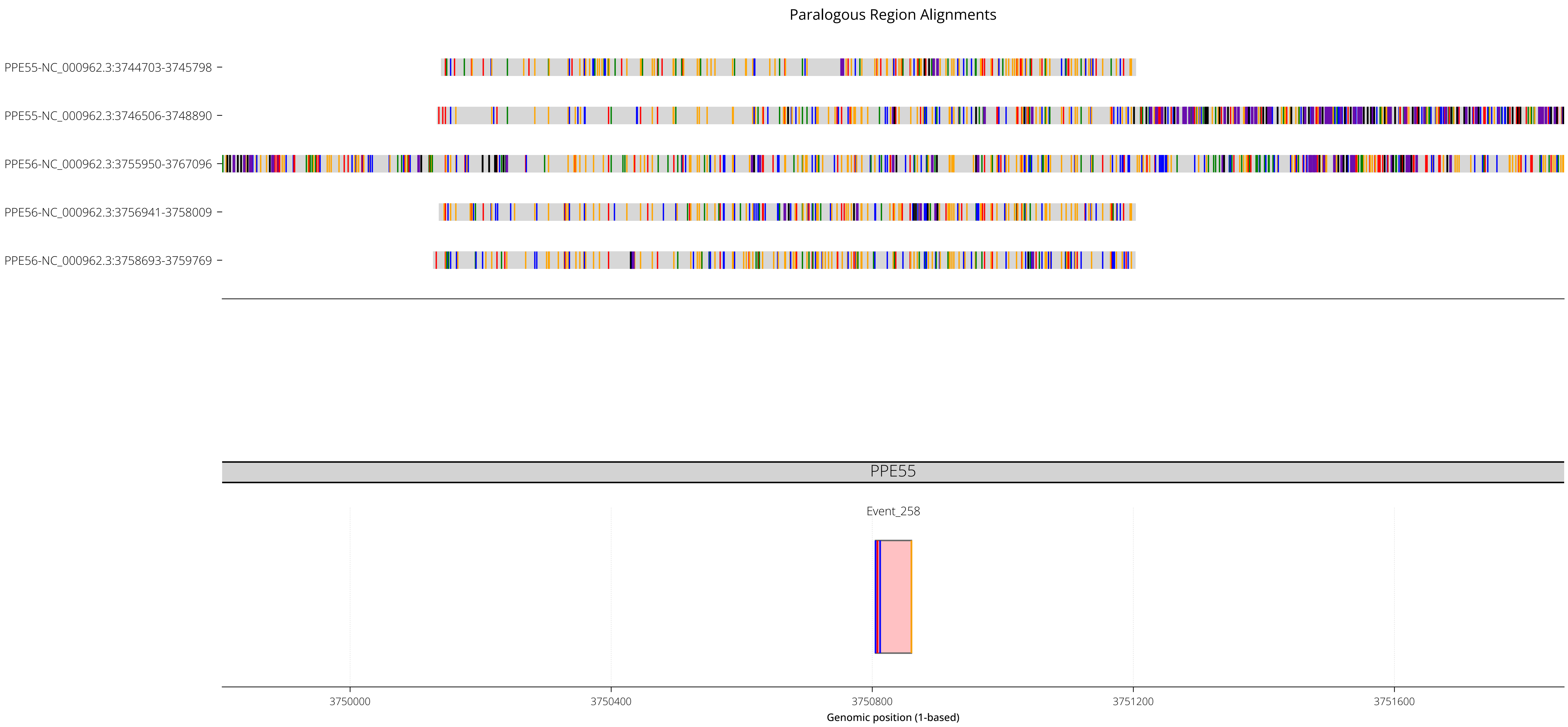

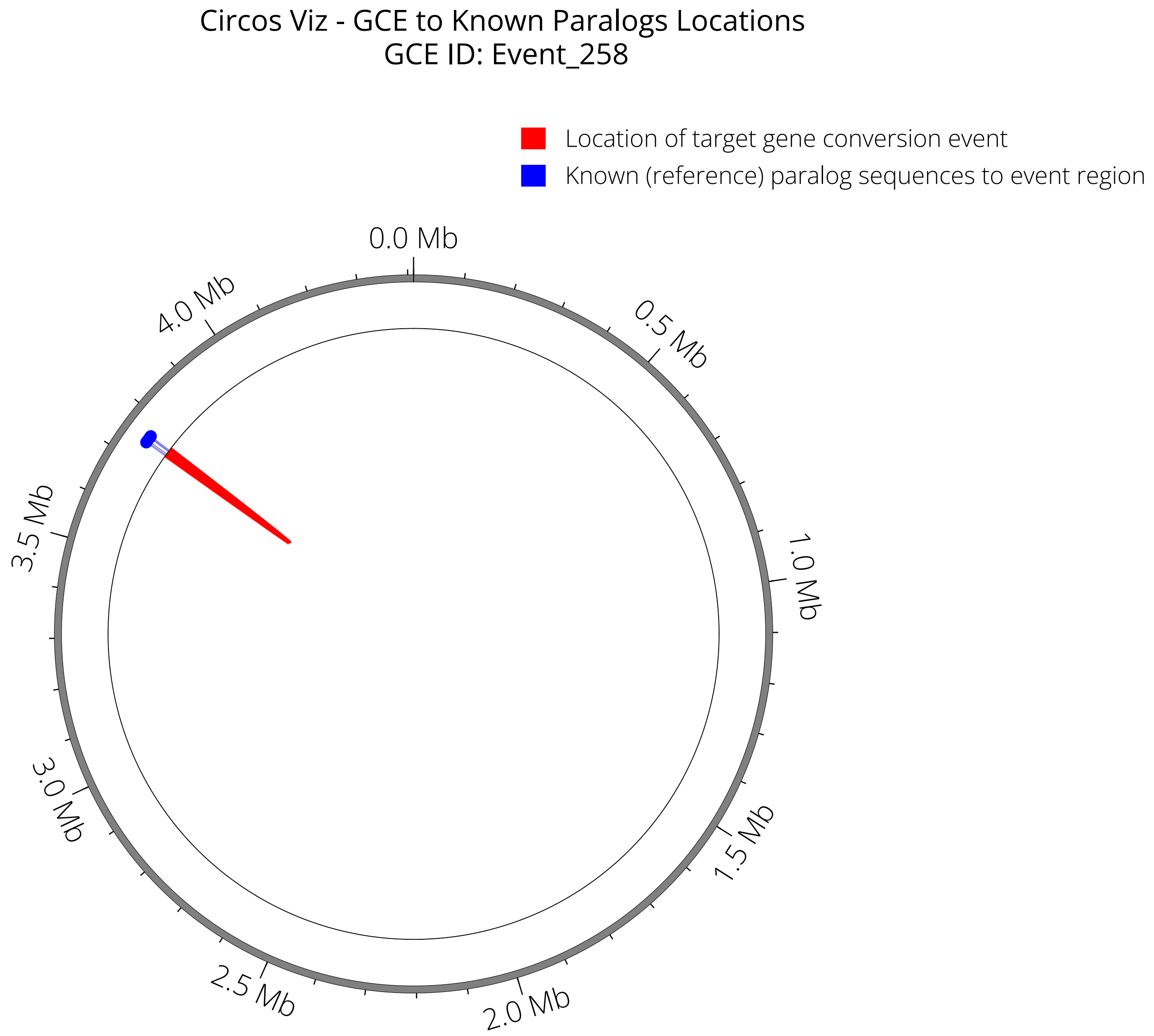
Circos Viz - GCE to Known Paralogs Locations
GCE ID: Event_258
Location of target gene conversion event
Known (reference) paralog sequences to event region
0.0 Mb
0.5 Mb
1.0 Mb
1.5 Mb
2.0 Mb
2.5 Mb
3.0 Mb
3.5 Mb
4.0 Mb

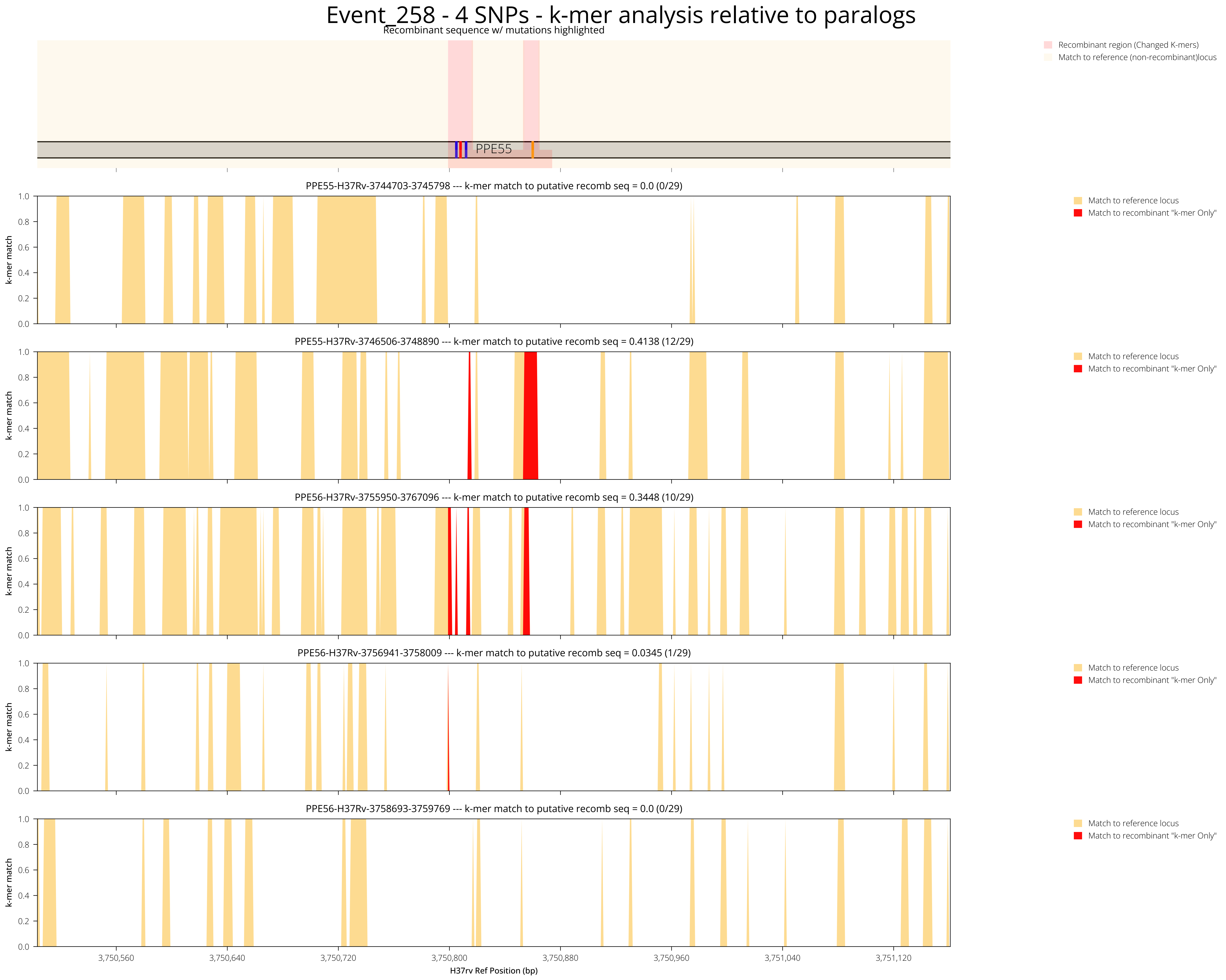

Event_258 - 4 SNPs - k-mer analysis relative to paralogs
Recombinant sequence w/ mutations highlighted
Recombinant region (Changed K-mers)
Match to reference (non-recombinant)locus
PPE55
PPE55-H37Rv-3744703-3745798 --- k-mer match to putative recomb seq = 0.0 (0/29)
PPE55-H37Rv-3746506-3748890 --- k-mer match to putative recomb seq = 0.4138 (12/29)
PPE56-H37Rv-3755950-3767096 --- k-mer match to putative recomb seq = 0.3448 (10/29)
PPE56-H37Rv-3756941-3758009 --- k-mer match to putative recomb seq = 0.0345 (1/29)
PPE56-H37Rv-3758693-3759769 --- k-mer match to putative recomb seq = 0.0 (0/29)
Match to reference locus
Match to recombinant "k-mer Only"
k-mer match
0.0
0.2
0.4
0.6
0.8
1.0
3,750,560
3,750,640
3,750,720
3,750,800
3,750,880
3,750,960
3,751,040
3,751,120
H37rv Ref Position (bp)

Sub-phylogeny Viz + Paralog Alignments
GCE ID: Event_258

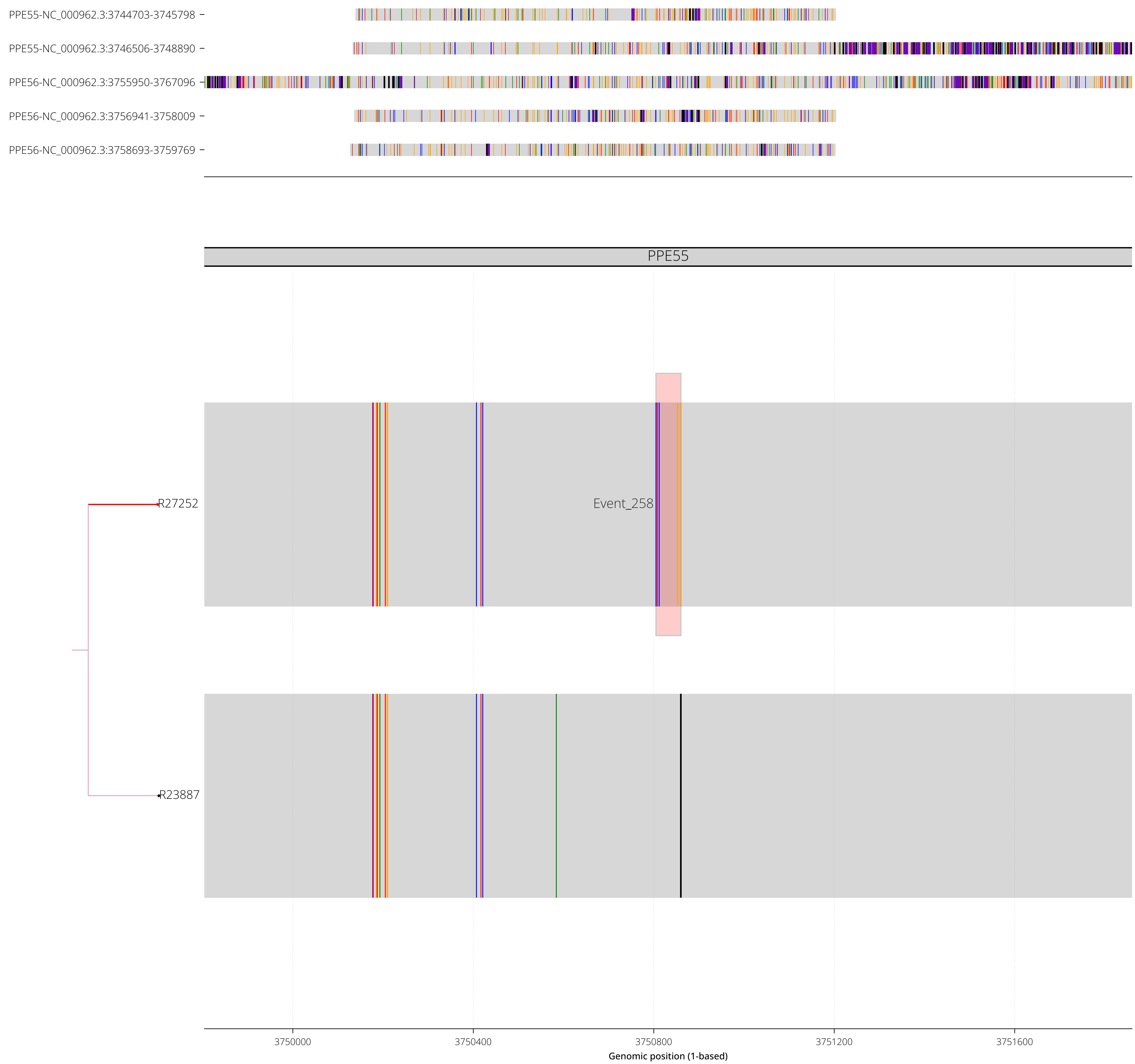

Full Phylogeny Viz + Paralog Alignments
GCE ID: Event_258

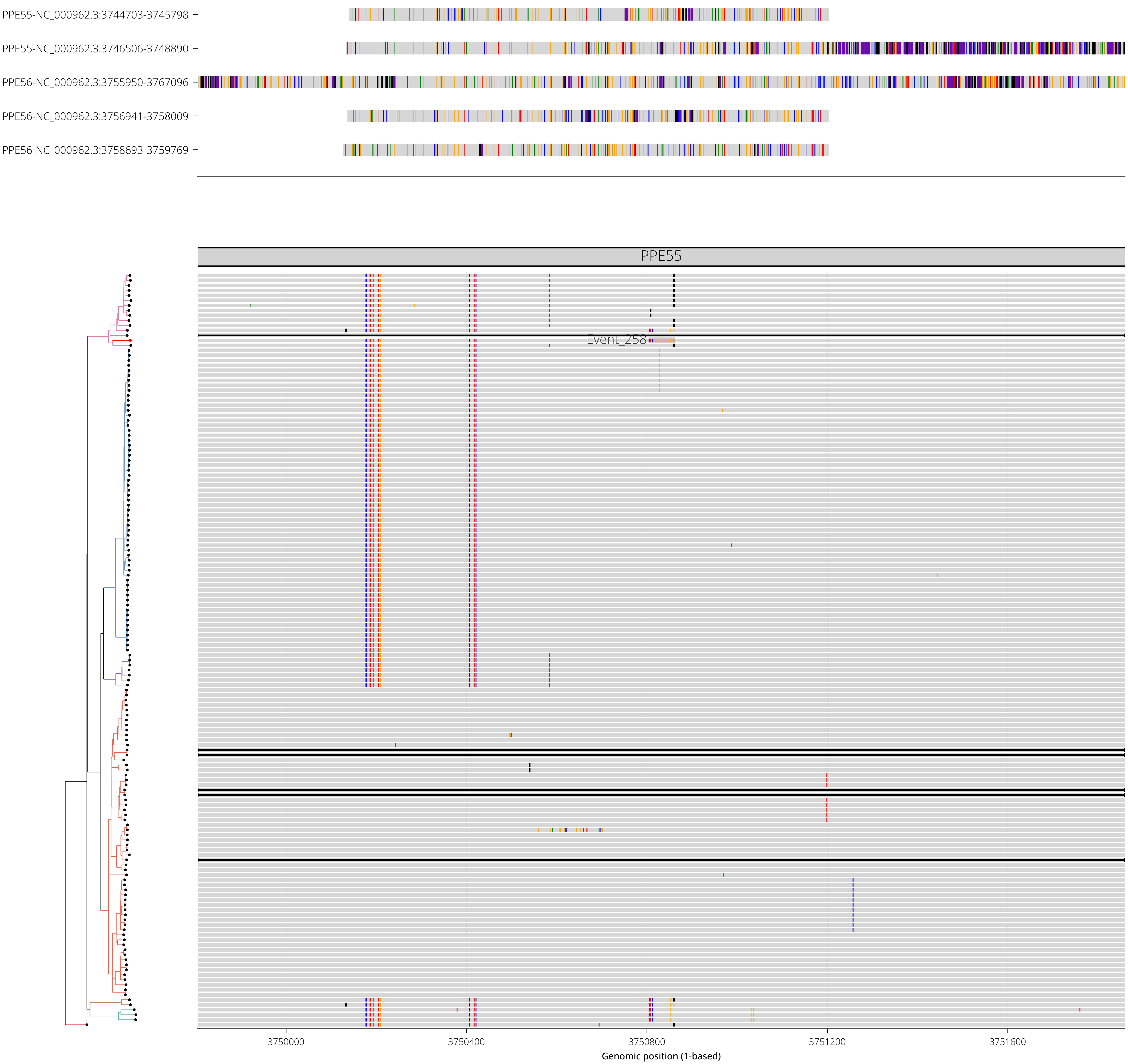

Individual GCE + Known Paralogs
GCE ID: Event_259

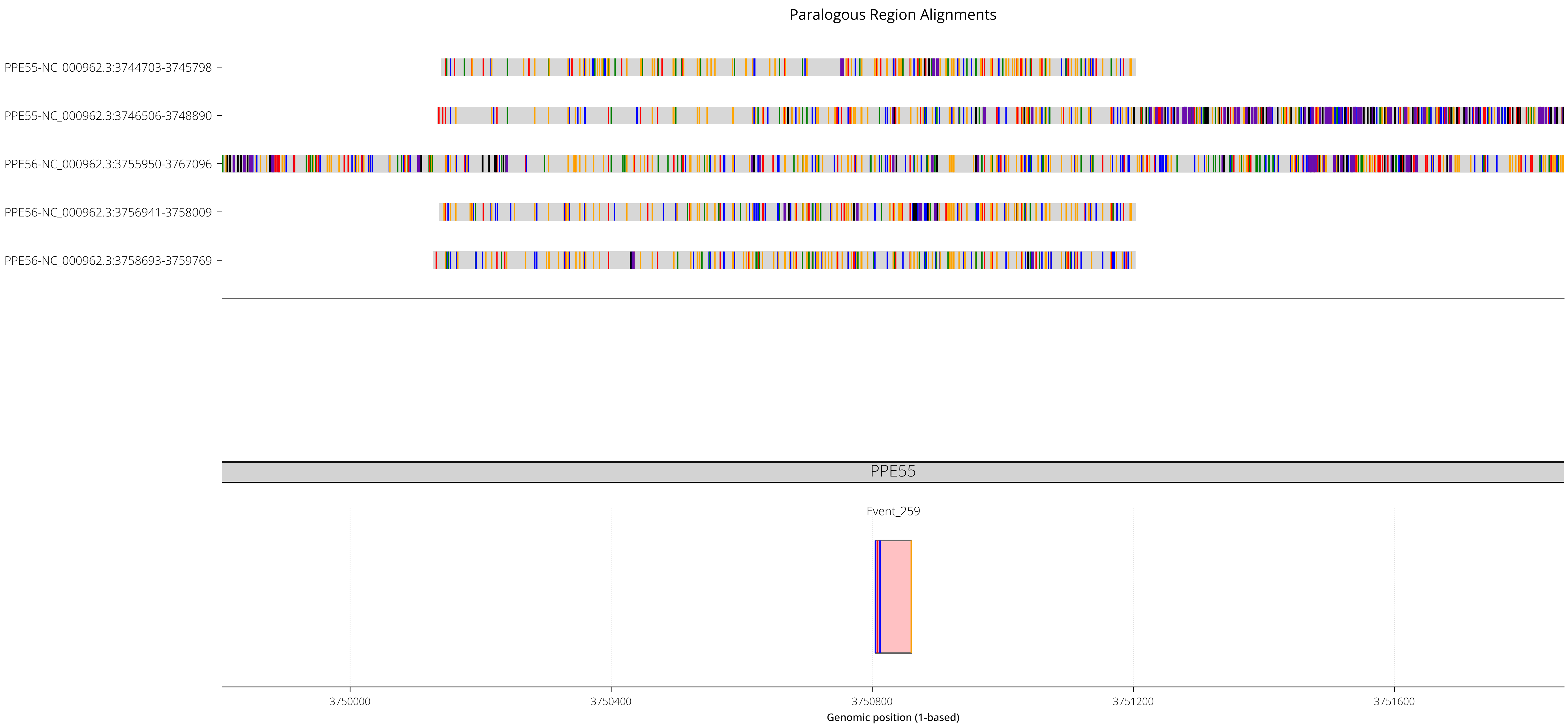

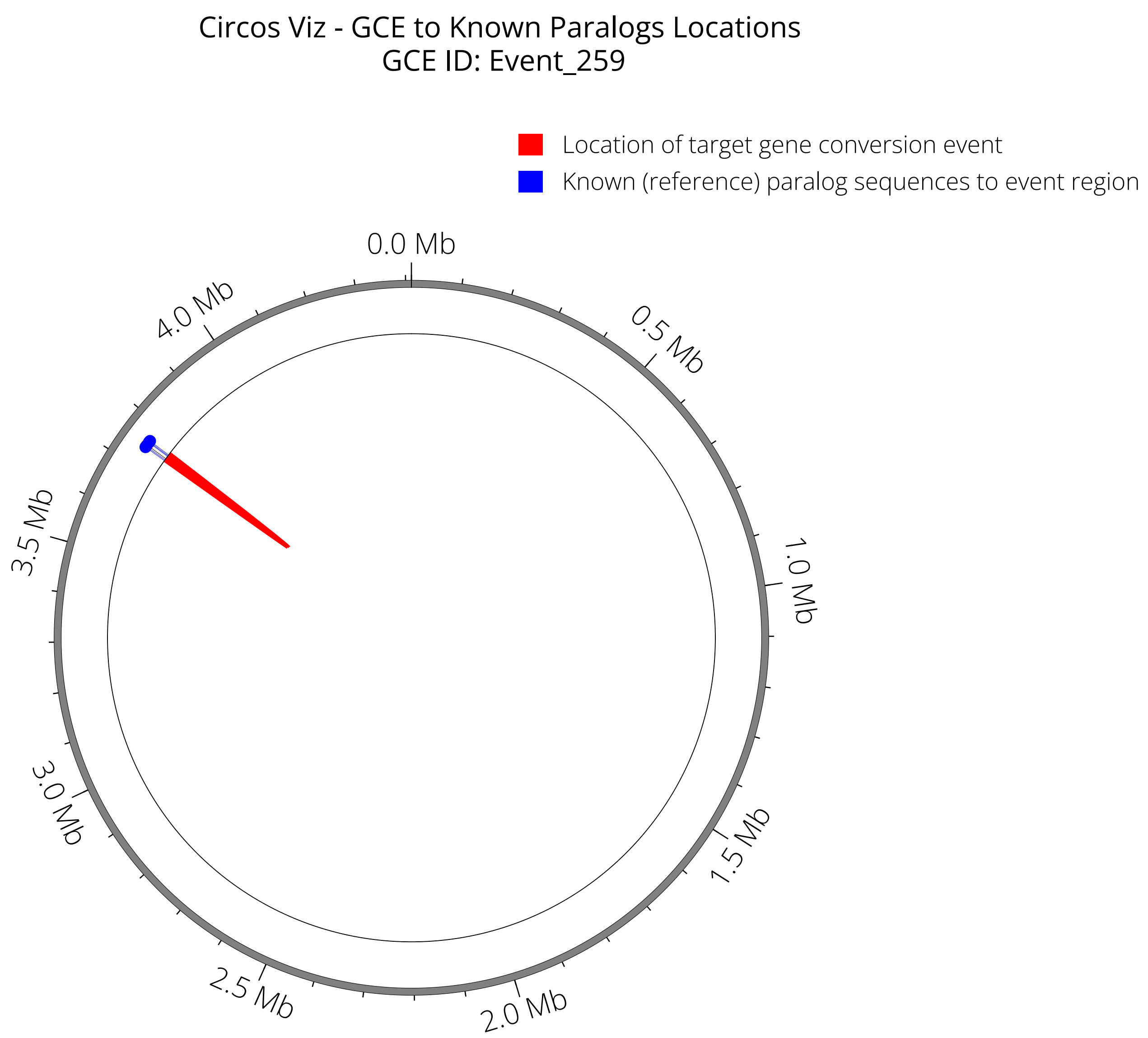

Circos Viz - GCE to Known Paralogs Locations
GCE ID: Event_259
Location of target gene conversion event
Known (reference) paralog sequences to event region
0.0 Mb
0.5 Mb
1.0 Mb
1.5 Mb
2.0 Mb
2.5 Mb
3.0 Mb
3.5 Mb
4.0 Mb

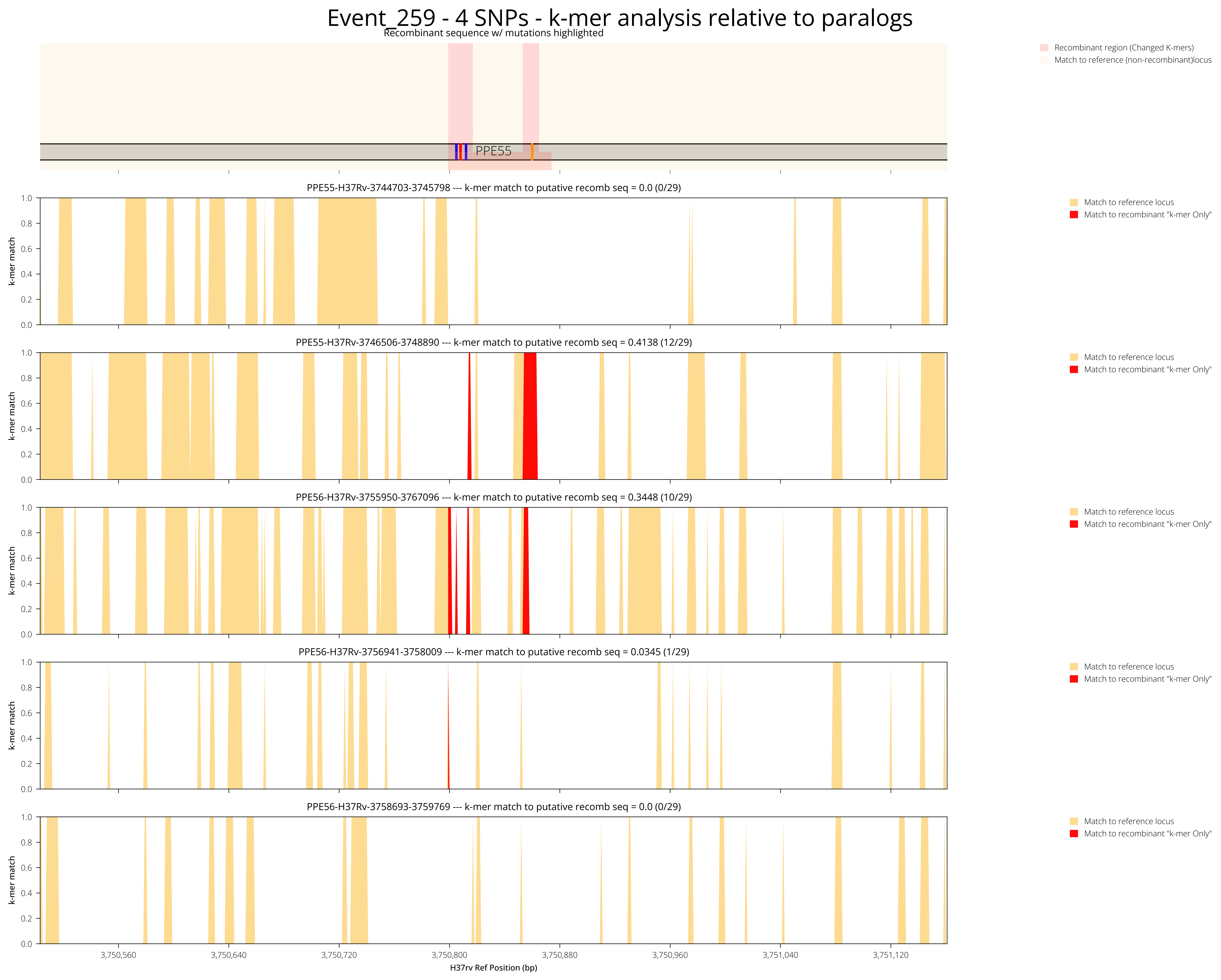
Event_259 - 4 SNPs - k-mer analysis relative to paralogs
Recombinant sequence w/ mutations highlighted
Recombinant region (Changed K-mers)
Match to reference (non-recombinant)locus
PPE55
PPE55-H37Rv-3744703-3745798 --- k-mer match to putative recomb seq = 0.0 (0/29)
PPE55-H37Rv-3746506-3748890 --- k-mer match to putative recomb seq = 0.4138 (12/29)
PPE56-H37Rv-3755950-3767096 --- k-mer match to putative recomb seq = 0.3448 (10/29)
PPE56-H37Rv-3756941-3758009 --- k-mer match to putative recomb seq = 0.0345 (1/29)
PPE56-H37Rv-3758693-3759769 --- k-mer match to putative recomb seq = 0.0 (0/29)
k-mer match
0.0
0.2
0.4
0.6
0.8
1.0
Match to reference locus
Match to recombinant "k-mer Only"
3,750,560
3,750,640
3,750,720
3,750,800
3,750,880
3,750,960
3,751,040
3,751,120
H37rv Ref Position (bp)

Sub-phylogeny Viz + Paralog Alignments
GCE ID: Event_259

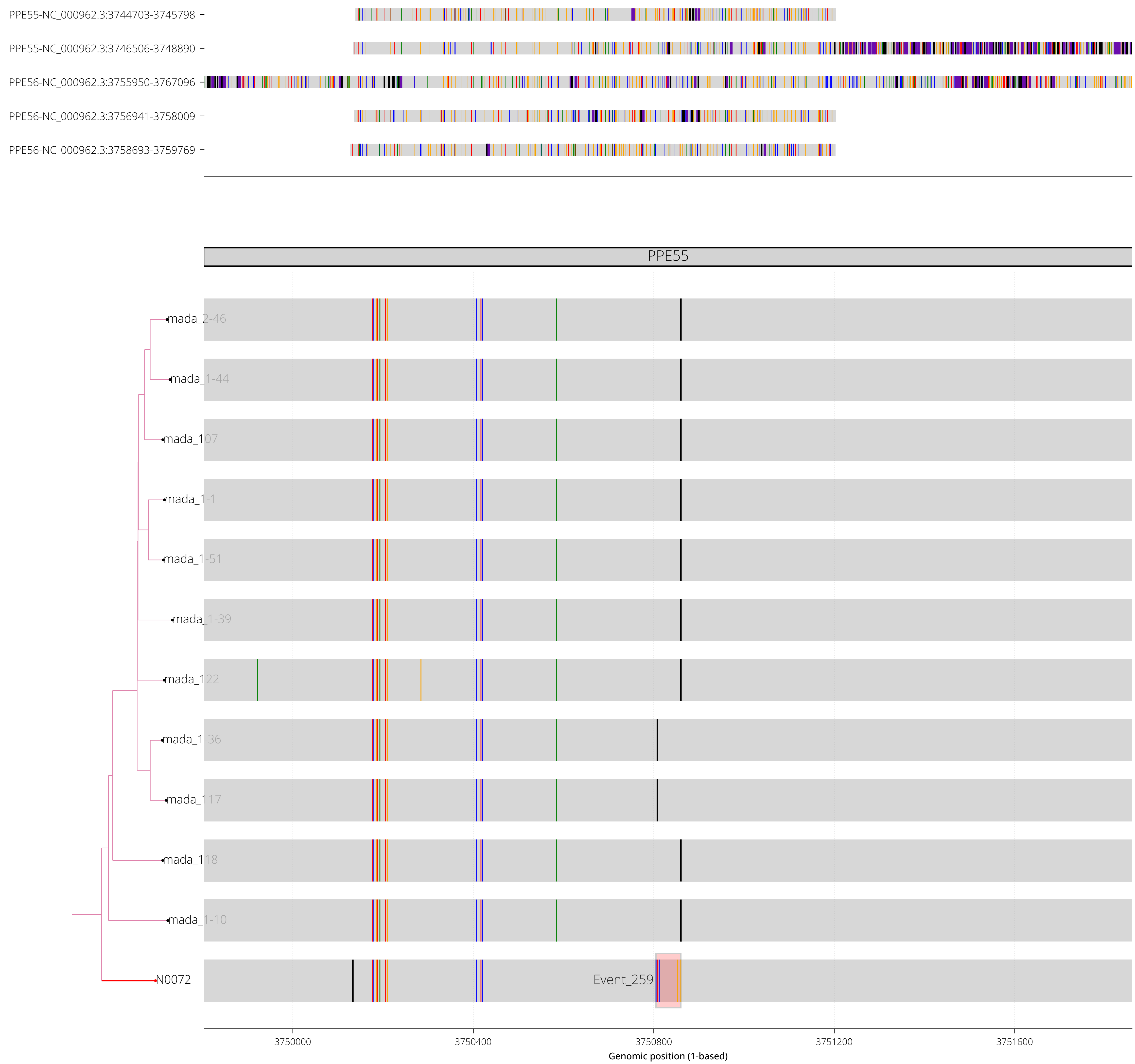

Full Phylogeny Viz + Paralog Alignments
GCE ID: Event_259

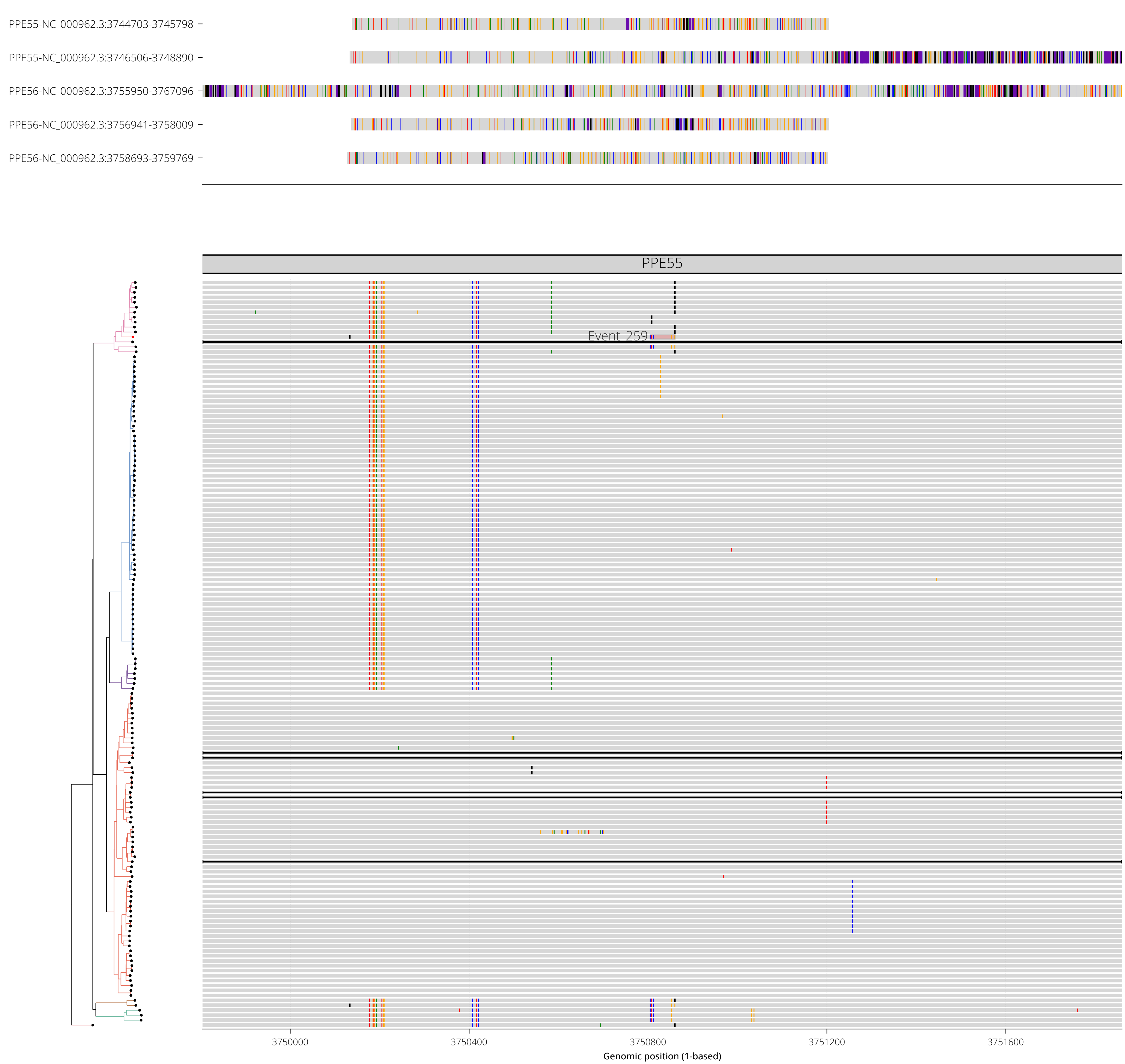

Individual GCE + Known Paralogs
GCE ID: Event_260

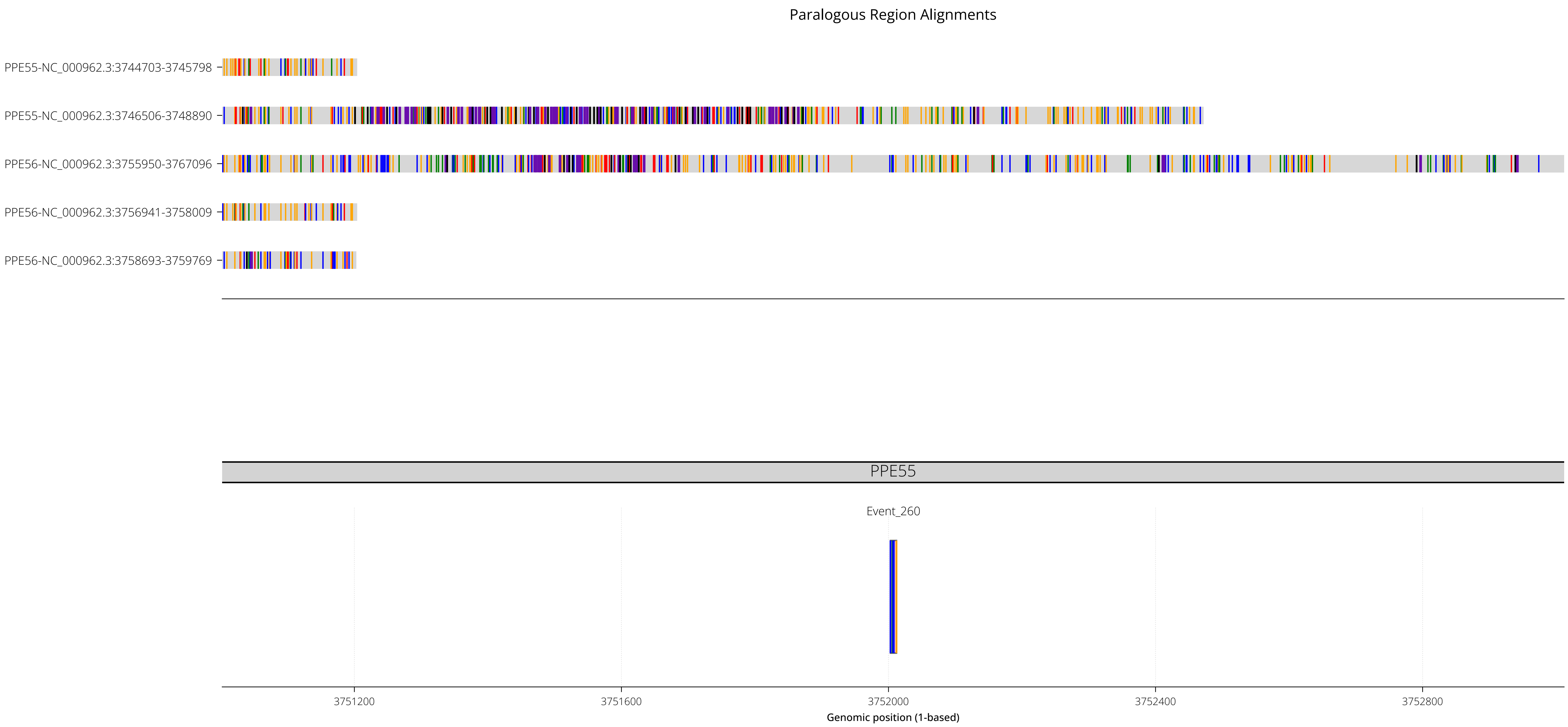

Circos Viz - GCE to Known Paralogs Locations
GCE ID: Event_260

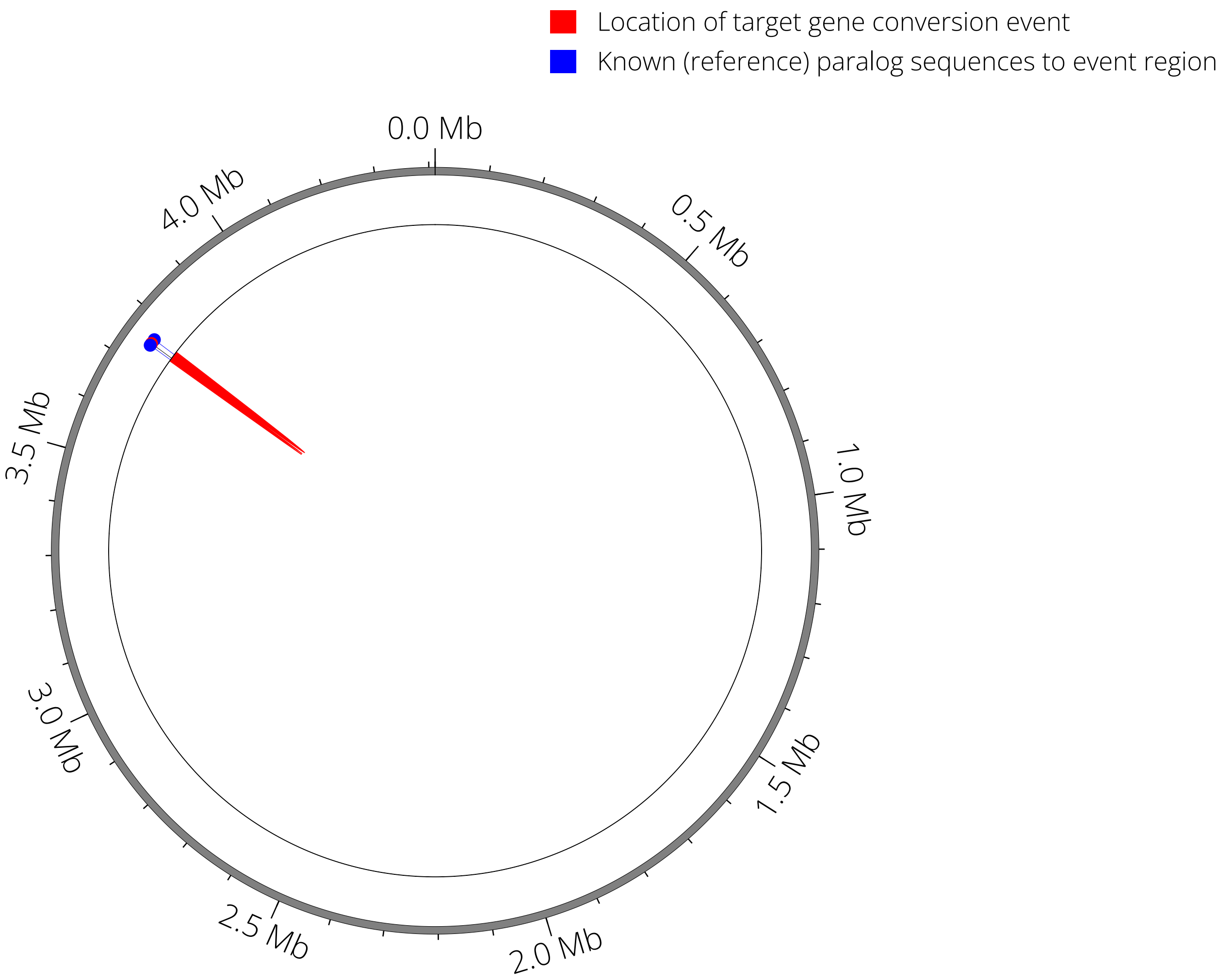

# Event_260 - 5 SNPs - k-mer analysis relative to paralogs

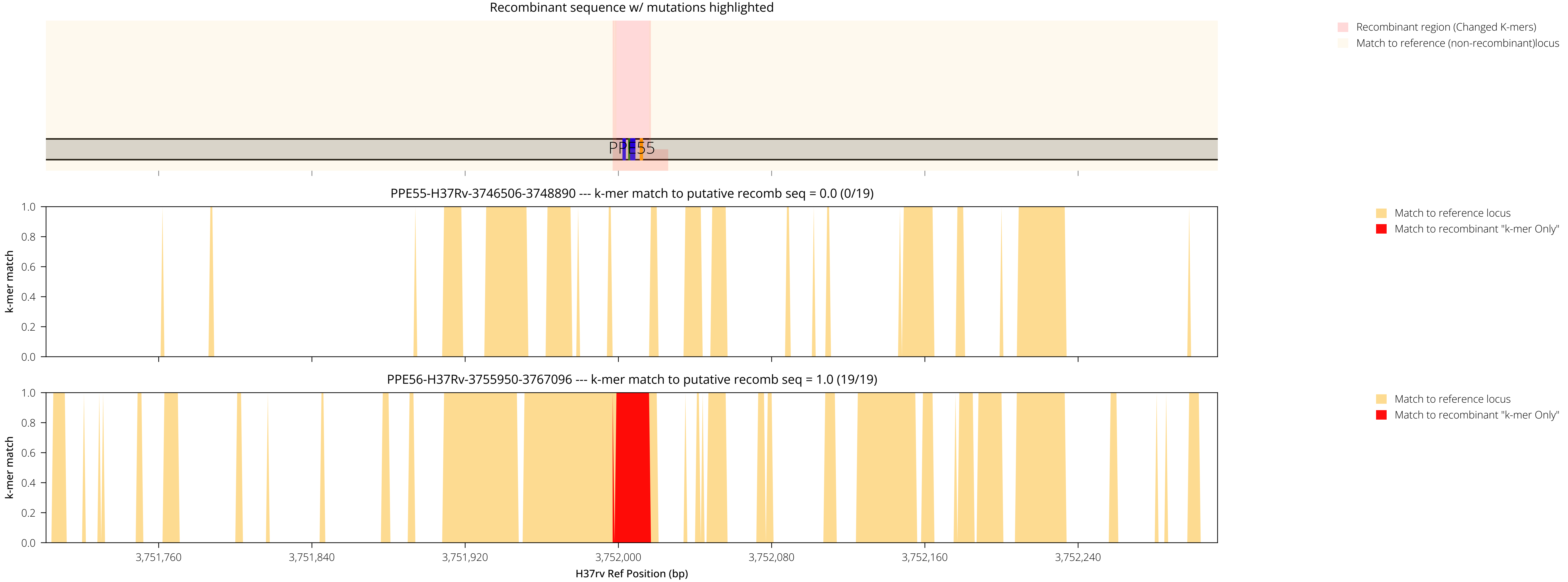

Sub-phylogeny Viz + Paralog Alignments
GCE ID: Event_260

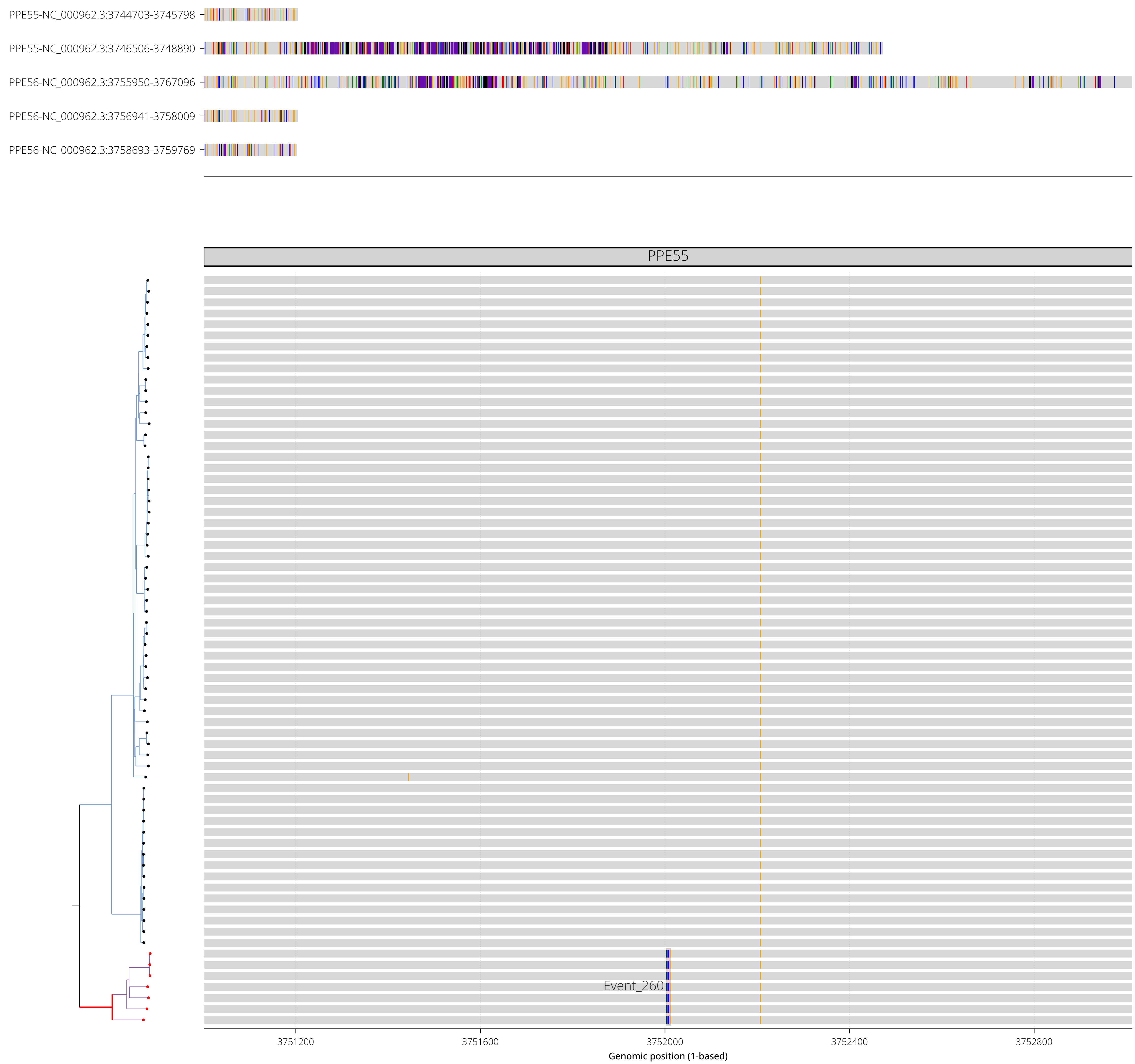

Full Phylogeny Viz + Paralog Alignments
GCE ID: Event_260

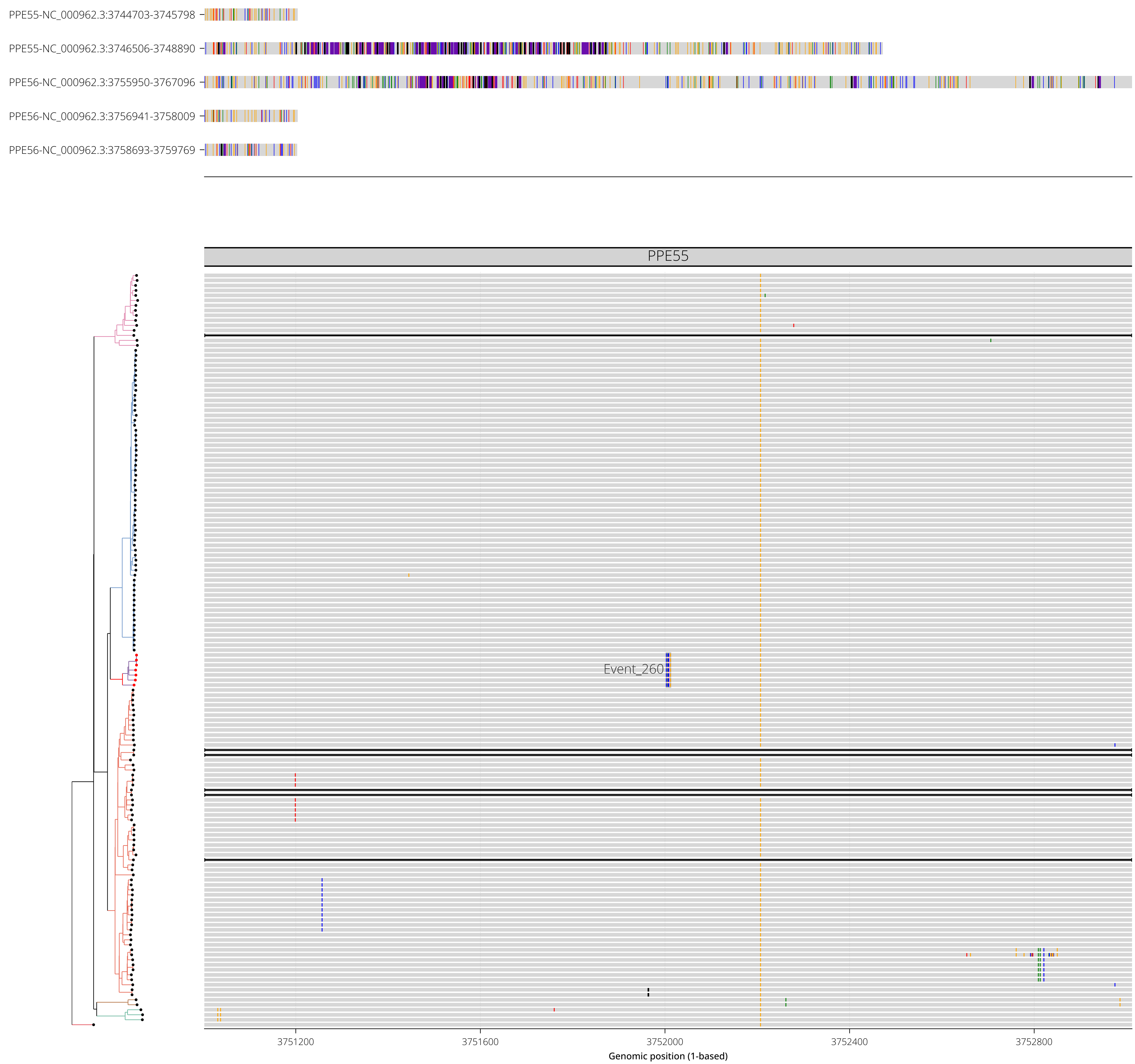

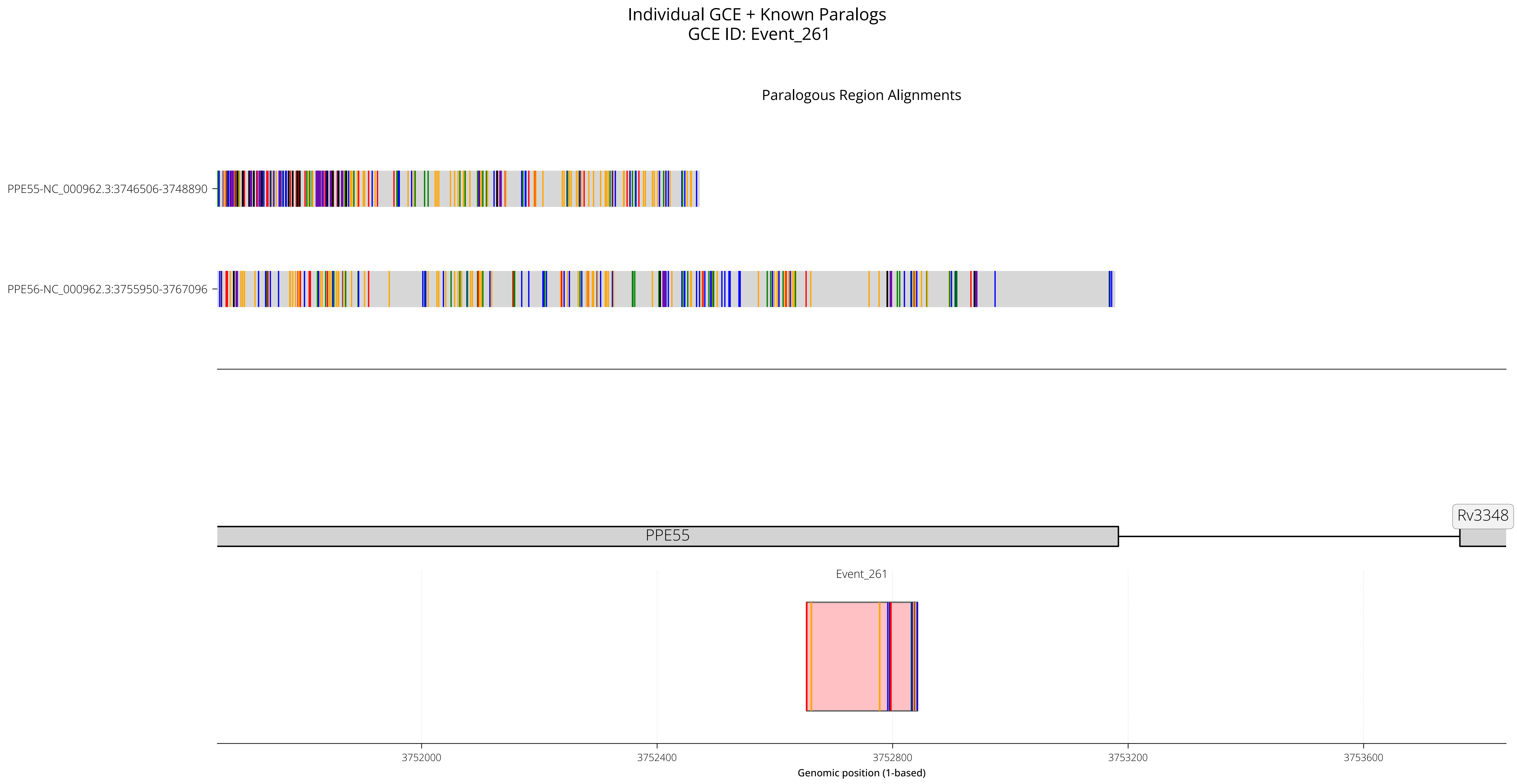

Individual GCE + Known Paralogs
GCE ID: Event_261
Paralogous Region Alignments
PPE55-NC_000962.3:3746506-3748890
PPE56-NC_000962.3:3755950-3767096
Rv3348
PPE55
Event_261
3752000
3752400
3752800
3753200
3753600
Genomic position (1-based)

Circos Viz - GCE to Known Paralogs Locations
GCE ID: Event_261

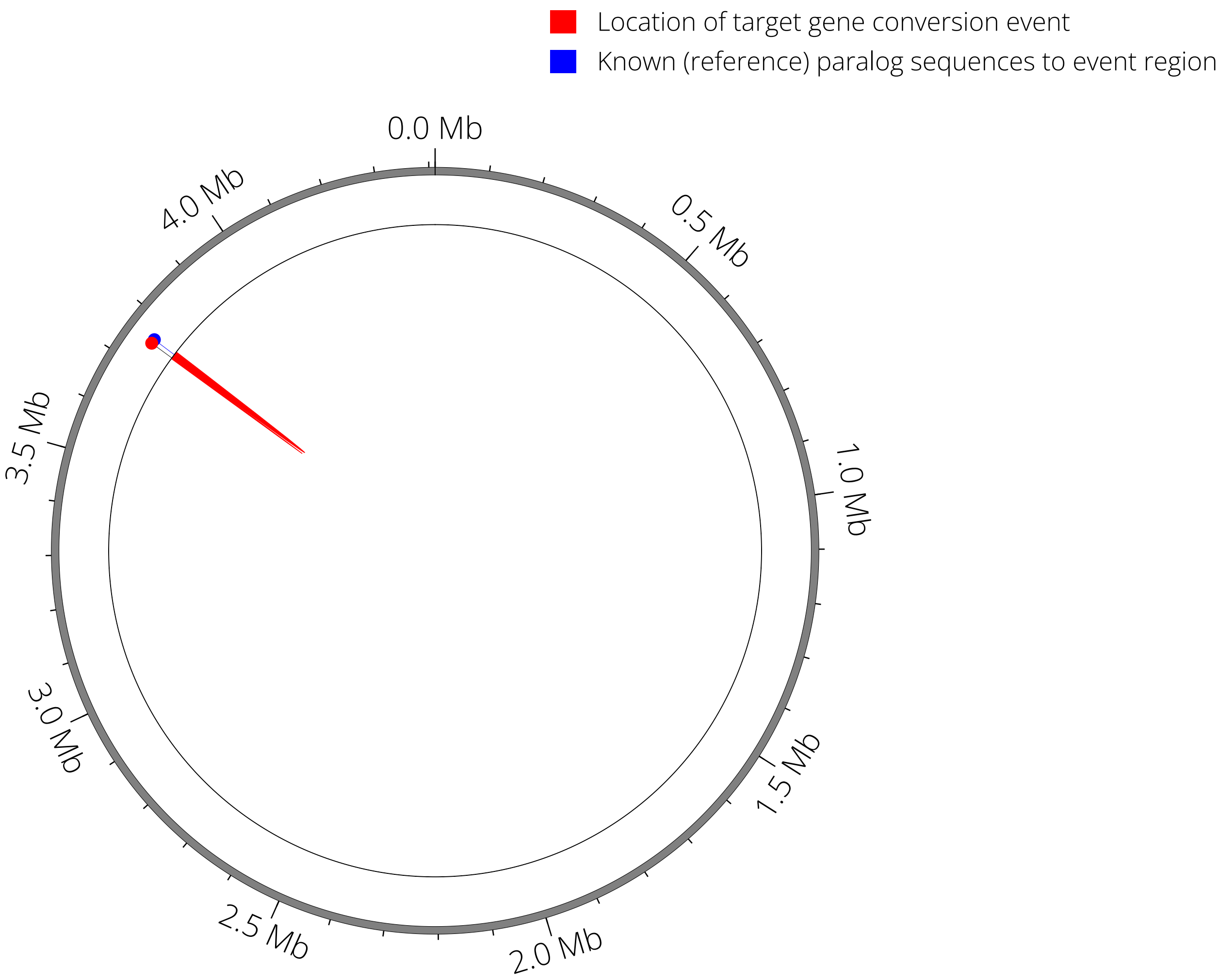

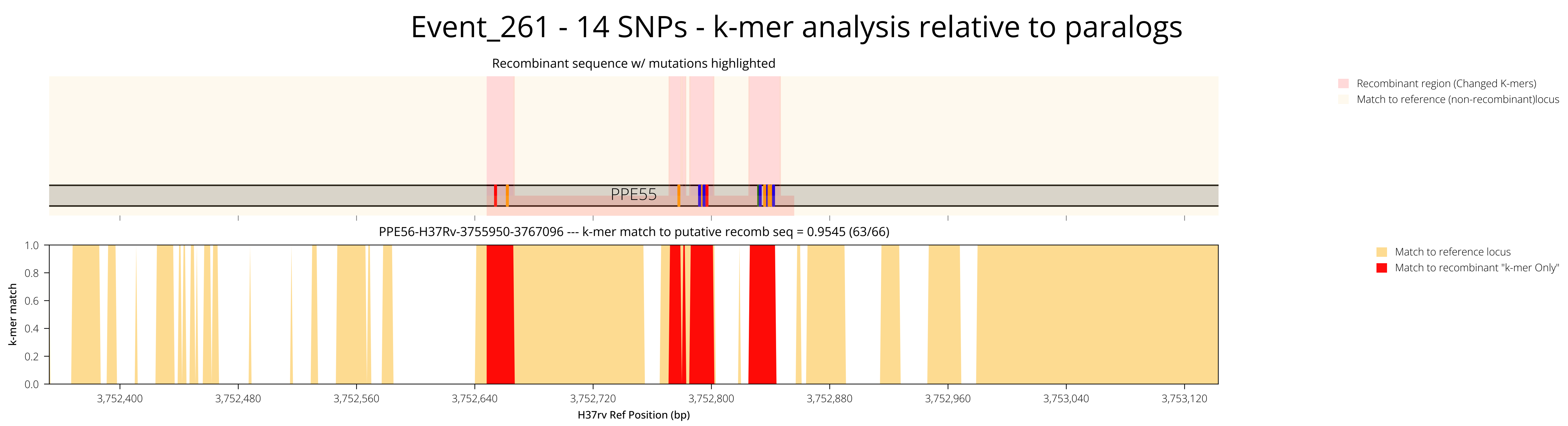

Event_261 - 14 SNPs - k-mer analysis relative to paralogs
Recombinant sequence w/ mutations highlighted
PPE55
Recombinant region (Changed K-mers)
Match to reference (non-recombinant)locus
PPE56-H37Rv-3755950-3767096 --- k-mer match to putative recomb seq = 0.9545 (63/66)
Match to reference locus
Match to recombinant "k-mer Only"
k-mer match
1.0
0.8
0.6
0.4
0.2
0.0
3,752,400
3,752,480
3,752,560
3,752,640
3,752,720
3,752,800
3,752,880
3,752,960
3,753,040
3,753,120
H37rv Ref Position (bp)

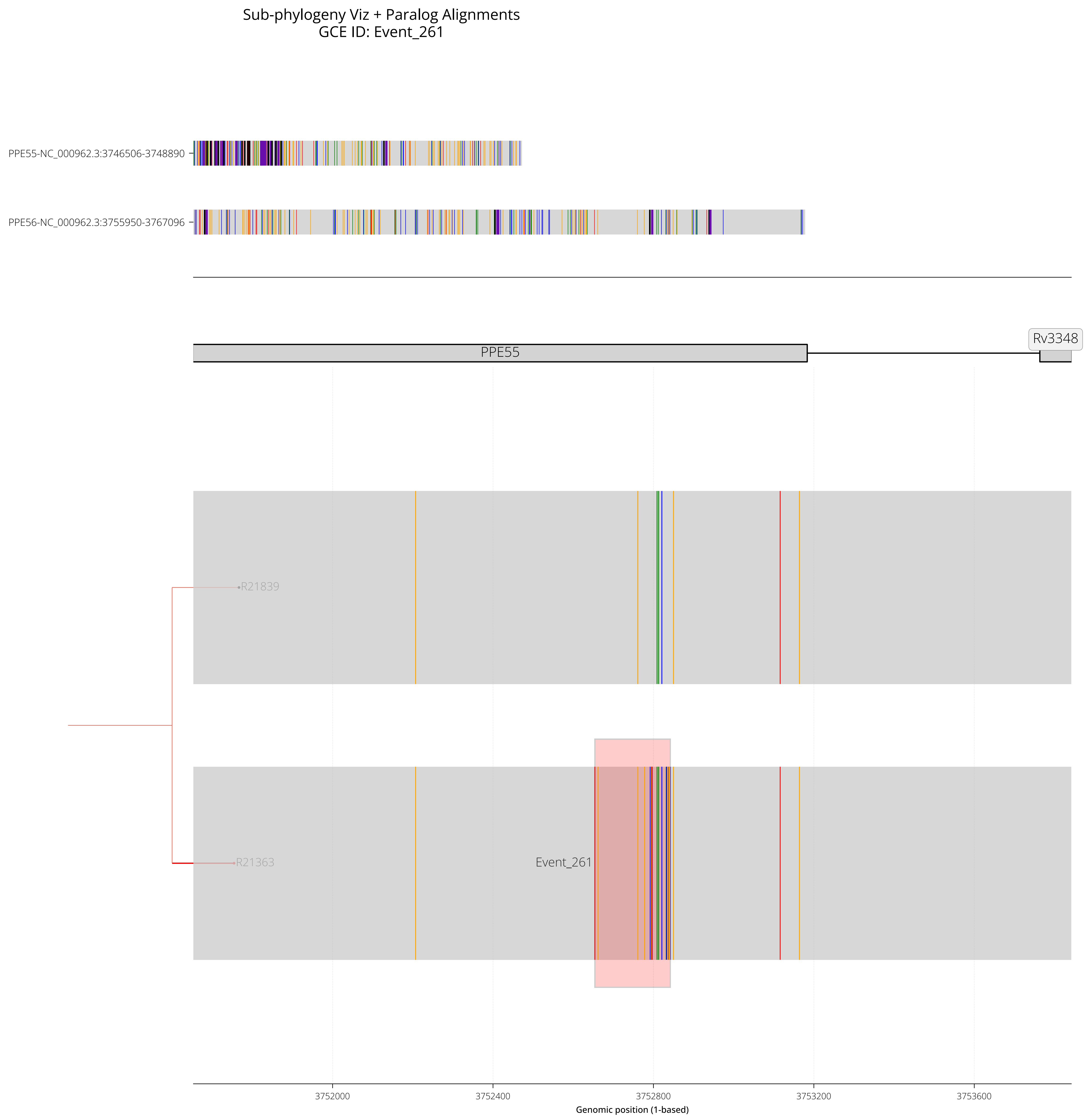

Sub-phylogeny Viz + Paralog Alignments
GCE ID: Event_261
PPE55-NC_000962.3:3746506-3748890
PPE56-NC_000962.3:3755950-3767096
PPE55
Rv3348
R21839
R21363
Event_261
3752000
3752400
3752800
3753200
3753600
Genomic position (1-based)

# Full Phylogeny Viz + Paralog Alignments
GCE ID: Event_261

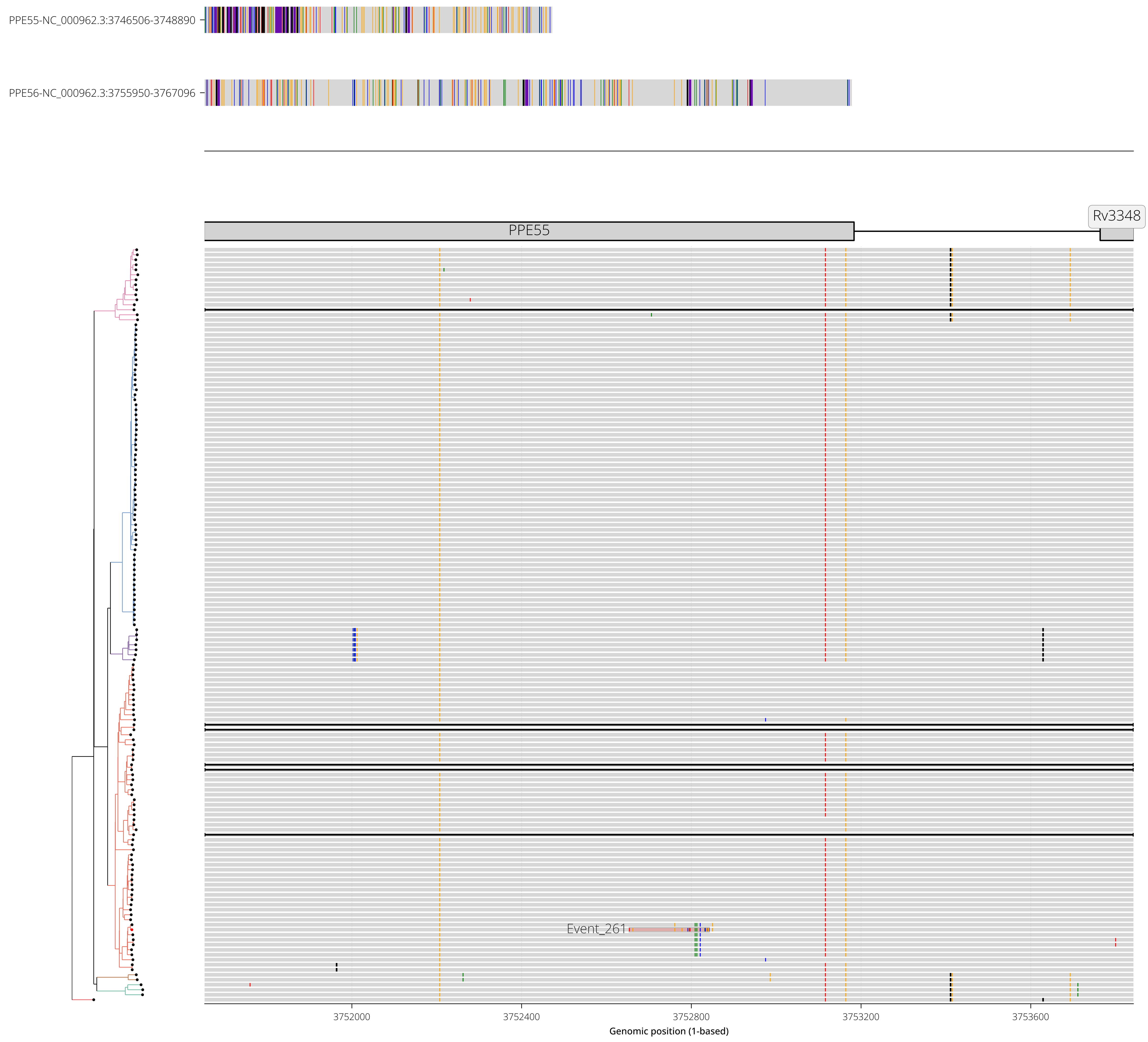

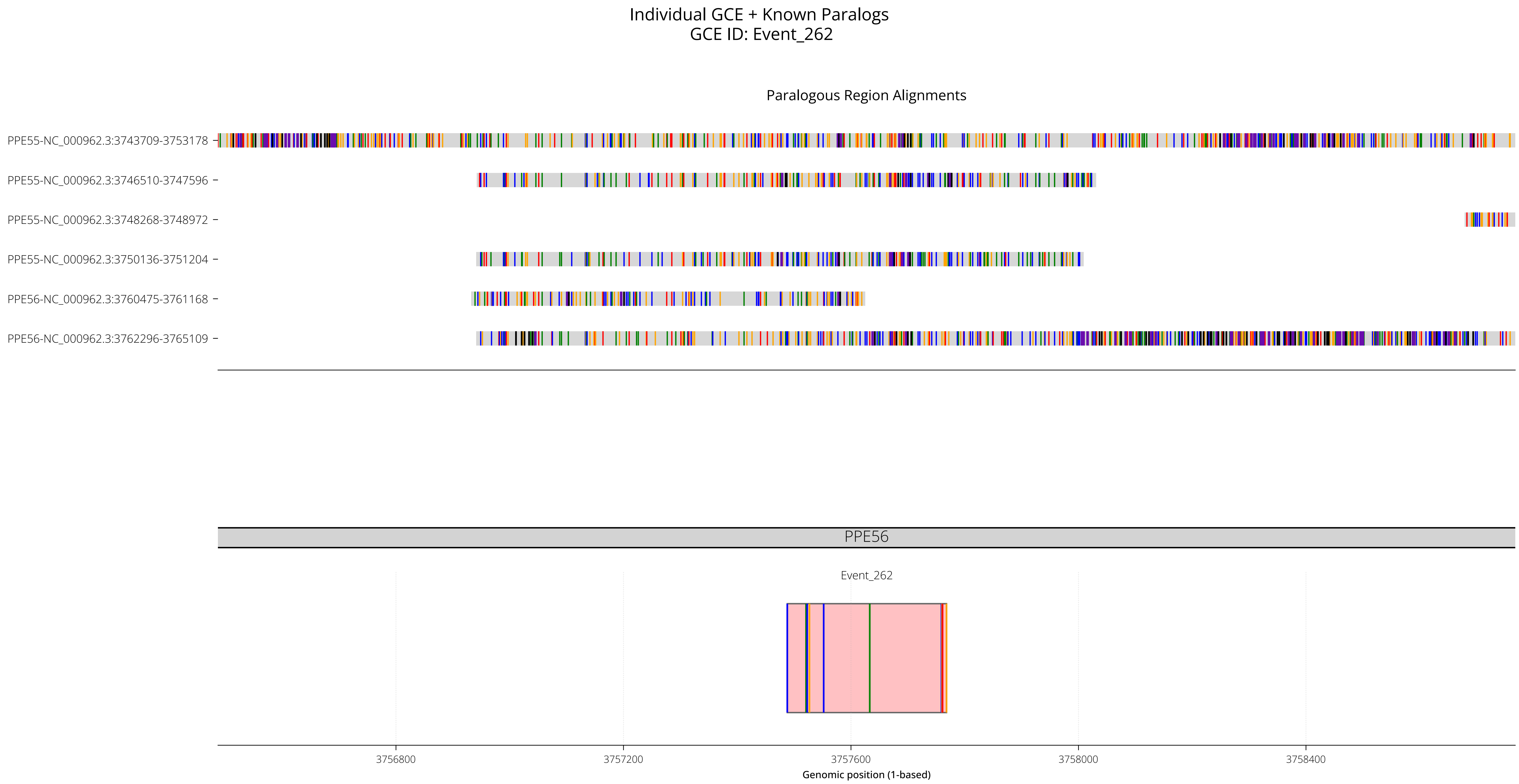
Individual GCE + Known Paralogs
GCE ID: Event_262
Paralogous Region Alignments
PPE55-NC_000962.3:3743709-3753178
PPE55-NC_000962.3:3746510-3747596
PPE55-NC_000962.3:3748268-3748972
PPE55-NC_000962.3:3750136-3751204
PPE56-NC_000962.3:3760475-3761168
PPE56-NC_000962.3:3762296-3765109
PPE56
Event_262
3756800
3757200
3757600
3758000
3758400
Genomic position (1-based)

Circos Viz - GCE to Known Paralogs Locations
GCE ID: Event_262

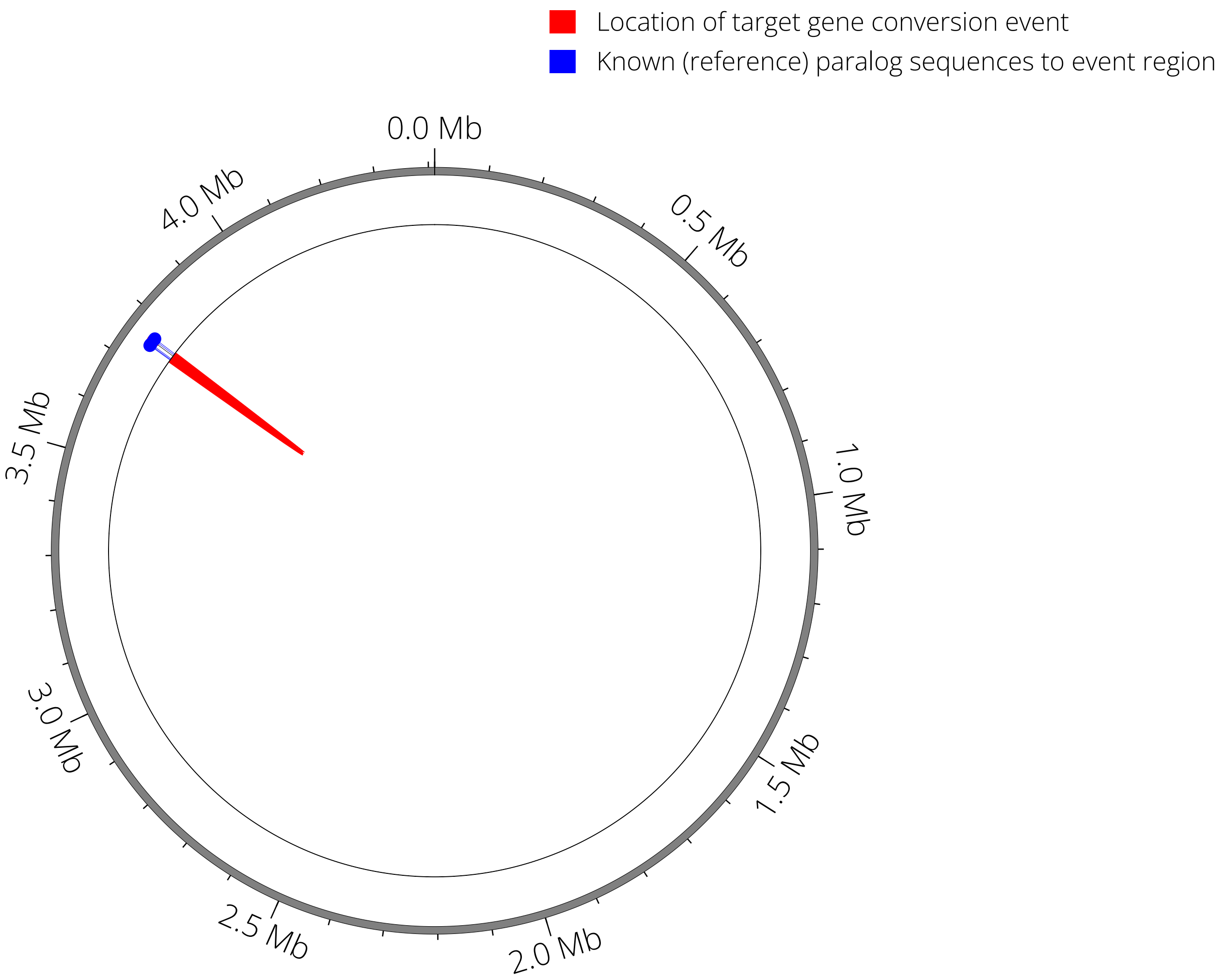

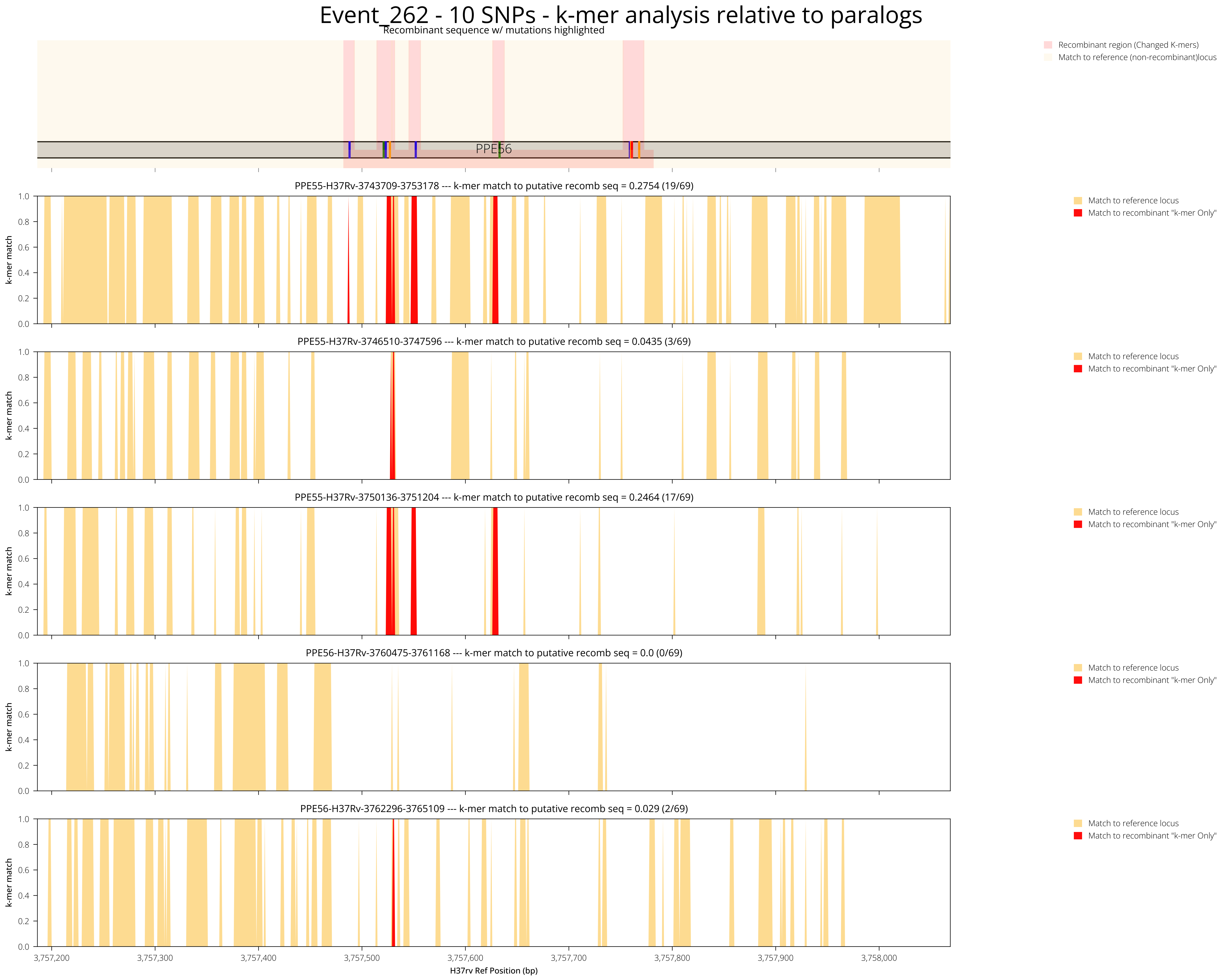
Event_262 - 10 SNPs - k-mer analysis relative to paralogs
Recombinant sequence w/ mutations highlighted
Recombinant region (Changed K-mers)
Match to reference (non-recombinant)locus
PPE56
PPE55-H37Rv-3743709-3753178 --- k-mer match to putative recomb seq = 0.2754 (19/69)
PPE55-H37Rv-3746510-3747596 --- k-mer match to putative recomb seq = 0.0435 (3/69)
PPE55-H37Rv-3750136-3751204 --- k-mer match to putative recomb seq = 0.2464 (17/69)
PPE56-H37Rv-3760475-3761168 --- k-mer match to putative recomb seq = 0.0 (0/69)
PPE56-H37Rv-3762296-3765109 --- k-mer match to putative recomb seq = 0.029 (2/69)
Match to reference locus
Match to recombinant "k-mer Only"
k-mer match
1.0
0.8
0.6
0.4
0.2
0.0
3,757,200
3,757,300
3,757,400
3,757,500
3,757,600
3,757,700
3,757,800
3,757,900
3,758,000
H37rv Ref Position (bp)

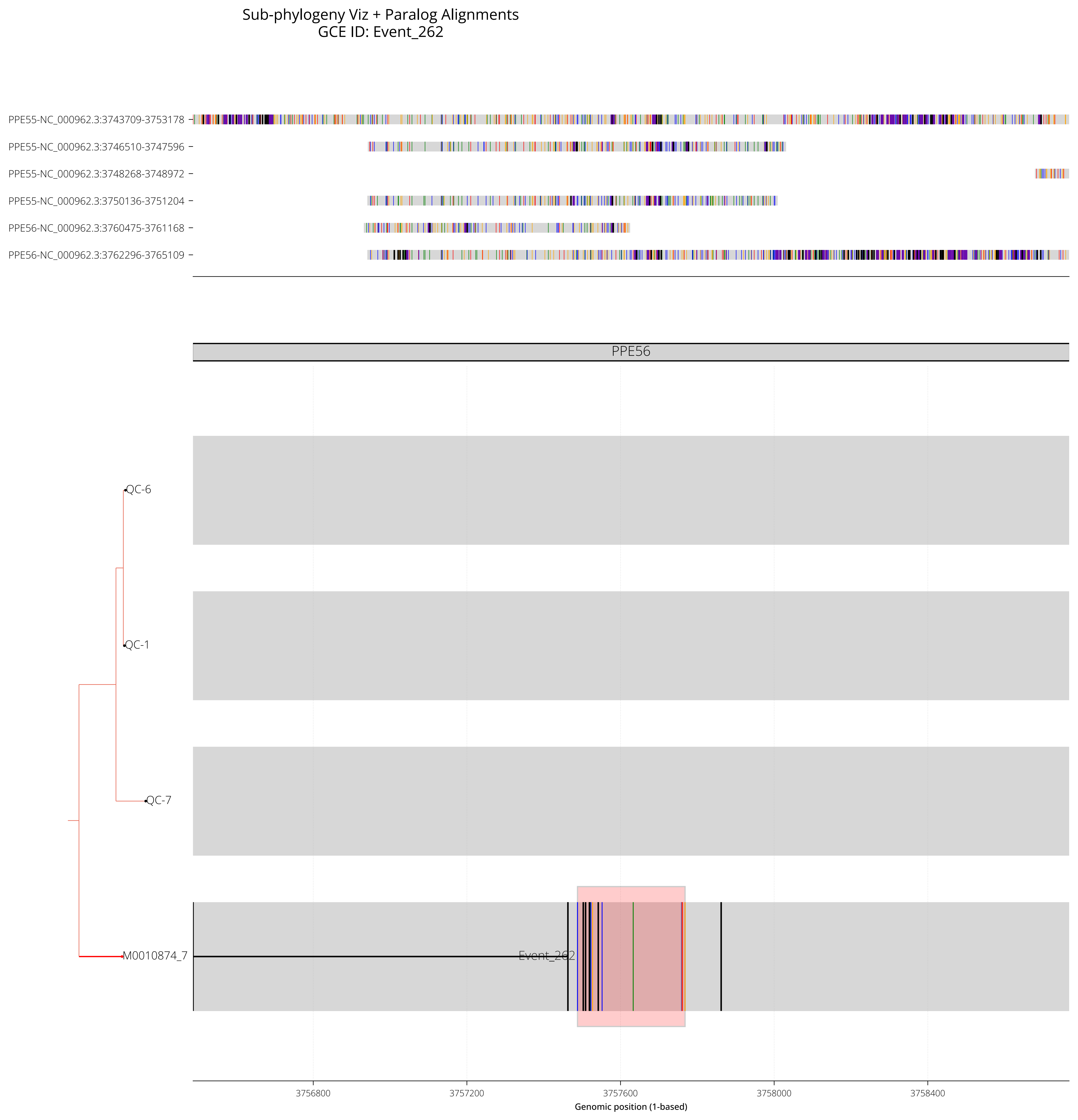
Sub-phylogeny Viz + Paralog Alignments
GCE ID: Event_262
PPE55-NC_000962.3:3743709-3753178
PPE55-NC_000962.3:3746510-3747596
PPE55-NC_000962.3:3748268-3748972
PPE55-NC_000962.3:3750136-3751204
PPE56-NC_000962.3:3760475-3761168
PPE56-NC_000962.3:3762296-3765109
PPE56
QC-6
QC-1
QC-7
M0010874_7
Event_262
3756800
3757200
3757600
3758000
3758400
Genomic position (1-based)

Full Phylogeny Viz + Paralog Alignments
GCE ID: Event_262

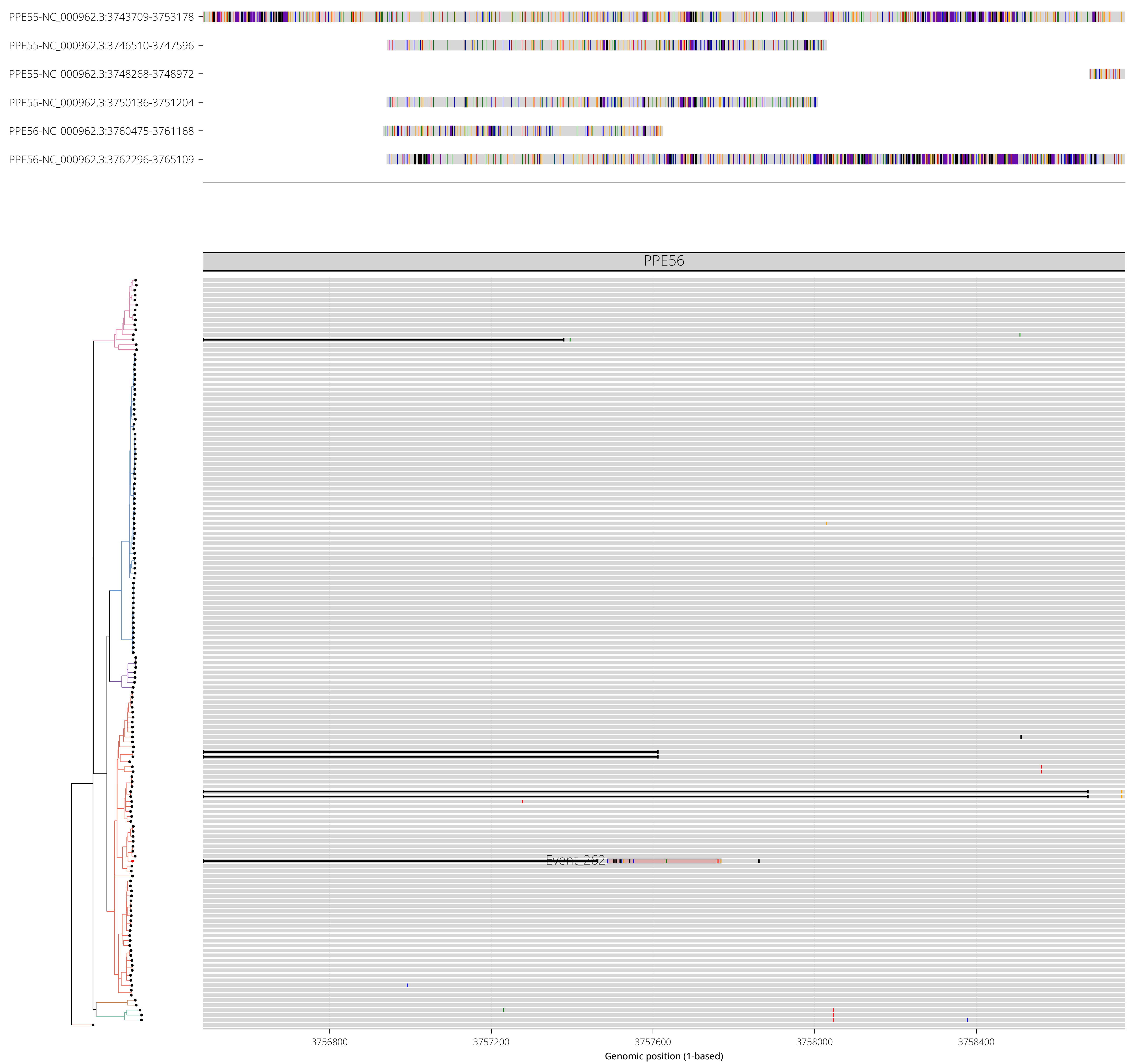

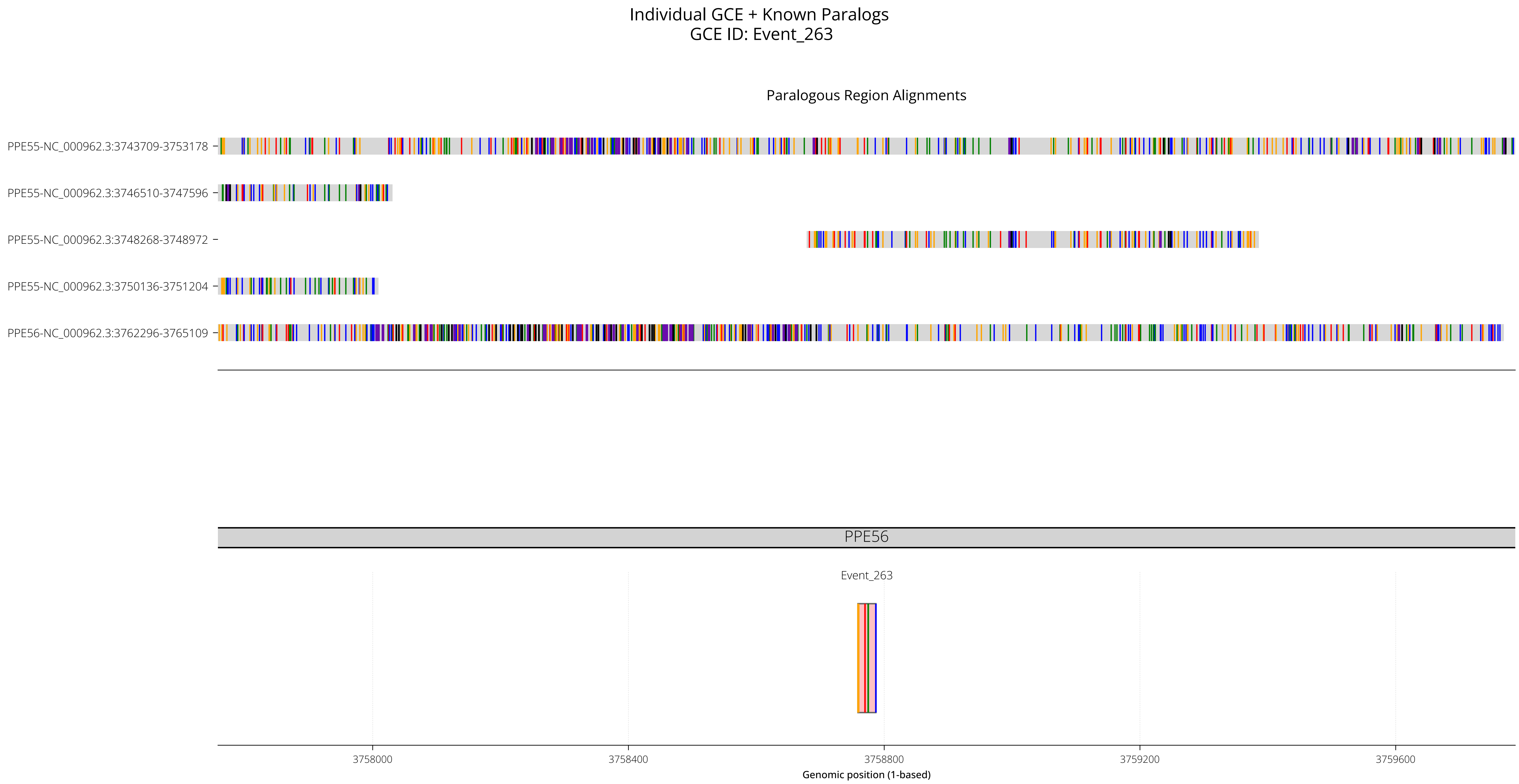

Individual GCE + Known Paralogs
GCE ID: Event_263
Paralogous Region Alignments
PPE55-NC_000962.3:3743709-3753178
PPE55-NC_000962.3:3746510-3747596
PPE55-NC_000962.3:3748268-3748972
PPE55-NC_000962.3:3750136-3751204
PPE56-NC_000962.3:3762296-3765109
PPE56
Event_263
3758000
3758400
3758800
3759200
3759600
Genomic position (1-based)

Circos Viz - GCE to Known Paralogs Locations
GCE ID: Event_263

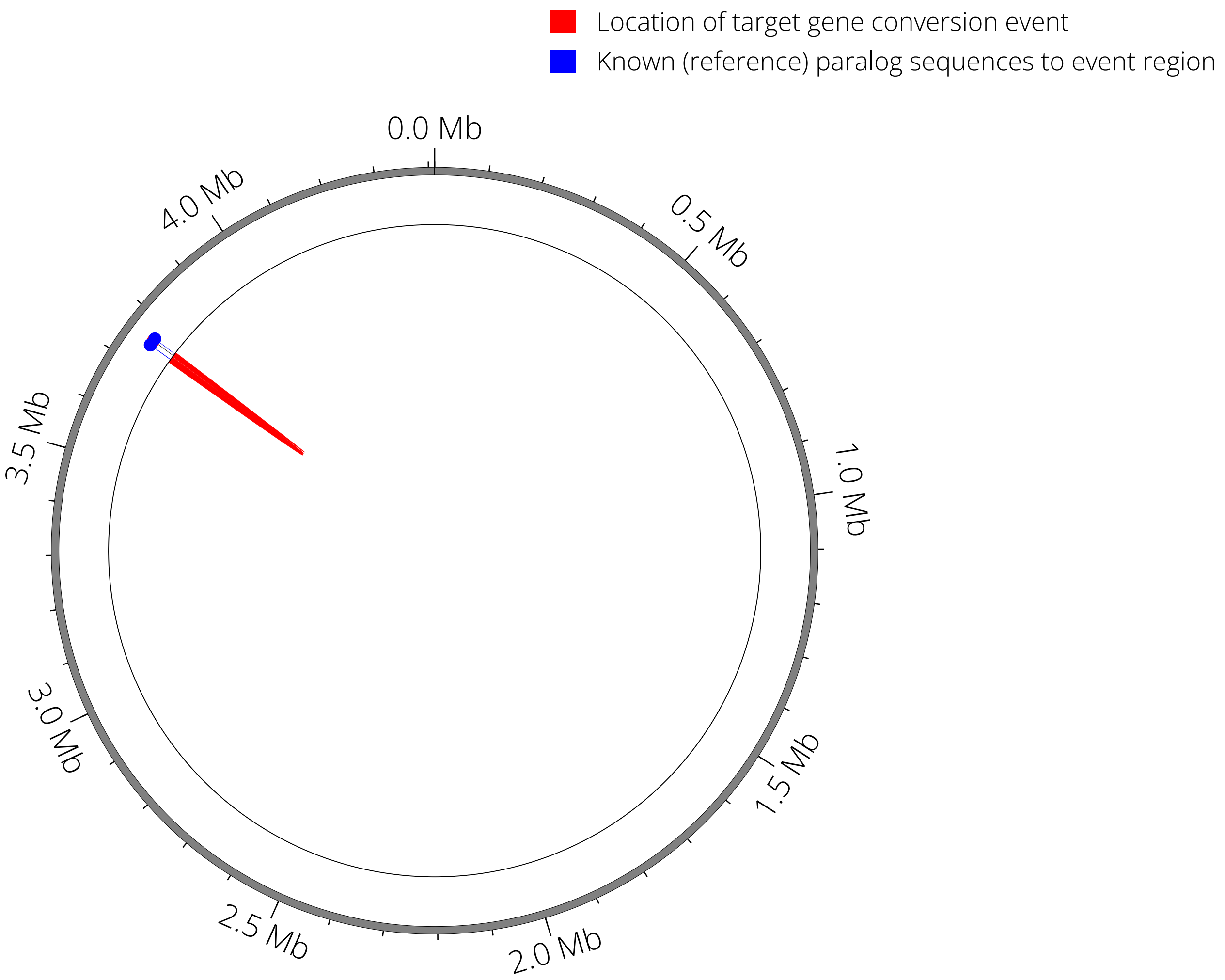

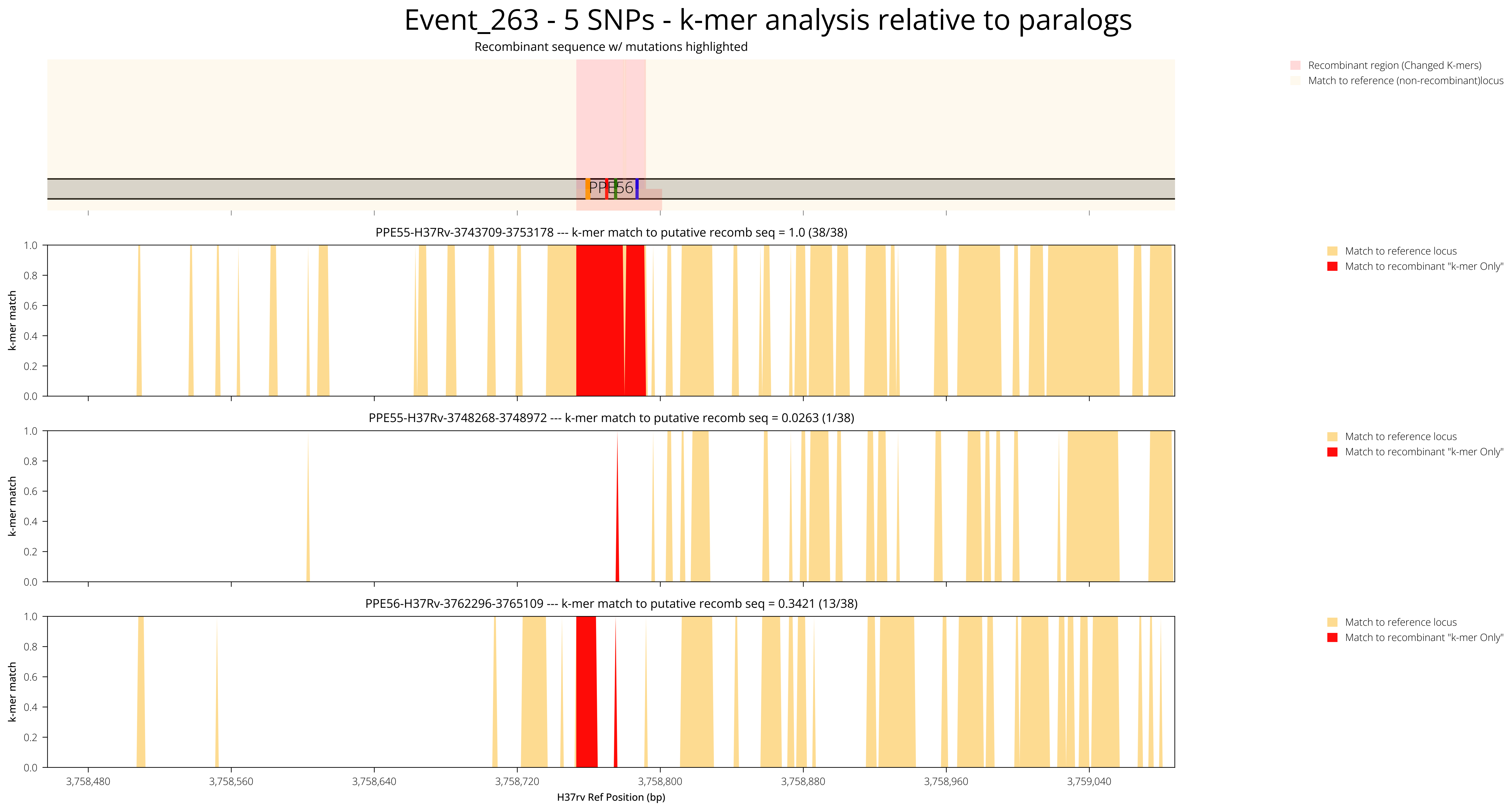
Event_263 - 5 SNPs - k-mer analysis relative to paralogs
Recombinant sequence w/ mutations highlighted
Recombinant region (Changed K-mers)
Match to reference (non-recombinant)locus
PPE56
PPE55-H37Rv-3743709-3753178 --- k-mer match to putative recomb seq = 1.0 (38/38)
Match to reference locus
Match to recombinant "k-mer Only"
PPE55-H37Rv-3748268-3748972 --- k-mer match to putative recomb seq = 0.0263 (1/38)
Match to reference locus
Match to recombinant "k-mer Only"
PPE56-H37Rv-3762296-3765109 --- k-mer match to putative recomb seq = 0.3421 (13/38)
Match to reference locus
Match to recombinant "k-mer Only"
k-mer match
1.0
0.8
0.6
0.4
0.2
0.0
3,758,480
3,758,560
3,758,640
3,758,720
3,758,800
3,758,880
3,758,960
3,759,040
H37rv Ref Position (bp)

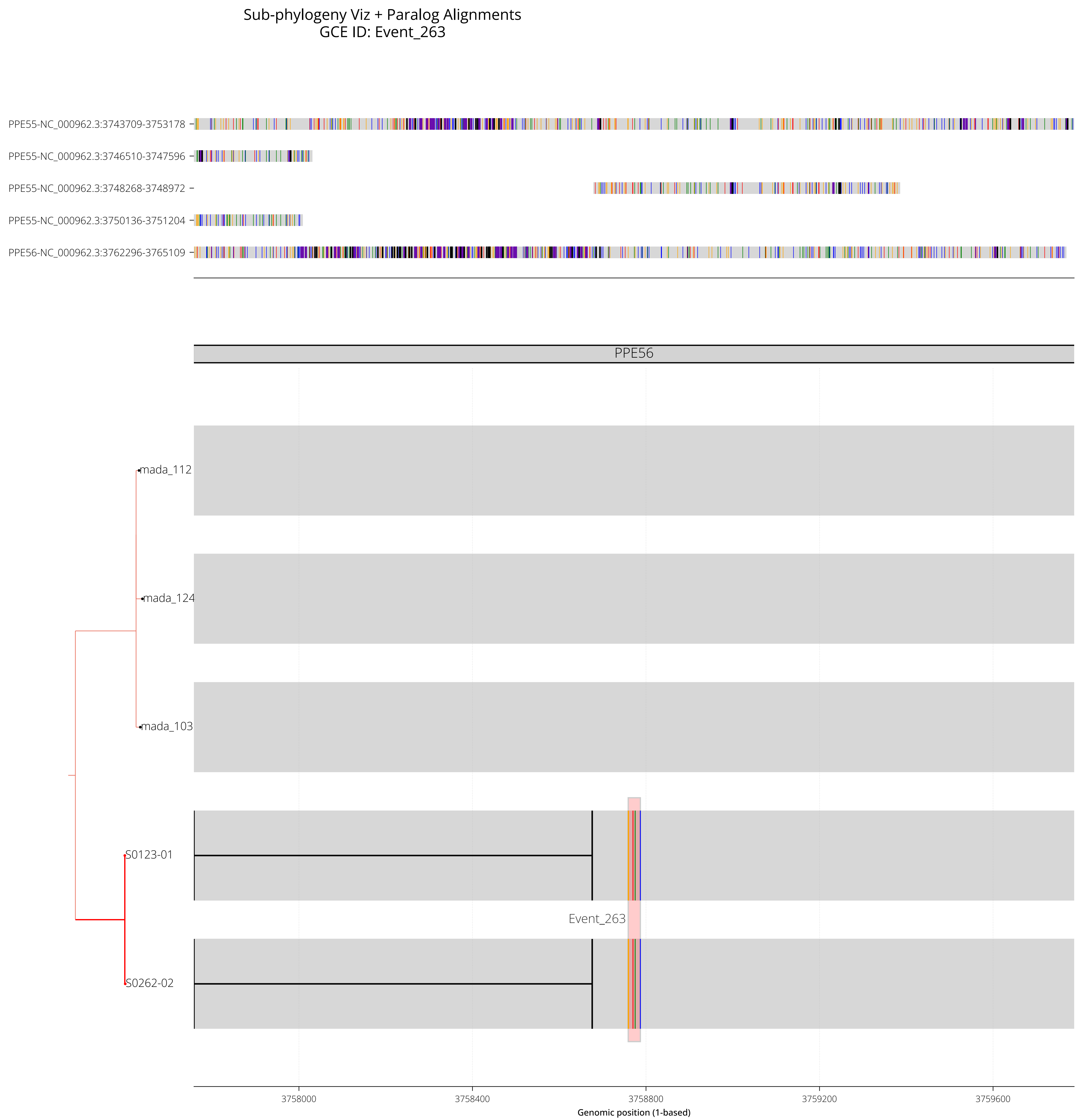
Sub-phylogeny Viz + Paralog Alignments
GCE ID: Event_263
PPE55-NC_000962.3:3743709-3753178
PPE55-NC_000962.3:3746510-3747596
PPE55-NC_000962.3:3748268-3748972
PPE55-NC_000962.3:3750136-3751204
PPE56-NC_000962.3:3762296-3765109
PPE56
mada_112
mada_124
mada_103
S0123-01
Event_263
S0262-02
3758000
3758400
3758800
3759200
3759600
Genomic position (1-based)

Full Phylogeny Viz + Paralog Alignments
GCE ID: Event_263

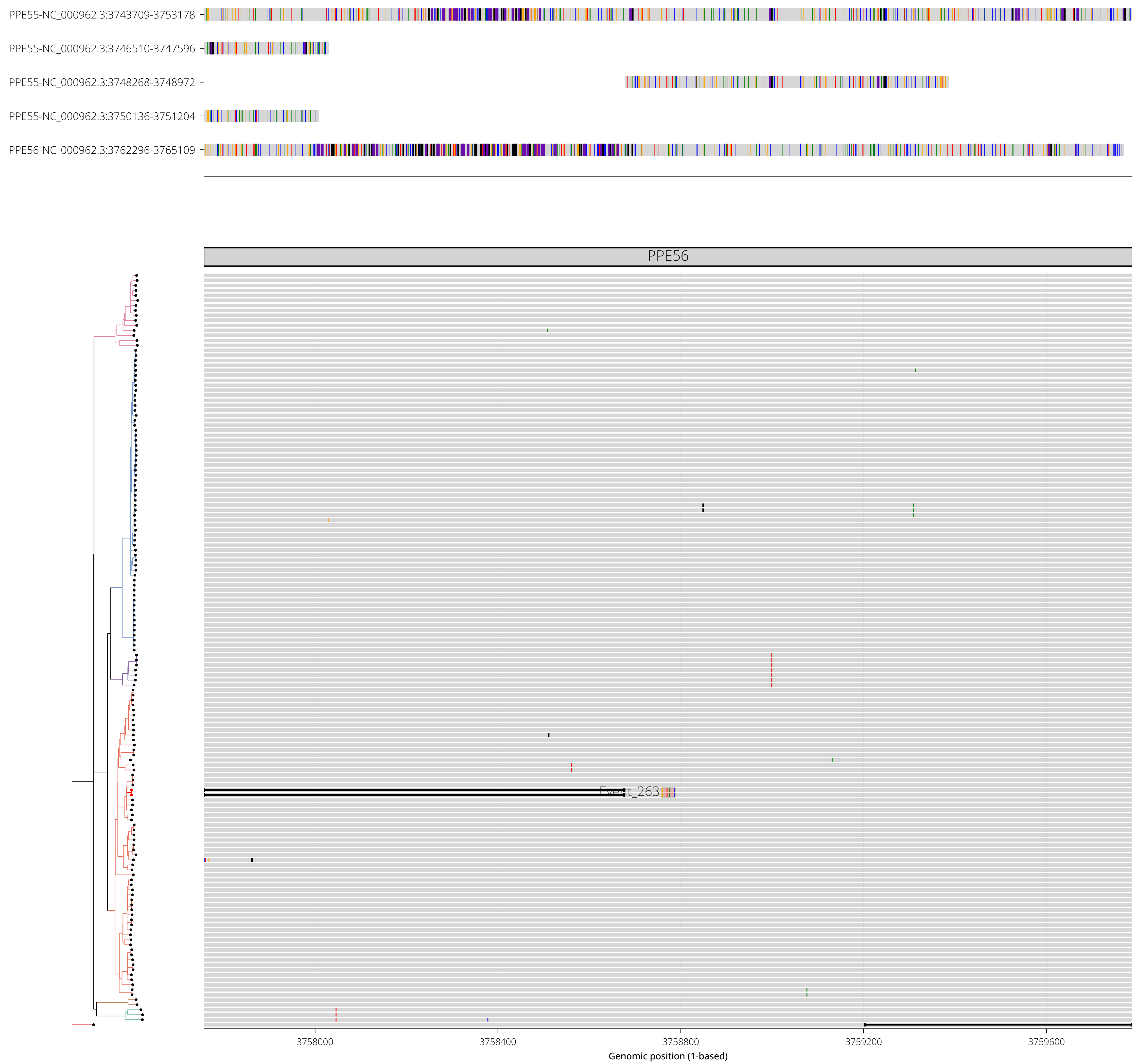

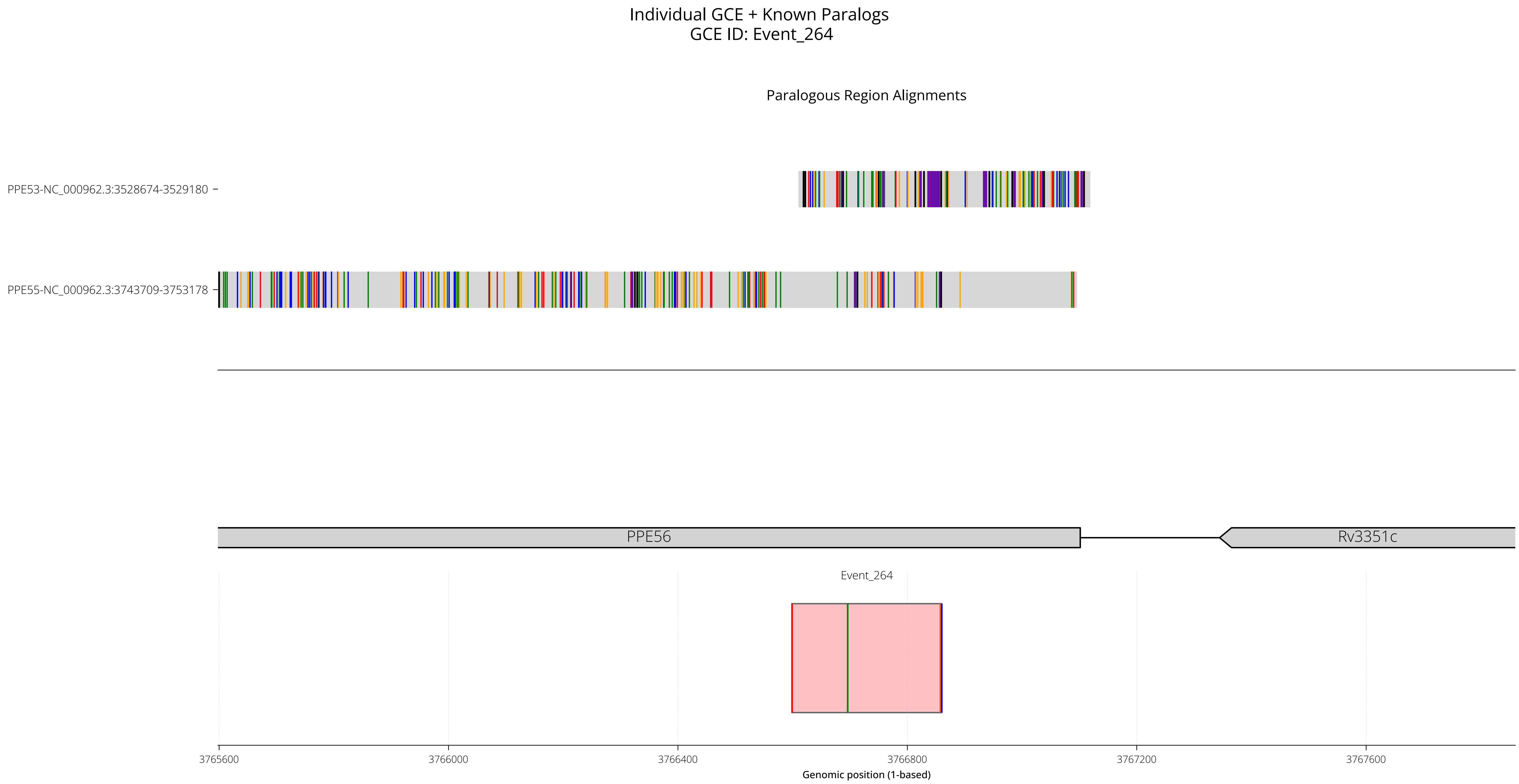

Individual GCE + Known Paralogs
GCE ID: Event_264
Paralogous Region Alignments
PPE53-NC_000962.3:3528674-3529180
PPE55-NC_000962.3:3743709-3753178
PPE56
Rv3351c
Event_264
3765600
3766000
3766400
3766800
3767200
3767600
Genomic position (1-based)

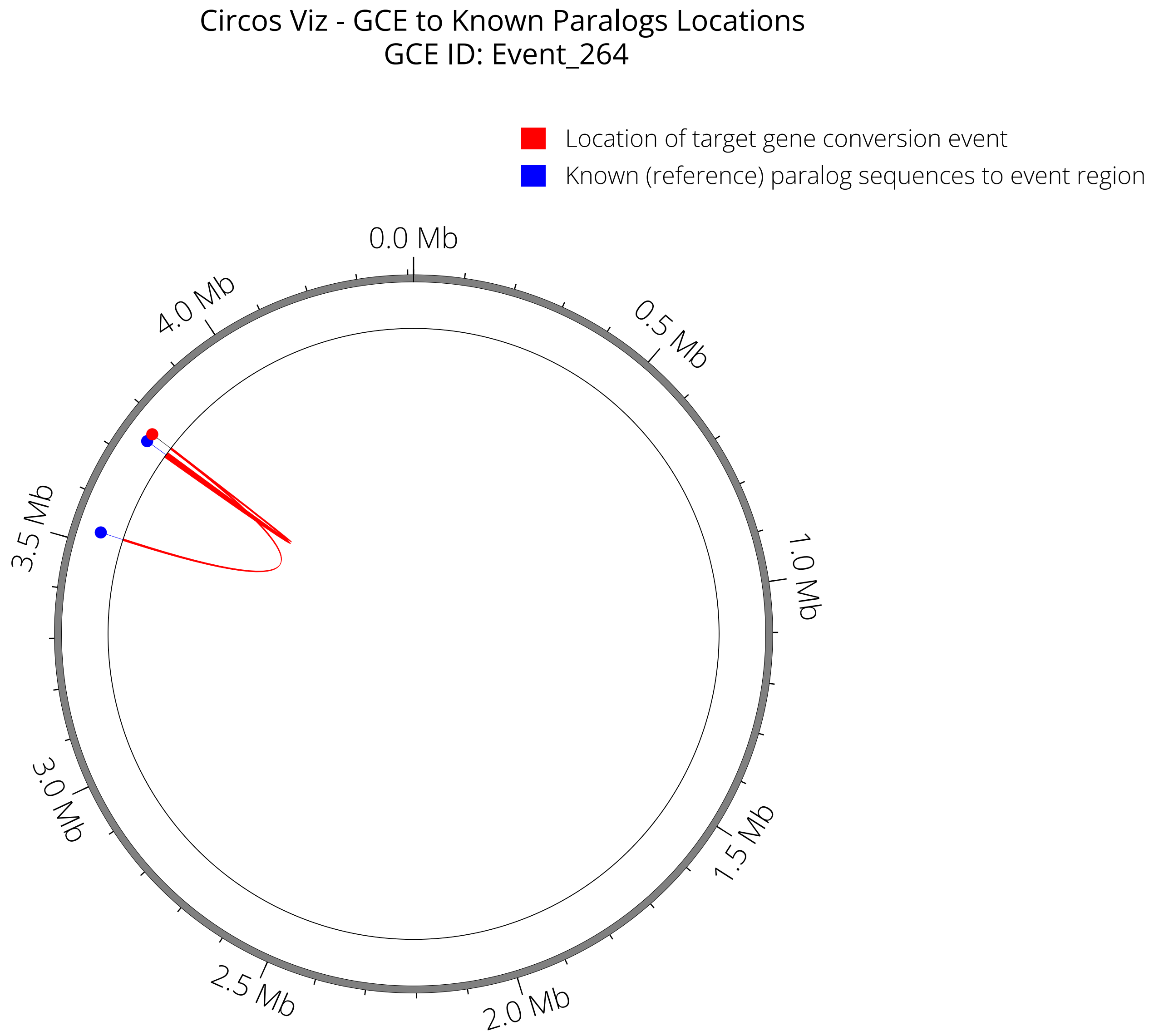
Circos Viz - GCE to Known Paralogs Locations
GCE ID: Event_264
Location of target gene conversion event
Known (reference) paralog sequences to event region
0.0 Mb
0.5 Mb
1.0 Mb
1.5 Mb
2.0 Mb
2.5 Mb
3.0 Mb
3.5 Mb
4.0 Mb

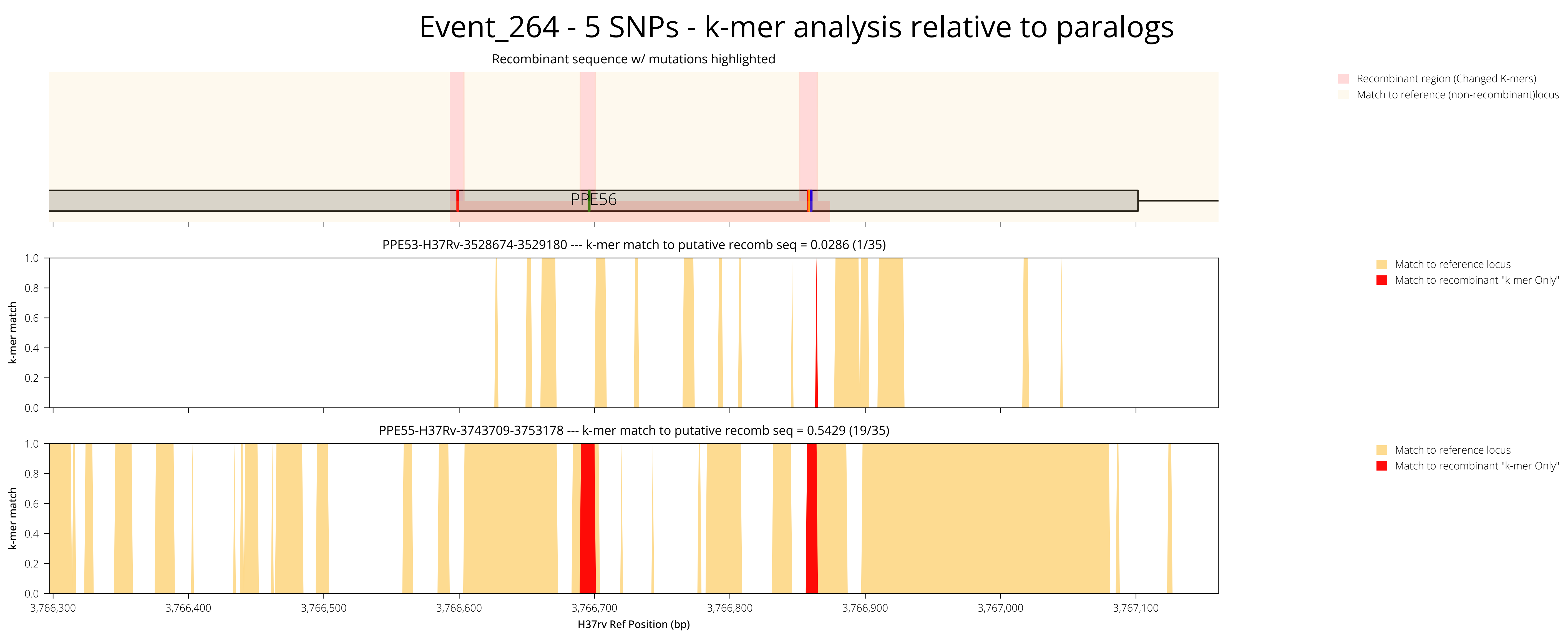

Event_264 - 5 SNPs - k-mer analysis relative to paralogs
Recombinant sequence w/ mutations highlighted
Recombinant region (Changed K-mers)
Match to reference (non-recombinant)locus
PPE56
PPE53-H37Rv-3528674-3529180 --- k-mer match to putative recomb seq = 0.0286 (1/35)
Match to reference locus
Match to recombinant "k-mer Only"
k-mer match
1.0
0.8
0.6
0.4
0.2
0.0
PPE55-H37Rv-3743709-3753178 --- k-mer match to putative recomb seq = 0.5429 (19/35)
Match to reference locus
Match to recombinant "k-mer Only"
k-mer match
3,766,300
3,766,400
3,766,500
3,766,600
3,766,700
3,766,800
3,766,900
3,767,000
3,767,100
H37rv Ref Position (bp)

Sub-phylogeny Viz + Paralog Alignments
GCE ID: Event_264

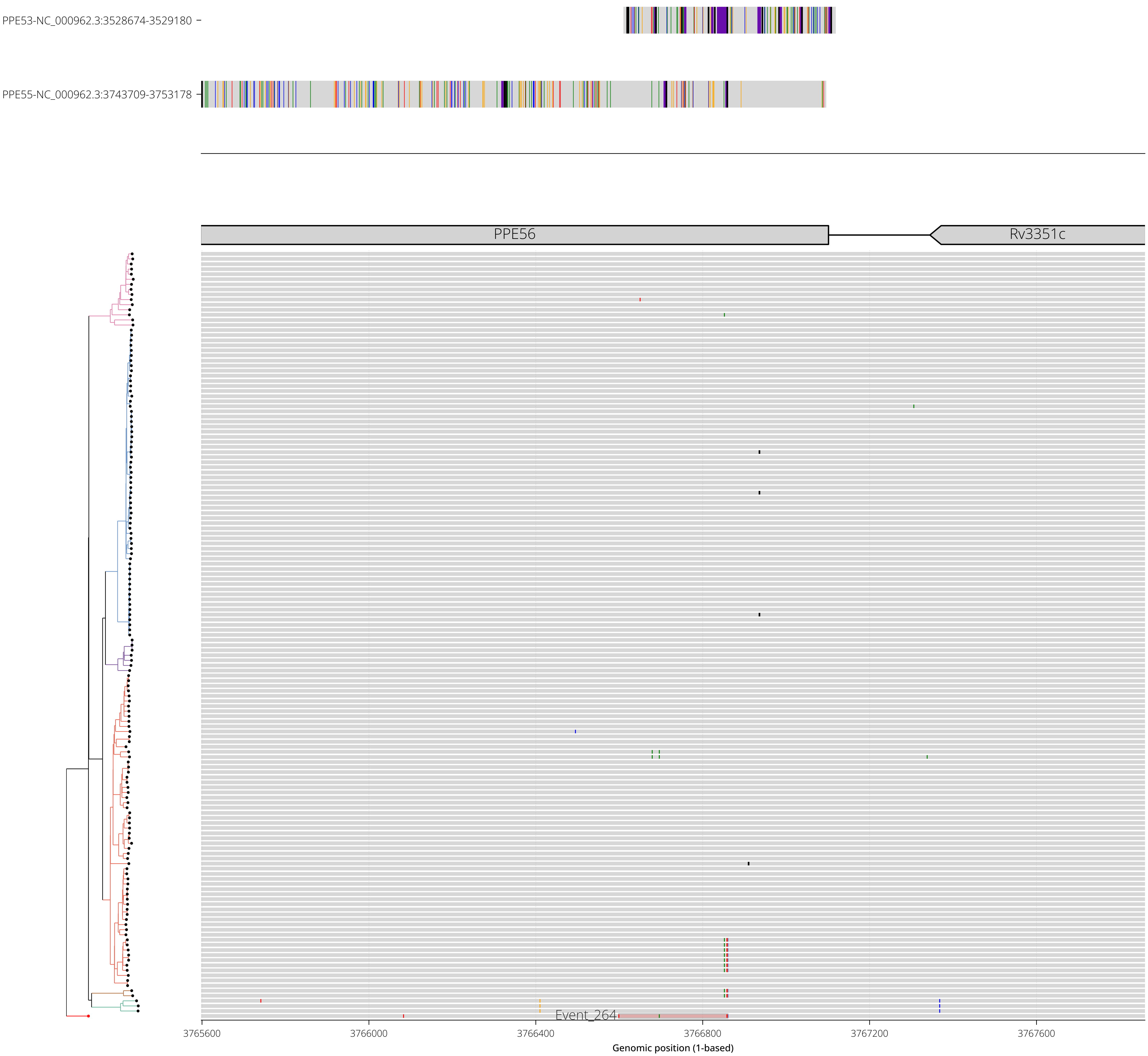

Full Phylogeny Viz + Paralog Alignments
GCE ID: Event_264

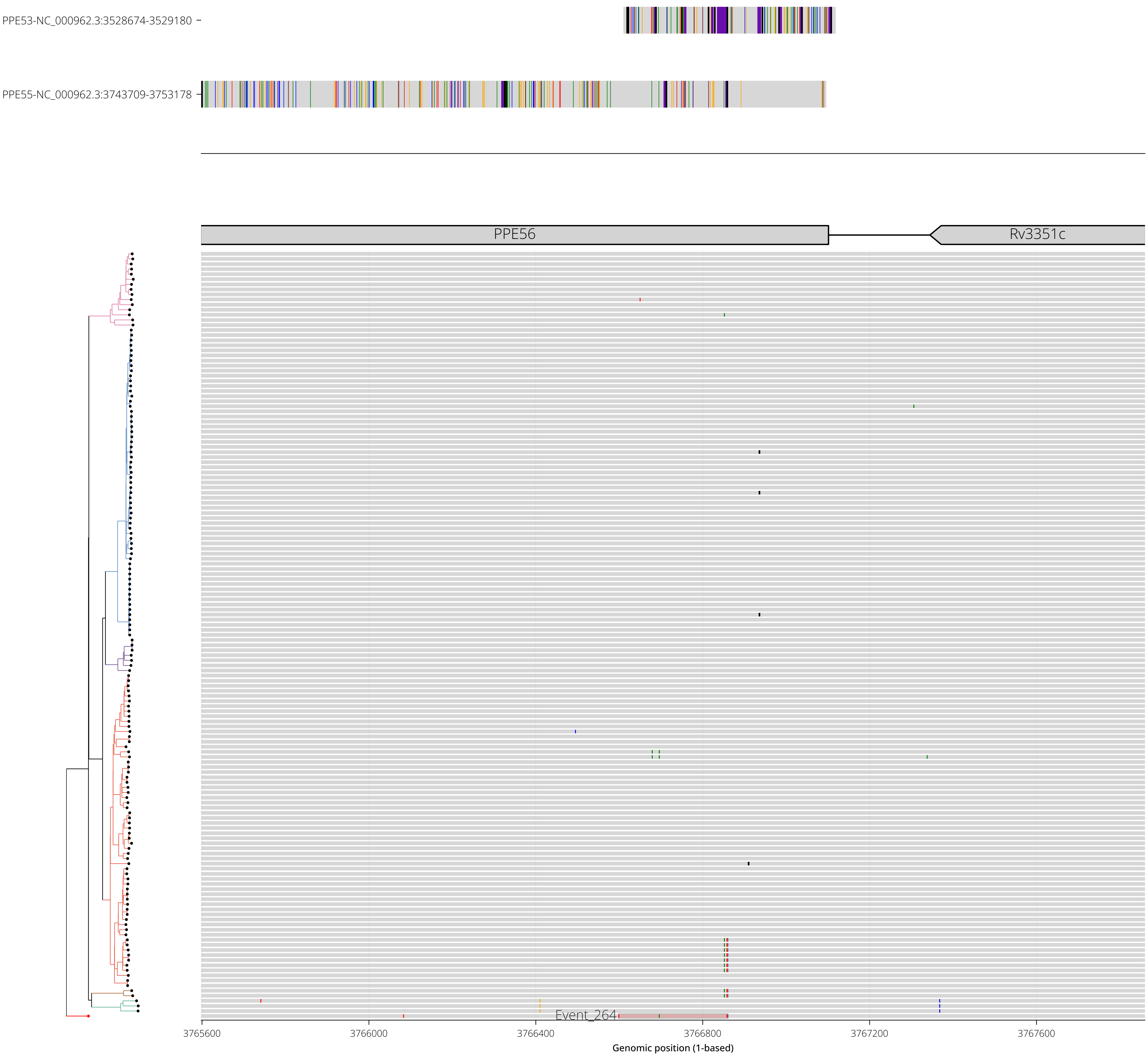

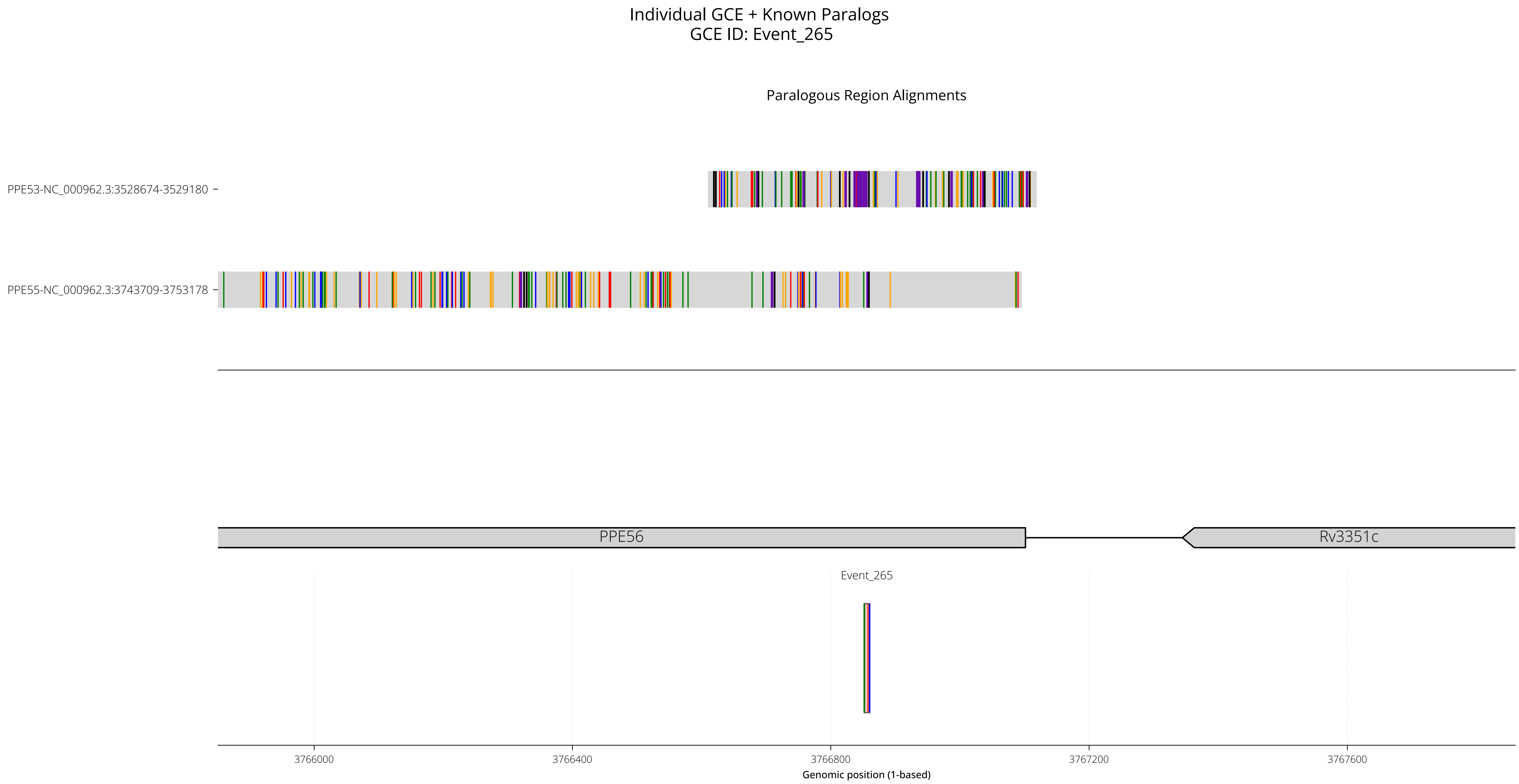

Individual GCE + Known Paralogs
GCE ID: Event_265
Paralogous Region Alignments
PPE53-NC_000962.3:3528674-3529180
PPE55-NC_000962.3:3743709-3753178
PPE56
Rv3351c
Event_265
3766000
3766400
3766800
3767200
3767600
Genomic position (1-based)

Circos Viz - GCE to Known Paralogs Locations
GCE ID: Event_265

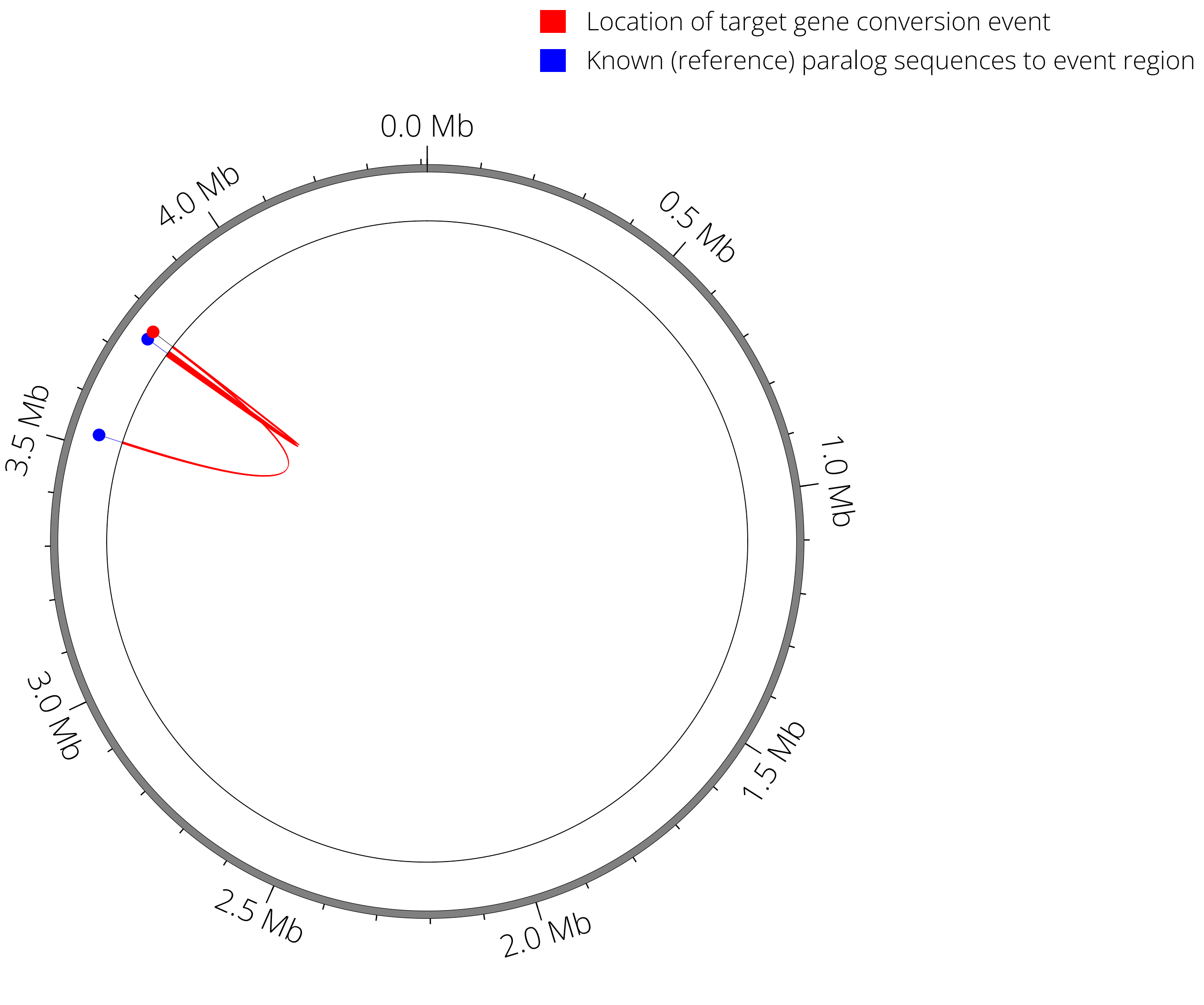

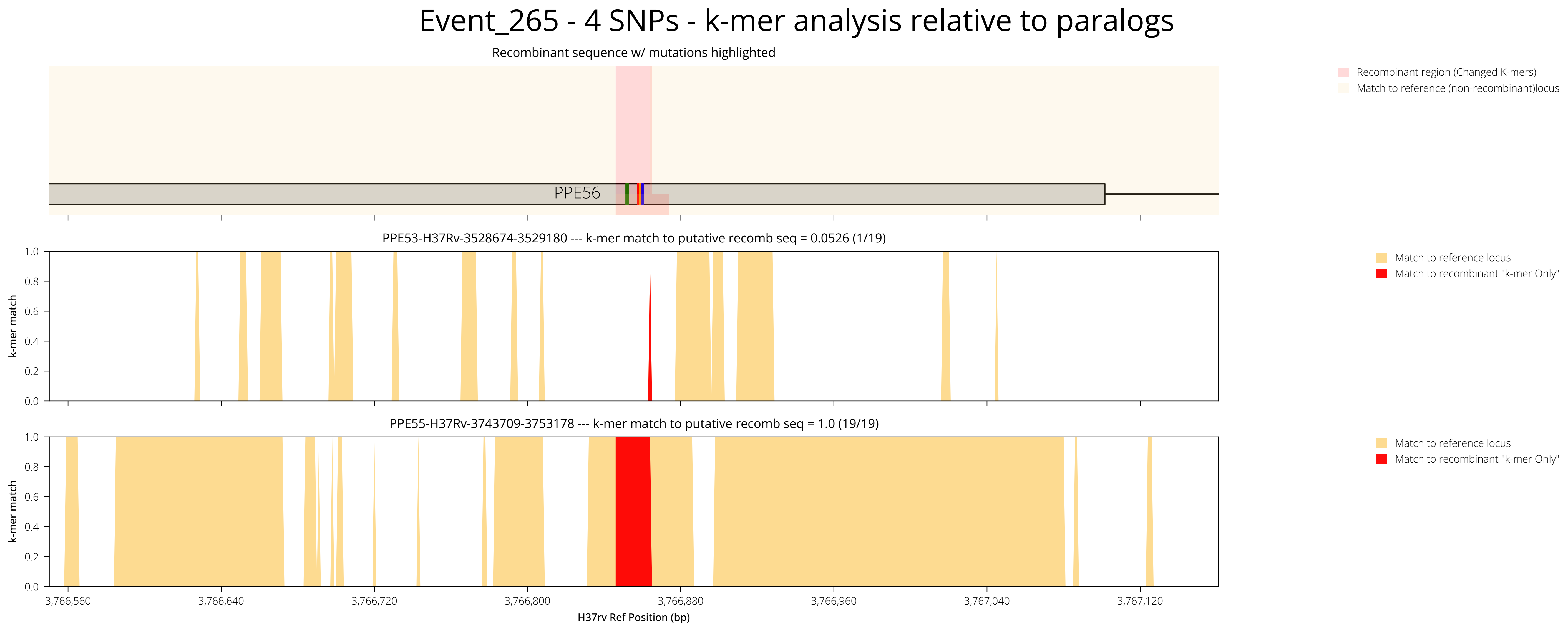

Event_265 - 4 SNPs - k-mer analysis relative to paralogs
Recombinant sequence w/ mutations highlighted
PPE56
Recombinant region (Changed K-mers)
Match to reference (non-recombinant)locus
PPE53-H37Rv-3528674-3529180 --- k-mer match to putative recomb seq = 0.0526 (1/19)
Match to reference locus
Match to recombinant "k-mer Only"
PPE55-H37Rv-3743709-3753178 --- k-mer match to putative recomb seq = 1.0 (19/19)
Match to reference locus
Match to recombinant "k-mer Only"
k-mer match
1.0
0.8
0.6
0.4
0.2
0.0
3,766,560
3,766,640
3,766,720
3,766,800
3,766,880
3,766,960
3,767,040
3,767,120
H37rv Ref Position (bp)

Sub-phylogeny Viz + Paralog Alignments
GCE ID: Event_265

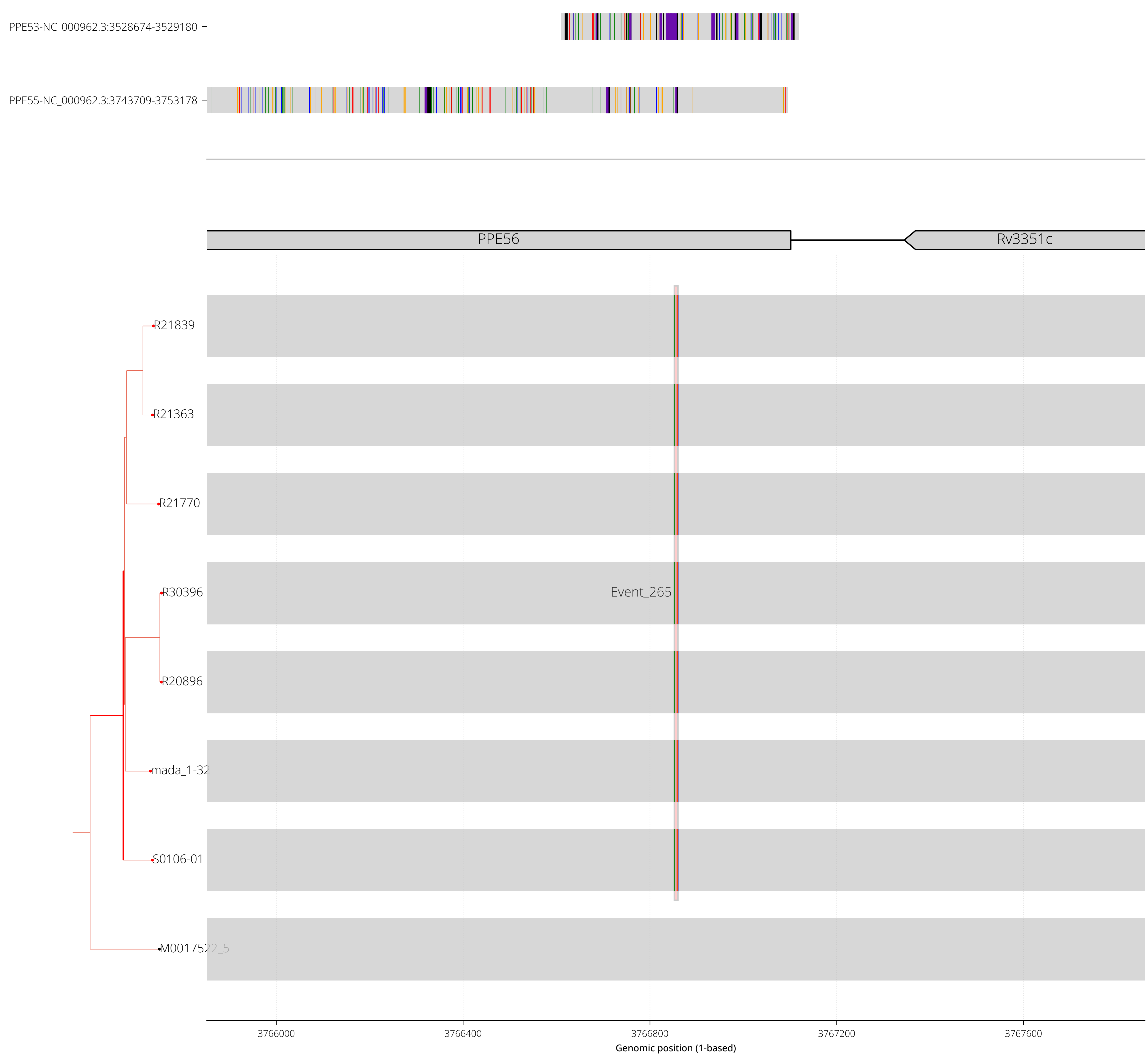

Full Phylogeny Viz + Paralog Alignments
GCE ID: Event_265

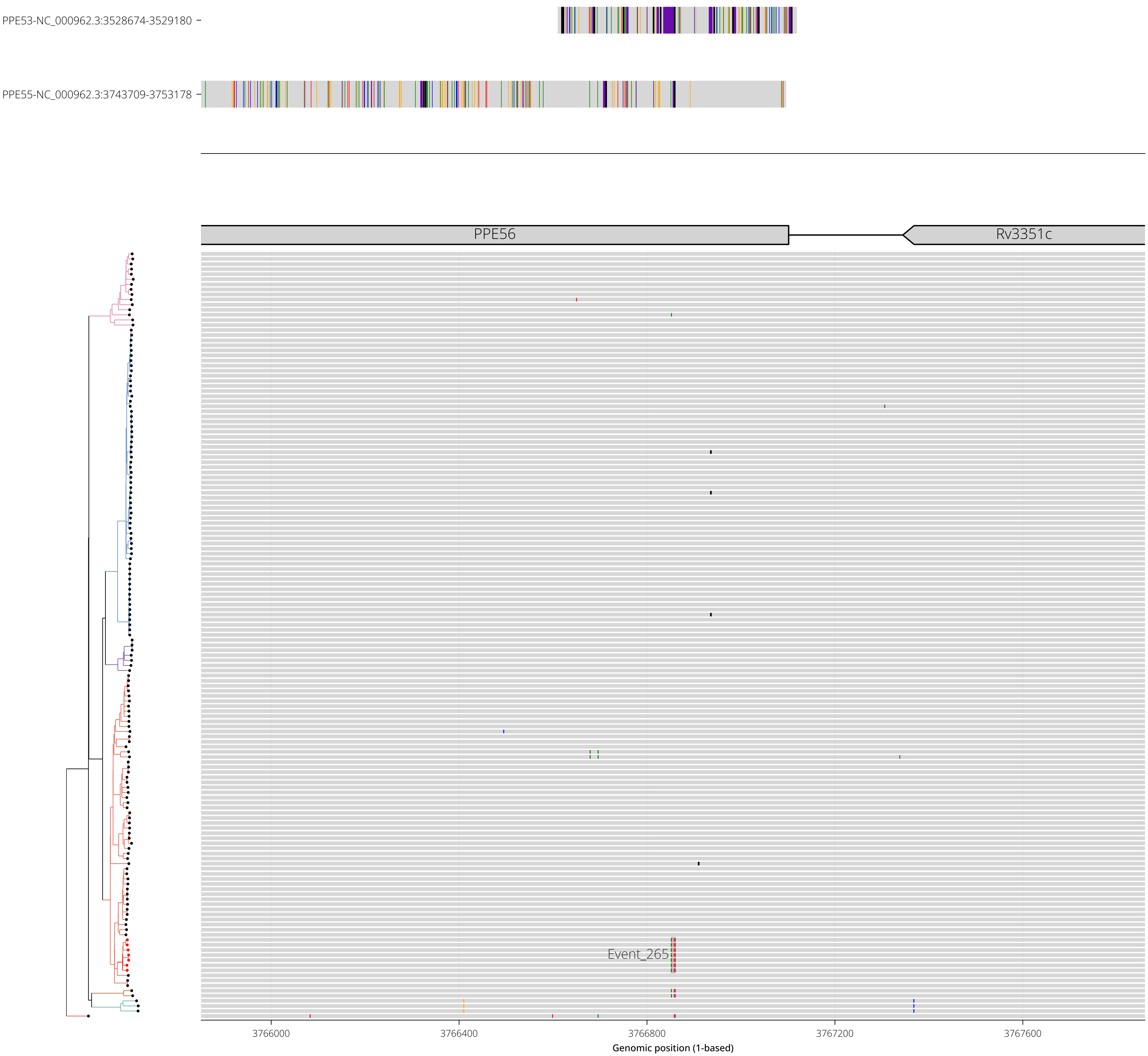

Individual GCE + Known Paralogs
GCE ID: Event_266

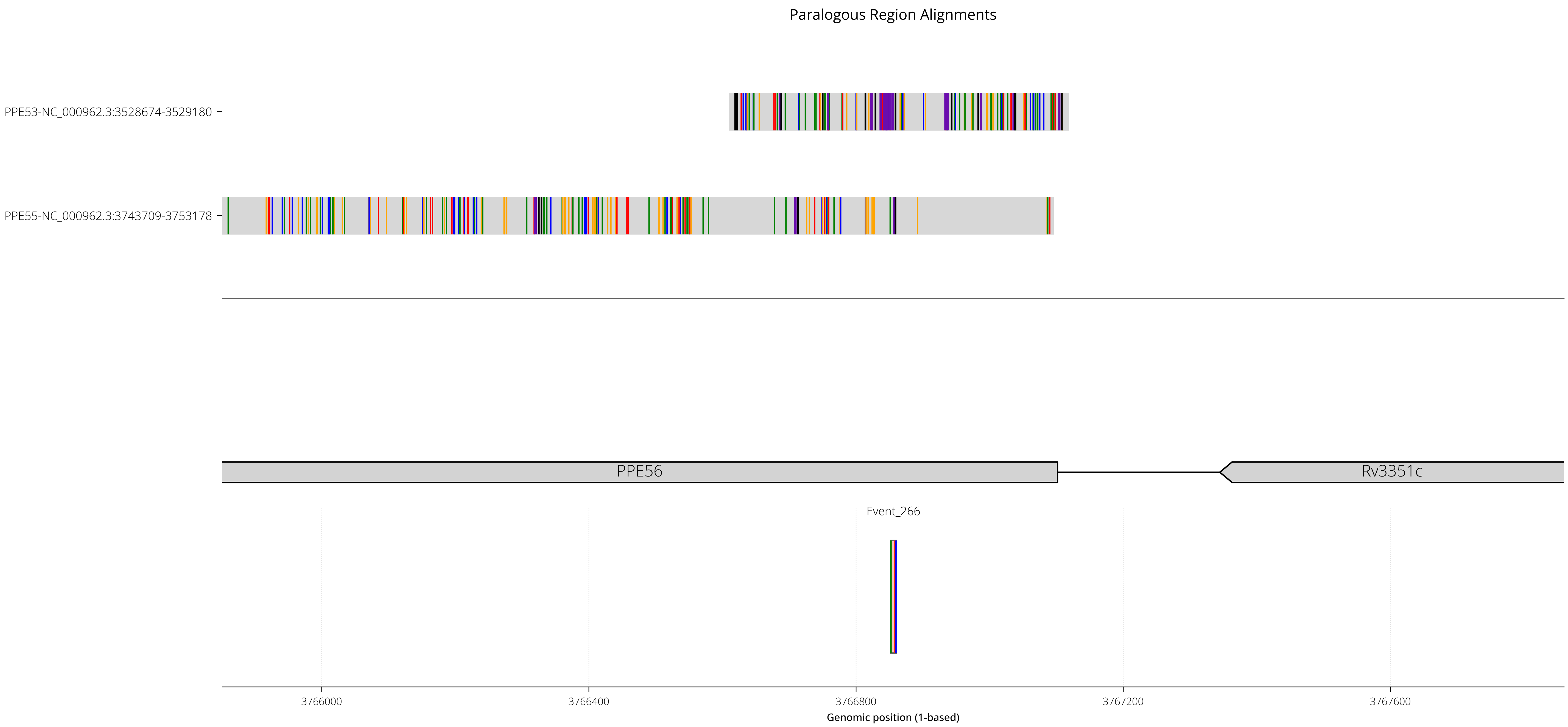

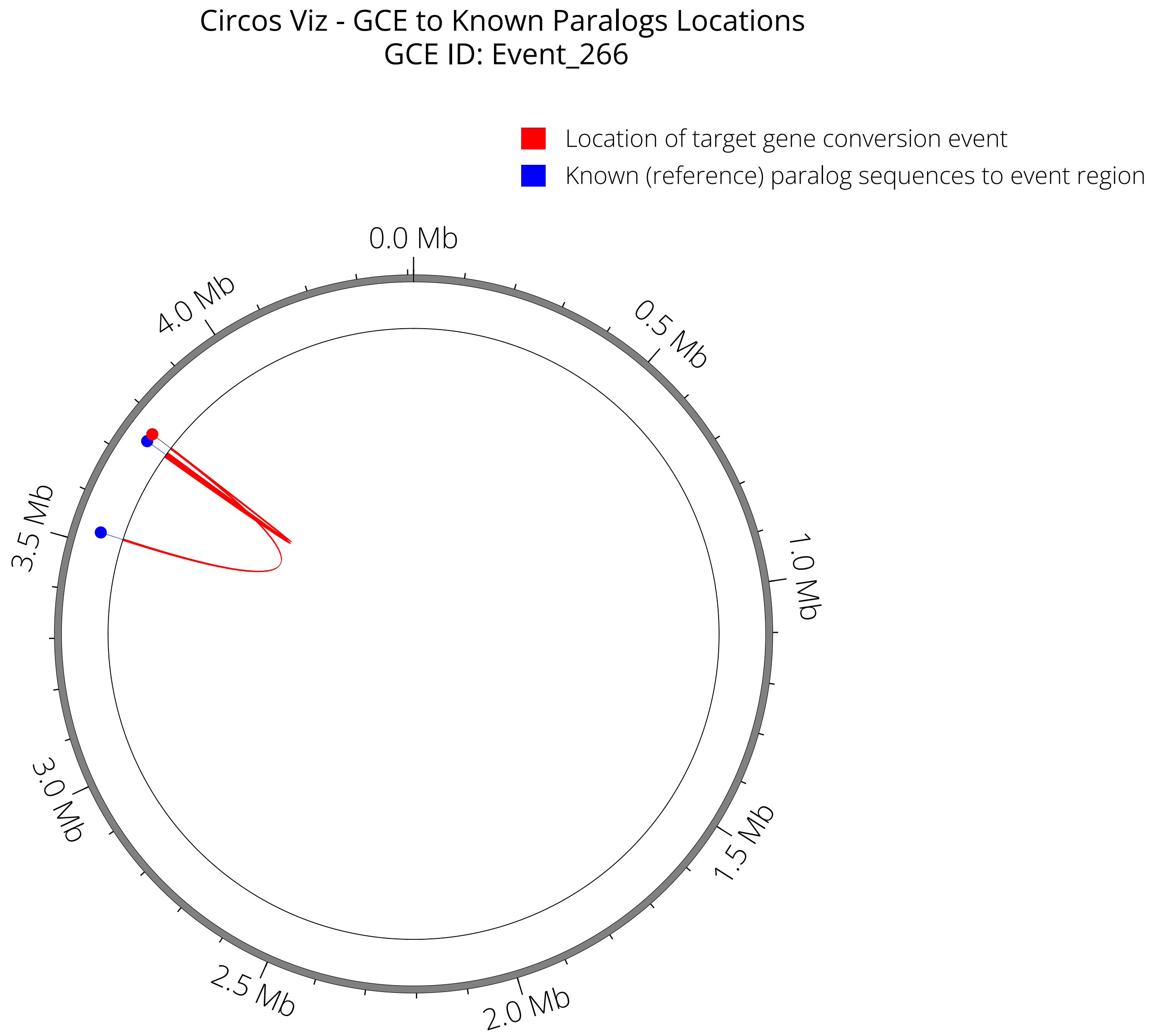
Circos Viz - GCE to Known Paralogs Locations
GCE ID: Event_266
Location of target gene conversion event
Known (reference) paralog sequences to event region
0.0 Mb
0.5 Mb
1.0 Mb
1.5 Mb
2.0 Mb
2.5 Mb
3.0 Mb
3.5 Mb
4.0 Mb

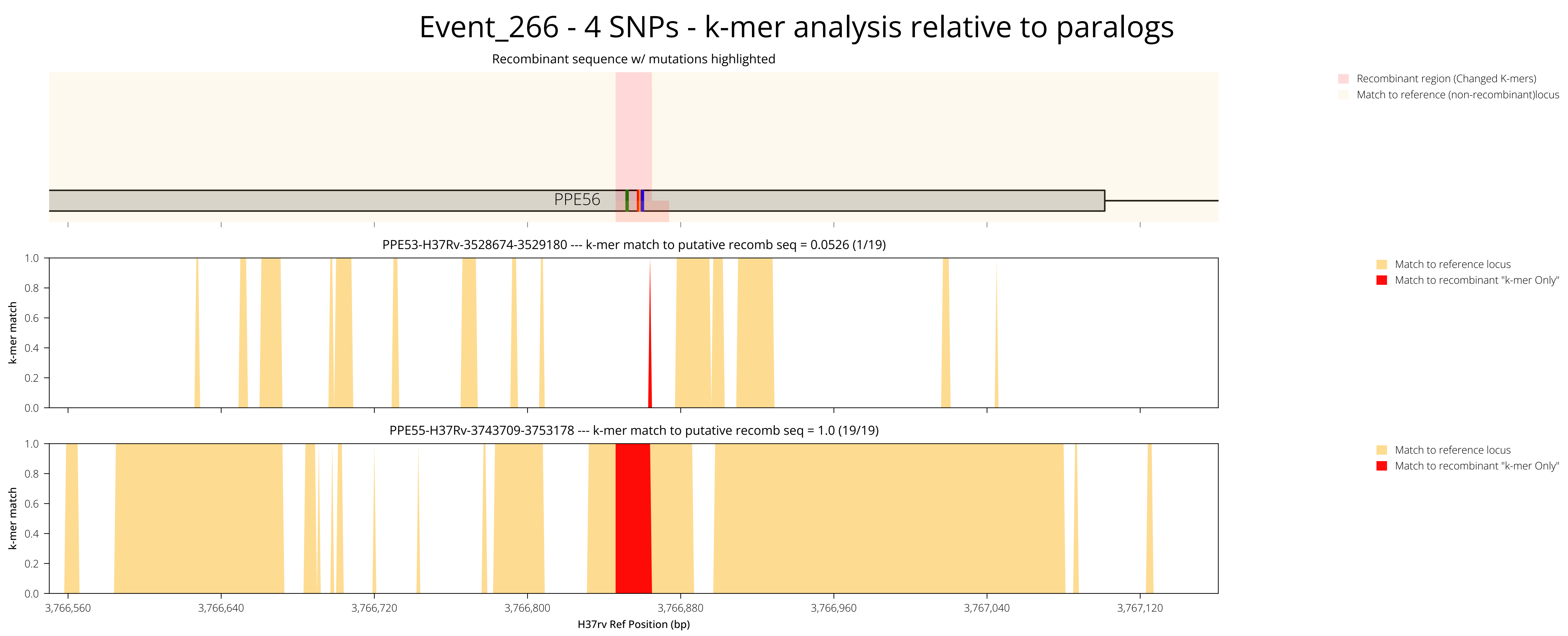
Event_266 - 4 SNPs - k-mer analysis relative to paralogs
Recombinant sequence w/ mutations highlighted
PPE56
Recombinant region (Changed K-mers)
Match to reference (non-recombinant)locus
PPE53-H37Rv-3528674-3529180 --- k-mer match to putative recomb seq = 0.0526 (1/19)
Match to reference locus
Match to recombinant "k-mer Only"
k-mer match
1.0
0.8
0.6
0.4
0.2
0.0
PPE55-H37Rv-3743709-3753178 --- k-mer match to putative recomb seq = 1.0 (19/19)
Match to reference locus
Match to recombinant "k-mer Only"
k-mer match
3,766,560
3,766,640
3,766,720
3,766,800
3,766,880
3,766,960
3,767,040
3,767,120
H37rv Ref Position (bp)

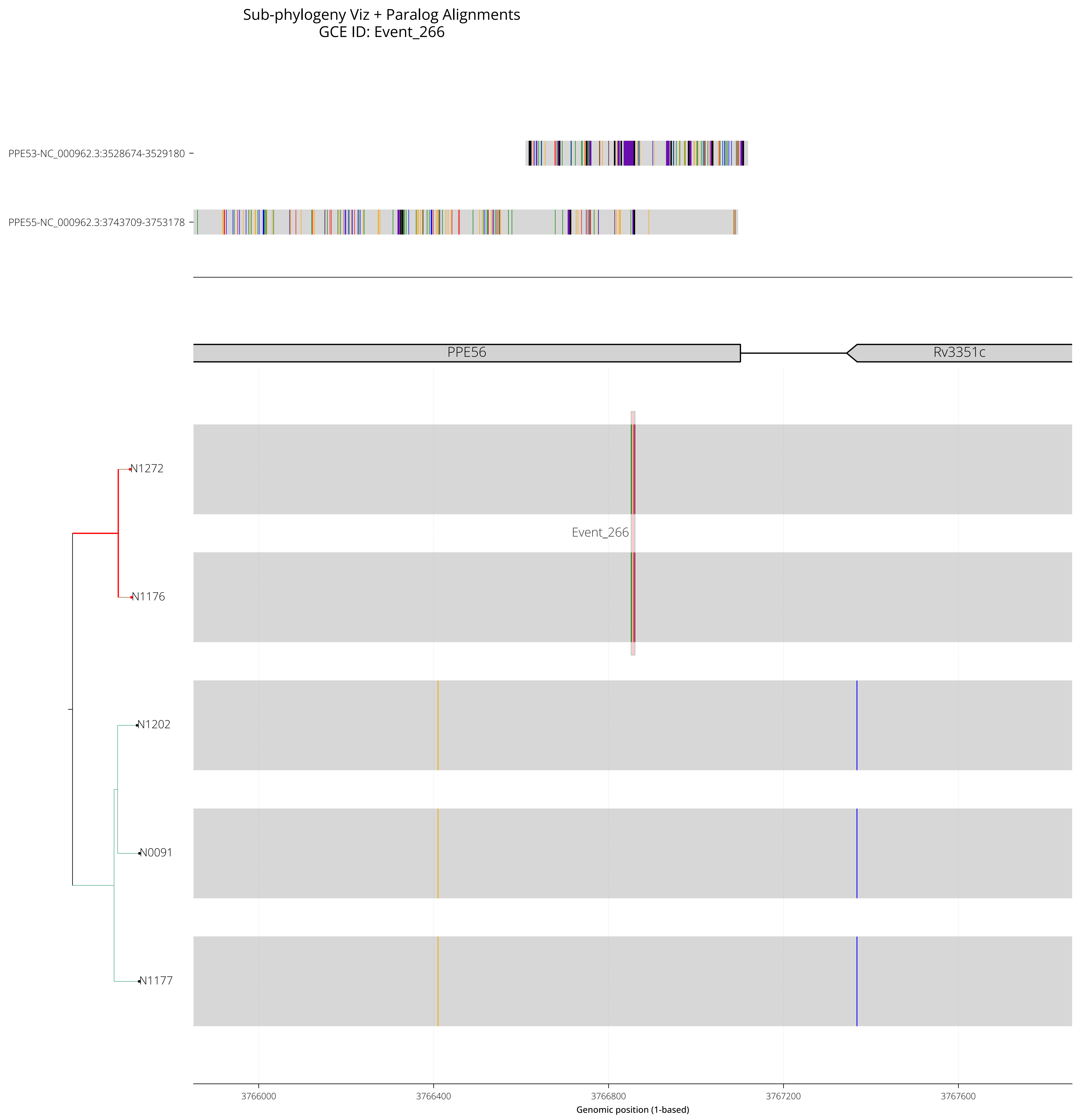

Sub-phylogeny Viz + Paralog Alignments
GCE ID: Event_266
PPE53-NC_000962.3:3528674-3529180
PPE55-NC_000962.3:3743709-3753178
PPE56
Rv3351c
N1272
Event_266
N1176
N1202
N0091
N1177
3766000
3766400
3766800
3767200
3767600
Genomic position (1-based)

Full Phylogeny Viz + Paralog Alignments
GCE ID: Event_266

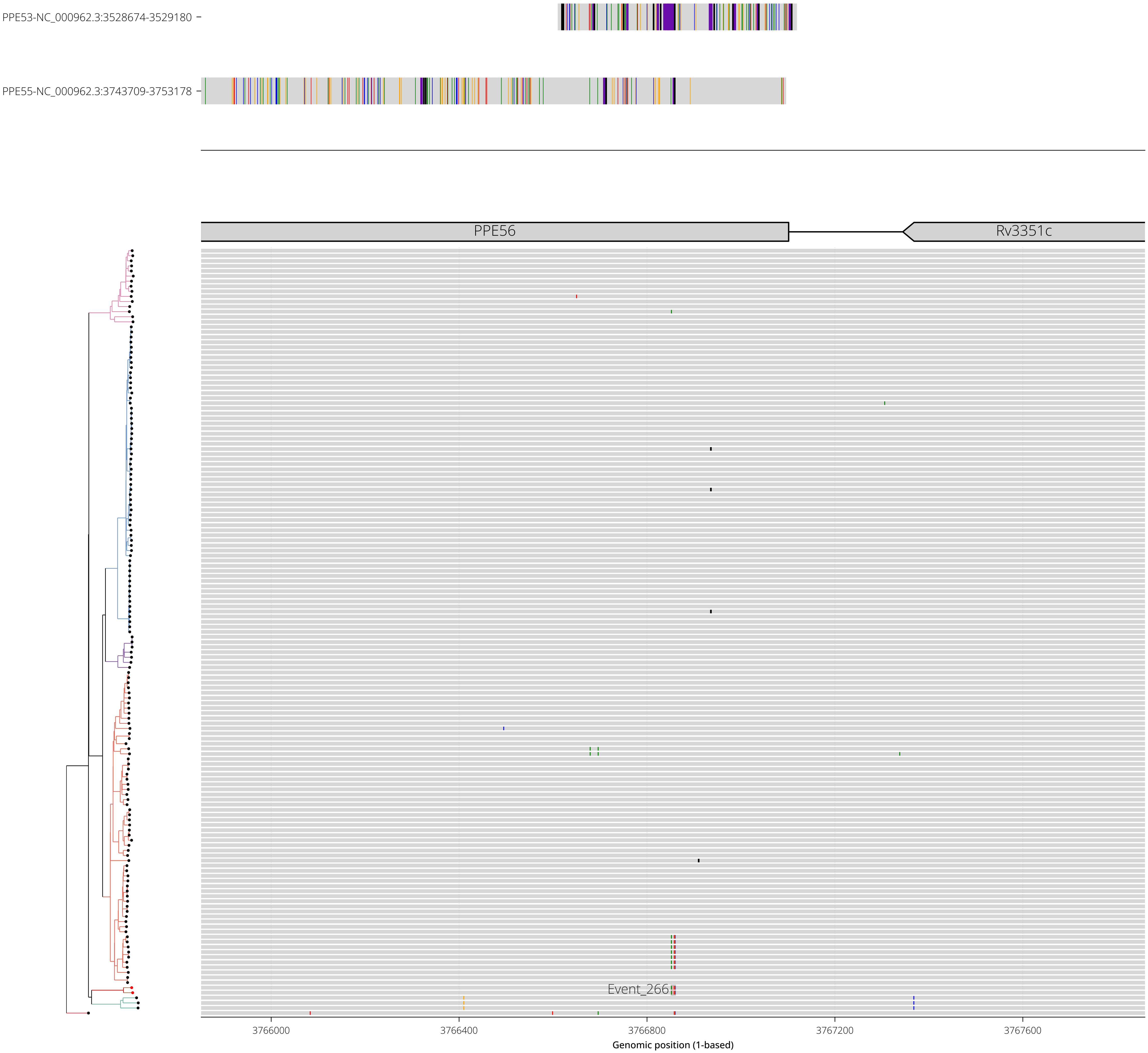

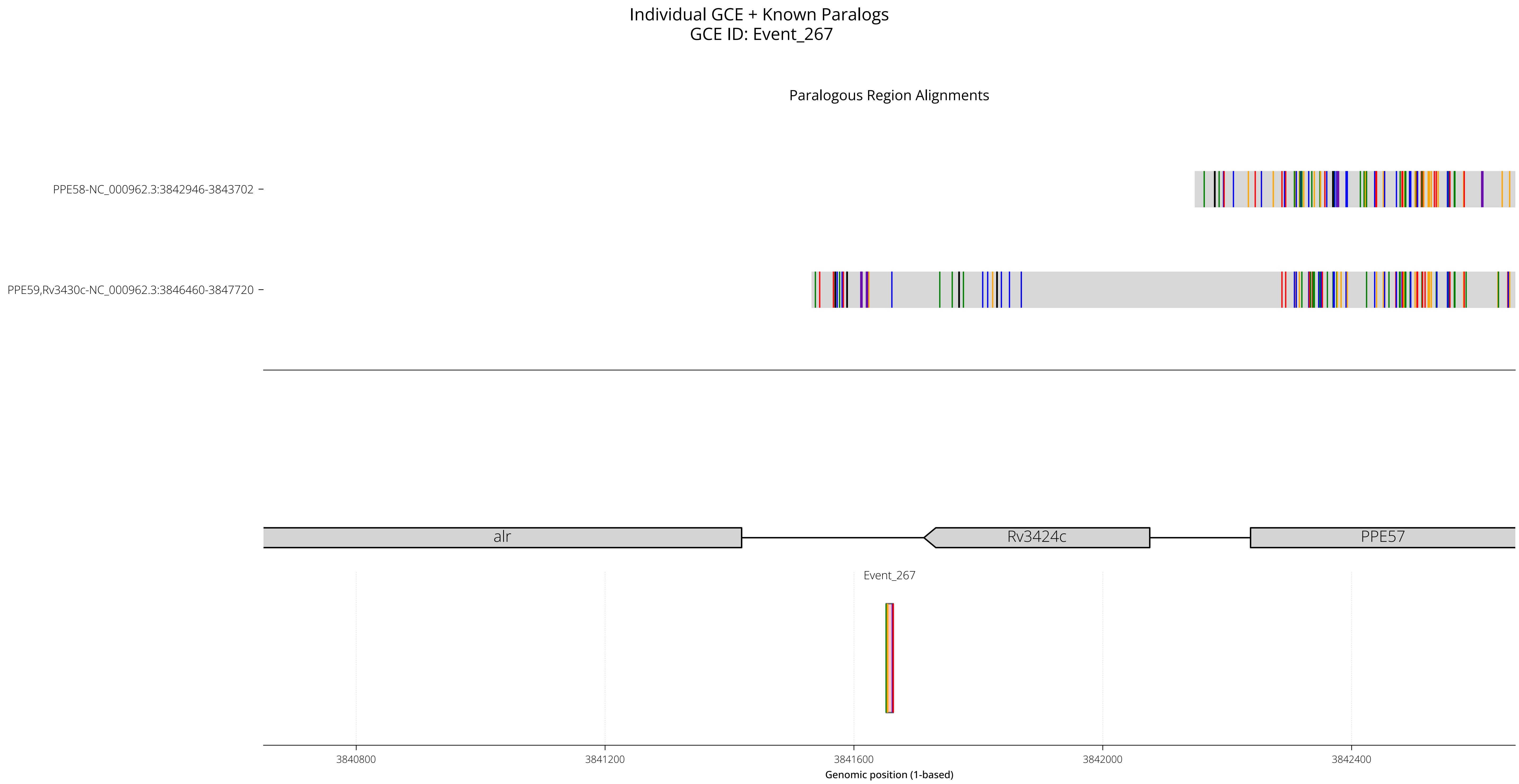

Individual GCE + Known Paralogs
GCE ID: Event_267
Paralogous Region Alignments
PPE58-NC_000962.3:3842946-3843702
PPE59,Rv3430c-NC_000962.3:3846460-3847720
alr
Rv3424c
PPE57
Event_267
3840800
3841200
3841600
3842000
3842400
Genomic position (1-based)

Circos Viz - GCE to Known Paralogs Locations
GCE ID: Event_267

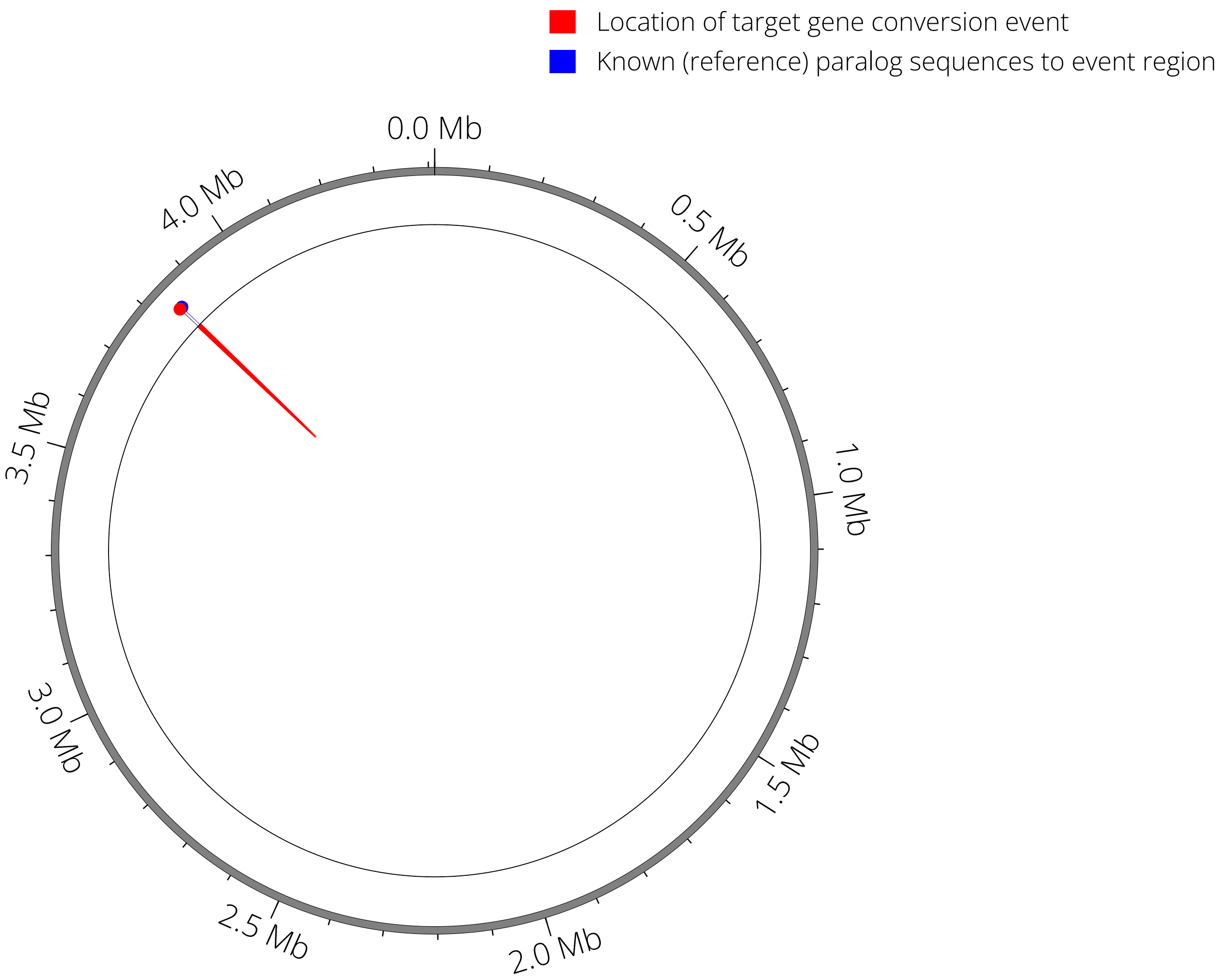

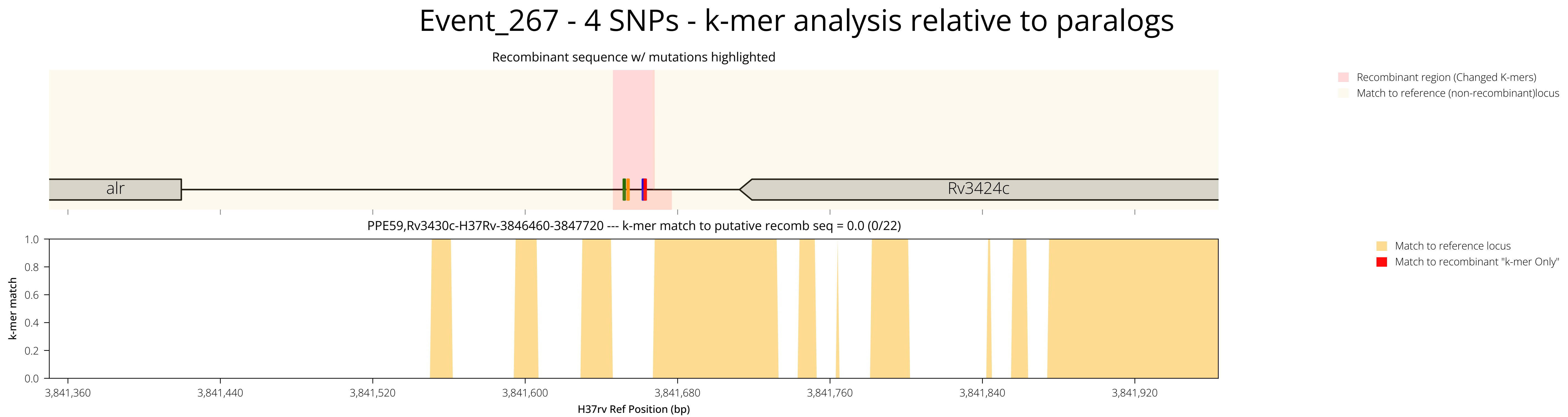

Event_267 - 4 SNPs - k-mer analysis relative to paralogs
Recombinant sequence w/ mutations highlighted
Recombinant region (Changed K-mers)
Match to reference (non-recombinant)locus
alr
Rv3424c
PPE59,Rv3430c-H37Rv-3846460-3847720 --- k-mer match to putative recomb seq = 0.0 (0/22)
Match to reference locus
Match to recombinant "k-mer Only"
k-mer match
1.0
0.8
0.6
0.4
0.2
0.0
3,841,360
3,841,440
3,841,520
3,841,600
3,841,680
3,841,760
3,841,840
3,841,920
H37rv Ref Position (bp)

Sub-phylogeny Viz + Paralog Alignments
GCE ID: Event_267

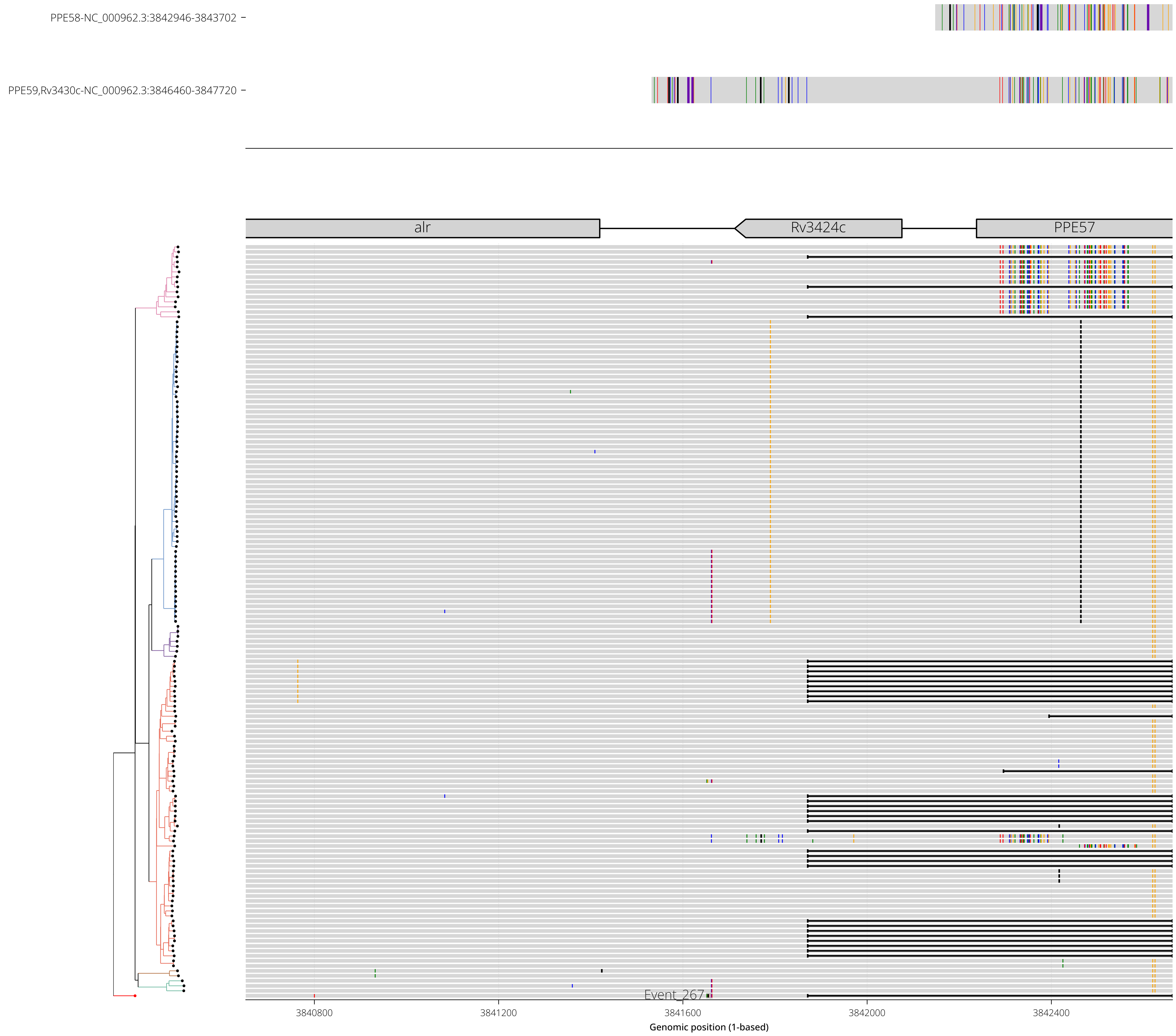

Full Phylogeny Viz + Paralog Alignments
GCE ID: Event_267

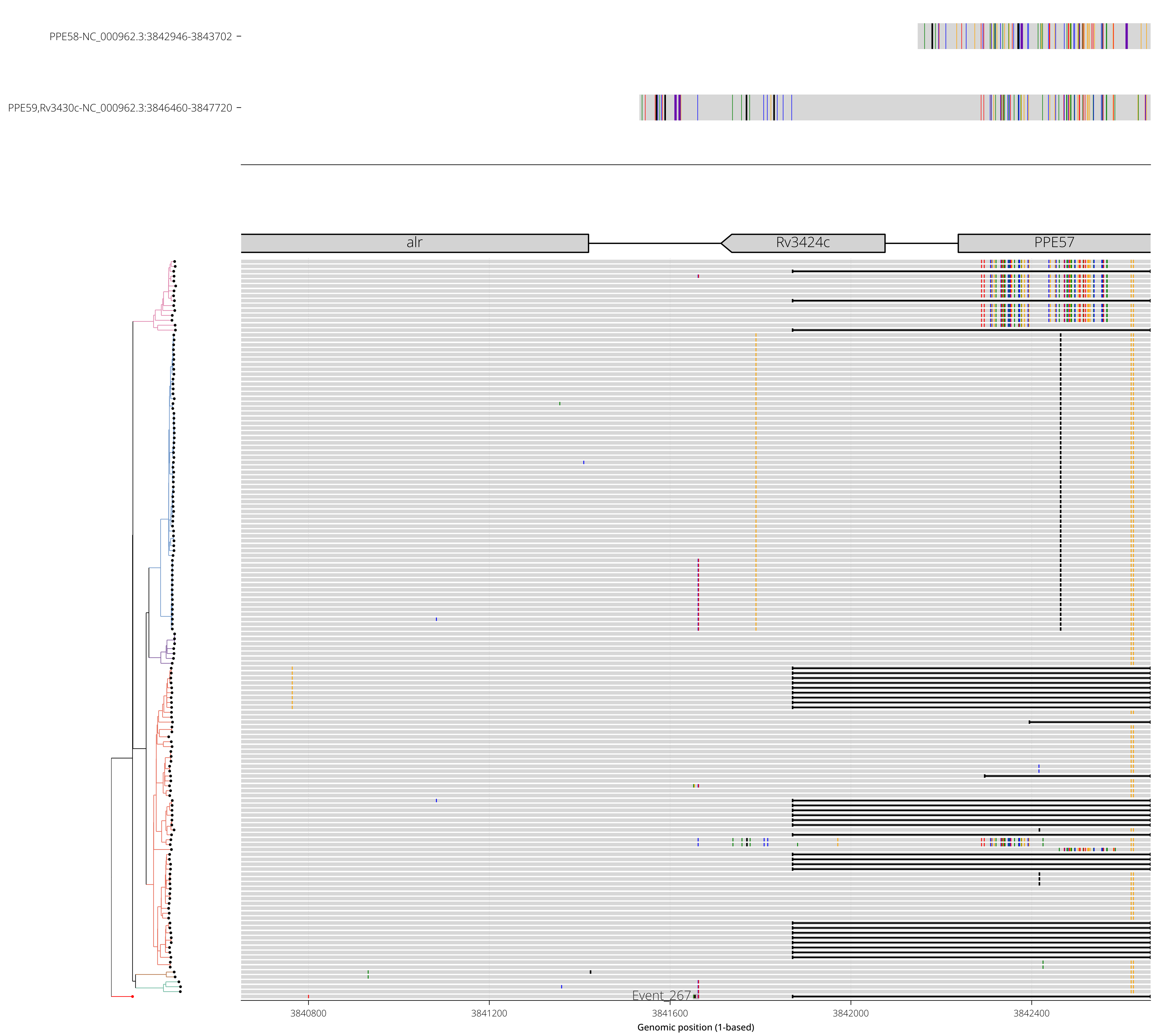

Individual GCE + Known Paralogs
GCE ID: Event_268

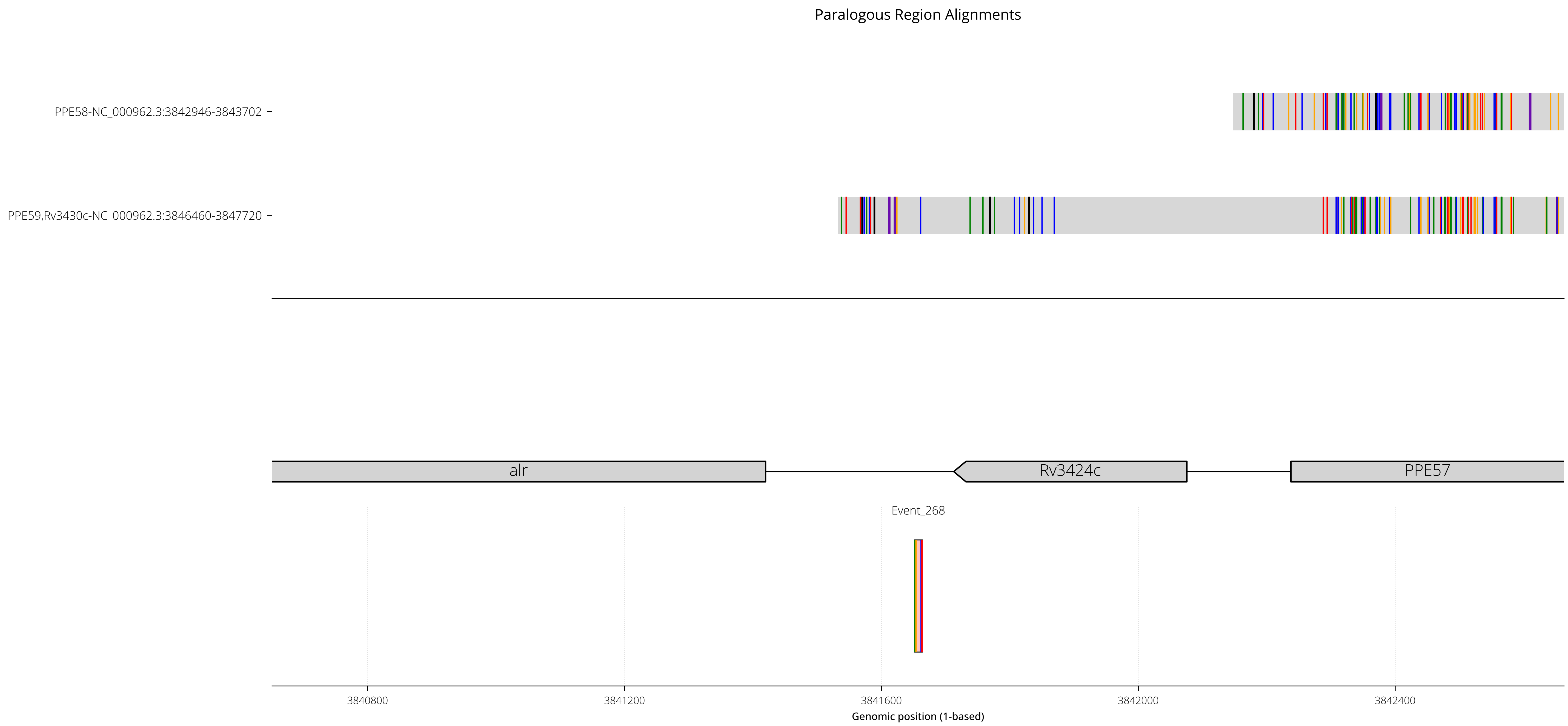

# Circos Viz - GCE to Known Paralogs Locations
GCE ID: Event_268

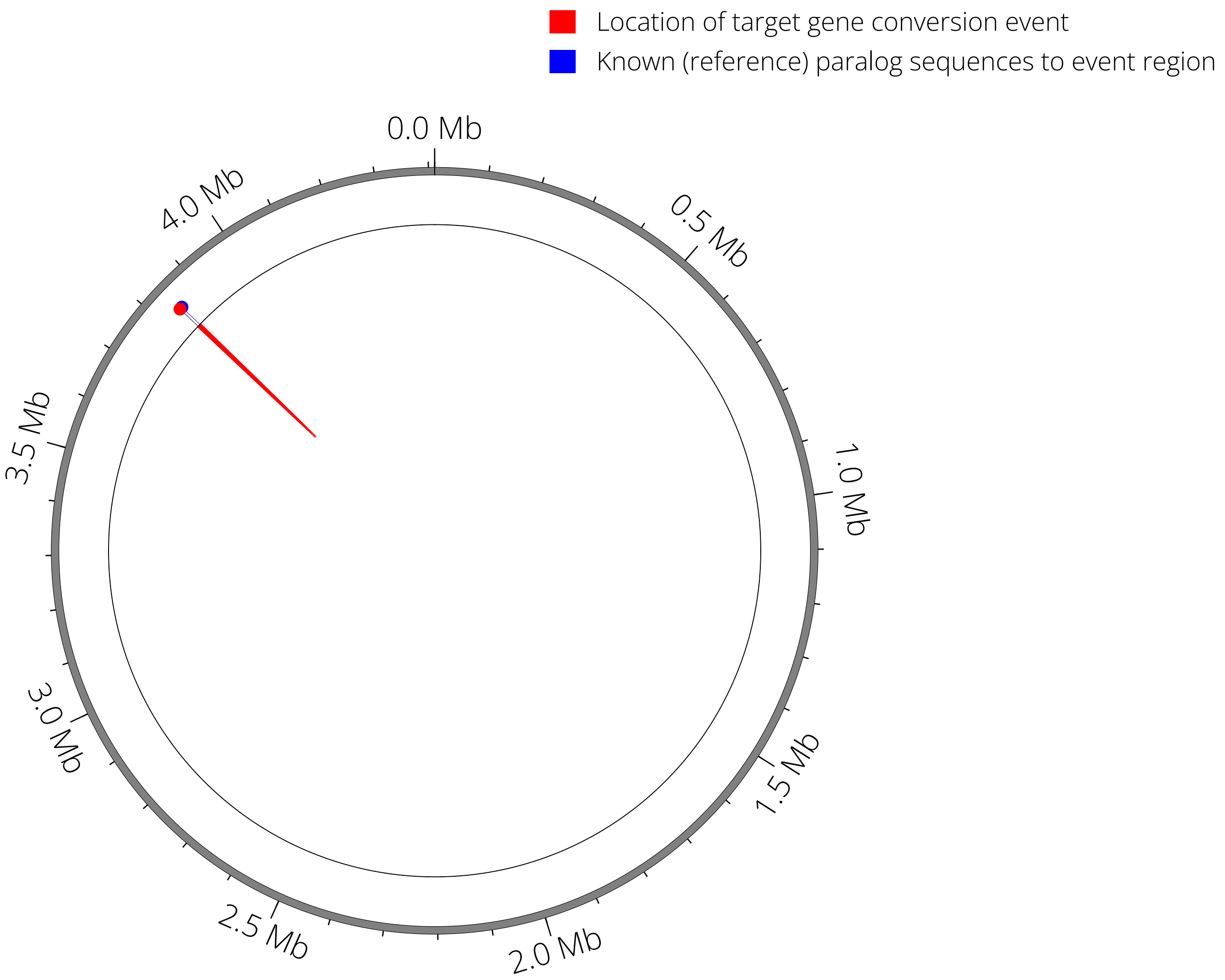

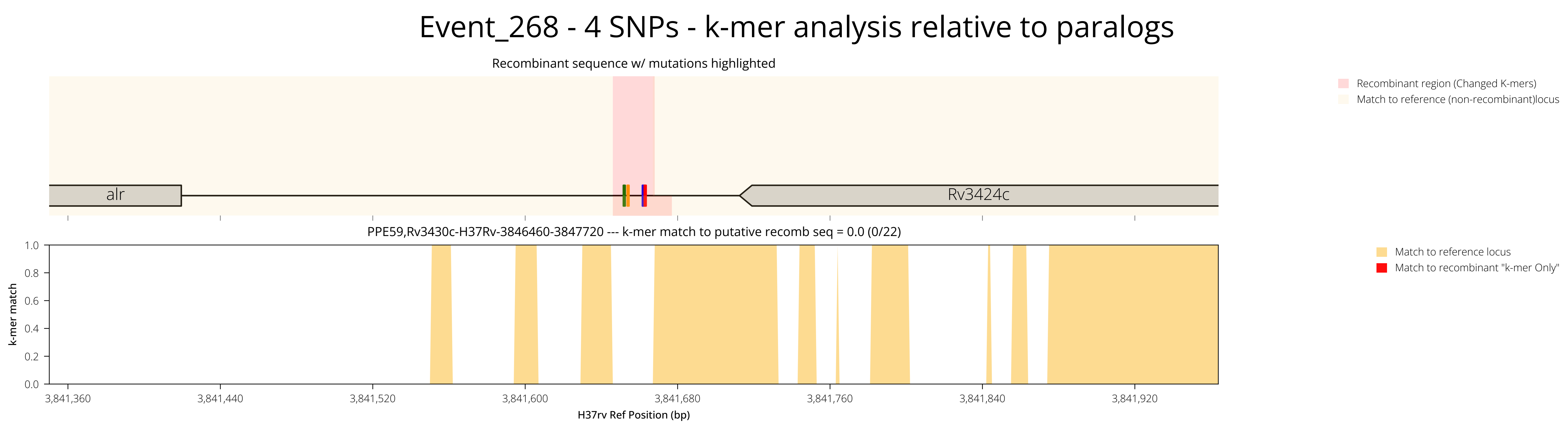

Event_268 - 4 SNPs - k-mer analysis relative to paralogs
Recombinant sequence w/ mutations highlighted
Recombinant region (Changed K-mers)
Match to reference (non-recombinant)locus
alr
Rv3424c
PPE59,Rv3430c-H37Rv-3846460-3847720 --- k-mer match to putative recomb seq = 0.0 (0/22)
Match to reference locus
Match to recombinant "k-mer Only"
k-mer match
1.0
0.8
0.6
0.4
0.2
0.0
3,841,360
3,841,440
3,841,520
3,841,600
3,841,680
3,841,760
3,841,840
3,841,920
H37rv Ref Position (bp)

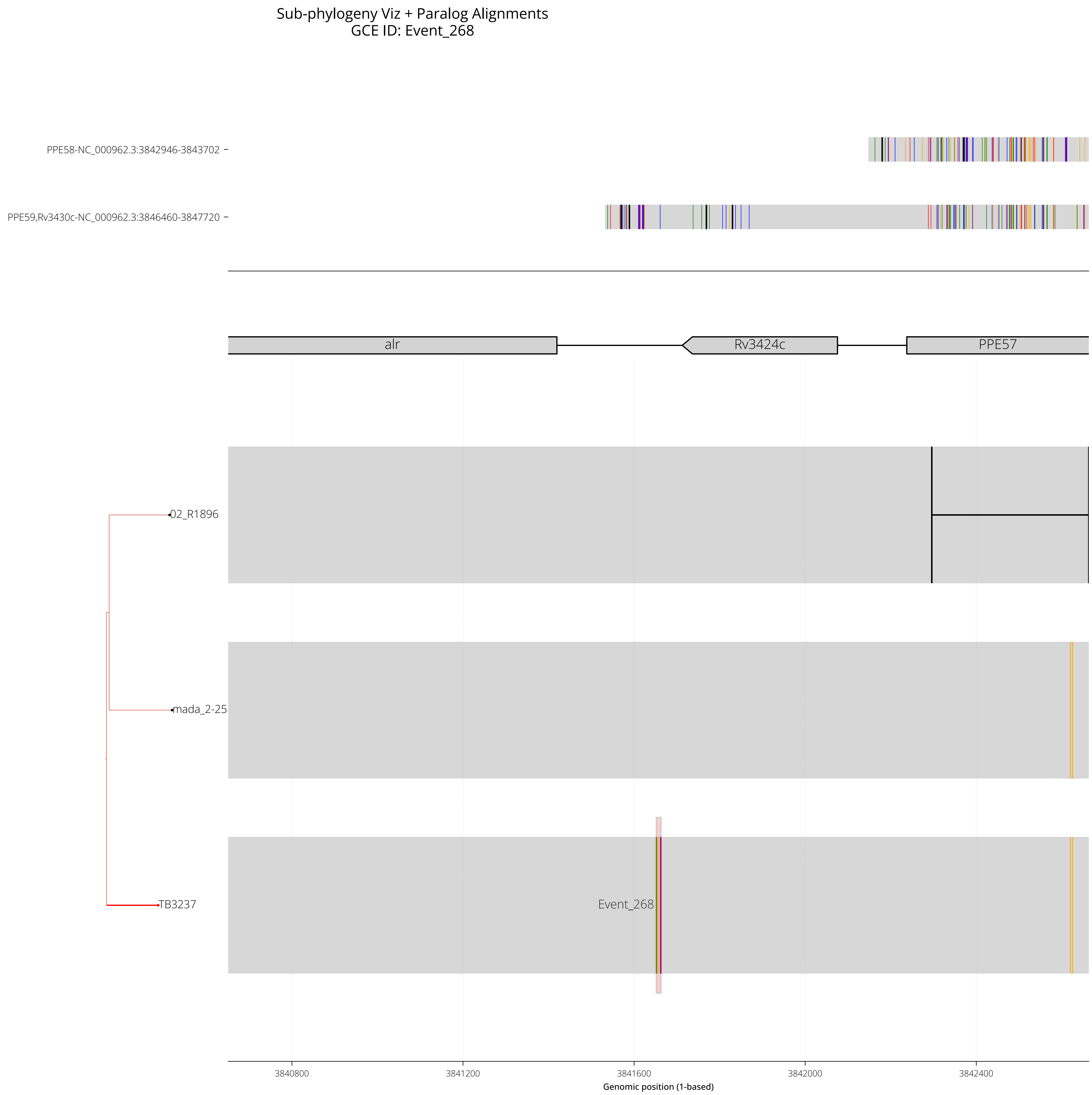

Sub-phylogeny Viz + Paralog Alignments
GCE ID: Event_268
PPE58-NC_000962.3:3842946-3843702
PPE59,Rv3430c-NC_000962.3:3846460-3847720
alr
Rv3424c
PPE57
02_R1896
mada_2-25
TB3237
Event_268
3840800
3841200
3841600
3842000
3842400
Genomic position (1-based)

Full Phylogeny Viz + Paralog Alignments
GCE ID: Event_268

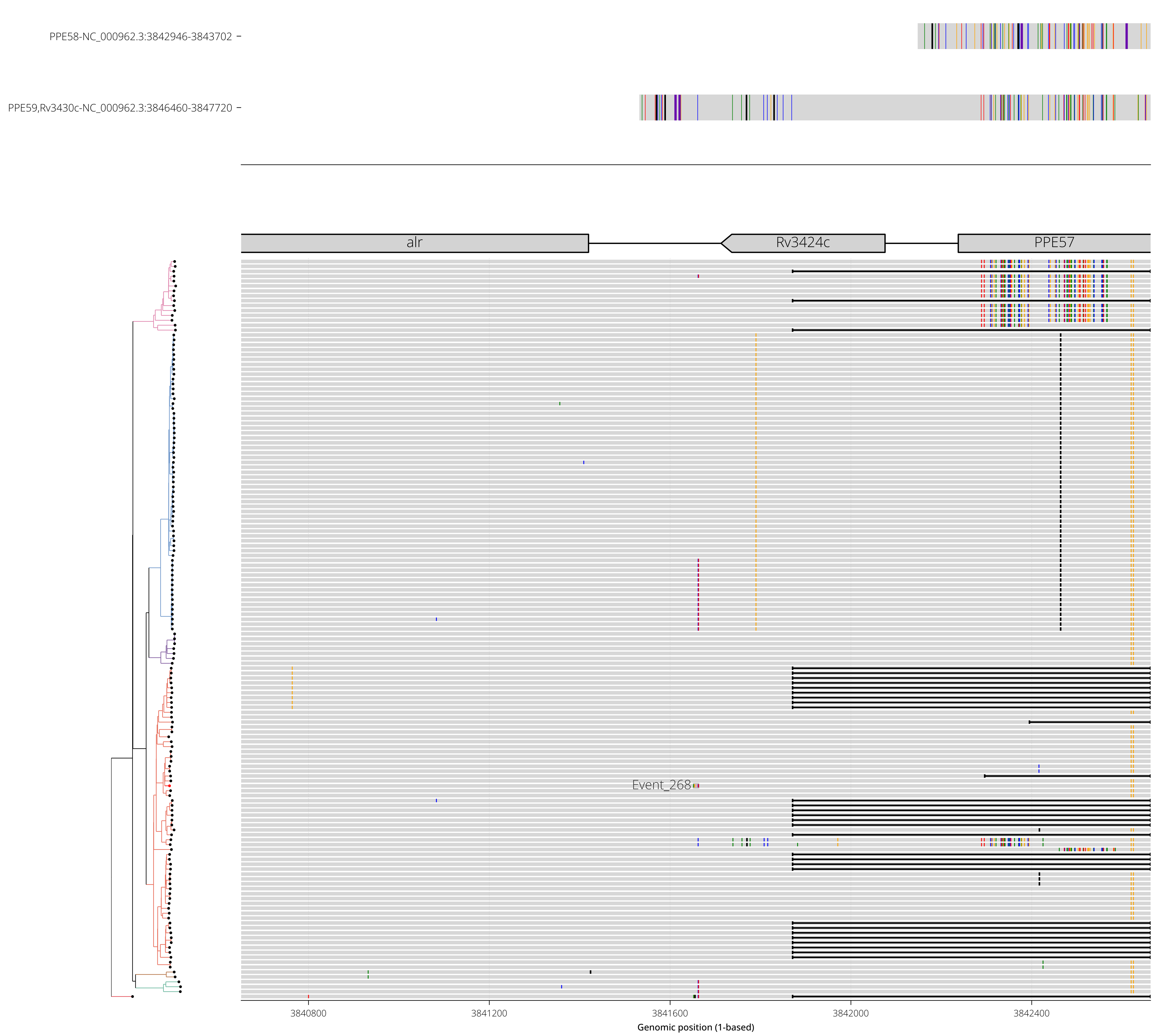

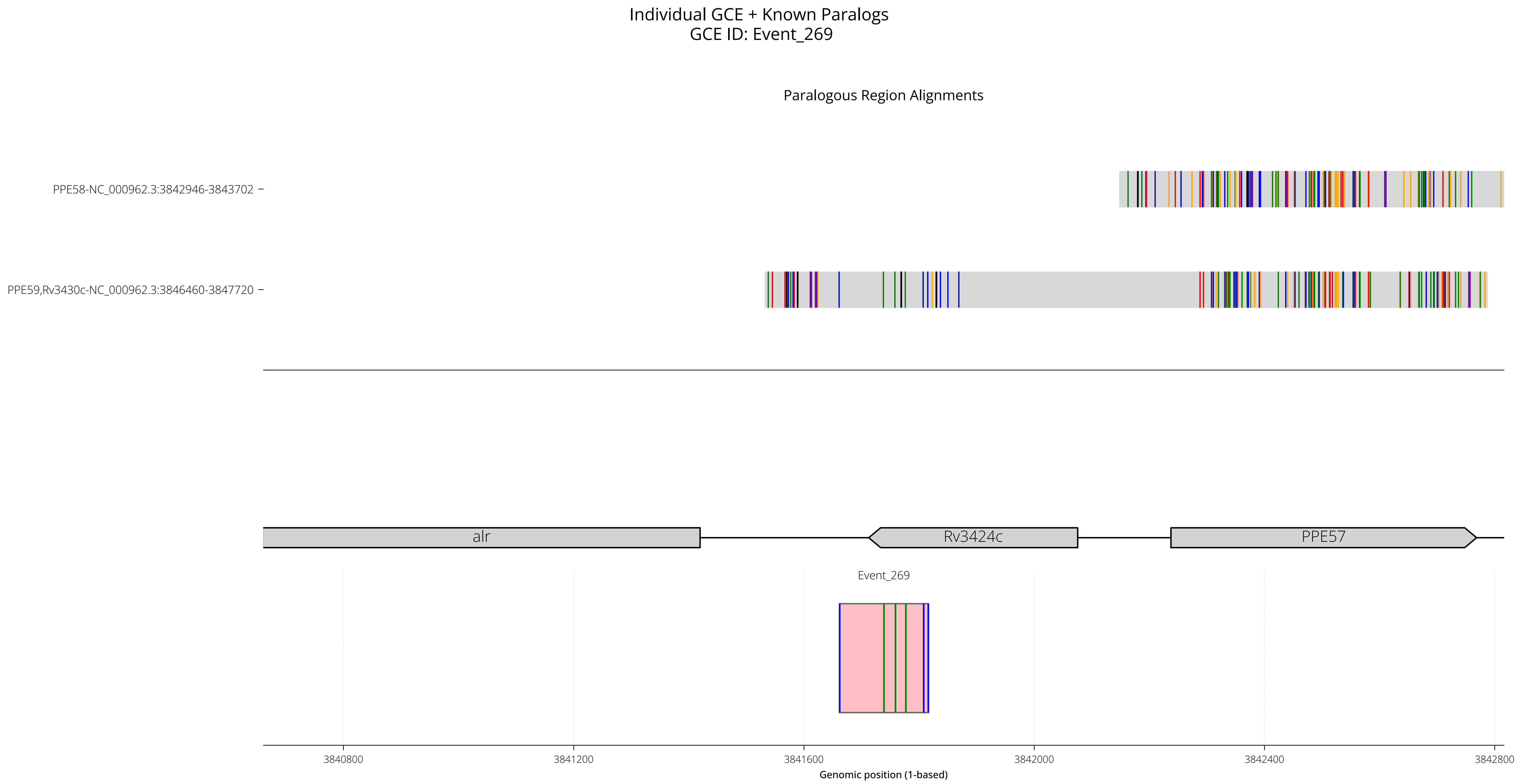

Individual GCE + Known Paralogs
GCE ID: Event_269
Paralogous Region Alignments
PPE58-NC_000962.3:3842946-3843702
PPE59,Rv3430c-NC_000962.3:3846460-3847720
alr
Rv3424c
PPE57
Event_269
3840800
3841200
3841600
3842000
3842400
3842800
Genomic position (1-based)

Circos Viz - GCE to Known Paralogs Locations
GCE ID: Event_269

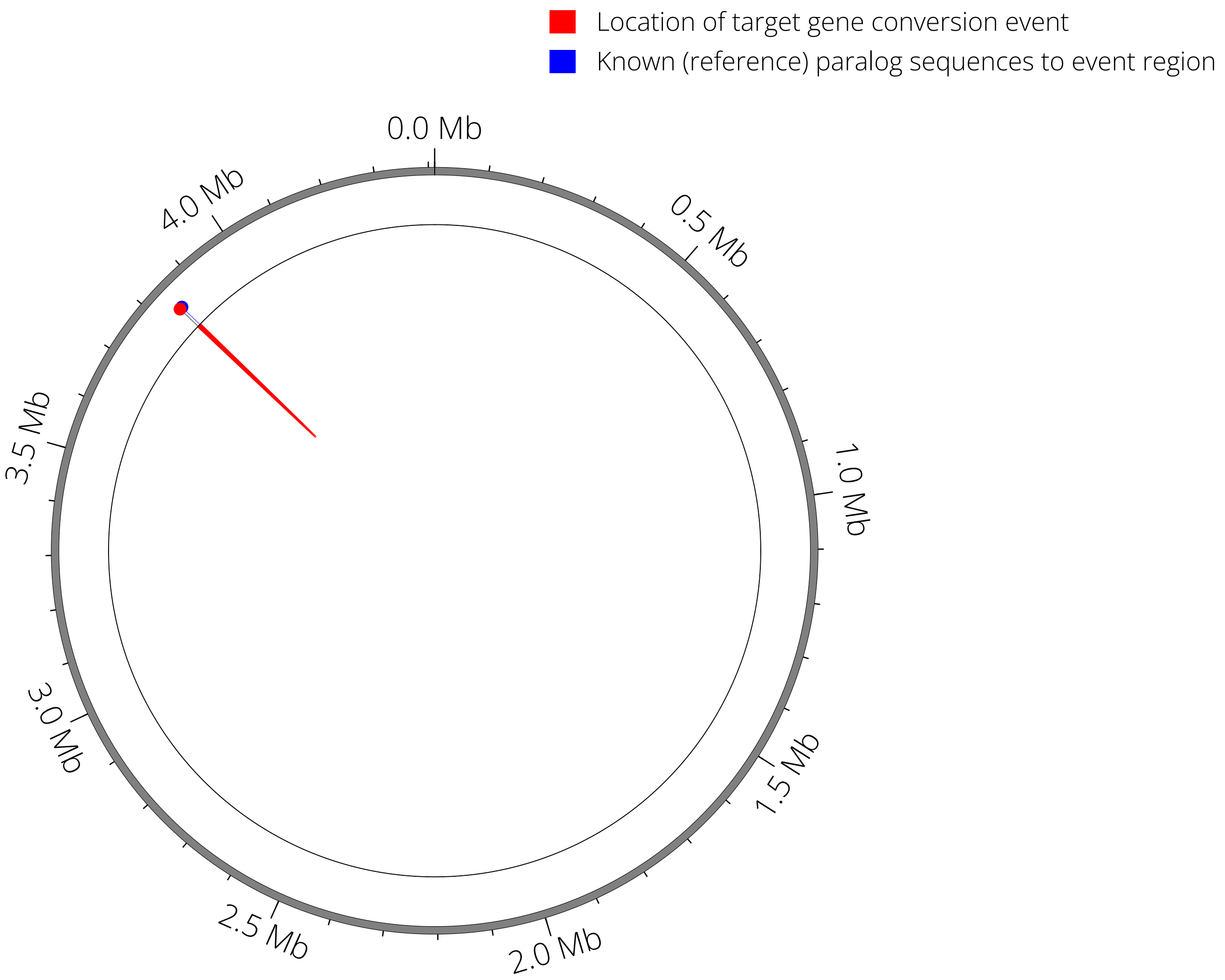

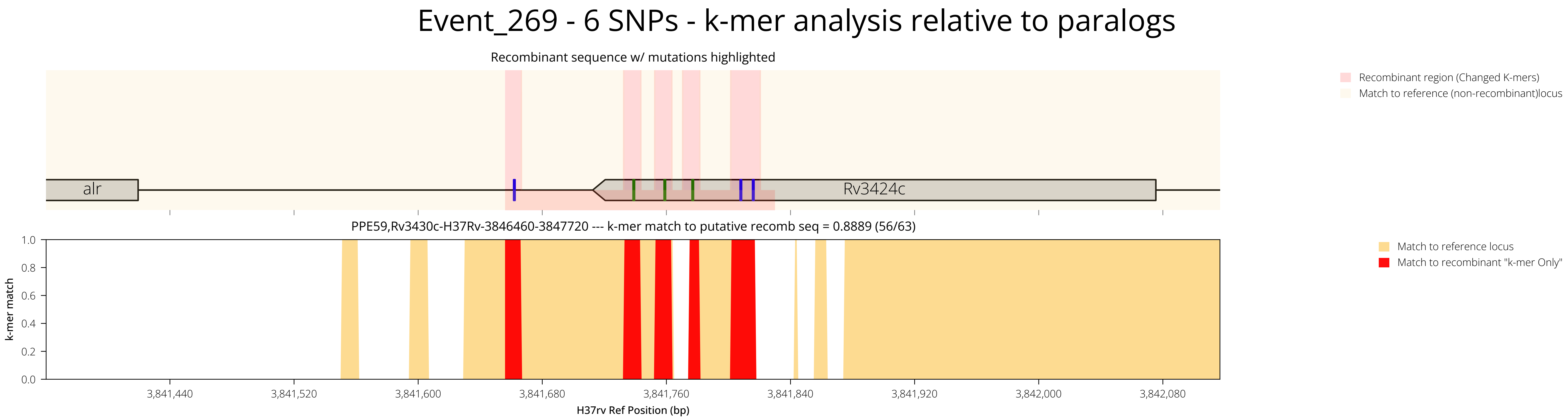
Event_269 - 6 SNPs - k-mer analysis relative to paralogs
Recombinant sequence w/ mutations highlighted
Recombinant region (Changed K-mers)
Match to reference (non-recombinant)locus
alr
Rv3424c
PPE59,Rv3430c-H37Rv-3846460-3847720 --- k-mer match to putative recomb seq = 0.8889 (56/63)
Match to reference locus
Match to recombinant "k-mer Only"
k-mer match
1.0
0.8
0.6
0.4
0.2
0.0
3,841,440
3,841,520
3,841,600
3,841,680
3,841,760
3,841,840
3,841,920
3,842,000
3,842,080
H37rv Ref Position (bp)

Sub-phylogeny Viz + Paralog Alignments
GCE ID: Event_269

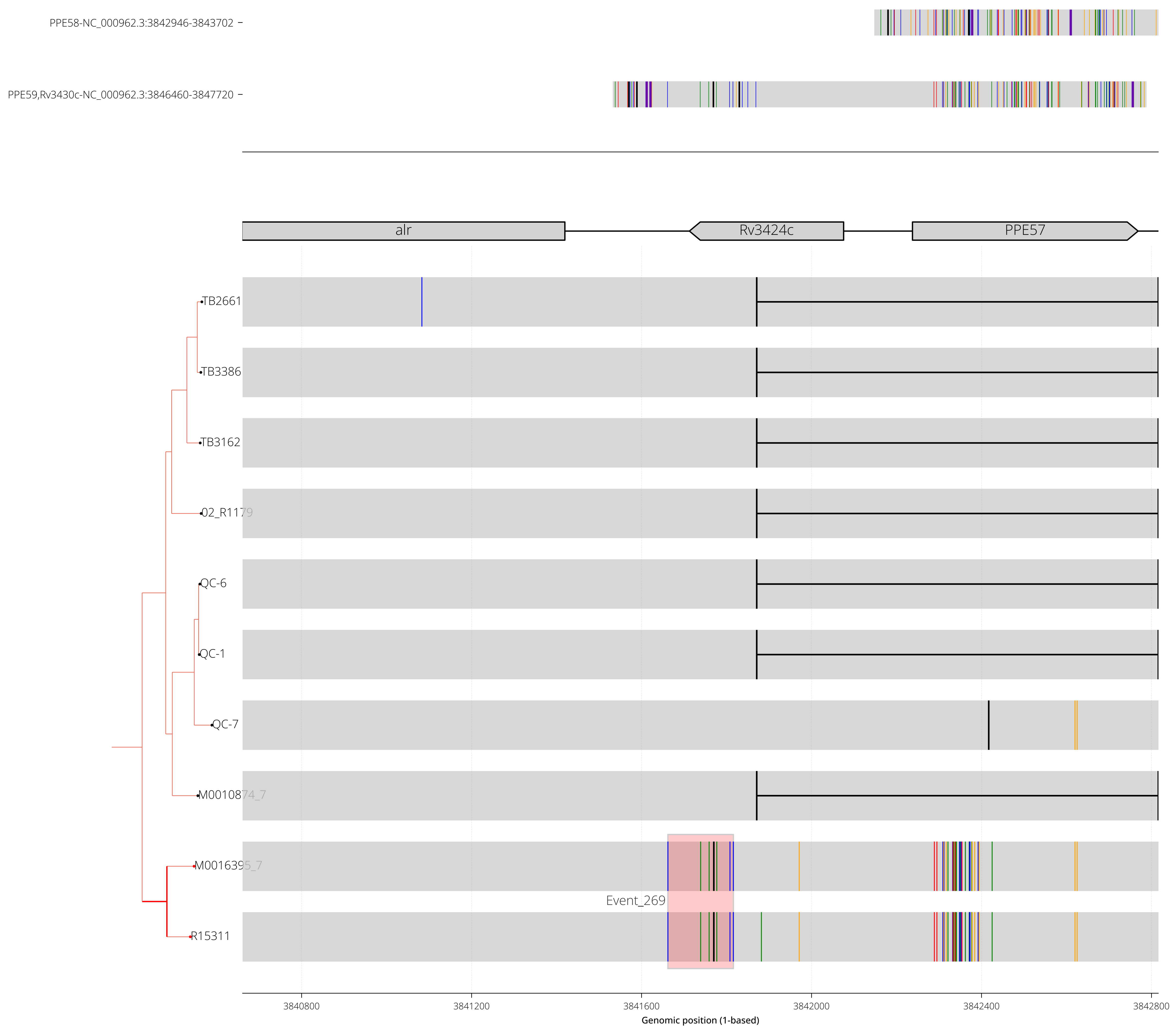

Full Phylogeny Viz + Paralog Alignments
GCE ID: Event_269

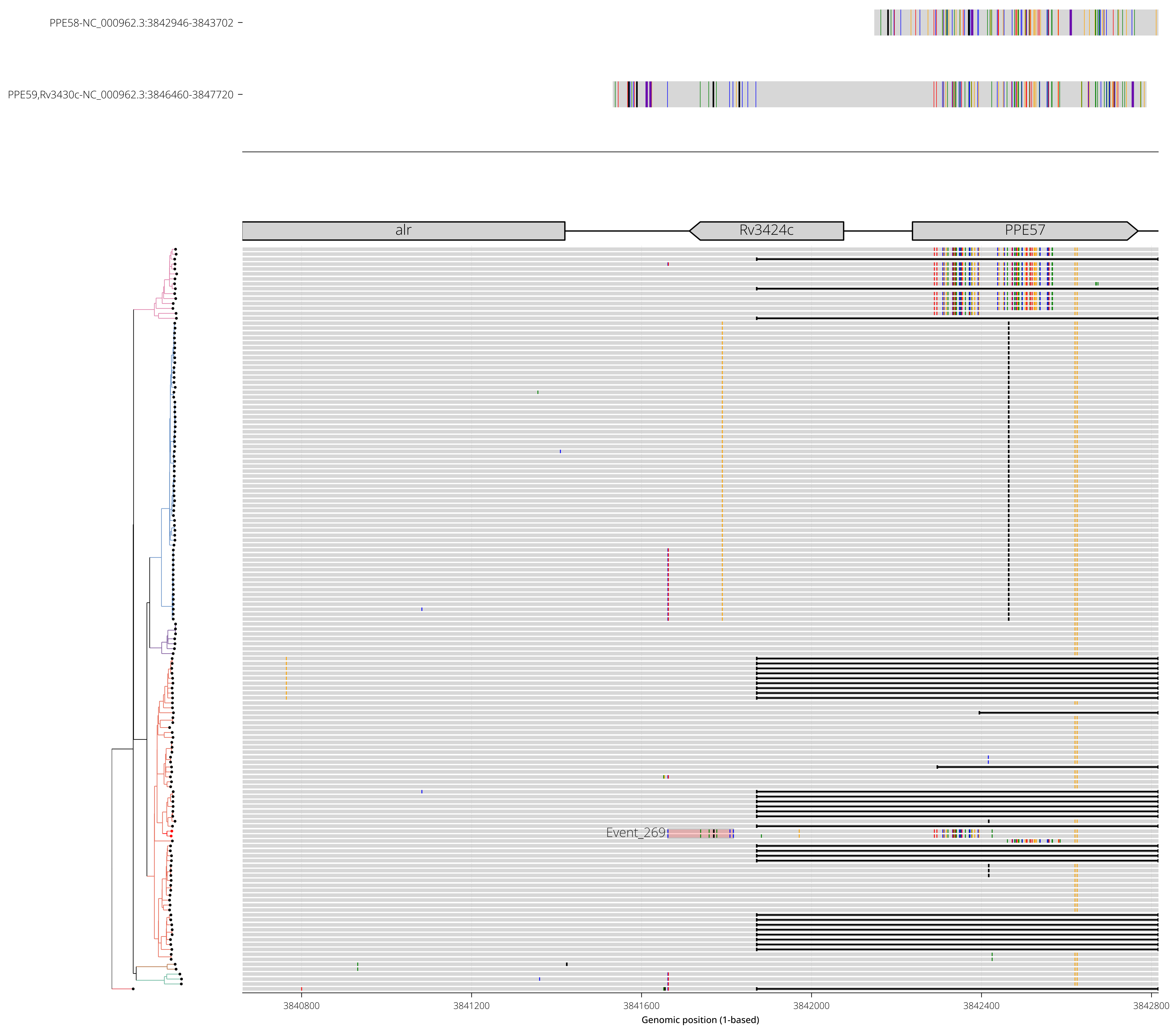

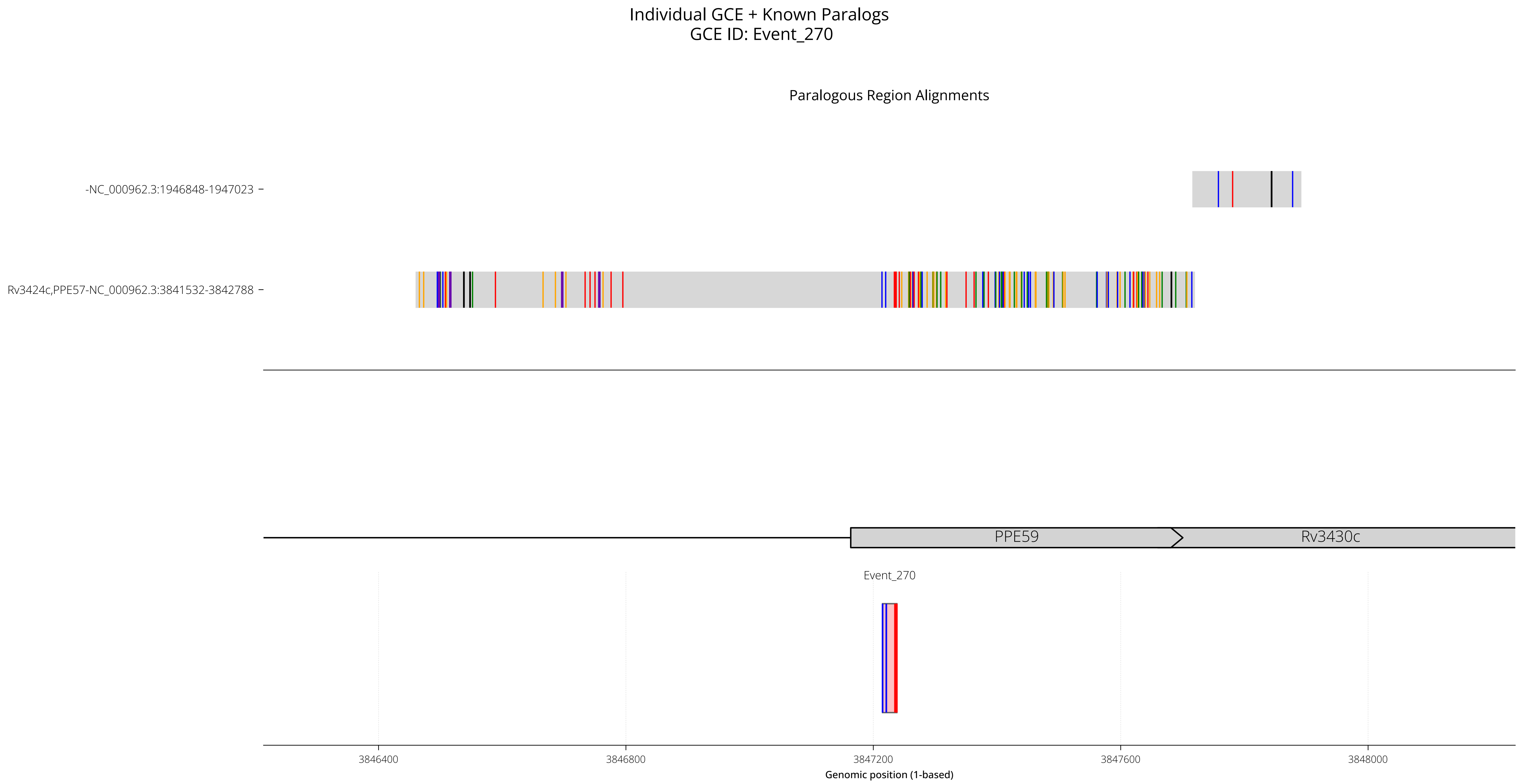
Individual GCE + Known Paralogs
GCE ID: Event_270
Paralogous Region Alignments
-NC_000962.3:1946848-1947023
Rv3424c,PPE57-NC_000962.3:3841532-3842788
PPE59
Rv3430c
Event_270
3846400
3846800
3847200
3847600
3848000
Genomic position (1-based)

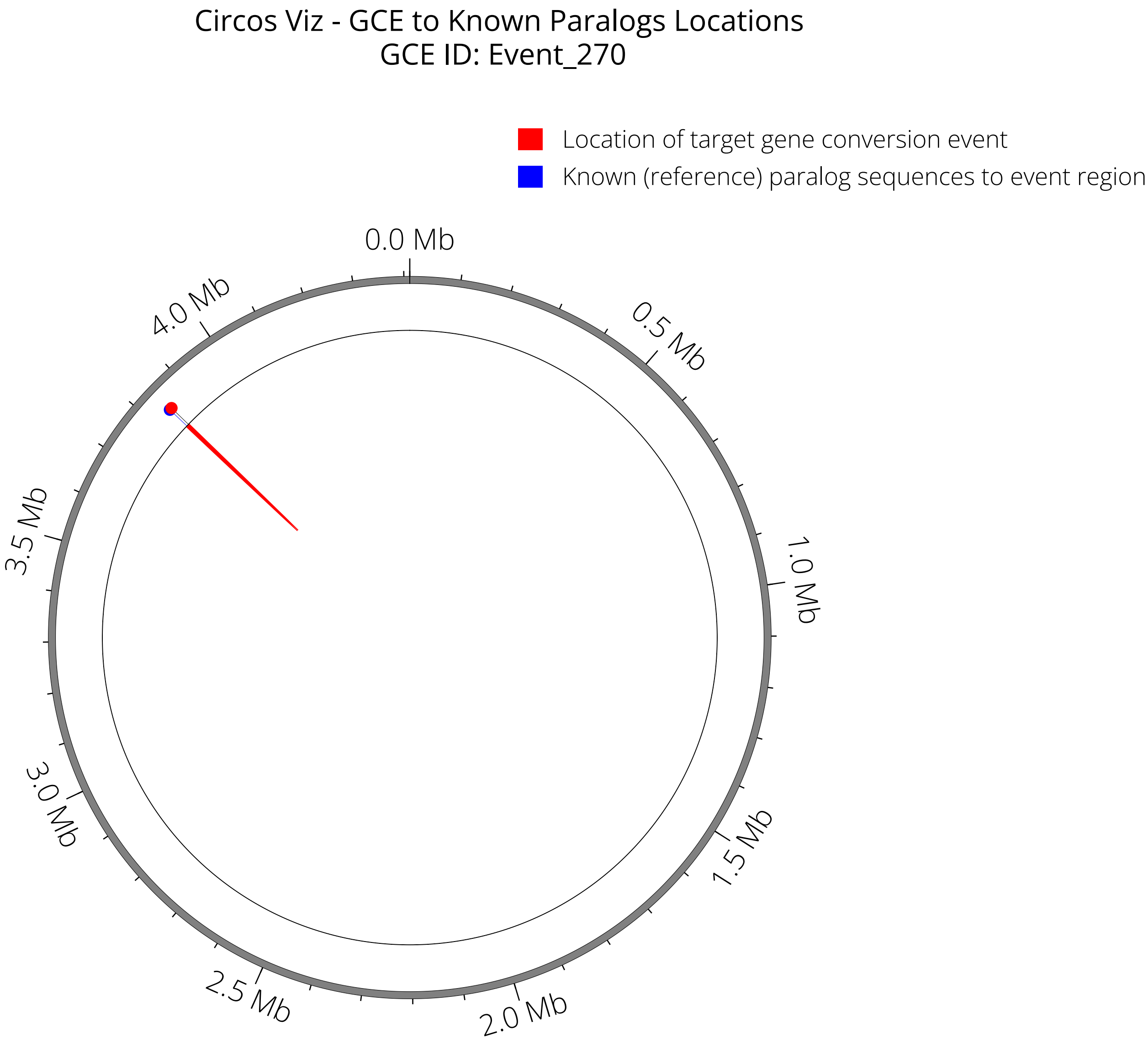
Circos Viz - GCE to Known Paralogs Locations
GCE ID: Event_270
Location of target gene conversion event
Known (reference) paralog sequences to event region
0.0 Mb
0.5 Mb
1.0 Mb
1.5 Mb
2.0 Mb
2.5 Mb
3.0 Mb
3.5 Mb
4.0 Mb

# Event_270 - 5 SNPs - k-mer analysis relative to paralogs

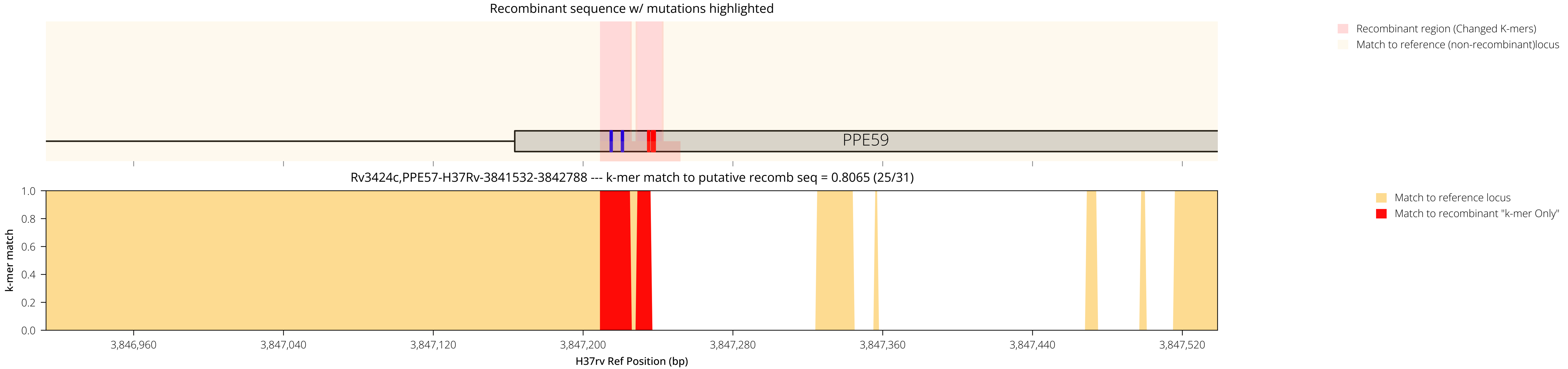

Sub-phylogeny Viz + Paralog Alignments
GCE ID: Event_270

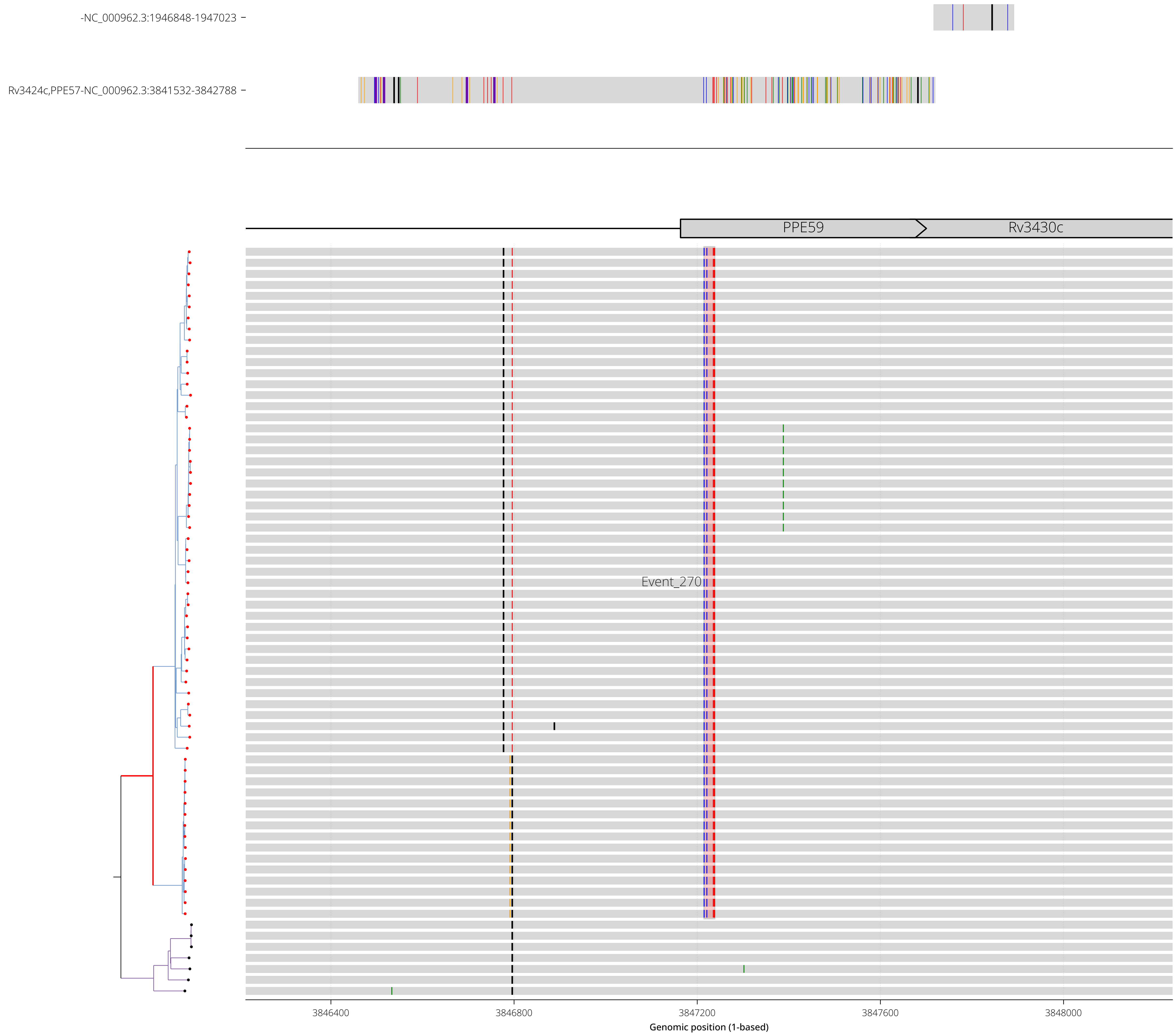

Full Phylogeny Viz + Paralog Alignments
GCE ID: Event_270

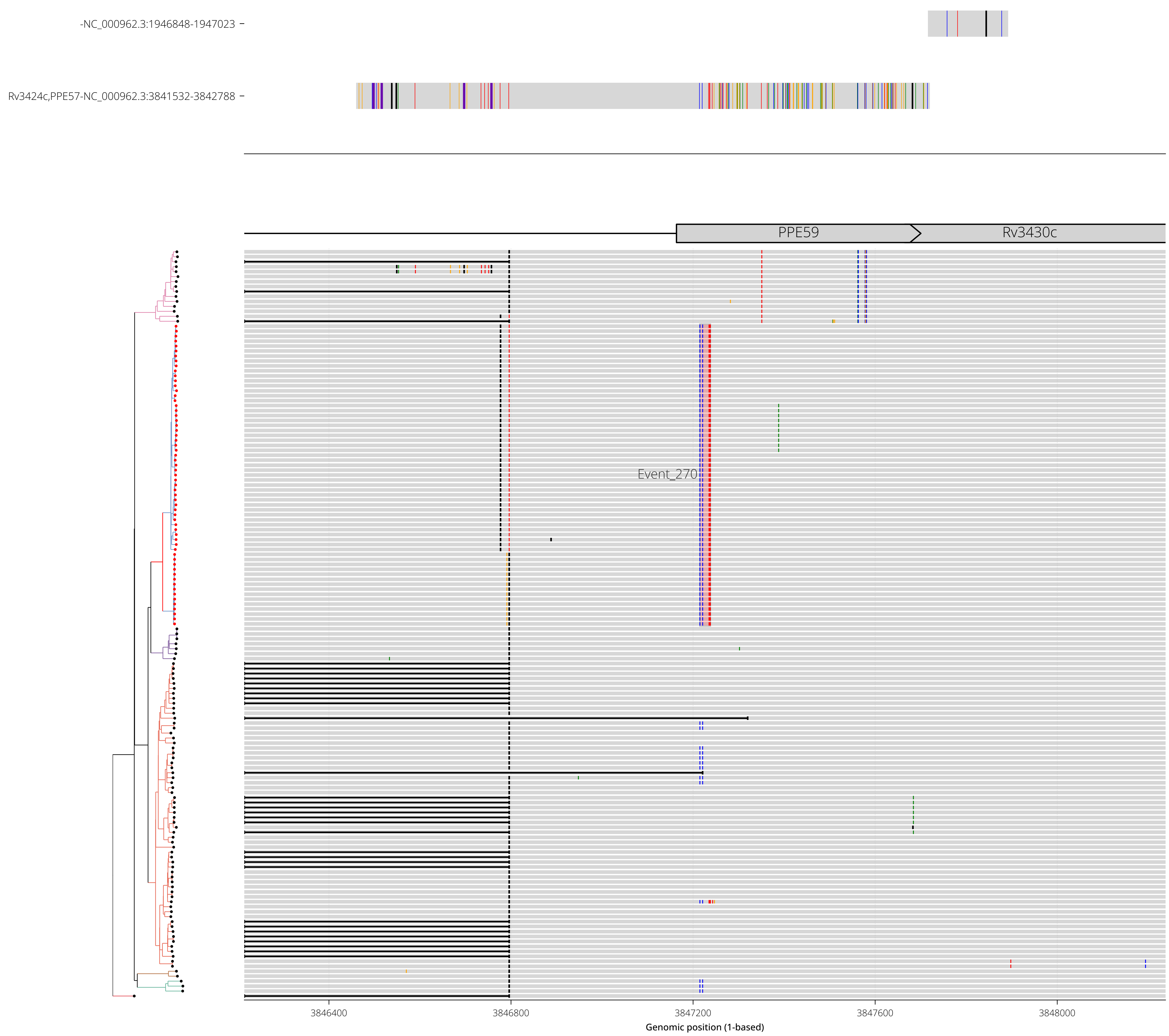

Individual GCE + Known Paralogs
GCE ID: Event_271

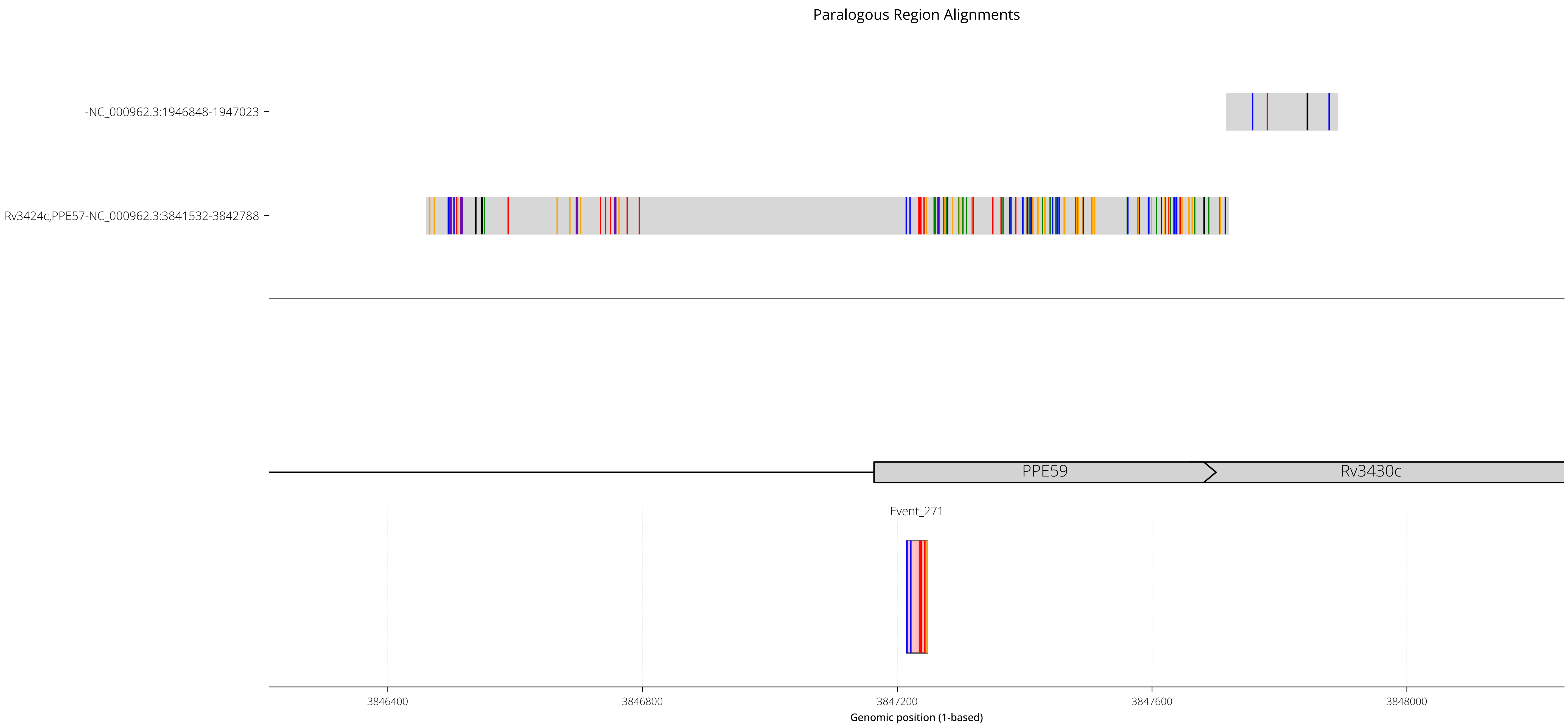

Circos Viz - GCE to Known Paralogs Locations
GCE ID: Event_271

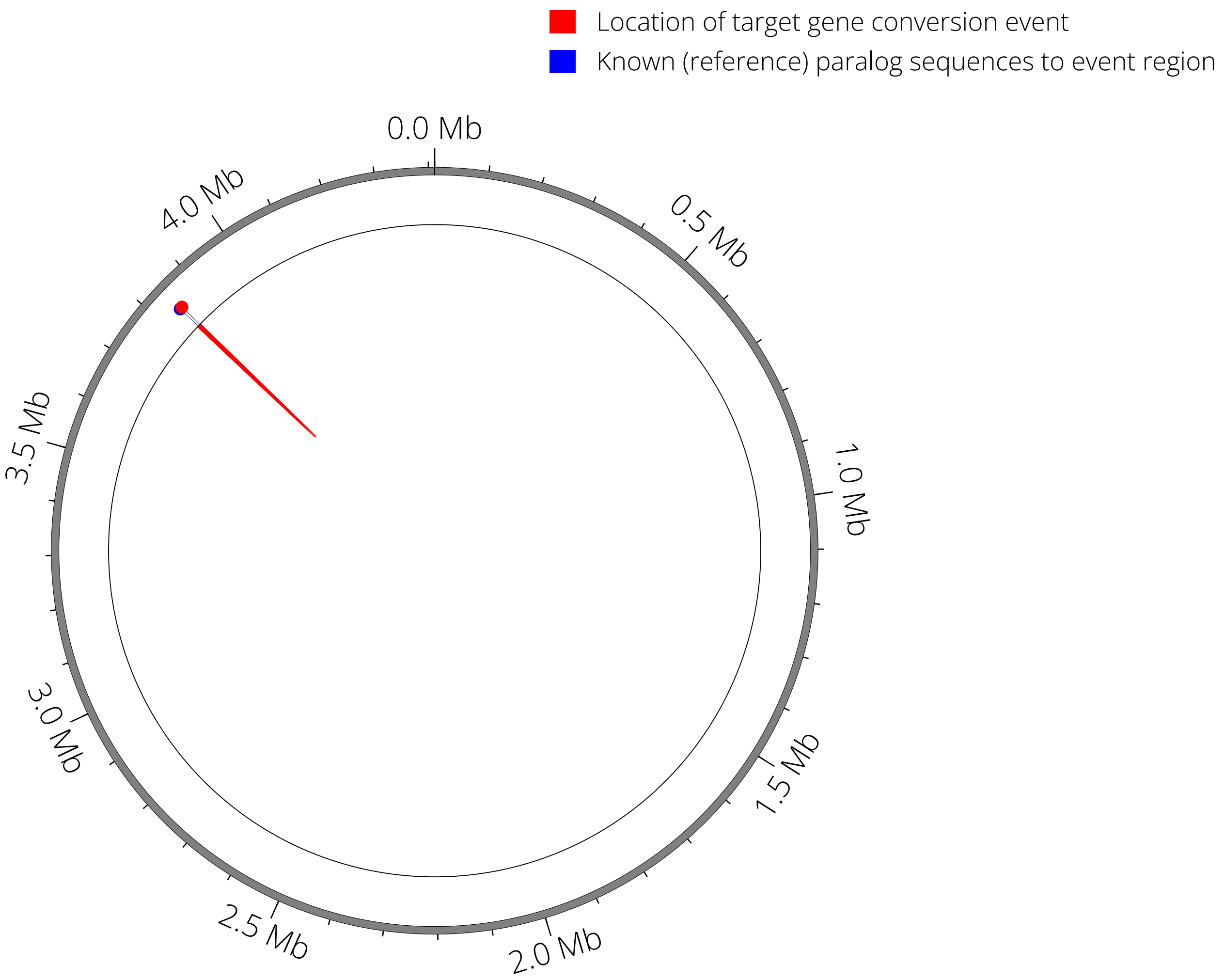

# Event_271 - 7 SNPs - k-mer analysis relative to paralogs

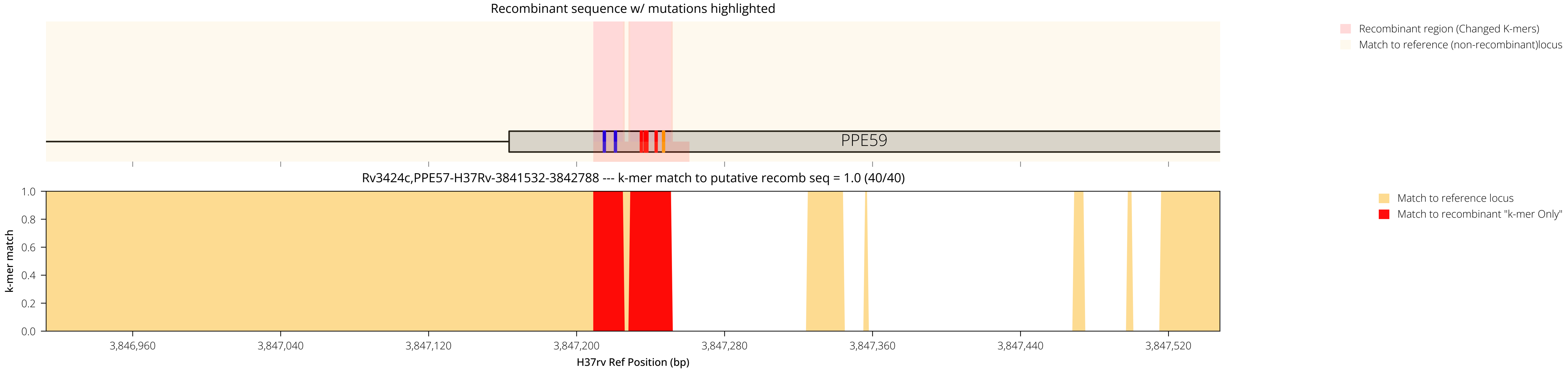

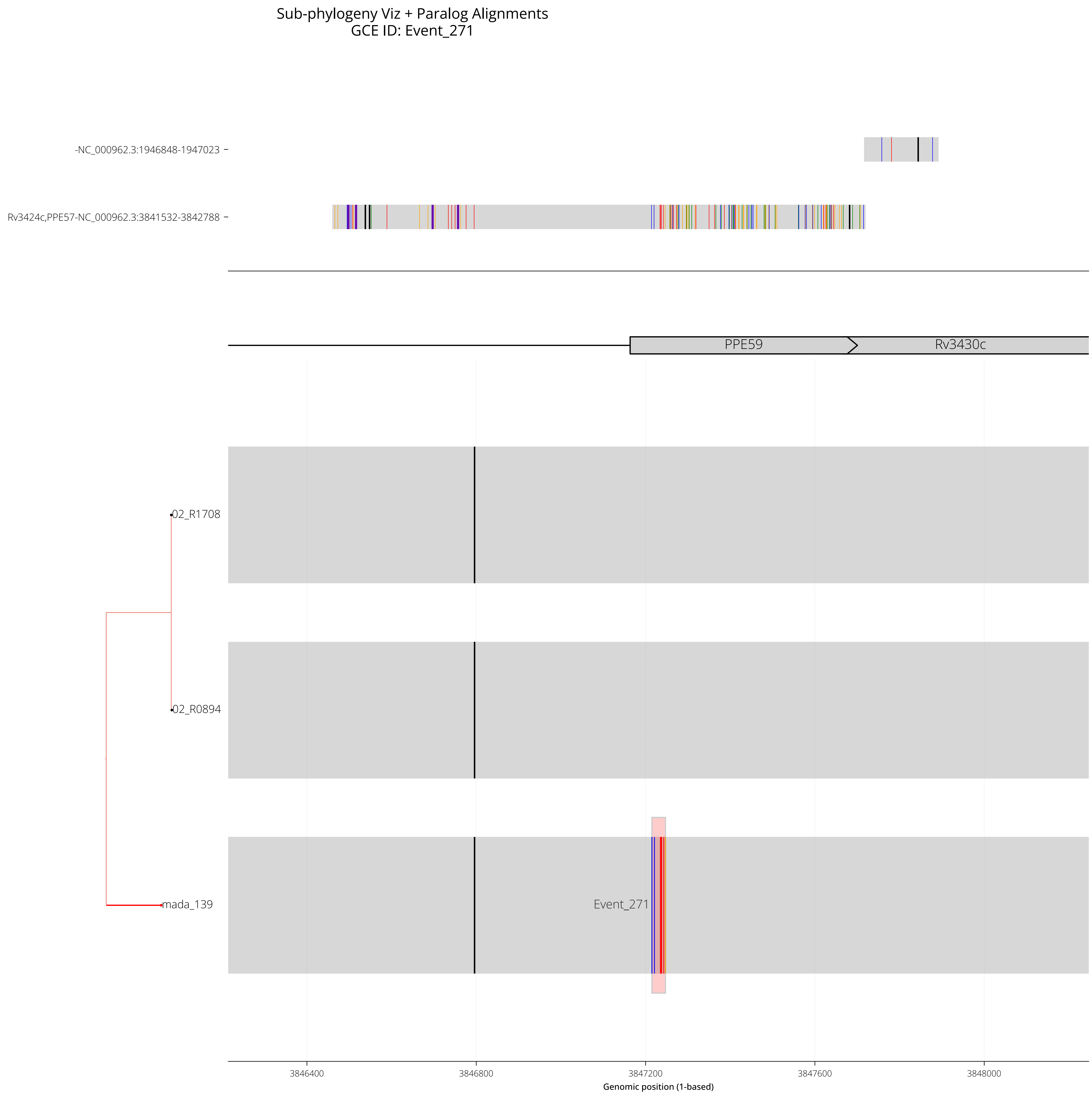
Sub-phylogeny Viz + Paralog Alignments
GCE ID: Event_271
-NC_000962.3:1946848-1947023
Rv3424c,PPE57-NC_000962.3:3841532-3842788
PPE59
Rv3430c
02_R1708
02_R0894
mada_139
Event_271
3846400
3846800
3847200
3847600
3848000
Genomic position (1-based)

Full Phylogeny Viz + Paralog Alignments
GCE ID: Event_271

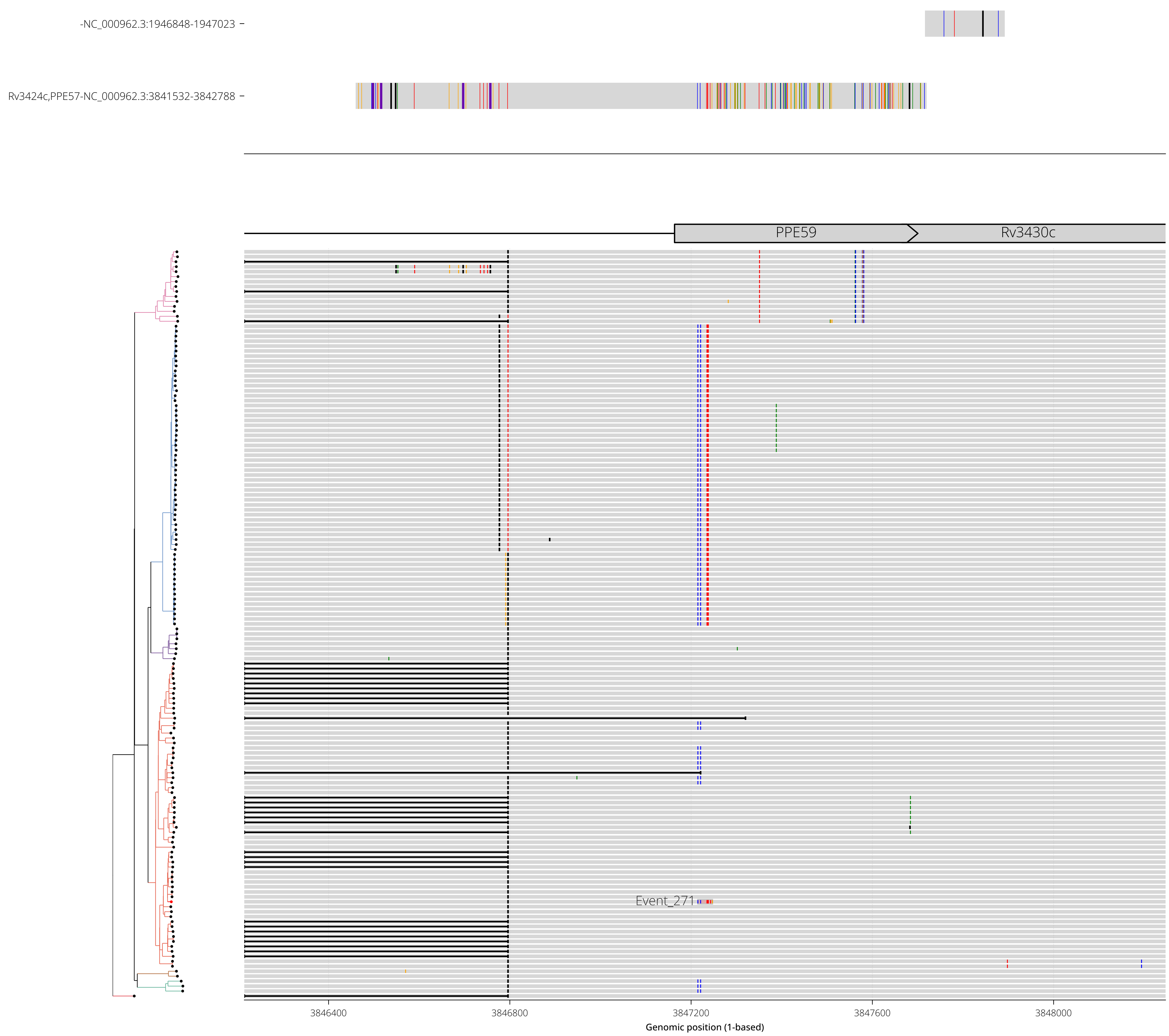

Individual GCE + Known Paralogs
GCE ID: Event_272

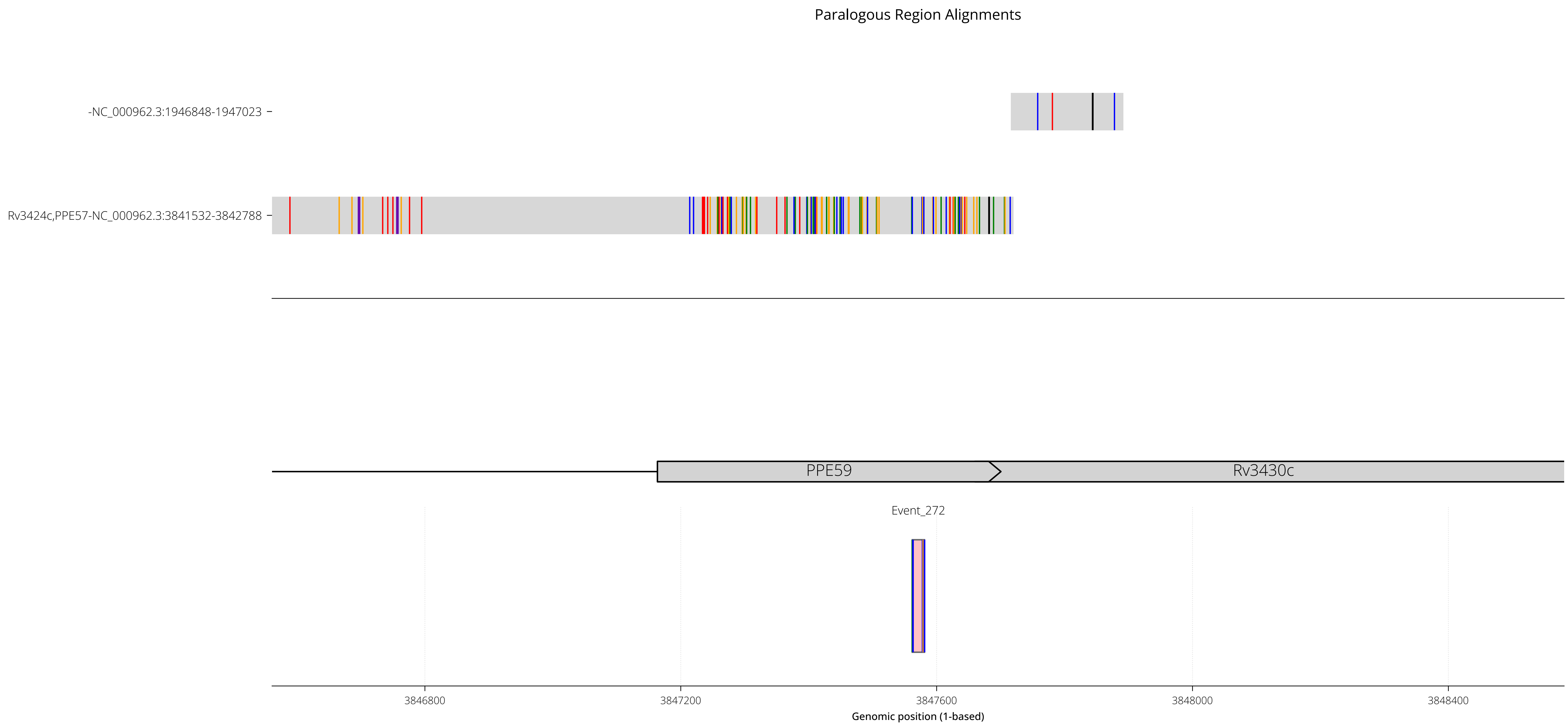

Circos Viz - GCE to Known Paralogs Locations
GCE ID: Event_272

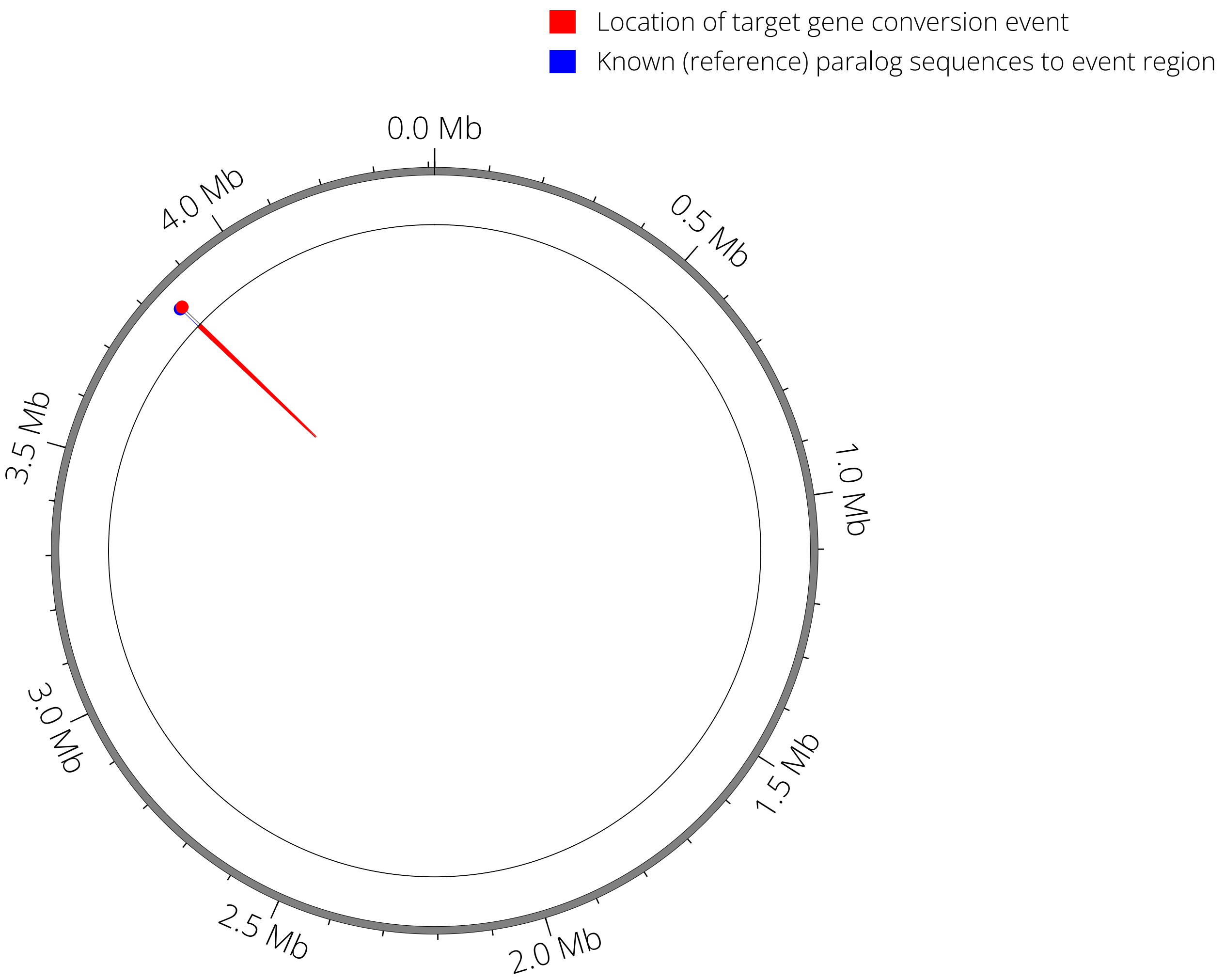

# Event_272 - 5 SNPs - k-mer analysis relative to paralogs

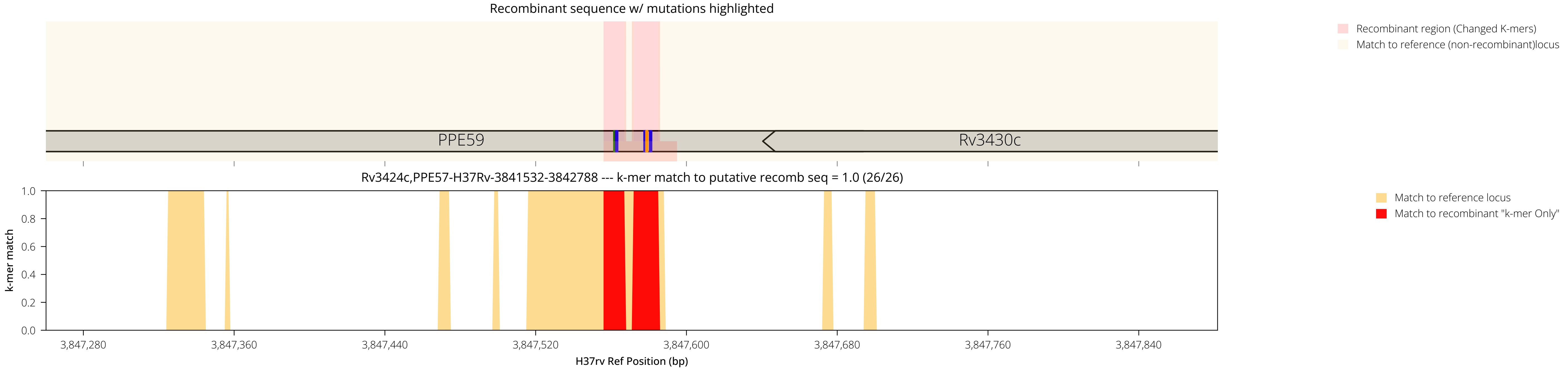

Sub-phylogeny Viz + Paralog Alignments
GCE ID: Event_272

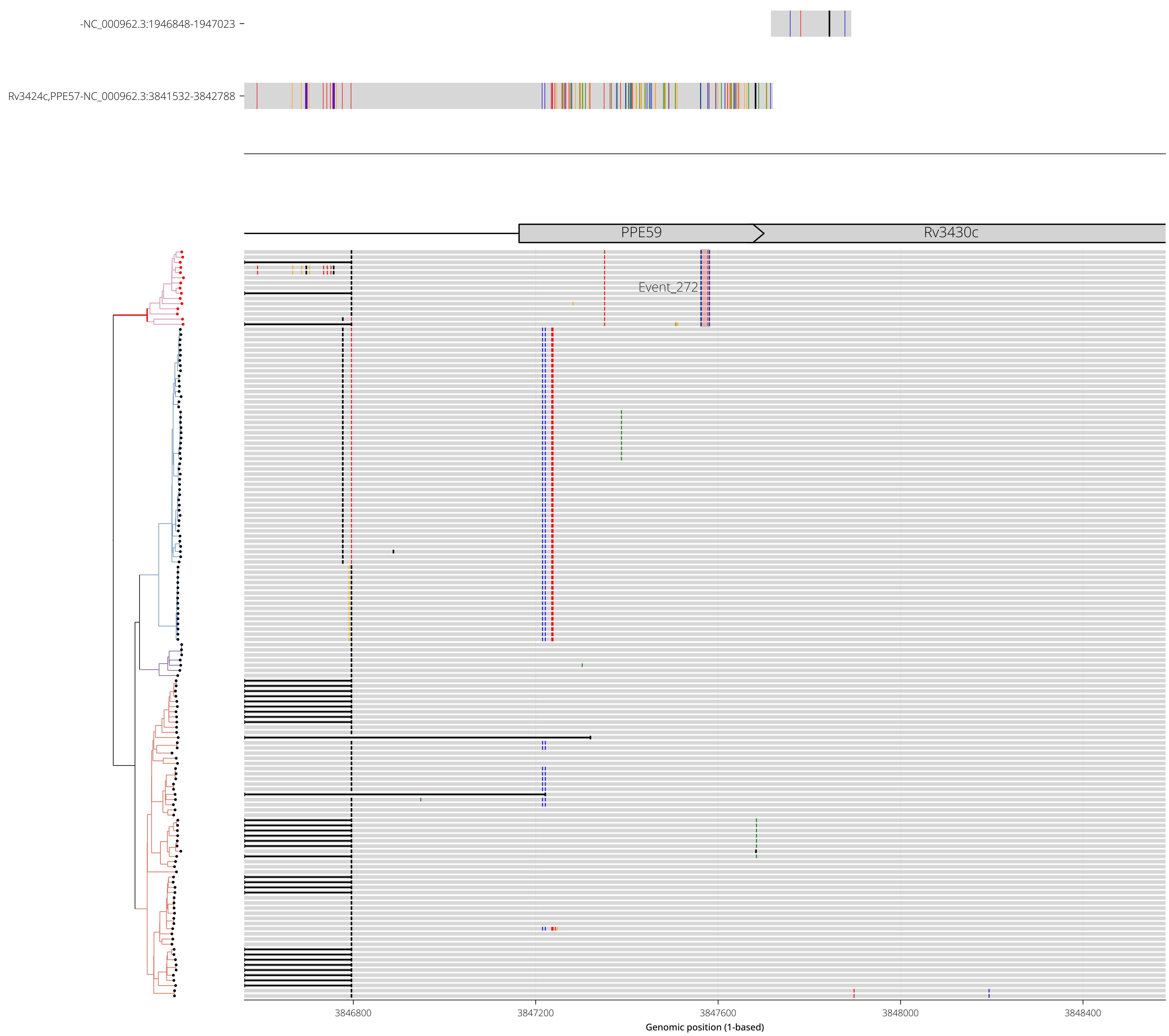

Full Phylogeny Viz + Paralog Alignments
GCE ID: Event_272

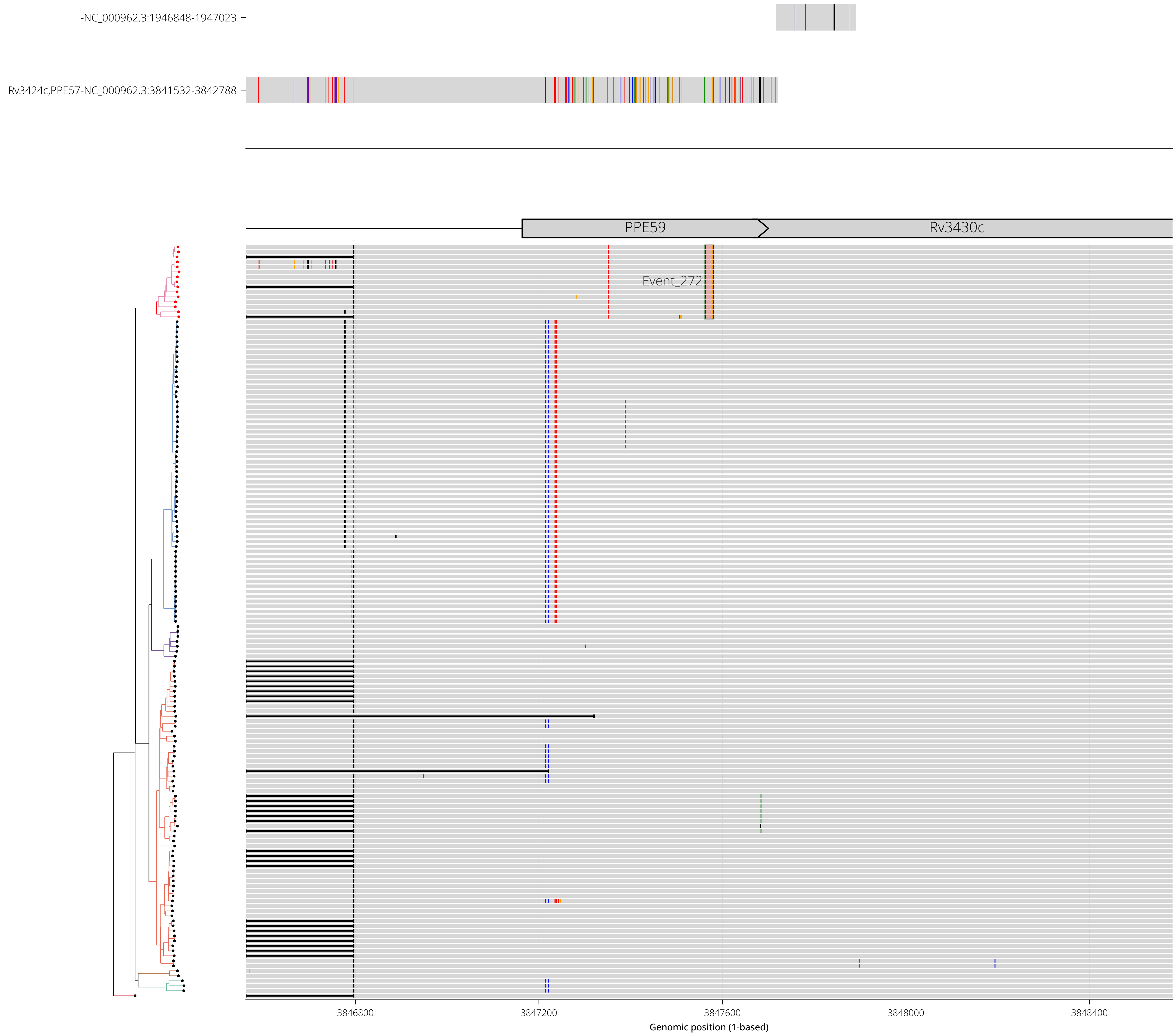

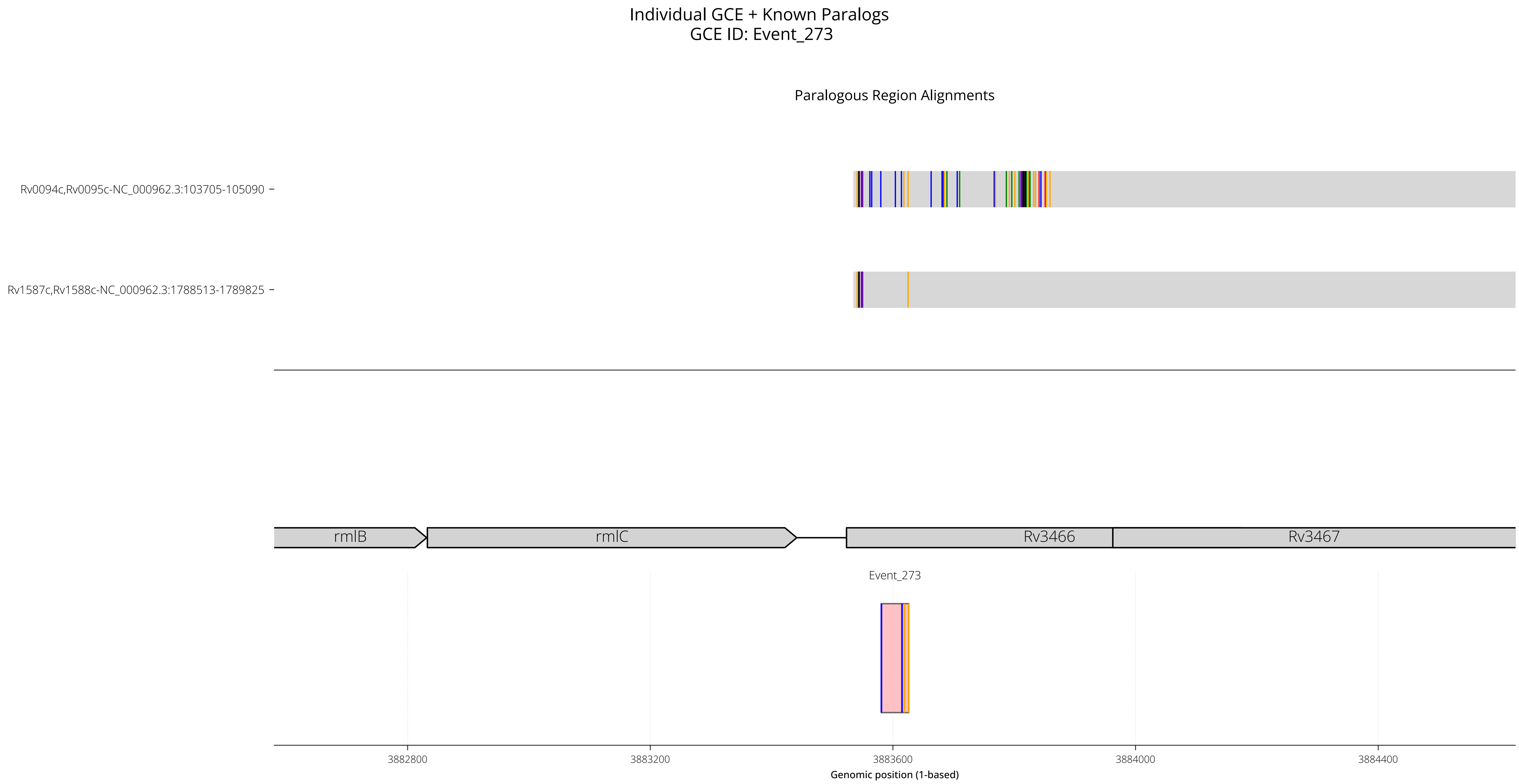

Individual GCE + Known Paralogs
GCE ID: Event_273
Paralogous Region Alignments
Rv0094c,Rv0095c-NC_000962.3:103705-105090
Rv1587c,Rv1588c-NC_000962.3:1788513-1789825
rmlB
rmlC
Rv3466
Rv3467
Event_273
3882800
3883200
3883600
3884000
3884400
Genomic position (1-based)

Circos Viz - GCE to Known Paralogs Locations
GCE ID: Event_273

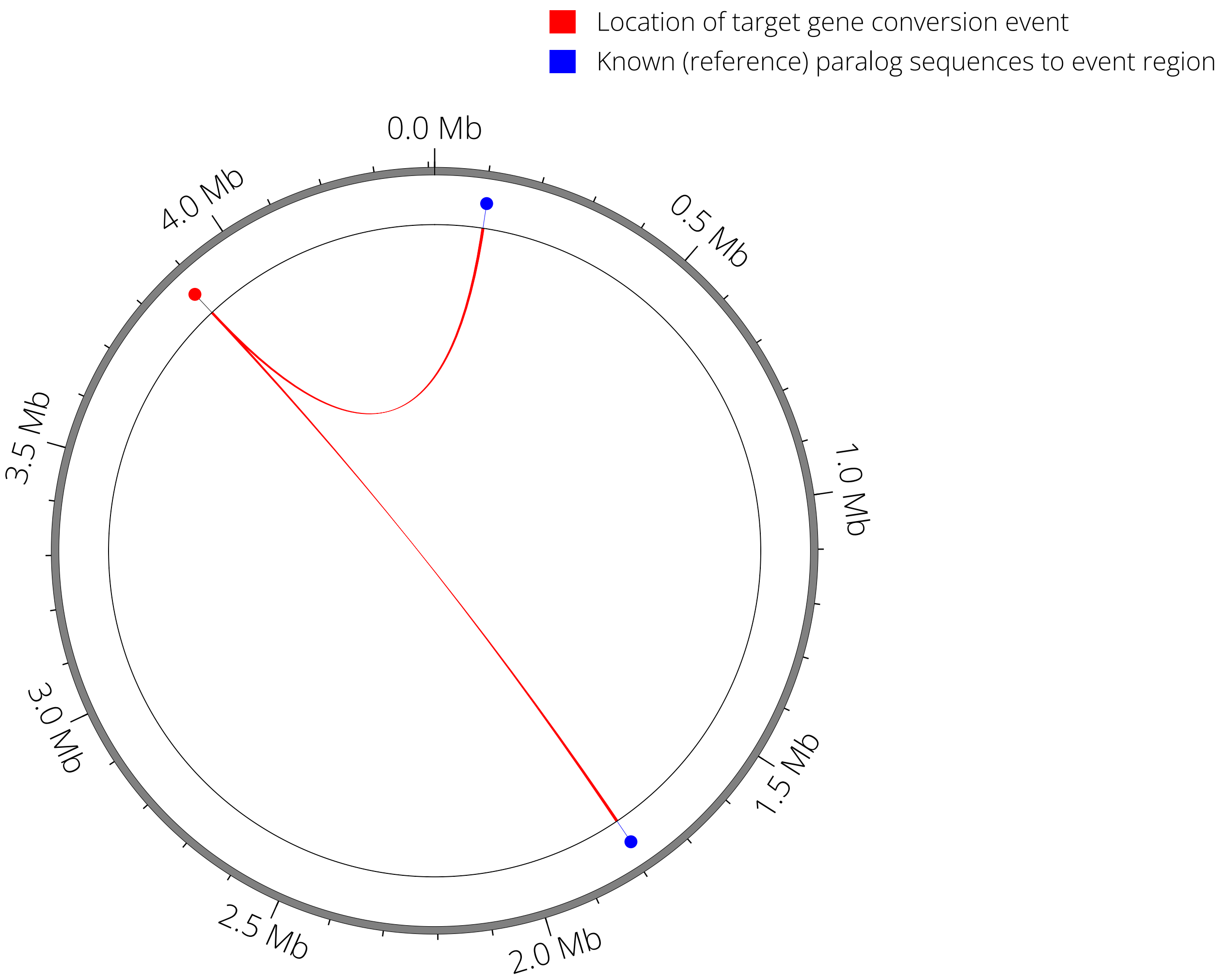

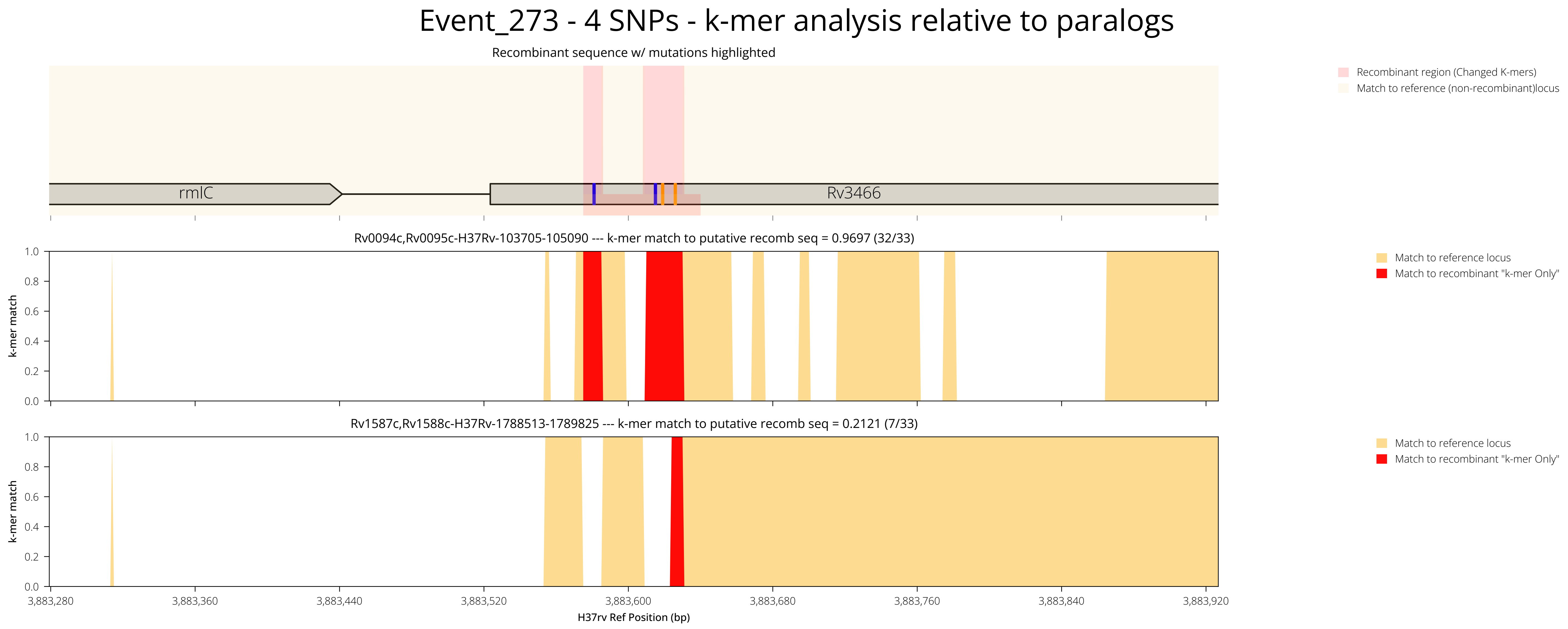

Event_273 - 4 SNPs - k-mer analysis relative to paralogs
Recombinant sequence w/ mutations highlighted
Recombinant region (Changed K-mers)
Match to reference (non-recombinant)locus
rmlC
Rv3466
Rv0094c,Rv0095c-H37Rv-103705-105090 --- k-mer match to putative recomb seq = 0.9697 (32/33)
Match to reference locus
Match to recombinant "k-mer Only"
k-mer match
1.0
0.8
0.6
0.4
0.2
0.0
Rv1587c,Rv1588c-H37Rv-1788513-1789825 --- k-mer match to putative recomb seq = 0.2121 (7/33)
Match to reference locus
Match to recombinant "k-mer Only"
k-mer match
1.0
0.8
0.6
0.4
0.2
0.0
3,883,280
3,883,360
3,883,440
3,883,520
3,883,600
3,883,680
3,883,760
3,883,840
3,883,920
H37rv Ref Position (bp)

Sub-phylogeny Viz + Paralog Alignments
GCE ID: Event_273

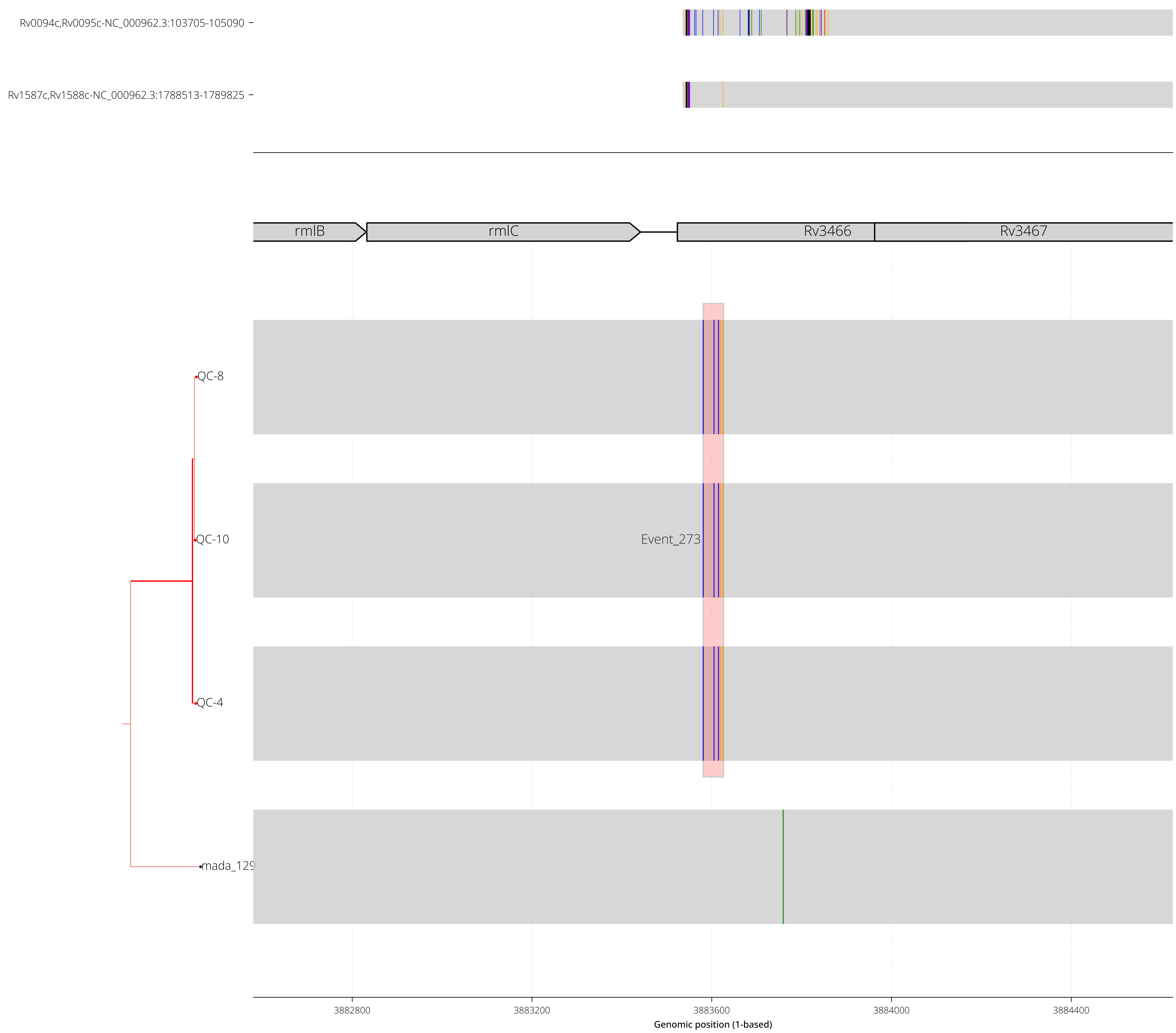

Full Phylogeny Viz + Paralog Alignments
GCE ID: Event_273

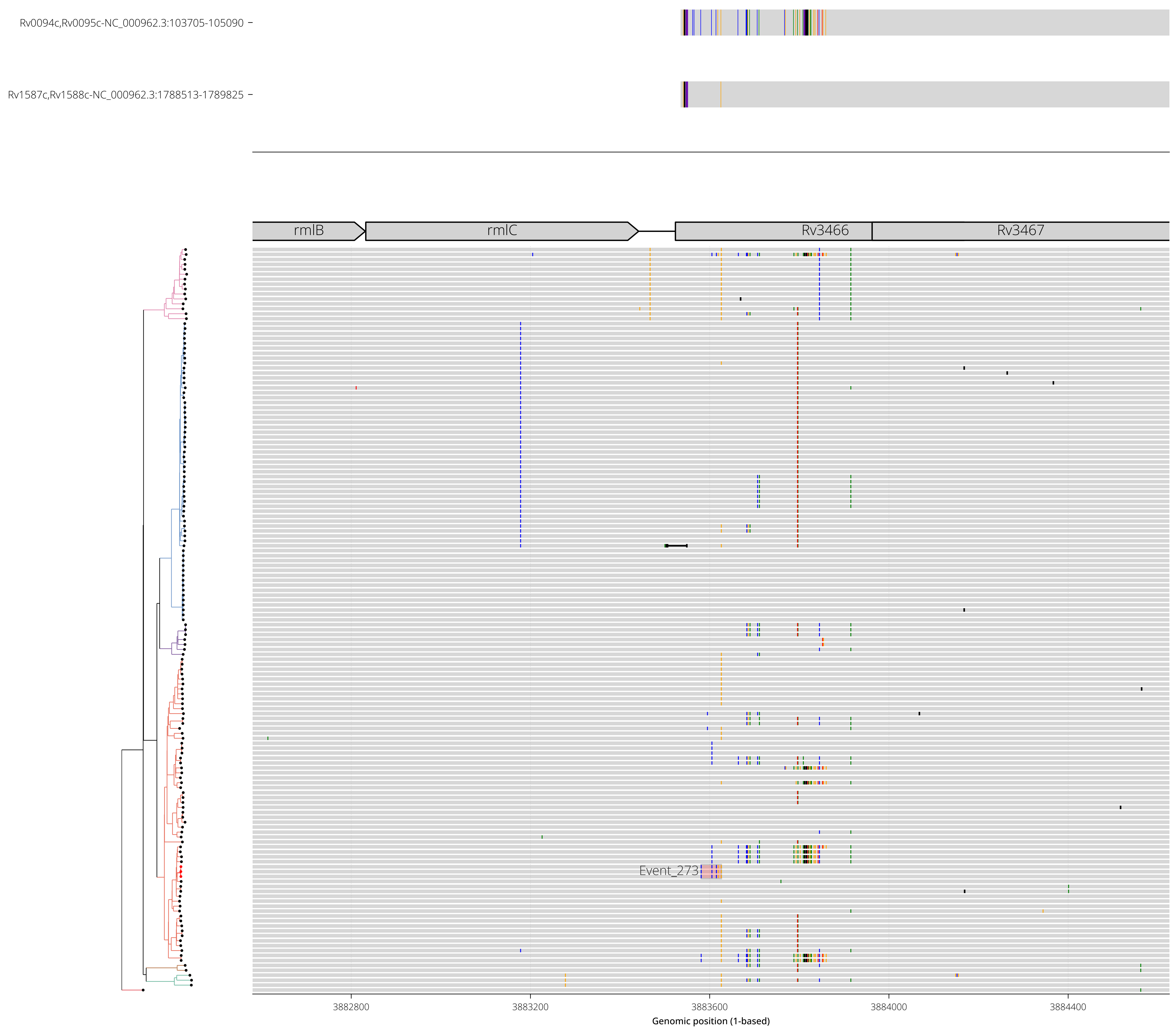

Individual GCE + Known Paralogs
GCE ID: Event_274

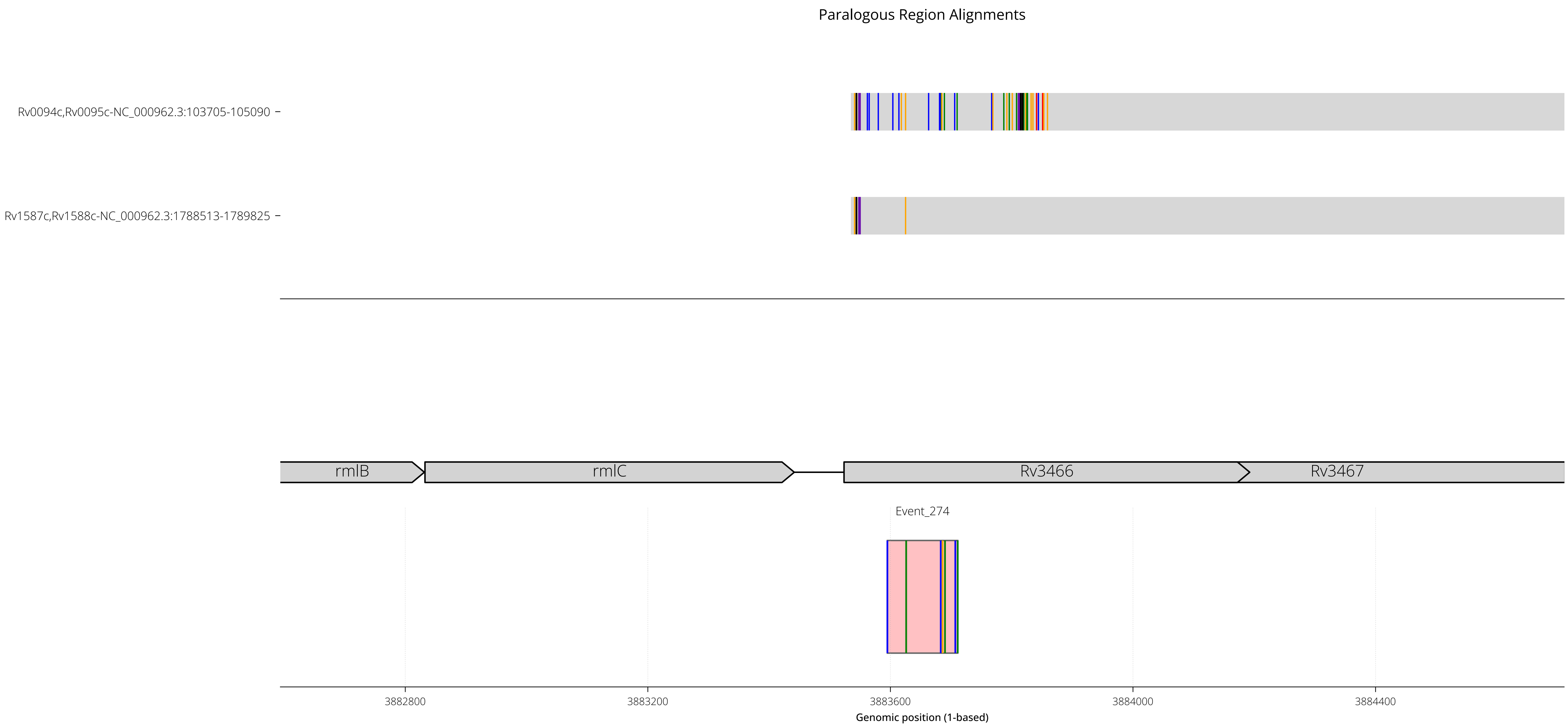

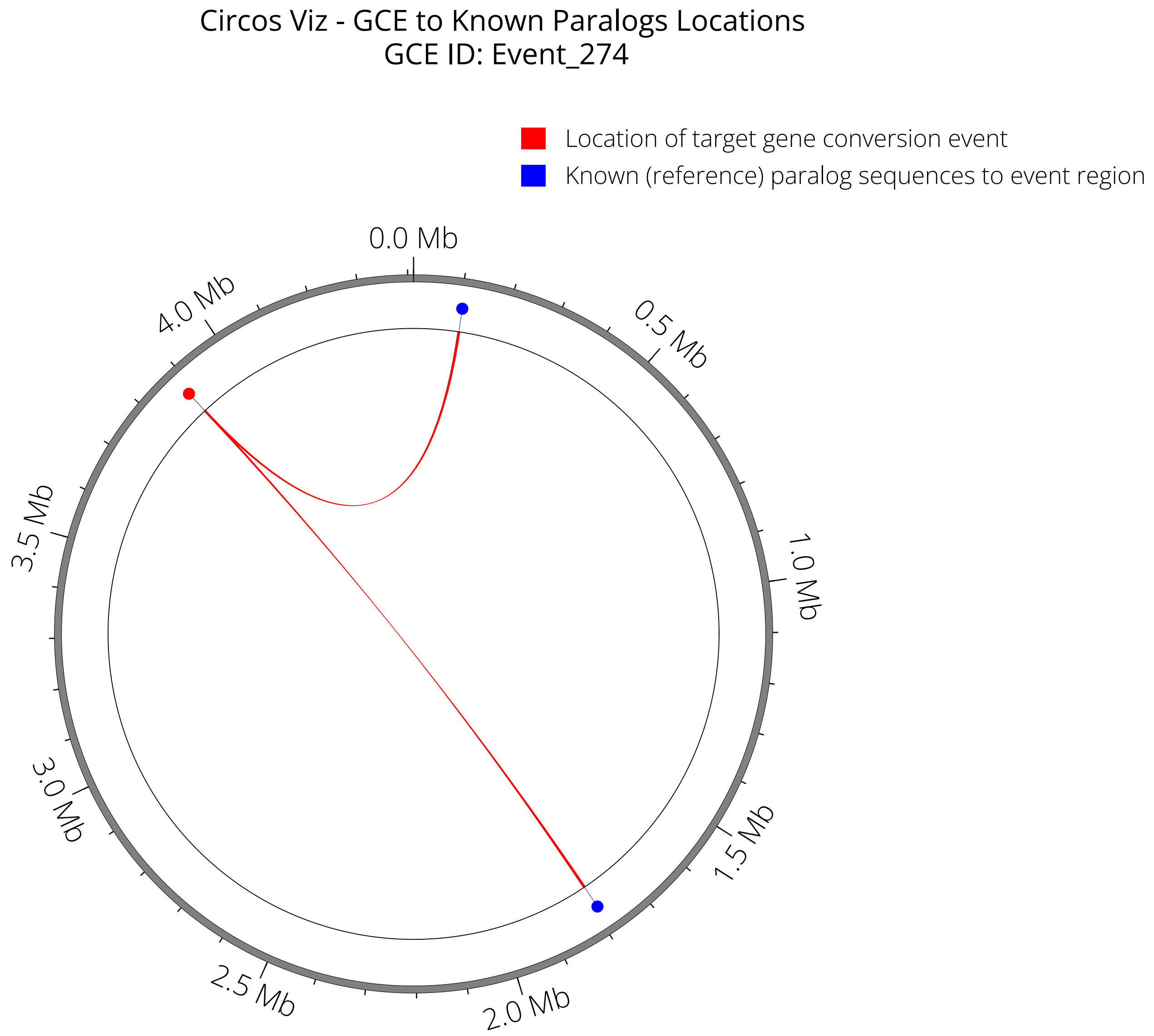

Circos Viz - GCE to Known Paralogs Locations
GCE ID: Event_274
Location of target gene conversion event
Known (reference) paralog sequences to event region
0.0 Mb
0.5 Mb
1.0 Mb
1.5 Mb
2.0 Mb
2.5 Mb
3.0 Mb
3.5 Mb
4.0 Mb

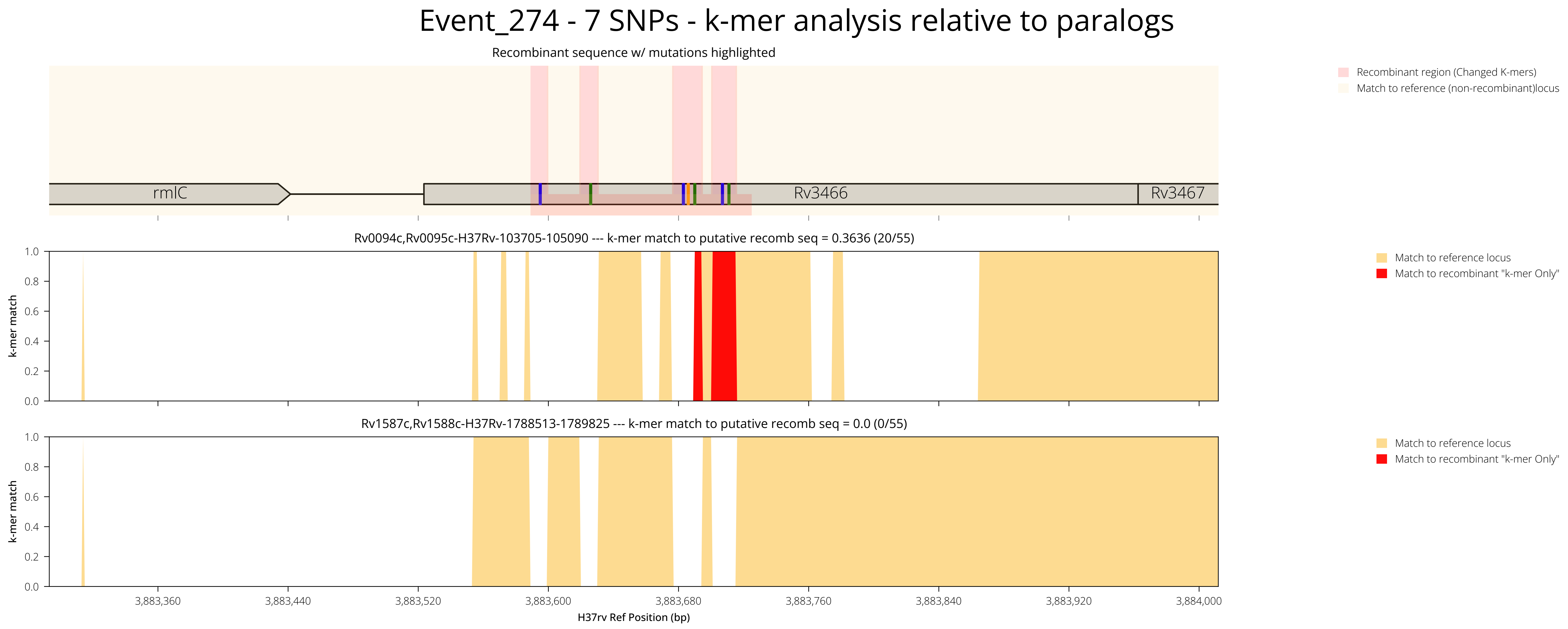

Event_274 - 7 SNPs - k-mer analysis relative to paralogs
Recombinant sequence w/ mutations highlighted
Recombinant region (Changed K-mers)
Match to reference (non-recombinant)locus
rmlC
Rv3466
Rv3467
Rv0094c,Rv0095c-H37Rv-103705-105090 --- k-mer match to putative recomb seq = 0.3636 (20/55)
Match to reference locus
Match to recombinant "k-mer Only"
k-mer match
Rv1587c,Rv1588c-H37Rv-1788513-1789825 --- k-mer match to putative recomb seq = 0.0 (0/55)
Match to reference locus
Match to recombinant "k-mer Only"
k-mer match
1.0
0.8
0.6
0.4
0.2
0.0
3,883,360
3,883,440
3,883,520
3,883,600
3,883,680
3,883,760
3,883,840
3,883,920
3,884,000
H37rv Ref Position (bp)

Sub-phylogeny Viz + Paralog Alignments
GCE ID: Event_274

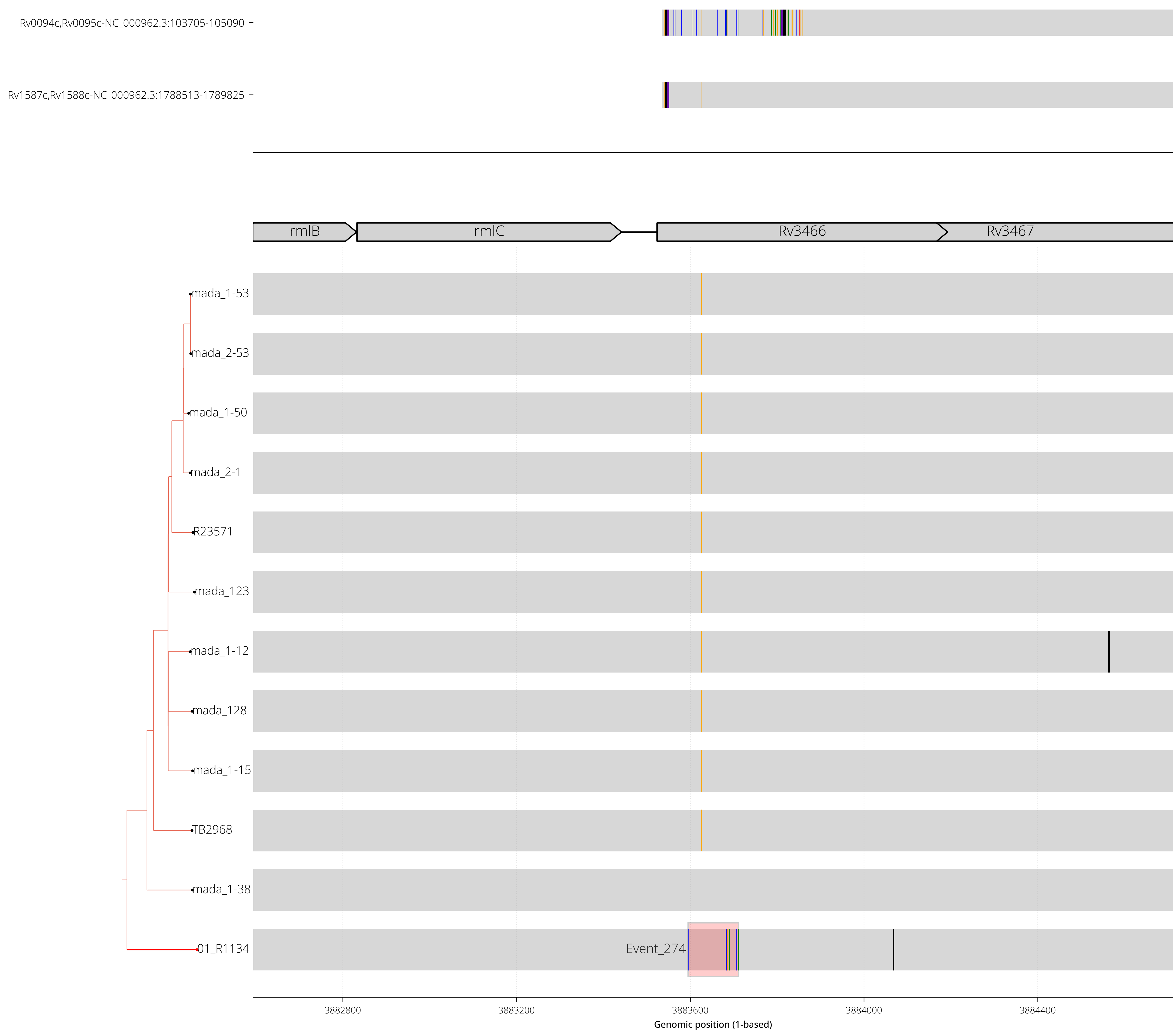

Full Phylogeny Viz + Paralog Alignments
GCE ID: Event_274

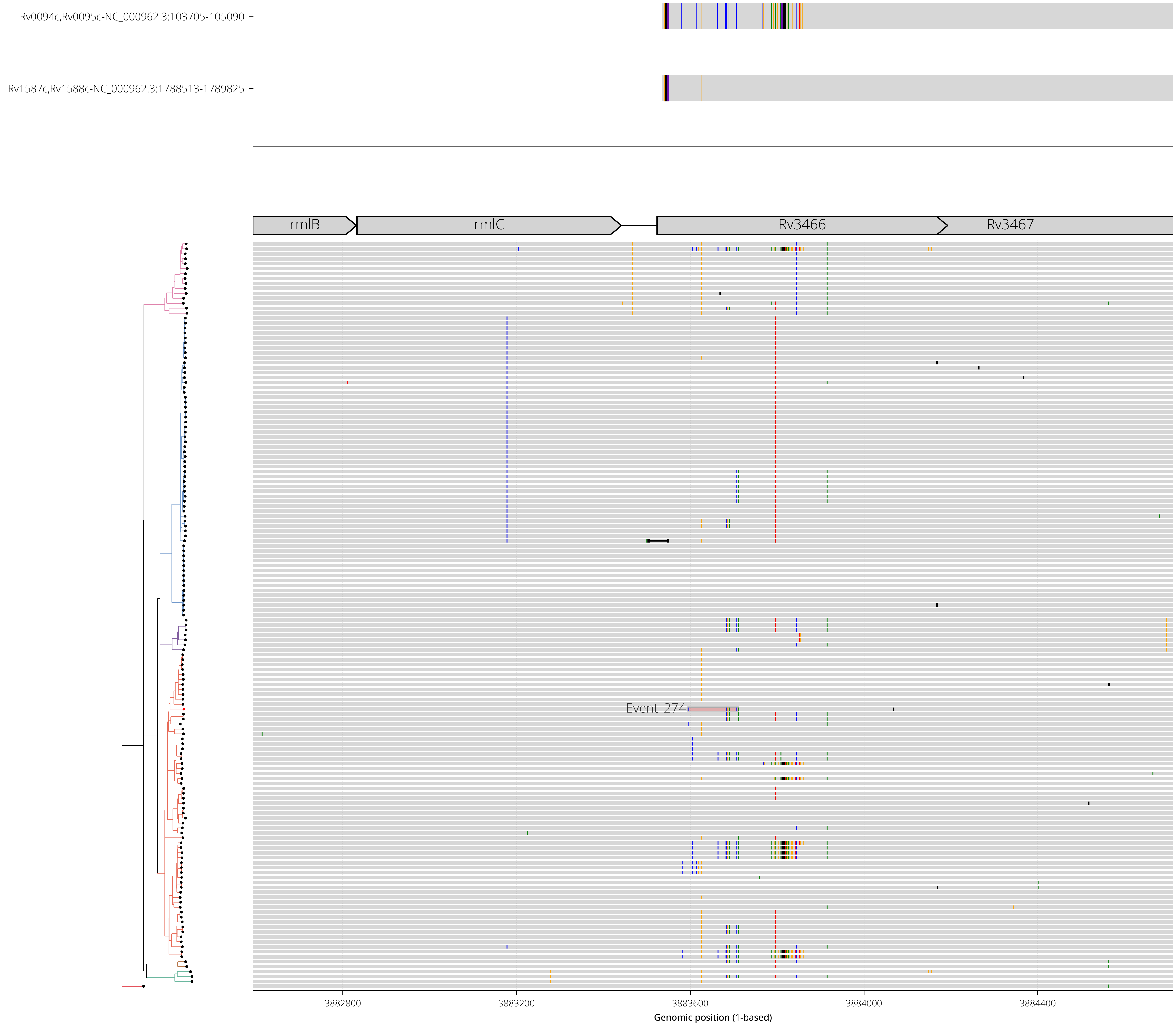

Individual GCE + Known Paralogs
GCE ID: Event_275

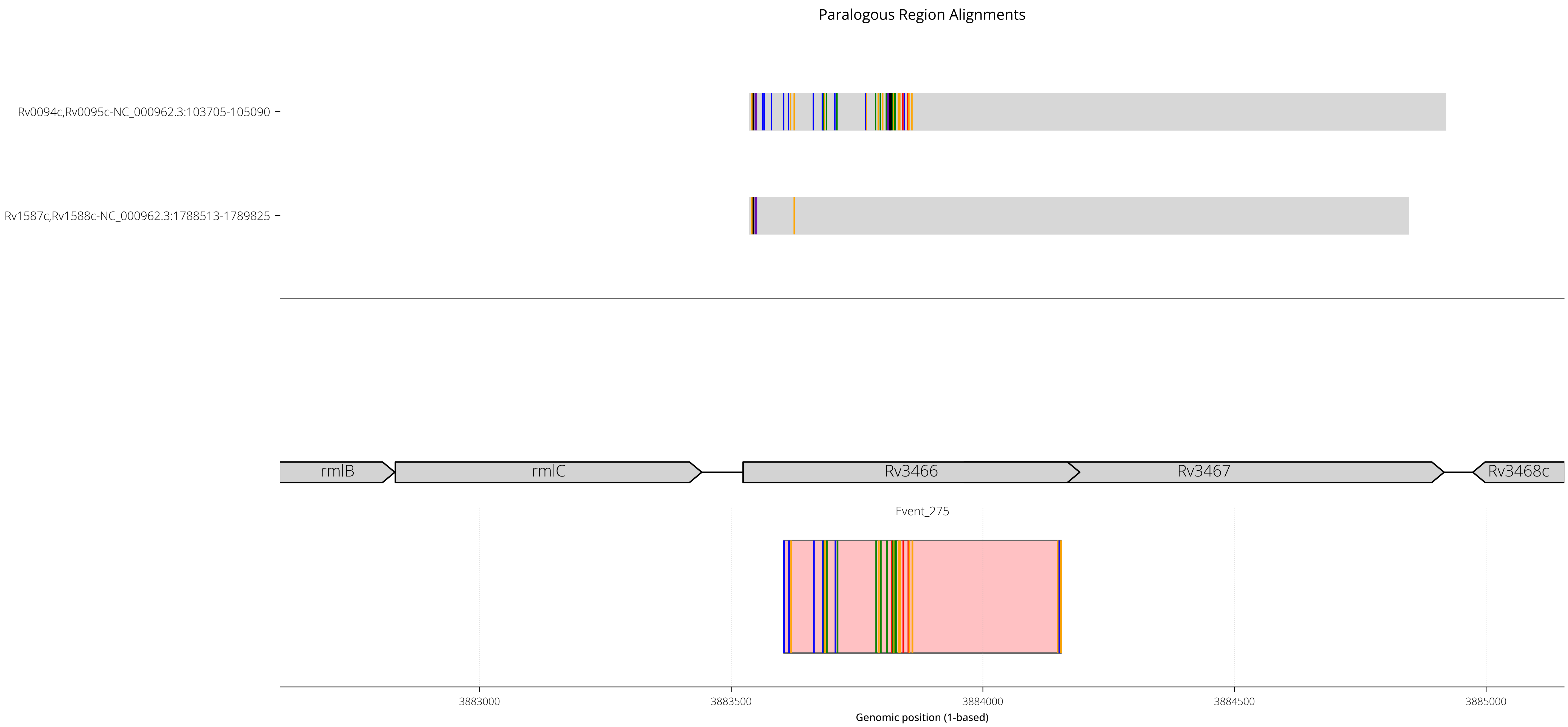

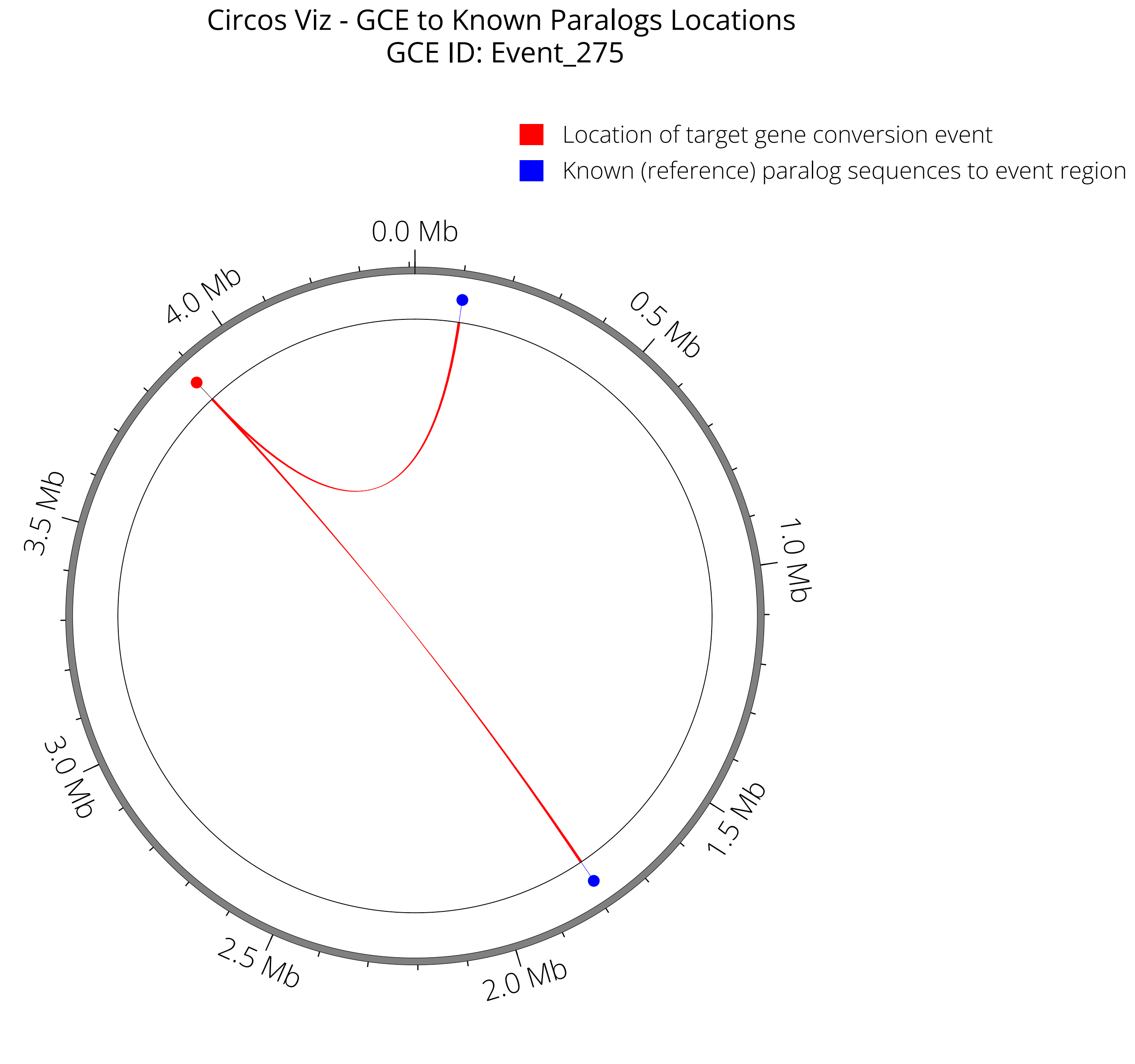
Circos Viz - GCE to Known Paralogs Locations
GCE ID: Event_275
Location of target gene conversion event
Known (reference) paralog sequences to event region
0.0 Mb
0.5 Mb
1.0 Mb
1.5 Mb
2.0 Mb
2.5 Mb
3.0 Mb
3.5 Mb
4.0 Mb

# Event_275 - 30 SNPs - k-mer analysis relative to paralogs

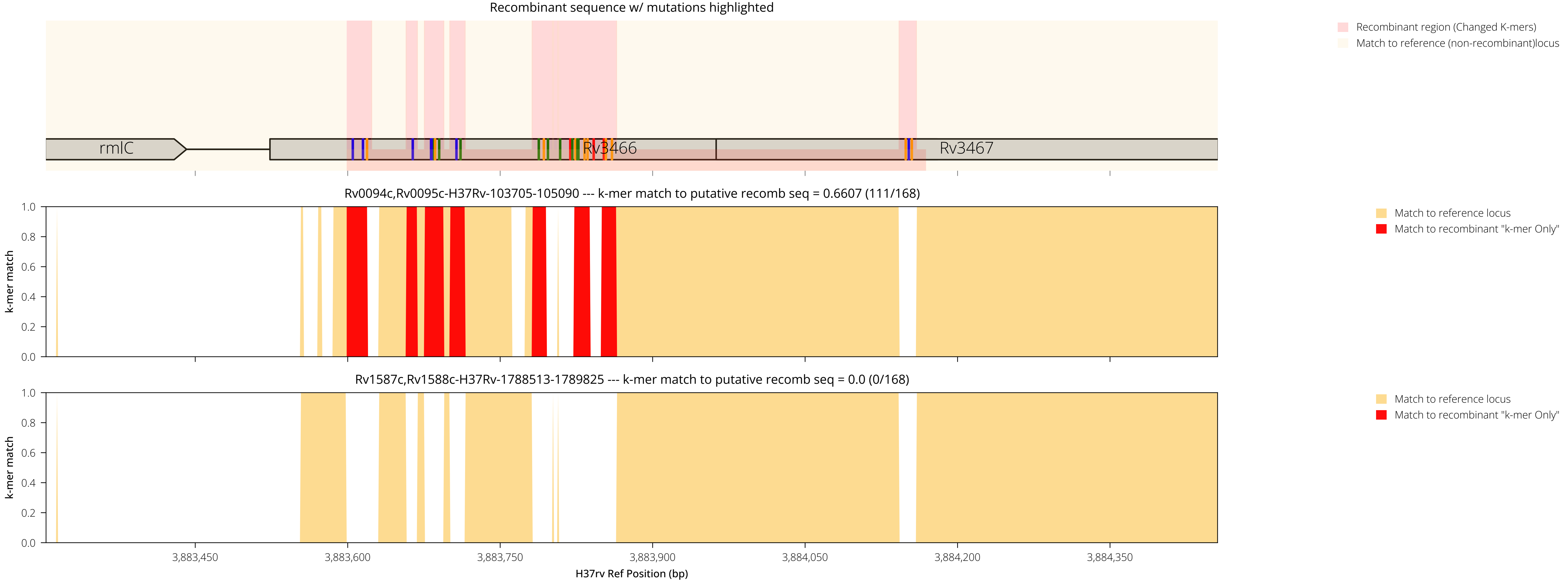

Sub-phylogeny Viz + Paralog Alignments
GCE ID: Event_275

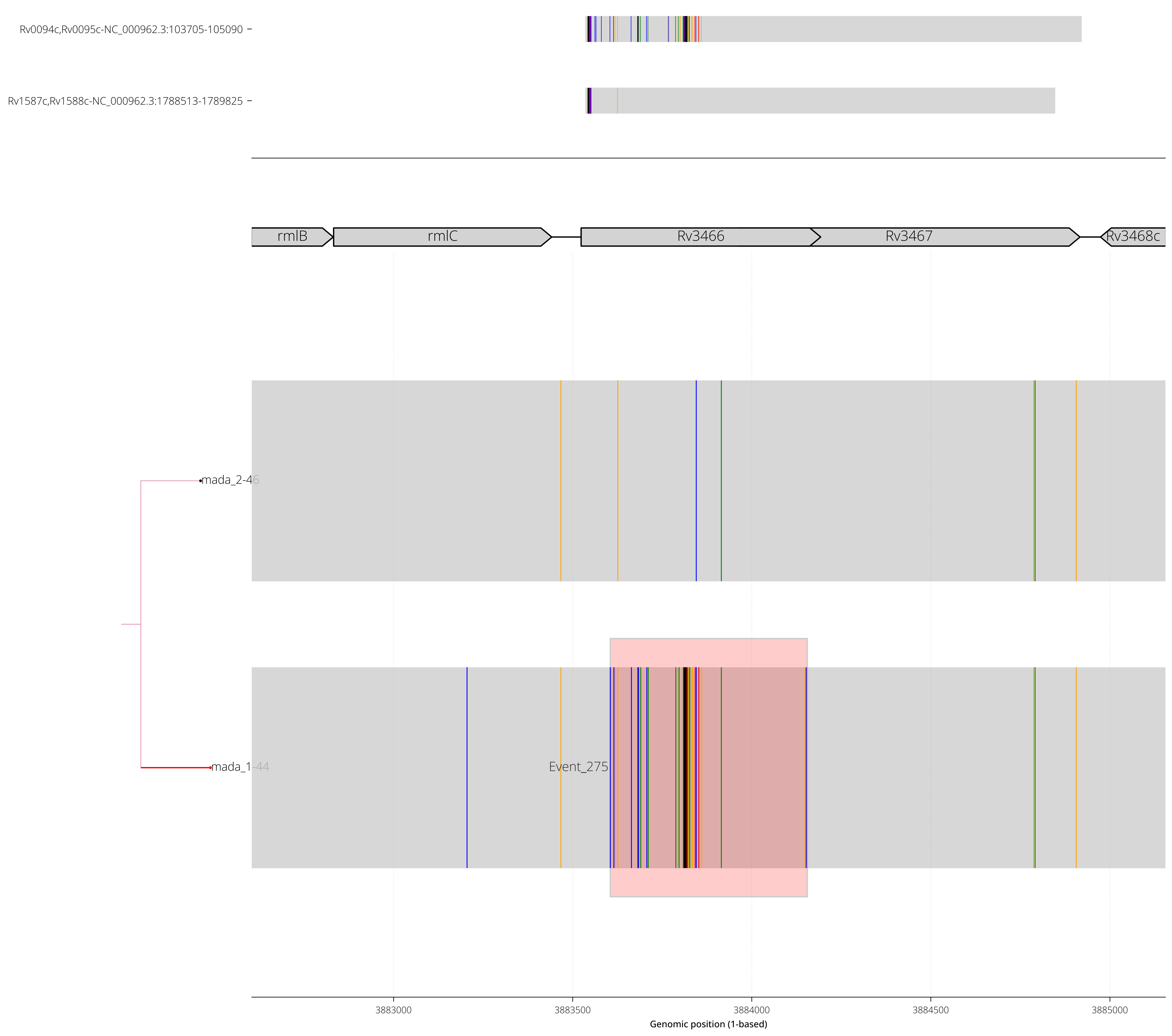

Full Phylogeny Viz + Paralog Alignments
GCE ID: Event_275

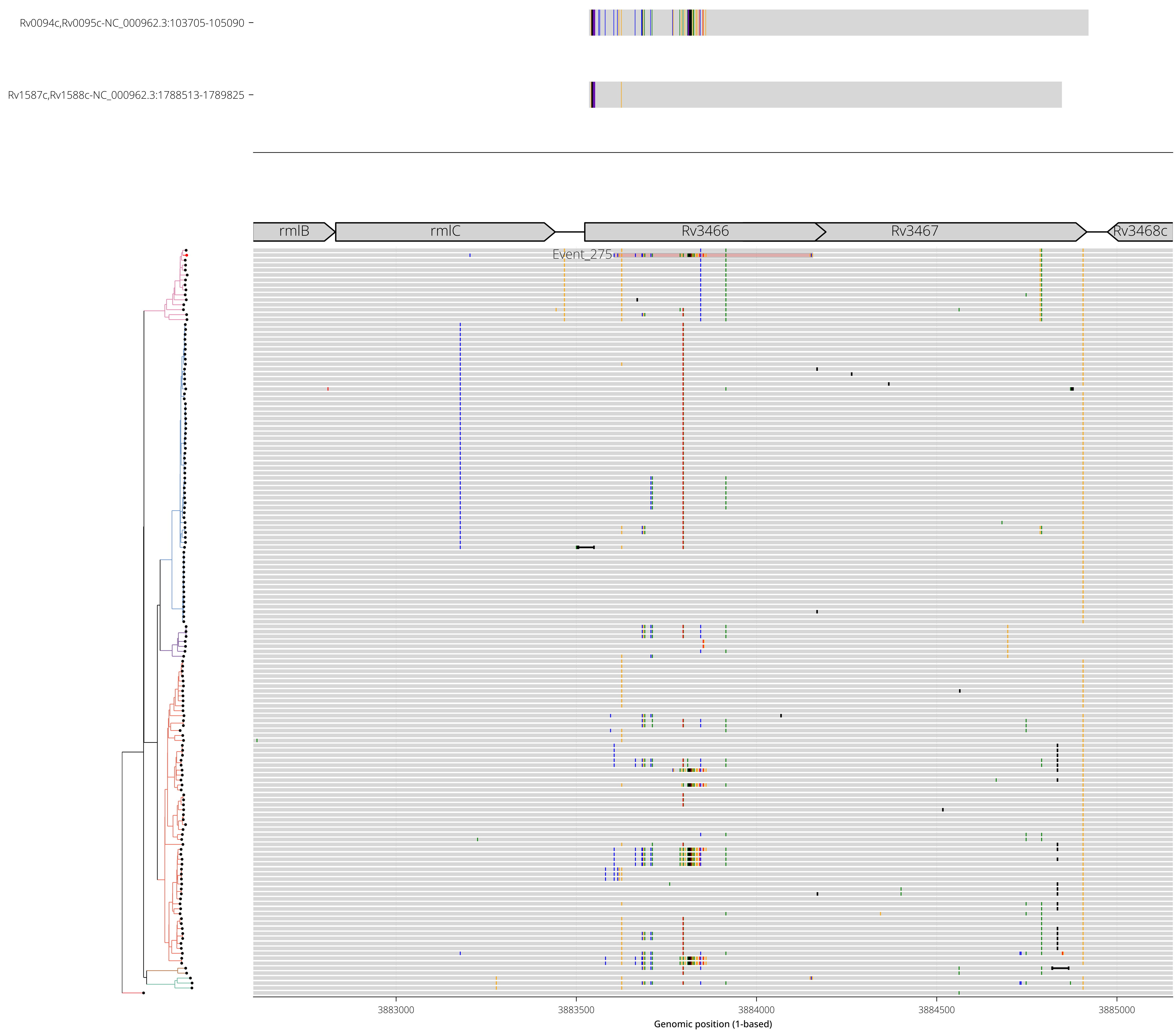

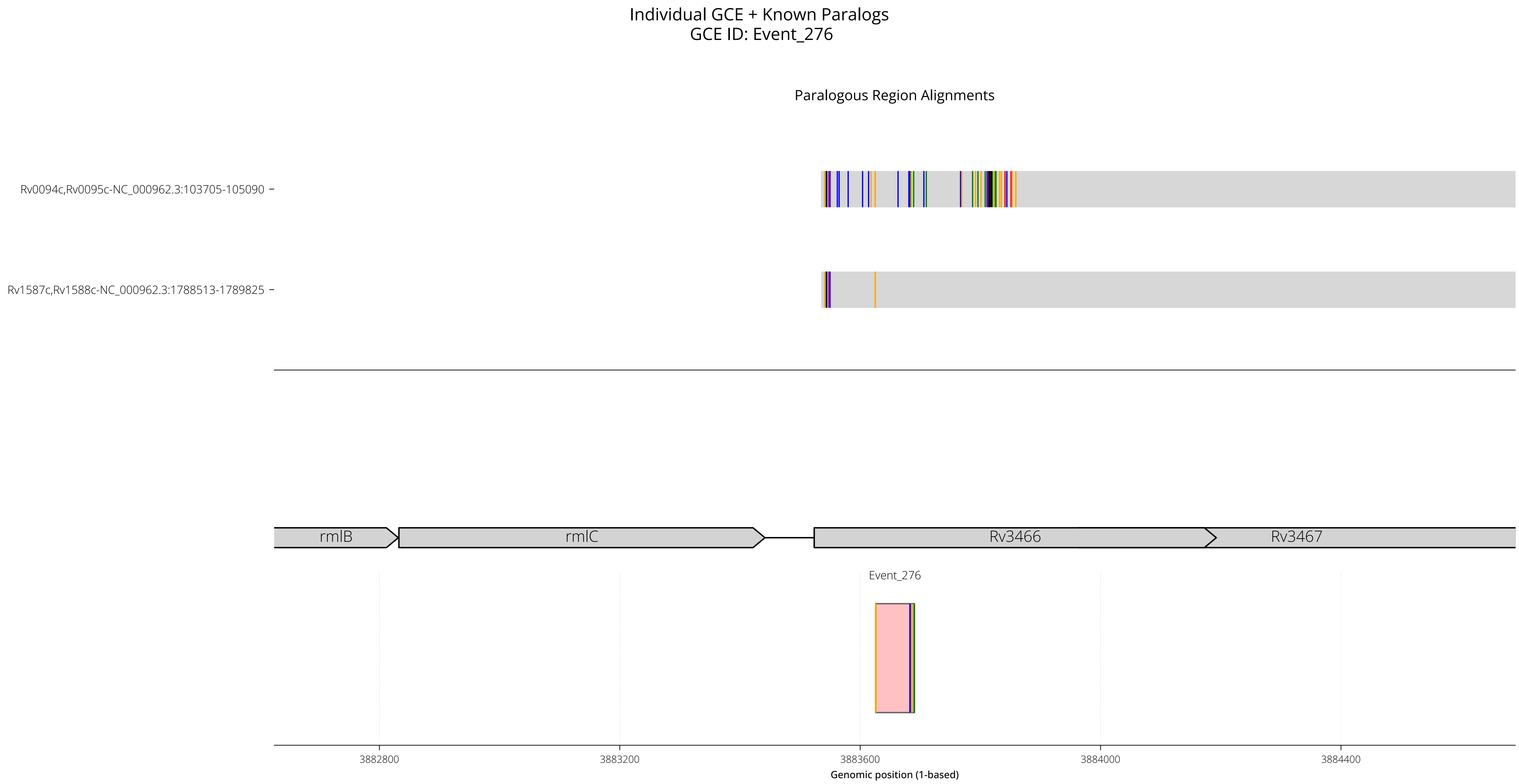

Individual GCE + Known Paralogs
GCE ID: Event_276
Paralogous Region Alignments
Rv0094c,Rv0095c-NC_000962.3:103705-105090
Rv1587c,Rv1588c-NC_000962.3:1788513-1789825
rmlB
rmlC
Rv3466
Rv3467
Event_276
3882800
3883200
3883600
3884000
3884400
Genomic position (1-based)

Circos Viz - GCE to Known Paralogs Locations
GCE ID: Event_276

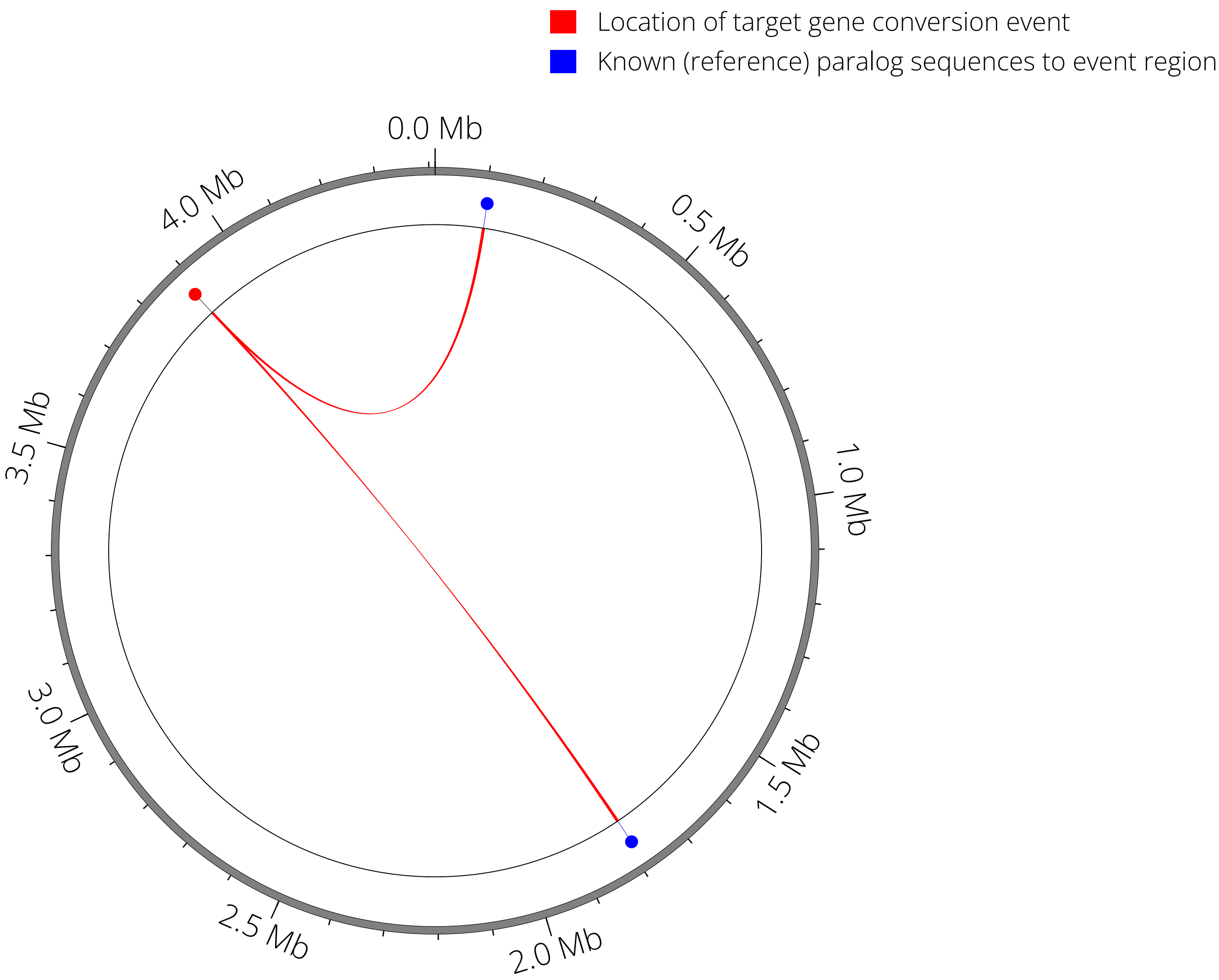

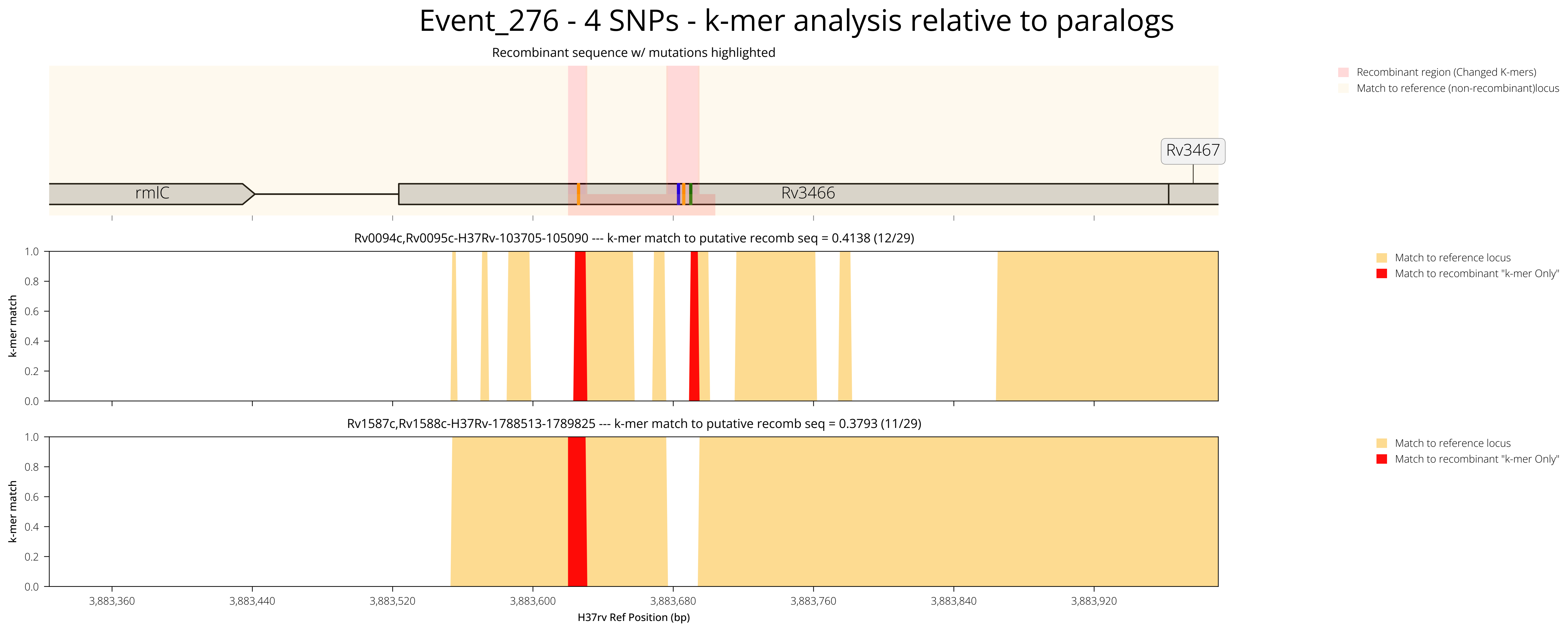

Event_276 - 4 SNPs - k-mer analysis relative to paralogs
Recombinant sequence w/ mutations highlighted
Recombinant region (Changed K-mers)
Match to reference (non-recombinant)locus
Rv3467
rmlC
Rv3466
Rv0094c,Rv0095c-H37Rv-103705-105090 --- k-mer match to putative recomb seq = 0.4138 (12/29)
Match to reference locus
Match to recombinant "k-mer Only"
k-mer match
1.0
0.8
0.6
0.4
0.2
0.0
Rv1587c,Rv1588c-H37Rv-1788513-1789825 --- k-mer match to putative recomb seq = 0.3793 (11/29)
Match to reference locus
Match to recombinant "k-mer Only"
k-mer match
1.0
0.8
0.6
0.4
0.2
0.0
3,883,360
3,883,440
3,883,520
3,883,600
3,883,680
3,883,760
3,883,840
3,883,920
H37rv Ref Position (bp)

Sub-phylogeny Viz + Paralog Alignments
GCE ID: Event_276

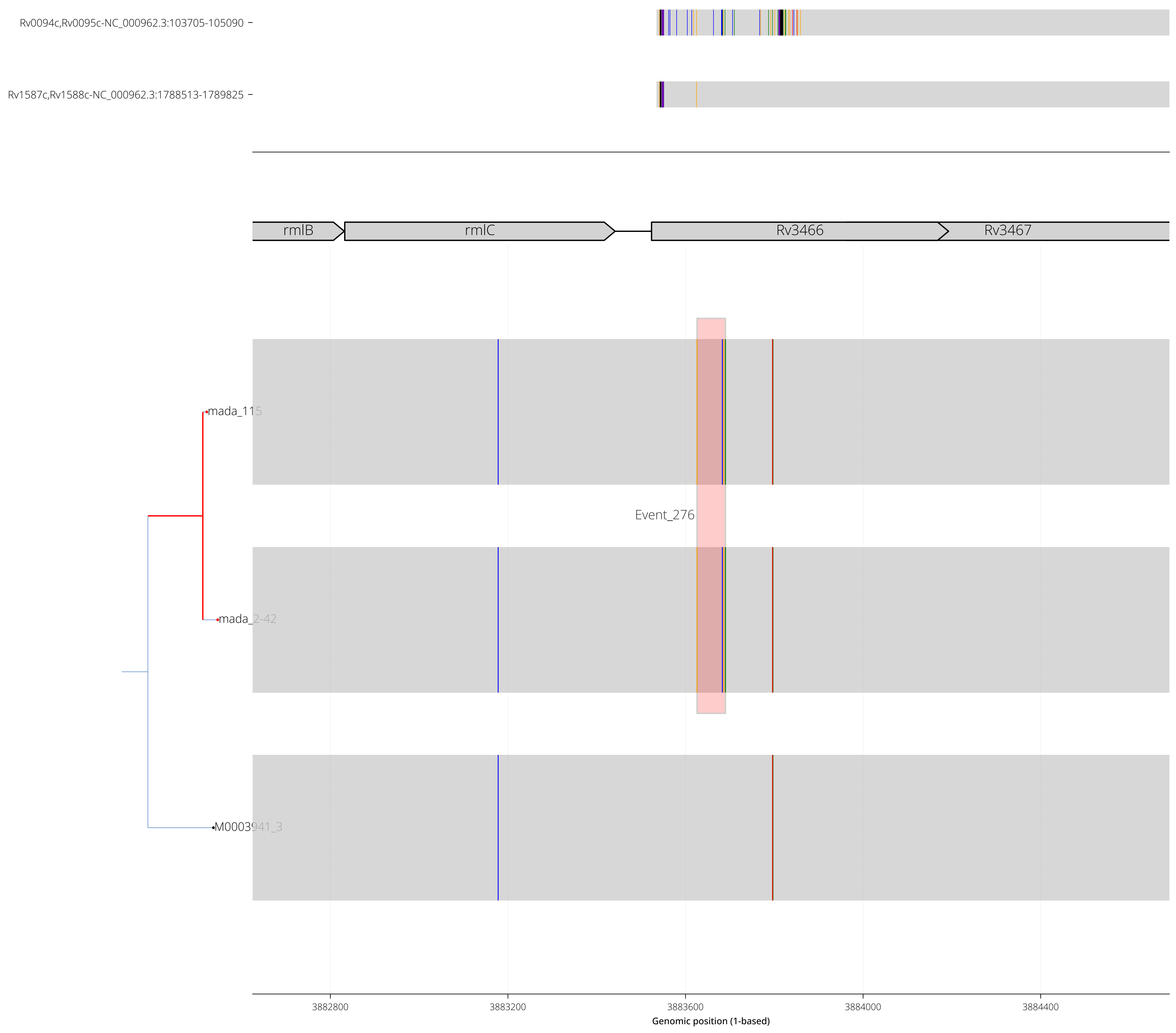

Full Phylogeny Viz + Paralog Alignments
GCE ID: Event_276

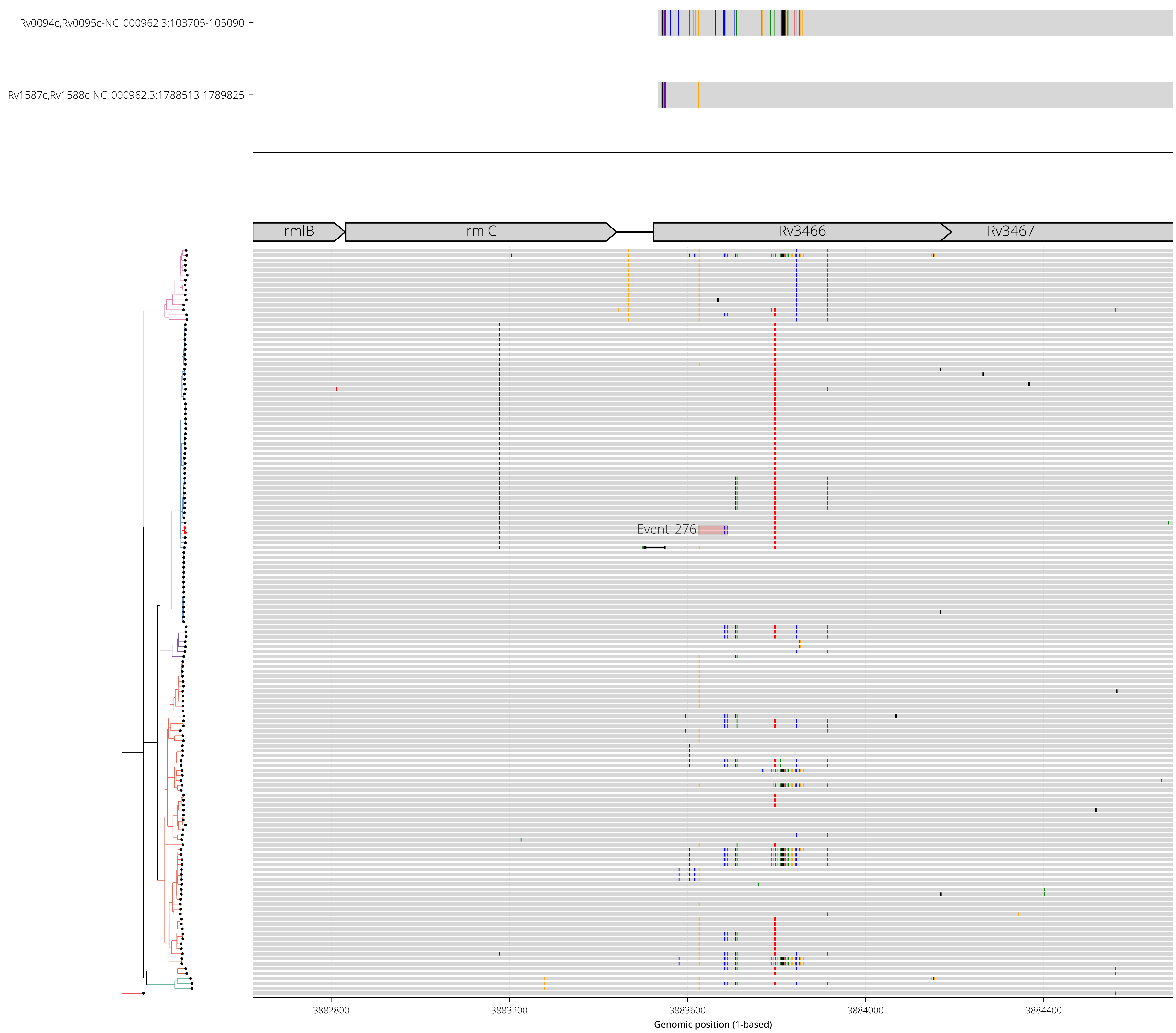

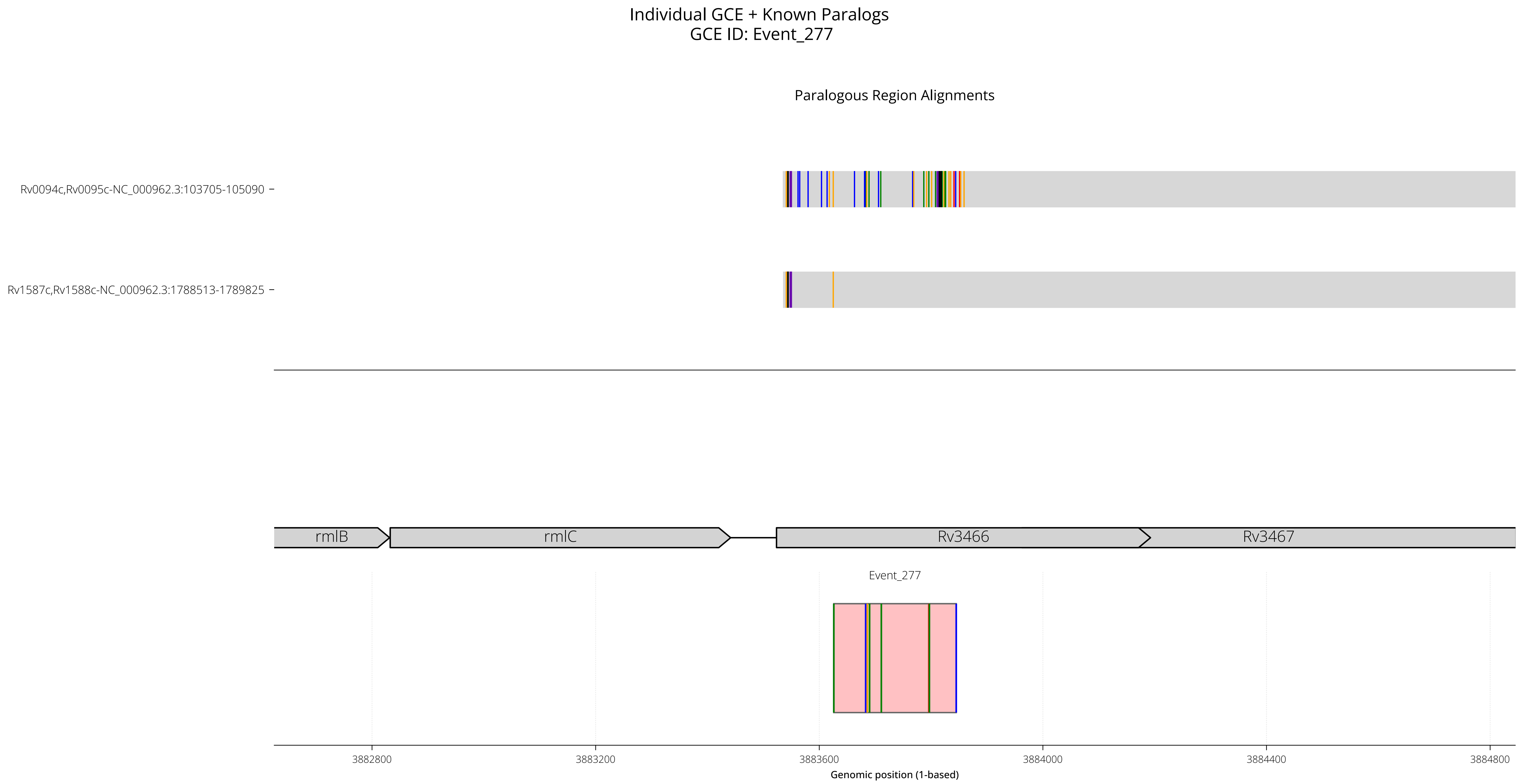
Individual GCE + Known Paralogs
GCE ID: Event_277
Paralogous Region Alignments
Rv0094c,Rv0095c-NC_000962.3:103705-105090
Rv1587c,Rv1588c-NC_000962.3:1788513-1789825
rmlB
rmlC
Rv3466
Rv3467
Event_277
3882800
3883200
3883600
3884000
3884400
3884800
Genomic position (1-based)

Circos Viz - GCE to Known Paralogs Locations
GCE ID: Event_277

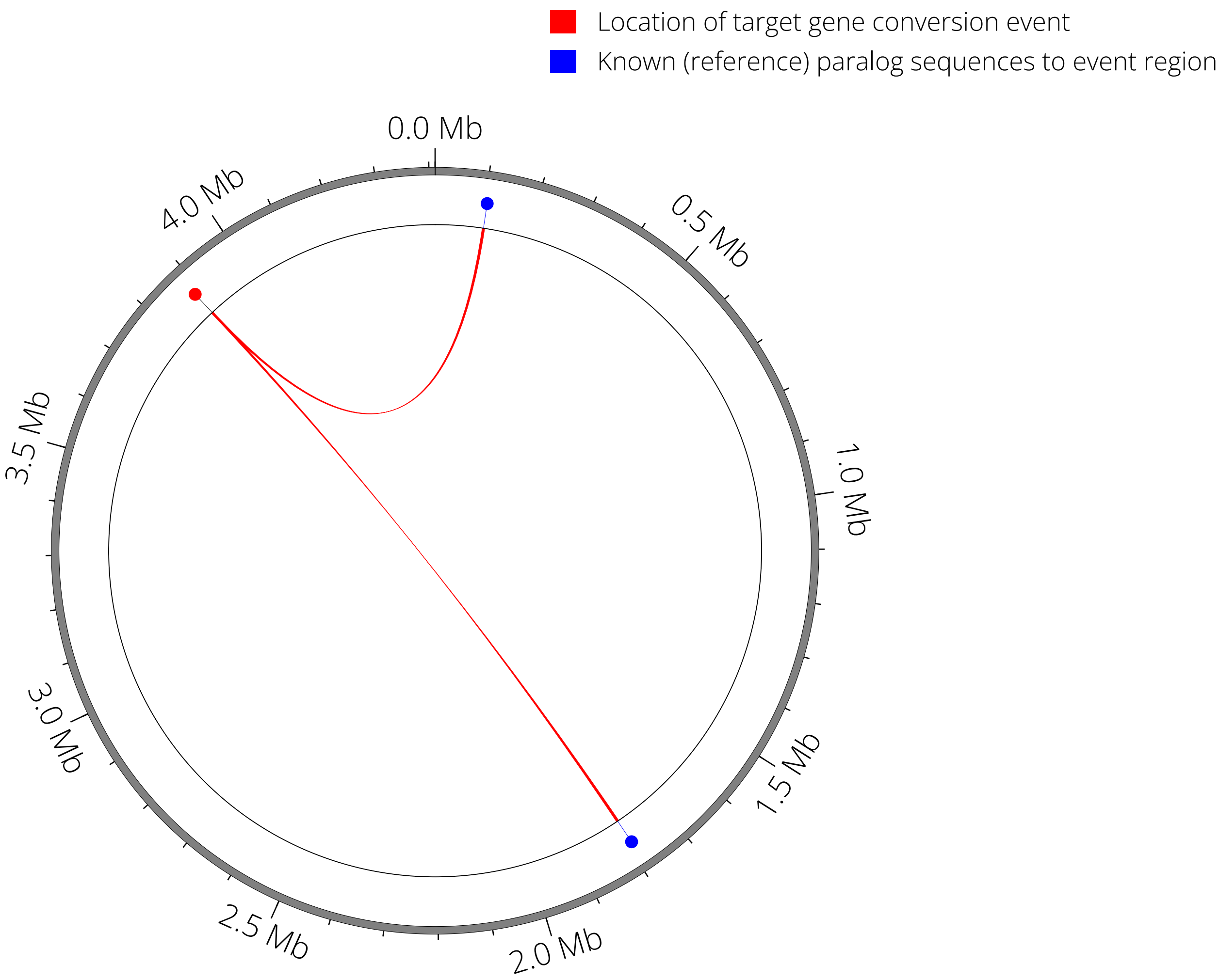

# Event_277 - 8 SNPs - k-mer analysis relative to paralogs

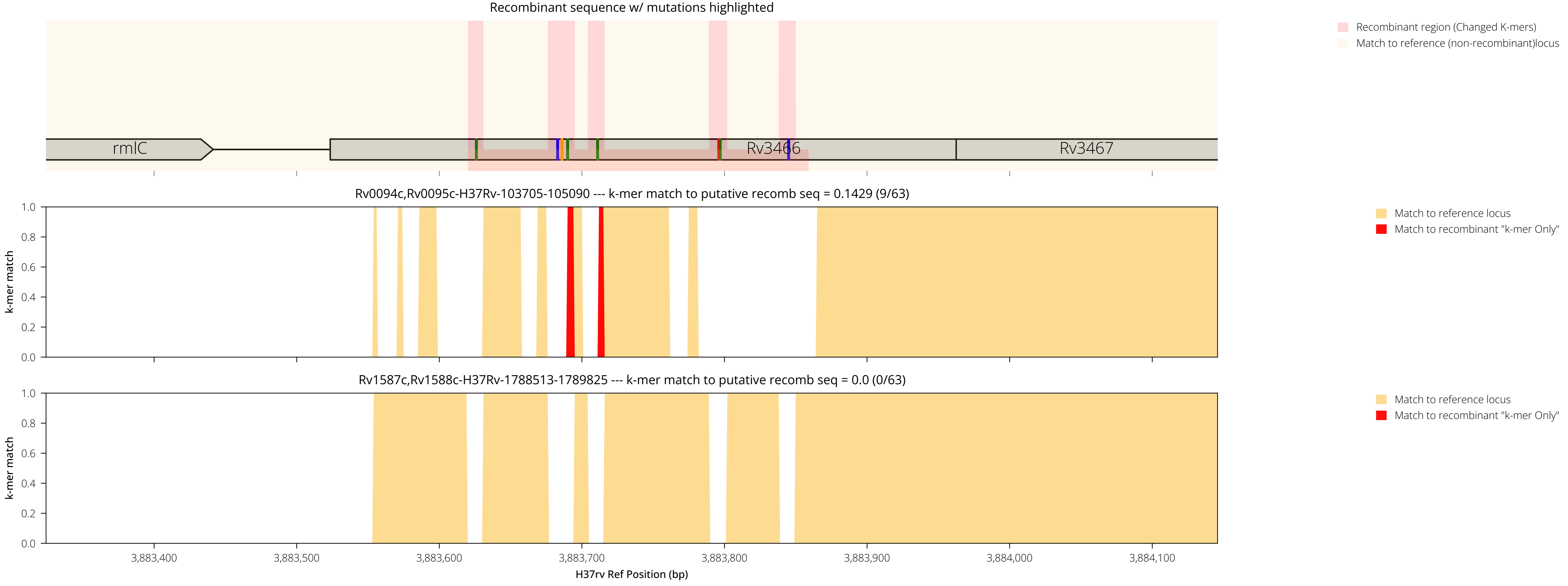

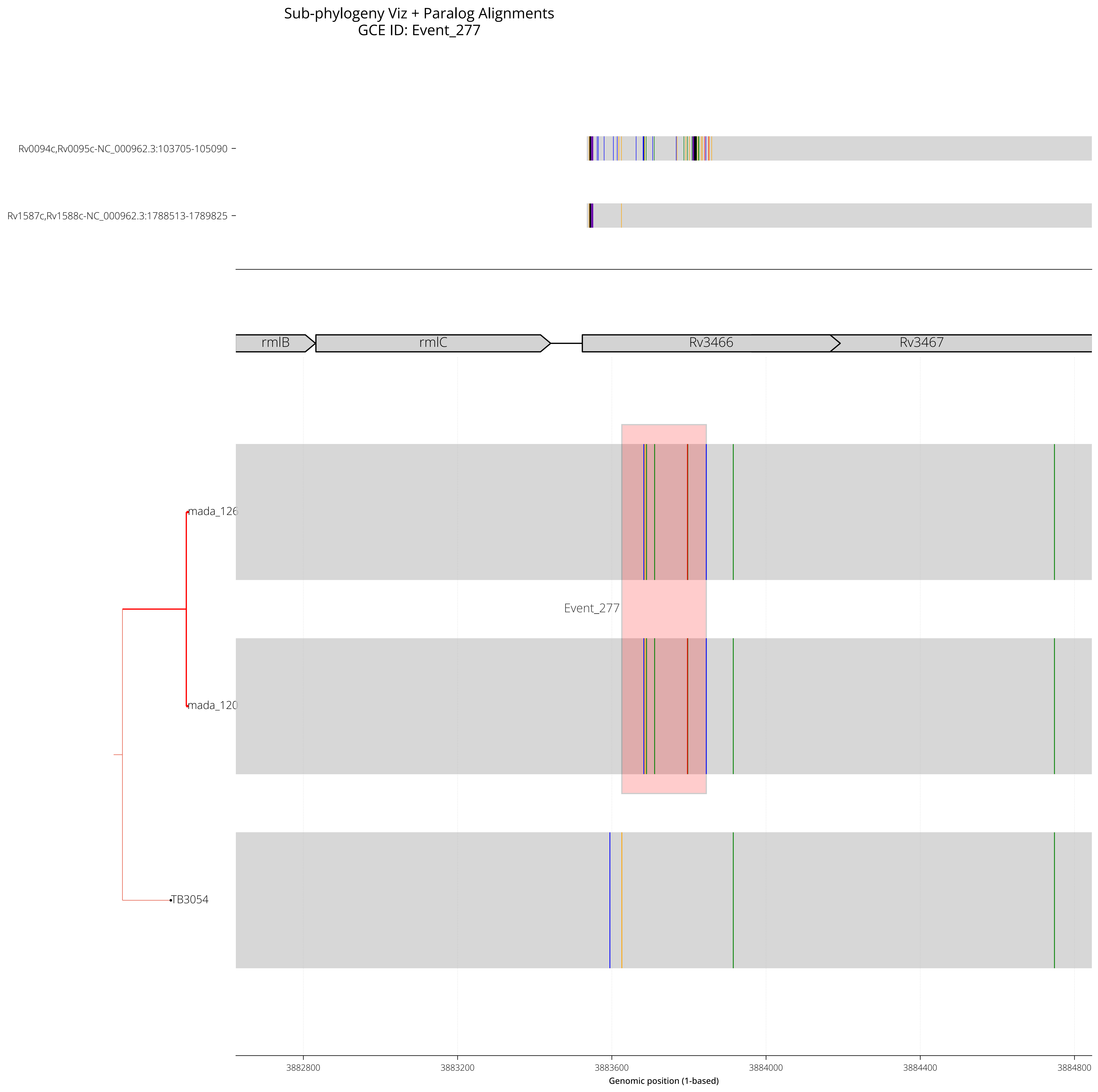
Sub-phylogeny Viz + Paralog Alignments
GCE ID: Event_277
Rv0094c,Rv0095c-NC_000962.3:103705-105090
Rv1587c,Rv1588c-NC_000962.3:1788513-1789825
rmlB
rmlC
Rv3466
Rv3467
mada_126
Event_277
mada_120
TB3054
3882800
3883200
3883600
3884000
3884400
3884800
Genomic position (1-based)

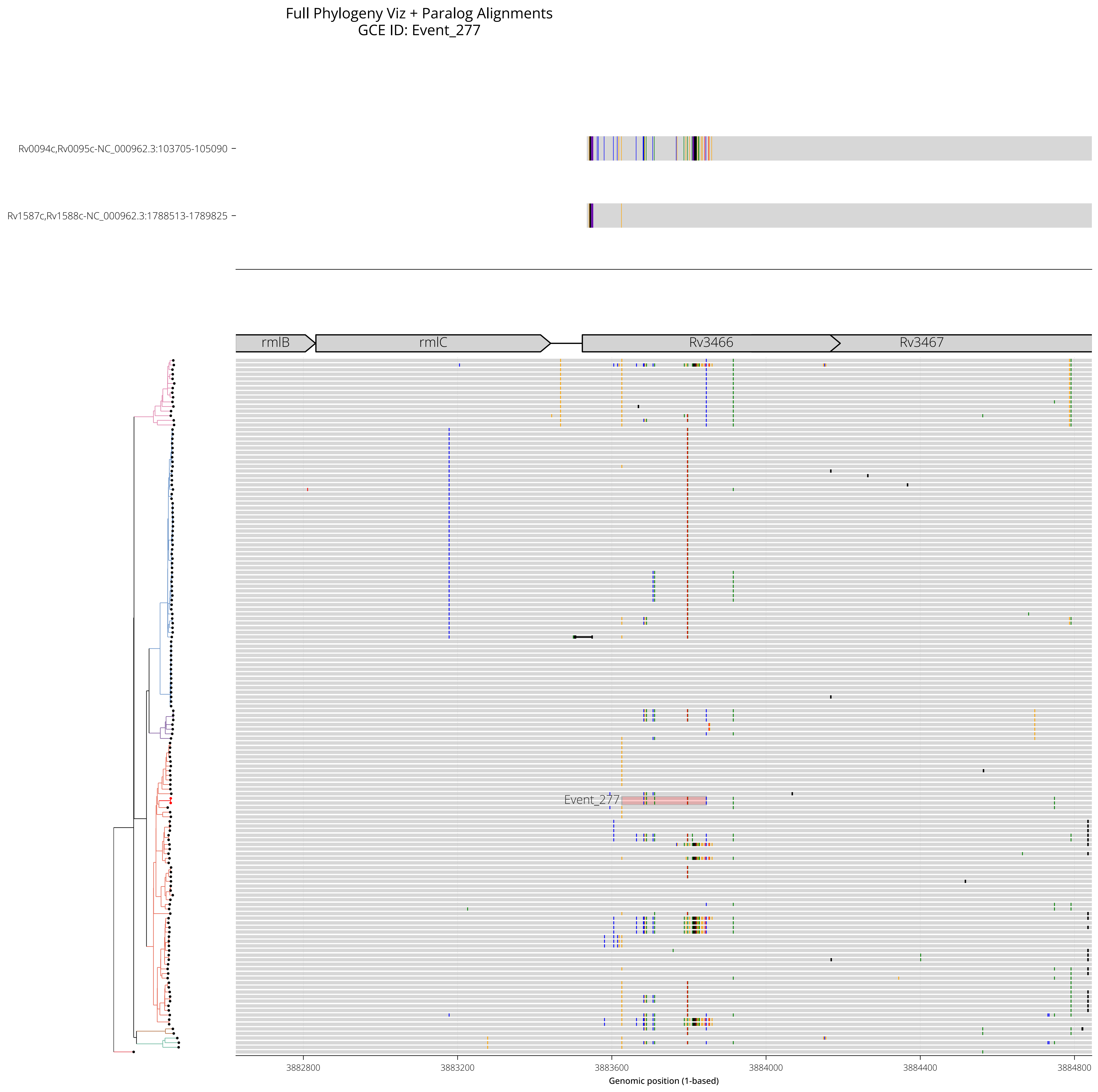

Full Phylogeny Viz + Paralog Alignments
GCE ID: Event_277
Rv0094c,Rv0095c-NC_000962.3:103705-105090
Rv1587c,Rv1588c-NC_000962.3:1788513-1789825
rmlB
rmlC
Rv3466
Rv3467
Event_277
3882800
3883200
3883600
3884000
3884400
3884800
Genomic position (1-based)

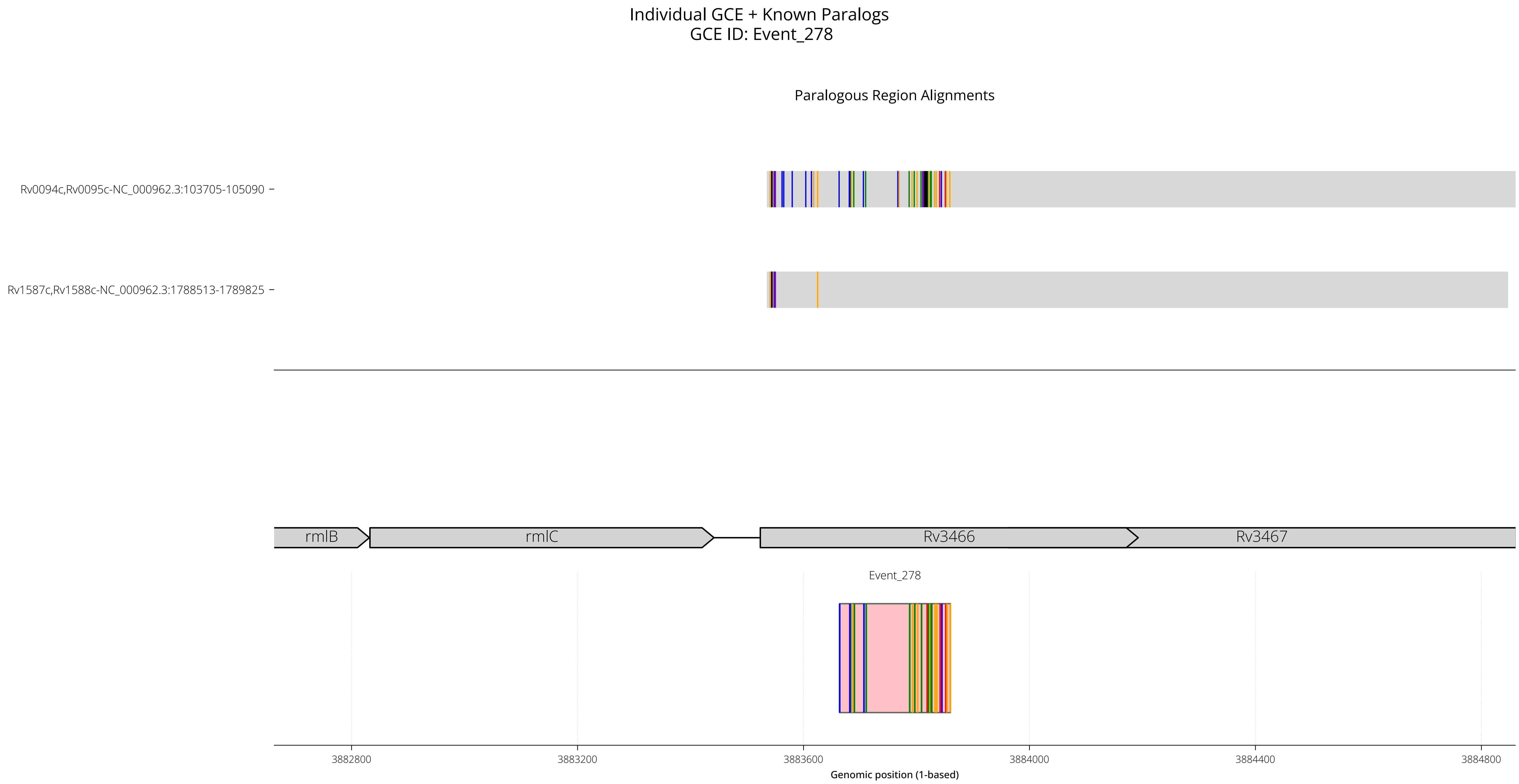
Individual GCE + Known Paralogs
GCE ID: Event_278
Paralogous Region Alignments
Rv0094c,Rv0095c-NC_000962.3:103705-105090
Rv1587c,Rv1588c-NC_000962.3:1788513-1789825
rmlB
rmlC
Rv3466
Rv3467
Event_278
3882800
3883200
3883600
3884000
3884400
3884800
Genomic position (1-based)

Circos Viz - GCE to Known Paralogs Locations
GCE ID: Event_278

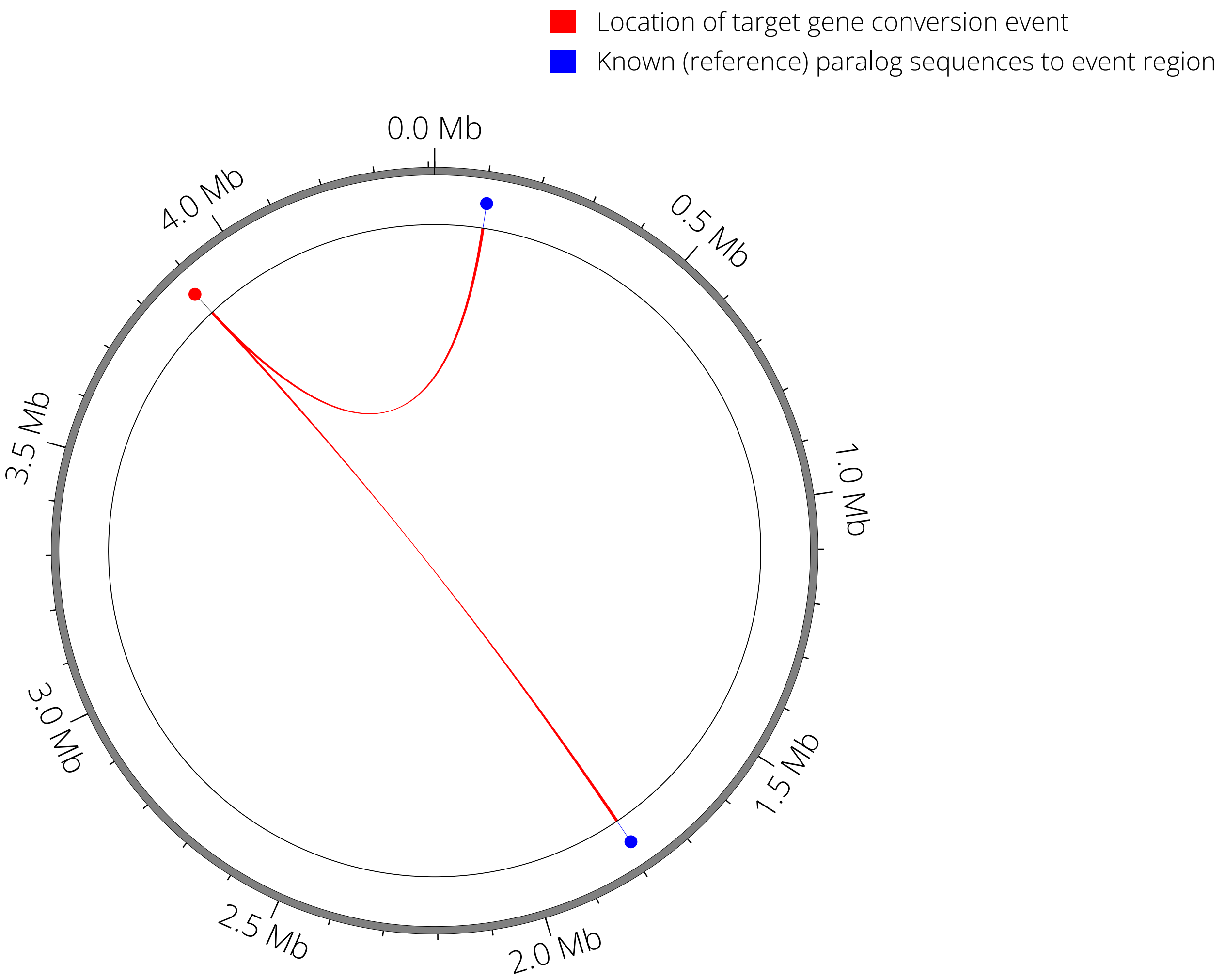

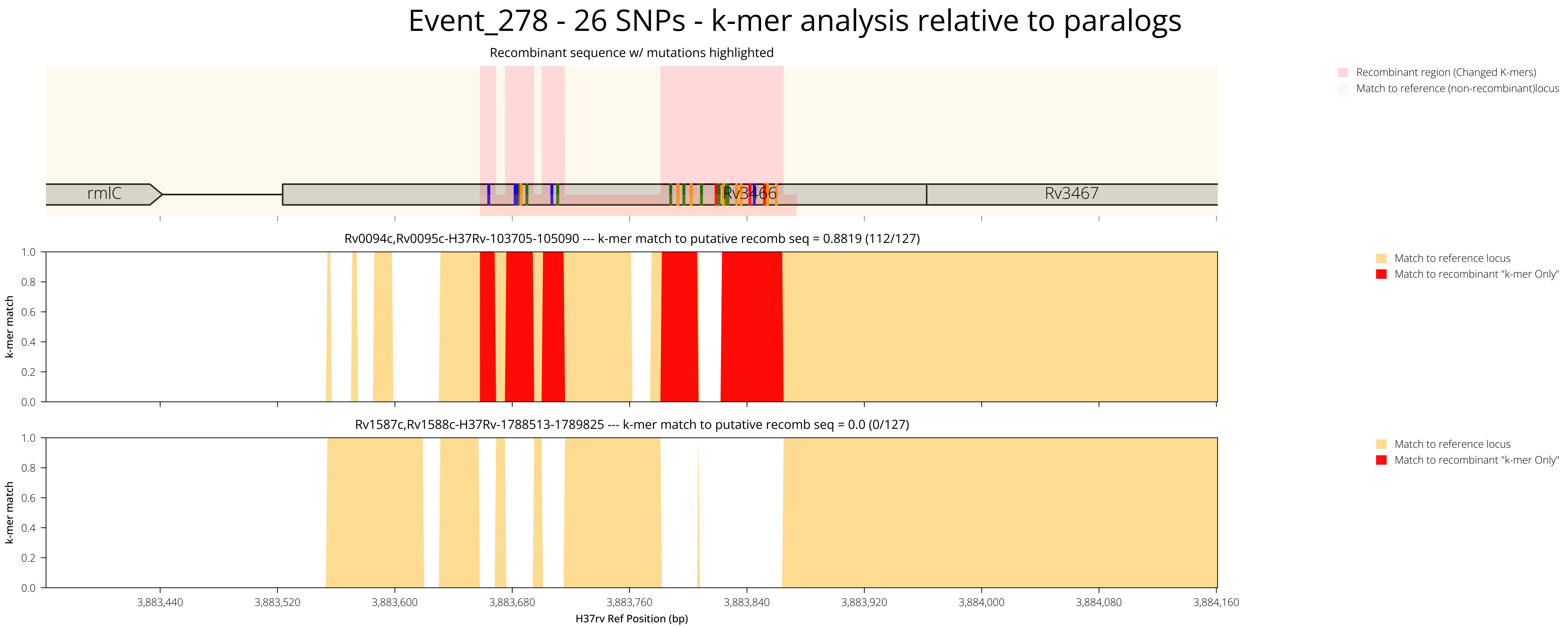

Event_278 - 26 SNPs - k-mer analysis relative to paralogs
Recombinant sequence w/ mutations highlighted
Recombinant region (Changed K-mers)
Match to reference (non-recombinant)locus
rmlC
Rv3466
Rv3467
Rv0094c,Rv0095c-H37Rv-103705-105090 --- k-mer match to putative recomb seq = 0.8819 (112/127)
Match to reference locus
Match to recombinant "k-mer Only"
Rv1587c,Rv1588c-H37Rv-1788513-1789825 --- k-mer match to putative recomb seq = 0.0 (0/127)
Match to reference locus
Match to recombinant "k-mer Only"
k-mer match
1.0
0.8
0.6
0.4
0.2
0.0
3,883,440
3,883,520
3,883,600
3,883,680
3,883,760
3,883,840
3,883,920
3,884,000
3,884,080
3,884,160
H37rv Ref Position (bp)

Sub-phylogeny Viz + Paralog Alignments
GCE ID: Event_278

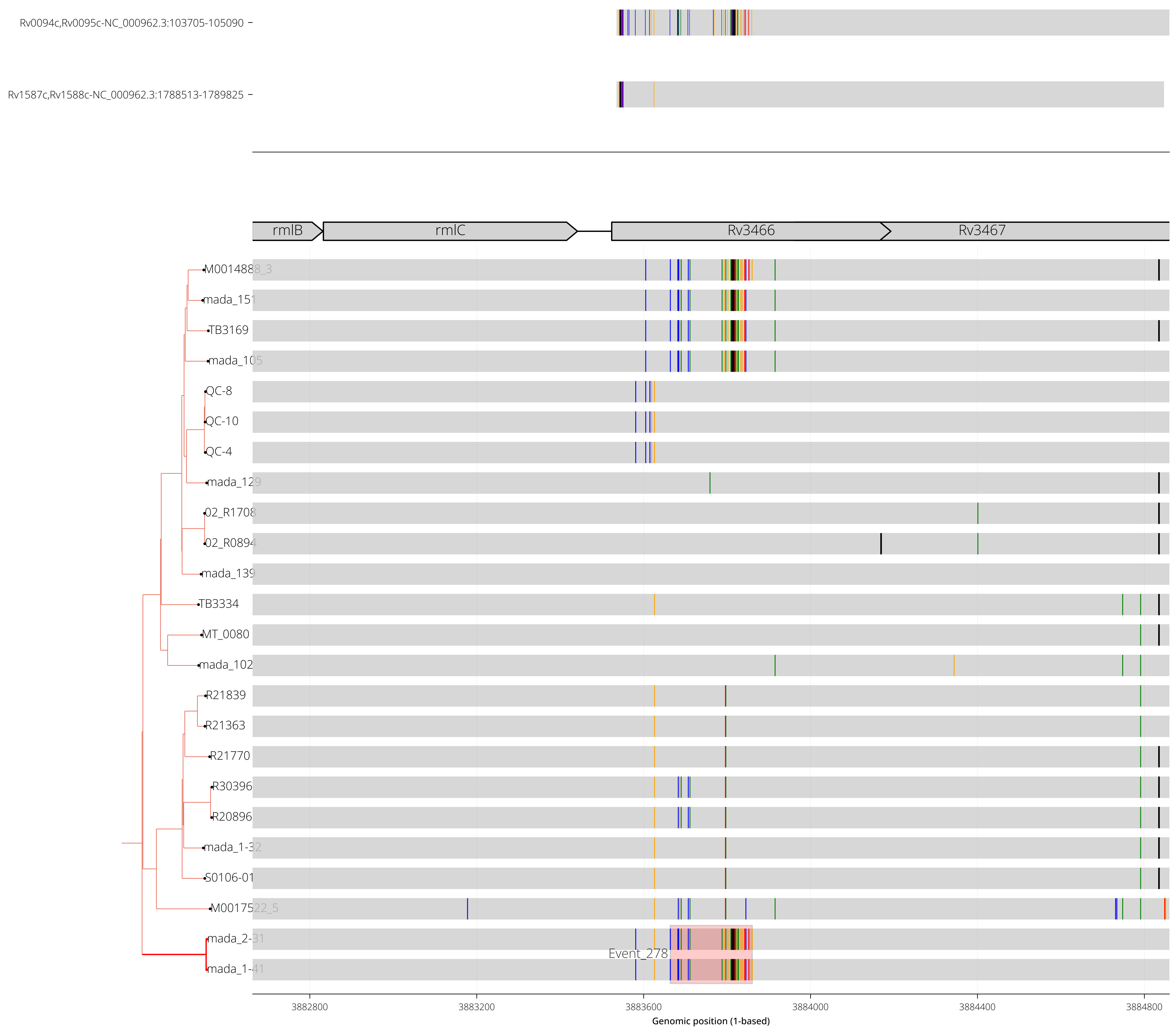

Full Phylogeny Viz + Paralog Alignments
GCE ID: Event_278

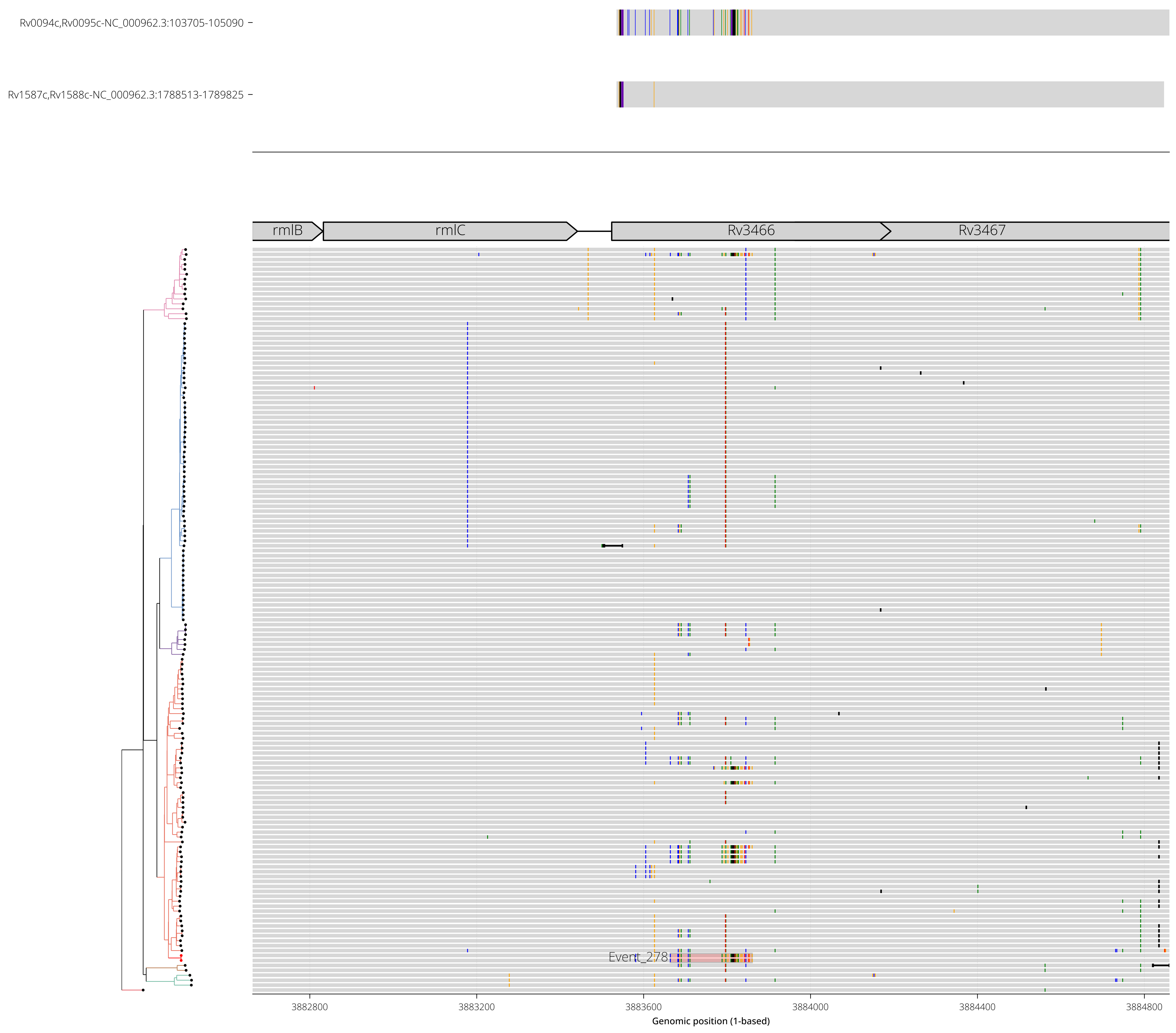

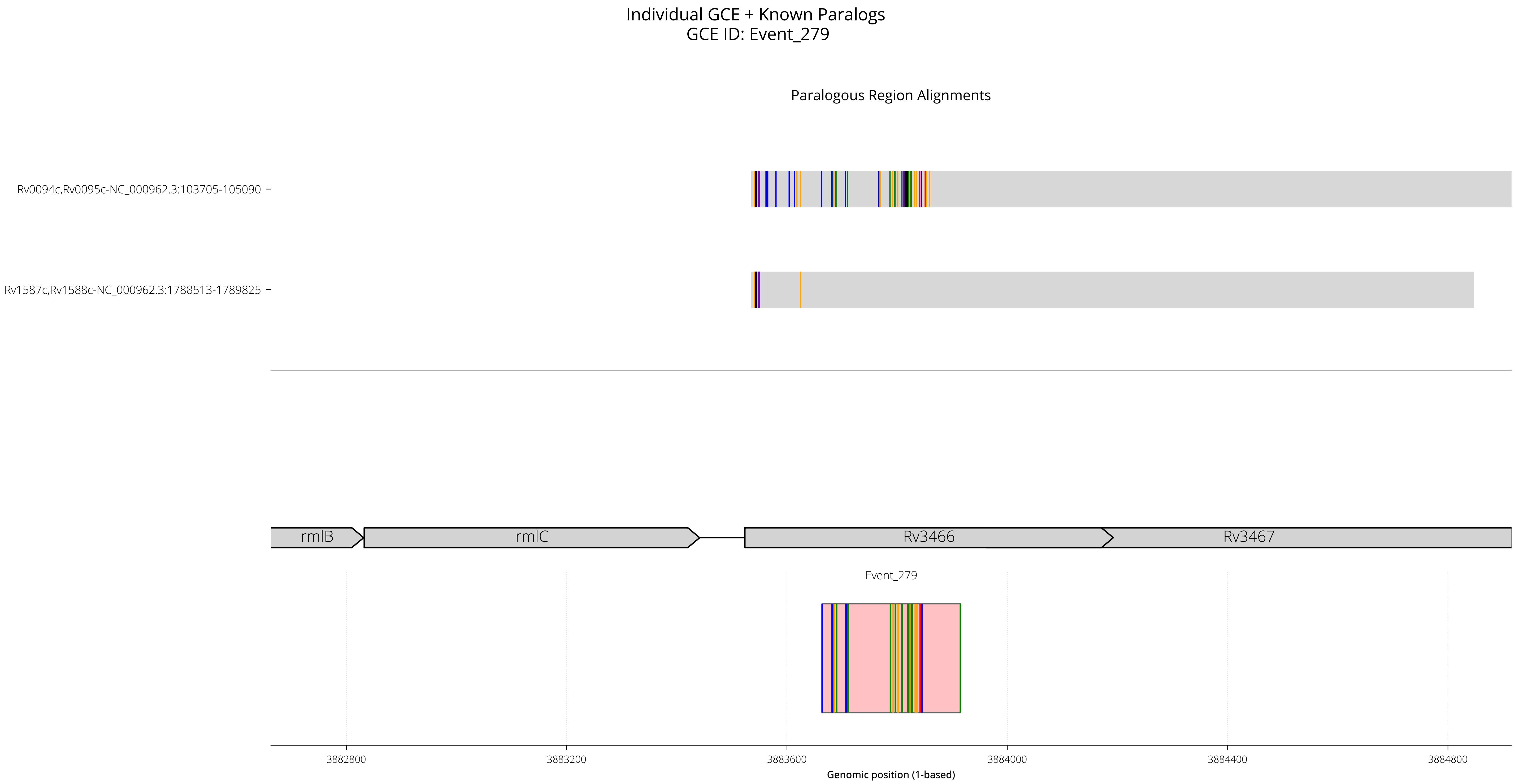

Individual GCE + Known Paralogs
GCE ID: Event_279
Paralogous Region Alignments
Rv0094c,Rv0095c-NC_000962.3:103705-105090
Rv1587c,Rv1588c-NC_000962.3:1788513-1789825
rmlB
rmlC
Rv3466
Rv3467
Event_279
3882800
3883200
3883600
3884000
3884400
3884800
Genomic position (1-based)

Circos Viz - GCE to Known Paralogs Locations
GCE ID: Event_279

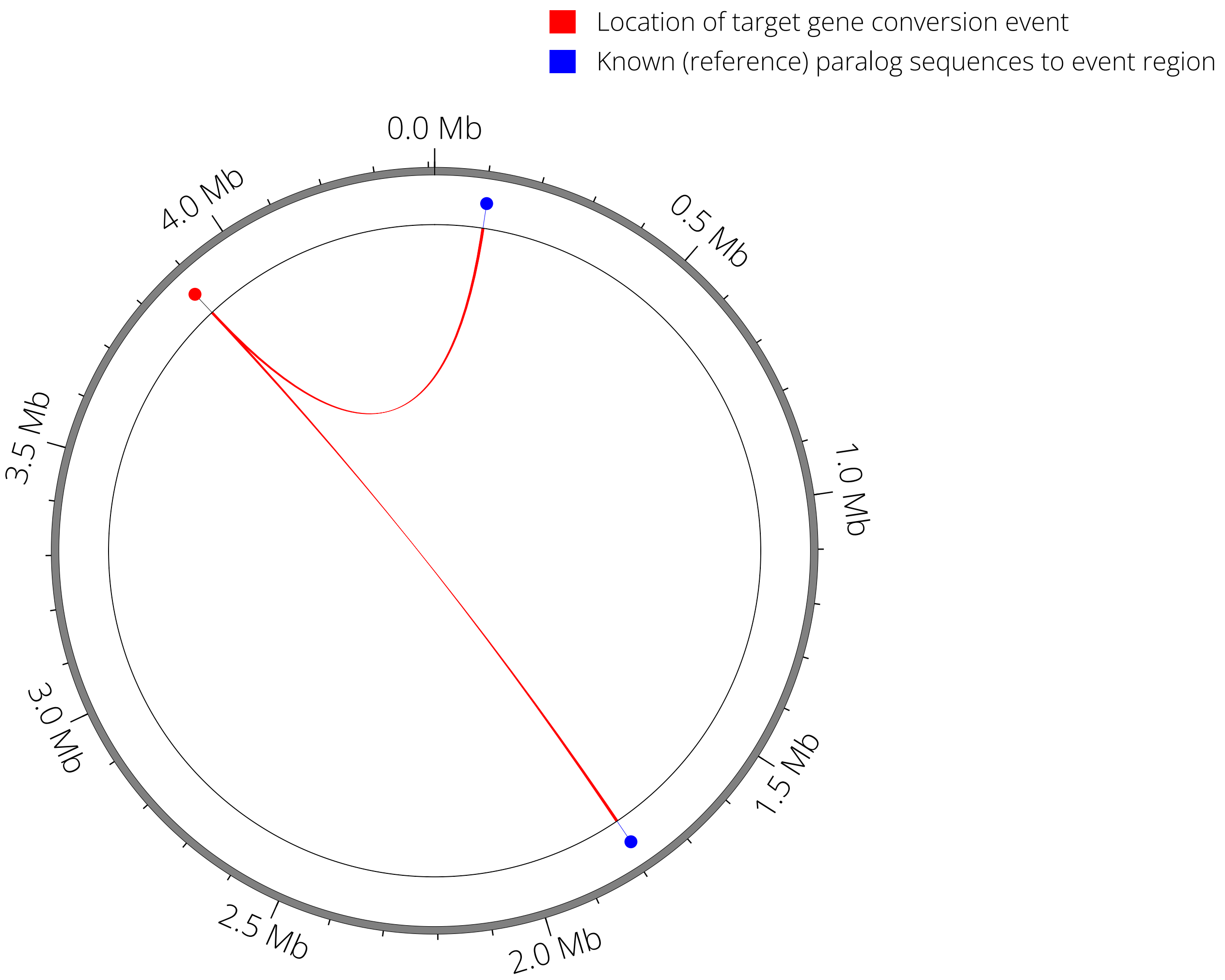

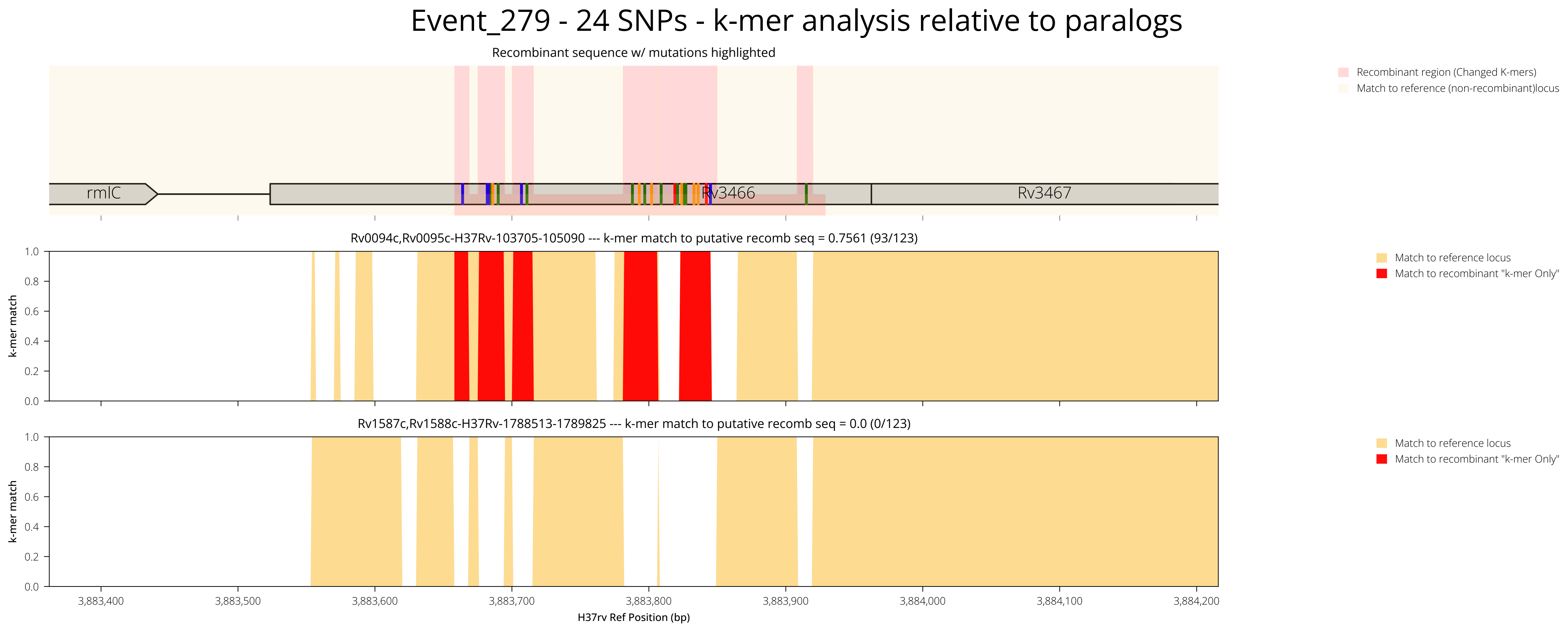

Event_279 - 24 SNPs - k-mer analysis relative to paralogs
Recombinant sequence w/ mutations highlighted
rmlC
Rv3466
Rv3467
Recombinant region (Changed K-mers)
Match to reference (non-recombinant)locus
Rv0094c,Rv0095c-H37Rv-103705-105090 --- k-mer match to putative recomb seq = 0.7561 (93/123)
Match to reference locus
Match to recombinant "k-mer Only"
Rv1587c,Rv1588c-H37Rv-1788513-1789825 --- k-mer match to putative recomb seq = 0.0 (0/123)
Match to reference locus
Match to recombinant "k-mer Only"
k-mer match
1.0
0.8
0.6
0.4
0.2
0.0
3,883,400
3,883,500
3,883,600
3,883,700
3,883,800
3,883,900
3,884,000
3,884,100
3,884,200
H37rv Ref Position (bp)

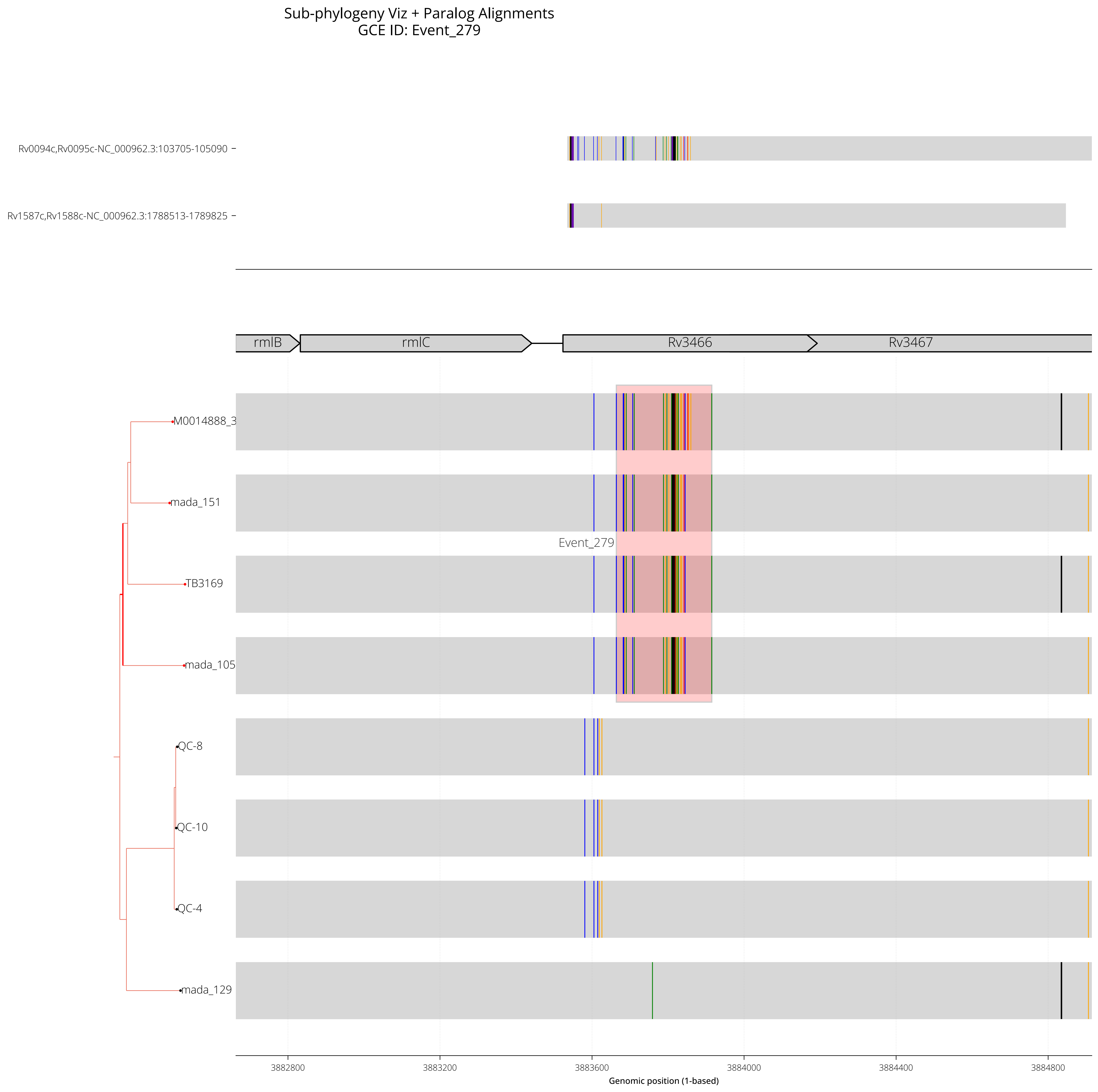

Sub-phylogeny Viz + Paralog Alignments
GCE ID: Event_279
Rv0094c,Rv0095c-NC_000962.3:103705-105090
Rv1587c,Rv1588c-NC_000962.3:1788513-1789825
rmlB
rmlC
Rv3466
Rv3467
M0014888_3
mada_151
Event_279
TB3169
mada_105
QC-8
QC-10
QC-4
mada_129
3882800
3883200
3883600
3884000
3884400
3884800
Genomic position (1-based)

Full Phylogeny Viz + Paralog Alignments
GCE ID: Event_279

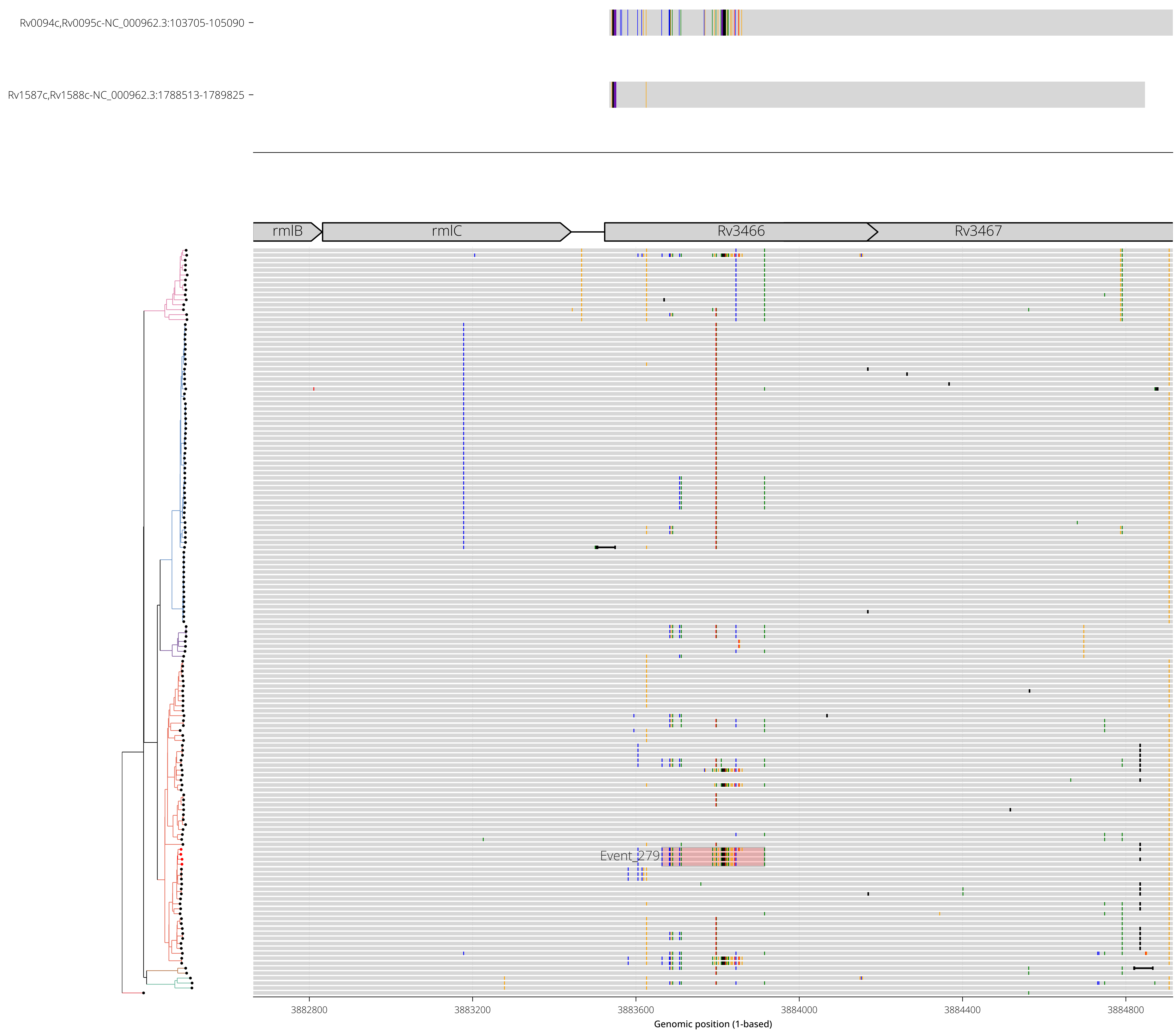

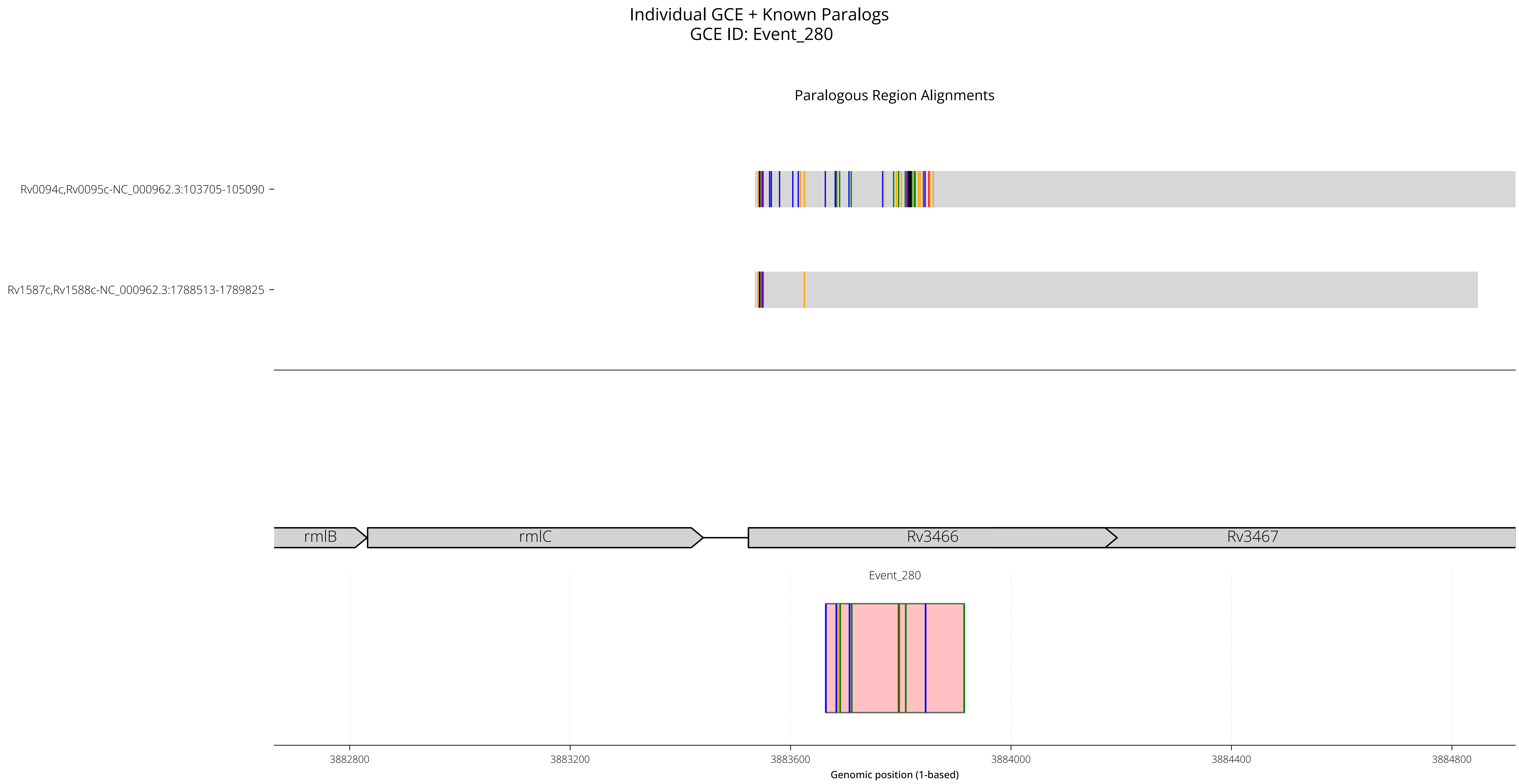
Individual GCE + Known Paralogs
GCE ID: Event_280
Paralogous Region Alignments
Rv0094c,Rv0095c-NC_000962.3:103705-105090
Rv1587c,Rv1588c-NC_000962.3:1788513-1789825
rmlB
rmlC
Rv3466
Rv3467
Event_280
3882800
3883200
3883600
3884000
3884400
3884800
Genomic position (1-based)

Circos Viz - GCE to Known Paralogs Locations
GCE ID: Event_280

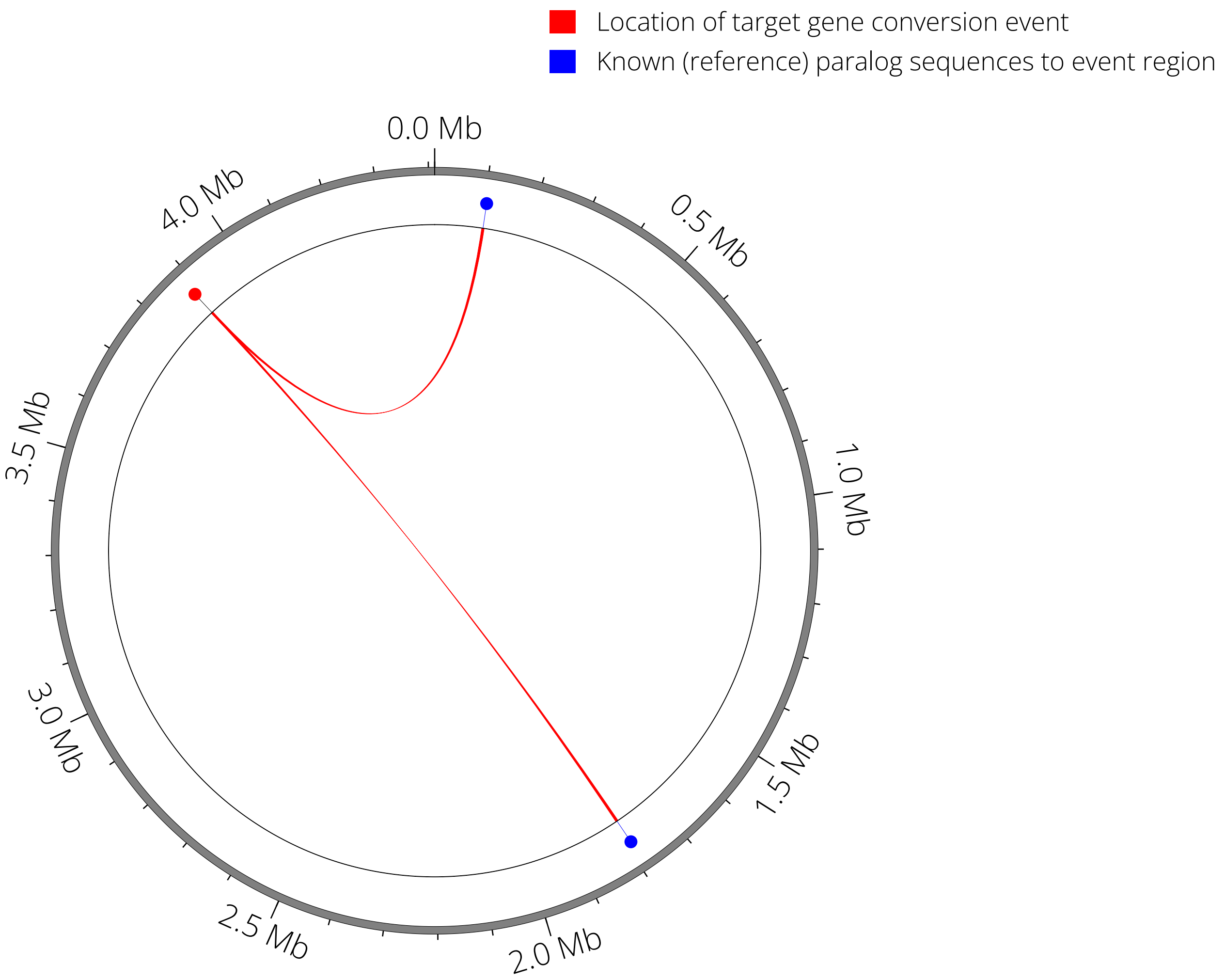

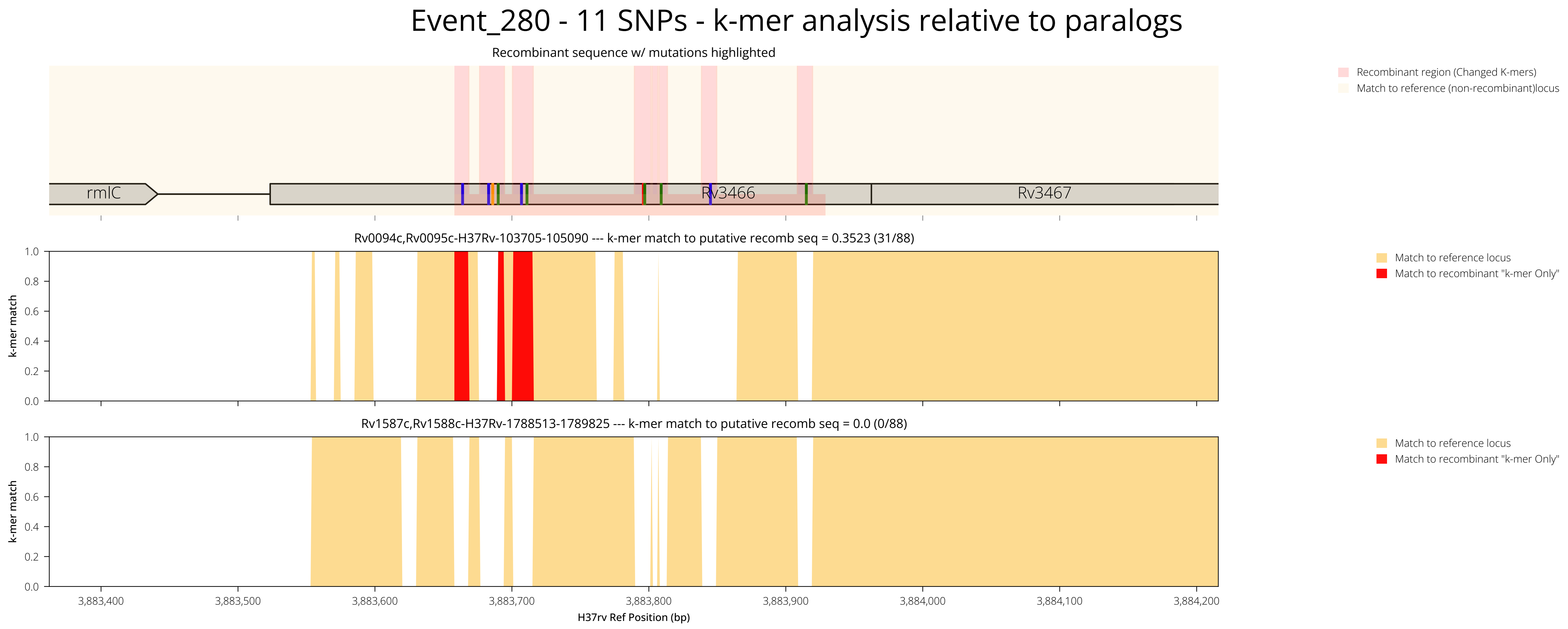

Event_280 - 11 SNPs - k-mer analysis relative to paralogs
Recombinant sequence w/ mutations highlighted
Recombinant region (Changed K-mers)
Match to reference (non-recombinant)locus
rmlC
Rv3466
Rv3467
Rv0094c,Rv0095c-H37Rv-103705-105090 --- k-mer match to putative recomb seq = 0.3523 (31/88)
Match to reference locus
Match to recombinant "k-mer Only"
k-mer match
Rv1587c,Rv1588c-H37Rv-1788513-1789825 --- k-mer match to putative recomb seq = 0.0 (0/88)
Match to reference locus
Match to recombinant "k-mer Only"
k-mer match
3,883,400
3,883,500
3,883,600
3,883,700
3,883,800
3,883,900
3,884,000
3,884,100
3,884,200
H37rv Ref Position (bp)

Sub-phylogeny Viz + Paralog Alignments
GCE ID: Event_280

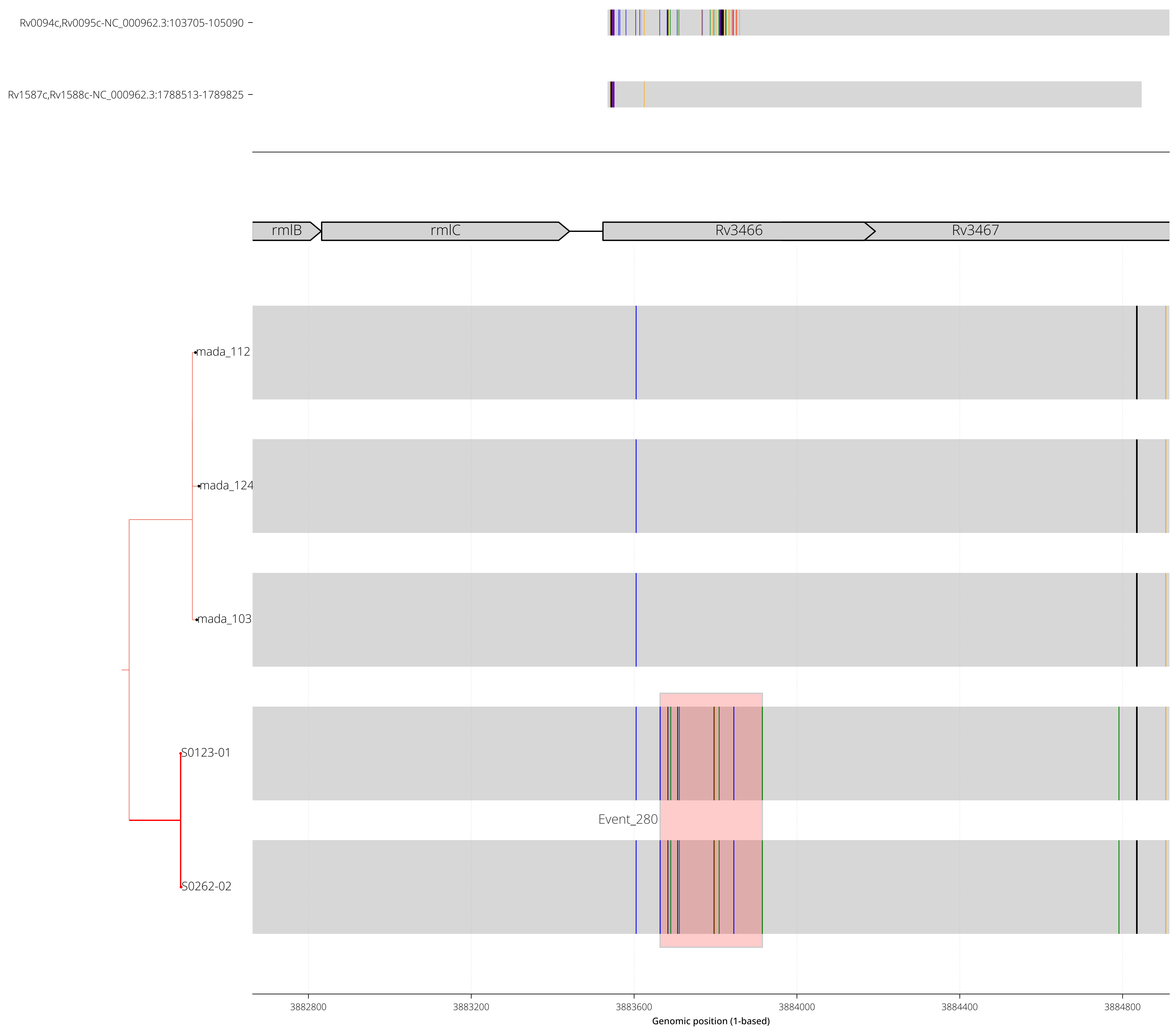

Full Phylogeny Viz + Paralog Alignments
GCE ID: Event_280

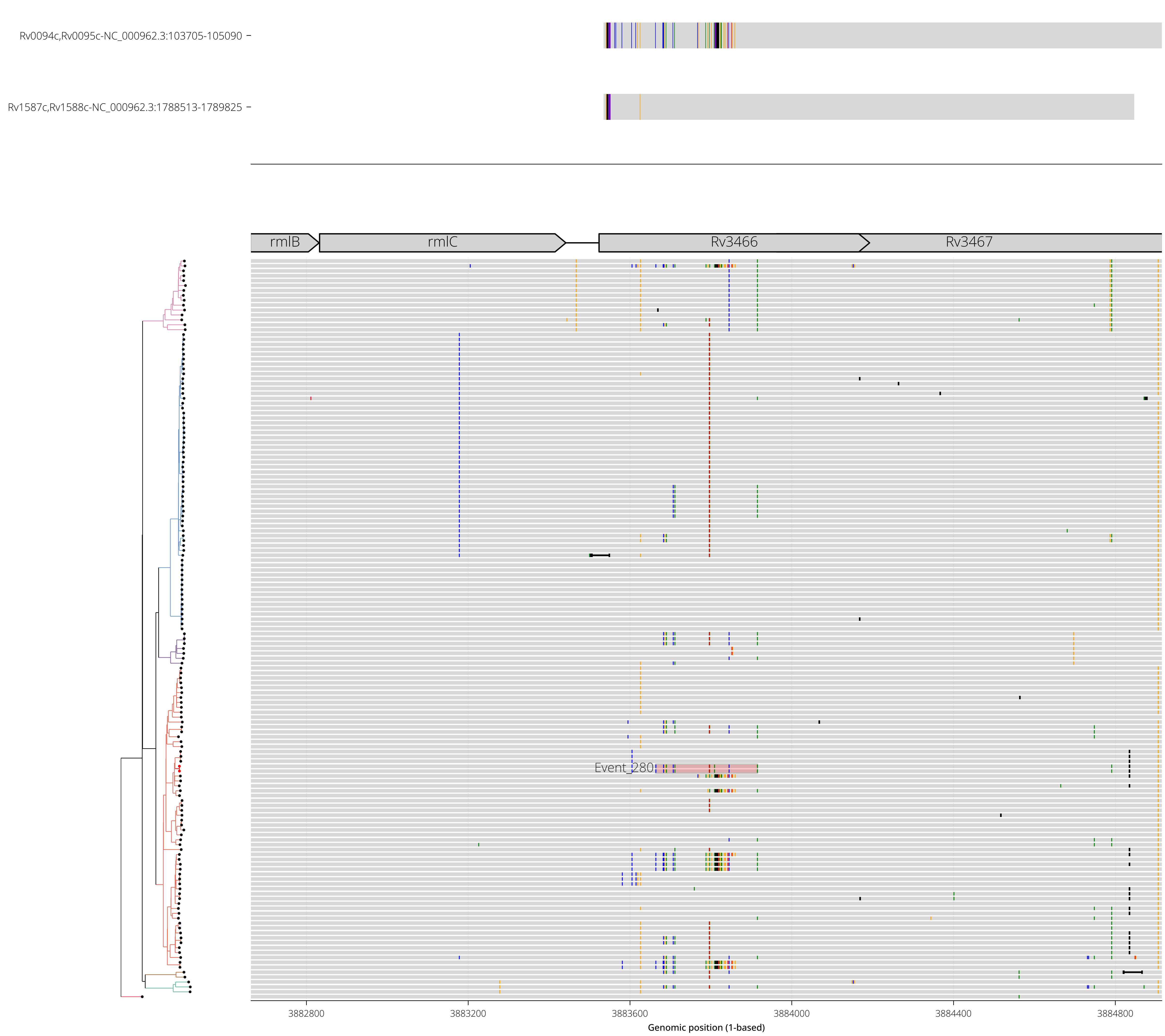

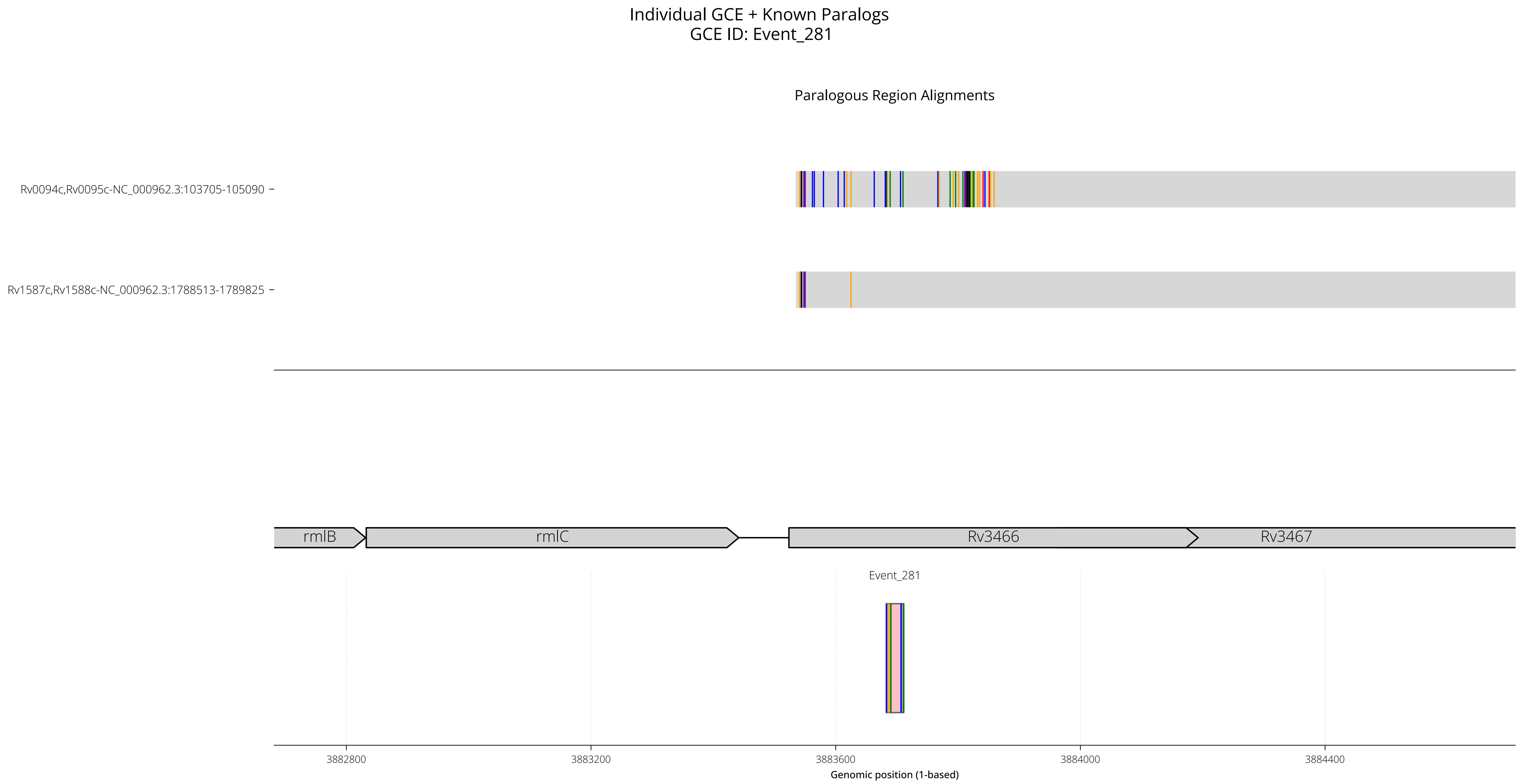
Individual GCE + Known Paralogs
GCE ID: Event_281
Paralogous Region Alignments
Rv0094c,Rv0095c-NC_000962.3:103705-105090
Rv1587c,Rv1588c-NC_000962.3:1788513-1789825
rmlB
rmlC
Rv3466
Rv3467
Event_281
3882800
3883200
3883600
3884000
3884400
Genomic position (1-based)

Circos Viz - GCE to Known Paralogs Locations
GCE ID: Event_281

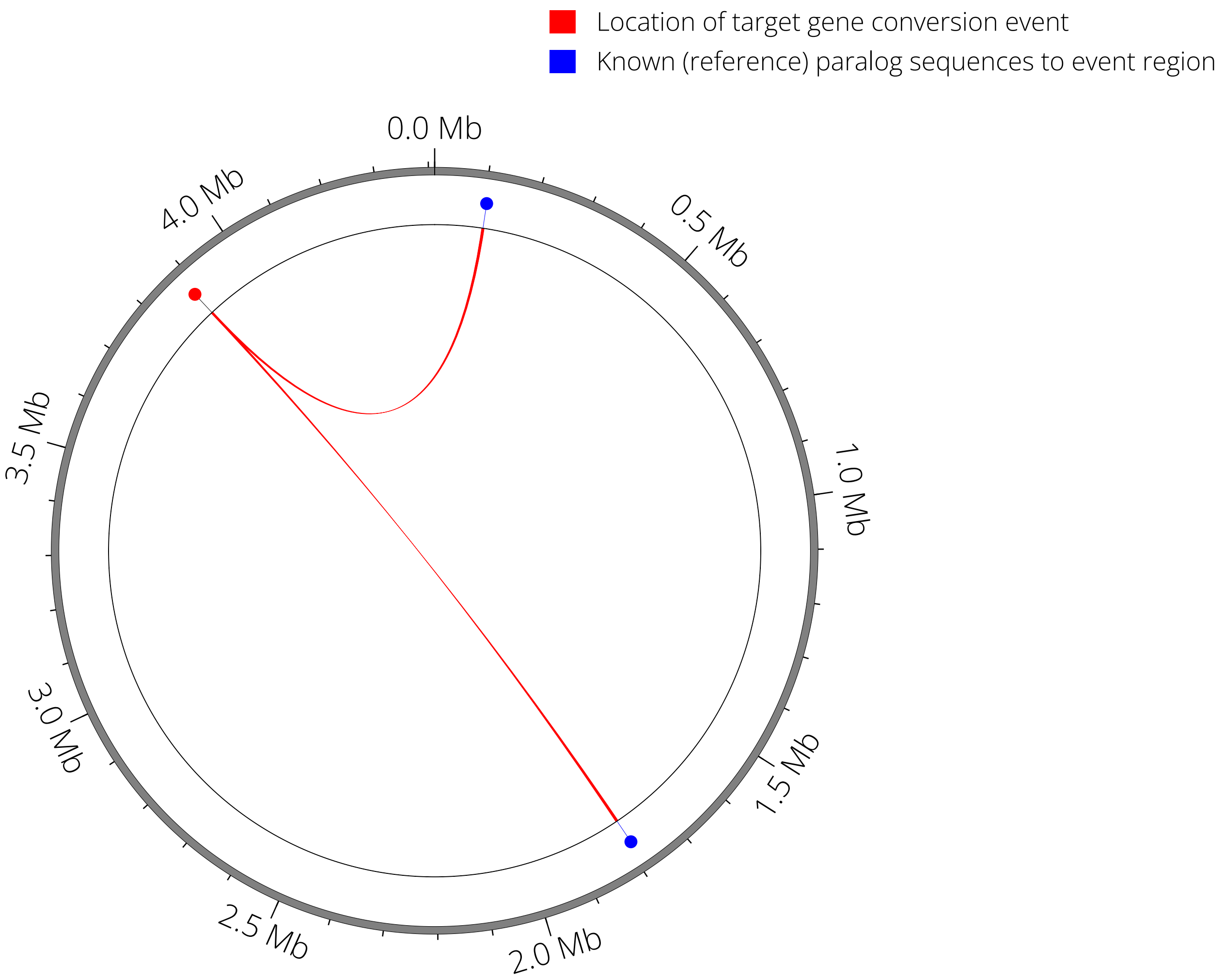

# Event_281 - 5 SNPs - k-mer analysis relative to paralogs

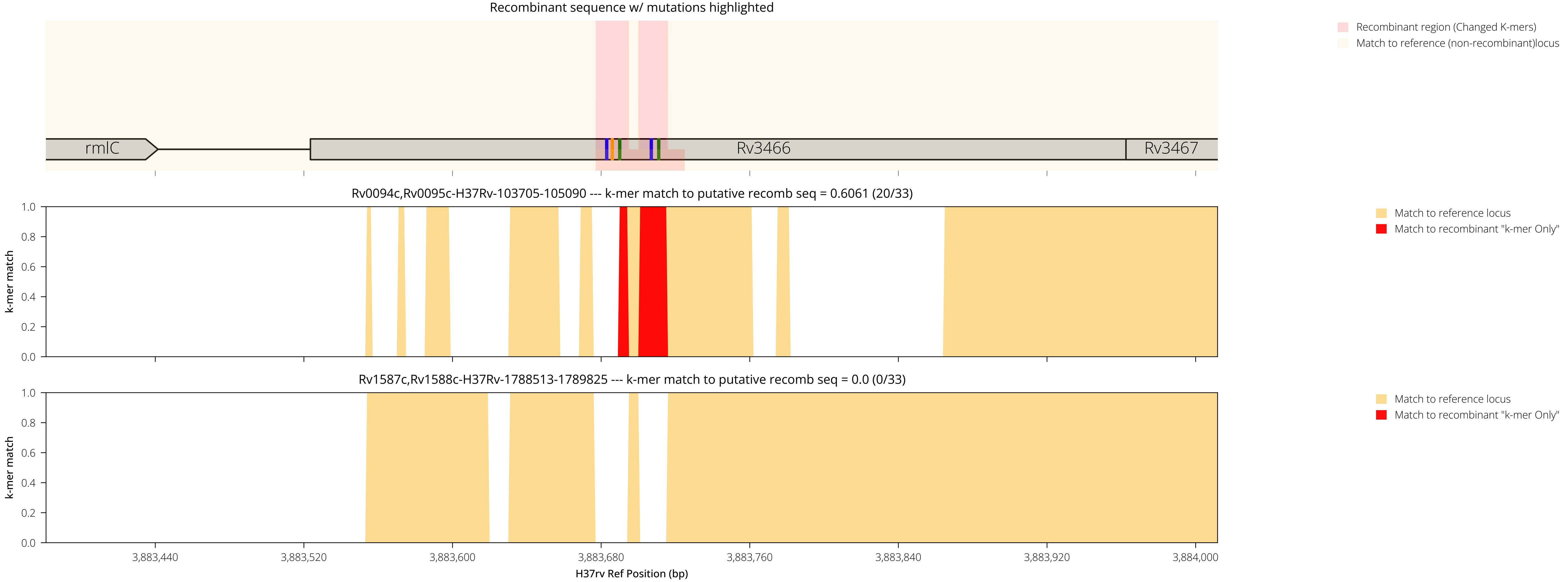

Sub-phylogeny Viz + Paralog Alignments
GCE ID: Event_281

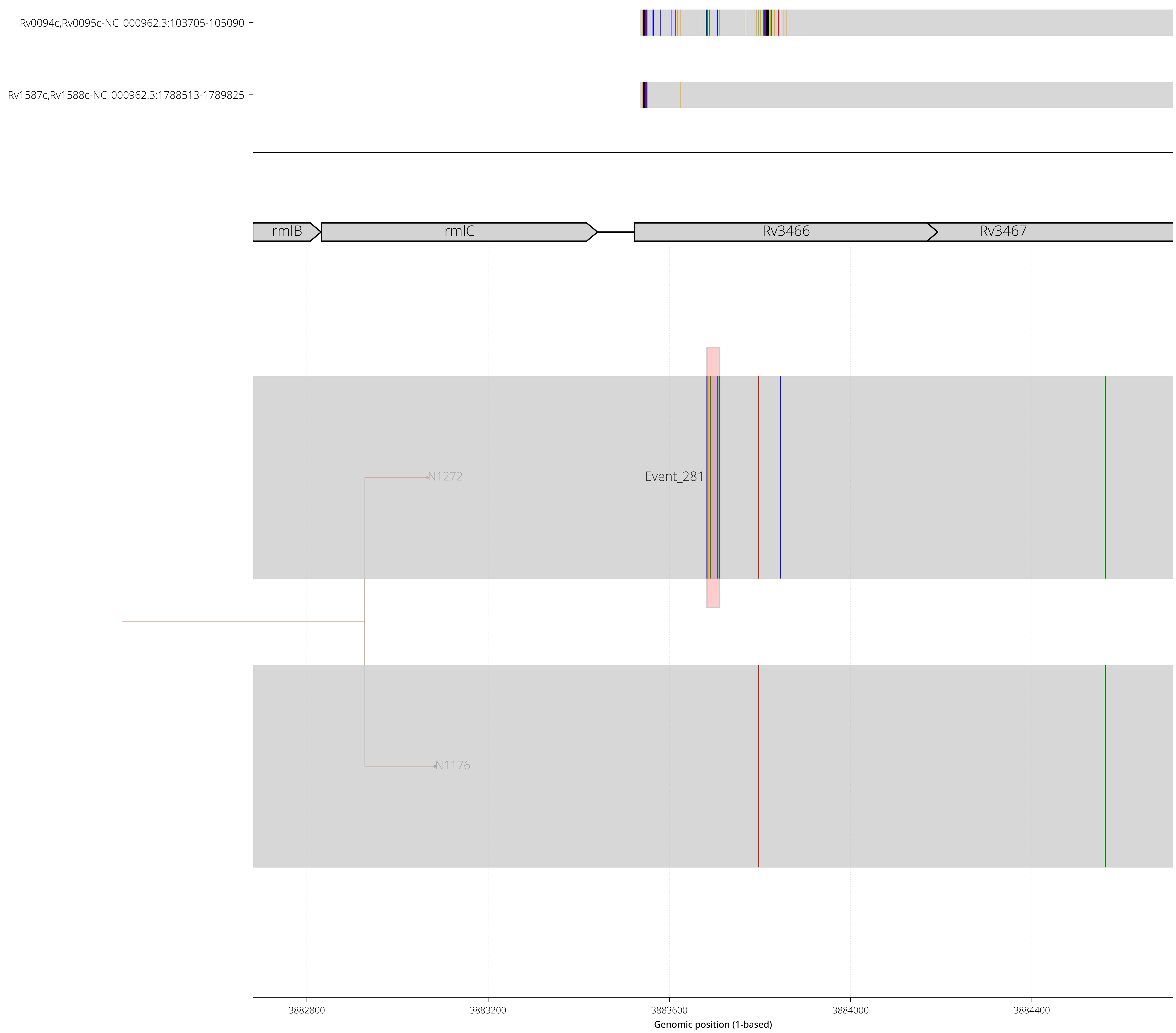

Full Phylogeny Viz + Paralog Alignments
GCE ID: Event_281

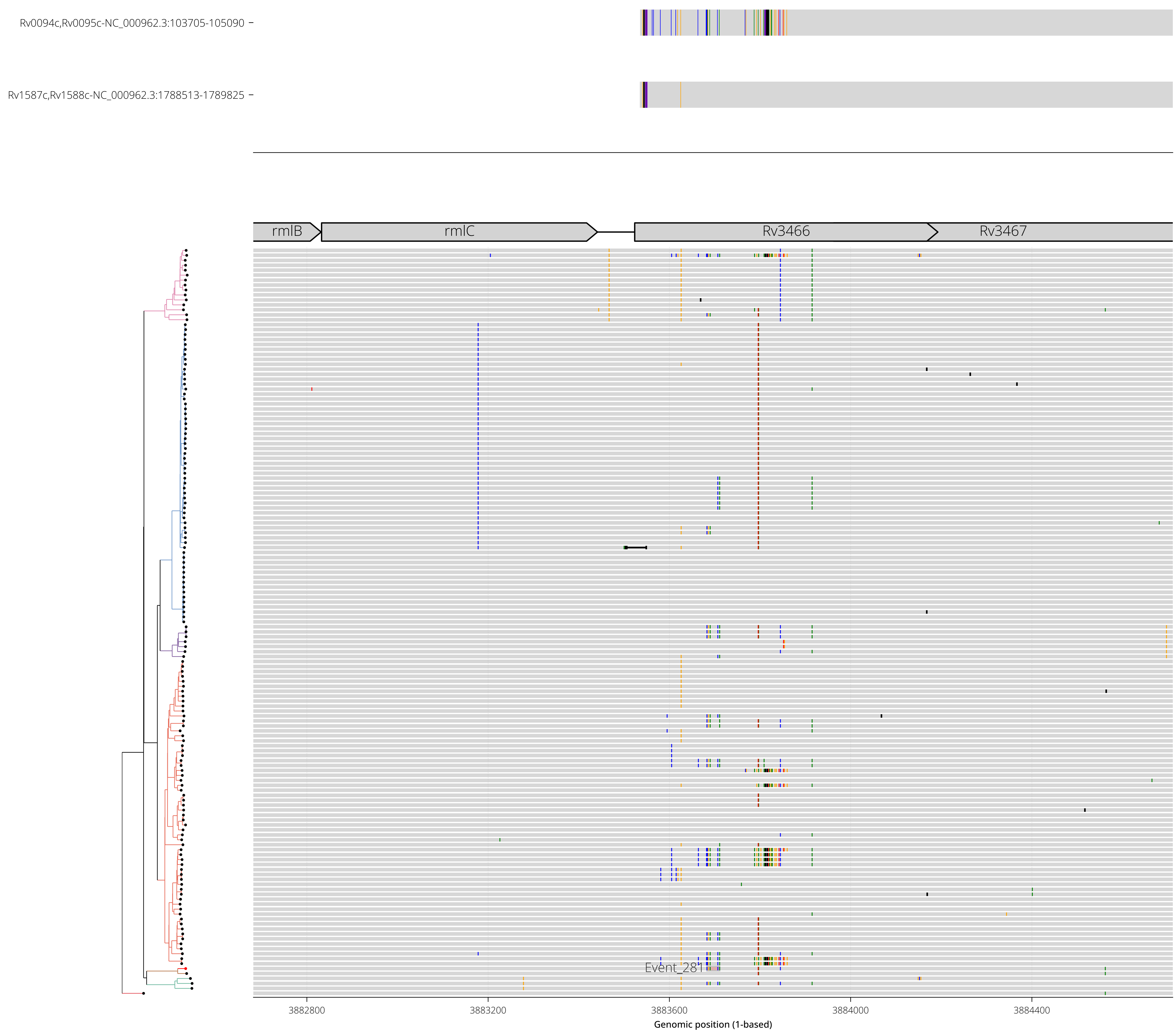

Individual GCE + Known Paralogs
GCE ID: Event_282

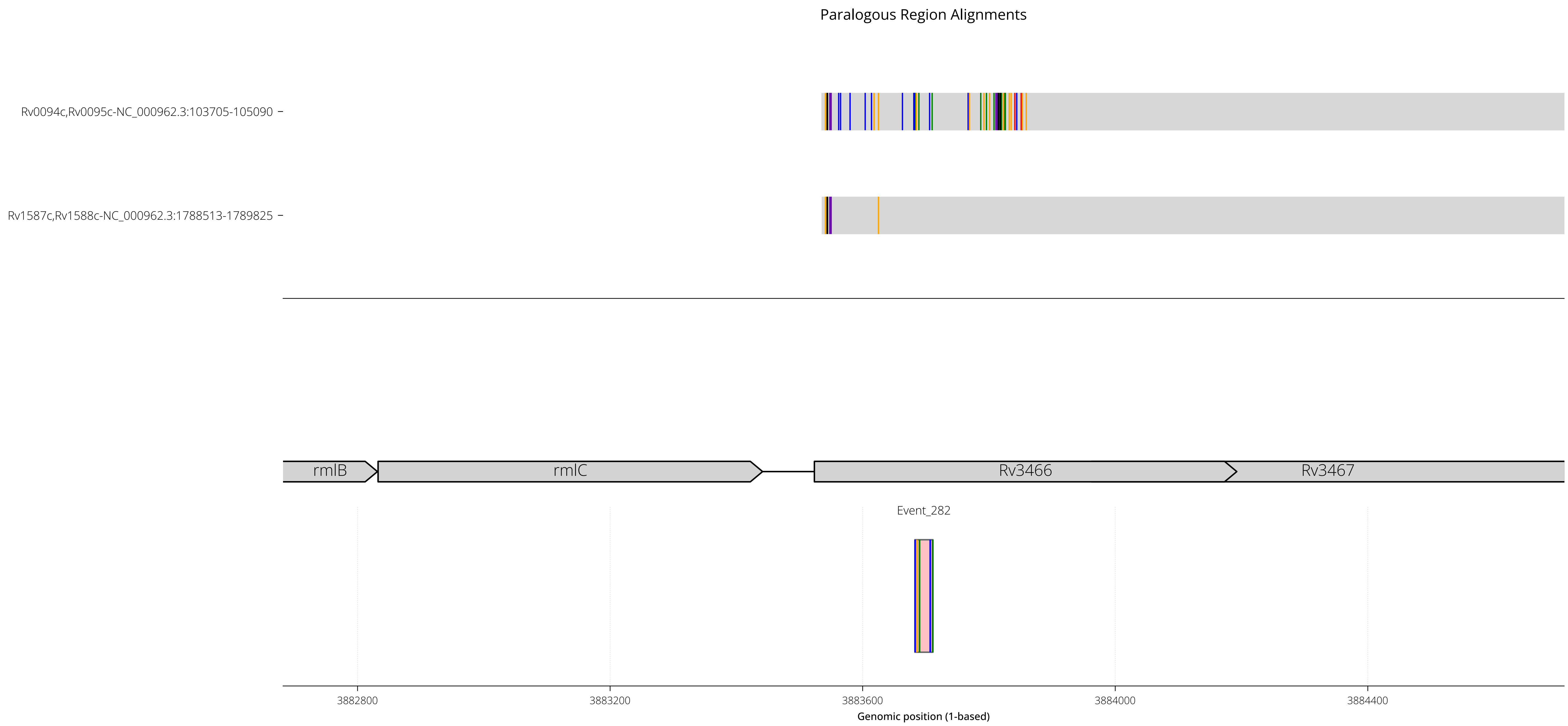

Circos Viz - GCE to Known Paralogs Locations
GCE ID: Event_282

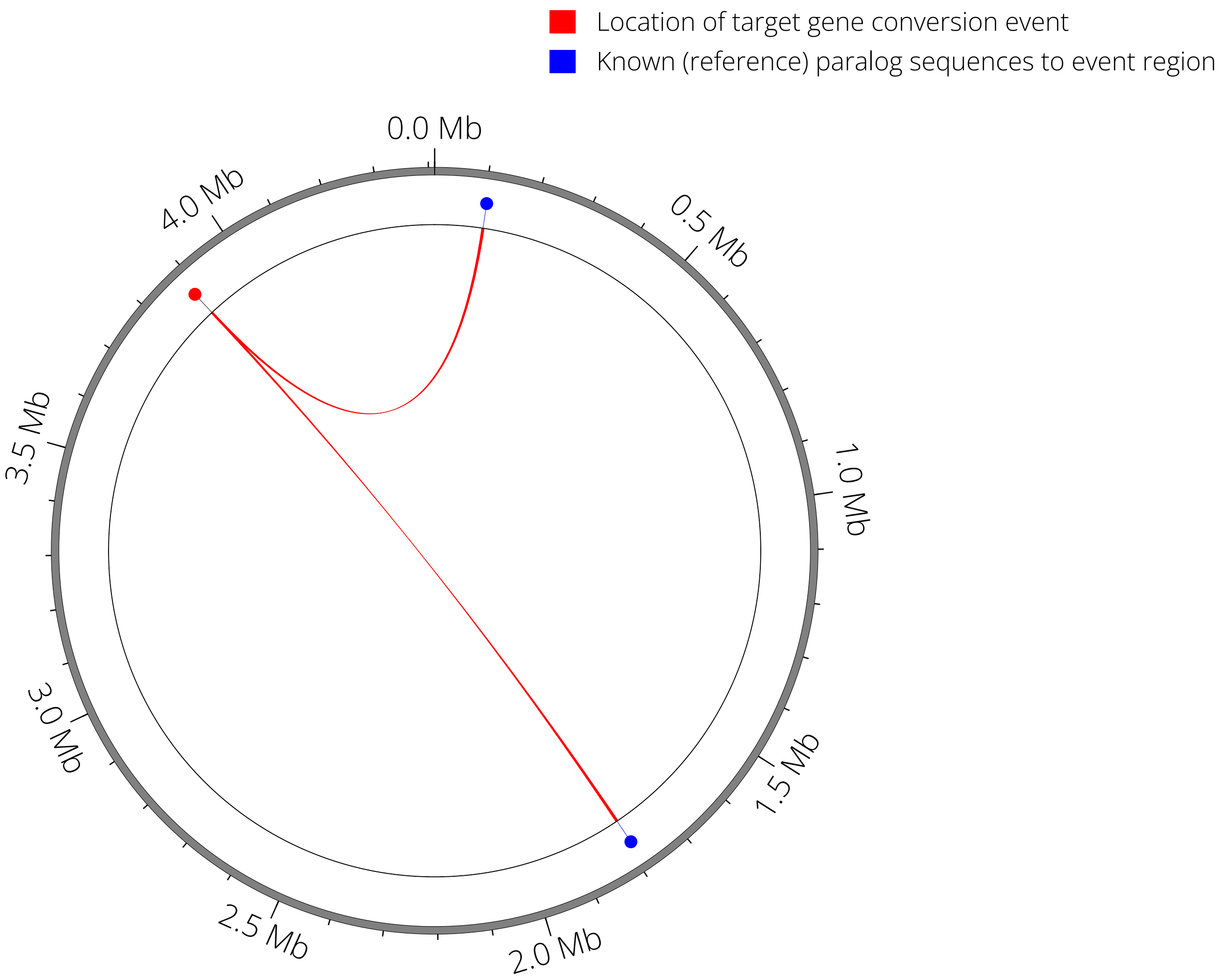

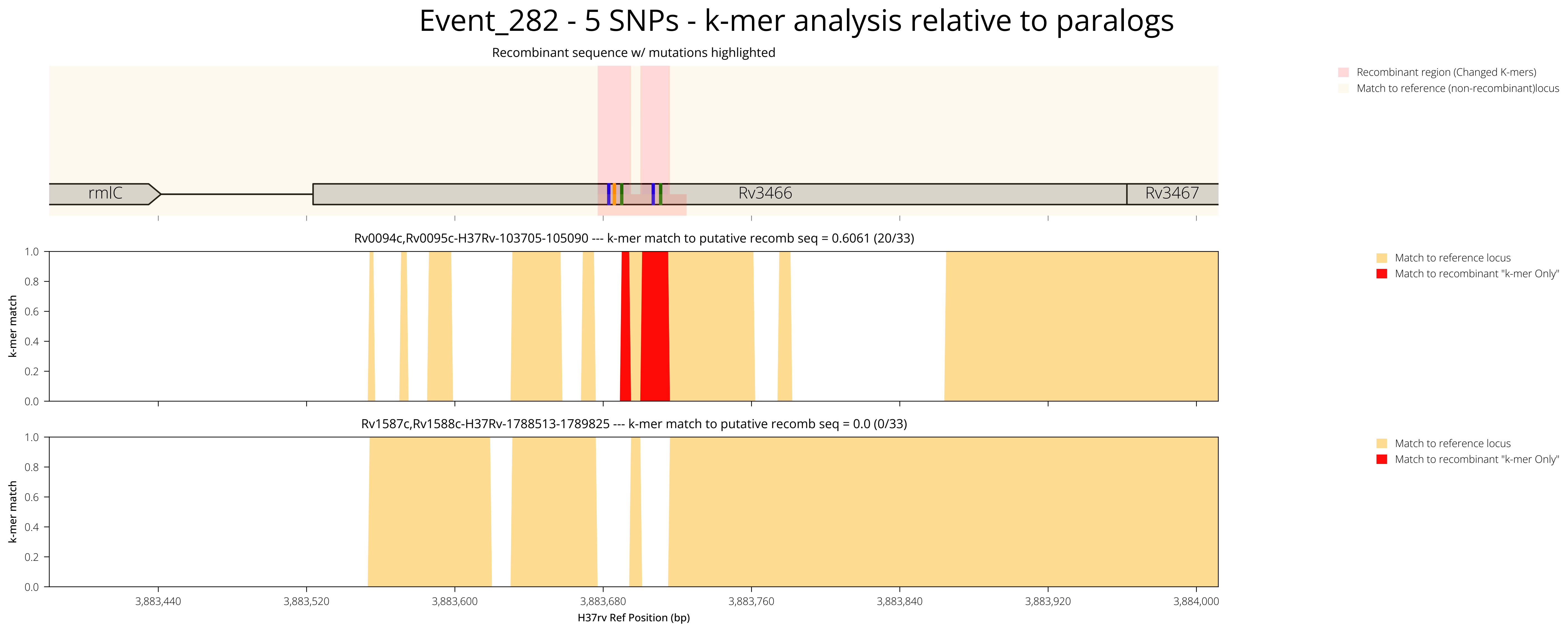

Event_282 - 5 SNPs - k-mer analysis relative to paralogs
Recombinant sequence w/ mutations highlighted
rmlC
Rv3466
Rv3467
Recombinant region (Changed K-mers)
Match to reference (non-recombinant)locus
Rv0094c,Rv0095c-H37Rv-103705-105090 --- k-mer match to putative recomb seq = 0.6061 (20/33)
k-mer match
1.0
0.8
0.6
0.4
0.2
0.0
Match to reference locus
Match to recombinant "k-mer Only"
Rv1587c,Rv1588c-H37Rv-1788513-1789825 --- k-mer match to putative recomb seq = 0.0 (0/33)
k-mer match
1.0
0.8
0.6
0.4
0.2
0.0
Match to reference locus
Match to recombinant "k-mer Only"
3,883,440
3,883,520
3,883,600
3,883,680
3,883,760
3,883,840
3,883,920
3,884,000
H37rv Ref Position (bp)

Sub-phylogeny Viz + Paralog Alignments
GCE ID: Event_282

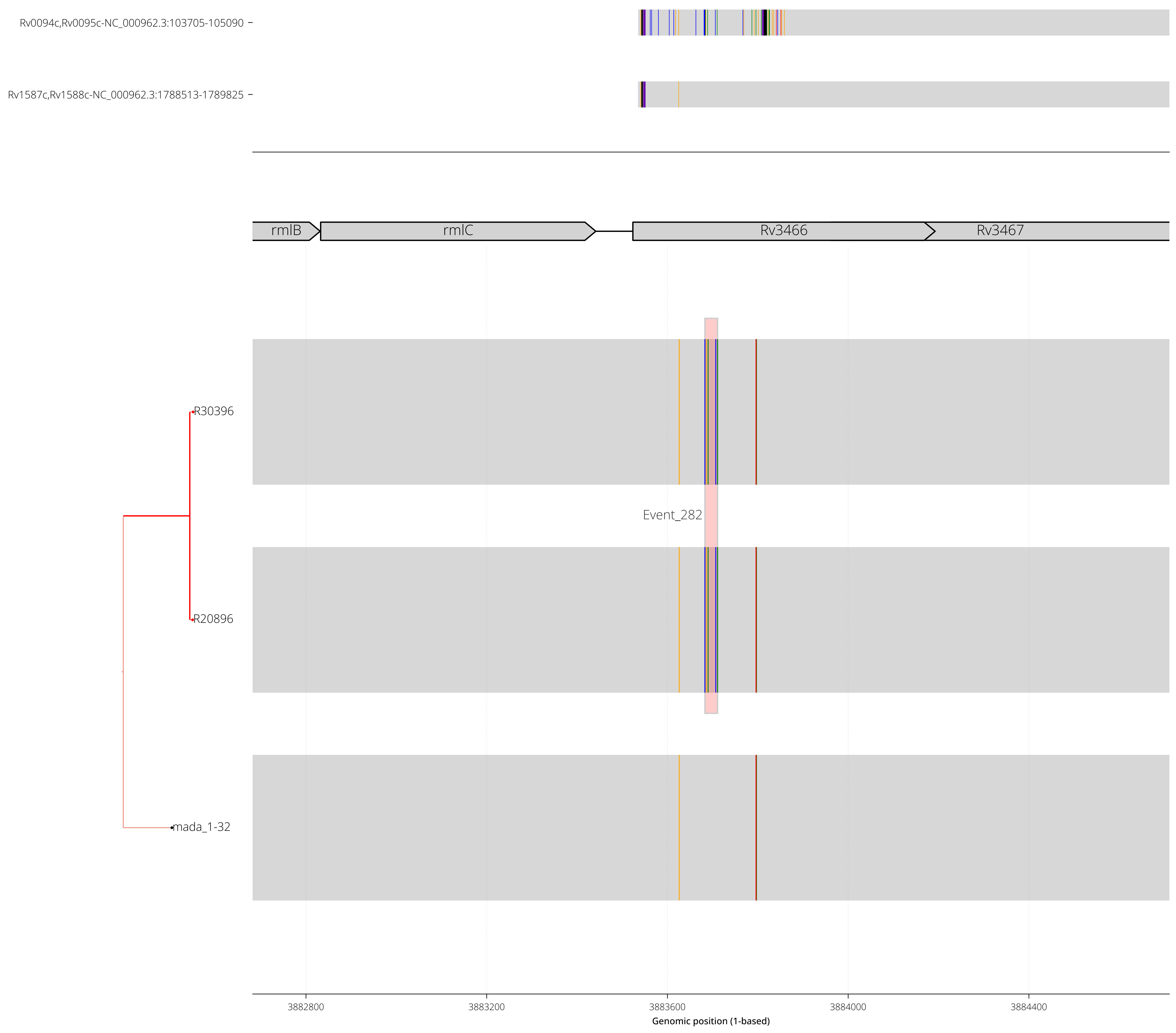

Full Phylogeny Viz + Paralog Alignments
GCE ID: Event_282

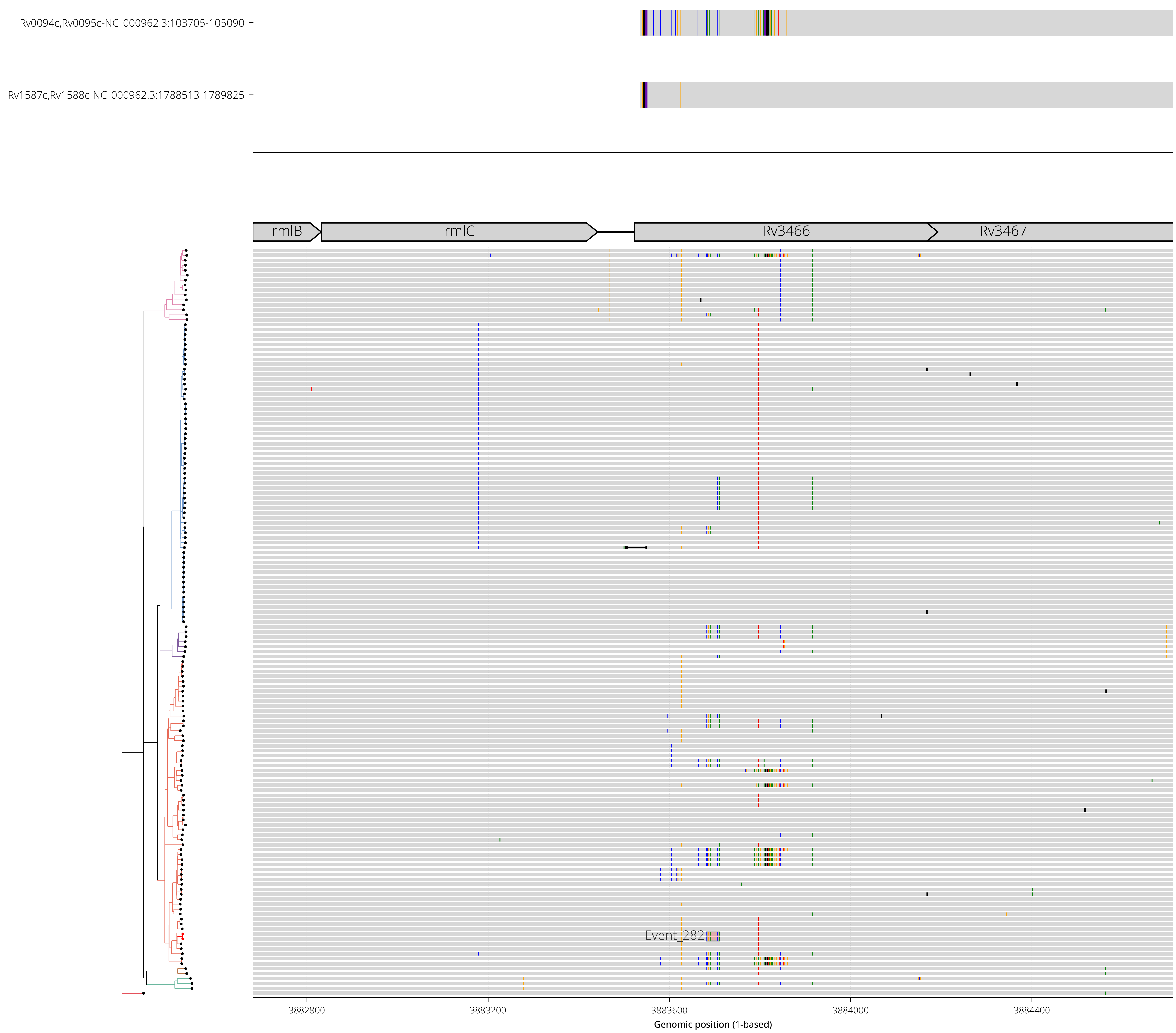

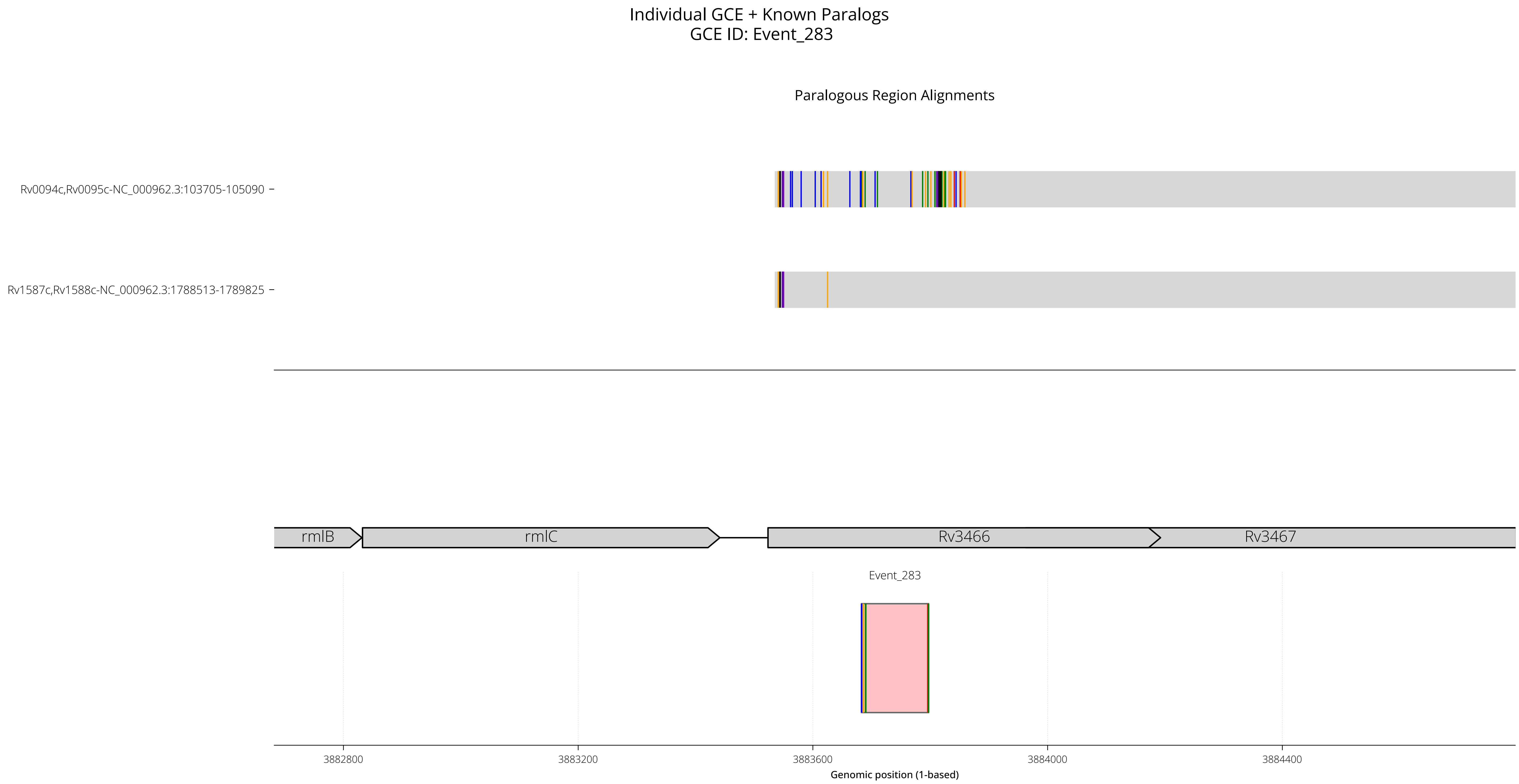
Individual GCE + Known Paralogs
GCE ID: Event_283
Paralogous Region Alignments
Rv0094c,Rv0095c-NC_000962.3:103705-105090
Rv1587c,Rv1588c-NC_000962.3:1788513-1789825
rmlB
rmlC
Rv3466
Rv3467
Event_283
3882800
3883200
3883600
3884000
3884400
Genomic position (1-based)

Circos Viz - GCE to Known Paralogs Locations
GCE ID: Event_283

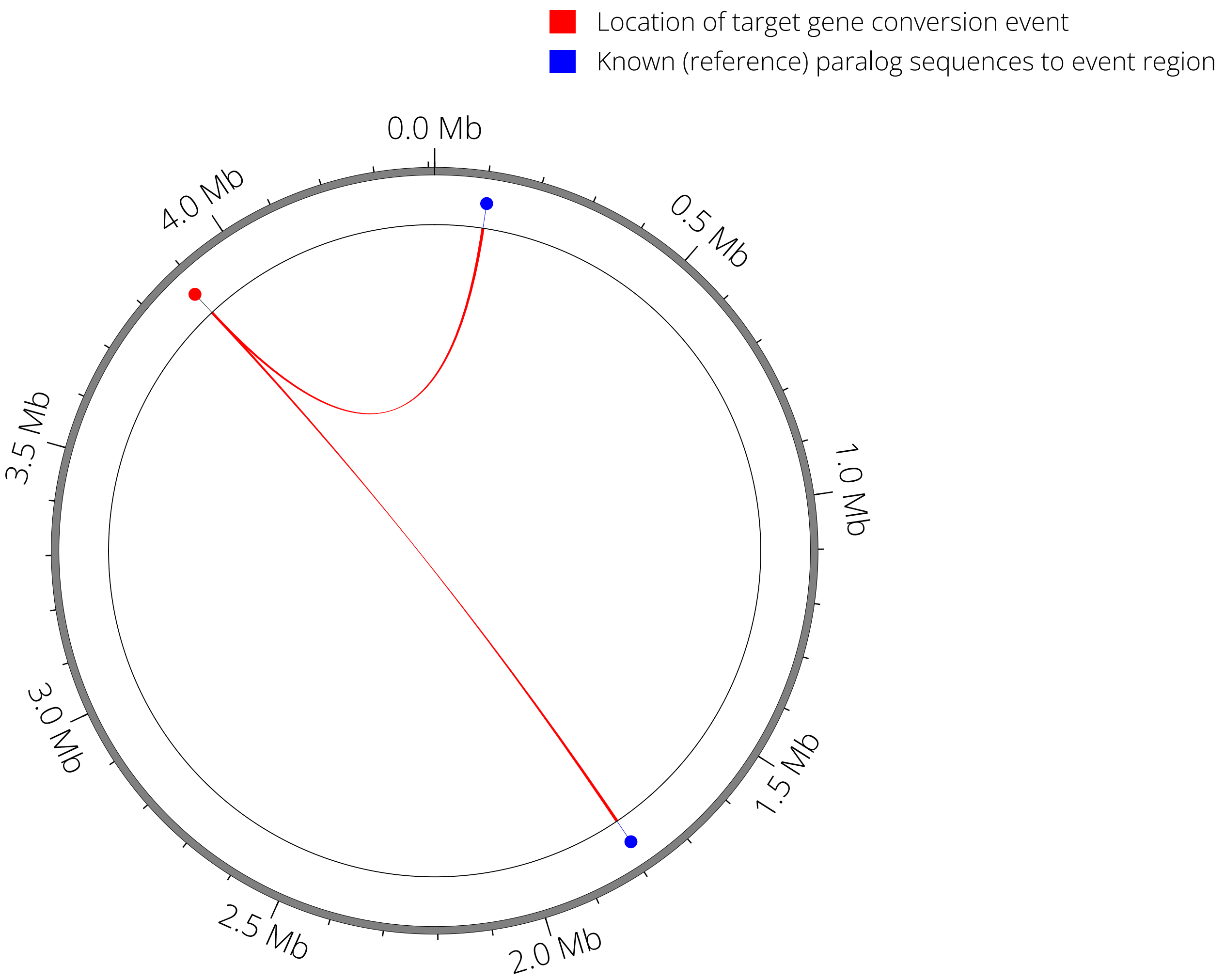

# Event_283 - 5 SNPs - k-mer analysis relative to paralogs

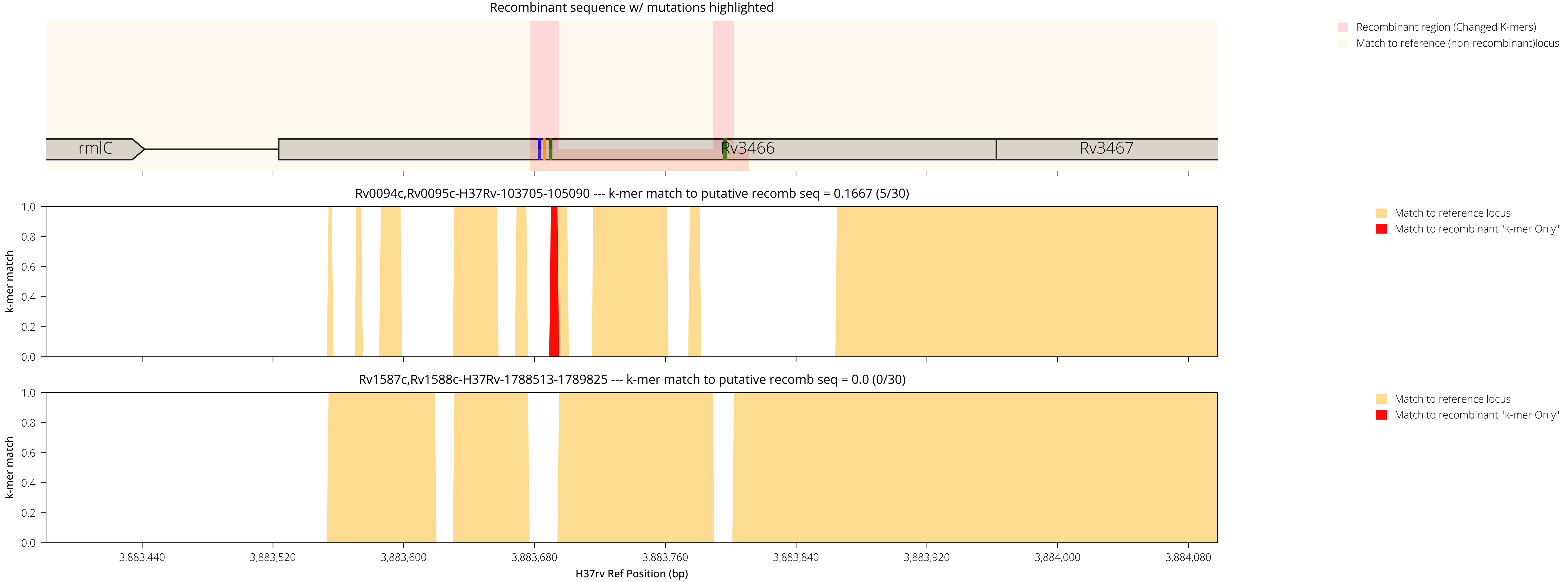

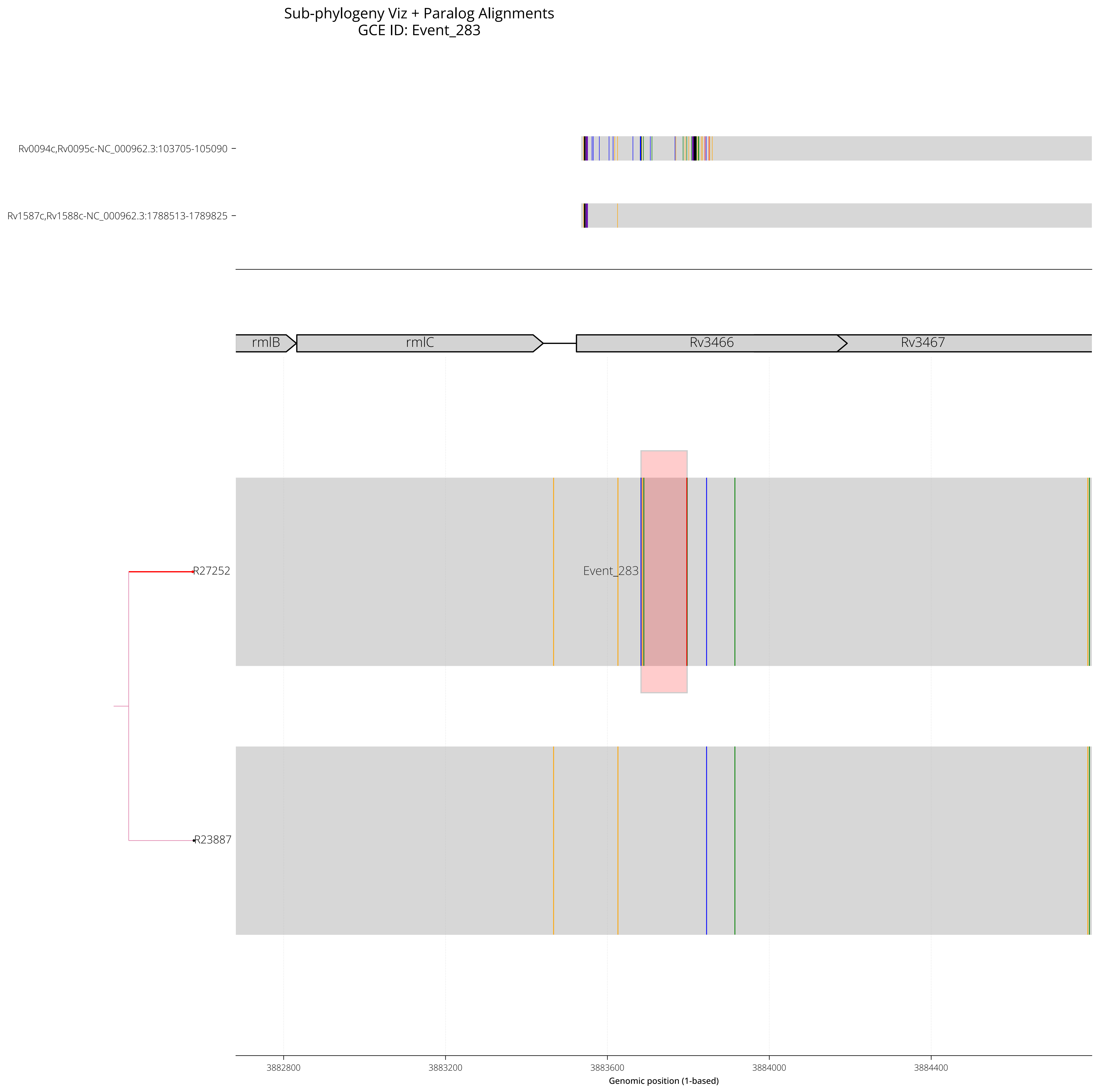

Sub-phylogeny Viz + Paralog Alignments
GCE ID: Event_283
Rv0094c,Rv0095c-NC_000962.3:103705-105090
Rv1587c,Rv1588c-NC_000962.3:1788513-1789825
rmlB
rmlC
Rv3466
Rv3467
R27252
Event_283
R23887
3882800
3883200
3883600
3884000
3884400
Genomic position (1-based)

Full Phylogeny Viz + Paralog Alignments
GCE ID: Event_283

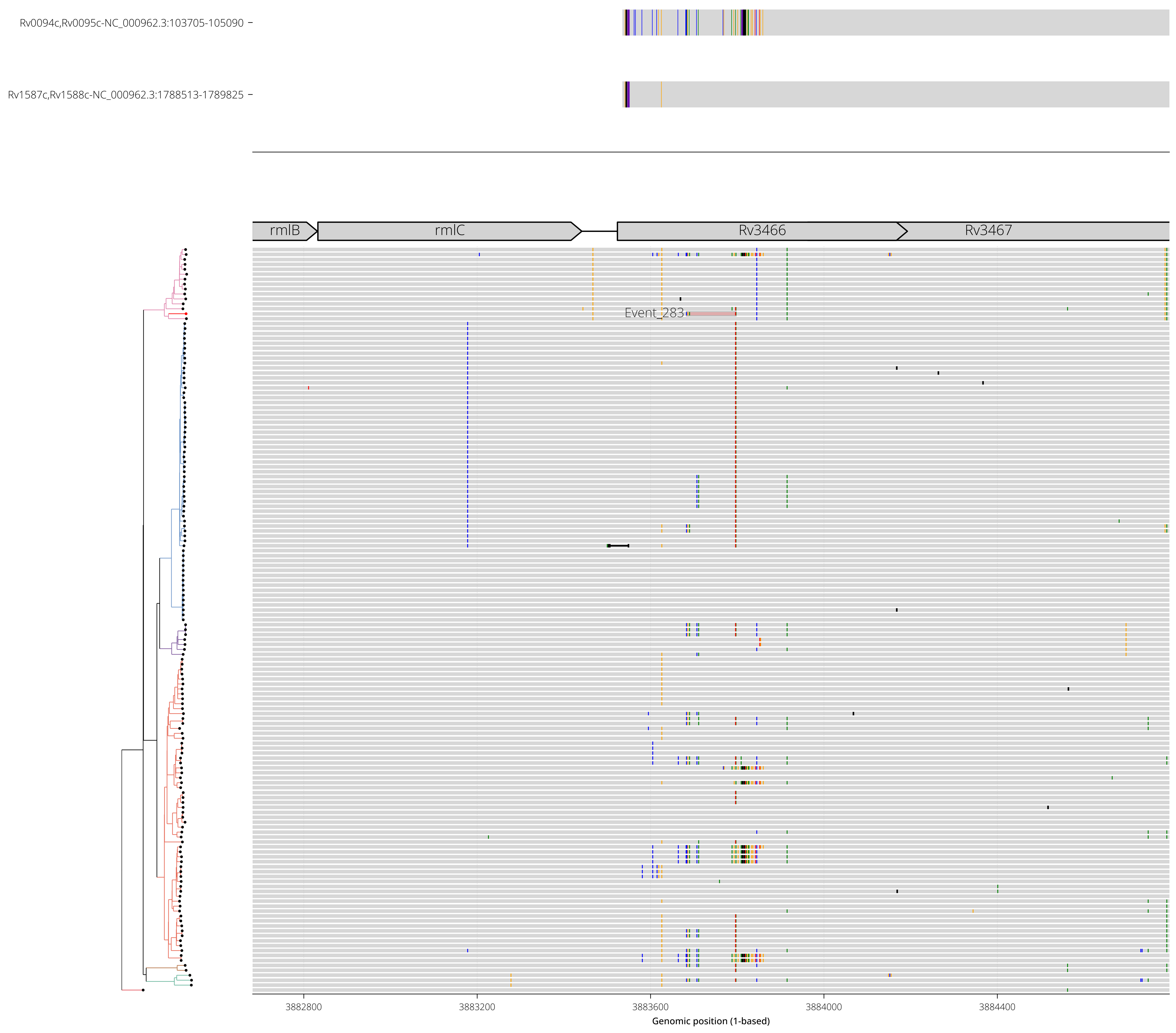

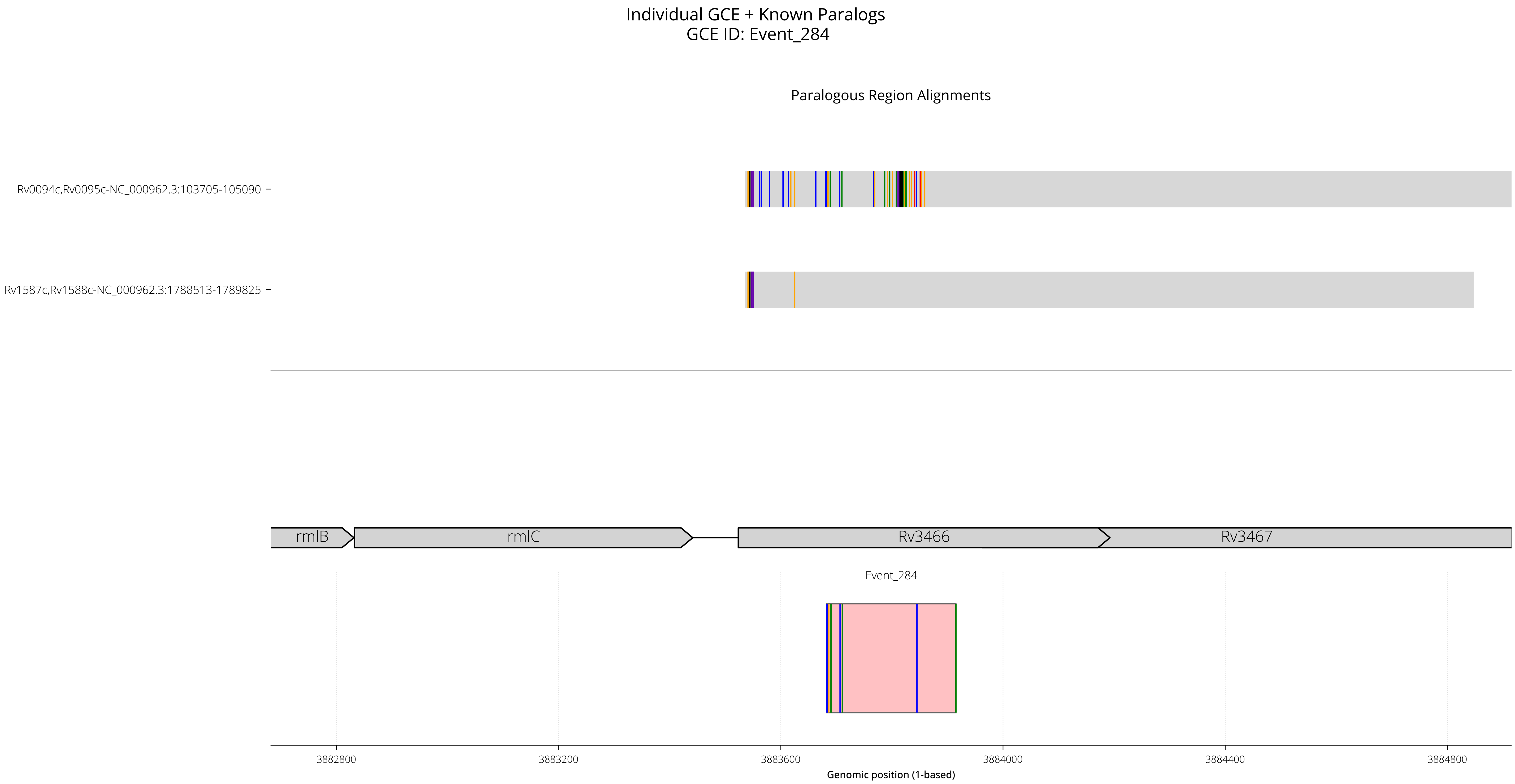

Individual GCE + Known Paralogs
GCE ID: Event_284
Paralogous Region Alignments
Rv0094c,Rv0095c-NC_000962.3:103705-105090
Rv1587c,Rv1588c-NC_000962.3:1788513-1789825
rmlB
rmlC
Rv3466
Rv3467
Event_284
3882800
3883200
3883600
3884000
3884400
3884800
Genomic position (1-based)

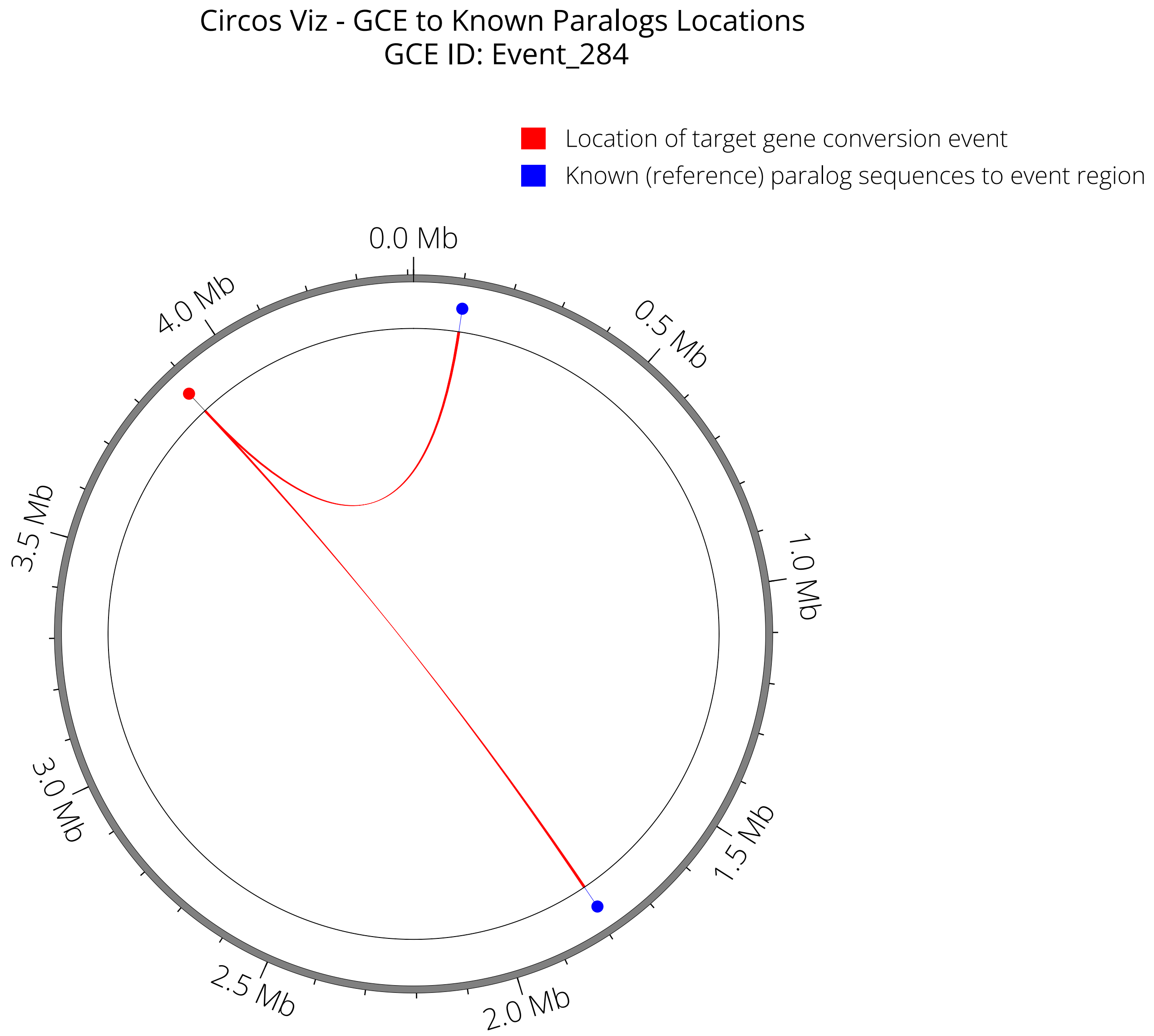

Circos Viz - GCE to Known Paralogs Locations
GCE ID: Event_284
Location of target gene conversion event
Known (reference) paralog sequences to event region
0.0 Mb
0.5 Mb
1.0 Mb
1.5 Mb
2.0 Mb
2.5 Mb
3.0 Mb
3.5 Mb
4.0 Mb

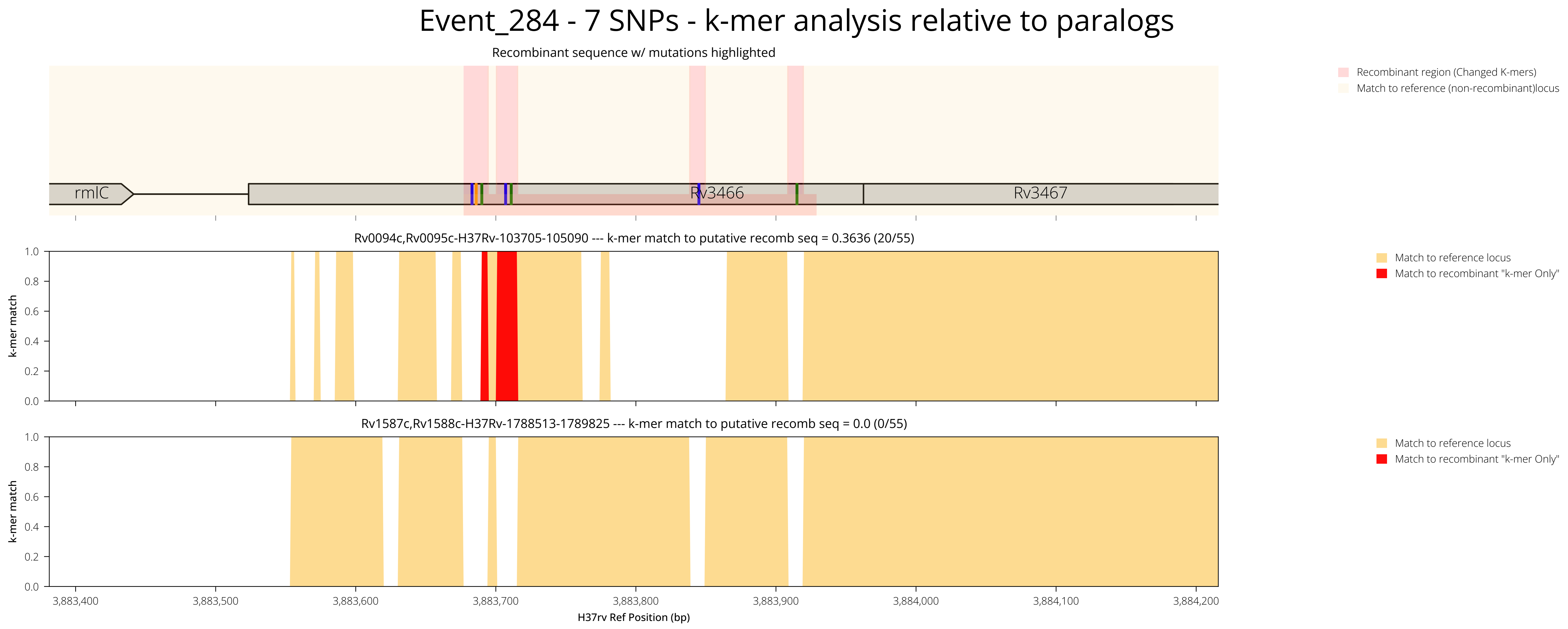
Event_284 - 7 SNPs - k-mer analysis relative to paralogs
Recombinant sequence w/ mutations highlighted
Recombinant region (Changed K-mers)
Match to reference (non-recombinant)locus
rmlC
Rv3466
Rv3467
Rv0094c,Rv0095c-H37Rv-103705-105090 --- k-mer match to putative recomb seq = 0.3636 (20/55)
Match to reference locus
Match to recombinant "k-mer Only"
k-mer match
Rv1587c,Rv1588c-H37Rv-1788513-1789825 --- k-mer match to putative recomb seq = 0.0 (0/55)
Match to reference locus
Match to recombinant "k-mer Only"
k-mer match
3,883,400
3,883,500
3,883,600
3,883,700
3,883,800
3,883,900
3,884,000
3,884,100
3,884,200
H37rv Ref Position (bp)

Sub-phylogeny Viz + Paralog Alignments
GCE ID: Event_284

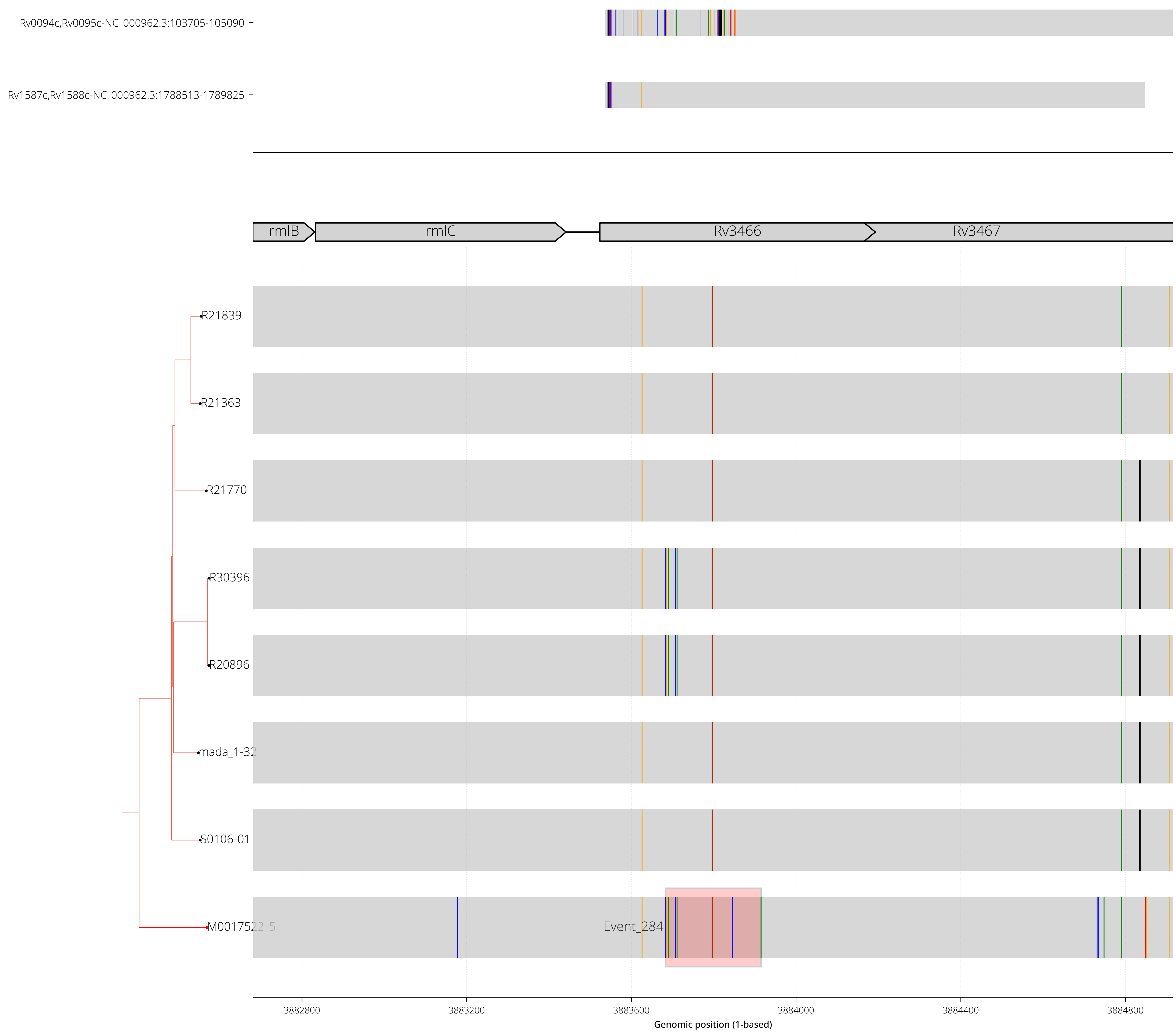

Full Phylogeny Viz + Paralog Alignments
GCE ID: Event_284

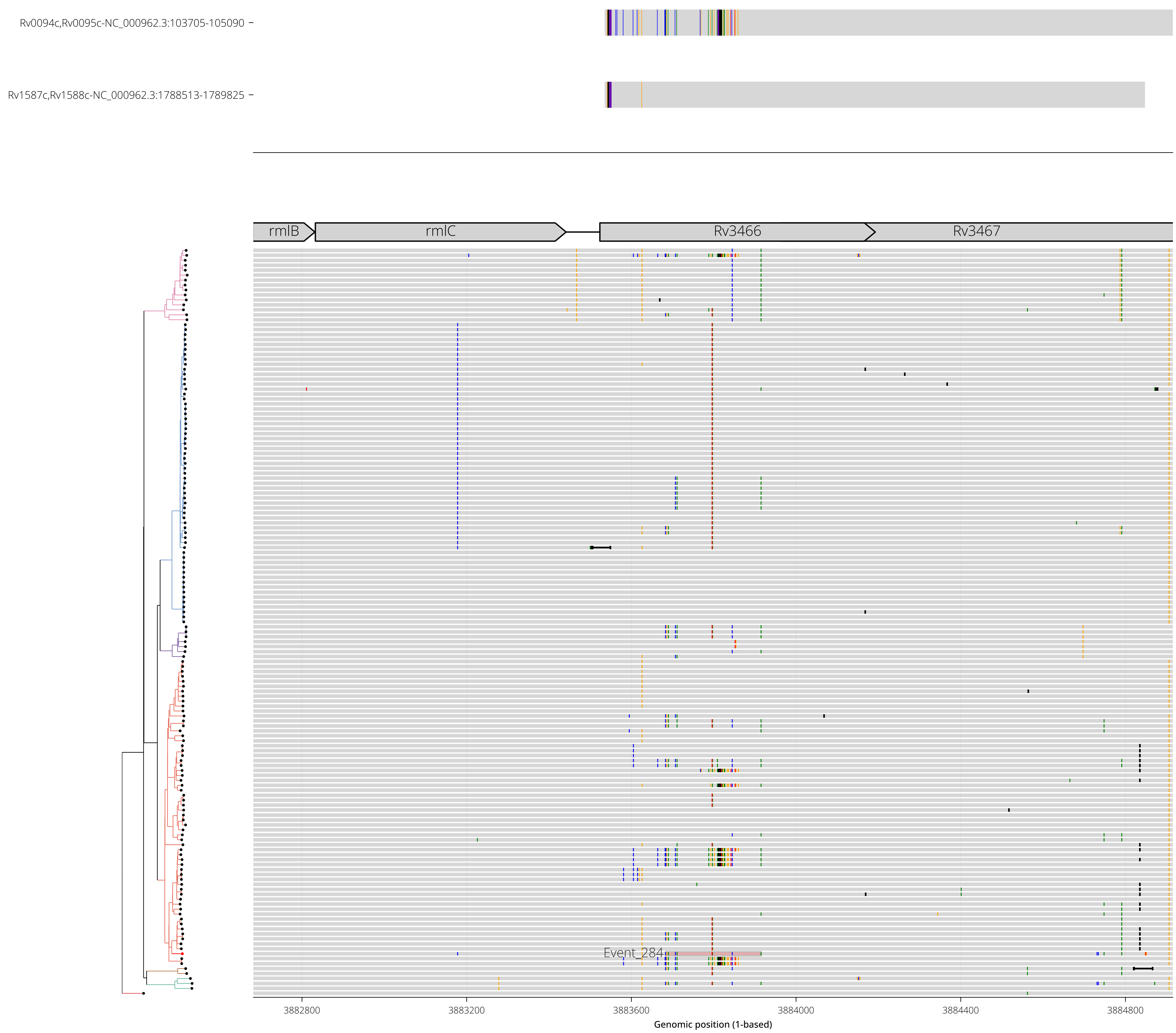

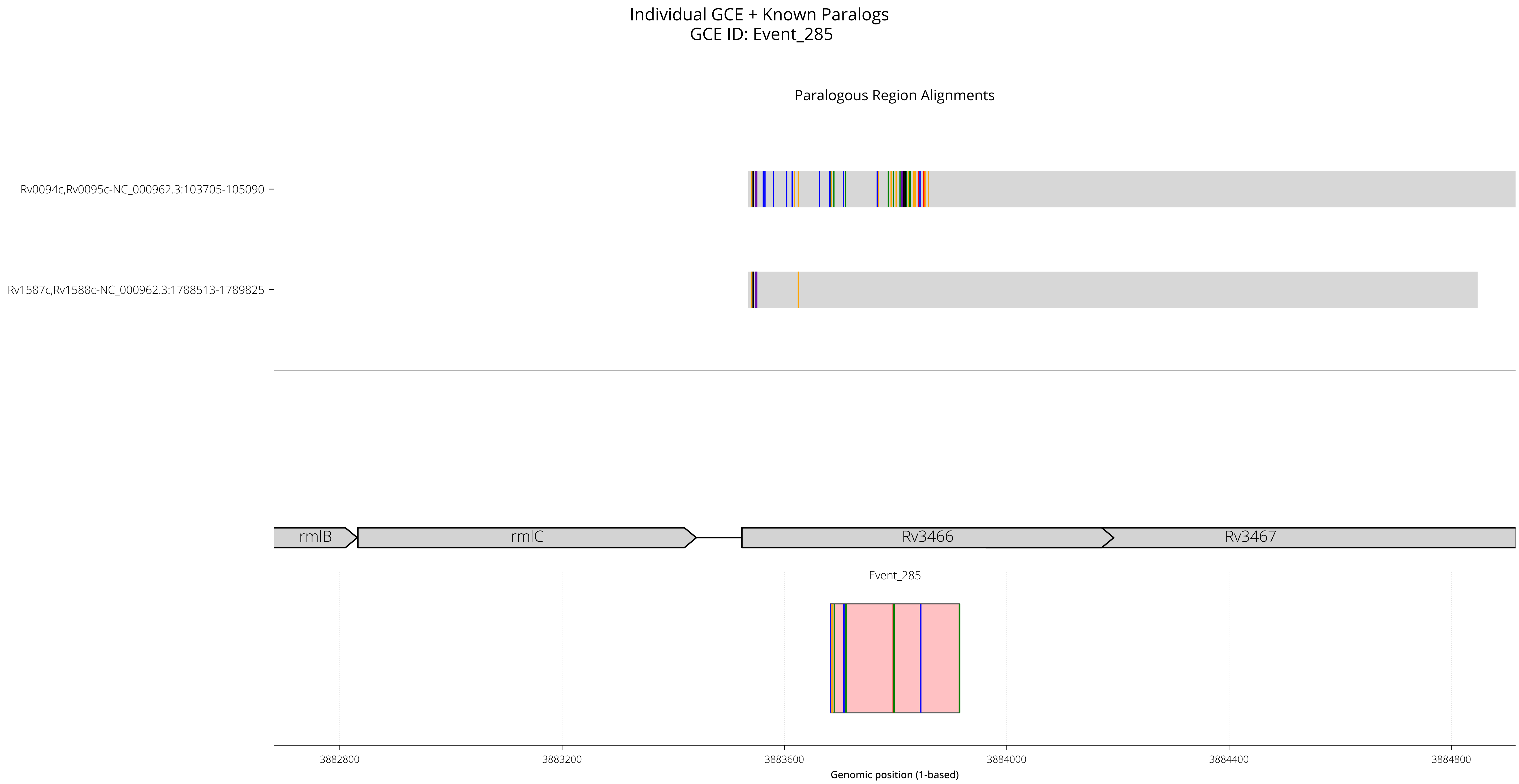

Individual GCE + Known Paralogs
GCE ID: Event_285
Paralogous Region Alignments
Rv0094c,Rv0095c-NC_000962.3:103705-105090
Rv1587c,Rv1588c-NC_000962.3:1788513-1789825
rmlB
rmlC
Rv3466
Rv3467
Event_285
3882800
3883200
3883600
3884000
3884400
3884800
Genomic position (1-based)

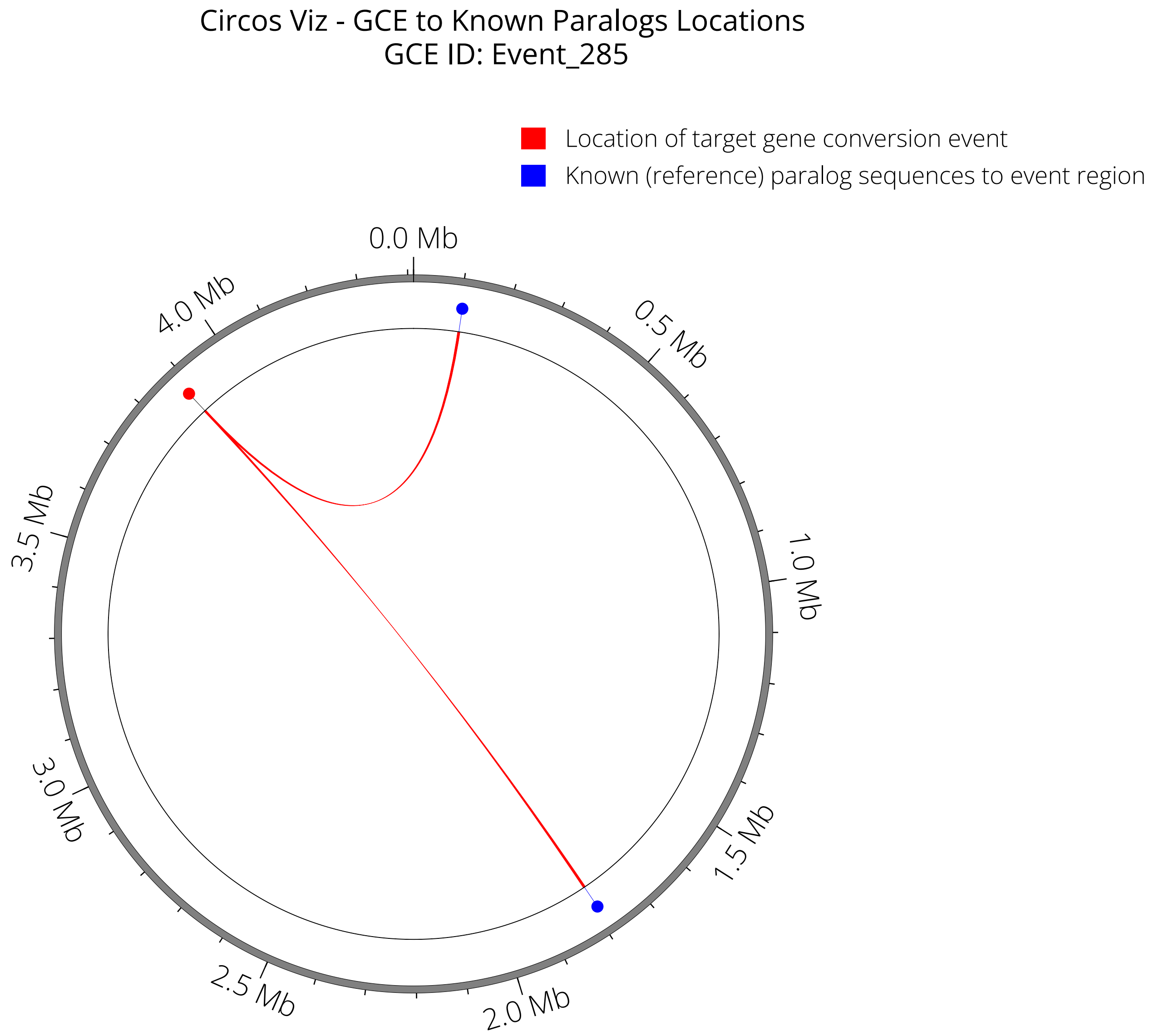

Circos Viz - GCE to Known Paralogs Locations
GCE ID: Event_285
Location of target gene conversion event
Known (reference) paralog sequences to event region
0.0 Mb
0.5 Mb
1.0 Mb
1.5 Mb
2.0 Mb
2.5 Mb
3.0 Mb
3.5 Mb
4.0 Mb

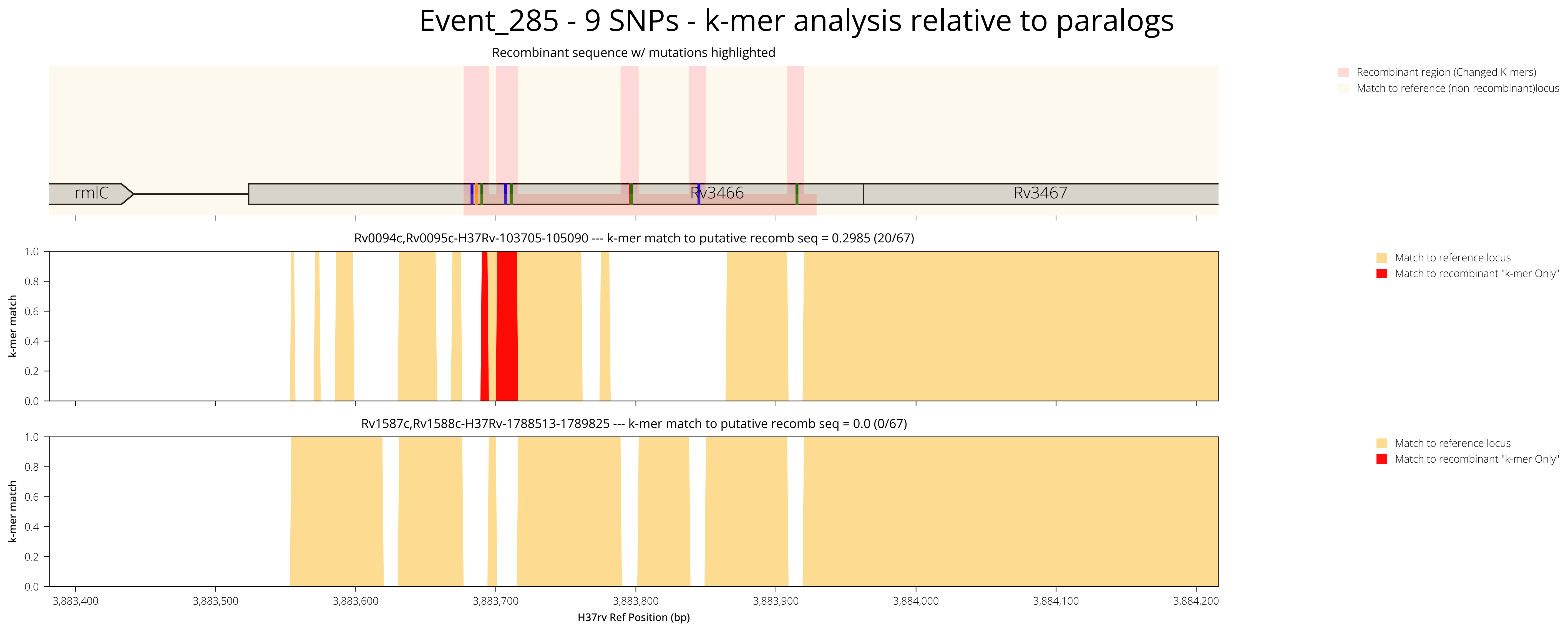

Event_285 - 9 SNPs - k-mer analysis relative to paralogs
Recombinant sequence w/ mutations highlighted
Recombinant region (Changed K-mers)
Match to reference (non-recombinant)locus
rmlC
Rv3466
Rv3467
Rv0094c,Rv0095c-H37Rv-103705-105090 --- k-mer match to putative recomb seq = 0.2985 (20/67)
Match to reference locus
Match to recombinant "k-mer Only"
k-mer match
Rv1587c,Rv1588c-H37Rv-1788513-1789825 --- k-mer match to putative recomb seq = 0.0 (0/67)
Match to reference locus
Match to recombinant "k-mer Only"
k-mer match
3,883,400
3,883,500
3,883,600
3,883,700
3,883,800
3,883,900
3,884,000
3,884,100
3,884,200
H37rv Ref Position (bp)

# Sub-phylogeny Viz + Paralog Alignments
GCE ID: Event_285

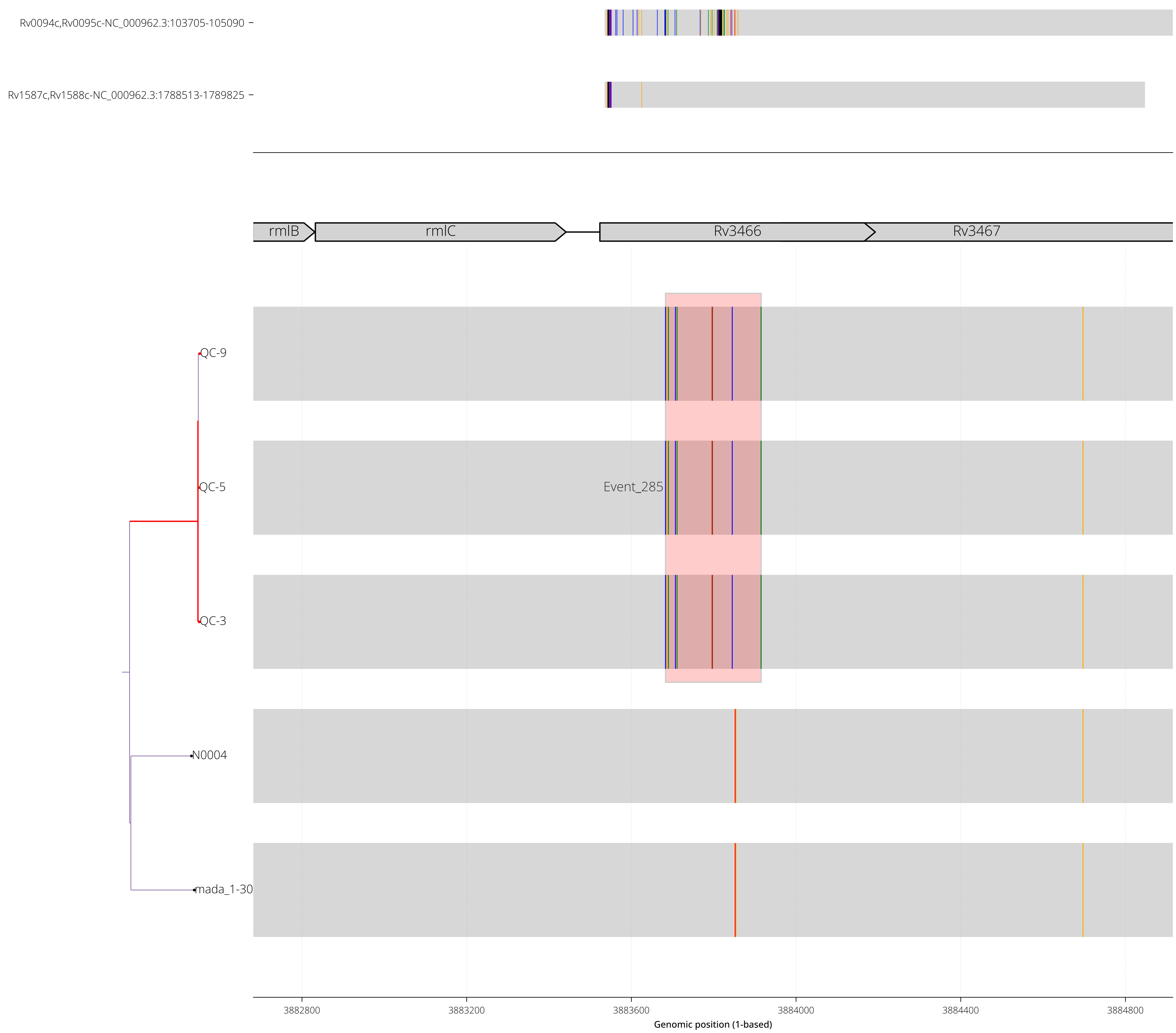

Full Phylogeny Viz + Paralog Alignments
GCE ID: Event_285

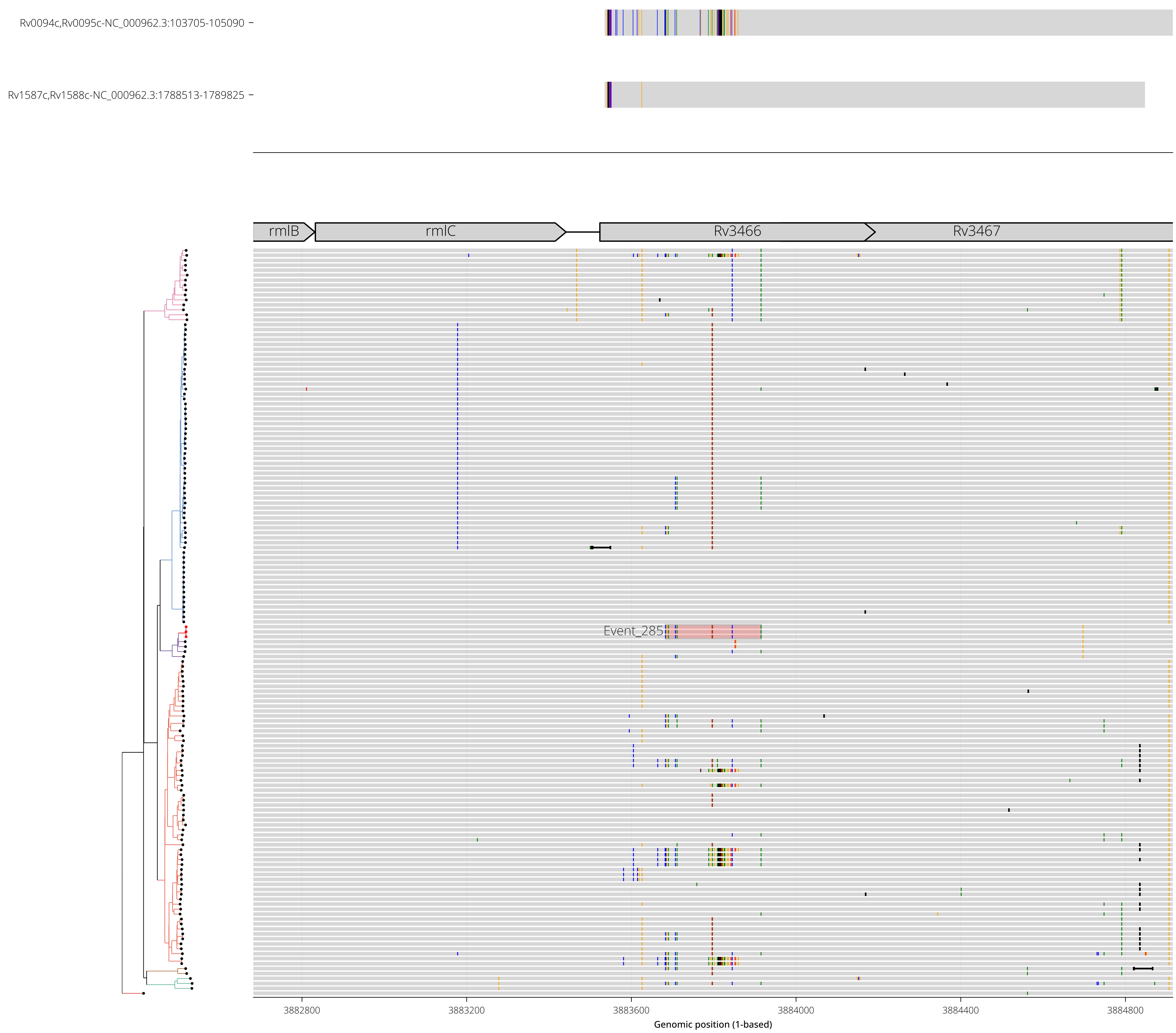

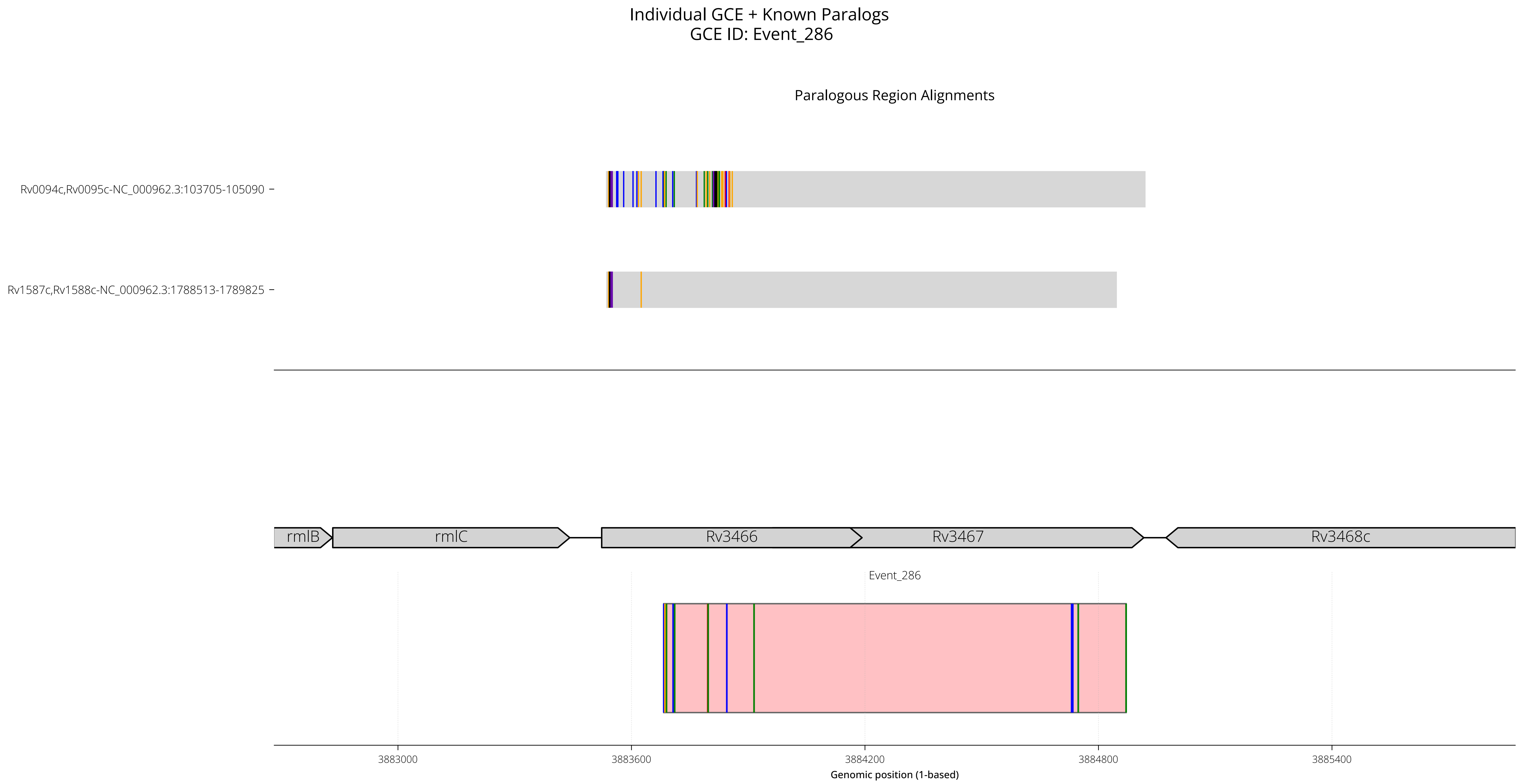
Individual GCE + Known Paralogs
GCE ID: Event_286
Paralogous Region Alignments
Rv0094c,Rv0095c-NC_000962.3:103705-105090
Rv1587c,Rv1588c-NC_000962.3:1788513-1789825
rmlB
rmlC
Rv3466
Rv3467
Rv3468c
Event_286
3883000
3883600
3884200
3884800
3885400
Genomic position (1-based)

Circos Viz - GCE to Known Paralogs Locations
GCE ID: Event_286

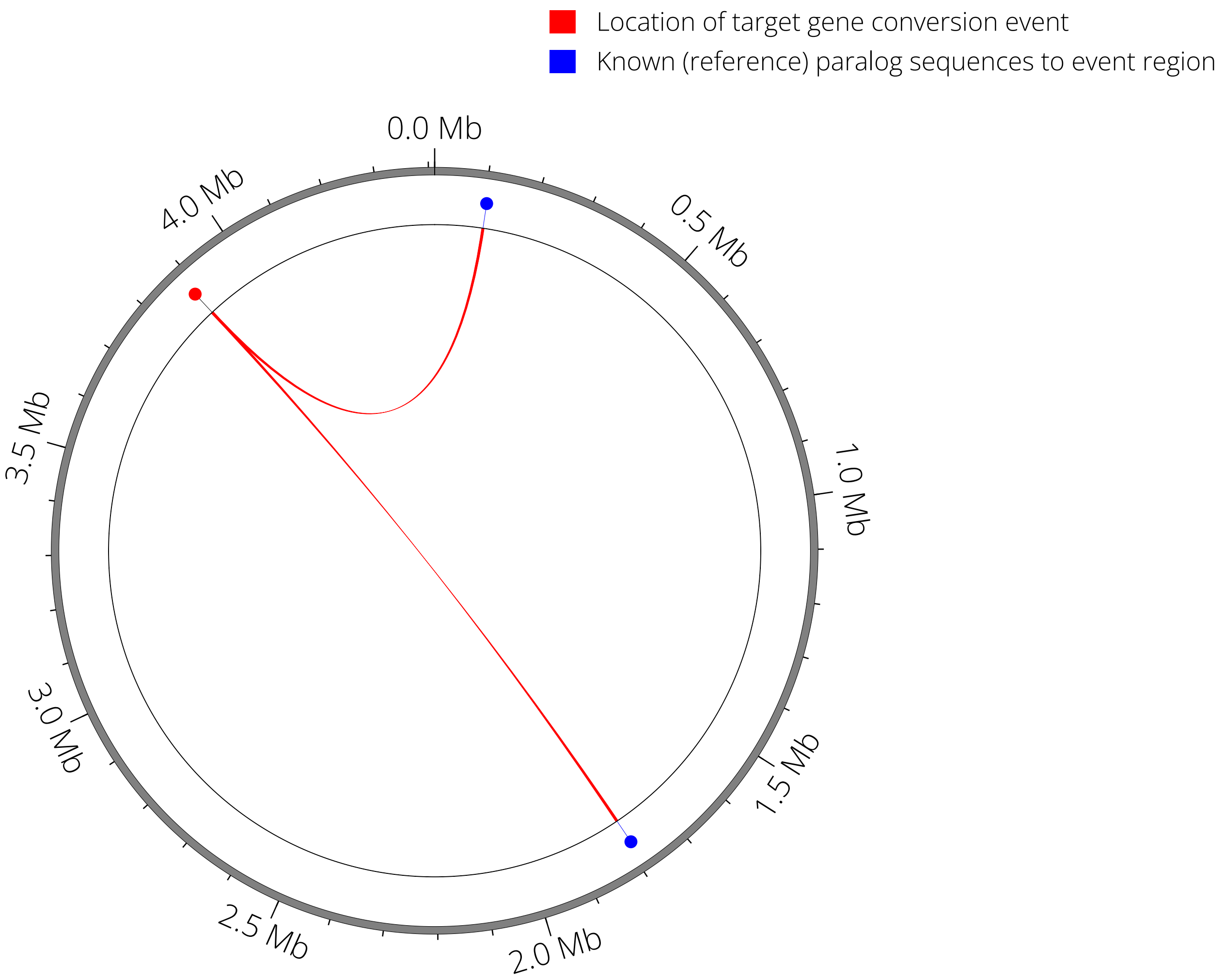

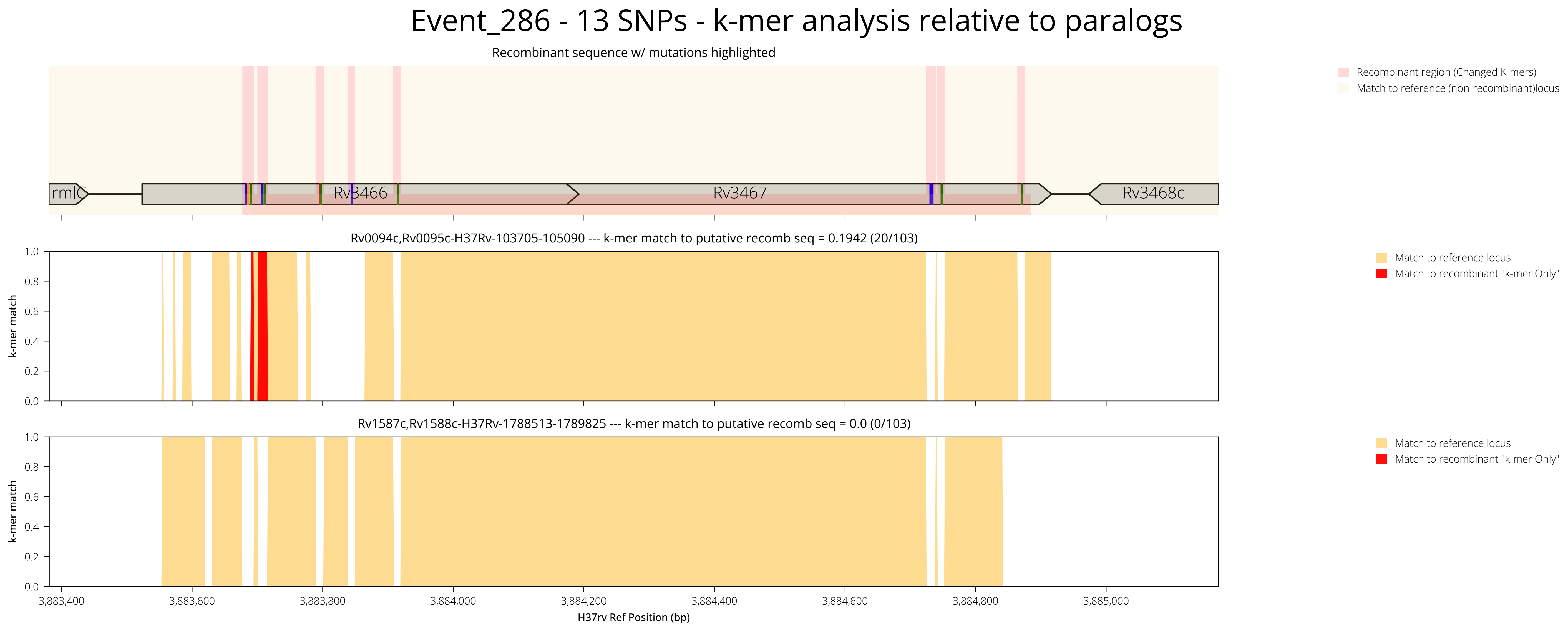

Event_286 - 13 SNPs - k-mer analysis relative to paralogs
Recombinant sequence w/ mutations highlighted
rmlC
Rv3466
Rv3467
Rv3468c
Recombinant region (Changed K-mers)
Match to reference (non-recombinant)locus
Rv0094c,Rv0095c-H37Rv-103705-105090 --- k-mer match to putative recomb seq = 0.1942 (20/103)
k-mer match
Match to reference locus
Match to recombinant "k-mer Only"
Rv1587c,Rv1588c-H37Rv-1788513-1789825 --- k-mer match to putative recomb seq = 0.0 (0/103)
k-mer match
Match to reference locus
Match to recombinant "k-mer Only"
0.0
0.2
0.4
0.6
0.8
1.0
3,883,400
3,883,600
3,883,800
3,884,000
3,884,200
3,884,400
3,884,600
3,884,800
3,885,000
H37rv Ref Position (bp)

Sub-phylogeny Viz + Paralog Alignments
GCE ID: Event_286

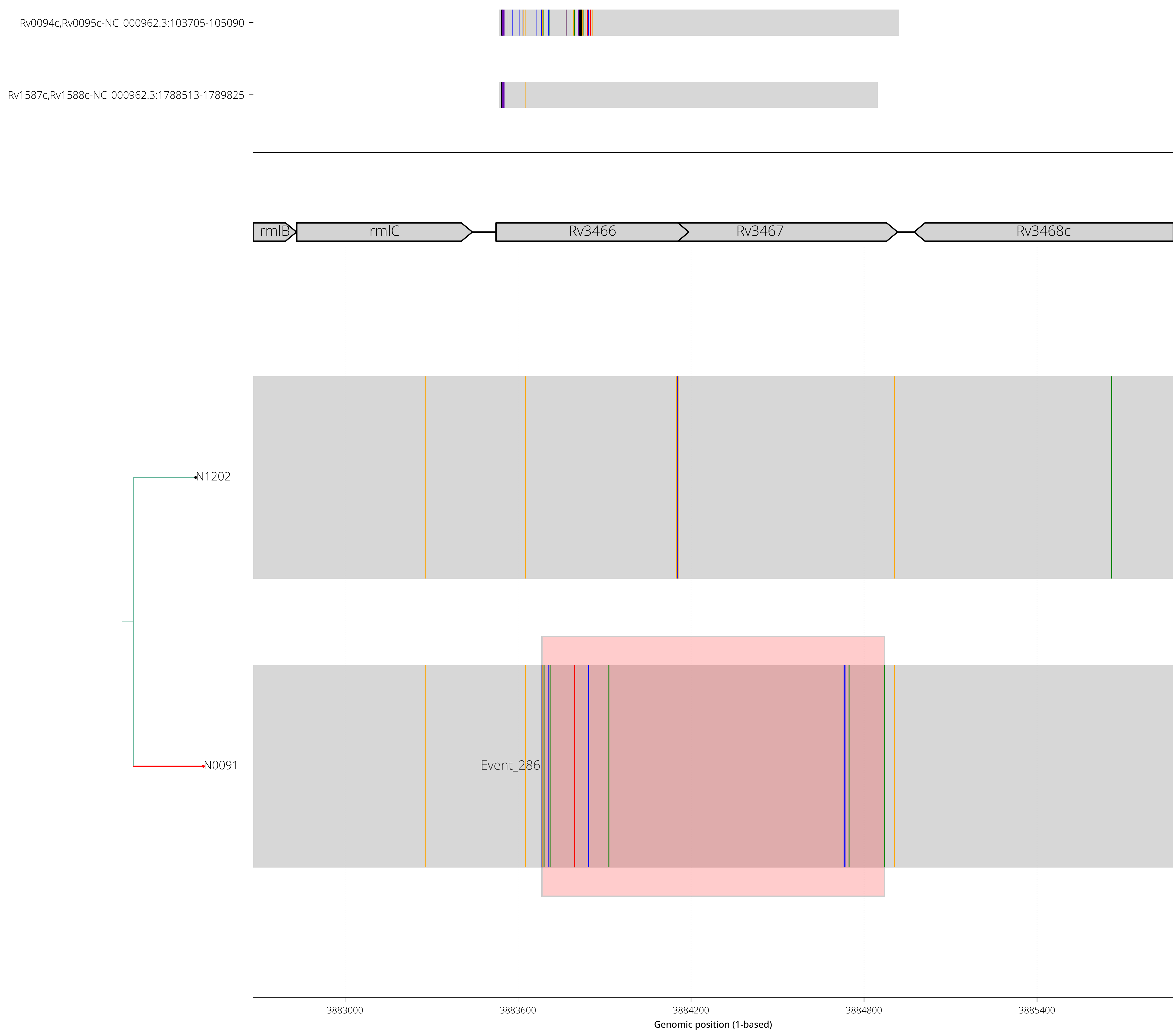

Full Phylogeny Viz + Paralog Alignments
GCE ID: Event_286

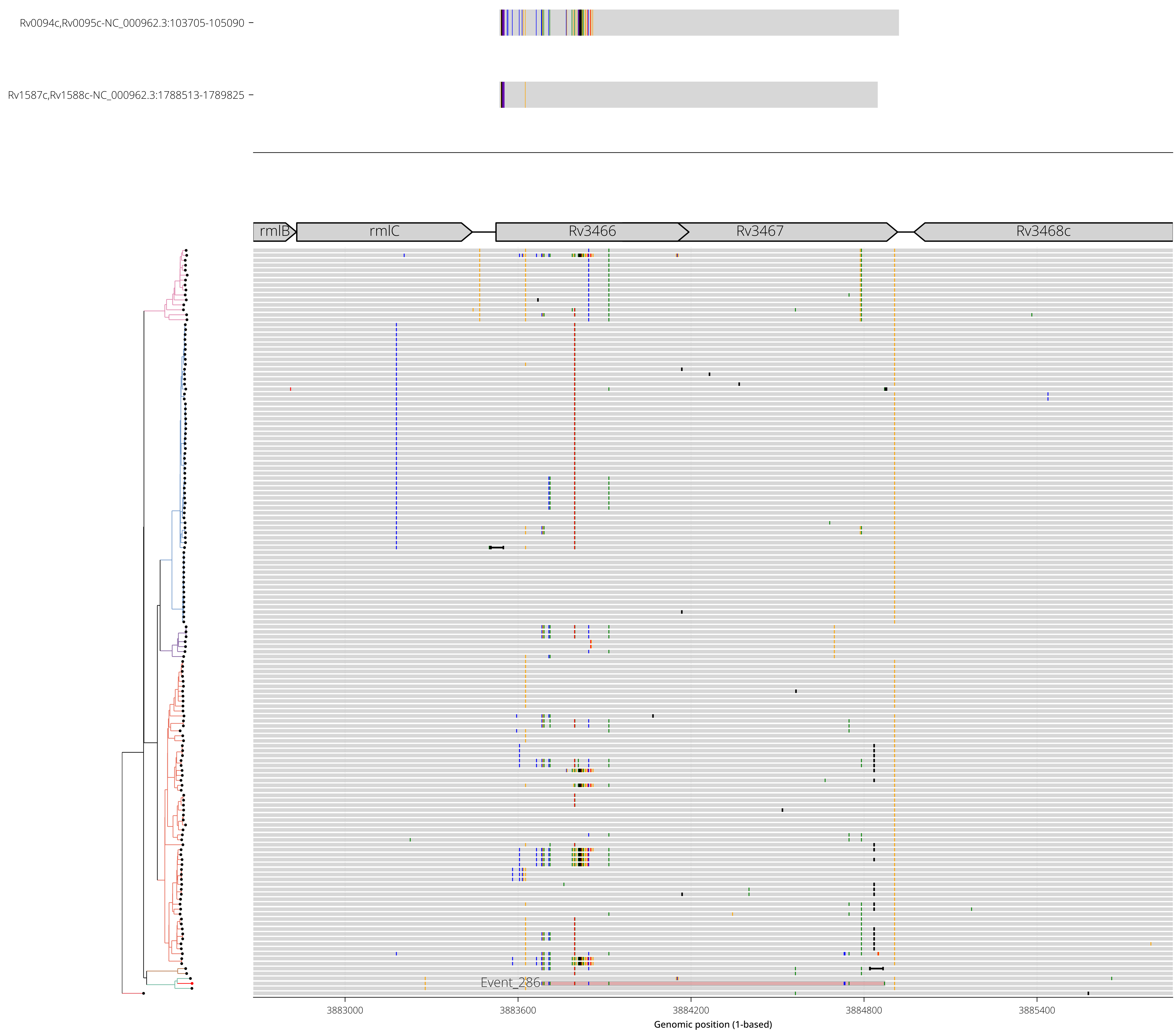

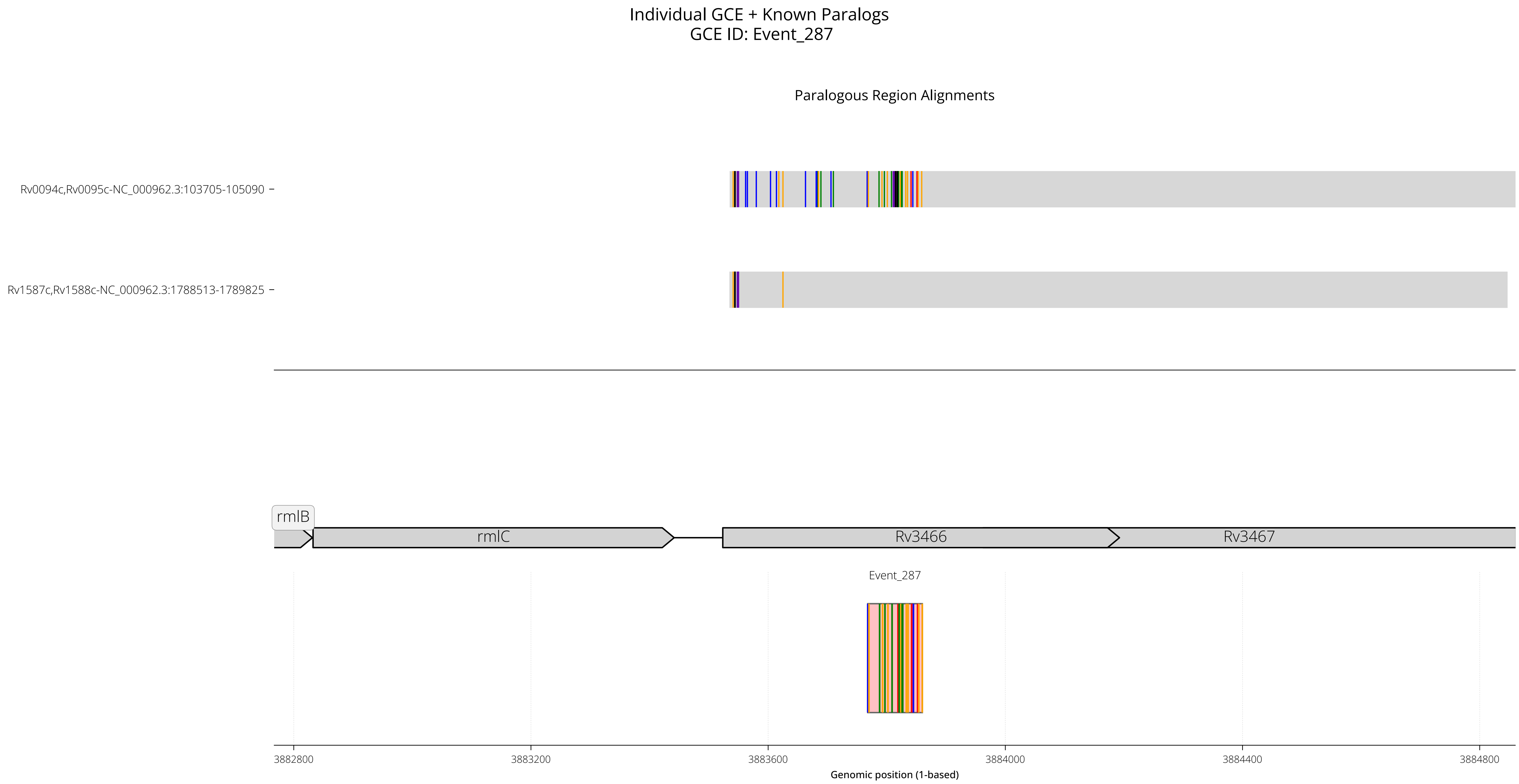

Individual GCE + Known Paralogs
GCE ID: Event_287
Paralogous Region Alignments
Rv0094c,Rv0095c-NC_000962.3:103705-105090
Rv1587c,Rv1588c-NC_000962.3:1788513-1789825
rmlB
rmlC
Rv3466
Rv3467
Event_287
3882800
3883200
3883600
3884000
3884400
3884800
Genomic position (1-based)

Circos Viz - GCE to Known Paralogs Locations
GCE ID: Event_287

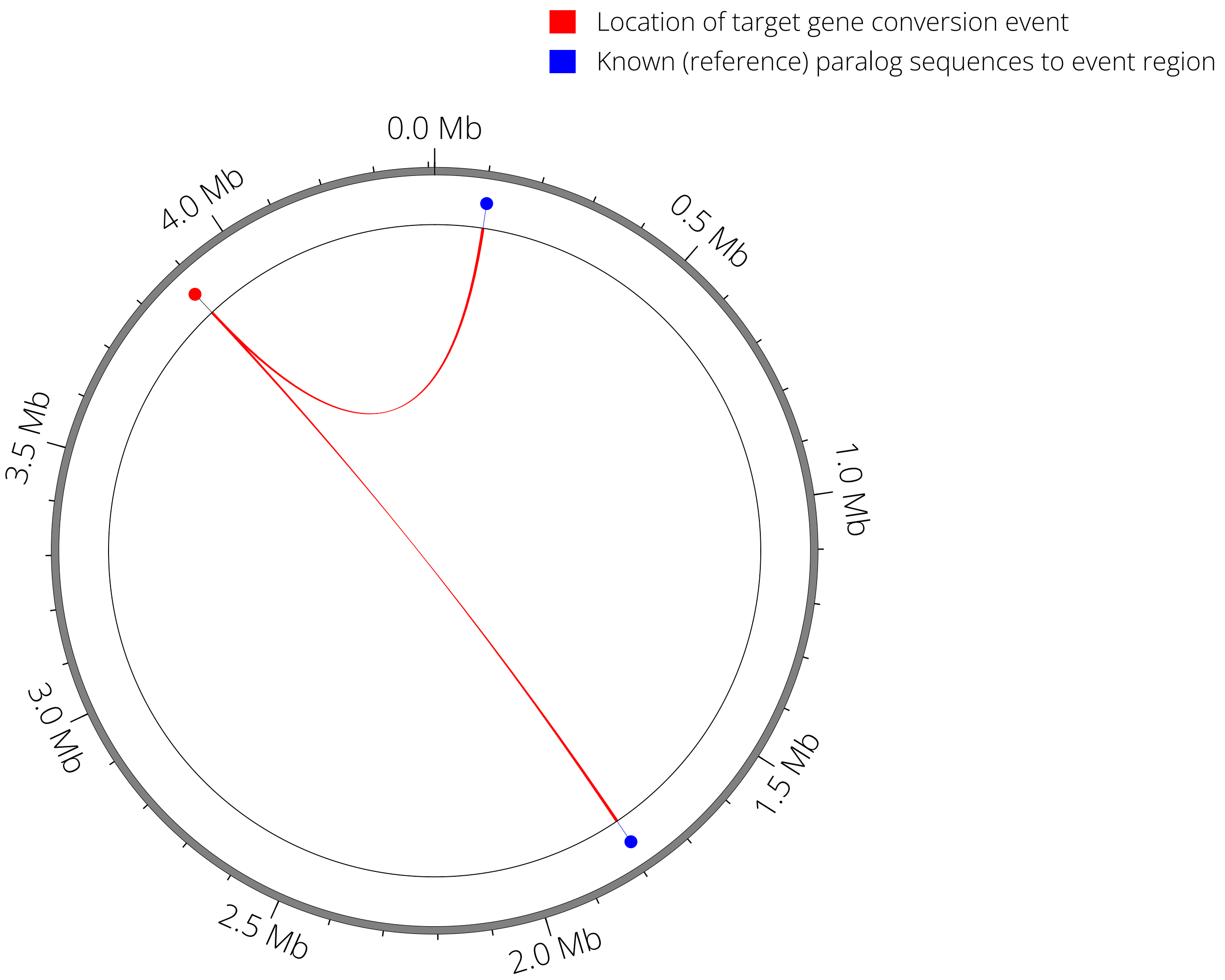

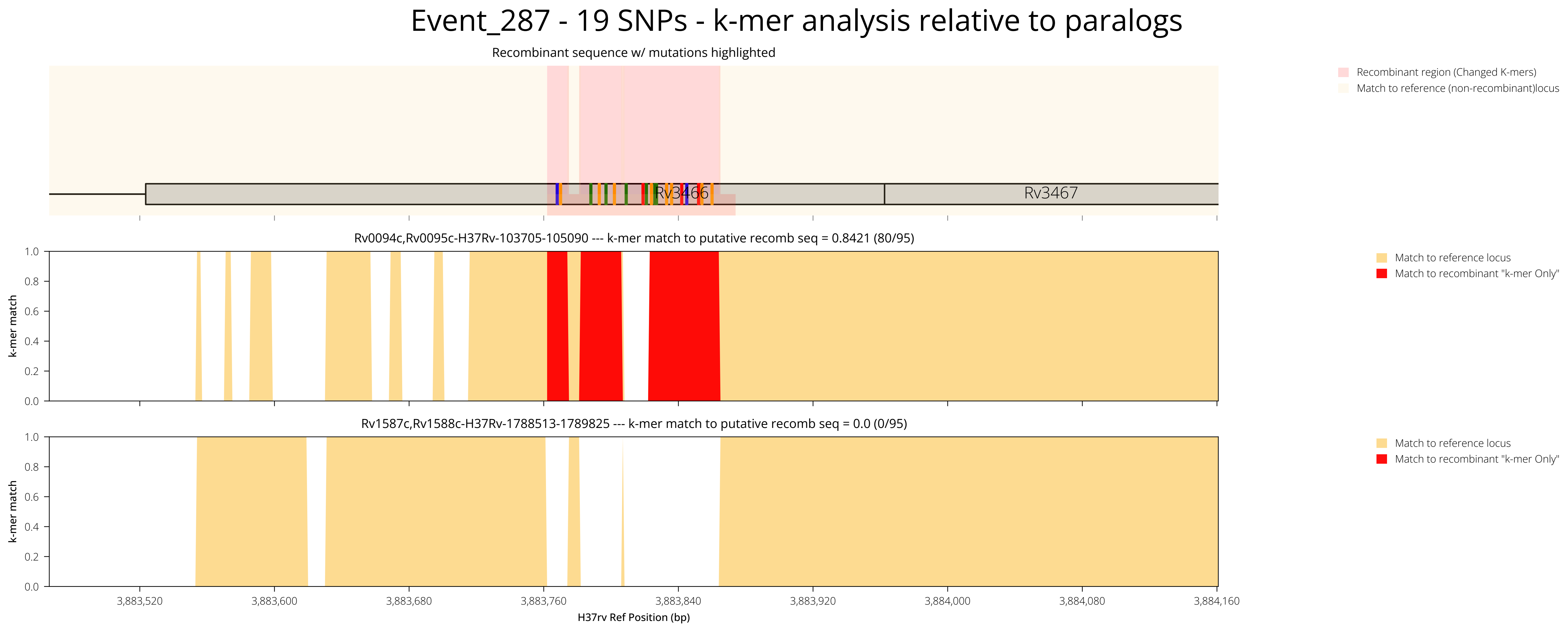
Event_287 - 19 SNPs - k-mer analysis relative to paralogs
Recombinant sequence w/ mutations highlighted
Recombinant region (Changed K-mers)
Match to reference (non-recombinant)locus
Rv3466
Rv3467
Rv0094c,Rv0095c-H37Rv-103705-105090 --- k-mer match to putative recomb seq = 0.8421 (80/95)
Match to reference locus
Match to recombinant "k-mer Only"
Rv1587c,Rv1588c-H37Rv-1788513-1789825 --- k-mer match to putative recomb seq = 0.0 (0/95)
Match to reference locus
Match to recombinant "k-mer Only"
k-mer match
1.0
0.8
0.6
0.4
0.2
0.0
3,883,520
3,883,600
3,883,680
3,883,760
3,883,840
3,883,920
3,884,000
3,884,080
3,884,160
H37rv Ref Position (bp)

Sub-phylogeny Viz + Paralog Alignments
GCE ID: Event_287

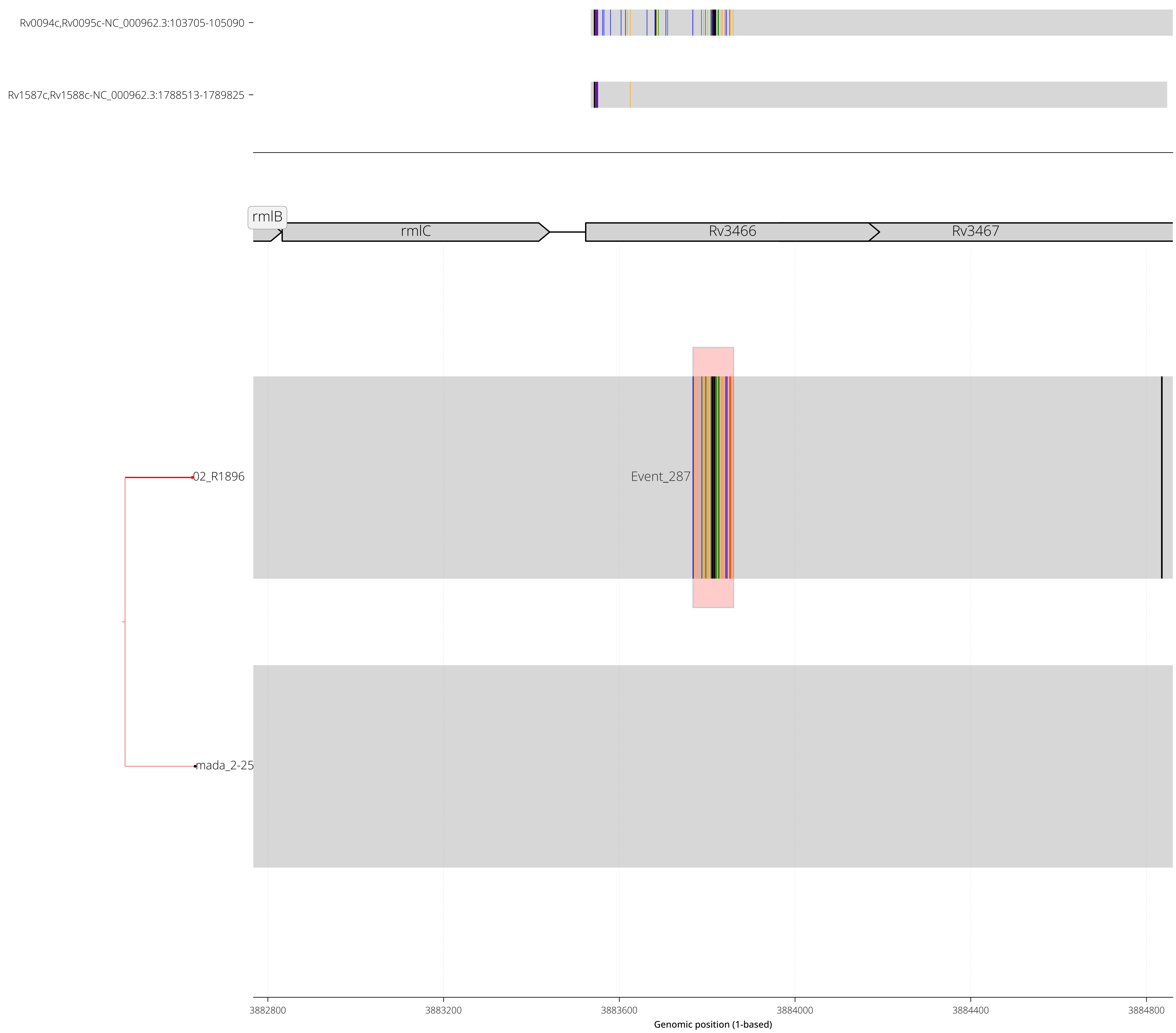

Full Phylogeny Viz + Paralog Alignments
GCE ID: Event_287

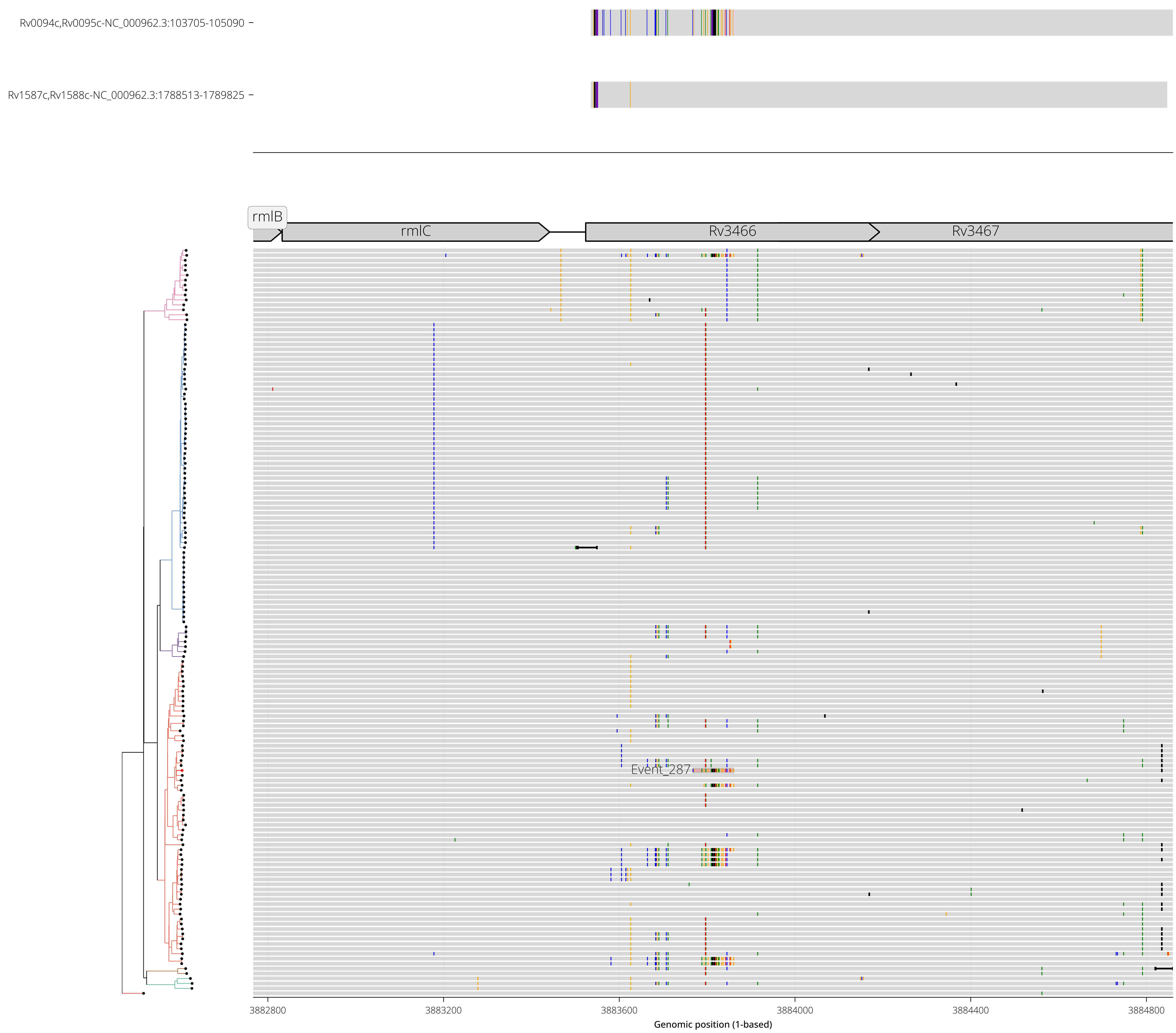

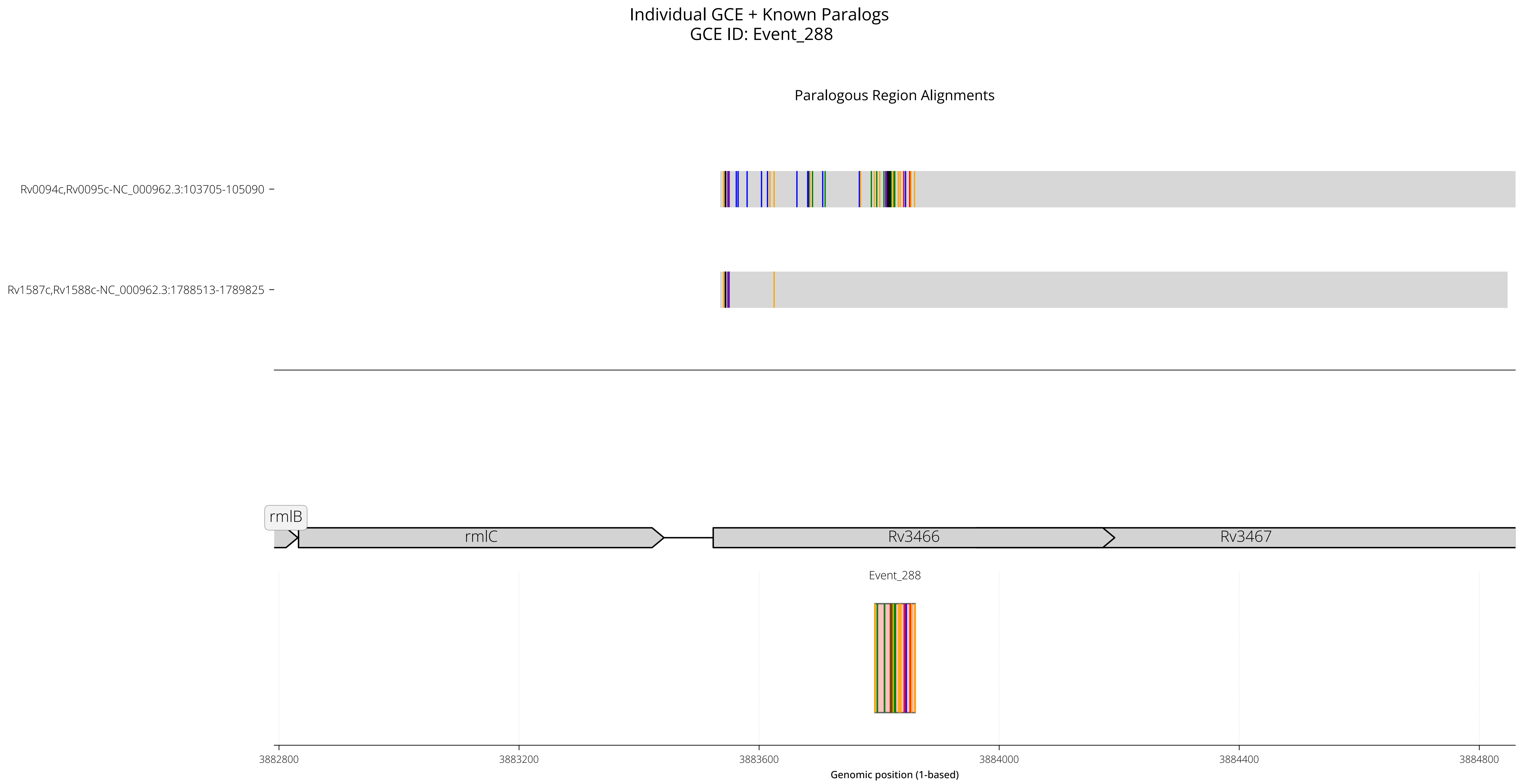

Individual GCE + Known Paralogs
GCE ID: Event_288
Paralogous Region Alignments
Rv0094c,Rv0095c-NC_000962.3:103705-105090
Rv1587c,Rv1588c-NC_000962.3:1788513-1789825
rmlB
rmlC
Rv3466
Rv3467
Event_288
3882800
3883200
3883600
3884000
3884400
3884800
Genomic position (1-based)

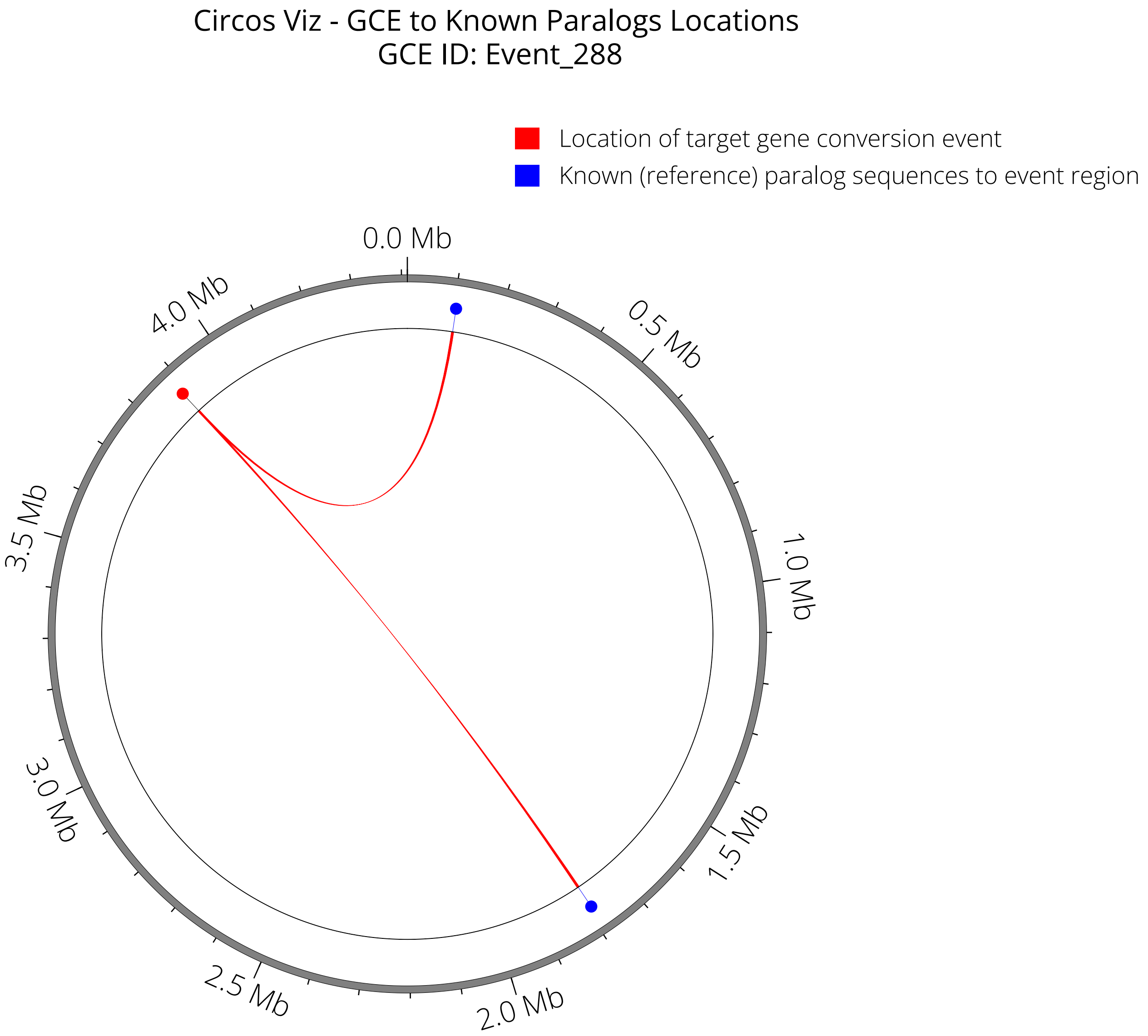
Circos Viz - GCE to Known Paralogs Locations
GCE ID: Event_288
Location of target gene conversion event
Known (reference) paralog sequences to event region
0.0 Mb
0.5 Mb
1.0 Mb
1.5 Mb
2.0 Mb
2.5 Mb
3.0 Mb
3.5 Mb
4.0 Mb

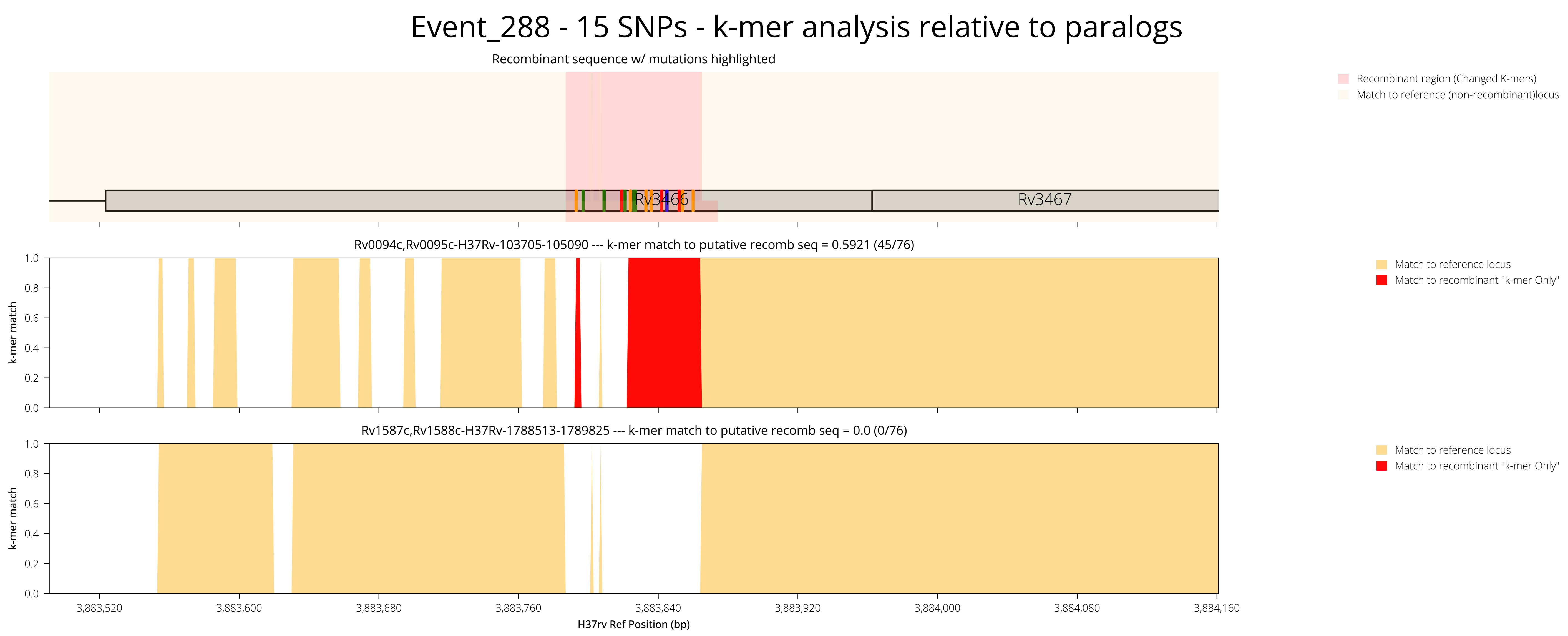
Event_288 - 15 SNPs - k-mer analysis relative to paralogs
Recombinant sequence w/ mutations highlighted
Recombinant region (Changed K-mers)
Match to reference (non-recombinant)locus
Rv3466
Rv3467
Rv0094c,Rv0095c-H37Rv-103705-105090 --- k-mer match to putative recomb seq = 0.5921 (45/76)
Match to reference locus
Match to recombinant "k-mer Only"
k-mer match
1.0
0.8
0.6
0.4
0.2
0.0
Rv1587c,Rv1588c-H37Rv-1788513-1789825 --- k-mer match to putative recomb seq = 0.0 (0/76)
Match to reference locus
Match to recombinant "k-mer Only"
k-mer match
3,883,520
3,883,600
3,883,680
3,883,760
3,883,840
3,883,920
3,884,000
3,884,080
3,884,160
H37rv Ref Position (bp)

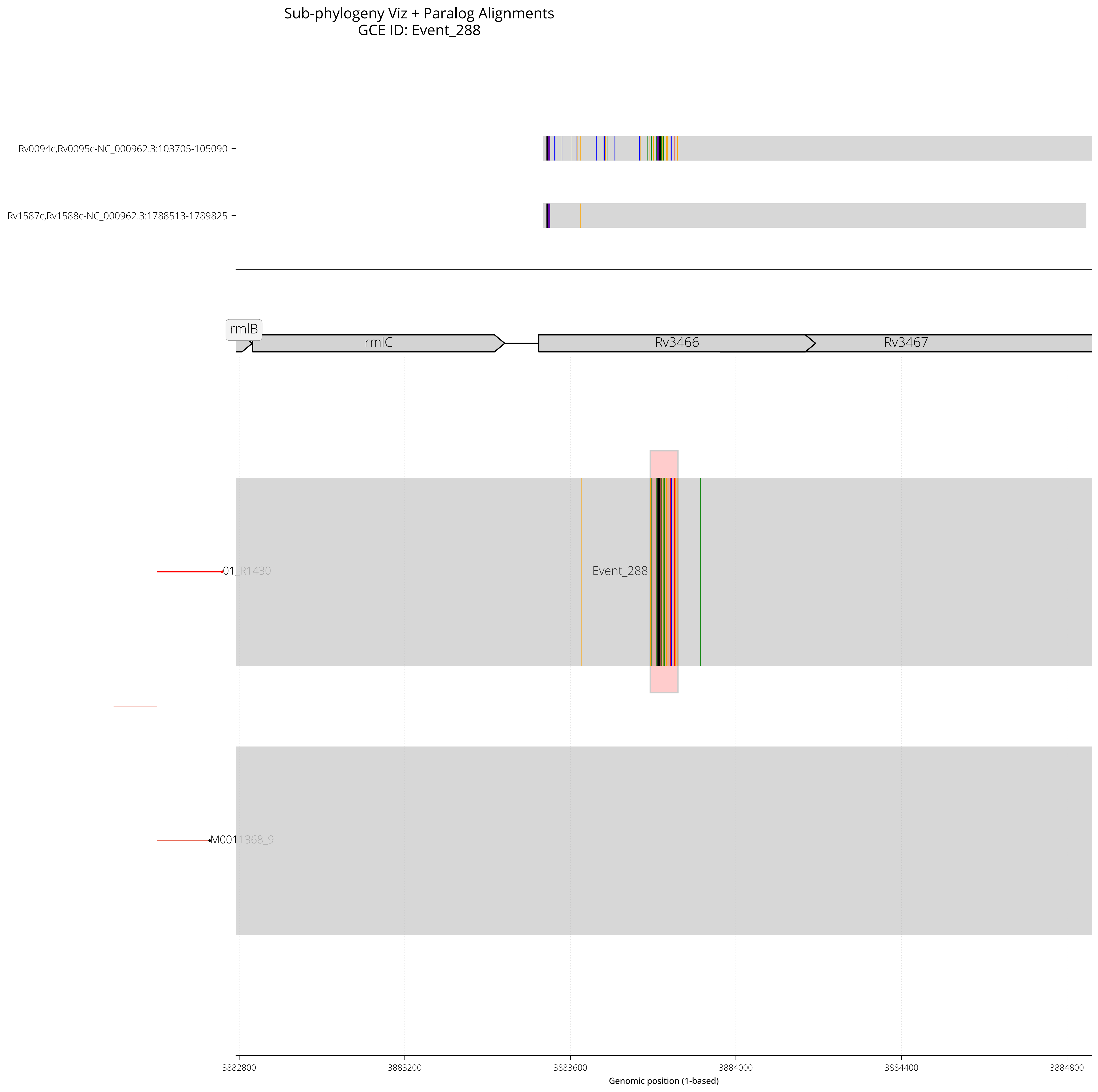
Sub-phylogeny Viz + Paralog Alignments
GCE ID: Event_288
Rv0094c,Rv0095c-NC_000962.3:103705-105090
Rv1587c,Rv1588c-NC_000962.3:1788513-1789825
rmlB
rmlC
Rv3466
Rv3467
01_R1430
Event_288
M0011368_9
3882800
3883200
3883600
3884000
3884400
3884800
Genomic position (1-based)

## Full Phylogeny Viz + Paralog Alignments
## GCE ID: Event_288

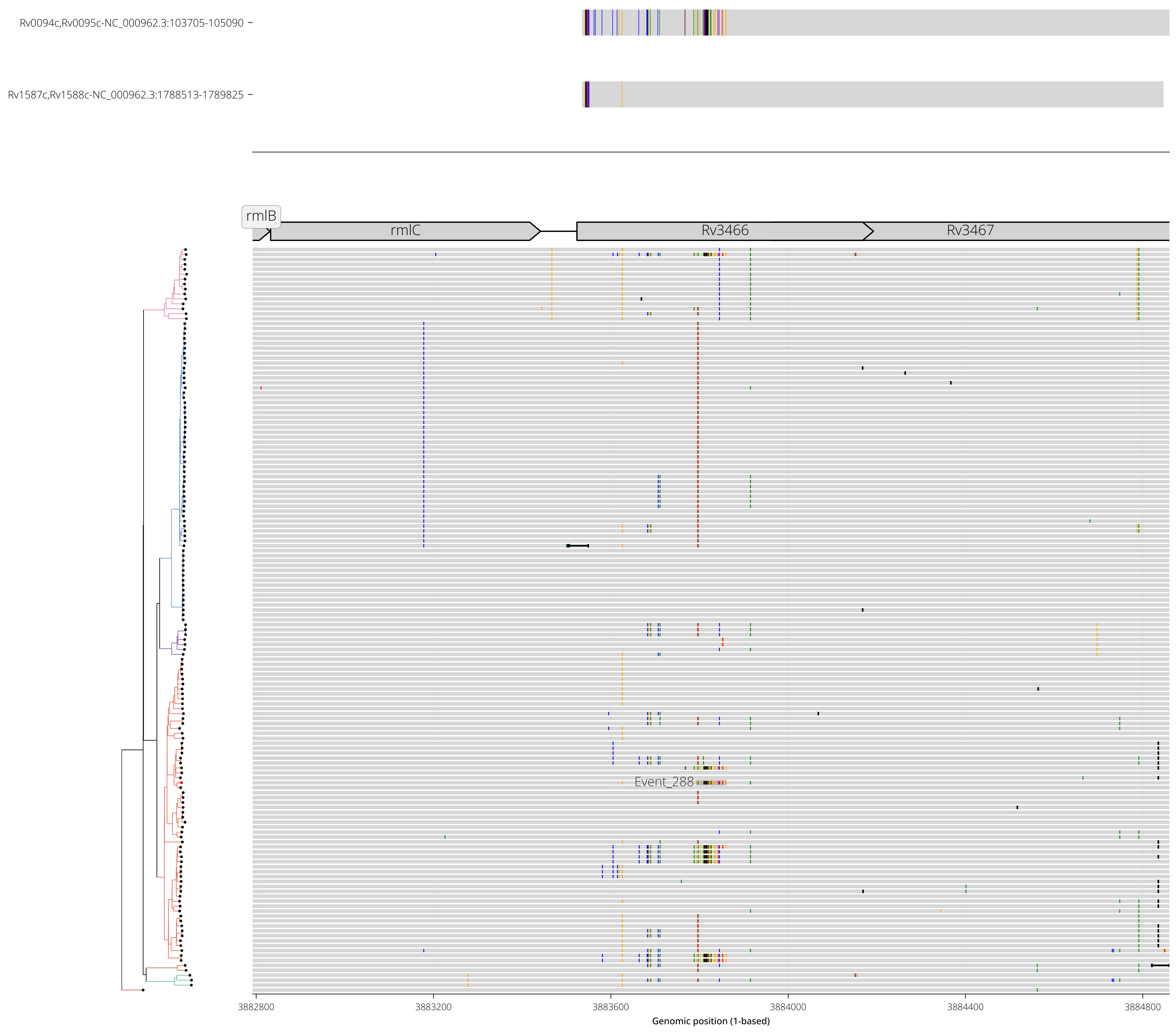

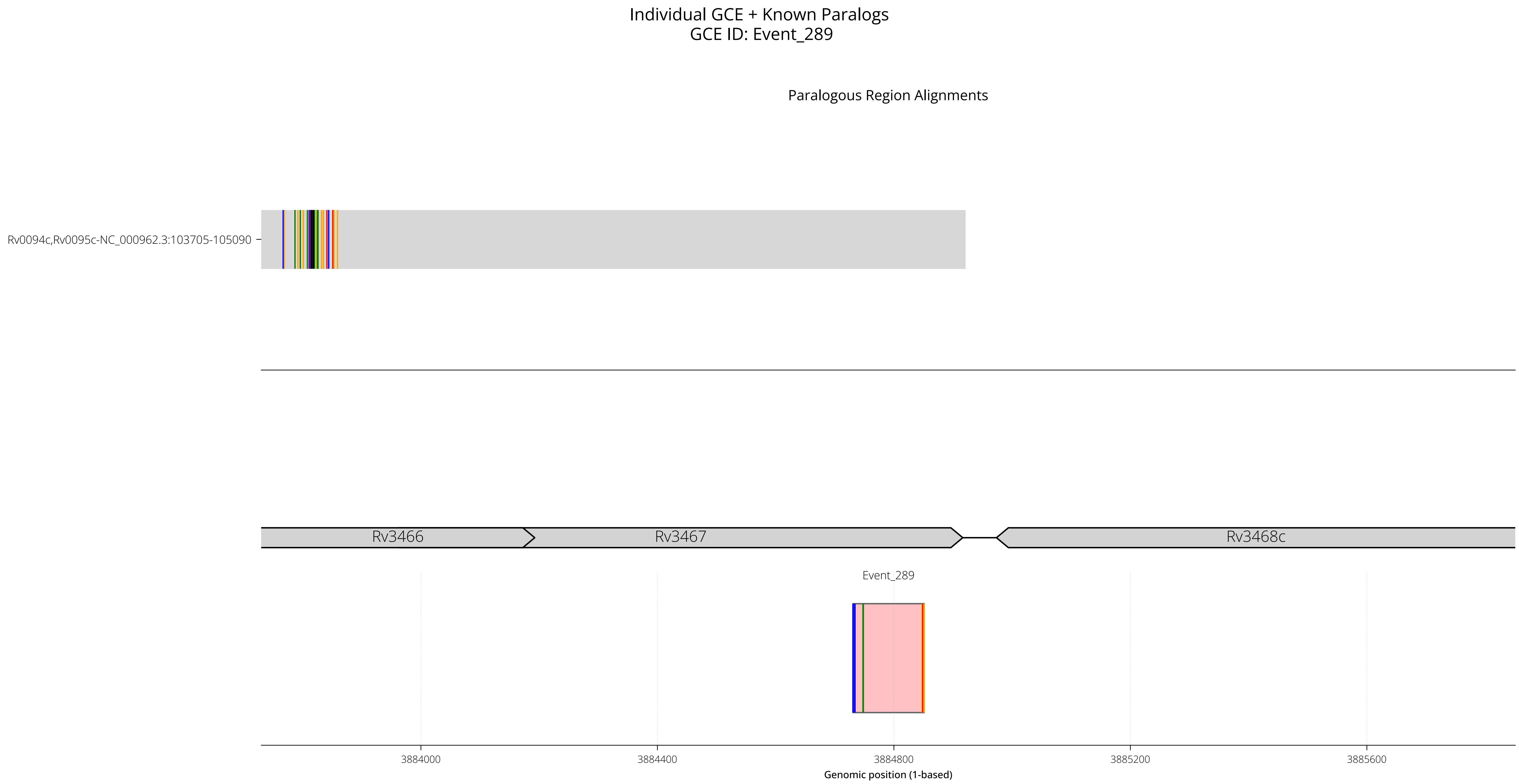

Individual GCE + Known Paralogs
GCE ID: Event_289
Paralogous Region Alignments
Rv0094c,Rv0095c-NC_000962.3:103705-105090
Rv3466
Rv3467
Rv3468c
Event_289
3884000
3884400
3884800
3885200
3885600
Genomic position (1-based)

Circos Viz - GCE to Known Paralogs Locations
GCE ID: Event_289

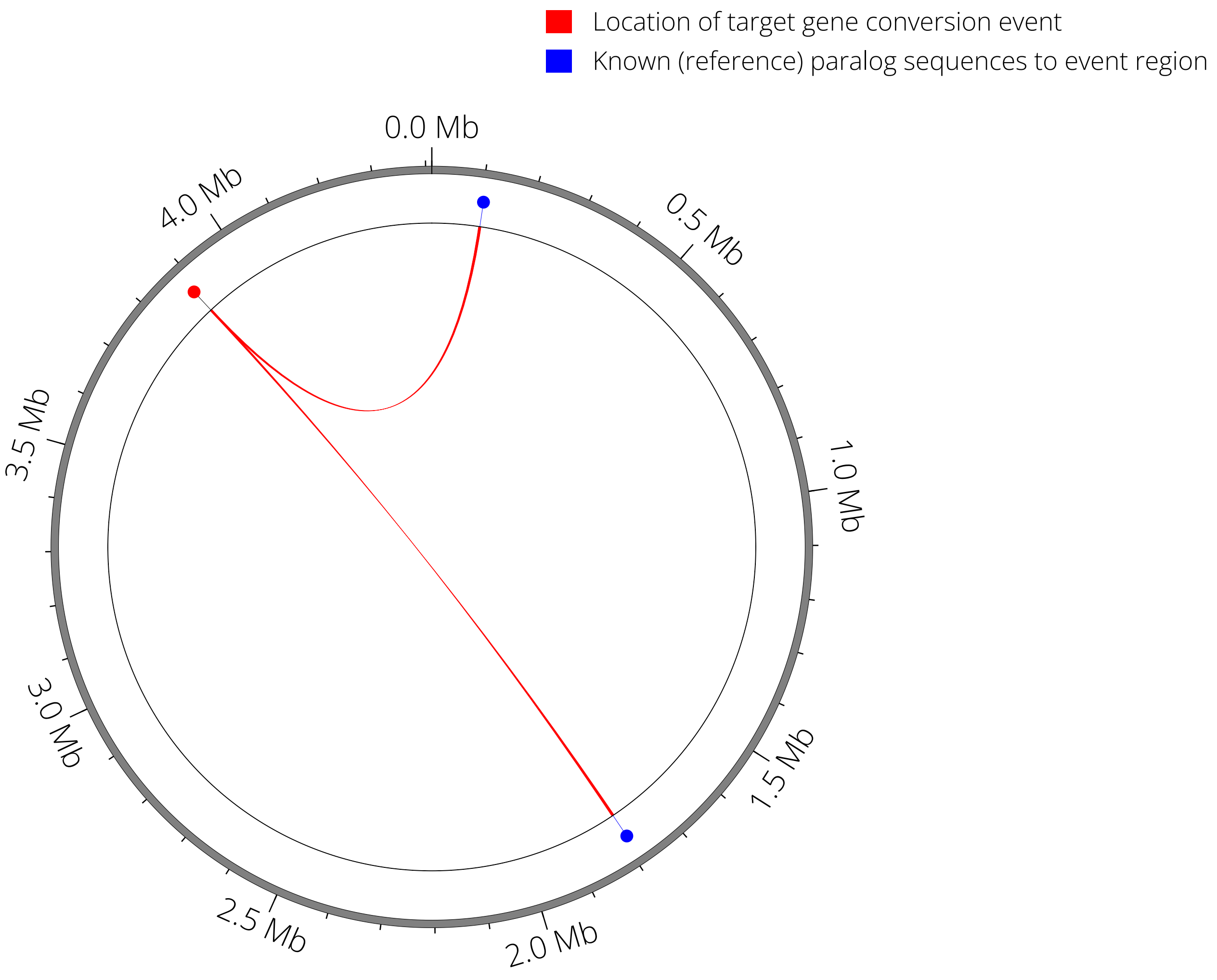

# Event_289 - 6 SNPs - k-mer analysis relative to paralogs

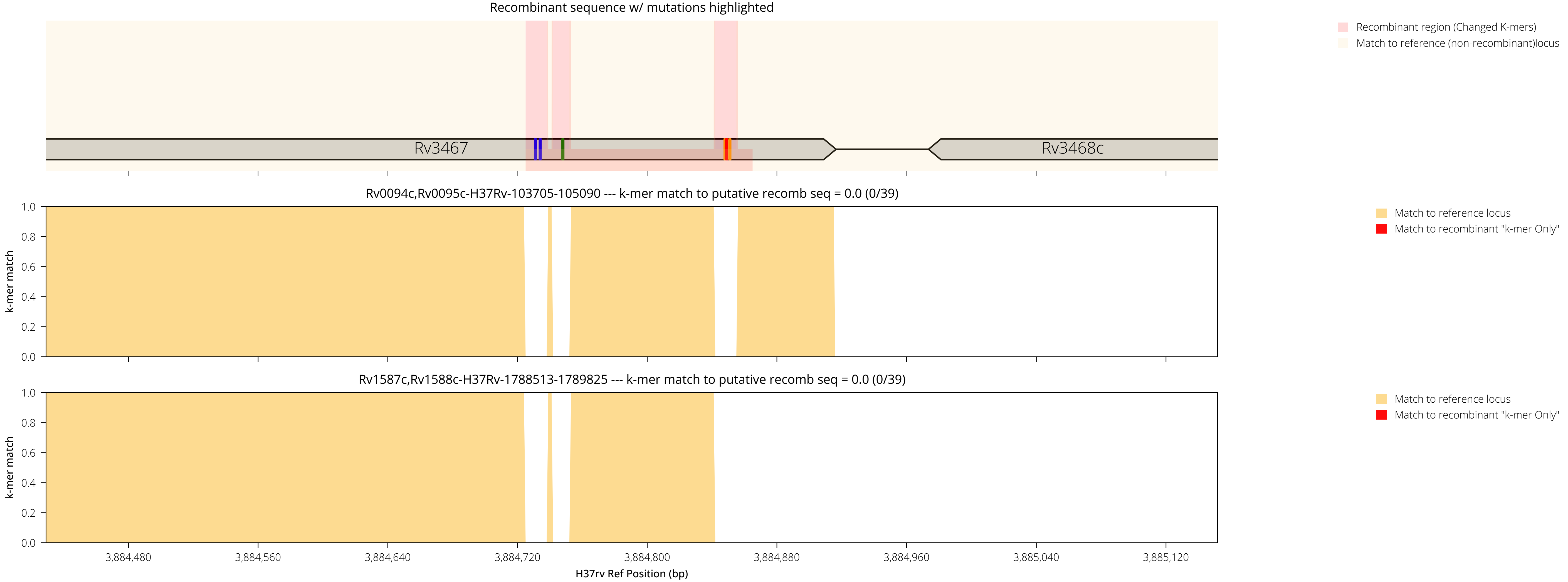

Sub-phylogeny Viz + Paralog Alignments
GCE ID: Event_289

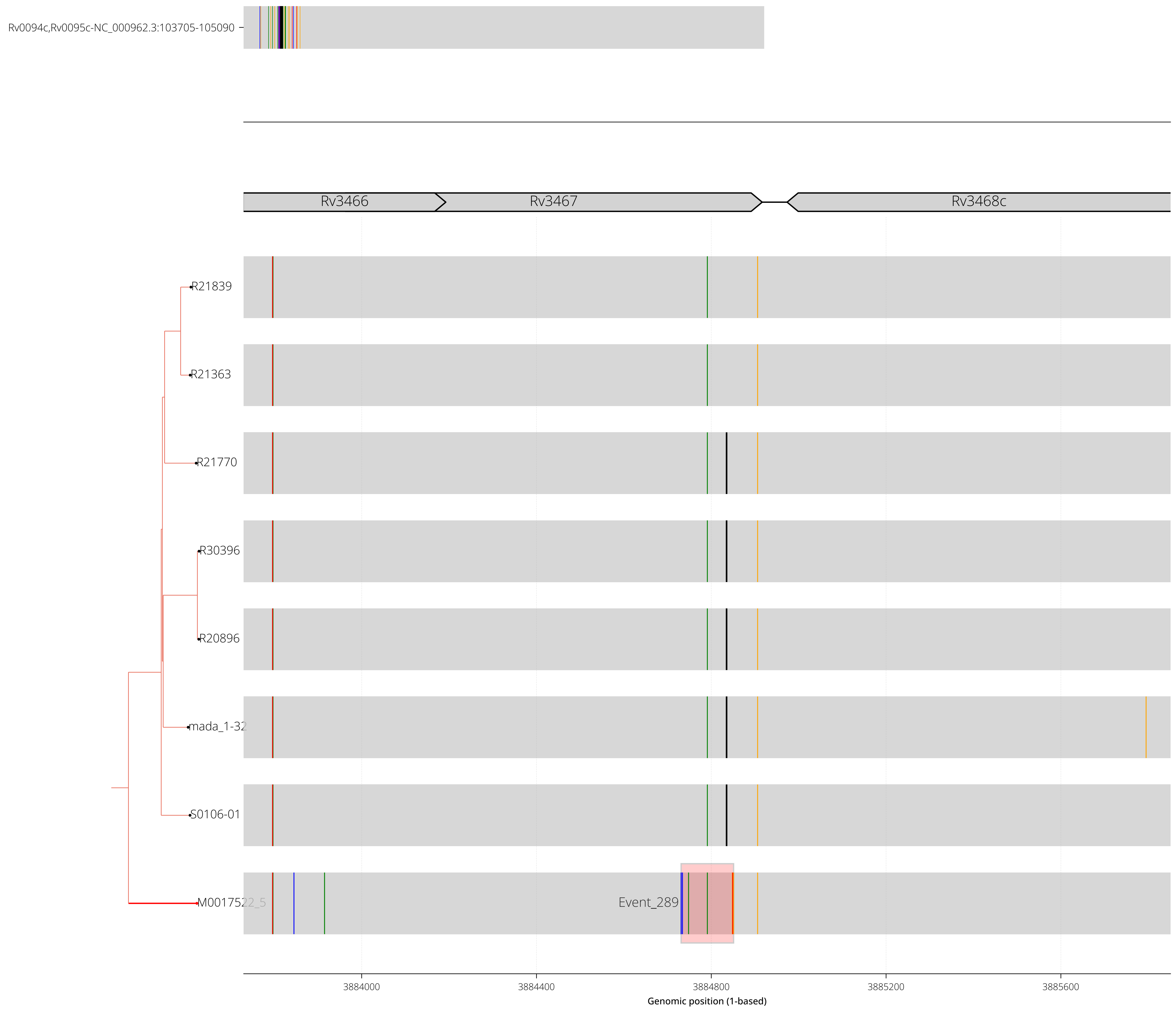

Full Phylogeny Viz + Paralog Alignments
GCE ID: Event_289

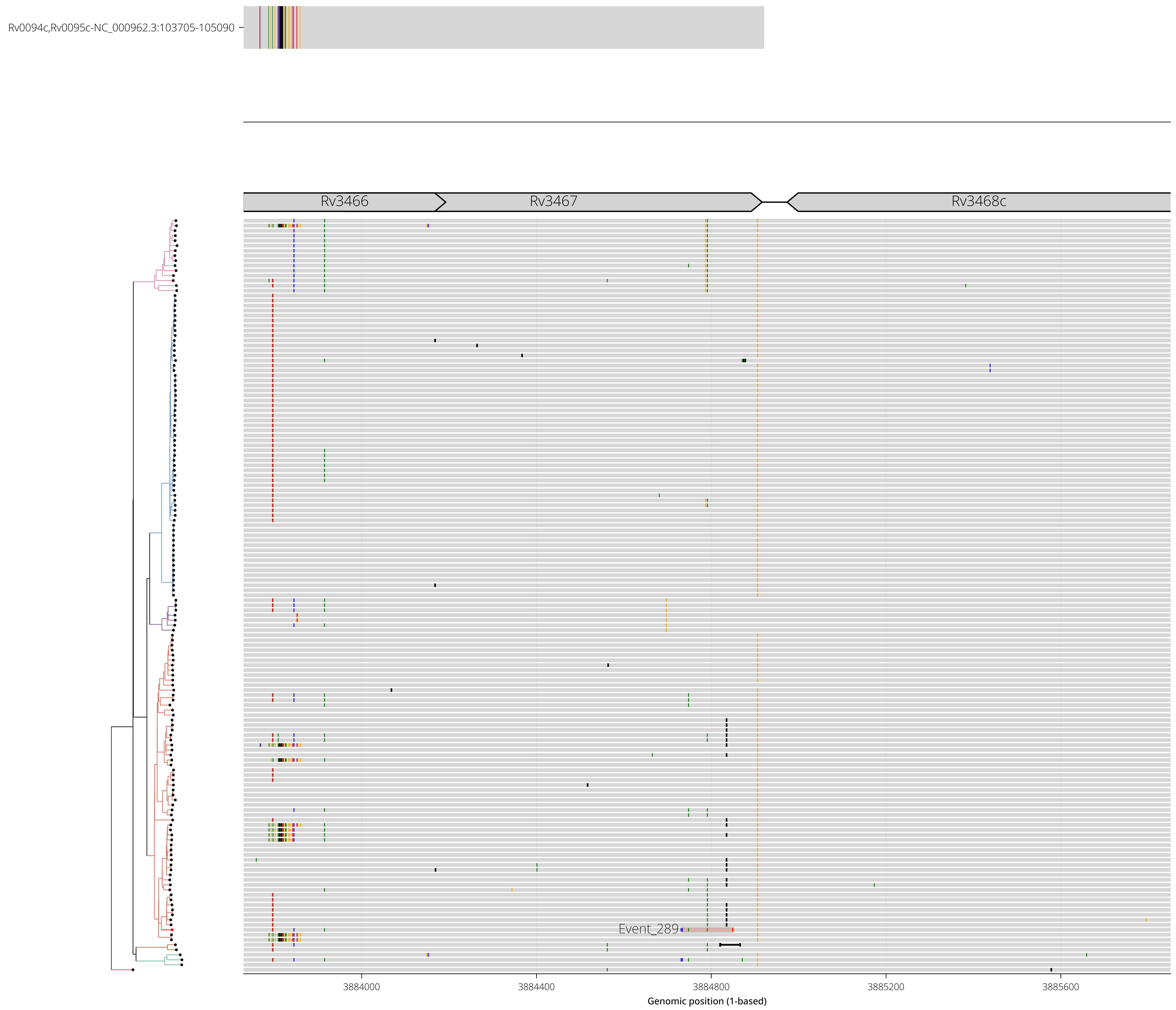

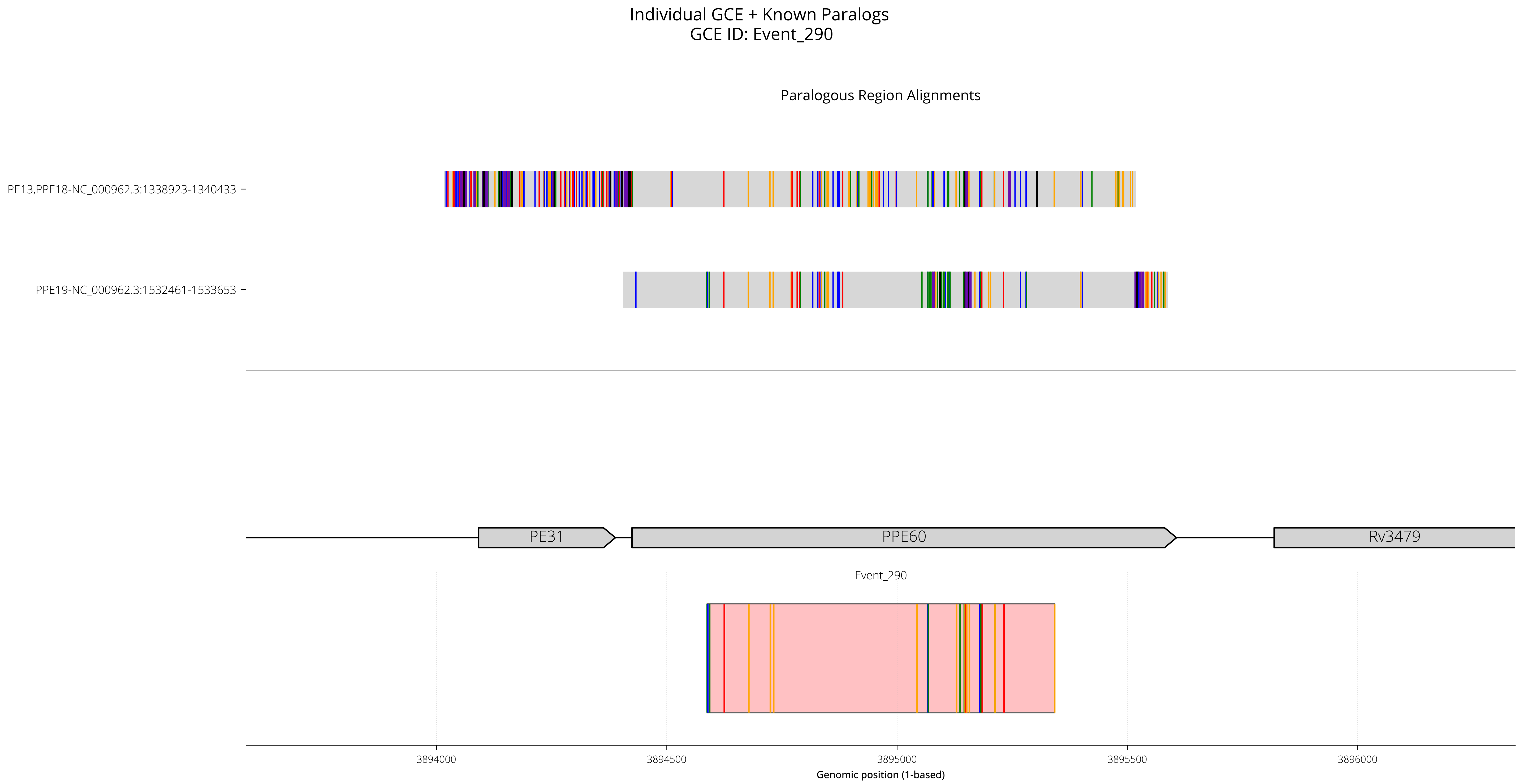

Individual GCE + Known Paralogs
GCE ID: Event_290
Paralogous Region Alignments
PE13,PPE18-NC_000962.3:1338923-1340433
PPE19-NC_000962.3:1532461-1533653
PE31
PPE60
Rv3479
Event_290
3894000
3894500
3895000
3895500
3896000
Genomic position (1-based)

Circos Viz - GCE to Known Paralogs Locations
GCE ID: Event_290

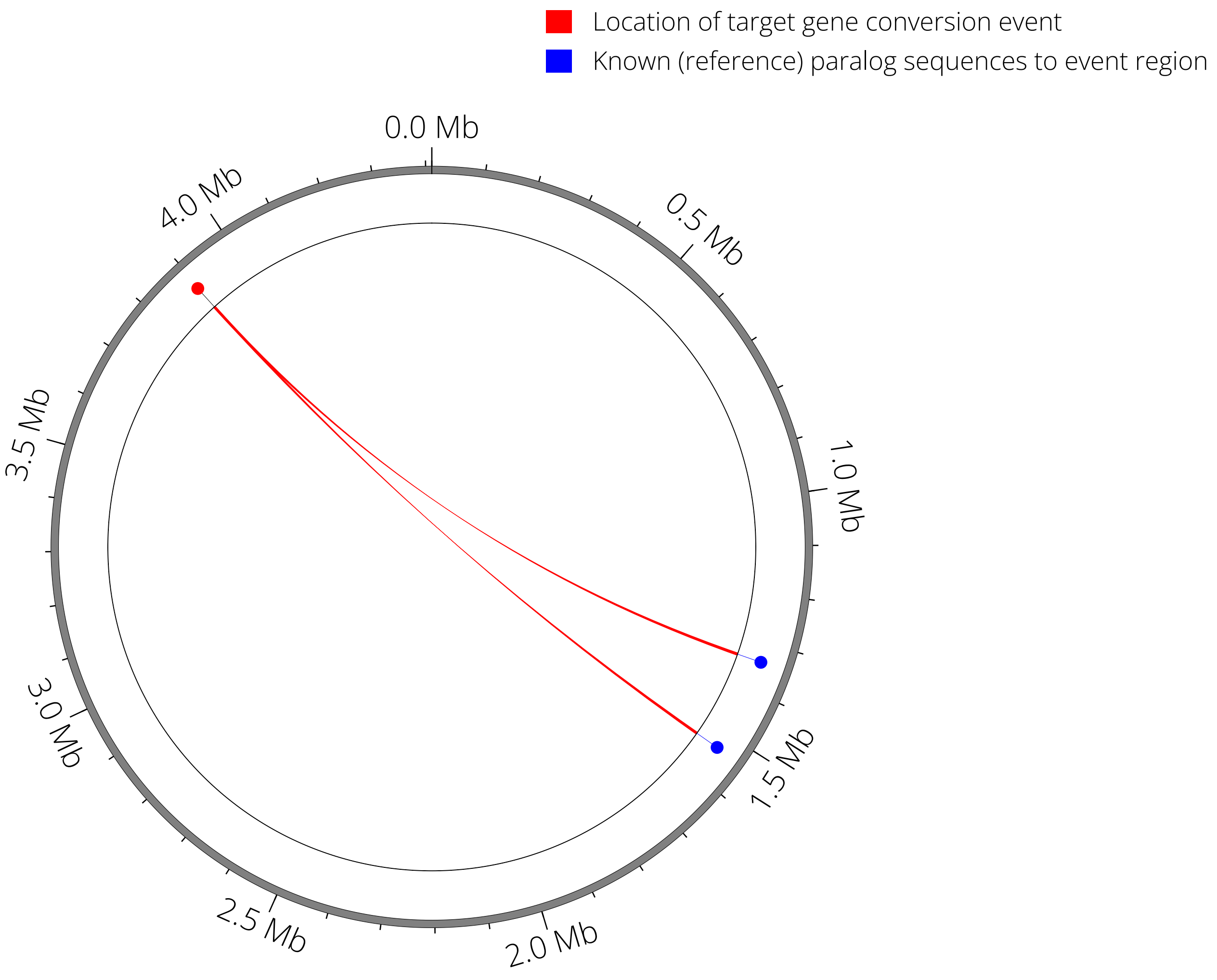

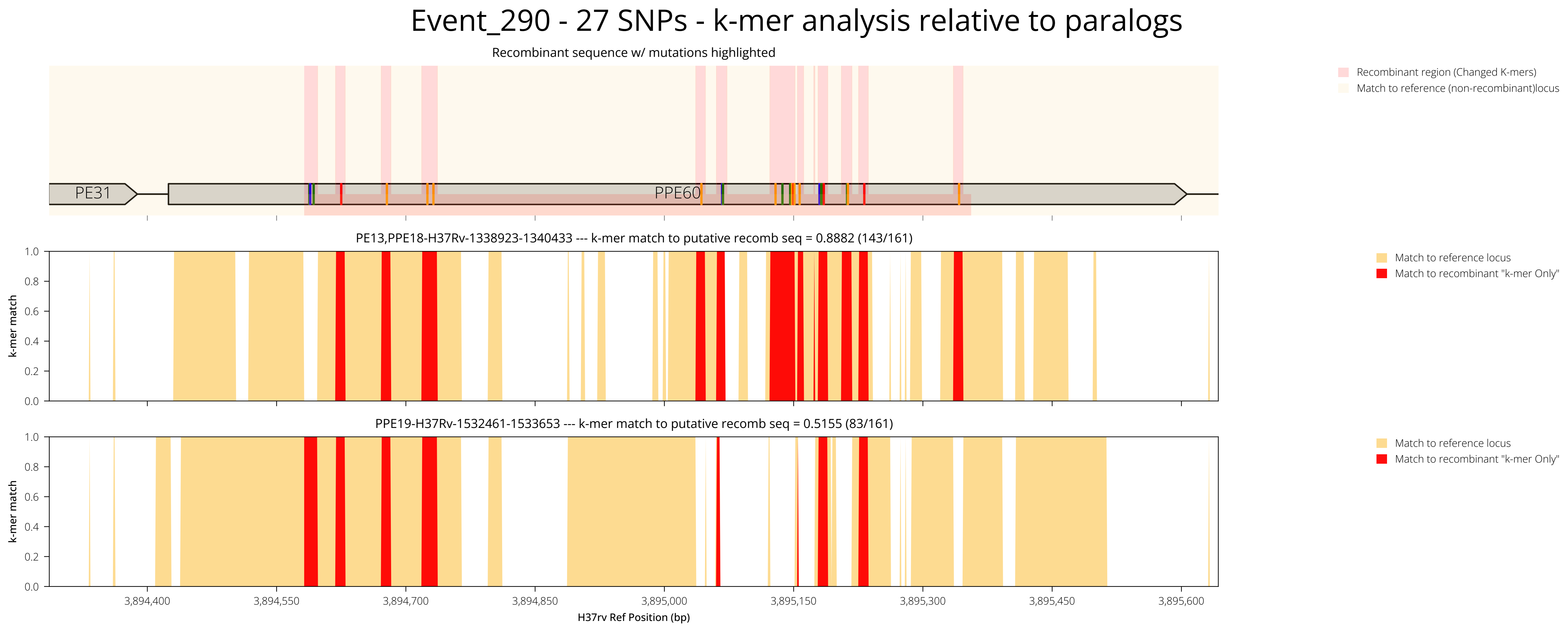

Event_290 - 27 SNPs - k-mer analysis relative to paralogs
Recombinant sequence w/ mutations highlighted
Recombinant region (Changed K-mers)
Match to reference (non-recombinant)locus
PE31
PPE60
PE13,PPE18-H37Rv-1338923-1340433 --- k-mer match to putative recomb seq = 0.8882 (143/161)
Match to reference locus
Match to recombinant "k-mer Only"
k-mer match
1.0
0.8
0.6
0.4
0.2
0.0
PPE19-H37Rv-1532461-1533653 --- k-mer match to putative recomb seq = 0.5155 (83/161)
Match to reference locus
Match to recombinant "k-mer Only"
k-mer match
3,894,400
3,894,550
3,894,700
3,894,850
3,895,000
3,895,150
3,895,300
3,895,450
3,895,600
H37rv Ref Position (bp)

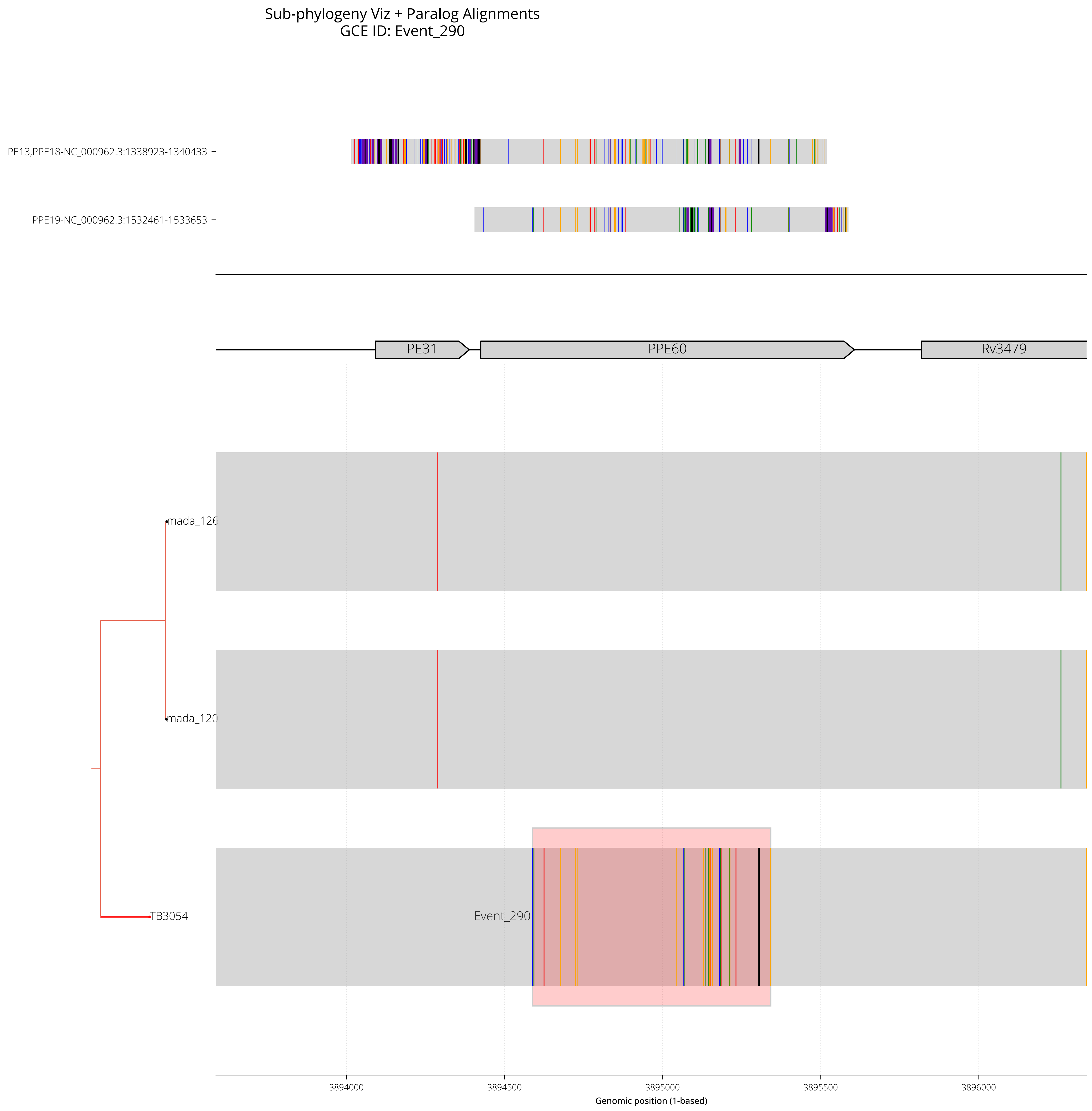
Sub-phylogeny Viz + Paralog Alignments
GCE ID: Event_290
PE13,PPE18-NC_000962.3:1338923-1340433
PPE19-NC_000962.3:1532461-1533653
PE31
PPE60
Rv3479
mada_126
mada_120
TB3054
Event_290
3894000
3894500
3895000
3895500
3896000
Genomic position (1-based)

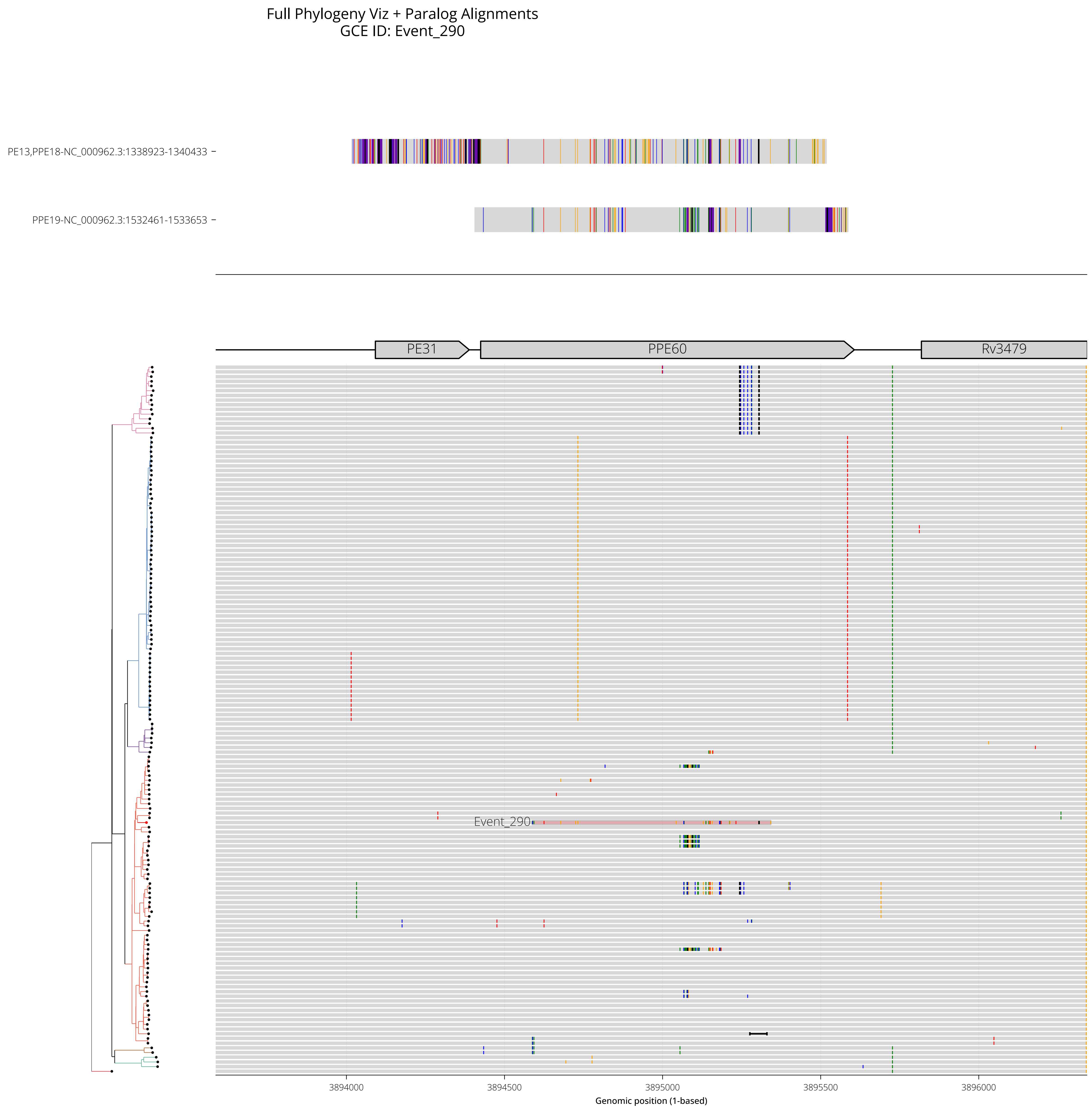
Full Phylogeny Viz + Paralog Alignments
GCE ID: Event_290
PE13,PPE18-NC_000962.3:1338923-1340433
PPE19-NC_000962.3:1532461-1533653
PE31
PPE60
Rv3479
Event_290
3894000
3894500
3895000
3895500
3896000
Genomic position (1-based)

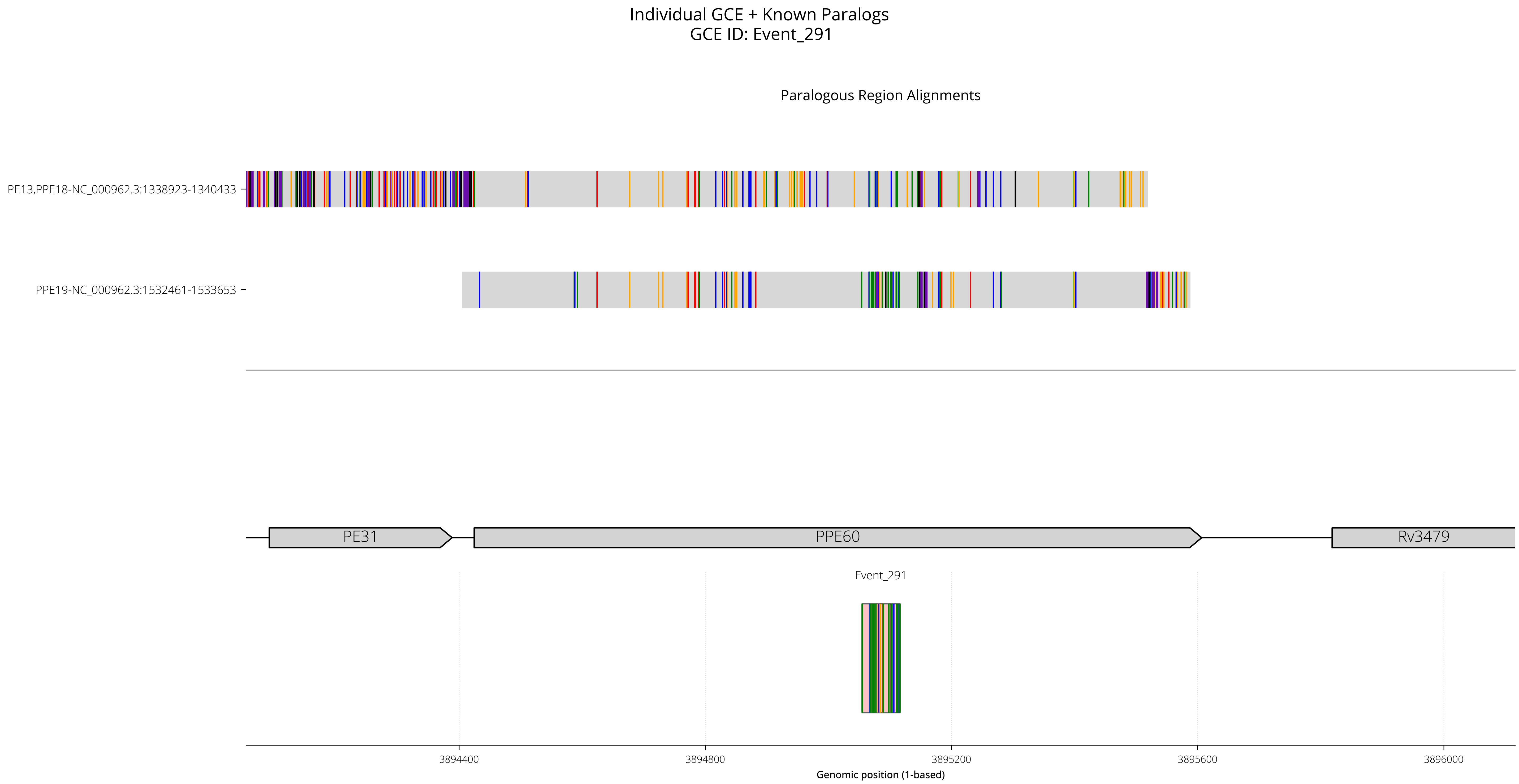

Individual GCE + Known Paralogs
GCE ID: Event_291
Paralogous Region Alignments
PE13,PPE18-NC_000962.3:1338923-1340433
PPE19-NC_000962.3:1532461-1533653
PE31
PPE60
Rv3479
Event_291
3894400
3894800
3895200
3895600
3896000
Genomic position (1-based)

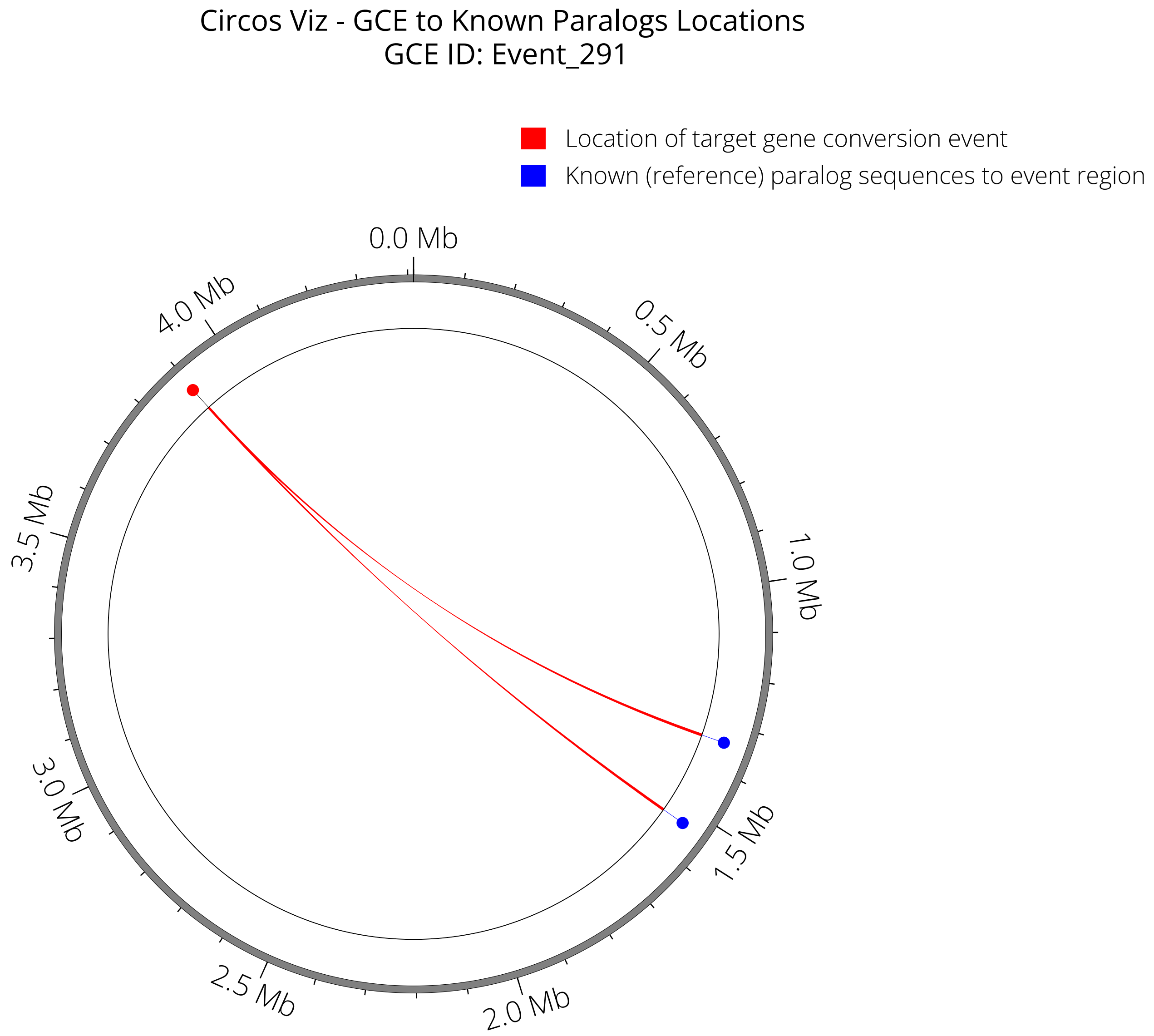

Circos Viz - GCE to Known Paralogs Locations
GCE ID: Event_291
Location of target gene conversion event
Known (reference) paralog sequences to event region
0.0 Mb
0.5 Mb
1.0 Mb
1.5 Mb
2.0 Mb
2.5 Mb
3.0 Mb
3.5 Mb
4.0 Mb

# Event_291 - 20 SNPs - k-mer analysis relative to paralogs

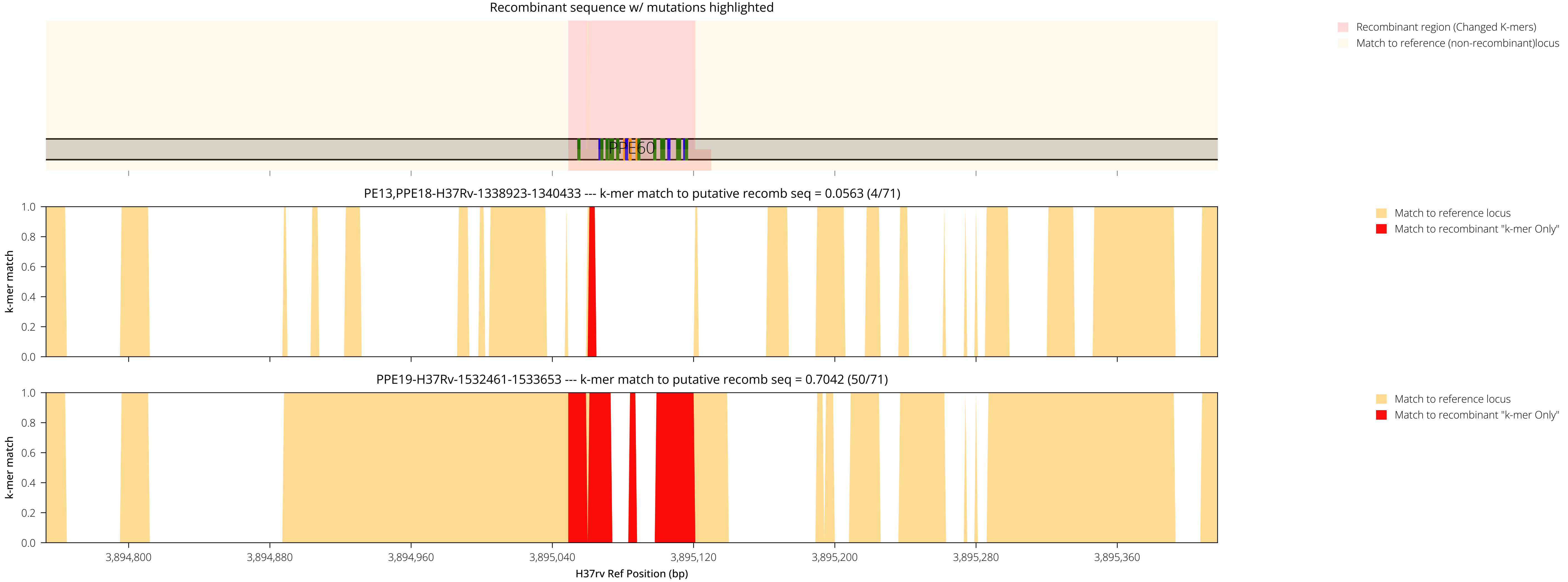

Sub-phylogeny Viz + Paralog Alignments
GCE ID: Event_291

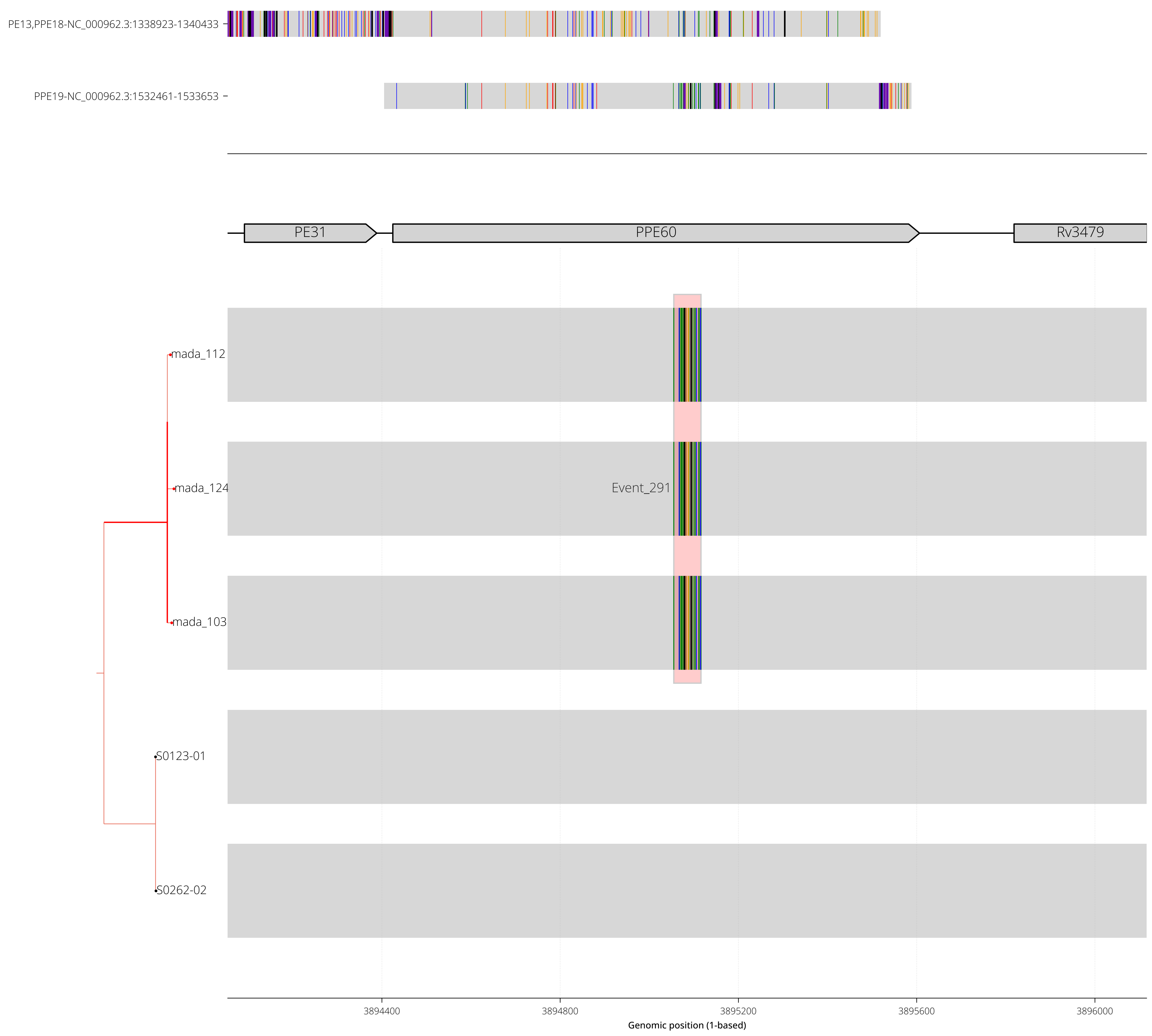

Full Phylogeny Viz + Paralog Alignments
GCE ID: Event_291

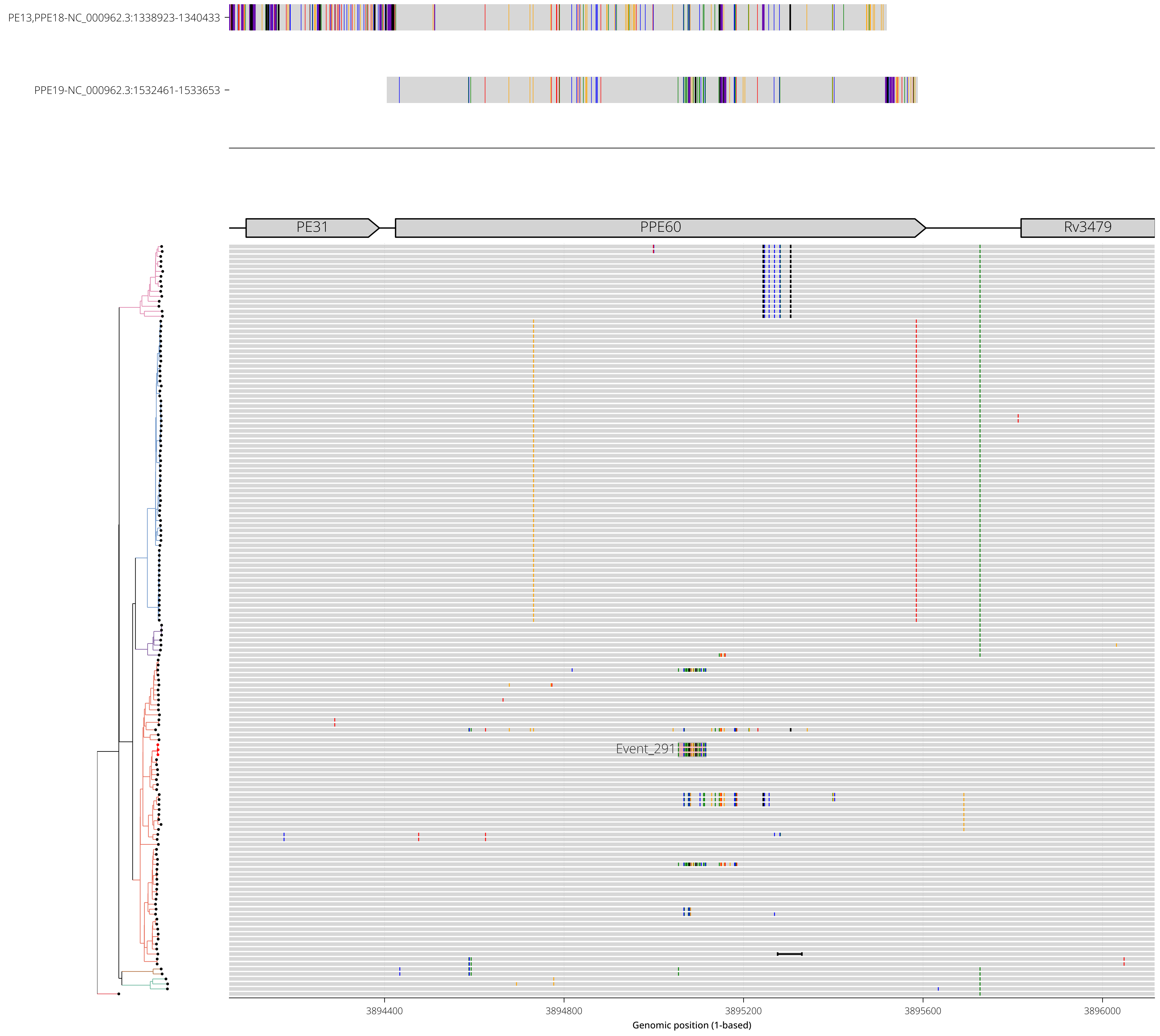

Individual GCE + Known Paralogs
GCE ID: Event_292

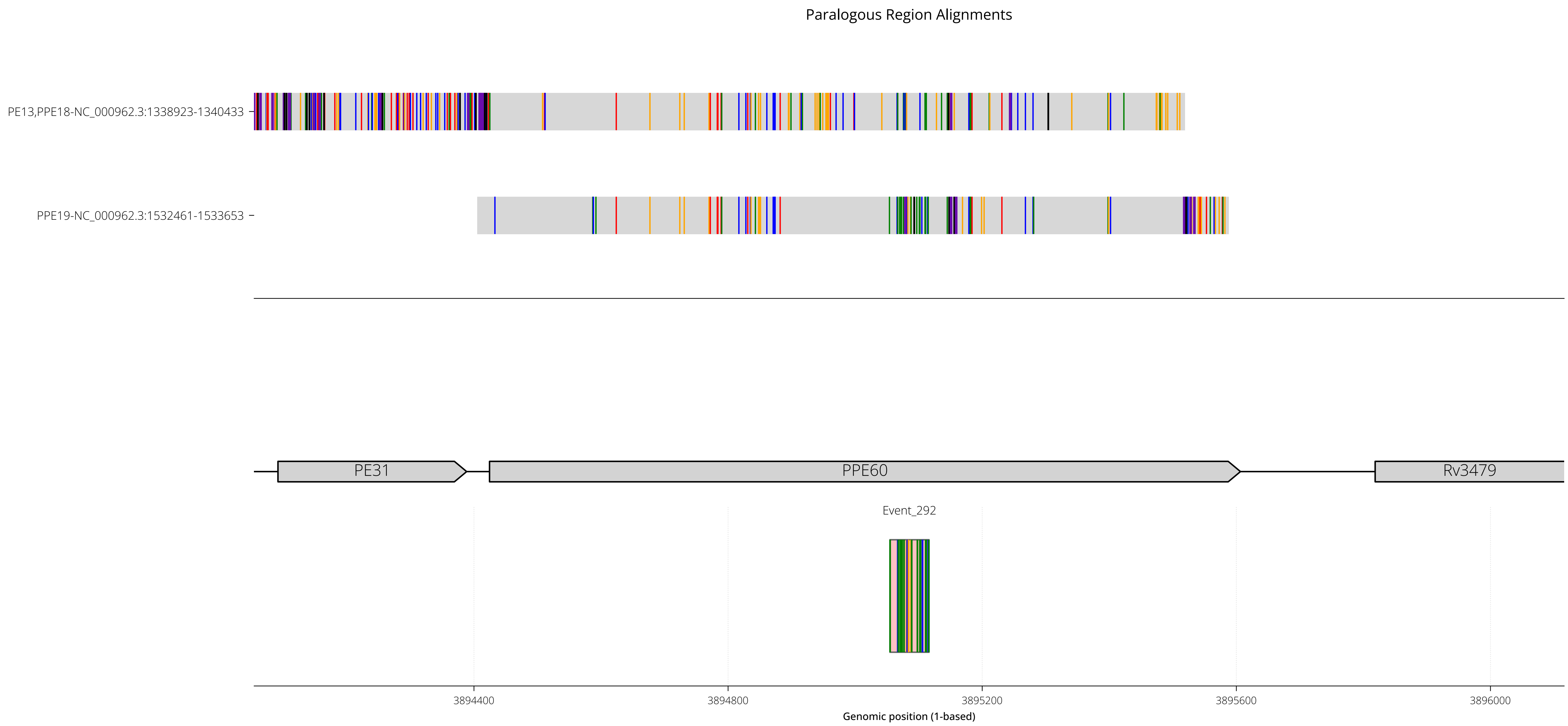

Circos Viz - GCE to Known Paralogs Locations
GCE ID: Event_292

Location of target gene conversion event
Known (reference) paralog sequences to event region

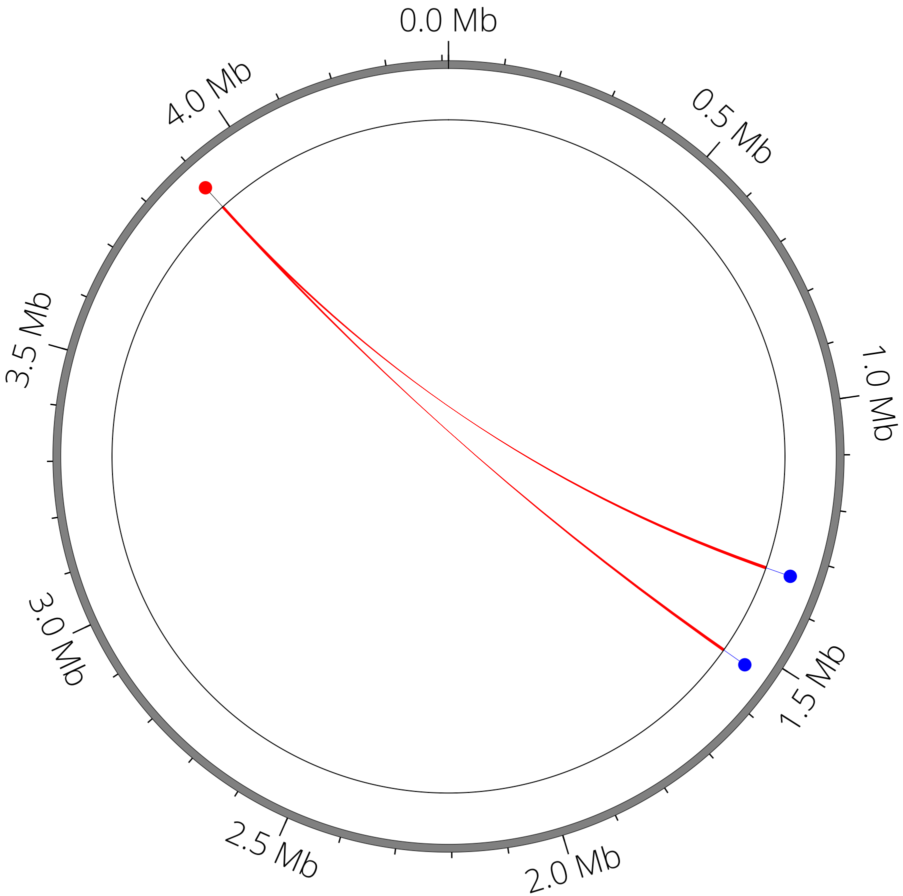

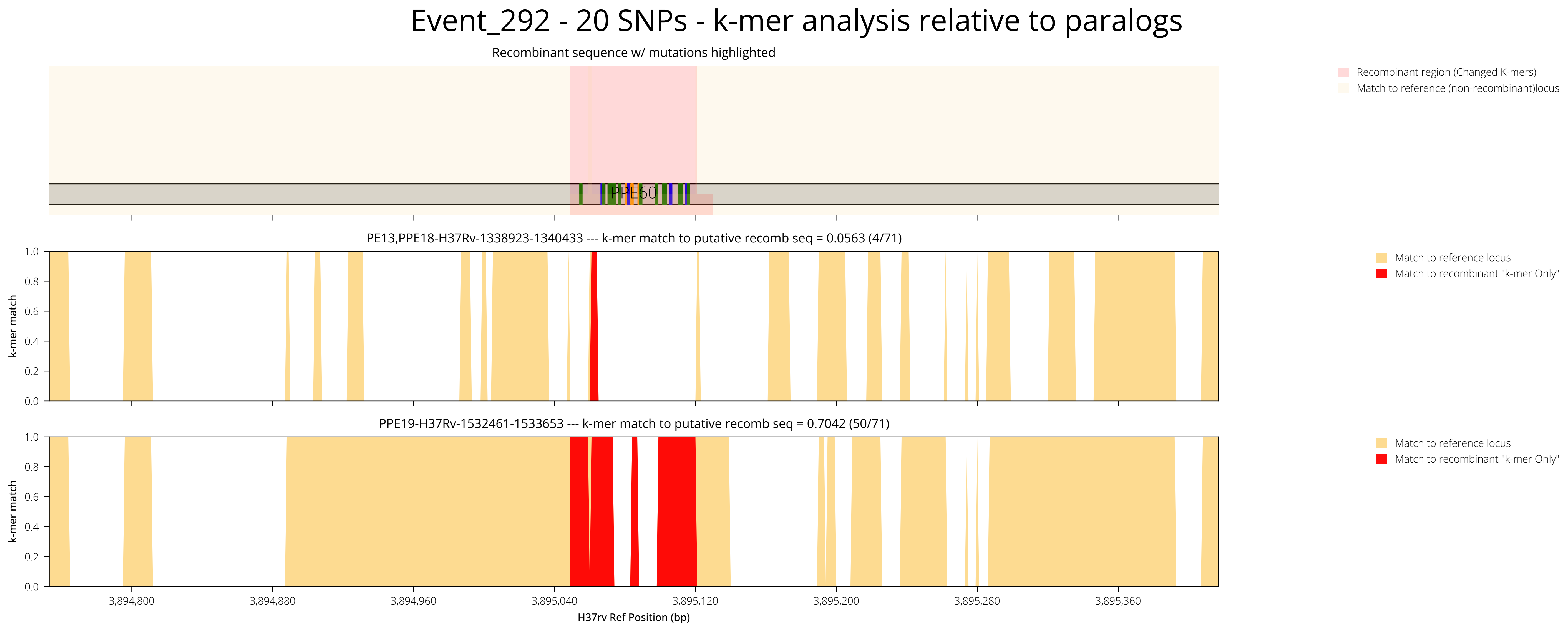
Event_292 - 20 SNPs - k-mer analysis relative to paralogs
Recombinant sequence w/ mutations highlighted
Recombinant region (Changed K-mers)
Match to reference (non-recombinant)locus
PPE60
PE13,PPE18-H37Rv-1338923-1340433 --- k-mer match to putative recomb seq = 0.0563 (4/71)
Match to reference locus
Match to recombinant "k-mer Only"
k-mer match
1.0
0.8
0.6
0.4
0.2
0.0
PPE19-H37Rv-1532461-1533653 --- k-mer match to putative recomb seq = 0.7042 (50/71)
Match to reference locus
Match to recombinant "k-mer Only"
k-mer match
1.0
0.8
0.6
0.4
0.2
0.0
3,894,800
3,894,880
3,894,960
3,895,040
3,895,120
3,895,200
3,895,280
3,895,360
H37rv Ref Position (bp)

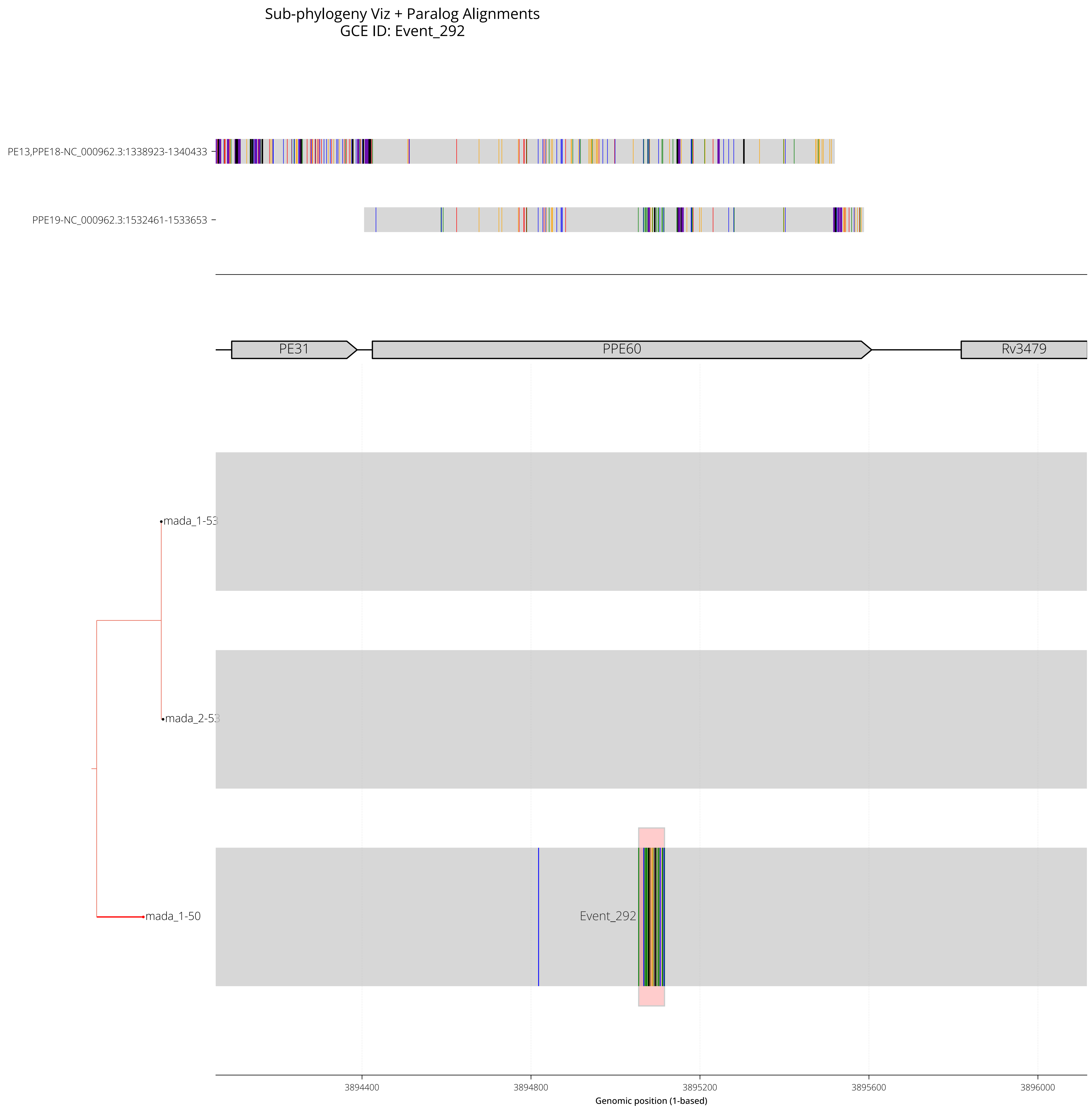
Sub-phylogeny Viz + Paralog Alignments
GCE ID: Event_292
PE13,PPE18-NC_000962.3:1338923-1340433
PPE19-NC_000962.3:1532461-1533653
PE31
PPE60
Rv3479
mada_1-53
mada_2-53
mada_1-50
Event_292
3894400
3894800
3895200
3895600
3896000
Genomic position (1-based)

Full Phylogeny Viz + Paralog Alignments
GCE ID: Event_292

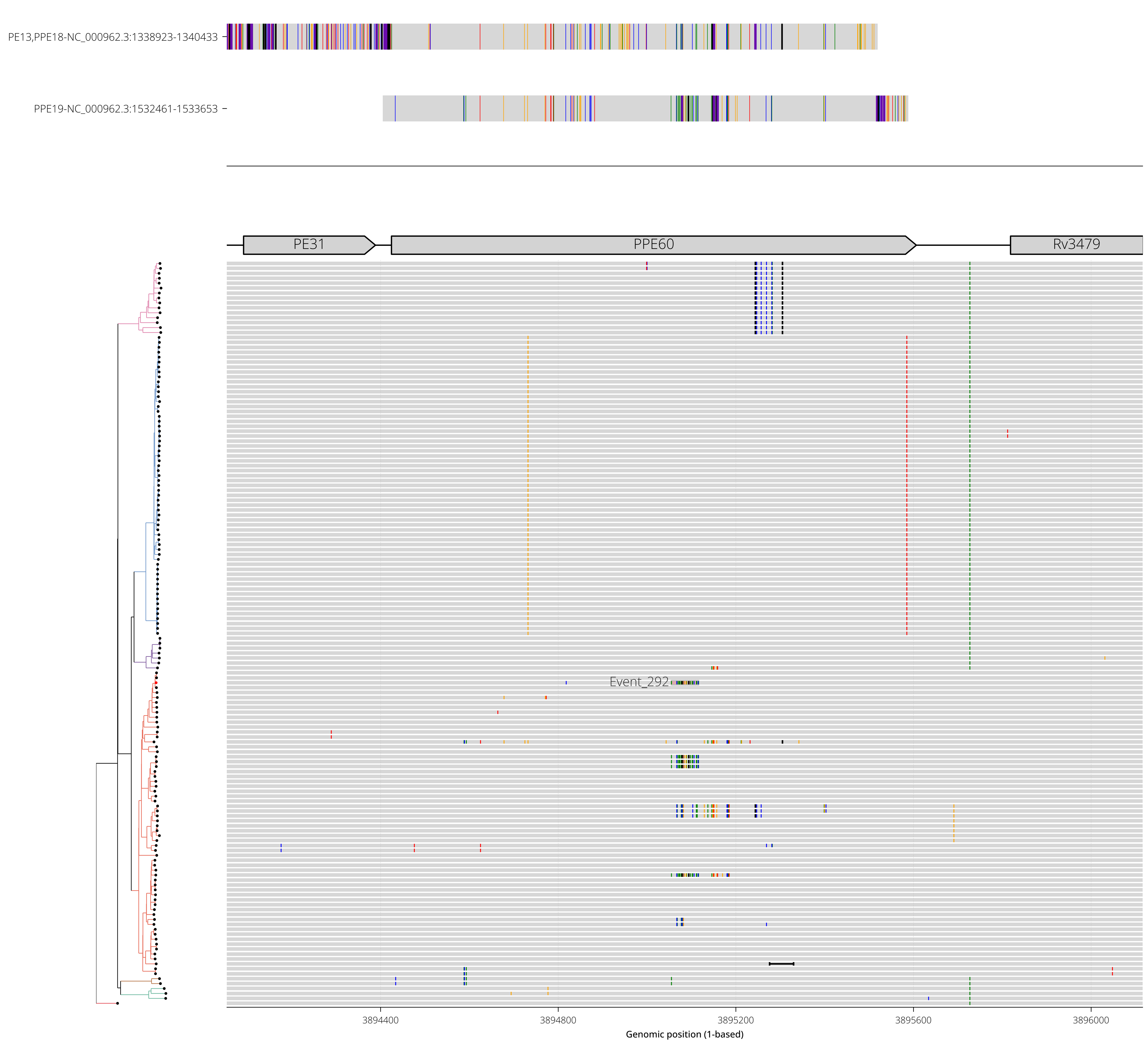

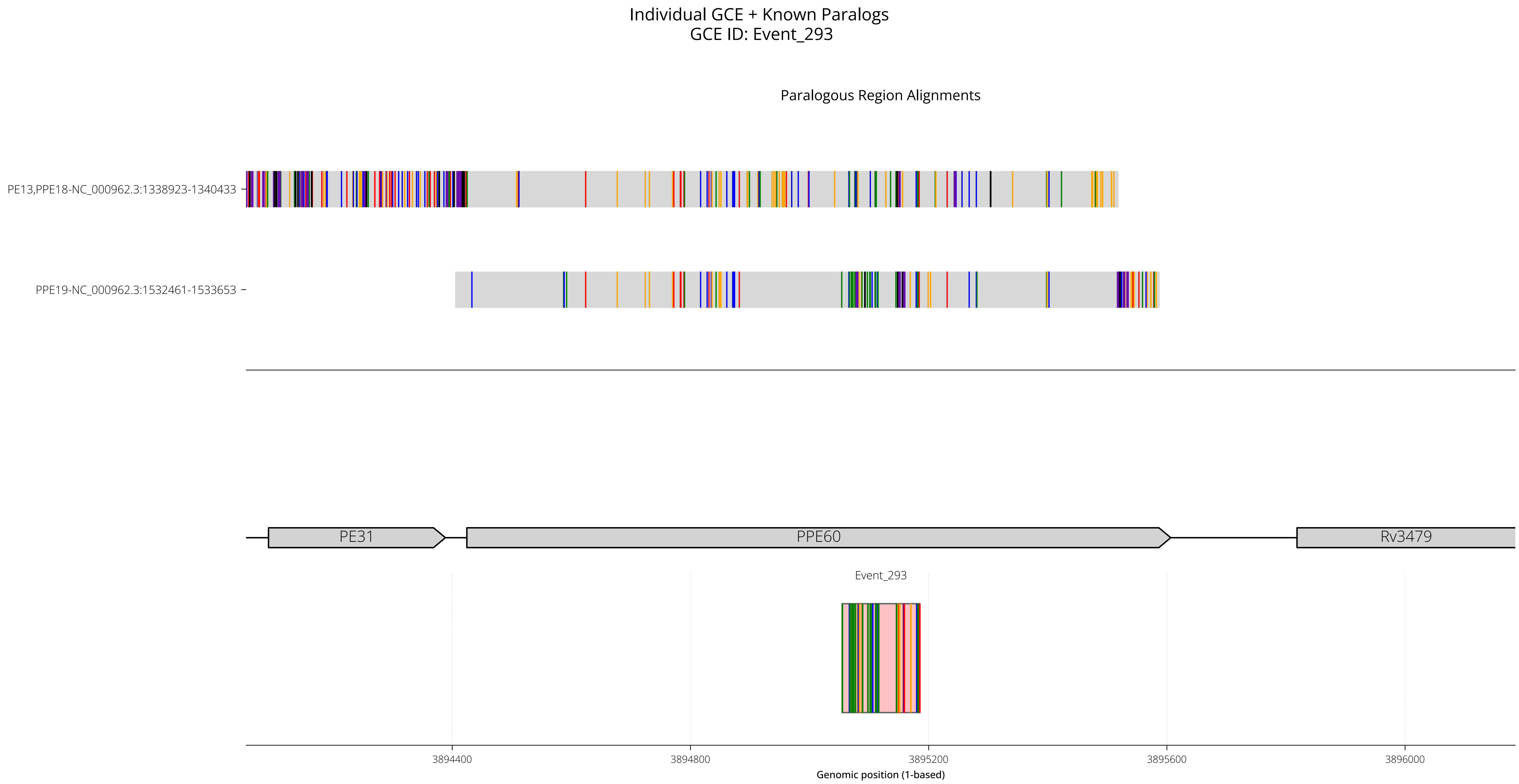
Individual GCE + Known Paralogs
GCE ID: Event_293
Paralogous Region Alignments
PE13,PPE18-NC_000962.3:1338923-1340433
PPE19-NC_000962.3:1532461-1533653
PE31
PPE60
Rv3479
Event_293
3894400
3894800
3895200
3895600
3896000
Genomic position (1-based)

Circos Viz - GCE to Known Paralogs Locations
GCE ID: Event_293

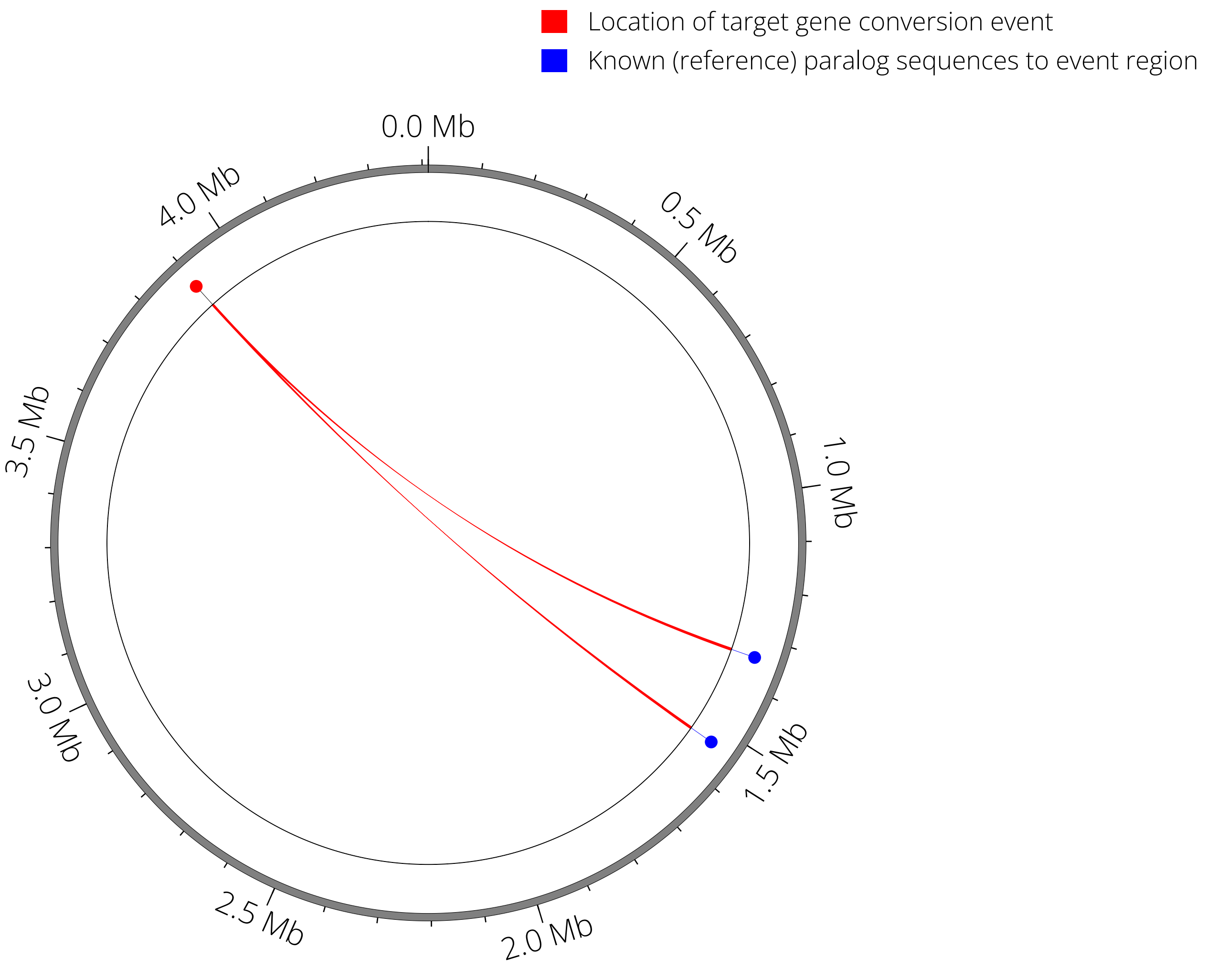

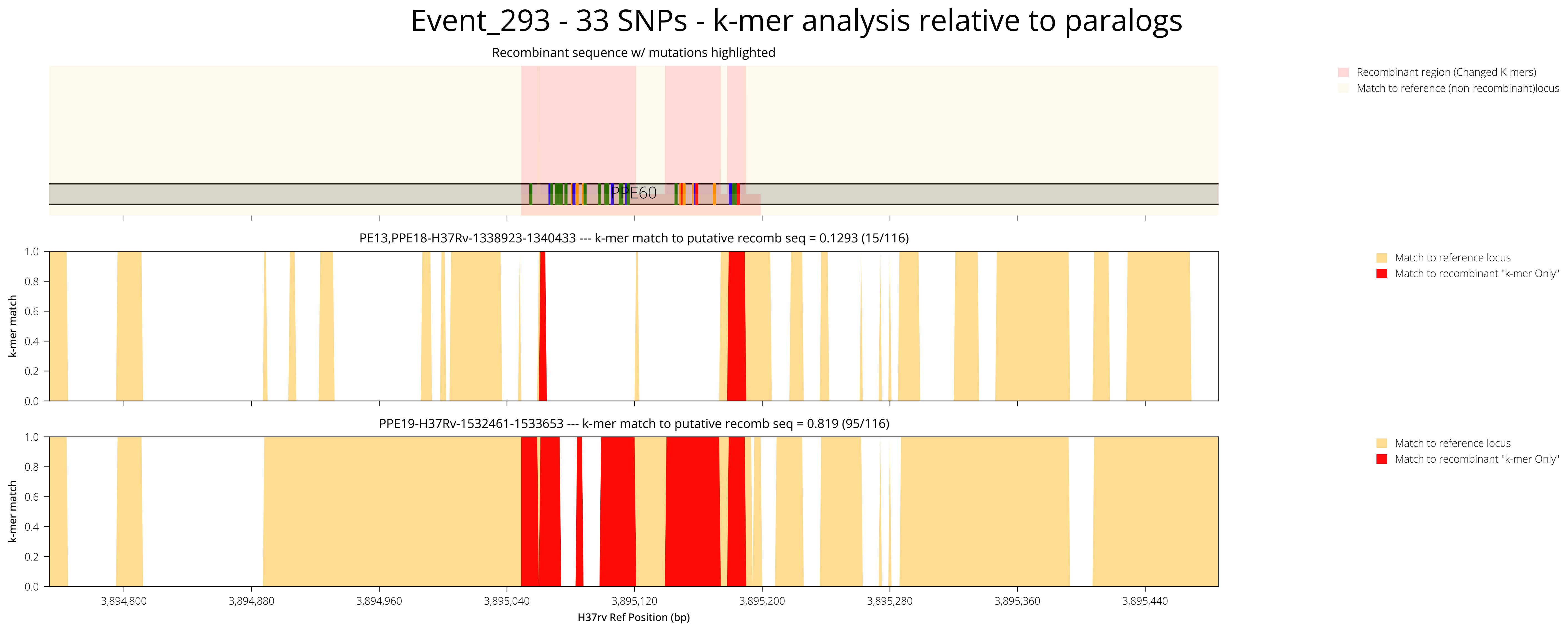
Event_293 - 33 SNPs - k-mer analysis relative to paralogs
Recombinant sequence w/ mutations highlighted
PPE60
Recombinant region (Changed K-mers)
Match to reference (non-recombinant)locus
PE13,PPE18-H37Rv-1338923-1340433 --- k-mer match to putative recomb seq = 0.1293 (15/116)
Match to reference locus
Match to recombinant "k-mer Only"
PPE19-H37Rv-1532461-1533653 --- k-mer match to putative recomb seq = 0.819 (95/116)
Match to reference locus
Match to recombinant "k-mer Only"
k-mer match
1.0
0.8
0.6
0.4
0.2
0.0
3,894,800
3,894,880
3,894,960
3,895,040
3,895,120
3,895,200
3,895,280
3,895,360
3,895,440
H37rv Ref Position (bp)

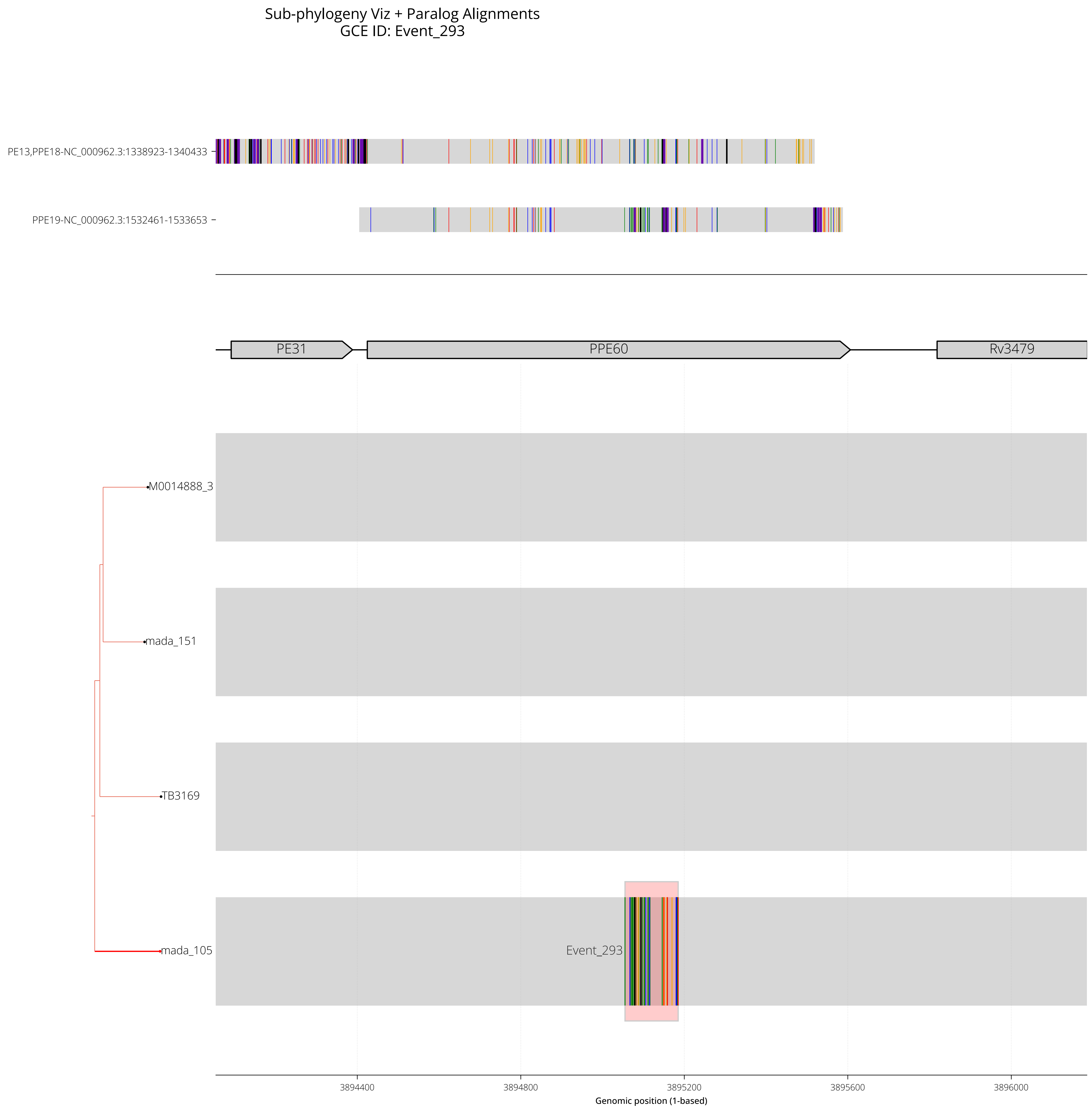

Sub-phylogeny Viz + Paralog Alignments
GCE ID: Event_293
PE13,PPE18-NC_000962.3:1338923-1340433
PPE19-NC_000962.3:1532461-1533653
PE31
PPE60
Rv3479
M0014888_3
mada_151
TB3169
mada_105
Event_293
3894400
3894800
3895200
3895600
3896000
Genomic position (1-based)

Full Phylogeny Viz + Paralog Alignments
GCE ID: Event_293

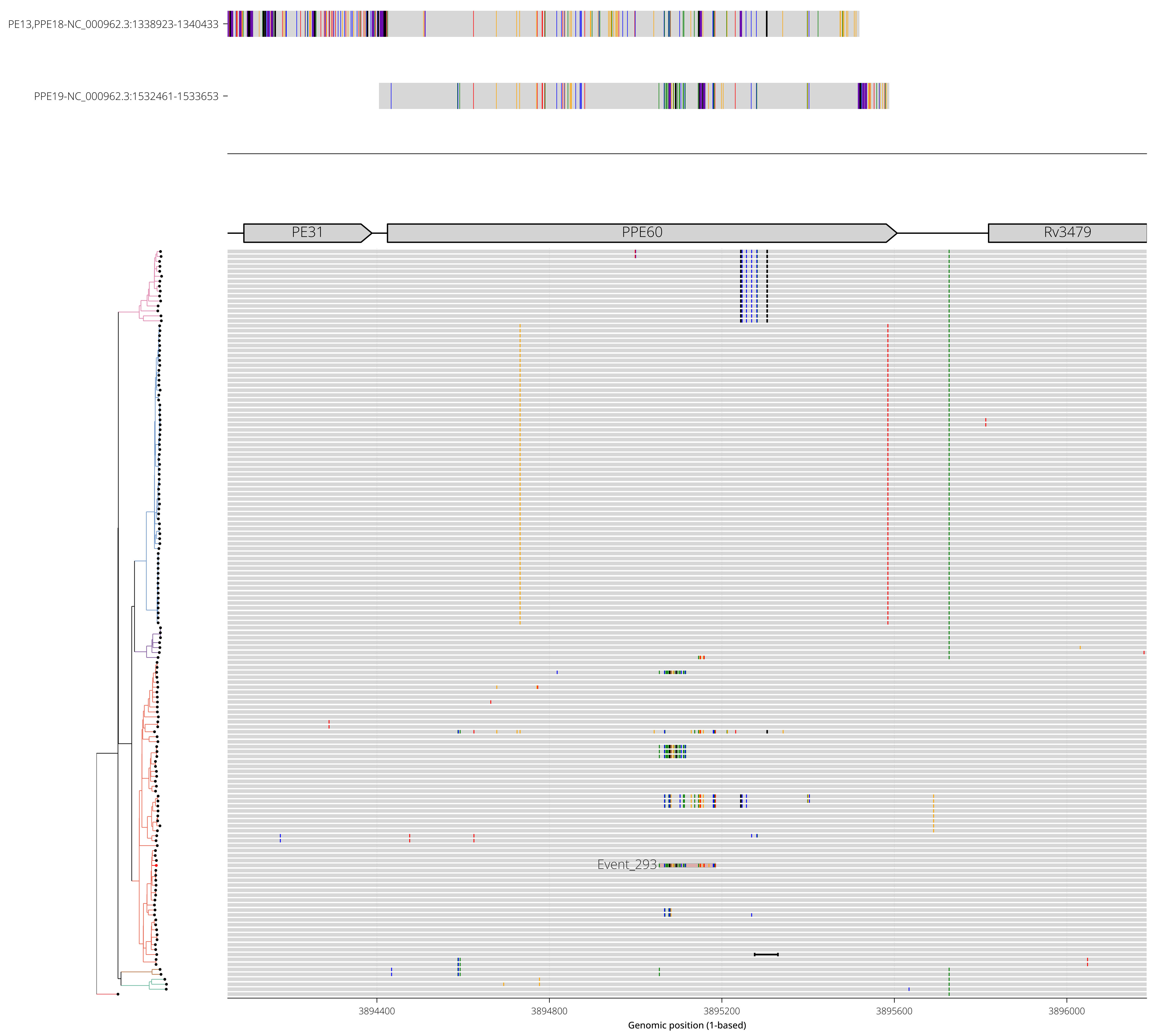

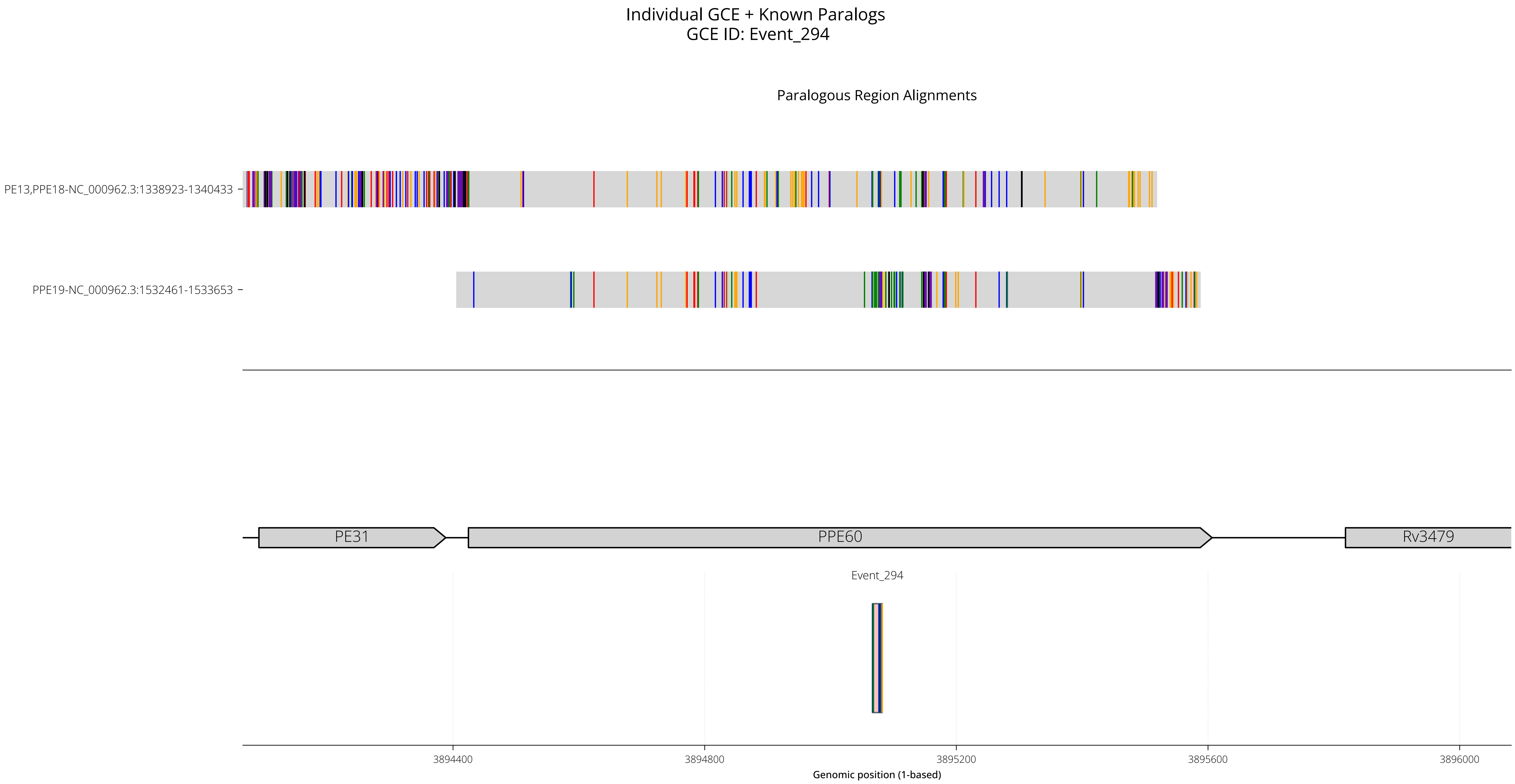
Individual GCE + Known Paralogs
GCE ID: Event_294
Paralogous Region Alignments
PE13,PPE18-NC_000962.3:1338923-1340433
PPE19-NC_000962.3:1532461-1533653
PE31
PPE60
Rv3479
Event_294
3894400
3894800
3895200
3895600
3896000
Genomic position (1-based)

Circos Viz - GCE to Known Paralogs Locations
GCE ID: Event_294

Location of target gene conversion event
Known (reference) paralog sequences to event region

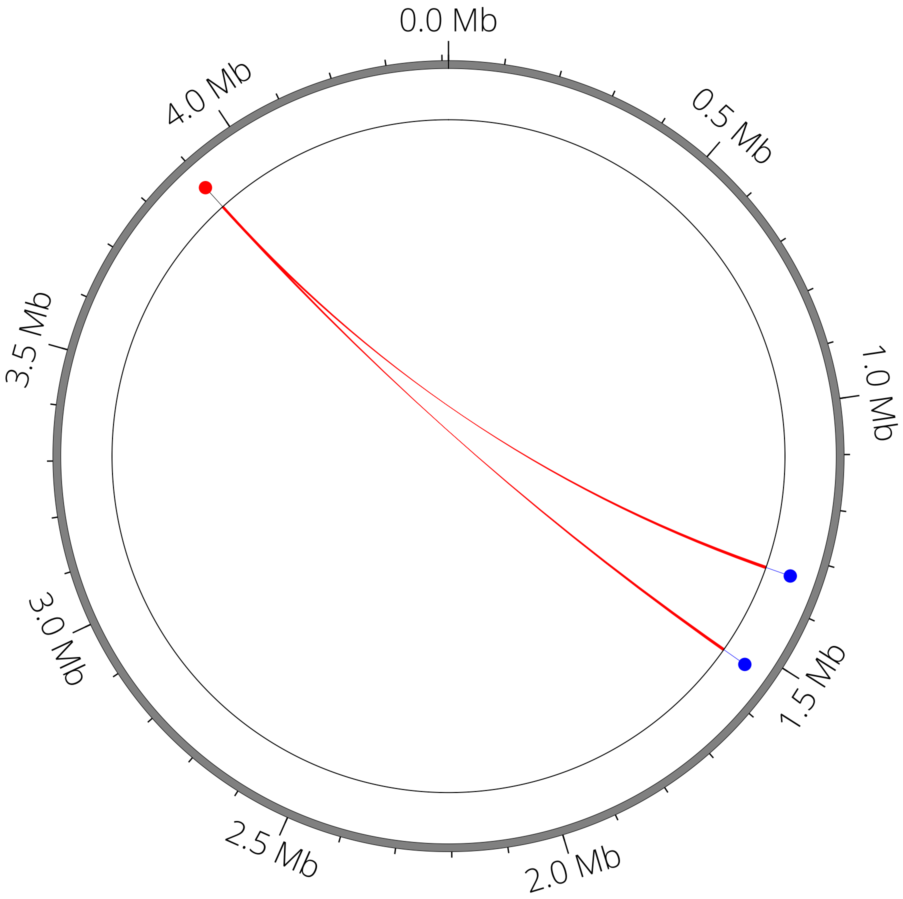

# Event_294 - 7 SNPs - k-mer analysis relative to paralogs

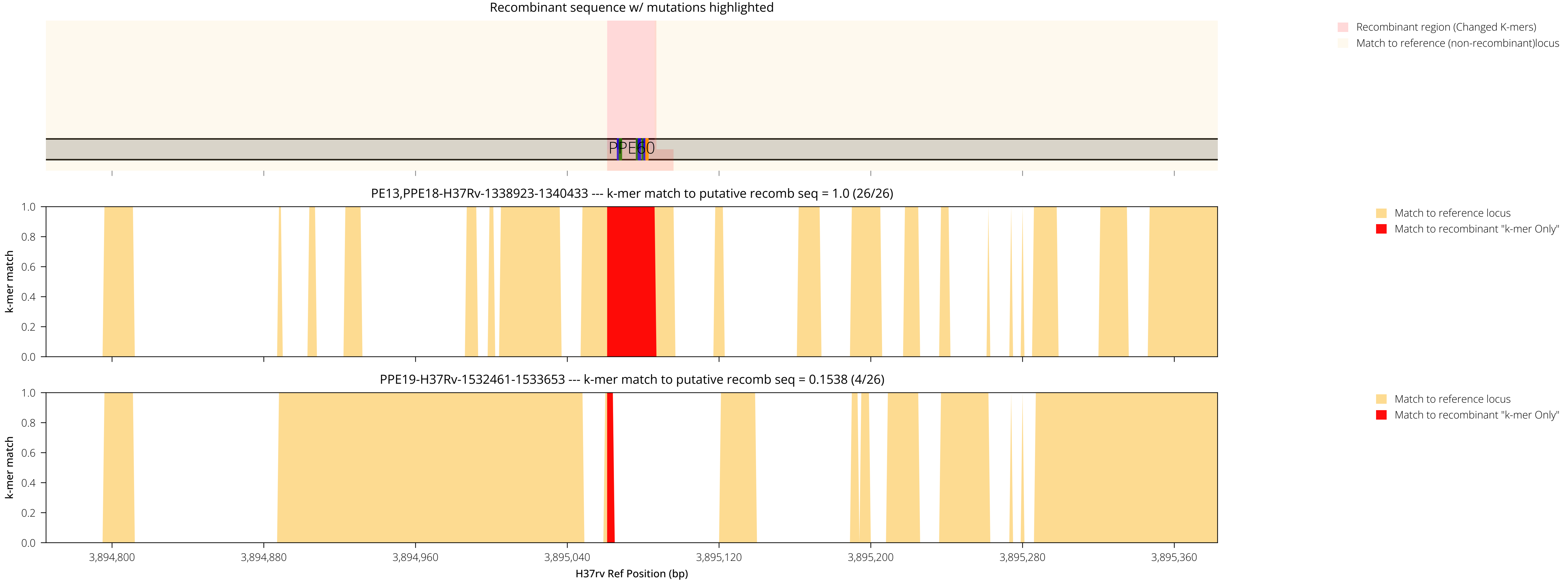

Sub-phylogeny Viz + Paralog Alignments
GCE ID: Event_294

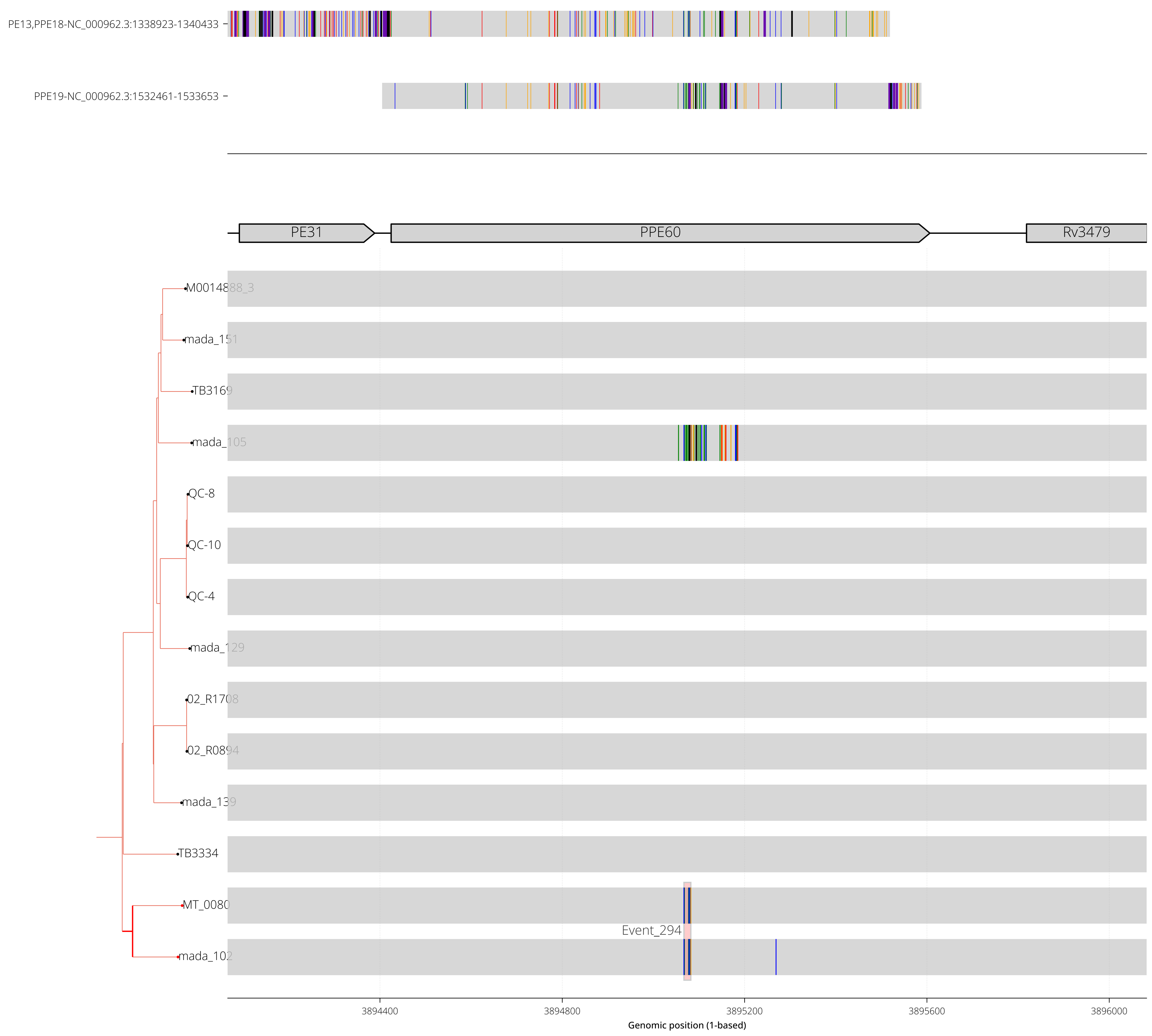

Full Phylogeny Viz + Paralog Alignments
GCE ID: Event_294

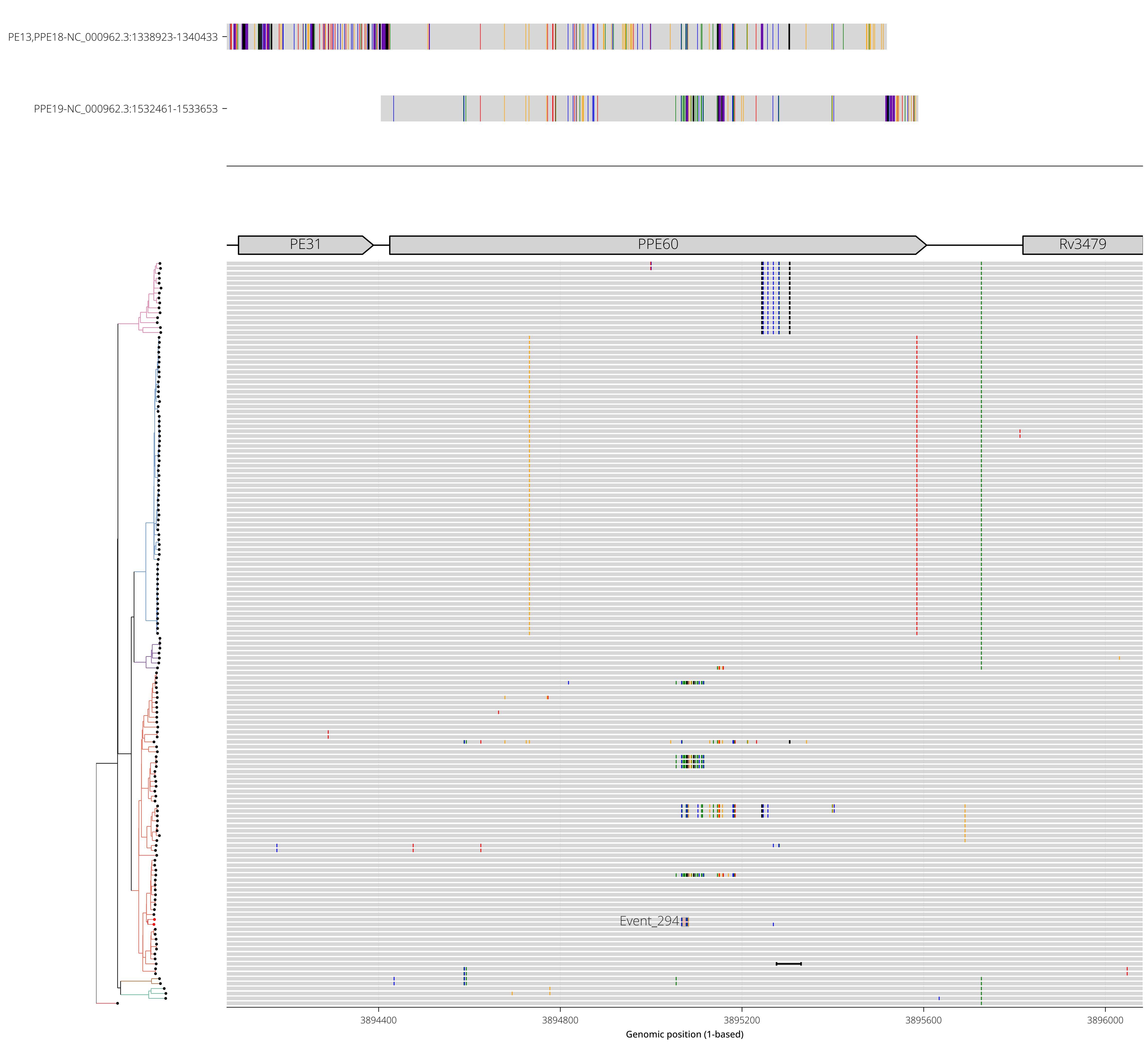

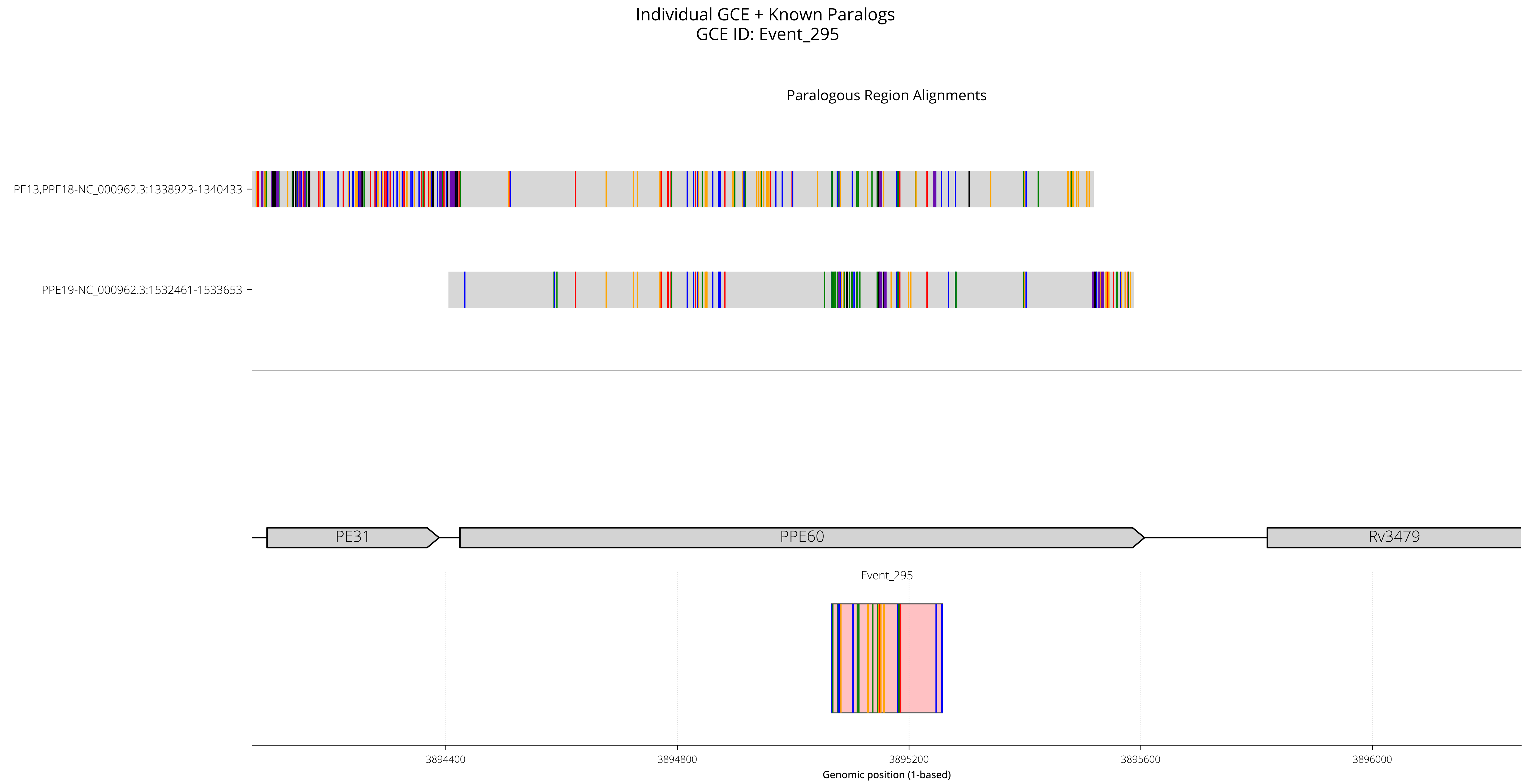

Individual GCE + Known Paralogs
GCE ID: Event_295
Paralogous Region Alignments
PE13,PPE18-NC_000962.3:1338923-1340433
PPE19-NC_000962.3:1532461-1533653
PE31
PPE60
Rv3479
Event_295
3894400
3894800
3895200
3895600
3896000
Genomic position (1-based)

Circos Viz - GCE to Known Paralogs Locations
GCE ID: Event_295

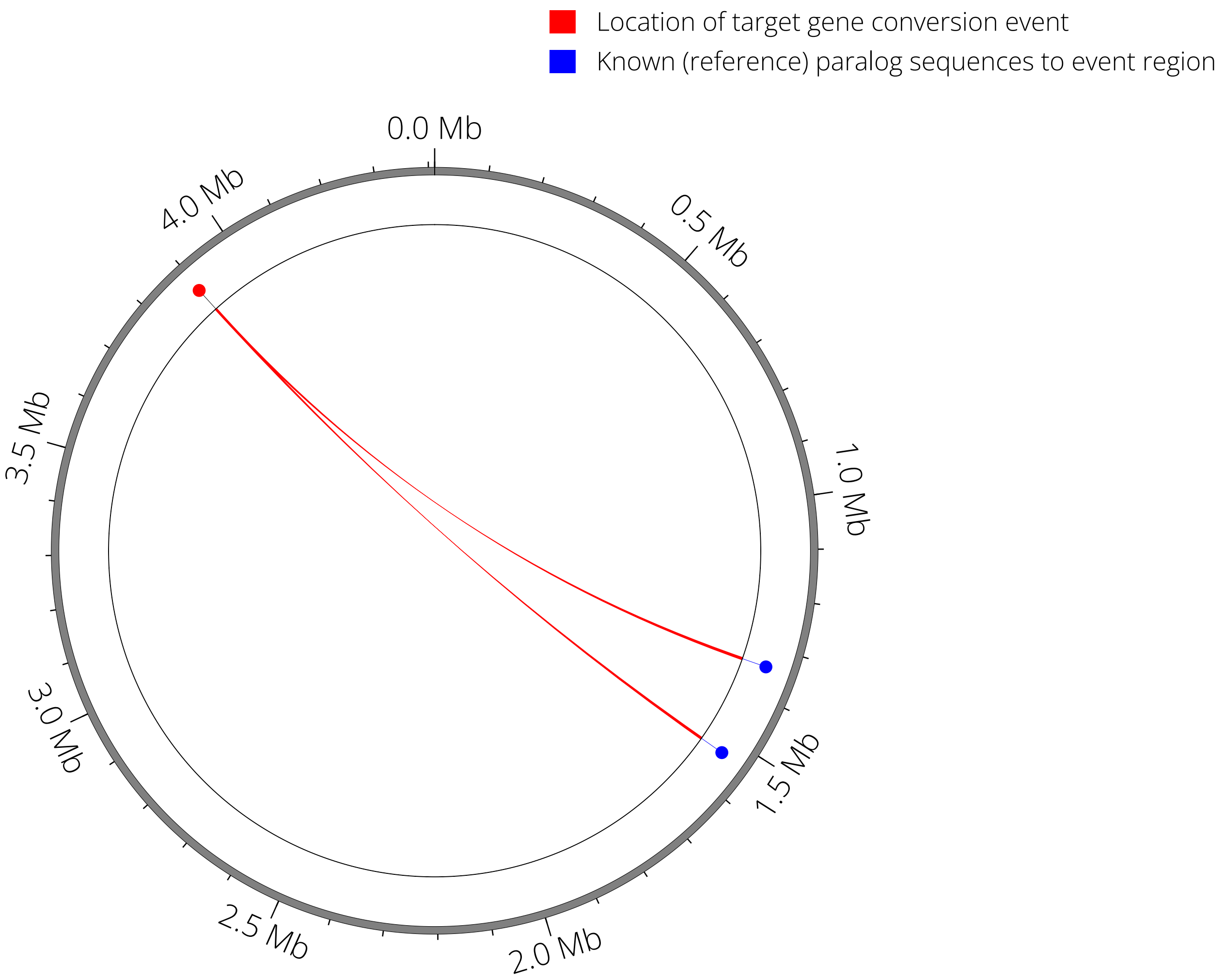

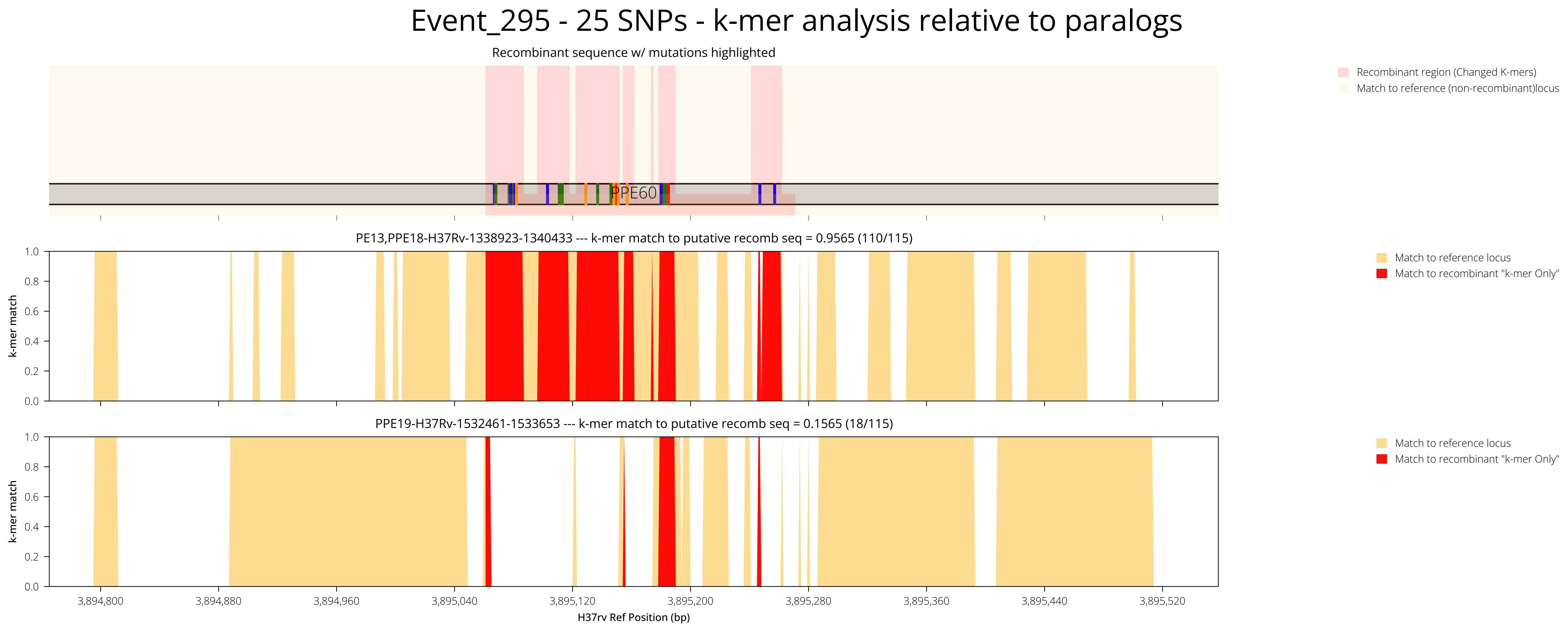

Event_295 - 25 SNPs - k-mer analysis relative to paralogs
Recombinant sequence w/ mutations highlighted
Recombinant region (Changed K-mers)
Match to reference (non-recombinant)locus
PPE60
PE13,PPE18-H37Rv-1338923-1340433 --- k-mer match to putative recomb seq = 0.9565 (110/115)
Match to reference locus
Match to recombinant "k-mer Only"
PPE19-H37Rv-1532461-1533653 --- k-mer match to putative recomb seq = 0.1565 (18/115)
Match to reference locus
Match to recombinant "k-mer Only"
k-mer match
1.0
0.8
0.6
0.4
0.2
0.0
3,894,800
3,894,880
3,894,960
3,895,040
3,895,120
3,895,200
3,895,280
3,895,360
3,895,440
3,895,520
H37rv Ref Position (bp)

Sub-phylogeny Viz + Paralog Alignments
GCE ID: Event_295

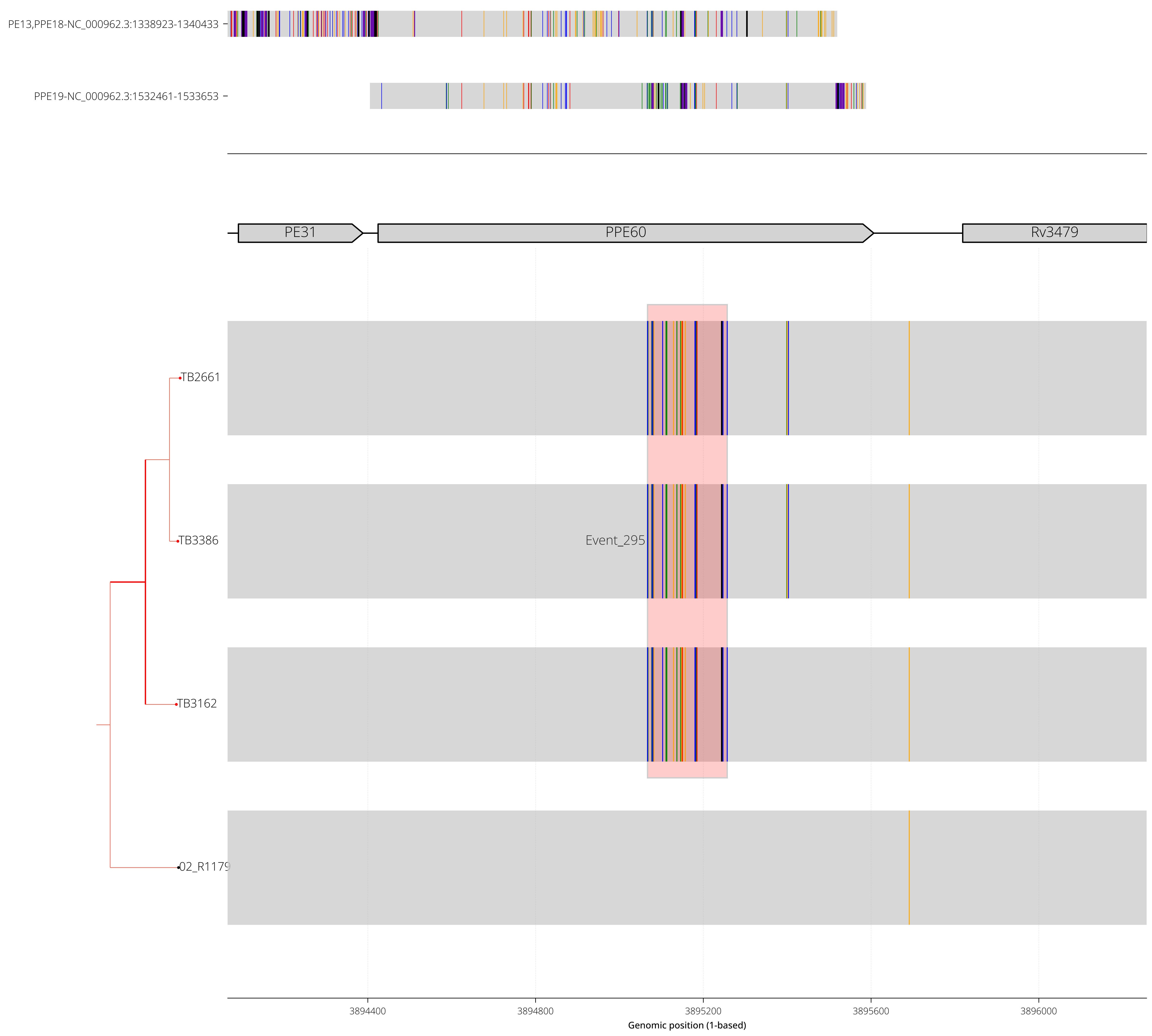

Full Phylogeny Viz + Paralog Alignments
GCE ID: Event_295

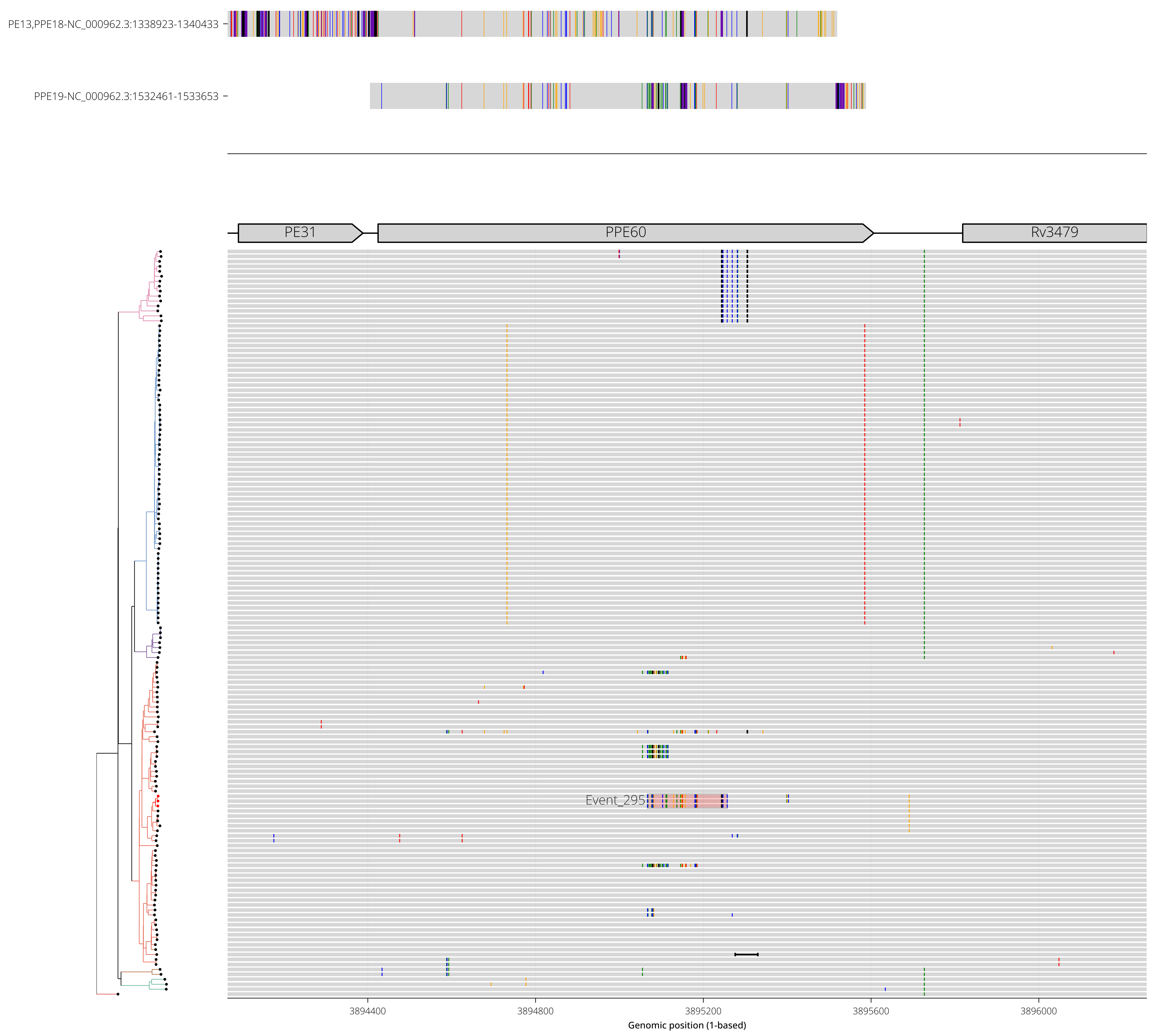

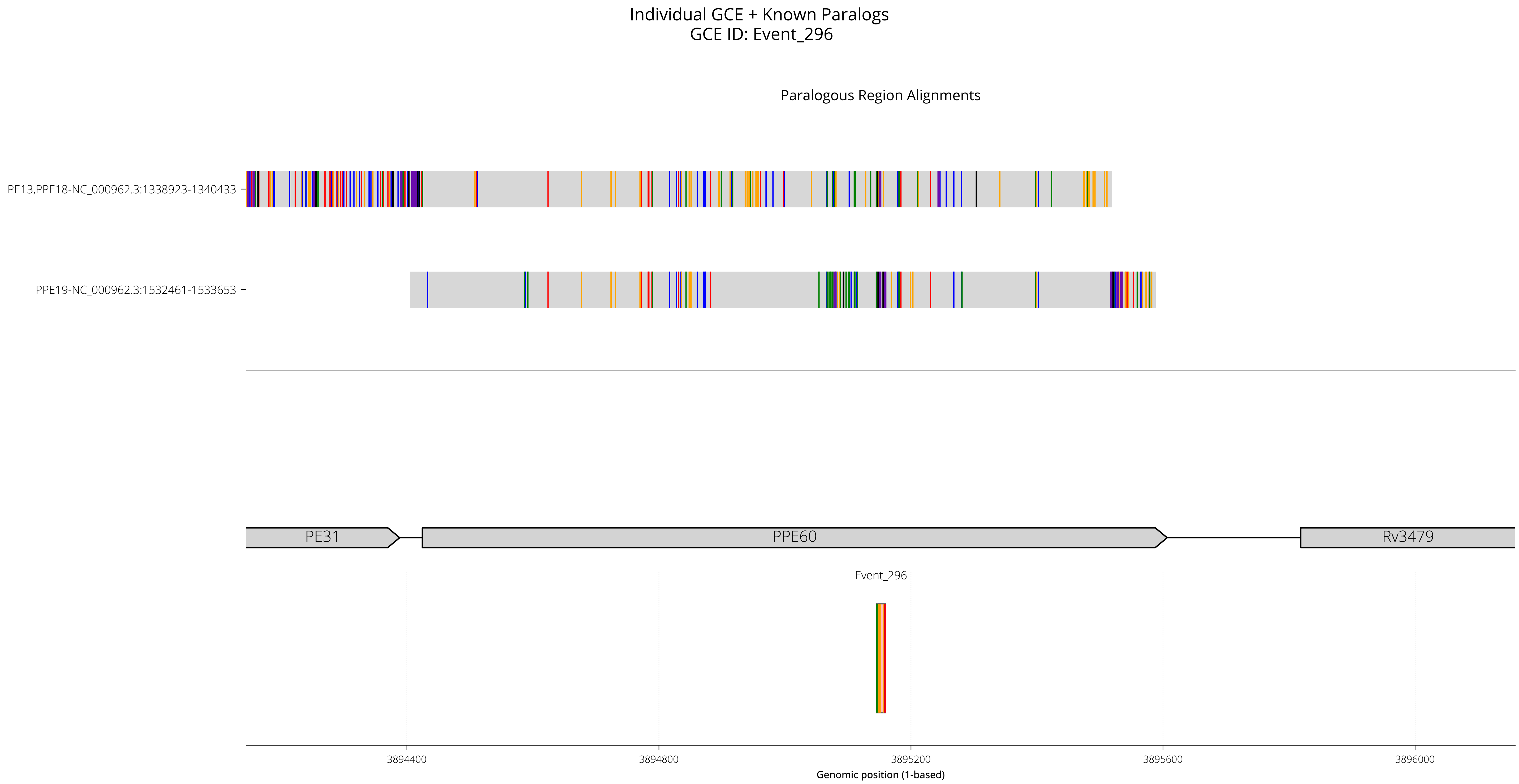
Individual GCE + Known Paralogs
GCE ID: Event_296
Paralogous Region Alignments
PE13,PPE18-NC_000962.3:1338923-1340433
PPE19-NC_000962.3:1532461-1533653
PE31
PPE60
Rv3479
Event_296
3894400
3894800
3895200
3895600
3896000
Genomic position (1-based)

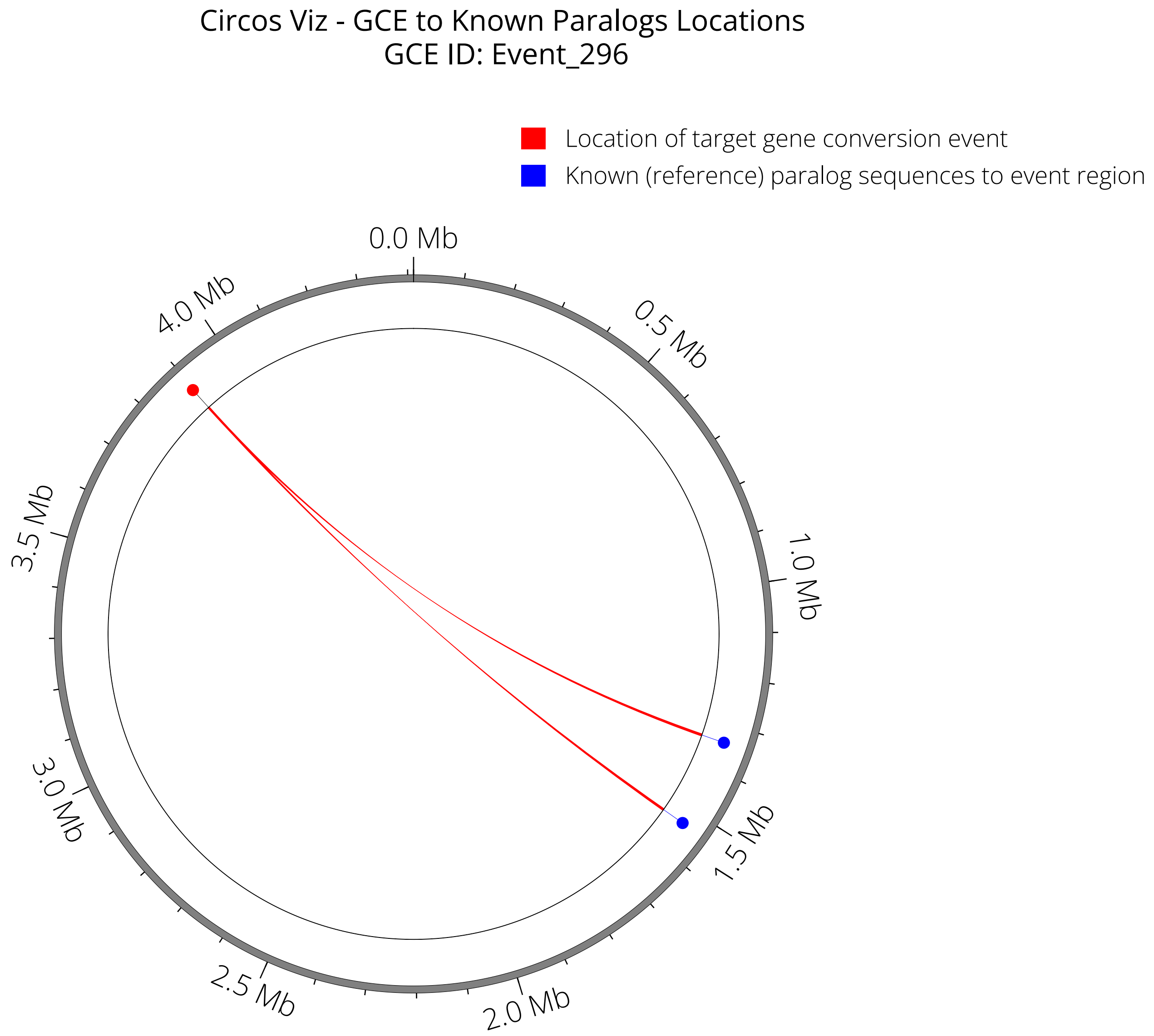

Circos Viz - GCE to Known Paralogs Locations
GCE ID: Event_296
Location of target gene conversion event
Known (reference) paralog sequences to event region
0.0 Mb
0.5 Mb
1.0 Mb
1.5 Mb
2.0 Mb
2.5 Mb
3.0 Mb
3.5 Mb
4.0 Mb

# Event_296 - 7 SNPs - k-mer analysis relative to paralogs

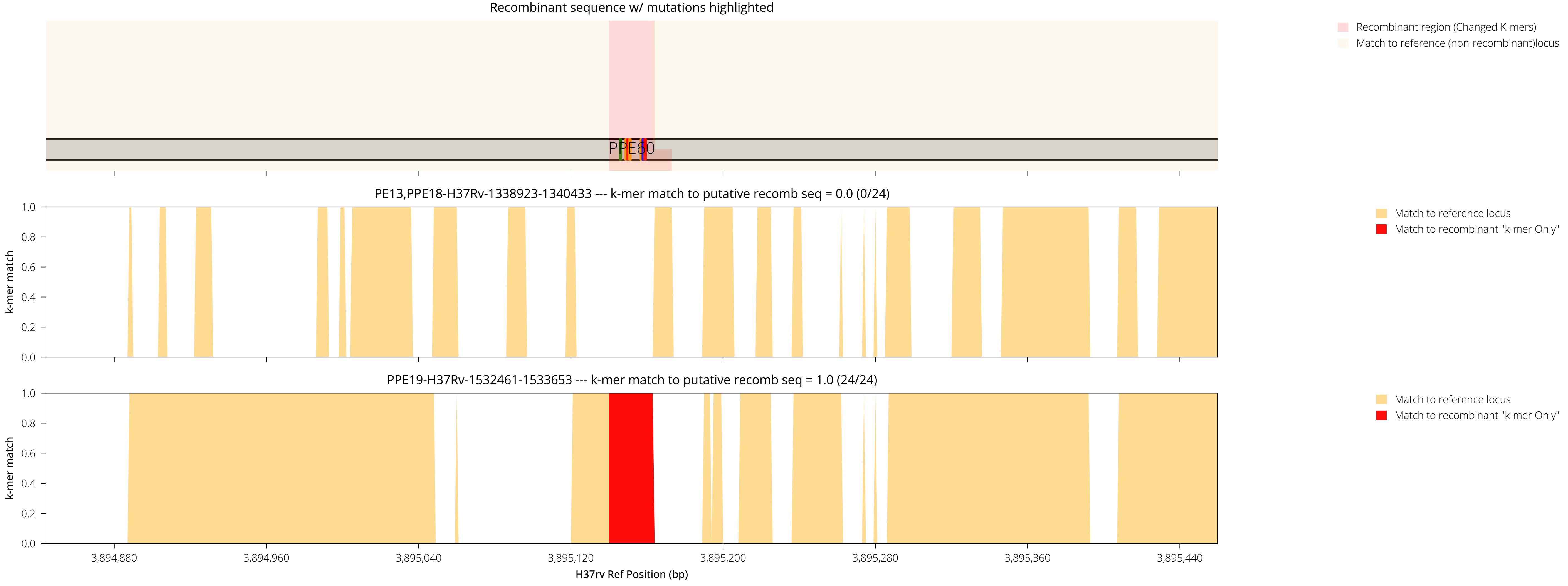

Sub-phylogeny Viz + Paralog Alignments
GCE ID: Event_296

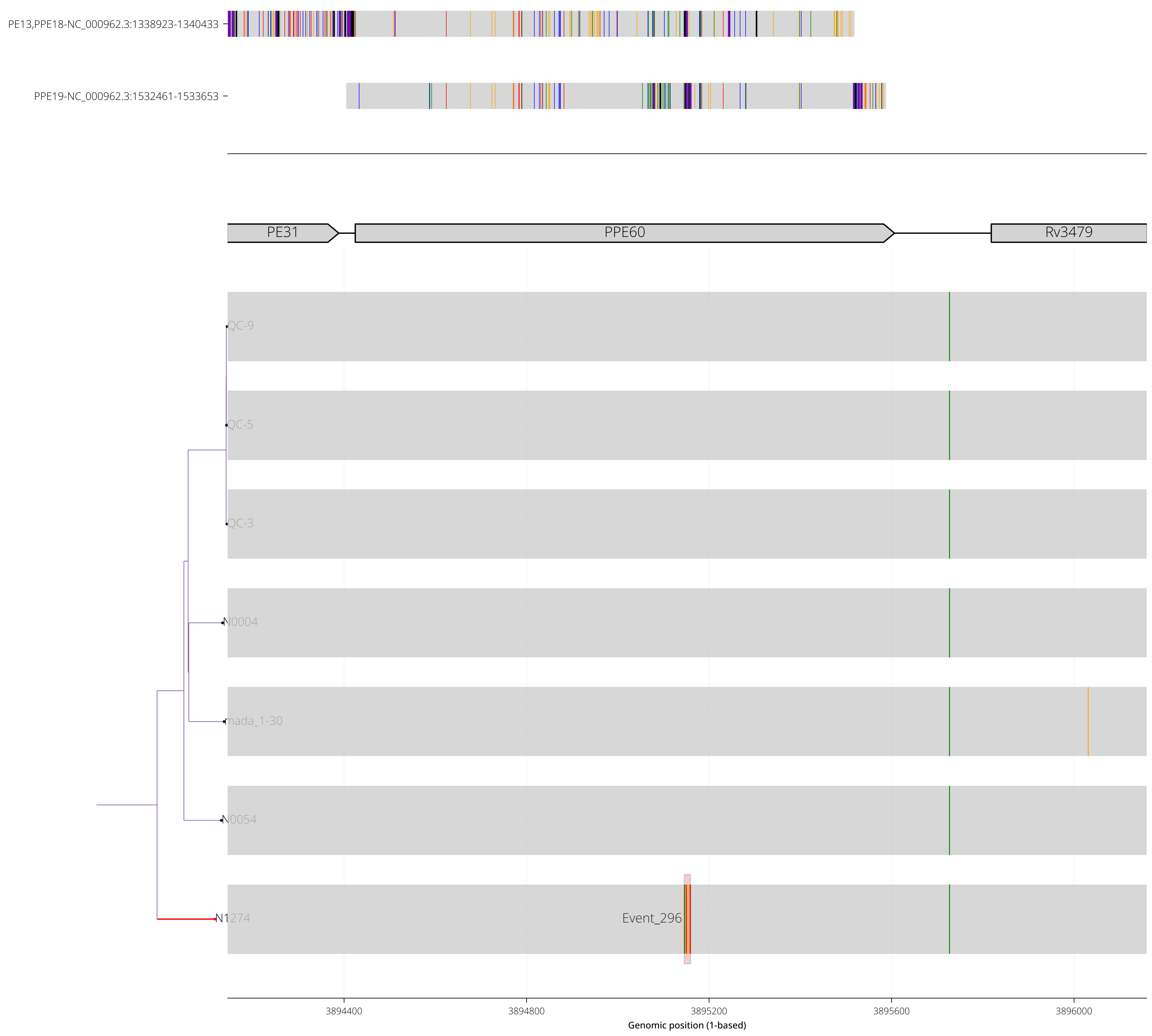

Full Phylogeny Viz + Paralog Alignments
GCE ID: Event_296

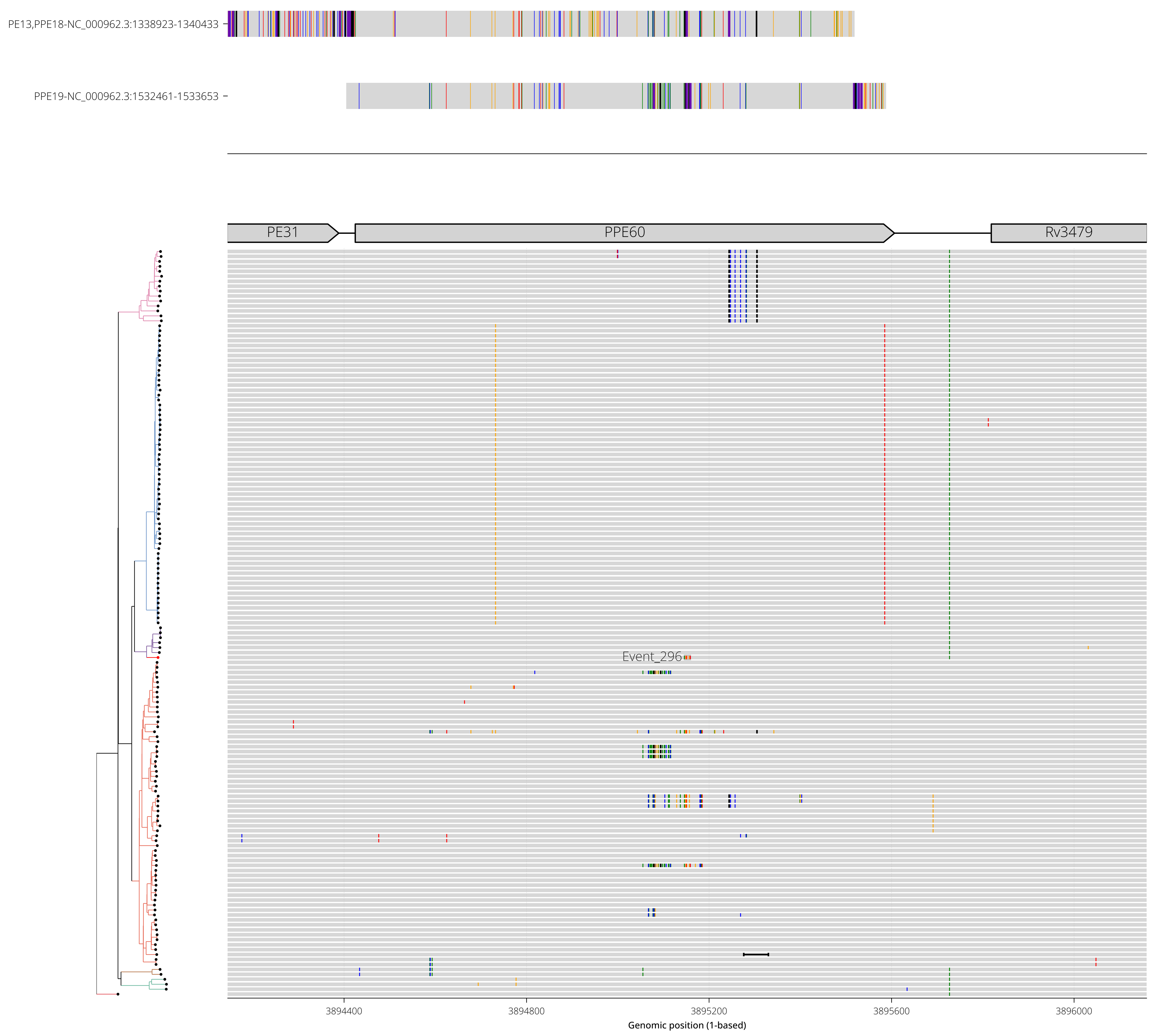

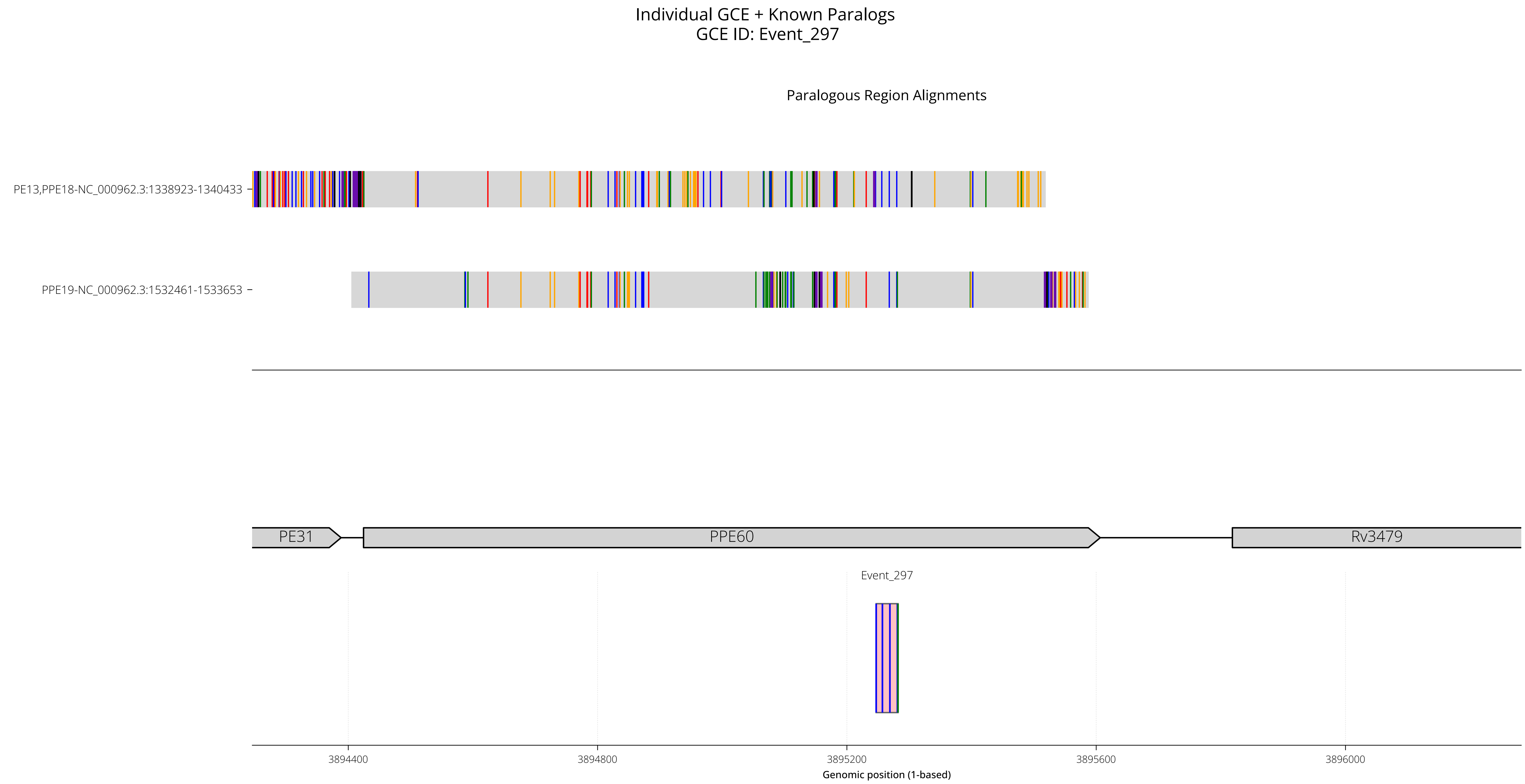

Individual GCE + Known Paralogs
GCE ID: Event_297
Paralogous Region Alignments
PE13,PPE18-NC_000962.3:1338923-1340433
PPE19-NC_000962.3:1532461-1533653
PE31
PPE60
Rv3479
Event_297
3894400
3894800
3895200
3895600
3896000
Genomic position (1-based)

Circos Viz - GCE to Known Paralogs Locations
GCE ID: Event_297

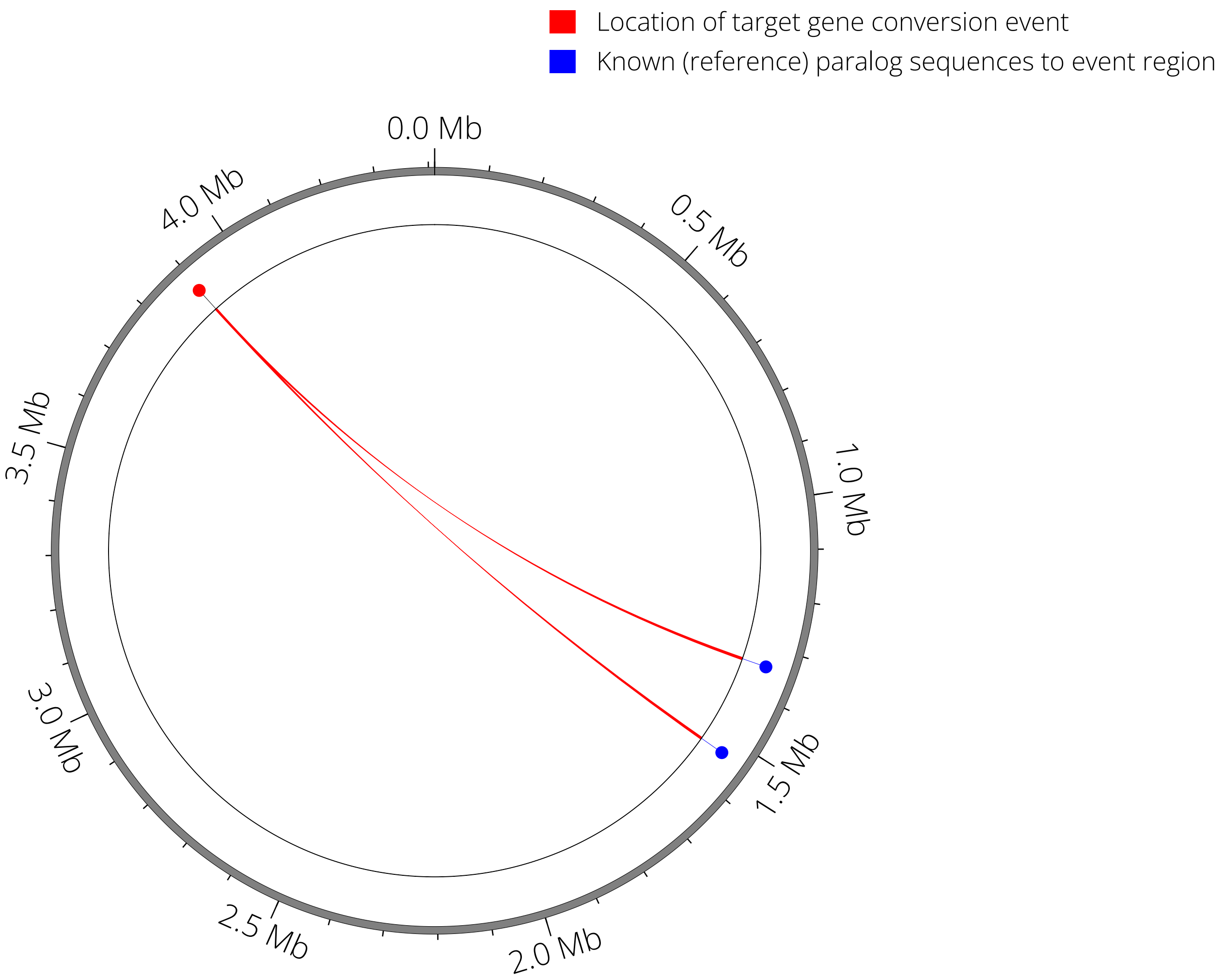

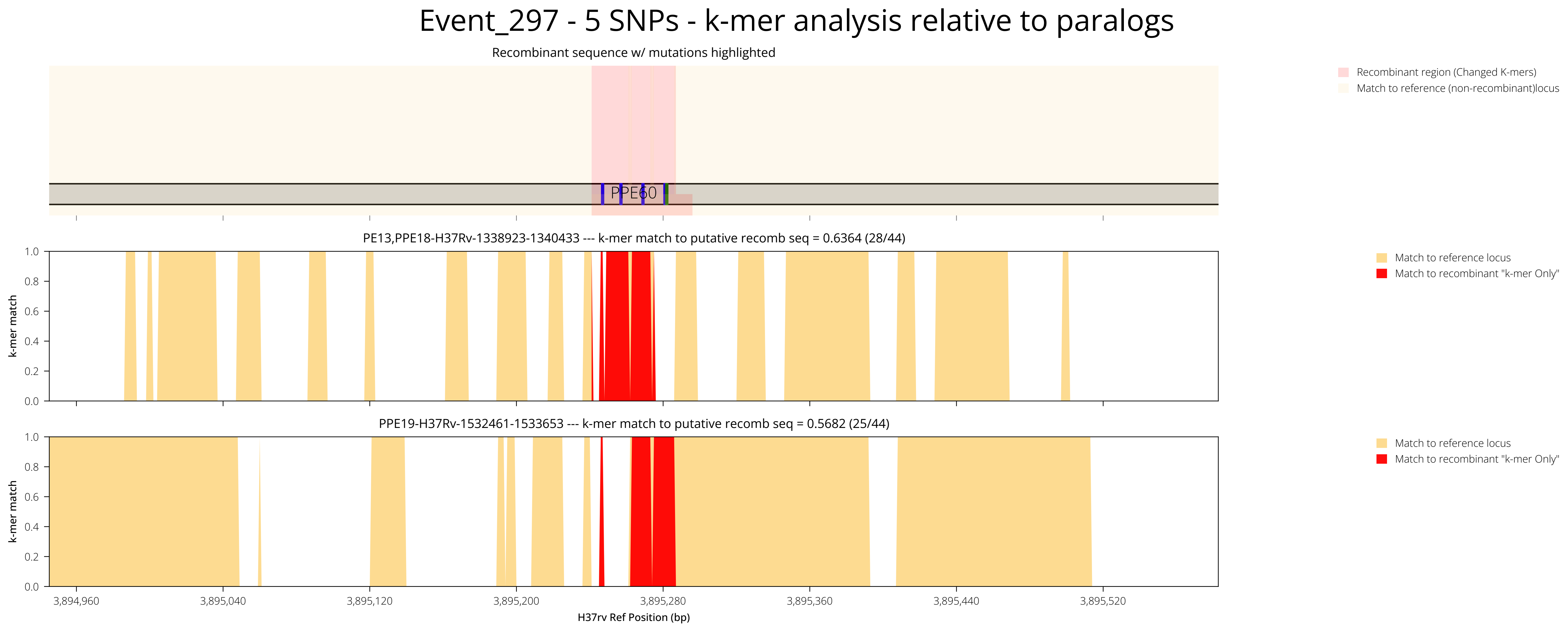
Event_297 - 5 SNPs - k-mer analysis relative to paralogs
Recombinant sequence w/ mutations highlighted
Recombinant region (Changed K-mers)
Match to reference (non-recombinant)locus
PPE60
PE13,PPE18-H37Rv-1338923-1340433 --- k-mer match to putative recomb seq = 0.6364 (28/44)
Match to reference locus
Match to recombinant "k-mer Only"
k-mer match
1.0
0.8
0.6
0.4
0.2
0.0
PPE19-H37Rv-1532461-1533653 --- k-mer match to putative recomb seq = 0.5682 (25/44)
Match to reference locus
Match to recombinant "k-mer Only"
k-mer match
3,894,960
3,895,040
3,895,120
3,895,200
3,895,280
3,895,360
3,895,440
3,895,520
H37rv Ref Position (bp)

Sub-phylogeny Viz + Paralog Alignments
GCE ID: Event_297

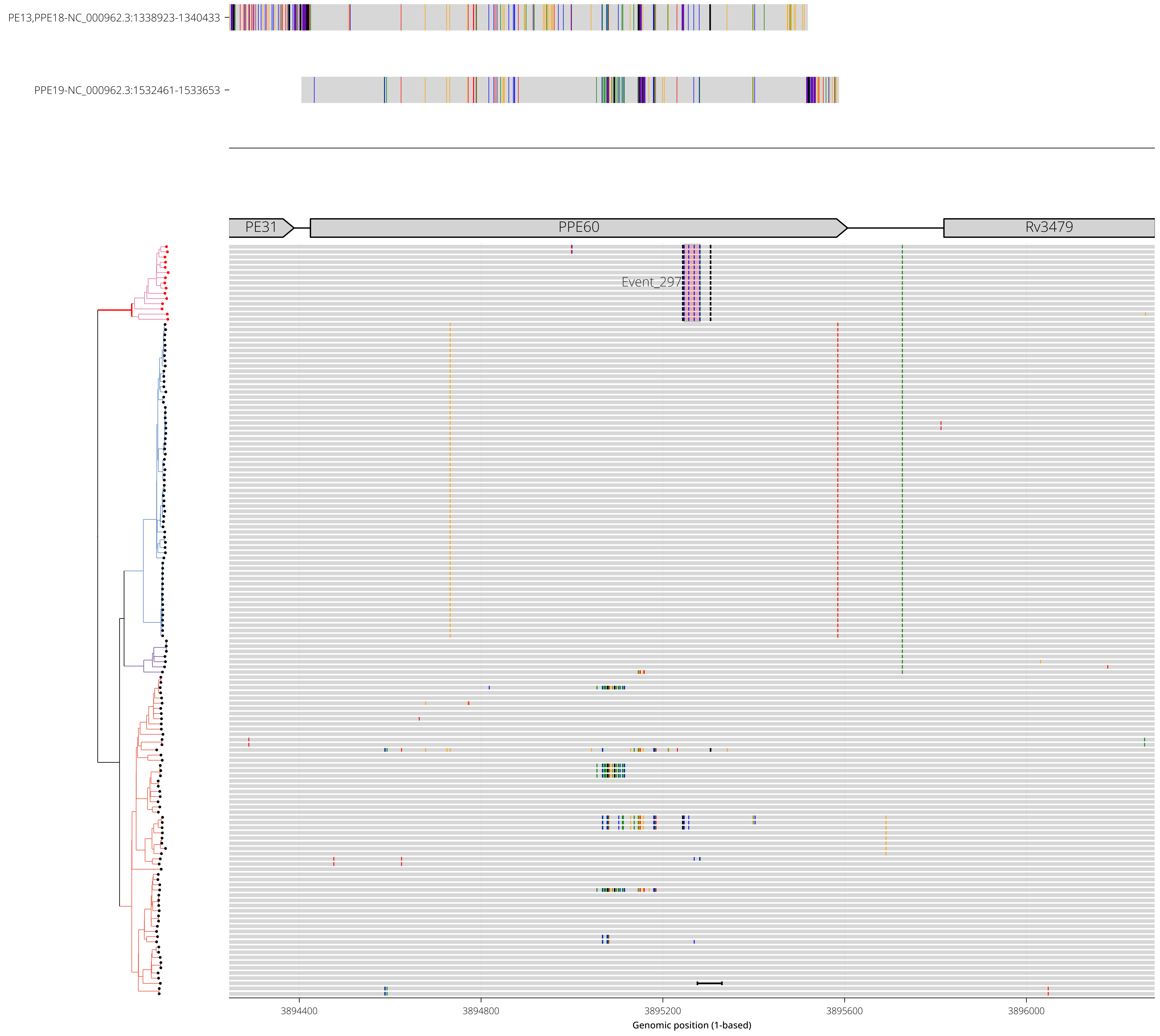

Full Phylogeny Viz + Paralog Alignments
GCE ID: Event_297

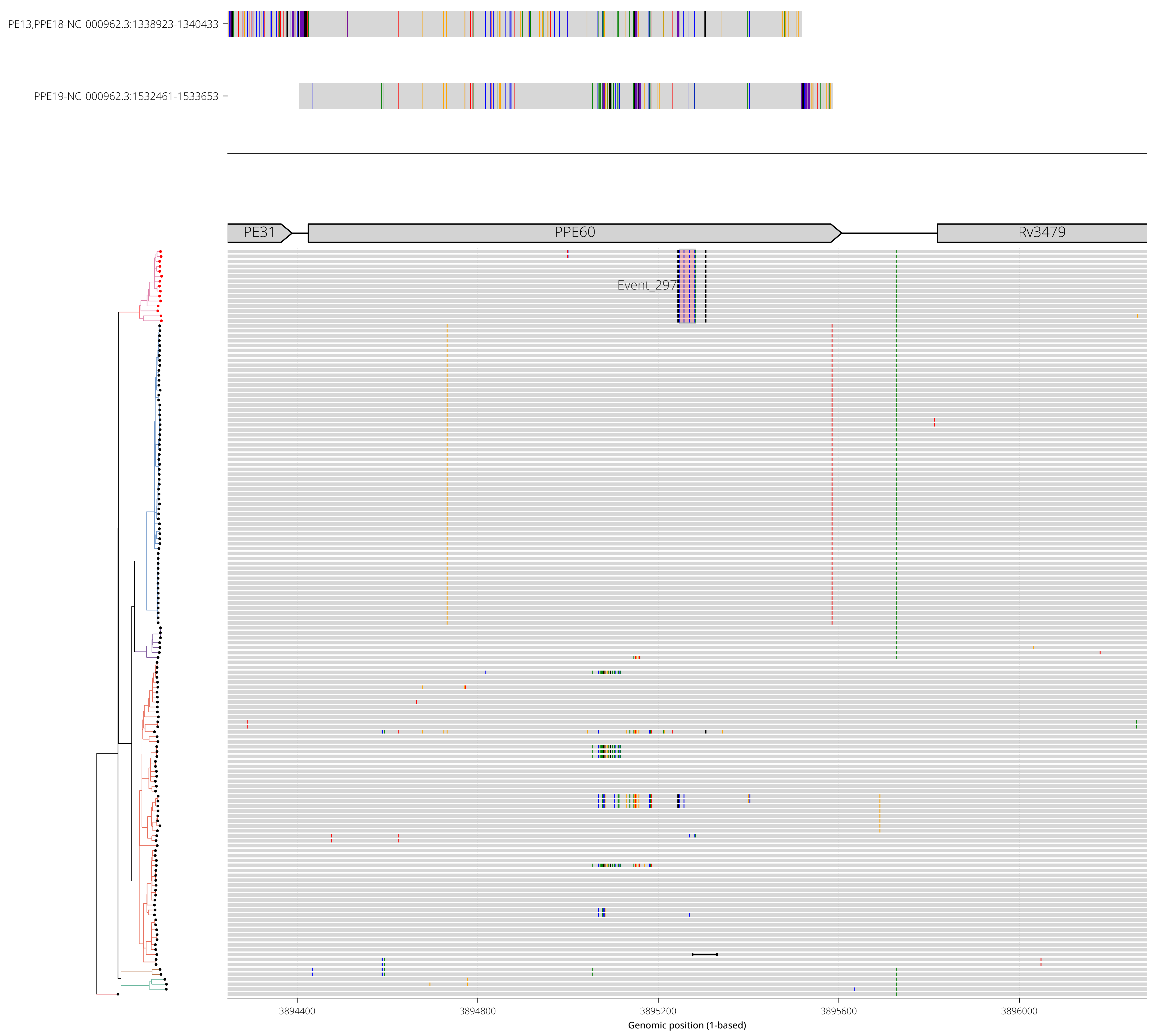

Individual GCE + Known Paralogs
GCE ID: Event_298

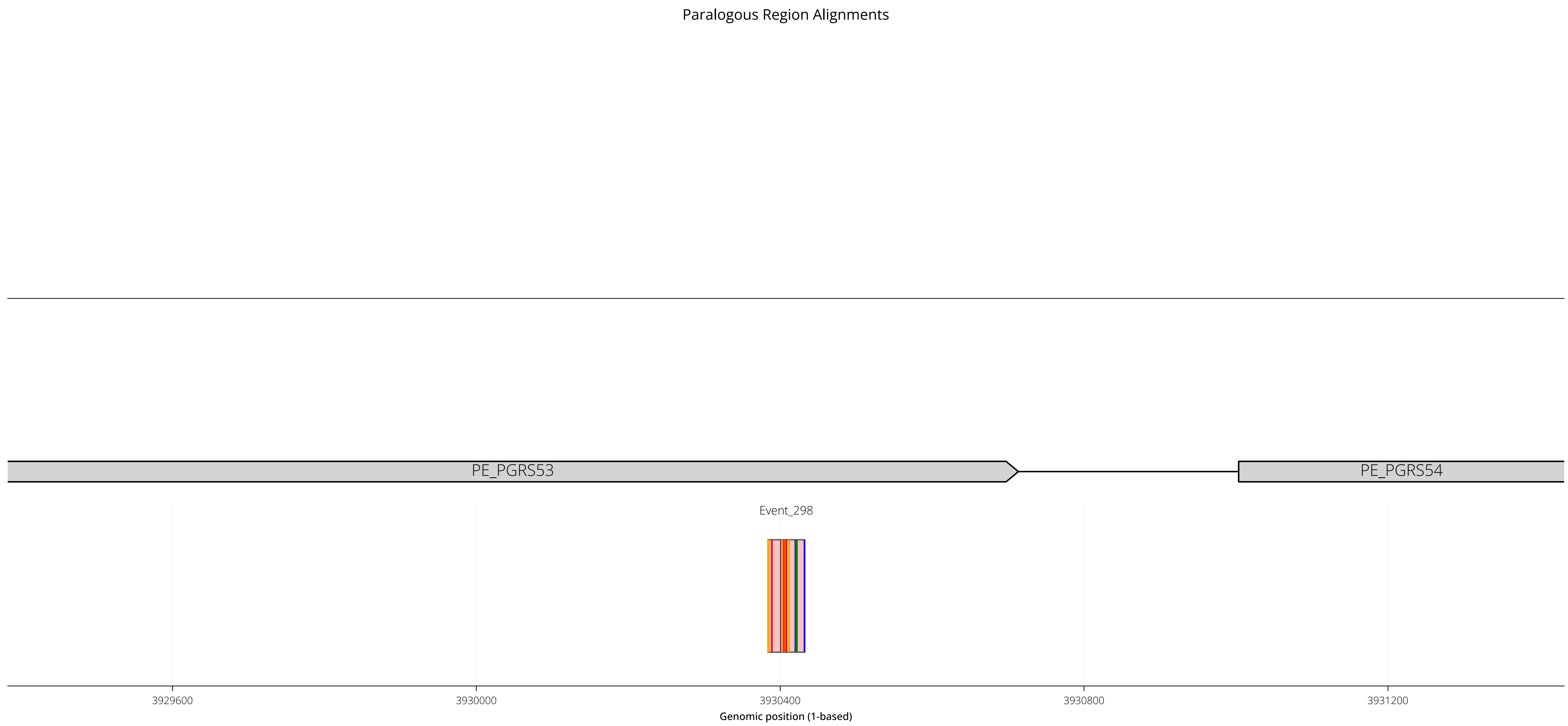

Sub-phylogeny Viz + Paralog Alignments
GCE ID: Event_298

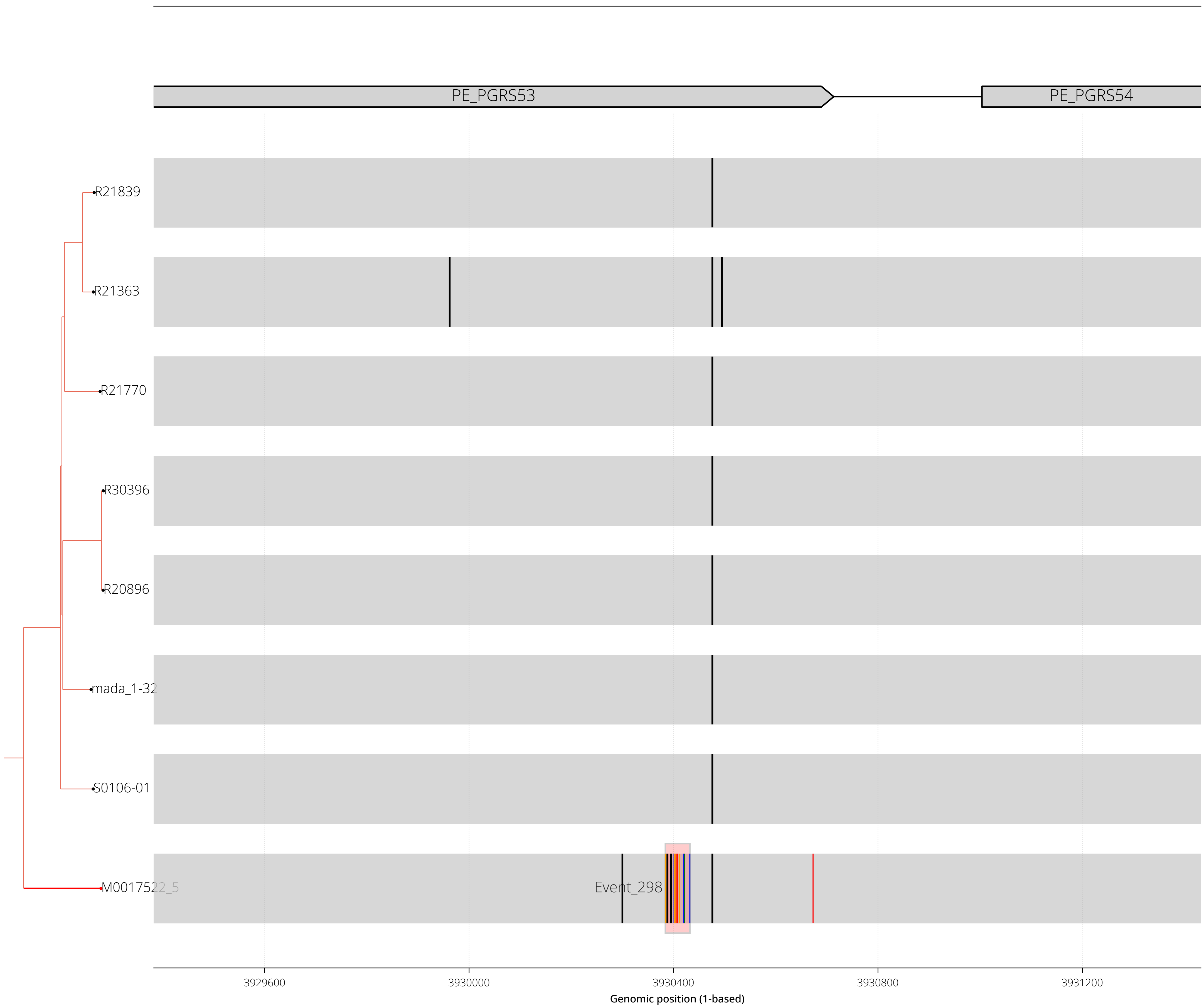

Full Phylogeny Viz + Paralog Alignments
GCE ID: Event_298

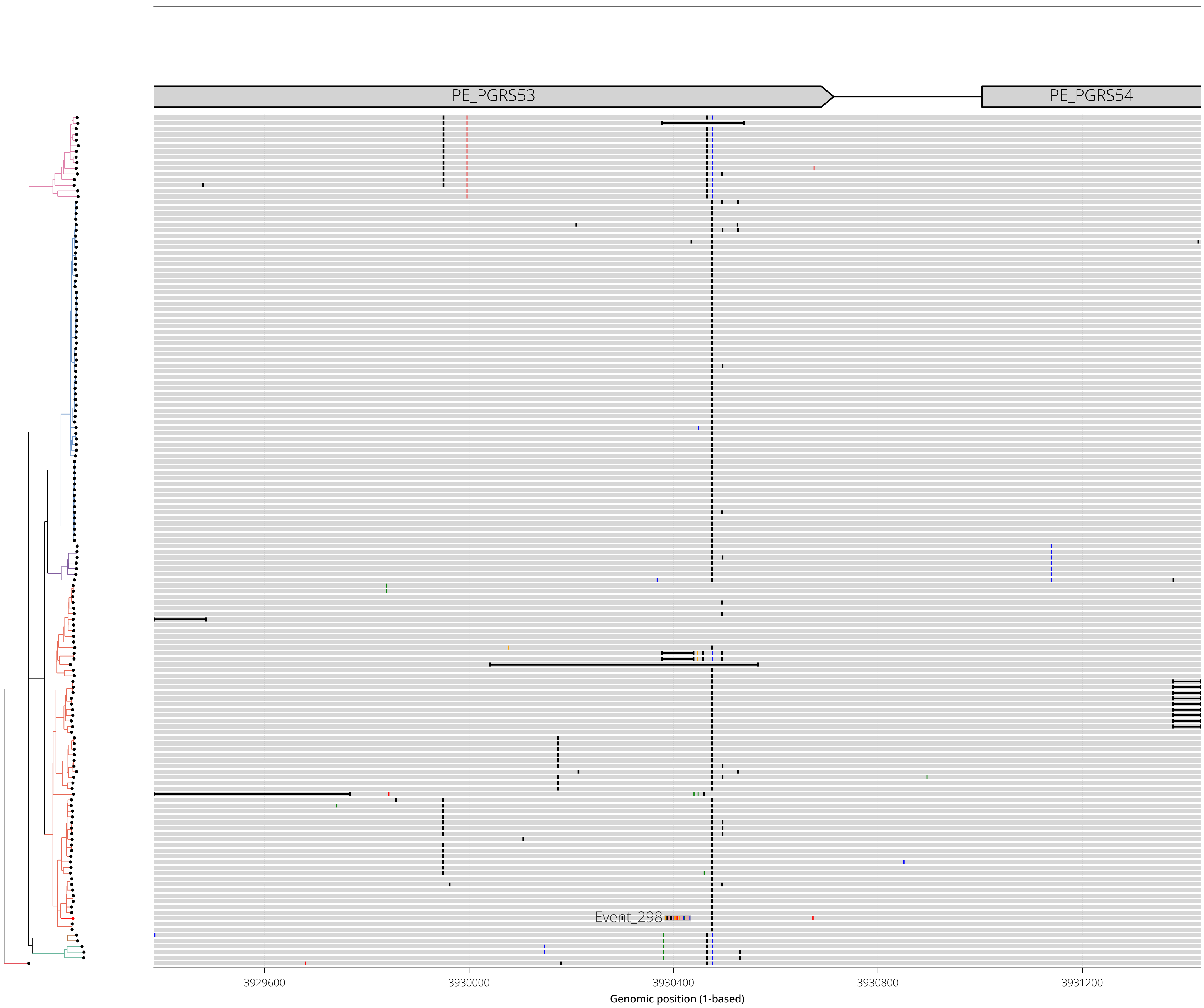

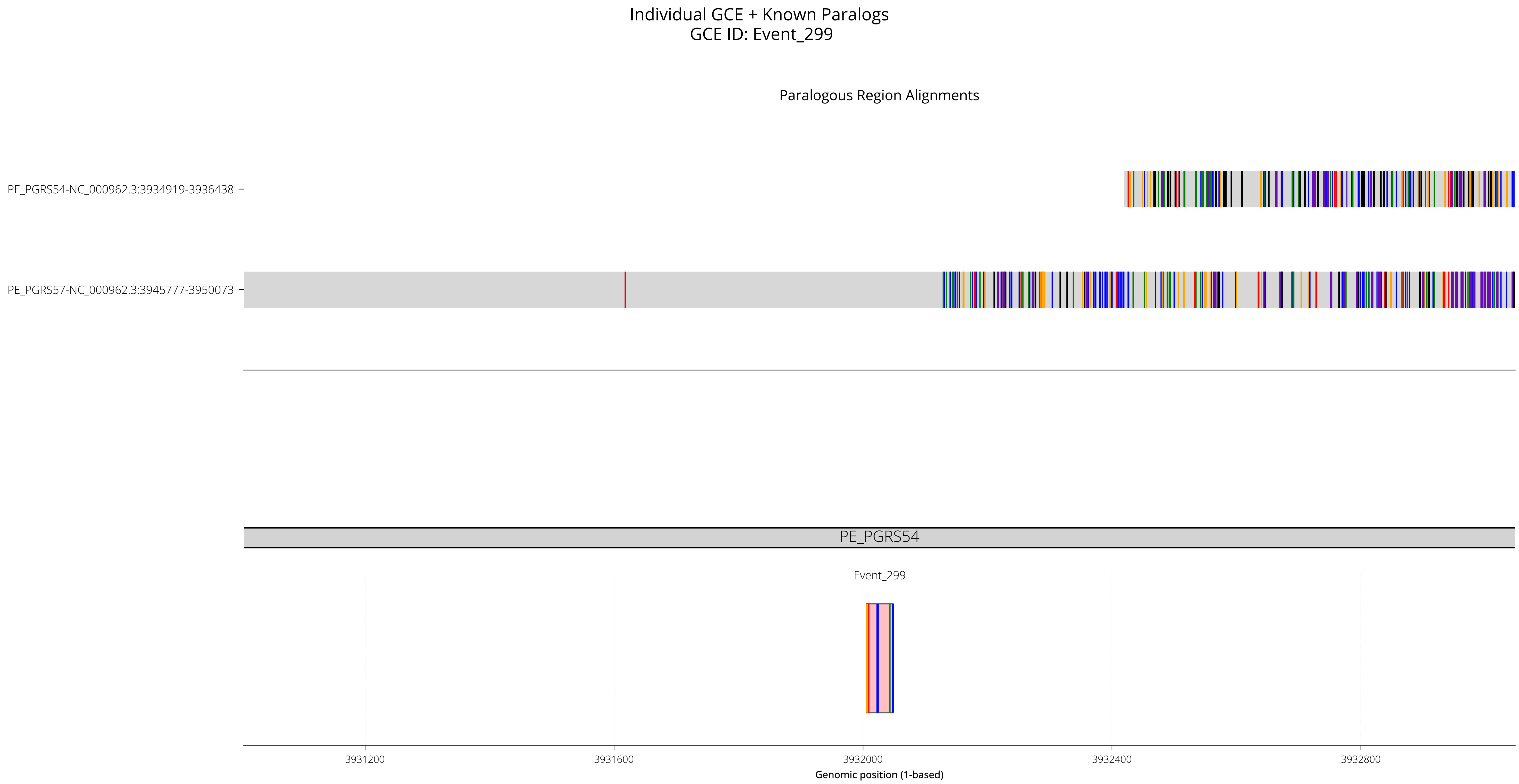
Individual GCE + Known Paralogs
GCE ID: Event_299
Paralogous Region Alignments
PE_PGRS54-NC_000962.3:3934919-3936438
PE_PGRS57-NC_000962.3:3945777-3950073
PE_PGRS54
Event_299
3931200
3931600
3932000
3932400
3932800
Genomic position (1-based)

Circos Viz - GCE to Known Paralogs Locations
GCE ID: Event_299

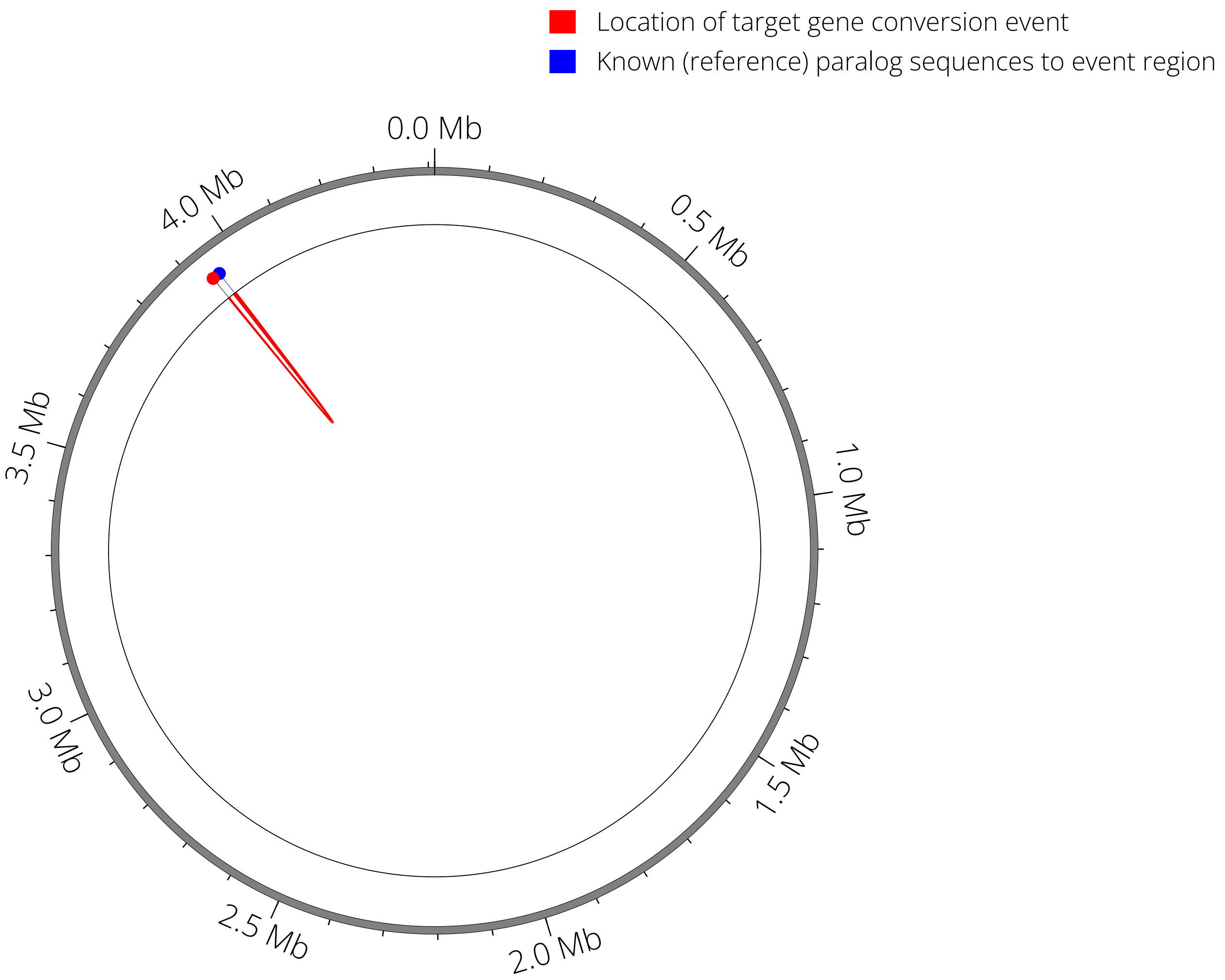

# Event_299 - 6 SNPs - k-mer analysis relative to paralogs

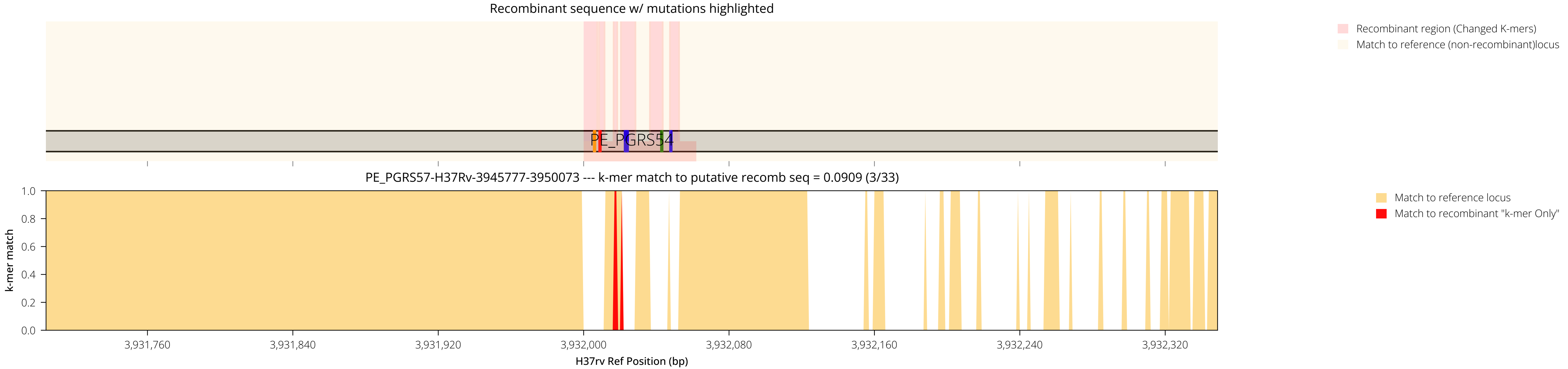

Sub-phylogeny Viz + Paralog Alignments
GCE ID: Event_299

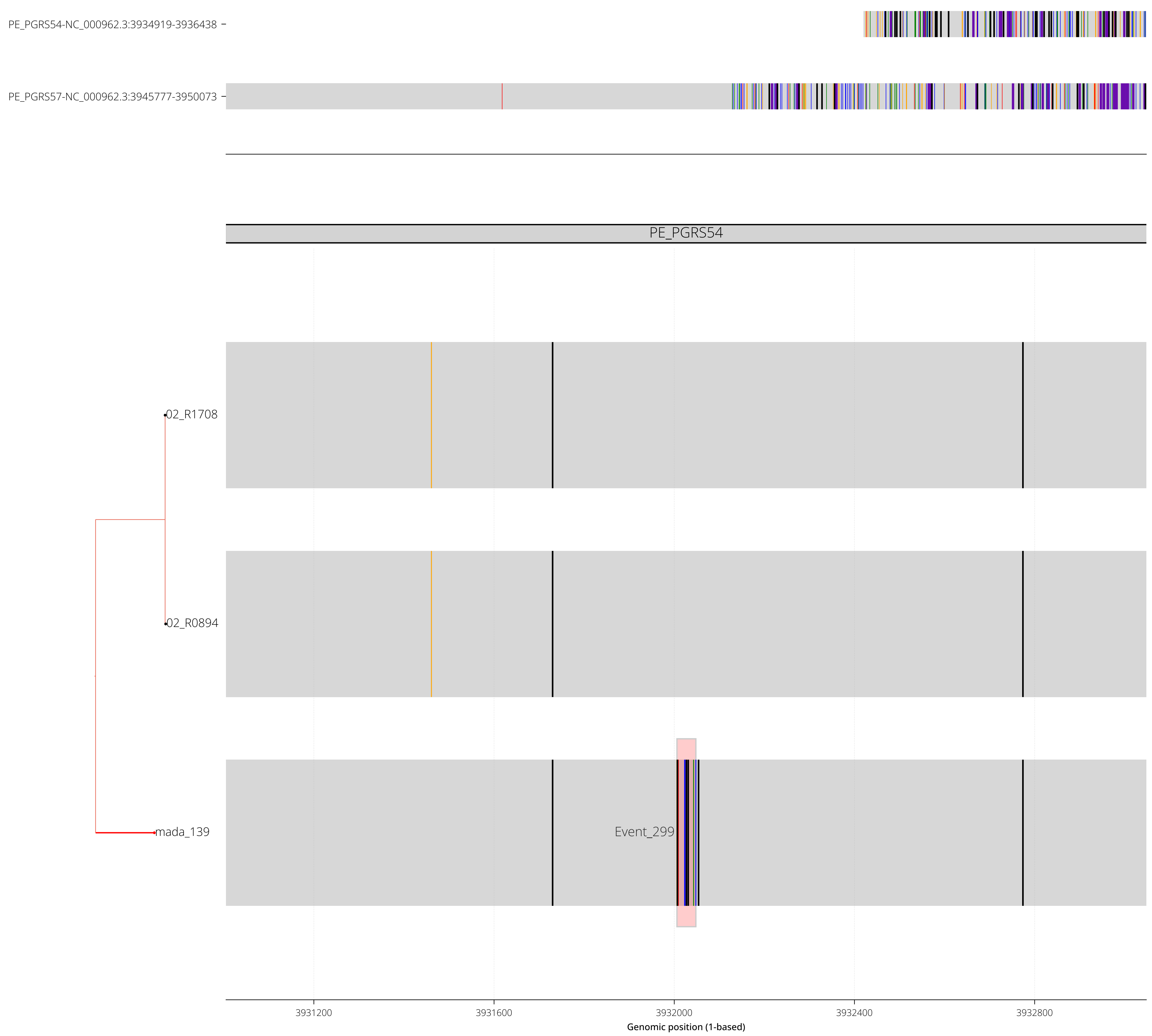

Full Phylogeny Viz + Paralog Alignments
GCE ID: Event_299

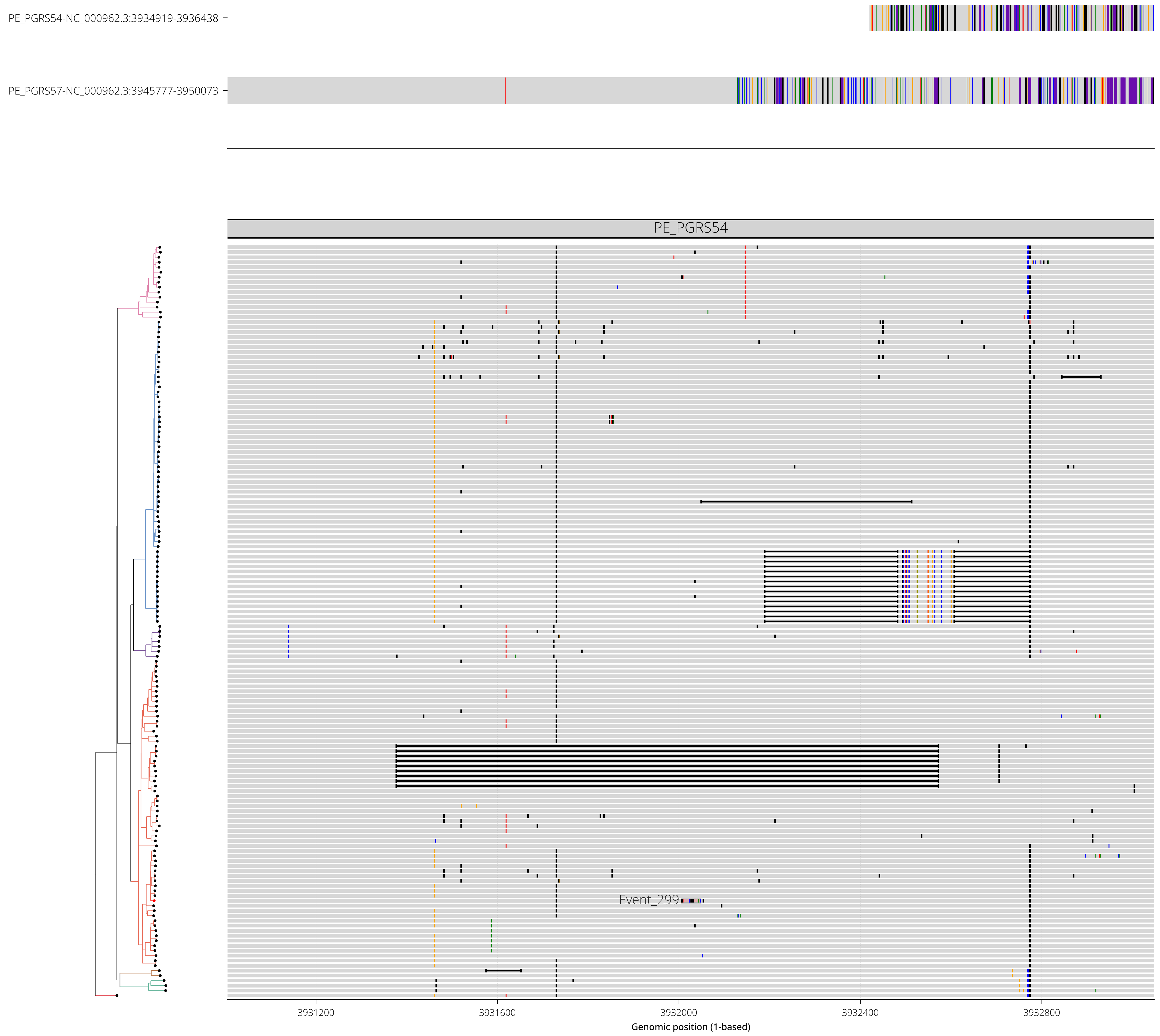

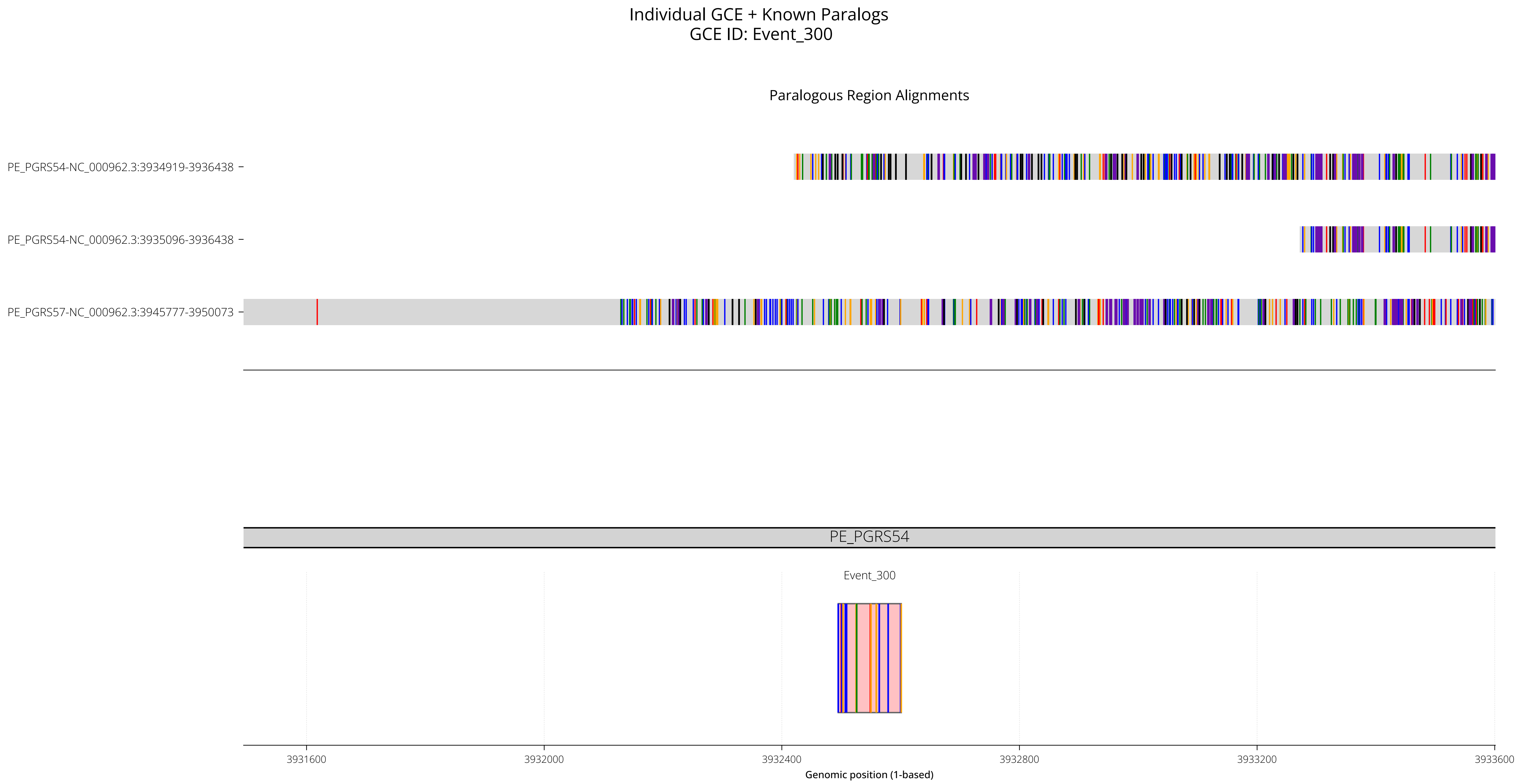
Individual GCE + Known Paralogs
GCE ID: Event_300
Paralogous Region Alignments
PE_PGRS54-NC_000962.3:3934919-3936438
PE_PGRS54-NC_000962.3:3935096-3936438
PE_PGRS57-NC_000962.3:3945777-3950073
PE_PGRS54
Event_300
3931600
3932000
3932400
3932800
3933200
3933600
Genomic position (1-based)

Circos Viz - GCE to Known Paralogs Locations
GCE ID: Event_300

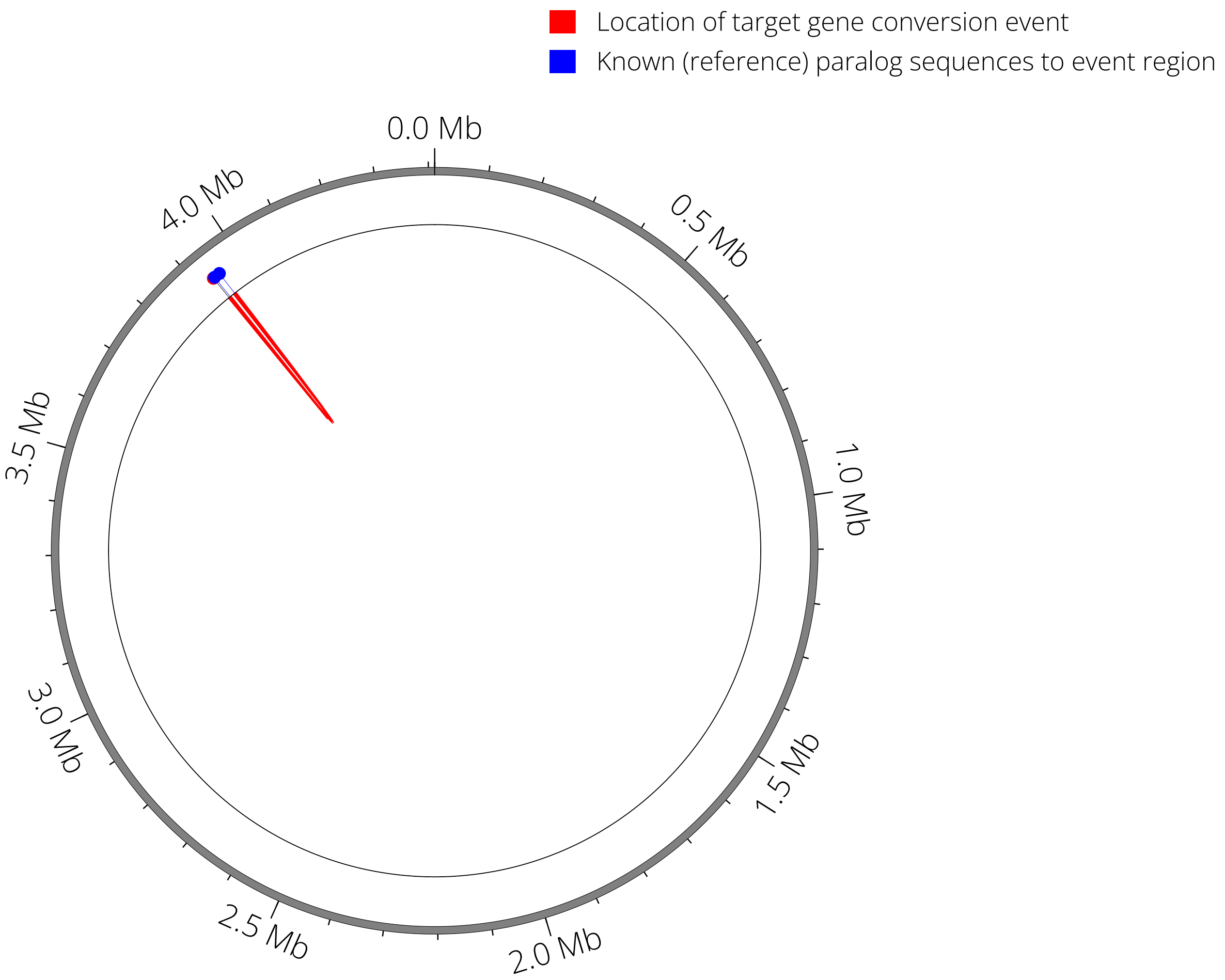

# Event_300 - 15 SNPs - k-mer analysis relative to paralogs

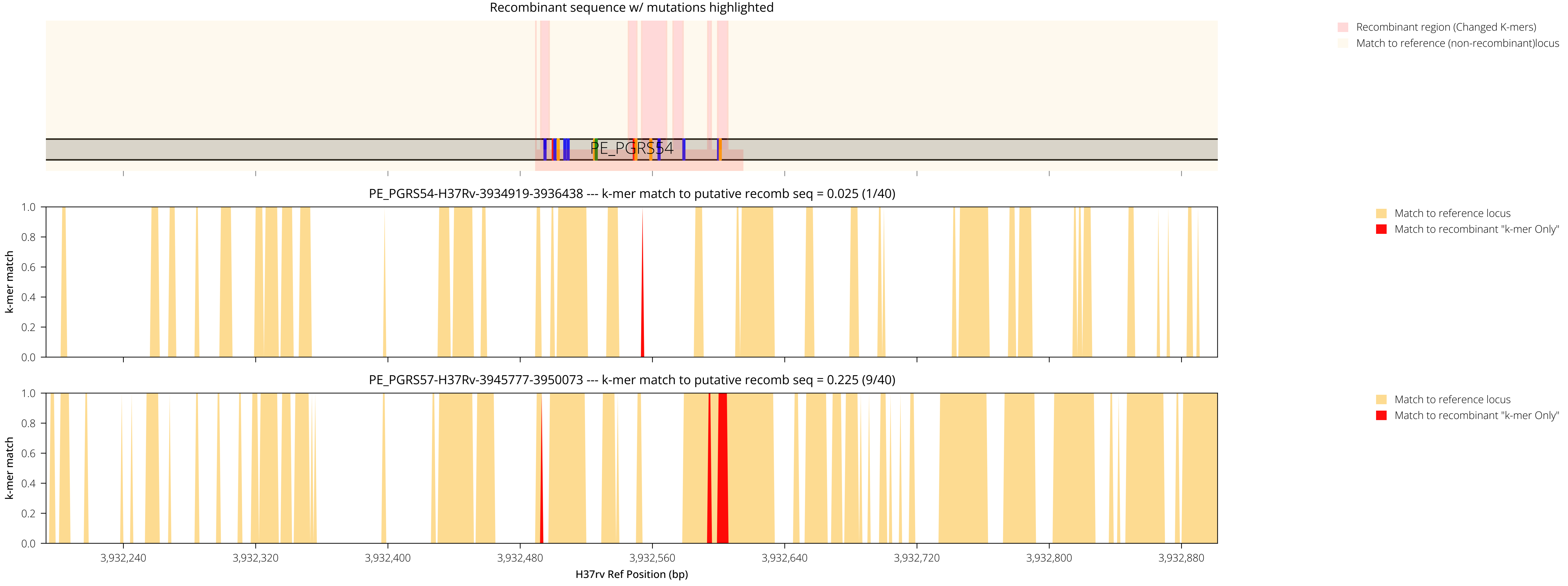

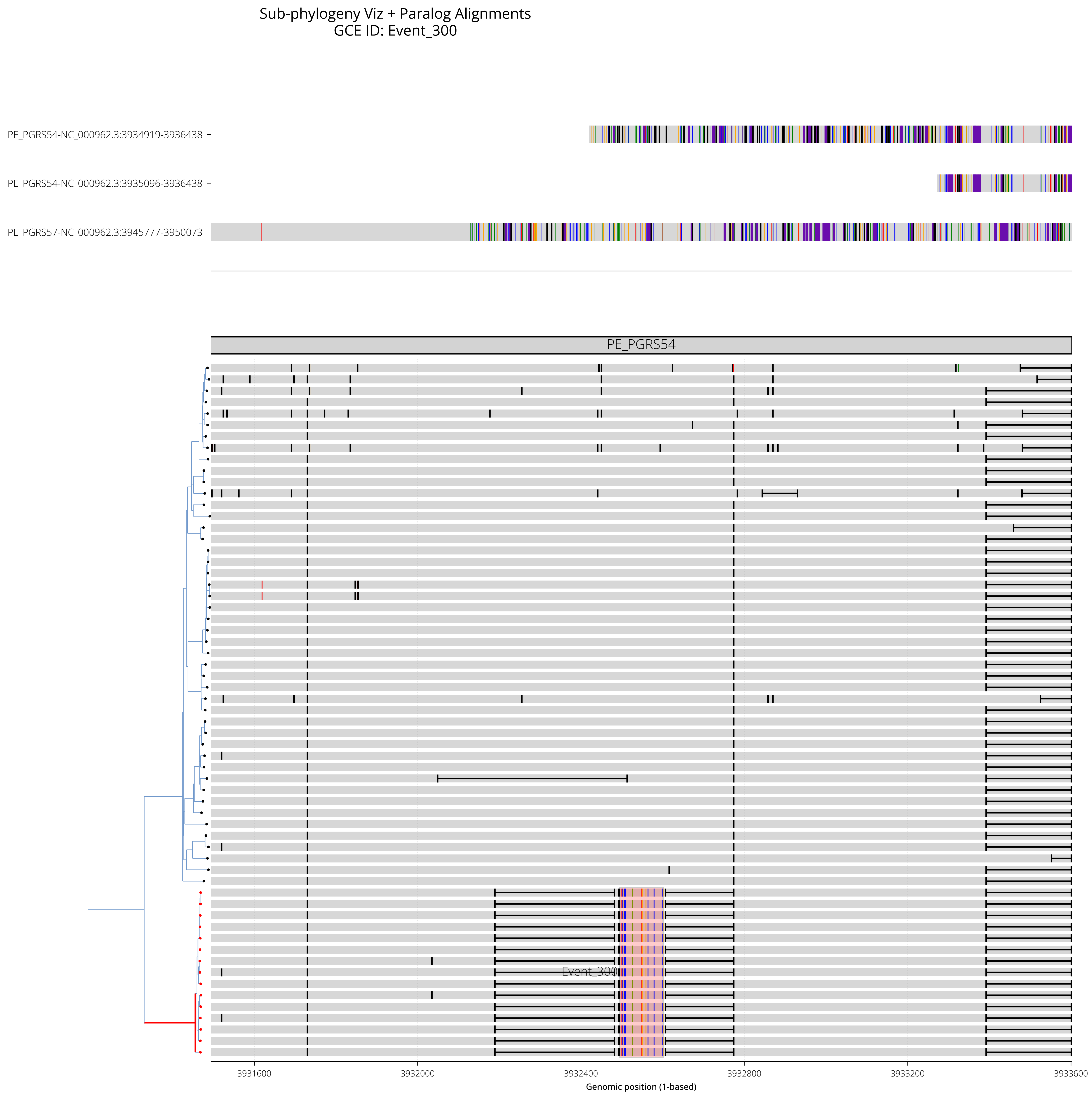

Sub-phylogeny Viz + Paralog Alignments
GCE ID: Event_300
PE_PGRS54-NC_000962.3:3934919-3936438
PE_PGRS54-NC_000962.3:3935096-3936438
PE_PGRS57-NC_000962.3:3945777-3950073
PE_PGRS54
Event_300
3931600
3932000
3932400
3932800
3933200
3933600
Genomic position (1-based)

Full Phylogeny Viz + Paralog Alignments
GCE ID: Event_300

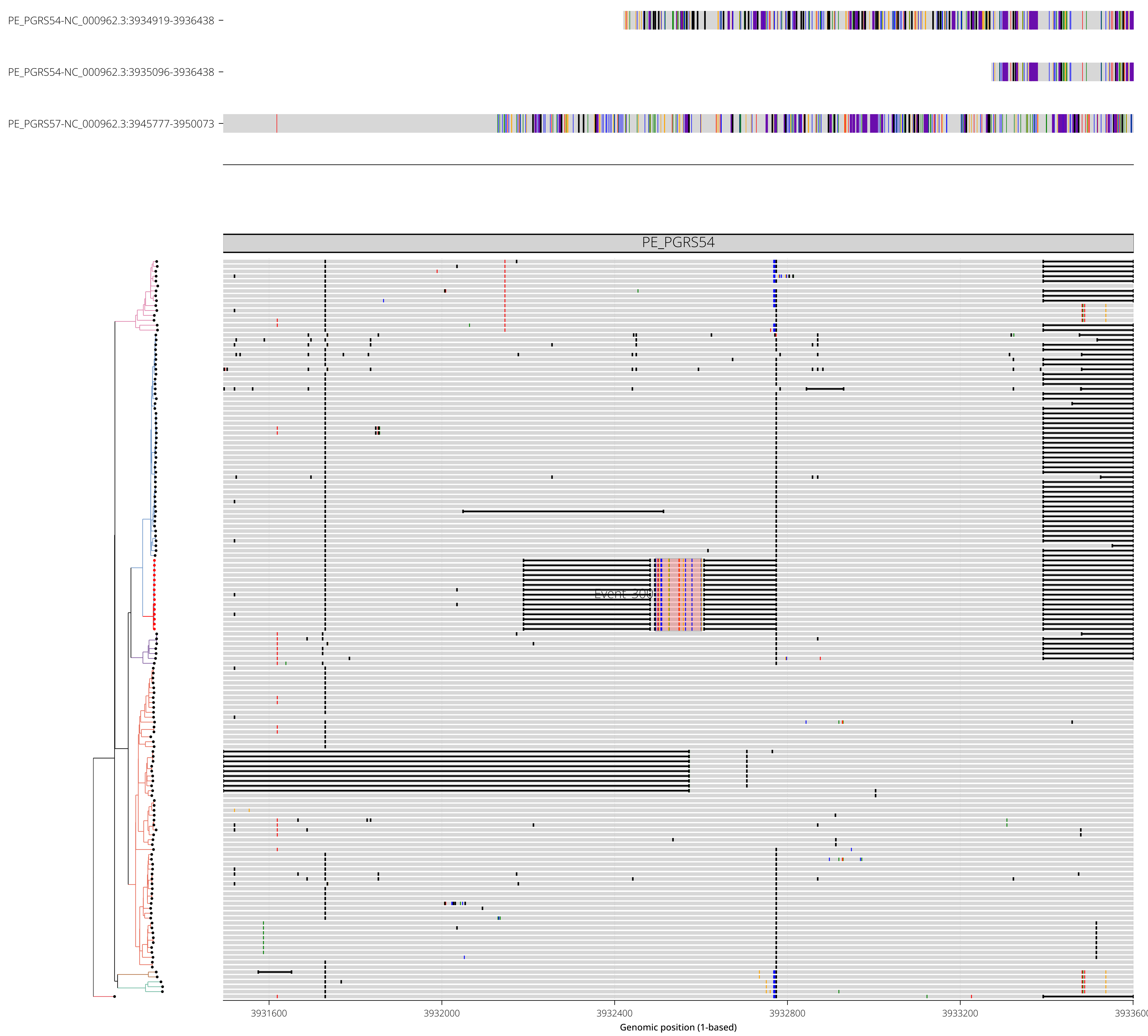

Individual GCE + Known Paralogs
GCE ID: Event_301

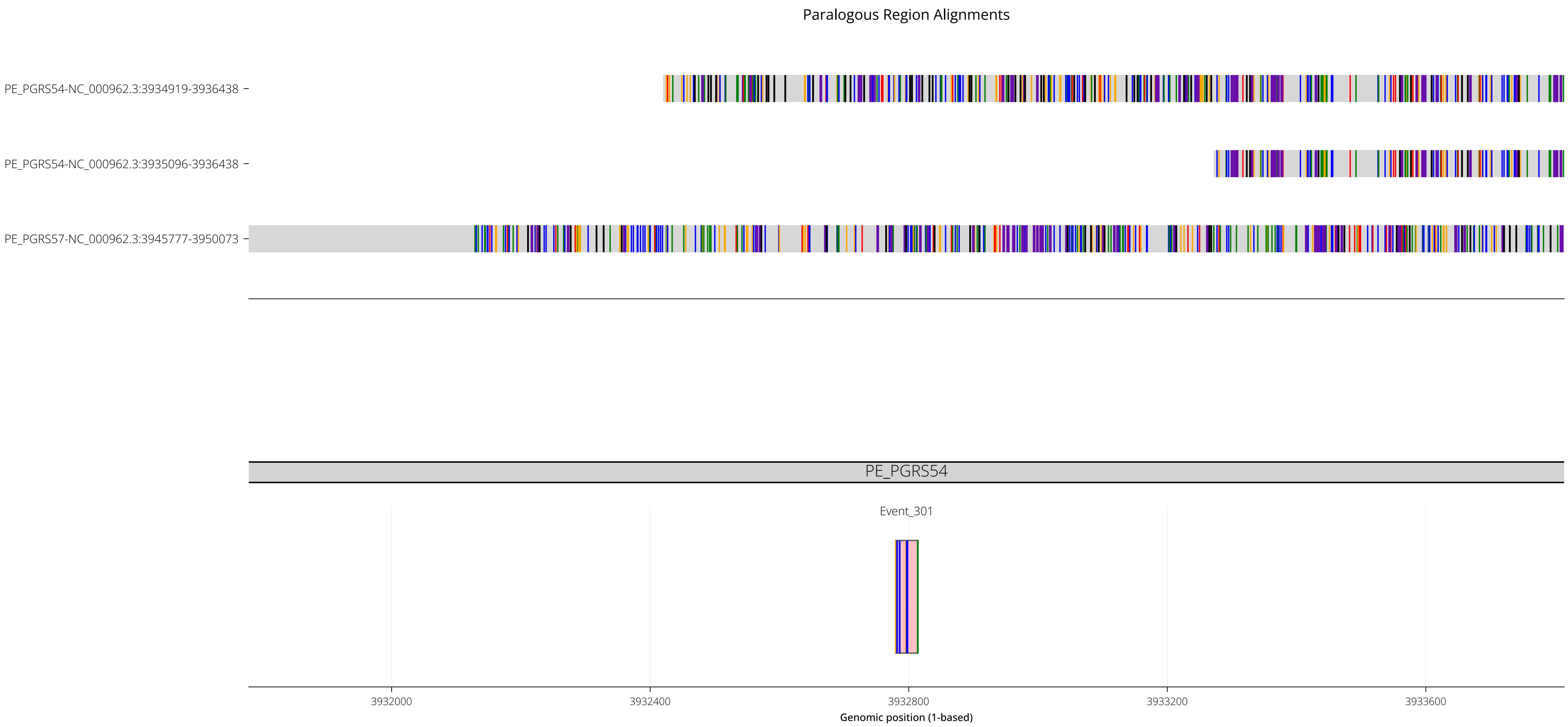

# Circos Viz - GCE to Known Paralogs Locations
GCE ID: Event_301

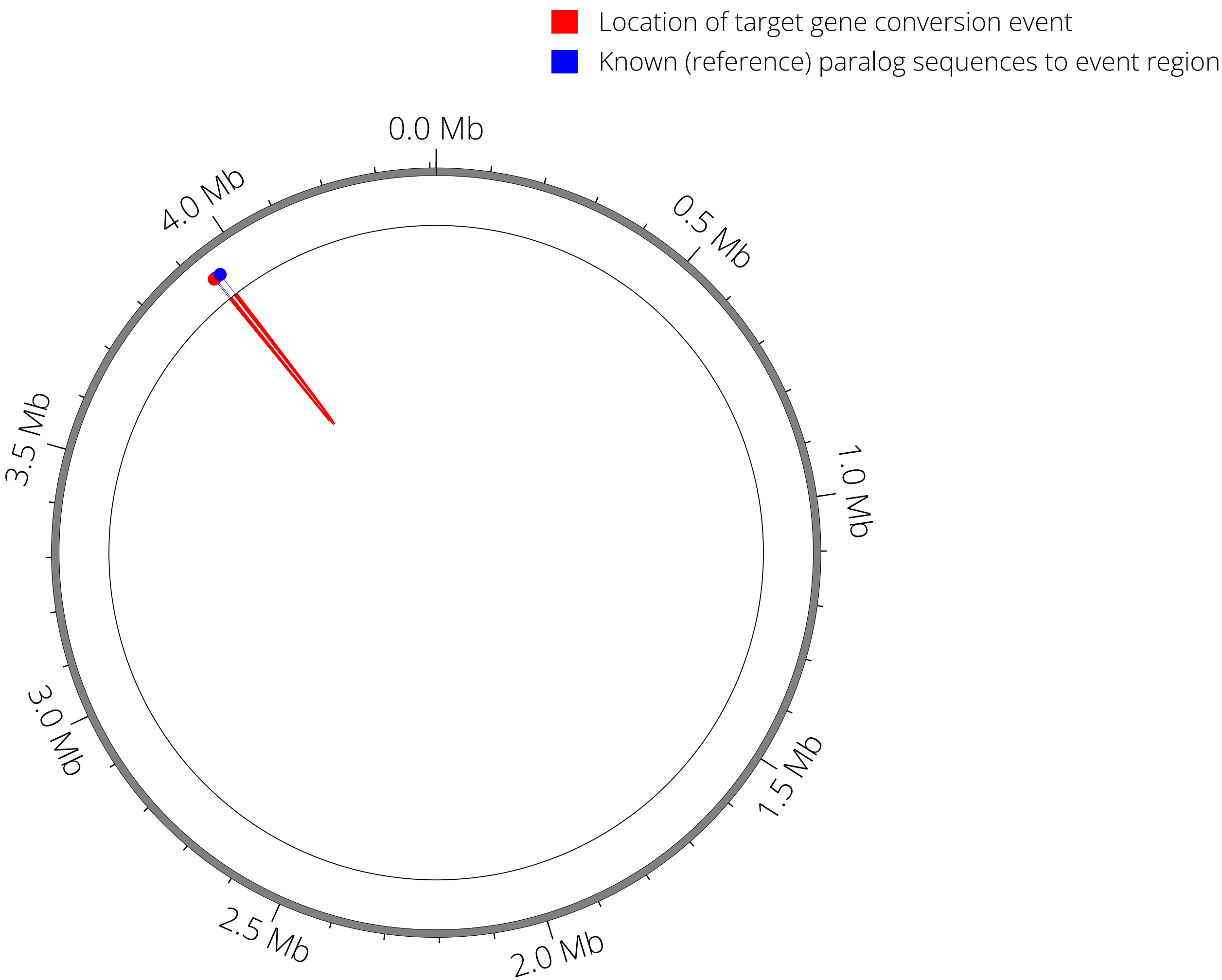

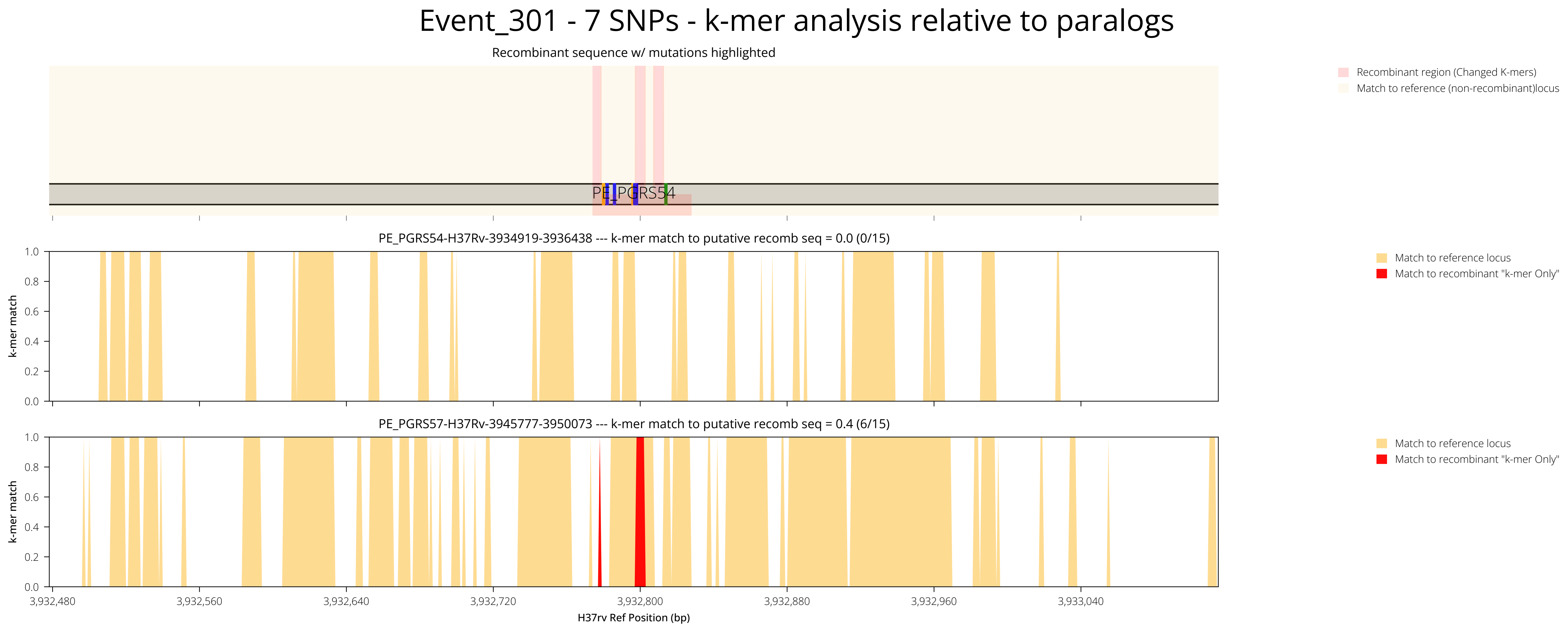

Event_301 - 7 SNPs - k-mer analysis relative to paralogs
Recombinant sequence w/ mutations highlighted
PE_PGRS54
Recombinant region (Changed K-mers)
Match to reference (non-recombinant)locus
PE_PGRS54-H37Rv-3934919-3936438 --- k-mer match to putative recomb seq = 0.0 (0/15)
PE_PGRS57-H37Rv-3945777-3950073 --- k-mer match to putative recomb seq = 0.4 (6/15)
k-mer match
Match to reference locus
Match to recombinant "k-mer Only"
1.0
0.8
0.6
0.4
0.2
0.0
3,932,480
3,932,560
3,932,640
3,932,720
3,932,800
3,932,880
3,932,960
3,933,040
H37rv Ref Position (bp)

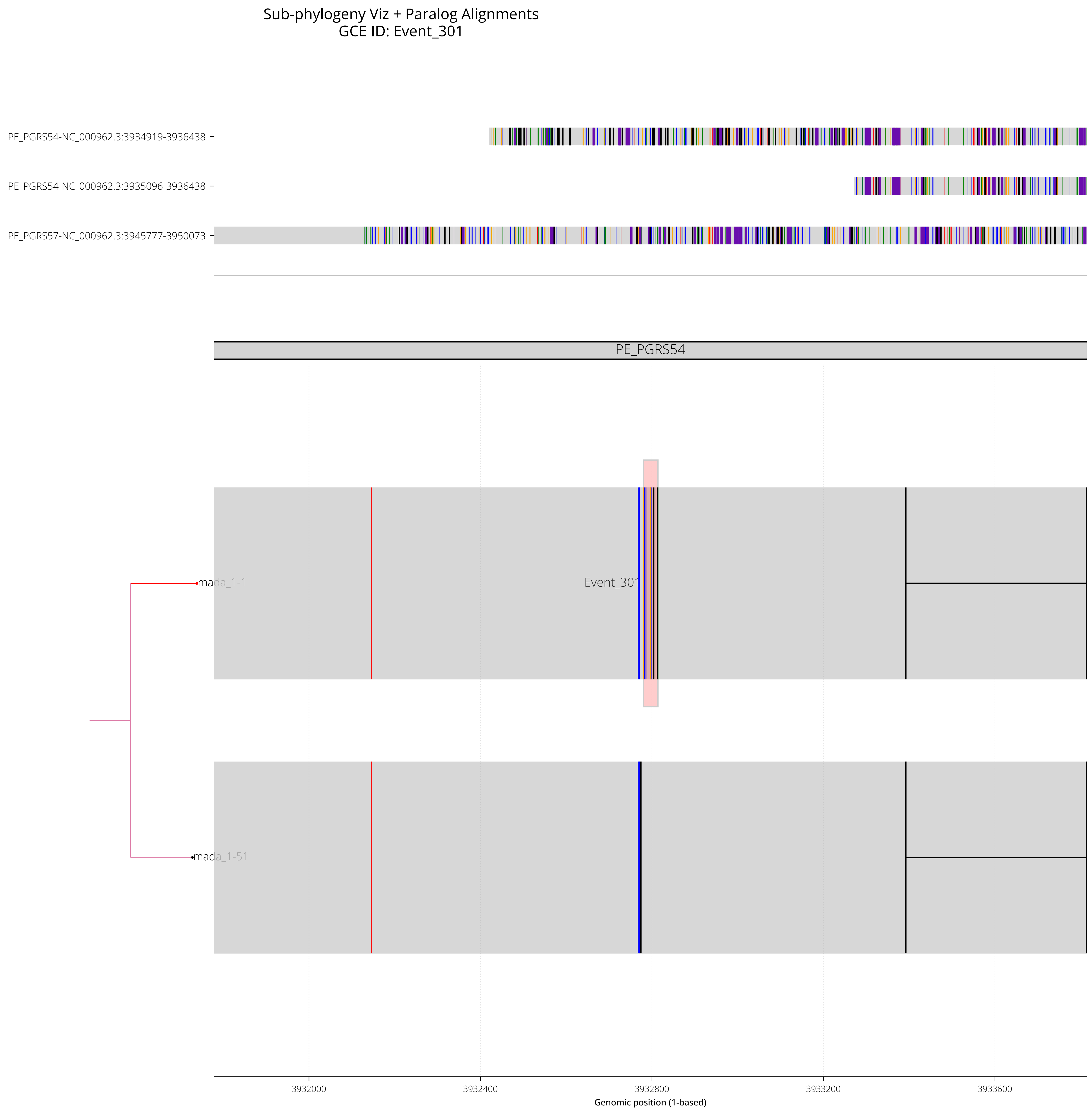

Sub-phylogeny Viz + Paralog Alignments
GCE ID: Event_301
PE_PGRS54-NC_000962.3:3934919-3936438
PE_PGRS54-NC_000962.3:3935096-3936438
PE_PGRS57-NC_000962.3:3945777-3950073
PE_PGRS54
mada_1-1
Event_301
mada_1-51
3932000
3932400
3932800
3933200
3933600
Genomic position (1-based)

Full Phylogeny Viz + Paralog Alignments
GCE ID: Event_301

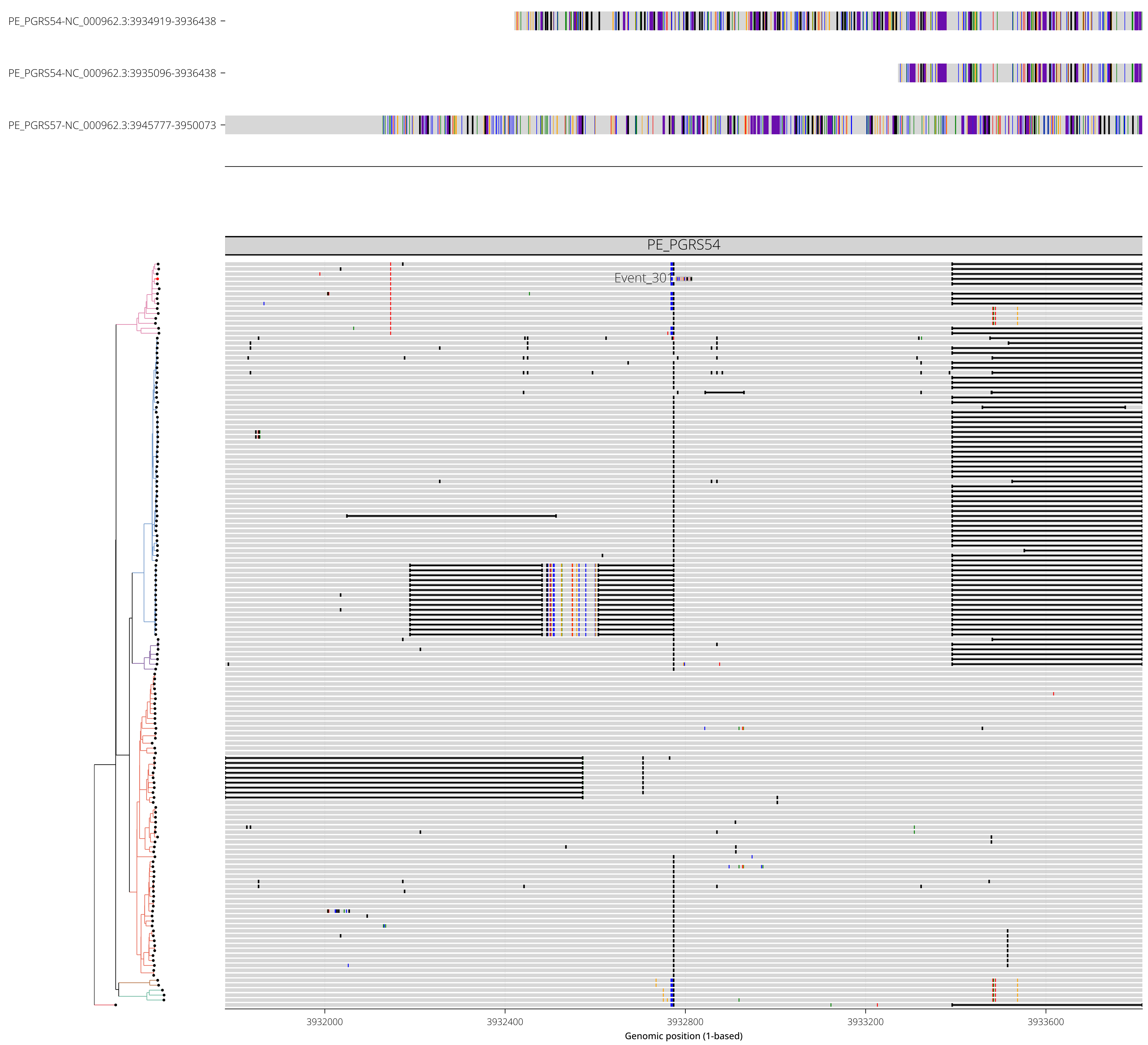

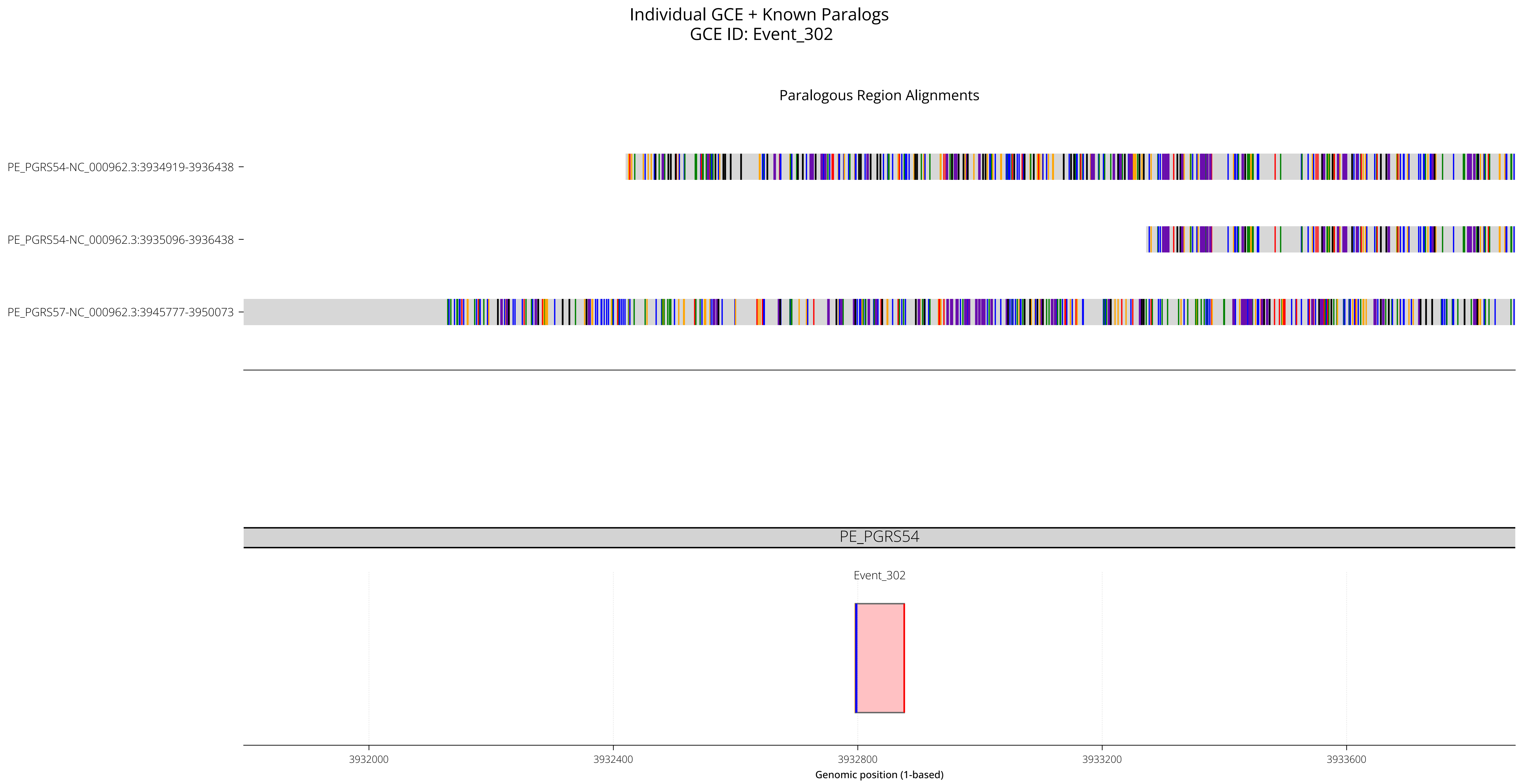

Individual GCE + Known Paralogs
GCE ID: Event_302
Paralogous Region Alignments
PE_PGRS54-NC_000962.3:3934919-3936438
PE_PGRS54-NC_000962.3:3935096-3936438
PE_PGRS57-NC_000962.3:3945777-3950073
PE_PGRS54
Event_302
3932000
3932400
3932800
3933200
3933600
Genomic position (1-based)

Circos Viz - GCE to Known Paralogs Locations
GCE ID: Event_302

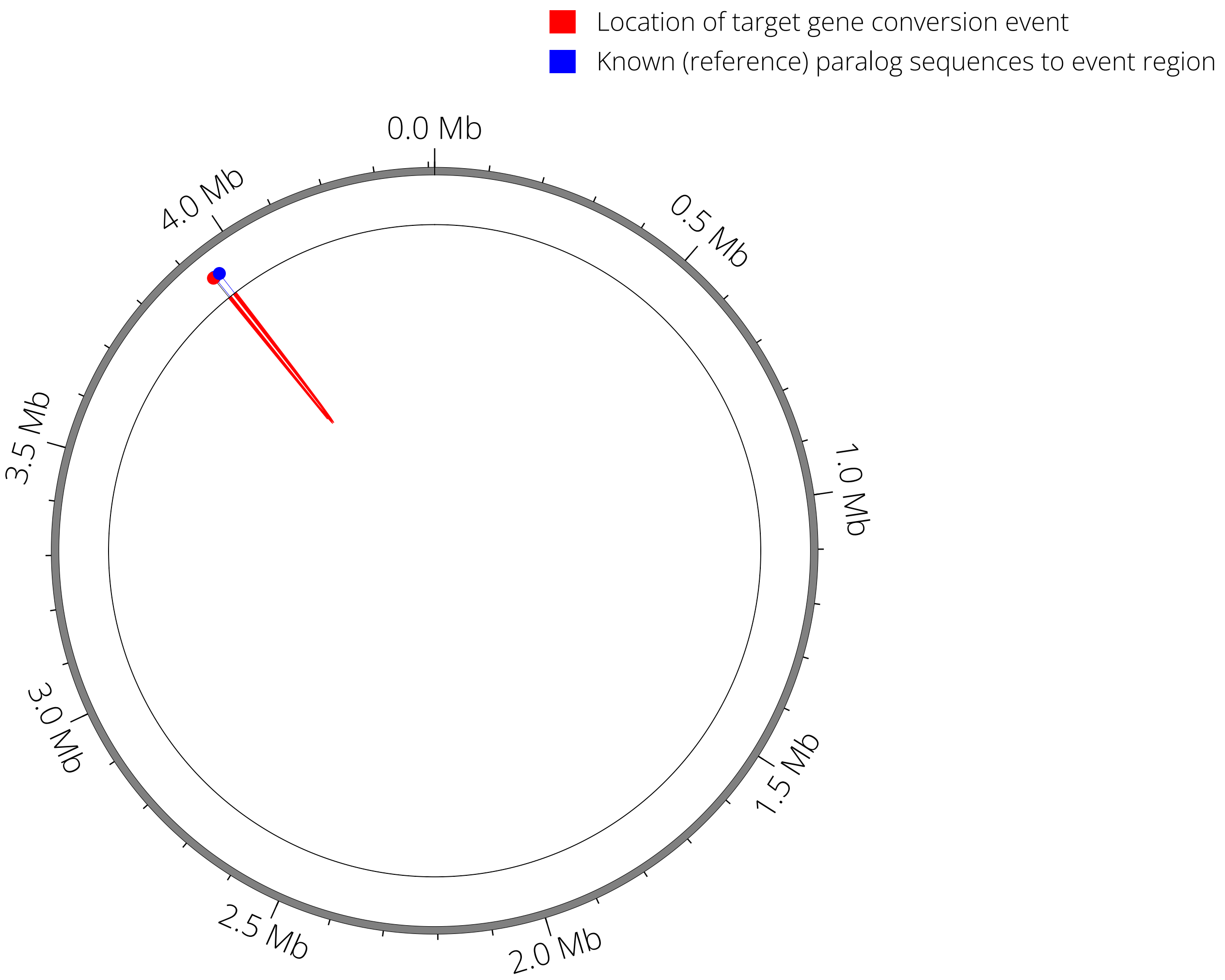

# Event_302 - 4 SNPs - k-mer analysis relative to paralogs

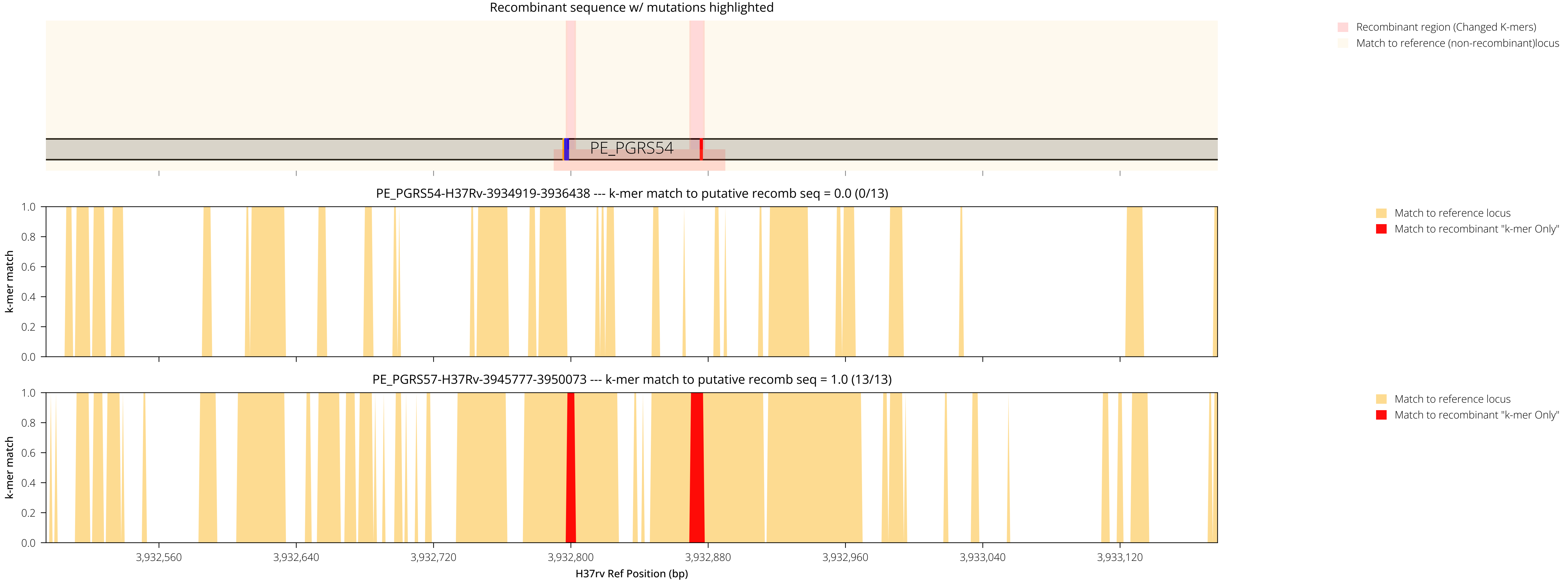

Sub-phylogeny Viz + Paralog Alignments
GCE ID: Event_302

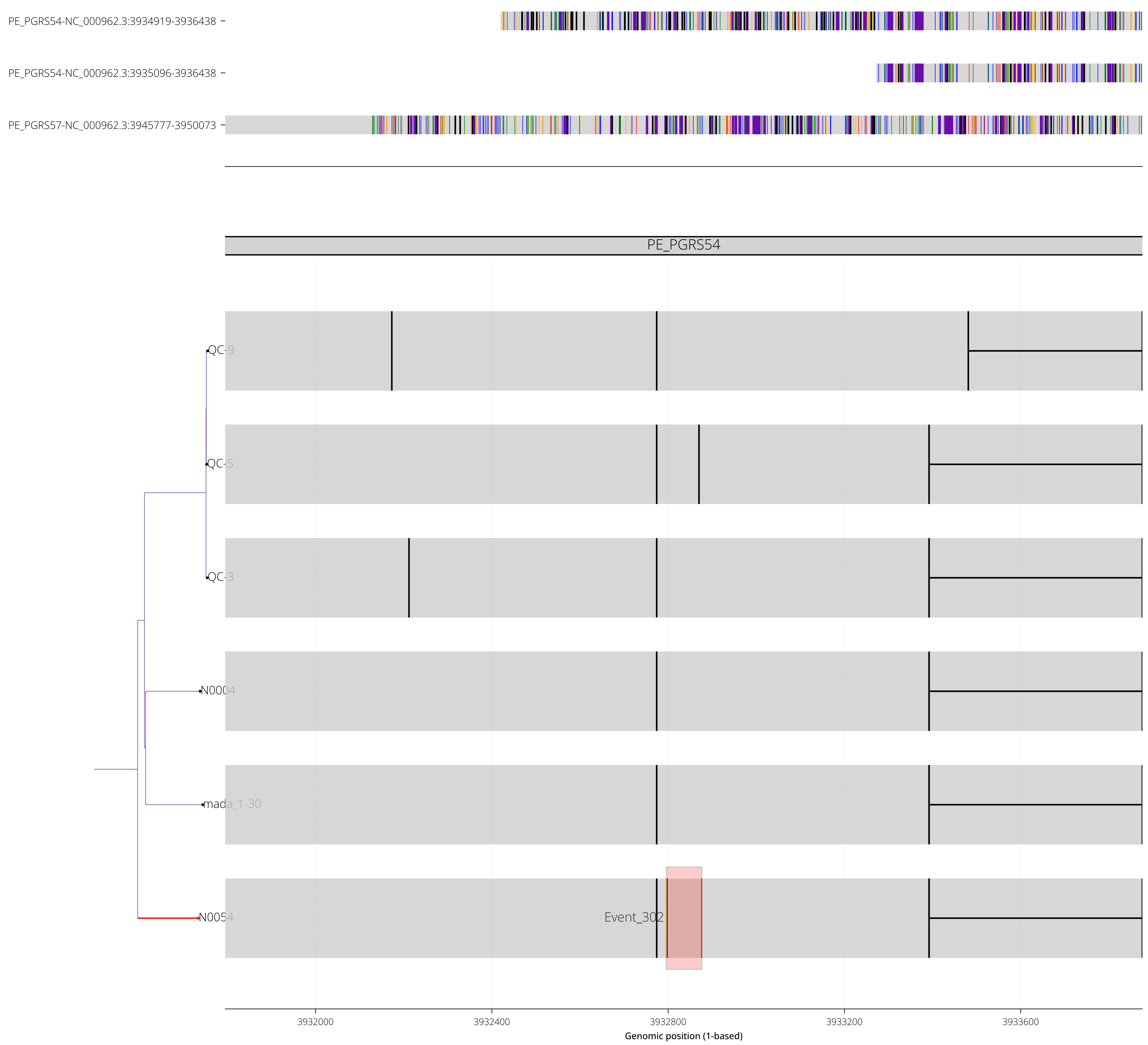

Full Phylogeny Viz + Paralog Alignments
GCE ID: Event_302

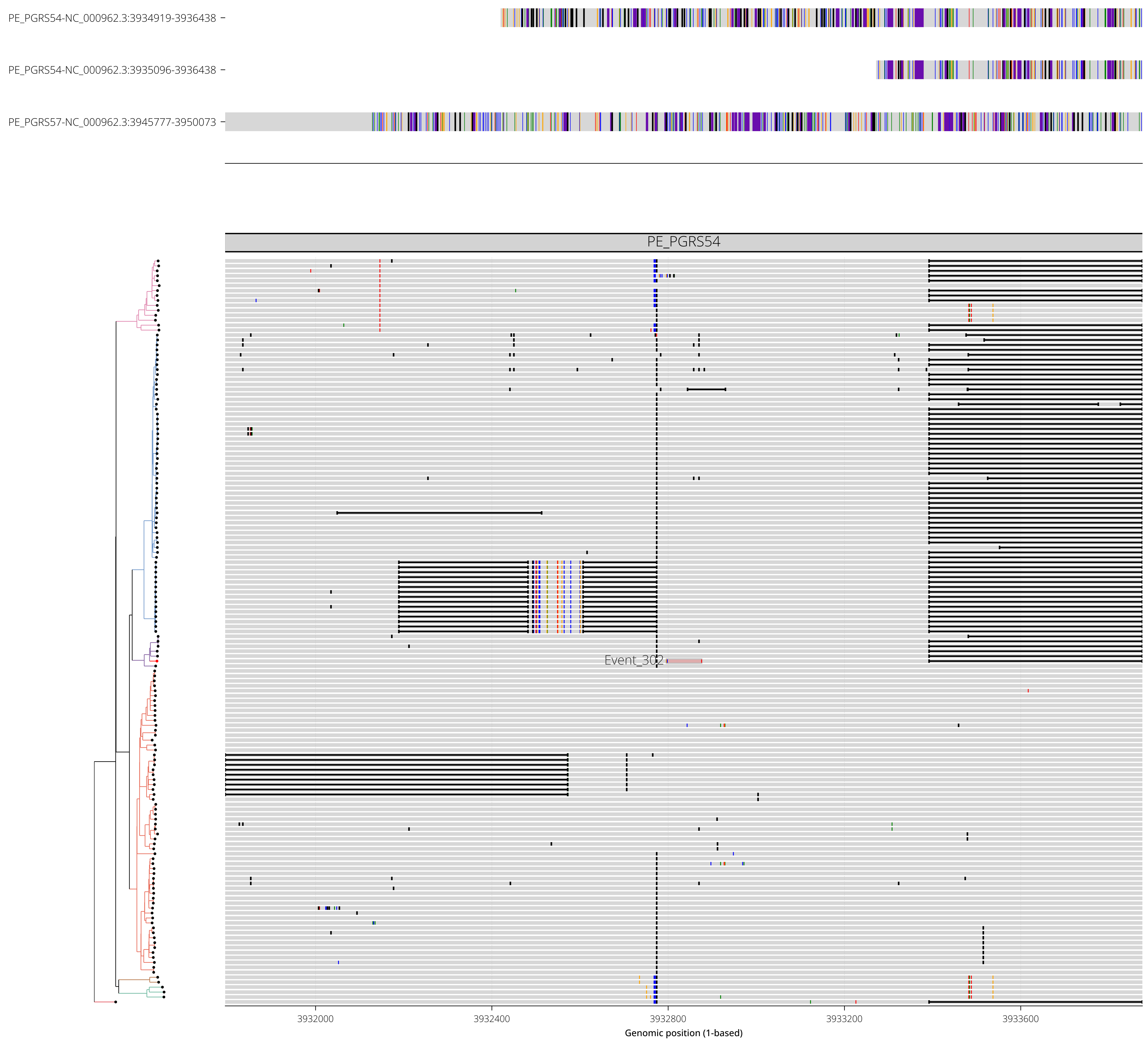

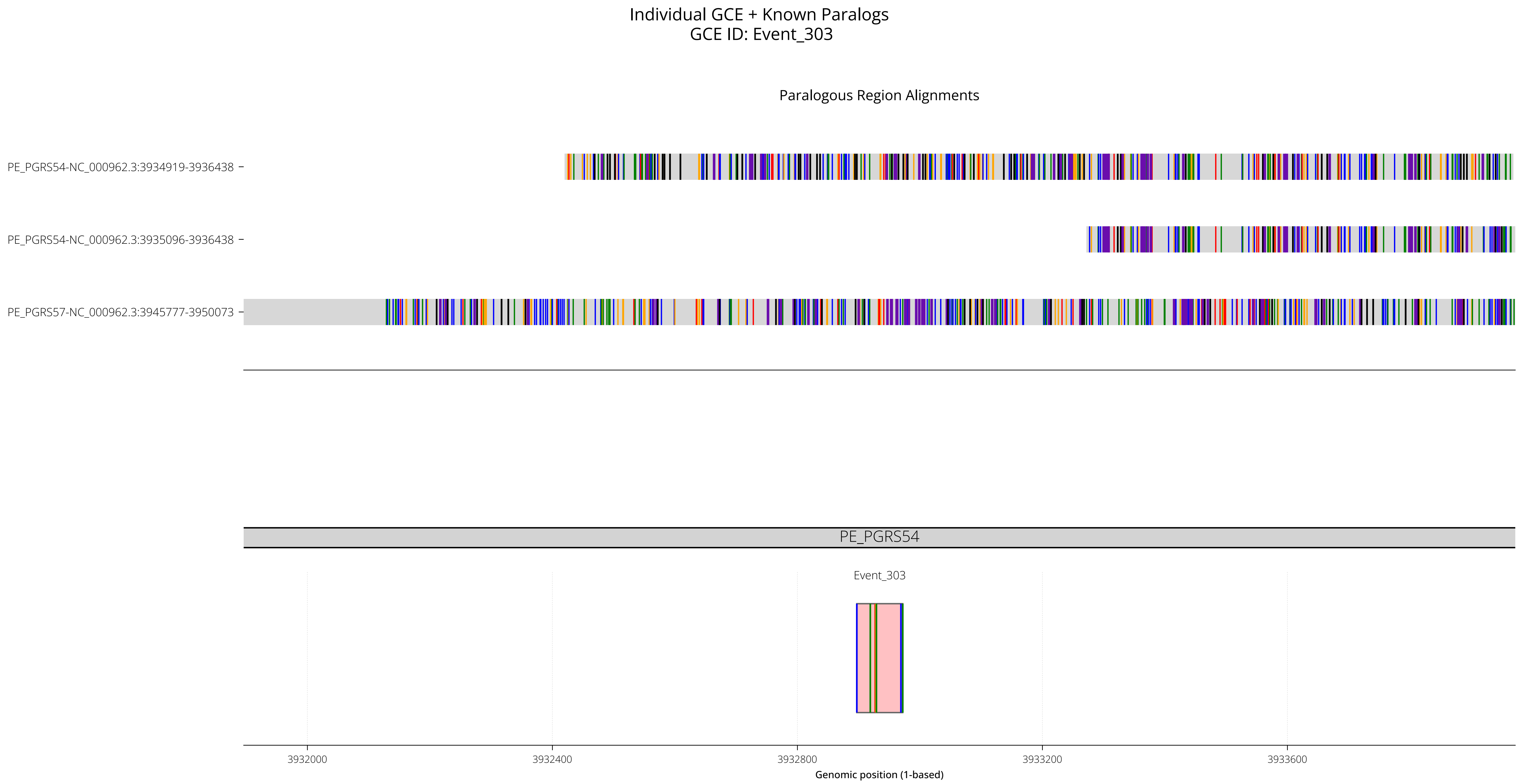
Individual GCE + Known Paralogs
GCE ID: Event_303
Paralogous Region Alignments
PE_PGRS54-NC_000962.3:3934919-3936438
PE_PGRS54-NC_000962.3:3935096-3936438
PE_PGRS57-NC_000962.3:3945777-3950073
PE_PGRS54
Event_303
3932000
3932400
3932800
3933200
3933600
Genomic position (1-based)

Circos Viz - GCE to Known Paralogs Locations
GCE ID: Event_303

Location of target gene conversion event
Known (reference) paralog sequences to event region

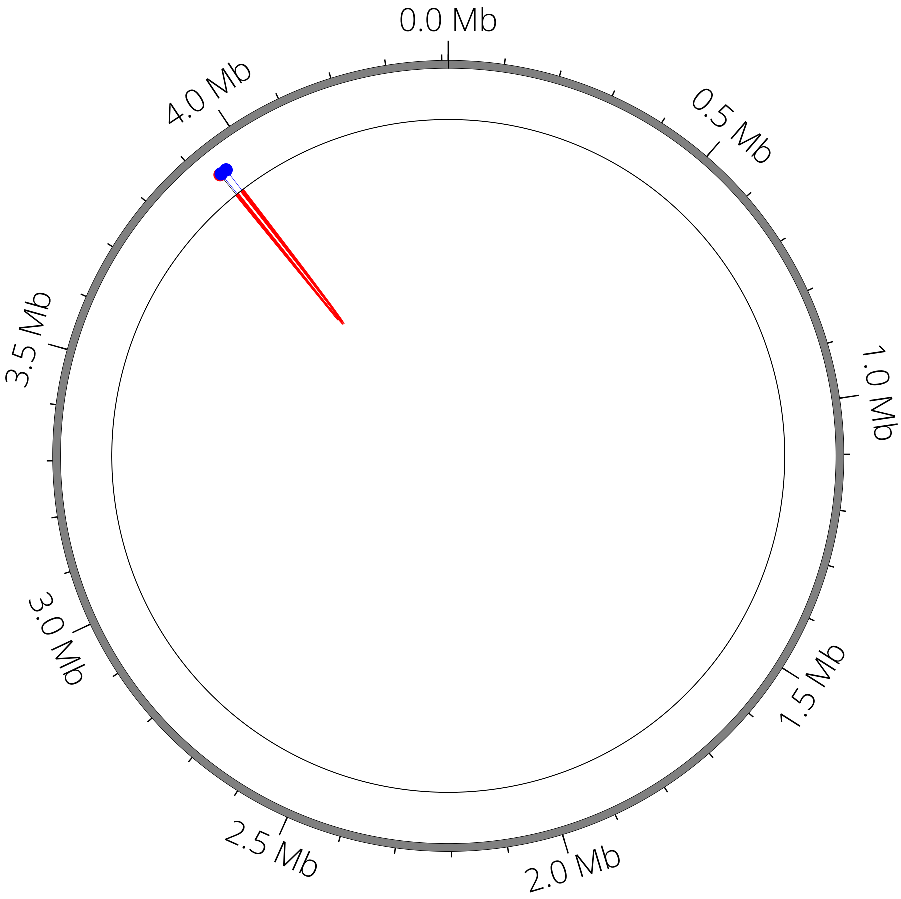

# Event_303 - 7 SNPs - k-mer analysis relative to paralogs

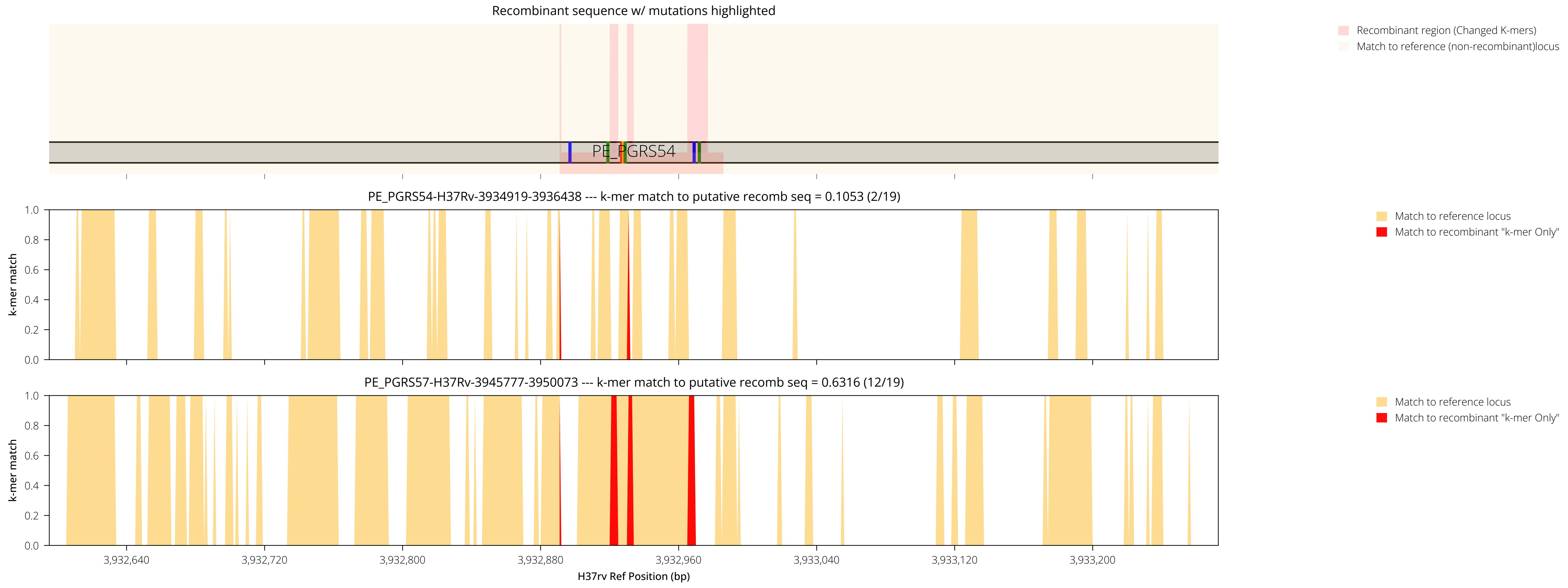

Sub-phylogeny Viz + Paralog Alignments
GCE ID: Event_303

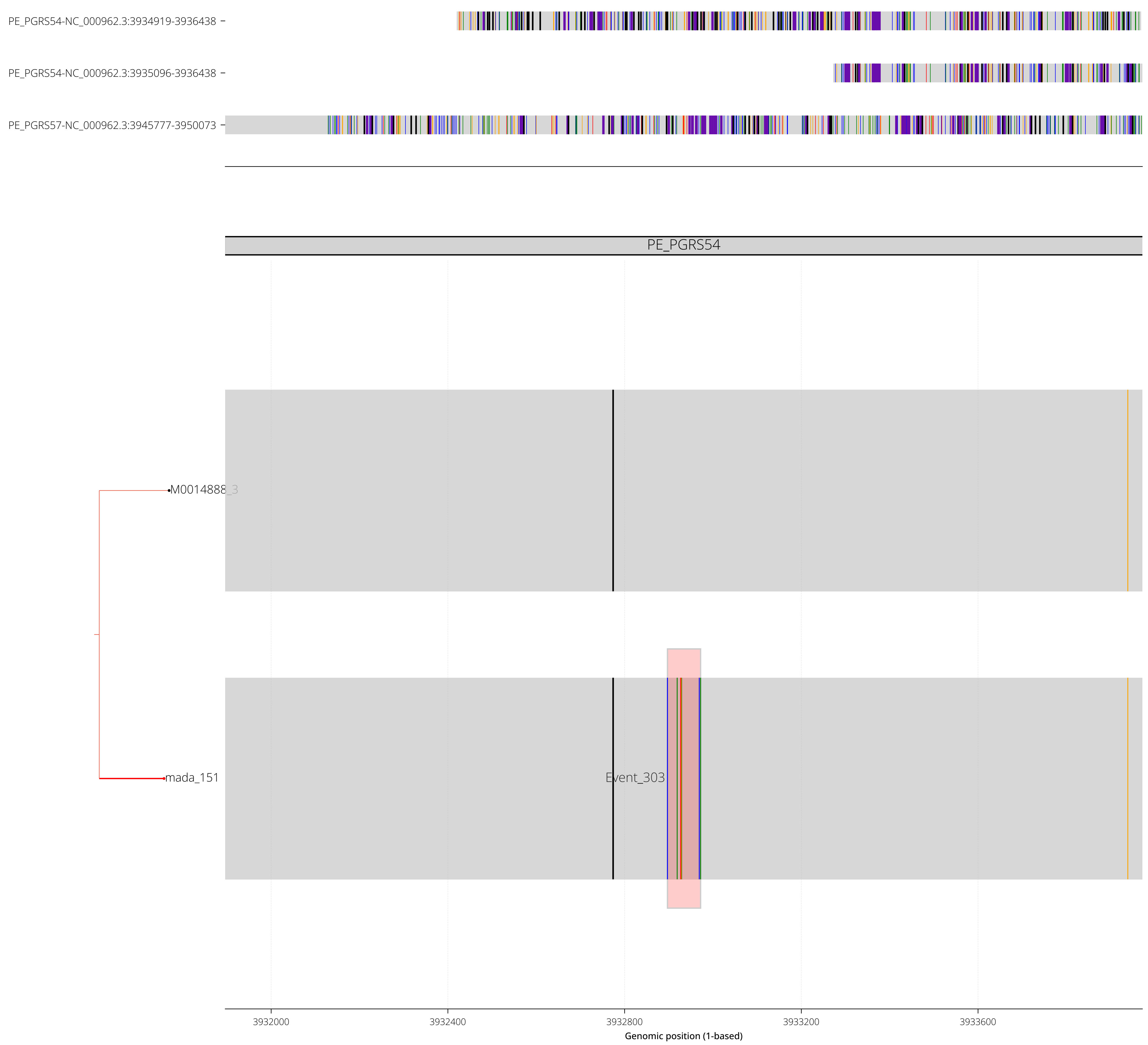

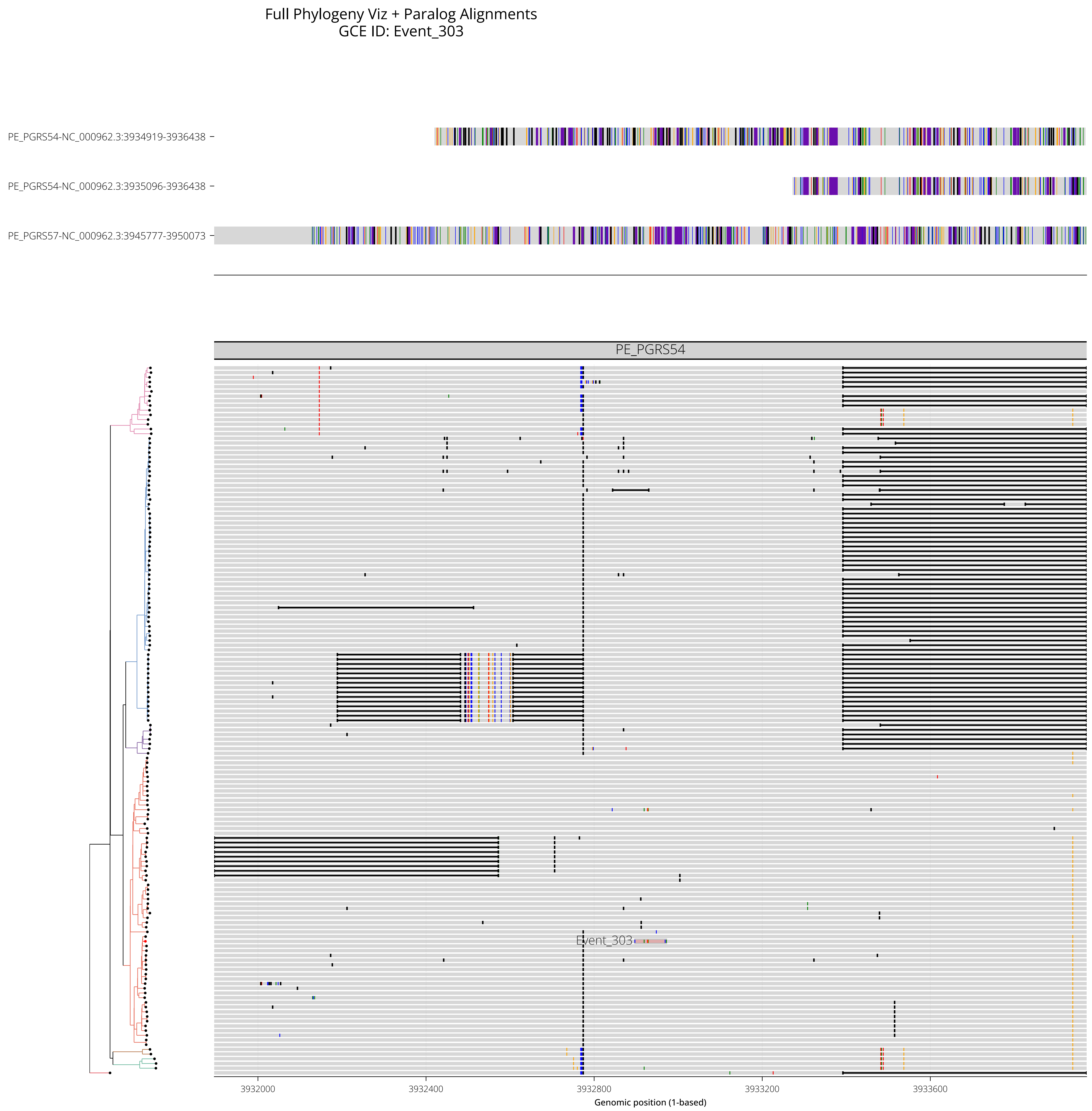
Full Phylogeny Viz + Paralog Alignments
GCE ID: Event_303
PE_PGRS54-NC_000962.3:3934919-3936438
PE_PGRS54-NC_000962.3:3935096-3936438
PE_PGRS57-NC_000962.3:3945777-3950073
PE_PGRS54
Event_303
3932000
3932400
3932800
3933200
3933600
Genomic position (1-based)

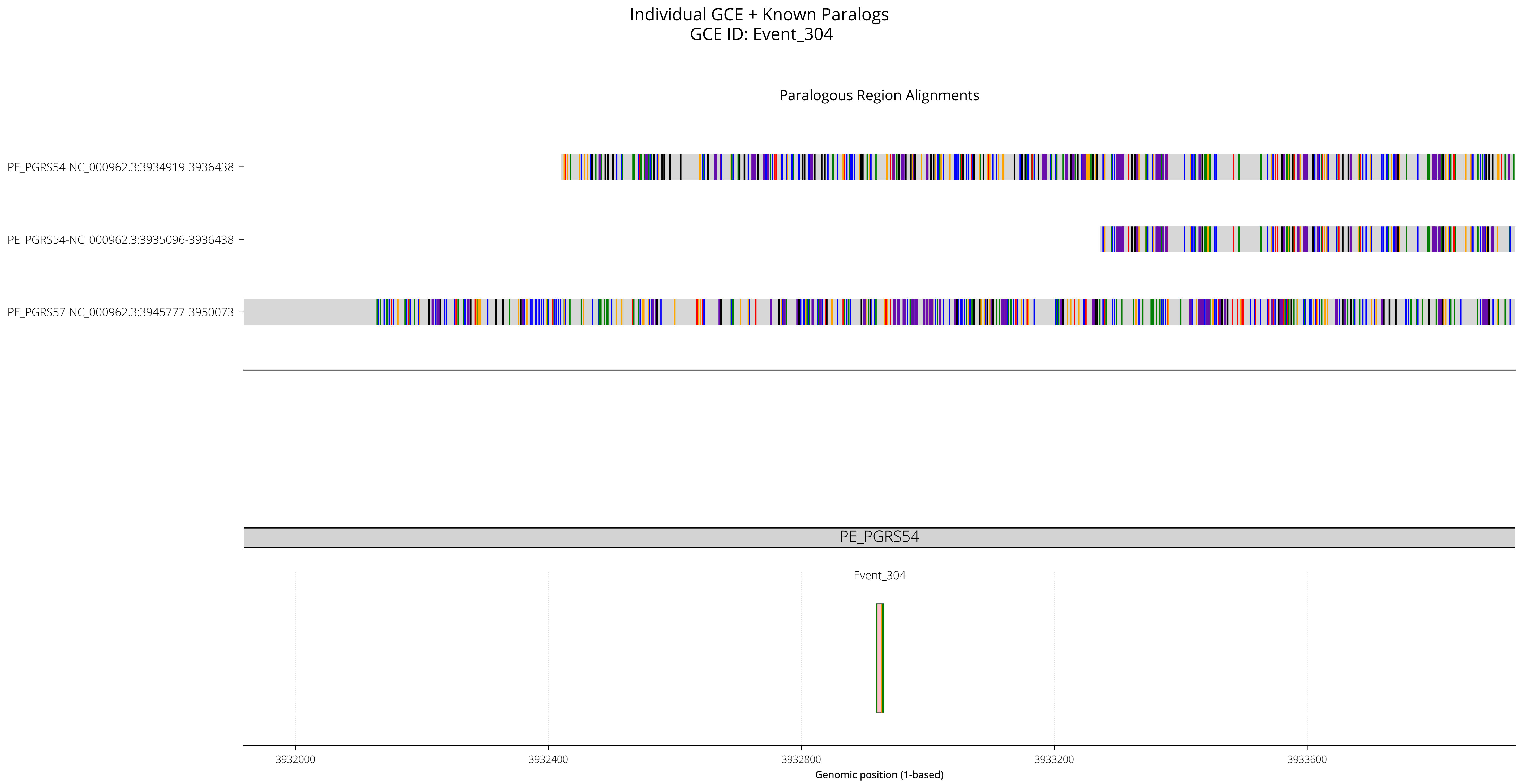
Individual GCE + Known Paralogs
GCE ID: Event_304
Paralogous Region Alignments
PE_PGRS54-NC_000962.3:3934919-3936438
PE_PGRS54-NC_000962.3:3935096-3936438
PE_PGRS57-NC_000962.3:3945777-3950073
PE_PGRS54
Event_304
3932000
3932400
3932800
3933200
3933600
Genomic position (1-based)

Circos Viz - GCE to Known Paralogs Locations
GCE ID: Event_304

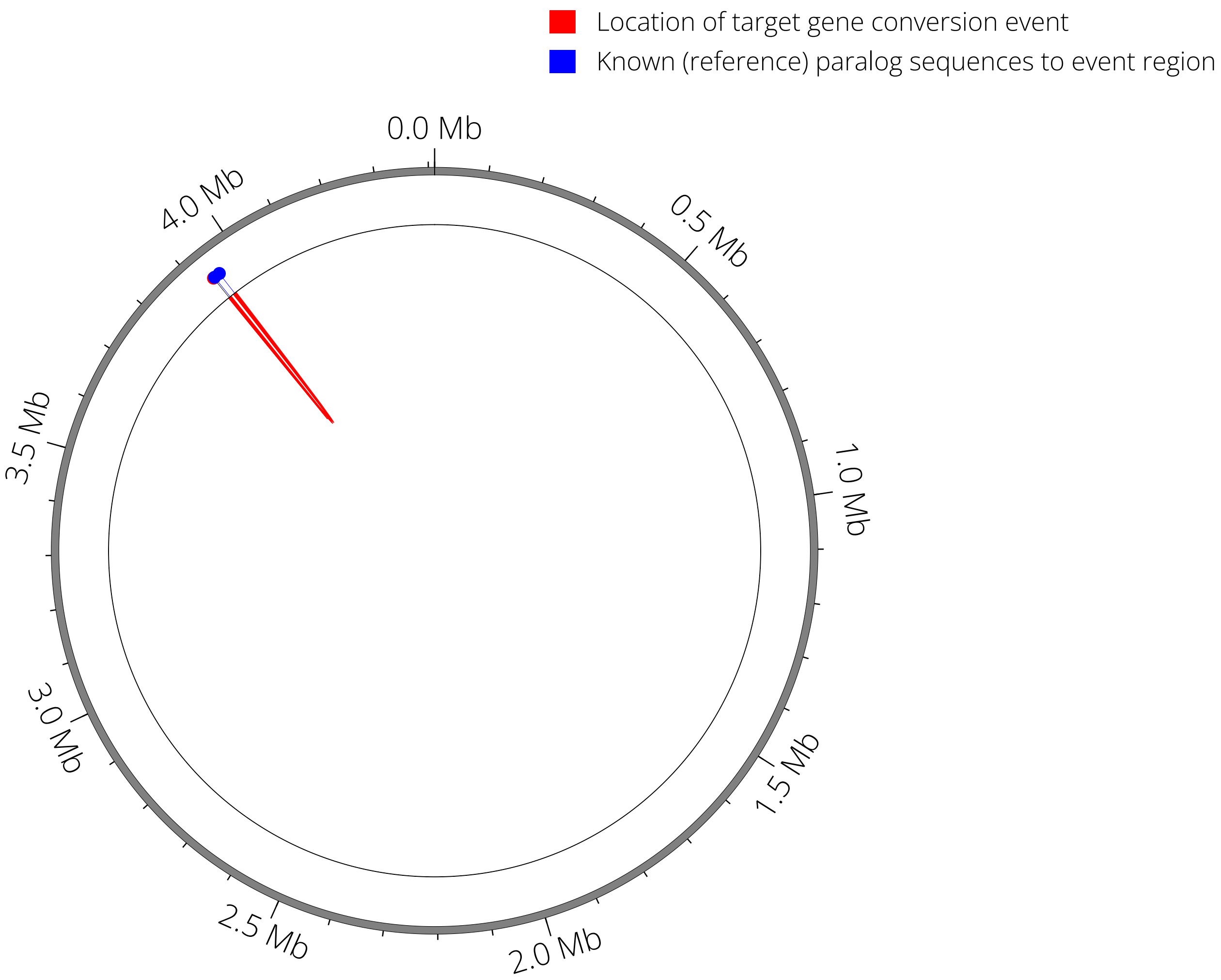

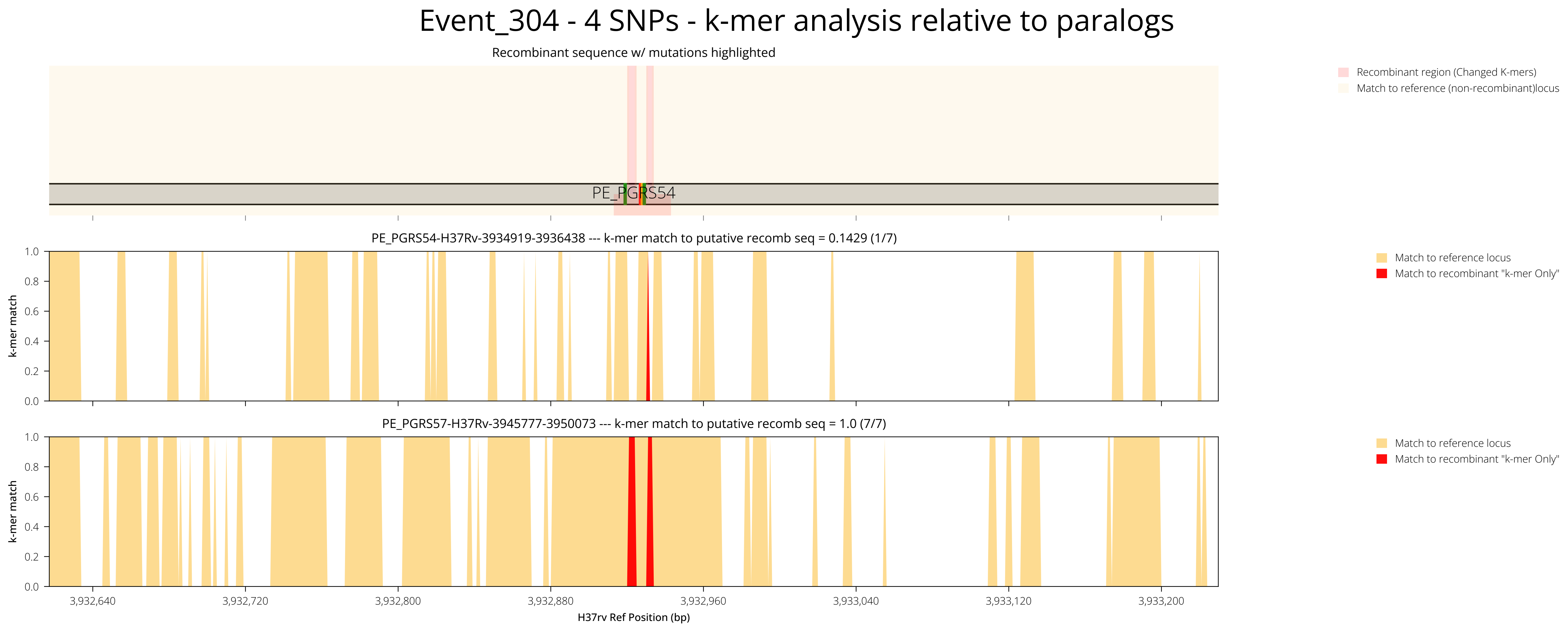

Event_304 - 4 SNPs - k-mer analysis relative to paralogs
Recombinant sequence w/ mutations highlighted
Recombinant region (Changed K-mers)
Match to reference (non-recombinant)locus
PE_PGRS54
PE_PGRS54-H37Rv-3934919-3936438 --- k-mer match to putative recomb seq = 0.1429 (1/7)
Match to reference locus
Match to recombinant "k-mer Only"
k-mer match
1.0
0.8
0.6
0.4
0.2
0.0
PE_PGRS57-H37Rv-3945777-3950073 --- k-mer match to putative recomb seq = 1.0 (7/7)
Match to reference locus
Match to recombinant "k-mer Only"
k-mer match
1.0
0.8
0.6
0.4
0.2
0.0
3,932,640
3,932,720
3,932,800
3,932,880
3,932,960
3,933,040
3,933,120
3,933,200
H37rv Ref Position (bp)

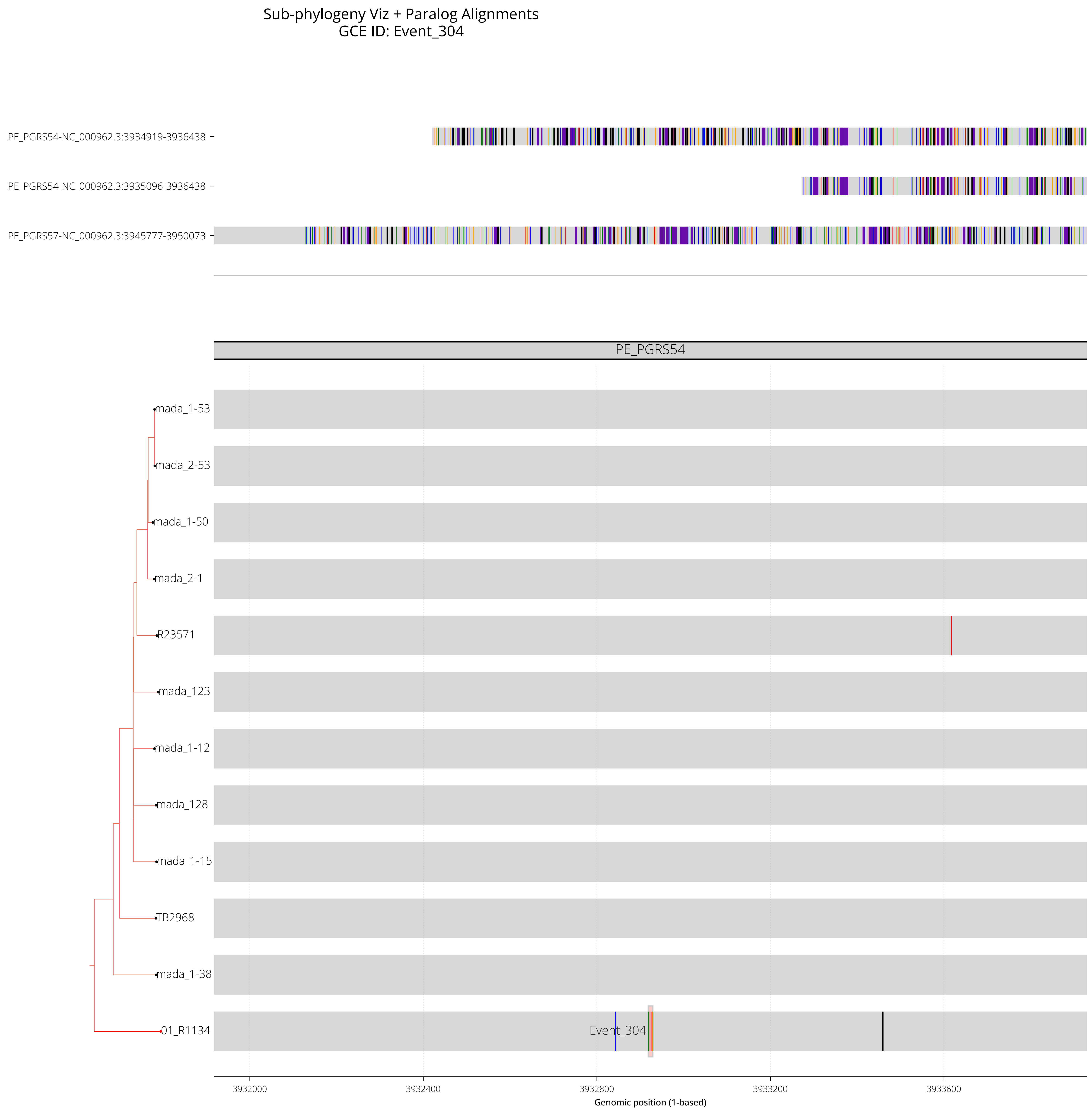

Sub-phylogeny Viz + Paralog Alignments
GCE ID: Event_304
PE_PGRS54-NC_000962.3:3934919-3936438
PE_PGRS54-NC_000962.3:3935096-3936438
PE_PGRS57-NC_000962.3:3945777-3950073
PE_PGRS54
mada_1-53
mada_2-53
mada_1-50
mada_2-1
R23571
mada_123
mada_1-12
mada_128
mada_1-15
TB2968
mada_1-38
01_R1134
Event_304
3932000
3932400
3932800
3933200
3933600
Genomic position (1-based)

Full Phylogeny Viz + Paralog Alignments
GCE ID: Event_304

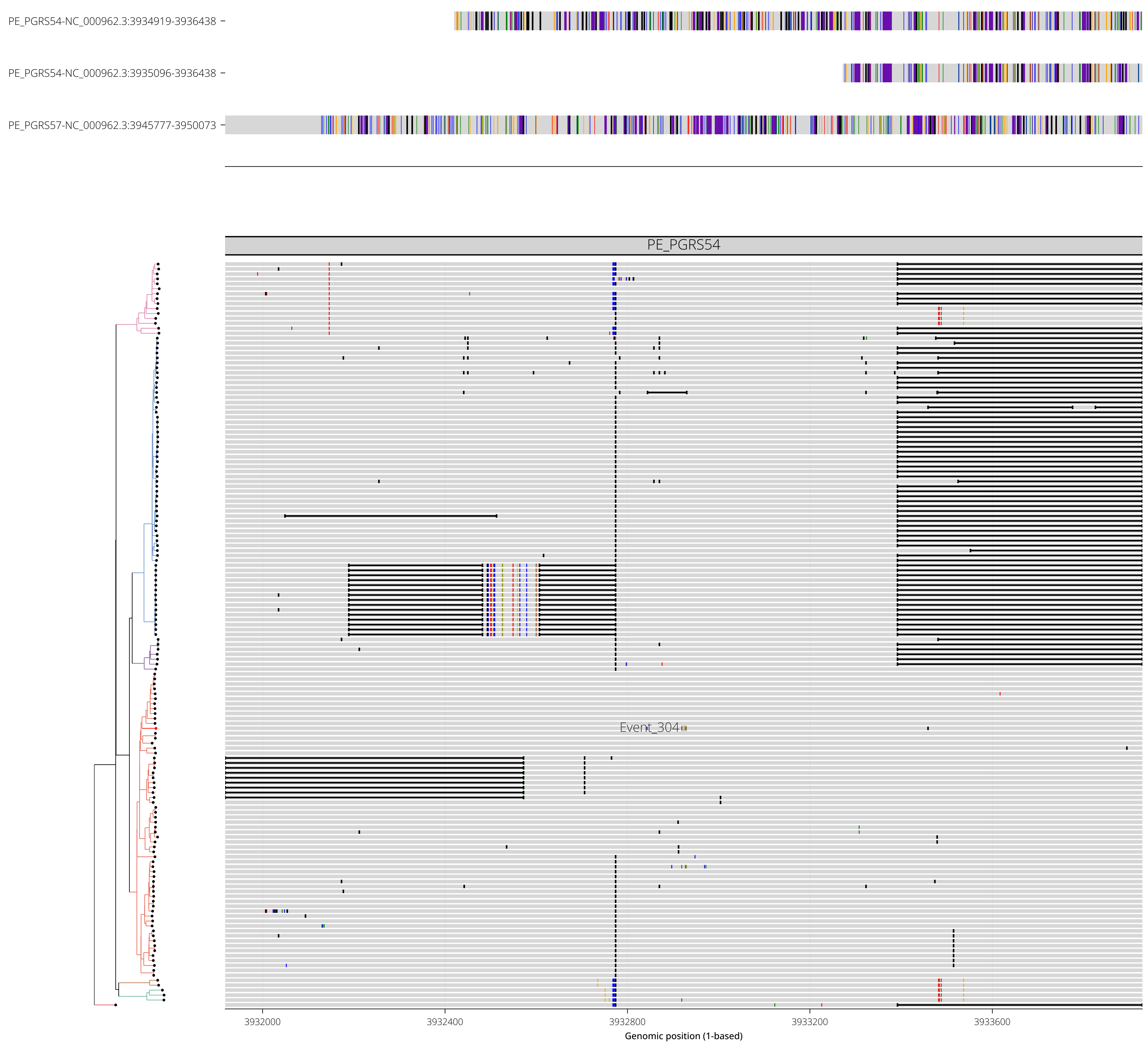

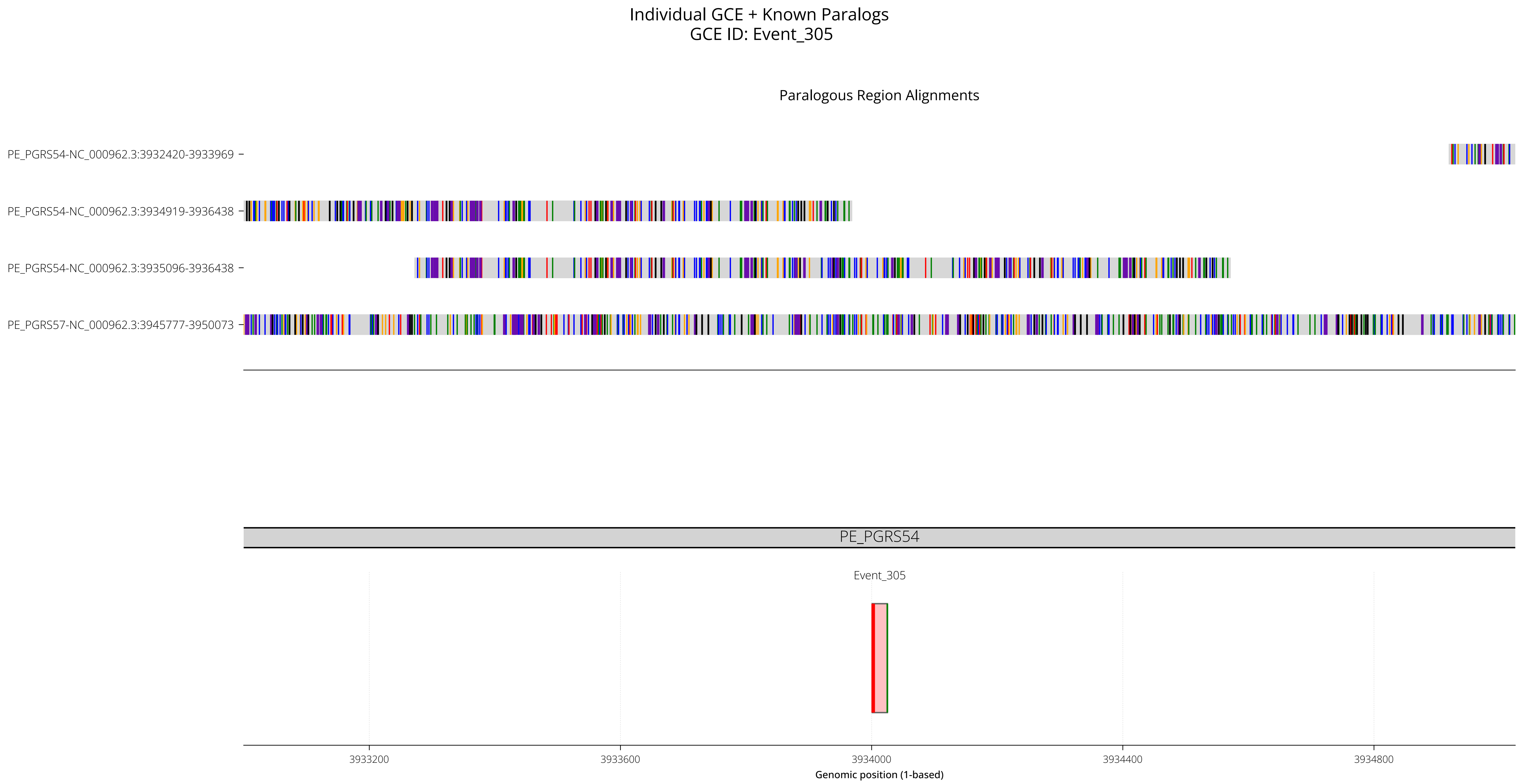
Individual GCE + Known Paralogs
GCE ID: Event_305
Paralogous Region Alignments
PE_PGRS54-NC_000962.3:3932420-3933969
PE_PGRS54-NC_000962.3:3934919-3936438
PE_PGRS54-NC_000962.3:3935096-3936438
PE_PGRS57-NC_000962.3:3945777-3950073
PE_PGRS54
Event_305
3933200
3933600
3934000
3934400
3934800
Genomic position (1-based)

# Circos Viz - GCE to Known Paralogs Locations
GCE ID: Event_305

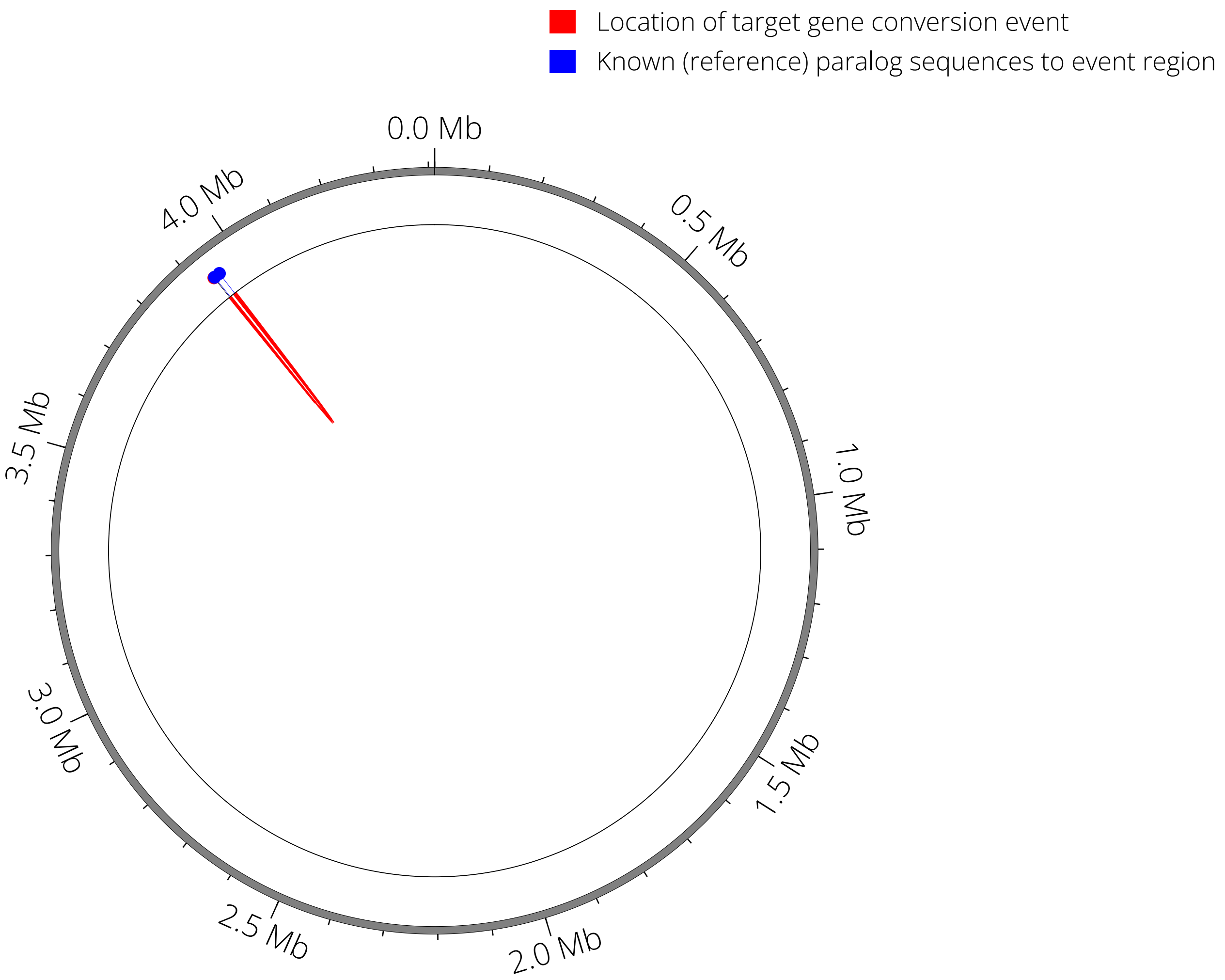

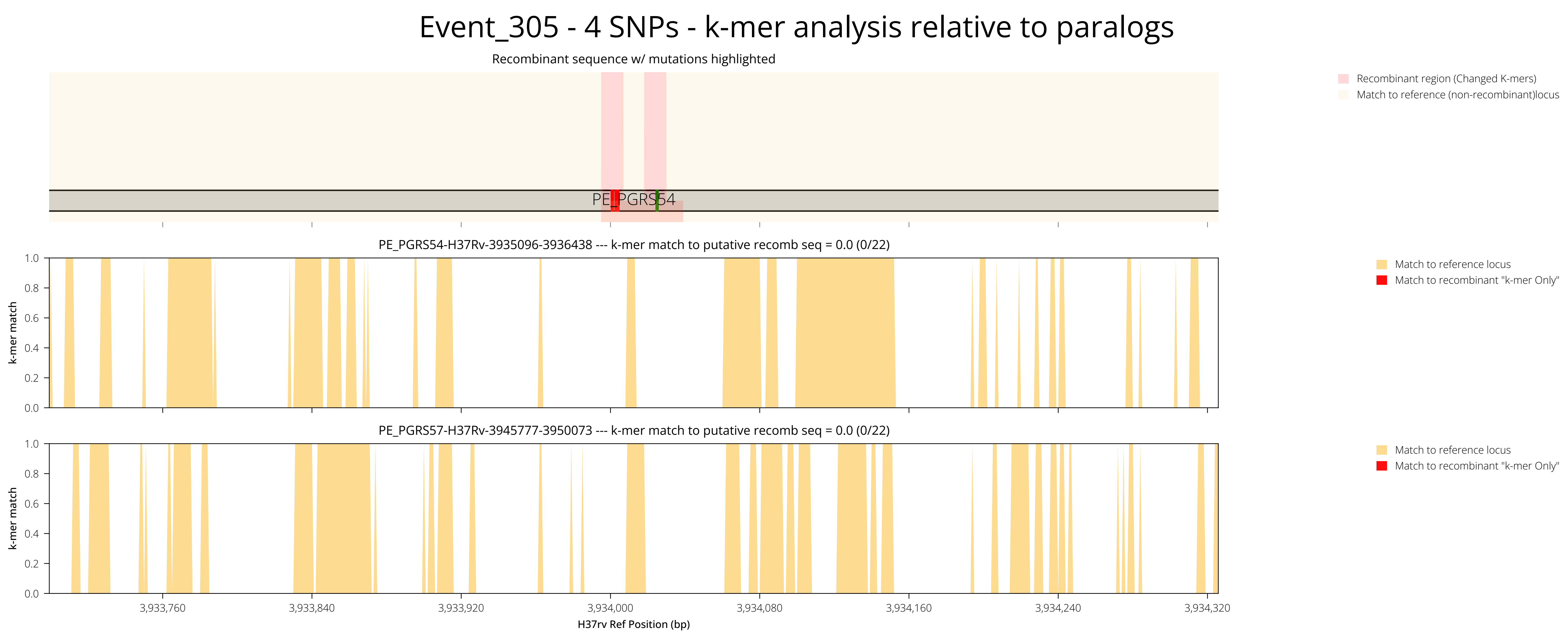

Event_305 - 4 SNPs - k-mer analysis relative to paralogs
Recombinant sequence w/ mutations highlighted
PE_PGRS54
Recombinant region (Changed K-mers)
Match to reference (non-recombinant)locus
PE_PGRS54-H37Rv-3935096-3936438 --- k-mer match to putative recomb seq = 0.0 (0/22)
Match to reference locus
Match to recombinant "k-mer Only"
k-mer match
1.0
0.8
0.6
0.4
0.2
0.0
PE_PGRS57-H37Rv-3945777-3950073 --- k-mer match to putative recomb seq = 0.0 (0/22)
Match to reference locus
Match to recombinant "k-mer Only"
k-mer match
1.0
0.8
0.6
0.4
0.2
0.0
3,933,760
3,933,840
3,933,920
3,934,000
3,934,080
3,934,160
3,934,240
3,934,320
H37rv Ref Position (bp)

## Sub-phylogeny Viz + Paralog Alignments
## GCE ID: Event_305

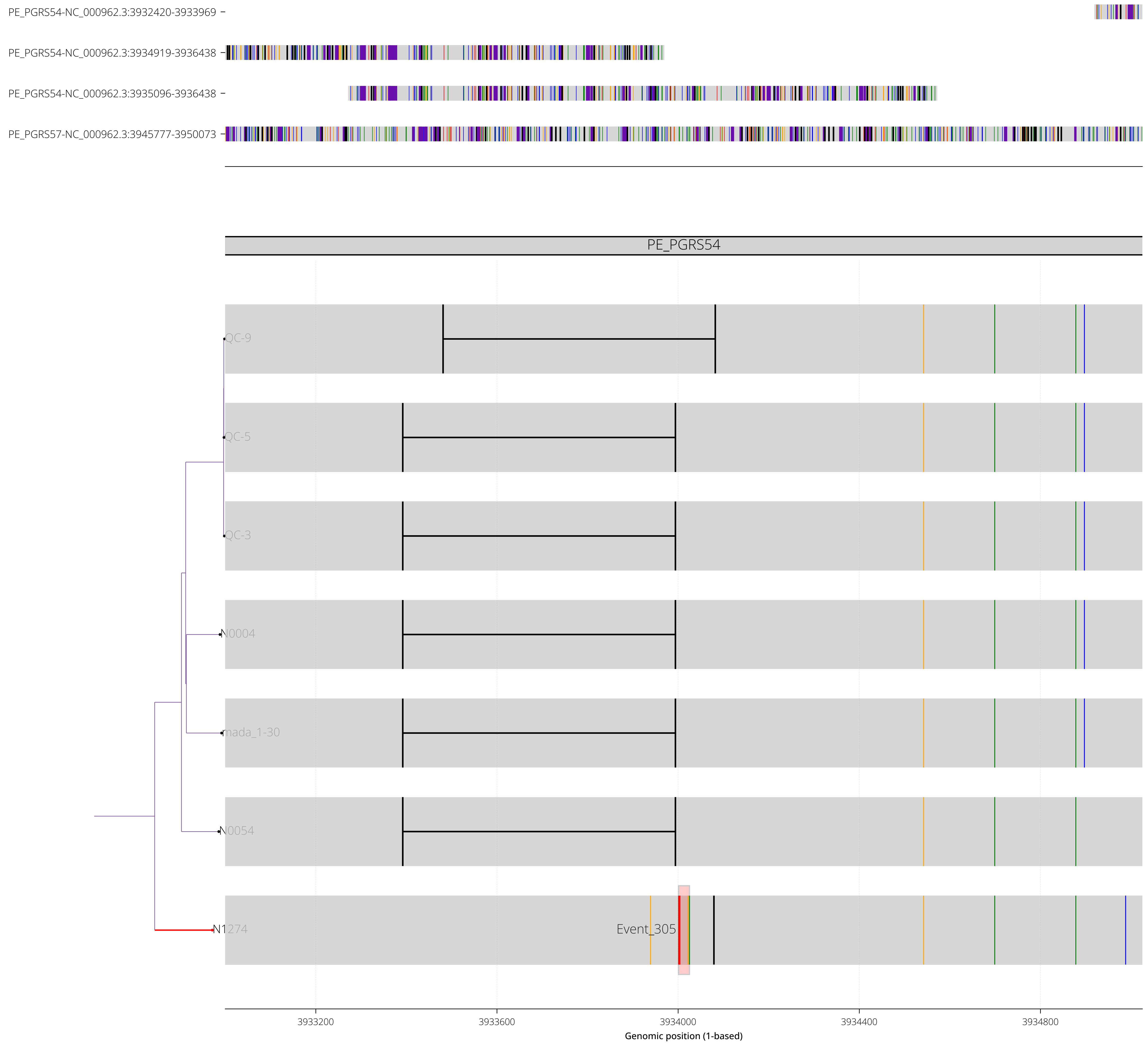

Full Phylogeny Viz + Paralog Alignments
GCE ID: Event_305

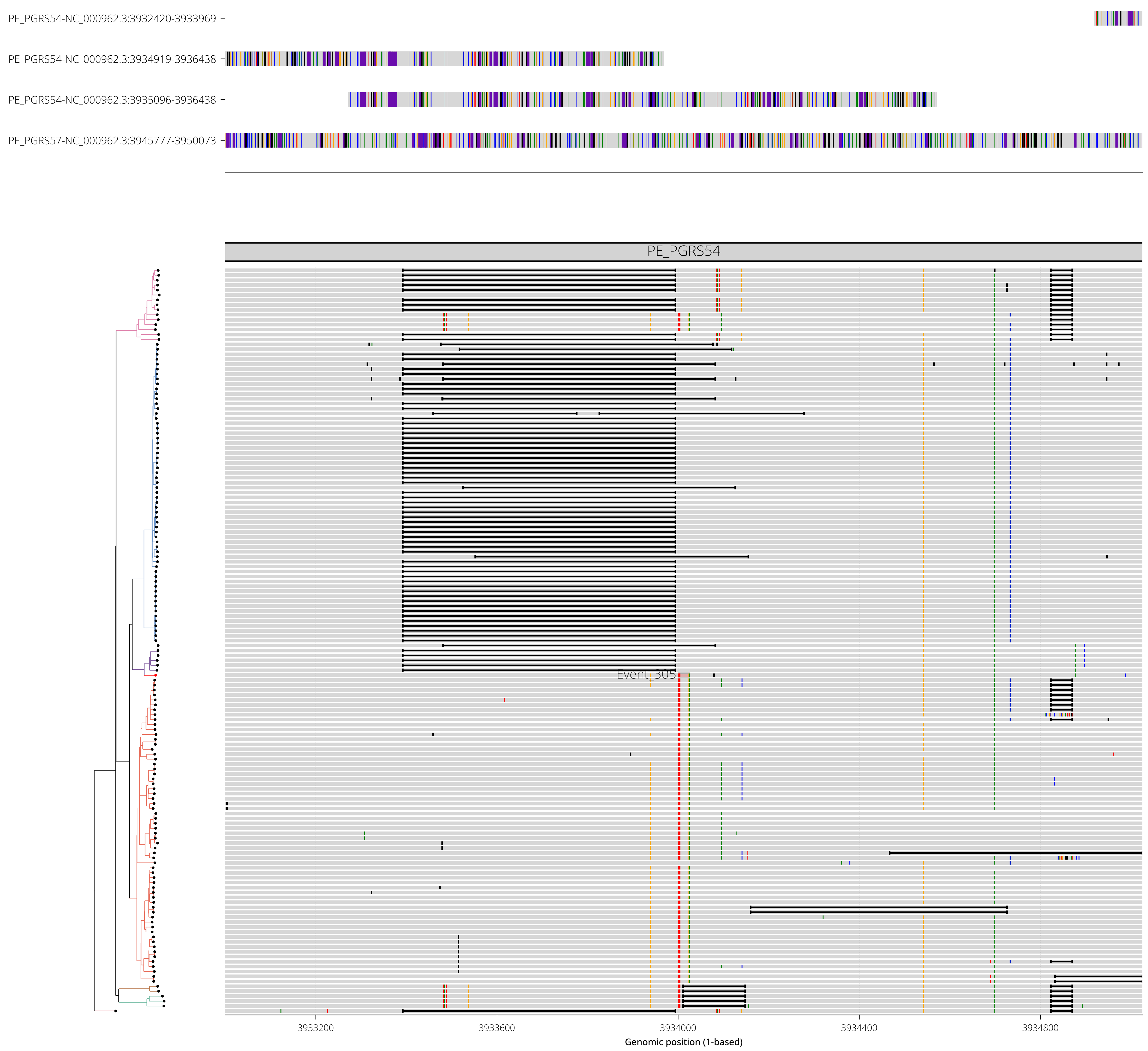

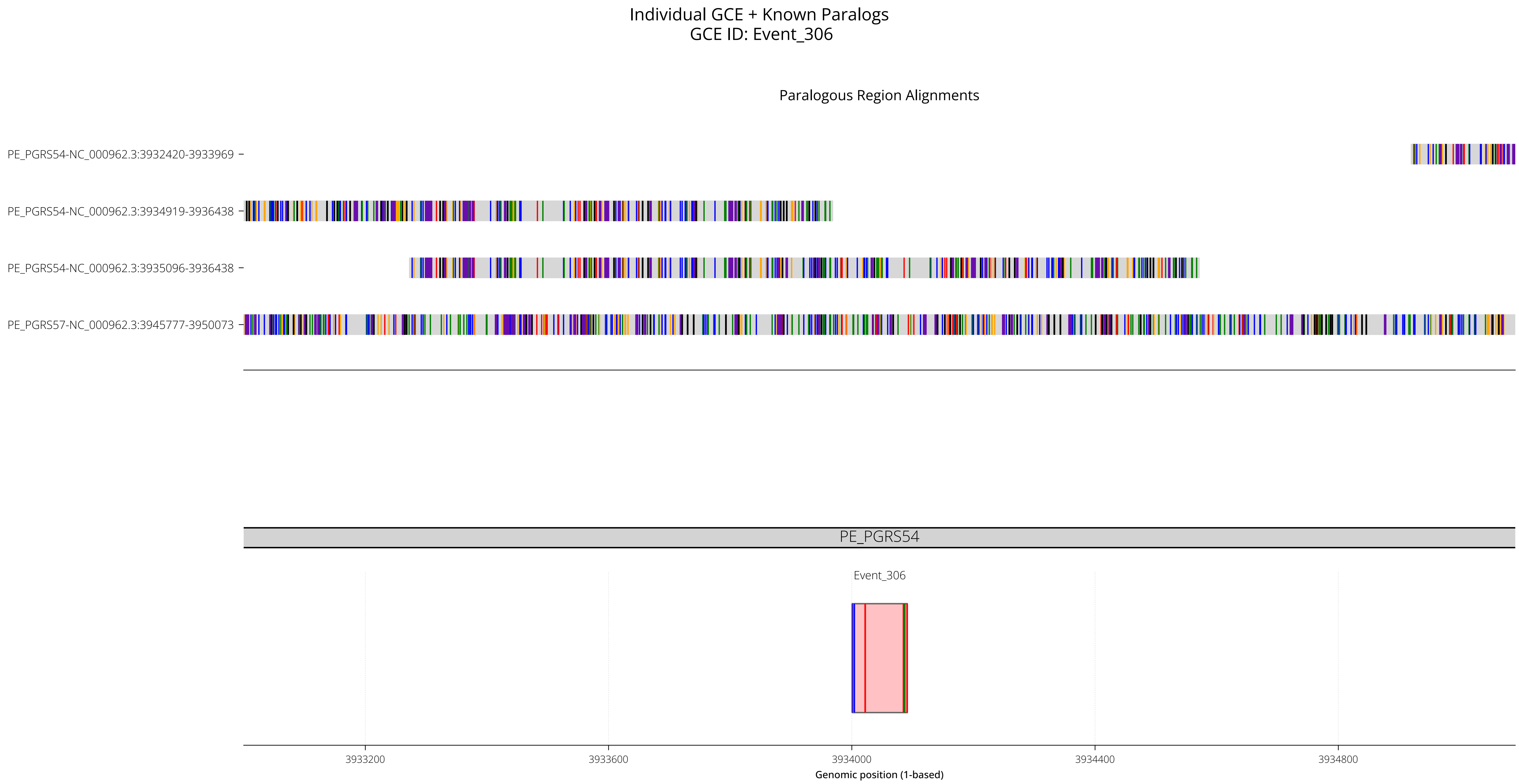

Individual GCE + Known Paralogs
GCE ID: Event_306
Paralogous Region Alignments
PE_PGRS54-NC_000962.3:3932420-3933969
PE_PGRS54-NC_000962.3:3934919-3936438
PE_PGRS54-NC_000962.3:3935096-3936438
PE_PGRS57-NC_000962.3:3945777-3950073
PE_PGRS54
Event_306
3933200
3933600
3934000
3934400
3934800
Genomic position (1-based)

Circos Viz - GCE to Known Paralogs Locations
GCE ID: Event_306

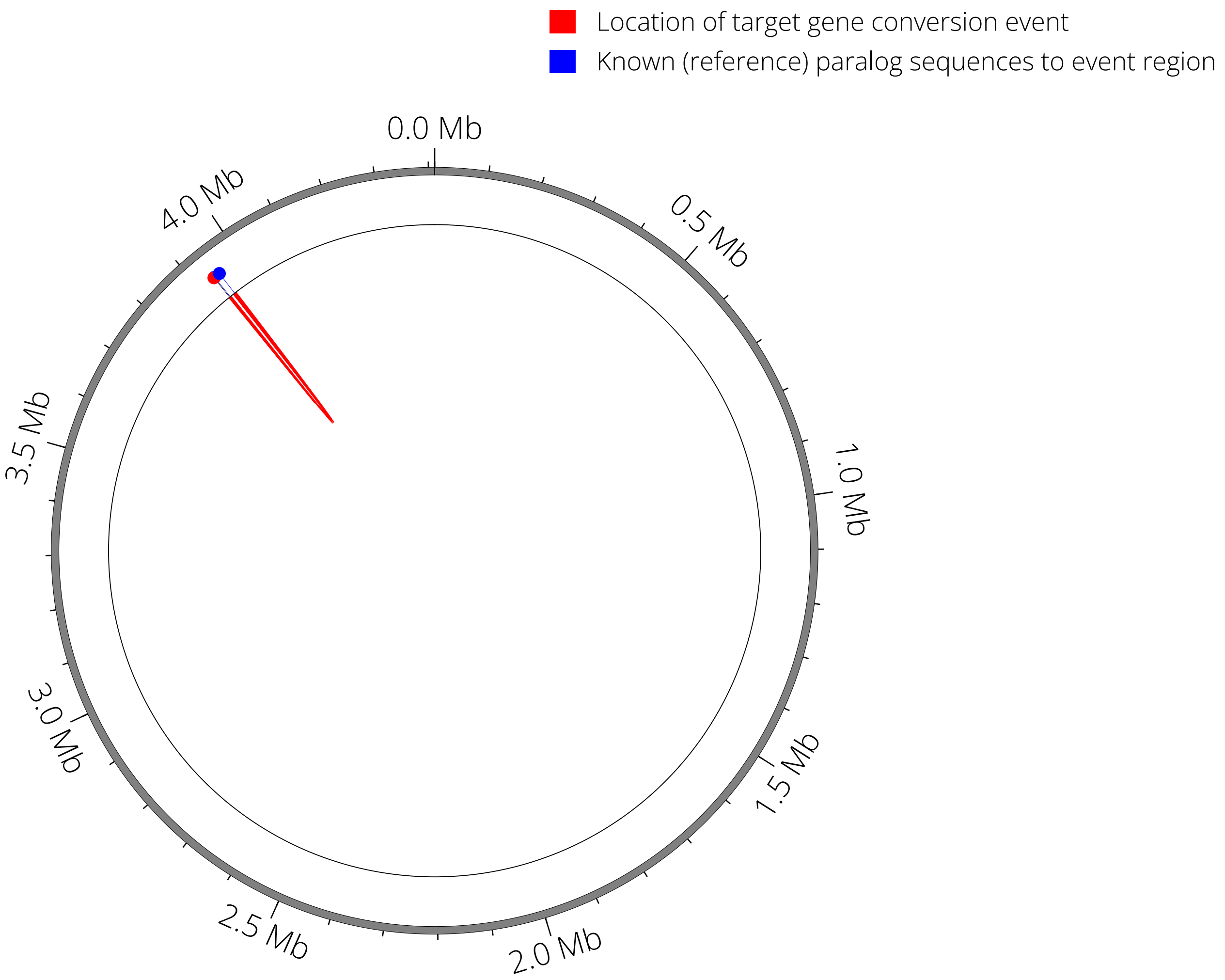

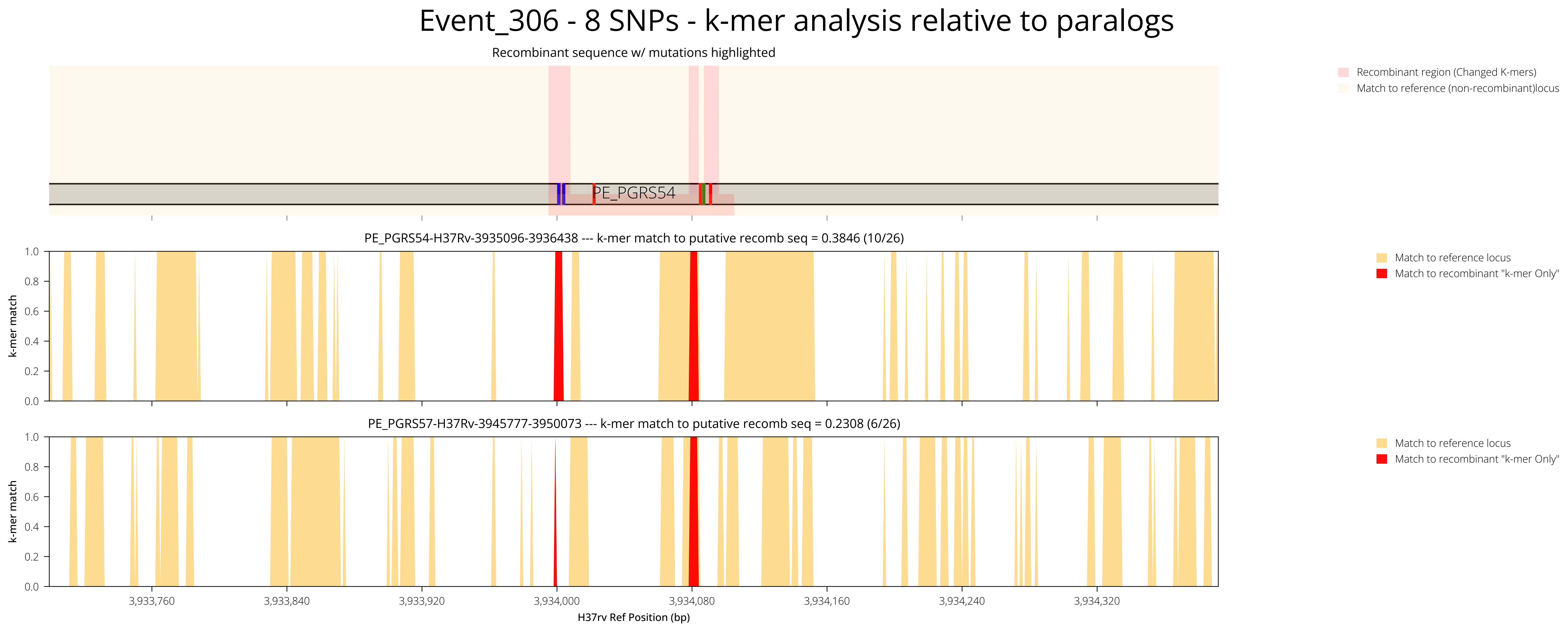

Event_306 - 8 SNPs - k-mer analysis relative to paralogs
Recombinant sequence w/ mutations highlighted
PE_PGRS54
Recombinant region (Changed K-mers)
Match to reference (non-recombinant)locus
PE_PGRS54-H37Rv-3935096-3936438 --- k-mer match to putative recomb seq = 0.3846 (10/26)
Match to reference locus
Match to recombinant "k-mer Only"
PE_PGRS57-H37Rv-3945777-3950073 --- k-mer match to putative recomb seq = 0.2308 (6/26)
Match to reference locus
Match to recombinant "k-mer Only"
k-mer match
1.0
0.8
0.6
0.4
0.2
0.0
3,933,760
3,933,840
3,933,920
3,934,000
3,934,080
3,934,160
3,934,240
3,934,320
H37rv Ref Position (bp)

Sub-phylogeny Viz + Paralog Alignments
GCE ID: Event_306

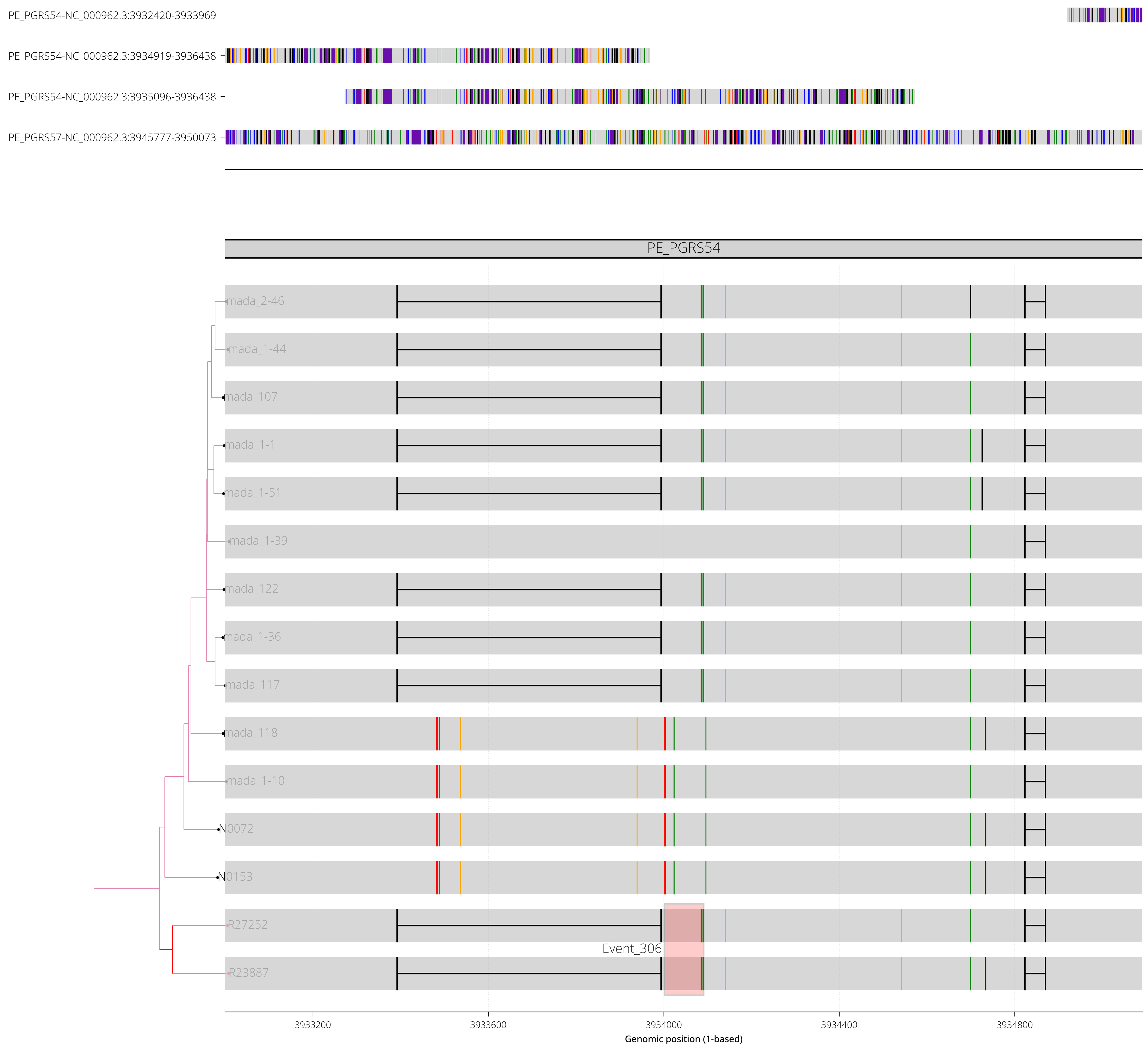

Full Phylogeny Viz + Paralog Alignments
GCE ID: Event_306

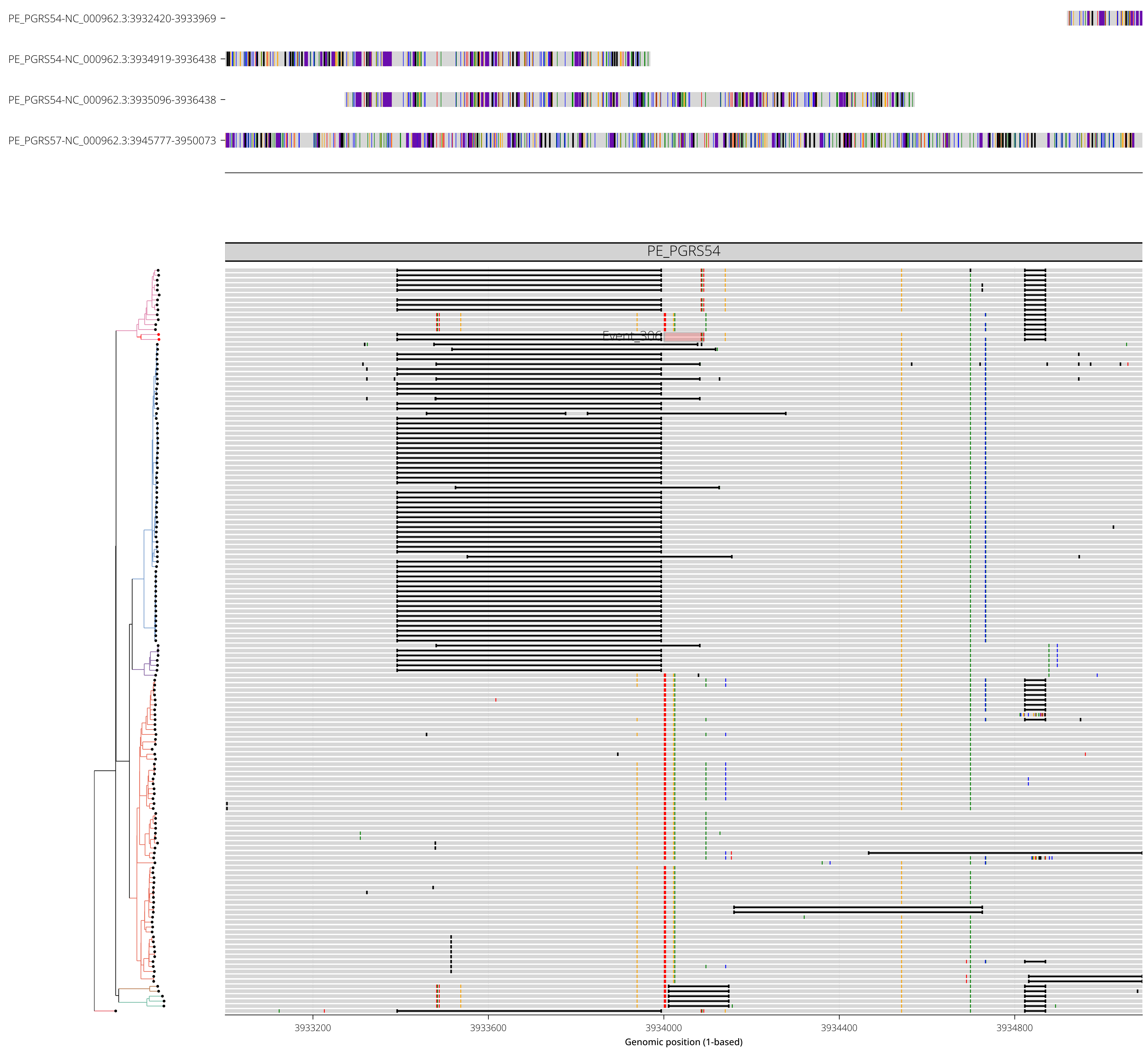

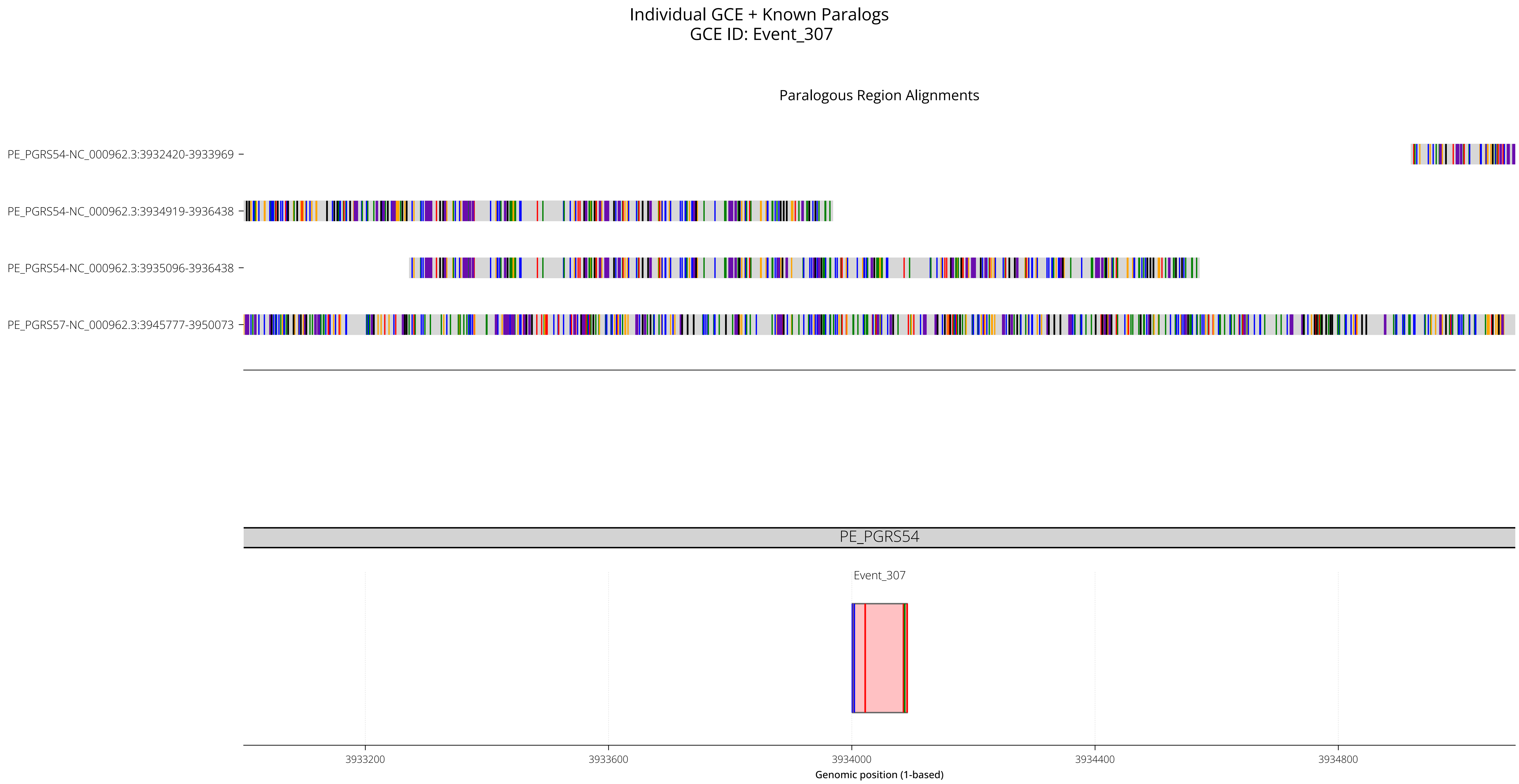
Individual GCE + Known Paralogs
GCE ID: Event_307
Paralogous Region Alignments
PE_PGRS54-NC_000962.3:3932420-3933969
PE_PGRS54-NC_000962.3:3934919-3936438
PE_PGRS54-NC_000962.3:3935096-3936438
PE_PGRS57-NC_000962.3:3945777-3950073
PE_PGRS54
Event_307
3933200
3933600
3934000
3934400
3934800
Genomic position (1-based)

# Circos Viz - GCE to Known Paralogs Locations
GCE ID: Event_307

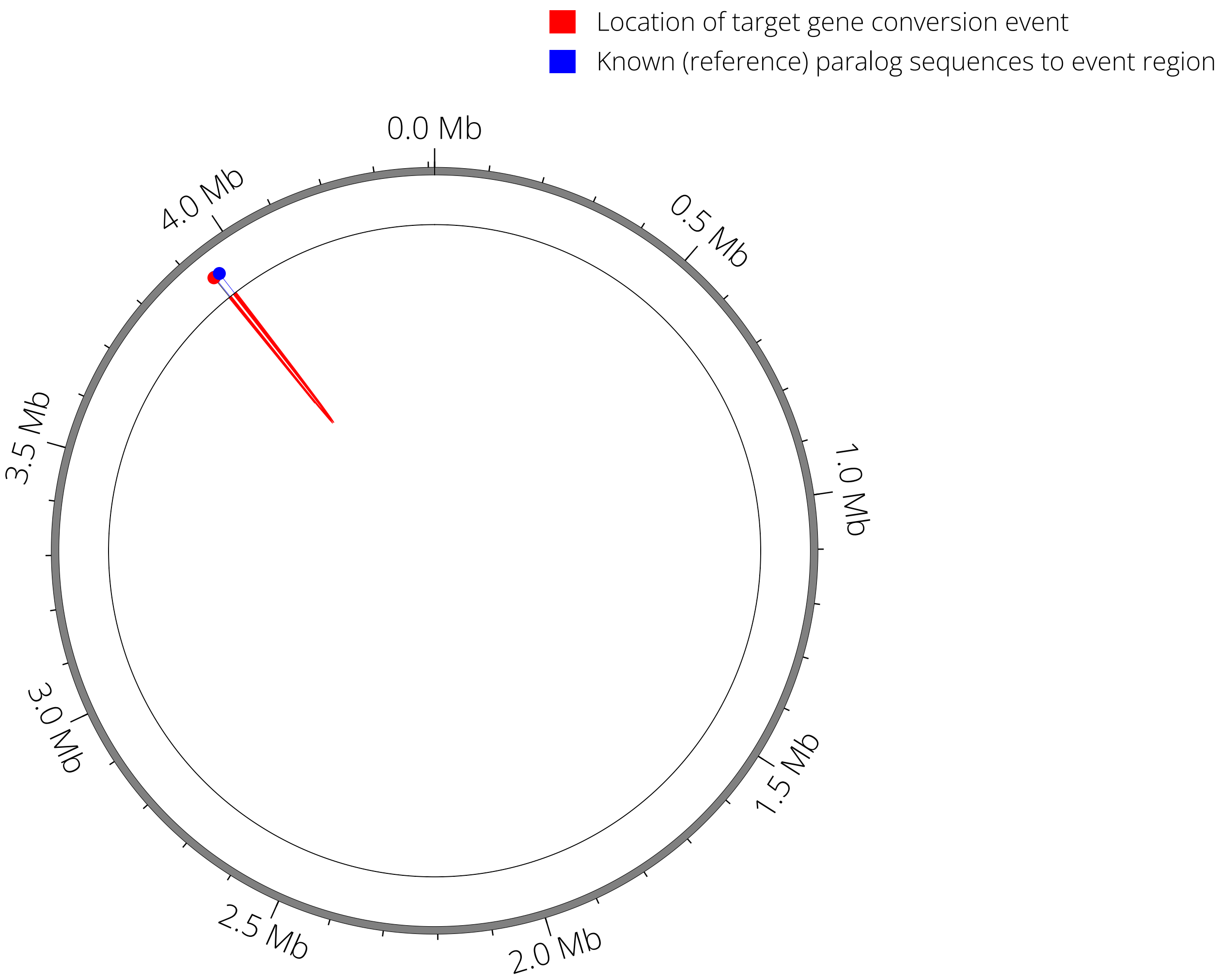

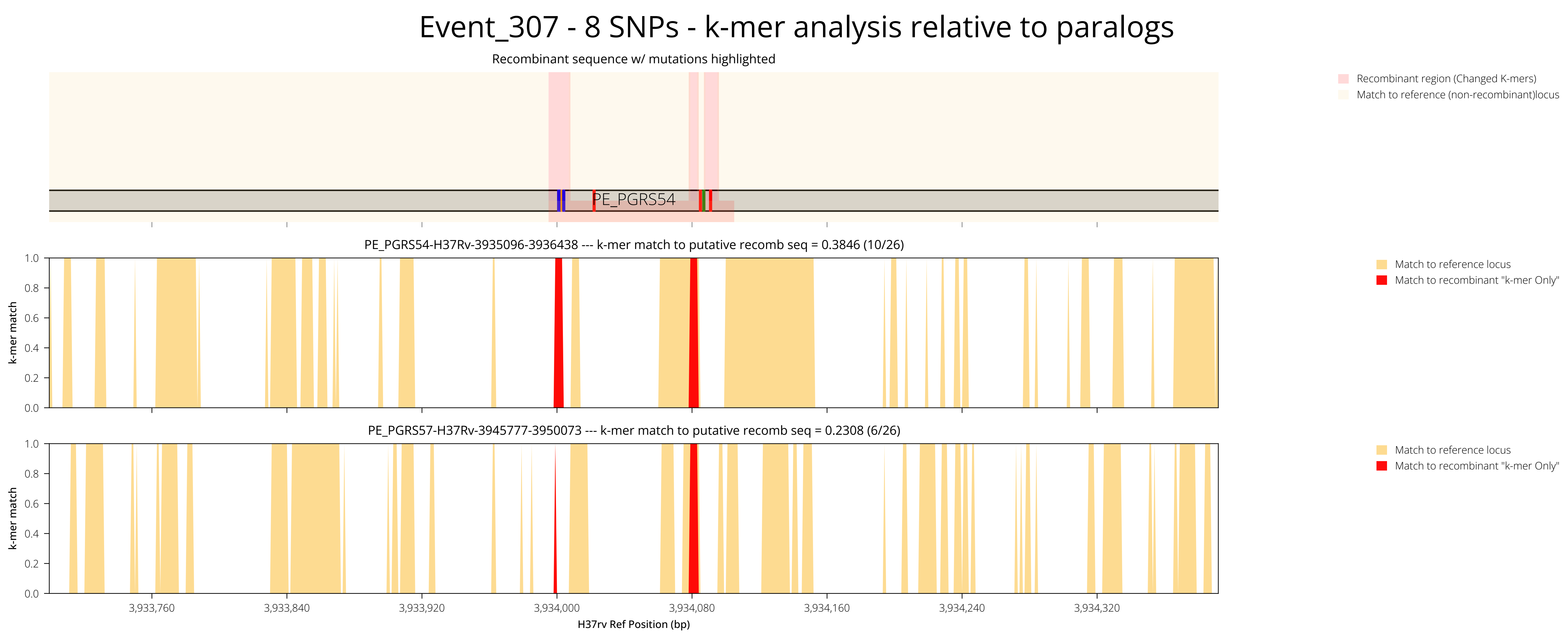
Event_307 - 8 SNPs - k-mer analysis relative to paralogs
Recombinant sequence w/ mutations highlighted
PE_PGRS54
Recombinant region (Changed K-mers)
Match to reference (non-recombinant)locus
PE_PGRS54-H37Rv-3935096-3936438 --- k-mer match to putative recomb seq = 0.3846 (10/26)
Match to reference locus
Match to recombinant "k-mer Only"
PE_PGRS57-H37Rv-3945777-3950073 --- k-mer match to putative recomb seq = 0.2308 (6/26)
Match to reference locus
Match to recombinant "k-mer Only"
k-mer match
1.0
0.8
0.6
0.4
0.2
0.0
3,933,760
3,933,840
3,933,920
3,934,000
3,934,080
3,934,160
3,934,240
3,934,320
H37rv Ref Position (bp)

Sub-phylogeny Viz + Paralog Alignments
GCE ID: Event_307

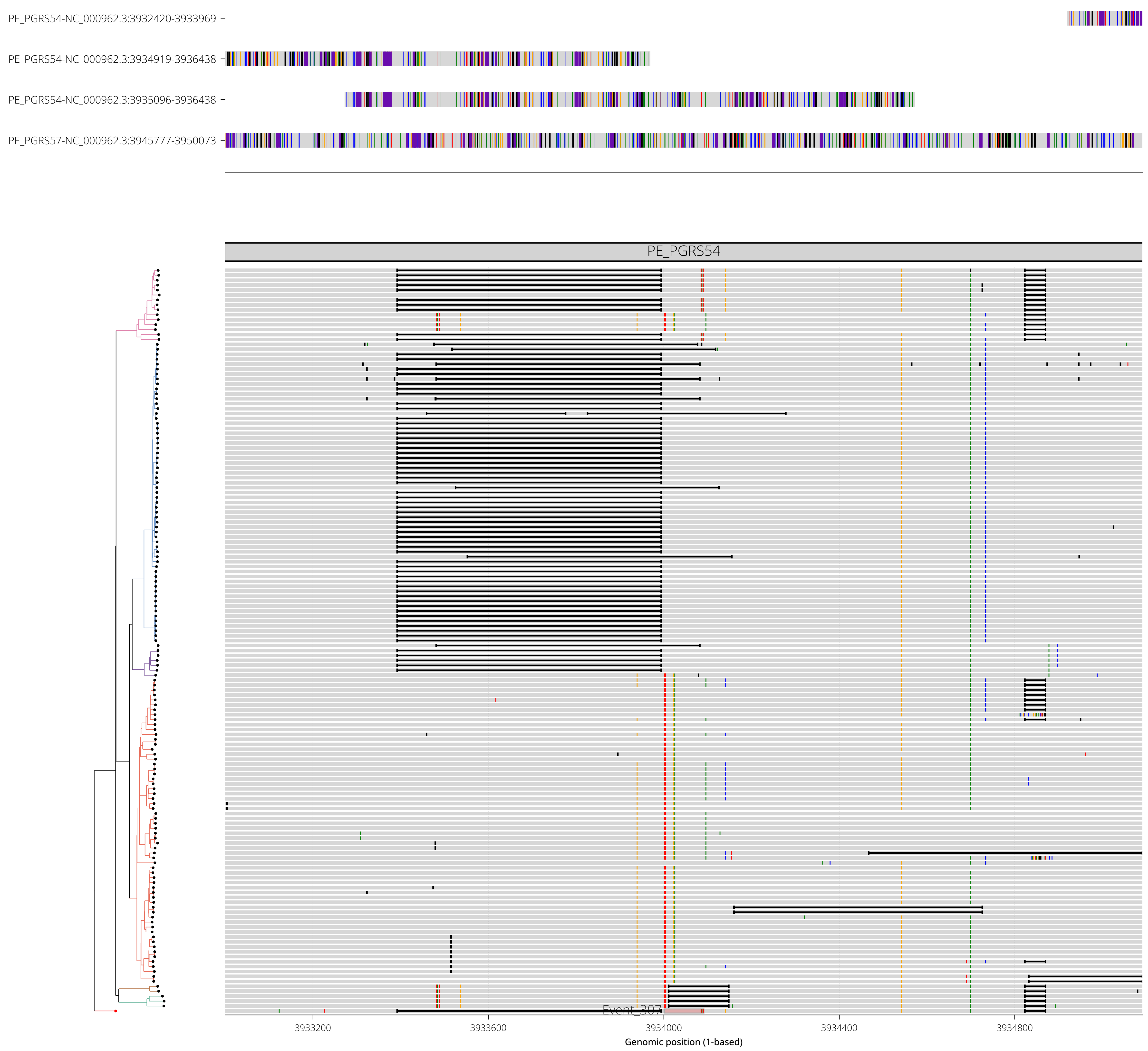

Full Phylogeny Viz + Paralog Alignments
GCE ID: Event_307

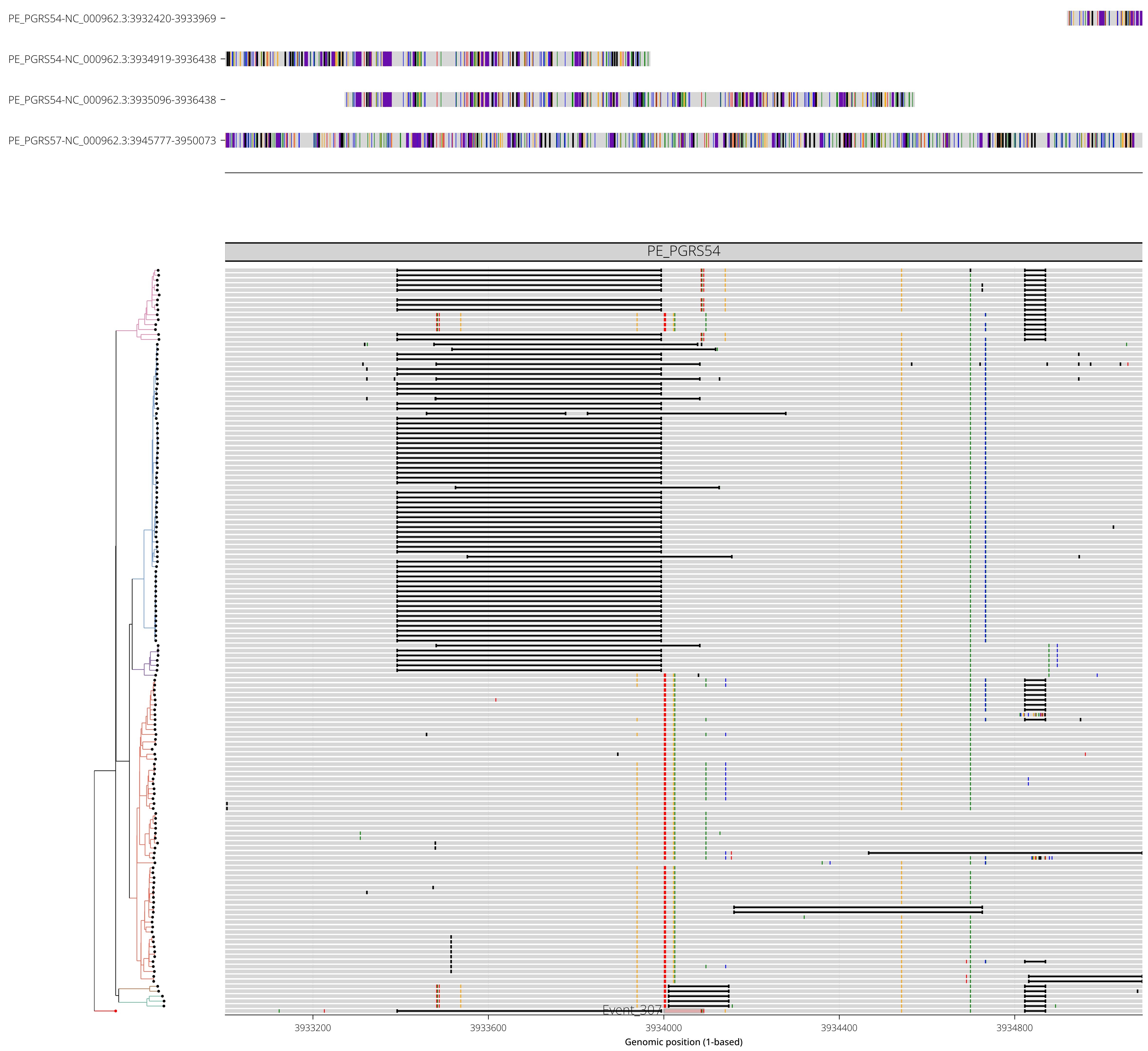

Individual GCE + Known Paralogs
GCE ID: Event_308

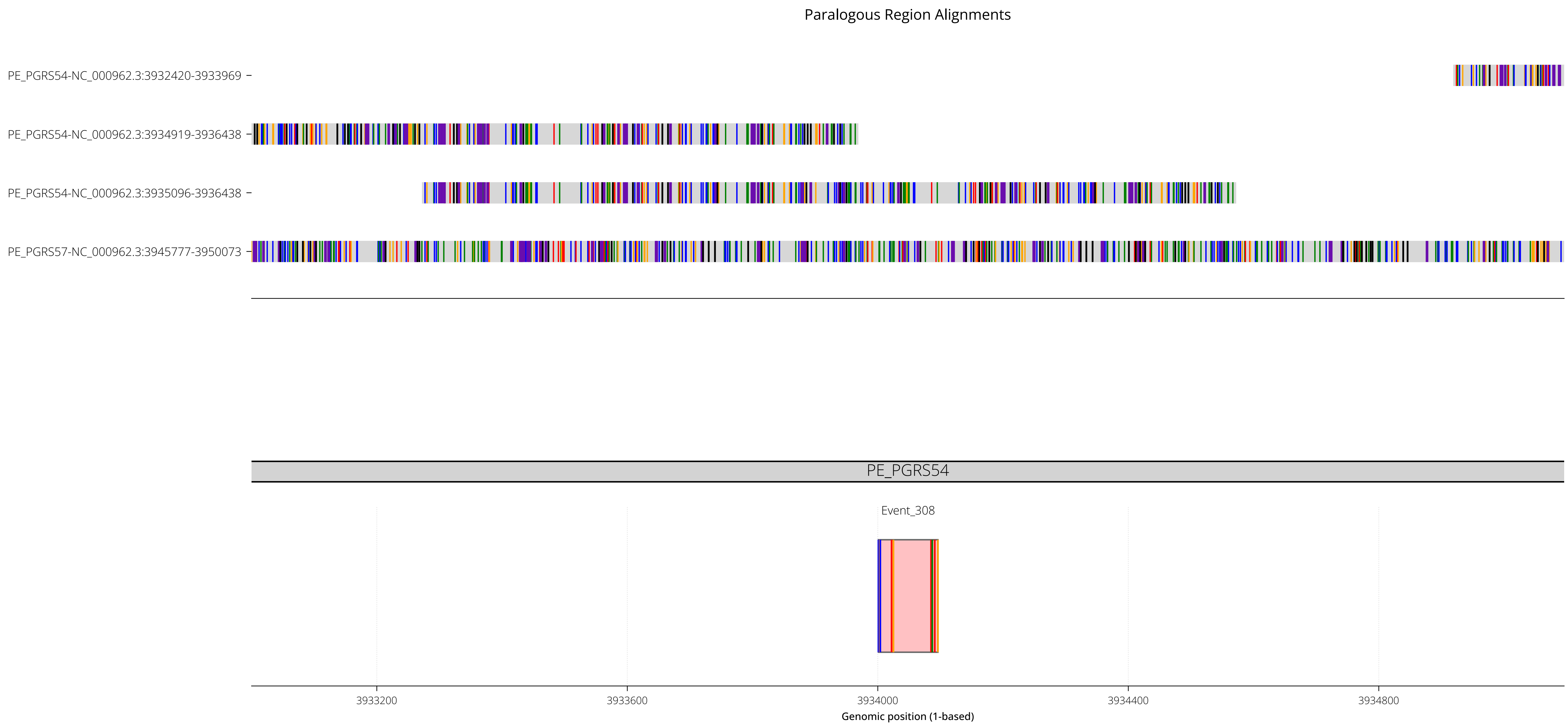

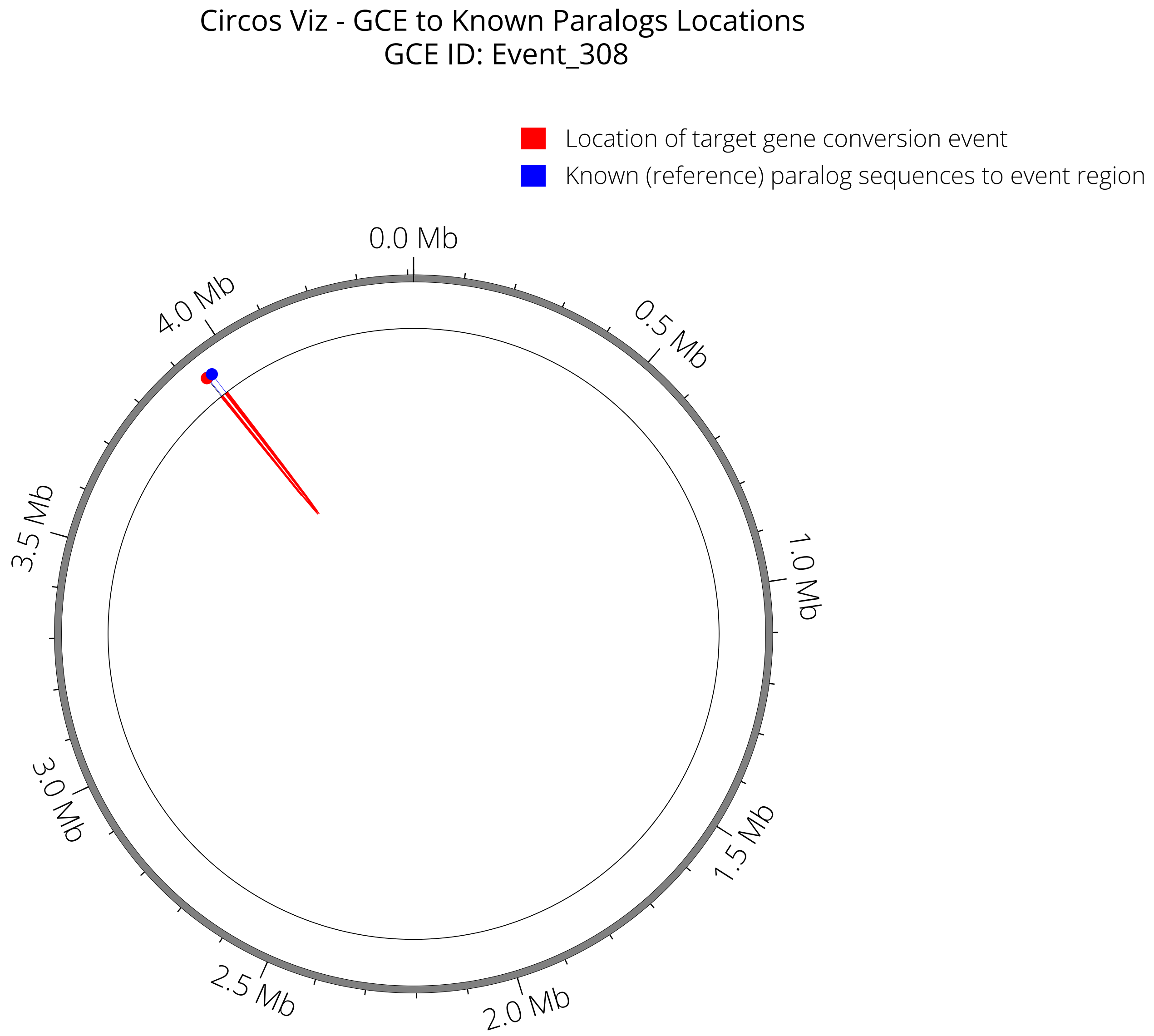
Circos Viz - GCE to Known Paralogs Locations
GCE ID: Event_308
Location of target gene conversion event
Known (reference) paralog sequences to event region
0.0 Mb
0.5 Mb
1.0 Mb
1.5 Mb
2.0 Mb
2.5 Mb
3.0 Mb
3.5 Mb
4.0 Mb

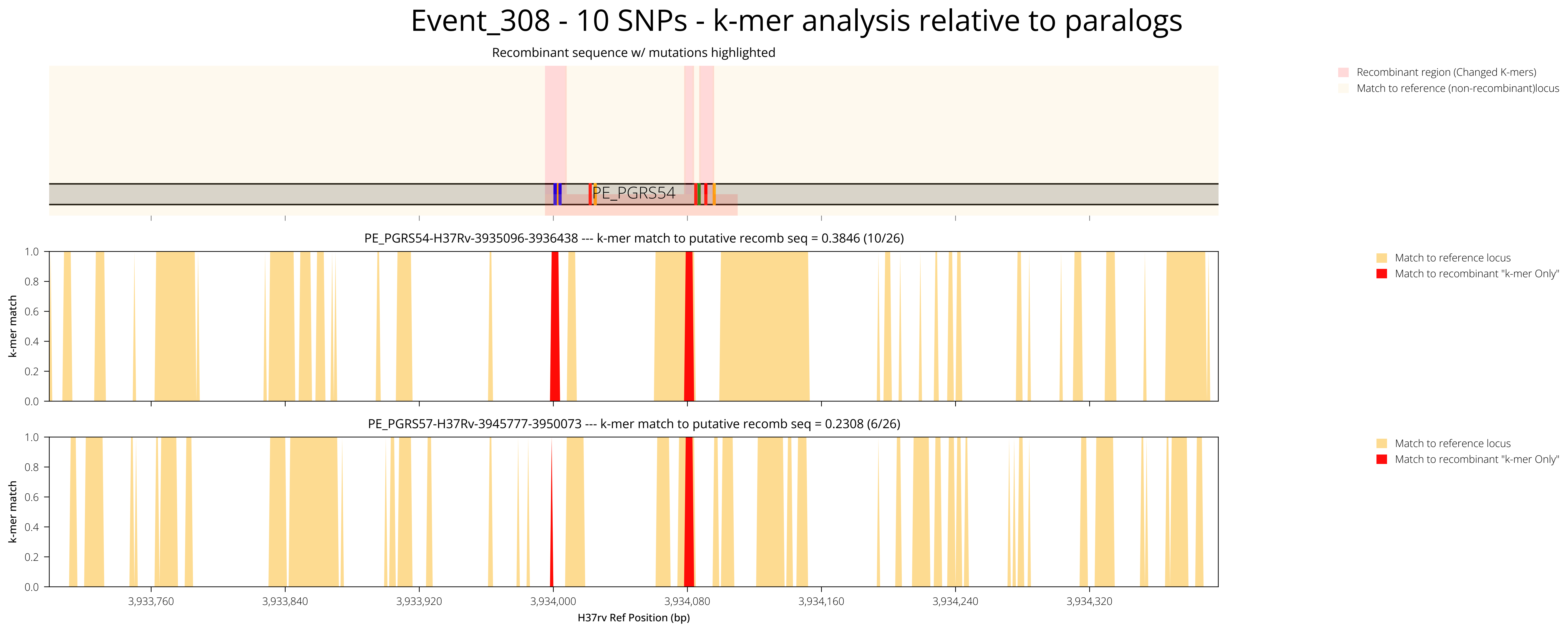

Event_308 - 10 SNPs - k-mer analysis relative to paralogs
Recombinant sequence w/ mutations highlighted
PE_PGRS54
Recombinant region (Changed K-mers)
Match to reference (non-recombinant)locus
PE_PGRS54-H37Rv-3935096-3936438 --- k-mer match to putative recomb seq = 0.3846 (10/26)
k-mer match
1.0
0.8
0.6
0.4
0.2
0.0
Match to reference locus
Match to recombinant "k-mer Only"
PE_PGRS57-H37Rv-3945777-3950073 --- k-mer match to putative recomb seq = 0.2308 (6/26)
k-mer match
1.0
0.8
0.6
0.4
0.2
0.0
Match to reference locus
Match to recombinant "k-mer Only"
3,933,760
3,933,840
3,933,920
3,934,000
3,934,080
3,934,160
3,934,240
3,934,320
H37rv Ref Position (bp)

Sub-phylogeny Viz + Paralog Alignments
GCE ID: Event_308

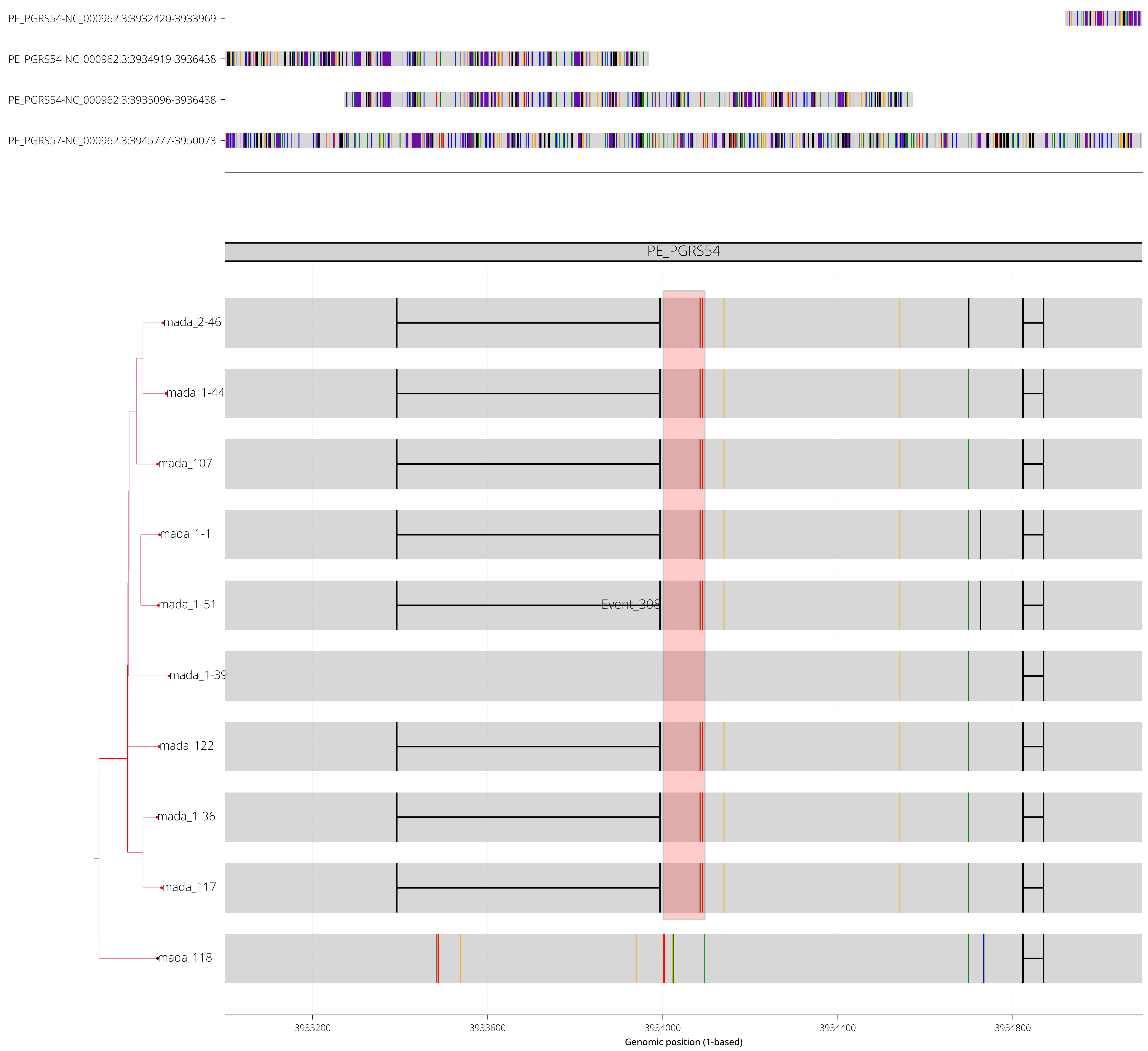

Full Phylogeny Viz + Paralog Alignments
GCE ID: Event_308

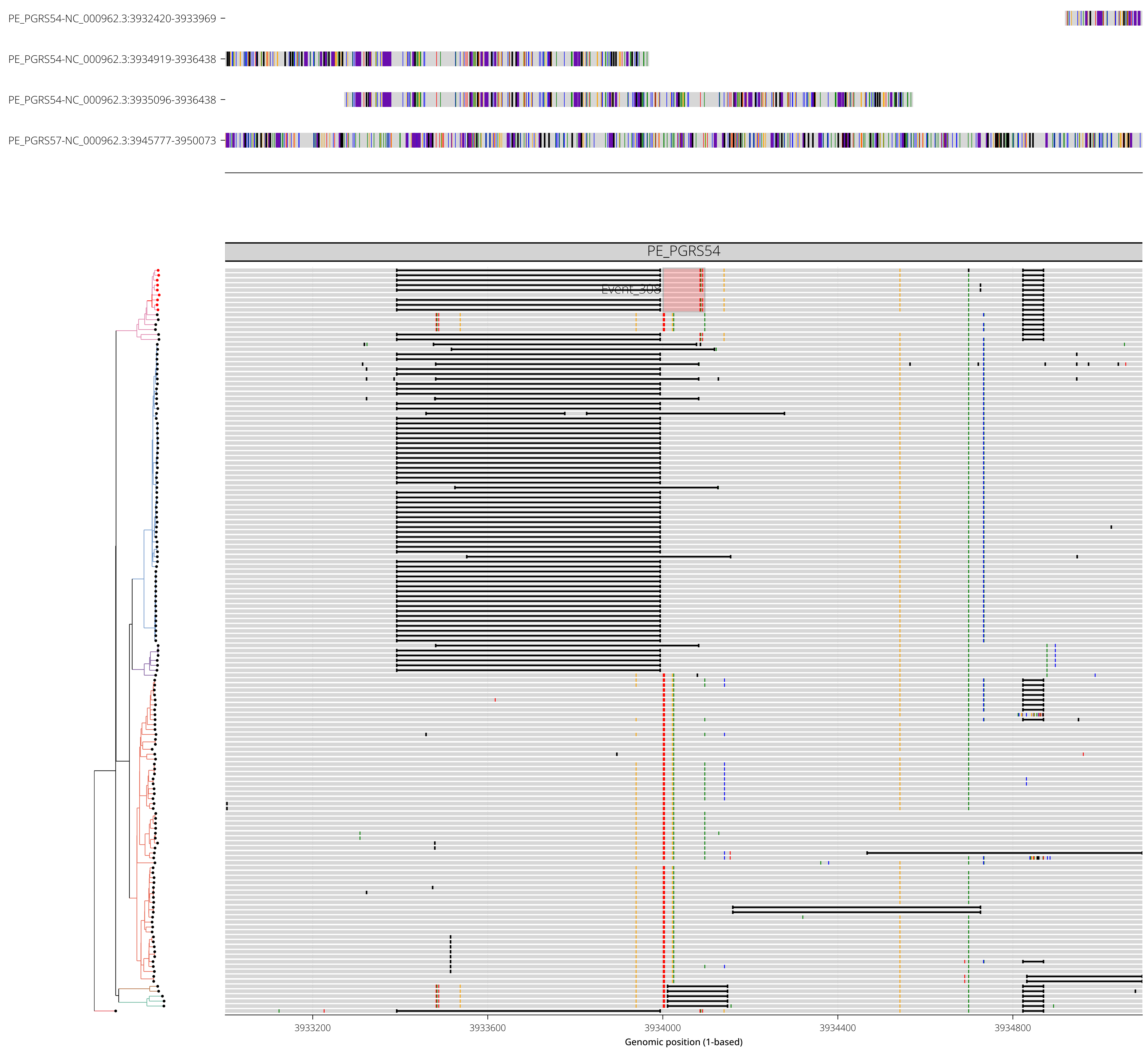

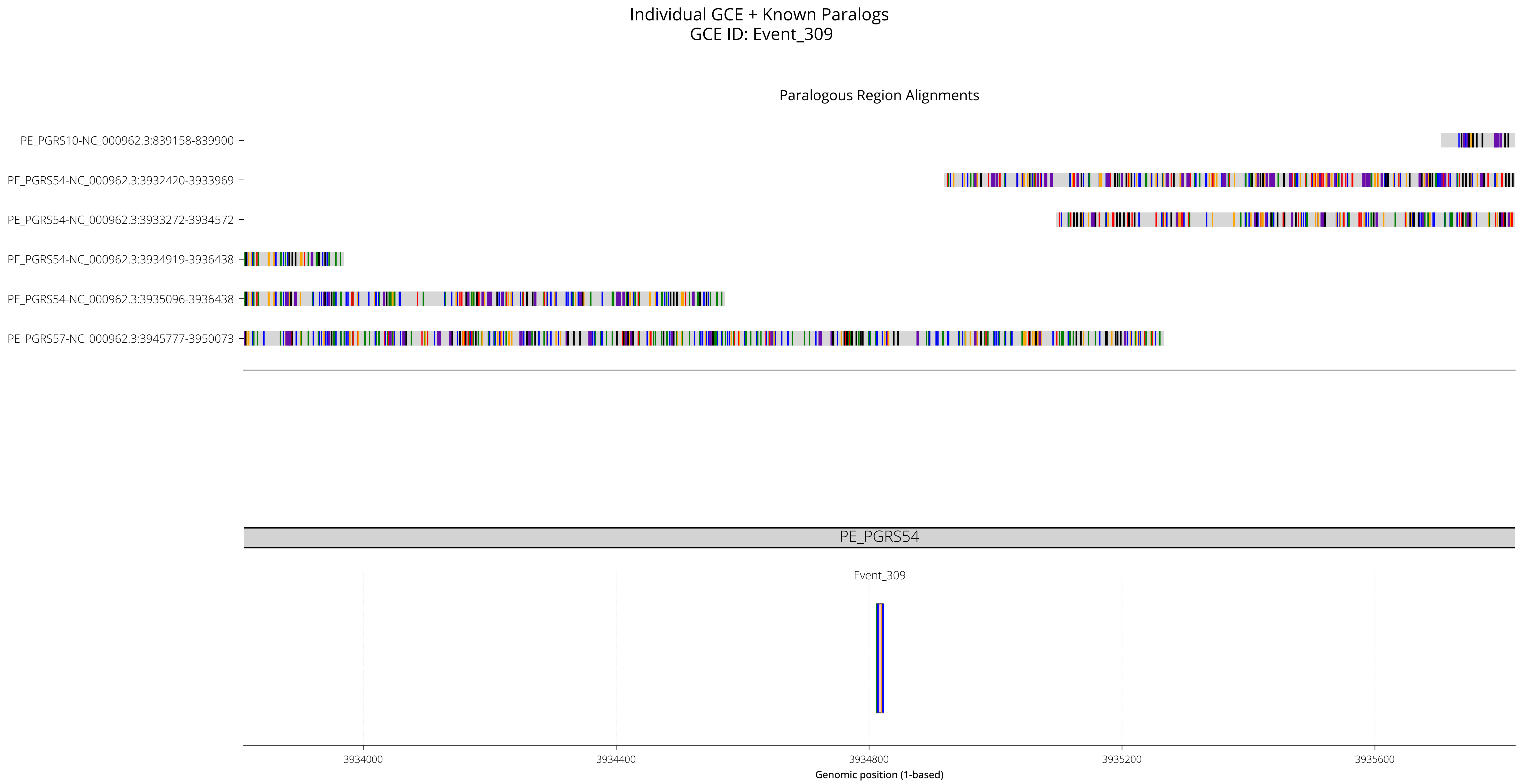

Individual GCE + Known Paralogs
GCE ID: Event_309
Paralogous Region Alignments
PE_PGRS10-NC_000962.3:839158-839900
PE_PGRS54-NC_000962.3:3932420-3933969
PE_PGRS54-NC_000962.3:3933272-3934572
PE_PGRS54-NC_000962.3:3934919-3936438
PE_PGRS54-NC_000962.3:3935096-3936438
PE_PGRS57-NC_000962.3:3945777-3950073
PE_PGRS54
Event_309
3934000
3934400
3934800
3935200
3935600
Genomic position (1-based)

Circos Viz - GCE to Known Paralogs Locations
GCE ID: Event_309

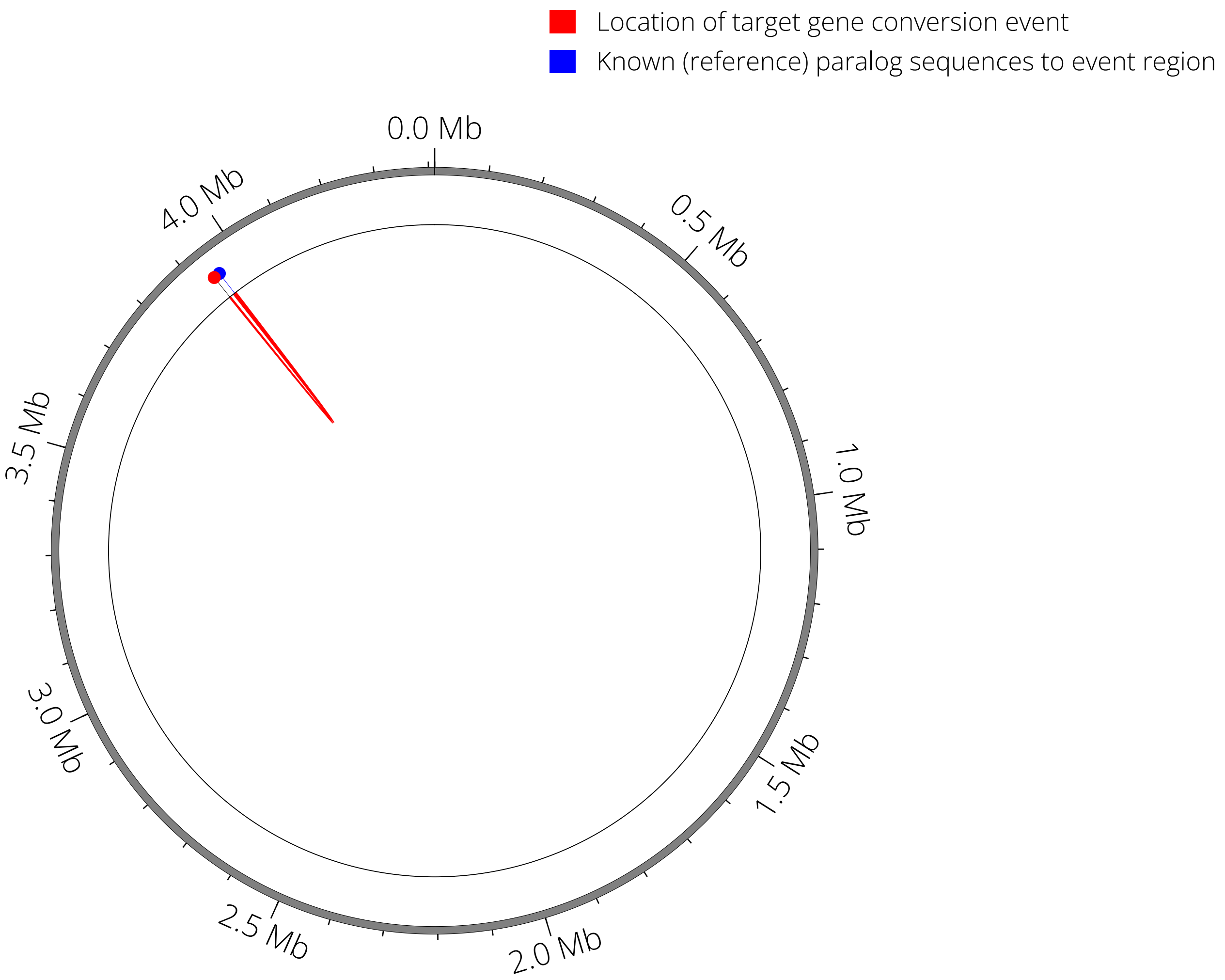

# Event_309 - 5 SNPs - k-mer analysis relative to paralogs

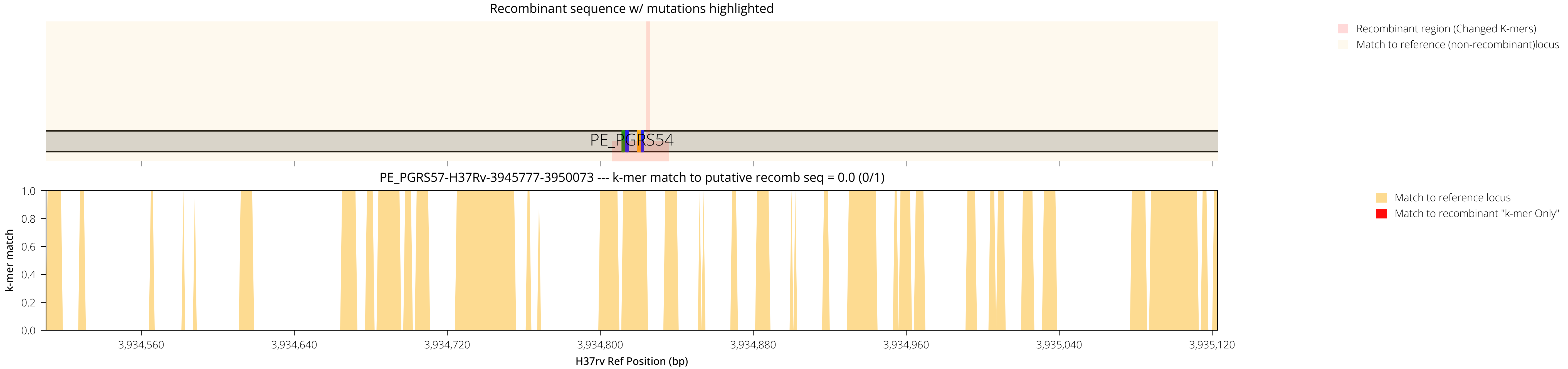

Sub-phylogeny Viz + Paralog Alignments
GCE ID: Event_309

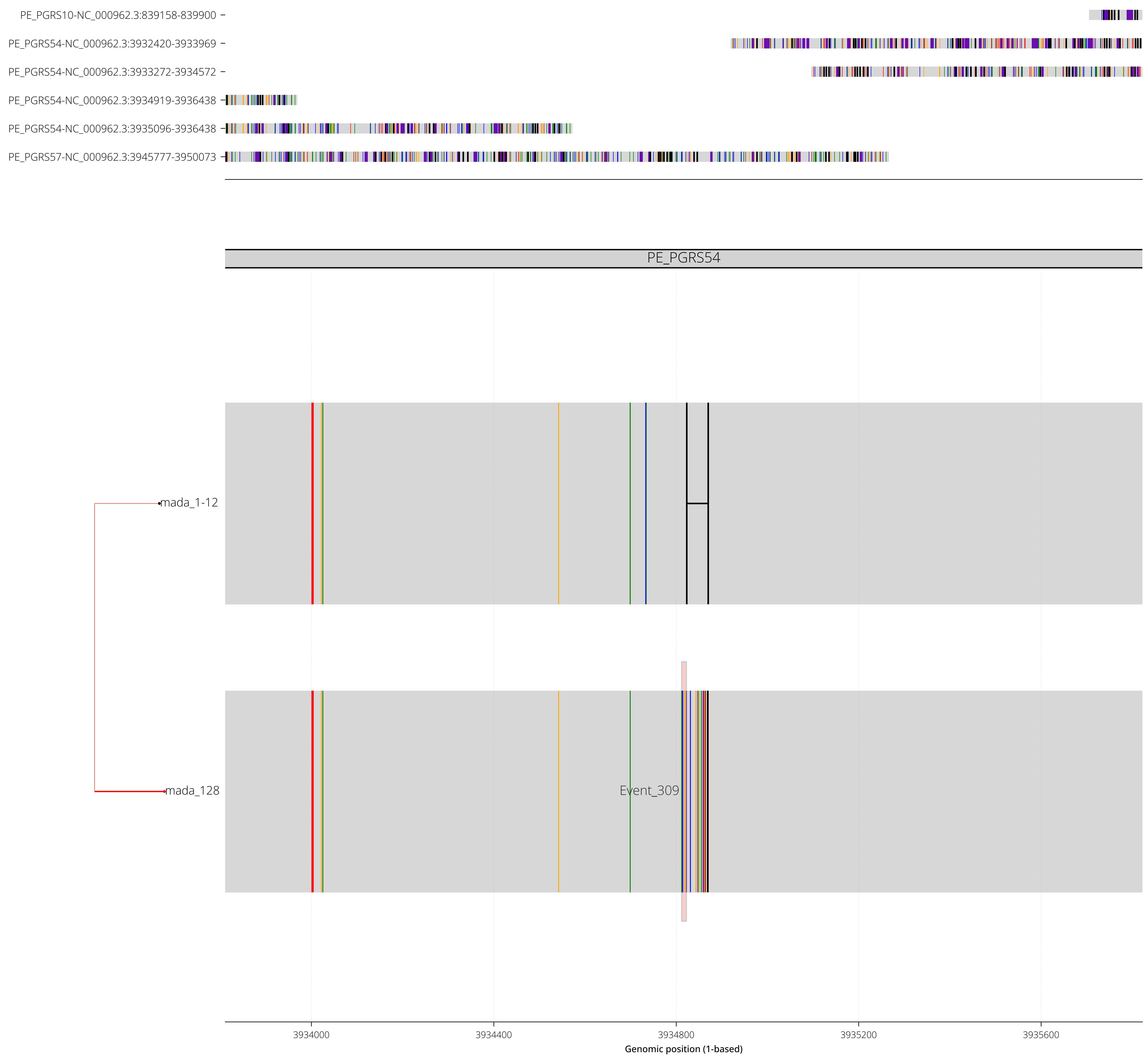

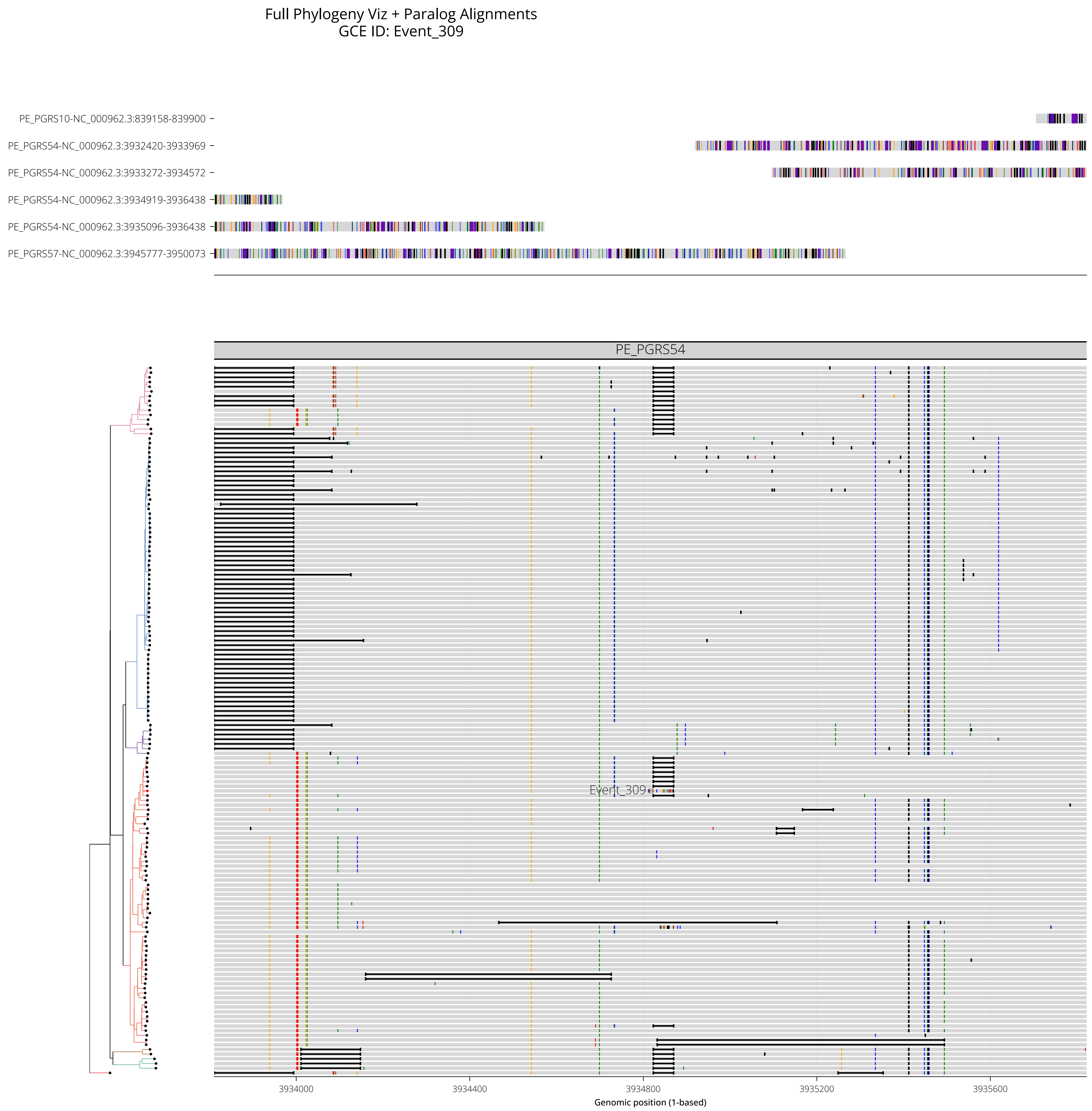

Full Phylogeny Viz + Paralog Alignments
GCE ID: Event_309
PE_PGRS10-NC_000962.3:839158-839900
PE_PGRS54-NC_000962.3:3932420-3933969
PE_PGRS54-NC_000962.3:3933272-3934572
PE_PGRS54-NC_000962.3:3934919-3936438
PE_PGRS54-NC_000962.3:3935096-3936438
PE_PGRS57-NC_000962.3:3945777-3950073
PE_PGRS54
Event_309
3934000
3934400
3934800
3935200
3935600
Genomic position (1-based)

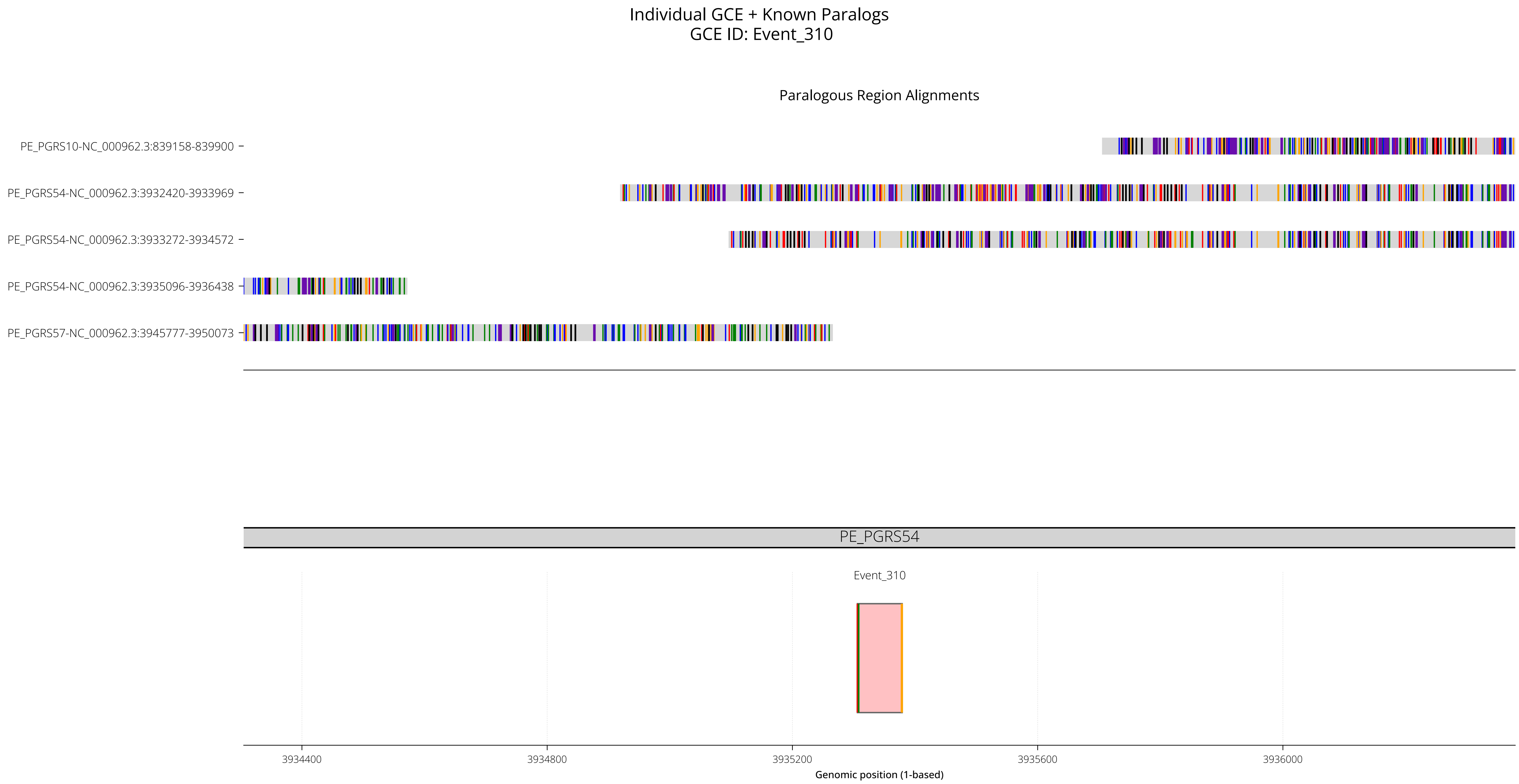
Individual GCE + Known Paralogs
GCE ID: Event_310
Paralogous Region Alignments
PE_PGRS10-NC_000962.3:839158-839900
PE_PGRS54-NC_000962.3:3932420-3933969
PE_PGRS54-NC_000962.3:3933272-3934572
PE_PGRS54-NC_000962.3:3935096-3936438
PE_PGRS57-NC_000962.3:3945777-3950073
PE_PGRS54
Event_310
3934400
3934800
3935200
3935600
3936000
Genomic position (1-based)

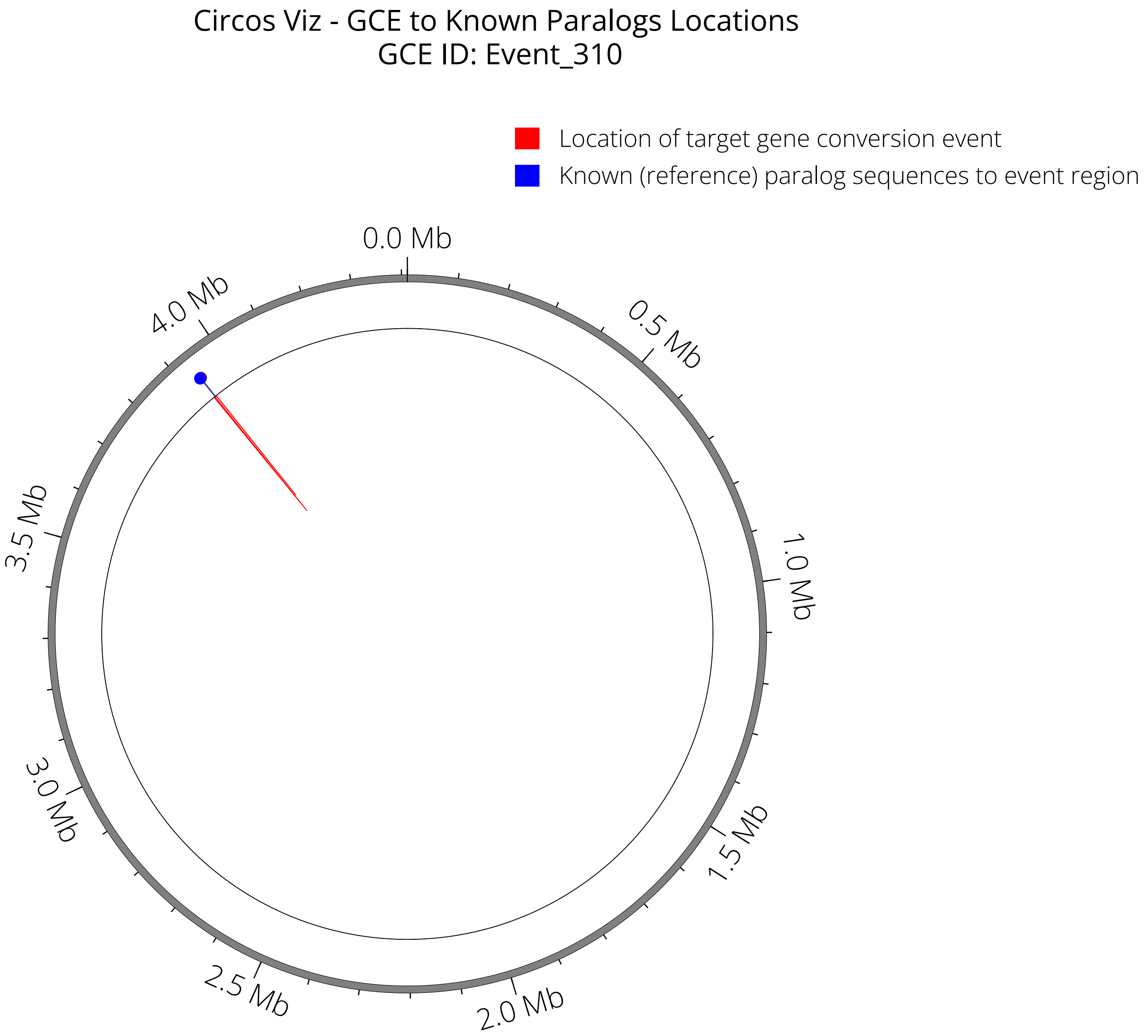

Circos Viz - GCE to Known Paralogs Locations
GCE ID: Event_310
Location of target gene conversion event
Known (reference) paralog sequences to event region
0.0 Mb
0.5 Mb
1.0 Mb
1.5 Mb
2.0 Mb
2.5 Mb
3.0 Mb
3.5 Mb
4.0 Mb

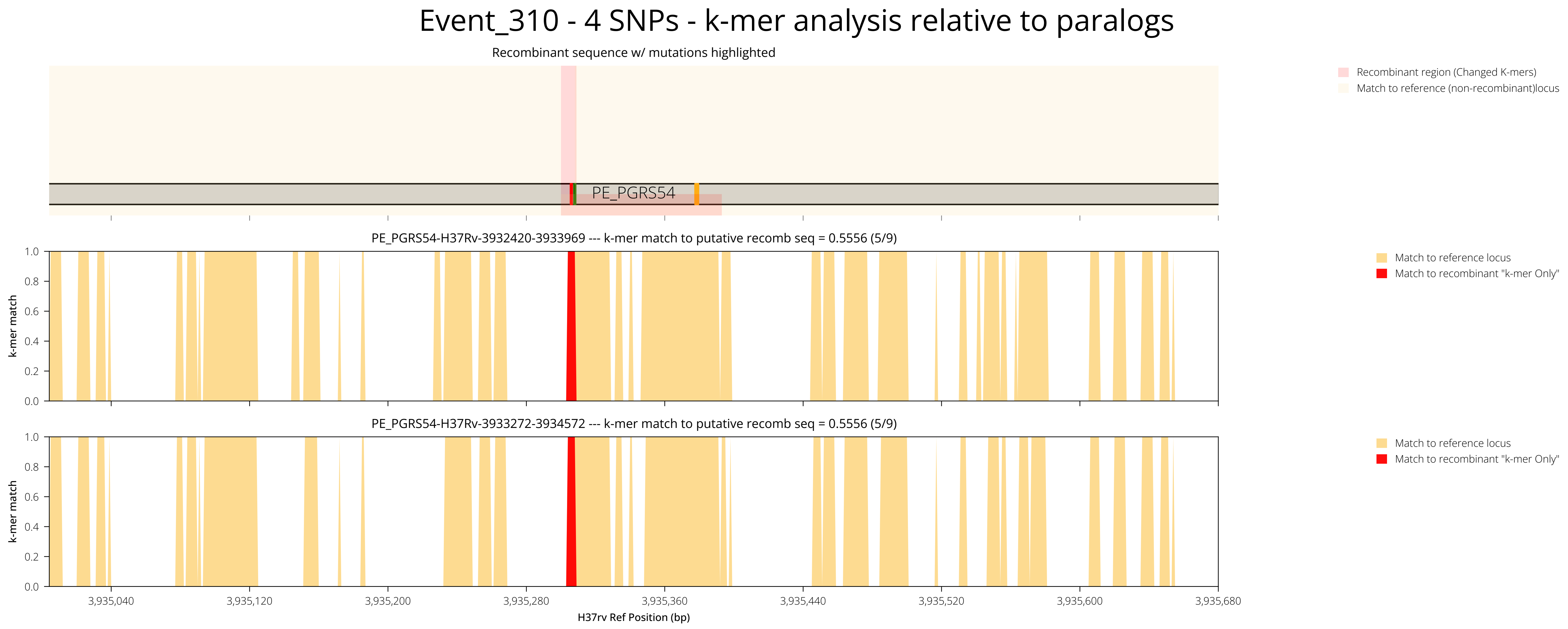
Event_310 - 4 SNPs - k-mer analysis relative to paralogs
Recombinant sequence w/ mutations highlighted
Recombinant region (Changed K-mers)
Match to reference (non-recombinant)locus
PE_PGRS54
PE_PGRS54-H37Rv-3932420-3933969 --- k-mer match to putative recomb seq = 0.5556 (5/9)
Match to reference locus
Match to recombinant "k-mer Only"
k-mer match
PE_PGRS54-H37Rv-3933272-3934572 --- k-mer match to putative recomb seq = 0.5556 (5/9)
Match to reference locus
Match to recombinant "k-mer Only"
k-mer match
3,935,040
3,935,120
3,935,200
3,935,280
3,935,360
3,935,440
3,935,520
3,935,600
3,935,680
H37rv Ref Position (bp)

Sub-phylogeny Viz + Paralog Alignments
GCE ID: Event_310

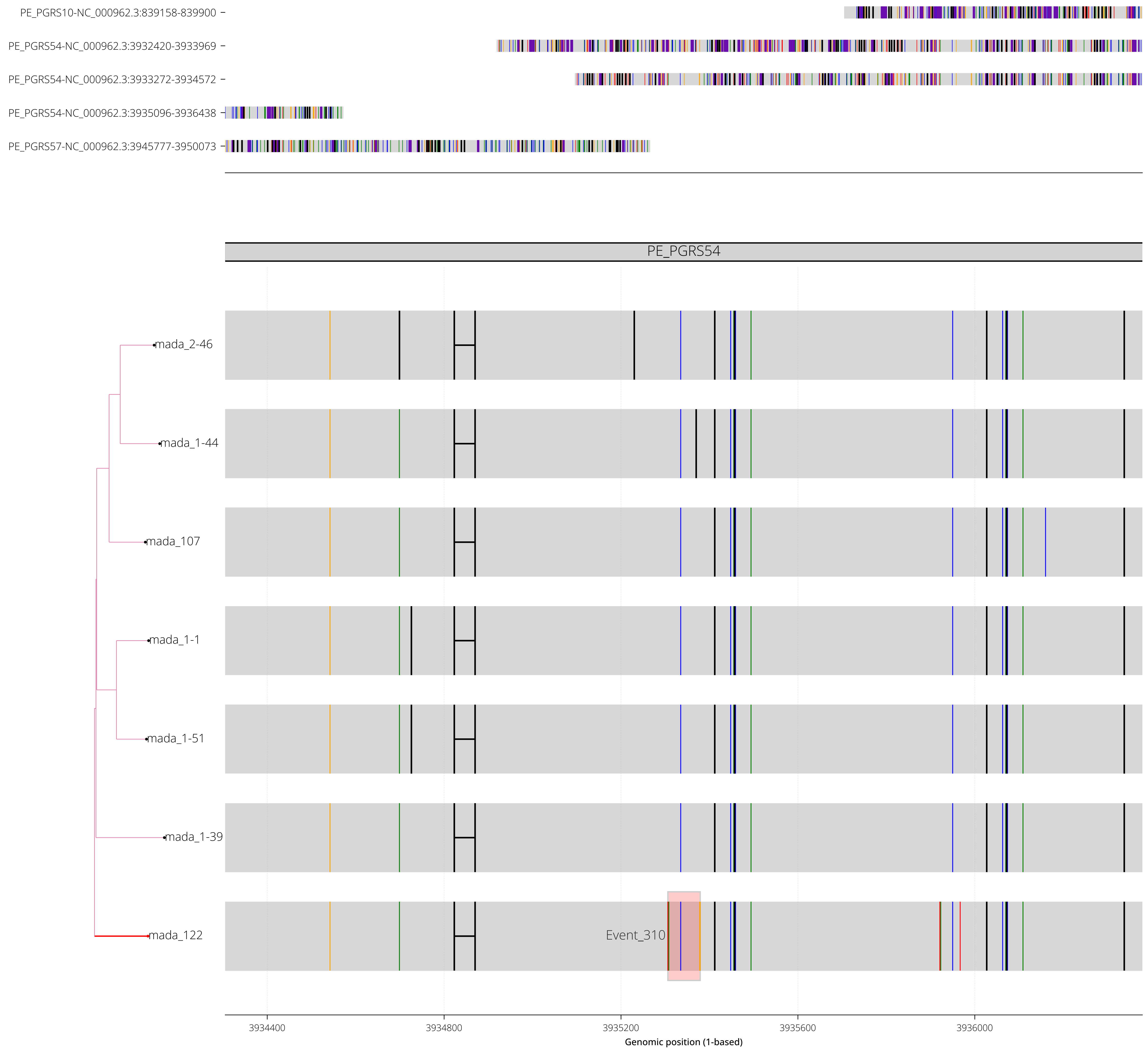

Full Phylogeny Viz + Paralog Alignments
GCE ID: Event_310

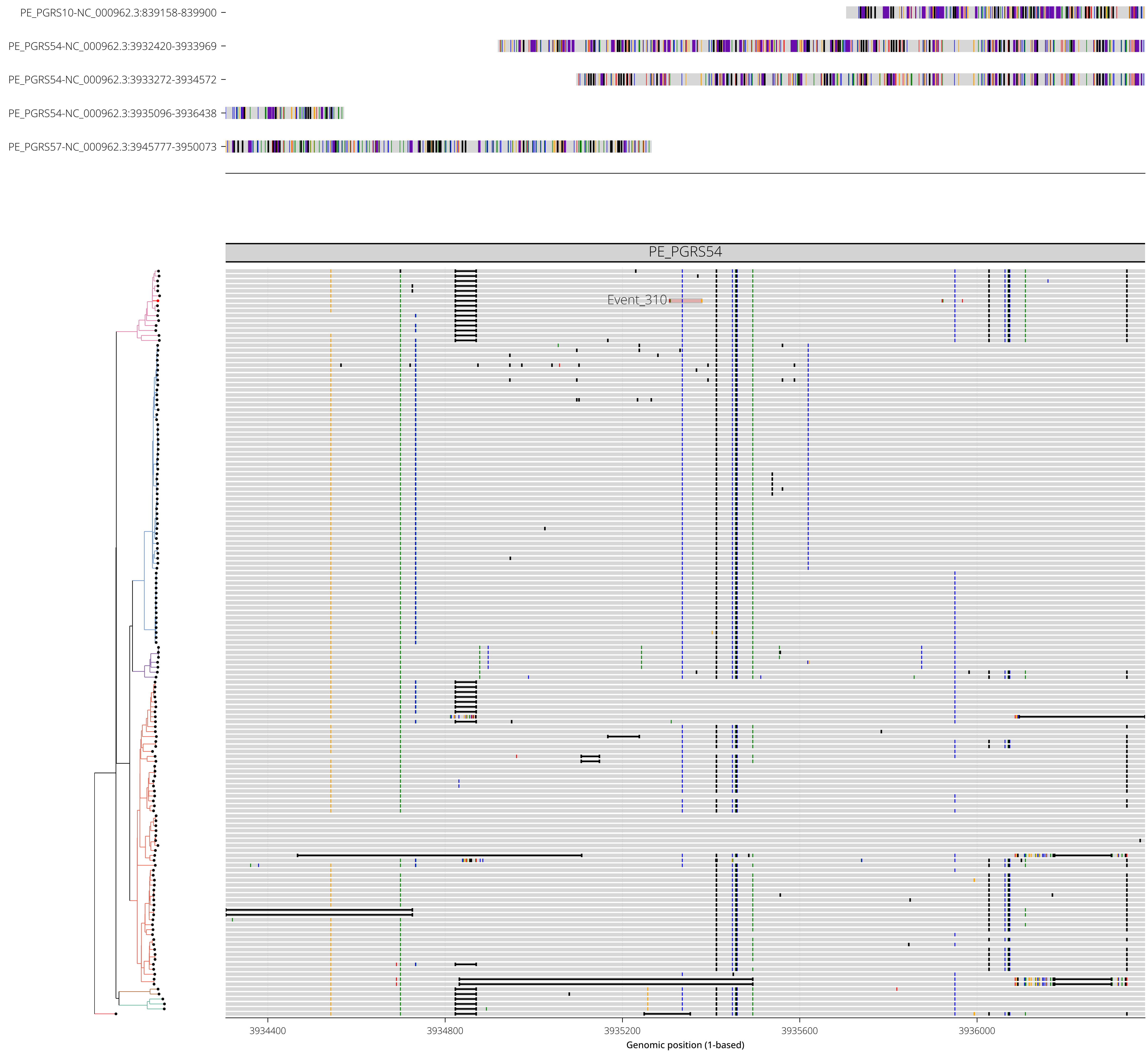

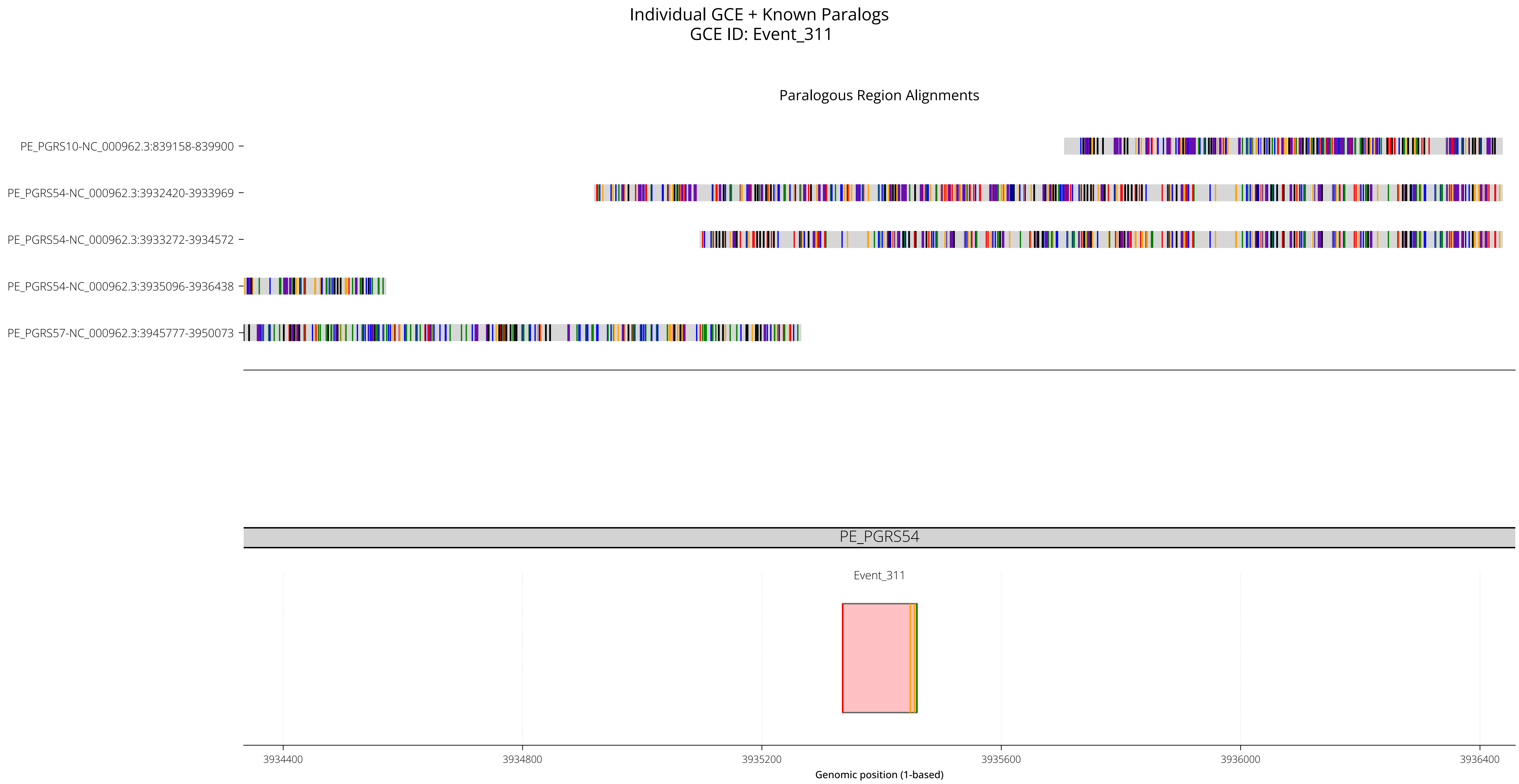

Individual GCE + Known Paralogs
GCE ID: Event_311
Paralogous Region Alignments
PE_PGRS10-NC_000962.3:839158-839900
PE_PGRS54-NC_000962.3:3932420-3933969
PE_PGRS54-NC_000962.3:3933272-3934572
PE_PGRS54-NC_000962.3:3935096-3936438
PE_PGRS57-NC_000962.3:3945777-3950073
PE_PGRS54
Event_311
3934400
3934800
3935200
3935600
3936000
3936400
Genomic position (1-based)

Circos Viz - GCE to Known Paralogs Locations
GCE ID: Event_311

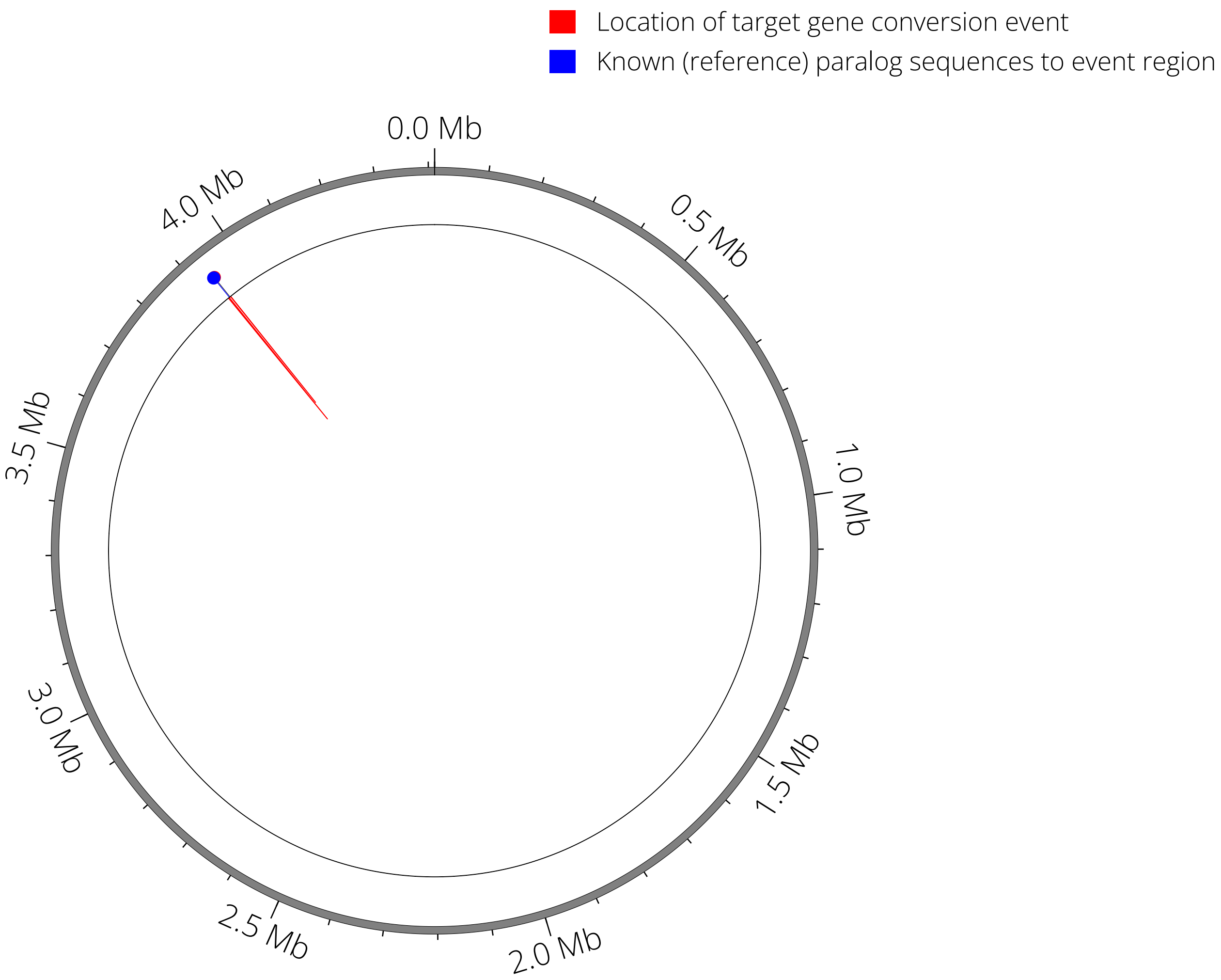

# Event_311 - 4 SNPs - k-mer analysis relative to paralogs

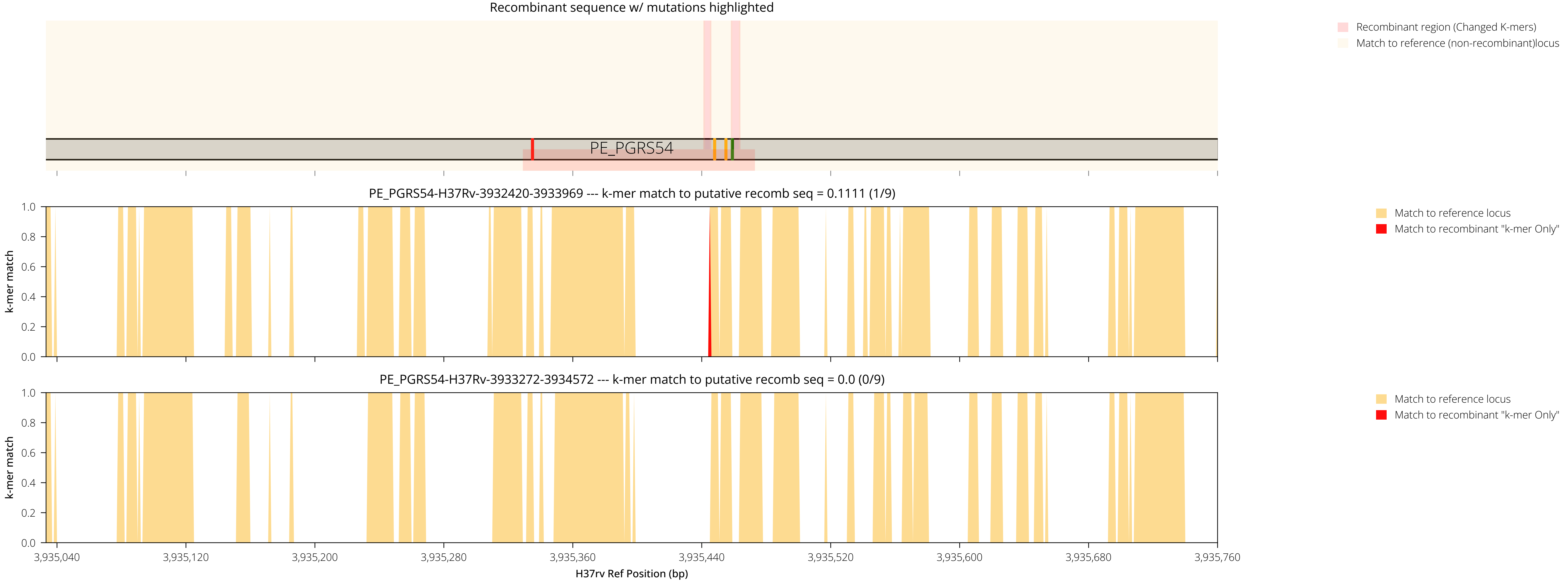

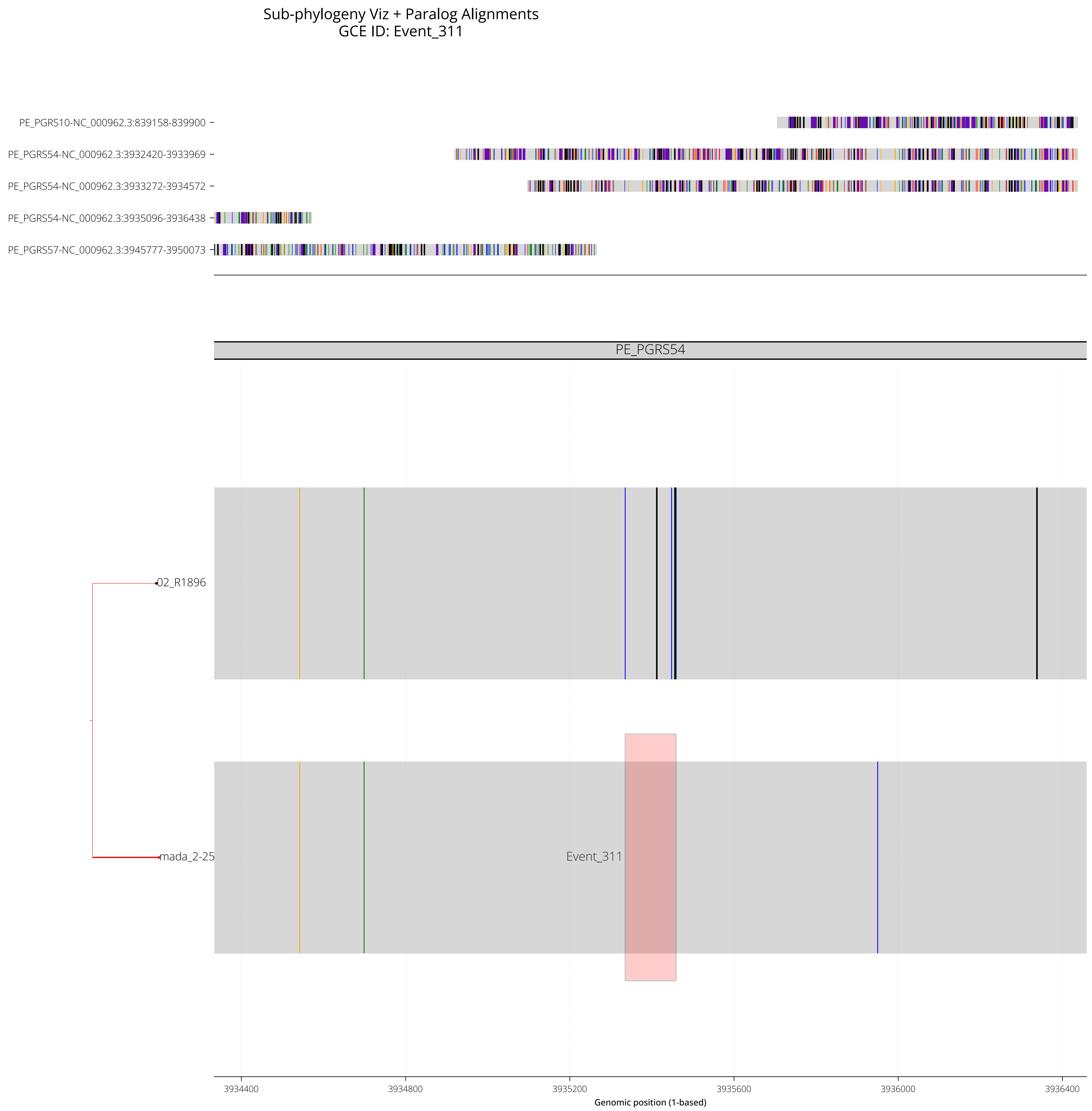

Sub-phylogeny Viz + Paralog Alignments
GCE ID: Event_311
PE_PGRS10-NC_000962.3:839158-839900
PE_PGRS54-NC_000962.3:3932420-3933969
PE_PGRS54-NC_000962.3:3933272-3934572
PE_PGRS54-NC_000962.3:3935096-3936438
PE_PGRS57-NC_000962.3:3945777-3950073
PE_PGRS54
02_R1896
mada_2-25
Event_311
3934400
3934800
3935200
3935600
3936000
3936400
Genomic position (1-based)

Full Phylogeny Viz + Paralog Alignments
GCE ID: Event_311

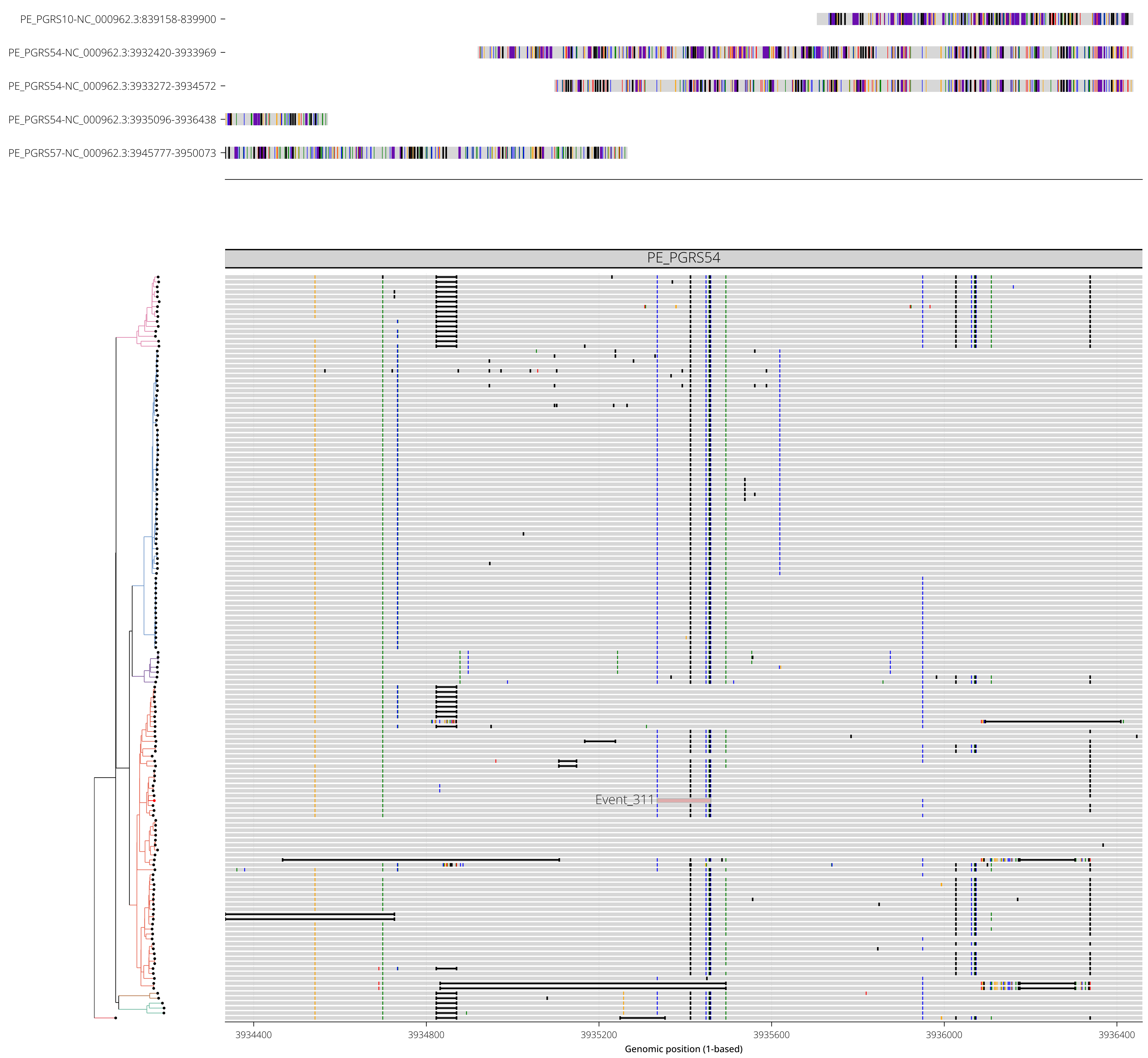

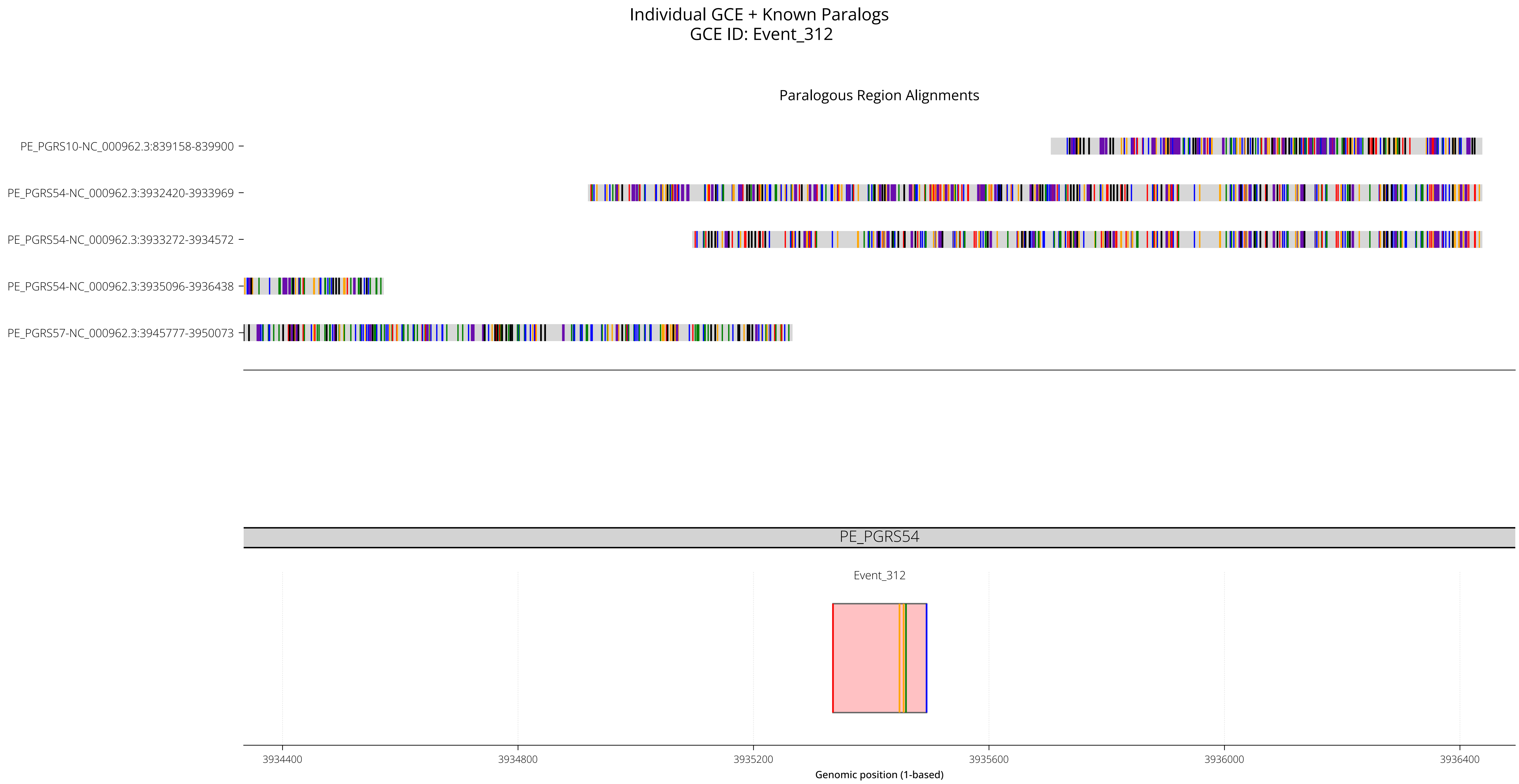

Individual GCE + Known Paralogs
GCE ID: Event_312
Paralogous Region Alignments
PE_PGRS10-NC_000962.3:839158-839900
PE_PGRS54-NC_000962.3:3932420-3933969
PE_PGRS54-NC_000962.3:3933272-3934572
PE_PGRS54-NC_000962.3:3935096-3936438
PE_PGRS57-NC_000962.3:3945777-3950073
PE_PGRS54
Event_312
3934400
3934800
3935200
3935600
3936000
3936400
Genomic position (1-based)

# Circos Viz - GCE to Known Paralogs Locations
GCE ID: Event_312

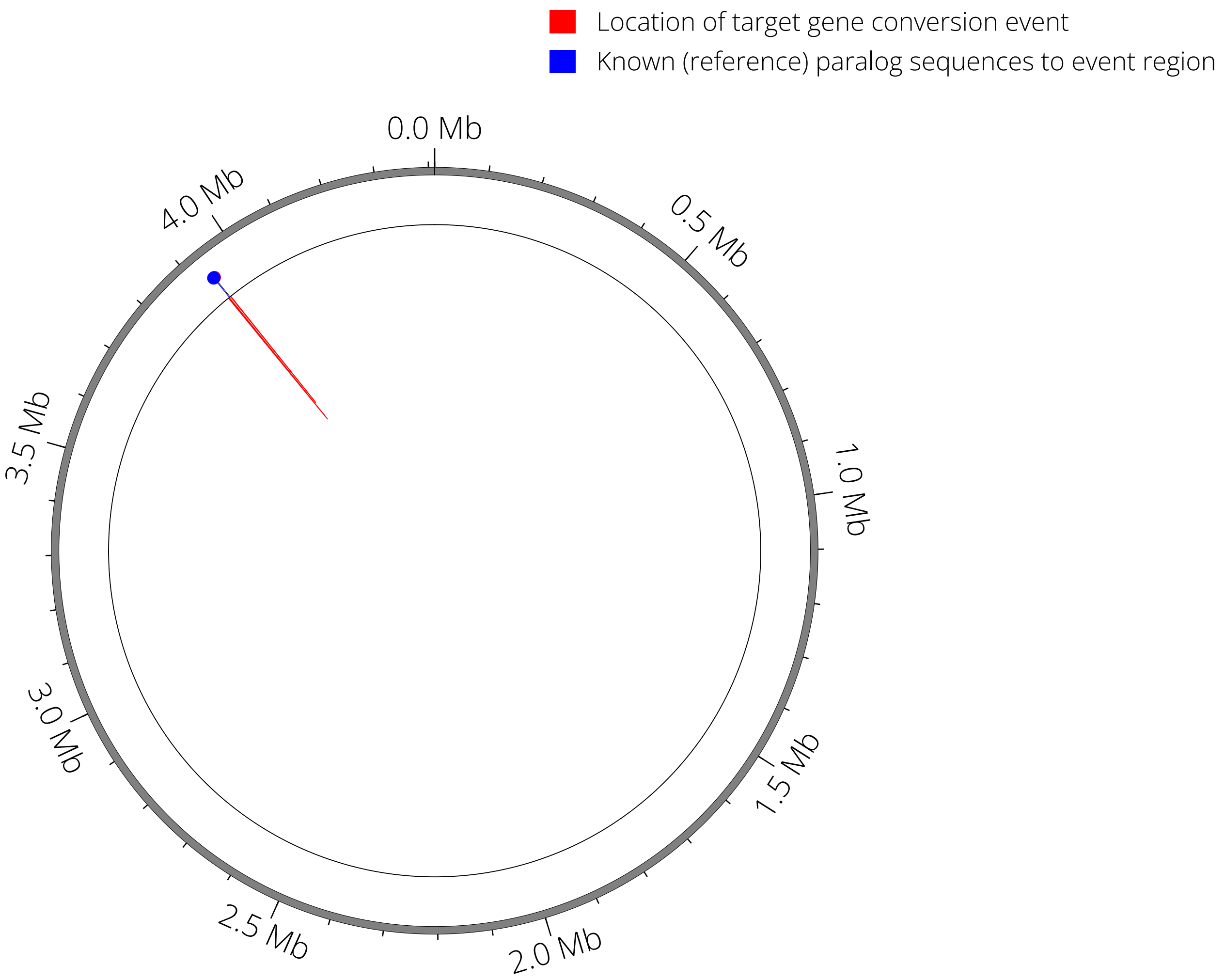

# Event_312 - 5 SNPs - k-mer analysis relative to paralogs

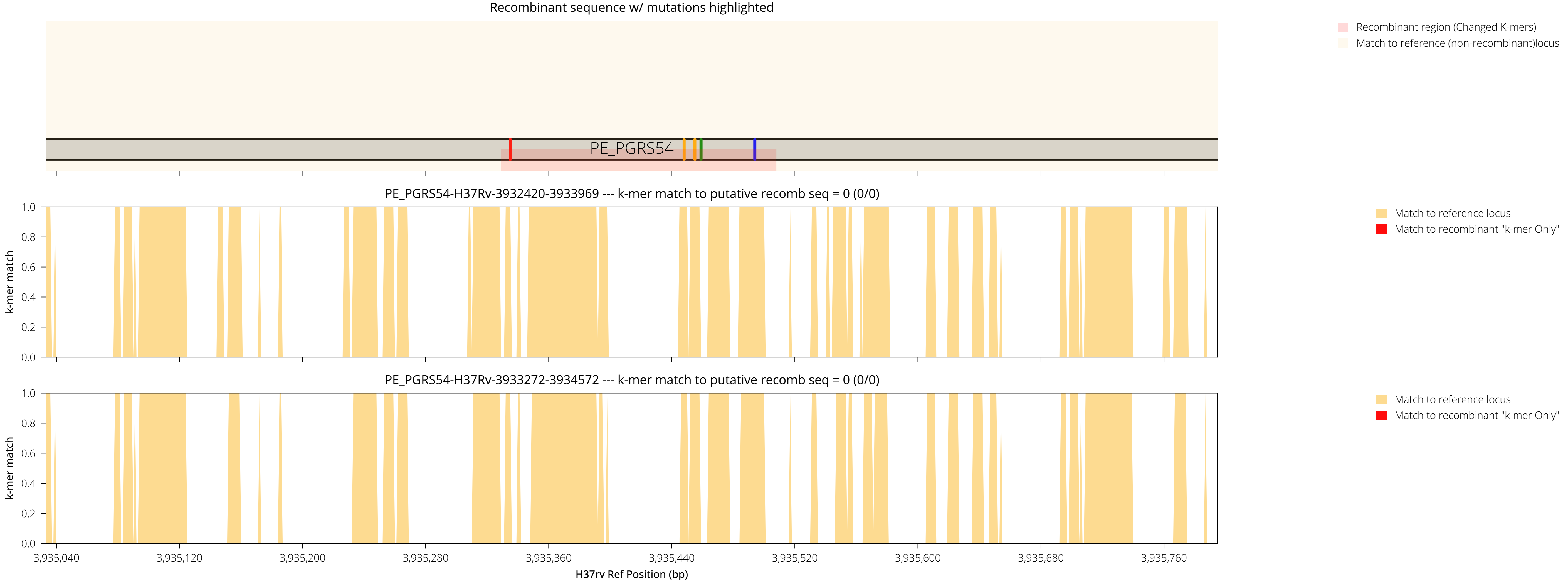

Sub-phylogeny Viz + Paralog Alignments
GCE ID: Event_312

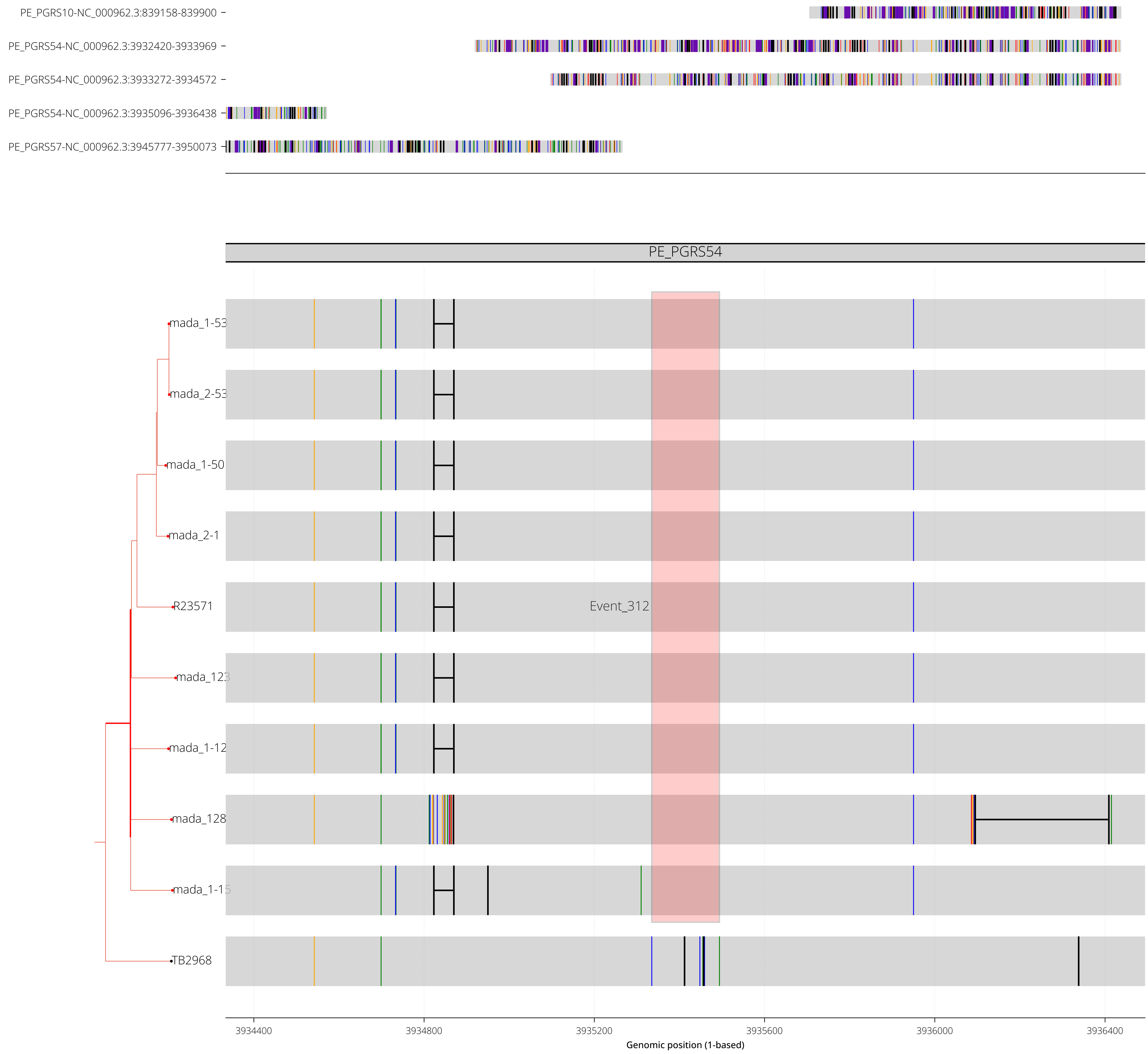

Full Phylogeny Viz + Paralog Alignments
GCE ID: Event_312

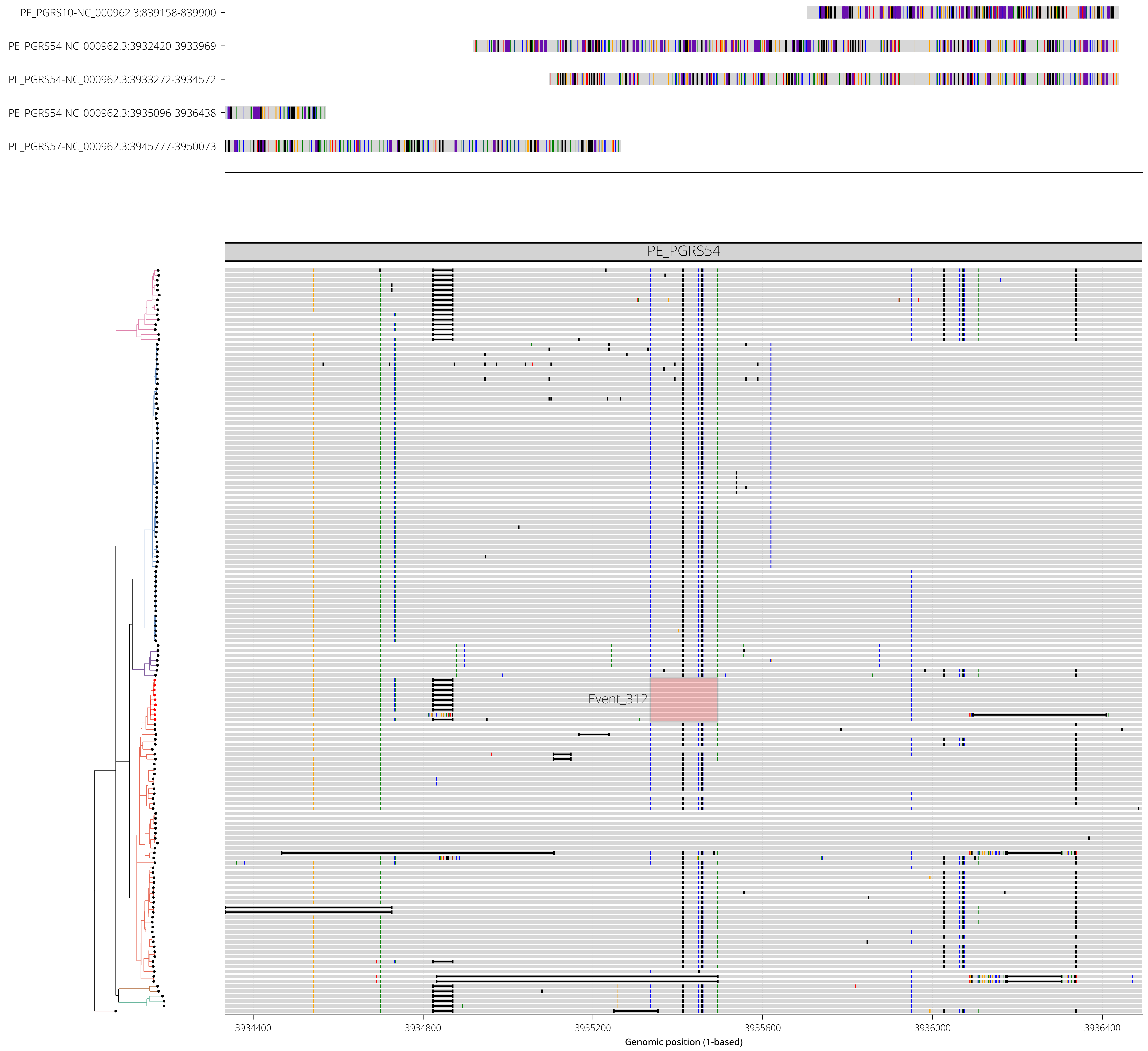

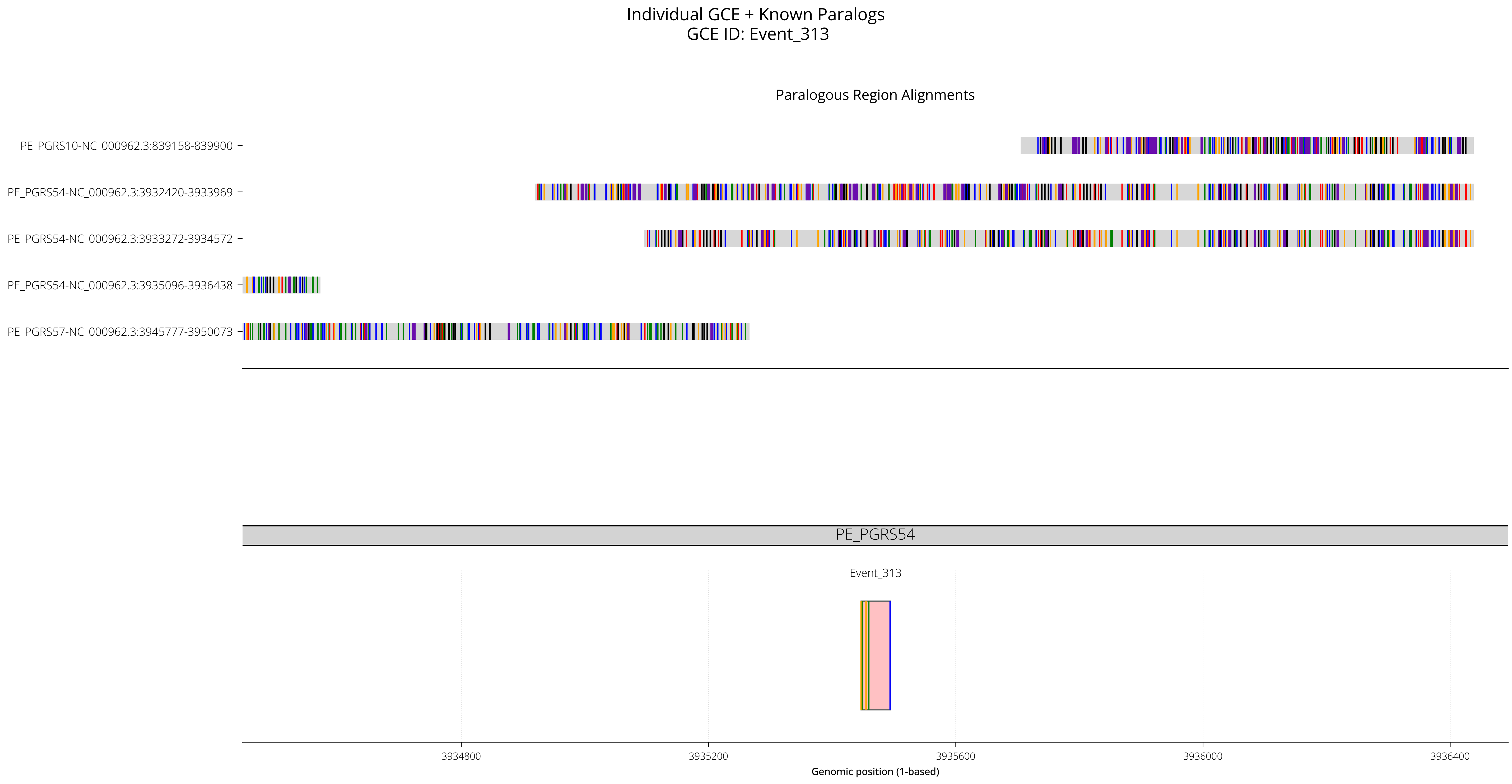

Individual GCE + Known Paralogs
GCE ID: Event_313
Paralogous Region Alignments
PE_PGRS10-NC_000962.3:839158-839900
PE_PGRS54-NC_000962.3:3932420-3933969
PE_PGRS54-NC_000962.3:3933272-3934572
PE_PGRS54-NC_000962.3:3935096-3936438
PE_PGRS57-NC_000962.3:3945777-3950073
PE_PGRS54
Event_313
3934800
3935200
3935600
3936000
3936400
Genomic position (1-based)

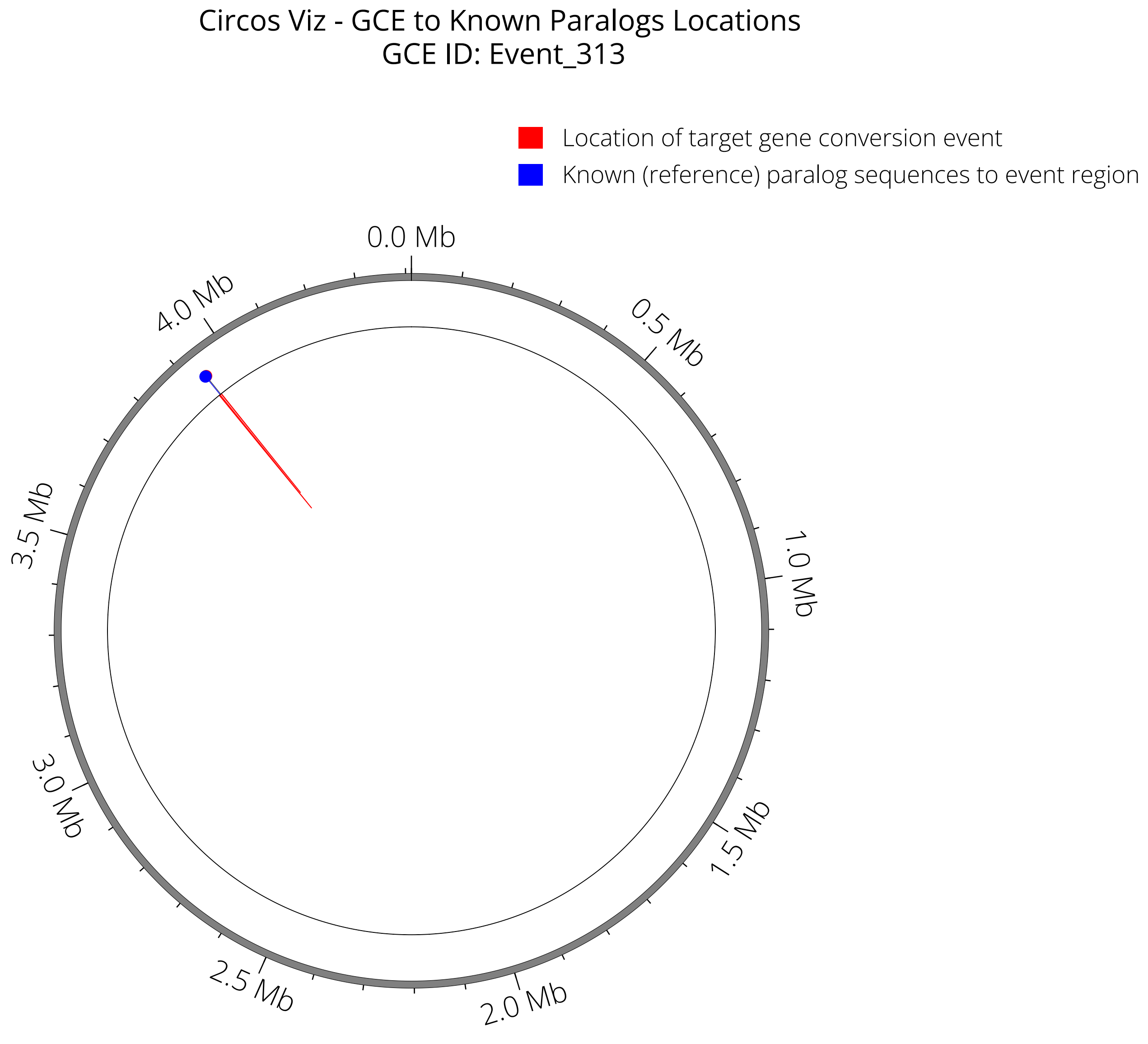
Circos Viz - GCE to Known Paralogs Locations
GCE ID: Event_313
Location of target gene conversion event
Known (reference) paralog sequences to event region
0.0 Mb
0.5 Mb
1.0 Mb
1.5 Mb
2.0 Mb
2.5 Mb
3.0 Mb
3.5 Mb
4.0 Mb

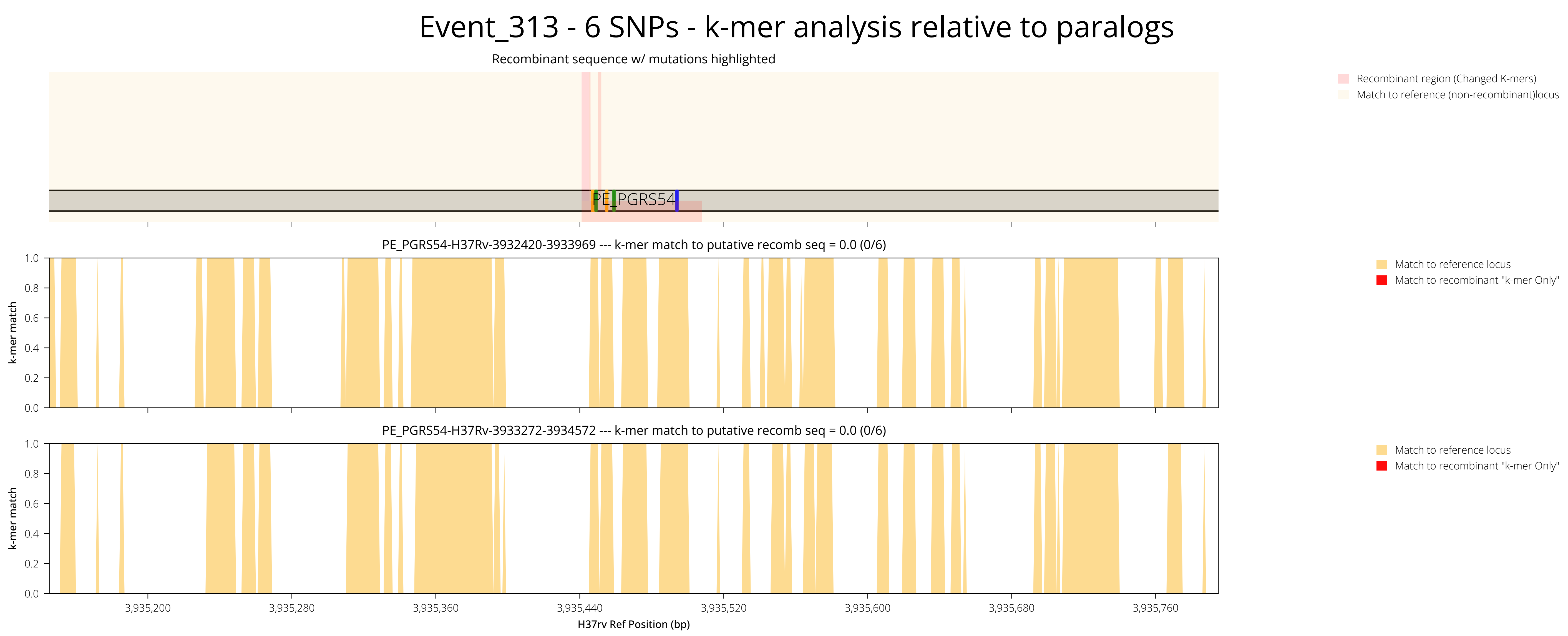

Event_313 - 6 SNPs - k-mer analysis relative to paralogs
Recombinant sequence w/ mutations highlighted
PE_PGRS54
Recombinant region (Changed K-mers)
Match to reference (non-recombinant)locus
PE_PGRS54-H37Rv-3932420-3933969 --- k-mer match to putative recomb seq = 0.0 (0/6)
Match to reference locus
Match to recombinant "k-mer Only"
PE_PGRS54-H37Rv-3933272-3934572 --- k-mer match to putative recomb seq = 0.0 (0/6)
Match to reference locus
Match to recombinant "k-mer Only"
k-mer match
1.0
0.8
0.6
0.4
0.2
0.0
3,935,200
3,935,280
3,935,360
3,935,440
3,935,520
3,935,600
3,935,680
3,935,760
H37rv Ref Position (bp)

# Sub-phylogeny Viz + Paralog Alignments
GCE ID: Event_313

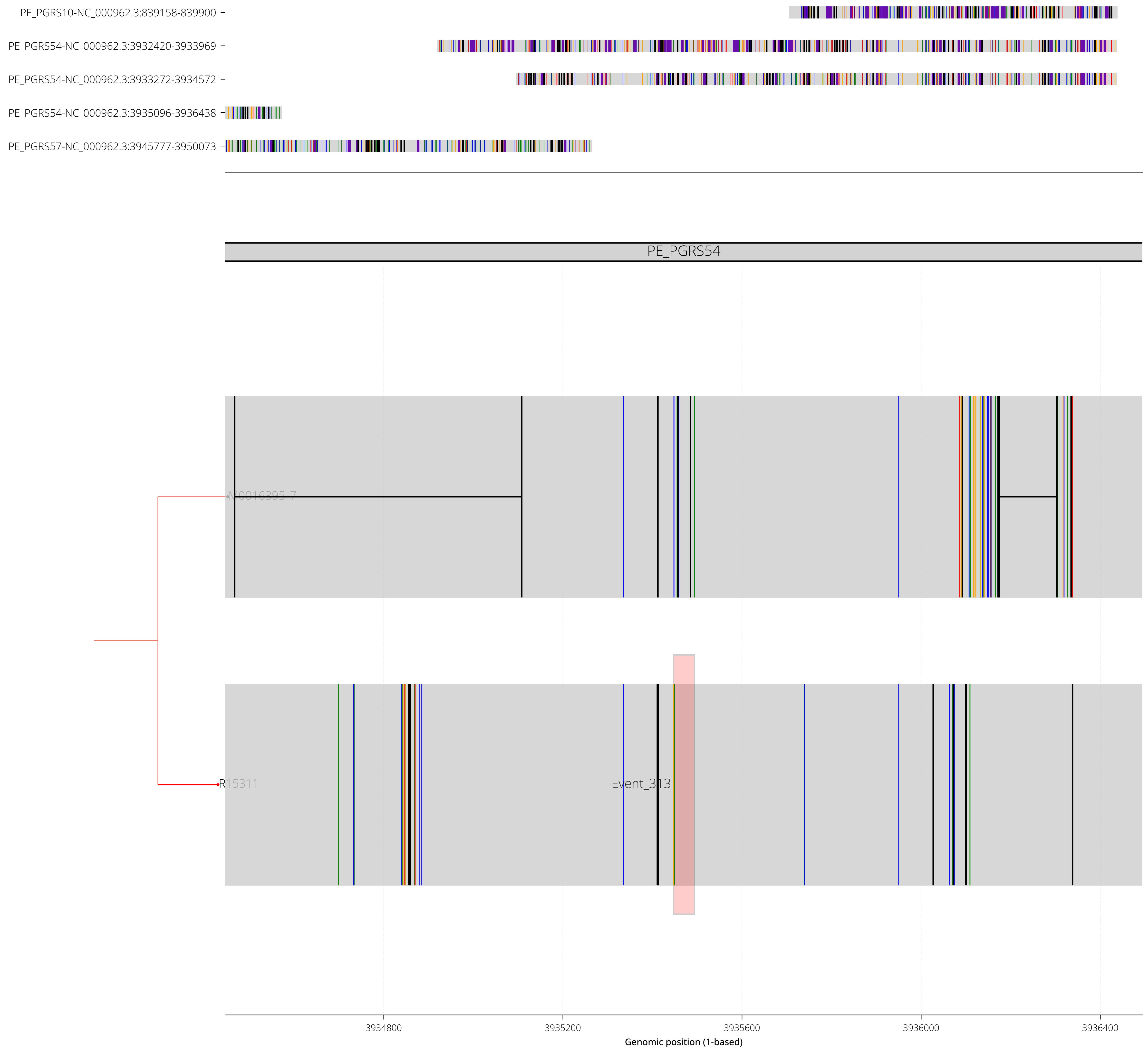

Full Phylogeny Viz + Paralog Alignments
GCE ID: Event_313

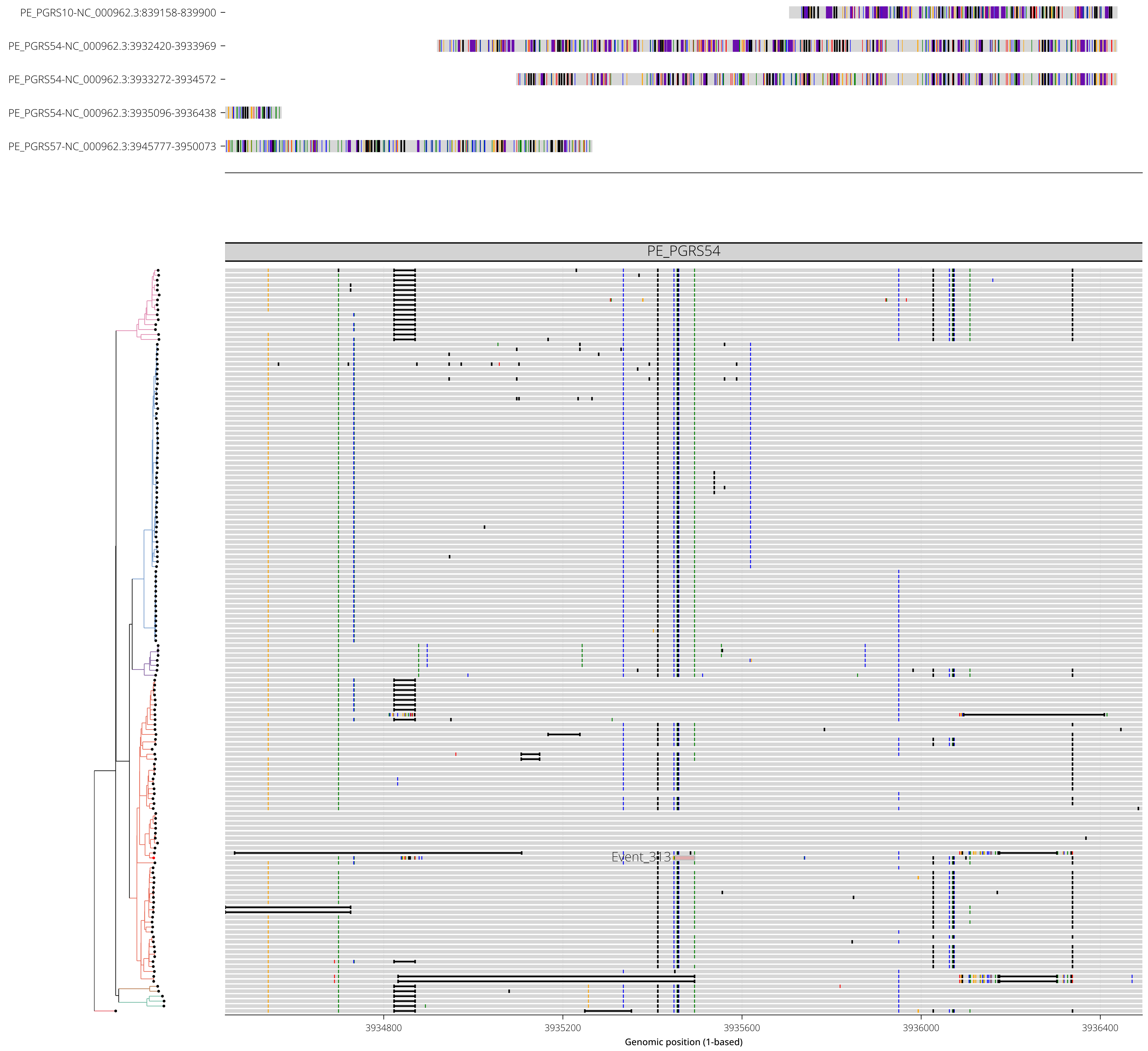

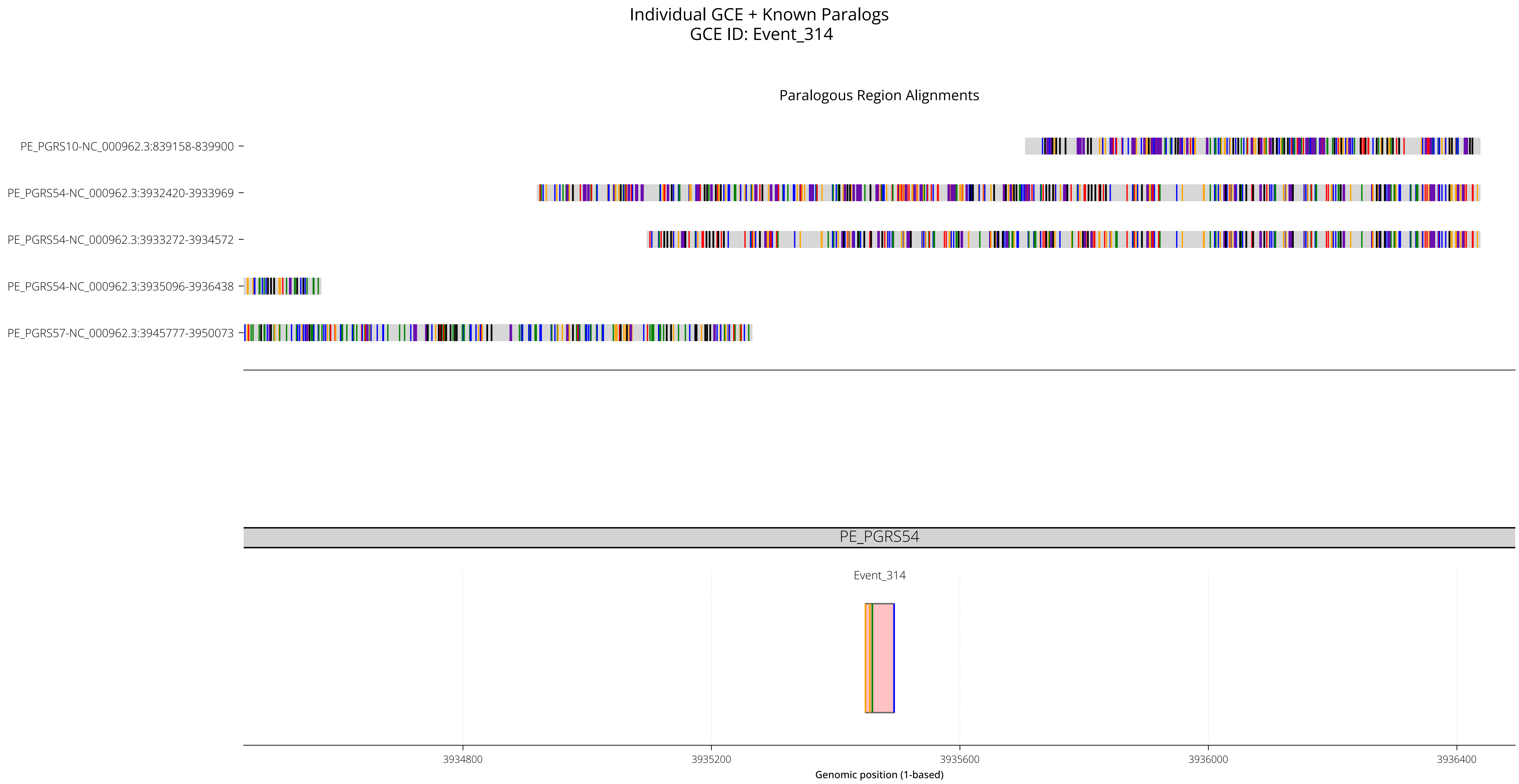

Individual GCE + Known Paralogs
GCE ID: Event_314
Paralogous Region Alignments
PE_PGRS10-NC_000962.3:839158-839900
PE_PGRS54-NC_000962.3:3932420-3933969
PE_PGRS54-NC_000962.3:3933272-3934572
PE_PGRS54-NC_000962.3:3935096-3936438
PE_PGRS57-NC_000962.3:3945777-3950073
PE_PGRS54
Event_314
3934800
3935200
3935600
3936000
3936400
Genomic position (1-based)

Circos Viz - GCE to Known Paralogs Locations
GCE ID: Event_314

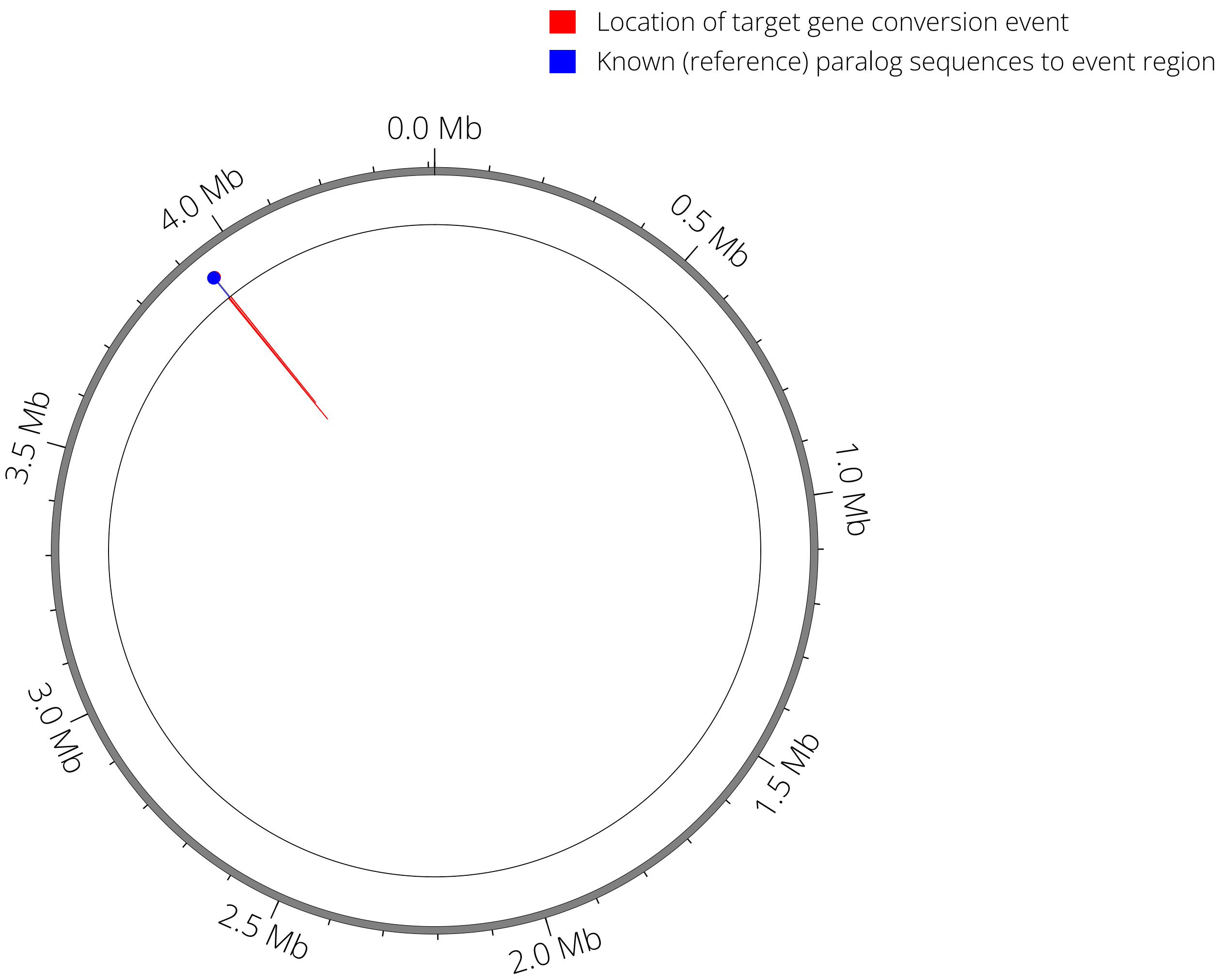

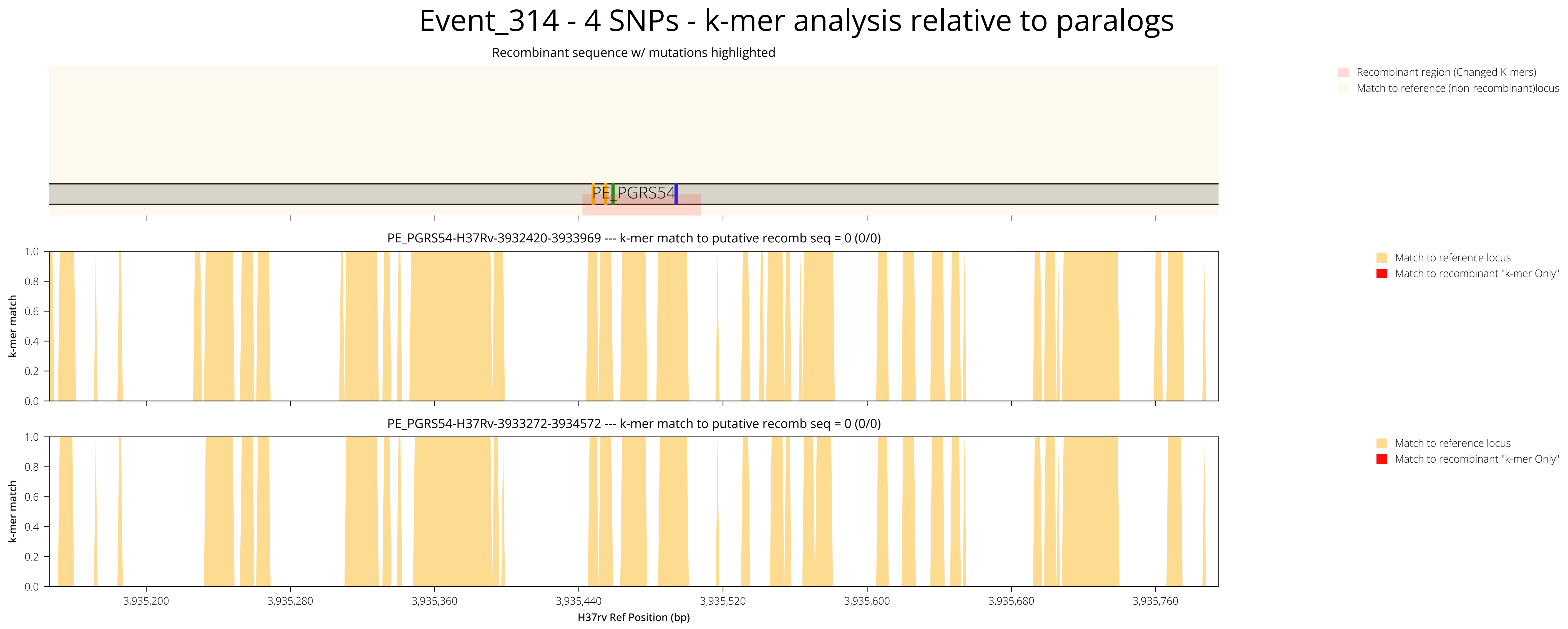

Event_314 - 4 SNPs - k-mer analysis relative to paralogs
Recombinant sequence w/ mutations highlighted
PE_PGRS54
Recombinant region (Changed K-mers)
Match to reference (non-recombinant)locus
PE_PGRS54-H37Rv-3932420-3933969 --- k-mer match to putative recomb seq = 0 (0/0)
Match to reference locus
Match to recombinant "k-mer Only"
k-mer match
1.0
0.8
0.6
0.4
0.2
0.0
PE_PGRS54-H37Rv-3933272-3934572 --- k-mer match to putative recomb seq = 0 (0/0)
Match to reference locus
Match to recombinant "k-mer Only"
k-mer match
3,935,200
3,935,280
3,935,360
3,935,440
3,935,520
3,935,600
3,935,680
3,935,760
H37rv Ref Position (bp)

Sub-phylogeny Viz + Paralog Alignments
GCE ID: Event_314

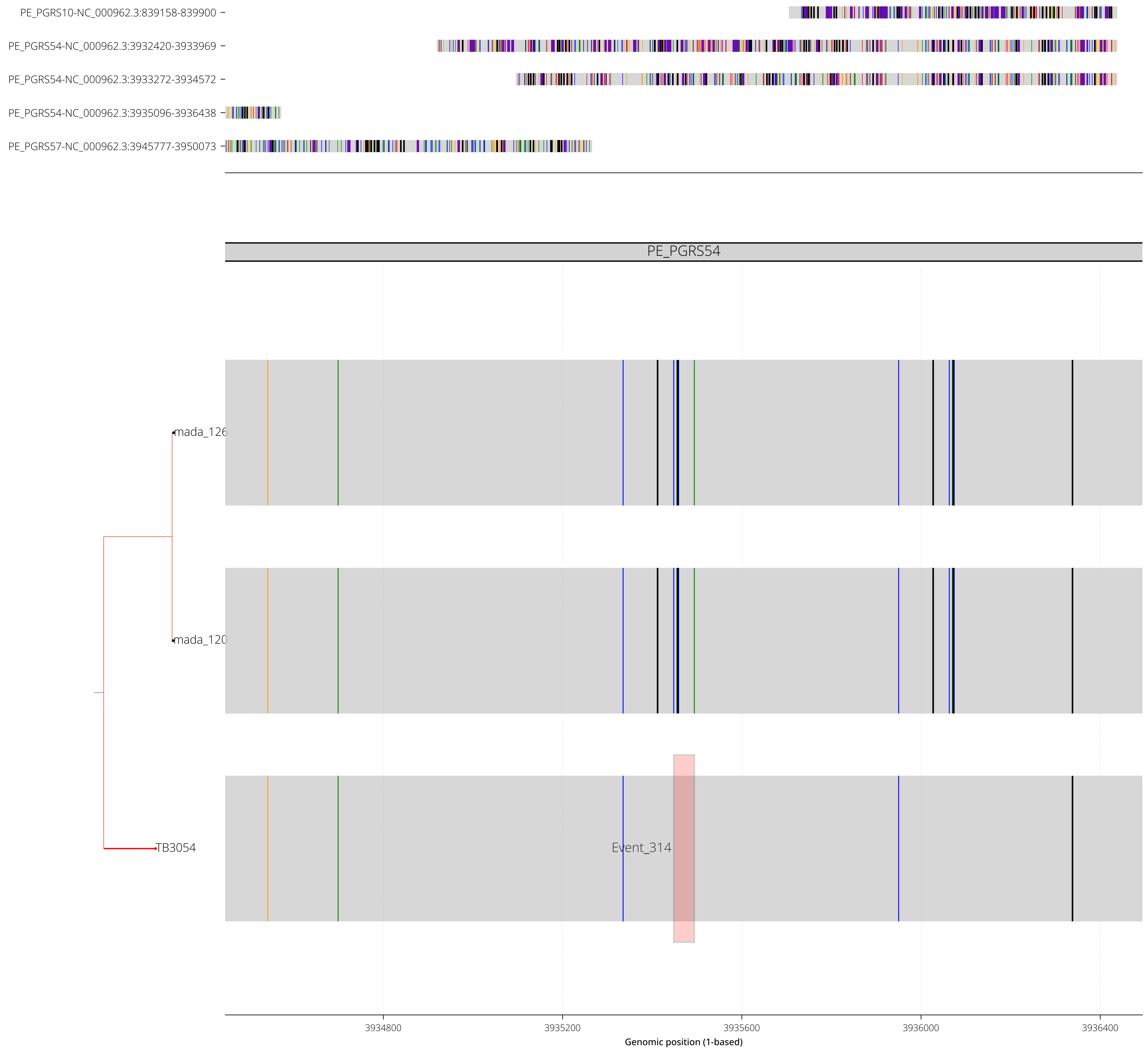

Full Phylogeny Viz + Paralog Alignments
GCE ID: Event_314

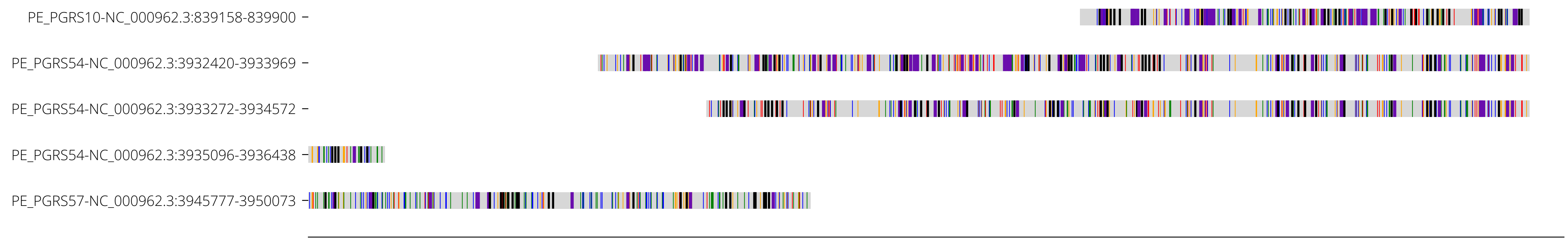


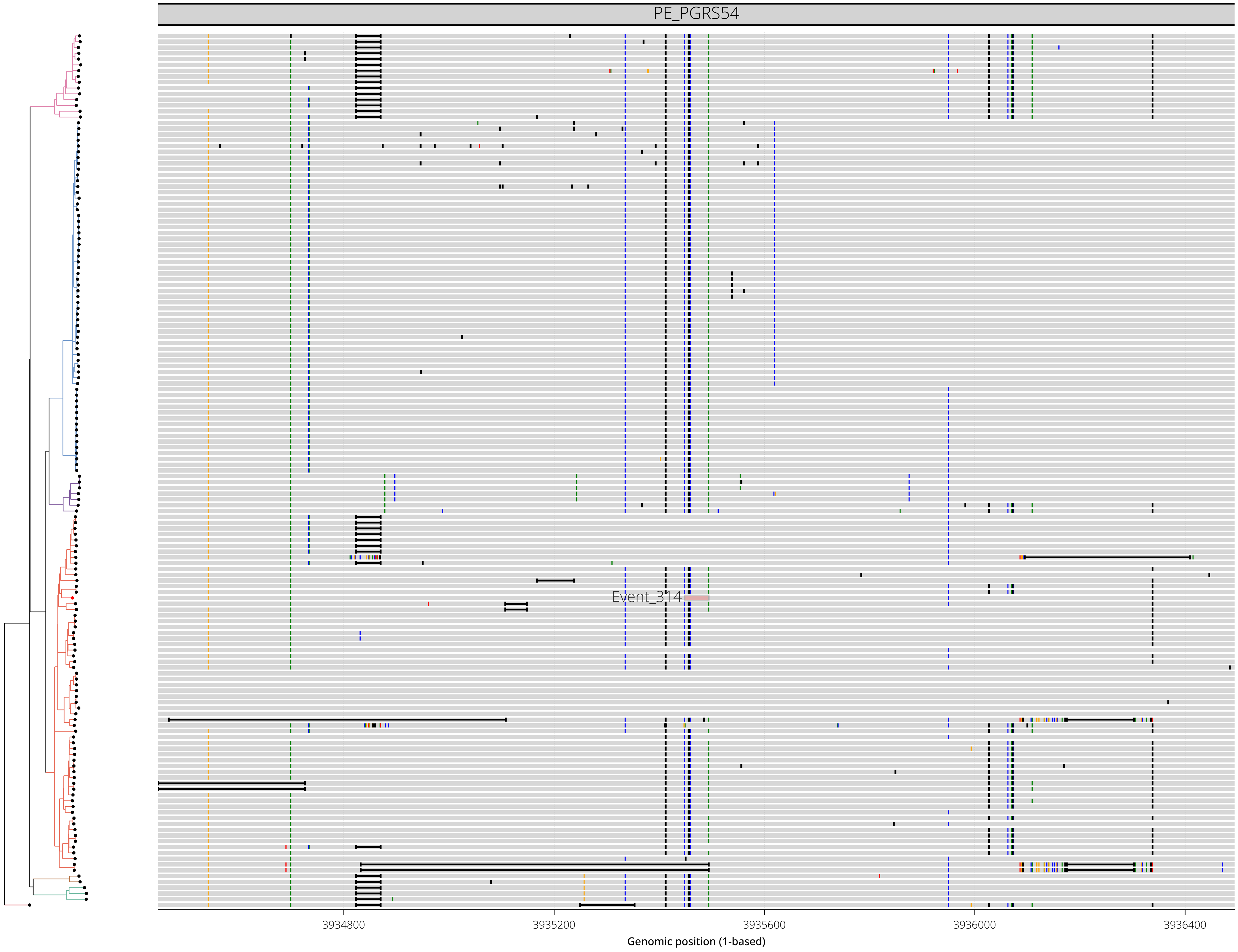

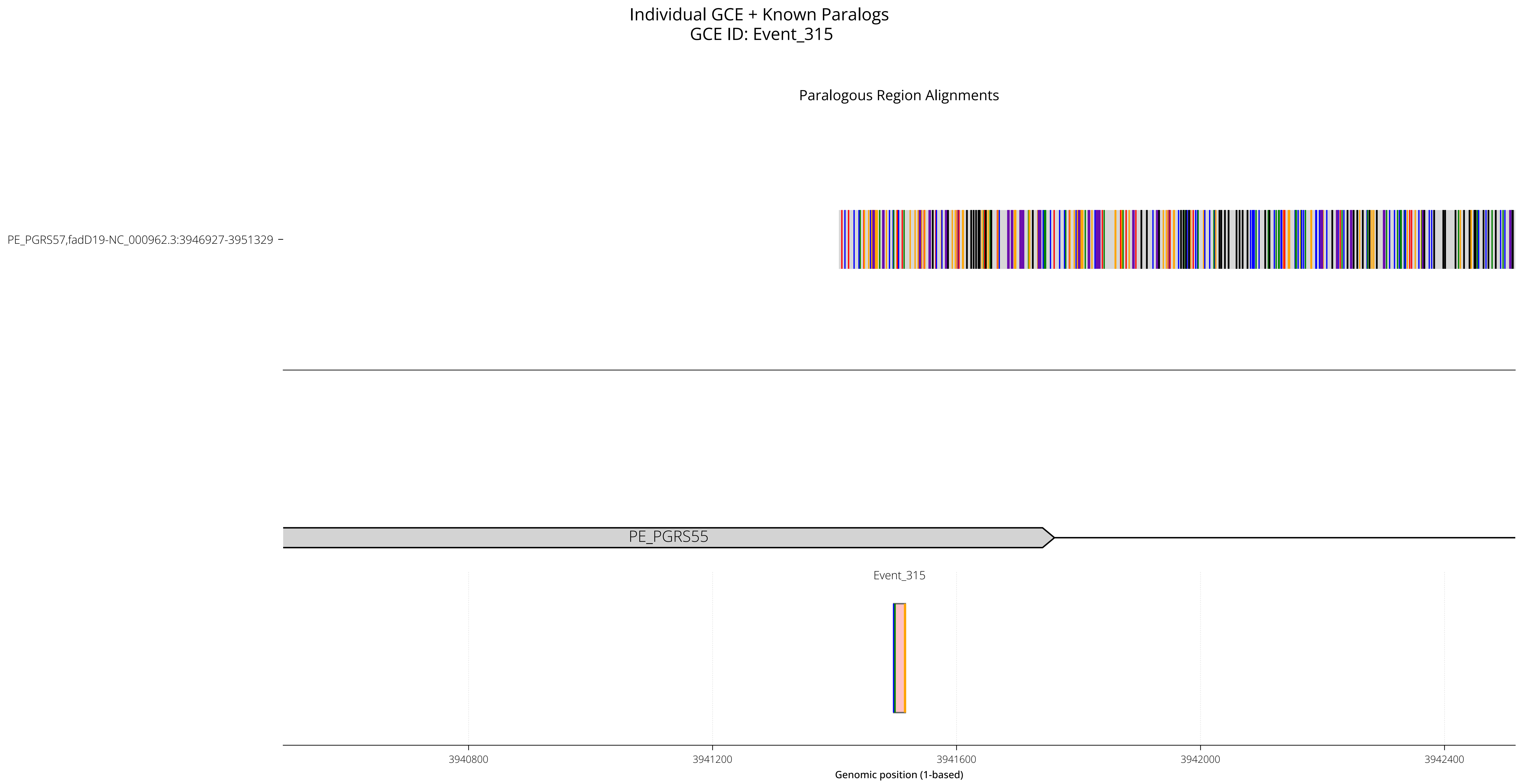
Individual GCE + Known Paralogs
GCE ID: Event_315
Paralogous Region Alignments
PE_PGRS57,fadD19-NC_000962.3:3946927-3951329
PE_PGRS55
Event_315
3940800
3941200
3941600
3942000
3942400
Genomic position (1-based)

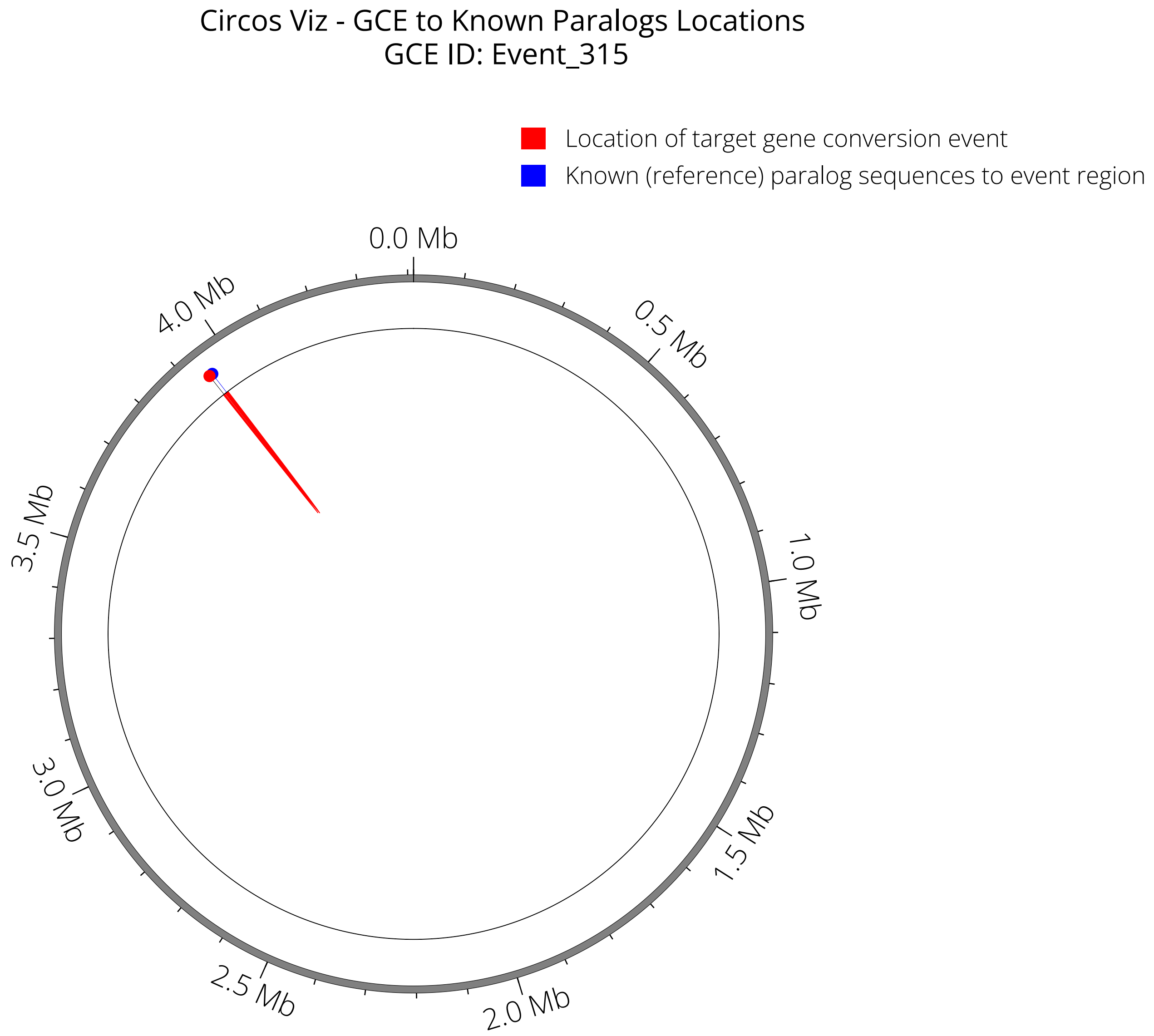
Circos Viz - GCE to Known Paralogs Locations
GCE ID: Event_315
Location of target gene conversion event
Known (reference) paralog sequences to event region
0.0 Mb
0.5 Mb
1.0 Mb
1.5 Mb
2.0 Mb
2.5 Mb
3.0 Mb
3.5 Mb
4.0 Mb

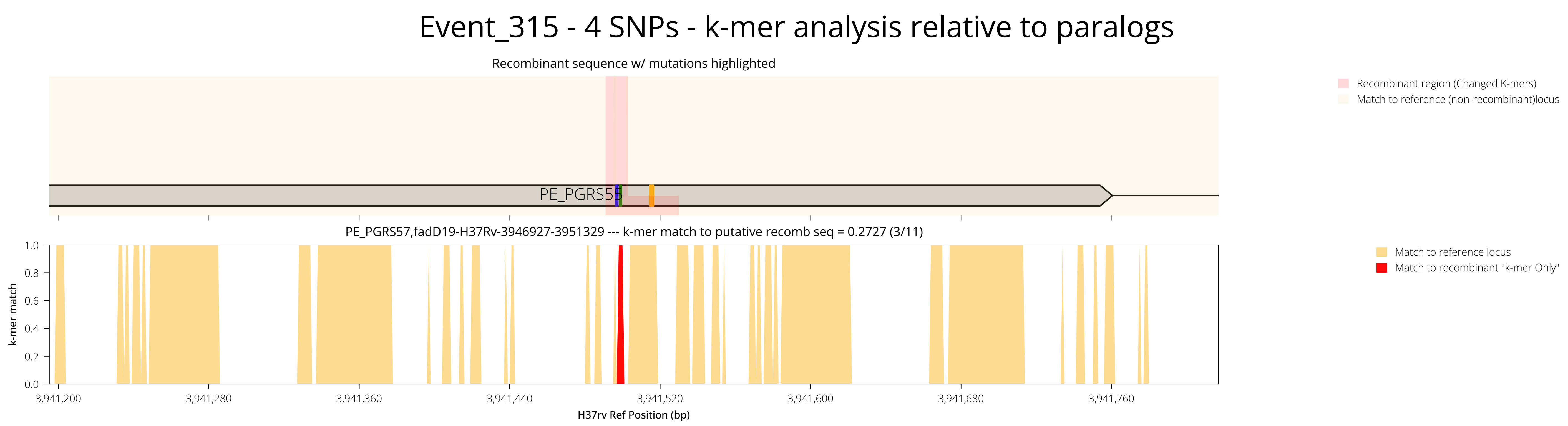

Event_315 - 4 SNPs - k-mer analysis relative to paralogs
Recombinant sequence w/ mutations highlighted
Recombinant region (Changed K-mers)
Match to reference (non-recombinant)locus
PE_PGRS55
PE_PGRS57,fadD19-H37Rv-3946927-3951329 --- k-mer match to putative recomb seq = 0.2727 (3/11)
Match to reference locus
Match to recombinant "k-mer Only"
k-mer match
1.0
0.8
0.6
0.4
0.2
0.0
3,941,200
3,941,280
3,941,360
3,941,440
3,941,520
3,941,600
3,941,680
3,941,760
H37rv Ref Position (bp)

Sub-phylogeny Viz + Paralog Alignments
GCE ID: Event_315

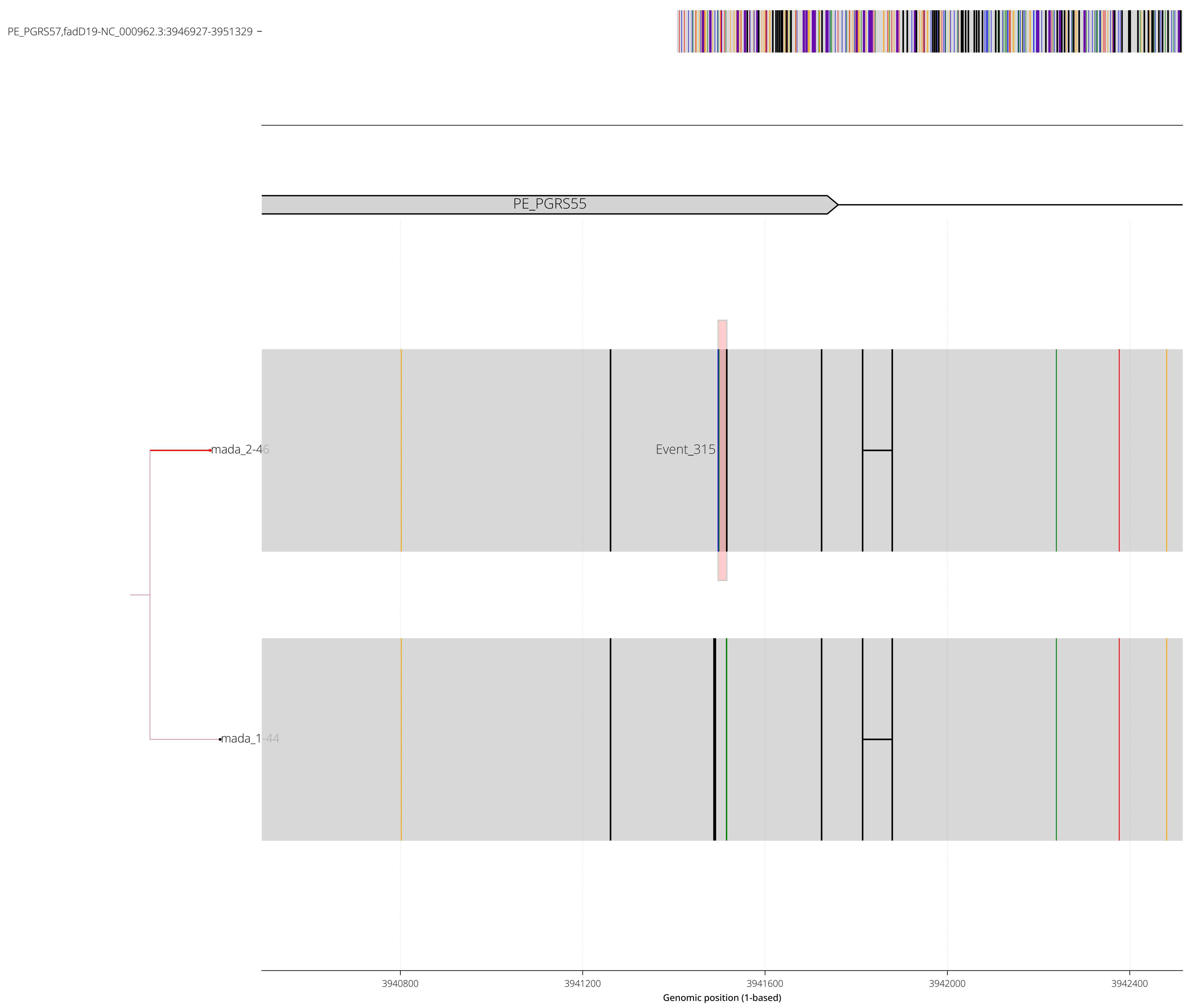

Full Phylogeny Viz + Paralog Alignments
GCE ID: Event_315

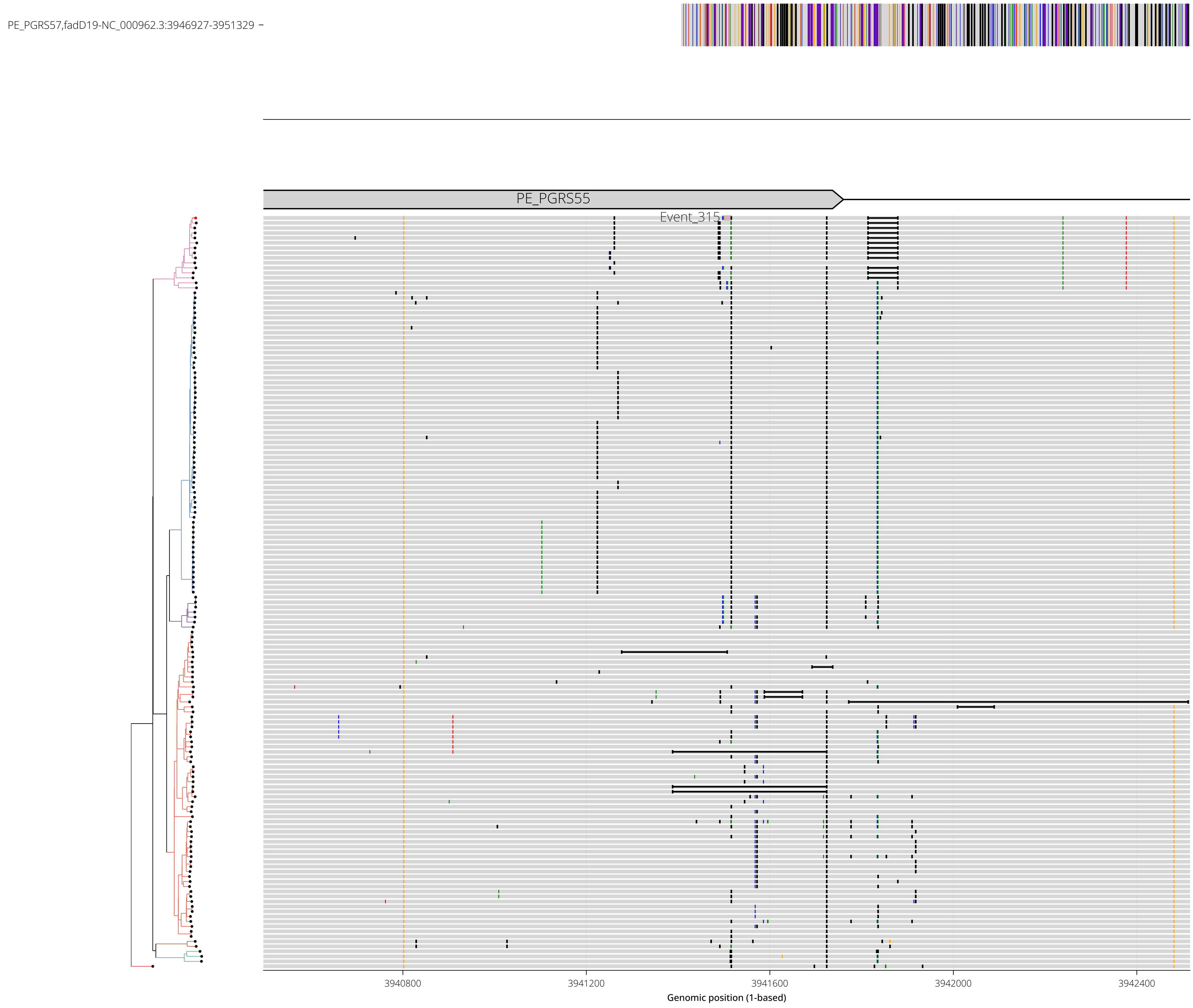

Individual GCE + Known Paralogs
GCE ID: Event_316

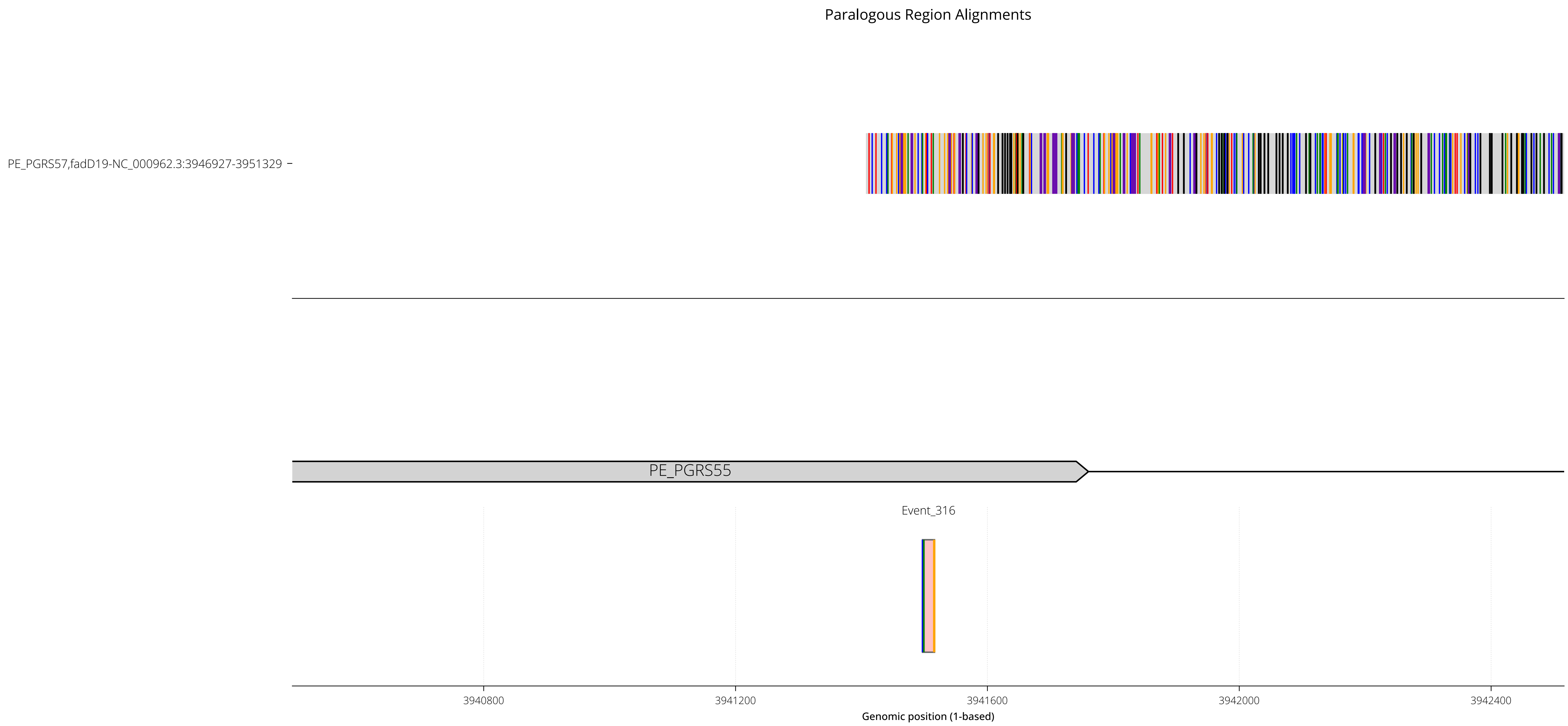

Circos Viz - GCE to Known Paralogs Locations
GCE ID: Event_316

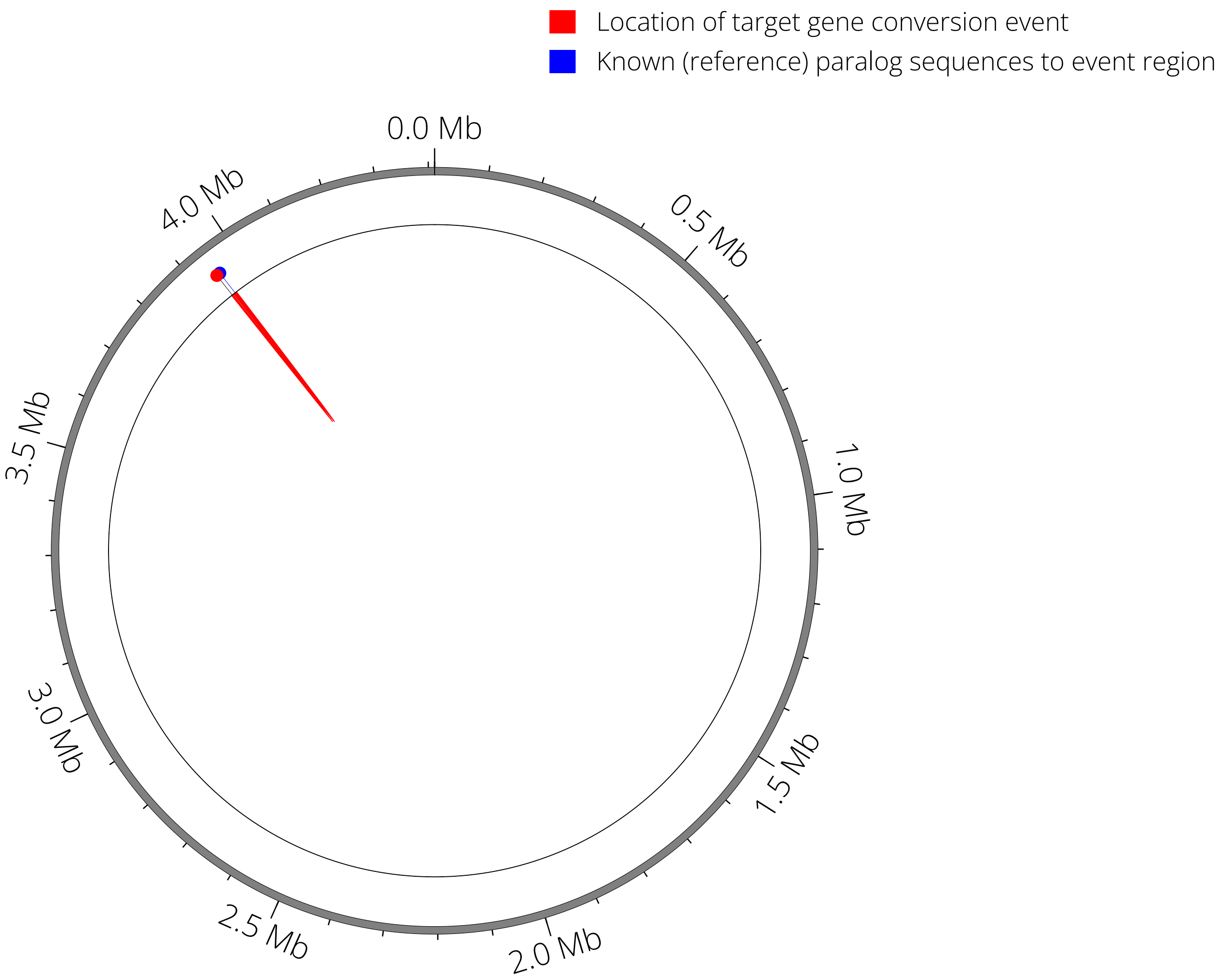

# Event_316 - 4 SNPs - k-mer analysis relative to paralogs

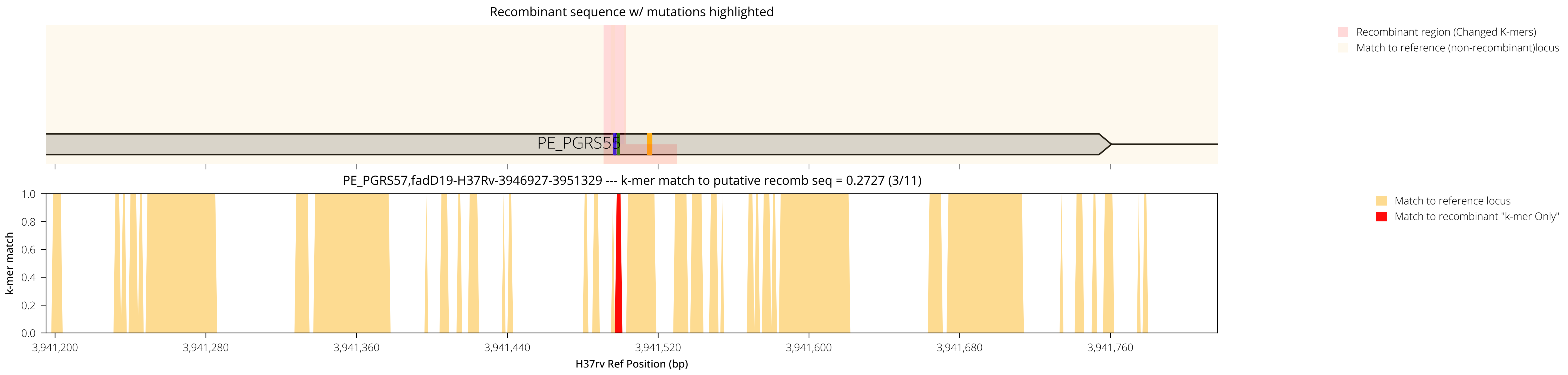

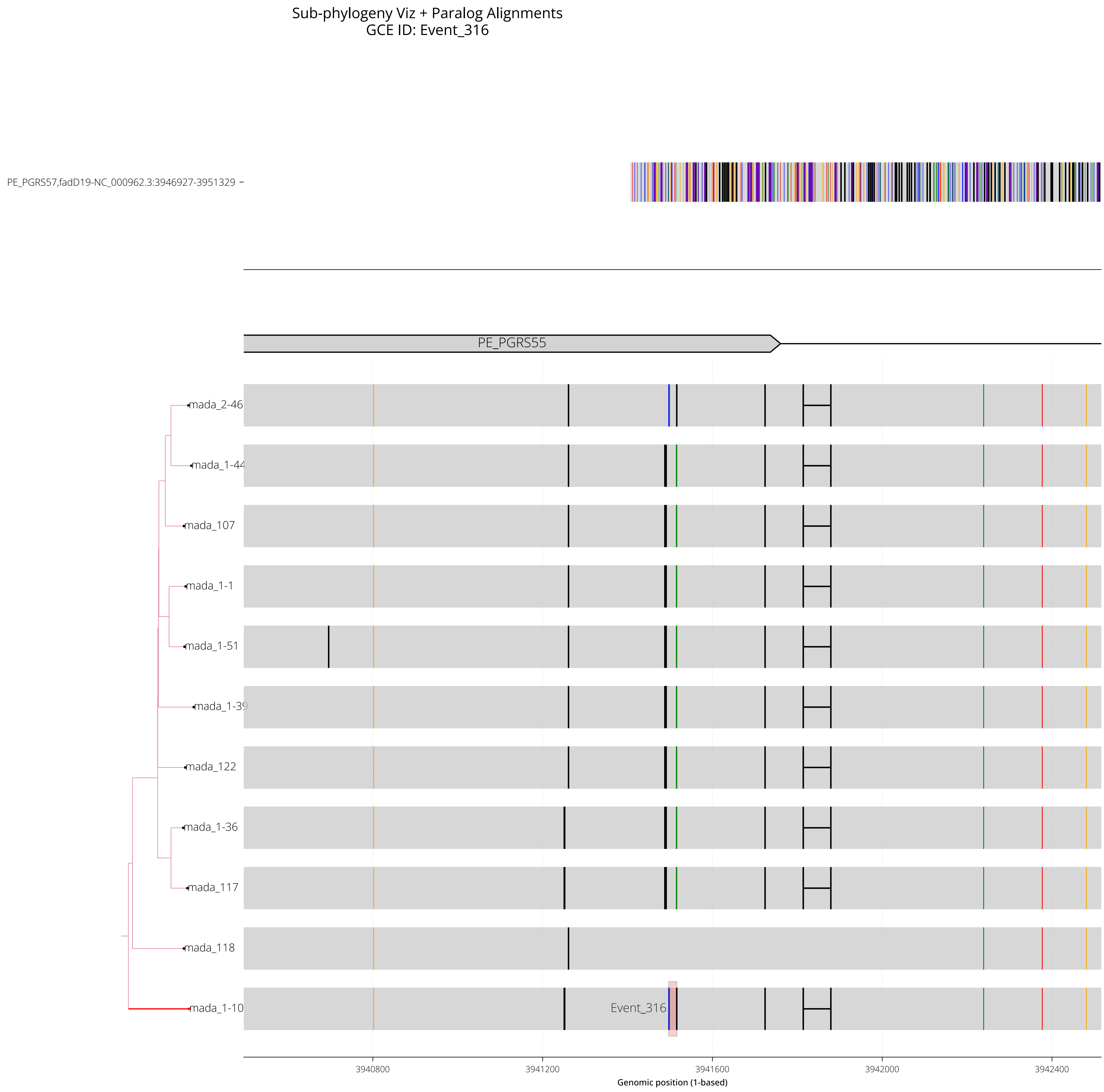

Sub-phylogeny Viz + Paralog Alignments
GCE ID: Event_316
PE_PGRS57,fadD19-NC_000962.3:3946927-3951329
PE_PGRS55
mada_2-46
mada_1-44
mada_107
mada_1-1
mada_1-51
mada_1-39
mada_122
mada_1-36
mada_117
mada_118
mada_1-10
Event_316
3940800
3941200
3941600
3942000
3942400
Genomic position (1-based)

Full Phylogeny Viz + Paralog Alignments
GCE ID: Event_316

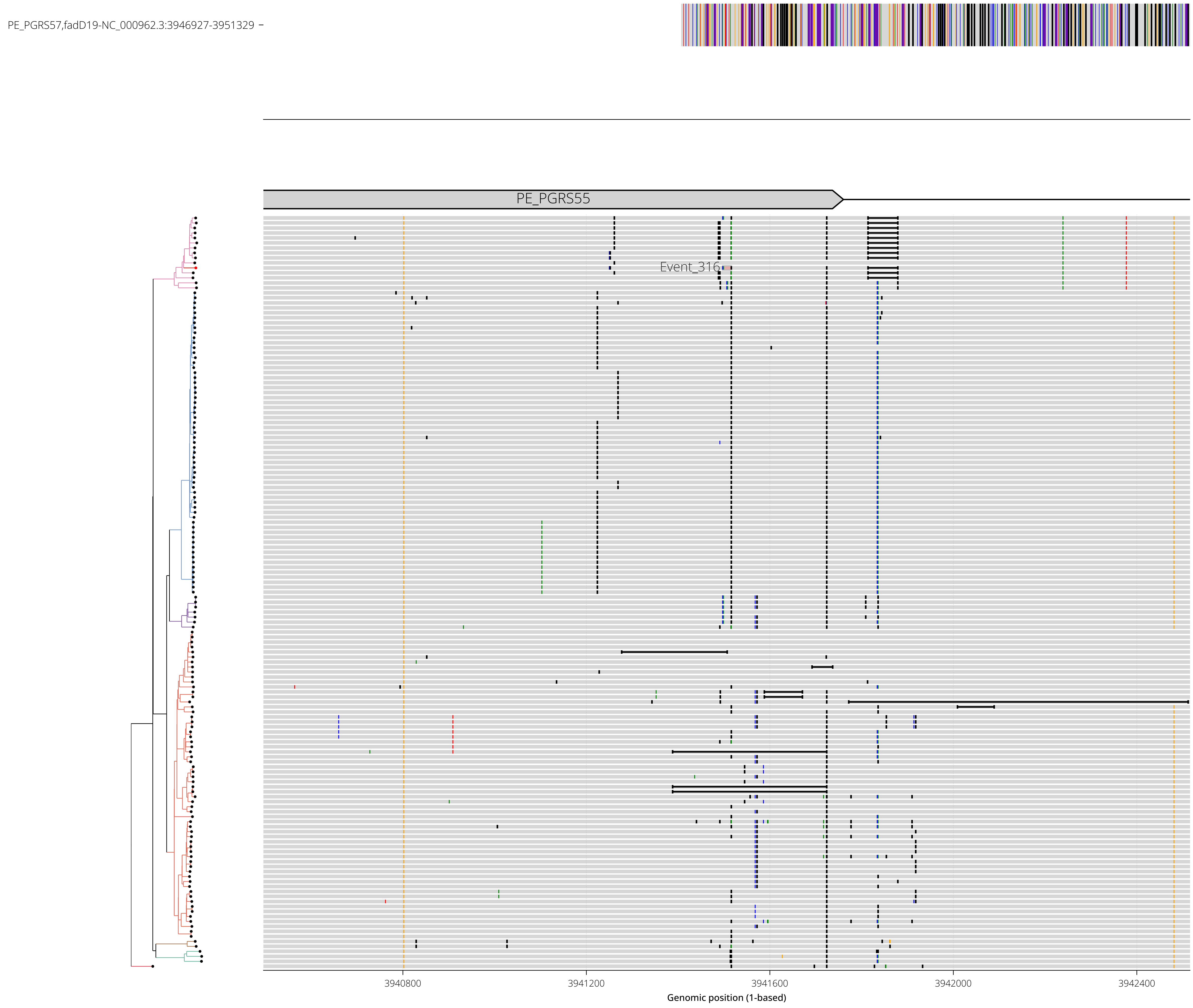

Individual GCE + Known Paralogs
GCE ID: Event_317

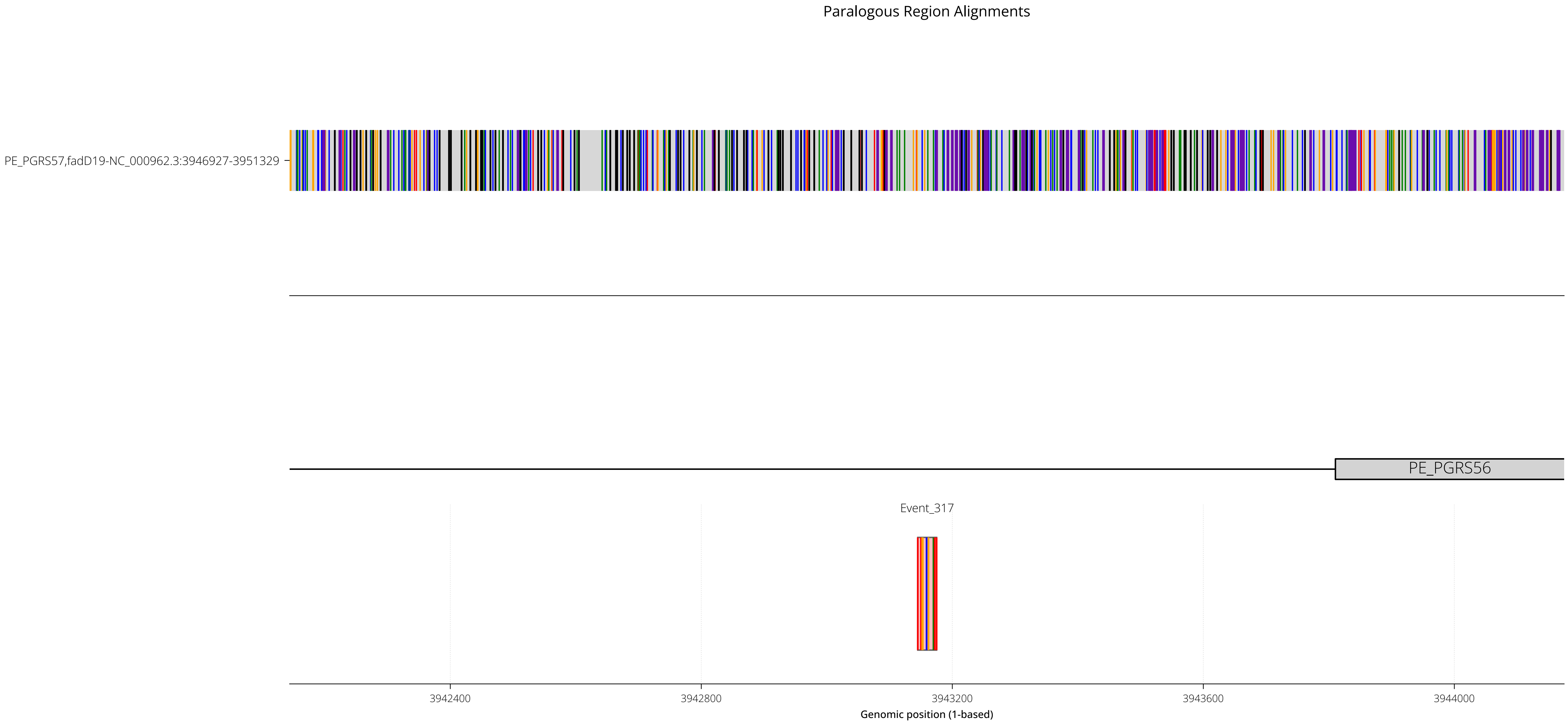

Circos Viz - GCE to Known Paralogs Locations
GCE ID: Event_317

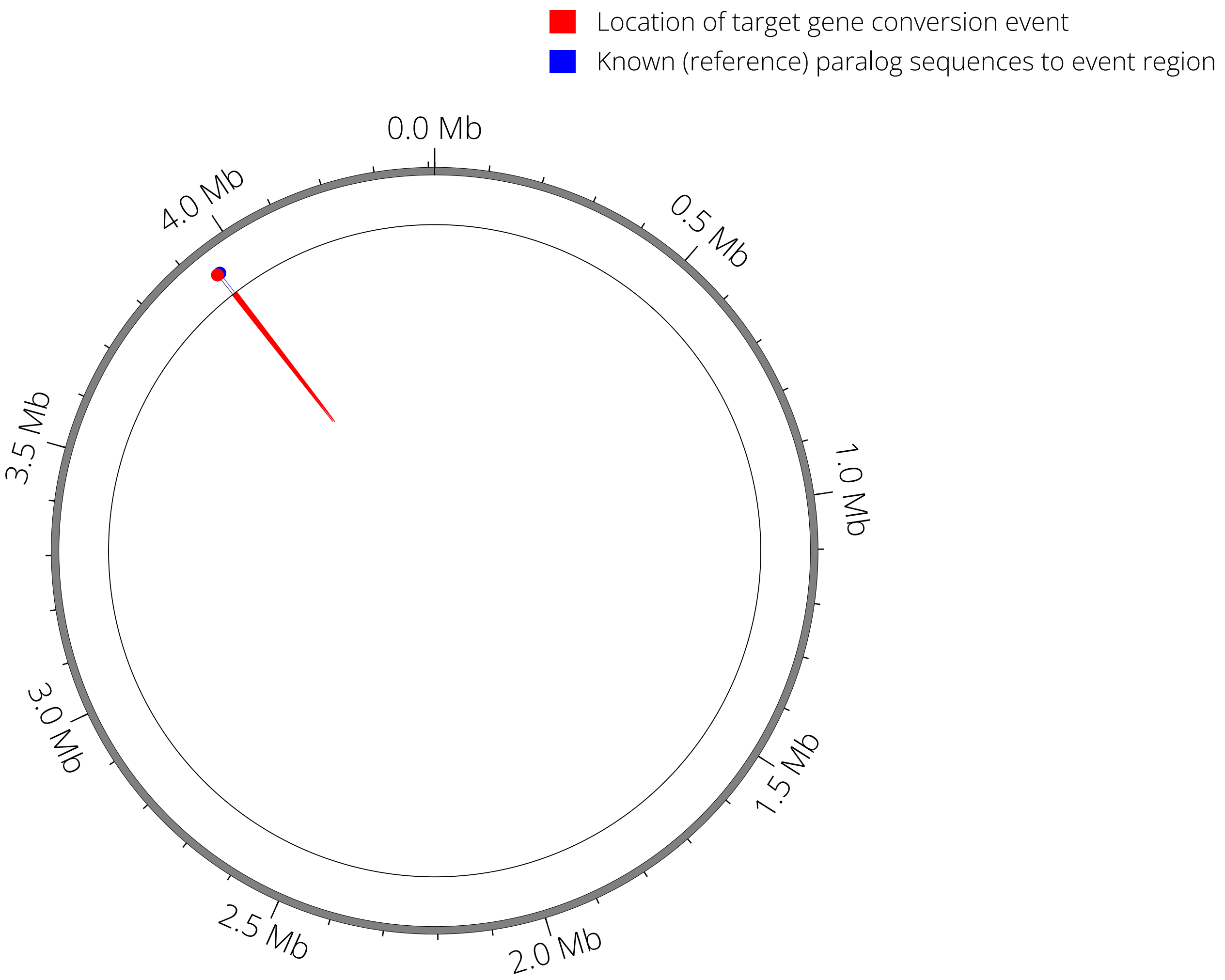

# Event_317 - 10 SNPs - k-mer analysis relative to paralogs

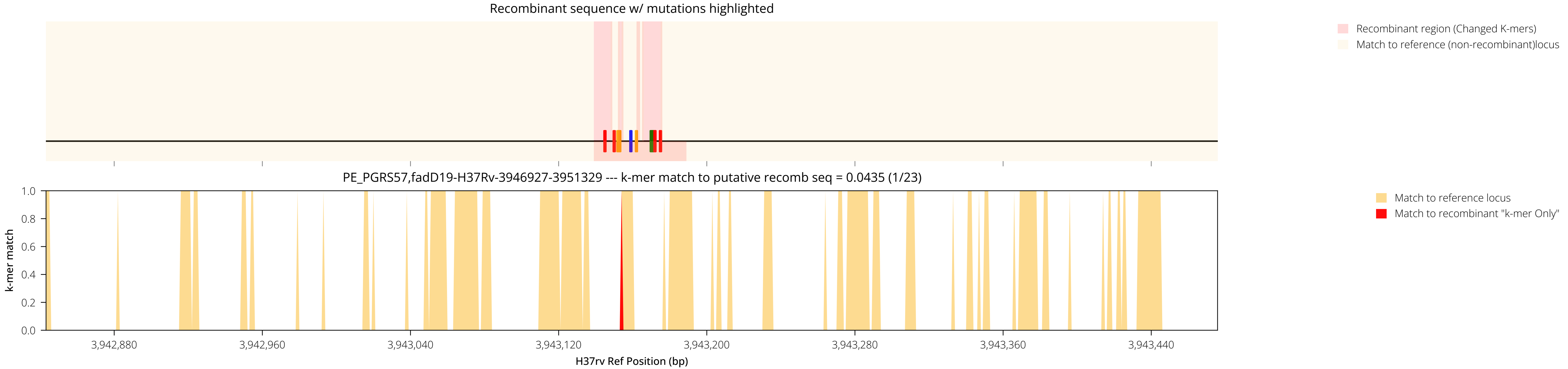

Sub-phylogeny Viz + Paralog Alignments
GCE ID: Event_317

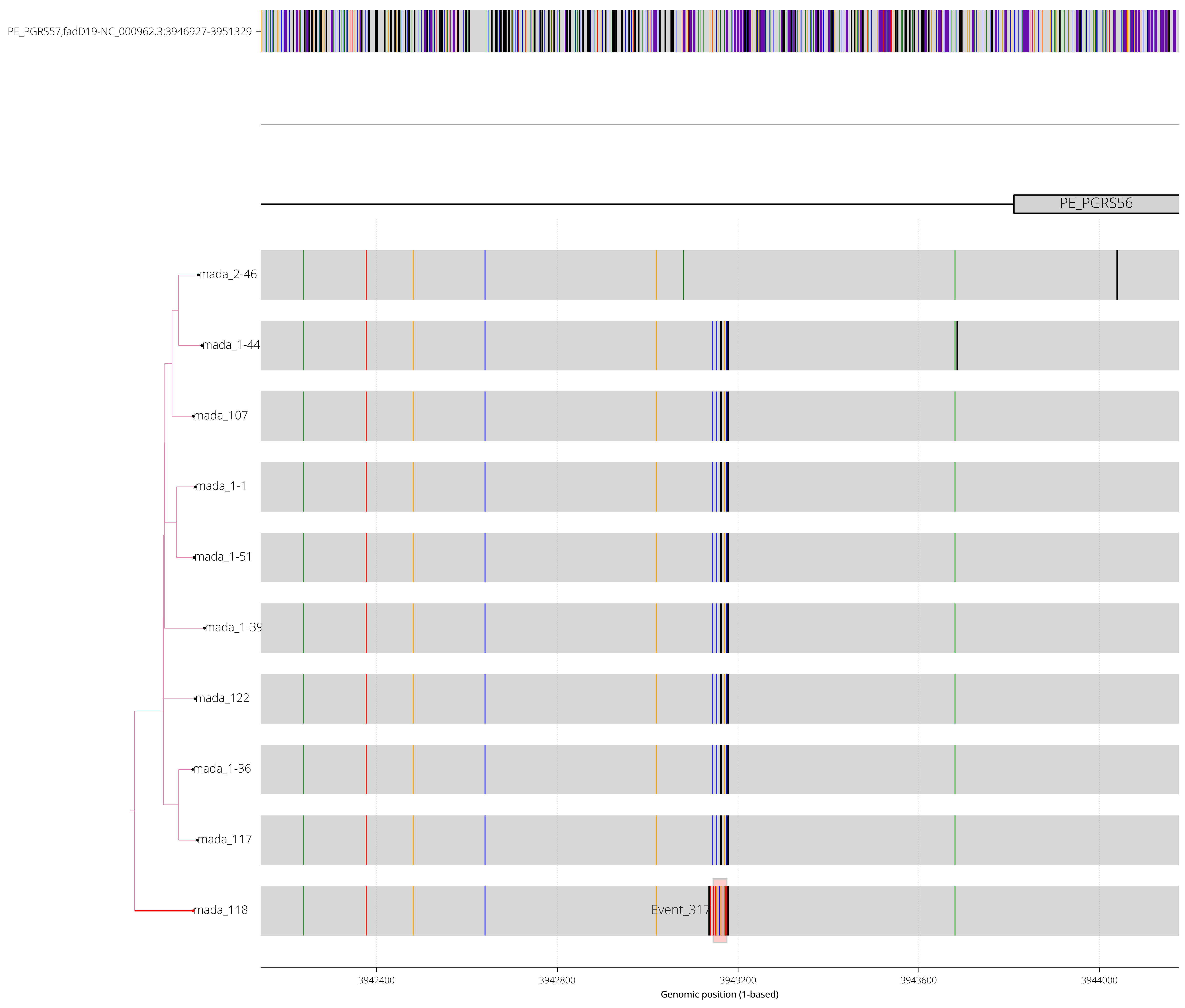

Full Phylogeny Viz + Paralog Alignments
GCE ID: Event_317

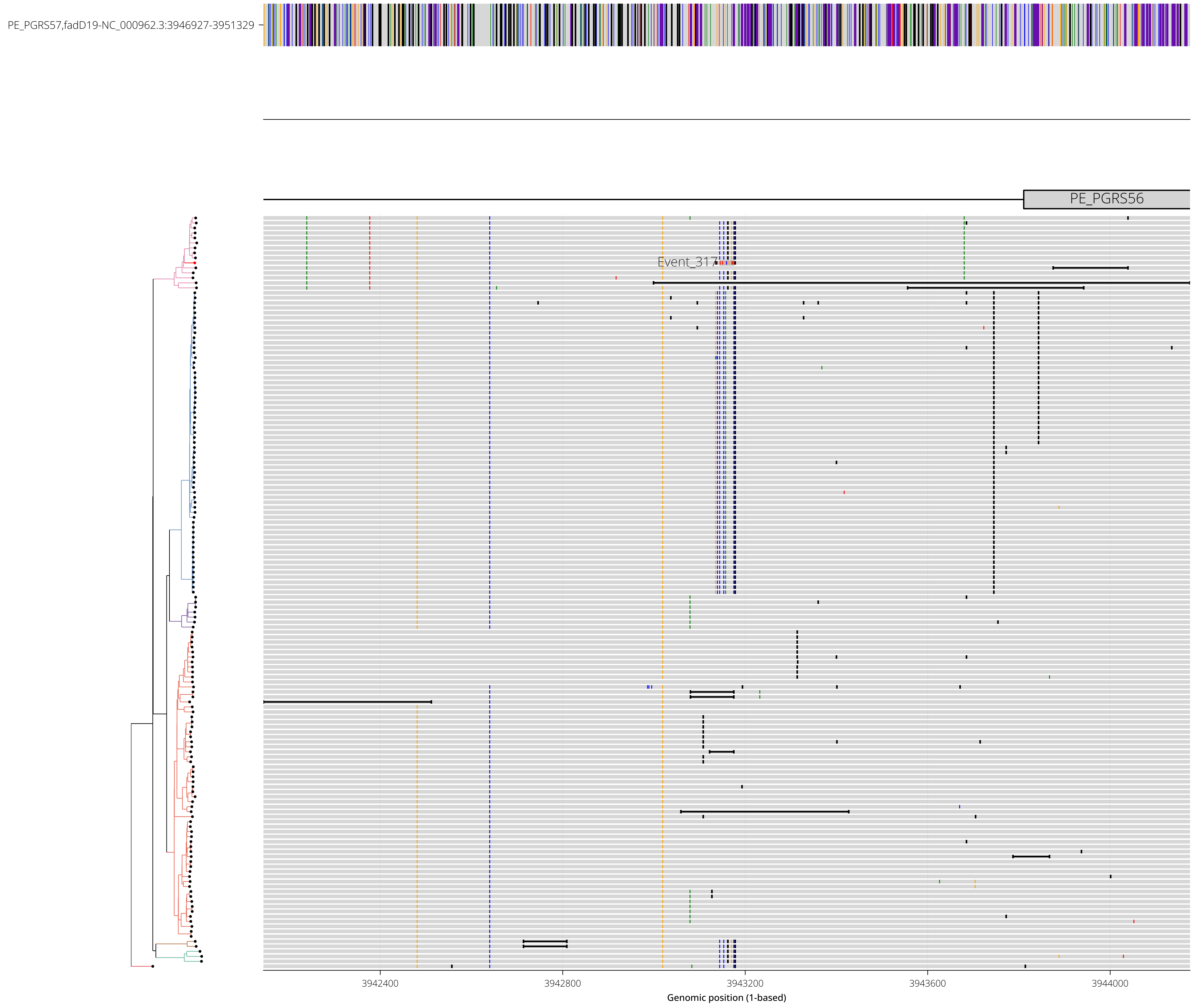

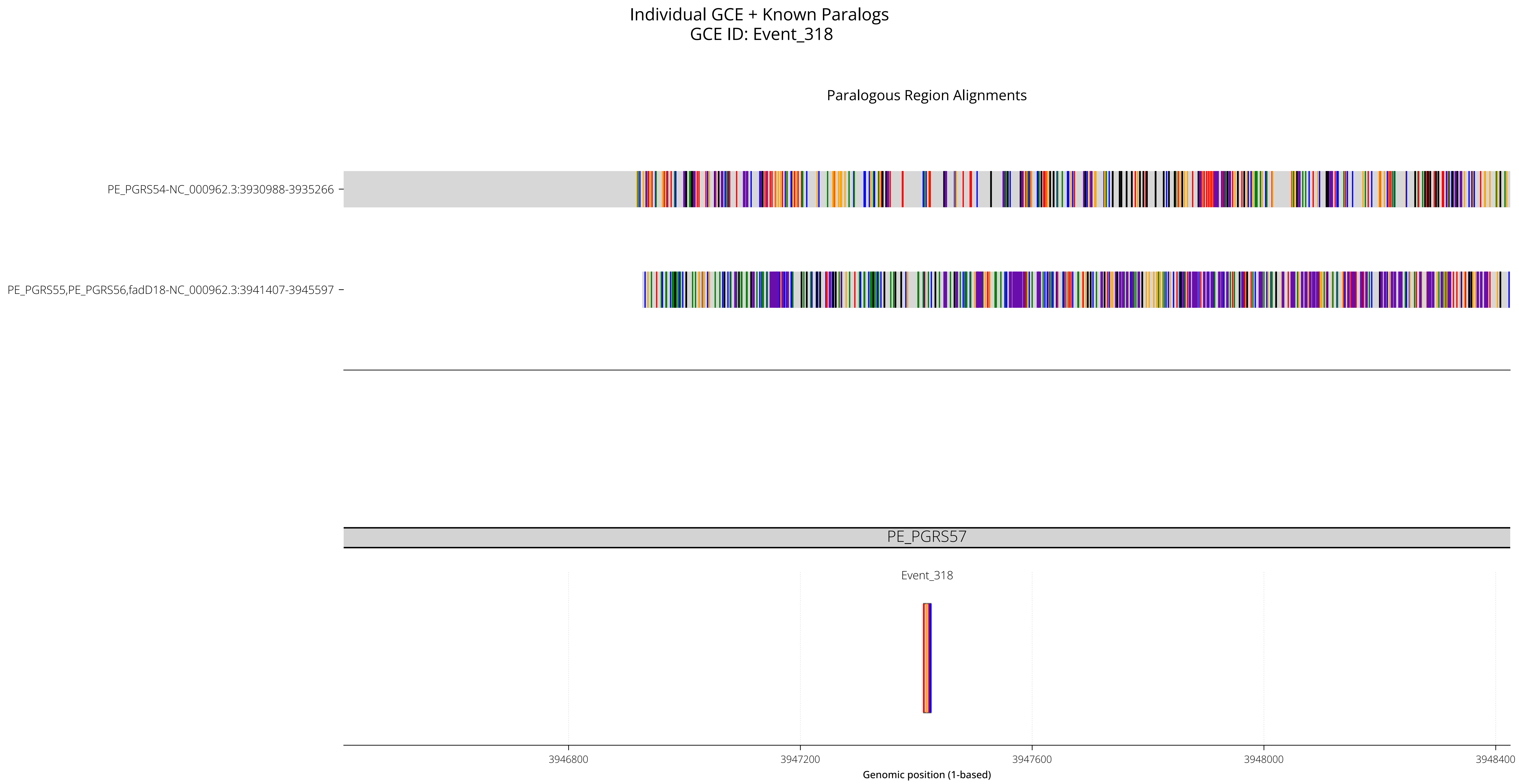

Individual GCE + Known Paralogs
GCE ID: Event_318
Paralogous Region Alignments
PE_PGRS54-NC_000962.3:3930988-3935266
PE_PGRS55,PE_PGRS56,fadD18-NC_000962.3:3941407-3945597
PE_PGRS57
Event_318
3946800
3947200
3947600
3948000
3948400
Genomic position (1-based)

Circos Viz - GCE to Known Paralogs Locations
GCE ID: Event_318

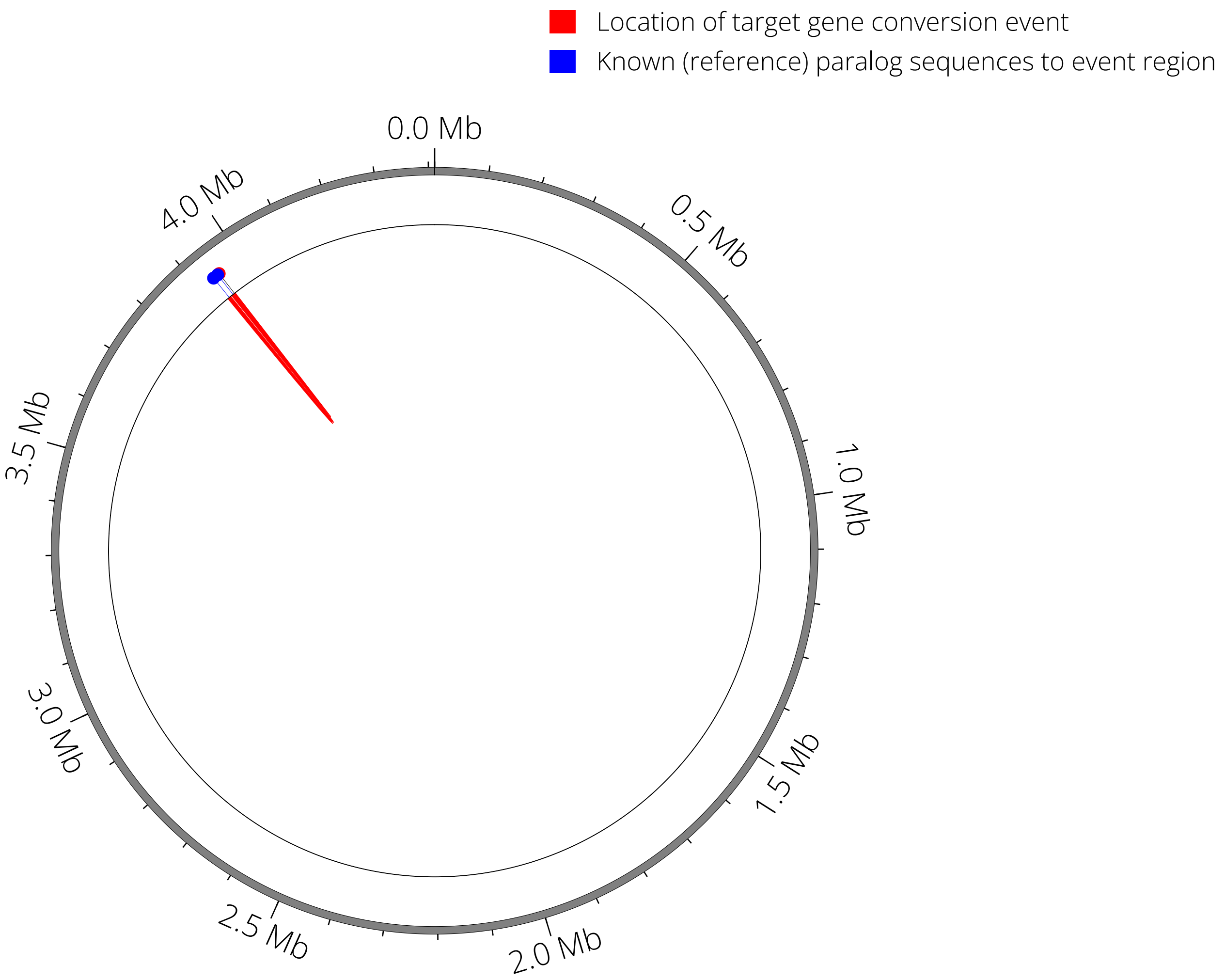

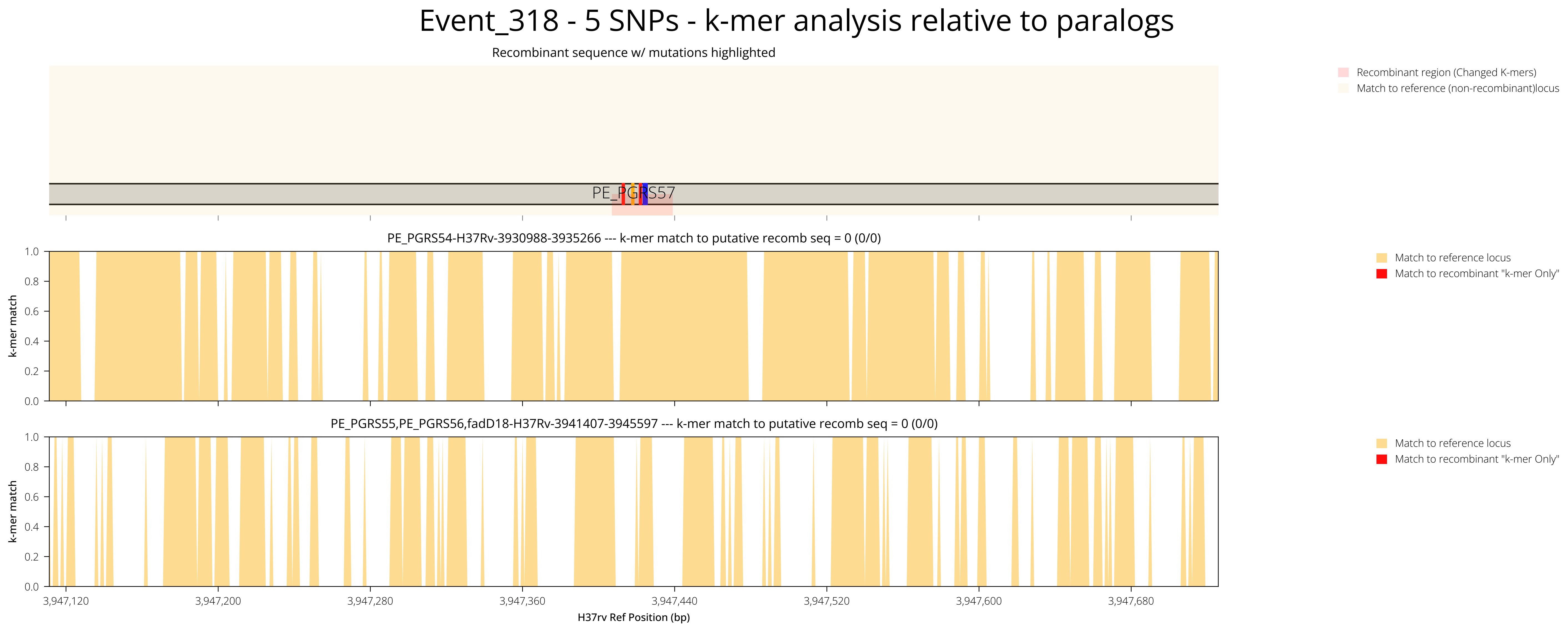
Event_318 - 5 SNPs - k-mer analysis relative to paralogs
Recombinant sequence w/ mutations highlighted
PE_PGRS57
Recombinant region (Changed K-mers)
Match to reference (non-recombinant)locus
PE_PGRS54-H37Rv-3930988-3935266 --- k-mer match to putative recomb seq = 0 (0/0)
Match to reference locus
Match to recombinant "k-mer Only"
k-mer match
1.0
0.8
0.6
0.4
0.2
0.0
PE_PGRS55,PE_PGRS56,fadD18-H37Rv-3941407-3945597 --- k-mer match to putative recomb seq = 0 (0/0)
3,947,120
3,947,200
3,947,280
3,947,360
3,947,440
3,947,520
3,947,600
3,947,680
H37rv Ref Position (bp)

Sub-phylogeny Viz + Paralog Alignments
GCE ID: Event_318

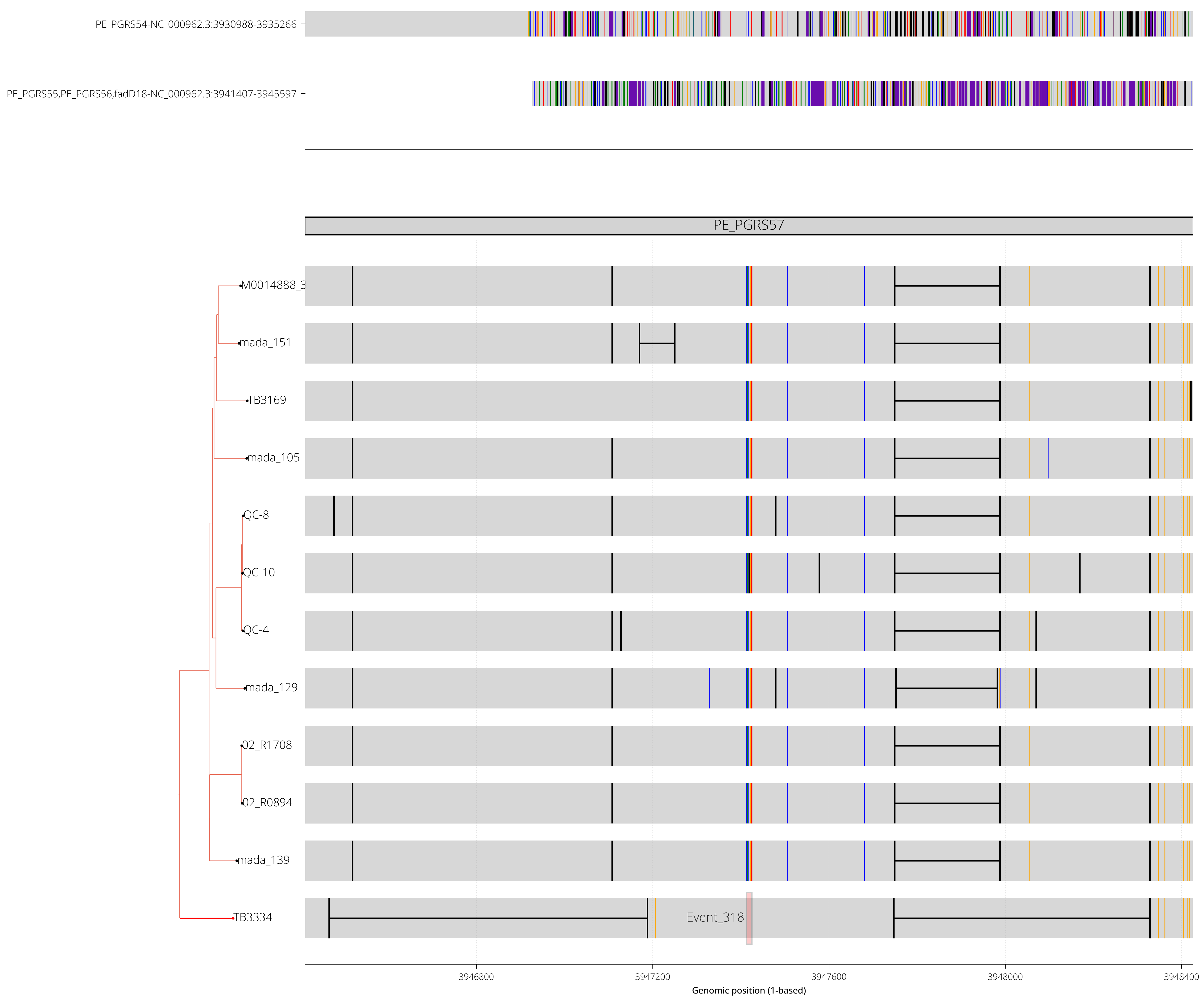

Full Phylogeny Viz + Paralog Alignments
GCE ID: Event_318

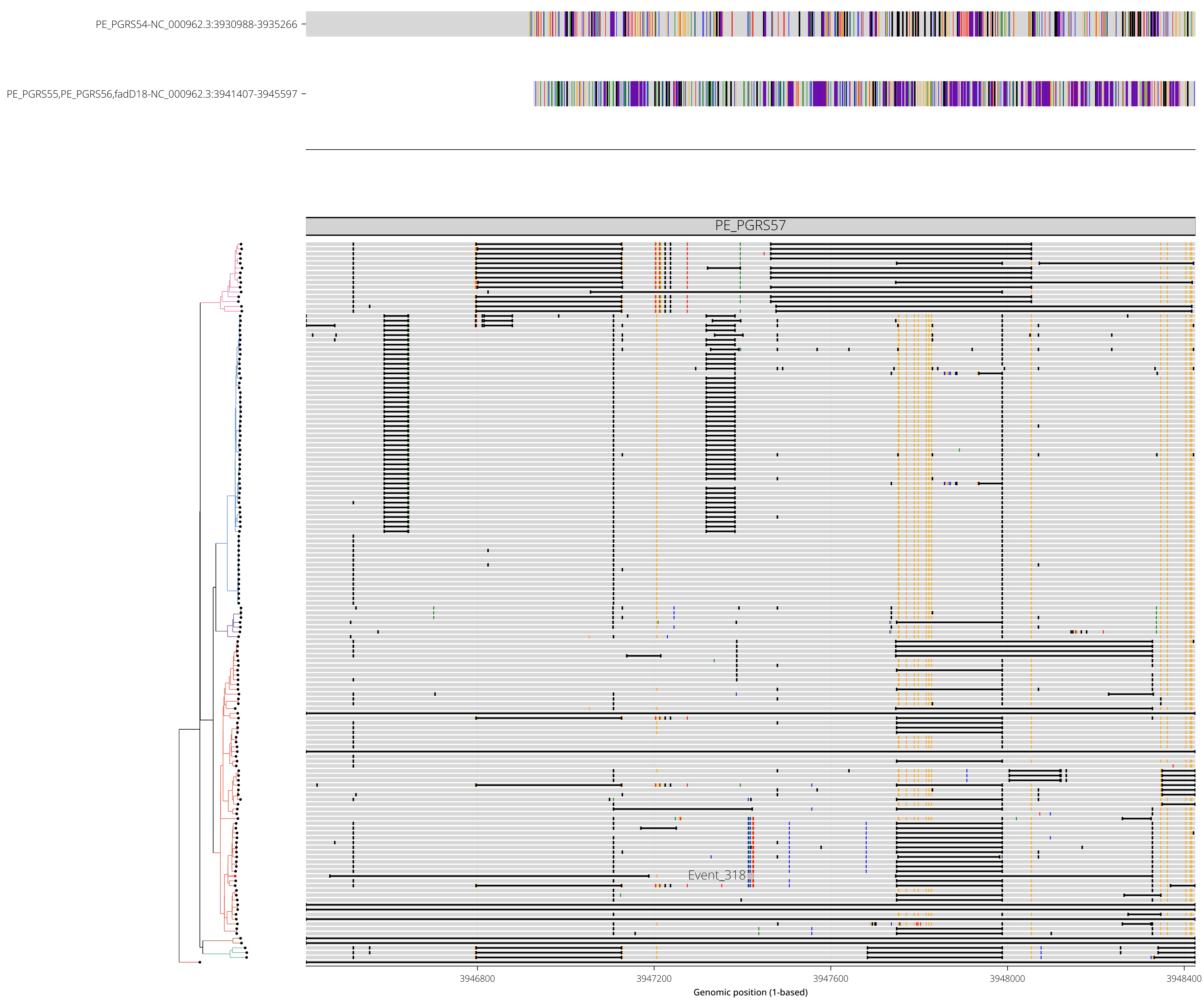

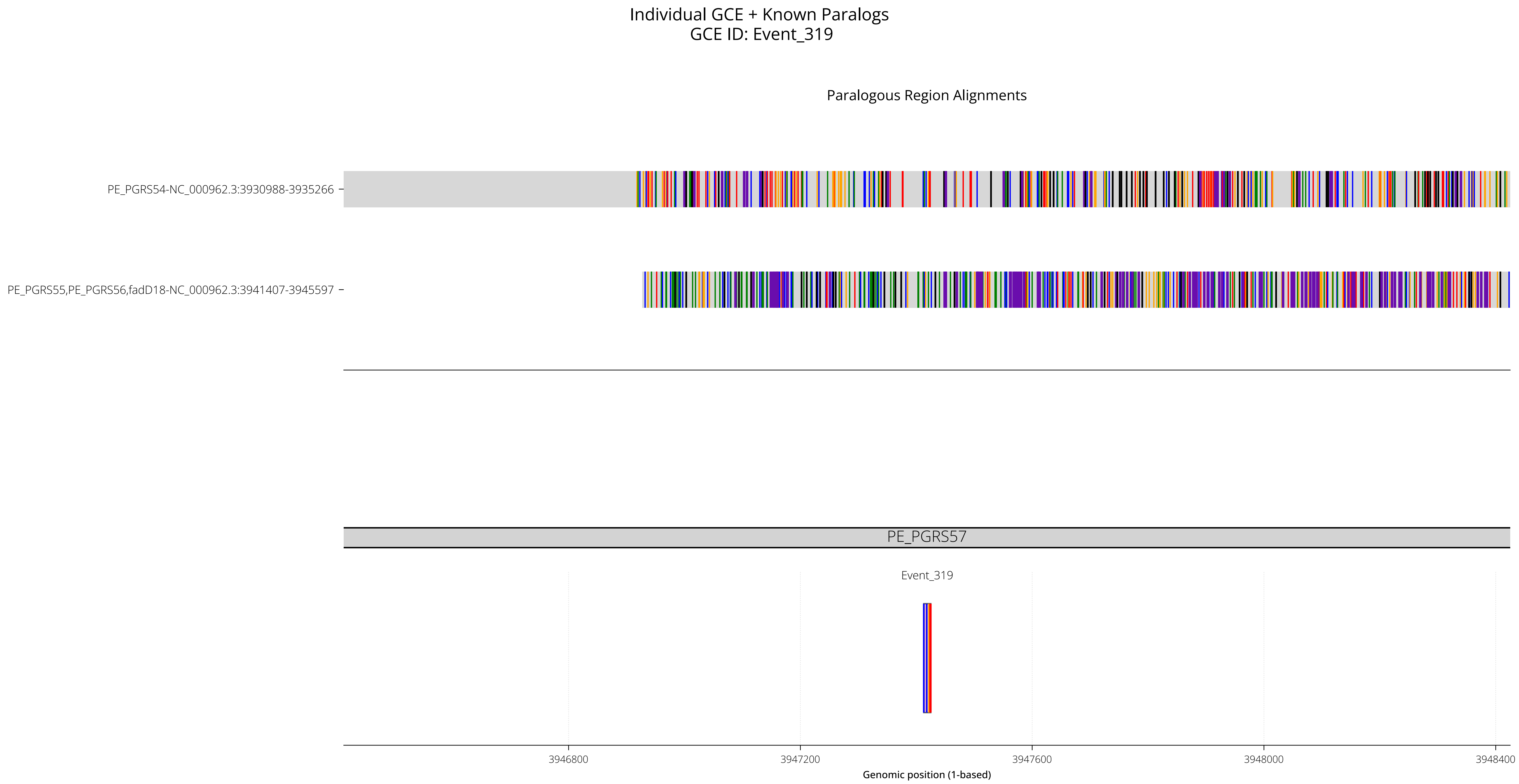

Individual GCE + Known Paralogs
GCE ID: Event_319
Paralogous Region Alignments
PE_PGRS54-NC_000962.3:3930988-3935266
PE_PGRS55,PE_PGRS56,fadD18-NC_000962.3:3941407-3945597
PE_PGRS57
Event_319
3946800
3947200
3947600
3948000
3948400
Genomic position (1-based)

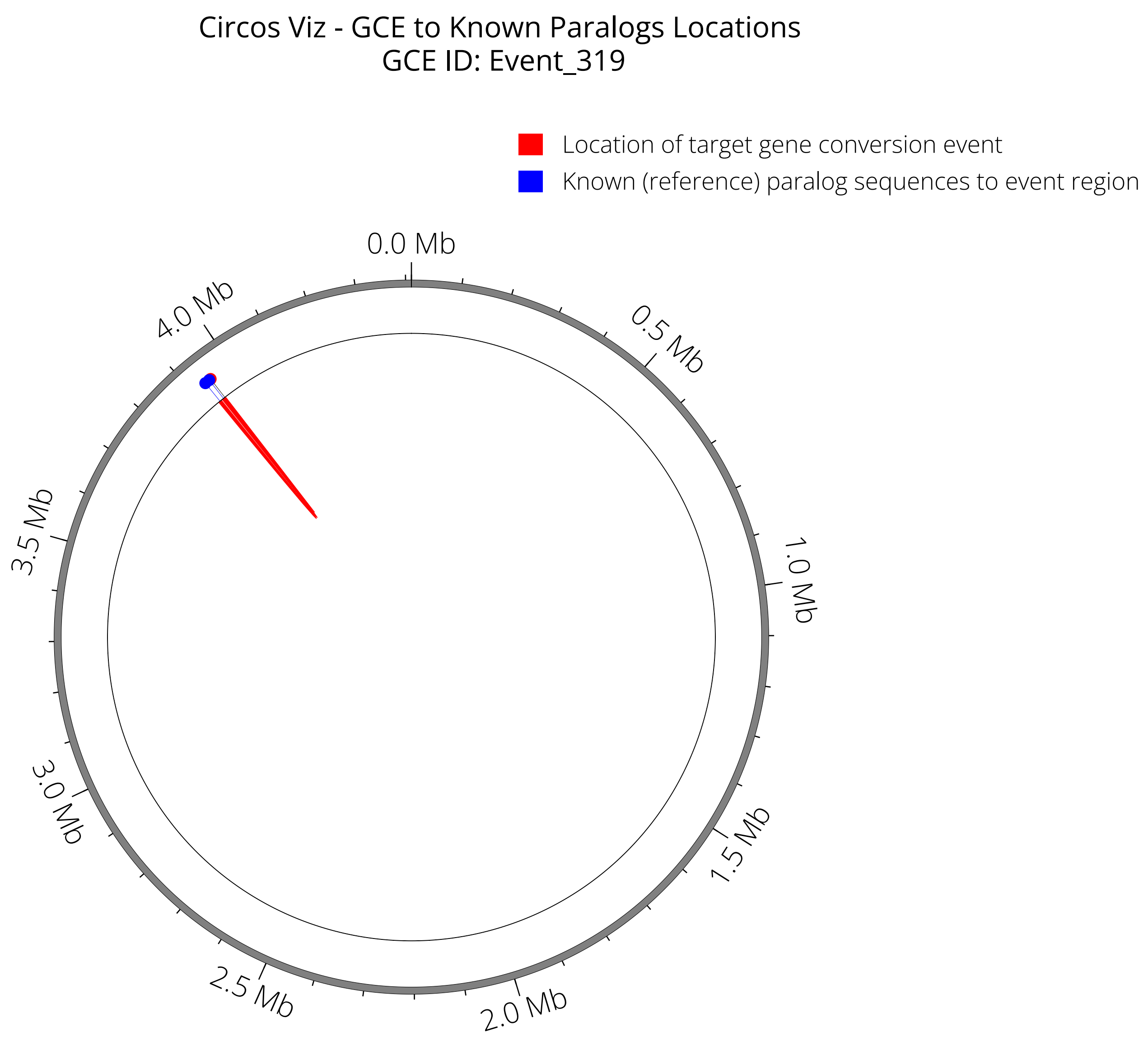
Circos Viz - GCE to Known Paralogs Locations
GCE ID: Event_319
Location of target gene conversion event
Known (reference) paralog sequences to event region
0.0 Mb
0.5 Mb
1.0 Mb
1.5 Mb
2.0 Mb
2.5 Mb
3.0 Mb
3.5 Mb
4.0 Mb

# Event_319 - 5 SNPs - k-mer analysis relative to paralogs

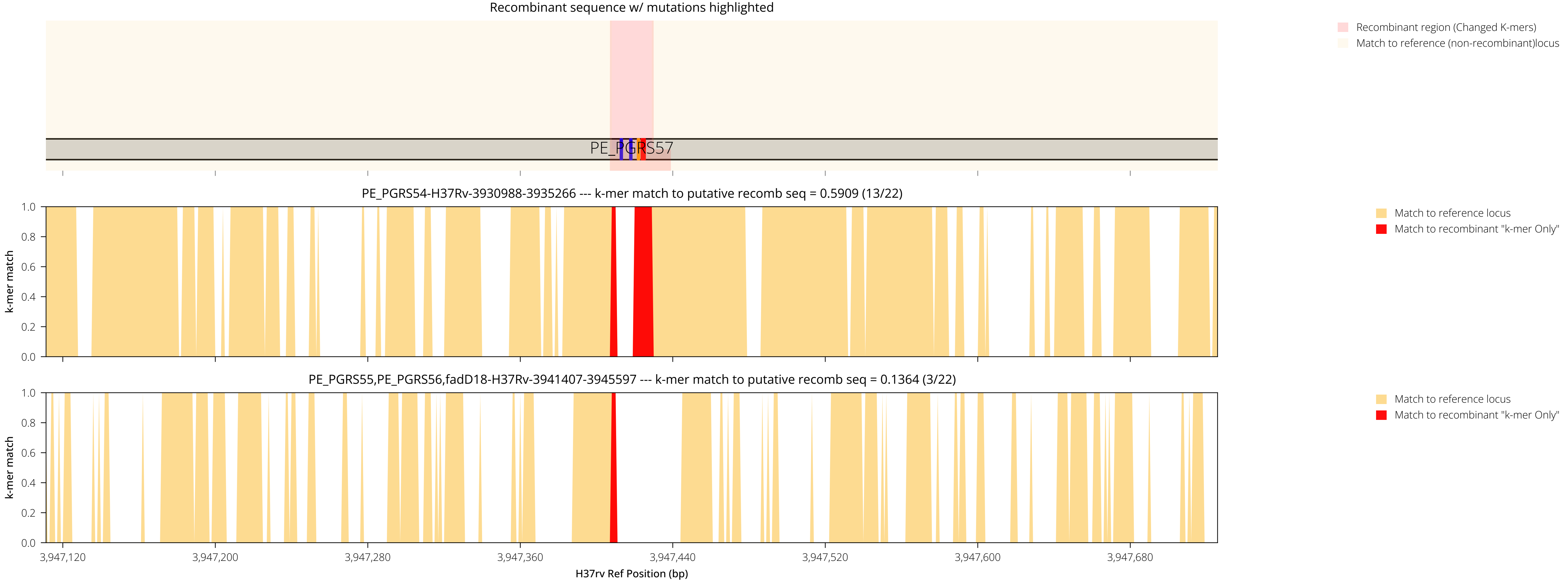

Sub-phylogeny Viz + Paralog Alignments
GCE ID: Event_319

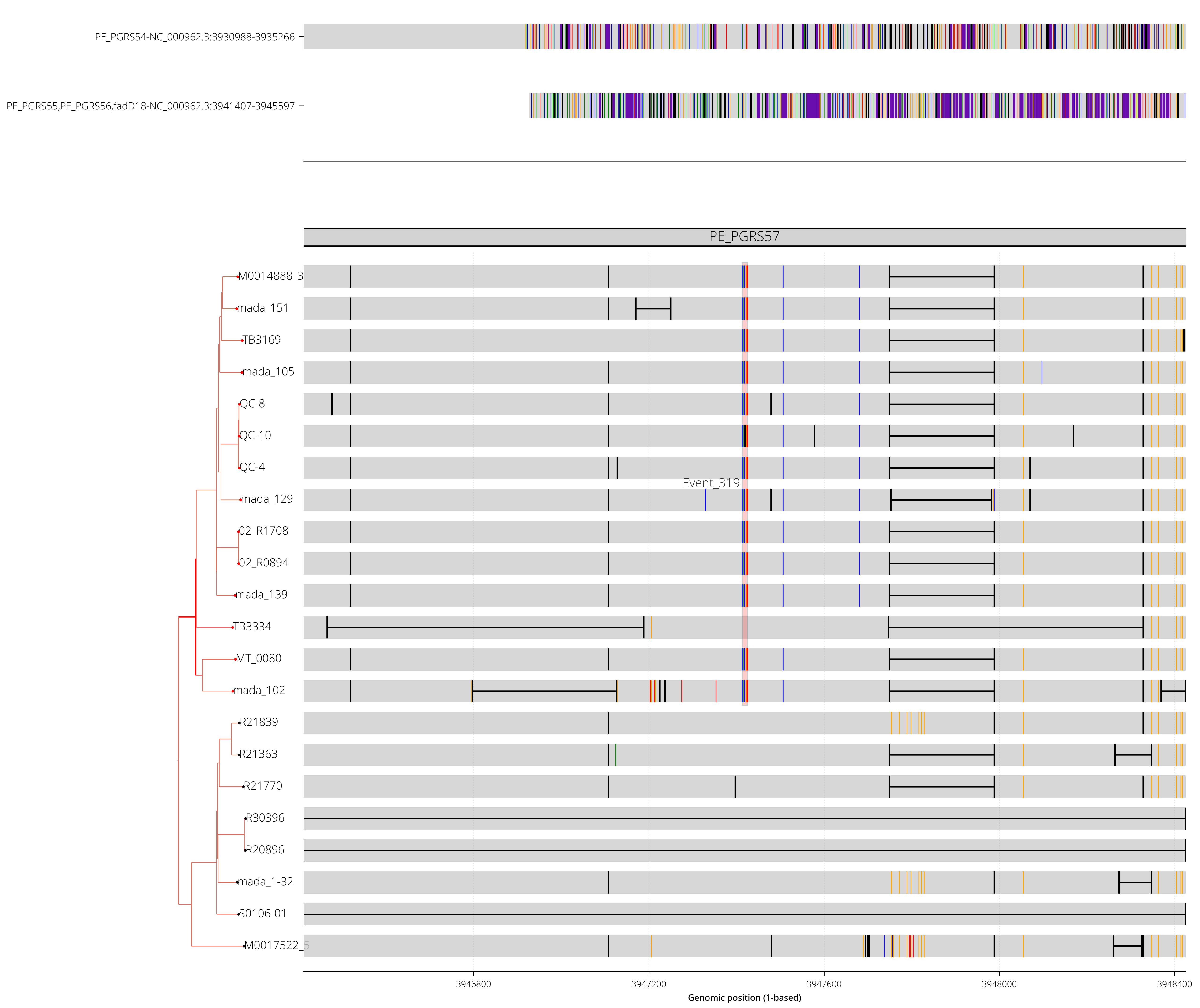

Full Phylogeny Viz + Paralog Alignments
GCE ID: Event_319

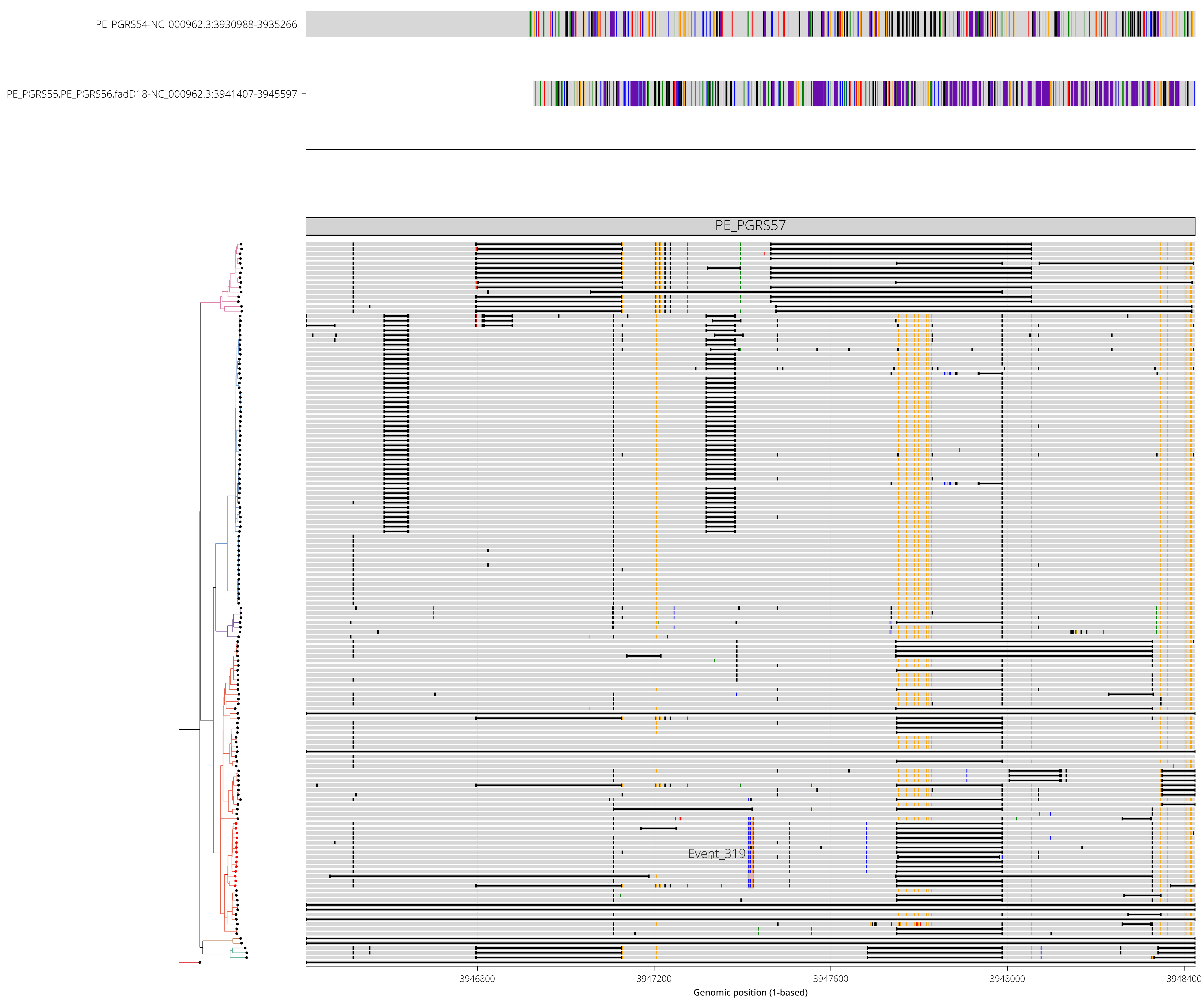

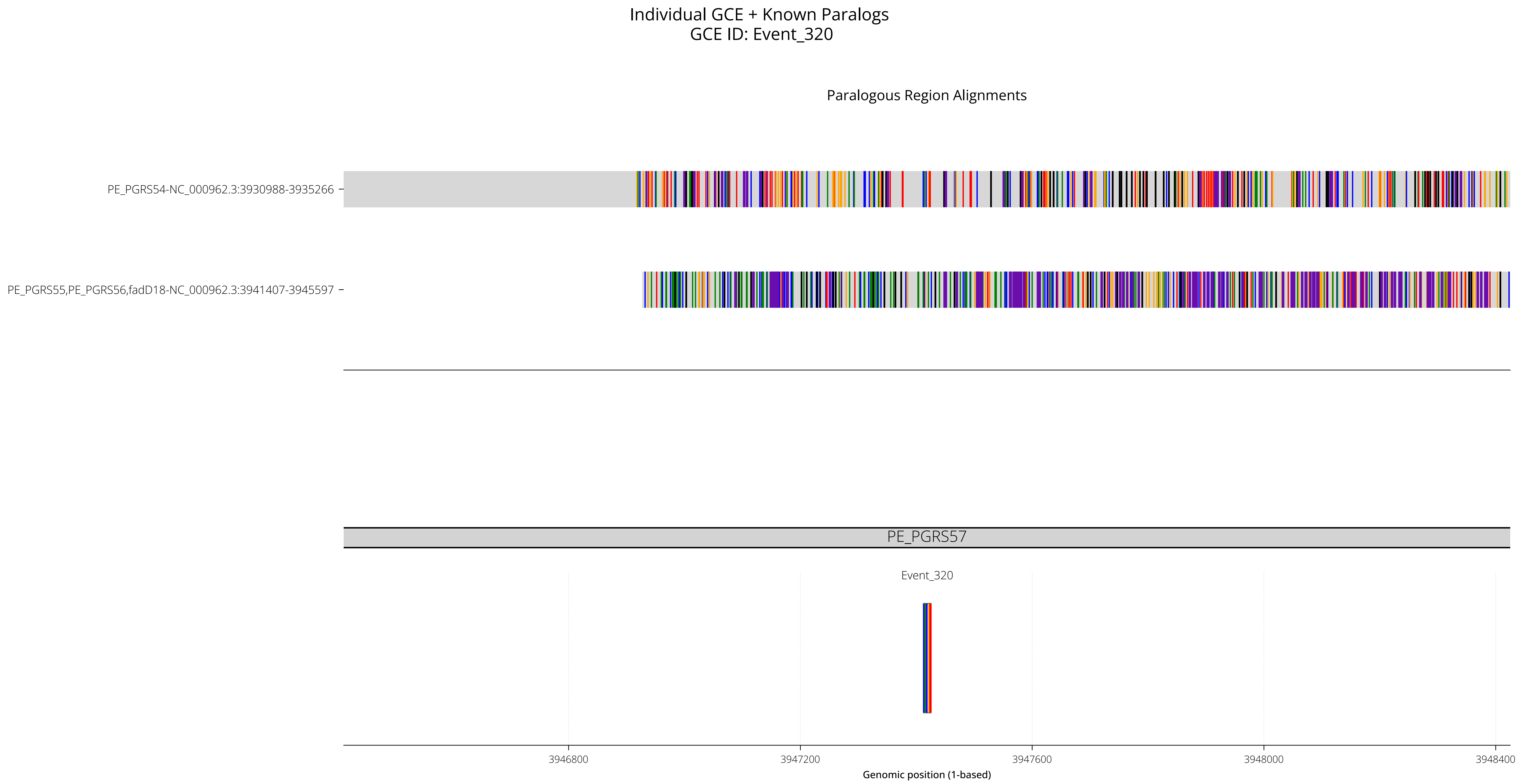

Individual GCE + Known Paralogs
GCE ID: Event_320
Paralogous Region Alignments
PE_PGRS54-NC_000962.3:3930988-3935266
PE_PGRS55,PE_PGRS56,fadD18-NC_000962.3:3941407-3945597
PE_PGRS57
Event_320
3946800
3947200
3947600
3948000
3948400
Genomic position (1-based)

Circos Viz - GCE to Known Paralogs Locations
GCE ID: Event_320

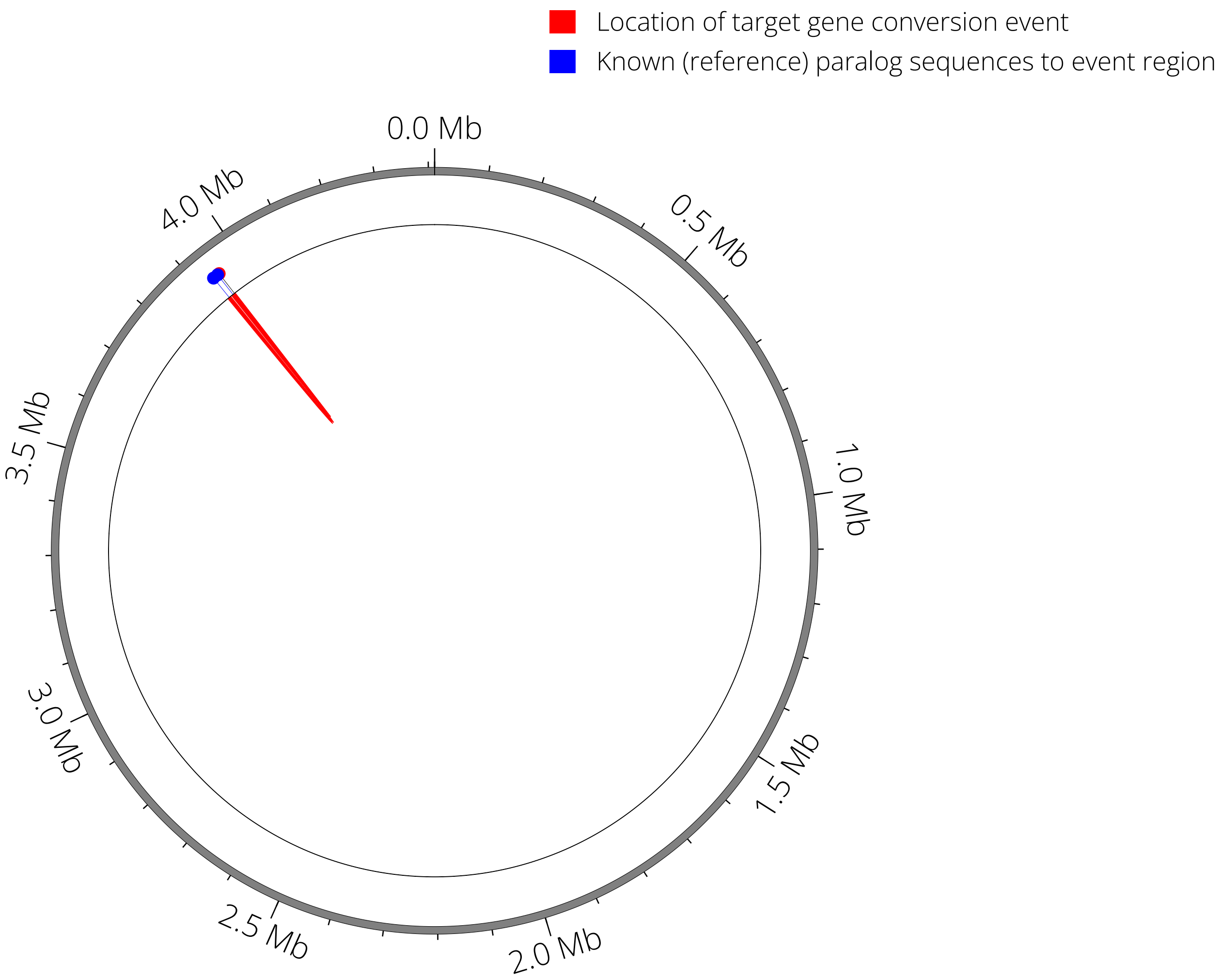

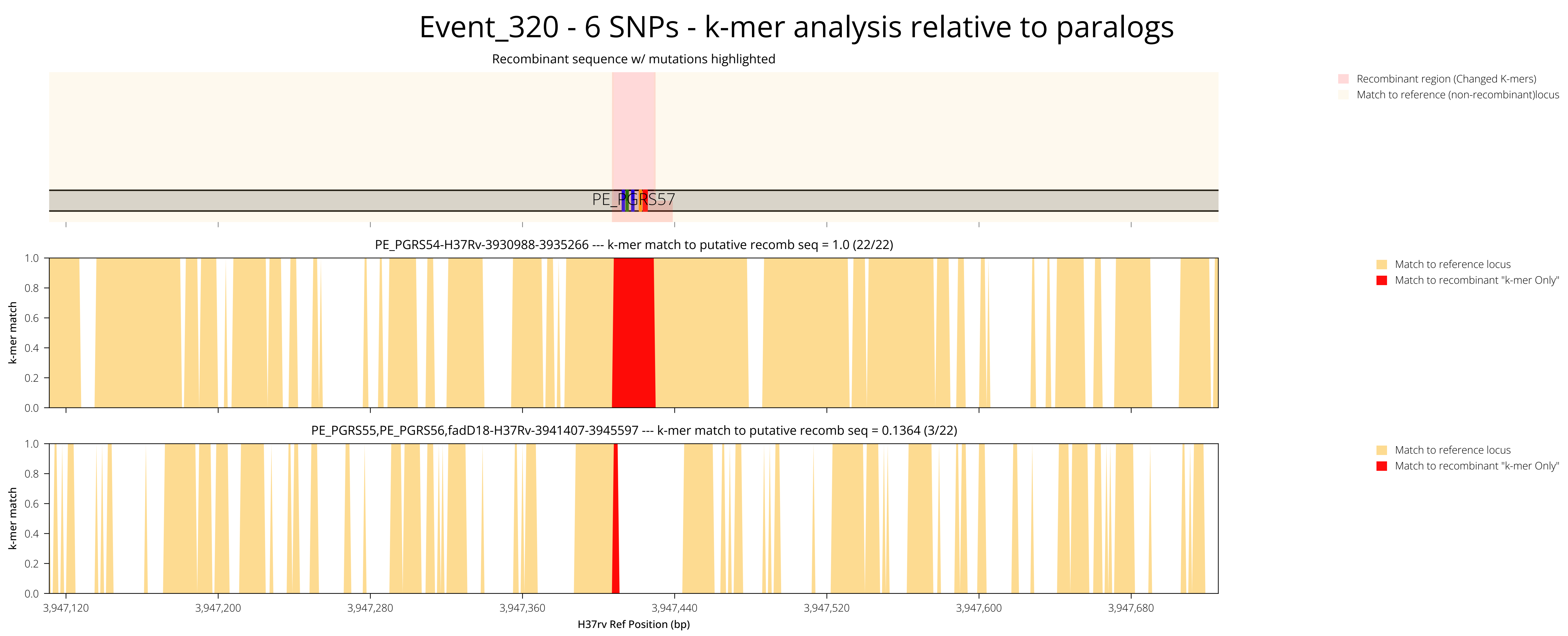
Event_320 - 6 SNPs - k-mer analysis relative to paralogs
Recombinant sequence w/ mutations highlighted
Recombinant region (Changed K-mers)
Match to reference (non-recombinant)locus
PE_PGRS57
PE_PGRS54-H37Rv-3930988-3935266 --- k-mer match to putative recomb seq = 1.0 (22/22)
Match to reference locus
Match to recombinant "k-mer Only"
k-mer match
1.0
0.8
0.6
0.4
0.2
0.0
PE_PGRS55,PE_PGRS56,fadD18-H37Rv-3941407-3945597 --- k-mer match to putative recomb seq = 0.1364 (3/22)
Match to reference locus
Match to recombinant "k-mer Only"
k-mer match
1.0
0.8
0.6
0.4
0.2
0.0
3,947,120
3,947,200
3,947,280
3,947,360
3,947,440
3,947,520
3,947,600
3,947,680
H37rv Ref Position (bp)

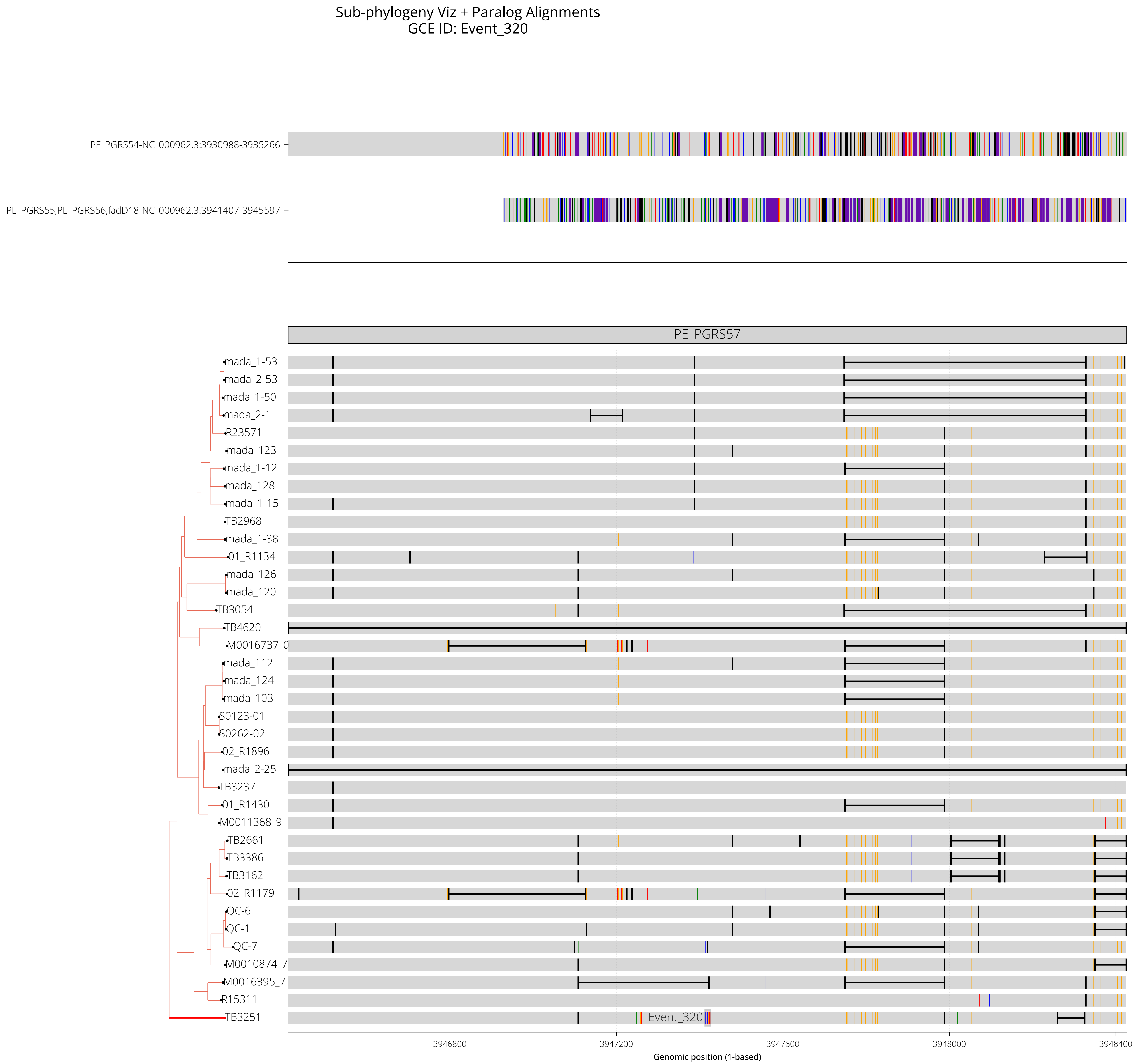

Sub-phylogeny Viz + Paralog Alignments
GCE ID: Event_320
PE_PGRS54-NC_000962.3:3930988-3935266
PE_PGRS55,PE_PGRS56,fadD18-NC_000962.3:3941407-3945597
PE_PGRS57
mada_1-53
mada_2-53
mada_1-50
mada_2-1
R23571
mada_123
mada_1-12
mada_128
mada_1-15
TB2968
mada_1-38
01_R1134
mada_126
mada_120
TB3054
TB4620
M0016737_0
mada_112
mada_124
mada_103
S0123-01
S0262-02
02_R1896
mada_2-25
TB3237
01_R1430
M0011368_9
TB2661
TB3386
TB3162
02_R1179
QC-6
QC-1
QC-7
M0010874_7
M0016395_7
R15311
TB3251
Event_320
3946800
3947200
3947600
3948000
3948400
Genomic position (1-based)

Full Phylogeny Viz + Paralog Alignments
GCE ID: Event_320

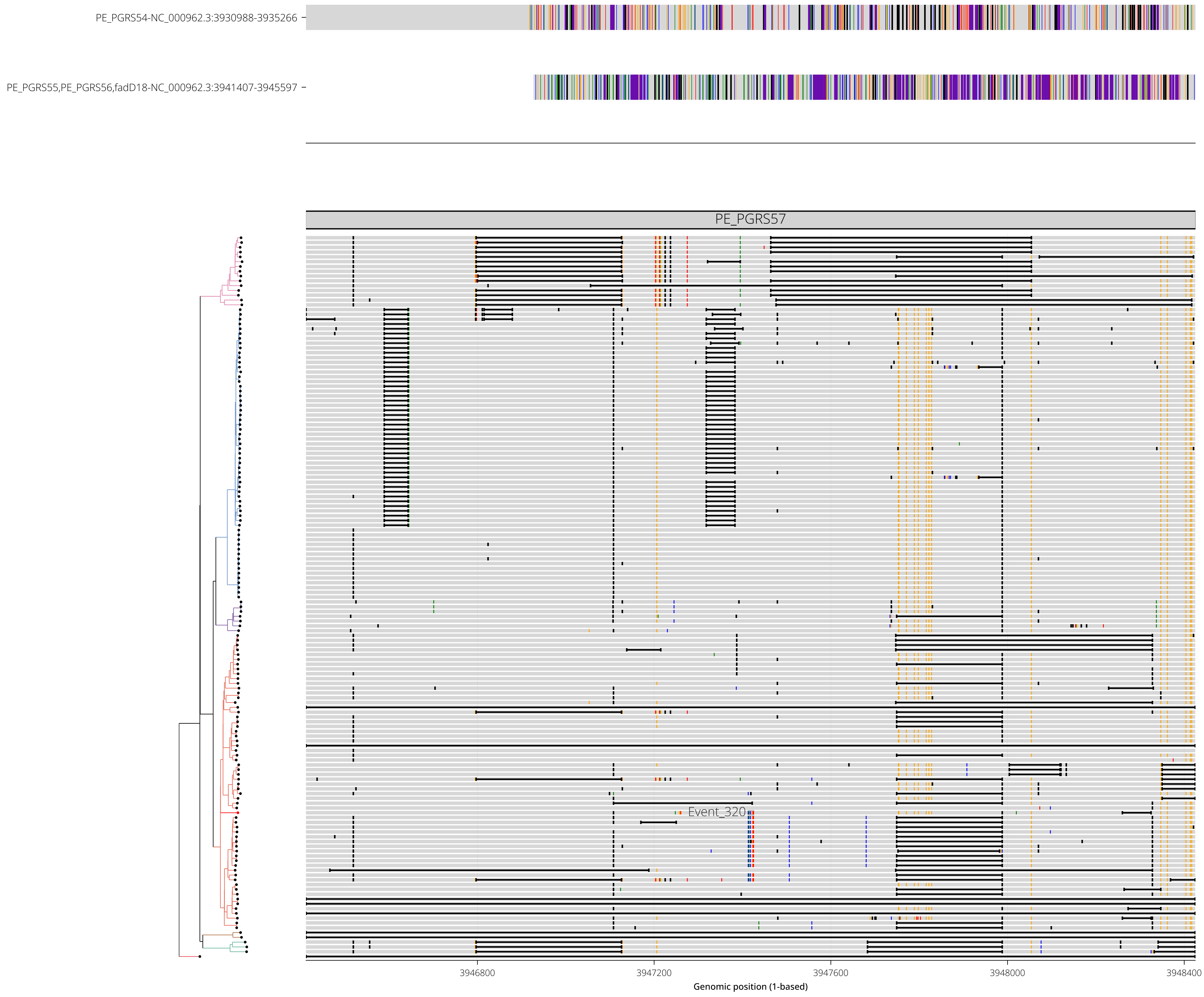

Individual GCE + Known Paralogs
GCE ID: Event_321

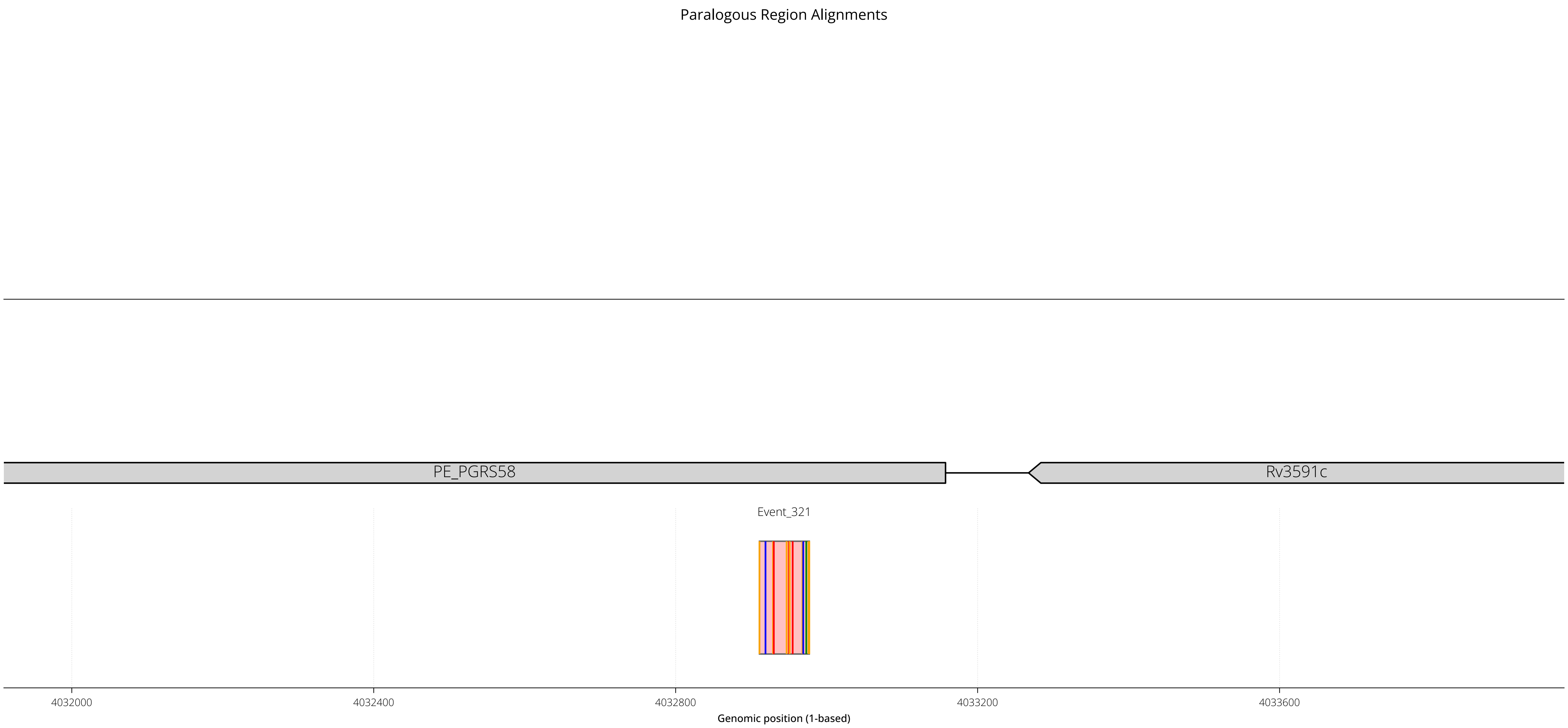

Sub-phylogeny Viz + Paralog Alignments
GCE ID: Event_321

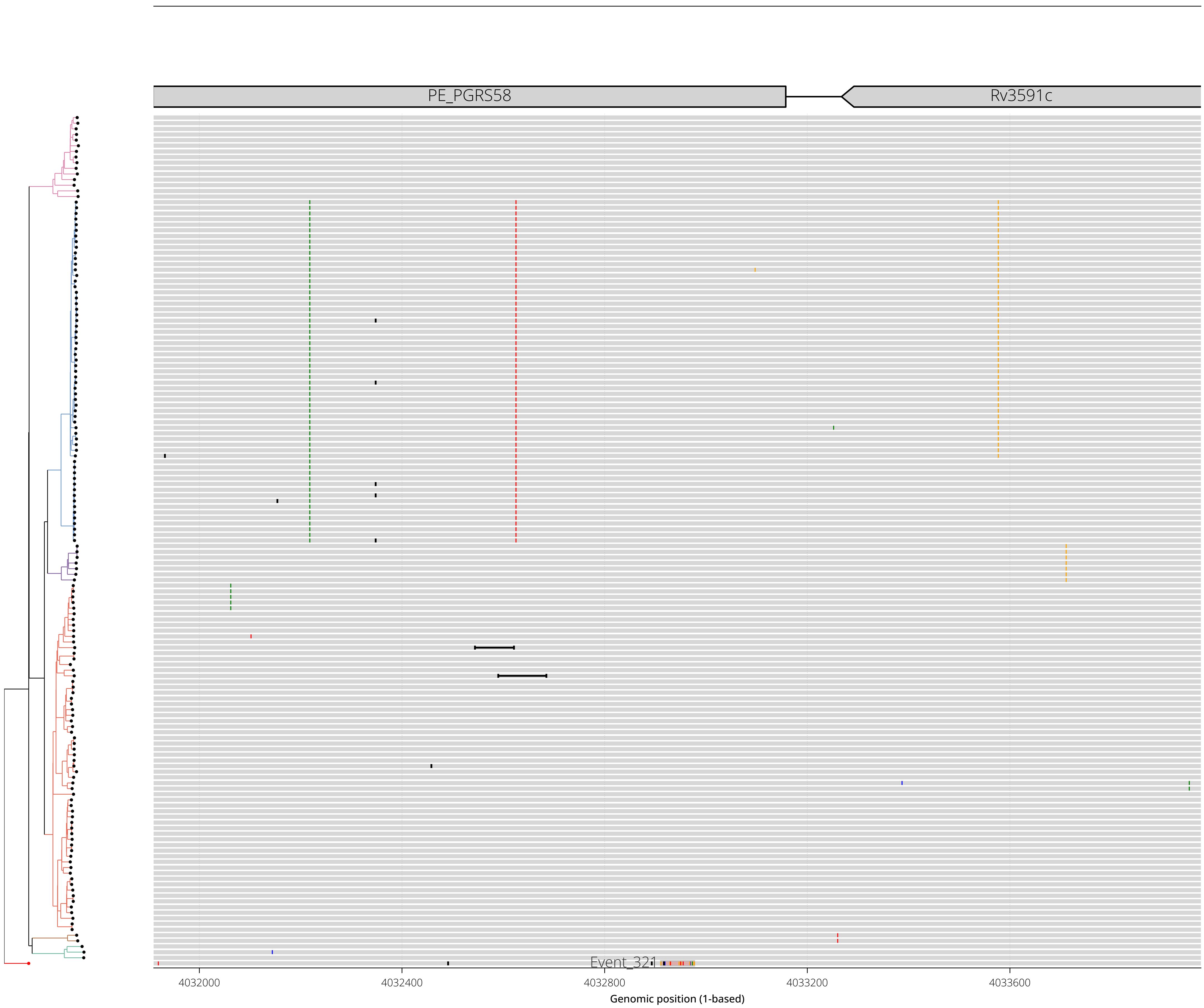

Full Phylogeny Viz + Paralog Alignments
GCE ID: Event_321

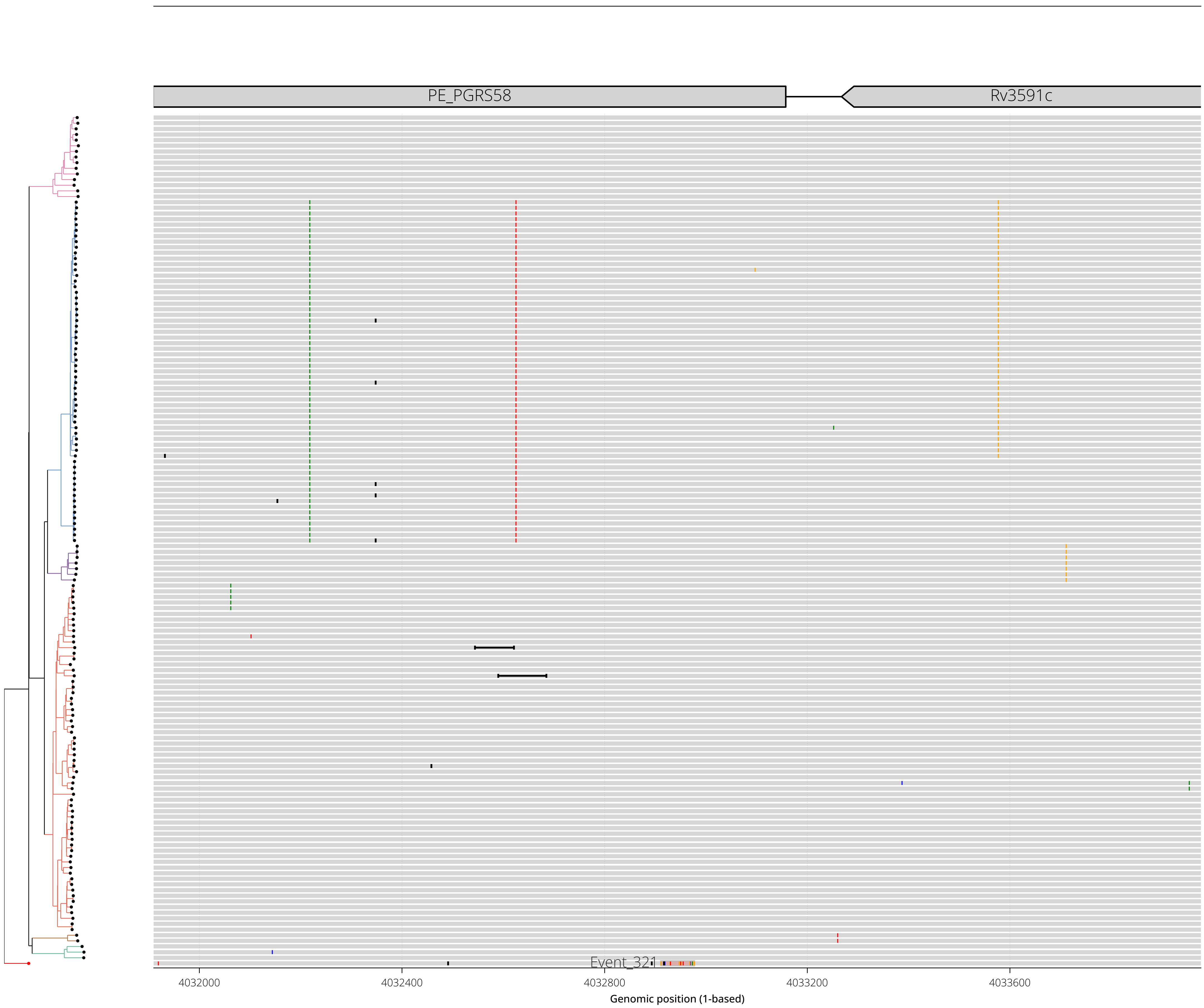

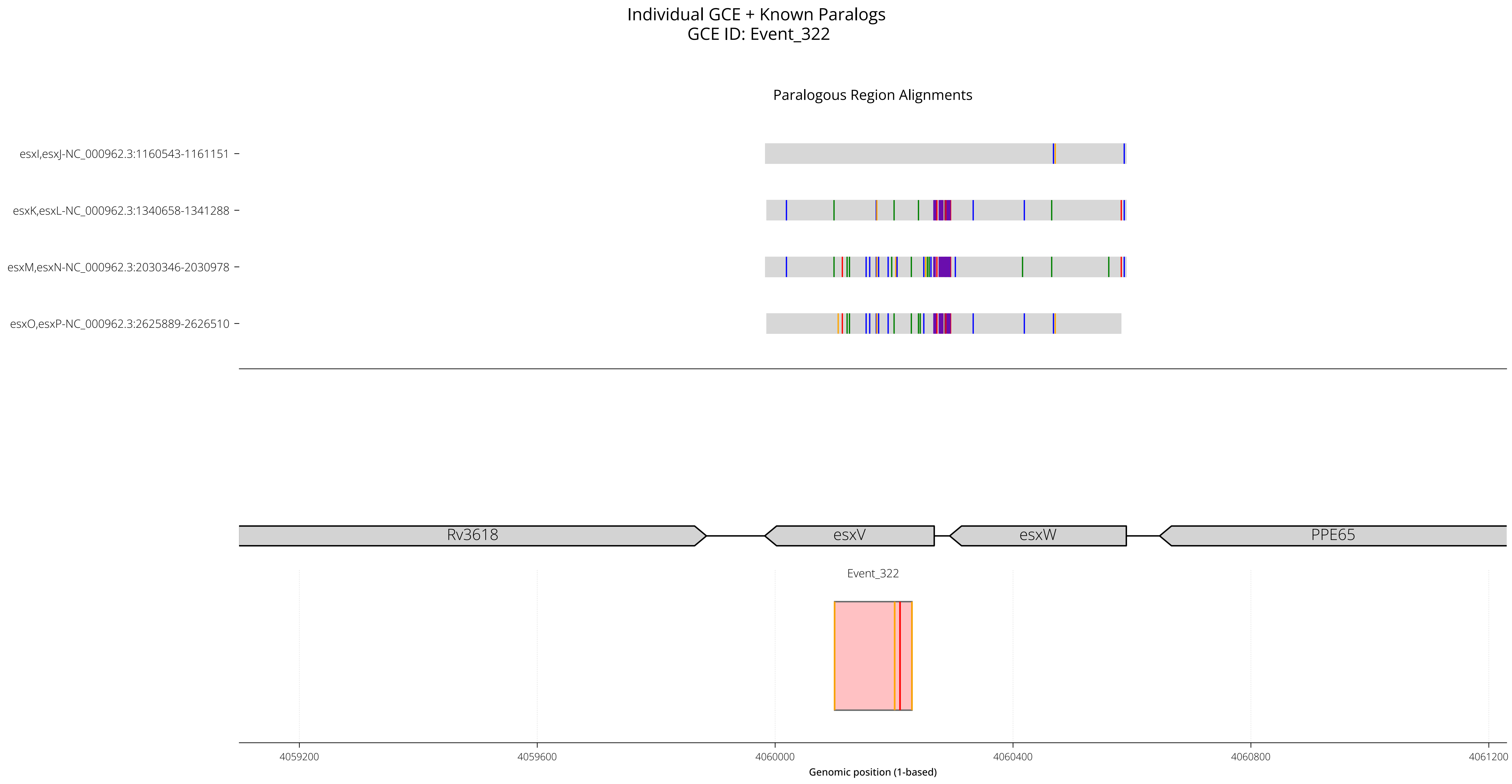

Individual GCE + Known Paralogs
GCE ID: Event_322
Paralogous Region Alignments
esxI,esxJ-NC_000962.3:1160543-1161151
esxK,esxL-NC_000962.3:1340658-1341288
esxM,esxN-NC_000962.3:2030346-2030978
esxO,esxP-NC_000962.3:2625889-2626510
Rv3618
esxV
esxW
PPE65
Event_322
4059200
4059600
4060000
4060400
4060800
4061200
Genomic position (1-based)

# Circos Viz - GCE to Known Paralogs Locations
GCE ID: Event_322

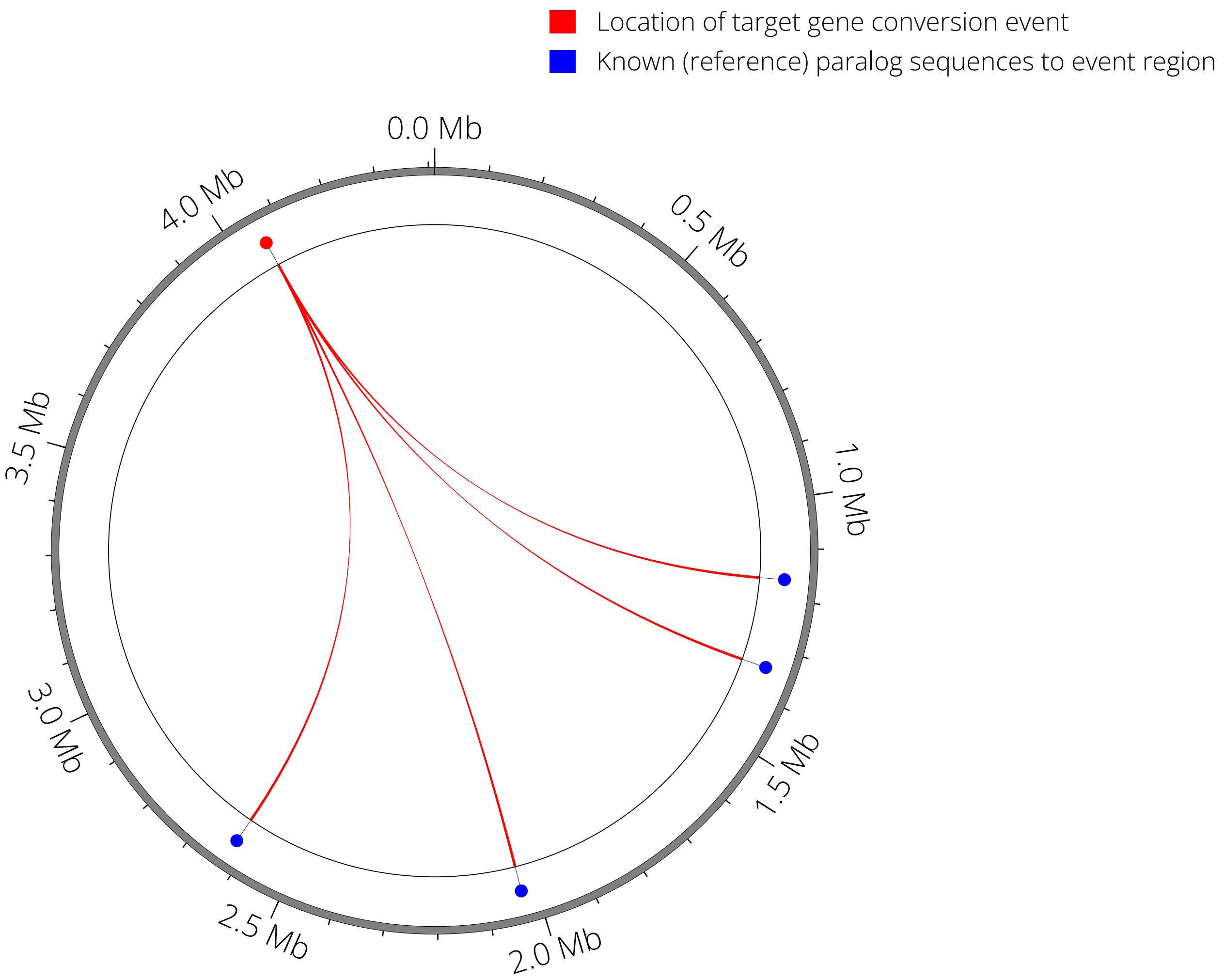

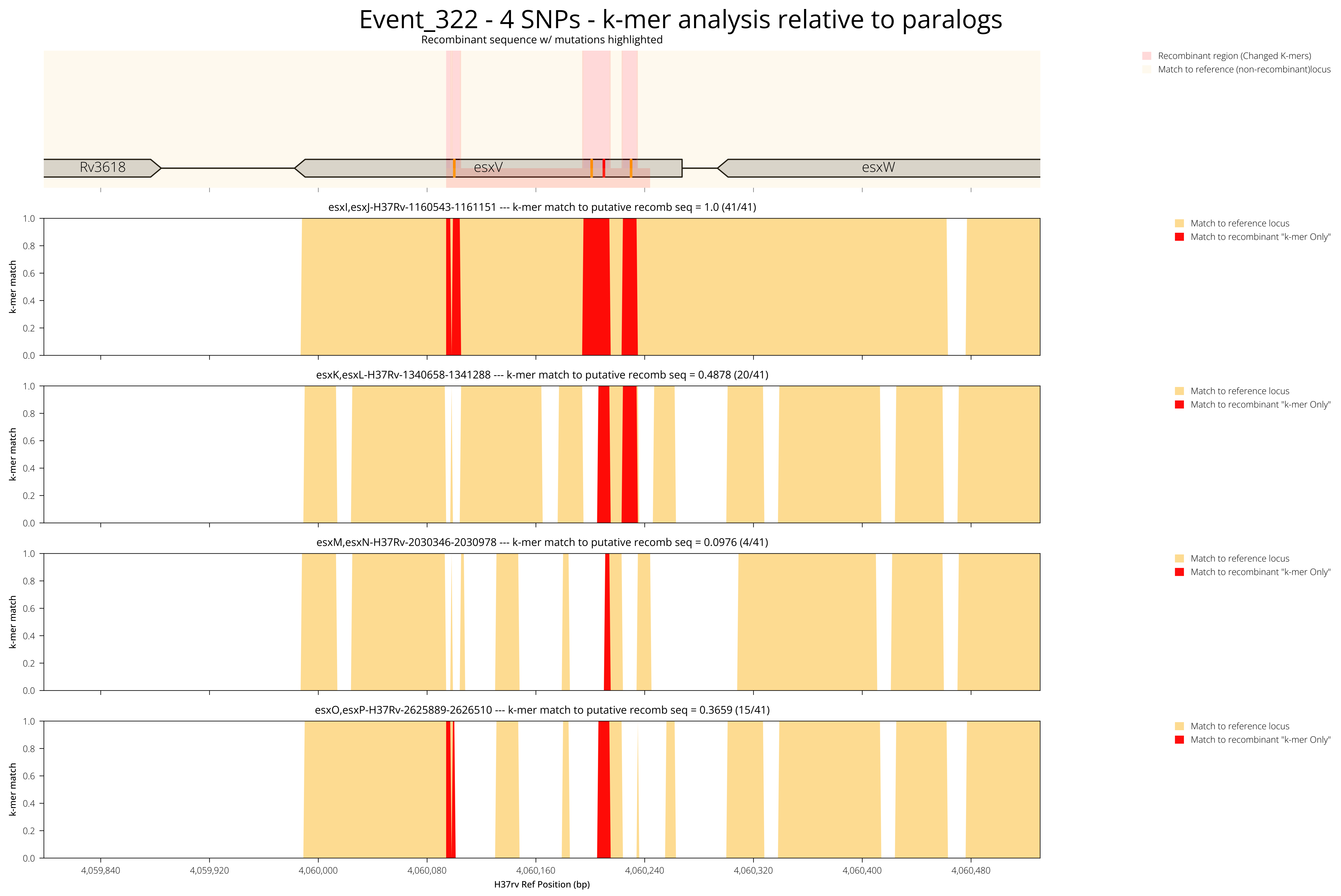

Event_322 - 4 SNPs - k-mer analysis relative to paralogs
Recombinant sequence w/ mutations highlighted
Recombinant region (Changed K-mers)
Match to reference (non-recombinant)locus
Rv3618
esxV
esxW
esxI,esxJ-H37Rv-1160543-1161151 --- k-mer match to putative recomb seq = 1.0 (41/41)
esxK,esxL-H37Rv-1340658-1341288 --- k-mer match to putative recomb seq = 0.4878 (20/41)
esxM,esxN-H37Rv-2030346-2030978 --- k-mer match to putative recomb seq = 0.0976 (4/41)
esxO,esxP-H37Rv-2625889-2626510 --- k-mer match to putative recomb seq = 0.3659 (15/41)
Match to reference locus
Match to recombinant "k-mer Only"
k-mer match
0.0
0.2
0.4
0.6
0.8
1.0
4,059,840
4,059,920
4,060,000
4,060,080
4,060,160
4,060,240
4,060,320
4,060,400
4,060,480
H37rv Ref Position (bp)

Sub-phylogeny Viz + Paralog Alignments
GCE ID: Event_322

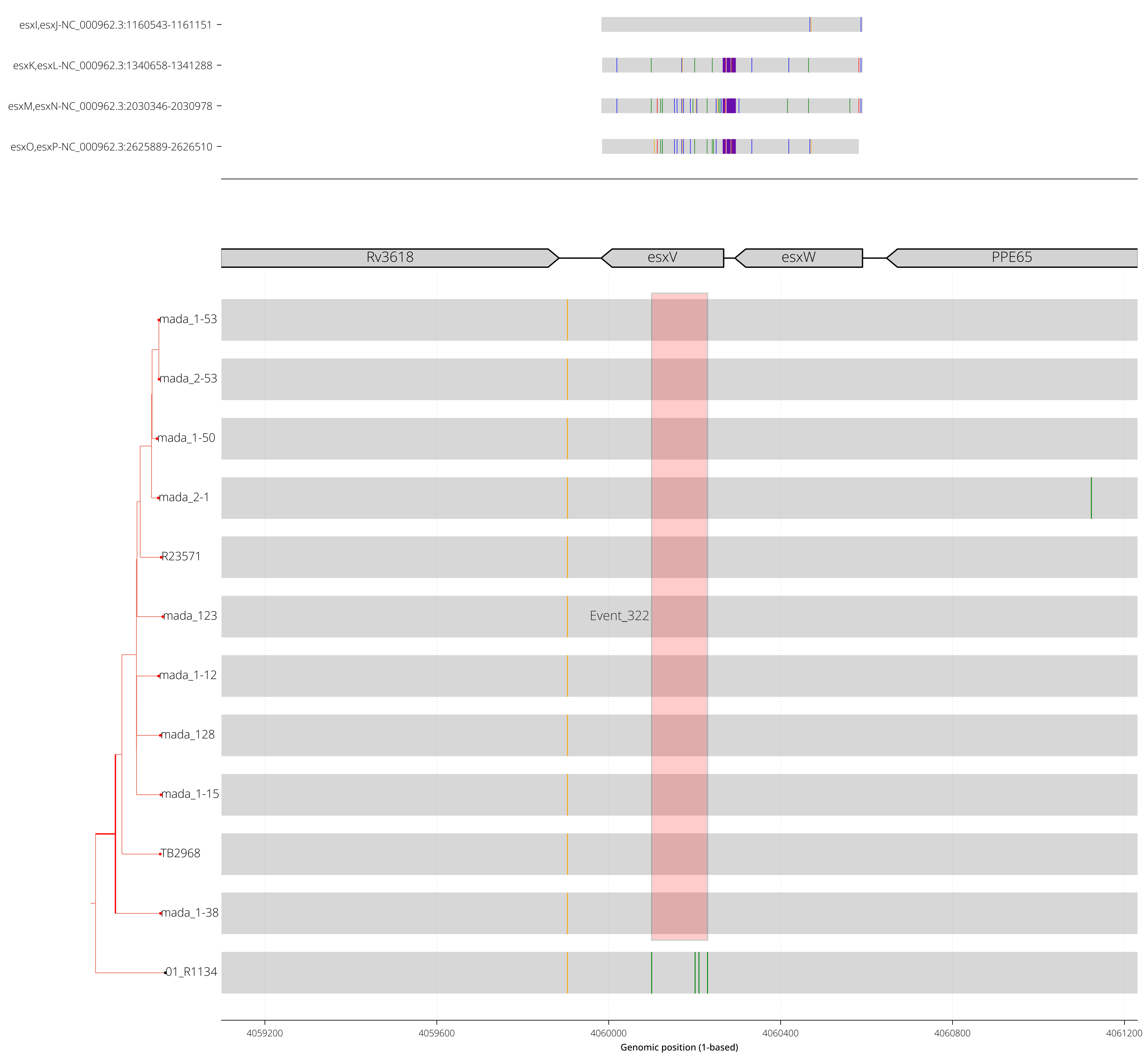

Full Phylogeny Viz + Paralog Alignments
GCE ID: Event_322

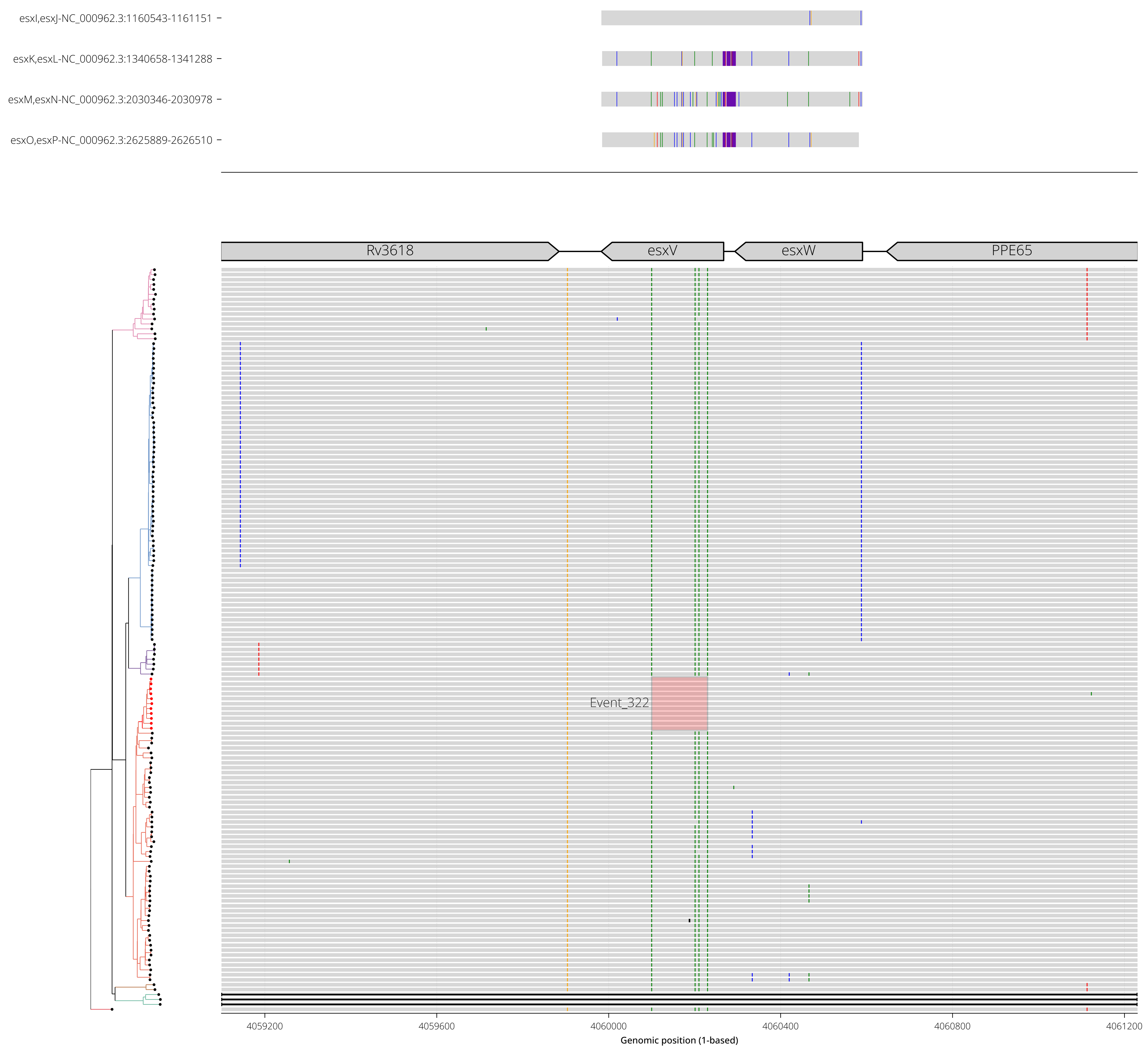

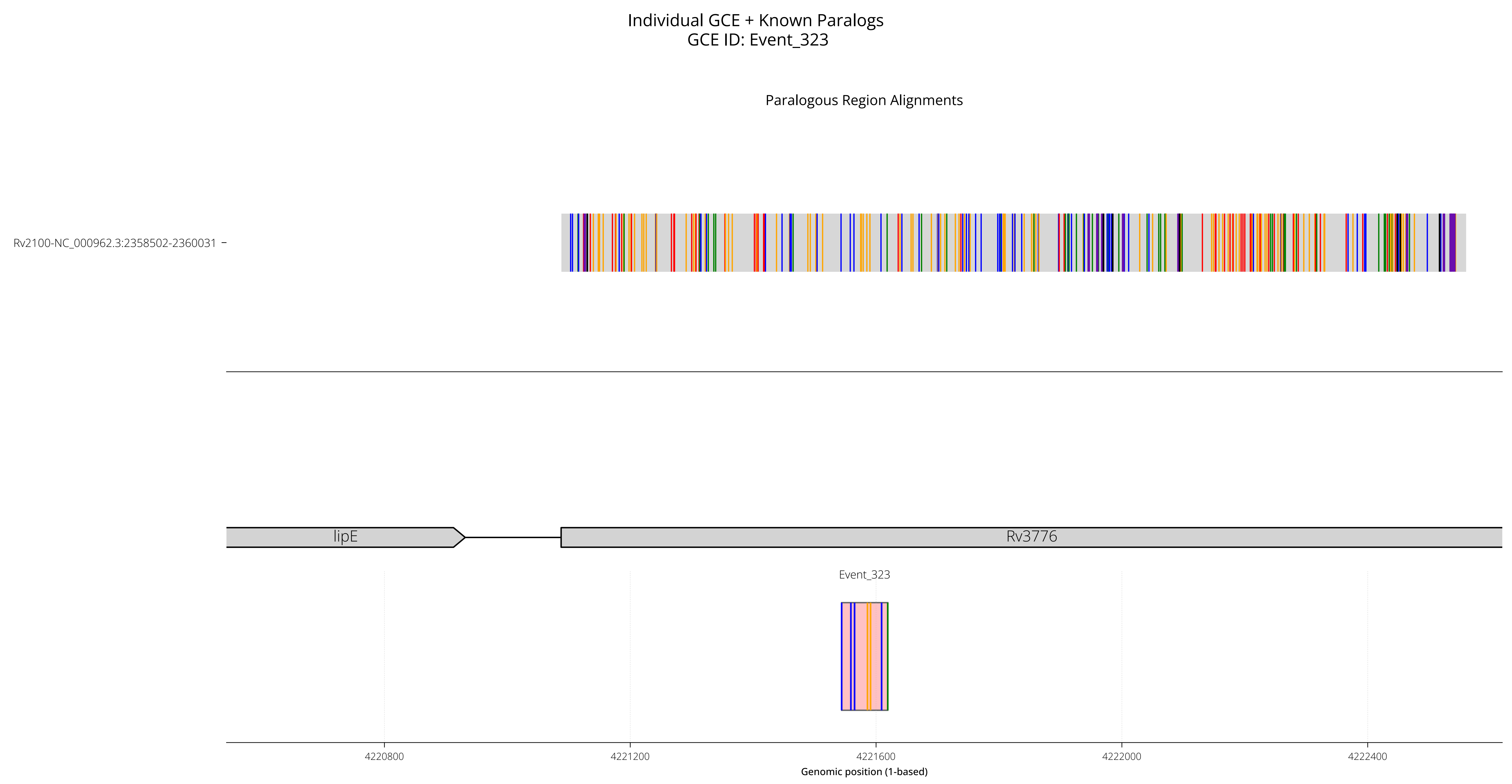
Individual GCE + Known Paralogs
GCE ID: Event_323
Paralogous Region Alignments
Rv2100-NC_000962.3:2358502-2360031
lipE
Rv3776
Event_323
4220800
4221200
4221600
4222000
4222400
Genomic position (1-based)

Circos Viz - GCE to Known Paralogs Locations
GCE ID: Event_323

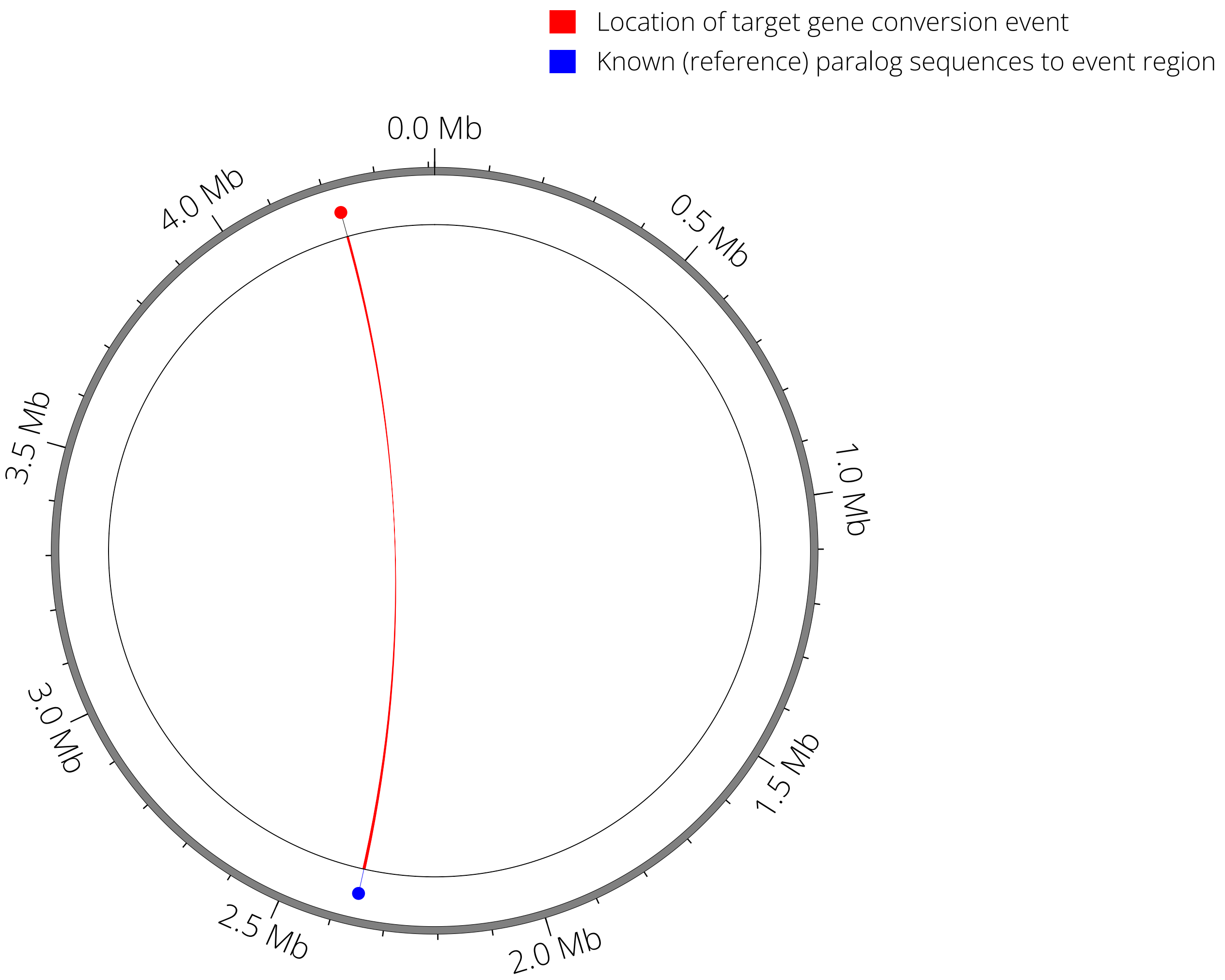

# Event_323 - 7 SNPs - k-mer analysis relative to paralogs

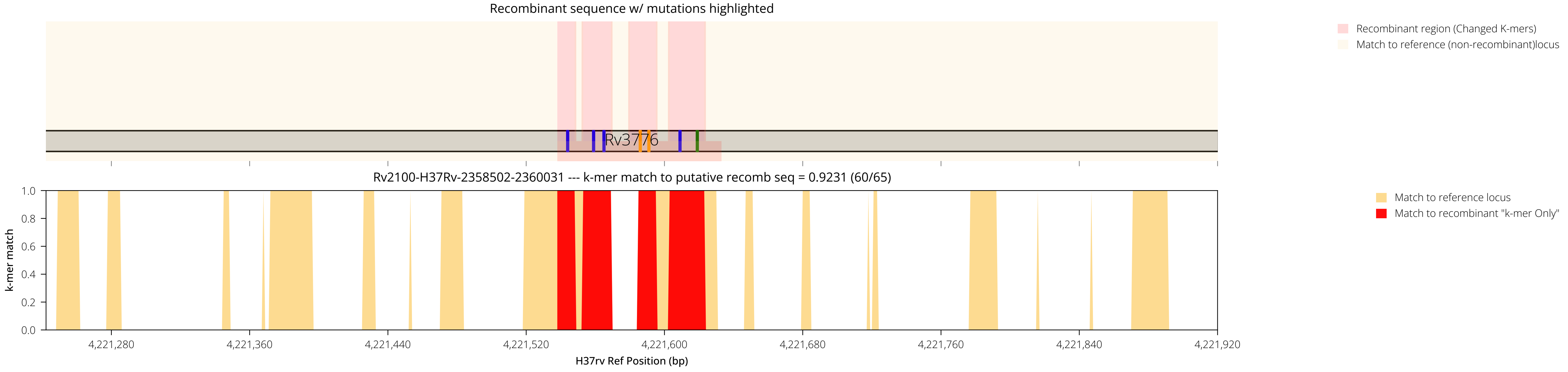

Sub-phylogeny Viz + Paralog Alignments
GCE ID: Event_323

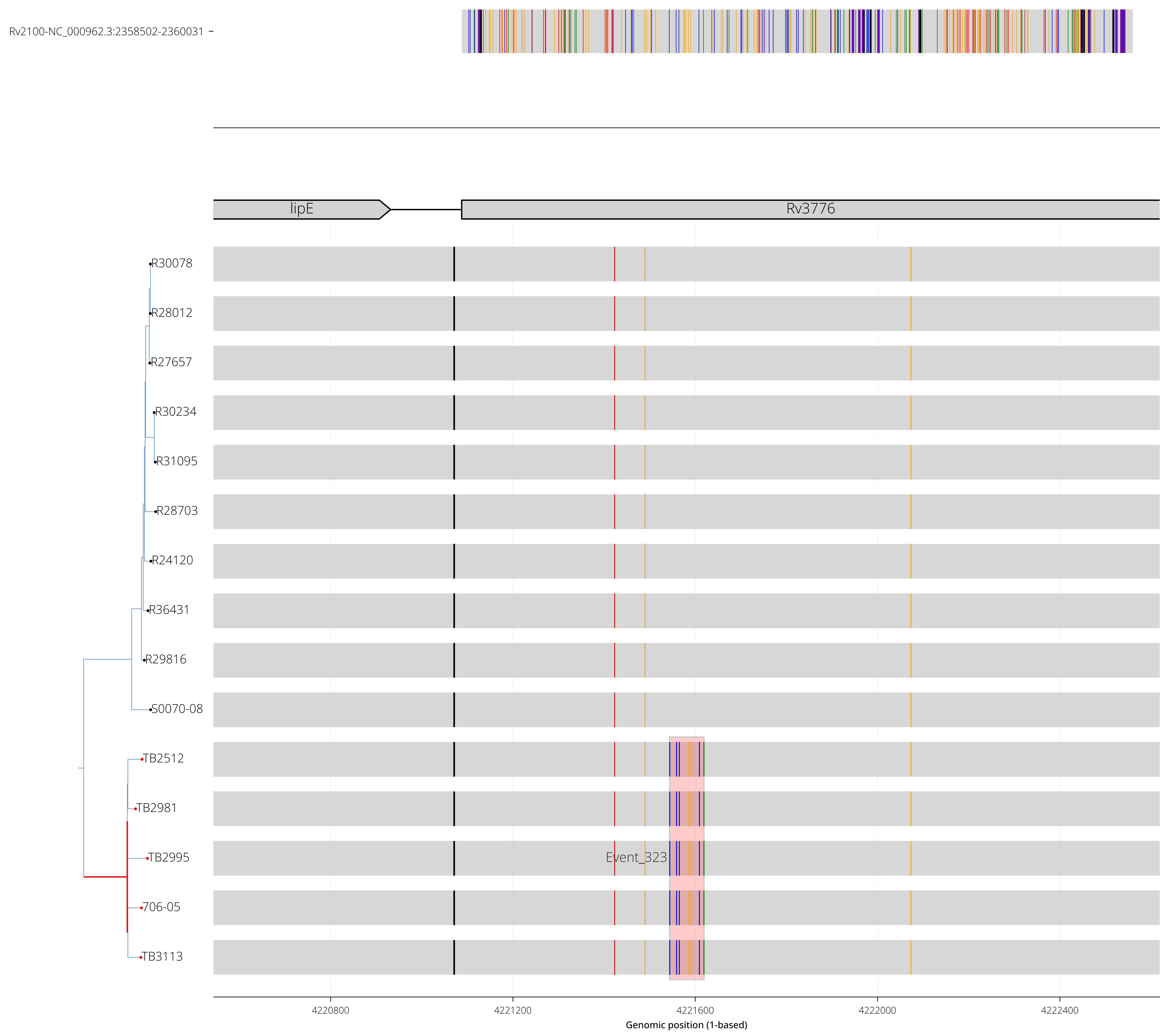

Full Phylogeny Viz + Paralog Alignments
GCE ID: Event_323

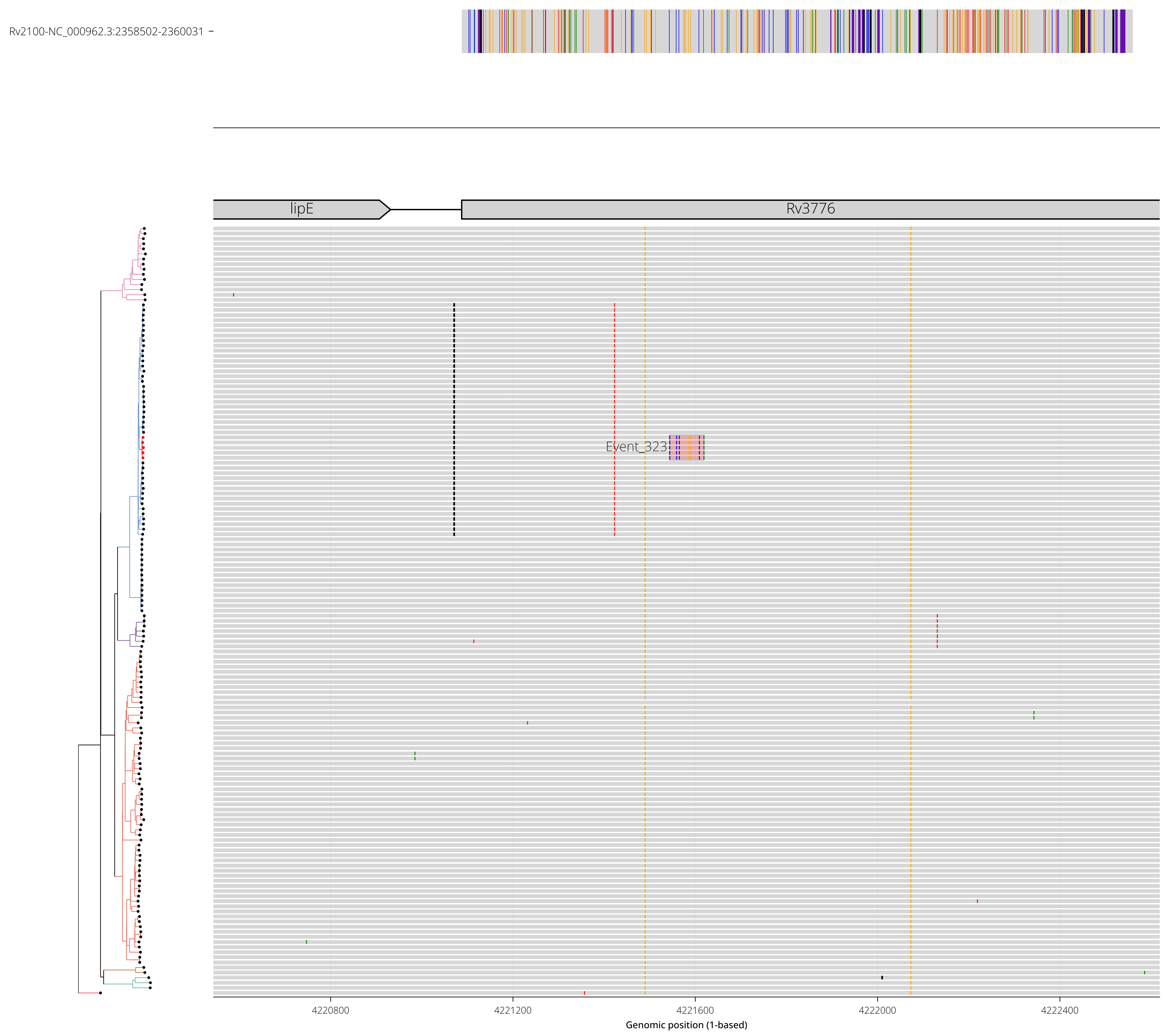

Individual GCE + Known Paralogs
GCE ID: Event_324

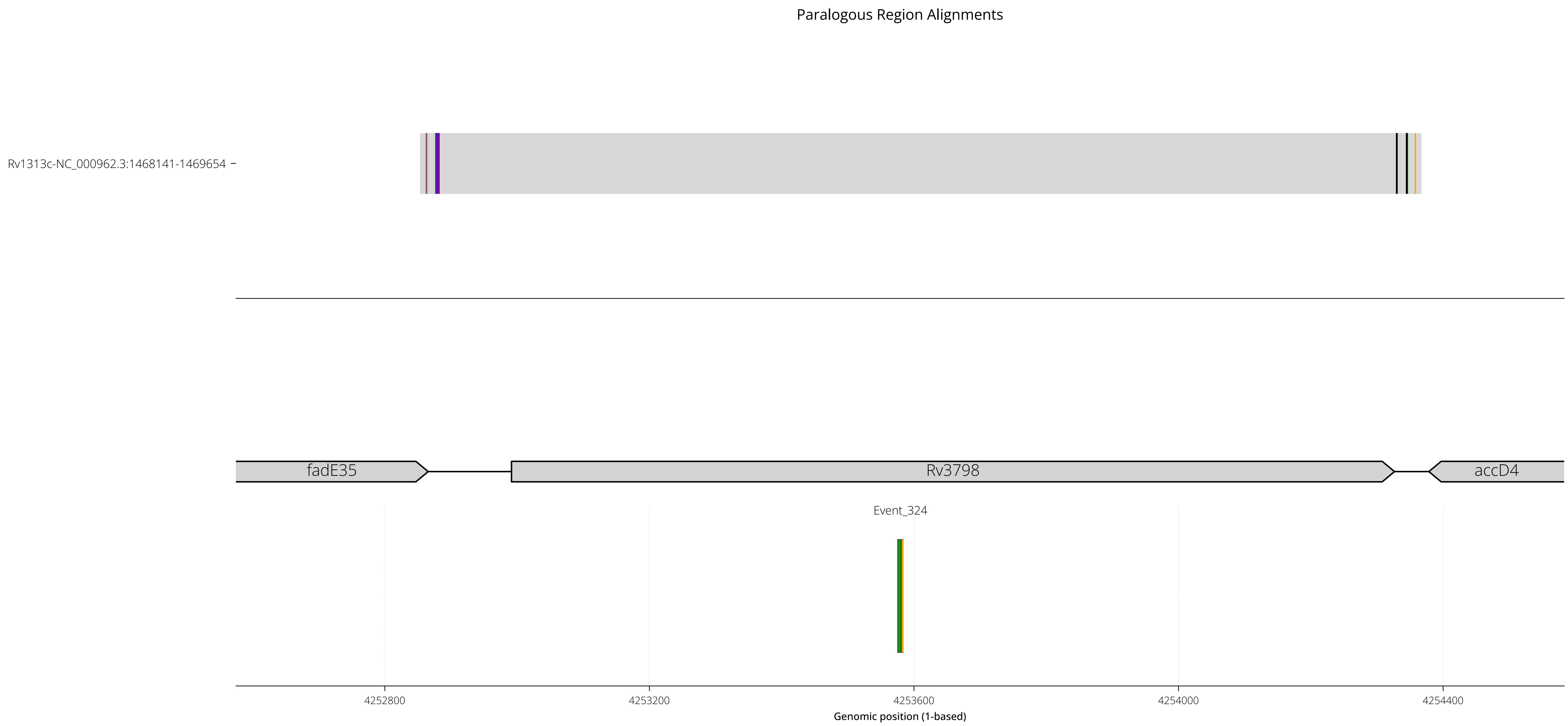

Circos Viz - GCE to Known Paralogs Locations
GCE ID: Event_324

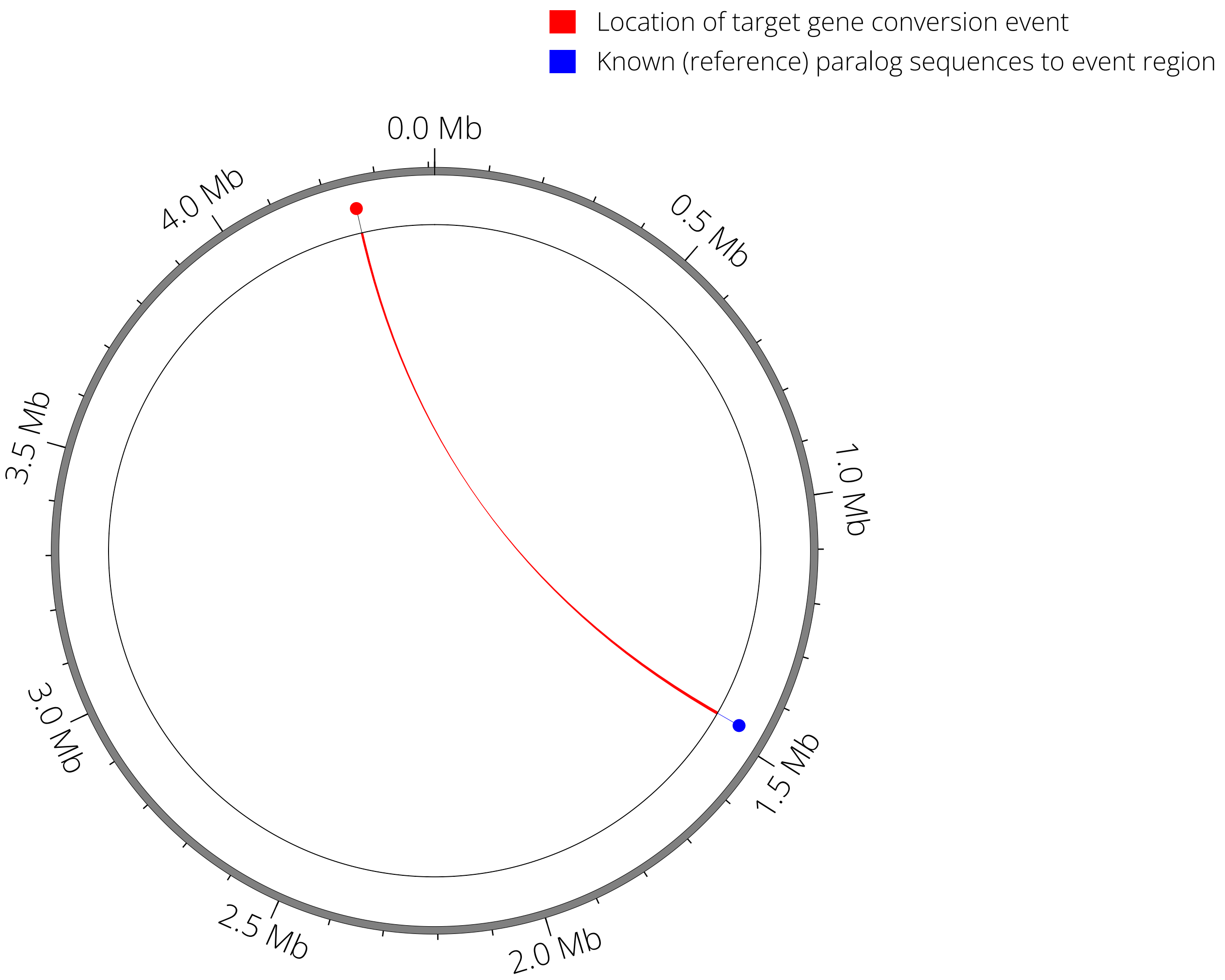

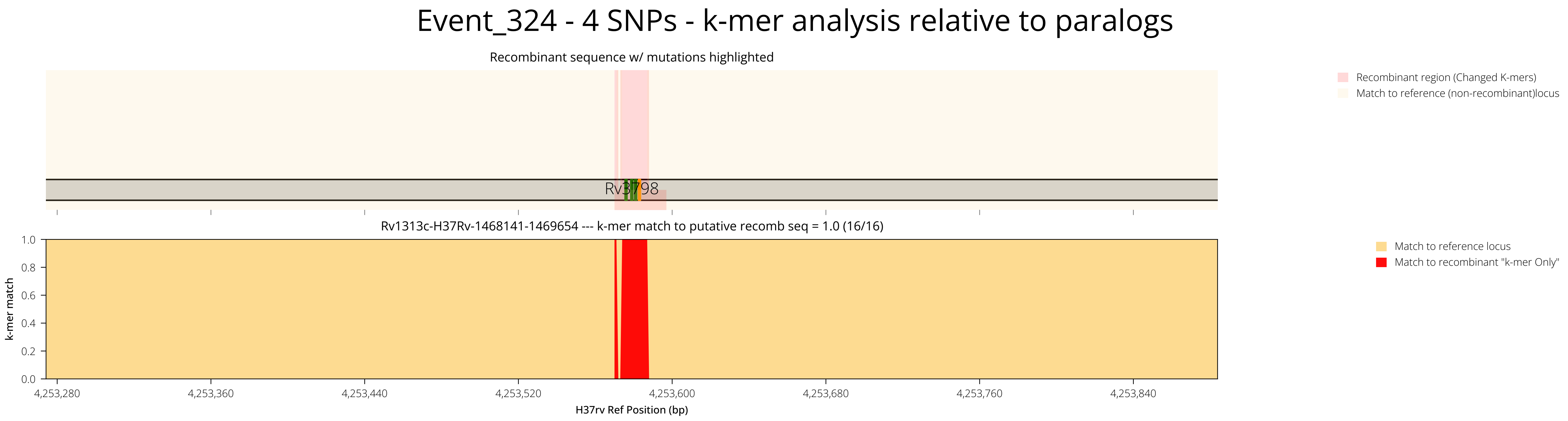

Event_324 - 4 SNPs - k-mer analysis relative to paralogs
Recombinant sequence w/ mutations highlighted
Rv3798
Recombinant region (Changed K-mers)
Match to reference (non-recombinant)locus
Rv1313c-H37Rv-1468141-1469654 --- k-mer match to putative recomb seq = 1.0 (16/16)
Match to reference locus
Match to recombinant "k-mer Only"
k-mer match
1.0
0.8
0.6
0.4
0.2
0.0
4,253,280
4,253,360
4,253,440
4,253,520
4,253,600
4,253,680
4,253,760
4,253,840
H37rv Ref Position (bp)

Sub-phylogeny Viz + Paralog Alignments
GCE ID: Event_324

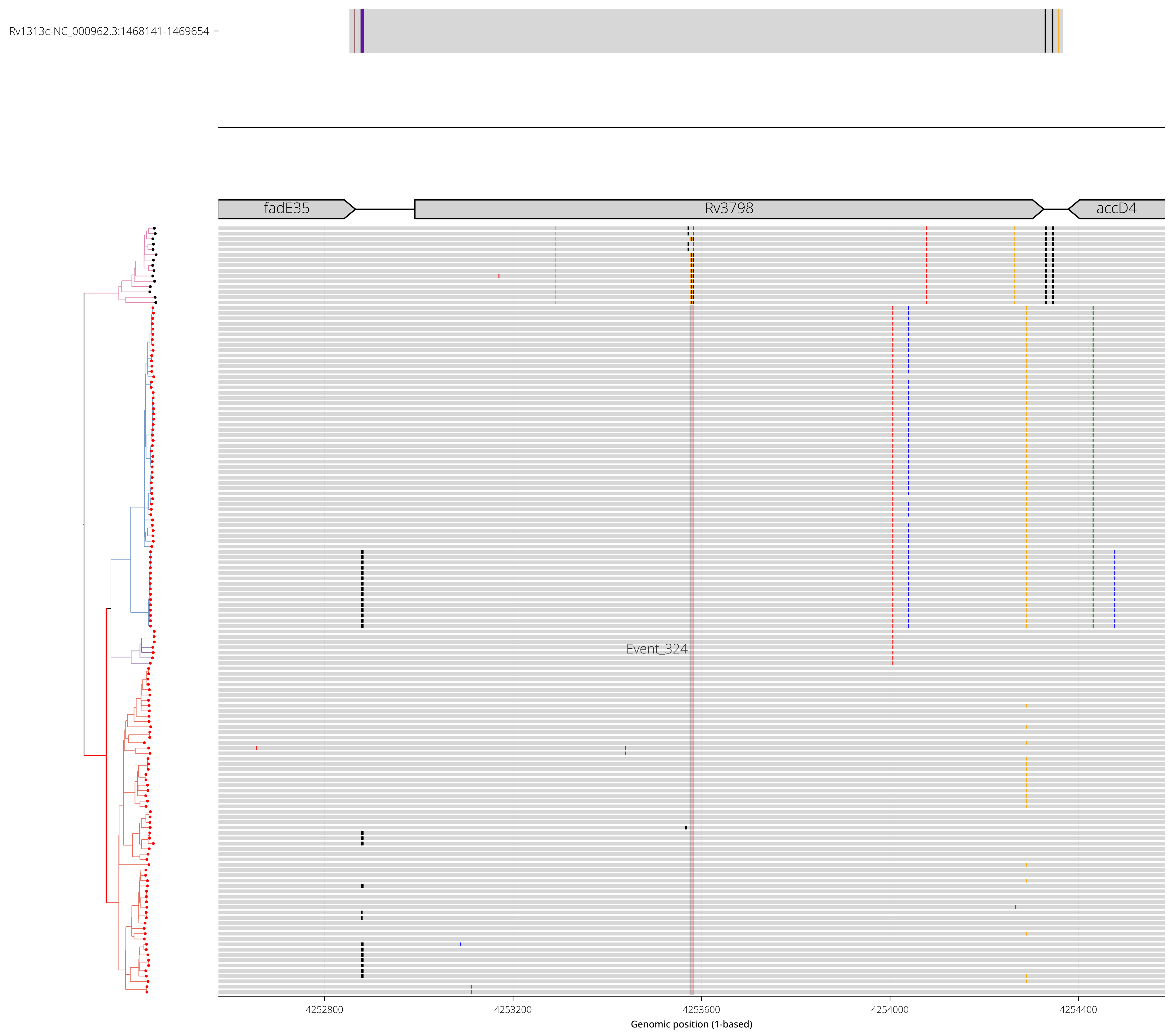

Full Phylogeny Viz + Paralog Alignments
GCE ID: Event_324

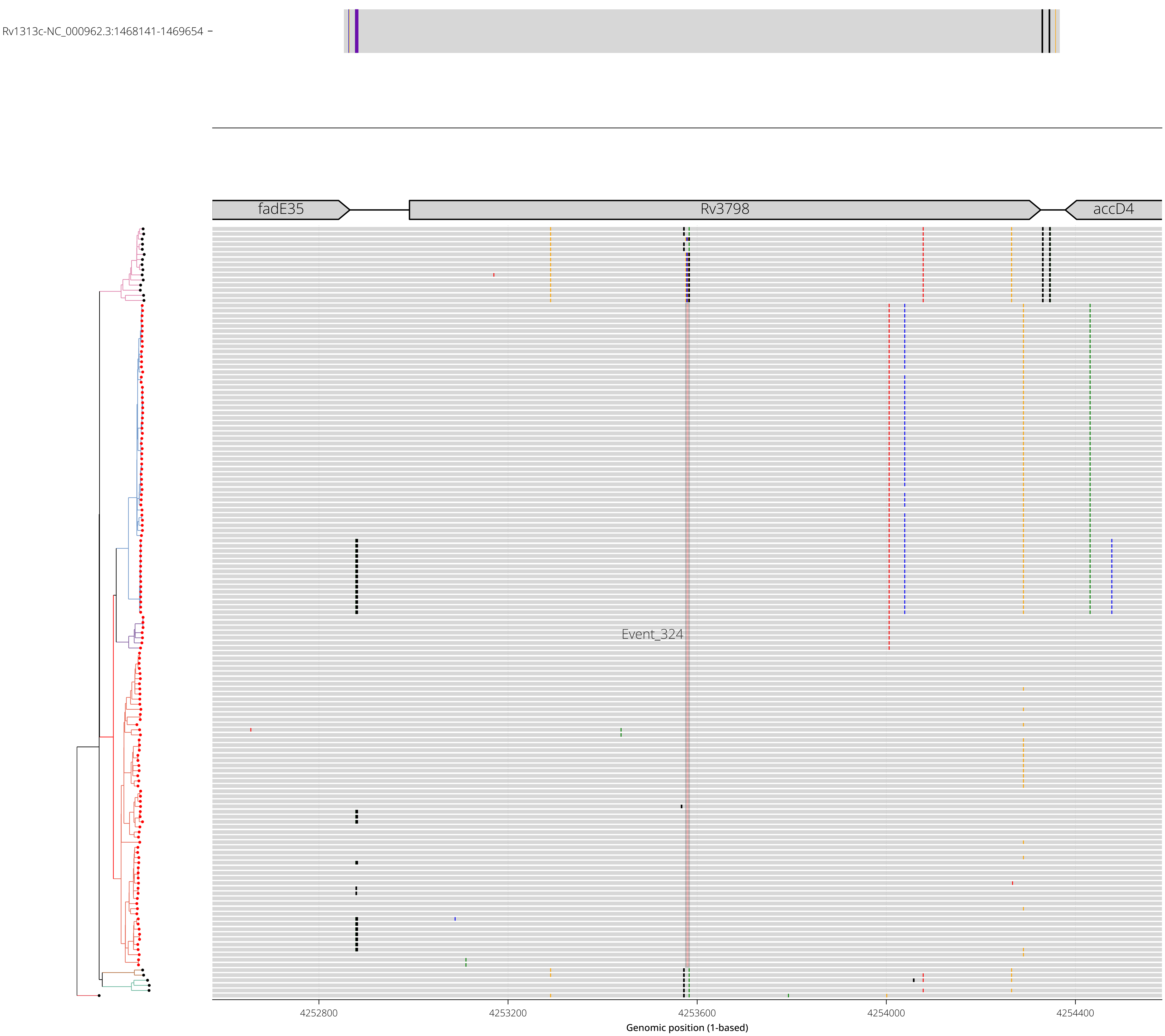

Supplement: Supplement 14 [file media-14.pdf]
